# Supplementary figures and images for: Extensive identification and analysis of conserved small ORFs in animals
Source: Genome Biol. 2015 Sep 14;16:179. doi: 10.1186/s13059-015-0742-x (PMC4568590; doi:10.1186/s13059-015-0742-x)

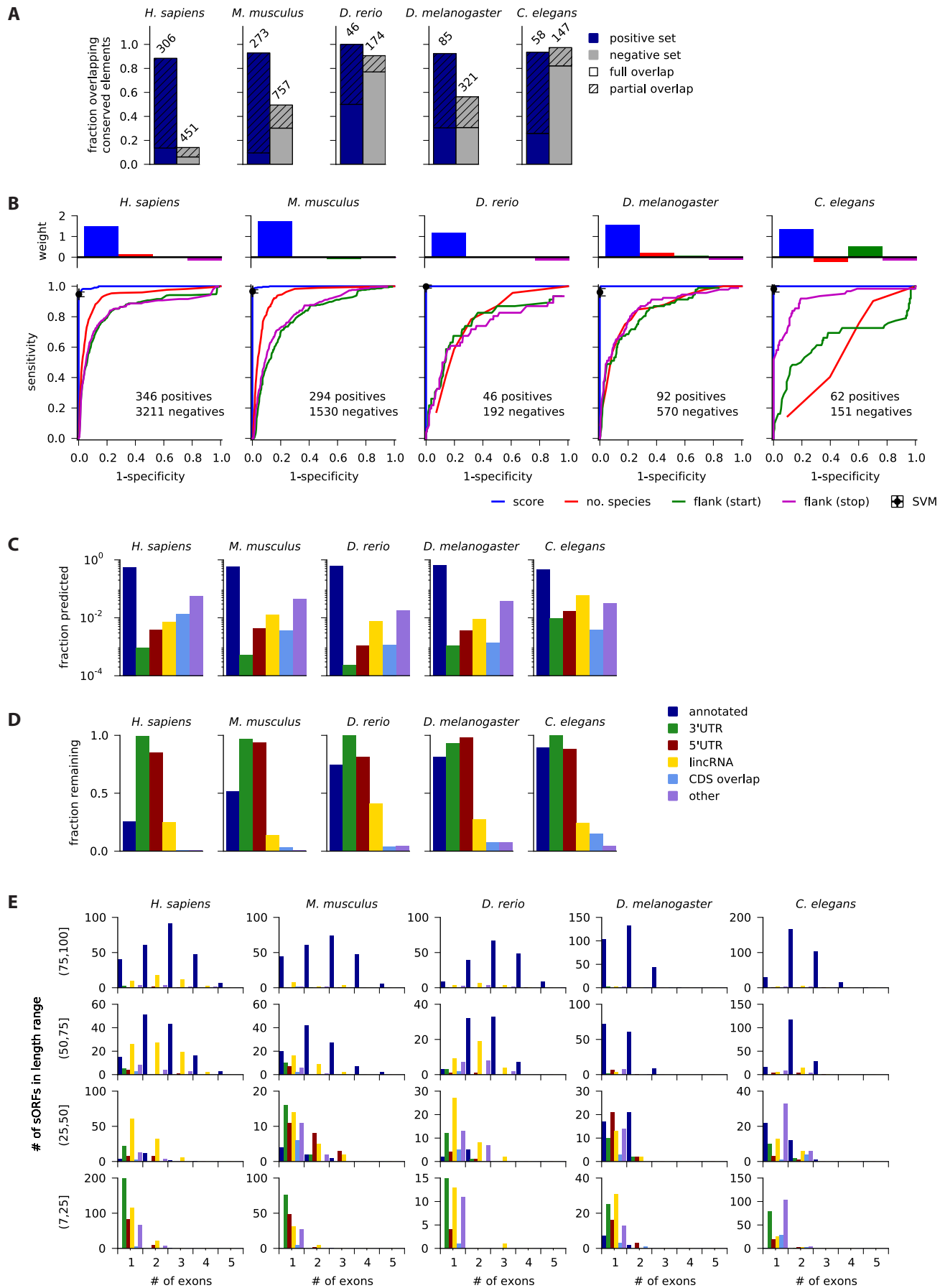

Supplement: Additional file 1: Figure S1. — Overview of the pipeline (relating to Fig. 1). A Many sORFs from the positive control and from the negative control overlap fully or partially with phastCons conserved elements. B The four conservation features all permit to separate positive from negative control (bottom panels); however, the phyloCSF score contributes most strongly to the SVM classifier. C Fraction of sORFs predicted as conserved (pre-overlap filter) for each category. D Fraction of sORFs retained after overlap filter in each category. E Number of exons spanned by sORFs in different length ranges. (PDF 1063 kb) [file 13059_2015_742_MOESM1_ESM.pdf]

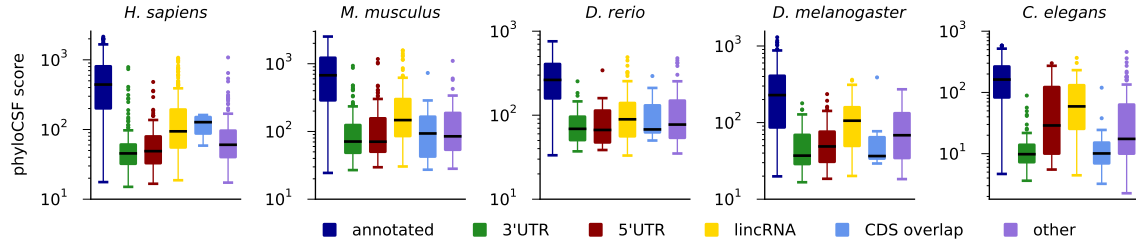

Supplement: Additional file 7: Figure S2. — (Relating to Fig. 2) non-adjusted phyloCSF scores for sORFs in different categories. (PDF 27 kb) [file 13059_2015_742_MOESM7_ESM.pdf]

**A: human transcriptome**

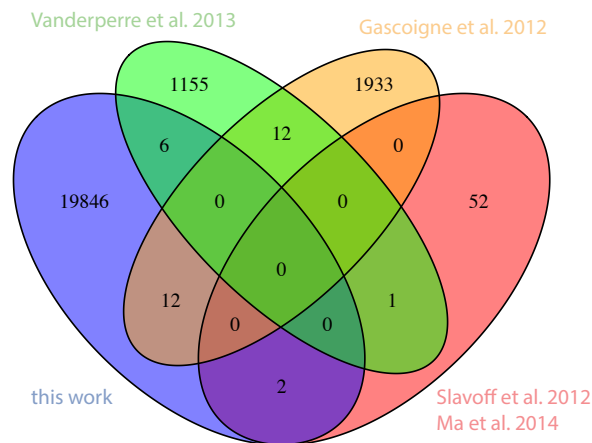

**B: human lincRNAs**

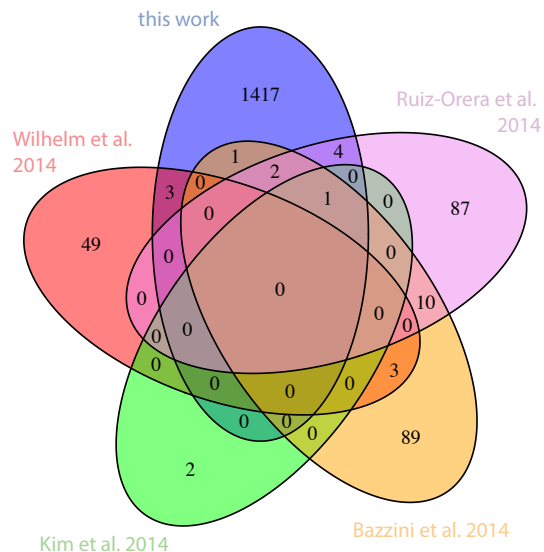

**C: mouse transcriptome**

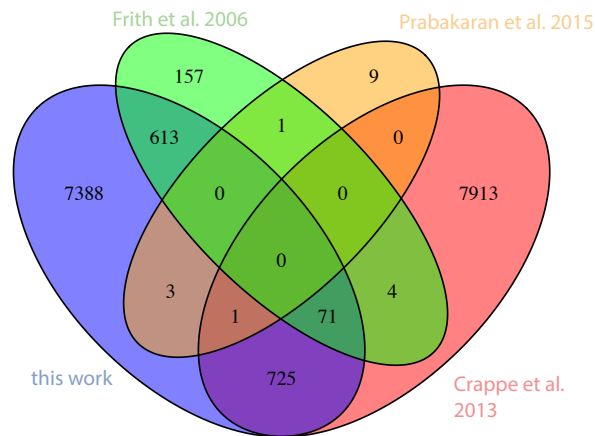

**D: zebrafish lincRNAs**

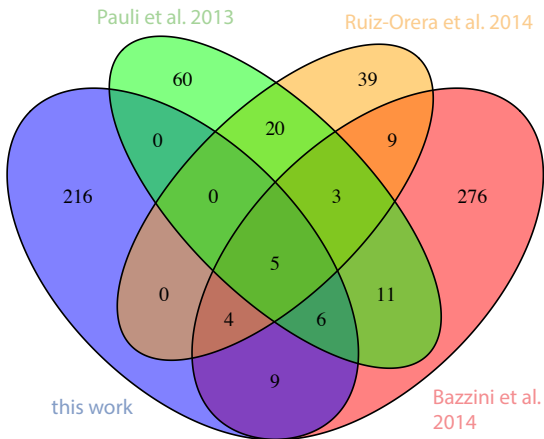

Supplement: Additional file 9: Figure S3. — Comparison between previous studies (relating to Fig. 3) by Venn diagrams for sORFs in the human transcriptome (A), in human lincRNAs (B), in the mouse transcriptome (C), and zebrafish lincRNAs (D). Results from this study are used before overlap filter. (PDF 999 kb) [file 13059_2015_742_MOESM9_ESM.pdf]

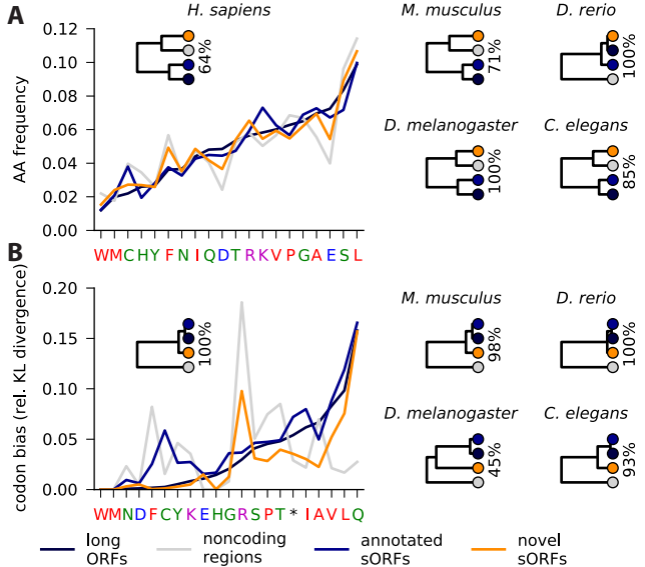

Supplement: Additional file 10: Figure S4. — Sequence features of novel peptides (relating to Fig. 4). A Amino acid frequencies in long annotated ORFs, ORFs from non-coding control regions, predicted annotated sORFs and novel predicted sORFs are compared (shown for H. sapiens), and a hierarchical clustering is performed. Percentage values indicate how often the same clusters are obtained in a re-sampling analysis. Hydrophobic, acidic, basic, and hydroxyl residues are colored red, blue, magenta, and green, respectively. B Codon bias is evaluated from the Kullback-Leibler divergence (Materials and methods). Clustering done as in A. (PDF 911 kb) [file 13059_2015_742_MOESM10_ESM.pdf]

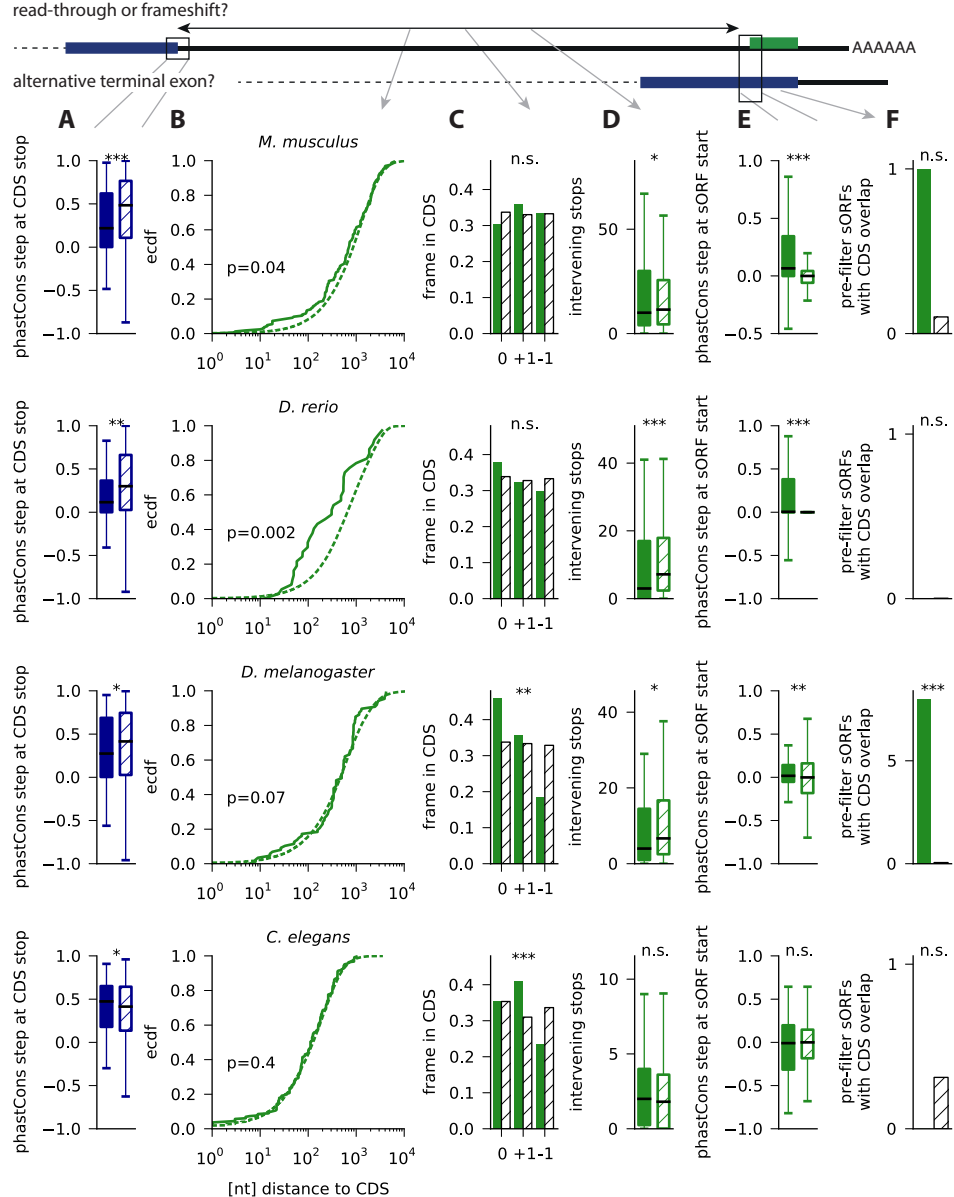

Supplement: Additional file 11: Figure S5. — Properties of 3′UTR sORFs (same as Fig. 5 for the other species). (PDF 1026 kb) [file 13059_2015_742_MOESM11_ESM.pdf]

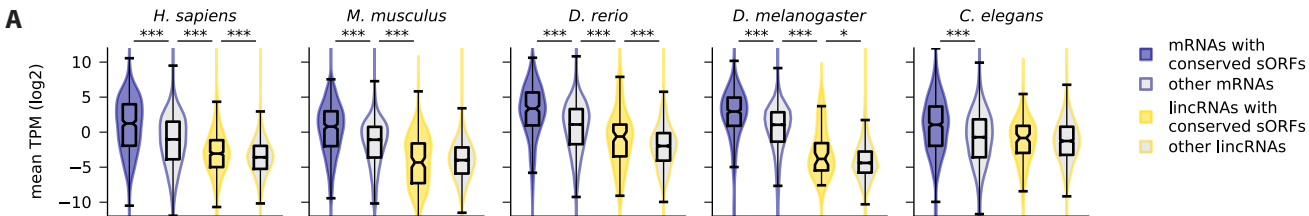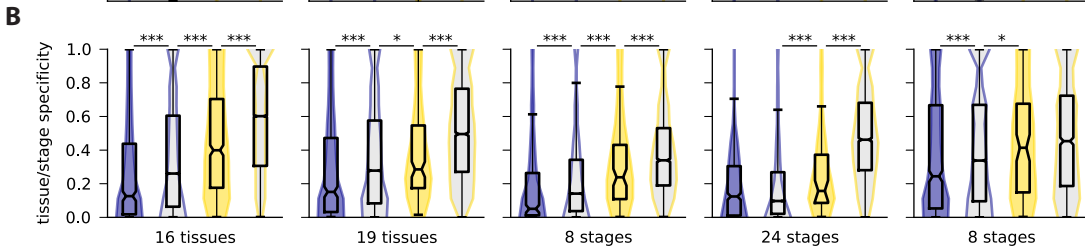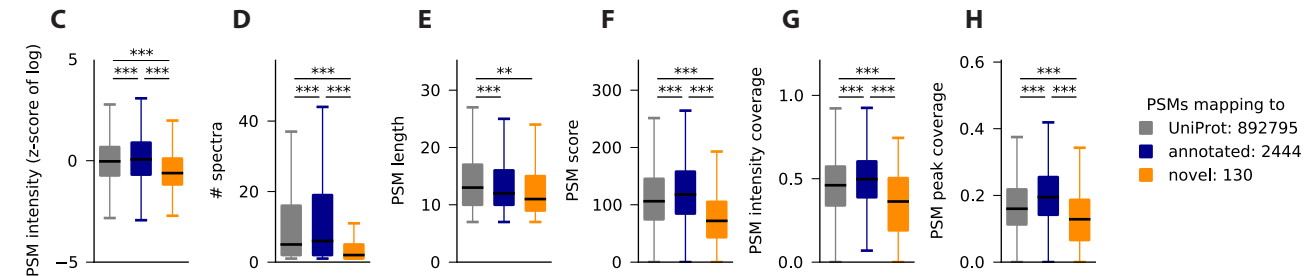

Supplement: Additional file 13: Figure S6. — Expression analysis (relating to Fig. 6). A Violin and box plots of mean TPM values for mRNAs hosting predicted annotated sORFs, other mRNAs, lincRNAs hosting predicted novel sORFs, and other lincRNAs, for 16 and 19 tissues in human and mouse, and eight, 24, and eight developmental stages in zebrafish, fruit fly, and C. elegans, respectively. B Violin and box plots of tissue or stage specificity for these transcripts. C Intensity for PSMs supporting annotated sORFs and peptides supporting novel predicted sORFs, aggregated over all datasets after log-transformation and normalization (z-score) relative to PSMs mapping to UniProt proteins. D Number of spectra, (E) PSM length, (F) Andromeda score, (G) peak intensity coverage, and (H) peak coverage for the PSMs shown in C. ***P <0.001; **P <0.01; *P <0.05 (Mann-Whitney tests). (PDF 1042 kb) [file 13059_2015_742_MOESM13_ESM.pdf]
