# Supplementary material for: Extensive identification and analysis of conserved small ORFs in animals
Source: Genome Biol. 2015 Sep 14;16:179. doi: 10.1186/s13059-015-0742-x (PMC4568590; doi:10.1186/s13059-015-0742-x)
Supplement: Additional file 16: Figure S7. — Spectra for the PMS from the human datasets. (PDF 1496 kb) [file 13059_2015_742_MOESM16_ESM.pdf]

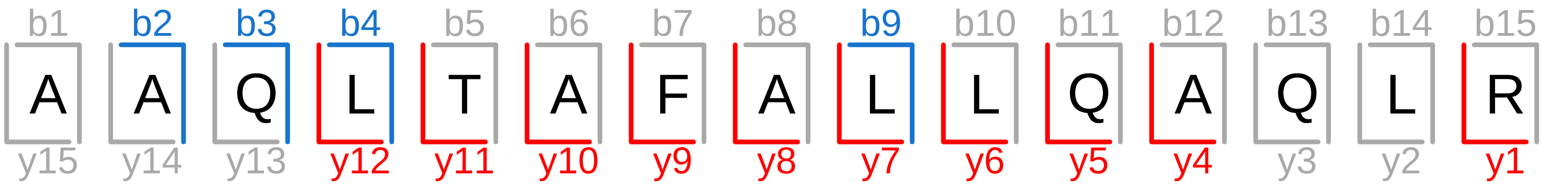

AAQLTAFALLQAQLR
Score: 57 ; 1613.9253 m/z; 807.96994 m/z; −0.96247 ppm; MULTI−MSMS

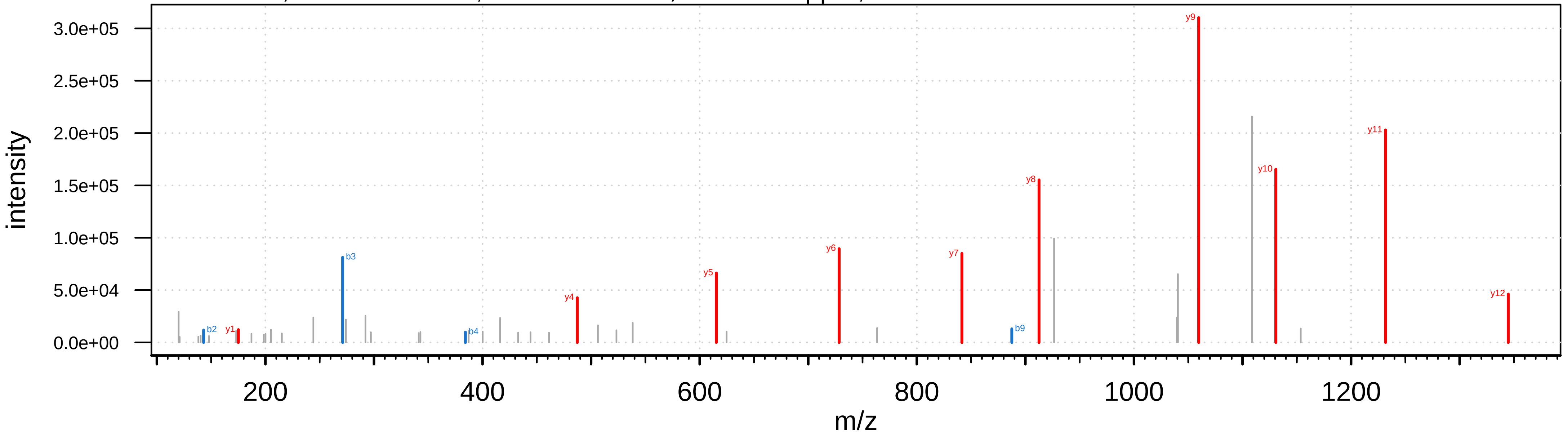

Raw File: Bibo_20130110_CHS_IEF100_20121129_3−10linear_Serva_7cm_250ug_06
Scan Number: 45895
Proteins:
sp|Q69YL0|NCAS2_HUMAN
ENST00000602845_chr3:196669588−196669887:+

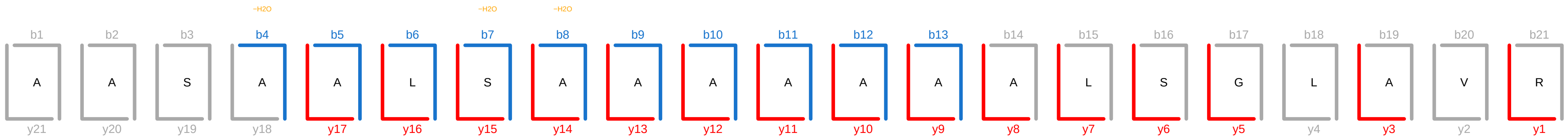

(ac)AASAALSAAAAAAALSGLAVR

Score: 121 ; 1825.0058 m/z; 913.51016 m/z; −0.24434 ppm; MULTI−MSMS

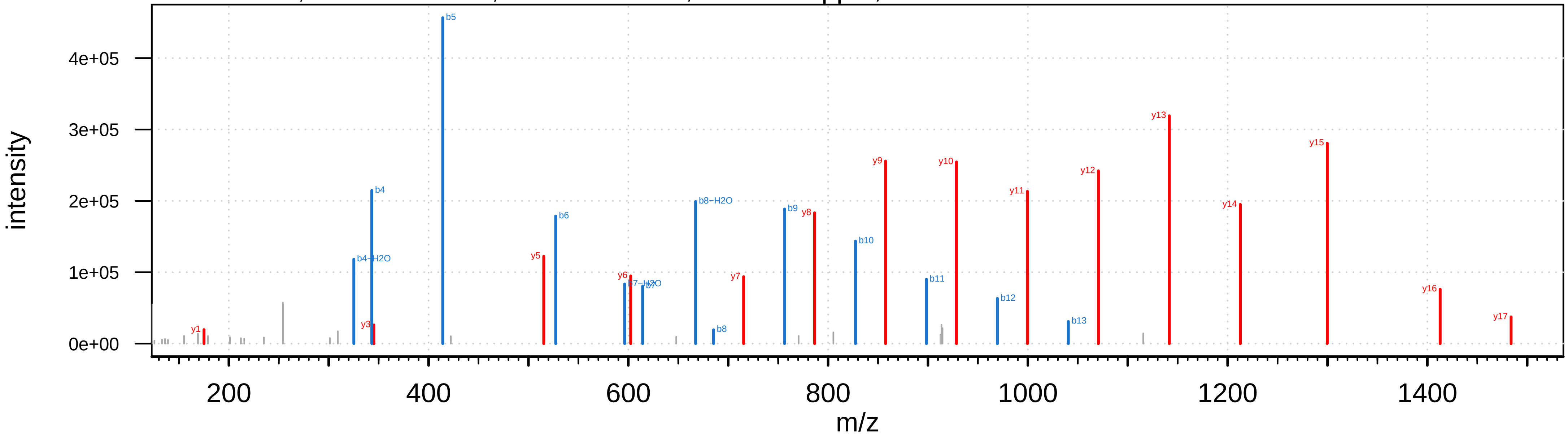

Raw File: Bibo_20130621_CHS_IEF_3−10linear_24slices_08
Scan Number: 16939
Proteins:
ENST00000433425_chrX:134232427−134232663:−
ENST00000417443_chrX:134556057−134556293:+

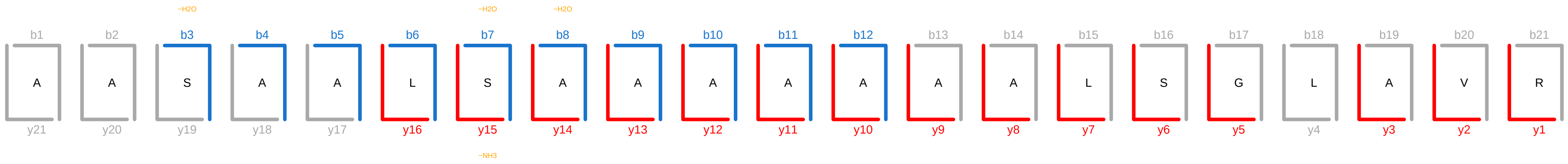

(ac)AASAALSAAAAAAALSGLAVR

Score: 89 ; 1825.0058 m/z; 913.51016 m/z; −1.3711 ppm; MULTI−MSMS

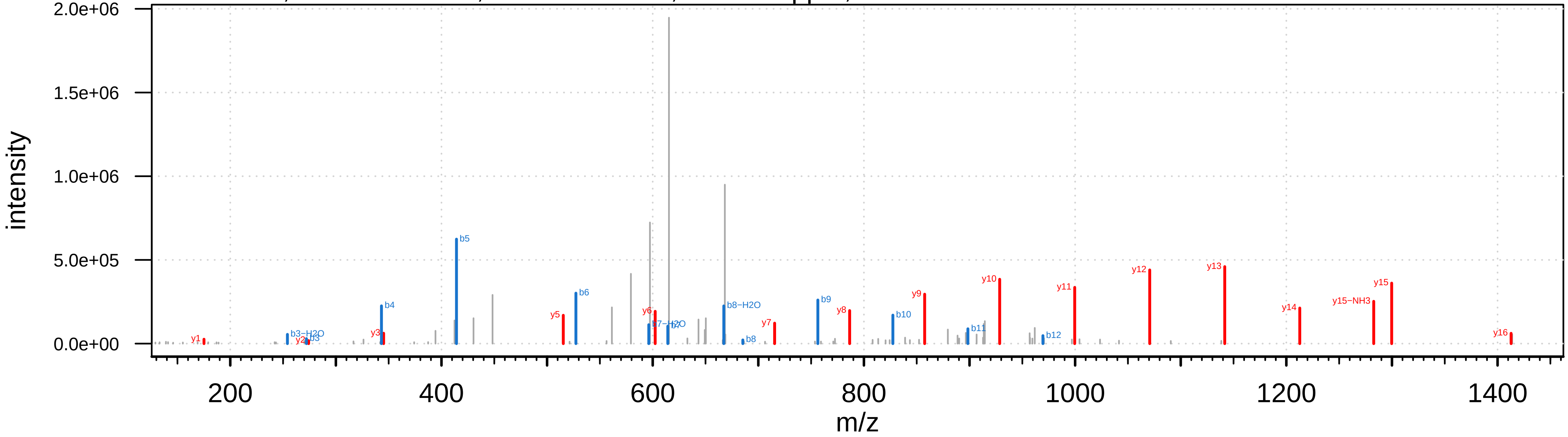

Raw File: Bibo_20130621_CHS_IEF_3−10linearpep_24slices_11

Scan Number: 17553

Proteins:

ENST00000433425_chrX:134232427−134232663:−

ENST00000417443_chrX:134556057−134556293:+

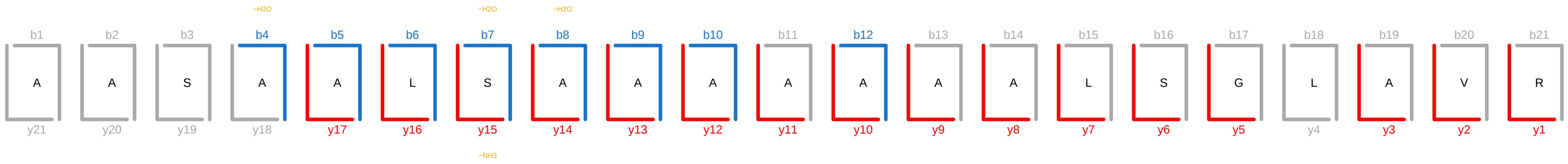

(ac)AASAALSAAAAAAALSGLAVR

Score: 94 ; 1825.0058 m/z; 913.51016 m/z; NaN ppm; MSMS

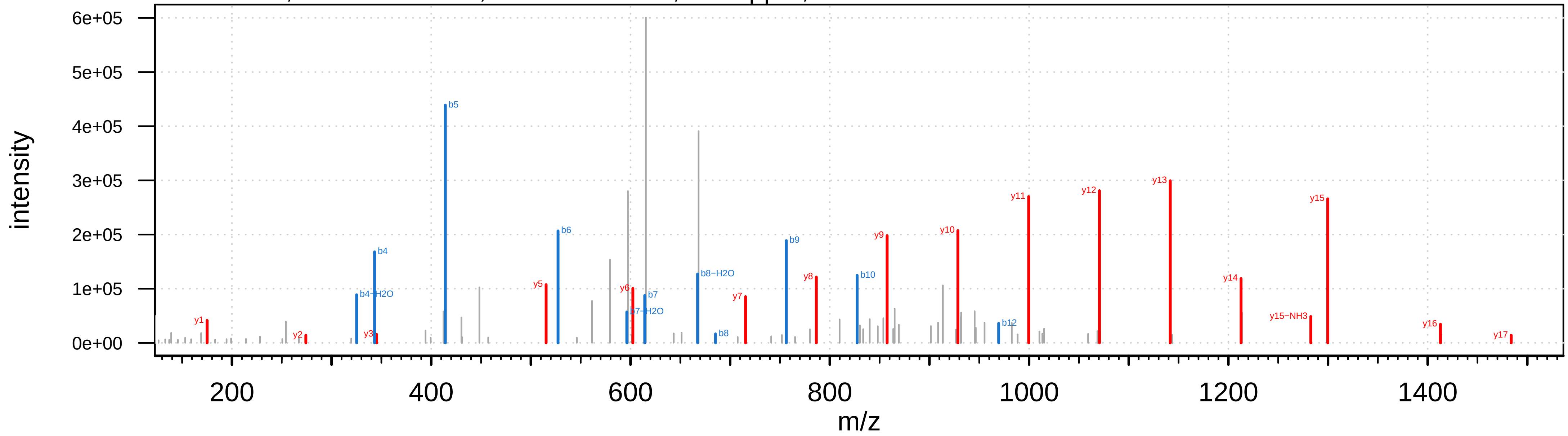

Raw File: Bibo_20130621_CHS_IEF_3−10linearpep_24slices_12

Scan Number: 18159

Proteins:

ENST00000433425_chrX:134232427−134232663:−

ENST00000417443_chrX:134556057−134556293:+

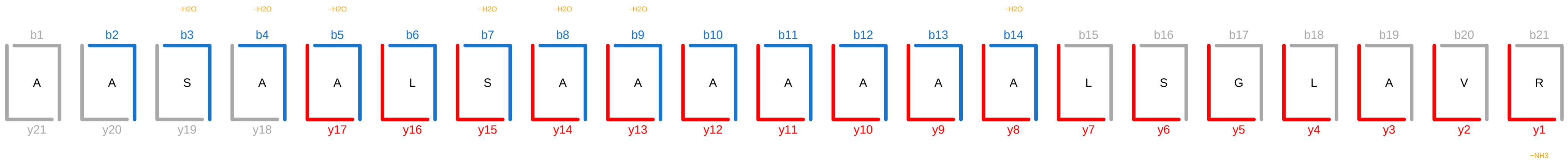

(ac)AASAALSAAAAAAALSGLAVR

Score: 163 ; 1825.0058 m/z; 913.51016 m/z; −0.35688 ppm; MULTI−MSMS

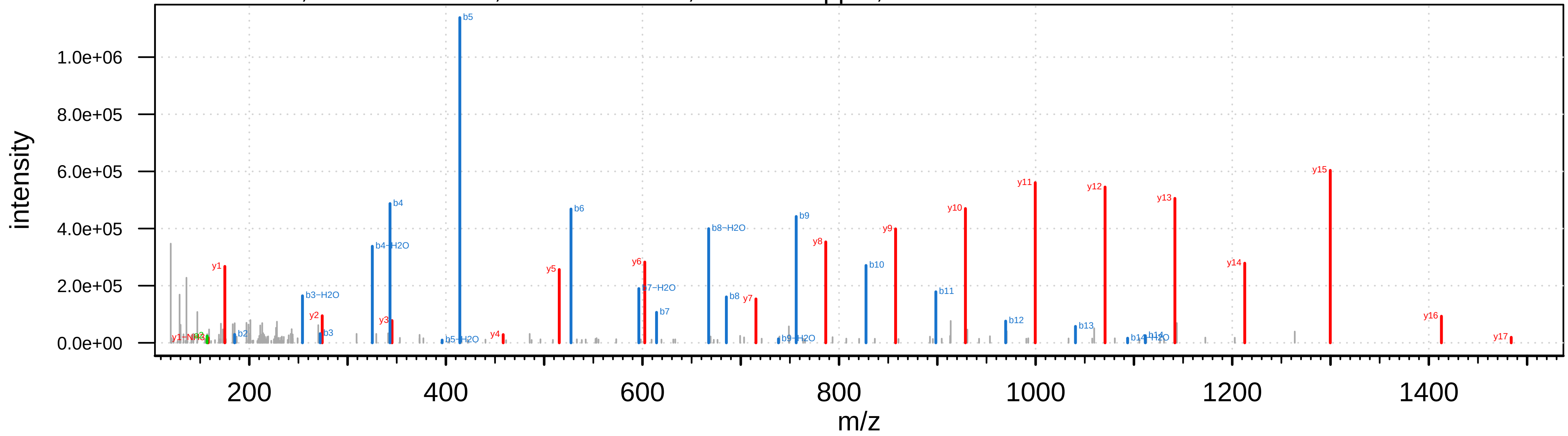

Raw File: Grobi_20121019_CHS_PIECEIEF_20121017_3−10_7cm_250ug_05

Scan Number: 102781

Proteins:

ENST00000433425_chrX:134232427−134232663:−

ENST00000417443_chrX:134556057−134556293:+

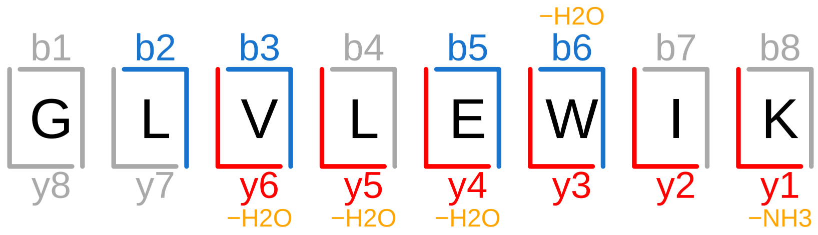

GLVLEWIK
Score: 96 ; 956.5695 m/z; 479.29203 m/z; 0.46632 ppm; MULTI−MSMS

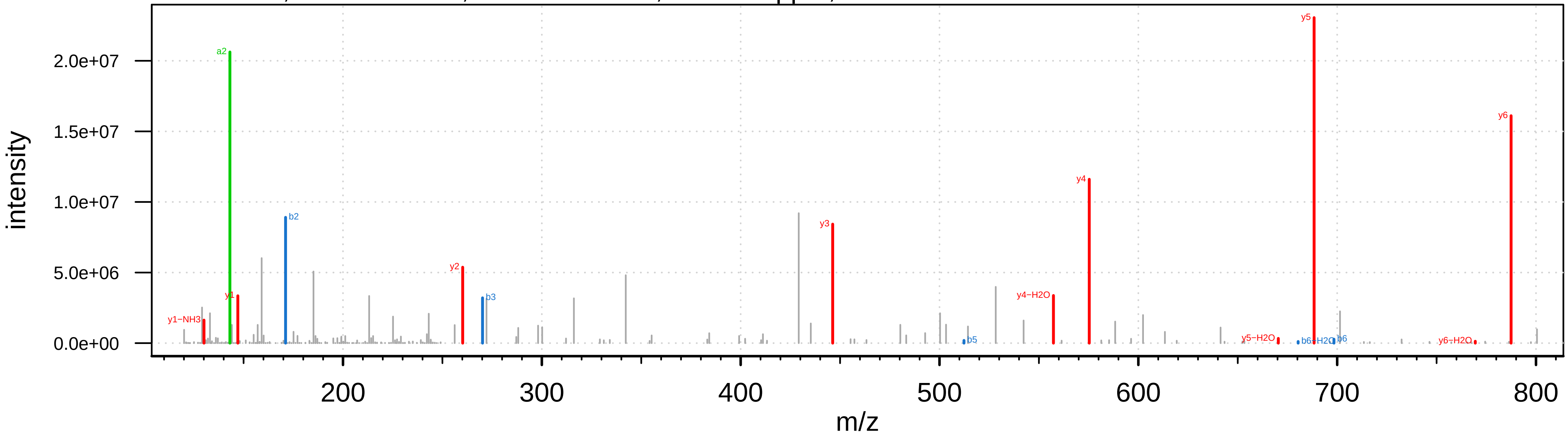

Raw File: Grobi_20121019_CHS_PIECEIEF_20121017_3−10_7cm_250ug_05
Scan Number: 63753
Proteins:
TCONS_l2_00001296_chr1:79520703−79520992:−

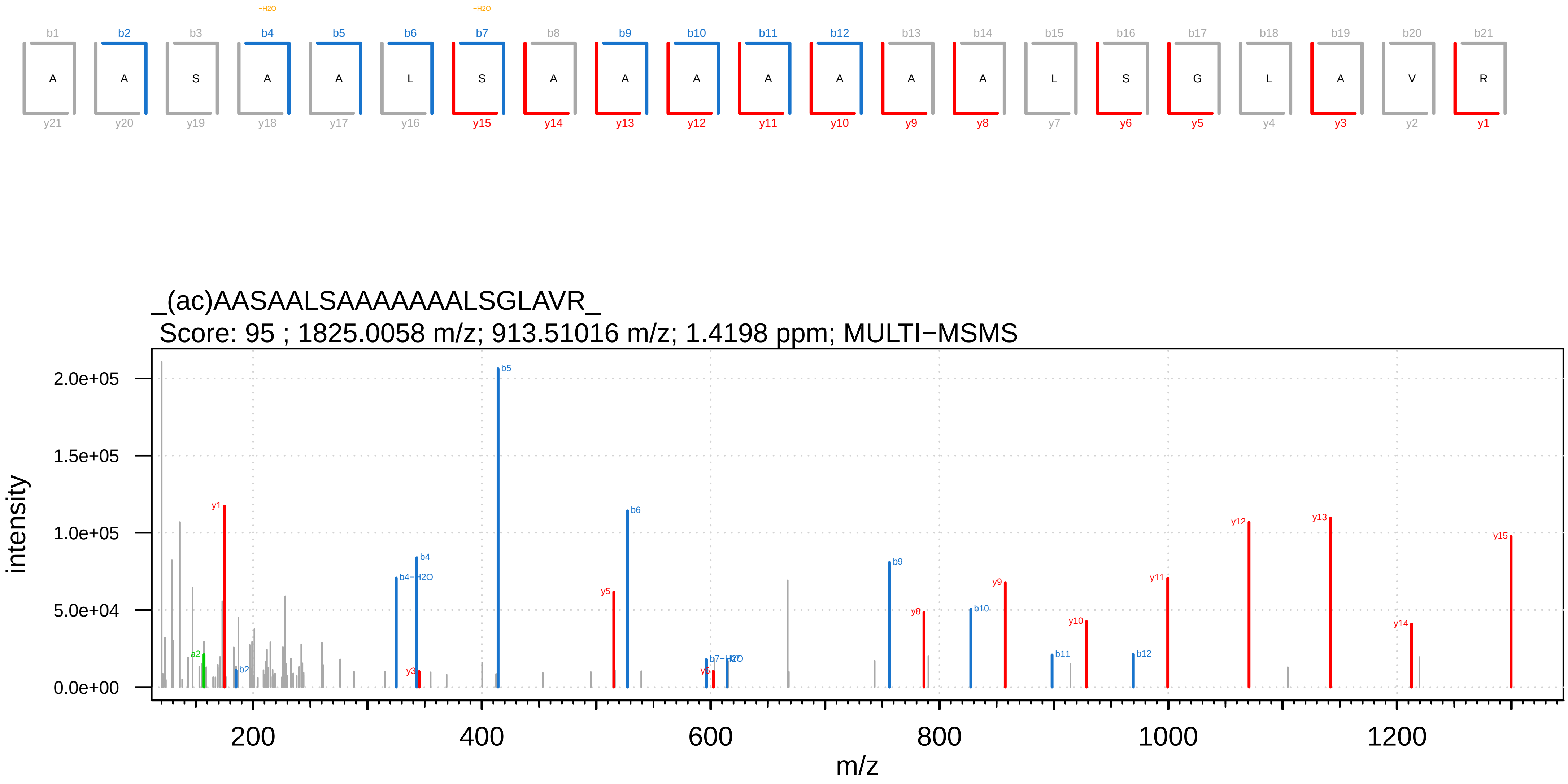

Raw File: Grobi_20130312_CHS_IEF100_20130309_3−10linear_Serva_7cm_1mg_1elution_02
Scan Number: 93471
Proteins:
ENST00000433425_chrX:134232427−134232663:−
ENST00000417443_chrX:134556057−134556293:+

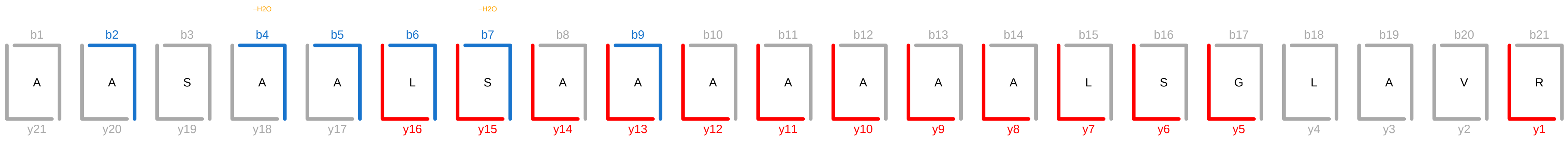

(ac)AASAALSAAAAAAALSGLAVR
Score: 57 ; 1825.0058 m/z; 913.51016 m/z; 0.27962 ppm; MULTI−MSMS

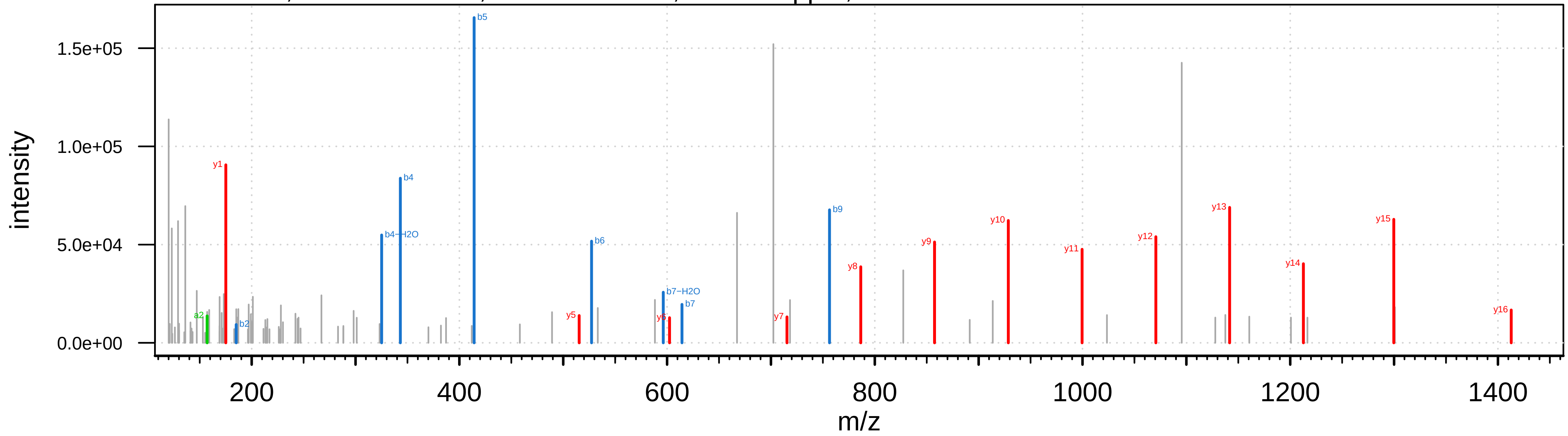

Raw File: Grobi_20130312_CHS_IEF100_20130309_3−10linear_Serva_7cm_1mg_1elution_04
Scan Number: 95039
Proteins:
ENST00000433425_chrX:134232427−134232663:−
ENST00000417443_chrX:134556057−134556293:+

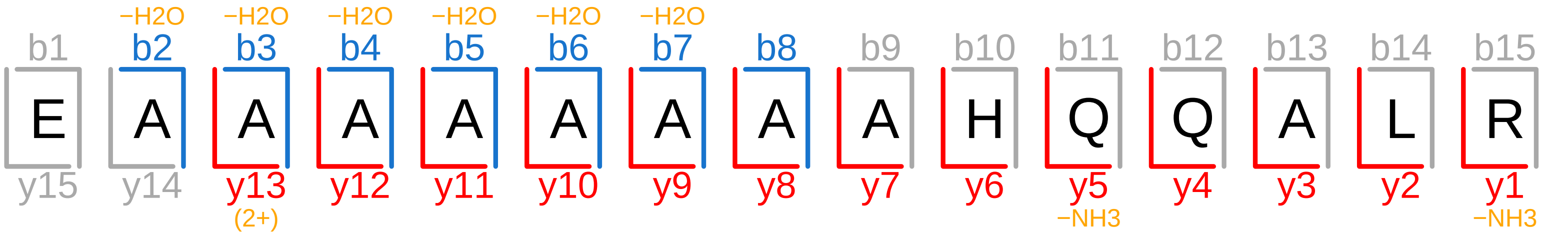

EAAAAAAAAHQQALR
Score: 130 ; 1448.7484 m/z; 725.38149 m/z; 1.8631 ppm; MULTI−MSMS

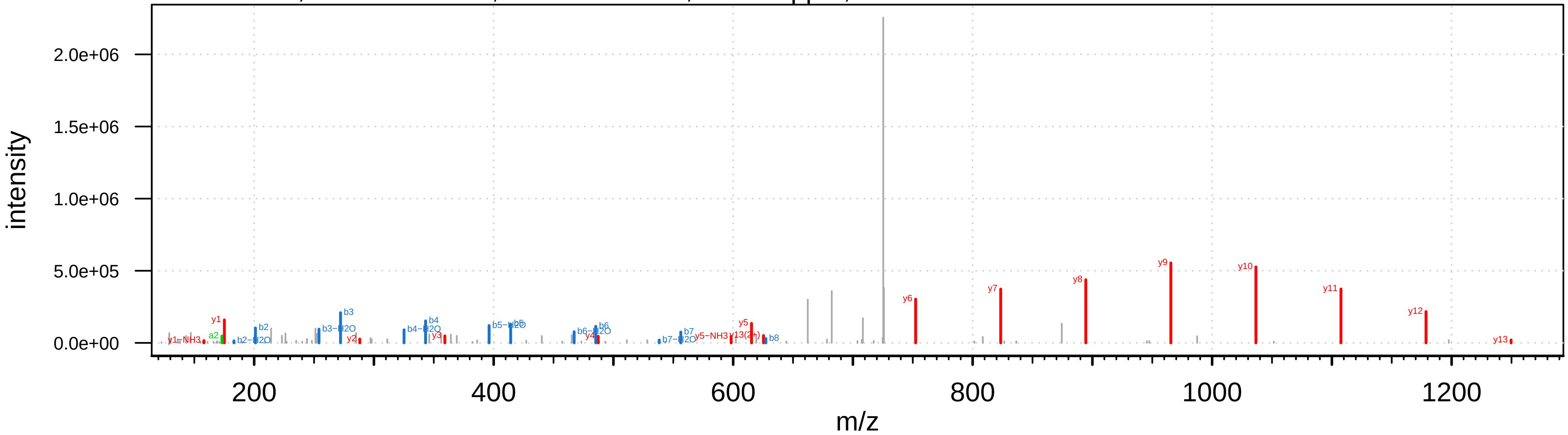

Raw File: Grobi_20130312_CHS_IEF100_20130309_3−10linear_Serva_7cm_1mg_1elution_04
Scan Number: 3952
Proteins:
ENST00000394071_chr14:74185546−74185677:−

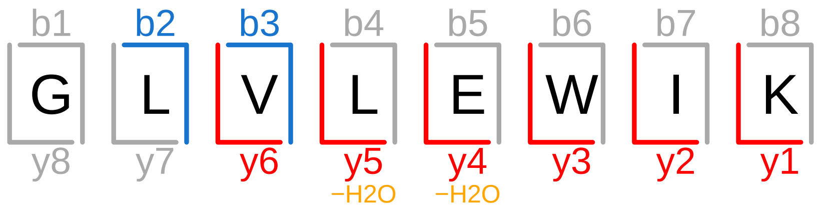

GLVLEWIK
Score: 62 ; 956.5695 m/z; 479.29203 m/z; 0.288 ppm; MULTI−MSMS

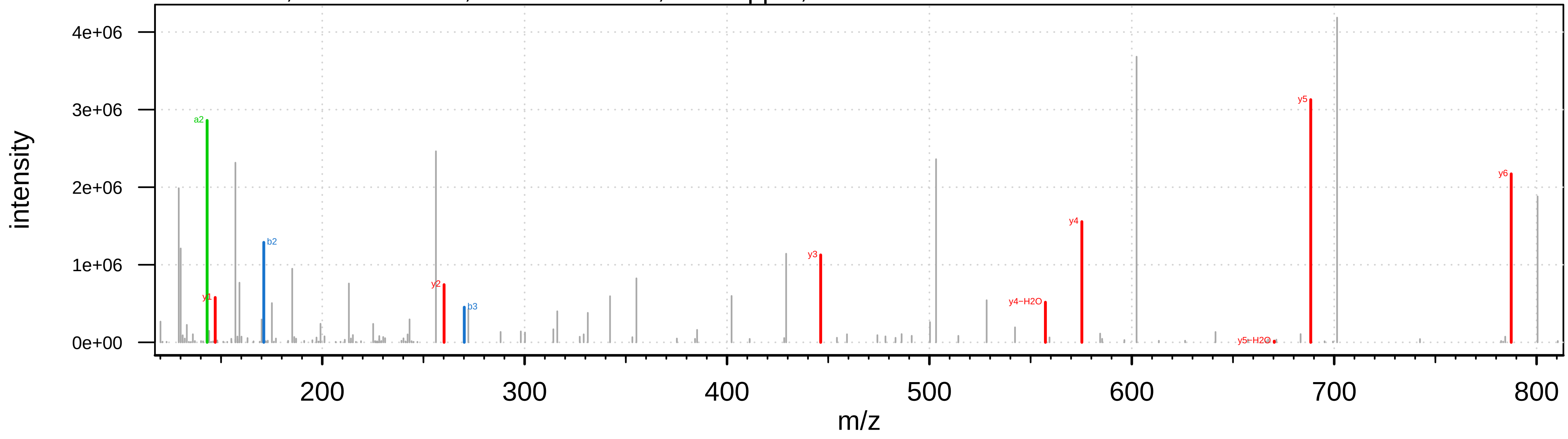

Raw File: Grobi_20130312_CHS_IEF100_20130309_3−10linear_Serva_7cm_1mg_1elution_04
Scan Number: 38409
Proteins:
TCONS_l2_00001296_chr1:79520703−79520992:−

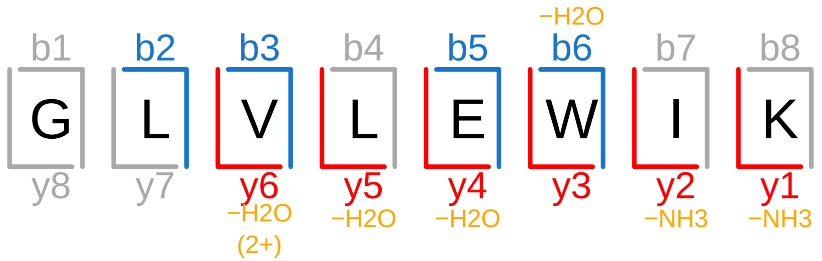

GLVLEWIK
Score: 76 ; 956.5695 m/z; 479.29203 m/z; 0.288 ppm; MULTI−MSMS

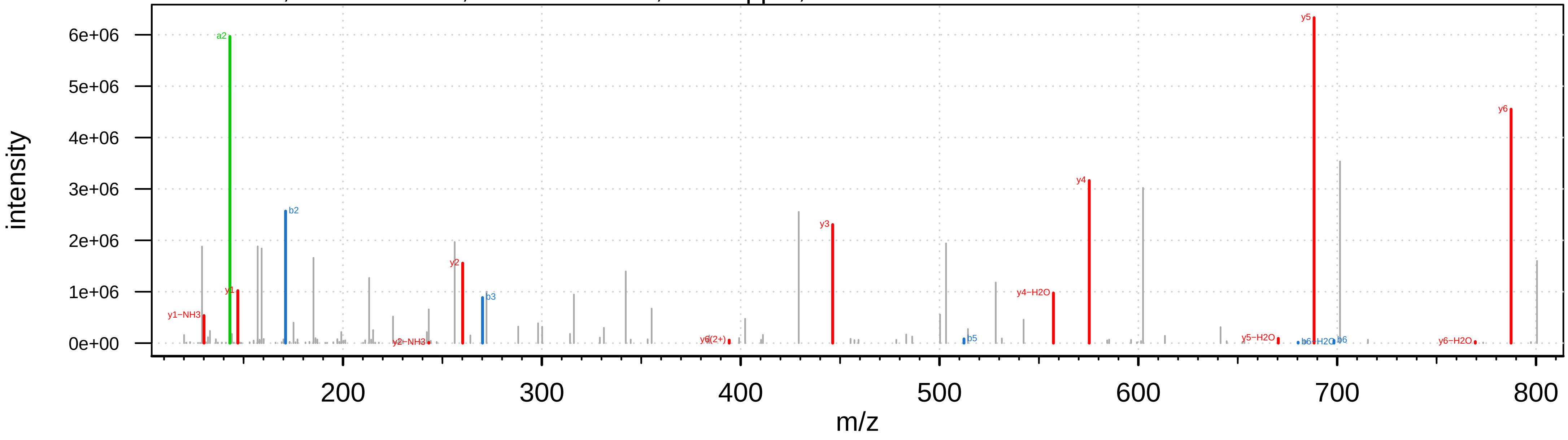

Raw File: Grobi_20130312_CHS_IEF100_20130309_3−10linear_Serva_7cm_1mg_1elution_04
Scan Number: 38654
Proteins:
TCONS_l2_00001296_chr1:79520703−79520992:−

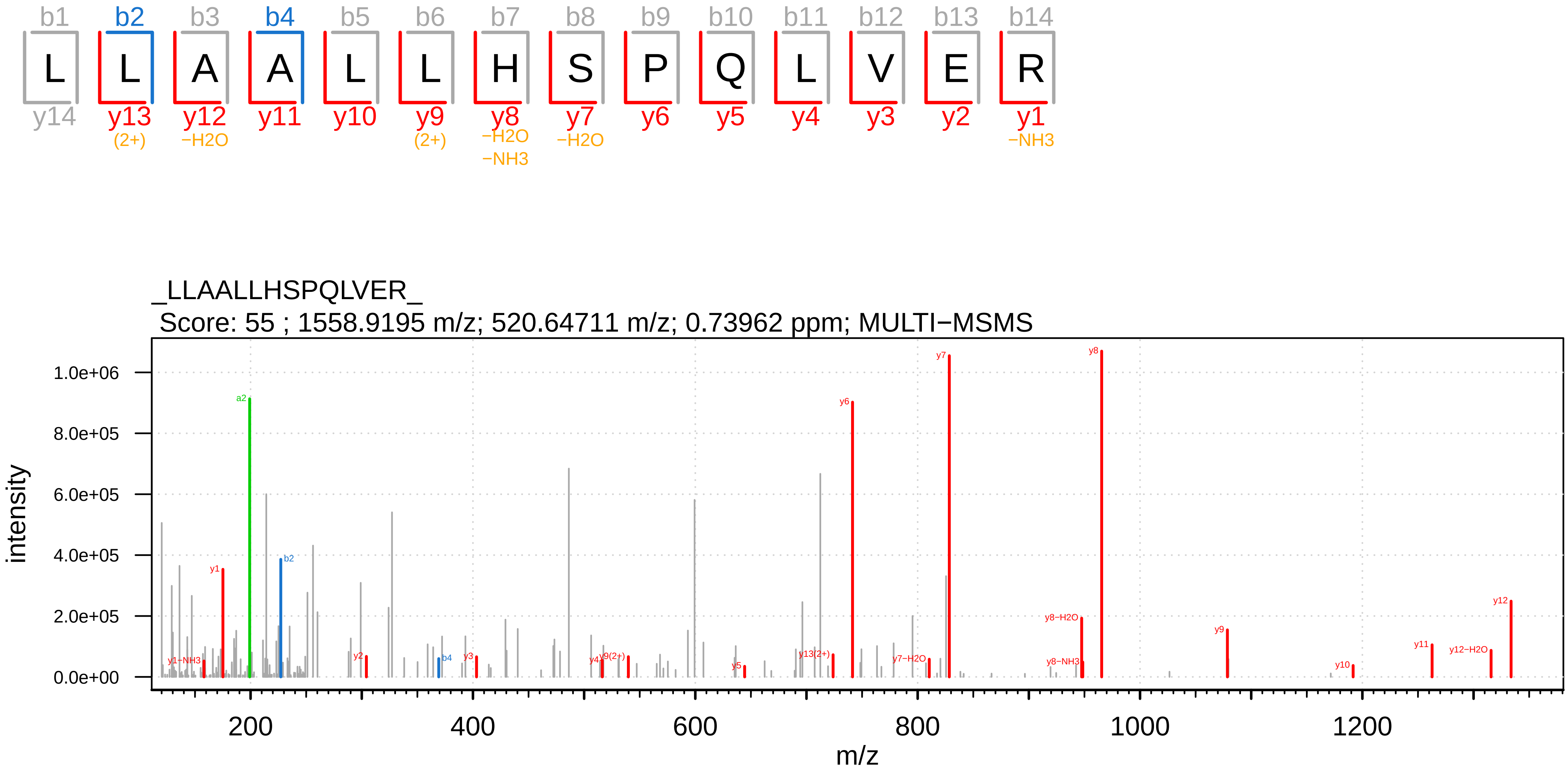

Raw File: Grobi_20130312_CHS_IEF100_20130309_3−10linear_Serva_7cm_1mg_1elution_04
Scan Number: 33266
Proteins:
sp|Q69YL0|NCAS2_HUMAN
ENST00000602845_chr3:196669588−196669887:+

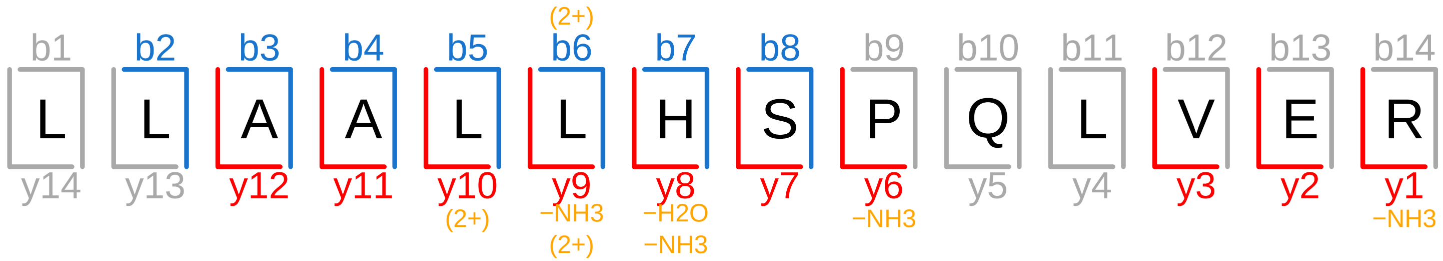

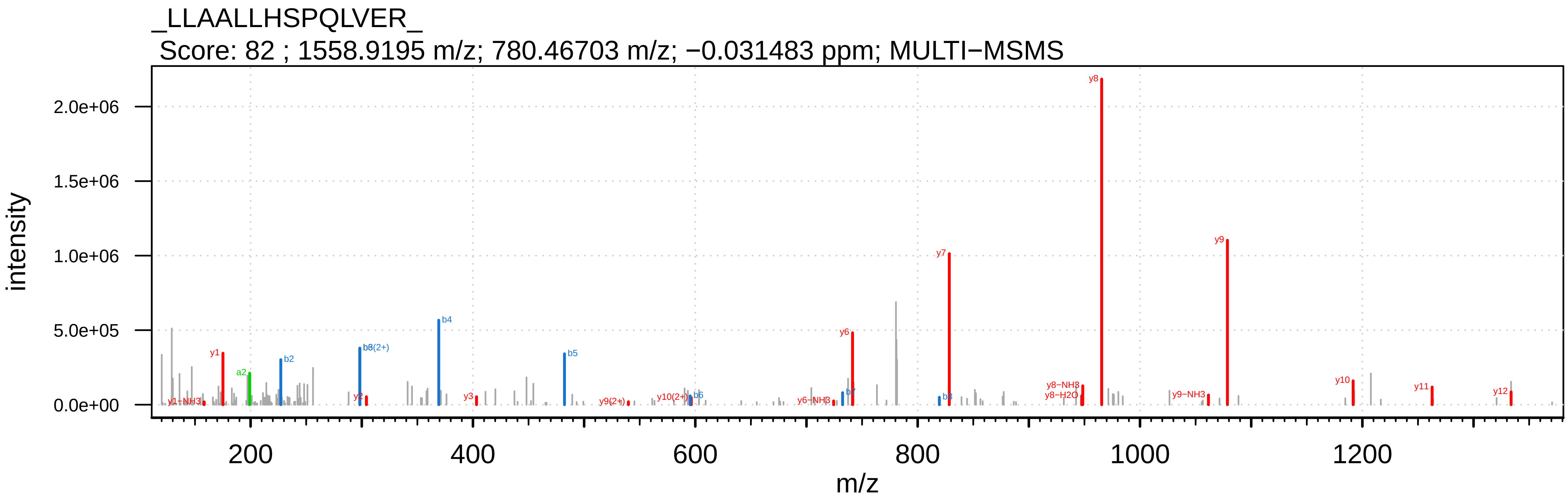

Raw File: Grobi_20130312_CHS_IEF100_20130309_3−10linear_Serva_7cm_1mg_1elution_04
Scan Number: 33305
Proteins:
sp|Q69YL0|NCAS2_HUMAN
ENST00000602845_chr3:196669588−196669887:+

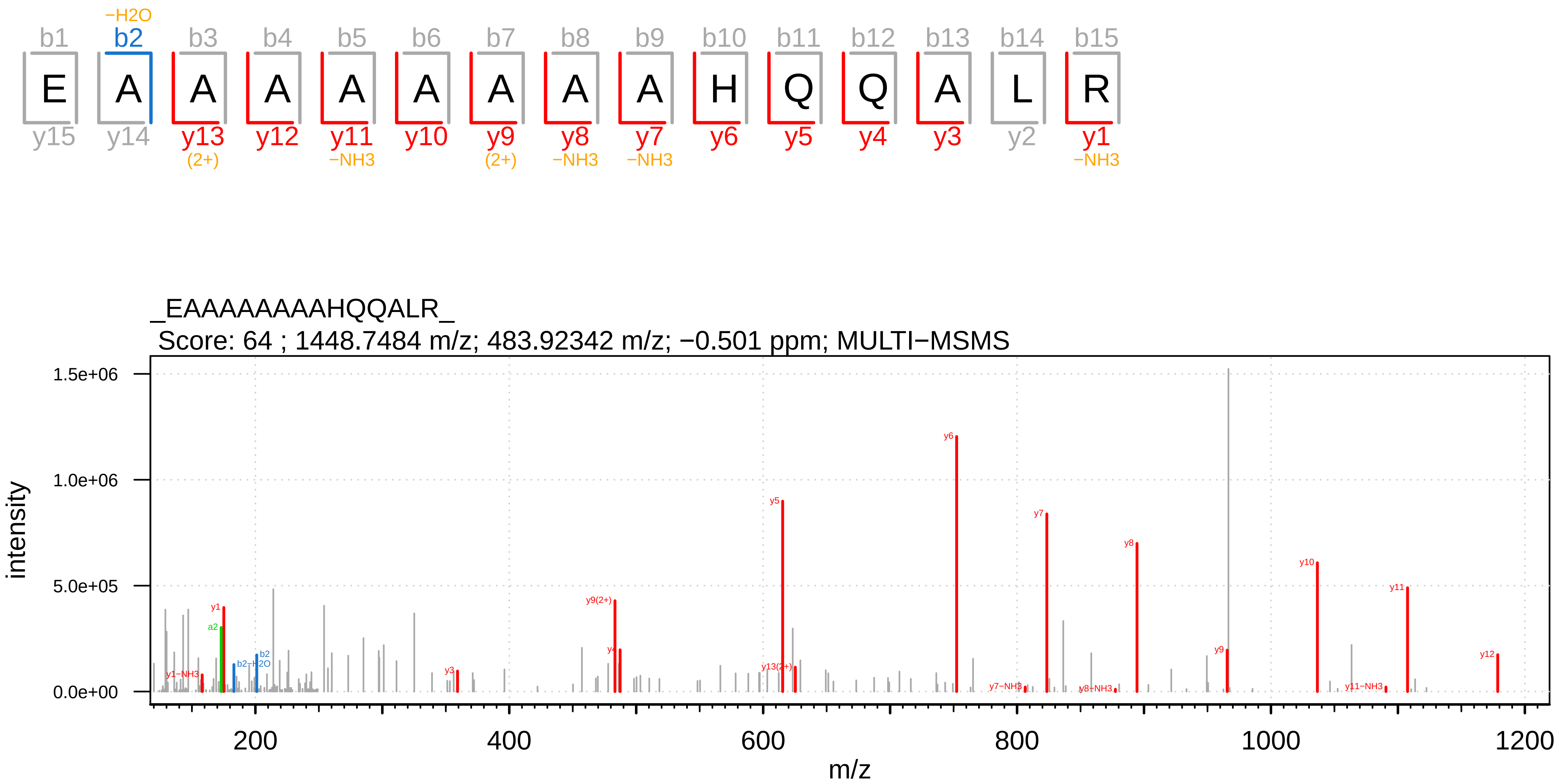

Raw File: Grobi_20121019_CHS_PIECEIEF_20121017_3−10_7cm_250ug_06
Scan Number: 17358
Proteins:
ENST00000394071_chr14:74185546−74185677:−

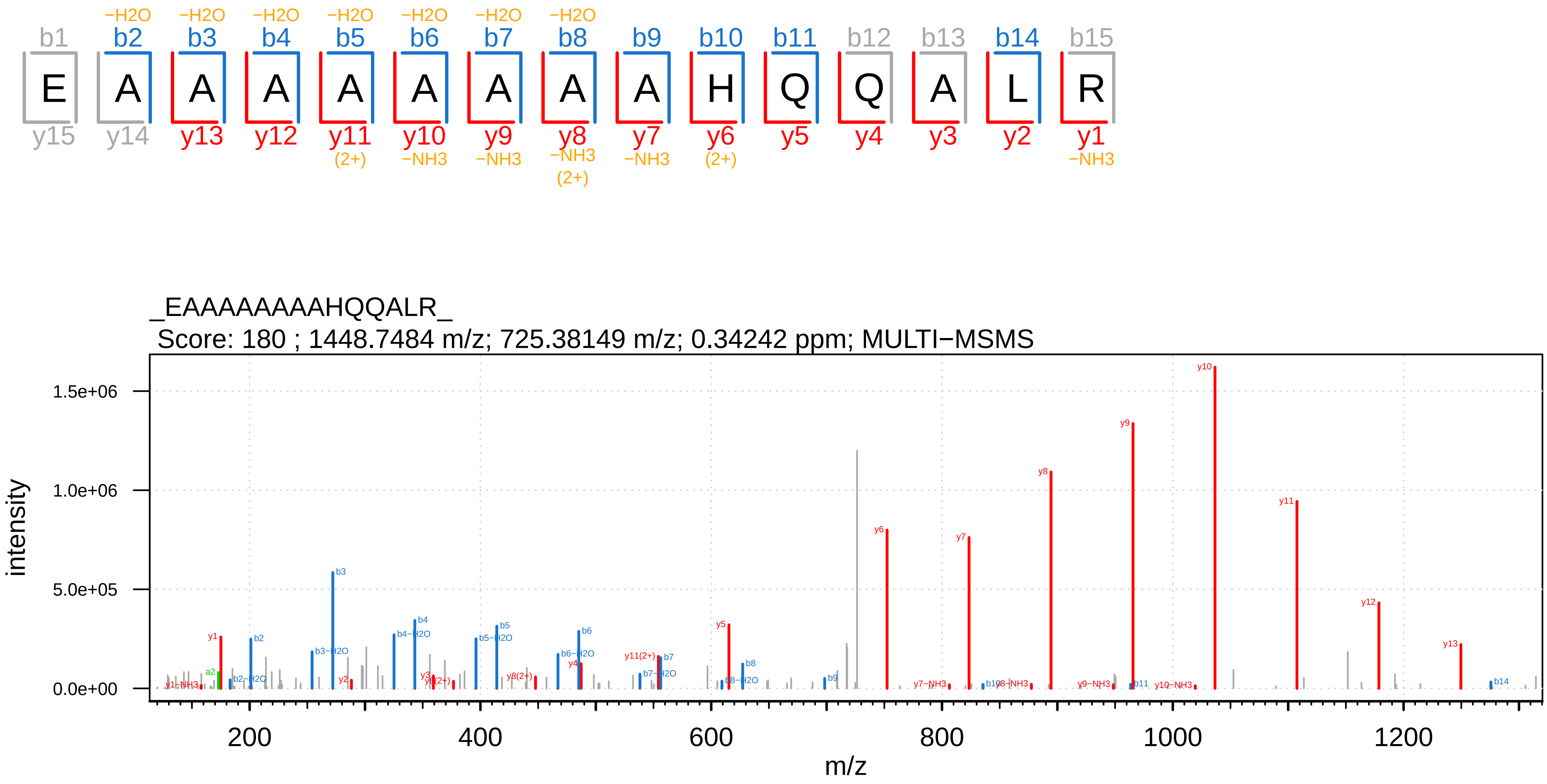

Raw File: Grobi_20121019_CHS_PIECEIEF_20121017_3−10_7cm_250ug_06
Scan Number: 17364
Proteins:
ENST00000394071_chr14:74185546−74185677:−

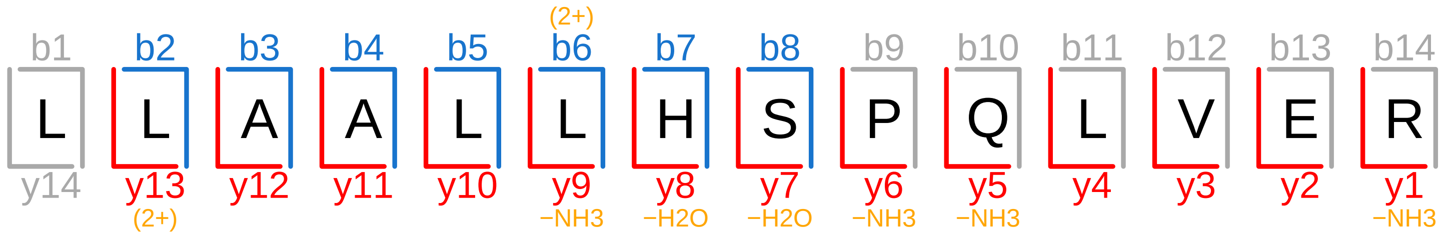

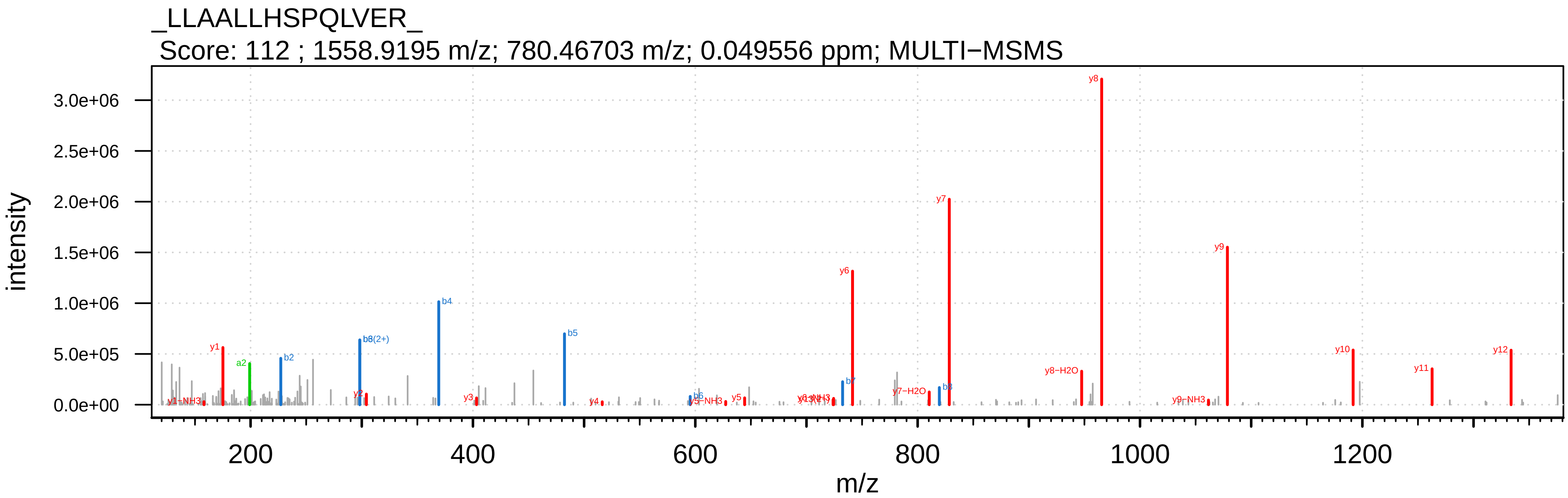

Raw File: Grobi_20121019_CHS_PIECEIEF_20121017_3−10_7cm_250ug_06
Scan Number: 57689
Proteins:
sp|Q69YL0|NCAS2_HUMAN
ENST00000602845_chr3:196669588−196669887:+

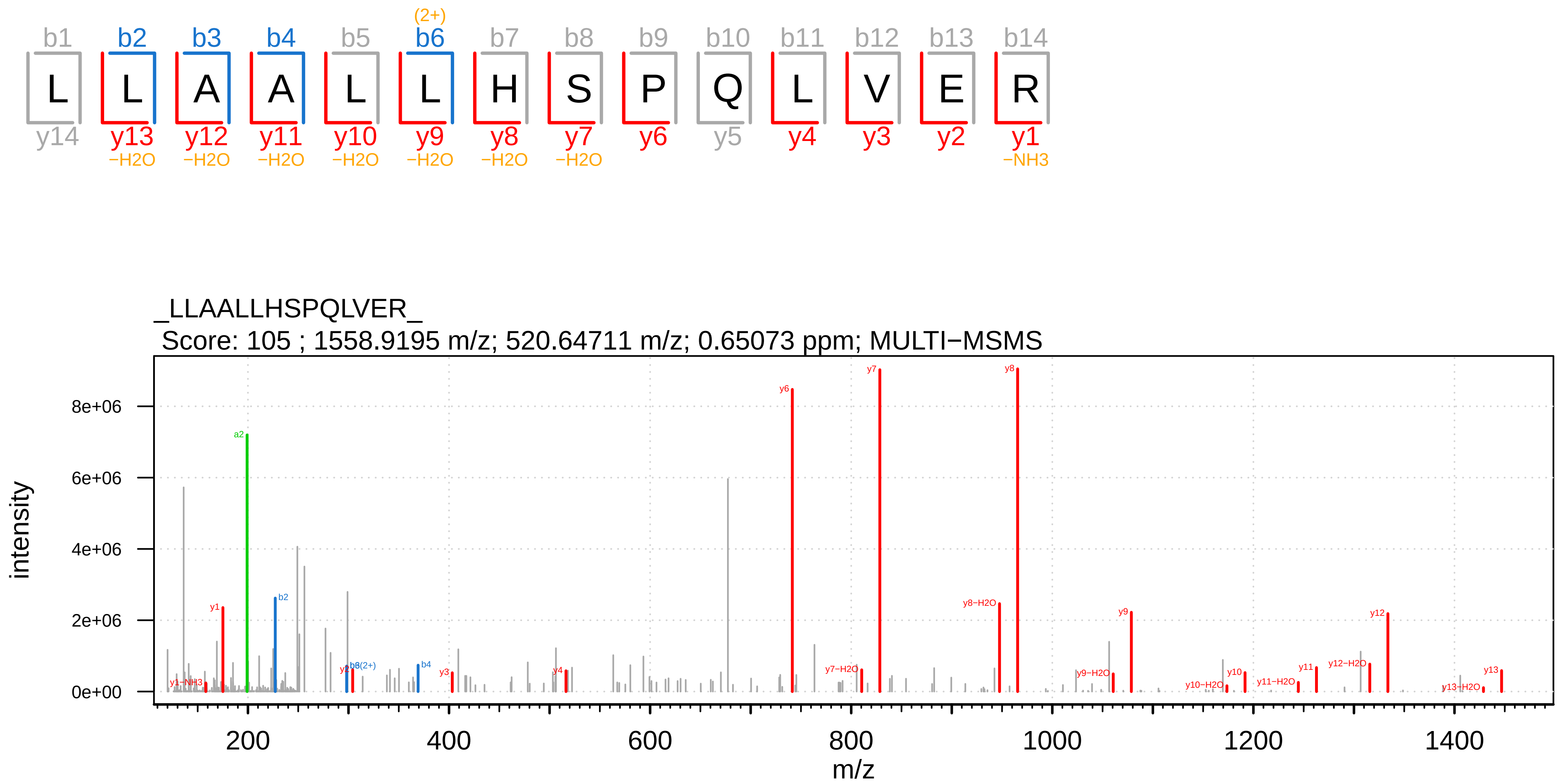

Raw File: Grobi_20121019_CHS_PIECEIEF_20121017_3−10_7cm_250ug_06
Scan Number: 57748
Proteins:
sp|Q69YL0|NCAS2_HUMAN
ENST00000602845_chr3:196669588−196669887:+

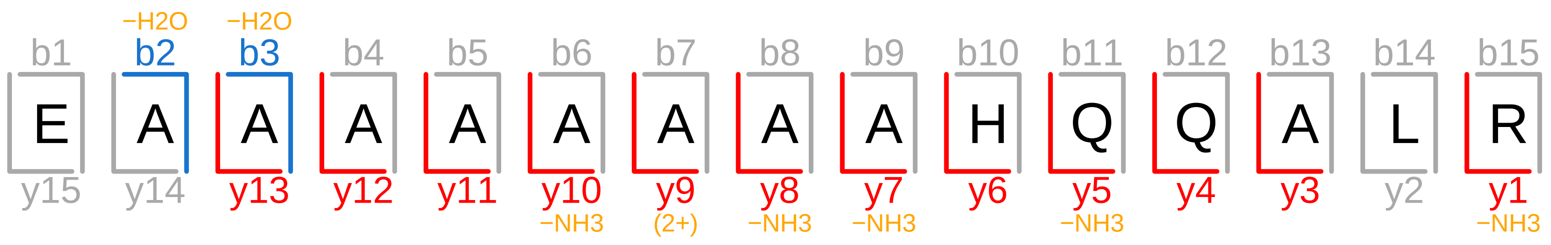

EAAAAAAAAHQQALR
Score: 84 ; 1448.7484 m/z; 483.92342 m/z; −0.98312 ppm; MULTI−MSMS

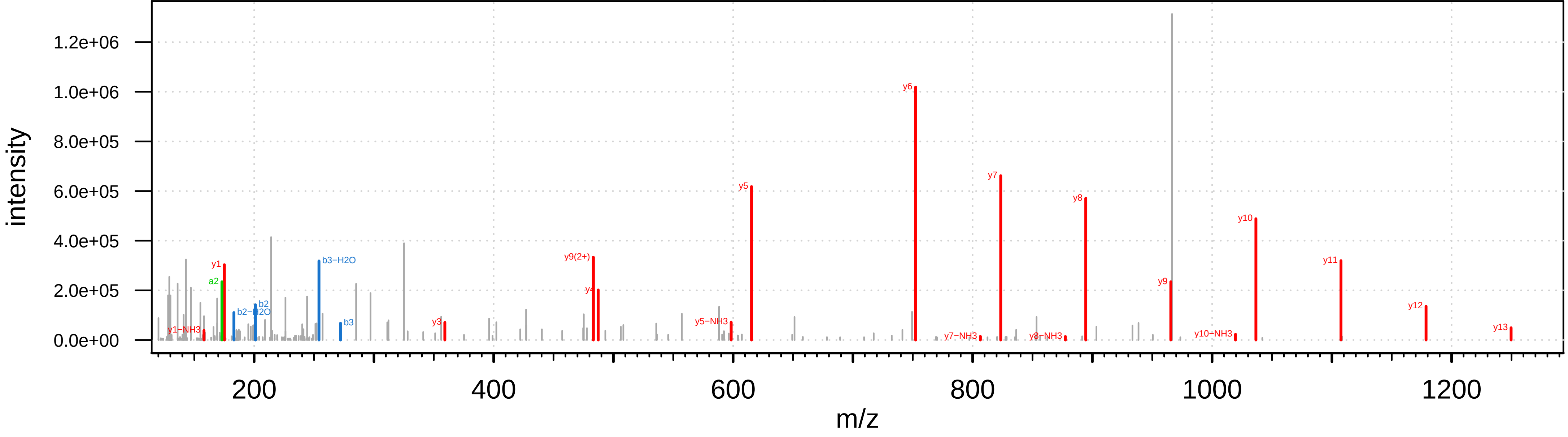

Raw File: Grobi_20121105_CHS_IEF_201210125_pep_gradient_7cm_250ug_06
Scan Number: 22066
Proteins:
ENST00000394071_chr14:74185546−74185677:−

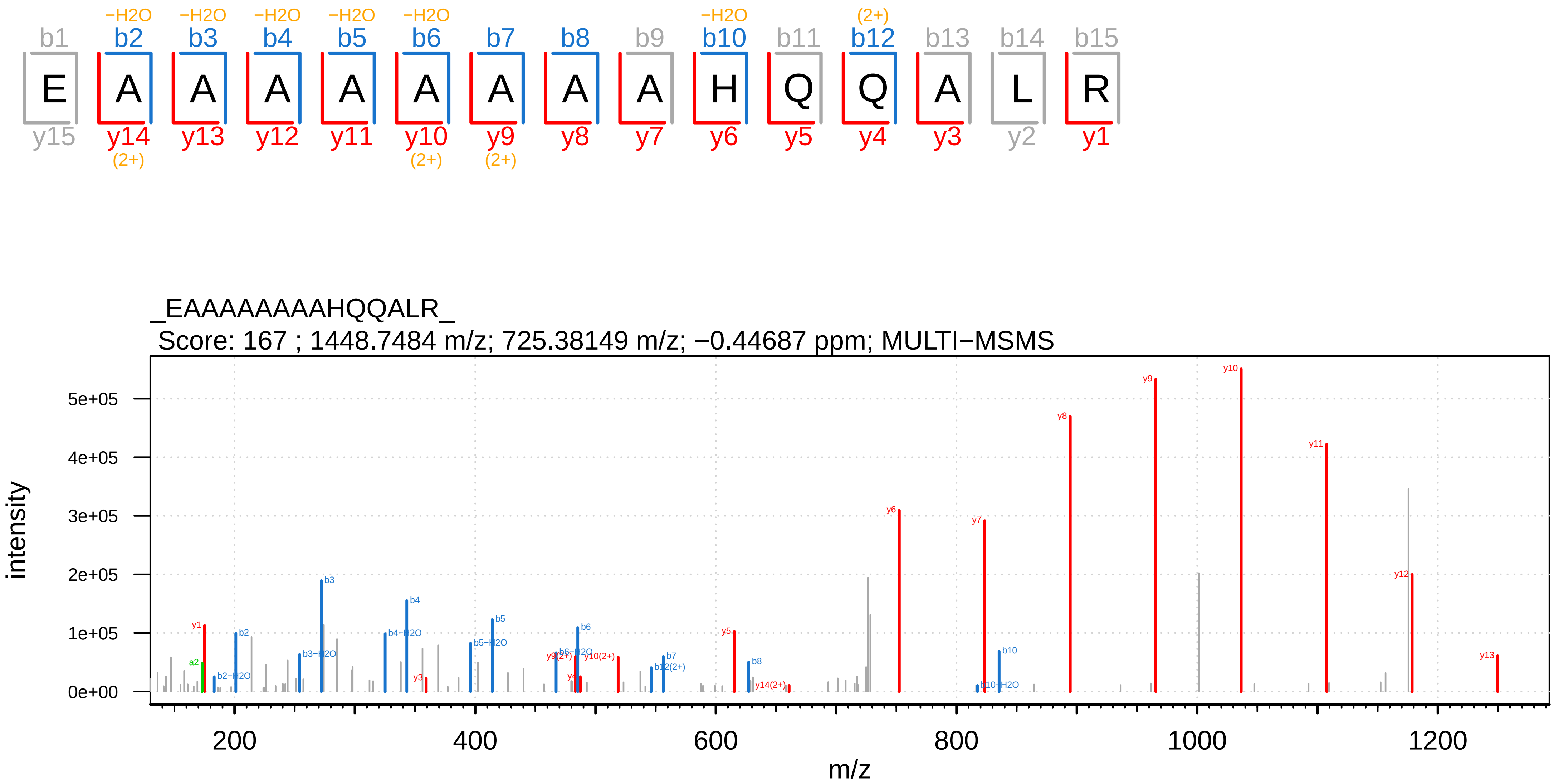

Raw File: Grobi_20121105_CHS_IEF_201210125_pep_gradient_7cm_250ug_06
Scan Number: 22080
Proteins:
ENST00000394071_chr14:74185546−74185677:−

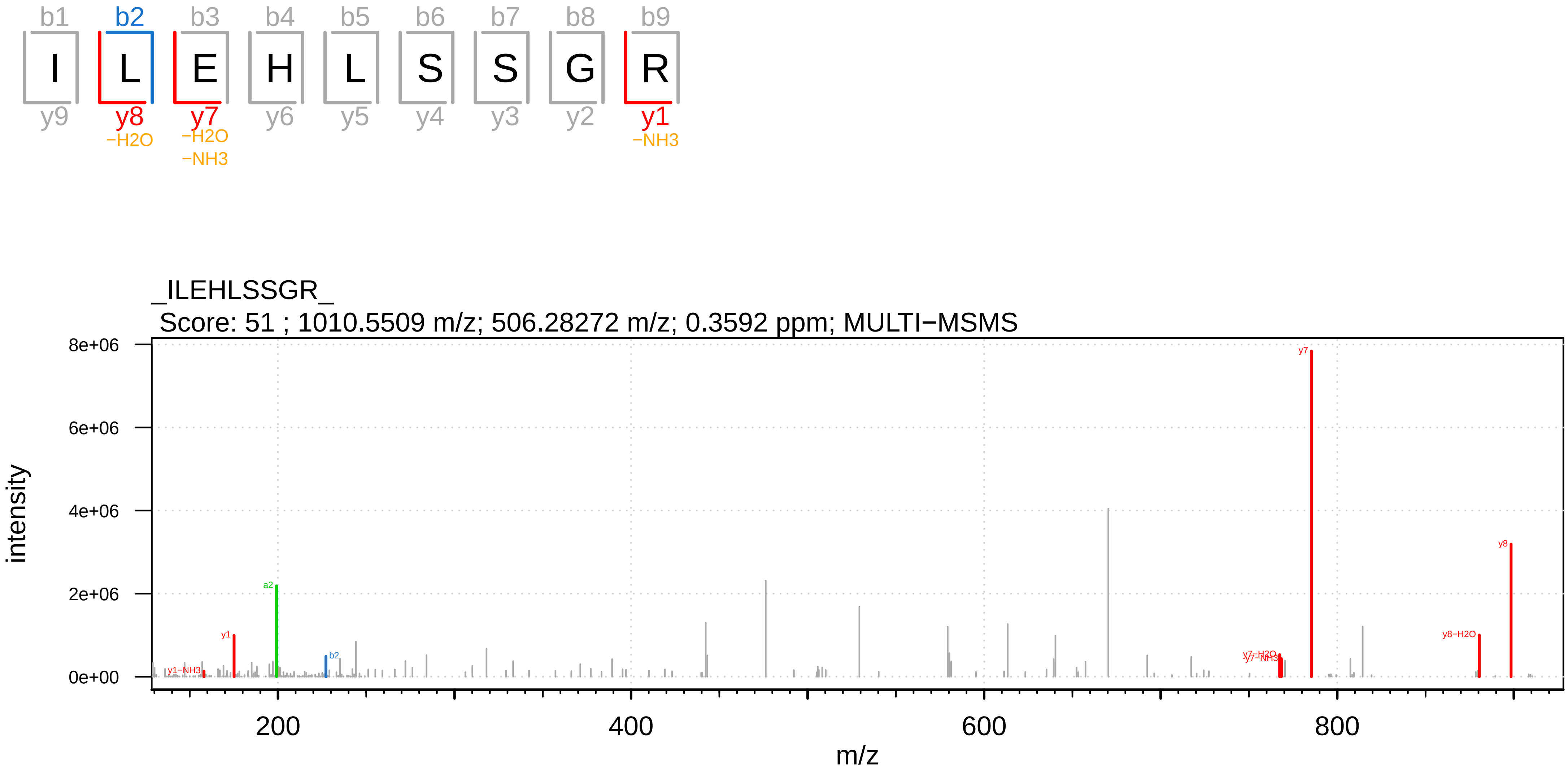

Raw File: Grobi_20121105_CHS_IEF_201210125_pep_gradient_7cm_250ug_06
Scan Number: 26406
Proteins:
ENST00000452079_chr1:3663088−3663306:−

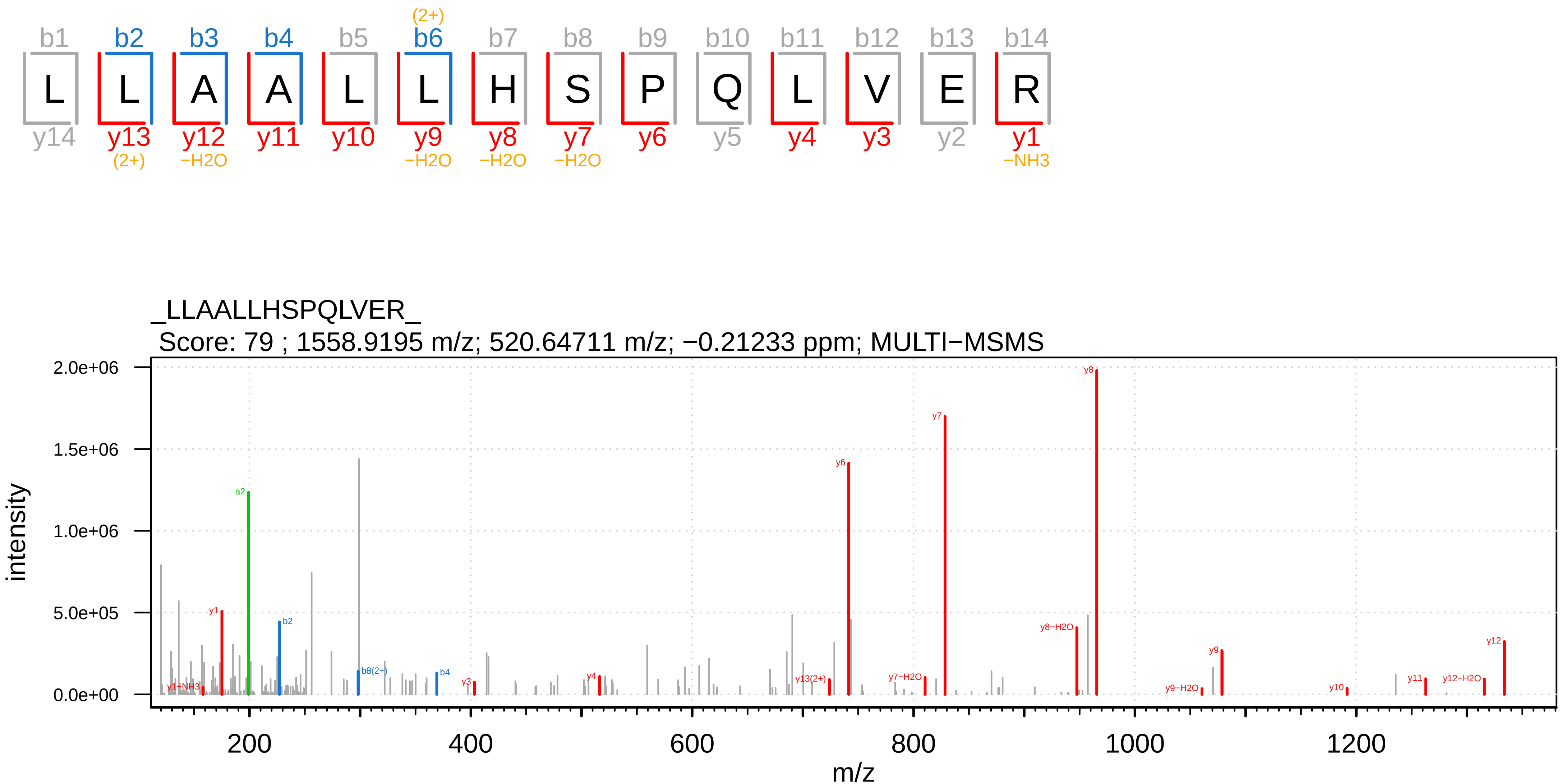

Raw File: Grobi_20121105_CHS_IEF_201210125_pep_gradient_7cm_250ug_06
Scan Number: 67878
Proteins:
sp|Q69YL0|NCAS2_HUMAN
ENST00000602845_chr3:196669588−196669887:+

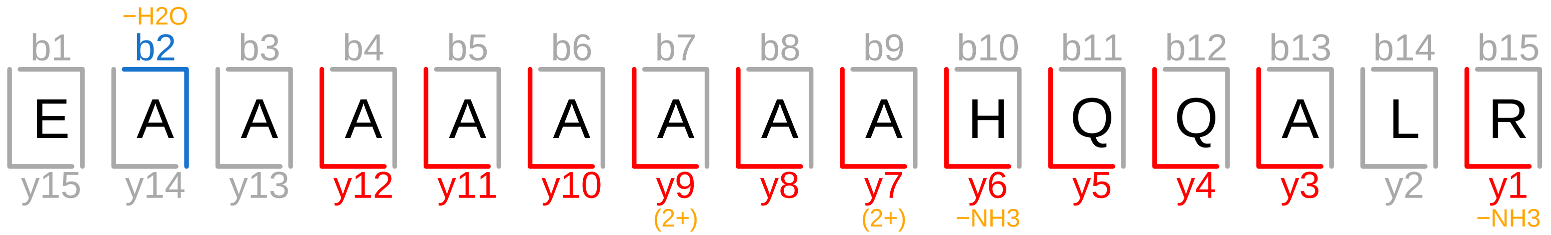

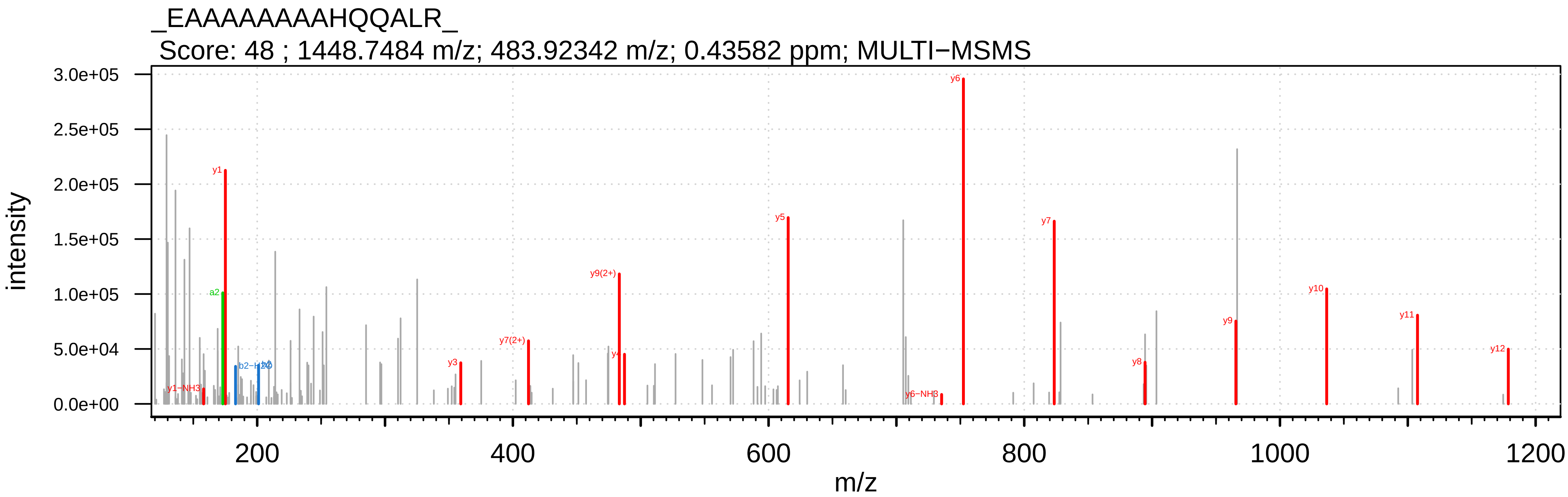

Raw File: Grobi_20121105_CHS_IEF100_20121025_pep_gradient_7cm_250ug_06
Scan Number: 21177
Proteins:
ENST00000394071_chr14:74185546−74185677:−

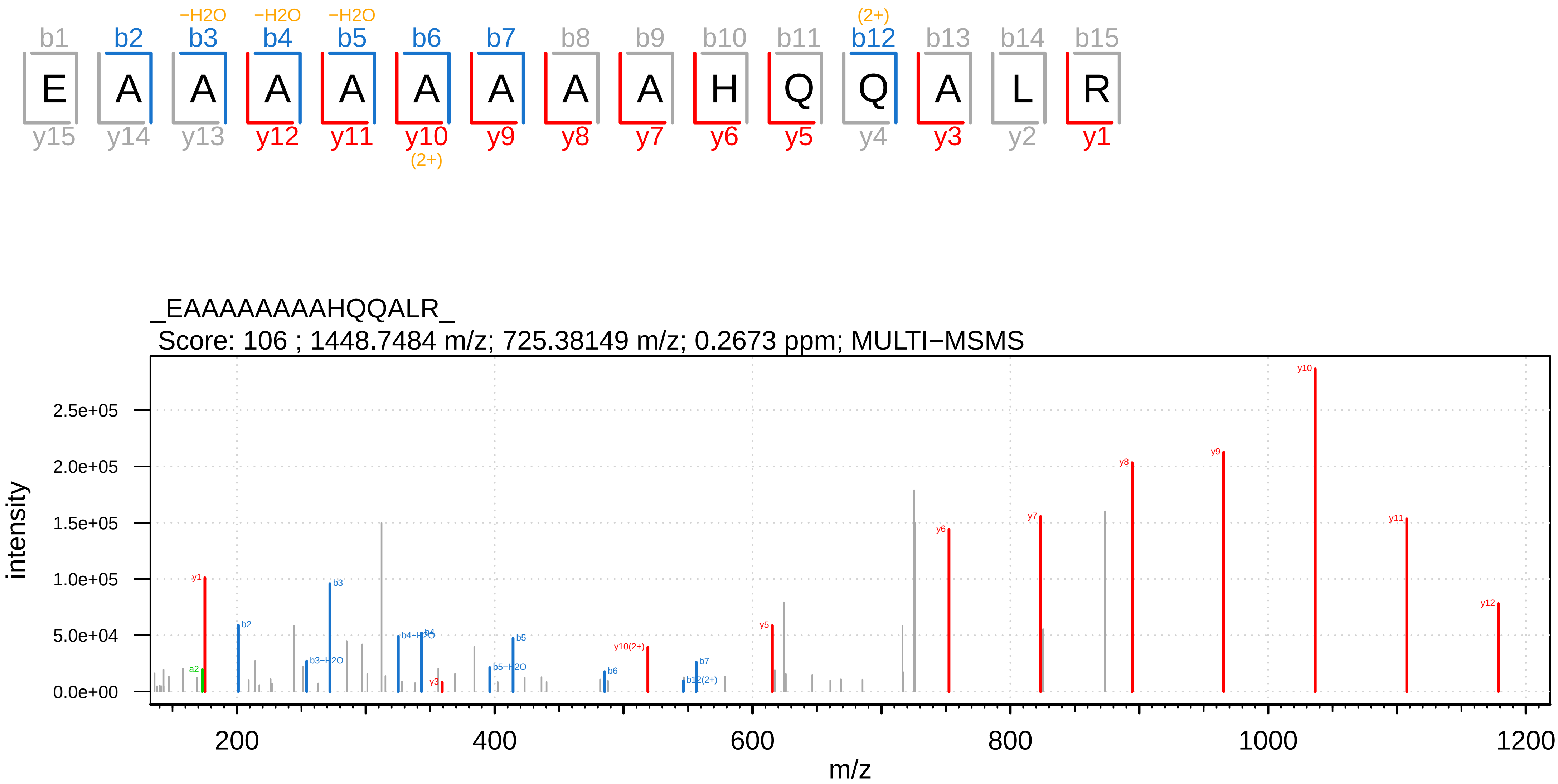

Raw File: Grobi_20121105_CHS_IEF100_20121025_pep_gradient_7cm_250ug_06
Scan Number: 21249
Proteins:
ENST00000394071_chr14:74185546−74185677:−

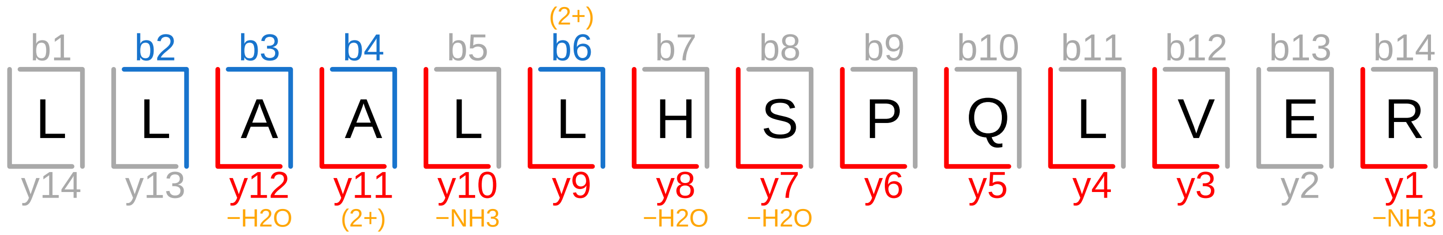

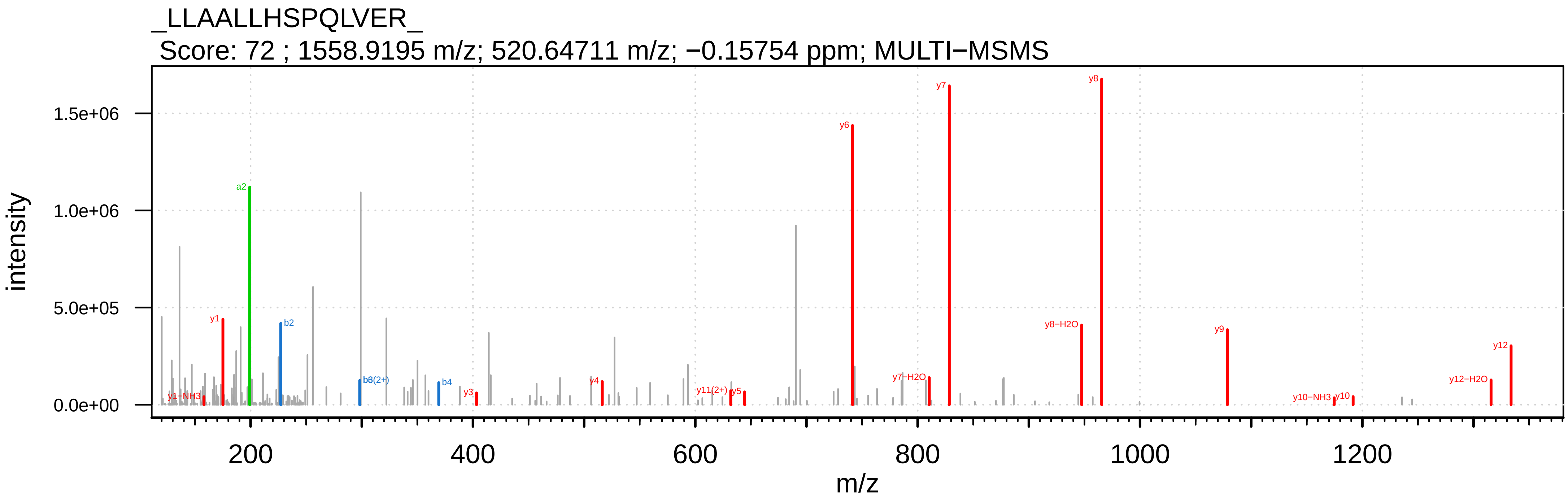

Raw File: Grobi_20121105_CHS_IEF100_20121025_pep_gradient_7cm_250ug_06
Scan Number: 67161
Proteins:
sp|Q69YL0|NCAS2_HUMAN
ENST00000602845_chr3:196669588−196669887:+

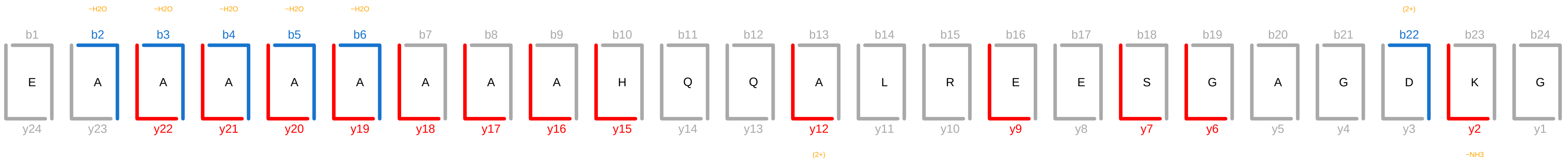

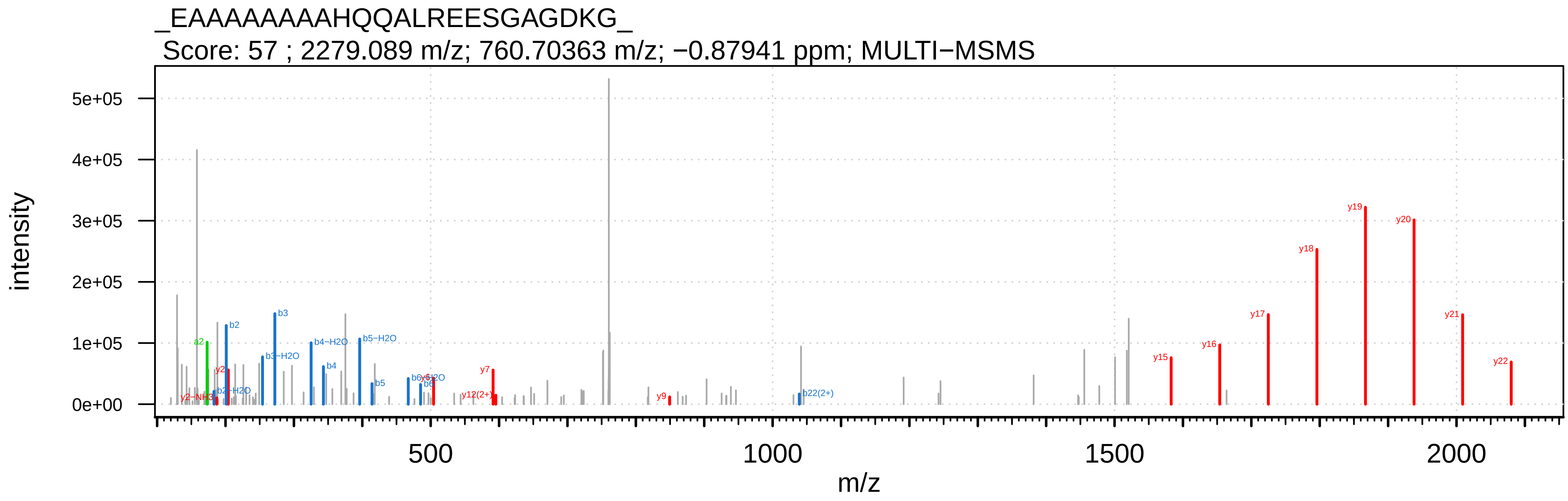

Raw File: Grobi_20121105_CHS_IEF100_20121025_pep_gradient_7cm_250ug_03
Scan Number: 25732
Proteins:
ENST00000394071_chr14:74185546−74185677:−

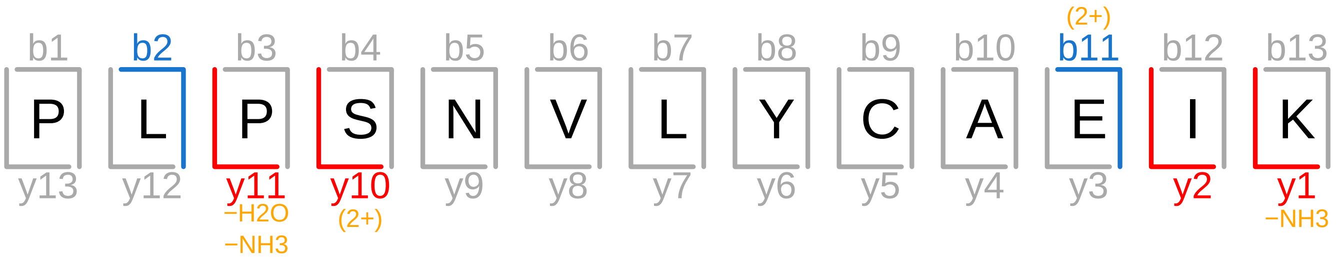

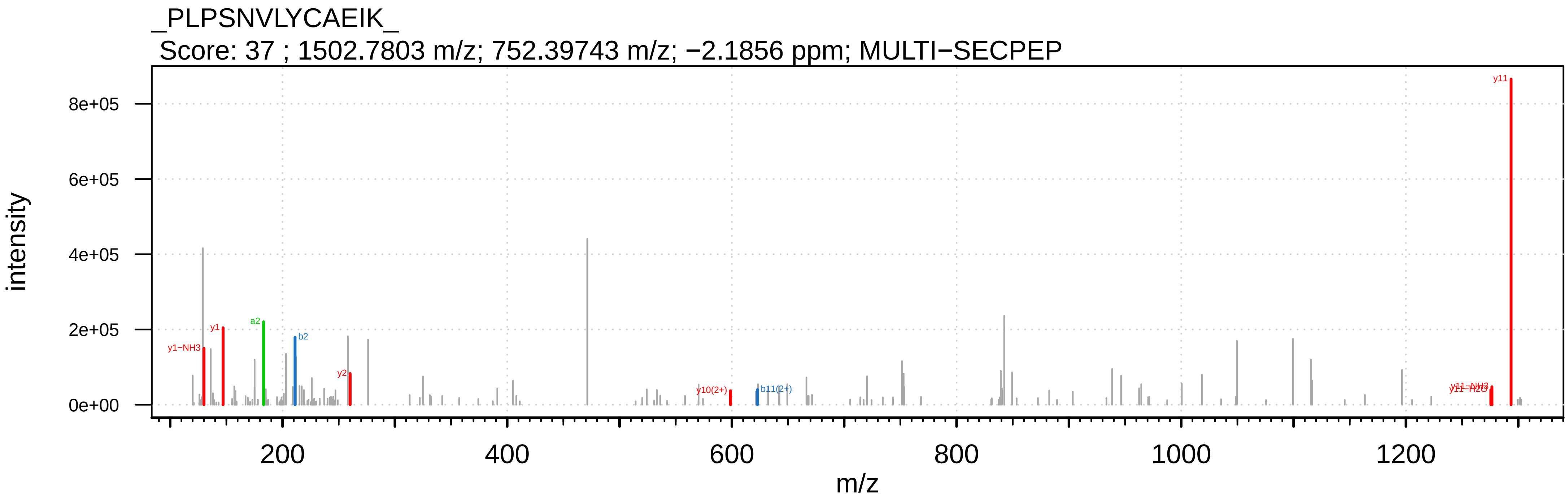

Raw File: Grobi_20121105_CHS_IEF100_20121025_pep_gradient_7cm_250ug_03
Scan Number: 38703
Proteins:
sp|A6NIN4|YQ014_HUMAN
ENST00000324348_chr17:7818840−7819234:−

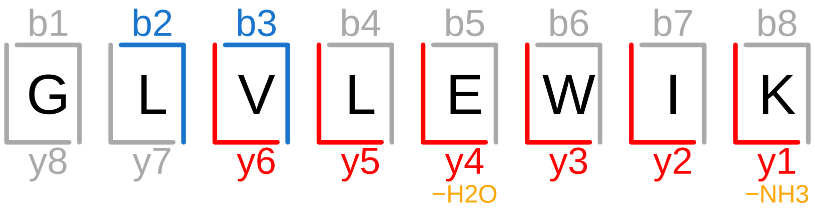

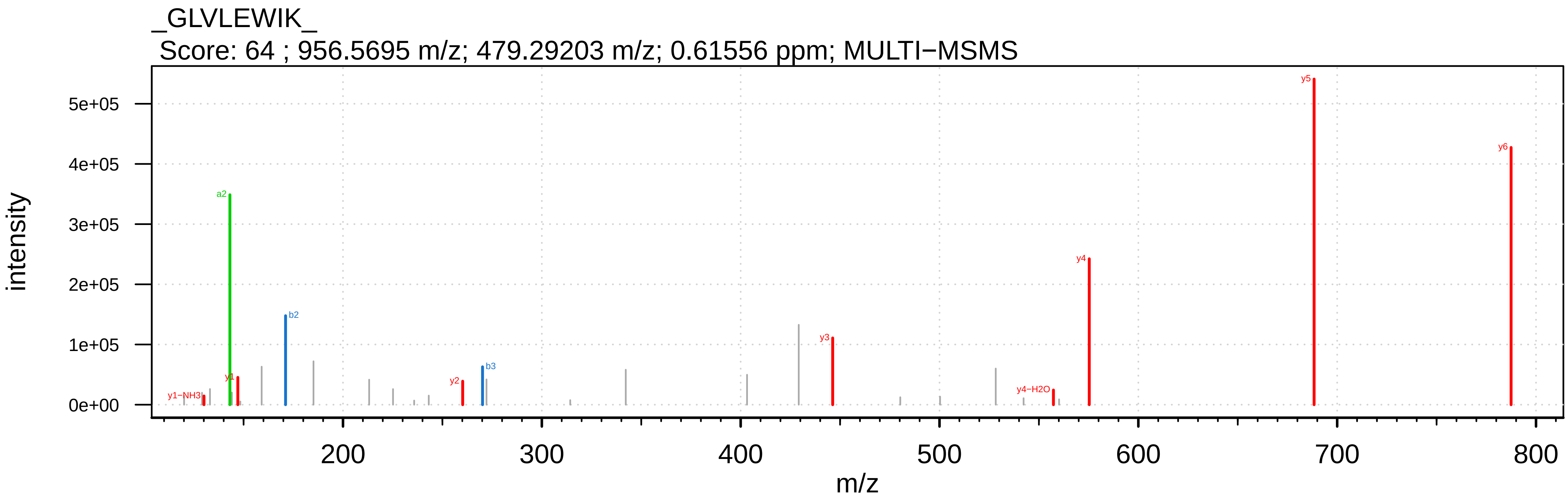

Raw File: Bibo_20121204_CHS_IEF100_20121129_3−10linear_Serva_7cm_250ug_03
Scan Number: 42970
Proteins:
TCONS_l2_00001296_chr1:79520703−79520992:−

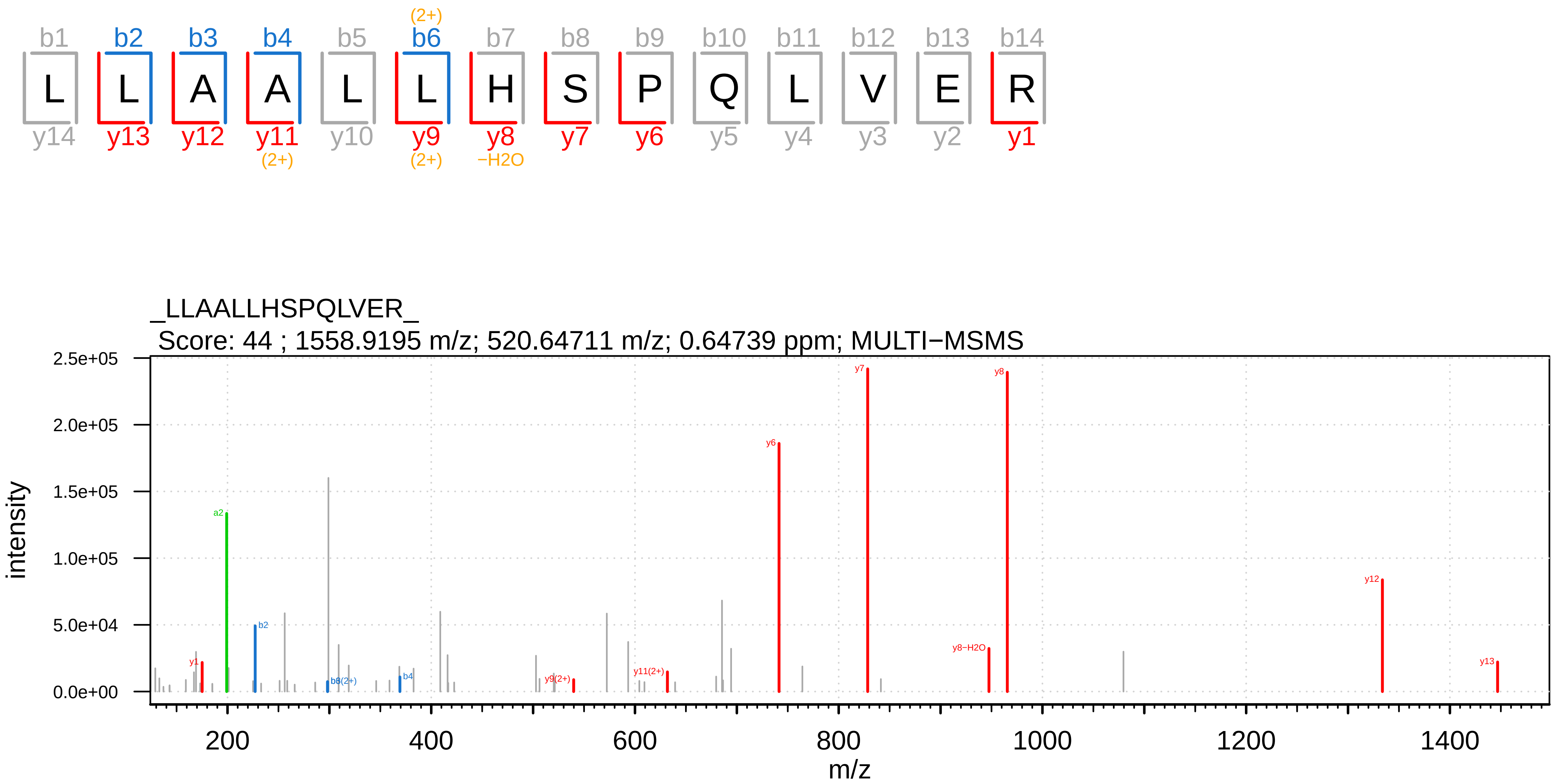

Raw File: Bibo_20130621_CHS_IEF_3−10linear_24slices_15
Scan Number: 12012
Proteins:
sp|Q69YL0|NCAS2_HUMAN
ENST00000602845_chr3:196669588−196669887:+

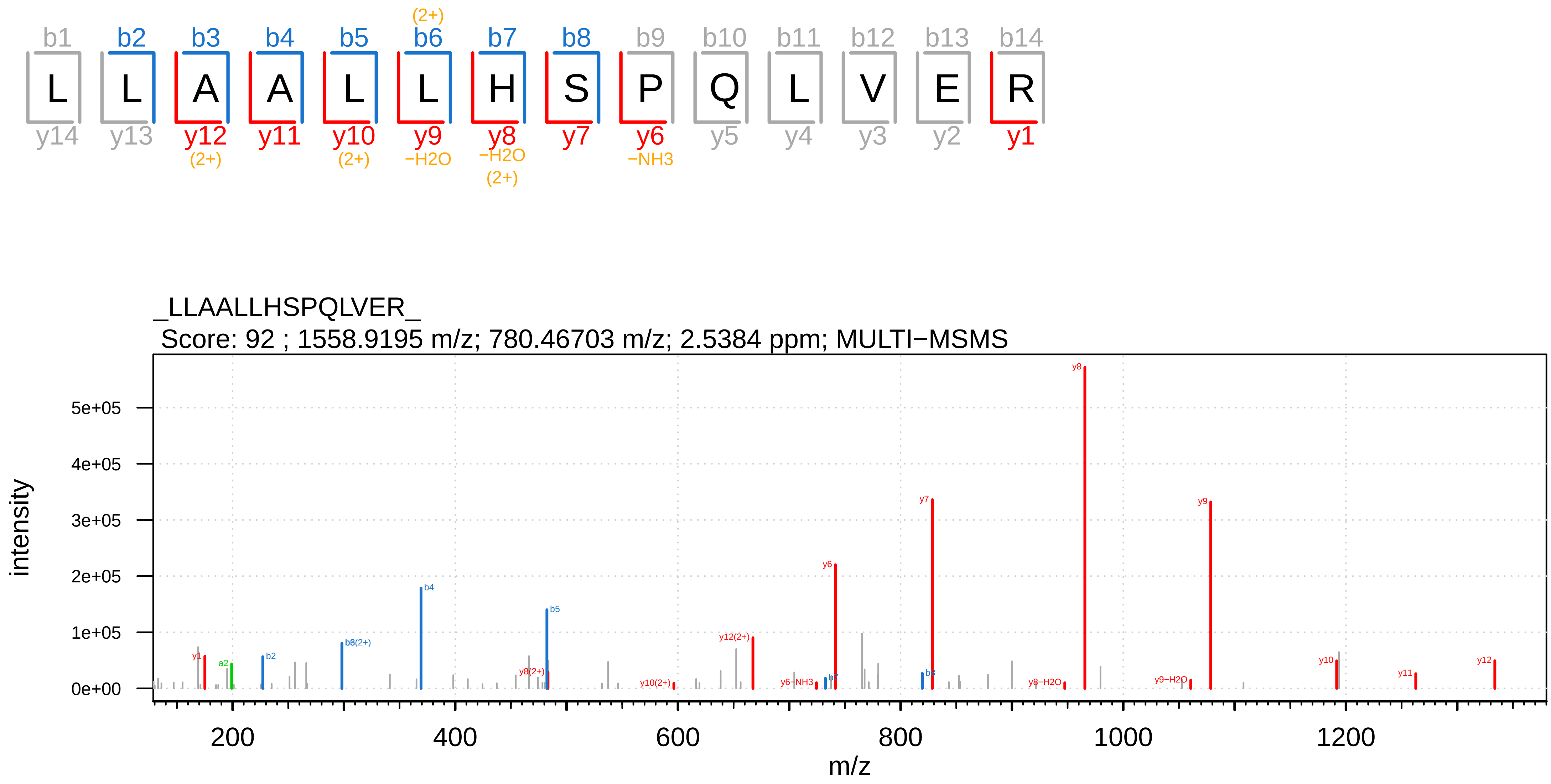

Raw File: Bibo_20130621_CHS_IEF_3−10linearpep_24slices_19
Scan Number: 11681
Proteins:
sp|Q69YL0|NCAS2_HUMAN
ENST00000602845_chr3:196669588−196669887:+

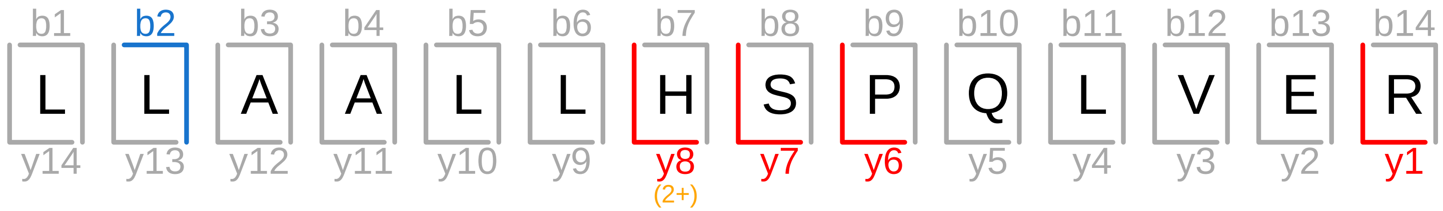

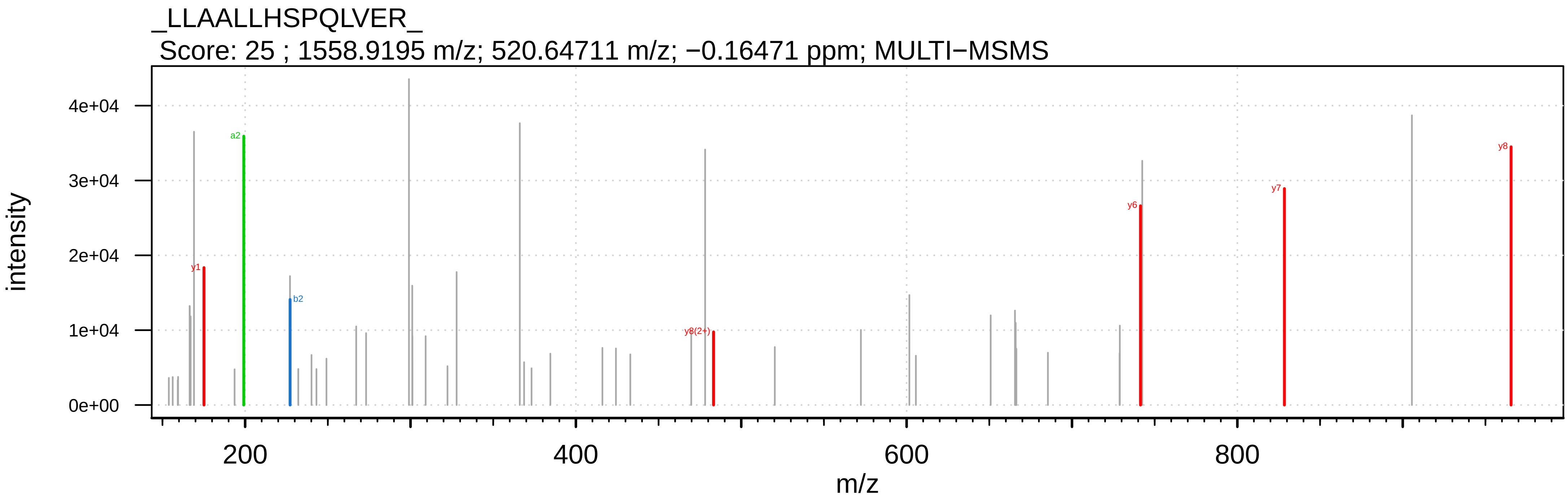

Raw File: Kermit_20130417_CHS_20130123_IEF100_201303xx_3−10linear_Serva_7cm_25ug_04
Scan Number: 36545
Proteins:
sp|Q69YL0|NCAS2_HUMAN
ENST00000602845_chr3:196669588−196669887:+

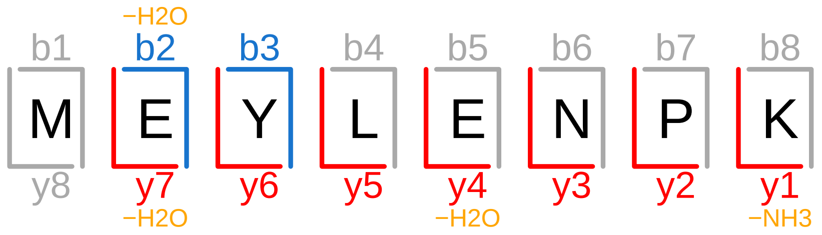

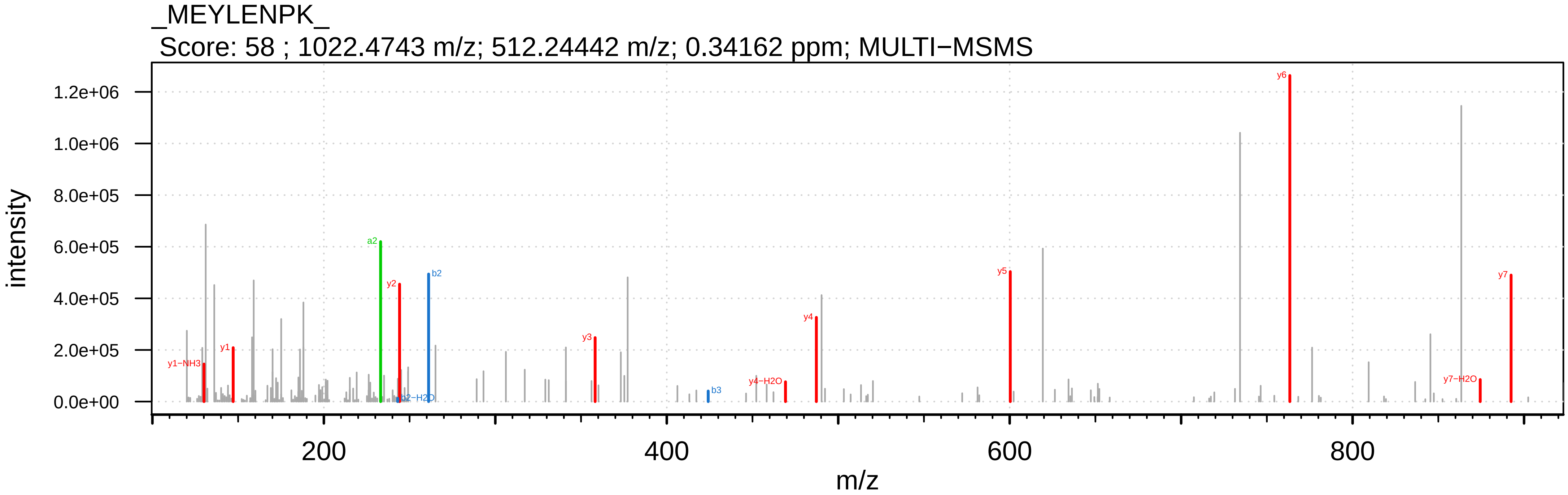

Raw File: Grobi_20121105_CHS_IEF_20121025_pep_gradient_7cm_250ug_01
Scan Number: 26967
Proteins:
ENST00000518958_chr8:120630400−120630519:−

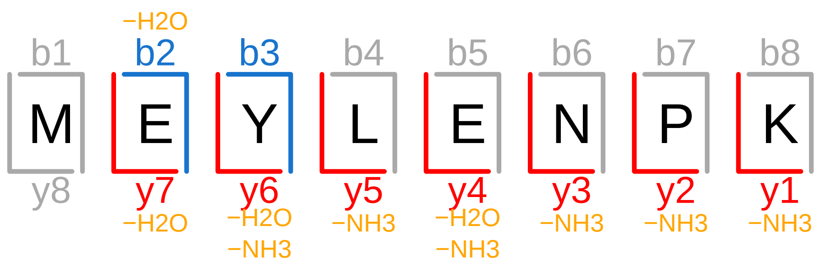

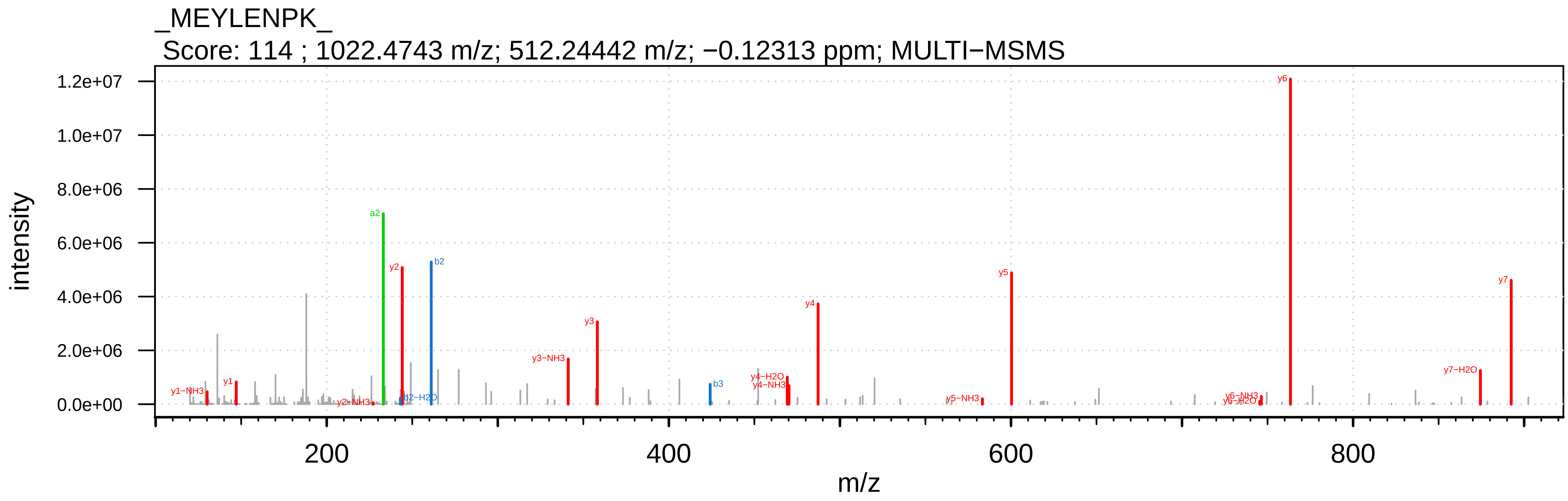

Raw File: Grobi_20121105_CHS_IEF_20121025_pep_gradient_7cm_250ug_02
Scan Number: 25840
Proteins:
ENST00000518958_chr8:120630400−120630519:−

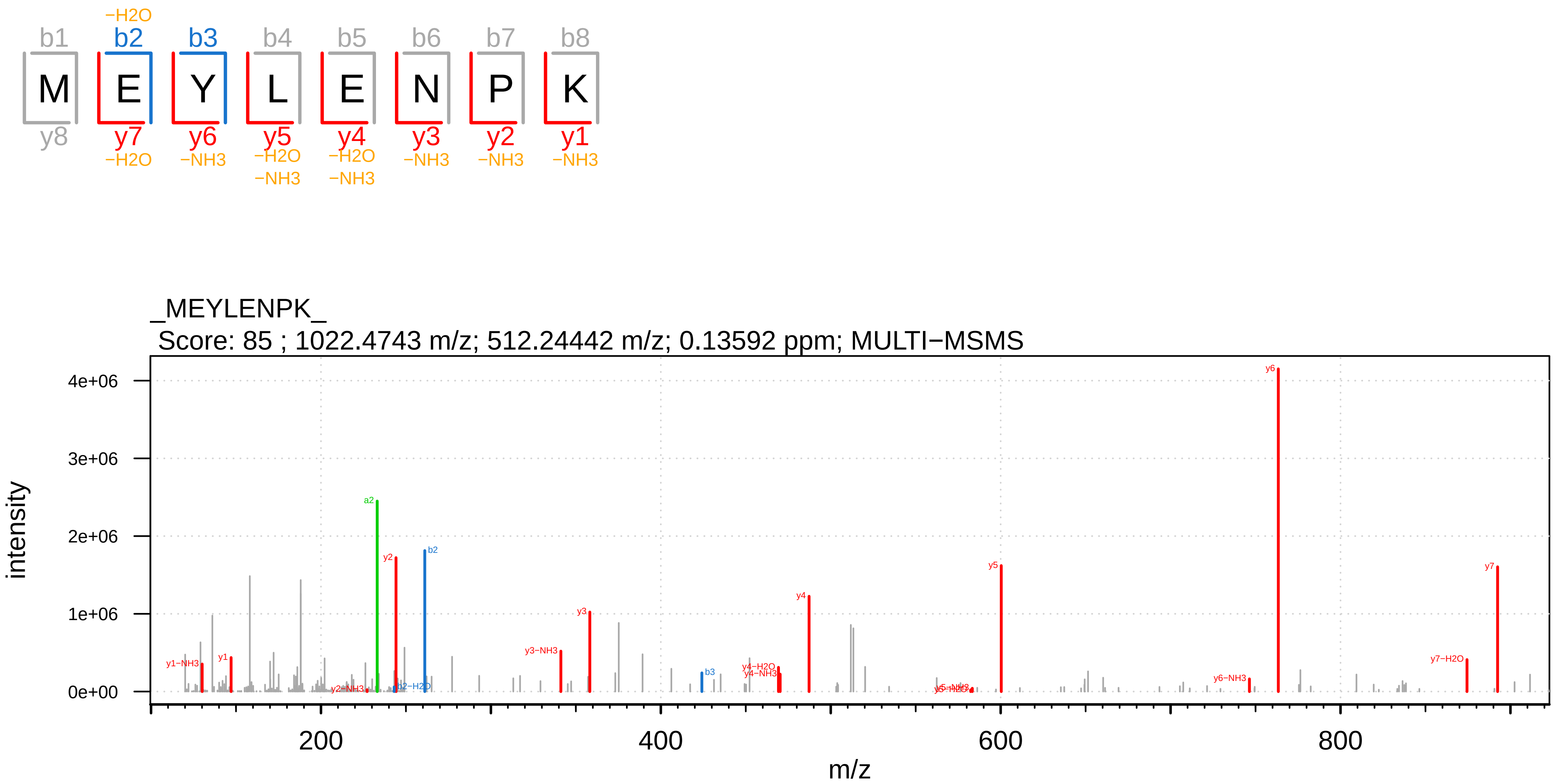

Raw File: Grobi_20121105_CHS_IEF_20121025_pep_gradient_7cm_250ug_03
Scan Number: 26964
Proteins:
ENST00000518958_chr8:120630400−120630519:−

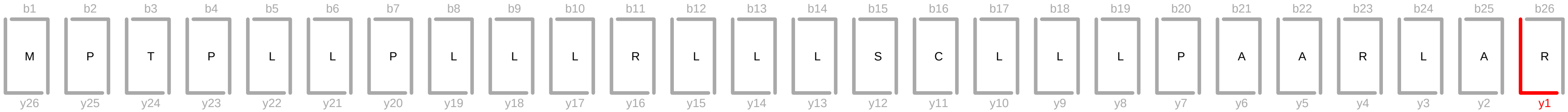

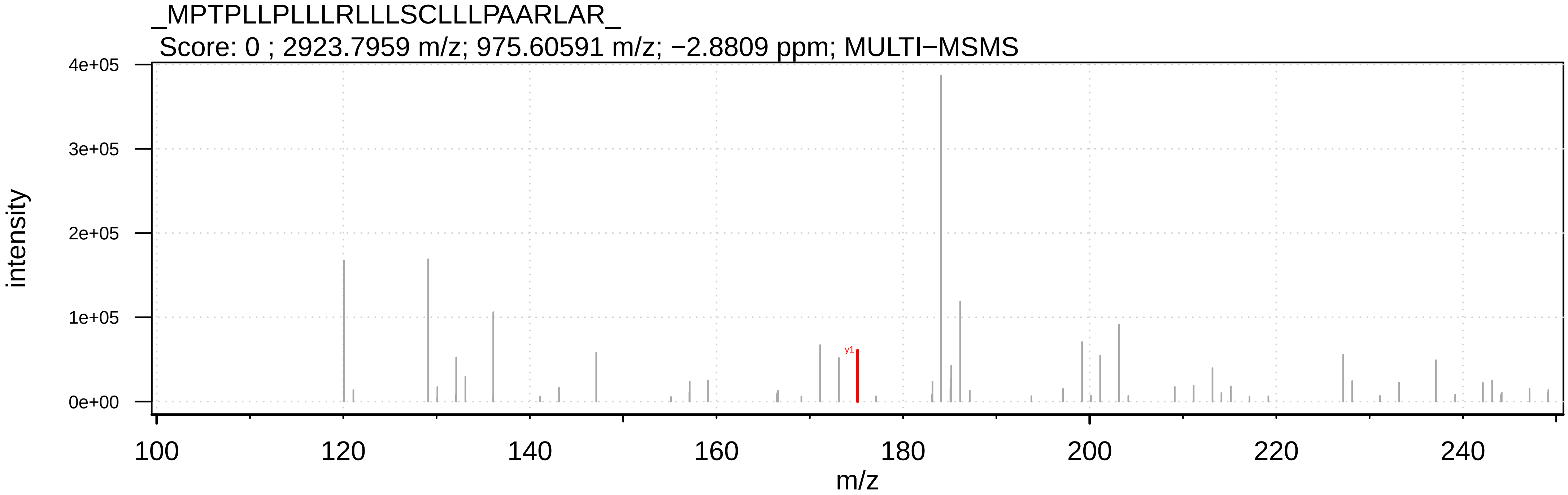

Raw File: Kermit_20130417_CHS_20130123_IEF100_201303xx_25ug
Scan Number: 103661
Proteins:
ENST00000573382_chr6:44185209−44185463:+

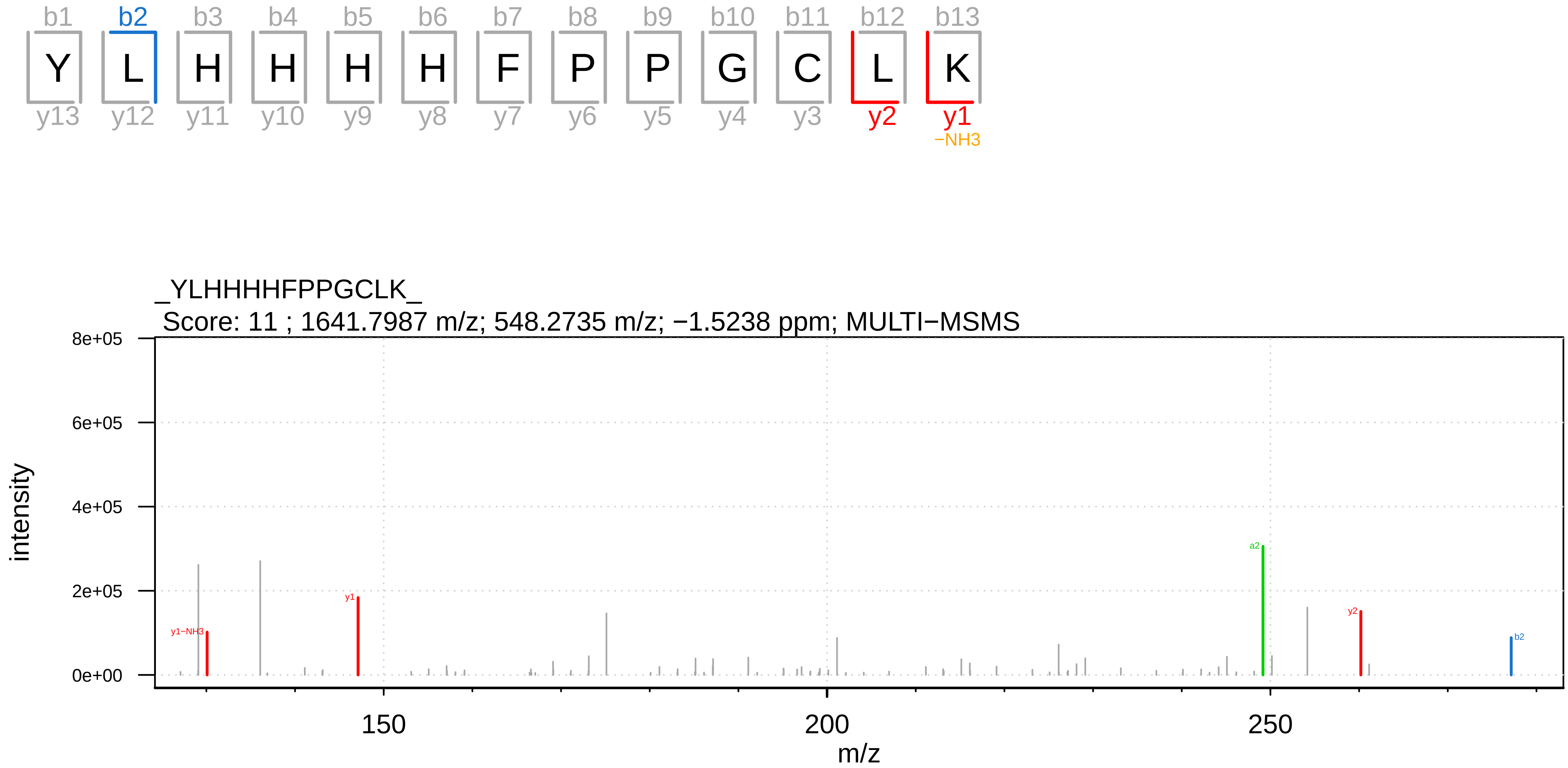

Raw File: Kermit_20130417_CHS_20130123_IEF100_201303xx_25ug
Scan Number: 13176
Proteins:
ENST00000525328_chr11:76423734−76423856:−

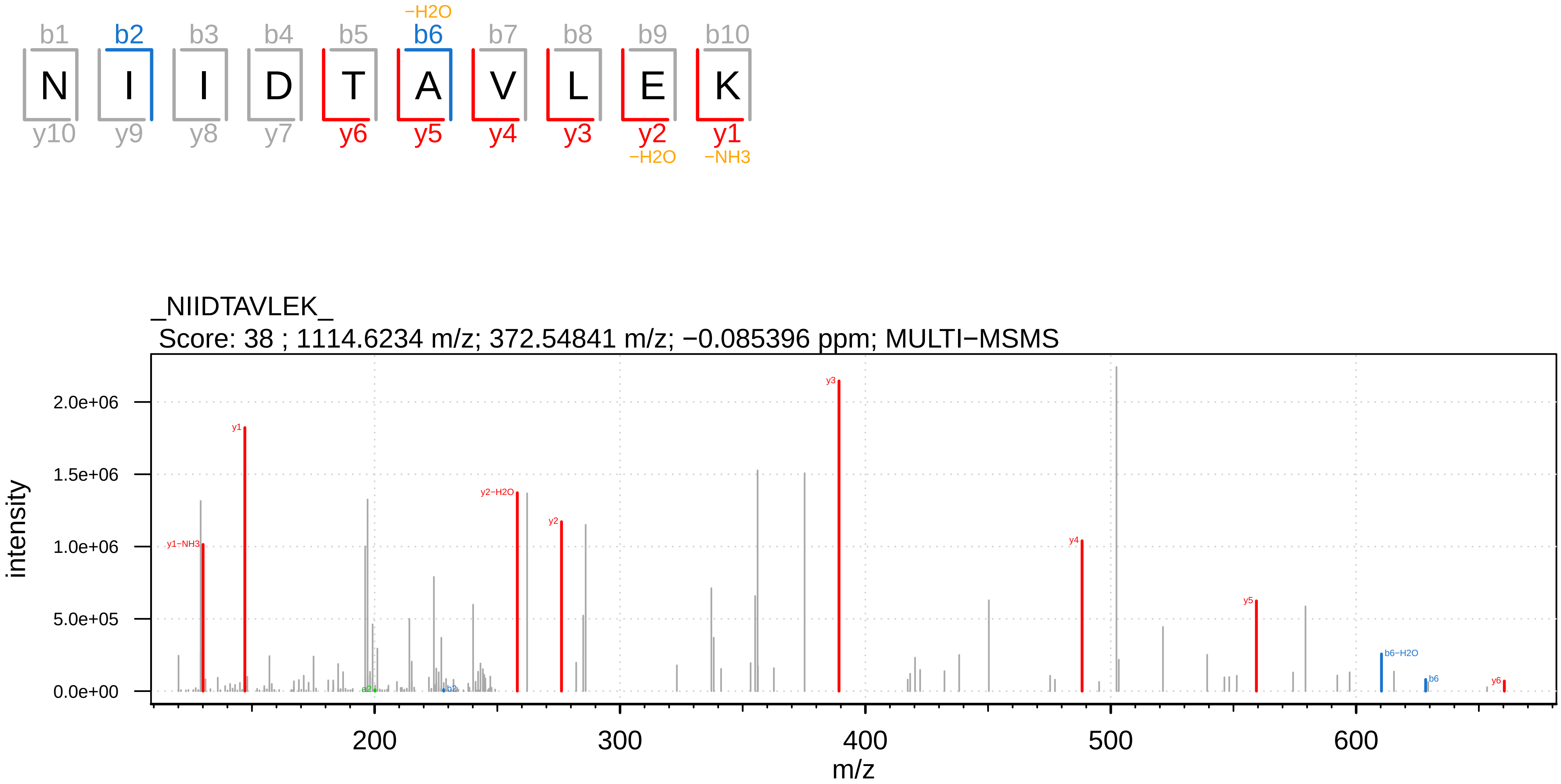

Raw File: Grobi_20121019_CHS_PIECEIEF_20121017_3−10_7cm_250ug_04
Scan Number: 19556
Proteins:
ENST00000497138_chr20:56806826−56807846:−

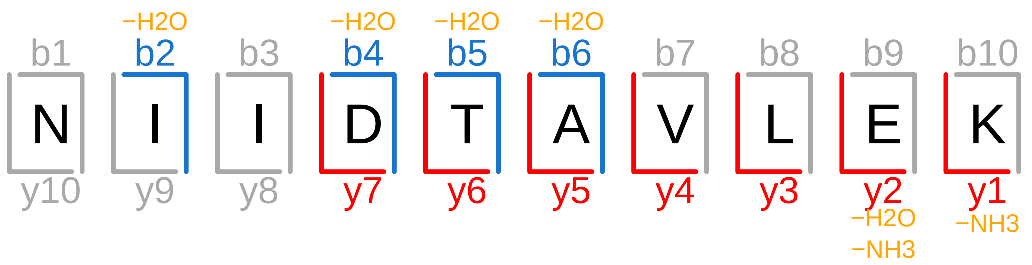

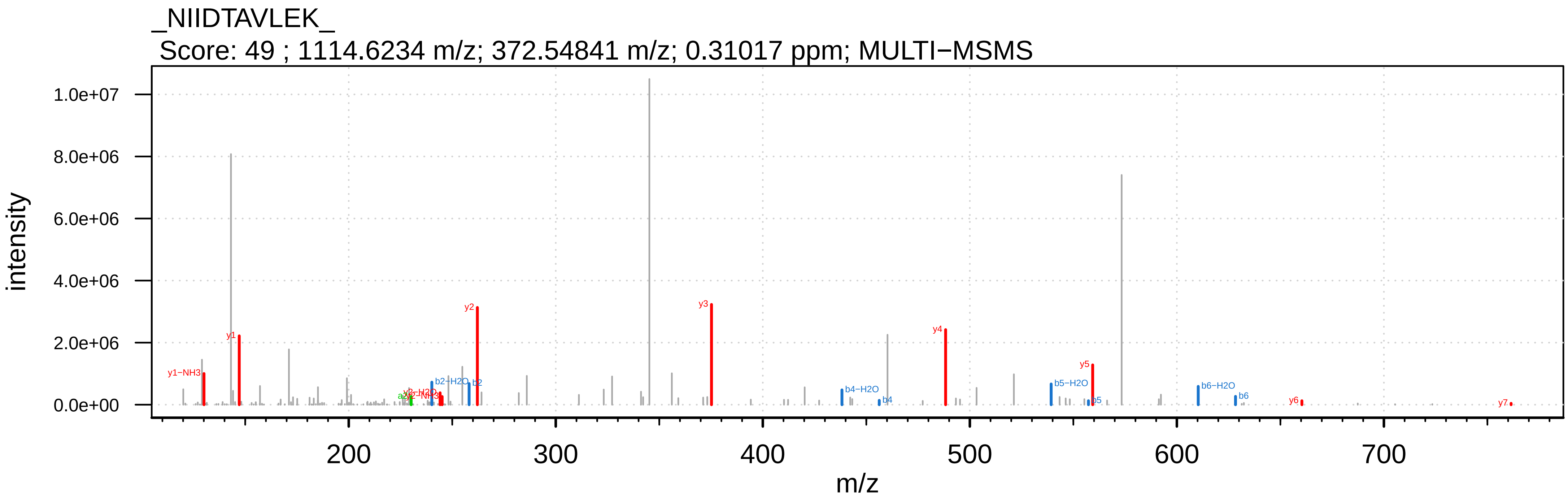

Raw File: Grobi_20130312_CHS_IEF100_20130309_3−10linear_Serva_7cm_1mg_1elution_03
Scan Number: 4098
Proteins:
ENST00000497138_chr20:56806826−56807846:−

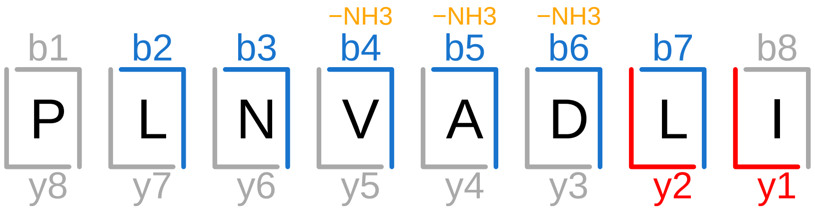

PLNVADLI
Score: 90 ; 853.49092 m/z; 427.75274 m/z; NaN ppm; MSMS

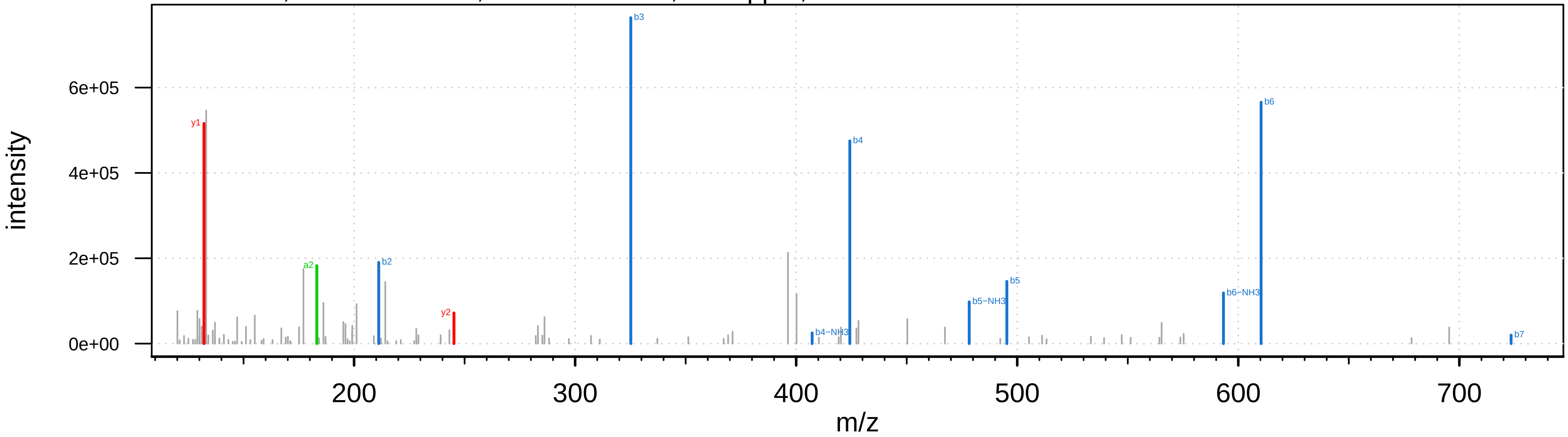

Raw File: Grobi_20121019_CHS_PIECEIEF_20121017_3−10_7cm_250ug_02
Scan Number: 75988
Proteins:
TCONS_l2_00005685_chr12:53548245−53548274:+

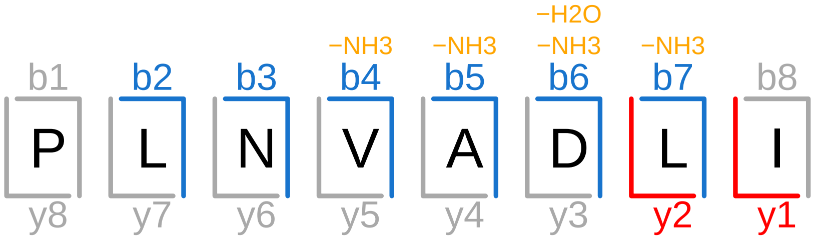

PLNVADLI
Score: 90 ; 853.49092 m/z; 427.75274 m/z; 0.4787 ppm; MULTI−MSMS

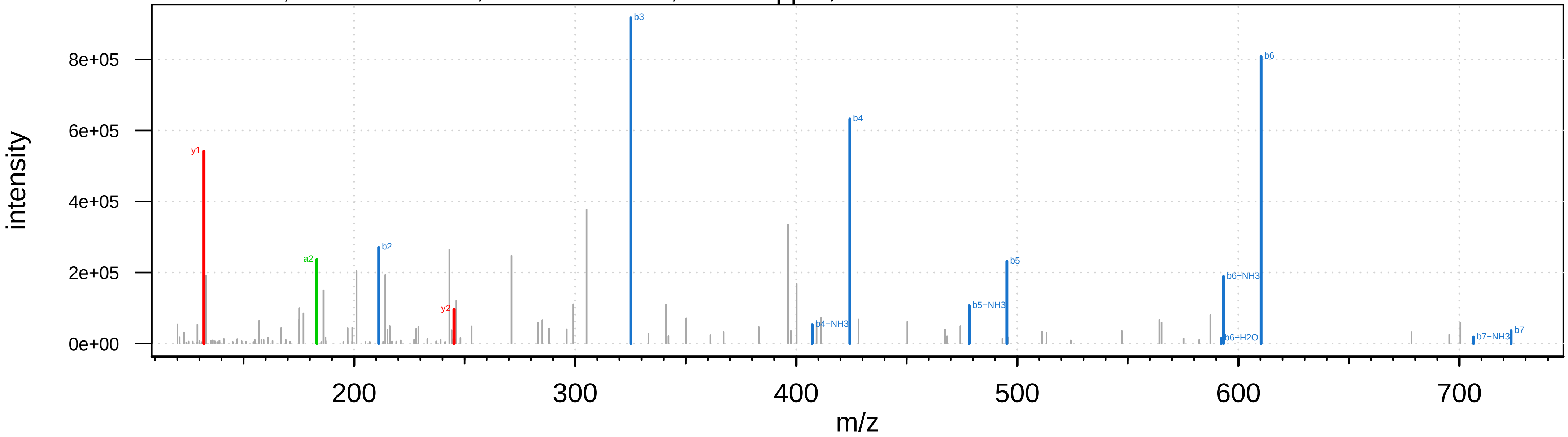

Raw File: Grobi_20121105_CHS_IEF100_20121025_pep_gradient_7cm_250ug_01
Scan Number: 83975
Proteins:
TCONS_l2_00005685_chr12:53548245−53548274:+

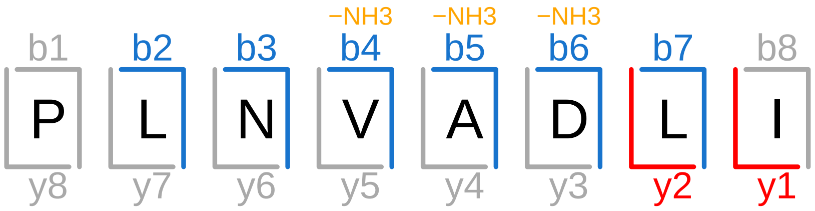

PLNVADLI
Score: 79 ; 853.49092 m/z; 427.75274 m/z; 0.28157 ppm; MULTI−MSMS

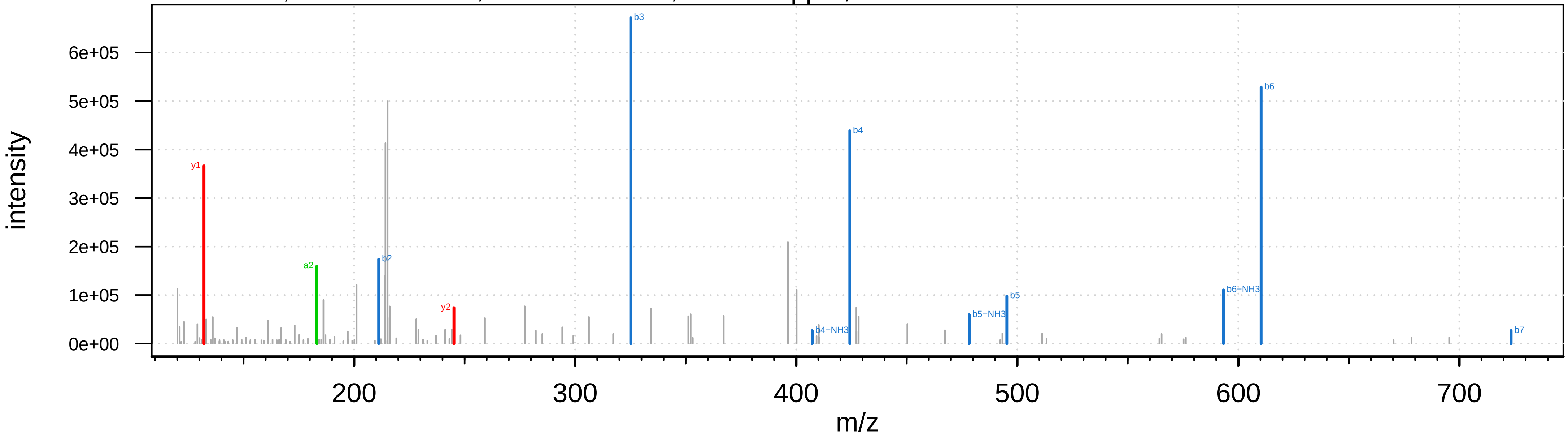

Raw File: Grobi_20121105_CHS_IEF100_20121025_pep_gradient_7cm_250ug_02
Scan Number: 84162
Proteins:
TCONS_l2_00005685_chr12:53548245−53548274:+

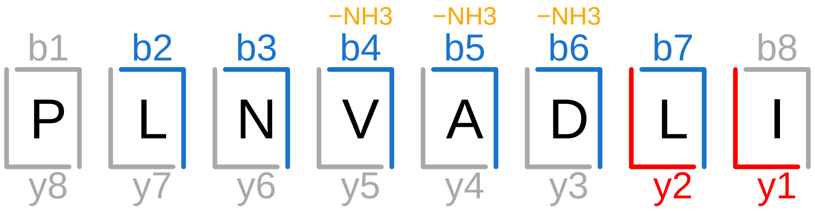

PLNVADLI

Score: 79 ; 853.49092 m/z; 427.75274 m/z; 0.074853 ppm; MULTI−MSMS

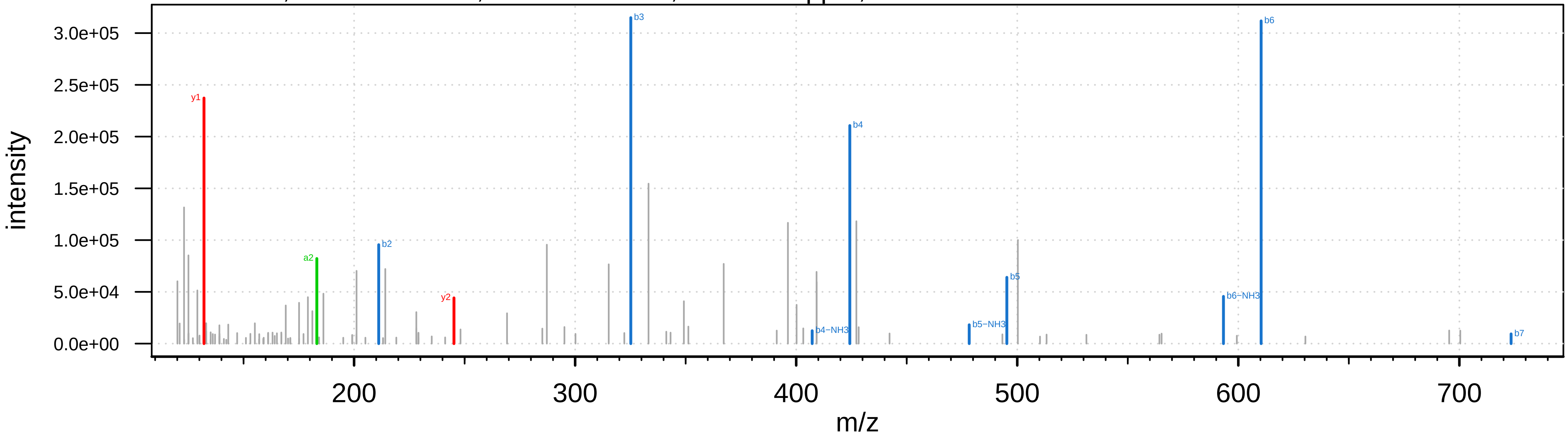

Raw File: Grobi_20130312_CHS_IEF100_20130309_3−10linear_Serva_7cm_1mg_1elution_01
Scan Number: 52743
Proteins:
TCONS_l2_00005685_chr12:53548245−53548274:+

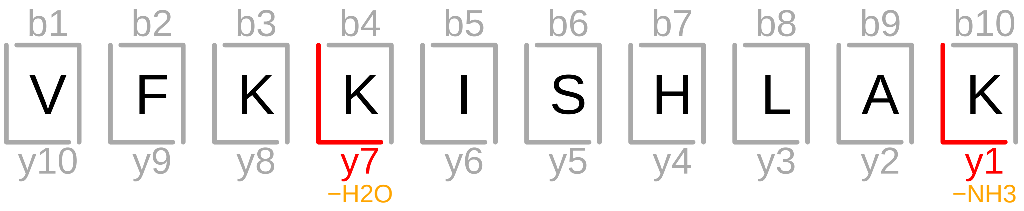

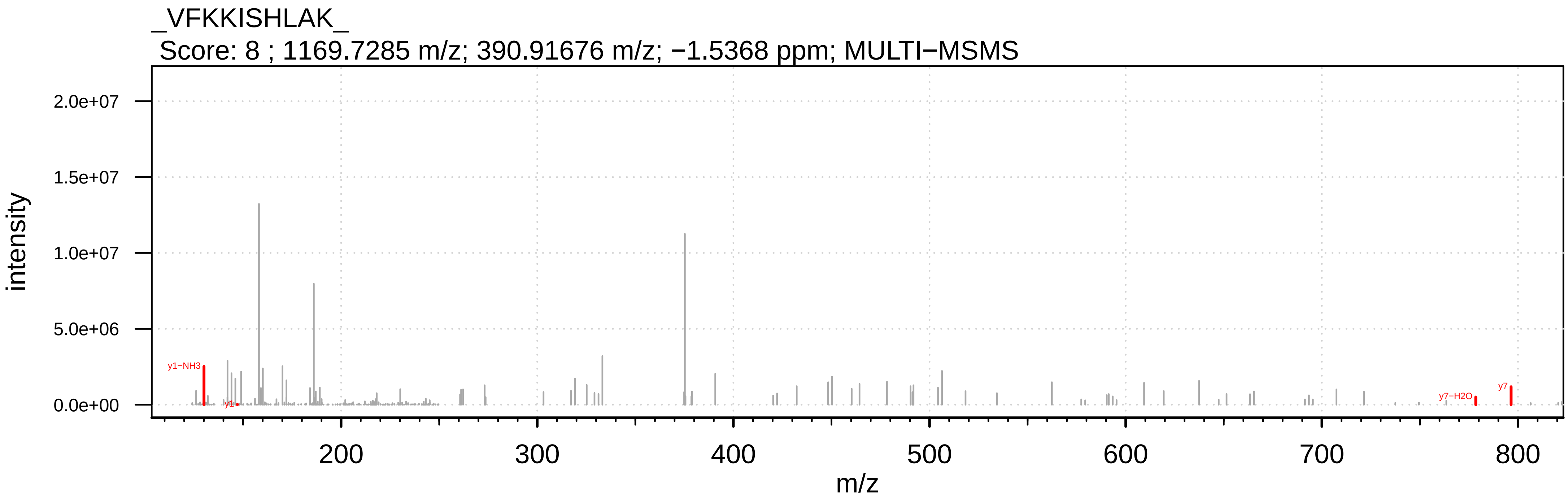

Raw File: Grobi_20121019_CHS_PIECEIEF_20121017_3−10_7cm_250ug_03
Scan Number: 8071
Proteins:
ENST00000490272_chr19:52826295−52826555:+

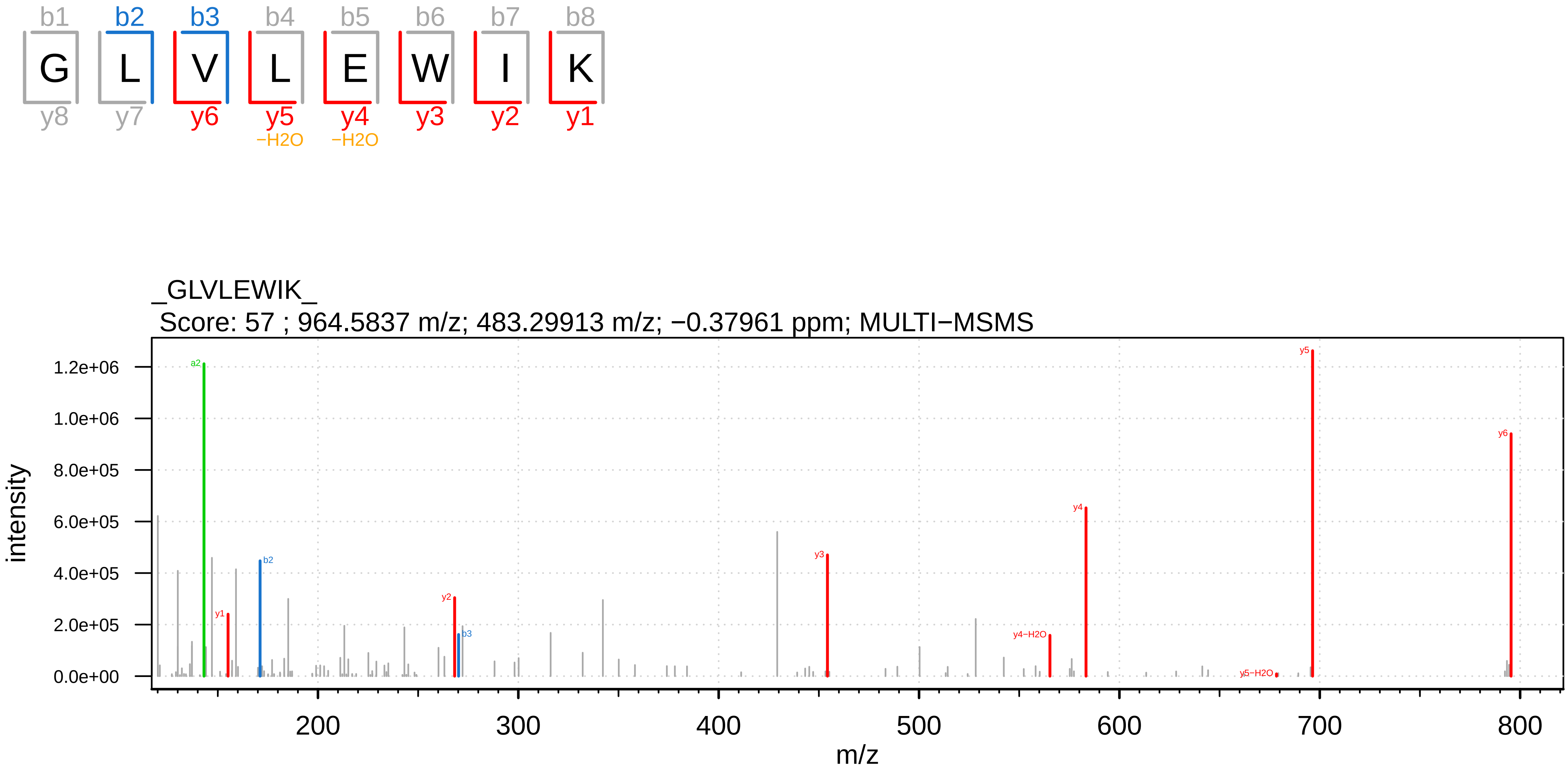

Raw File: Grobi_20140814_HZ_HS_A1L_MicroPeps_TriSilac
Scan Number: 73510
Proteins:
TCONS_l2_00001296_chr1:79520703−79520992:−

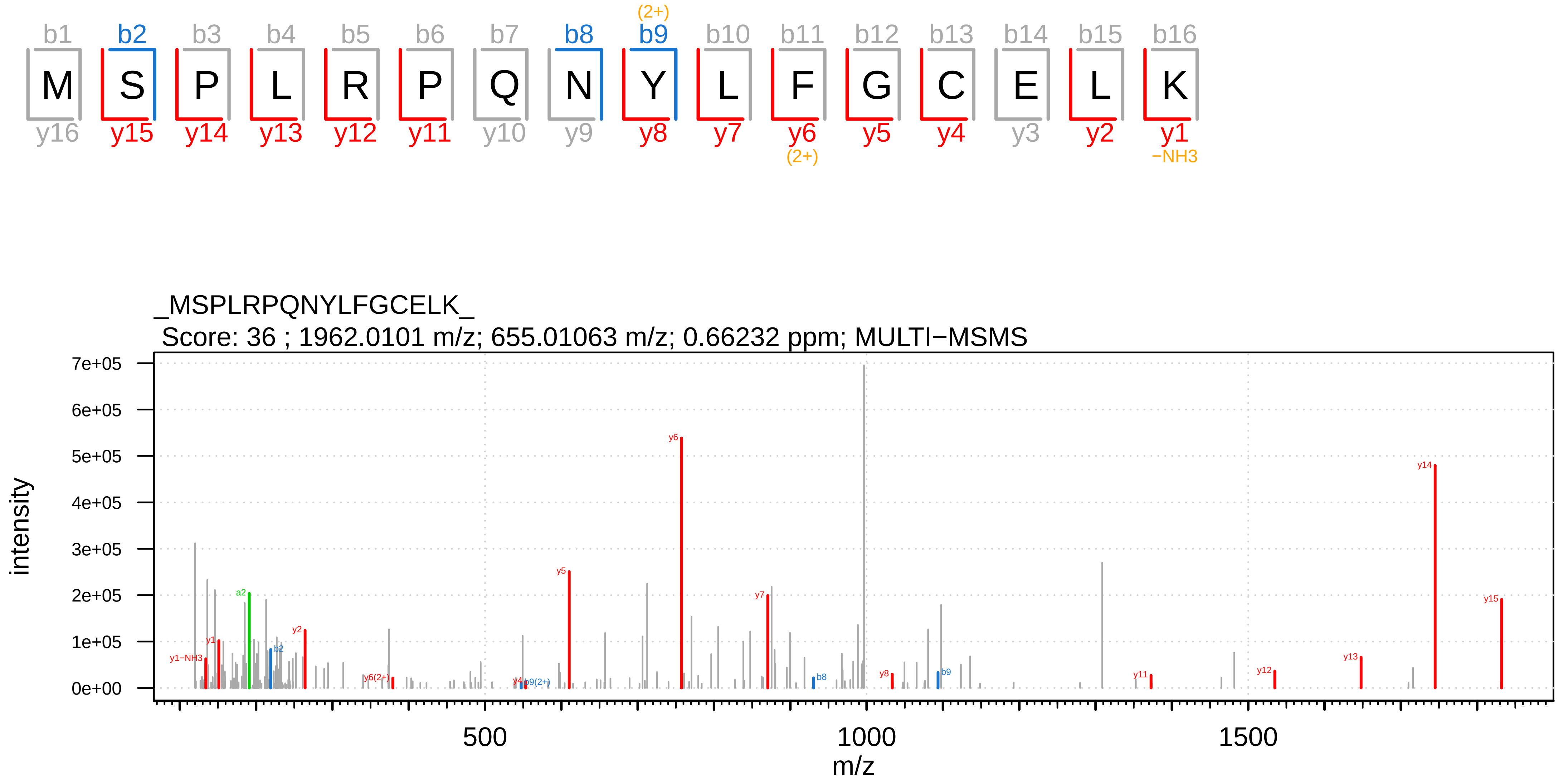

Raw File: Grobi_20140814_HZ_HS_A1L_MicroPeps_TriSilac
Scan Number: 64401
Proteins:
TCONS_l2_00008829_chr15:92829088−92829258:+

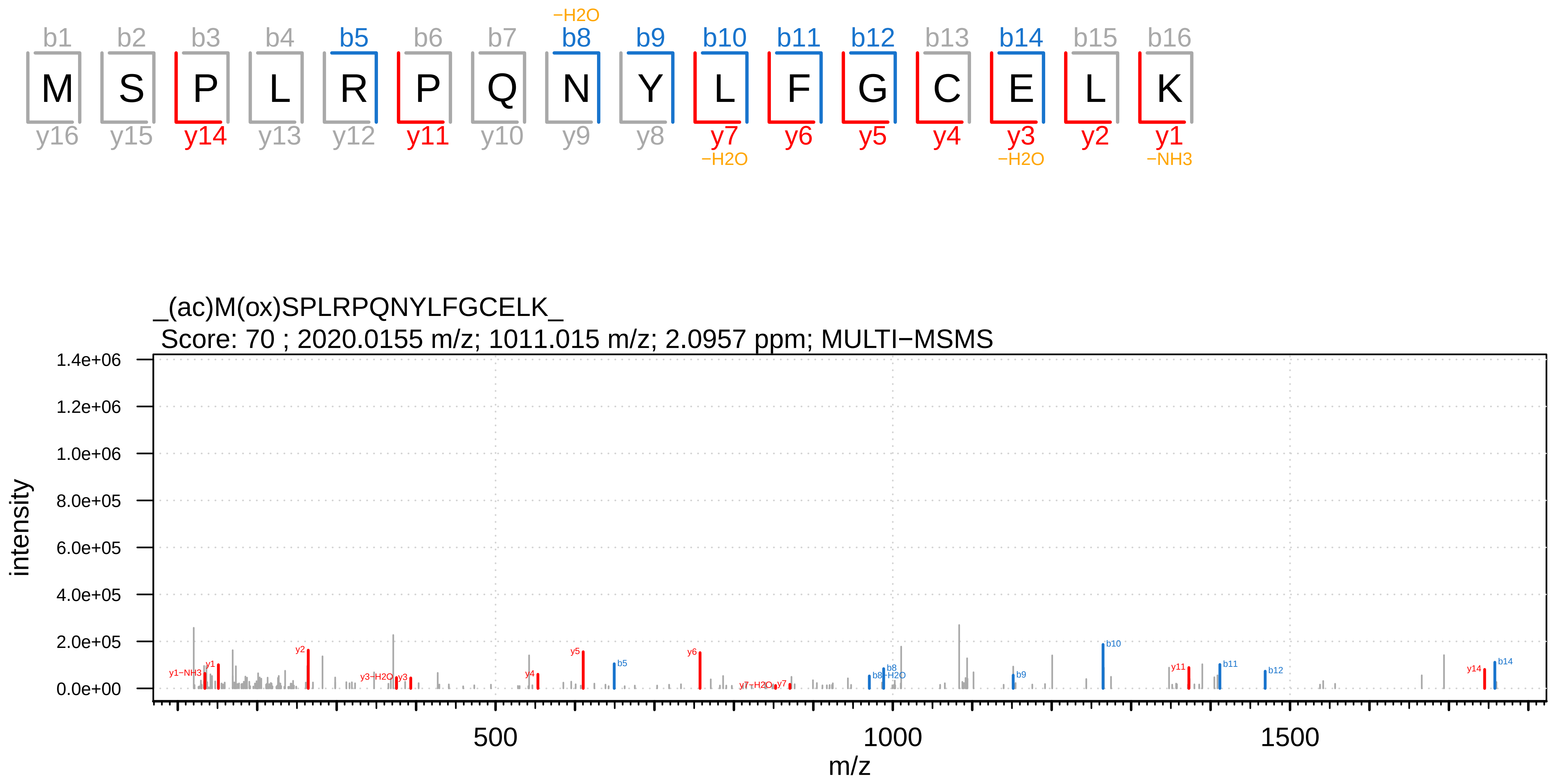

Raw File: Grobi_20140814_HZ_HS_A1L_MicroPeps_TriSilac
Scan Number: 72860
Proteins:
TCONS_l2_00008829_chr15:92829088−92829258:+

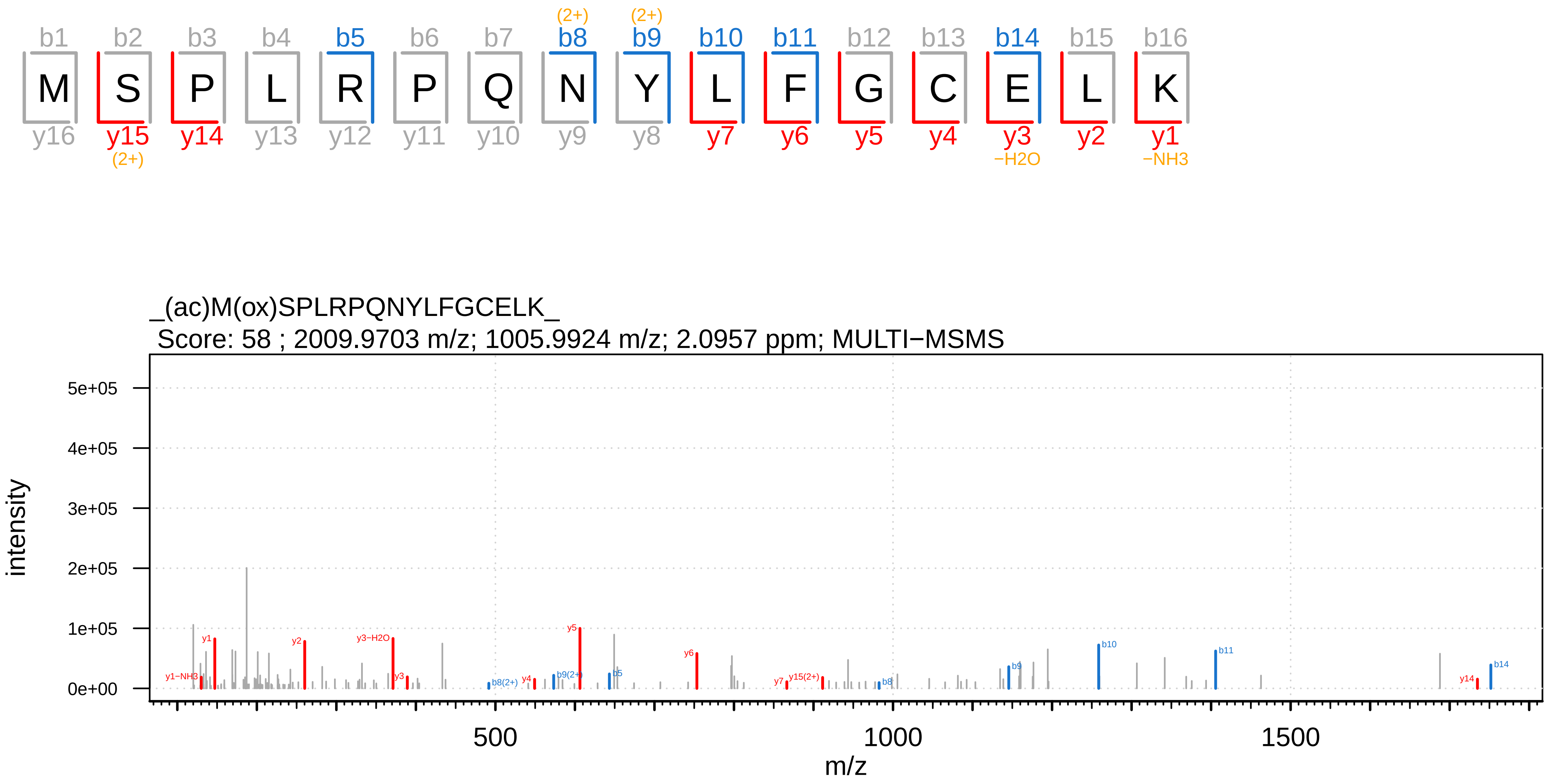

Raw File: Grobi_20140814_HZ_HS_A1L_MicroPeps_TriSilac
Scan Number: 72915
Proteins:
TCONS_l2_00008829_chr15:92829088−92829258:+

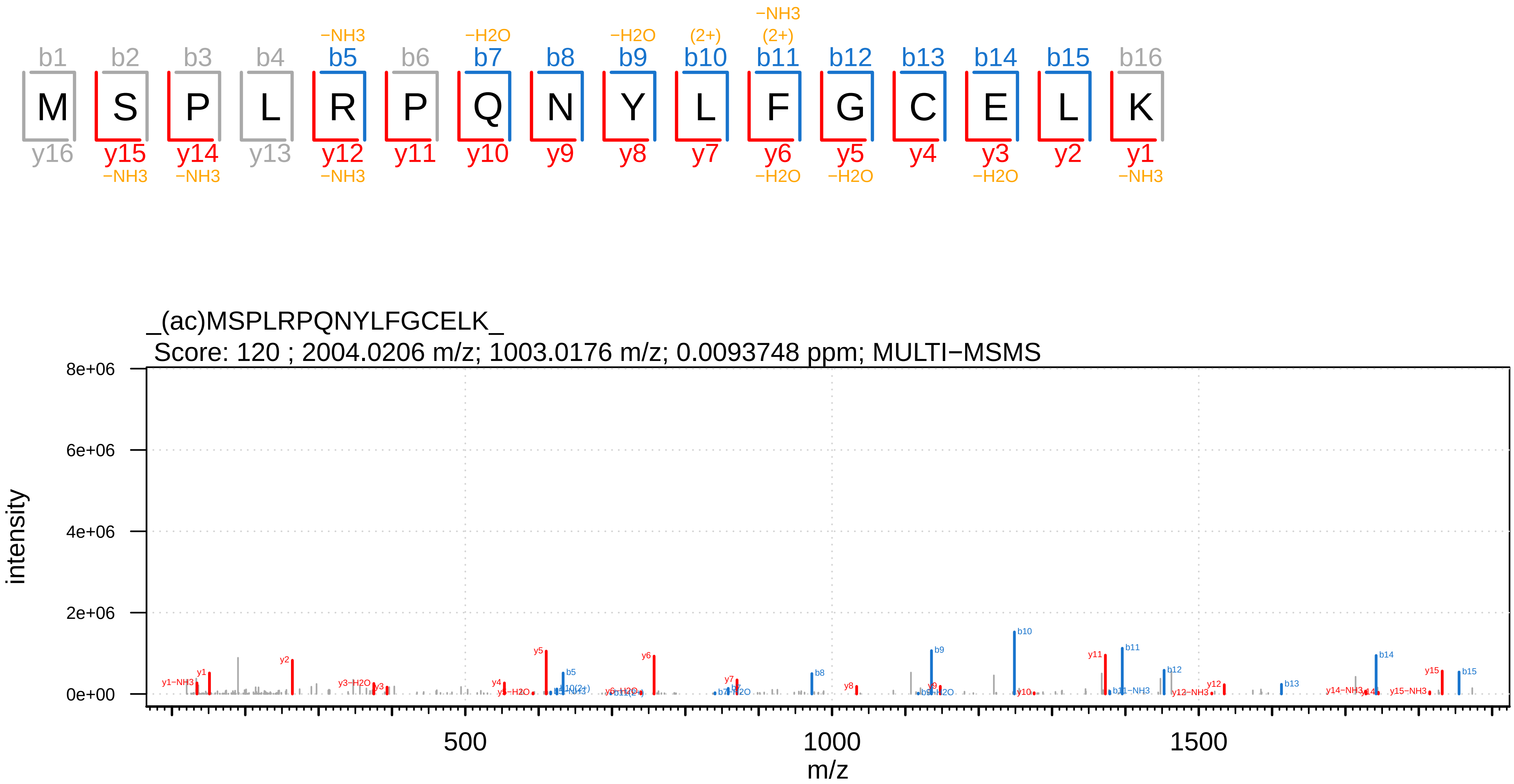

Raw File: Grobi_20140814_HZ_HS_A1L_MicroPeps_TriSilac
Scan Number: 79460
Proteins:
TCONS_l2_00008829_chr15:92829088−92829258:+

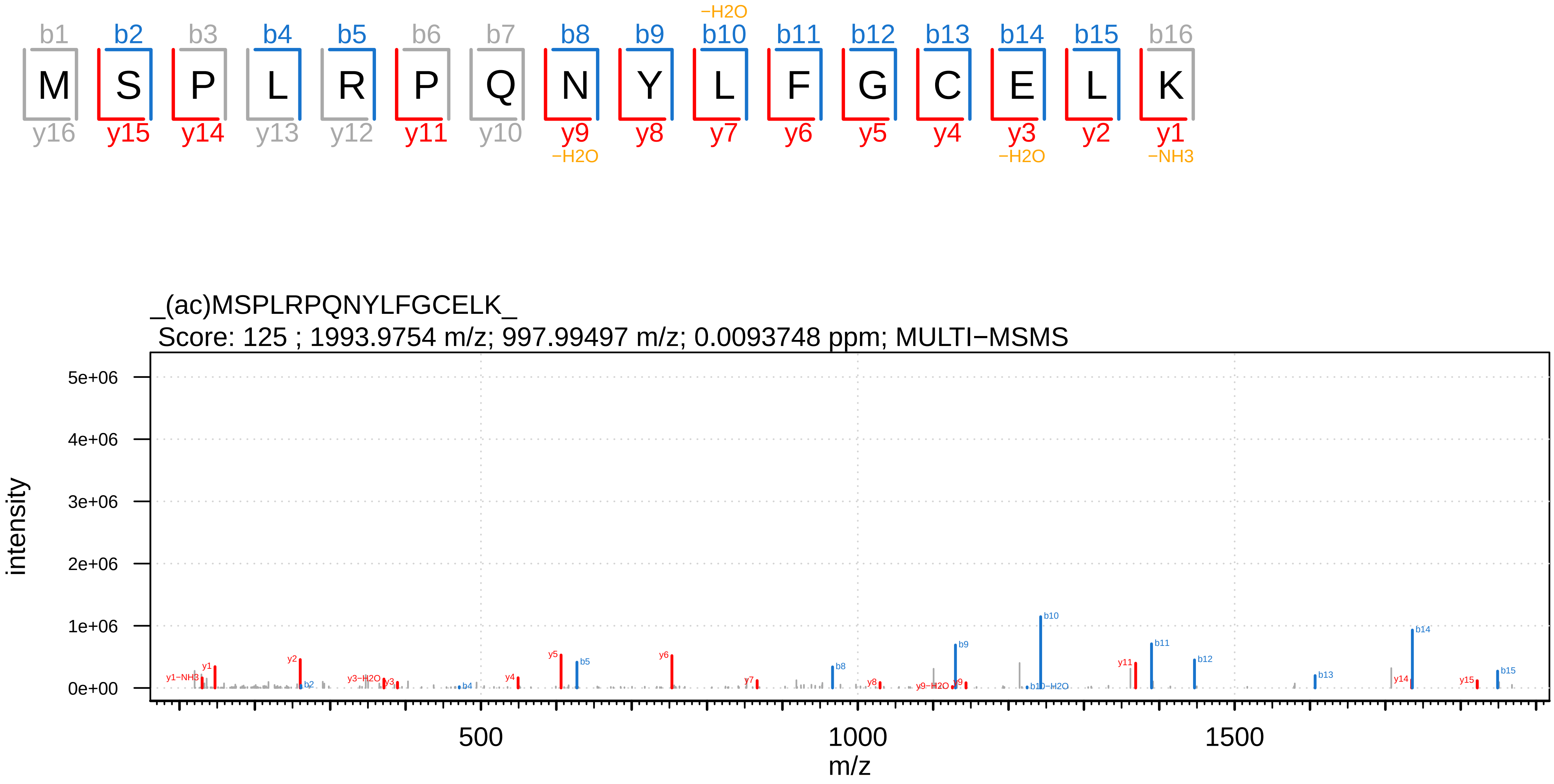

Raw File: Grobi_20140814_HZ_HS_A1L_MicroPeps_TriSilac
Scan Number: 79472
Proteins:
TCONS_l2_00008829_chr15:92829088−92829258:+

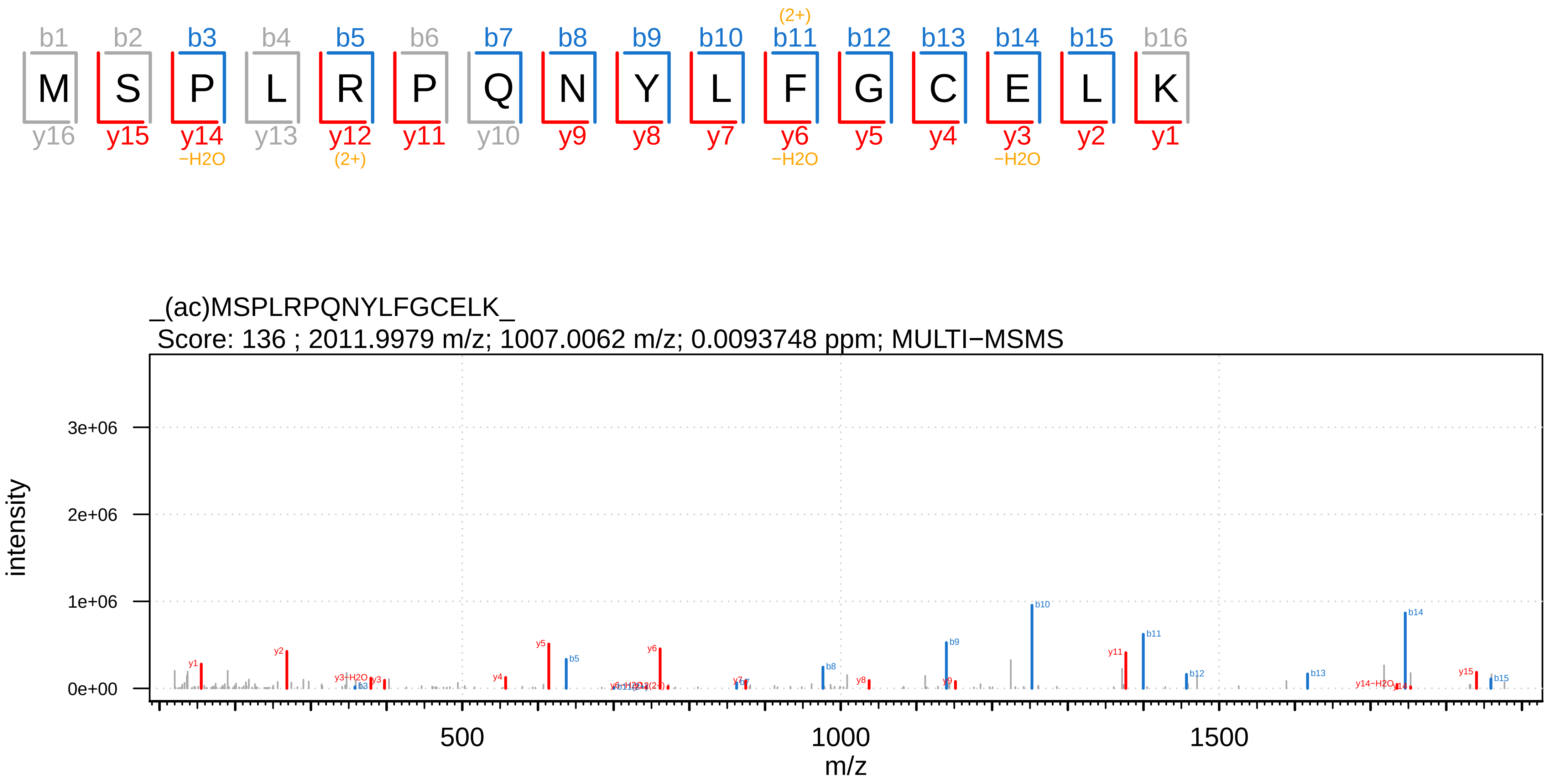

Raw File: Grobi_20140814_HZ_HS_A1L_MicroPeps_TriSilac
Scan Number: 79493
Proteins:
TCONS_l2_00008829_chr15:92829088−92829258:+

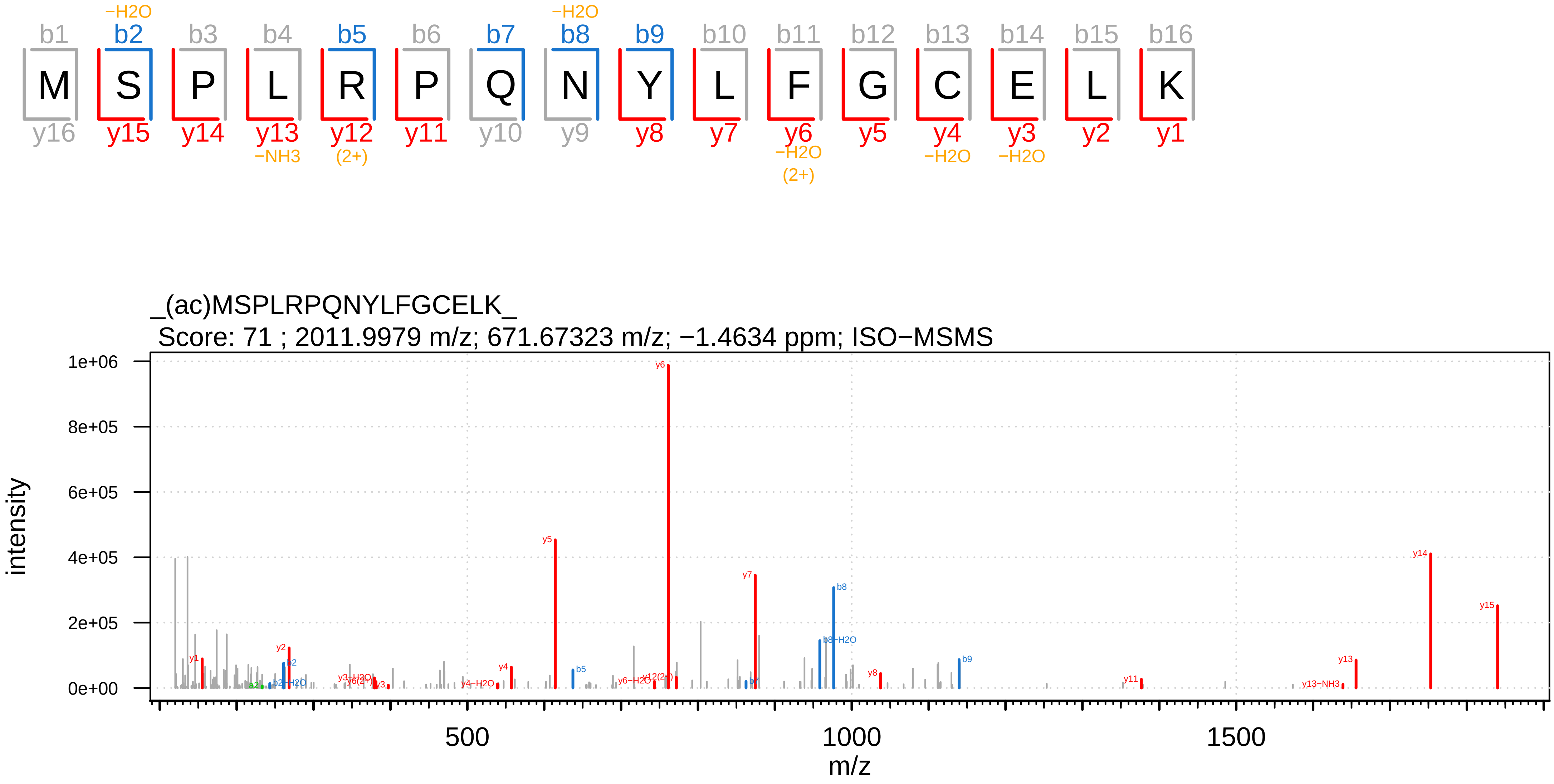

Raw File: Grobi_20140814_HZ_HS_A1L_MicroPeps_TriSilac
Scan Number: 79520
Proteins:
TCONS_l2_00008829_chr15:92829088−92829258:+

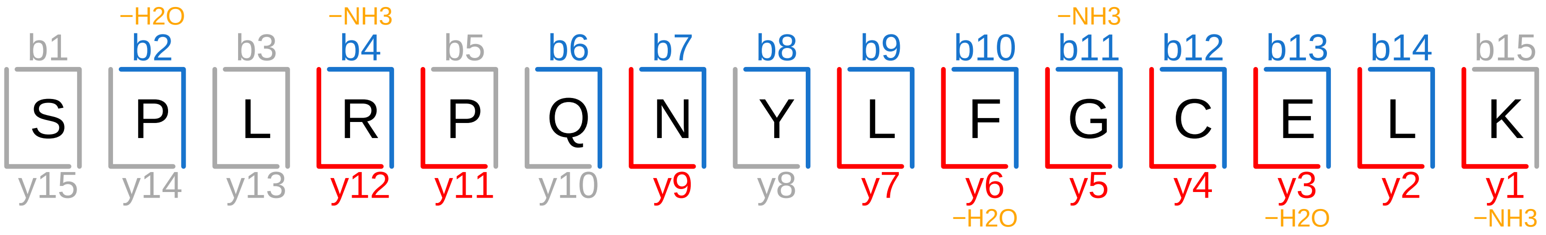

SPLRPQNYLFGCELK
Score: 84 ; 1830.9696 m/z; 916.49206 m/z; 0.24756 ppm; MULTI−MSMS

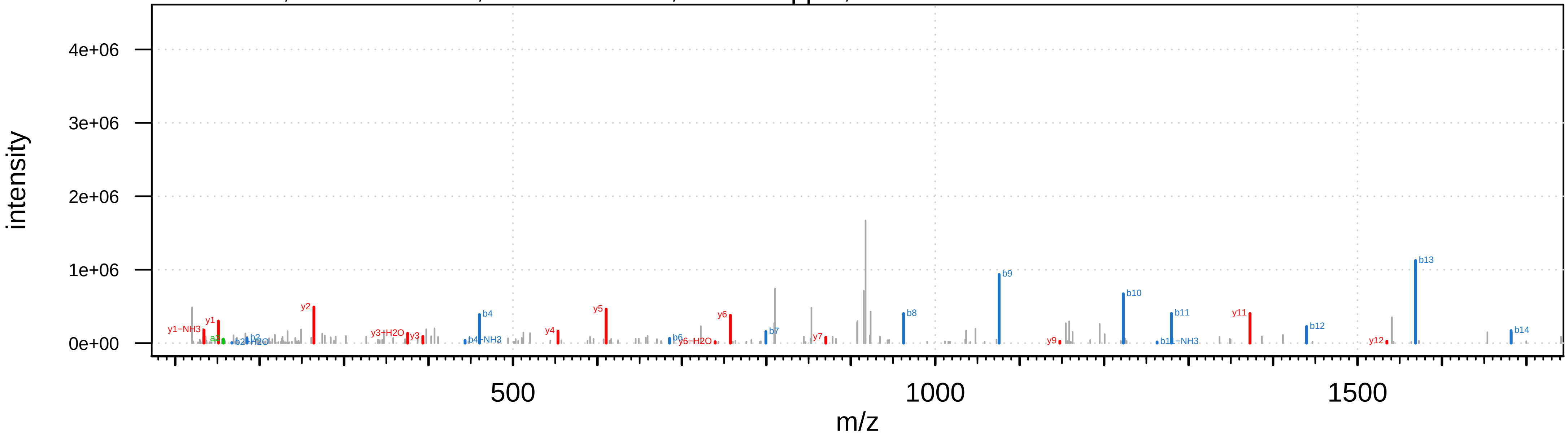

Raw File: Grobi_20140814_HZ_HS_A1L_MicroPeps_TriSilac
Scan Number: 60226
Proteins:
TCONS_l2_00008829_chr15:92829088−92829258:+

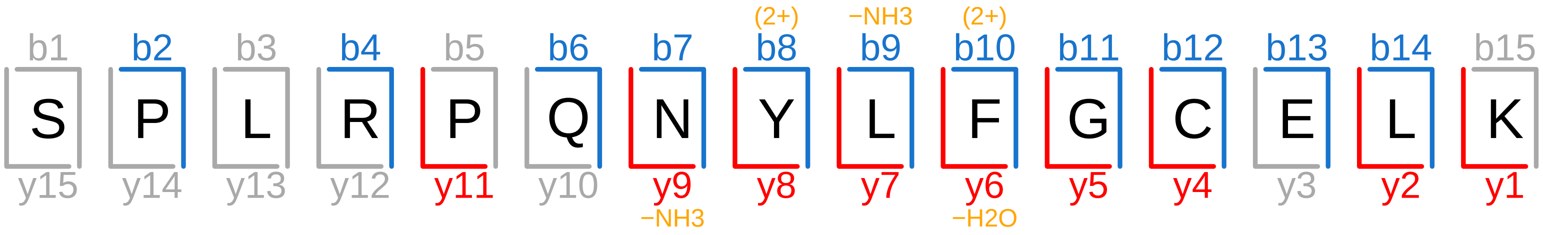

SPLRPQNYLFGCELK
Score: 64 ; 1838.9468 m/z; 920.48068 m/z; 0.24756 ppm; MULTI−MSMS

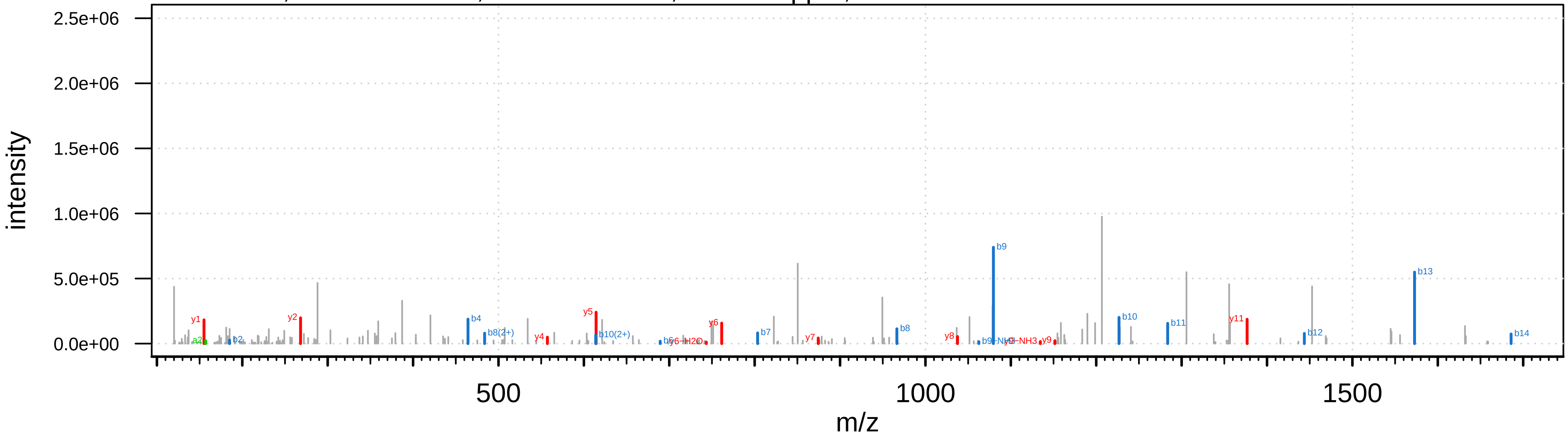

Raw File: Grobi_20140814_HZ_HS_A1L_MicroPeps_TriSilac
Scan Number: 60231
Proteins:
TCONS_l2_00008829_chr15:92829088−92829258:+

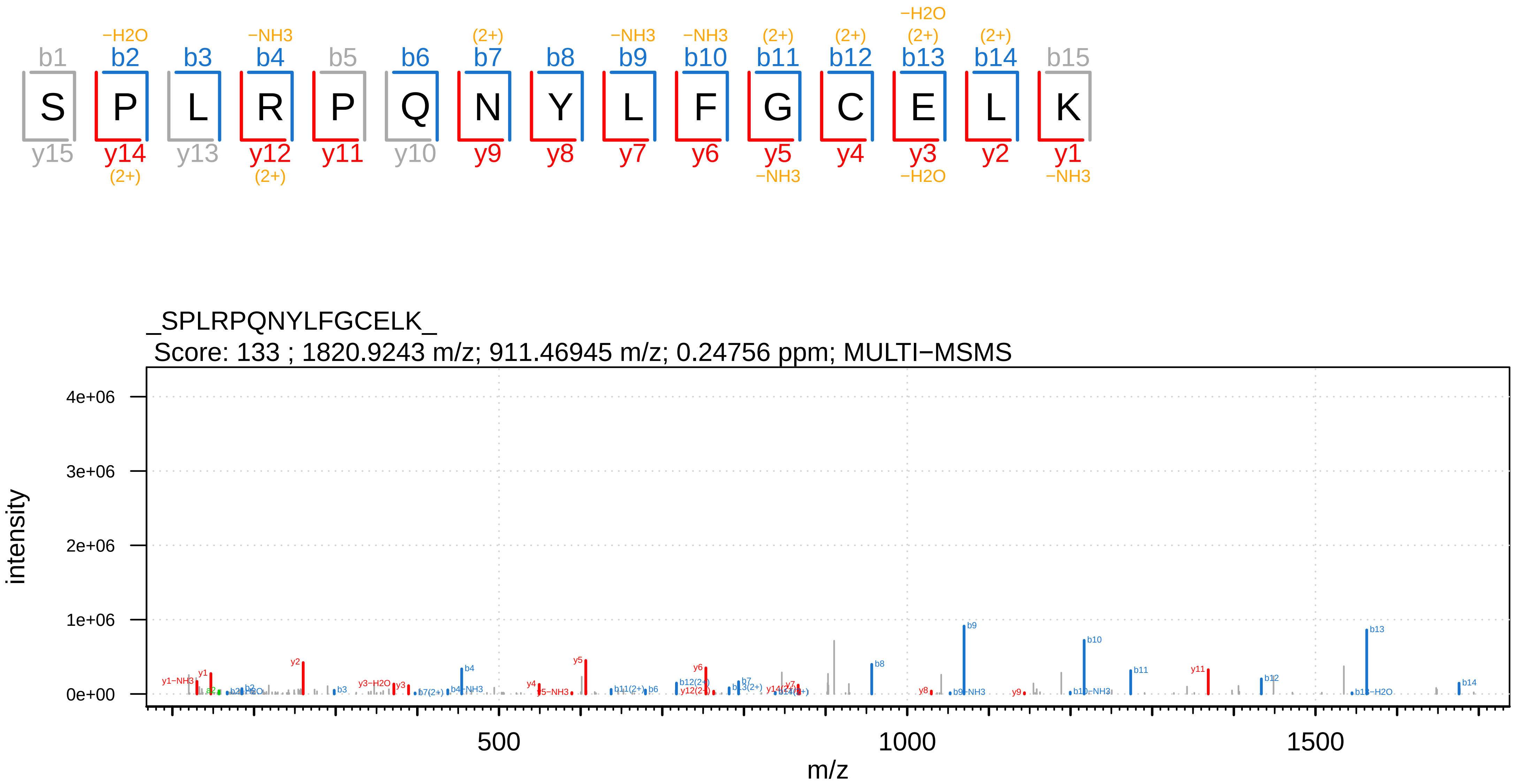

SPLRPQNYLFGCELK
Score: 133 ; 1820.9243 m/z; 911.46945 m/z; 0.24756 ppm; MULTI−MSMS

Raw File: Grobi_20140814_HZ_HS_A1L_MicroPeps_TriSilac
Scan Number: 60271
Proteins:
TCONS_l2_00008829_chr15:92829088−92829258:+

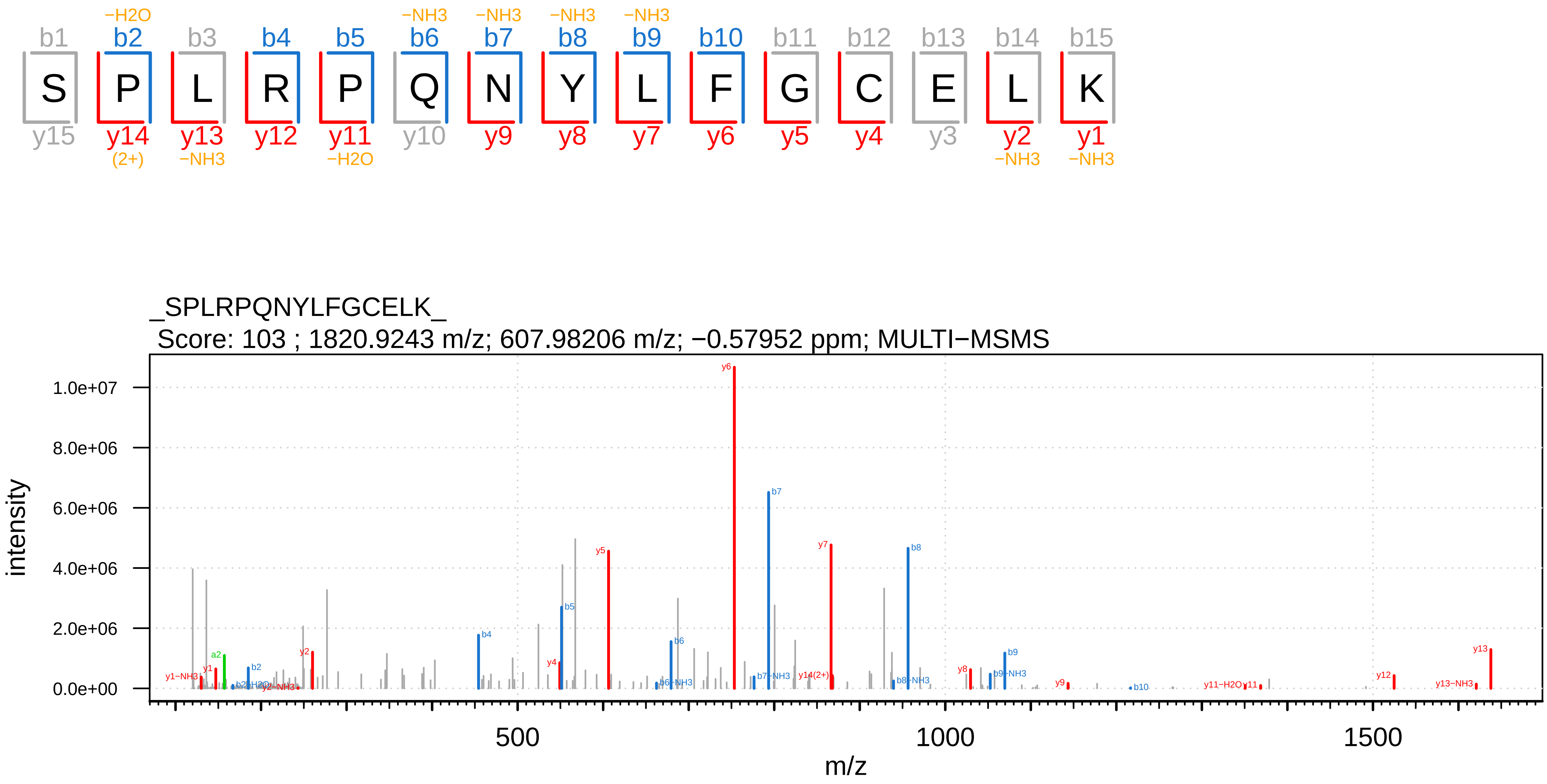

Raw File: Grobi_20140814_HZ_HS_A1L_MicroPeps_TriSilac

Scan Number: 60270

Proteins:

TCONS_l2_00008829_chr15:92829088−92829258:+

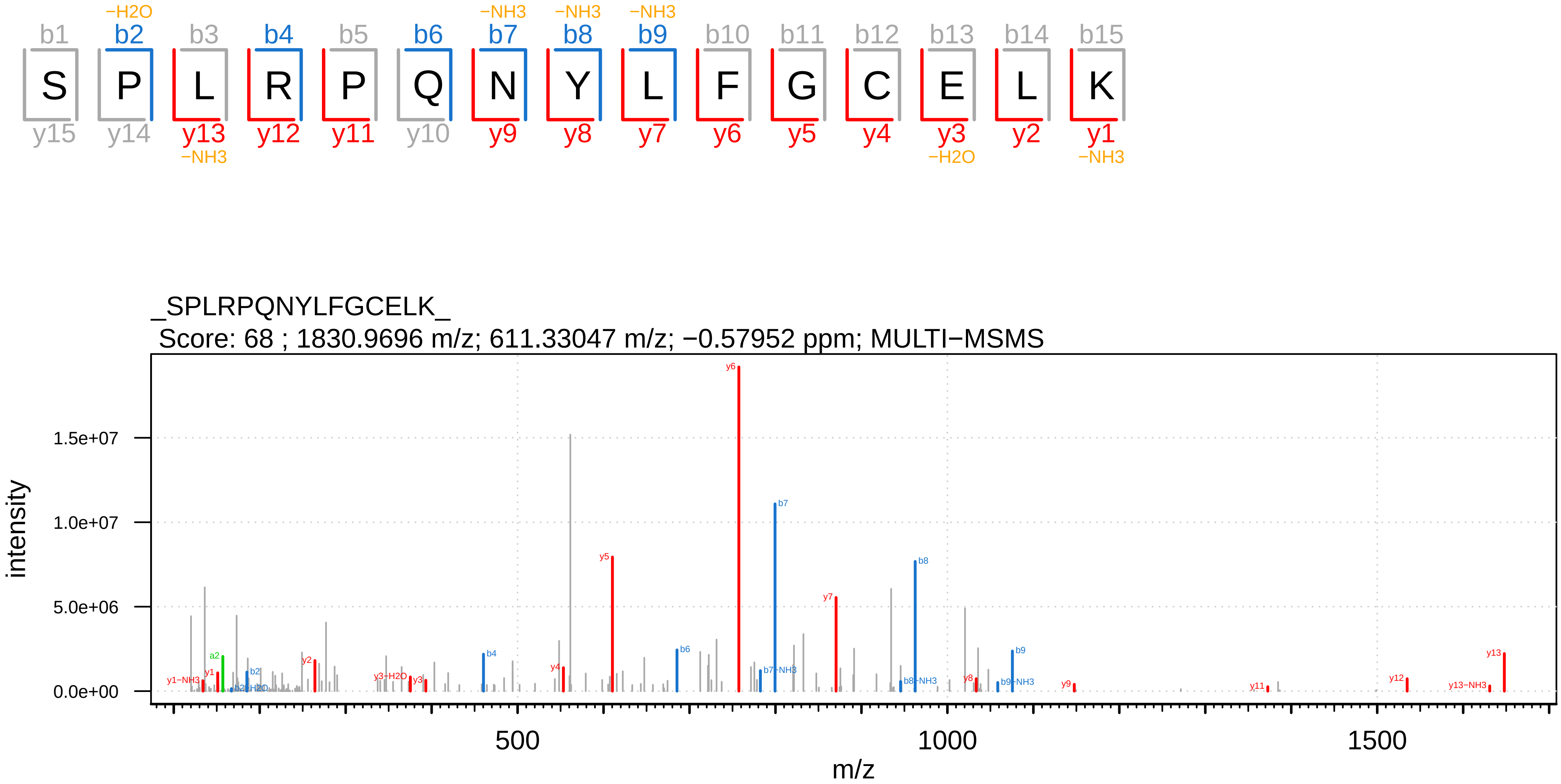

Raw File: Grobi_20140814_HZ_HS_A1L_MicroPeps_TriSilac
Scan Number: 60303
Proteins:
TCONS_l2_00008829_chr15:92829088−92829258:+

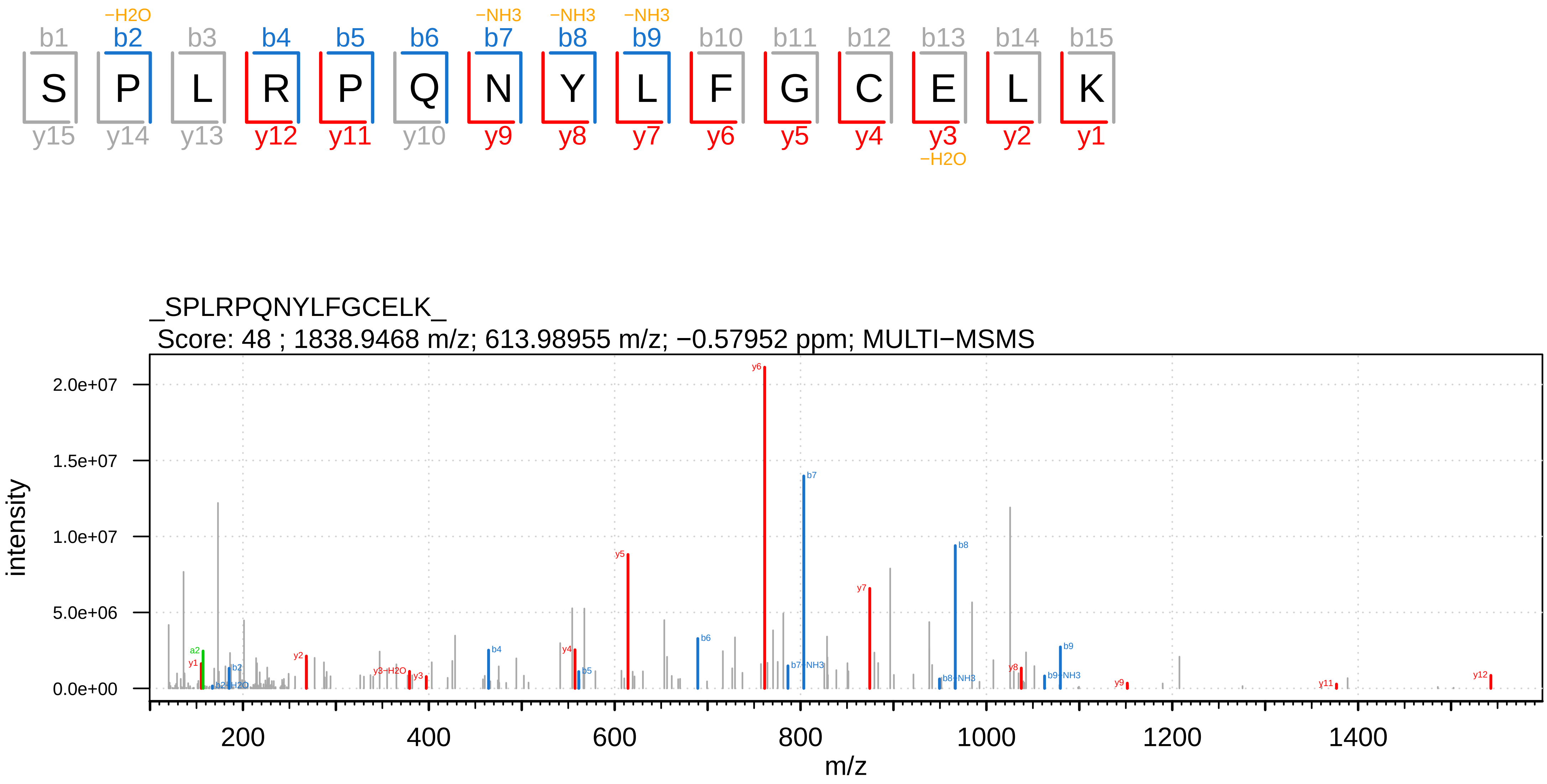

Raw File: Grobi_20140814_HZ_HS_A1L_MicroPeps_TriSilac
Scan Number: 60304
Proteins:
TCONS_l2_00008829_chr15:92829088−92829258:+

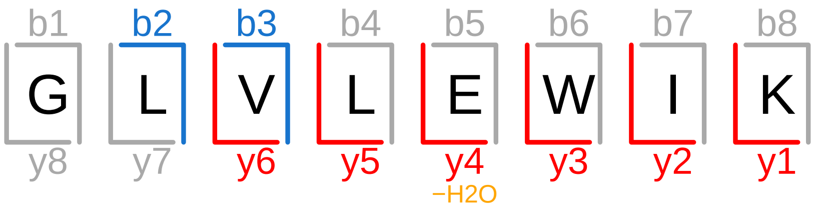

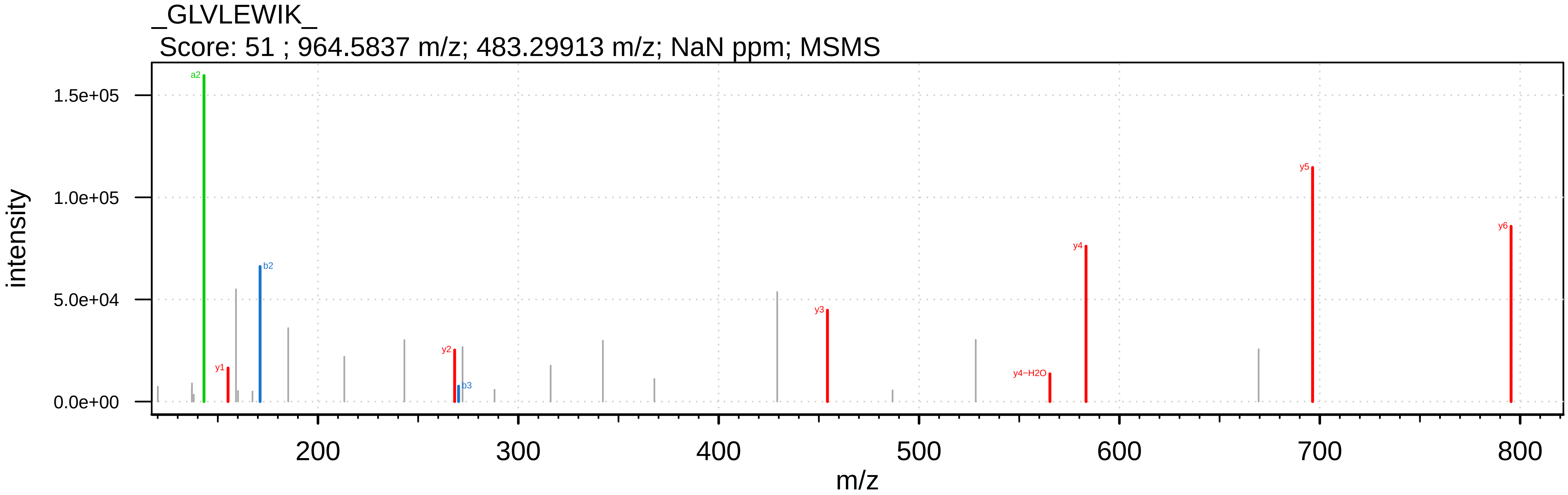

Raw File: Kermit_20140724_KK_HS_B1large
Scan Number: 39837
Proteins:
TCONS_l2_00001296_chr1:79520703−79520992:−

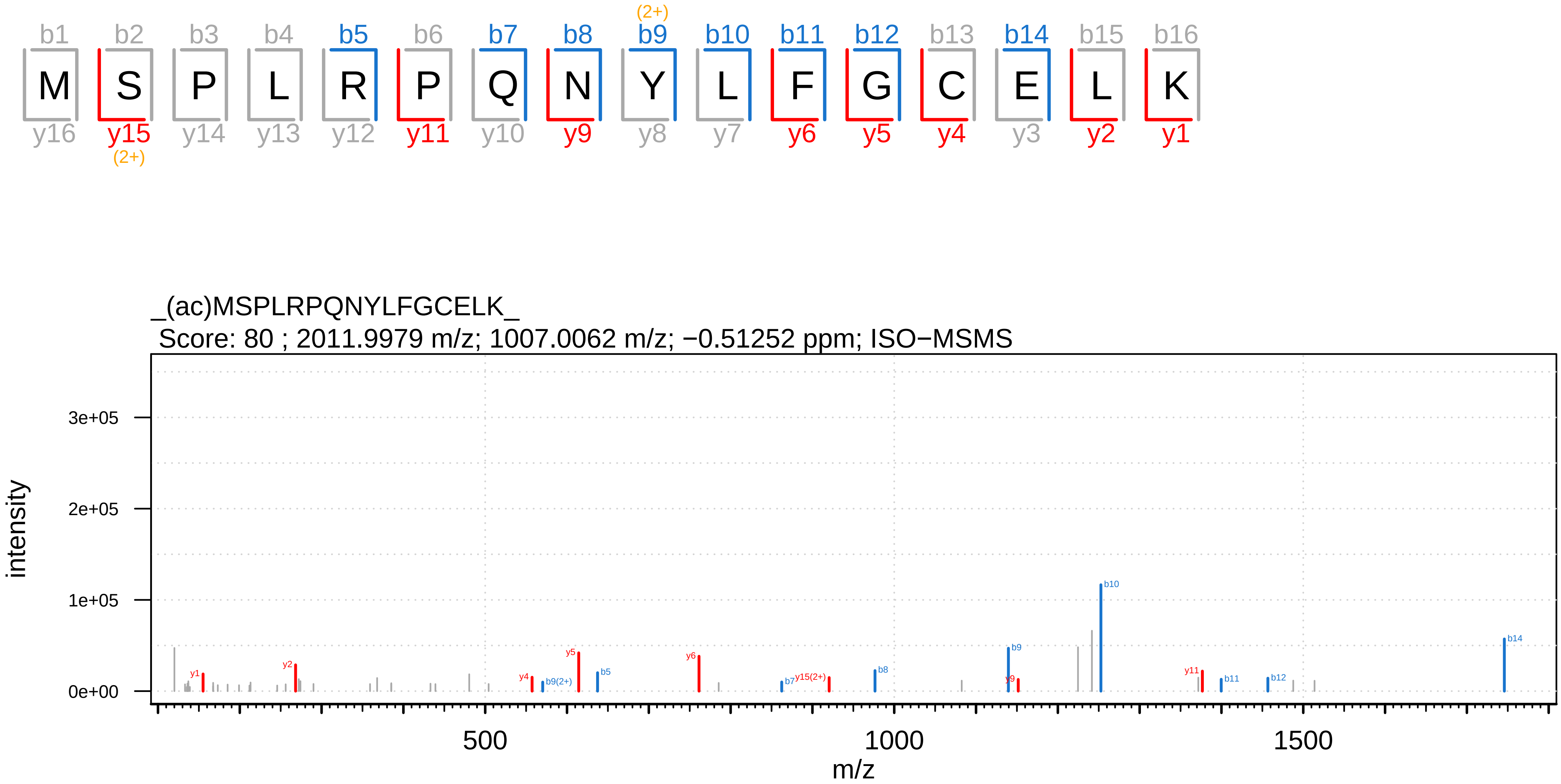

Raw File: Kermit_20140724_KK_HS_B1large
Scan Number: 45019
Proteins:
TCONS_l2_00008829_chr15:92829088−92829258:+

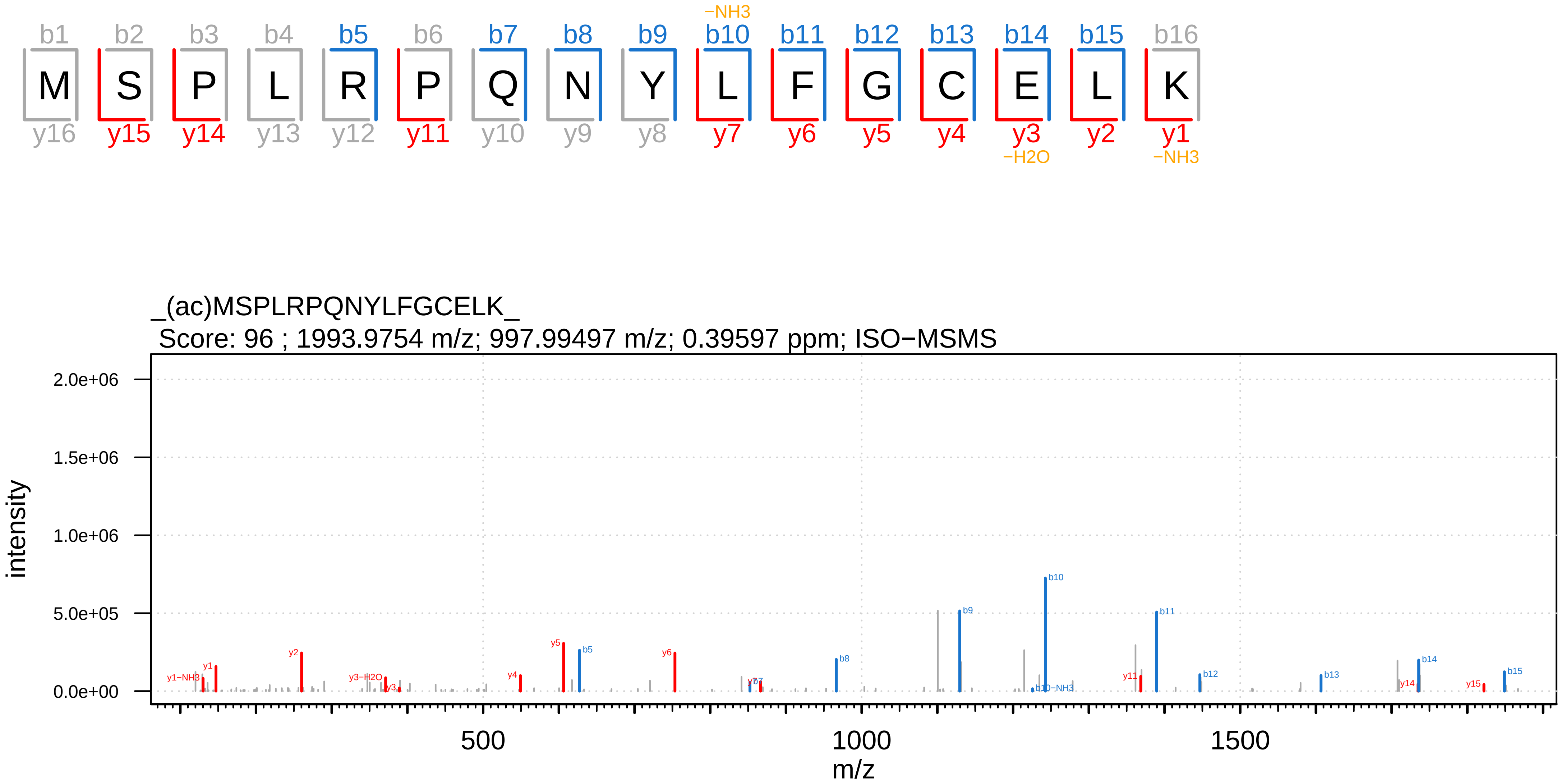

Raw File: Kermit_20140724_KK_HS_B1large
Scan Number: 45048
Proteins:
TCONS_l2_00008829_chr15:92829088−92829258:+

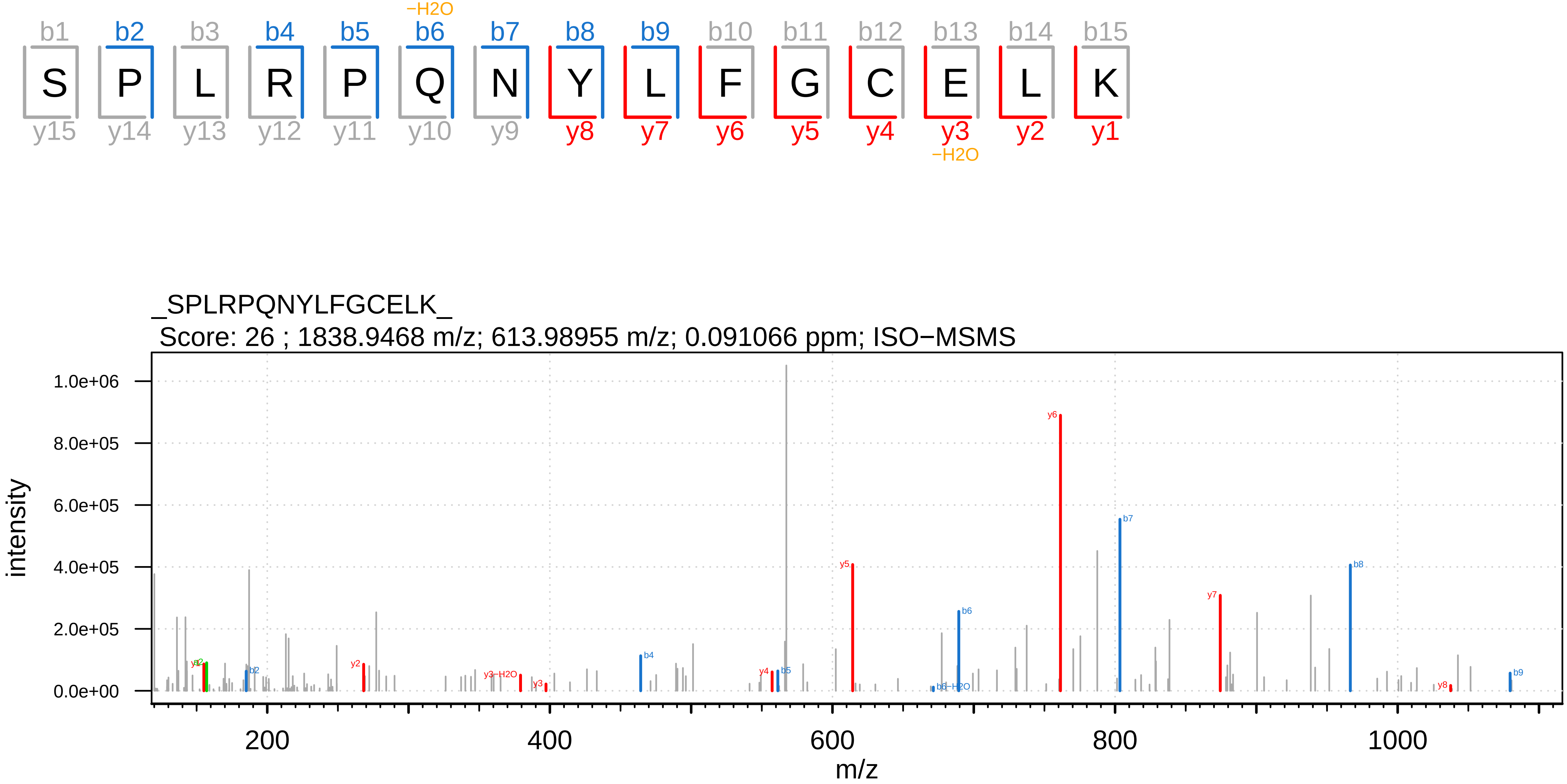

Raw File: Kermit_20140724_KK_HS_B1large
Scan Number: 30986
Proteins:
TCONS_l2_00008829_chr15:92829088−92829258:+

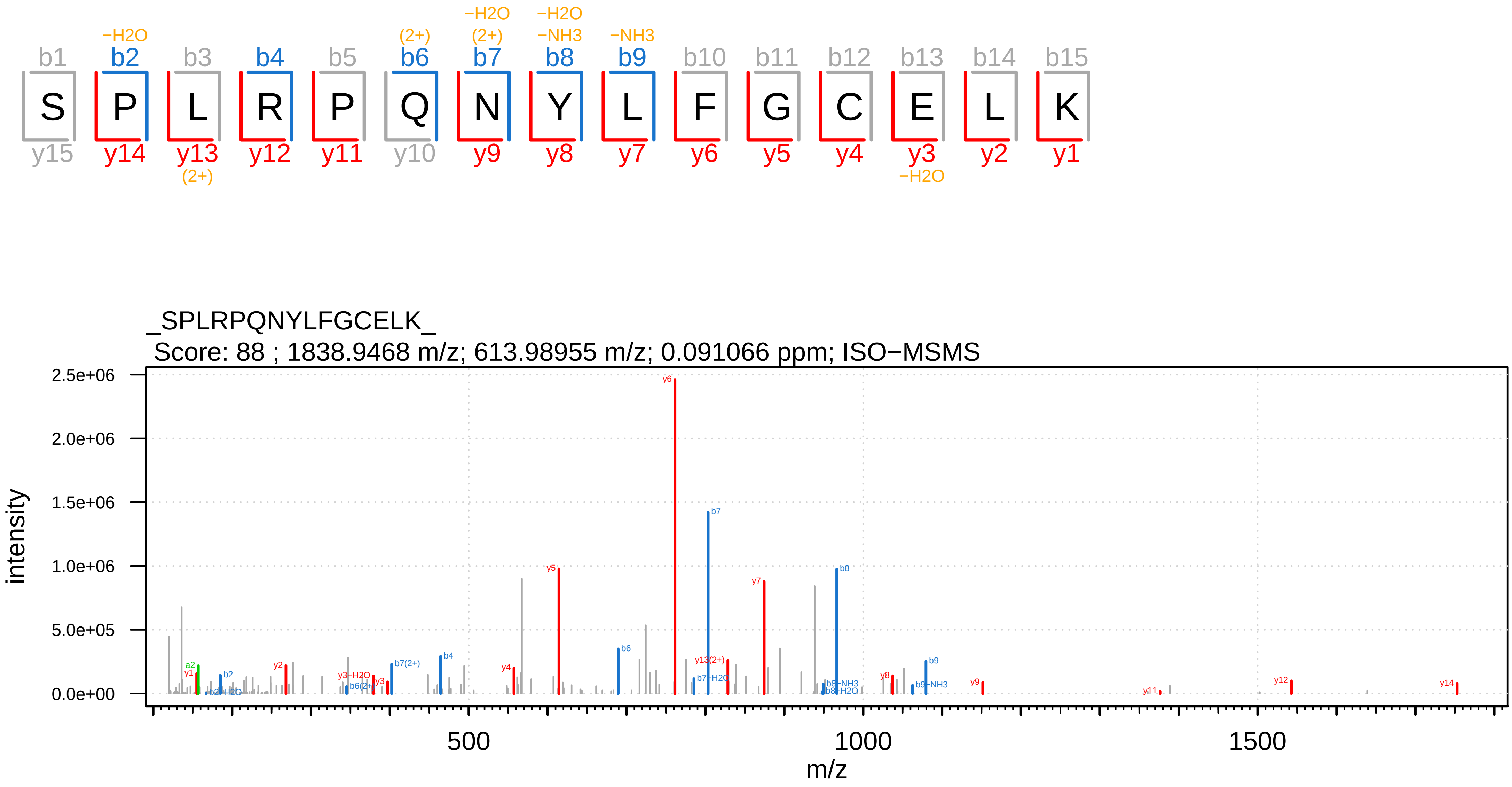

Raw File: Kermit_20140724_KK_HS_B1large
Scan Number: 31258
Proteins:
TCONS_l2_00008829_chr15:92829088−92829258:+

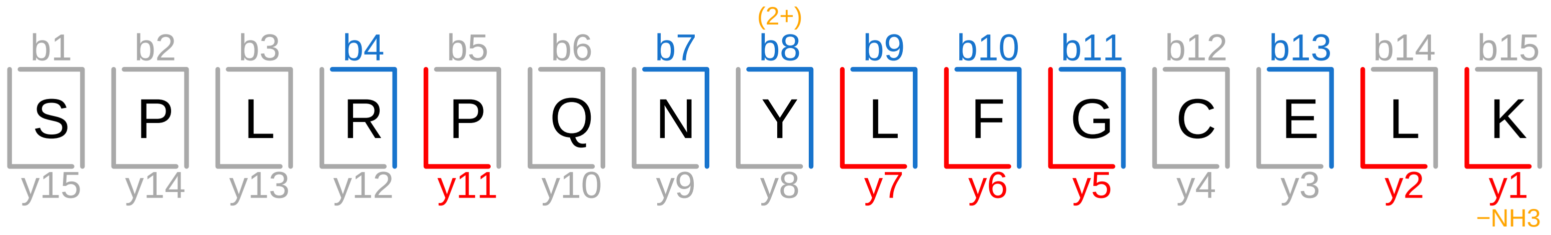

SPLRPQNYLFGCELK
Score: 62 ; 1820.9243 m/z; 911.46945 m/z; 0.30349 ppm; ISO−MSMS

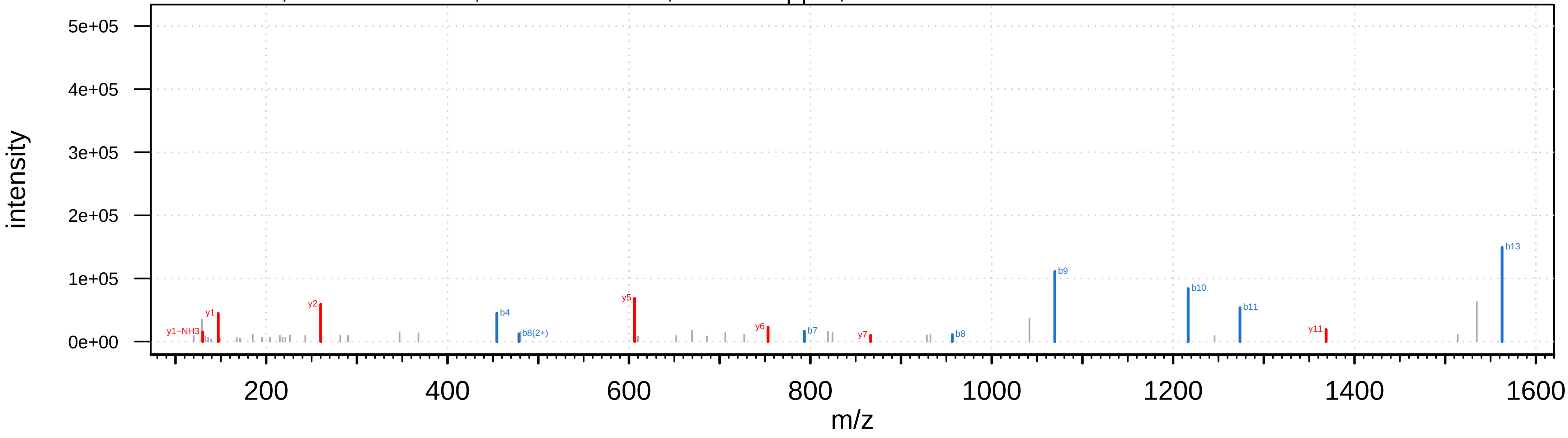

Raw File: Kermit_20140724_KK_HS_B1large
Scan Number: 31007
Proteins:
TCONS_l2_00008829_chr15:92829088−92829258:+

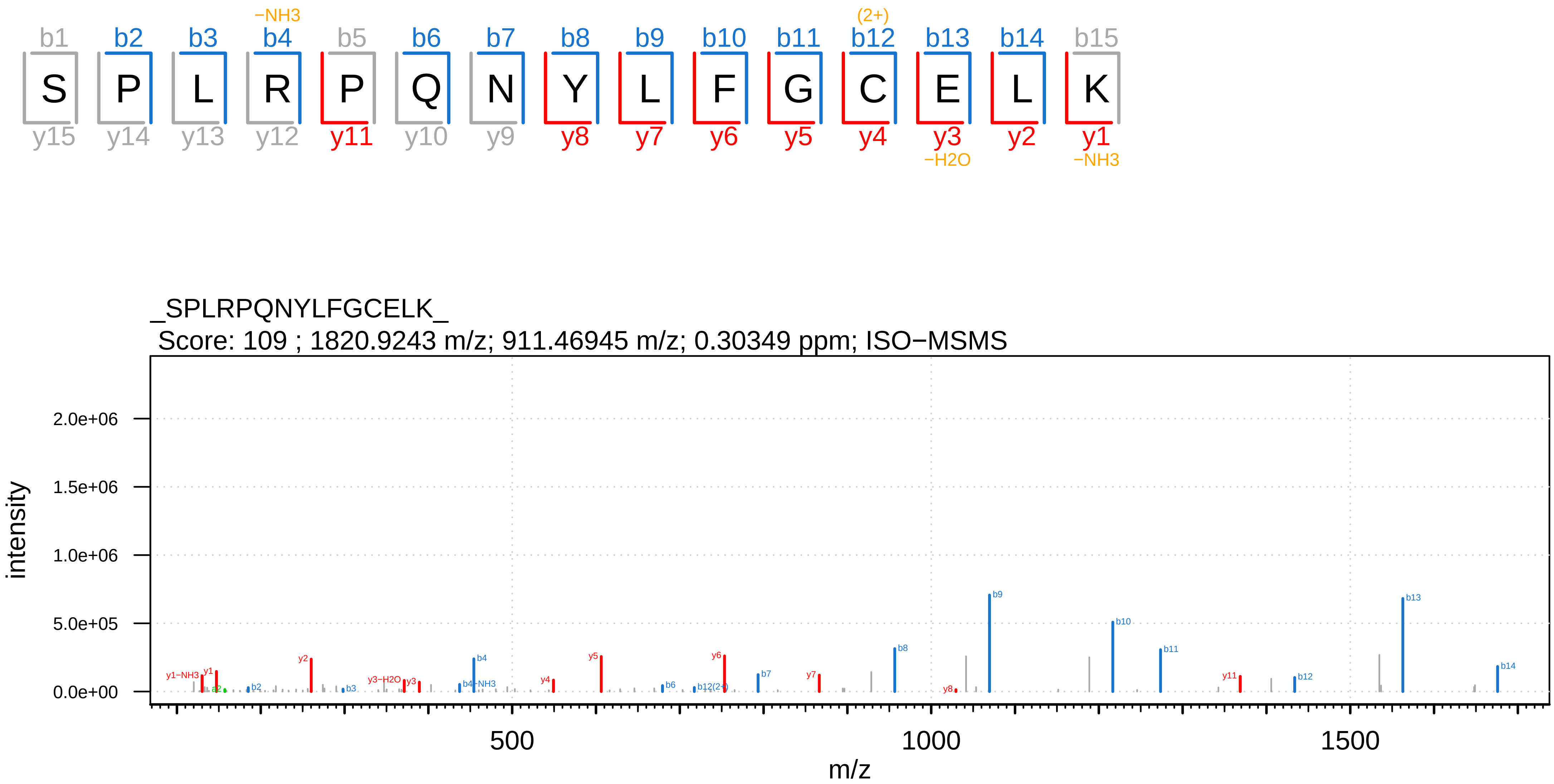

Raw File: Kermit_20140724_KK_HS_B1large
Scan Number: 31280
Proteins:
TCONS_l2_00008829_chr15:92829088−92829258:+

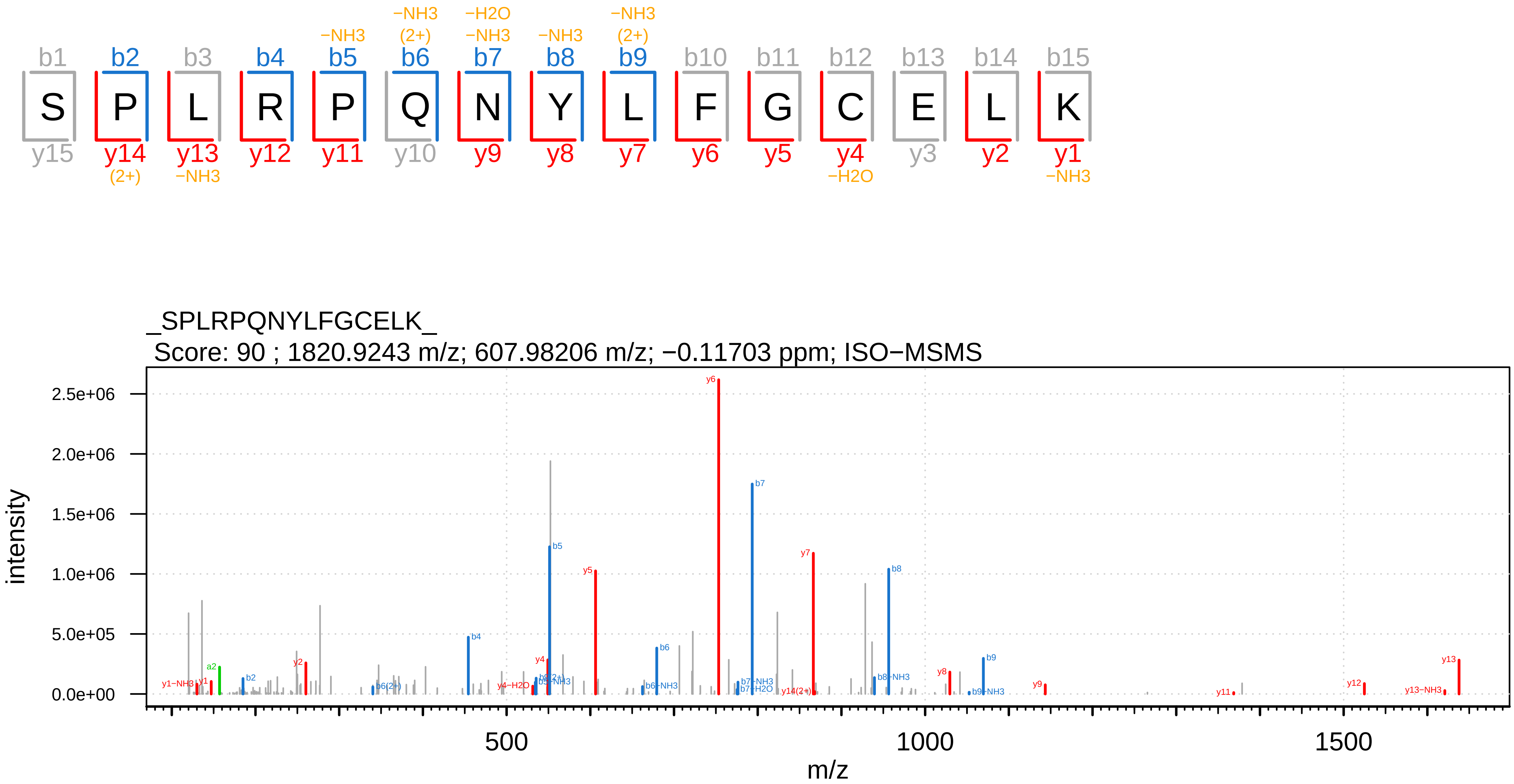

Raw File: Kermit_20140724_KK_HS_B1large
Scan Number: 31096
Proteins:
TCONS_l2_00008829_chr15:92829088−92829258:+

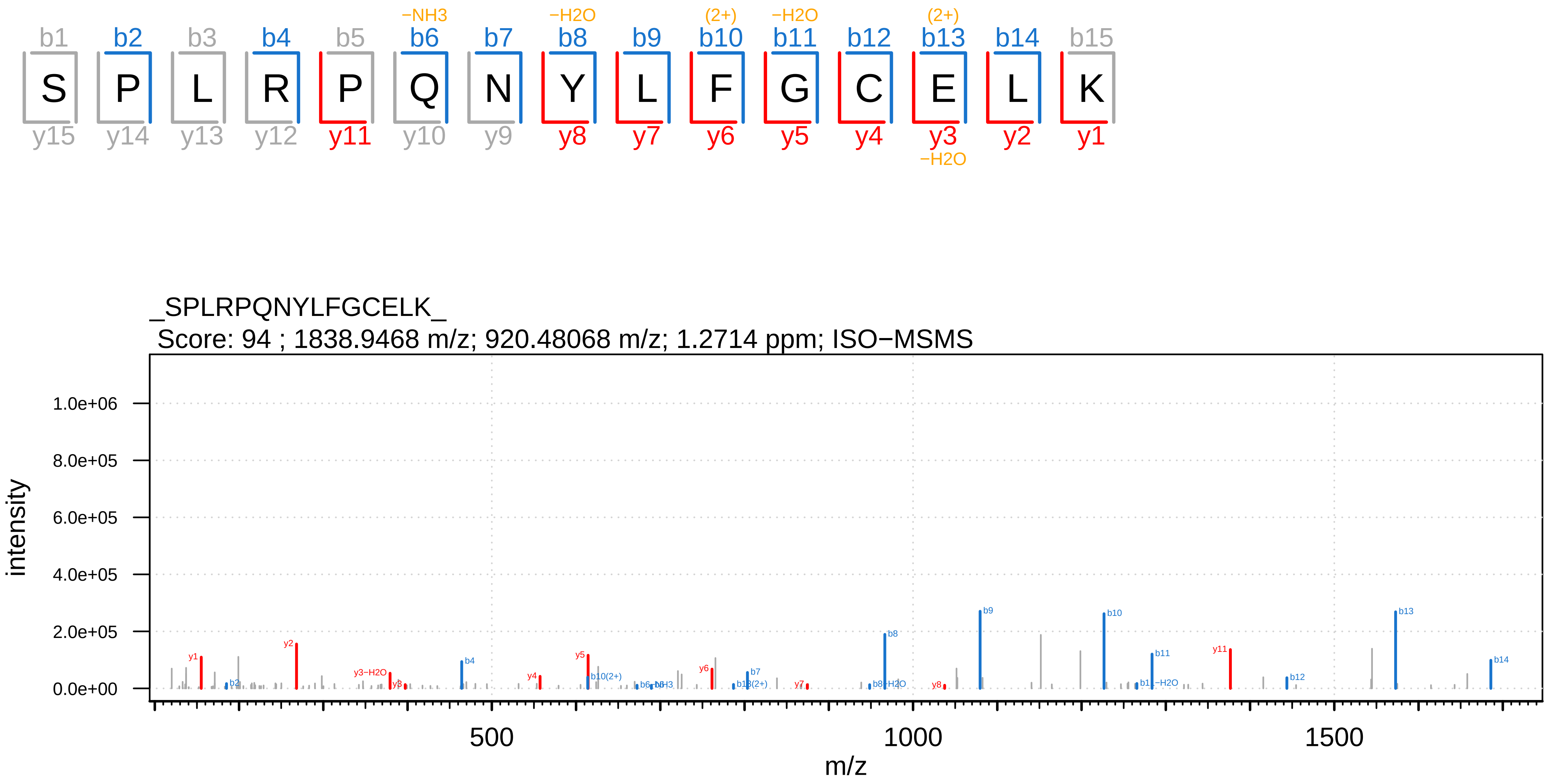

Raw File: Kermit_20140724_KK_HS_B1large
Scan Number: 31270
Proteins:
TCONS_l2_00008829_chr15:92829088−92829258:+

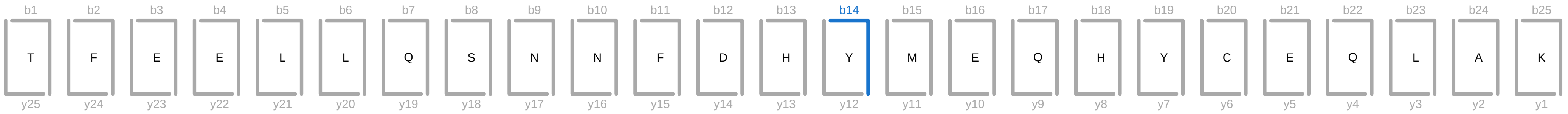

TFEELLQSNNFDHYMEQHYCEQLAK
Score: 0 ; 3177.411 m/z; 1060.1443 m/z; 4.1528 ppm; ISO−MSMS

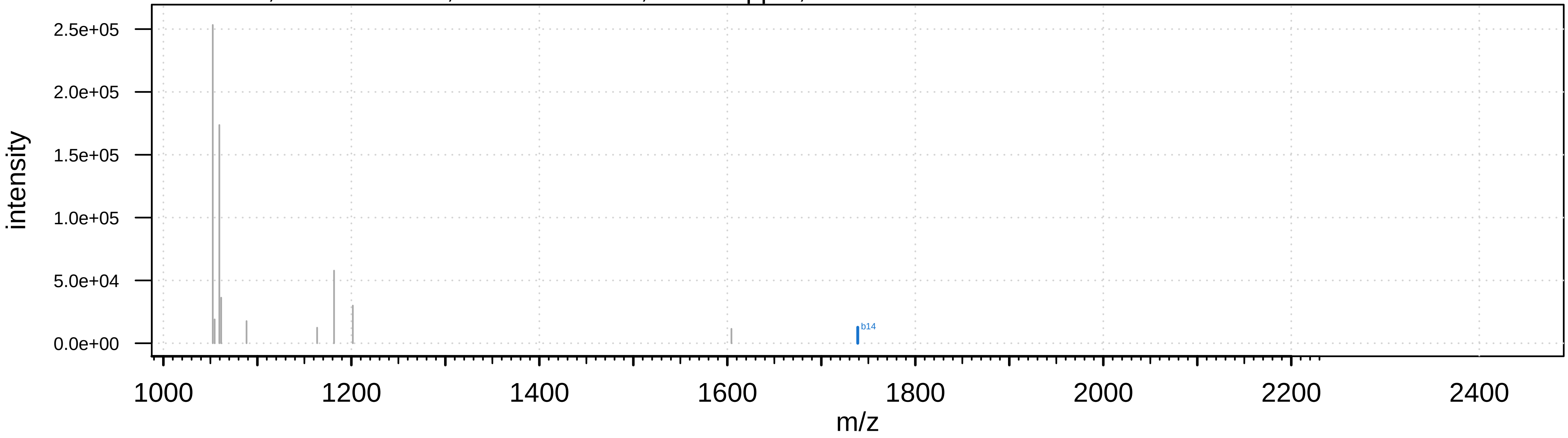

Raw File: Kermit_20140724_KK_HS_B1large
Scan Number: 19842
Proteins:
ENST00000608683_chr1:149649167−149701093:−

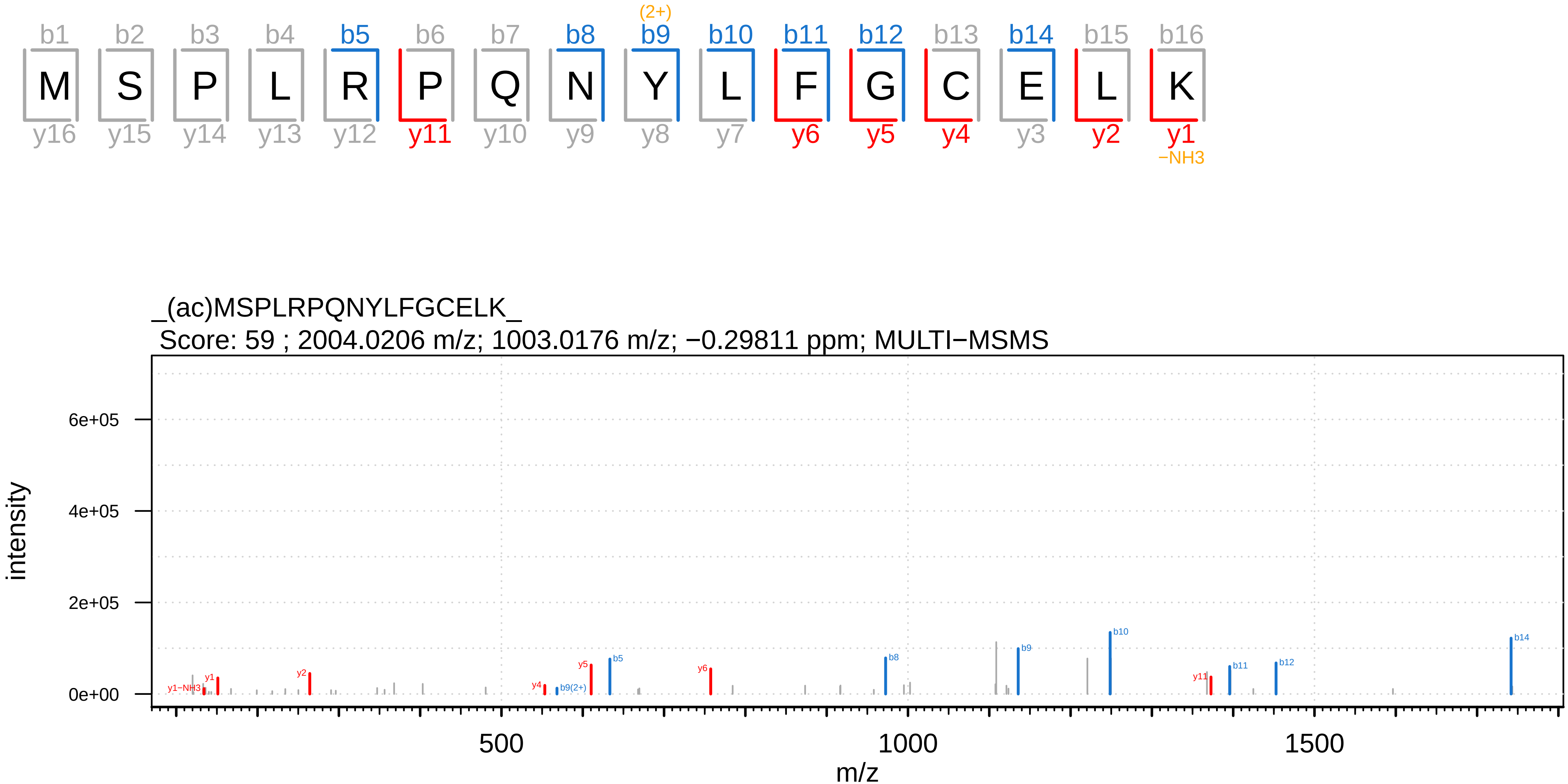

Raw File: Kermit_20140724_KK_HS_A1large
Scan Number: 48480
Proteins:
TCONS_l2_00008829_chr15:92829088−92829258:+

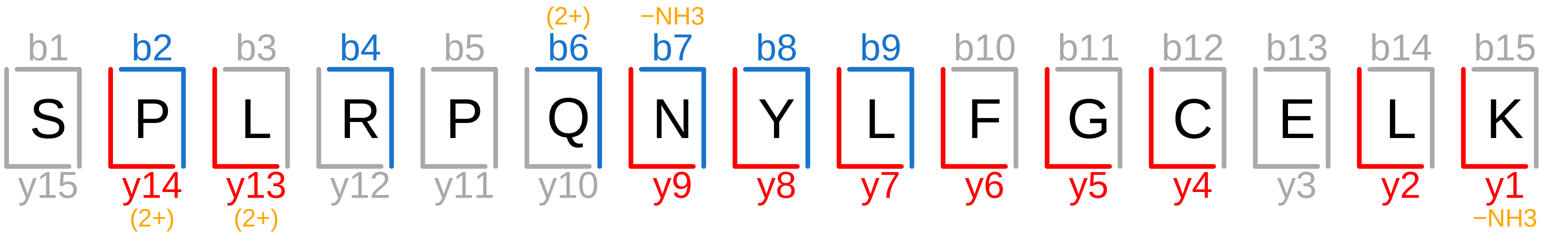

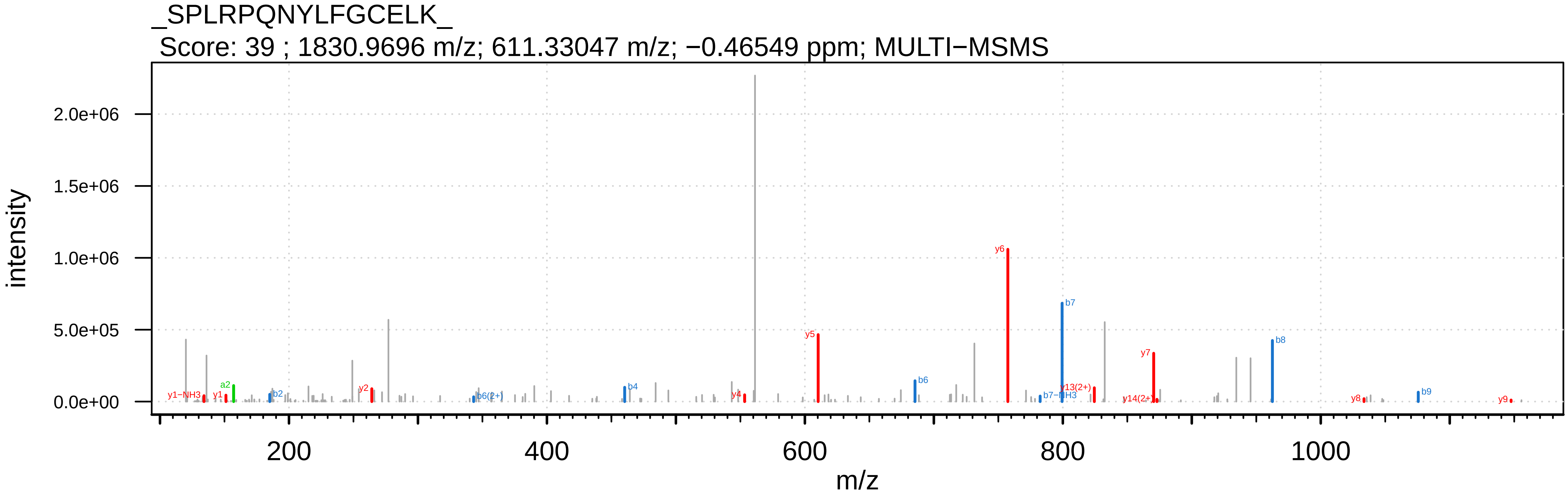

Raw File: Kermit_20140724_KK_HS_A1large
Scan Number: 33272
Proteins:
TCONS_l2_00008829_chr15:92829088−92829258:+

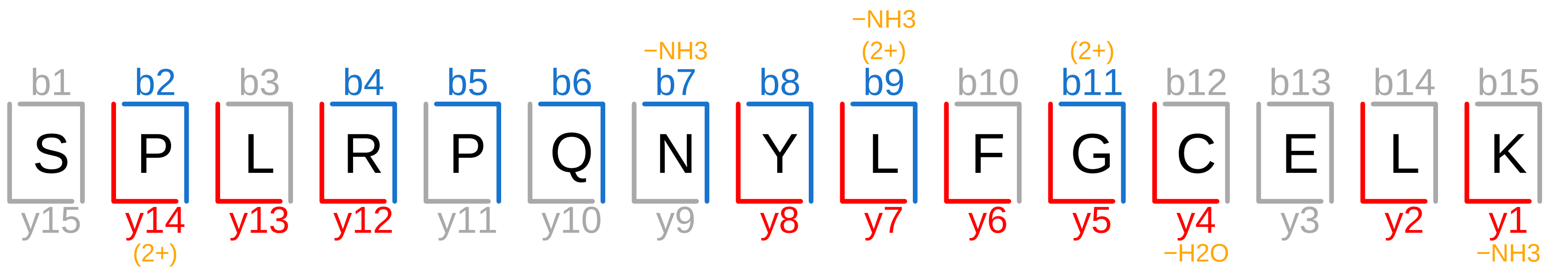

SPLRPQNYLFGCELK
Score: 72 ; 1820.9243 m/z; 607.98206 m/z; −0.46549 ppm; MULTI−MSMS

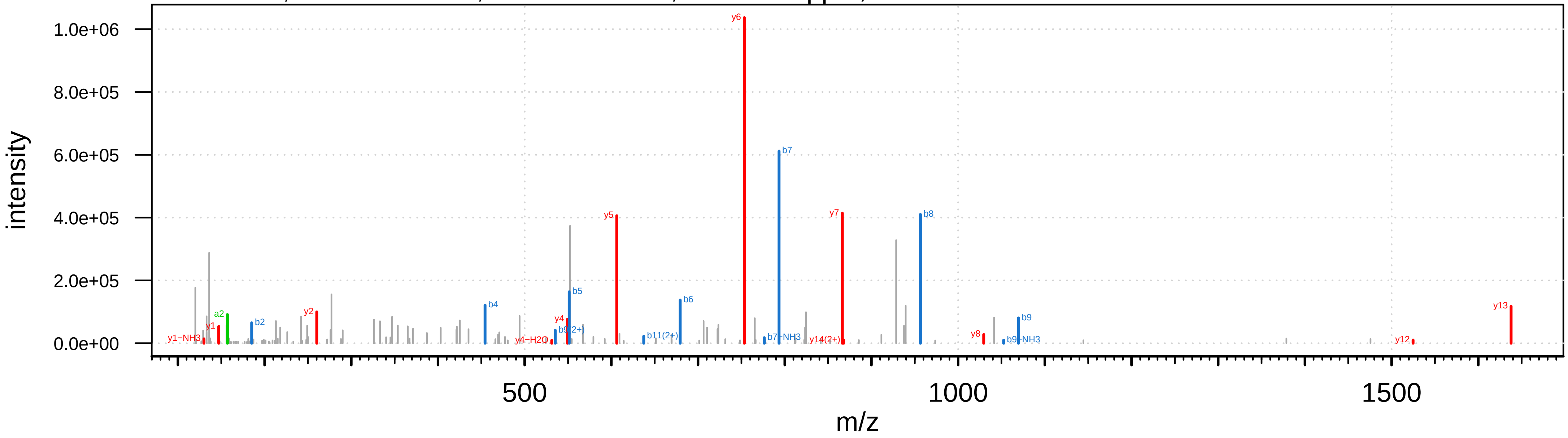

Raw File: Kermit_20140724_KK_HS_A1large
Scan Number: 33303
Proteins:
TCONS_l2_00008829_chr15:92829088−92829258:+

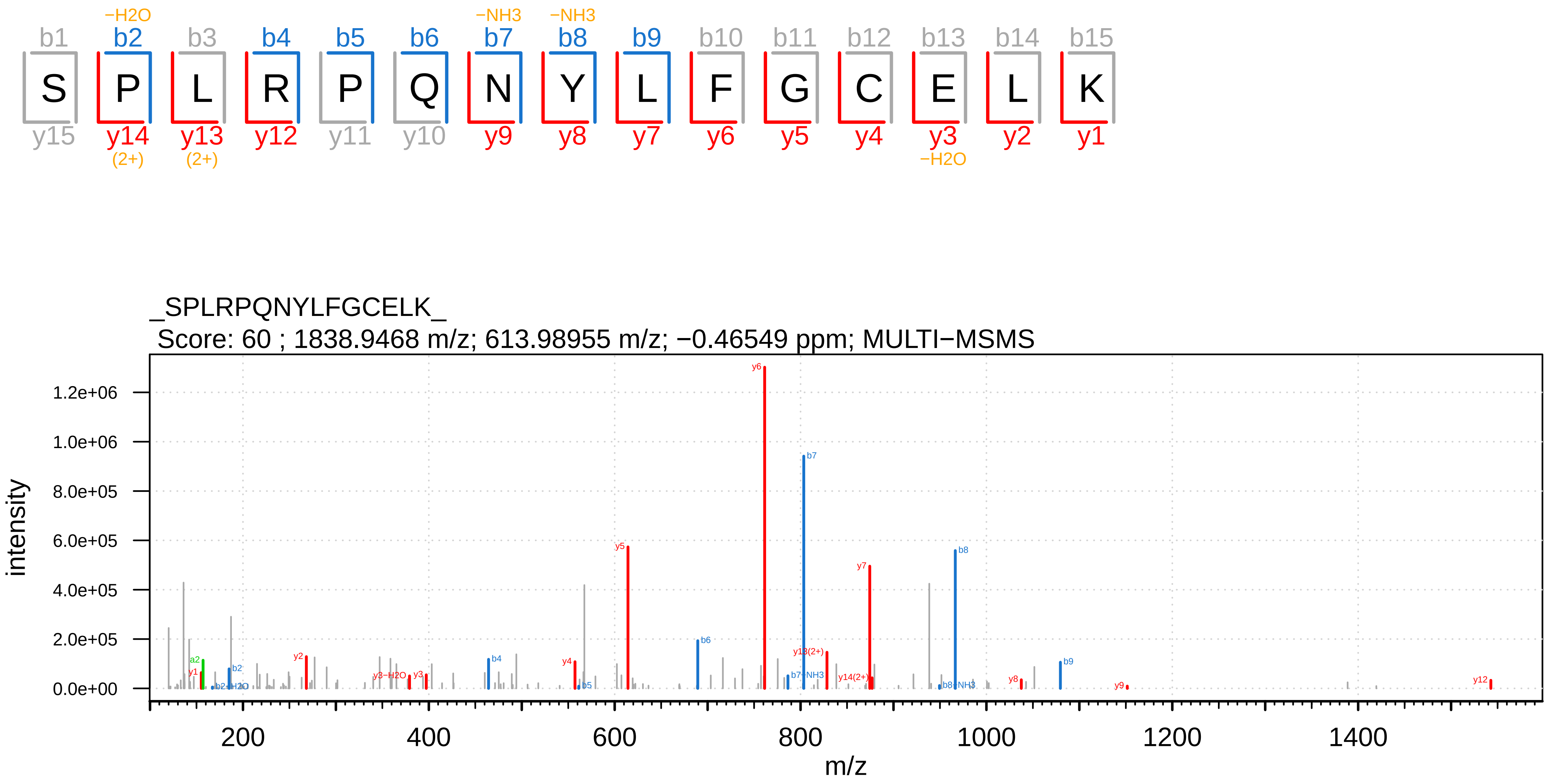

Raw File: Kermit_20140724_KK_HS_A1large
Scan Number: 33314
Proteins:
TCONS_l2_00008829_chr15:92829088−92829258:+

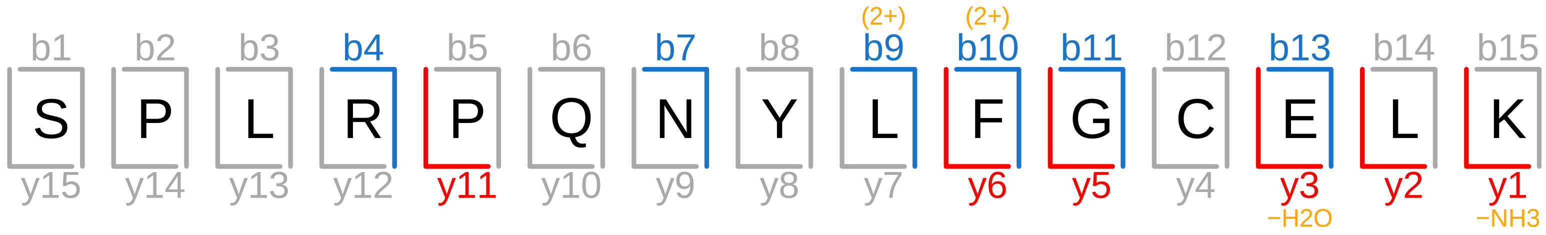

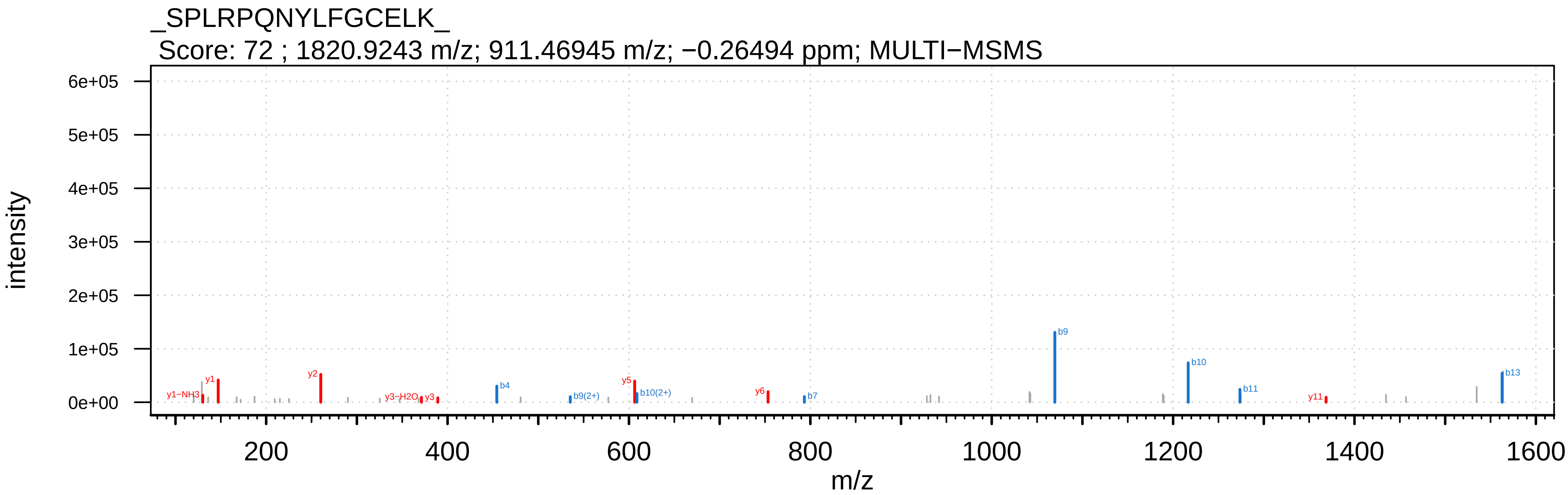

Raw File: Kermit_20140724_KK_HS_A1large
Scan Number: 33286
Proteins:
TCONS_l2_00008829_chr15:92829088−92829258:+

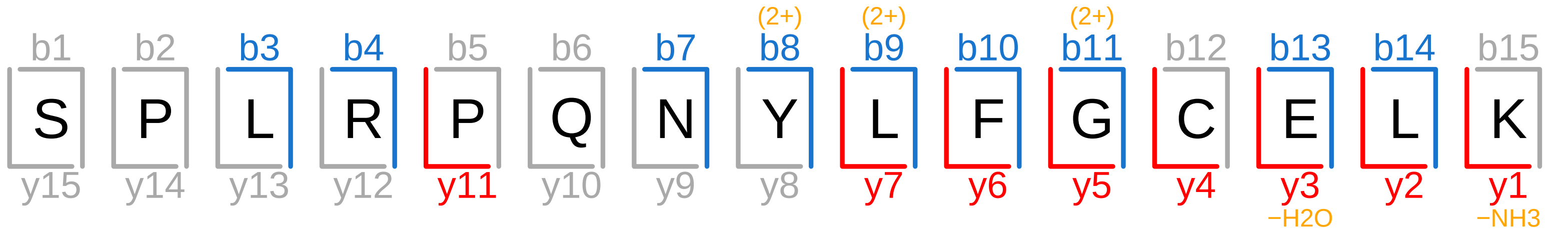

SPLRPQNYLFGCELK
Score: 91 ; 1830.9696 m/z; 916.49206 m/z; −0.26494 ppm; MULTI−MSMS

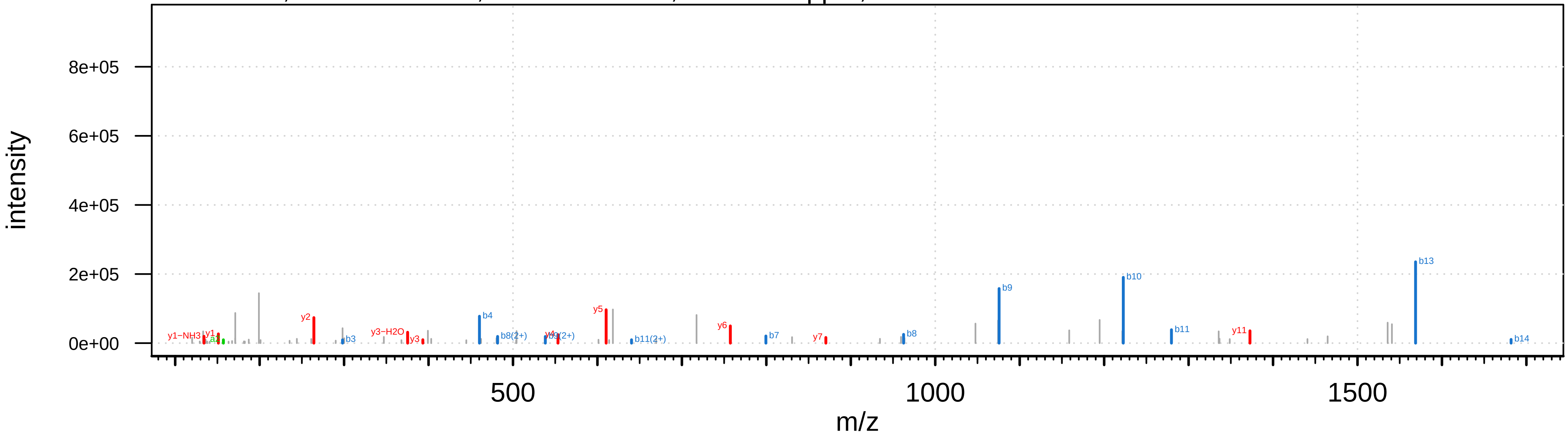

Raw File: Kermit_20140724_KK_HS_A1large
Scan Number: 33316
Proteins:
TCONS_l2_00008829_chr15:92829088−92829258:+

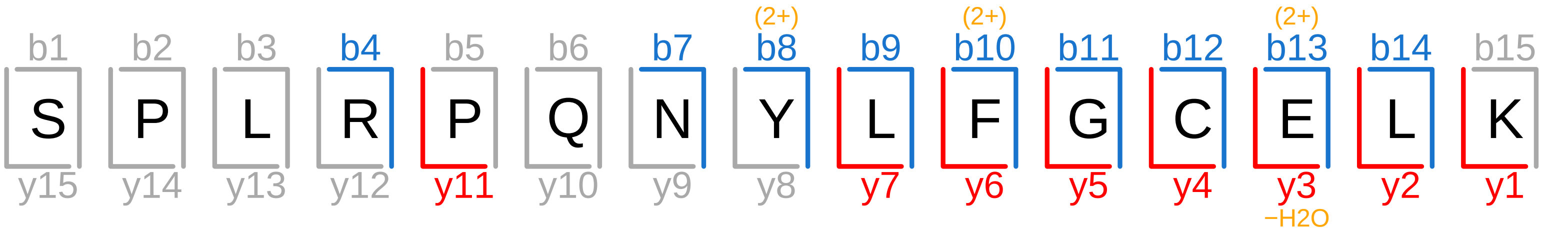

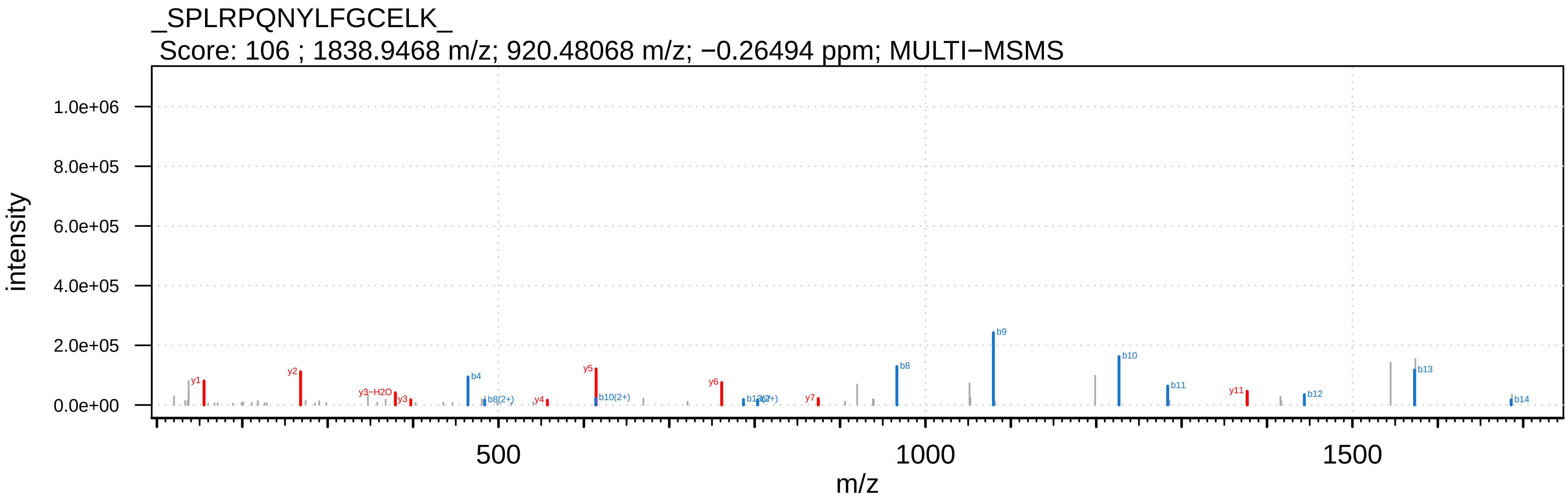

Raw File: Kermit_20140724_KK_HS_A1large
Scan Number: 33392
Proteins:
TCONS_l2_00008829_chr15:92829088−92829258:+

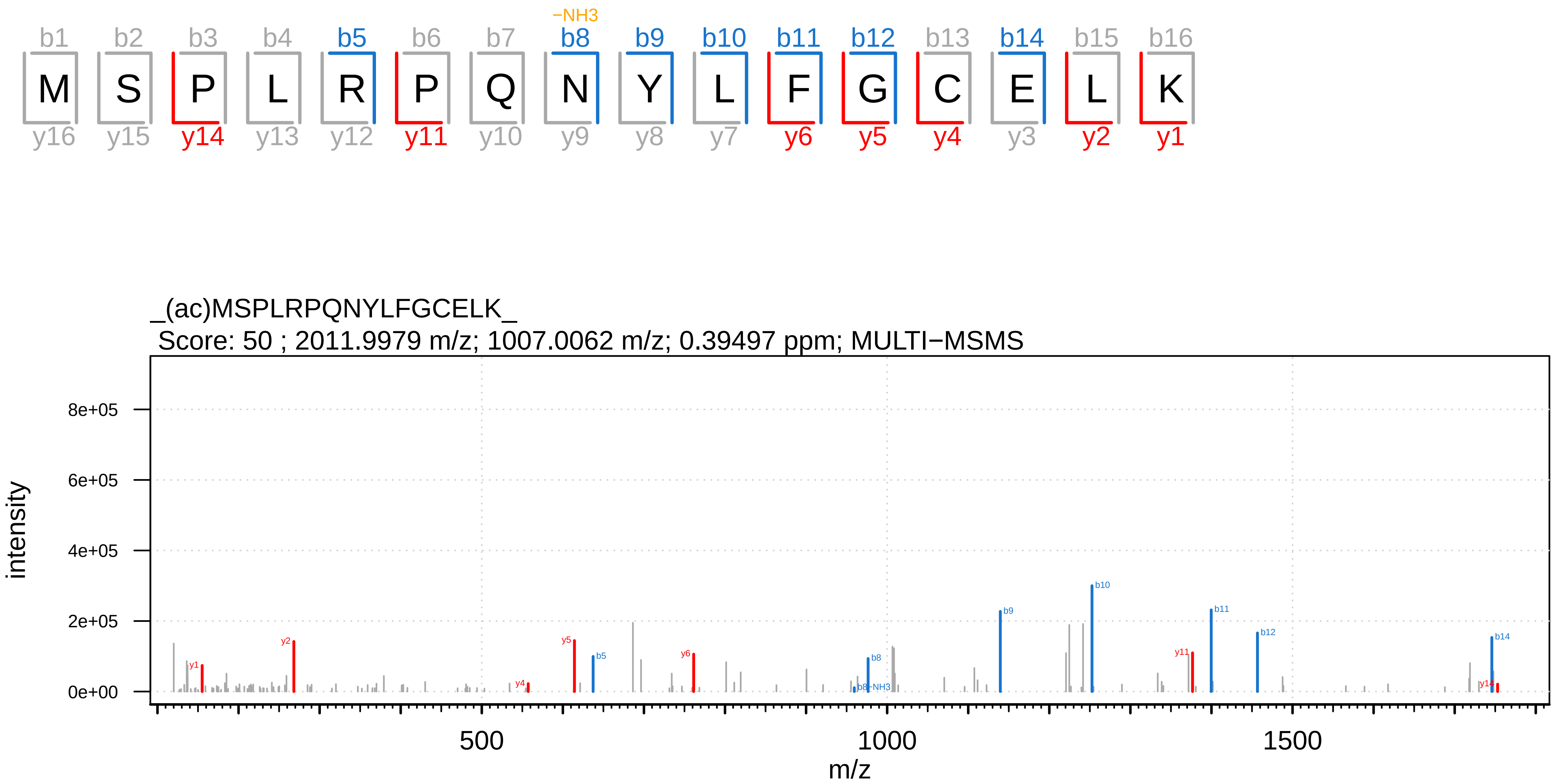

Raw File: Kermit_20140724_KK_HS_large
Scan Number: 47640
Proteins:
TCONS_l2_00008829_chr15:92829088−92829258:+

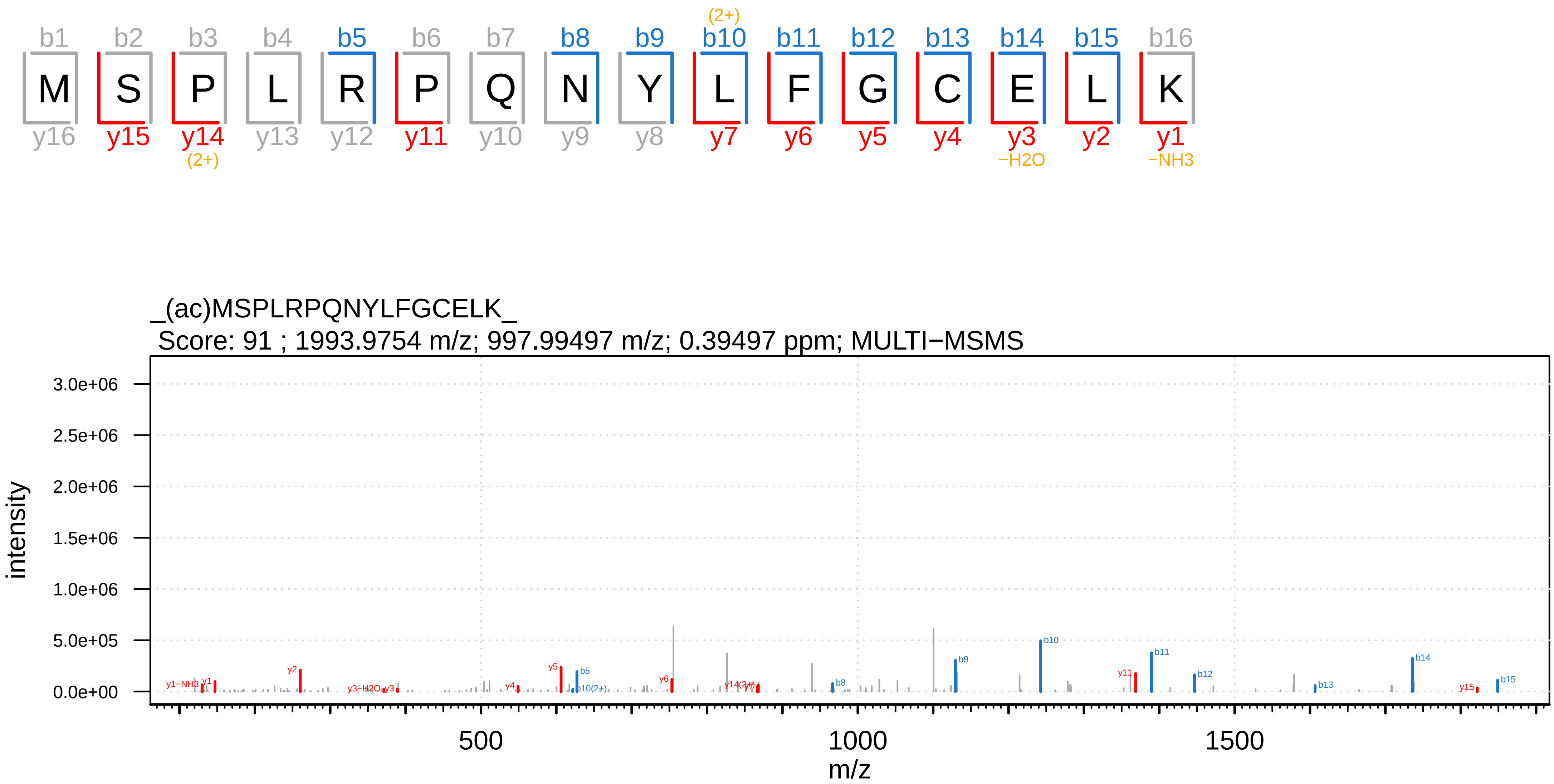

Raw File: Kermit_20140724_KK_HS_large
Scan Number: 47656
Proteins:
TCONS_l2_00008829_chr15:92829088−92829258:+

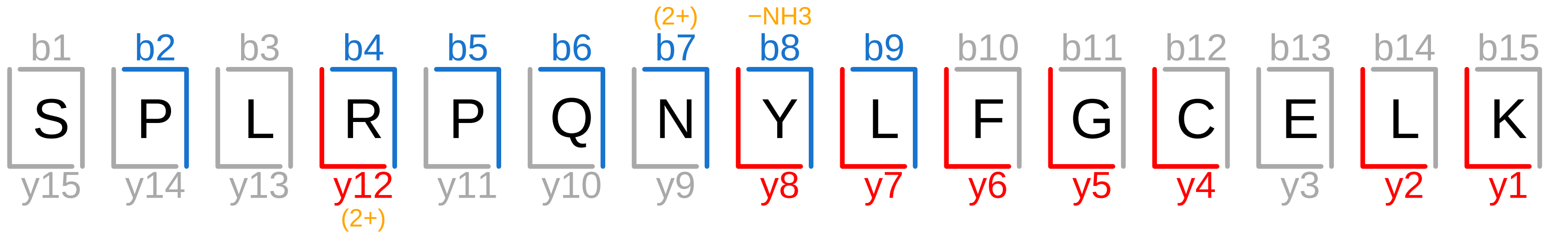

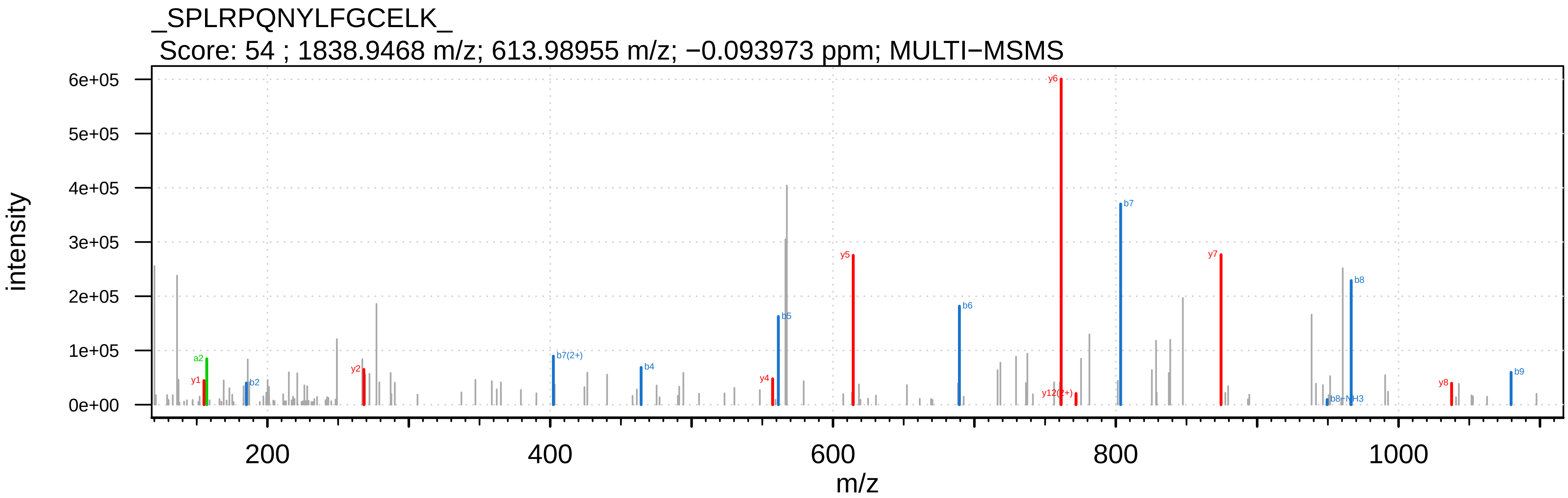

Raw File: Kermit_20140724_KK_HS_large
Scan Number: 31899
Proteins:
TCONS_l2_00008829_chr15:92829088−92829258:+

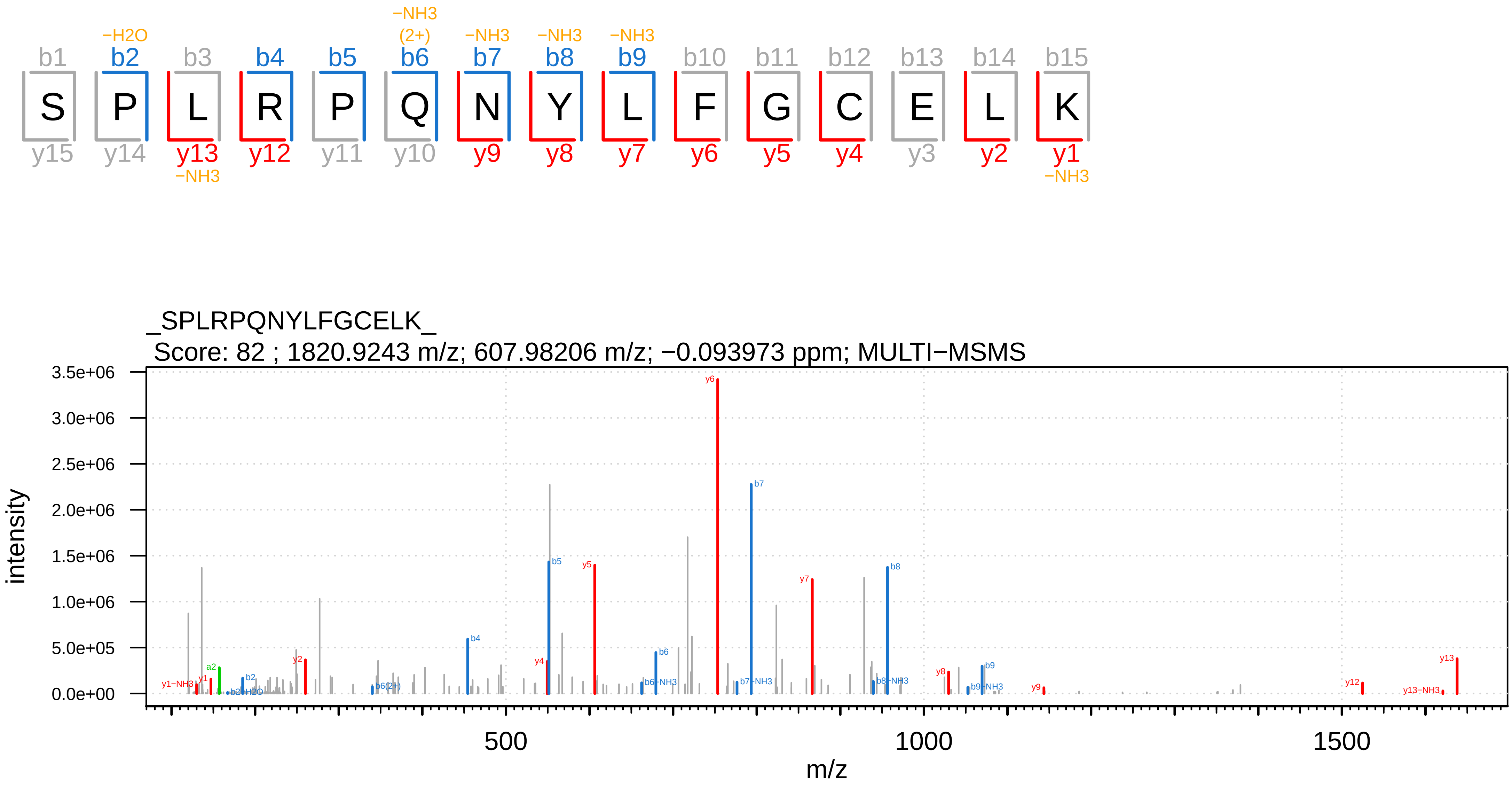

Raw File: Kermit_20140724_KK_HS_large
Scan Number: 32052
Proteins:
TCONS_l2_00008829_chr15:92829088−92829258:+

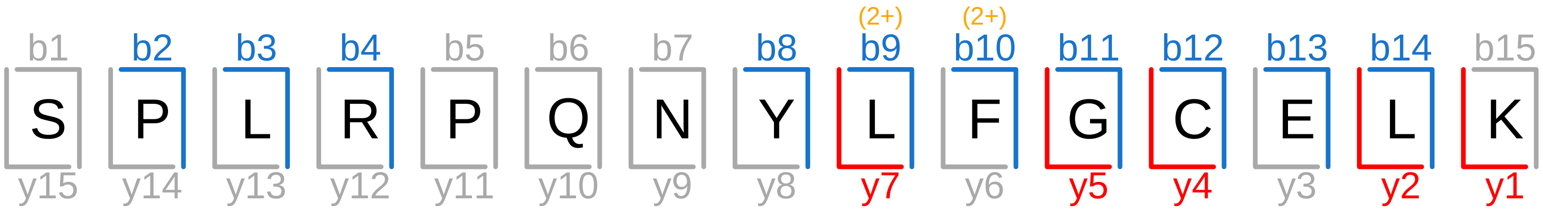

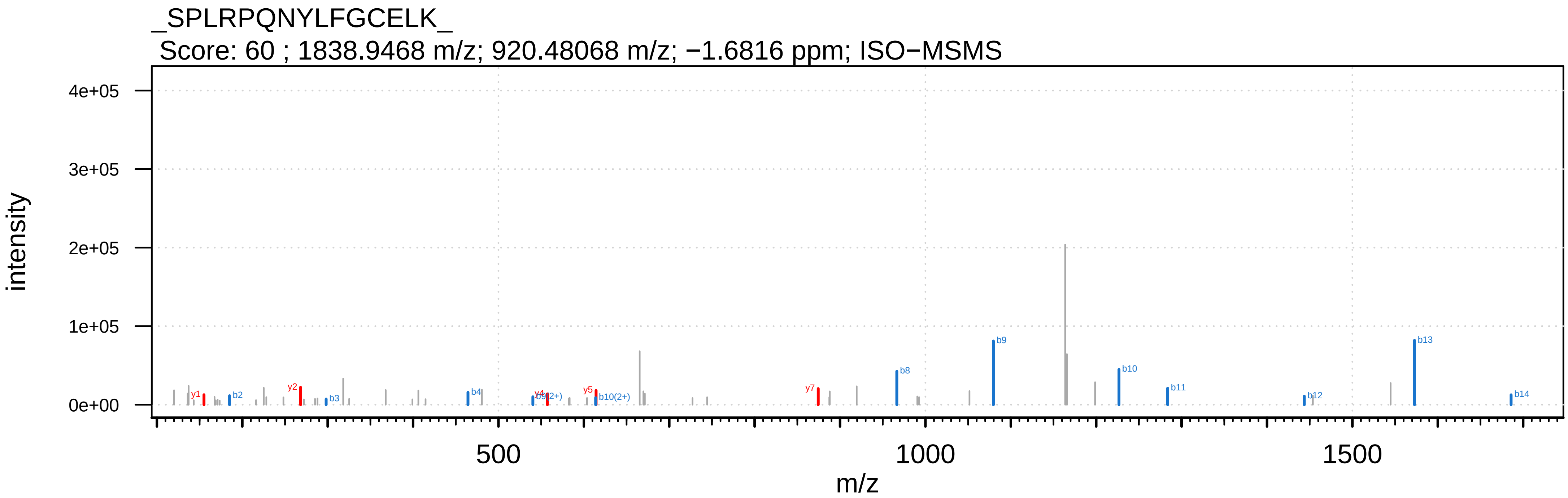

Raw File: Kermit_20140724_KK_HS_large
Scan Number: 31859
Proteins:
TCONS_l2_00008829_chr15:92829088−92829258:+

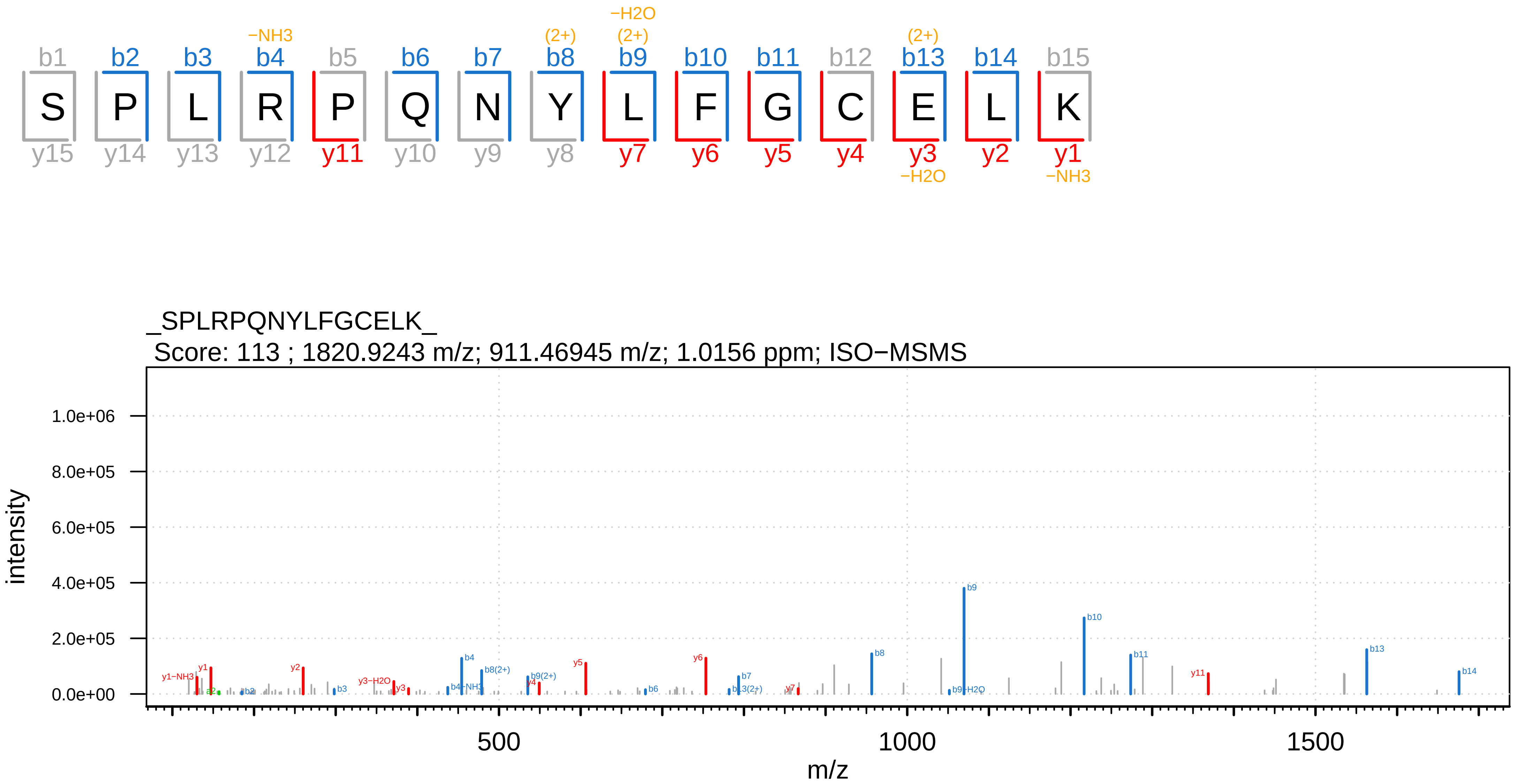

Raw File: Kermit_20140724_KK_HS_large
Scan Number: 31900
Proteins:
TCONS_l2_00008829_chr15:92829088−92829258:+

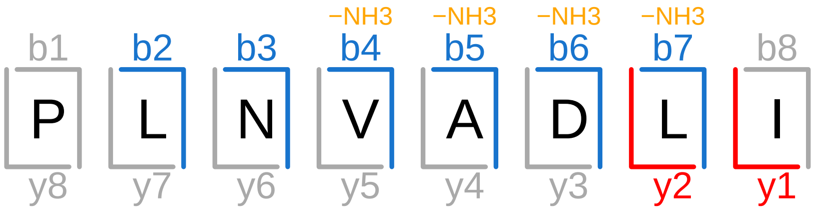

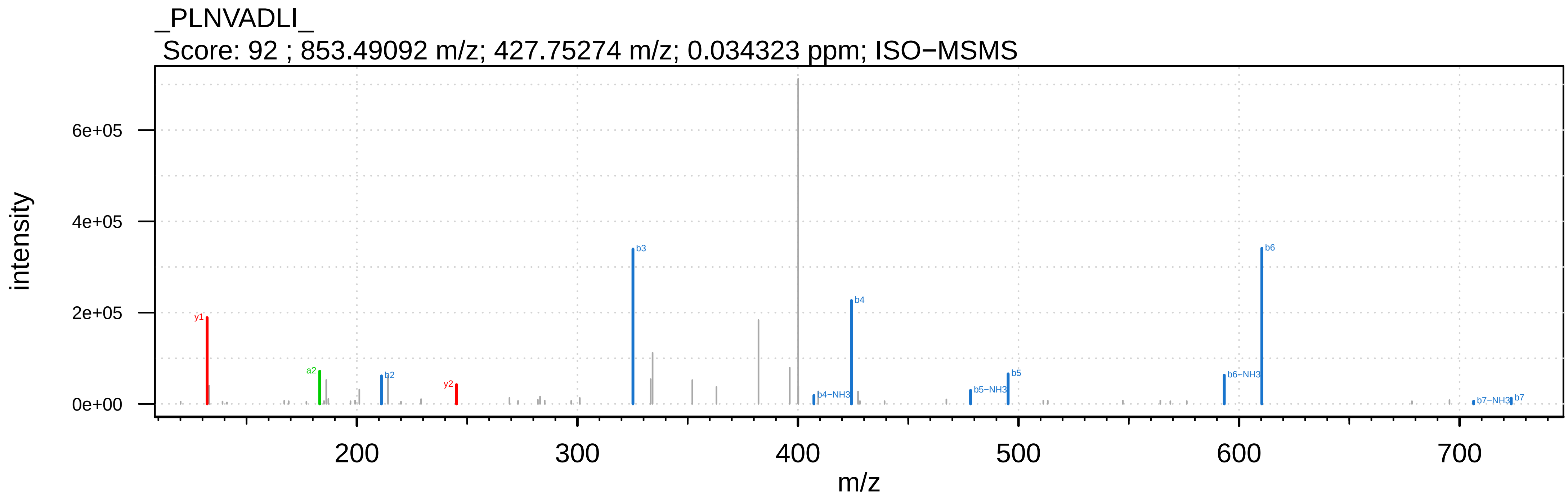

Raw File: Grobi_20140814_HZ_HS_A1S_MicroPeps_TriSilac
Scan Number: 75836
Proteins:
TCONS_l2_00005685_chr12:53548245−53548274:+

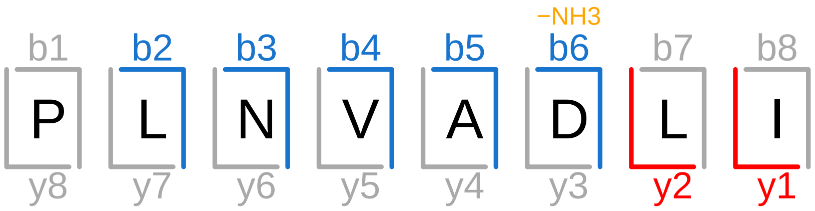

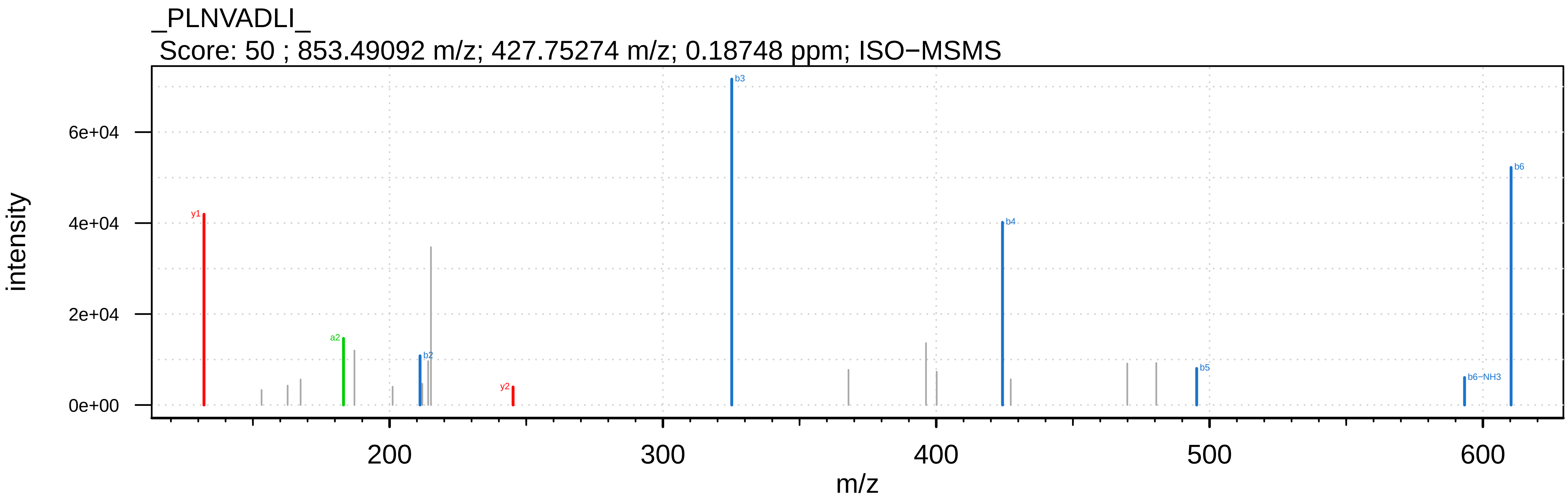

Raw File: Kermit_20140724_KK_HS_B1small
Scan Number: 15593
Proteins:
TCONS_l2_00005685_chr12:53548245−53548274:+

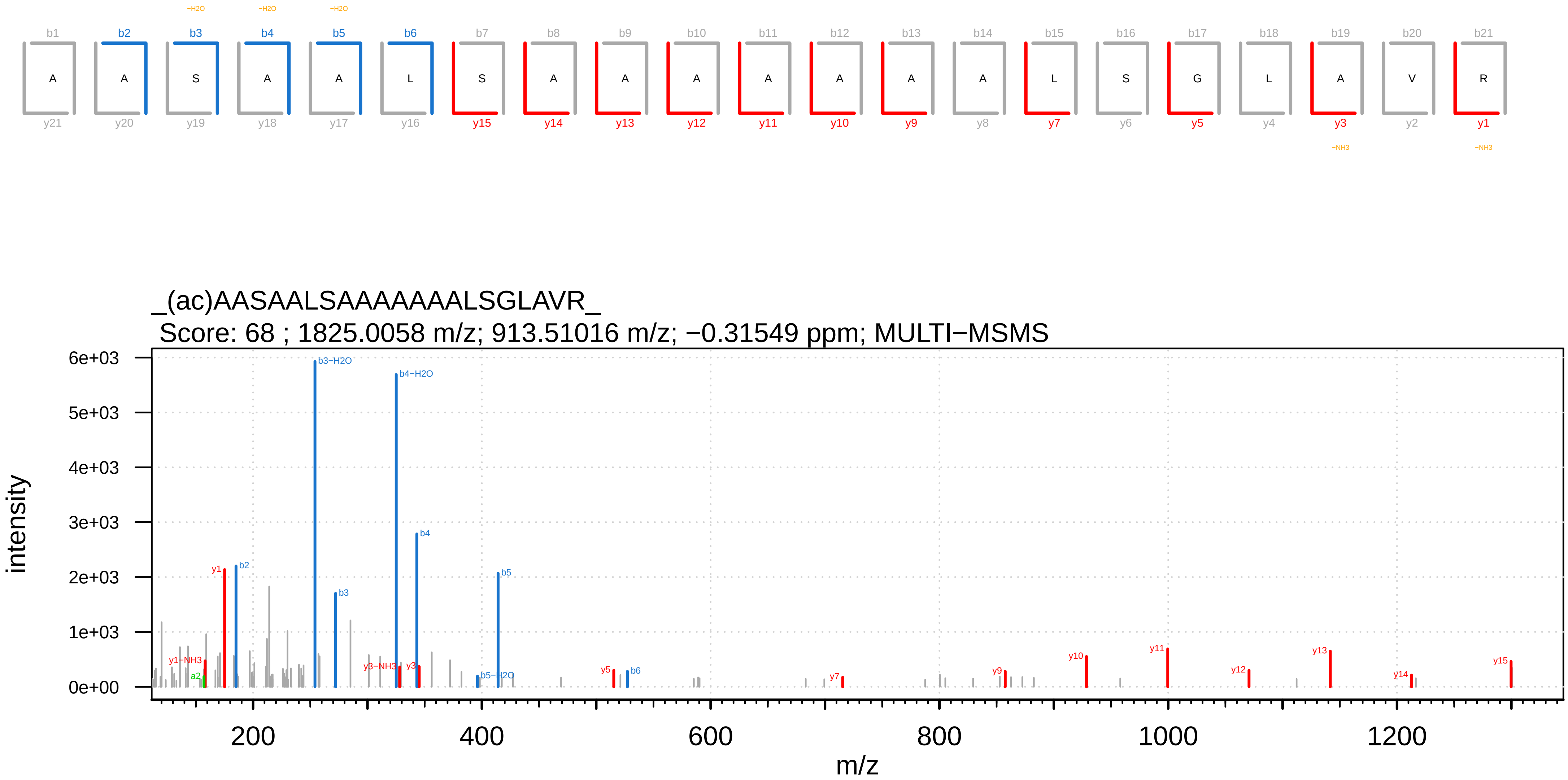

Raw File: 20100719_Velos1_TaGe_SA_LnCap_5
Scan Number: 47630
Proteins:
ENST00000433425_chrX:134232427−134232663:−
ENST00000417443_chrX:134556057−134556293:+

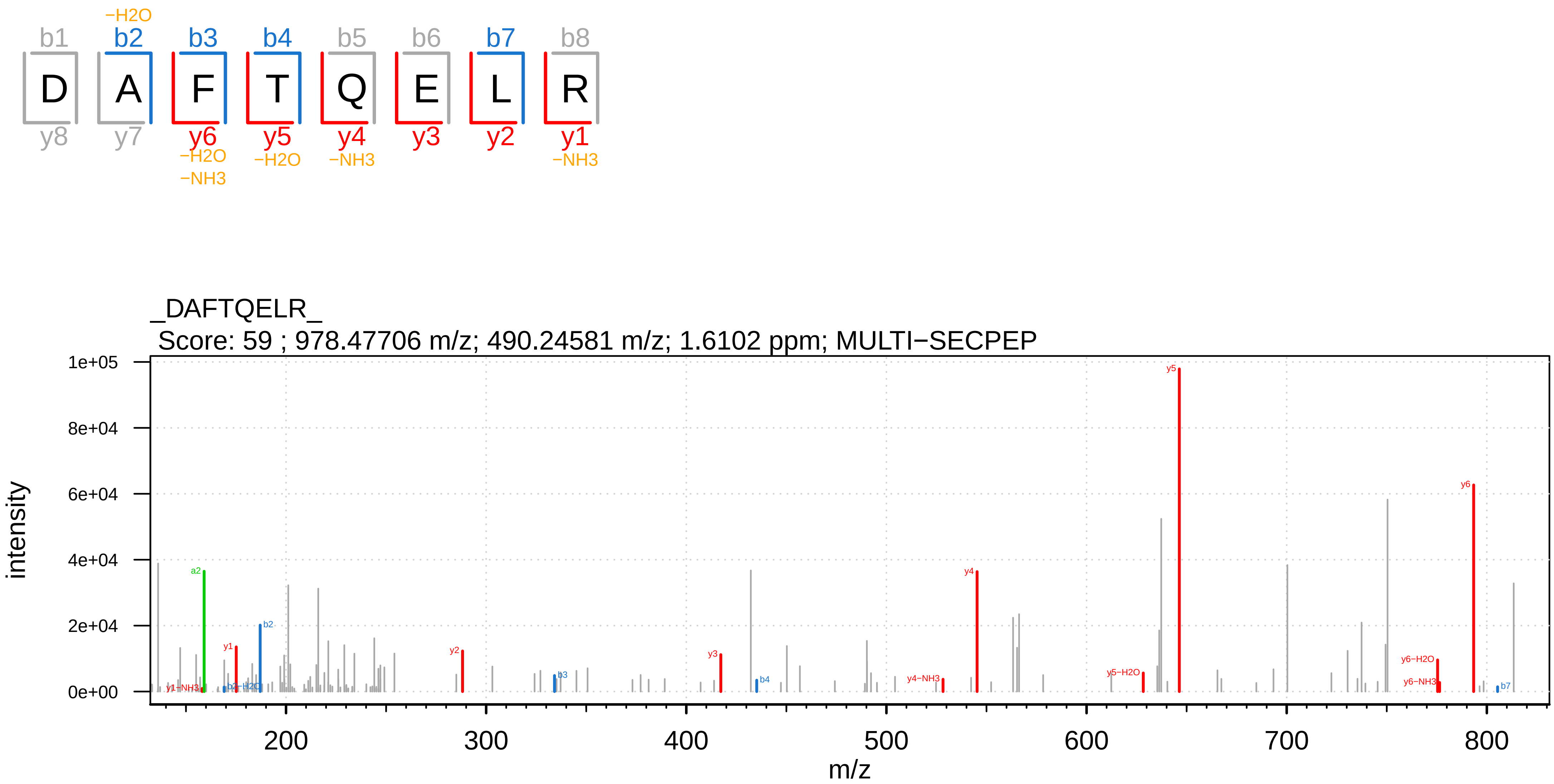

Raw File: 20100611_Velos1_TaGe_SA_Hela_1
Scan Number: 12547
Proteins:
ENST00000602845_chr3:196669588−196669887:+

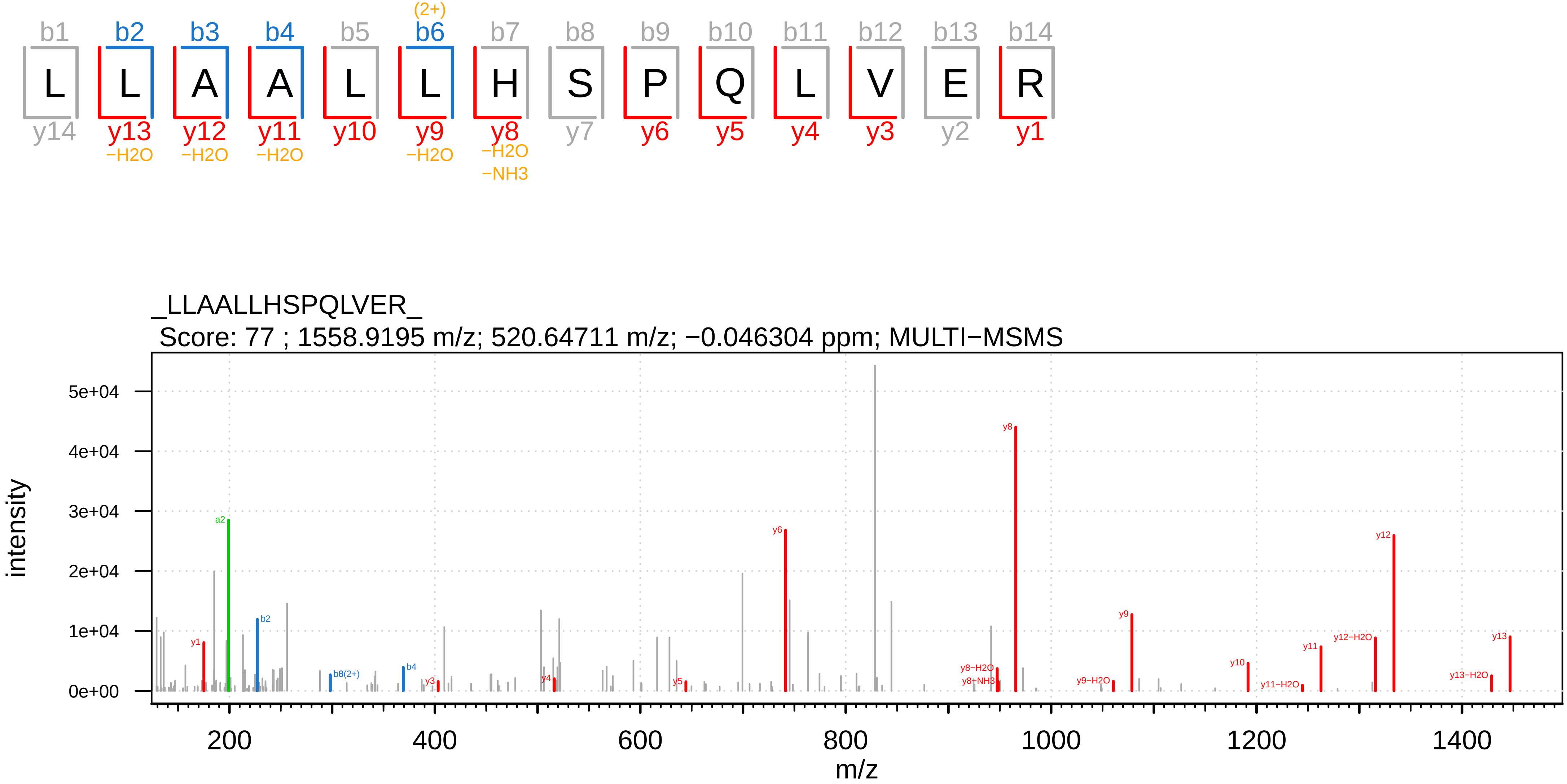

Raw File: 20100611_Velos1_TaGe_SA_Hela_1
Scan Number: 26961
Proteins:
ENST00000602845_chr3:196669588−196669887:+

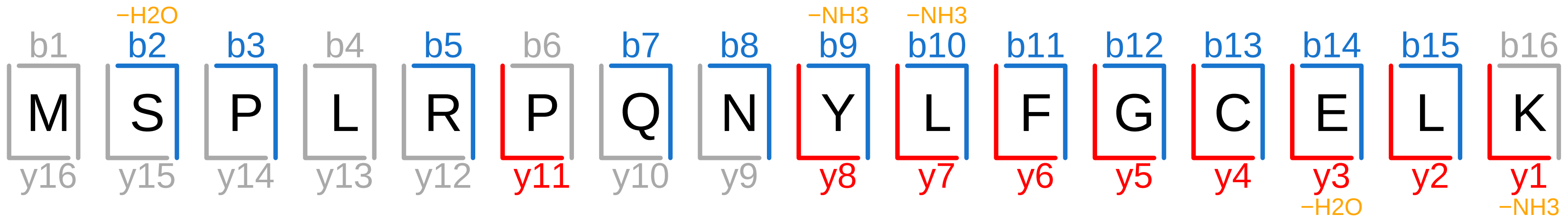

(ac)MSPLRPQNYLFGCELK
Score: 96 ; 1993.9754 m/z; 997.99497 m/z; 0.23821 ppm; MULTI−MSMS

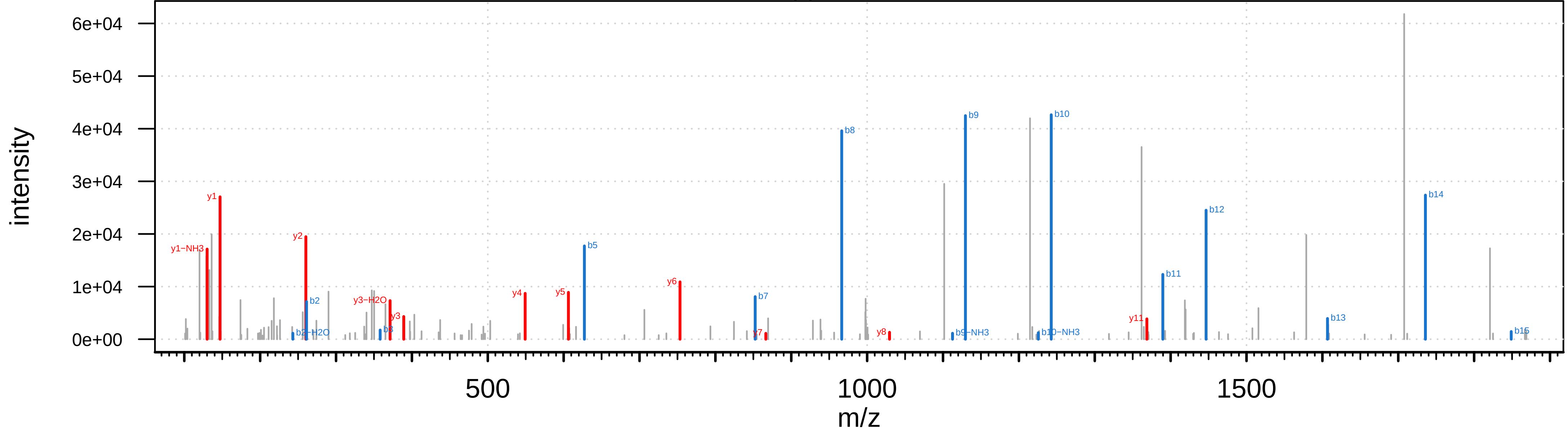

Raw File: 20100611_Velos1_TaGe_SA_Hela_1
Scan Number: 35799
Proteins:
TCONS_l2_00008829_chr15:92829088−92829258:+

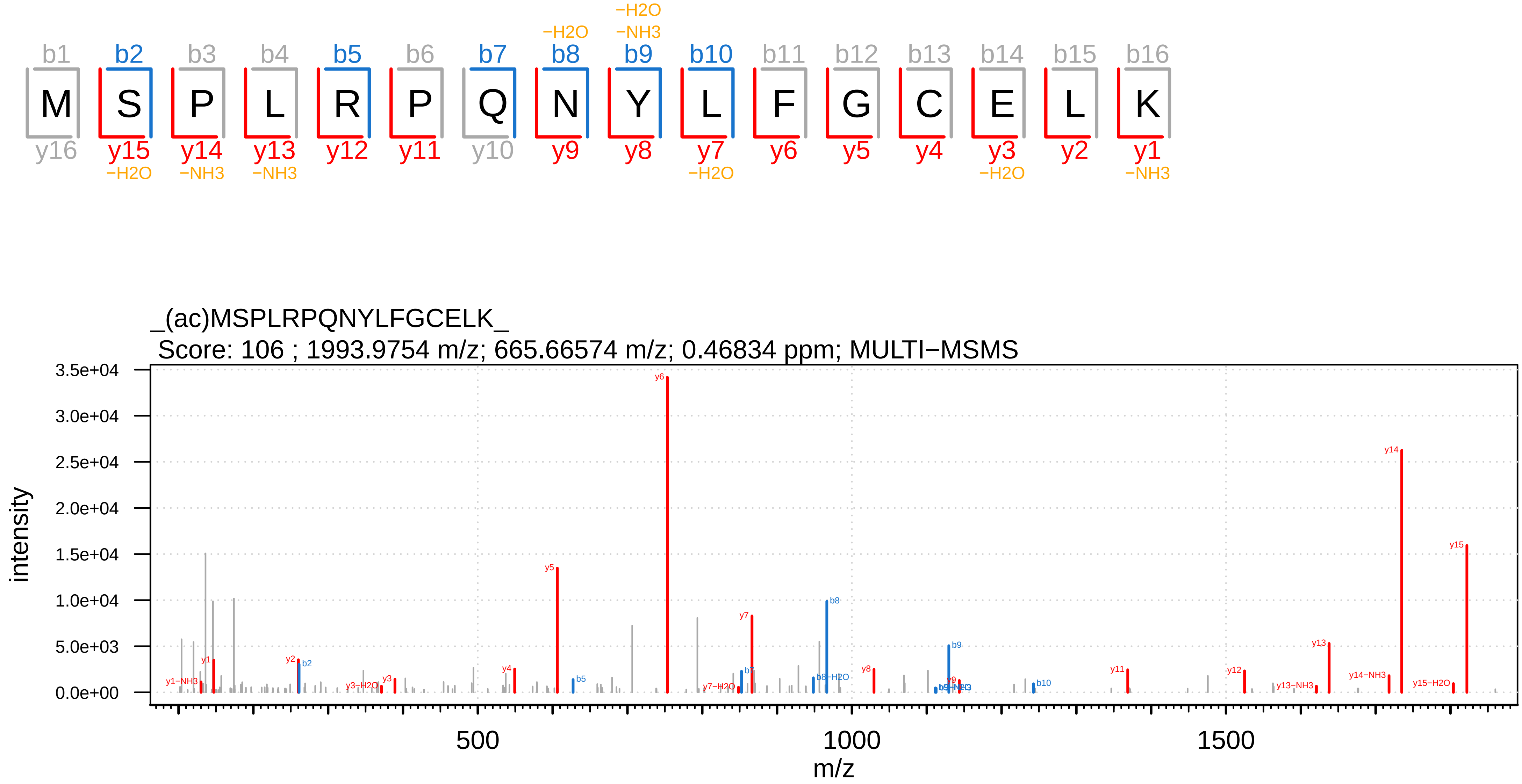

Raw File: 20100611_Velos1_TaGe_SA_Hela_1
Scan Number: 35813
Proteins:
TCONS_l2_00008829_chr15:92829088−92829258:+

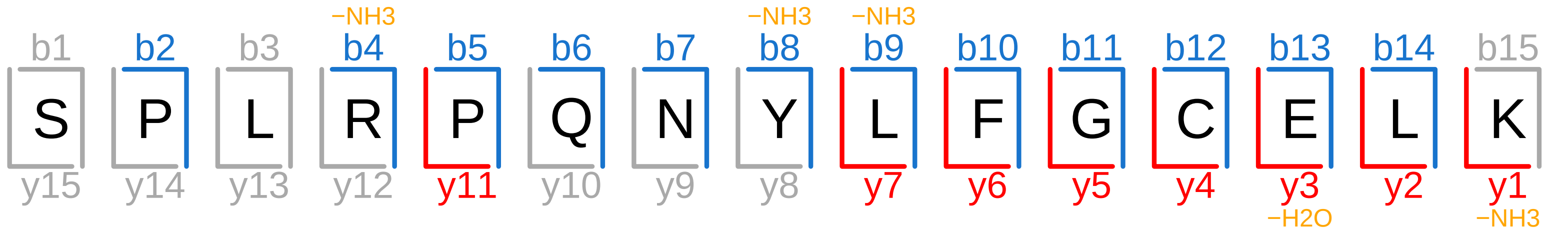

SPLRPQNYLFGCELK
Score: 110 ; 1820.9243 m/z; 911.46945 m/z; −0.67673 ppm; MULTI−MSMS

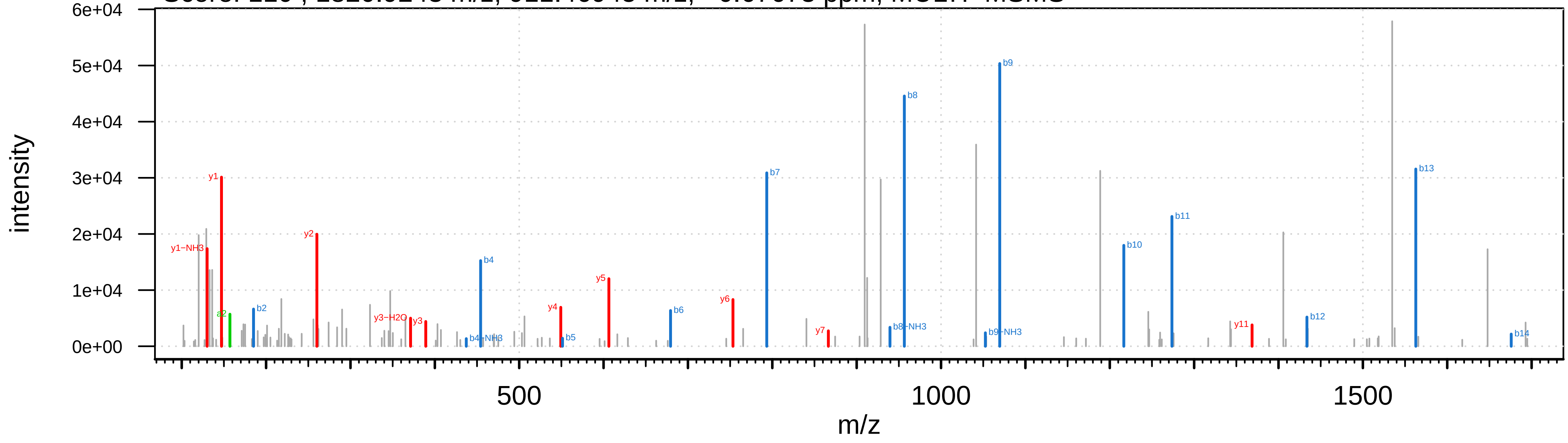

Raw File: 20100611_Velos1_TaGe_SA_Hela_1
Scan Number: 22310
Proteins:
TCONS_l2_00008829_chr15:92829088−92829258:+

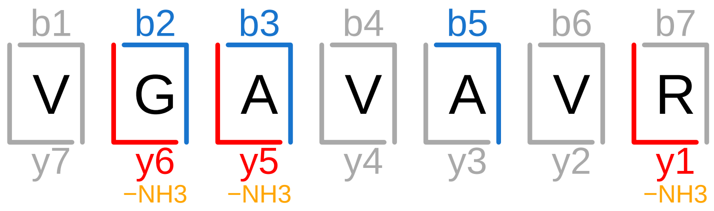

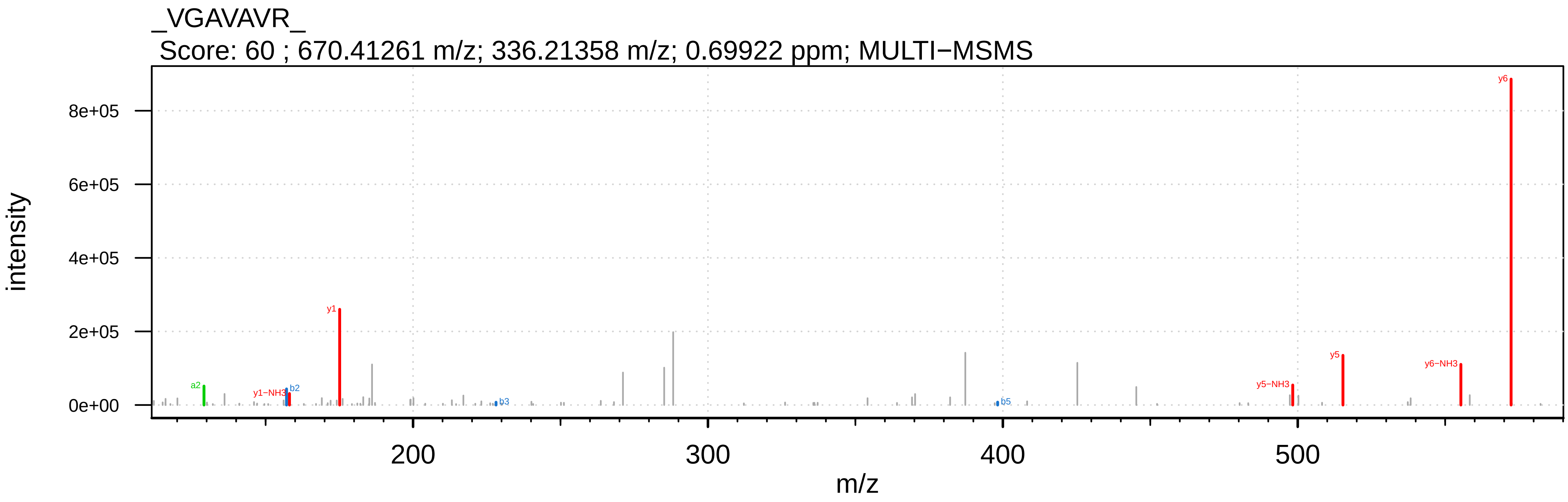

Raw File: 20100611_Velos1_TaGe_SA_Hela_1
Scan Number: 2321
Proteins:
TCONS_l2_00030545_chrX:79544539−79546436:−

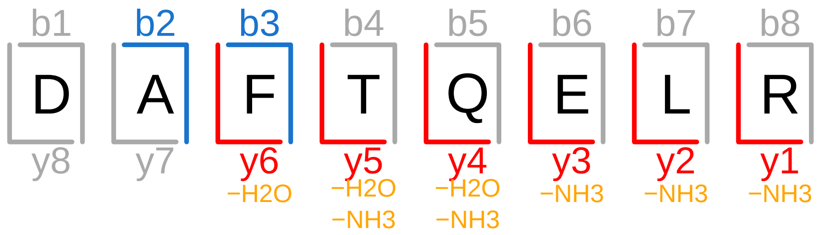

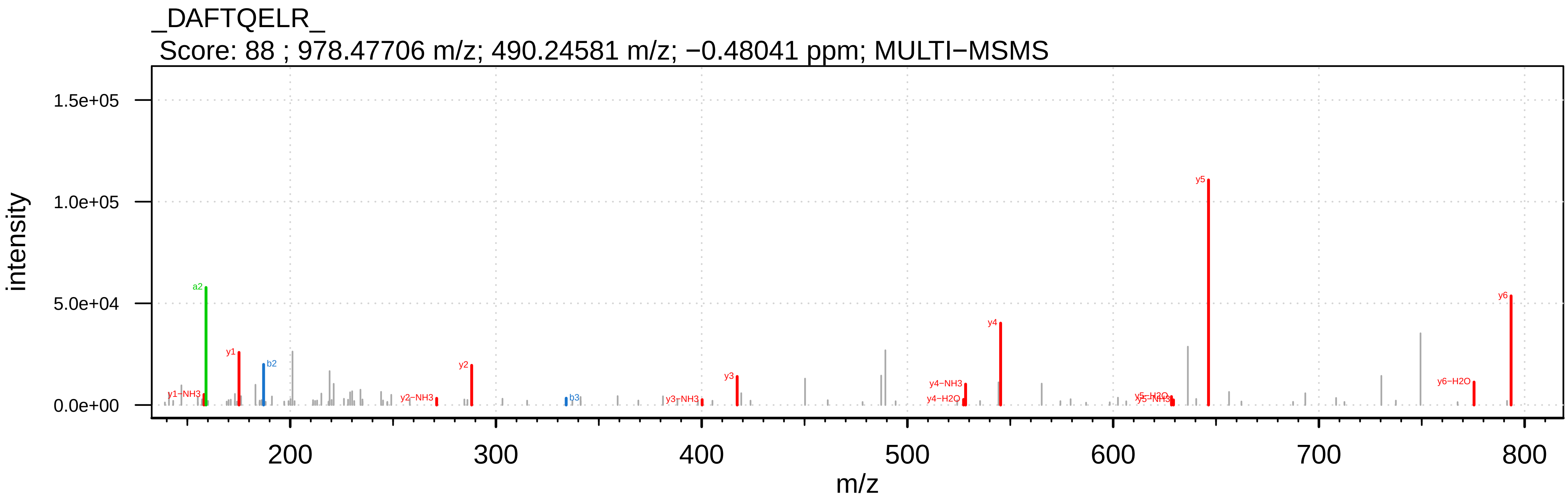

Raw File: 20100616_Velos1_TaGe_SA_MCF7_1
Scan Number: 13313
Proteins:
ENST00000602845_chr3:196669588−196669887:+

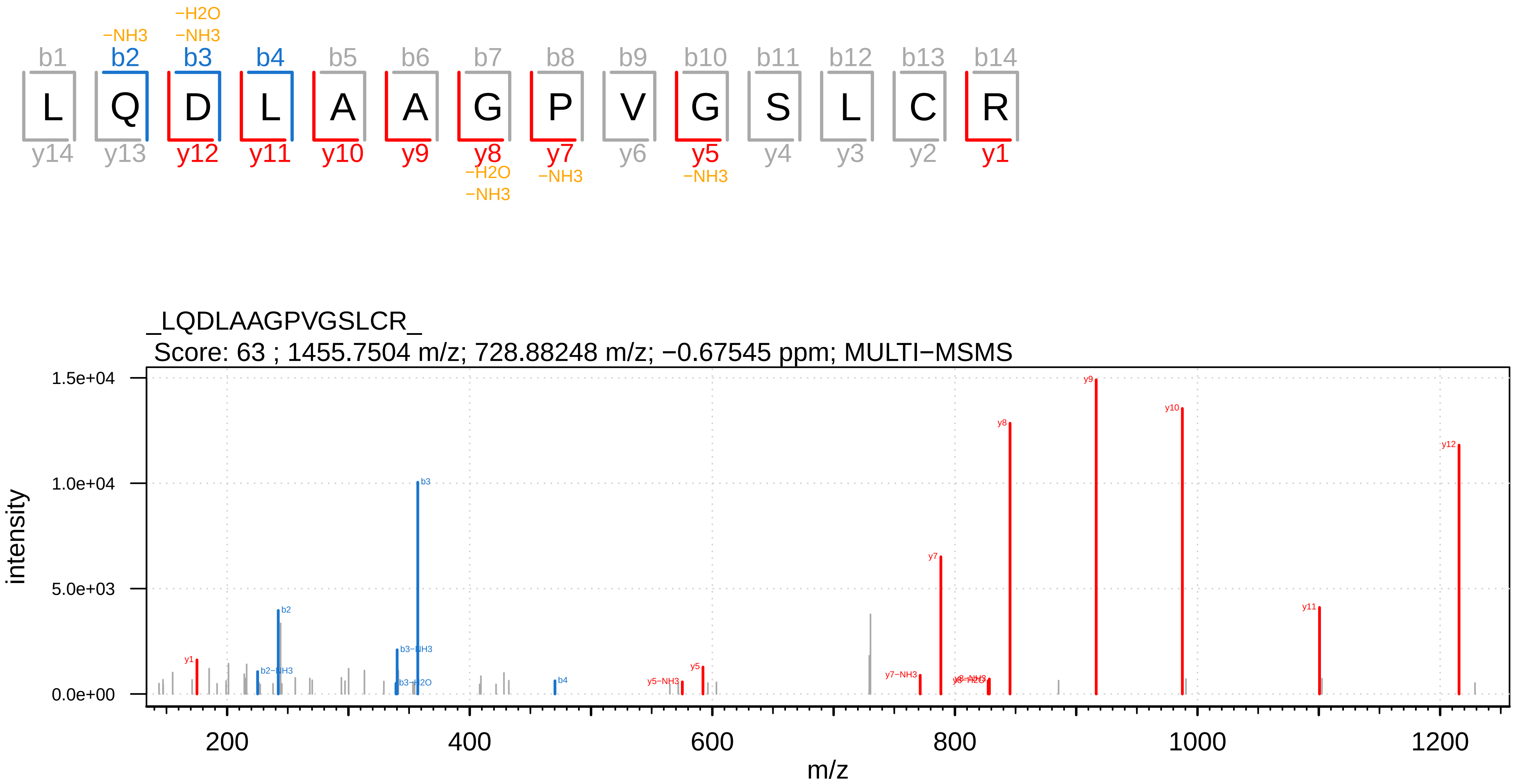

Raw File: 20100616_Velos1_TaGe_SA_MCF7_1
Scan Number: 21903
Proteins:
ENST00000602845_chr3:196669588−196669887:+

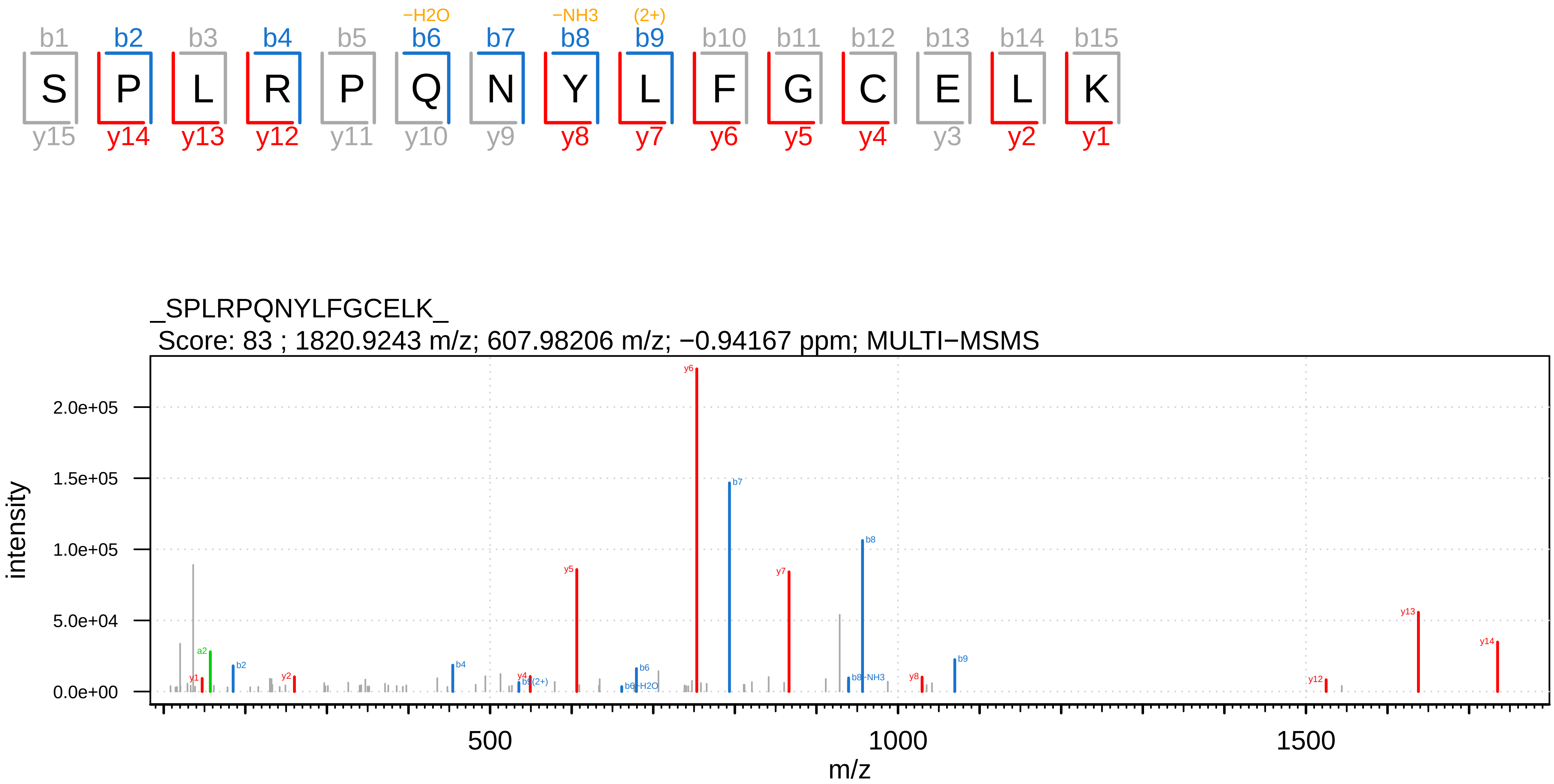

Raw File: 20100616_Velos1_TaGe_SA_MCF7_1
Scan Number: 23529
Proteins:
TCONS_l2_00008829_chr15:92829088−92829258:+

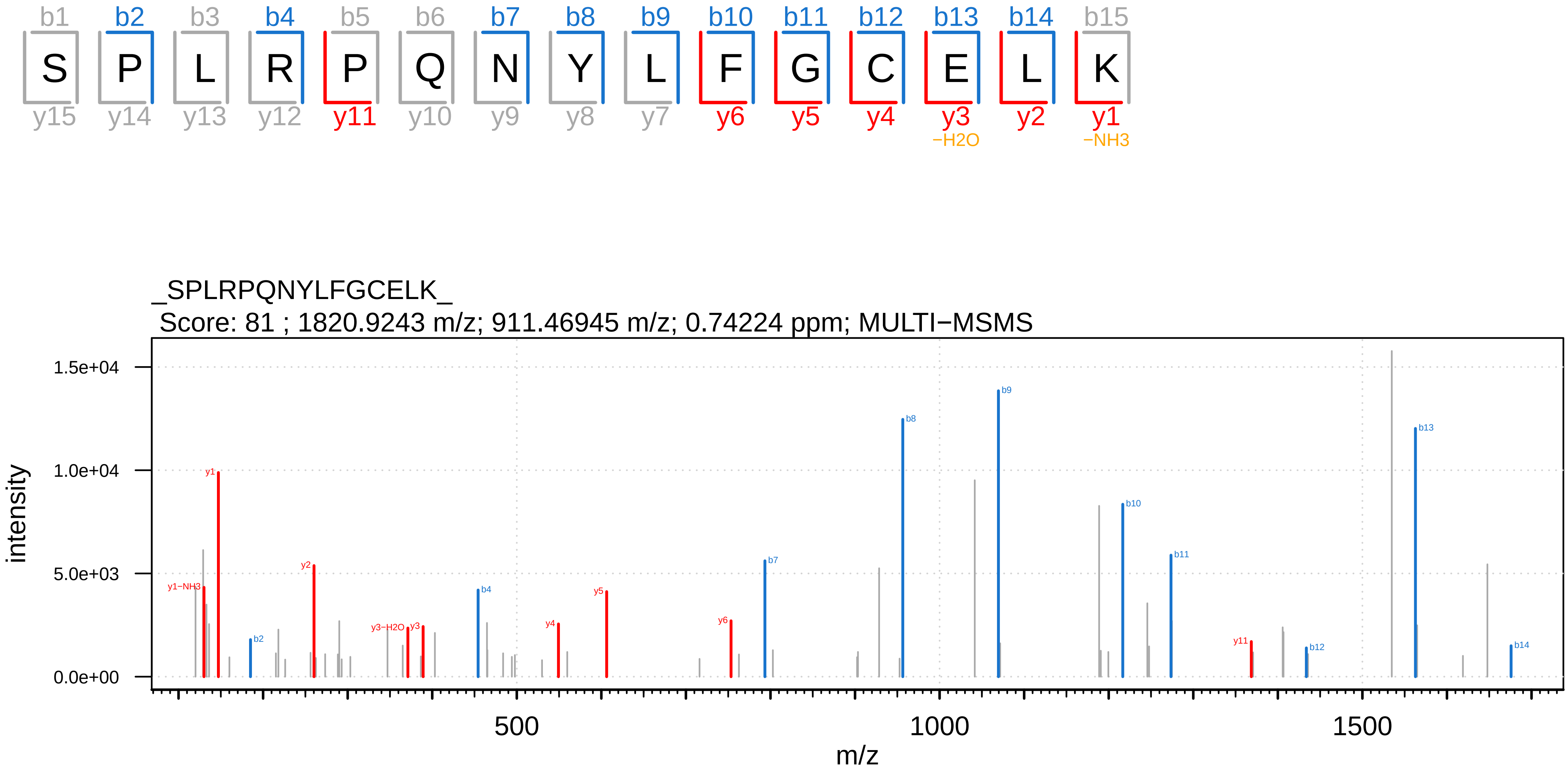

Raw File: 20100616_Velos1_TaGe_SA_MCF7_1
Scan Number: 23544
Proteins:
TCONS_l2_00008829_chr15:92829088−92829258:+

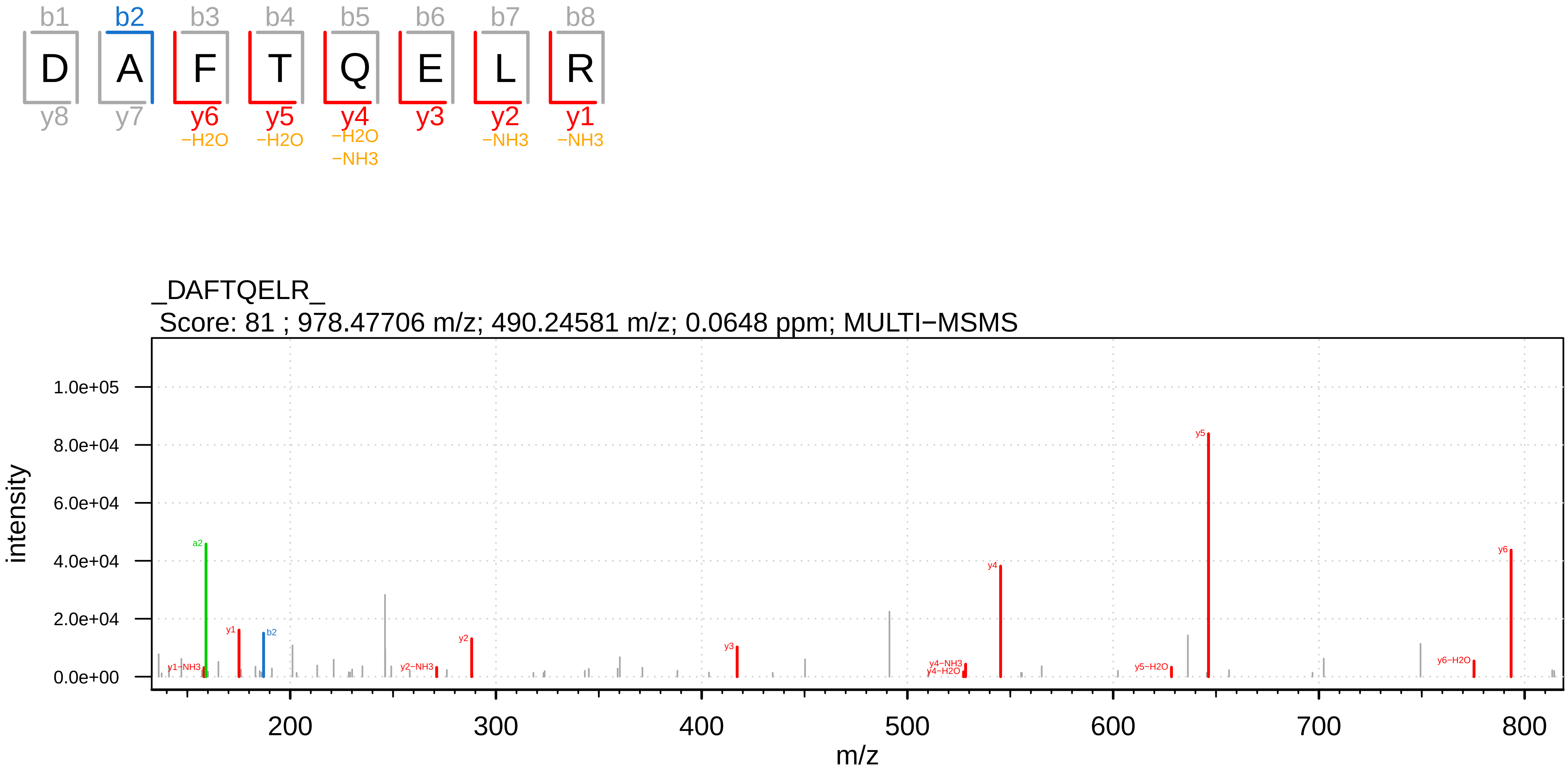

Raw File: 20100616_Velos1_TaGe_SA_MCF7_2
Scan Number: 12441
Proteins:
ENST00000602845_chr3:196669588−196669887:+

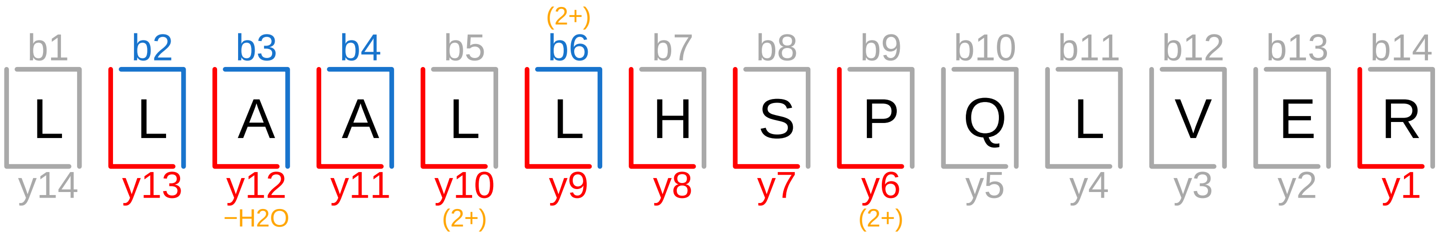

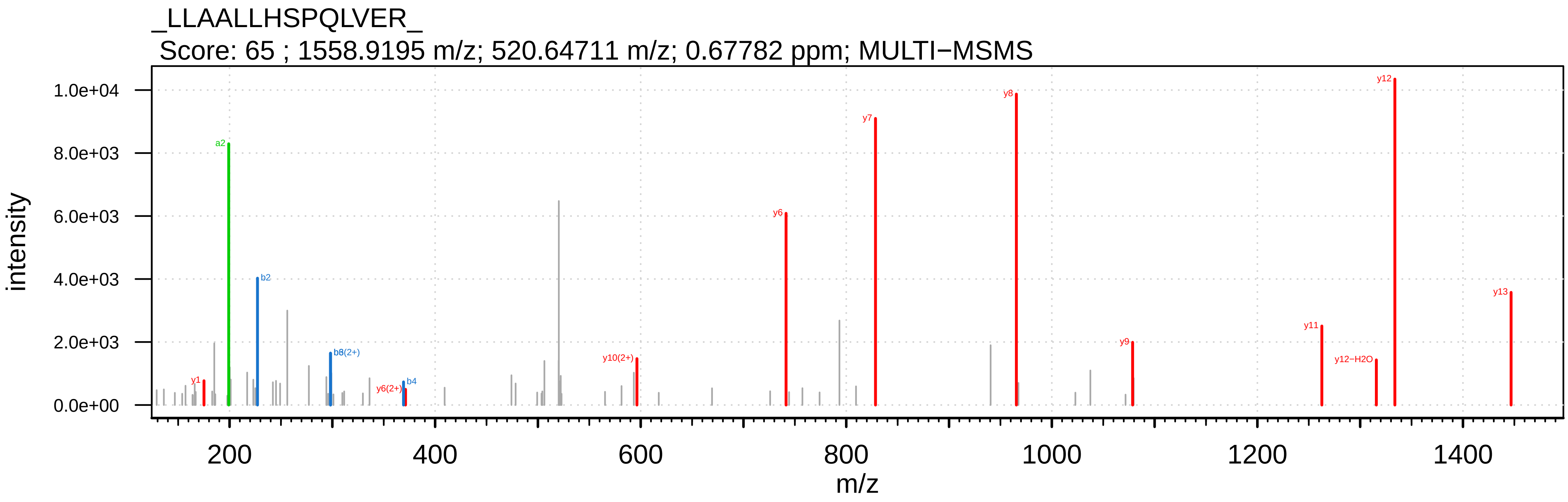

Raw File: 20100616_Velos1_TaGe_SA_MCF7_2
Scan Number: 24215
Proteins:
ENST00000602845_chr3:196669588−196669887:+

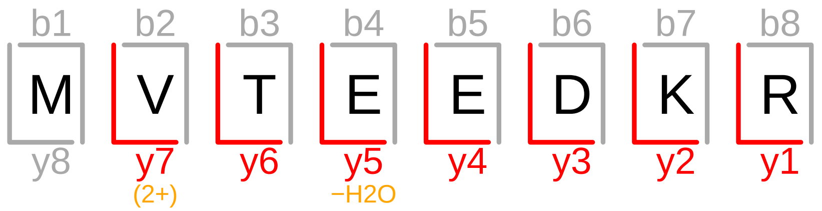

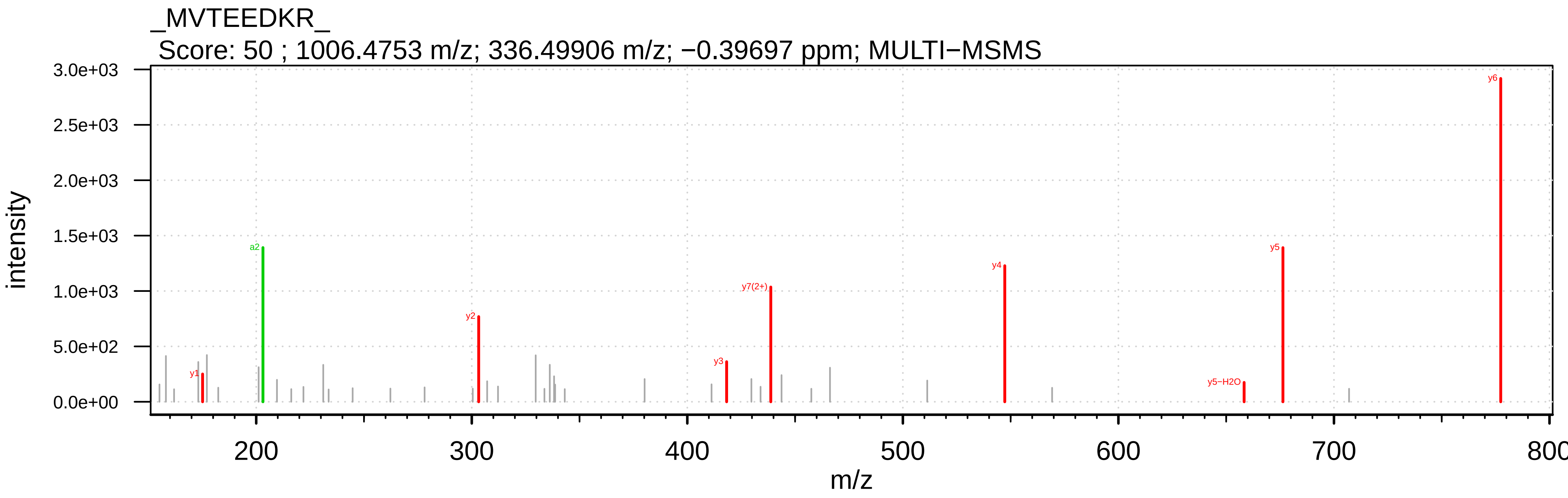

Raw File: 20100616_Velos1_TaGe_SA_MCF7_2
Scan Number: 1495
Proteins:
TCONS_l2_00006768_chr13:29174091−29174123:+

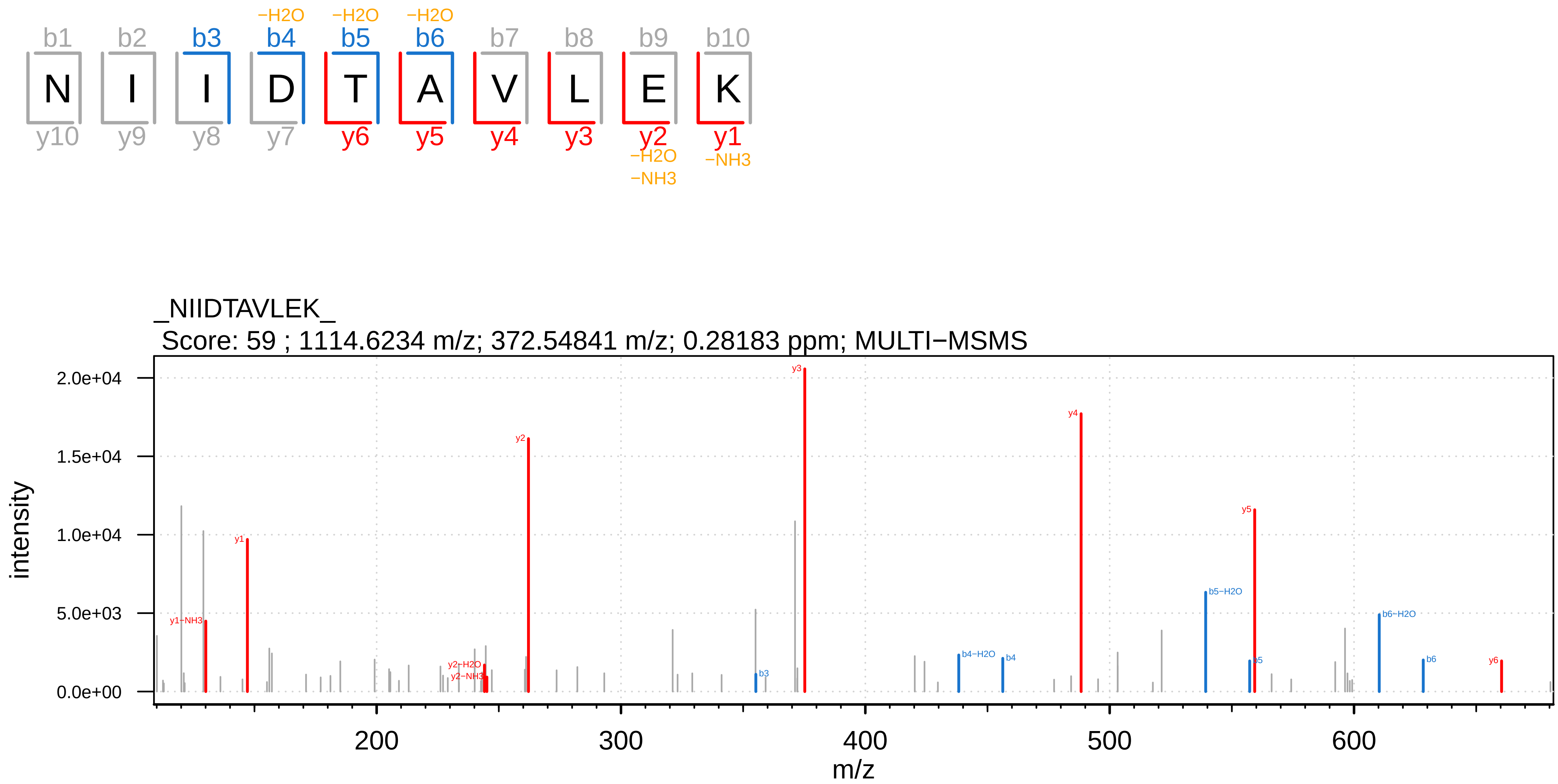

Raw File: 20100616_Velos1_TaGe_SA_MCF7_2
Scan Number: 7680
Proteins:
ENST00000497138_chr20:56806826−56807846:−

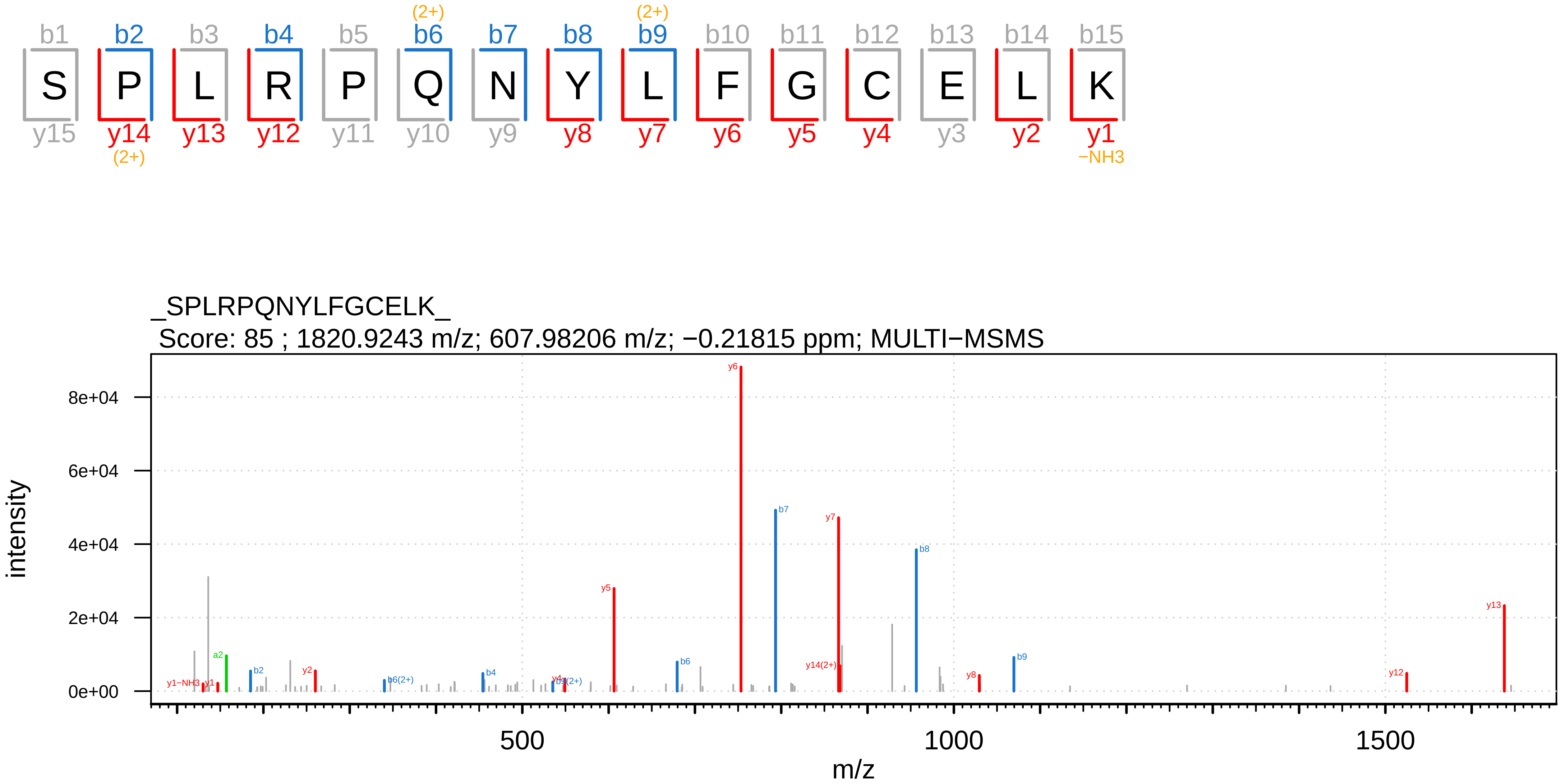

Raw File: 20100616_Velos1_TaGe_SA_MCF7_2
Scan Number: 21019
Proteins:
TCONS_l2_00008829_chr15:92829088−92829258:+

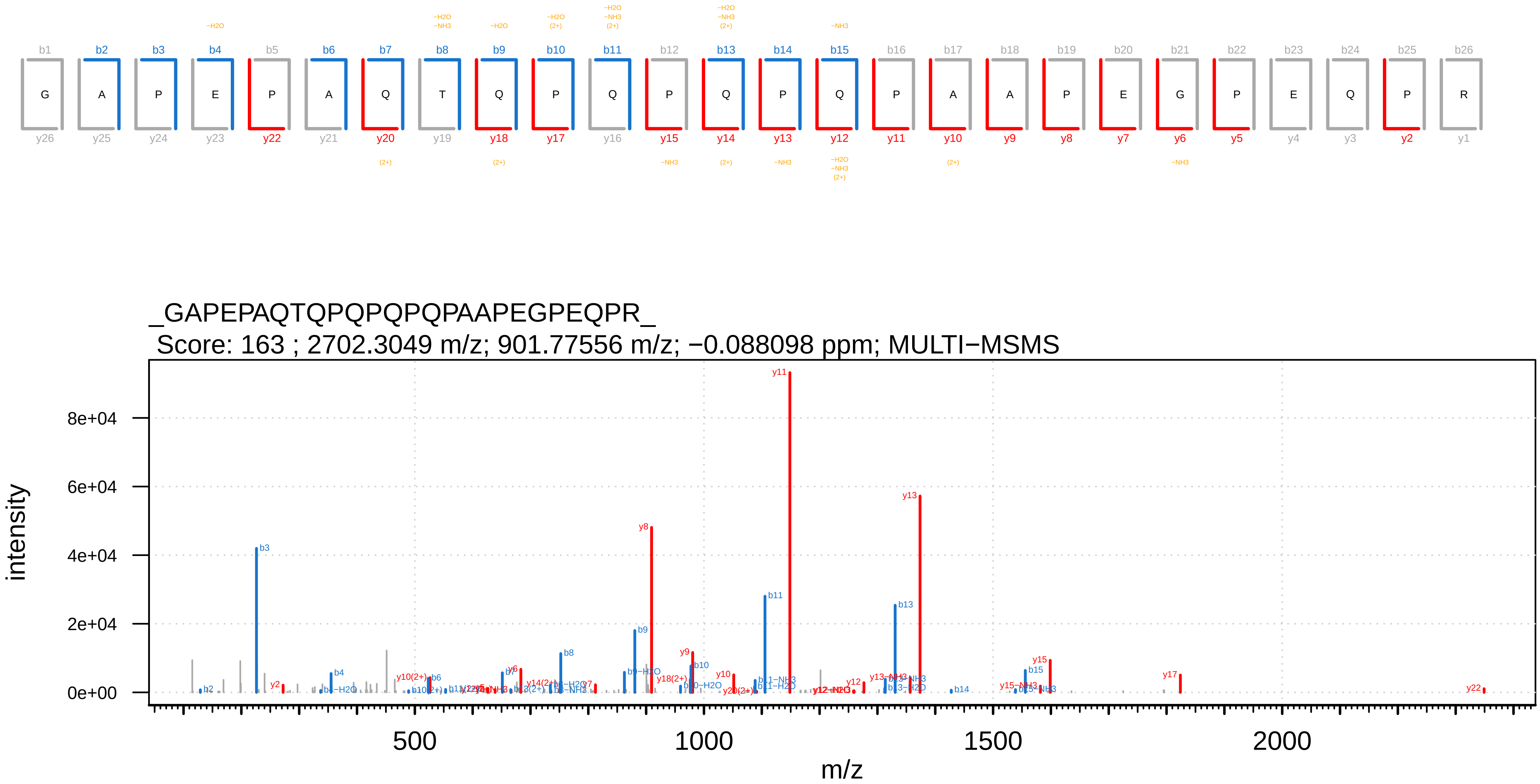

Raw File: 20101215_Velos1_TaGe_SA_A549_01
Scan Number: 11582
Proteins:
ENST00000424358_chr20:33865517−33865732:−

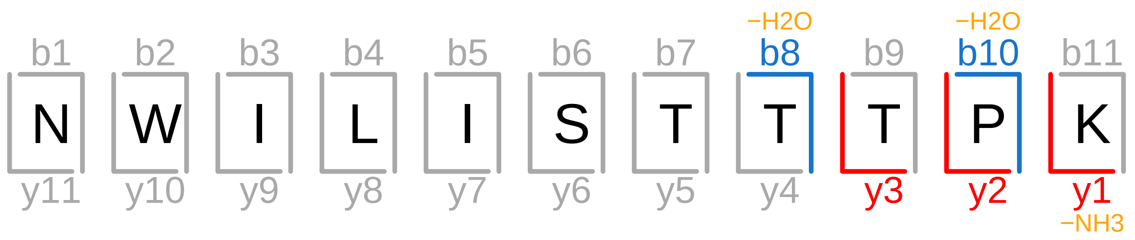

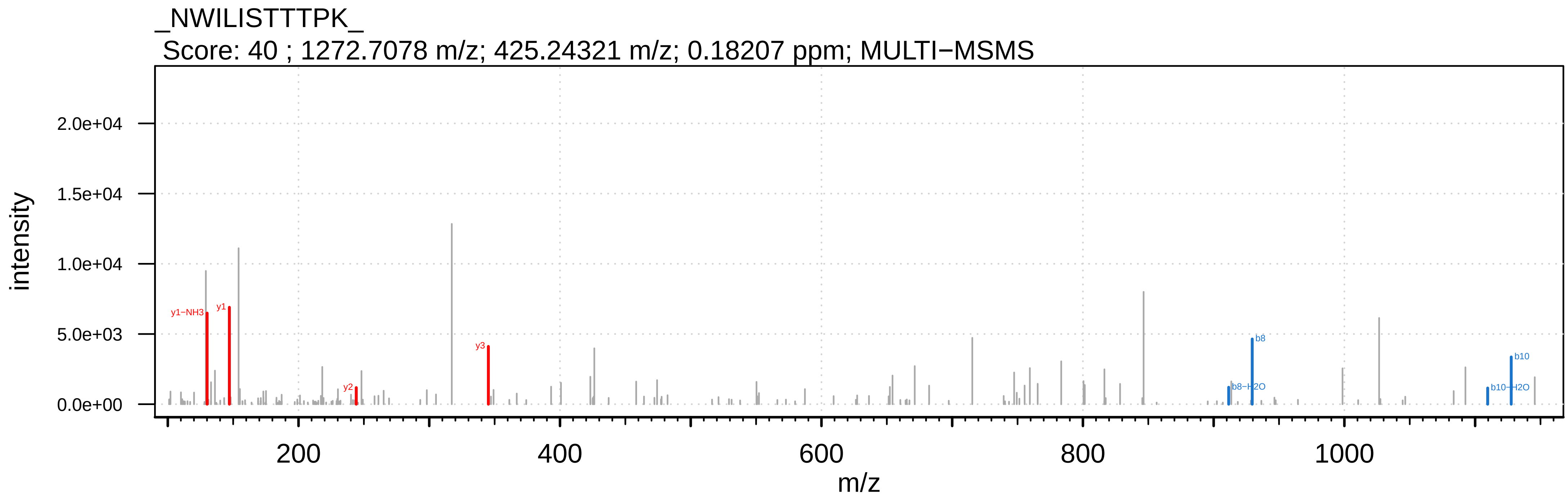

Raw File: 20101215_Velos1_TaGe_SA_A549_01
Scan Number: 23826
Proteins:
ENST00000430431_chr10:61496818−61496958:−

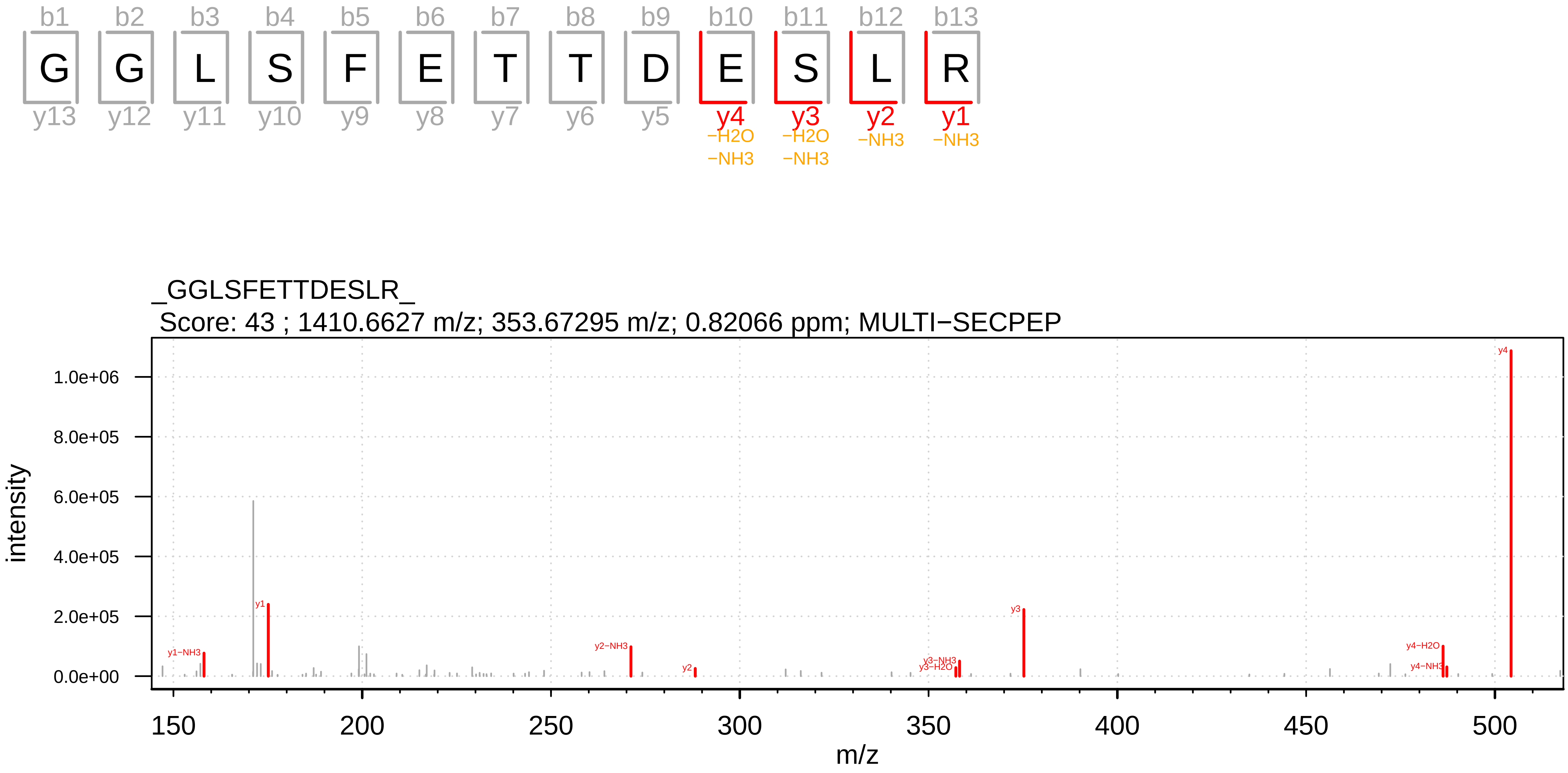

Raw File: 20101210_Velos1_AnWe_SA_MCF7_1
Scan Number: 6288
Proteins:
ENST00000511590_chr4:113802003−113803254:−

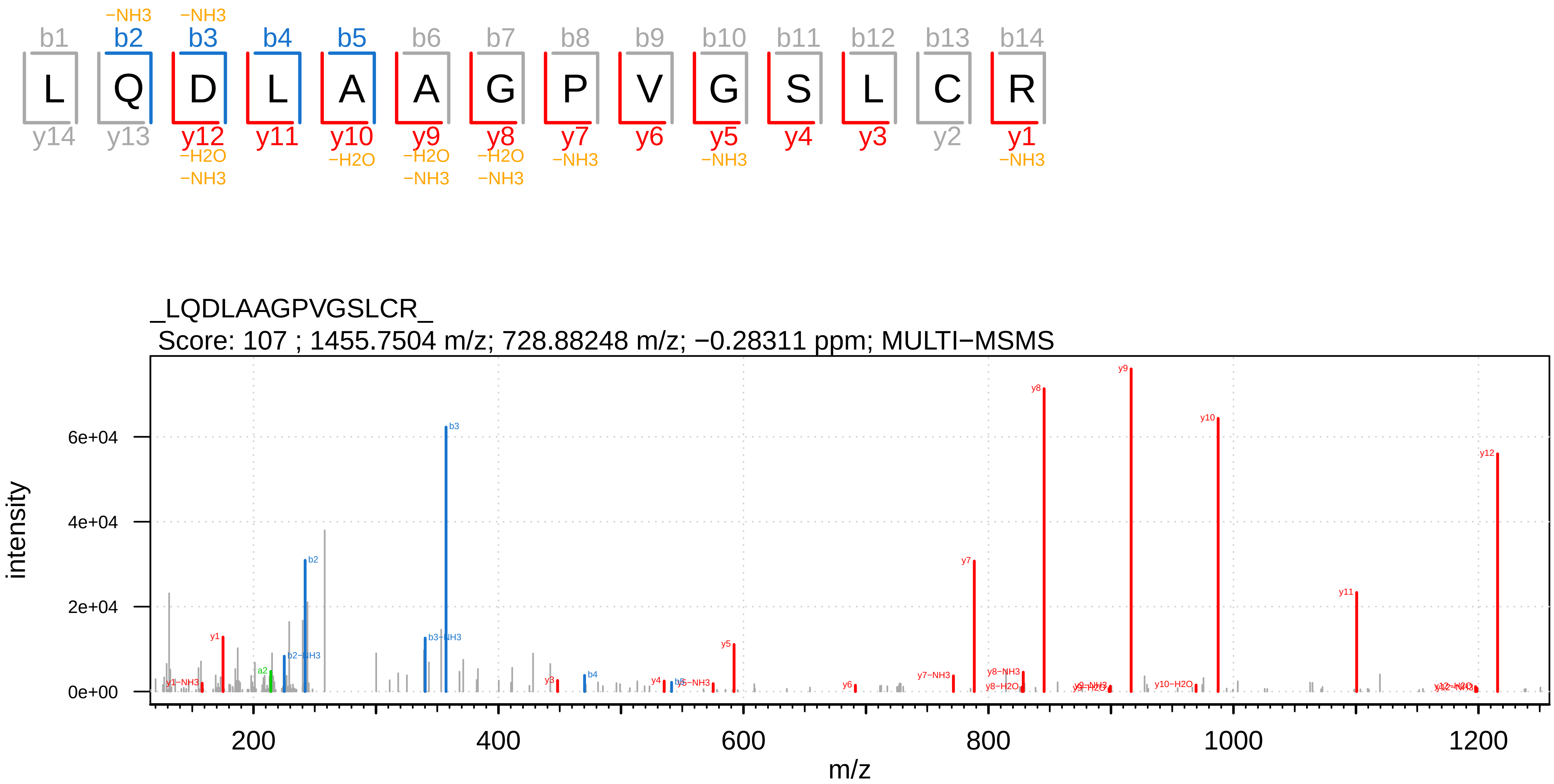

Raw File: 20101210_Velos1_AnWe_SA_MCF7_1
Scan Number: 21471
Proteins:
ENST00000602845_chr3:196669588−196669887:+

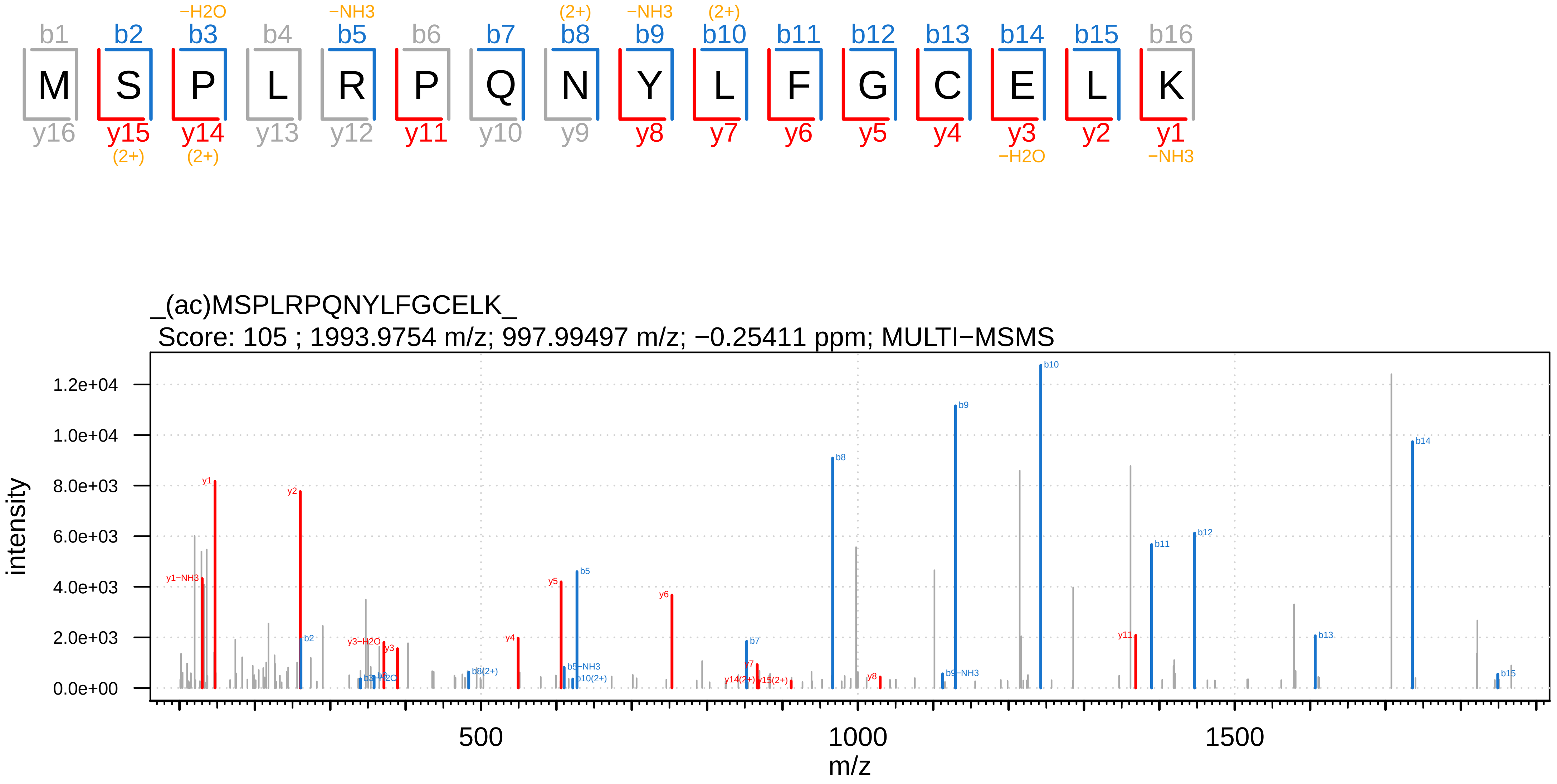

Raw File: 20101210_Velos1_AnWe_SA_MCF7_1
Scan Number: 36201
Proteins:
TCONS_l2_00008829_chr15:92829088−92829258:+

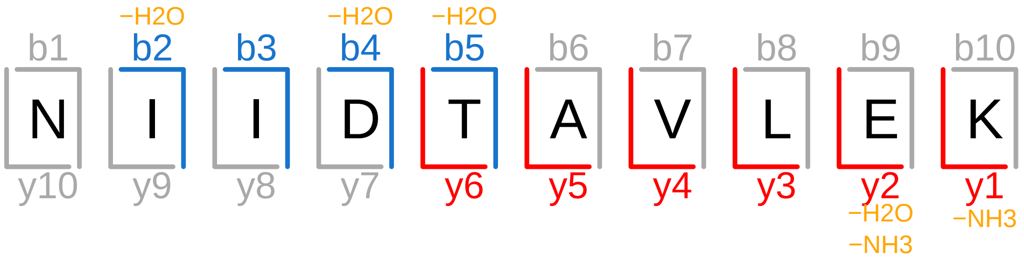

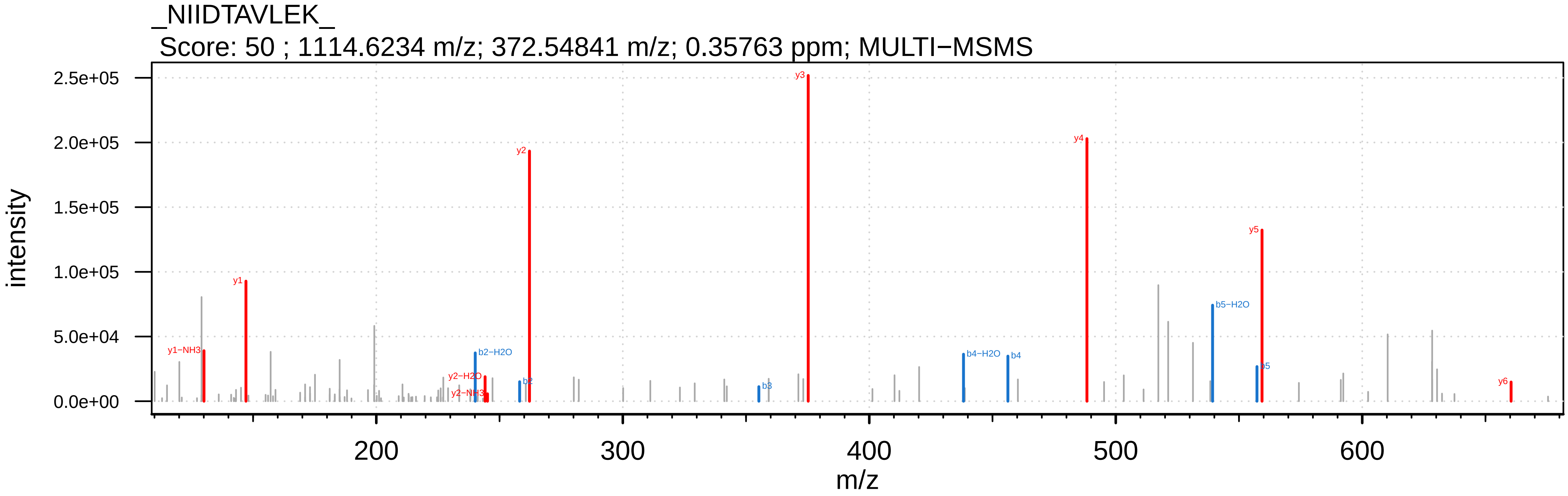

Raw File: 20101210_Velos1_AnWe_SA_MCF7_1
Scan Number: 9268
Proteins:
ENST00000497138_chr20:56806826−56807846:−

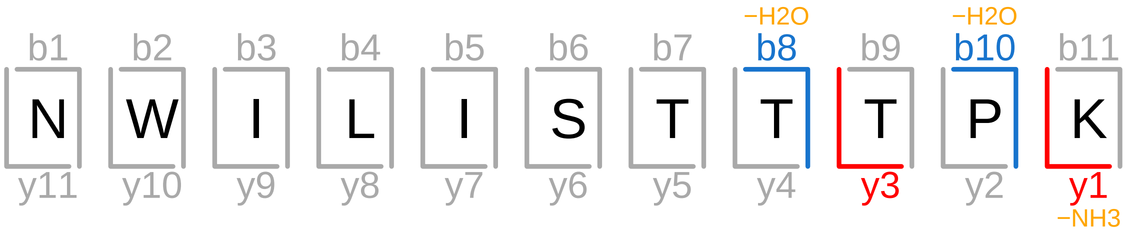

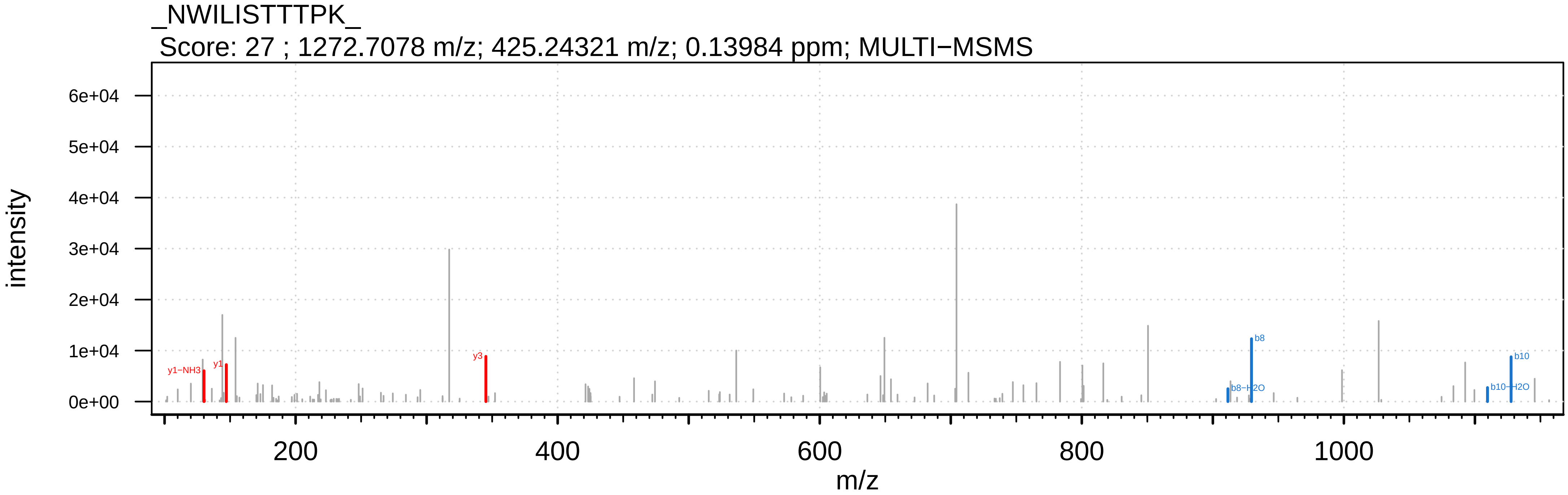

Raw File: 20101210_Velos1_AnWe_SA_MCF7_1
Scan Number: 26493
Proteins:
ENST00000430431_chr10:61496818−61496958:−

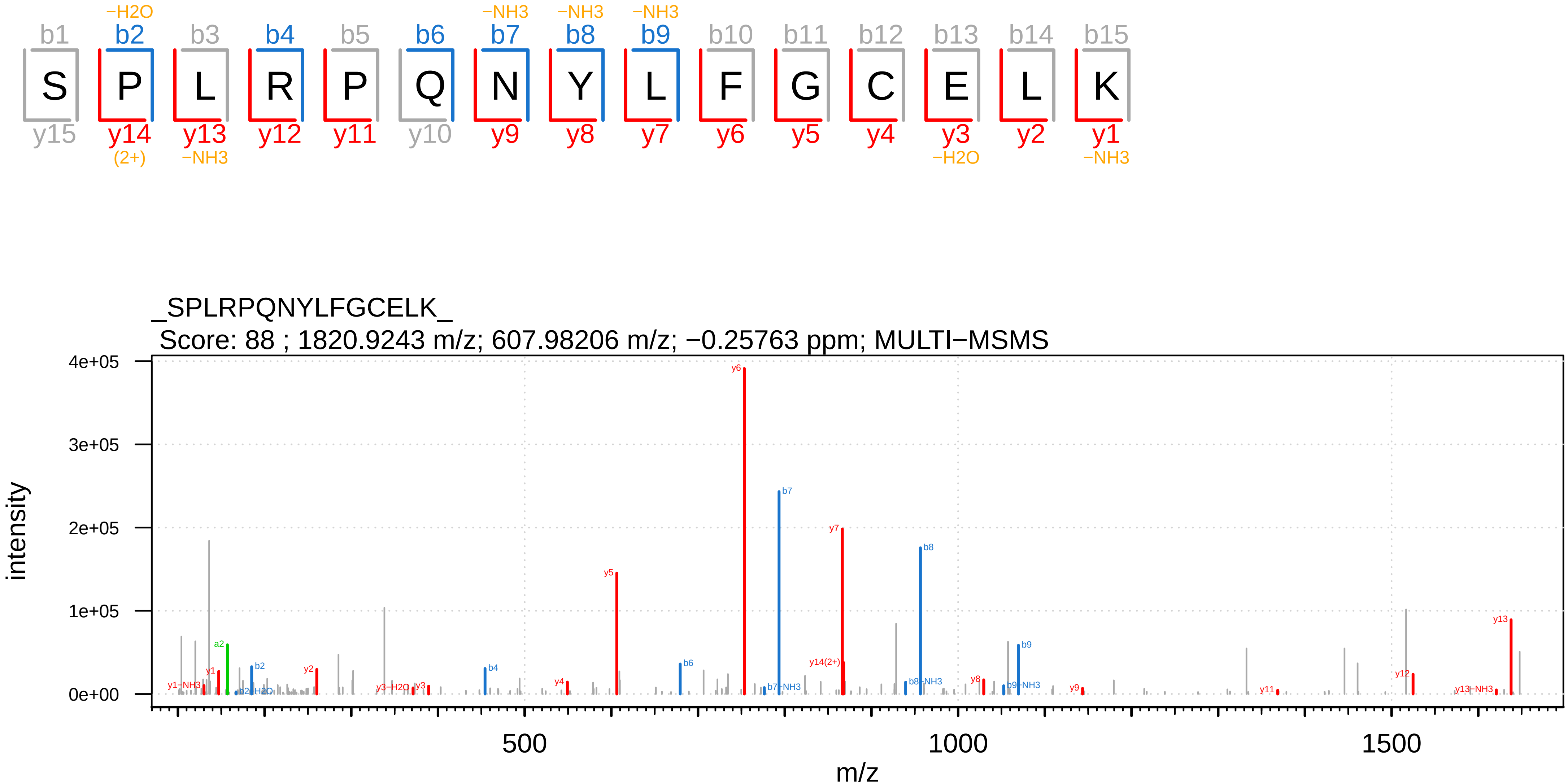

Raw File: 20101210_Velos1_AnWe_SA_MCF7_1
Scan Number: 23299
Proteins:
TCONS_l2_00008829_chr15:92829088−92829258:+

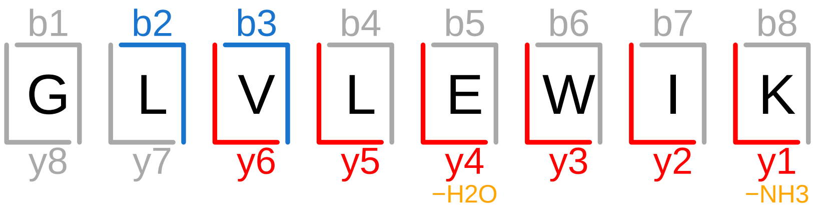

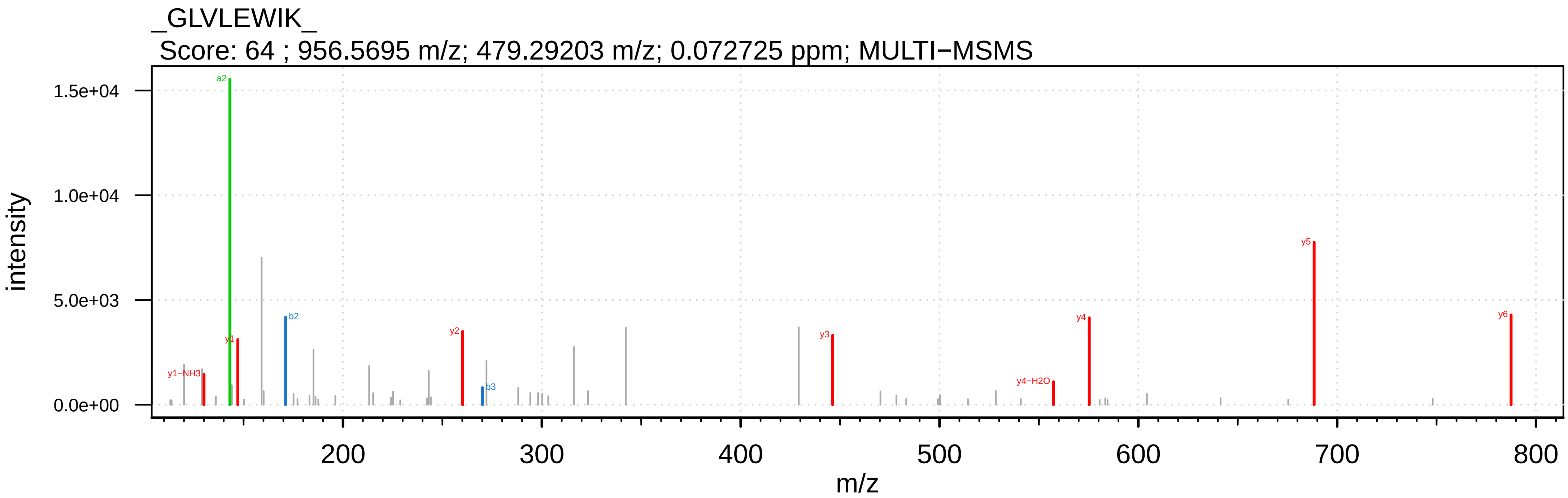

Raw File: 20100618_Velos1_TaGe_SA_LanCap_2
Scan Number: 25327
Proteins:
TCONS_l2_00001296_chr1:79520703−79520992:−

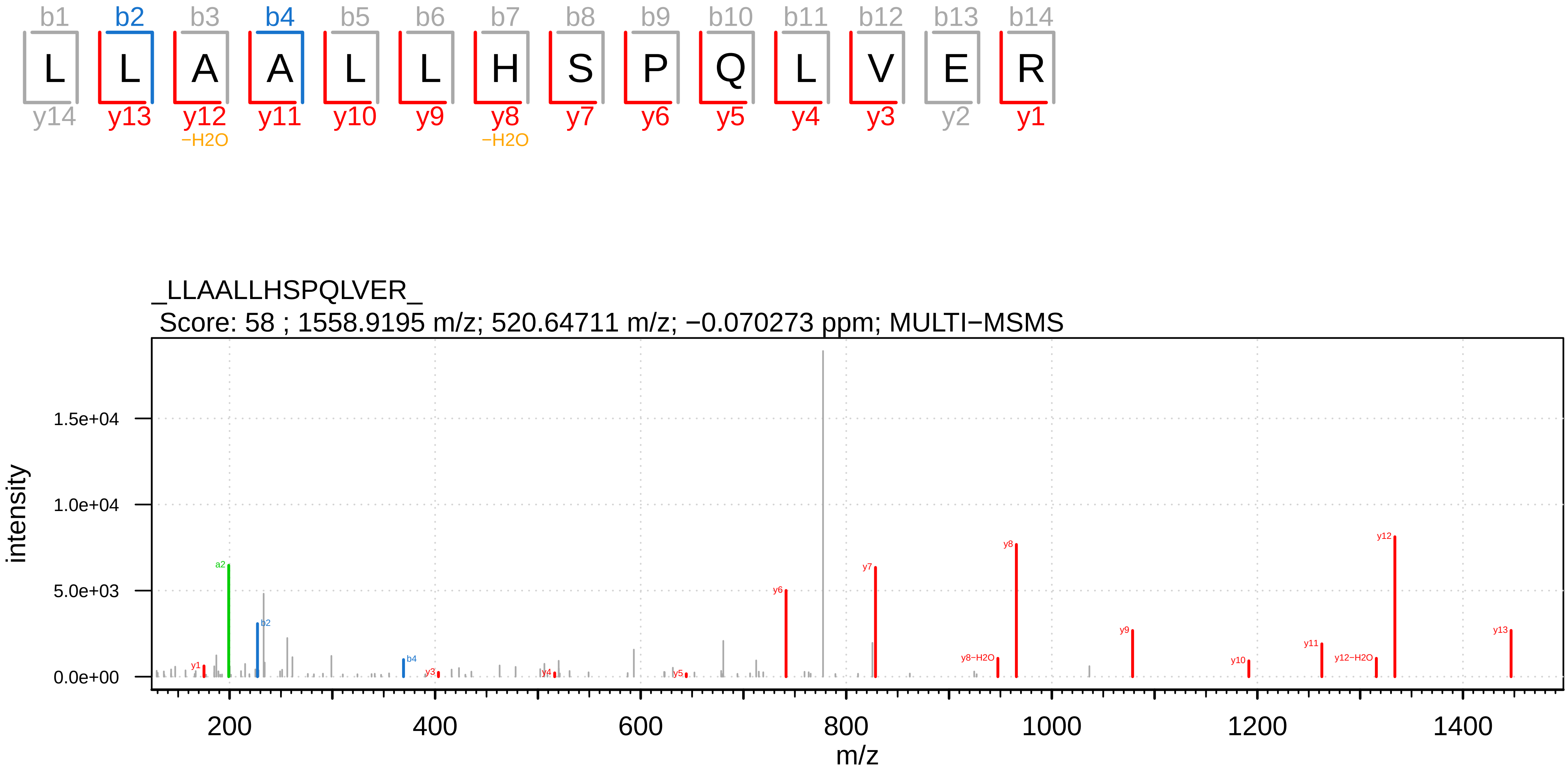

Raw File: 20100618_Velos1_TaGe_SA_LanCap_2
Scan Number: 23130
Proteins:
ENST00000602845_chr3:196669588−196669887:+

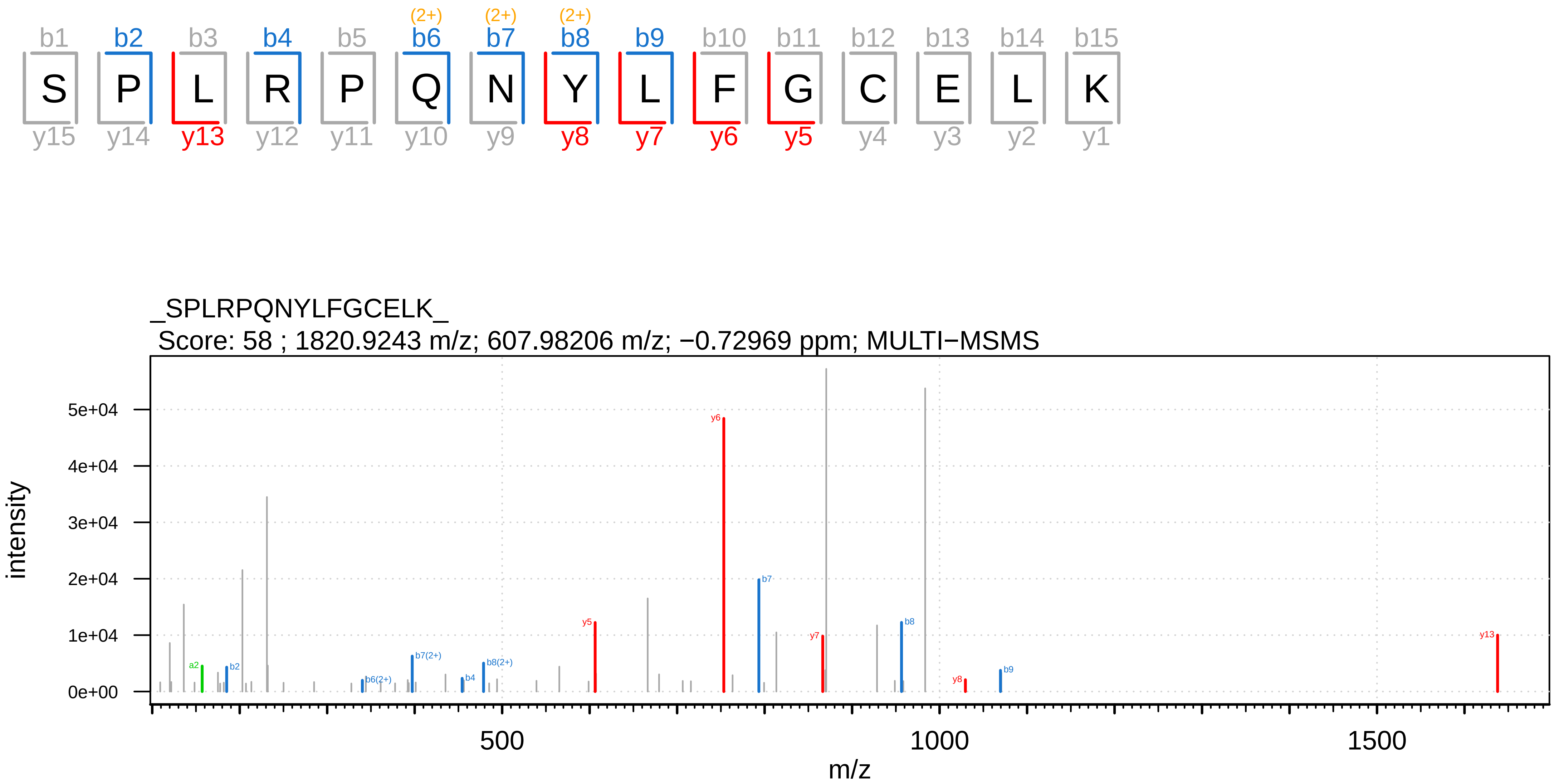

Raw File: 20100618_Velos1_TaGe_SA_LanCap_2
Scan Number: 19882
Proteins:
TCONS_l2_00008829_chr15:92829088−92829258:+

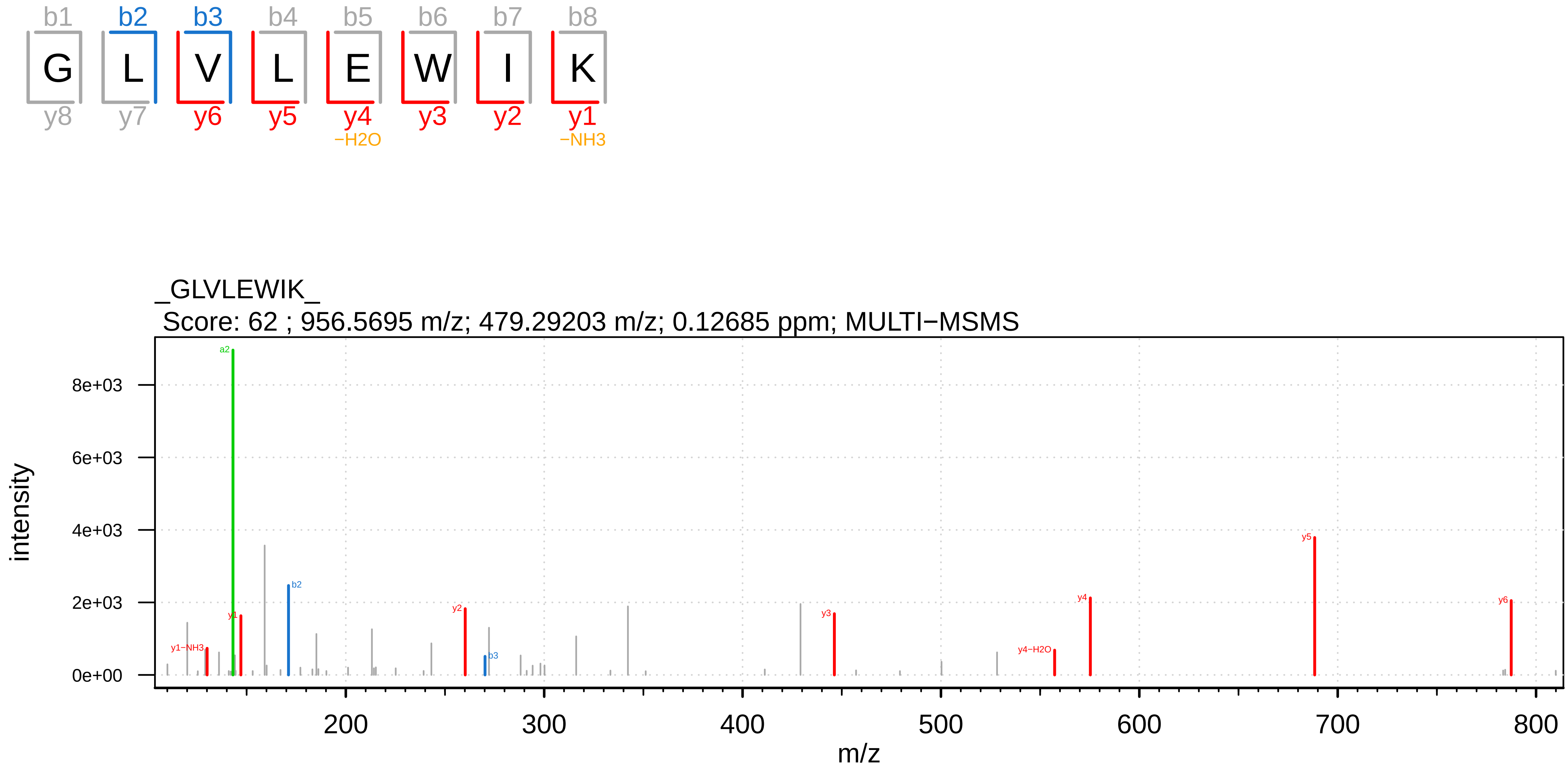

Raw File: 20100618_Velos1_TaGe_SA_LanCap_3
Scan Number: 23438
Proteins:
TCONS_l2_00001296_chr1:79520703−79520992:−

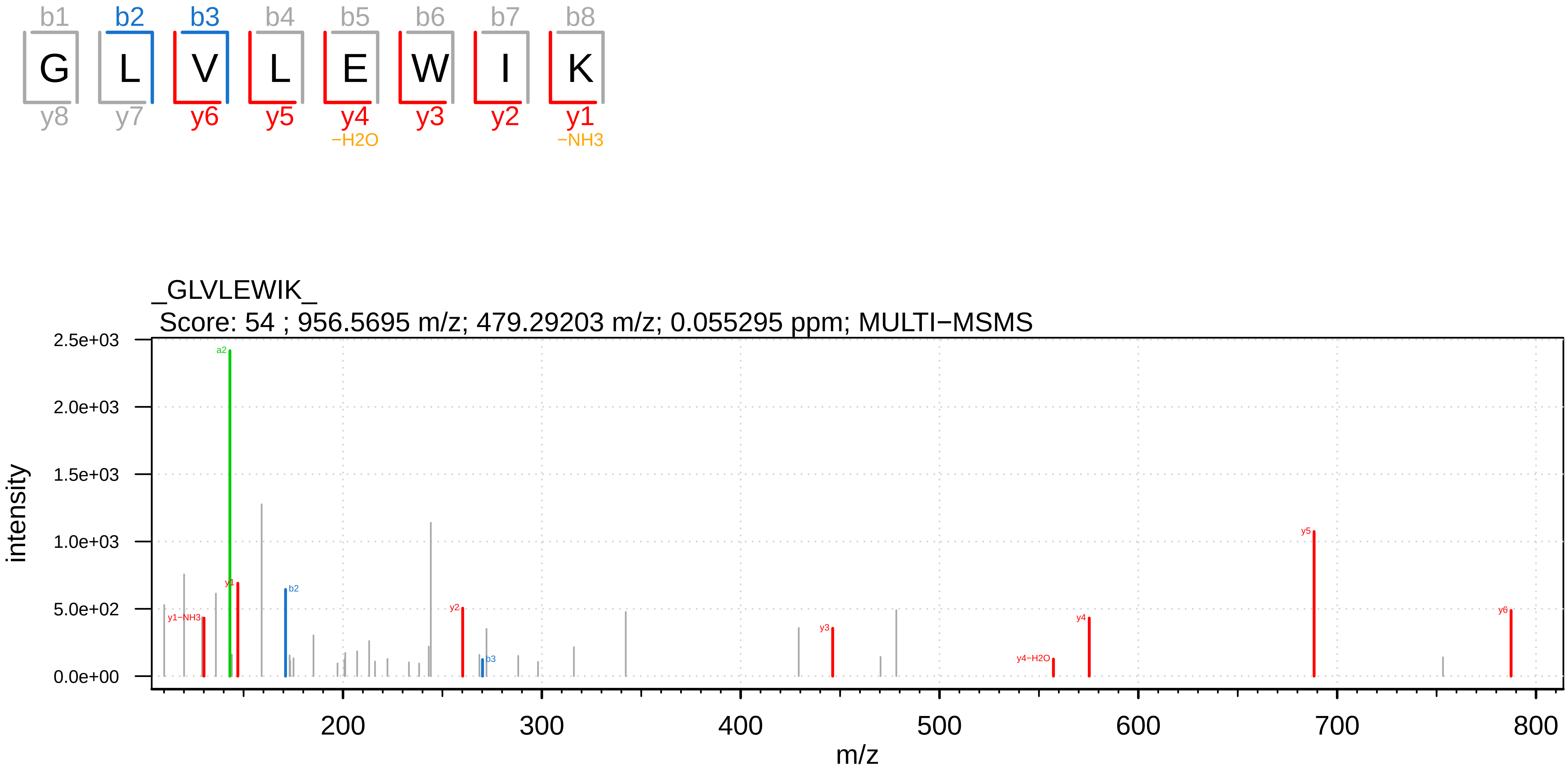

Raw File: 20100618_Velos1_TaGe_SA_LanCap_4
Scan Number: 20191
Proteins:
TCONS_l2_00001296_chr1:79520703−79520992:−

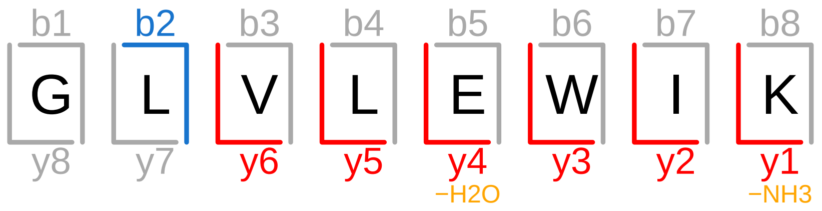

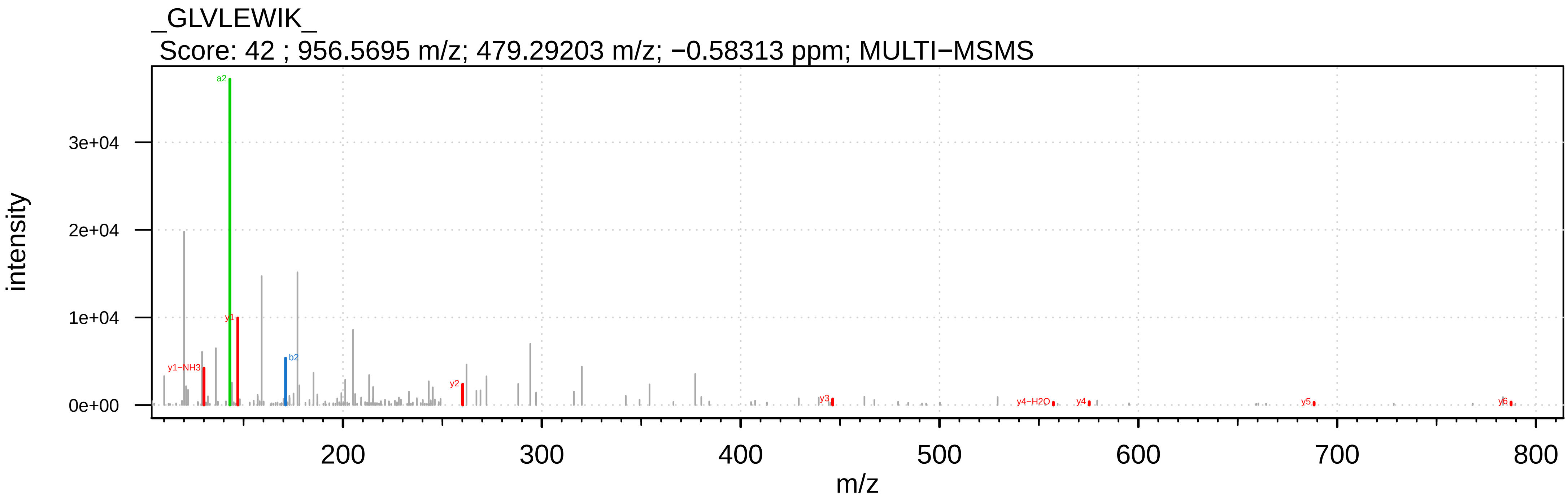

Raw File: 20100726_Velos1_TaGe_SA_HepG2_2
Scan Number: 22614
Proteins:
TCONS_l2_00001296_chr1:79520703−79520992:−

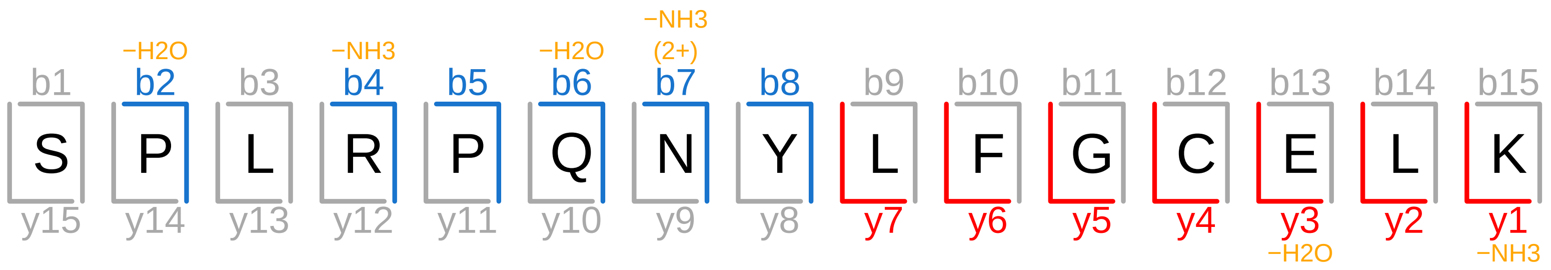

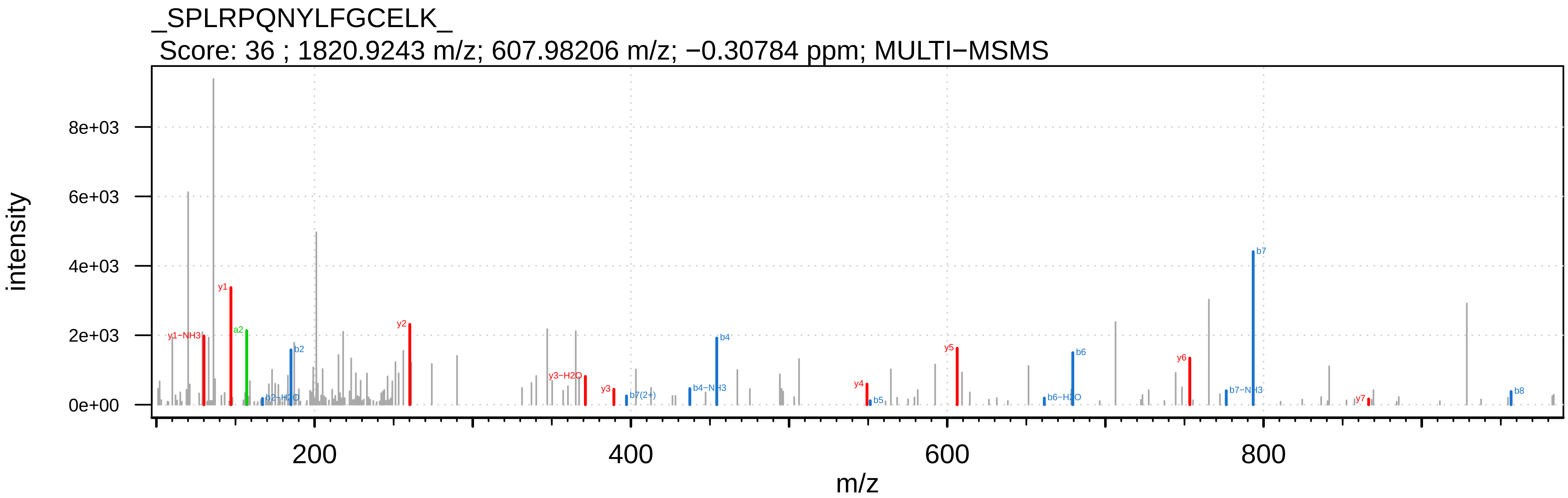

Raw File: 20100726_Velos1_TaGe_SA_HepG2_2
Scan Number: 16923
Proteins:
TCONS_l2_00008829_chr15:92829088−92829258:+

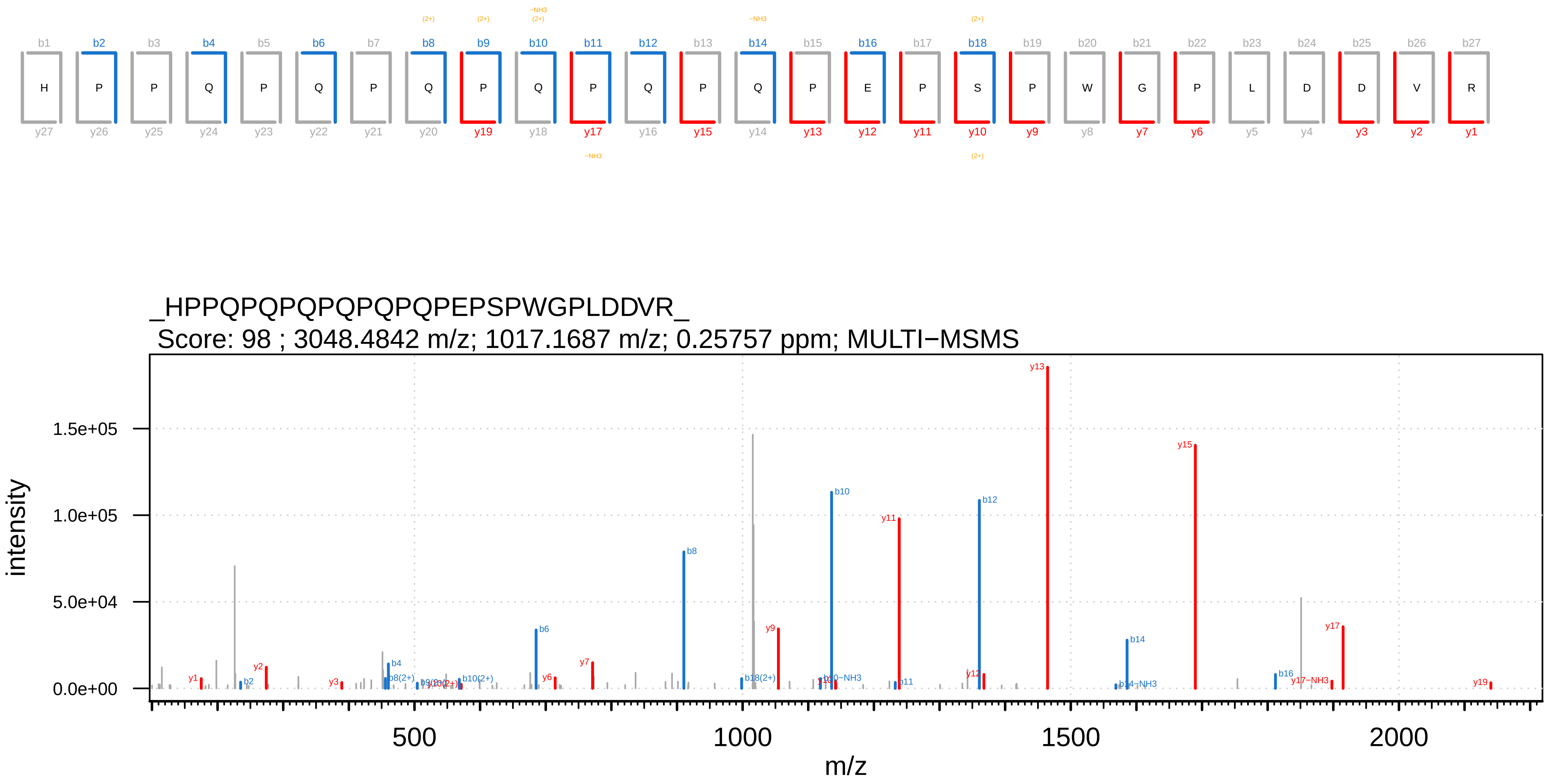

Raw File: 20100609_Velos1_TaGe_SA_GAMG_1
Scan Number: 22989
Proteins:
ENST00000424358_chr20:33865517−33865732:−

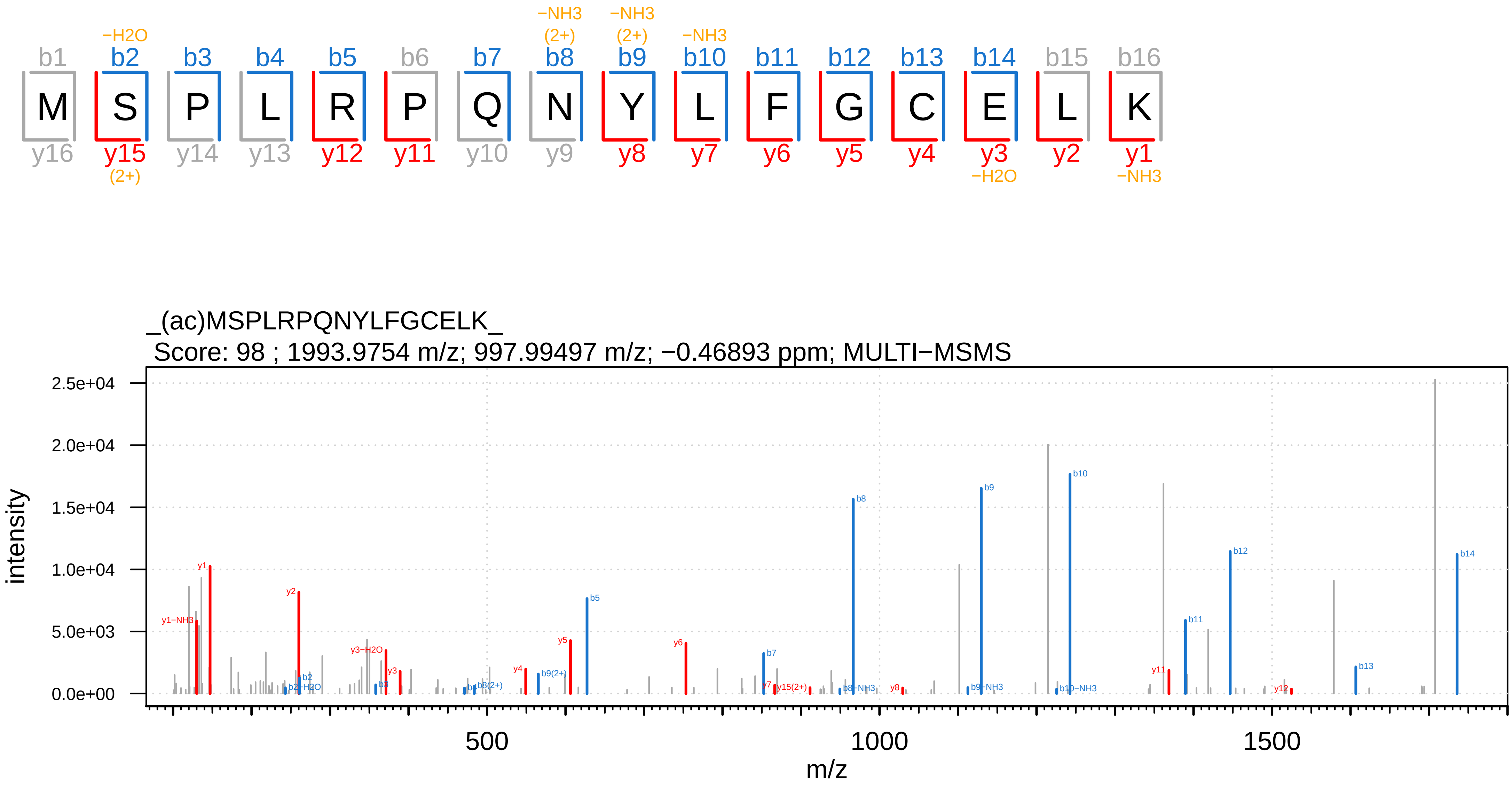

Raw File: 20100609_Velos1_TaGe_SA_GAMG_1
Scan Number: 36079
Proteins:
TCONS_l2_00008829_chr15:92829088−92829258:+

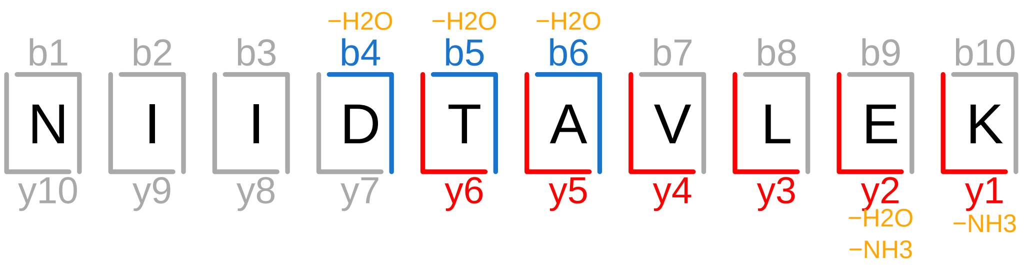

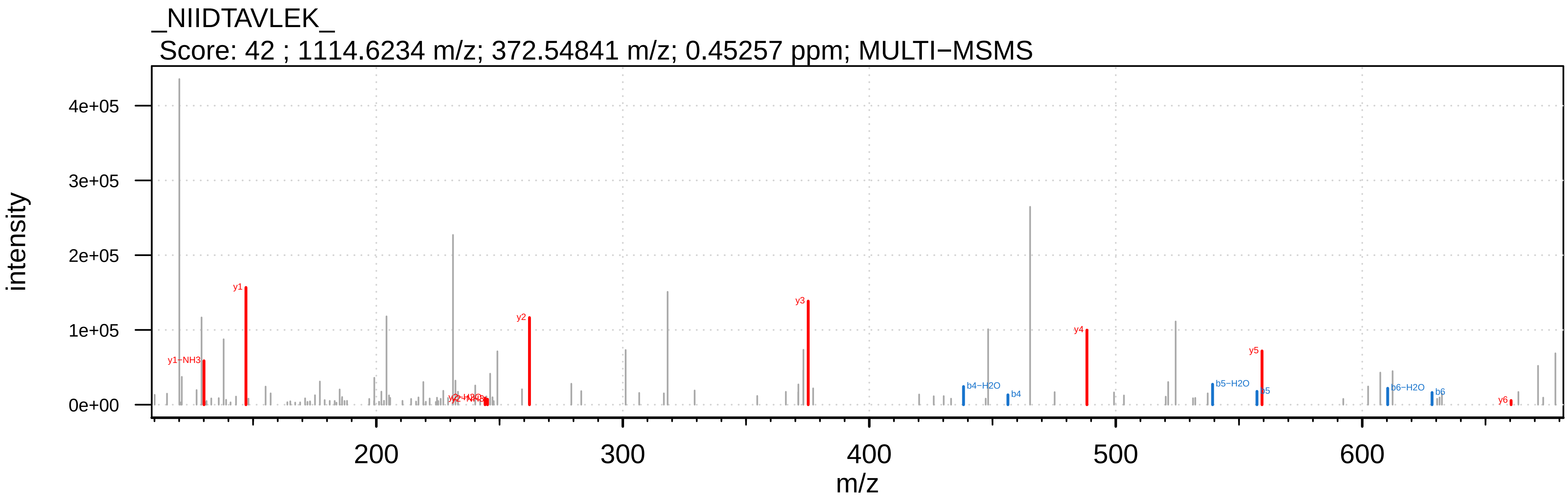

Raw File: 20100609_Velos1_TaGe_SA_GAMG_1
Scan Number: 4709
Proteins:
ENST00000497138_chr20:56806826−56807846:−

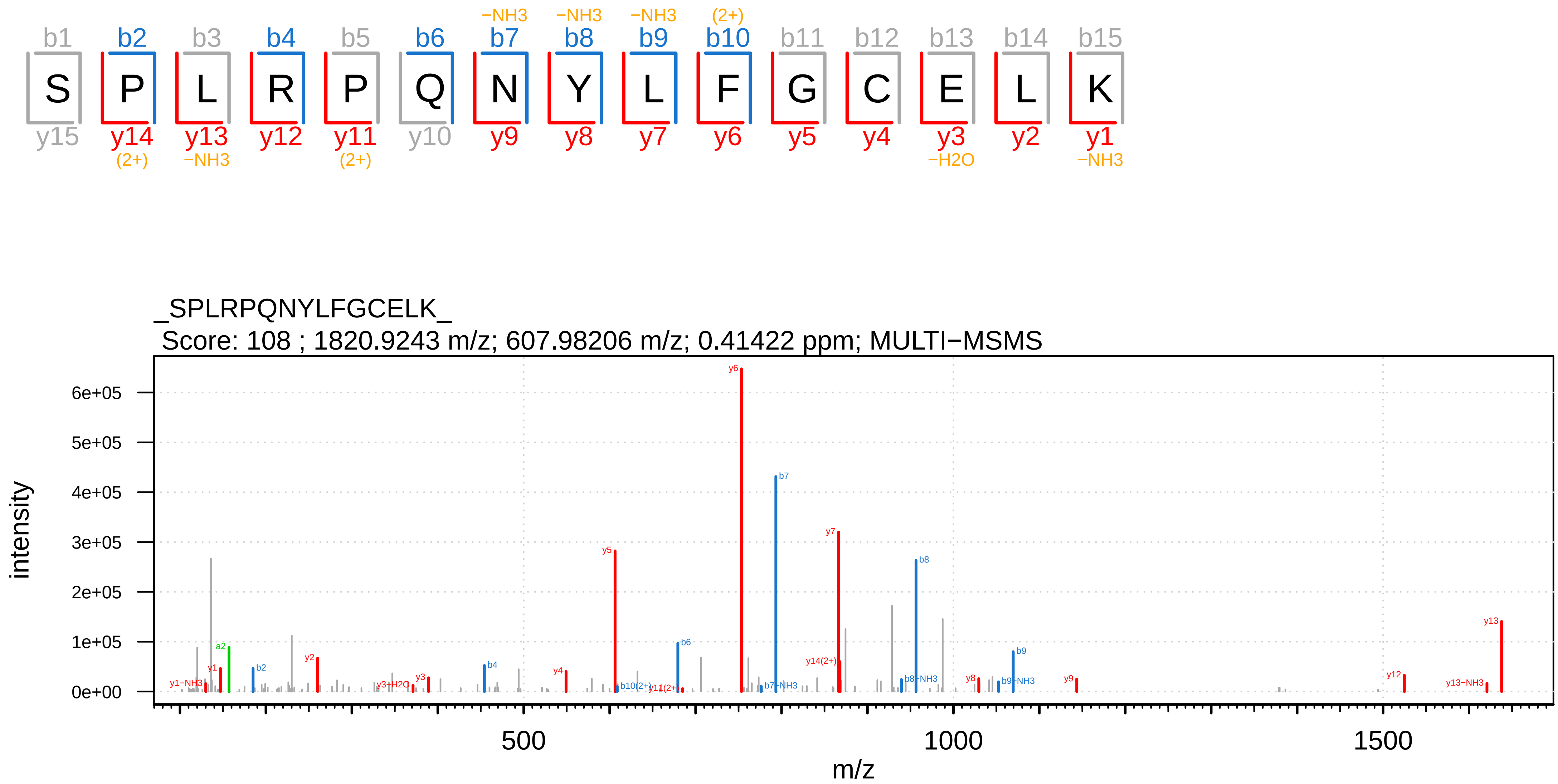

Raw File: 20100609_Velos1_TaGe_SA_GAMG_1
Scan Number: 20632
Proteins:
TCONS_l2_00008829_chr15:92829088−92829258:+

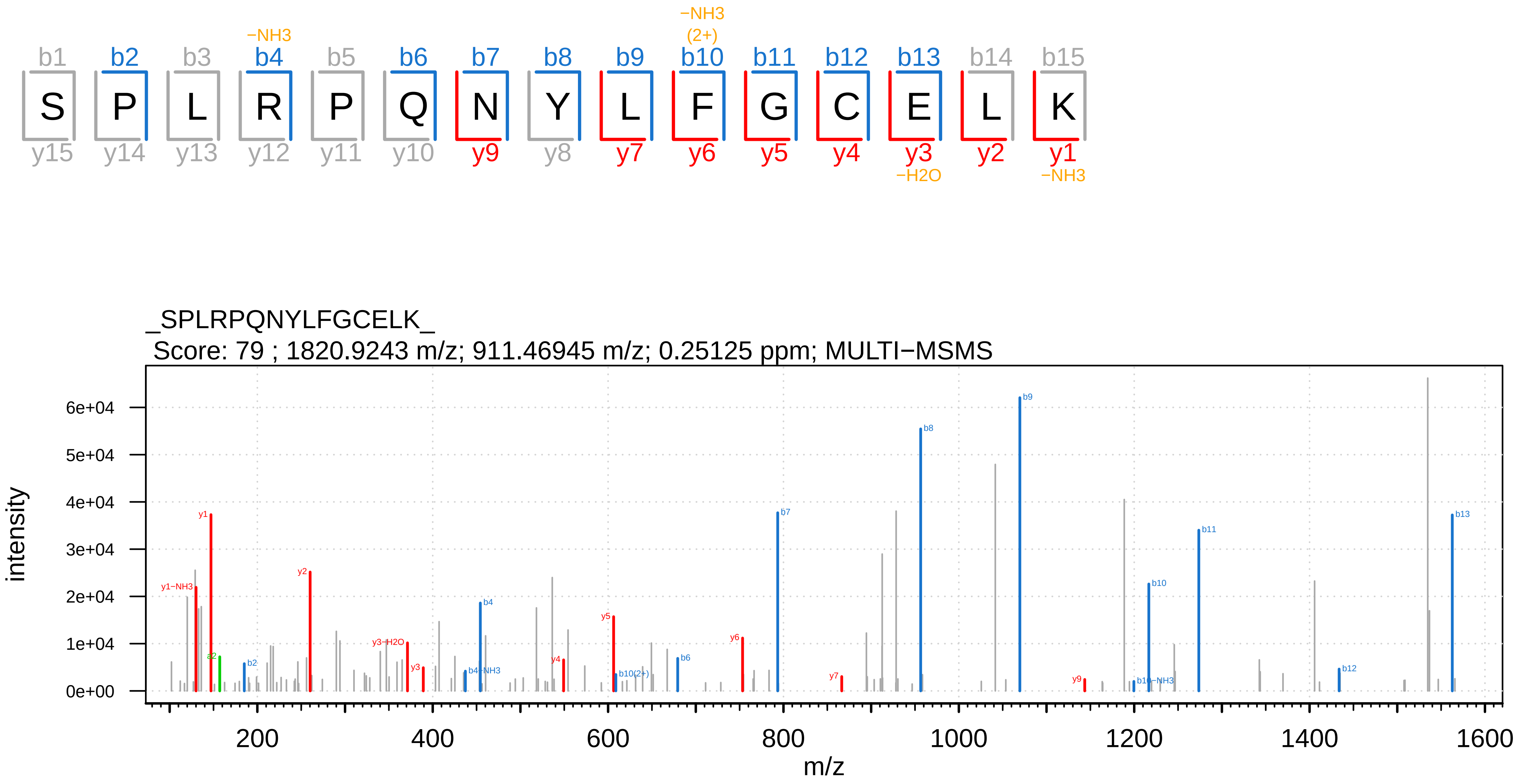

Raw File: 20100609_Velos1_TaGe_SA_GAMG_1
Scan Number: 20645
Proteins:
TCONS_l2_00008829_chr15:92829088−92829258:+

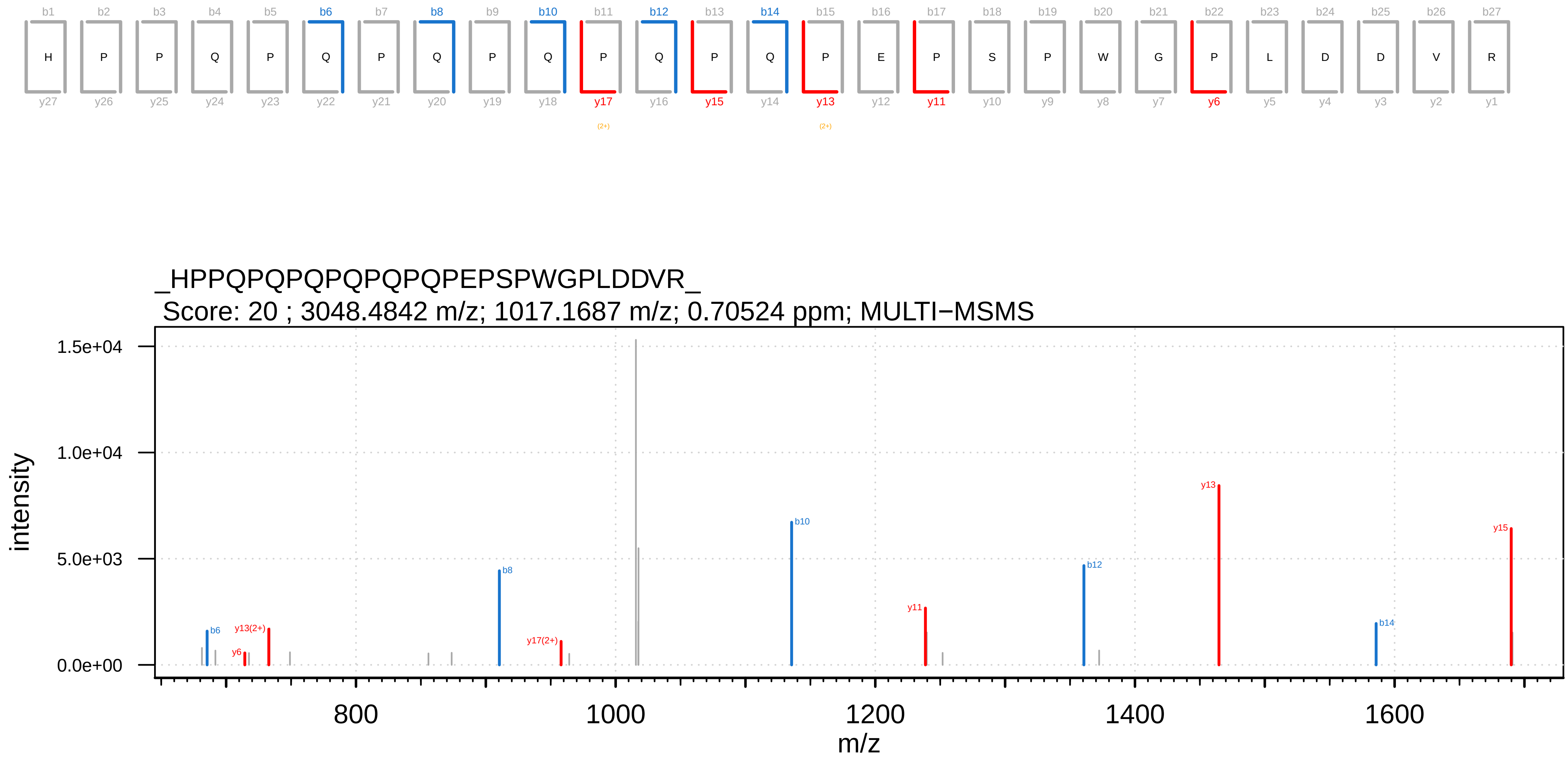

Raw File: 20100618_Velos1_TaGe_SA_LanCap_1
Scan Number: 24132
Proteins:
ENST00000424358_chr20:33865517−33865732:−

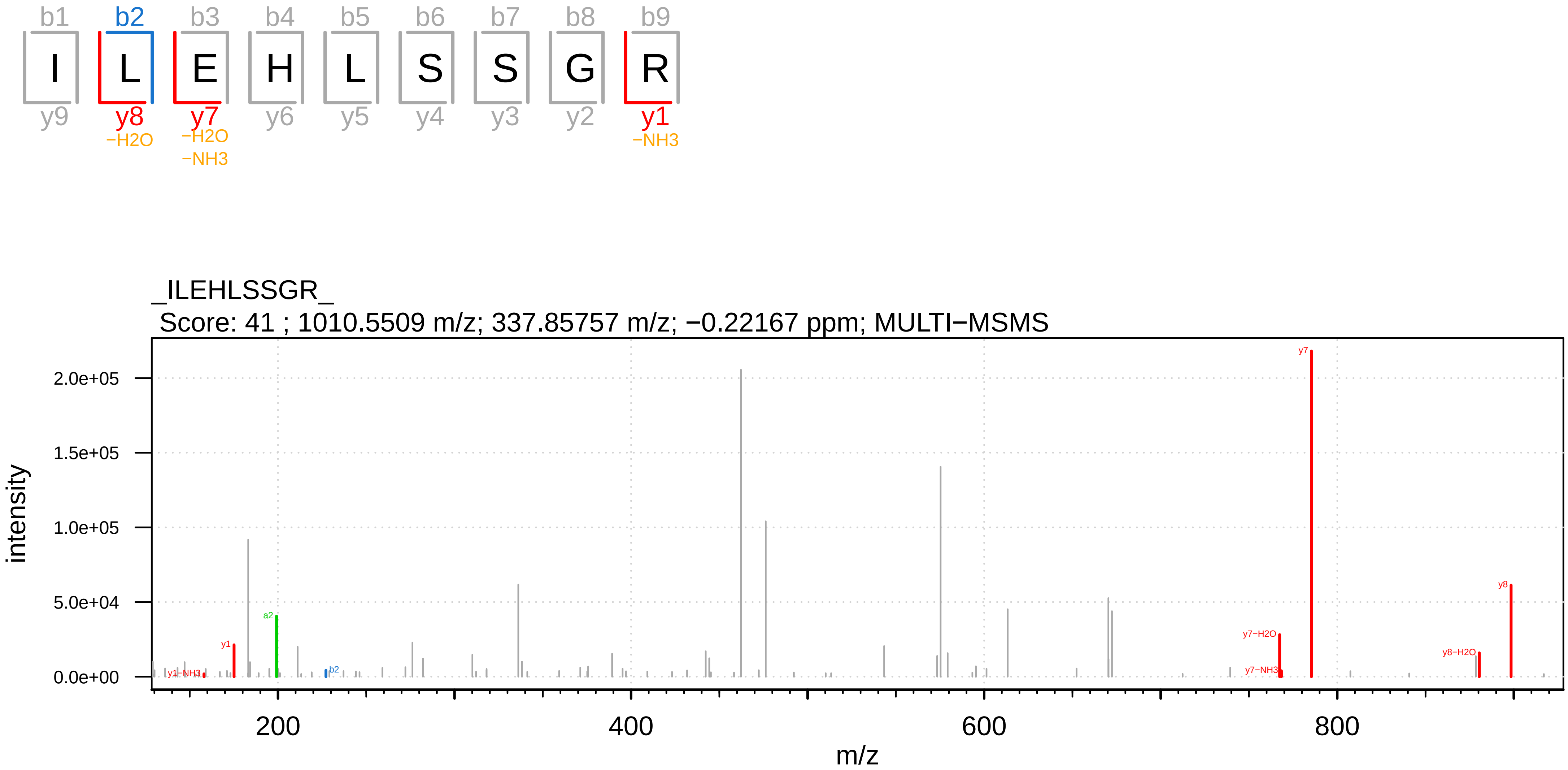

Raw File: 20100618_Velos1_TaGe_SA_LanCap_1
Scan Number: 6018
Proteins:
ENST00000452079_chr1:3663088−3663306:−

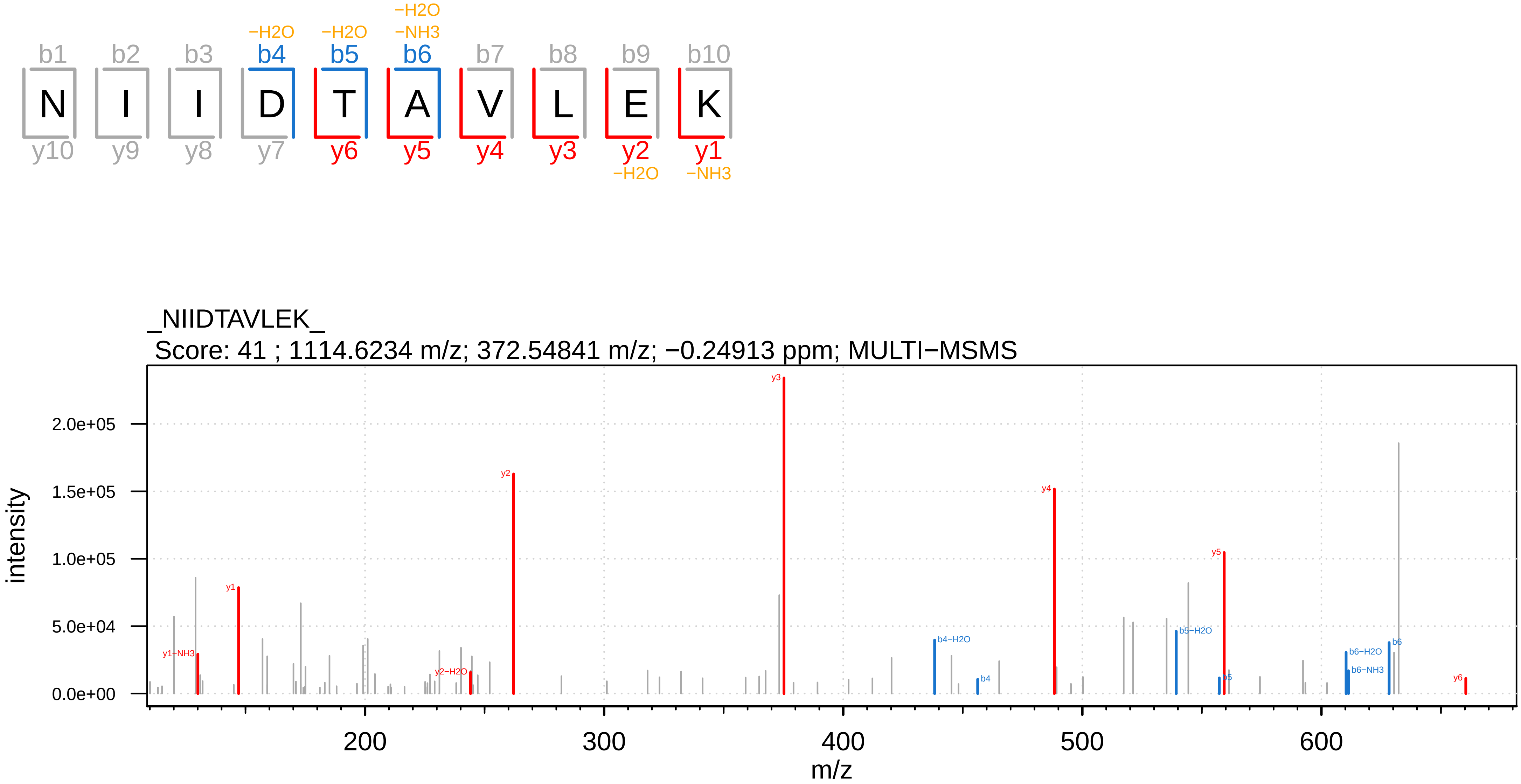

Raw File: 20100618_Velos1_TaGe_SA_LanCap_1

Scan Number: 5368

Proteins:

ENST00000497138_chr20:56806826−56807846:−

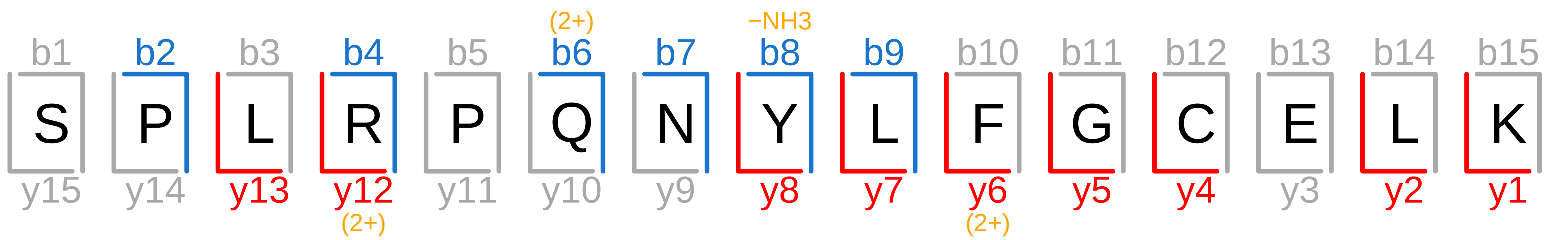

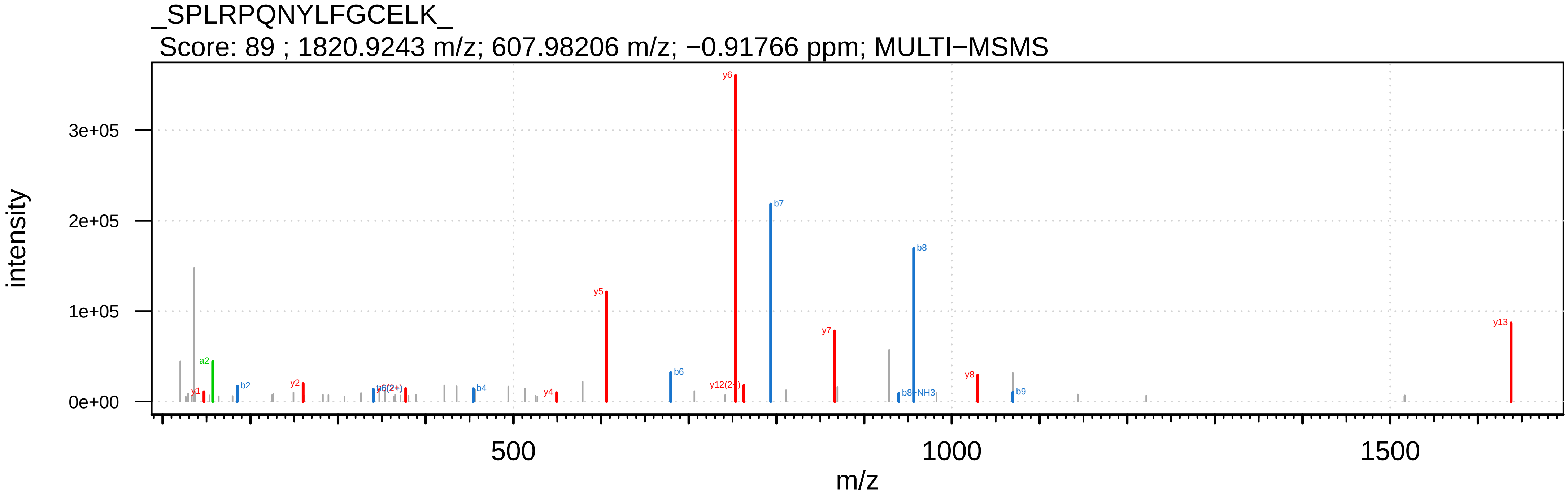

Raw File: 20100618_Velos1_TaGe_SA_LanCap_1
Scan Number: 22913
Proteins:
TCONS_l2_00008829_chr15:92829088−92829258:+

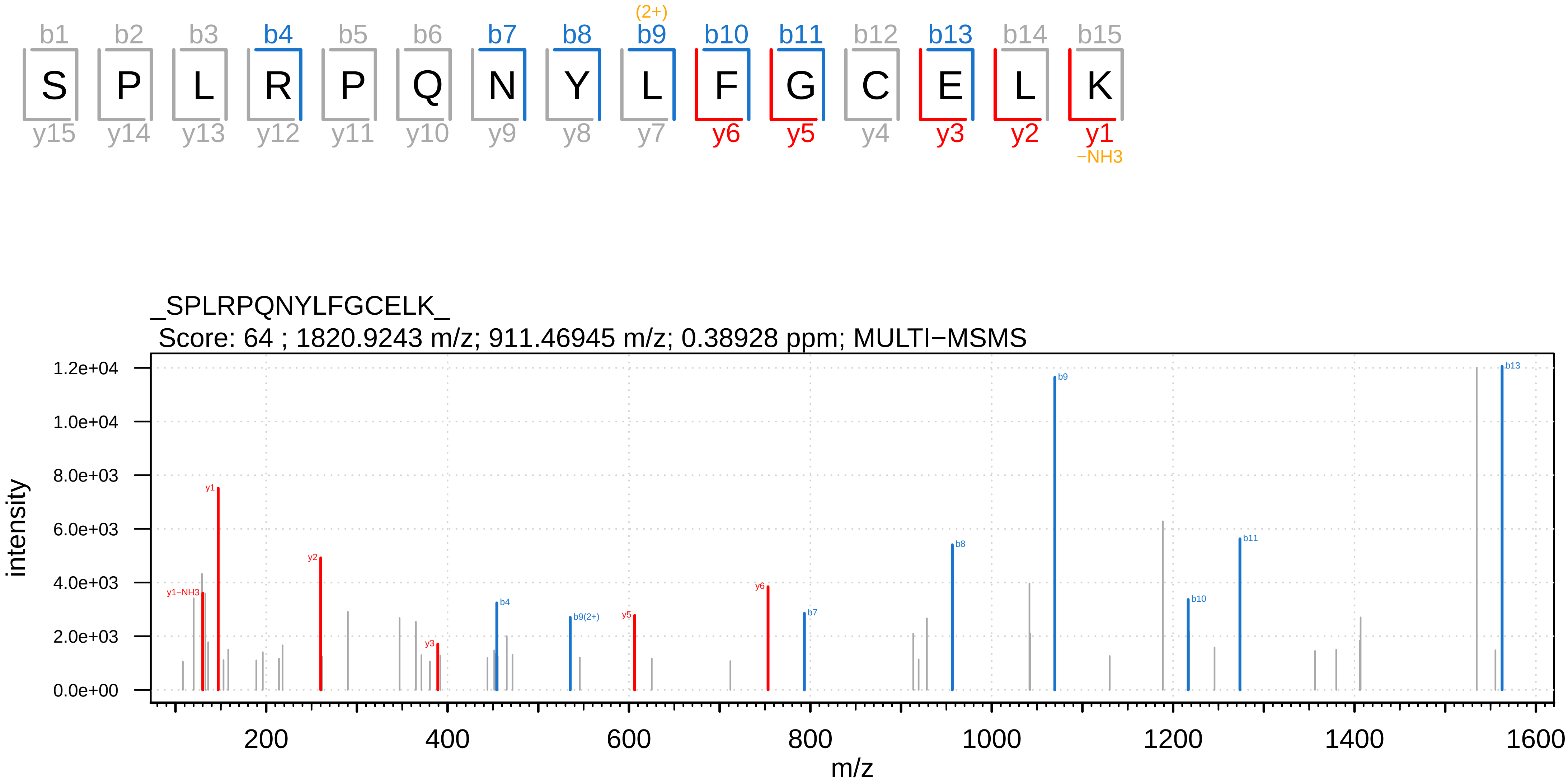

Raw File: 20100618_Velos1_TaGe_SA_LanCap_1
Scan Number: 22917
Proteins:
TCONS_l2_00008829_chr15:92829088−92829258:+

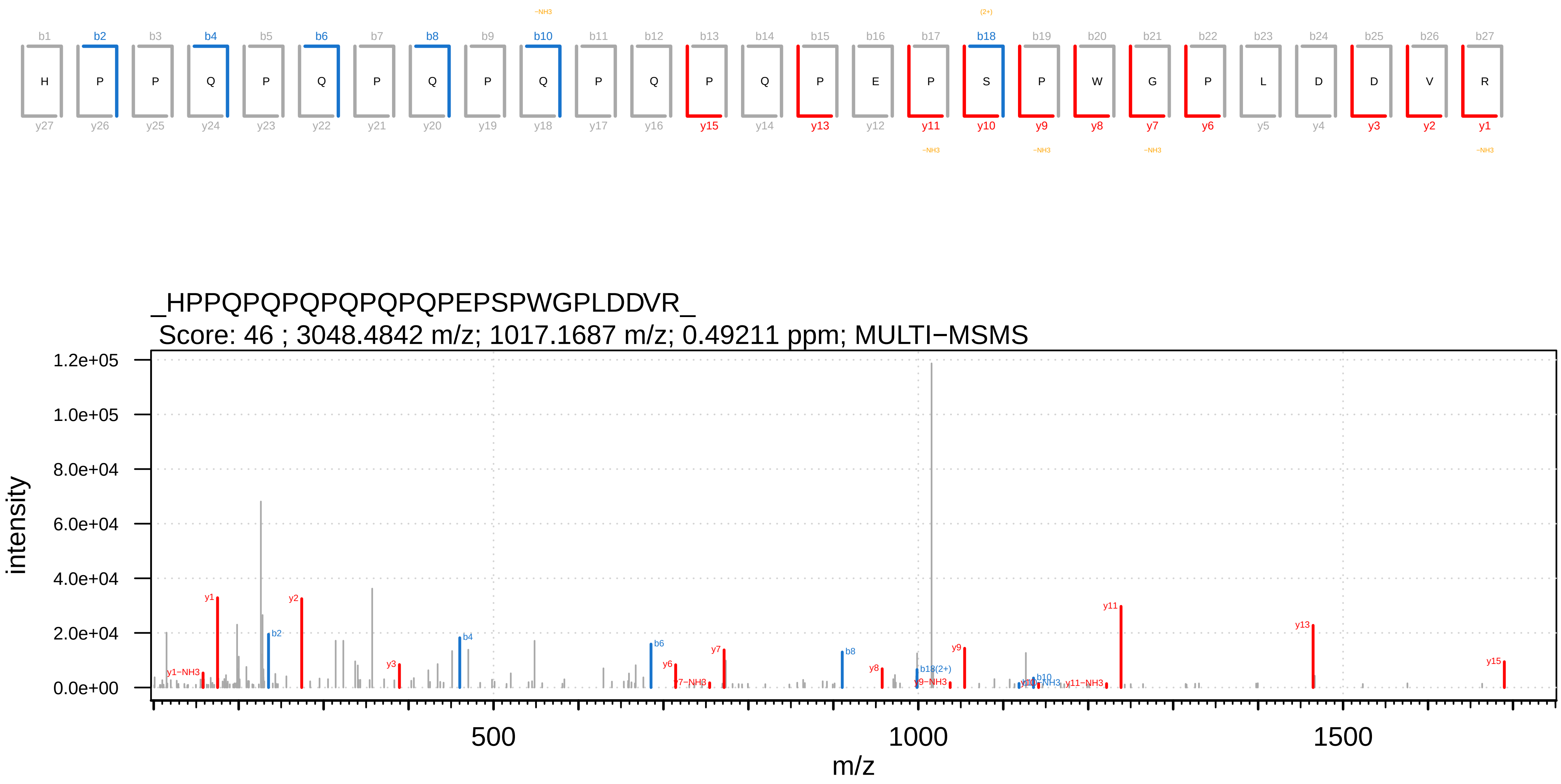

Raw File: 20100719_Velos1_TaGe_SA_LnCap_1
Scan Number: 18918
Proteins:
ENST00000424358_chr20:33865517−33865732:−

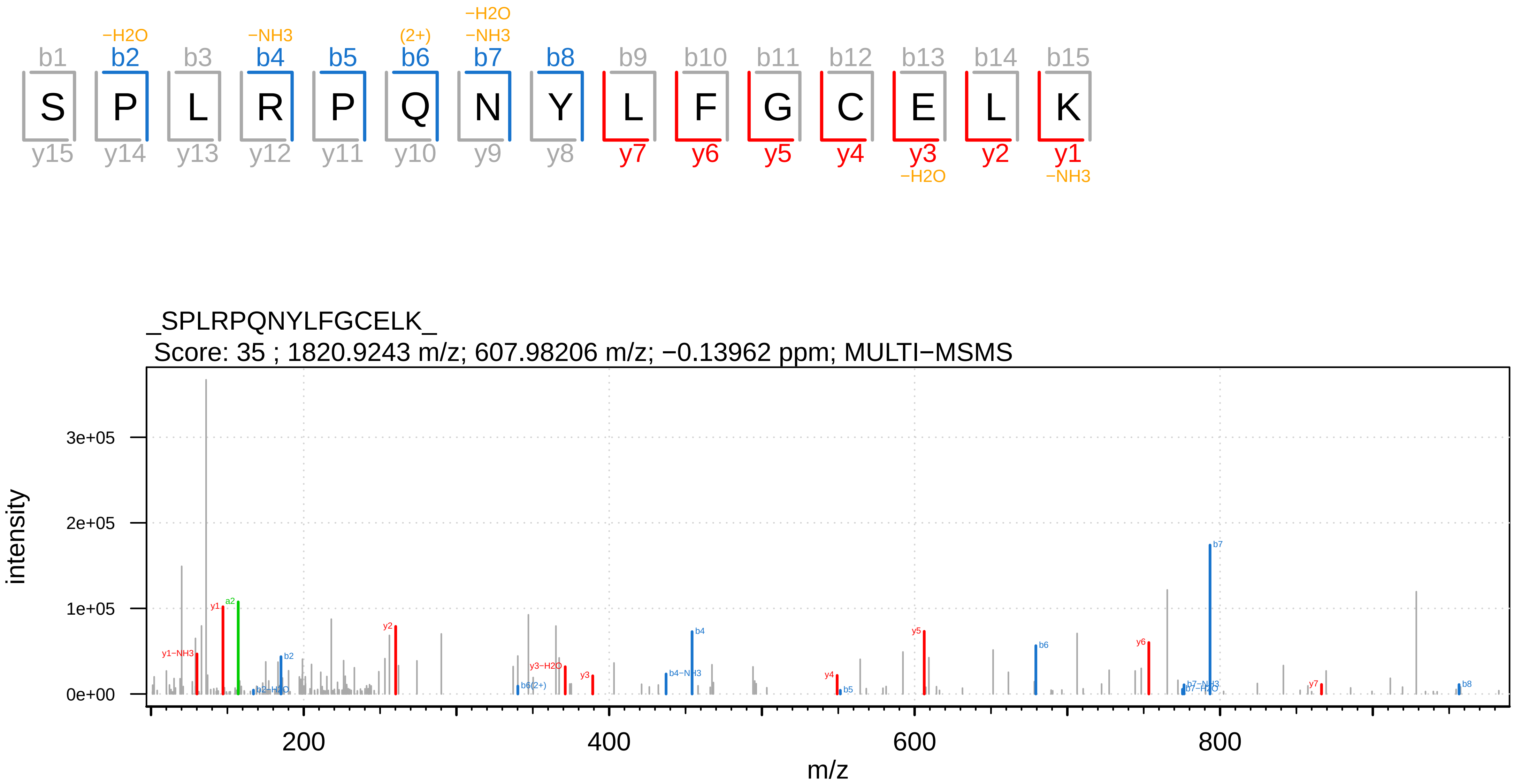

Raw File: 20100719_Velos1_TaGe_SA_LnCap_1
Scan Number: 18162
Proteins:
TCONS_l2_00008829_chr15:92829088−92829258:+

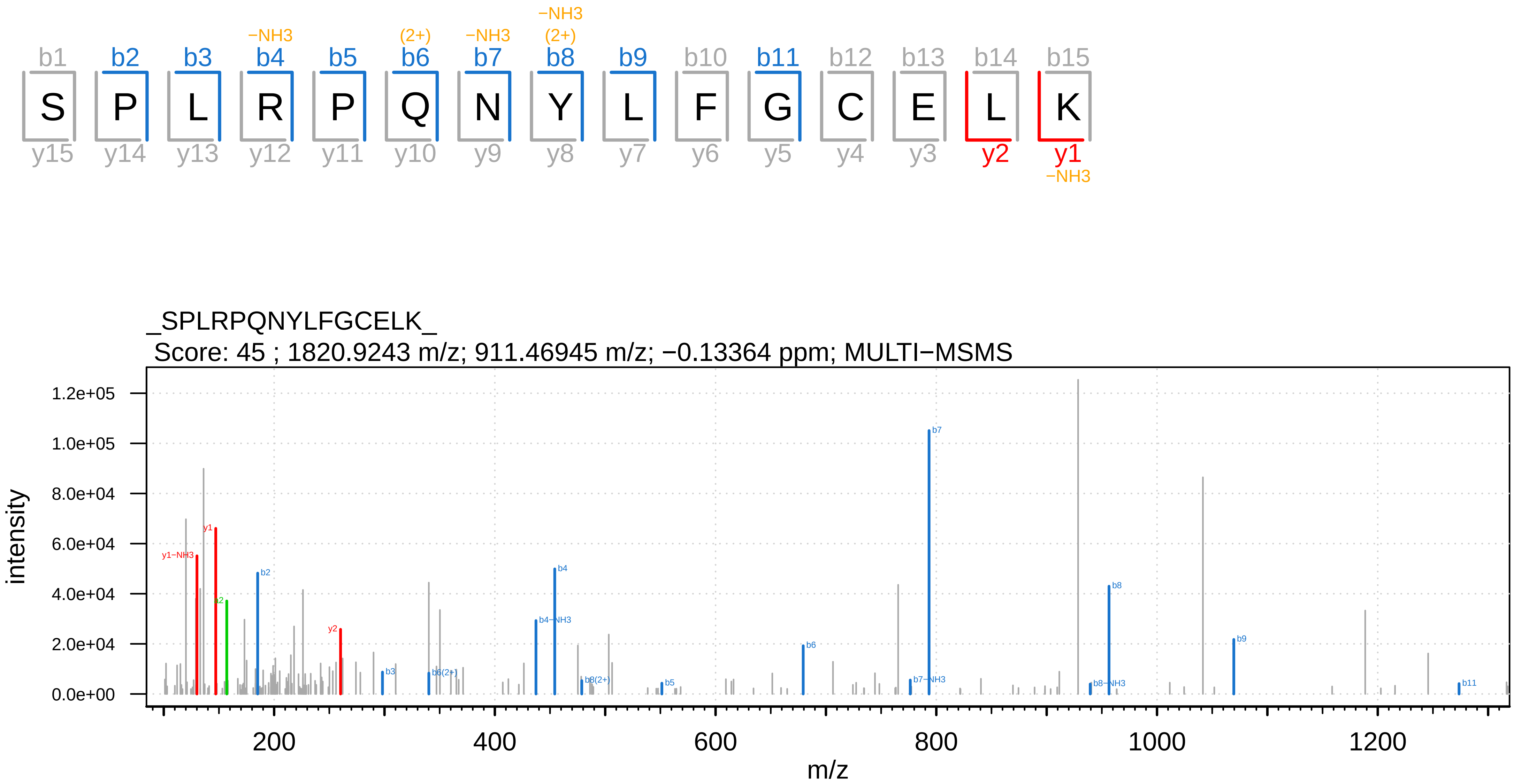

Raw File: 20100719_Velos1_TaGe_SA_LnCap_1
Scan Number: 18173
Proteins:
TCONS_l2_00008829_chr15:92829088−92829258:+

HPPQPQPQPQPQPQPEPSPWGPLDDVR
Score: 83 ; 3048.4842 m/z; 1017.1687 m/z; 0.31452 ppm; MULTI−MSMS

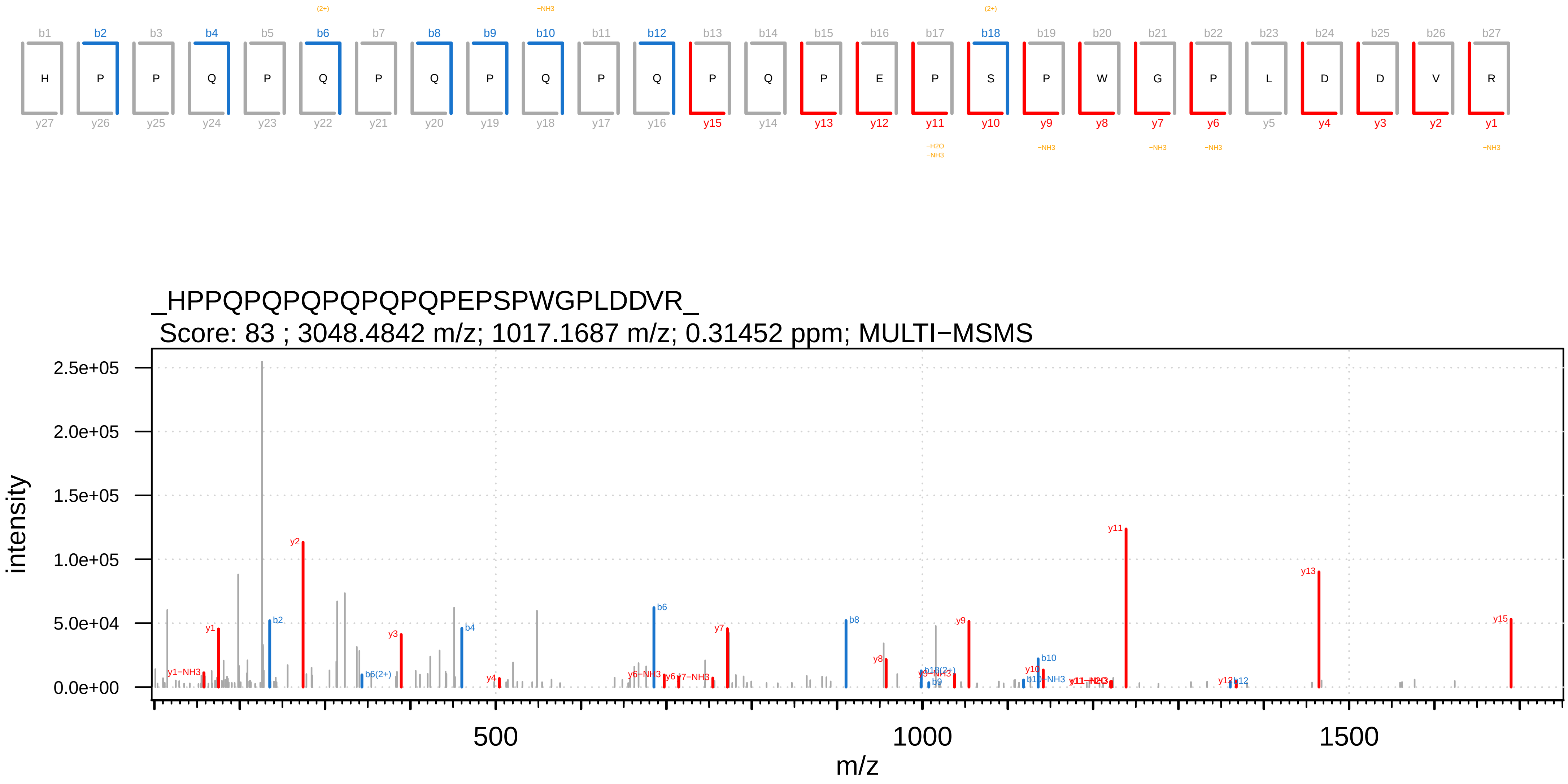

Raw File: 20100723_Velos1_TaGe_SA_Gamg_1
Scan Number: 19123
Proteins:
ENST00000424358_chr20:33865517−33865732:−

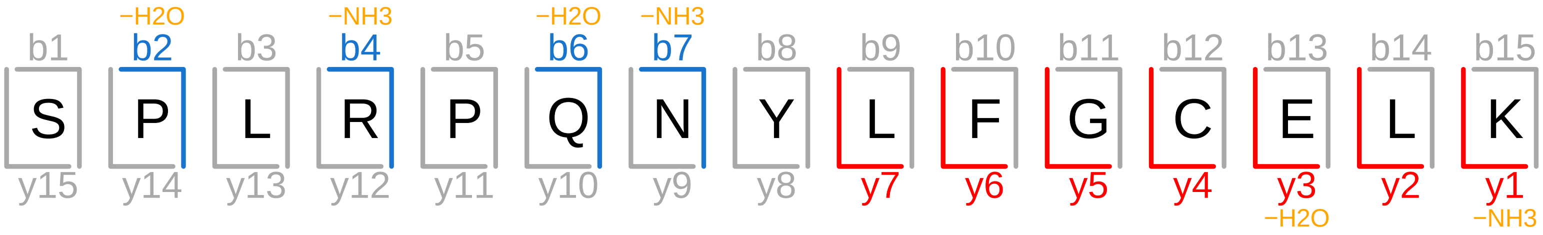

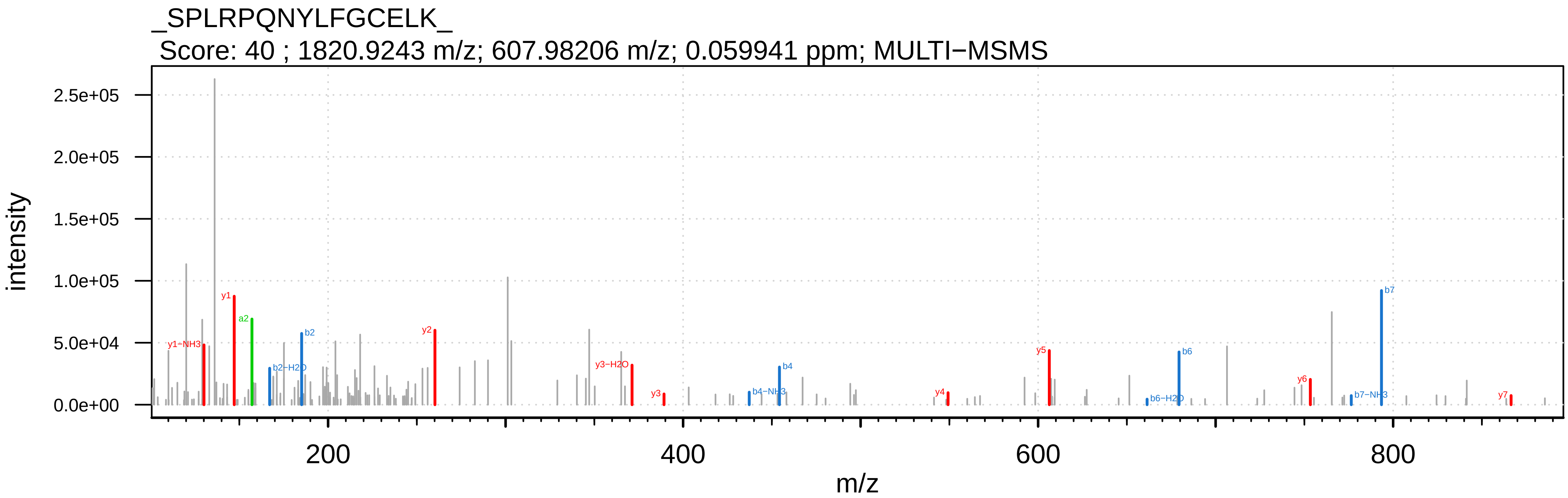

Raw File: 20100723_Velos1_TaGe_SA_Gamg_1
Scan Number: 18354
Proteins:
TCONS_l2_00008829_chr15:92829088−92829258:+

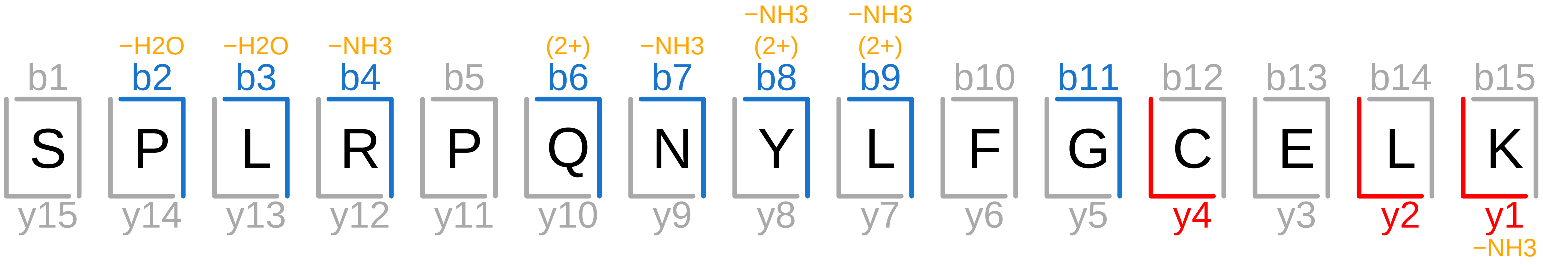

SPLRPQNYLFGCELK
Score: 56 ; 1820.9243 m/z; 911.46945 m/z; −0.22532 ppm; MULTI−MSMS

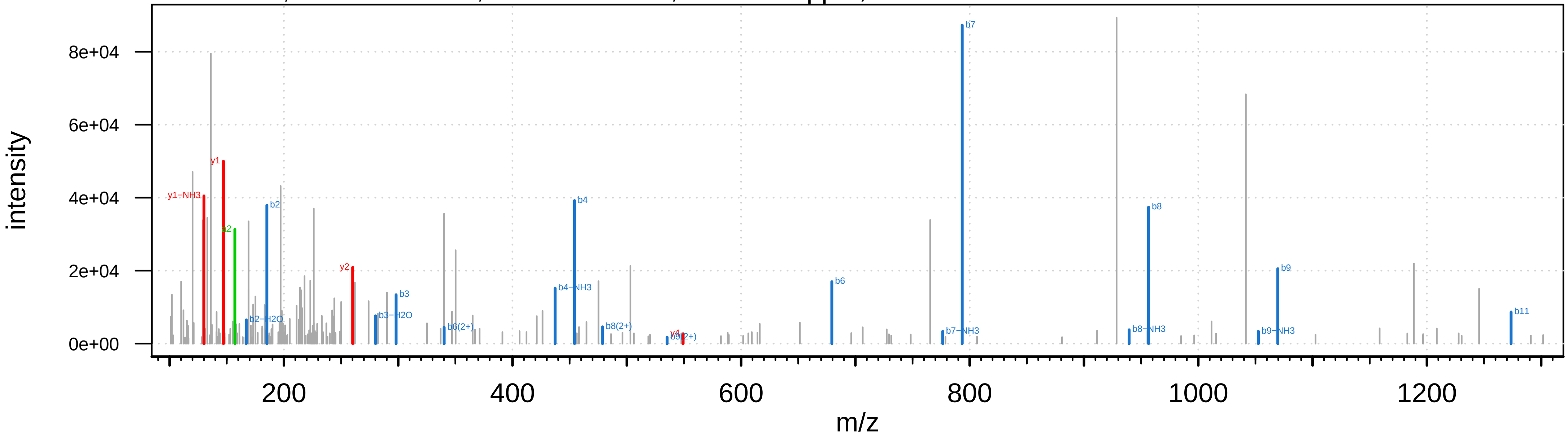

Raw File: 20100723_Velos1_TaGe_SA_Gamg_1
Scan Number: 18363
Proteins:
TCONS_l2_00008829_chr15:92829088−92829258:+

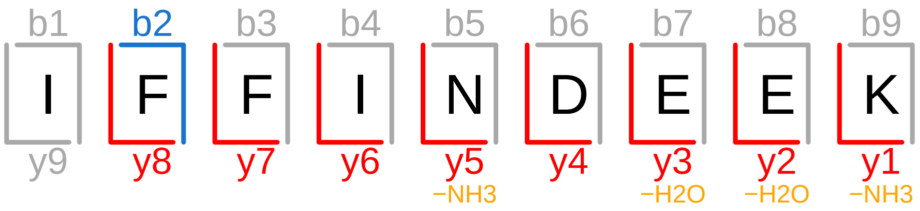

IFFINDEEK
Score: 69 ; 1153.5655 m/z; 577.79005 m/z; −0.30868 ppm; MULTI−MSMS

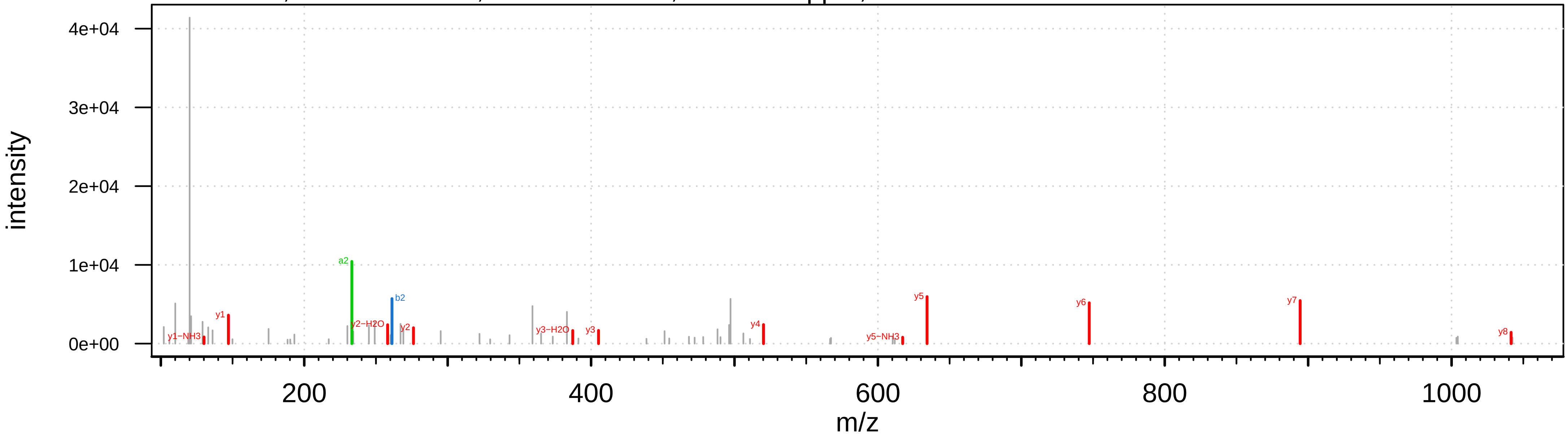

Raw File: 20100604_Velos1_TaGe_SA_A549_5
Scan Number: 11134
Proteins:
TCONS_l2_00001718_chr1:204336923−204346793:−

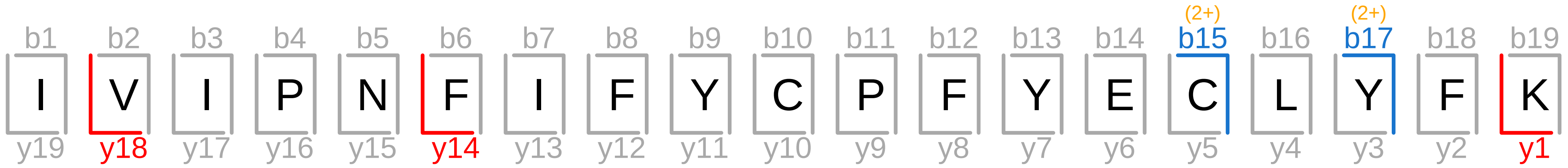

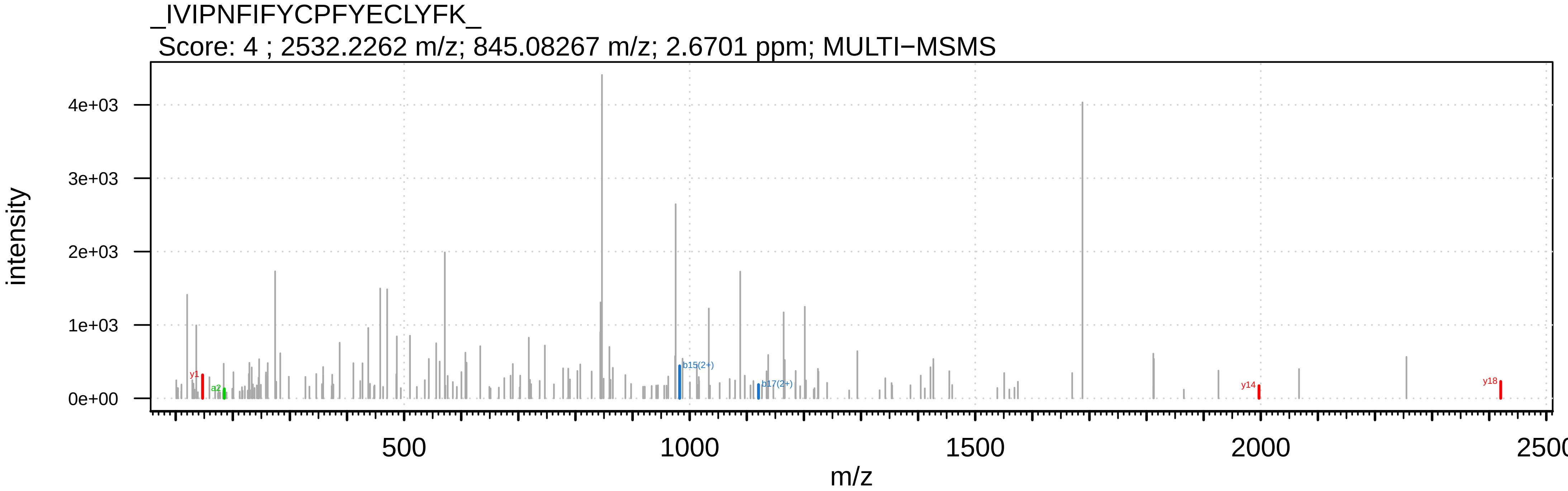

Raw File: 20101227_Velos1_TaGe_SA_GAMG6
Scan Number: 35131
Proteins:
ENST00000291592_chr21:45878569−45878637:+

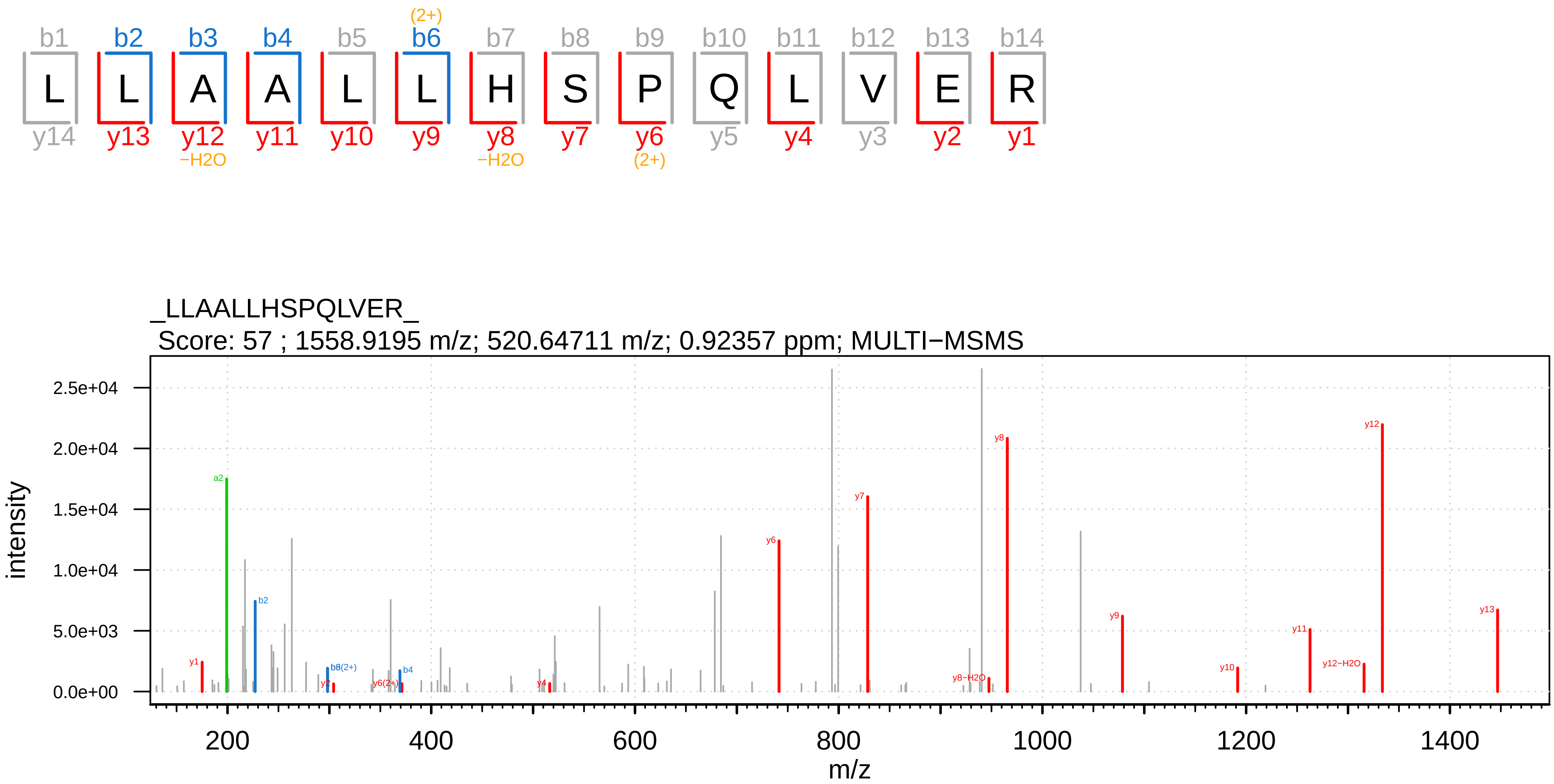

Raw File: 20100616_Velos1_TaGe_SA_MCF7_3
Scan Number: 23325
Proteins:
ENST00000602845_chr3:196669588−196669887:+

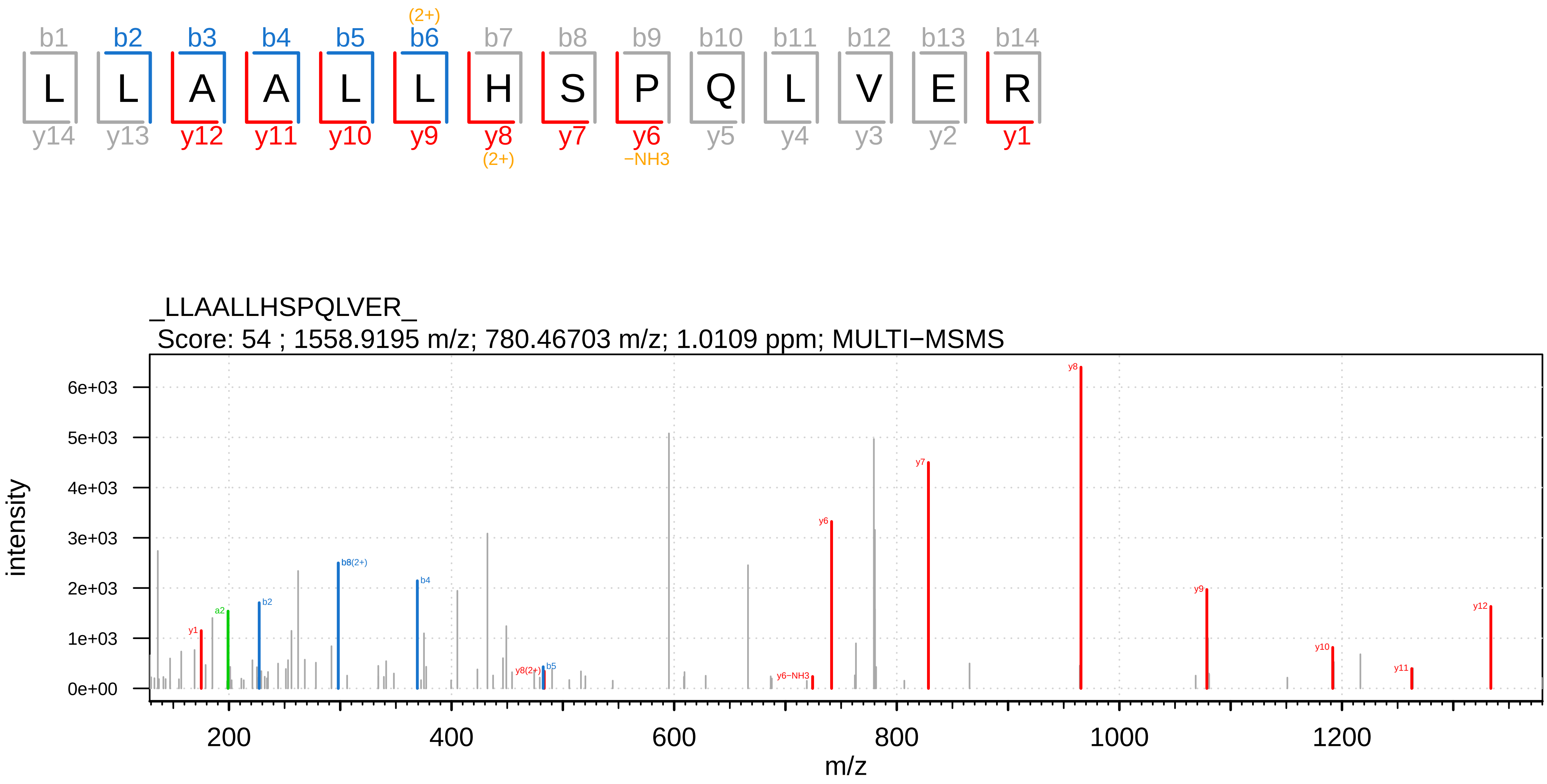

Raw File: 20100616_Velos1_TaGe_SA_MCF7_3
Scan Number: 23362
Proteins:
ENST00000602845_chr3:196669588−196669887:+

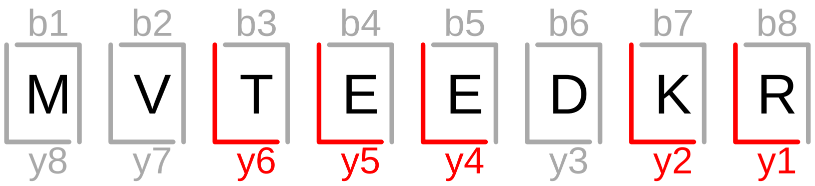

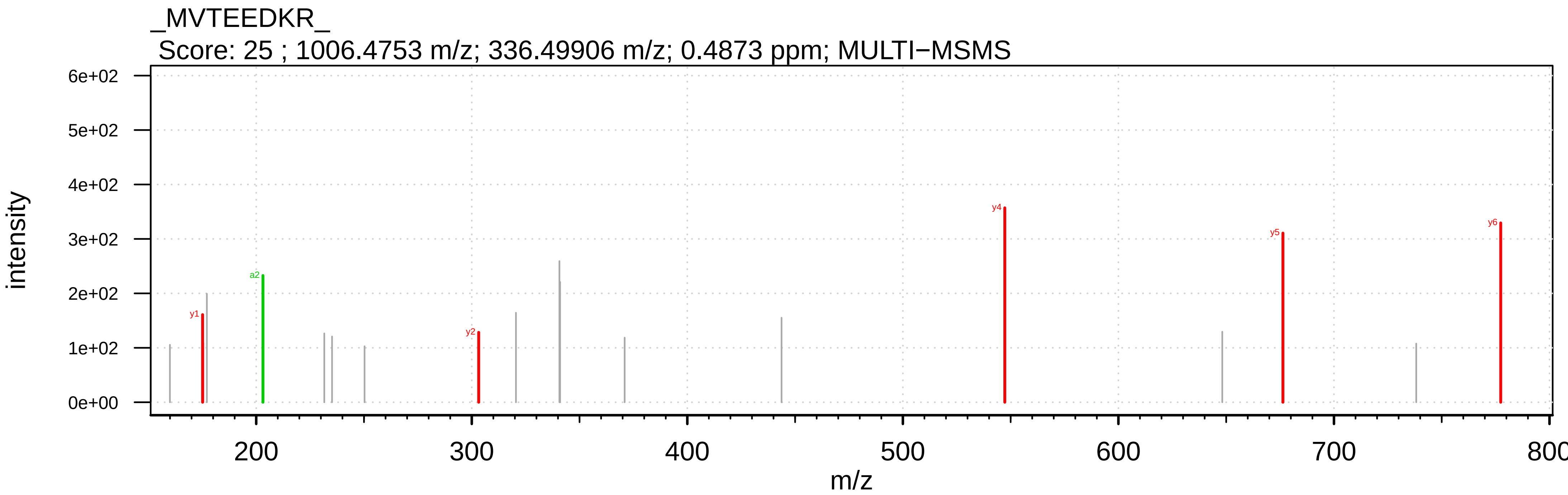

Raw File: 20100616_Velos1_TaGe_SA_MCF7_3
Scan Number: 1594
Proteins:
TCONS_l2_00006768_chr13:29174091−29174123:+

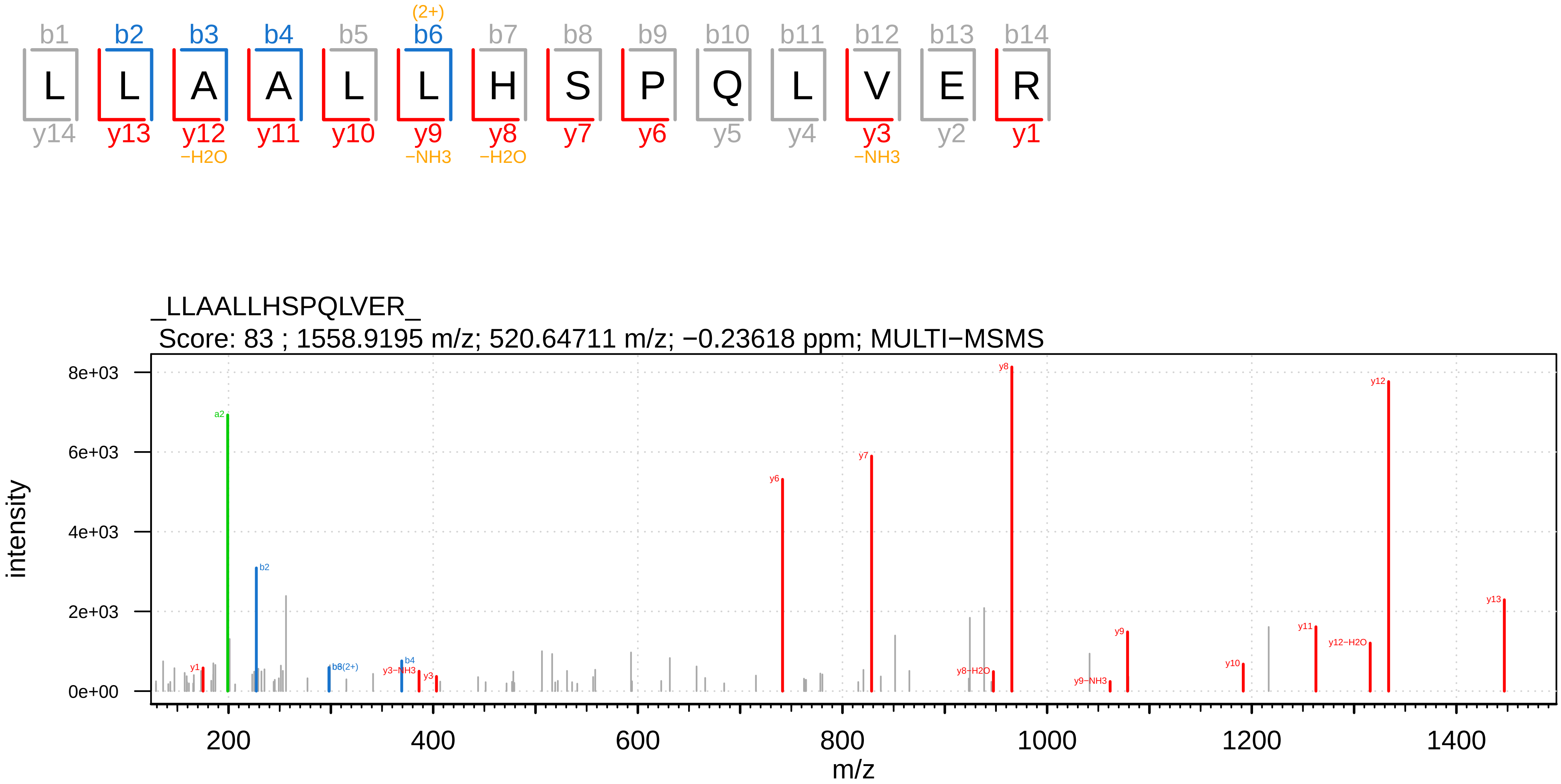

Raw File: 20100616_Velos1_TaGe_SA_MCF7_4
Scan Number: 19997
Proteins:
ENST00000602845_chr3:196669588−196669887:+

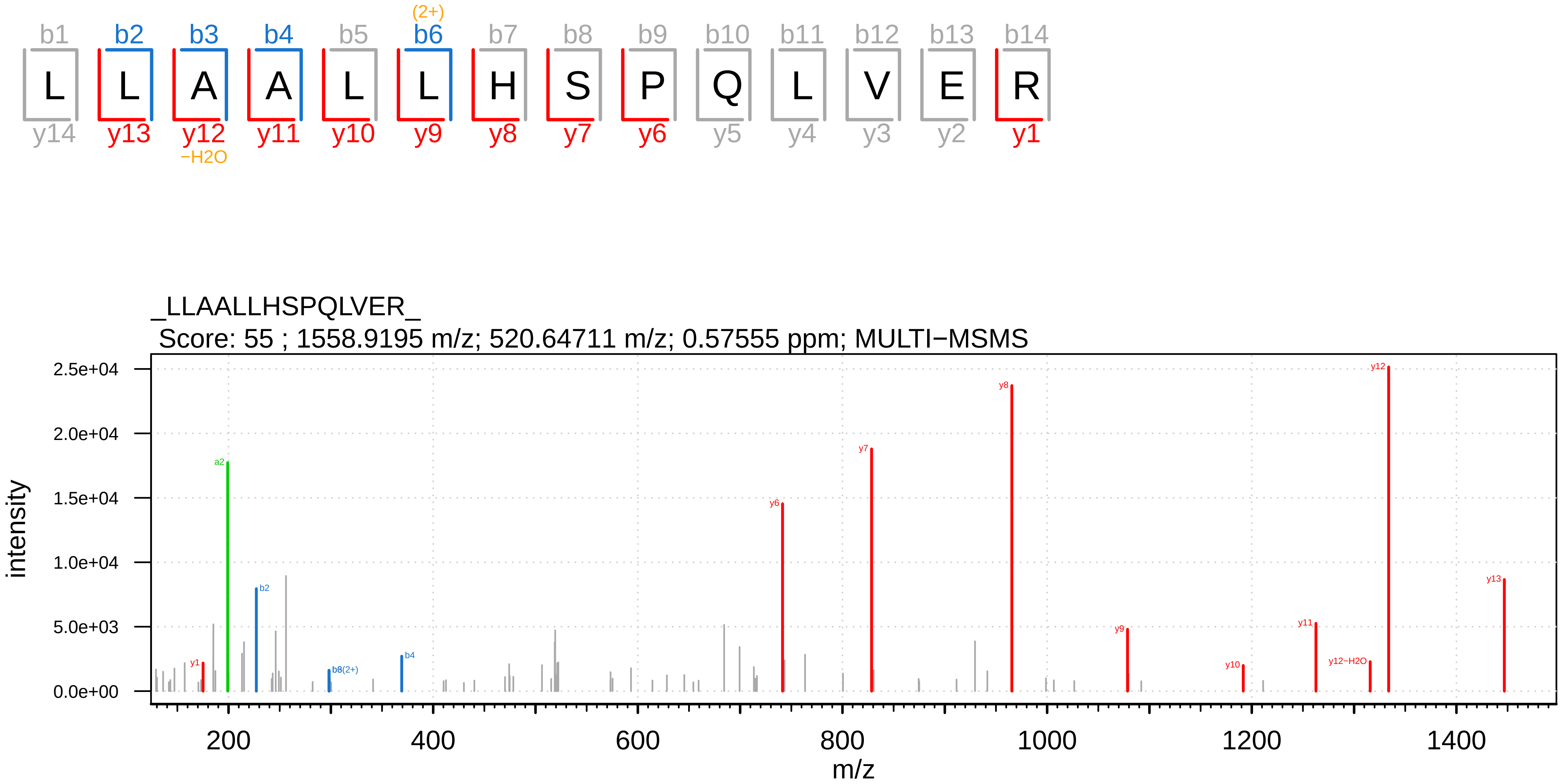

Raw File: 20100616_Velos1_TaGe_SA_RKO_1
Scan Number: 27484
Proteins:
ENST00000602845_chr3:196669588−196669887:+

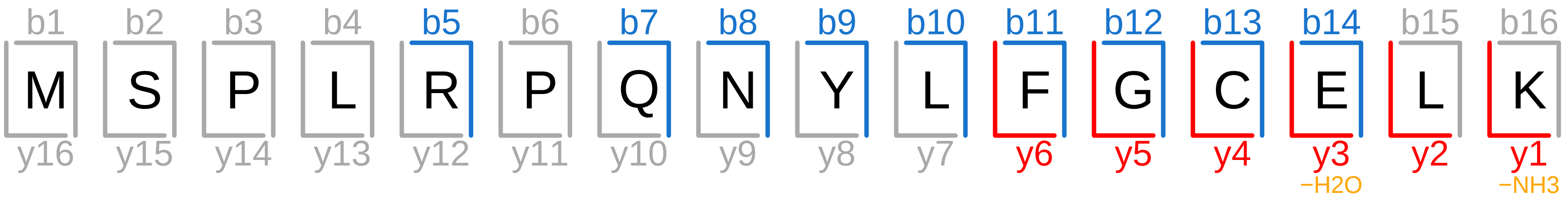

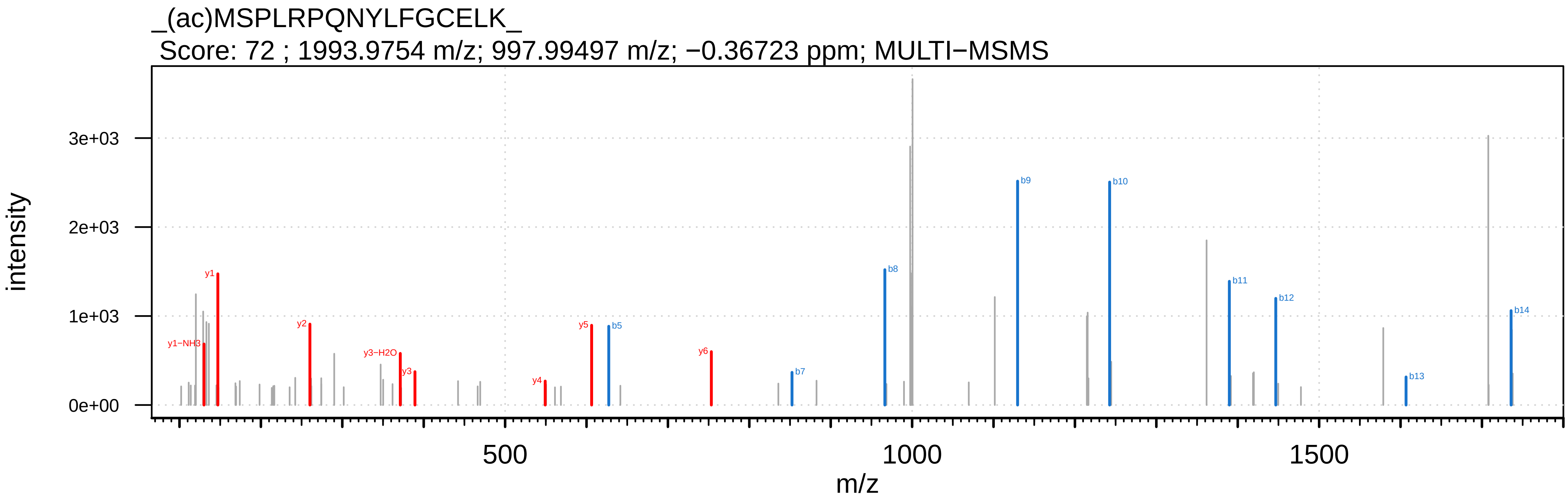

Raw File: 20100616_Velos1_TaGe_SA_RKO_1
Scan Number: 36901
Proteins:
TCONS_l2_00008829_chr15:92829088−92829258:+

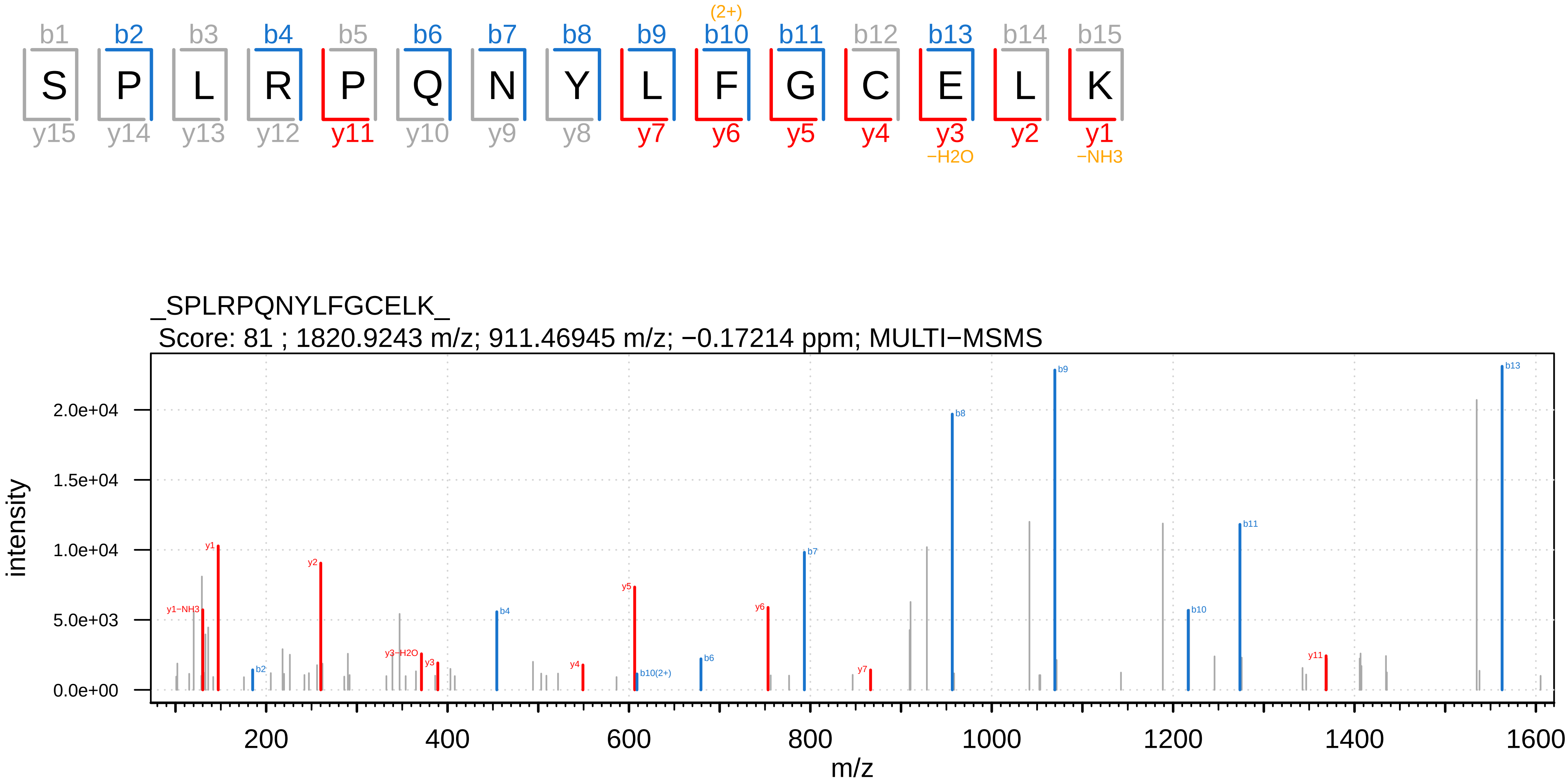

Raw File: 20100616_Velos1_TaGe_SA_RKO_1
Scan Number: 22671
Proteins:
TCONS_l2_00008829_chr15:92829088−92829258:+

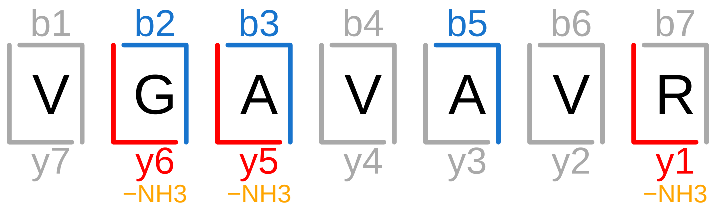

VGAVAVR
Score: 68 ; 670.41261 m/z; 336.21358 m/z; 0.64034 ppm; MULTI−MSMS

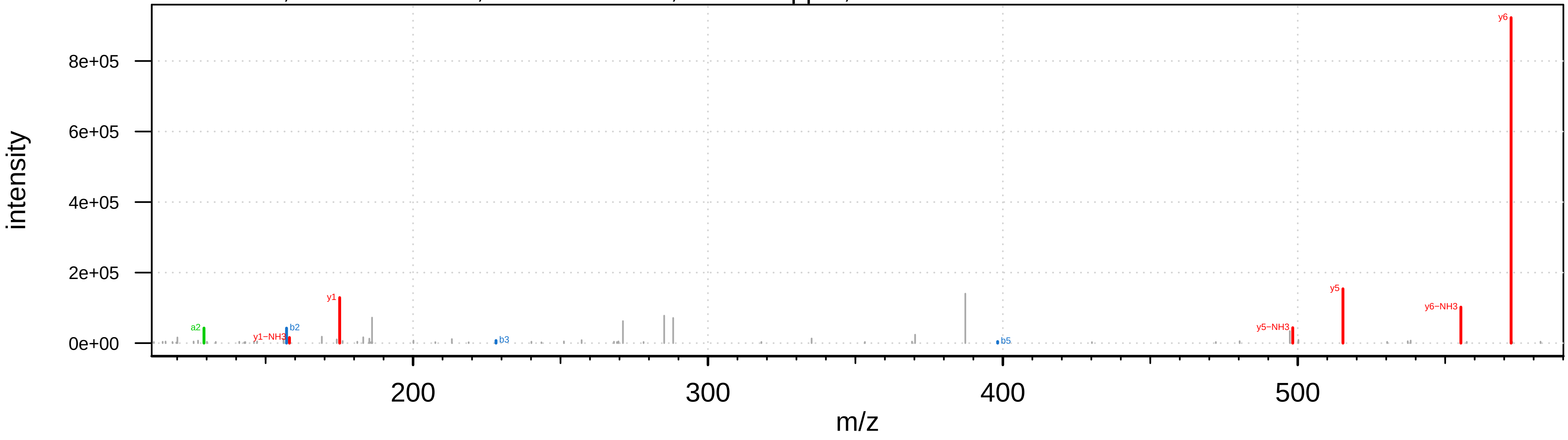

Raw File: 20100616_Velos1_TaGe_SA_RKO_1
Scan Number: 2969
Proteins:
TCONS_l2_00030545_chrX:79544539−79546436:−

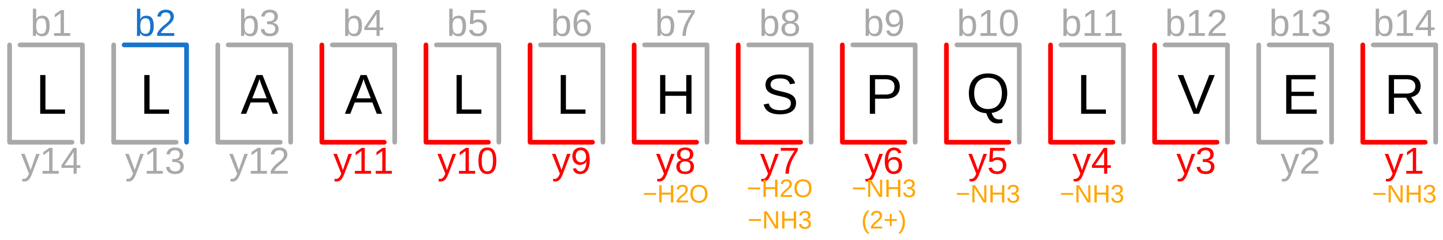

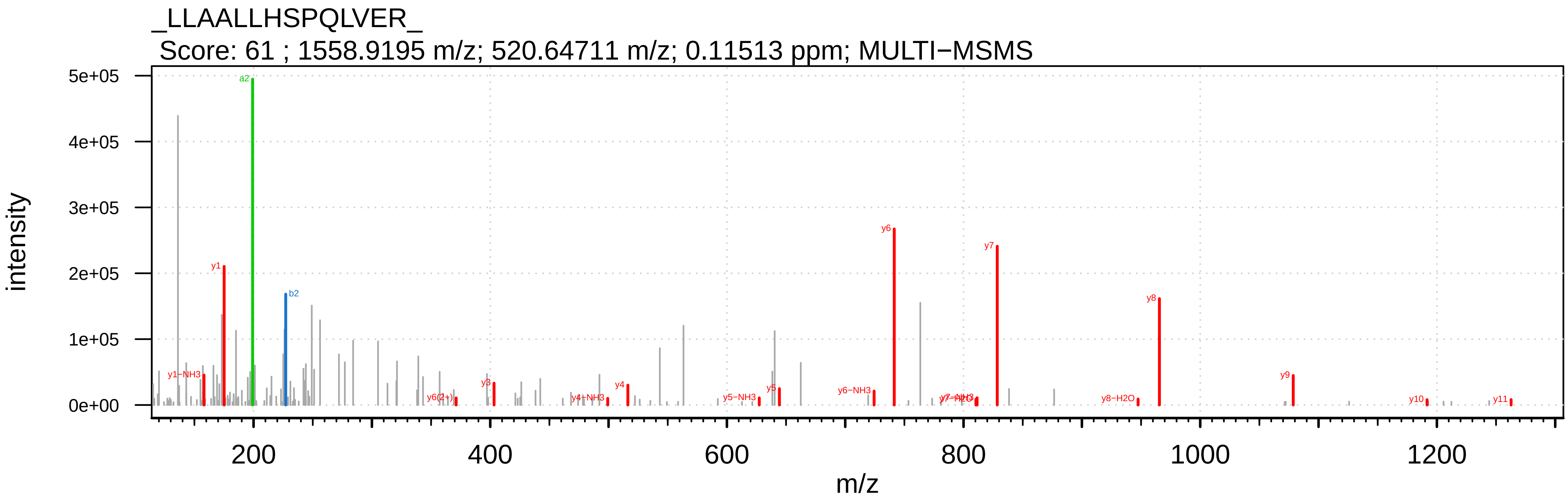

Raw File: 20100719_Velos1_TaGe_SA_MCF7_01
Scan Number: 26418
Proteins:
ENST00000602845_chr3:196669588−196669887:+

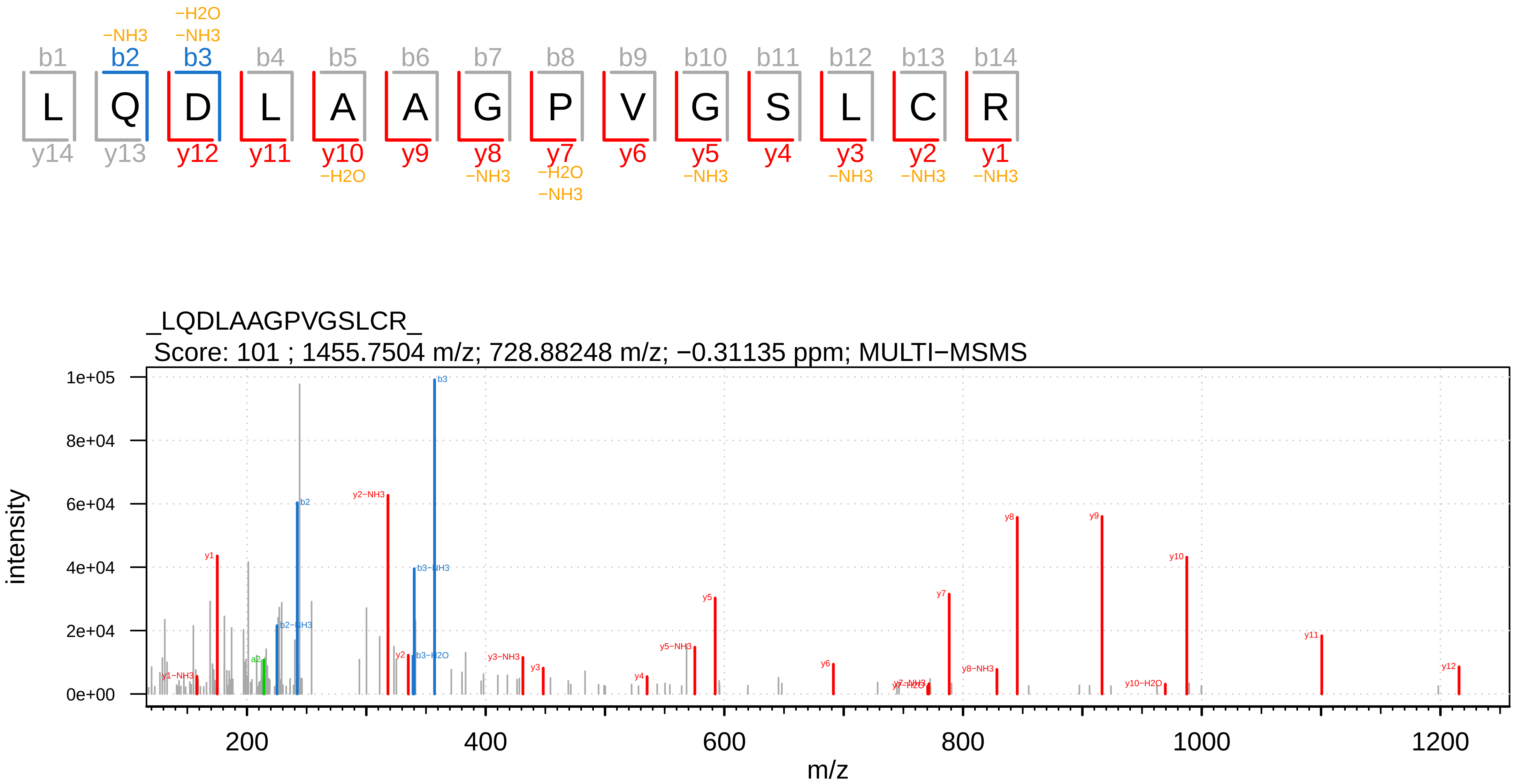

Raw File: 20100719_Velos1_TaGe_SA_MCF7_01
Scan Number: 22824
Proteins:
ENST00000602845_chr3:196669588−196669887:+

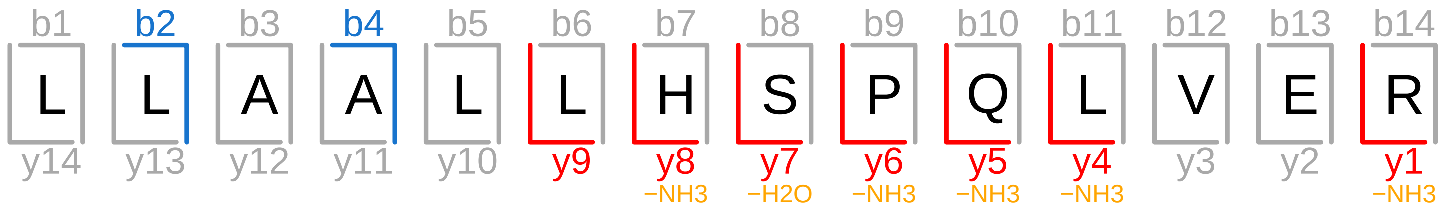

LLAALLHSPQLVER
Score: 48 ; 1558.9195 m/z; 520.64711 m/z; 0.10264 ppm; MULTI−MSMS

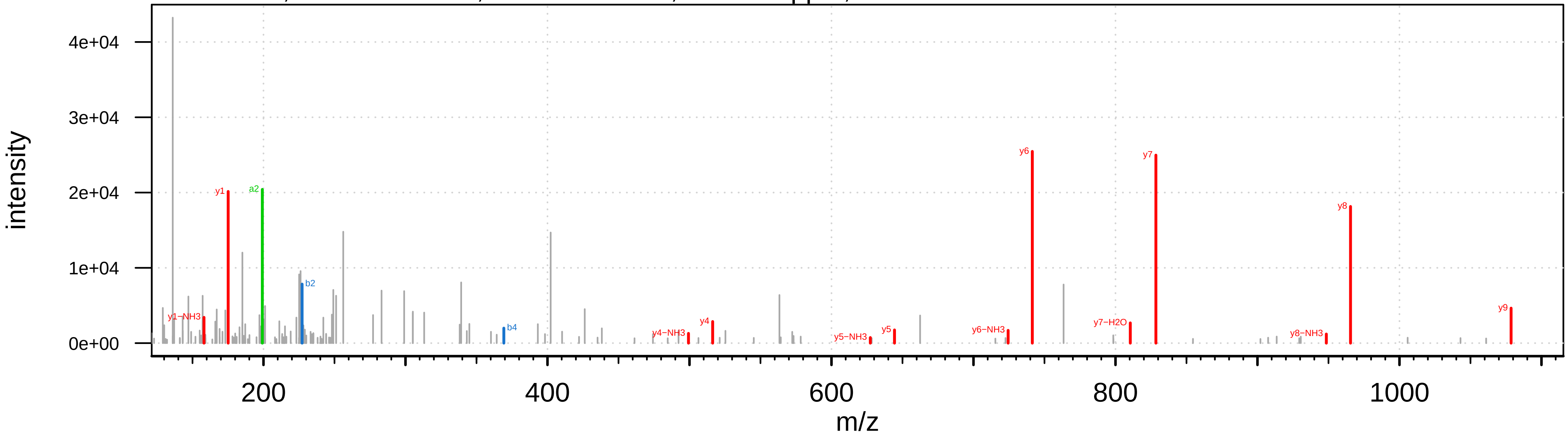

Raw File: 20100719_Velos1_TaGe_SA_MCF7_02
Scan Number: 23454
Proteins:
ENST00000602845_chr3:196669588−196669887:+

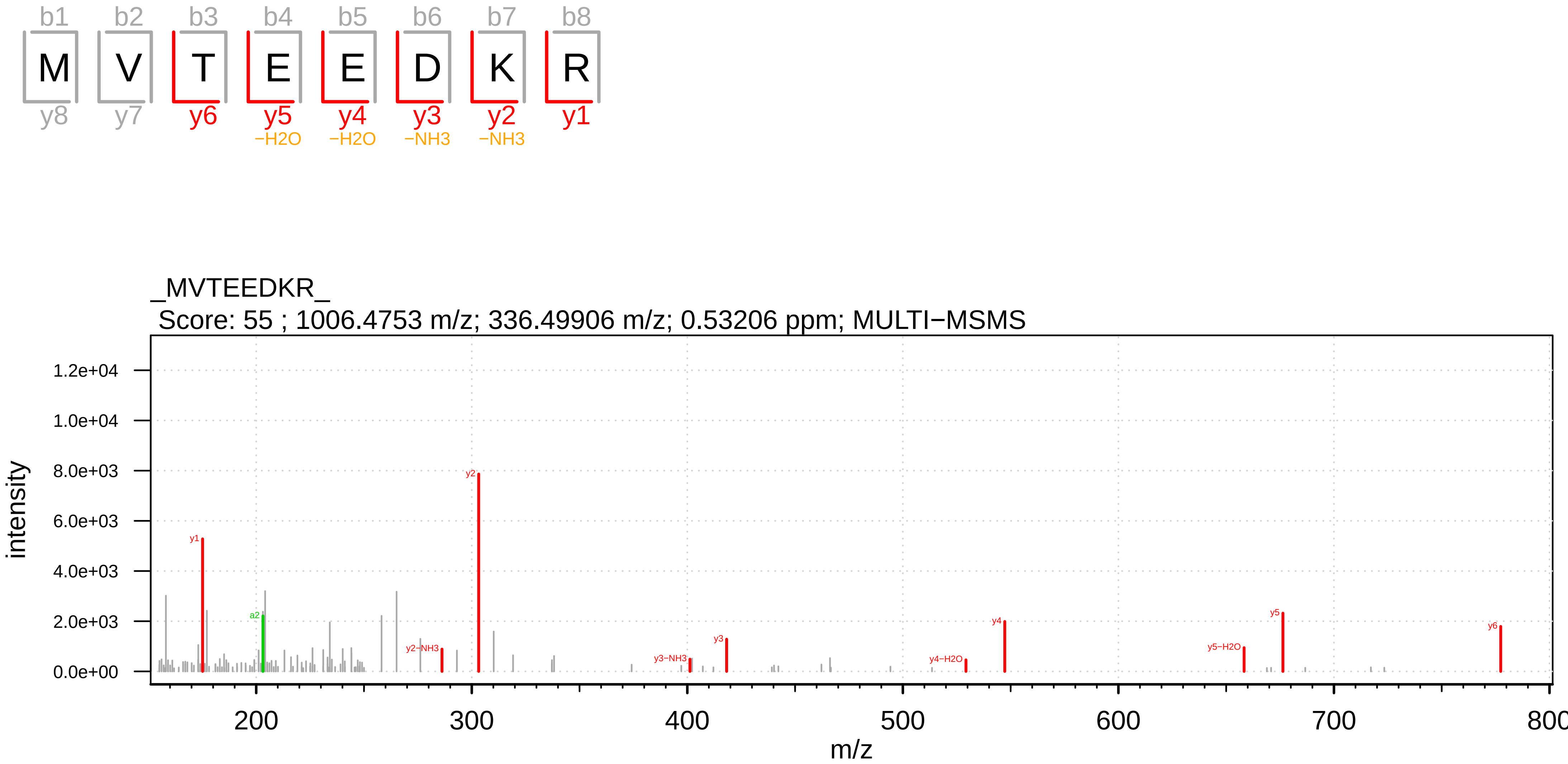

Raw File: 20100719_Velos1_TaGe_SA_MCF7_02
Scan Number: 4195
Proteins:
TCONS_l2_00006768_chr13:29174091−29174123:+

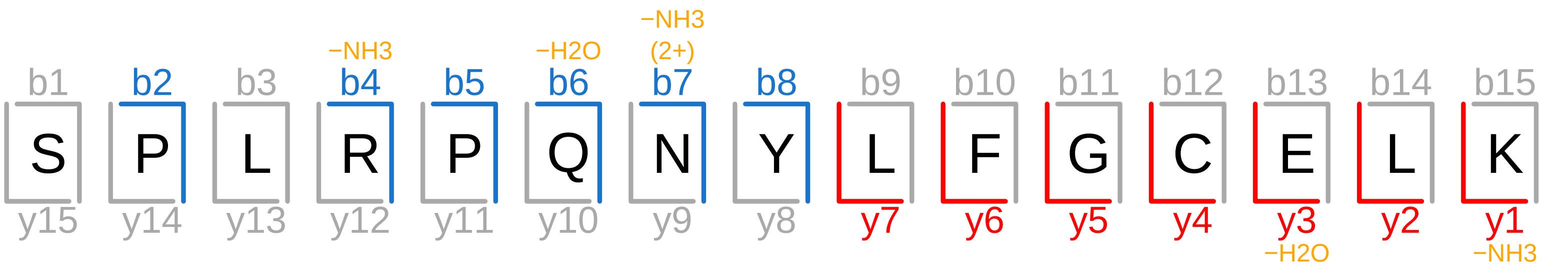

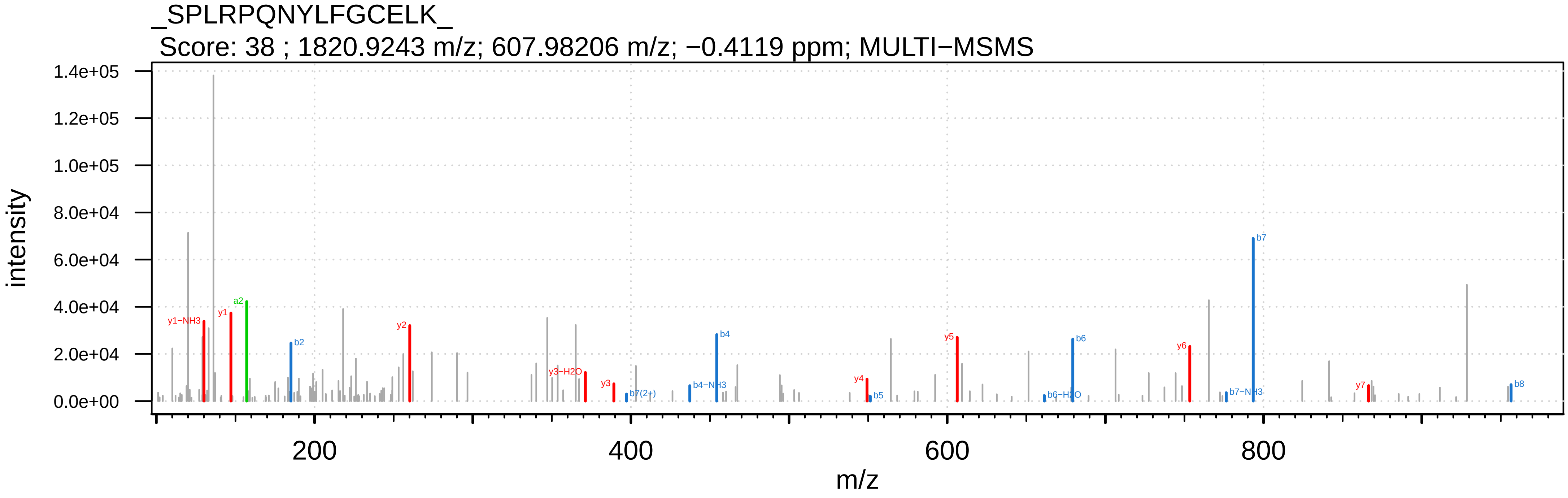

Raw File: 20100719_Velos1_TaGe_SA_MCF7_02

Scan Number: 19997

Proteins:

TCONS_l2_00008829_chr15:92829088−92829258:+

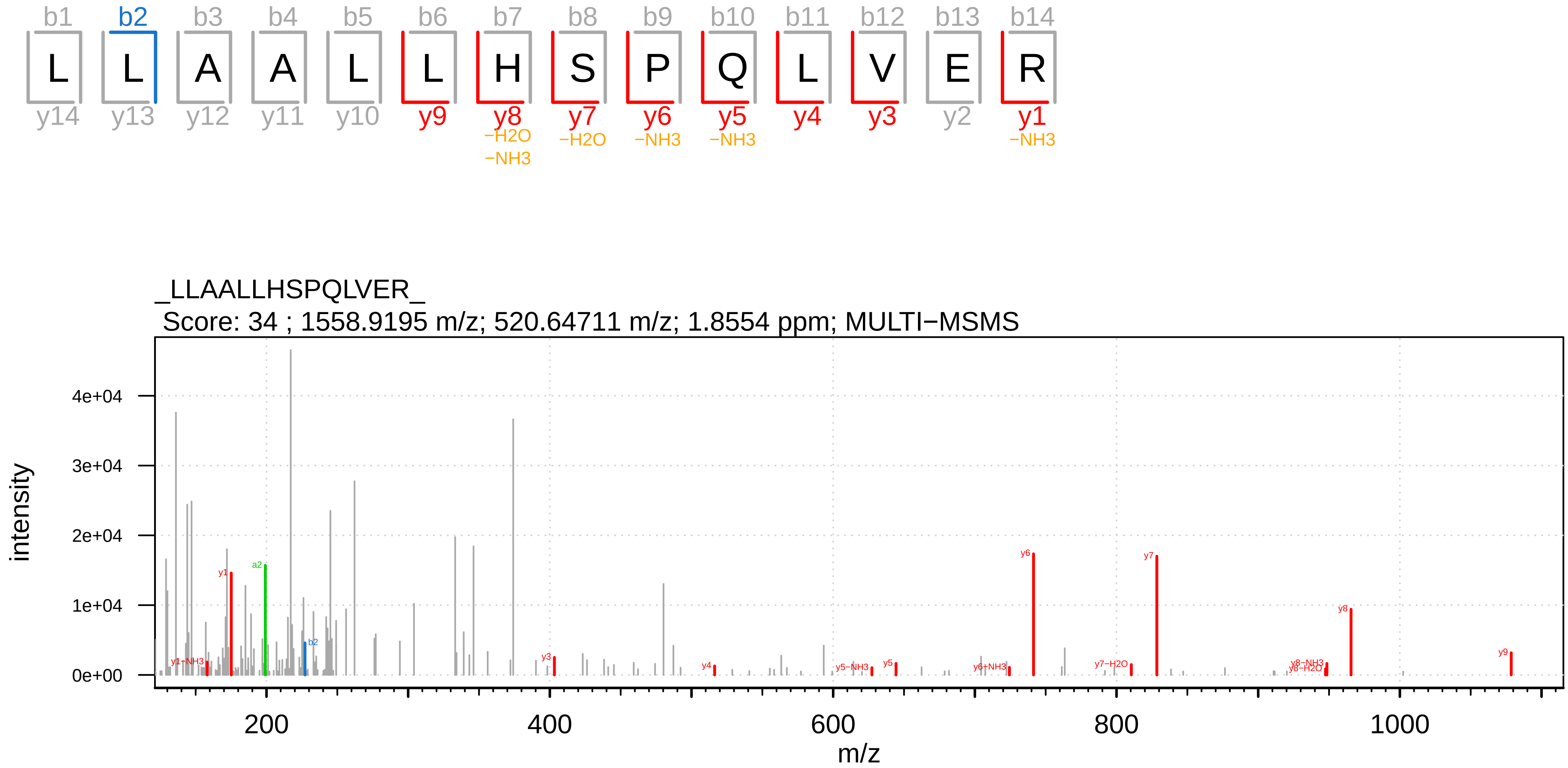

Raw File: 20100719_Velos1_TaGe_SA_MCF7_03
Scan Number: 22752
Proteins:
ENST00000602845_chr3:196669588−196669887:+

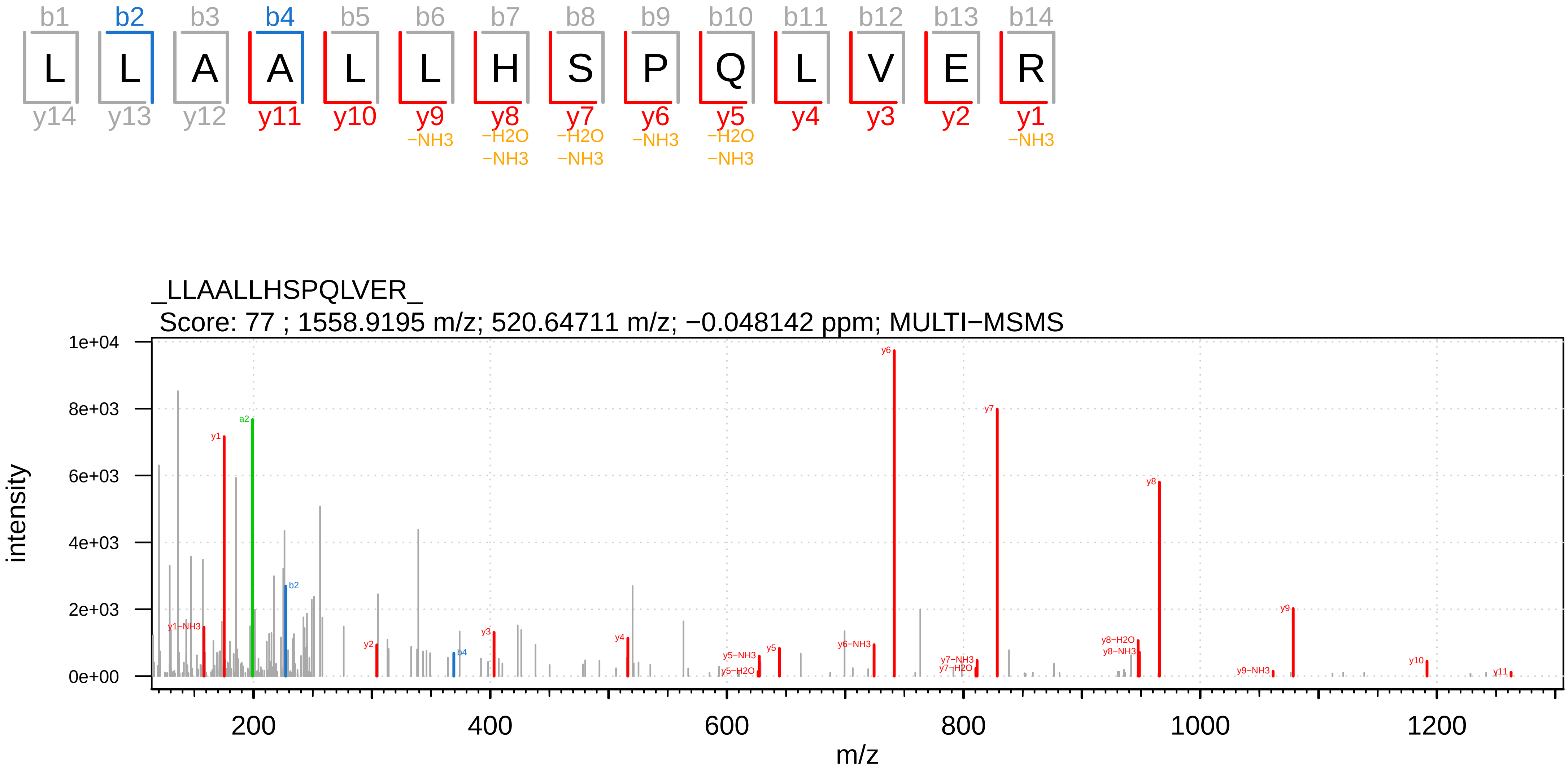

Raw File: 20100726_Velos1_TaGe_SA_HeLa_2
Scan Number: 21111
Proteins:
ENST00000602845_chr3:196669588−196669887:+

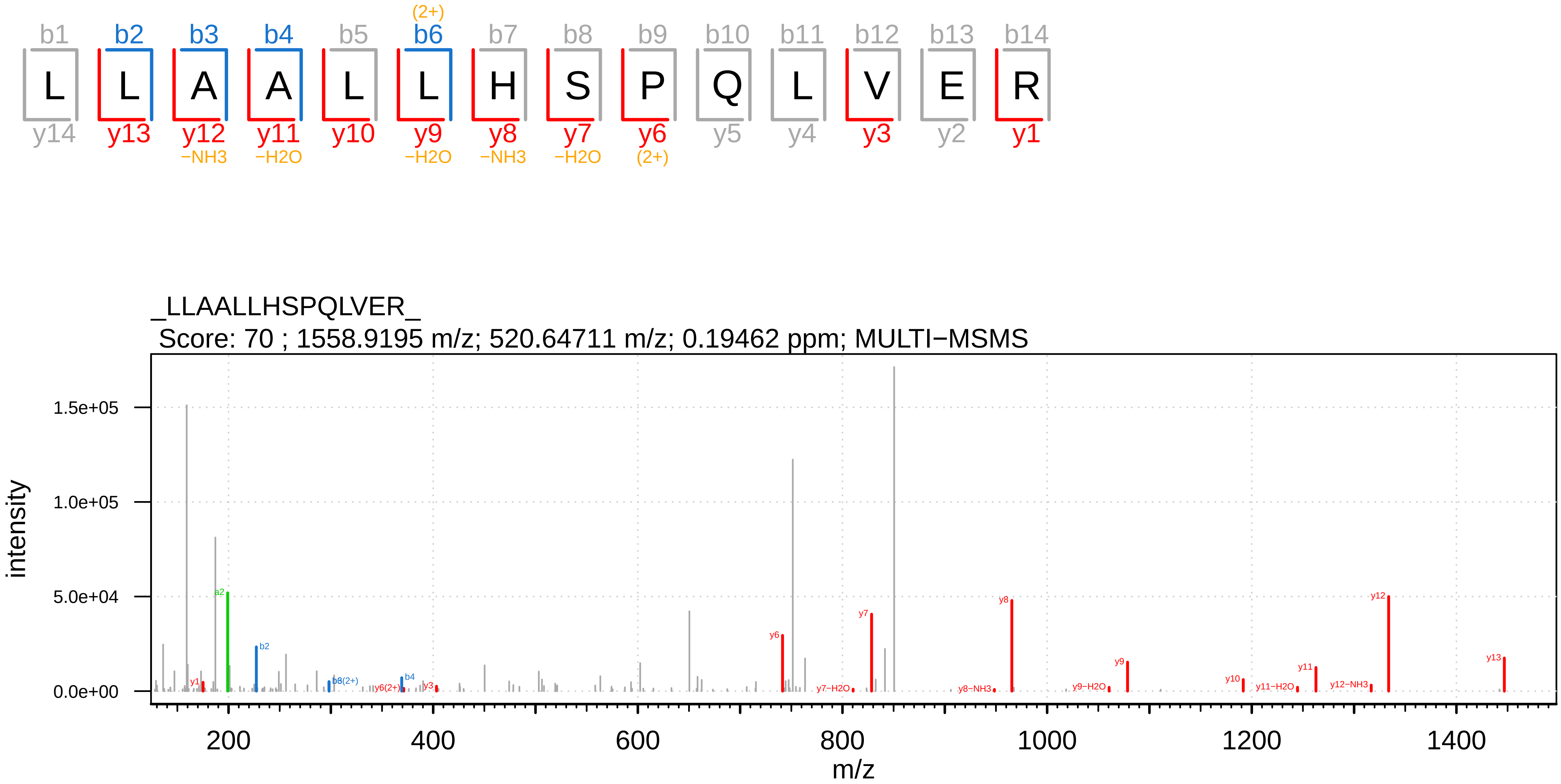

Raw File: 20101210_Velos1_AnWe_SA_MCF7_2
Scan Number: 22820
Proteins:
ENST00000602845_chr3:196669588−196669887:+

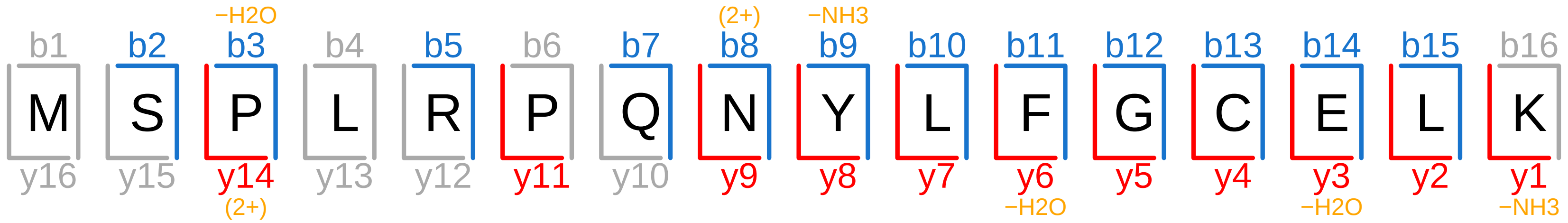

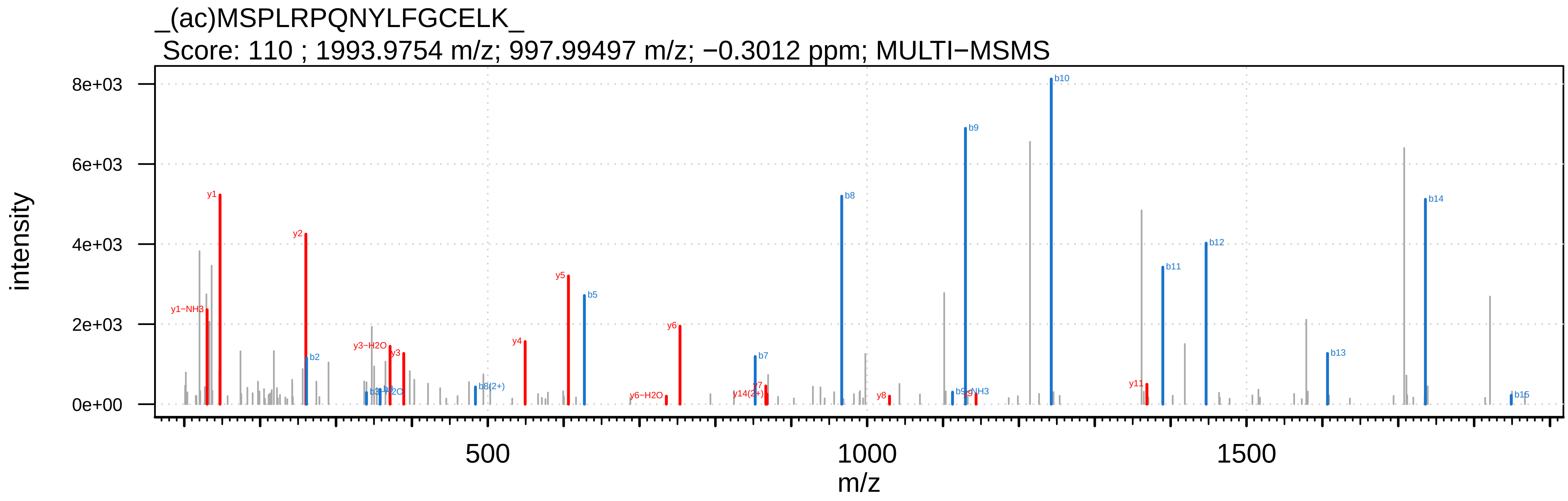

Raw File: 20101210_Velos1_AnWe_SA_MCF7_2
Scan Number: 30652
Proteins:
TCONS_l2_00008829_chr15:92829088−92829258:+

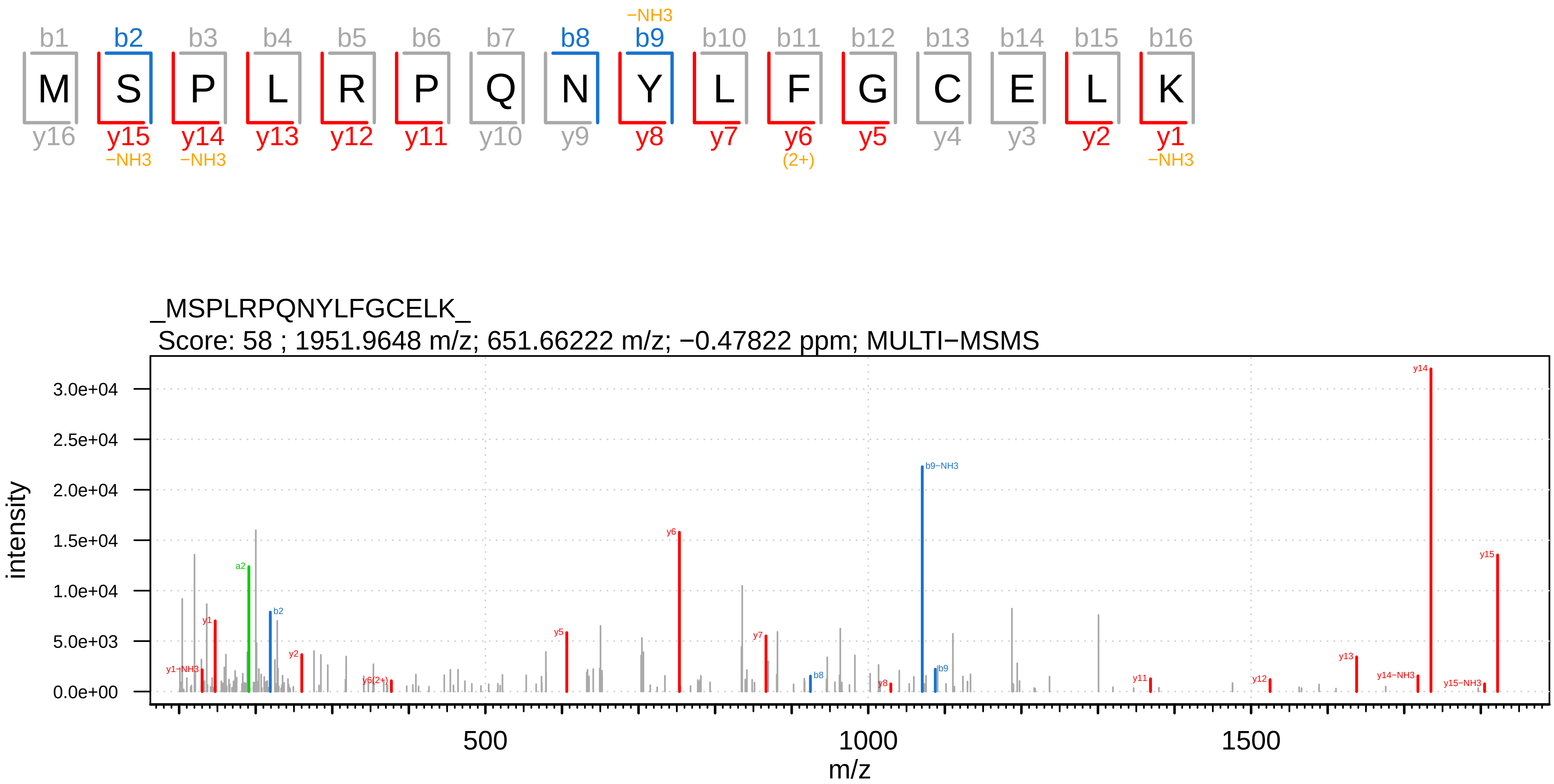

Raw File: 20101210_Velos1_AnWe_SA_MCF7_2
Scan Number: 22814
Proteins:
TCONS_l2_00008829_chr15:92829088−92829258:+

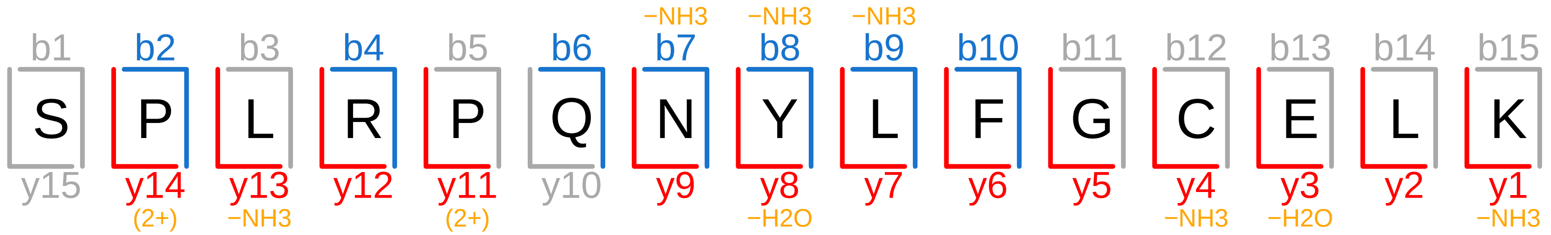

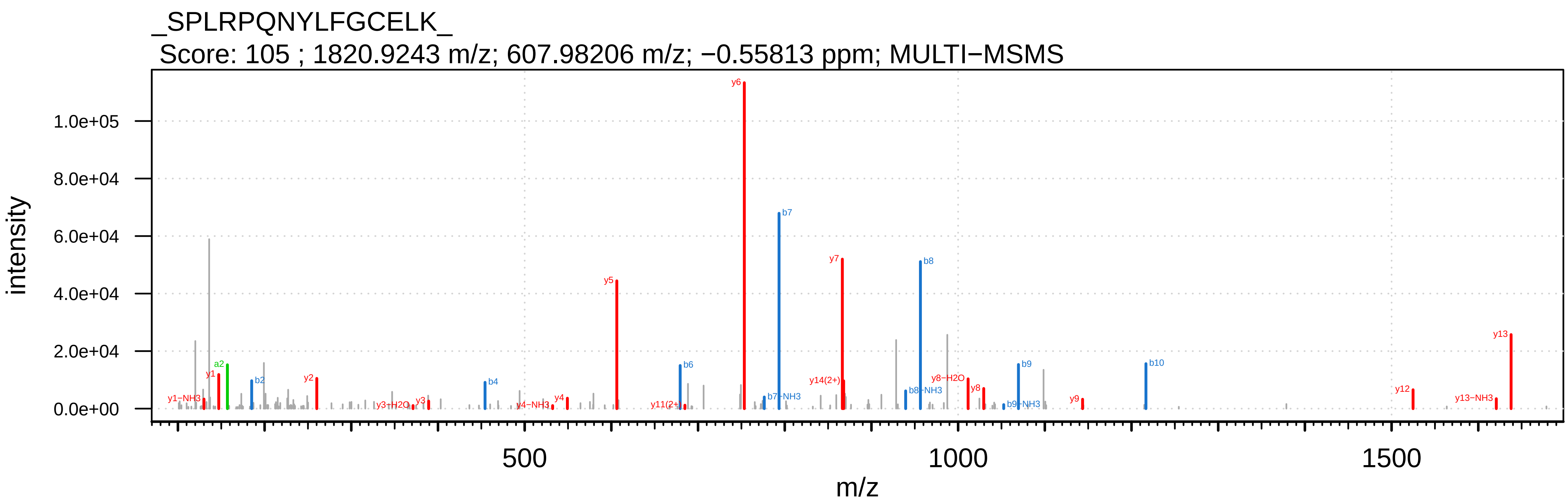

Raw File: 20101210_Velos1_AnWe_SA_MCF7_2
Scan Number: 20040
Proteins:
TCONS_l2_00008829_chr15:92829088−92829258:+

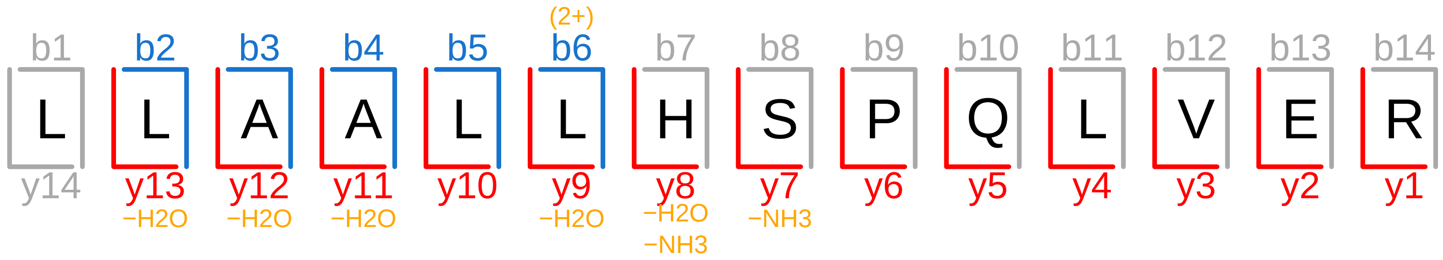

LLAALLHSPQLVER
Score: 100 ; 1558.9195 m/z; 520.64711 m/z; 0.073501 ppm; MULTI−MSMS

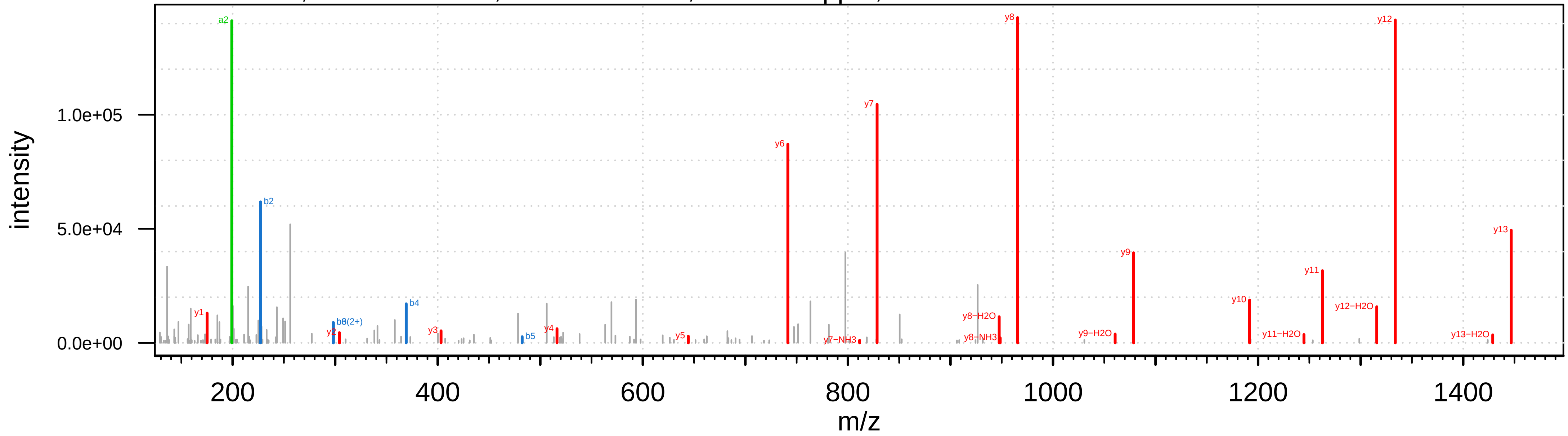

Raw File: 20101210_Velos1_AnWe_SA_MCF7_3
Scan Number: 22064
Proteins:
ENST00000602845_chr3:196669588−196669887:+

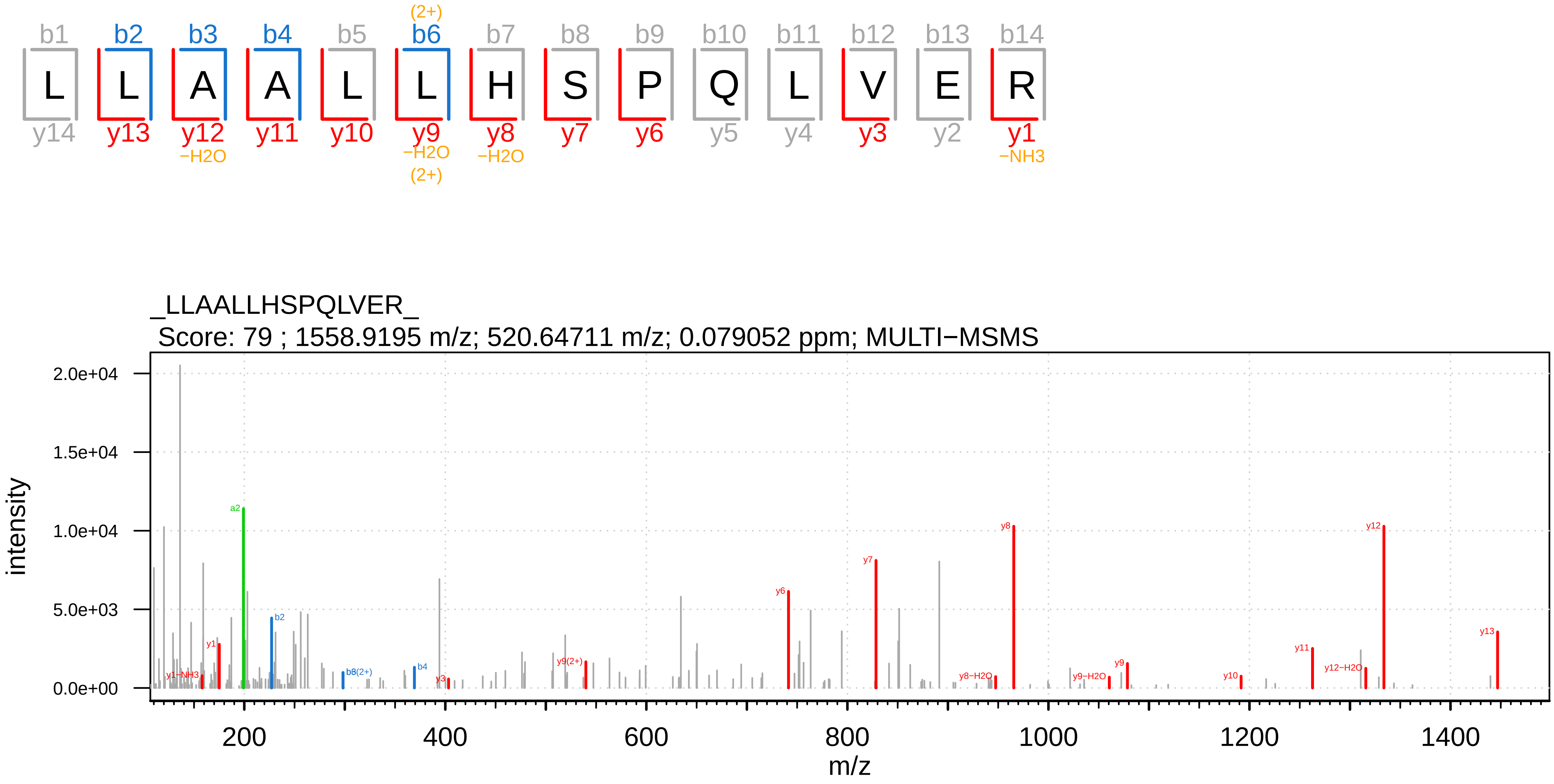

Raw File: 20101210_Velos1_AnWe_SA_MCF7_4

Scan Number: 20013

Proteins:

ENST00000602845_chr3:196669588−196669887:+

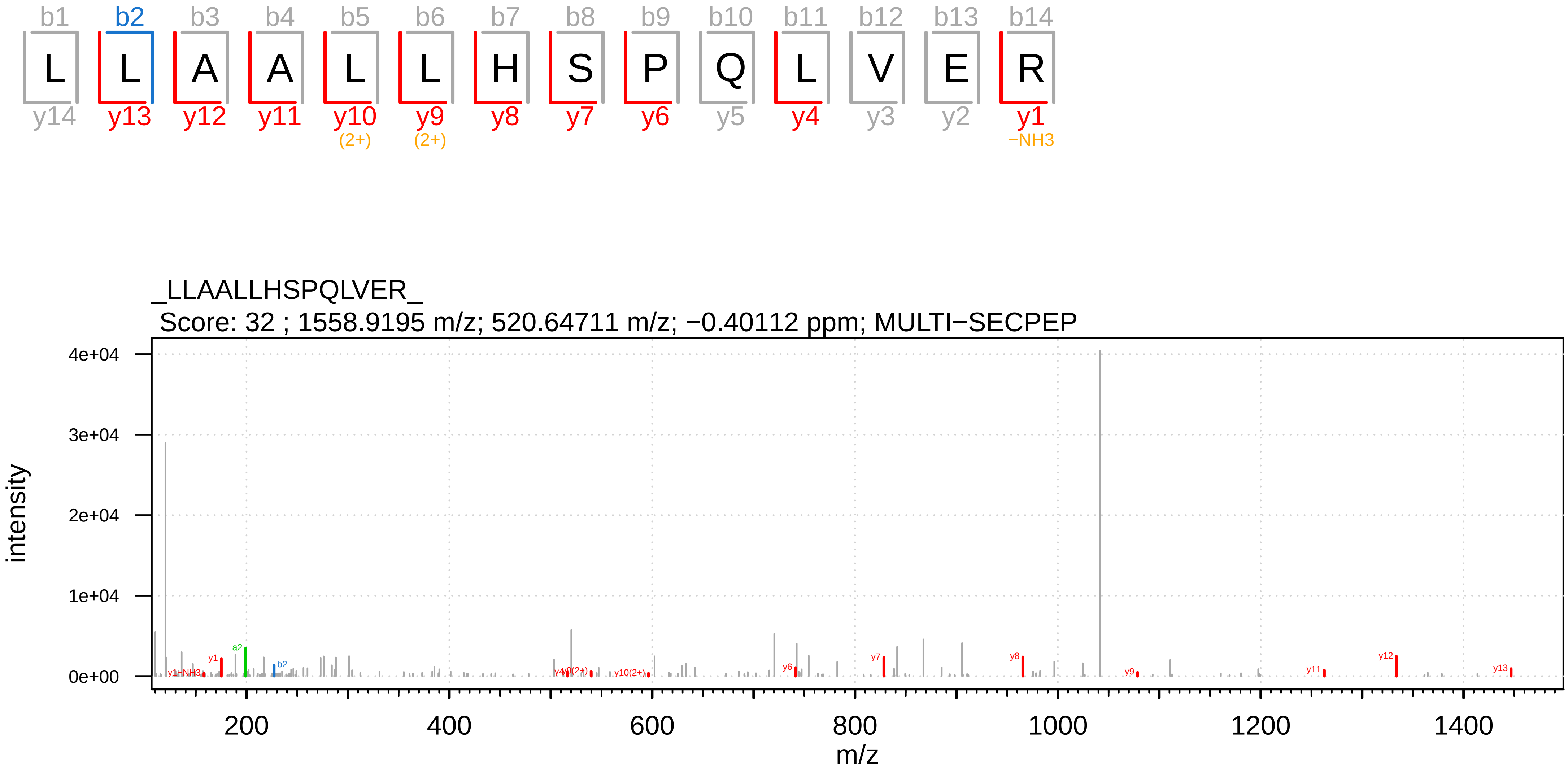

Raw File: 20101224_Velos1_TaGe_SA_HepG2_3
Scan Number: 24144
Proteins:
ENST00000602845_chr3:196669588−196669887:+

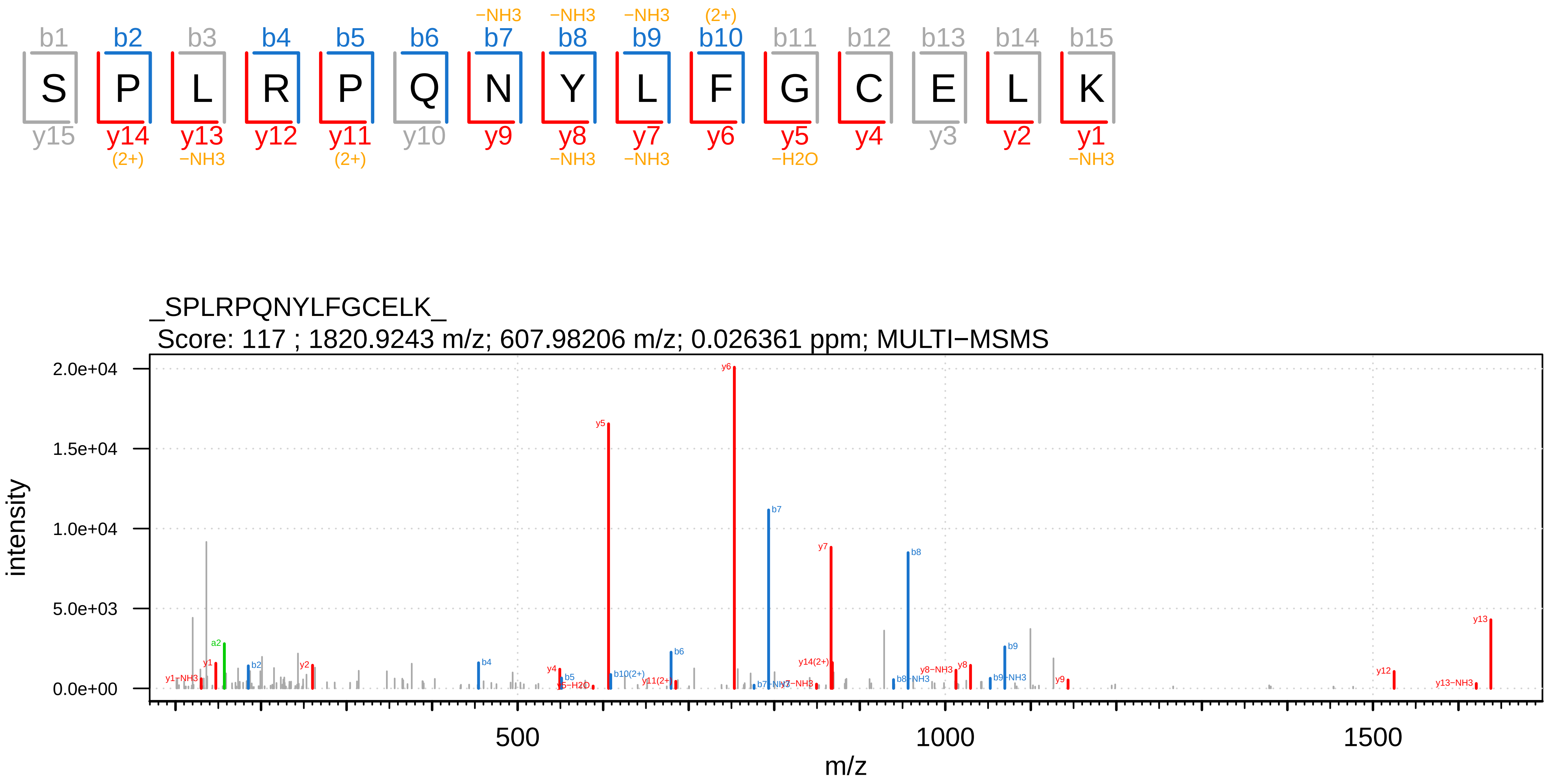

Raw File: 20101224_Velos1_TaGe_SA_HepG2_3
Scan Number: 20547
Proteins:
TCONS_l2_00008829_chr15:92829088−92829258:+

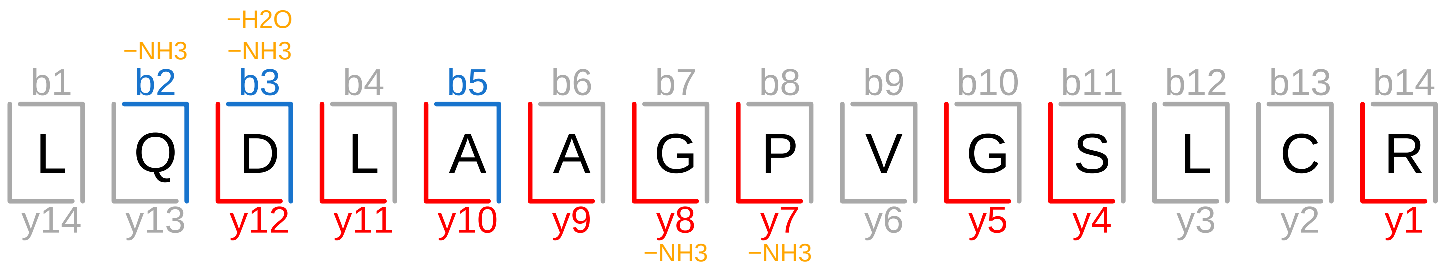

LQDLAAGPVGSLCR

Score: 51 ; 1455.7504 m/z; 728.88248 m/z; −0.27203 ppm; MULTI−MSMS

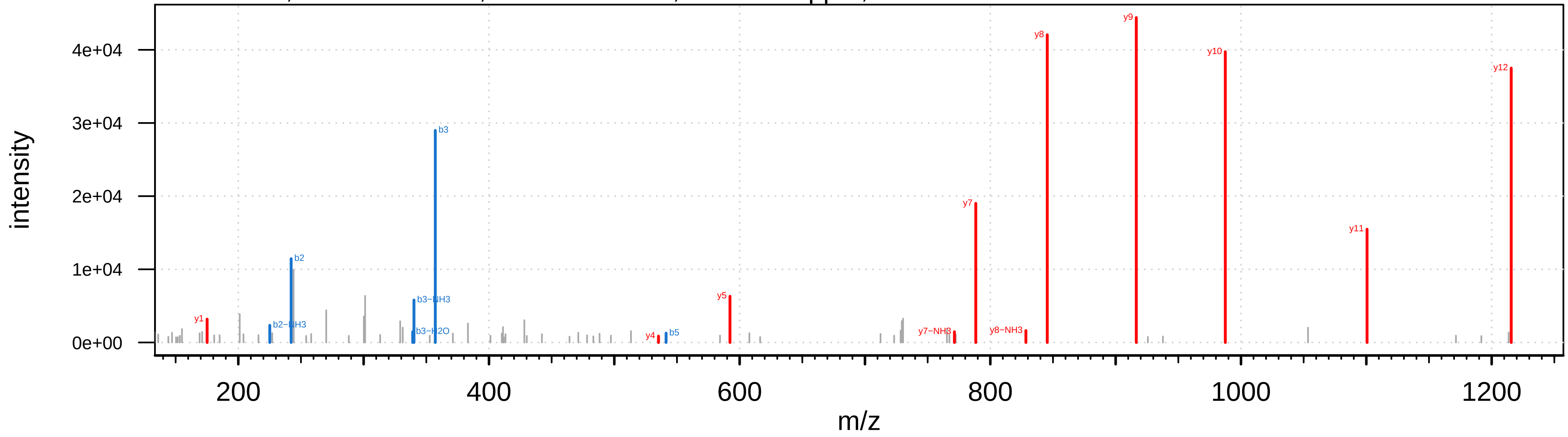

Raw File: 20100618_Velos1_TaGe_SA_U2OS_1
Scan Number: 21570
Proteins:
ENST00000602845_chr3:196669588−196669887:+

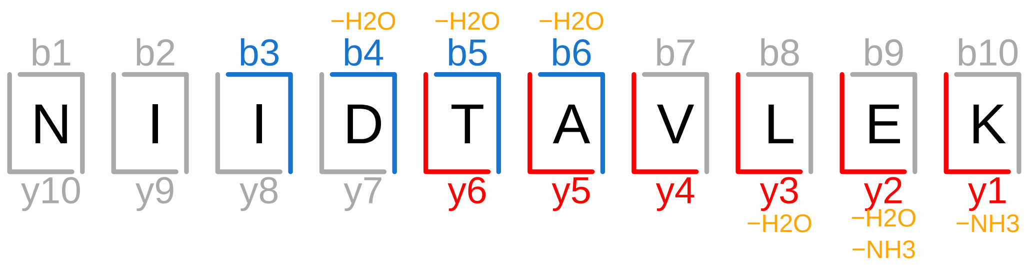

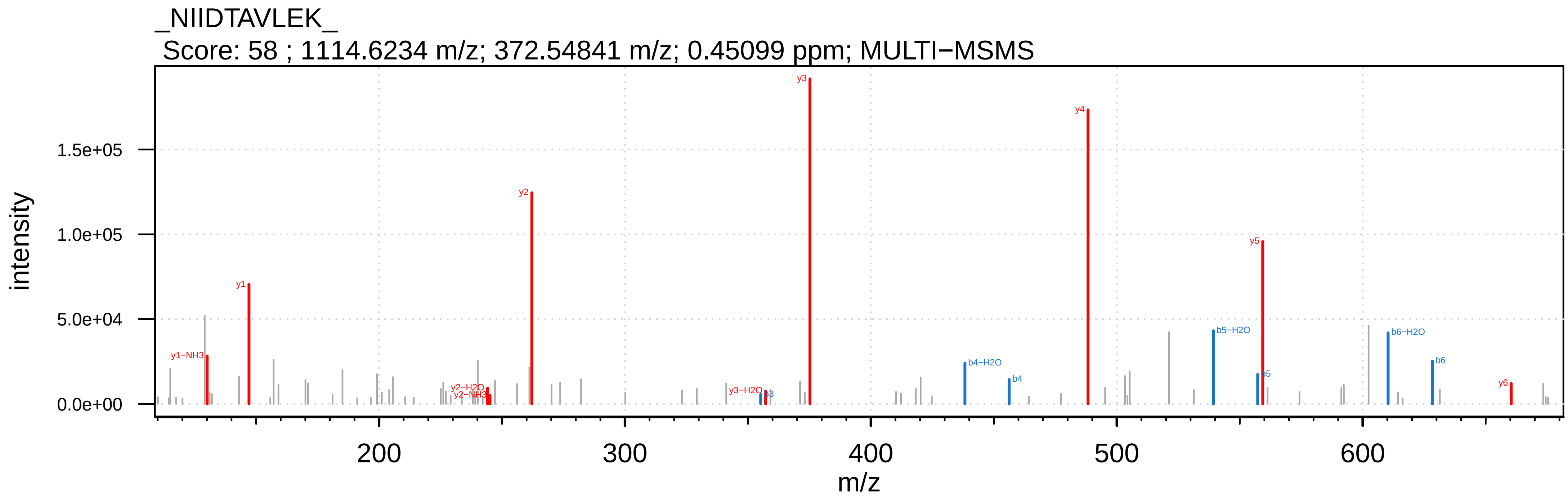

Raw File: 20100618_Velos1_TaGe_SA_U2OS_1
Scan Number: 5637
Proteins:
ENST00000497138_chr20:56806826−56807846:−

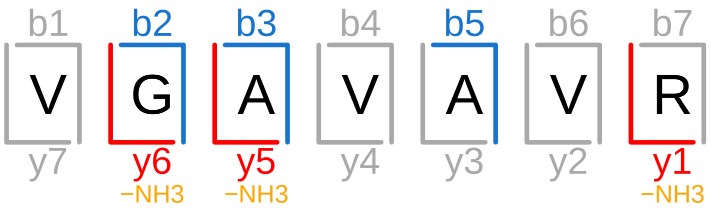

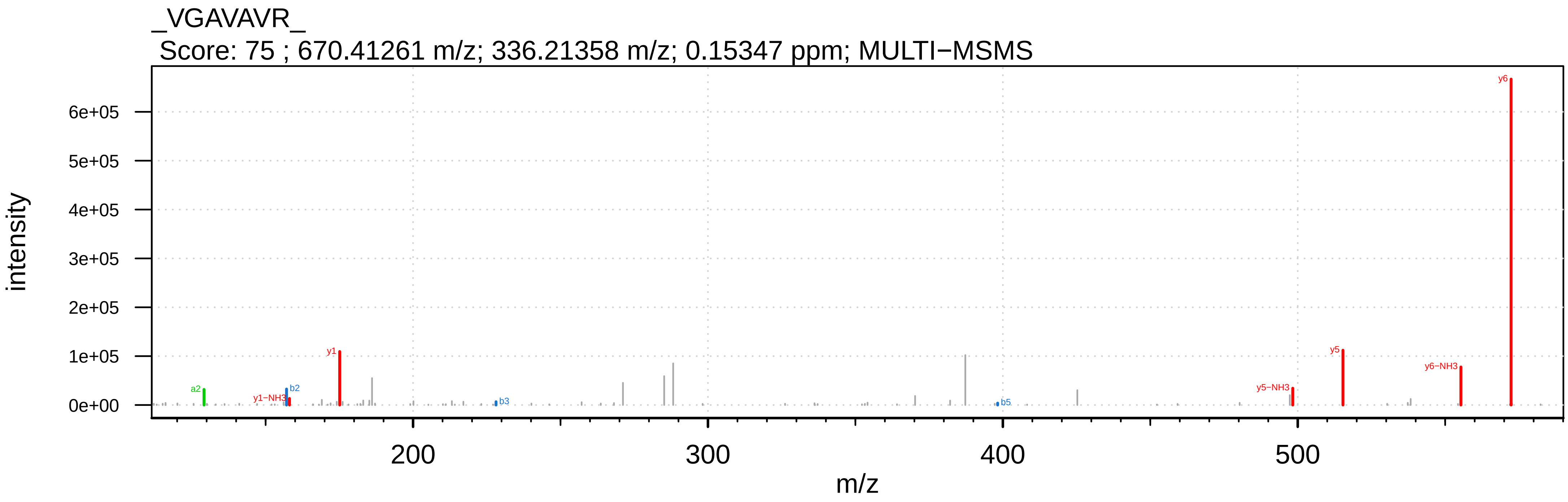

Raw File: 20100618_Velos1_TaGe_SA_U2OS_1
Scan Number: 2825
Proteins:
TCONS_l2_00030545_chrX:79544539−79546436:−

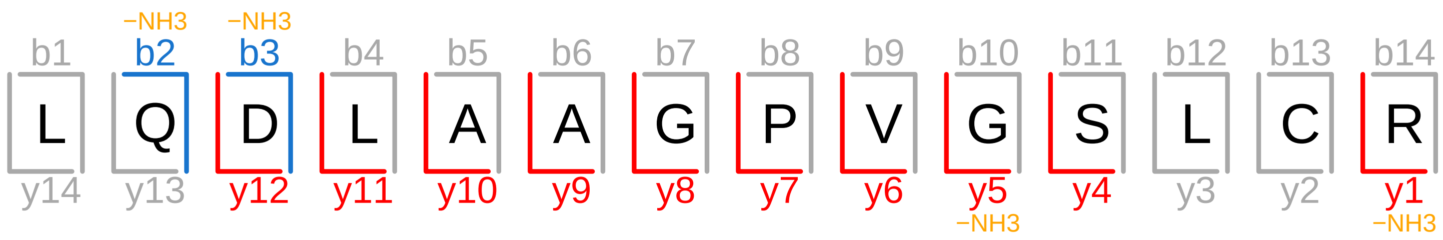

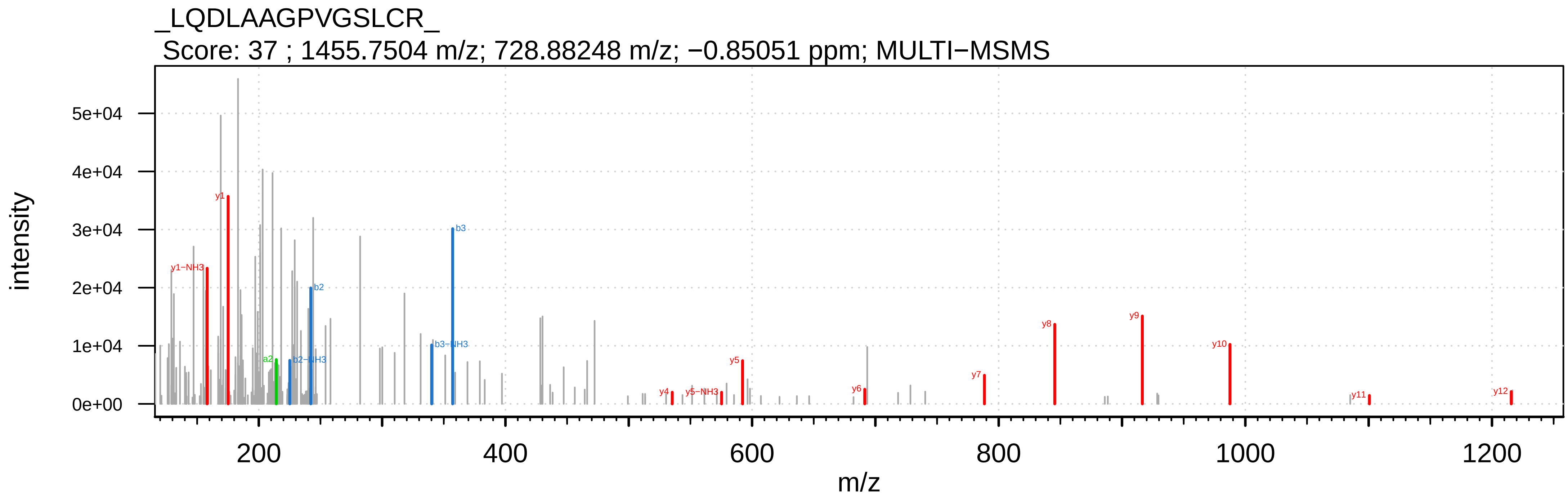

Raw File: 20100721_Velos1_TaGe_SA_U2OS_1
Scan Number: 17691
Proteins:
ENST00000602845_chr3:196669588−196669887:+

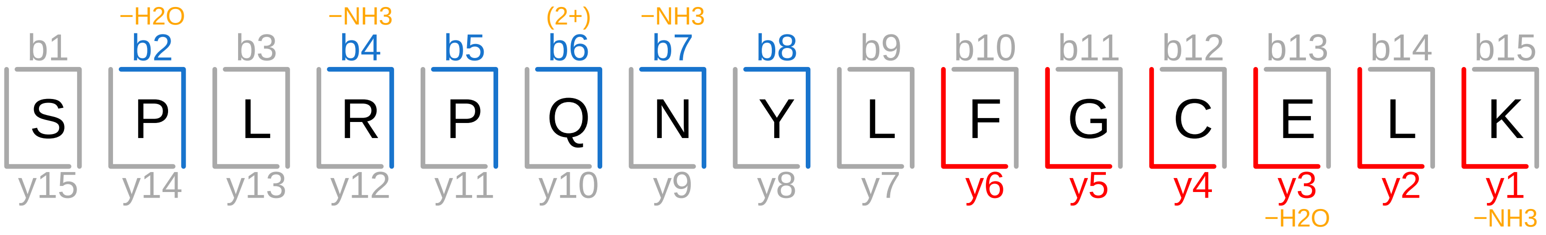

SPLRPQNYLFGCELK
Score: 35 ; 1820.9243 m/z; 607.98206 m/z; 0.021342 ppm; MULTI−MSMS

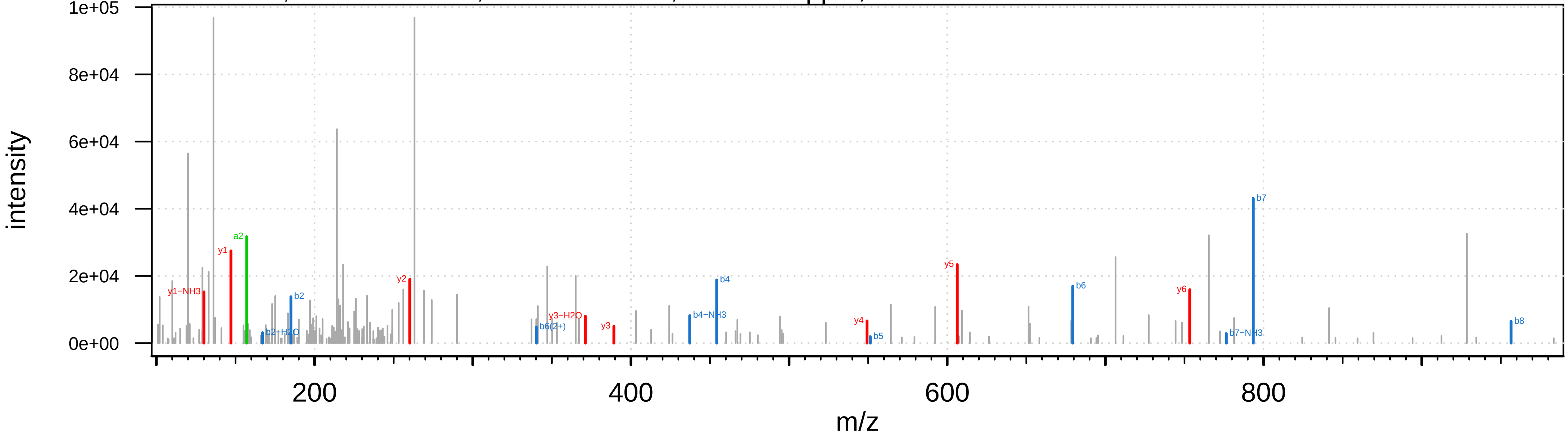

Raw File: 20100721_Velos1_TaGe_SA_U2OS_1
Scan Number: 19584
Proteins:
TCONS_l2_00008829_chr15:92829088−92829258:+

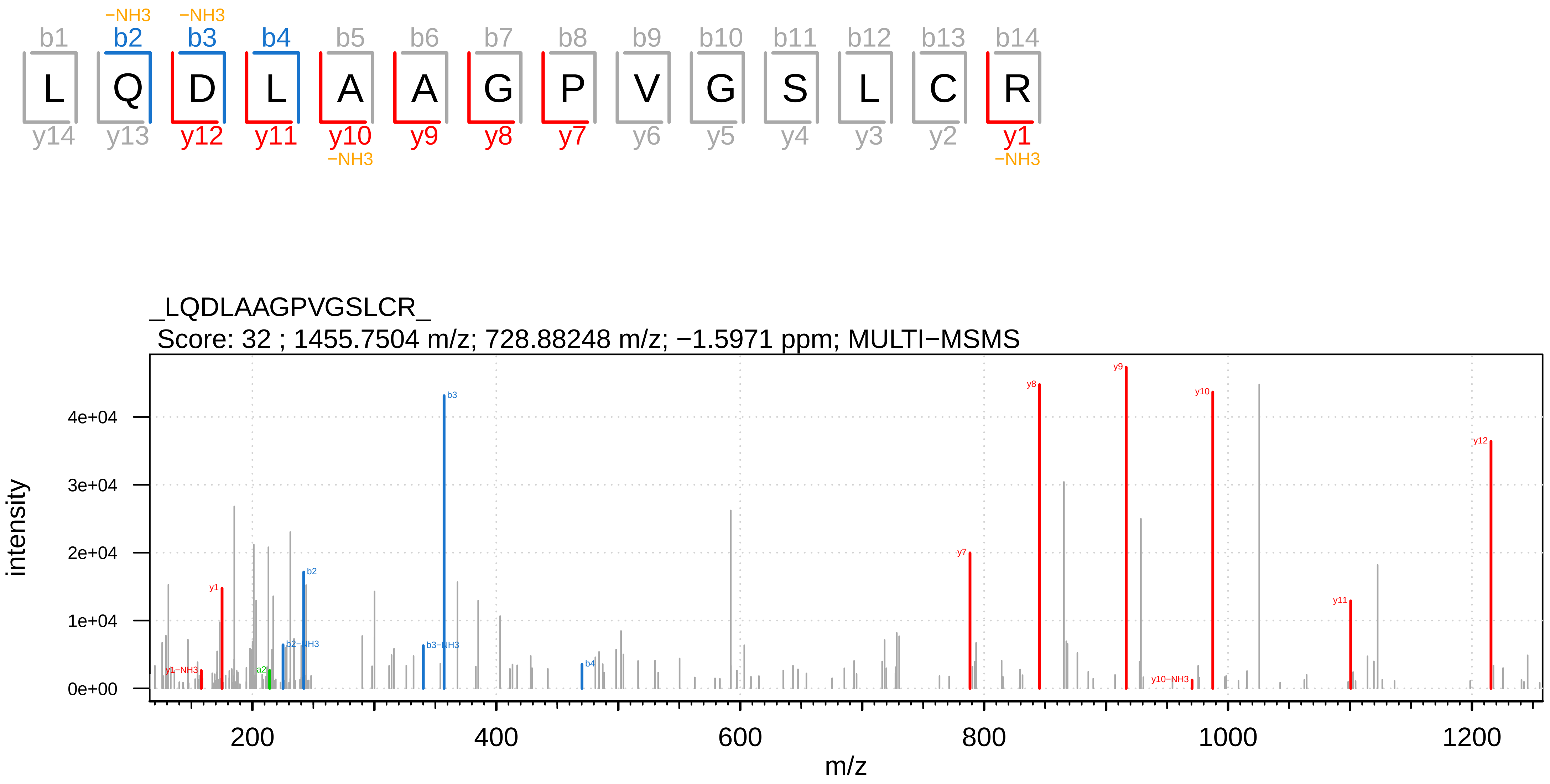

Raw File: 20101210_Velos1_AnWe_SA_LnCap_1
Scan Number: 20166
Proteins:
ENST00000602845_chr3:196669588−196669887:+

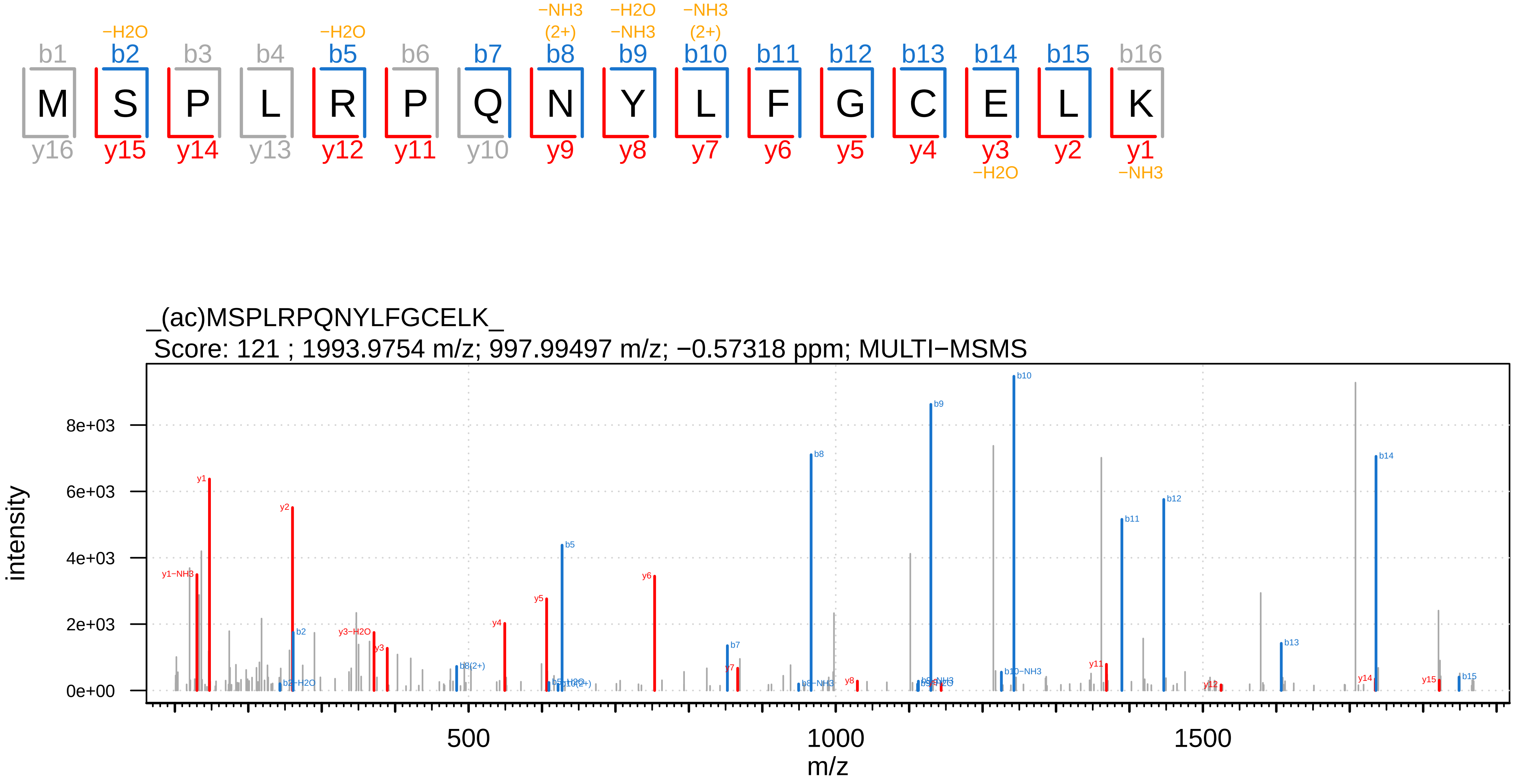

Raw File: 20101210_Velos1_AnWe_SA_LnCap_1
Scan Number: 36029
Proteins:
TCONS_l2_00008829_chr15:92829088−92829258:+

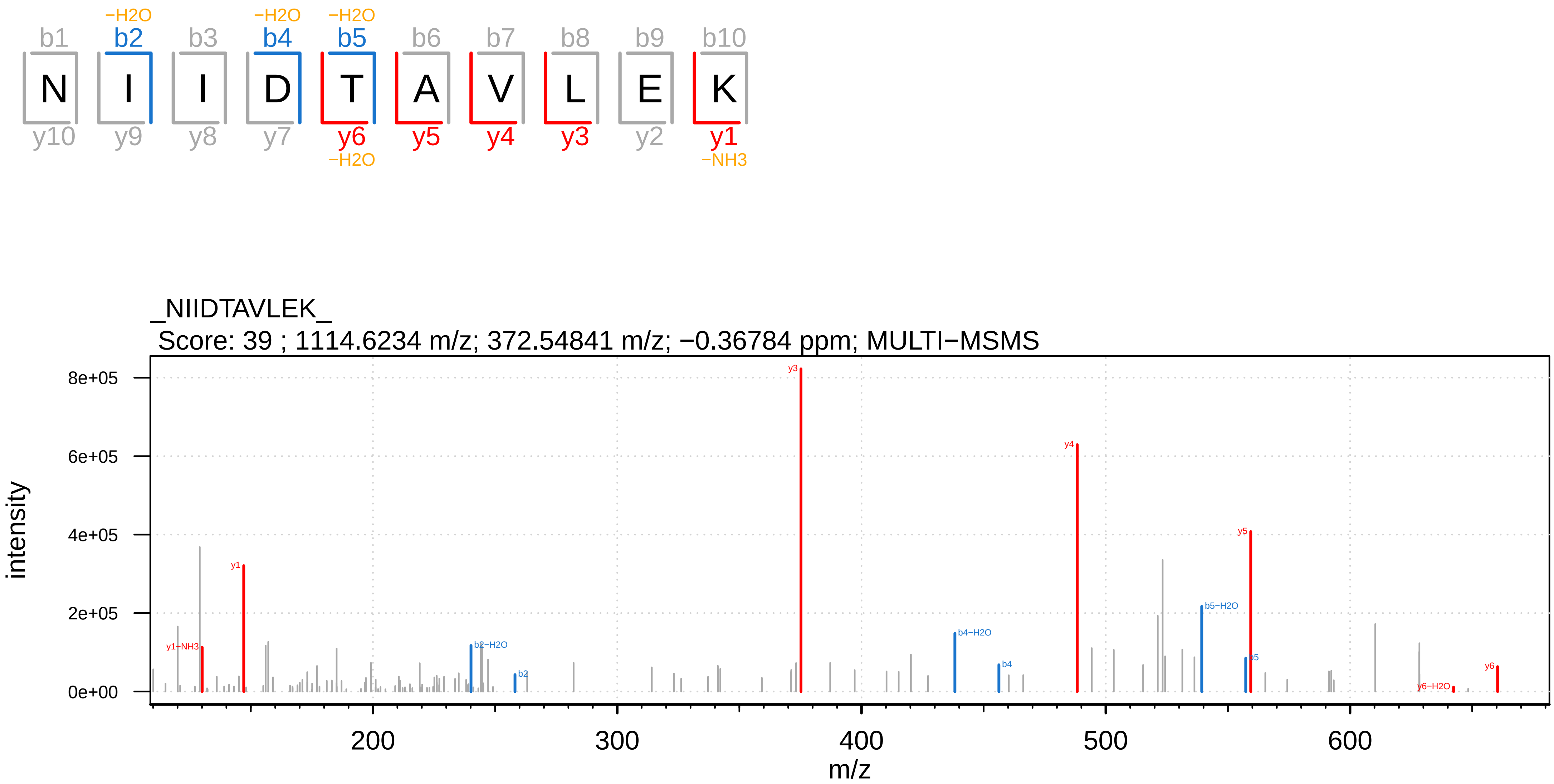

Raw File: 20101210_Velos1_AnWe_SA_LnCap_1
Scan Number: 5308
Proteins:
ENST00000497138_chr20:56806826−56807846:−

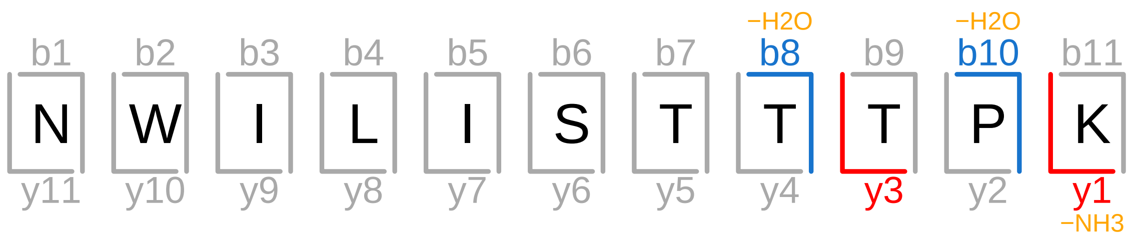

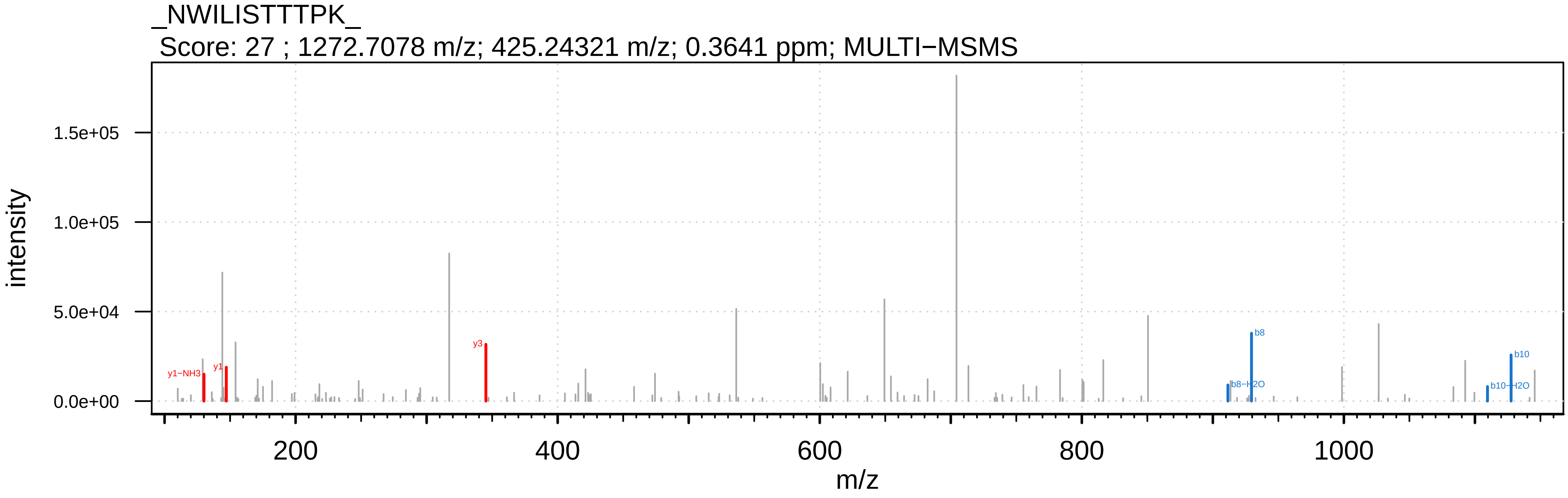

Raw File: 20101210_Velos1_AnWe_SA_LnCap_1
Scan Number: 25645
Proteins:
ENST00000430431_chr10:61496818−61496958:−

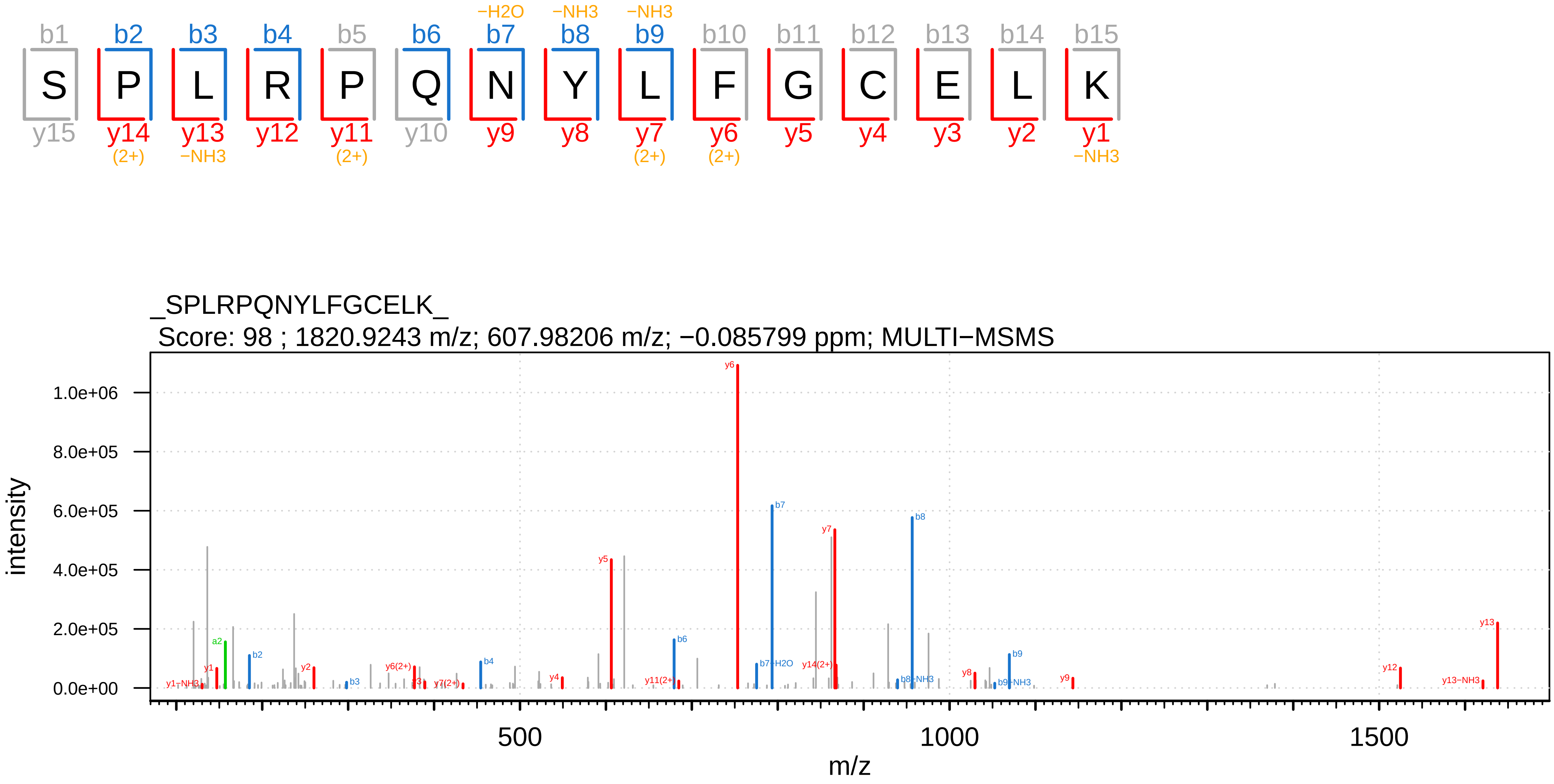

Raw File: 20101210_Velos1_AnWe_SA_LnCap_1
Scan Number: 22329
Proteins:
TCONS_l2_00008829_chr15:92829088−92829258:+

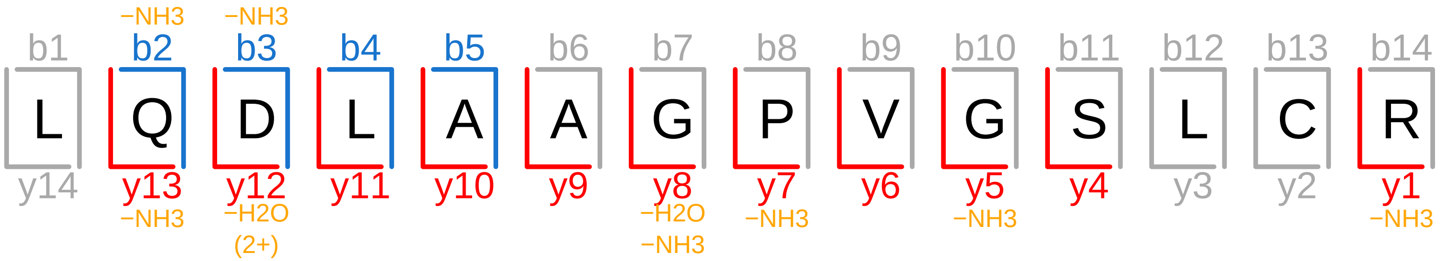

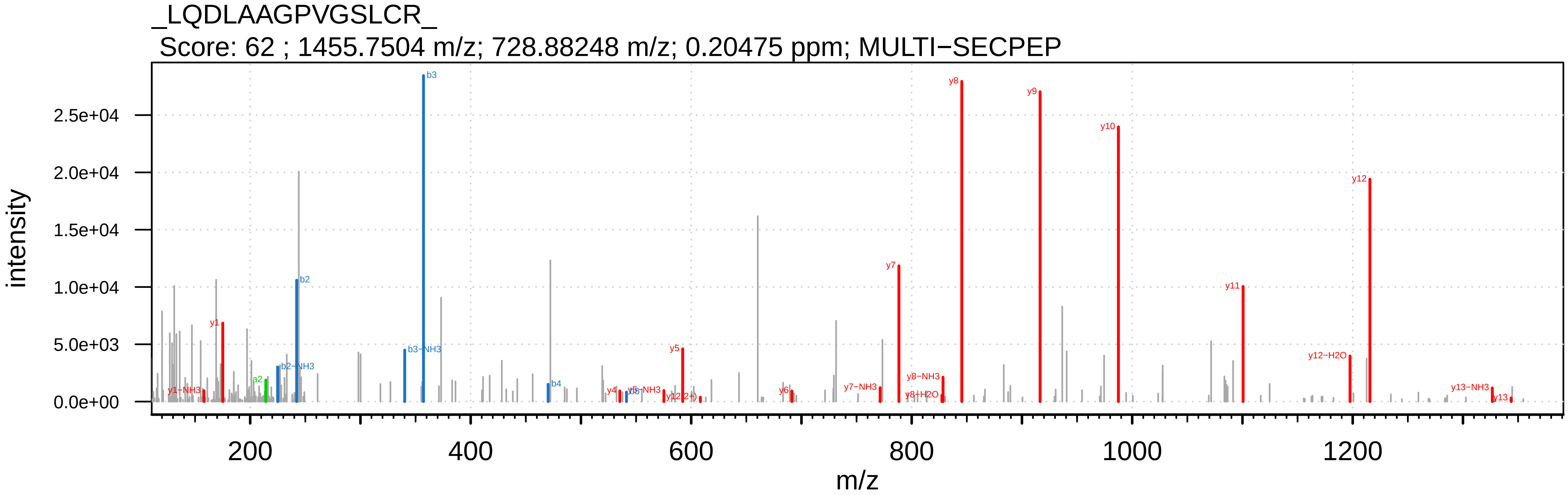

Raw File: 20101224_Velos1_TaGe_SA_HeLa_01
Scan Number: 20424
Proteins:
ENST00000602845_chr3:196669588−196669887:+

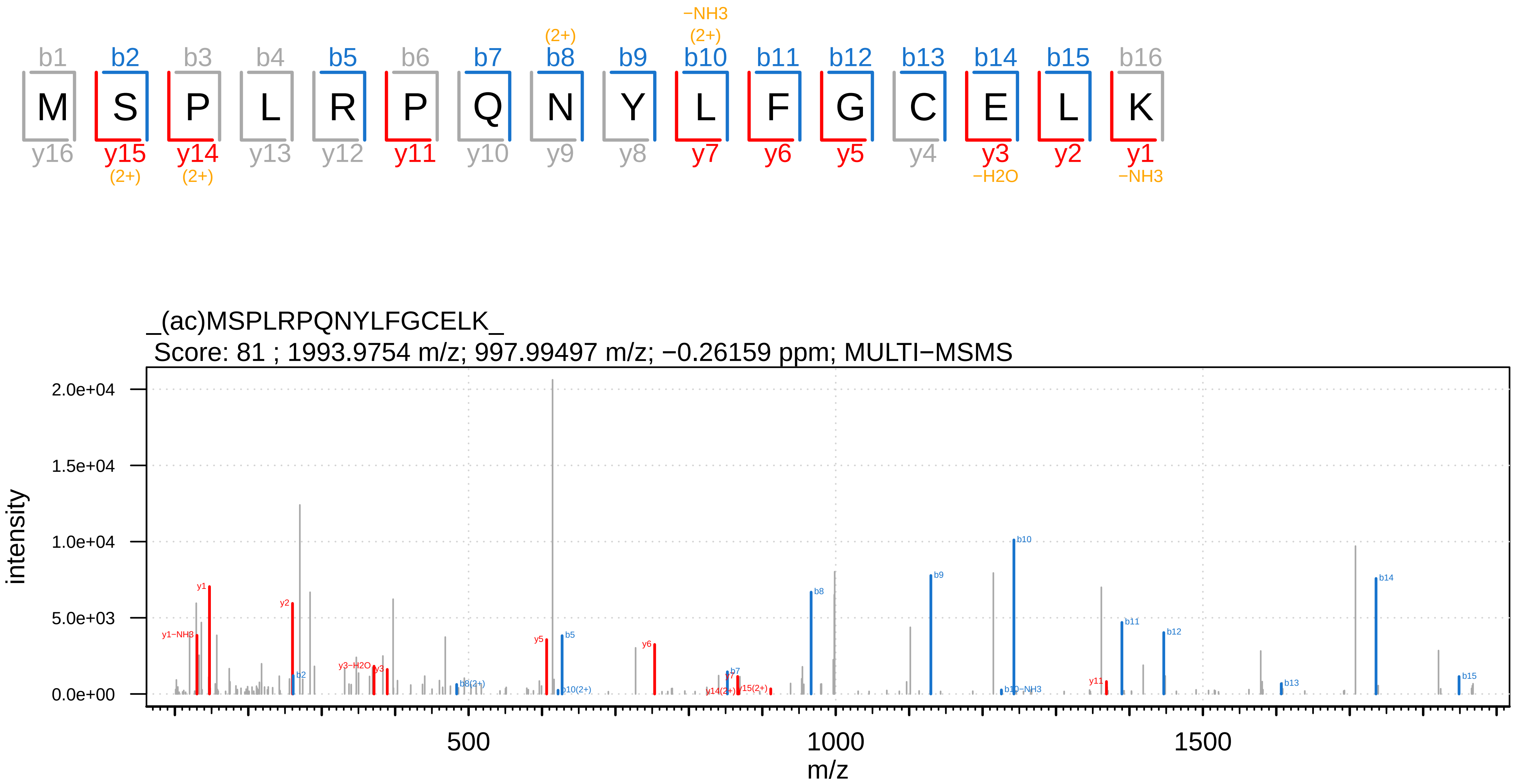

Raw File: 20101224_Velos1_TaGe_SA_HeLa_01
Scan Number: 37071
Proteins:
TCONS_l2_00008829_chr15:92829088−92829258:+

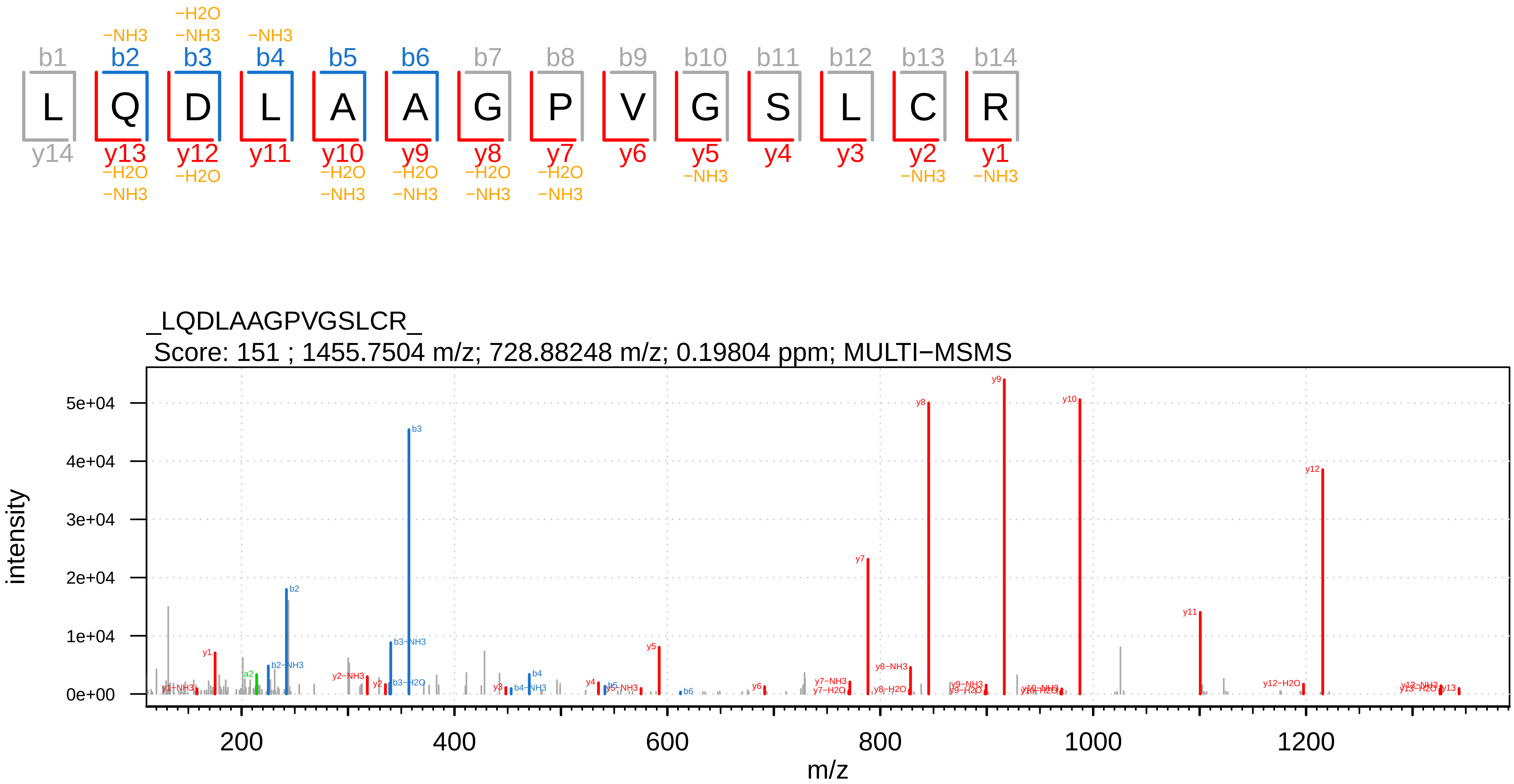

Raw File: 20101224_Velos1_TaGe_SA_HepG2_1
Scan Number: 20233
Proteins:
ENST00000602845_chr3:196669588−196669887:+

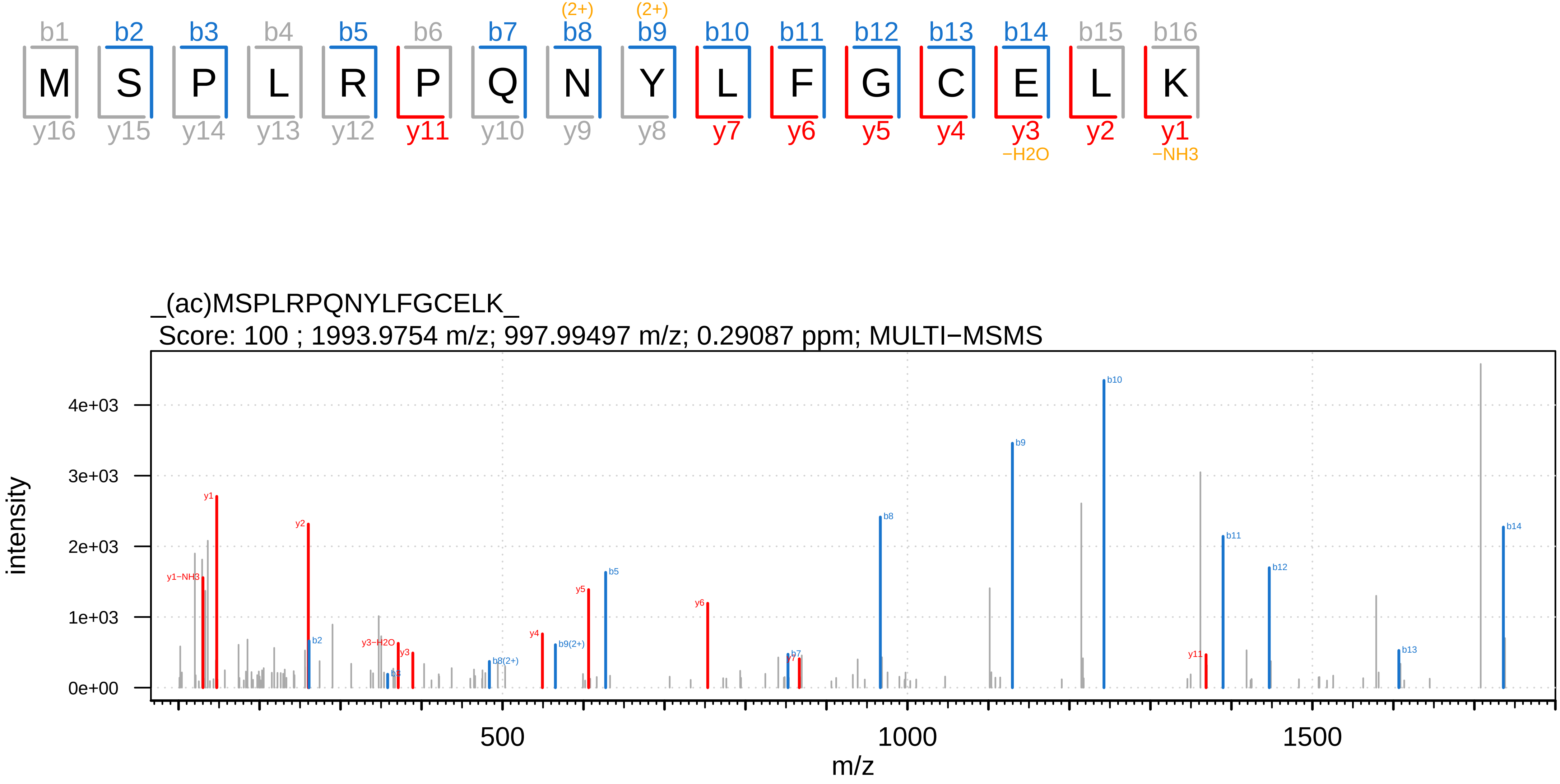

Raw File: 20101224_Velos1_TaGe_SA_HepG2_1
Scan Number: 37022
Proteins:
TCONS_l2_00008829_chr15:92829088−92829258:+

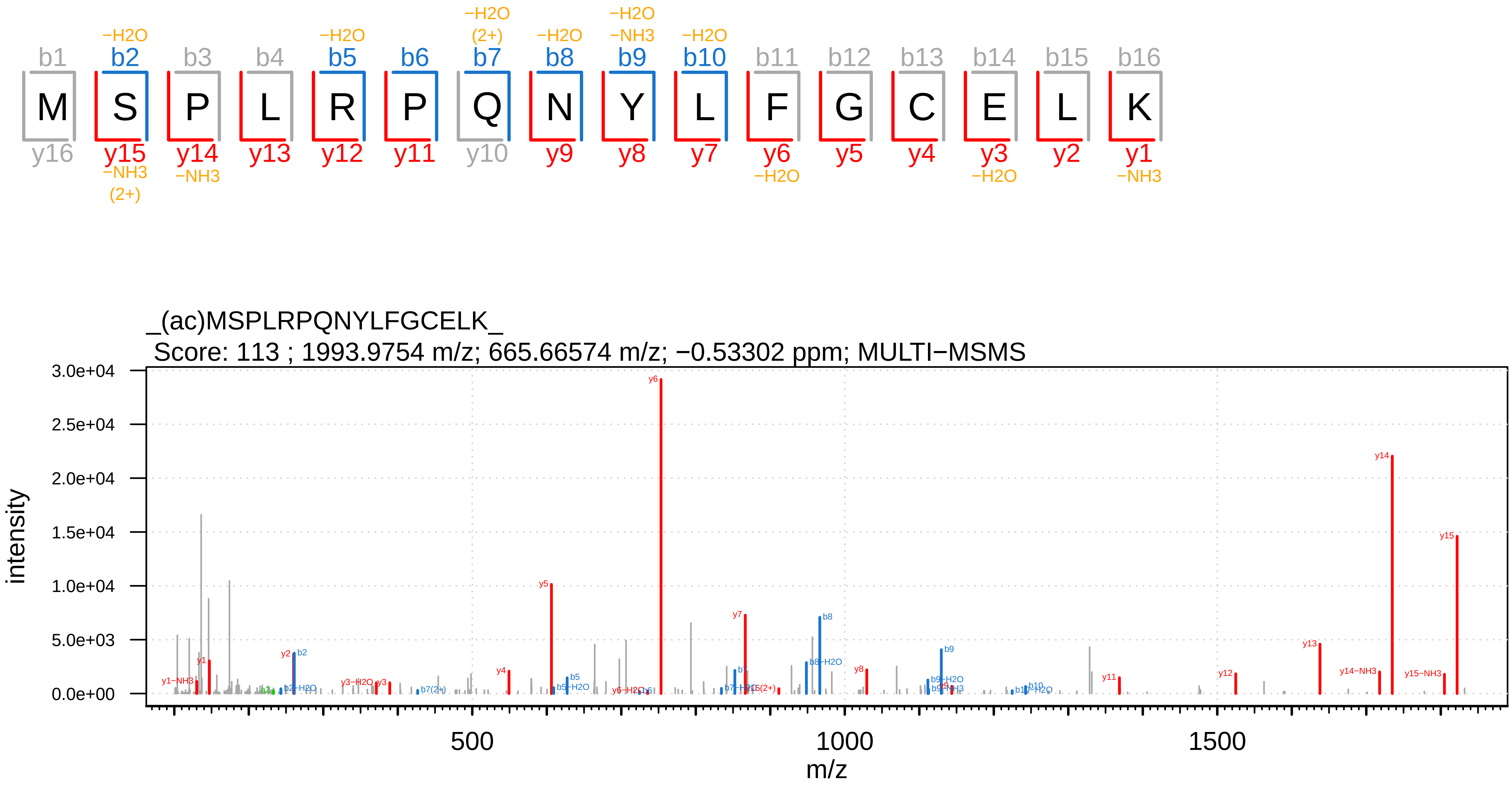

Raw File: 20101224_Velos1_TaGe_SA_HepG2_1
Scan Number: 37032
Proteins:
TCONS_l2_00008829_chr15:92829088−92829258:+

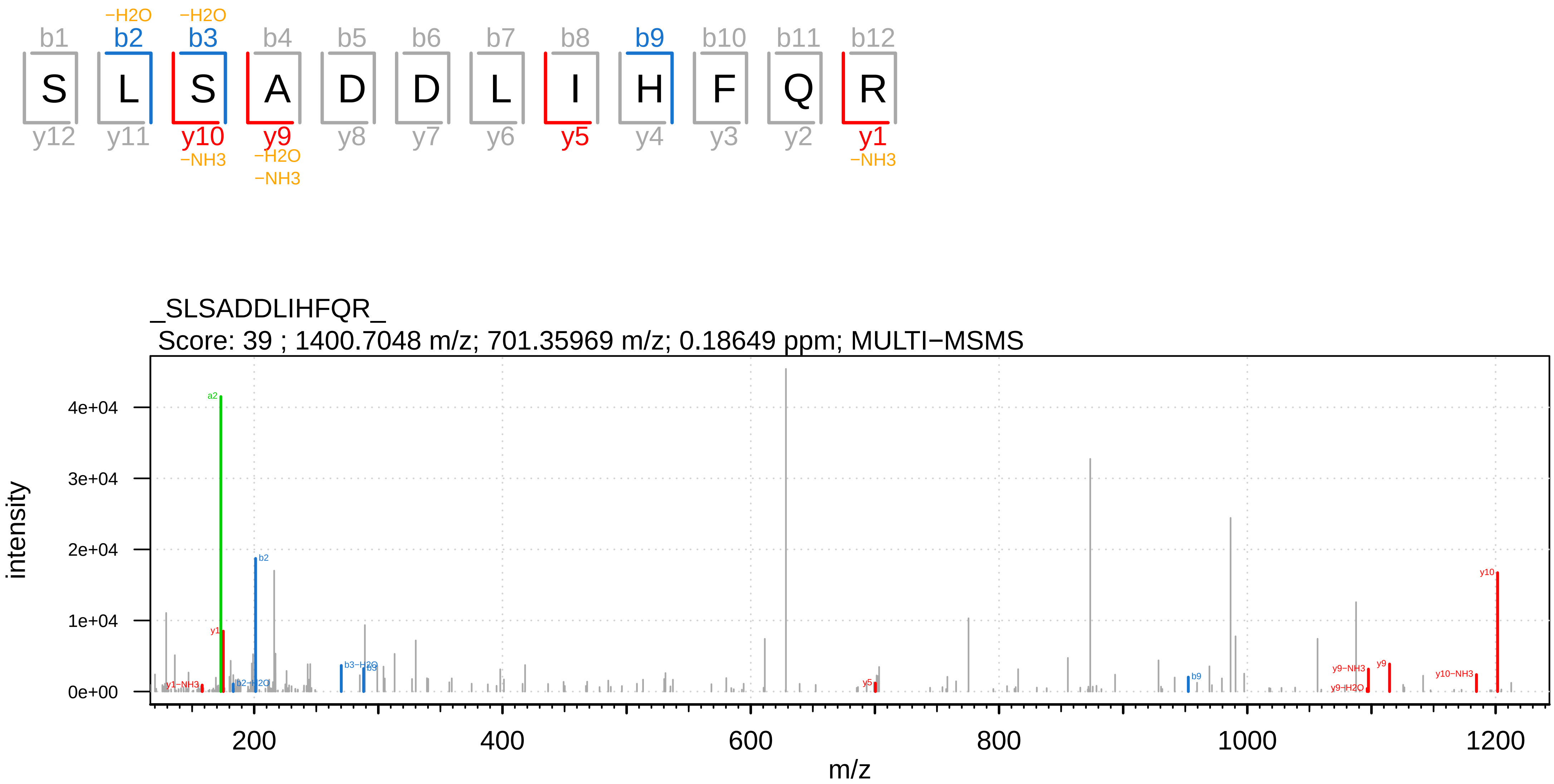

Raw File: 20101224_Velos1_TaGe_SA_HepG2_1
Scan Number: 17037
Proteins:
TCONS_l2_00002988_chr10:38499673−38501345:+

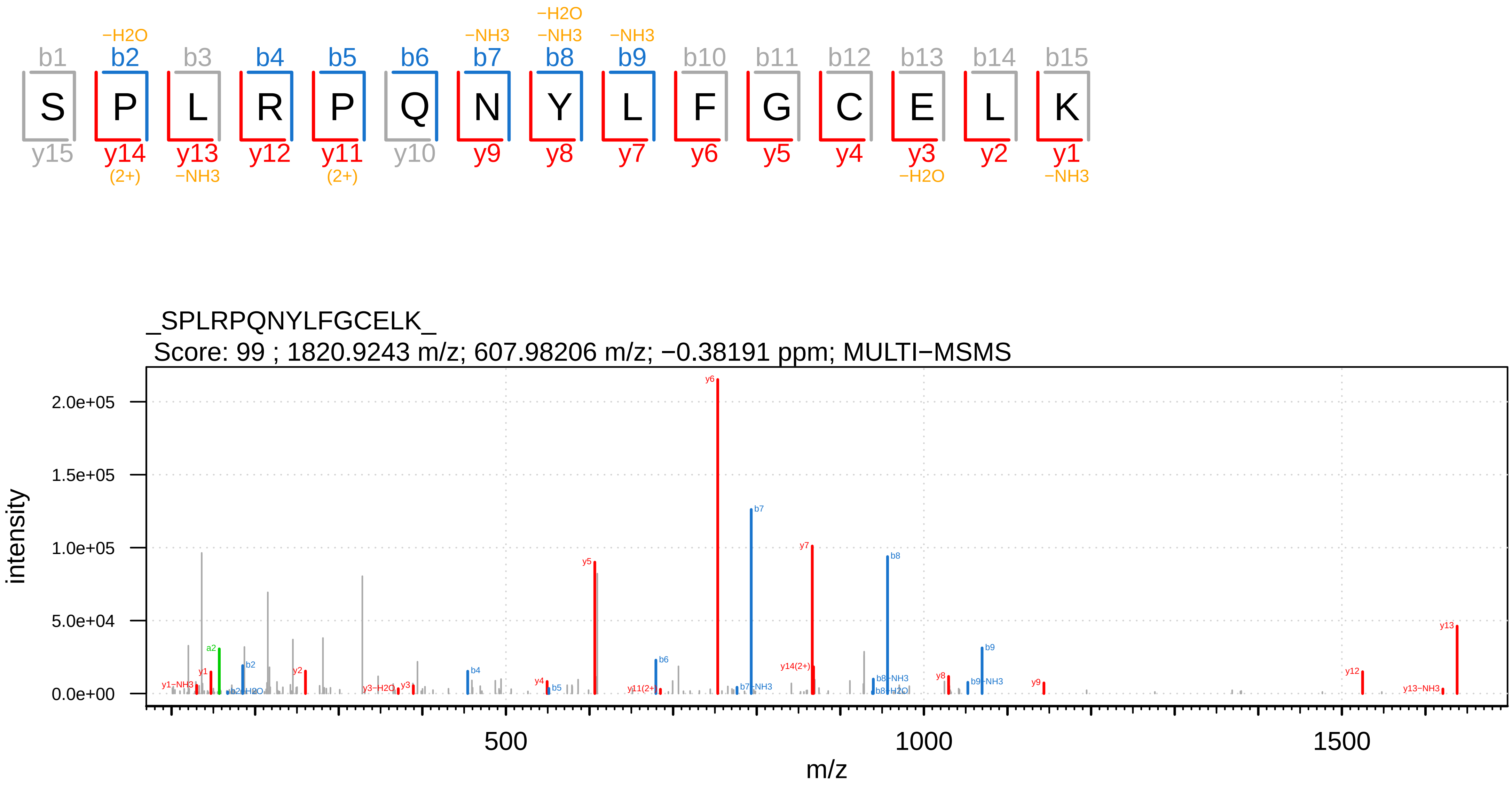

Raw File: 20101224_Velos1_TaGe_SA_HepG2_1
Scan Number: 23847
Proteins:
TCONS_l2_00008829_chr15:92829088−92829258:+

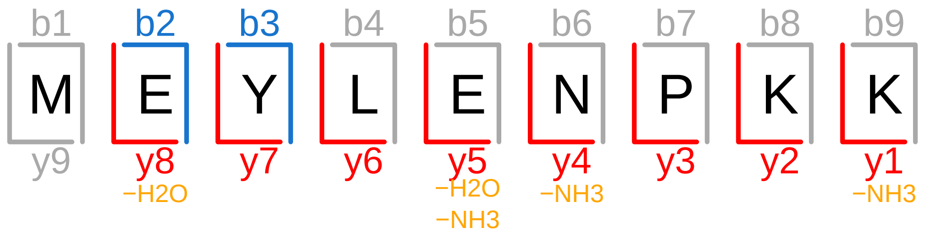

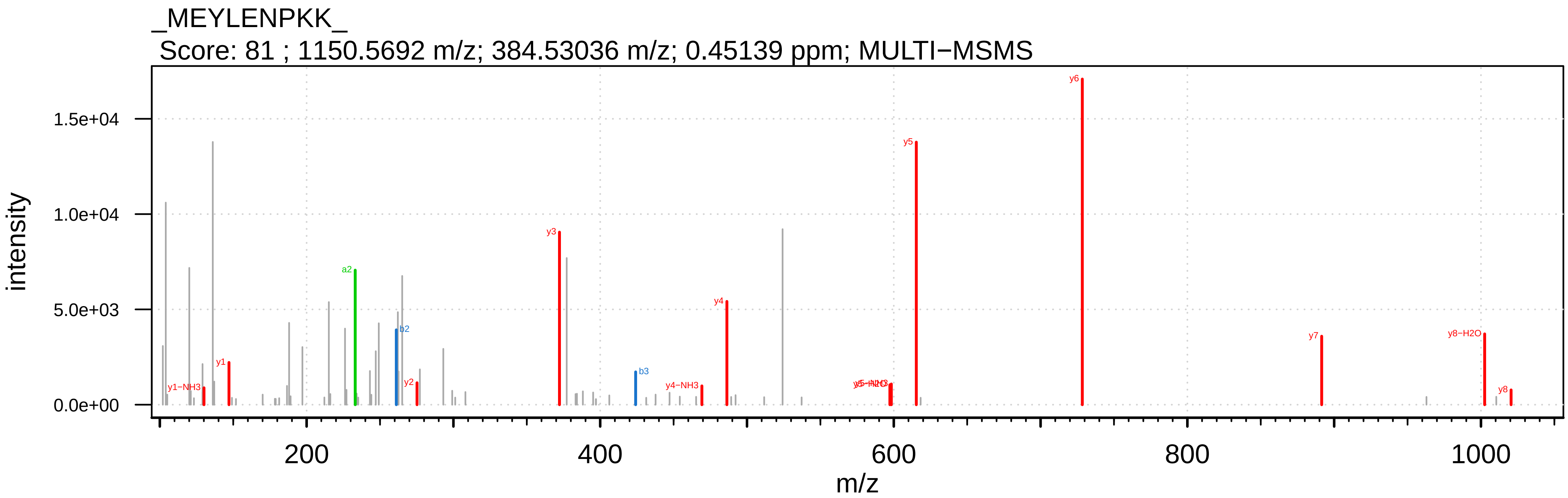

Raw File: 20100618_Velos1_TaGe_SA_U2OS_6
Scan Number: 5833
Proteins:
ENST00000518958_chr8:120630400−120630519:−

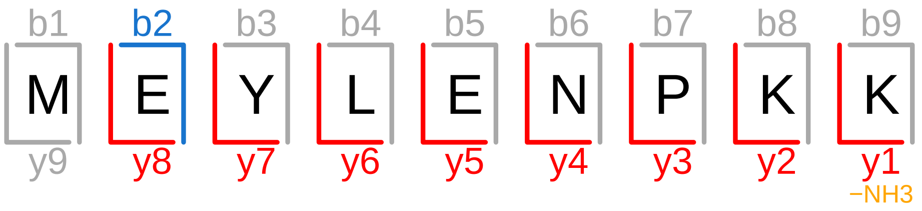

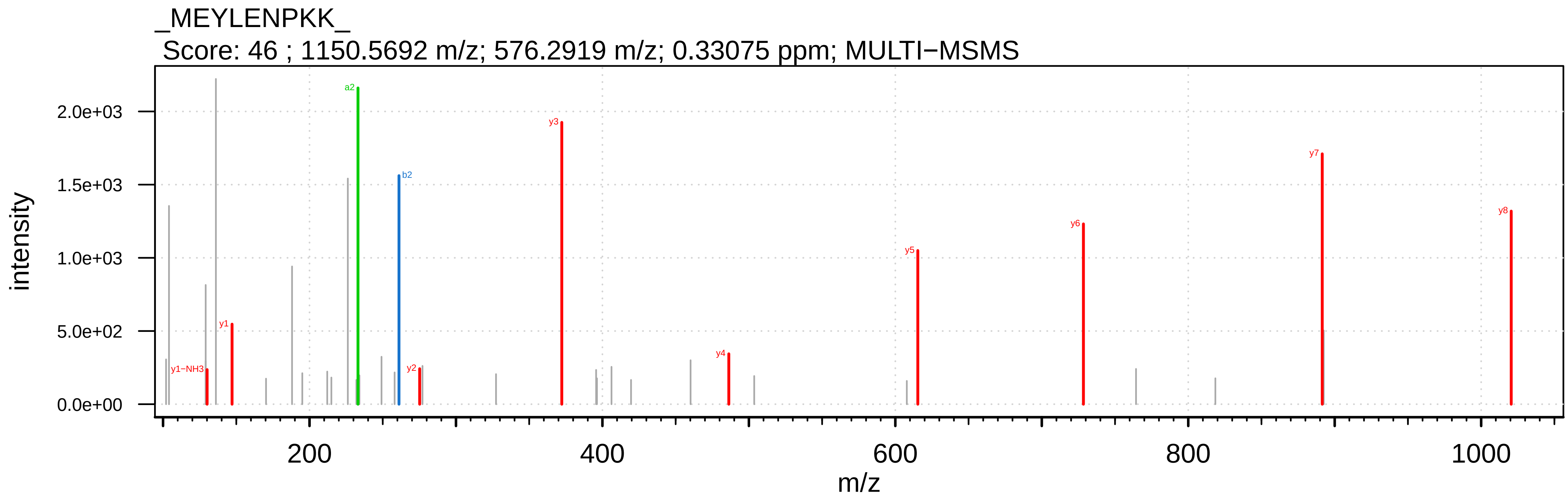

Raw File: 20100618_Velos1_TaGe_SA_U2OS_6
Scan Number: 5845
Proteins:
ENST00000518958_chr8:120630400−120630519:−

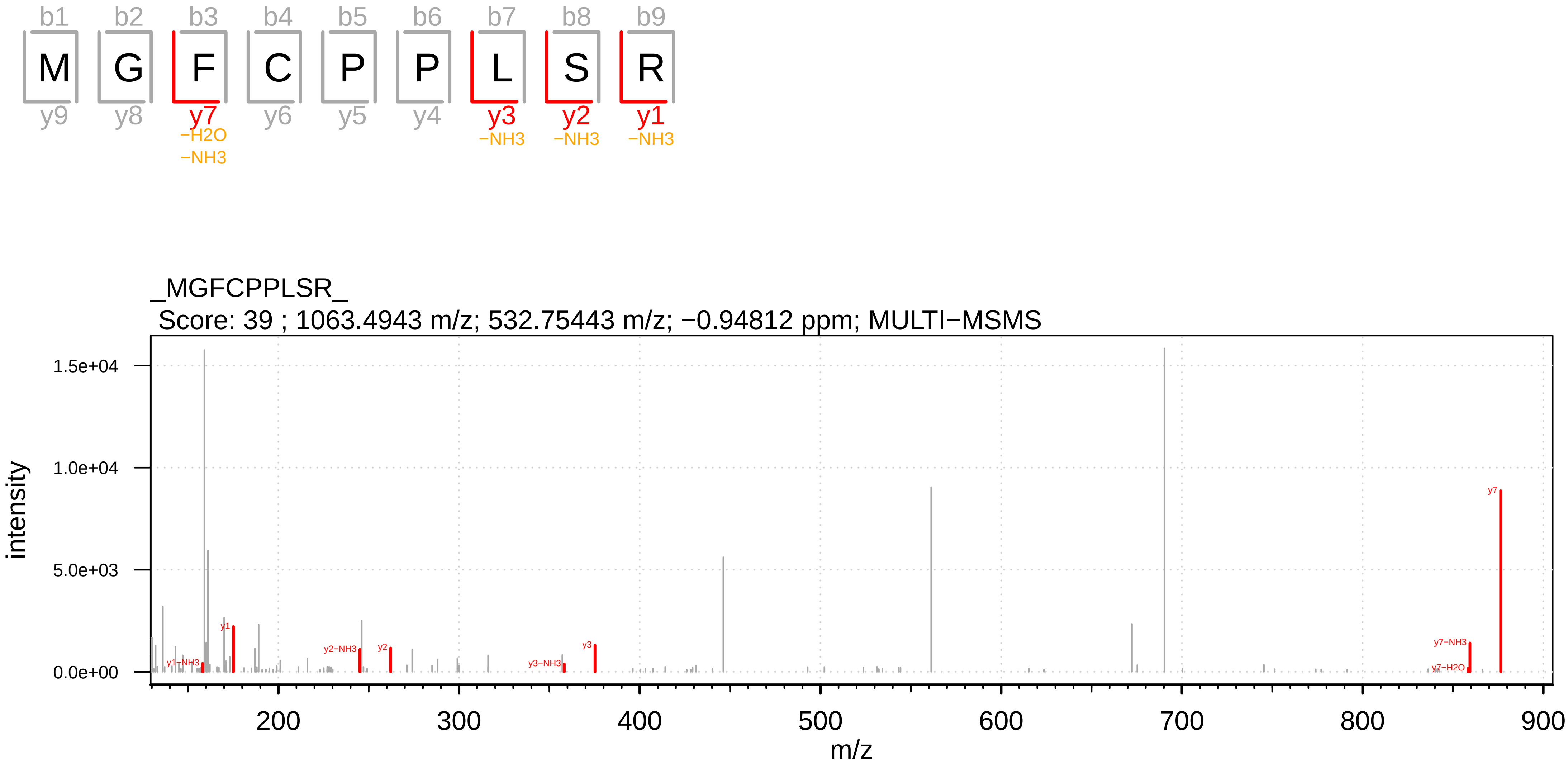

Raw File: 20101224_Velos1_TaGe_SA_HepG2_4
Scan Number: 13185
Proteins:
ENST00000381213_chr5:56218114−56218146:−

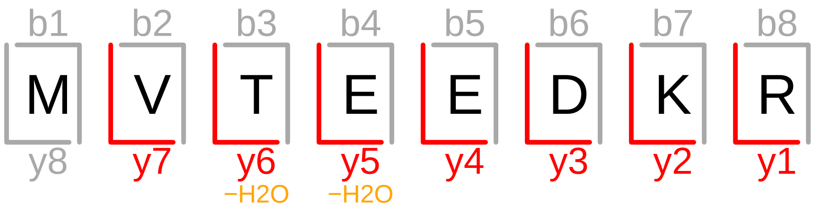

MVTEEDKR

Score: 50 ; 1006.4753 m/z; 336.49906 m/z; −0.24202 ppm; MULTI−MSMS

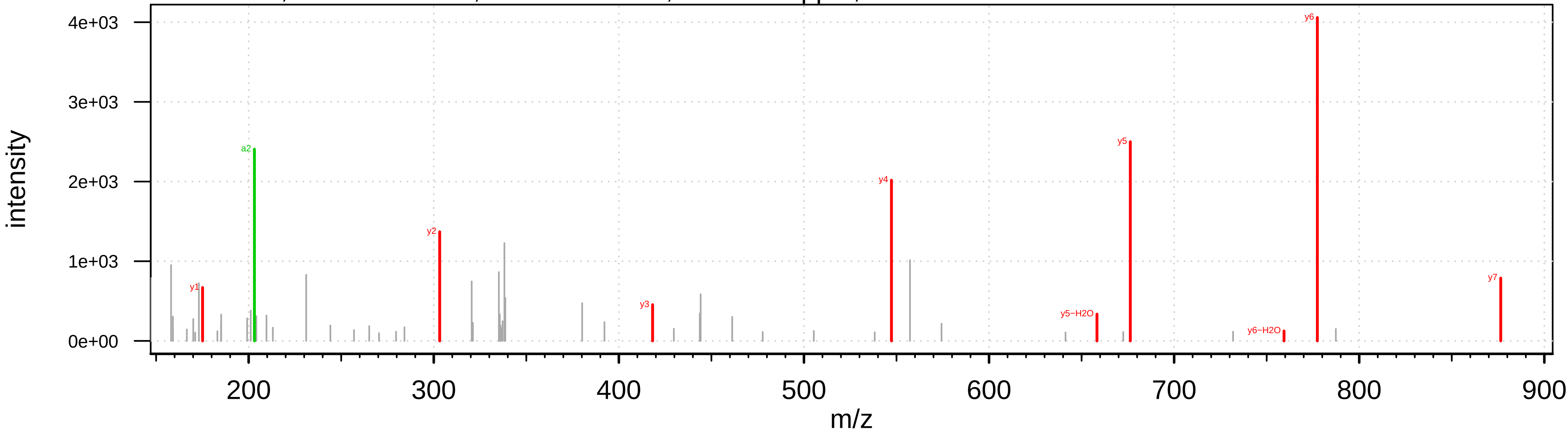

Raw File: 20101224_Velos1_TaGe_SA_HepG2_4
Scan Number: 929
Proteins:
TCONS_l2_00006768_chr13:29174091−29174123:+

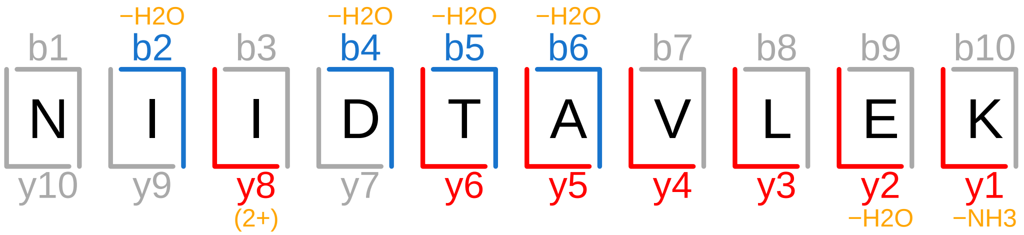

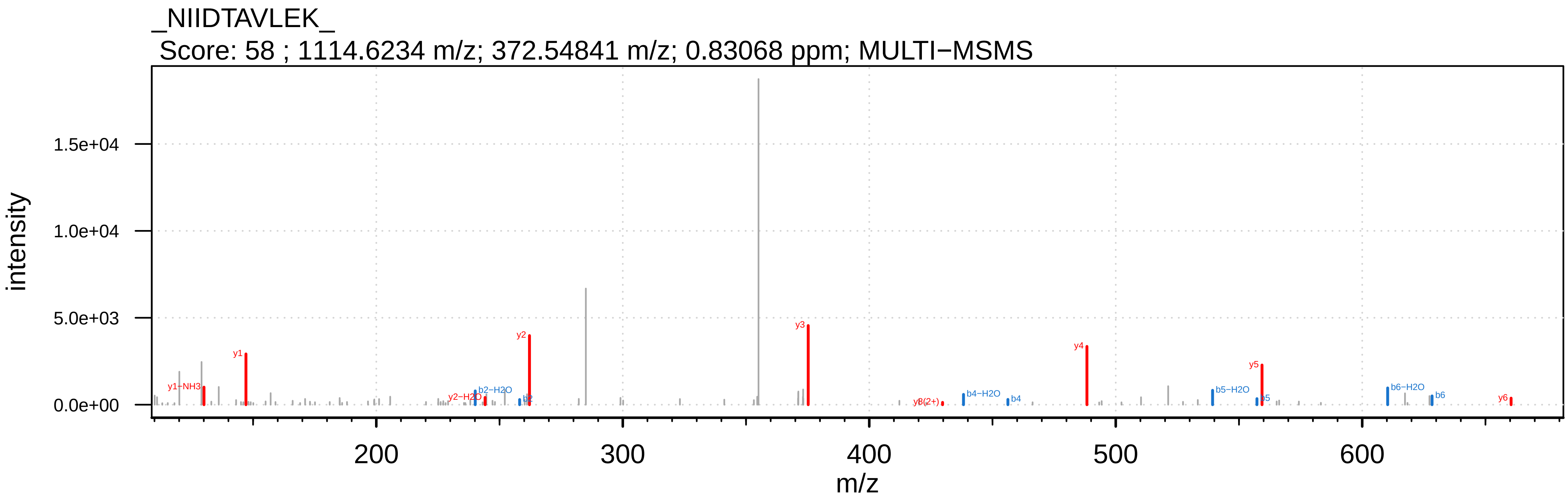

Raw File: 20101224_Velos1_TaGe_SA_HepG2_4
Scan Number: 6466
Proteins:
ENST00000497138_chr20:56806826−56807846:−

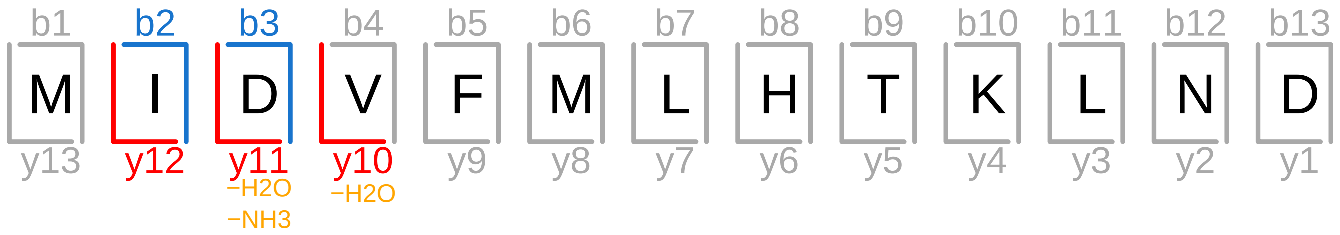

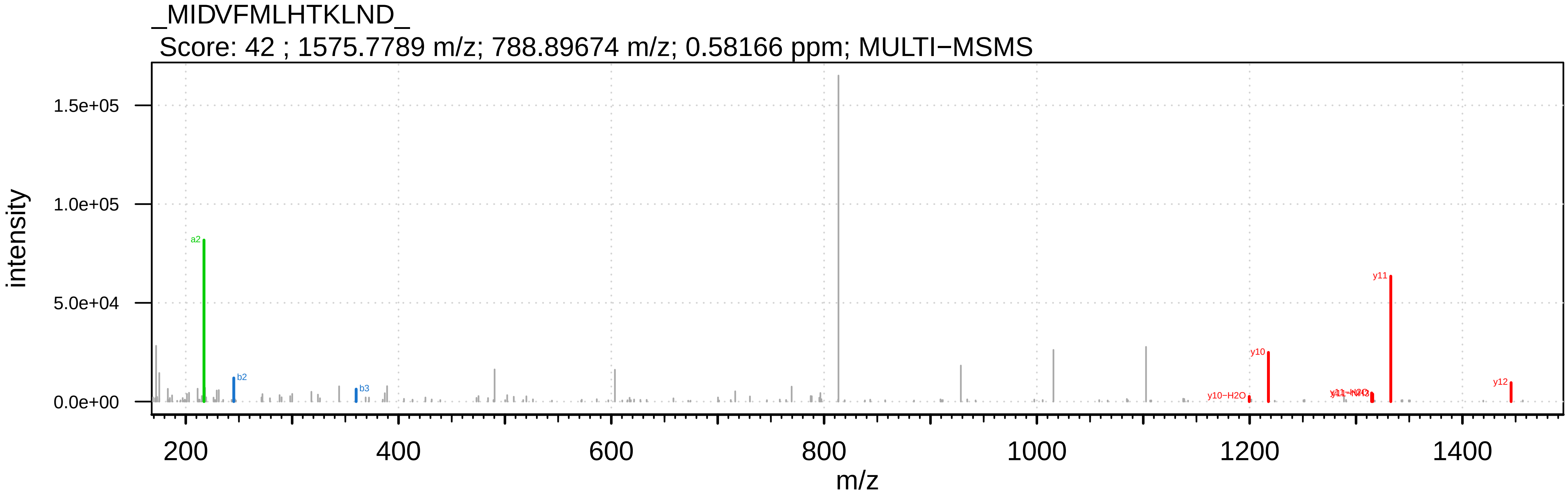

Raw File: 20100609_Velos1_TaGe_SA_293_1
Scan Number: 30776
Proteins:
ENST00000335670_chr15:60788697−60788738:−

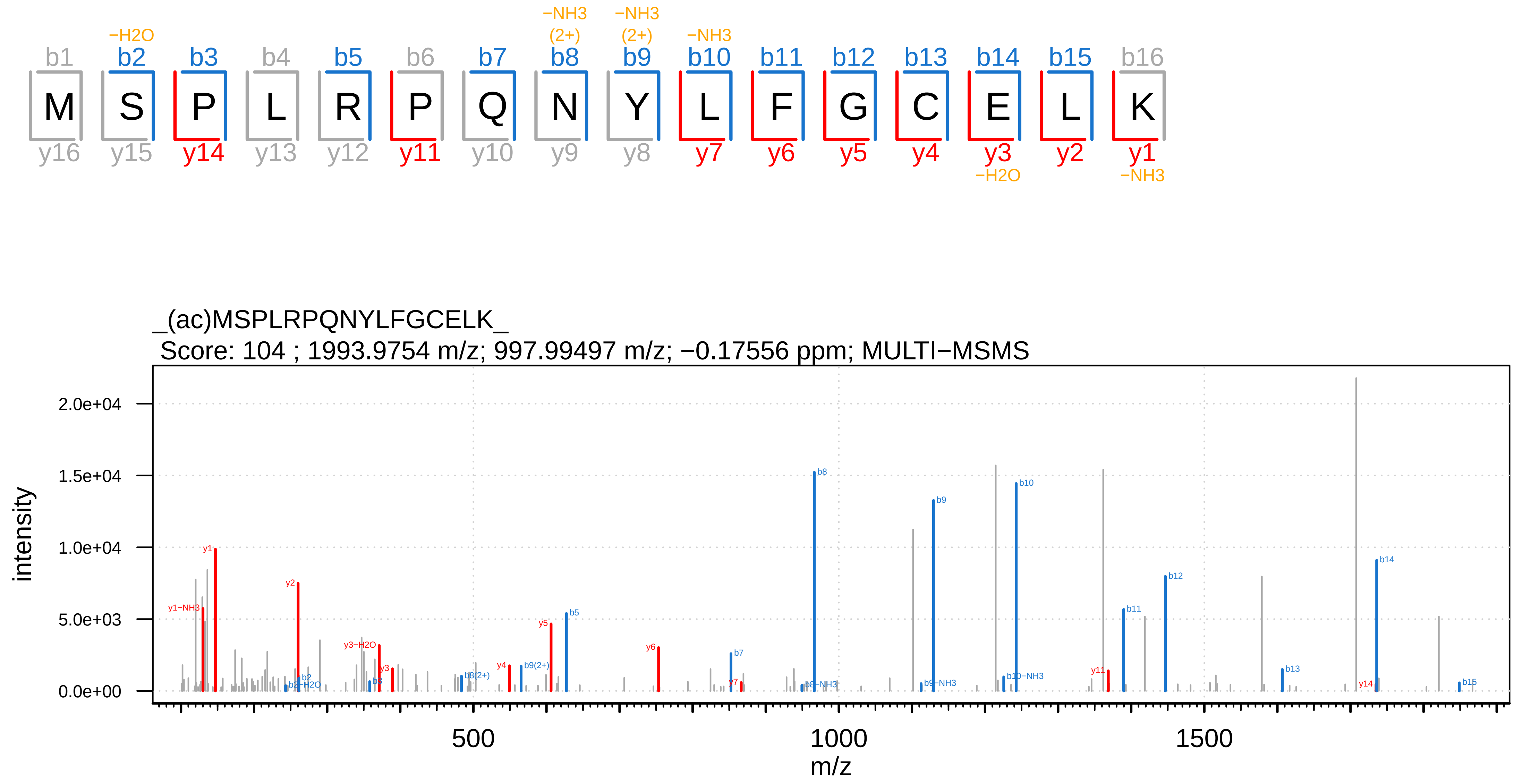

Raw File: 20100609_Velos1_TaGe_SA_293_1
Scan Number: 36580
Proteins:
TCONS_l2_00008829_chr15:92829088−92829258:+

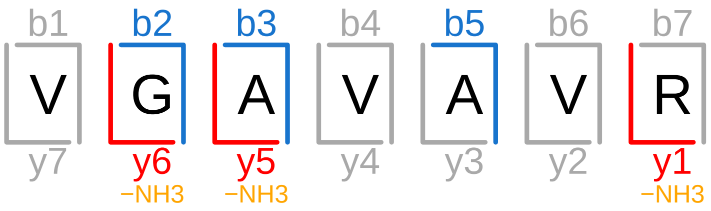

VGAVAVR
Score: 69 ; 670.41261 m/z; 336.21358 m/z; 0.2178 ppm; MULTI−MSMS

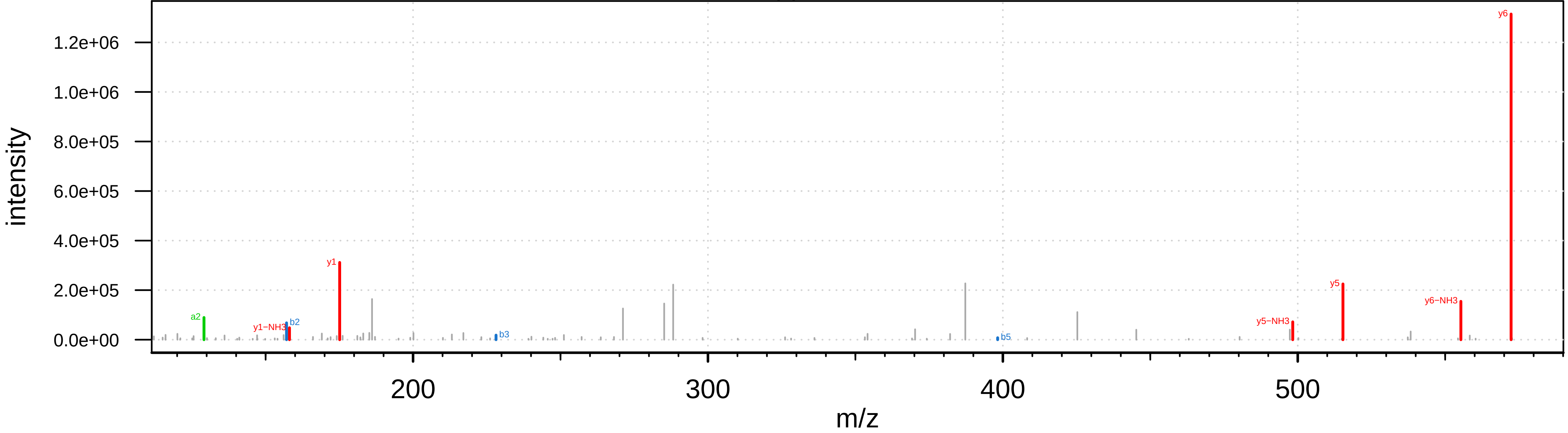

Raw File: 20100609_Velos1_TaGe_SA_293_1
Scan Number: 2254
Proteins:
TCONS_l2_00030545_chrX:79544539−79546436:−

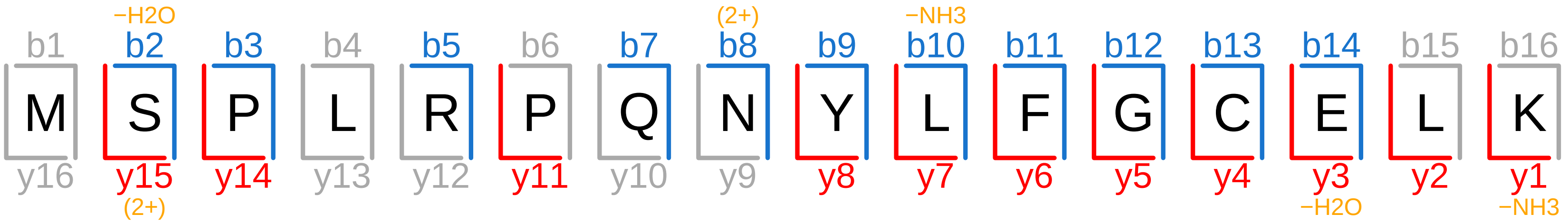

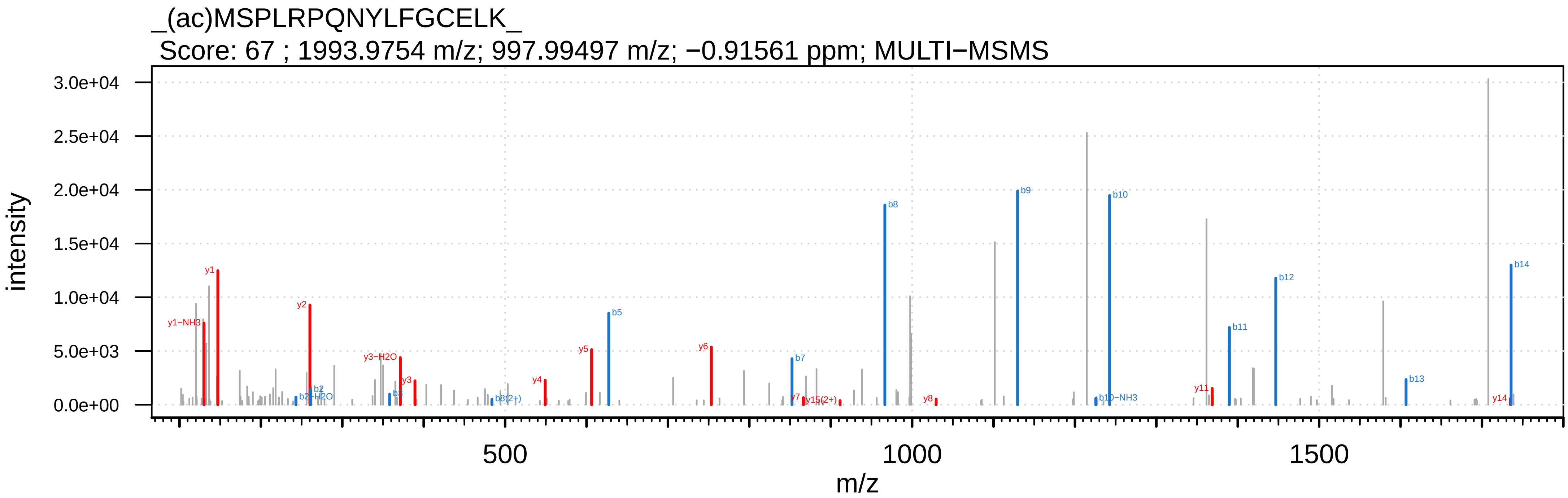

Raw File: 20100604_Velos1_TaGe_SA_A549_1
Scan Number: 34597
Proteins:
TCONS_l2_00008829_chr15:92829088−92829258:+

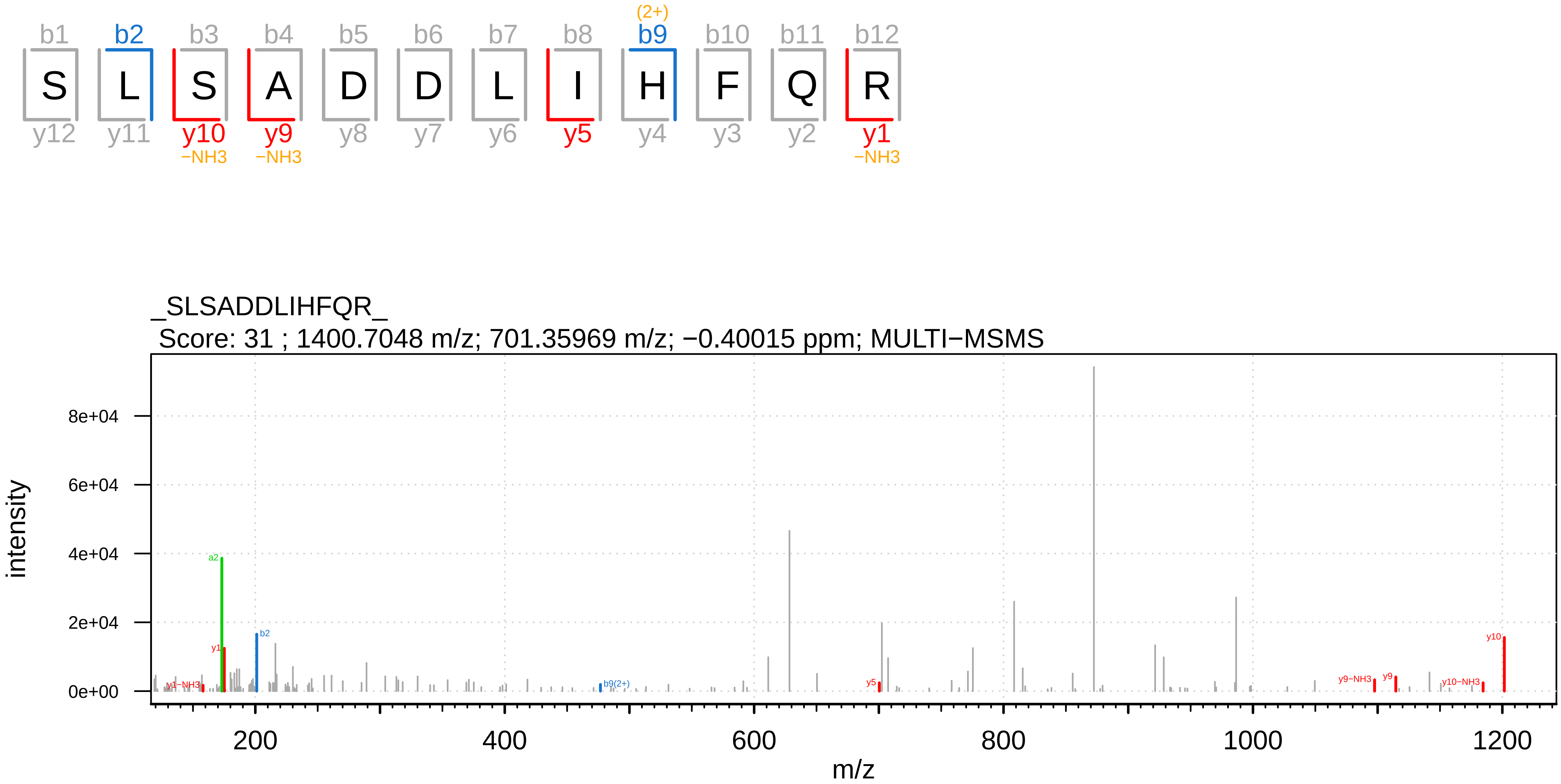

Raw File: 20100604_Velos1_TaGe_SA_A549_1
Scan Number: 16586
Proteins:
TCONS_l2_00002988_chr10:38499673−38501345:+

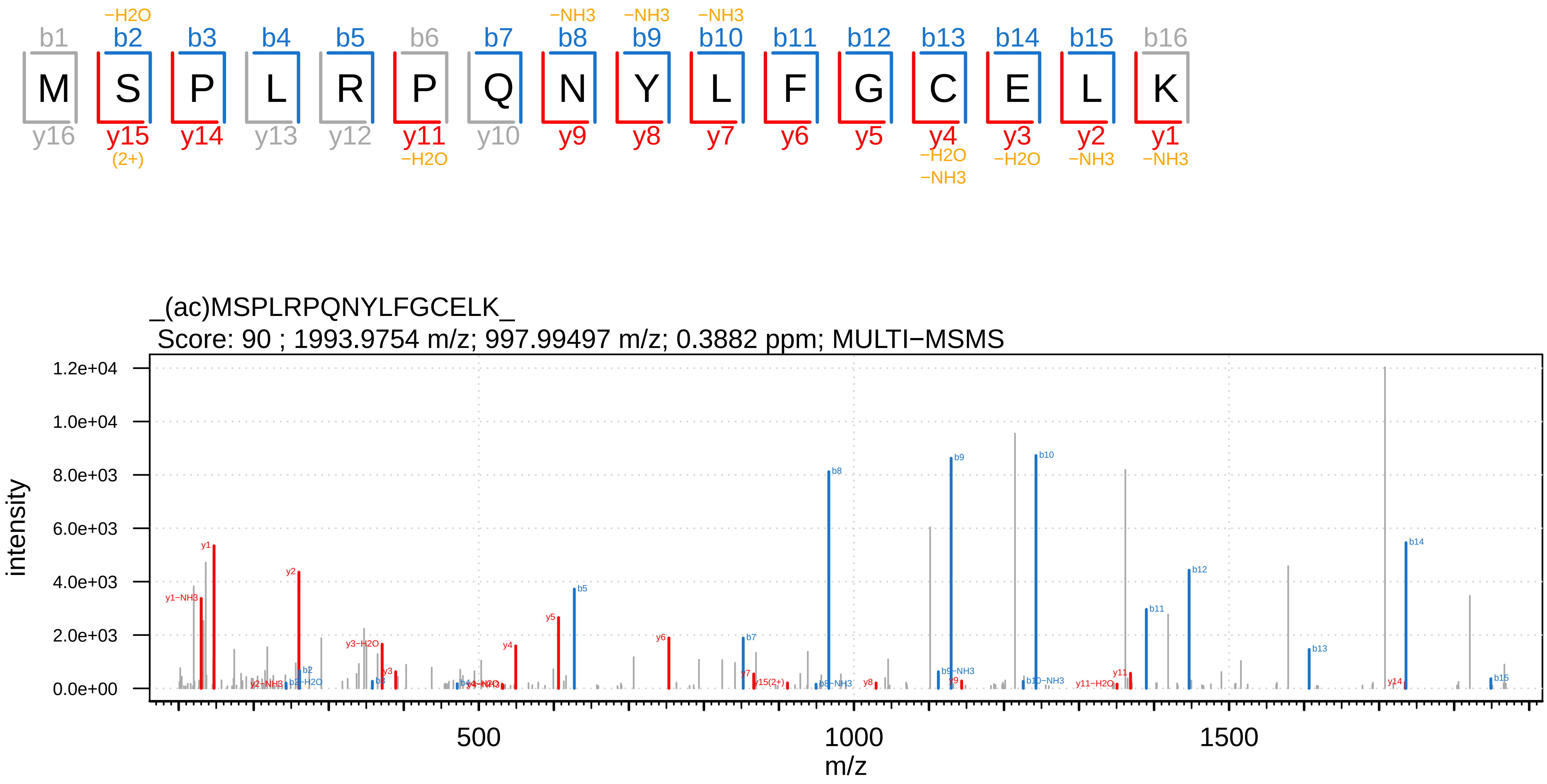

Raw File: 20100611_Velos1_TaGe_SA_Hela_2
Scan Number: 25725
Proteins:
TCONS_l2_00008829_chr15:92829088−92829258:+

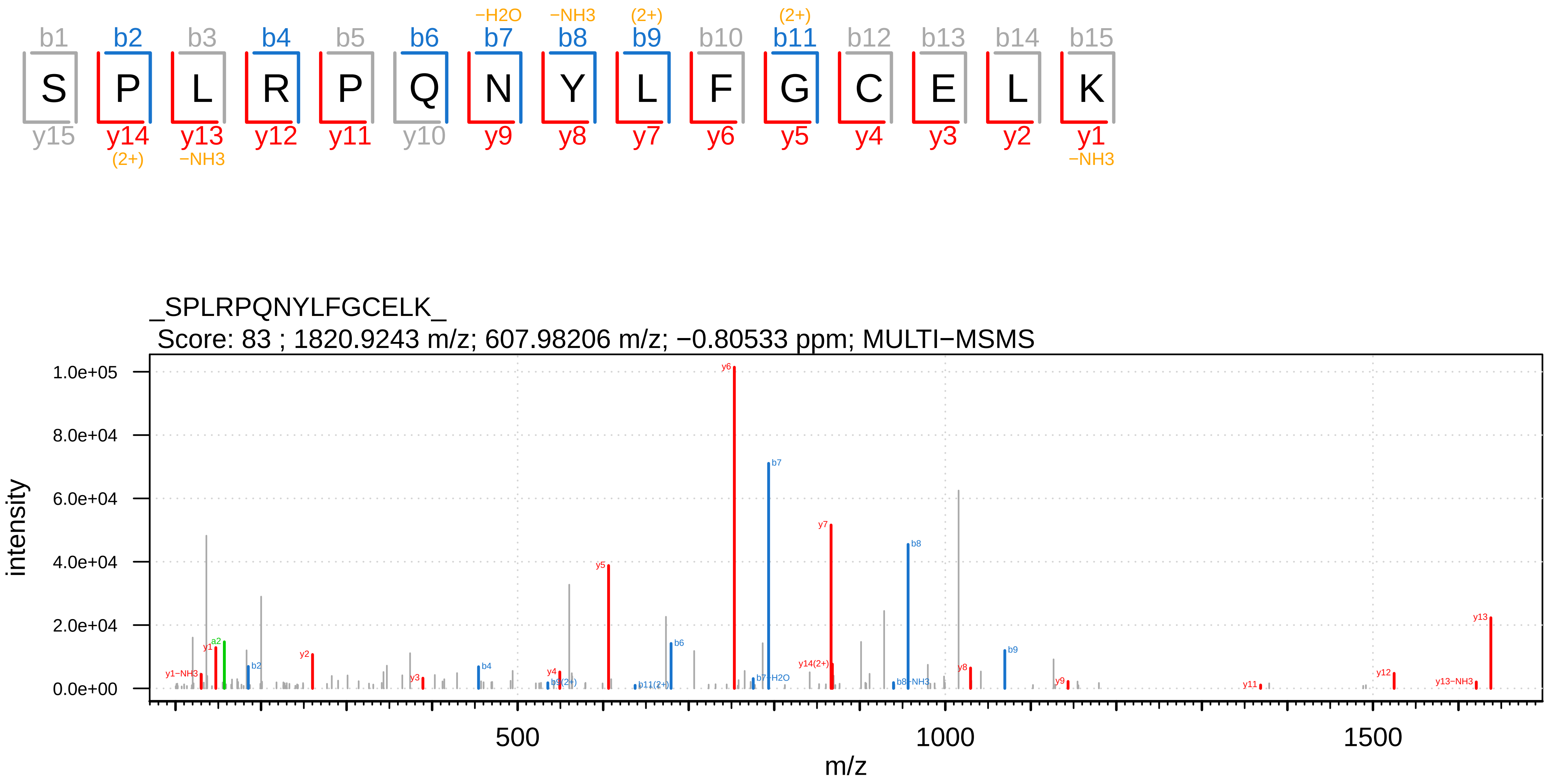

Raw File: 20100611_Velos1_TaGe_SA_Hela_2
Scan Number: 16815
Proteins:
TCONS_l2_00008829_chr15:92829088−92829258:+

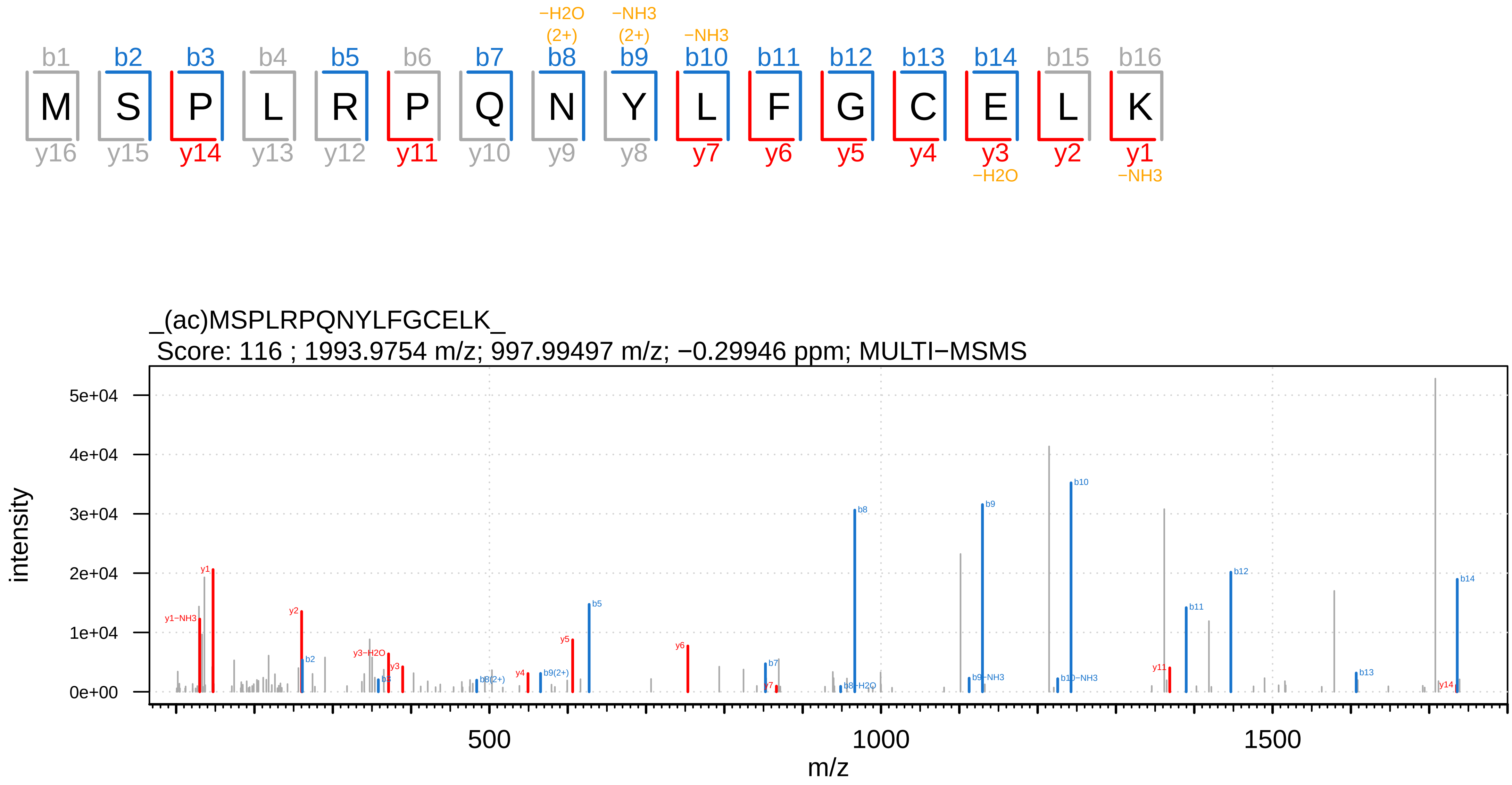

Raw File: 20100611_Velos1_TaGe_SA_HepG2_1
Scan Number: 36267
Proteins:
TCONS_l2_00008829_chr15:92829088−92829258:+

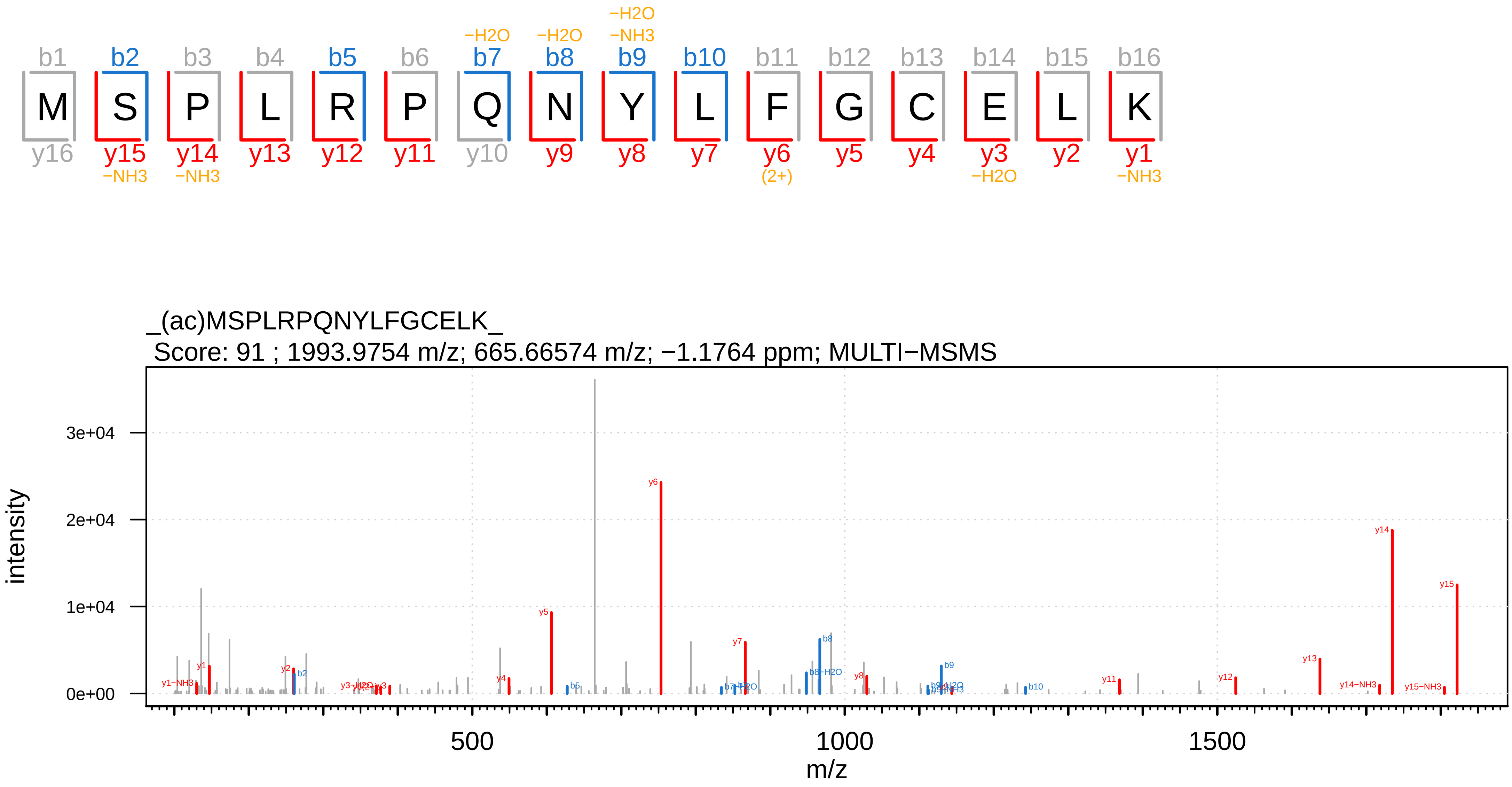

Raw File: 20100611_Velos1_TaGe_SA_HepG2_1
Scan Number: 36292
Proteins:
TCONS_l2_00008829_chr15:92829088−92829258:+

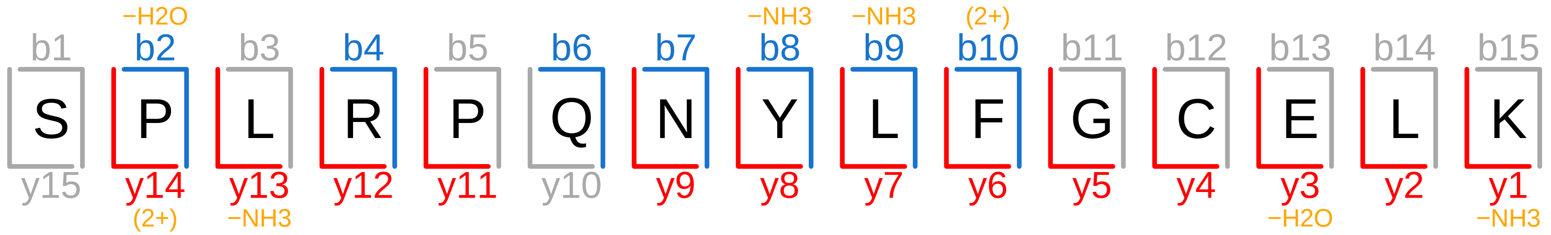

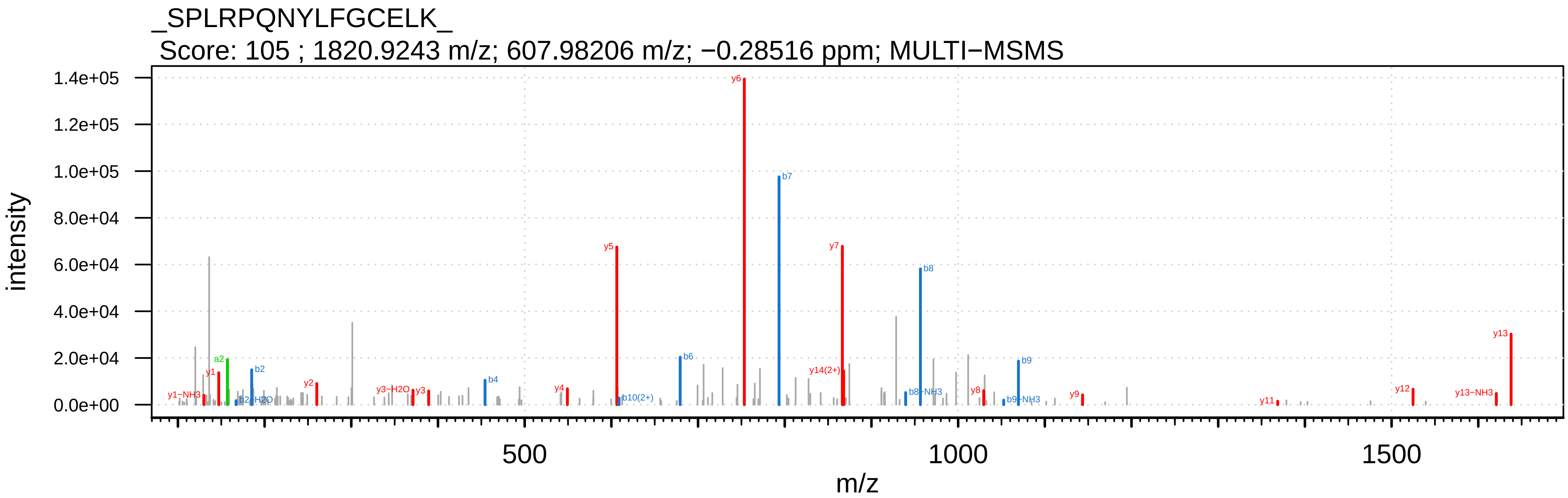

Raw File: 20100611_Velos1_TaGe_SA_HepG2_1
Scan Number: 21108
Proteins:
TCONS_l2_00008829_chr15:92829088−92829258:+

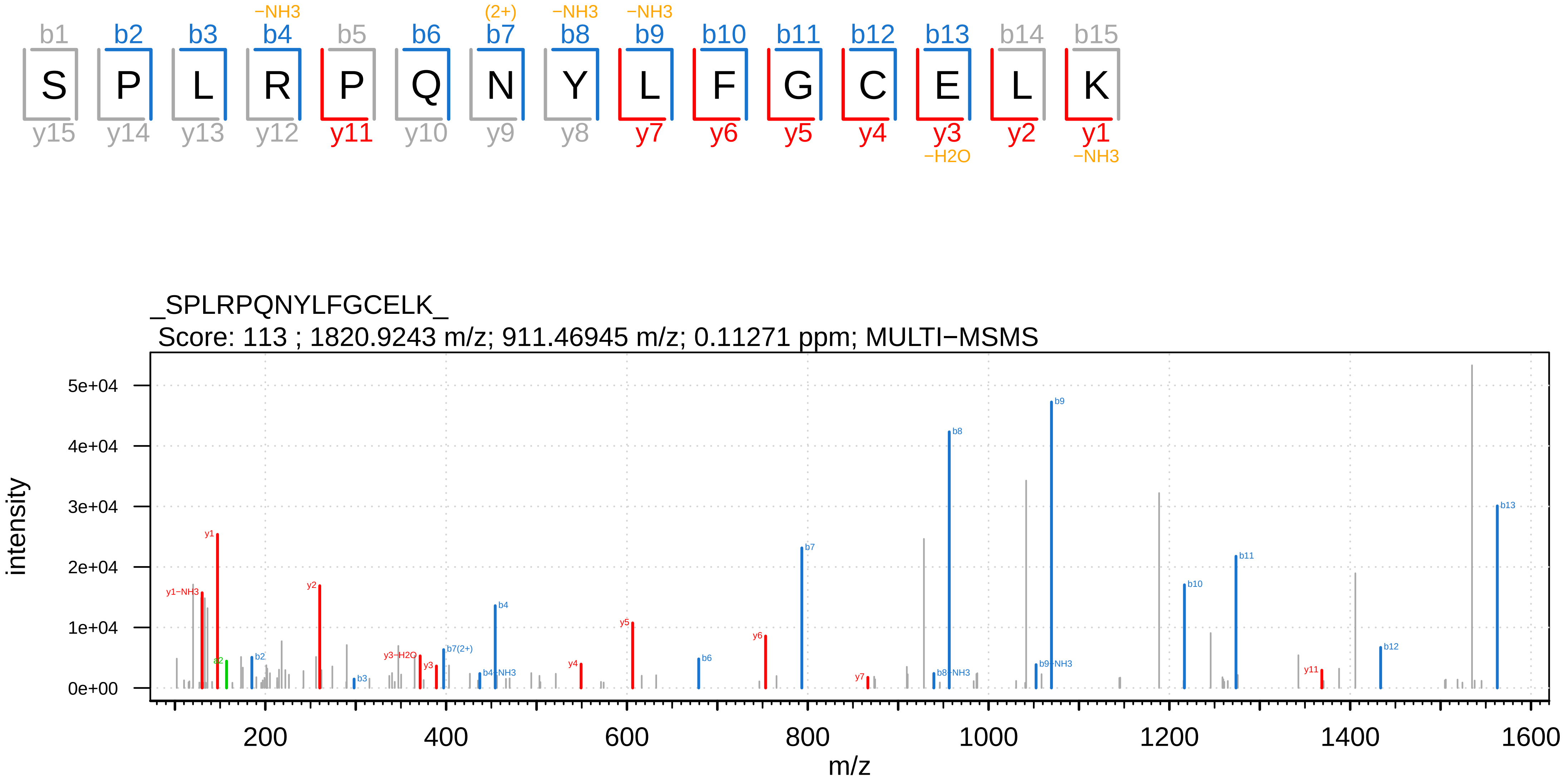

Raw File: 20100611_Velos1_TaGe_SA_HepG2_1
Scan Number: 21134
Proteins:
TCONS_l2_00008829_chr15:92829088−92829258:+

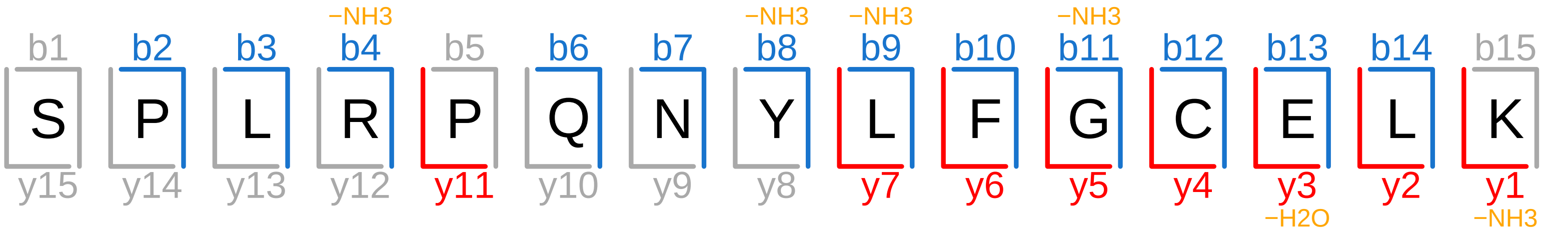

SPLRPQNYLFGCELK
Score: 68 ; 1820.9243 m/z; 911.46945 m/z; 0.11271 ppm; MULTI−MSMS

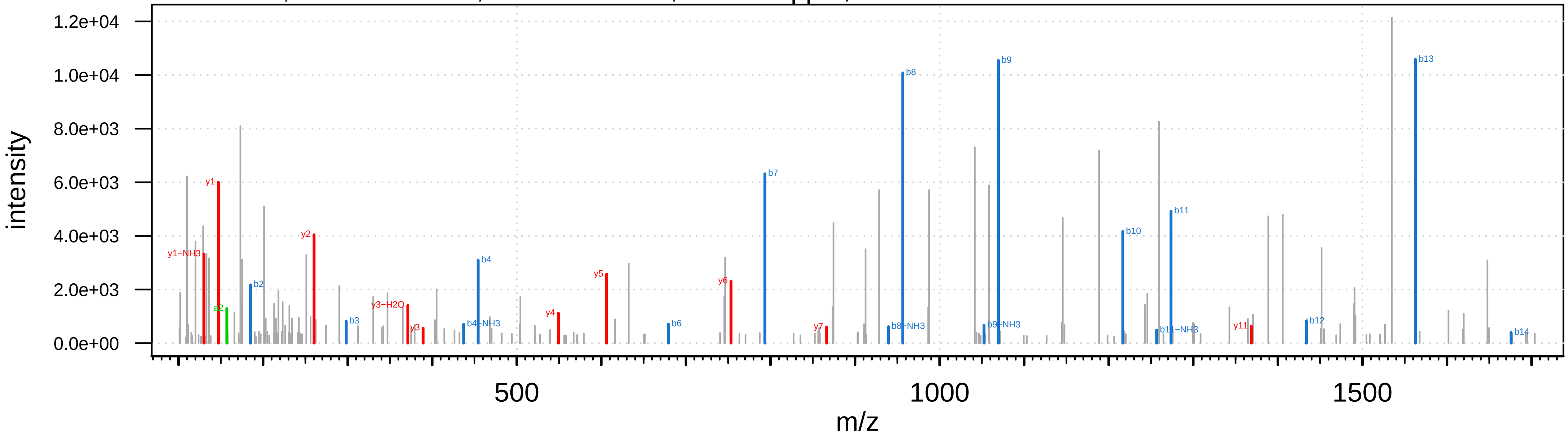

Raw File: 20100611_Velos1_TaGe_SA_HepG2_1
Scan Number: 21265
Proteins:
TCONS_l2_00008829_chr15:92829088−92829258:+

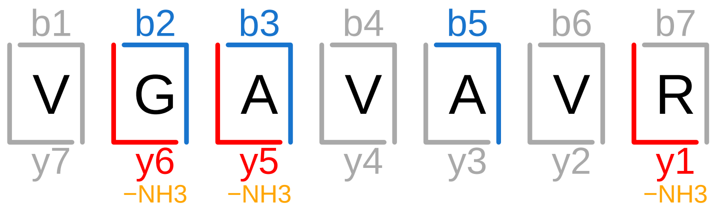

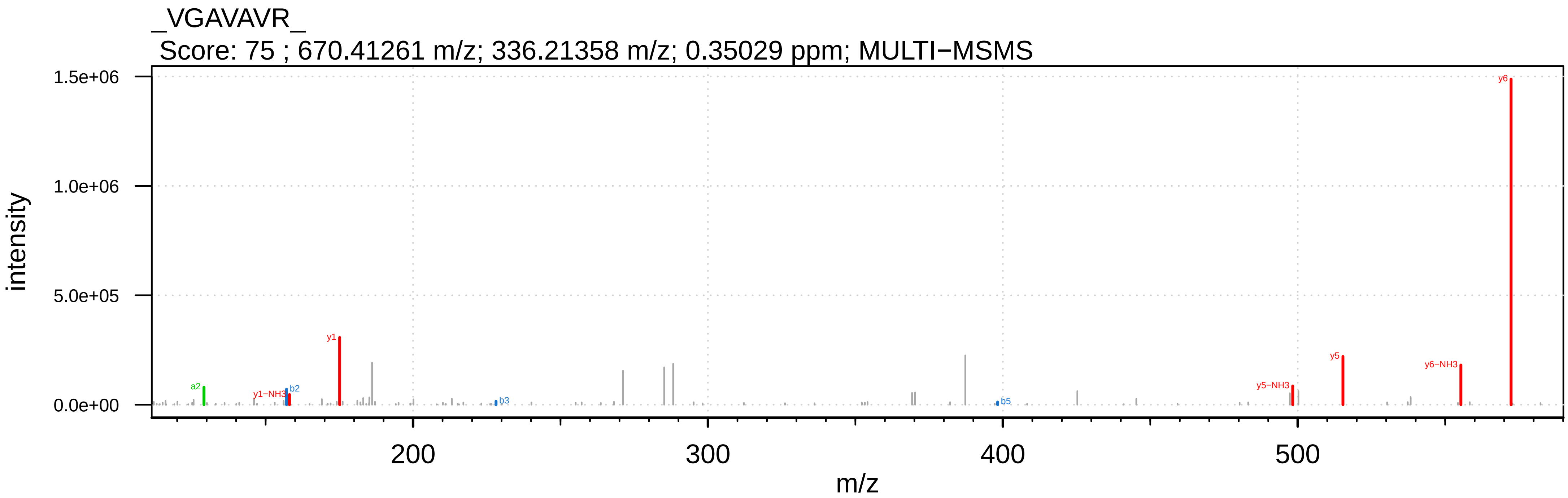

Raw File: 20100611_Velos1_TaGe_SA_HepG2_1
Scan Number: 2142
Proteins:
TCONS_l2_00030545_chrX:79544539−79546436:−

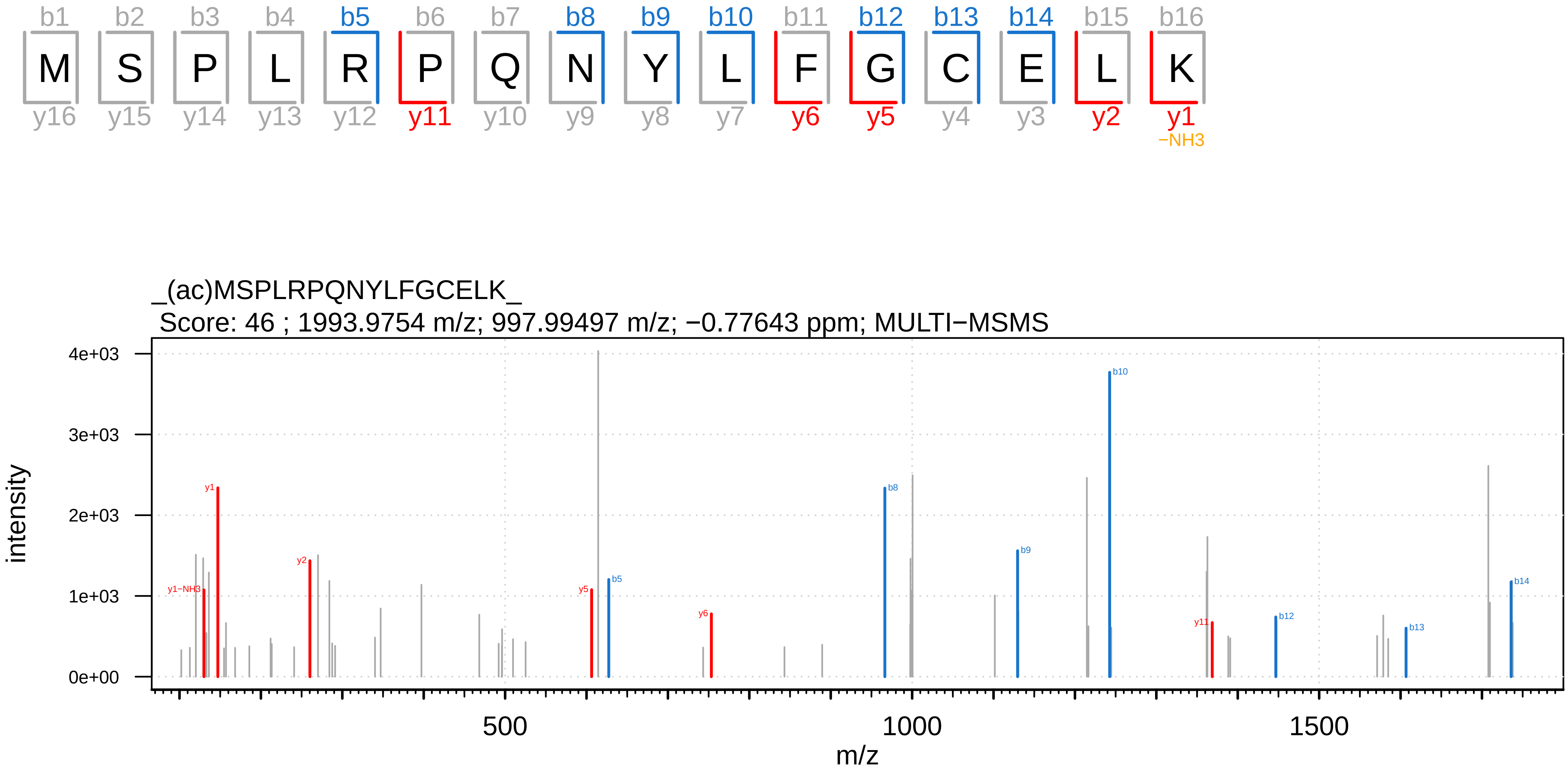

Raw File: 20100614_Velos1_TaGe_SA_Jurkat_1
Scan Number: 36266
Proteins:
TCONS_l2_00008829_chr15:92829088−92829258:+

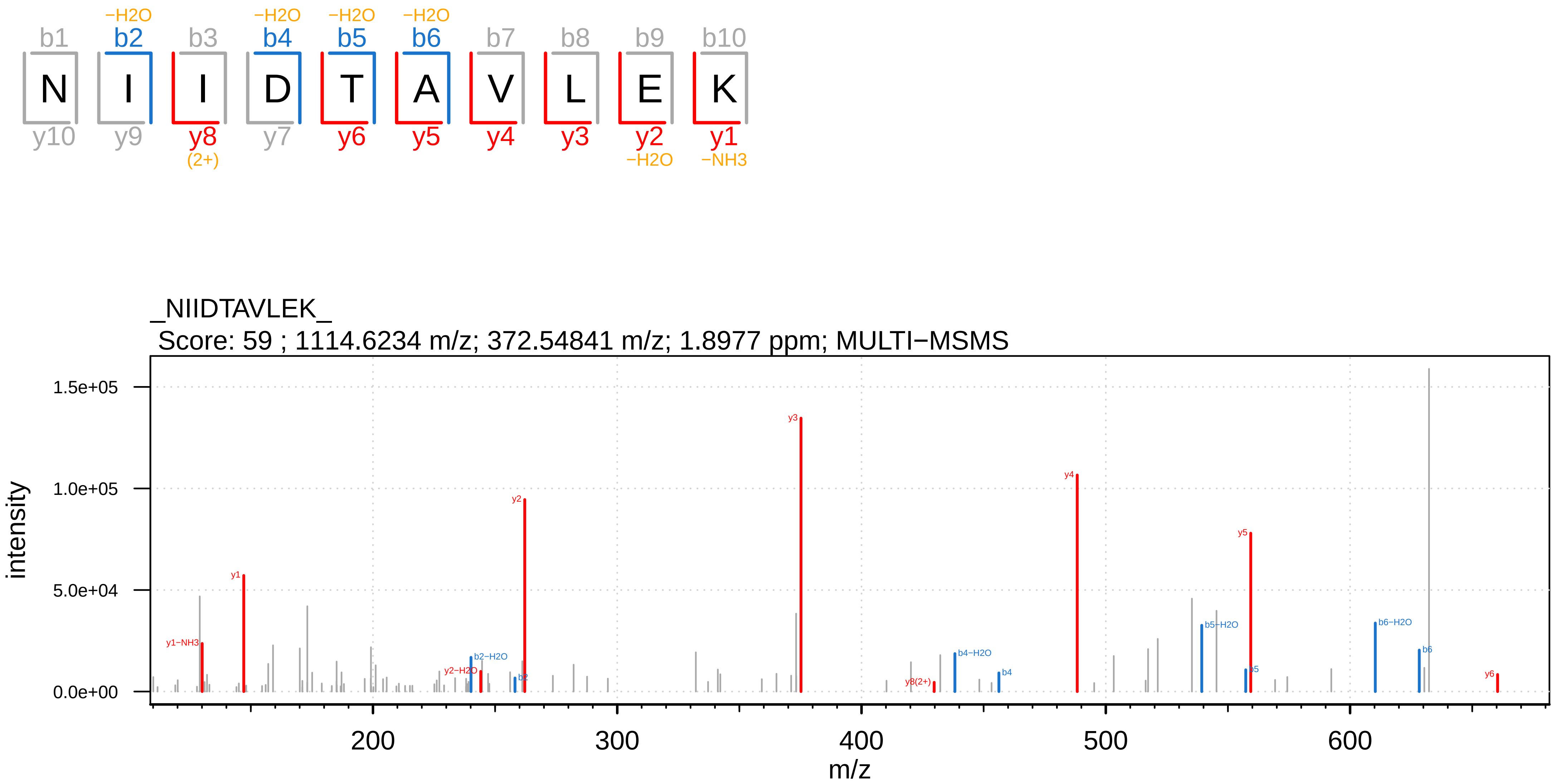

Raw File: 20100614_Velos1_TaGe_SA_Jurkat_1
Scan Number: 5508
Proteins:
ENST00000497138_chr20:56806826−56807846:−

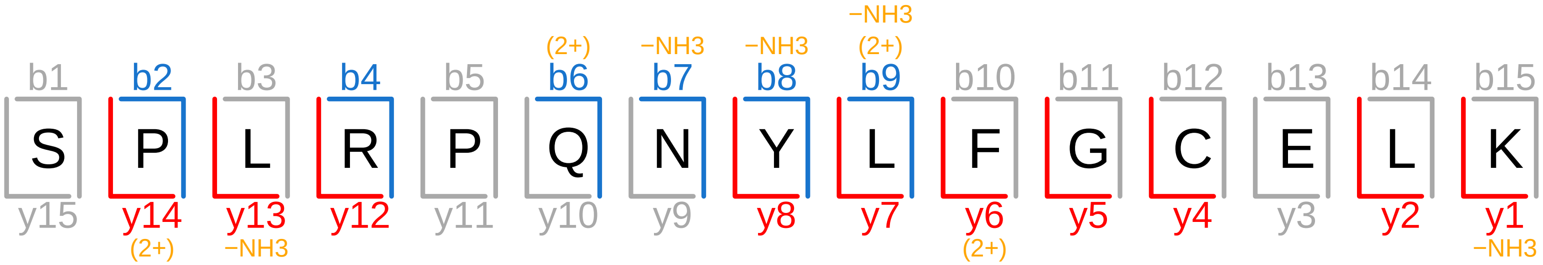

SPLRPQNYLFGCELK
Score: 85 ; 1820.9243 m/z; 607.98206 m/z; −1.3631 ppm; MULTI−MSMS

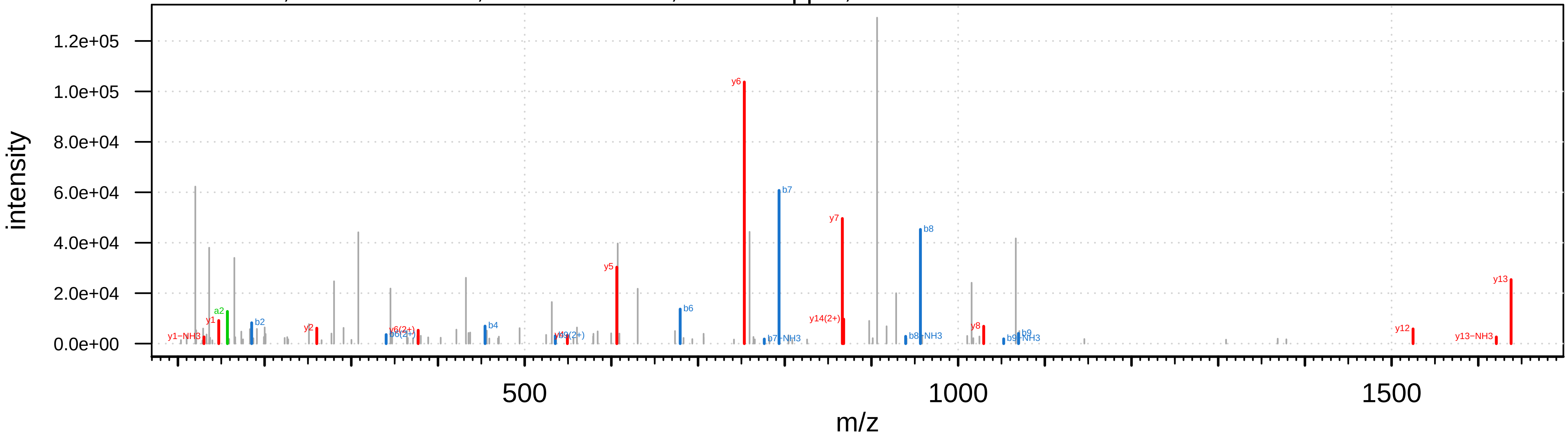

Raw File: 20100614_Velos1_TaGe_SA_Jurkat_1
Scan Number: 22522
Proteins:
TCONS_l2_00008829_chr15:92829088−92829258:+

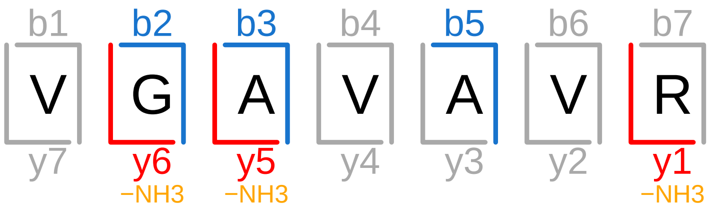

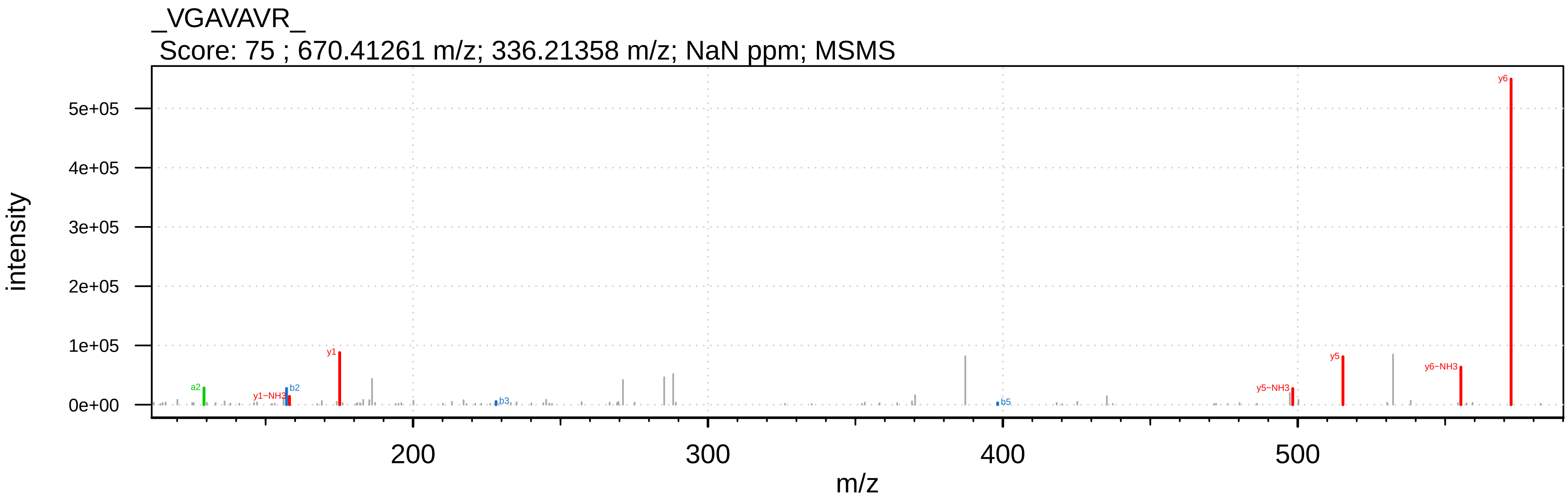

Raw File: 20100614_Velos1_TaGe_SA_Jurkat_1
Scan Number: 2948
Proteins:
TCONS_l2_00030545_chrX:79544539−79546436:−

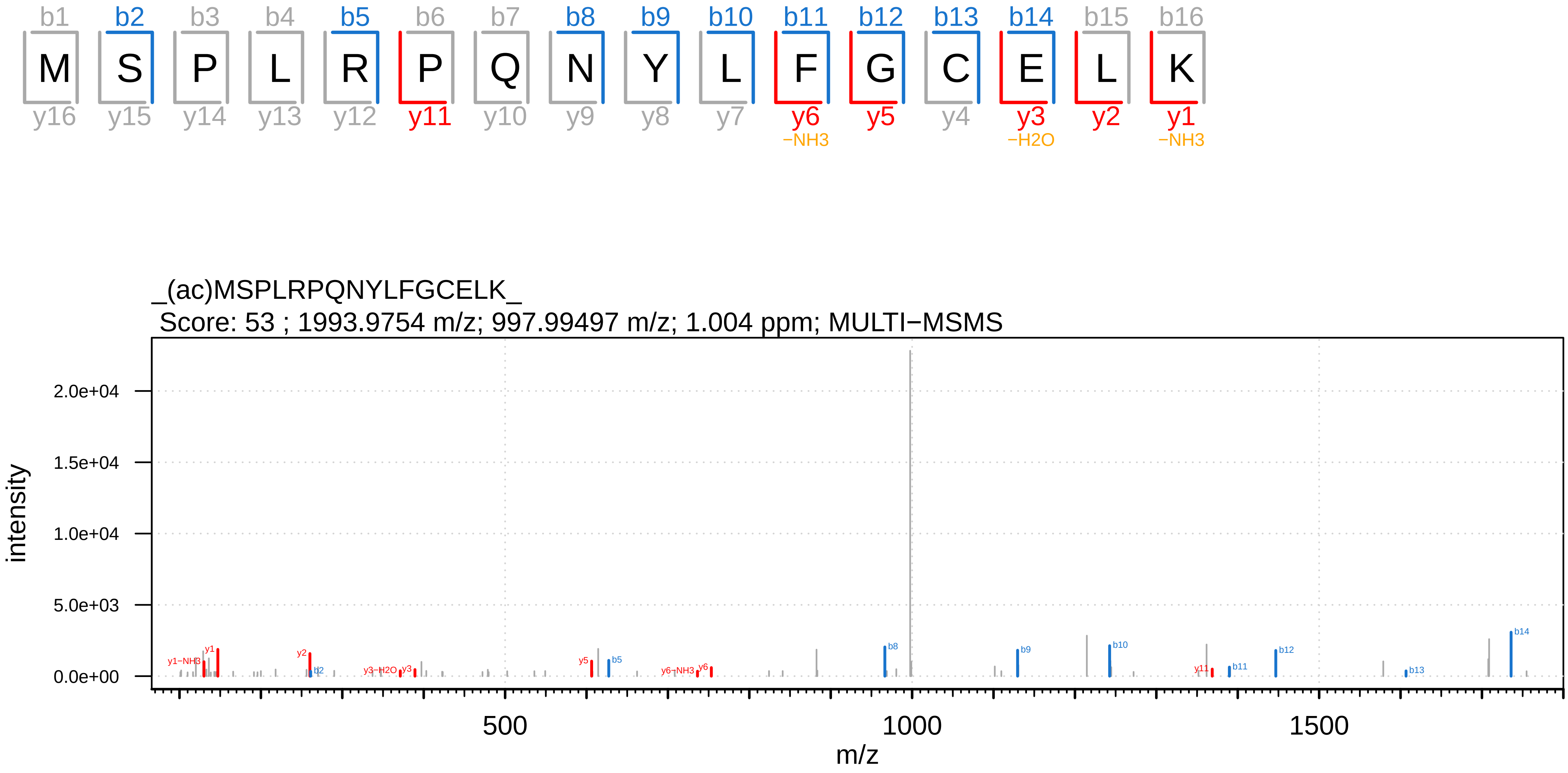

Raw File: 20100614_Velos1_TaGe_SA_K562_1
Scan Number: 37185
Proteins:
TCONS_l2_00008829_chr15:92829088−92829258:+

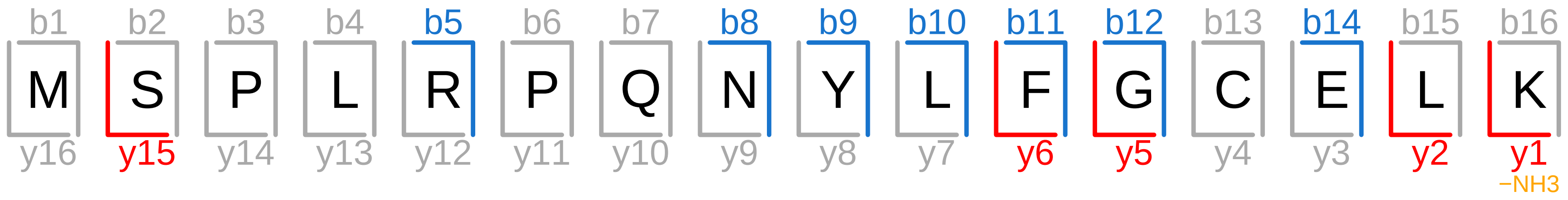

(ac)MSPLRPQNYLFGCELK
Score: 46 ; 1993.9754 m/z; 997.99497 m/z; 1.004 ppm; MULTI−MSMS

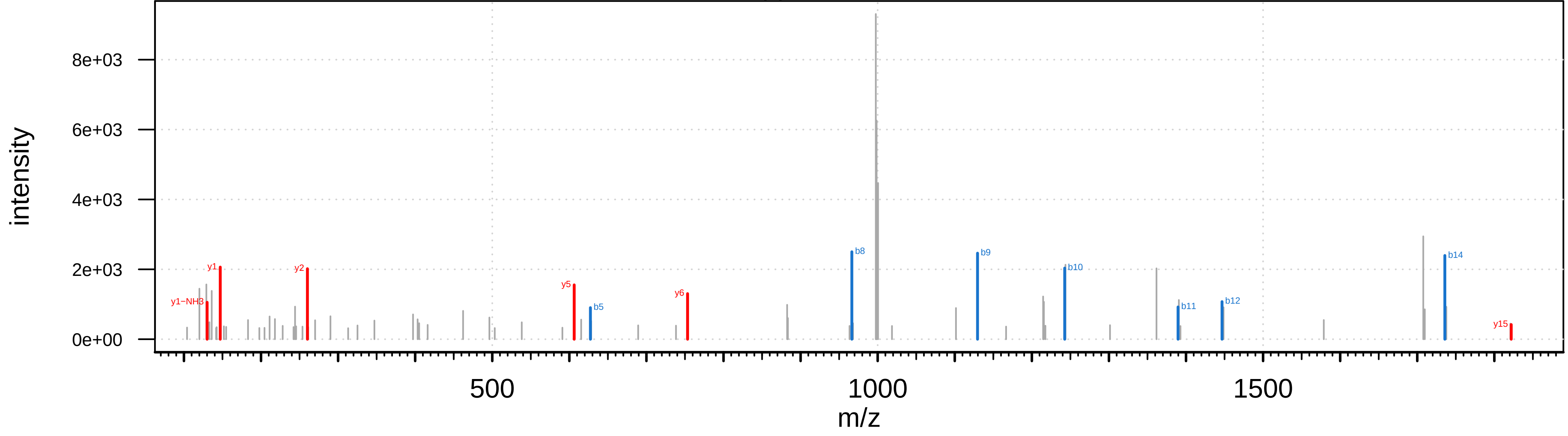

Raw File: 20100614_Velos1_TaGe_SA_K562_1
Scan Number: 37223
Proteins:
TCONS_l2_00008829_chr15:92829088−92829258:+

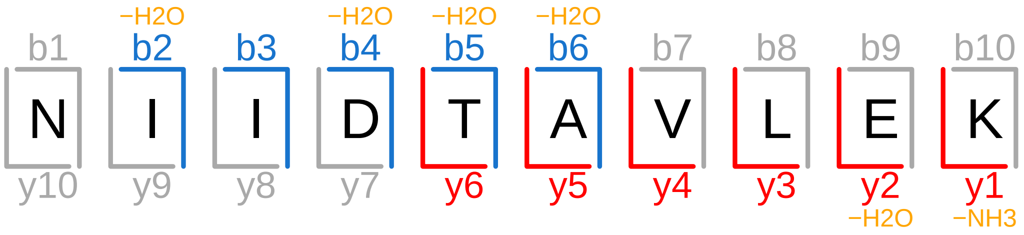

NIIDTAVLEK
Score: 42 ; 1114.6234 m/z; 372.54841 m/z; 1.0444 ppm; MULTI−MSMS

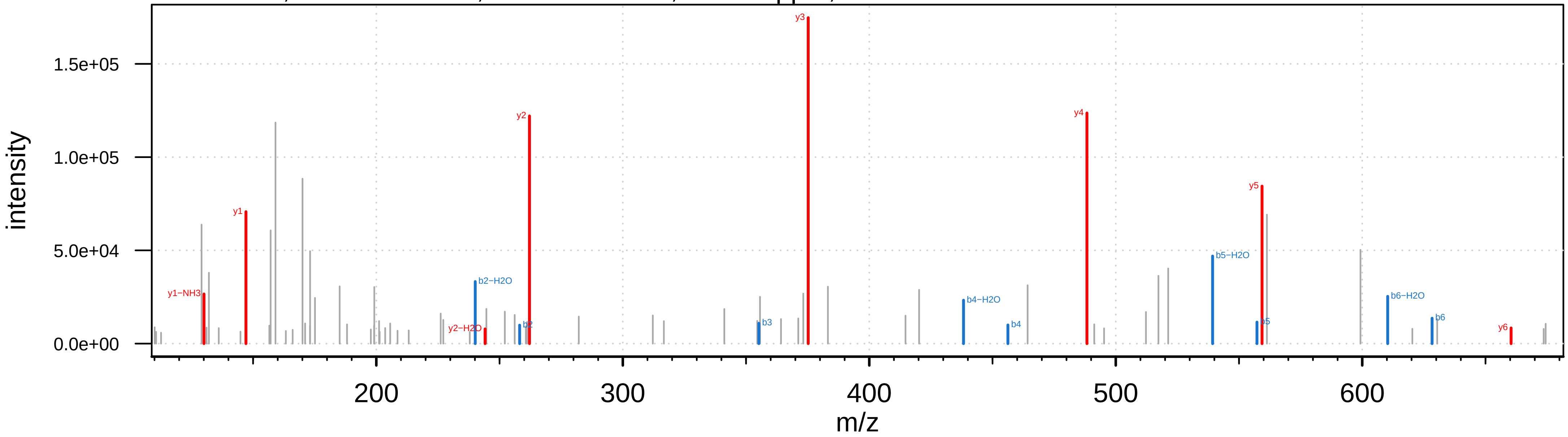

Raw File: 20100614_Velos1_TaGe_SA_K562_1
Scan Number: 5533
Proteins:
ENST00000497138_chr20:56806826−56807846:−

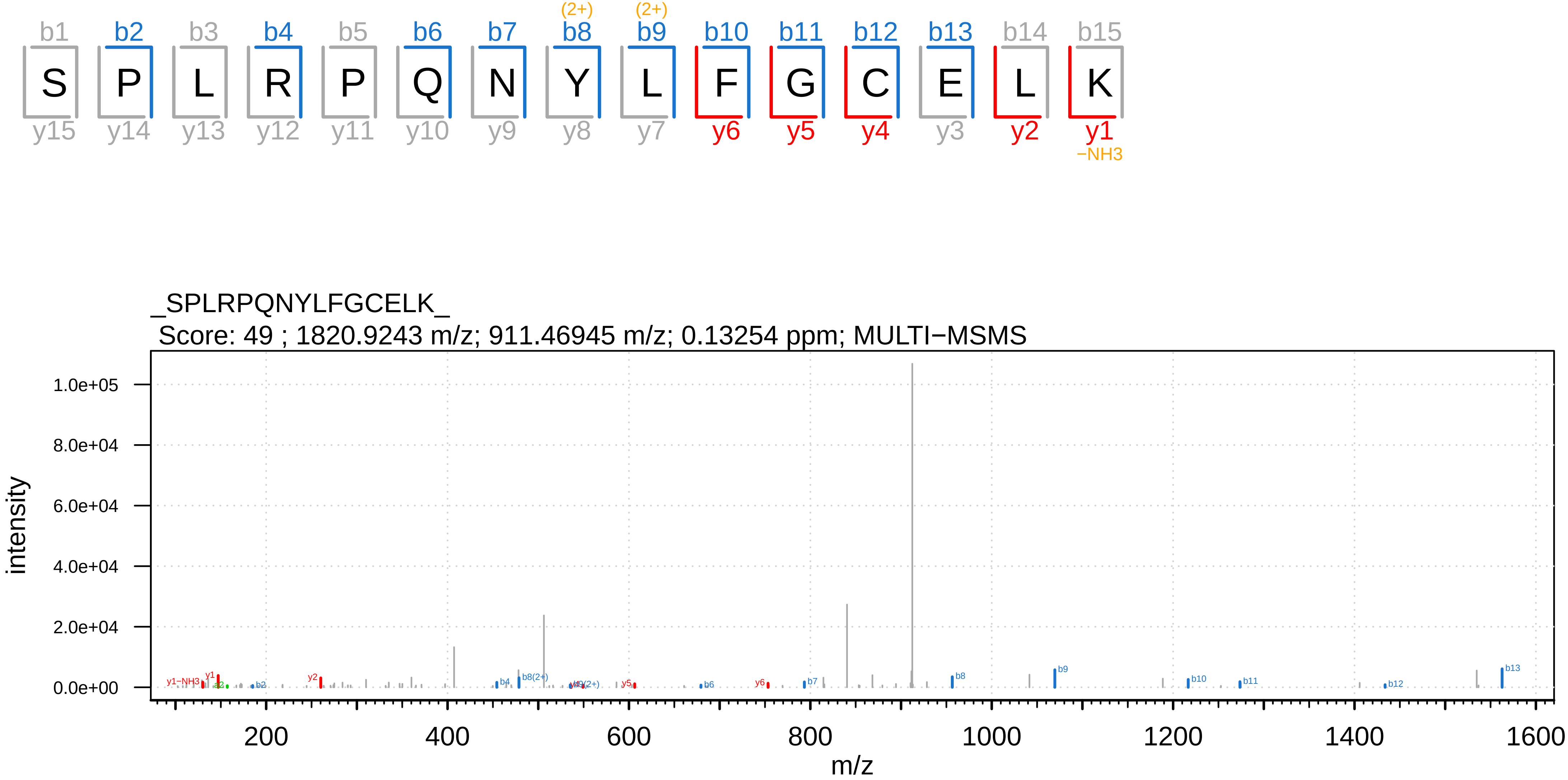

Raw File: 20100614_Velos1_TaGe_SA_K562_1
Scan Number: 22646
Proteins:
TCONS_l2_00008829_chr15:92829088−92829258:+

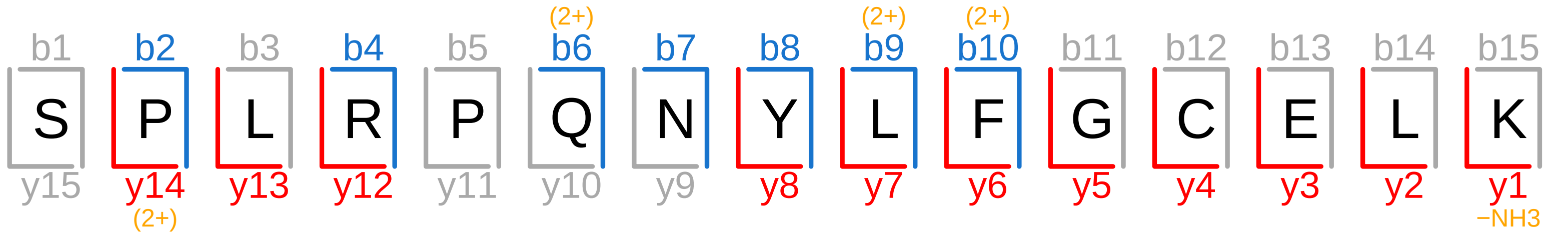

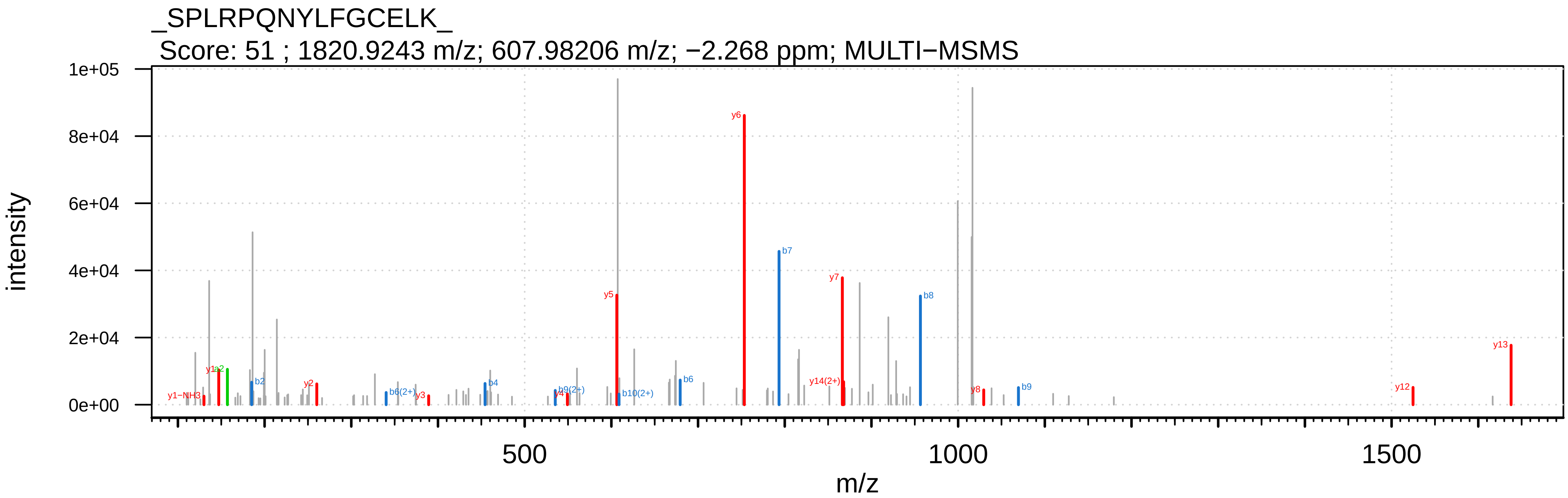

Raw File: 20100614_Velos1_TaGe_SA_K562_1
Scan Number: 22656
Proteins:
TCONS_l2_00008829_chr15:92829088−92829258:+

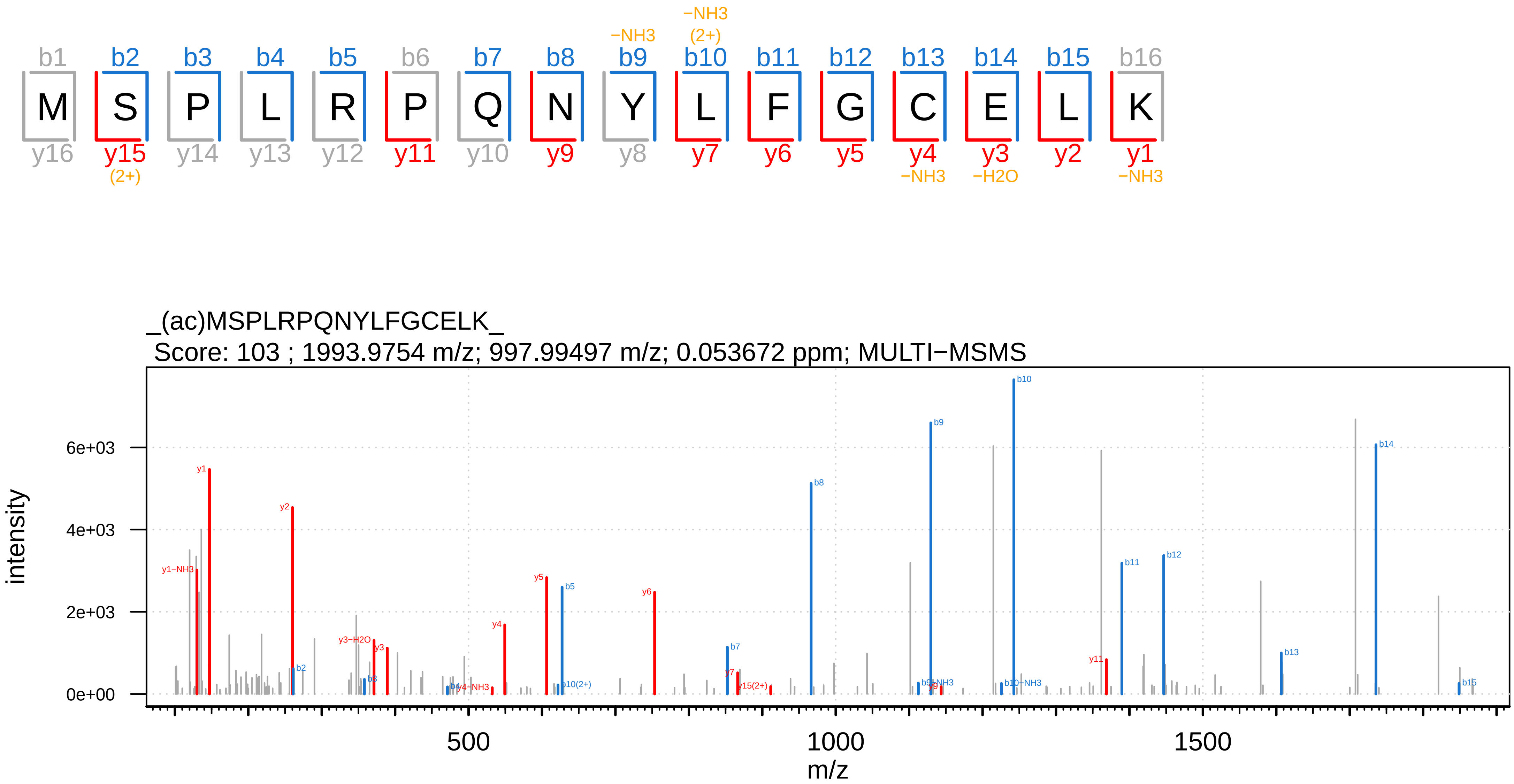

Raw File: 20101210_Velos1_AnWe_SA_LnCap_2
Scan Number: 32137
Proteins:
TCONS_l2_00008829_chr15:92829088−92829258:+

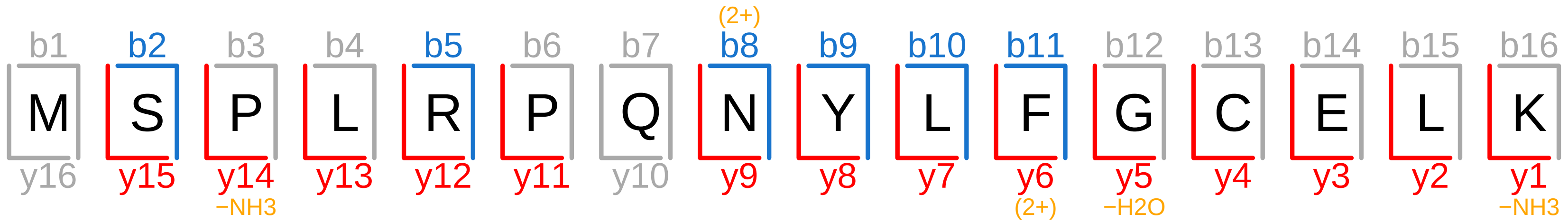

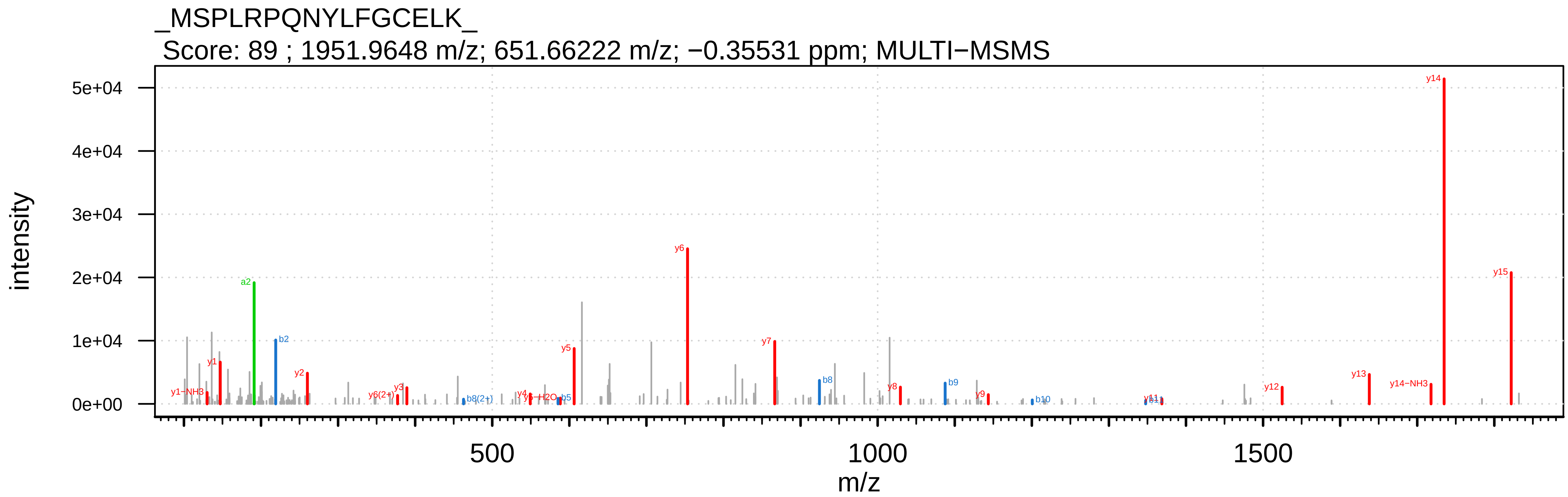

Raw File: 20101210_Velos1_AnWe_SA_LnCap_2
Scan Number: 23919
Proteins:
TCONS_l2_00008829_chr15:92829088−92829258:+

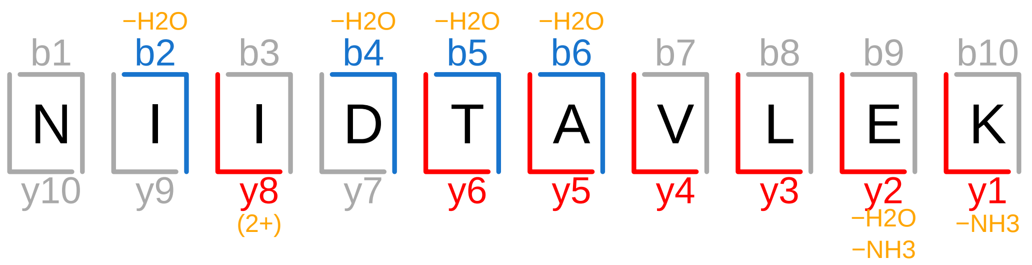

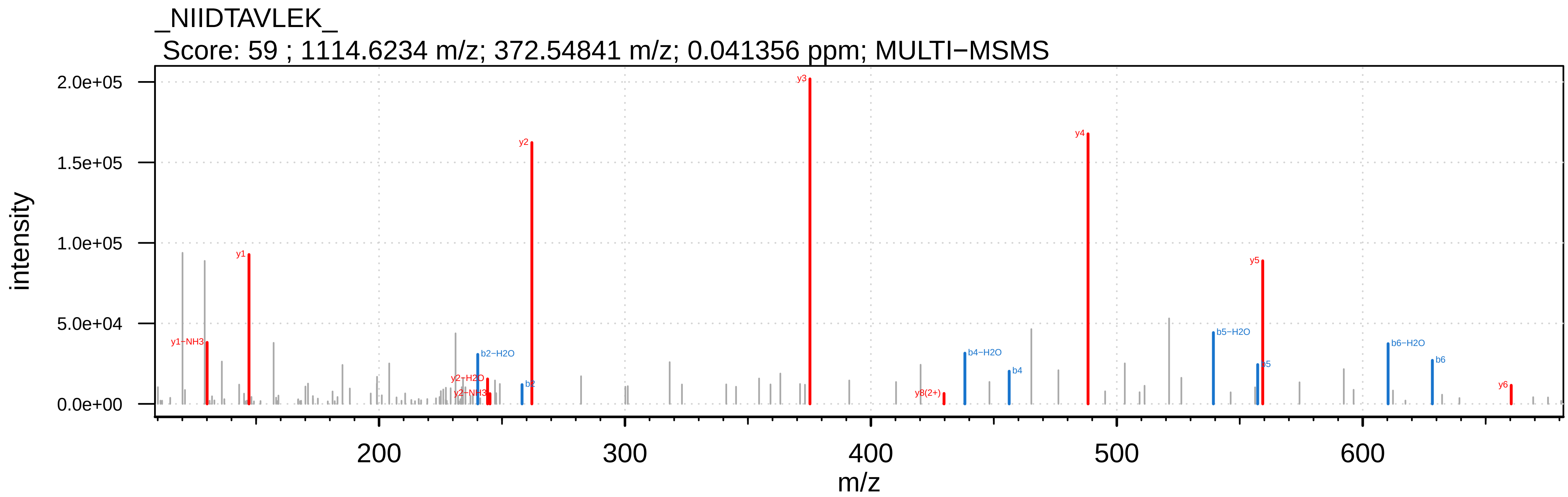

Raw File: 20101210_Velos1_AnWe_SA_LnCap_2
Scan Number: 9707
Proteins:
ENST00000497138_chr20:56806826−56807846:−

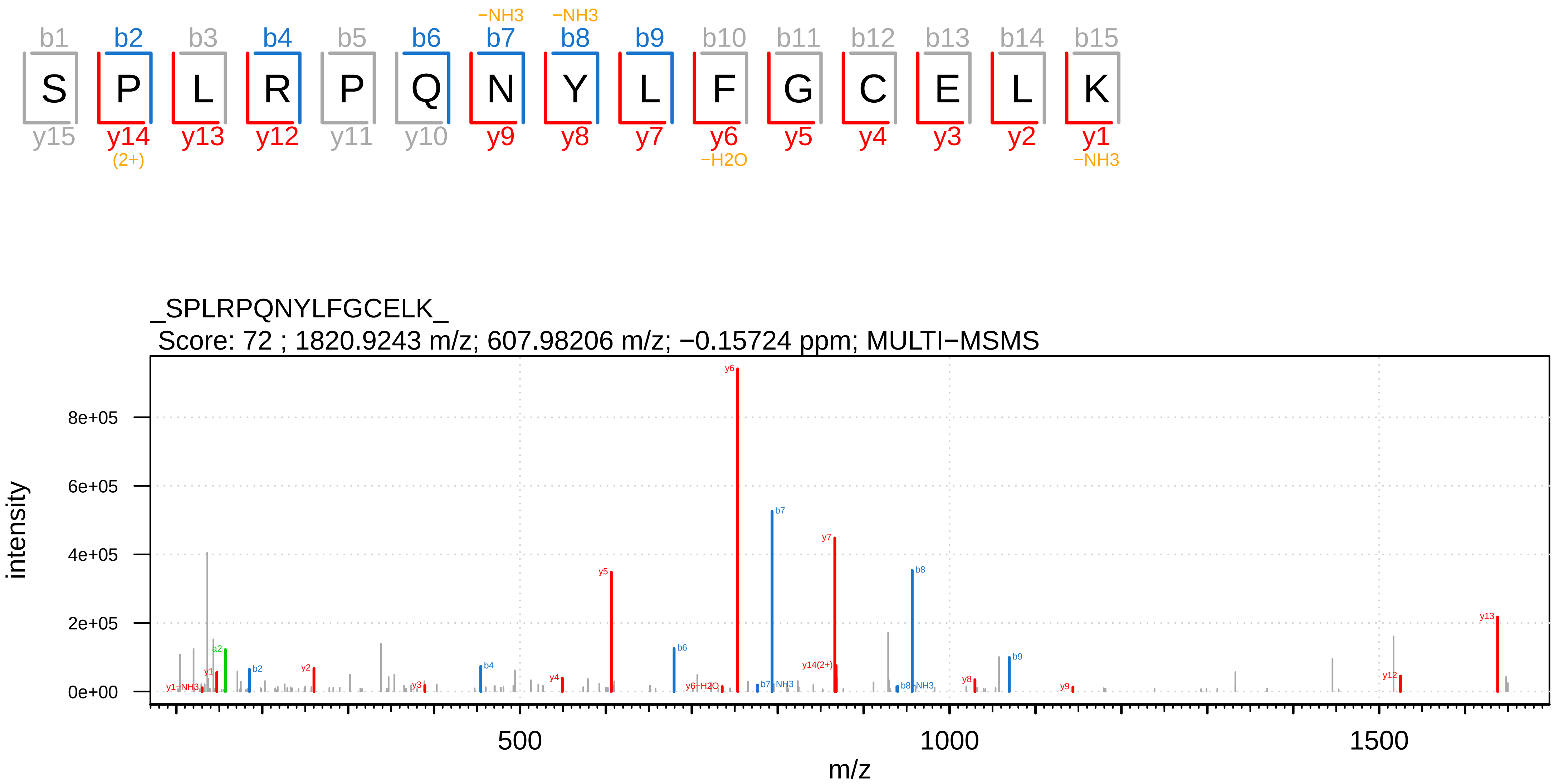

Raw File: 20101210_Velos1_AnWe_SA_LnCap_2
Scan Number: 21025
Proteins:
TCONS_l2_00008829_chr15:92829088−92829258:+

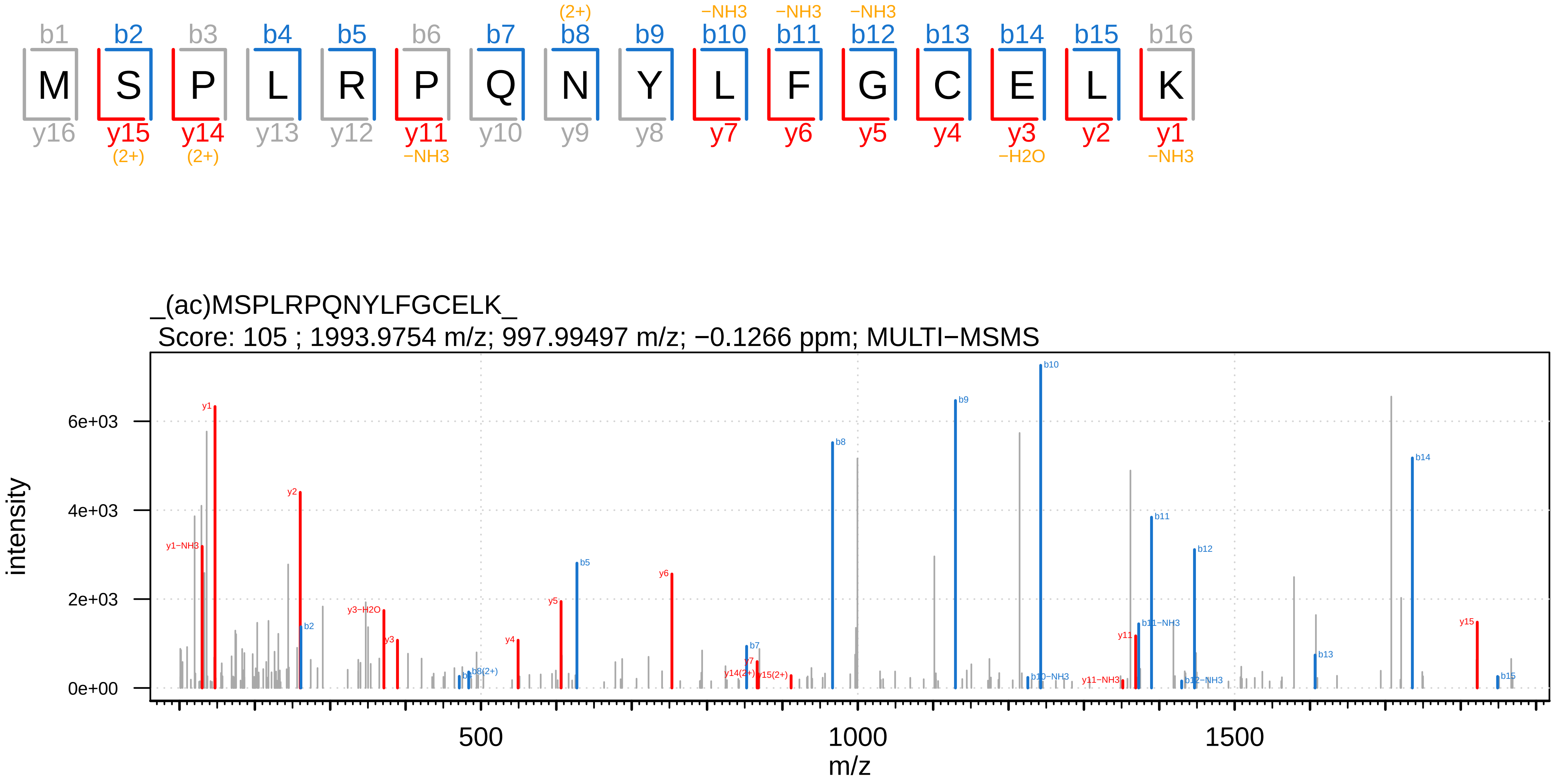

Raw File: 20101210_Velos1_AnWe_SA_U2OS_1
Scan Number: 38163
Proteins:
TCONS_l2_00008829_chr15:92829088−92829258:+

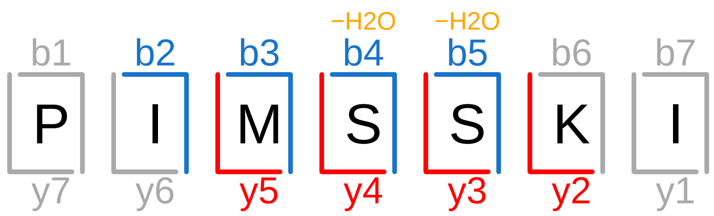

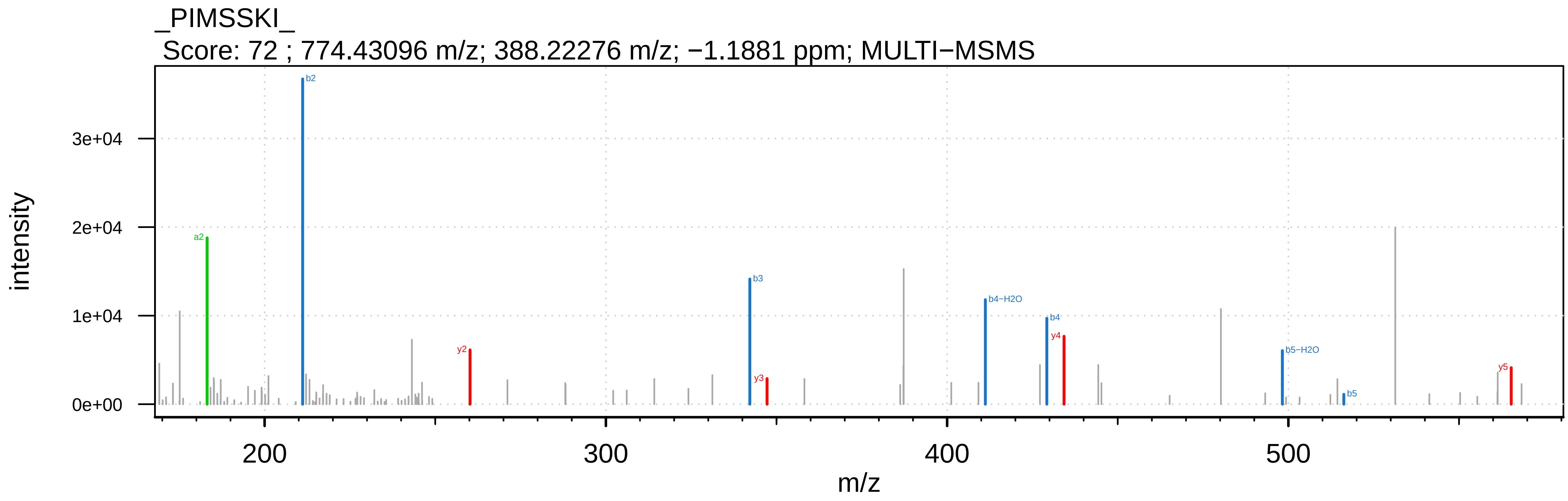

Raw File: 20101210_Velos1_AnWe_SA_U2OS_1
Scan Number: 22625
Proteins:
ENST00000490272_chr19:52826295−52826555:+

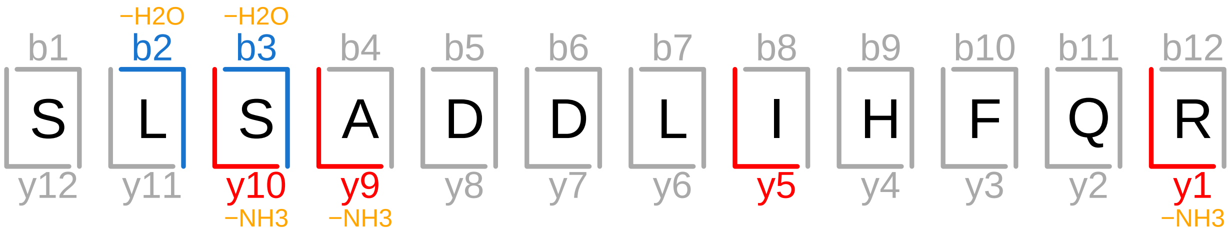

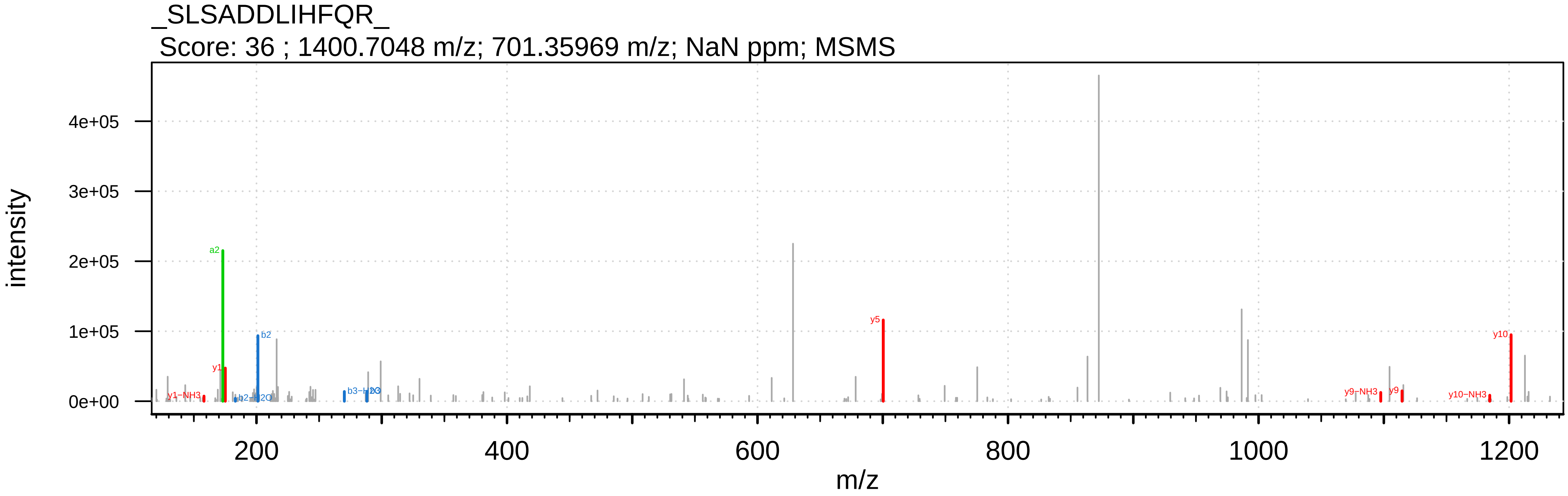

Raw File: 20101210_Velos1_AnWe_SA_U2OS_1
Scan Number: 19671
Proteins:
TCONS_l2_00002988_chr10:38499673−38501345:+

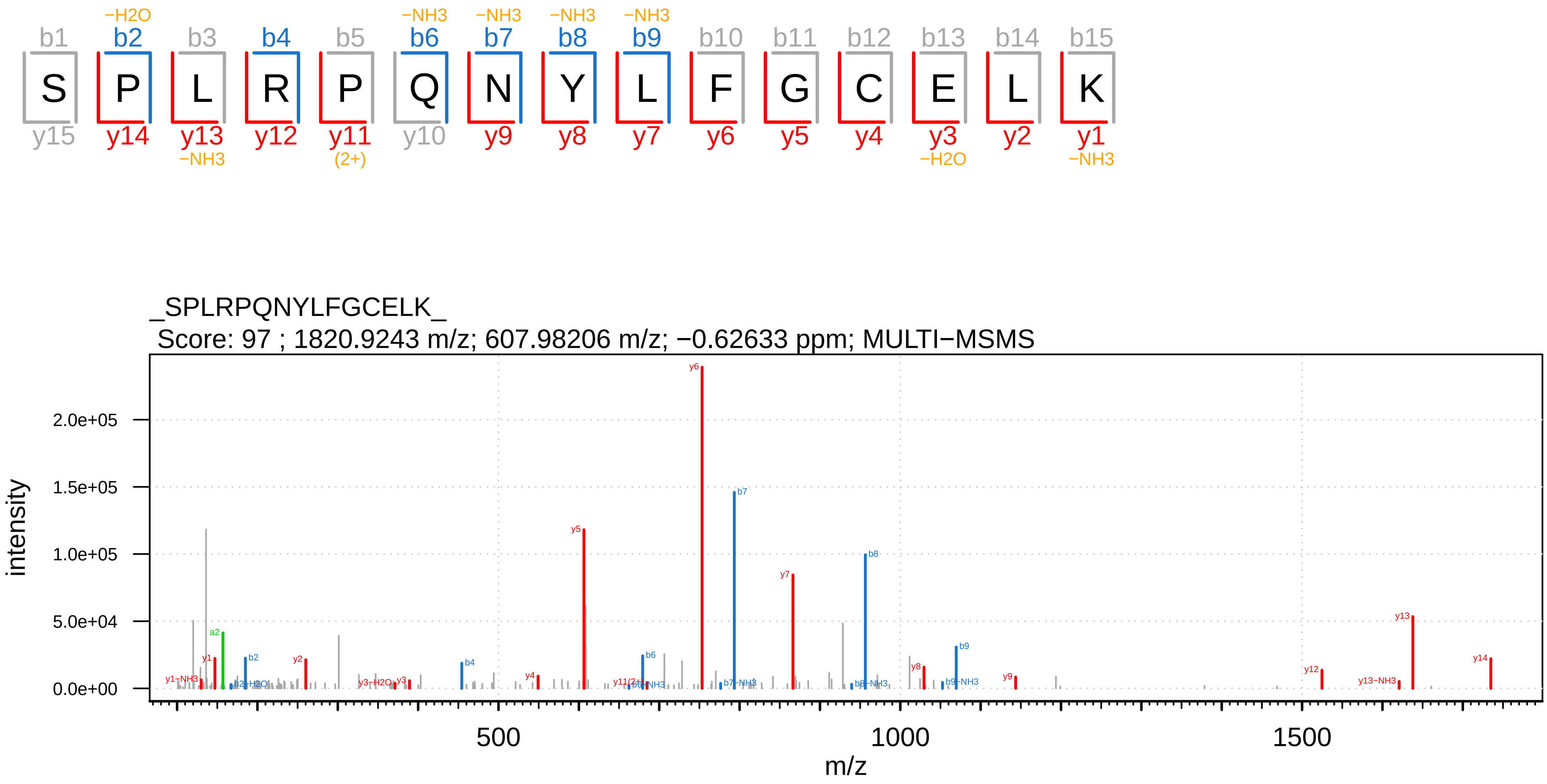

Raw File: 20101210_Velos1_AnWe_SA_U2OS_1
Scan Number: 23828
Proteins:
TCONS_l2_00008829_chr15:92829088−92829258:+

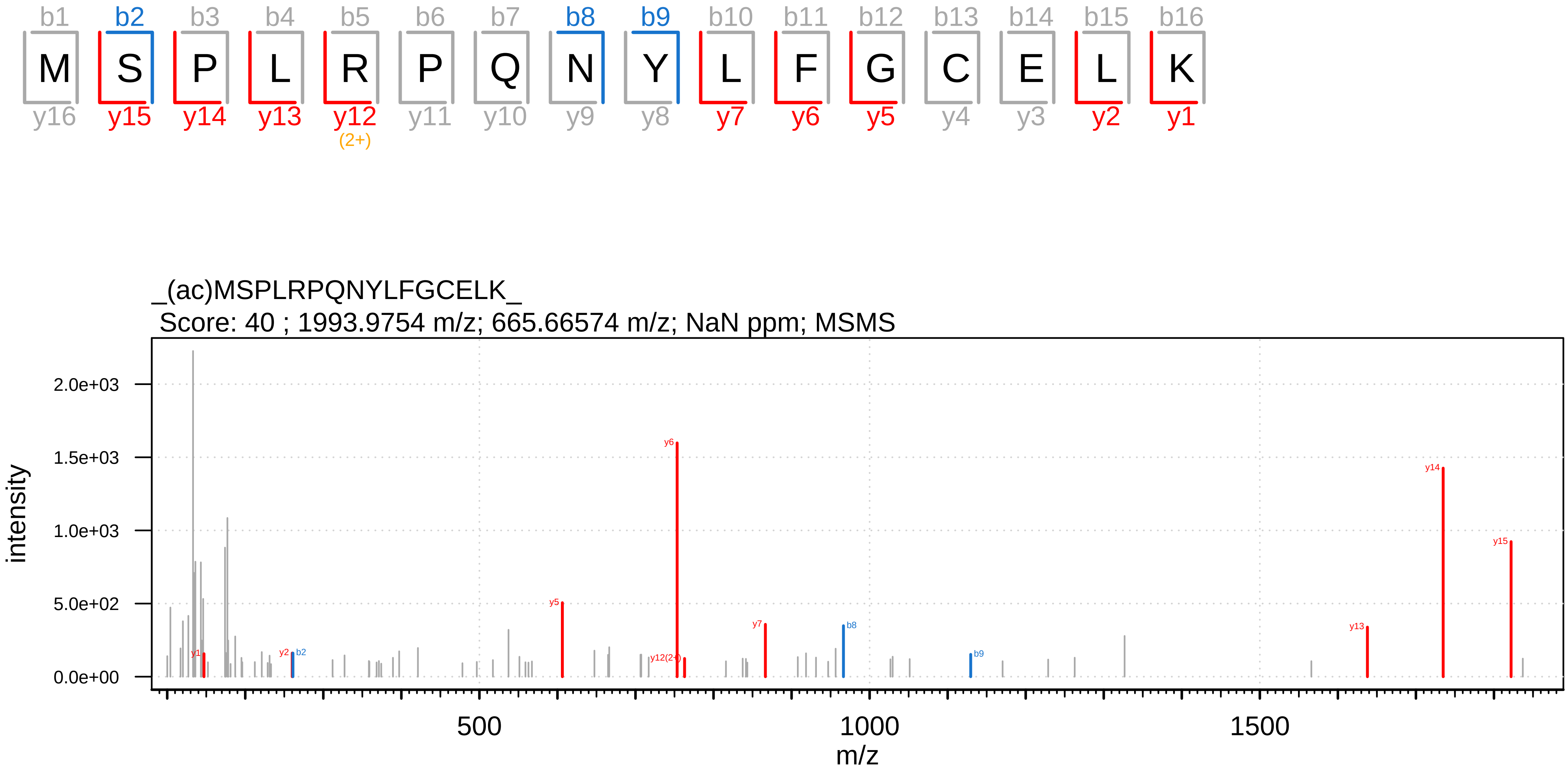

Raw File: 20101222_Velos1_TaGe_SA_K562_01
Scan Number: 22570
Proteins:
TCONS_l2_00008829_chr15:92829088−92829258:+

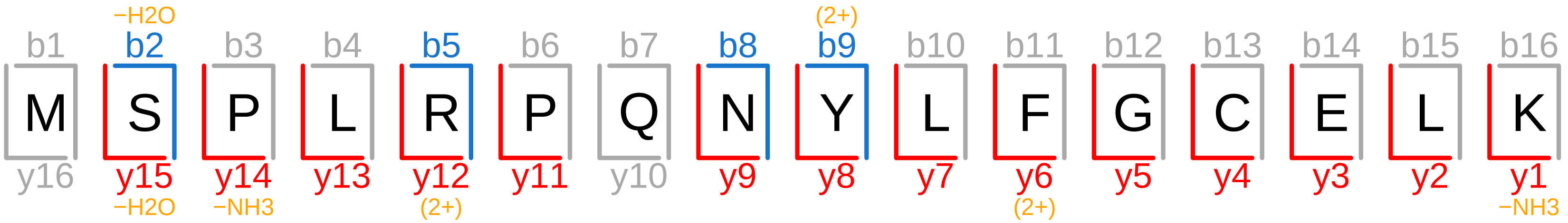

(ac)M(ox)SPLRPQNYLFGCELK
Score: 73 ; 2009.9703 m/z; 670.99738 m/z; −0.1032 ppm; MULTI−MSMS

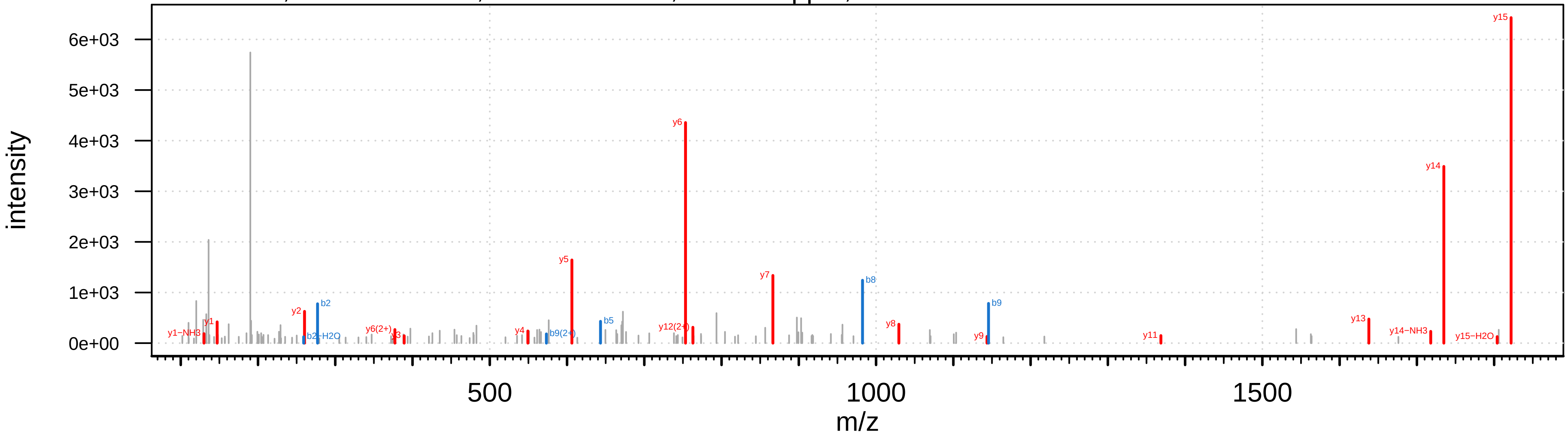

Raw File: 20101222_Velos1_TaGe_SA_K562_01
Scan Number: 19383
Proteins:
TCONS_l2_00008829_chr15:92829088−92829258:+

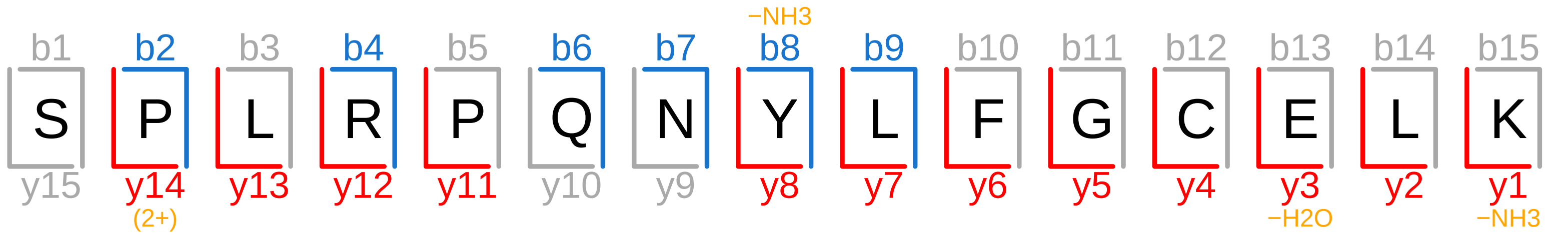

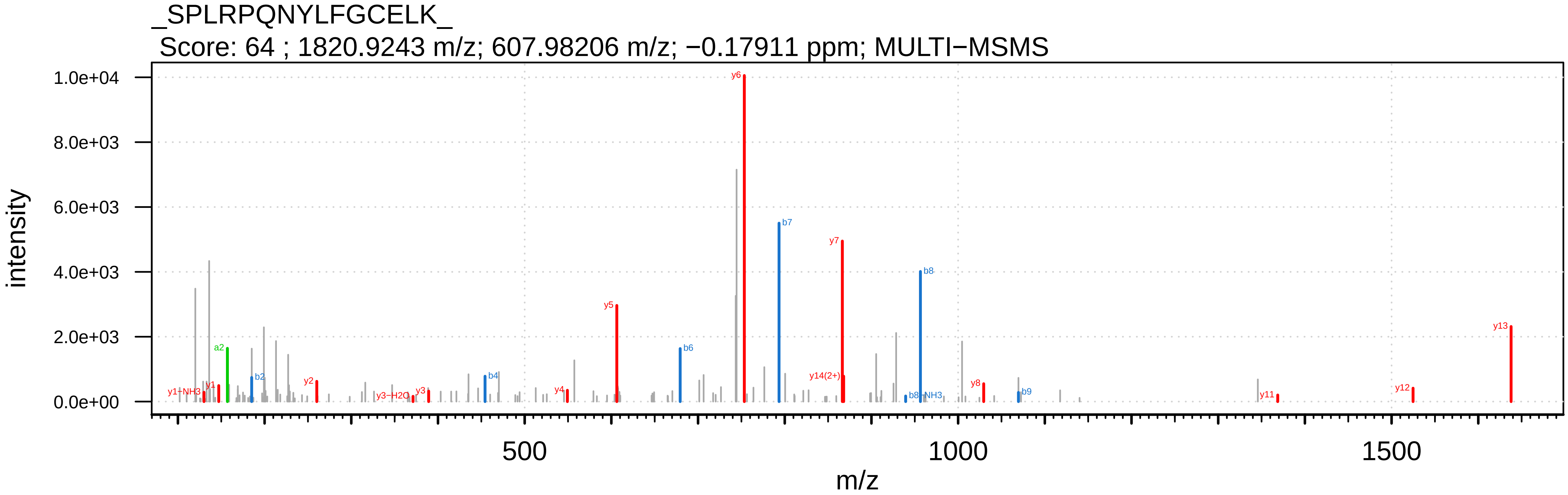

Raw File: 20101222_Velos1_TaGe_SA_K562_01
Scan Number: 14066
Proteins:
TCONS_l2_00008829_chr15:92829088−92829258:+

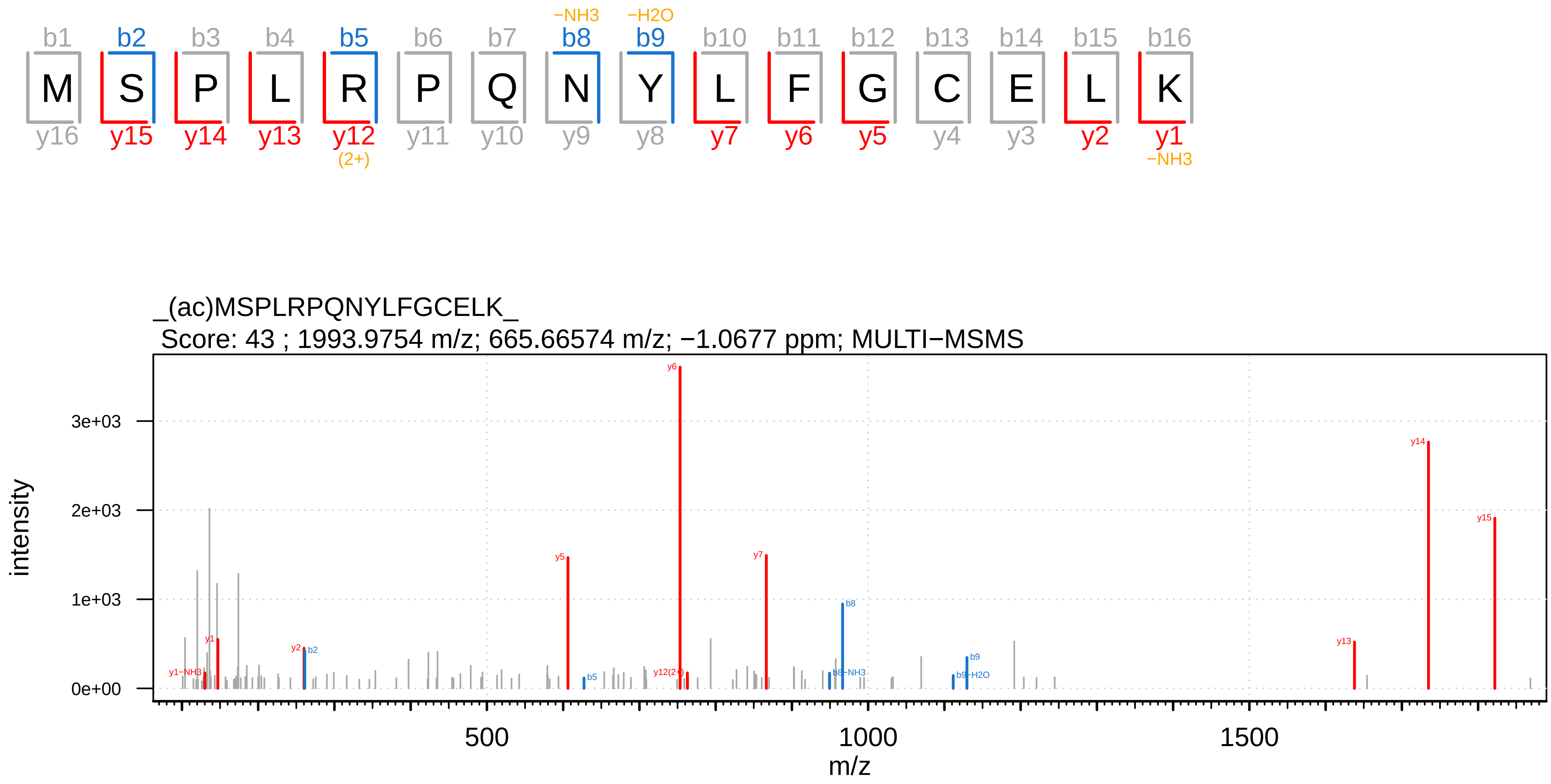

Raw File: 20101222_Velos1_TaGe_SA_K562_02
Scan Number: 20965
Proteins:
TCONS_l2_00008829_chr15:92829088−92829258:+

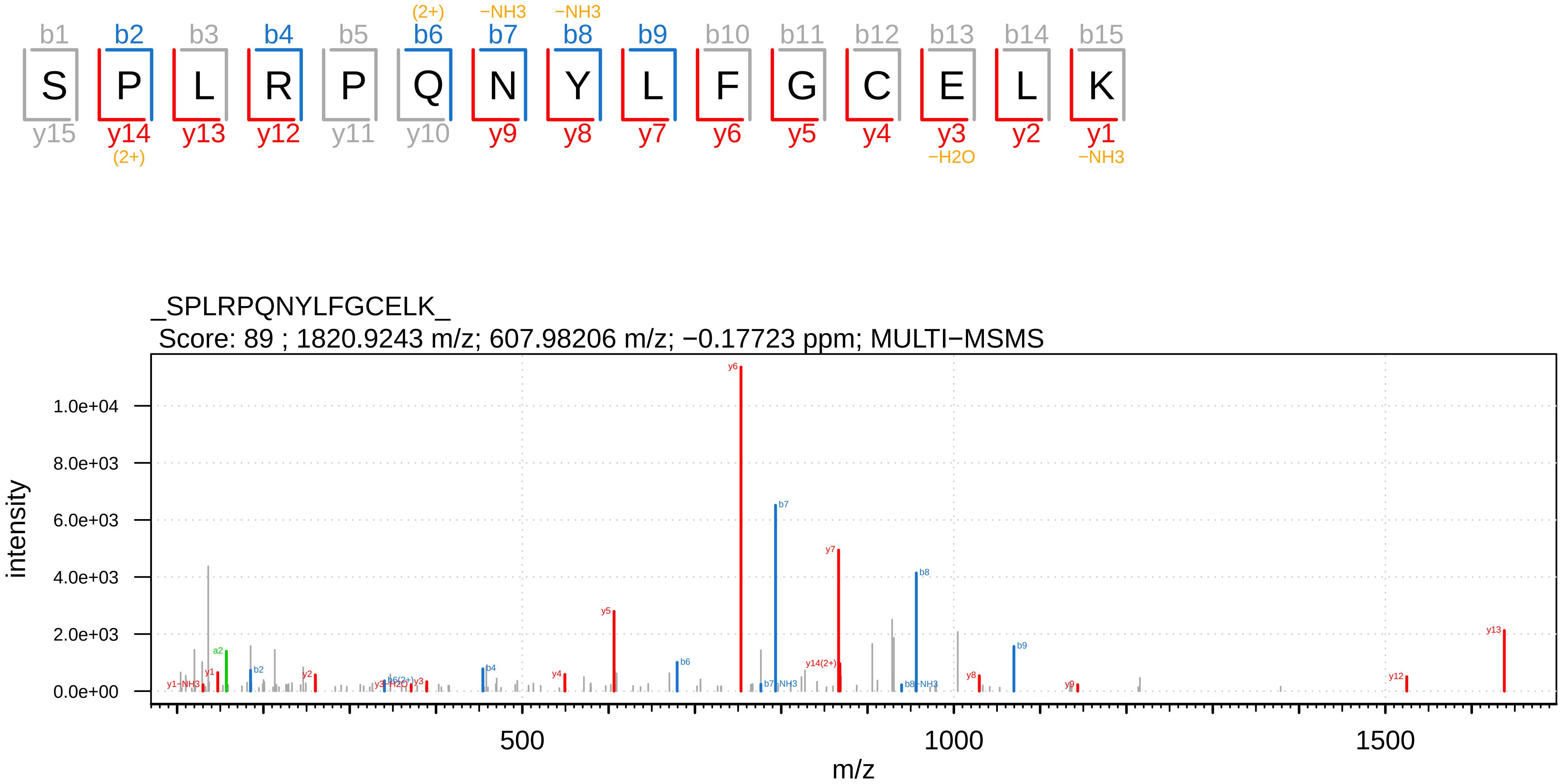

Raw File: 20101222_Velos1_TaGe_SA_K562_02
Scan Number: 12418
Proteins:
TCONS_l2_00008829_chr15:92829088−92829258:+

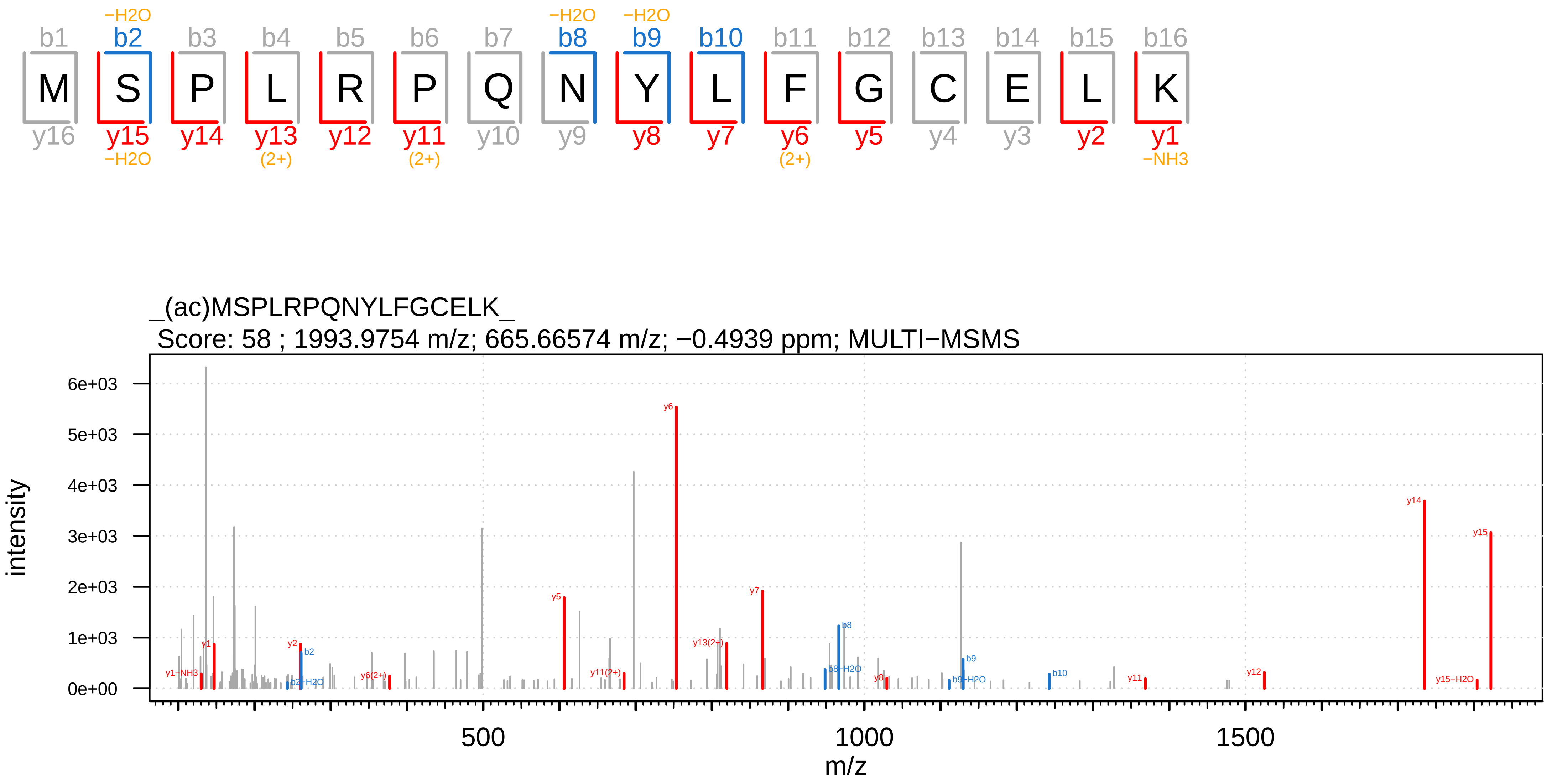

Raw File: 20101224_Velos1_TaGe_SA_HepG2_2
Scan Number: 33012
Proteins:
TCONS_l2_00008829_chr15:92829088−92829258:+

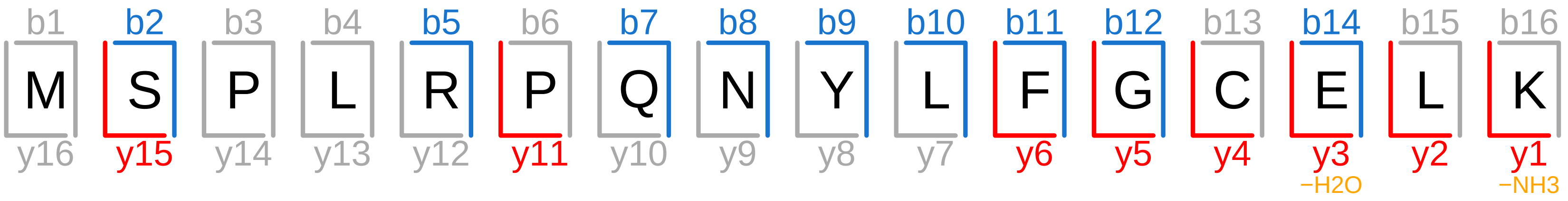

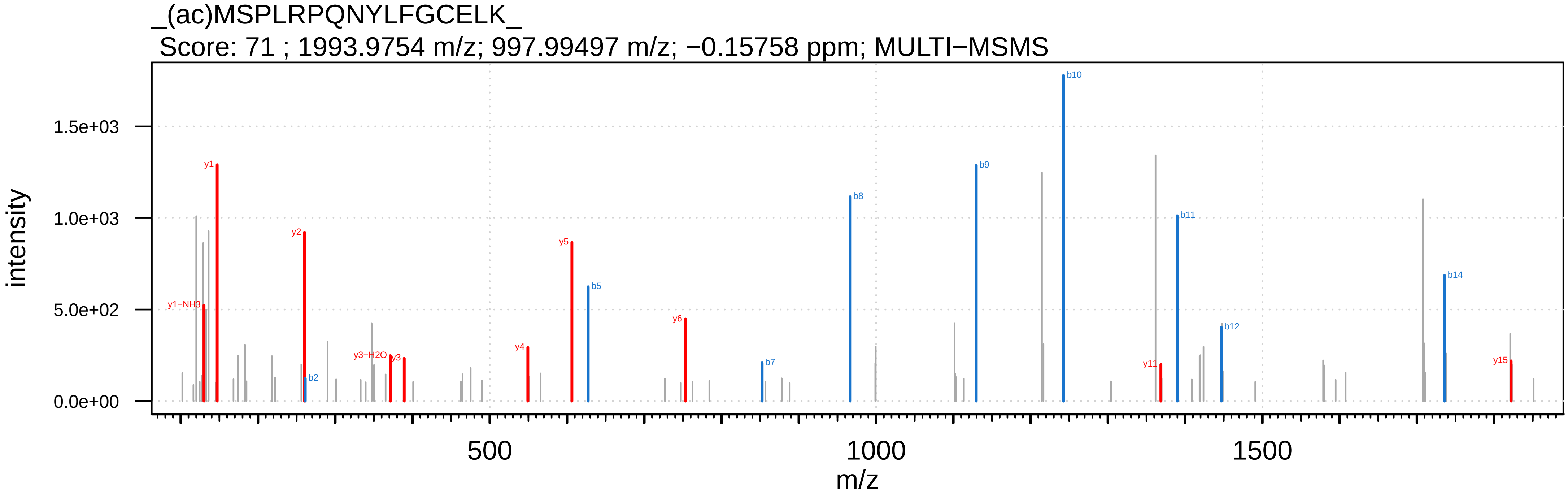

Raw File: 20101224_Velos1_TaGe_SA_HepG2_2
Scan Number: 33013
Proteins:
TCONS_l2_00008829_chr15:92829088−92829258:+

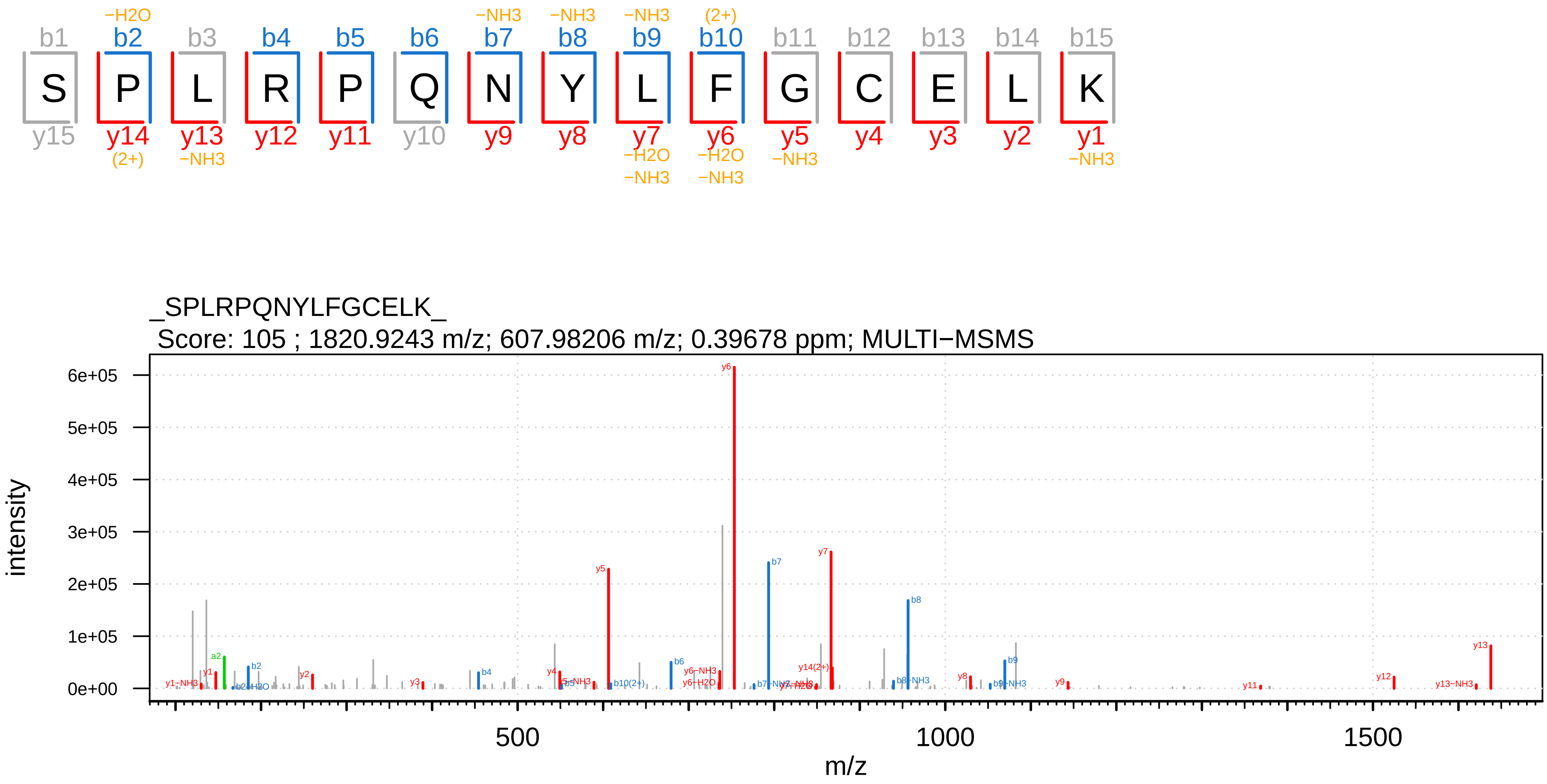

Raw File: 20101224_Velos1_TaGe_SA_HepG2_2
Scan Number: 22244
Proteins:
TCONS_l2_00008829_chr15:92829088−92829258:+

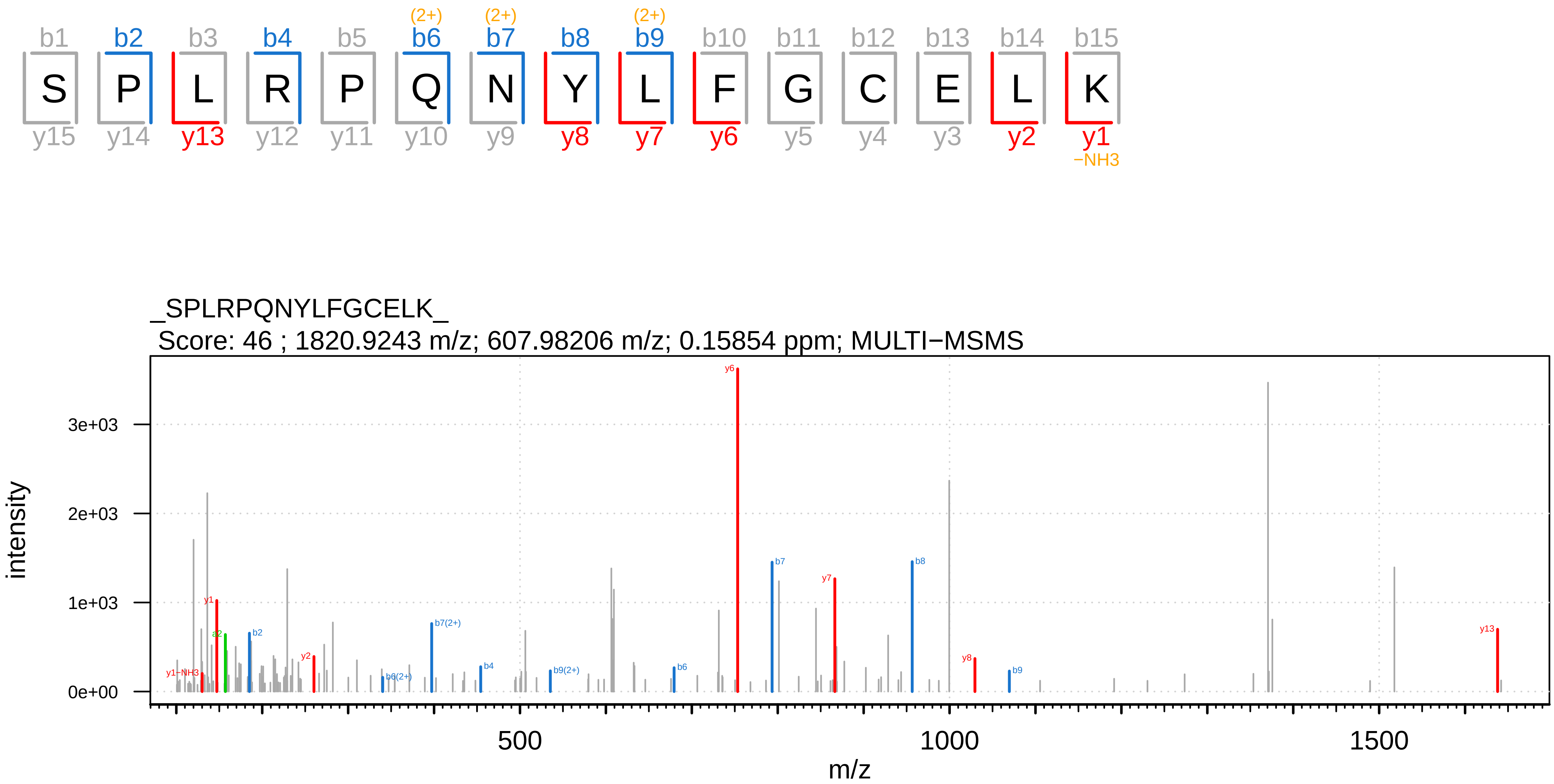

Raw File: 20101224_Velos1_TaGe_SA_HepG2_2
Scan Number: 33935
Proteins:
TCONS_l2_00008829_chr15:92829088−92829258:+

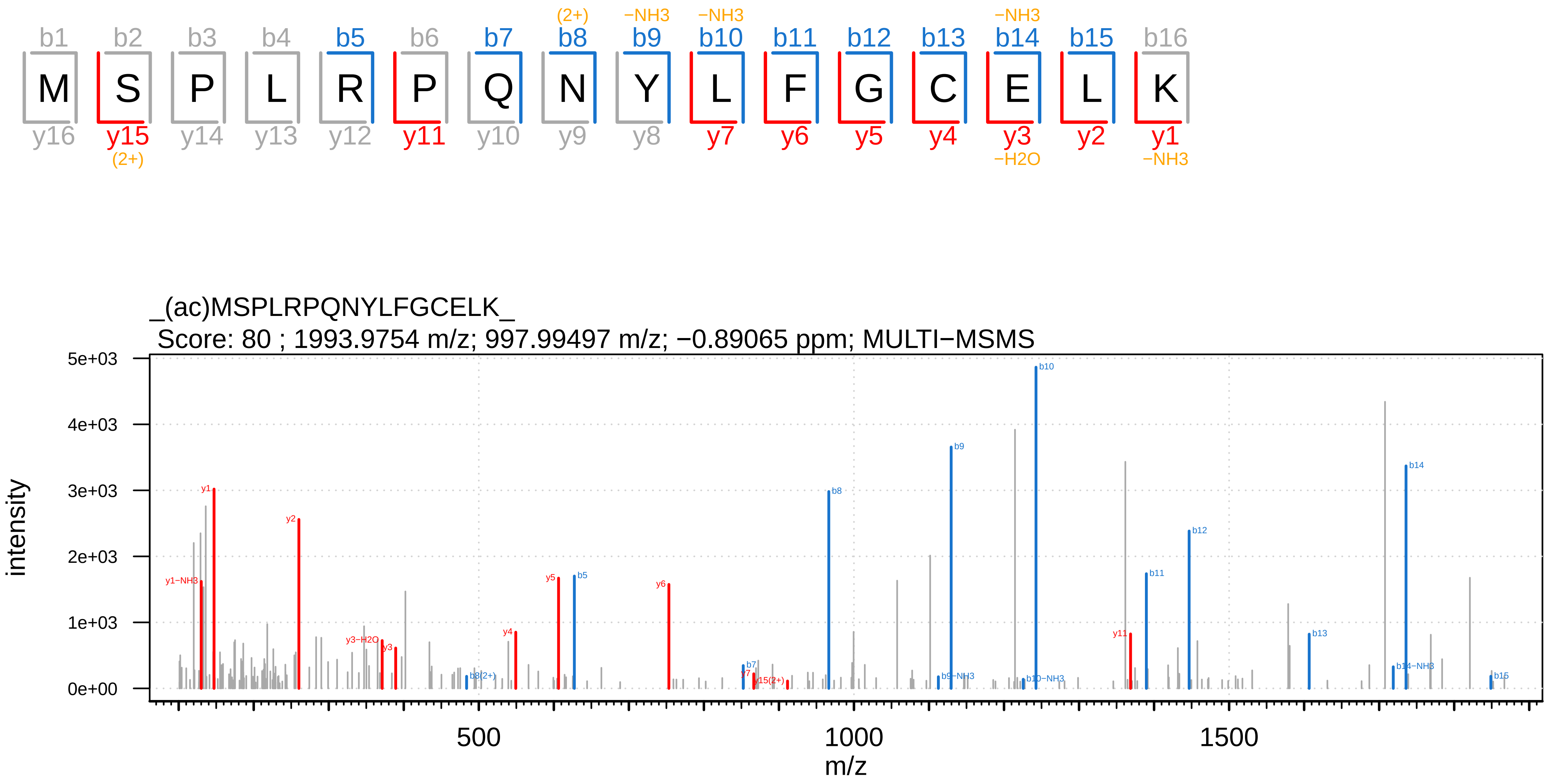

Raw File: 20101227_Velos1_TaGe_SA_GAMG_101230100451
Scan Number: 38847
Proteins:
TCONS_l2_00008829_chr15:92829088−92829258:+

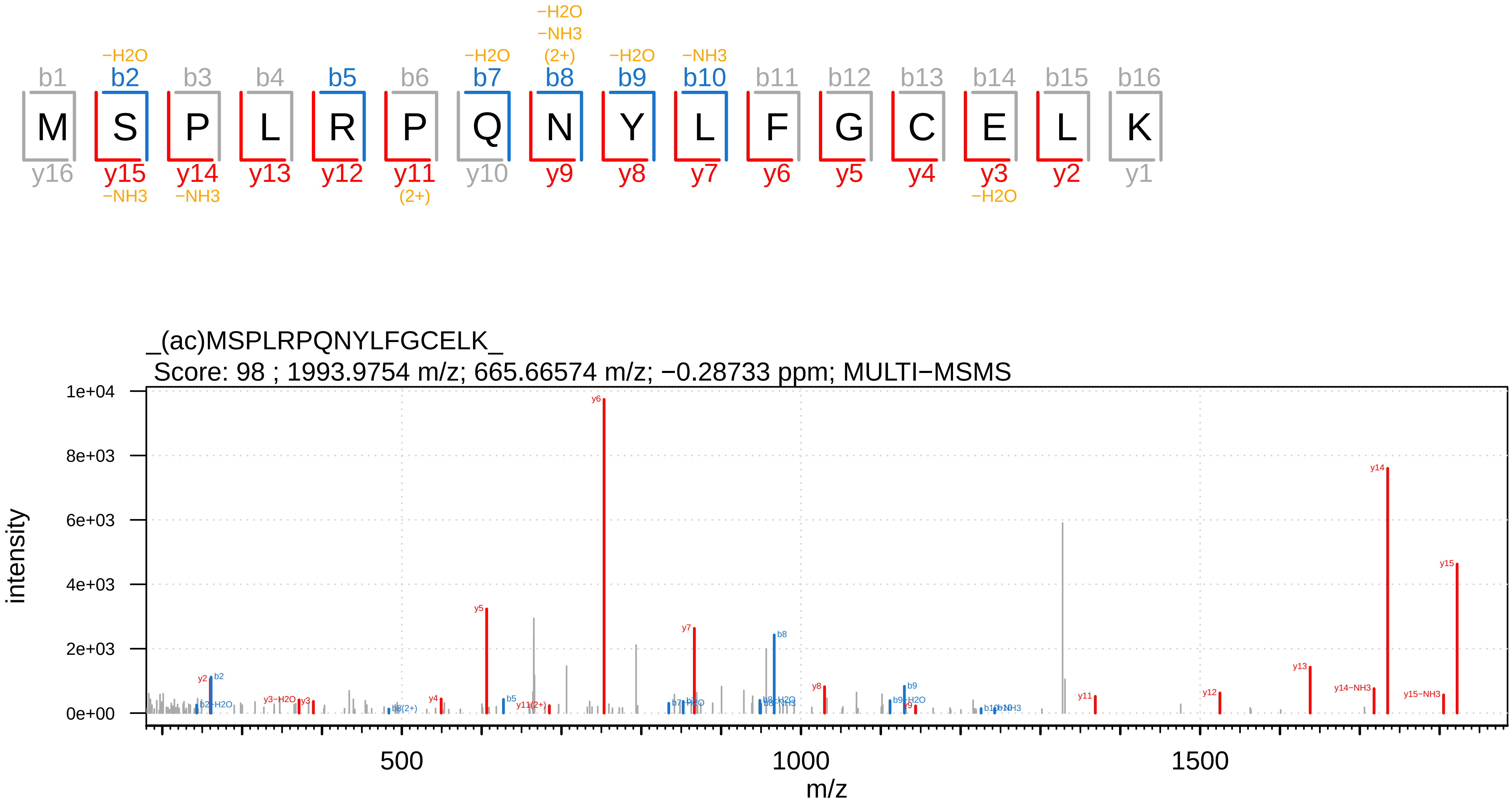

Raw File: 20101227_Velos1_TaGe_SA_GAMG_101230100451
Scan Number: 38898
Proteins:
TCONS_l2_00008829_chr15:92829088−92829258:+

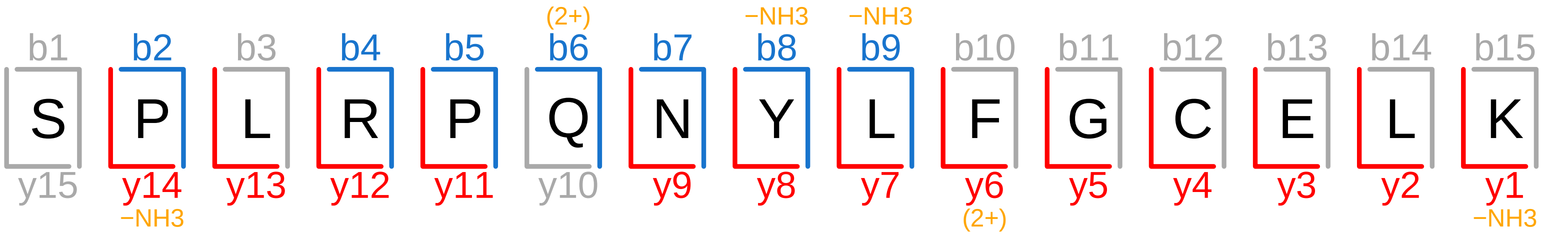

SPLRPQNYLFGCELK

Score: 92 ; 1820.9243 m/z; 607.98206 m/z; 0.66713 ppm; MULTI−MSMS

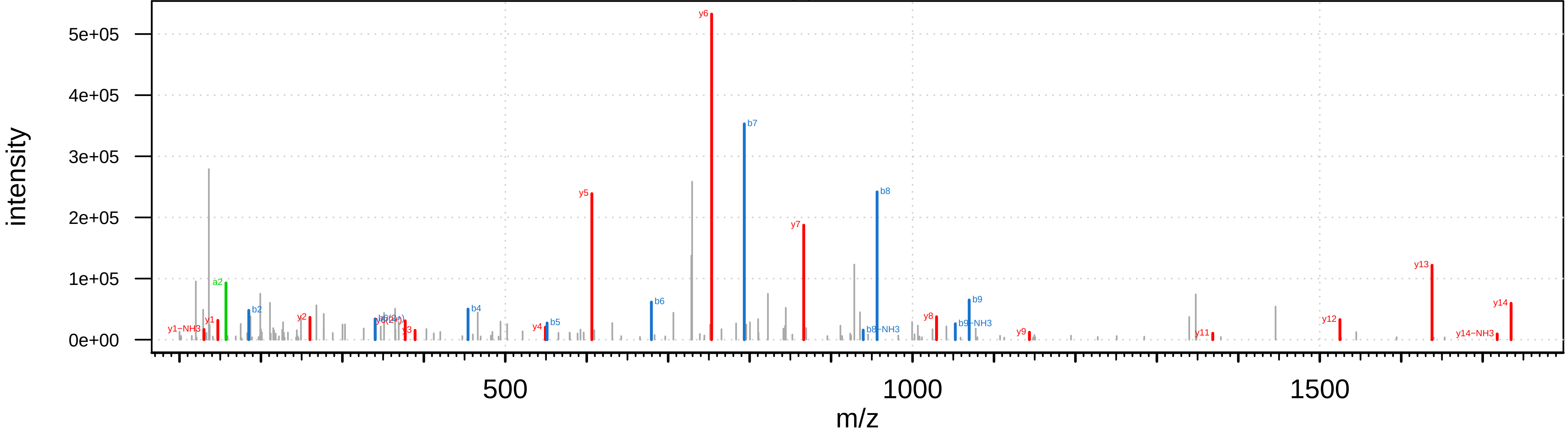

Raw File: 20101227_Velos1_TaGe_SA_GAMG_101230100451

Scan Number: 25043

Proteins:

TCONS_l2_00008829_chr15:92829088−92829258:+

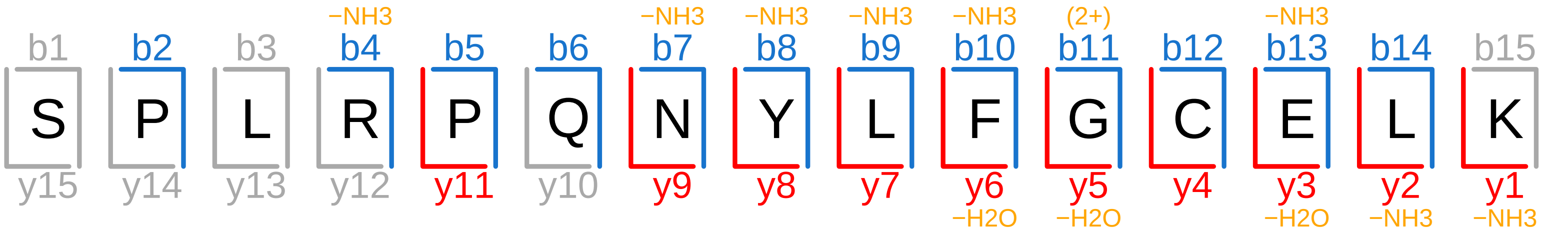

SPLRPQNYLFGCELK
Score: 106 ; 1820.9243 m/z; 911.46945 m/z; 0.351 ppm; MULTI−MSMS

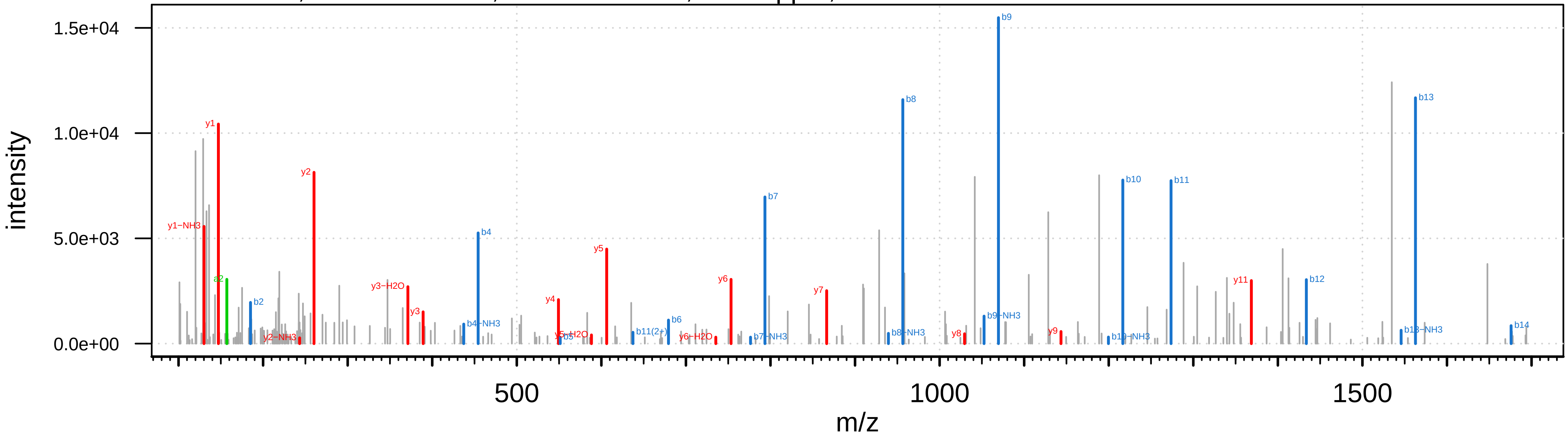

Raw File: 20101227_Velos1_TaGe_SA_GAMG_101230100451
Scan Number: 25047
Proteins:
TCONS_l2_00008829_chr15:92829088−92829258:+

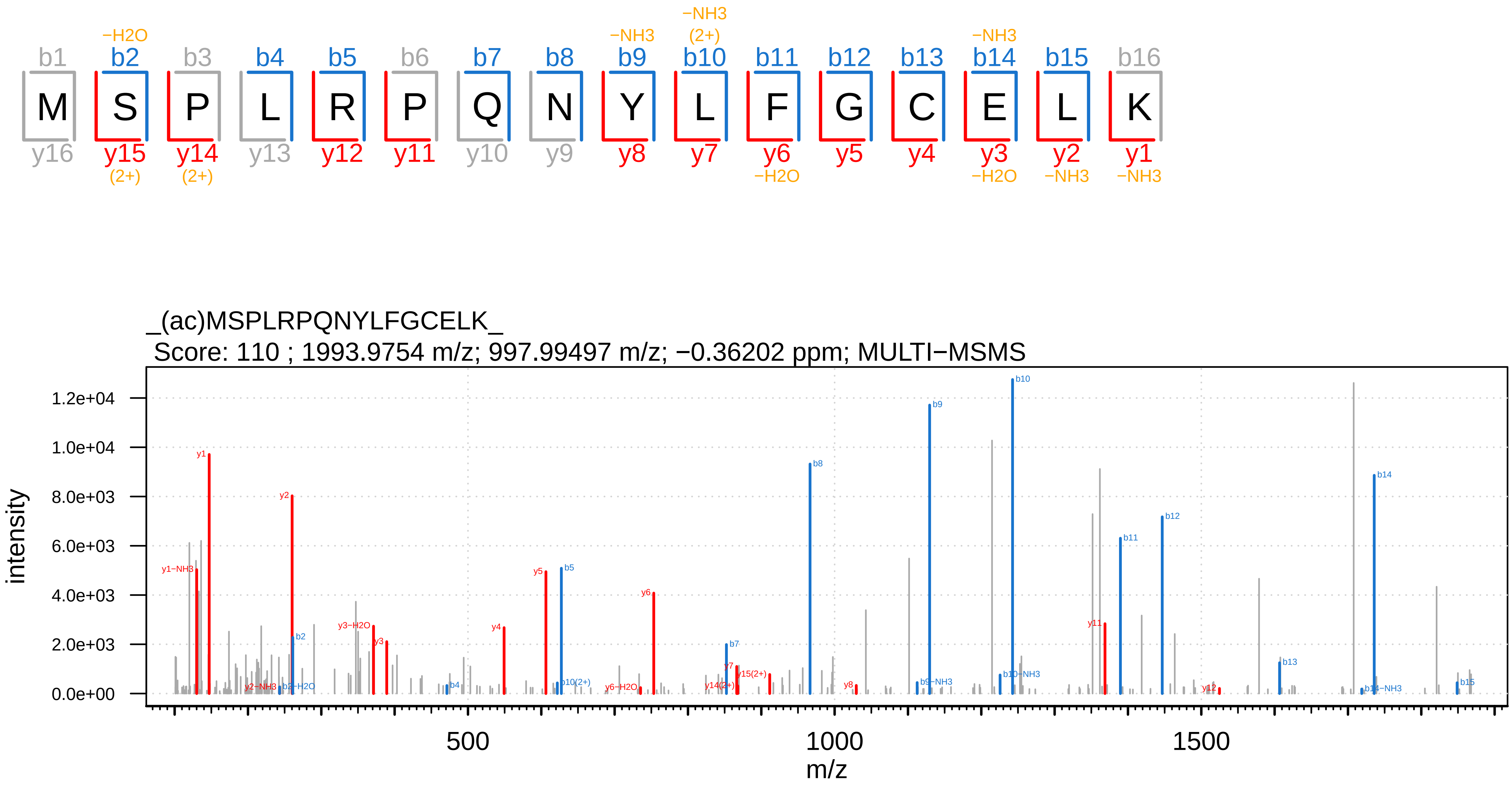

Raw File: 20101227_Velos1_TaGe_SA_HEK293_01
Scan Number: 36838
Proteins:
TCONS_l2_00008829_chr15:92829088−92829258:+

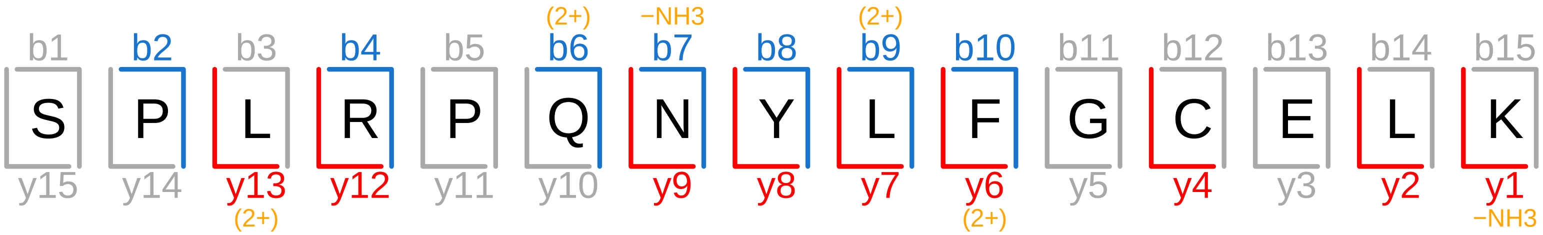

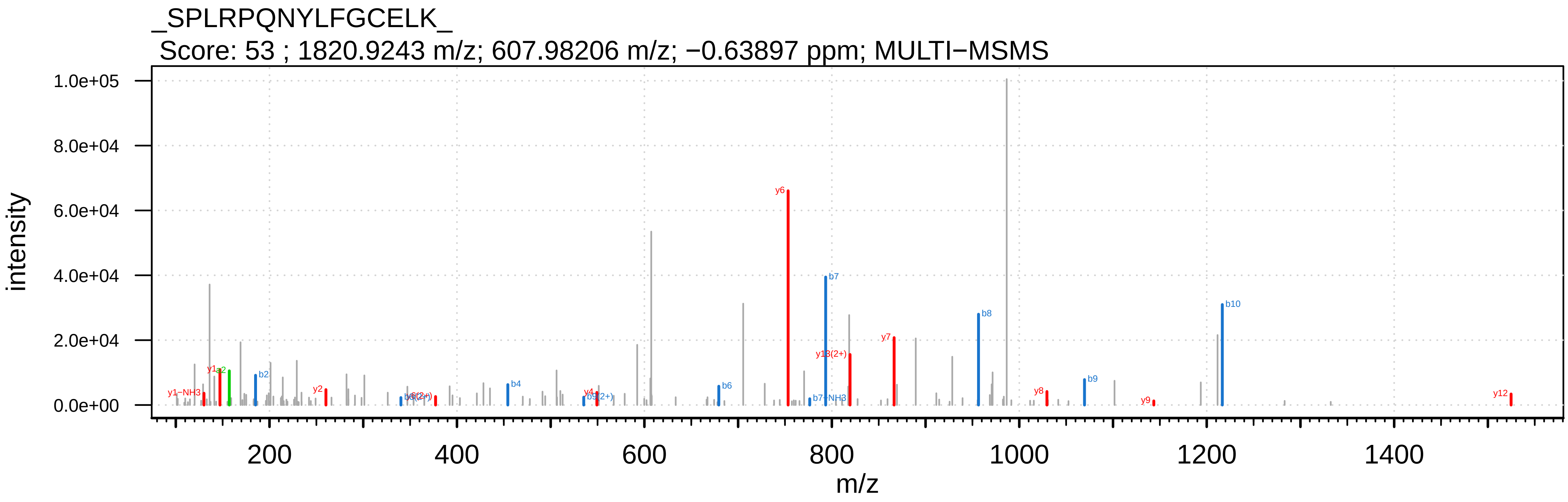

Raw File: 20101227_Velos1_TaGe_SA_HEK293_01
Scan Number: 21139
Proteins:
TCONS_l2_00008829_chr15:92829088−92829258:+

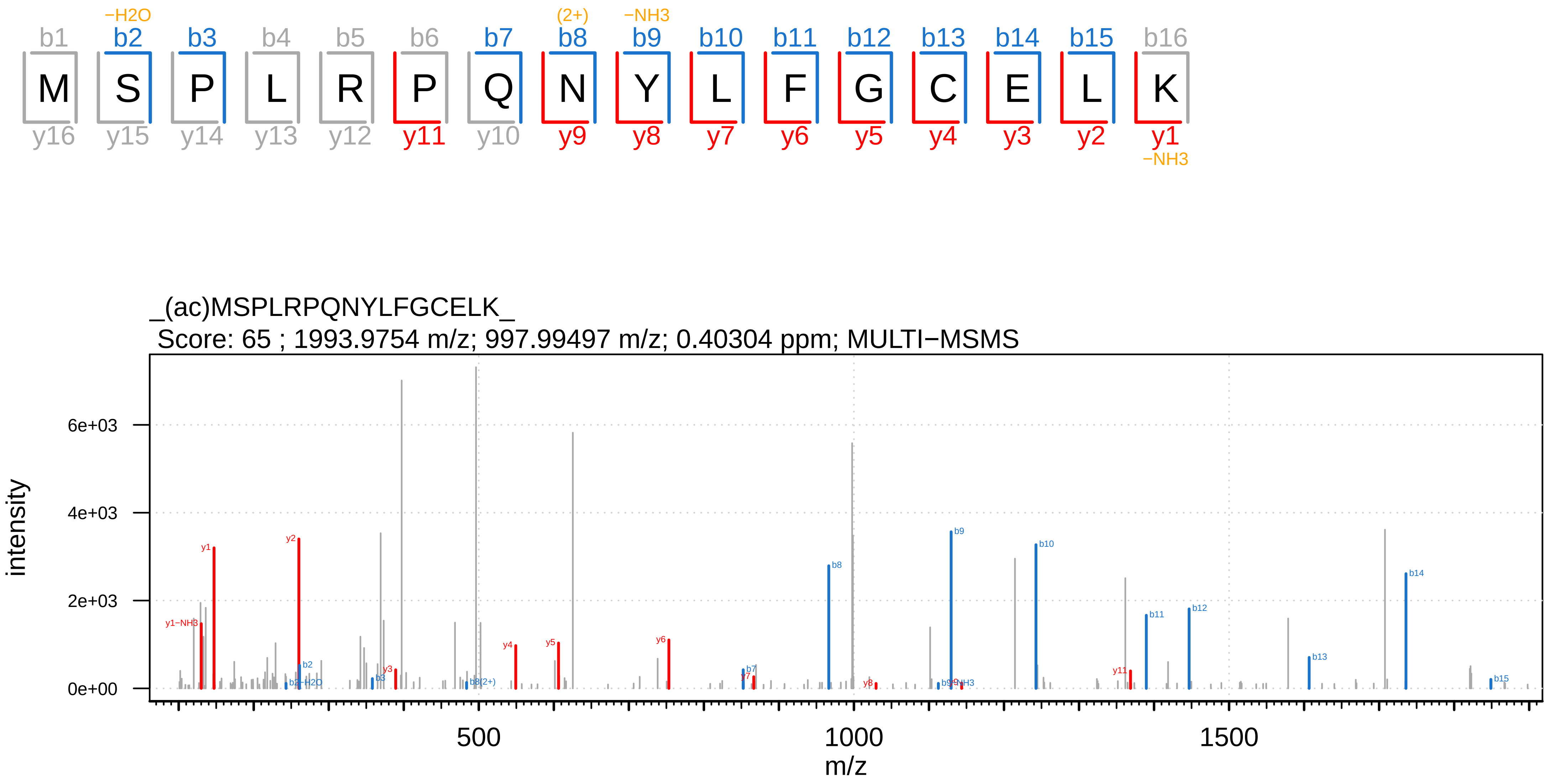

Raw File: 20101230_Velos1_TaGe_SA_Jurkat1
Scan Number: 39660
Proteins:
TCONS_l2_00008829_chr15:92829088−92829258:+

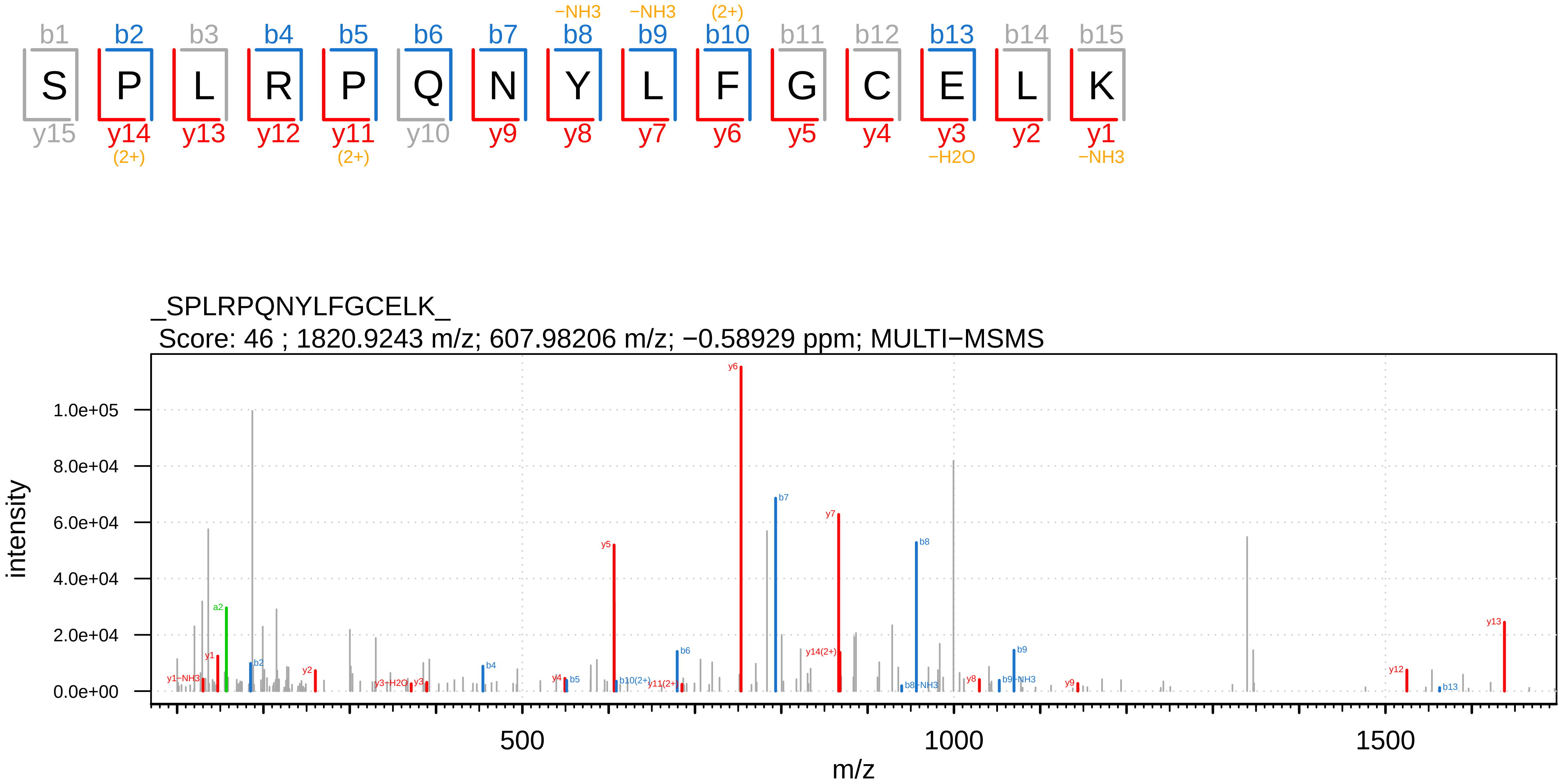

Raw File: 20101230_Velos1_TaGe_SA_Jurkat1
Scan Number: 24939
Proteins:
TCONS_l2_00008829_chr15:92829088−92829258:+

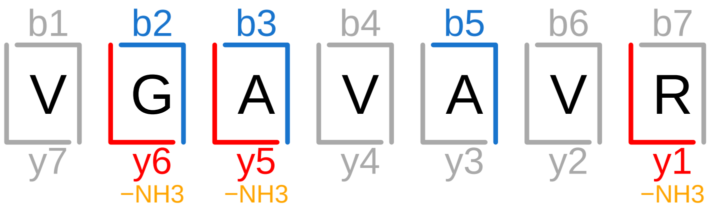

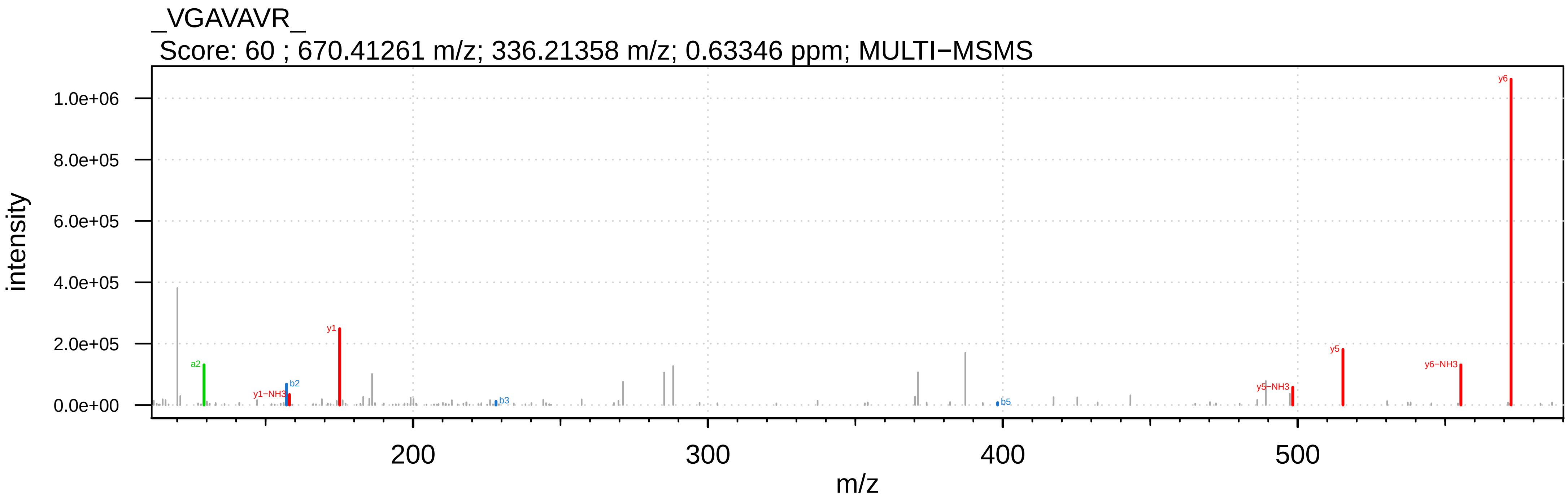

Raw File: 20101230_Velos1_TaGe_SA_Jurkat1
Scan Number: 3081
Proteins:
TCONS_l2_00030545_chrX:79544539−79546436:−

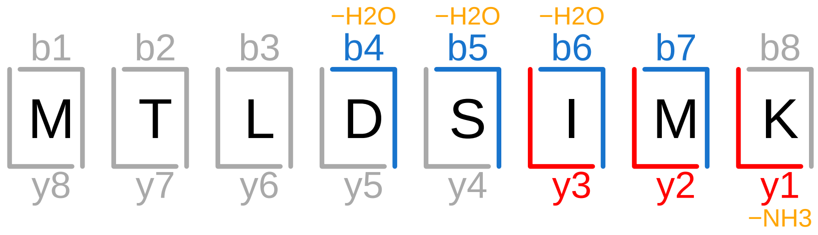

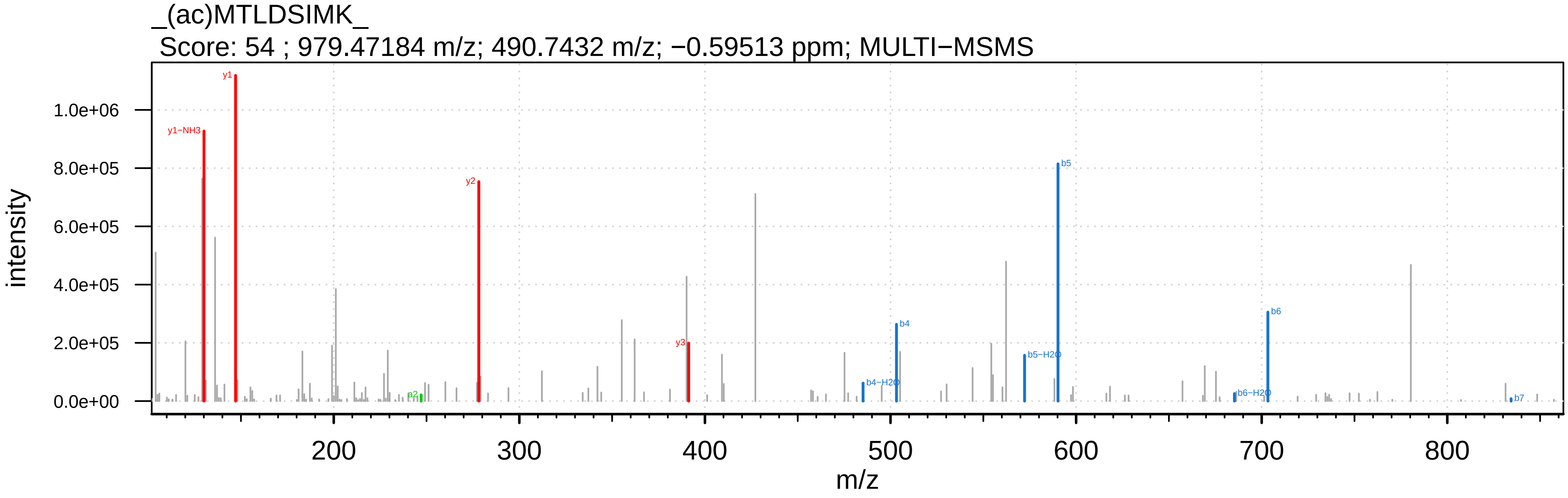

Raw File: 20101210_Velos1_AnWe_SA_U2OS_5
Scan Number: 17472
Proteins:
TCONS_l2_00001211_chr1:47348822−47348905:−

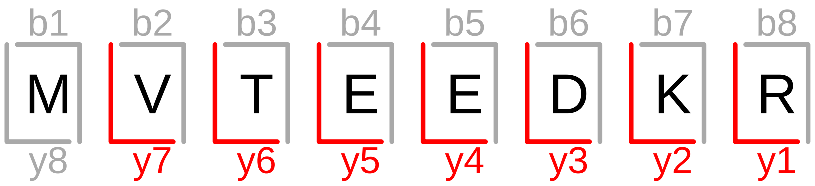

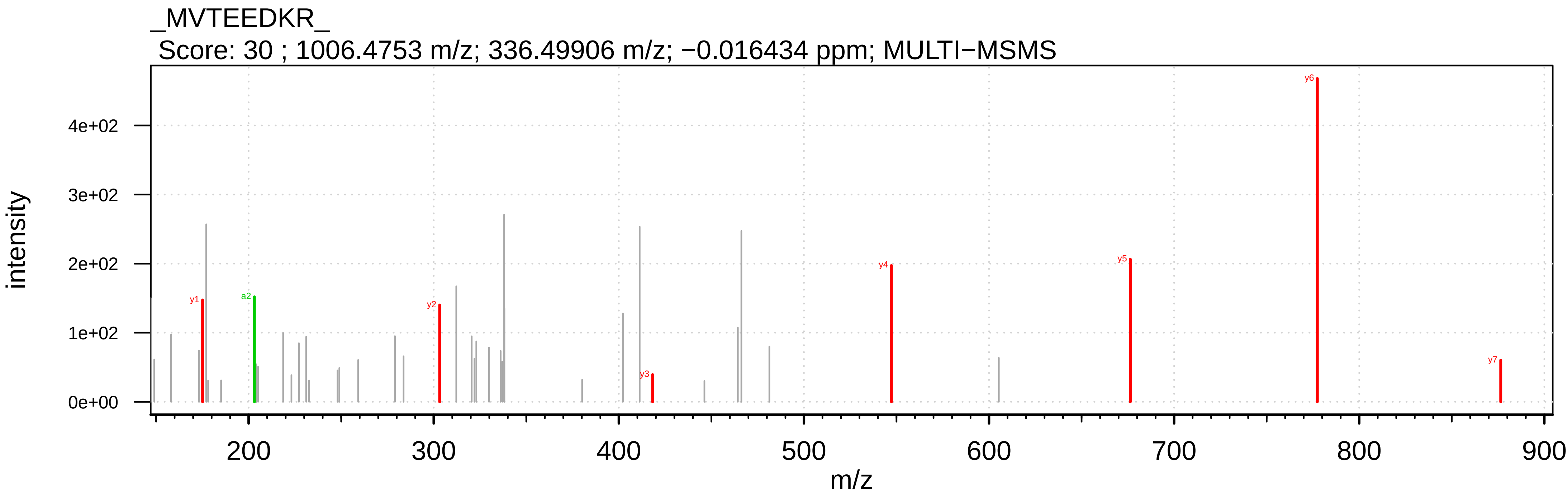

Raw File: 20100611_Velos1_TaGe_SA_Hela_3
Scan Number: 1127
Proteins:
TCONS_l2_00006768_chr13:29174091−29174123:+

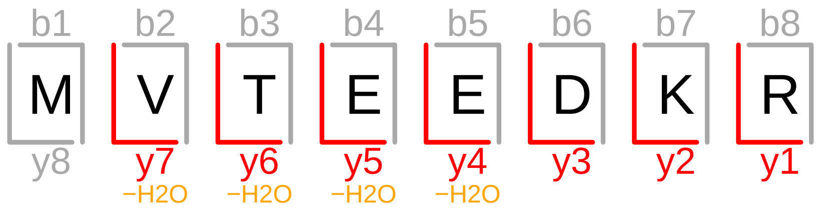

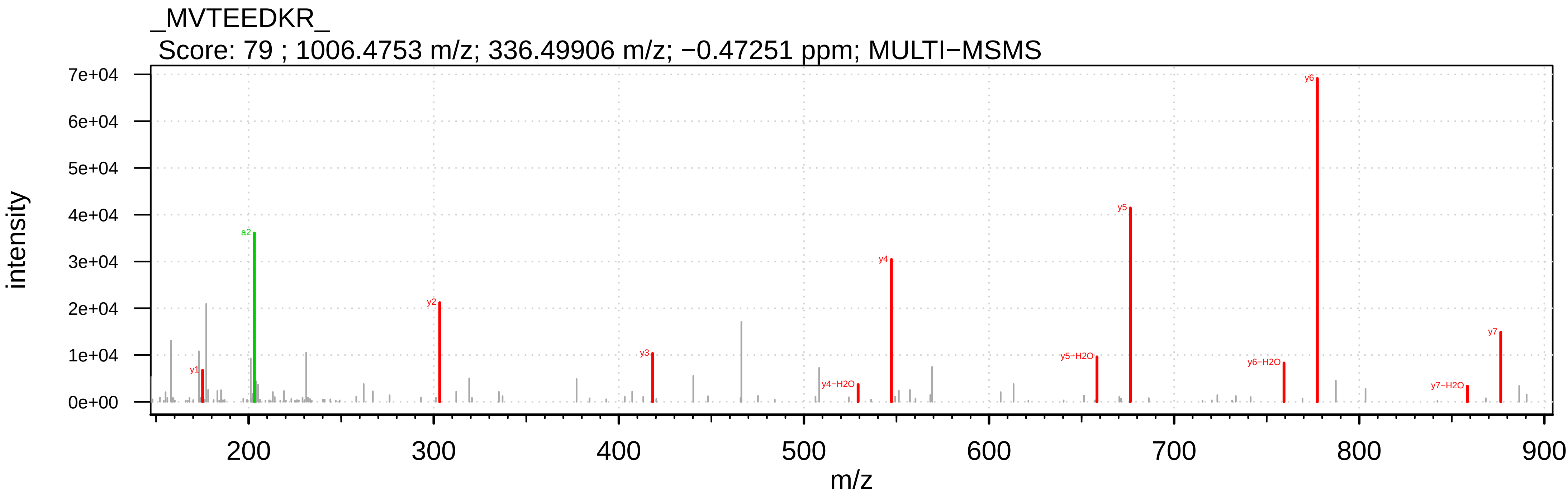

Raw File: 20101210_Velos1_AnWe_SA_U2OS_3
Scan Number: 1563
Proteins:
TCONS_l2_00006768_chr13:29174091−29174123:+

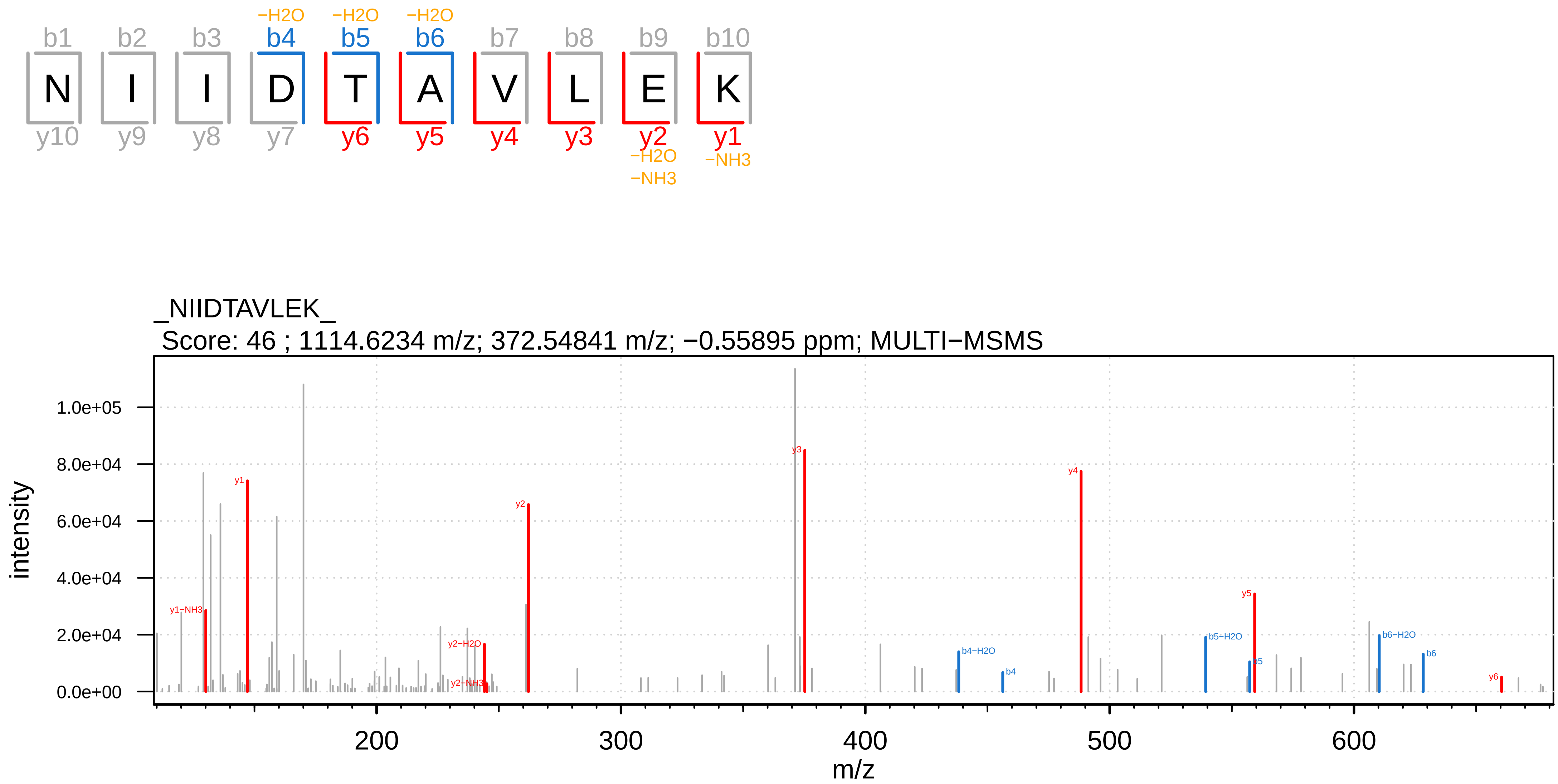

Raw File: 20101210_Velos1_AnWe_SA_U2OS_3
Scan Number: 8671
Proteins:
ENST00000497138_chr20:56806826−56807846:−

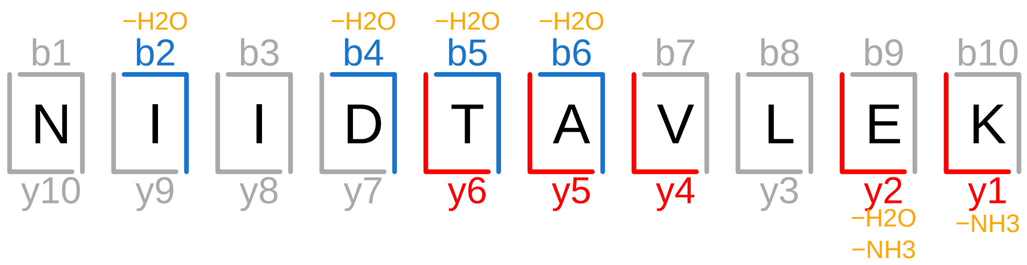

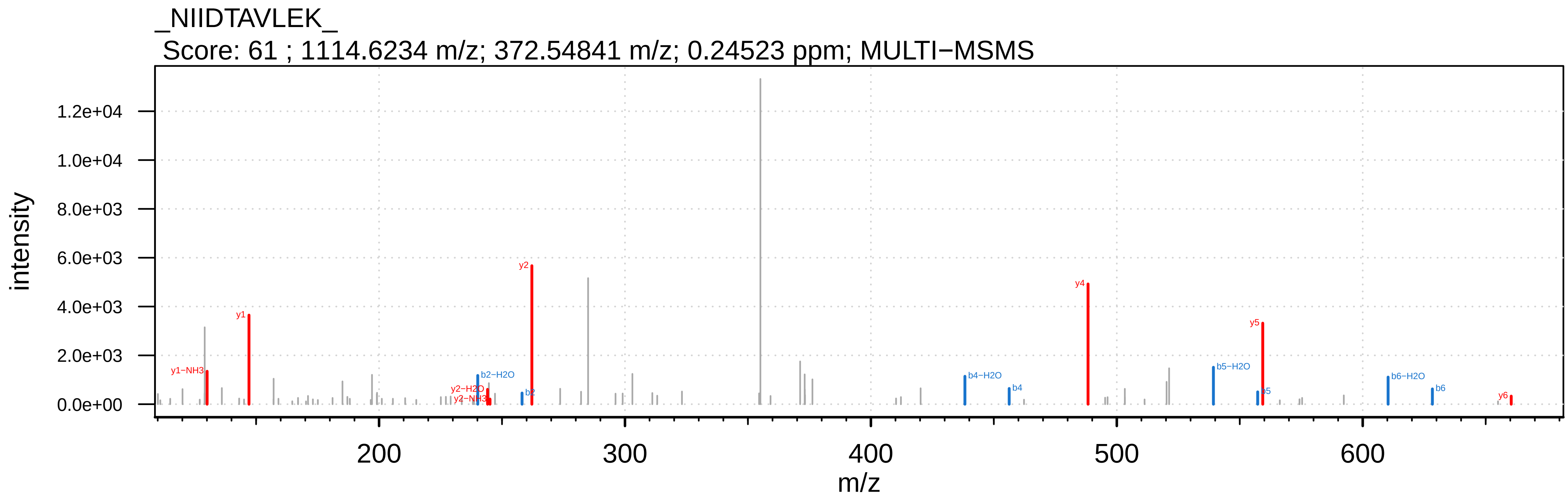

Raw File: 20100609_Velos1_TaGe_SA_293_5
Scan Number: 4694
Proteins:
ENST00000497138_chr20:56806826−56807846:−

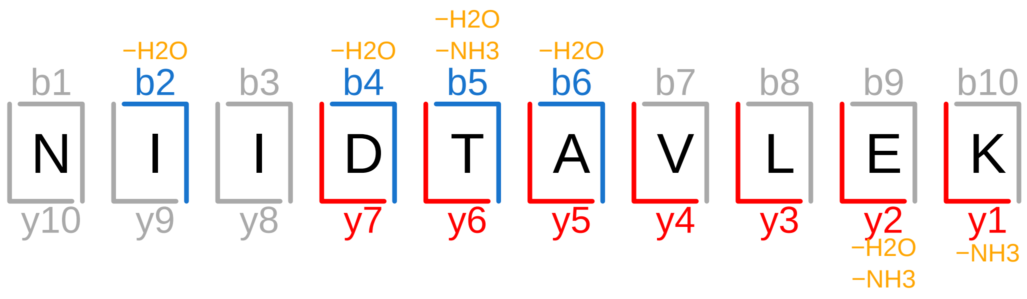

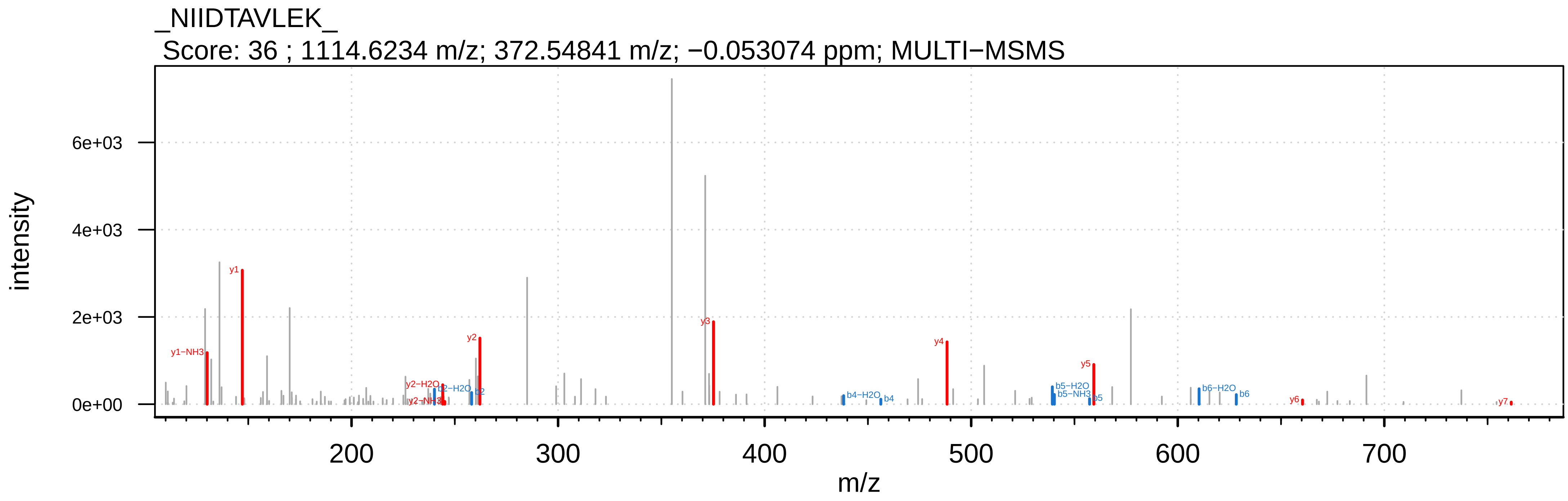

Raw File: 20100609_Velos1_TaGe_SA_GAMG_5
Scan Number: 4529
Proteins:
ENST00000497138_chr20:56806826−56807846:−

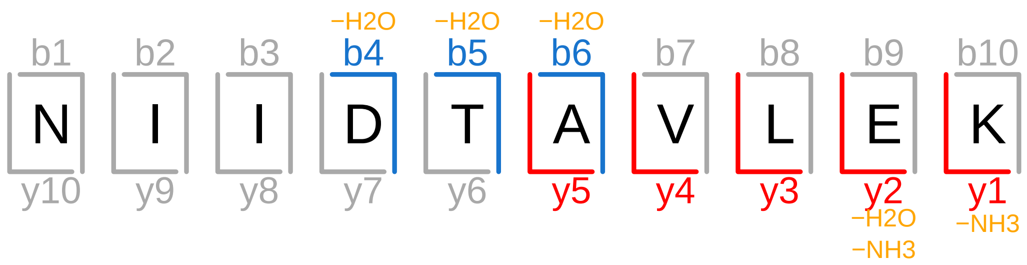

NIIDTAVLEK

Score: 41 ; 1114.6234 m/z; 372.54841 m/z; 0.64168 ppm; MULTI−MSMS

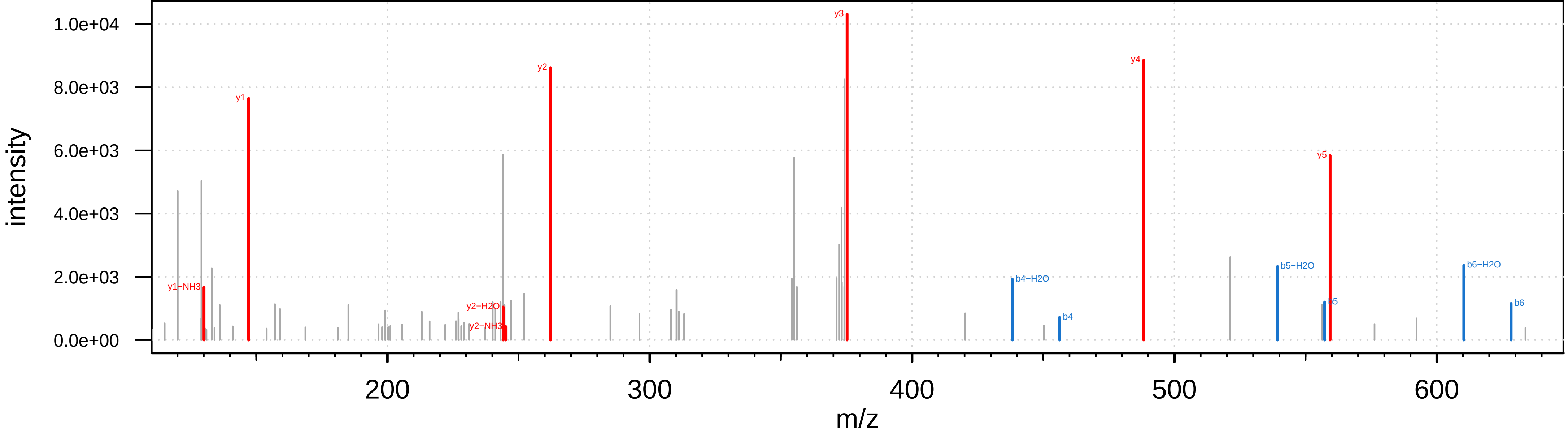

Raw File: 20100616_Velos1_TaGe_SA_RKO_3

Scan Number: 7681

Proteins:

ENST00000497138_chr20:56806826−56807846:−

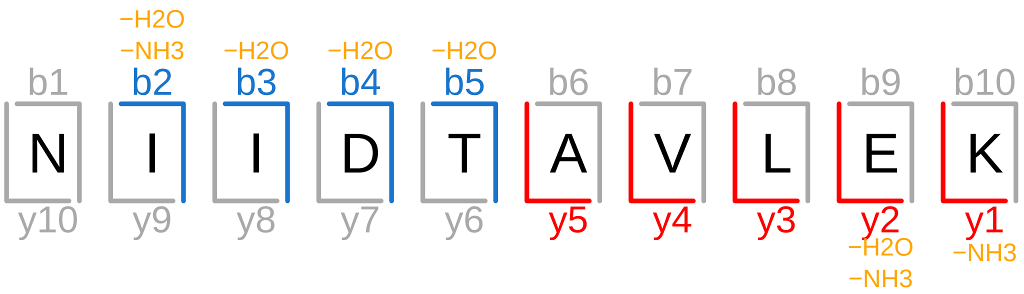

NIIDTAVLEK
Score: 52 ; 1114.6234 m/z; 372.54841 m/z; 0.14773 ppm; MULTI−MSMS

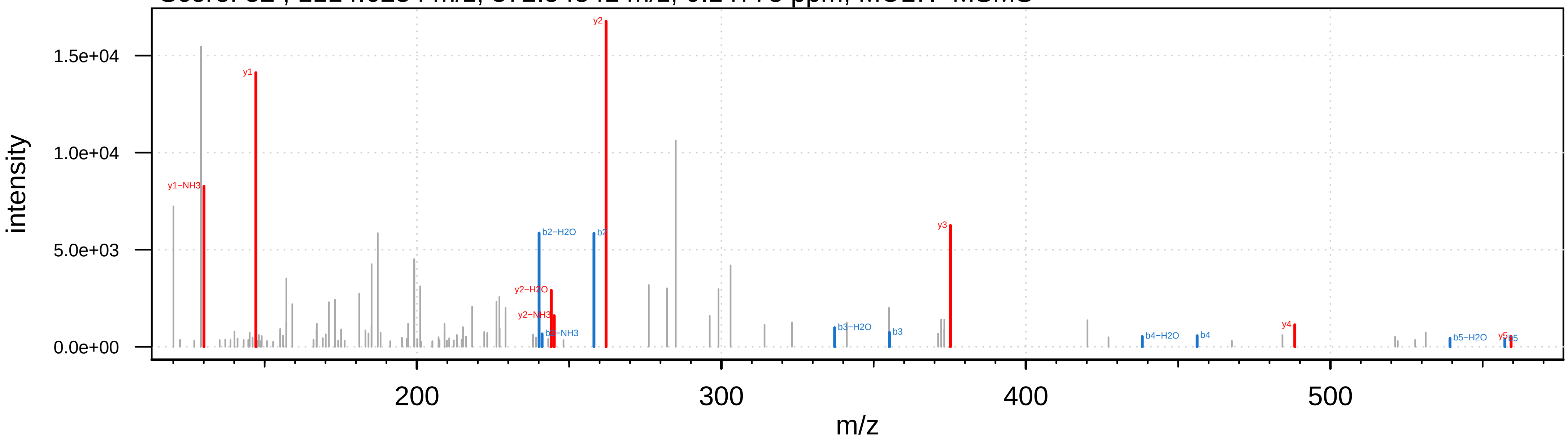

Raw File: 20100721_Velos1_TaGe_SA_A549_04
Scan Number: 7276
Proteins:
ENST00000497138_chr20:56806826−56807846:−

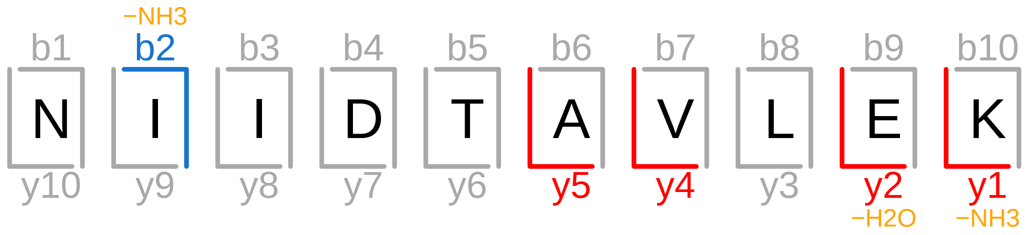

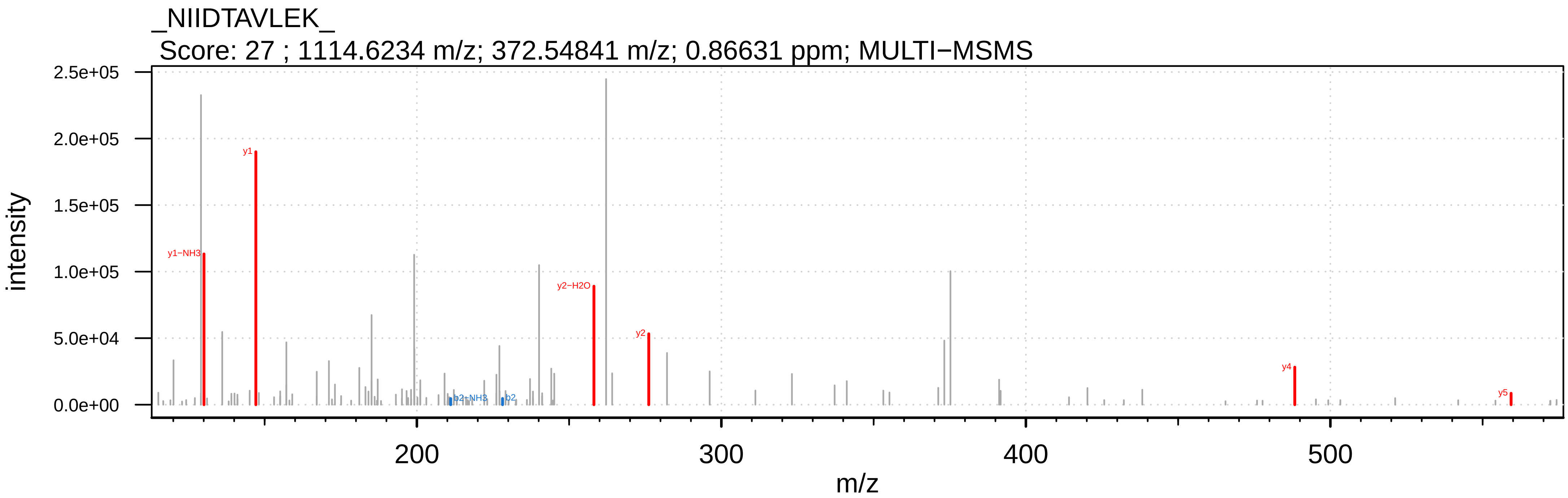

Raw File: 20100723_Velos1_TaGe_SA_Hek293_02
Scan Number: 7568
Proteins:
ENST00000497138_chr20:56806826−56807846:−

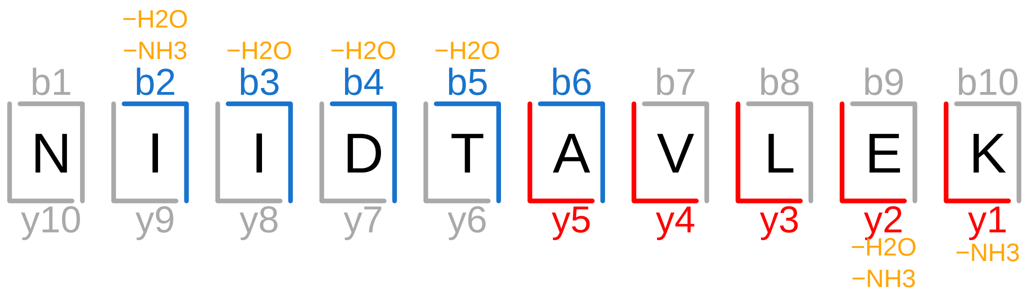

NIIDTAVLEK
Score: 59 ; 1114.6234 m/z; 372.54841 m/z; −0.15321 ppm; MULTI−MSMS

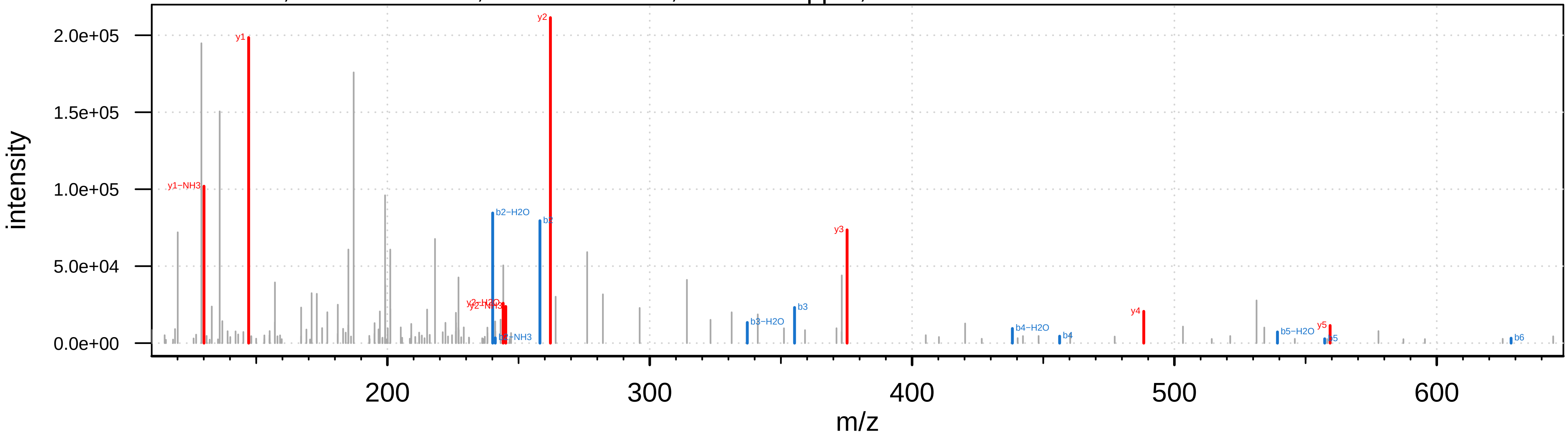

Raw File: 20100723_Velos1_TaGe_SA_Hek293_03
Scan Number: 7807
Proteins:
ENST00000497138_chr20:56806826−56807846:−

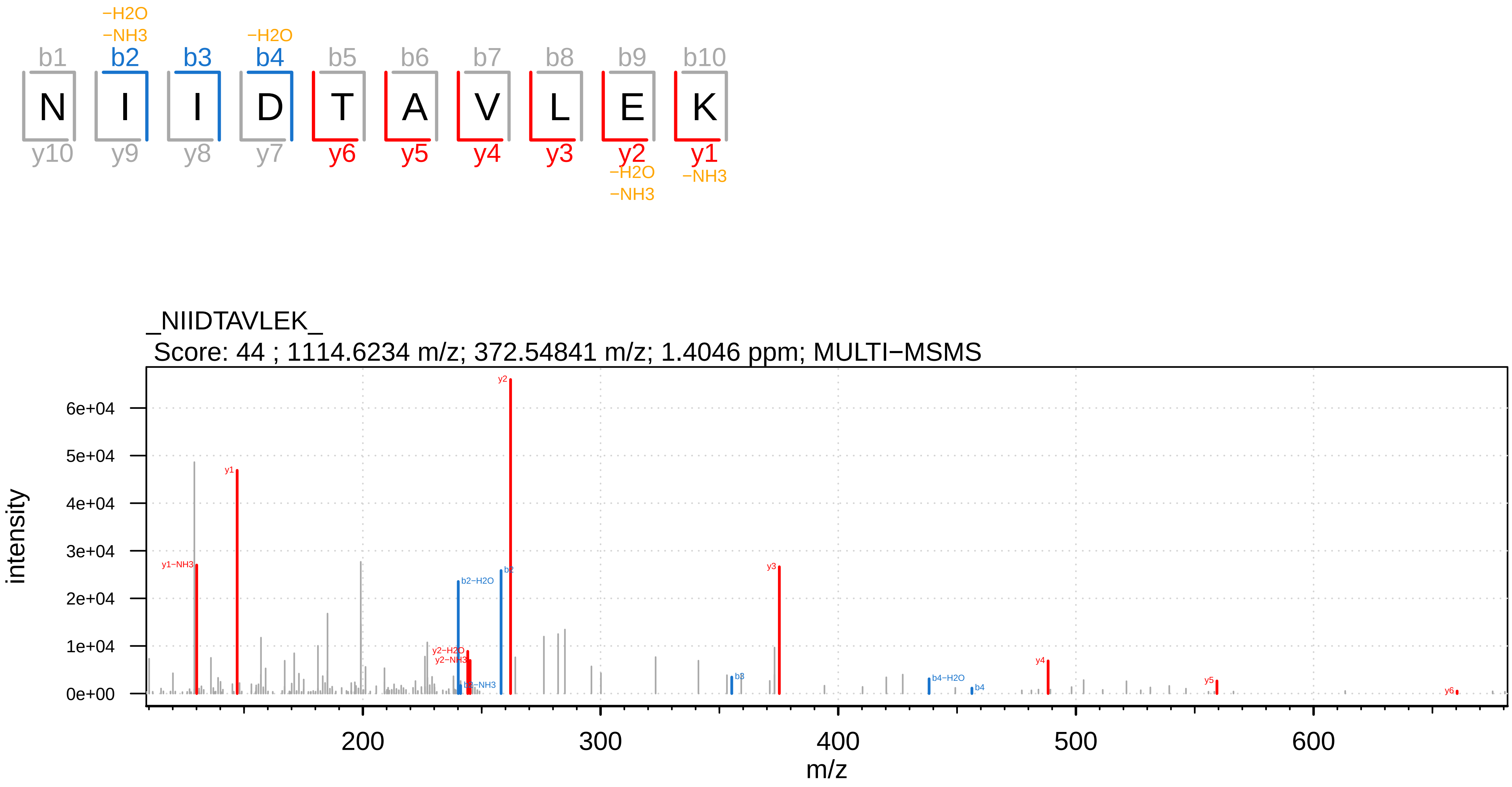

Raw File: 20100723_Velos1_TaGe_SA_Hek293_04
Scan Number: 7858
Proteins:
ENST00000497138_chr20:56806826−56807846:−

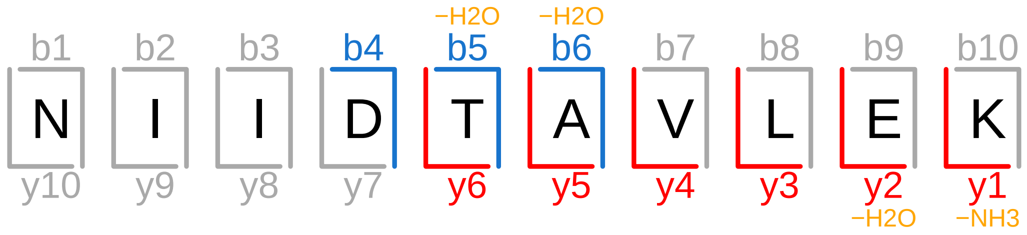

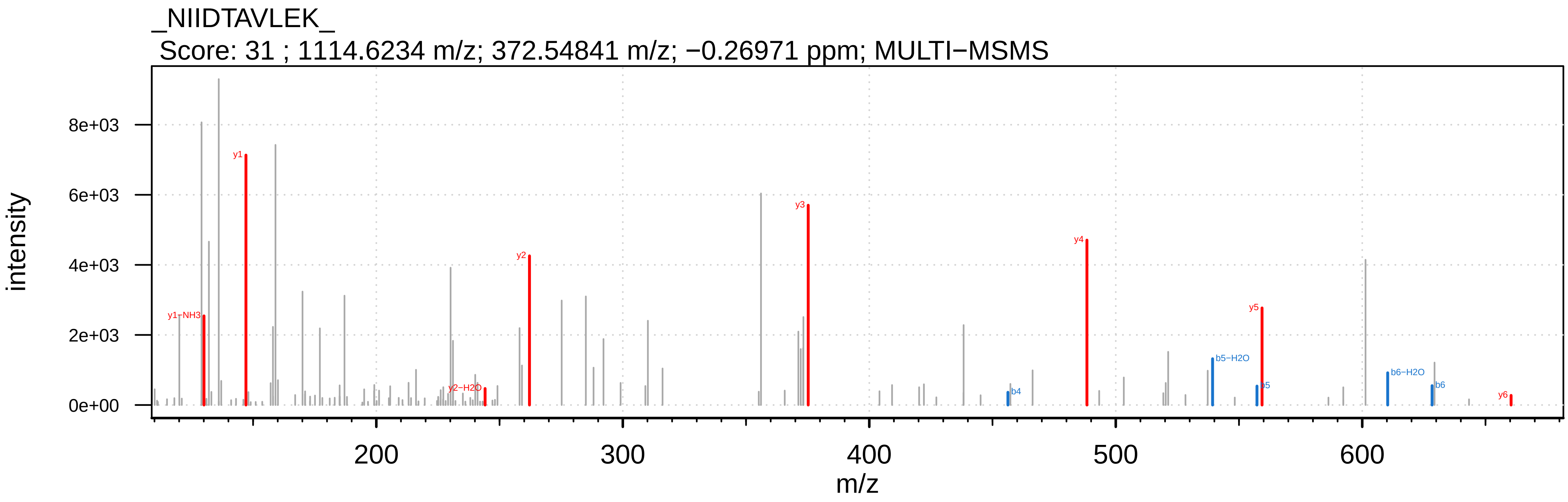

Raw File: 20101230_Velos1_TaGe_SA_Jurkat4
Scan Number: 6642
Proteins:
ENST00000497138_chr20:56806826−56807846:−

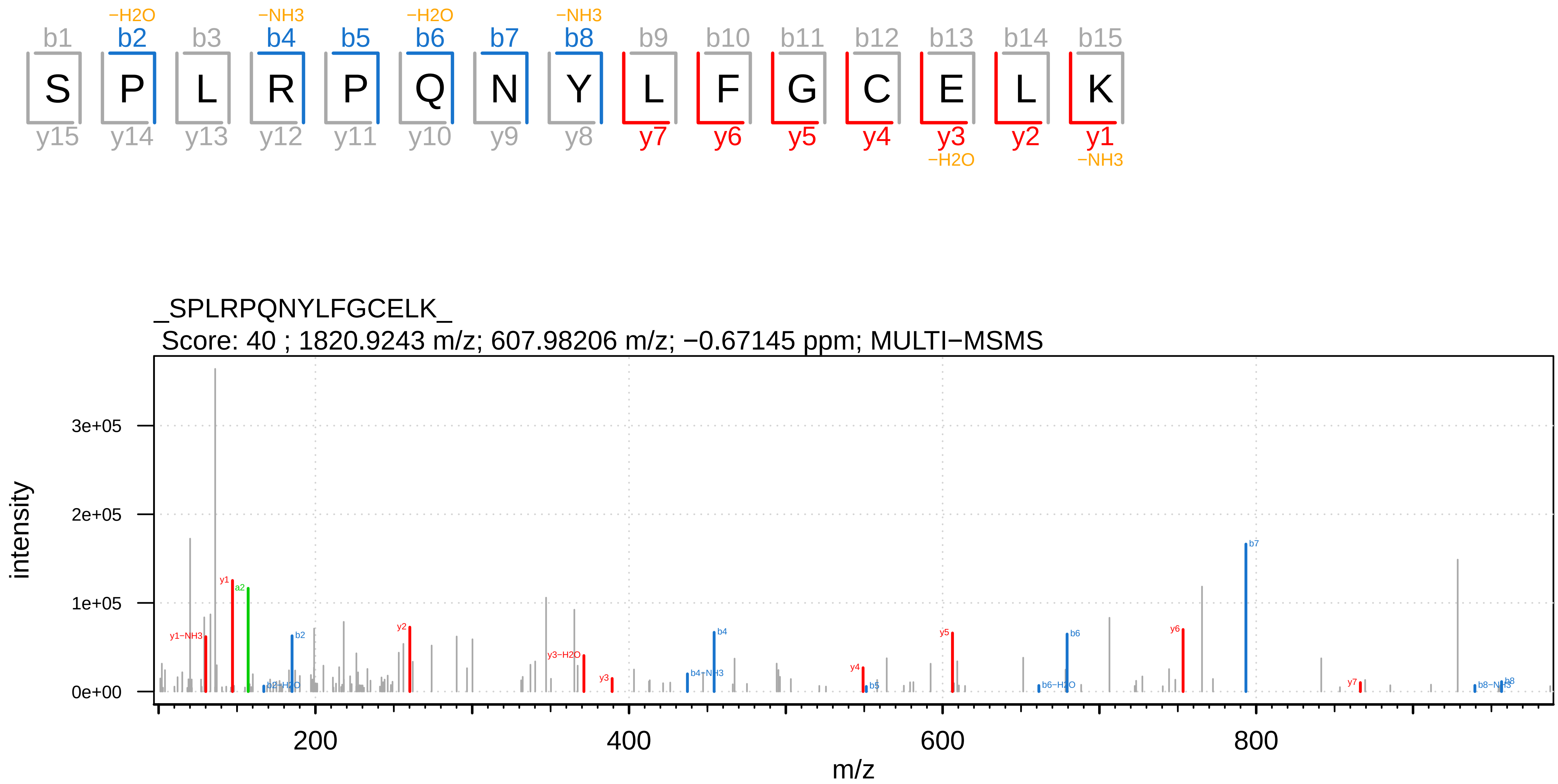

Raw File: 20100719_Velos1_TaGe_SA_LnCap_2
Scan Number: 20183
Proteins:
TCONS_l2_00008829_chr15:92829088−92829258:+

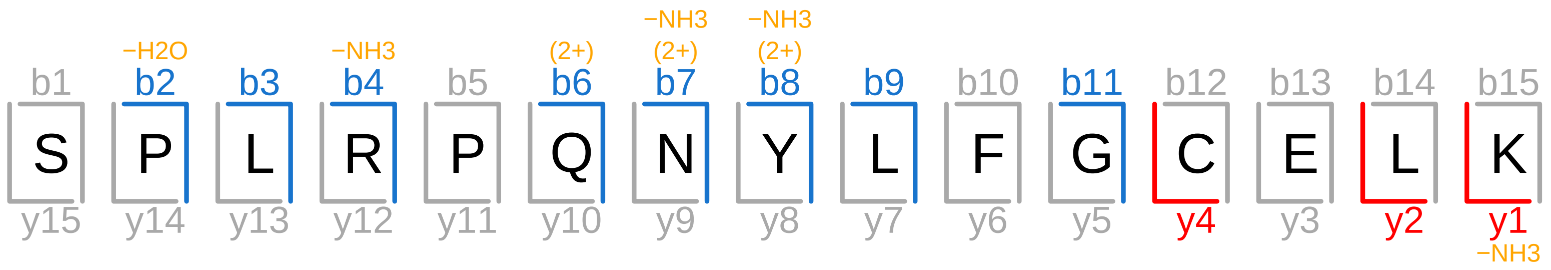

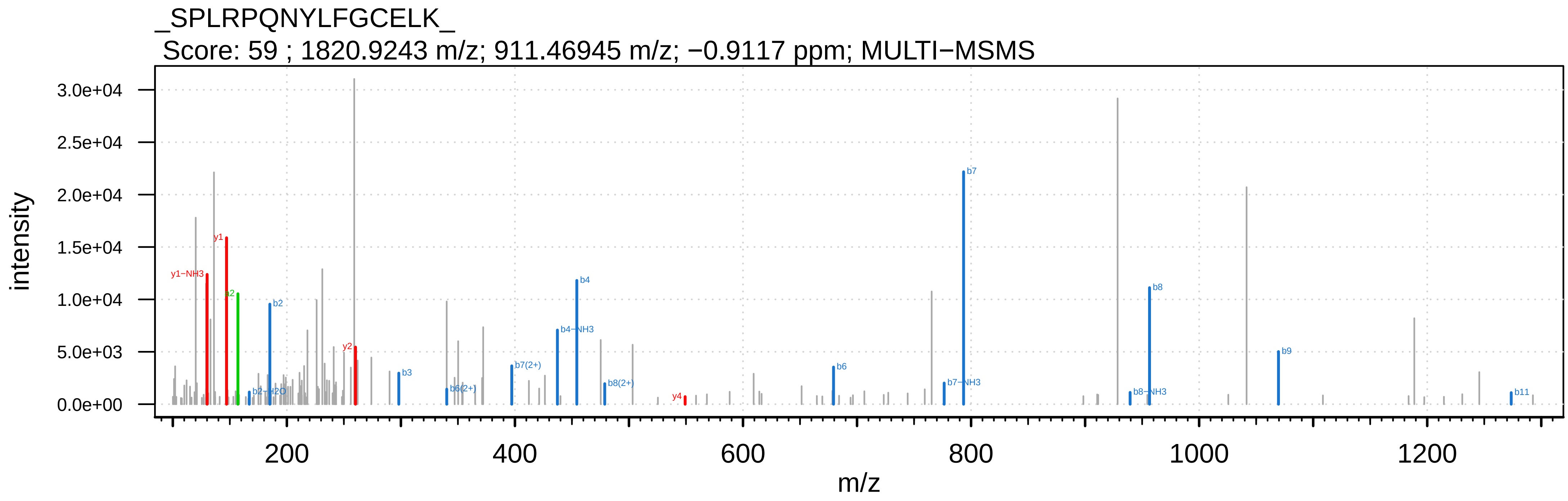

Raw File: 20100719_Velos1_TaGe_SA_LnCap_2
Scan Number: 20212
Proteins:
TCONS_l2_00008829_chr15:92829088−92829258:+

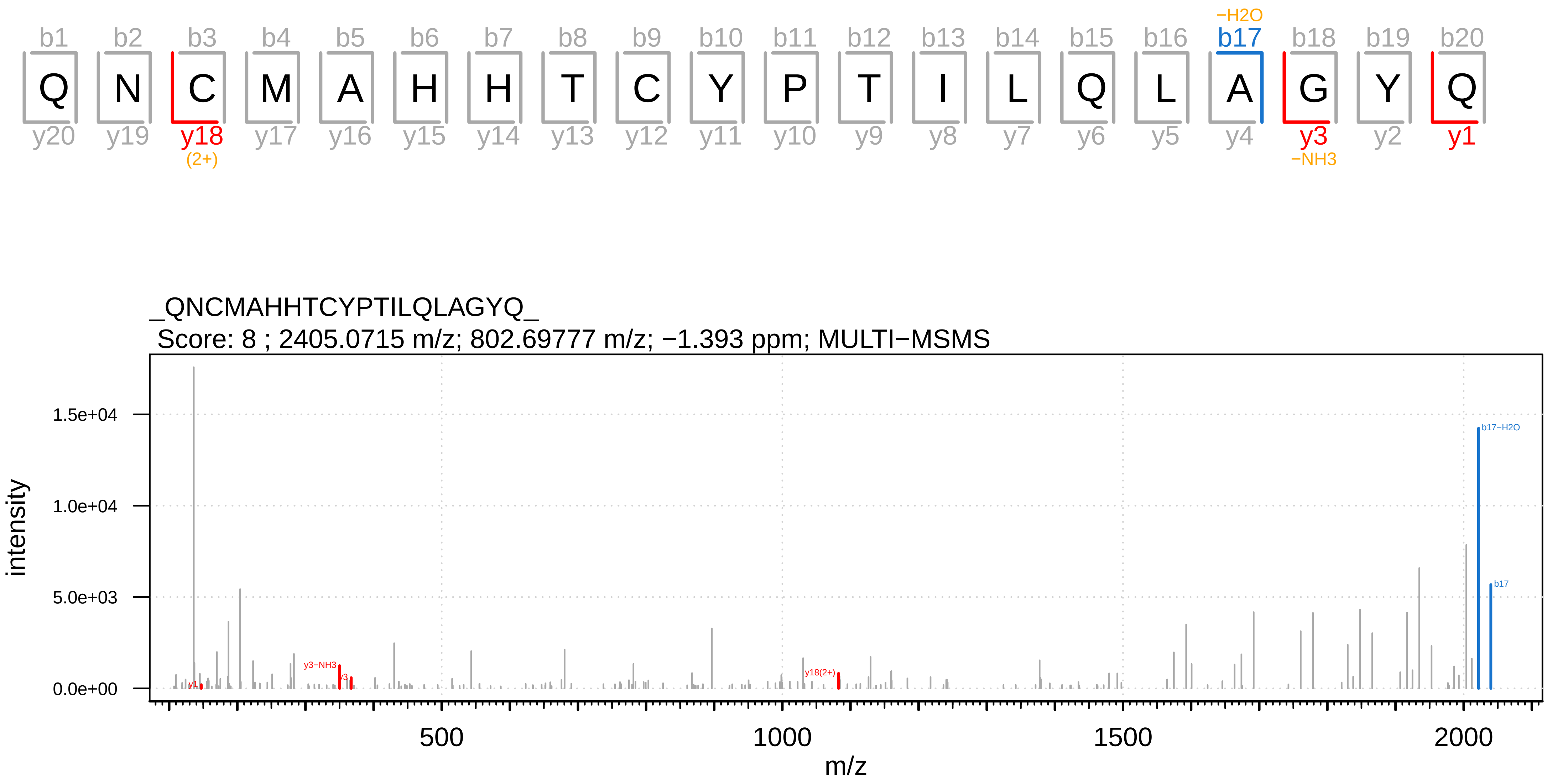

Raw File: 20100604_Velos1_TaGe_SA_A549_3
Scan Number: 12478
Proteins:
TCONS_l2_00030931_chrY:13309531−13309647:−

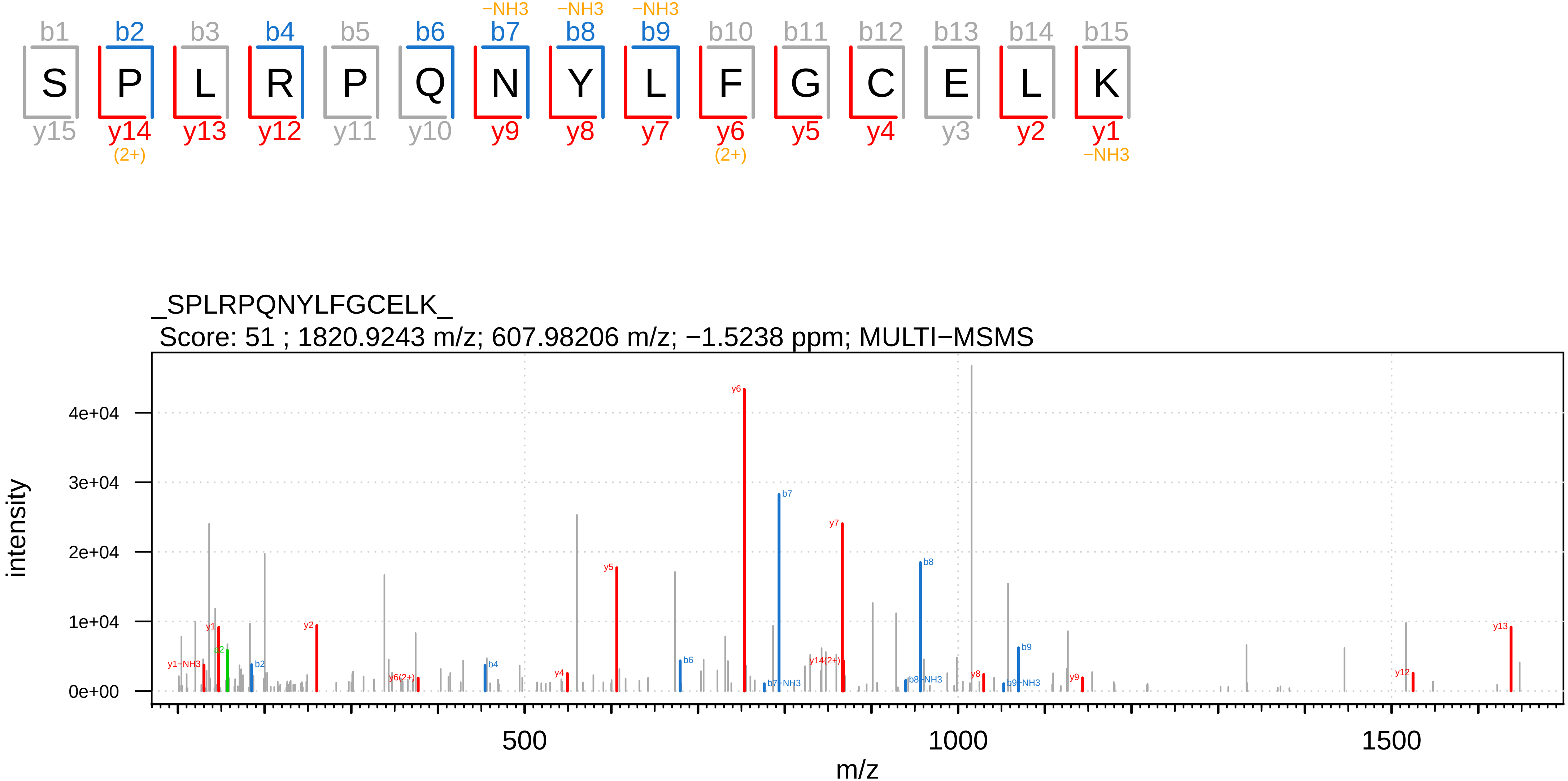

Raw File: 20100604_Velos1_TaGe_SA_A549_2
Scan Number: 15896
Proteins:
TCONS_l2_00008829_chr15:92829088−92829258:+

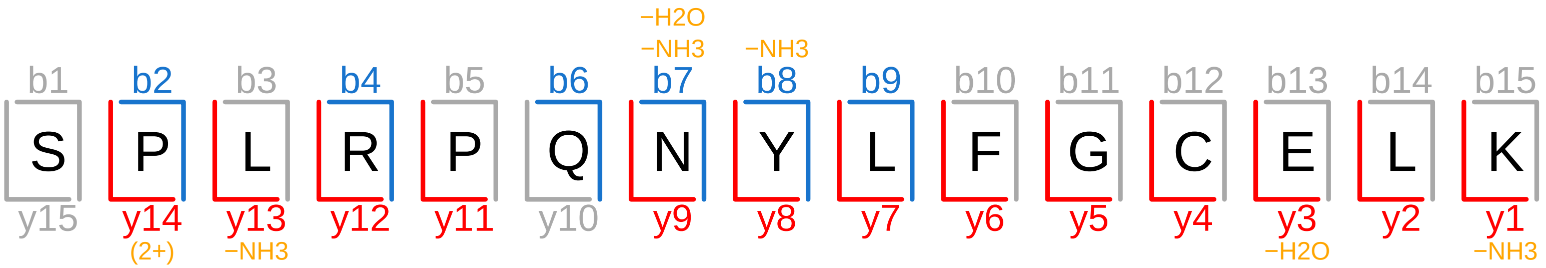

SPLRPQNYLFGCELK
Score: 88 ; 1820.9243 m/z; 607.98206 m/z; −0.56293 ppm; MULTI−MSMS

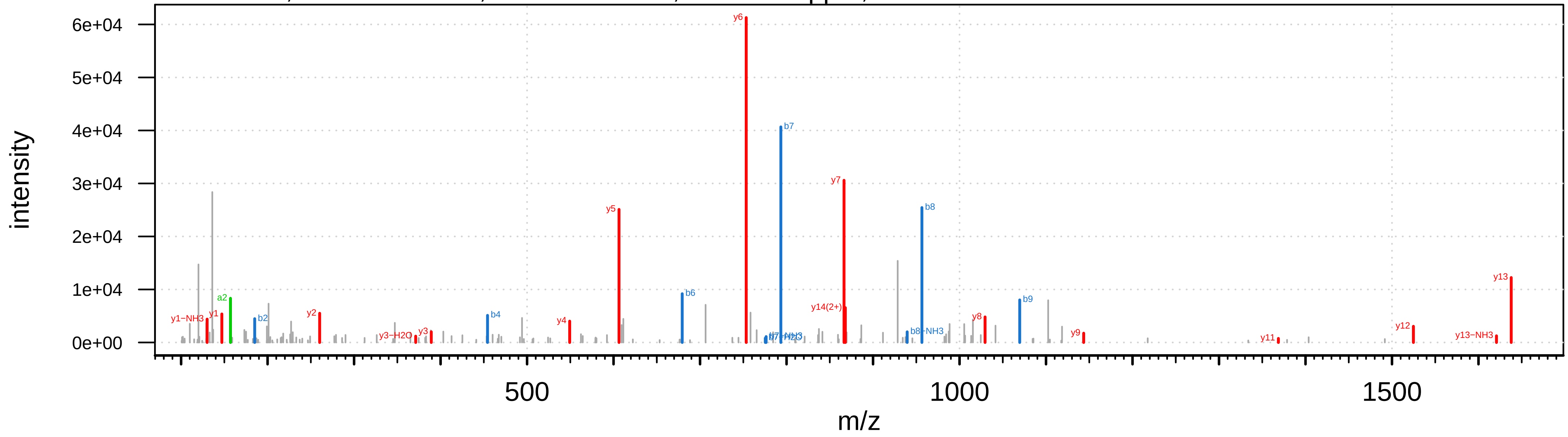

Raw File: 20100609_Velos1_TaGe_SA_GAMG_2
Scan Number: 16160
Proteins:
TCONS_l2_00008829_chr15:92829088−92829258:+

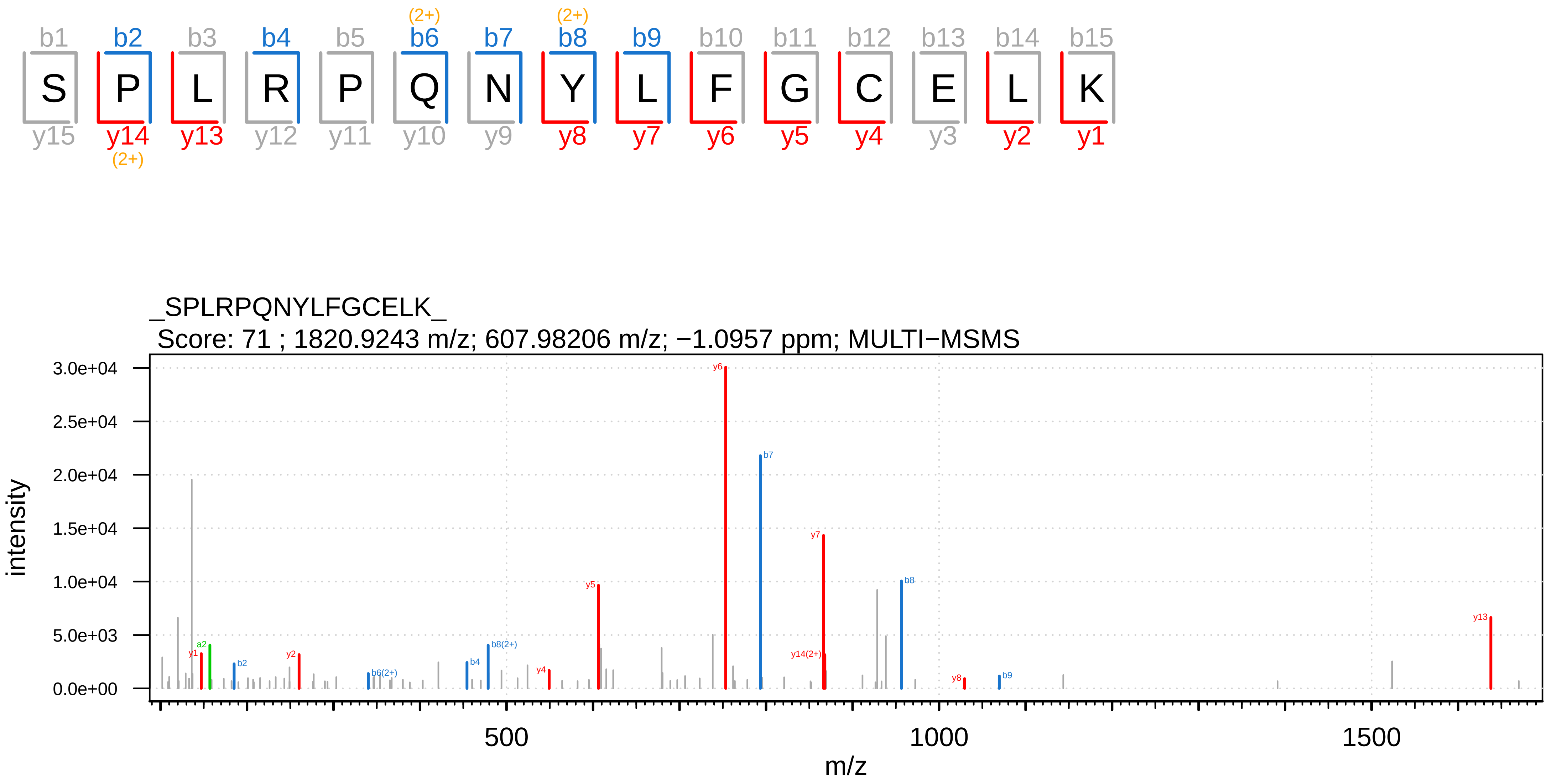

Raw File: 20100611_Velos1_TaGe_SA_Hela_6
Scan Number: 13703
Proteins:
TCONS_l2_00008829_chr15:92829088−92829258:+

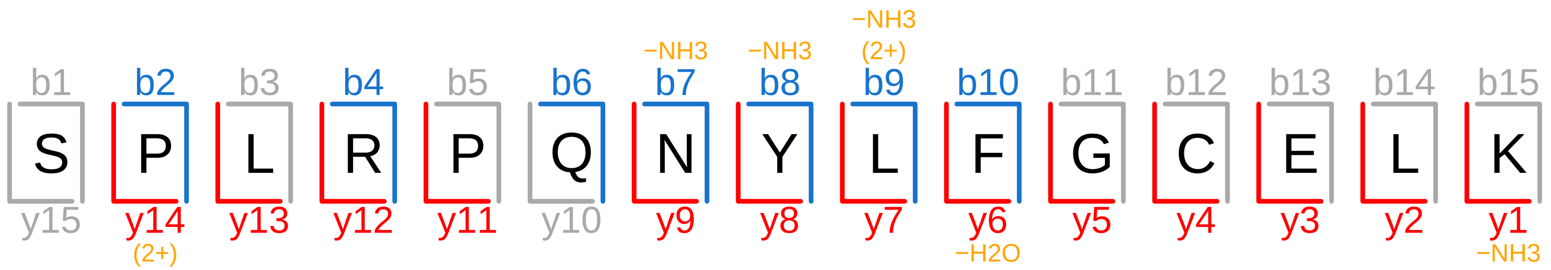

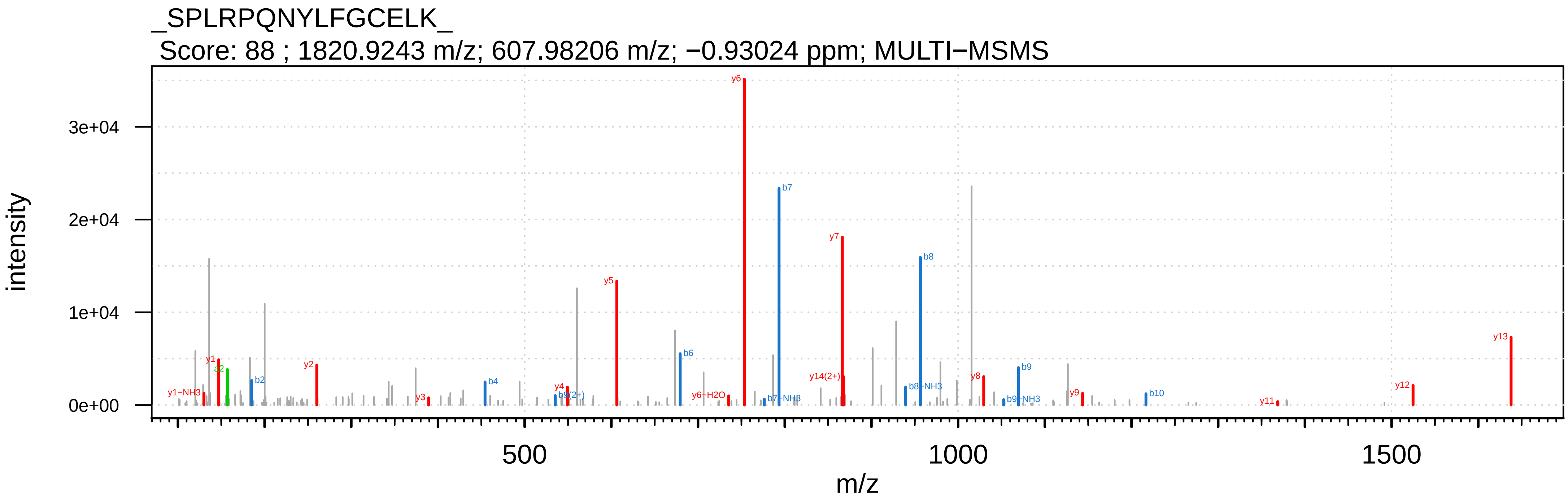

Raw File: 20100611_Velos1_TaGe_SA_HepG2_2
Scan Number: 17273
Proteins:
TCONS_l2_00008829_chr15:92829088−92829258:+

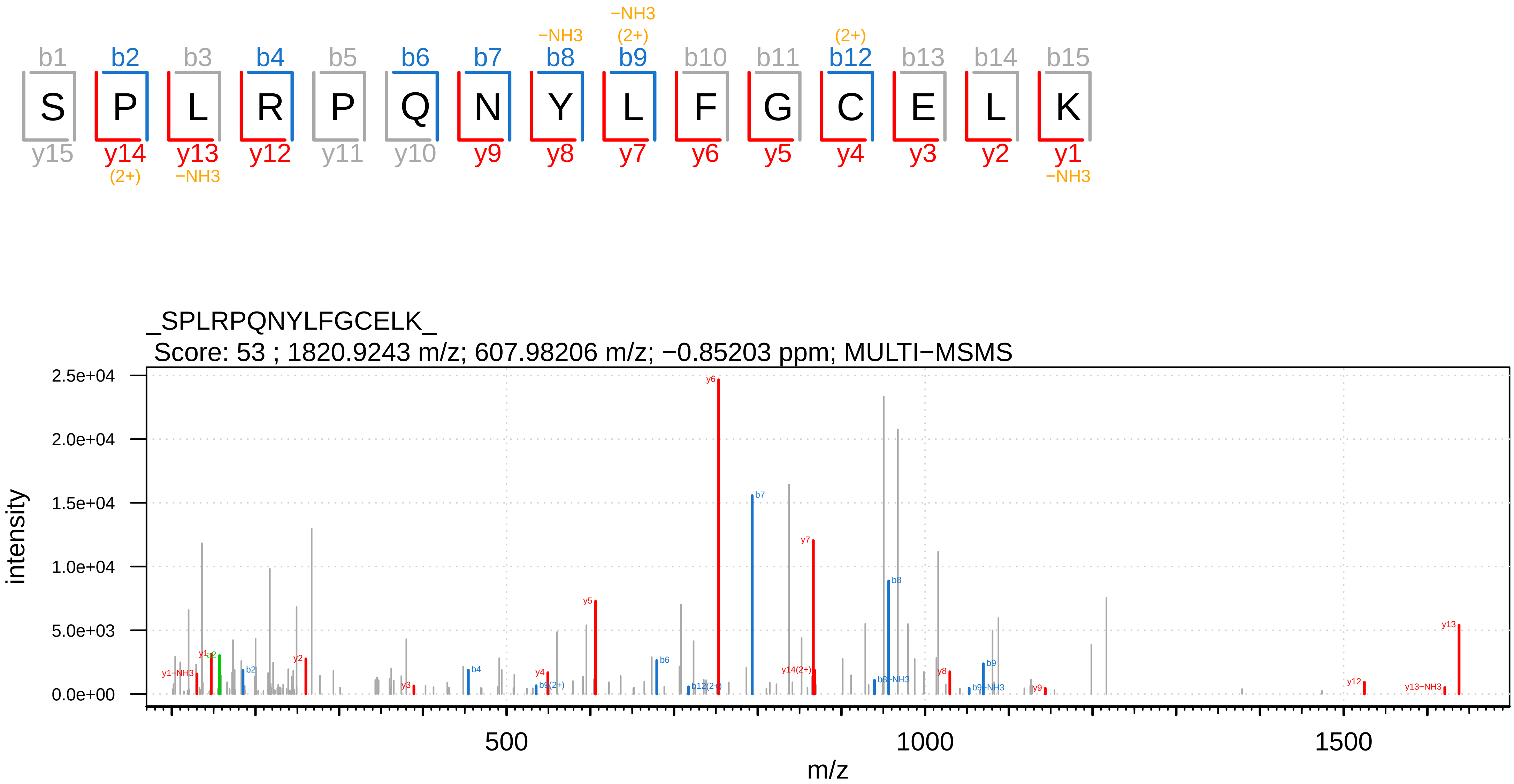

Raw File: 20100611_Velos1_TaGe_SA_HepG2_3
Scan Number: 16508
Proteins:
TCONS_l2_00008829_chr15:92829088−92829258:+

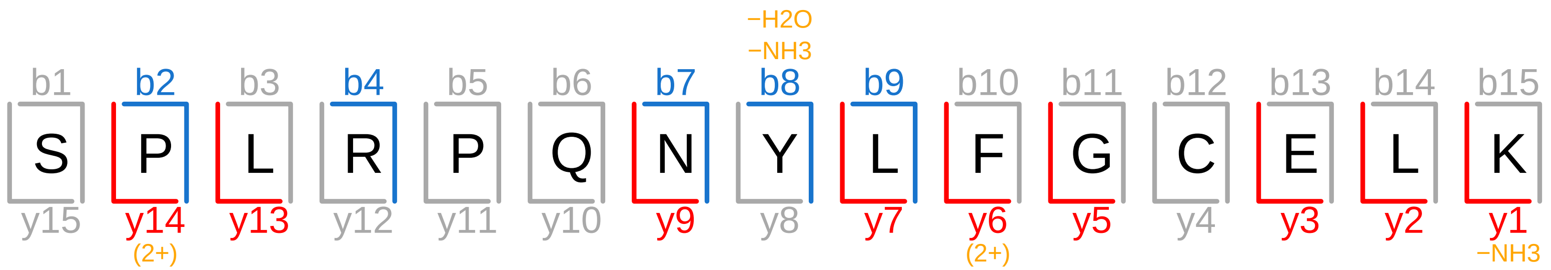

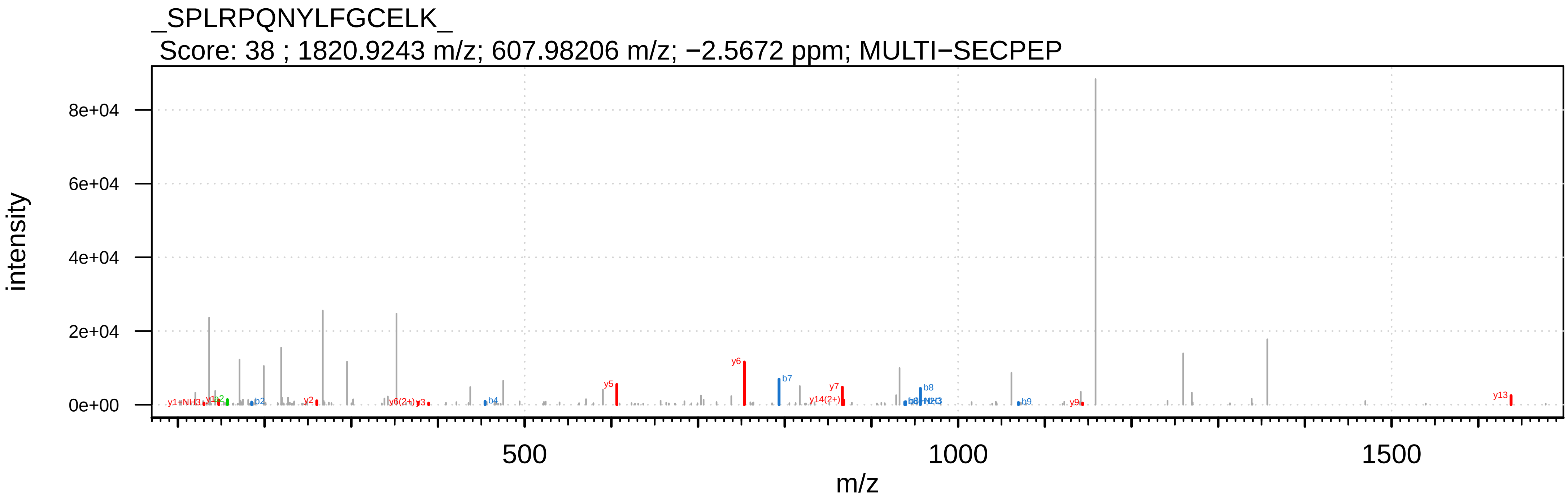

Raw File: 20100611_Velos1_TaGe_SA_HepG2_6
Scan Number: 11162
Proteins:
TCONS_l2_00008829_chr15:92829088−92829258:+

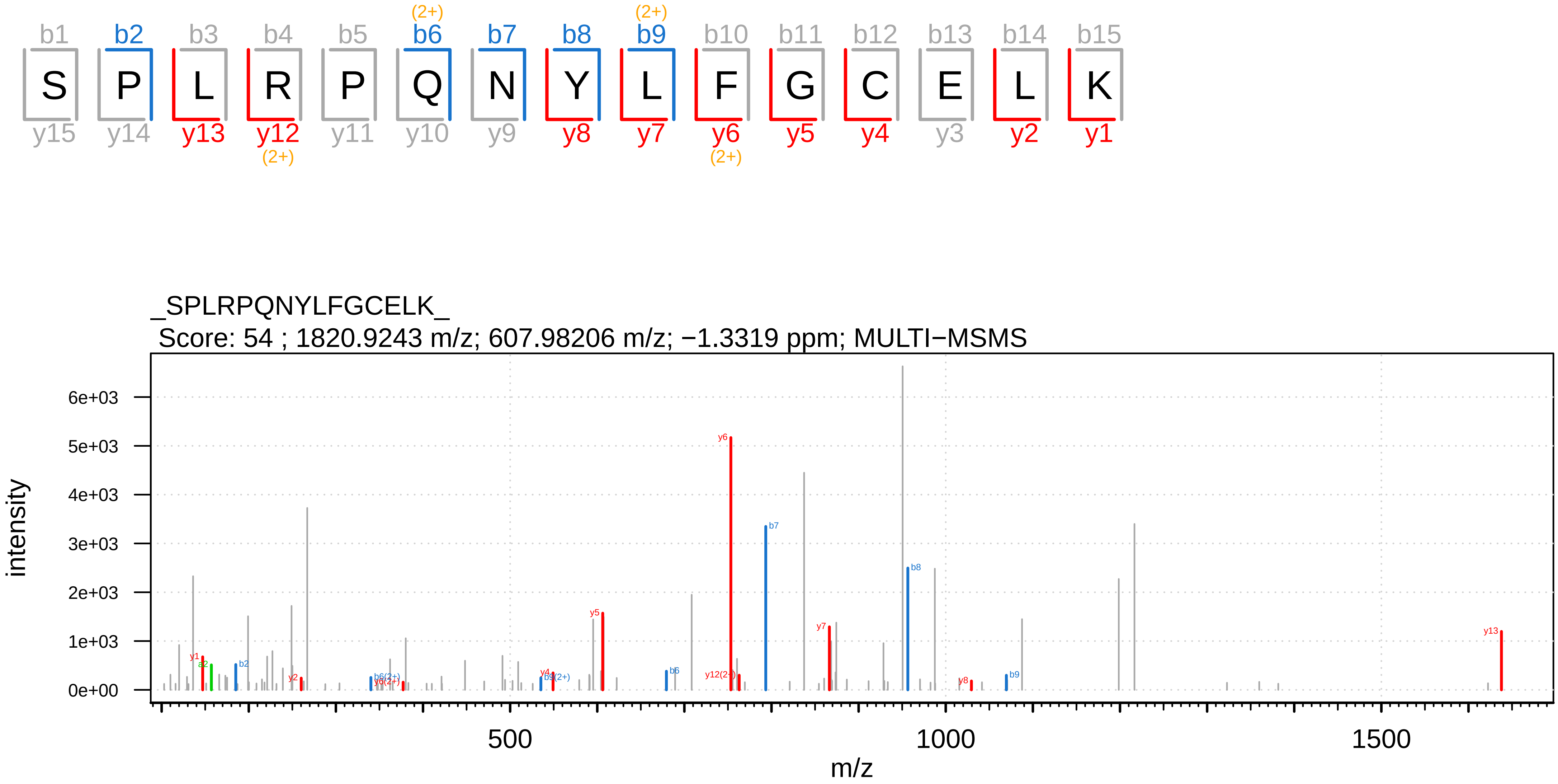

Raw File: 20100614_Velos1_TaGe_SA_Jurkat_2
Scan Number: 18378
Proteins:
TCONS_l2_00008829_chr15:92829088−92829258:+

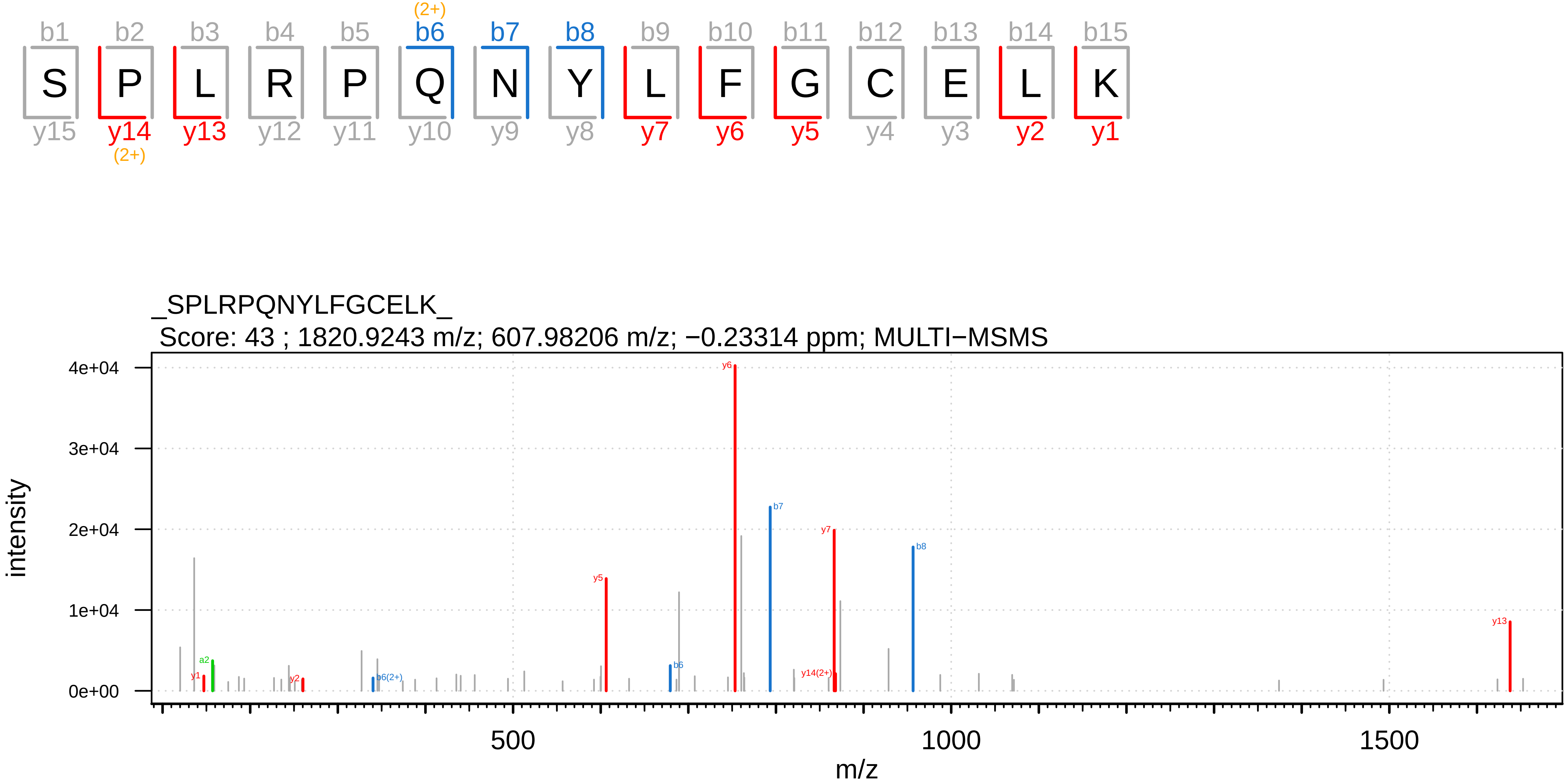

Raw File: 20100616_Velos1_TaGe_SA_RKO_2
Scan Number: 19011
Proteins:
TCONS_l2_00008829_chr15:92829088−92829258:+

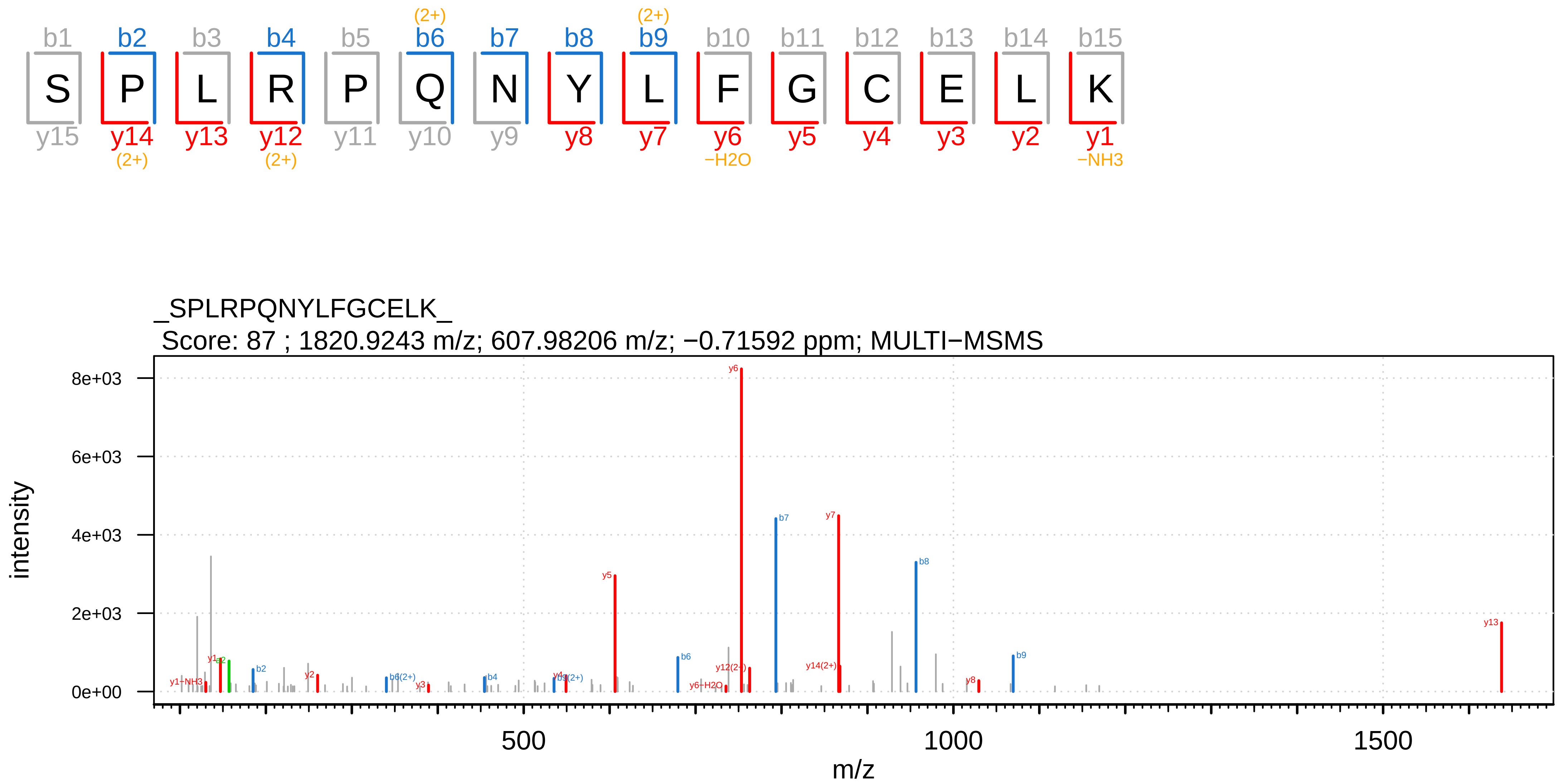

Raw File: 20100616_Velos1_TaGe_SA_RKO_6
Scan Number: 15140
Proteins:
TCONS_l2_00008829_chr15:92829088−92829258:+

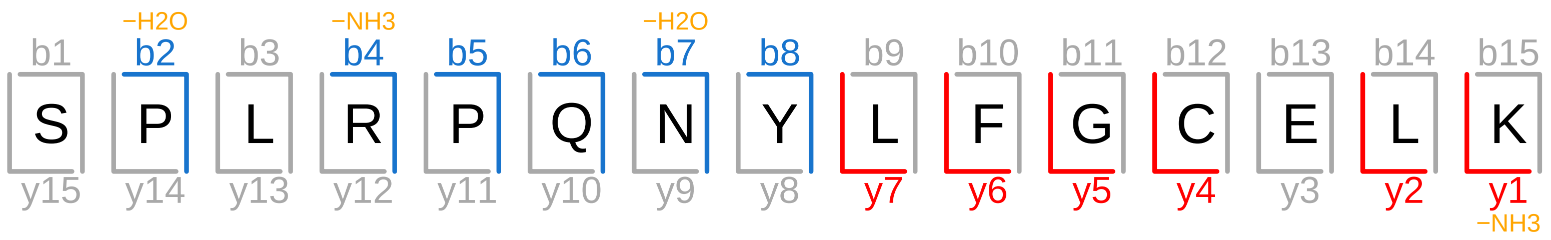

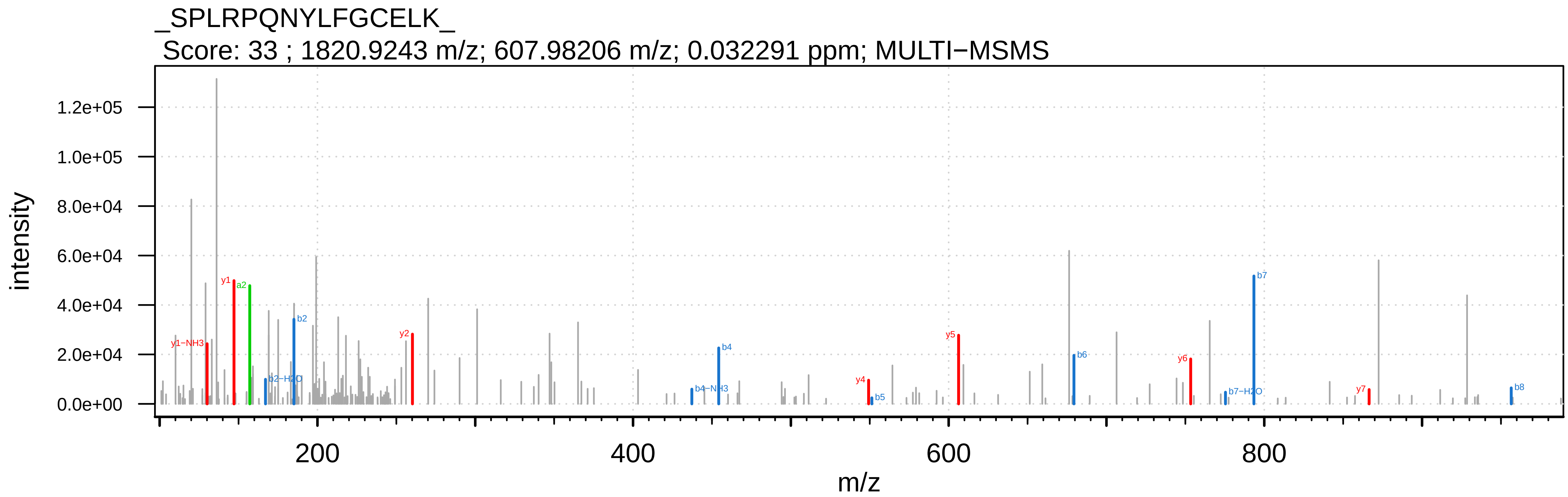

Raw File: 20100721_Velos1_TaGe_SA_A549_01
Scan Number: 18501
Proteins:
TCONS_l2_00008829_chr15:92829088−92829258:+

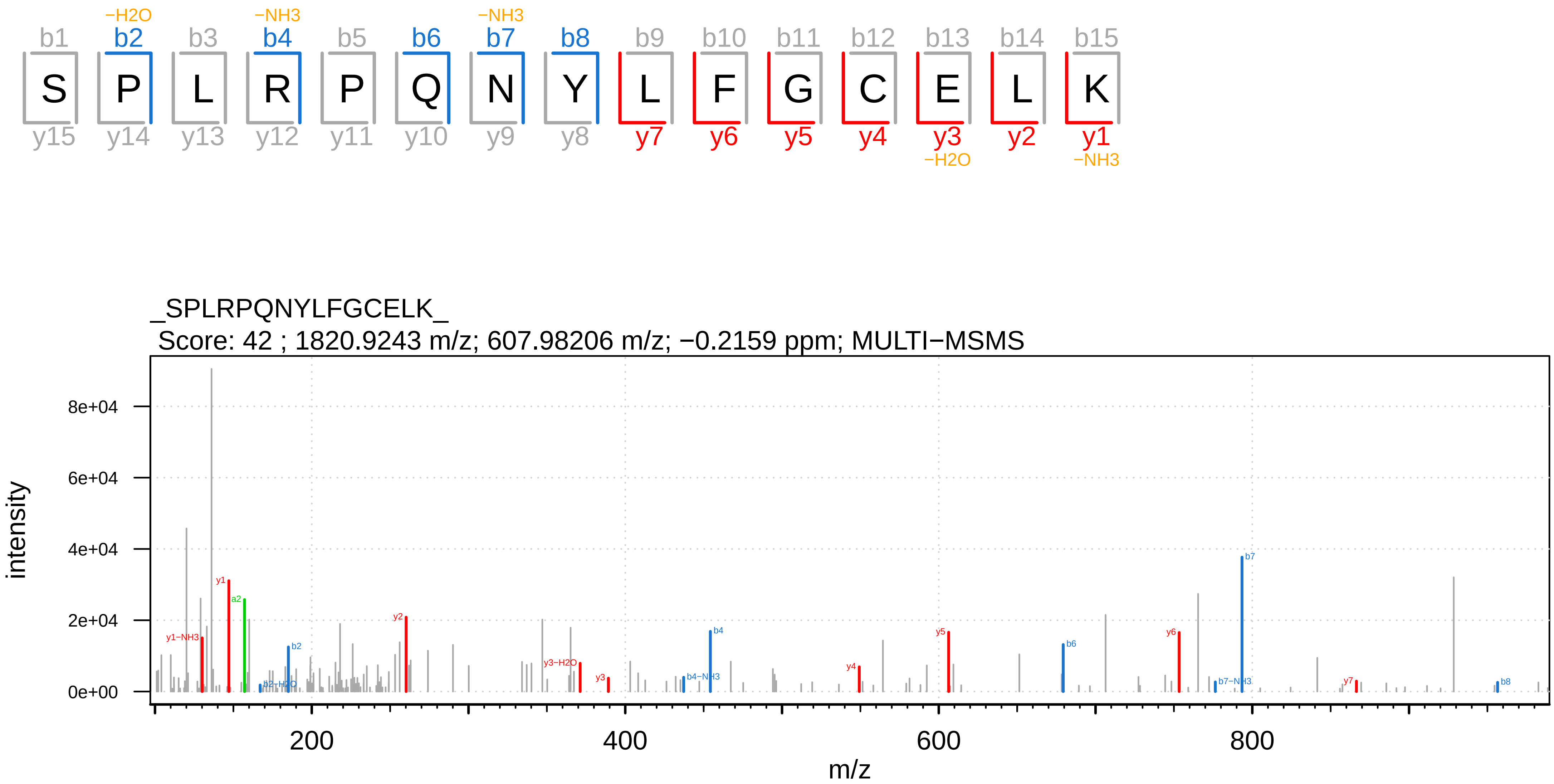

Raw File: 20100721_Velos1_TaGe_SA_A549_02

Scan Number: 19876

Proteins:

TCONS_l2_00008829_chr15:92829088−92829258:+

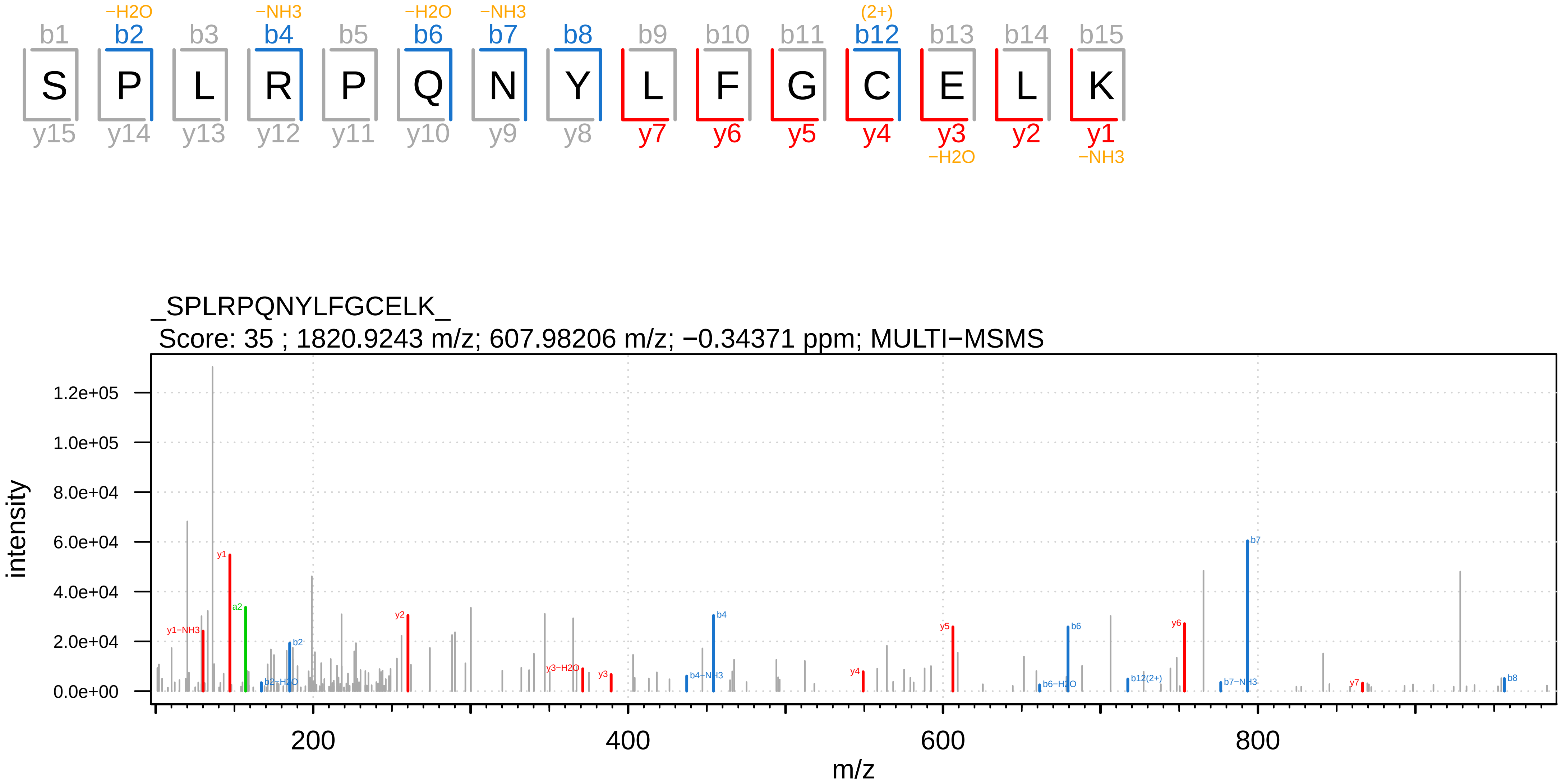

Raw File: 20100723_Velos1_TaGe_SA_Gamg_2
Scan Number: 20646
Proteins:
TCONS_l2_00008829_chr15:92829088−92829258:+

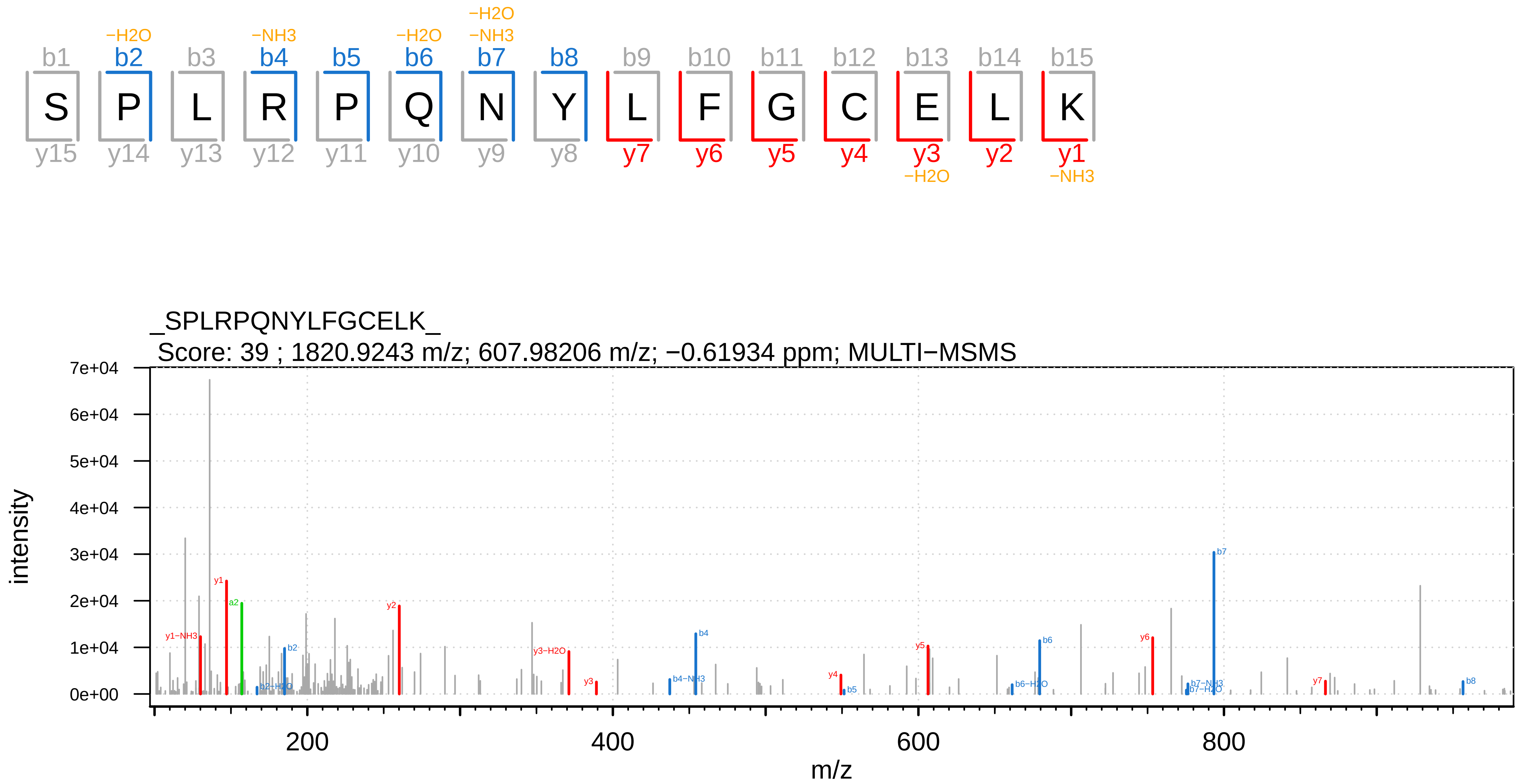

Raw File: 20100726_Velos1_TaGe_SA_HeLa_1
Scan Number: 17509
Proteins:
TCONS_l2_00008829_chr15:92829088−92829258:+

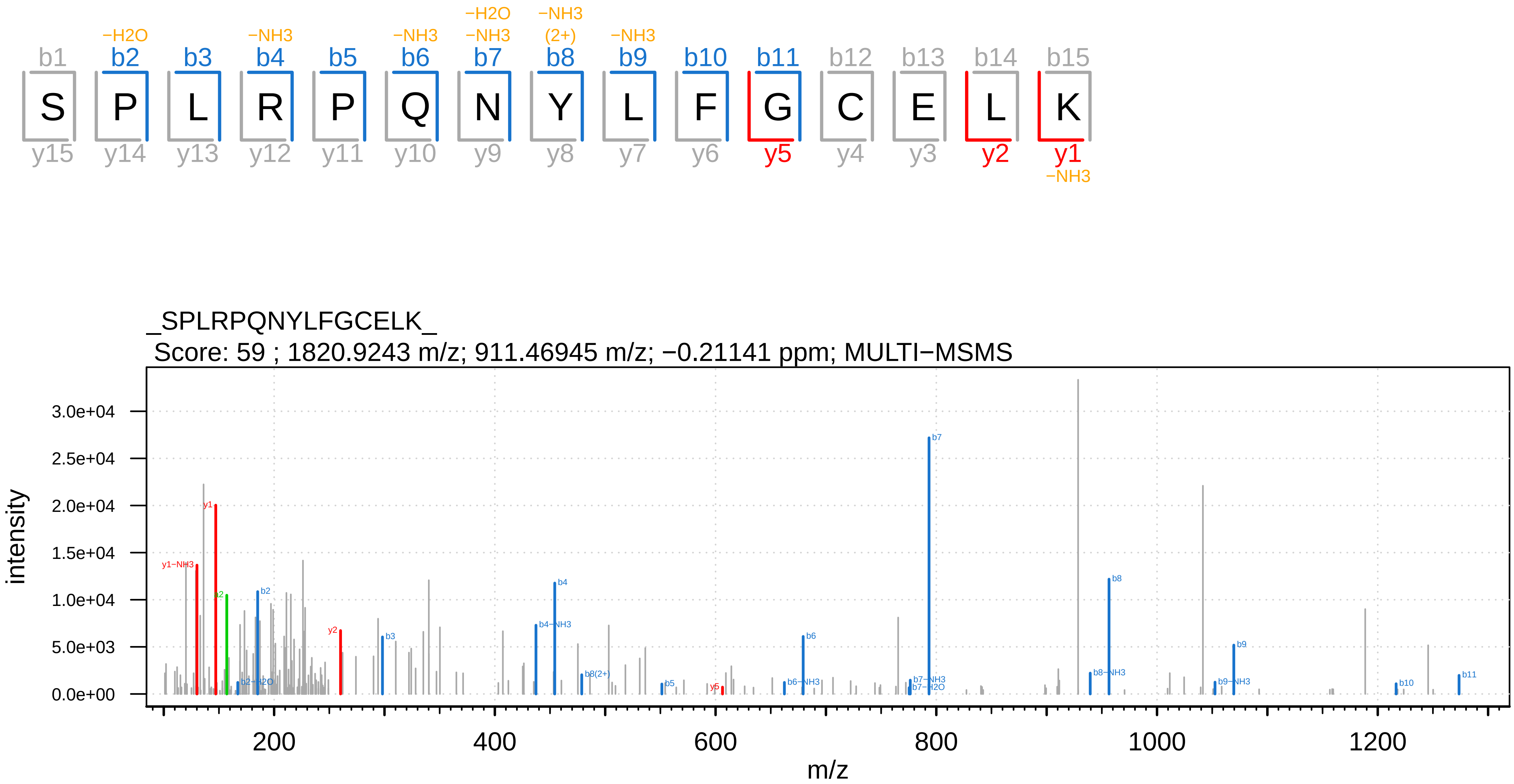

Raw File: 20100726_Velos1_TaGe_SA_HeLa_1
Scan Number: 17529
Proteins:
TCONS_l2_00008829_chr15:92829088−92829258:+

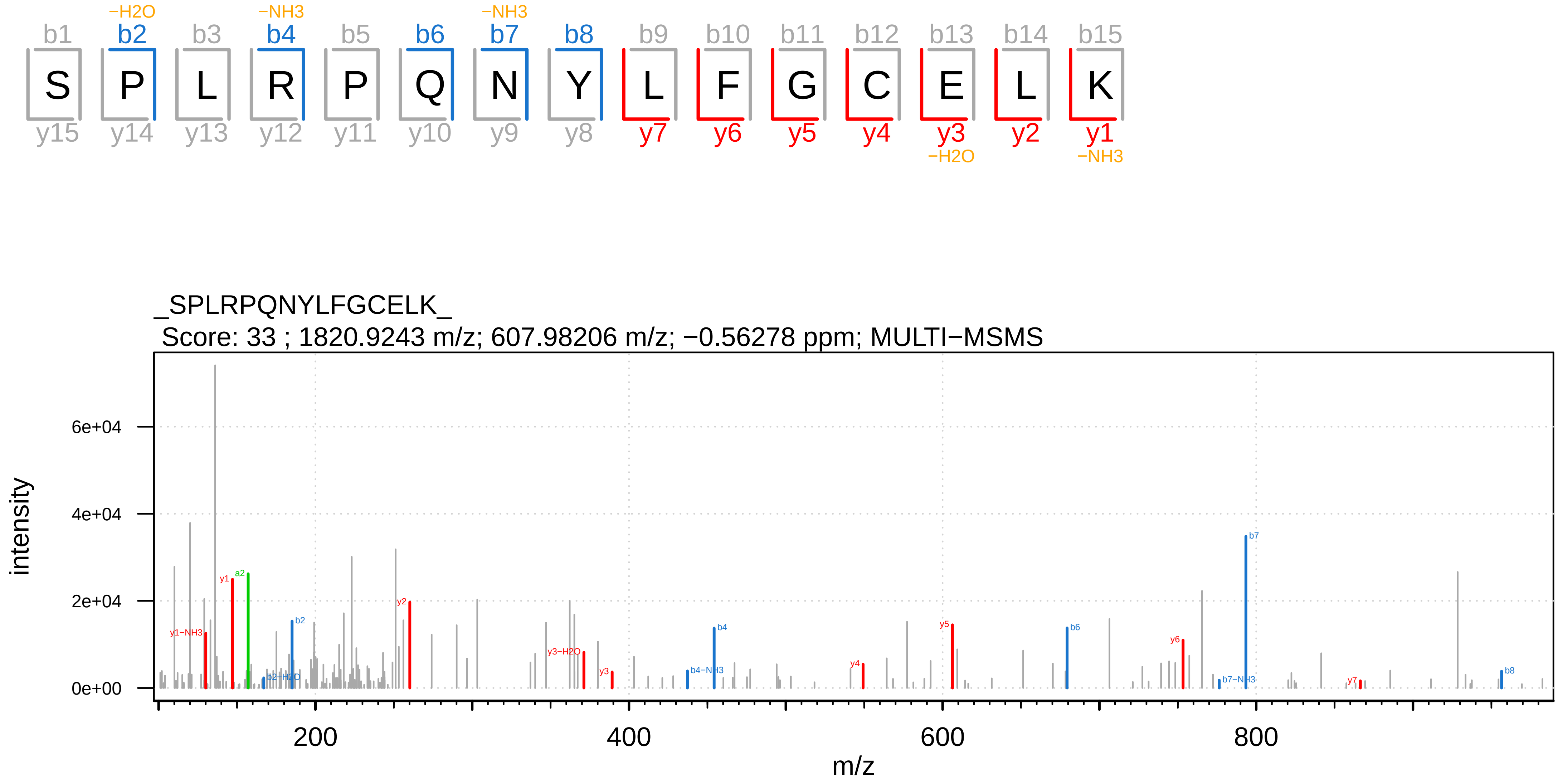

Raw File: 20100726_Velos1_TaGe_SA_HepG2_1
Scan Number: 17794
Proteins:
TCONS_l2_00008829_chr15:92829088−92829258:+

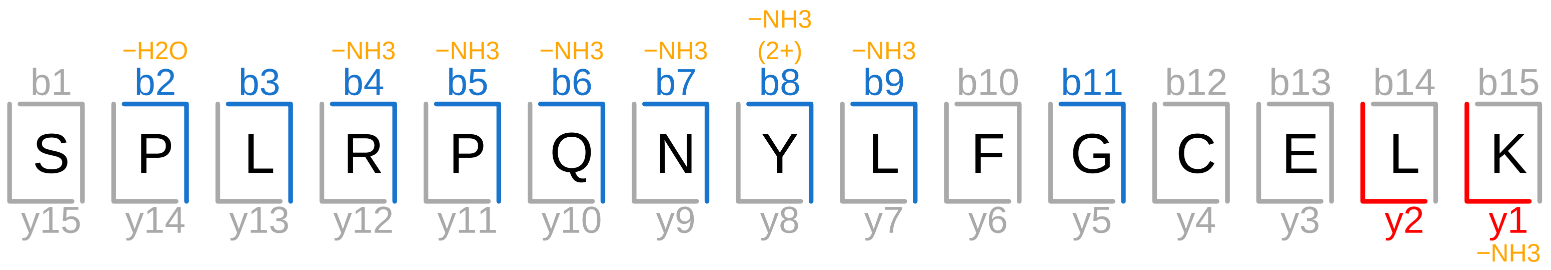

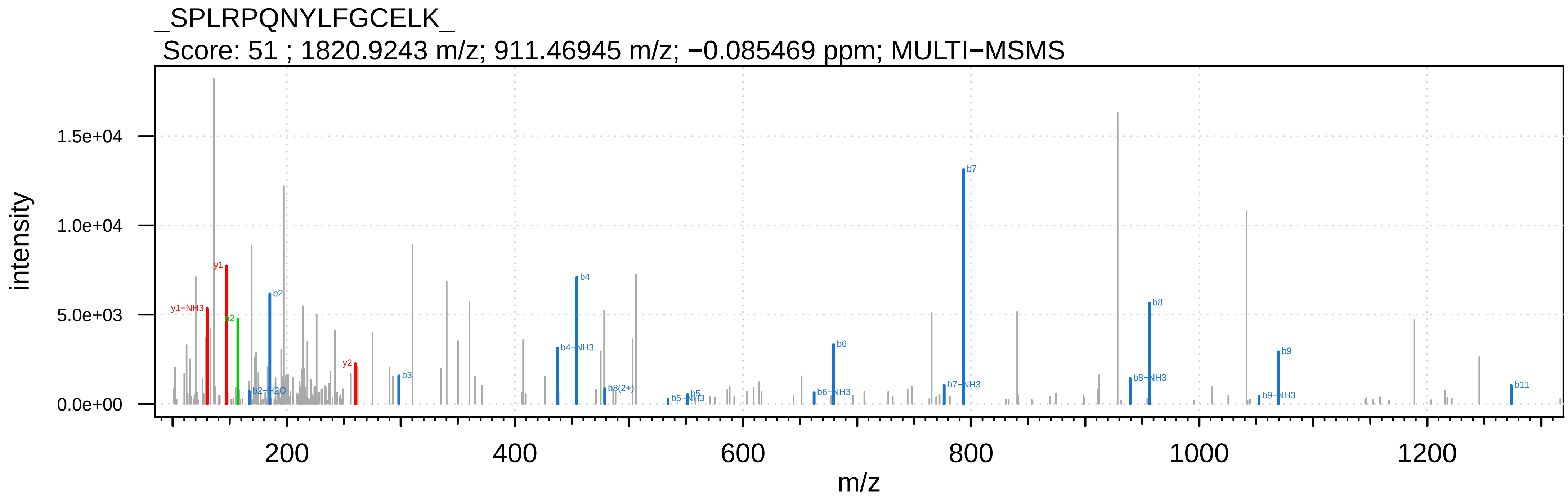

Raw File: 20100726_Velos1_TaGe_SA_HepG2_1
Scan Number: 17800
Proteins:
TCONS_l2_00008829_chr15:92829088−92829258:+

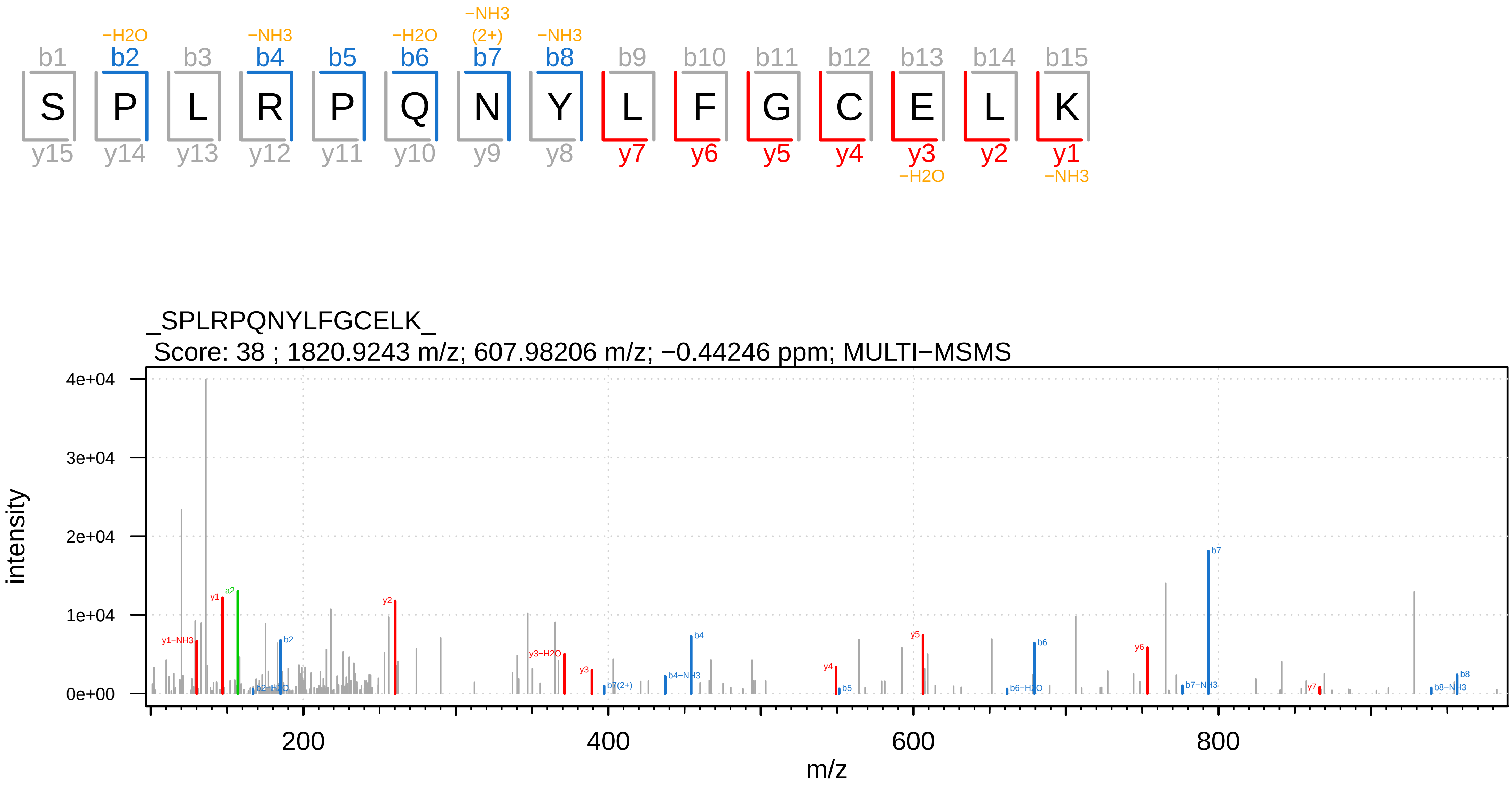

Raw File: 20100801_Velos1_TaGe_SA_RKO_01
Scan Number: 15215
Proteins:
TCONS_l2_00008829_chr15:92829088−92829258:+

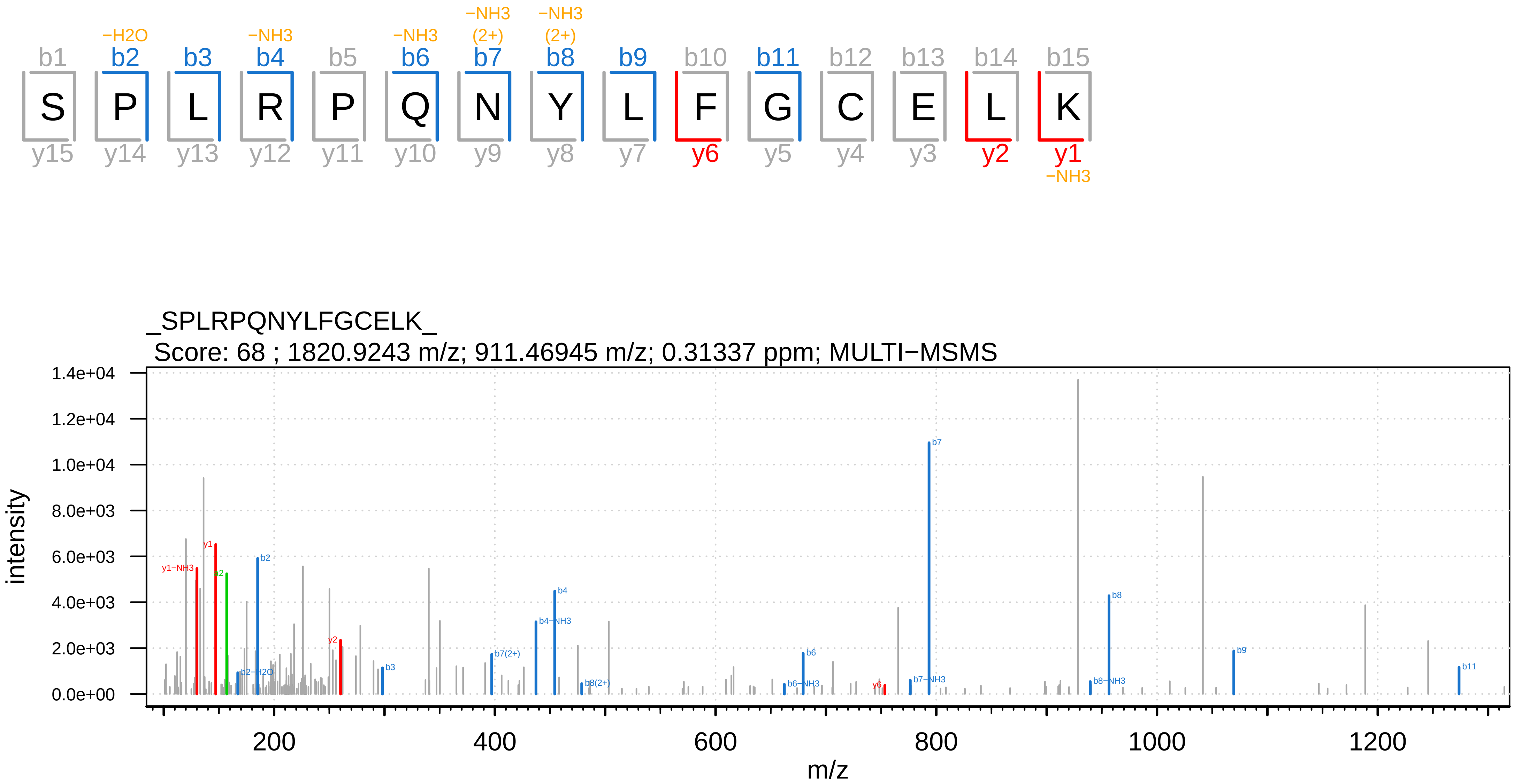

Raw File: 20100801_Velos1_TaGe_SA_RKO_01
Scan Number: 15226
Proteins:
TCONS_l2_00008829_chr15:92829088−92829258:+

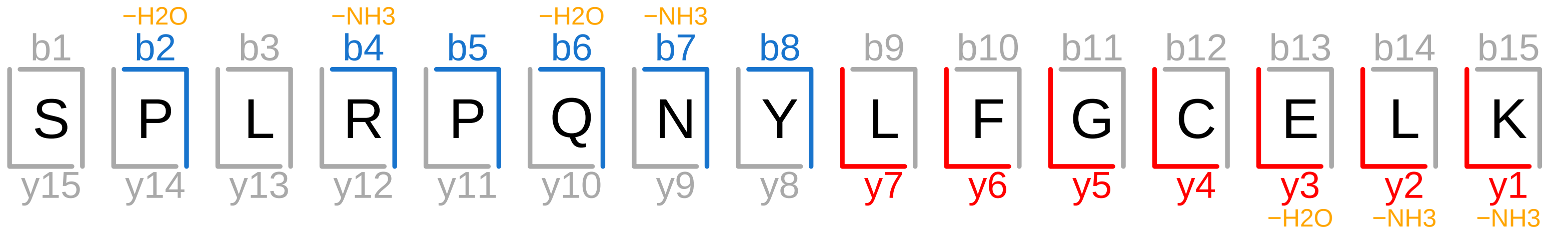

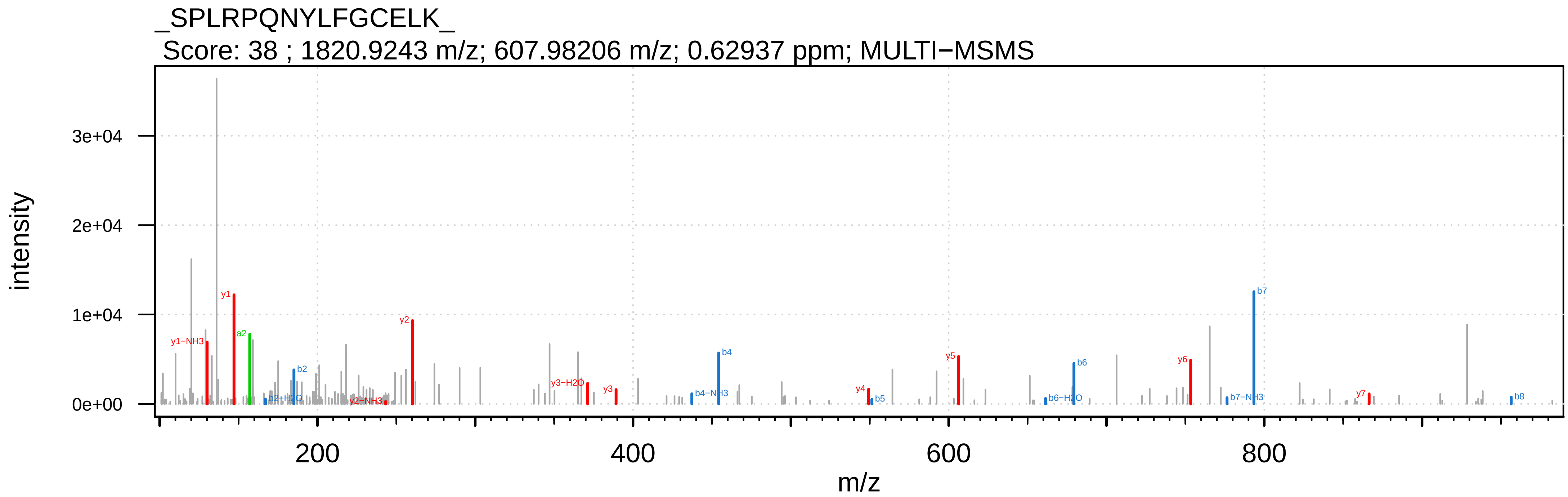

Raw File: 20100805_Velos1_TaGe_SA_RKO_06
Scan Number: 15209
Proteins:
TCONS_l2_00008829_chr15:92829088−92829258:+

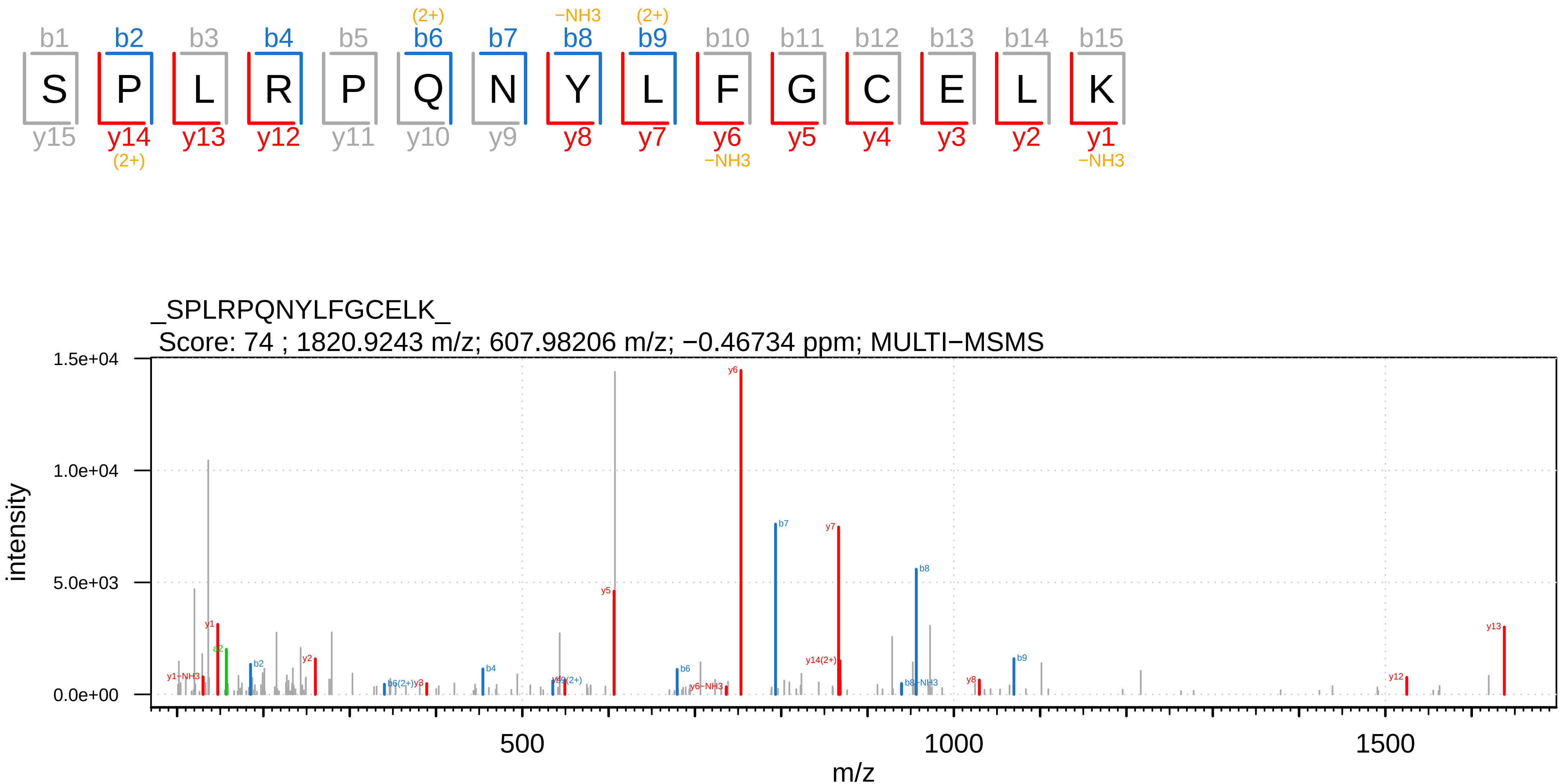

Raw File: 20101210_Velos1_AnWe_SA_LnCap_6
Scan Number: 15445
Proteins:
TCONS_l2_00008829_chr15:92829088−92829258:+

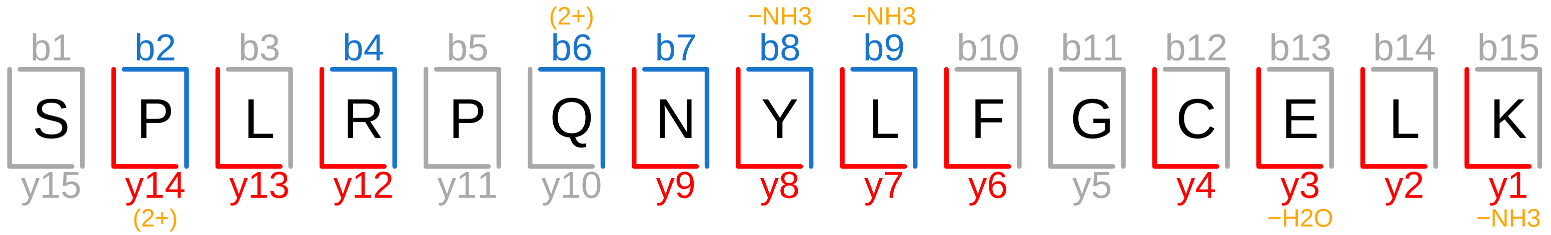

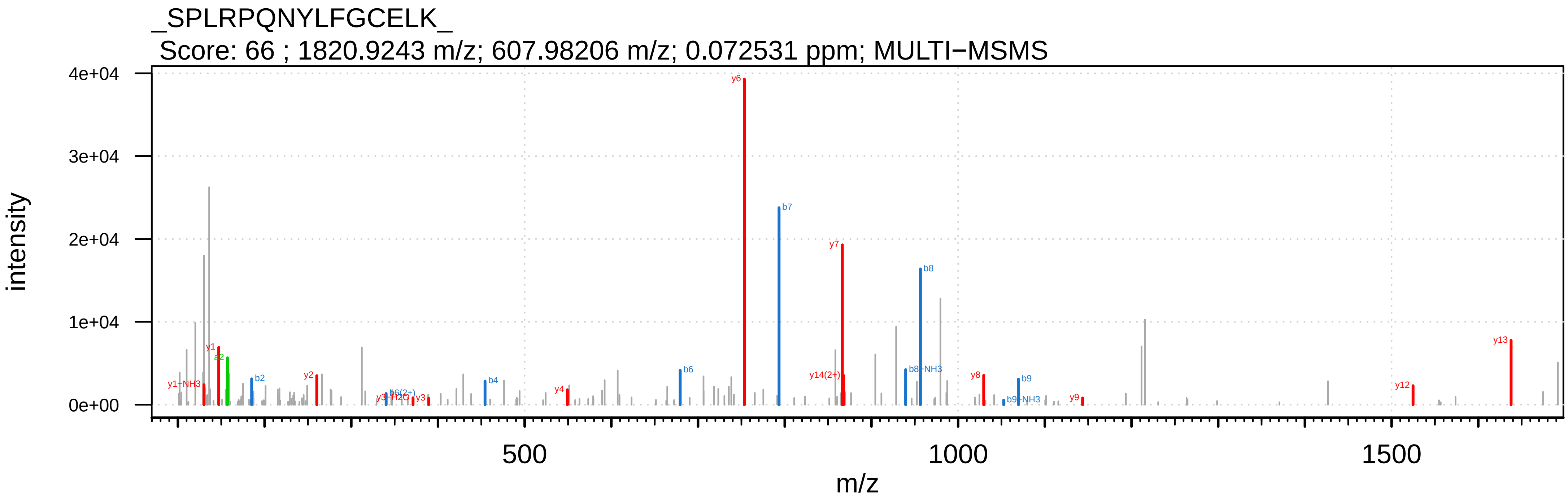

Raw File: 20101210_Velos1_AnWe_SA_MCF7_6
Scan Number: 16375
Proteins:
TCONS_l2_00008829_chr15:92829088−92829258:+

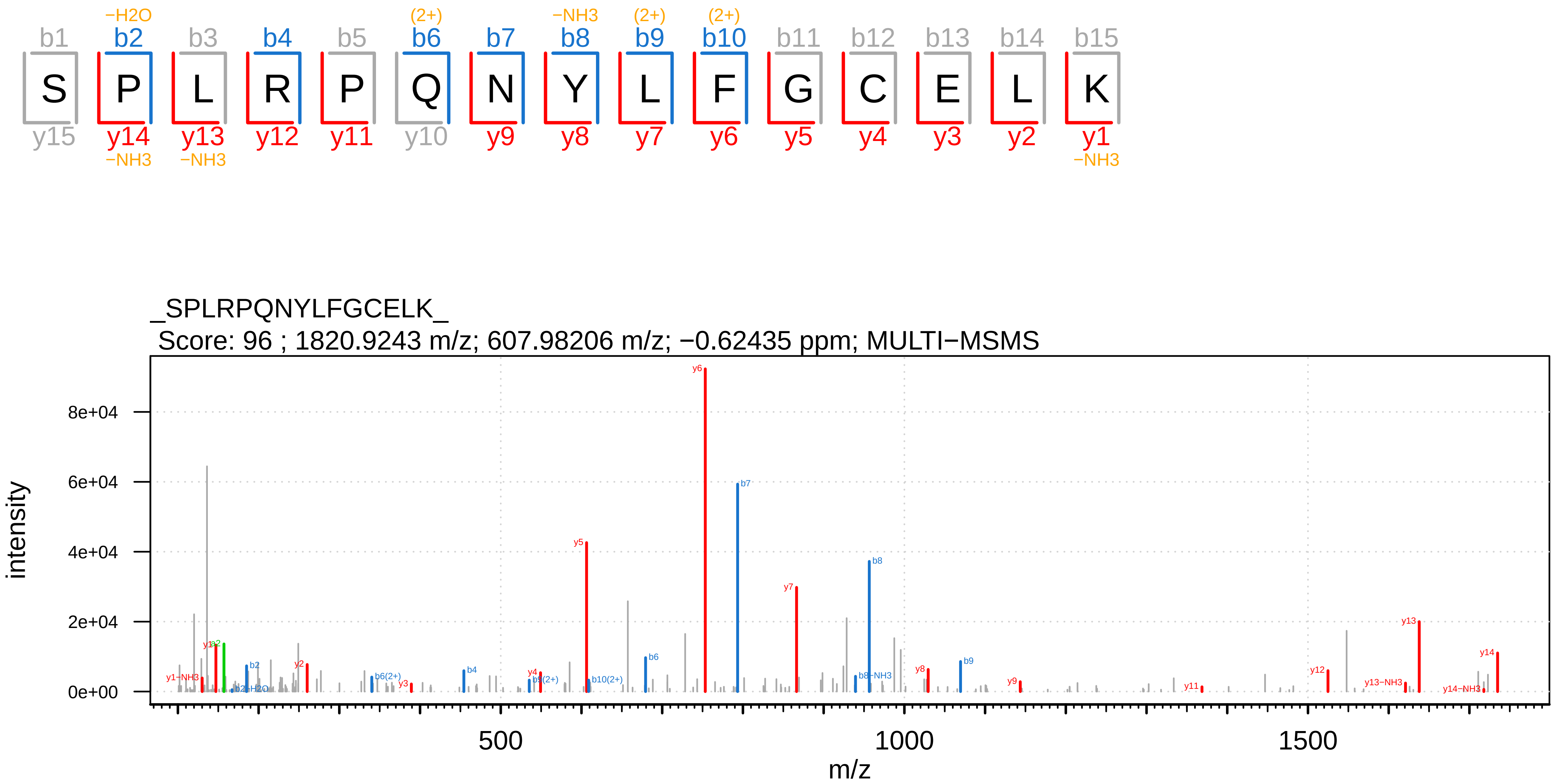

Raw File: 20101210_Velos1_AnWe_SA_U2OS_6
Scan Number: 17394
Proteins:
TCONS_l2_00008829_chr15:92829088−92829258:+

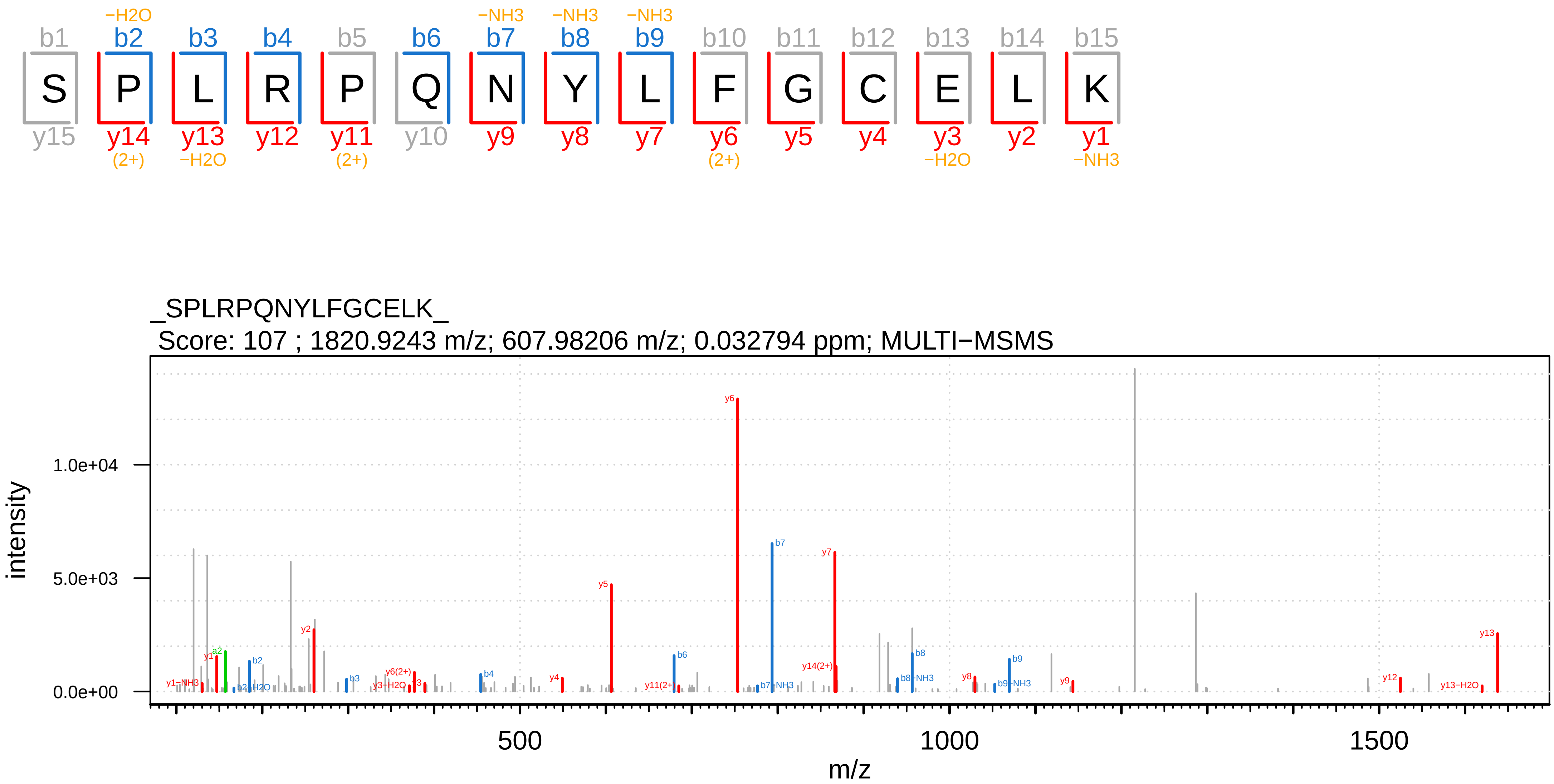

Raw File: 20101222_Velos1_TaGe_SA_K562_03
Scan Number: 12933
Proteins:
TCONS_l2_00008829_chr15:92829088−92829258:+

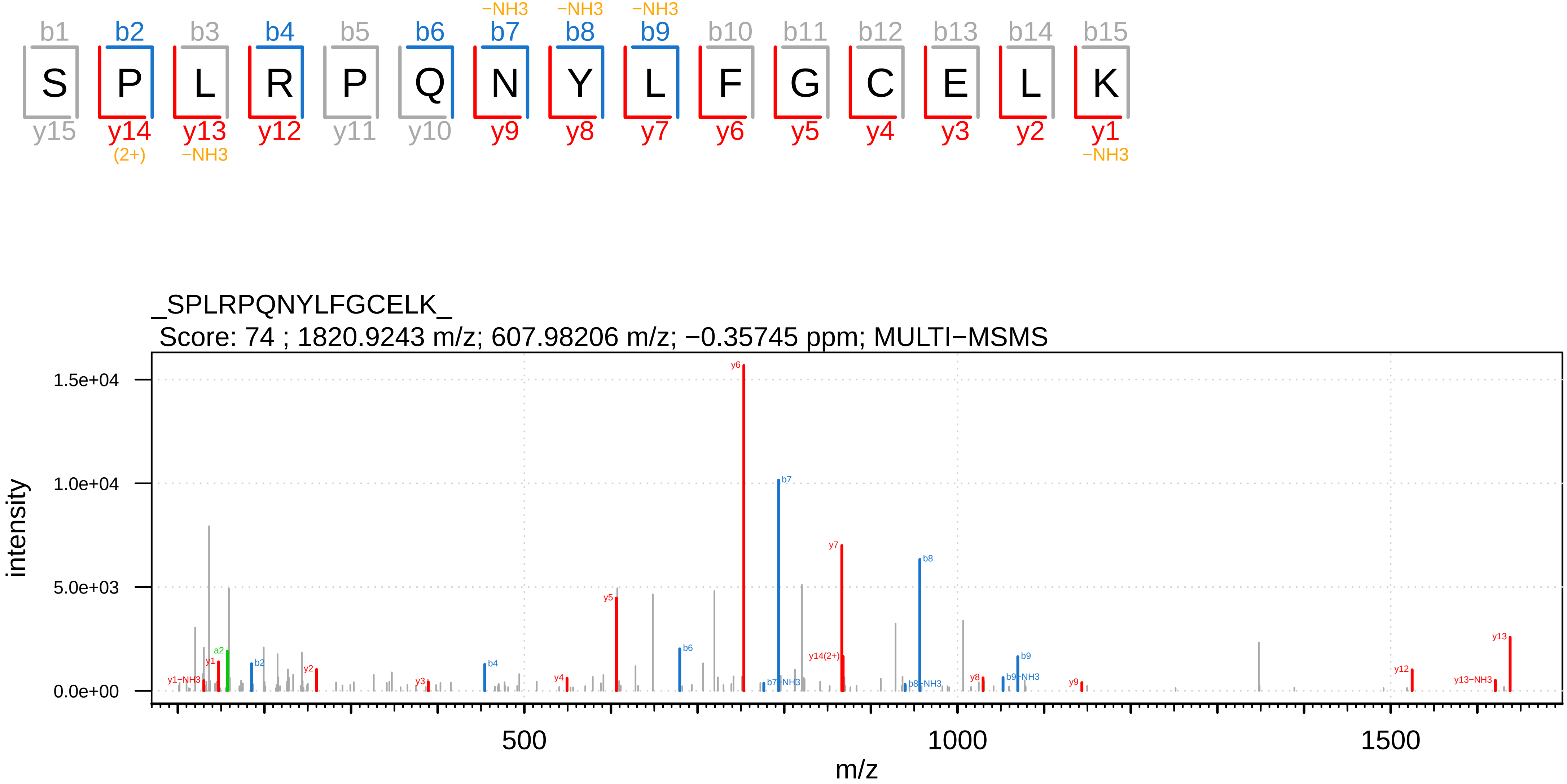

Raw File: 20101222_Velos1_TaGe_SA_K562_05
Scan Number: 12446
Proteins:
TCONS_l2_00008829_chr15:92829088−92829258:+

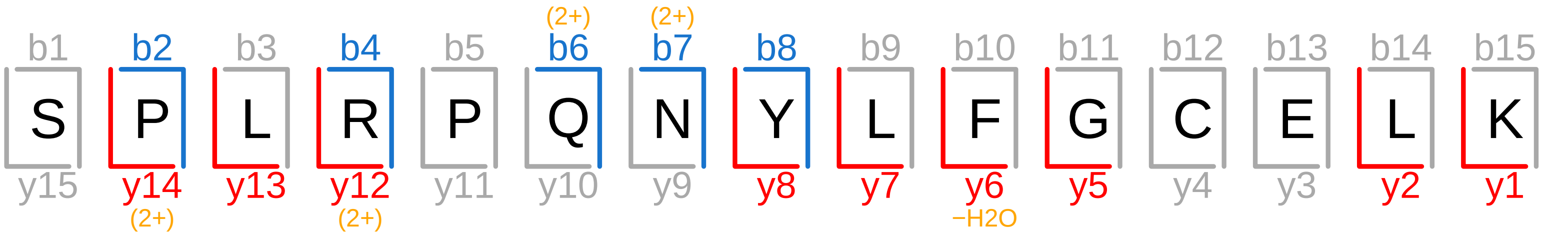

SPLRPQNYLFGCELK
Score: 53 ; 1820.9243 m/z; 607.98206 m/z; −2.0612 ppm; MULTI−MSMS

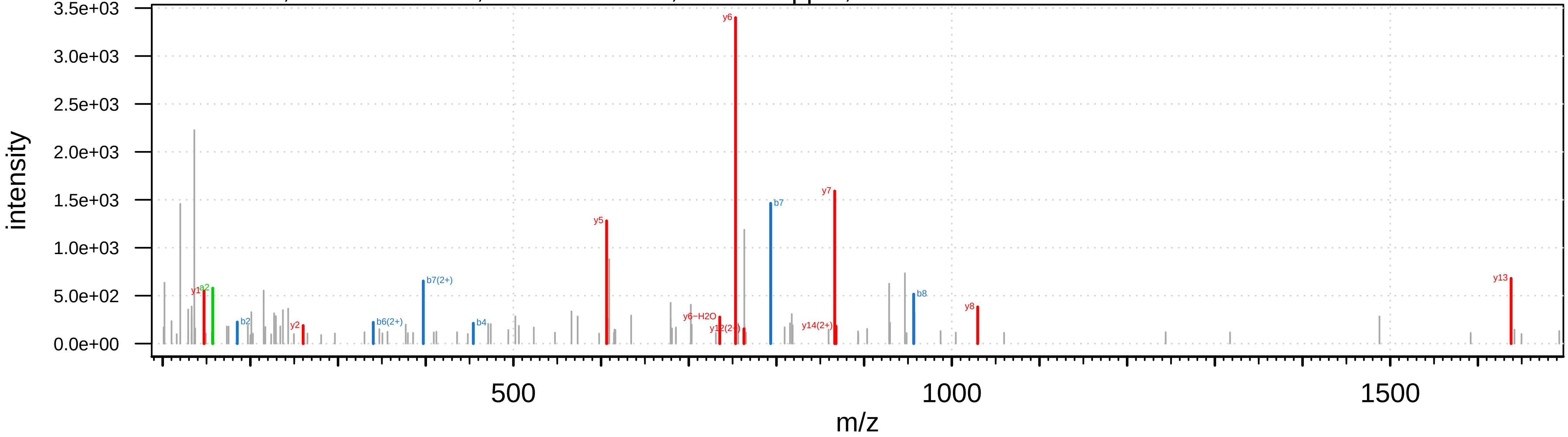

Raw File: 20101222_Velos1_TaGe_SA_K562_06
Scan Number: 11938
Proteins:
TCONS_l2_00008829_chr15:92829088−92829258:+

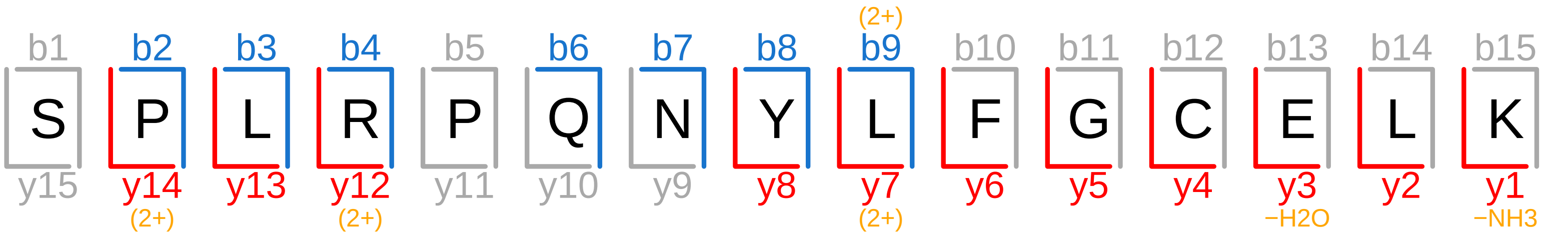

SPLRPQNYLFGCELK
Score: 58 ; 1820.9243 m/z; 607.98206 m/z; −0.11495 ppm; MULTI−MSMS

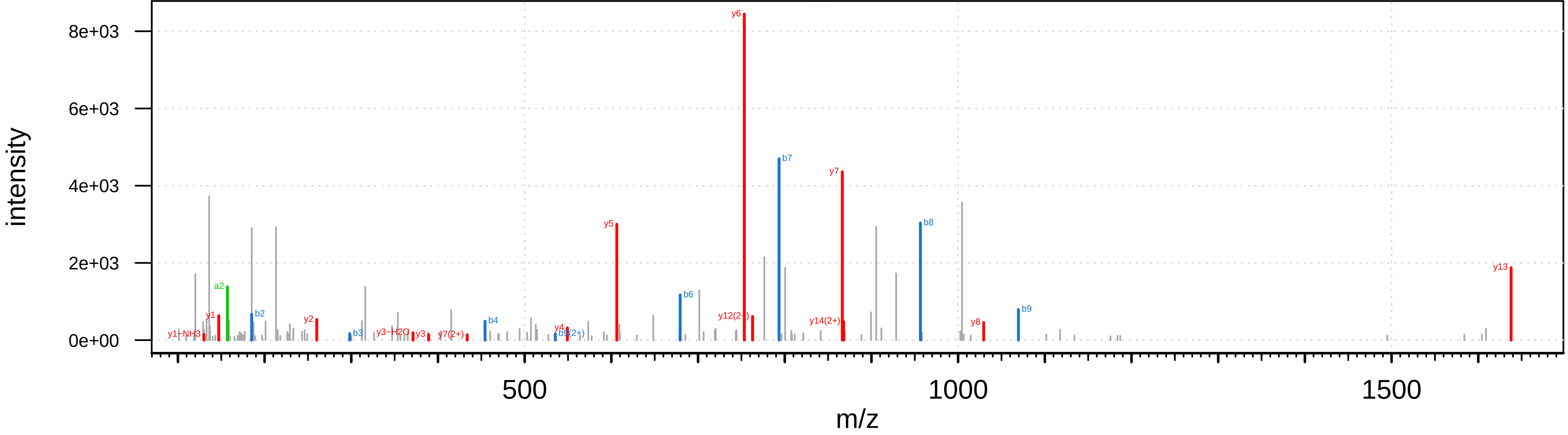

Raw File: 20101223_Velos1_TaGe_SA_RKO_01
Scan Number: 14825
Proteins:
TCONS_l2_00008829_chr15:92829088−92829258:+

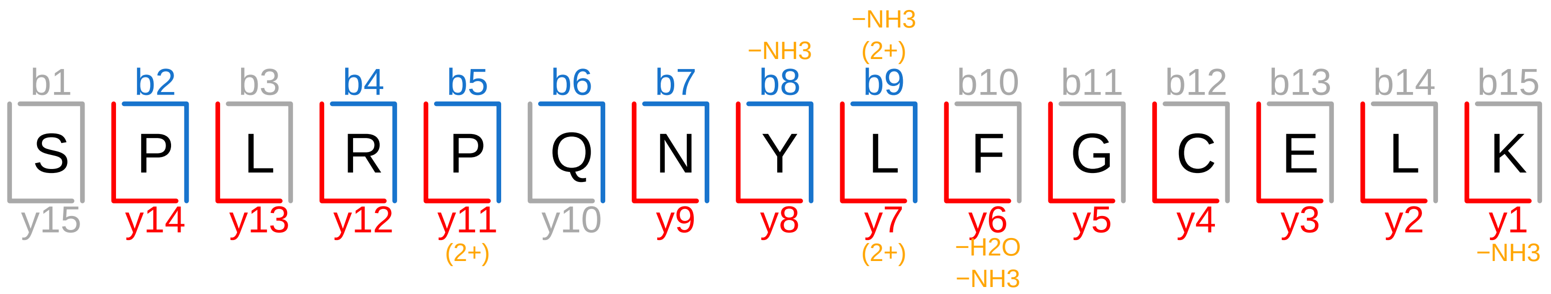

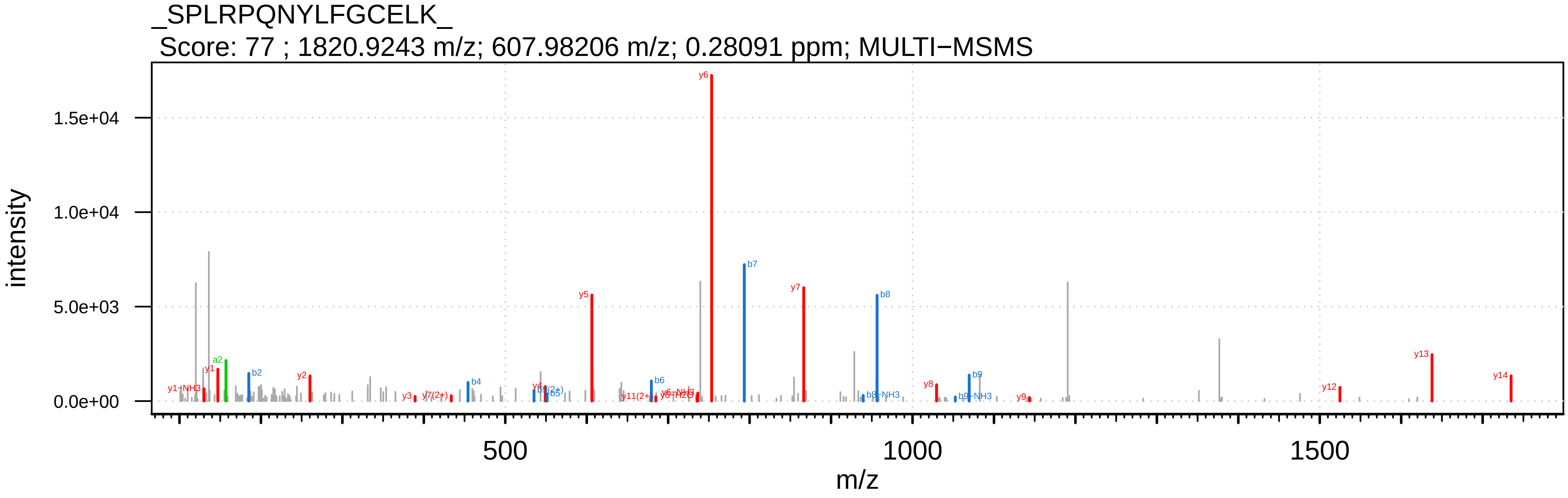

Raw File: 20101224_Velos1_TaGe_SA_HeLa_04
Scan Number: 19194
Proteins:
TCONS_l2_00008829_chr15:92829088−92829258:+

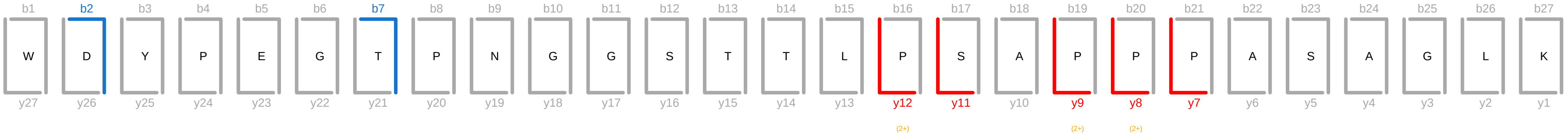

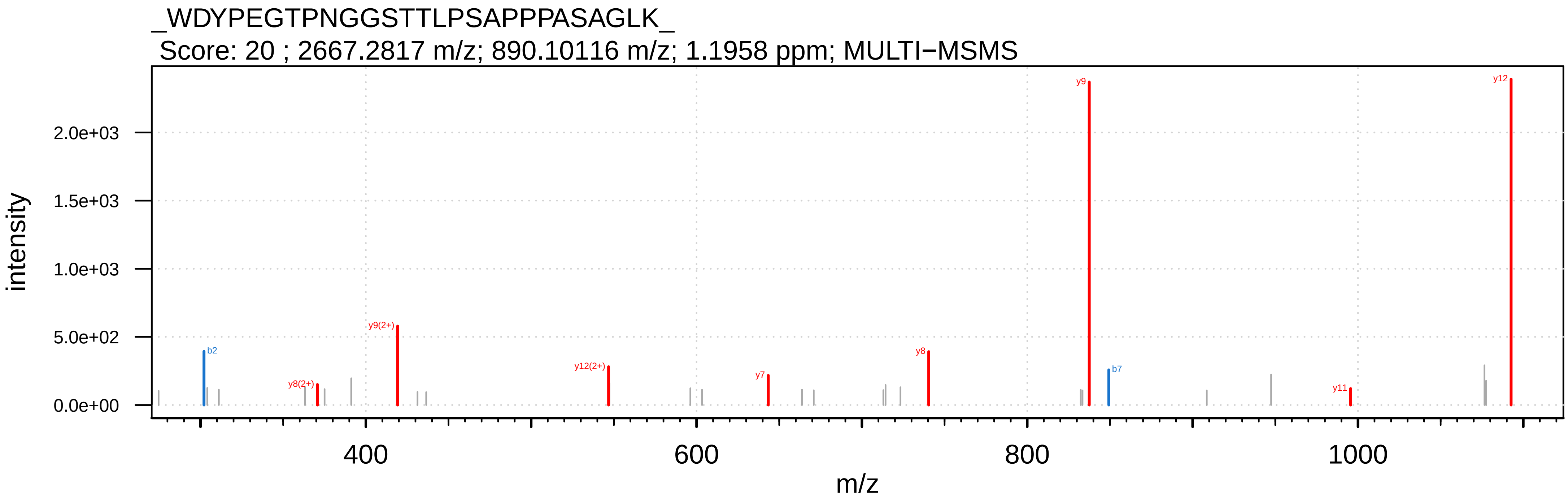

Raw File: 20101215_Velos1_TaGe_SA_A549_04
Scan Number: 19320
Proteins:
ENST00000374922_chrX:56755787−56755993:+

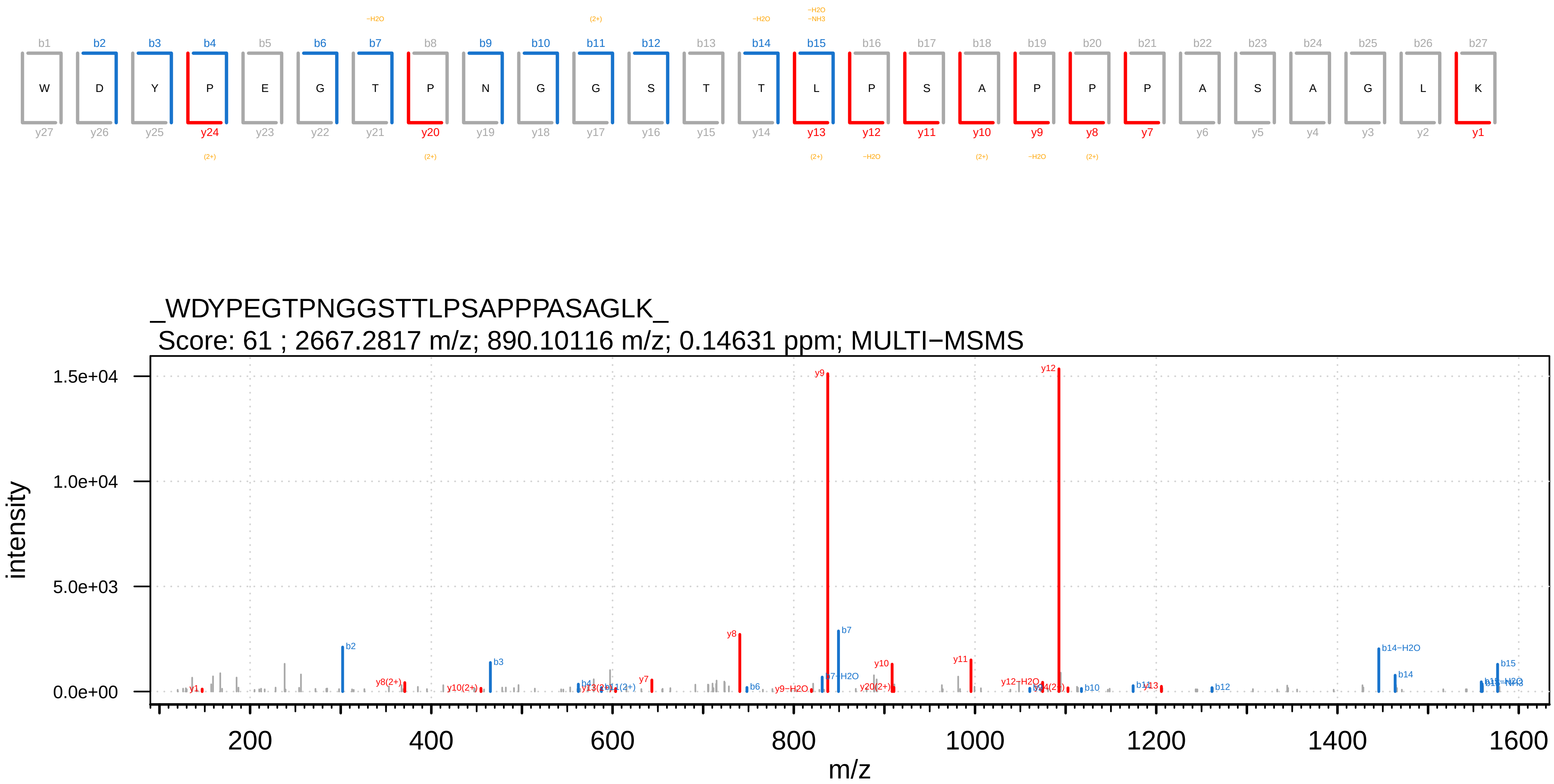

Raw File: 20101230_Velos1_TaGe_SA_Jurkat3
Scan Number: 22281
Proteins:
ENST00000374922_chrX:56755787−56755993:+

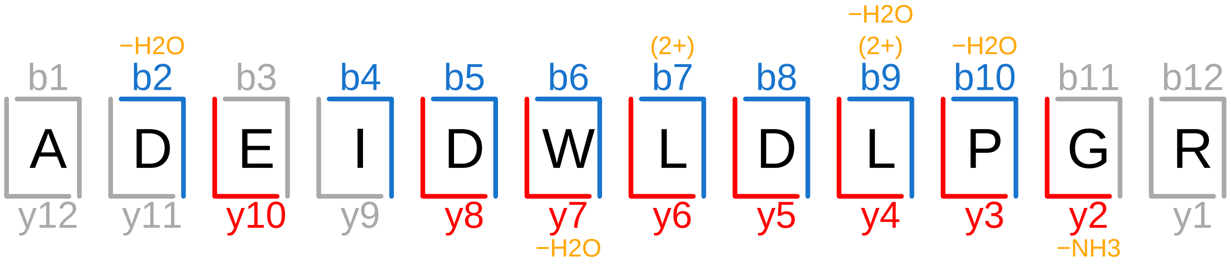

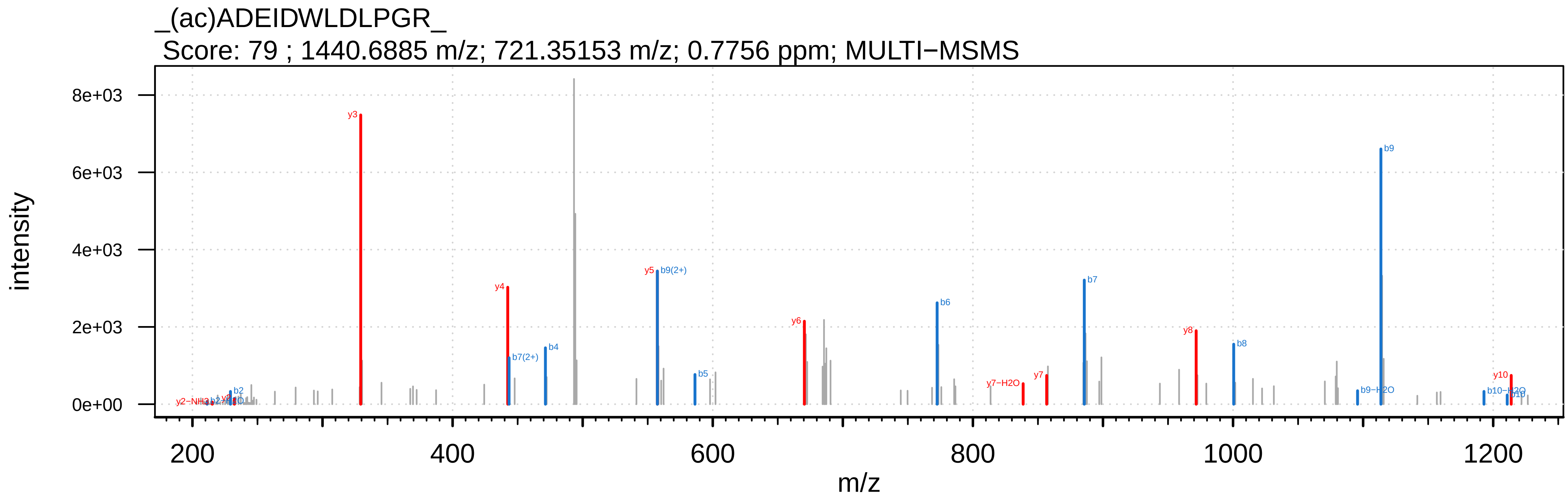

Raw File: Toni_20110714_FB_HepG2_2dot5_uROTO_F3_01
Scan Number: 31694
Proteins:
TCONS_l2_00001718_chr1:204336923−204346793:−

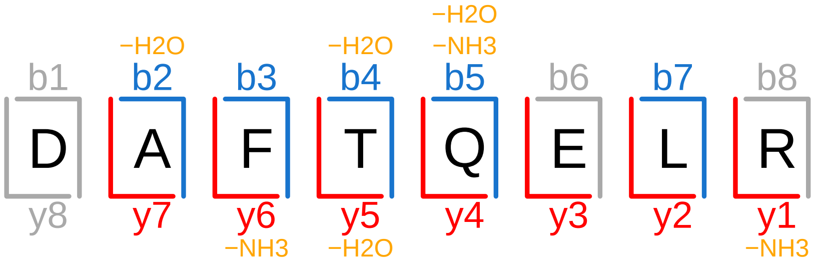

DAFTQELR
Score: 82 ; 978.47706 m/z; 490.24581 m/z; −1.9705 ppm; MULTI−MSMS

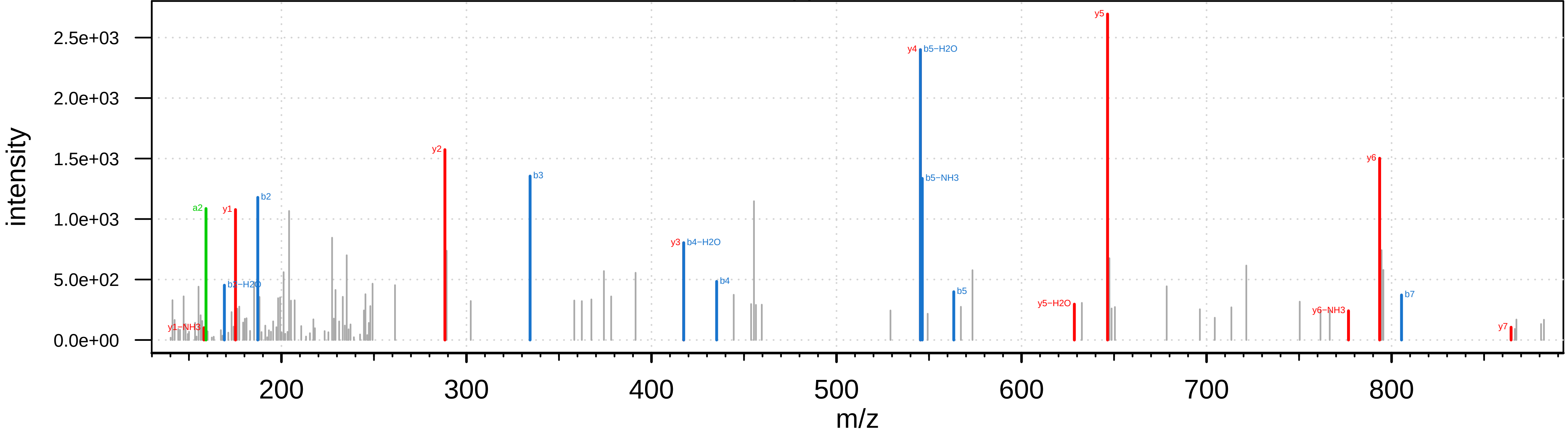

Raw File: Toni_20111125_FB_MCF7_F4_2
Scan Number: 30437
Proteins:
Q69YL0
ENST00000602845_chr3:196669588−196669887:+

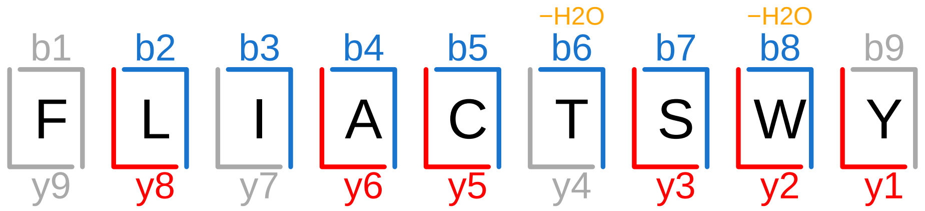

FLIACTSWY
Score: 102 ; 1159.5372 m/z; 580.77588 m/z; −0.53794 ppm; MULTI−MSMS

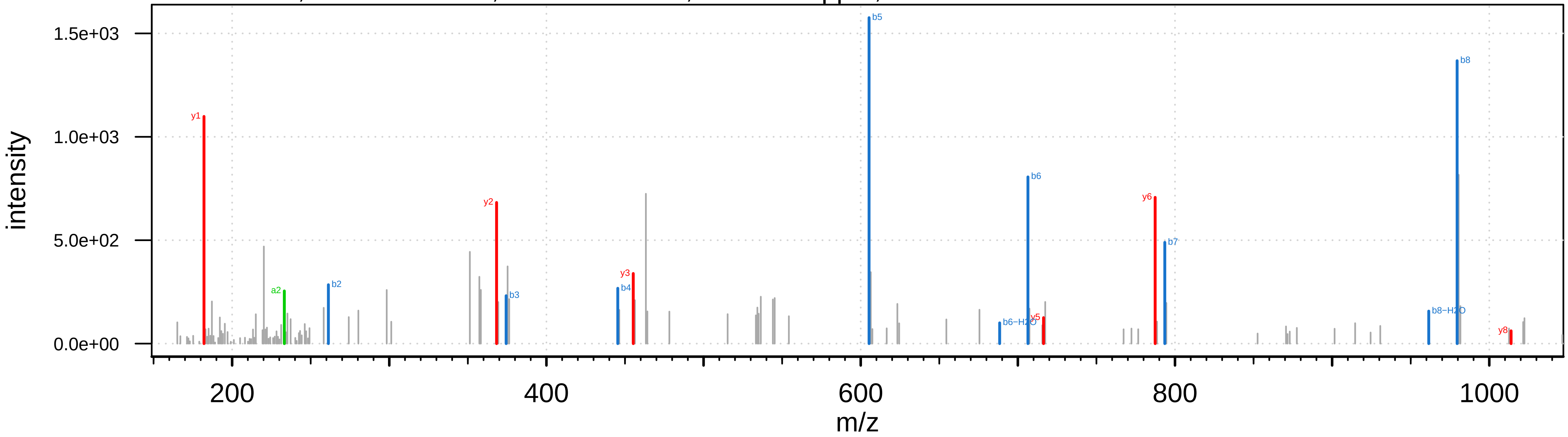

Raw File: Toni_20111114_FB_MDA−MB−231_F6_2
Scan Number: 53029
Proteins:
ENST00000424358_chr20:33865517−33865732:−

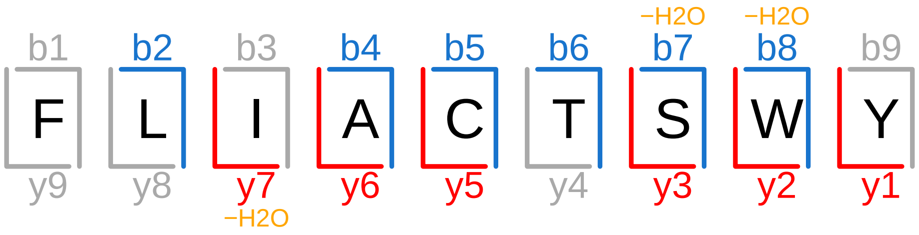

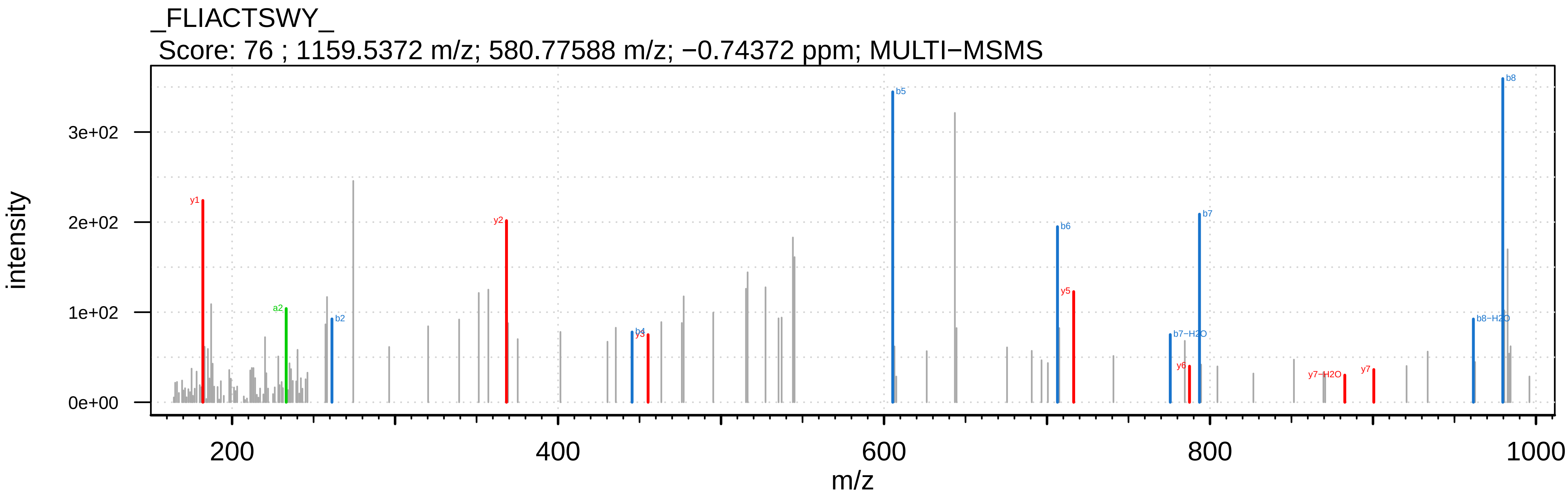

Raw File: Toni_20111114_FB_MDA−MB−231_F7_2
Scan Number: 51019
Proteins:
ENST00000424358_chr20:33865517−33865732:−

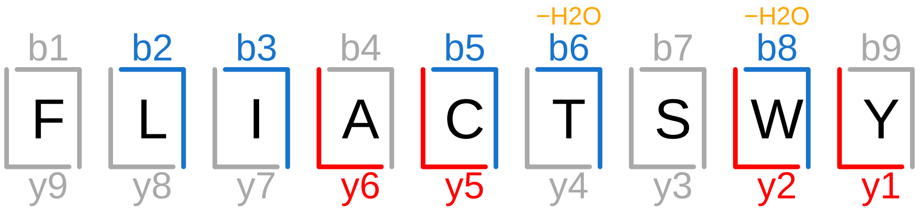

FLIACTSWY
Score: 57 ; 1159.5372 m/z; 580.77588 m/z; −1.231 ppm; MULTI−MSMS

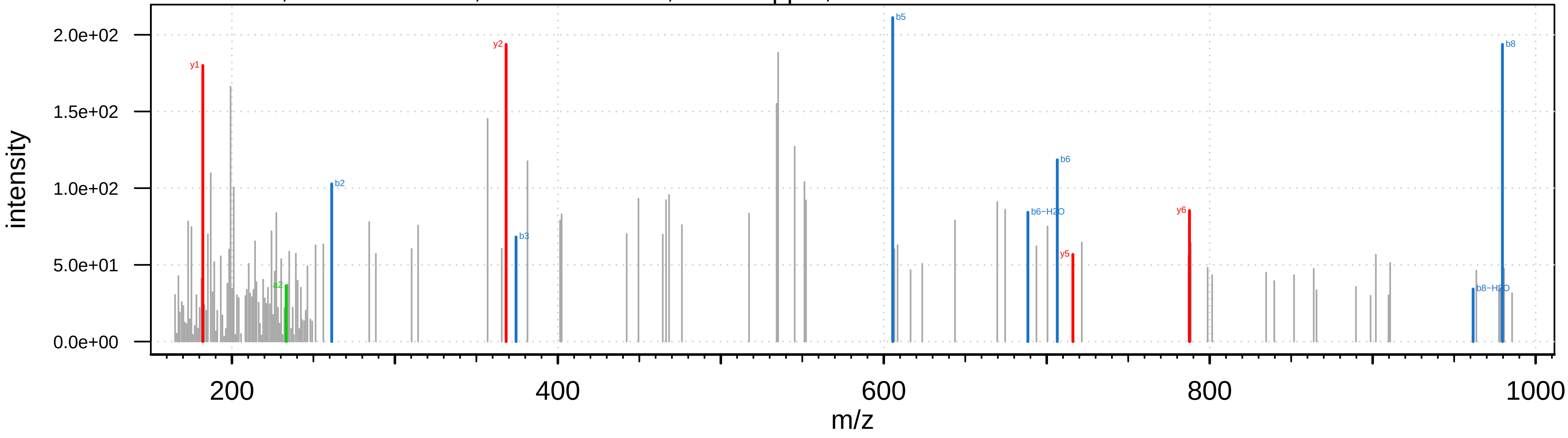

Raw File: Toni_20111125_FB_MCF7_F7_1
Scan Number: 56501
Proteins:
ENST00000424358_chr20:33865517−33865732:−

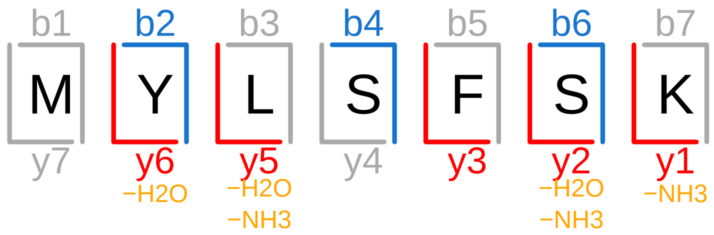

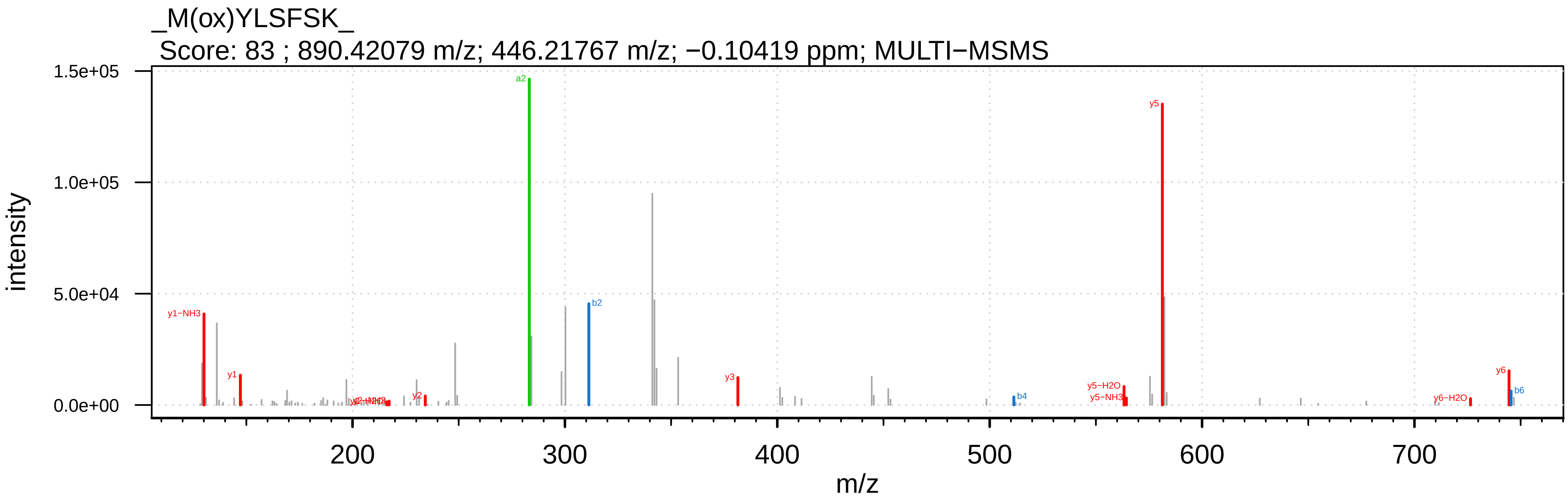

Raw File: Toni_20111125_FB_MCF7_F7_1
Scan Number: 26760
Proteins:
ENST00000392145_chr6:160874572−160874622:+

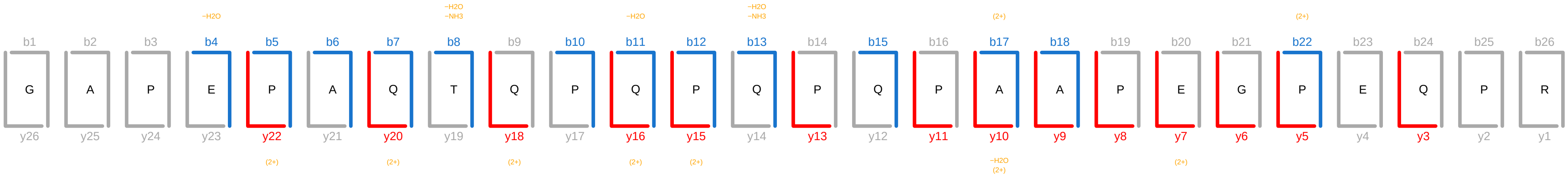

GAPEPAQTQPQPQPQPAAPEGPEQPR

Score: 105 ; 2702.3049 m/z; 901.77556 m/z; 0.21536 ppm; MULTI−MSMS

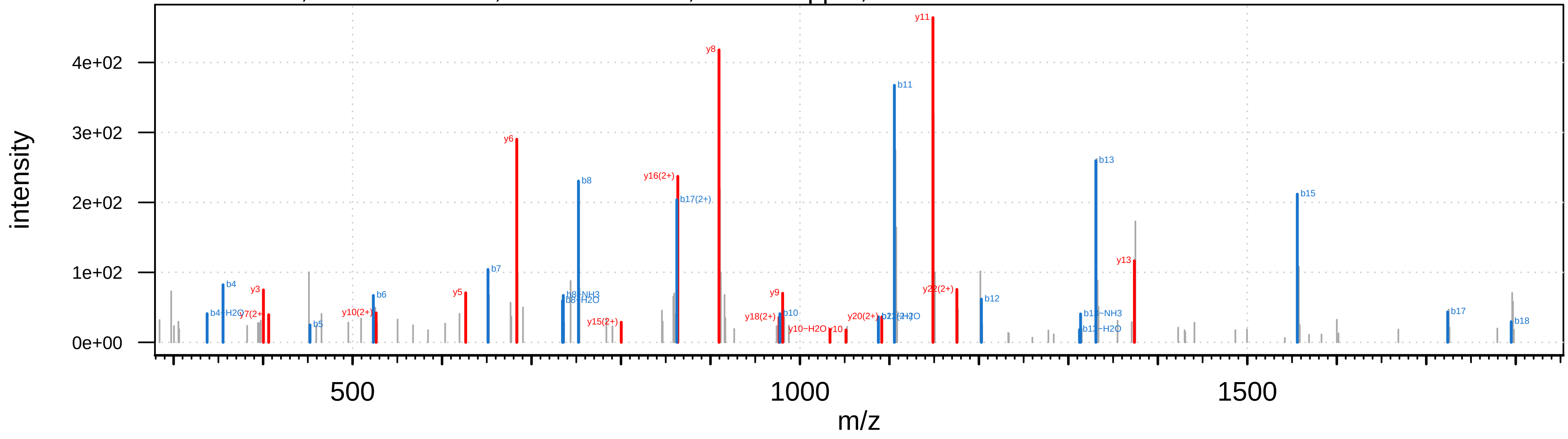

Raw File: Toni_20111114_FB_MDA−MB−231_F5_1
Scan Number: 22679
Proteins:
ENST00000424358_chr20:33865517−33865732:−

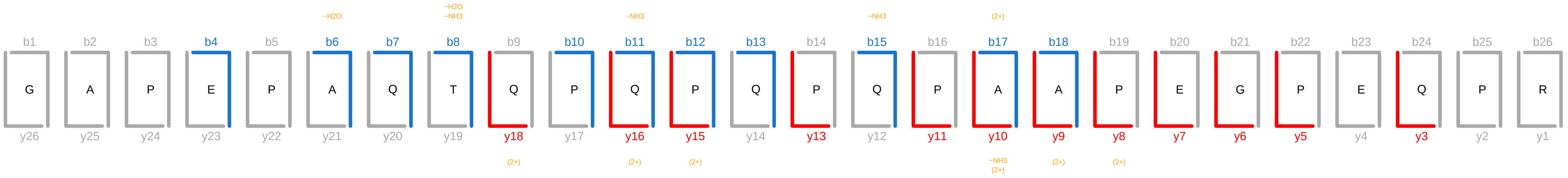

GAPEPAQTQPQPQPQPAAPEGPEQPR
Score: 90 ; 2702.3049 m/z; 901.77556 m/z; 0.14638 ppm; MULTI−MSMS

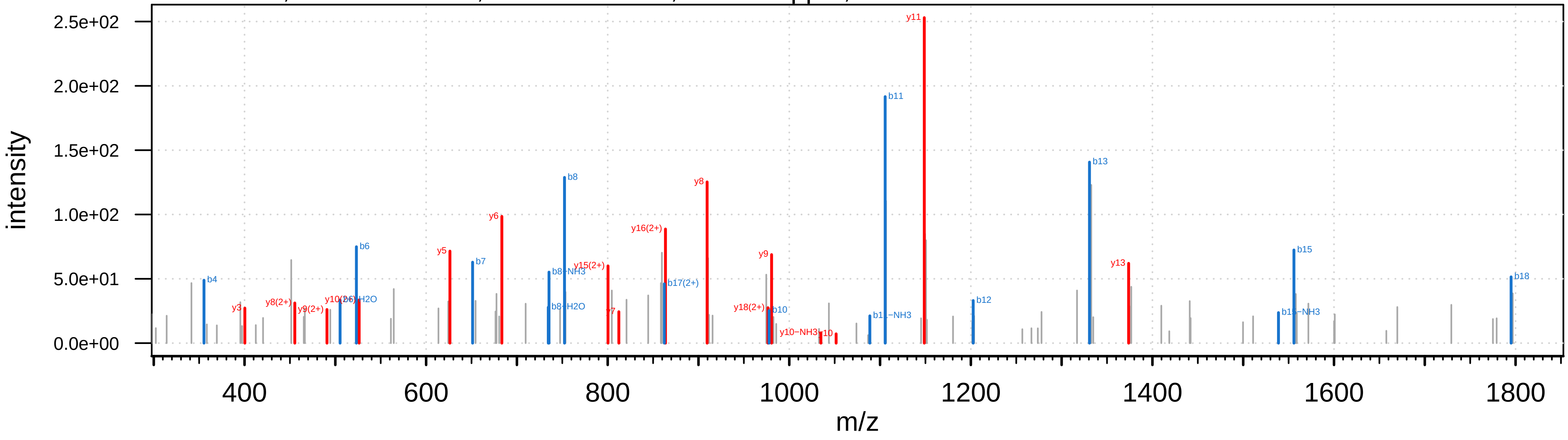

Raw File: Toni_20111114_FB_MDA−MB−231_F5_2
Scan Number: 22692
Proteins:
ENST00000424358_chr20:33865517−33865732:−

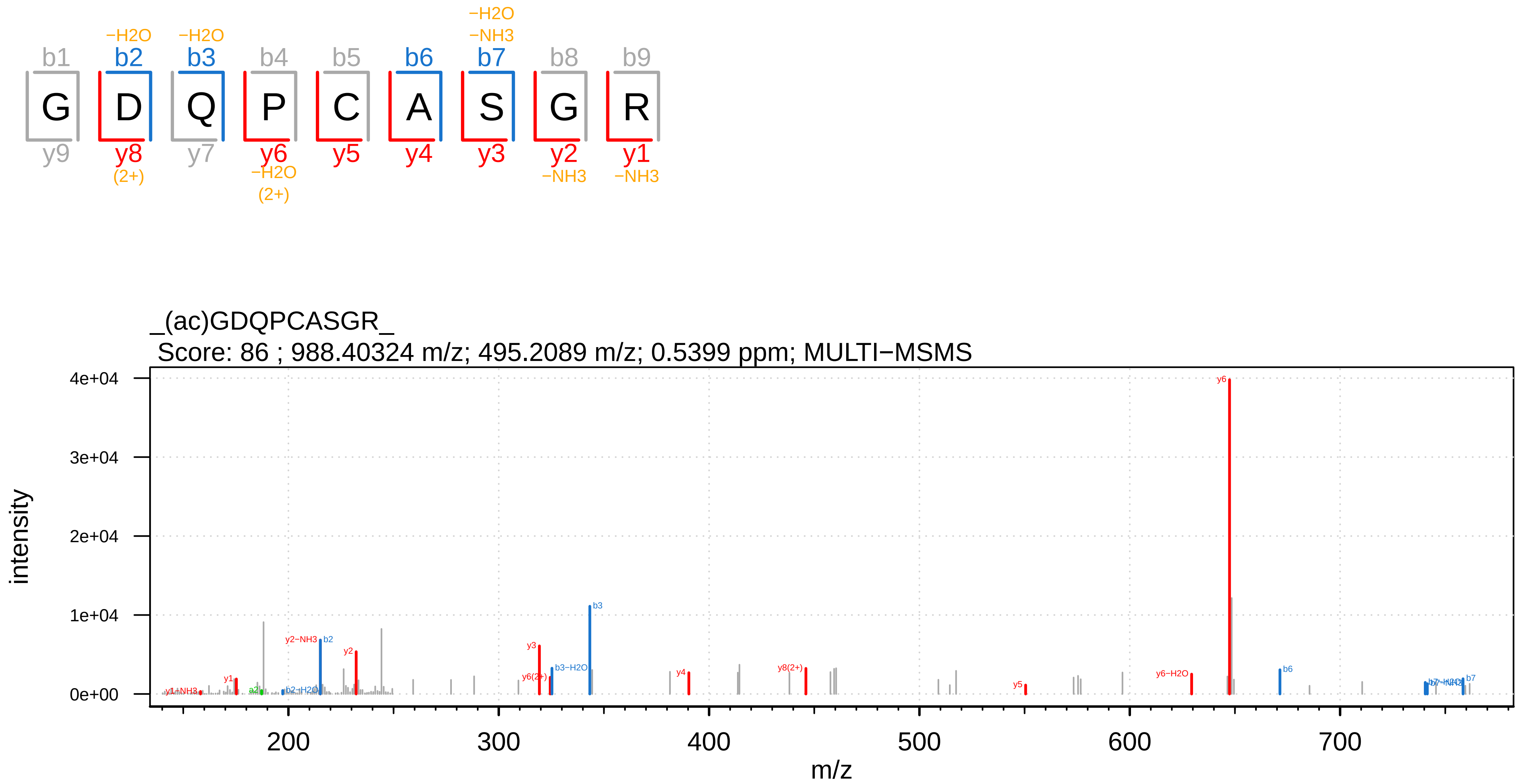

Raw File: Toni_20110714_FB_HepG2_2dot5_uROTO_F4_01
Scan Number: 6359
Proteins:
ENST00000374922_chrX:56755787−56755993:+

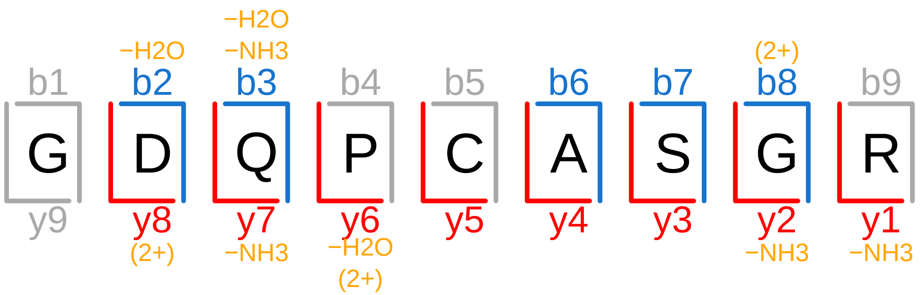

(ac)GDQPCASGR
Score: 71 ; 988.40324 m/z; 495.2089 m/z; −0.20786 ppm; MULTI−MSMS

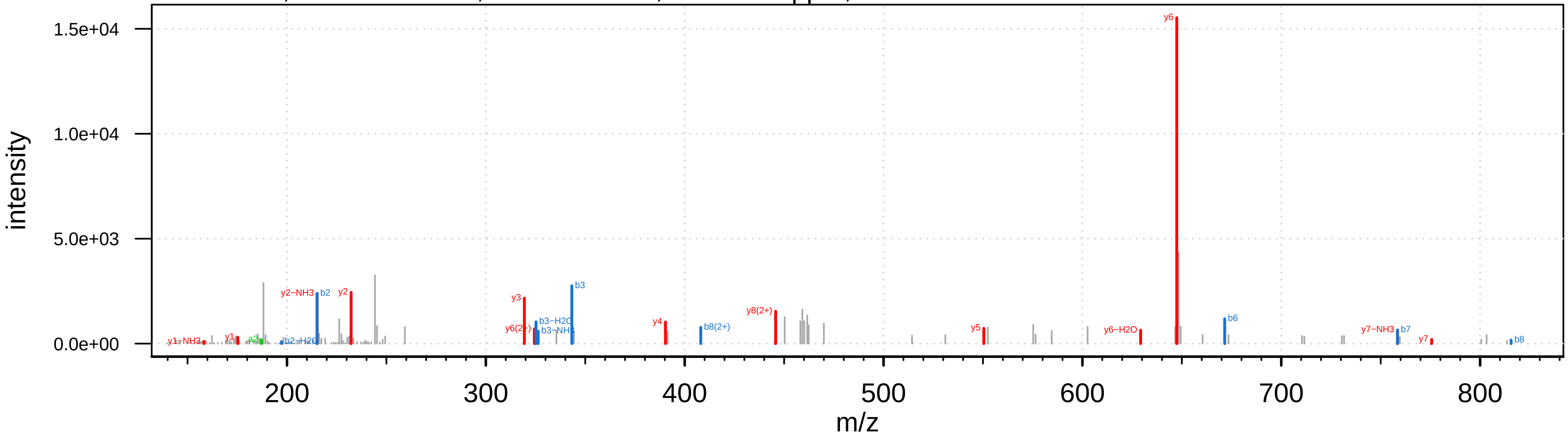

Raw File: Toni_20110714_FB_HepG2_2dot5_uROTO_F4_02
Scan Number: 4047
Proteins:
ENST00000374922_chrX:56755787−56755993:+

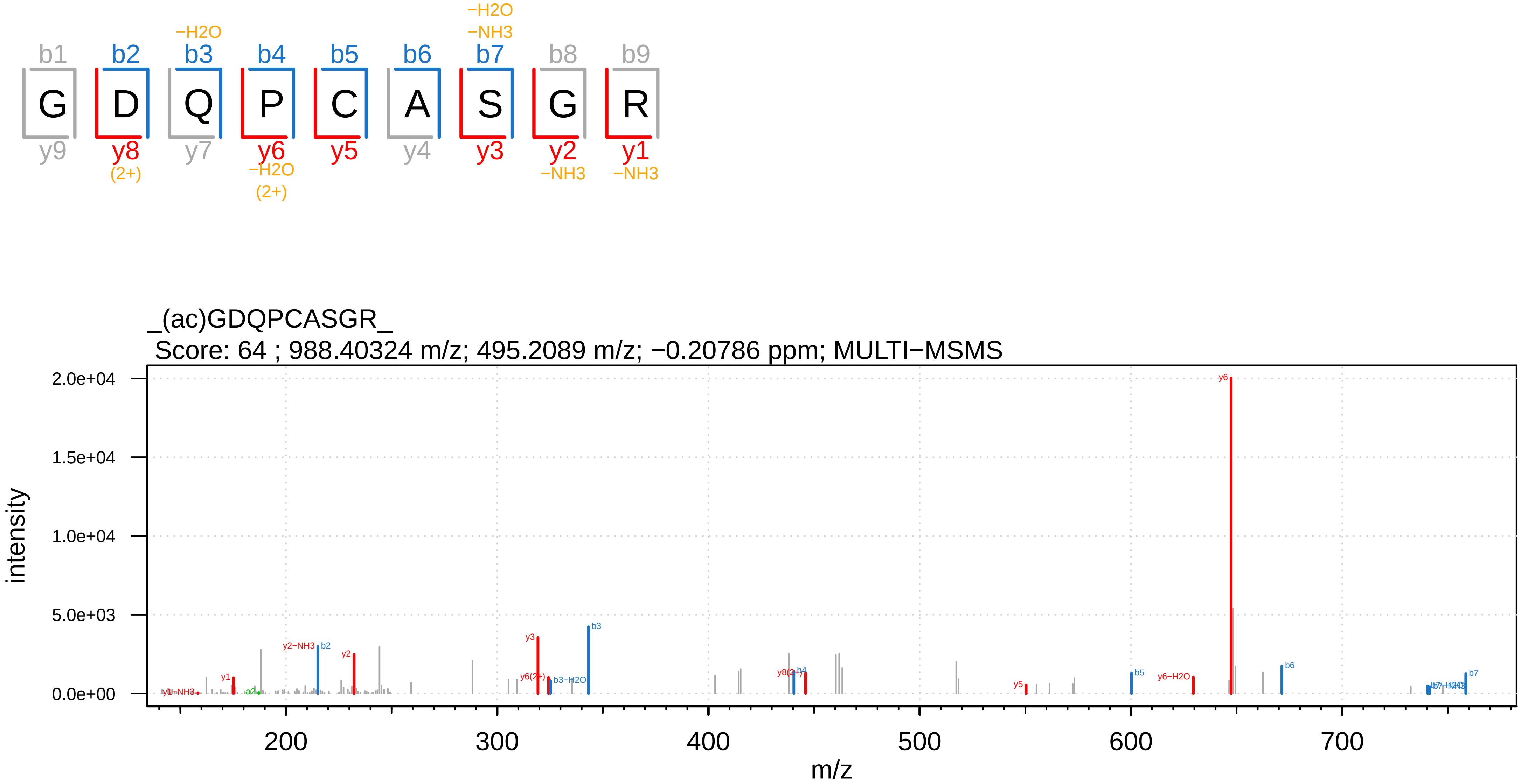

Raw File: Toni_20110714_FB_HepG2_2dot5_uROTO_F4_02
Scan Number: 4154
Proteins:
ENST00000374922_chrX:56755787−56755993:+

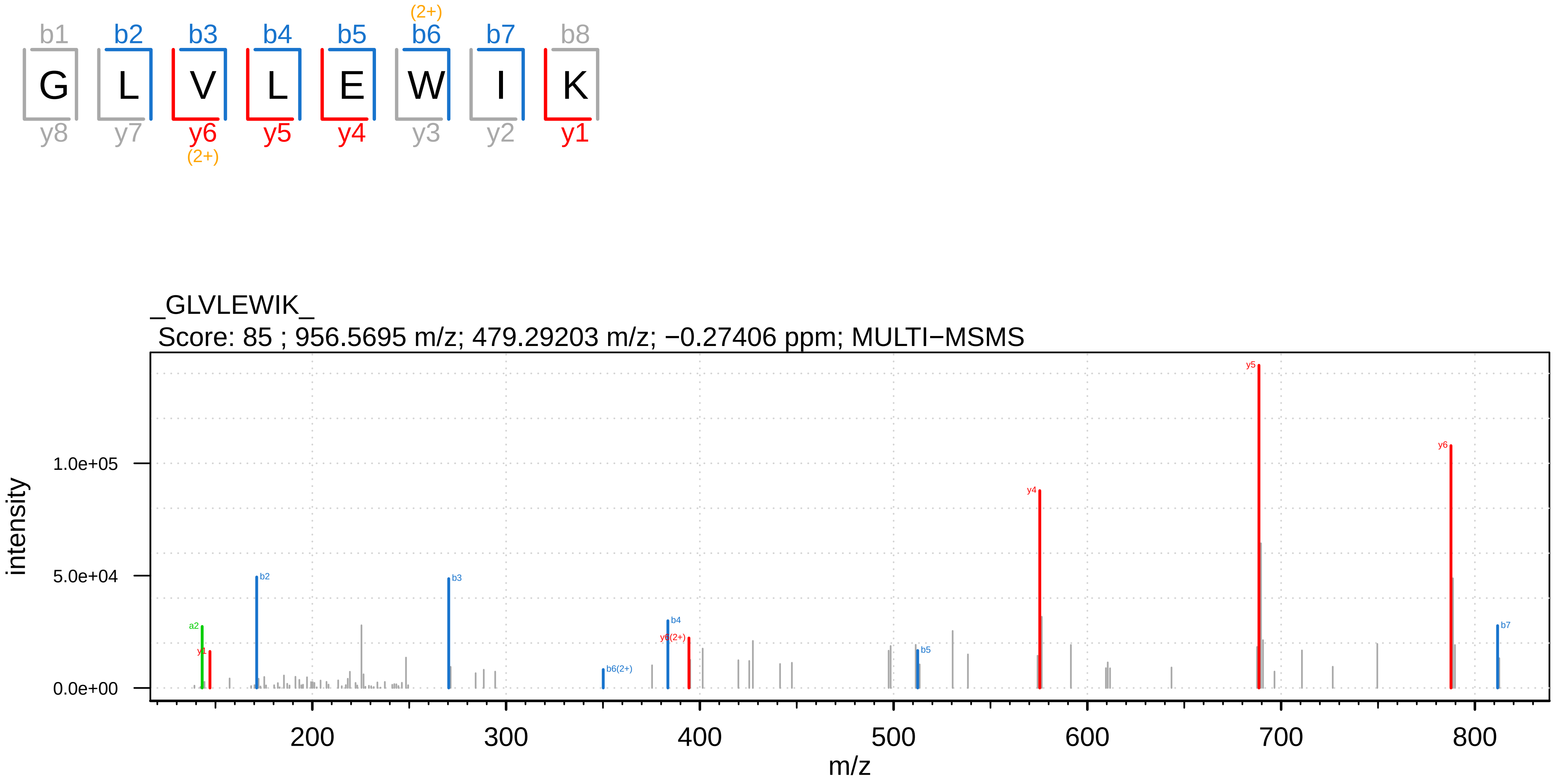

Raw File: Toni_20110714_FB_HepG2_2dot5_uROTO_F6_01
Scan Number: 36545
Proteins:
TCONS_l2_00001296_chr1:79520703−79520992:−

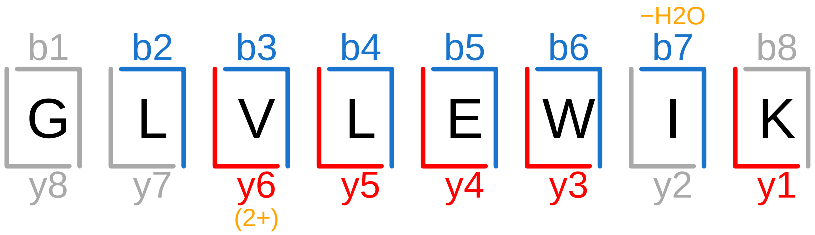

GLVLEWIK
Score: 93 ; 956.5695 m/z; 479.29203 m/z; −0.22202 ppm; MULTI−MSMS

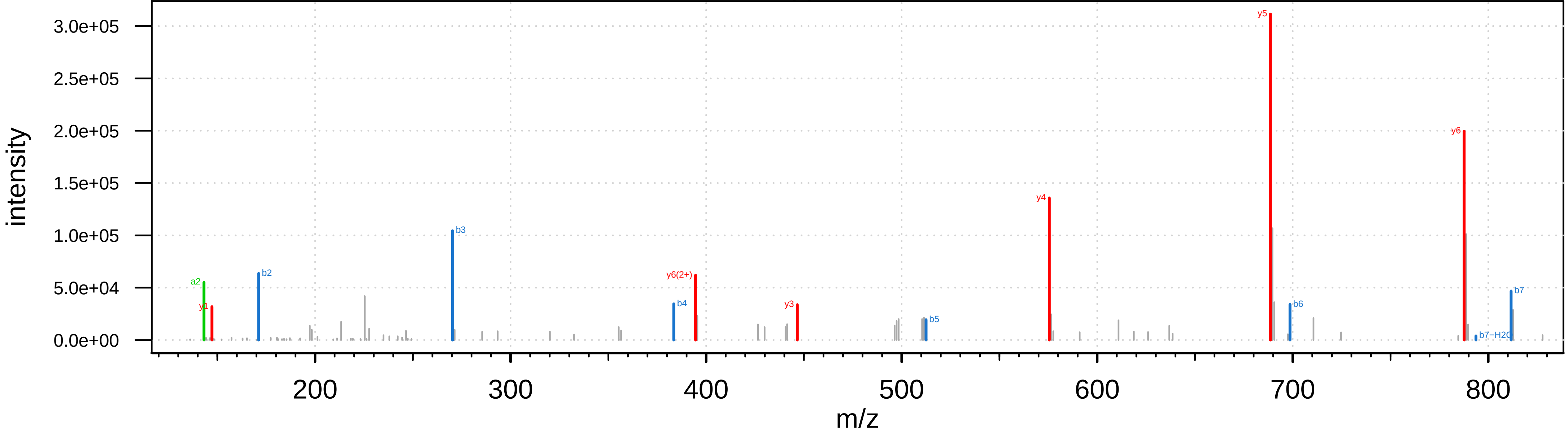

Raw File: Toni_20110714_FB_HepG2_2dot5_uROTO_F7_01
Scan Number: 35694
Proteins:
TCONS_l2_00001296_chr1:79520703−79520992:−

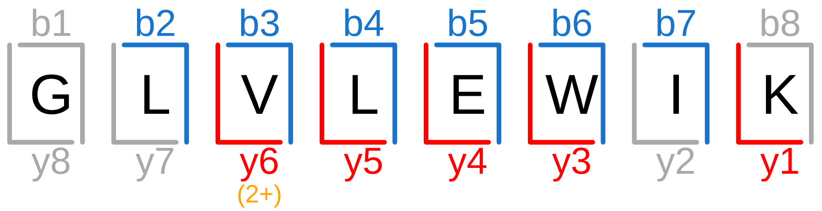

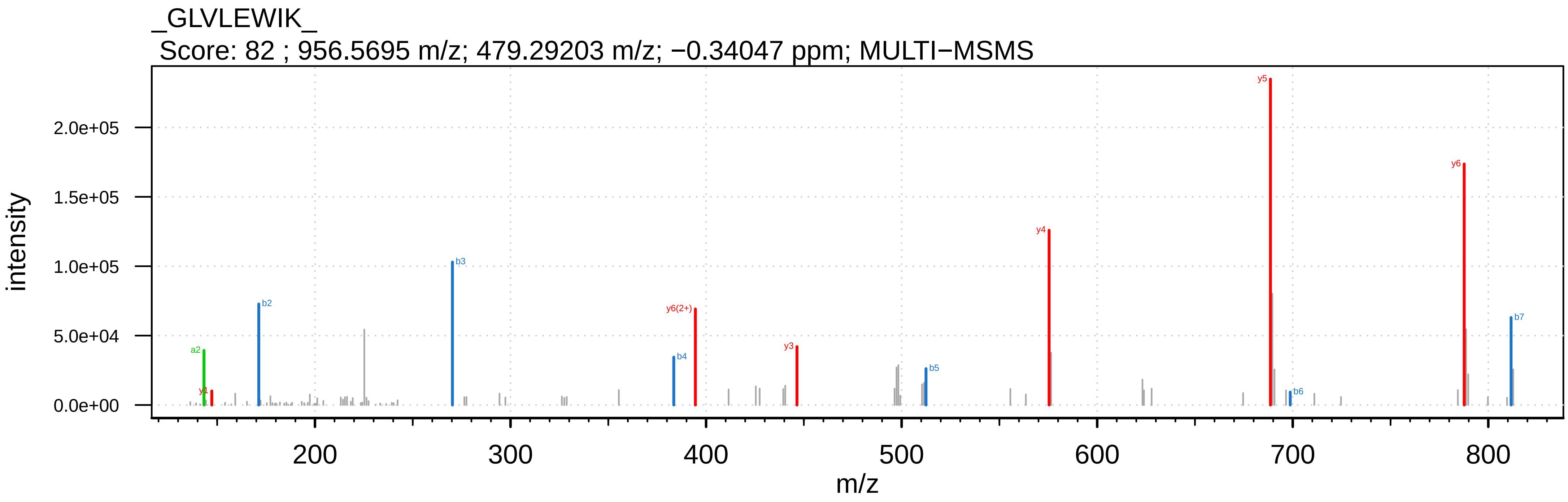

Raw File: Toni_20110714_FB_HepG2_2dot5_uROTO_F7_02
Scan Number: 35780
Proteins:
TCONS_l2_00001296_chr1:79520703−79520992:−

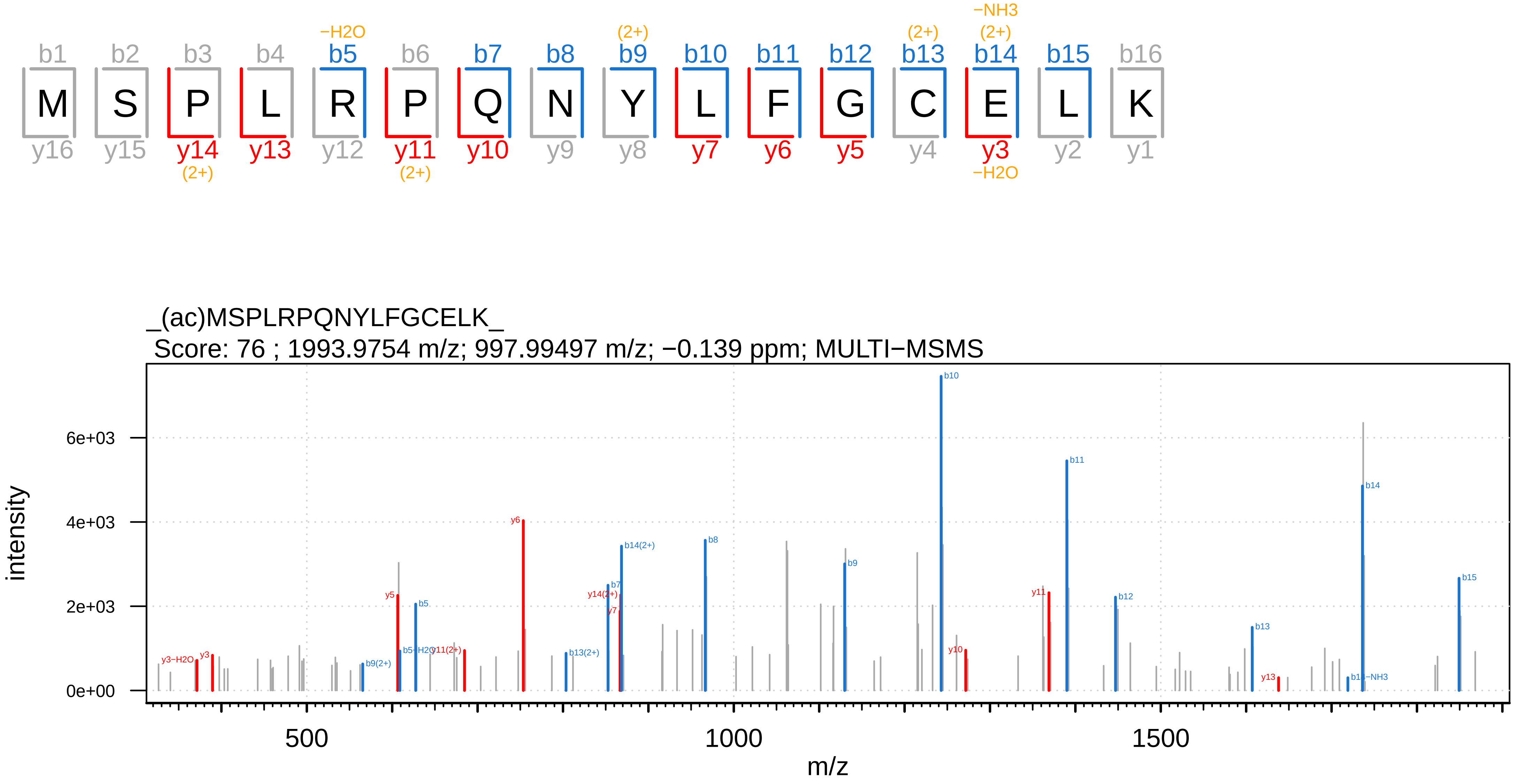

Raw File: Toni_20110714_FB_HepG2_2dot5_uROTO_F7_02
Scan Number: 37820
Proteins:
TCONS_l2_00008829_chr15:92829088−92829258:+

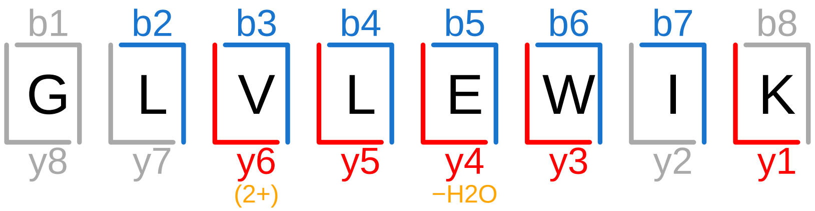

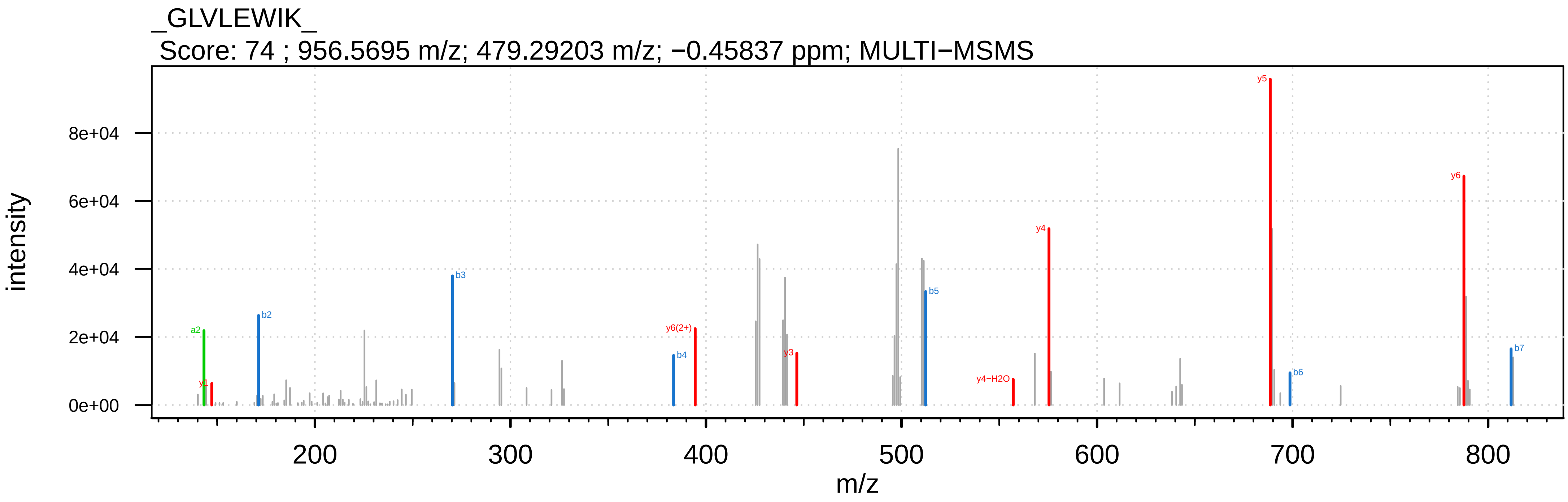

Raw File: Toni_20110714_FB_HepG2_2dot5_uROTO_F8_01
Scan Number: 33753
Proteins:
TCONS_l2_00001296_chr1:79520703−79520992:−

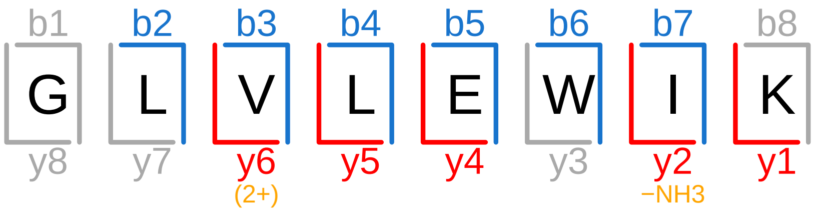

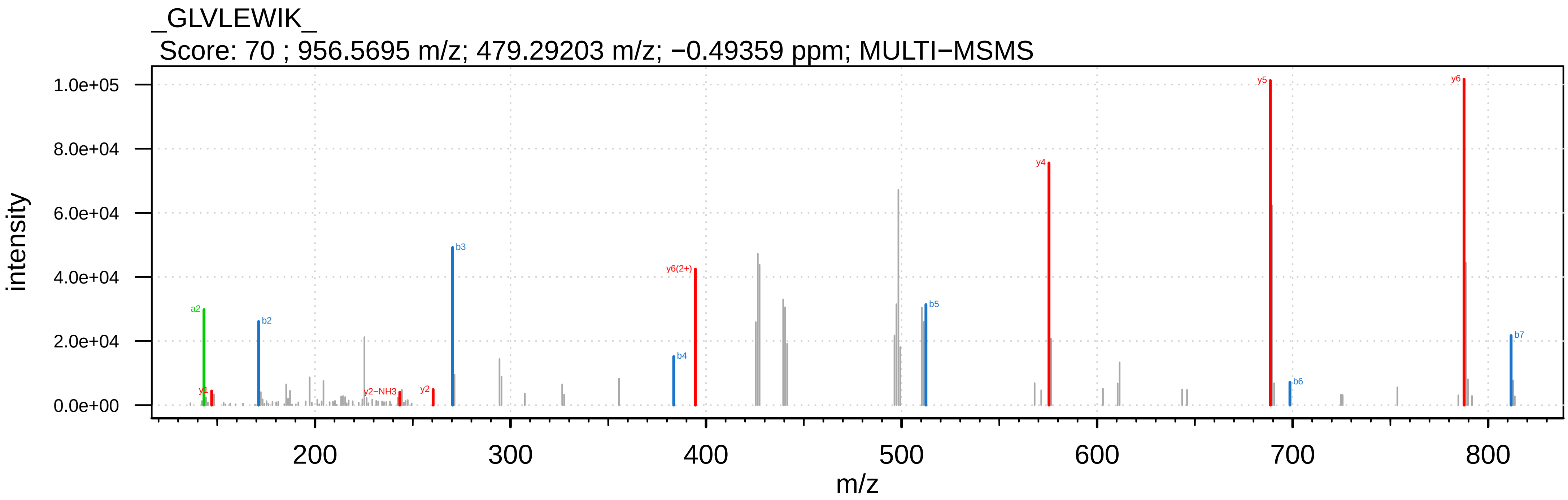

Raw File: Toni_20110714_FB_HepG2_2dot5_uROTO_F8_02
Scan Number: 33679
Proteins:
TCONS_l2_00001296_chr1:79520703−79520992:−

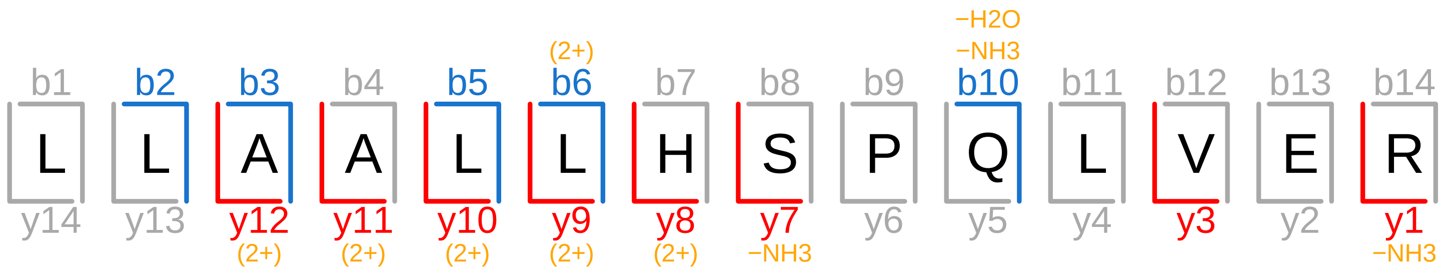

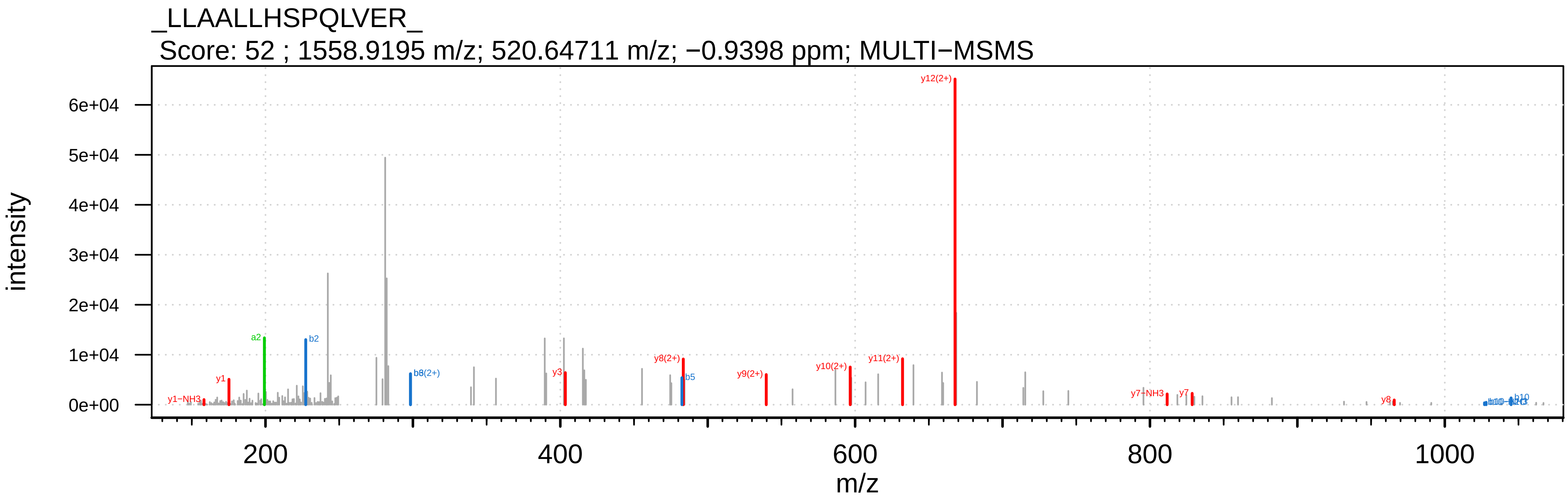

Raw File: Toni_20110714_FB_HepG2_2dot5_uROTO_F8_02
Scan Number: 30072
Proteins:
Q69YL0
ENST00000602845_chr3:196669588−196669887:+

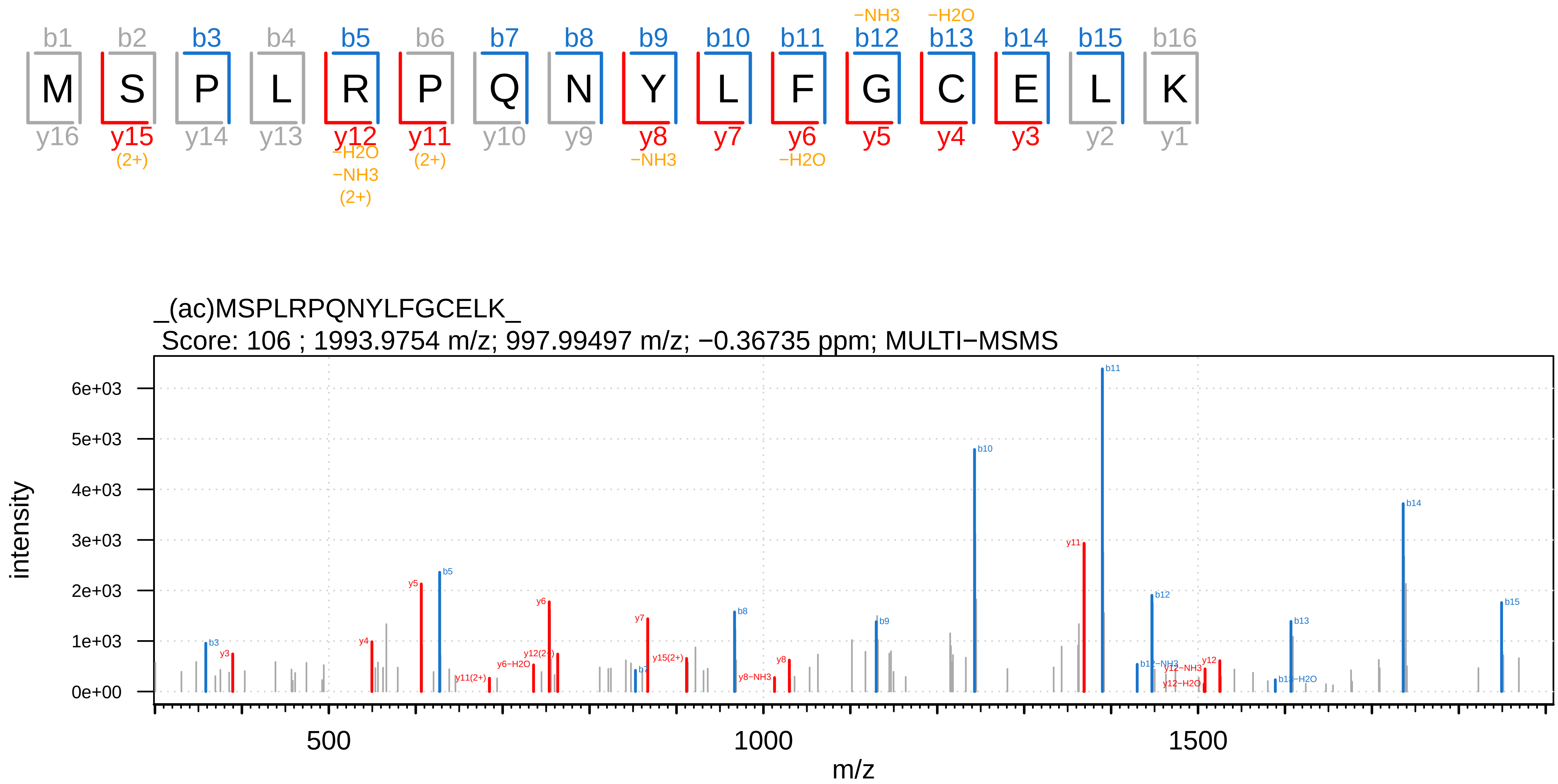

Raw File: Toni_20110714_FB_HepG2_2dot5_uROTO_F8_02
Scan Number: 35611
Proteins:
TCONS_l2_00008829_chr15:92829088−92829258:+

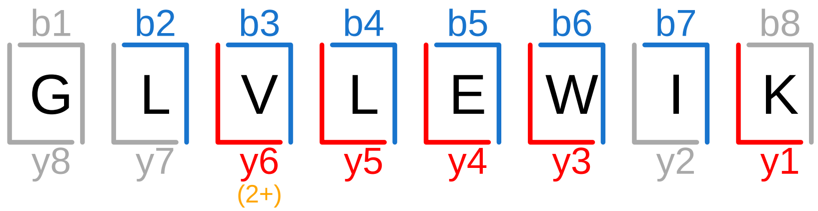

GLVLEWIK
Score: 84 ; 956.5695 m/z; 479.29203 m/z; −1.0659 ppm; MULTI−MSMS

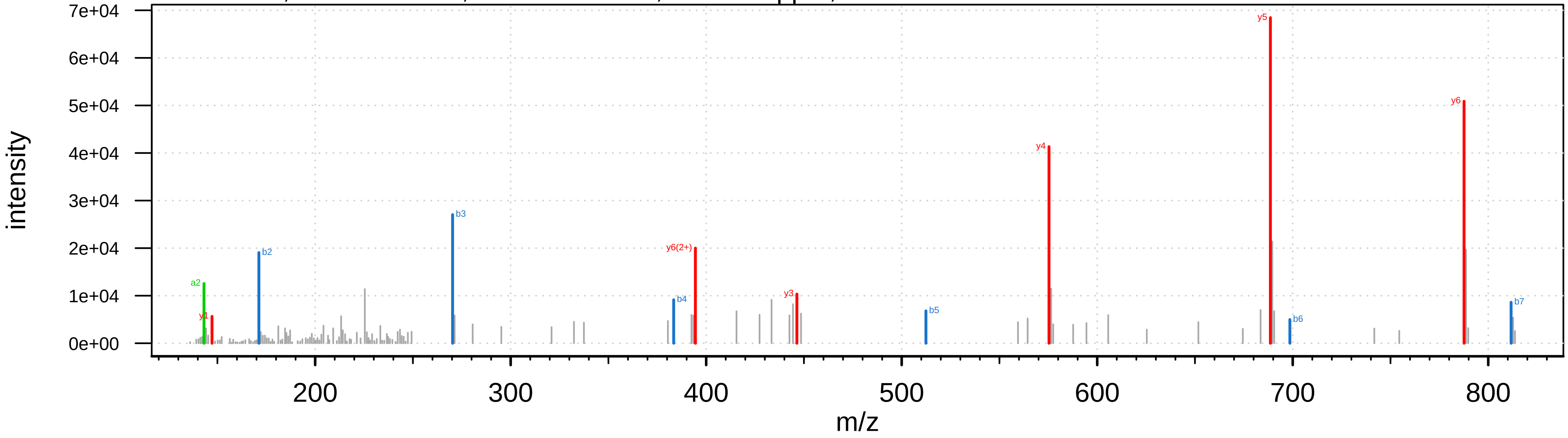

Raw File: Toni_20110714_FB_HepG2_2dot5_uROTO_F9_01
Scan Number: 33104
Proteins:
TCONS_l2_00001296_chr1:79520703−79520992:−

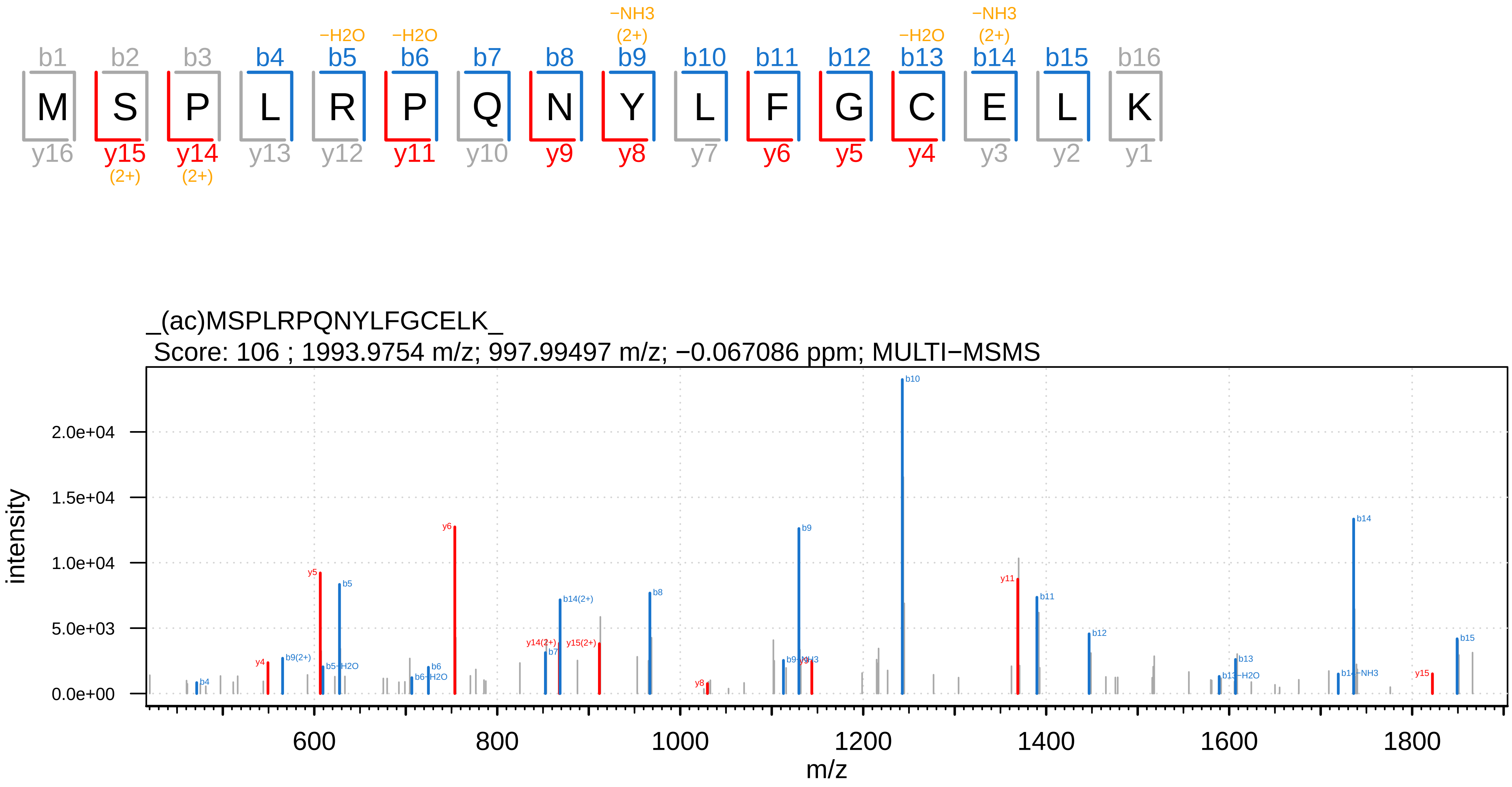

Raw File: Toni_20110714_FB_HepG2_2dot5_uROTO_F9_01

Scan Number: 35025

Proteins:

TCONS_l2_00008829_chr15:92829088−92829258:+

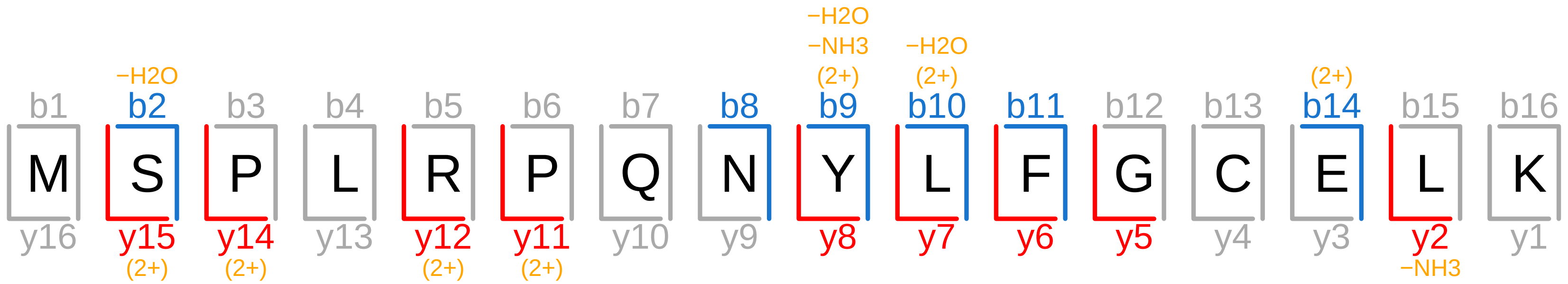

(ac)MSPLRPQNYLFGCELK
Score: 70 ; 1993.9754 m/z; 665.66574 m/z; −0.52943 ppm; MULTI−MSMS

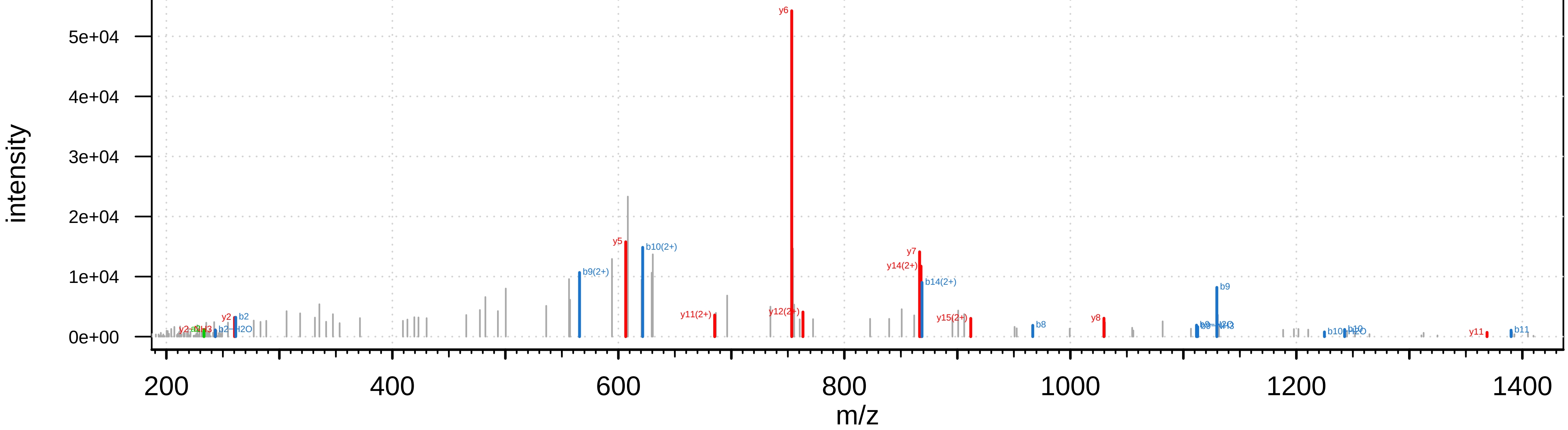

Raw File: Toni_20110714_FB_HepG2_2dot5_uROTO_F9_01
Scan Number: 35027
Proteins:
TCONS_l2_00008829_chr15:92829088−92829258:+

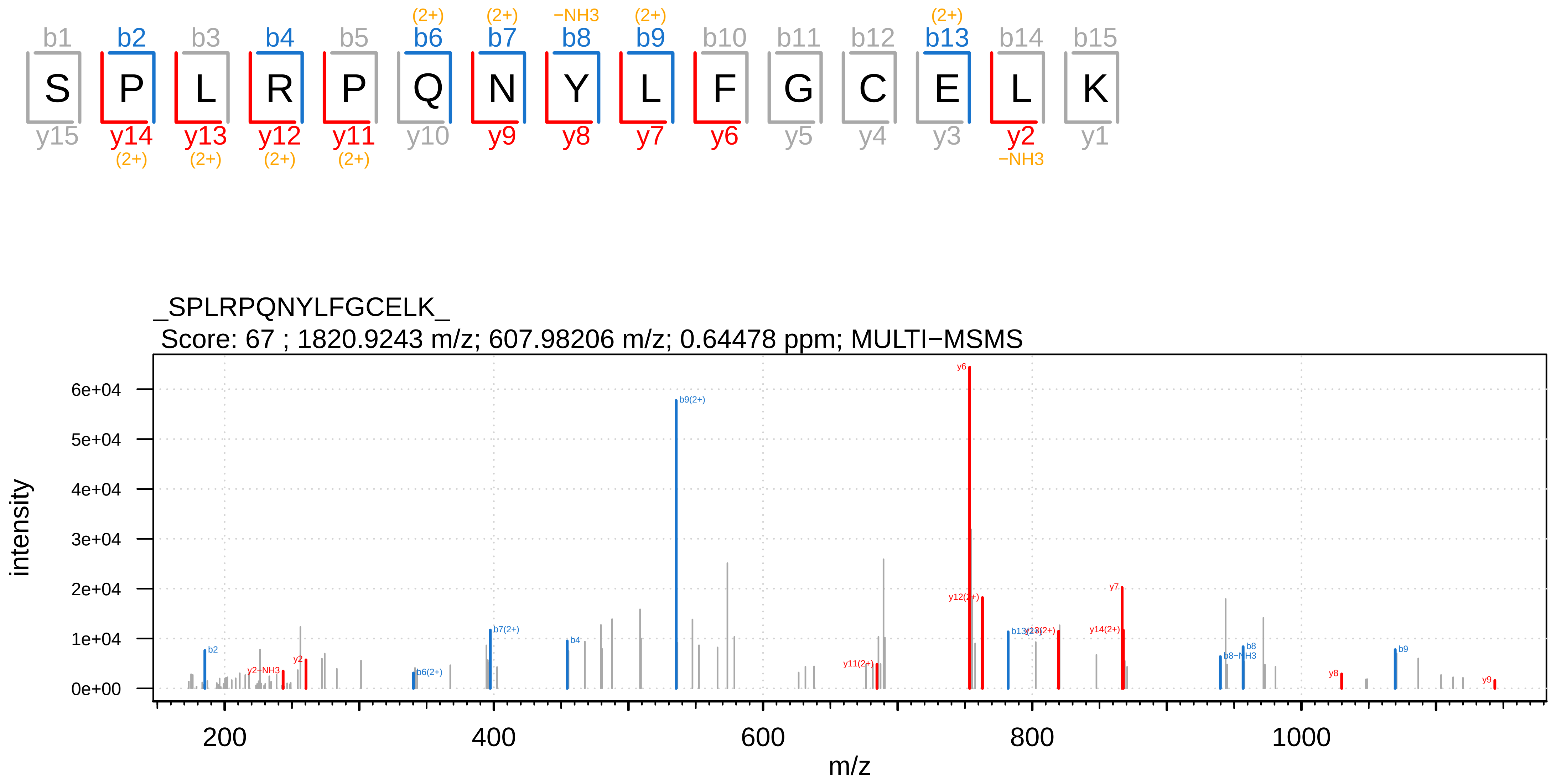

Raw File: Toni_20110714_FB_HepG2_2dot5_uROTO_F9_01
Scan Number: 28225
Proteins:
TCONS_l2_00008829_chr15:92829088−92829258:+

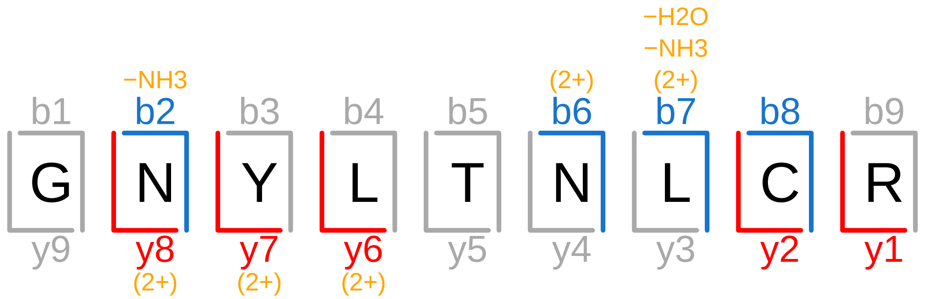

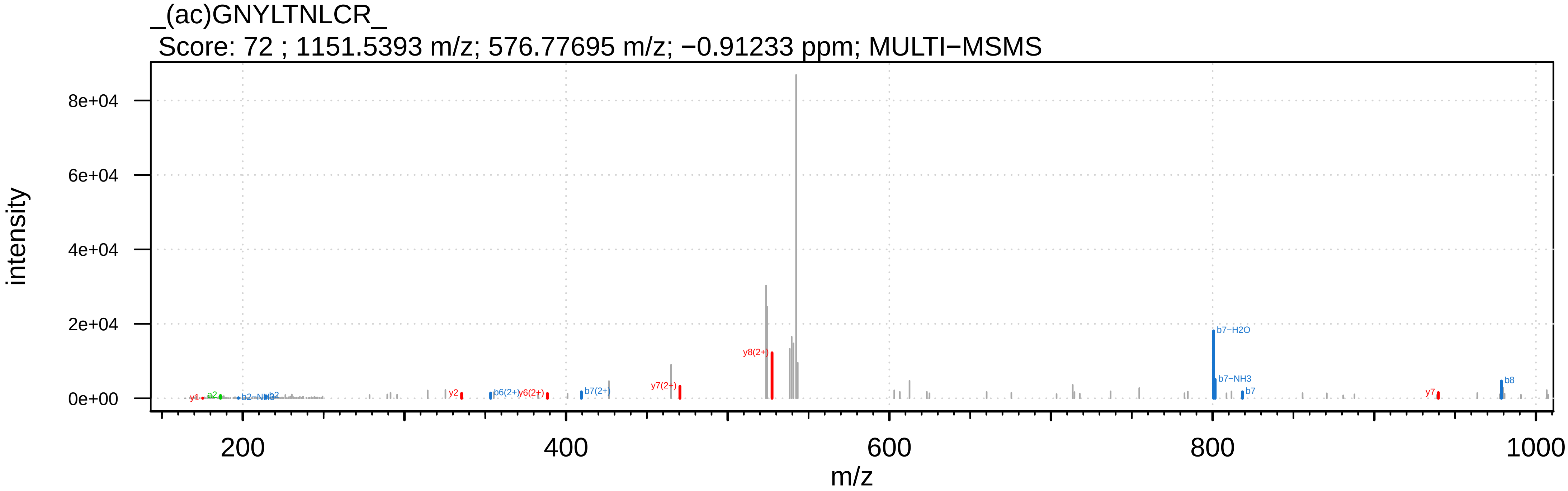

Raw File: Toni_20110506_FB_25_roto_WI38_F7_2
Scan Number: 8295
Proteins:
TCONS_l2_00030467_chrX:38343975−38344091:−

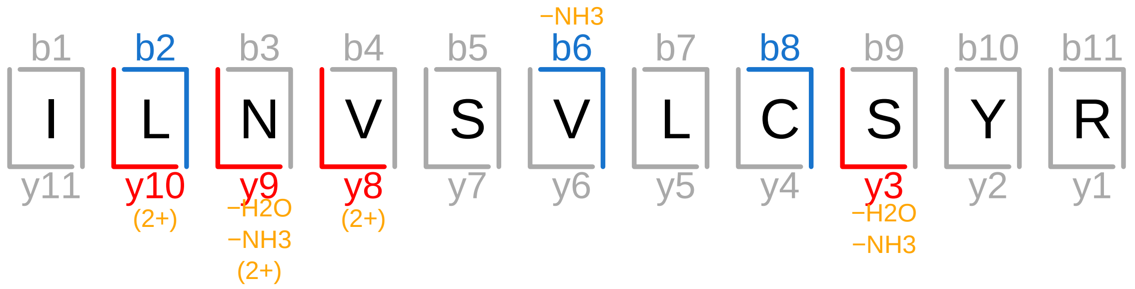

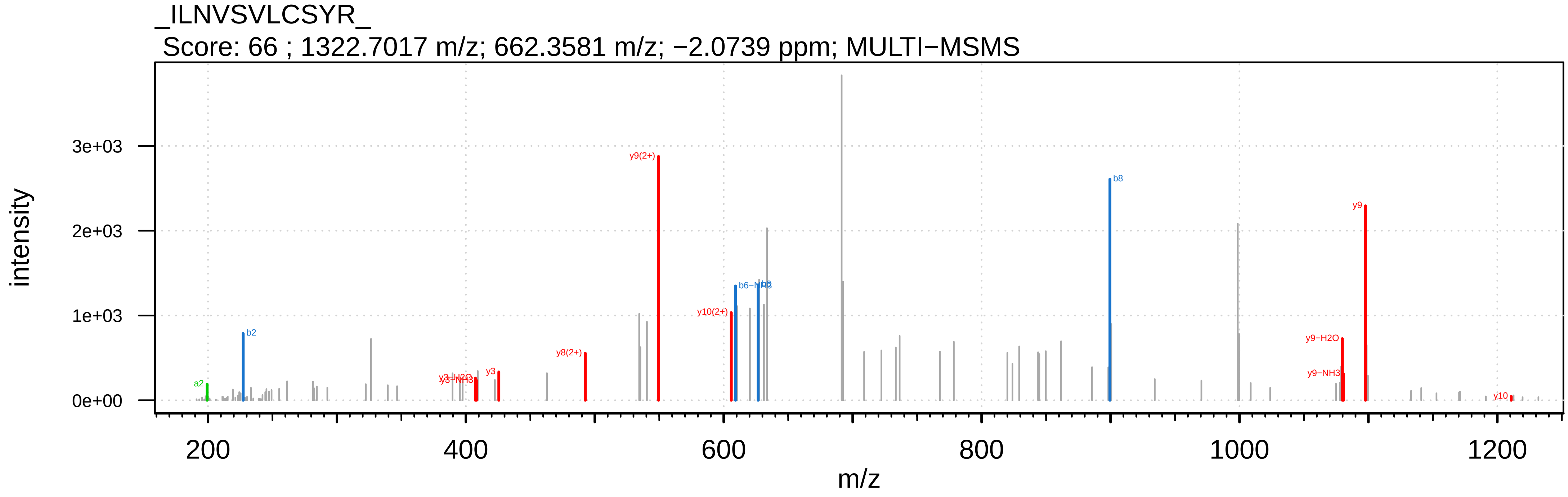

Raw File: Toni_20110506_FB_25_roto_WI38_F7_2
Scan Number: 30370
Proteins:
ENST00000297293_chr7:97835505−97835555:+

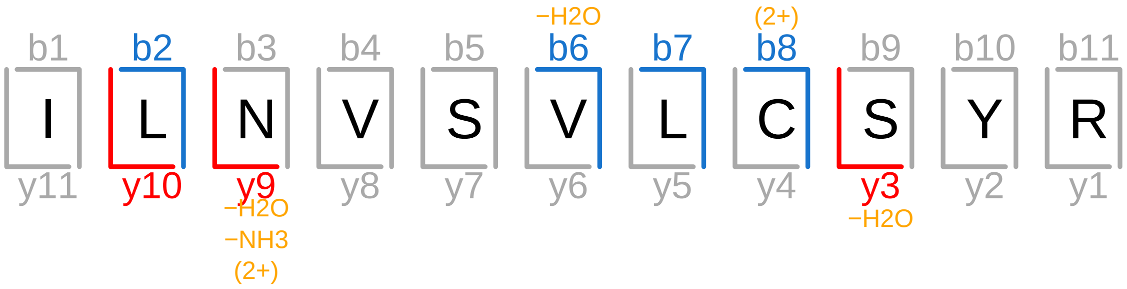

ILNVSVLCSYR
Score: 72 ; 1322.7017 m/z; 662.3581 m/z; −2.6873 ppm; MULTI−MSMS

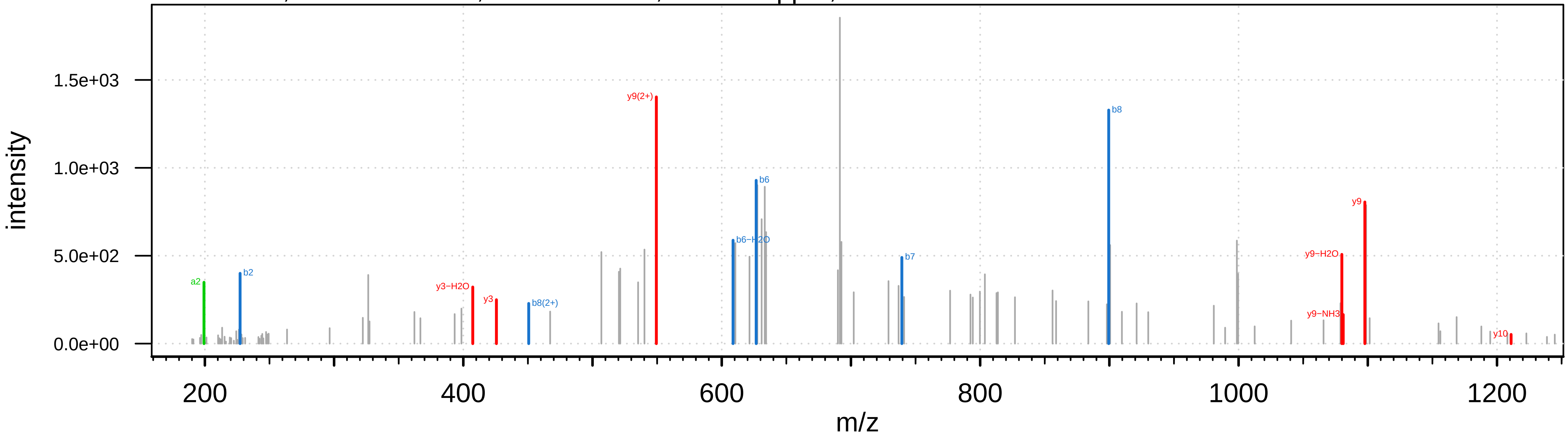

Raw File: Toni_20110506_FB_25_roto_WI38_F6_2
Scan Number: 29288
Proteins:
ENST00000297293_chr7:97835505−97835555:+

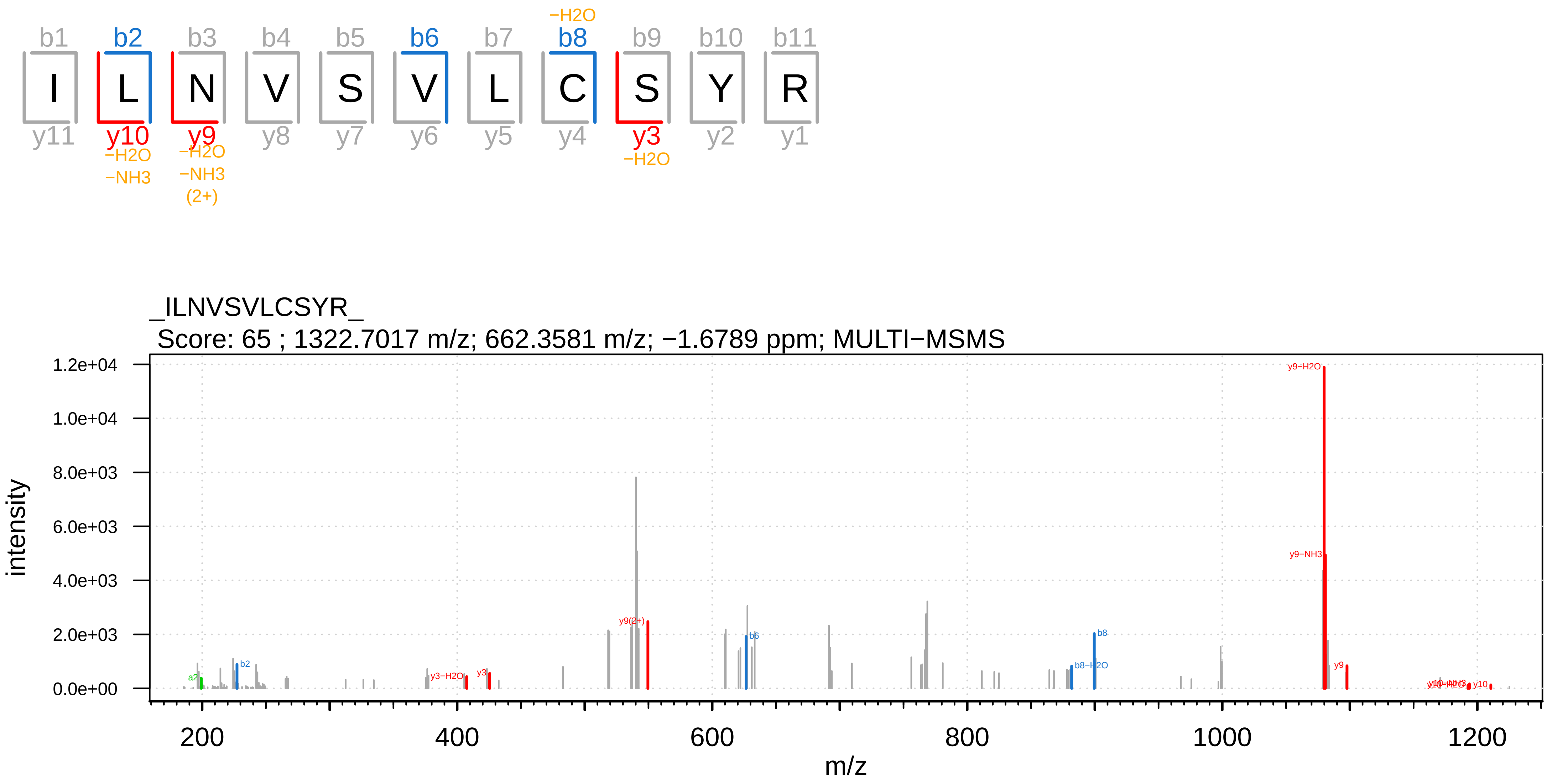

Raw File: Toni_20110506_FB_25_roto_WI38_F9_2
Scan Number: 29385
Proteins:
ENST00000297293_chr7:97835505−97835555:+

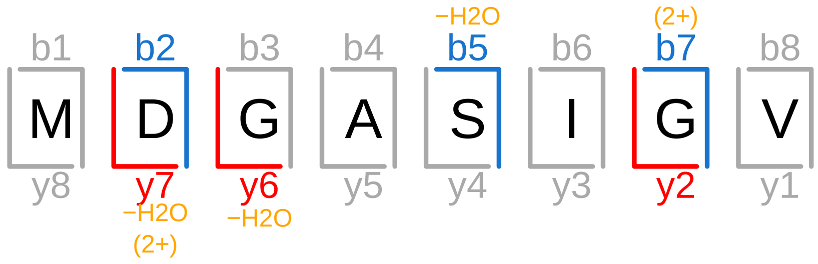

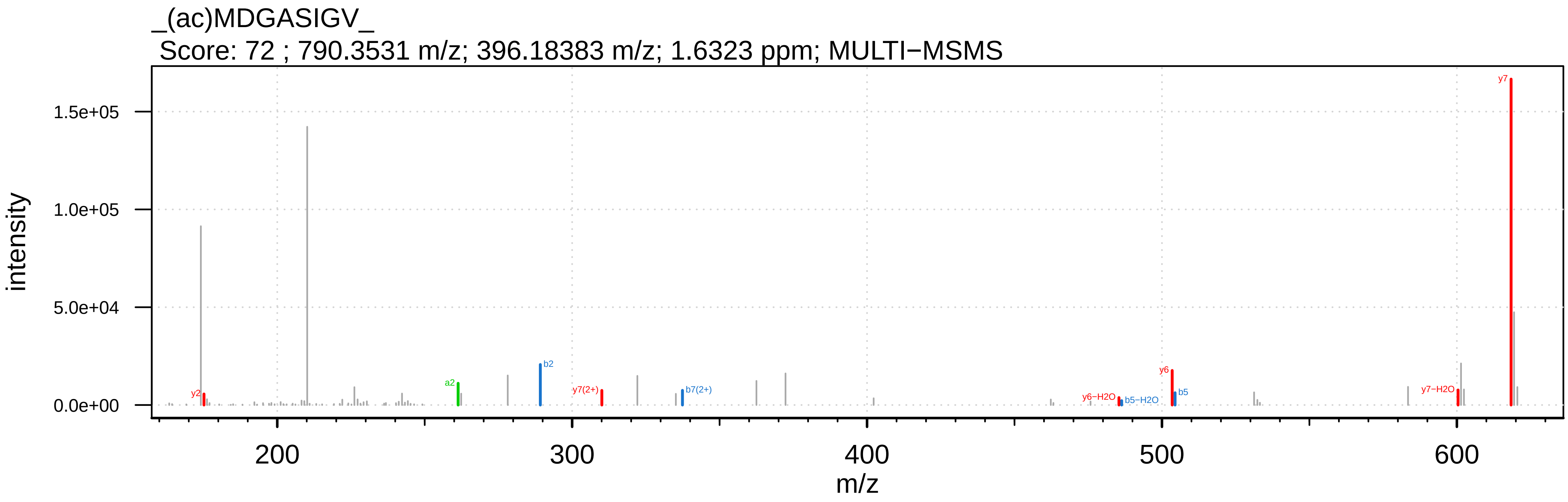

Raw File: Toni_20110714_FB_HepG2_2dot5_uROTO_F3_02
Scan Number: 17796
Proteins:
ENST00000529489_chr1:236681646−236681672:+

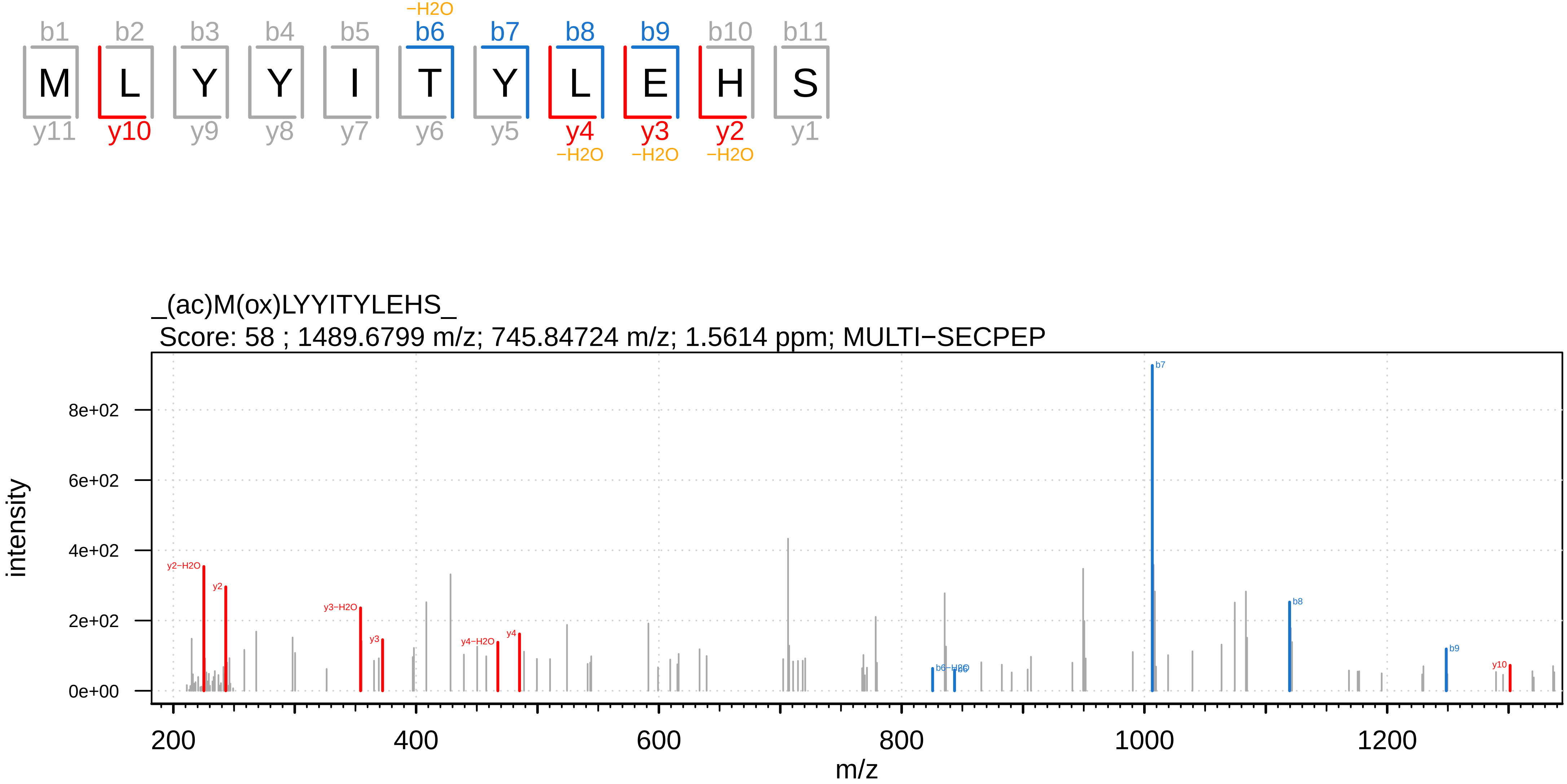

Raw File: Toni_20111125_FB_MCF7_F4_1
Scan Number: 39180
Proteins:
ENST00000317881_chrX:118605057−118605092:+

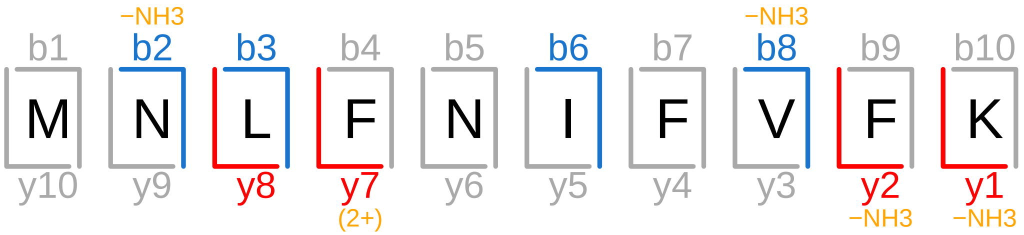

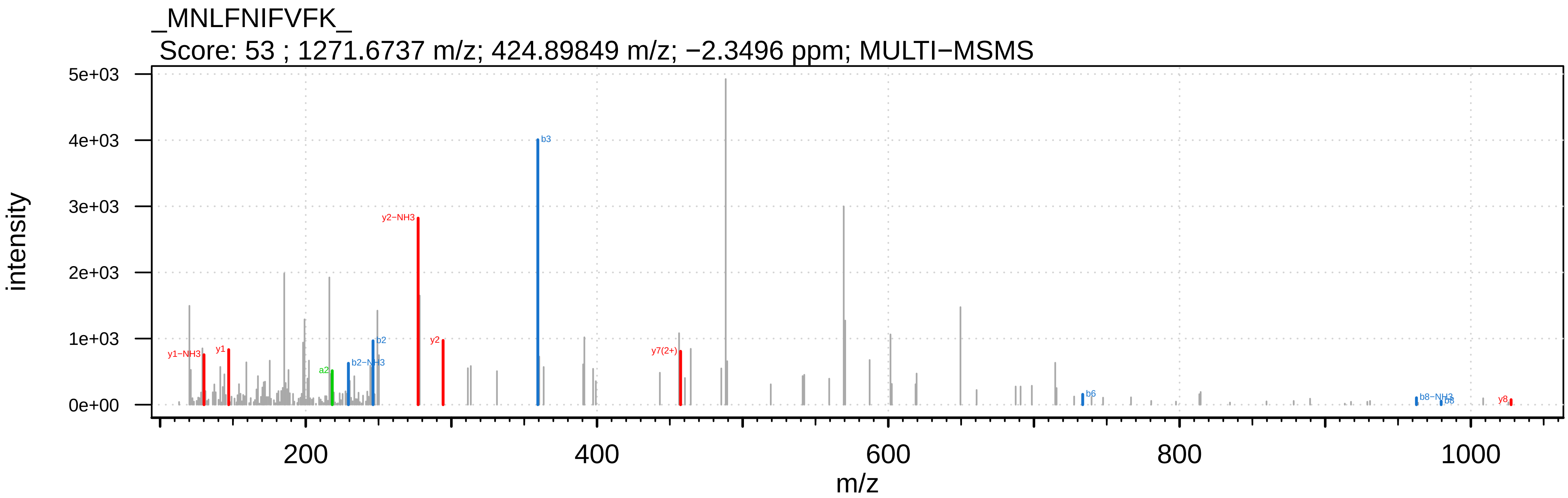

Raw File: Toni_20111125_FB_MCF7_F6_2
Scan Number: 33622
Proteins:
ENST00000274364_chr5:76003185−76003223:+

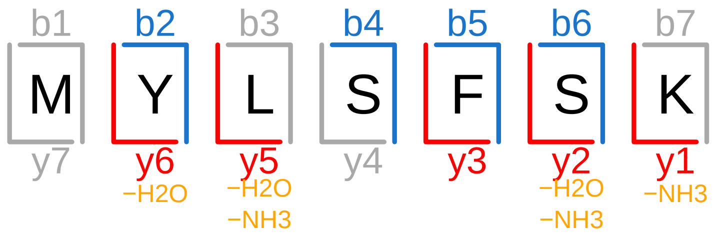

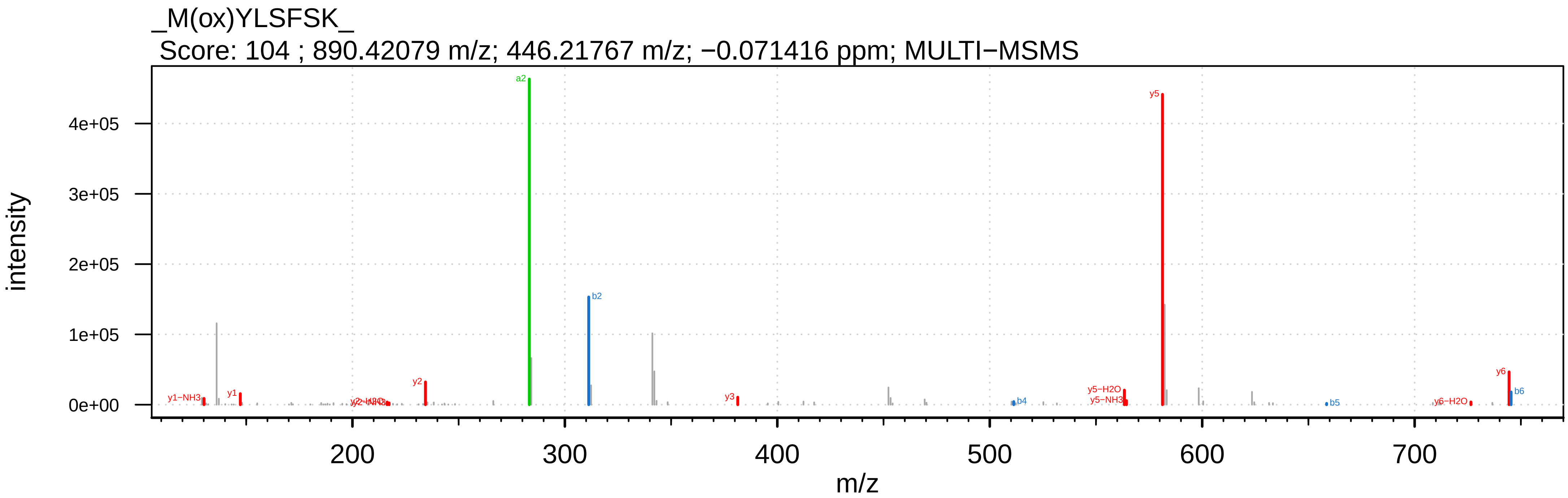

Raw File: Toni_20111125_FB_MCF7_F6_2
Scan Number: 27380
Proteins:
ENST00000392145_chr6:160874572−160874622:+

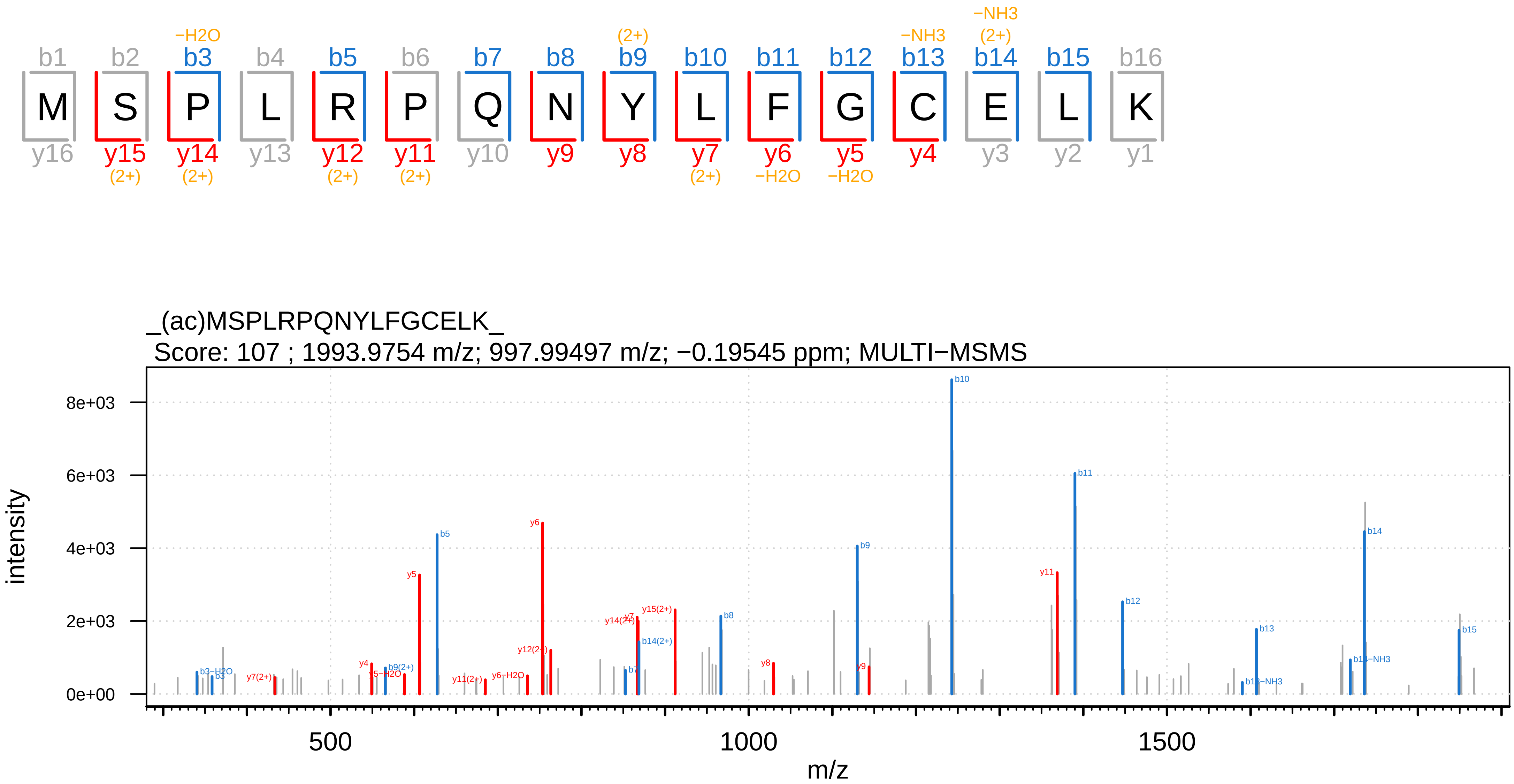

Raw File: Toni_20110714_FB_HepG2_2dot5_uROTO_F9_02
Scan Number: 35058
Proteins:
TCONS_l2_00008829_chr15:92829088−92829258:+

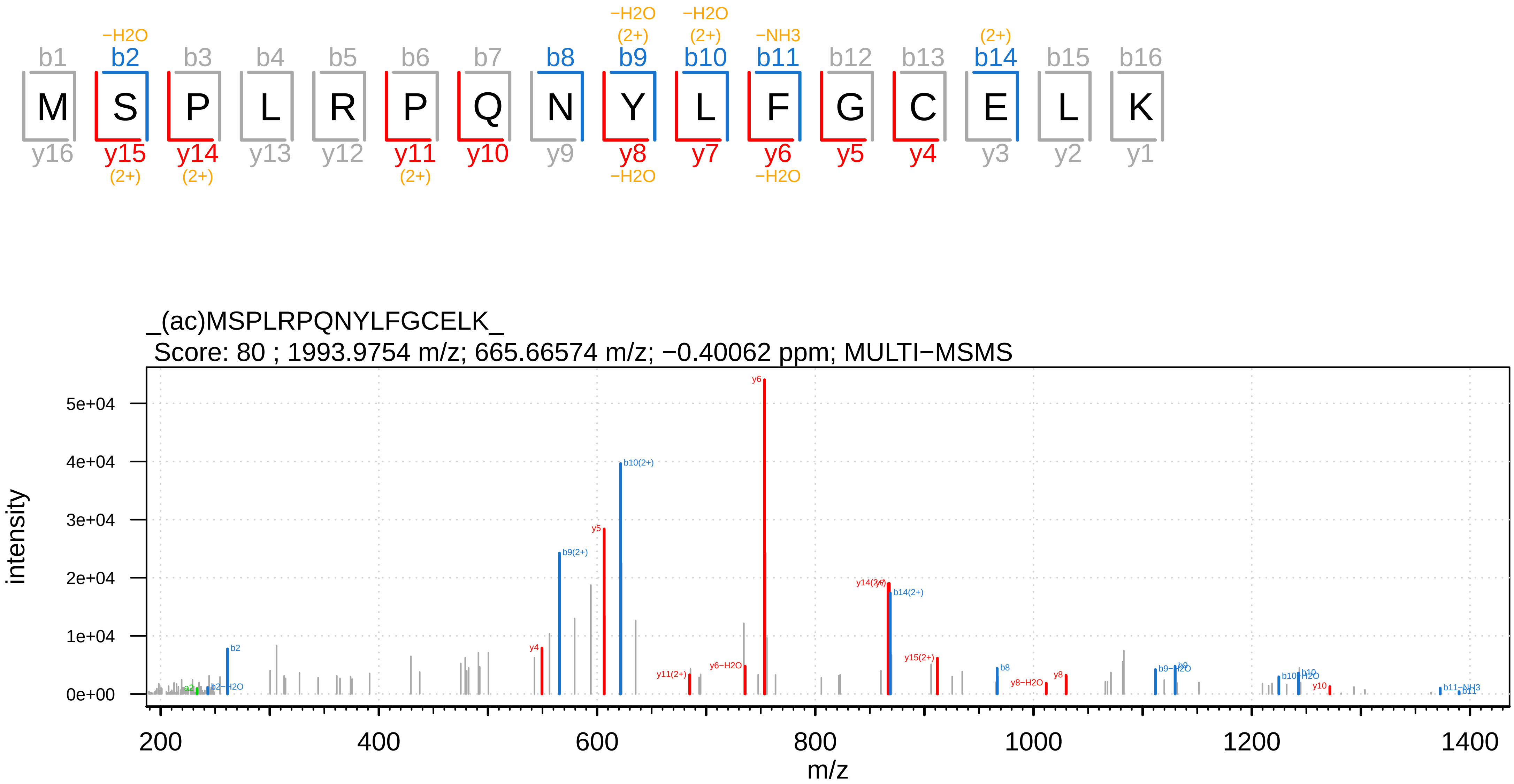

Raw File: Toni_20110714_FB_HepG2_2dot5_uROTO_F9_02

Scan Number: 35089

Proteins:

TCONS_l2_00008829_chr15:92829088−92829258:+

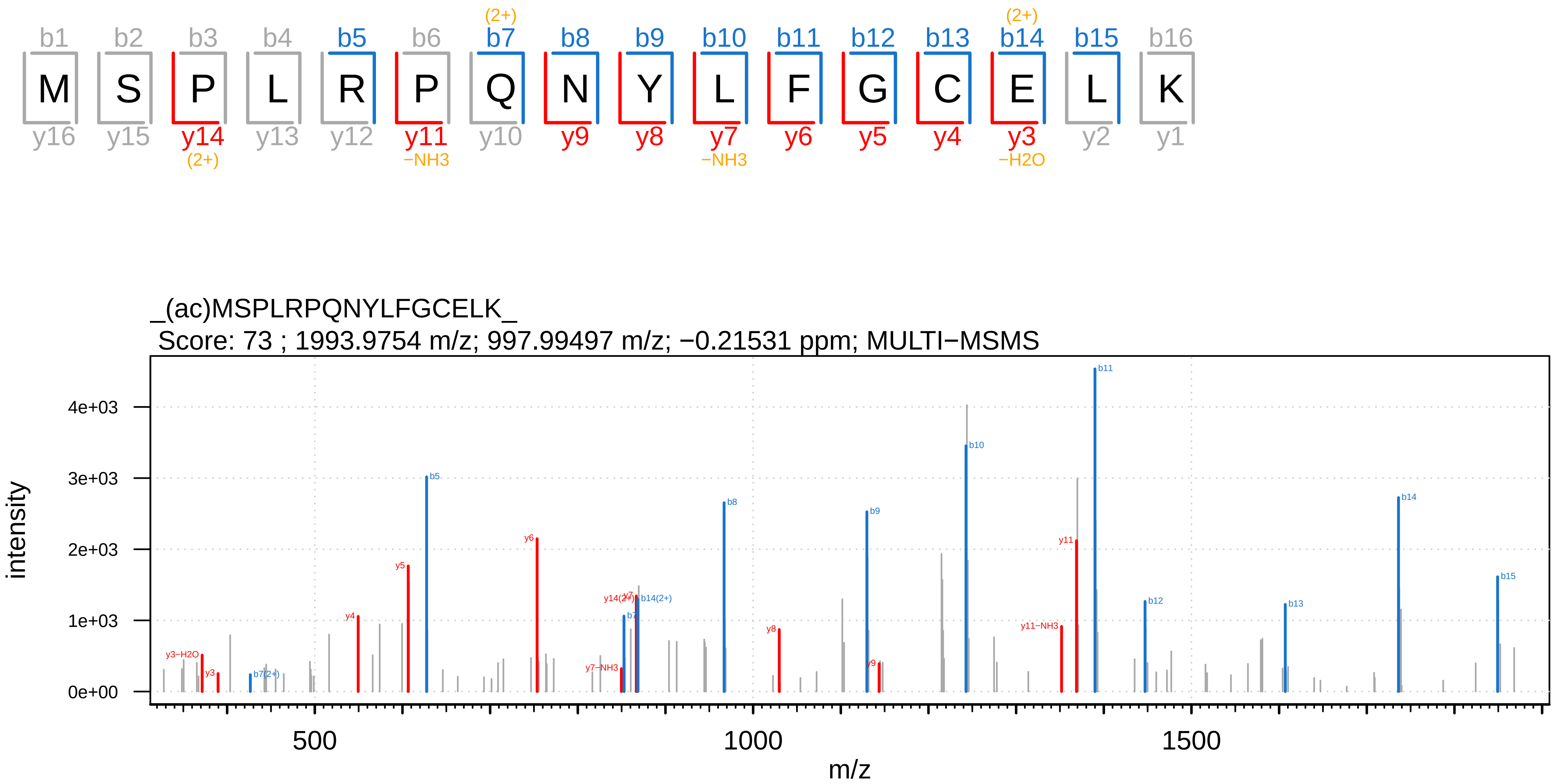

Raw File: Toni_20110714_FB_HepG2_2dot5_uROTO_F10_01
Scan Number: 34770
Proteins:
TCONS_l2_00008829_chr15:92829088−92829258:+

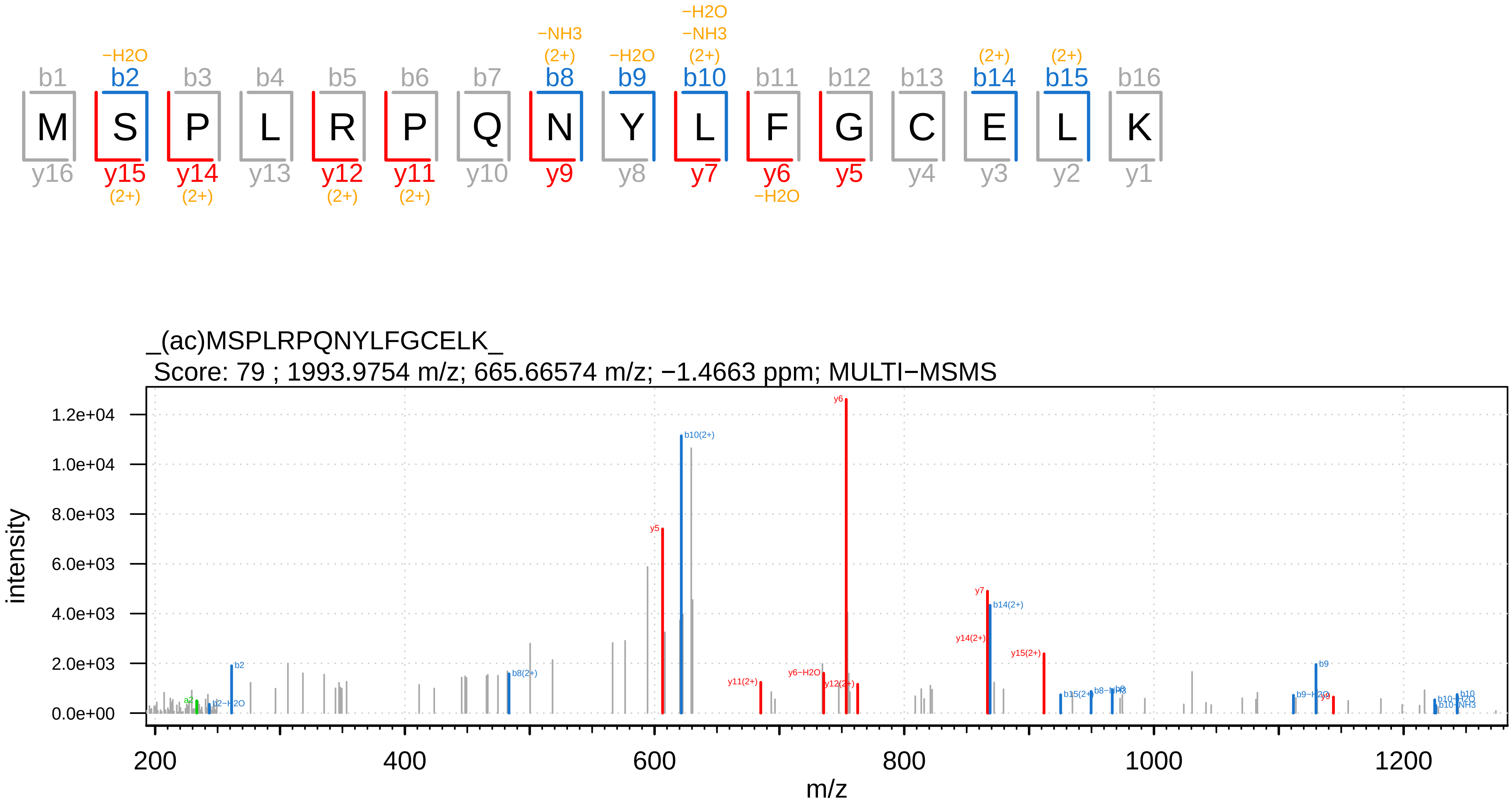

Raw File: Toni_20110714_FB_HepG2_2dot5_uROTO_F10_01
Scan Number: 34780
Proteins:
TCONS_l2_00008829_chr15:92829088−92829258:+

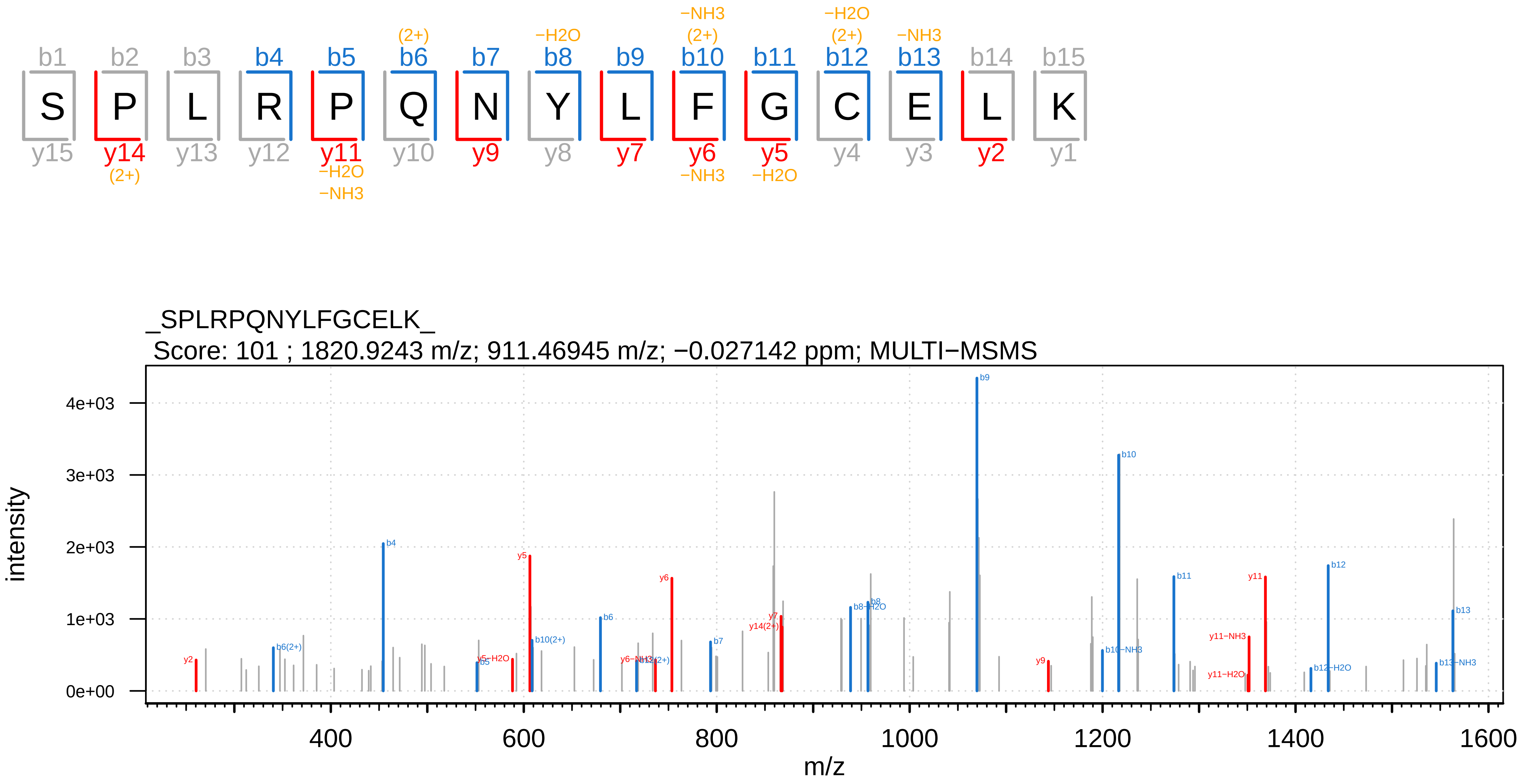

Raw File: Toni_20110714_FB_HepG2_2dot5_uROTO_F10_01
Scan Number: 28299
Proteins:
TCONS_l2_00008829_chr15:92829088−92829258:+

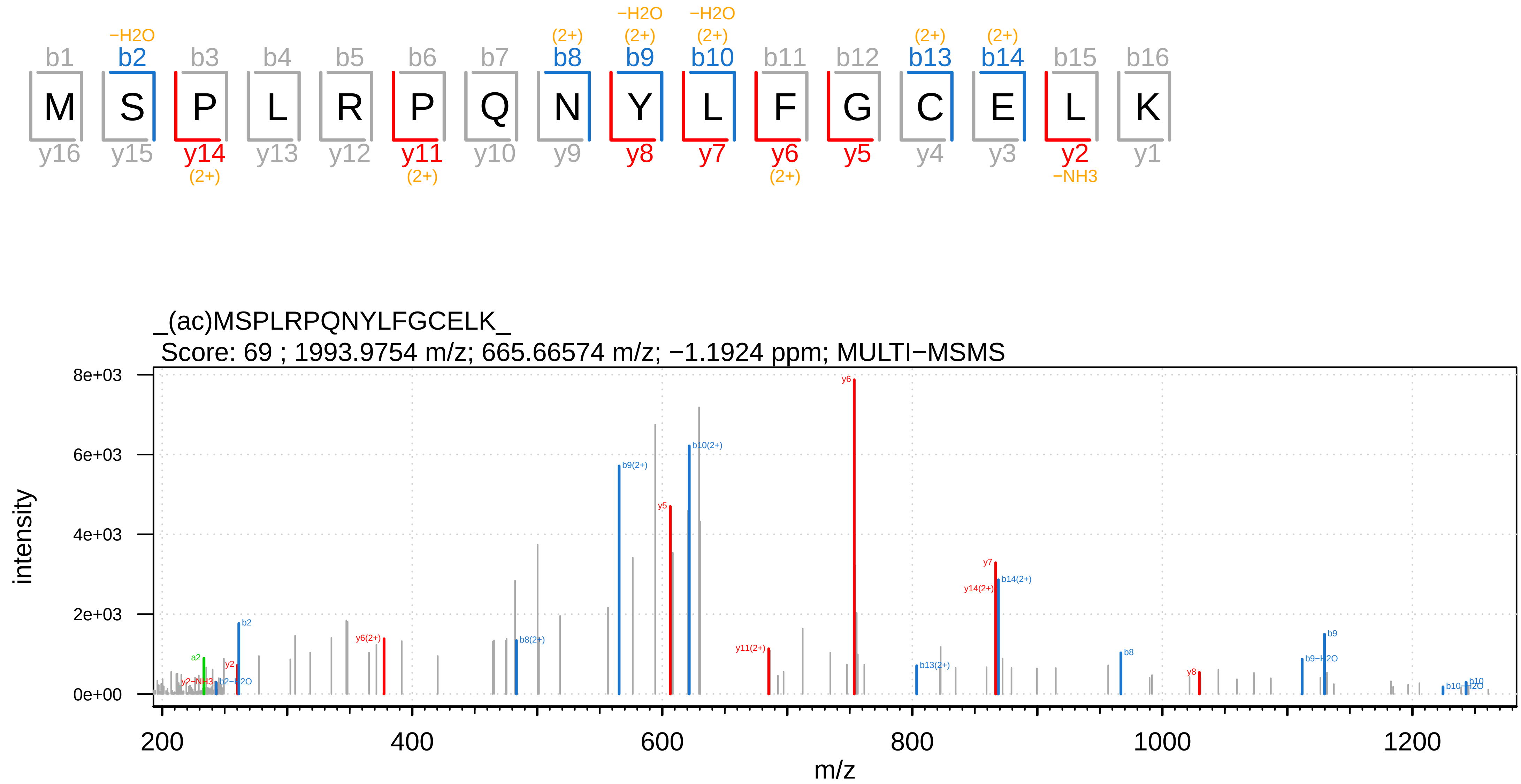

Raw File: Toni_20110714_FB_HepG2_2dot5_uROTO_F10_02
Scan Number: 34946
Proteins:
TCONS_l2_00008829_chr15:92829088−92829258:+

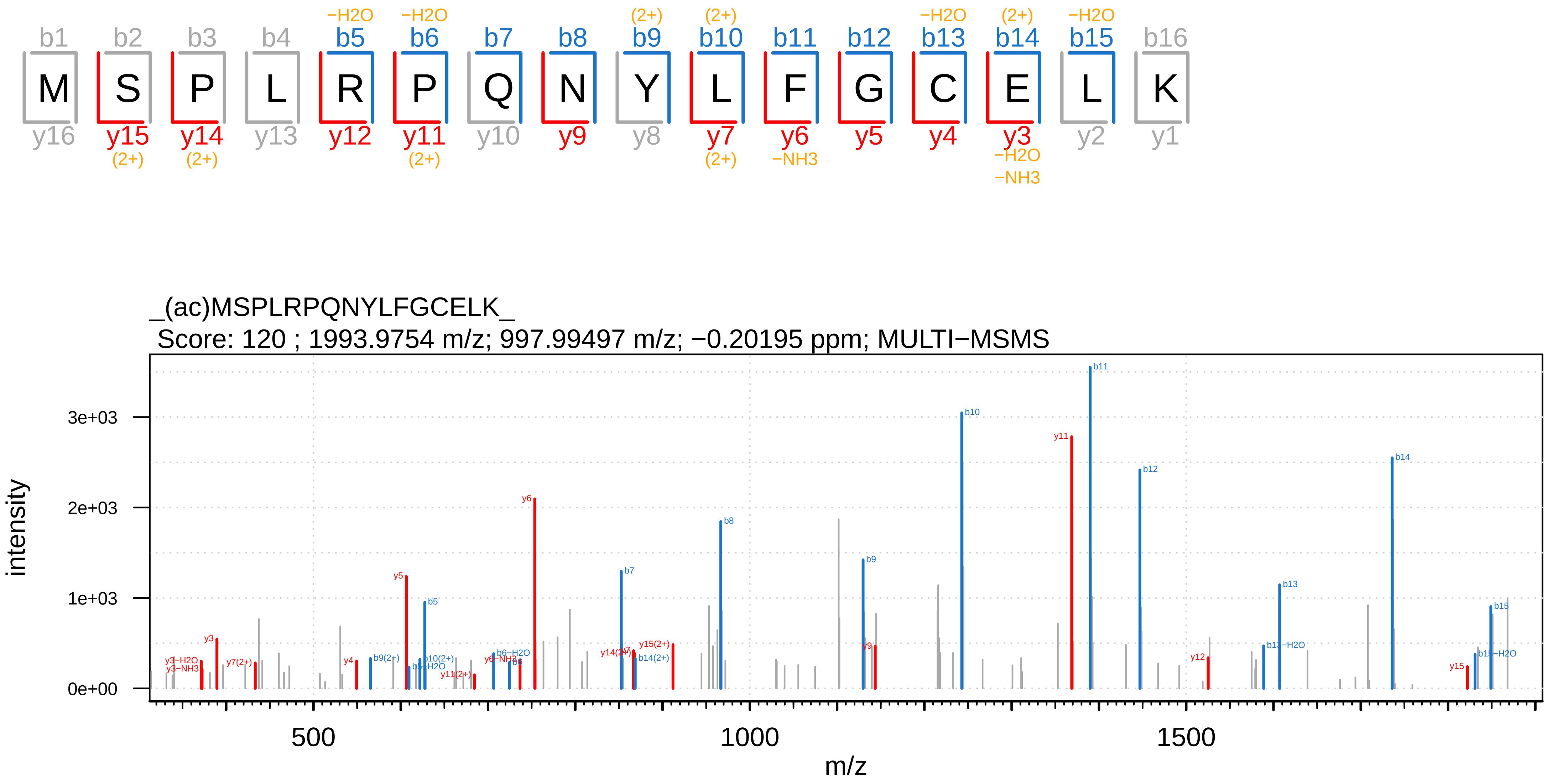

Raw File: Toni_20110714_FB_HepG2_2dot5_uROTO_F10_02
Scan Number: 34948
Proteins:
TCONS_l2_00008829_chr15:92829088−92829258:+

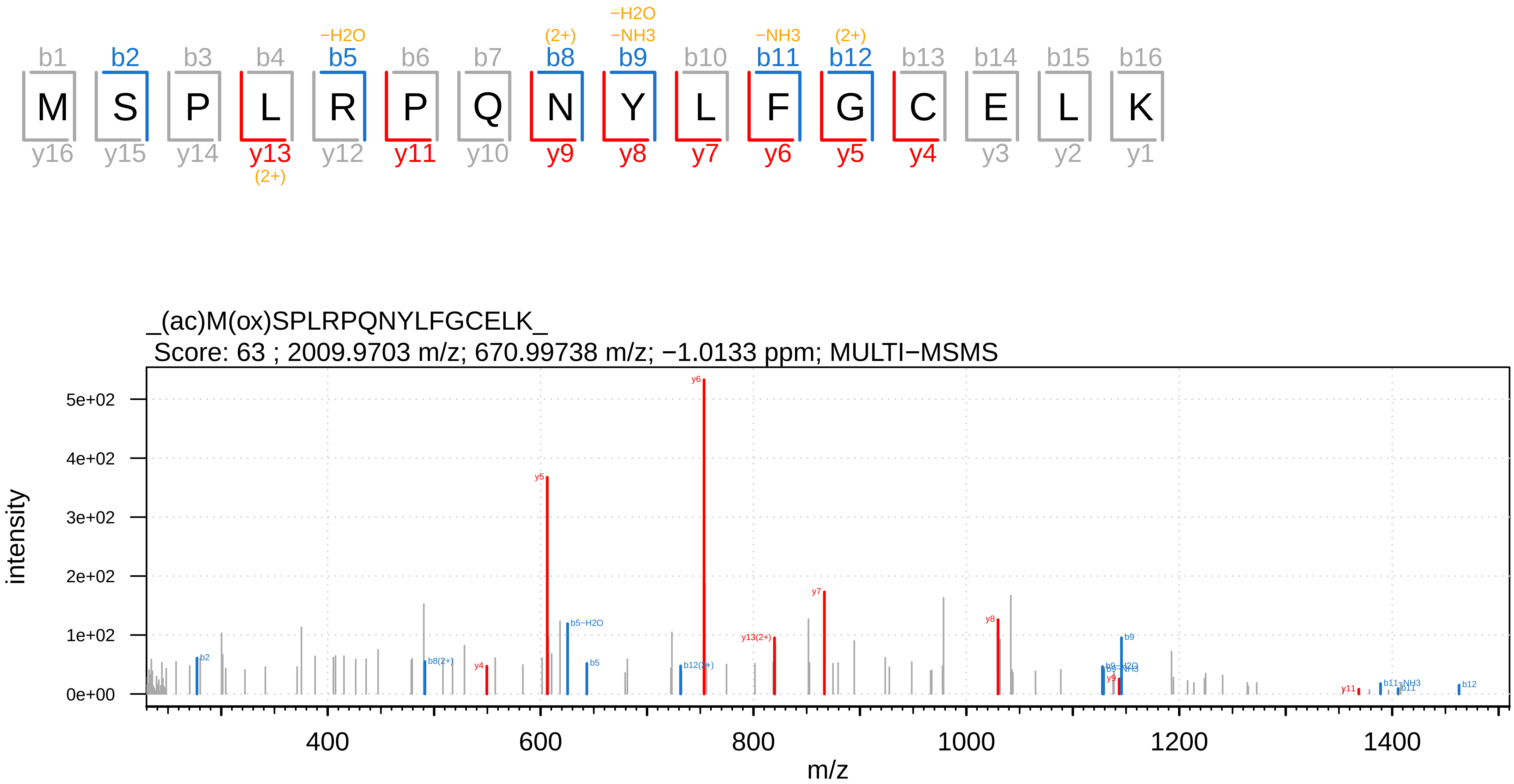

Raw File: Toni_20111125_FB_MCF7_F9_2
Scan Number: 42305
Proteins:
TCONS_l2_00008829_chr15:92829088−92829258:+

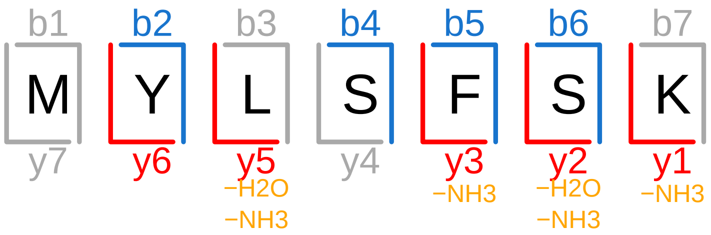

M(ox)YLSFSK
Score: 104 ; 890.42079 m/z; 446.21767 m/z; 0.50371 ppm; MULTI−MSMS

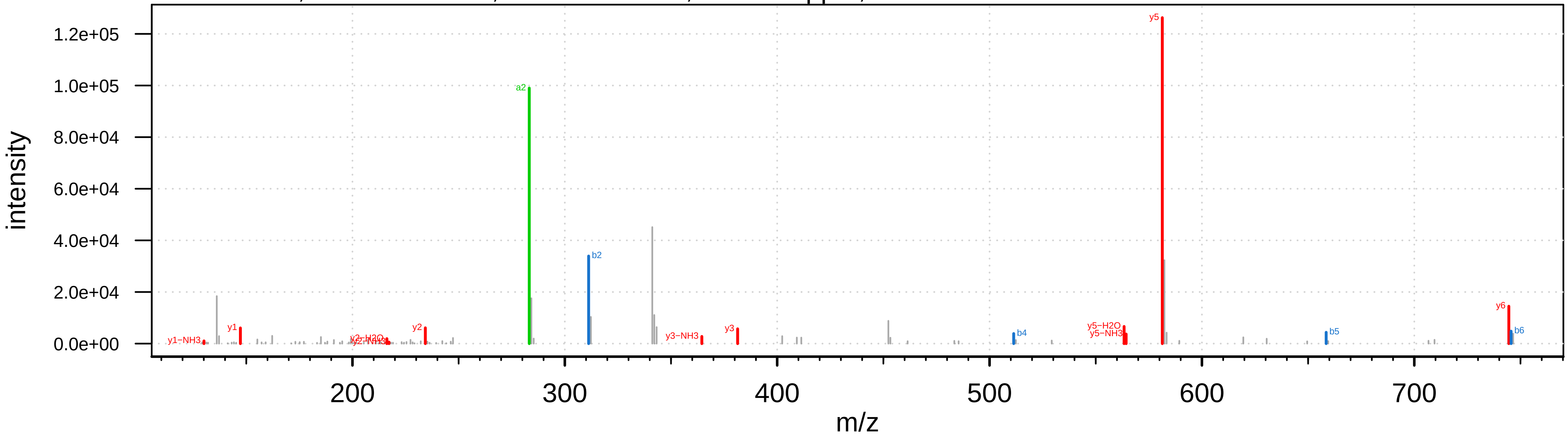

Raw File: Toni_20111114_FB_MDA−MB−231_F7_1
Scan Number: 23407
Proteins:
ENST00000392145_chr6:160874572−160874622:+

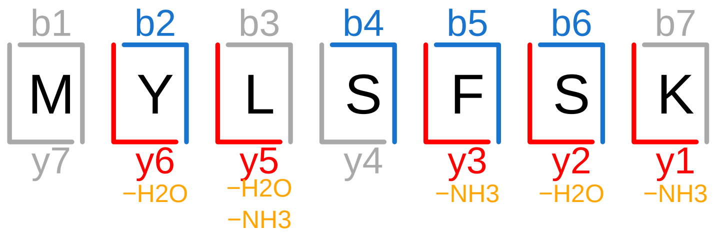

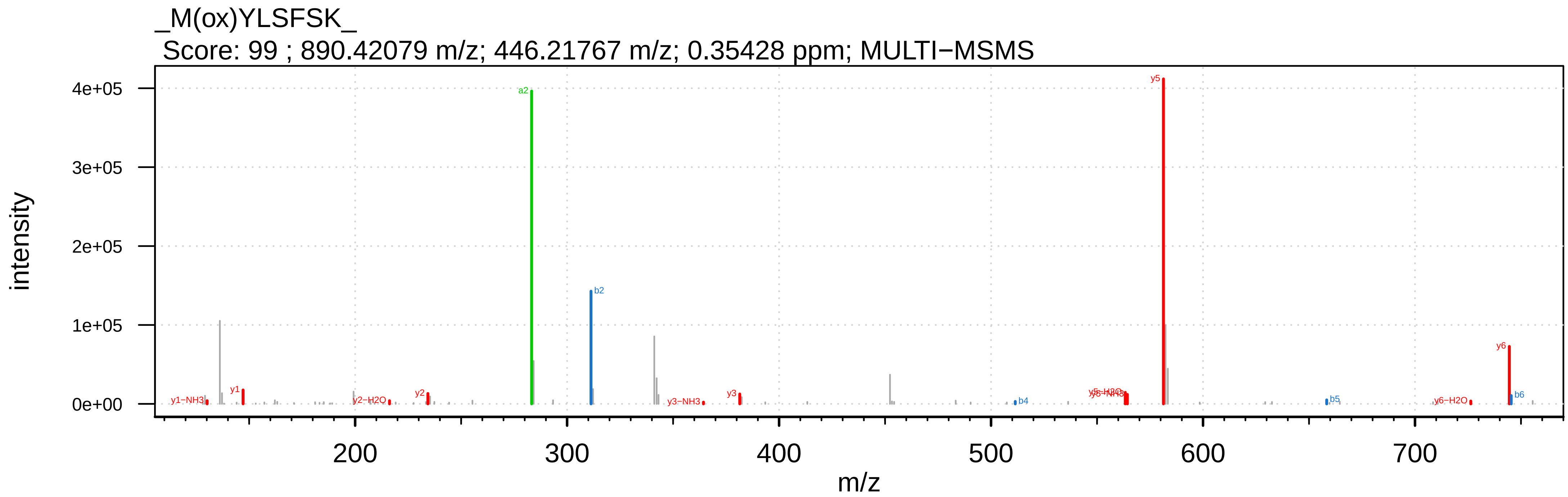

Raw File: Toni_20111125_FB_MCF7_F7_2
Scan Number: 27408
Proteins:
ENST00000392145_chr6:160874572−160874622:+

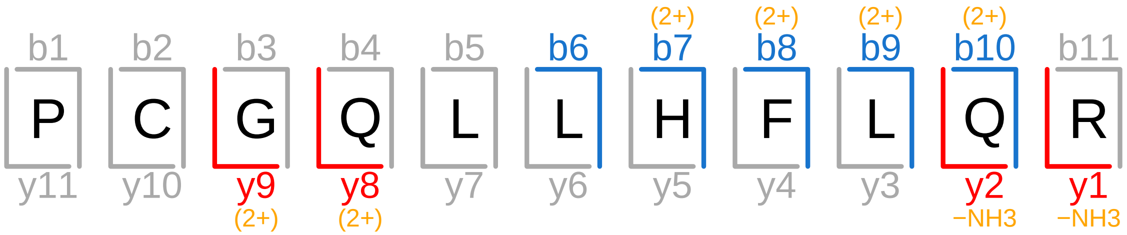

(ac)PCGQLLHFLQR
Score: 52 ; 1409.7238 m/z; 470.91521 m/z; −2.5999 ppm; MULTI−MSMS

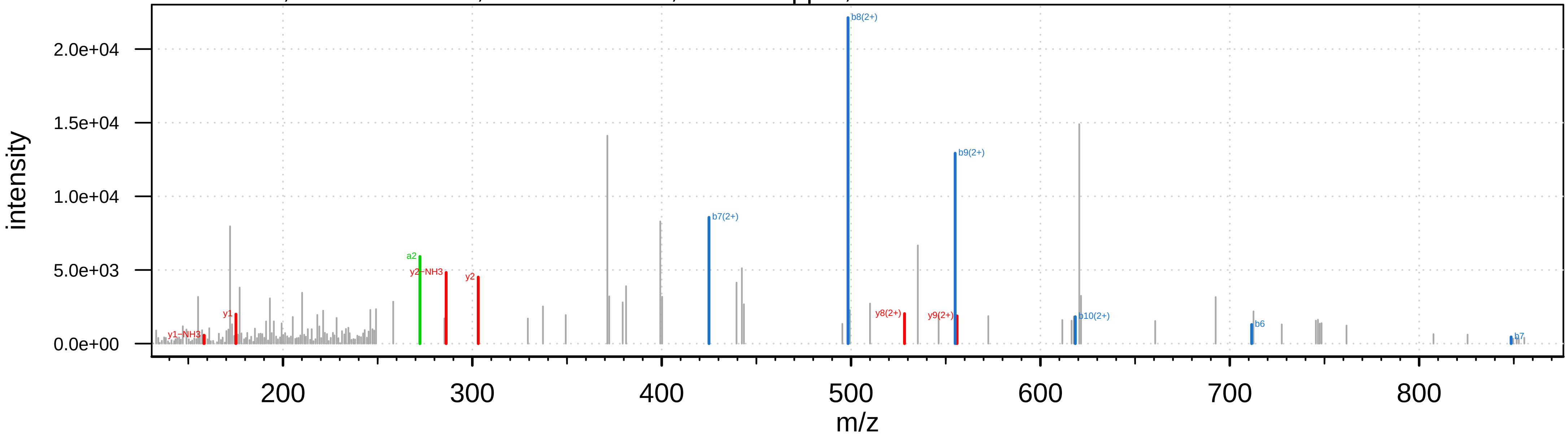

Raw File: Toni_20111109_FB_10A_F8_2
Scan Number: 42791
Proteins:
ENST00000605331_chr2:98317634−98318539:+

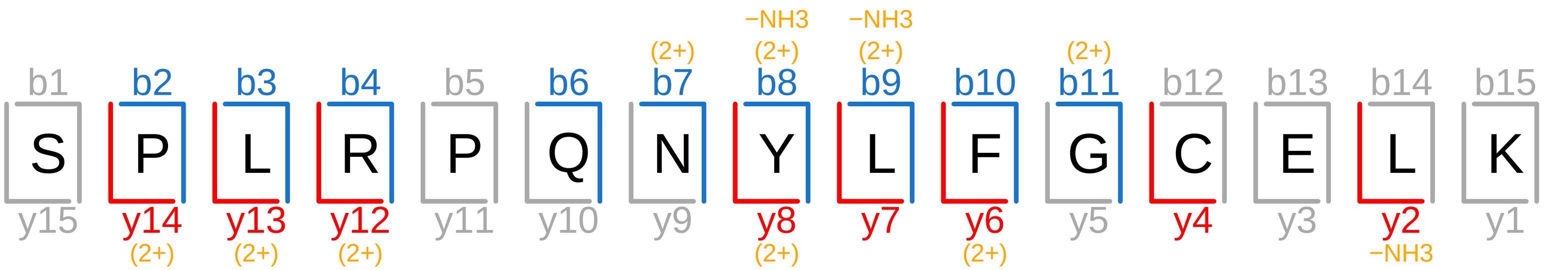

SPLRPQNYLFGCELK
Score: 78 ; 1820.9243 m/z; 607.98206 m/z; 0.60707 ppm; MULTI−MSMS

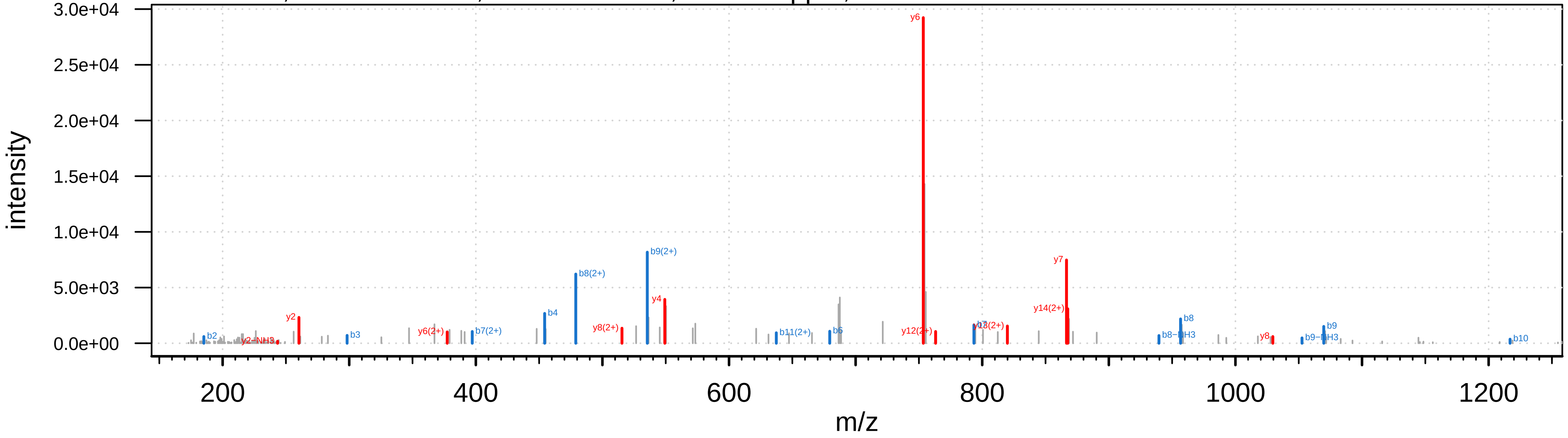

Raw File: Toni_20111109_FB_10A_F9_1
Scan Number: 37732
Proteins:
TCONS_l2_00008829_chr15:92829088−92829258:+

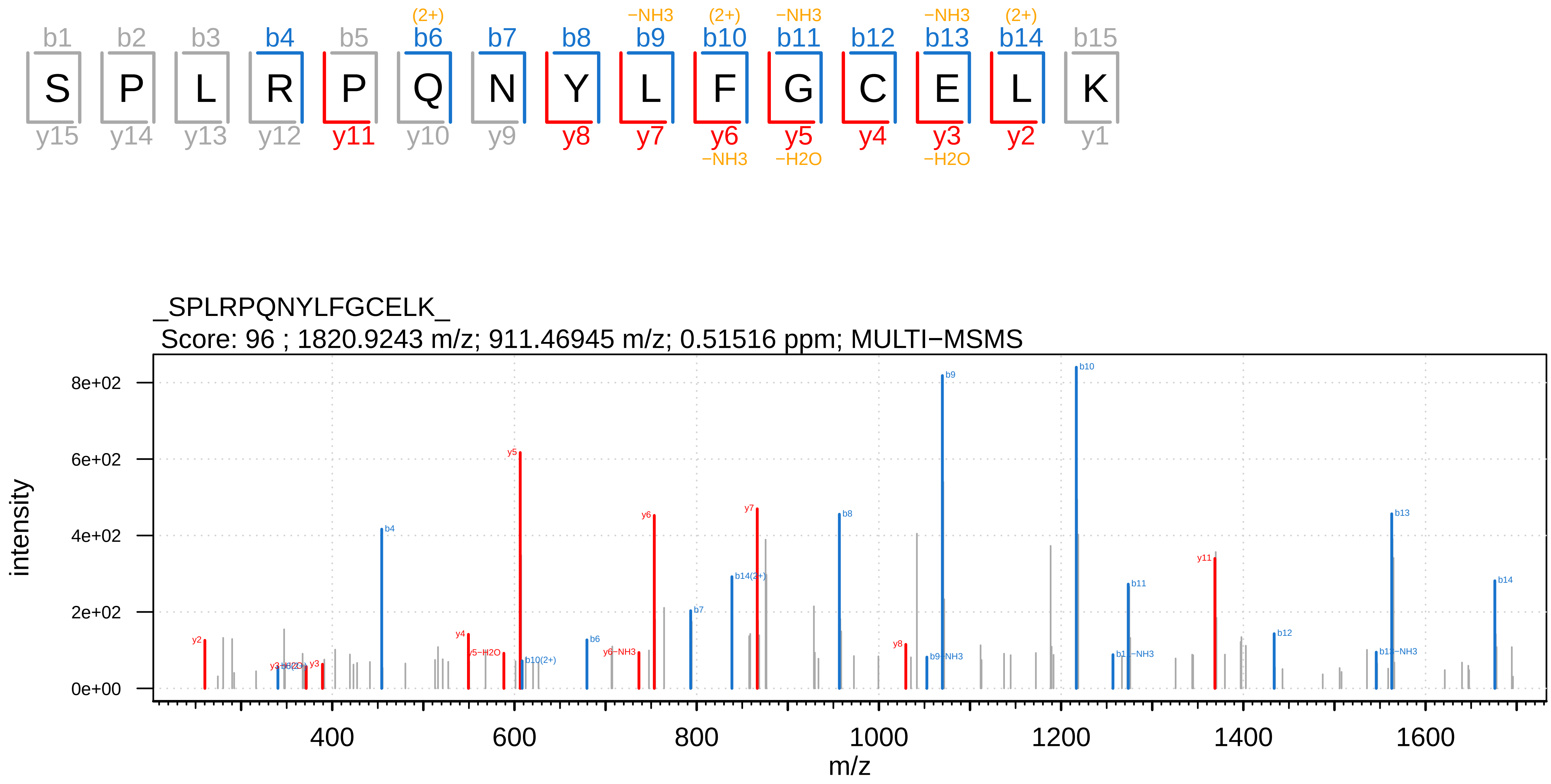

Raw File: Toni_20111109_FB_10A_F9_1
Scan Number: 37764
Proteins:
TCONS_l2_00008829_chr15:92829088−92829258:+

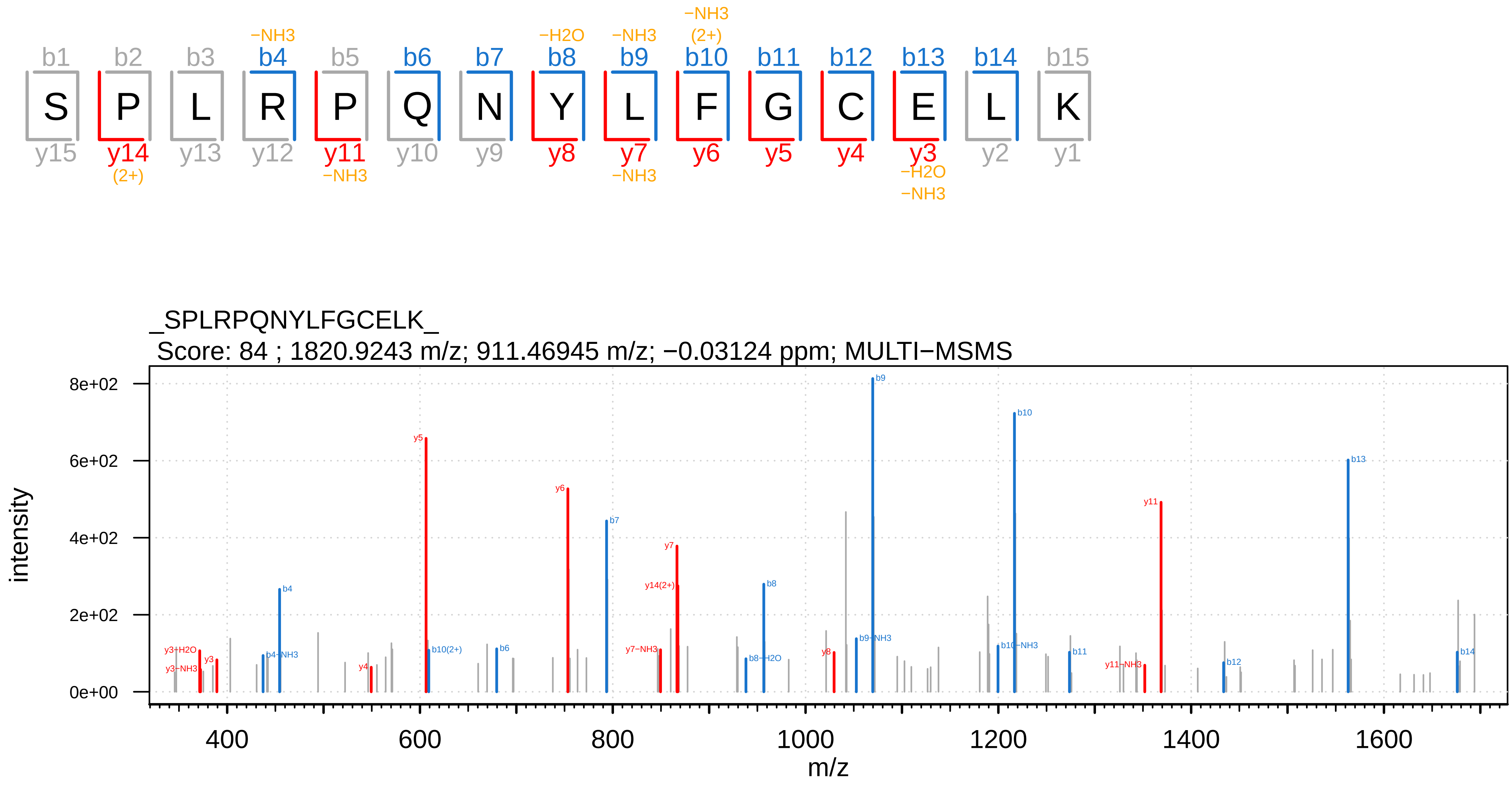

Raw File: Toni_20111109_FB_10A_F9_2
Scan Number: 37890
Proteins:
TCONS_l2_00008829_chr15:92829088−92829258:+

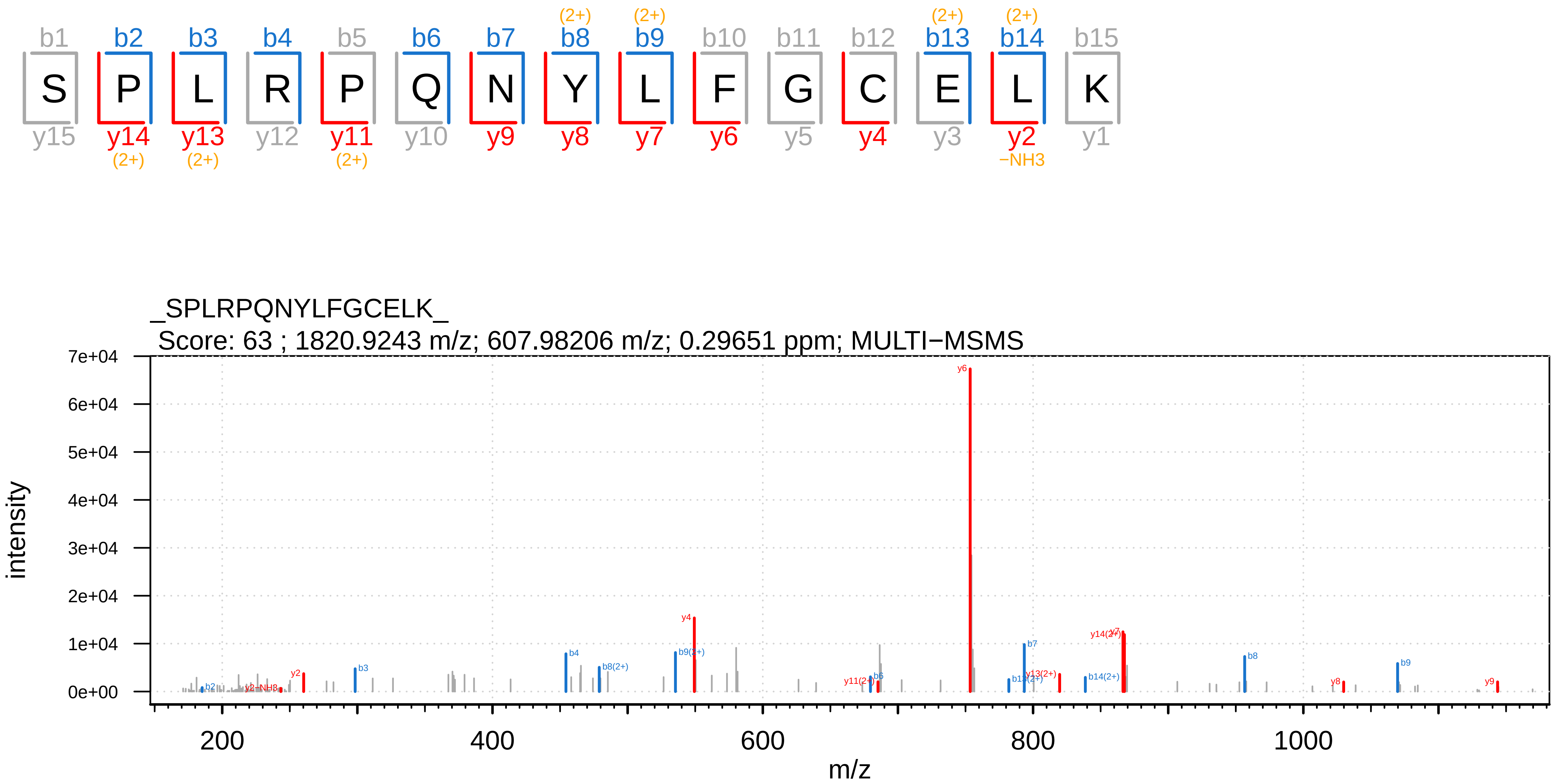

Raw File: Toni_20111109_FB_10A_F10_1
Scan Number: 38286
Proteins:
TCONS_l2_00008829_chr15:92829088−92829258:+

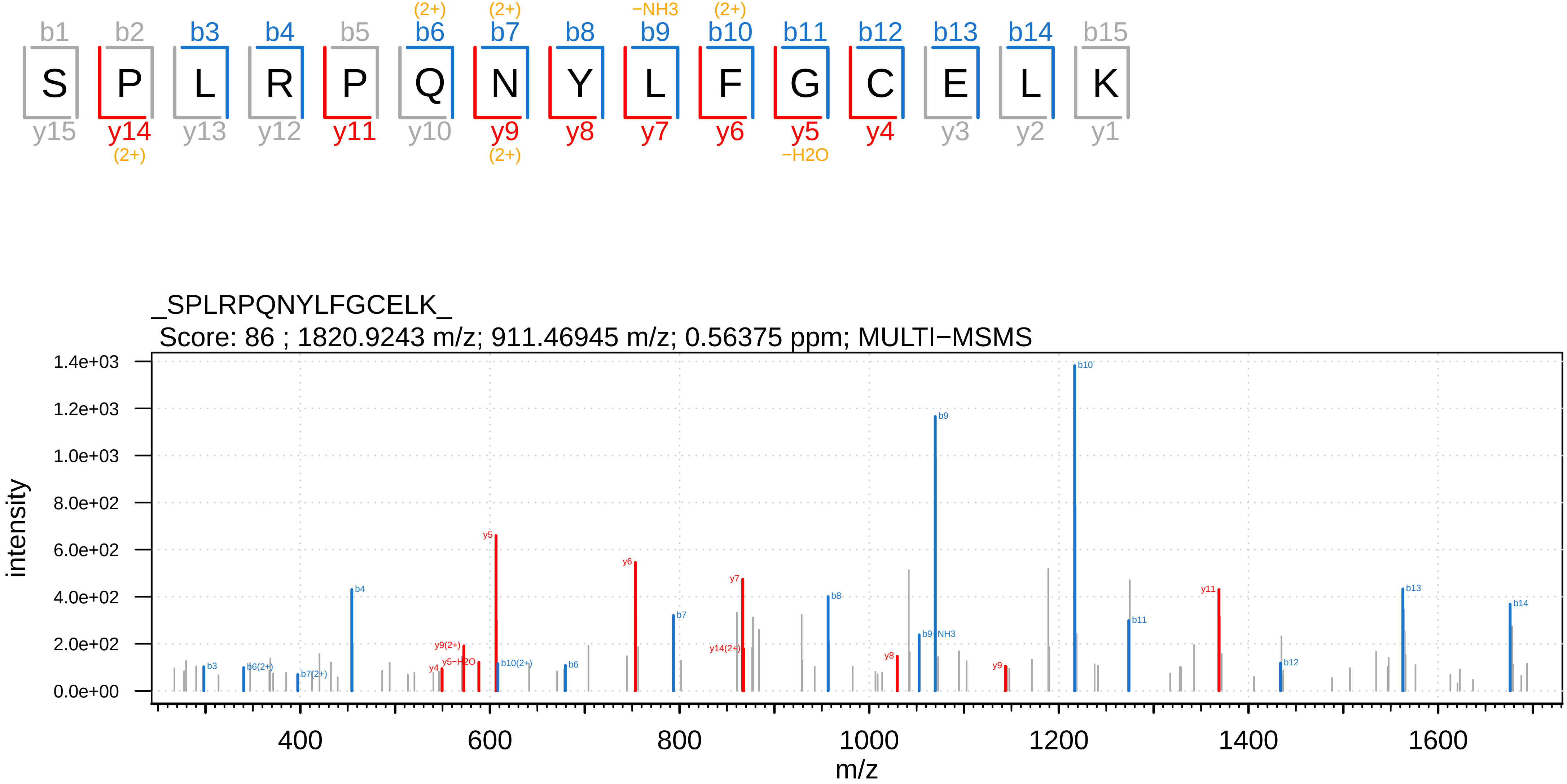

Raw File: Toni_20111109_FB_10A_F10_1
Scan Number: 38299
Proteins:
TCONS_l2_00008829_chr15:92829088−92829258:+

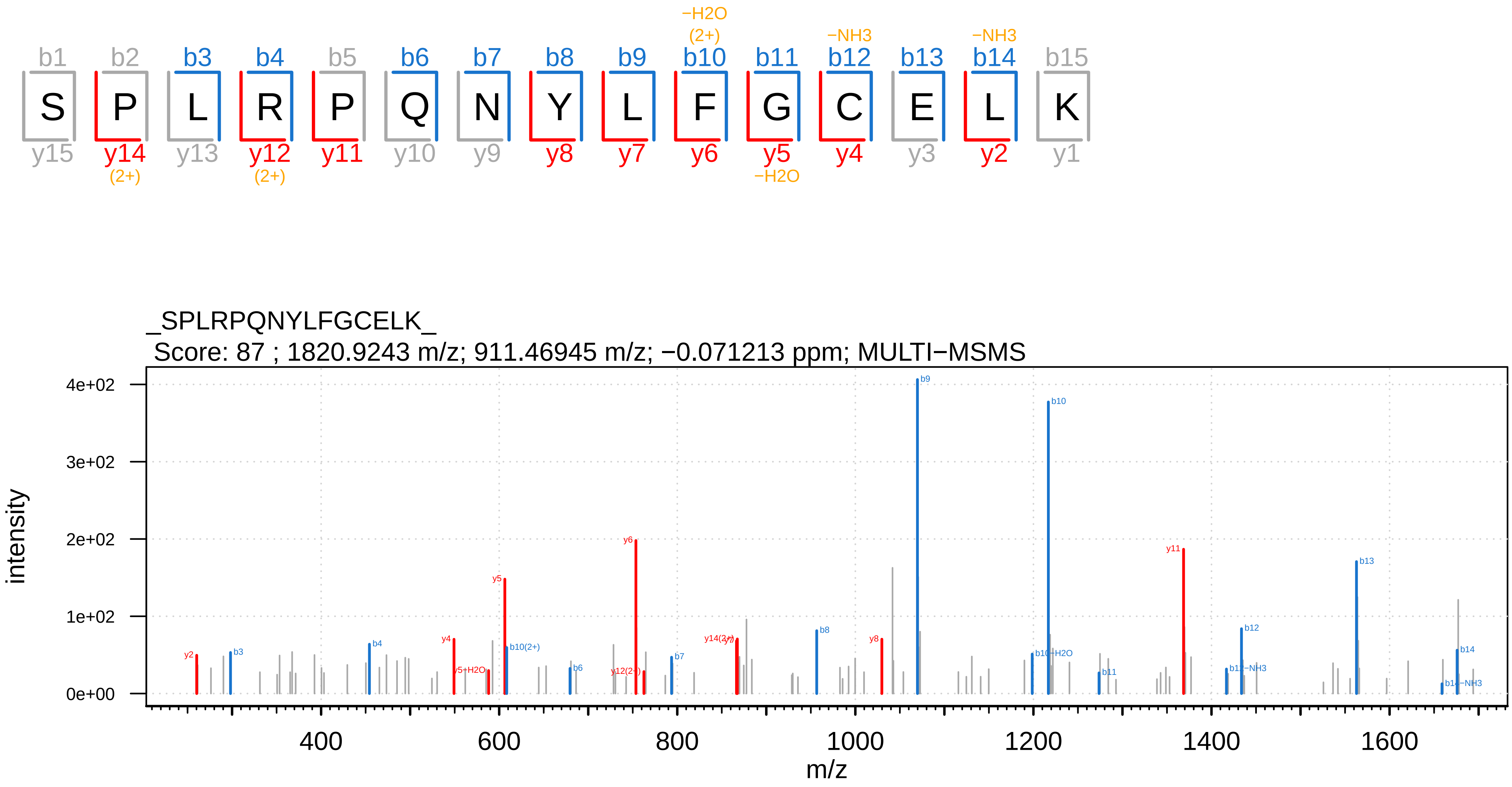

Raw File: Toni_20111114_FB_MDA−MB−231_F10_1
Scan Number: 41050
Proteins:
TCONS_l2_00008829_chr15:92829088−92829258:+

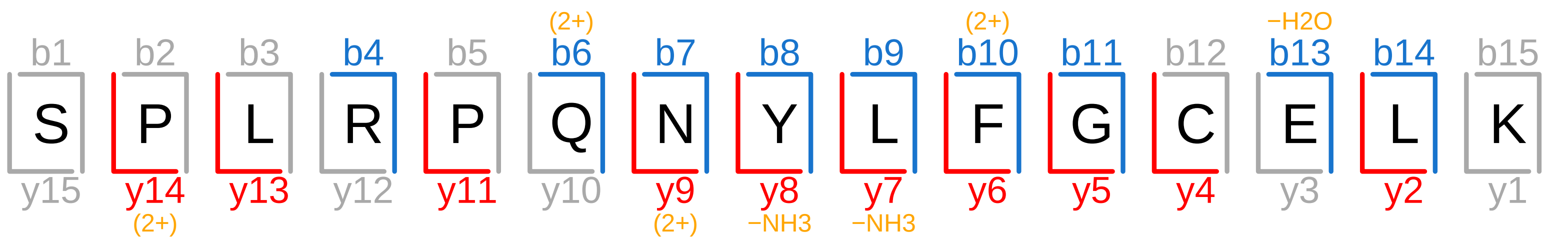

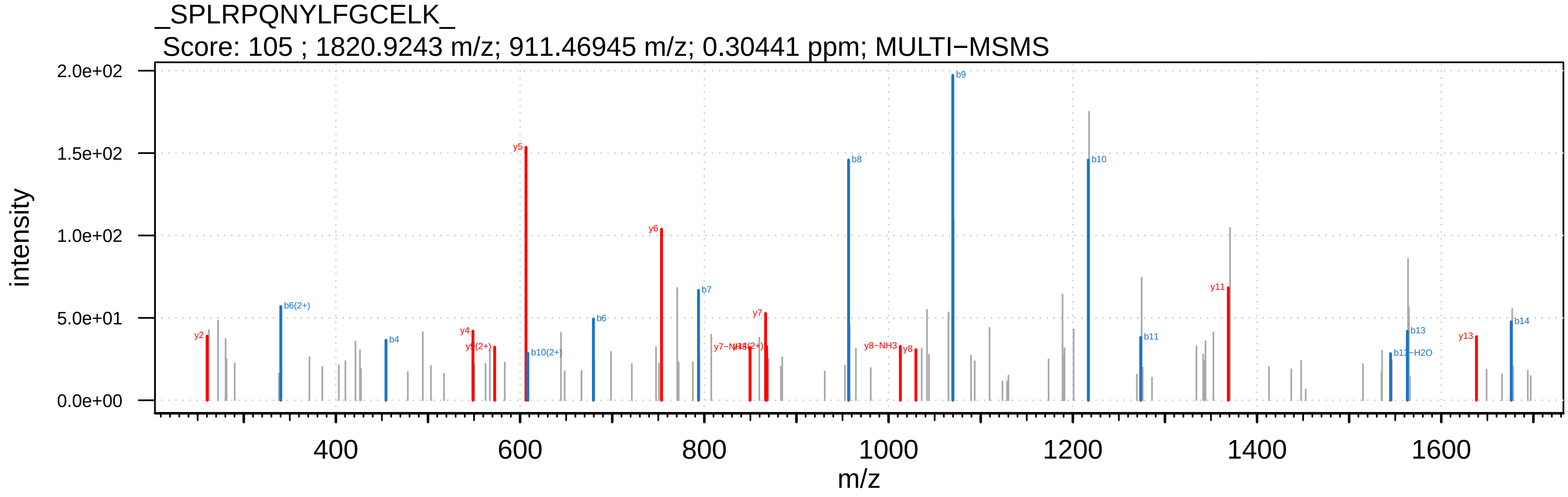

Raw File: Toni_20111114_FB_MDA−MB−231_F10_2
Scan Number: 41088
Proteins:
TCONS_l2_00008829_chr15:92829088−92829258:+
