# Supplementary material for: Extensive identification and analysis of conserved small ORFs in animals
Source: Genome Biol. 2015 Sep 14;16:179. doi: 10.1186/s13059-015-0742-x (PMC4568590; doi:10.1186/s13059-015-0742-x)
Supplement: Additional file 18: Figure S9. — Spectra for the PMS from the zebrafish datasets. (PDF 19 kb) [file 13059_2015_742_MOESM18_ESM.pdf]

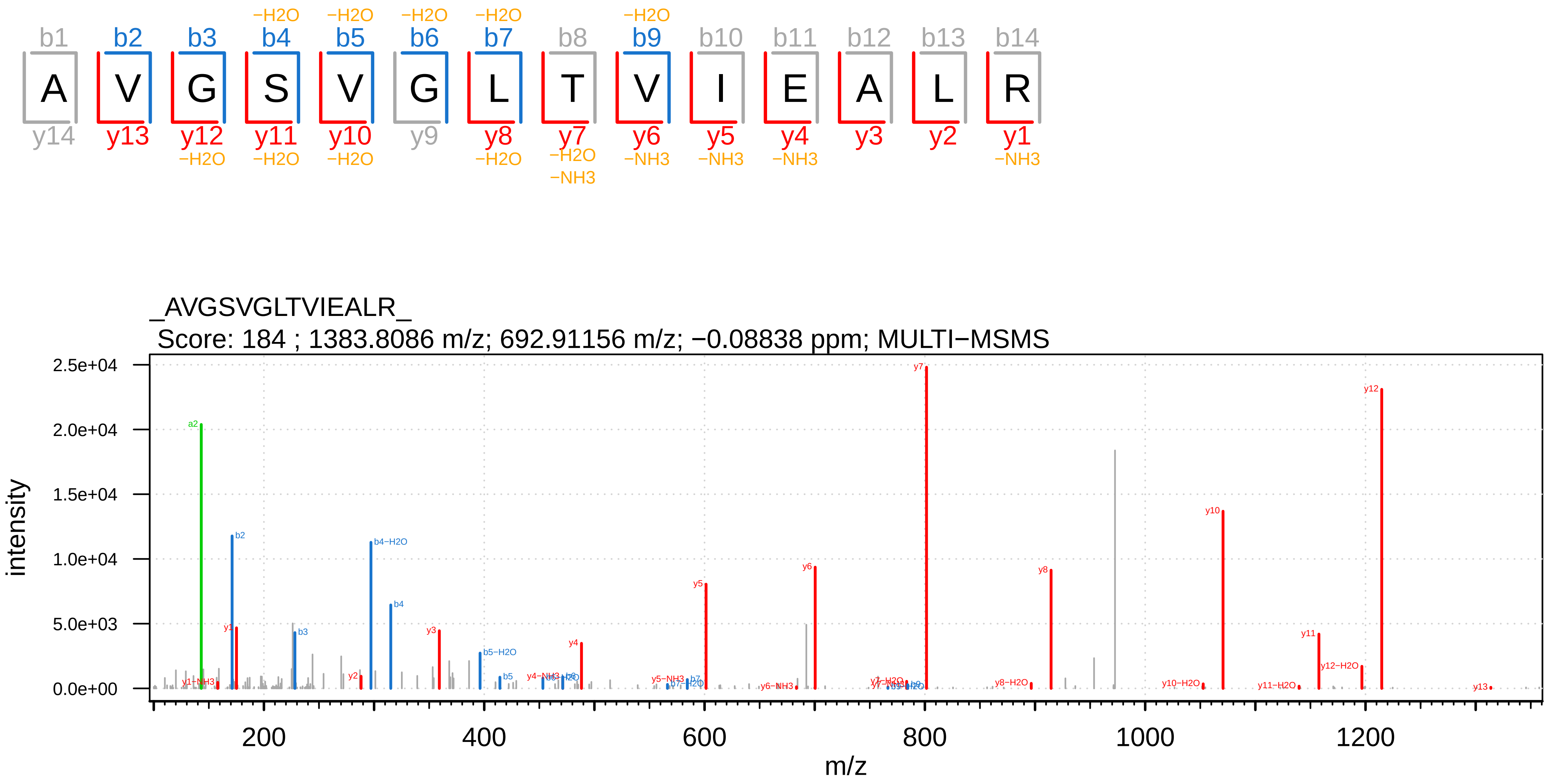

Raw File: ZF_Testis19
Scan Number: 3949
Proteins:
ENSDART00000115742_chr12:49033803−49035390:−

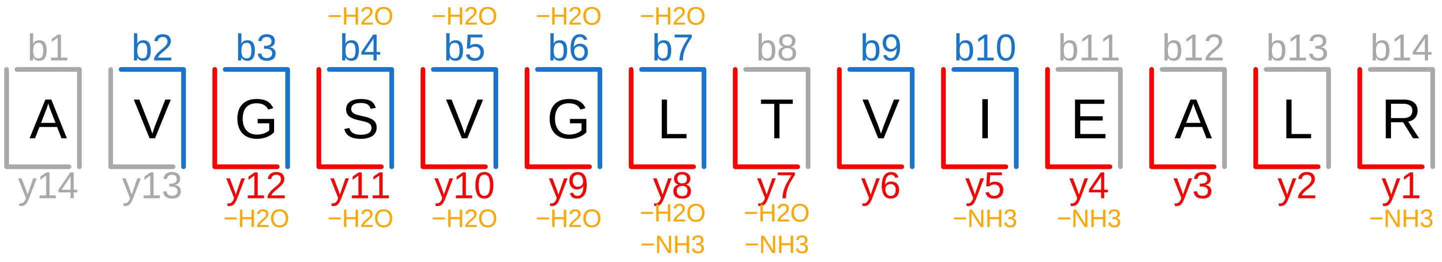

AVGSVGLTVIEALR
Score: 144 ; 1383.8086 m/z; 692.91156 m/z; −0.18849 ppm; MULTI−MSMS

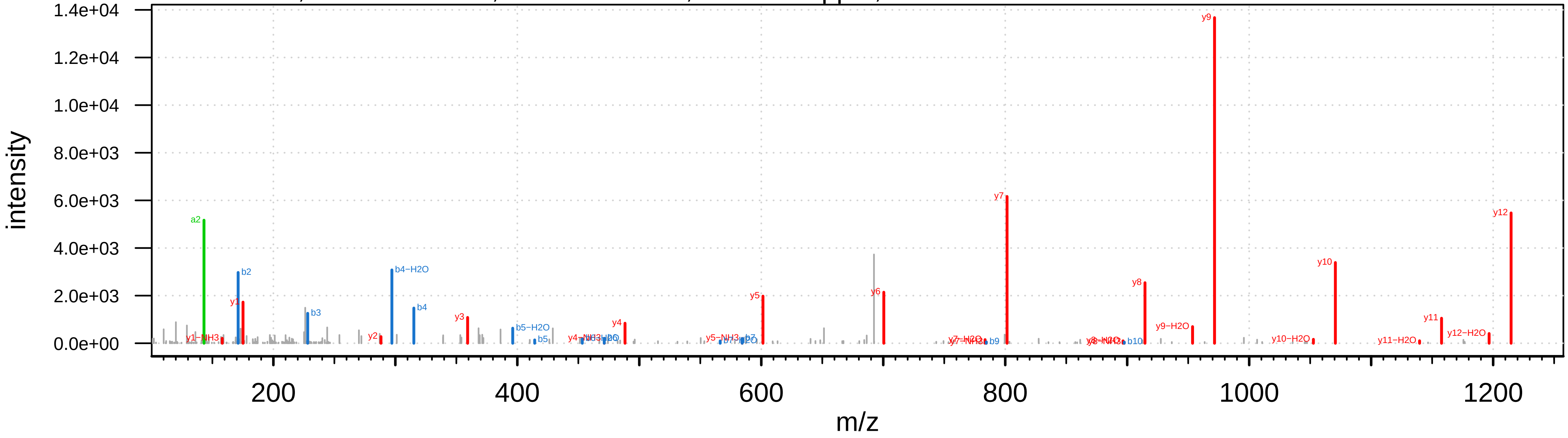

Raw File: ZF_Testis20
Scan Number: 3824
Proteins:
ENSDART00000115742_chr12:49033803−49035390:−

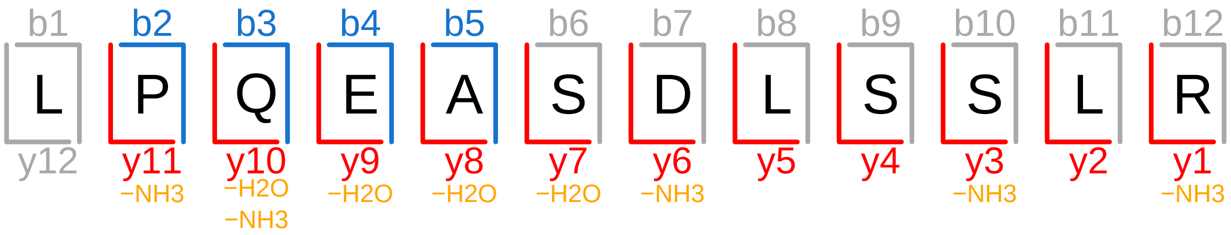

LPQEASDLSSLR

Score: 100 ; 1314.6779 m/z; 658.34625 m/z; 0.12786 ppm; MULTI−MSMS

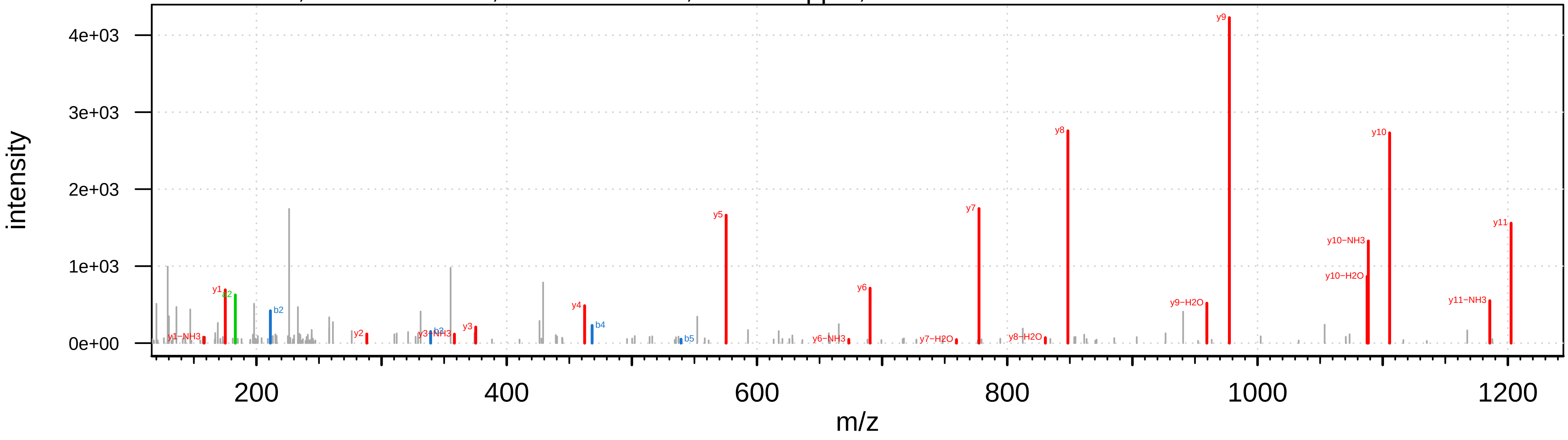

Raw File: ZF_Testis26
Scan Number: 1978
Proteins:
ENSDART00000129489_chr1:644897−645591:+
