# Supplementary material for: Extensive identification and analysis of conserved small ORFs in animals
Source: Genome Biol. 2015 Sep 14;16:179. doi: 10.1186/s13059-015-0742-x (PMC4568590; doi:10.1186/s13059-015-0742-x)
Supplement: Additional file 19: Figure S10. — Spectra for the PMS from the fly datasets. (PDF 70 kb) [file 13059_2015_742_MOESM19_ESM.pdf]

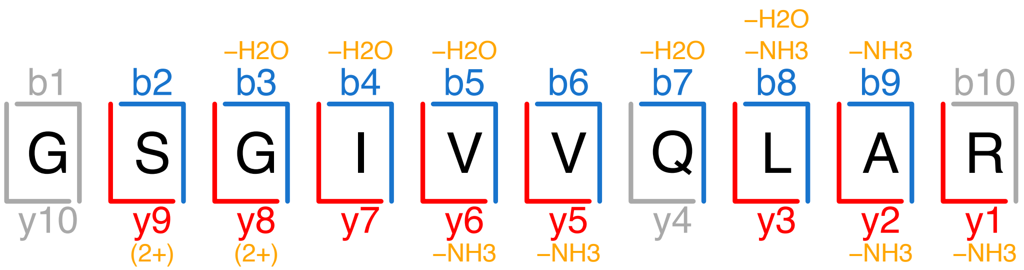

GSGIVVQLAR
Score: 177 ; 998.58728 m/z; 500.30092 m/z; 0.87561 ppm; MULTI−SECPEP

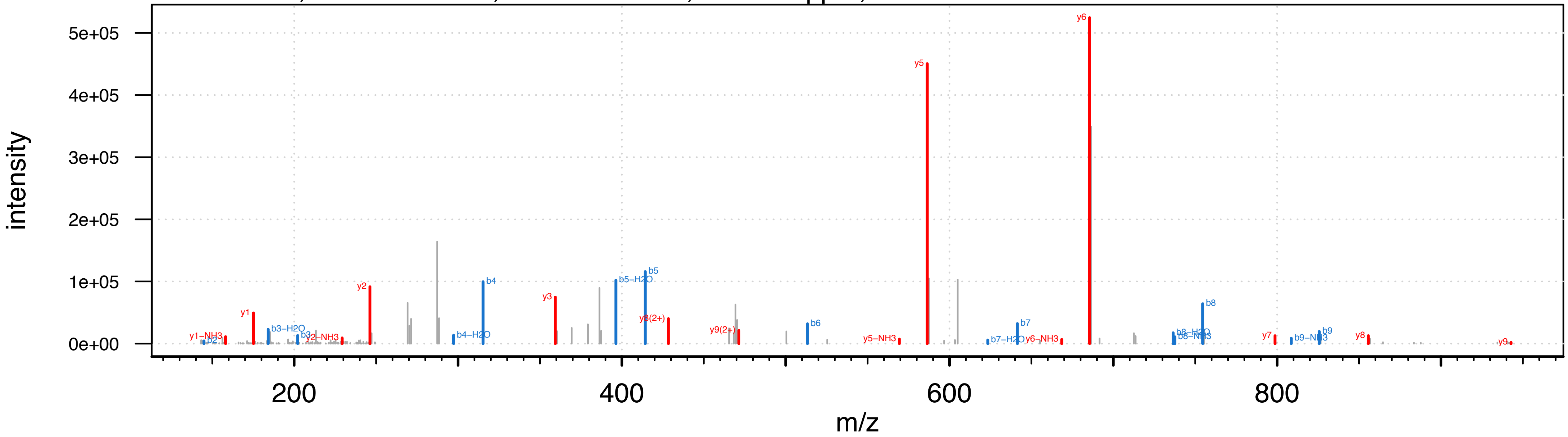

Raw File: 7040
Scan Number: 12711
Proteins:
FBtr0300900_chrX:7787355−7787519:−

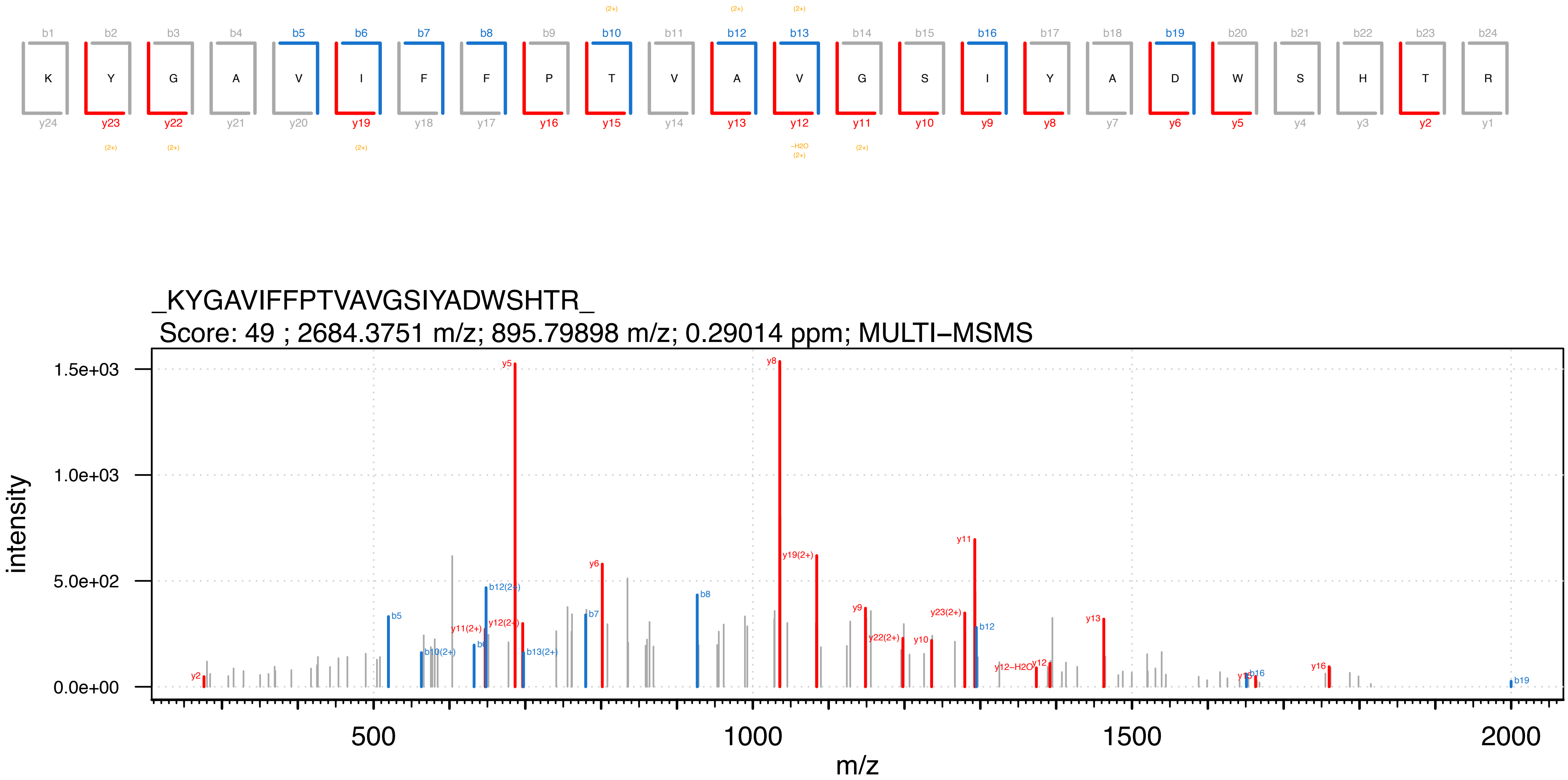

Raw File: 7070
Scan Number: 22287
Proteins:
FBtr0300900_chrX:7787355–7787519:–

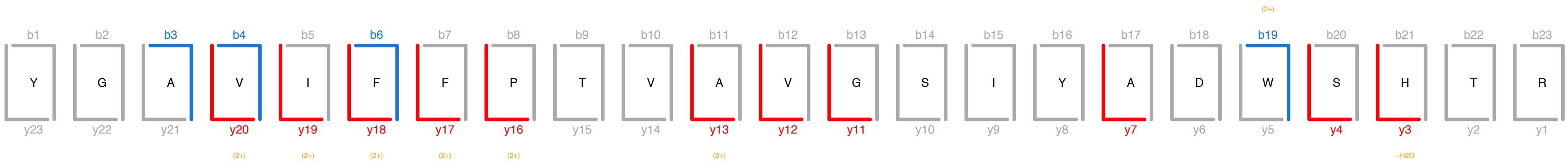

YGAVIFFPTVAVGSIYADWSHTR

Score: 43 ; 2556.2801 m/z; 853.10066 m/z; 0.12843 ppm; MULTI–MSMS

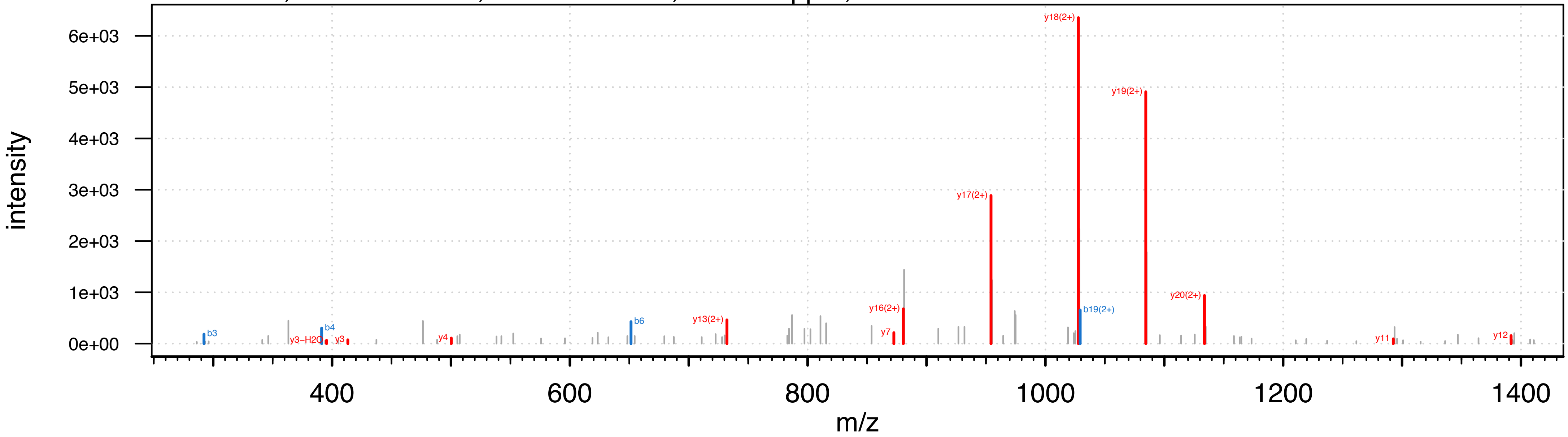

Raw File: 7070
Scan Number: 24382
Proteins:
FBtr0300900_chrX:7787355–7787519:–

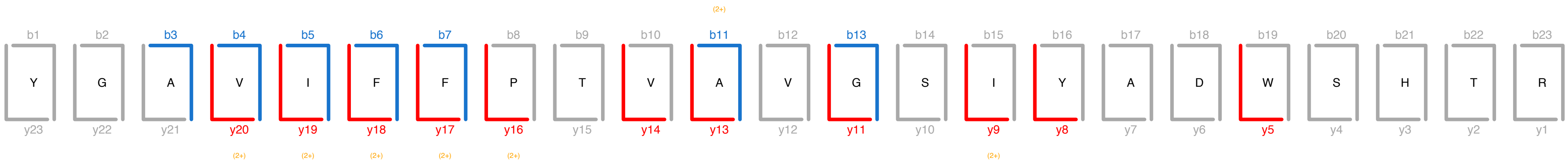

YGAVIFFPTVAVGSIYADWSHTR

Score: 43 ; 2556.2801 m/z; 853.10066 m/z; 0.12843 ppm; MULTI–MSMS

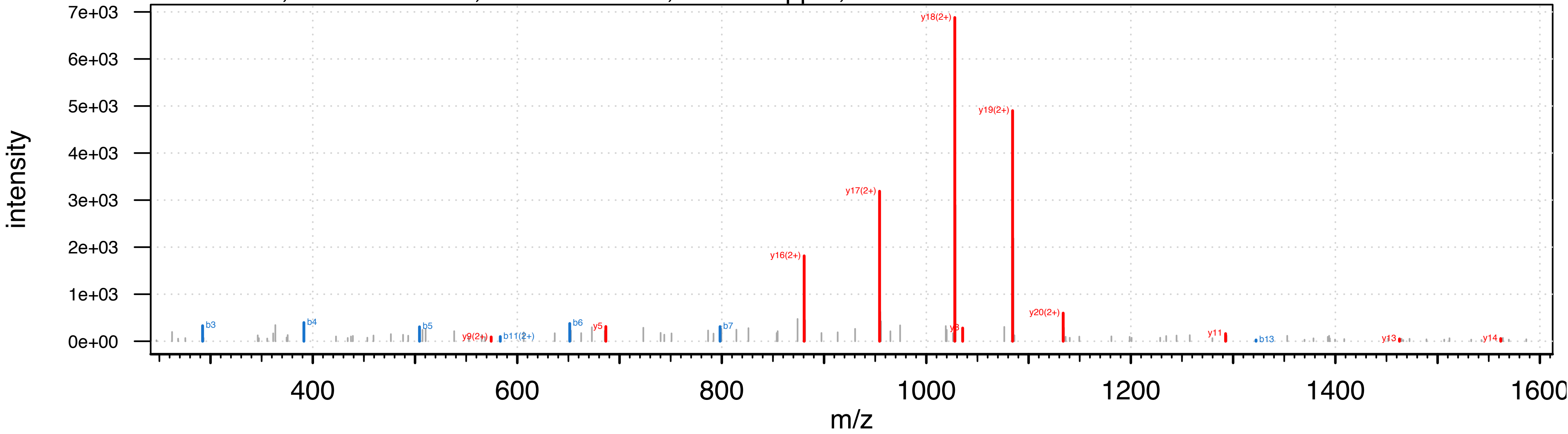

Raw File: 7070

Scan Number: 24414

Proteins:

FBtr0300900_chrX:7787355–7787519:–

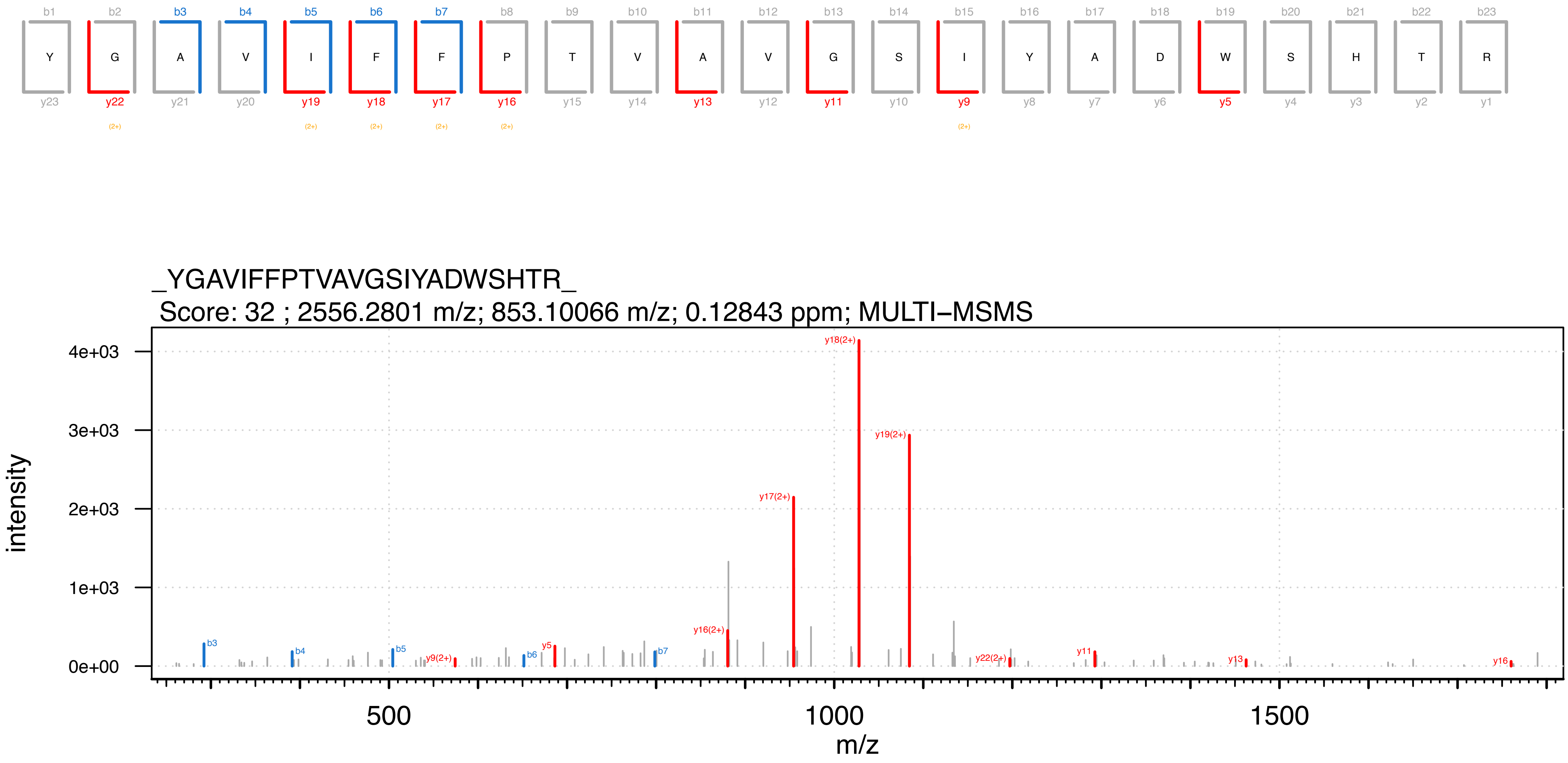

Raw File: 7070
Scan Number: 24443
Proteins:
FBtr0300900_chrX:7787355−7787519:−

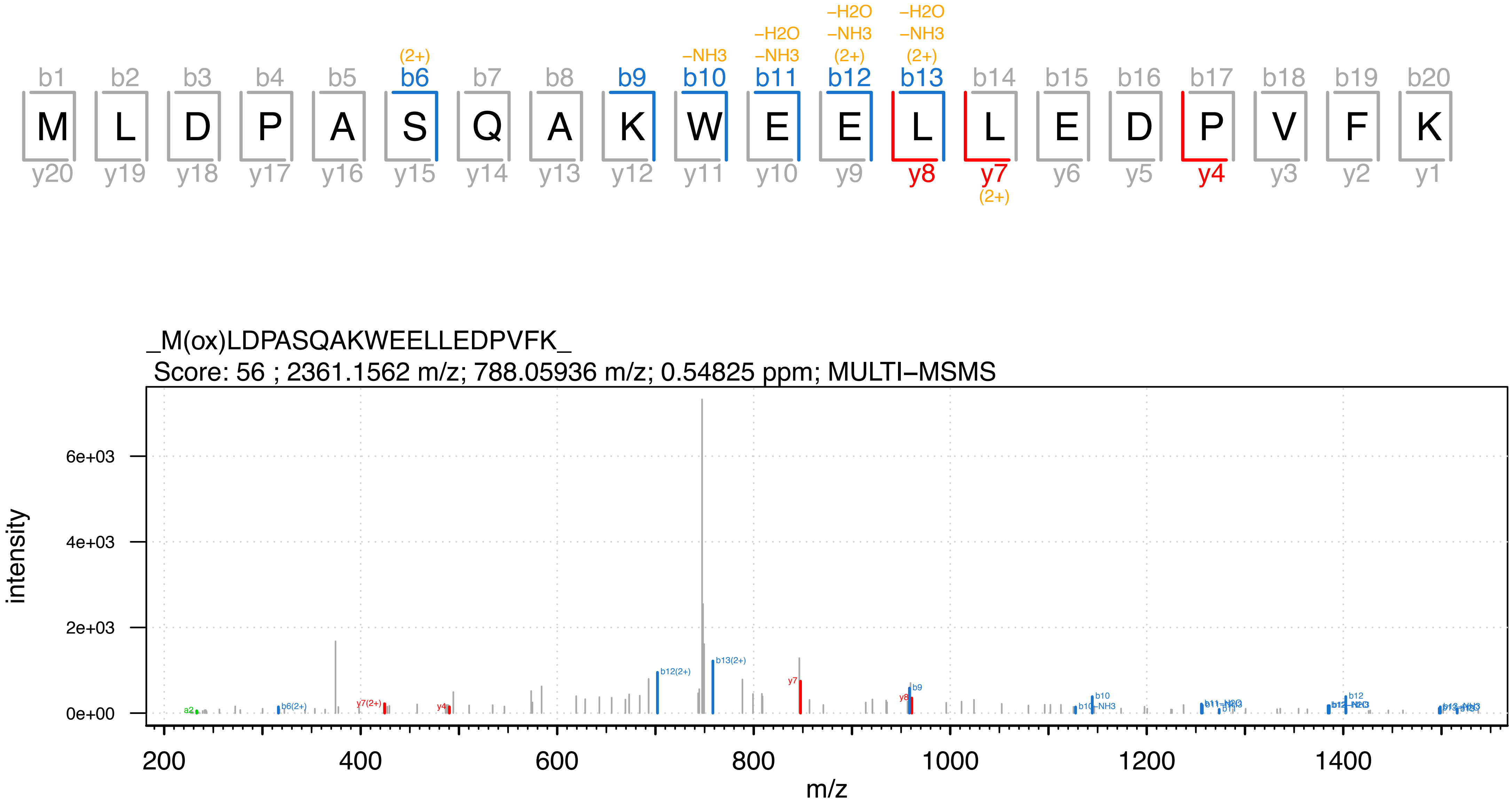

Raw File: 7080
Scan Number: 24063
Proteins:
FBtr0331968_chr2R:1552984−1553094:+

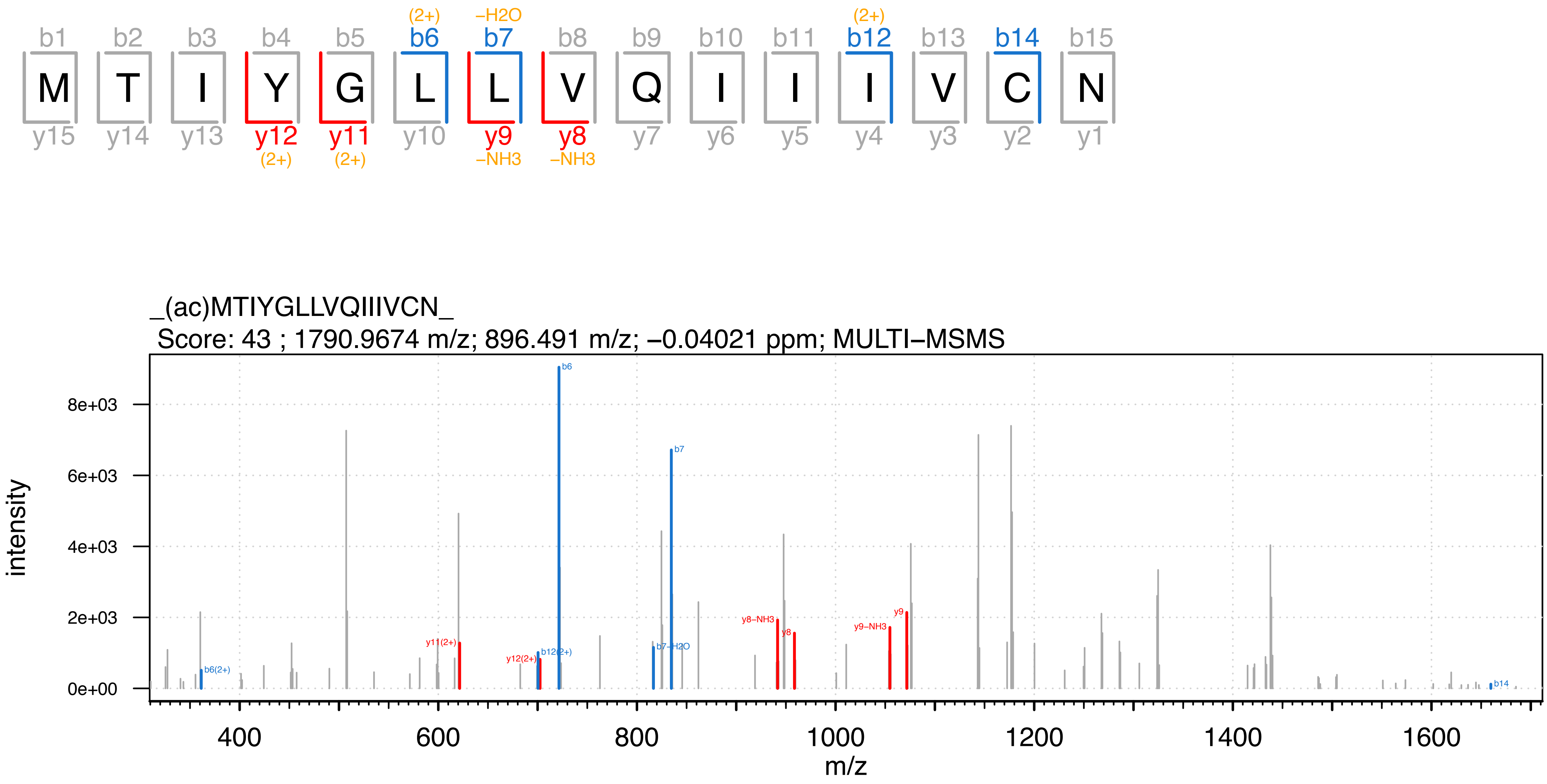

Raw File: 7011b
Scan Number: 31852
Proteins:
FBtr0091949_chrX:19068677–19068724:–
