# Supplementary material for: Extensive identification and analysis of conserved small ORFs in animals
Source: Genome Biol. 2015 Sep 14;16:179. doi: 10.1186/s13059-015-0742-x (PMC4568590; doi:10.1186/s13059-015-0742-x)
Supplement: Additional file 20: Figure S11. — Spectra for the PMS from the worm datasets. (PDF 65 kb) [file 13059_2015_742_MOESM20_ESM.pdf]

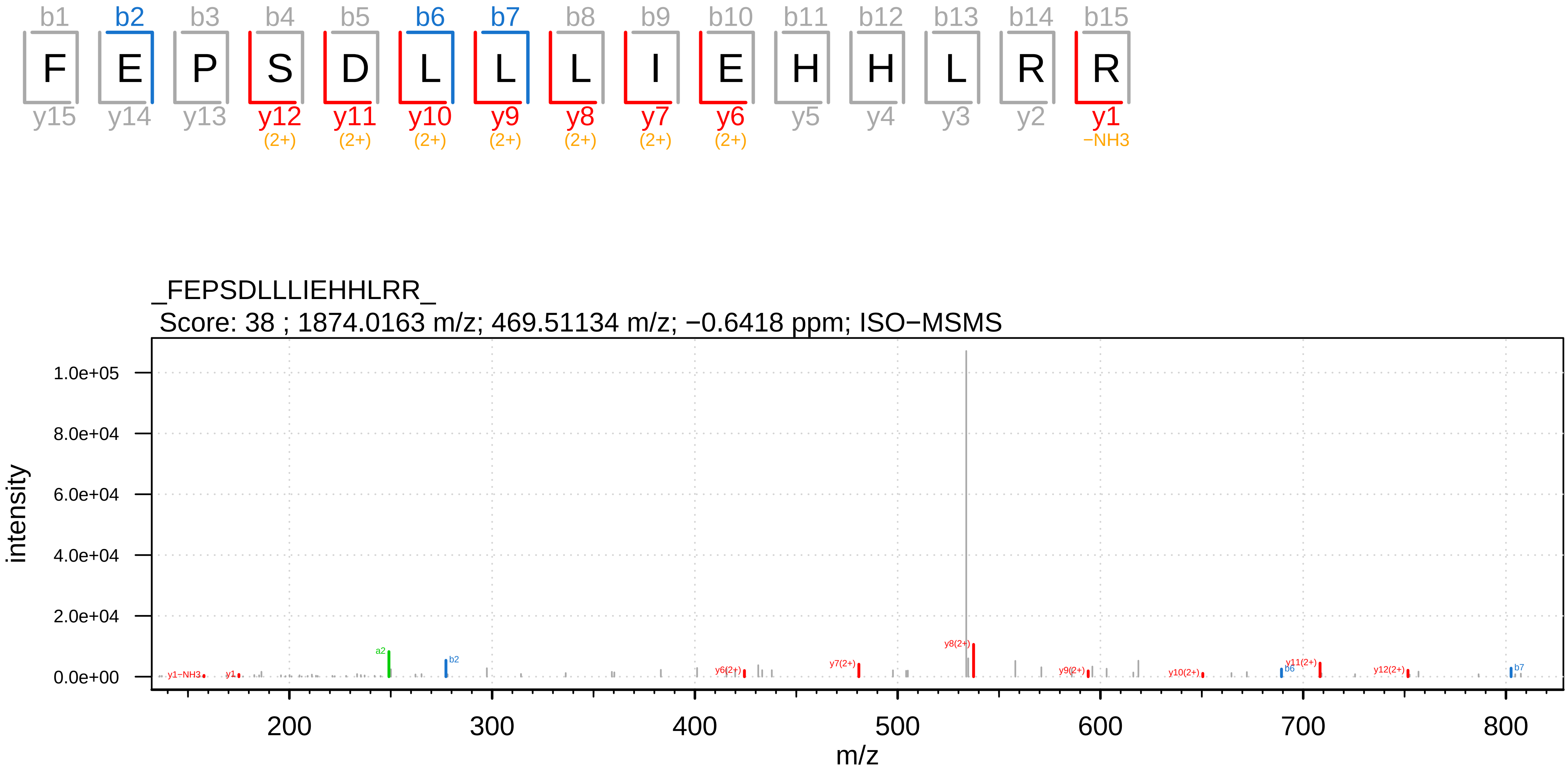

Raw File: Tiffy_20100416_mk_ce_trypsin_ref_13
Scan Number: 26809
Proteins:
AG1003_K08F11.3.T1_chrIV:4710473−4710730:−

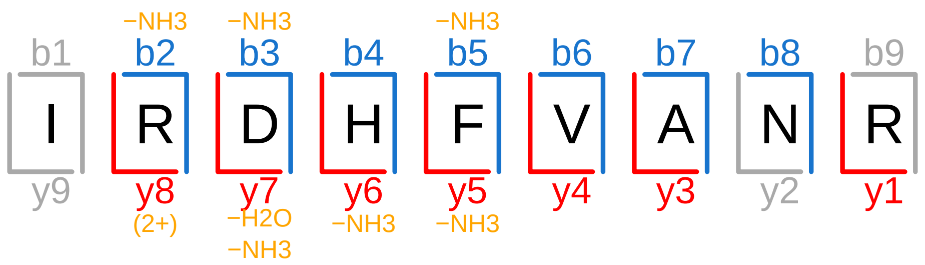

IRDHFVANR
Score: 110 ; 1126.5996 m/z; 564.30706 m/z; 0.32836 ppm; ISO−MSMS

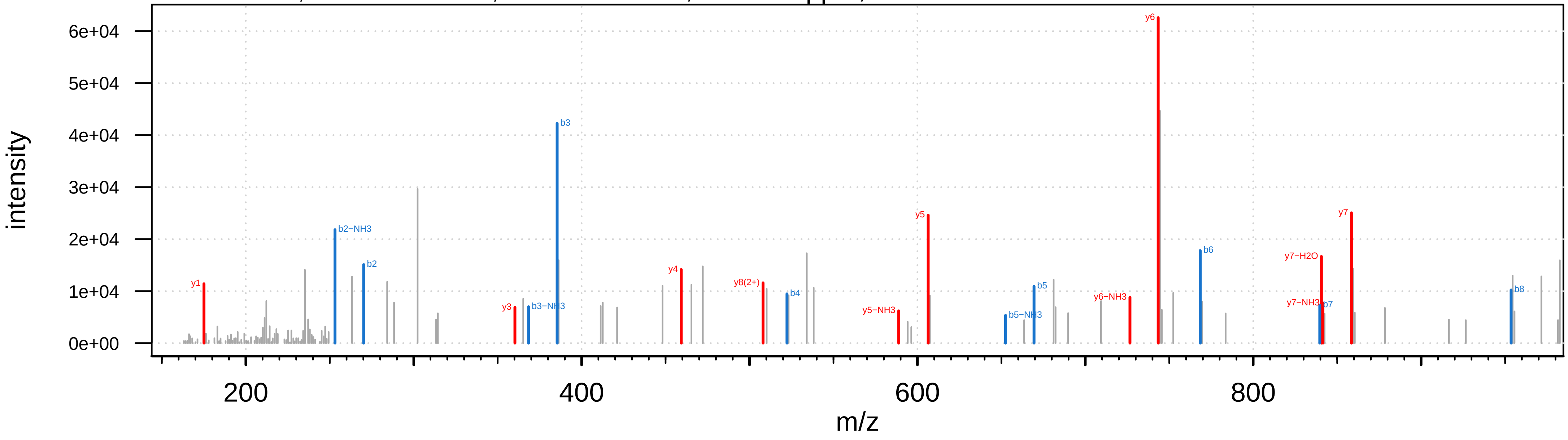

Raw File: Tiffy_20100416_mk_ce_trypsin_ref_13
Scan Number: 6685
Proteins:
F53G2.7.1_chrII:2478322−2479077:−

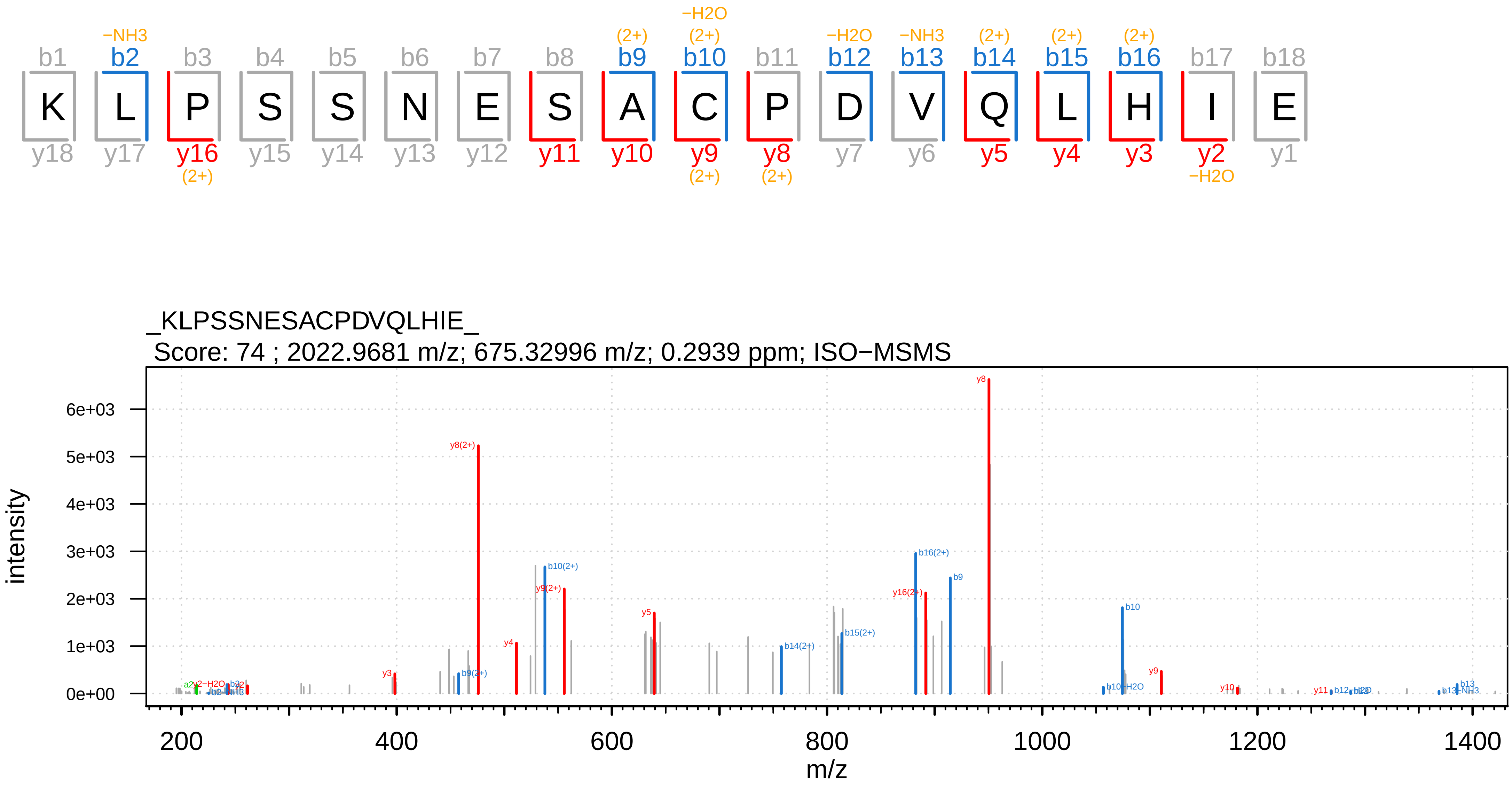

Raw File: Tiffy_20091028_MK_ce_embryo_a_16
Scan Number: 23207
Proteins:
AG1003_T23F2.1.T15_chrX:5493071−5493378:+

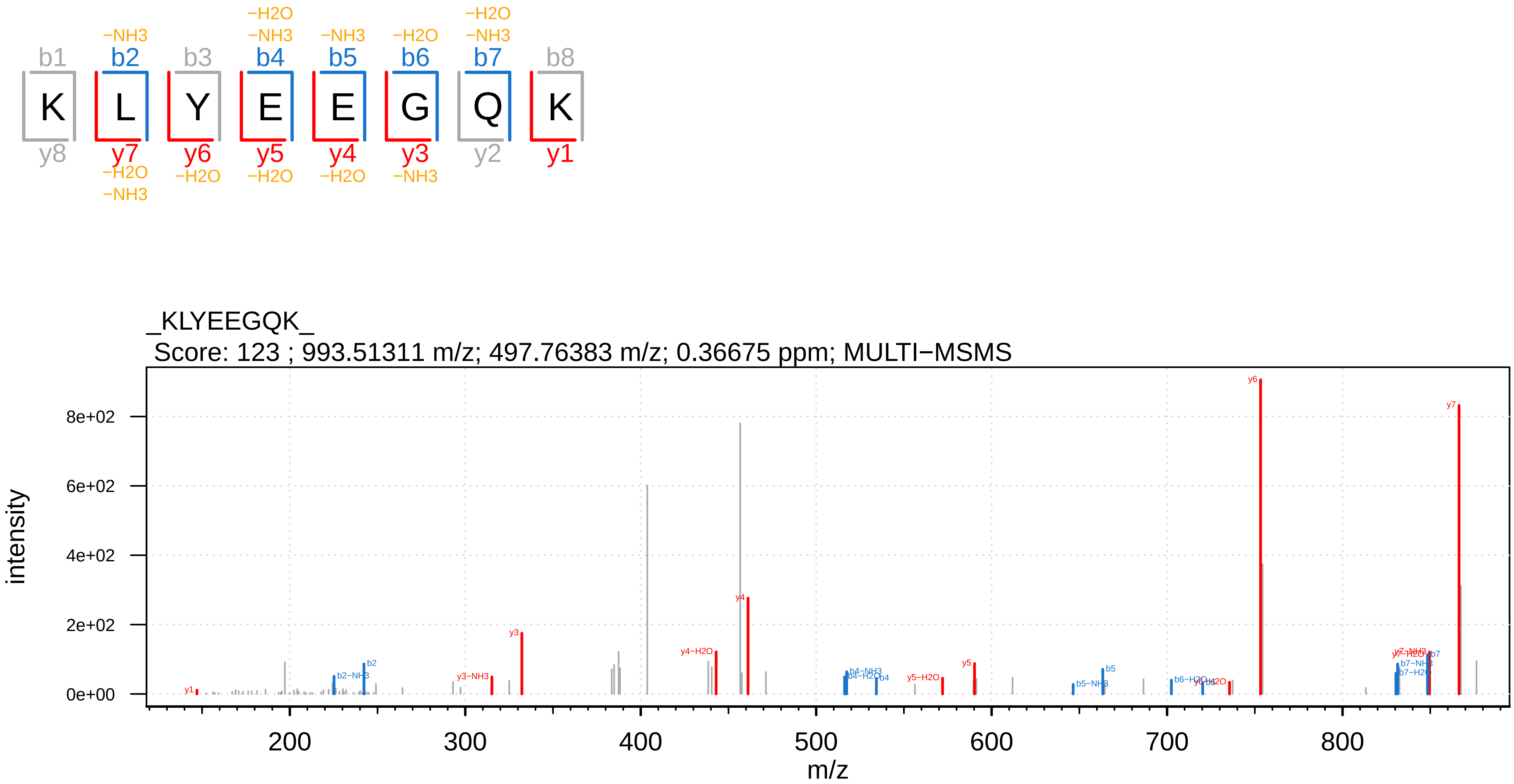

Raw File: Ernie_20100902_mk_ce_ref_e_re_13a
Scan Number: 1693
Proteins:
F53G2.7.1_chrII:2478322−2479077:−

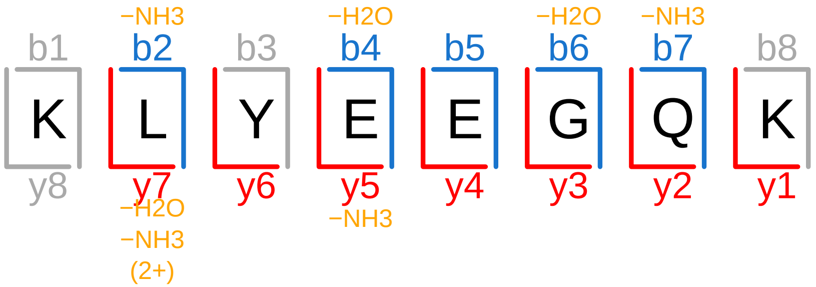

KLYEEGQK
Score: 109 ; 1009.5415 m/z; 505.77803 m/z; NaN ppm; MSMS

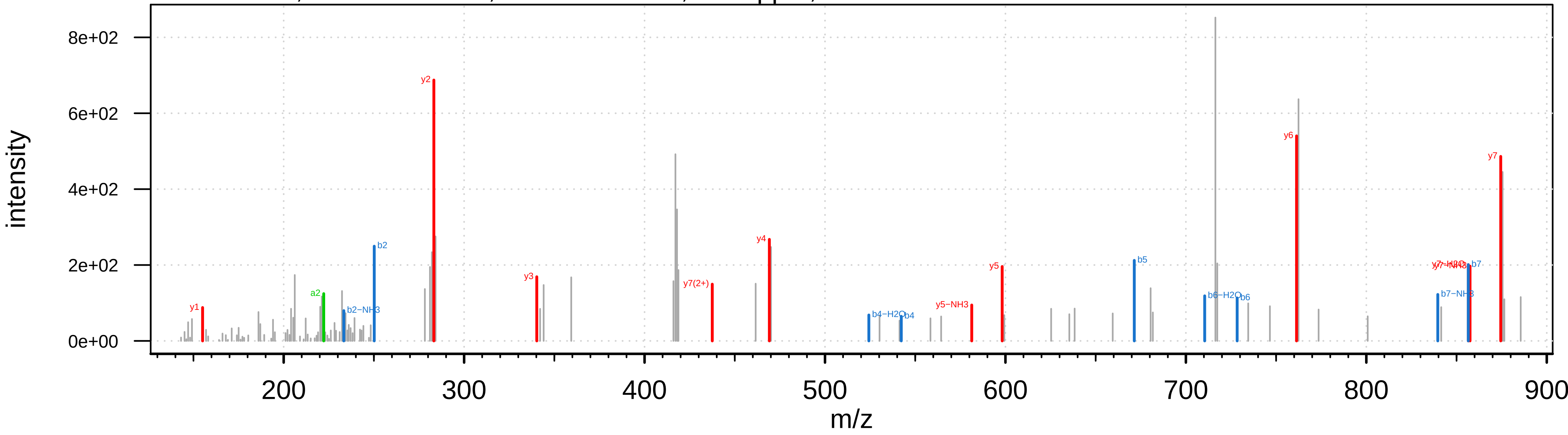

Raw File: Tiffy_20101115_mk_ce_biolrepl_b_embryo_12
Scan Number: 3469
Proteins:
F53G2.7.1_chrII:2478322−2479077:−

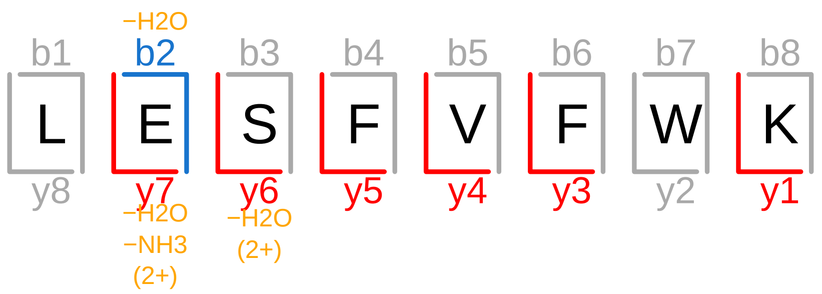

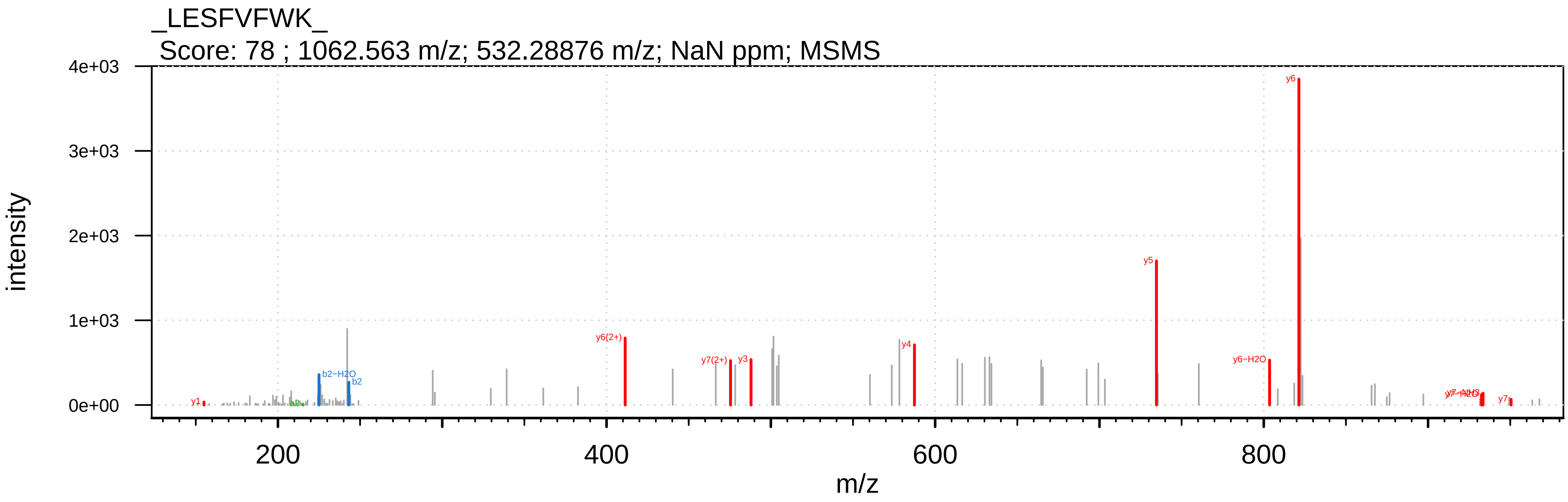

Raw File: Tiffy_20091028_MK_ce_embryo_a_13
Scan Number: 23632
Proteins:
AG1003_RIT1M.332.T1_chrI:12708133−12708494:−

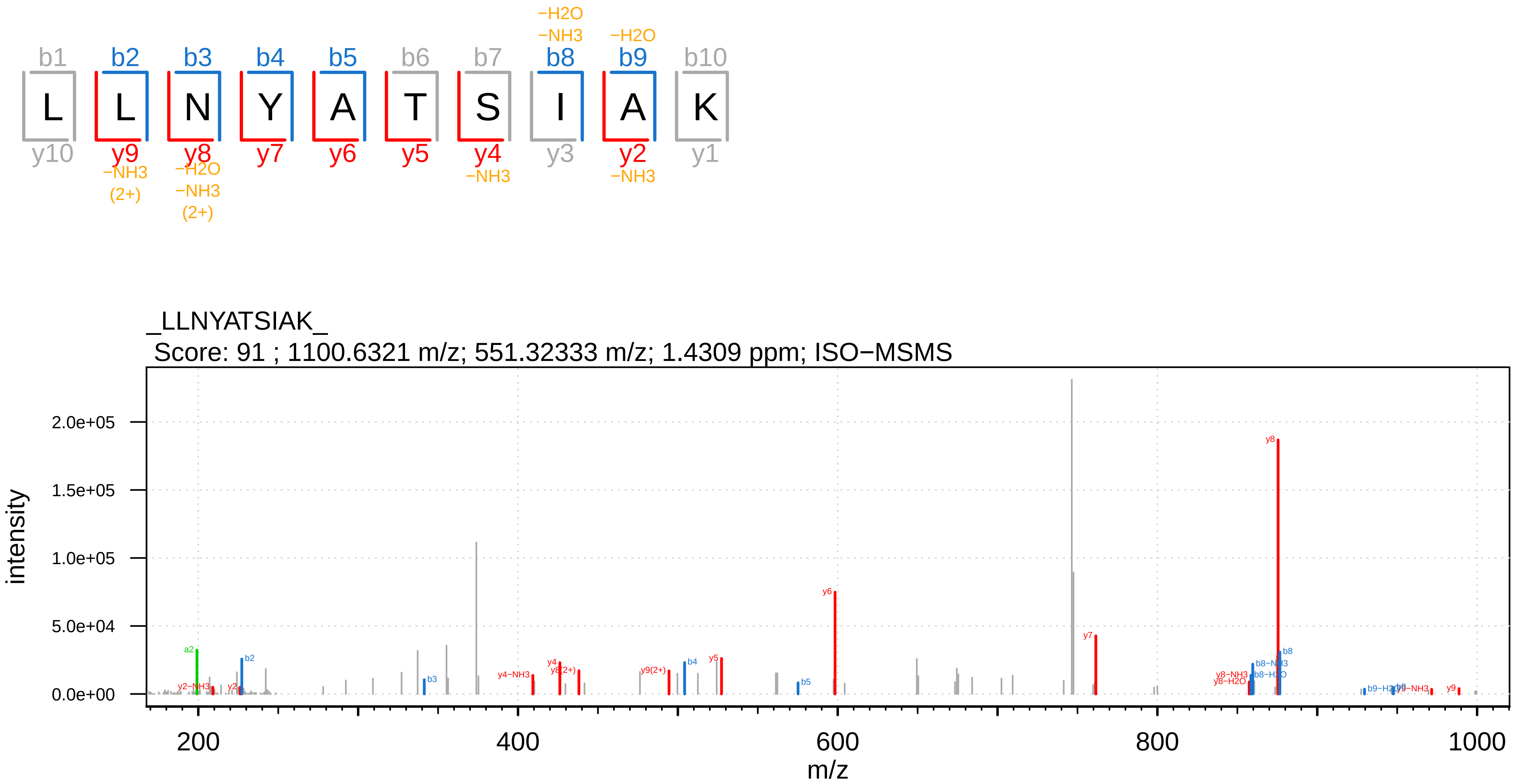

Raw File: Tiffy_20100416_mk_ce_trypsin_ref_14
Scan Number: 13863
Proteins:
AG1003_C34E10.10.T2_chrIII:5227661−5227840:−

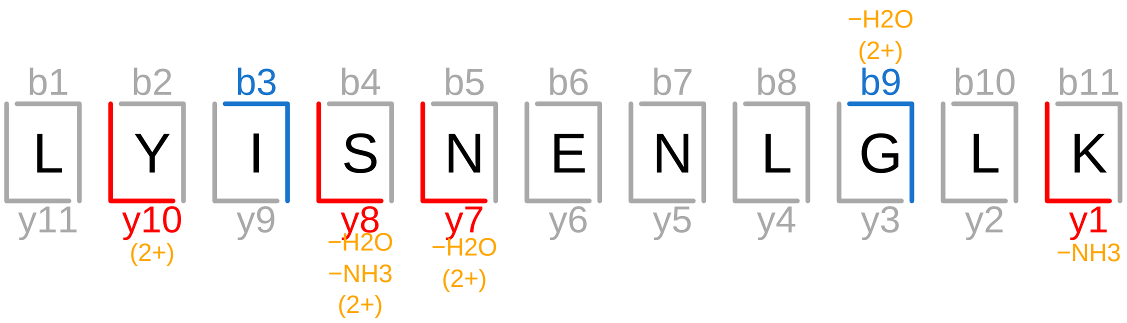

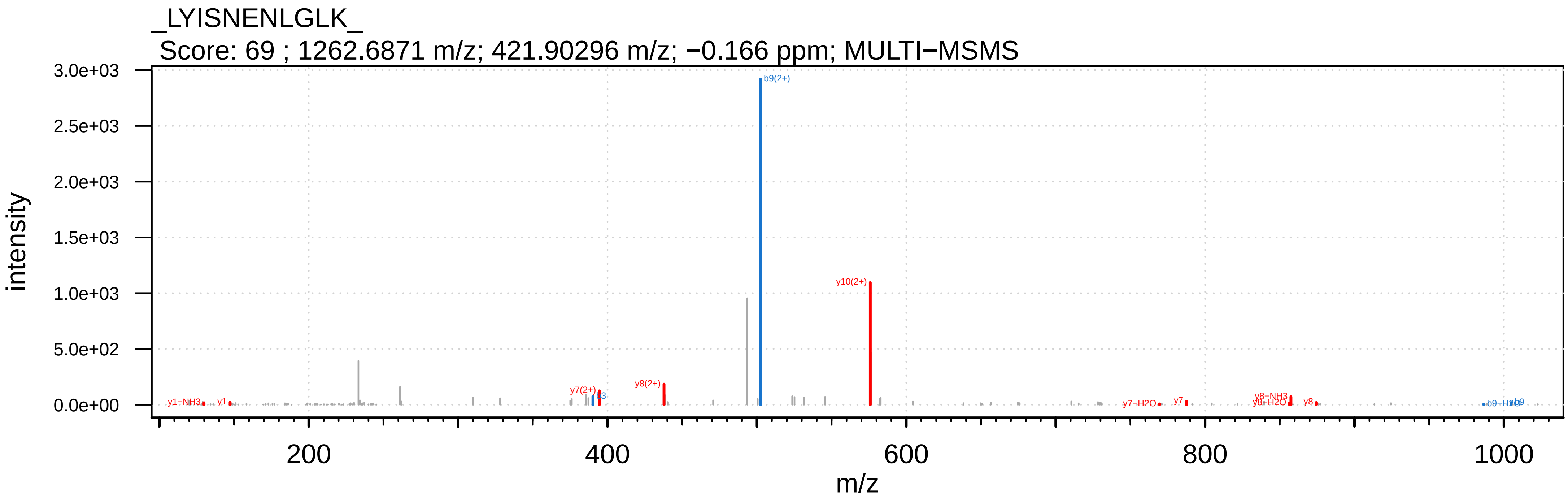

Raw File: Bert_20091026_MK_ce_embryo_a_05a
Scan Number: 13154
Proteins:
AG1003_F10G7.6.T1_chrII:4691683−4691923:+

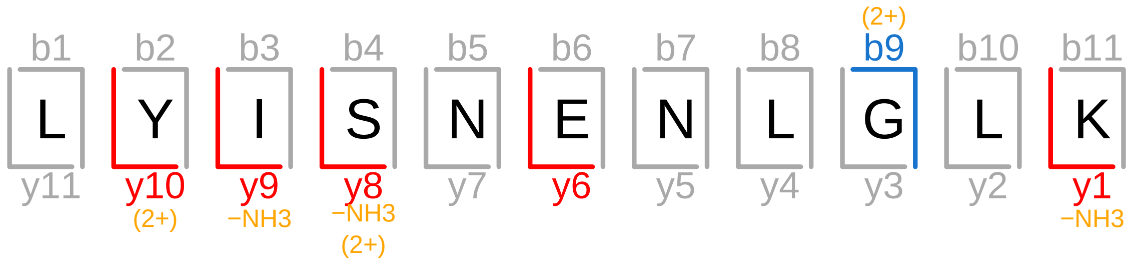

LYISNENLGLK
Score: 49 ; 1262.6871 m/z; 421.90296 m/z; −0.22825 ppm; MULTI−MSMS

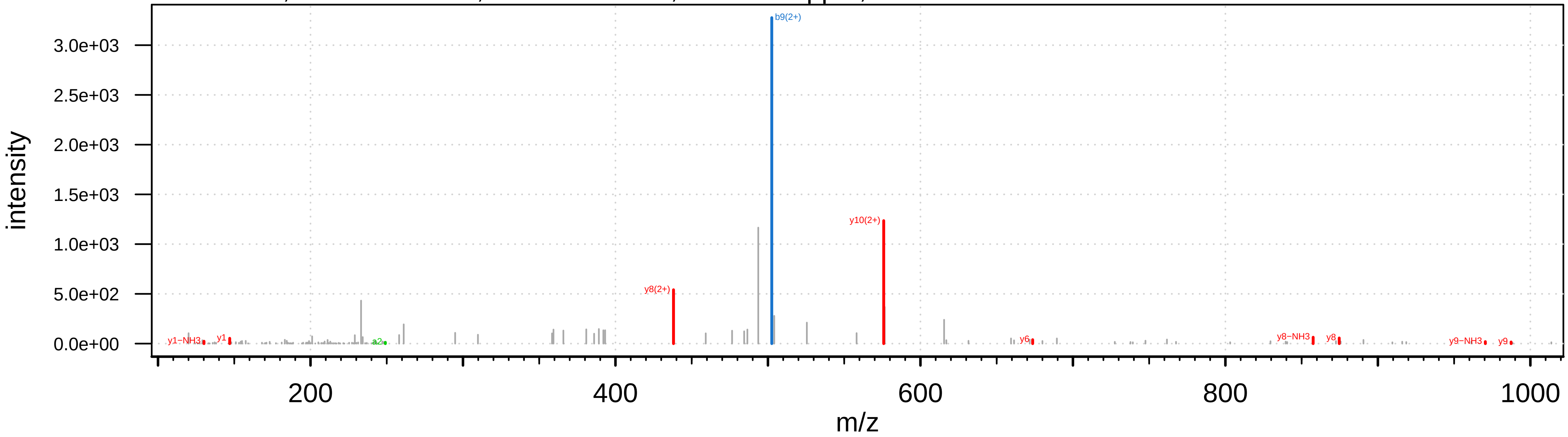

Raw File: Bert_20091026_MK_ce_embryo_a_09a
Scan Number: 14200
Proteins:
AG1003_F10G7.6.T1_chrII:4691683−4691923:+

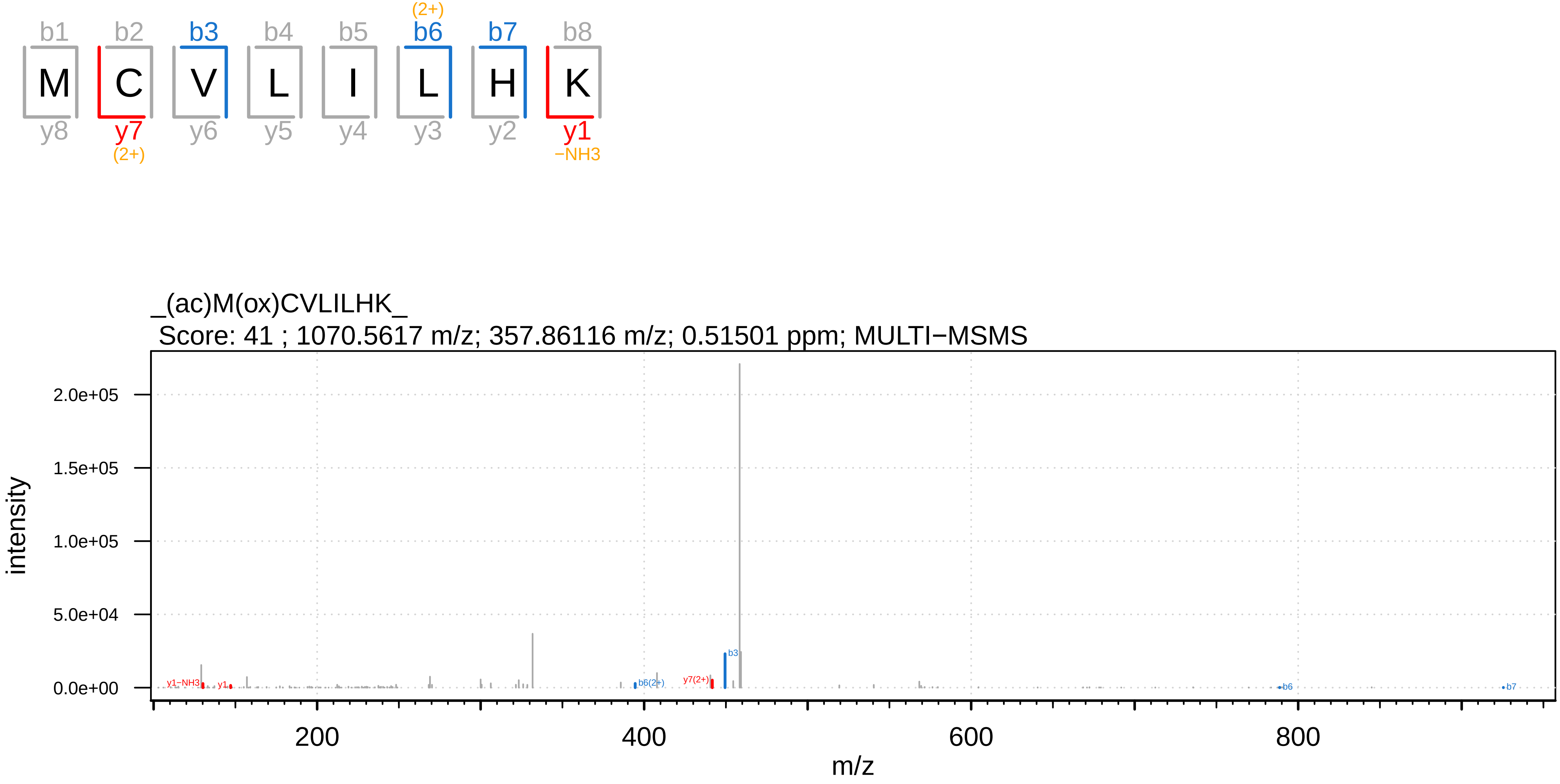

Raw File: Tiffy_20091104_MK_L1_a_13
Scan Number: 8227
Proteins:
AG1003_F48C5.1.T2_chrX:11444295−11444321:−

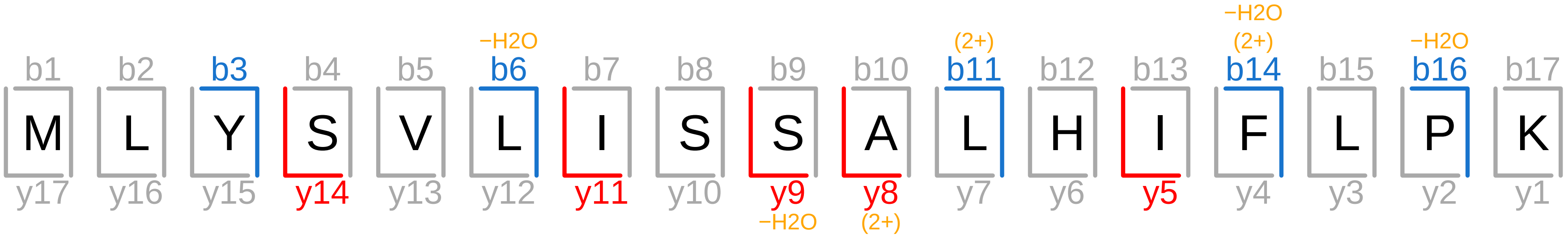

MLYSVLISSALHIFLPK
Score: 37 ; 1931.0954 m/z; 966.55499 m/z; 0.05538 ppm; MULTI−MSMS

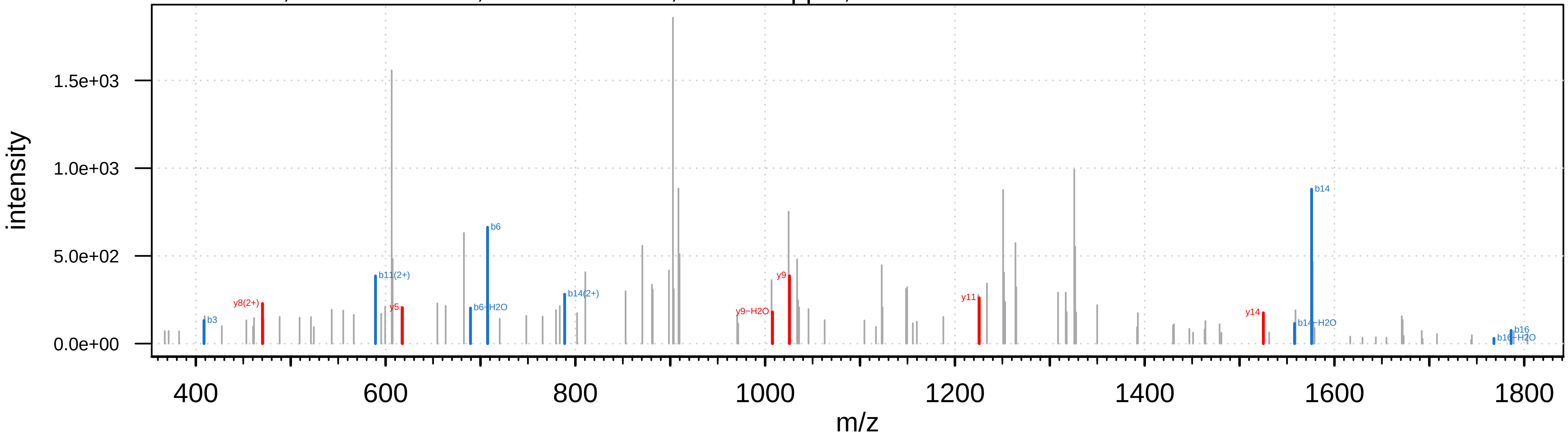

Raw File: Tiffy_20101115_mk_ce_EL1_8_101121140708
Scan Number: 10427
Proteins:
AG1003_K06A1.3.T1_chrII:6443651−6443719:+

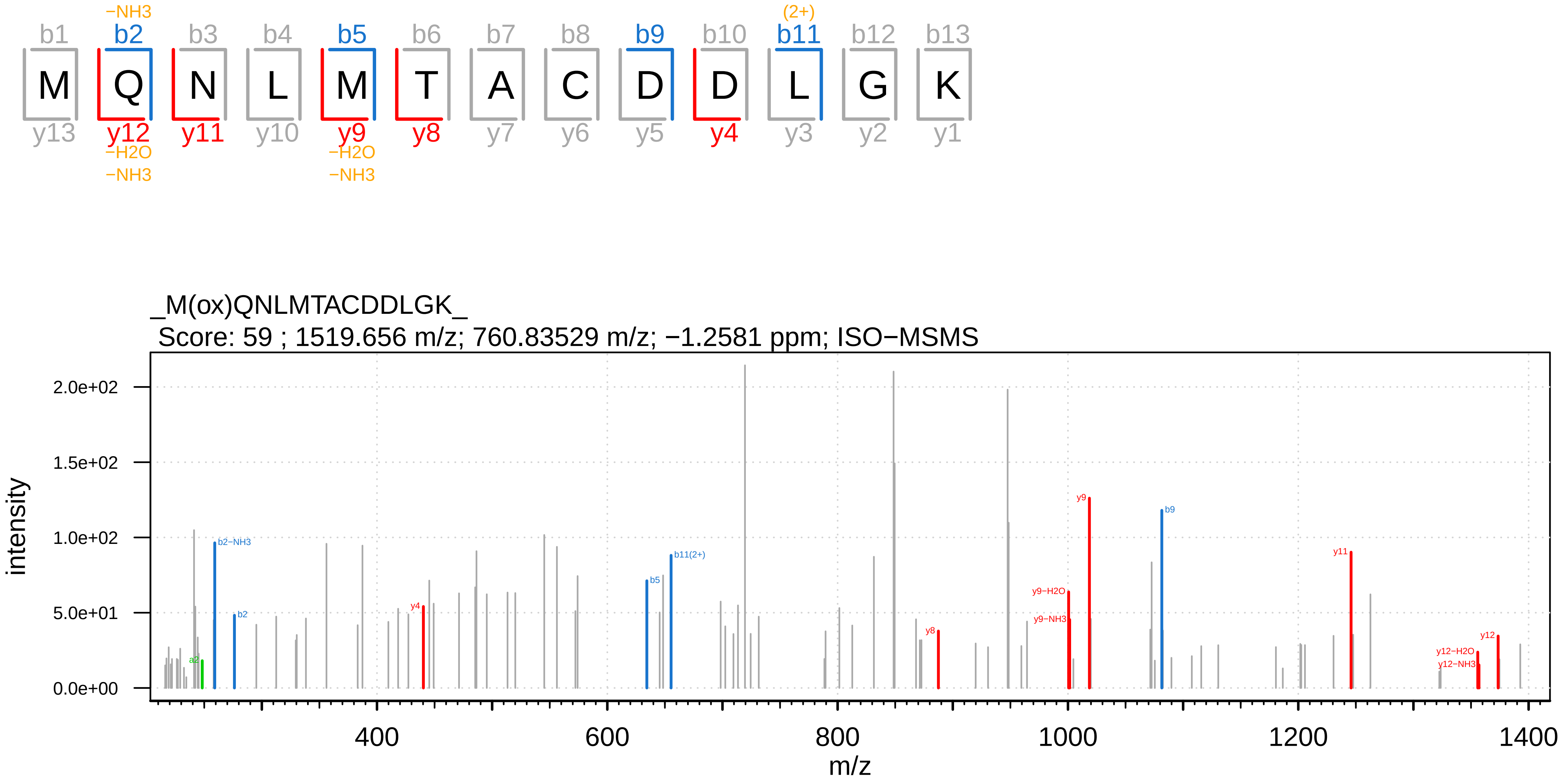

Raw File: Tiffy_20101115_mk_ce_L1E_3
Scan Number: 7506
Proteins:
AG1003_F15D3.1.T6_chrI:11527067−11527165:−

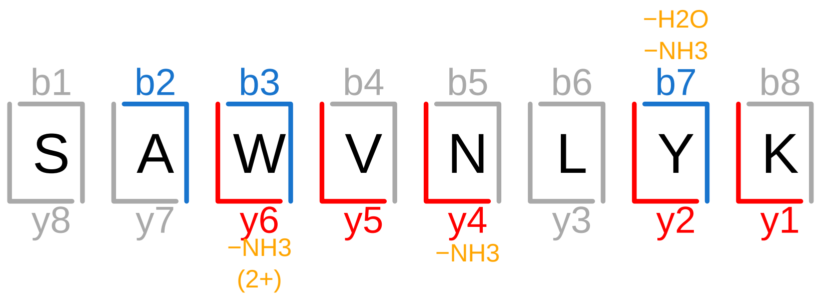

SAWVNLYK
Score: 67 ; 979.51272 m/z; 490.76363 m/z; 0.25179 ppm; MULTI−MSMS

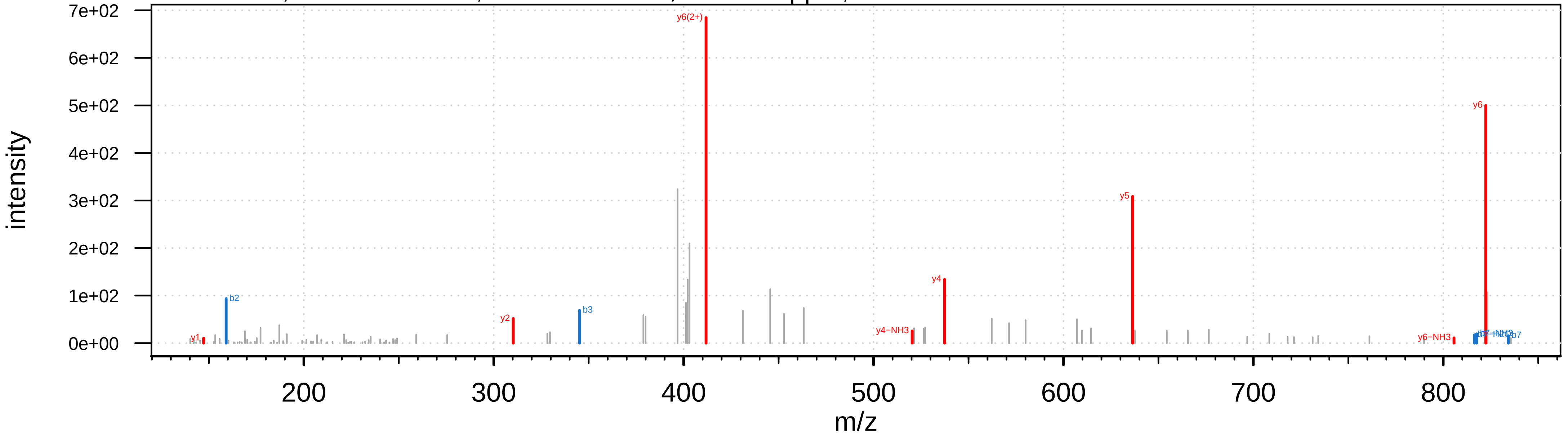

Raw File: Ernie_20100915_MK_ce_l1_repl_12a
Scan Number: 7764
Proteins:
F53G2.7.1_chrII:2478322−2479077:−

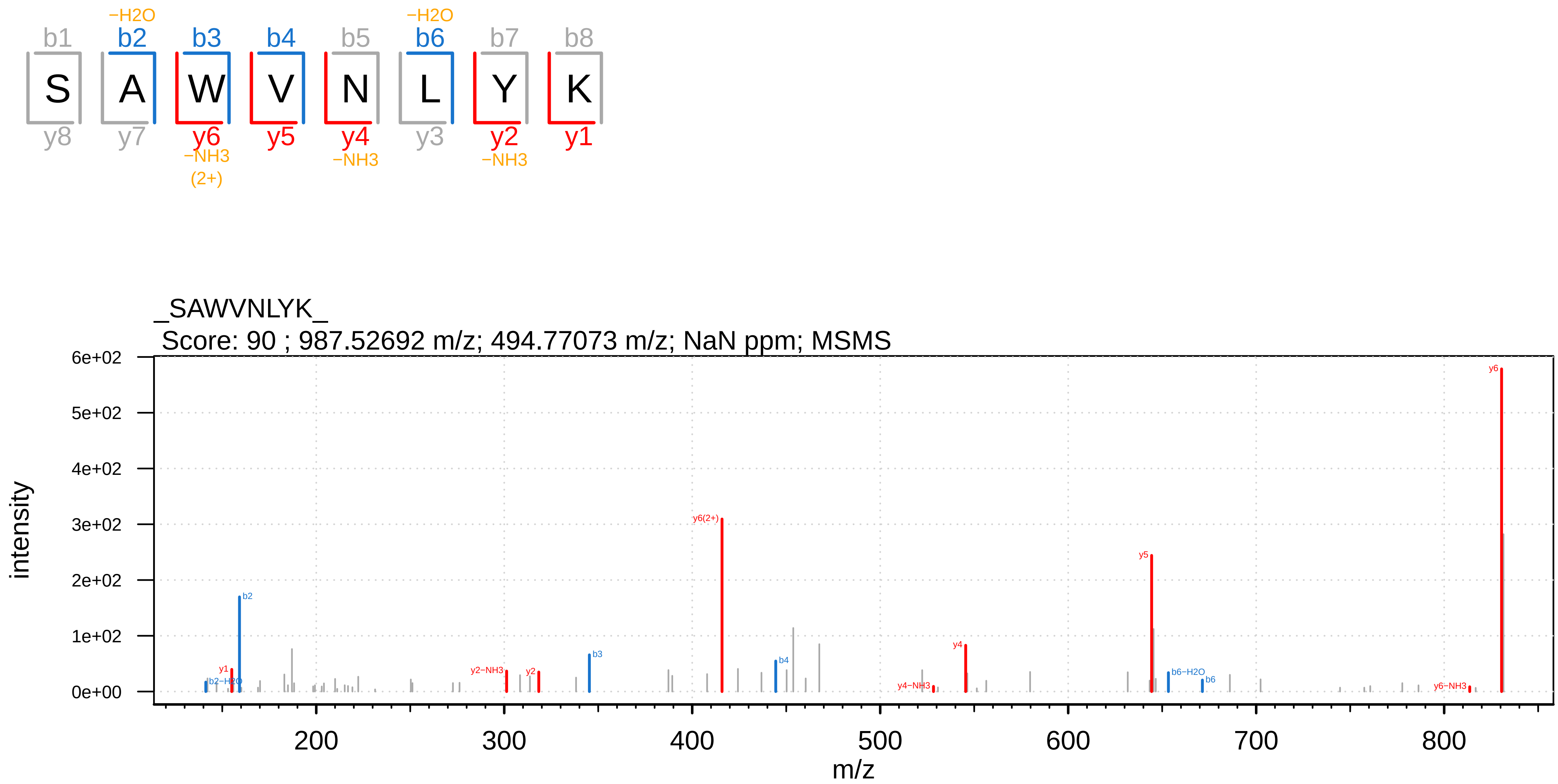

Raw File: Tiffy_20101115_mk_ce_biolrepl_b_embryo_13

Scan Number: 13585

Proteins:

F53G2.7.1_chrII:2478322−2479077:−

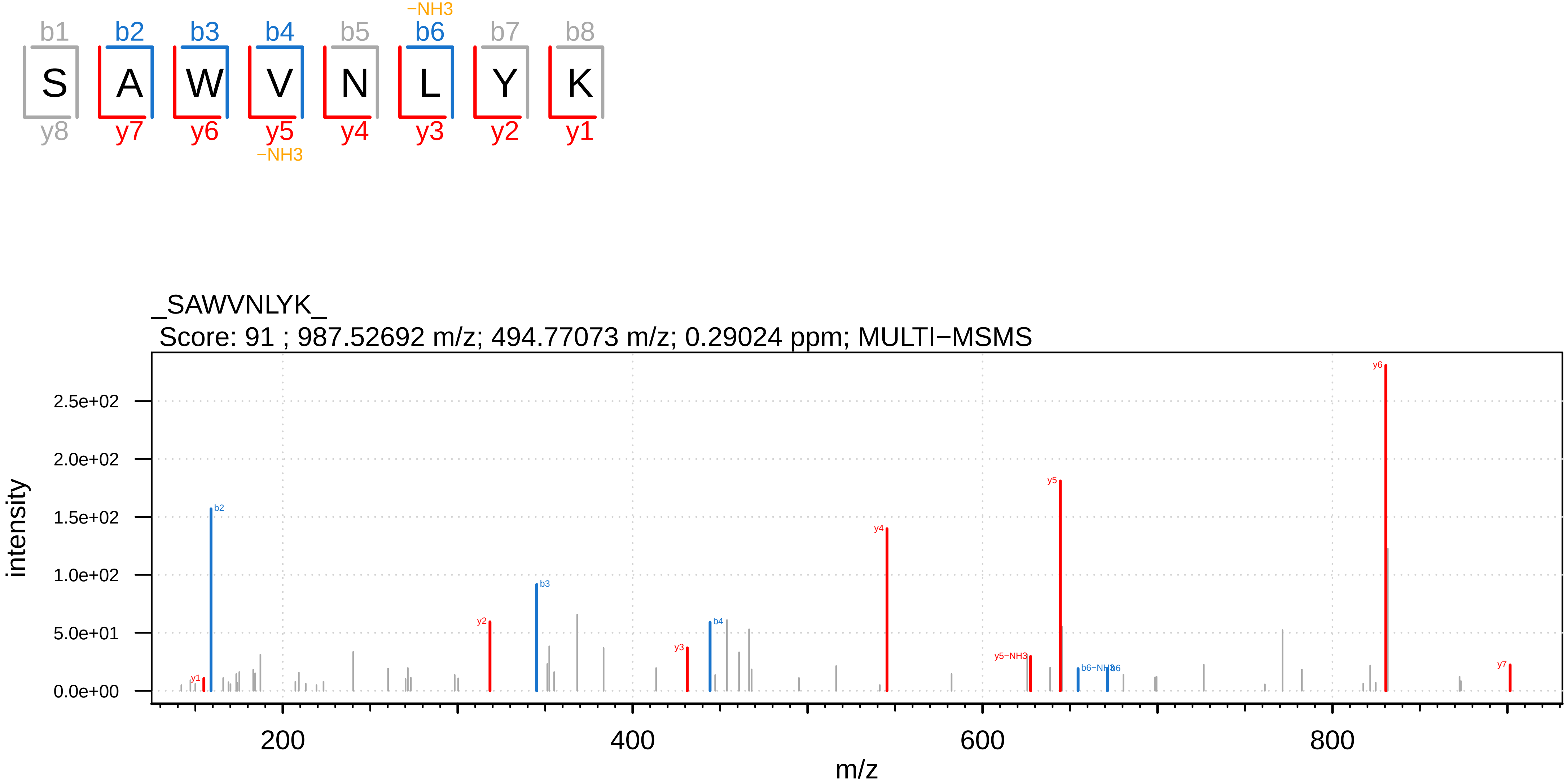

Raw File: Tiffy_20101115_mk_ce_biolrepl_b_l1_12_101201160041
Scan Number: 11031
Proteins:
F53G2.7.1_chrII:2478322−2479077:−

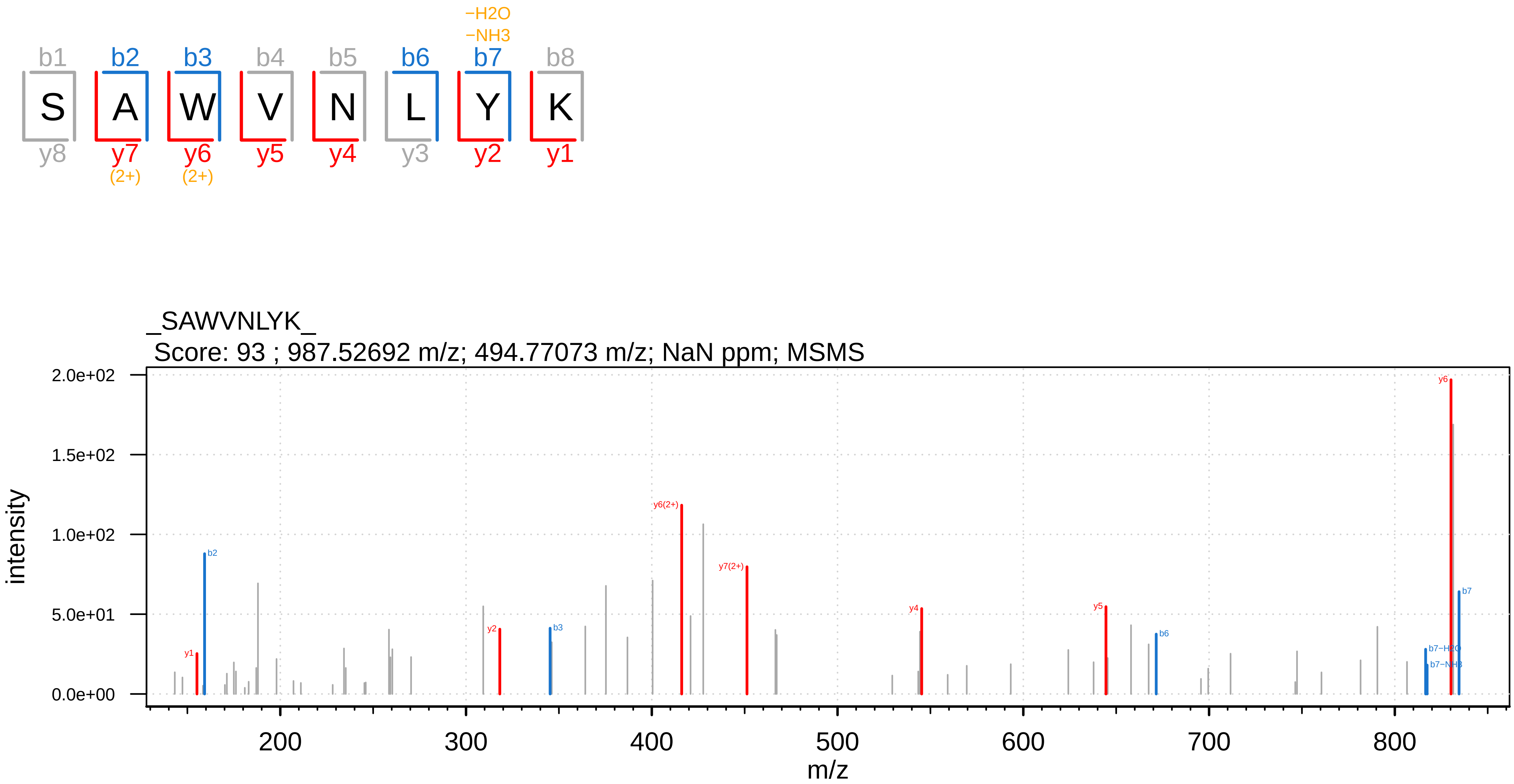

Raw File: Tiffy_20101115_mk_ce_EL1_12
Scan Number: 14258
Proteins:
F53G2.7.1_chrII:2478322−2479077:−
