# Supplementary material for: Extensive identification and analysis of conserved small ORFs in animals
Source: Genome Biol. 2015 Sep 14;16:179. doi: 10.1186/s13059-015-0742-x (PMC4568590; doi:10.1186/s13059-015-0742-x)
Supplement: Additional file 17: Figure S8. — Spectra for the PMS from the mouse datasets. (PDF 3586 kb) [file 13059_2015_742_MOESM17_ESM.pdf]

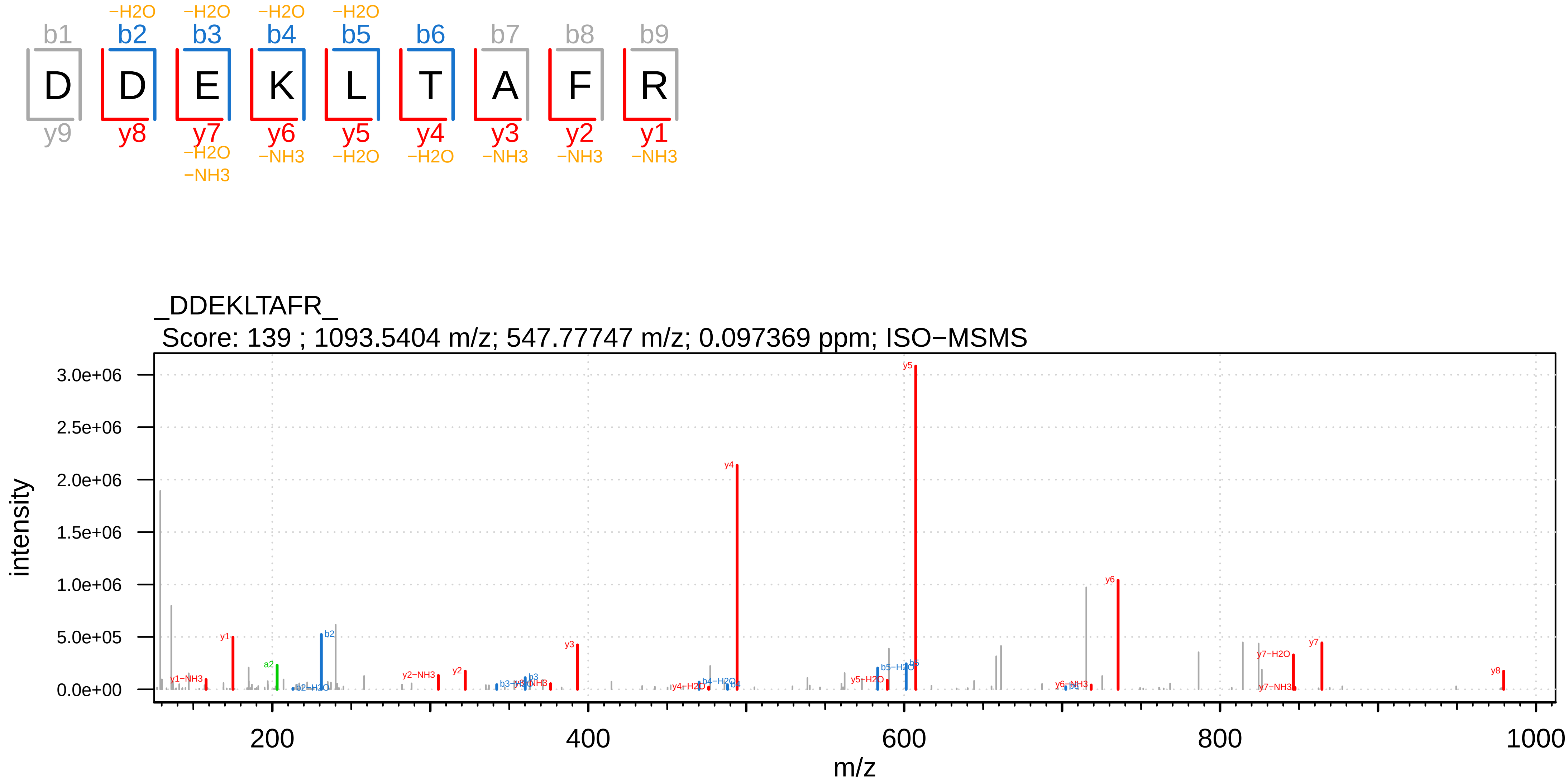

Raw File: Kermit_20111104_MSU_tube_18h_12
Scan Number: 12945
Proteins:
chr12_103206313−103214181.T2_chr12:101968174−101969629:−

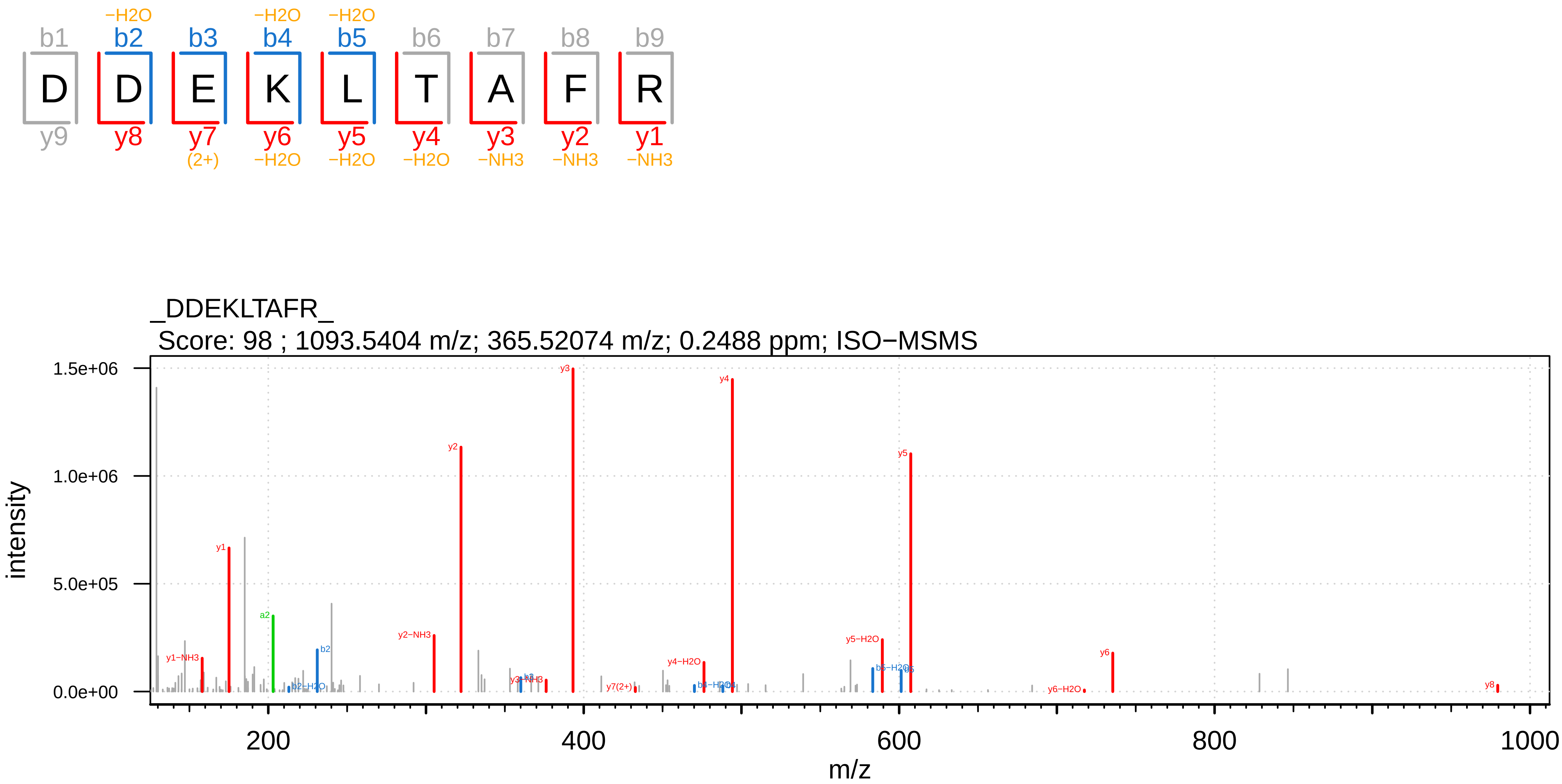

Raw File: Kermit_20111104_MSU_tube_18h_12
Scan Number: 12999
Proteins:
chr12_103206313−103214181.T2_chr12:101968174−101969629:−

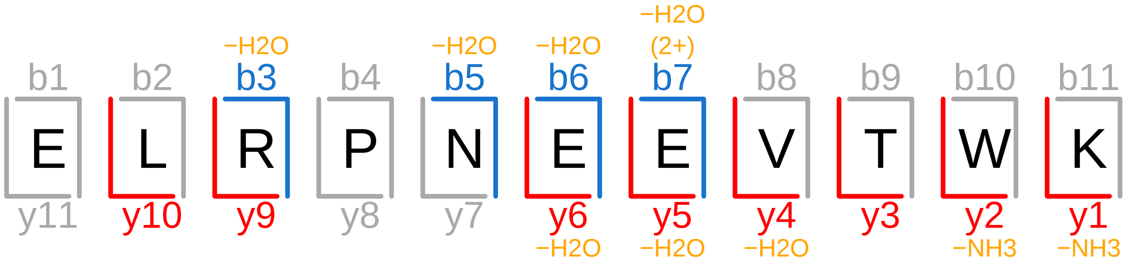

ELRPNEEVTWK
Score: 92 ; 1399.7096 m/z; 467.57714 m/z; 0.67466 ppm; ISO−MSMS

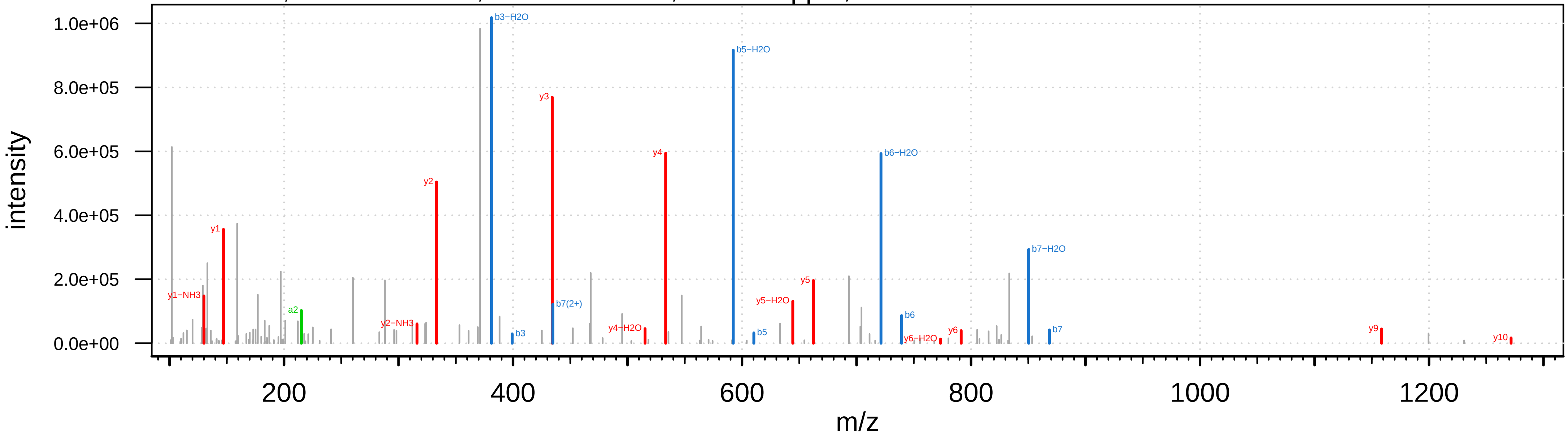

Raw File: Kermit_20111104_MSU_tube_18h_12
Scan Number: 17929
Proteins:
chr12_103206313−103214181.T2_chr12:101968174−101969629:−

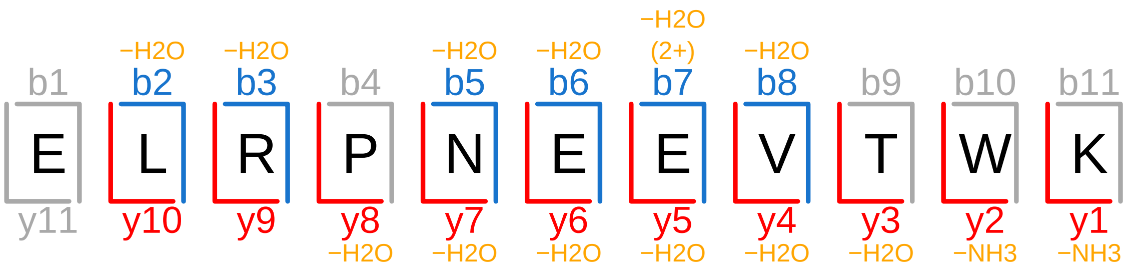

ELRPNEEVTWK

Score: 140 ; 1399.7096 m/z; 467.57714 m/z; 0.67466 ppm; ISO−MSMS

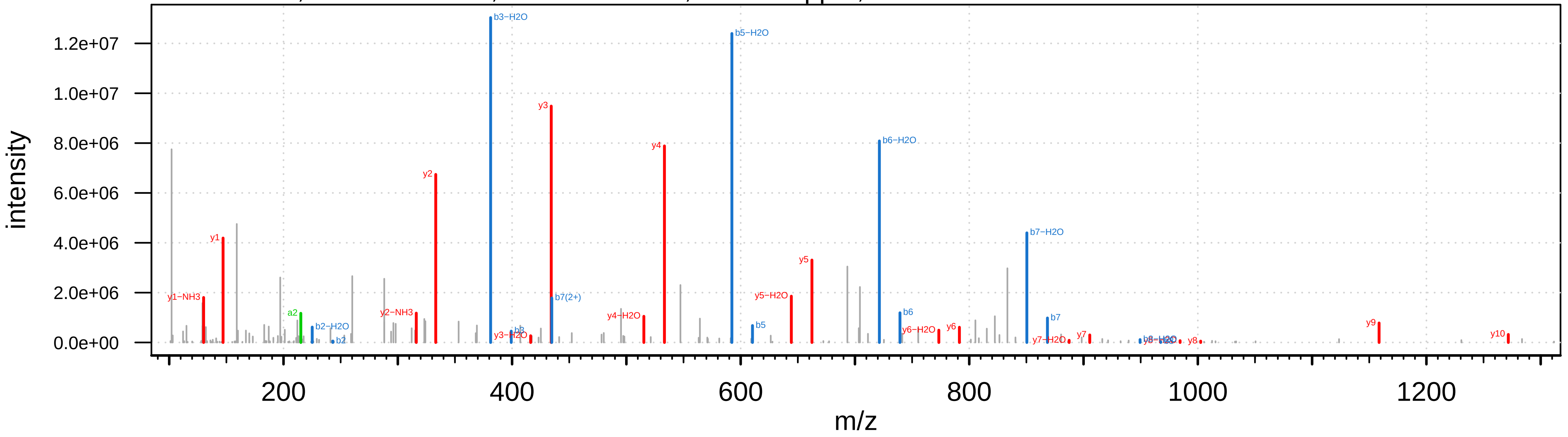

Raw File: Kermit_20111104_MSU_tube_18h_12
Scan Number: 18126
Proteins:
chr12_103206313−103214181.T2_chr12:101968174−101969629:−

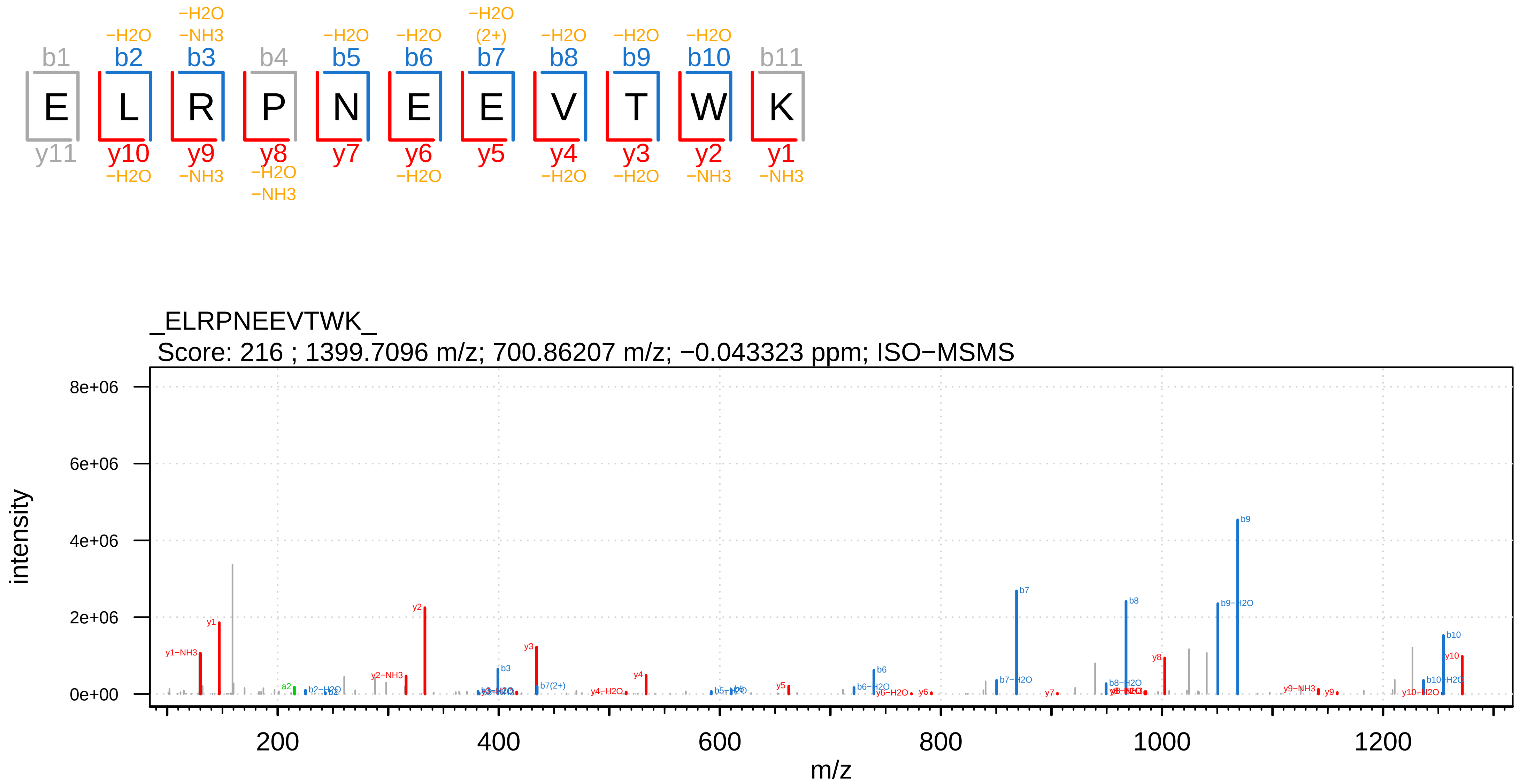

Raw File: Kermit_20111104_MSU_tube_18h_12
Scan Number: 18110
Proteins:
chr12_103206313−103214181.T2_chr12:101968174−101969629:−

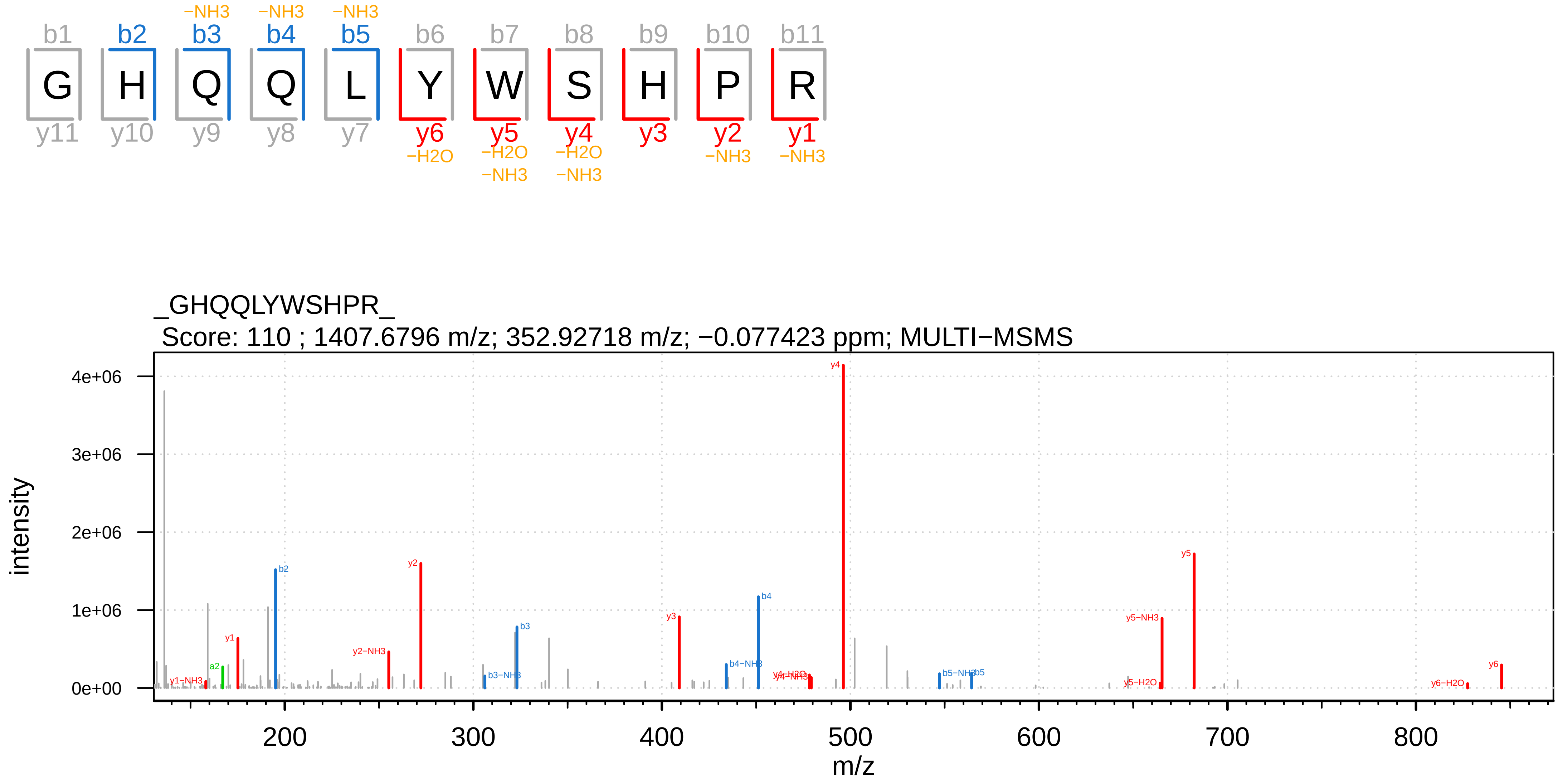

Raw File: Kermit_20111104_MSU_tube_18h_12
Scan Number: 8868
Proteins:
sp|P62274|RS29_MOUSE
ENSMUST00000037023_chr12:69157813−69159058:−
ENSMUST00000071737_chr13:73988963−73989133:−
tr|D3YYL7|D3YYL7_MOUSE

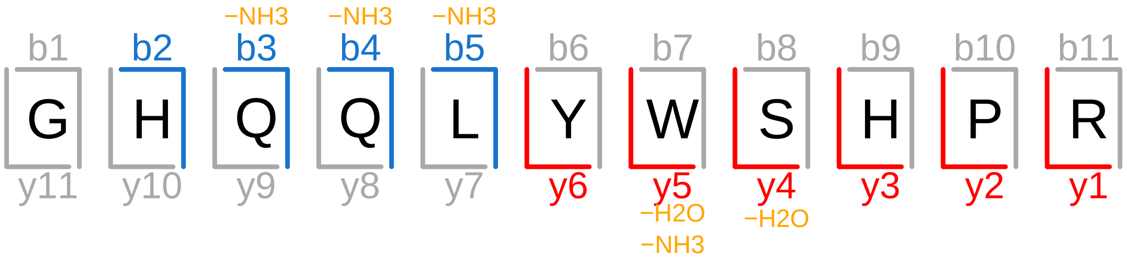

GHQQLYWSHPR
Score: 64 ; 1417.6879 m/z; 355.42925 m/z; −0.077423 ppm; MULTI−MSMS

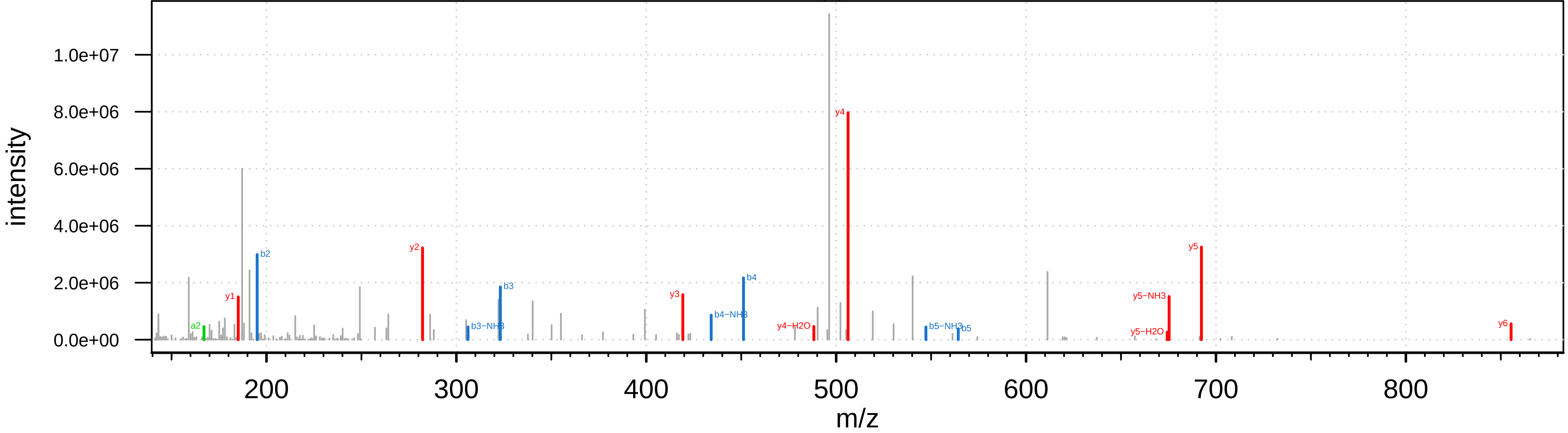

Raw File: Kermit_20111104_MSU_tube_18h_12
Scan Number: 8890
Proteins:
sp|P62274|RS29_MOUSE
ENSMUST00000037023_chr12:69157813−69159058:−
ENSMUST00000071737_chr13:73988963−73989133:−
tr|D3YYL7|D3YYL7_MOUSE

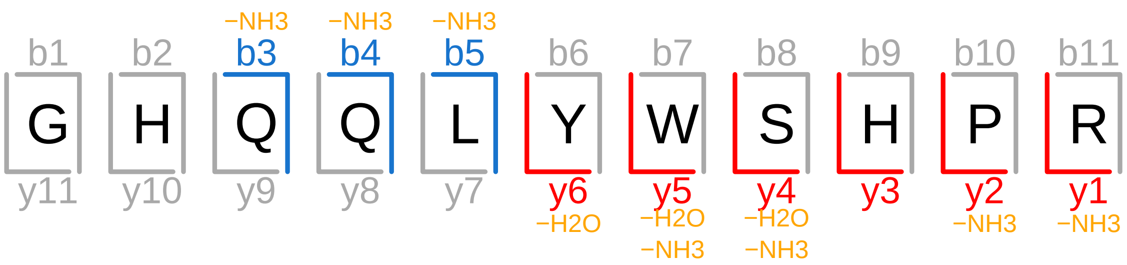

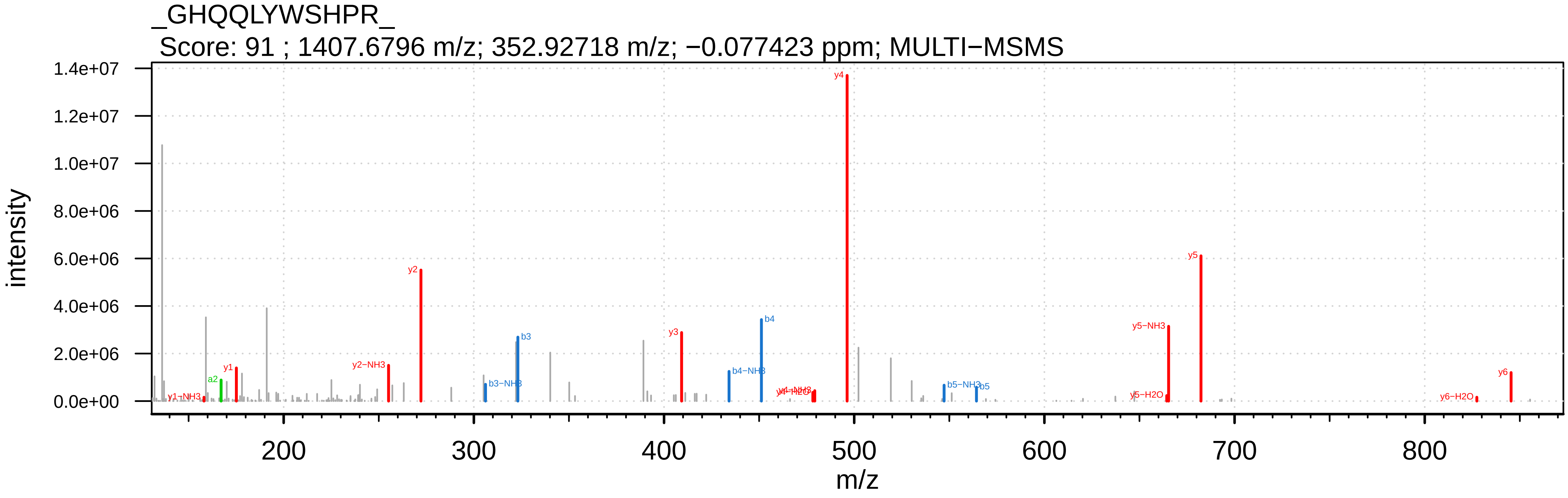

Raw File: Kermit_20111104_MSU_tube_18h_12
Scan Number: 9079
Proteins:
sp|P62274|RS29_MOUSE
ENSMUST00000037023_chr12:69157813−69159058:−
ENSMUST00000071737_chr13:73988963−73989133:−
tr|D3YYL7|D3YYL7_MOUSE

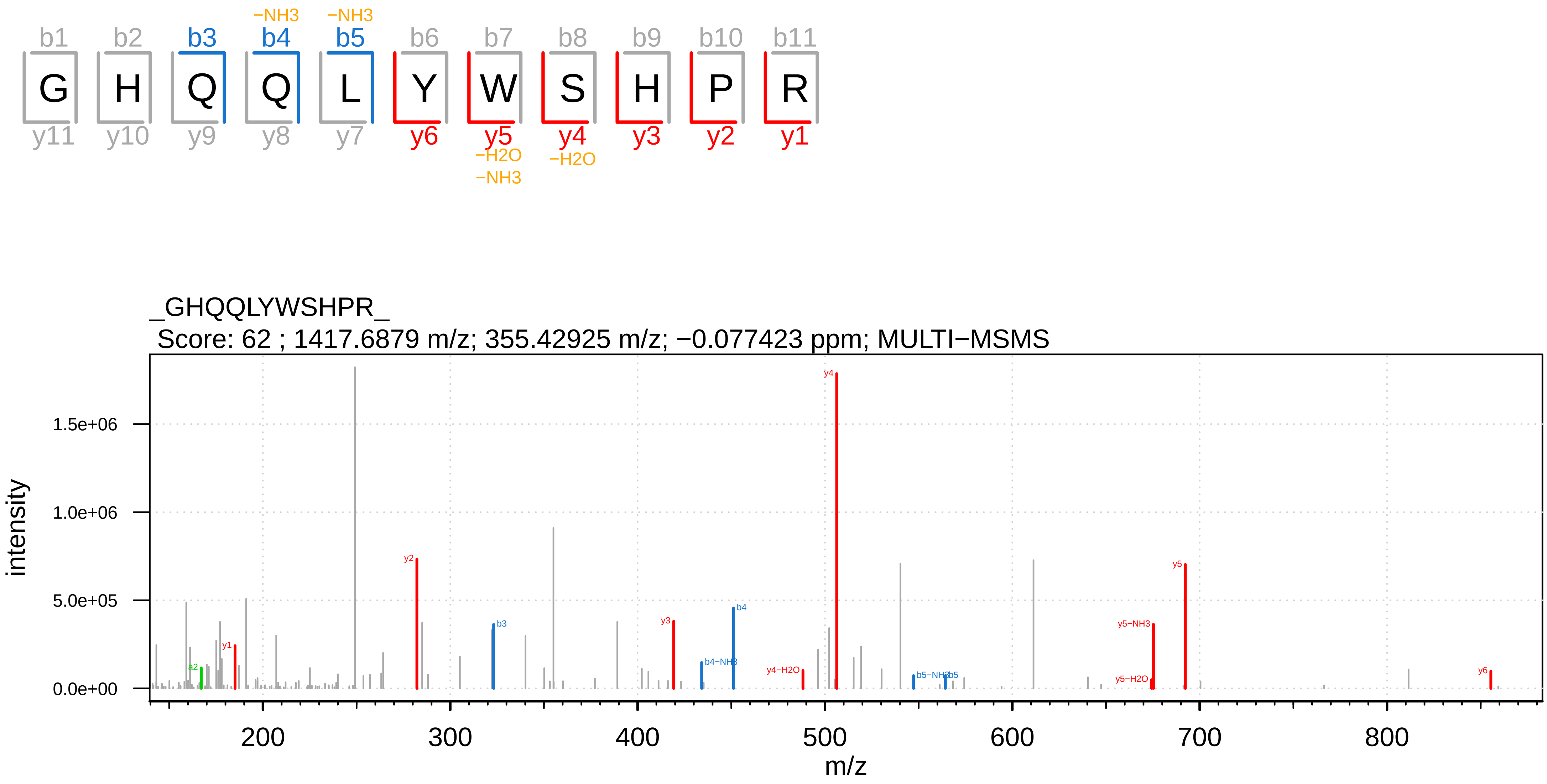

Raw File: Kermit_20111104_MSU_tube_18h_12
Scan Number: 9100
Proteins:
sp|P62274|RS29_MOUSE
ENSMUST00000037023_chr12:69157813−69159058:−
ENSMUST00000071737_chr13:73988963−73989133:−
tr|D3YYL7|D3YYL7_MOUSE

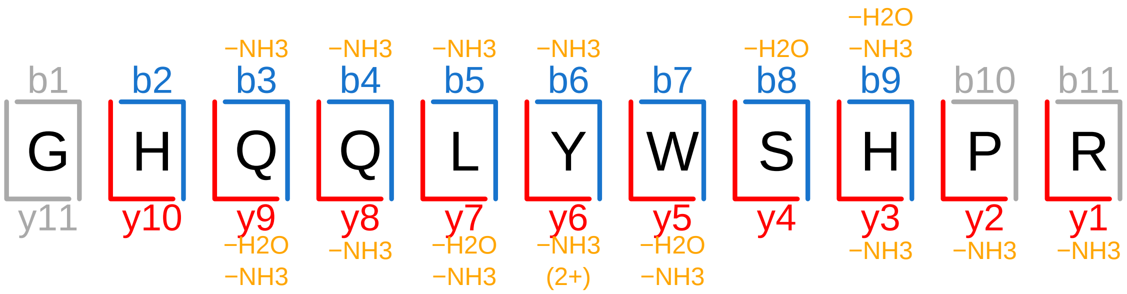

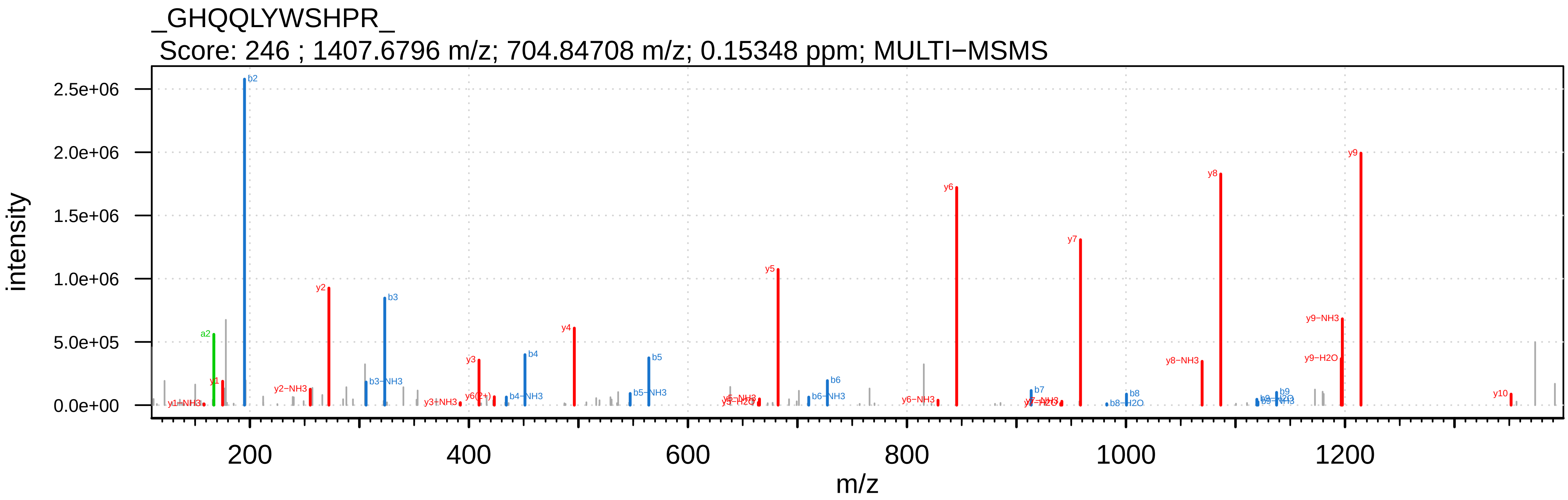

Raw File: Kermit_20111104_MSU_tube_18h_12
Scan Number: 8882
Proteins:
sp|P62274|RS29_MOUSE
ENSMUST00000037023_chr12:69157813−69159058:−
ENSMUST00000071737_chr13:73988963−73989133:−
tr|D3YYL7|D3YYL7_MOUSE

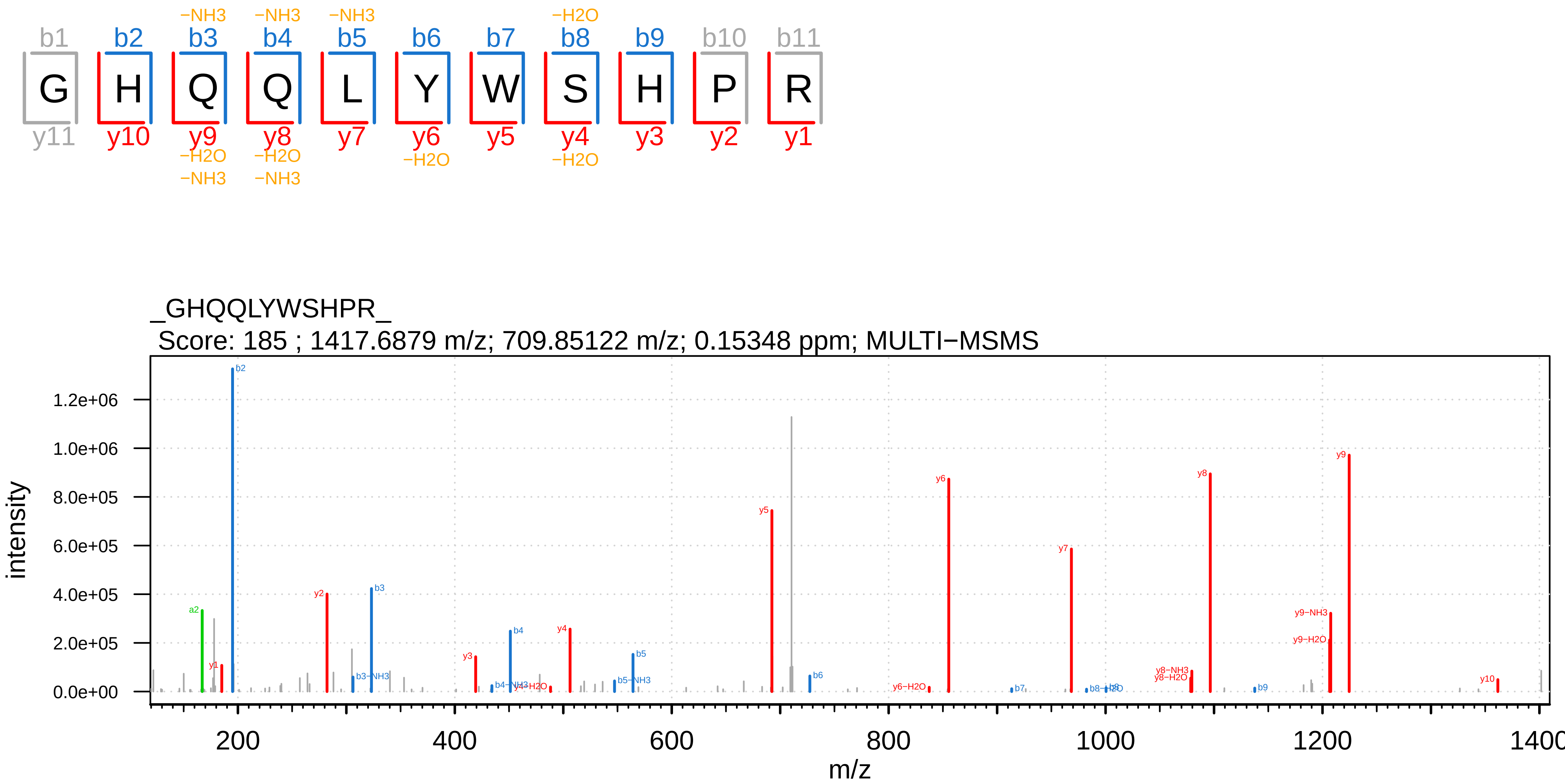

Raw File: Kermit_20111104_MSU_tube_18h_12
Scan Number: 8888
Proteins:
sp|P62274|RS29_MOUSE
ENSMUST00000037023_chr12:69157813−69159058:−
ENSMUST00000071737_chr13:73988963−73989133:−
tr|D3YYL7|D3YYL7_MOUSE

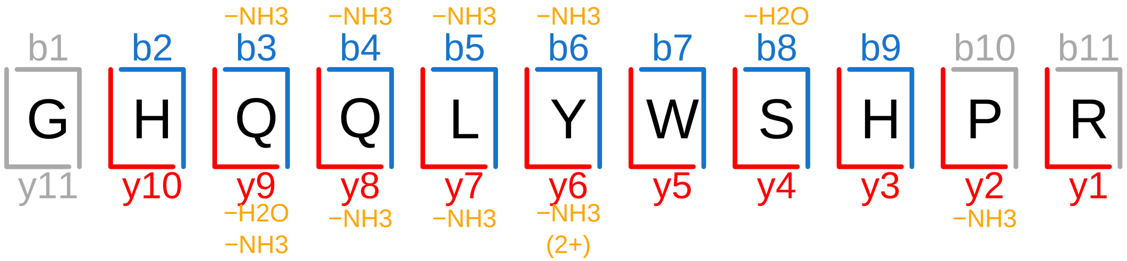

GHQQLYWSHPR
Score: 191 ; 1407.6796 m/z; 704.84708 m/z; 0.15348 ppm; MULTI−MSMS

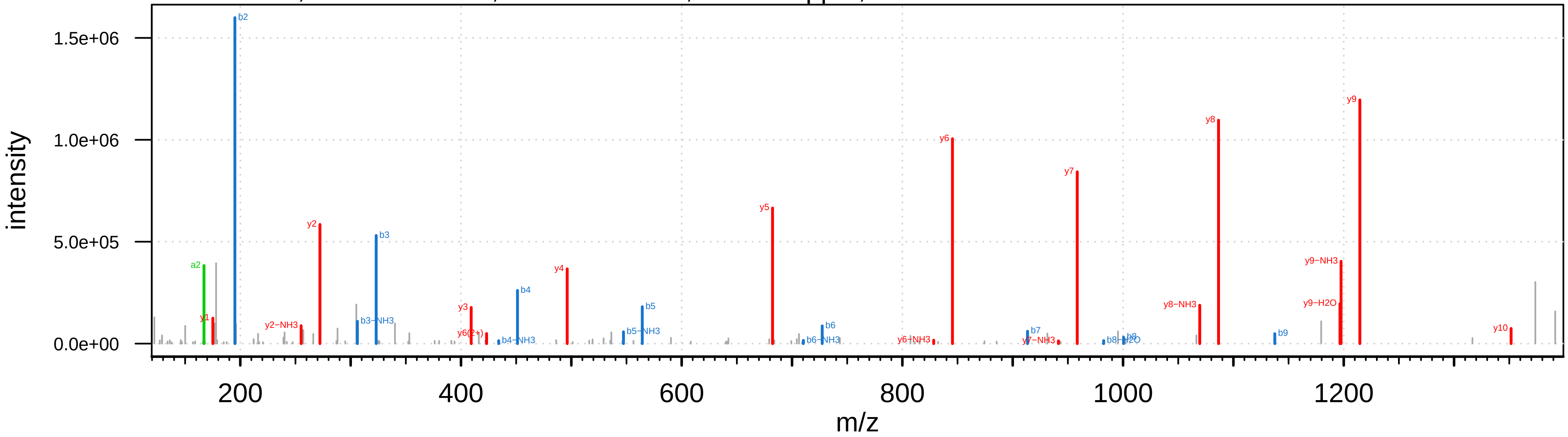

Raw File: Kermit_20111104_MSU_tube_18h_12
Scan Number: 9099
Proteins:
sp|P62274|RS29_MOUSE
ENSMUST00000037023_chr12:69157813−69159058:−
ENSMUST00000071737_chr13:73988963−73989133:−
tr|D3YYL7|D3YYL7_MOUSE

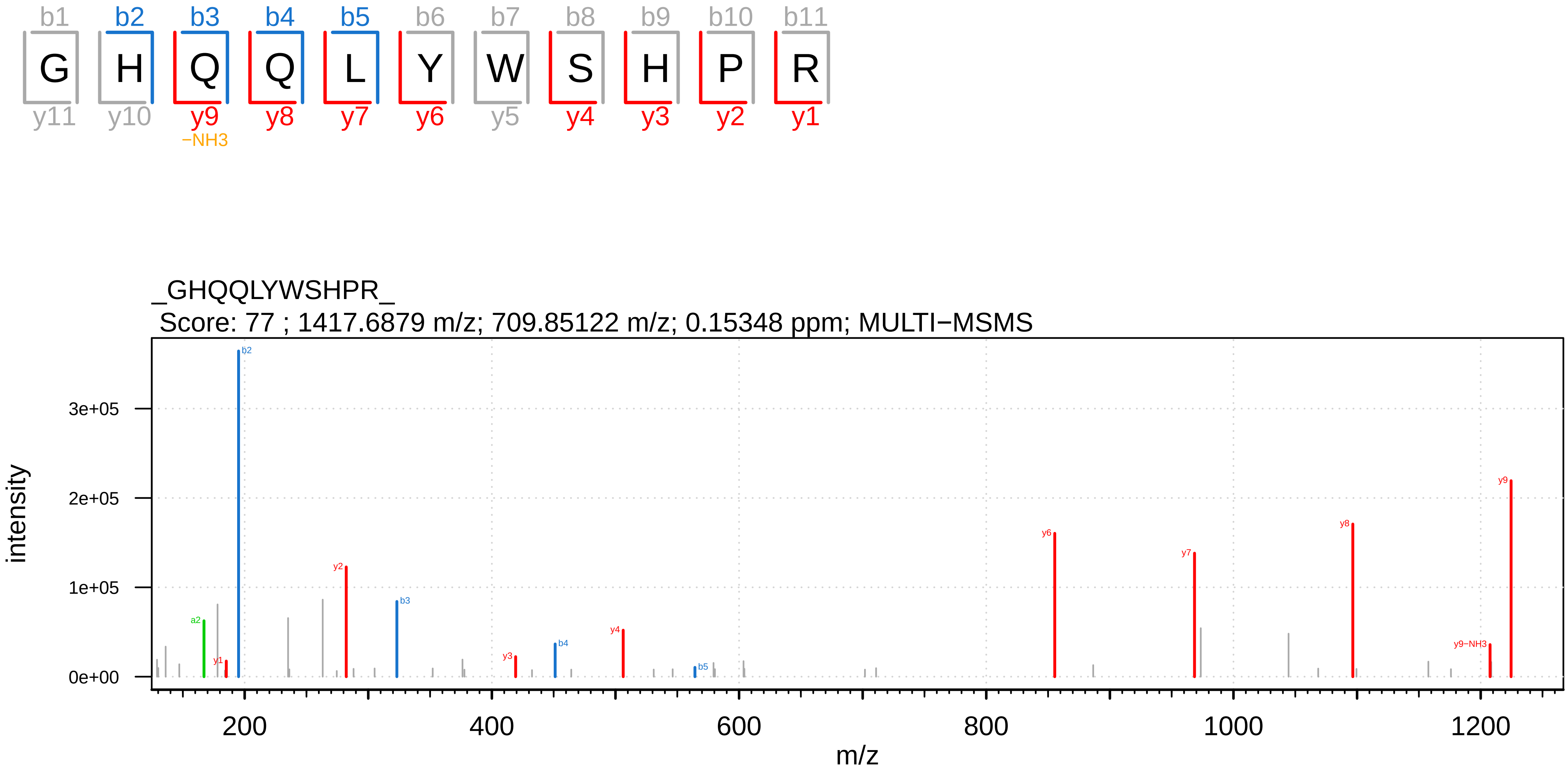

Raw File: Kermit_20111104_MSU_tube_18h_12
Scan Number: 9101
Proteins:
sp|P62274|RS29_MOUSE
ENSMUST00000037023_chr12:69157813−69159058:−
ENSMUST00000071737_chr13:73988963−73989133:−
tr|D3YYL7|D3YYL7_MOUSE

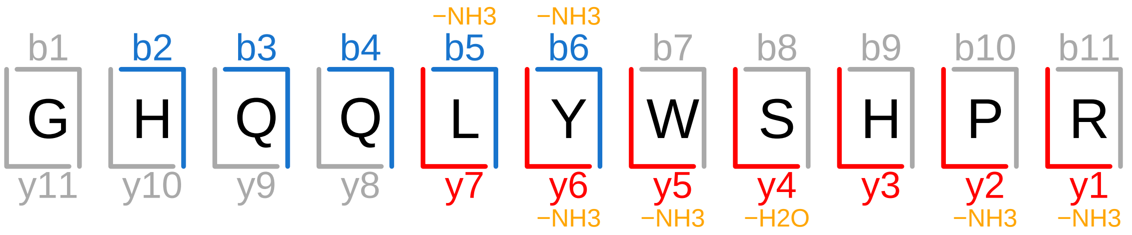

GHQQLYWSHPR
Score: 76 ; 1407.6796 m/z; 470.23382 m/z; 0.50869 ppm; MULTI−MSMS

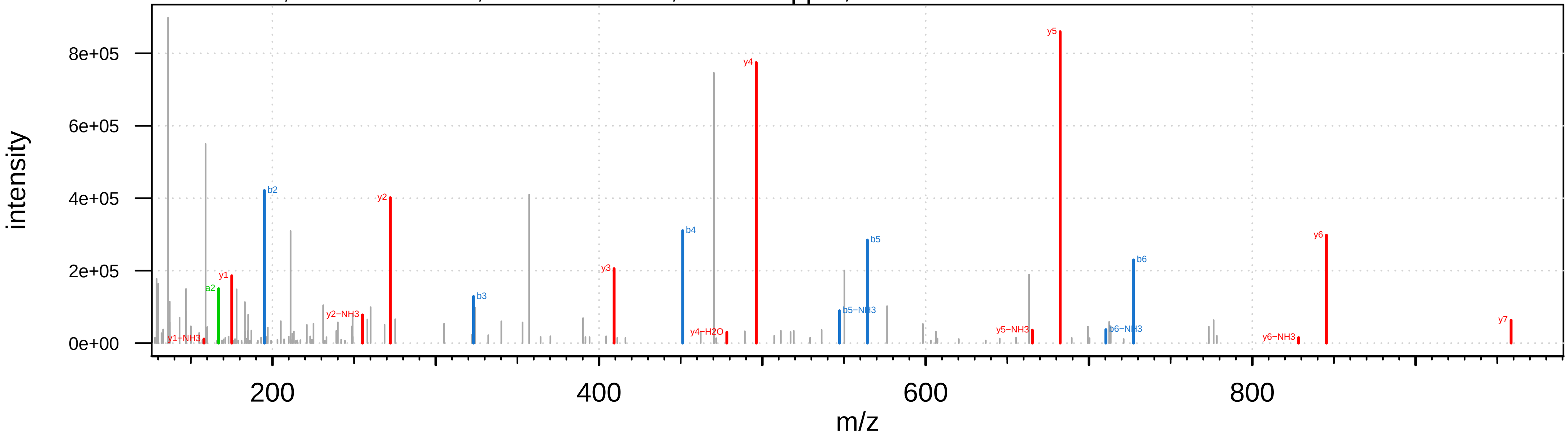

Raw File: Kermit_20111104_MSU_tube_18h_12
Scan Number: 9512
Proteins:
sp|P62274|RS29_MOUSE
ENSMUST00000037023_chr12:69157813−69159058:−
ENSMUST00000071737_chr13:73988963−73989133:−
tr|D3YYL7|D3YYL7_MOUSE

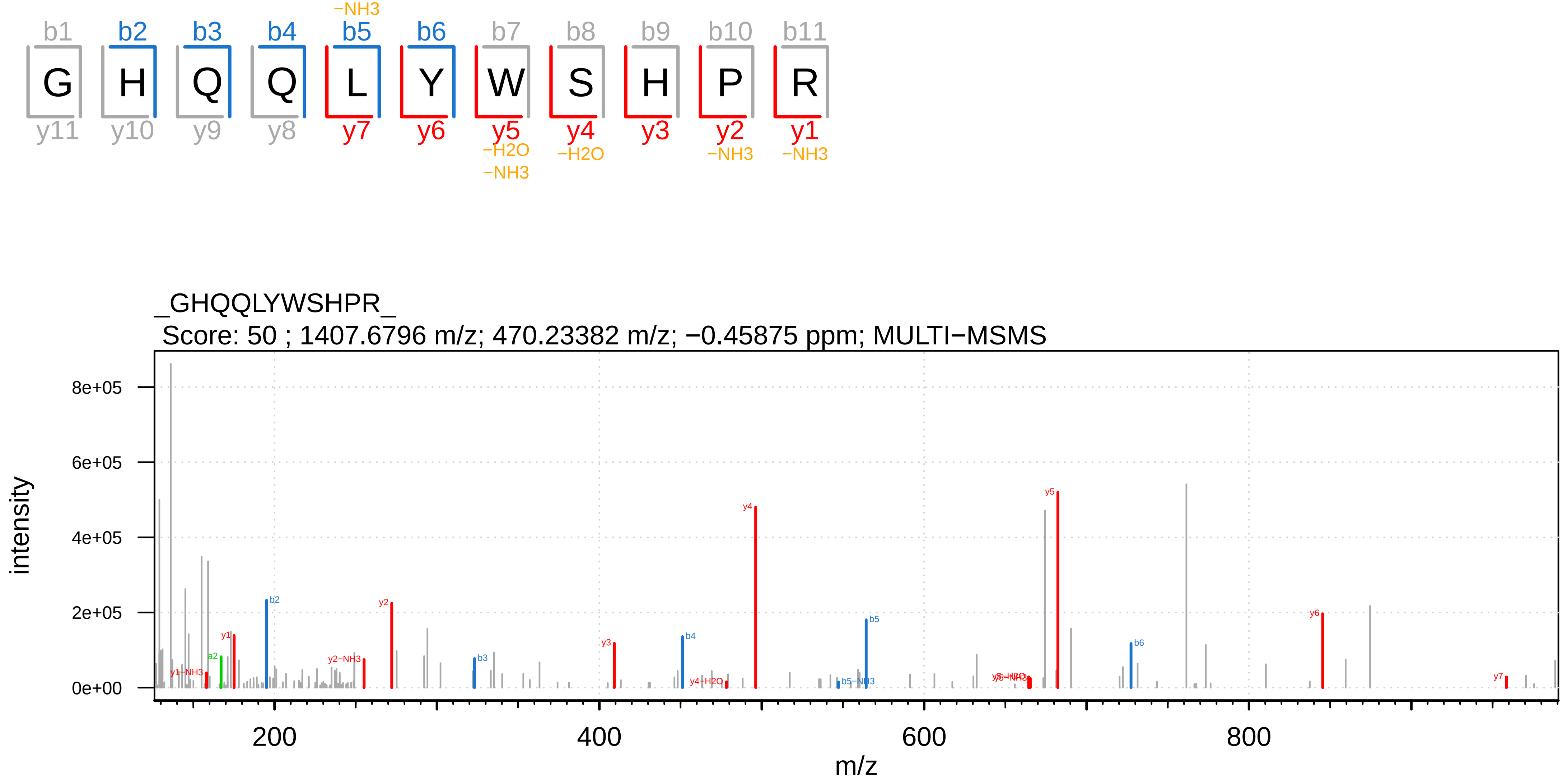

Raw File: Kermit_20111104_MSU_tube_18h_12
Scan Number: 10707
Proteins:
sp|P62274|RS29_MOUSE
ENSMUST00000037023_chr12:69157813−69159058:−
ENSMUST00000071737_chr13:73988963−73989133:−
tr|D3YYL7|D3YYL7_MOUSE

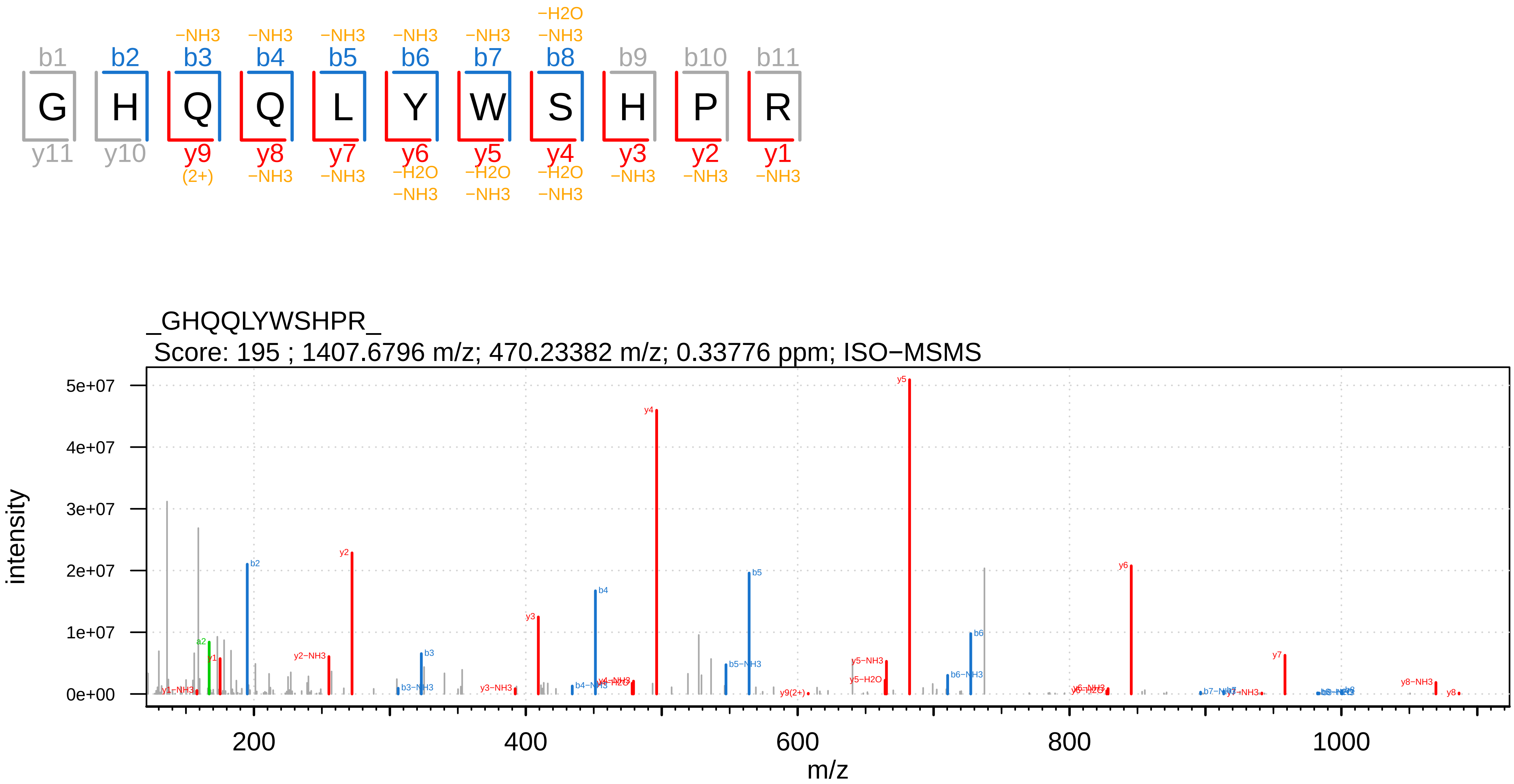

Raw File: Kermit_20111104_MSU_tube_18h_12
Scan Number: 8879
Proteins:
sp|P62274|RS29_MOUSE
ENSMUST00000037023_chr12:69157813−69159058:−
ENSMUST00000071737_chr13:73988963−73989133:−
tr|D3YYL7|D3YYL7_MOUSE

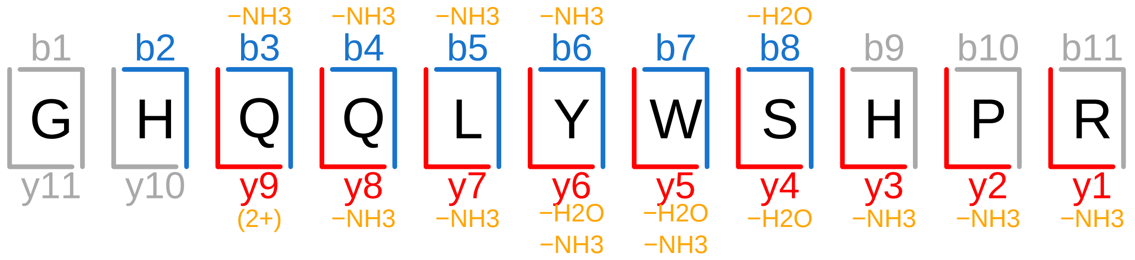

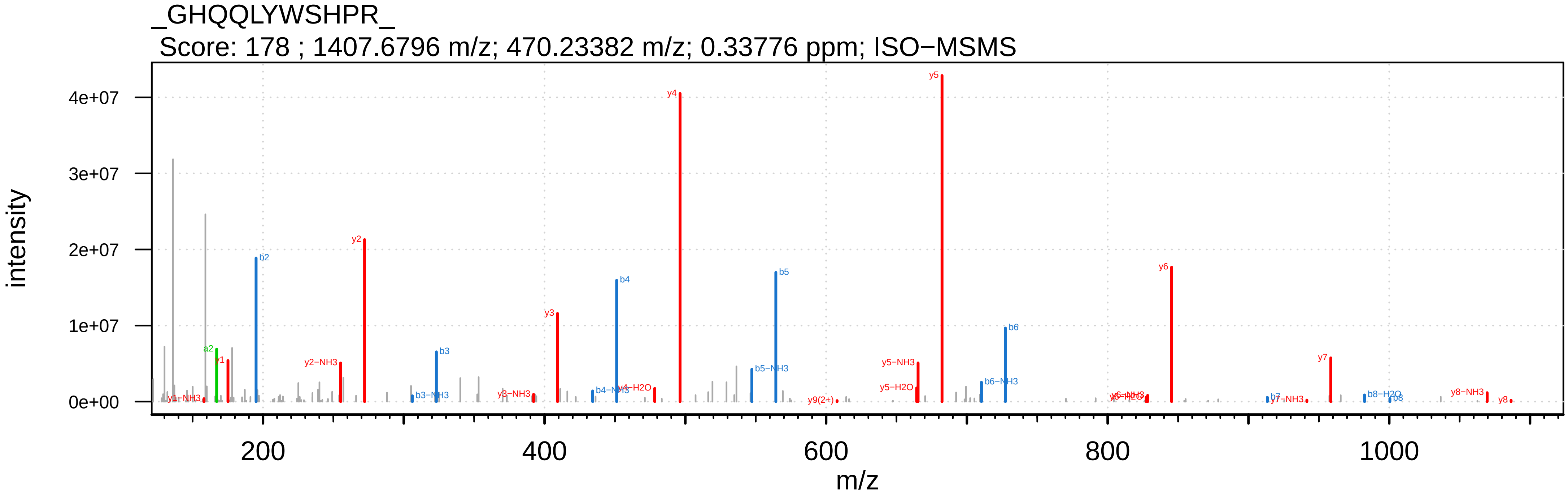

Raw File: Kermit_20111104_MSU_tube_18h_12
Scan Number: 9090
Proteins:
sp|P62274|RS29_MOUSE
ENSMUST00000037023_chr12:69157813−69159058:−
ENSMUST00000071737_chr13:73988963−73989133:−
tr|D3YYL7|D3YYL7_MOUSE

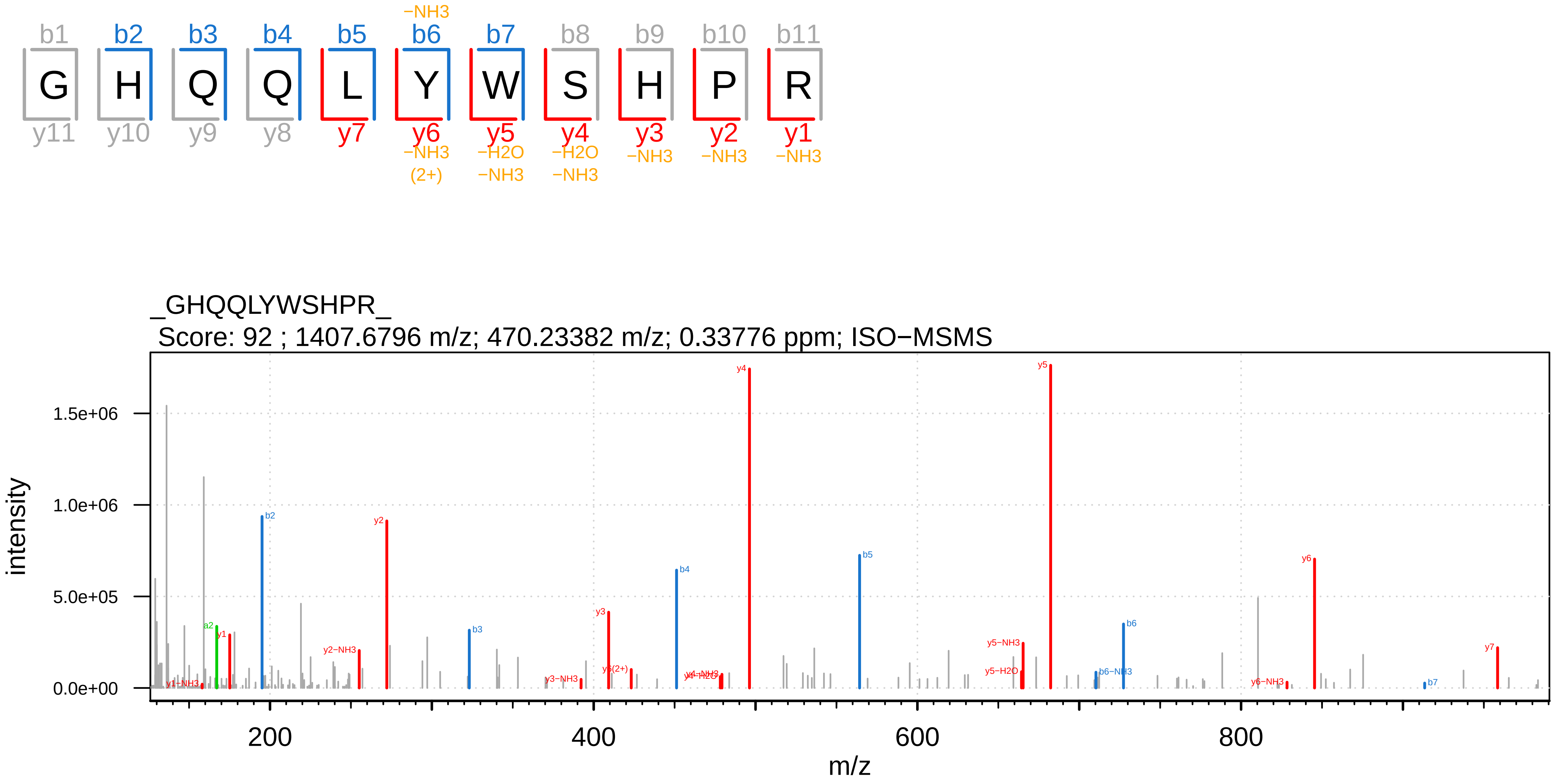

Raw File: Kermit_20111104_MSU_tube_18h_12
Scan Number: 9303
Proteins:
sp|P62274|RS29_MOUSE
ENSMUST00000037023_chr12:69157813−69159058:−
ENSMUST00000071737_chr13:73988963−73989133:−
tr|D3YYL7|D3YYL7_MOUSE

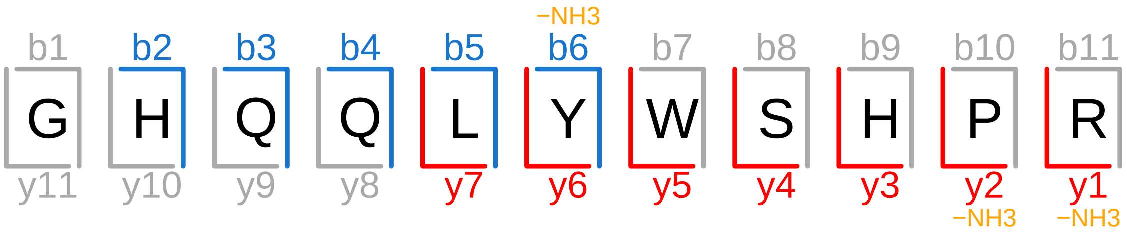

GHQQLYWSHPR
Score: 44 ; 1407.6796 m/z; 470.23382 m/z; −0.42364 ppm; ISO−MSMS

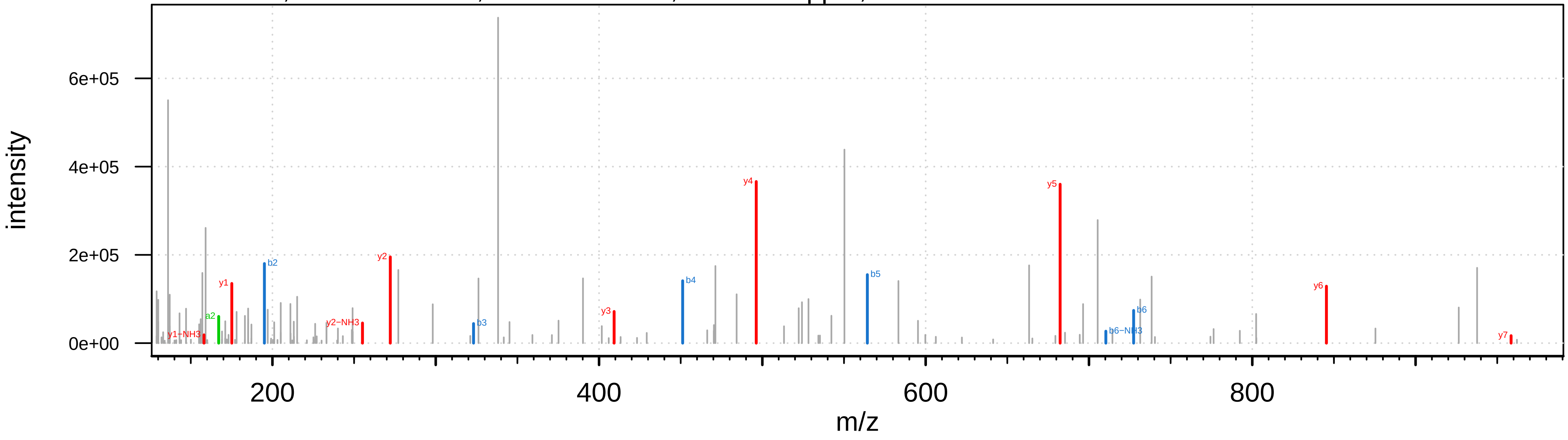

Raw File: Kermit_20111104_MSU_tube_18h_12
Scan Number: 11737
Proteins:
sp|P62274|RS29_MOUSE
ENSMUST00000037023_chr12:69157813−69159058:−
ENSMUST00000071737_chr13:73988963−73989133:−
tr|D3YYL7|D3YYL7_MOUSE

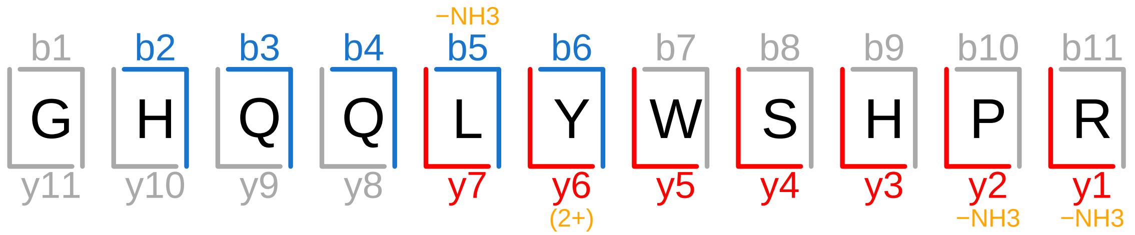

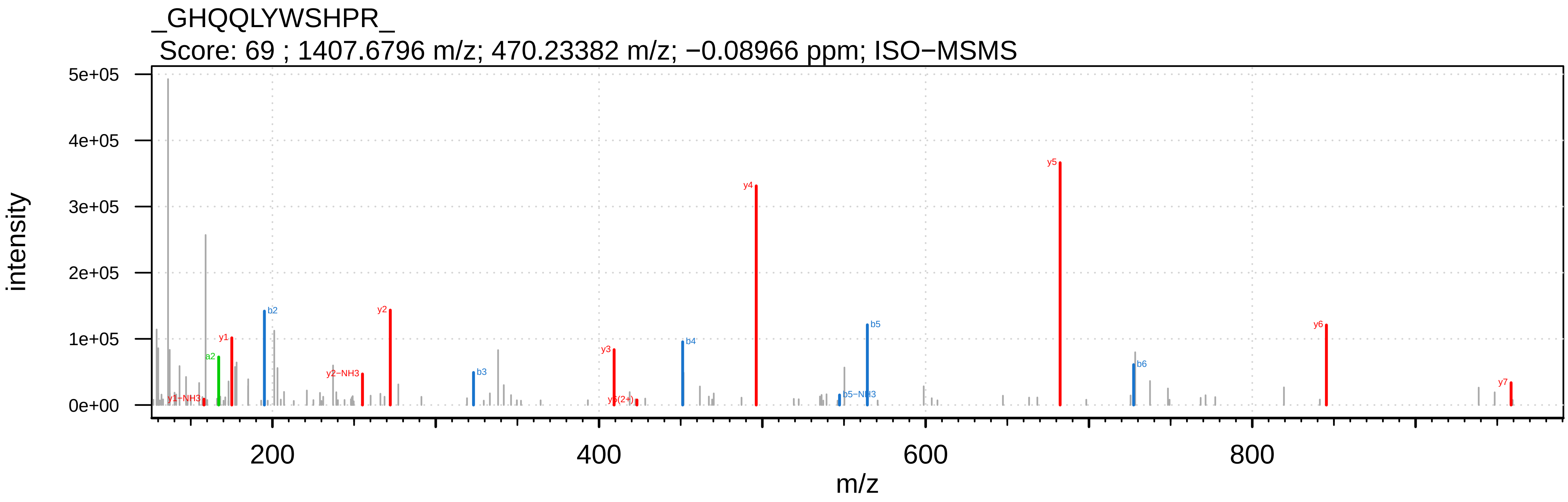

Raw File: Kermit_20111104_MSU_tube_18h_12
Scan Number: 12979
Proteins:
sp|P62274|RS29_MOUSE
ENSMUST00000037023_chr12:69157813−69159058:−
ENSMUST00000071737_chr13:73988963−73989133:−
tr|D3YYL7|D3YYL7_MOUSE

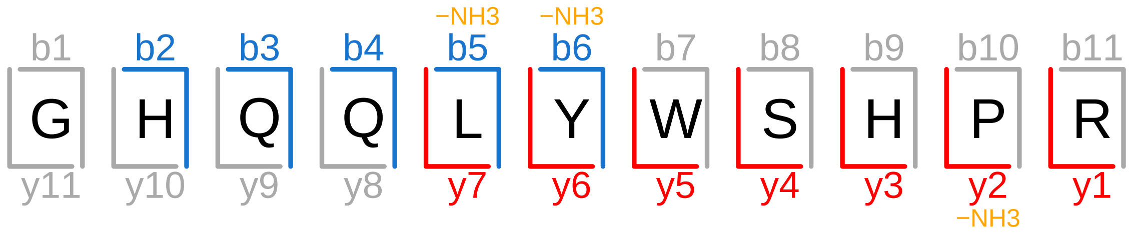

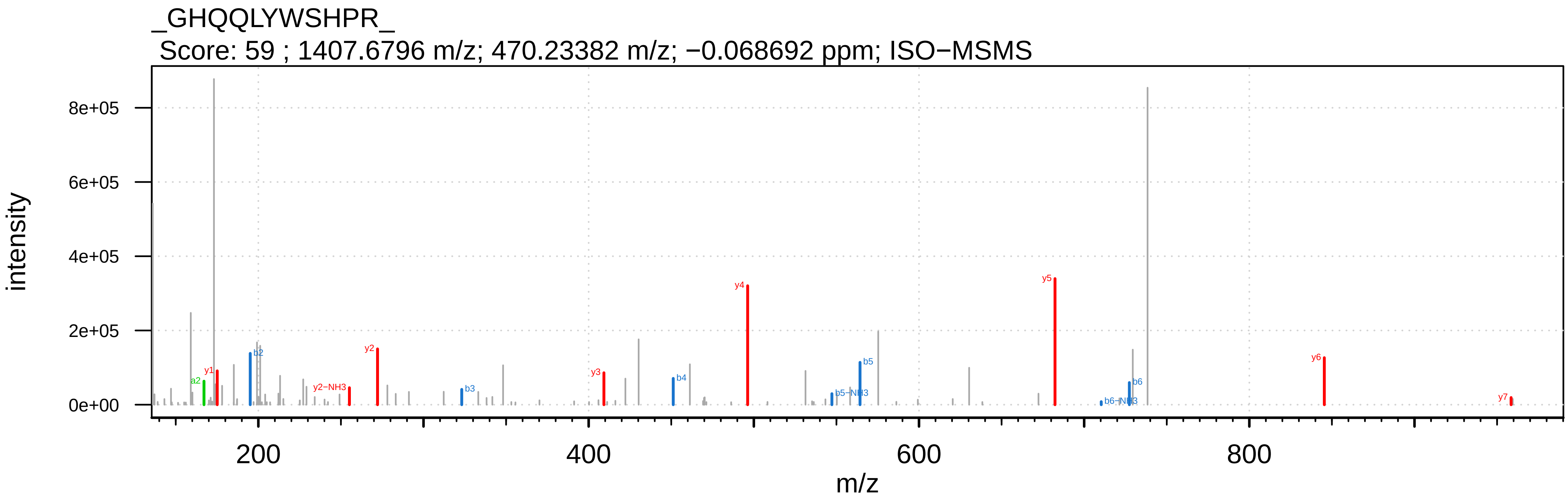

Raw File: Kermit_20111104_MSU_tube_18h_12
Scan Number: 14778
Proteins:
sp|P62274|RS29_MOUSE
ENSMUST00000037023_chr12:69157813−69159058:−
ENSMUST00000071737_chr13:73988963−73989133:−
tr|D3YYL7|D3YYL7_MOUSE

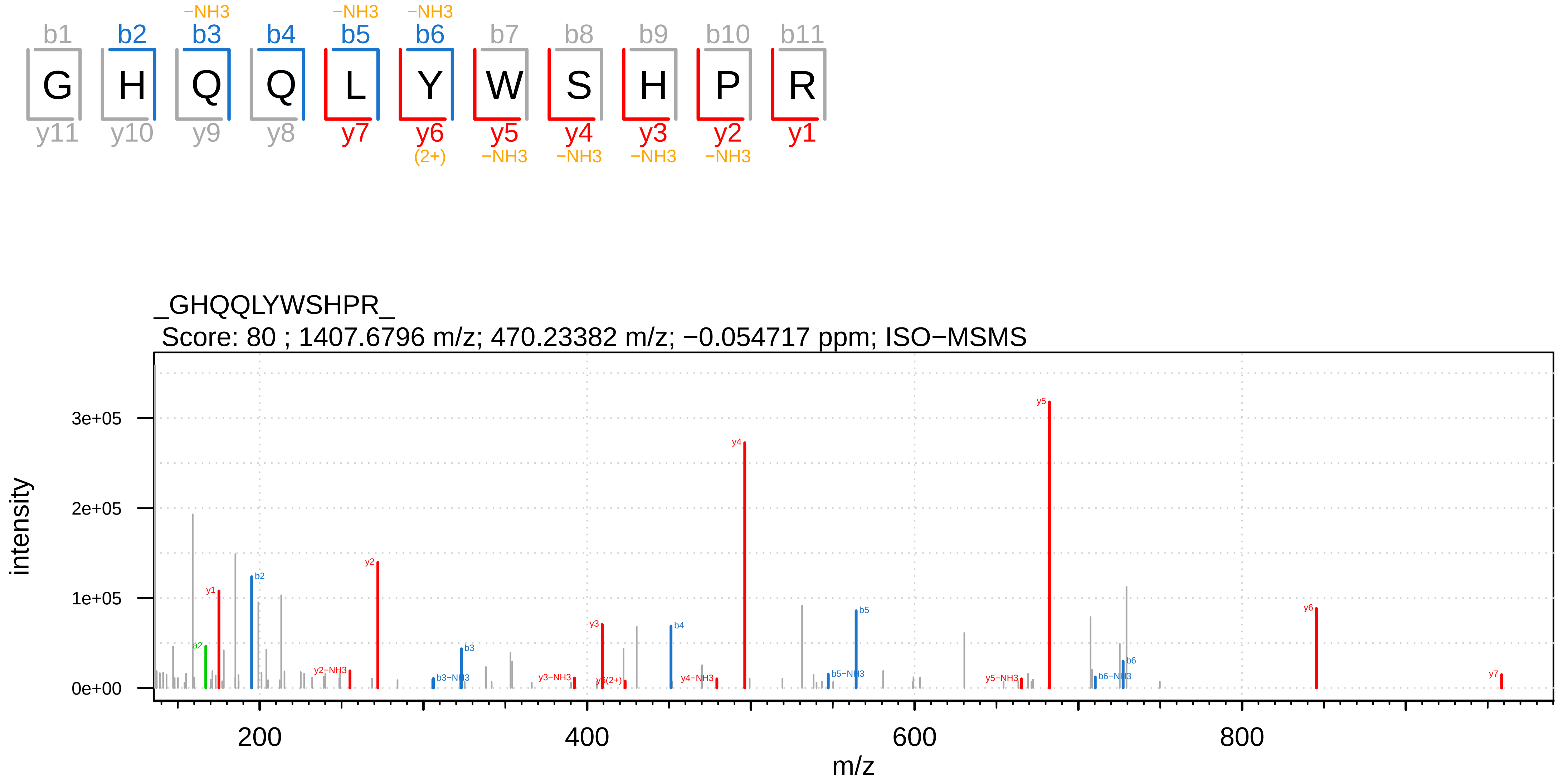

Raw File: Kermit_20111104_MSU_tube_18h_12
Scan Number: 14951
Proteins:
sp|P62274|RS29_MOUSE
ENSMUST00000037023_chr12:69157813−69159058:−
ENSMUST00000071737_chr13:73988963−73989133:−
tr|D3YYL7|D3YYL7_MOUSE

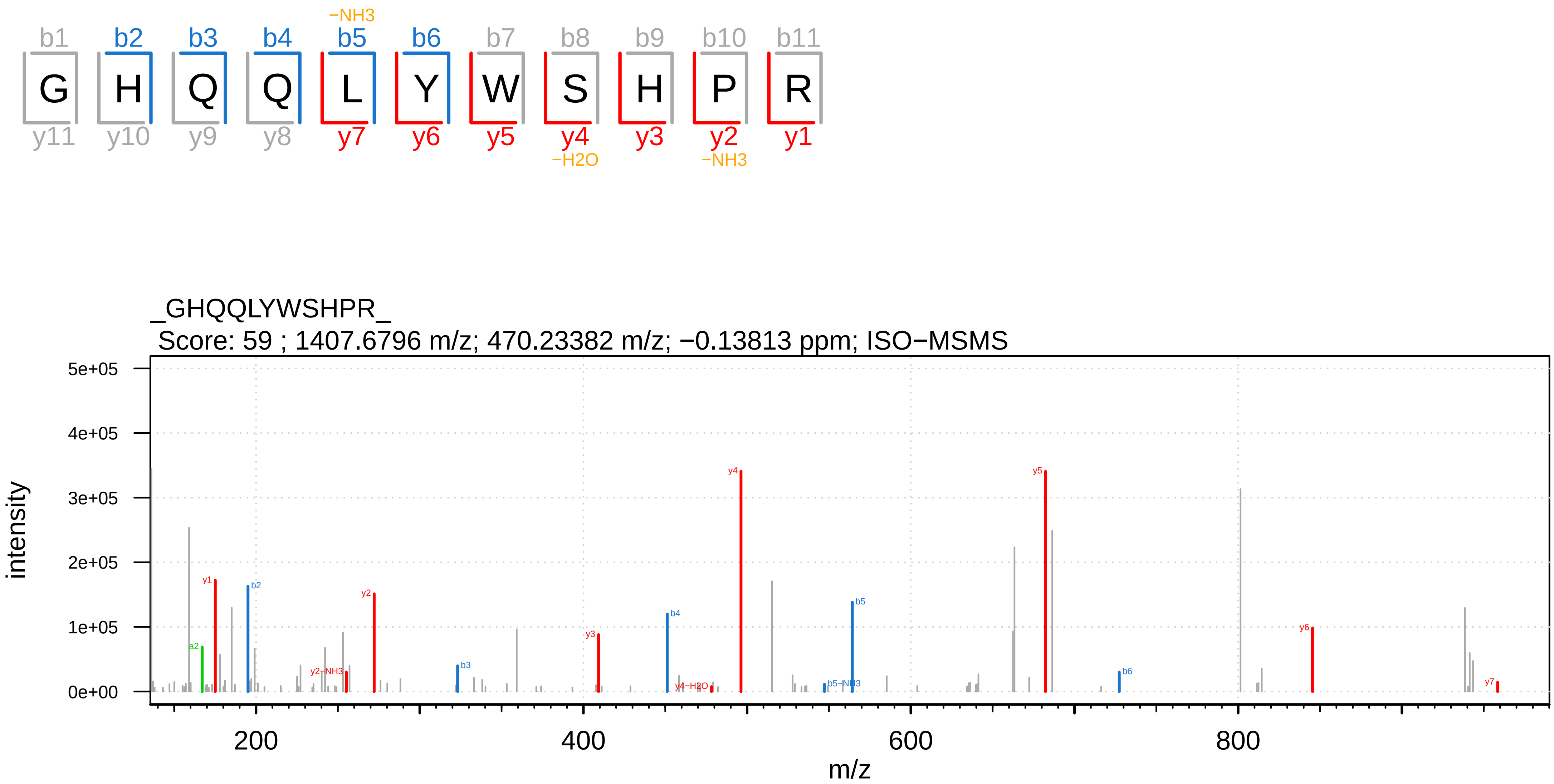

Raw File: Kermit_20111104_MSU_tube_18h_12
Scan Number: 15392
Proteins:
sp|P62274|RS29_MOUSE
ENSMUST00000037023_chr12:69157813−69159058:−
ENSMUST00000071737_chr13:73988963−73989133:−
tr|D3YYL7|D3YYL7_MOUSE

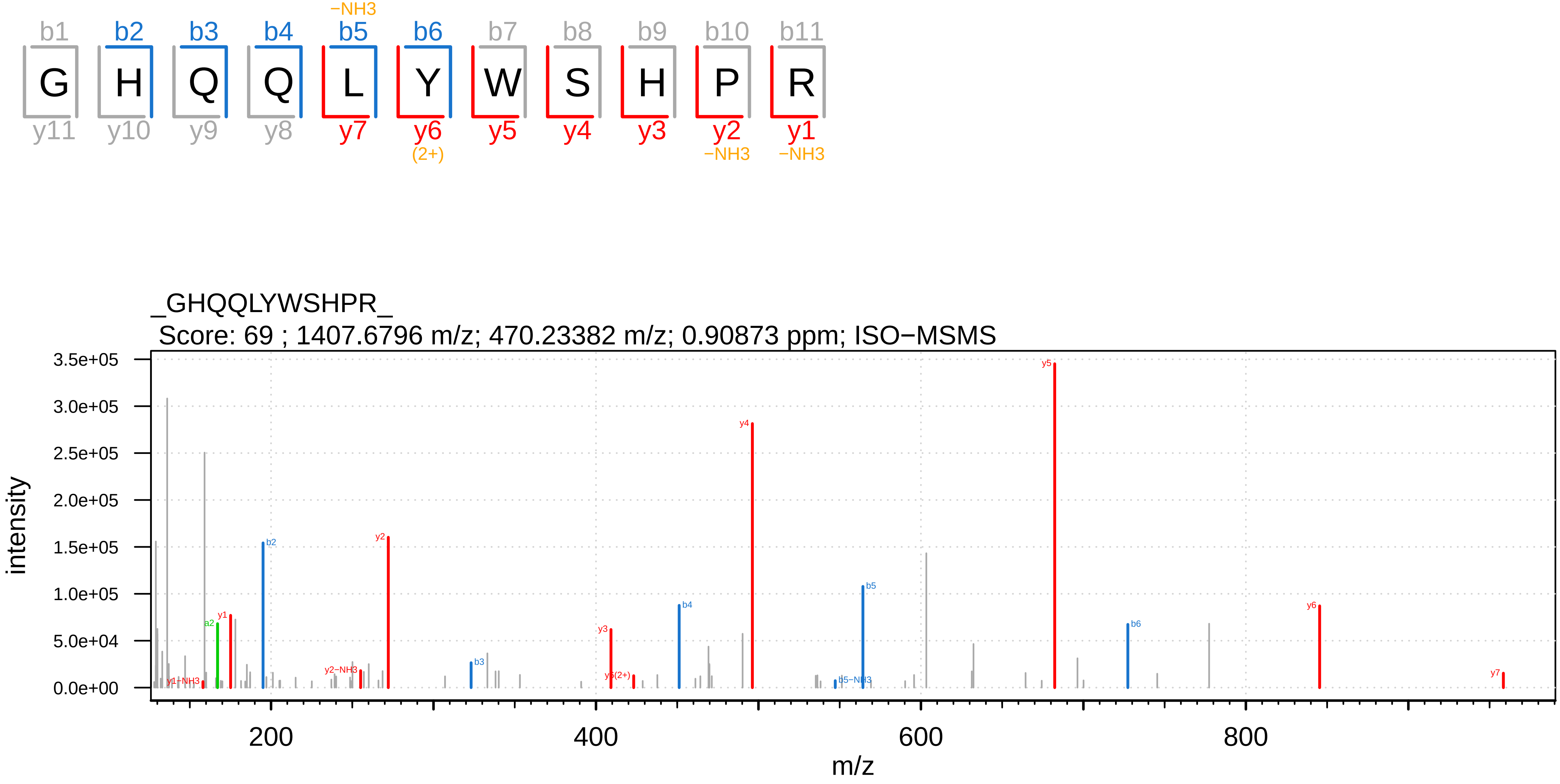

Raw File: Kermit_20111104_MSU_tube_18h_12
Scan Number: 16828
Proteins:
sp|P62274|RS29_MOUSE
ENSMUST00000037023_chr12:69157813−69159058:−
ENSMUST00000071737_chr13:73988963−73989133:−
tr|D3YYL7|D3YYL7_MOUSE

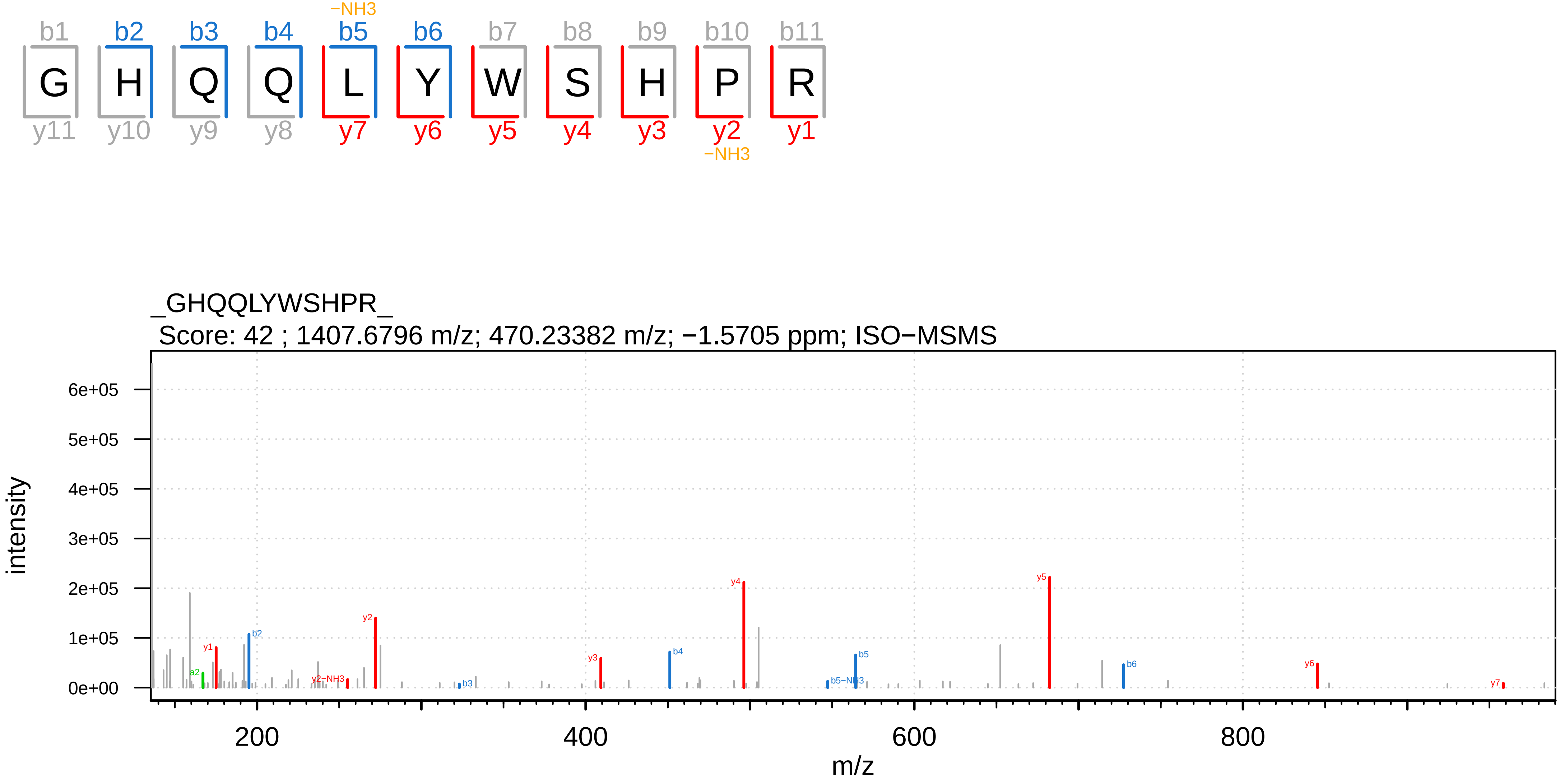

Raw File: Kermit_20111104_MSU_tube_18h_12
Scan Number: 20016
Proteins:
sp|P62274|RS29_MOUSE
ENSMUST00000037023_chr12:69157813−69159058:−
ENSMUST00000071737_chr13:73988963−73989133:−
tr|D3YYL7|D3YYL7_MOUSE

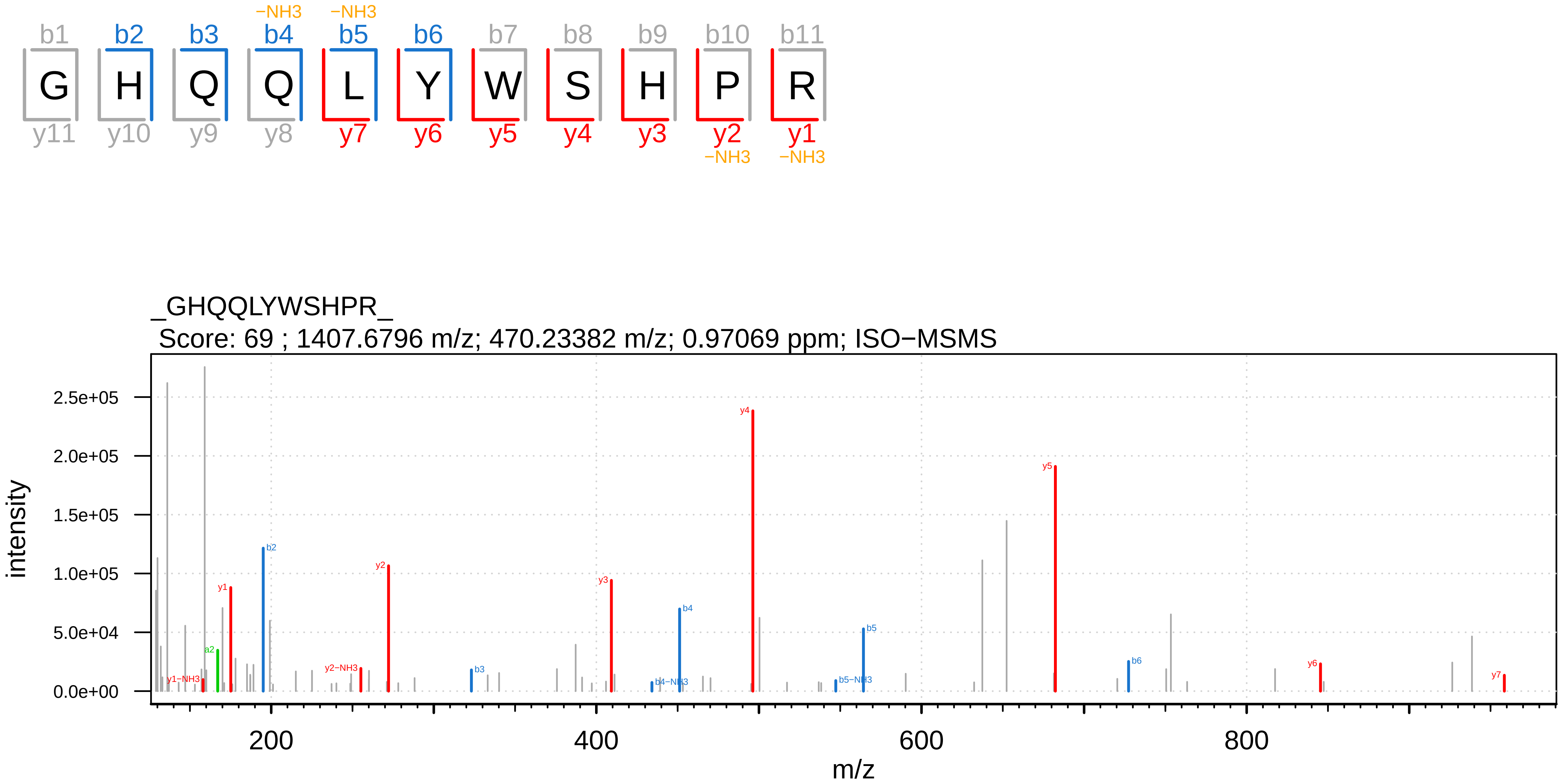

Raw File: Kermit_20111104_MSU_tube_18h_12
Scan Number: 21167
Proteins:
sp|P62274|RS29_MOUSE
ENSMUST00000037023_chr12:69157813−69159058:−
ENSMUST00000071737_chr13:73988963−73989133:−
tr|D3YYL7|D3YYL7_MOUSE

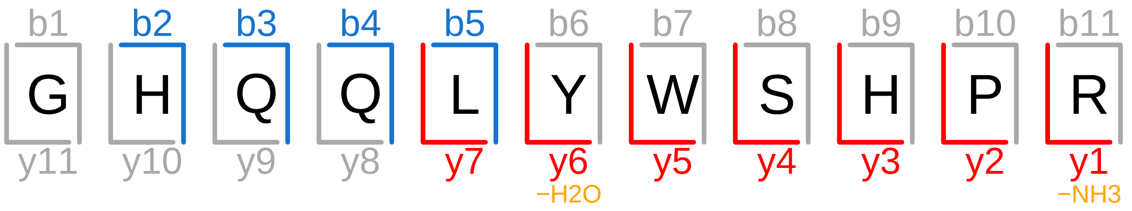

GHQQLYWSHPR
Score: 45 ; 1407.6796 m/z; 470.23382 m/z; NaN ppm; MSMS

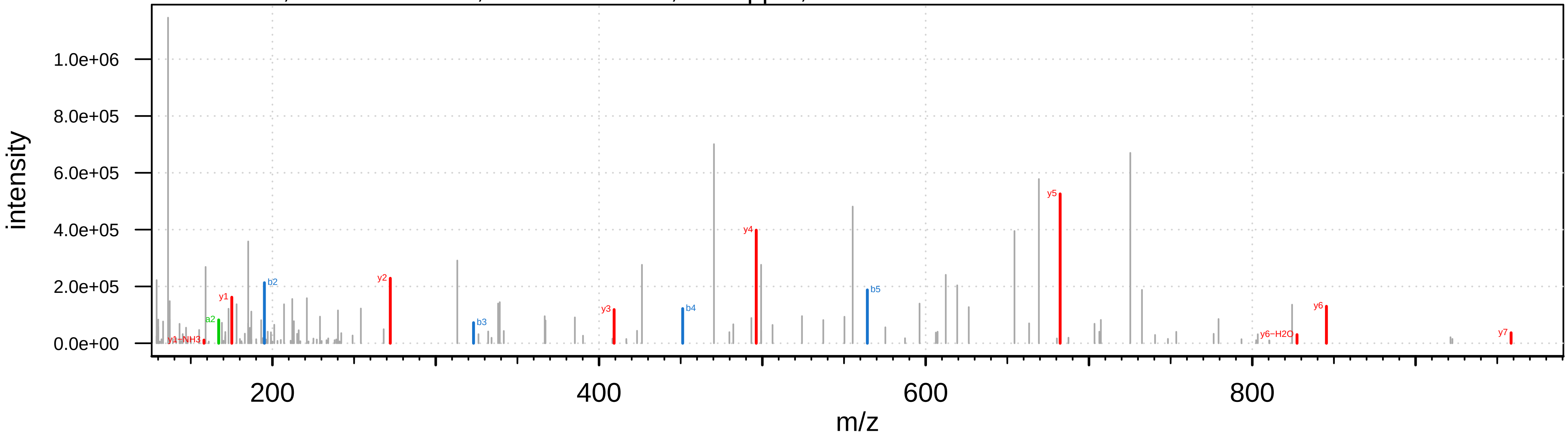

Raw File: Kermit_20111104_MSU_tube_18h_12
Scan Number: 12015
Proteins:
sp|P62274|RS29_MOUSE
ENSMUST00000037023_chr12:69157813−69159058:−
ENSMUST00000071737_chr13:73988963−73989133:−
tr|D3YYL7|D3YYL7_MOUSE

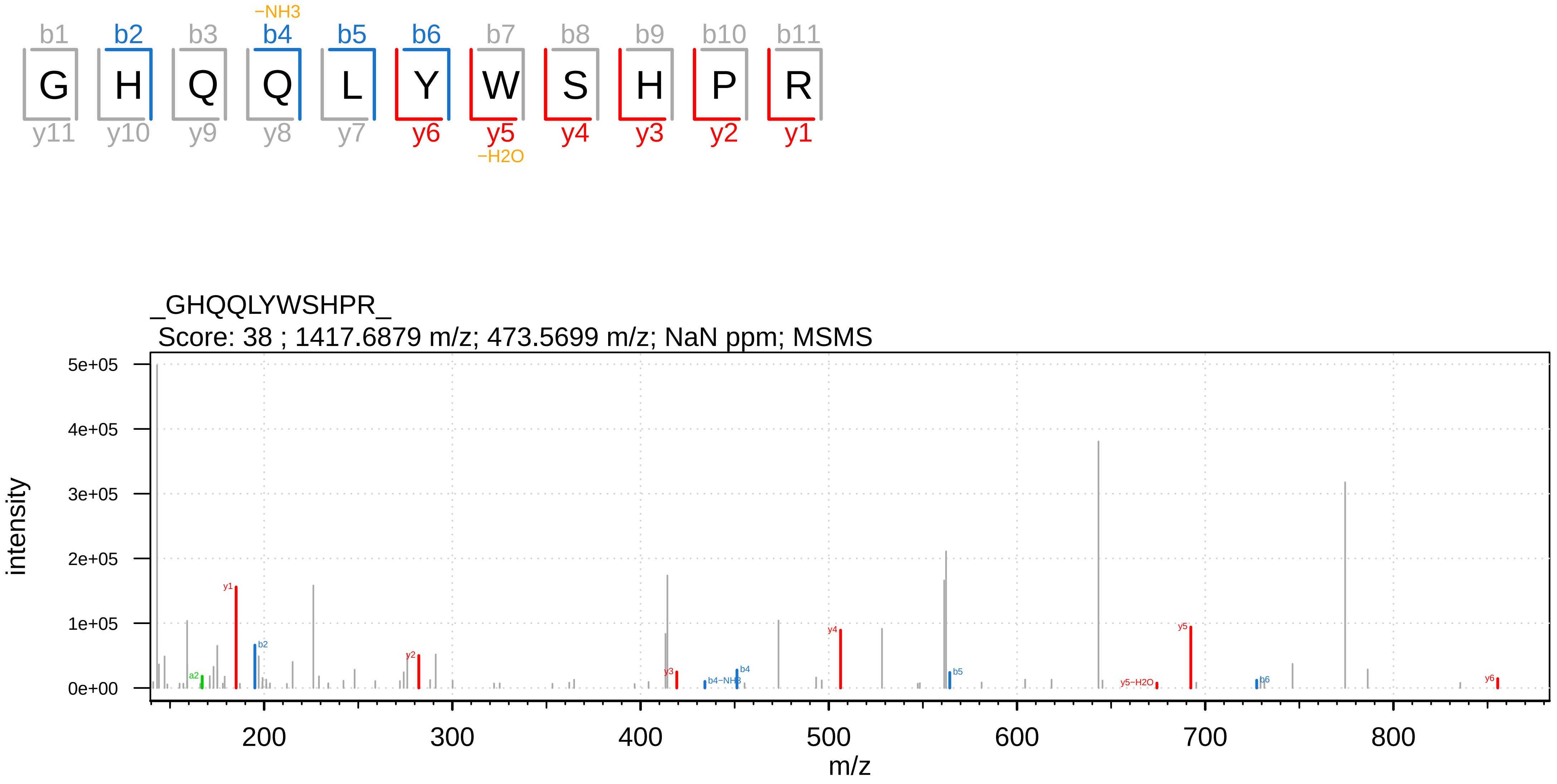

Raw File: Kermit_20111104_MSU_tube_18h_12
Scan Number: 14279
Proteins:
sp|P62274|RS29_MOUSE
ENSMUST00000037023_chr12:69157813−69159058:−
ENSMUST00000071737_chr13:73988963−73989133:−
tr|D3YYL7|D3YYL7_MOUSE

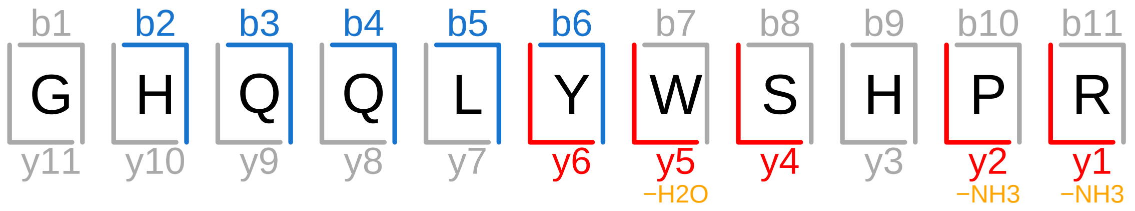

GHQQLYWSHPR
Score: 45 ; 1407.6796 m/z; 470.23382 m/z; NaN ppm; MSMS

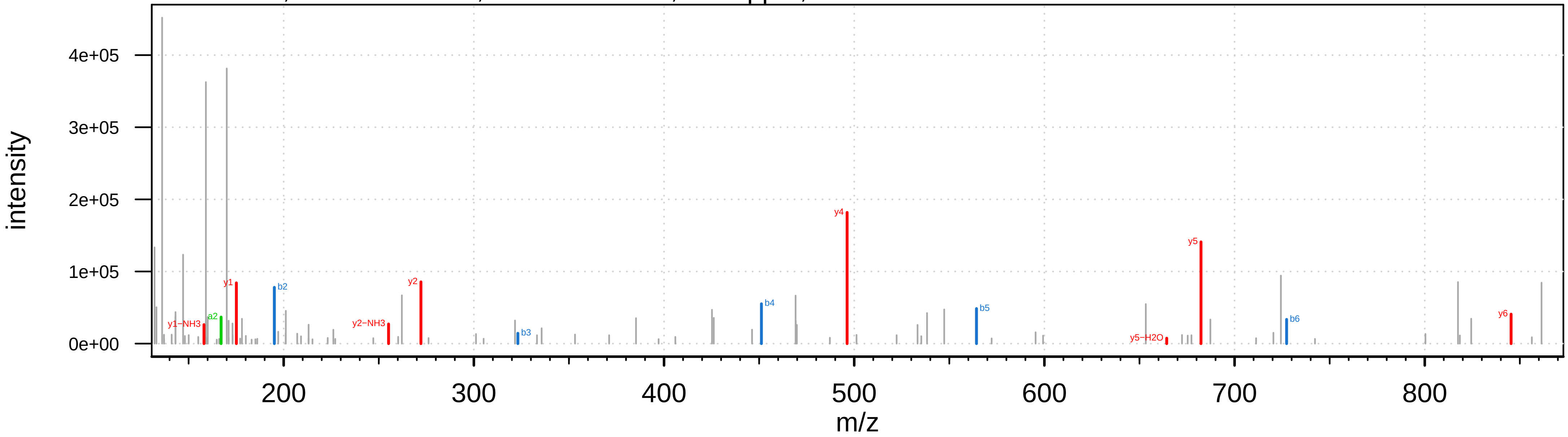

Raw File: Kermit_20111104_MSU_tube_18h_12
Scan Number: 20735
Proteins:
sp|P62274|RS29_MOUSE
ENSMUST00000037023_chr12:69157813−69159058:−
ENSMUST00000071737_chr13:73988963−73989133:−
tr|D3YYL7|D3YYL7_MOUSE

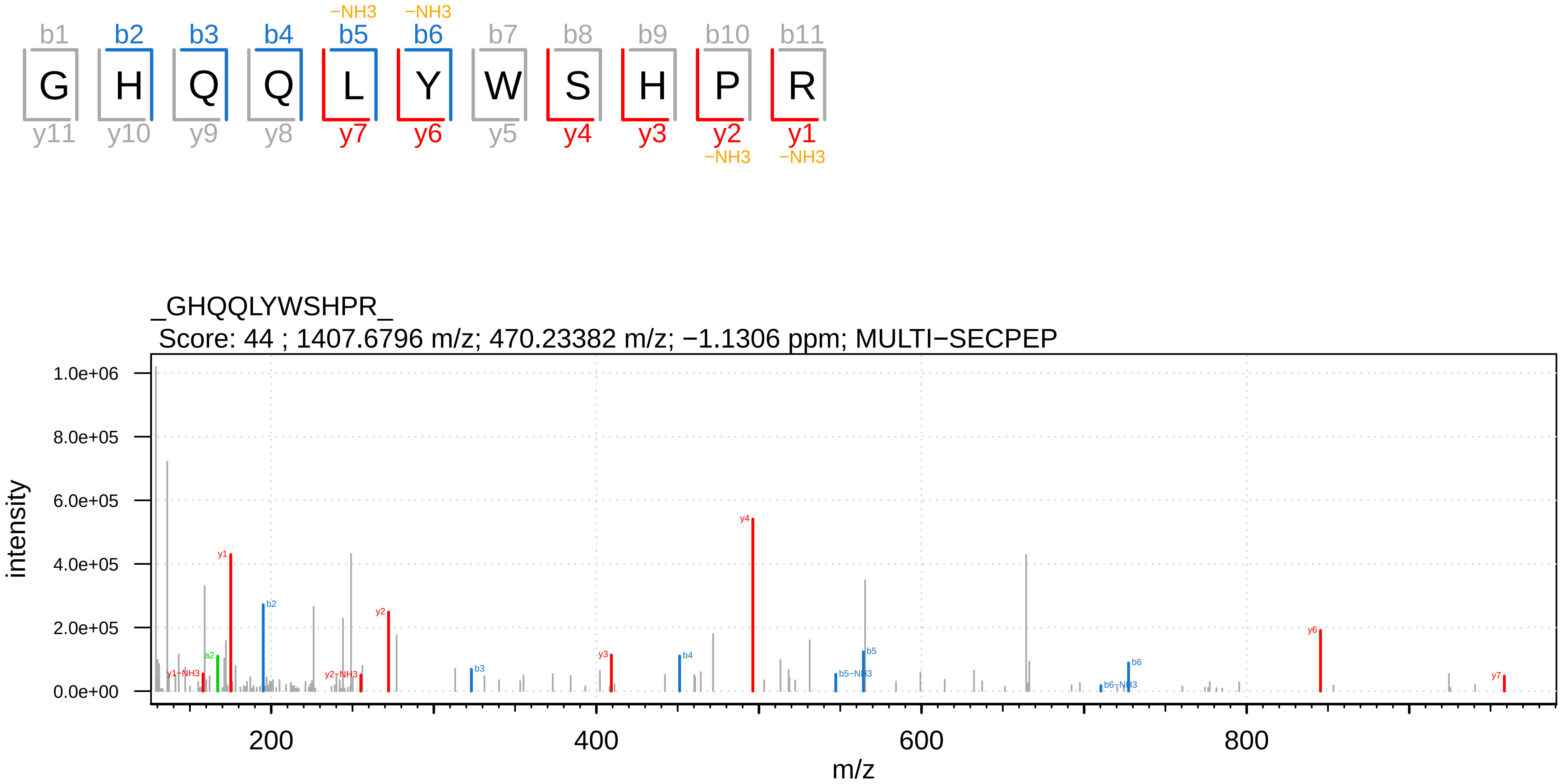

Raw File: Kermit_20111104_MSU_tube_18h_12
Scan Number: 10537
Proteins:
sp|P62274|RS29_MOUSE
ENSMUST00000037023_chr12:69157813−69159058:−
ENSMUST00000071737_chr13:73988963−73989133:−
tr|D3YYL7|D3YYL7_MOUSE

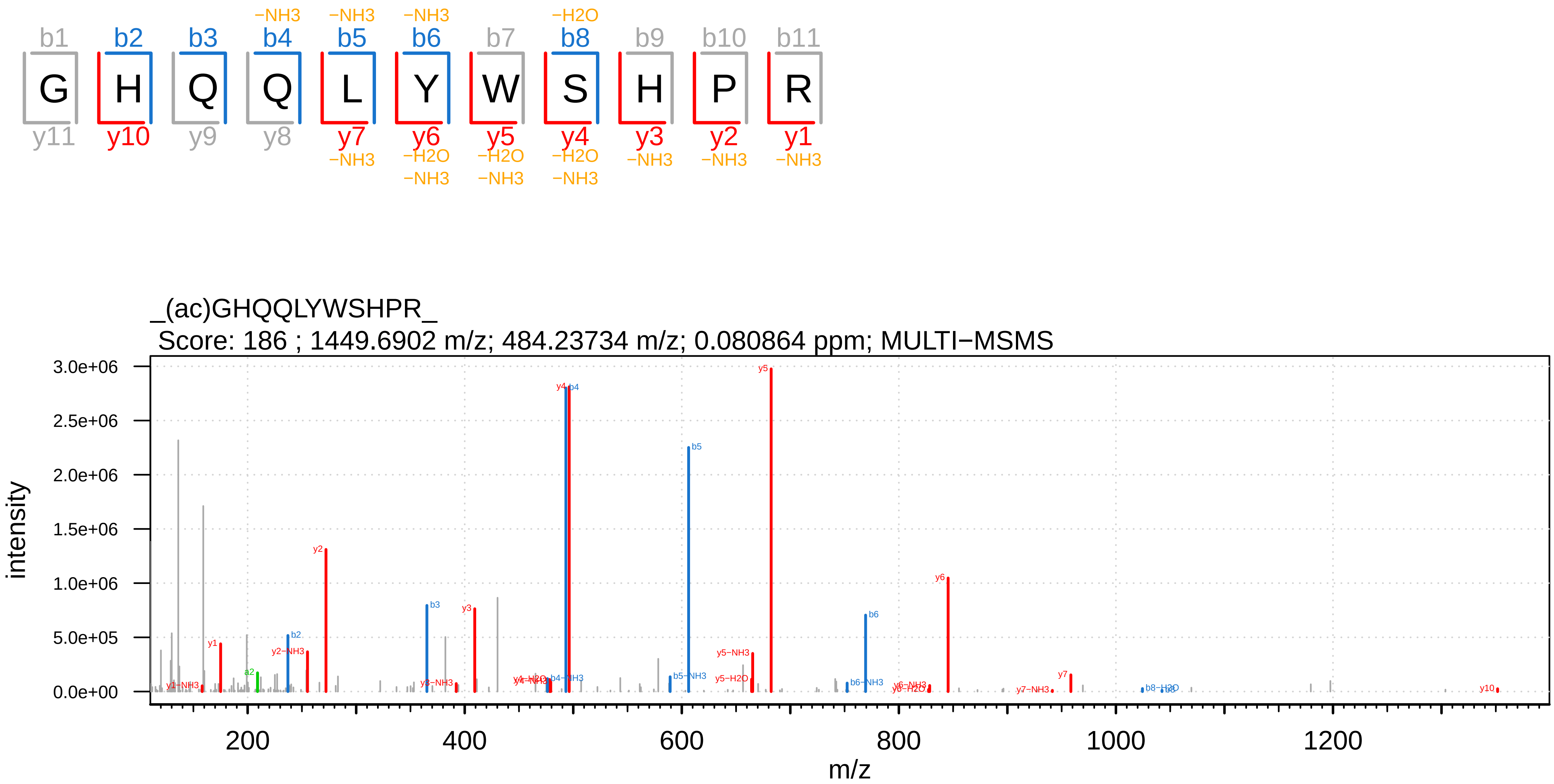

Raw File: Kermit_20111104_MSU_tube_18h_12
Scan Number: 14544
Proteins:
sp|P62274|RS29_MOUSE
ENSMUST00000037023_chr12:69157813−69159058:−
ENSMUST00000071737_chr13:73988963−73989133:−
tr|D3YYL7|D3YYL7_MOUSE

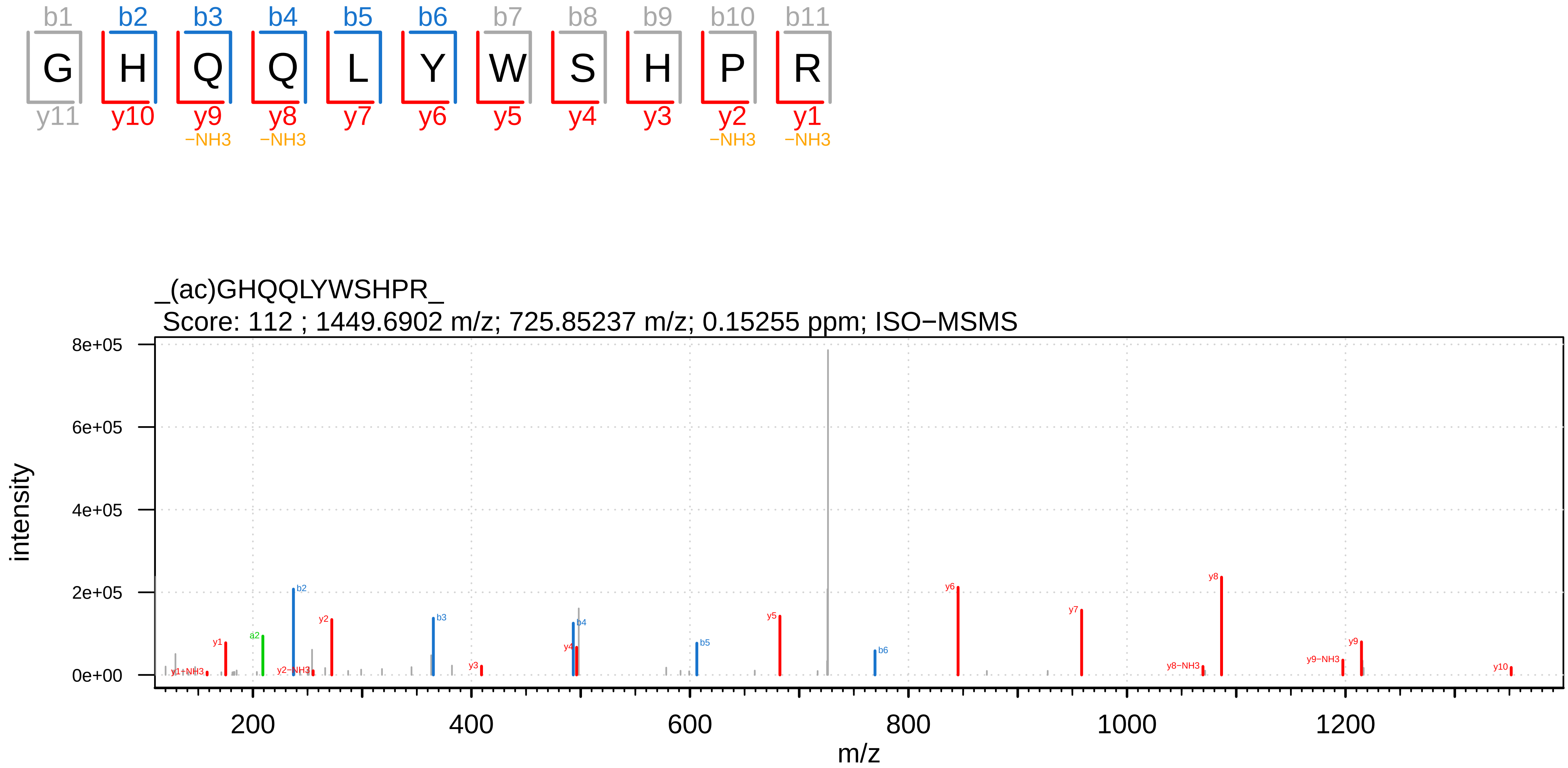

Raw File: Kermit_20111104_MSU_tube_18h_12
Scan Number: 14530
Proteins:
sp|P62274|RS29_MOUSE
ENSMUST00000037023_chr12:69157813−69159058:−
ENSMUST00000071737_chr13:73988963−73989133:−
tr|D3YYL7|D3YYL7_MOUSE

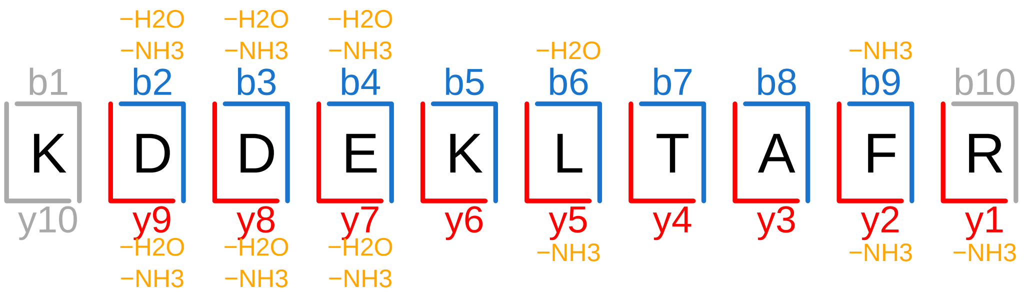

KDDEKLTAFR

Score: 217 ; 1221.6354 m/z; 611.82495 m/z; −0.27454 ppm; ISO−MSMS

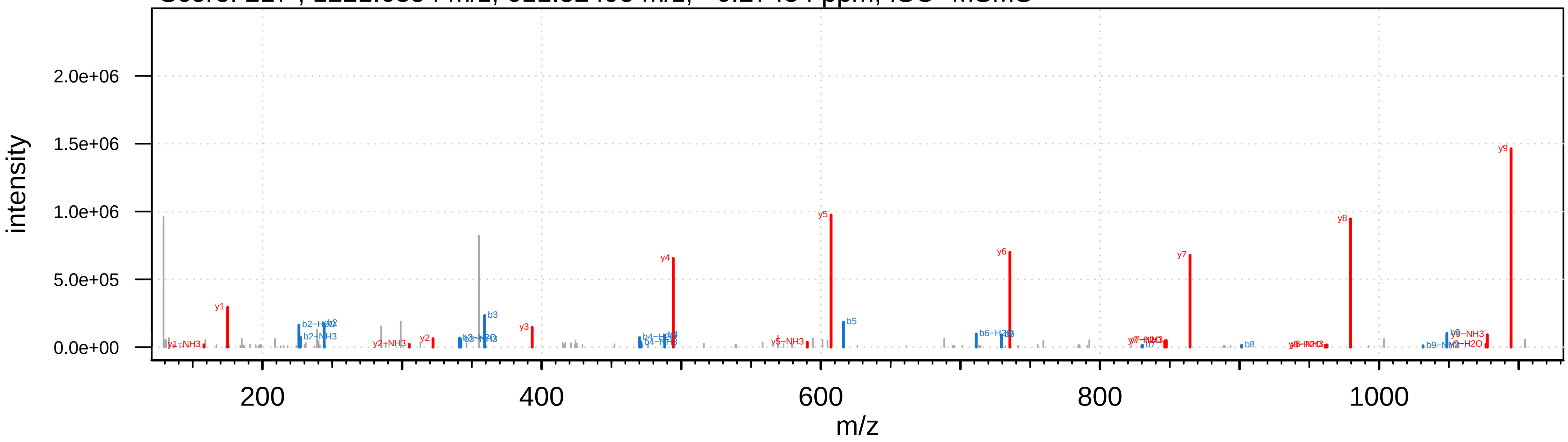

Raw File: Kermit_20111104_MSU_tube_18h_12
Scan Number: 8414
Proteins:
chr12_103206313−103214181.T2_chr12:101968174−101969629:−

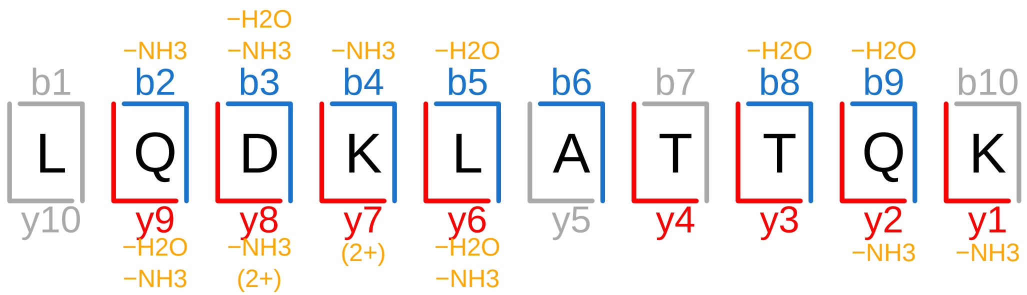

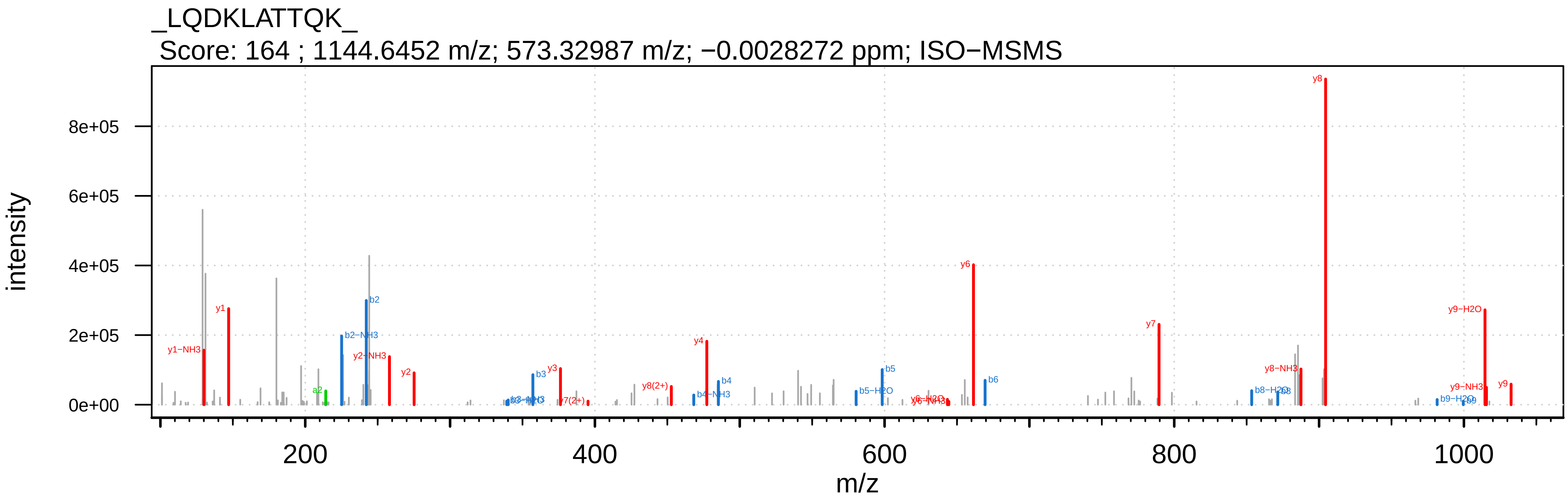

Raw File: Kermit_20111104_MSU_tube_18h_12
Scan Number: 5206
Proteins:
sp|Q8BT35|YB039_MOUSE
ENSMUST00000135091_chr2:127792297−127792467:−

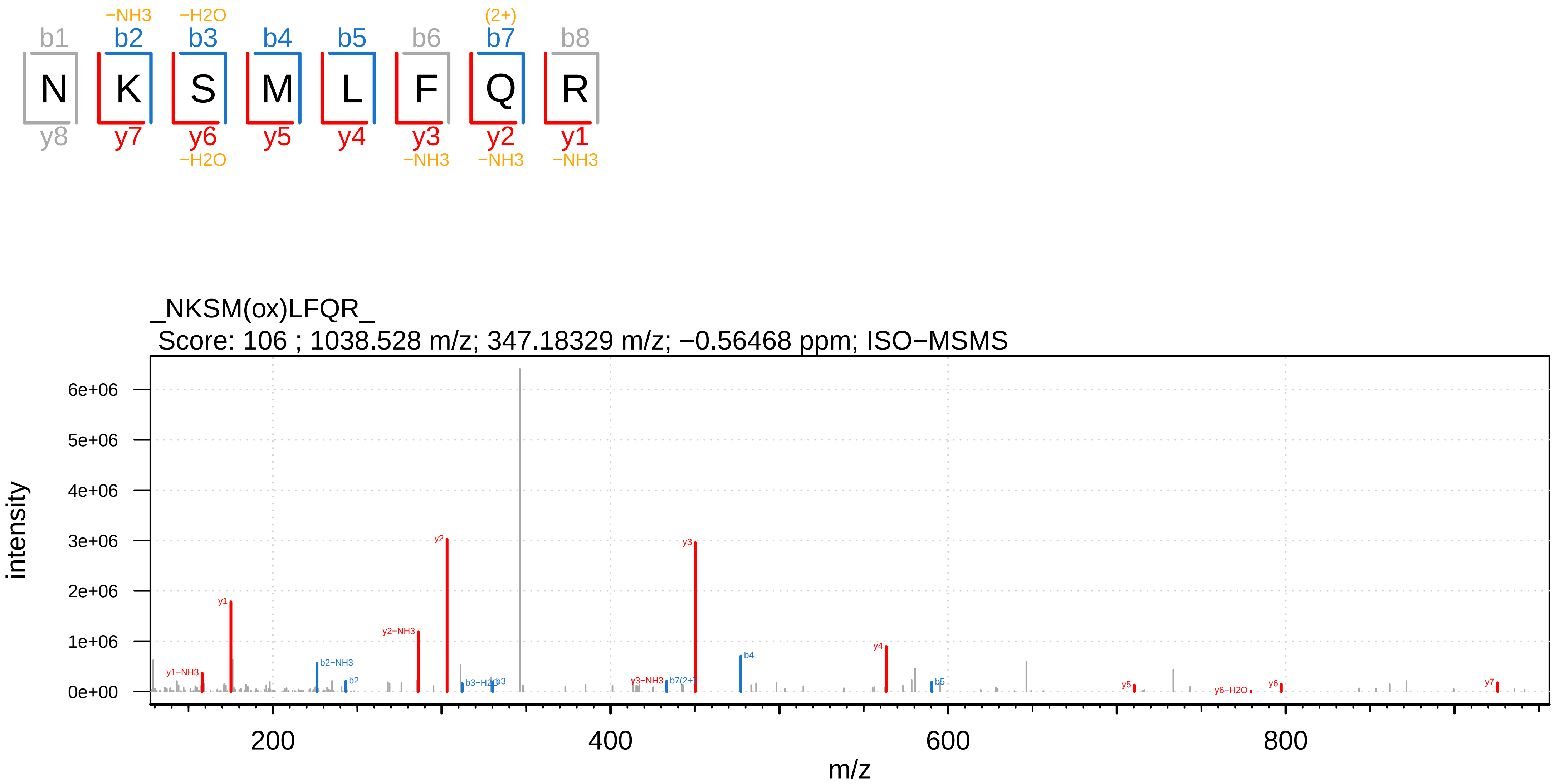

Raw File: Kermit_20111104_MSU_tube_18h_12
Scan Number: 6530
Proteins:
chr12_103206313−103214181.T2_chr12:101968174−101969629:−

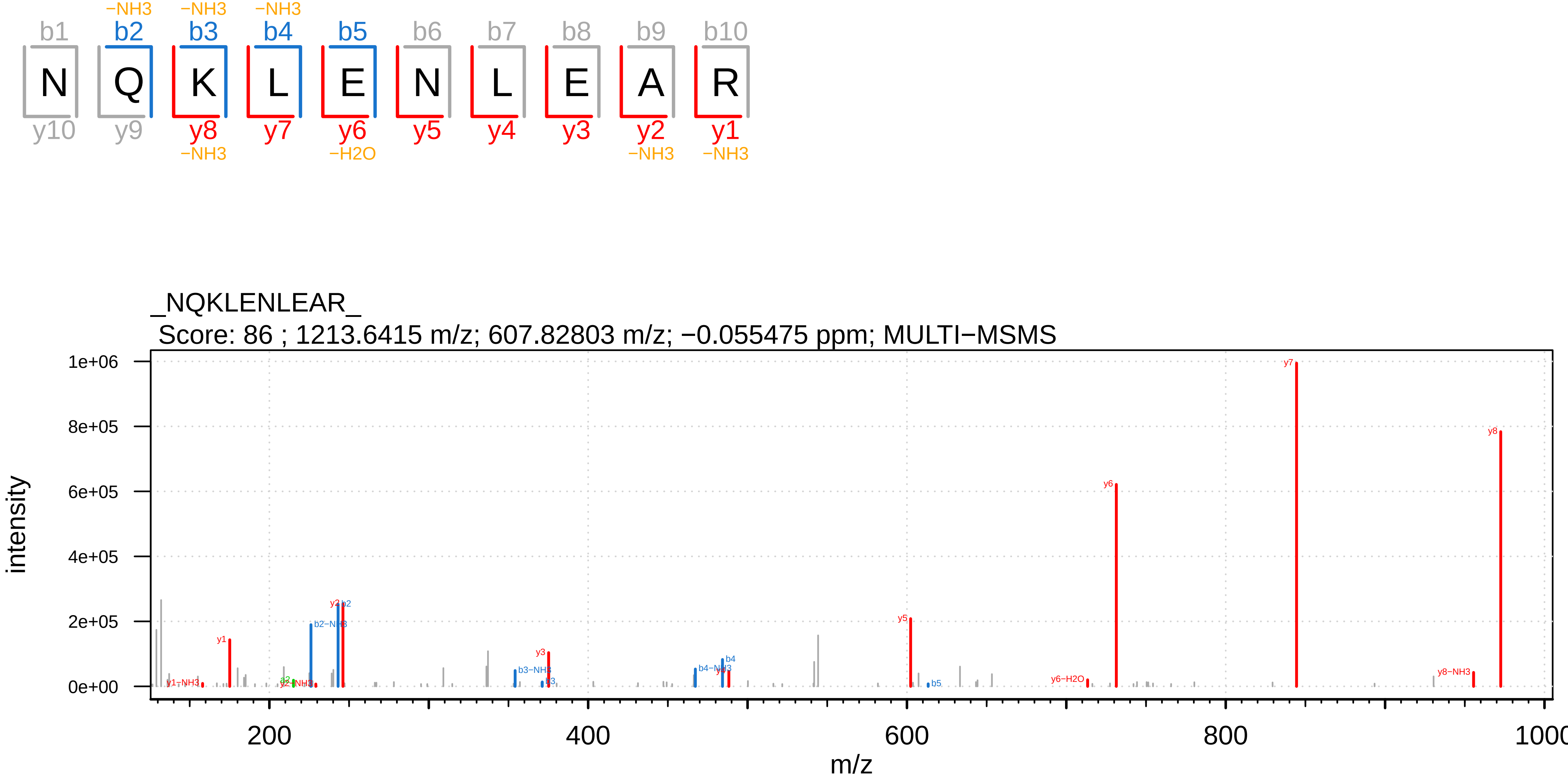

Raw File: Kermit_20111104_MSU_tube_18h_12

Scan Number: 7415

Proteins:

ENSMUST00000023048_chr15:80235835−80236047:+

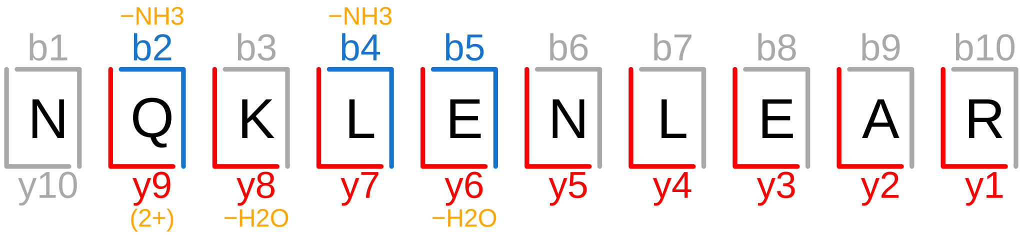

NQKLENLEAR

Score: 78 ; 1231.664 m/z; 616.83926 m/z; −0.055475 ppm; MULTI−MSMS

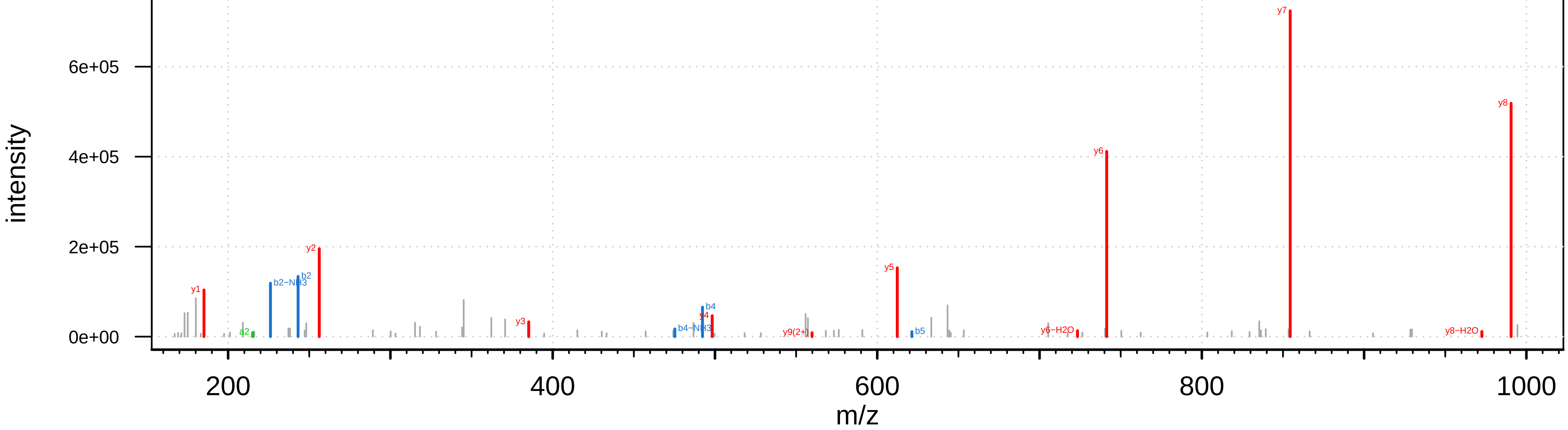

Raw File: Kermit_20111104_MSU_tube_18h_12

Scan Number: 7419

Proteins:

ENSMUST00000023048_chr15:80235835−80236047:+

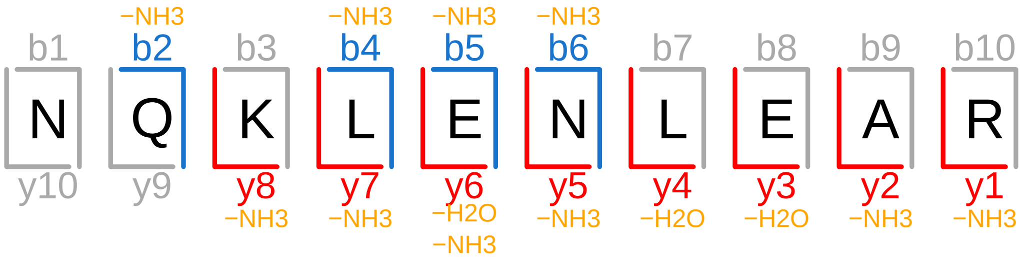

NQKLENLEAR

Score: 153 ; 1213.6415 m/z; 405.55444 m/z; 0.047931 ppm; MULTI−MSMS

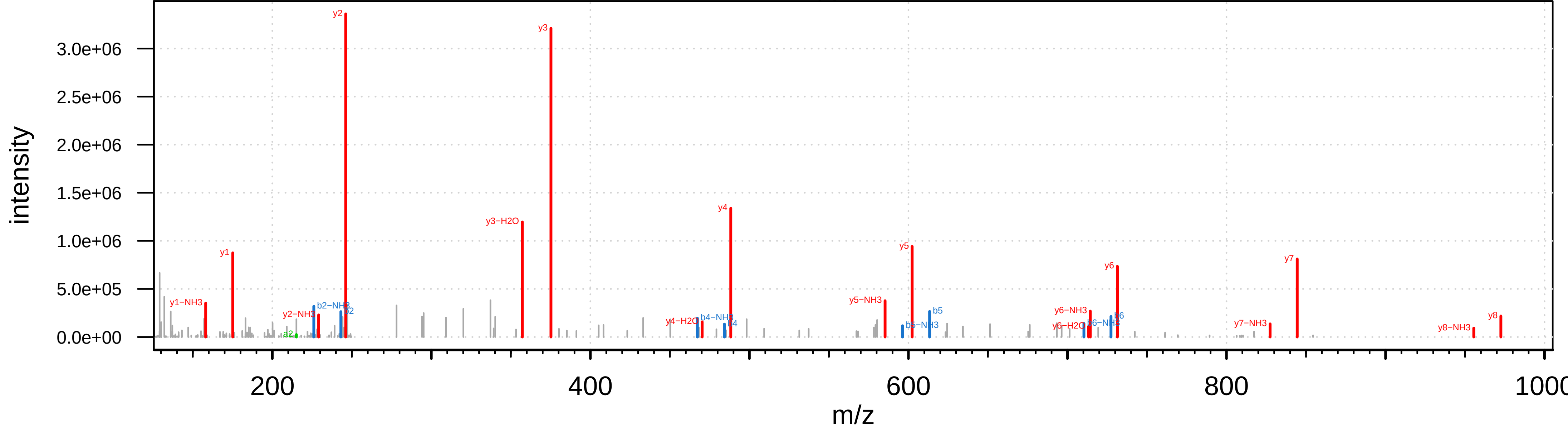

Raw File: Kermit_20111104_MSU_tube_18h_12

Scan Number: 7448

Proteins:

ENSMUST00000023048_chr15:80235835−80236047:+

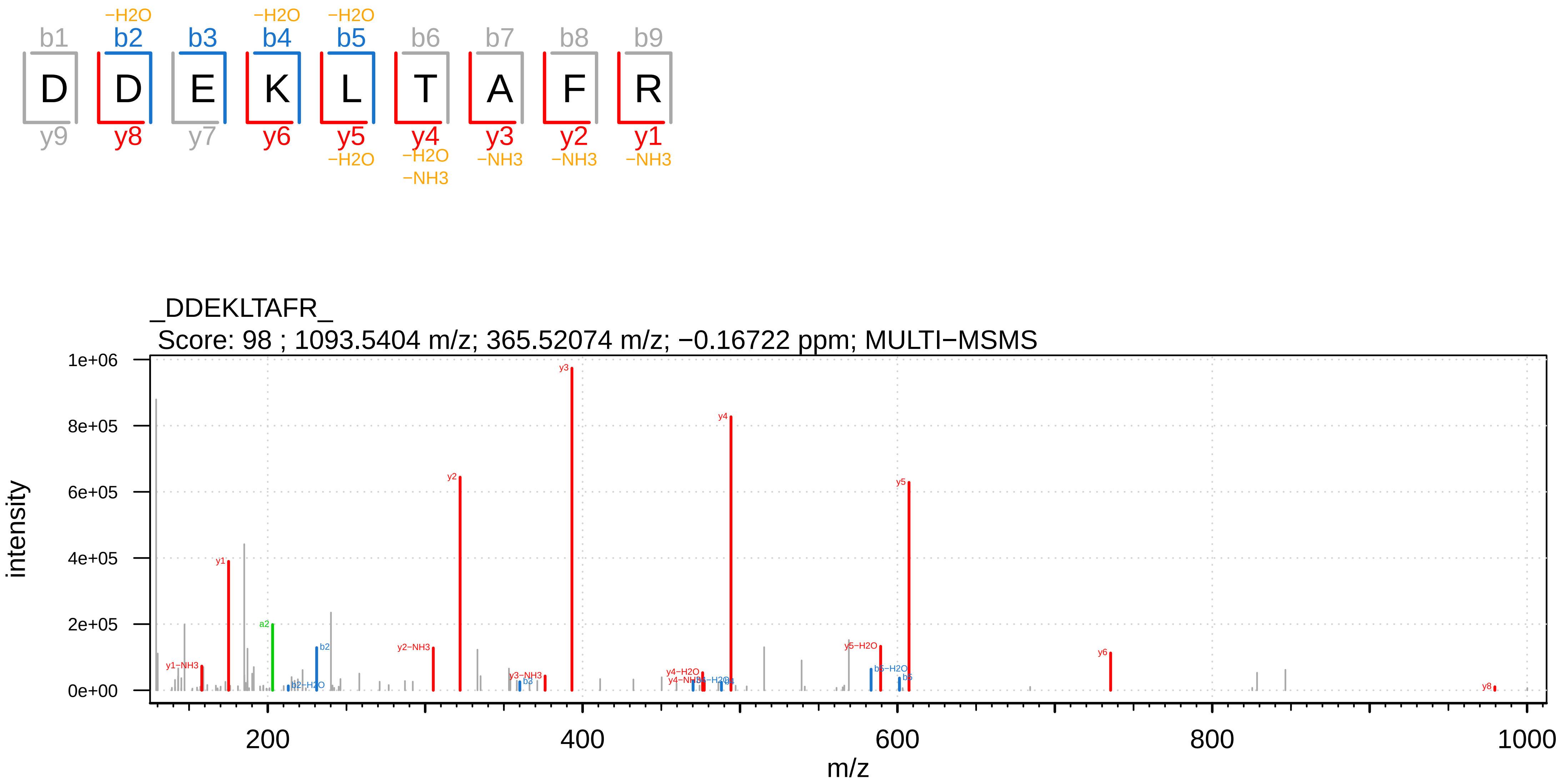

Raw File: Kermit_20111104_MSU_tube_6h_12
Scan Number: 12503
Proteins:
chr12_103206313−103214181.T2_chr12:101968174−101969629:−

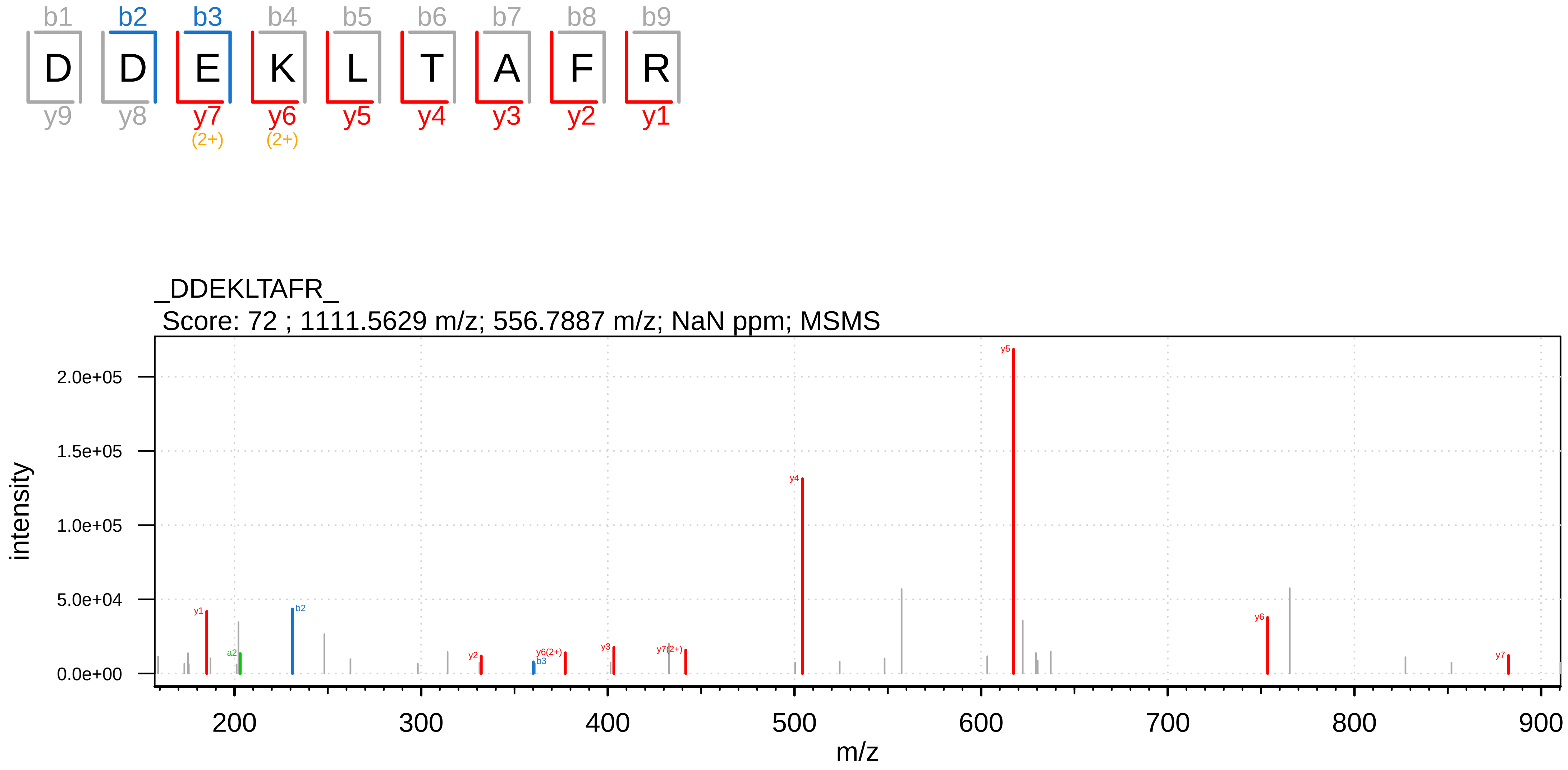

Raw File: Kermit_20111104_MSU_tube_6h_12
Scan Number: 12494
Proteins:
chr12_103206313−103214181.T2_chr12:101968174−101969629:−

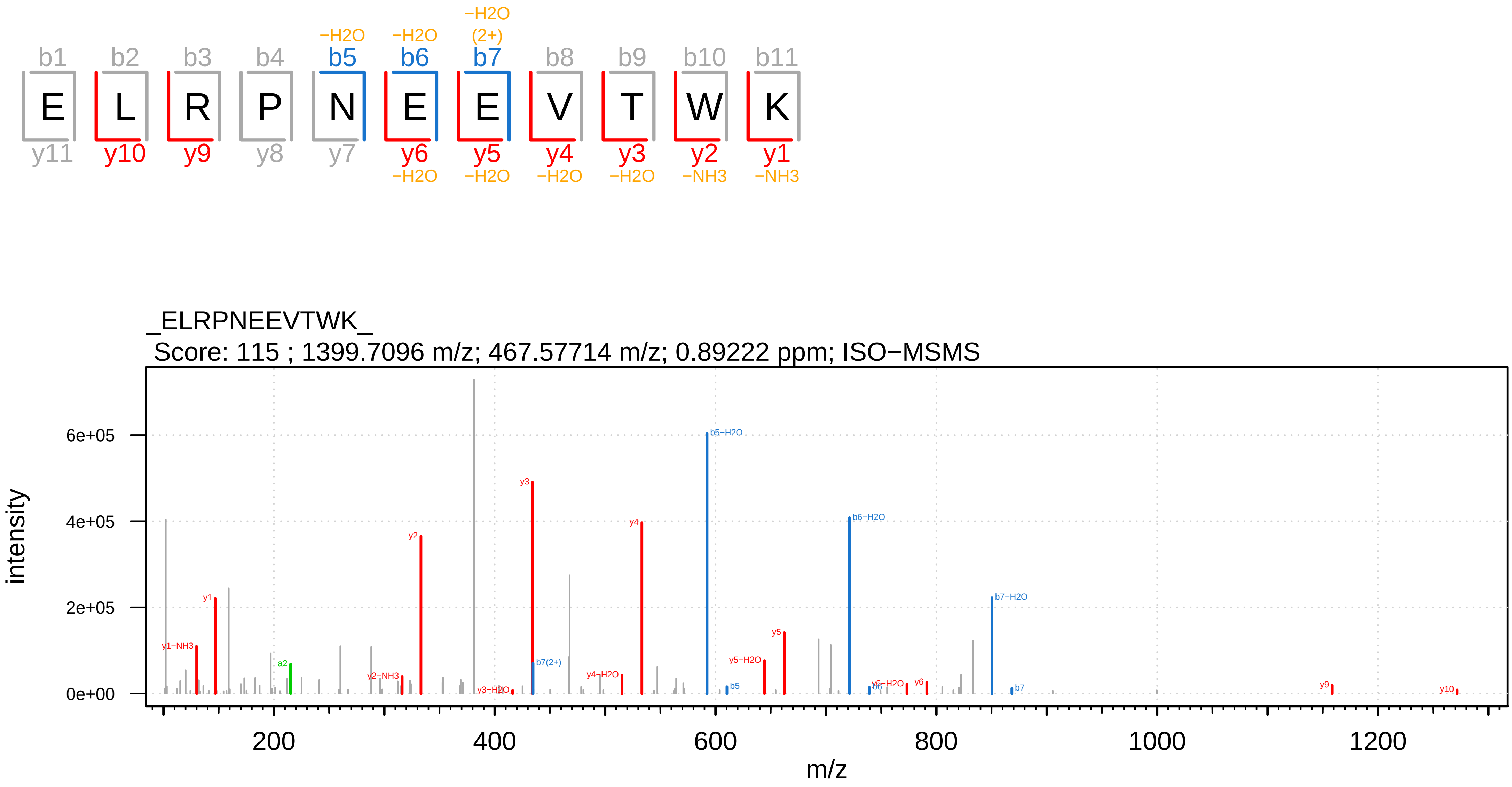

Raw File: Kermit_20111104_MSU_tube_6h_12
Scan Number: 16570
Proteins:
chr12_103206313−103214181.T2_chr12:101968174−101969629:−

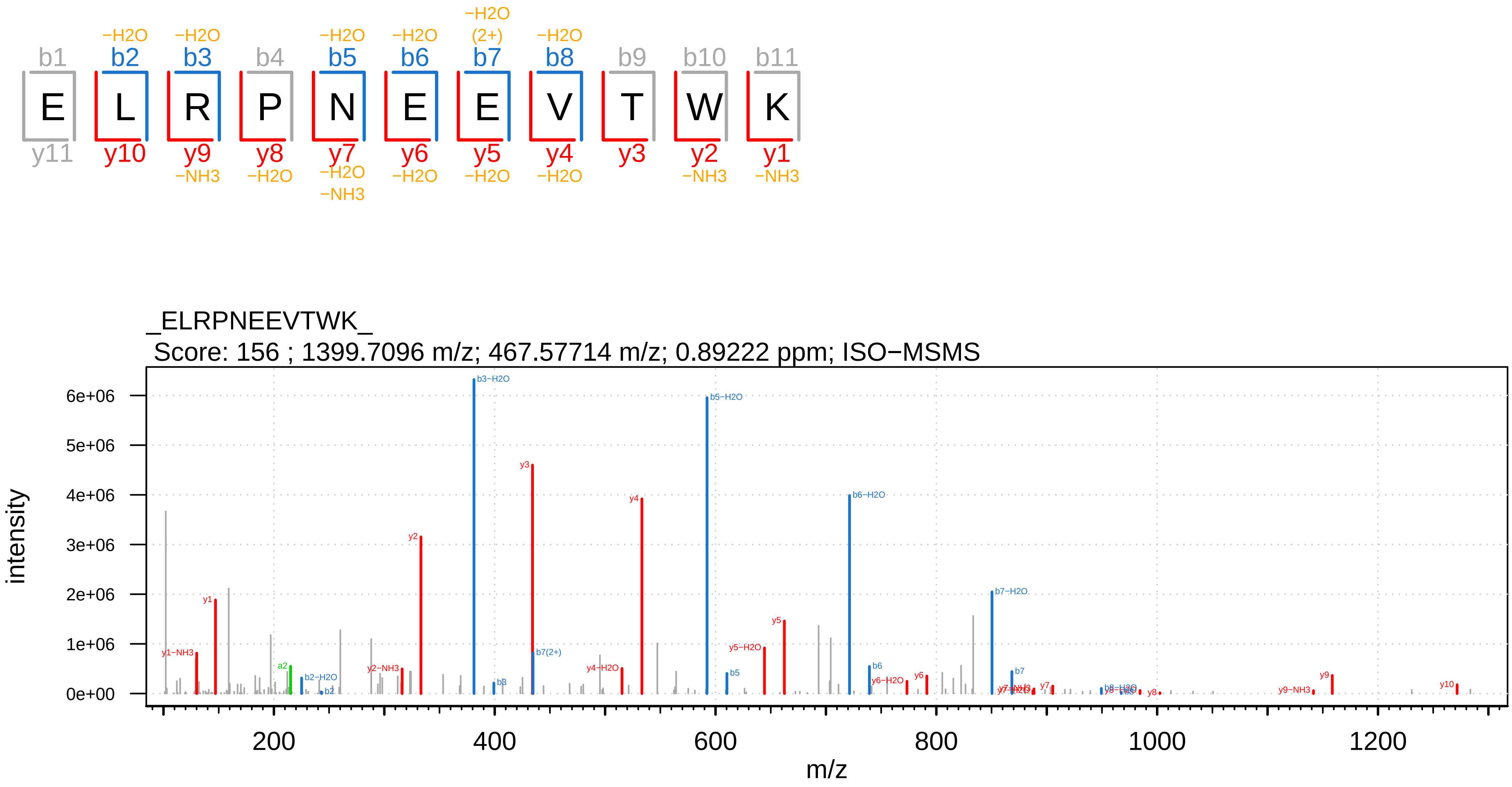

Raw File: Kermit_20111104_MSU_tube_6h_12
Scan Number: 16744
Proteins:
chr12_103206313−103214181.T2_chr12:101968174−101969629:−

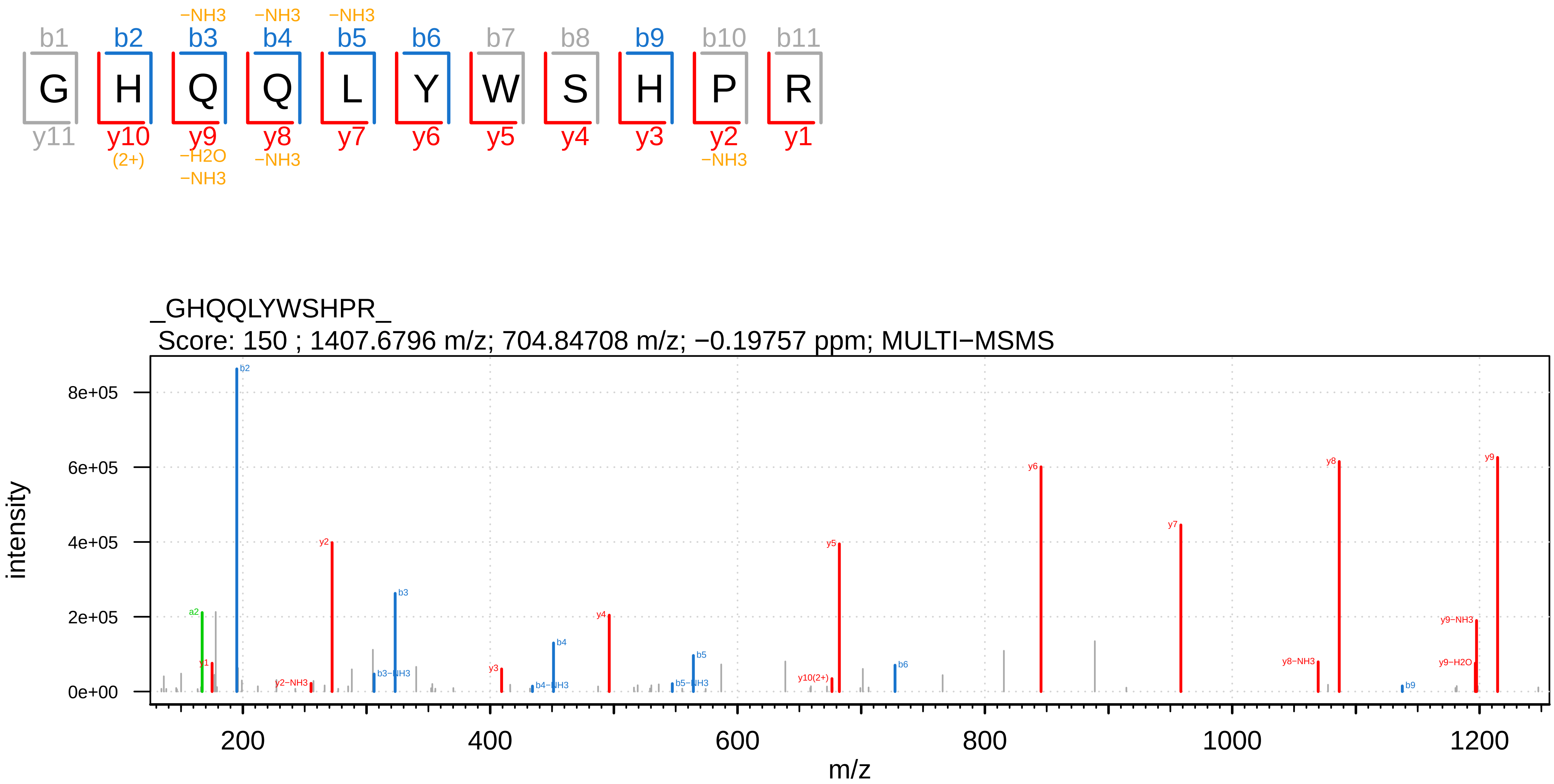

Raw File: Kermit_20111104_MSU_tube_6h_12
Scan Number: 8372
Proteins:
sp|P62274|RS29_MOUSE
ENSMUST00000037023_chr12:69157813−69159058:−
ENSMUST00000071737_chr13:73988963−73989133:−
tr|D3YYL7|D3YYL7_MOUSE

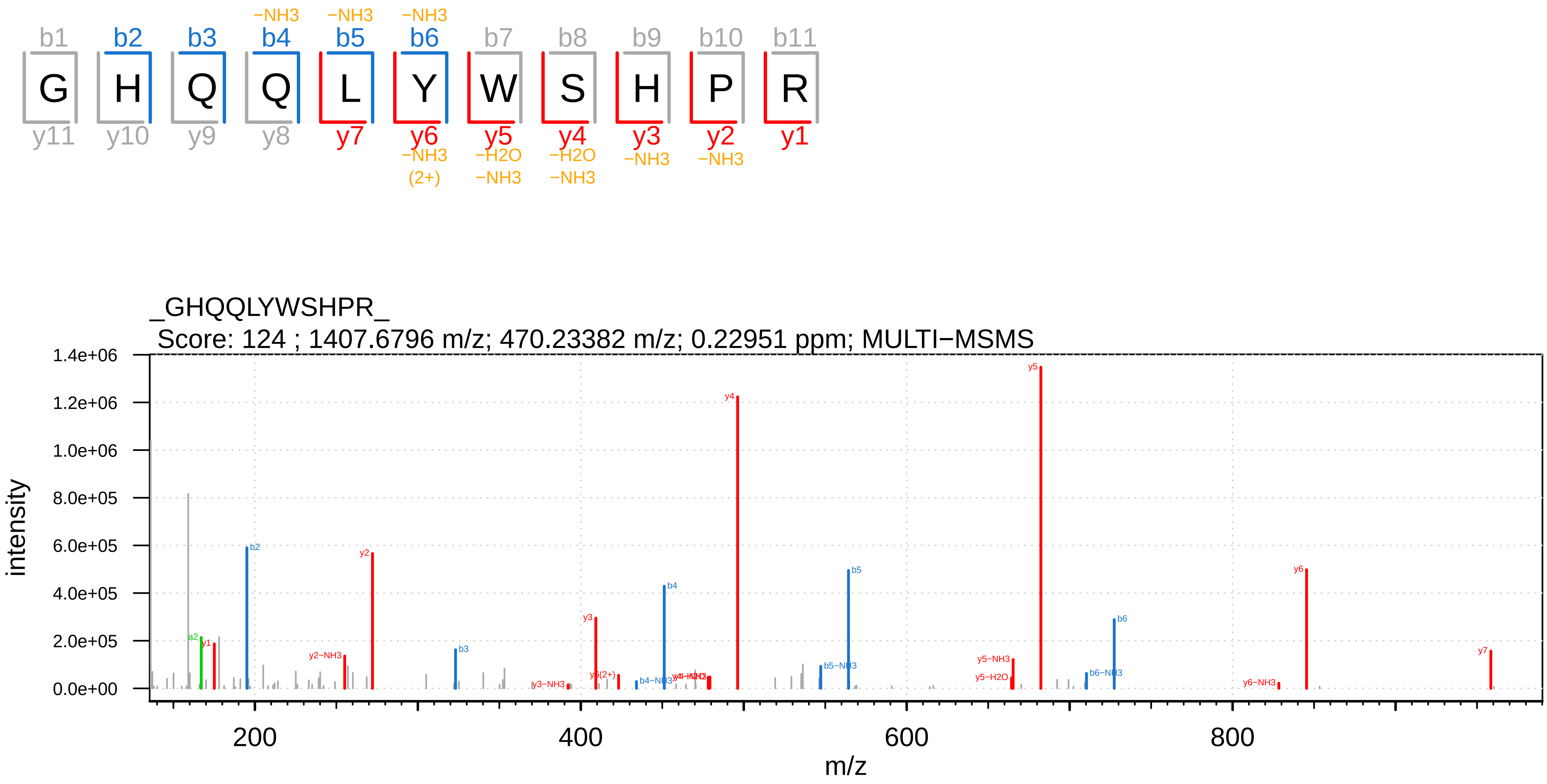

Raw File: Kermit_20111104_MSU_tube_6h_12
Scan Number: 49528
Proteins:
sp|P62274|RS29_MOUSE
ENSMUST00000037023_chr12:69157813−69159058:−
ENSMUST00000071737_chr13:73988963−73989133:−
tr|D3YYL7|D3YYL7_MOUSE

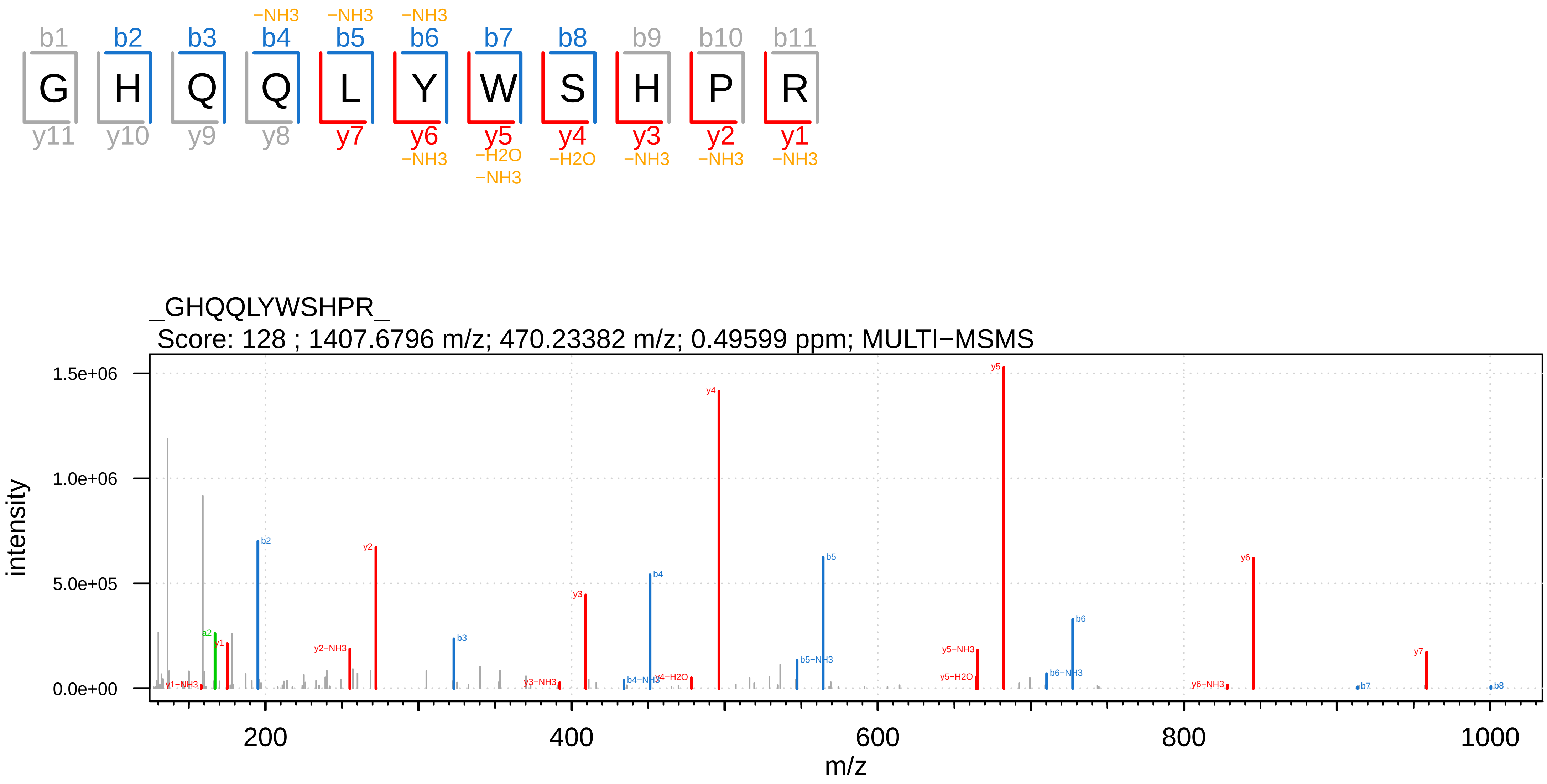

Raw File: Kermit_20111104_MSU_tube_6h_12
Scan Number: 49711
Proteins:
sp|P62274|RS29_MOUSE
ENSMUST00000037023_chr12:69157813−69159058:−
ENSMUST00000071737_chr13:73988963−73989133:−
tr|D3YYL7|D3YYL7_MOUSE

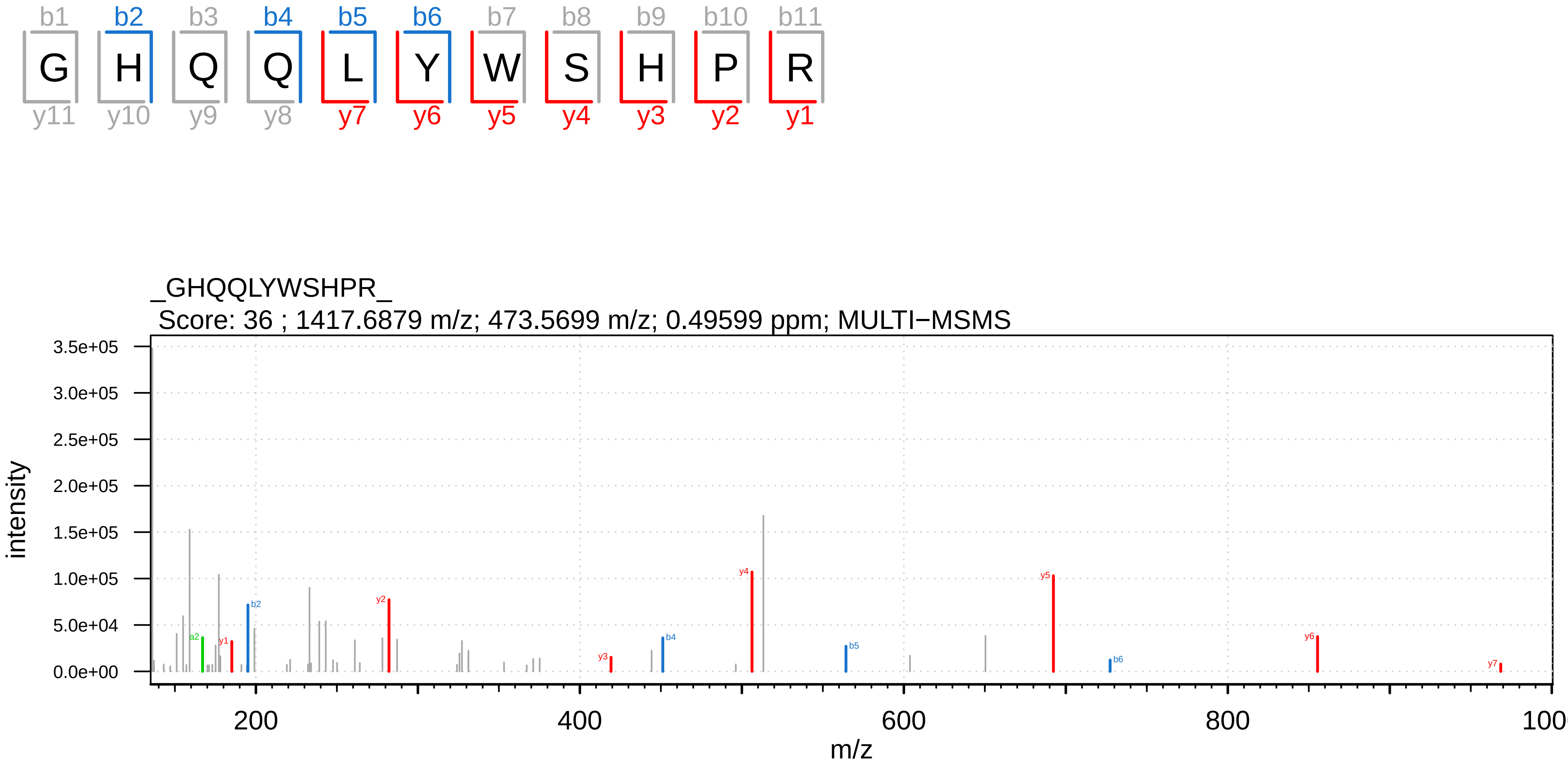

Raw File: Kermit_20111104_MSU_tube_6h_12
Scan Number: 49763
Proteins:
sp|P62274|RS29_MOUSE
ENSMUST00000037023_chr12:69157813−69159058:−
ENSMUST00000071737_chr13:73988963−73989133:−
tr|D3YYL7|D3YYL7_MOUSE

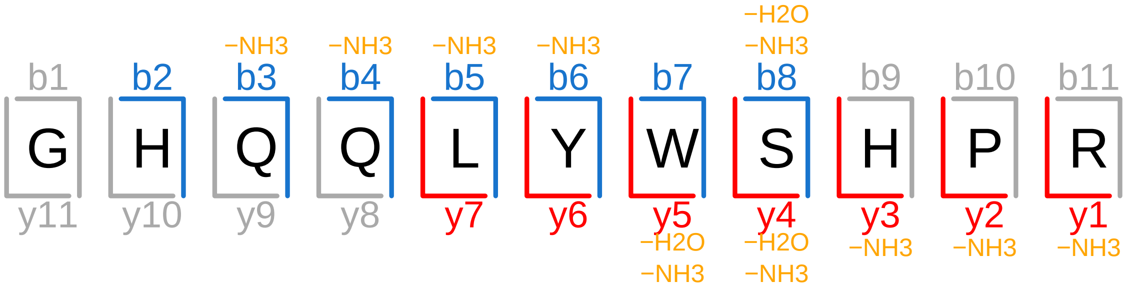

GHQQLYWSHPR

Score: 141 ; 1407.6796 m/z; 470.23382 m/z; 0.46128 ppm; MULTI−MSMS

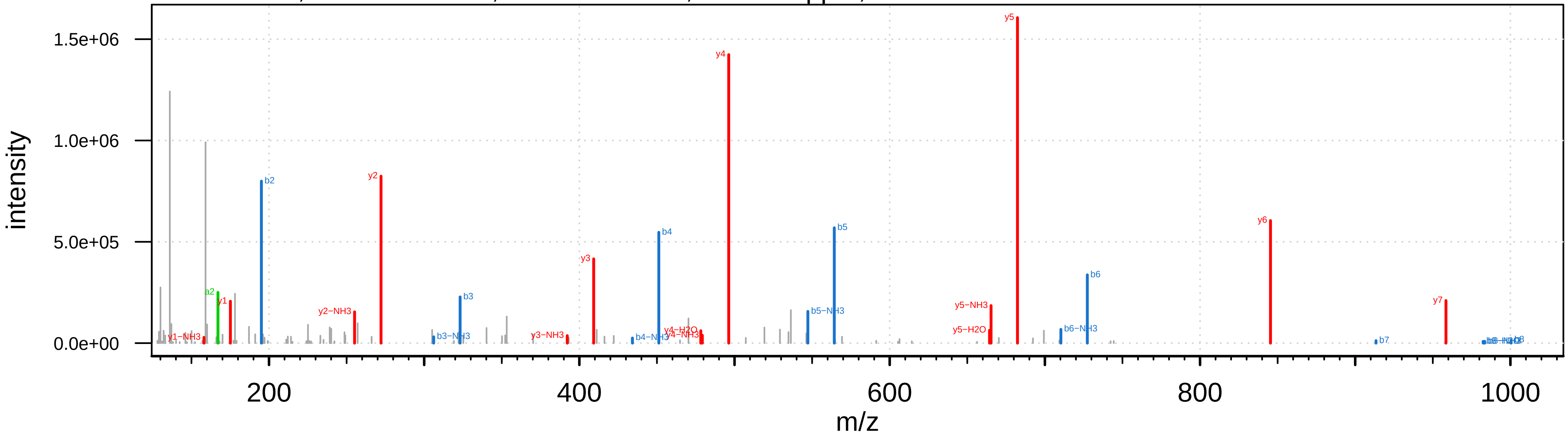

Raw File: Kermit_20111104_MSU_tube_6h_12
Scan Number: 49899
Proteins:
sp|P62274|RS29_MOUSE
ENSMUST00000037023_chr12:69157813−69159058:−
ENSMUST00000071737_chr13:73988963−73989133:−
tr|D3YYL7|D3YYL7_MOUSE

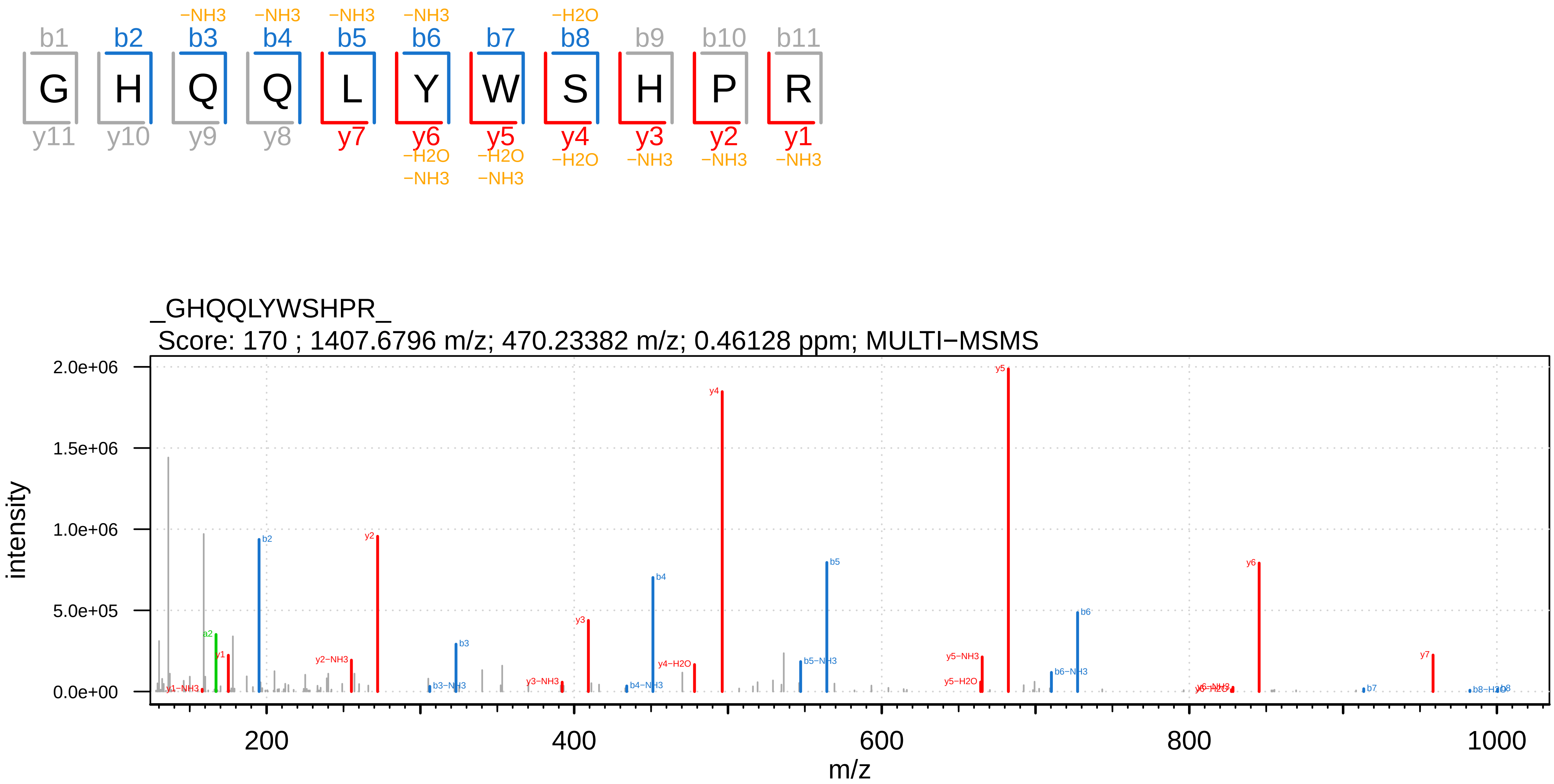

Raw File: Kermit_20111104_MSU_tube_6h_12
Scan Number: 50089
Proteins:
sp|P62274|RS29_MOUSE
ENSMUST00000037023_chr12:69157813−69159058:−
ENSMUST00000071737_chr13:73988963−73989133:−
tr|D3YYL7|D3YYL7_MOUSE

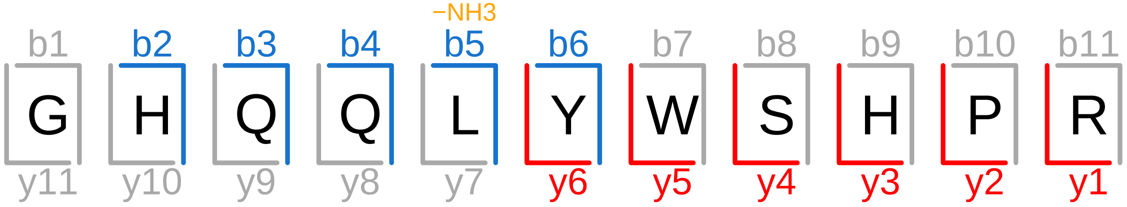

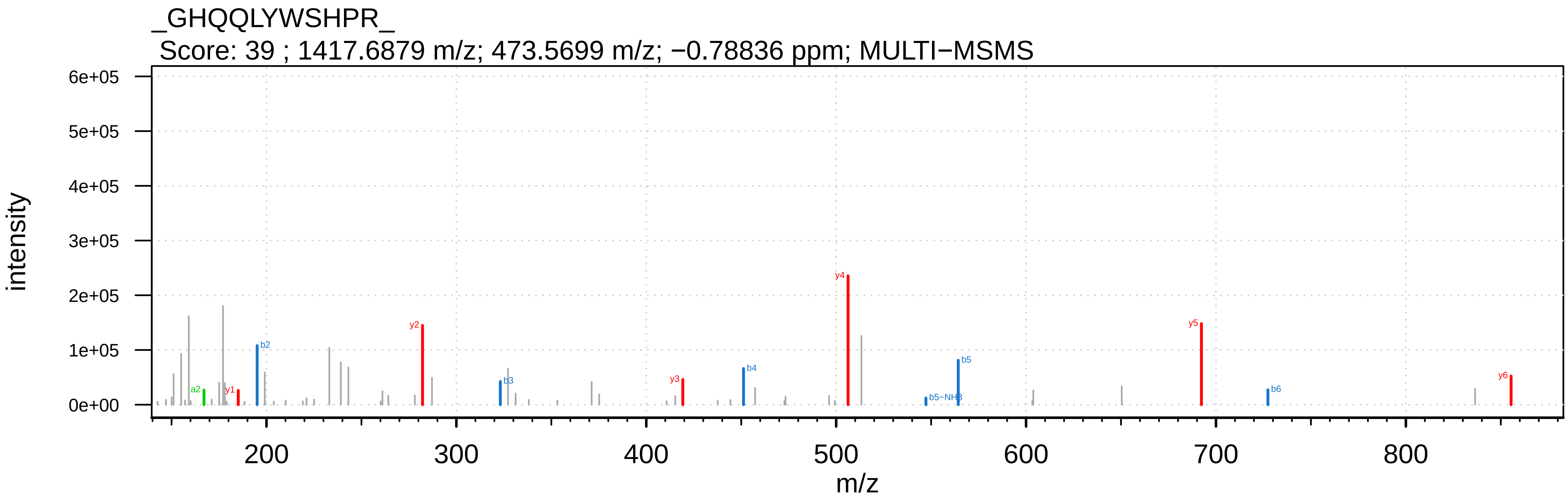

Raw File: Kermit_20111104_MSU_tube_6h_12
Scan Number: 50183
Proteins:
sp|P62274|RS29_MOUSE
ENSMUST00000037023_chr12:69157813−69159058:−
ENSMUST00000071737_chr13:73988963−73989133:−
tr|D3YYL7|D3YYL7_MOUSE

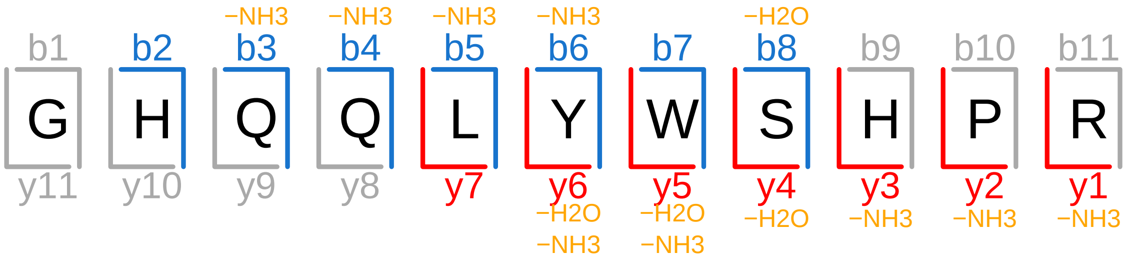

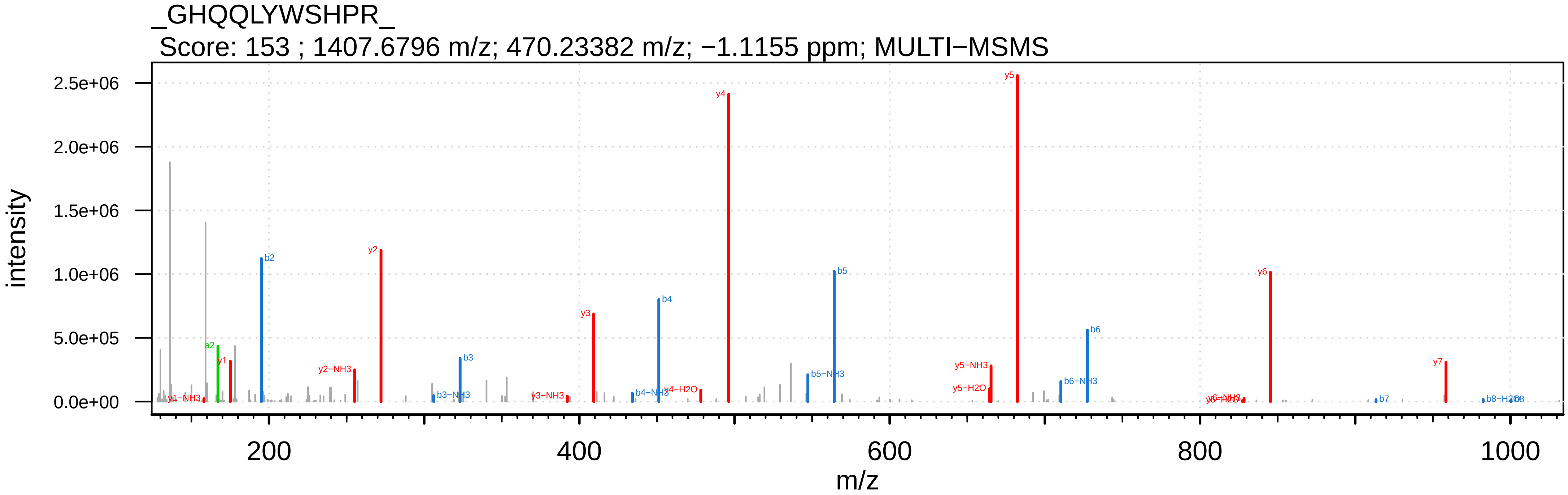

Raw File: Kermit_20111104_MSU_tube_6h_12
Scan Number: 50277
Proteins:
sp|P62274|RS29_MOUSE
ENSMUST00000037023_chr12:69157813−69159058:−
ENSMUST00000071737_chr13:73988963−73989133:−
tr|D3YYL7|D3YYL7_MOUSE

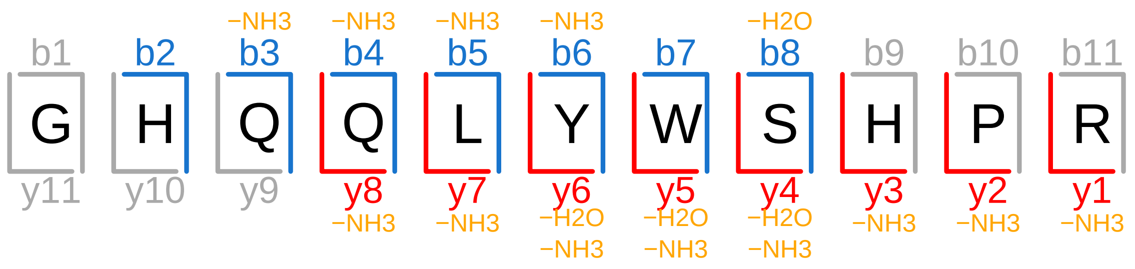

GHQQLYWSHPR

Score: 177 ; 1407.6796 m/z; 470.23382 m/z; 0.2182 ppm; MULTI−MSMS

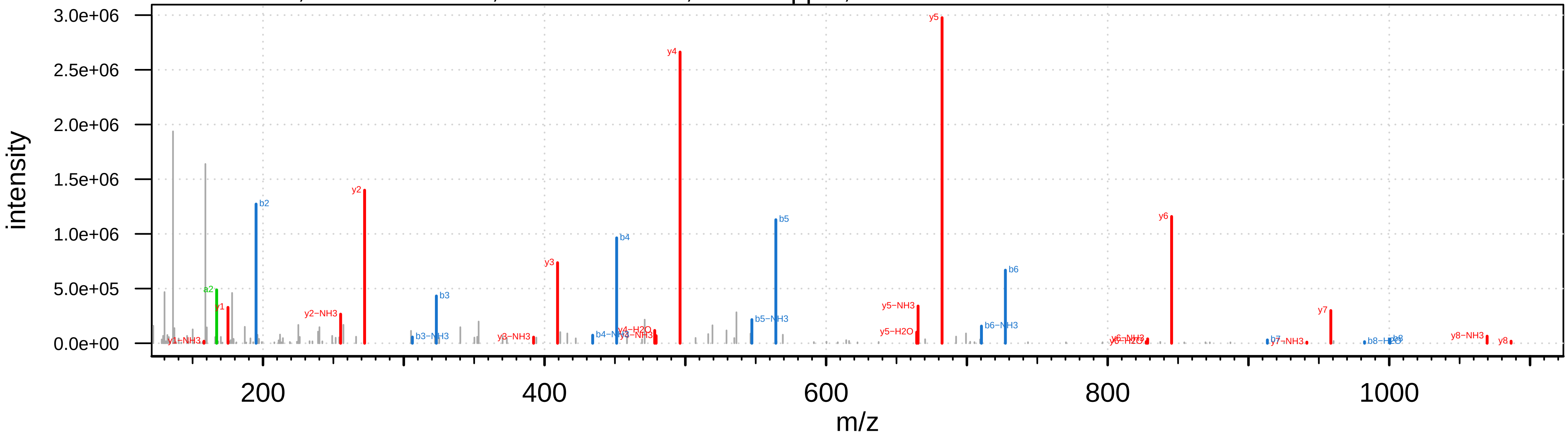

Raw File: Kermit_20111104_MSU_tube_6h_12

Scan Number: 50835

Proteins:

sp|P62274|RS29_MOUSE

ENSMUST00000037023_chr12:69157813−69159058:−

ENSMUST00000071737_chr13:73988963−73989133:−

tr|D3YYL7|D3YYL7_MOUSE

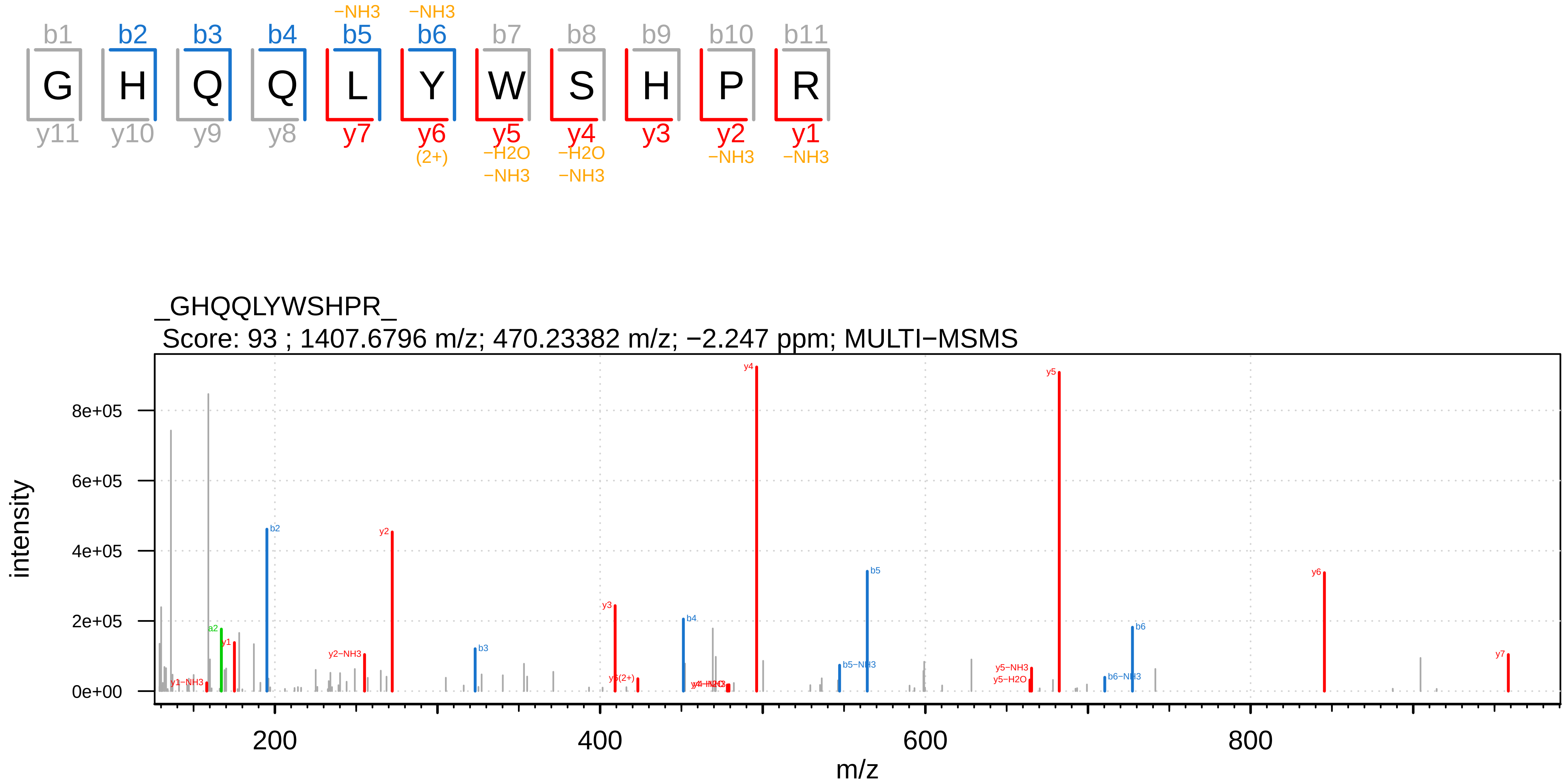

Raw File: Kermit_20111104_MSU_tube_6h_12
Scan Number: 52913
Proteins:
sp|P62274|RS29_MOUSE
ENSMUST00000037023_chr12:69157813−69159058:−
ENSMUST00000071737_chr13:73988963−73989133:−
tr|D3YYL7|D3YYL7_MOUSE

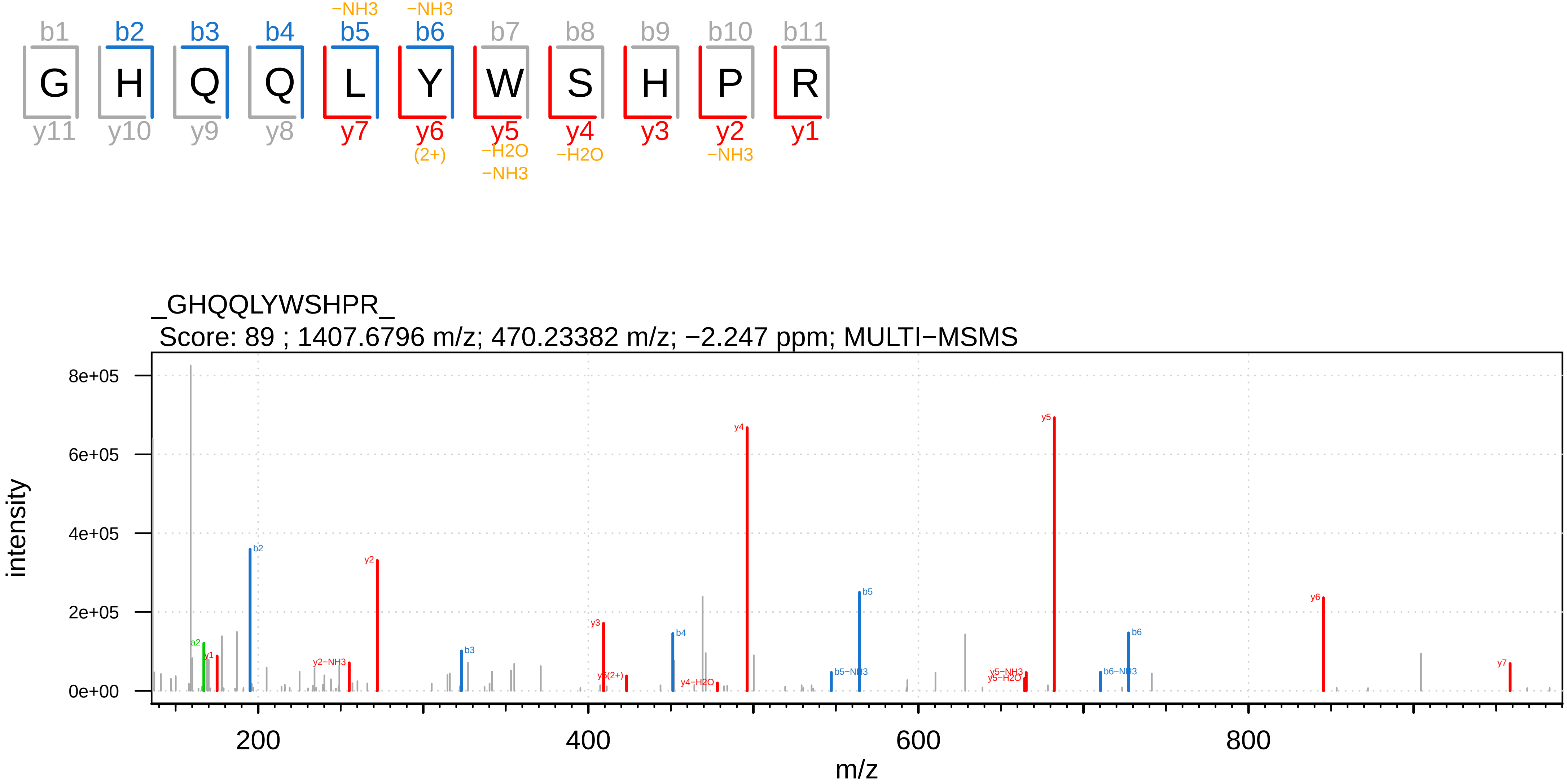

Raw File: Kermit_20111104_MSU_tube_6h_12
Scan Number: 53105
Proteins:
sp|P62274|RS29_MOUSE
ENSMUST00000037023_chr12:69157813−69159058:−
ENSMUST00000071737_chr13:73988963−73989133:−
tr|D3YYL7|D3YYL7_MOUSE

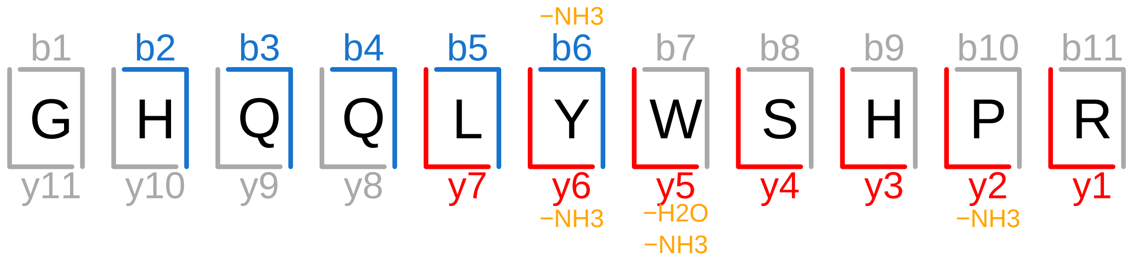

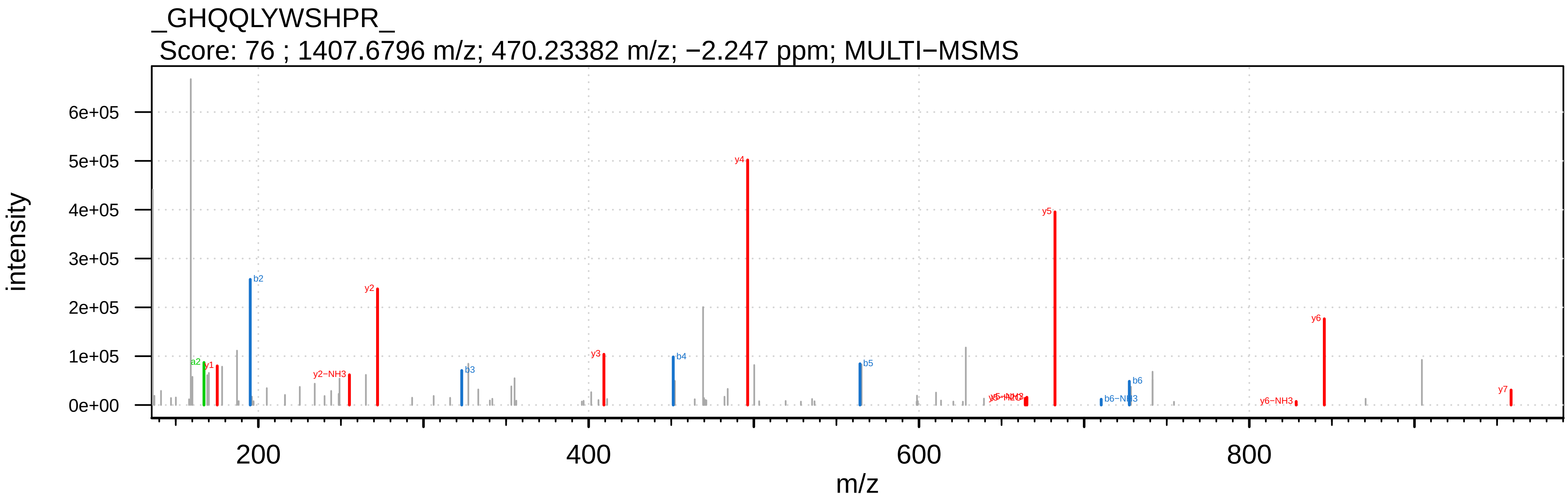

Raw File: Kermit_20111104_MSU_tube_6h_12
Scan Number: 53320
Proteins:
sp|P62274|RS29_MOUSE
ENSMUST00000037023_chr12:69157813−69159058:−
ENSMUST00000071737_chr13:73988963−73989133:−
tr|D3YYL7|D3YYL7_MOUSE

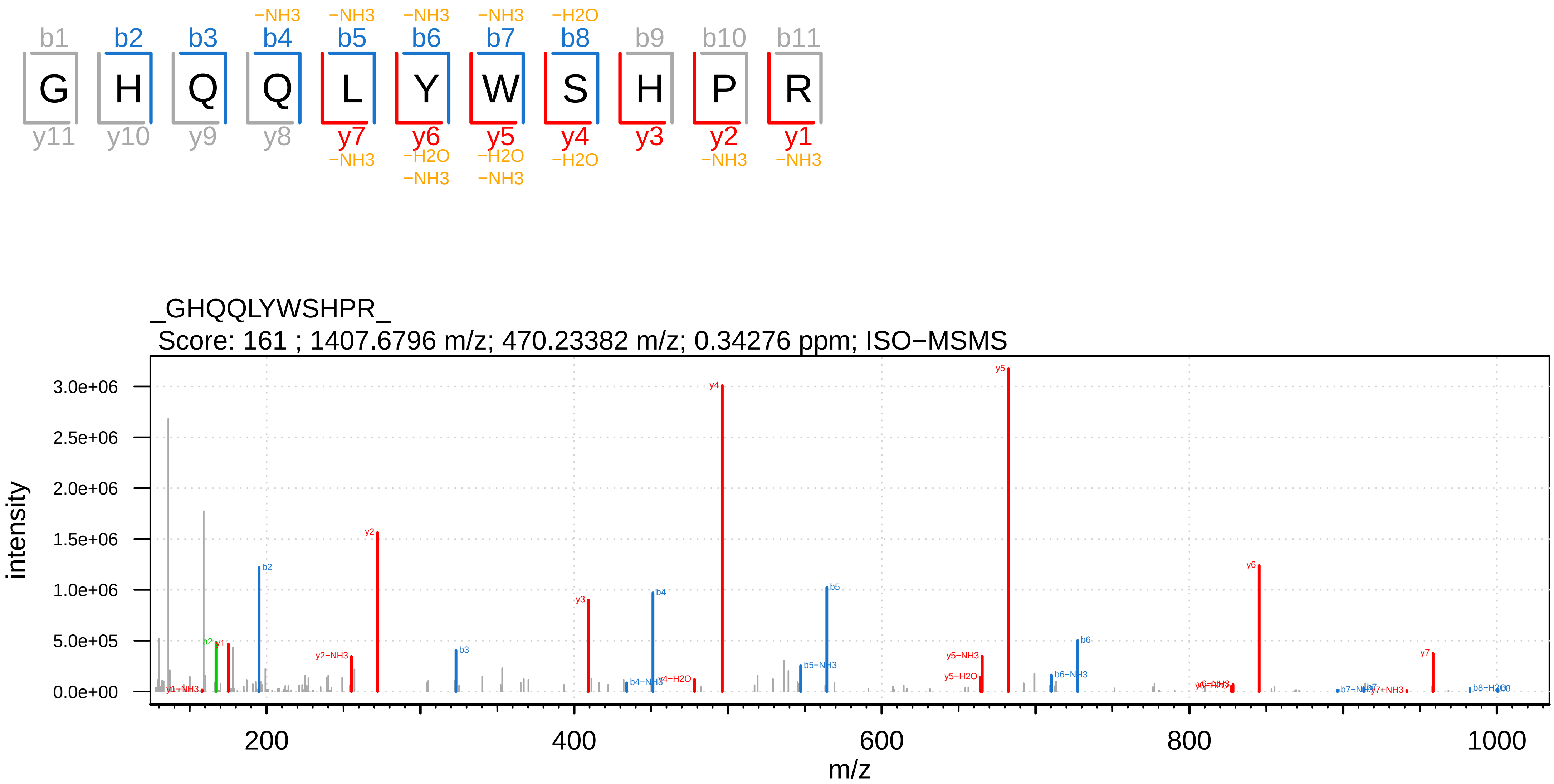

Raw File: Kermit_20111104_MSU_tube_6h_12
Scan Number: 8347
Proteins:
sp|P62274|RS29_MOUSE
ENSMUST00000037023_chr12:69157813−69159058:−
ENSMUST00000071737_chr13:73988963−73989133:−
tr|D3YYL7|D3YYL7_MOUSE

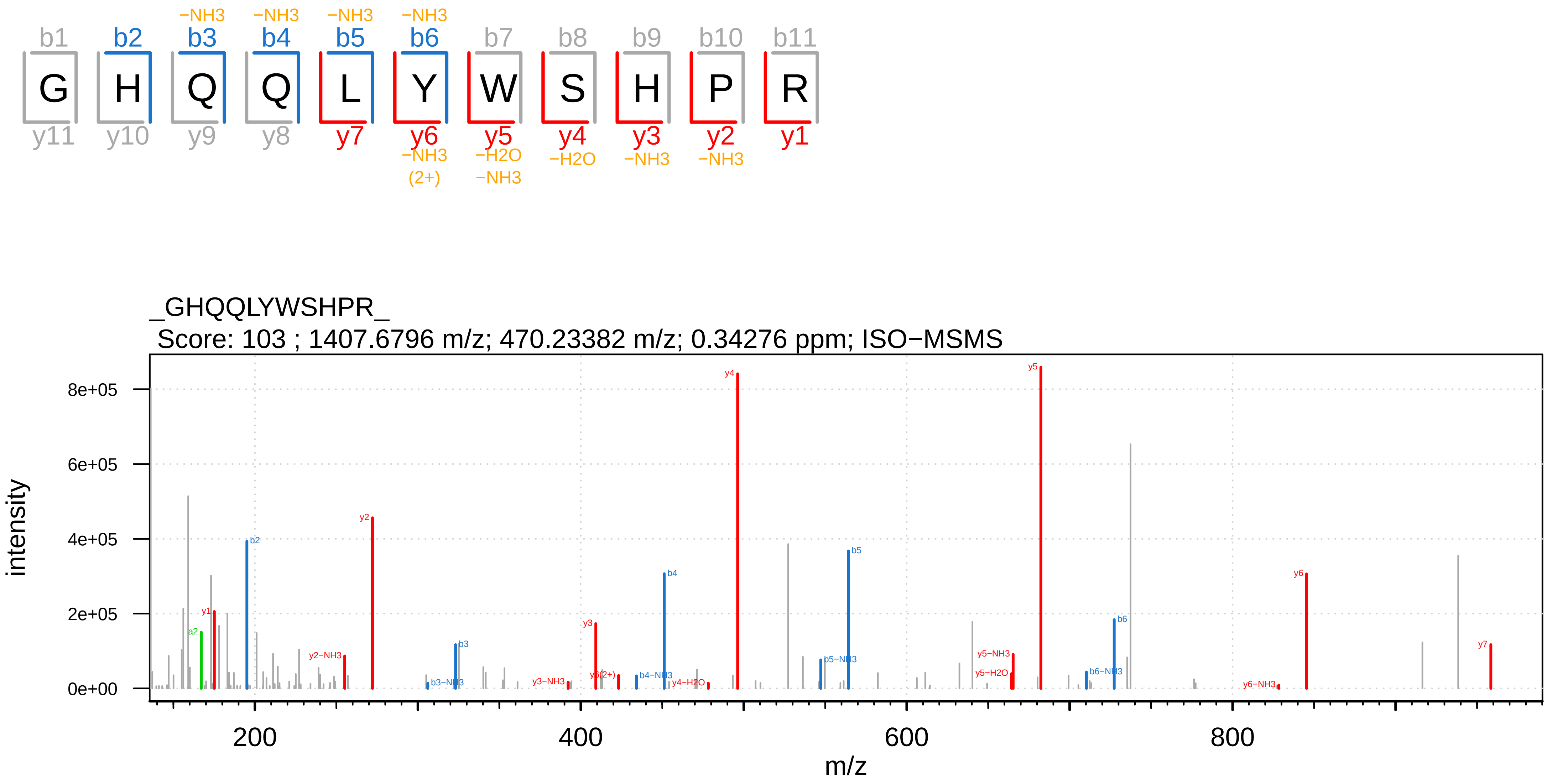

Raw File: Kermit_20111104_MSU_tube_6h_12
Scan Number: 8566
Proteins:
sp|P62274|RS29_MOUSE
ENSMUST00000037023_chr12:69157813−69159058:−
ENSMUST00000071737_chr13:73988963−73989133:−
tr|D3YYL7|D3YYL7_MOUSE

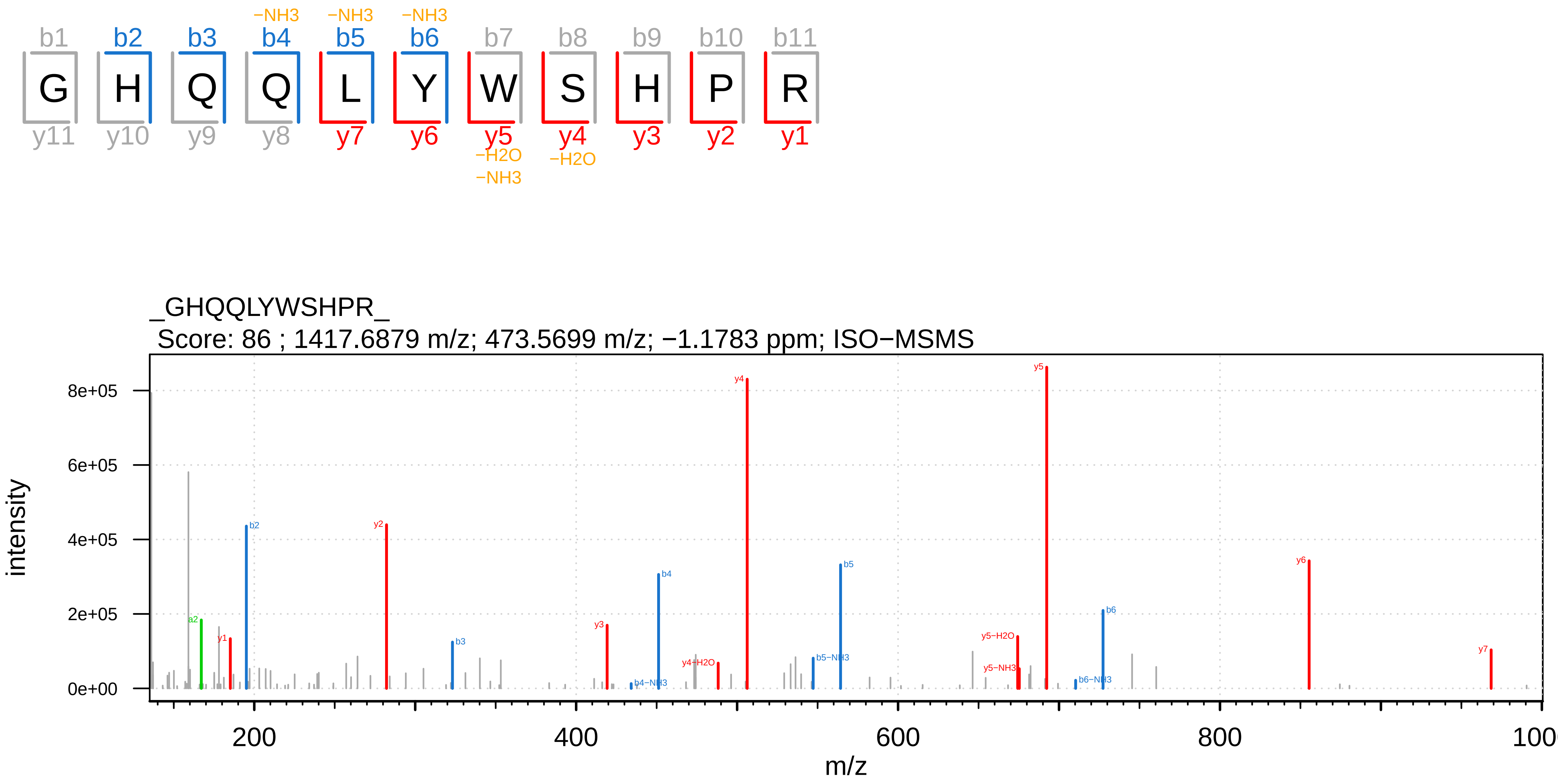

Raw File: Kermit_20111104_MSU_tube_6h_12
Scan Number: 8362
Proteins:
sp|P62274|RS29_MOUSE
ENSMUST00000037023_chr12:69157813−69159058:−
ENSMUST00000071737_chr13:73988963−73989133:−
tr|D3YYL7|D3YYL7_MOUSE

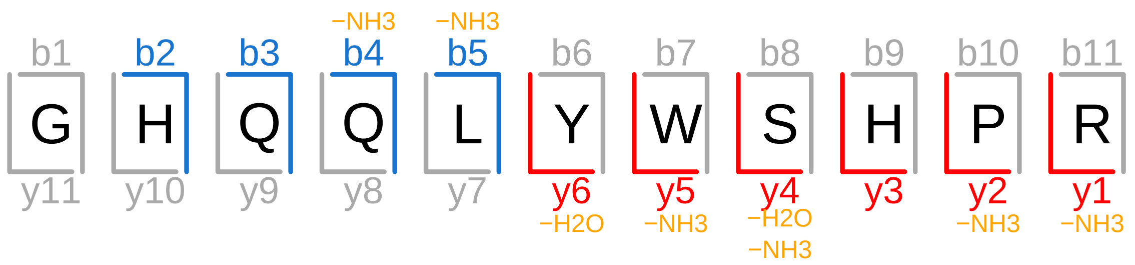

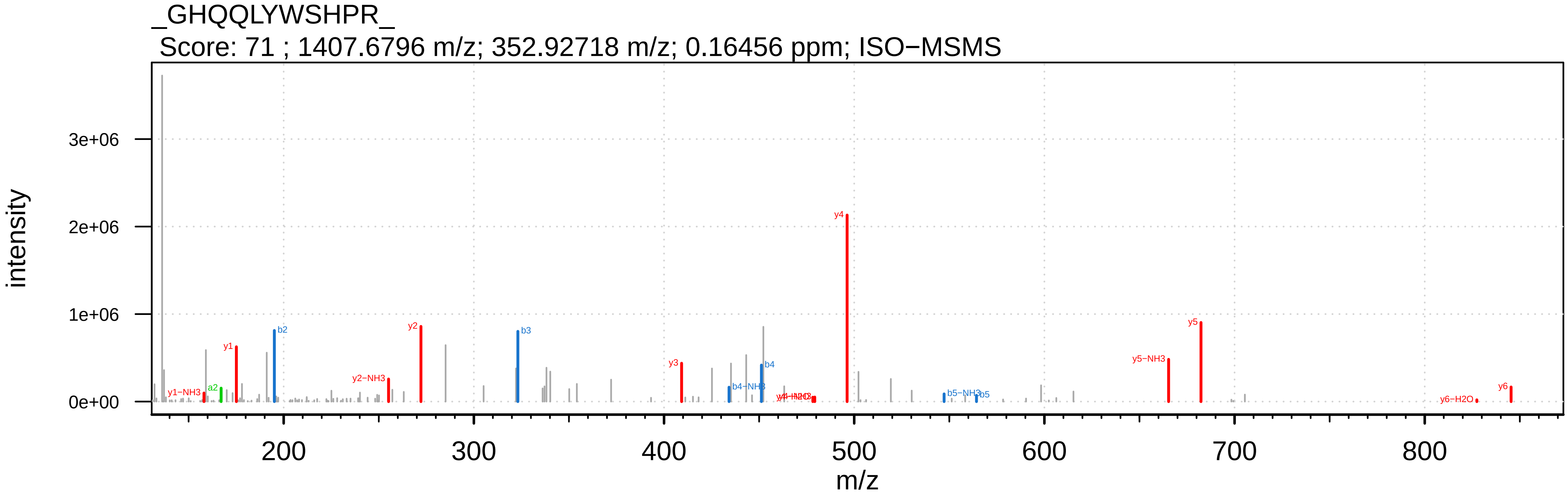

Raw File: Kermit_20111104_MSU_tube_6h_12
Scan Number: 8363
Proteins:
sp|P62274|RS29_MOUSE
ENSMUST00000037023_chr12:69157813−69159058:−
ENSMUST00000071737_chr13:73988963−73989133:−
tr|D3YYL7|D3YYL7_MOUSE

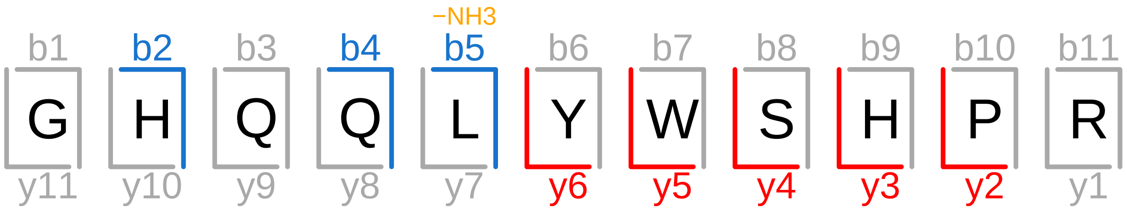

GHQQLYWSHPR
Score: 39 ; 1407.6796 m/z; 470.23382 m/z; −0.80337 ppm; ISO−MSMS

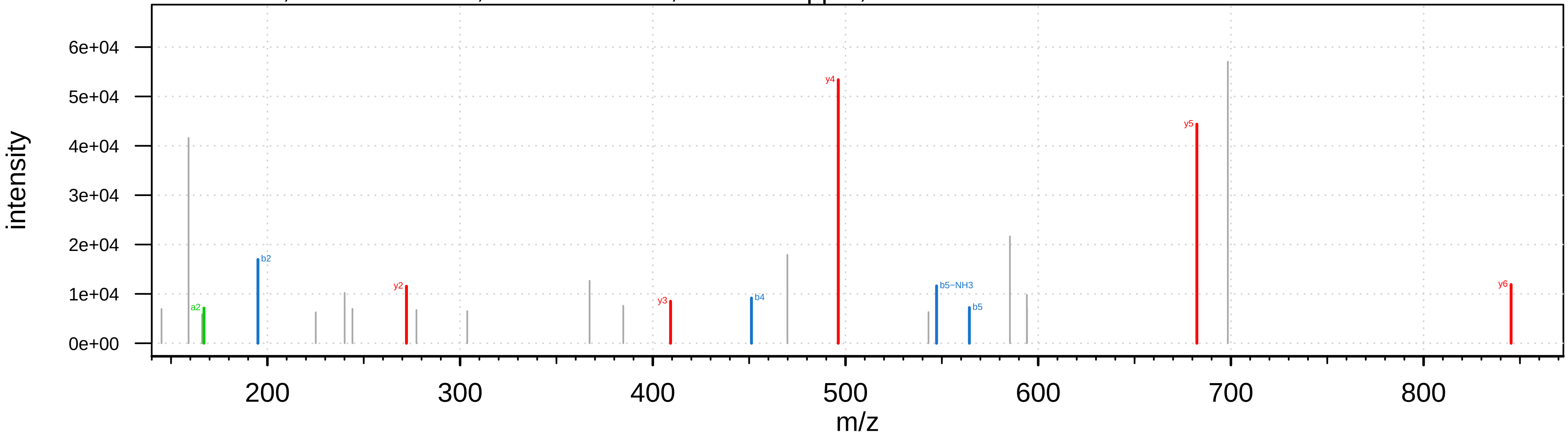

Raw File: Kermit_20111104_MSU_tube_6h_12
Scan Number: 23869
Proteins:
sp|P62274|RS29_MOUSE
ENSMUST00000037023_chr12:69157813−69159058:−
ENSMUST00000071737_chr13:73988963−73989133:−
tr|D3YYL7|D3YYL7_MOUSE

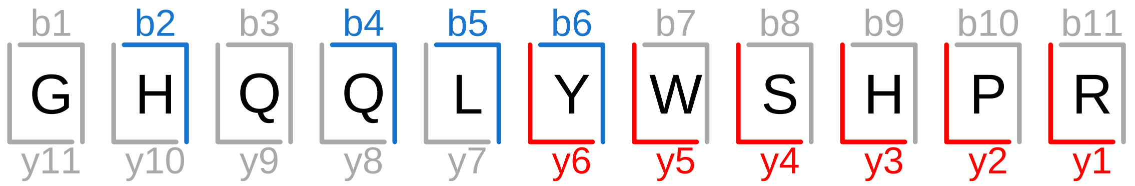

GHQQLYWSHPR
Score: 45 ; 1407.6796 m/z; 470.23382 m/z; 1.3932 ppm; ISO−MSMS

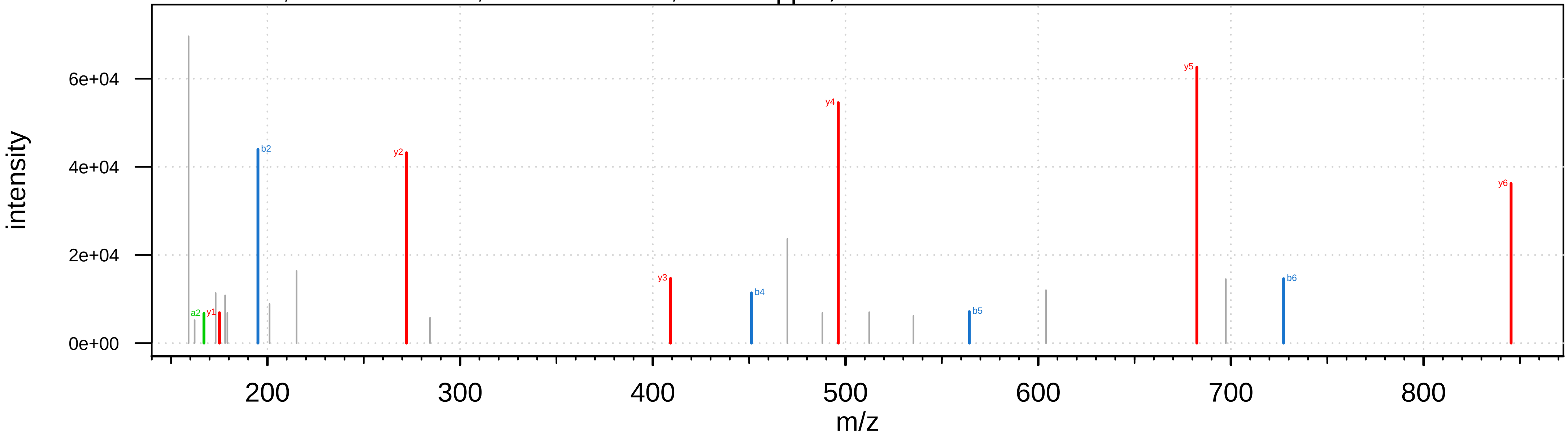

Raw File: Kermit_20111104_MSU_tube_6h_12
Scan Number: 24208
Proteins:
sp|P62274|RS29_MOUSE
ENSMUST00000037023_chr12:69157813−69159058:−
ENSMUST00000071737_chr13:73988963−73989133:−
tr|D3YYL7|D3YYL7_MOUSE

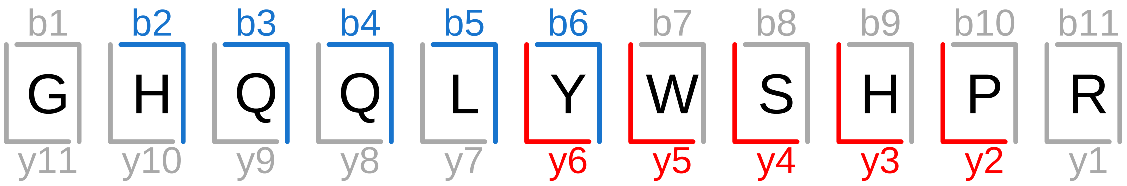

GHQQLYWSHPR
Score: 33 ; 1407.6796 m/z; 470.23382 m/z; 1.4676 ppm; ISO−MSMS

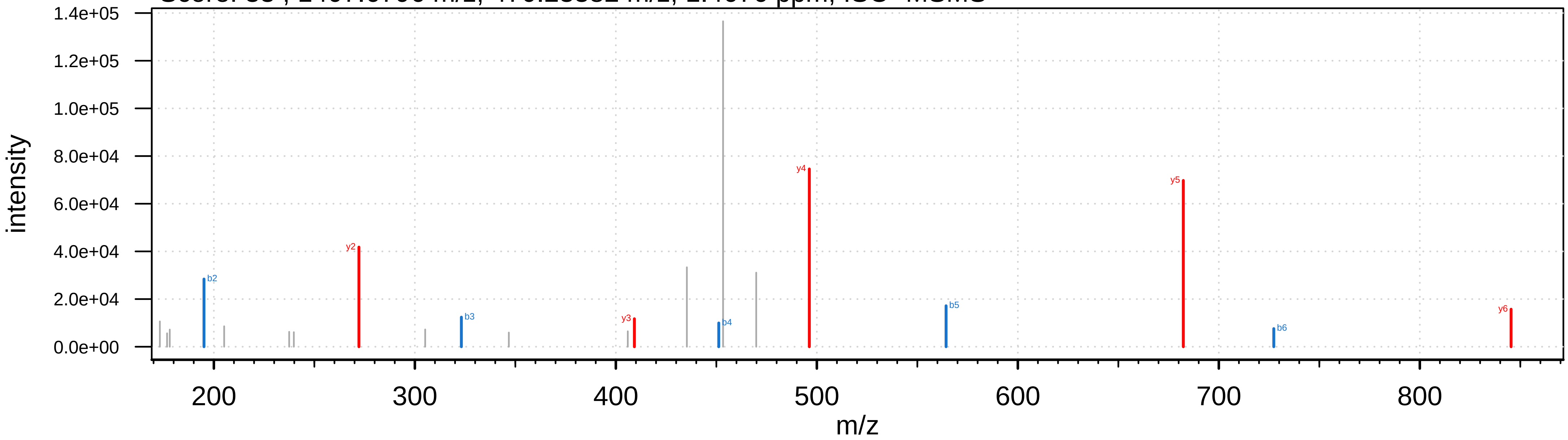

Raw File: Kermit_20111104_MSU_tube_6h_12
Scan Number: 24378
Proteins:
sp|P62274|RS29_MOUSE
ENSMUST00000037023_chr12:69157813−69159058:−
ENSMUST00000071737_chr13:73988963−73989133:−
tr|D3YYL7|D3YYL7_MOUSE

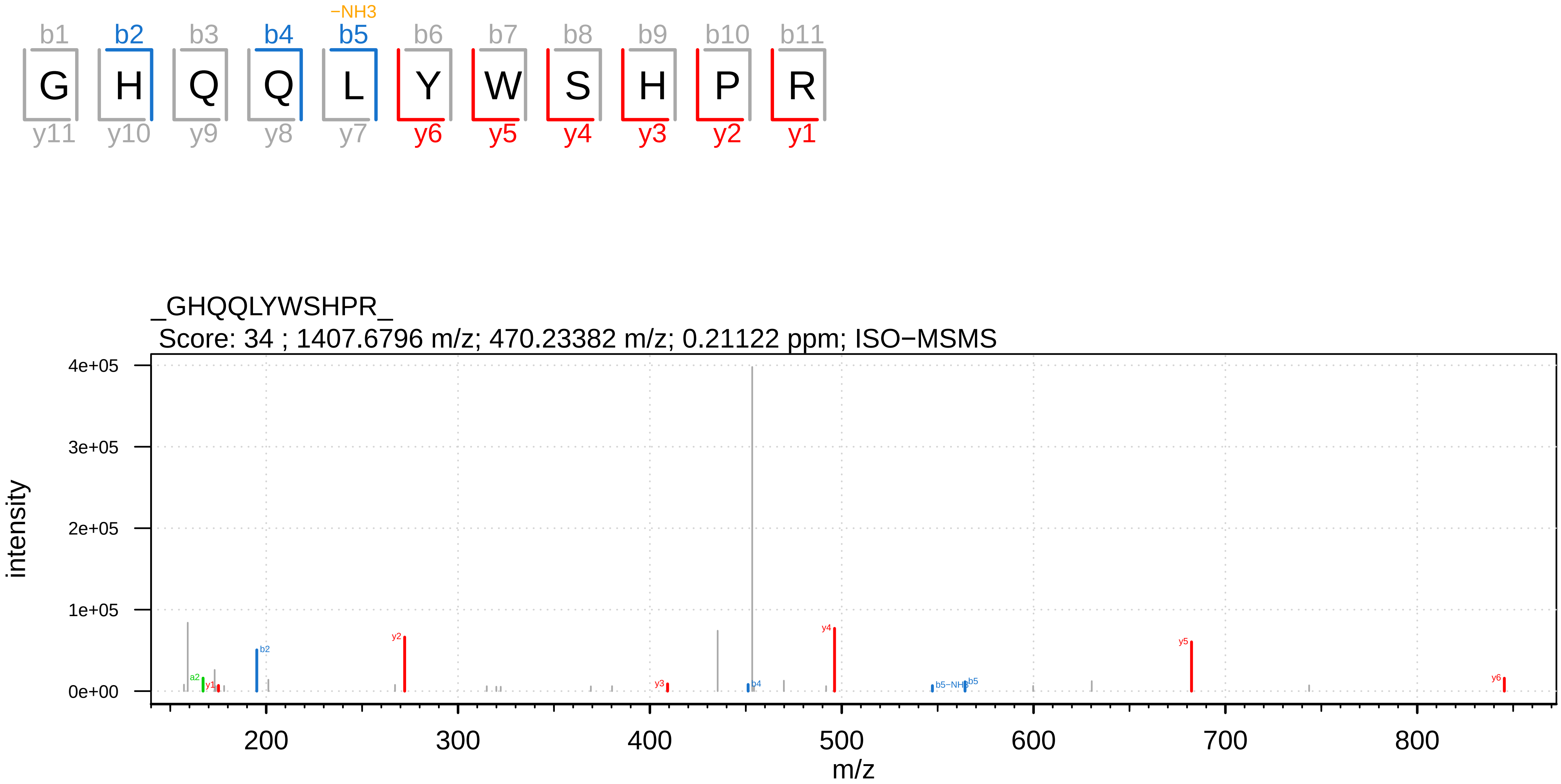

Raw File: Kermit_20111104_MSU_tube_6h_12
Scan Number: 24548
Proteins:
sp|P62274|RS29_MOUSE
ENSMUST00000037023_chr12:69157813−69159058:−
ENSMUST00000071737_chr13:73988963−73989133:−
tr|D3YYL7|D3YYL7_MOUSE

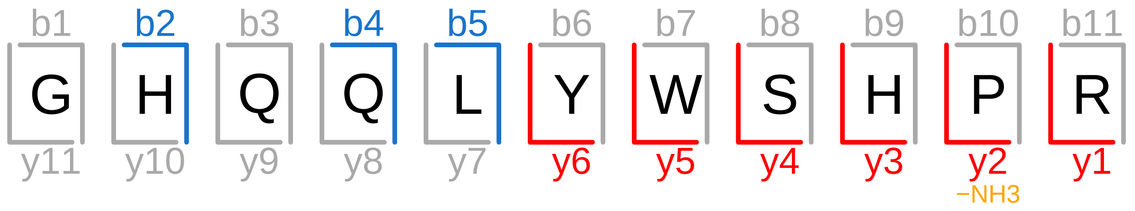

GHQQLYWSHPR
Score: 38 ; 1407.6796 m/z; 470.23382 m/z; −1.5783 ppm; ISO−MSMS

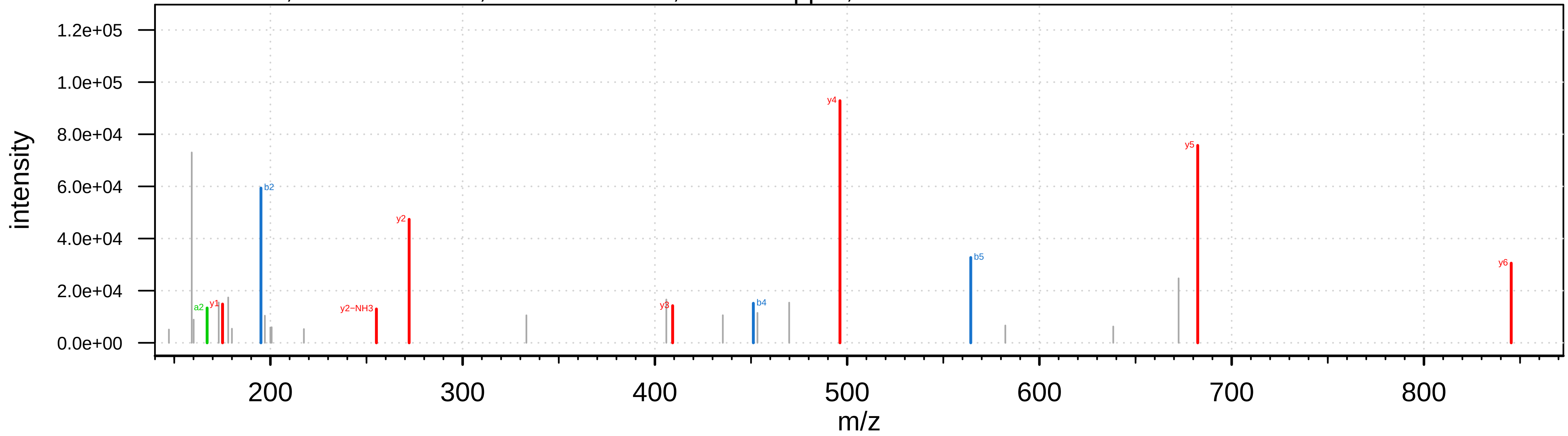

Raw File: Kermit_20111104_MSU_tube_6h_12
Scan Number: 24728
Proteins:
sp|P62274|RS29_MOUSE
ENSMUST00000037023_chr12:69157813−69159058:−
ENSMUST00000071737_chr13:73988963−73989133:−
tr|D3YYL7|D3YYL7_MOUSE

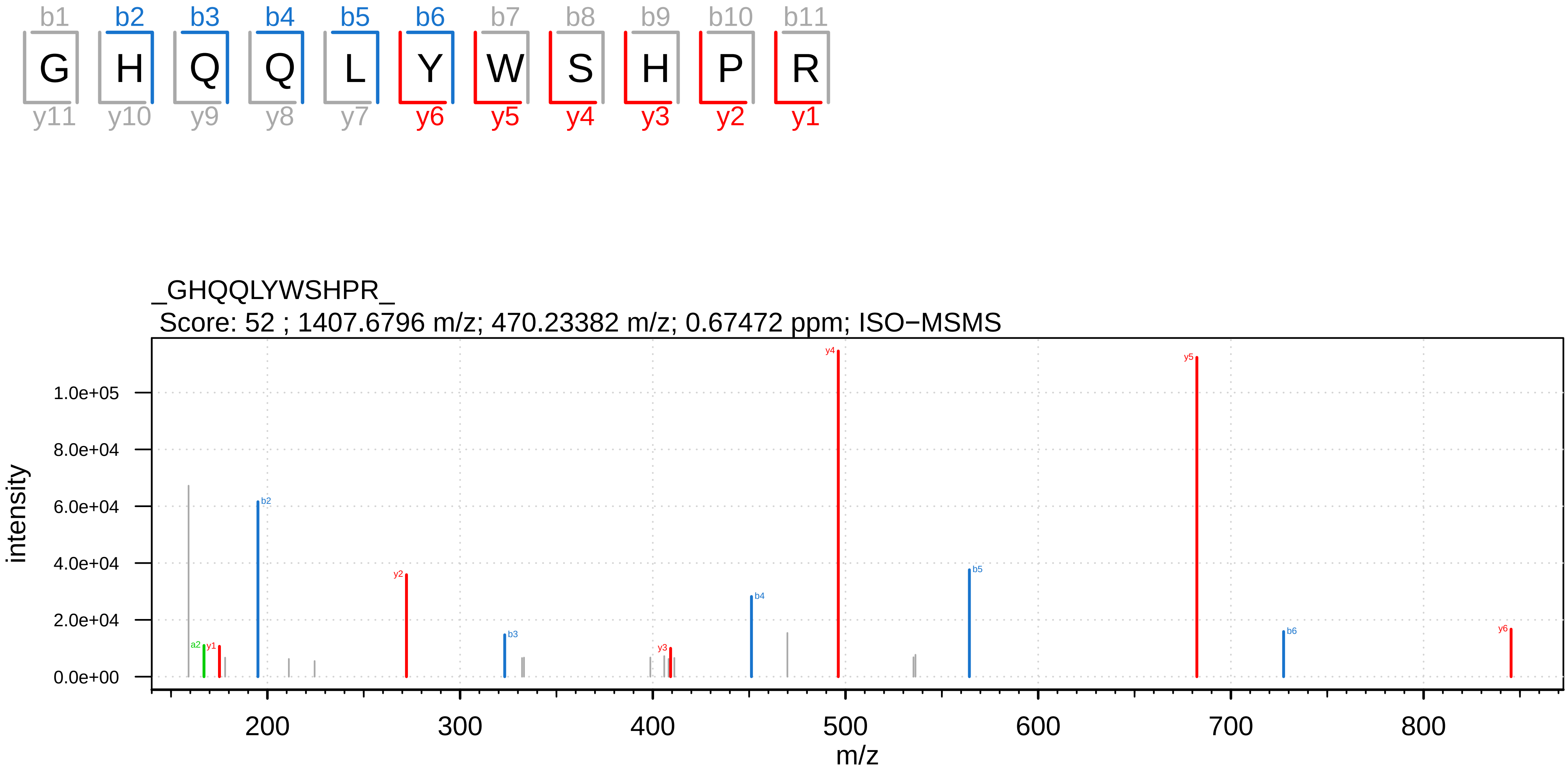

Raw File: Kermit_20111104_MSU_tube_6h_12
Scan Number: 25081
Proteins:
sp|P62274|RS29_MOUSE
ENSMUST00000037023_chr12:69157813−69159058:−
ENSMUST00000071737_chr13:73988963−73989133:−
tr|D3YYL7|D3YYL7_MOUSE

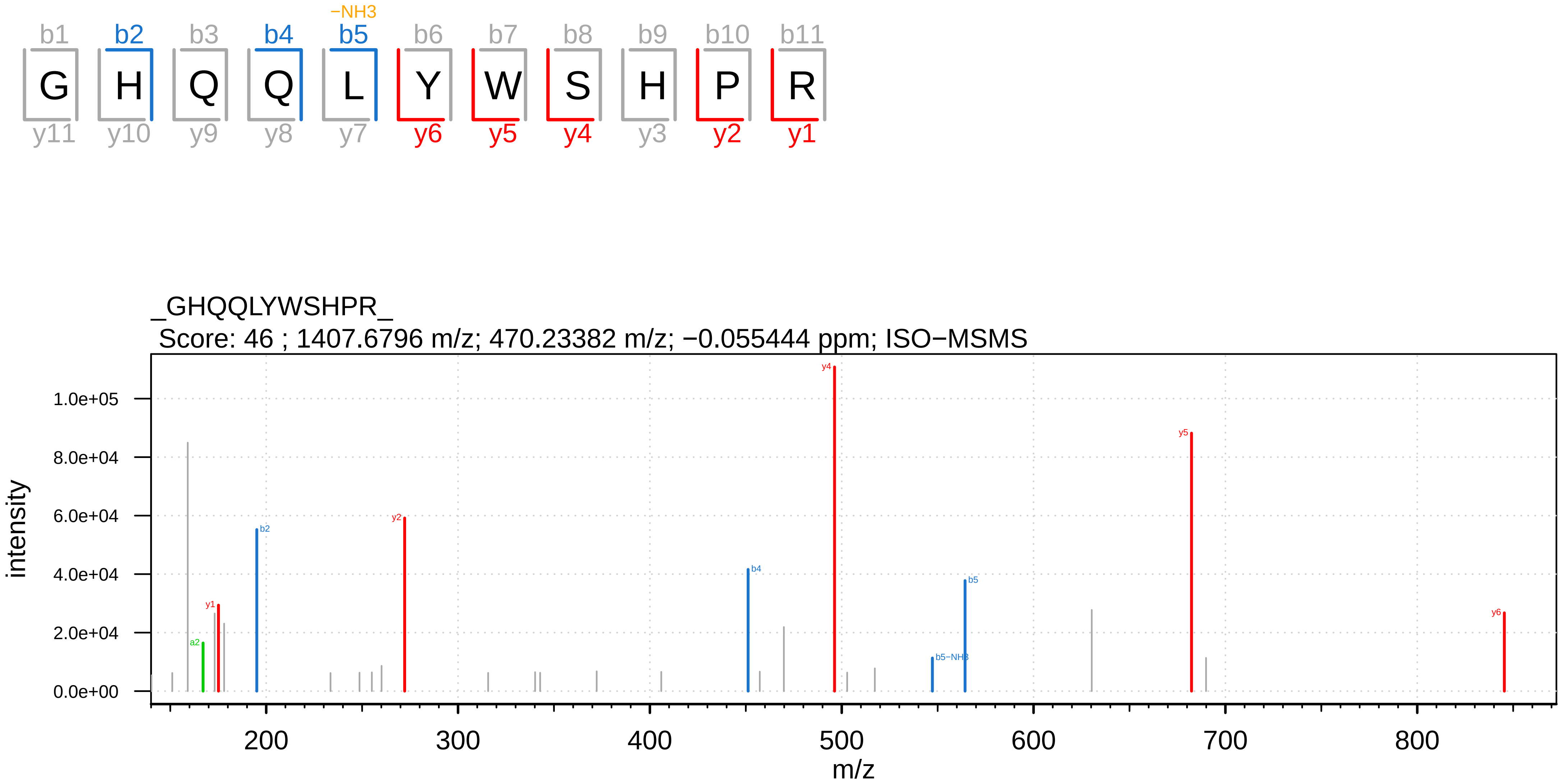

Raw File: Kermit_20111104_MSU_tube_6h_12
Scan Number: 25245
Proteins:
sp|P62274|RS29_MOUSE
ENSMUST00000037023_chr12:69157813−69159058:−
ENSMUST00000071737_chr13:73988963−73989133:−
tr|D3YYL7|D3YYL7_MOUSE

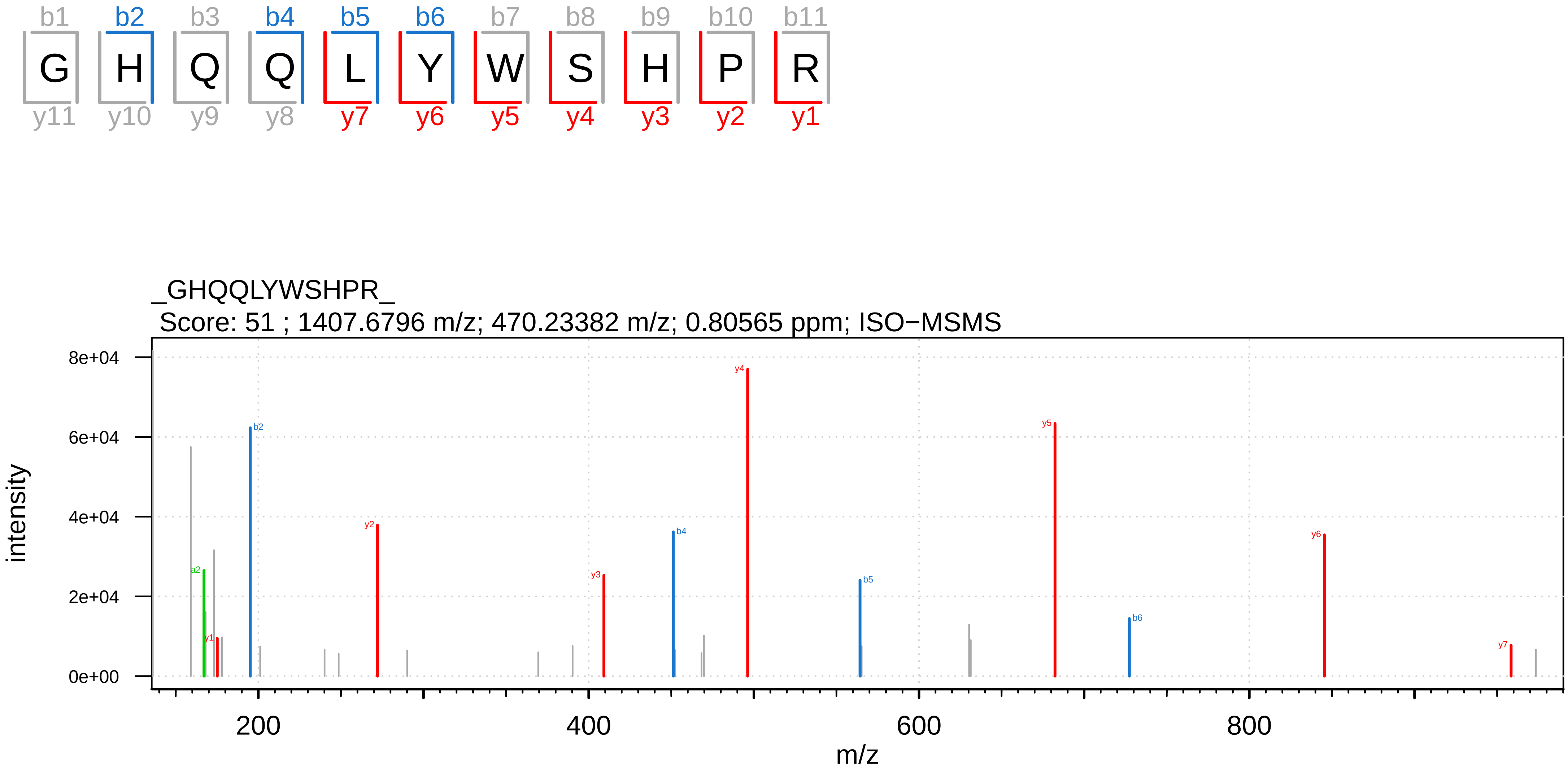

Raw File: Kermit_20111104_MSU_tube_6h_12
Scan Number: 25410
Proteins:
sp|P62274|RS29_MOUSE
ENSMUST00000037023_chr12:69157813−69159058:−
ENSMUST00000071737_chr13:73988963−73989133:−
tr|D3YYL7|D3YYL7_MOUSE

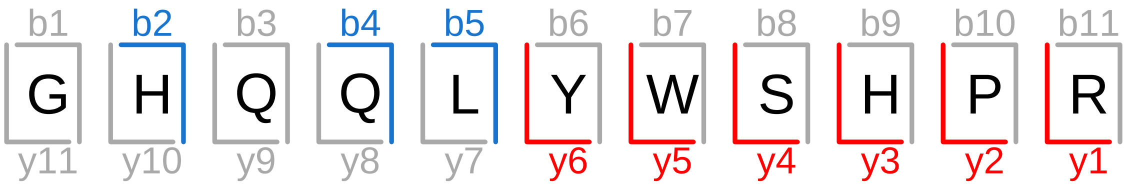

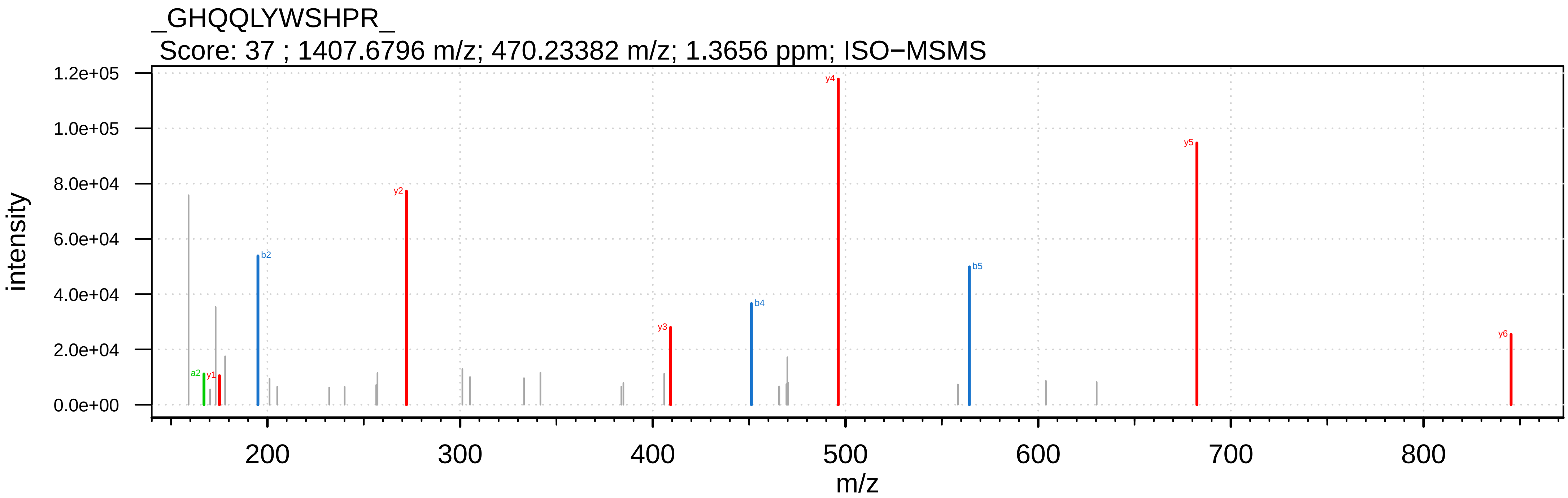

Raw File: Kermit_20111104_MSU_tube_6h_12
Scan Number: 25586
Proteins:
sp|P62274|RS29_MOUSE
ENSMUST00000037023_chr12:69157813−69159058:−
ENSMUST00000071737_chr13:73988963−73989133:−
tr|D3YYL7|D3YYL7_MOUSE

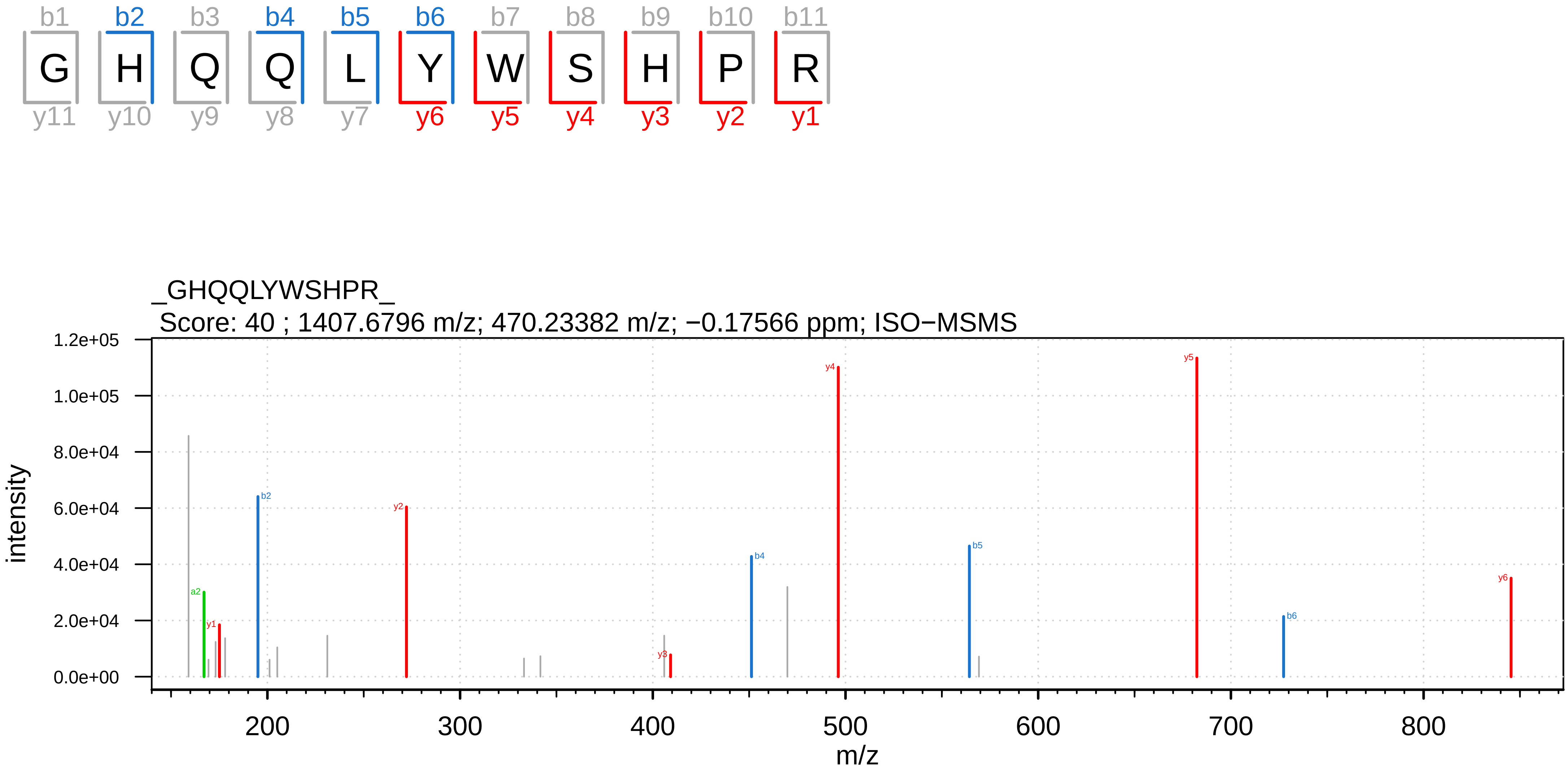

Raw File: Kermit_20111104_MSU_tube_6h_12
Scan Number: 25754
Proteins:
sp|P62274|RS29_MOUSE
ENSMUST00000037023_chr12:69157813−69159058:−
ENSMUST00000071737_chr13:73988963−73989133:−
tr|D3YYL7|D3YYL7_MOUSE

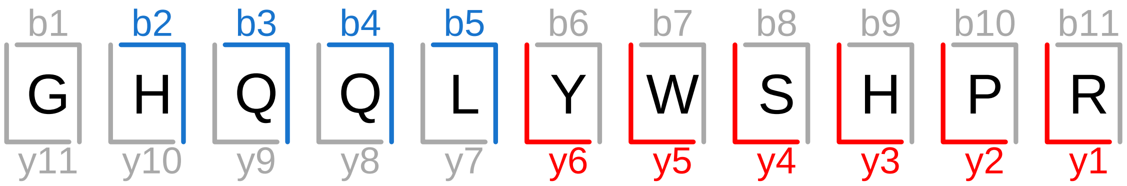

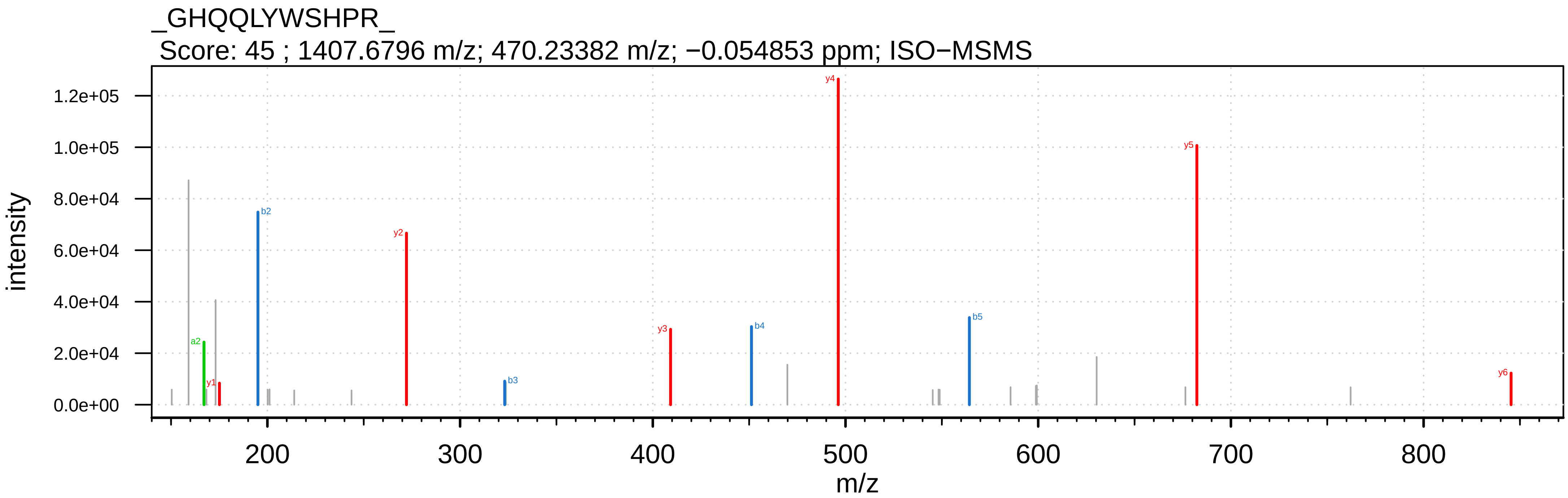

Raw File: Kermit_20111104_MSU_tube_6h_12
Scan Number: 25913
Proteins:
sp|P62274|RS29_MOUSE
ENSMUST00000037023_chr12:69157813−69159058:−
ENSMUST00000071737_chr13:73988963−73989133:−
tr|D3YYL7|D3YYL7_MOUSE

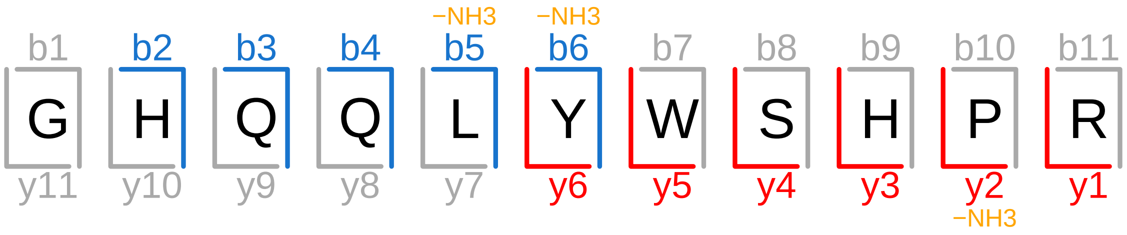

GHQQLYWSHPR
Score: 70 ; 1407.6796 m/z; 470.23382 m/z; −0.16993 ppm; ISO−MSMS

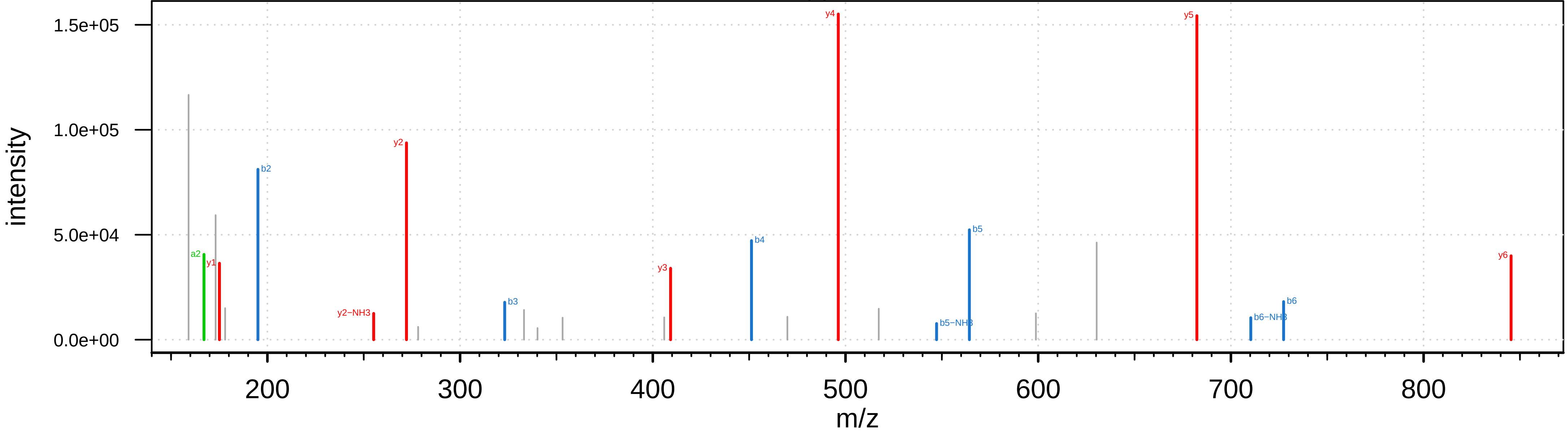

Raw File: Kermit_20111104_MSU_tube_6h_12
Scan Number: 26076
Proteins:
sp|P62274|RS29_MOUSE
ENSMUST00000037023_chr12:69157813−69159058:−
ENSMUST00000071737_chr13:73988963−73989133:−
tr|D3YYL7|D3YYL7_MOUSE

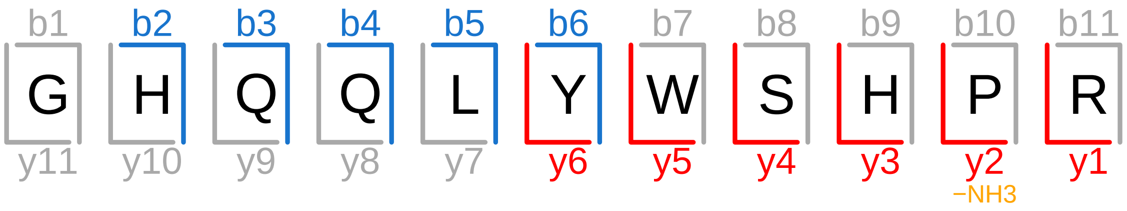

GHQQLYWSHPR
Score: 52 ; 1407.6796 m/z; 470.23382 m/z; −0.088962 ppm; ISO−MSMS

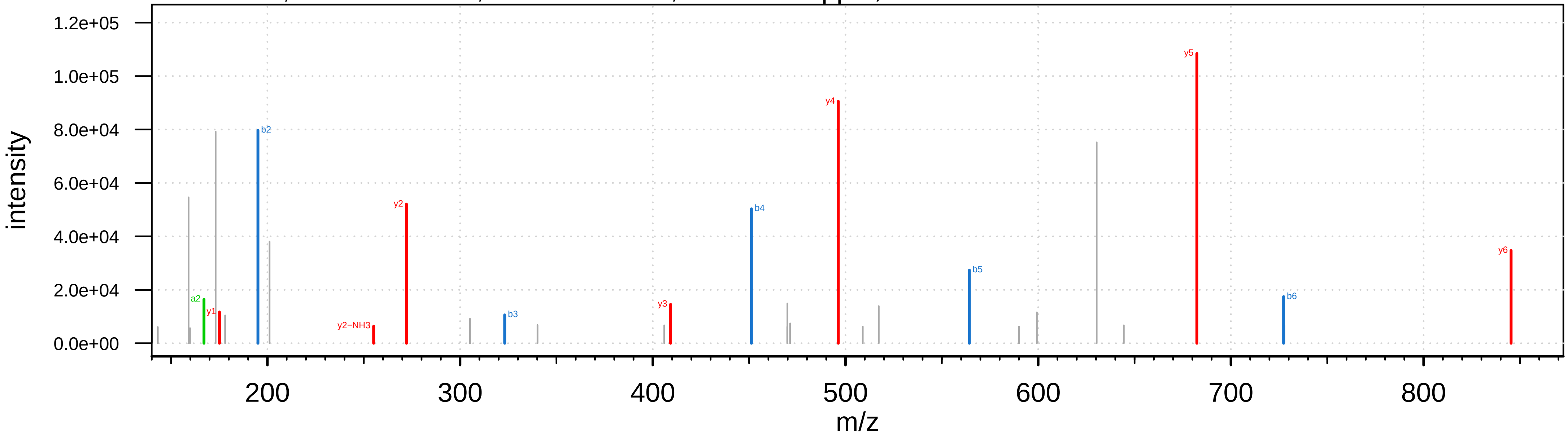

Raw File: Kermit_20111104_MSU_tube_6h_12
Scan Number: 26249
Proteins:
sp|P62274|RS29_MOUSE
ENSMUST00000037023_chr12:69157813−69159058:−
ENSMUST00000071737_chr13:73988963−73989133:−
tr|D3YYL7|D3YYL7_MOUSE

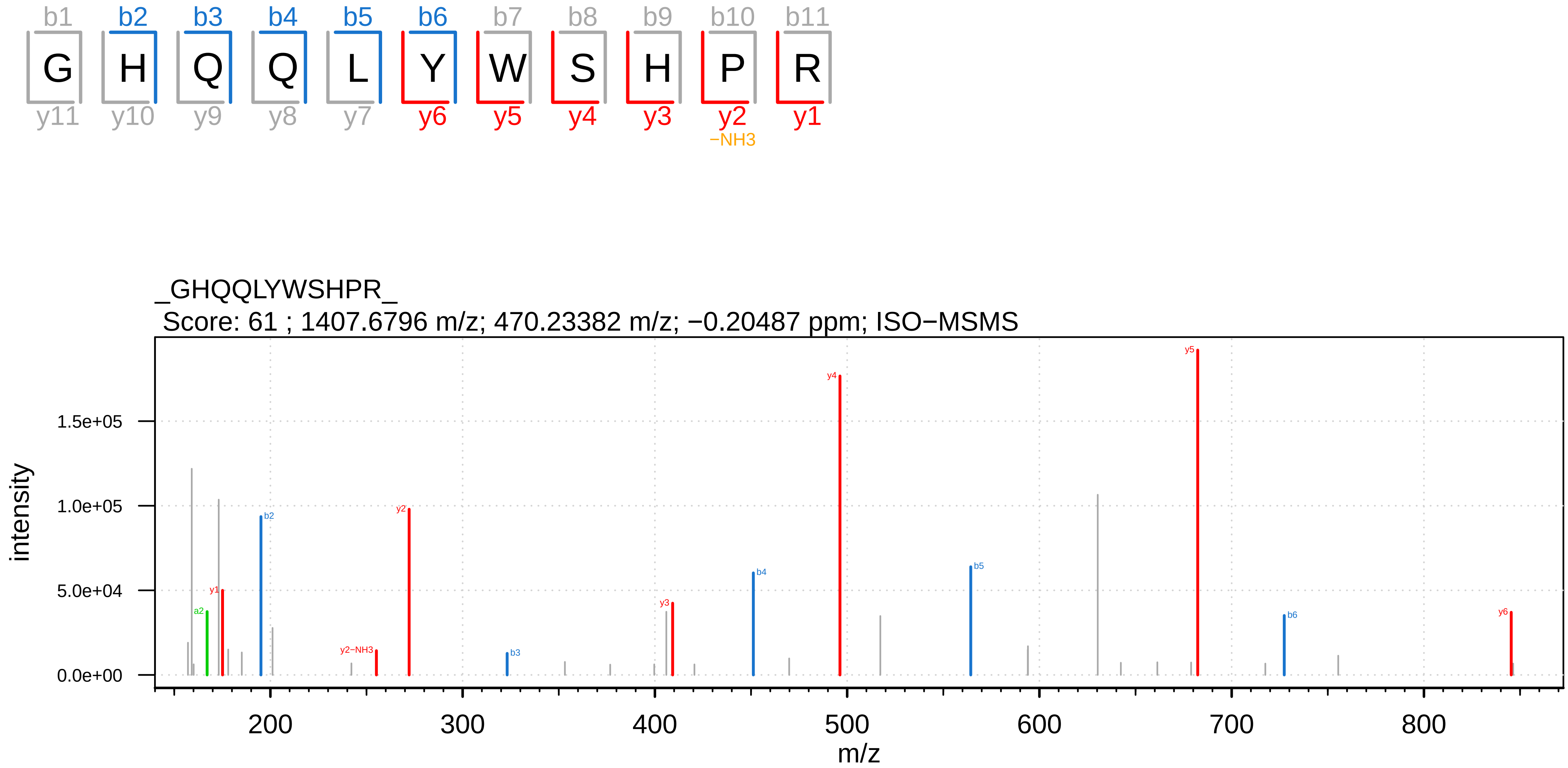

Raw File: Kermit_20111104_MSU_tube_6h_12
Scan Number: 26412
Proteins:
sp|P62274|RS29_MOUSE
ENSMUST00000037023_chr12:69157813−69159058:−
ENSMUST00000071737_chr13:73988963−73989133:−
tr|D3YYL7|D3YYL7_MOUSE

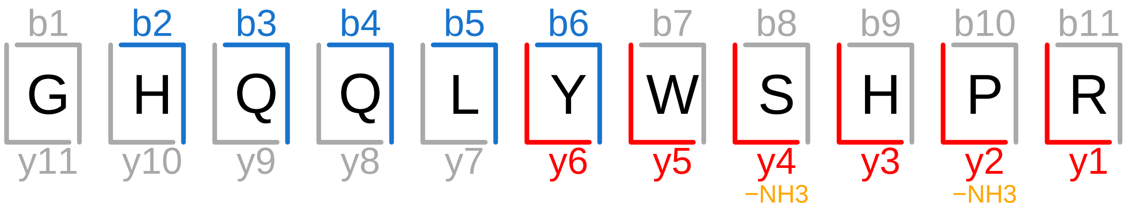

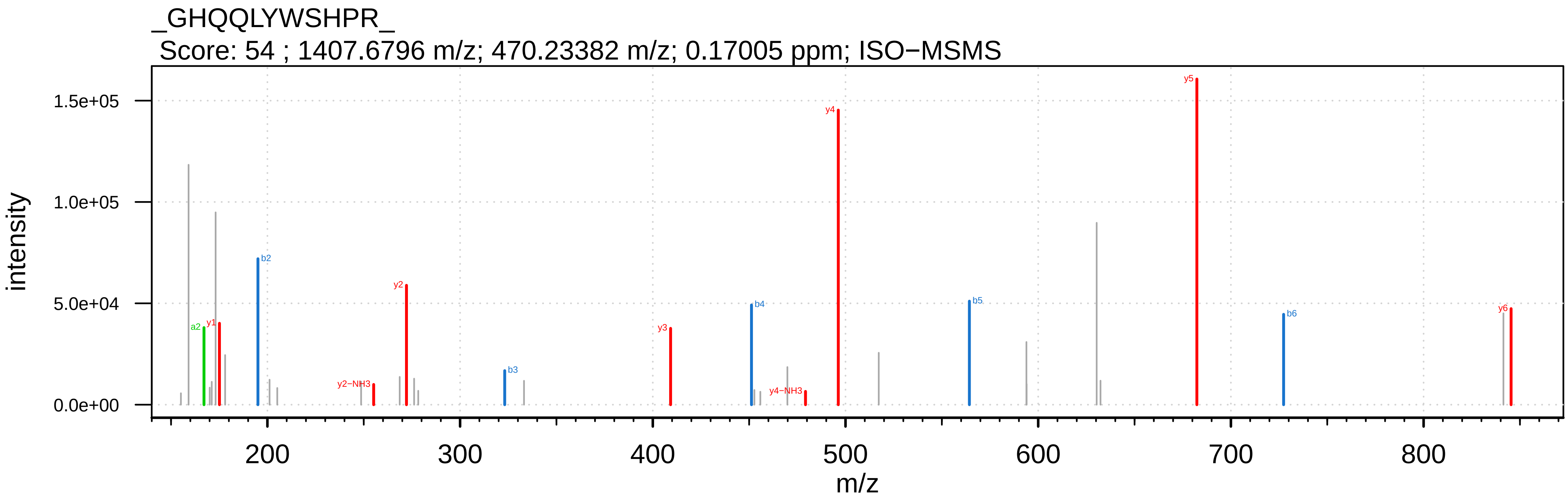

Raw File: Kermit_20111104_MSU_tube_6h_12
Scan Number: 26744
Proteins:
sp|P62274|RS29_MOUSE
ENSMUST00000037023_chr12:69157813−69159058:−
ENSMUST00000071737_chr13:73988963−73989133:−
tr|D3YYL7|D3YYL7_MOUSE

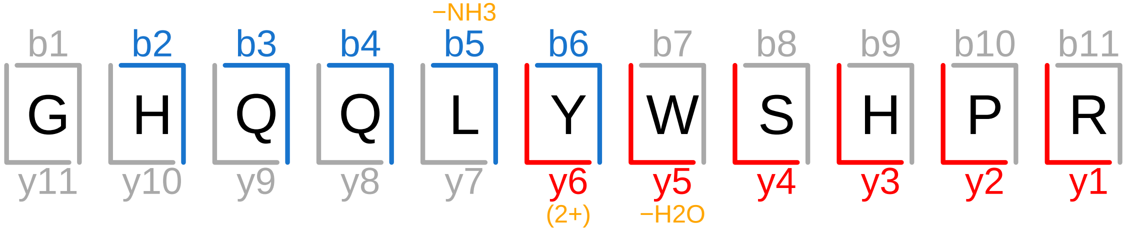

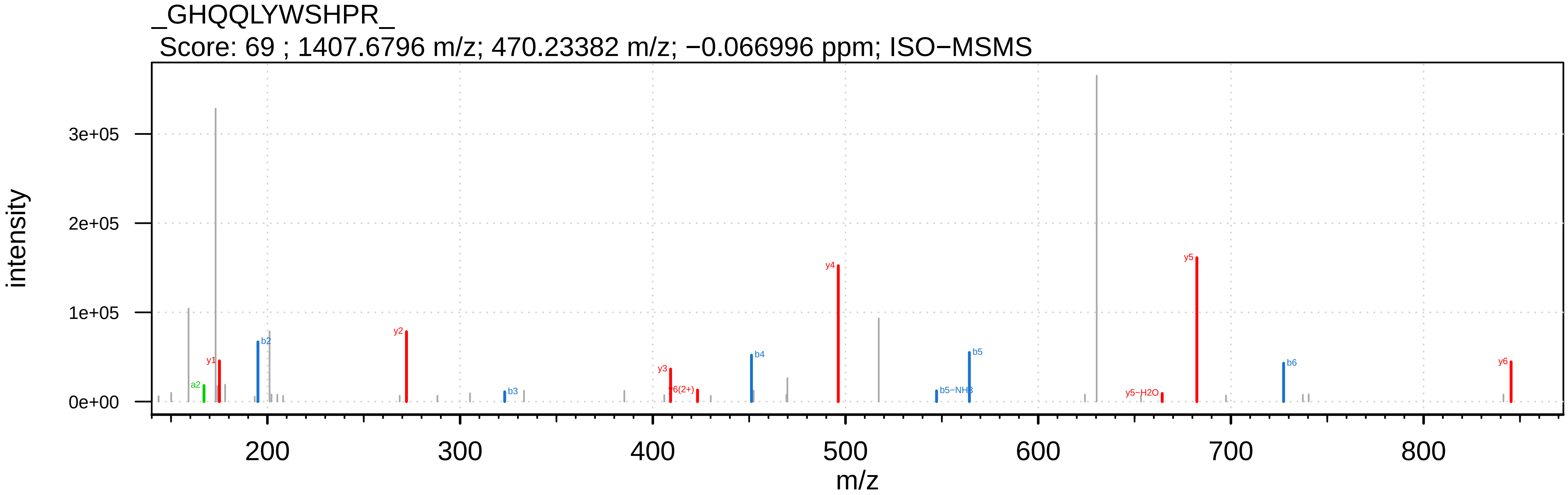

Raw File: Kermit_20111104_MSU_tube_6h_12
Scan Number: 26906
Proteins:
sp|P62274|RS29_MOUSE
ENSMUST00000037023_chr12:69157813−69159058:−
ENSMUST00000071737_chr13:73988963−73989133:−
tr|D3YYL7|D3YYL7_MOUSE

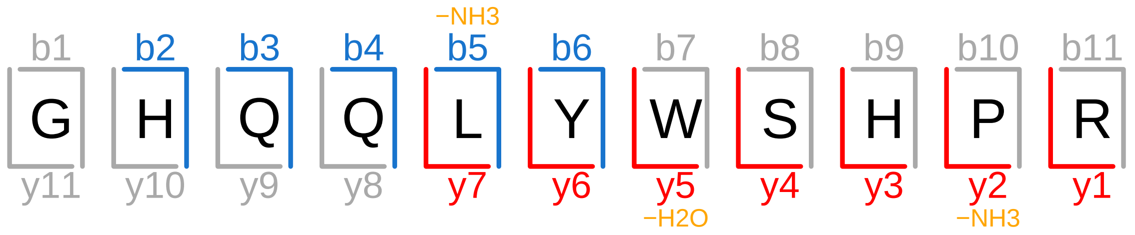

GHQQLYWSHPR
Score: 78 ; 1407.6796 m/z; 470.23382 m/z; 1.2317 ppm; ISO−MSMS

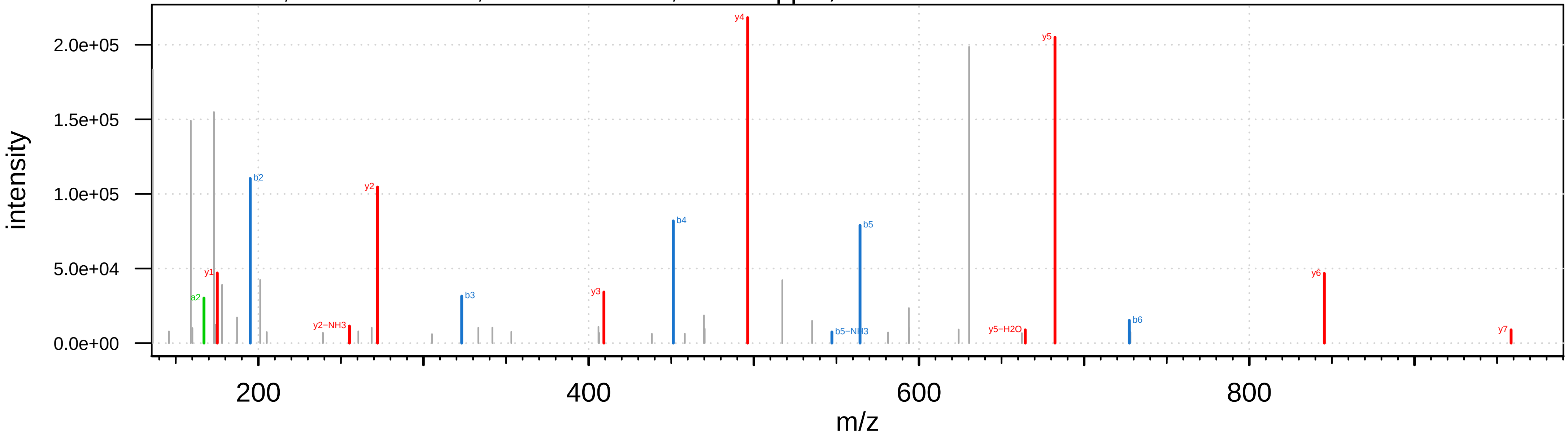

Raw File: Kermit_20111104_MSU_tube_6h_12
Scan Number: 27072
Proteins:
sp|P62274|RS29_MOUSE
ENSMUST00000037023_chr12:69157813−69159058:−
ENSMUST00000071737_chr13:73988963−73989133:−
tr|D3YYL7|D3YYL7_MOUSE

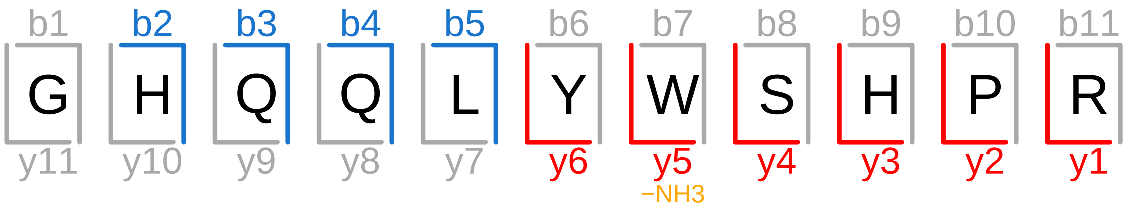

GHQQLYWSHPR

Score: 53 ; 1407.6796 m/z; 470.23382 m/z; 1.0189 ppm; ISO−MSMS

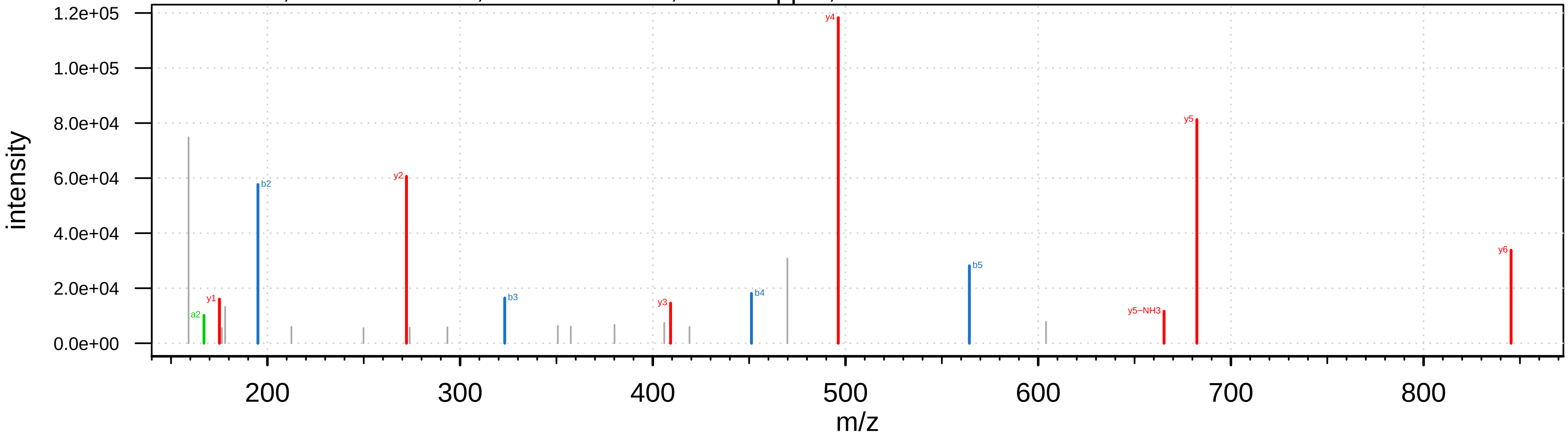

Raw File: Kermit_20111104_MSU_tube_6h_12
Scan Number: 27234
Proteins:
sp|P62274|RS29_MOUSE
ENSMUST00000037023_chr12:69157813−69159058:−
ENSMUST00000071737_chr13:73988963−73989133:−
tr|D3YYL7|D3YYL7_MOUSE

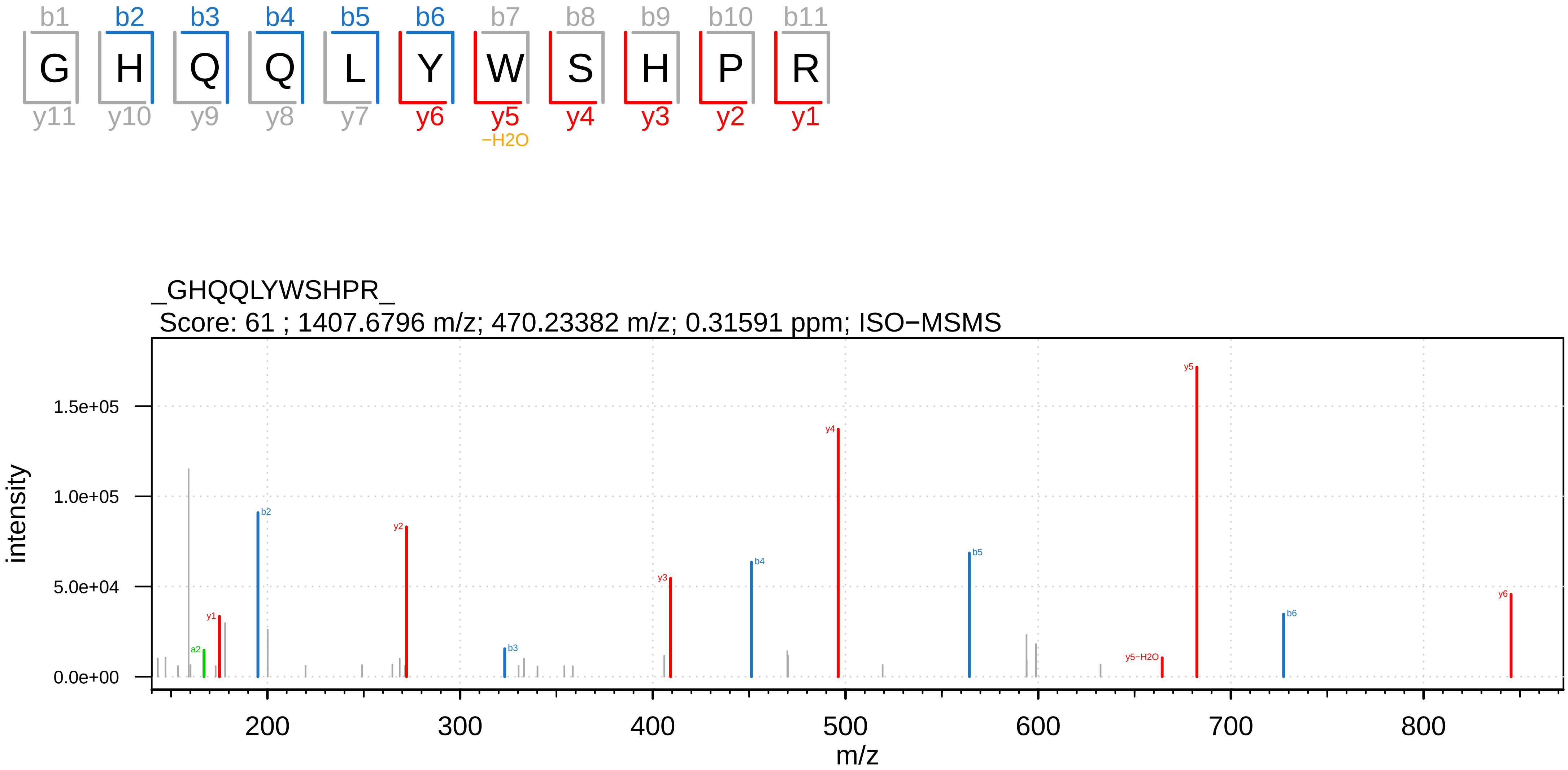

Raw File: Kermit_20111104_MSU_tube_6h_12
Scan Number: 27401
Proteins:
sp|P62274|RS29_MOUSE
ENSMUST00000037023_chr12:69157813−69159058:−
ENSMUST00000071737_chr13:73988963−73989133:−
tr|D3YYL7|D3YYL7_MOUSE

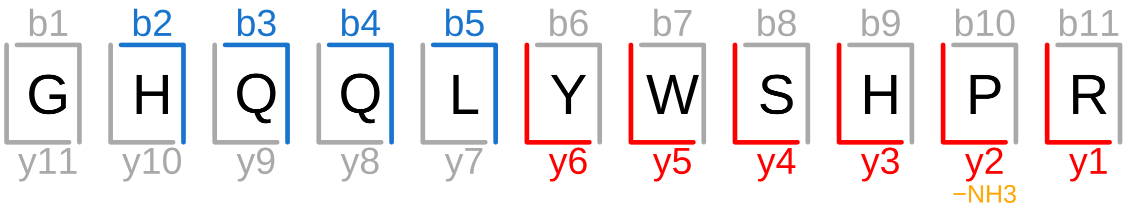

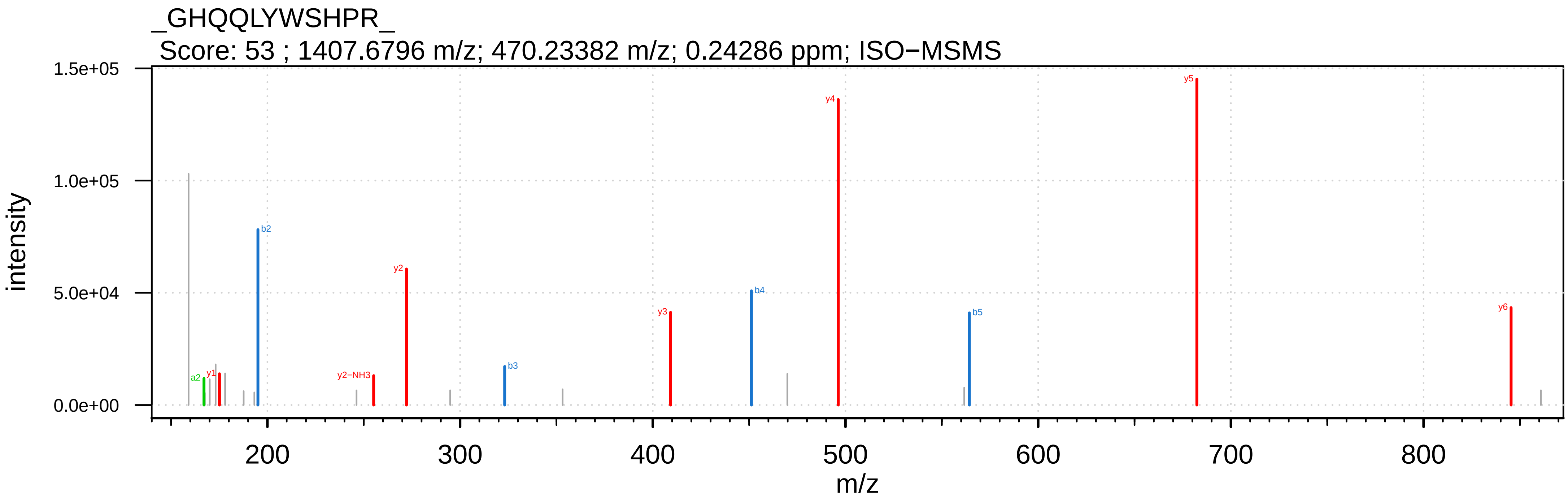

Raw File: Kermit_20111104_MSU_tube_6h_12
Scan Number: 27567
Proteins:
sp|P62274|RS29_MOUSE
ENSMUST00000037023_chr12:69157813−69159058:−
ENSMUST00000071737_chr13:73988963−73989133:−
tr|D3YYL7|D3YYL7_MOUSE

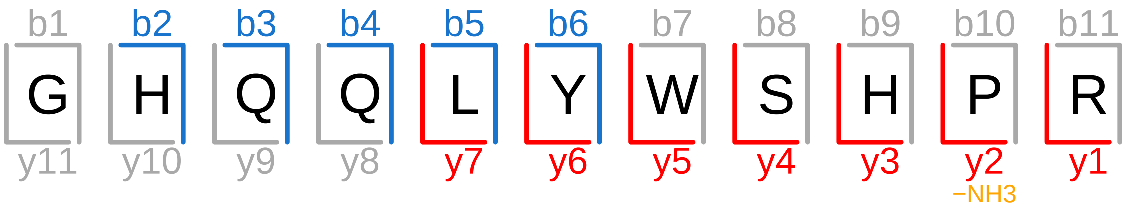

GHQQLYWSHPR
Score: 59 ; 1407.6796 m/z; 470.23382 m/z; 0.48538 ppm; ISO−MSMS

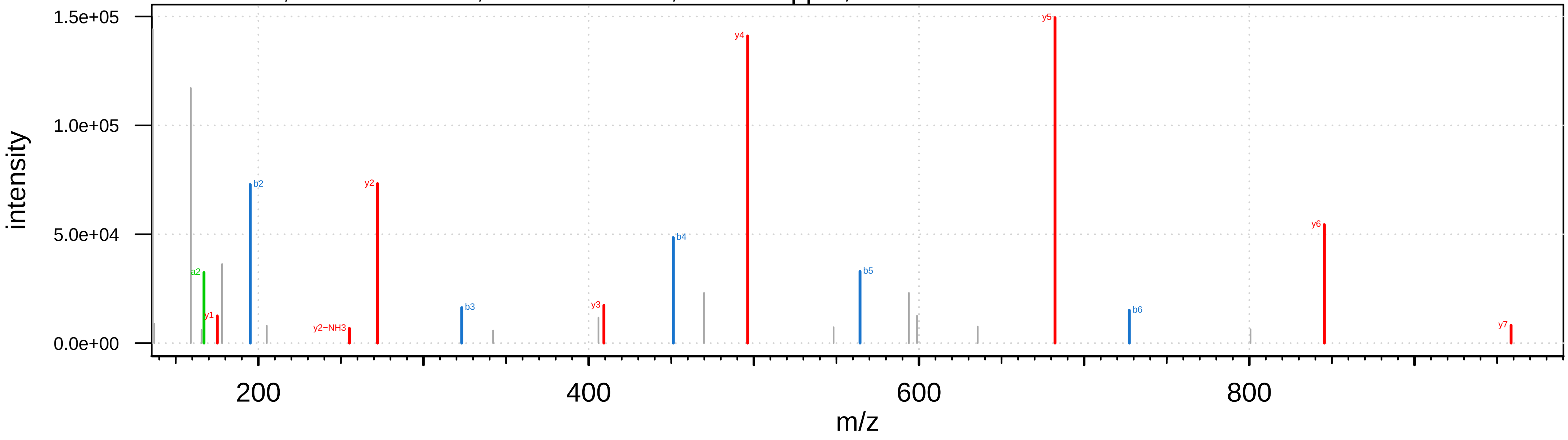

Raw File: Kermit_20111104_MSU_tube_6h_12
Scan Number: 27731
Proteins:
sp|P62274|RS29_MOUSE
ENSMUST00000037023_chr12:69157813−69159058:−
ENSMUST00000071737_chr13:73988963−73989133:−
tr|D3YYL7|D3YYL7_MOUSE

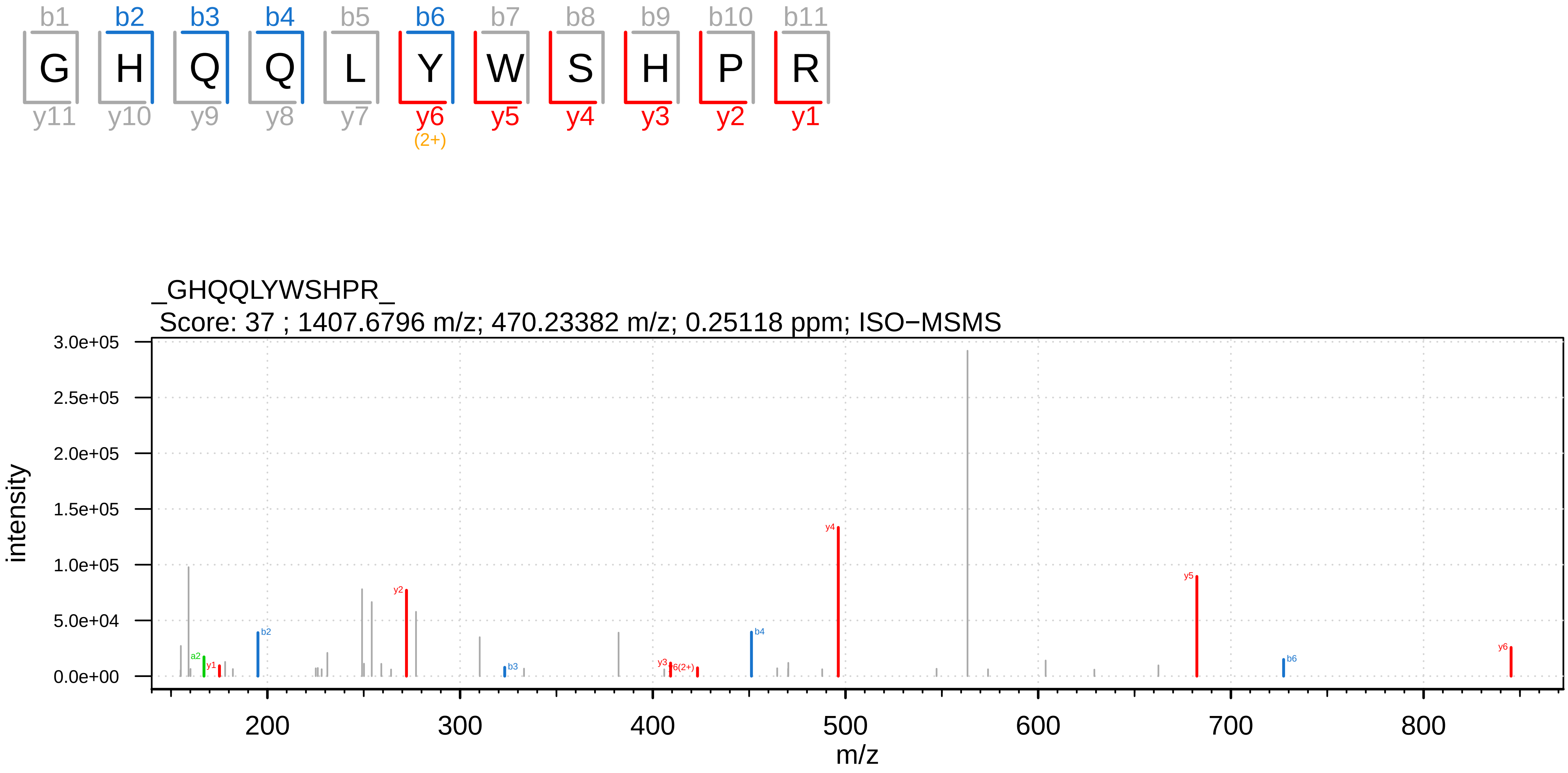

Raw File: Kermit_20111104_MSU_tube_6h_12
Scan Number: 28296
Proteins:
sp|P62274|RS29_MOUSE
ENSMUST00000037023_chr12:69157813−69159058:−
ENSMUST00000071737_chr13:73988963−73989133:−
tr|D3YYL7|D3YYL7_MOUSE

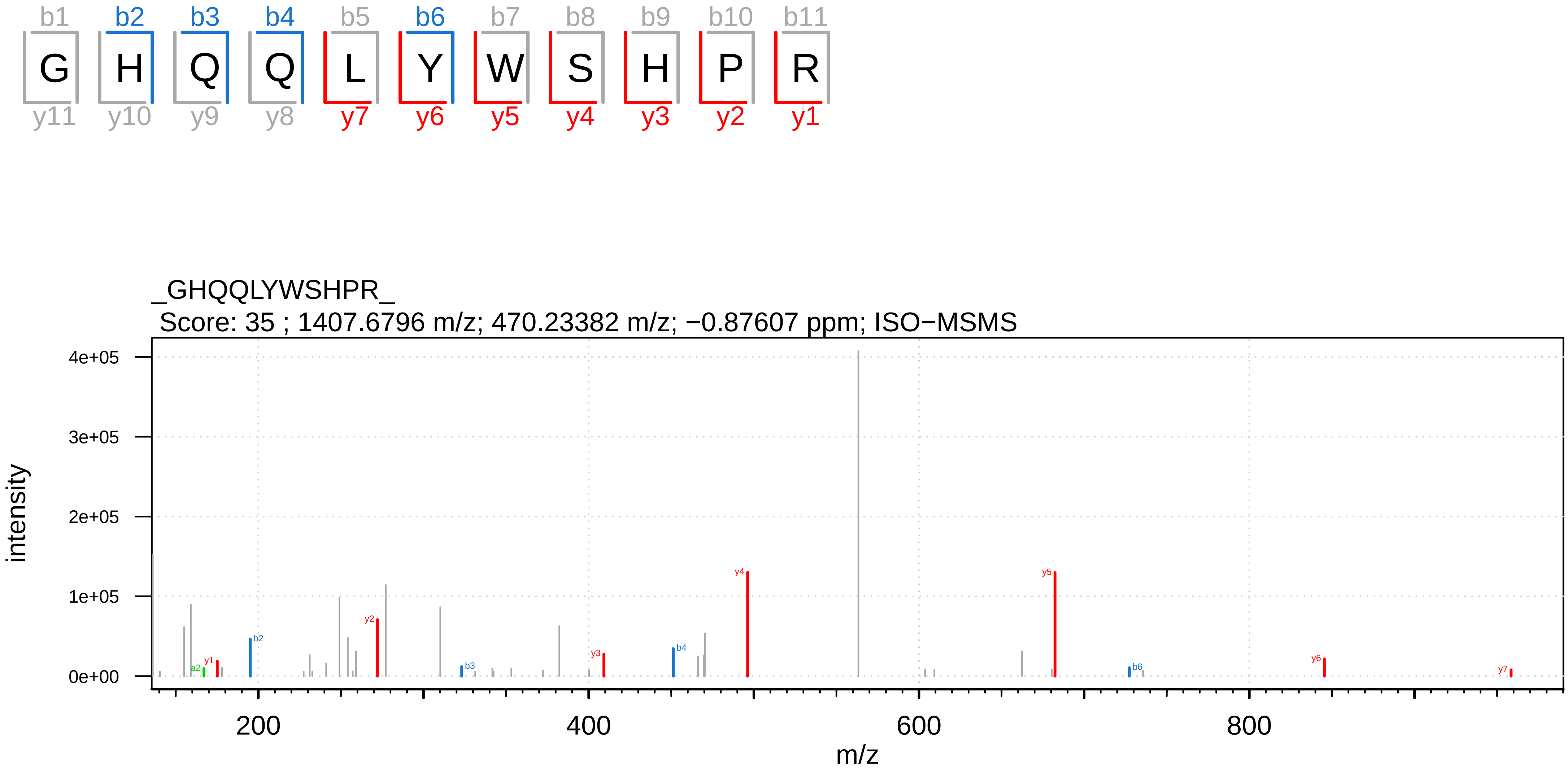

Raw File: Kermit_20111104_MSU_tube_6h_12
Scan Number: 28514
Proteins:
sp|P62274|RS29_MOUSE
ENSMUST00000037023_chr12:69157813−69159058:−
ENSMUST00000071737_chr13:73988963−73989133:−
tr|D3YYL7|D3YYL7_MOUSE

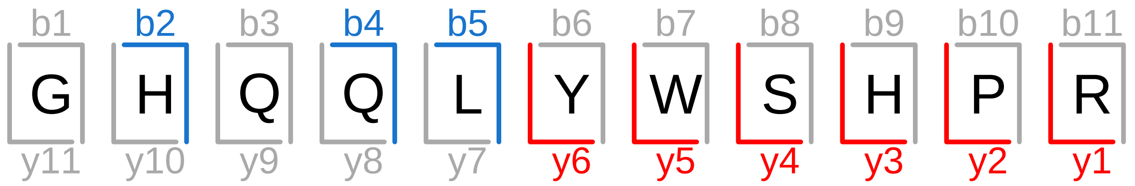

GHQQLYWSHPR
Score: 32 ; 1407.6796 m/z; 470.23382 m/z; −0.63854 ppm; ISO−MSMS

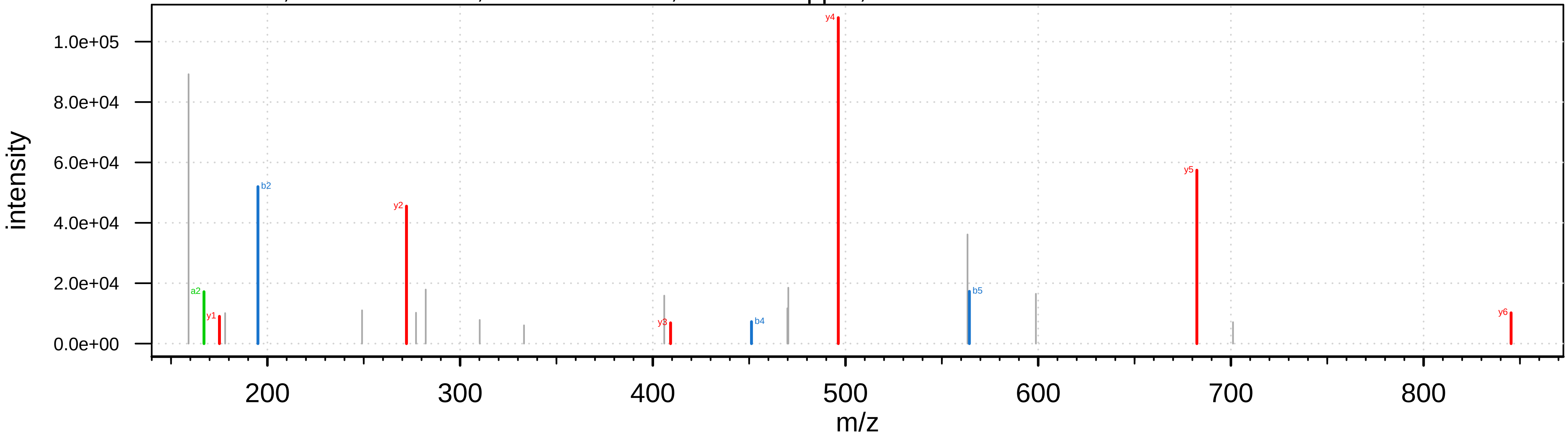

Raw File: Kermit_20111104_MSU_tube_6h_12
Scan Number: 28682
Proteins:
sp|P62274|RS29_MOUSE
ENSMUST00000037023_chr12:69157813−69159058:−
ENSMUST00000071737_chr13:73988963−73989133:−
tr|D3YYL7|D3YYL7_MOUSE

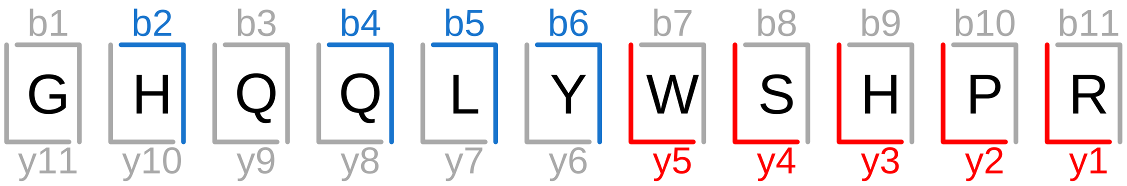

GHQQLYWSHPR
Score: 39 ; 1407.6796 m/z; 470.23382 m/z; 0.95949 ppm; ISO−MSMS

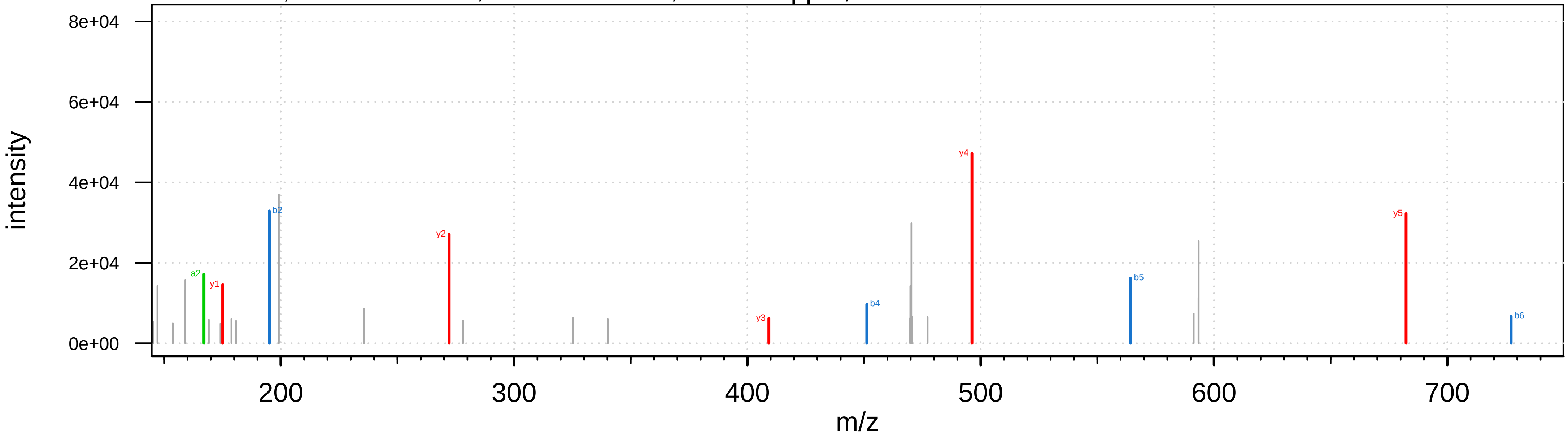

Raw File: Kermit_20111104_MSU_tube_6h_12
Scan Number: 46038
Proteins:
sp|P62274|RS29_MOUSE
ENSMUST00000037023_chr12:69157813−69159058:−
ENSMUST00000071737_chr13:73988963−73989133:−
tr|D3YYL7|D3YYL7_MOUSE

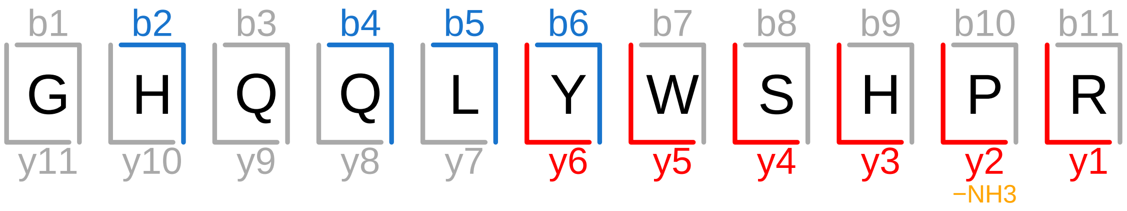

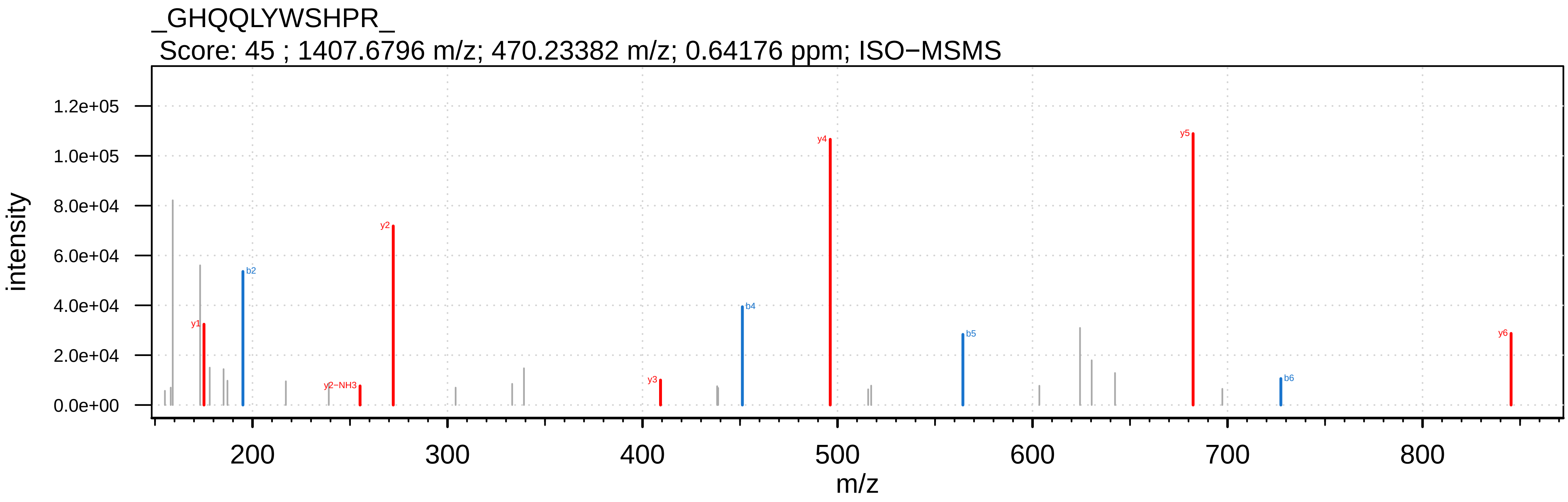

Raw File: Kermit_20111104_MSU_tube_6h_12
Scan Number: 46753
Proteins:
sp|P62274|RS29_MOUSE
ENSMUST00000037023_chr12:69157813−69159058:−
ENSMUST00000071737_chr13:73988963−73989133:−
tr|D3YYL7|D3YYL7_MOUSE

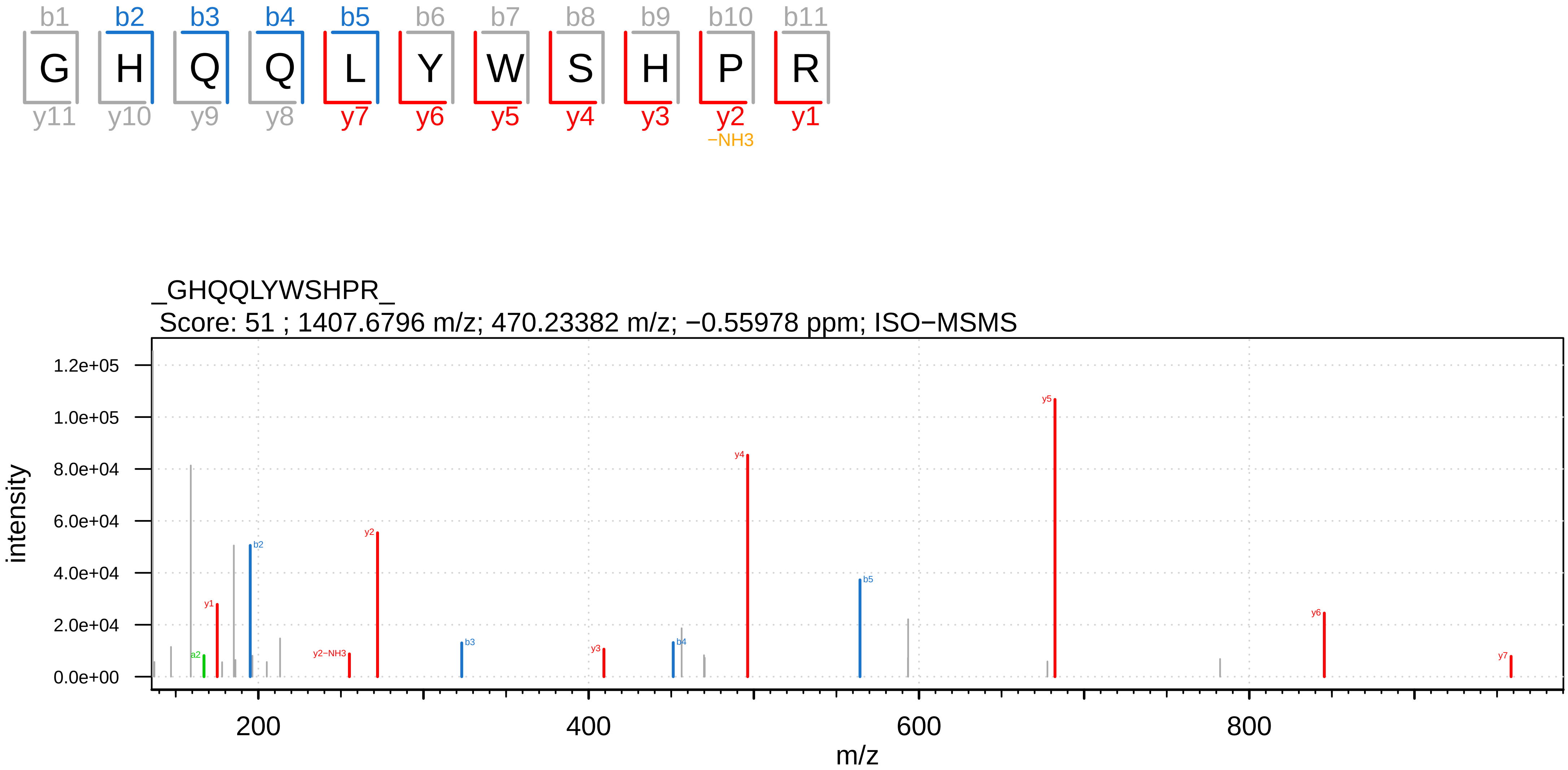

Raw File: Kermit_20111104_MSU_tube_6h_12
Scan Number: 46997
Proteins:
sp|P62274|RS29_MOUSE
ENSMUST00000037023_chr12:69157813−69159058:−
ENSMUST00000071737_chr13:73988963−73989133:−
tr|D3YYL7|D3YYL7_MOUSE

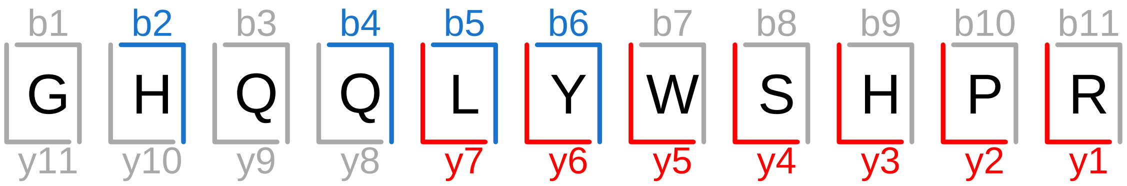

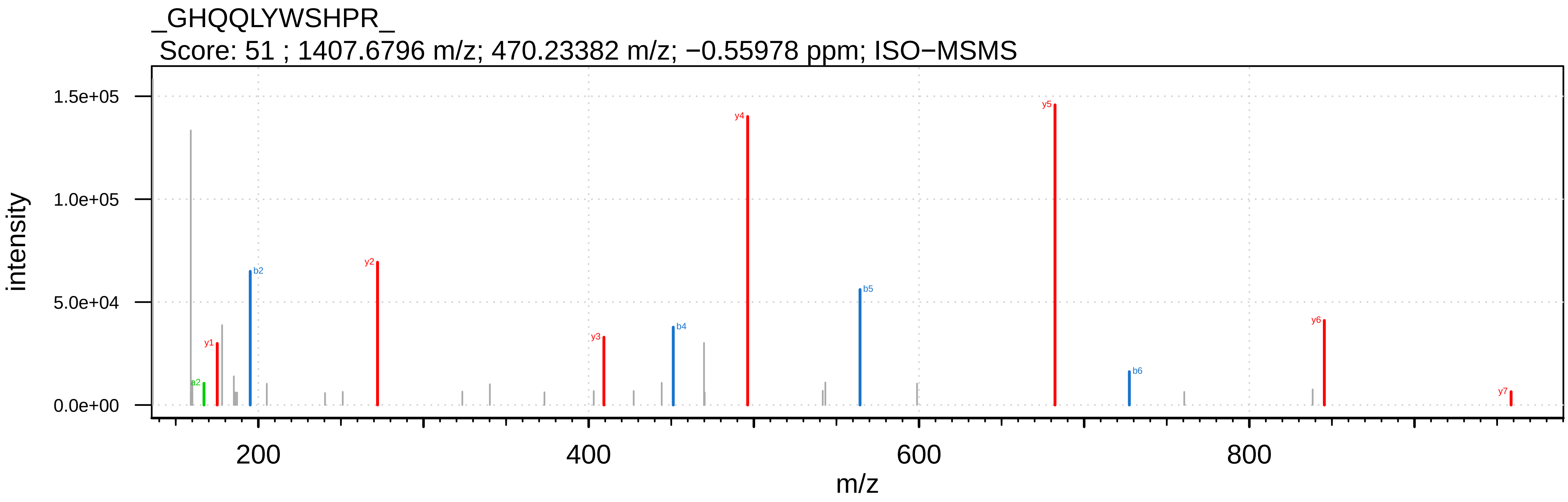

Raw File: Kermit_20111104_MSU_tube_6h_12
Scan Number: 47182
Proteins:
sp|P62274|RS29_MOUSE
ENSMUST00000037023_chr12:69157813−69159058:−
ENSMUST00000071737_chr13:73988963−73989133:−
tr|D3YYL7|D3YYL7_MOUSE

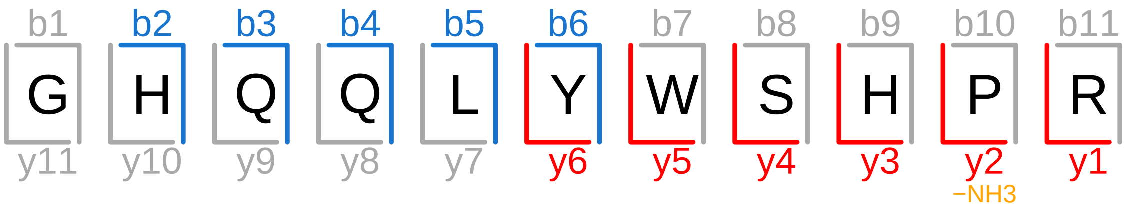

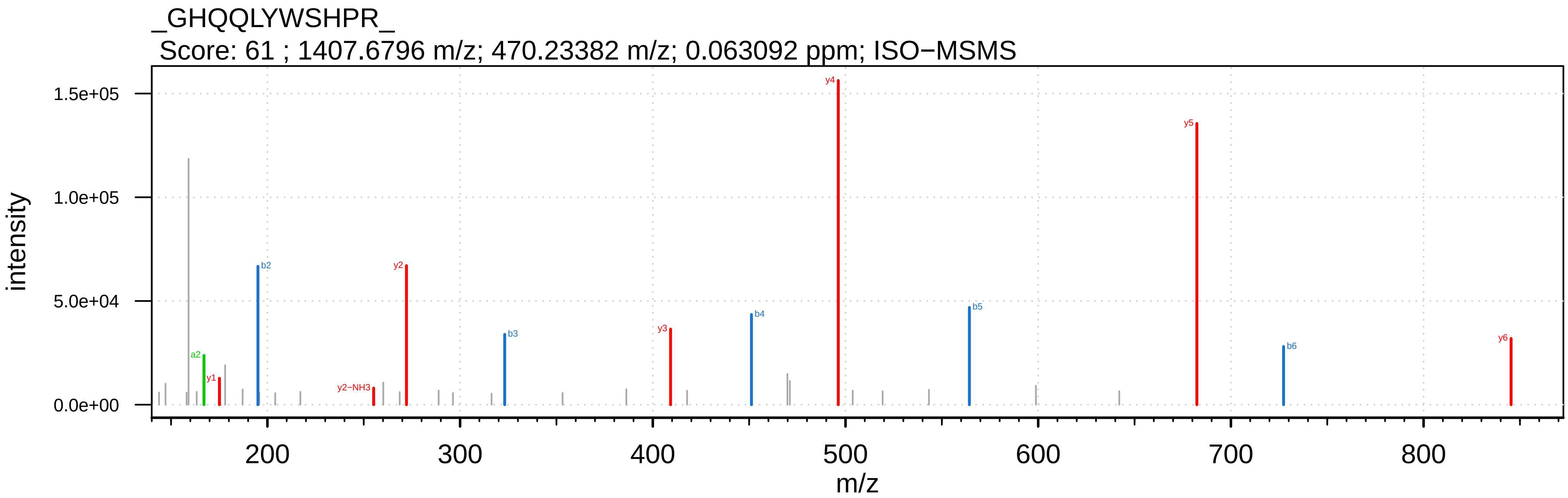

Raw File: Kermit_20111104_MSU_tube_6h_12
Scan Number: 47367
Proteins:
sp|P62274|RS29_MOUSE
ENSMUST00000037023_chr12:69157813−69159058:−
ENSMUST00000071737_chr13:73988963−73989133:−
tr|D3YYL7|D3YYL7_MOUSE

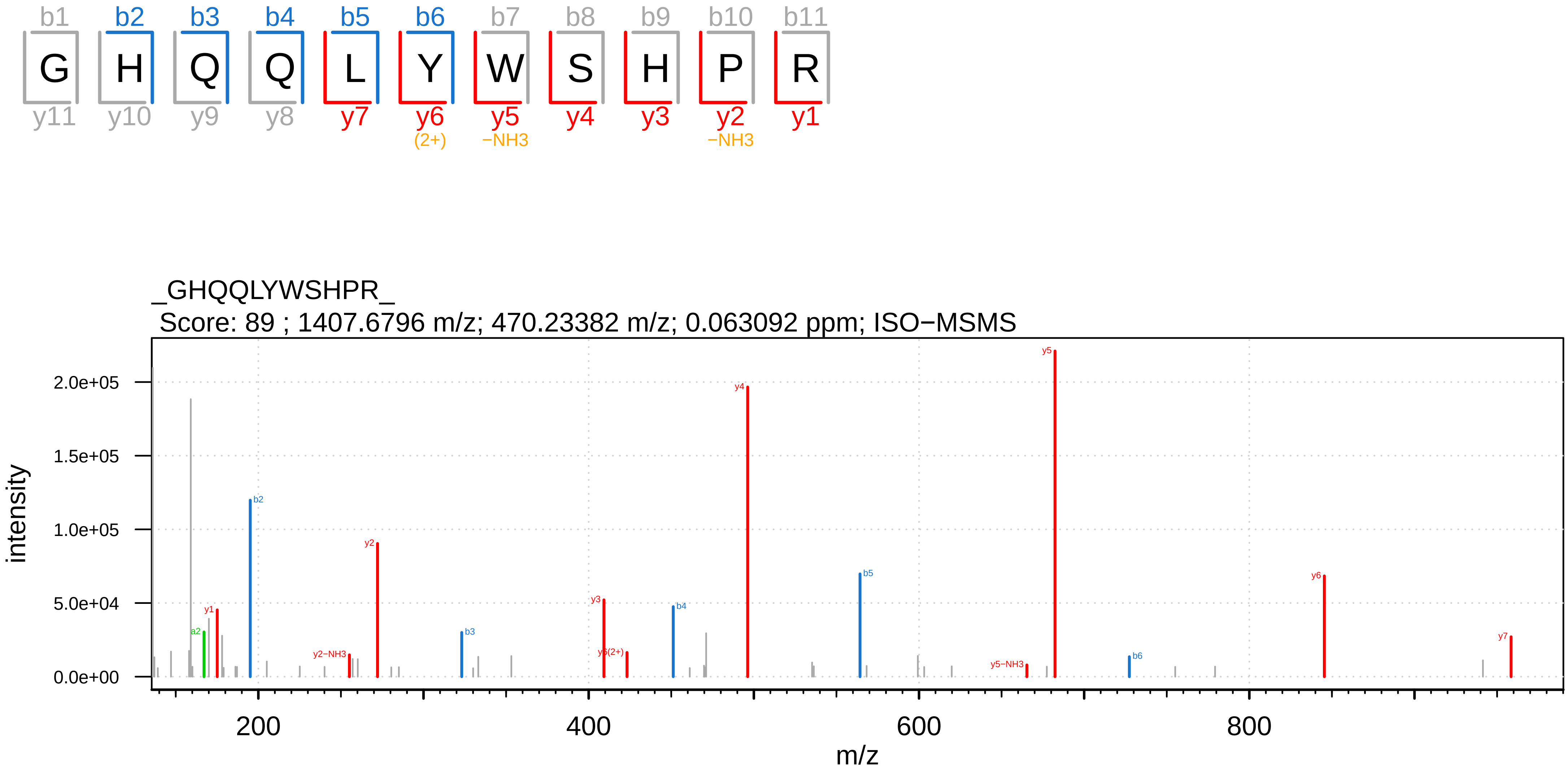

Raw File: Kermit_20111104_MSU_tube_6h_12
Scan Number: 47549
Proteins:
sp|P62274|RS29_MOUSE
ENSMUST00000037023_chr12:69157813−69159058:−
ENSMUST00000071737_chr13:73988963−73989133:−
tr|D3YYL7|D3YYL7_MOUSE

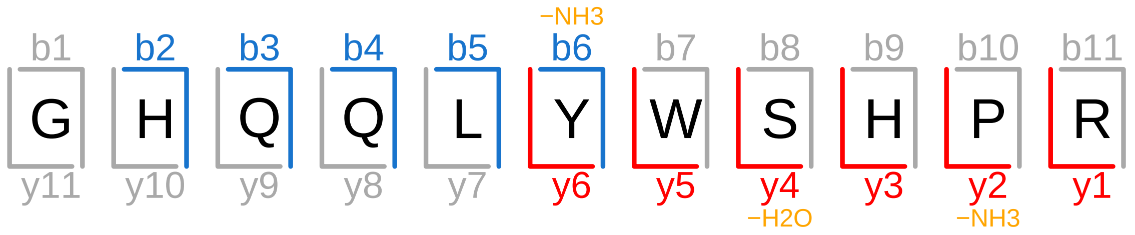

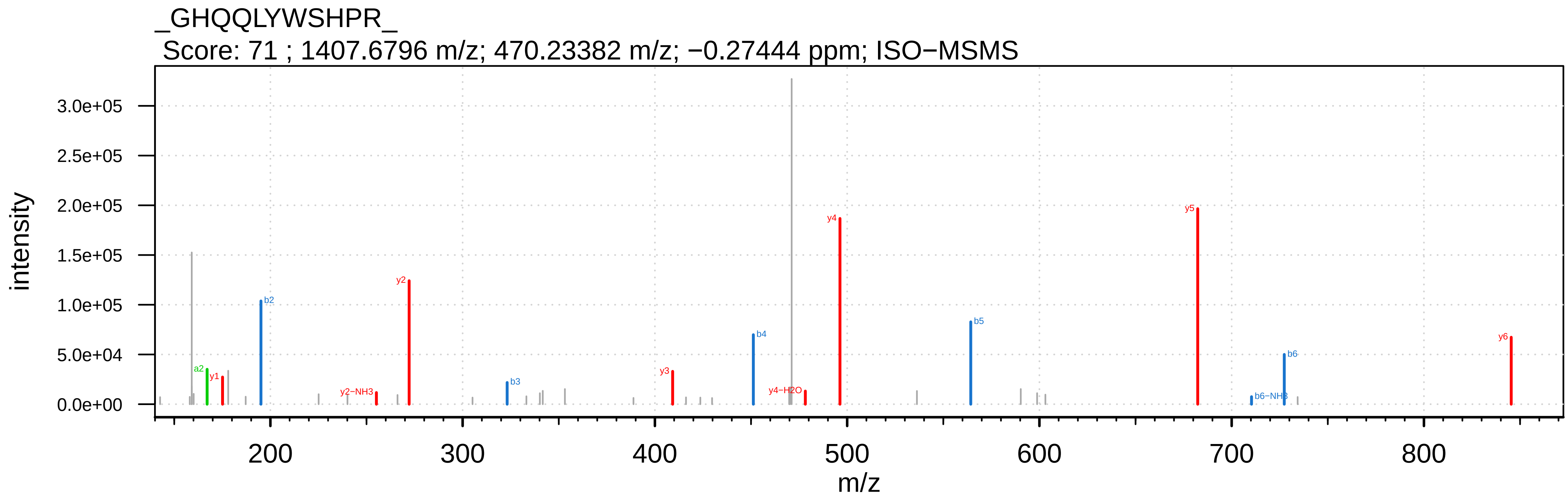

Raw File: Kermit_20111104_MSU_tube_6h_12
Scan Number: 47729
Proteins:
sp|P62274|RS29_MOUSE
ENSMUST00000037023_chr12:69157813−69159058:−
ENSMUST00000071737_chr13:73988963−73989133:−
tr|D3YYL7|D3YYL7_MOUSE

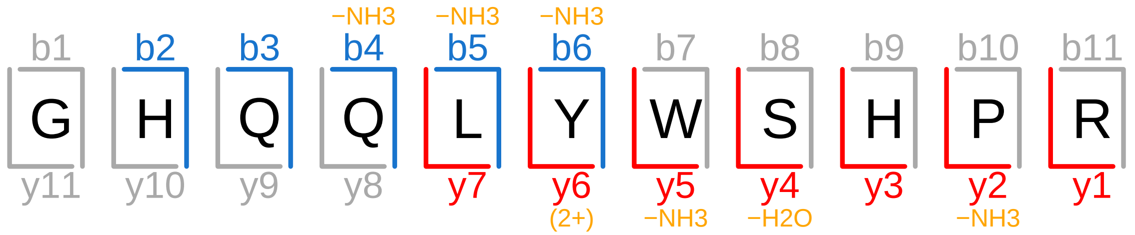

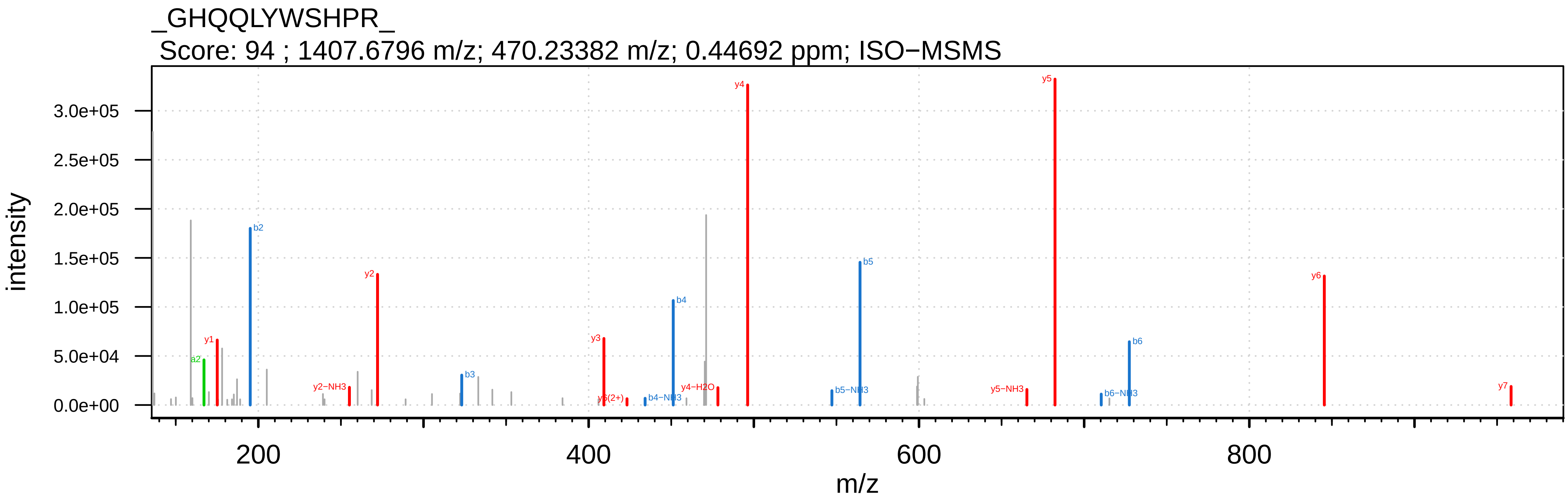

Raw File: Kermit_20111104_MSU_tube_6h_12
Scan Number: 47908
Proteins:
sp|P62274|RS29_MOUSE
ENSMUST00000037023_chr12:69157813−69159058:−
ENSMUST00000071737_chr13:73988963−73989133:−
tr|D3YYL7|D3YYL7_MOUSE

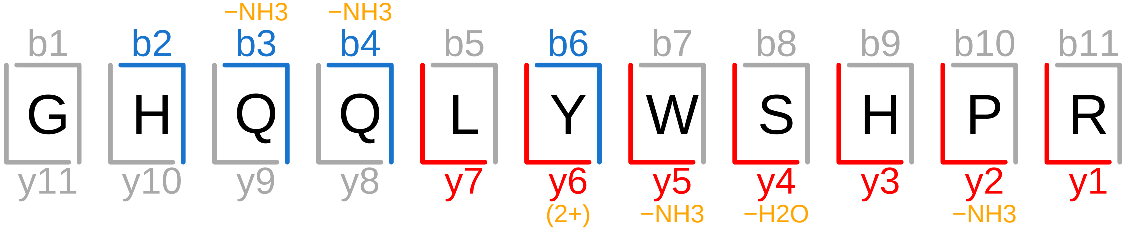

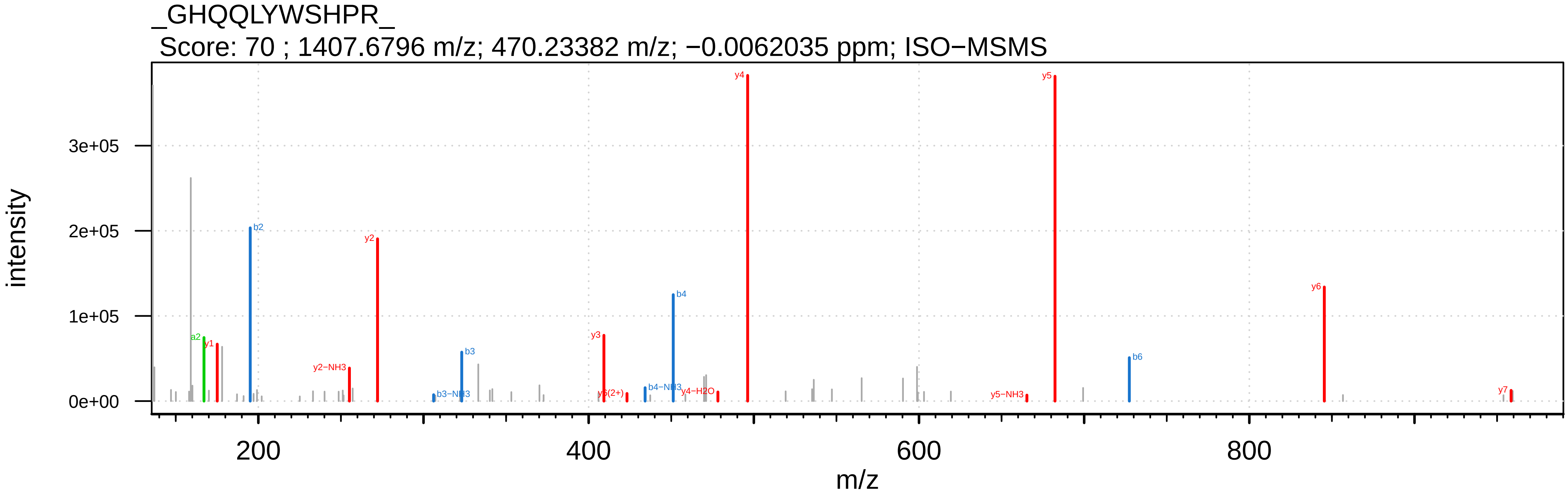

Raw File: Kermit_20111104_MSU_tube_6h_12
Scan Number: 48091
Proteins:
sp|P62274|RS29_MOUSE
ENSMUST00000037023_chr12:69157813−69159058:−
ENSMUST00000071737_chr13:73988963−73989133:−
tr|D3YYL7|D3YYL7_MOUSE

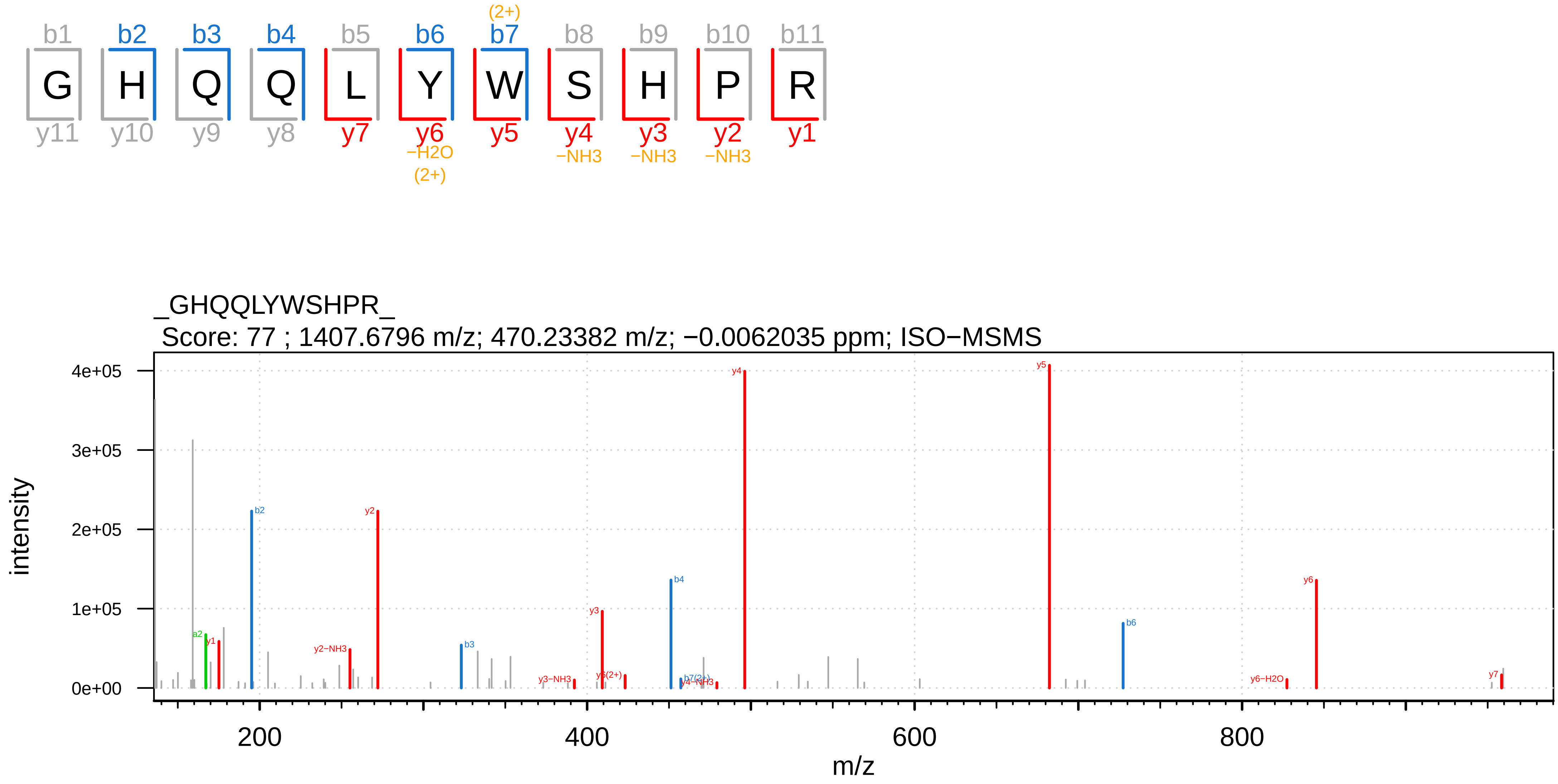

Raw File: Kermit_20111104_MSU_tube_6h_12
Scan Number: 48275
Proteins:
sp|P62274|RS29_MOUSE
ENSMUST00000037023_chr12:69157813−69159058:−
ENSMUST00000071737_chr13:73988963−73989133:−
tr|D3YYL7|D3YYL7_MOUSE

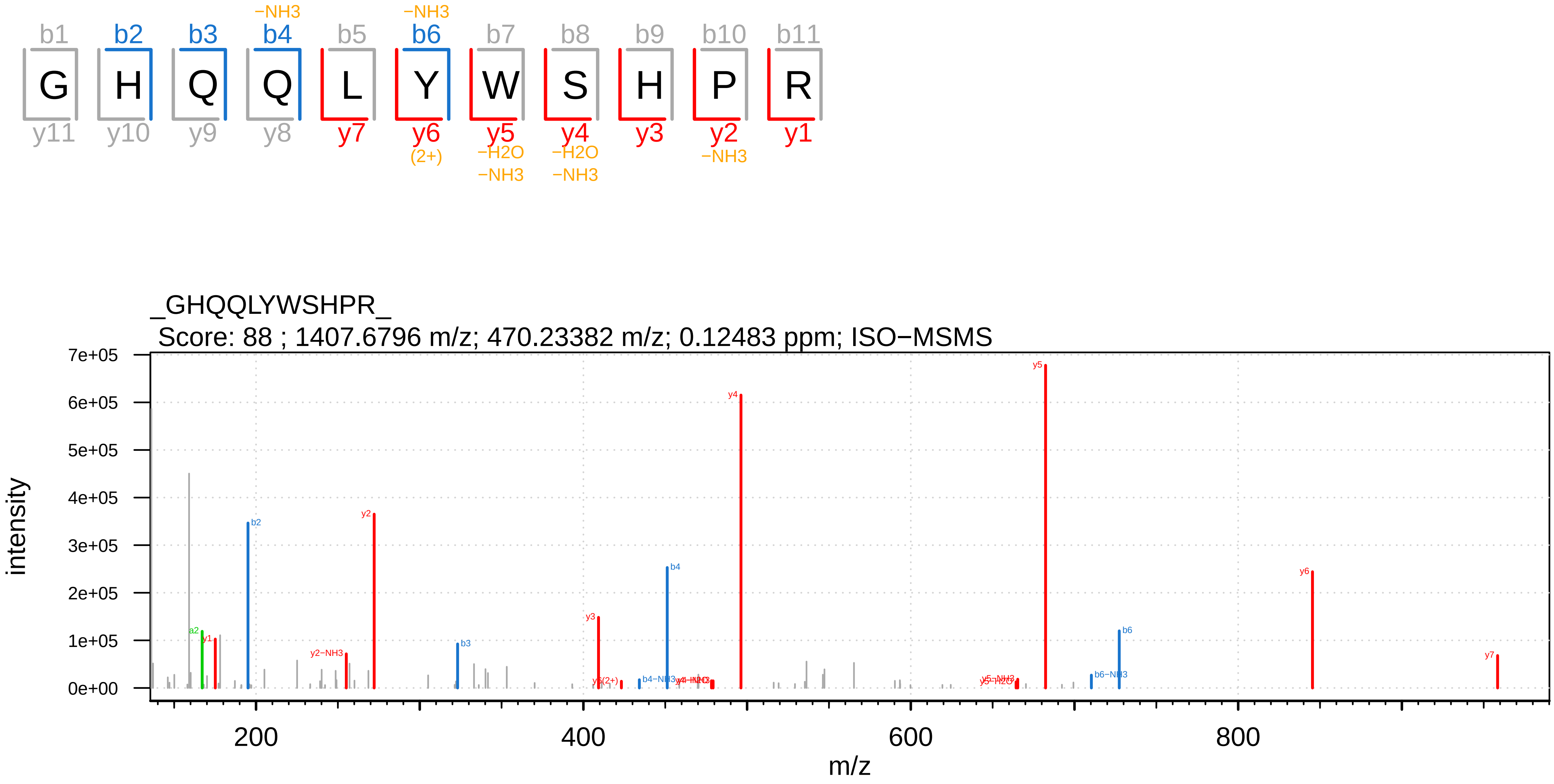

Raw File: Kermit_20111104_MSU_tube_6h_12
Scan Number: 48620
Proteins:
sp|P62274|RS29_MOUSE
ENSMUST00000037023_chr12:69157813−69159058:−
ENSMUST00000071737_chr13:73988963−73989133:−
tr|D3YYL7|D3YYL7_MOUSE

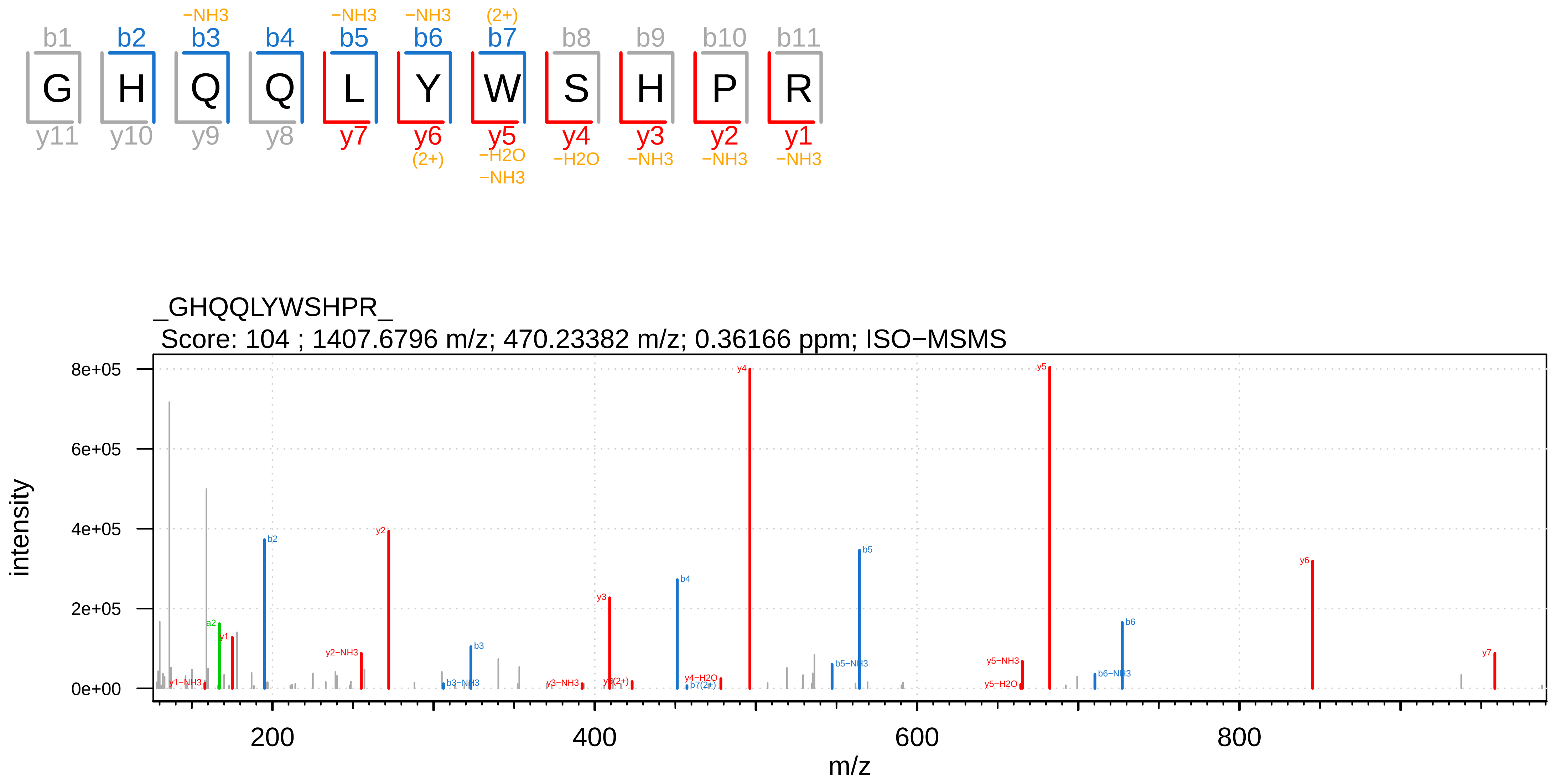

Raw File: Kermit_20111104_MSU_tube_6h_12
Scan Number: 48800
Proteins:
sp|P62274|RS29_MOUSE
ENSMUST00000037023_chr12:69157813−69159058:−
ENSMUST00000071737_chr13:73988963−73989133:−
tr|D3YYL7|D3YYL7_MOUSE

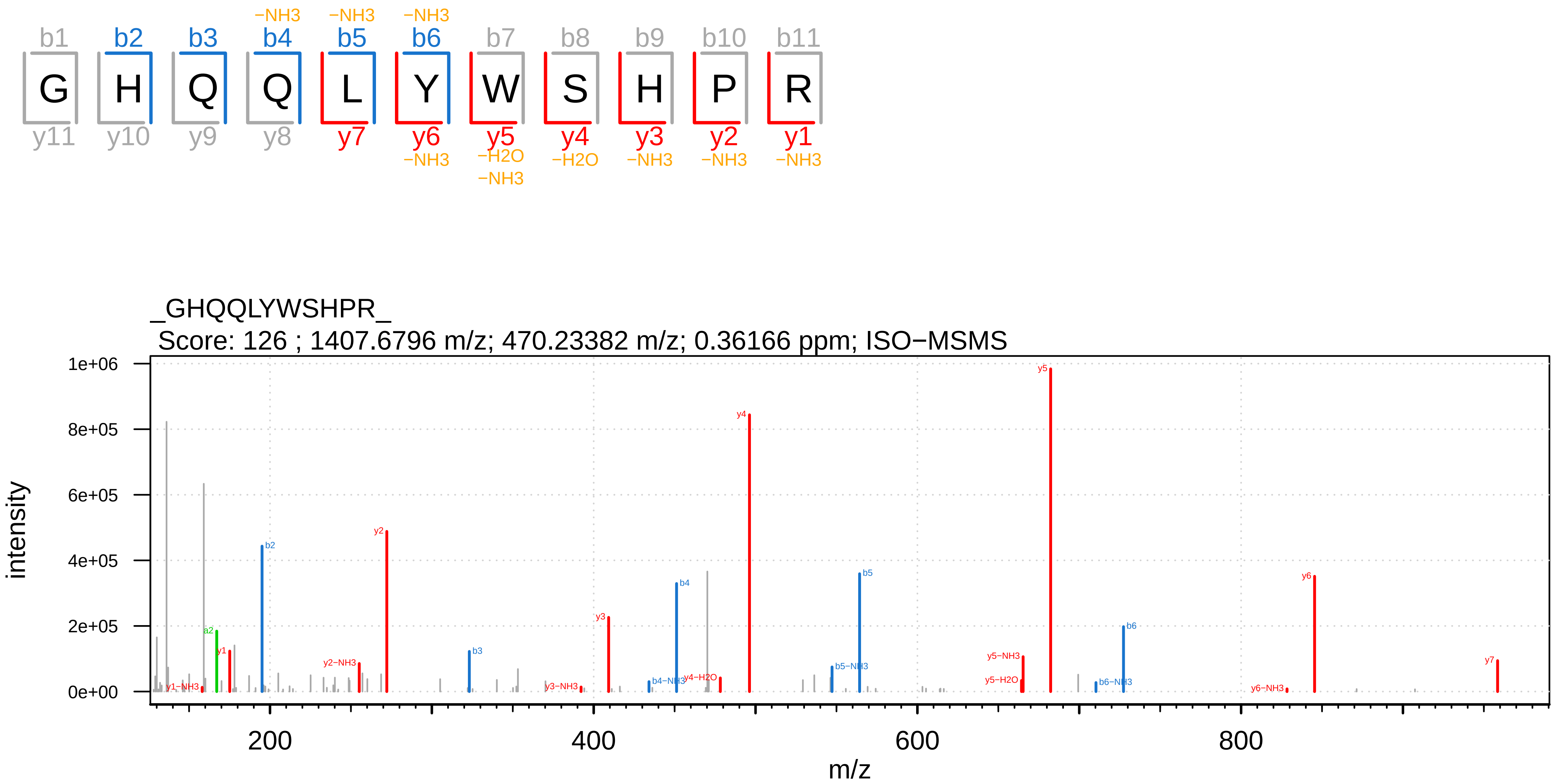

Raw File: Kermit_20111104_MSU_tube_6h_12
Scan Number: 48983
Proteins:
sp|P62274|RS29_MOUSE
ENSMUST00000037023_chr12:69157813−69159058:−
ENSMUST00000071737_chr13:73988963−73989133:−
tr|D3YYL7|D3YYL7_MOUSE

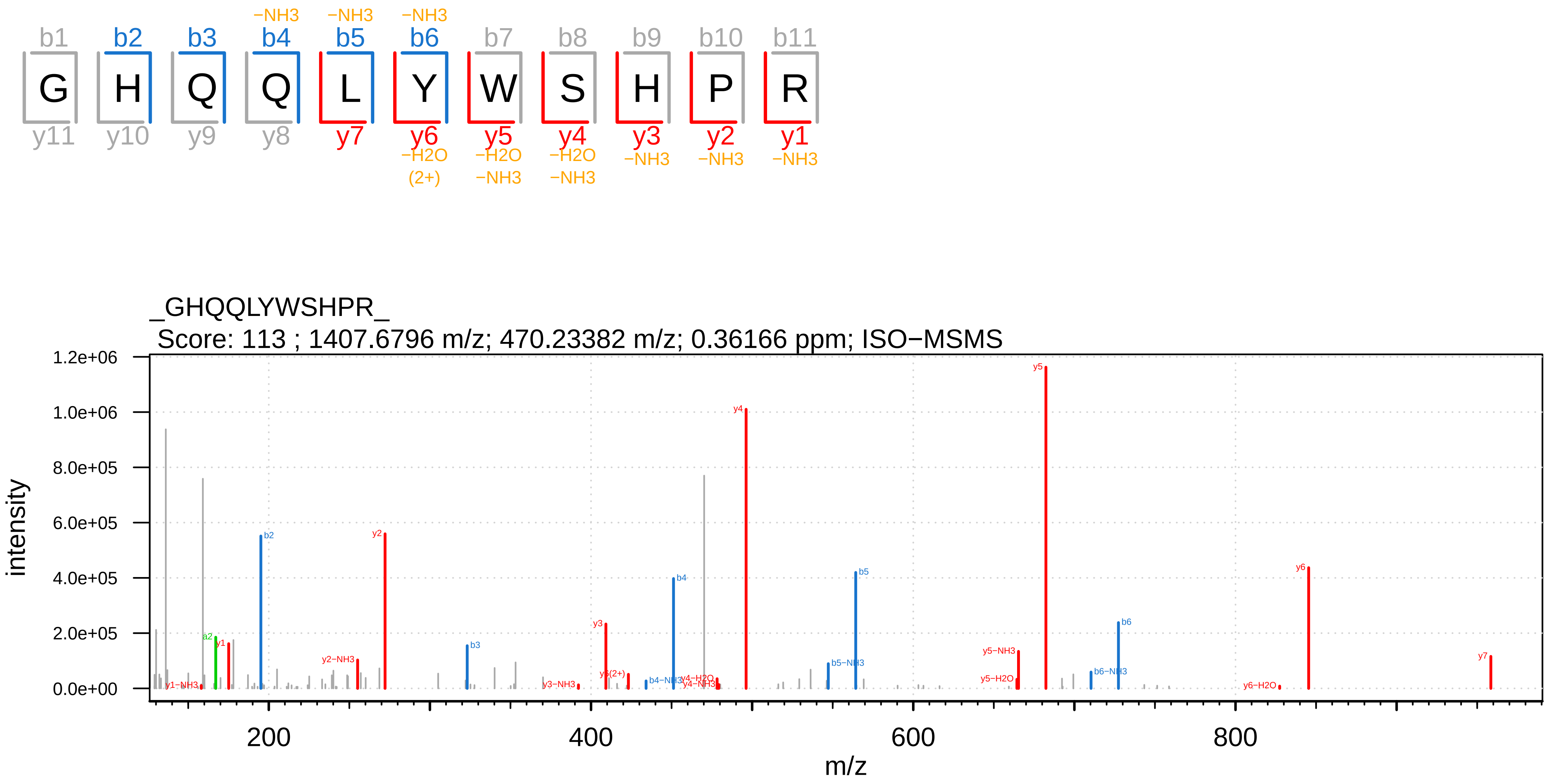

Raw File: Kermit_20111104_MSU_tube_6h_12
Scan Number: 49169
Proteins:
sp|P62274|RS29_MOUSE
ENSMUST00000037023_chr12:69157813−69159058:−
ENSMUST00000071737_chr13:73988963−73989133:−
tr|D3YYL7|D3YYL7_MOUSE

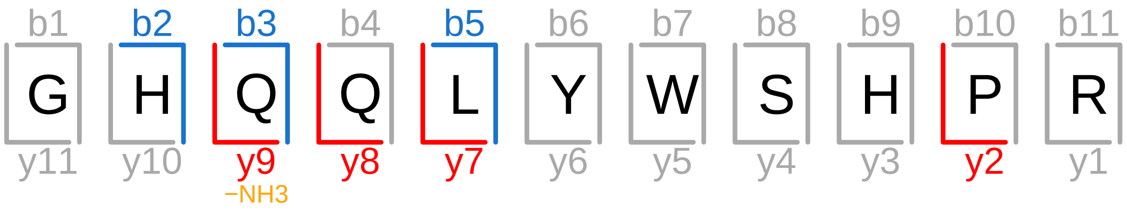

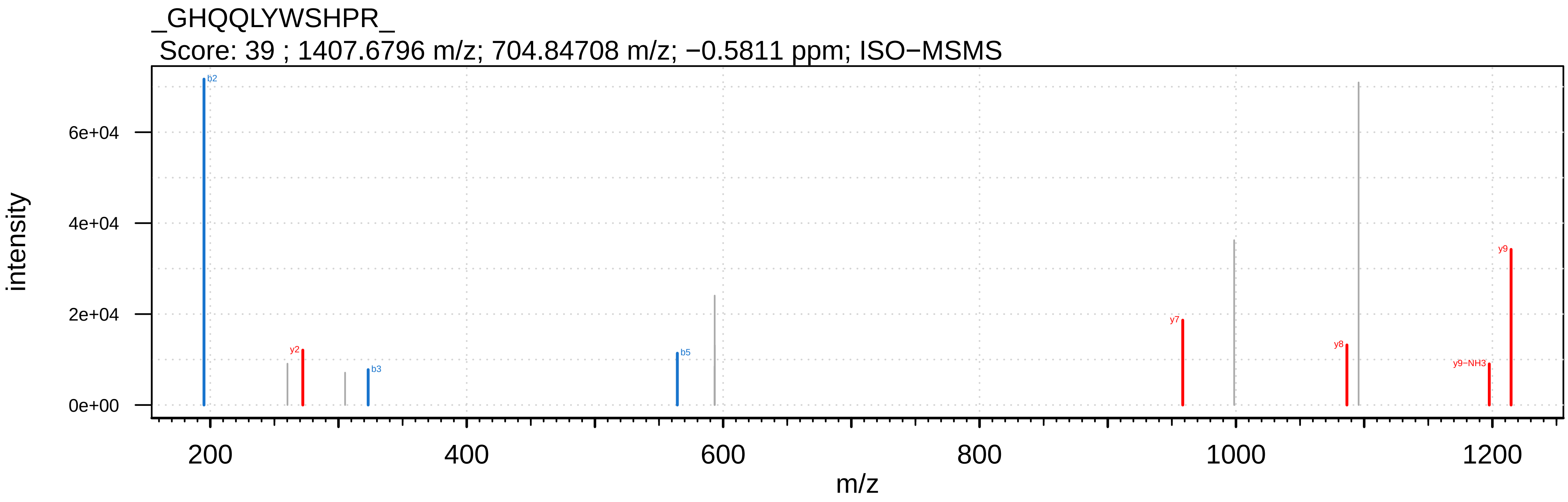

Raw File: Kermit_20111104_MSU_tube_6h_12
Scan Number: 49484
Proteins:
sp|P62274|RS29_MOUSE
ENSMUST00000037023_chr12:69157813−69159058:−
ENSMUST00000071737_chr13:73988963−73989133:−
tr|D3YYL7|D3YYL7_MOUSE

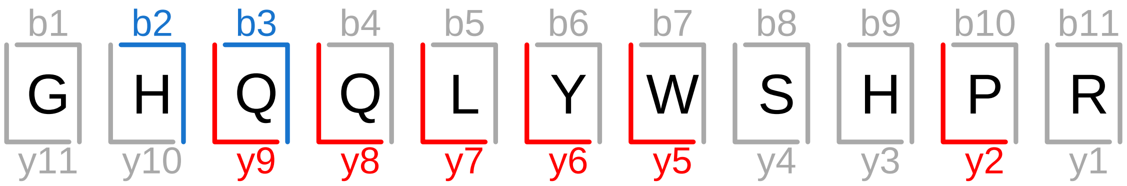

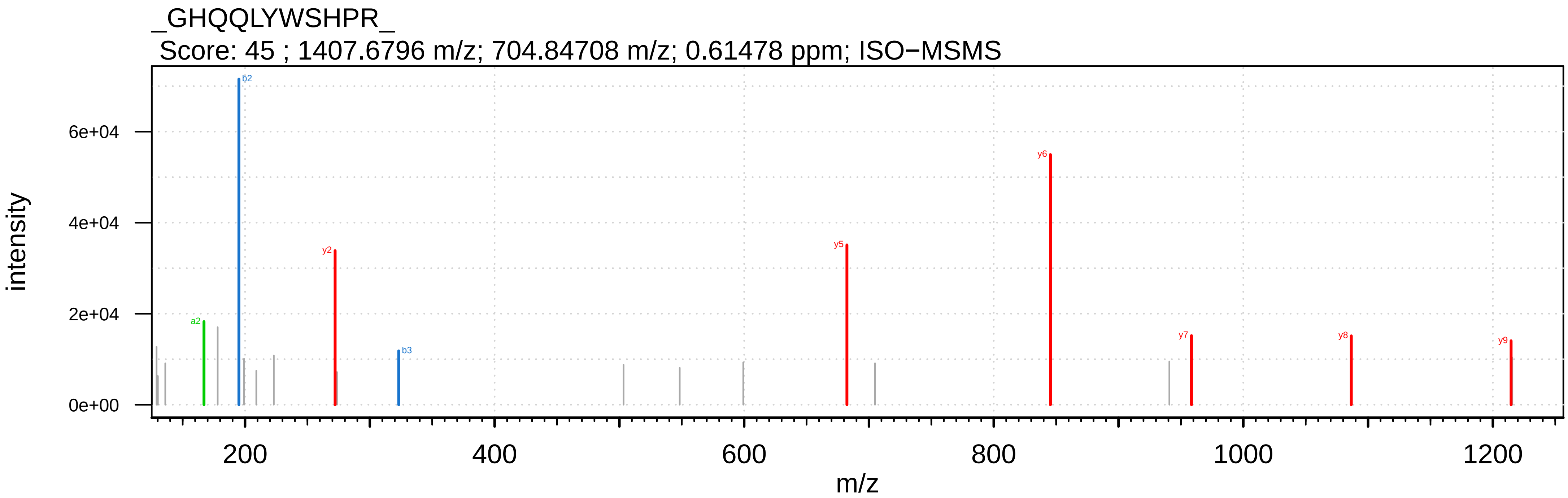

Raw File: Kermit_20111104_MSU_tube_6h_12
Scan Number: 49682
Proteins:
sp|P62274|RS29_MOUSE
ENSMUST00000037023_chr12:69157813−69159058:−
ENSMUST00000071737_chr13:73988963−73989133:−
tr|D3YYL7|D3YYL7_MOUSE

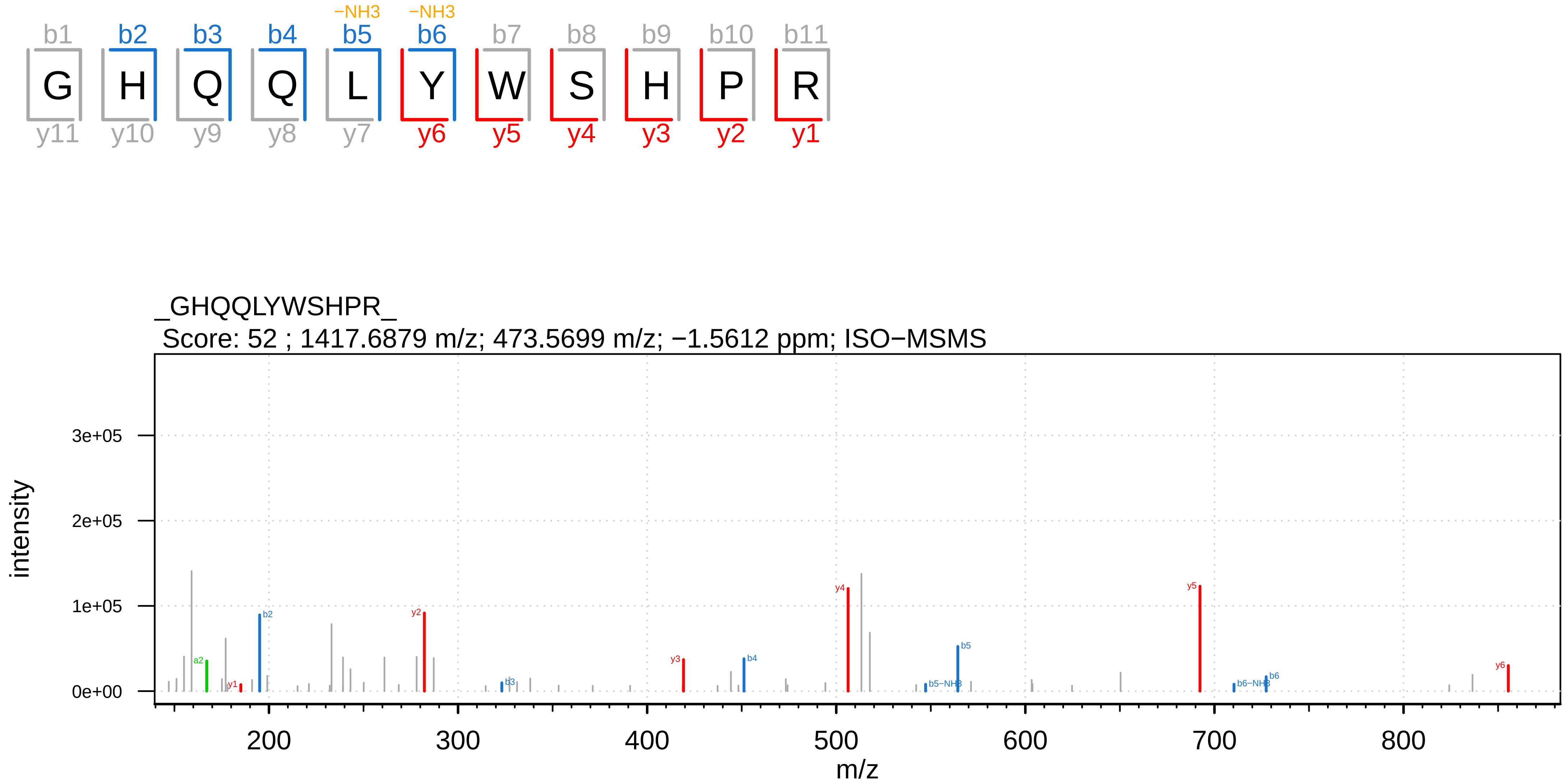

Raw File: Kermit_20111104_MSU_tube_6h_12
Scan Number: 49968
Proteins:
sp|P62274|RS29_MOUSE
ENSMUST00000037023_chr12:69157813−69159058:−
ENSMUST00000071737_chr13:73988963−73989133:−
tr|D3YYL7|D3YYL7_MOUSE

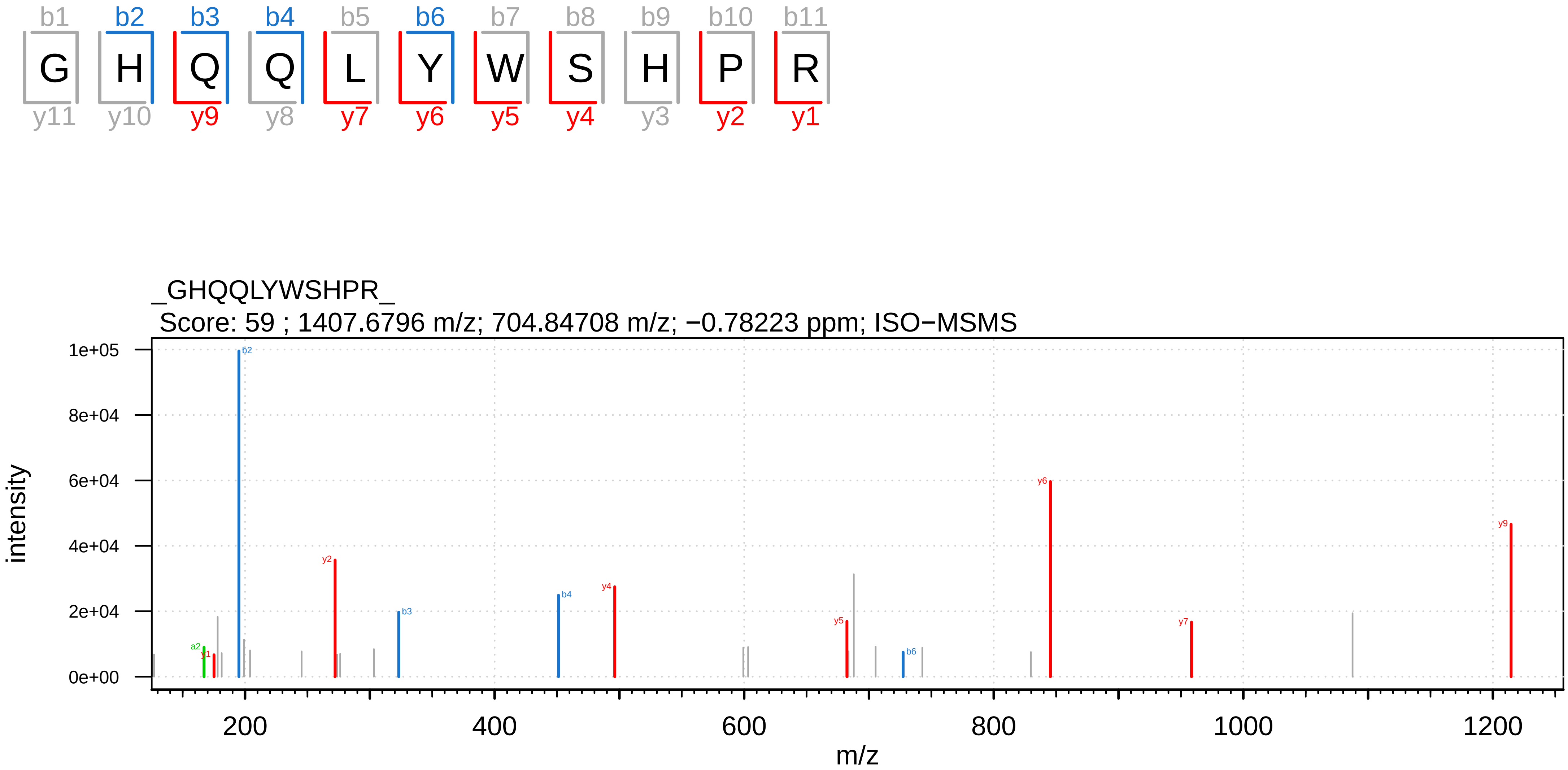

Raw File: Kermit_20111104_MSU_tube_6h_12
Scan Number: 50238
Proteins:
sp|P62274|RS29_MOUSE
ENSMUST00000037023_chr12:69157813−69159058:−
ENSMUST00000071737_chr13:73988963−73989133:−
tr|D3YYL7|D3YYL7_MOUSE

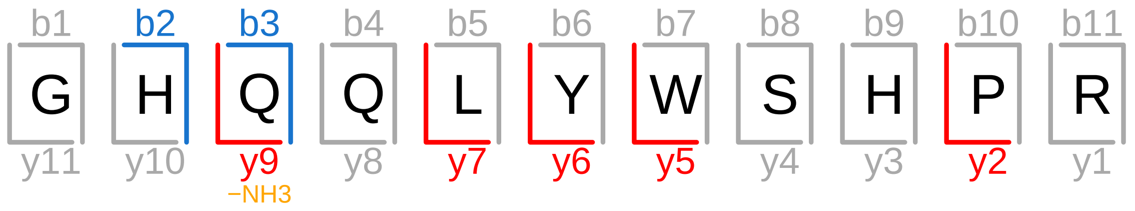

GHQQLYWSHPR
Score: 39 ; 1407.6796 m/z; 704.84708 m/z; 0.11708 ppm; ISO−MSMS

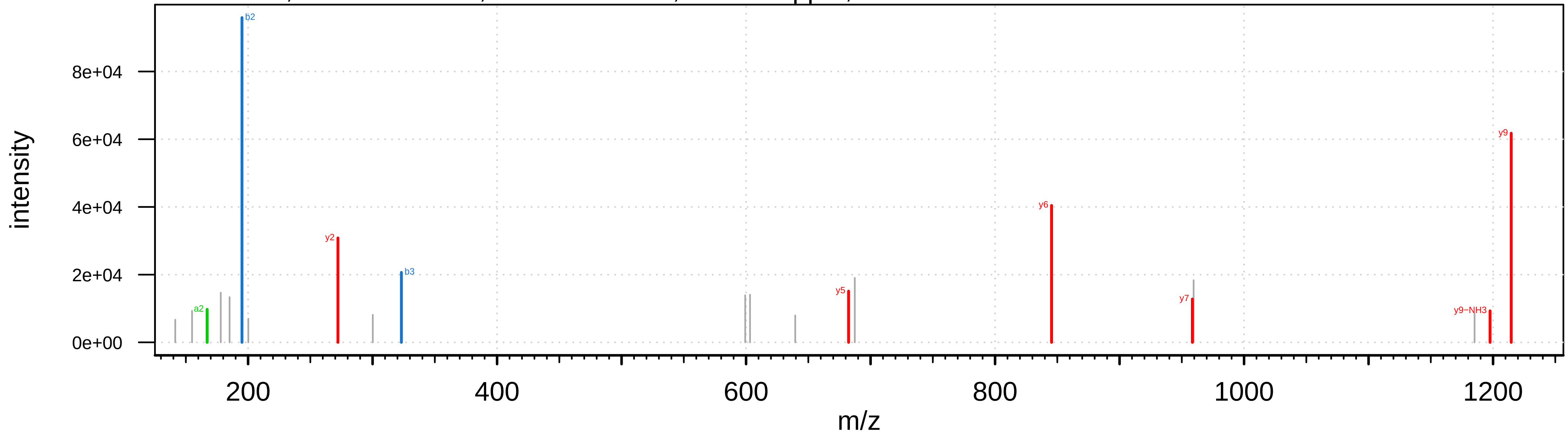

Raw File: Kermit_20111104_MSU_tube_6h_12
Scan Number: 50437
Proteins:
sp|P62274|RS29_MOUSE
ENSMUST00000037023_chr12:69157813−69159058:−
ENSMUST00000071737_chr13:73988963−73989133:−
tr|D3YYL7|D3YYL7_MOUSE

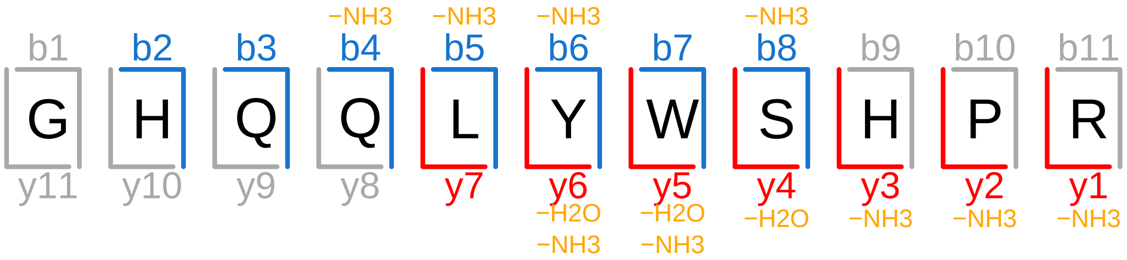

GHQQLYWSHPR
Score: 145 ; 1407.6796 m/z; 470.23382 m/z; −0.39683 ppm; ISO−MSMS

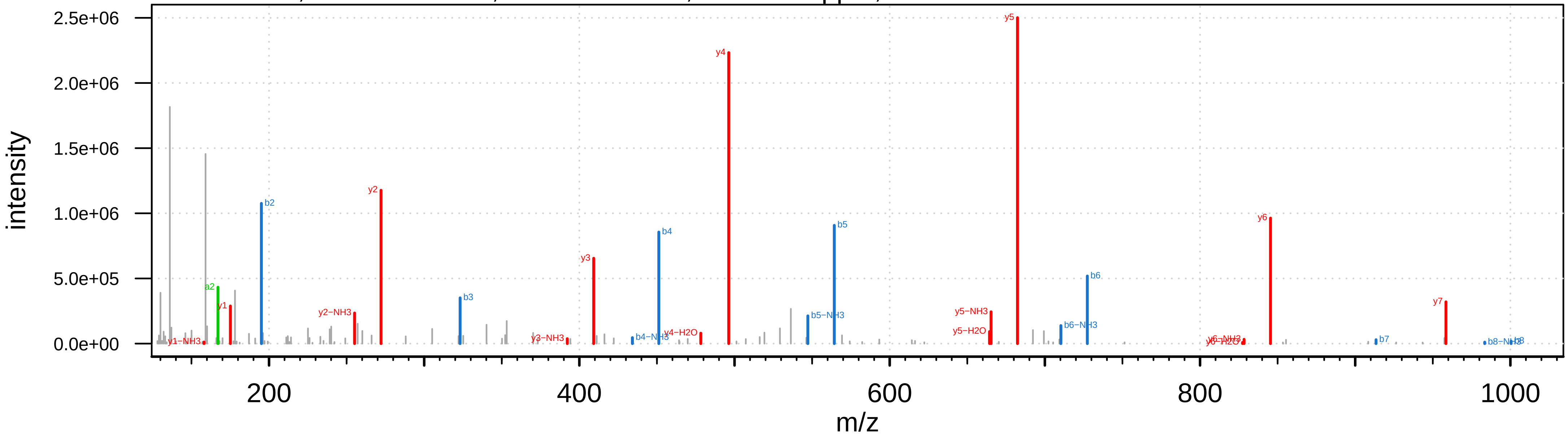

Raw File: Kermit_20111104_MSU_tube_6h_12
Scan Number: 50466
Proteins:
sp|P62274|RS29_MOUSE
ENSMUST00000037023_chr12:69157813−69159058:−
ENSMUST00000071737_chr13:73988963−73989133:−
tr|D3YYL7|D3YYL7_MOUSE

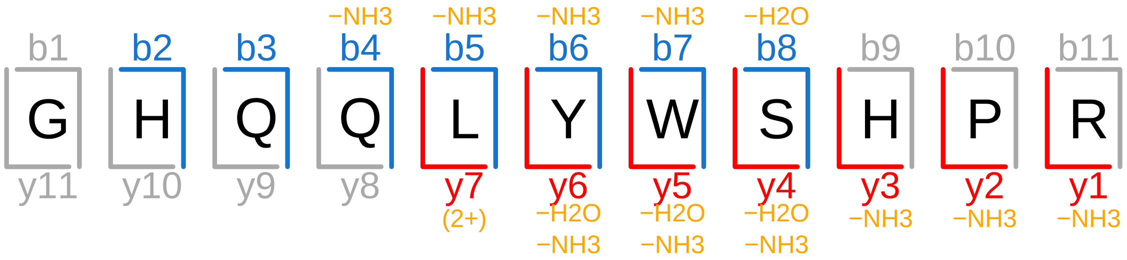

GHQQLYWSHPR
Score: 147 ; 1407.6796 m/z; 470.23382 m/z; 0.38202 ppm; ISO−MSMS

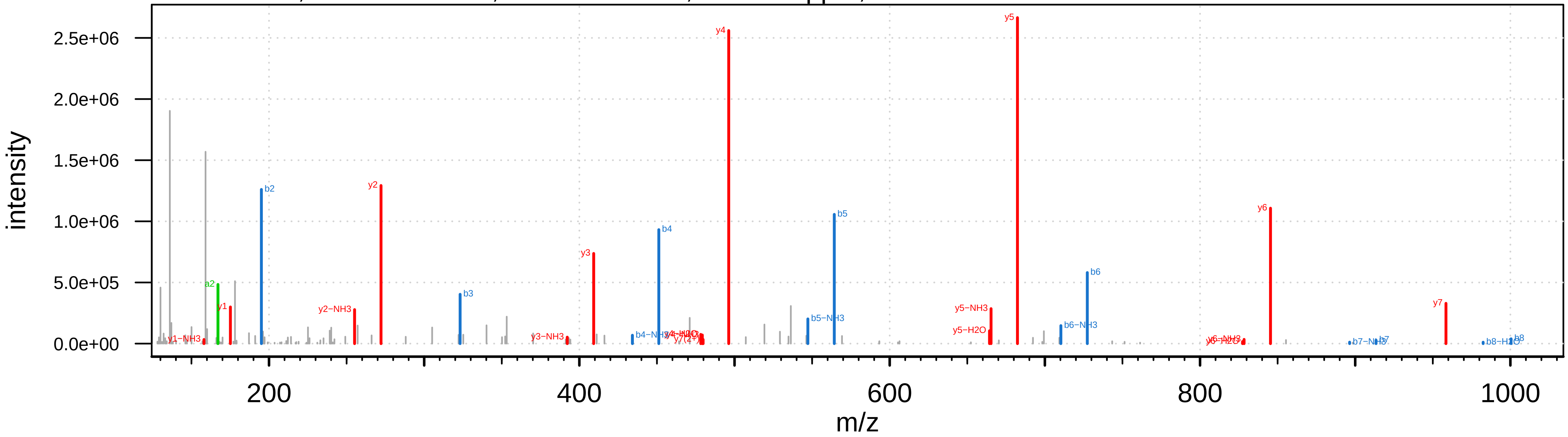

Raw File: Kermit_20111104_MSU_tube_6h_12
Scan Number: 50657
Proteins:
sp|P62274|RS29_MOUSE
ENSMUST00000037023_chr12:69157813−69159058:−
ENSMUST00000071737_chr13:73988963−73989133:−
tr|D3YYL7|D3YYL7_MOUSE

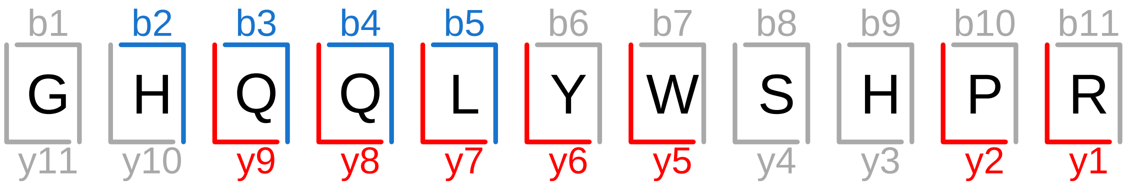

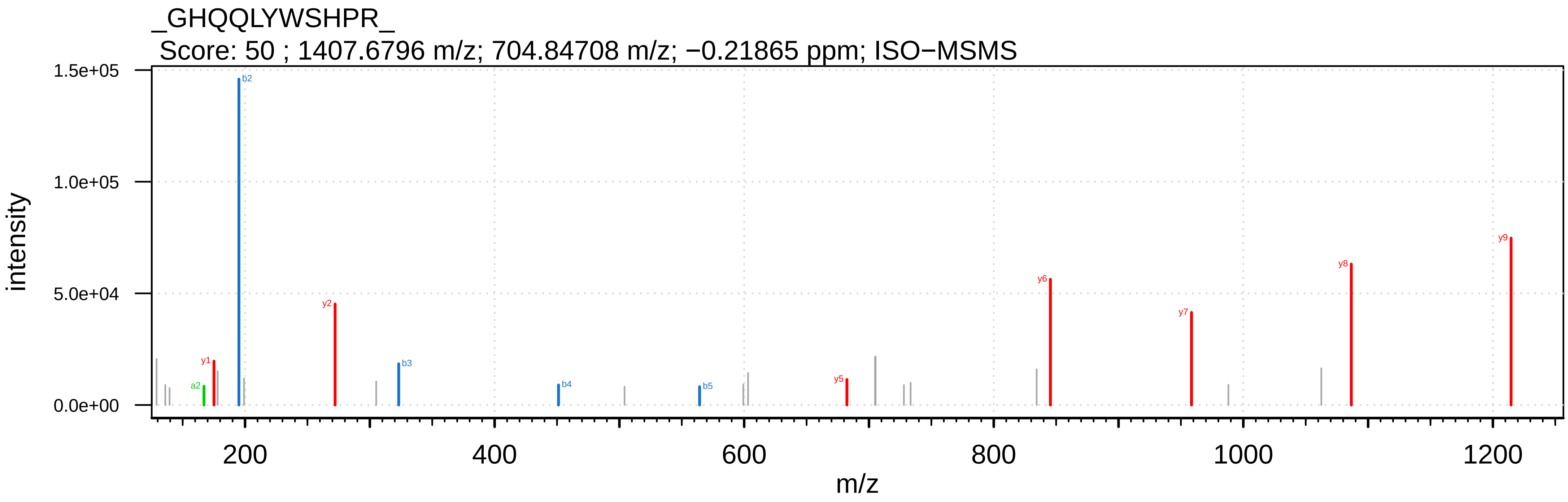

Raw File: Kermit_20111104_MSU_tube_6h_12
Scan Number: 50794
Proteins:
sp|P62274|RS29_MOUSE
ENSMUST00000037023_chr12:69157813−69159058:−
ENSMUST00000071737_chr13:73988963−73989133:−
tr|D3YYL7|D3YYL7_MOUSE

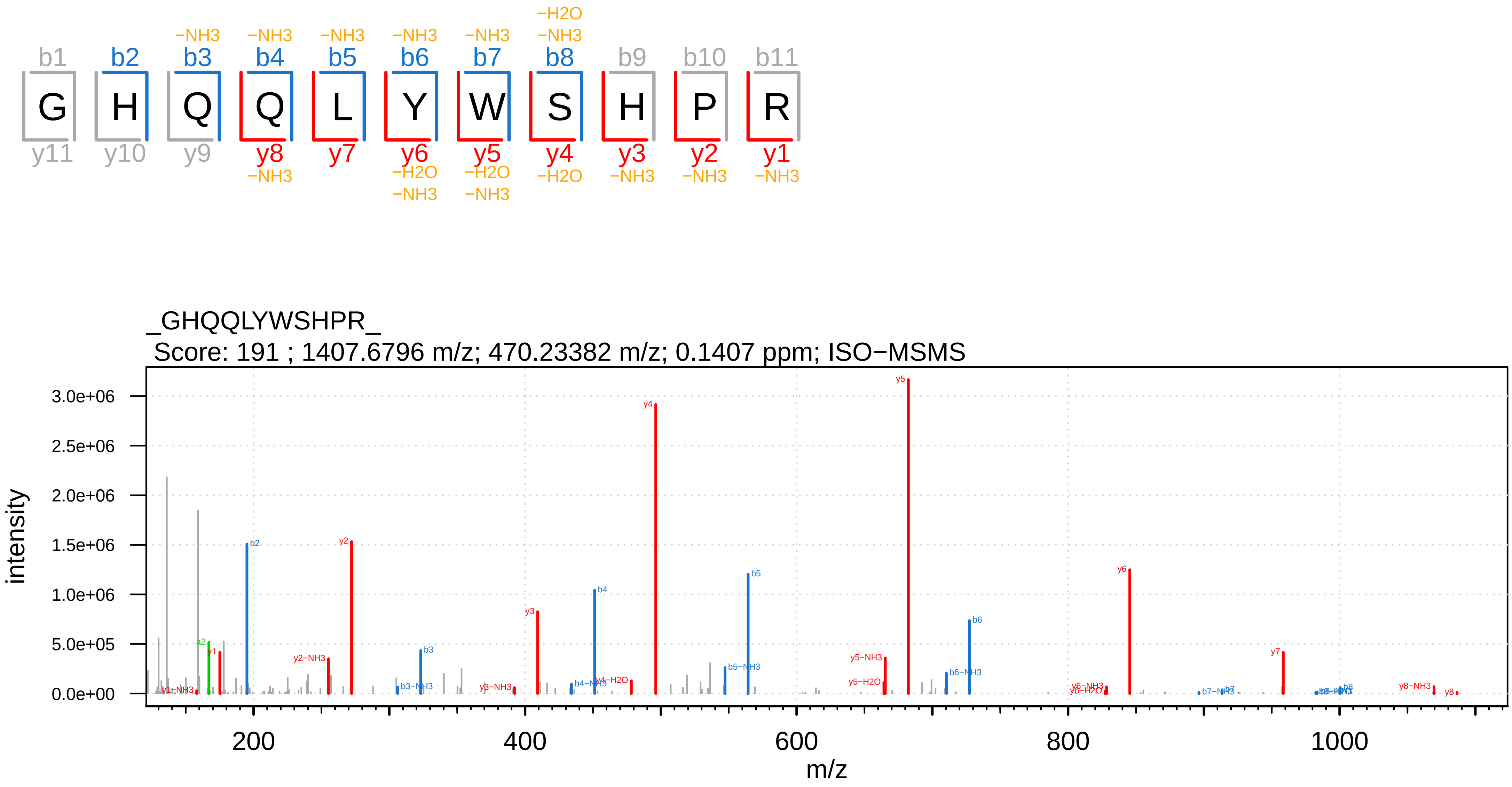

Raw File: Kermit_20111104_MSU_tube_6h_12
Scan Number: 51024
Proteins:
sp|P62274|RS29_MOUSE
ENSMUST00000037023_chr12:69157813−69159058:−
ENSMUST00000071737_chr13:73988963−73989133:−
tr|D3YYL7|D3YYL7_MOUSE

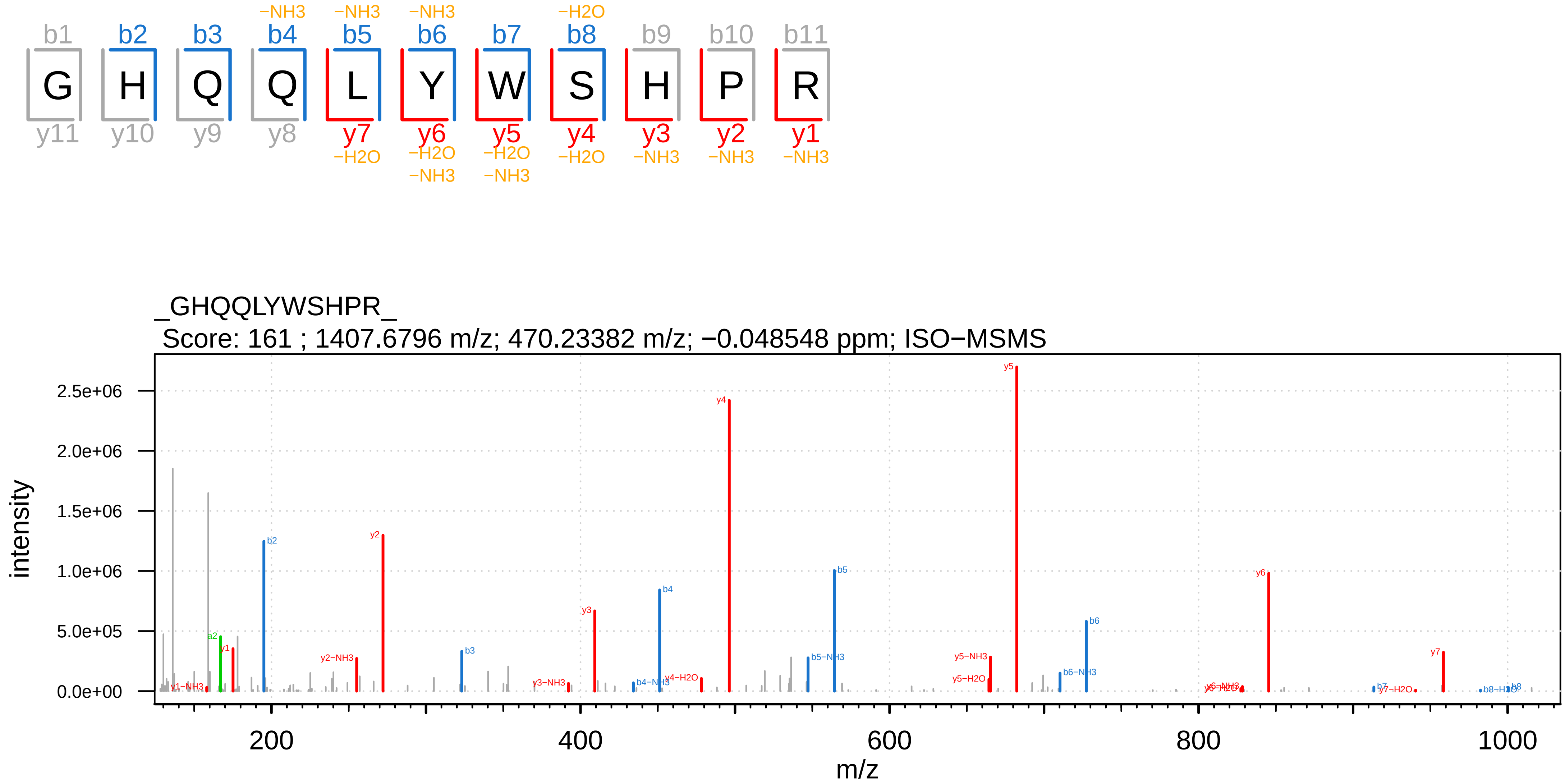

Raw File: Kermit_20111104_MSU_tube_6h_12
Scan Number: 51768
Proteins:
sp|P62274|RS29_MOUSE
ENSMUST00000037023_chr12:69157813−69159058:−
ENSMUST00000071737_chr13:73988963−73989133:−
tr|D3YYL7|D3YYL7_MOUSE

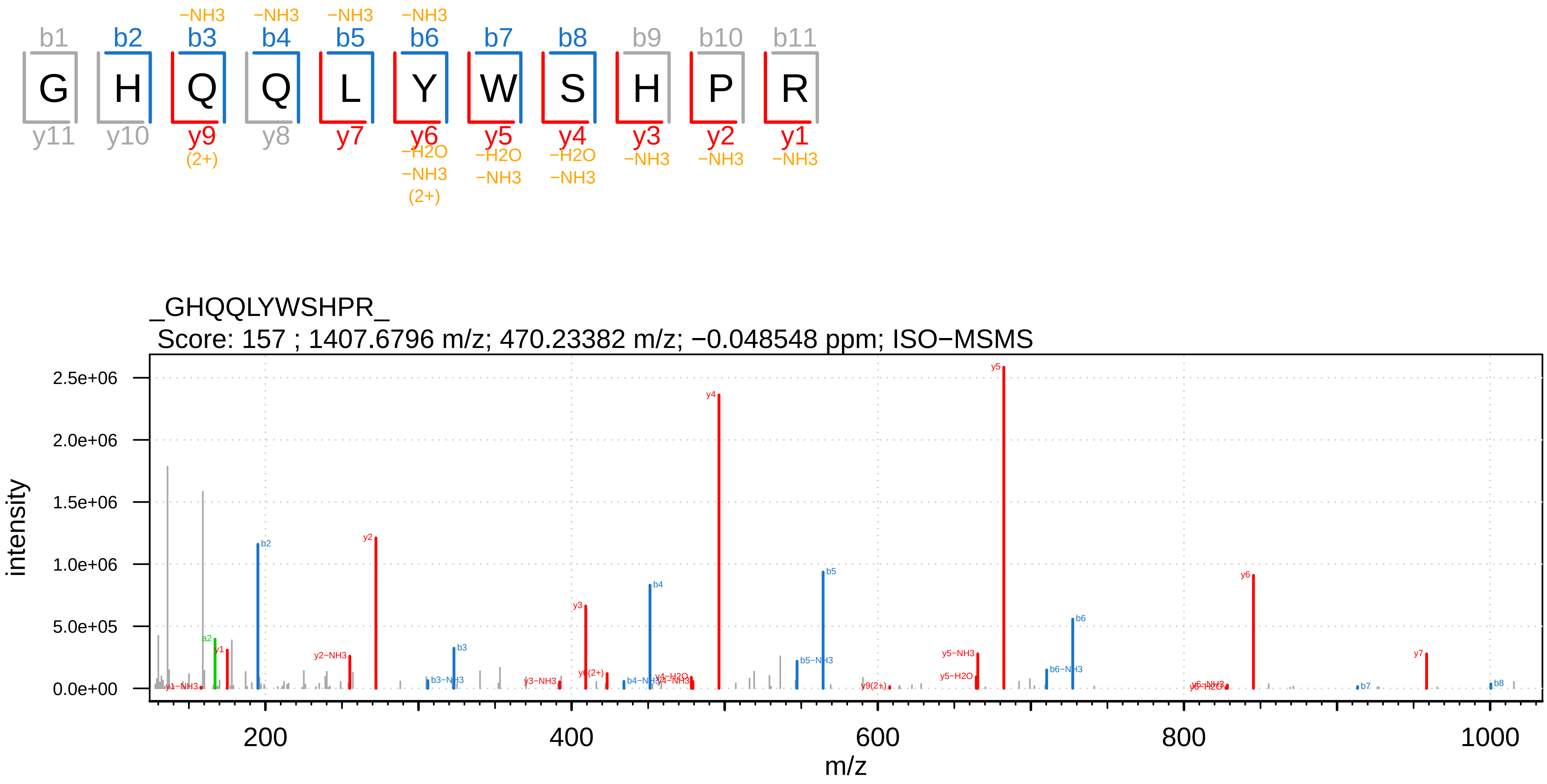

Raw File: Kermit_20111104_MSU_tube_6h_12
Scan Number: 51960
Proteins:
sp|P62274|RS29_MOUSE
ENSMUST00000037023_chr12:69157813−69159058:−
ENSMUST00000071737_chr13:73988963−73989133:−
tr|D3YYL7|D3YYL7_MOUSE

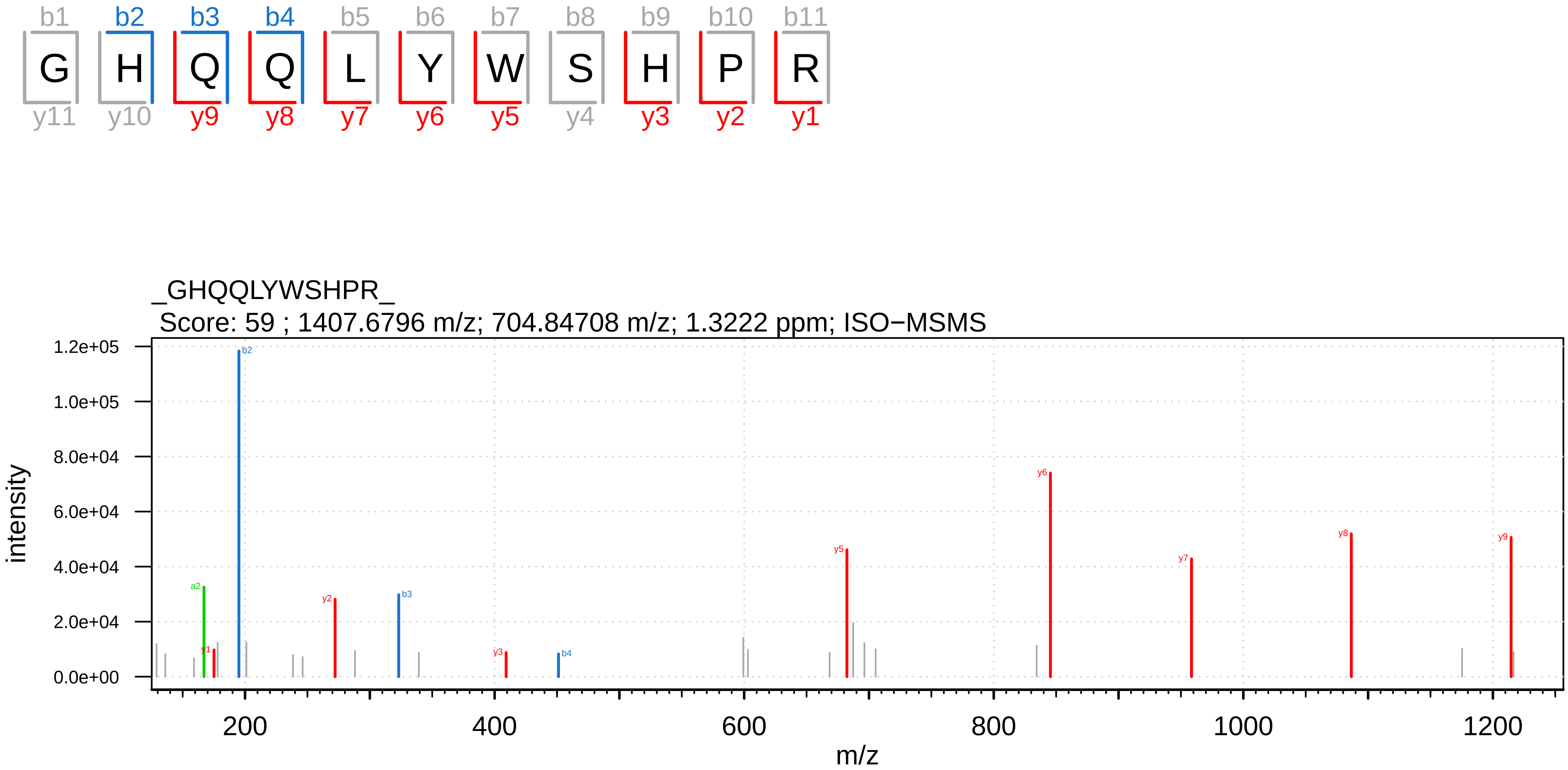

Raw File: Kermit_20111104_MSU_tube_6h_12
Scan Number: 51783
Proteins:
sp|P62274|RS29_MOUSE
ENSMUST00000037023_chr12:69157813−69159058:−
ENSMUST00000071737_chr13:73988963−73989133:−
tr|D3YYL7|D3YYL7_MOUSE

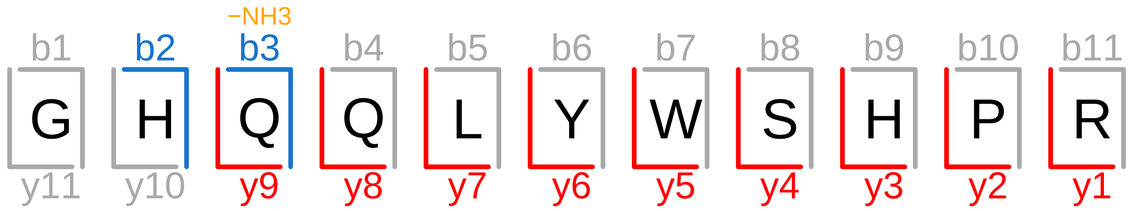

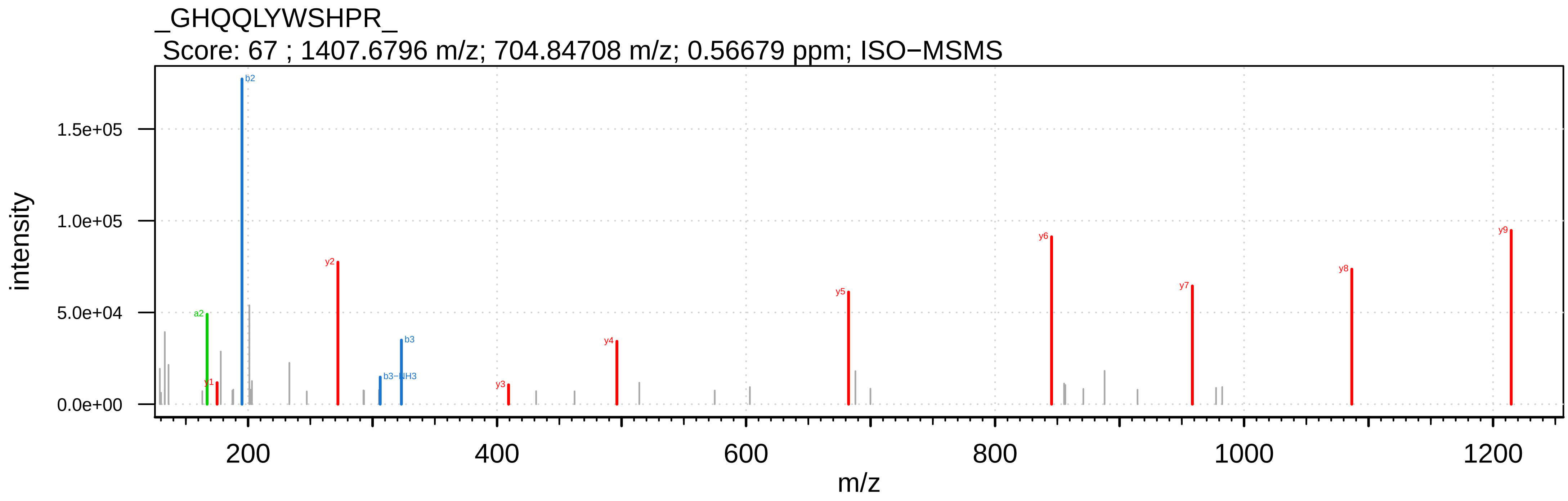

Raw File: Kermit_20111104_MSU_tube_6h_12
Scan Number: 51977
Proteins:
sp|P62274|RS29_MOUSE
ENSMUST00000037023_chr12:69157813−69159058:−
ENSMUST00000071737_chr13:73988963−73989133:−
tr|D3YYL7|D3YYL7_MOUSE

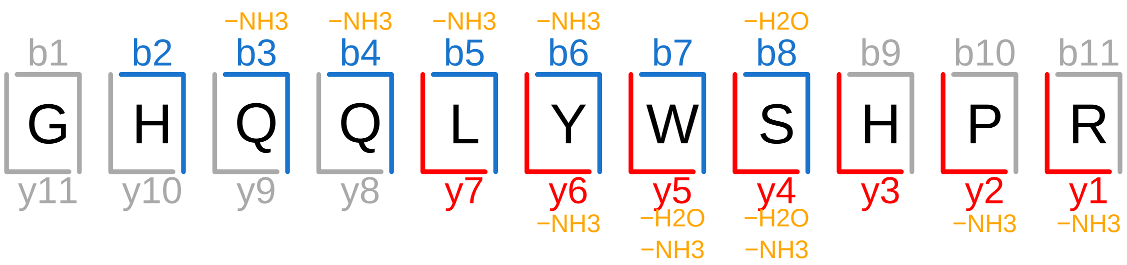

GHQQLYWSHPR
Score: 136 ; 1407.6796 m/z; 470.23382 m/z; −1.287 ppm; ISO−MSMS

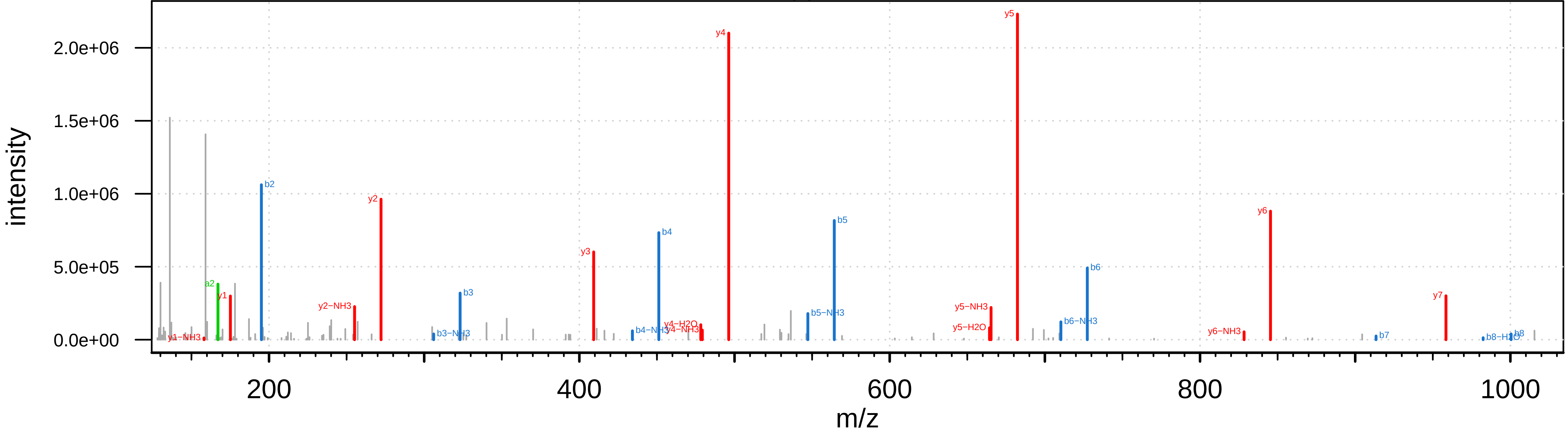

Raw File: Kermit_20111104_MSU_tube_6h_12
Scan Number: 52135
Proteins:
sp|P62274|RS29_MOUSE
ENSMUST00000037023_chr12:69157813−69159058:−
ENSMUST00000071737_chr13:73988963−73989133:−
tr|D3YYL7|D3YYL7_MOUSE

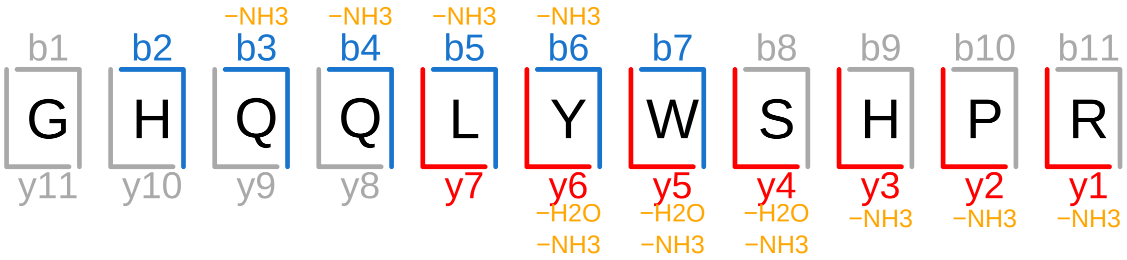

GHQQLYWSHPR
Score: 131 ; 1407.6796 m/z; 470.23382 m/z; −1.287 ppm; ISO−MSMS

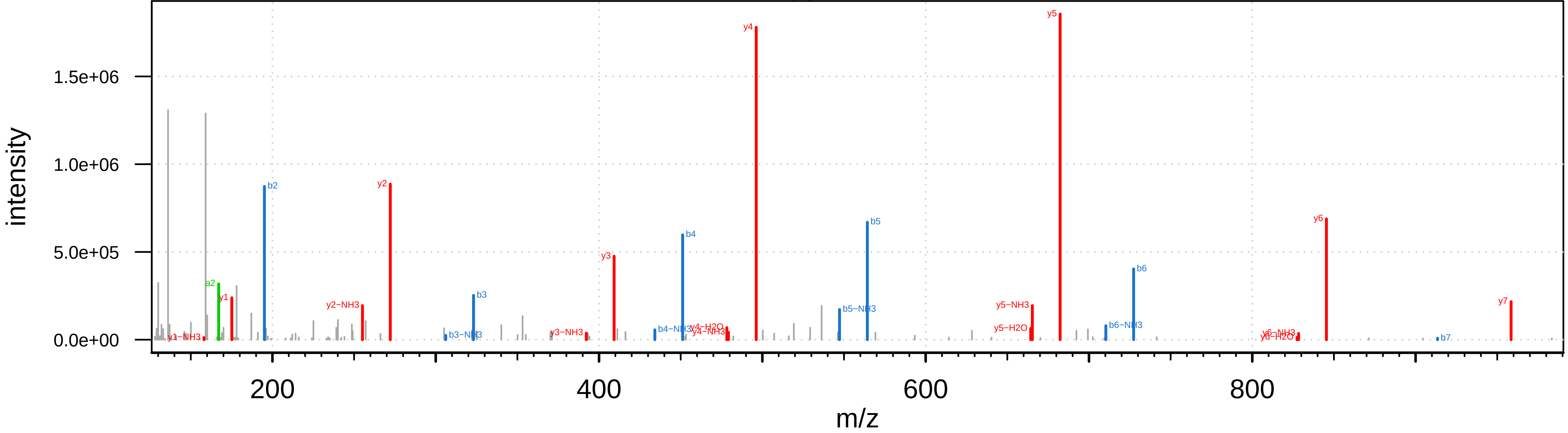

Raw File: Kermit_20111104_MSU_tube_6h_12
Scan Number: 52330
Proteins:
sp|P62274|RS29_MOUSE
ENSMUST00000037023_chr12:69157813−69159058:−
ENSMUST00000071737_chr13:73988963−73989133:−
tr|D3YYL7|D3YYL7_MOUSE

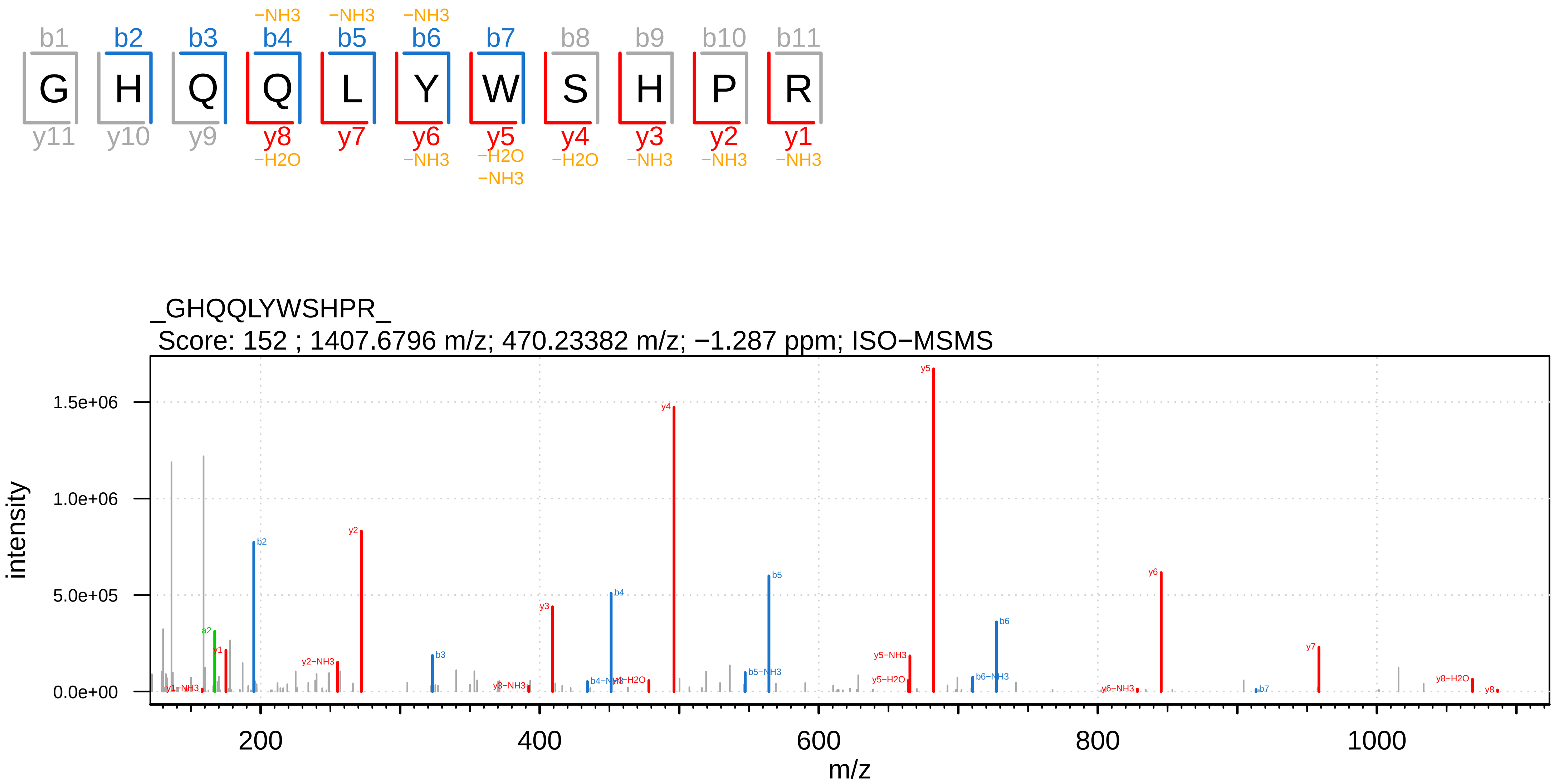

Raw File: Kermit_20111104_MSU_tube_6h_12
Scan Number: 52528
Proteins:
sp|P62274|RS29_MOUSE
ENSMUST00000037023_chr12:69157813−69159058:−
ENSMUST00000071737_chr13:73988963−73989133:−
tr|D3YYL7|D3YYL7_MOUSE

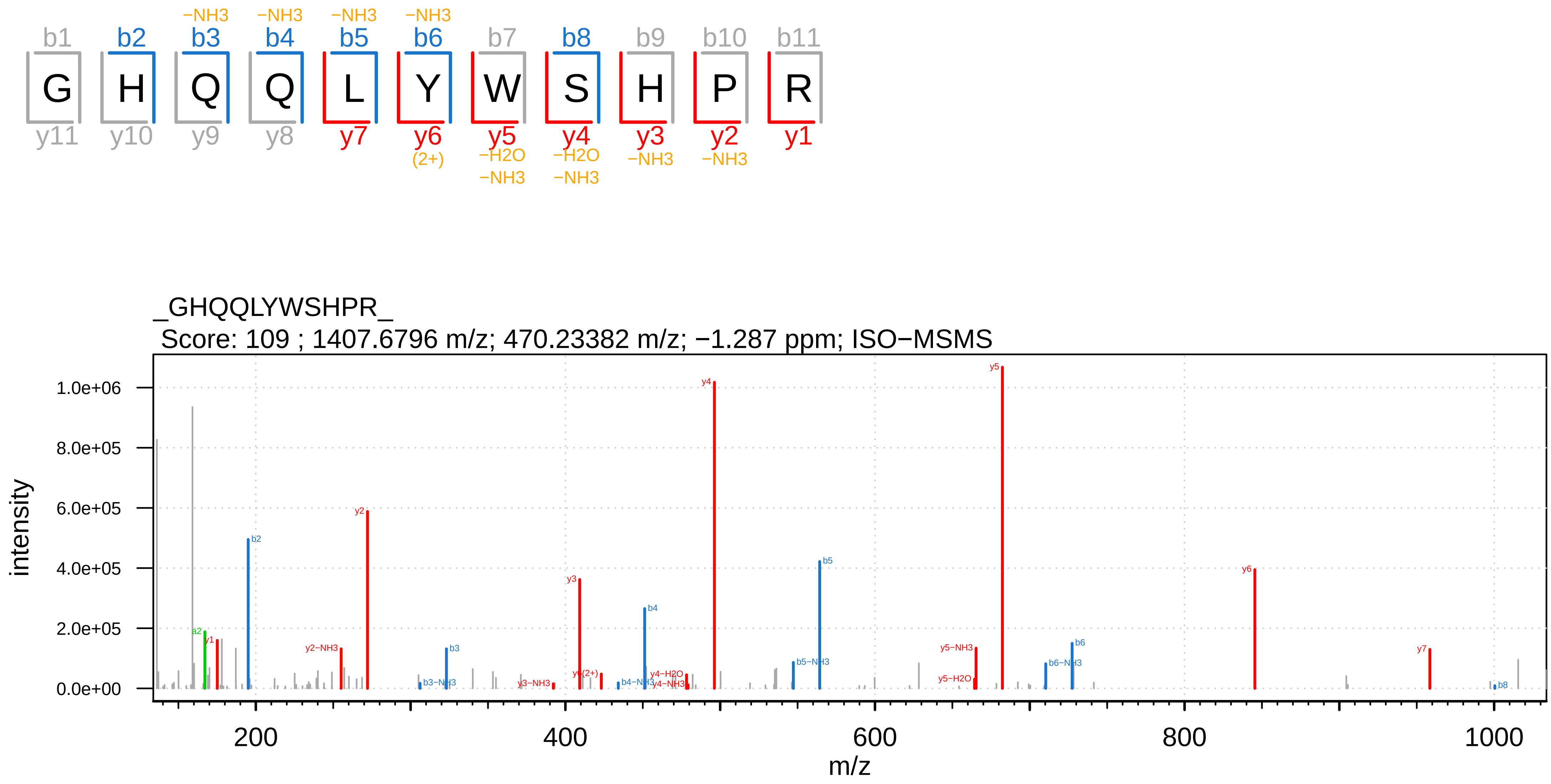

Raw File: Kermit_20111104_MSU_tube_6h_12
Scan Number: 52724
Proteins:
sp|P62274|RS29_MOUSE
ENSMUST00000037023_chr12:69157813−69159058:−
ENSMUST00000071737_chr13:73988963−73989133:−
tr|D3YYL7|D3YYL7_MOUSE

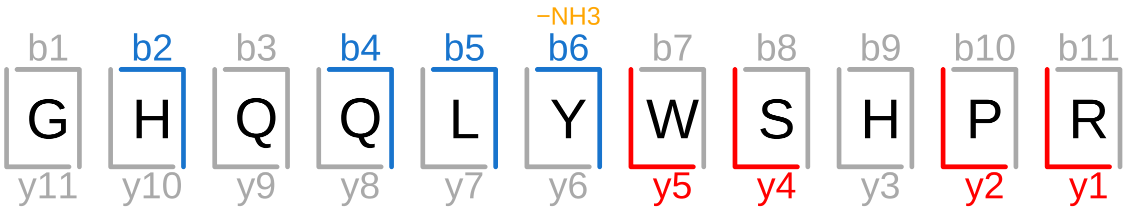

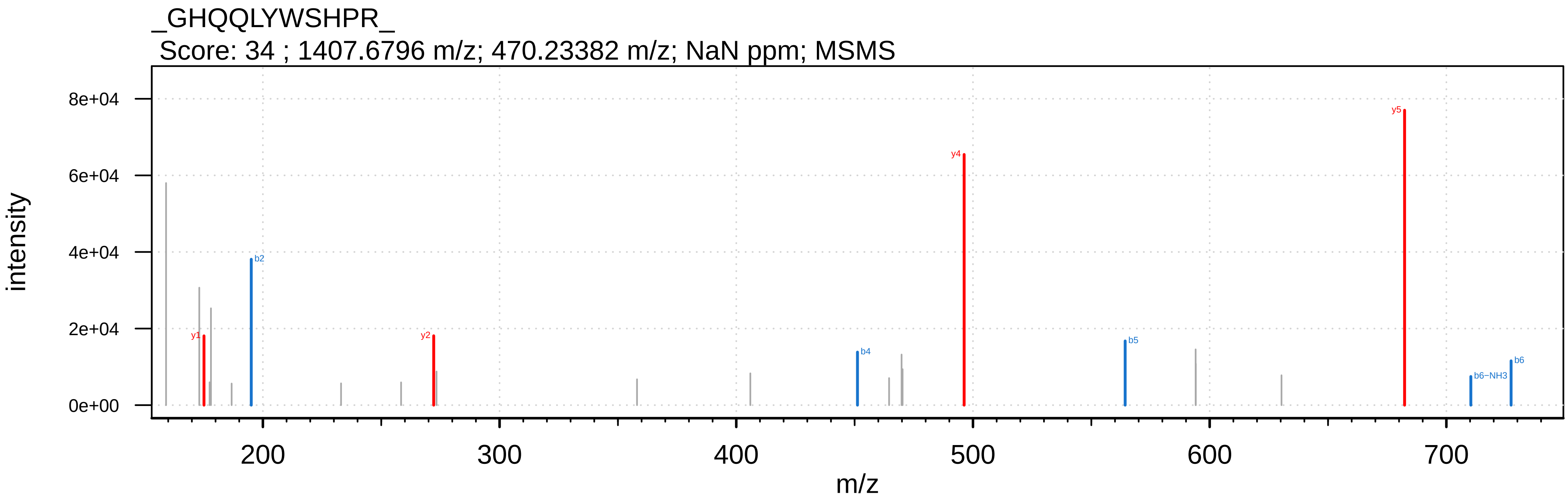

Raw File: Kermit_20111104_MSU_tube_6h_12
Scan Number: 24040
Proteins:
sp|P62274|RS29_MOUSE
ENSMUST00000037023_chr12:69157813−69159058:−
ENSMUST00000071737_chr13:73988963−73989133:−
tr|D3YYL7|D3YYL7_MOUSE

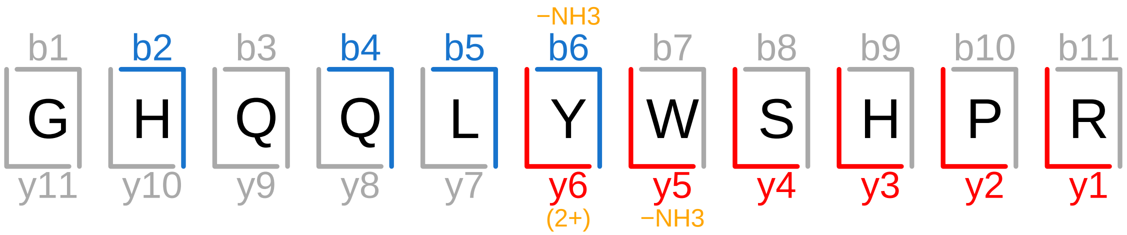

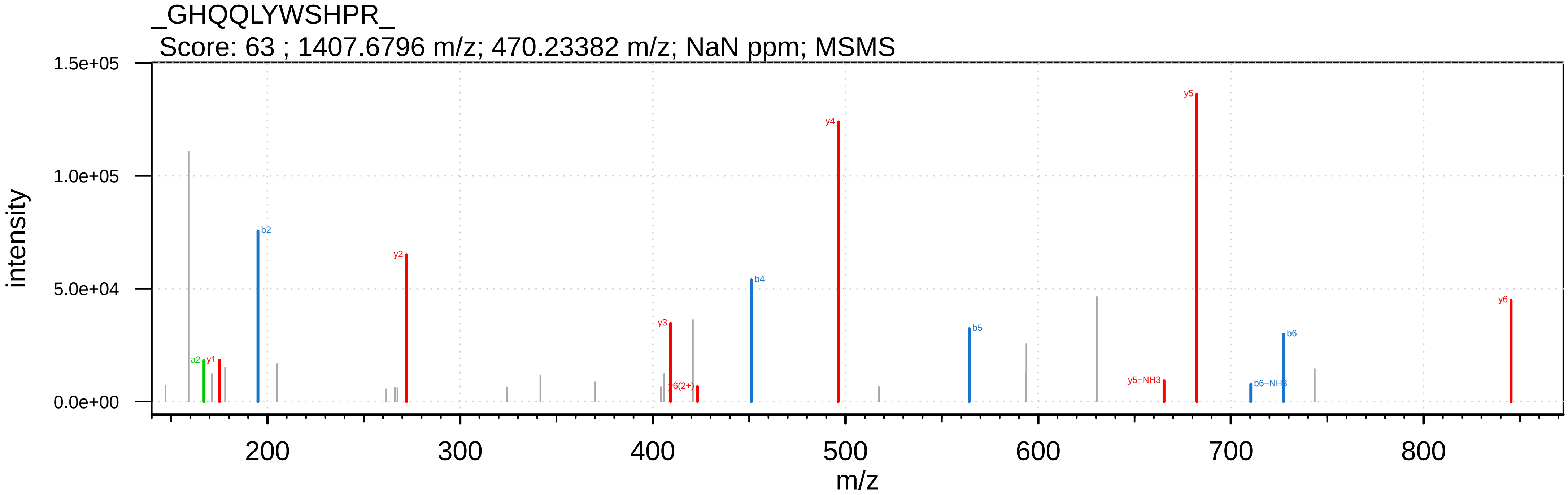

Raw File: Kermit_20111104_MSU_tube_6h_12
Scan Number: 27908
Proteins:
sp|P62274|RS29_MOUSE
ENSMUST00000037023_chr12:69157813−69159058:−
ENSMUST00000071737_chr13:73988963−73989133:−
tr|D3YYL7|D3YYL7_MOUSE

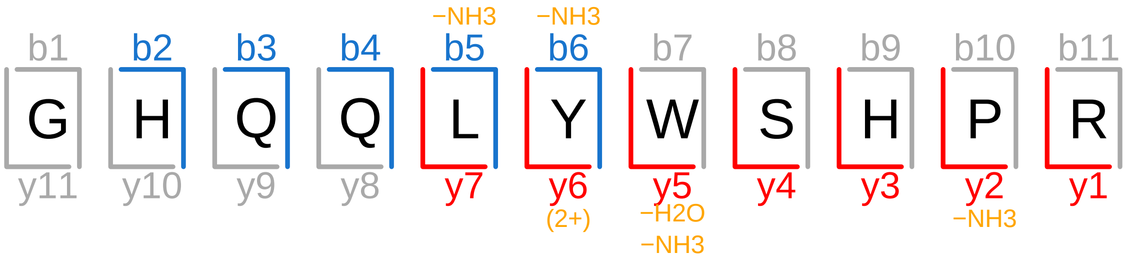

GHQQLYWSHPR

Score: 103 ; 1407.6796 m/z; 470.23382 m/z; NaN ppm; MSMS

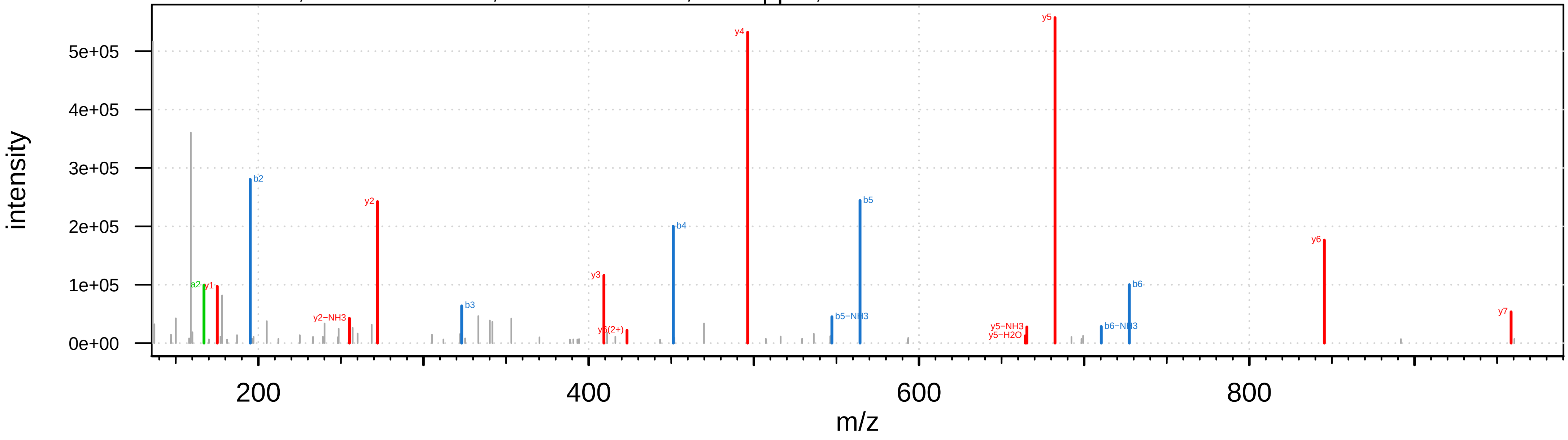

Raw File: Kermit_20111104_MSU_tube_6h_12
Scan Number: 48446
Proteins:
sp|P62274|RS29_MOUSE
ENSMUST00000037023_chr12:69157813−69159058:−
ENSMUST00000071737_chr13:73988963−73989133:−
tr|D3YYL7|D3YYL7_MOUSE

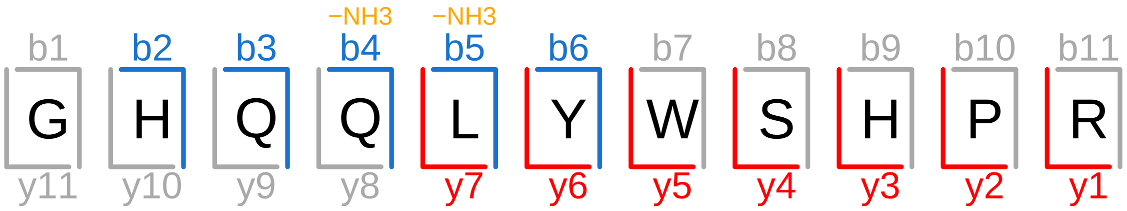

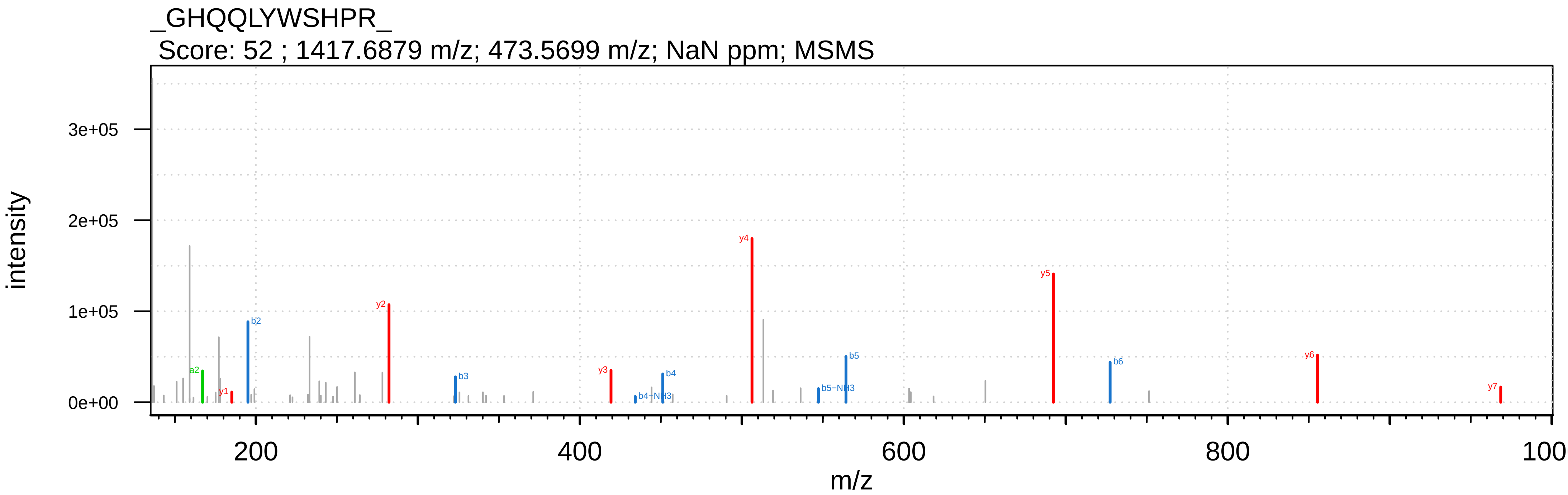

Raw File: Kermit_20111104_MSU_tube_6h_12
Scan Number: 50371
Proteins:
sp|P62274|RS29_MOUSE
ENSMUST00000037023_chr12:69157813−69159058:−
ENSMUST00000071737_chr13:73988963−73989133:−
tr|D3YYL7|D3YYL7_MOUSE

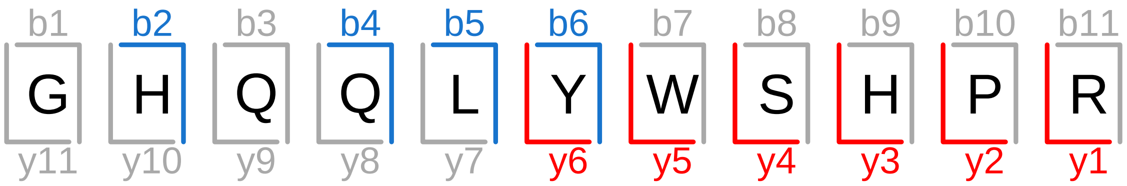

GHQQLYWSHPR
Score: 45 ; 1417.6879 m/z; 473.5699 m/z; NaN ppm; MSMS

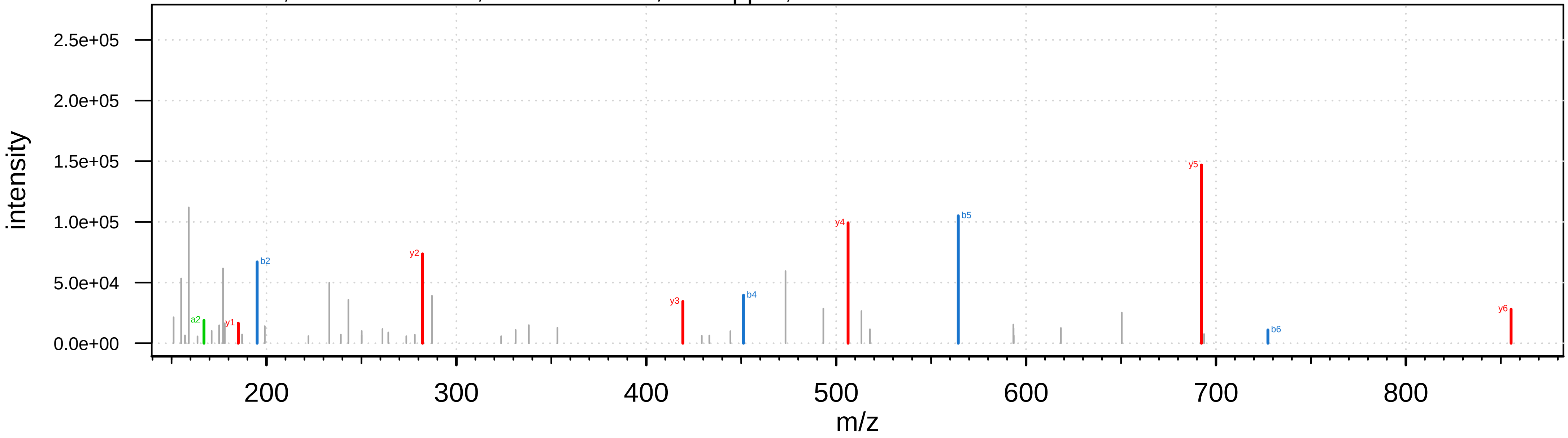

Raw File: Kermit_20111104_MSU_tube_6h_12
Scan Number: 50581
Proteins:
sp|P62274|RS29_MOUSE
ENSMUST00000037023_chr12:69157813−69159058:−
ENSMUST00000071737_chr13:73988963−73989133:−
tr|D3YYL7|D3YYL7_MOUSE

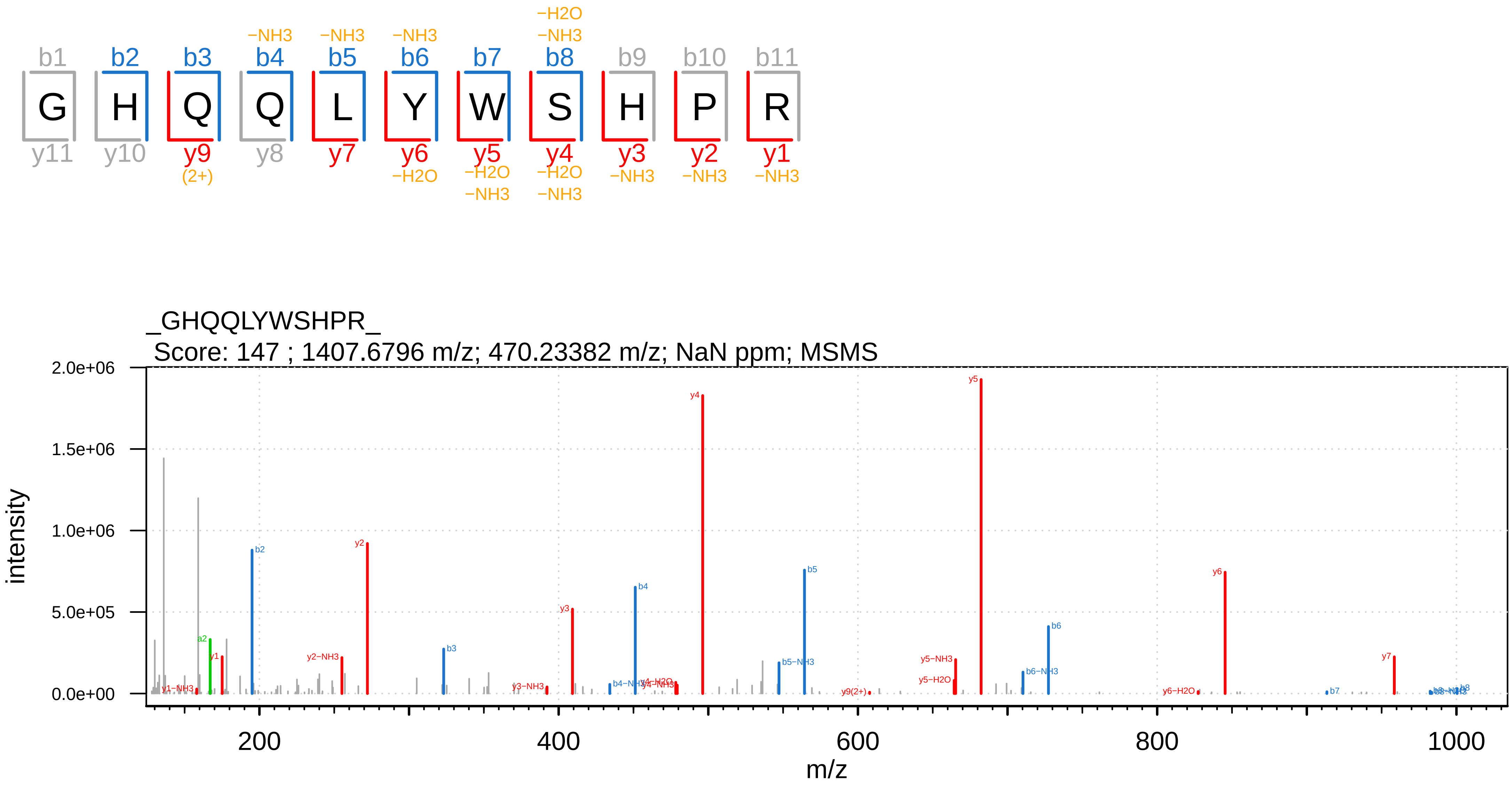

Raw File: Kermit_20111104_MSU_tube_6h_12
Scan Number: 51205
Proteins:
sp|P62274|RS29_MOUSE
ENSMUST00000037023_chr12:69157813−69159058:−
ENSMUST00000071737_chr13:73988963−73989133:−
tr|D3YYL7|D3YYL7_MOUSE

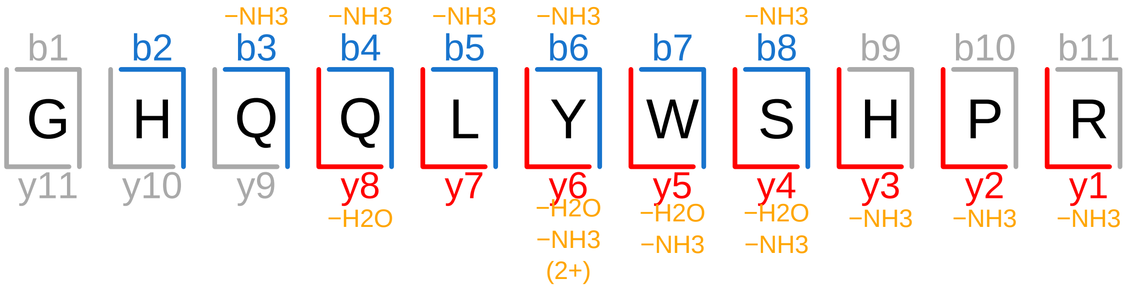

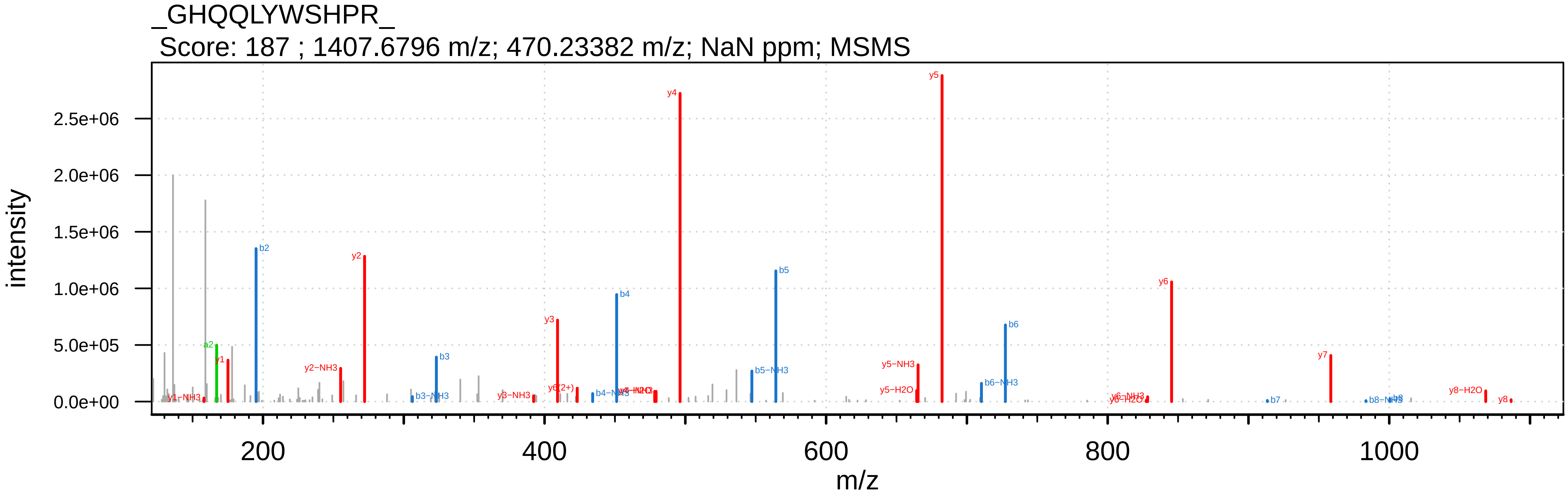

Raw File: Kermit_20111104_MSU_tube_6h_12
Scan Number: 51383
Proteins:
sp|P62274|RS29_MOUSE
ENSMUST00000037023_chr12:69157813−69159058:−
ENSMUST00000071737_chr13:73988963−73989133:−
tr|D3YYL7|D3YYL7_MOUSE

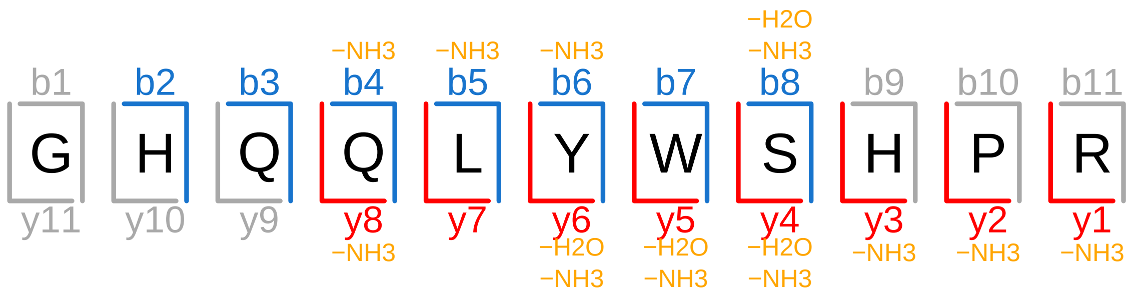

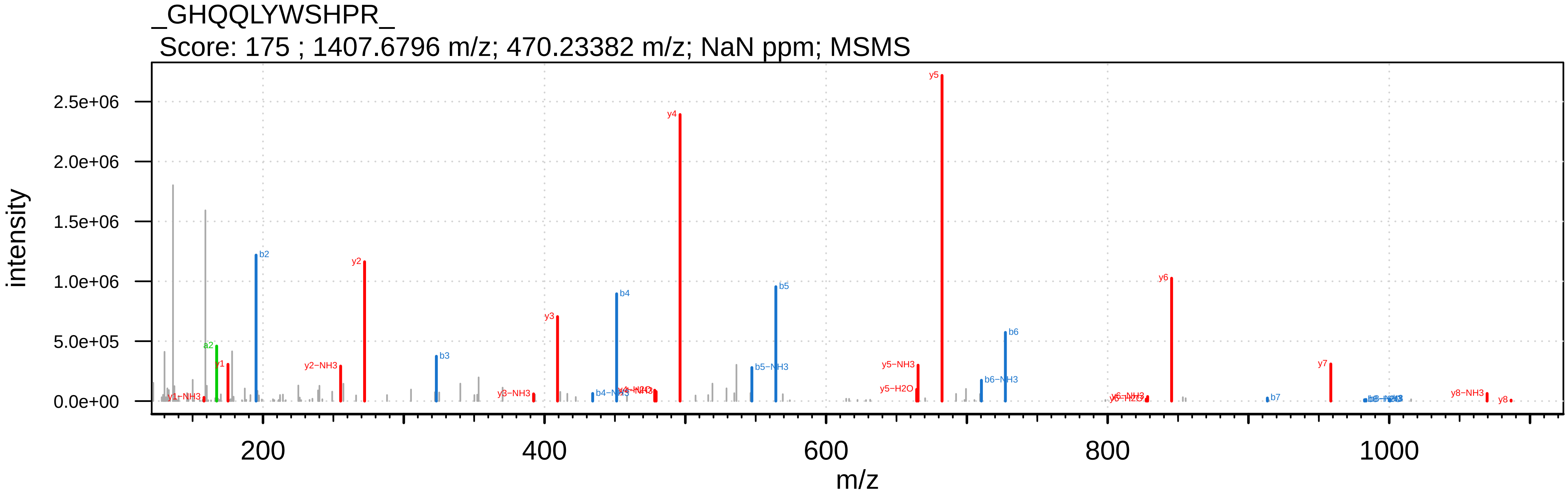

Raw File: Kermit_20111104_MSU_tube_6h_12
Scan Number: 51573
Proteins:
sp|P62274|RS29_MOUSE
ENSMUST00000037023_chr12:69157813−69159058:−
ENSMUST00000071737_chr13:73988963−73989133:−
tr|D3YYL7|D3YYL7_MOUSE

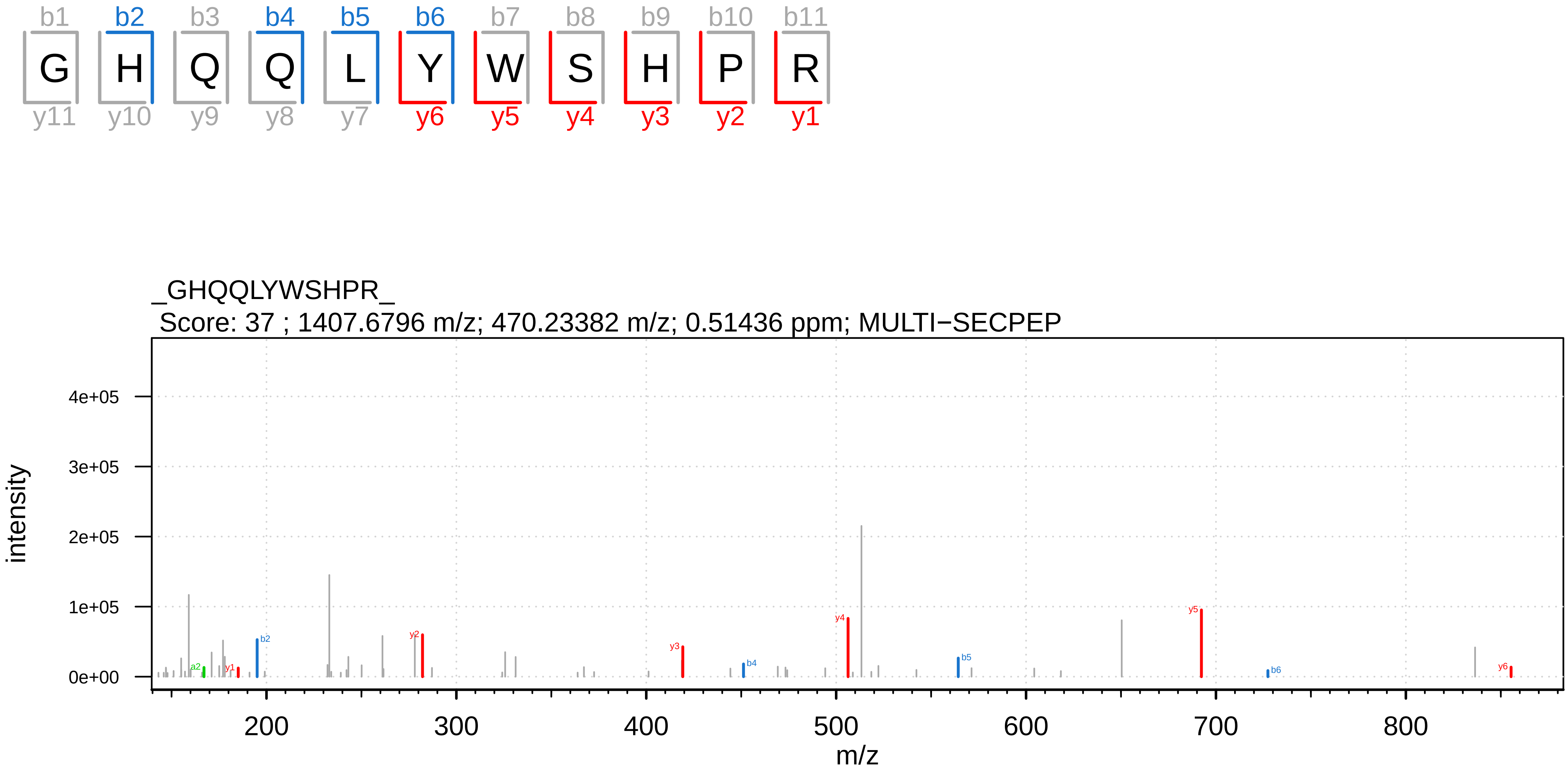

Raw File: Kermit_20111104_MSU_tube_6h_12
Scan Number: 49775
Proteins:
sp|P62274|RS29_MOUSE
ENSMUST00000037023_chr12:69157813−69159058:−
ENSMUST00000071737_chr13:73988963−73989133:−
tr|D3YYL7|D3YYL7_MOUSE

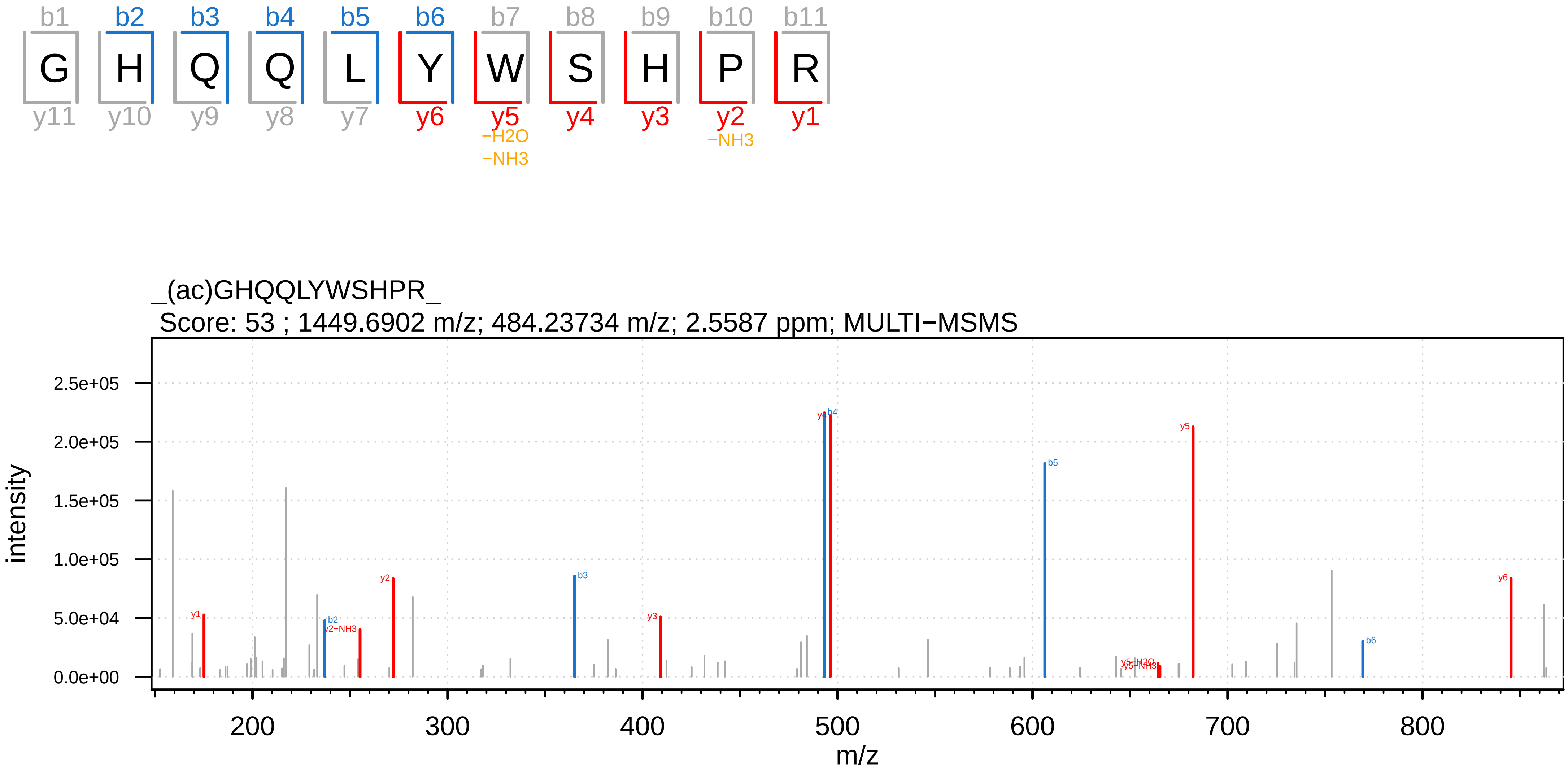

Raw File: Kermit_20111104_MSU_tube_6h_12
Scan Number: 13447
Proteins:
sp|P62274|RS29_MOUSE
ENSMUST00000037023_chr12:69157813−69159058:−
ENSMUST00000071737_chr13:73988963−73989133:−
tr|D3YYL7|D3YYL7_MOUSE

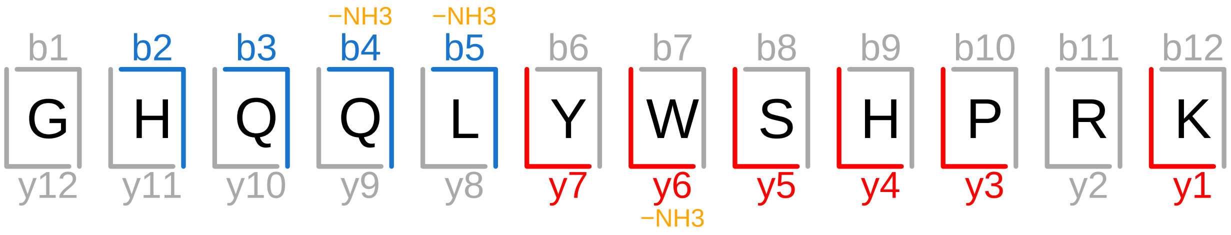

GHQQLYWSHPRK
Score: 42 ; 1535.7746 m/z; 384.95092 m/z; 0.31304 ppm; ISO−MSMS

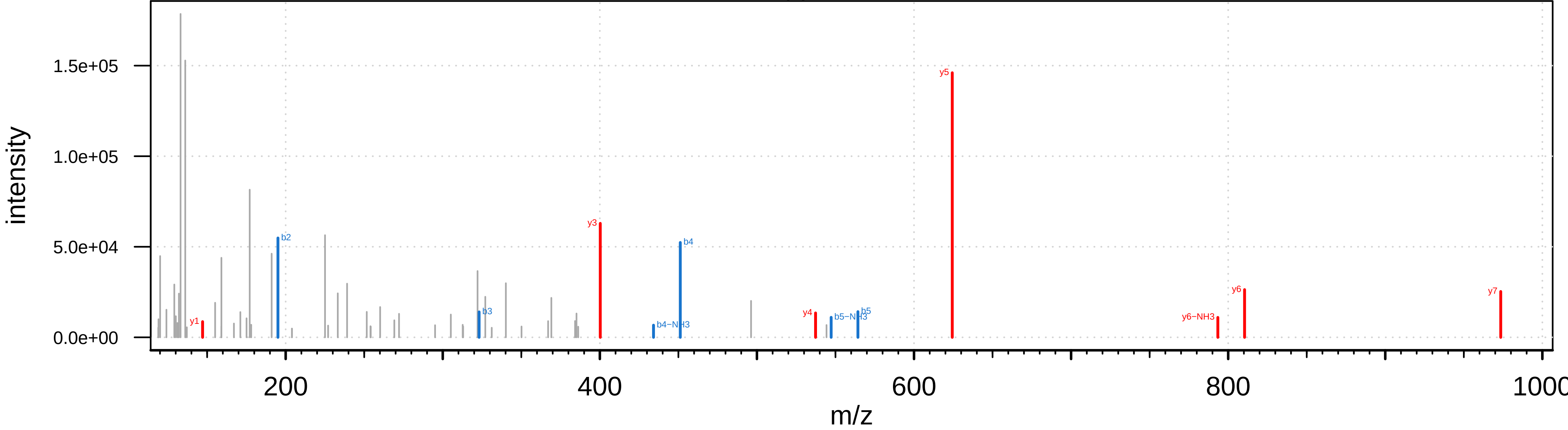

Raw File: Kermit_20111104_MSU_tube_6h_12
Scan Number: 59246
Proteins:
sp|P62274|RS29_MOUSE
ENSMUST00000037023_chr12:69157813−69159058:−
ENSMUST00000071737_chr13:73988963−73989133:−
tr|D3YYL7|D3YYL7_MOUSE

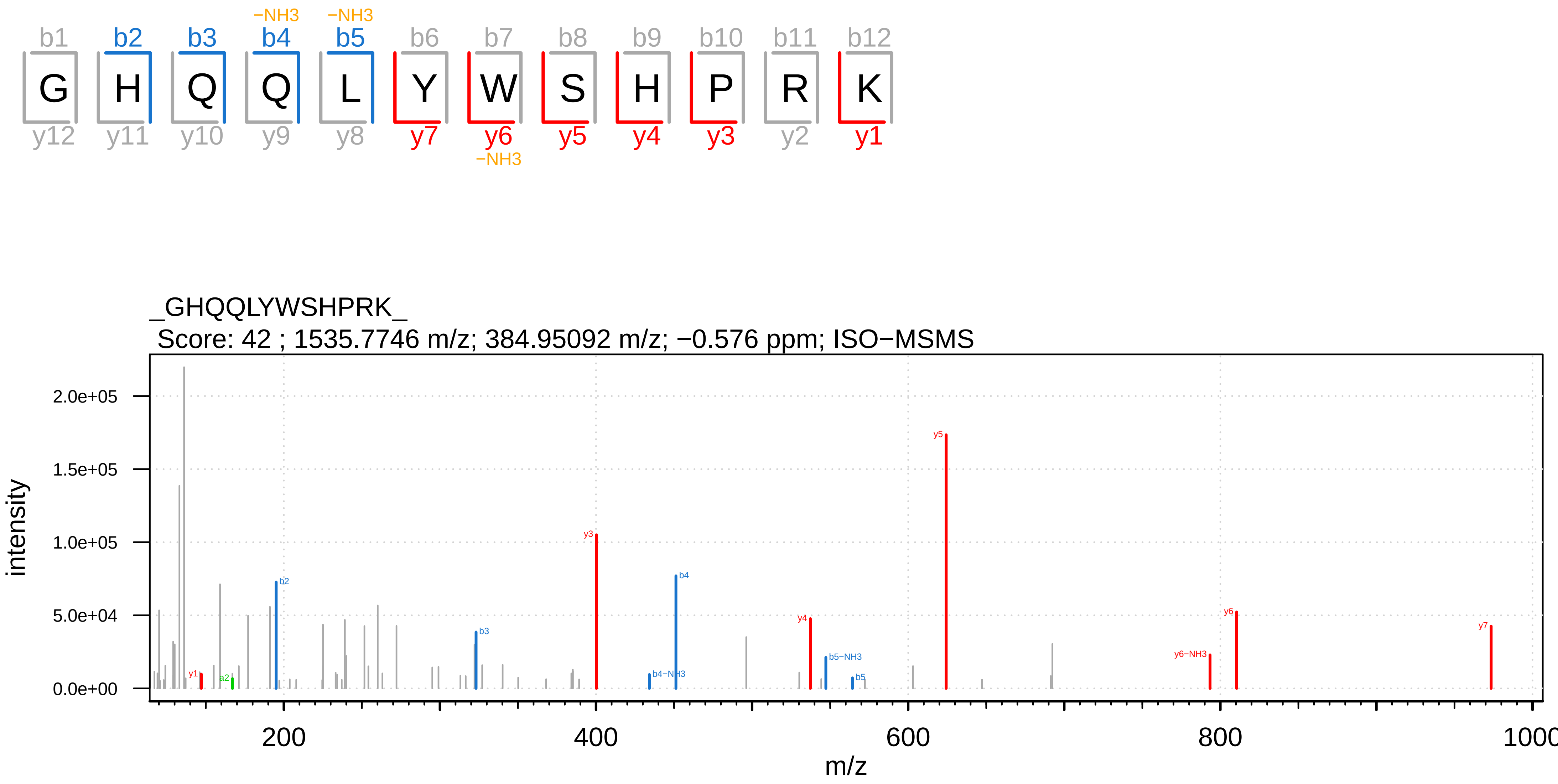

Raw File: Kermit_20111104_MSU_tube_6h_12
Scan Number: 59441
Proteins:
sp|P62274|RS29_MOUSE
ENSMUST00000037023_chr12:69157813−69159058:−
ENSMUST00000071737_chr13:73988963−73989133:−
tr|D3YYL7|D3YYL7_MOUSE

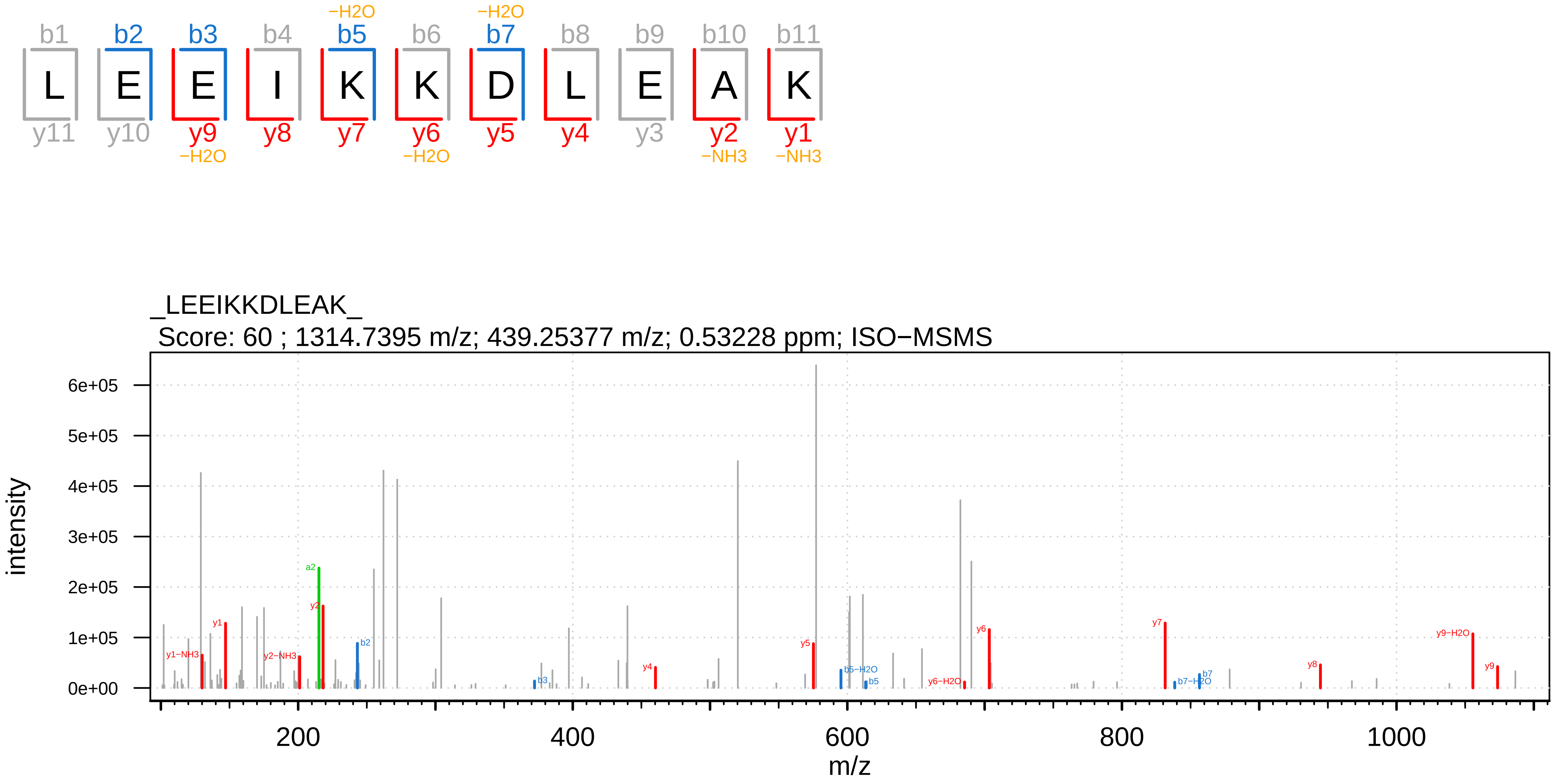

Raw File: Kermit_20111104_MSU_tube_6h_12

Scan Number: 8538

Proteins:

ENSMUST00000152147_chr6:35252768−35261233:+

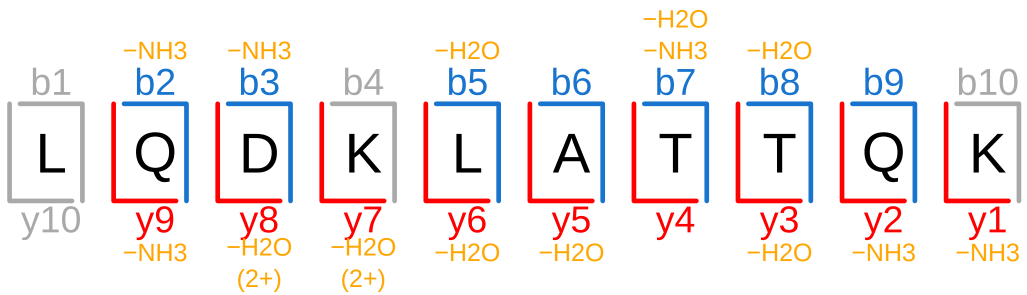

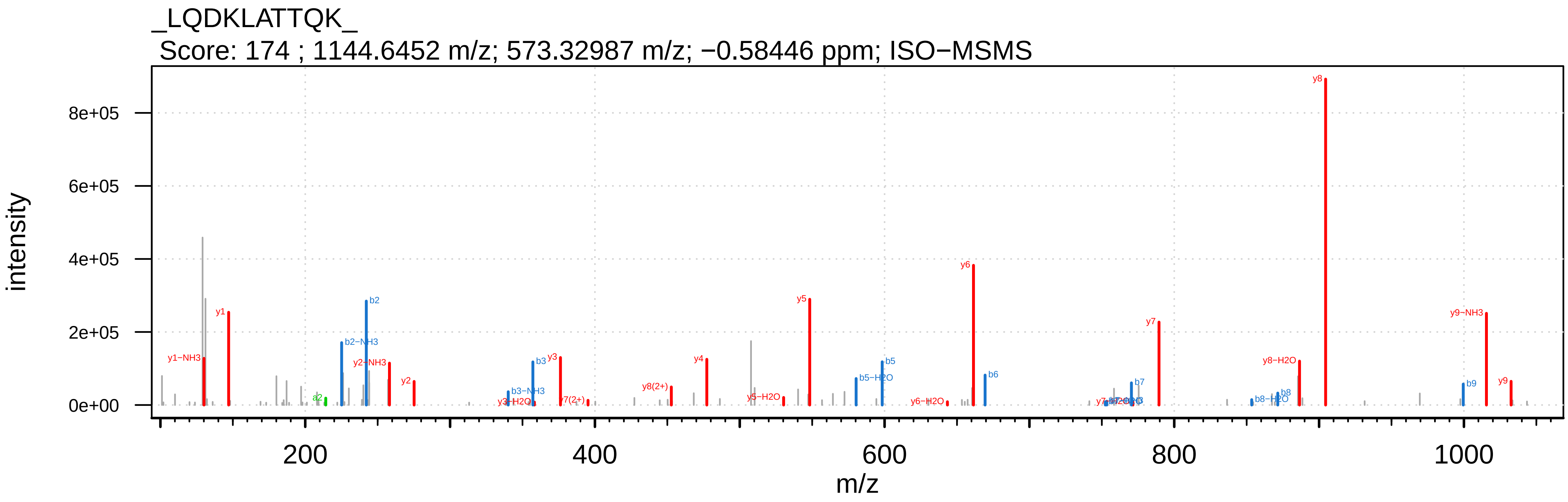

Raw File: Kermit_20111104_MSU_tube_6h_12
Scan Number: 5031
Proteins:
sp|Q8BT35|YB039_MOUSE
ENSMUST00000135091_chr2:127792297−127792467:−

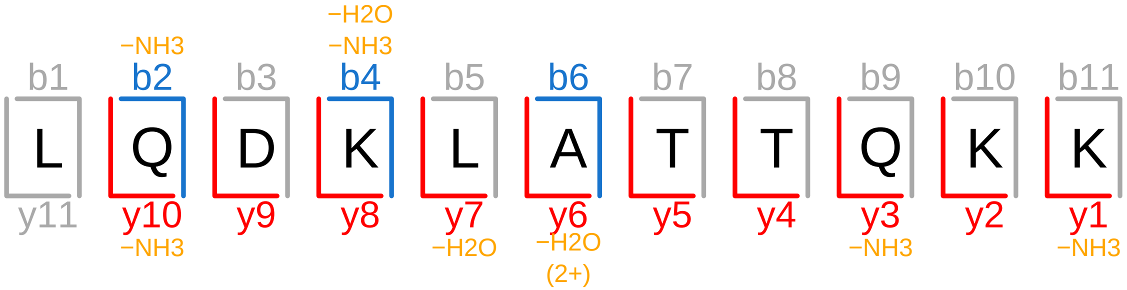

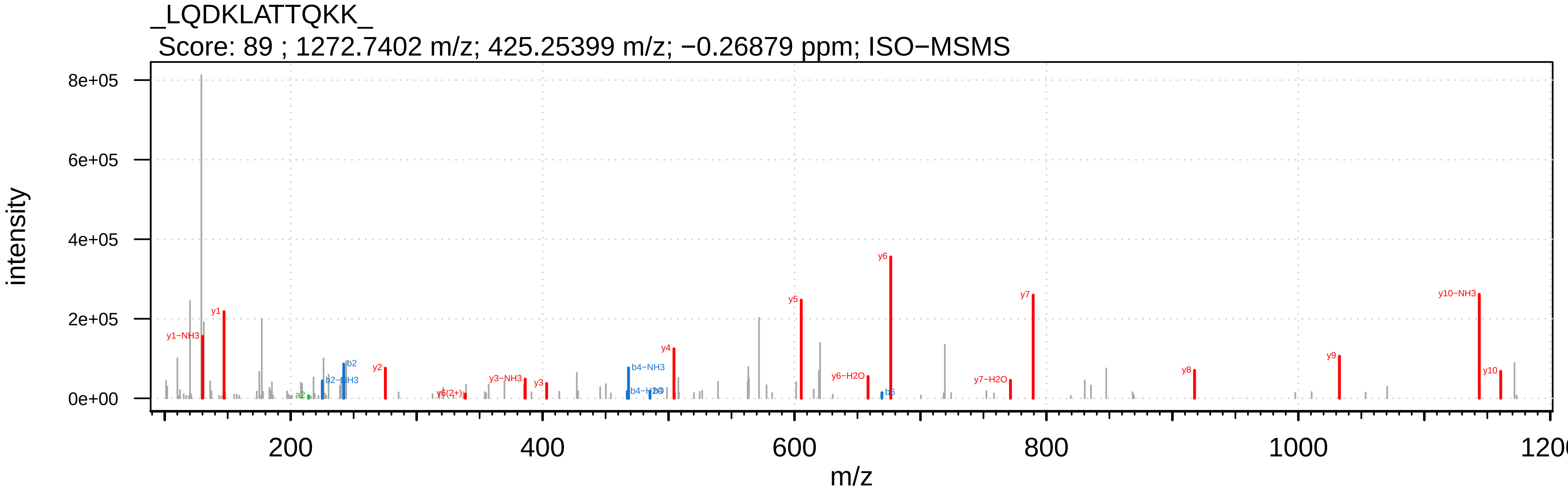

Raw File: Kermit_20111104_MSU_tube_6h_12
Scan Number: 3744
Proteins:
sp|Q8BT35|YB039_MOUSE
ENSMUST00000135091_chr2:127792297−127792467:−

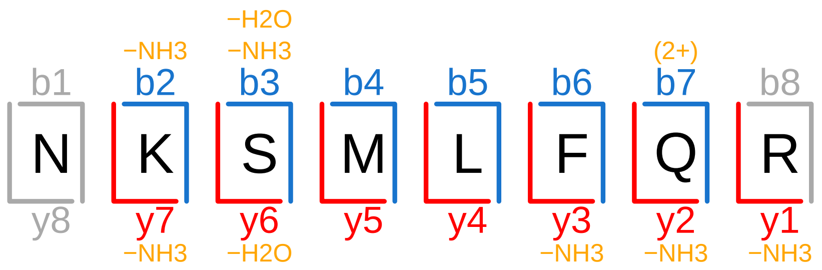

NKSM(ox)LFQR

Score: 76 ; 1038.528 m/z; 520.2713 m/z; −0.95352 ppm; MULTI−MSMS

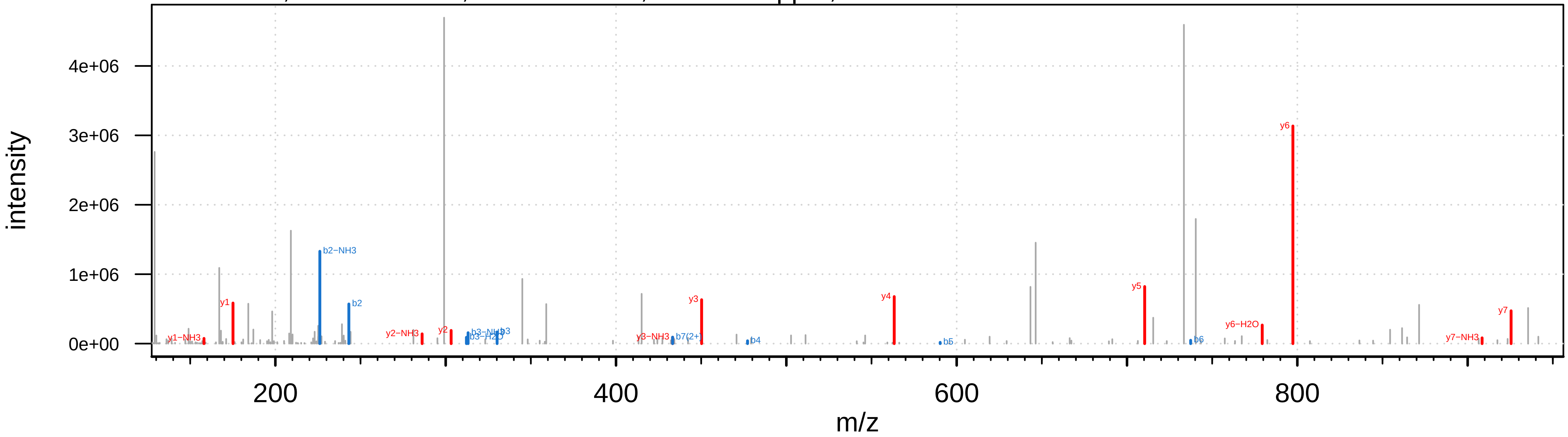

Raw File: Kermit_20111104_MSU_tube_6h_12

Scan Number: 6217

Proteins:

chr12_103206313−103214181.T2_chr12:101968174−101969629:−

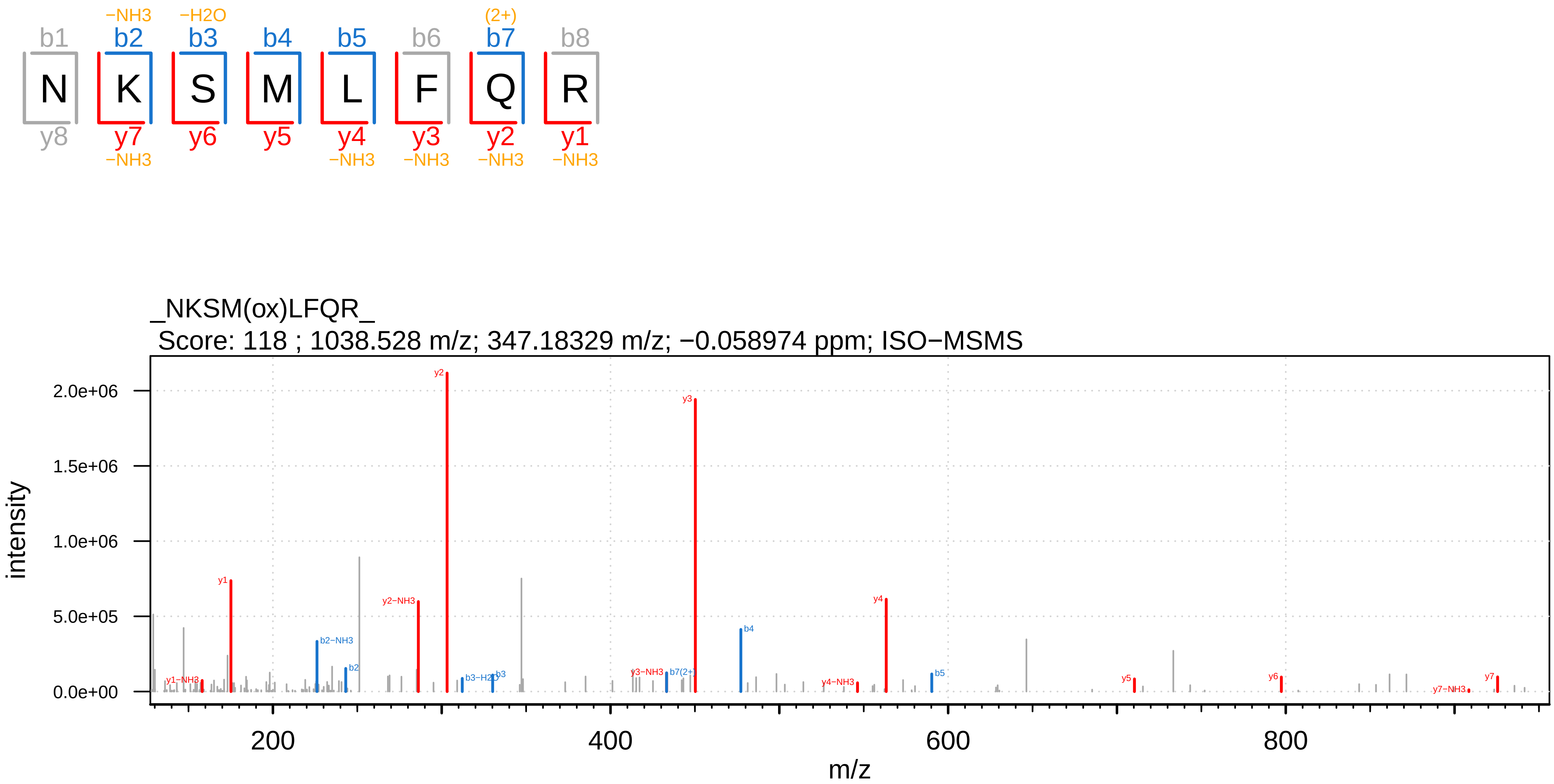

Raw File: Kermit_20111104_MSU_tube_6h_12
Scan Number: 6218
Proteins:
chr12_103206313−103214181.T2_chr12:101968174−101969629:−

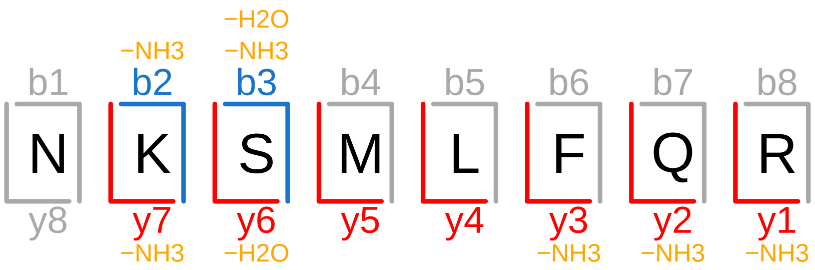

NKSMLFQR
Score: 85 ; 1022.5331 m/z; 512.27384 m/z; 0.12807 ppm; ISO−MSMS

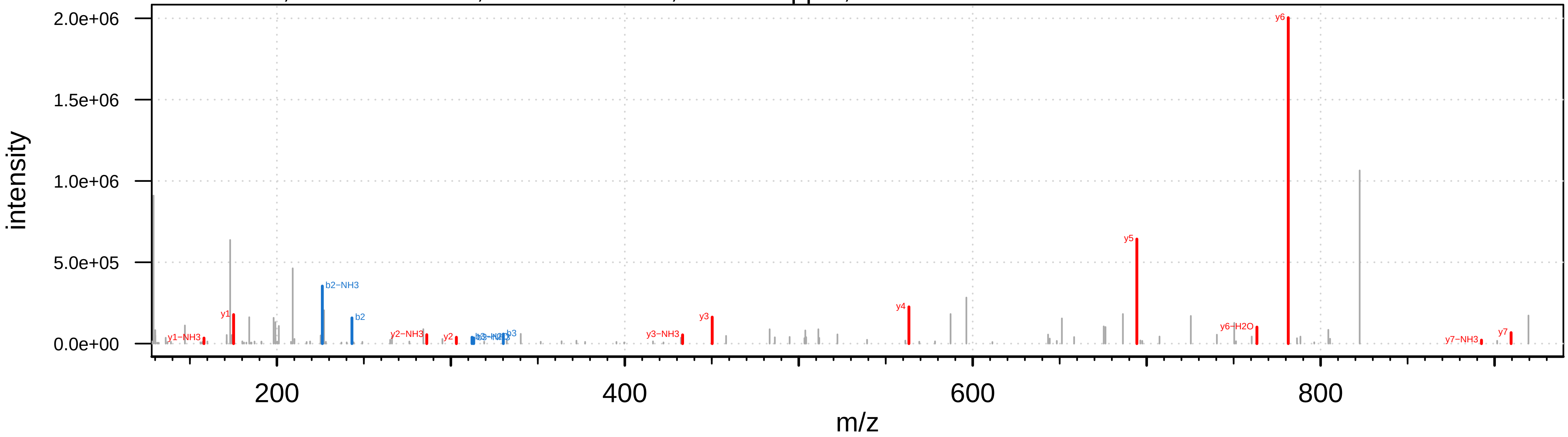

Raw File: Kermit_20111104_MSU_tube_6h_12
Scan Number: 9842
Proteins:
chr12_103206313−103214181.T2_chr12:101968174−101969629:−

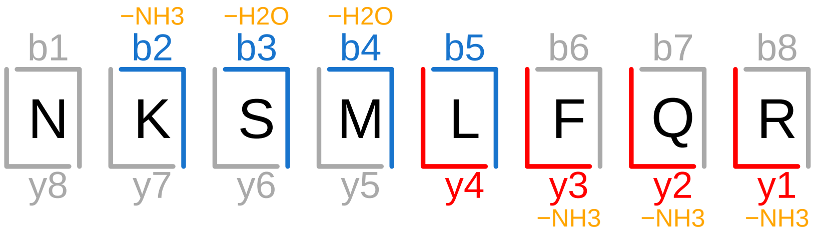

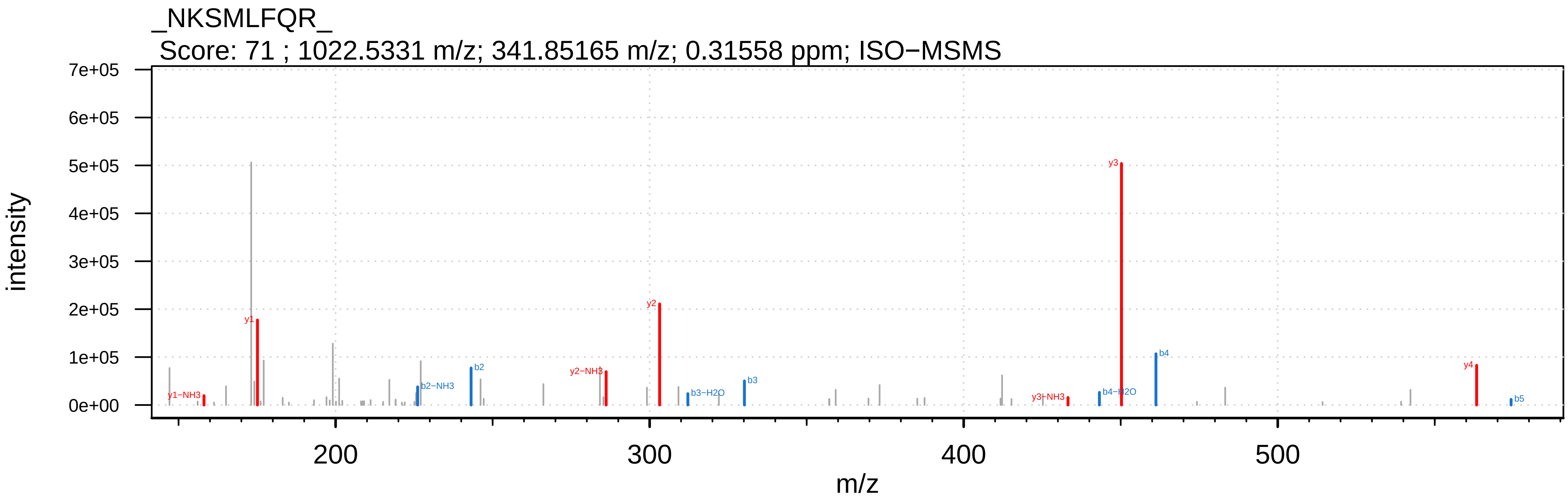

Raw File: Kermit_20111104_MSU_tube_6h_12
Scan Number: 9848
Proteins:
chr12_103206313−103214181.T2_chr12:101968174−101969629:−

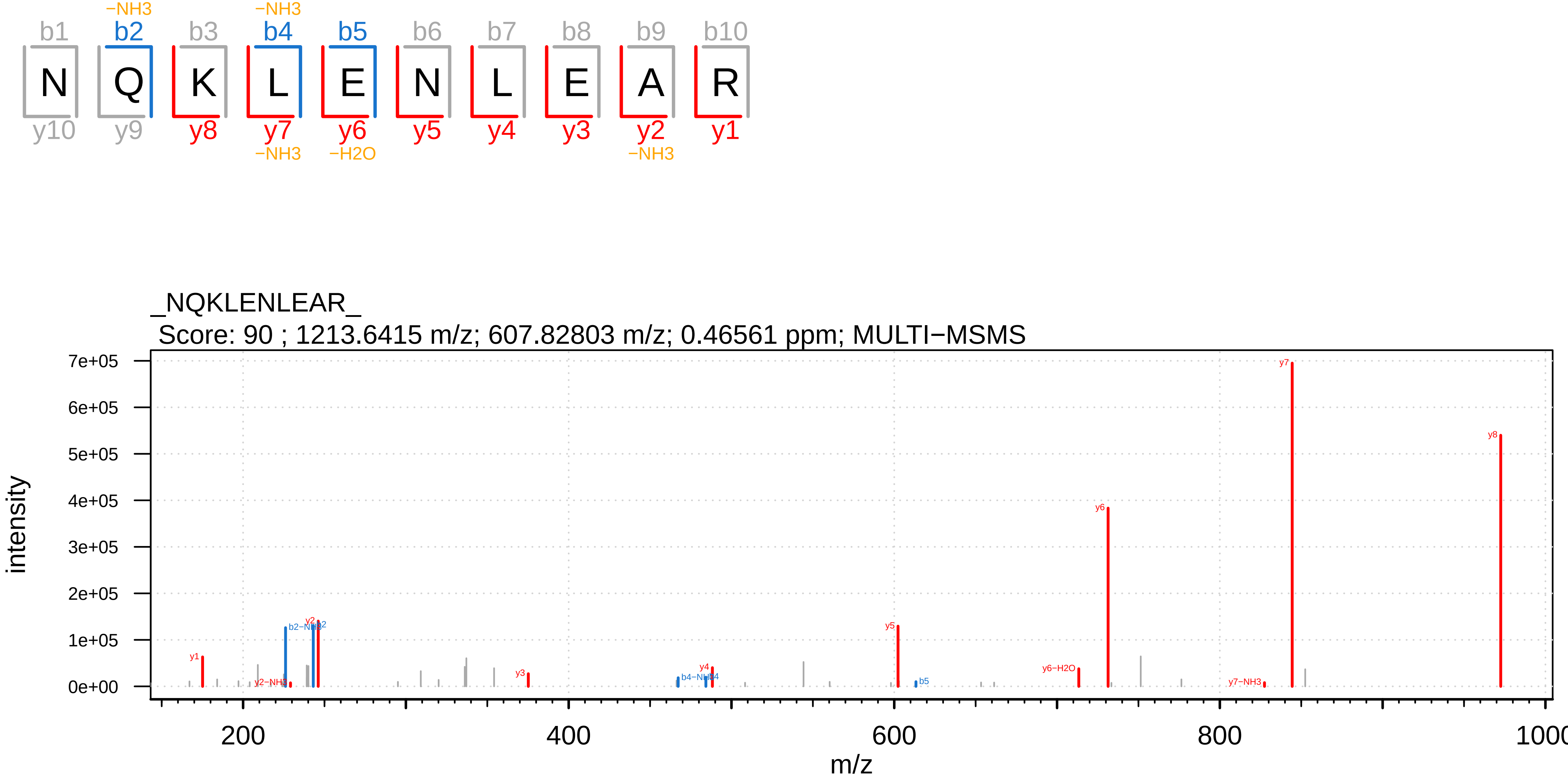

Raw File: Kermit_20111104_MSU_tube_6h_12
Scan Number: 7081
Proteins:
ENSMUST00000023048_chr15:80235835−80236047:+

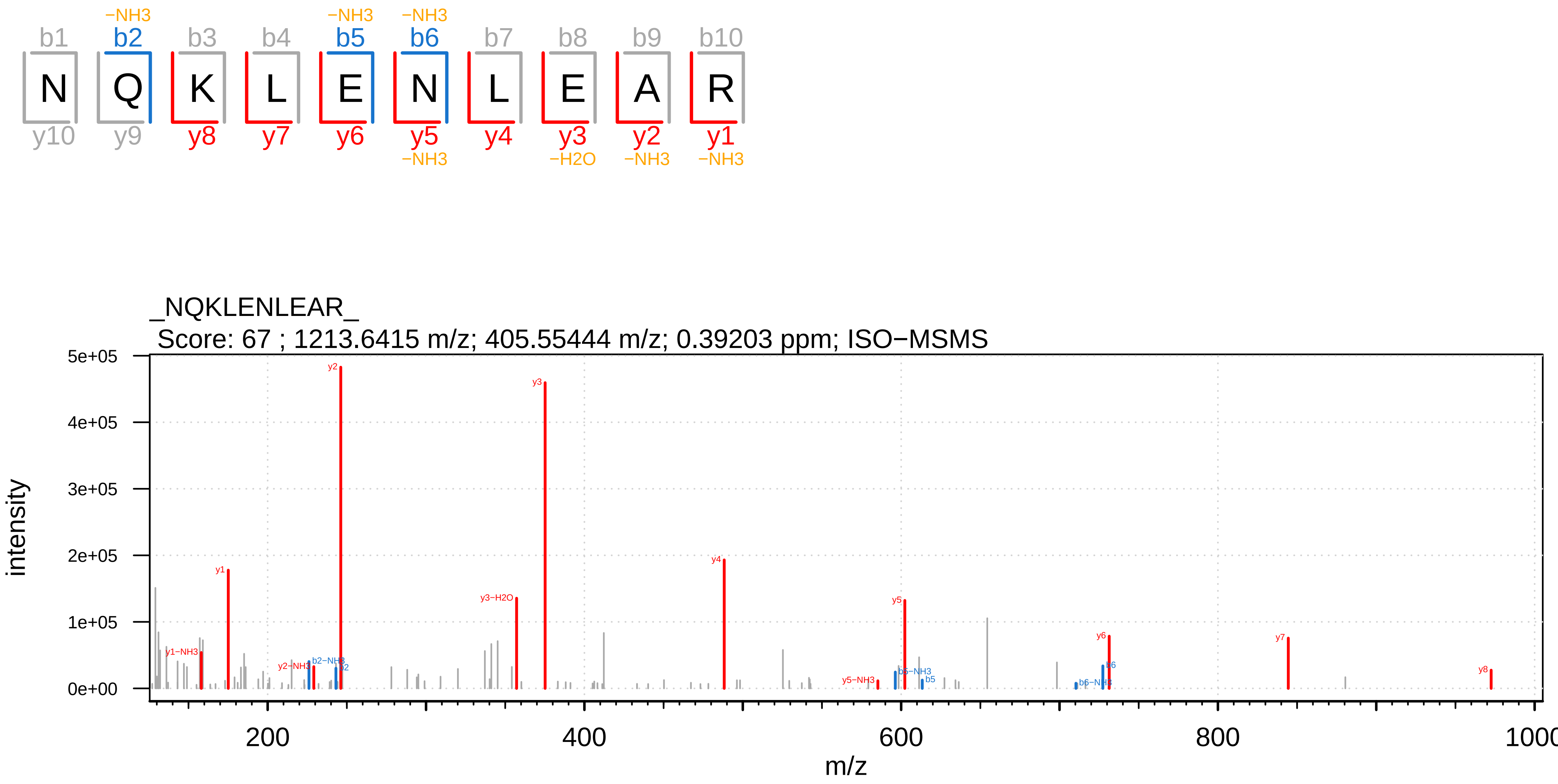

Raw File: Kermit_20111104_MSU_tube_6h_12
Scan Number: 7066
Proteins:
ENSMUST00000023048_chr15:80235835−80236047:+

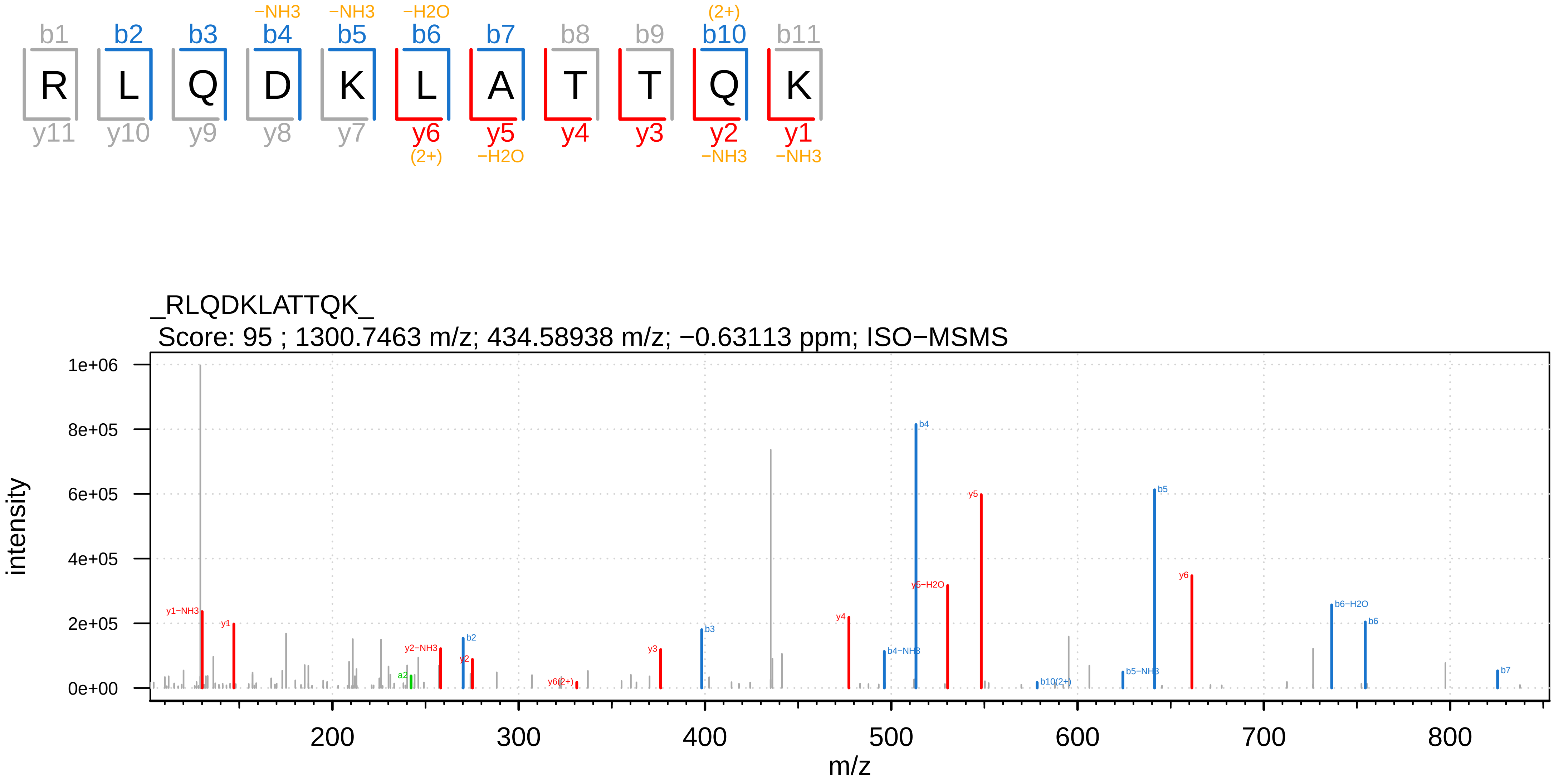

Raw File: Kermit_20111104_MSU_tube_6h_12
Scan Number: 4971
Proteins:
sp|Q8BT35|YB039_MOUSE
ENSMUST00000135091_chr2:127792297−127792467:−

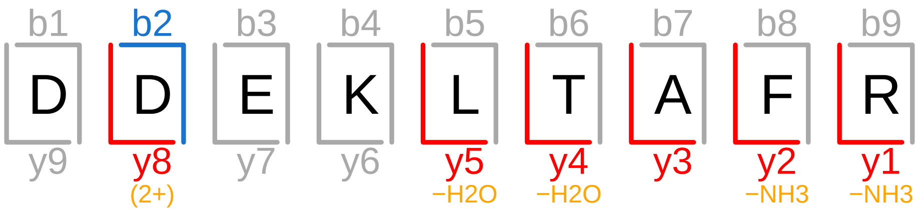

DDEKLTAFR
Score: 38 ; 1093.5404 m/z; 365.52074 m/z; 0.10176 ppm; ISO−MSMS

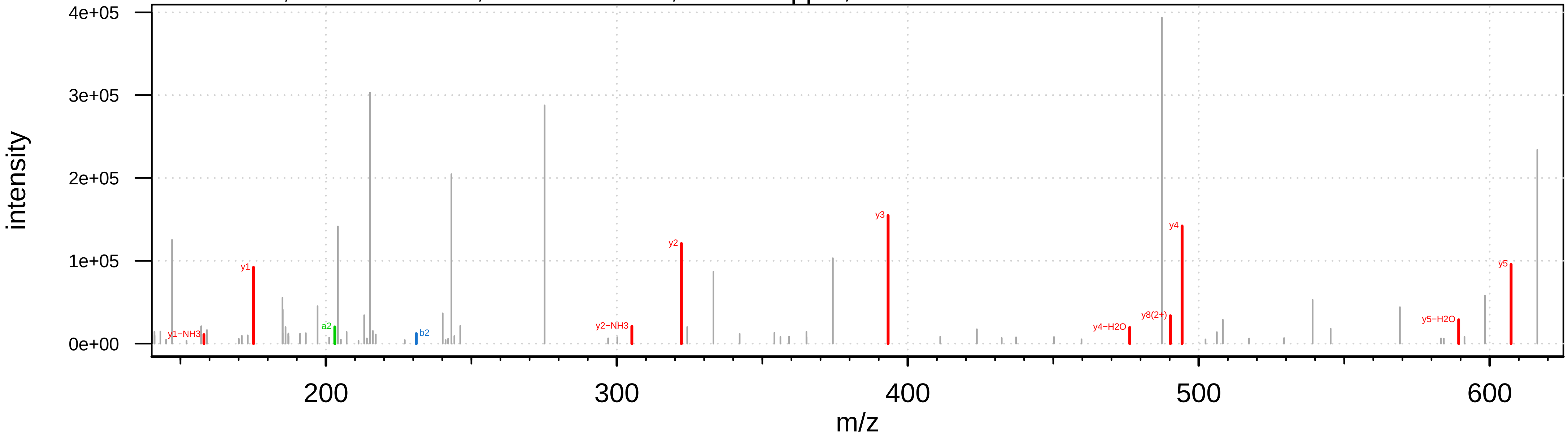

Raw File: Kermit_20111110_MSU_blast_3h_12
Scan Number: 7321
Proteins:
chr12_103206313−103214181.T2_chr12:101968174−101969629:−

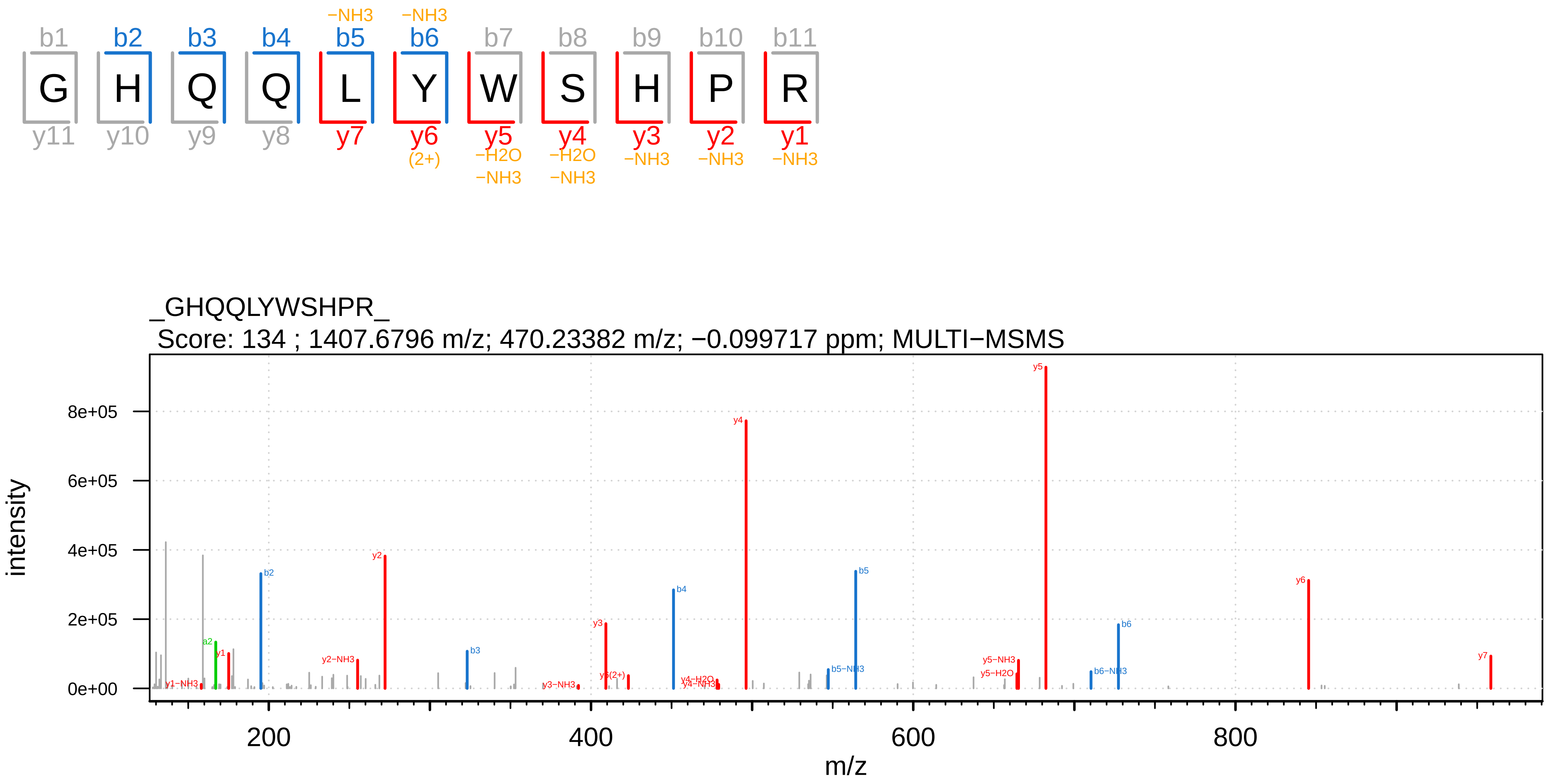

Raw File: Kermit_20111110_MSU_blast_3h_12
Scan Number: 57597
Proteins:
sp|P62274|RS29_MOUSE
ENSMUST00000037023_chr12:69157813−69159058:−
ENSMUST00000071737_chr13:73988963−73989133:−
tr|D3YYL7|D3YYL7_MOUSE

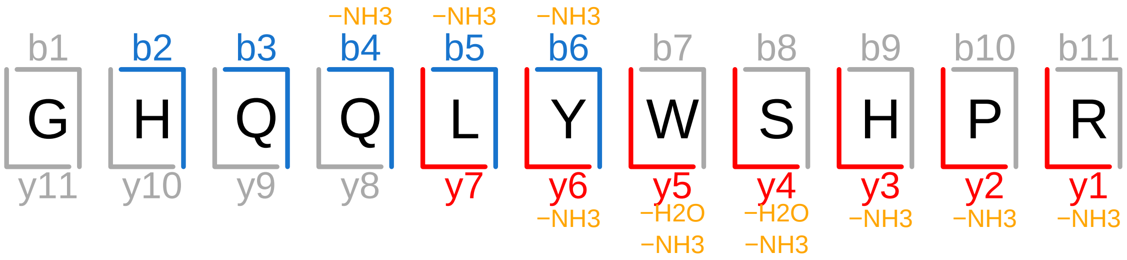

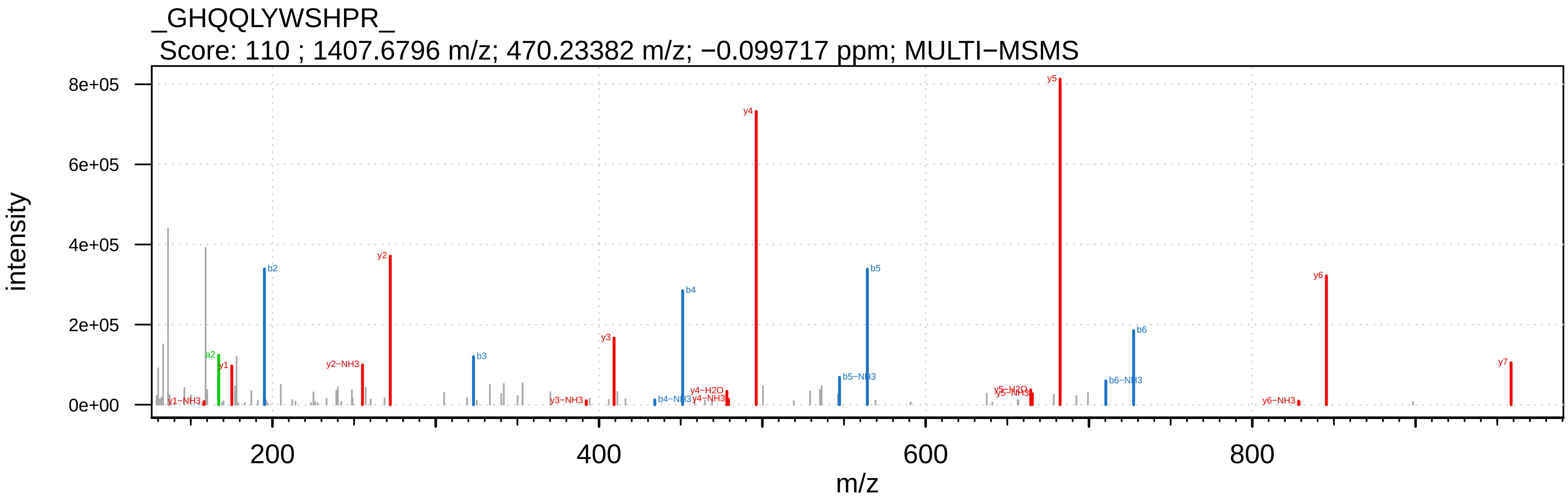

Raw File: Kermit_20111110_MSU_blast_3h_12
Scan Number: 57825
Proteins:
sp|P62274|RS29_MOUSE
ENSMUST00000037023_chr12:69157813−69159058:−
ENSMUST00000071737_chr13:73988963−73989133:−
tr|D3YYL7|D3YYL7_MOUSE

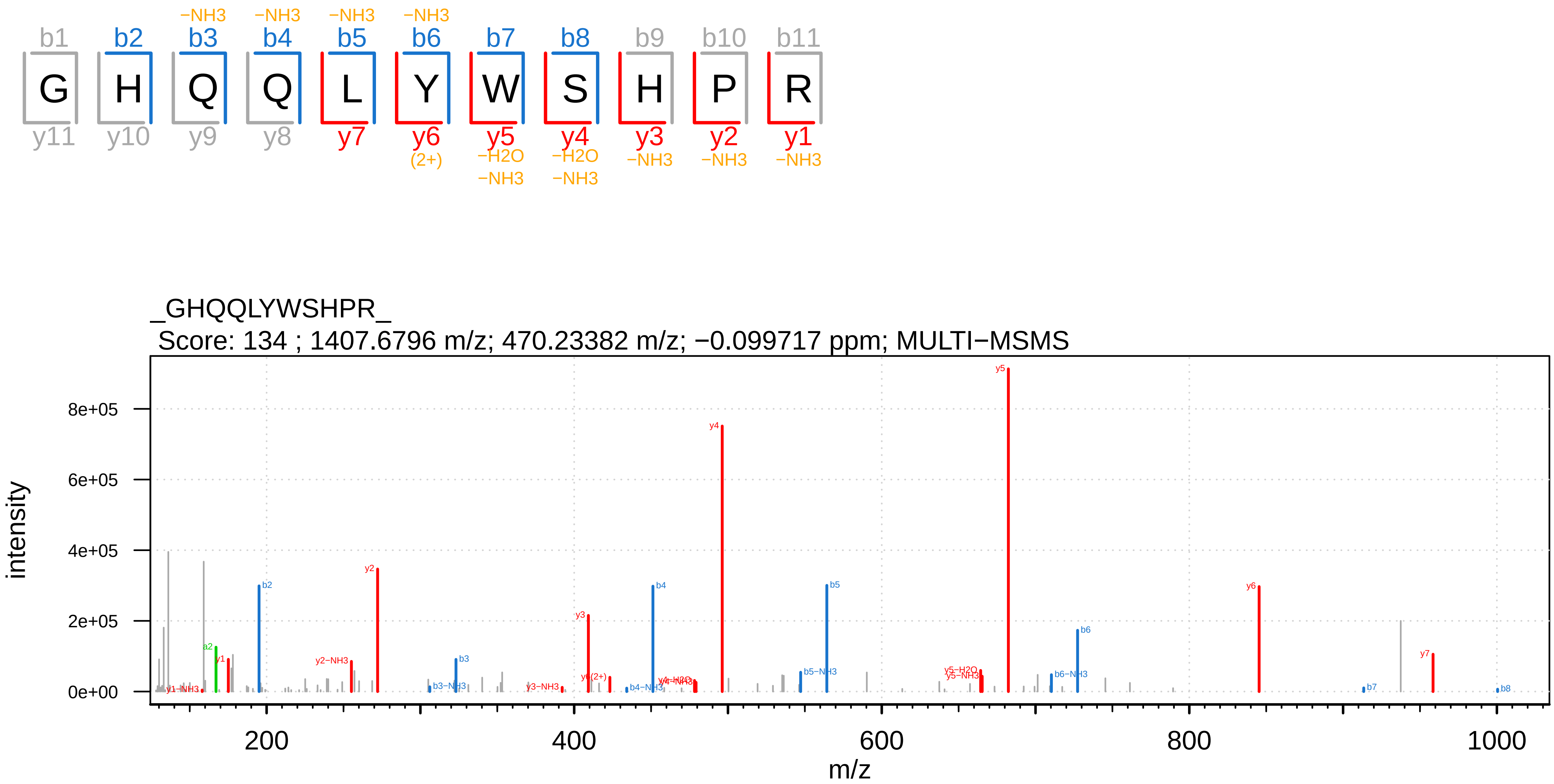

Raw File: Kermit_20111110_MSU_blast_3h_12
Scan Number: 58042
Proteins:
sp|P62274|RS29_MOUSE
ENSMUST00000037023_chr12:69157813−69159058:−
ENSMUST00000071737_chr13:73988963−73989133:−
tr|D3YYL7|D3YYL7_MOUSE

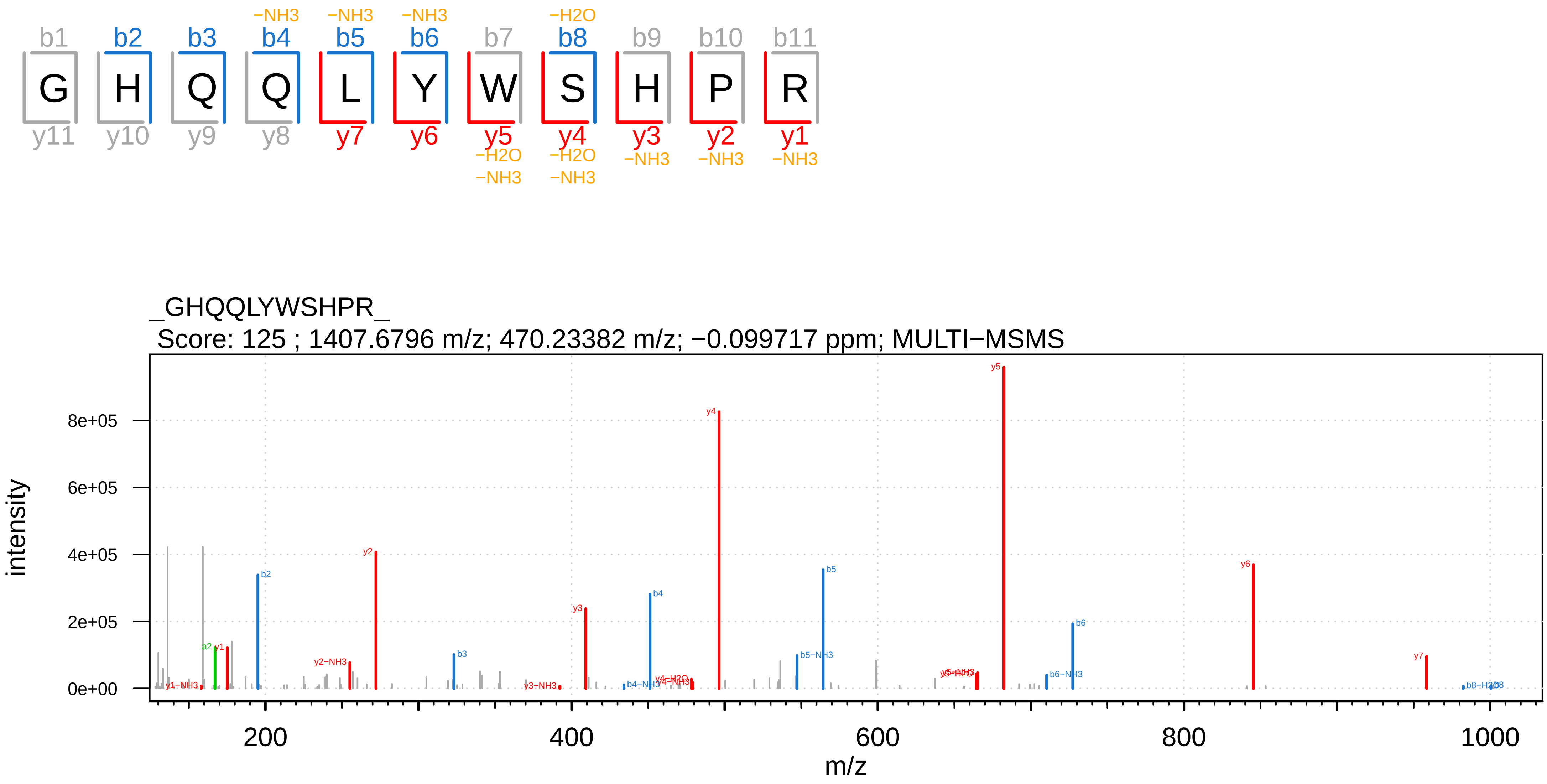

Raw File: Kermit_20111110_MSU_blast_3h_12
Scan Number: 58272
Proteins:
sp|P62274|RS29_MOUSE
ENSMUST00000037023_chr12:69157813−69159058:−
ENSMUST00000071737_chr13:73988963−73989133:−
tr|D3YYL7|D3YYL7_MOUSE

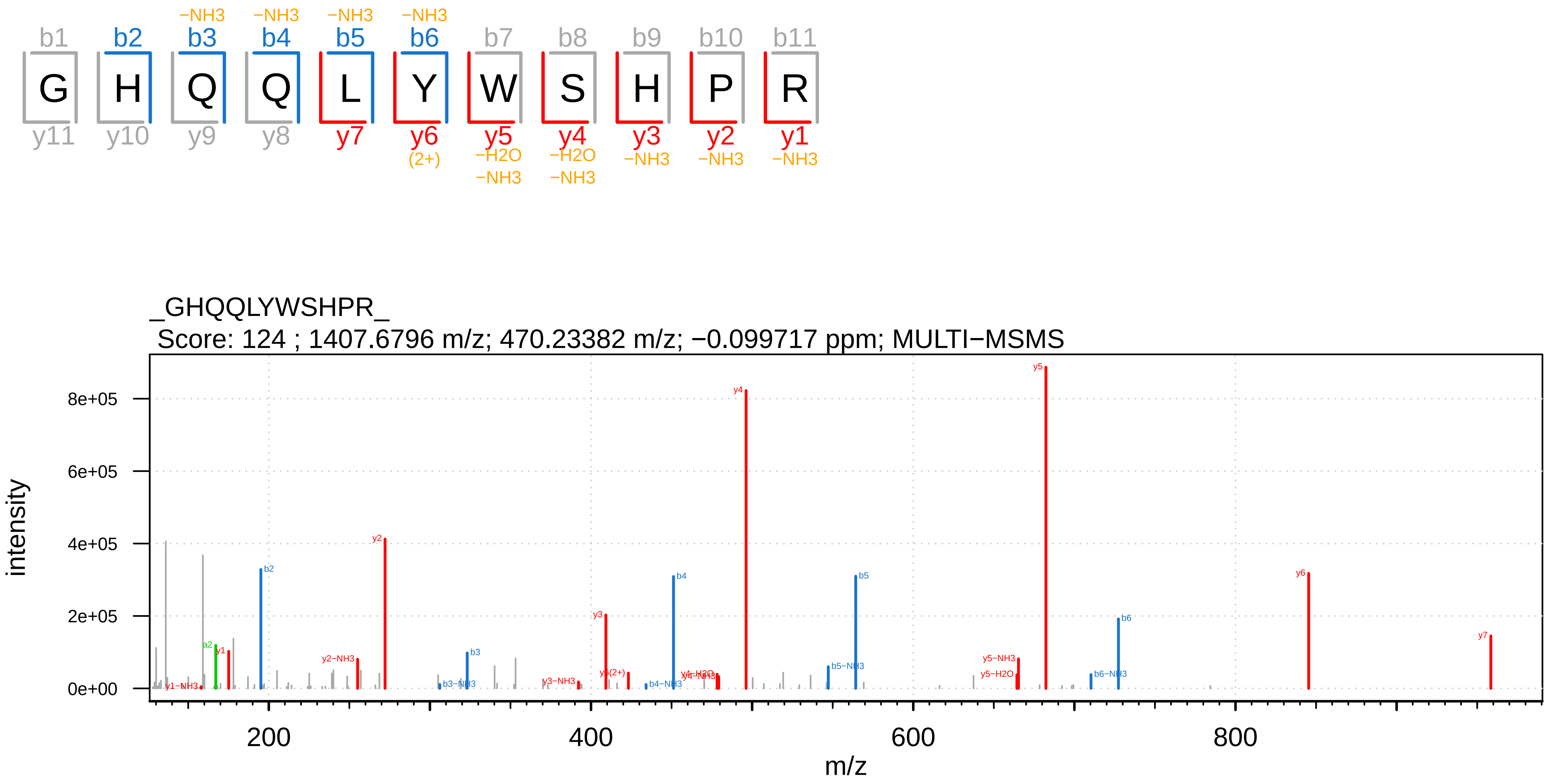

Raw File: Kermit_20111110_MSU_blast_3h_12
Scan Number: 58501
Proteins:
sp|P62274|RS29_MOUSE
ENSMUST00000037023_chr12:69157813−69159058:−
ENSMUST00000071737_chr13:73988963−73989133:−
tr|D3YYL7|D3YYL7_MOUSE

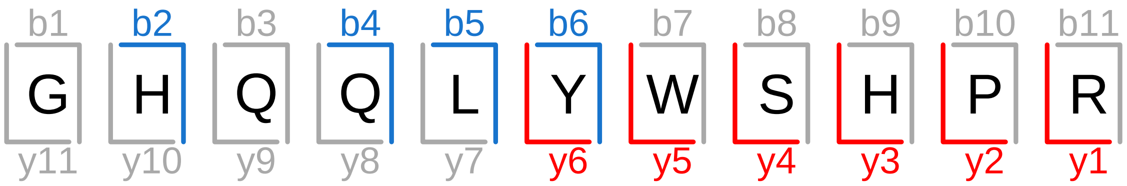

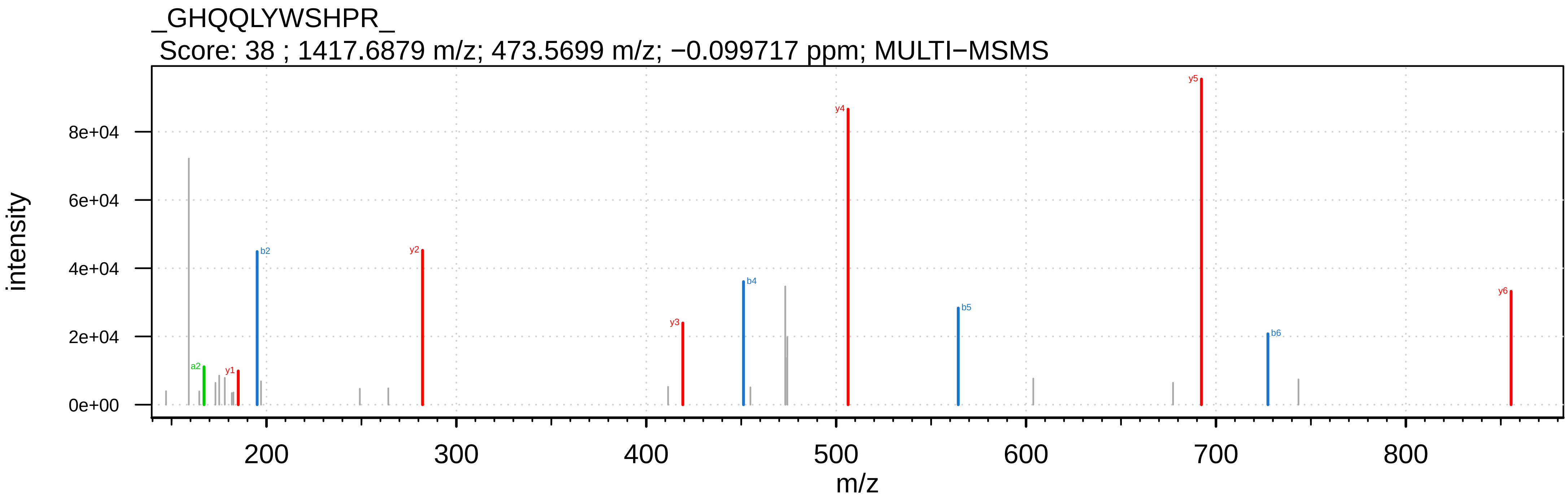

Raw File: Kermit_20111110_MSU_blast_3h_12
Scan Number: 58579
Proteins:
sp|P62274|RS29_MOUSE
ENSMUST00000037023_chr12:69157813−69159058:−
ENSMUST00000071737_chr13:73988963−73989133:−
tr|D3YYL7|D3YYL7_MOUSE

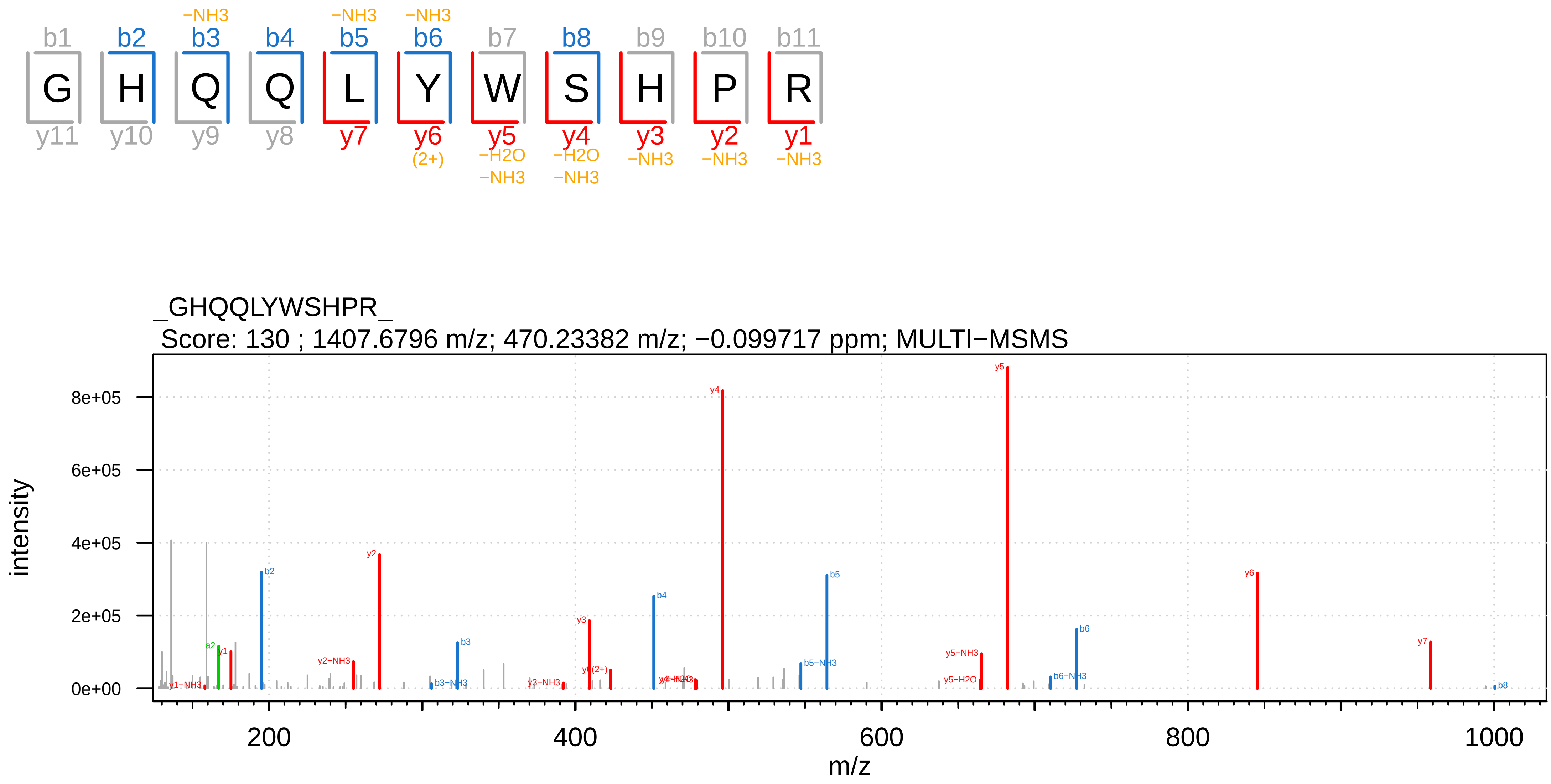

Raw File: Kermit_20111110_MSU_blast_3h_12
Scan Number: 58730
Proteins:
sp|P62274|RS29_MOUSE
ENSMUST00000037023_chr12:69157813−69159058:−
ENSMUST00000071737_chr13:73988963−73989133:−
tr|D3YYL7|D3YYL7_MOUSE

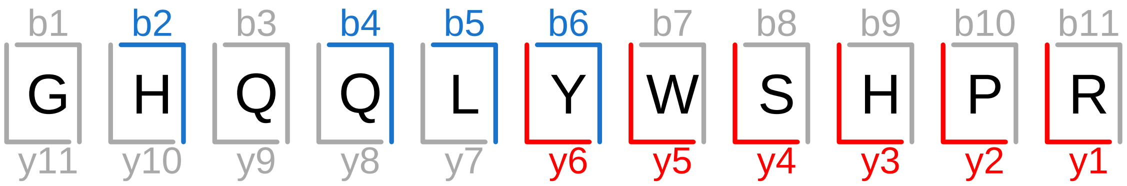

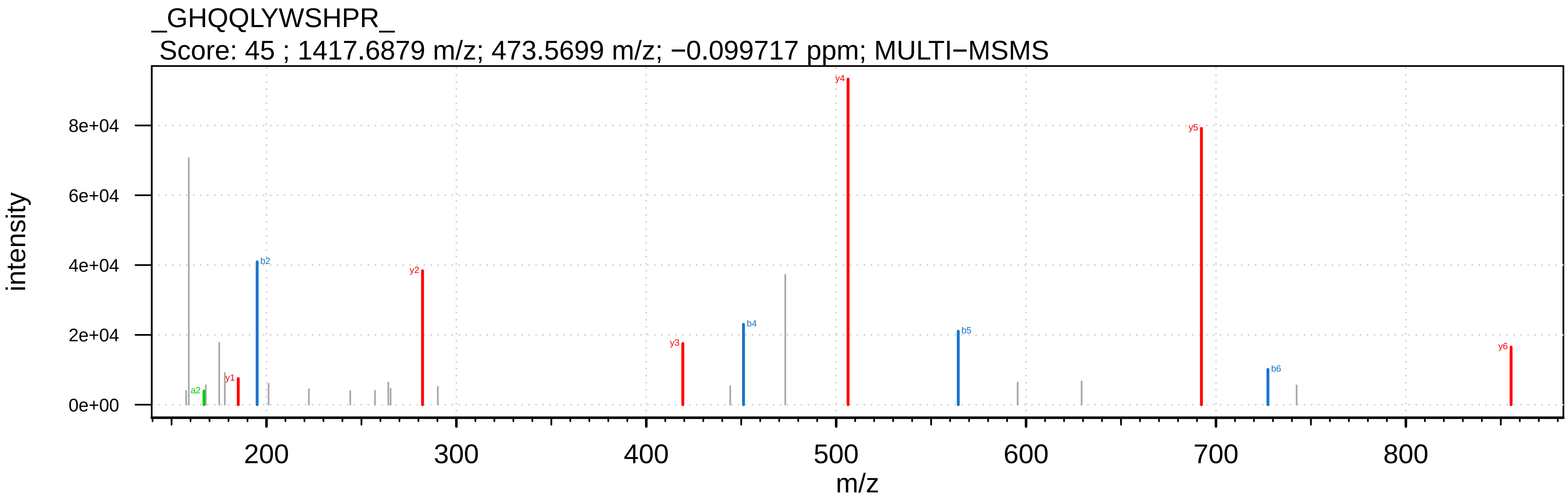

Raw File: Kermit_20111110_MSU_blast_3h_12
Scan Number: 58804
Proteins:
sp|P62274|RS29_MOUSE
ENSMUST00000037023_chr12:69157813−69159058:−
ENSMUST00000071737_chr13:73988963−73989133:−
tr|D3YYL7|D3YYL7_MOUSE

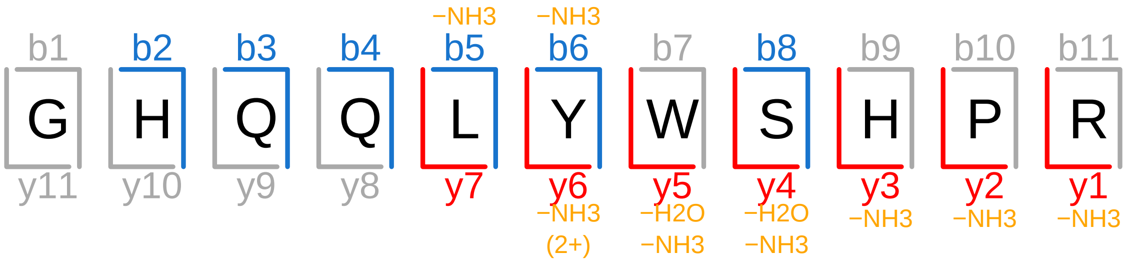

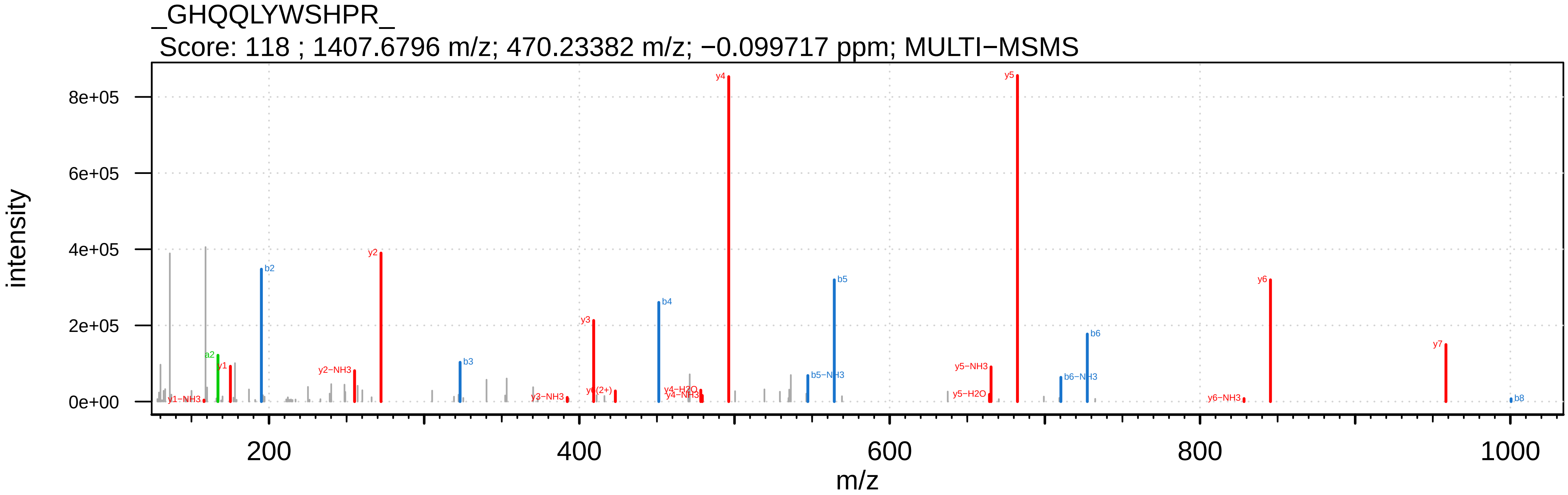

Raw File: Kermit_20111110_MSU_blast_3h_12
Scan Number: 58964
Proteins:
sp|P62274|RS29_MOUSE
ENSMUST00000037023_chr12:69157813−69159058:−
ENSMUST00000071737_chr13:73988963−73989133:−
tr|D3YYL7|D3YYL7_MOUSE

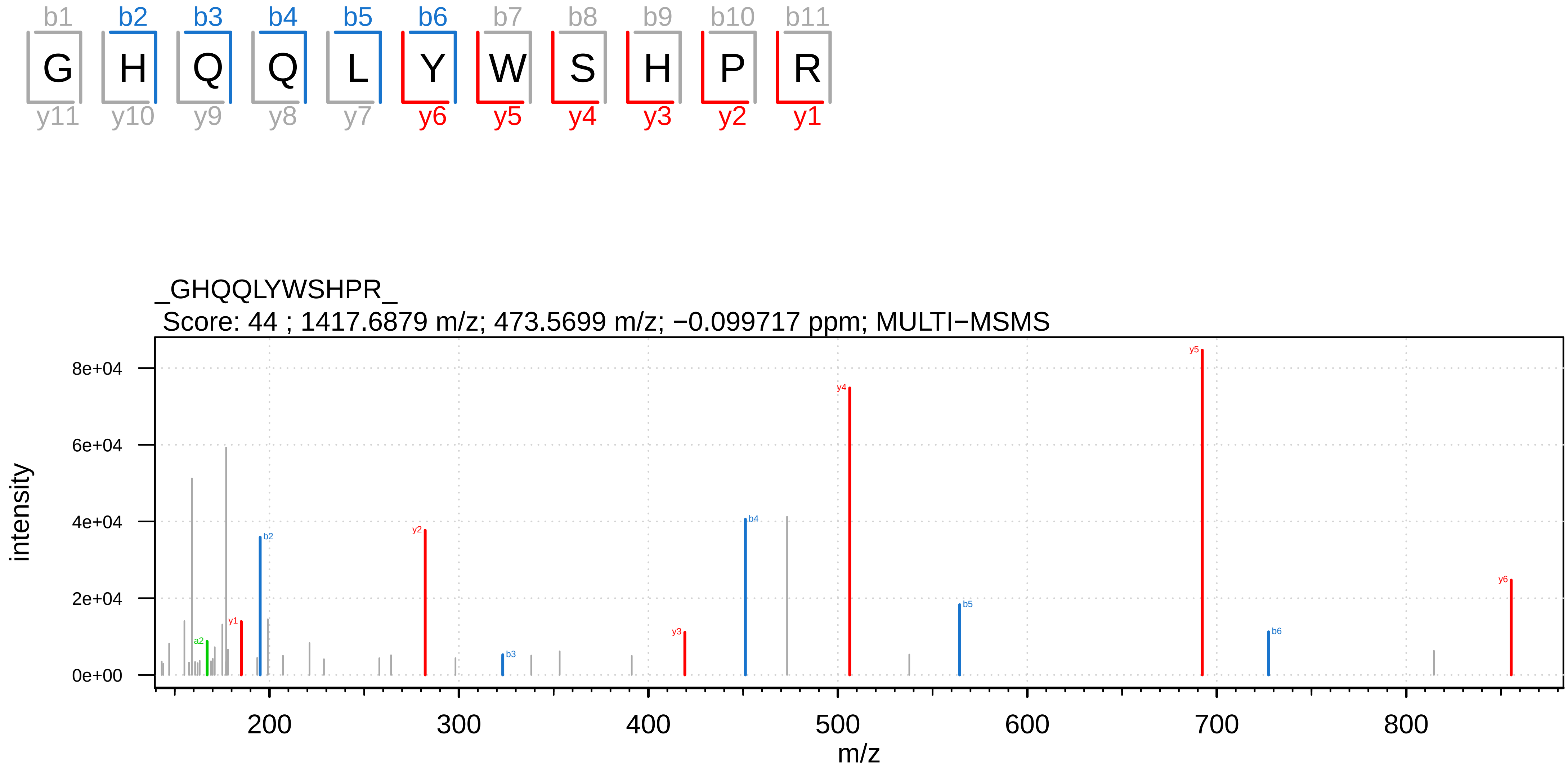

Raw File: Kermit_20111110_MSU_blast_3h_12
Scan Number: 59038
Proteins:
sp|P62274|RS29_MOUSE
ENSMUST00000037023_chr12:69157813−69159058:−
ENSMUST00000071737_chr13:73988963−73989133:−
tr|D3YYL7|D3YYL7_MOUSE

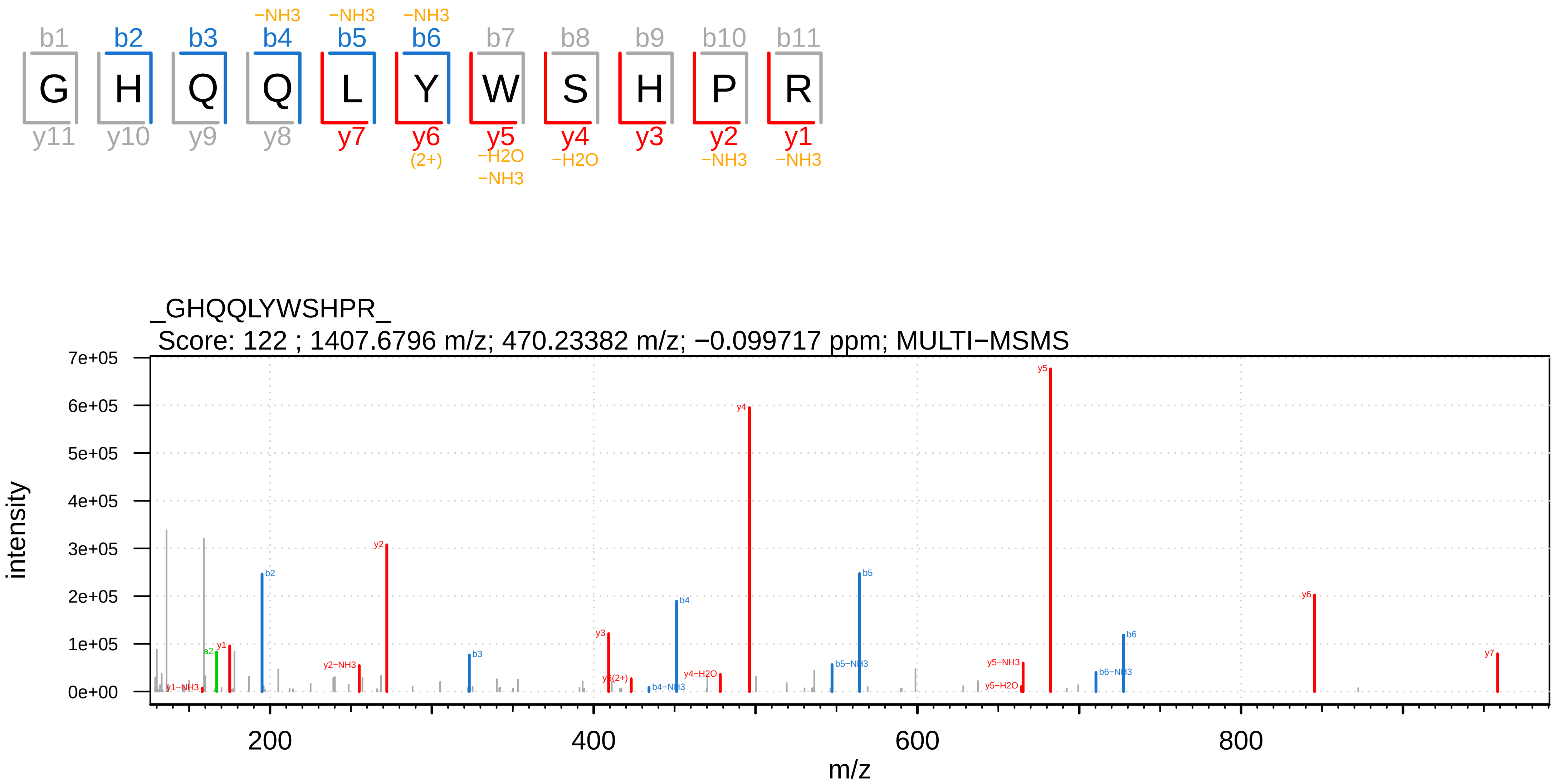

Raw File: Kermit_20111110_MSU_blast_3h_12
Scan Number: 59193
Proteins:
sp|P62274|RS29_MOUSE
ENSMUST00000037023_chr12:69157813−69159058:−
ENSMUST00000071737_chr13:73988963−73989133:−
tr|D3YYL7|D3YYL7_MOUSE

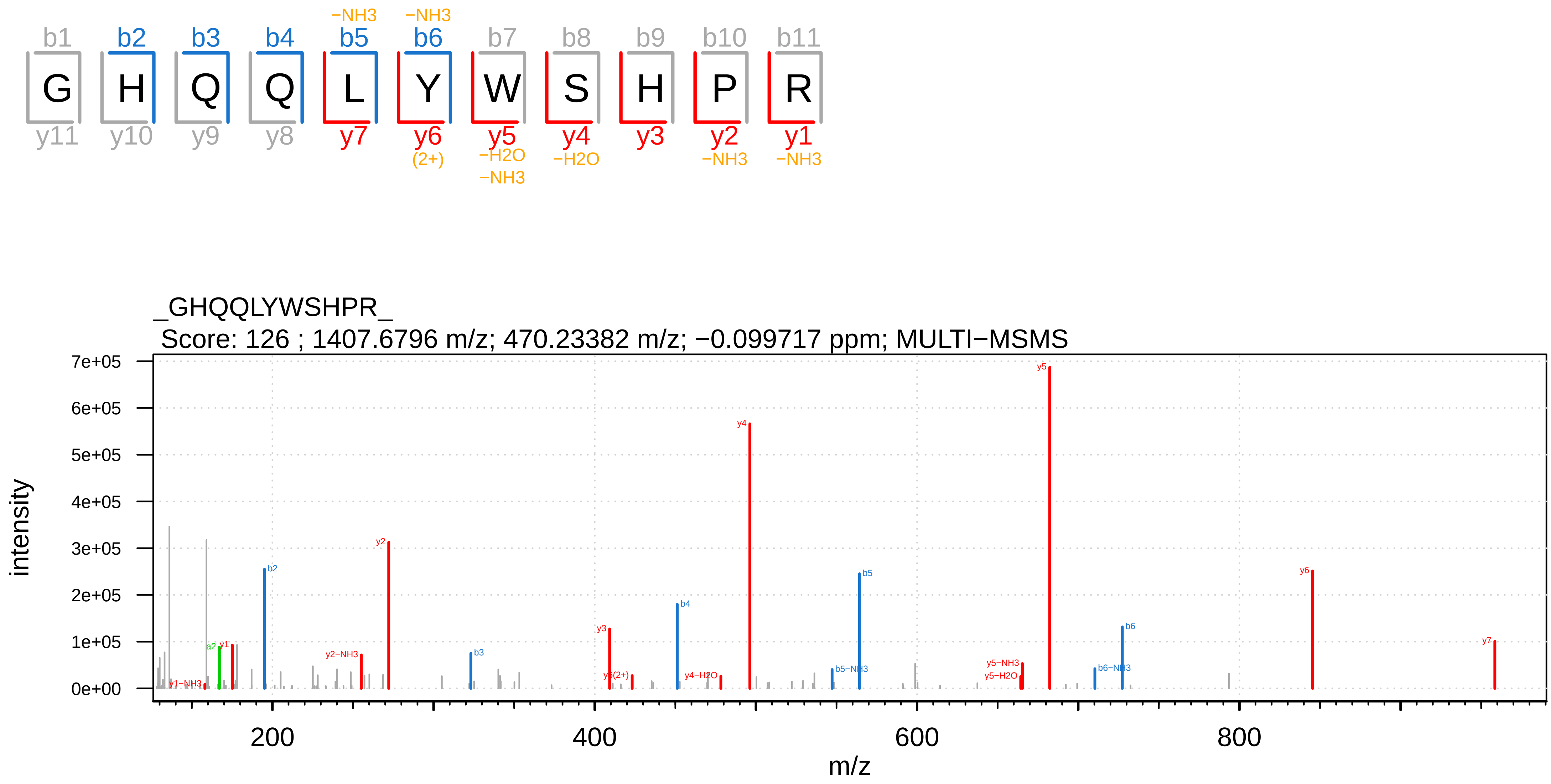

Raw File: Kermit_20111110_MSU_blast_3h_12
Scan Number: 59413
Proteins:
sp|P62274|RS29_MOUSE
ENSMUST00000037023_chr12:69157813−69159058:−
ENSMUST00000071737_chr13:73988963−73989133:−
tr|D3YYL7|D3YYL7_MOUSE

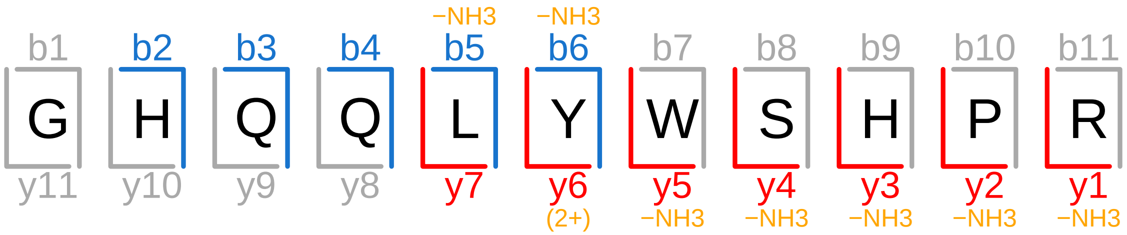

GHQQLYWSHPR
Score: 78 ; 1407.6796 m/z; 470.23382 m/z; −0.099717 ppm; MULTI−MSMS

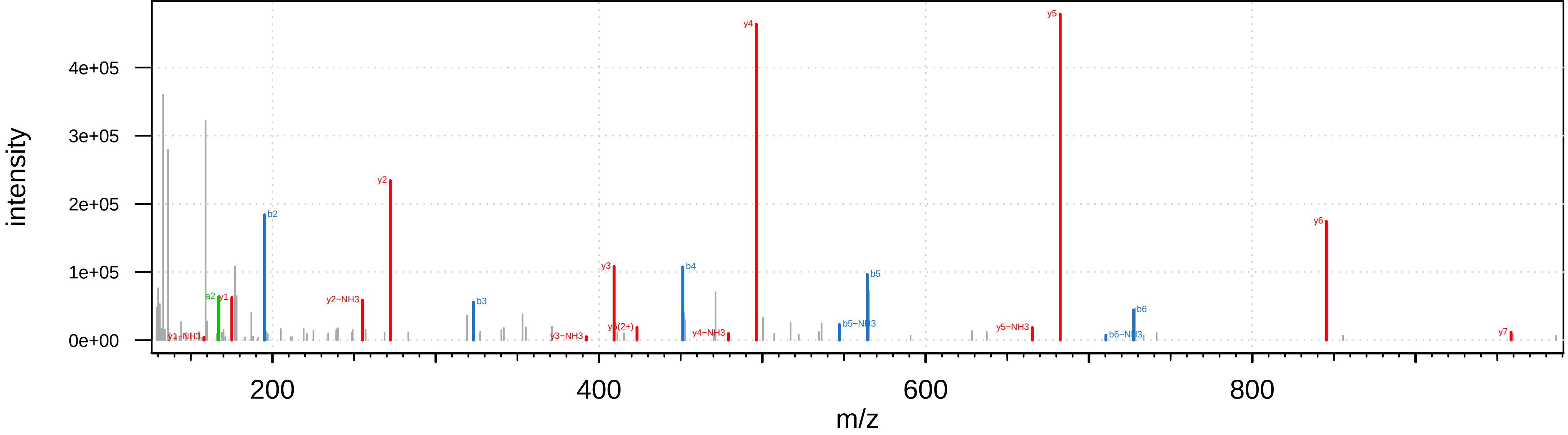

Raw File: Kermit_20111110_MSU_blast_3h_12
Scan Number: 59654
Proteins:
sp|P62274|RS29_MOUSE
ENSMUST00000037023_chr12:69157813−69159058:−
ENSMUST00000071737_chr13:73988963−73989133:−
tr|D3YYL7|D3YYL7_MOUSE

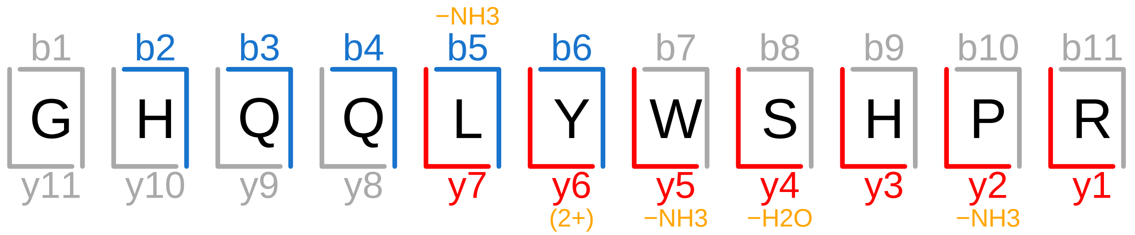

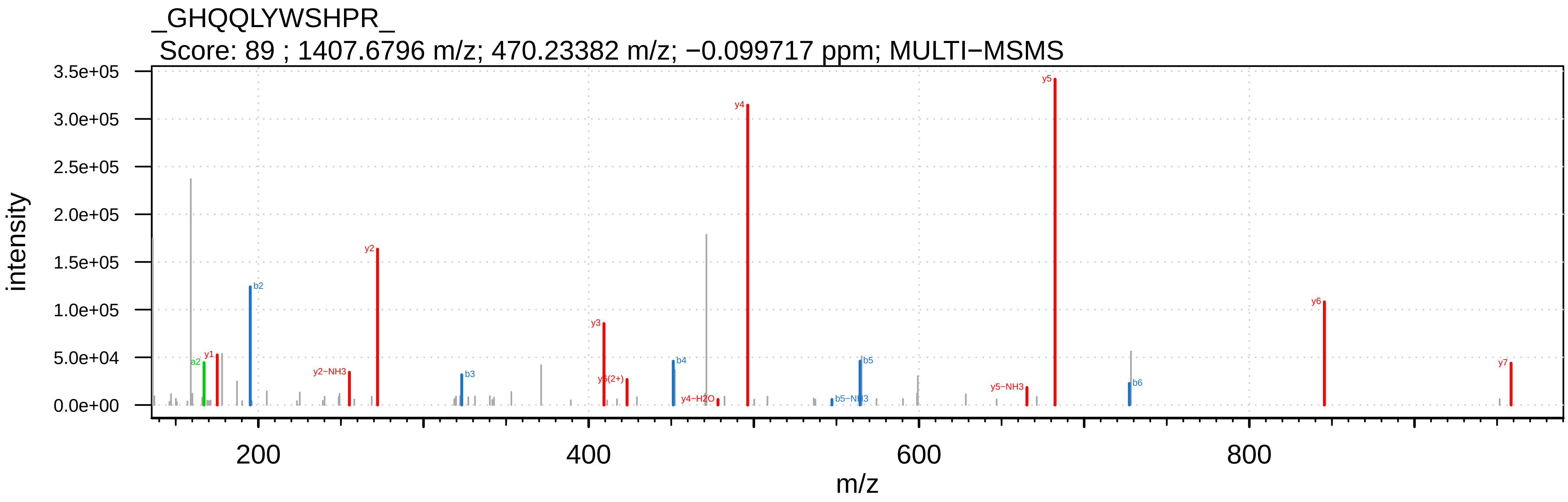

Raw File: Kermit_20111110_MSU_blast_3h_12
Scan Number: 59889
Proteins:
sp|P62274|RS29_MOUSE
ENSMUST00000037023_chr12:69157813−69159058:−
ENSMUST00000071737_chr13:73988963−73989133:−
tr|D3YYL7|D3YYL7_MOUSE

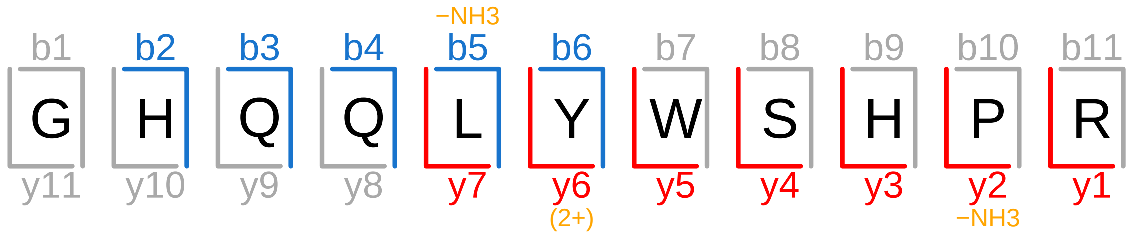

GHQQLYWSHPR

Score: 59 ; 1407.6796 m/z; 470.23382 m/z; −0.4971 ppm; ISO−MSMS

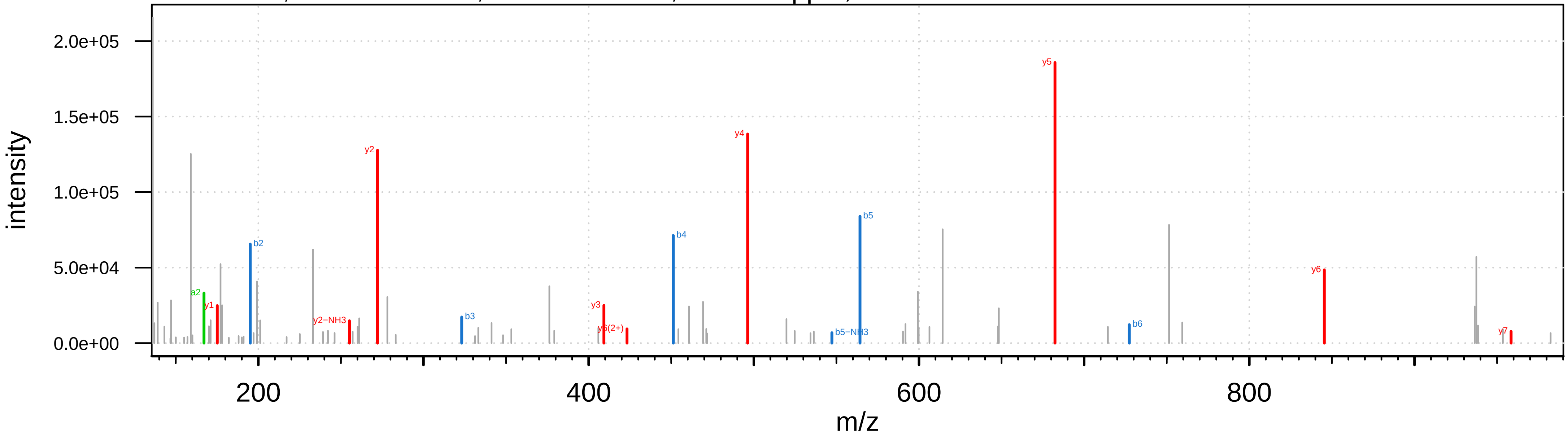

Raw File: Kermit_20111110_MSU_blast_3h_12

Scan Number: 54881

Proteins:

sp|P62274|RS29_MOUSE

ENSMUST00000037023_chr12:69157813−69159058:−

ENSMUST00000071737_chr13:73988963−73989133:−

tr|D3YYL7|D3YYL7_MOUSE

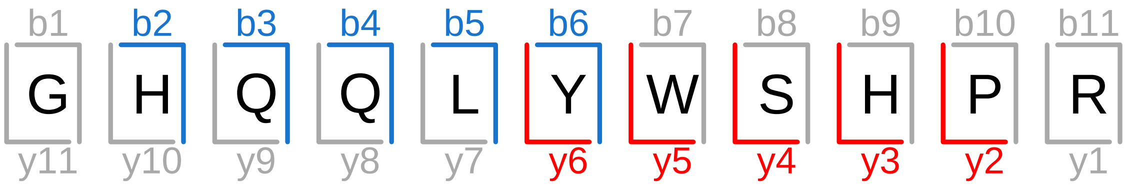

GHQQLYWSHPR
Score: 30 ; 1417.6879 m/z; 473.5699 m/z; −0.19052 ppm; ISO−MSMS

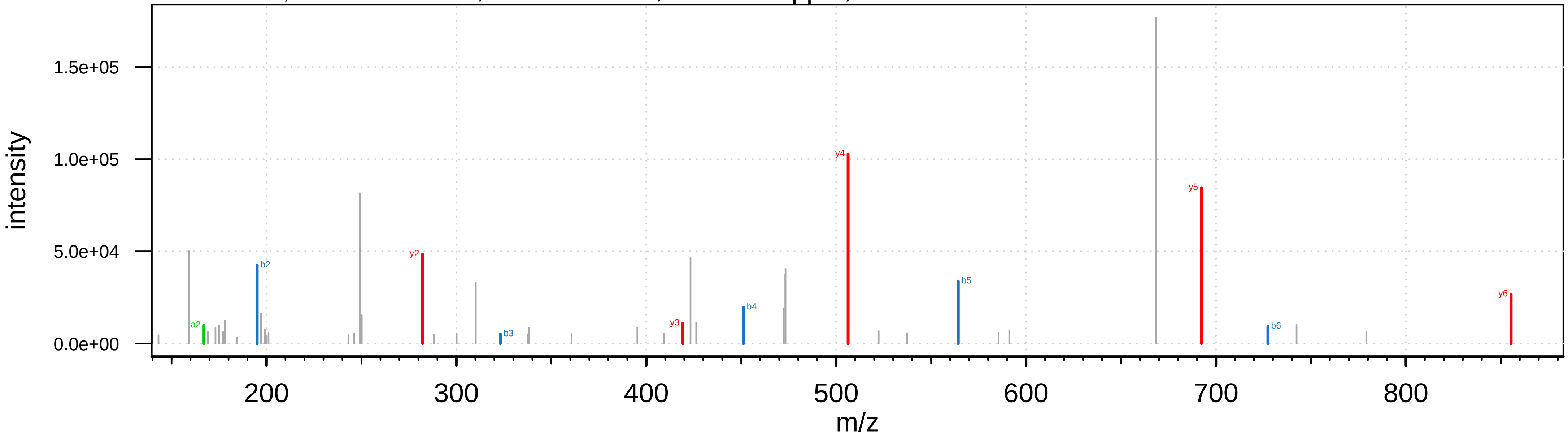

Raw File: Kermit_20111110_MSU_blast_3h_12
Scan Number: 57662
Proteins:
sp|P62274|RS29_MOUSE
ENSMUST00000037023_chr12:69157813−69159058:−
ENSMUST00000071737_chr13:73988963−73989133:−
tr|D3YYL7|D3YYL7_MOUSE

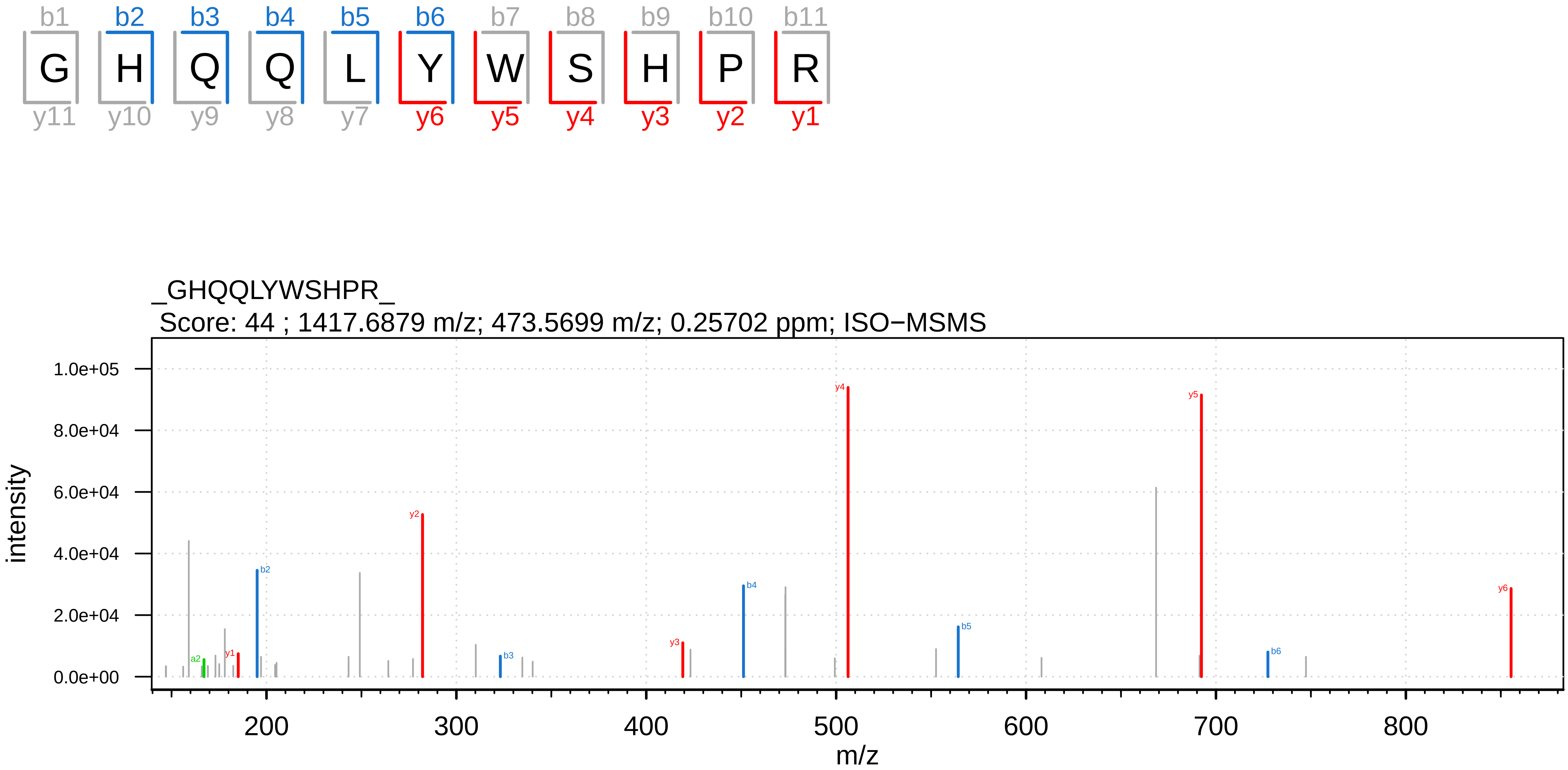

Raw File: Kermit_20111110_MSU_blast_3h_12
Scan Number: 57890
Proteins:
sp|P62274|RS29_MOUSE
ENSMUST00000037023_chr12:69157813−69159058:−
ENSMUST00000071737_chr13:73988963−73989133:−
tr|D3YYL7|D3YYL7_MOUSE

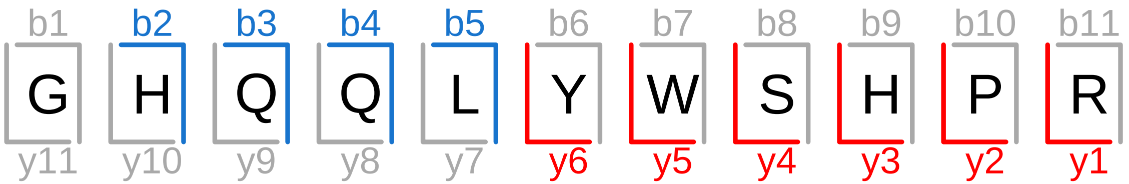

GHQQLYWSHPR
Score: 45 ; 1417.6879 m/z; 473.5699 m/z; 0.41859 ppm; ISO−MSMS

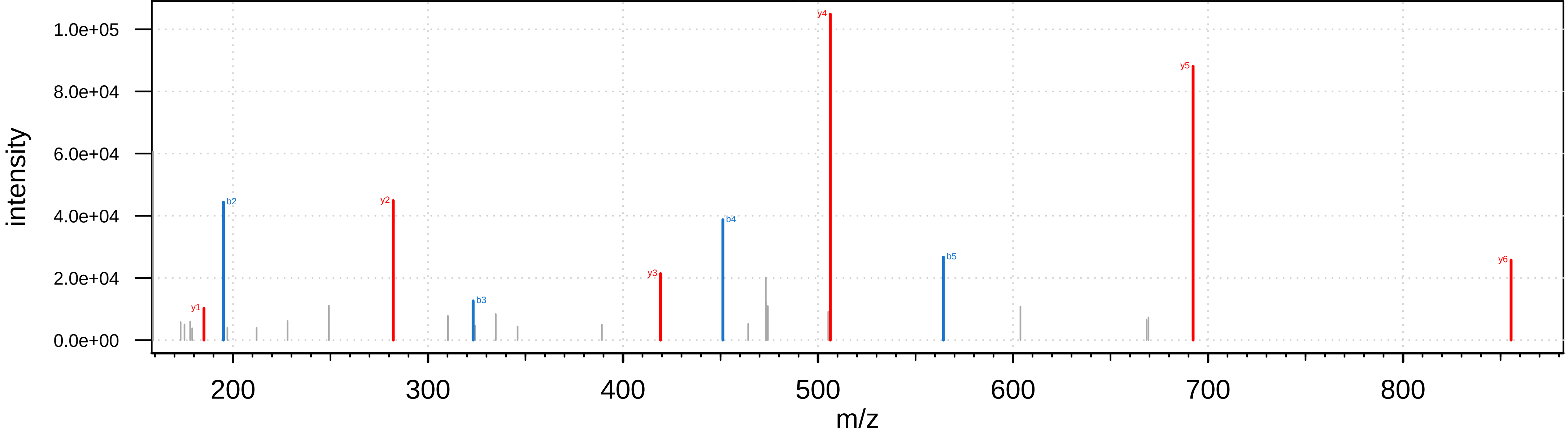

Raw File: Kermit_20111110_MSU_blast_3h_12
Scan Number: 58110
Proteins:
sp|P62274|RS29_MOUSE
ENSMUST00000037023_chr12:69157813−69159058:−
ENSMUST00000071737_chr13:73988963−73989133:−
tr|D3YYL7|D3YYL7_MOUSE

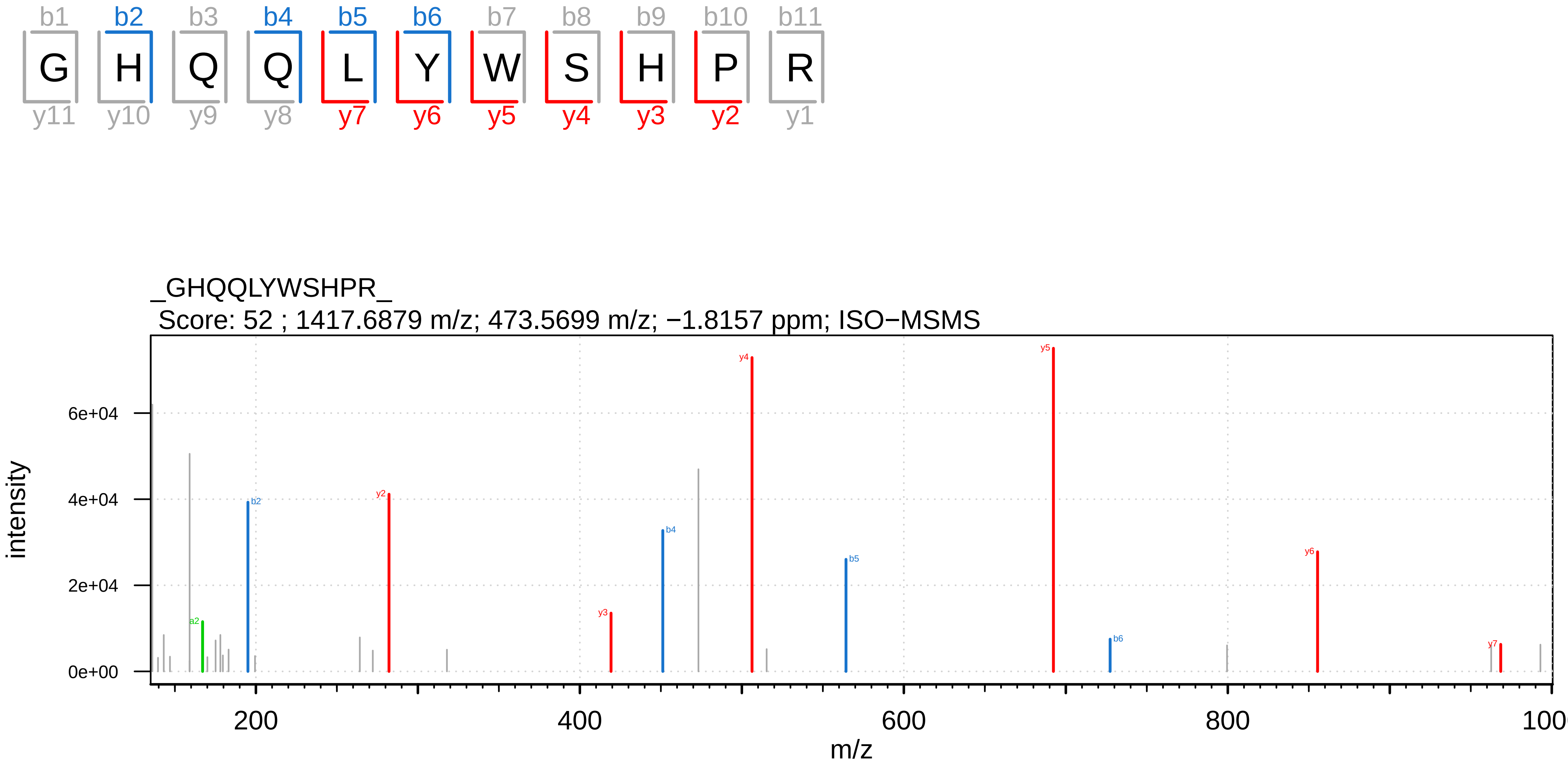

Raw File: Kermit_20111110_MSU_blast_3h_12
Scan Number: 59268
Proteins:
sp|P62274|RS29_MOUSE
ENSMUST00000037023_chr12:69157813−69159058:−
ENSMUST00000071737_chr13:73988963−73989133:−
tr|D3YYL7|D3YYL7_MOUSE

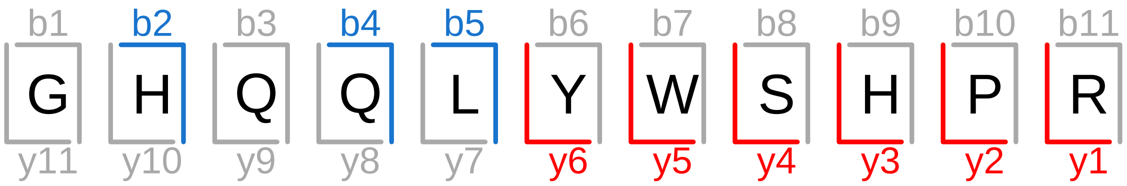

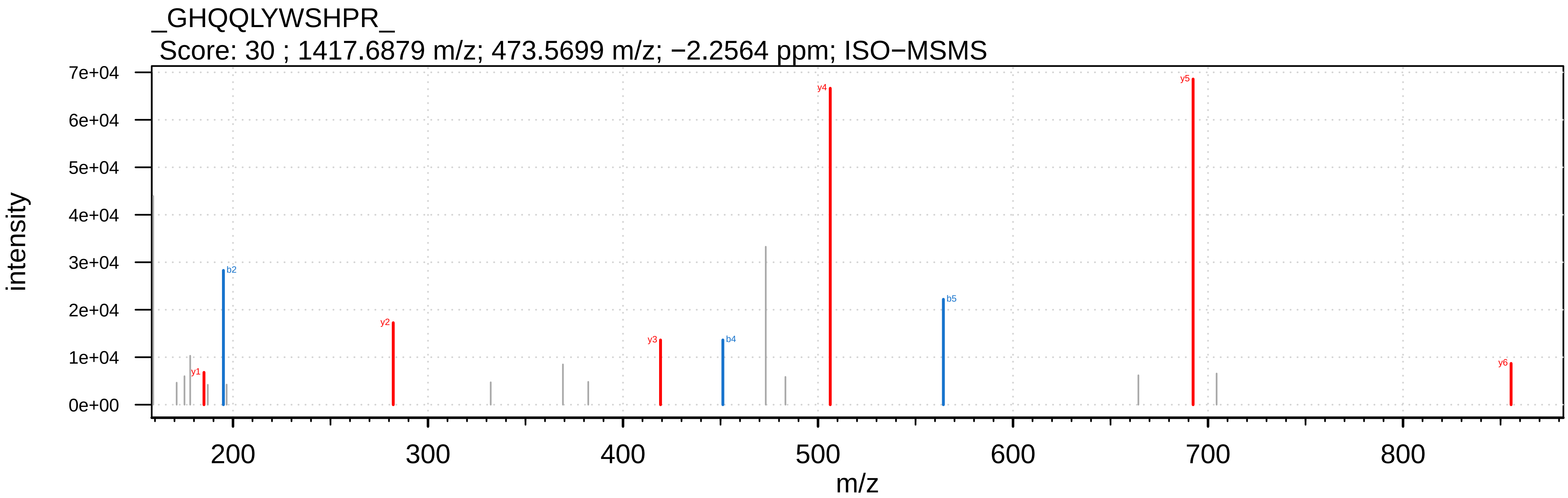

Raw File: Kermit_20111110_MSU_blast_3h_12
Scan Number: 59491
Proteins:
sp|P62274|RS29_MOUSE
ENSMUST00000037023_chr12:69157813−69159058:−
ENSMUST00000071737_chr13:73988963−73989133:−
tr|D3YYL7|D3YYL7_MOUSE

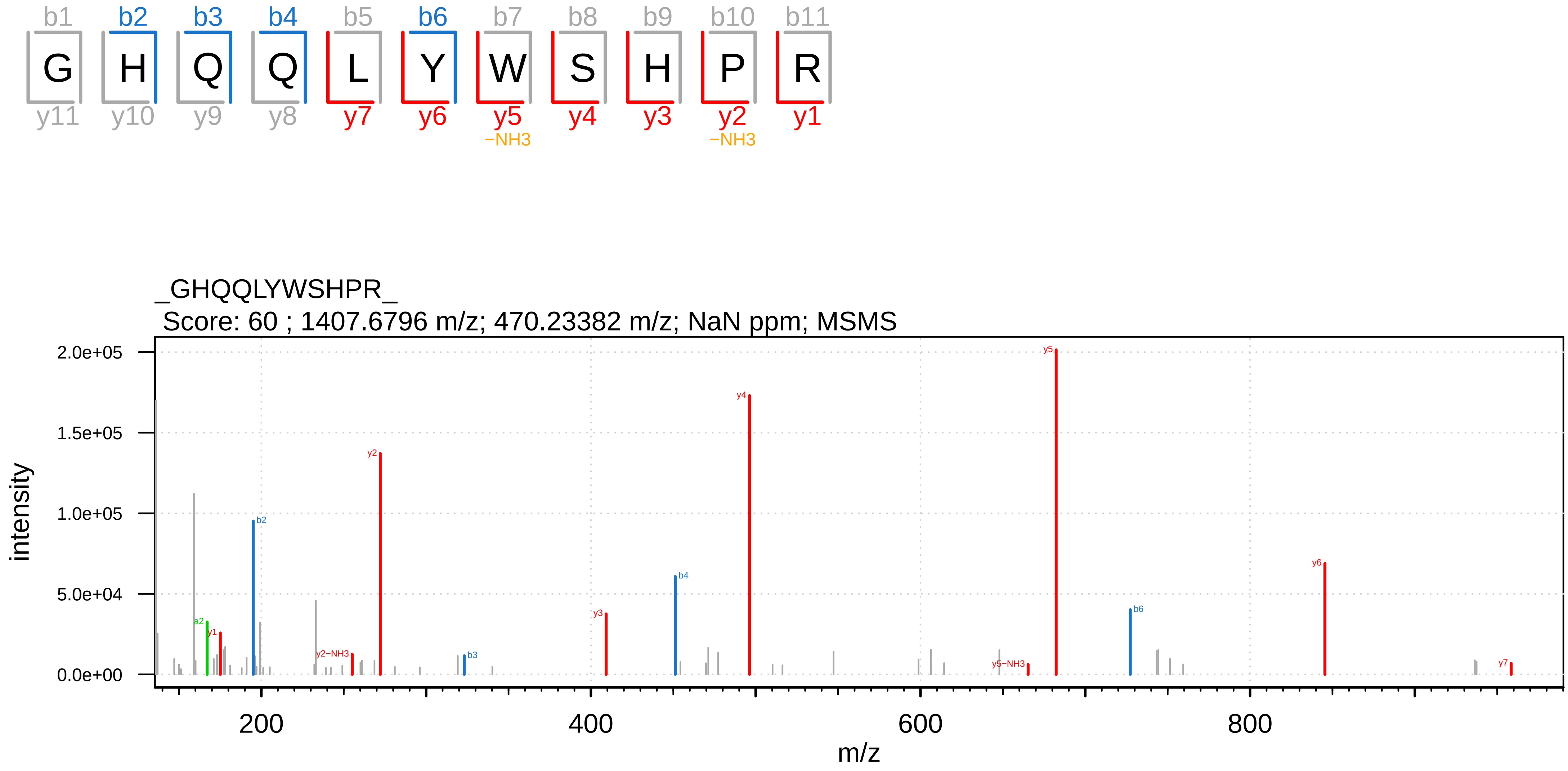

Raw File: Kermit_20111110_MSU_blast_3h_12
Scan Number: 55306
Proteins:
sp|P62274|RS29_MOUSE
ENSMUST00000037023_chr12:69157813−69159058:−
ENSMUST00000071737_chr13:73988963−73989133:−
tr|D3YYL7|D3YYL7_MOUSE

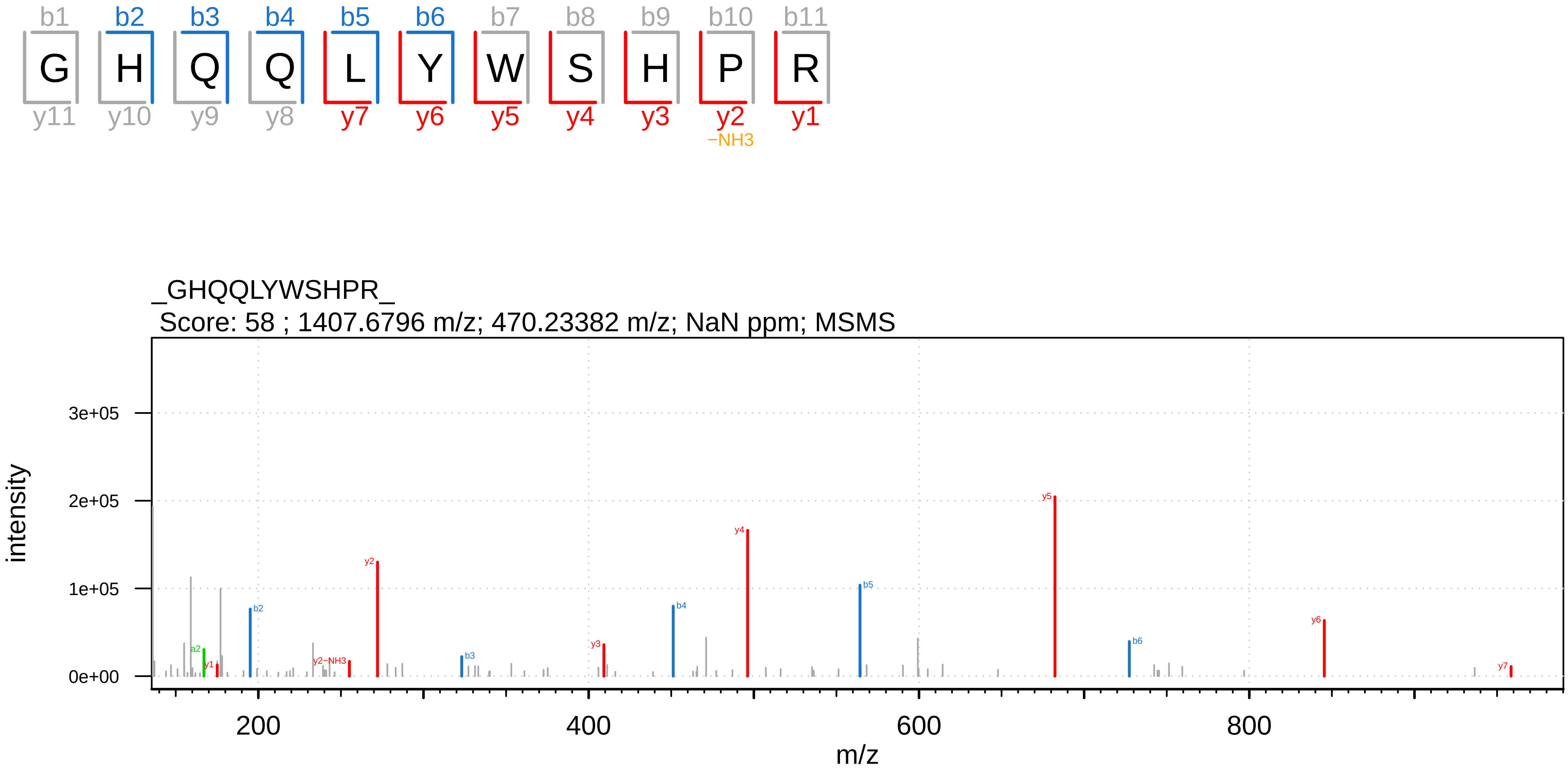

Raw File: Kermit_20111110_MSU_blast_3h_12
Scan Number: 55536
Proteins:
sp|P62274|RS29_MOUSE
ENSMUST00000037023_chr12:69157813−69159058:−
ENSMUST00000071737_chr13:73988963−73989133:−
tr|D3YYL7|D3YYL7_MOUSE

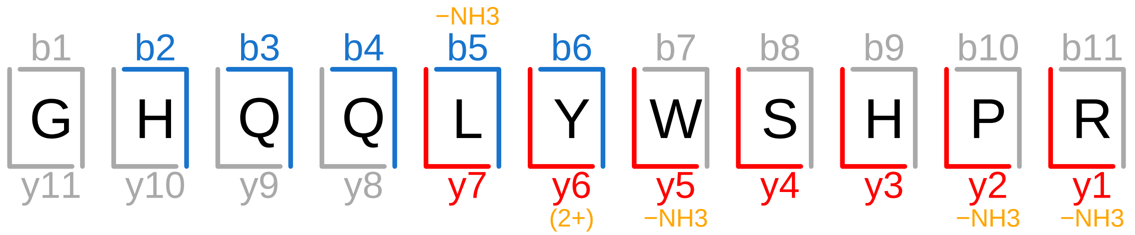

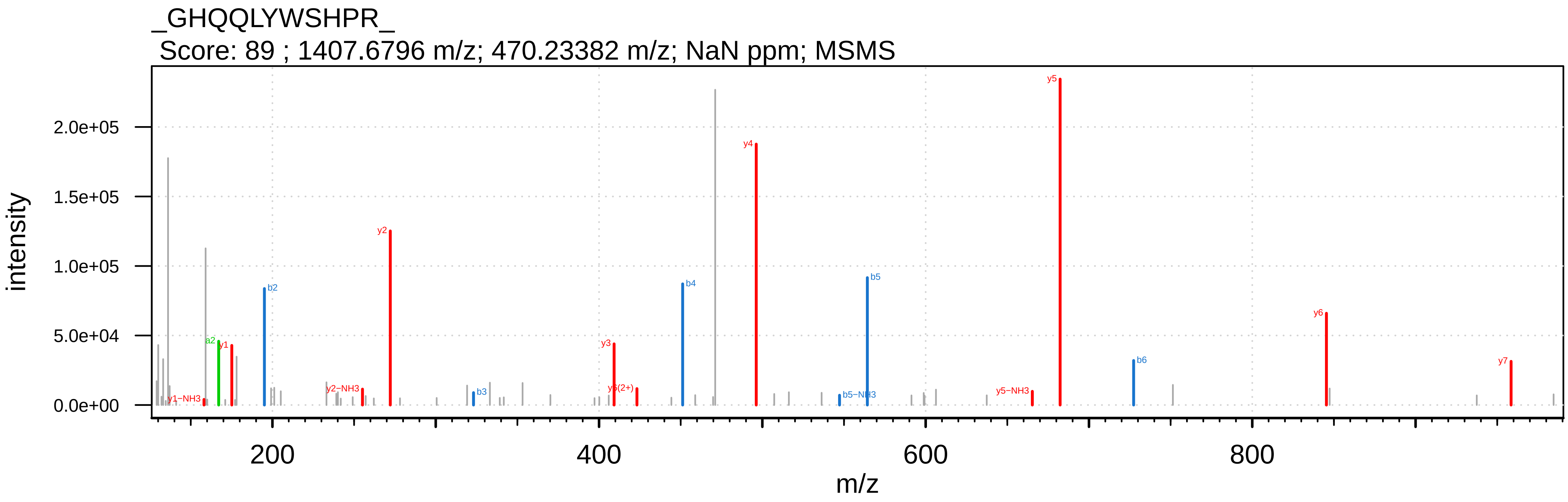

Raw File: Kermit_20111110_MSU_blast_3h_12
Scan Number: 55762
Proteins:
sp|P62274|RS29_MOUSE
ENSMUST00000037023_chr12:69157813−69159058:−
ENSMUST00000071737_chr13:73988963−73989133:−
tr|D3YYL7|D3YYL7_MOUSE

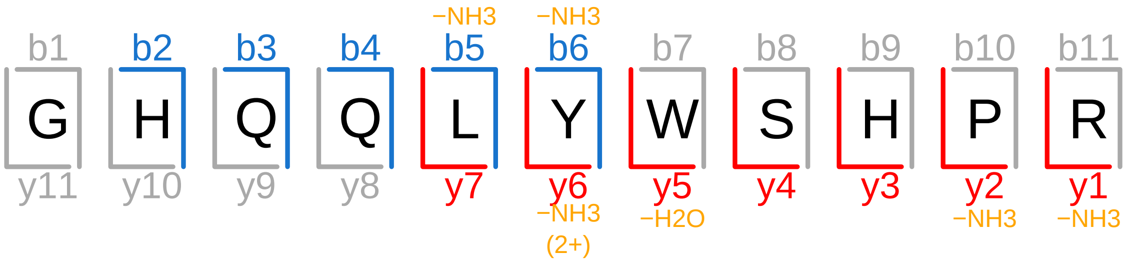

GHQQLYWSHPR

Score: 107 ; 1407.6796 m/z; 470.23382 m/z; NaN ppm; MSMS

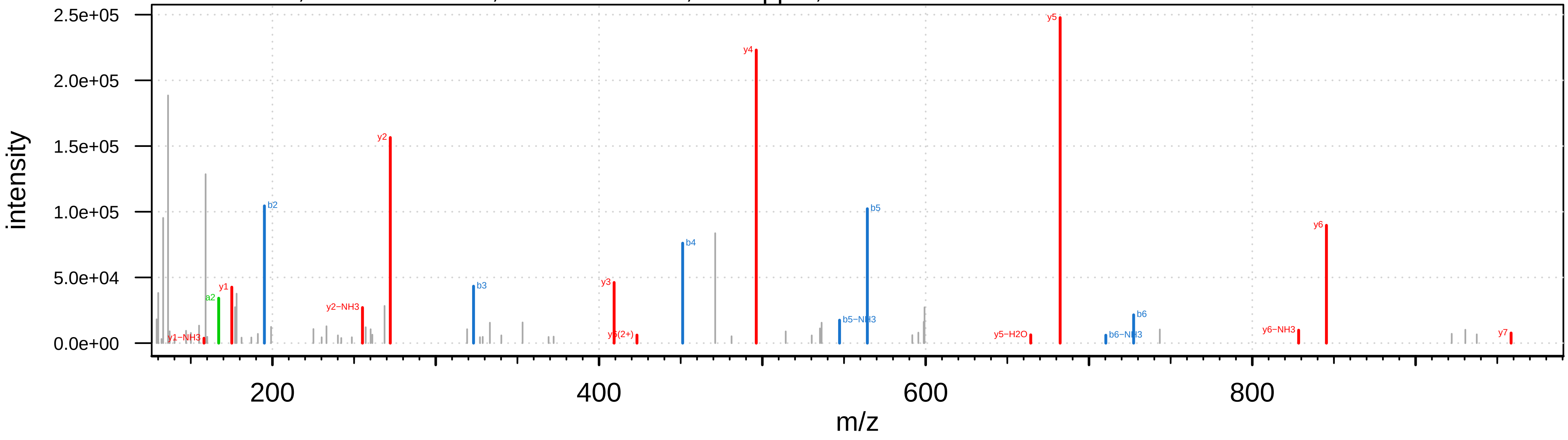

Raw File: Kermit_20111110_MSU_blast_3h_12
Scan Number: 55992
Proteins:
sp|P62274|RS29_MOUSE
ENSMUST00000037023_chr12:69157813−69159058:−
ENSMUST00000071737_chr13:73988963−73989133:−
tr|D3YYL7|D3YYL7_MOUSE

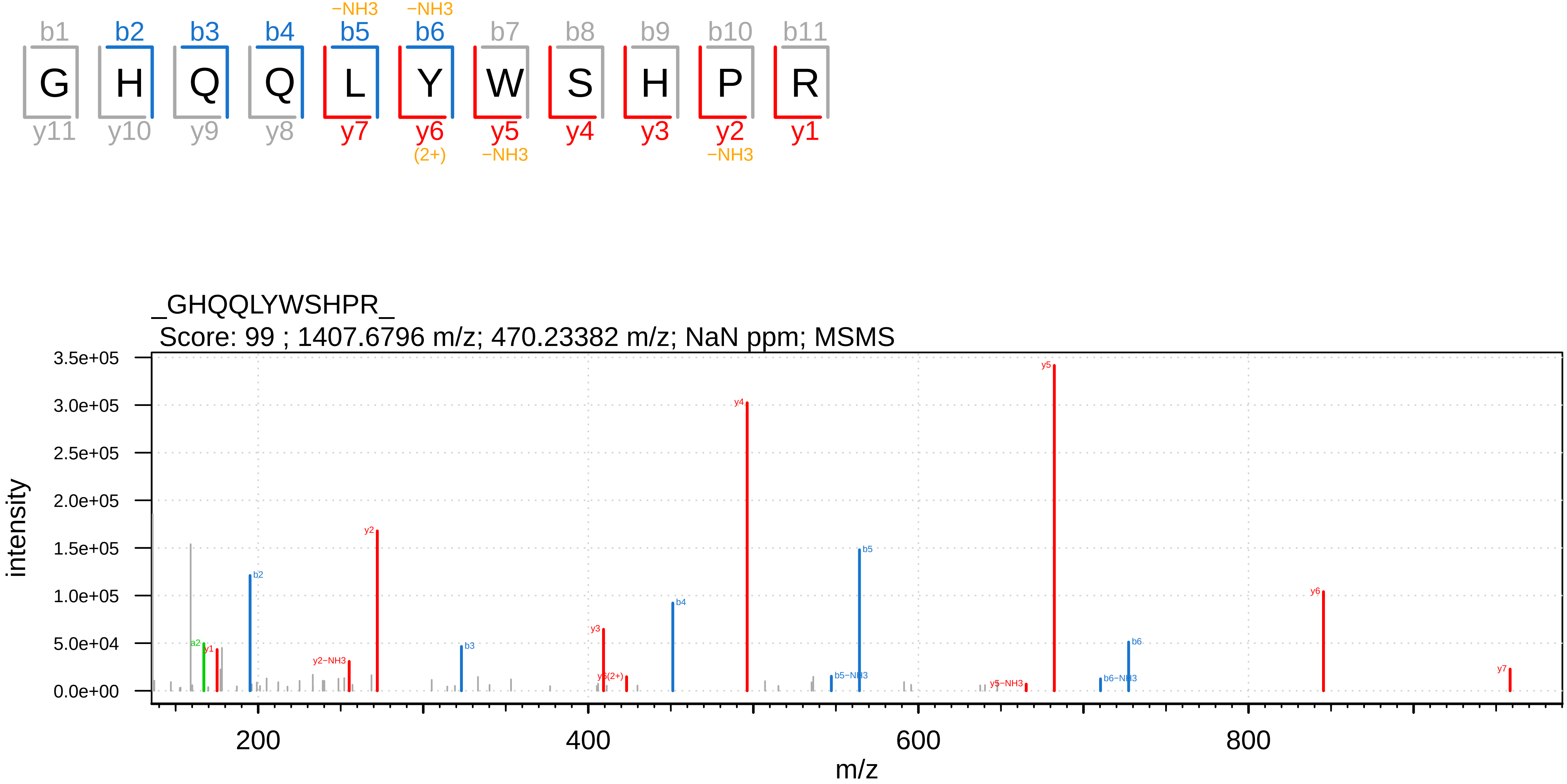

Raw File: Kermit_20111110_MSU_blast_3h_12
Scan Number: 56221
Proteins:
sp|P62274|RS29_MOUSE
ENSMUST00000037023_chr12:69157813−69159058:−
ENSMUST00000071737_chr13:73988963−73989133:−
tr|D3YYL7|D3YYL7_MOUSE

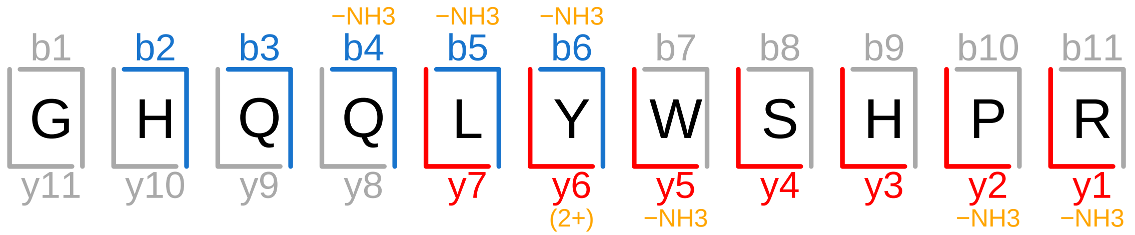

GHQQLYWSHPR
Score: 104 ; 1407.6796 m/z; 470.23382 m/z; NaN ppm; MSMS

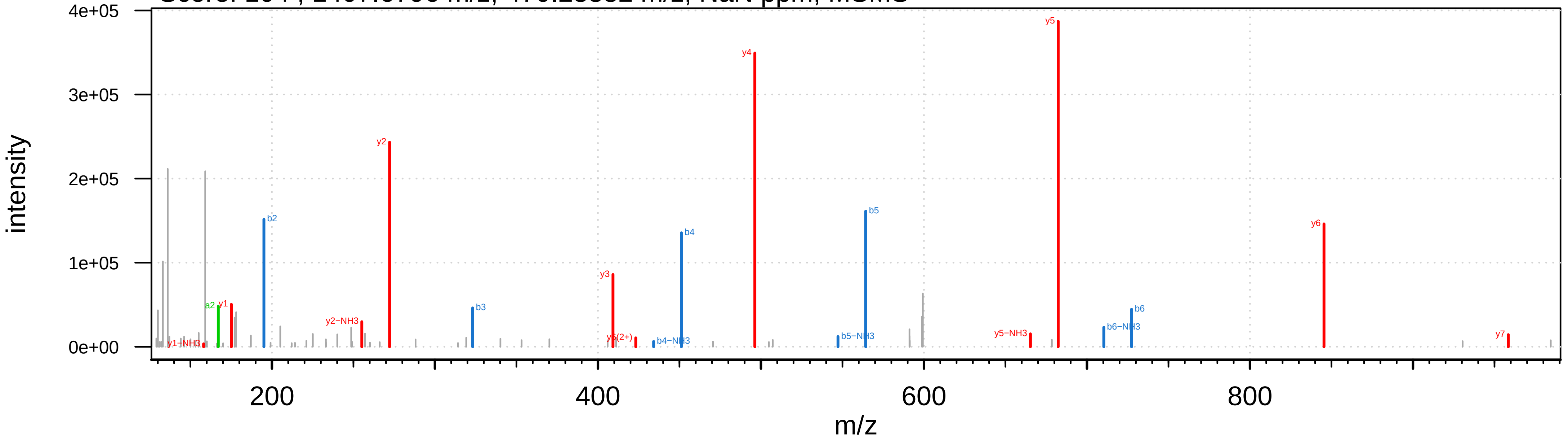

Raw File: Kermit_20111110_MSU_blast_3h_12
Scan Number: 56449
Proteins:
sp|P62274|RS29_MOUSE
ENSMUST00000037023_chr12:69157813−69159058:−
ENSMUST00000071737_chr13:73988963−73989133:−
tr|D3YYL7|D3YYL7_MOUSE

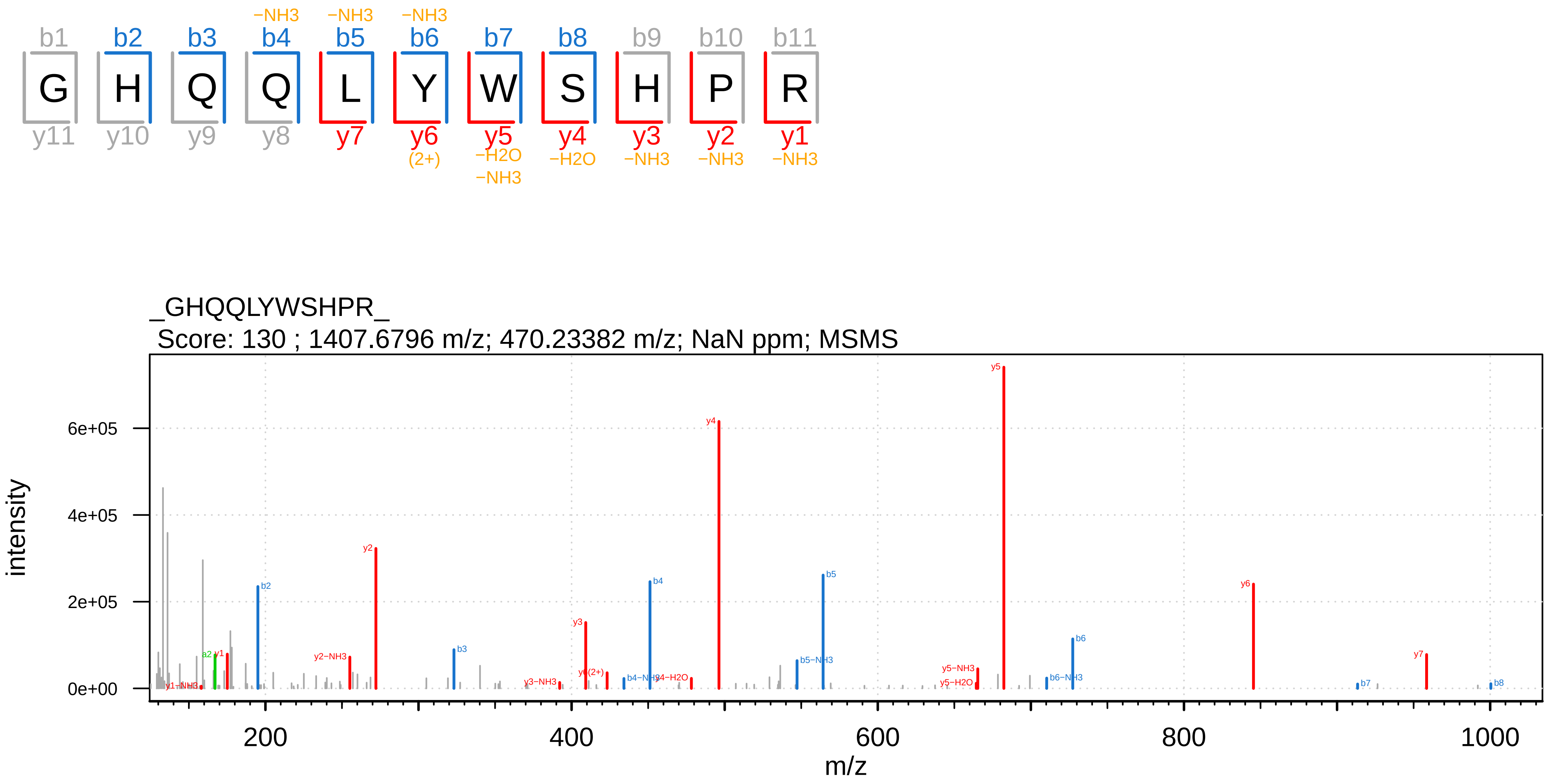

Raw File: Kermit_20111110_MSU_blast_3h_12
Scan Number: 56925
Proteins:
sp|P62274|RS29_MOUSE
ENSMUST00000037023_chr12:69157813−69159058:−
ENSMUST00000071737_chr13:73988963−73989133:−
tr|D3YYL7|D3YYL7_MOUSE

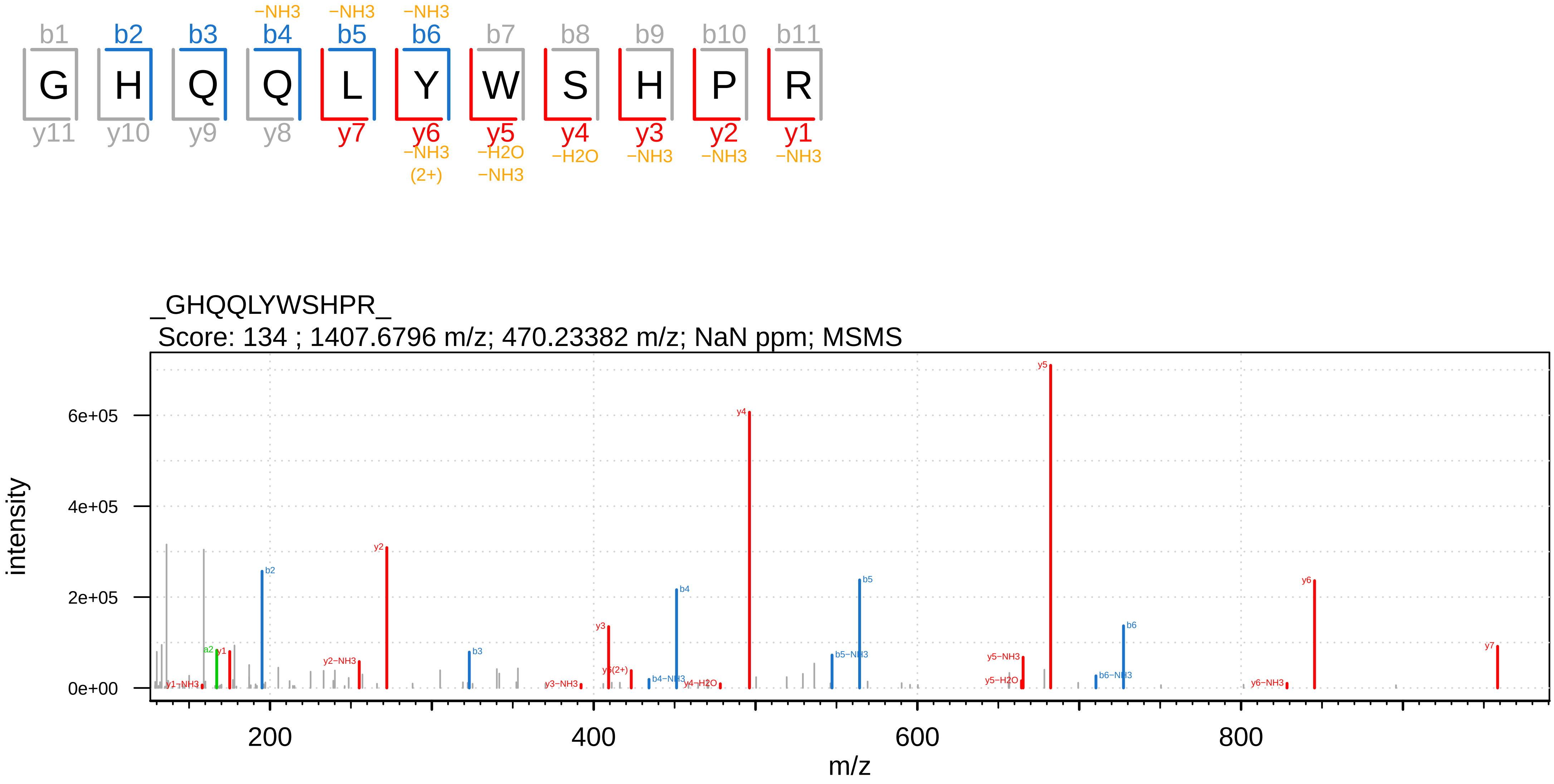

Raw File: Kermit_20111110_MSU_blast_3h_12
Scan Number: 57152
Proteins:
sp|P62274|RS29_MOUSE
ENSMUST00000037023_chr12:69157813−69159058:−
ENSMUST00000071737_chr13:73988963−73989133:−
tr|D3YYL7|D3YYL7_MOUSE

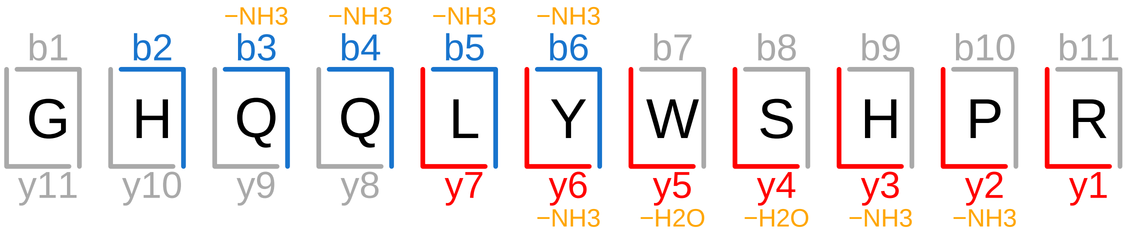

GHQQLYWSHPR
Score: 107 ; 1407.6796 m/z; 470.23382 m/z; NaN ppm; MSMS

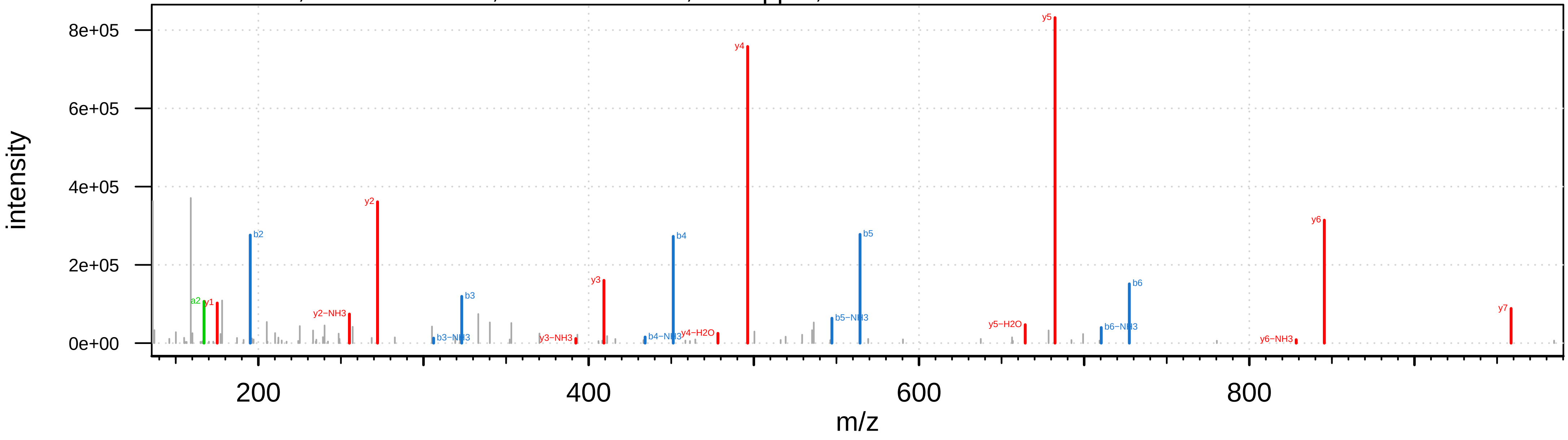

Raw File: Kermit_20111110_MSU_blast_3h_12
Scan Number: 57367
Proteins:
sp|P62274|RS29_MOUSE
ENSMUST00000037023_chr12:69157813−69159058:−
ENSMUST00000071737_chr13:73988963−73989133:−
tr|D3YYL7|D3YYL7_MOUSE

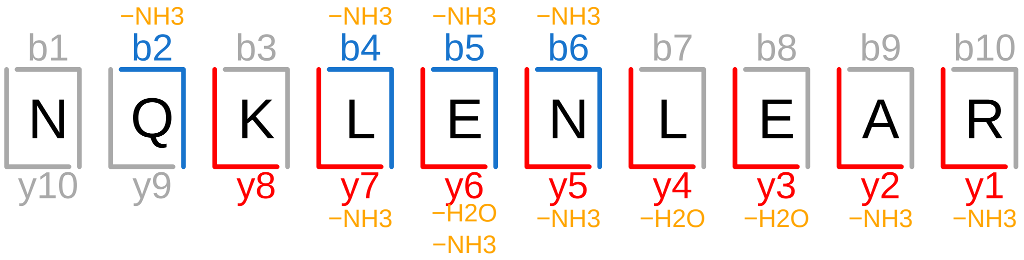

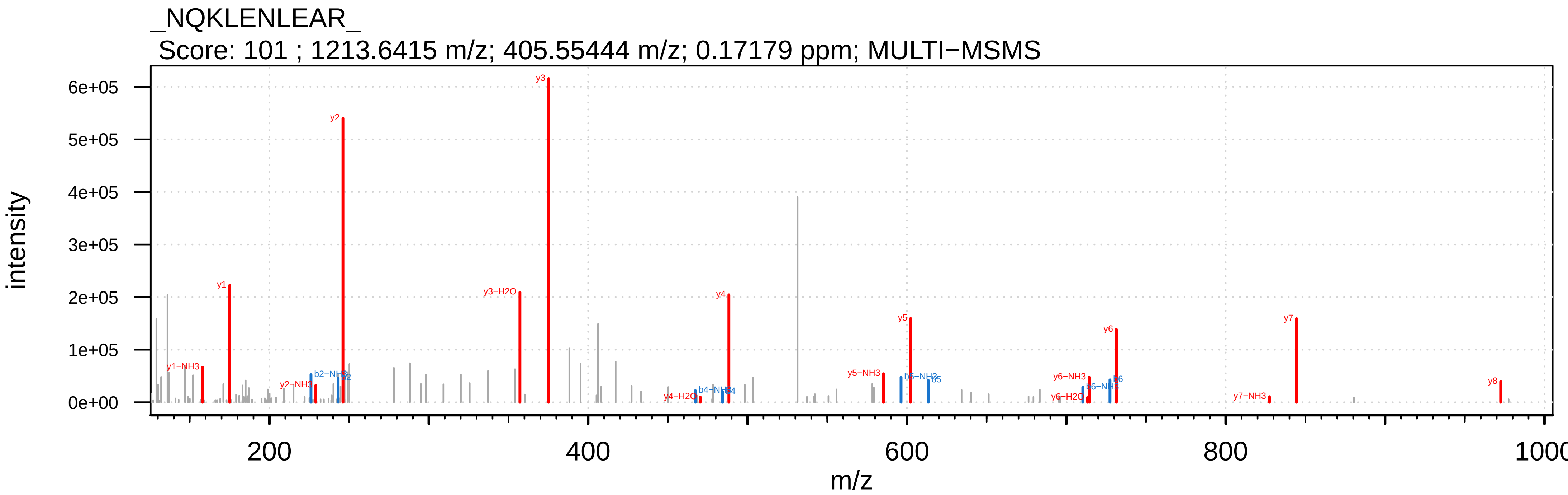

Raw File: Kermit_20111110_MSU_blast_3h_12
Scan Number: 3927
Proteins:
ENSMUST00000023048_chr15:80235835−80236047:+

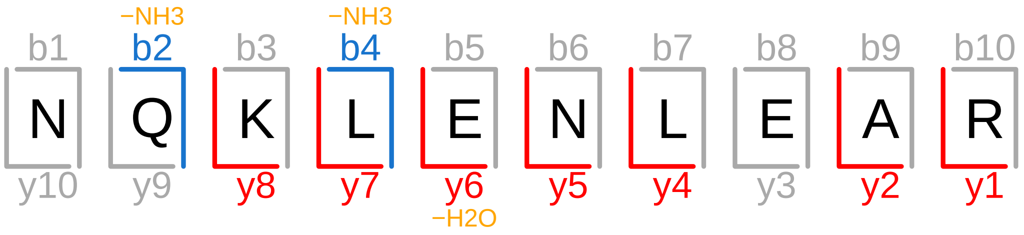

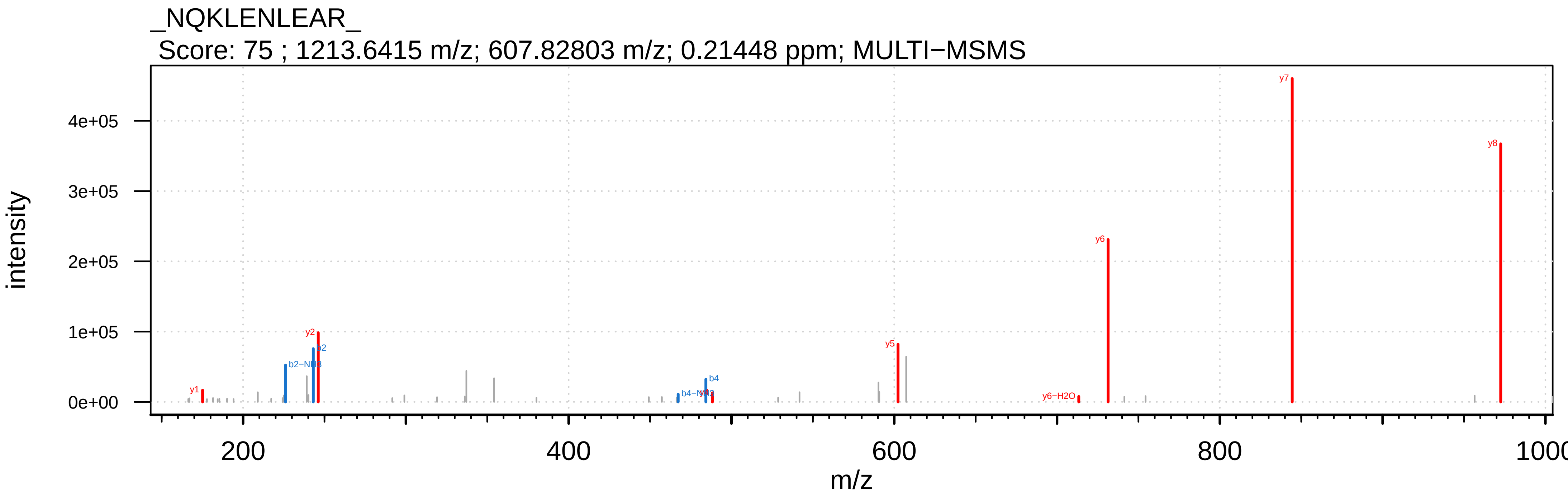

Raw File: Kermit_20111110_MSU_blast_3h_12
Scan Number: 3929
Proteins:
ENSMUST00000023048_chr15:80235835−80236047:+

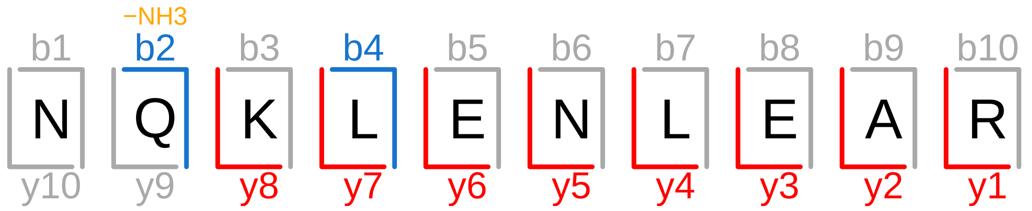

NQKLENLEAR
Score: 62 ; 1231.664 m/z; 616.83926 m/z; 0.21448 ppm; MULTI−MSMS

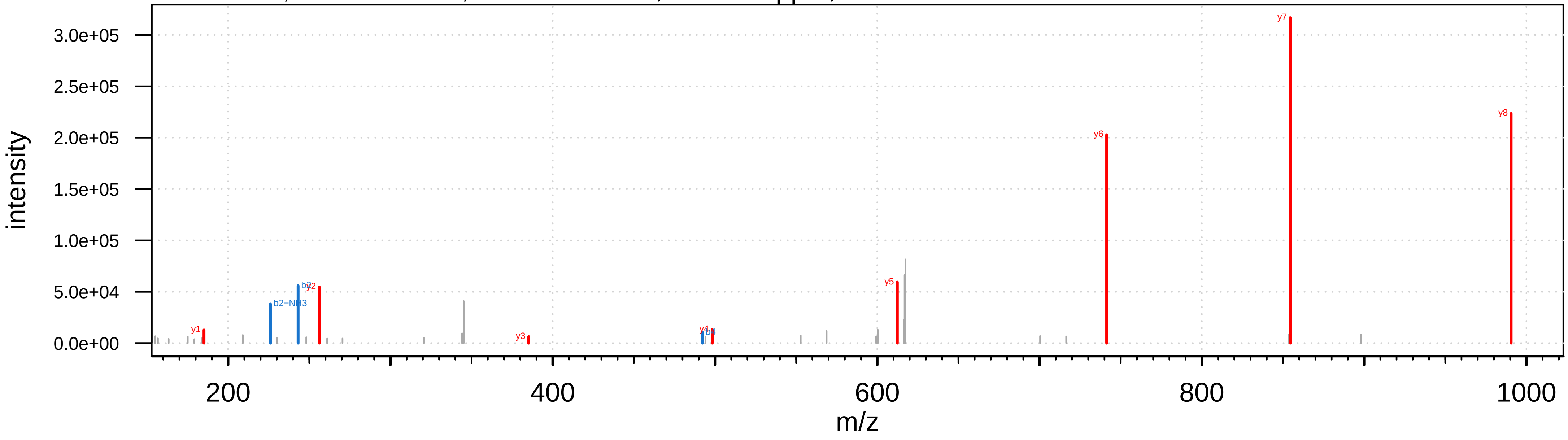

Raw File: Kermit_20111110_MSU_blast_3h_12
Scan Number: 3960
Proteins:
ENSMUST00000023048_chr15:80235835−80236047:+

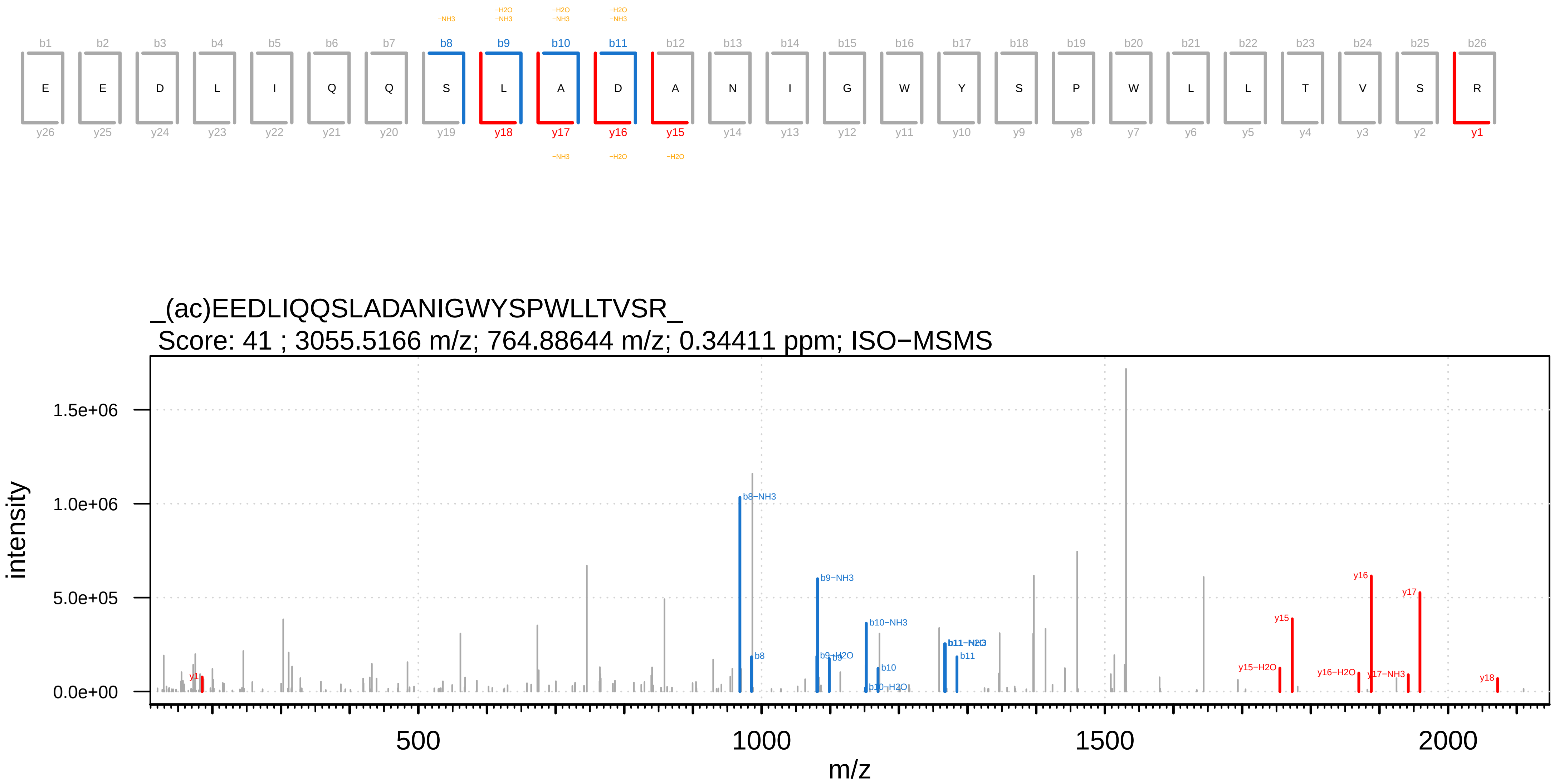

Raw File: Kermit_20111110_MSU_diff1_02
Scan Number: 23273
Proteins:
ENSMUST00000098653_chr8:70932294−70932404:+

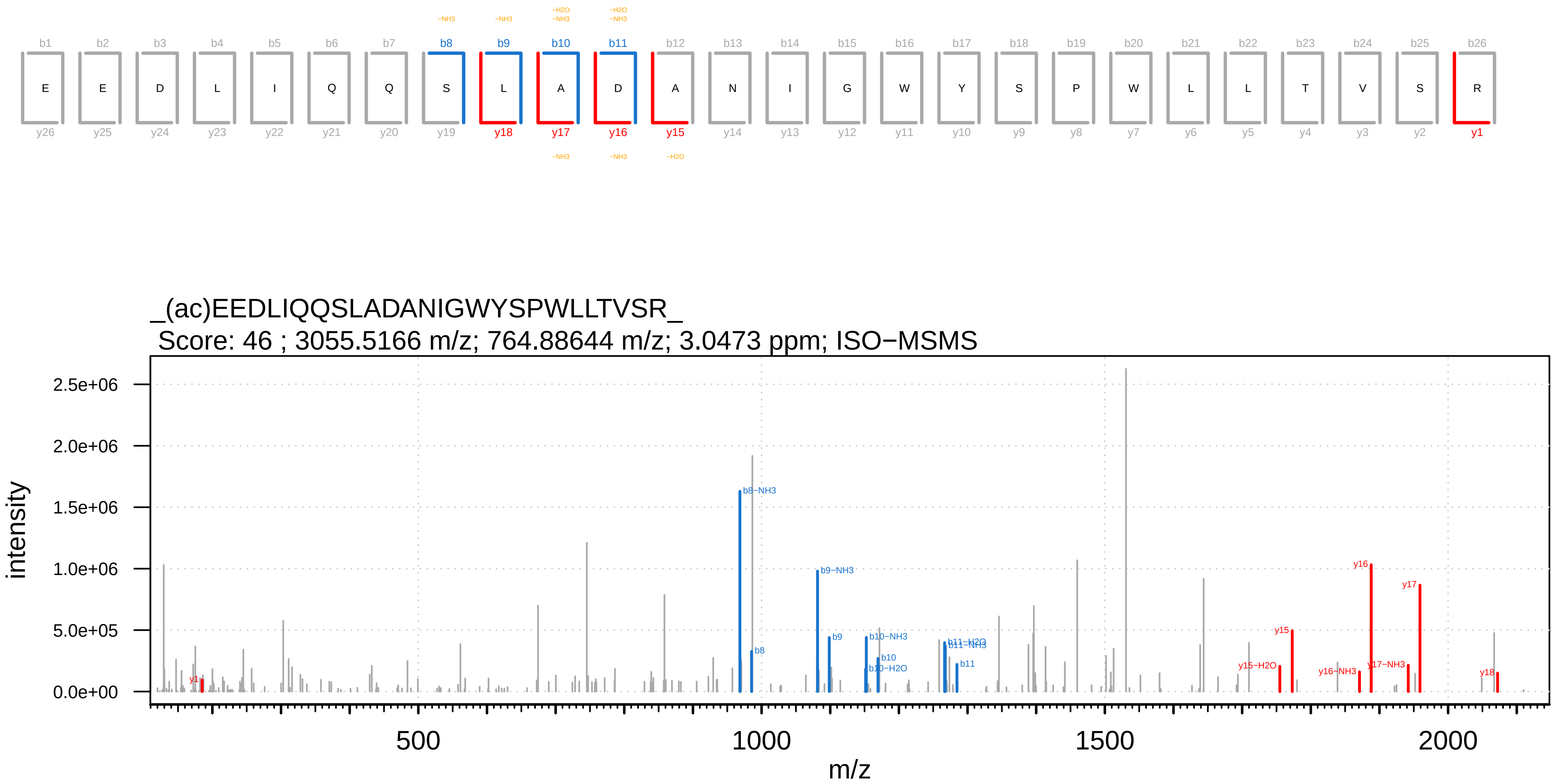

Raw File: Kermit_20111110_MSU_diff2_02
Scan Number: 27080
Proteins:
ENSMUST00000098653_chr8:70932294−70932404:+

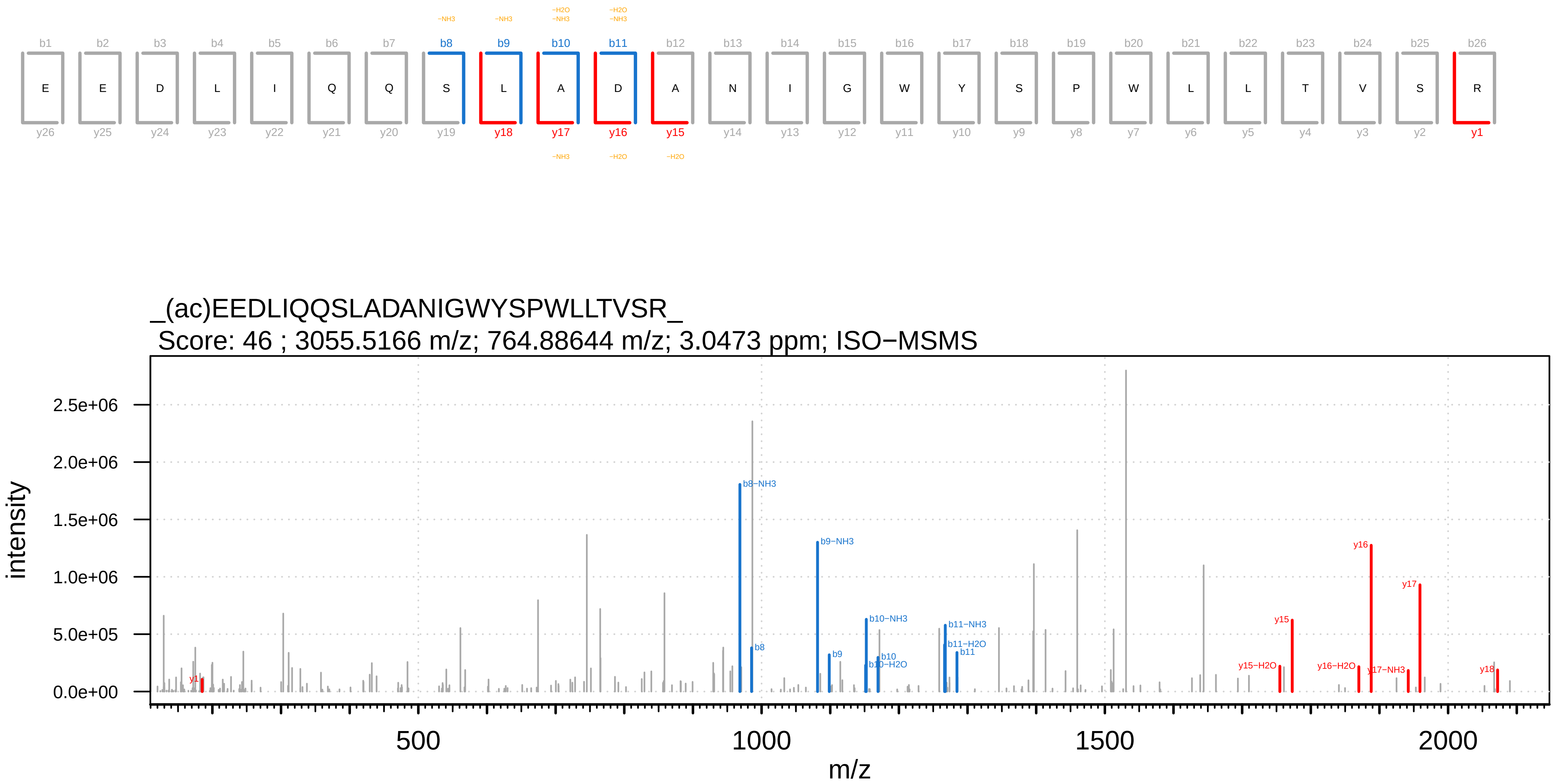

Raw File: Kermit_20111110_MSU_diff2_02
Scan Number: 27319
Proteins:
ENSMUST00000098653_chr8:70932294−70932404:+

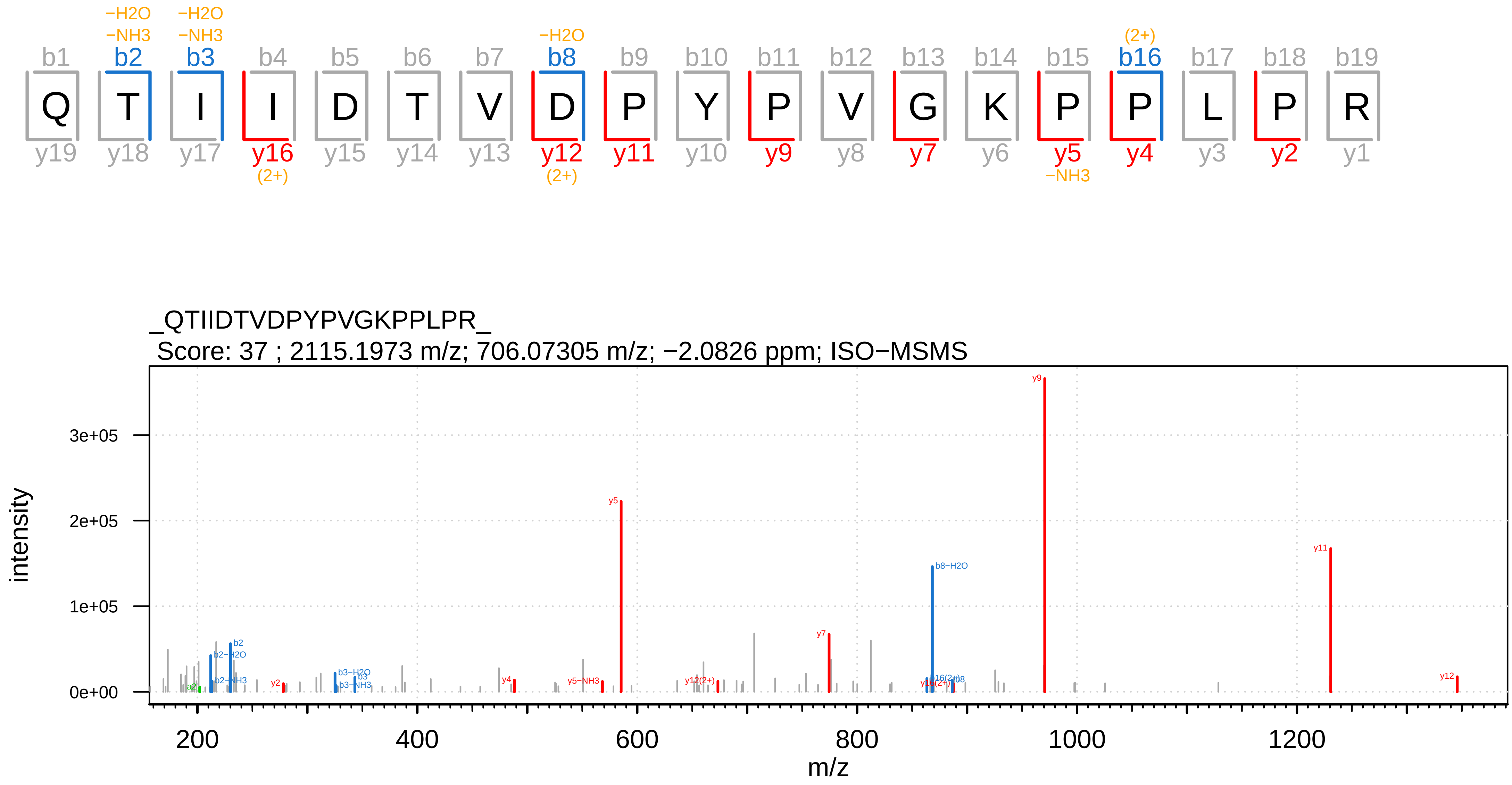

Raw File: Kermit_20111110_MSU_diff2_02
Scan Number: 52984
Proteins:
ENSMUST00000136126_chr3:79172848−79174982:−

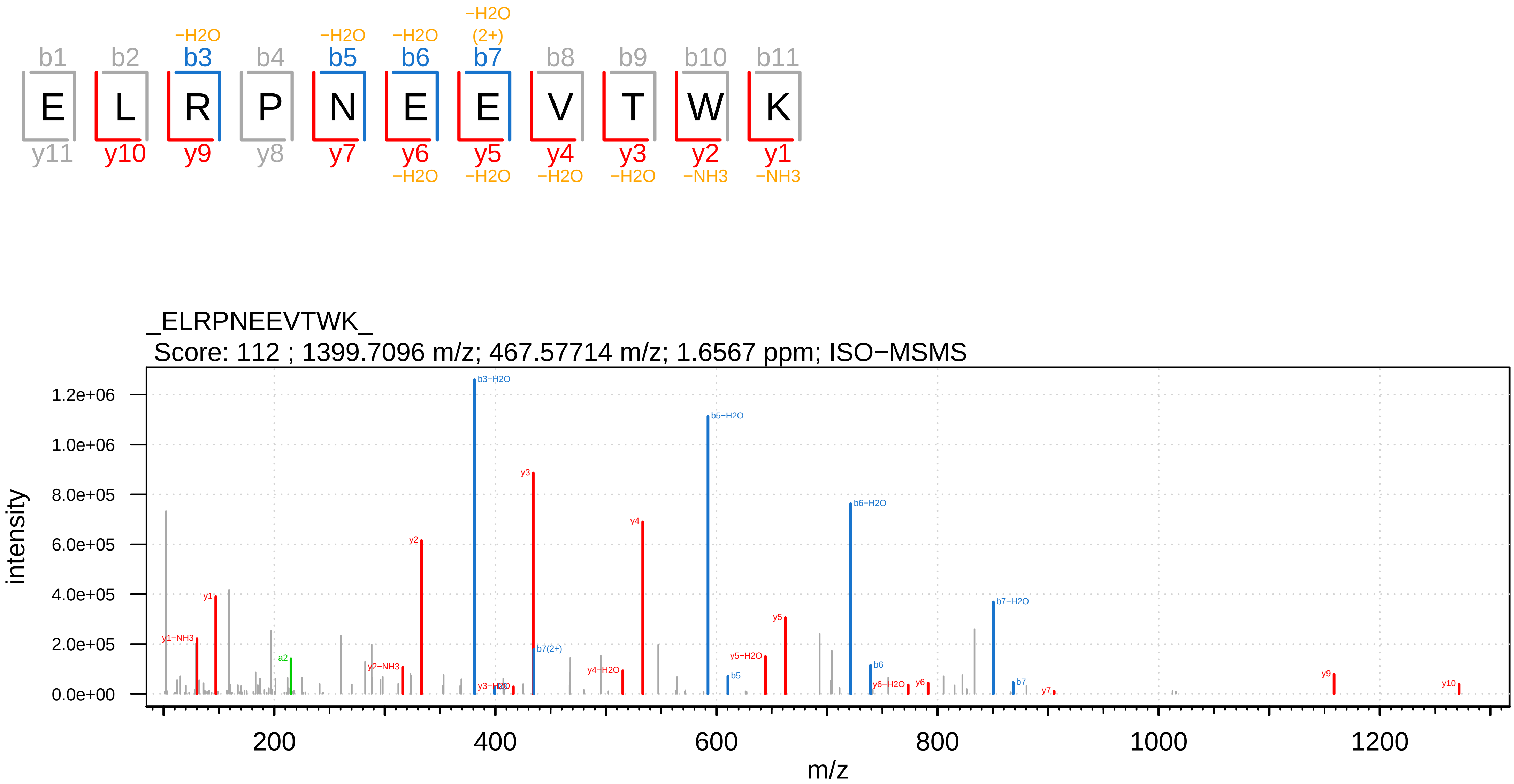

Raw File: Kermit_20111028_MSU_tube_54h_12

Scan Number: 19188

Proteins:

chr12_103206313−103214181.T2_chr12:101968174−101969629:−

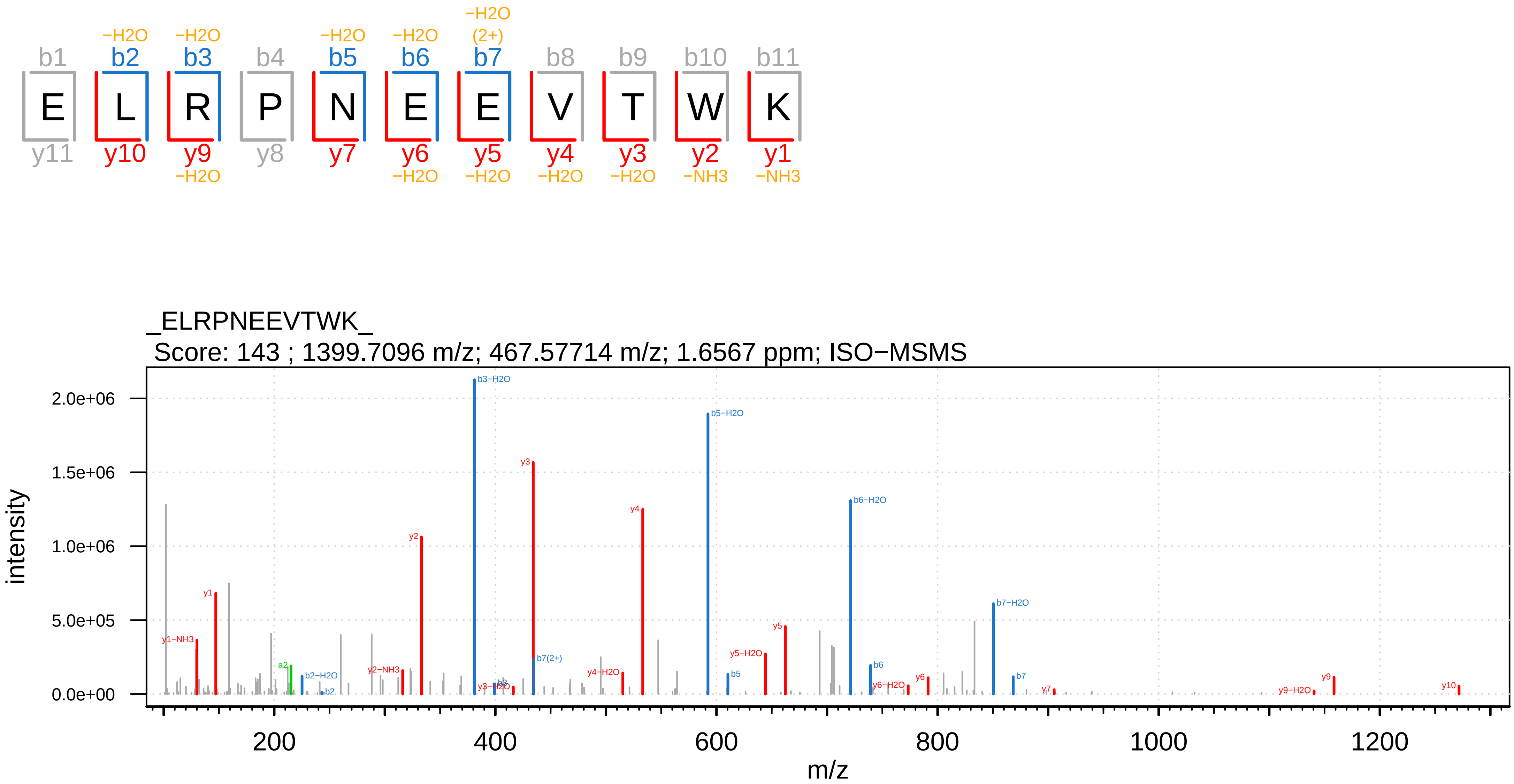

Raw File: Kermit_20111028_MSU_tube_54h_12

Scan Number: 19377

Proteins:

chr12_103206313−103214181.T2_chr12:101968174−101969629:−

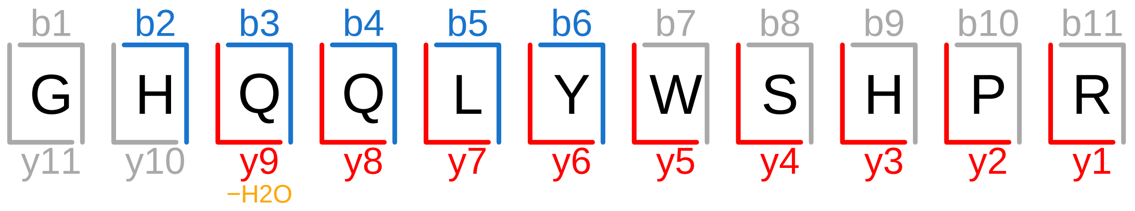

GHQQLYWSHPR

Score: 83 ; 1407.6796 m/z; 704.84708 m/z; 1.4249 ppm; MULTI−MSMS

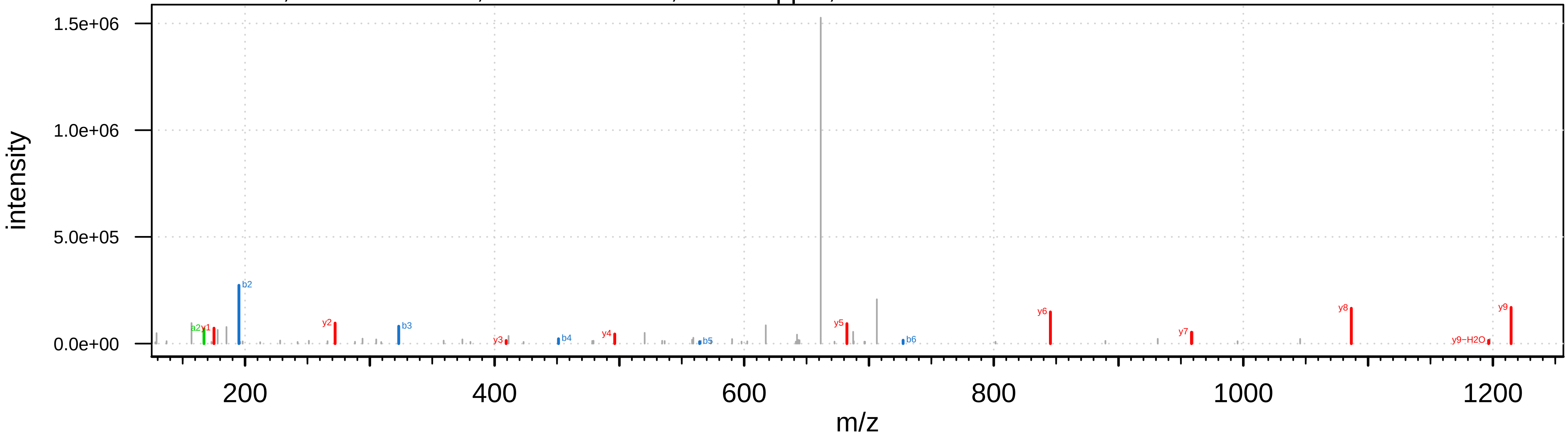

Raw File: Kermit_20111028_MSU_tube_54h_12
Scan Number: 11580
Proteins:
sp|P62274|RS29_MOUSE
ENSMUST00000037023_chr12:69157813−69159058:−
ENSMUST00000071737_chr13:73988963−73989133:−
tr|D3YYL7|D3YYL7_MOUSE

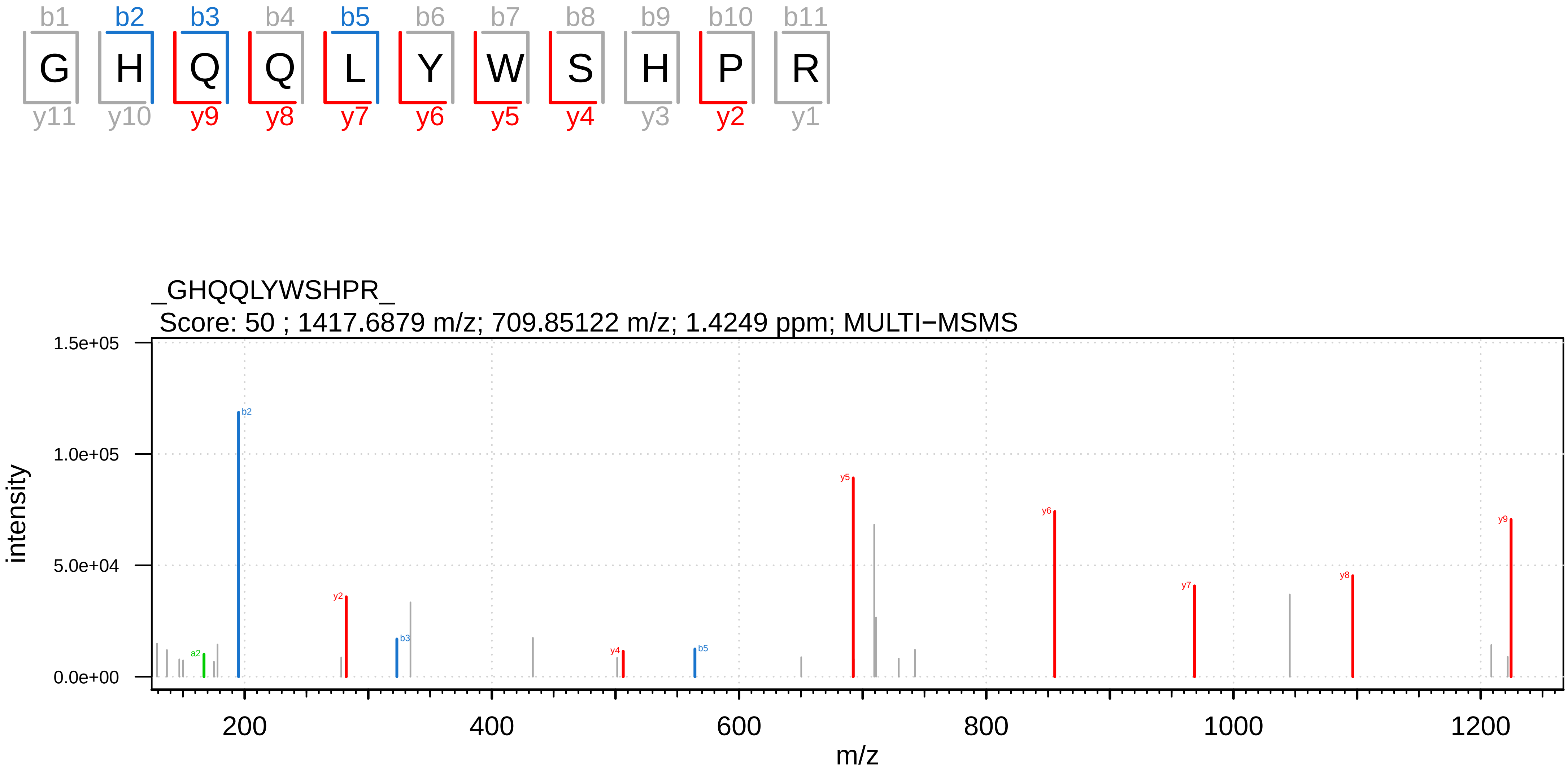

Raw File: Kermit_20111028_MSU_tube_54h_12
Scan Number: 11595
Proteins:
sp|P62274|RS29_MOUSE
ENSMUST00000037023_chr12:69157813−69159058:−
ENSMUST00000071737_chr13:73988963−73989133:−
tr|D3YYL7|D3YYL7_MOUSE

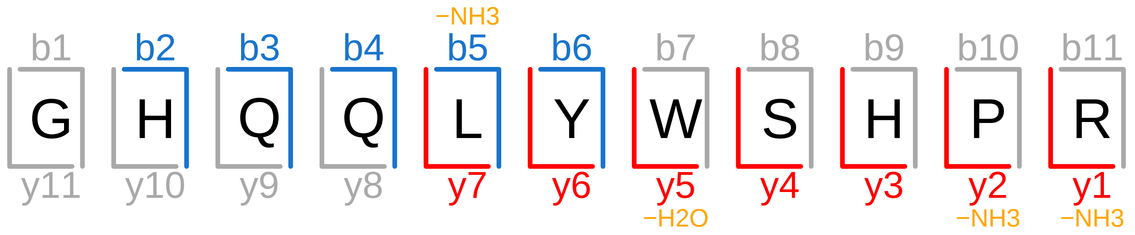

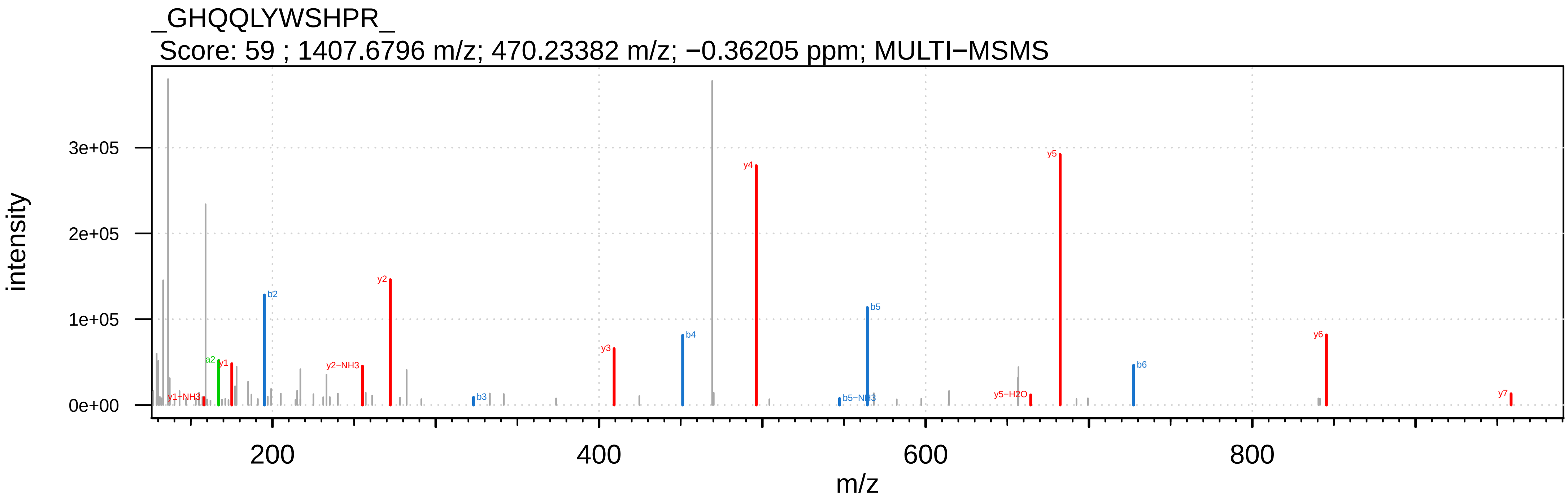

Raw File: Kermit_20111028_MSU_tube_54h_12
Scan Number: 49854
Proteins:
sp|P62274|RS29_MOUSE
ENSMUST00000037023_chr12:69157813−69159058:−
ENSMUST00000071737_chr13:73988963−73989133:−
tr|D3YYL7|D3YYL7_MOUSE

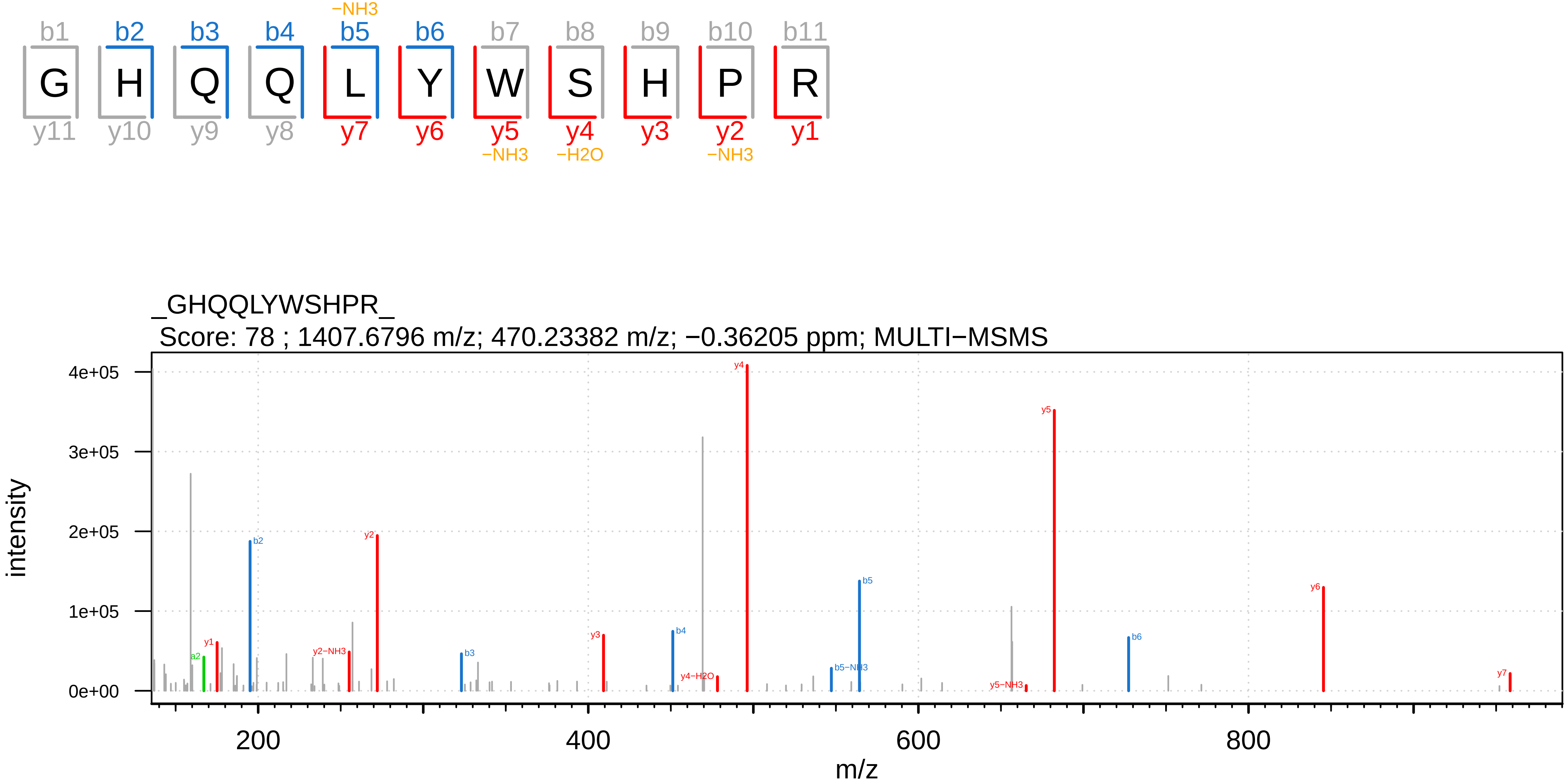

Raw File: Kermit_20111028_MSU_tube_54h_12
Scan Number: 50042
Proteins:
sp|P62274|RS29_MOUSE
ENSMUST00000037023_chr12:69157813−69159058:−
ENSMUST00000071737_chr13:73988963−73989133:−
tr|D3YYL7|D3YYL7_MOUSE

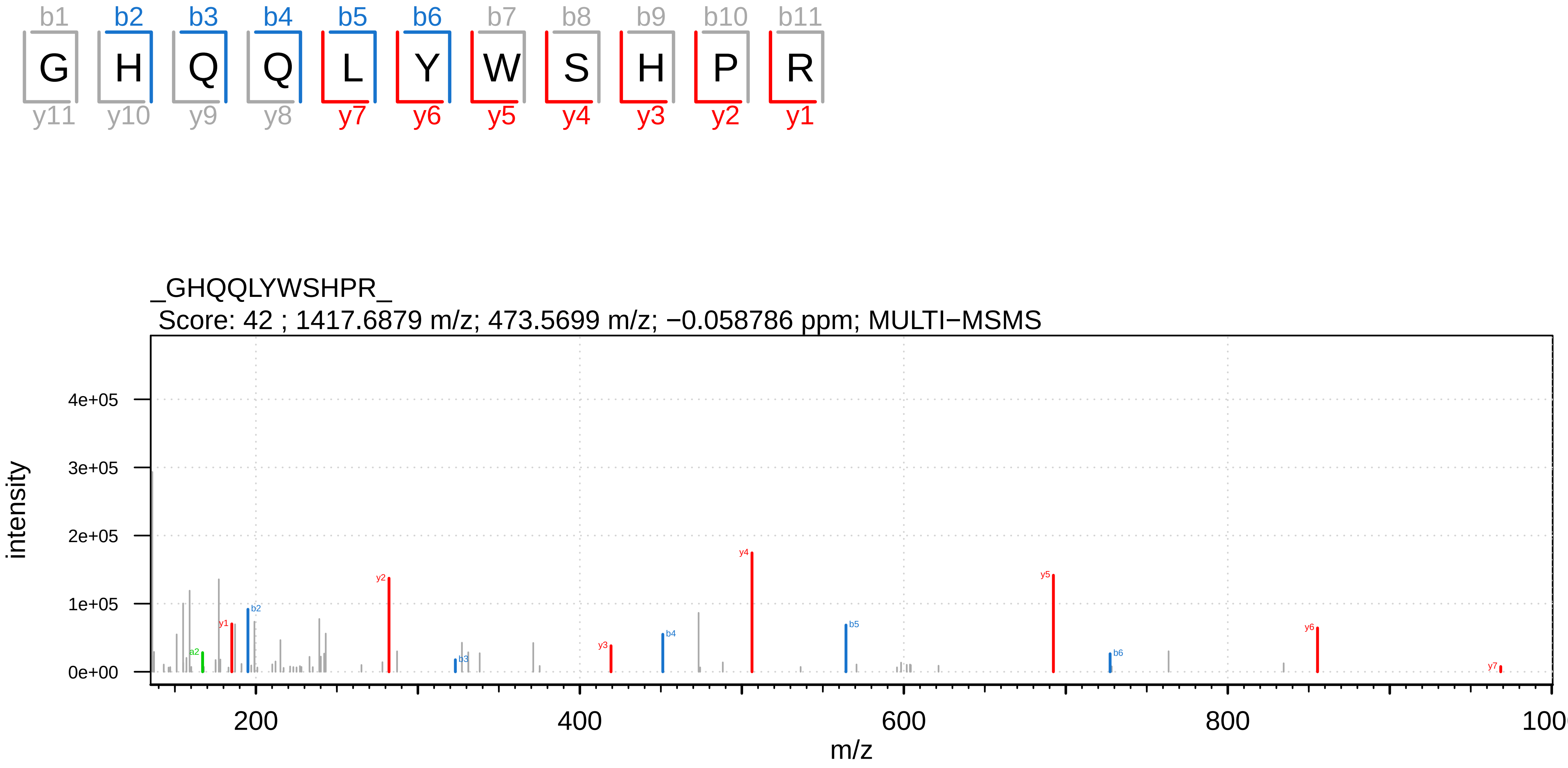

Raw File: Kermit_20111028_MSU_tube_54h_12
Scan Number: 50107
Proteins:
sp|P62274|RS29_MOUSE
ENSMUST00000037023_chr12:69157813−69159058:−
ENSMUST00000071737_chr13:73988963−73989133:−
tr|D3YYL7|D3YYL7_MOUSE

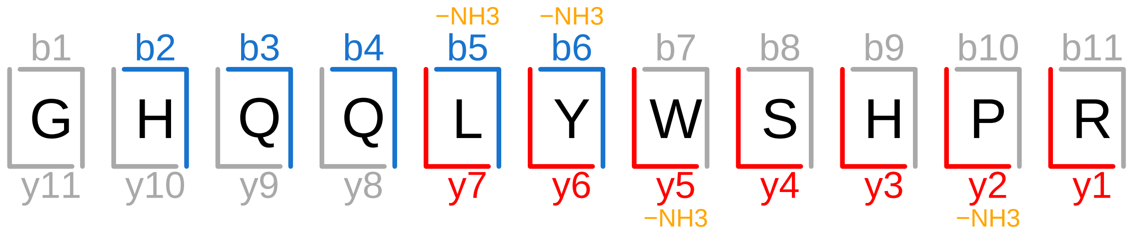

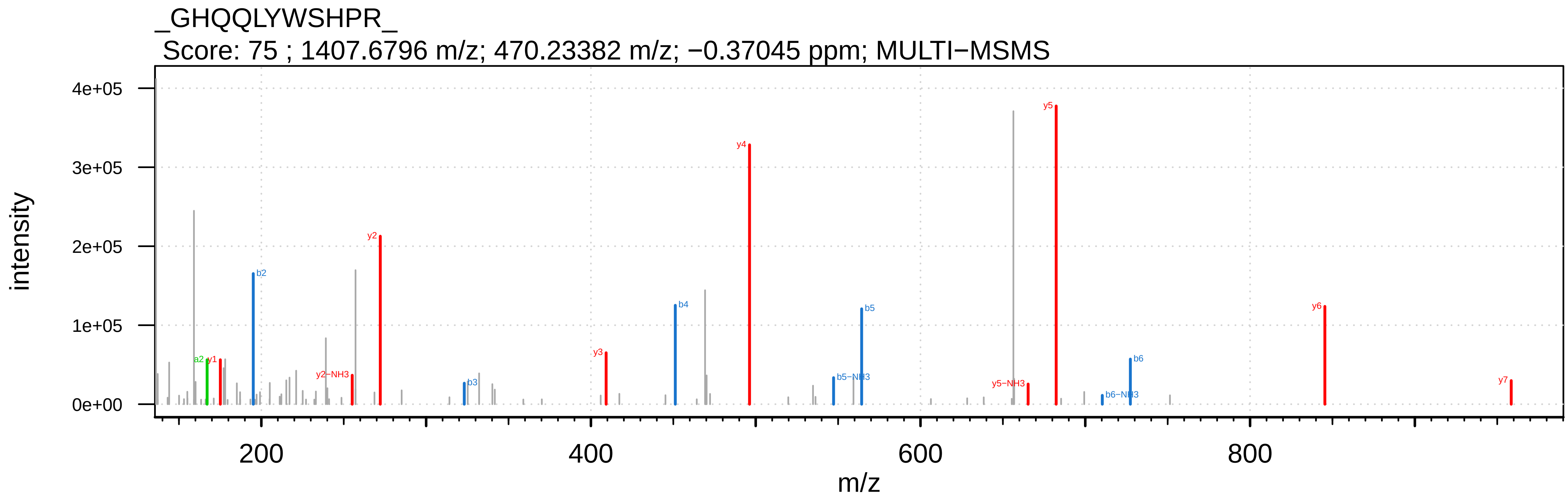

Raw File: Kermit_20111028_MSU_tube_54h_12
Scan Number: 50228
Proteins:
sp|P62274|RS29_MOUSE
ENSMUST00000037023_chr12:69157813−69159058:−
ENSMUST00000071737_chr13:73988963−73989133:−
tr|D3YYL7|D3YYL7_MOUSE

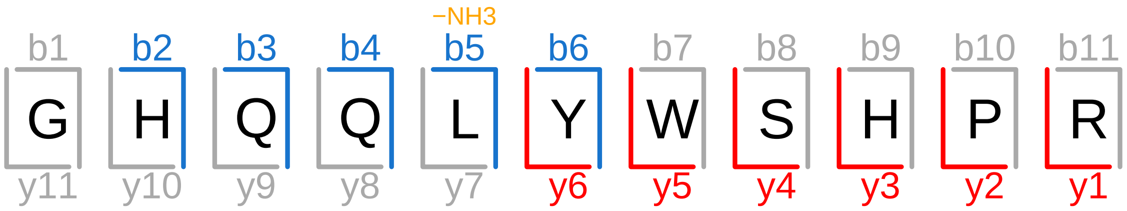

GHQQLYWSHPR
Score: 44 ; 1417.6879 m/z; 473.5699 m/z; −0.37045 ppm; MULTI−MSMS

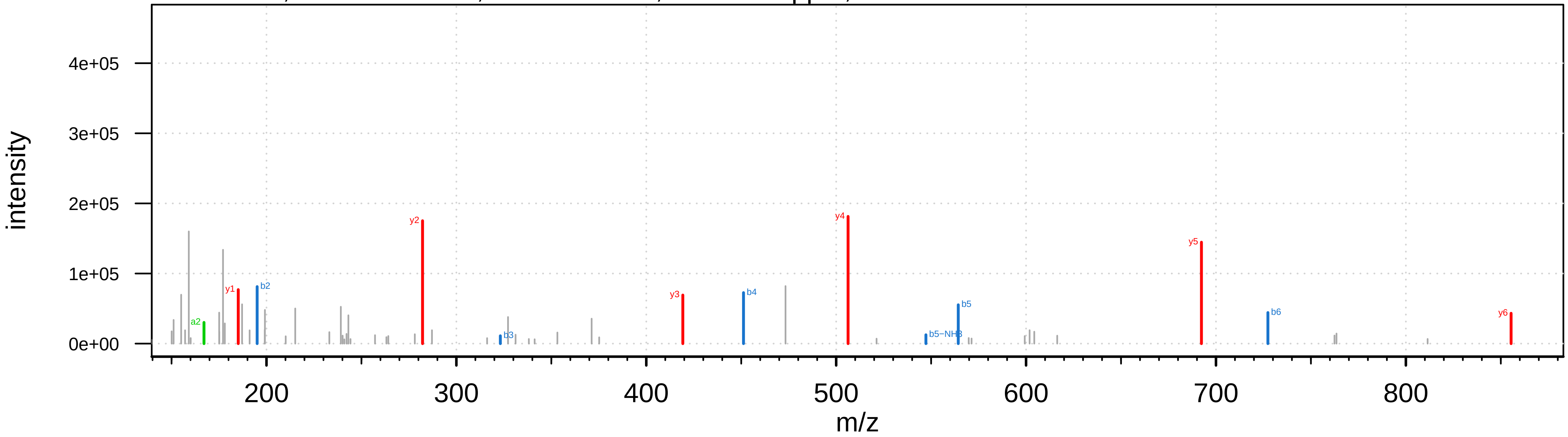

Raw File: Kermit_20111028_MSU_tube_54h_12
Scan Number: 50292
Proteins:
sp|P62274|RS29_MOUSE
ENSMUST00000037023_chr12:69157813−69159058:−
ENSMUST00000071737_chr13:73988963−73989133:−
tr|D3YYL7|D3YYL7_MOUSE

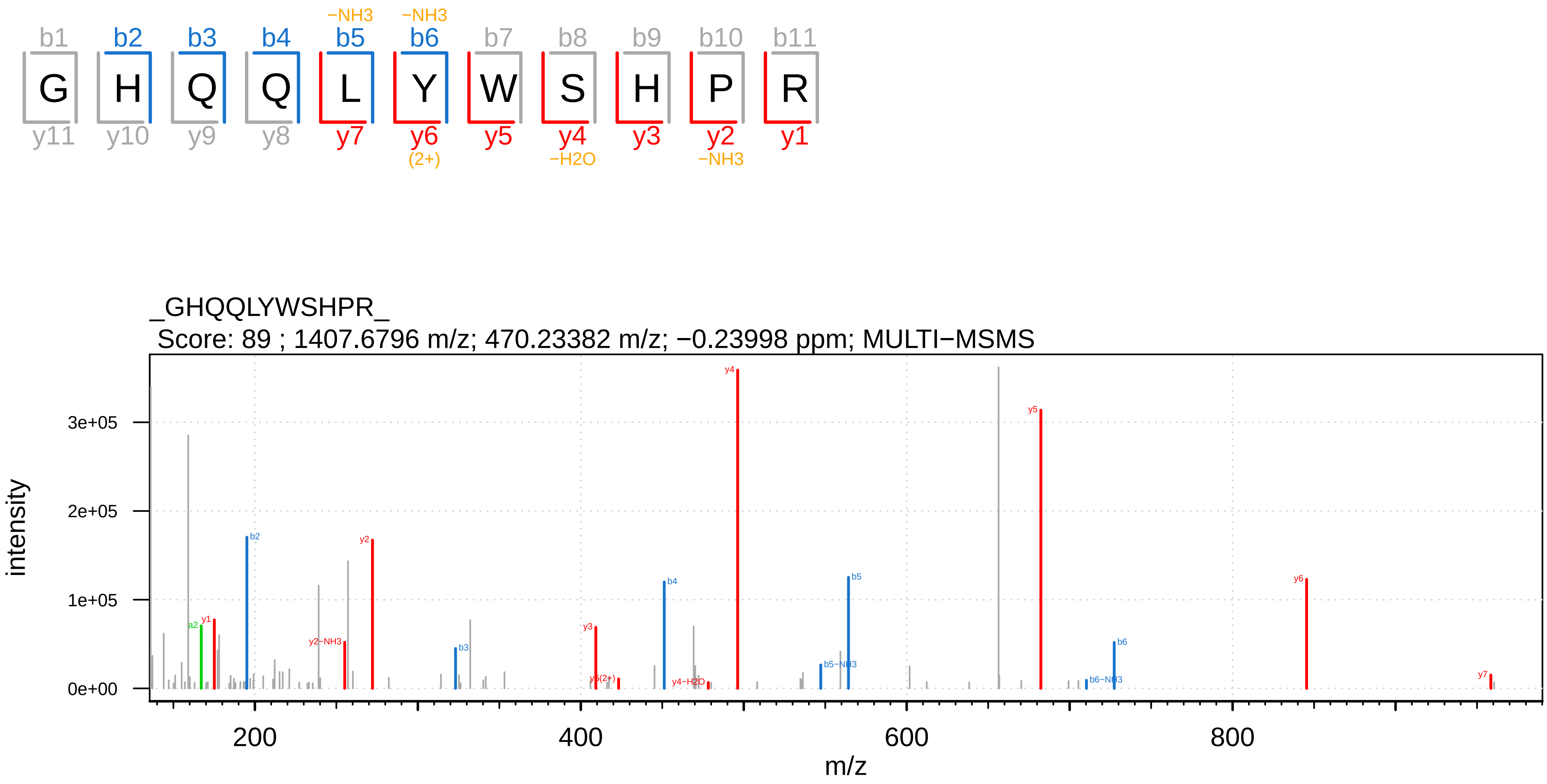

Raw File: Kermit_20111028_MSU_tube_54h_12
Scan Number: 50416
Proteins:
sp|P62274|RS29_MOUSE
ENSMUST00000037023_chr12:69157813−69159058:−
ENSMUST00000071737_chr13:73988963−73989133:−
tr|D3YYL7|D3YYL7_MOUSE

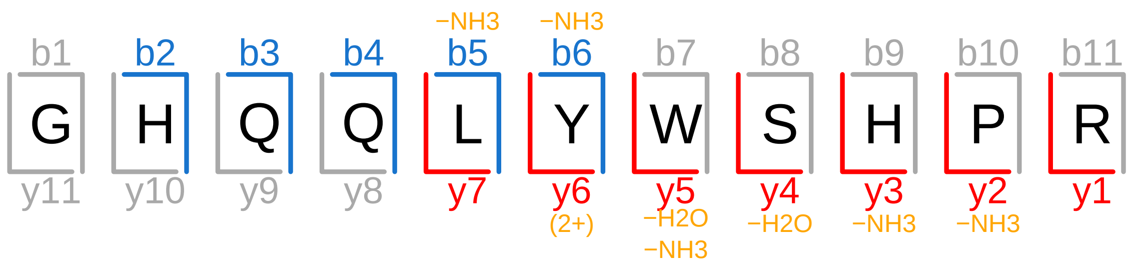

GHQQLYWSHPR
Score: 85 ; 1407.6796 m/z; 470.23382 m/z; −0.60672 ppm; MULTI−MSMS

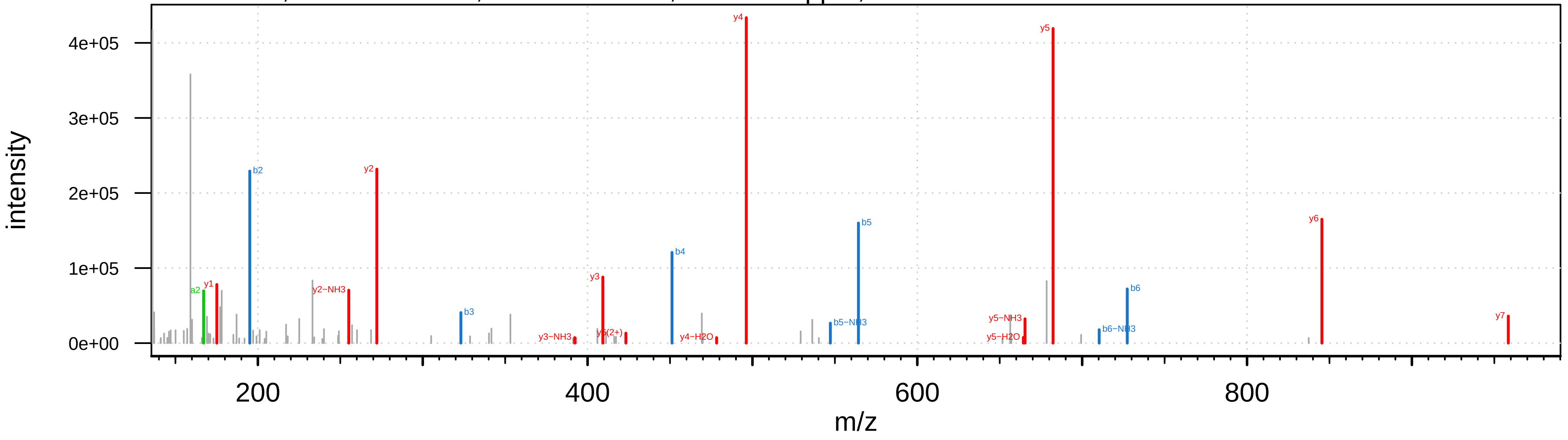

Raw File: Kermit_20111028_MSU_tube_54h_12
Scan Number: 51142
Proteins:
sp|P62274|RS29_MOUSE
ENSMUST00000037023_chr12:69157813−69159058:−
ENSMUST00000071737_chr13:73988963−73989133:−
tr|D3YYL7|D3YYL7_MOUSE

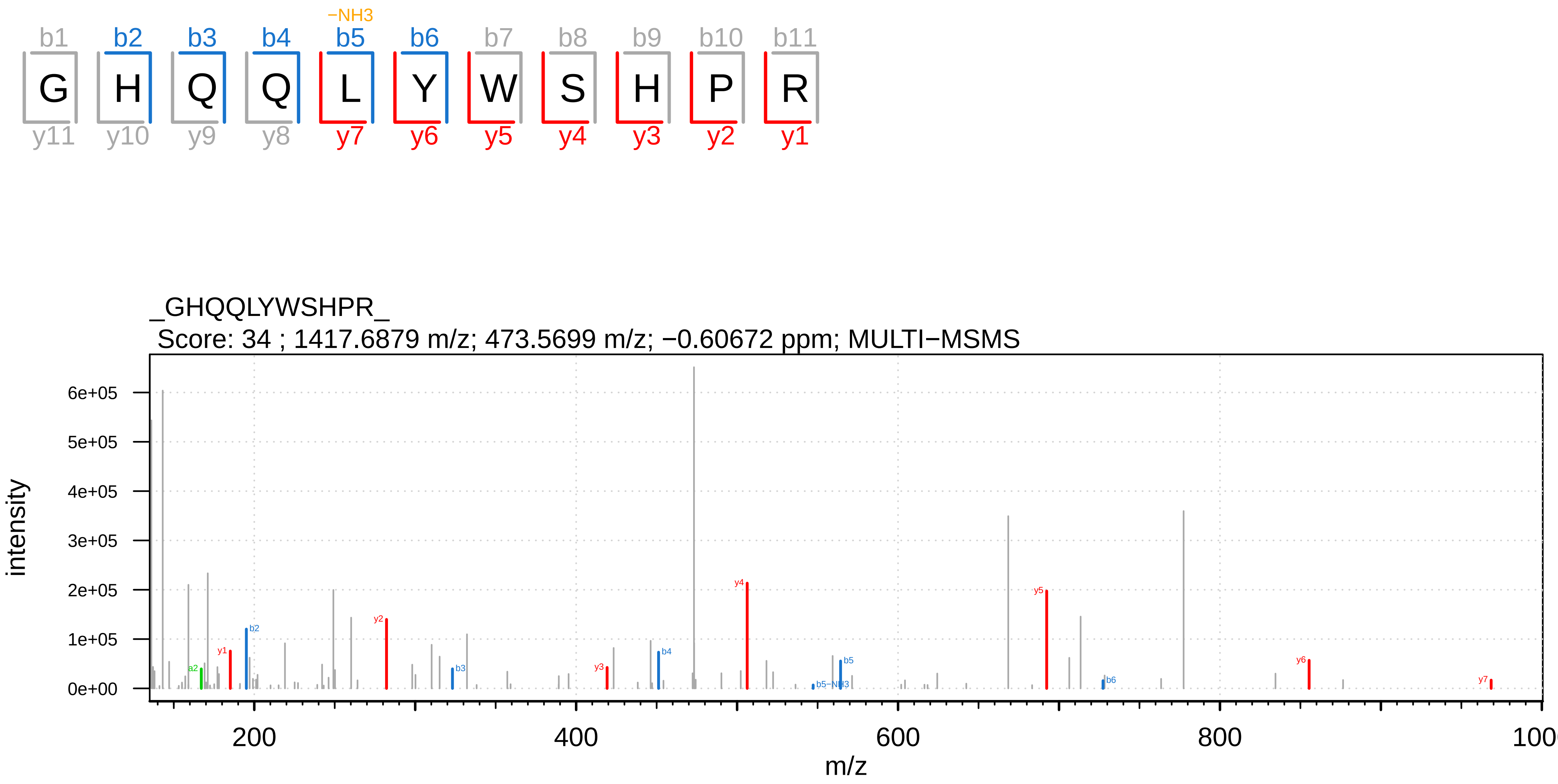

Raw File: Kermit_20111028_MSU_tube_54h_12
Scan Number: 51207
Proteins:
sp|P62274|RS29_MOUSE
ENSMUST00000037023_chr12:69157813−69159058:−
ENSMUST00000071737_chr13:73988963−73989133:−
tr|D3YYL7|D3YYL7_MOUSE

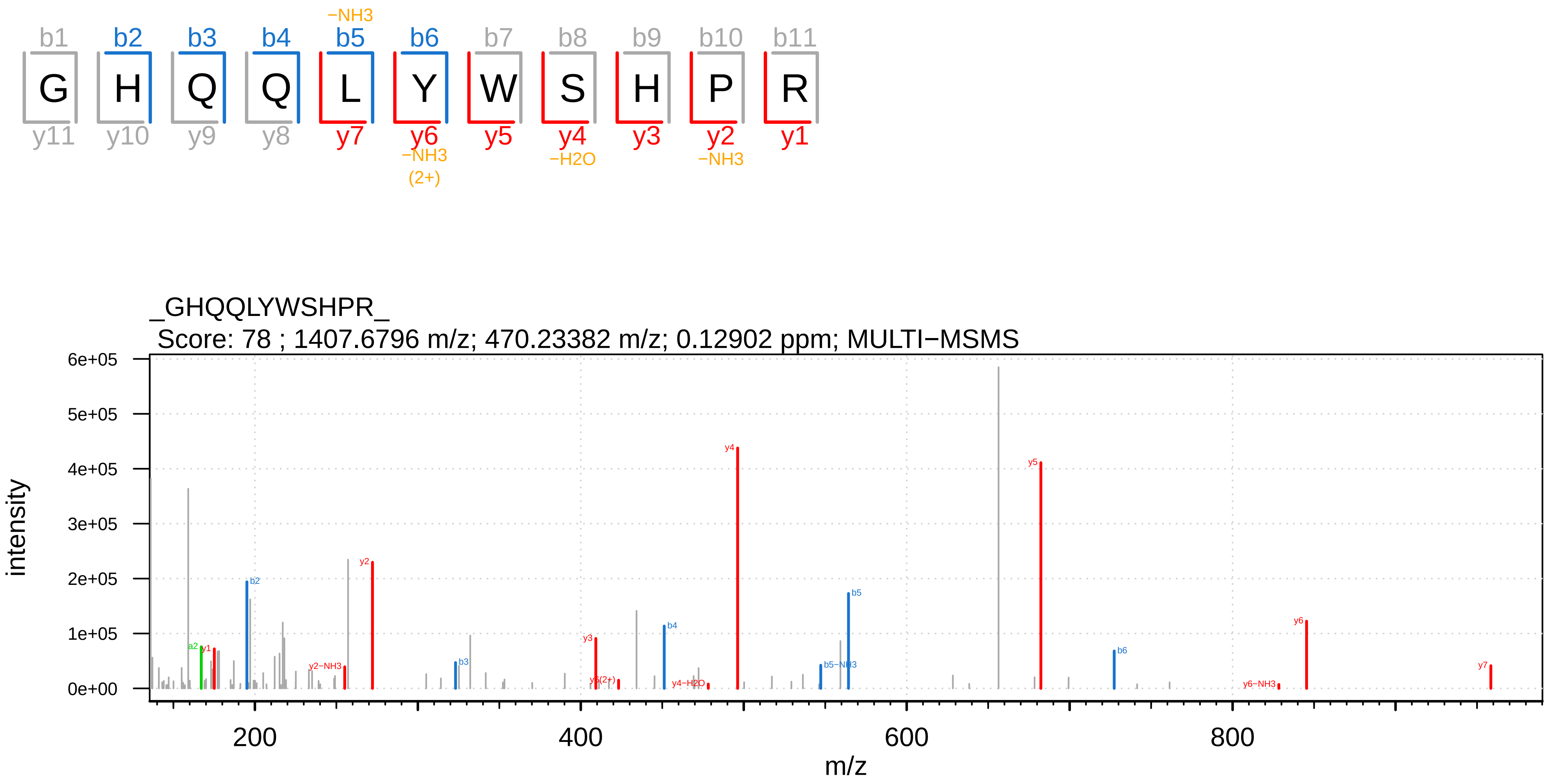

Raw File: Kermit_20111028_MSU_tube_54h_12
Scan Number: 51330
Proteins:
sp|P62274|RS29_MOUSE
ENSMUST00000037023_chr12:69157813−69159058:−
ENSMUST00000071737_chr13:73988963−73989133:−
tr|D3YYL7|D3YYL7_MOUSE

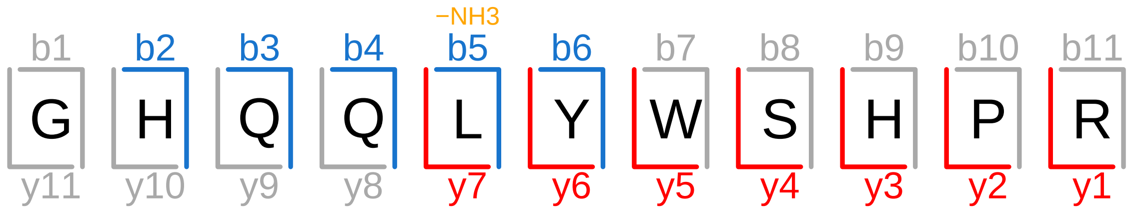

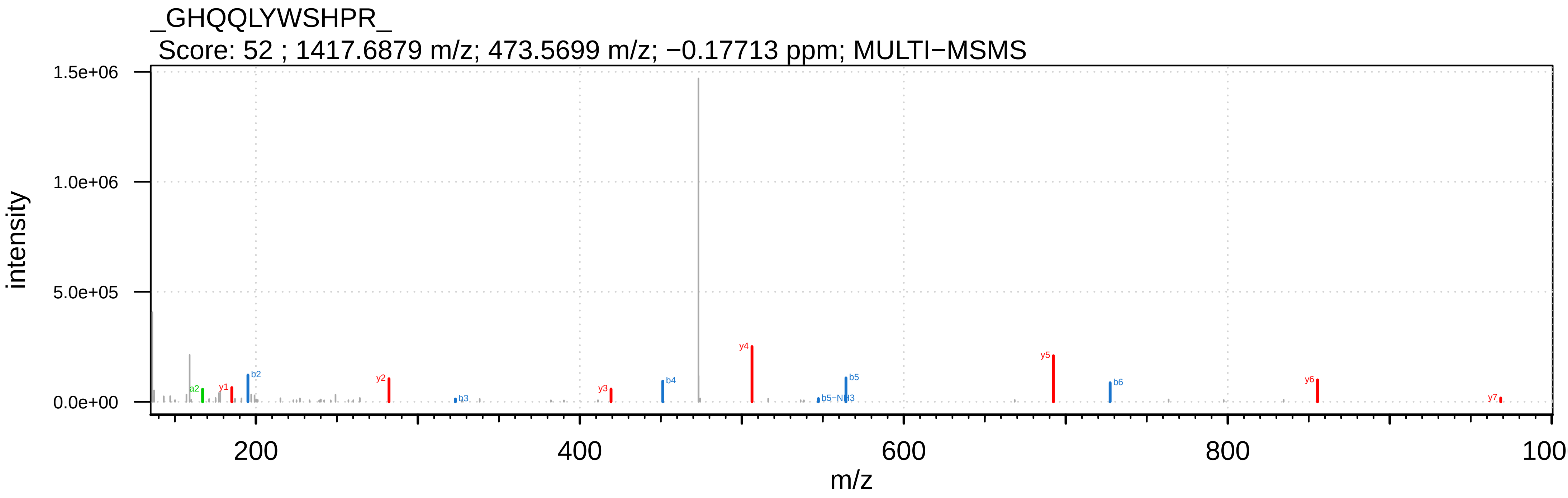

Raw File: Kermit_20111028_MSU_tube_54h_12
Scan Number: 51408
Proteins:
sp|P62274|RS29_MOUSE
ENSMUST00000037023_chr12:69157813−69159058:−
ENSMUST00000071737_chr13:73988963−73989133:−
tr|D3YYL7|D3YYL7_MOUSE

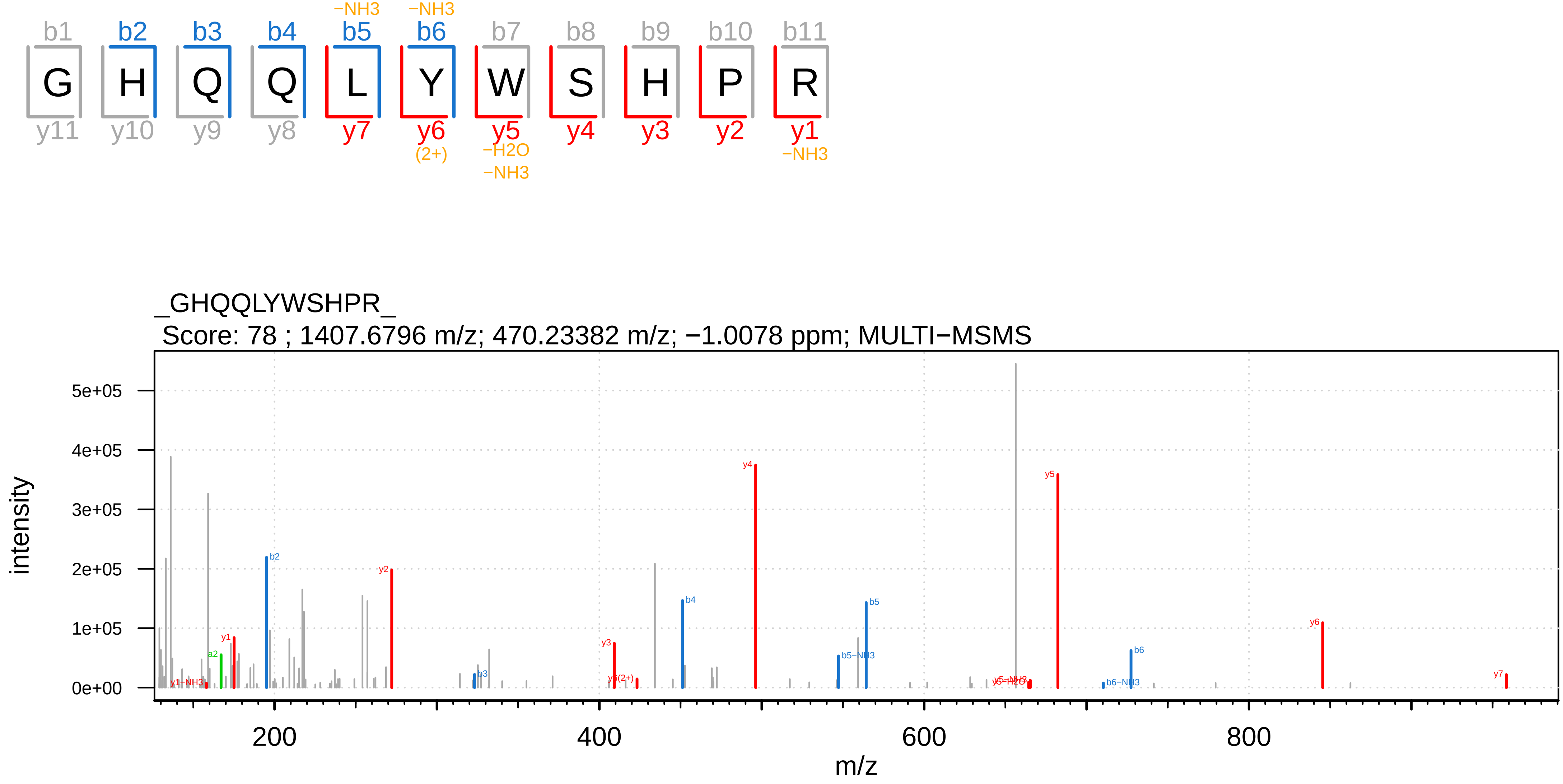

Raw File: Kermit_20111028_MSU_tube_54h_12
Scan Number: 51522
Proteins:
sp|P62274|RS29_MOUSE
ENSMUST00000037023_chr12:69157813−69159058:−
ENSMUST00000071737_chr13:73988963−73989133:−
tr|D3YYL7|D3YYL7_MOUSE

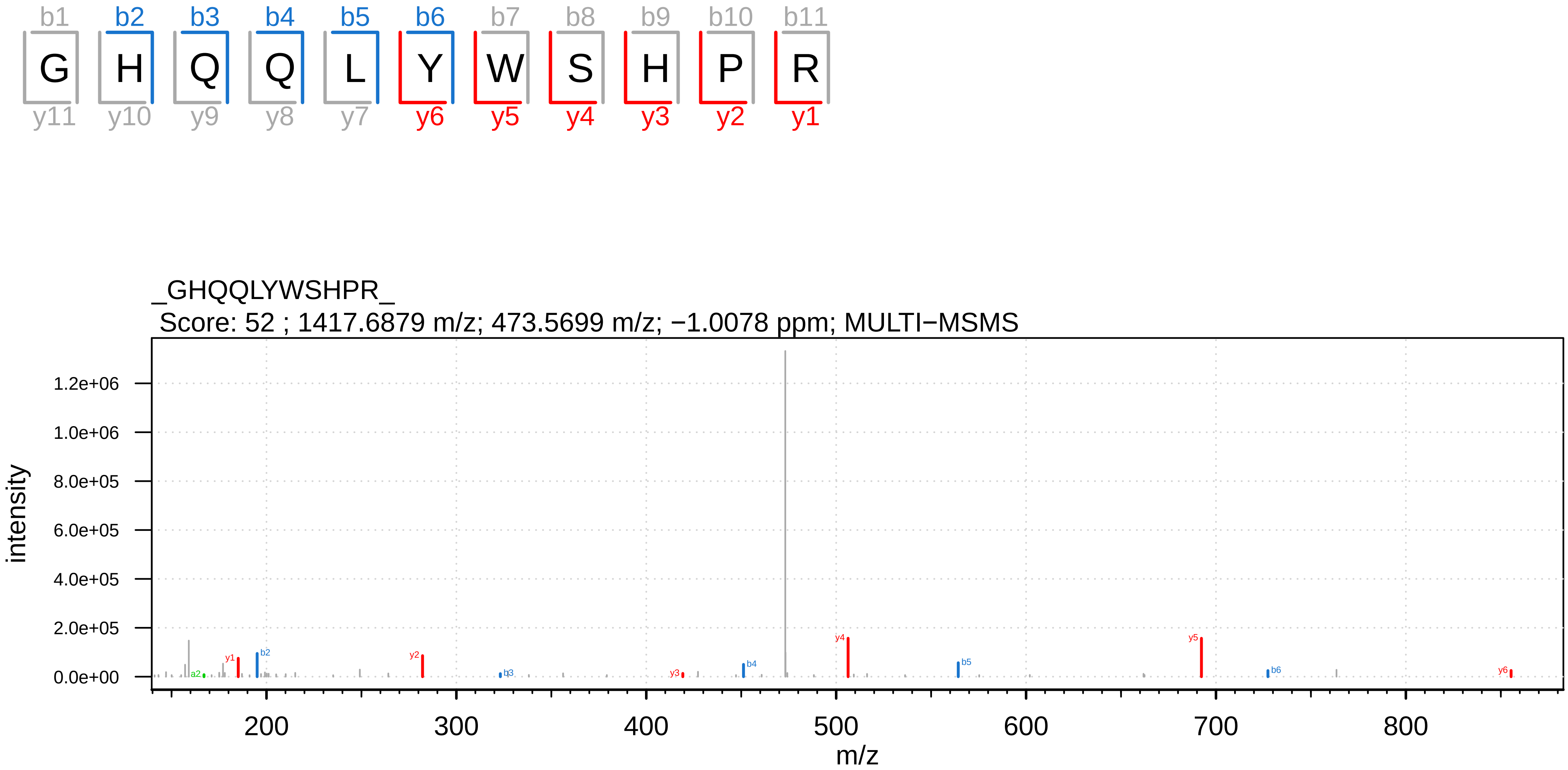

Raw File: Kermit_20111028_MSU_tube_54h_12
Scan Number: 51592
Proteins:
sp|P62274|RS29_MOUSE
ENSMUST00000037023_chr12:69157813−69159058:−
ENSMUST00000071737_chr13:73988963−73989133:−
tr|D3YYL7|D3YYL7_MOUSE

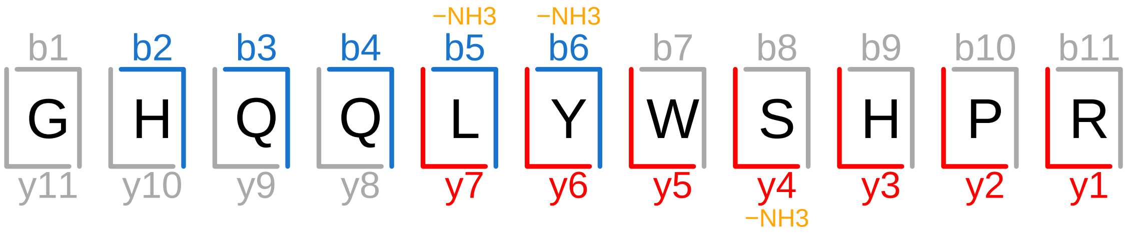

GHQQLYWSHPR
Score: 59 ; 1407.6796 m/z; 470.23382 m/z; −0.52604 ppm; MULTI−MSMS

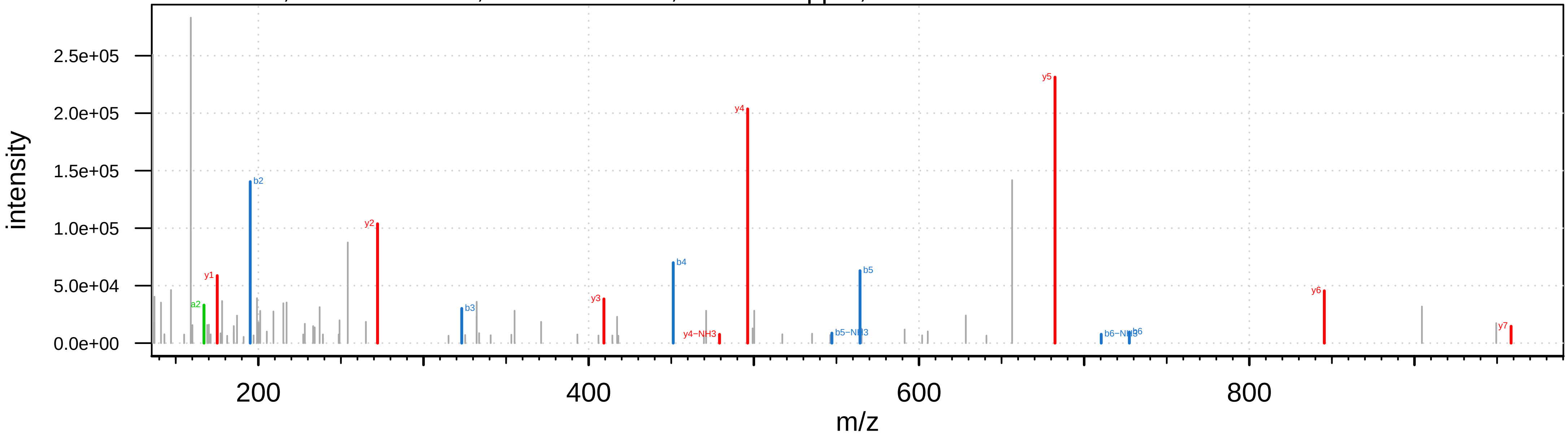

Raw File: Kermit_20111028_MSU_tube_54h_12
Scan Number: 52089
Proteins:
sp|P62274|RS29_MOUSE
ENSMUST00000037023_chr12:69157813−69159058:−
ENSMUST00000071737_chr13:73988963−73989133:−
tr|D3YYL7|D3YYL7_MOUSE

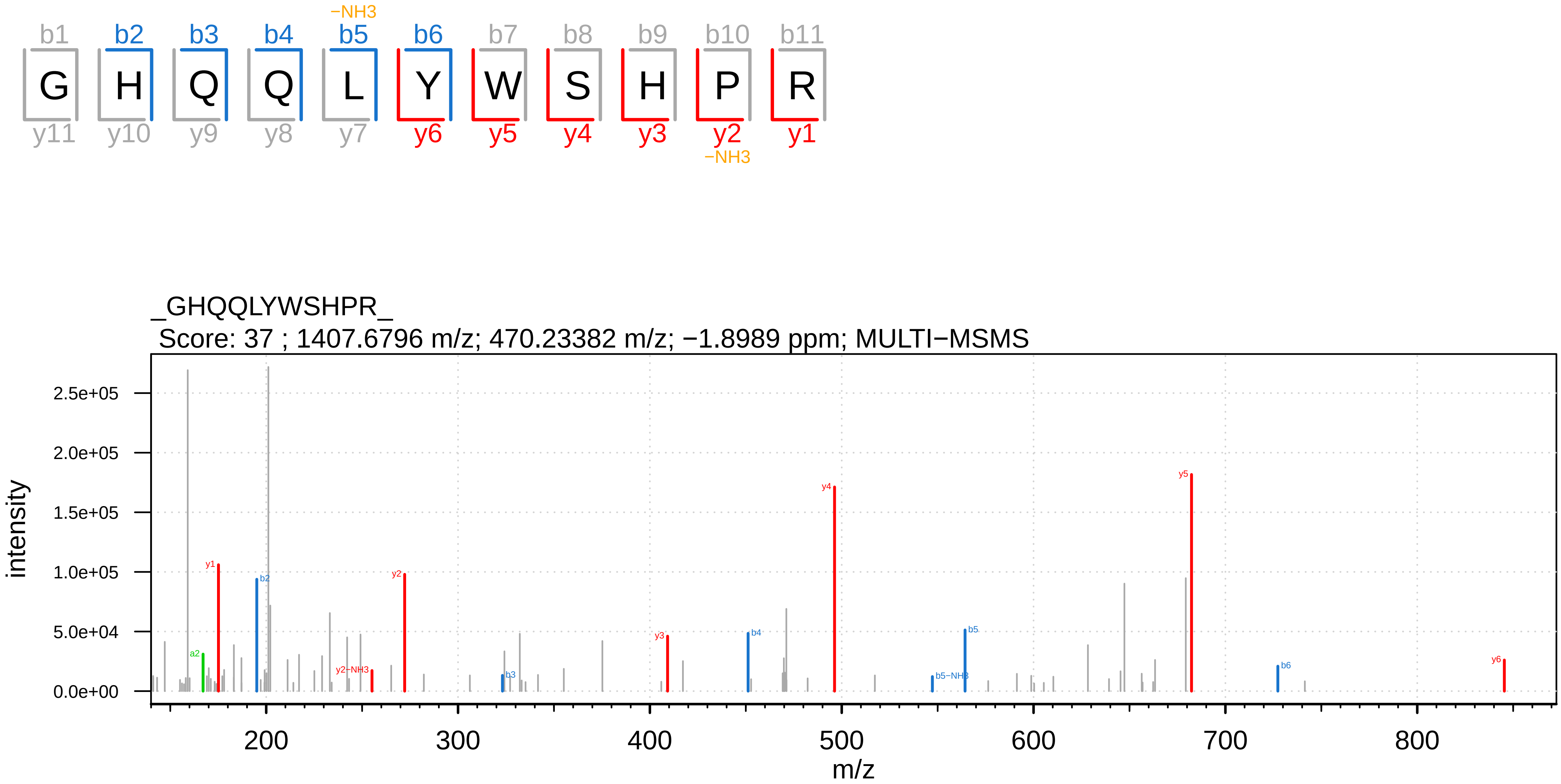

Raw File: Kermit_20111028_MSU_tube_54h_12
Scan Number: 52291
Proteins:
sp|P62274|RS29_MOUSE
ENSMUST00000037023_chr12:69157813−69159058:−
ENSMUST00000071737_chr13:73988963−73989133:−
tr|D3YYL7|D3YYL7_MOUSE

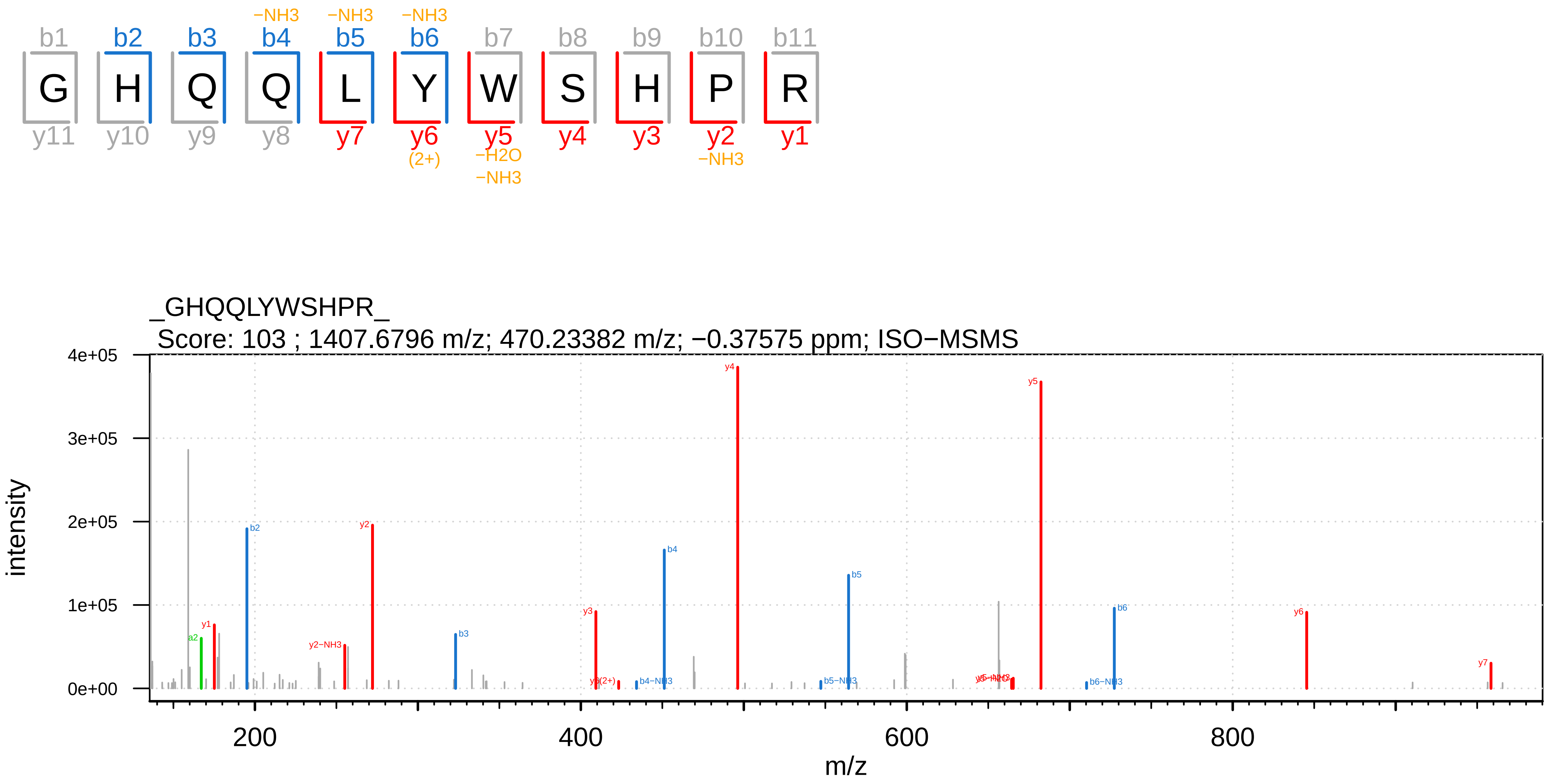

Raw File: Kermit_20111028_MSU_tube_54h_12
Scan Number: 50594
Proteins:
sp|P62274|RS29_MOUSE
ENSMUST00000037023_chr12:69157813−69159058:−
ENSMUST00000071737_chr13:73988963−73989133:−
tr|D3YYL7|D3YYL7_MOUSE

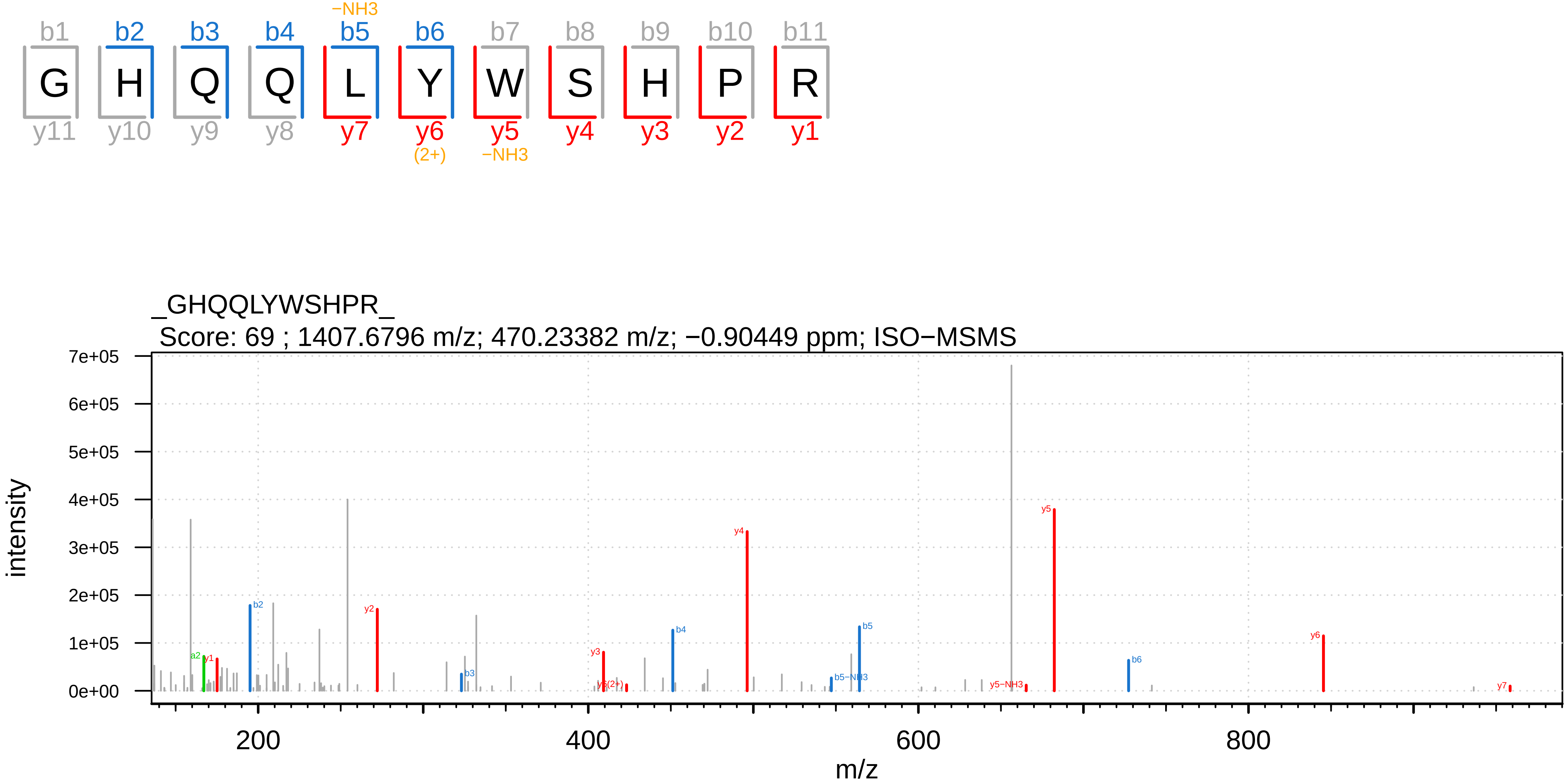

Raw File: Kermit_20111028_MSU_tube_54h_12
Scan Number: 51706
Proteins:
sp|P62274|RS29_MOUSE
ENSMUST00000037023_chr12:69157813−69159058:−
ENSMUST00000071737_chr13:73988963−73989133:−
tr|D3YYL7|D3YYL7_MOUSE

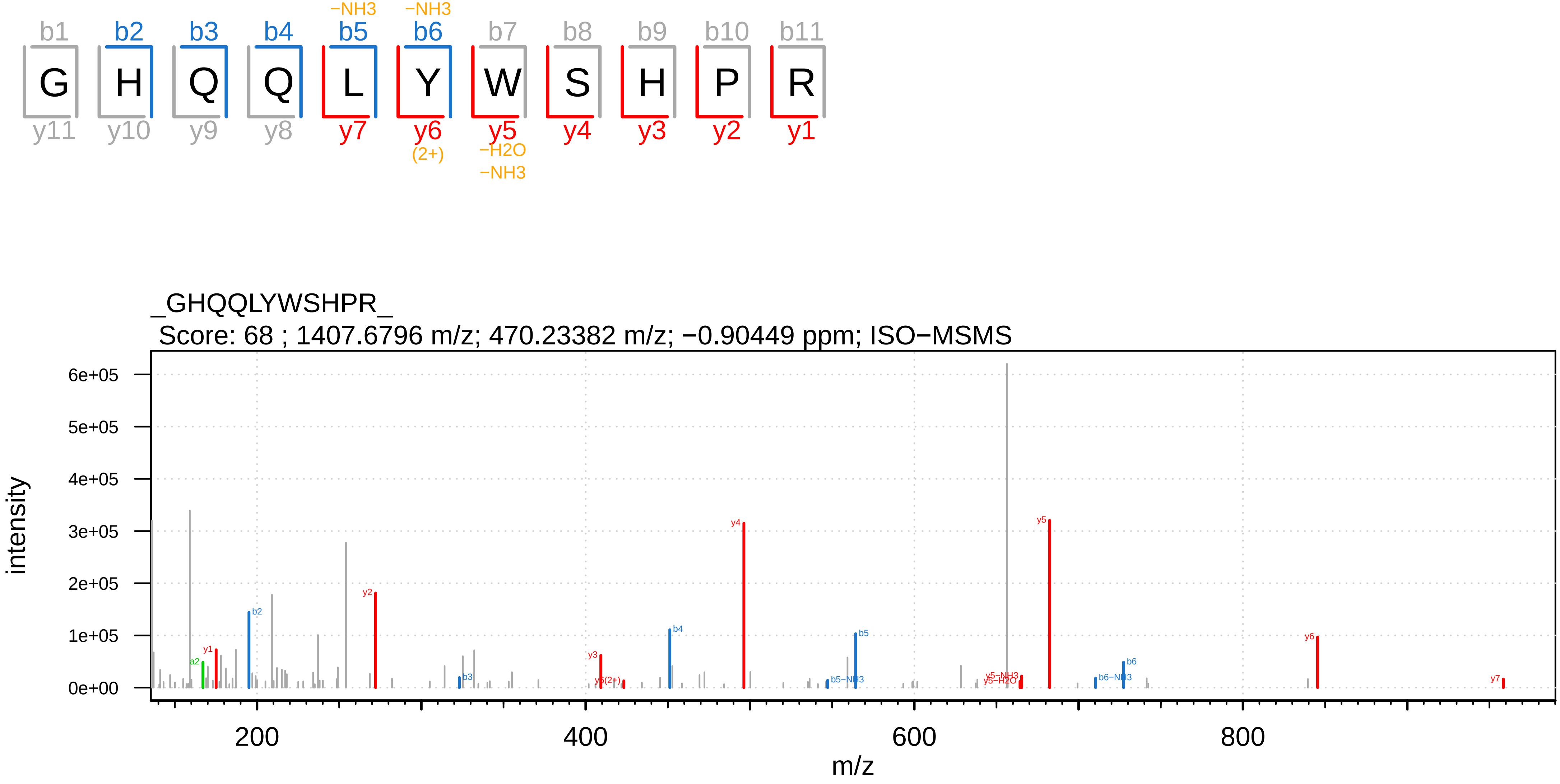

Raw File: Kermit_20111028_MSU_tube_54h_12
Scan Number: 51894
Proteins:
sp|P62274|RS29_MOUSE
ENSMUST00000037023_chr12:69157813−69159058:−
ENSMUST00000071737_chr13:73988963−73989133:−
tr|D3YYL7|D3YYL7_MOUSE

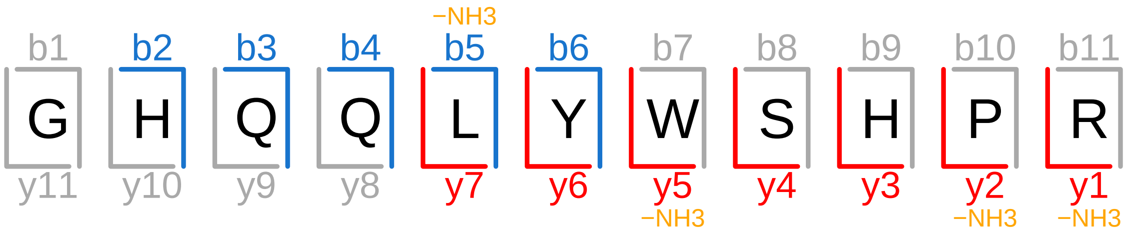

GHQQLYWSHPR
Score: 53 ; 1407.6796 m/z; 470.23382 m/z; NaN ppm; MSMS

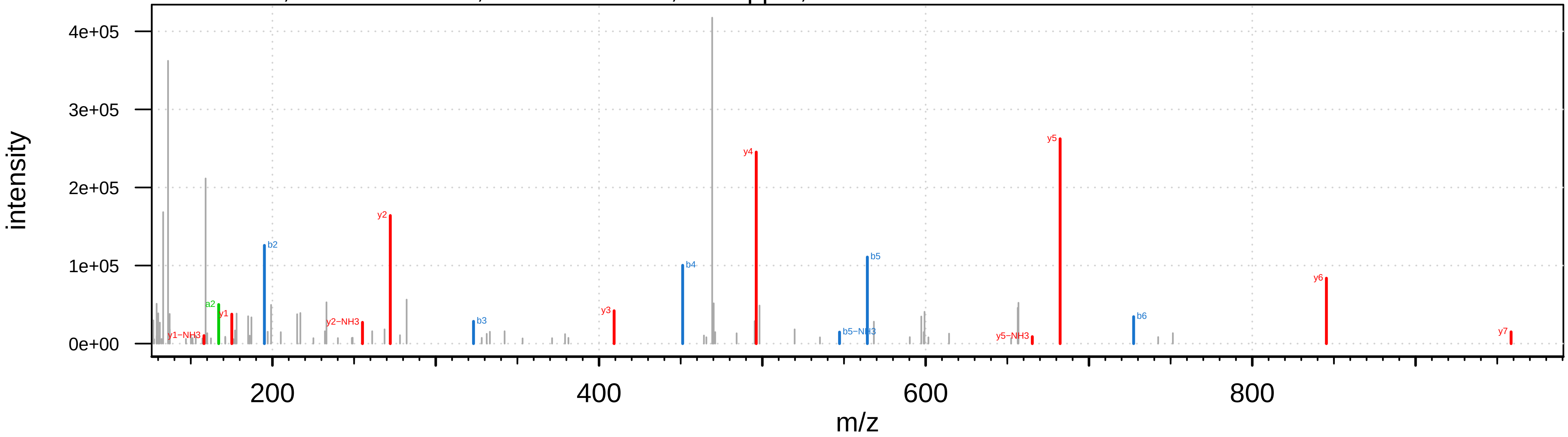

Raw File: Kermit_20111028_MSU_tube_54h_12
Scan Number: 49667
Proteins:
sp|P62274|RS29_MOUSE
ENSMUST00000037023_chr12:69157813−69159058:−
ENSMUST00000071737_chr13:73988963−73989133:−
tr|D3YYL7|D3YYL7_MOUSE

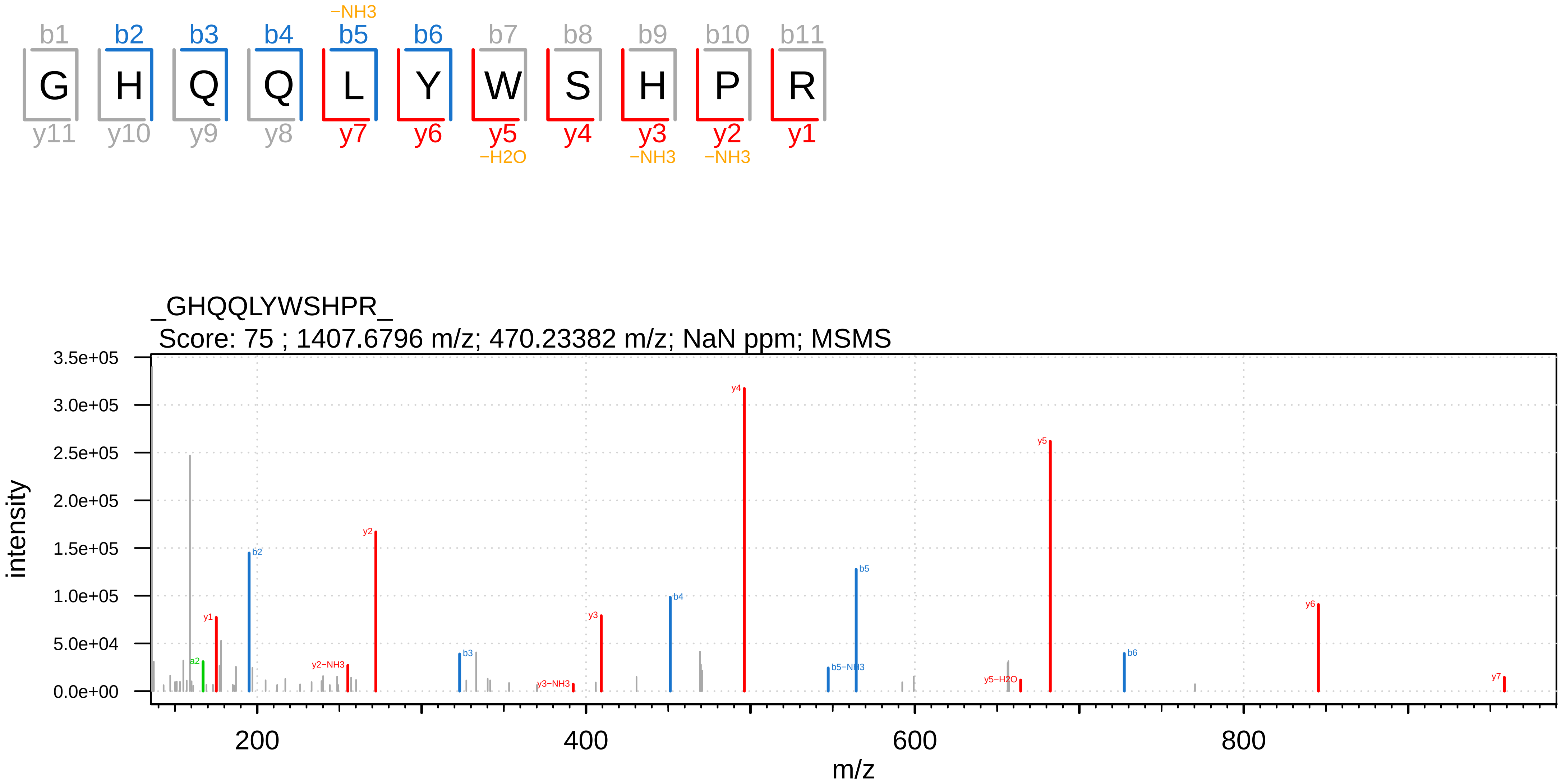

Raw File: Kermit_20111028_MSU_tube_54h_12
Scan Number: 50766
Proteins:
sp|P62274|RS29_MOUSE
ENSMUST00000037023_chr12:69157813−69159058:−
ENSMUST00000071737_chr13:73988963−73989133:−
tr|D3YYL7|D3YYL7_MOUSE

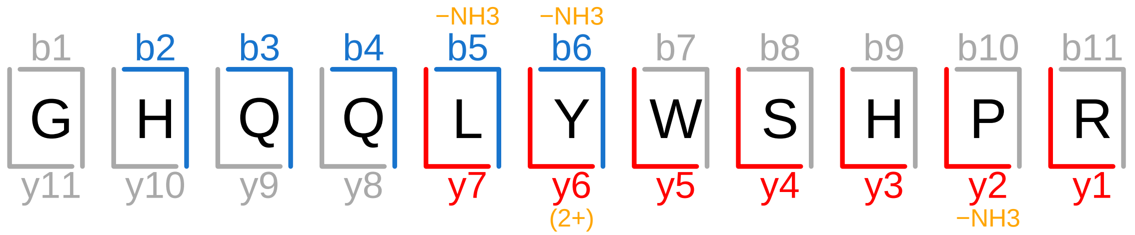

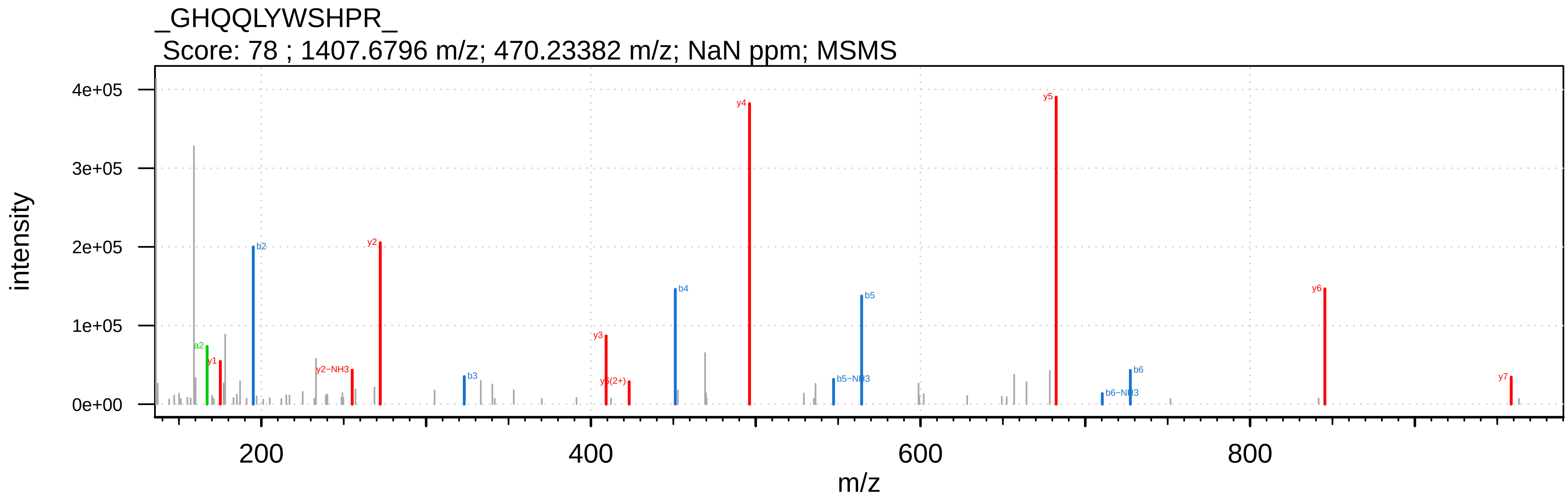

Raw File: Kermit_20111028_MSU_tube_54h_12
Scan Number: 50952
Proteins:
sp|P62274|RS29_MOUSE
ENSMUST00000037023_chr12:69157813−69159058:−
ENSMUST00000071737_chr13:73988963−73989133:−
tr|D3YYL7|D3YYL7_MOUSE

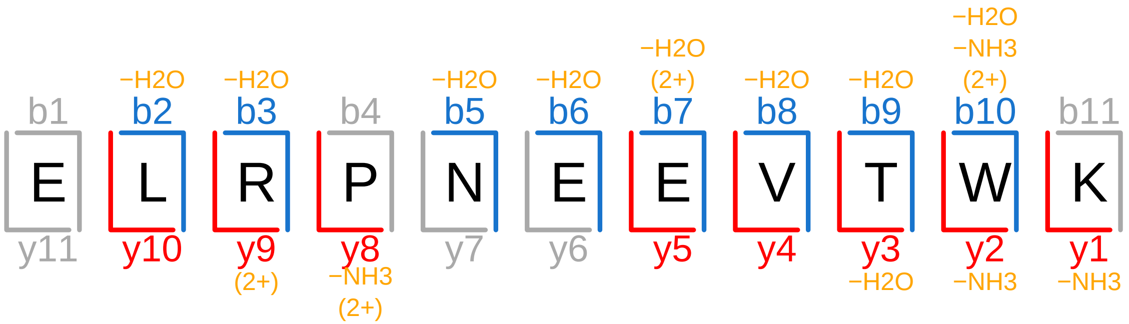

ELRPNEEVTWK
Score: 163 ; 1399.7096 m/z; 700.86207 m/z; −0.5505 ppm; MULTI−MSMS

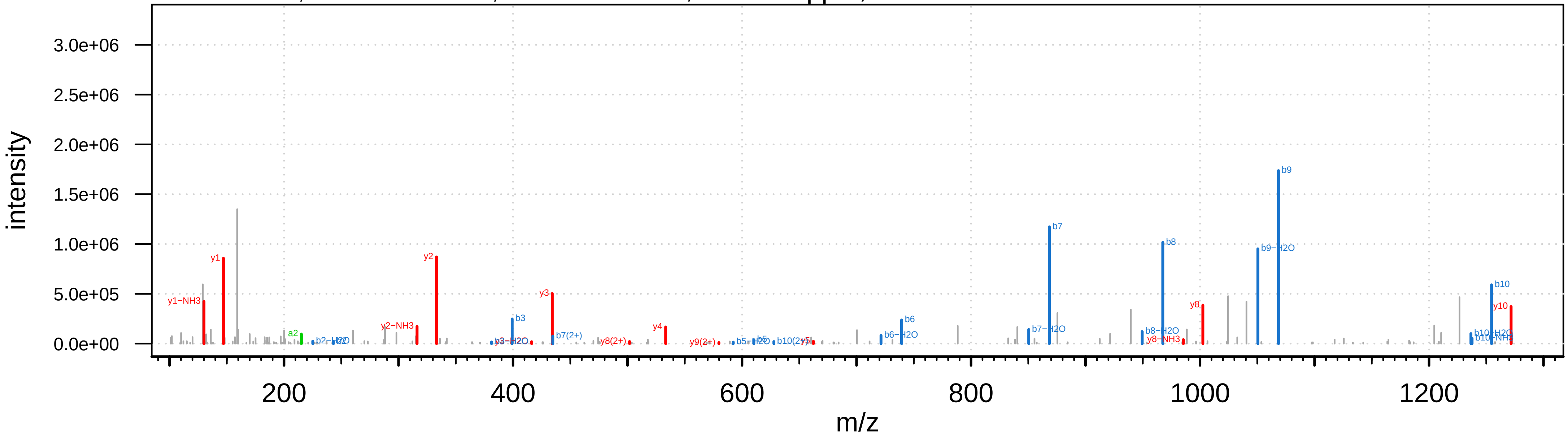

Raw File: Kermit_20111104_MSU_tube_18h_11
Scan Number: 17255
Proteins:
chr12_103206313−103214181.T2_chr12:101968174−101969629:−

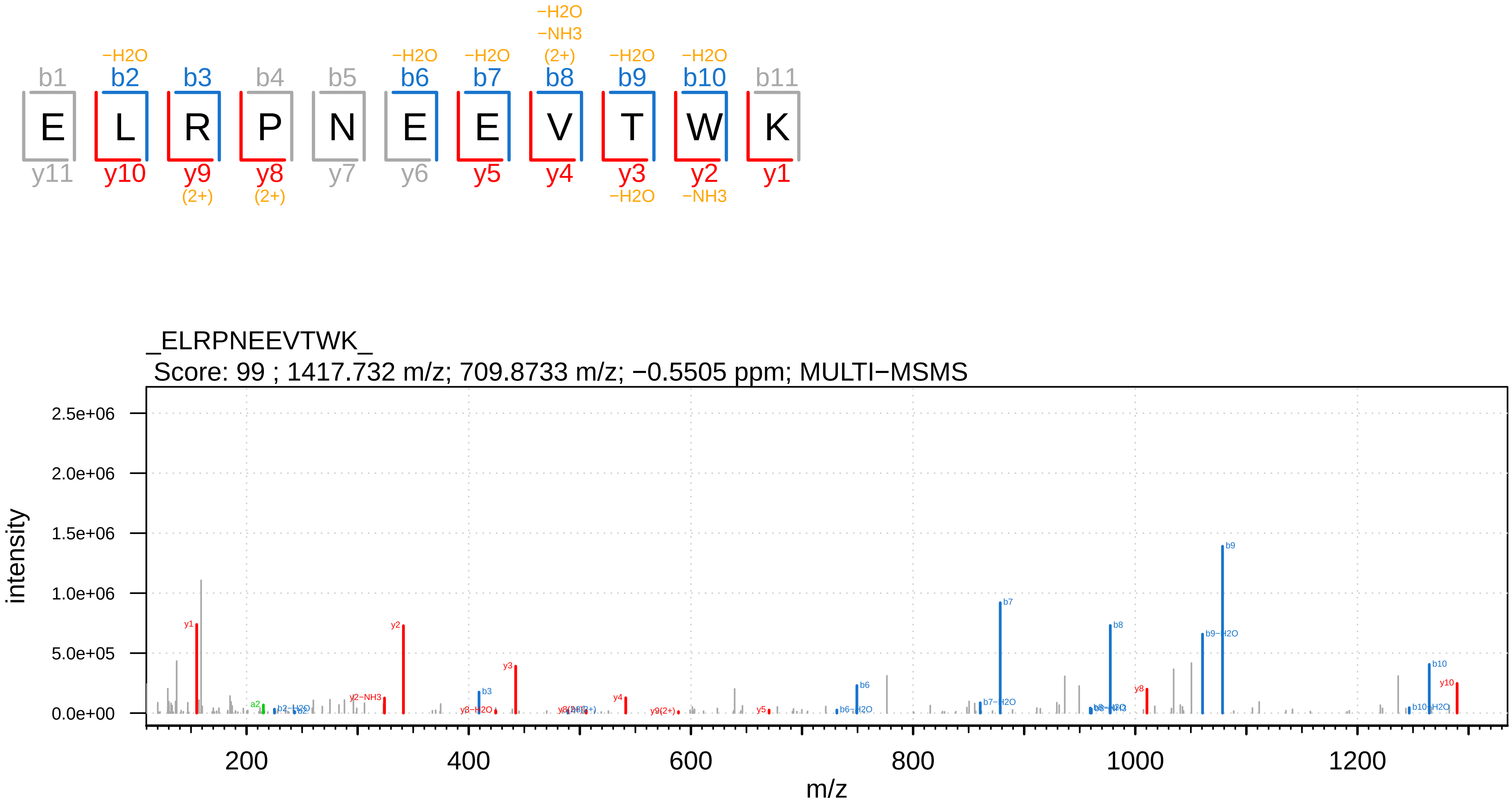

Raw File: Kermit_20111104_MSU_tube_18h_11
Scan Number: 17324
Proteins:
chr12_103206313−103214181.T2_chr12:101968174−101969629:−

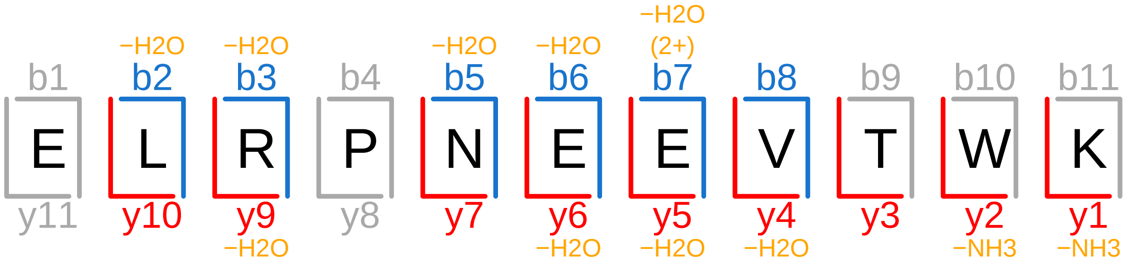

ELRPNEEVTWK
Score: 132 ; 1399.7096 m/z; 467.57714 m/z; 1.2198 ppm; ISO−MSMS

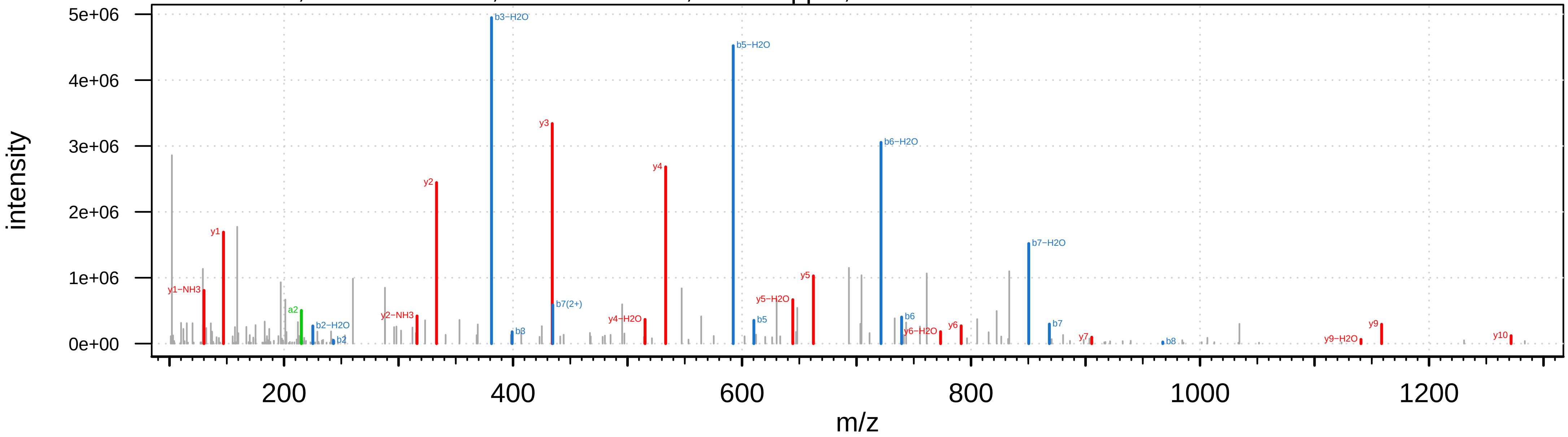

Raw File: Kermit_20111104_MSU_tube_18h_11
Scan Number: 17264
Proteins:
chr12_103206313−103214181.T2_chr12:101968174−101969629:−

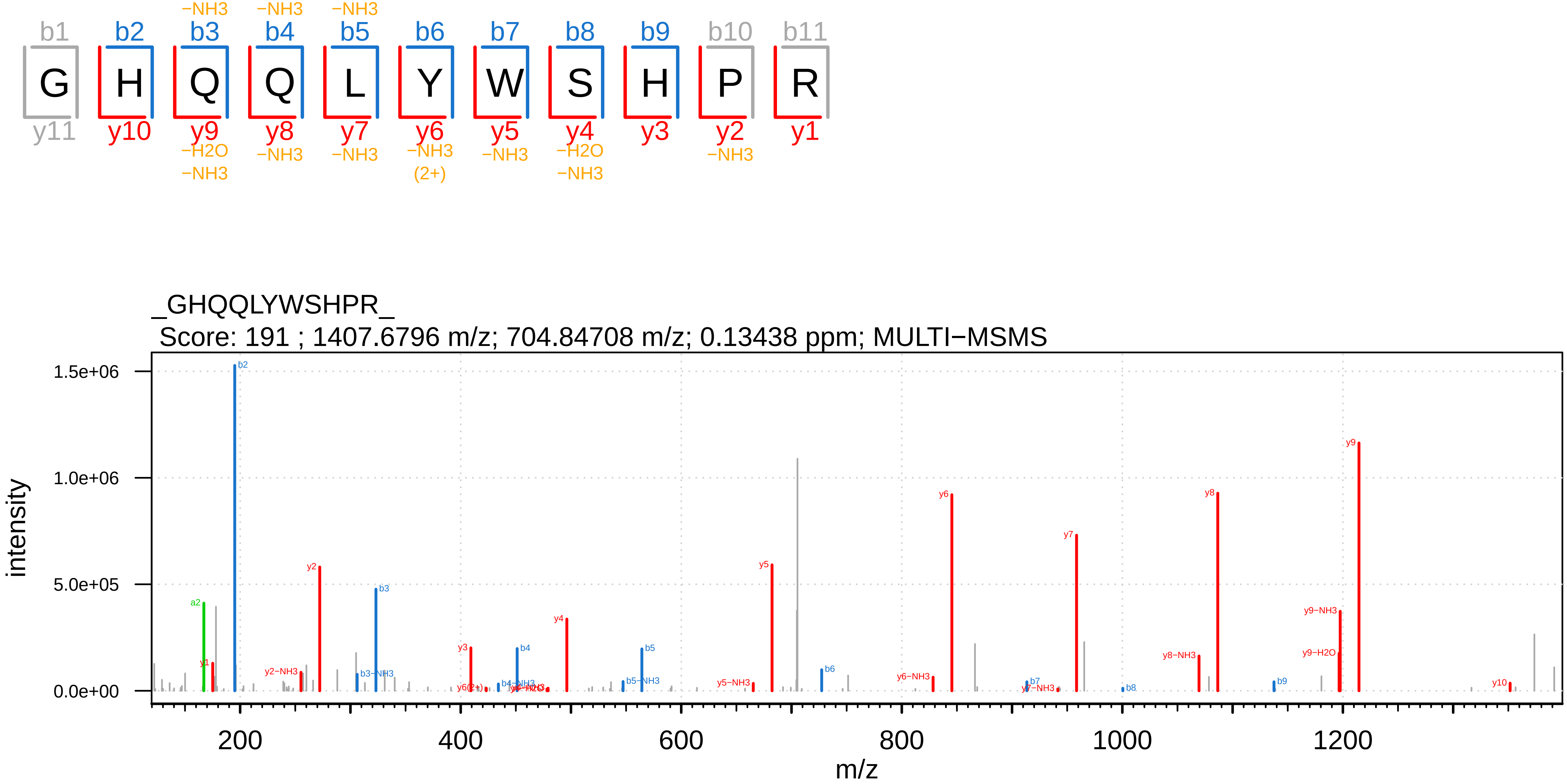

Raw File: Kermit_20111104_MSU_tube_18h_11
Scan Number: 8468
Proteins:
sp|P62274|RS29_MOUSE
ENSMUST00000037023_chr12:69157813−69159058:−
ENSMUST00000071737_chr13:73988963−73989133:−
tr|D3YYL7|D3YYL7_MOUSE

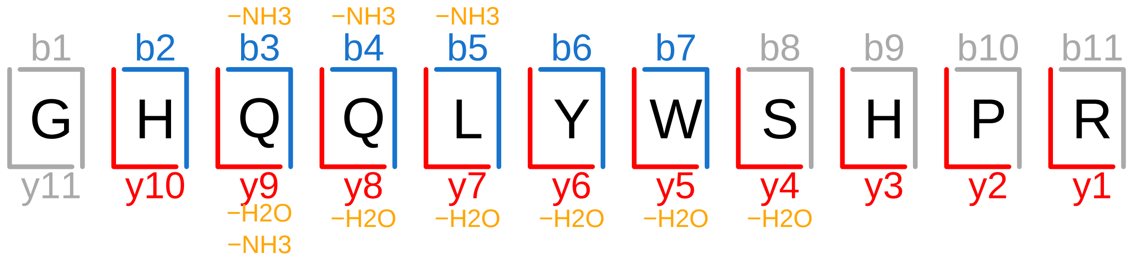

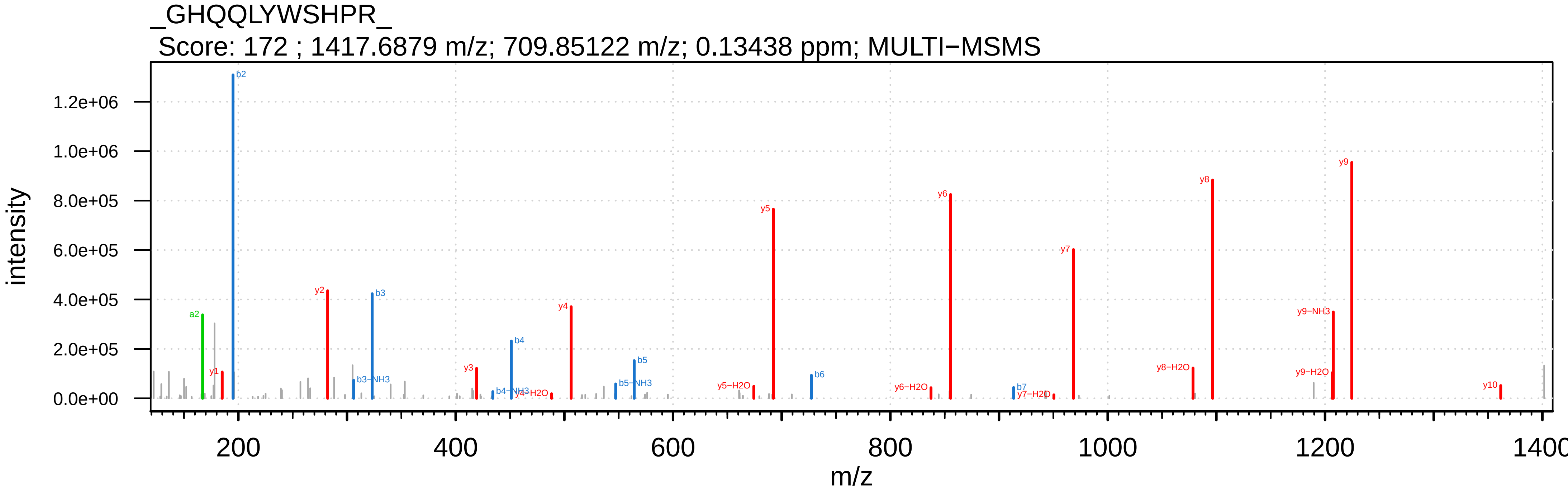

Raw File: Kermit_20111104_MSU_tube_18h_11
Scan Number: 8487
Proteins:
sp|P62274|RS29_MOUSE
ENSMUST00000037023_chr12:69157813−69159058:−
ENSMUST00000071737_chr13:73988963−73989133:−
tr|D3YYL7|D3YYL7_MOUSE

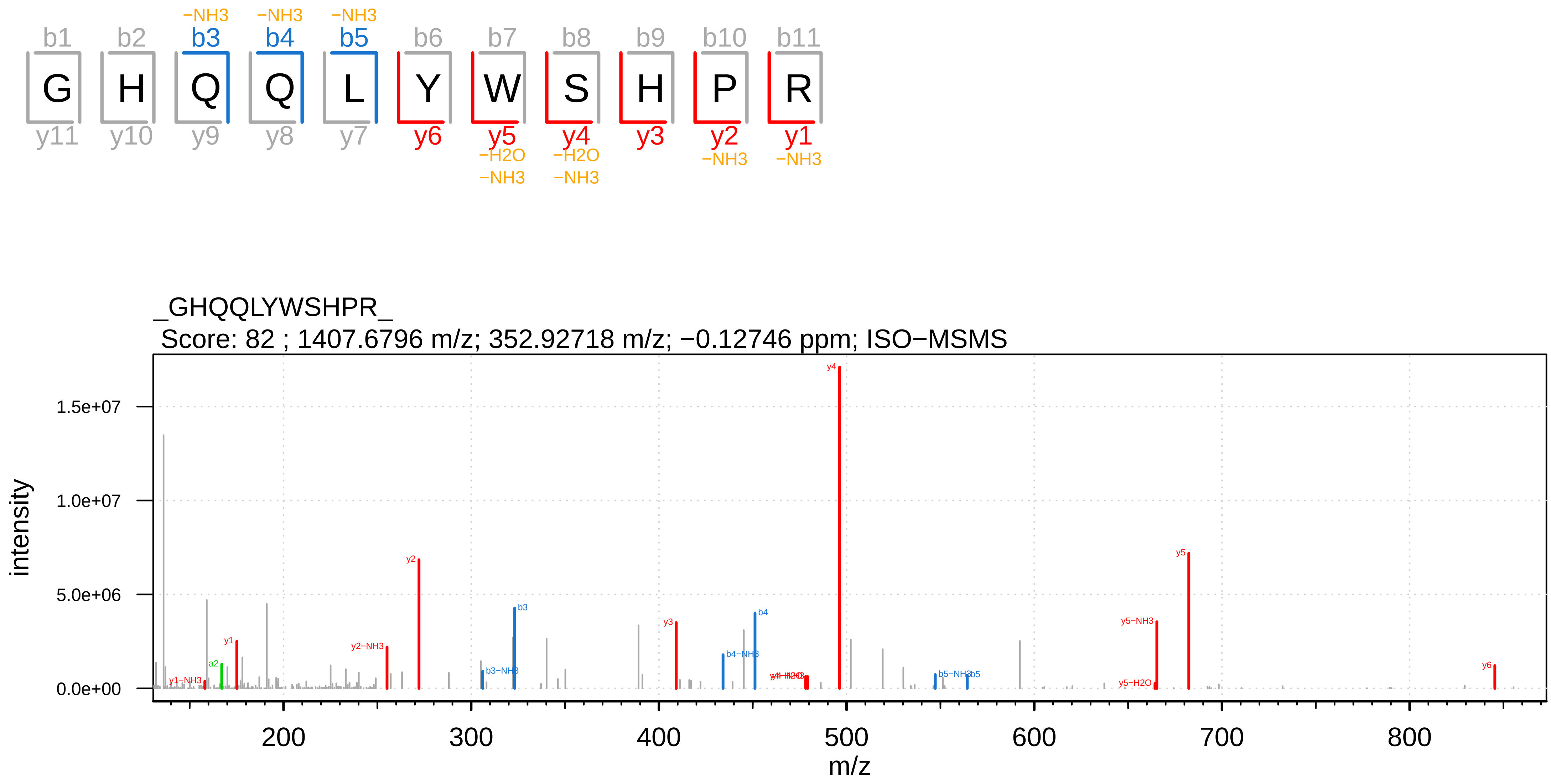

Raw File: Kermit_20111104_MSU_tube_18h_11
Scan Number: 8475
Proteins:
sp|P62274|RS29_MOUSE
ENSMUST00000037023_chr12:69157813−69159058:−
ENSMUST00000071737_chr13:73988963−73989133:−
tr|D3YYL7|D3YYL7_MOUSE

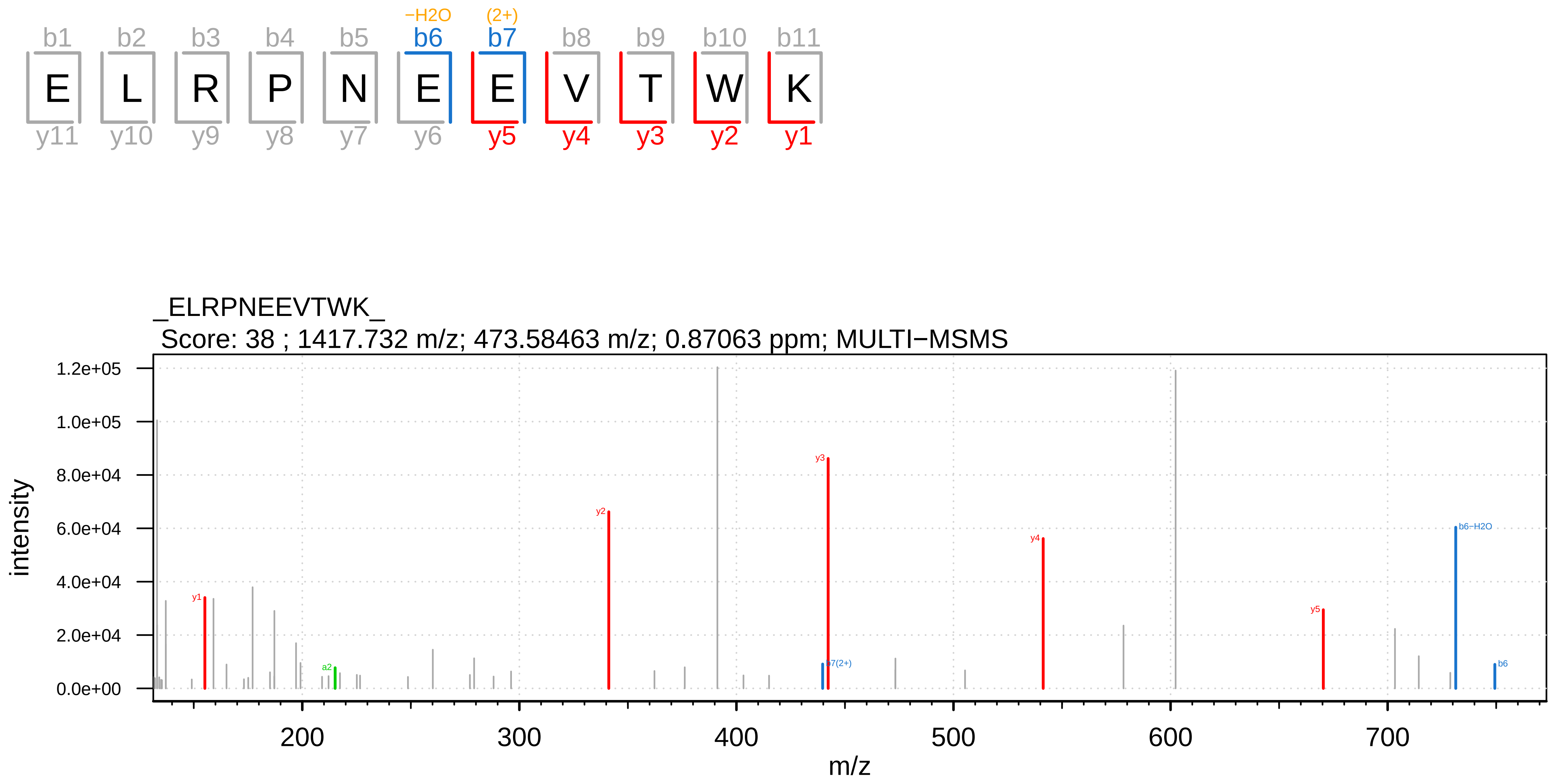

Raw File: Kermit_20111110_MSU_diff1_12
Scan Number: 55497
Proteins:
chr12_103206313−103214181.T2_chr12:101968174−101969629:−

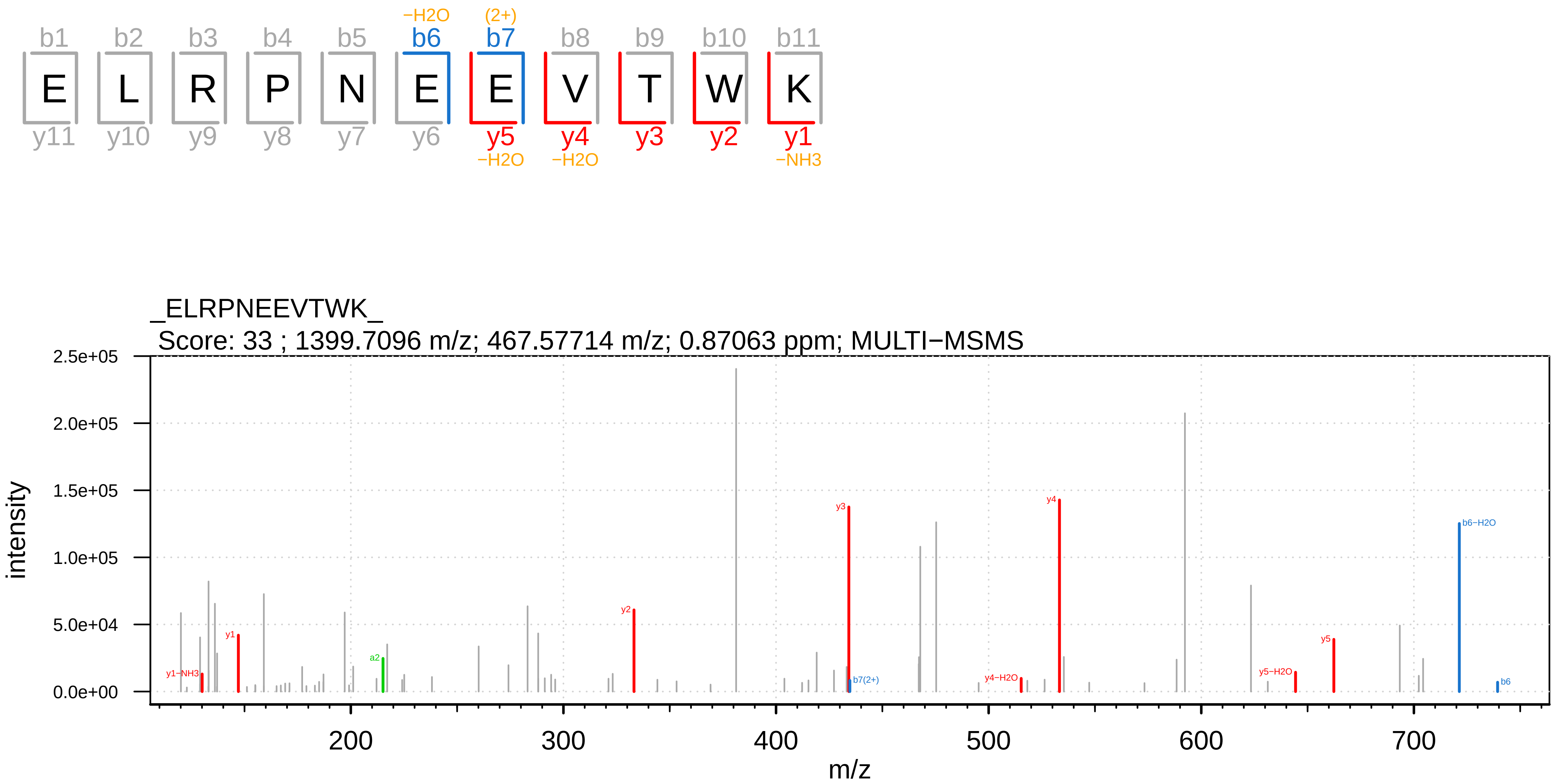

Raw File: Kermit_20111110_MSU_diff1_12
Scan Number: 55704
Proteins:
chr12_103206313−103214181.T2_chr12:101968174−101969629:−

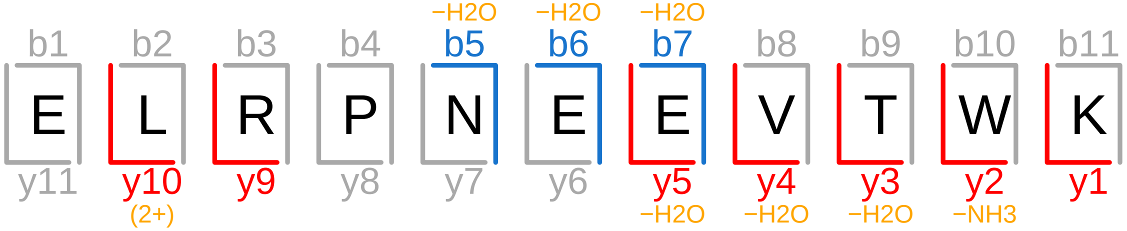

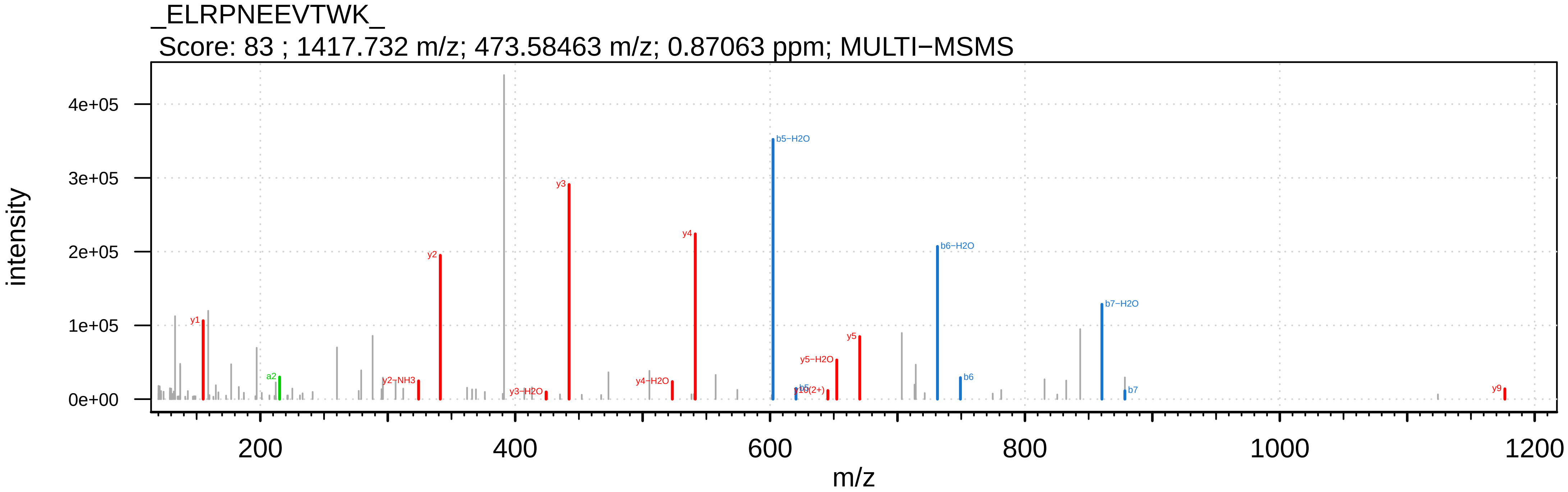

Raw File: Kermit_20111110_MSU_diff1_12
Scan Number: 55723
Proteins:
chr12_103206313−103214181.T2_chr12:101968174−101969629:−

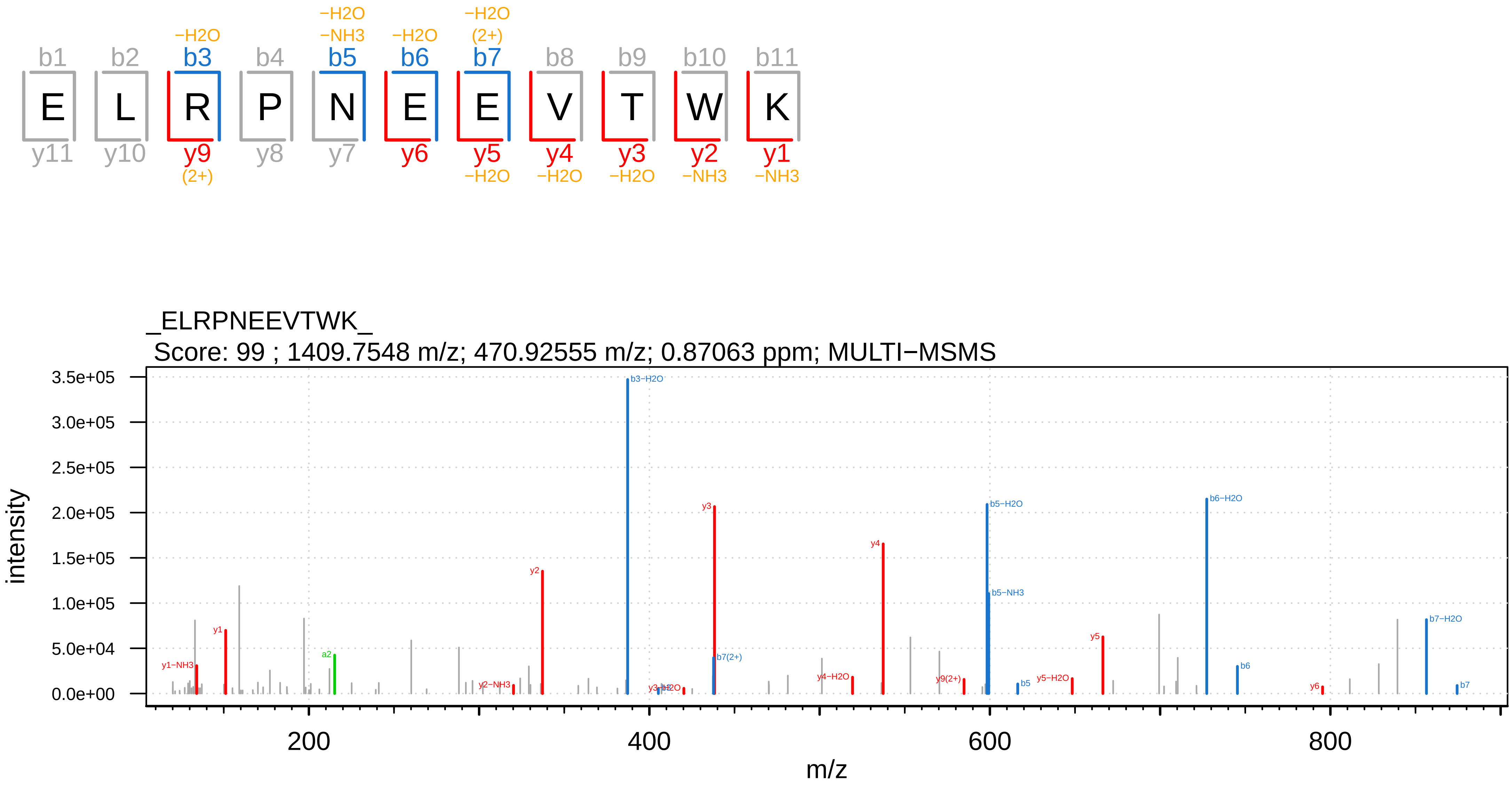

Raw File: Kermit_20111110_MSU_diff1_12
Scan Number: 55735
Proteins:
chr12_103206313−103214181.T2_chr12:101968174−101969629:−

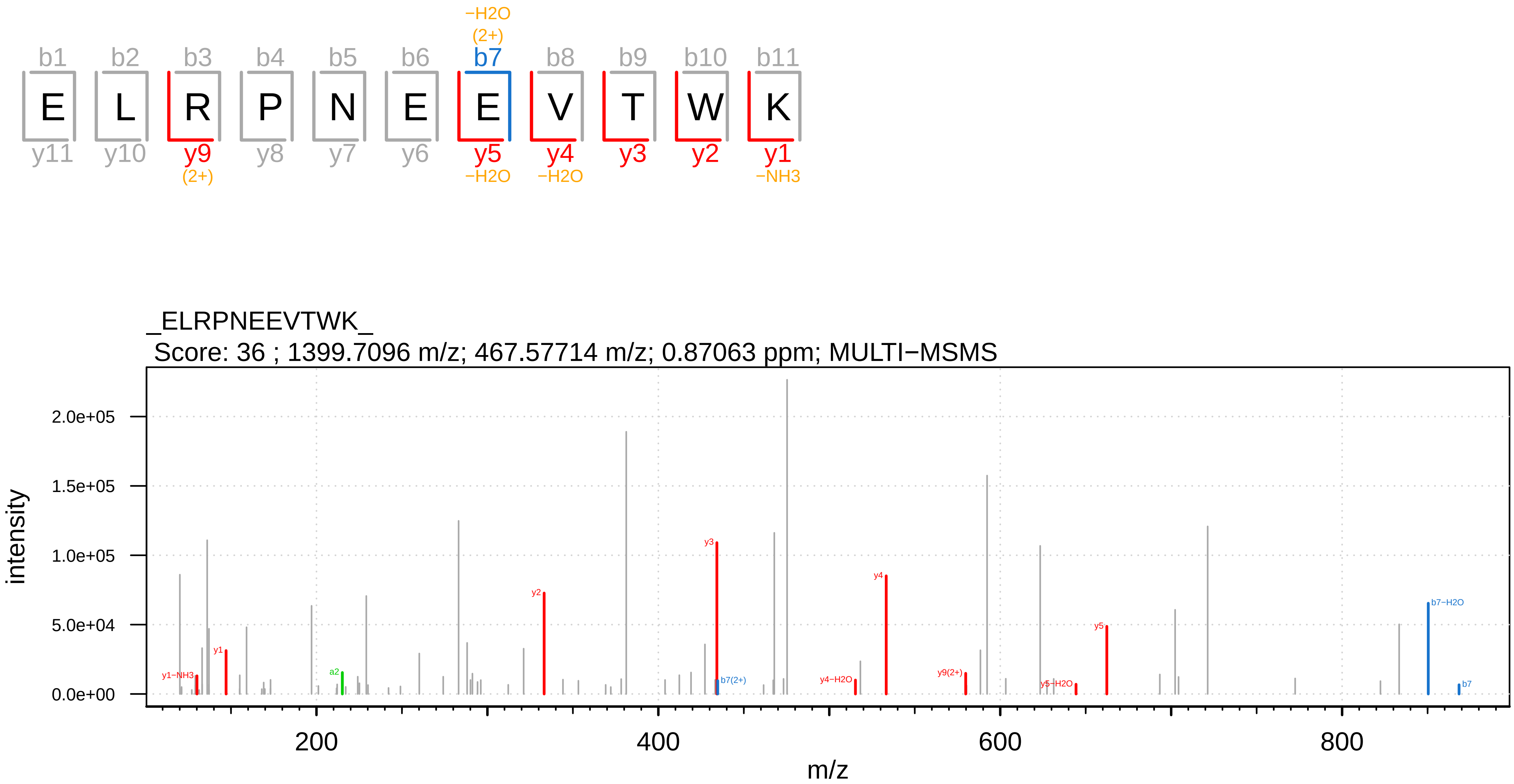

Raw File: Kermit_20111110_MSU_diff1_12
Scan Number: 55935
Proteins:
chr12_103206313−103214181.T2_chr12:101968174−101969629:−

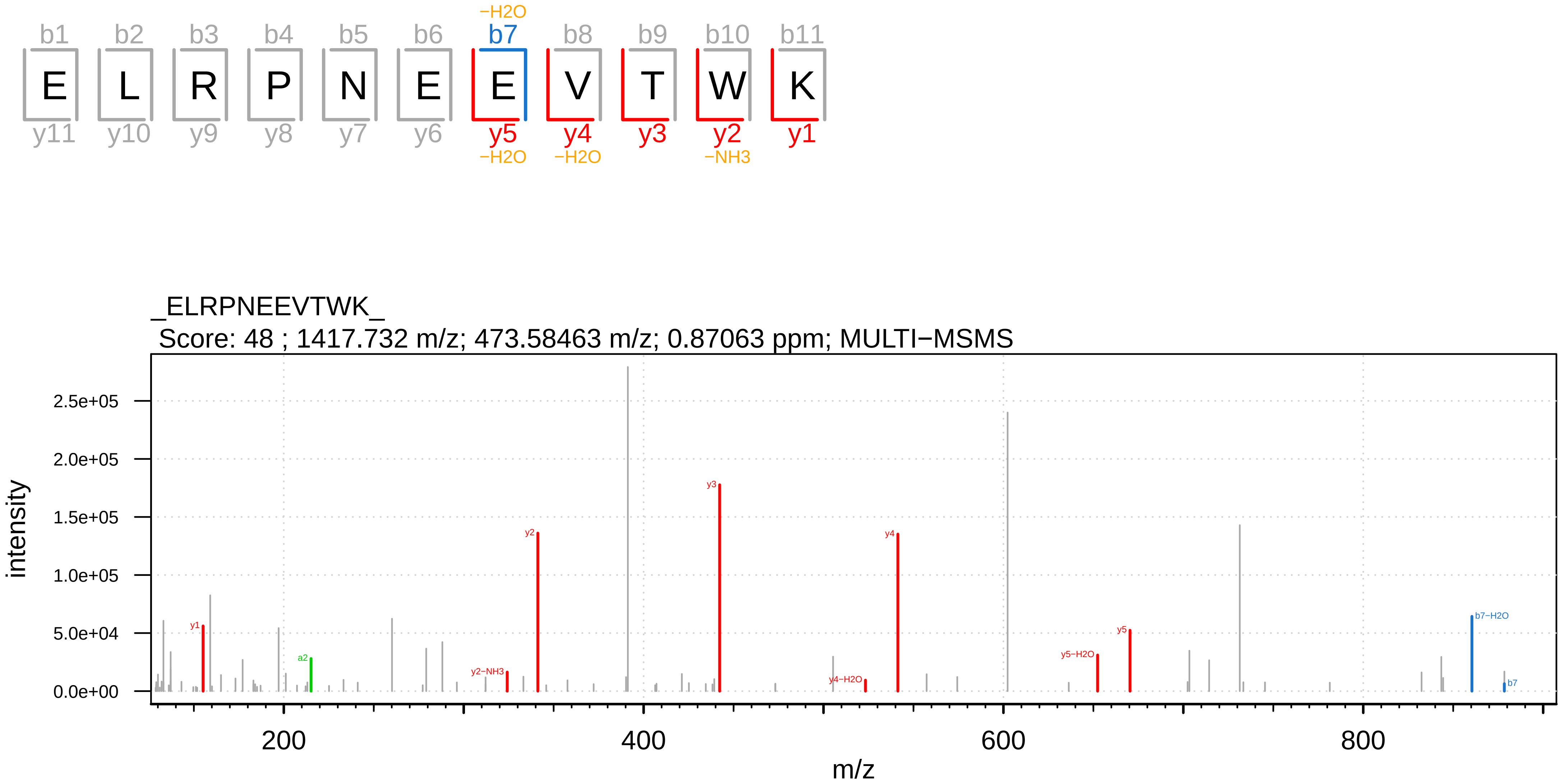

Raw File: Kermit_20111110_MSU_diff1_12
Scan Number: 55942
Proteins:
chr12_103206313−103214181.T2_chr12:101968174−101969629:−

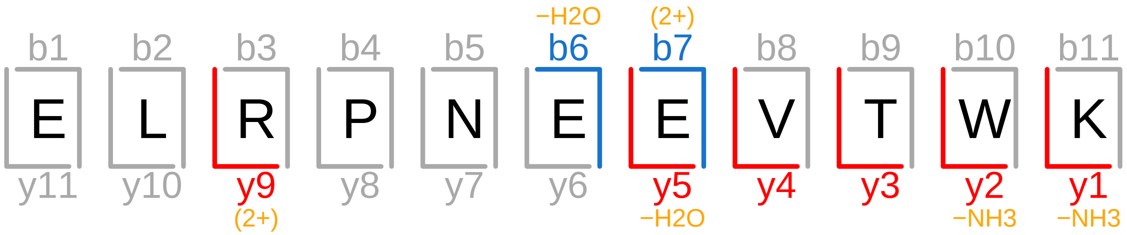

ELRPNEEVTWK

Score: 64 ; 1409.7548 m/z; 470.92555 m/z; 0.87063 ppm; MULTI−MSMS

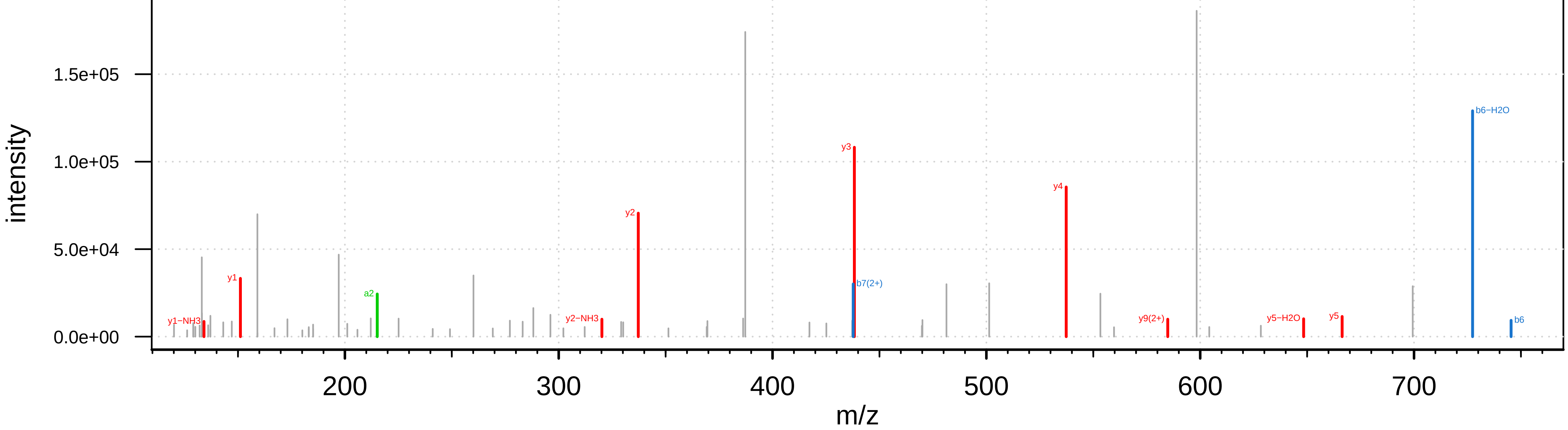

Raw File: Kermit_20111110_MSU_diff1_12

Scan Number: 55963

Proteins:

chr12_103206313−103214181.T2_chr12:101968174−101969629:−

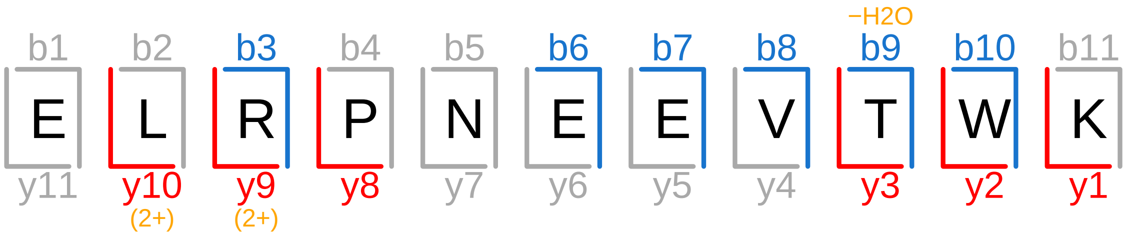

ELRPNEEVTWK
Score: 65 ; 1417.732 m/z; 709.8733 m/z; 0.50658 ppm; MULTI−MSMS

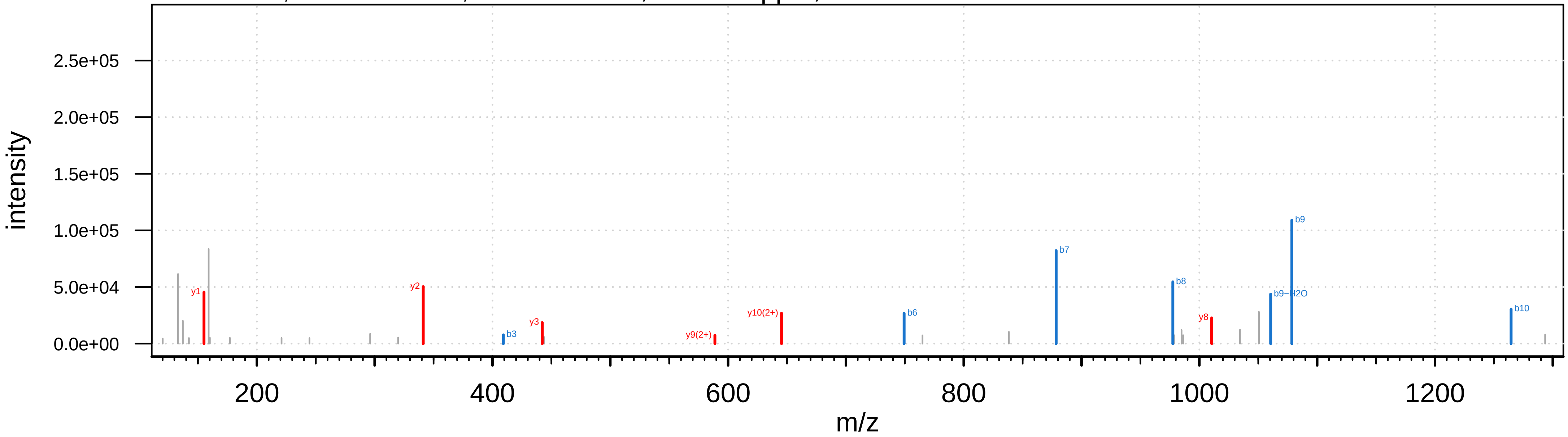

Raw File: Kermit_20111110_MSU_diff1_12
Scan Number: 55749
Proteins:
chr12_103206313−103214181.T2_chr12:101968174−101969629:−

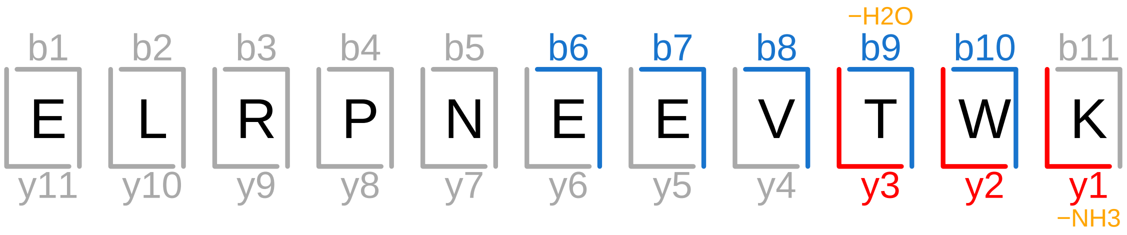

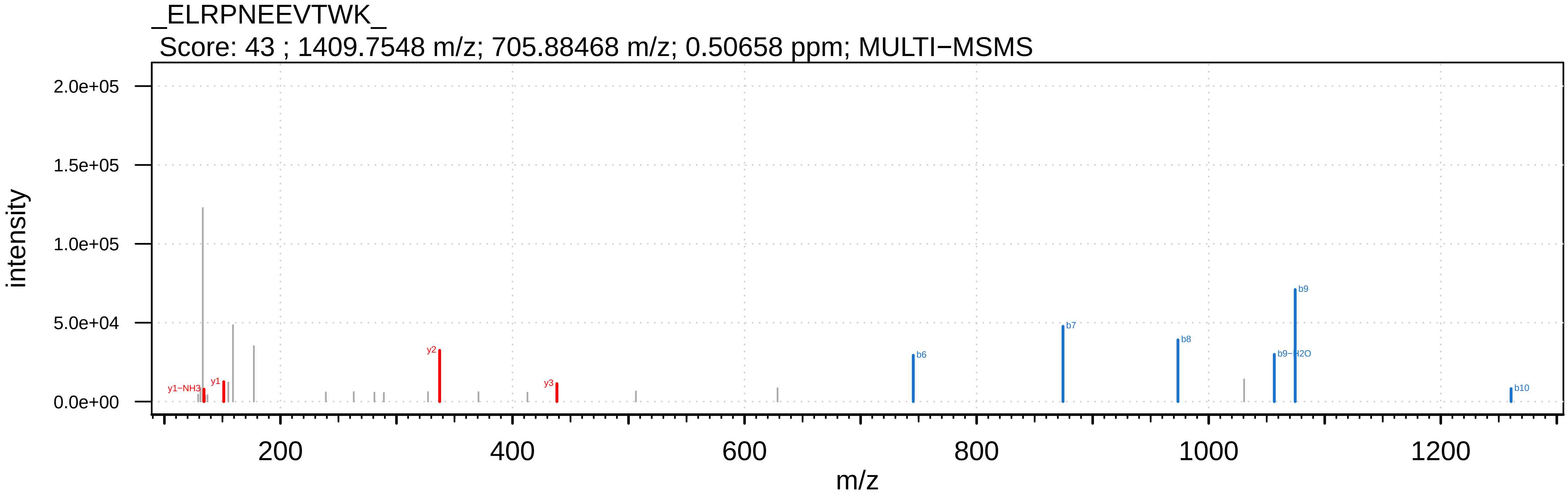

Raw File: Kermit_20111110_MSU_diff1_12
Scan Number: 55750
Proteins:
chr12_103206313−103214181.T2_chr12:101968174−101969629:−

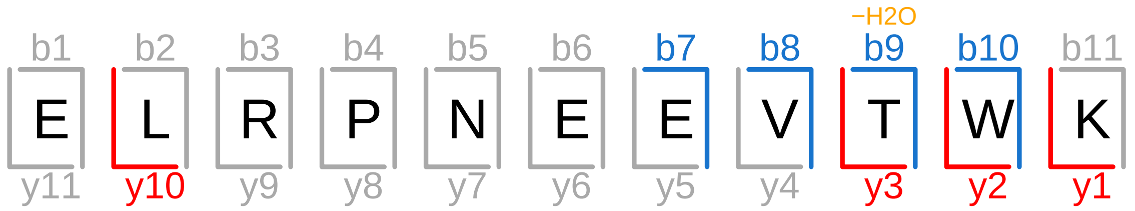

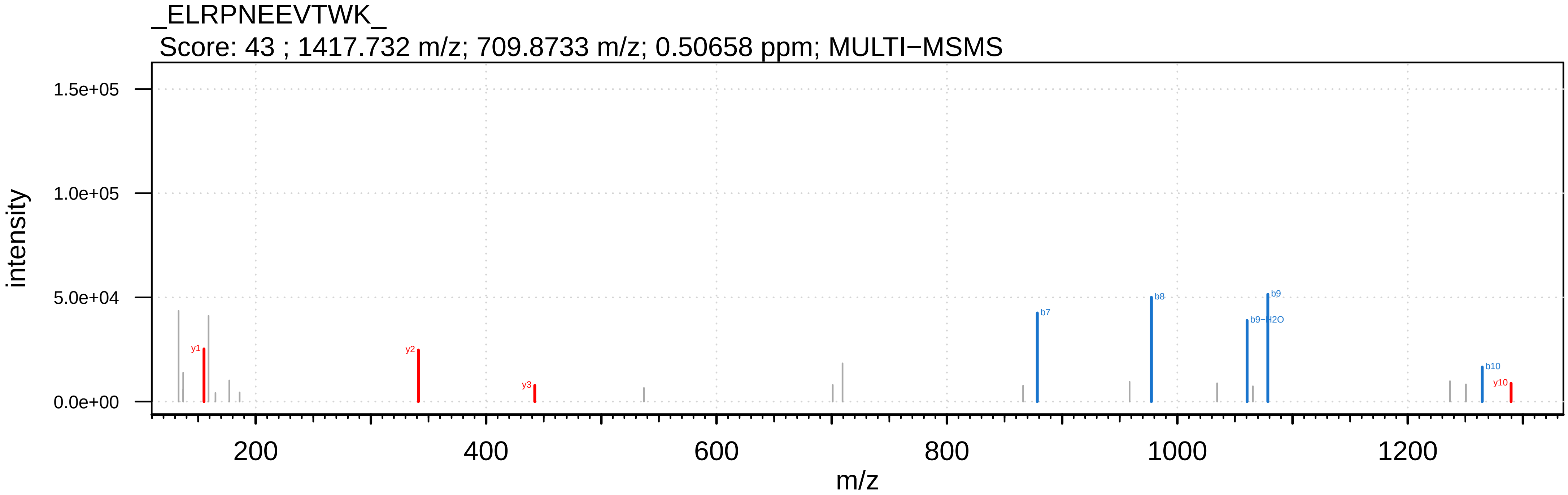

Raw File: Kermit_20111110_MSU_diff1_12
Scan Number: 55972
Proteins:
chr12_103206313−103214181.T2_chr12:101968174−101969629:−

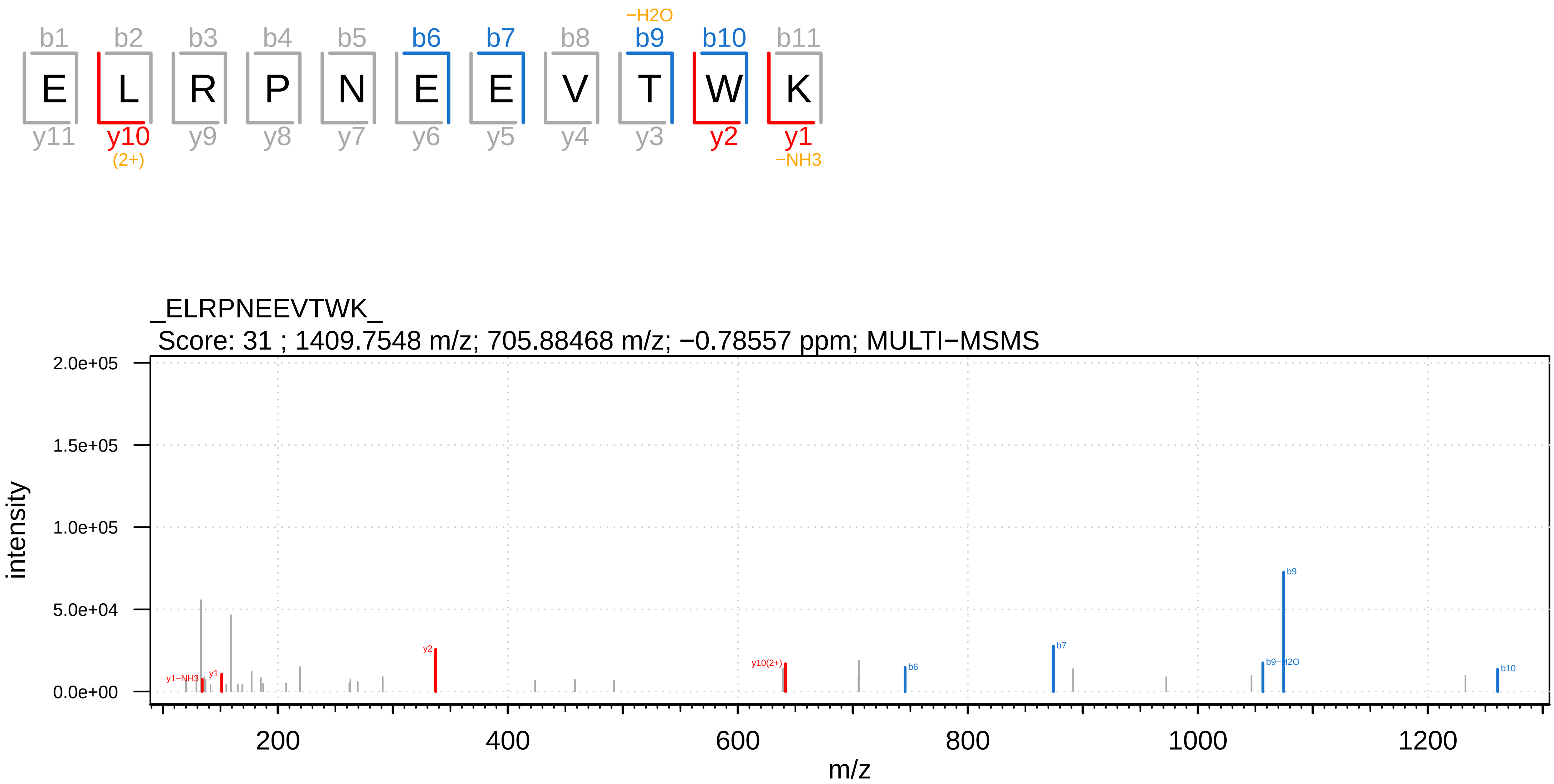

Raw File: Kermit_20111118_MSU_diff2_12
Scan Number: 52276
Proteins:
chr12_103206313−103214181.T2_chr12:101968174−101969629:−

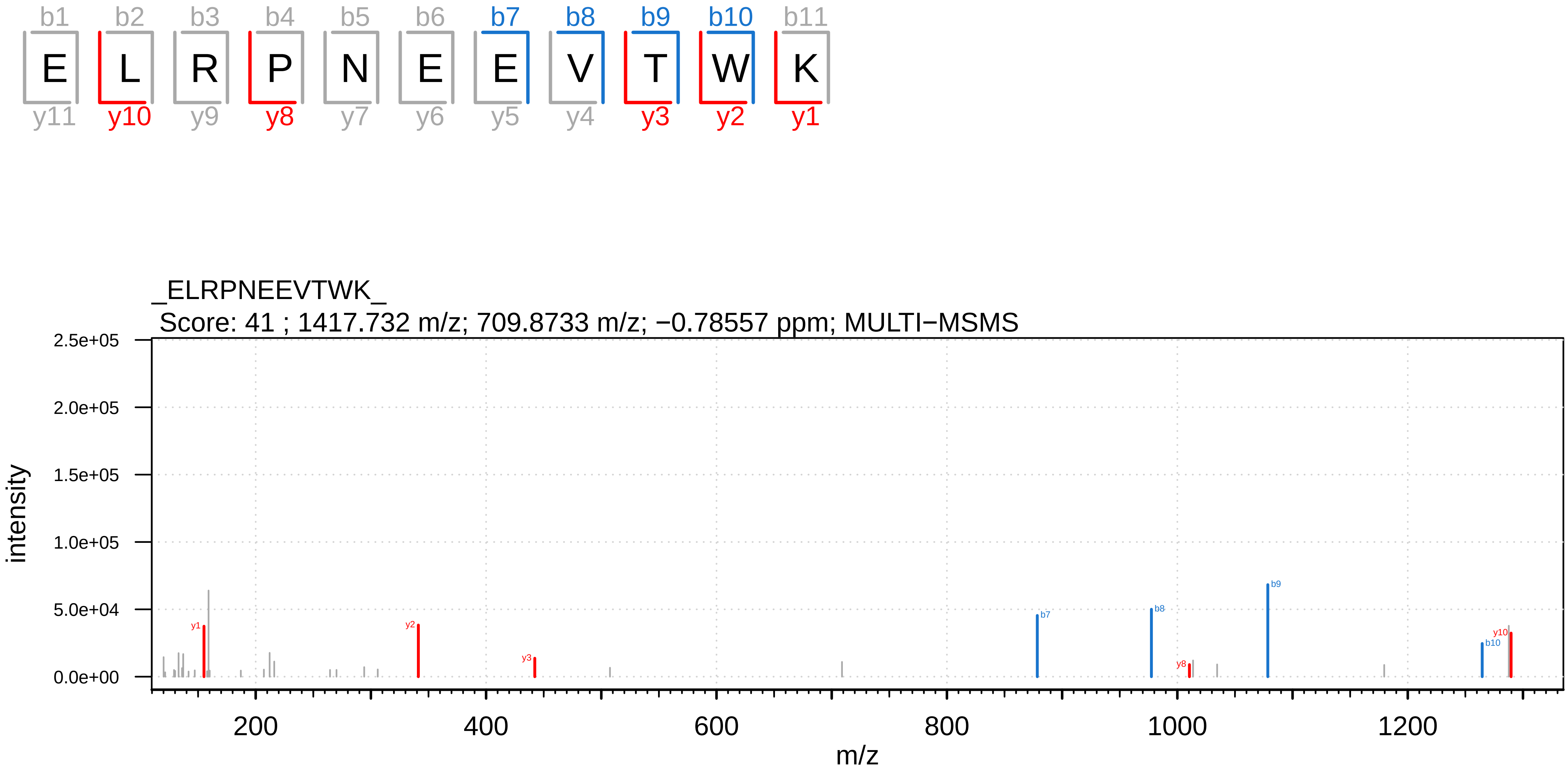

Raw File: Kermit_20111118_MSU_diff2_12
Scan Number: 52305
Proteins:
chr12_103206313−103214181.T2_chr12:101968174−101969629:−

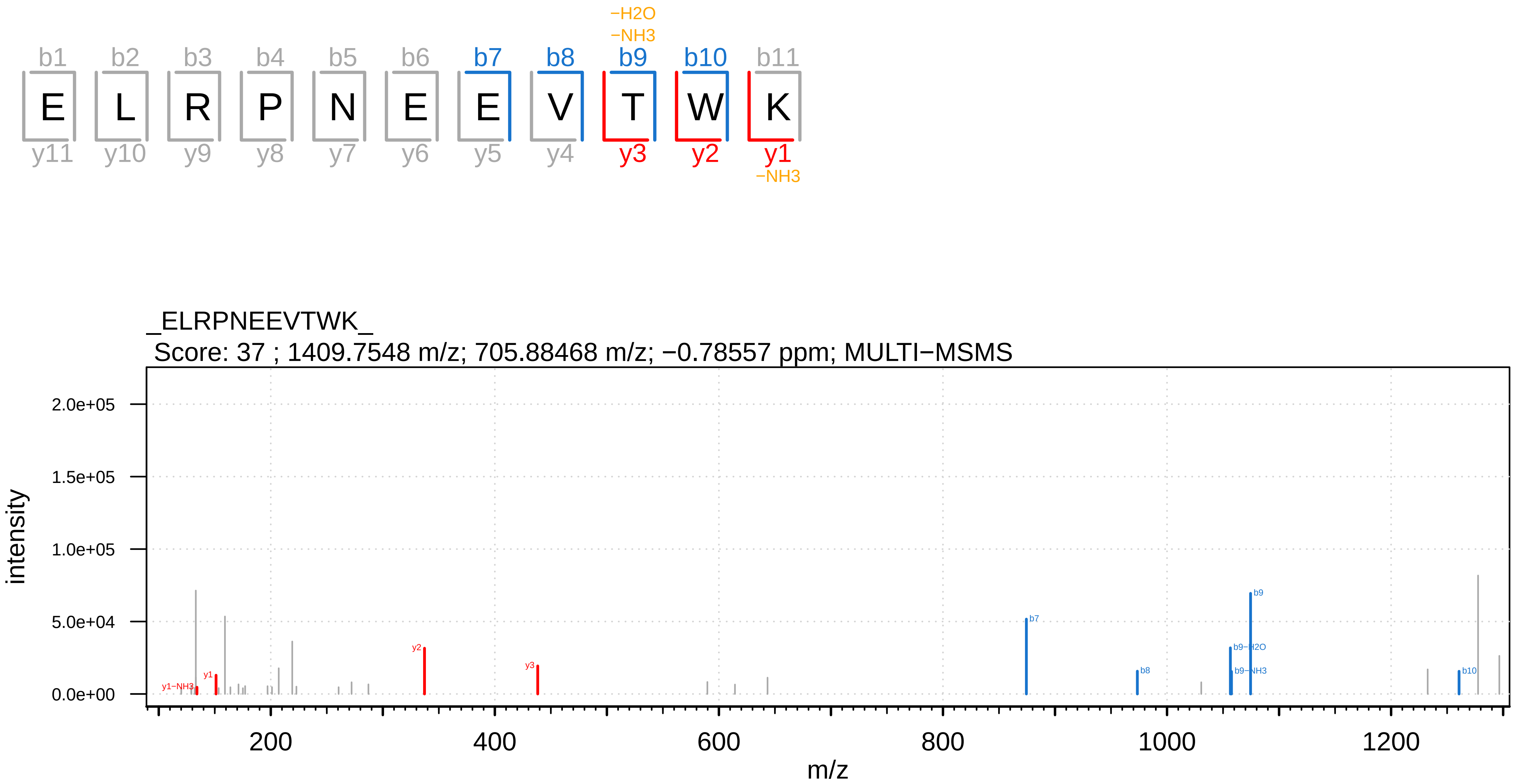

Raw File: Kermit_20111118_MSU_diff2_12
Scan Number: 52512
Proteins:
chr12_103206313−103214181.T2_chr12:101968174−101969629:−

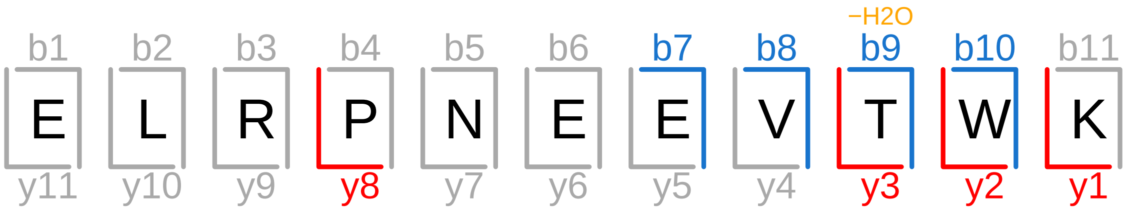

ELRPNEEVTWK
Score: 30 ; 1417.732 m/z; 709.8733 m/z; −0.78557 ppm; MULTI−MSMS

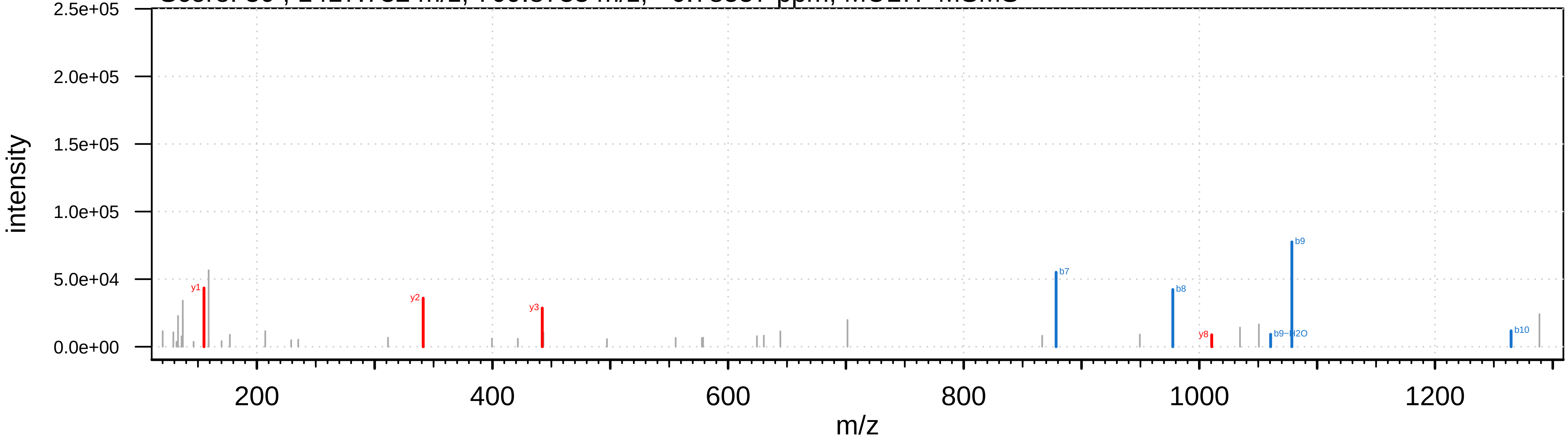

Raw File: Kermit_20111118_MSU_diff2_12
Scan Number: 52540
Proteins:
chr12_103206313−103214181.T2_chr12:101968174−101969629:−

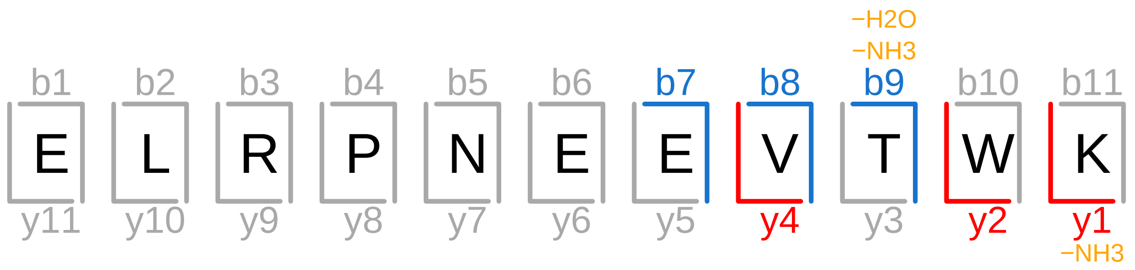

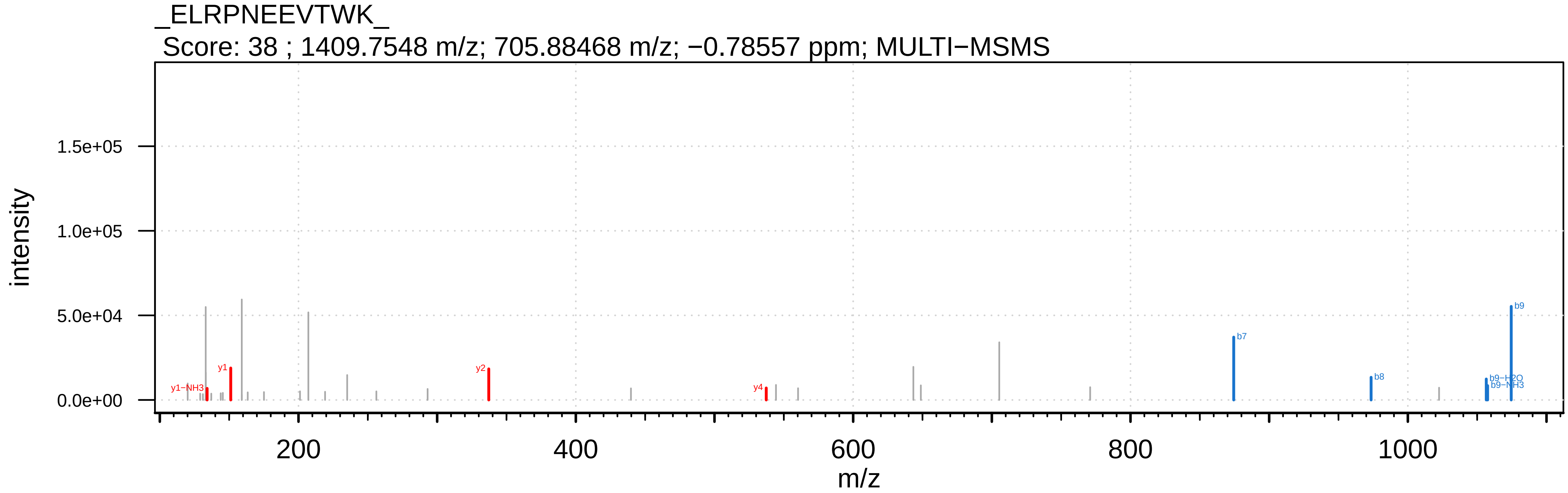

Raw File: Kermit_20111118_MSU_diff2_12
Scan Number: 52751
Proteins:
chr12_103206313−103214181.T2_chr12:101968174−101969629:−

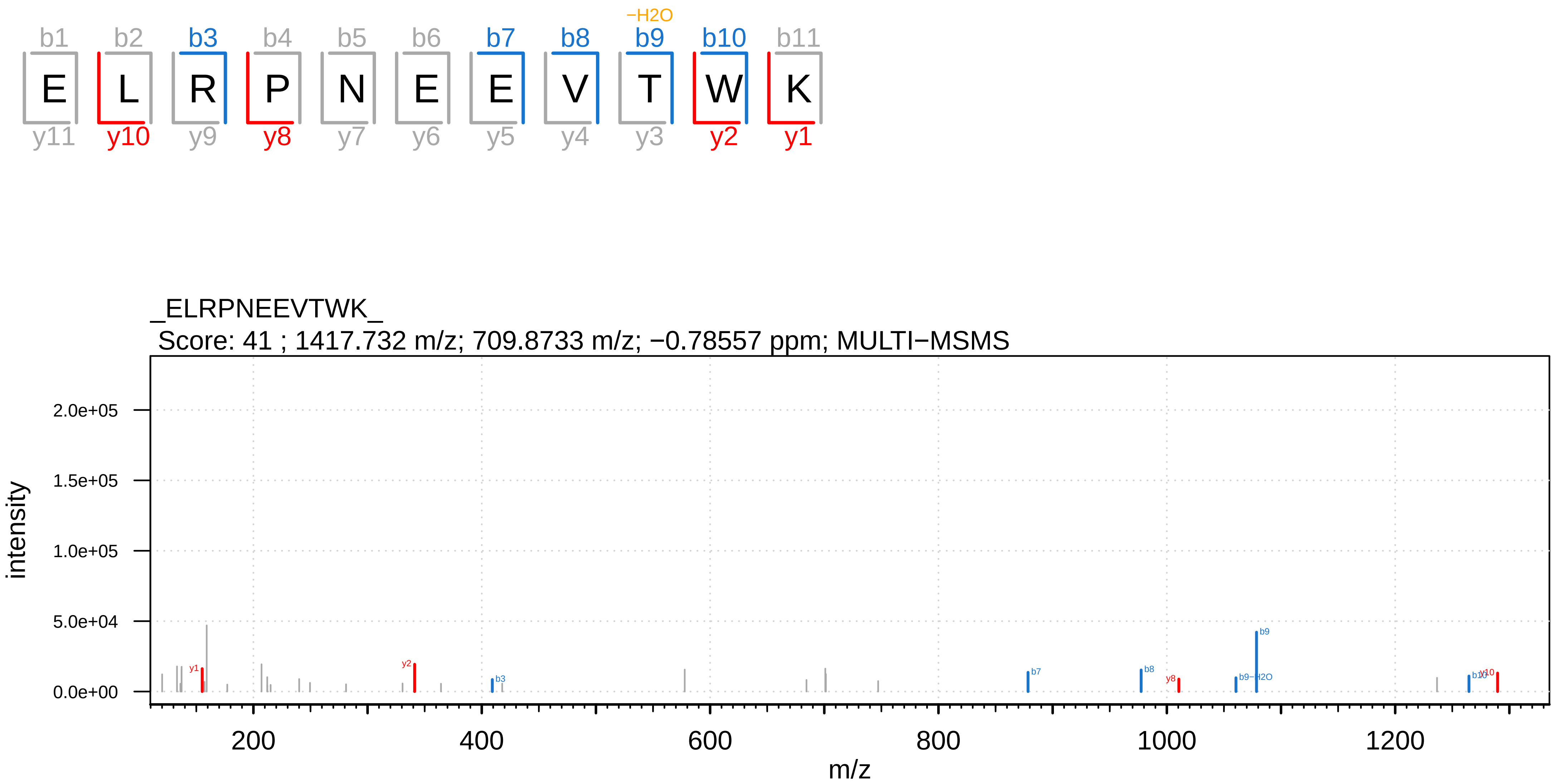

Raw File: Kermit_20111118_MSU_diff2_12
Scan Number: 52777
Proteins:
chr12_103206313−103214181.T2_chr12:101968174−101969629:−

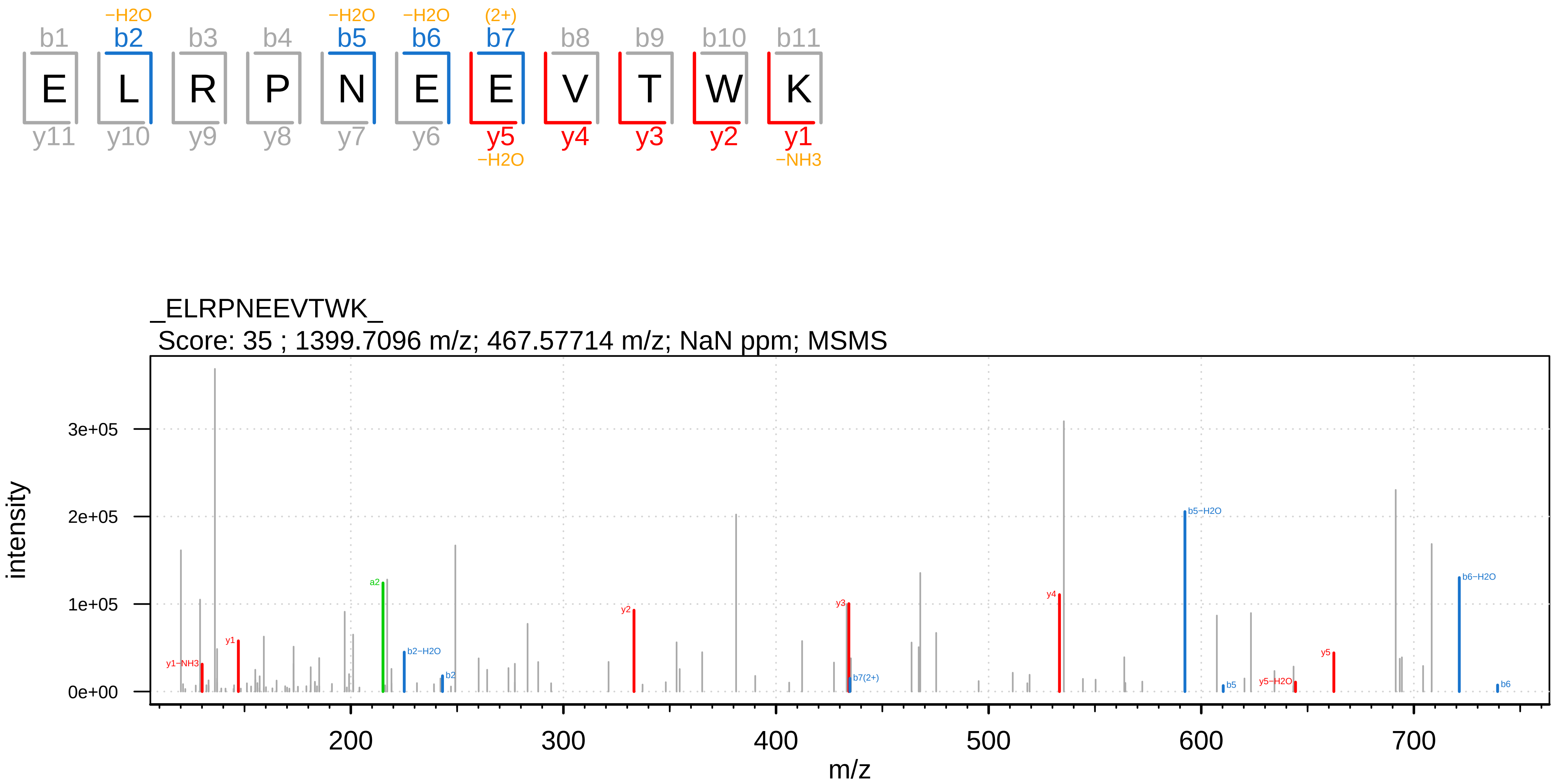

Raw File: Kermit_20111118_MSU_diff2_12
Scan Number: 52516
Proteins:
chr12_103206313−103214181.T2_chr12:101968174−101969629:−

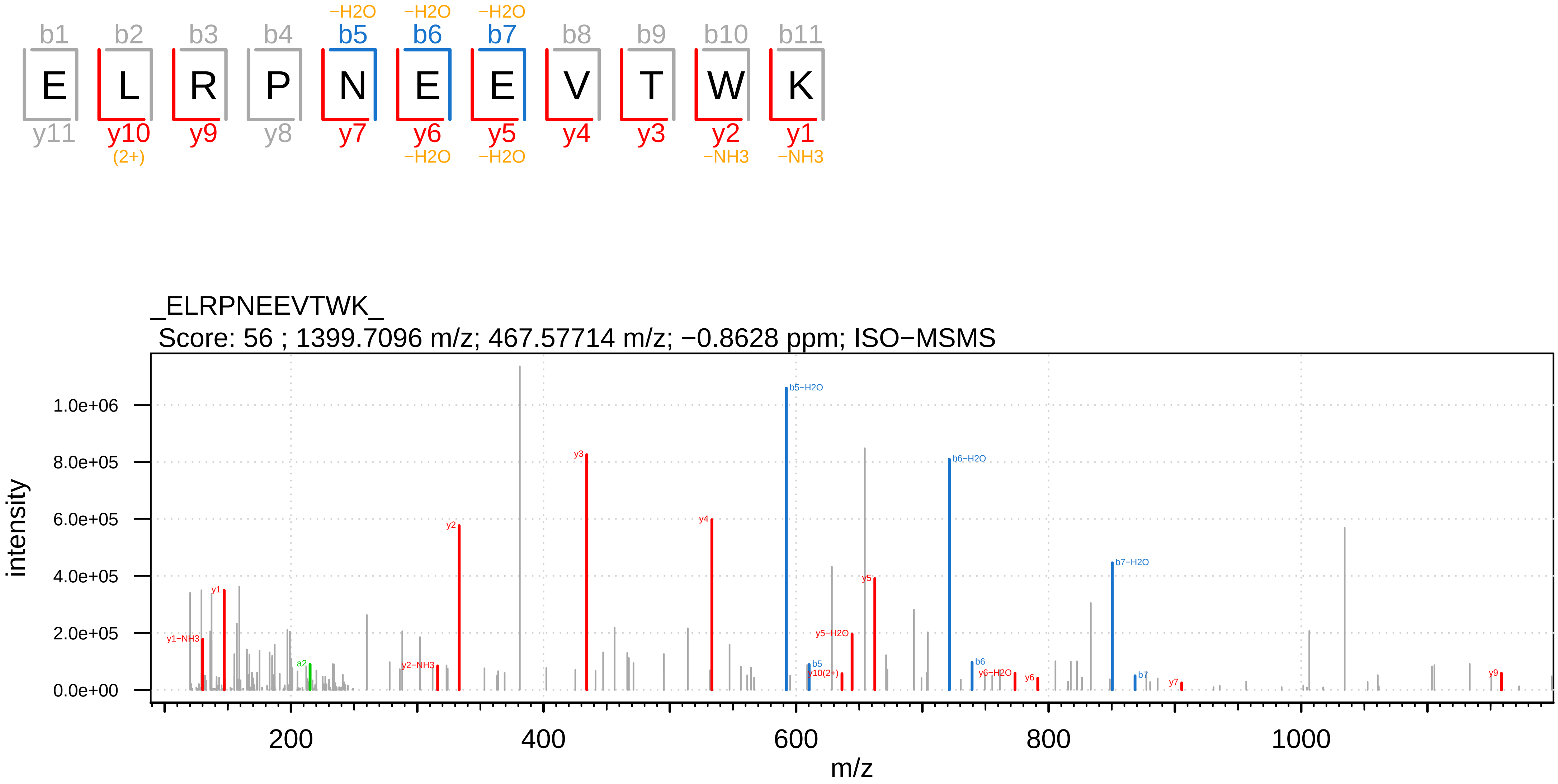

Raw File: Kermit_20111122_MSU_tube_54h_11
Scan Number: 10649
Proteins:
chr12_103206313−103214181.T2_chr12:101968174−101969629:−

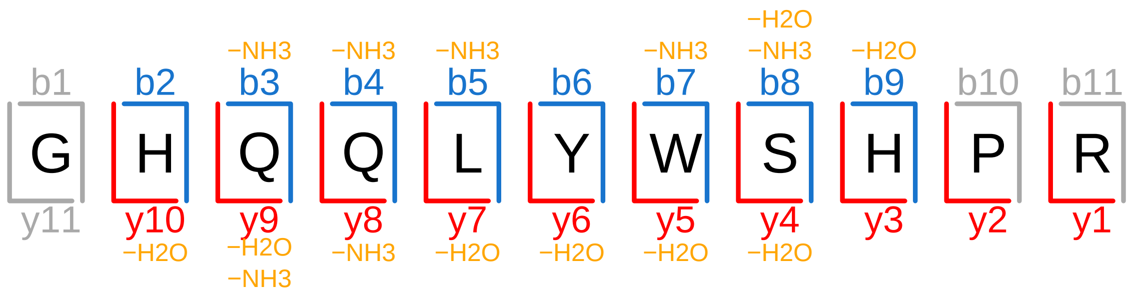

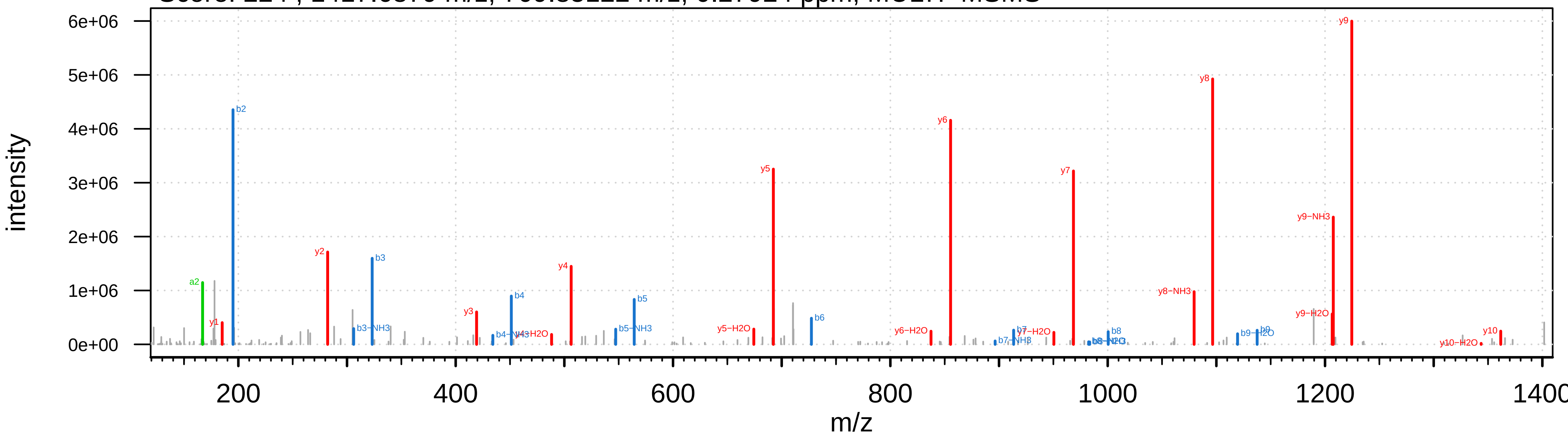

Raw File: Kermit_20111122_MSU_tube_54h_11
Scan Number: 5522
Proteins:
sp|P62274|RS29_MOUSE
ENSMUST00000037023_chr12:69157813−69159058:−
ENSMUST00000071737_chr13:73988963−73989133:−
tr|D3YYL7|D3YYL7_MOUSE

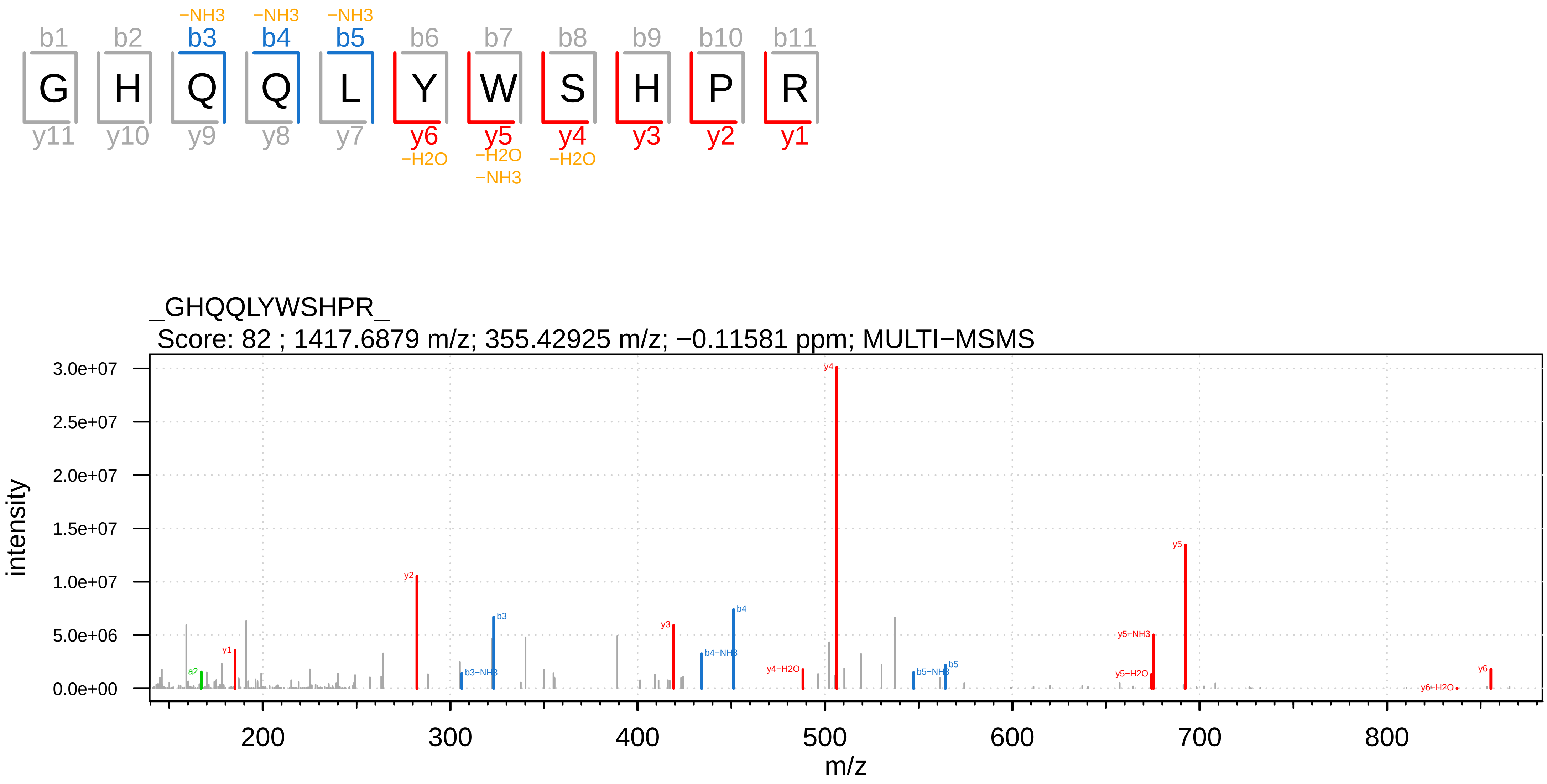

Raw File: Kermit_20111122_MSU_tube_54h_11

Scan Number: 5533

Proteins:

sp|P62274|RS29_MOUSE

ENSMUST00000037023_chr12:69157813−69159058:−

ENSMUST00000071737_chr13:73988963−73989133:−

tr|D3YYL7|D3YYL7_MOUSE

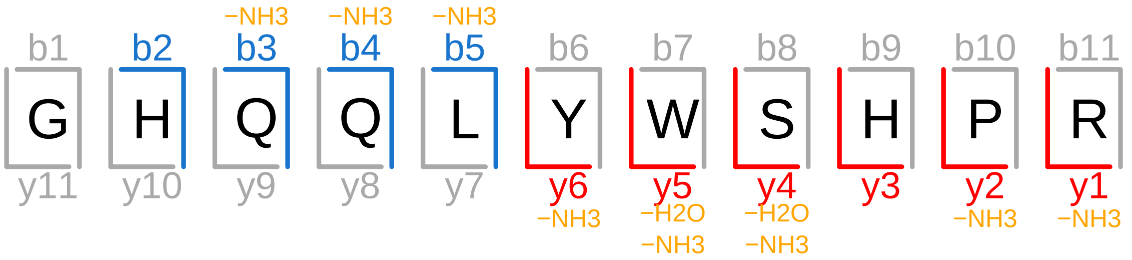

GHQQLYWSHPR
Score: 101 ; 1407.6796 m/z; 352.92718 m/z; −0.11581 ppm; MULTI−MSMS

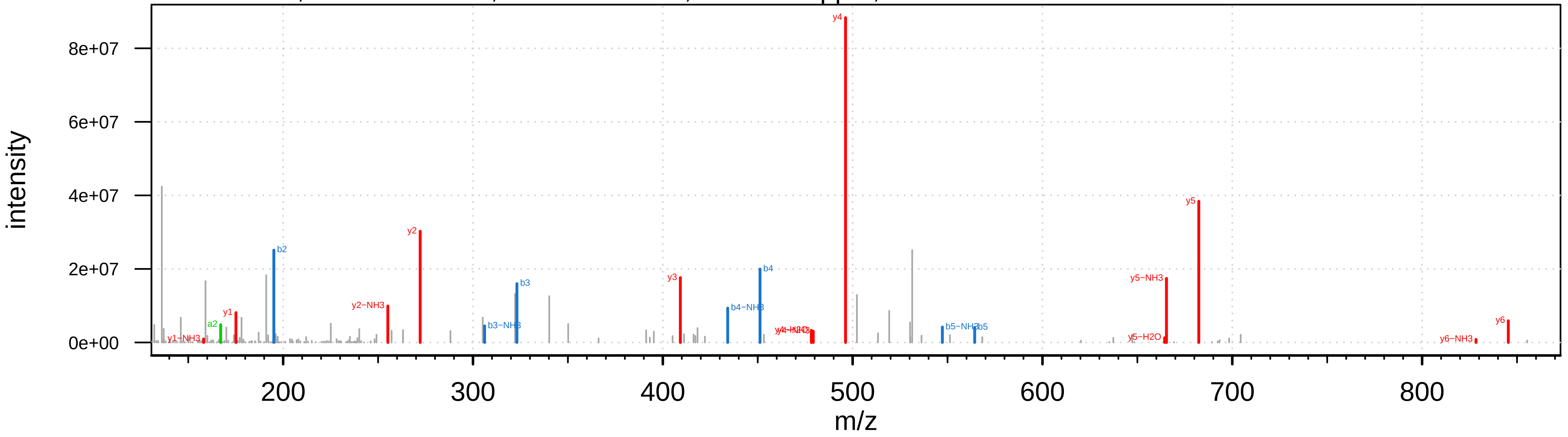

Raw File: Kermit_20111122_MSU_tube_54h_11
Scan Number: 5563
Proteins:
sp|P62274|RS29_MOUSE
ENSMUST00000037023_chr12:69157813−69159058:−
ENSMUST00000071737_chr13:73988963−73989133:−
tr|D3YYL7|D3YYL7_MOUSE

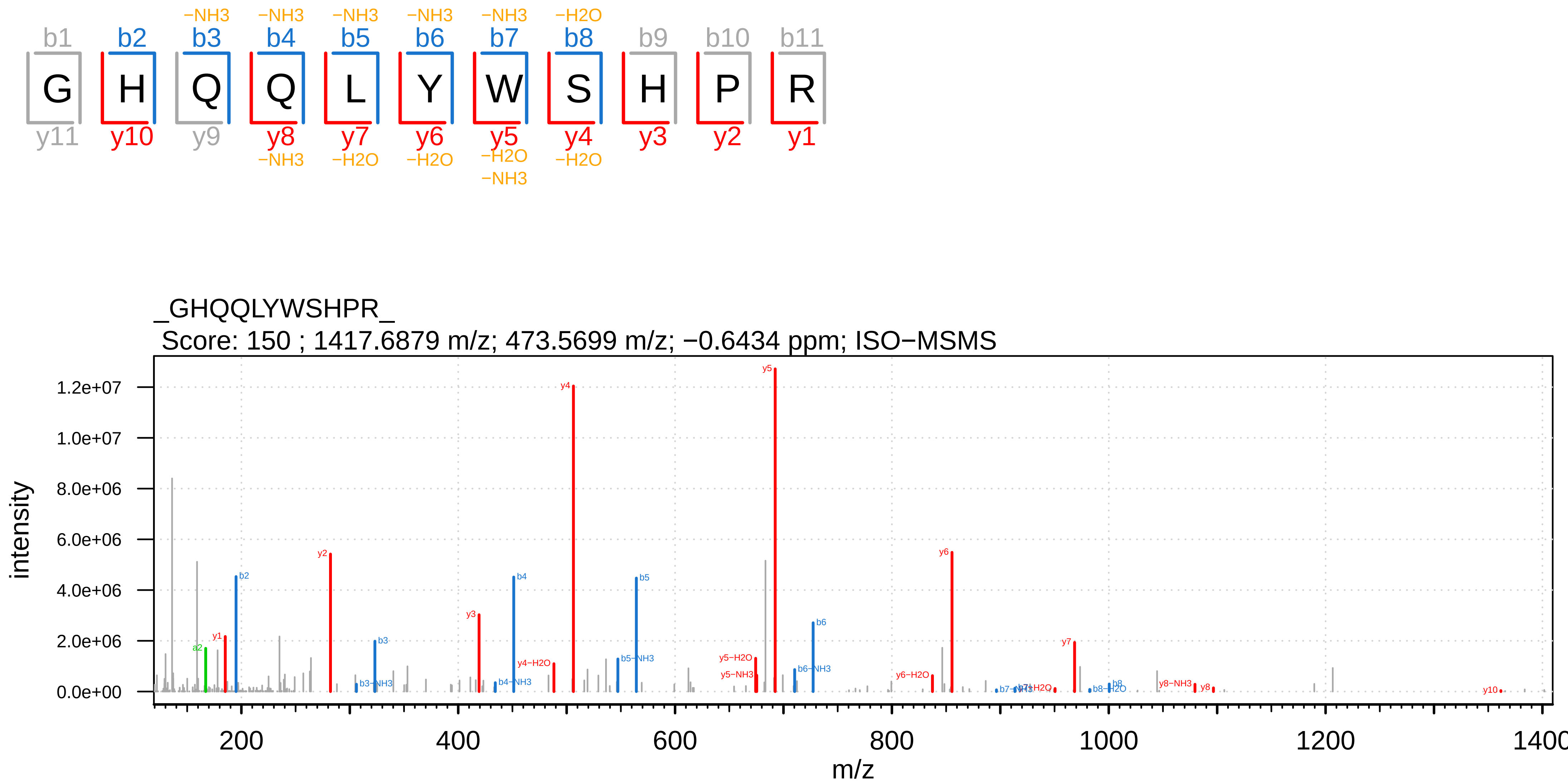

Raw File: Kermit_20111122_MSU_tube_54h_11
Scan Number: 5502
Proteins:
sp|P62274|RS29_MOUSE
ENSMUST00000037023_chr12:69157813−69159058:−
ENSMUST00000071737_chr13:73988963−73989133:−
tr|D3YYL7|D3YYL7_MOUSE

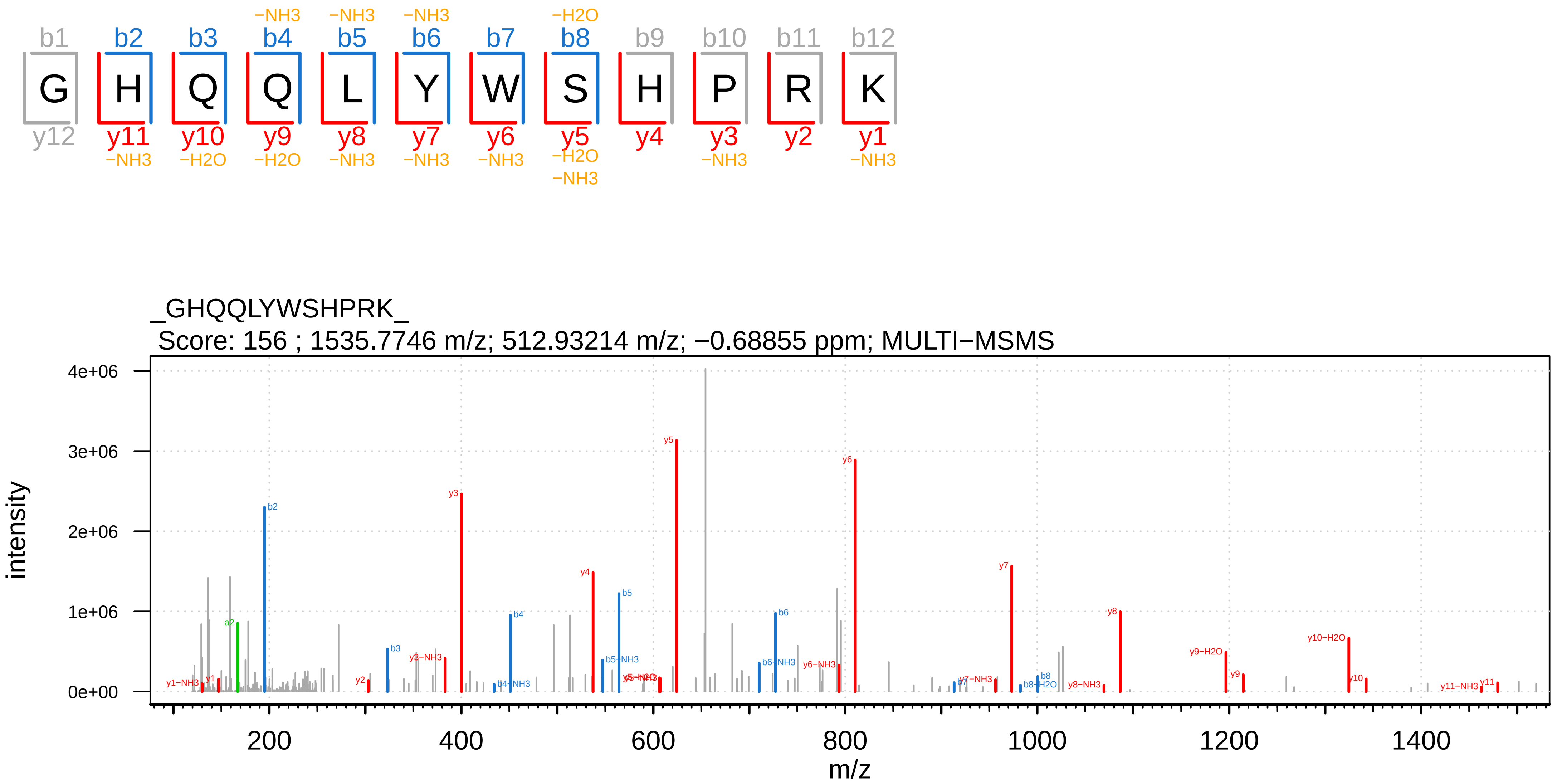

Raw File: Kermit_20111122_MSU_tube_54h_11
Scan Number: 4138
Proteins:
sp|P62274|RS29_MOUSE
ENSMUST00000037023_chr12:69157813−69159058:−
ENSMUST00000071737_chr13:73988963−73989133:−
tr|D3YYL7|D3YYL7_MOUSE

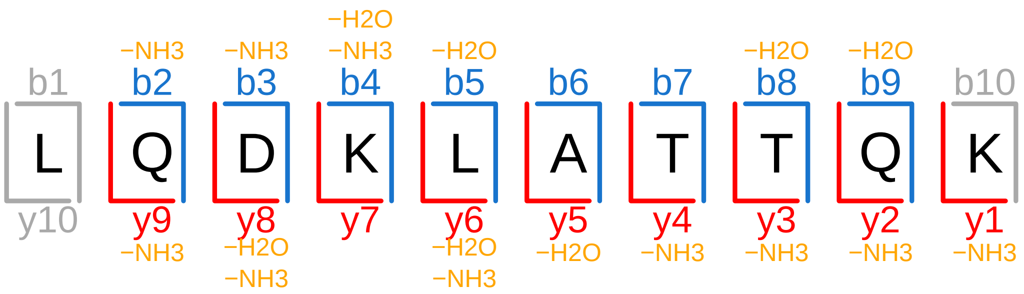

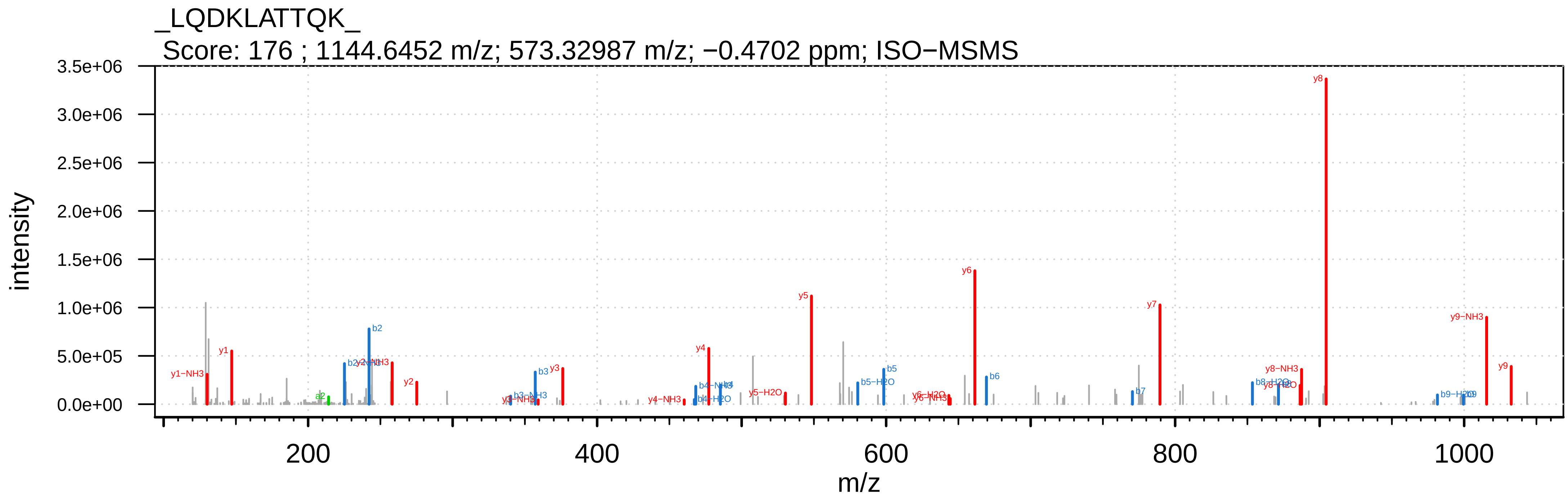

Raw File: Kermit_20111122_MSU_tube_54h_11
Scan Number: 3495
Proteins:
sp|Q8BT35|YB039_MOUSE
ENSMUST00000135091_chr2:127792297−127792467:−

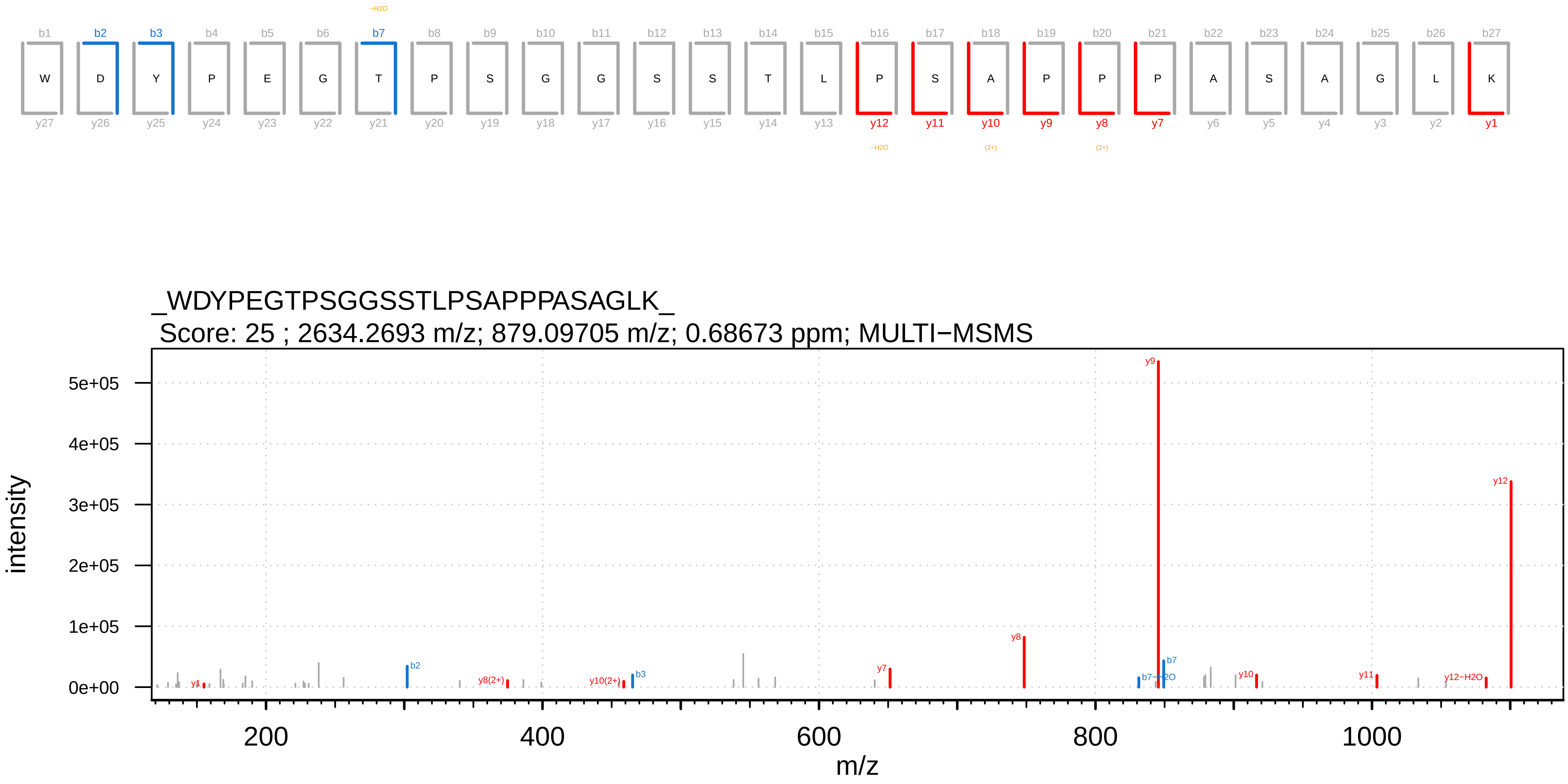

Raw File: Kermit_20111122_MSU_tube_54h_11
Scan Number: 46550
Proteins:
ENSMUST00000149514_chrX:153723700−153723906:+

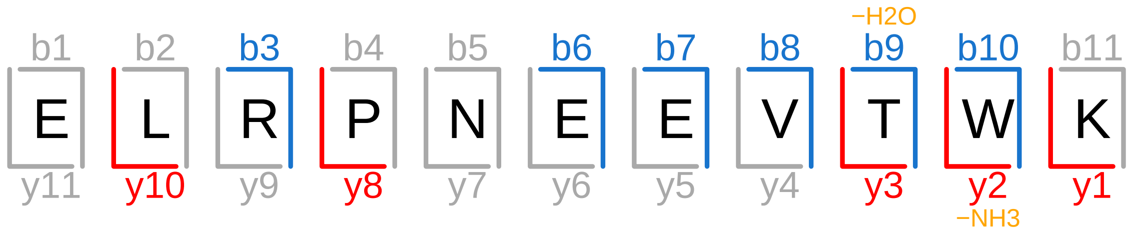

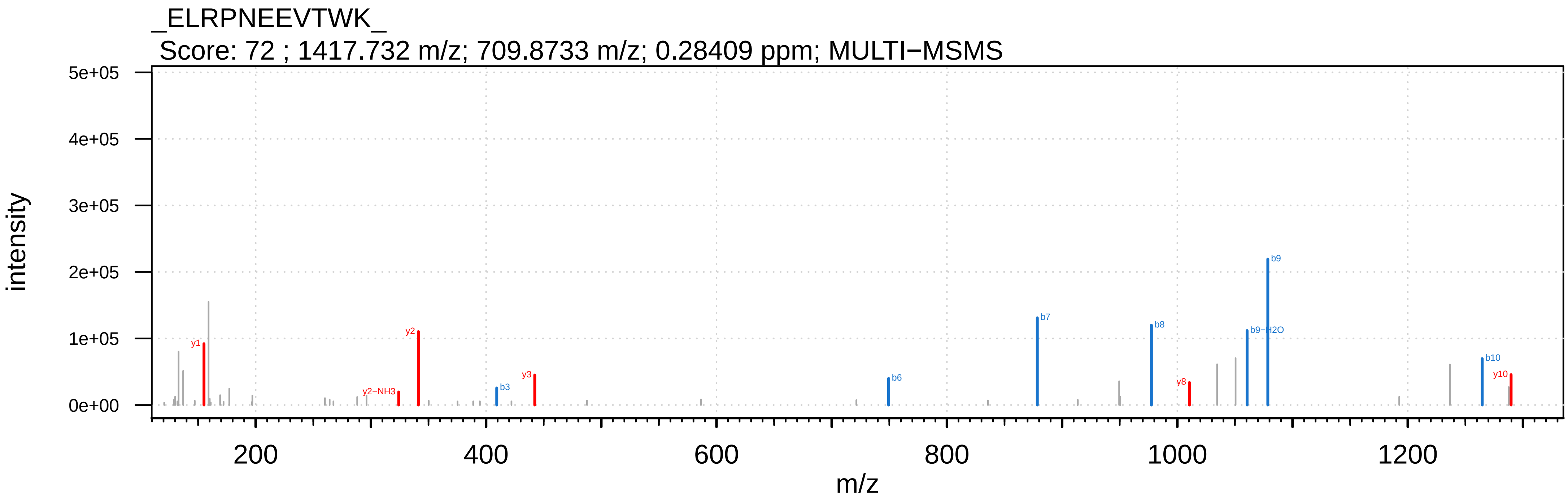

Raw File: Kermit_20111122_MSU_tube_54h_12
Scan Number: 45412
Proteins:
chr12_103206313−103214181.T2_chr12:101968174−101969629:−

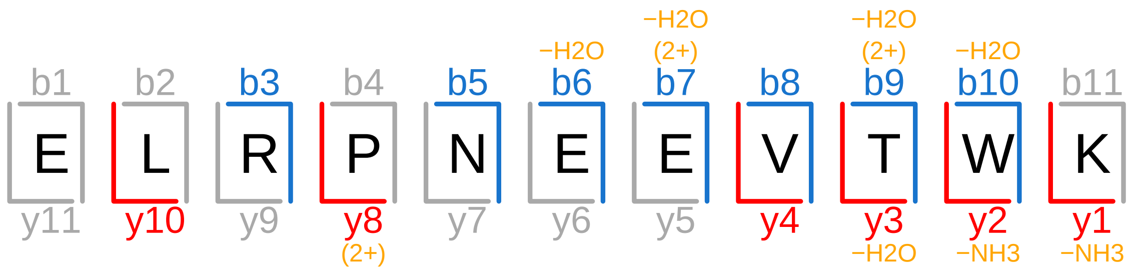

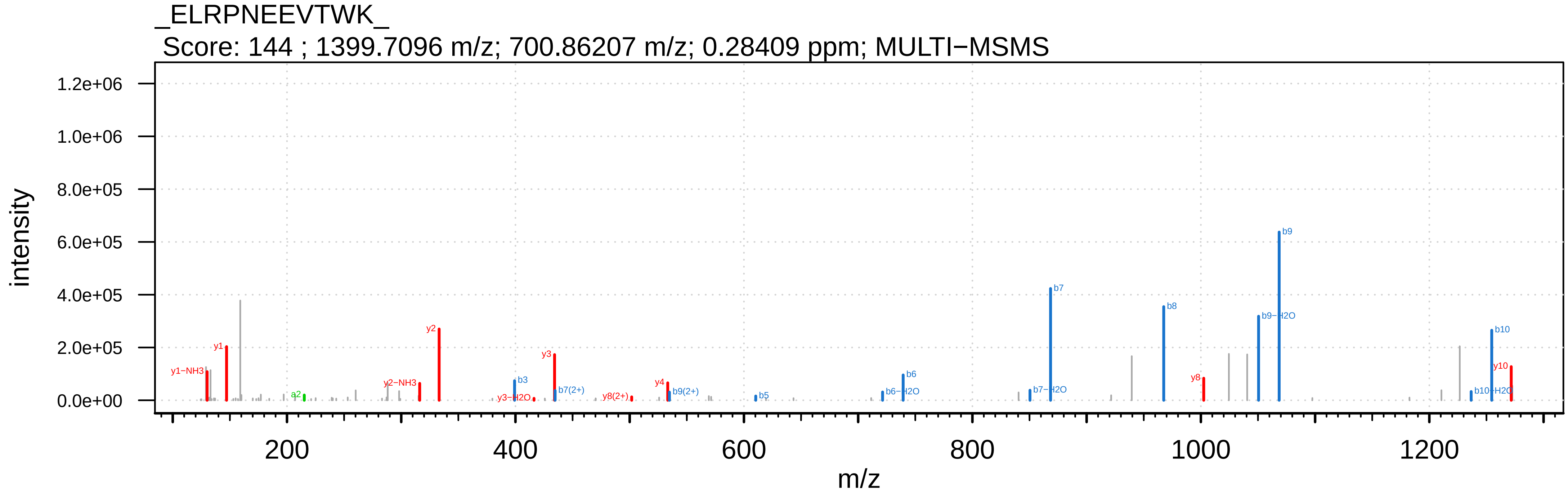

Raw File: Kermit_20111122_MSU_tube_54h_12
Scan Number: 45483
Proteins:
chr12_103206313−103214181.T2_chr12:101968174−101969629:−

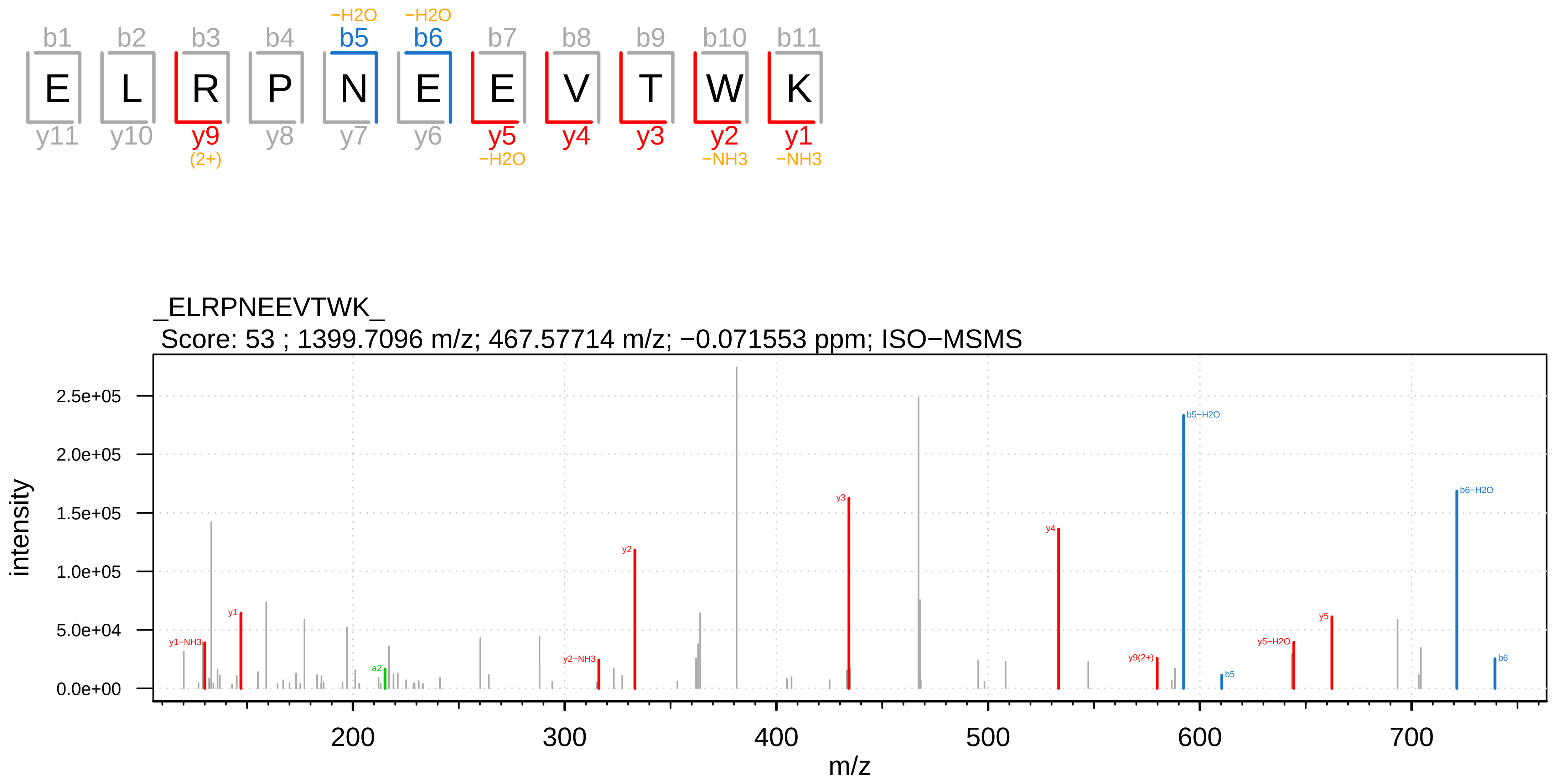

Raw File: Kermit_20111122_MSU_tube_54h_12
Scan Number: 44796
Proteins:
chr12_103206313−103214181.T2_chr12:101968174−101969629:−

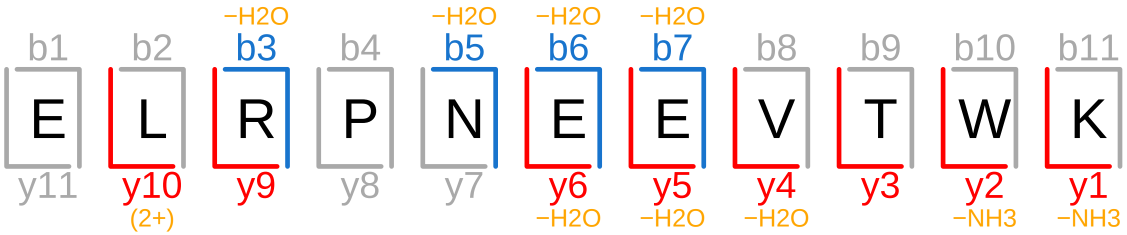

ELRPNEEVTWK

Score: 103 ; 1399.7096 m/z; 467.57714 m/z; 0.49805 ppm; ISO−MSMS

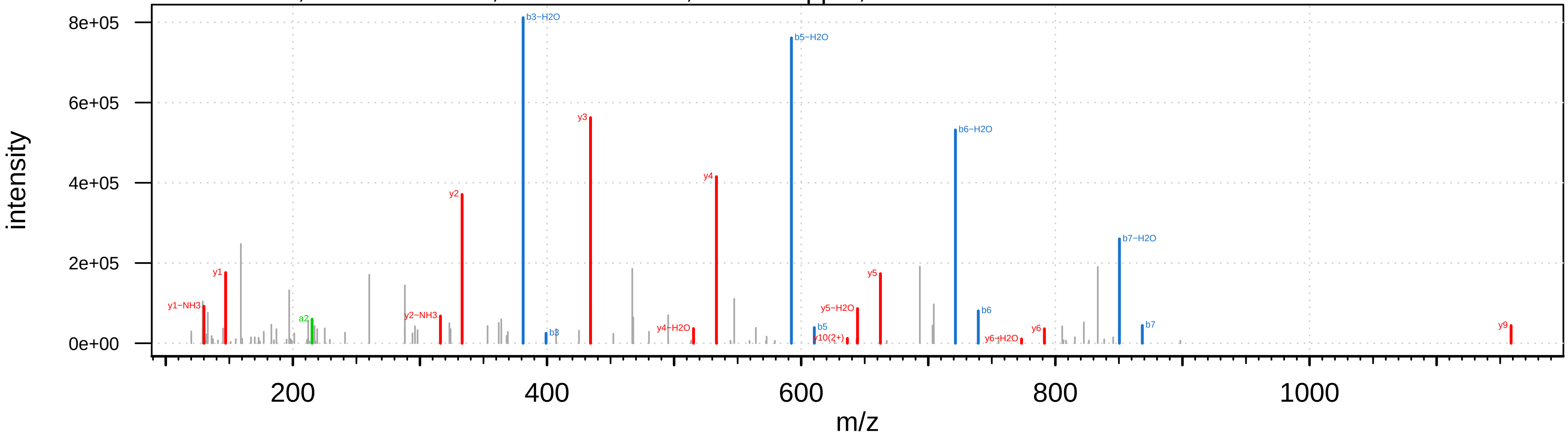

Raw File: Kermit_20111122_MSU_tube_54h_12

Scan Number: 44984

Proteins:

chr12_103206313−103214181.T2_chr12:101968174−101969629:−

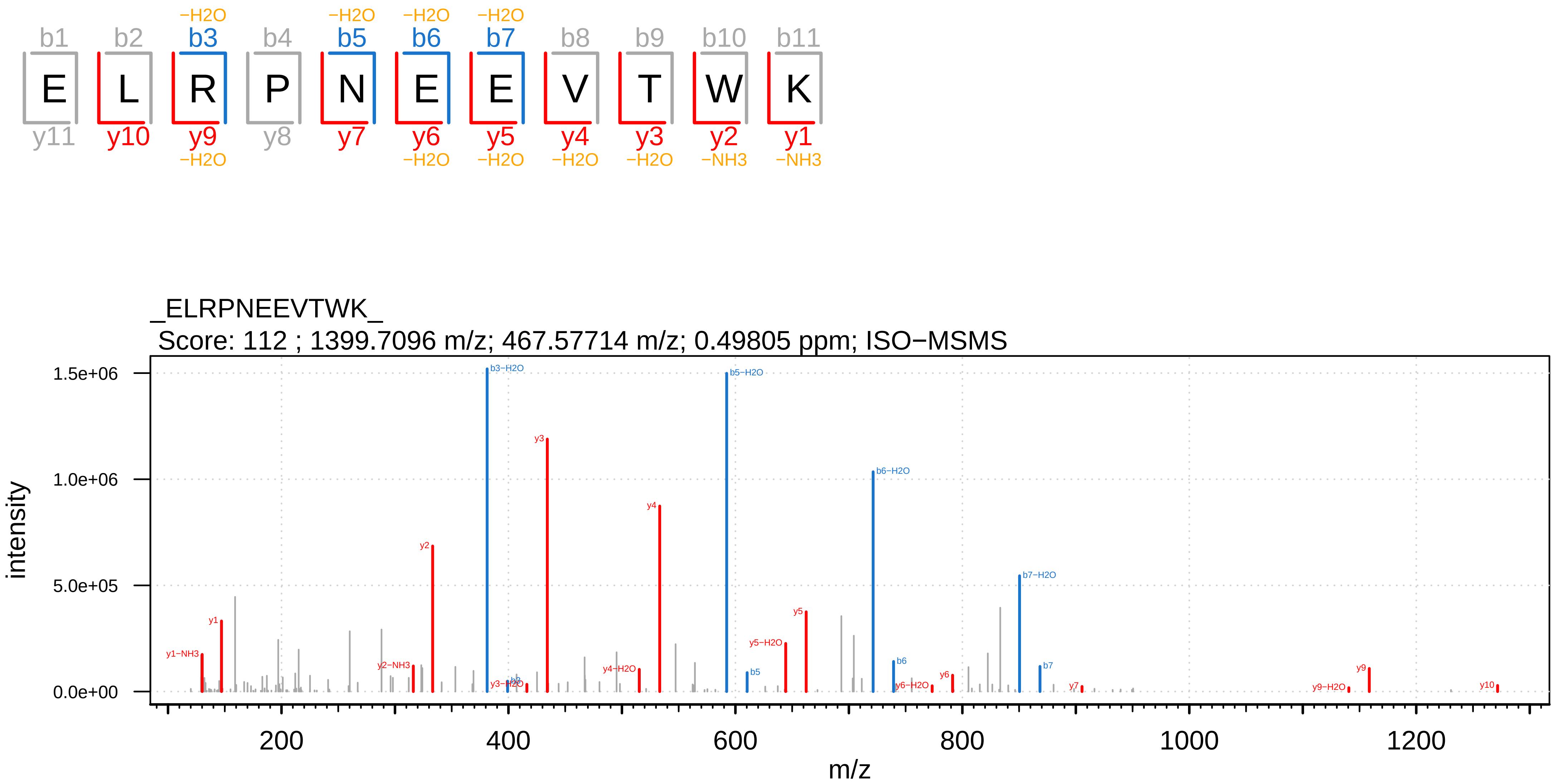

Raw File: Kermit_20111122_MSU_tube_54h_12
Scan Number: 45179
Proteins:
chr12_103206313−103214181.T2_chr12:101968174−101969629:−

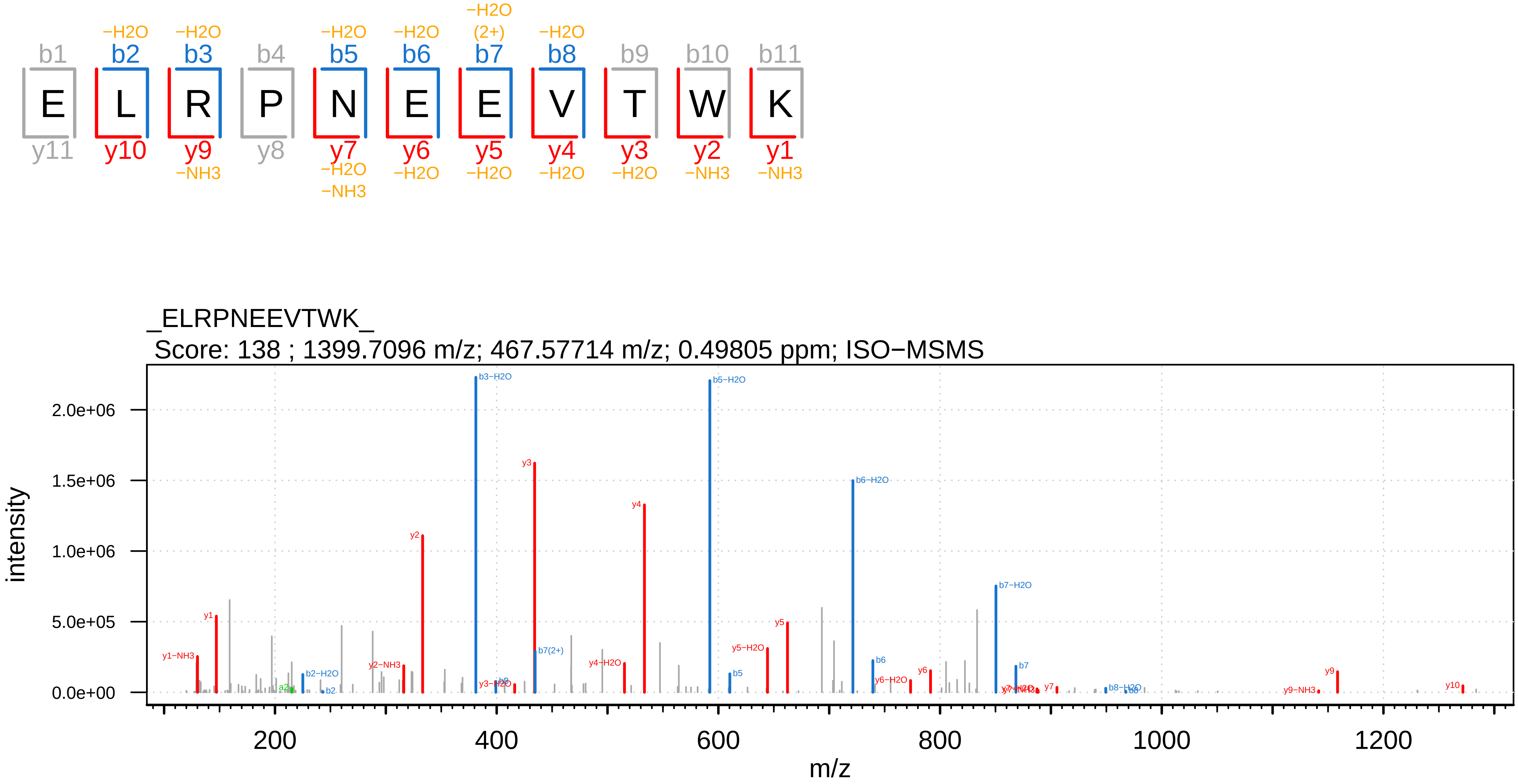

Raw File: Kermit_20111122_MSU_tube_54h_12
Scan Number: 45374
Proteins:
chr12_103206313−103214181.T2_chr12:101968174−101969629:−

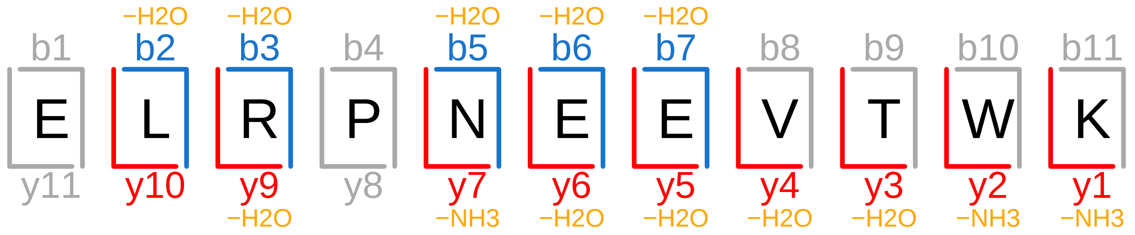

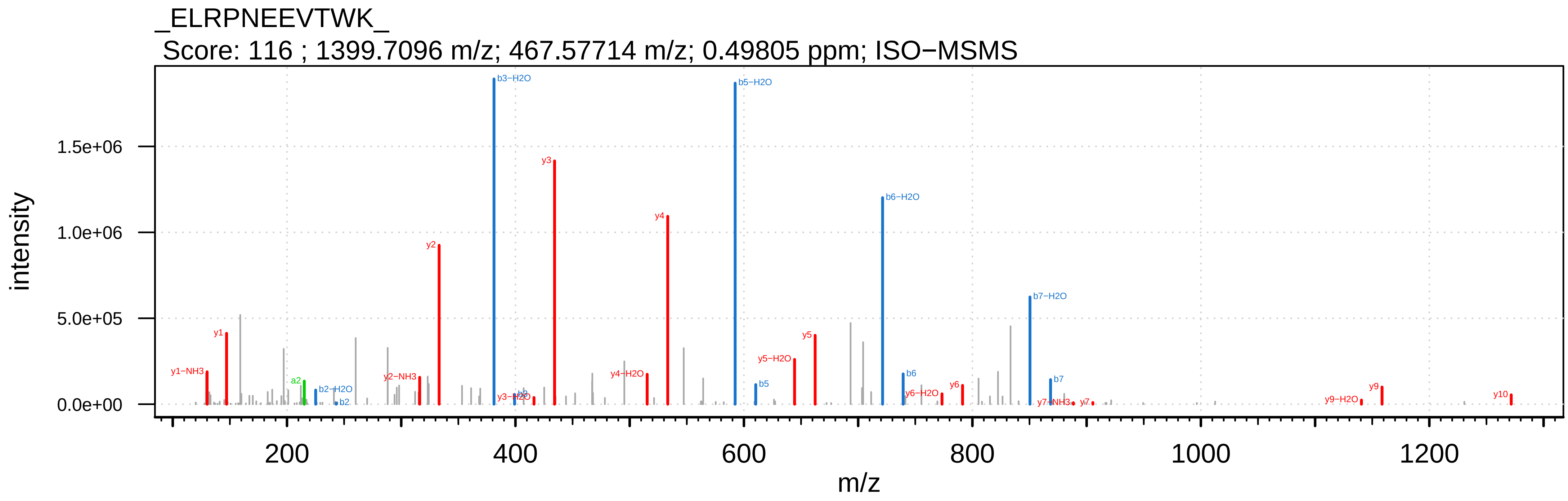

Raw File: Kermit_20111122_MSU_tube_54h_12
Scan Number: 45570
Proteins:
chr12_103206313−103214181.T2_chr12:101968174−101969629:−

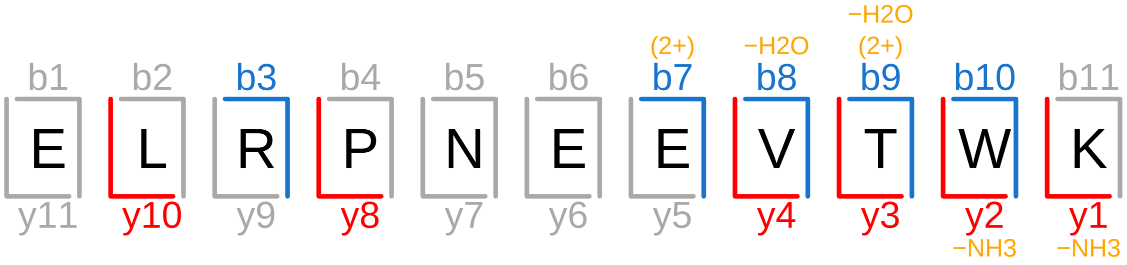

ELRPNEEVTWK
Score: 92 ; 1399.7096 m/z; 700.86207 m/z; 0.29455 ppm; ISO−MSMS

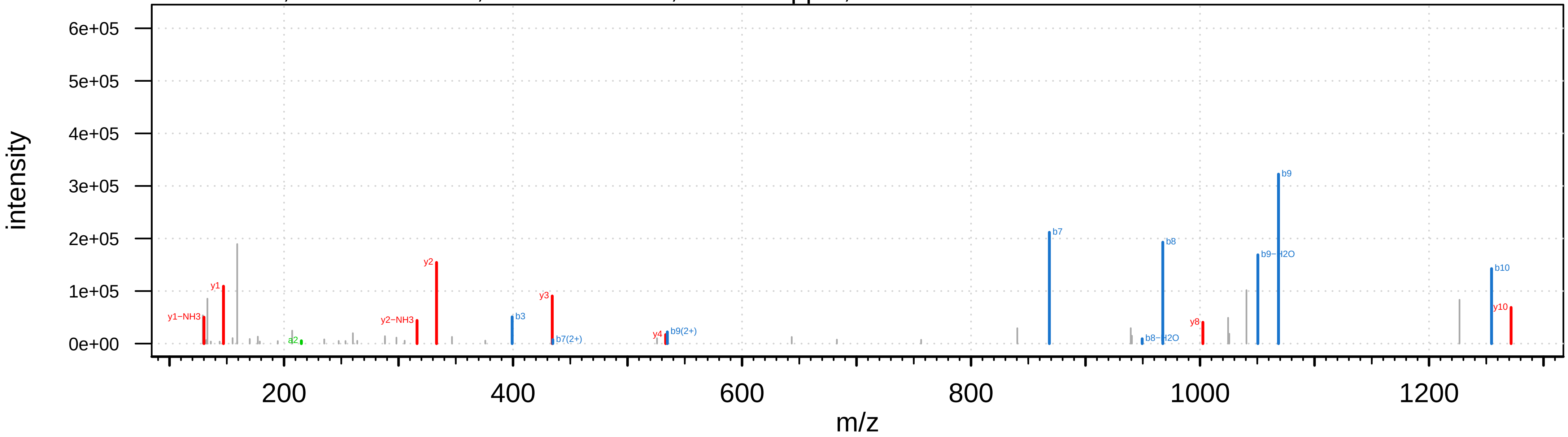

Raw File: Kermit_20111122_MSU_tube_54h_12
Scan Number: 45287
Proteins:
chr12_103206313−103214181.T2_chr12:101968174−101969629:−

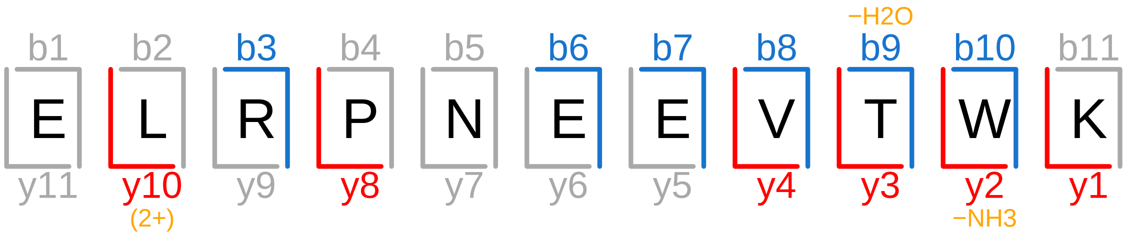

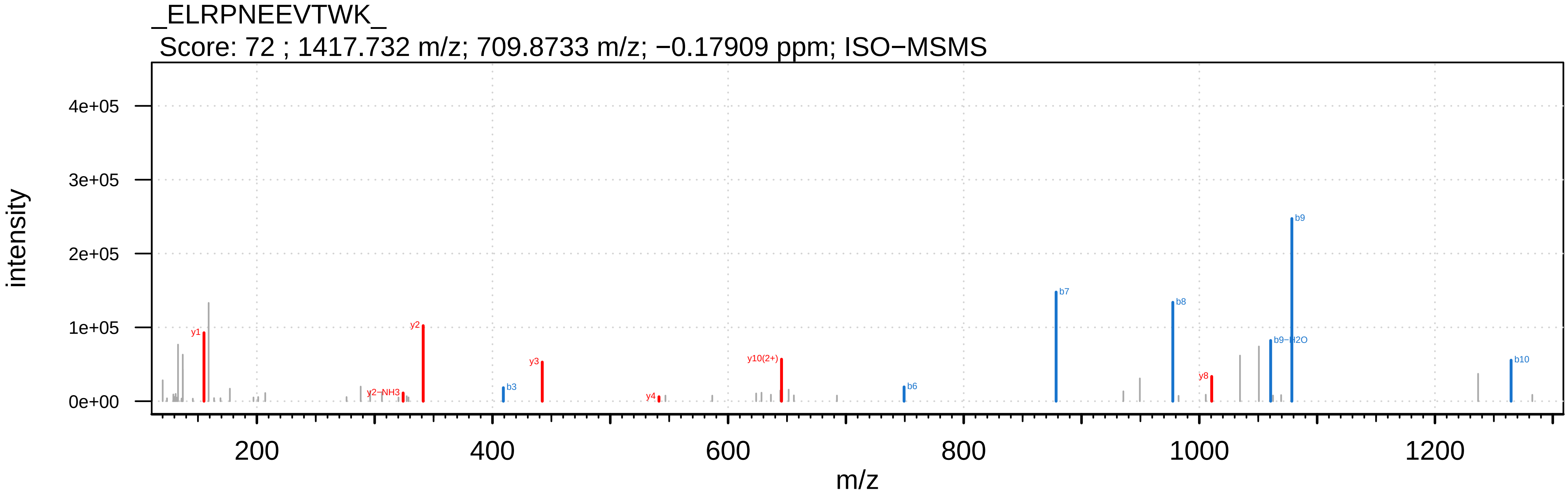

Raw File: Kermit_20111122_MSU_tube_54h_12
Scan Number: 45610
Proteins:
chr12_103206313−103214181.T2_chr12:101968174−101969629:−

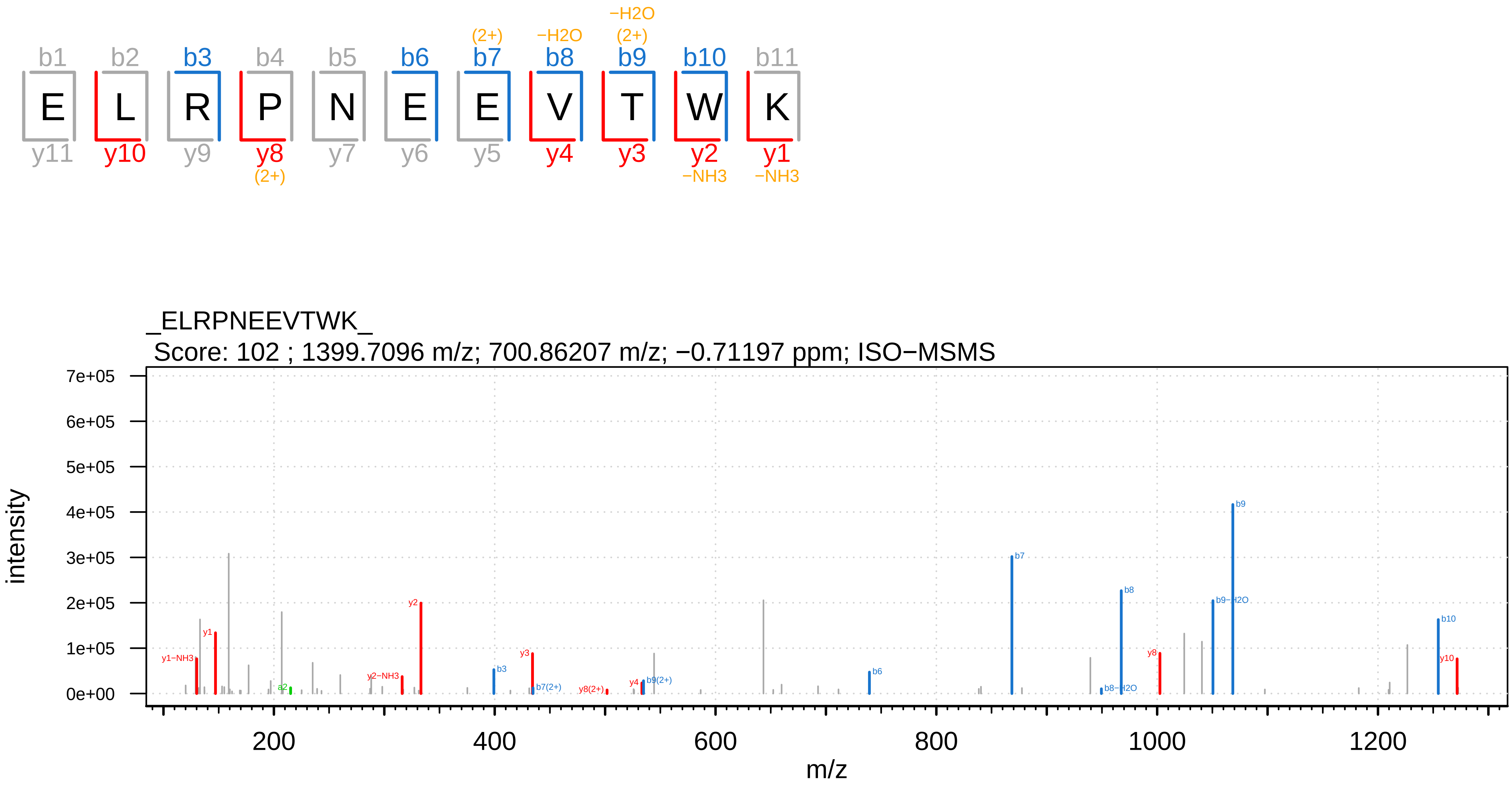

Raw File: Kermit_20111122_MSU_tube_54h_12
Scan Number: 45677
Proteins:
chr12_103206313−103214181.T2_chr12:101968174−101969629:−

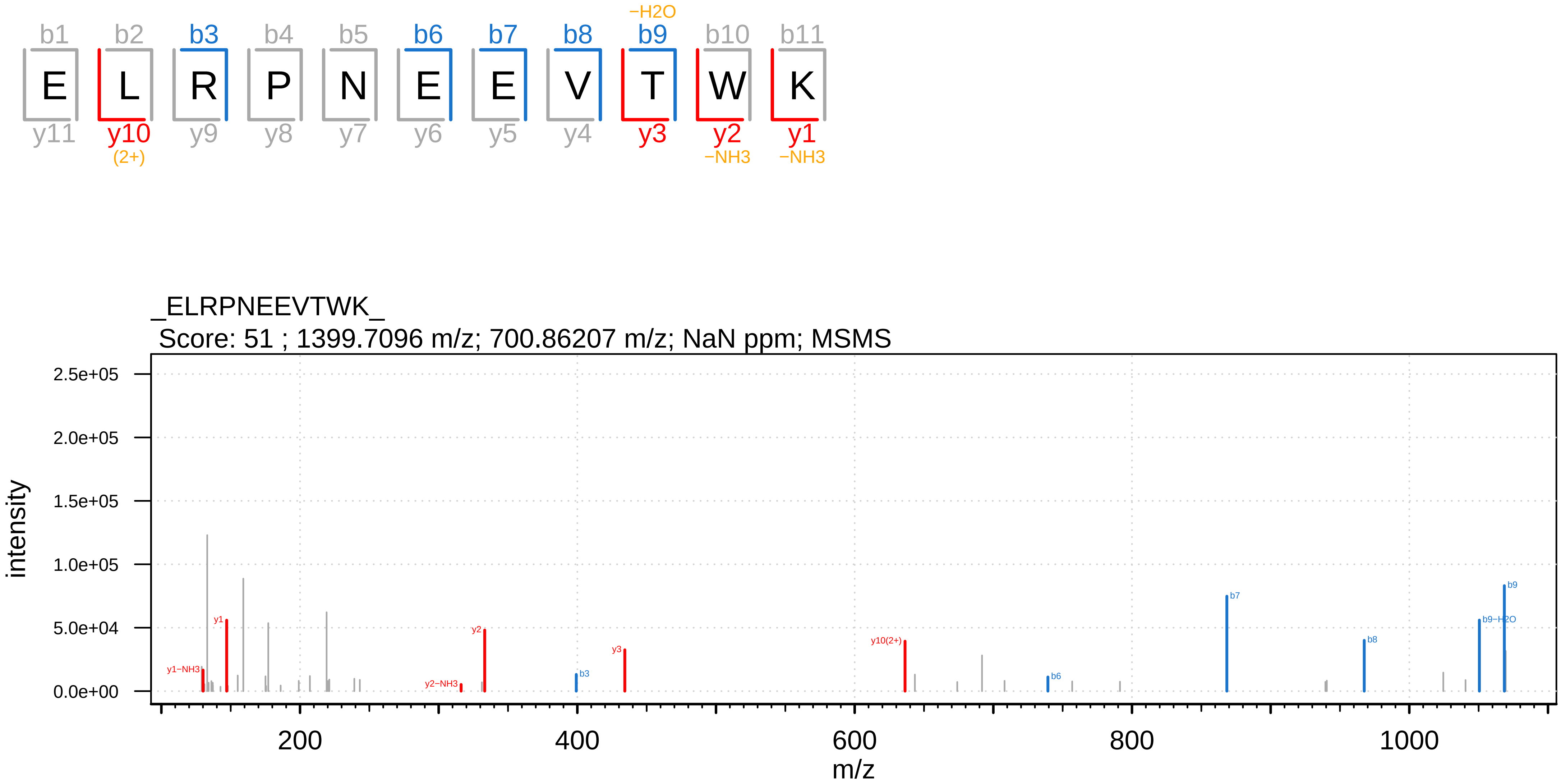

Raw File: Kermit_20111122_MSU_tube_54h_12
Scan Number: 44910
Proteins:
chr12_103206313−103214181.T2_chr12:101968174−101969629:−

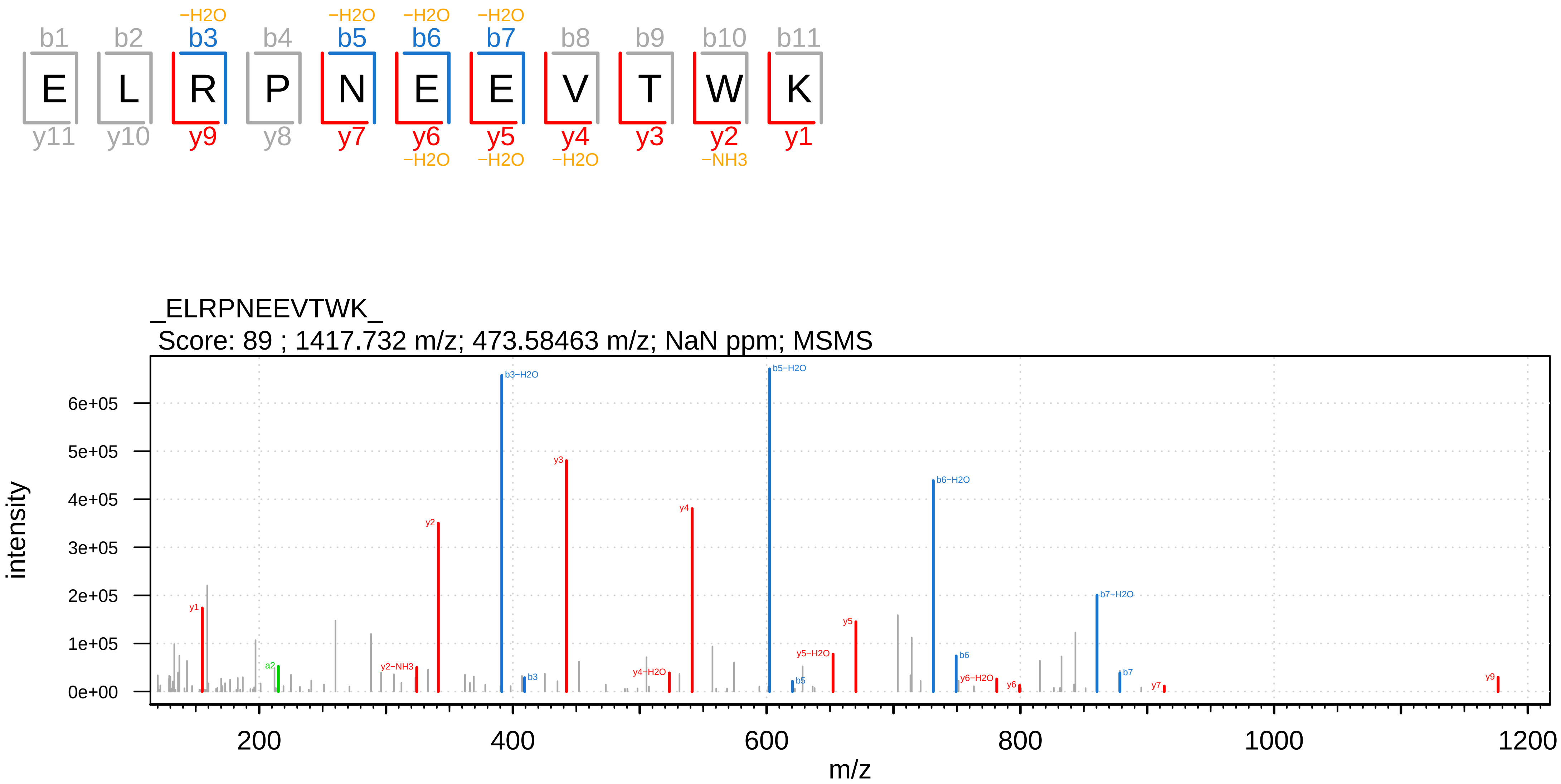

Raw File: Kermit_20111122_MSU_tube_54h_12
Scan Number: 45154
Proteins:
chr12_103206313−103214181.T2_chr12:101968174−101969629:−

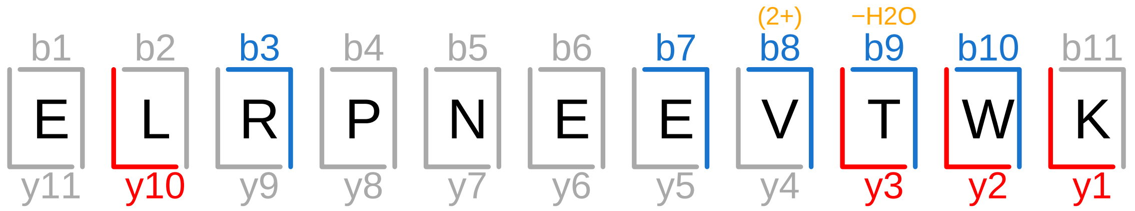

ELRPNEEVTWK
Score: 49 ; 1417.732 m/z; 709.8733 m/z; NaN ppm; MSMS

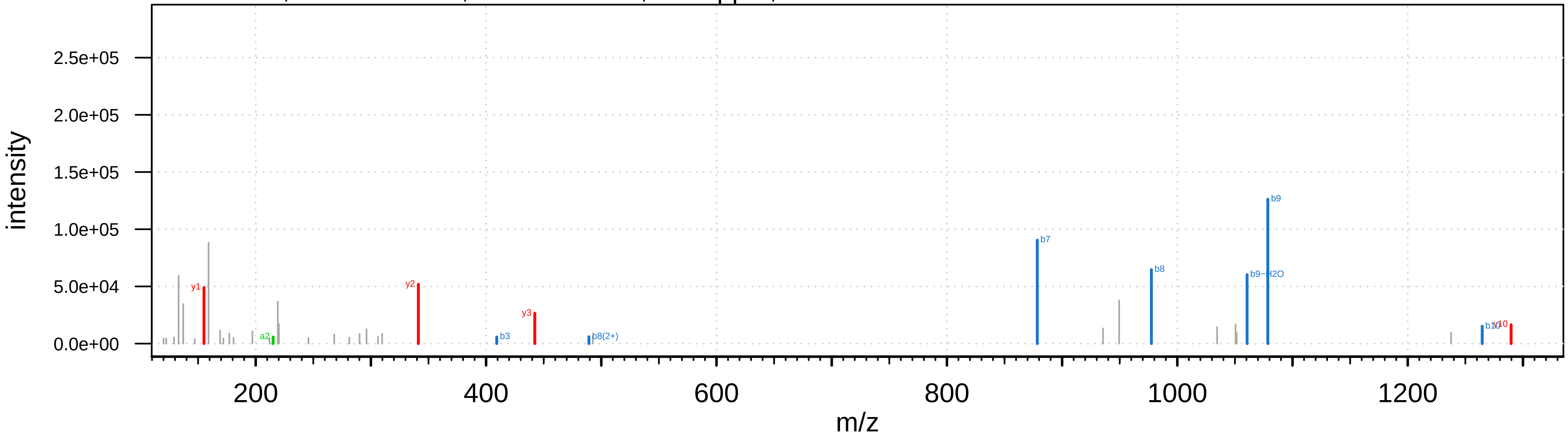

Raw File: Kermit_20111122_MSU_tube_54h_12
Scan Number: 45208
Proteins:
chr12_103206313−103214181.T2_chr12:101968174−101969629:−

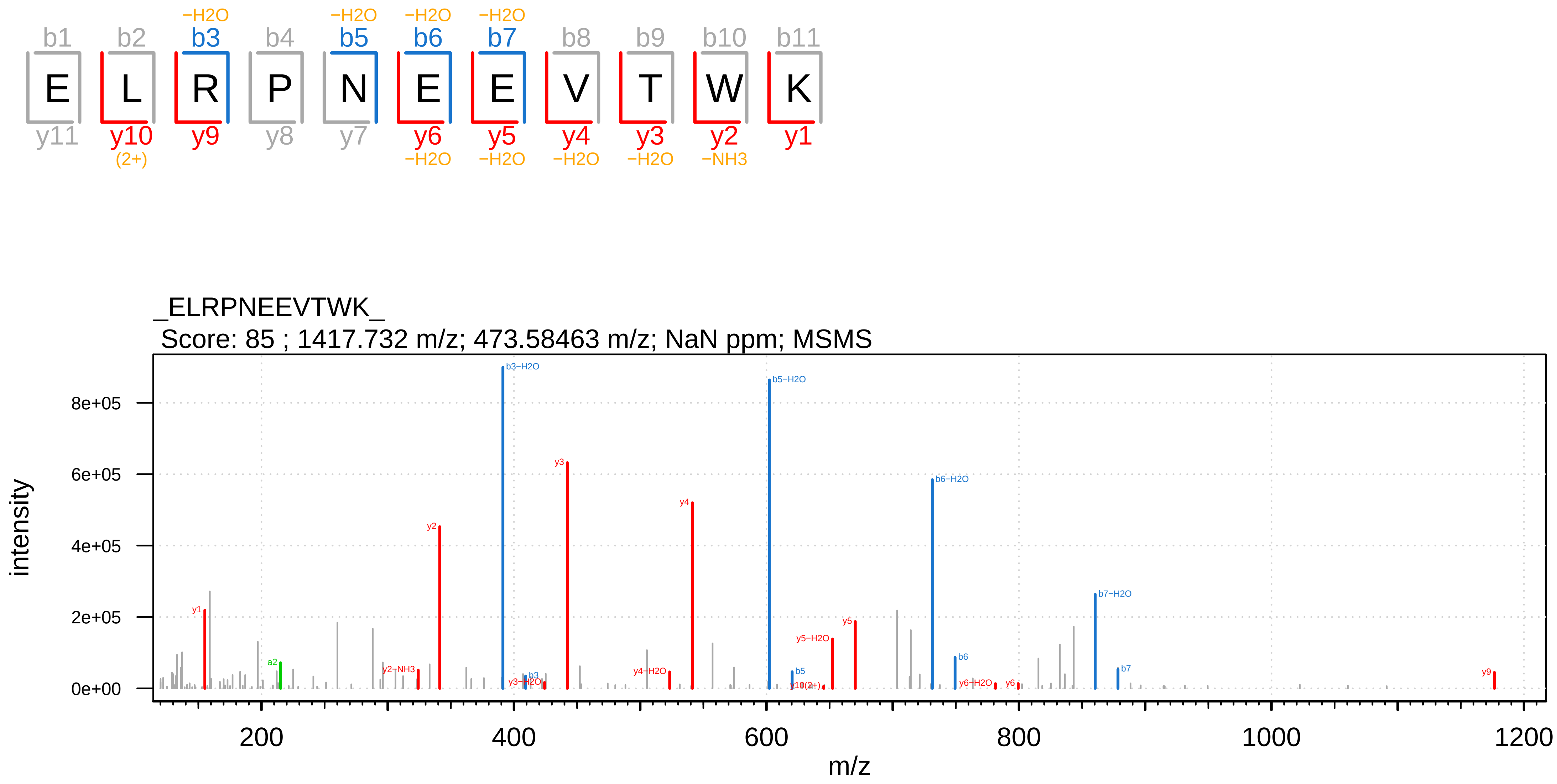

Raw File: Kermit_20111122_MSU_tube_54h_12
Scan Number: 45552
Proteins:
chr12_103206313−103214181.T2_chr12:101968174−101969629:−

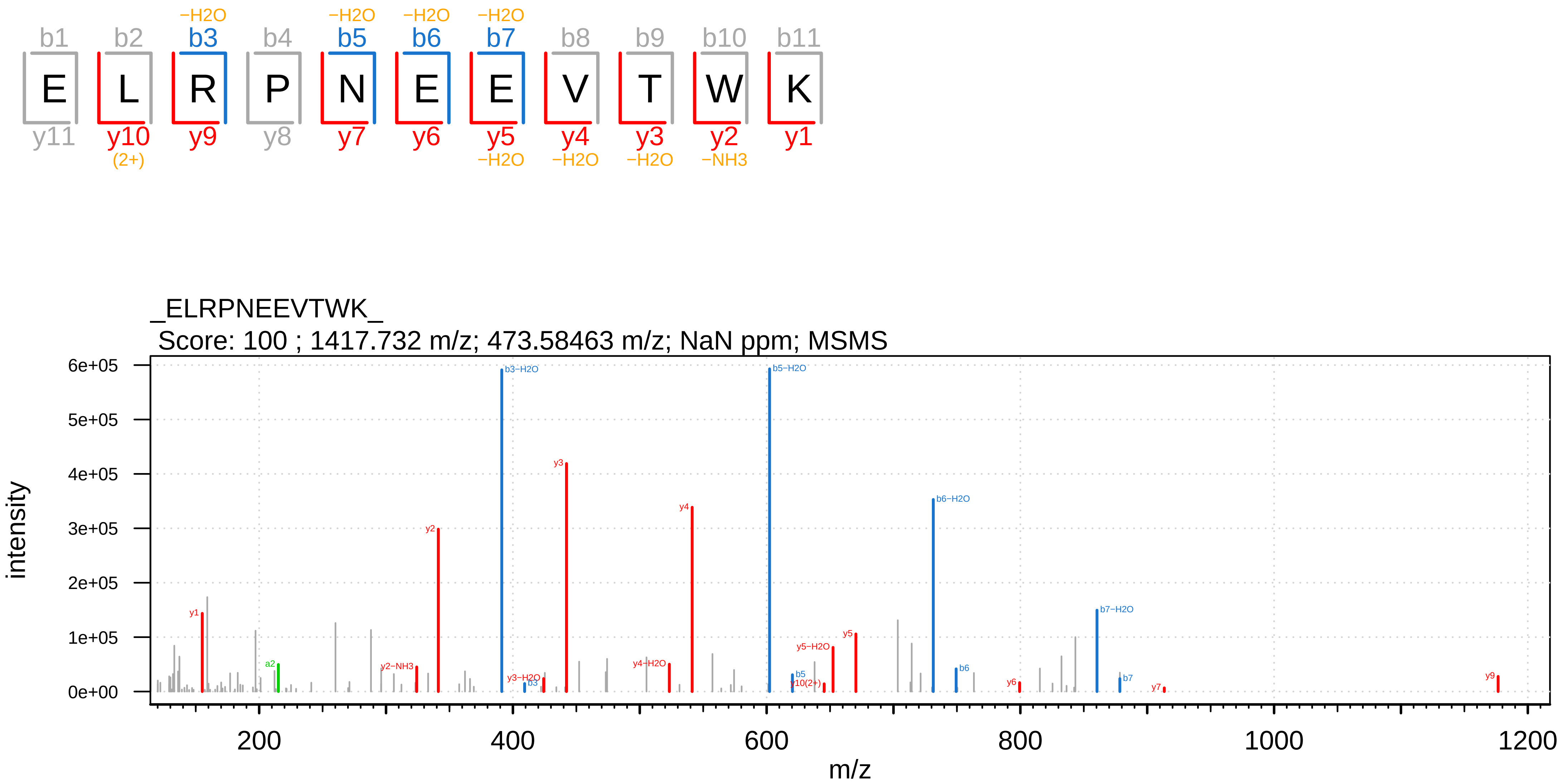

Raw File: Kermit_20111122_MSU_tube_54h_12
Scan Number: 45743
Proteins:
chr12_103206313−103214181.T2_chr12:101968174−101969629:−

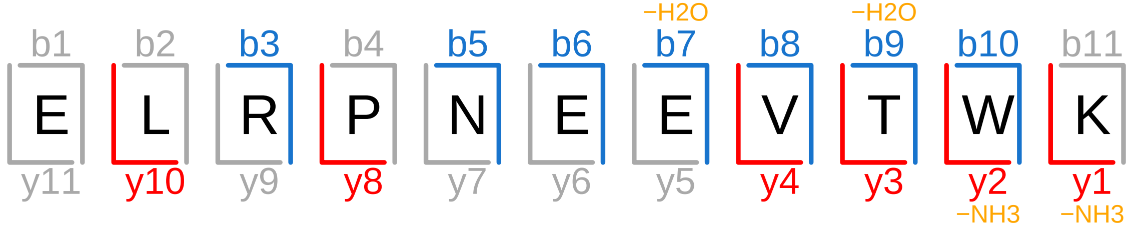

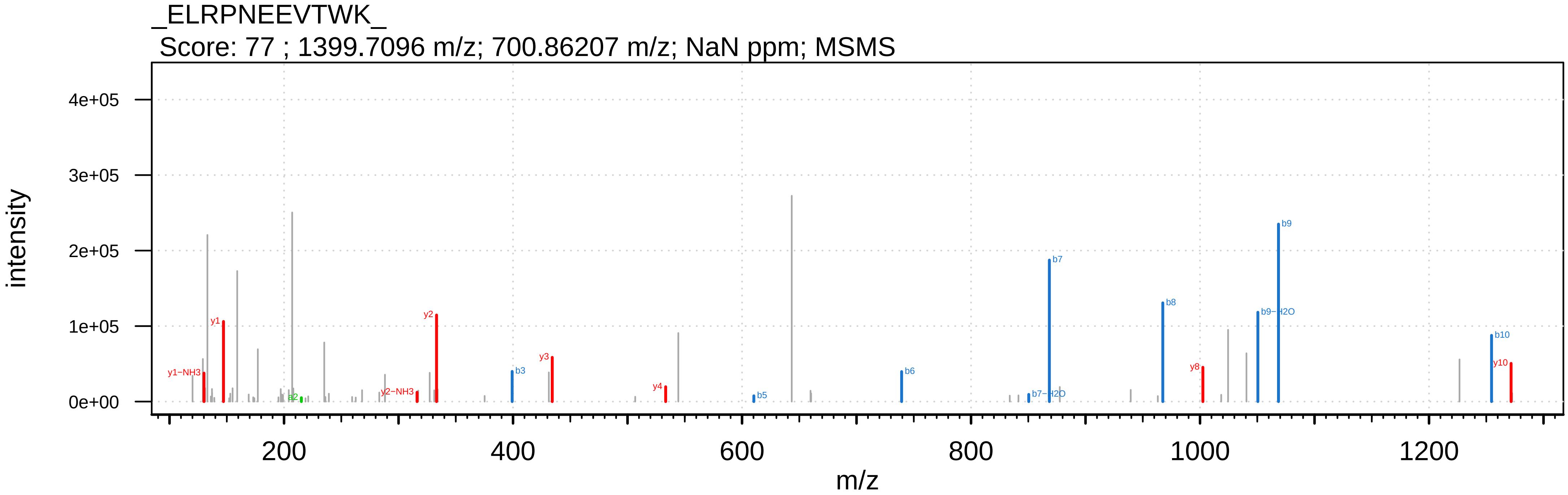

Raw File: Kermit_20111122_MSU_tube_54h_12
Scan Number: 45869
Proteins:
chr12_103206313−103214181.T2_chr12:101968174−101969629:−

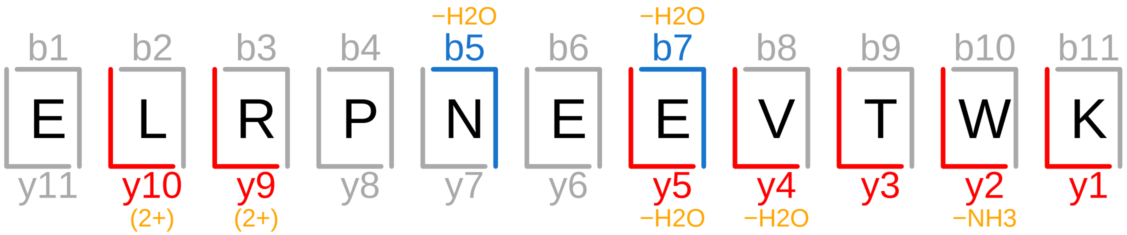

ELRPNEEVTWK
Score: 54 ; 1417.732 m/z; 473.58463 m/z; NaN ppm; MSMS

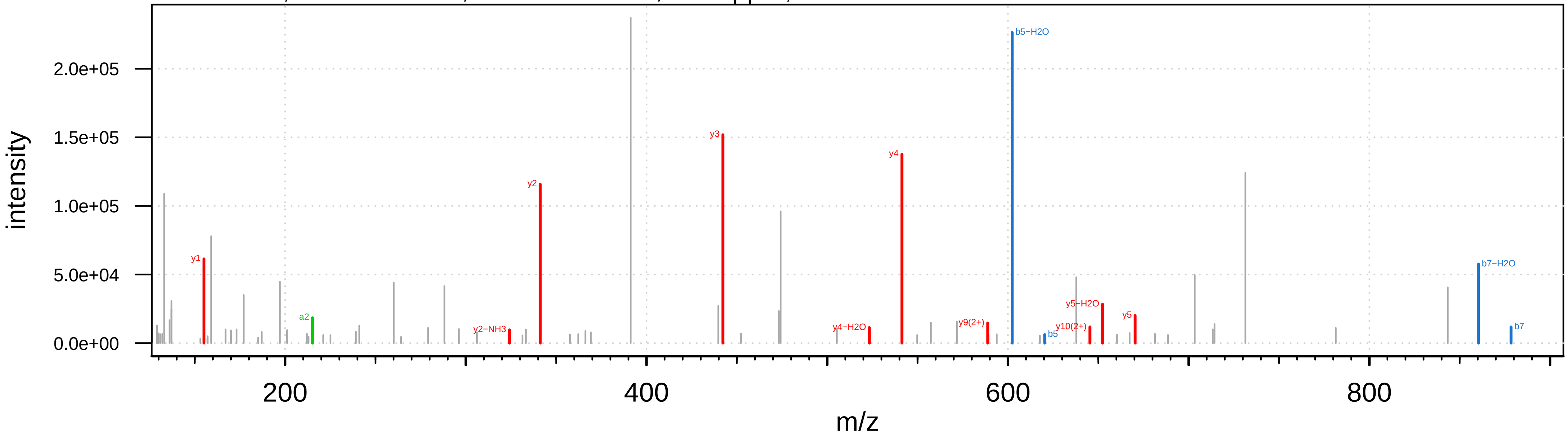

Raw File: Kermit_20111122_MSU_tube_54h_12
Scan Number: 45931
Proteins:
chr12_103206313−103214181.T2_chr12:101968174−101969629:−

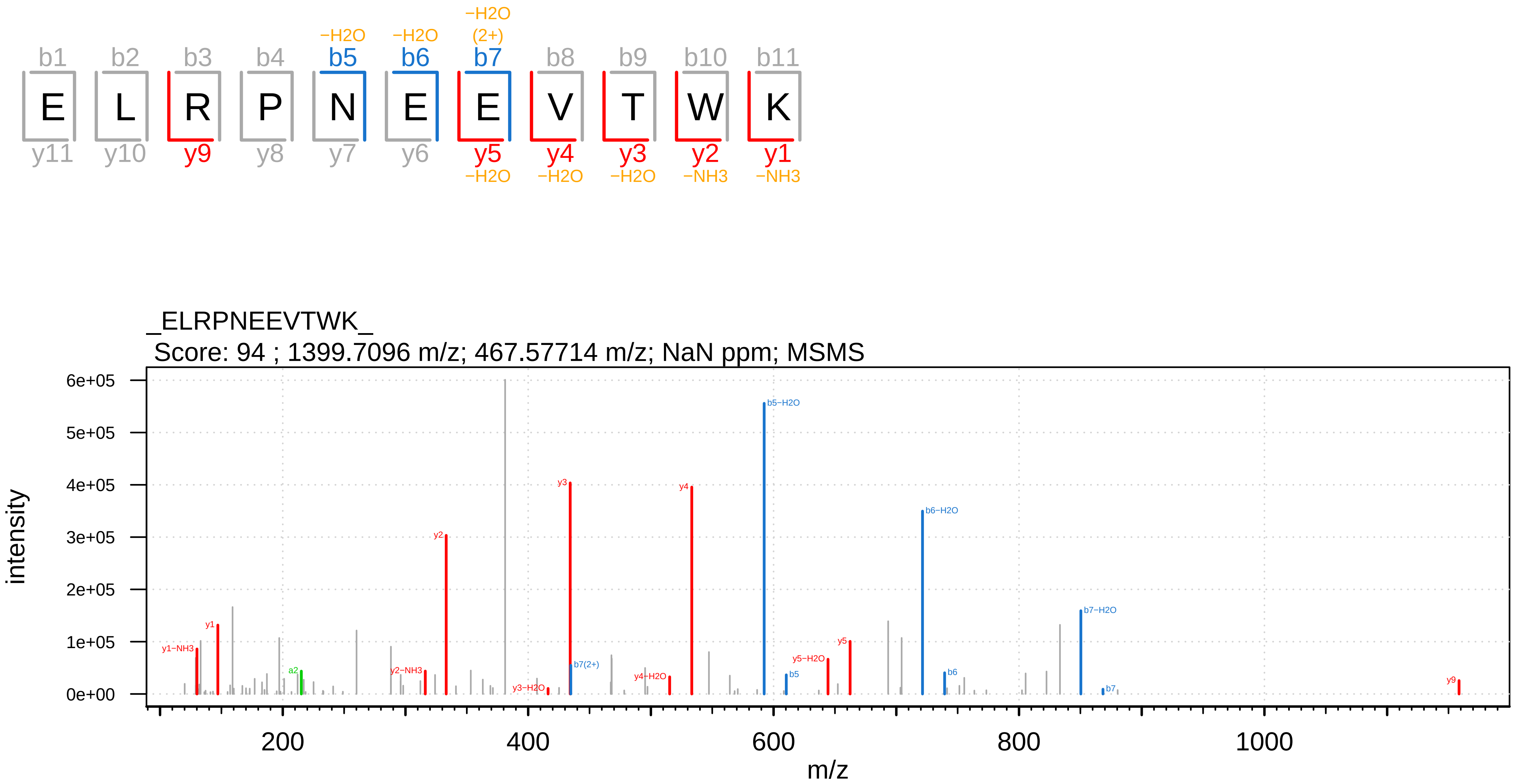

Raw File: Kermit_20111122_MSU_tube_54h_12
Scan Number: 45952
Proteins:
chr12_103206313−103214181.T2_chr12:101968174−101969629:−

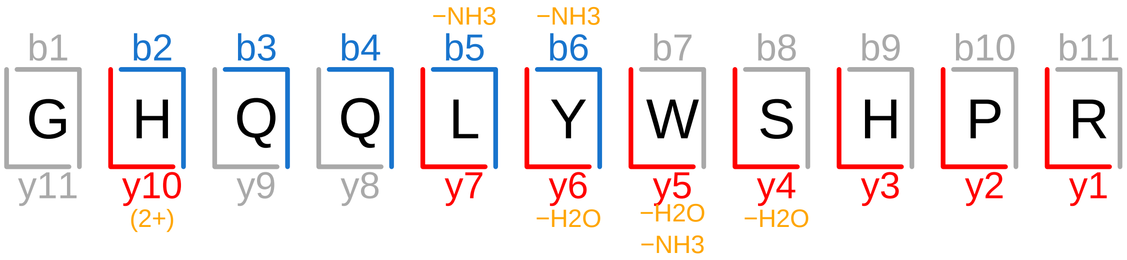

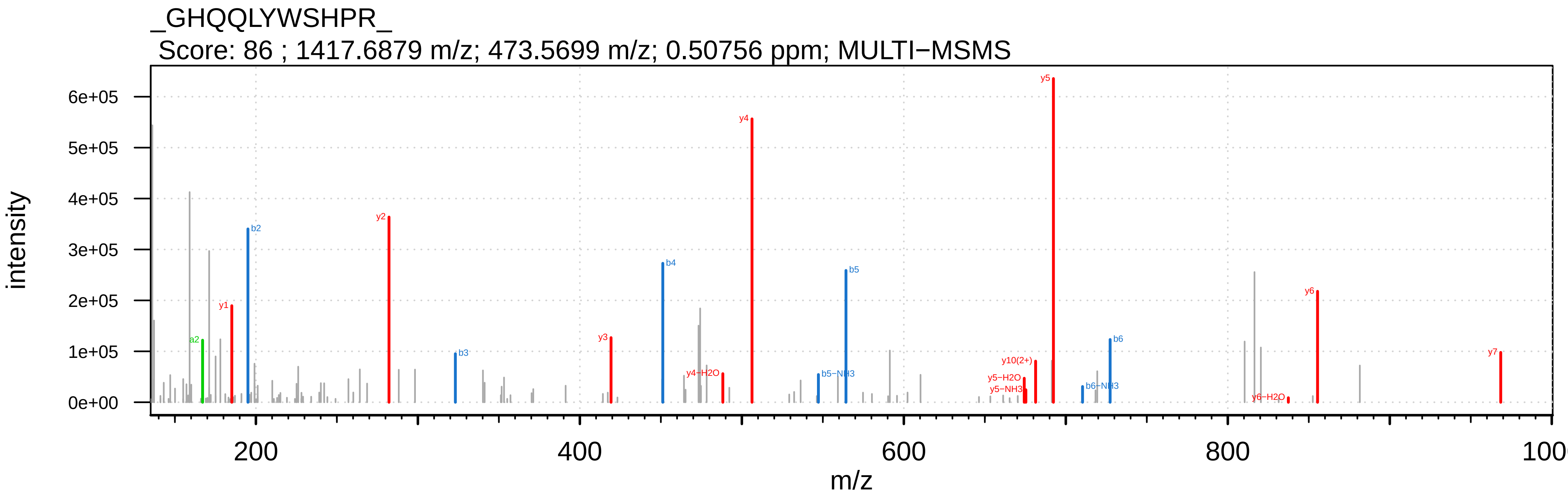

Raw File: Kermit_20111028_MSU_blast_9h_11
Scan Number: 13413
Proteins:
sp|P62274|RS29_MOUSE
ENSMUST00000037023_chr12:69157813−69159058:−
ENSMUST00000071737_chr13:73988963−73989133:−
tr|D3YYL7|D3YYL7_MOUSE

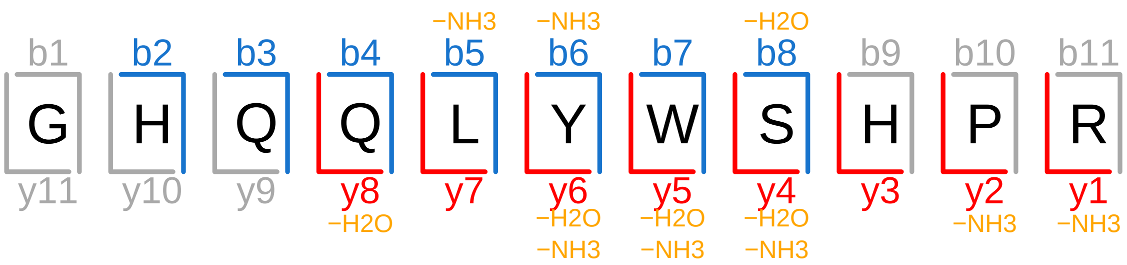

GHQQLYWSHPR

Score: 121 ; 1407.6796 m/z; 470.23382 m/z; 0.50756 ppm; MULTI−MSMS

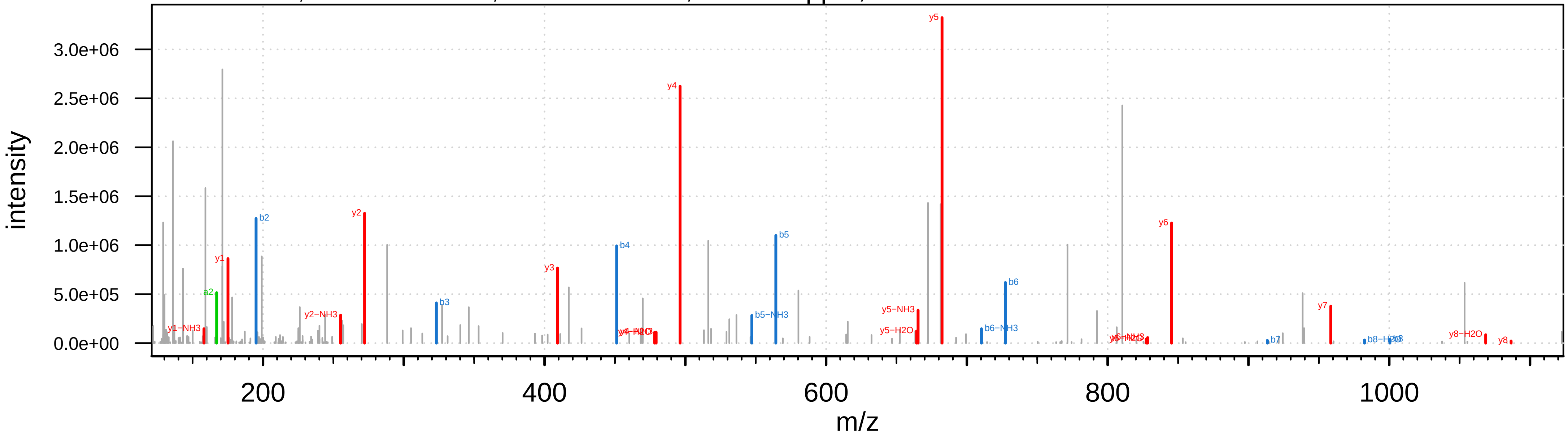

Raw File: Kermit_20111028_MSU_blast_9h_11

Scan Number: 13433

Proteins:

sp|P62274|RS29_MOUSE

ENSMUST00000037023_chr12:69157813−69159058:−

ENSMUST00000071737_chr13:73988963−73989133:−

tr|D3YYL7|D3YYL7_MOUSE

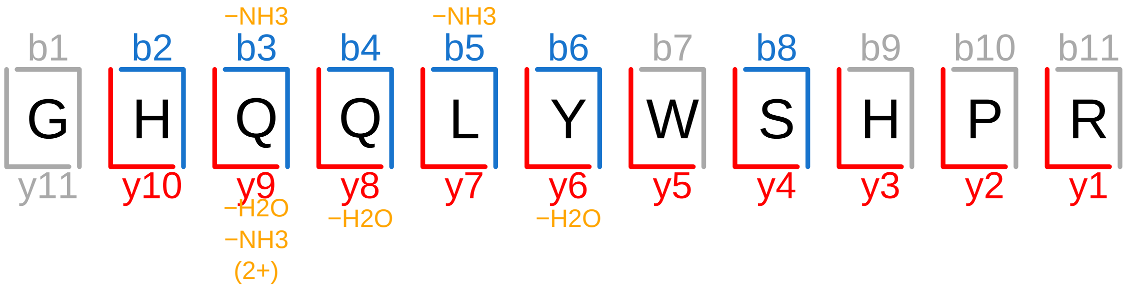

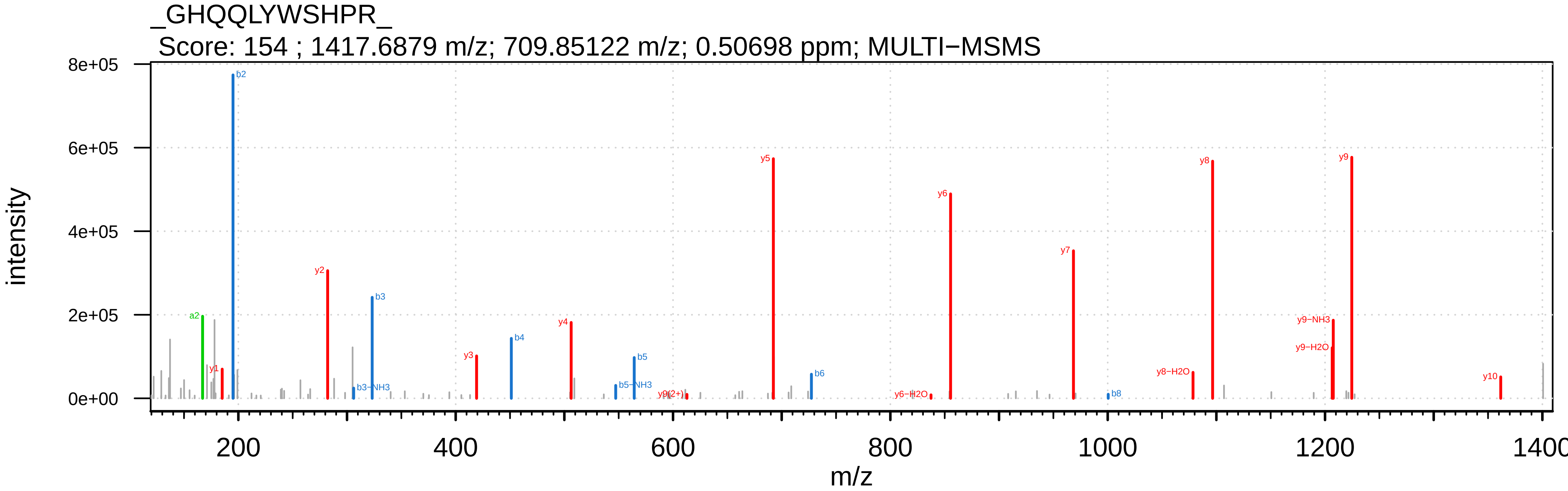

Raw File: Kermit_20111028_MSU_blast_9h_11
Scan Number: 13446
Proteins:
sp|P62274|RS29_MOUSE
ENSMUST00000037023_chr12:69157813−69159058:−
ENSMUST00000071737_chr13:73988963−73989133:−
tr|D3YYL7|D3YYL7_MOUSE

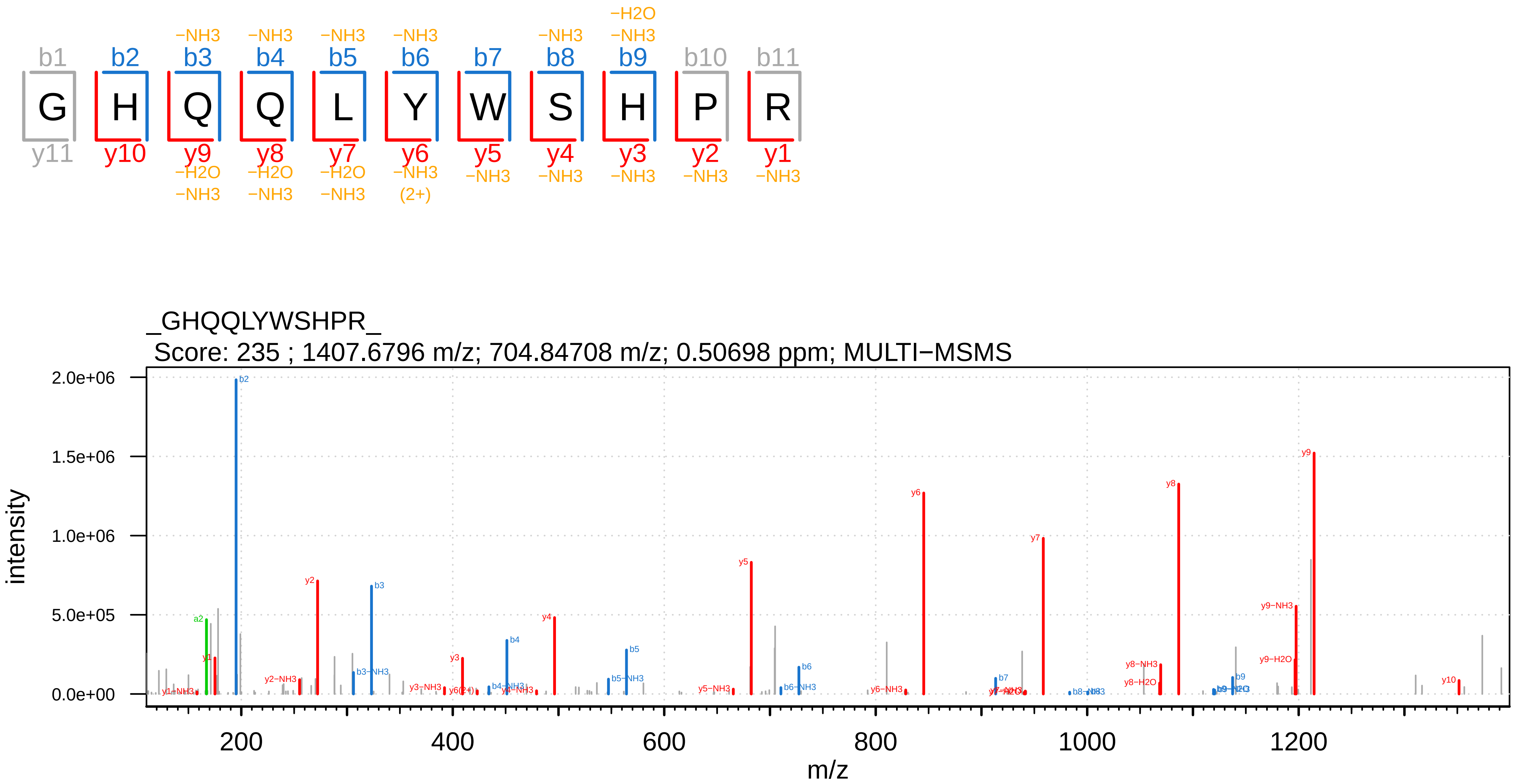

Raw File: Kermit_20111028_MSU_blast_9h_11
Scan Number: 13459
Proteins:
sp|P62274|RS29_MOUSE
ENSMUST00000037023_chr12:69157813−69159058:−
ENSMUST00000071737_chr13:73988963−73989133:−
tr|D3YYL7|D3YYL7_MOUSE

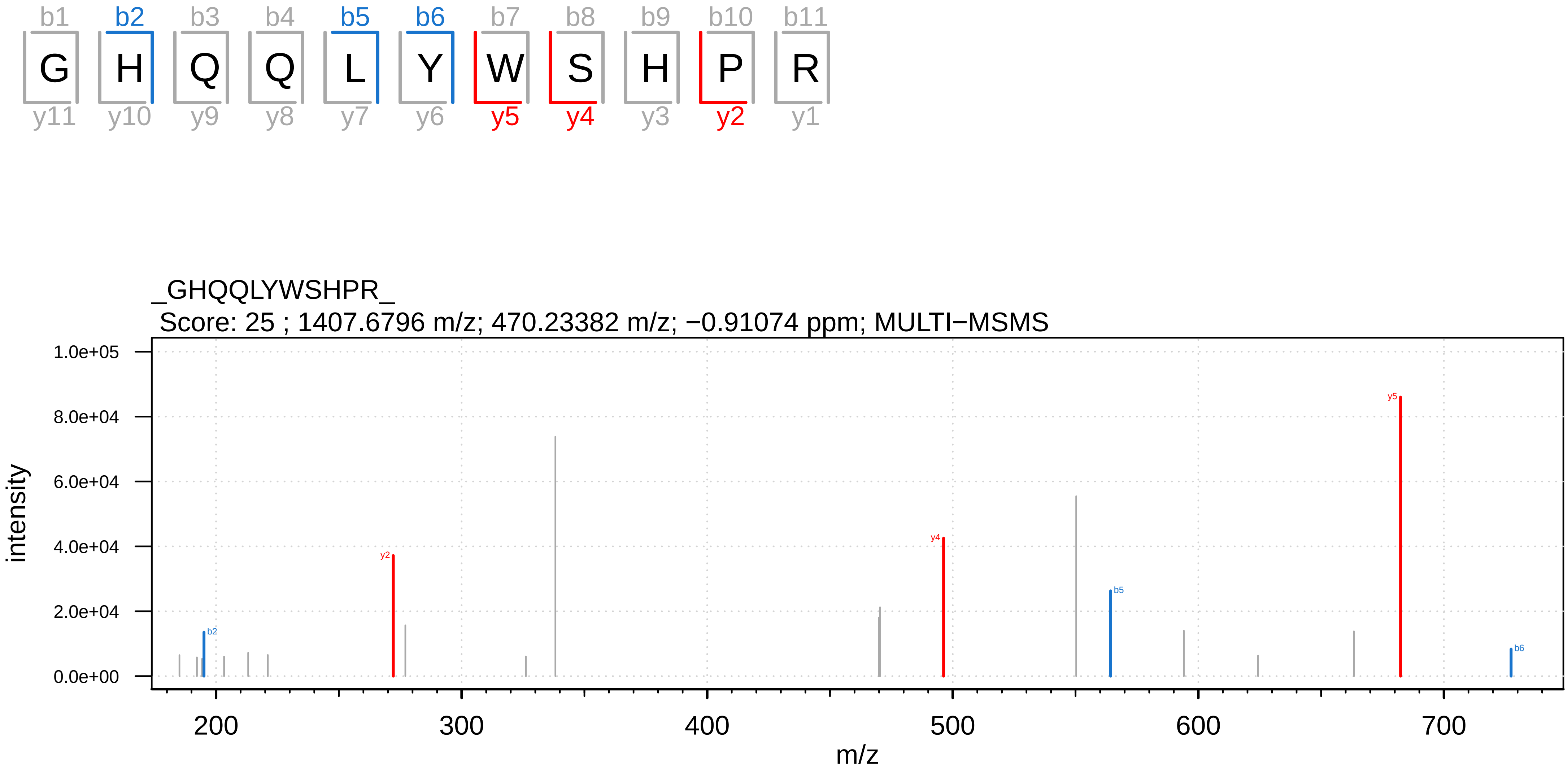

Raw File: Kermit_20111028_MSU_blast_9h_12
Scan Number: 12517
Proteins:
sp|P62274|RS29_MOUSE
ENSMUST00000037023_chr12:69157813−69159058:−
ENSMUST00000071737_chr13:73988963−73989133:−
tr|D3YYL7|D3YYL7_MOUSE

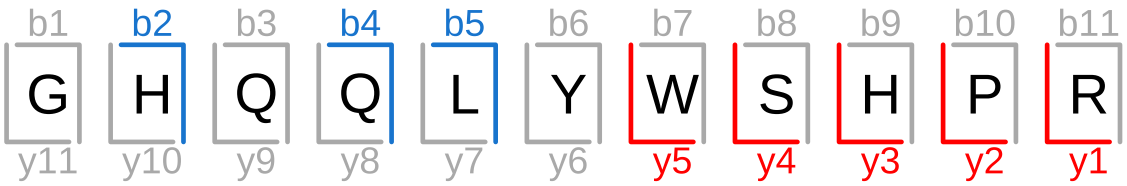

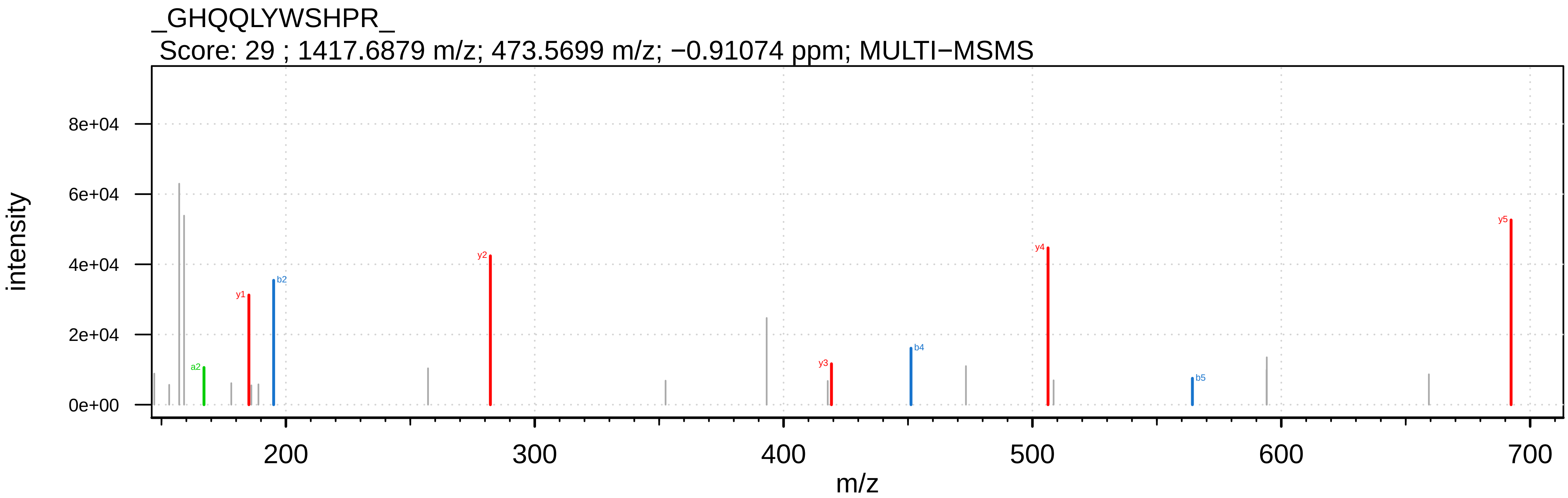

Raw File: Kermit_20111028_MSU_blast_9h_12
Scan Number: 12564
Proteins:
sp|P62274|RS29_MOUSE
ENSMUST00000037023_chr12:69157813−69159058:−
ENSMUST00000071737_chr13:73988963−73989133:−
tr|D3YYL7|D3YYL7_MOUSE

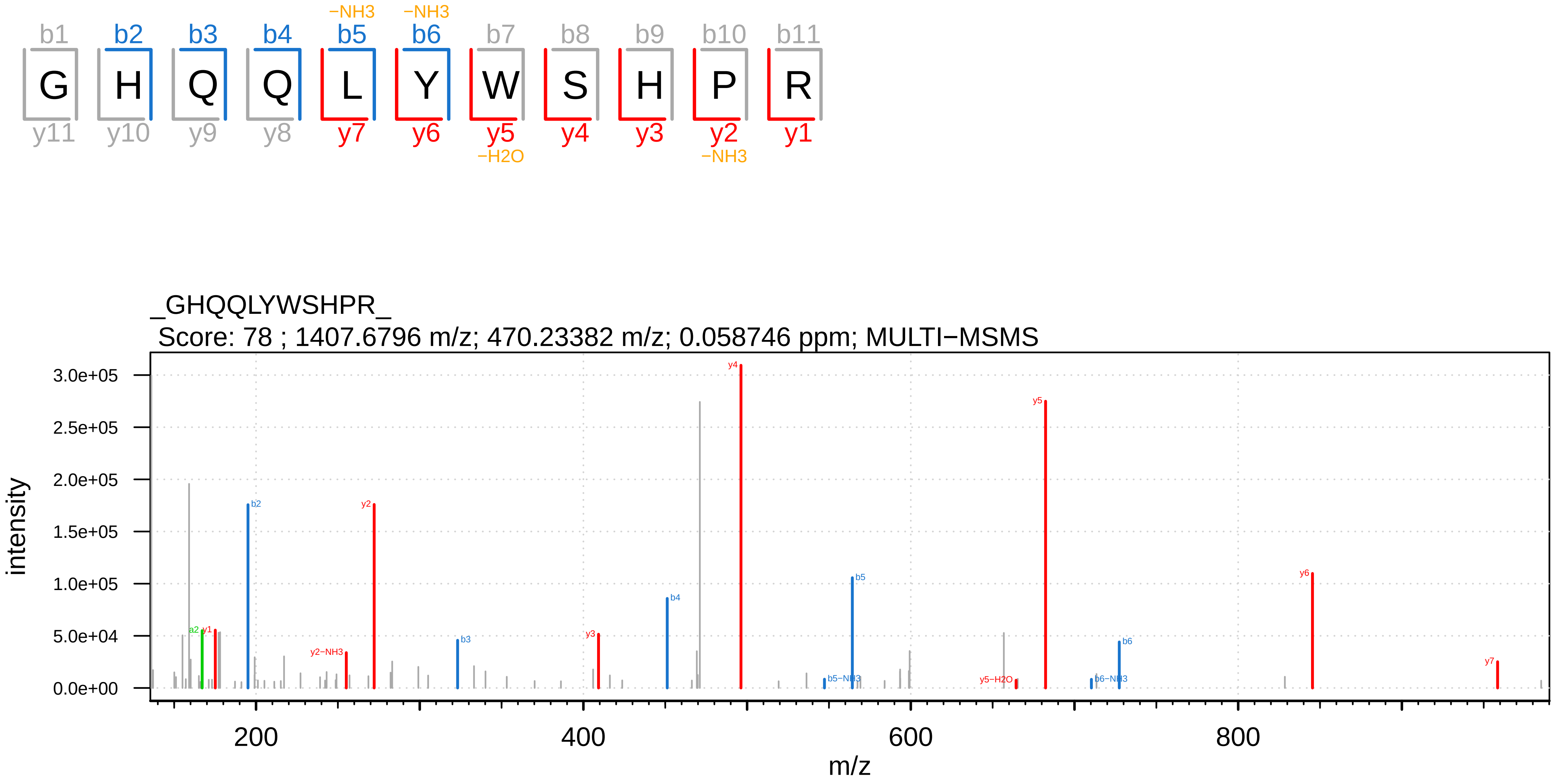

Raw File: Kermit_20111028_MSU_blast_9h_12
Scan Number: 47873
Proteins:
sp|P62274|RS29_MOUSE
ENSMUST00000037023_chr12:69157813−69159058:−
ENSMUST00000071737_chr13:73988963−73989133:−
tr|D3YYL7|D3YYL7_MOUSE

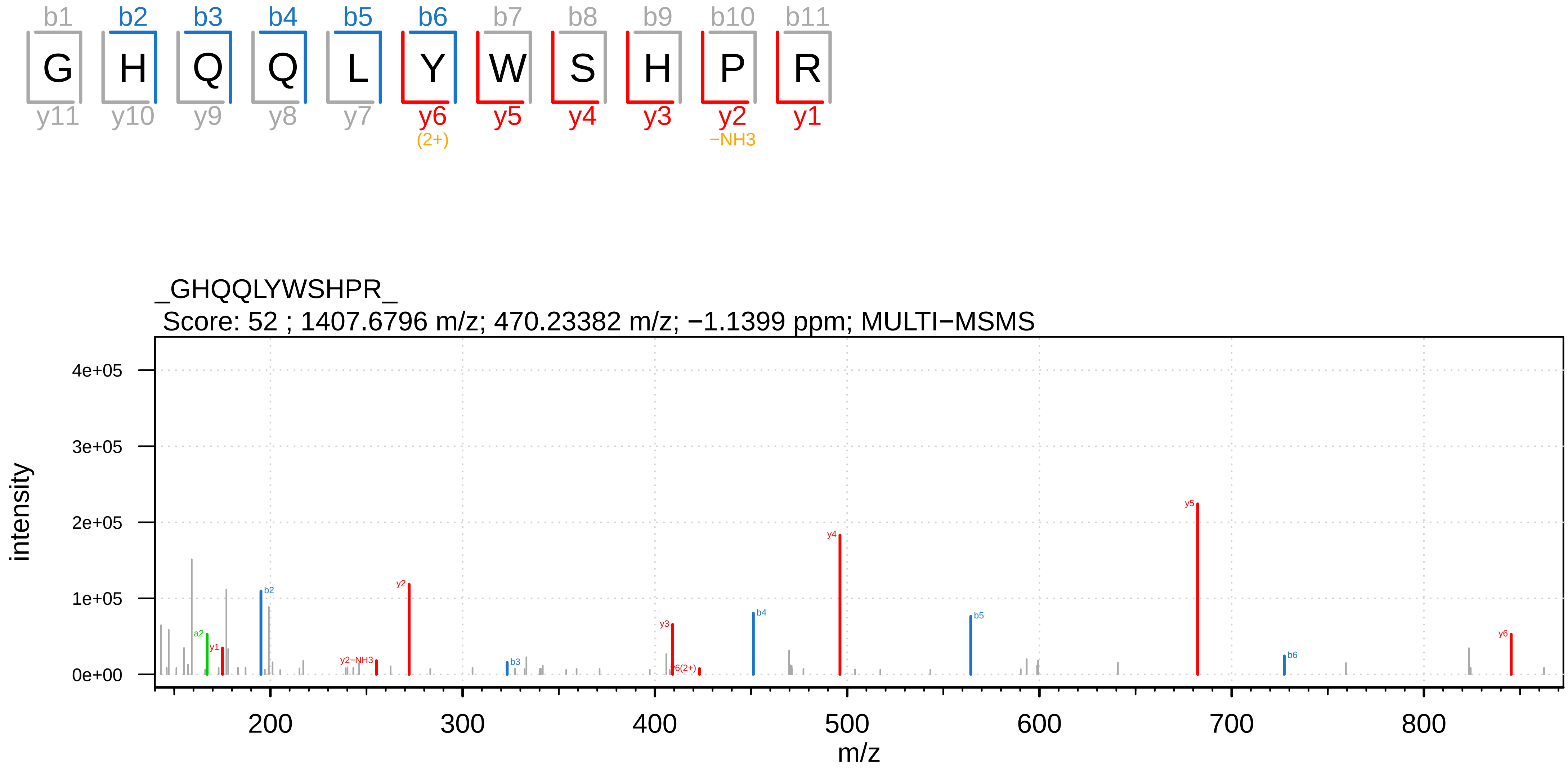

Raw File: Kermit_20111028_MSU_blast_9h_12
Scan Number: 48767
Proteins:
sp|P62274|RS29_MOUSE
ENSMUST00000037023_chr12:69157813−69159058:−
ENSMUST00000071737_chr13:73988963−73989133:−
tr|D3YYL7|D3YYL7_MOUSE

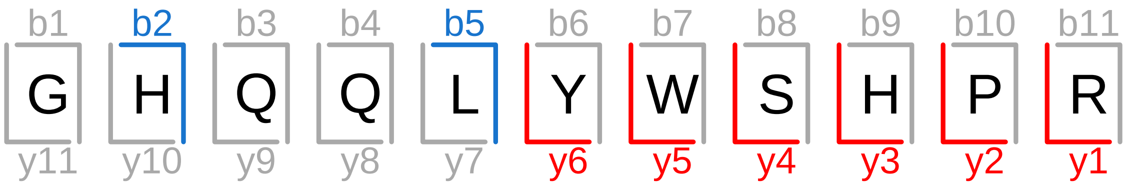

GHQQLYWSHPR
Score: 23 ; 1417.6879 m/z; 473.5699 m/z; −1.7678 ppm; MULTI−MSMS

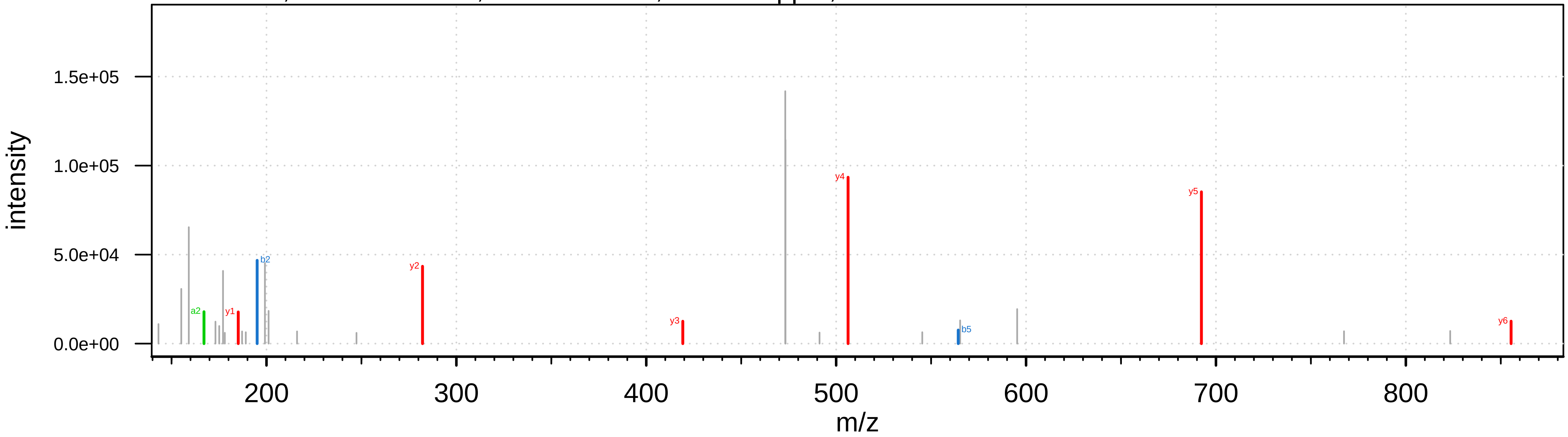

Raw File: Kermit_20111028_MSU_blast_9h_12
Scan Number: 48883
Proteins:
sp|P62274|RS29_MOUSE
ENSMUST00000037023_chr12:69157813−69159058:−
ENSMUST00000071737_chr13:73988963−73989133:−
tr|D3YYL7|D3YYL7_MOUSE

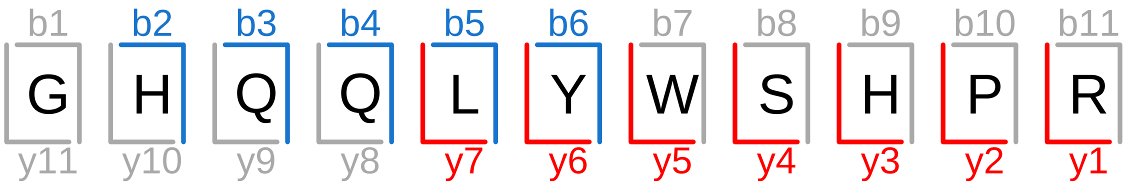

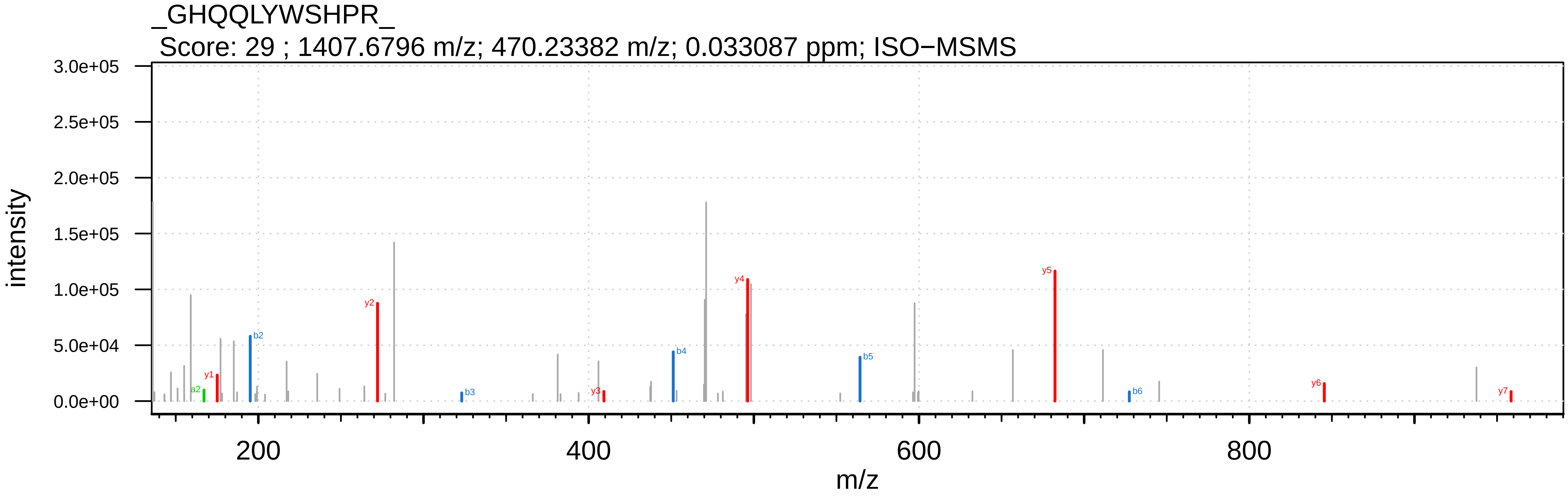

Raw File: Kermit_20111028_MSU_blast_9h_12
Scan Number: 47518
Proteins:
sp|P62274|RS29_MOUSE
ENSMUST00000037023_chr12:69157813−69159058:−
ENSMUST00000071737_chr13:73988963−73989133:−
tr|D3YYL7|D3YYL7_MOUSE

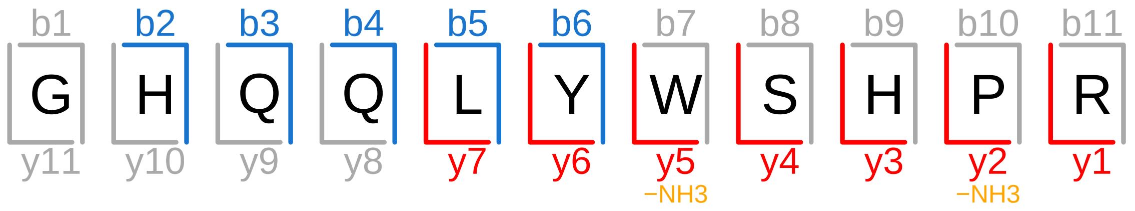

GHQQLYWSHPR
Score: 59 ; 1407.6796 m/z; 470.23382 m/z; 0.033087 ppm; ISO−MSMS

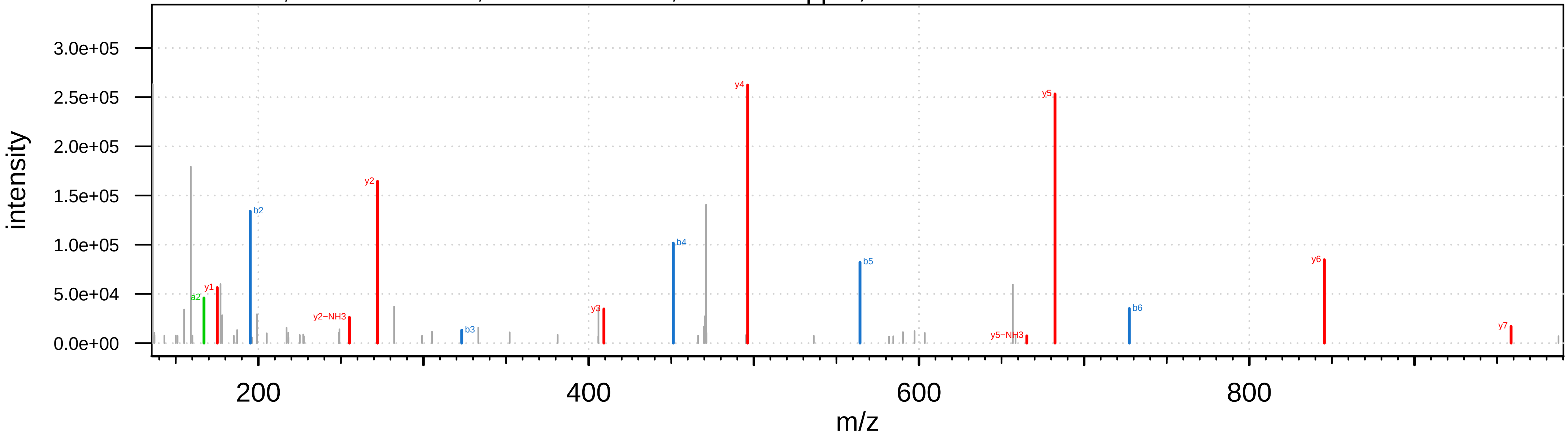

Raw File: Kermit_20111028_MSU_blast_9h_12
Scan Number: 47696
Proteins:
sp|P62274|RS29_MOUSE
ENSMUST00000037023_chr12:69157813−69159058:−
ENSMUST00000071737_chr13:73988963−73989133:−
tr|D3YYL7|D3YYL7_MOUSE

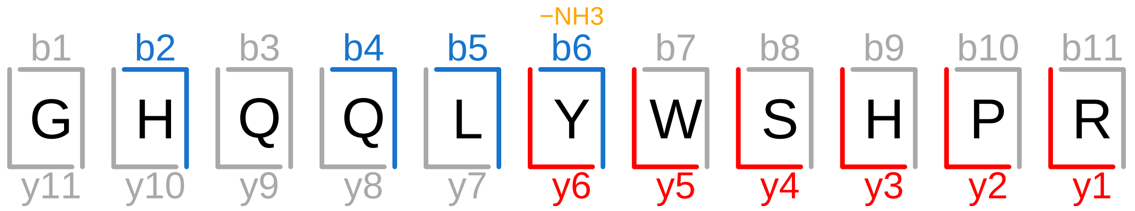

GHQQLYWSHPR

Score: 45 ; 1417.6879 m/z; 473.5699 m/z; 0.0058145 ppm; ISO−MSMS

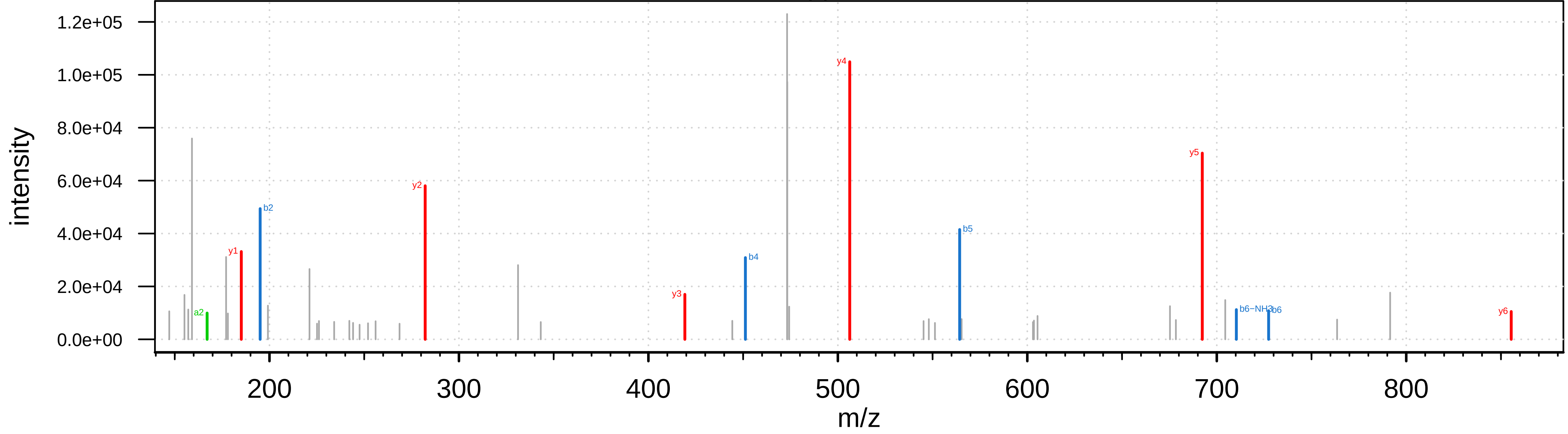

Raw File: Kermit_20111028_MSU_blast_9h_12

Scan Number: 47682

Proteins:

sp|P62274|RS29_MOUSE

ENSMUST00000037023_chr12:69157813−69159058:−

ENSMUST00000071737_chr13:73988963−73989133:−

tr|D3YYL7|D3YYL7_MOUSE

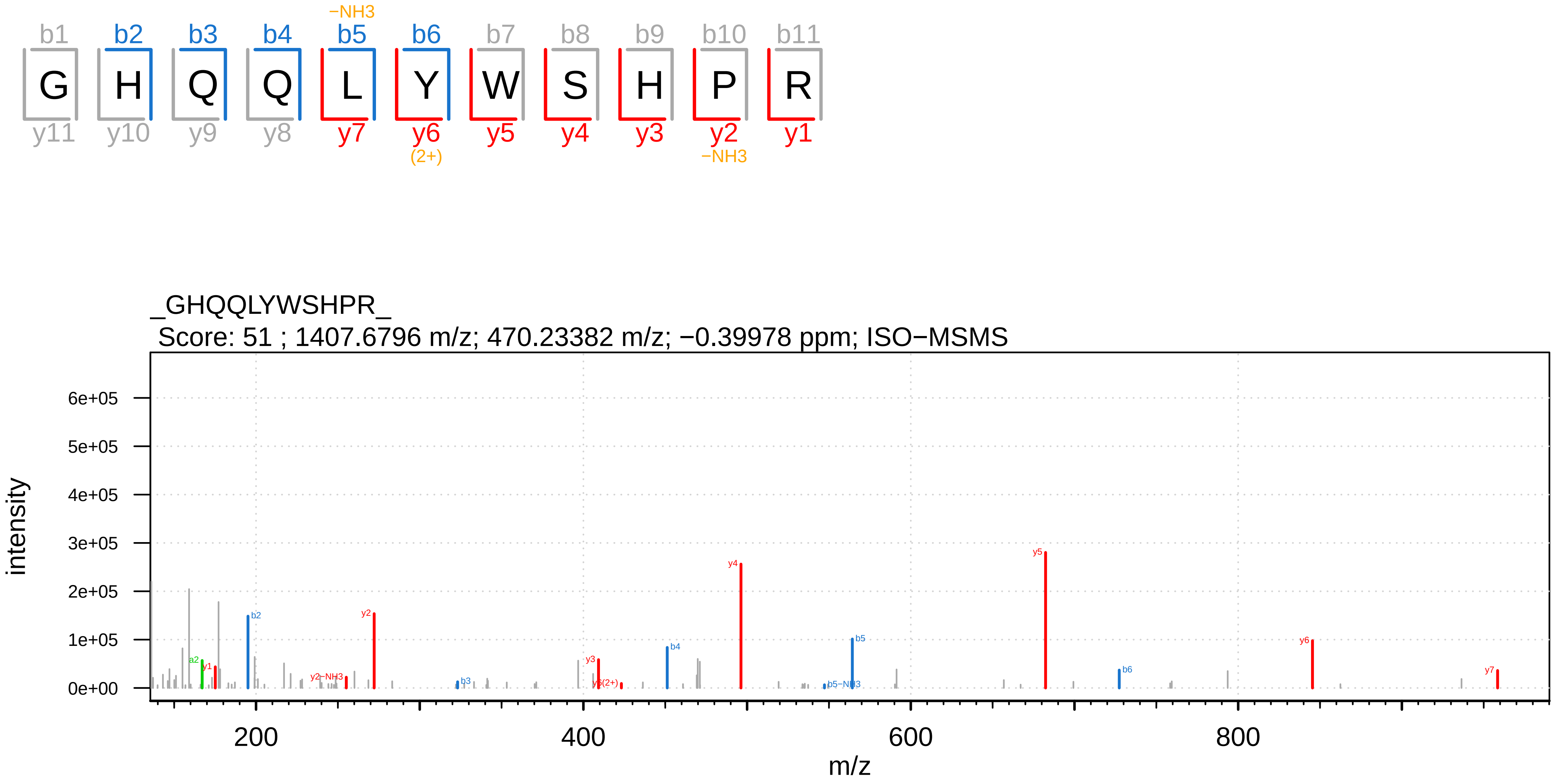

Raw File: Kermit_20111028_MSU_blast_9h_12
Scan Number: 48595
Proteins:
sp|P62274|RS29_MOUSE
ENSMUST00000037023_chr12:69157813−69159058:−
ENSMUST00000071737_chr13:73988963−73989133:−
tr|D3YYL7|D3YYL7_MOUSE

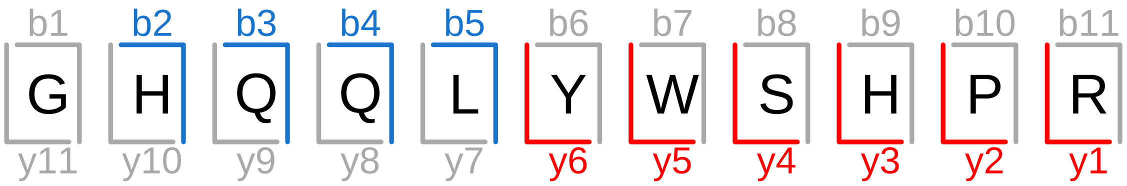

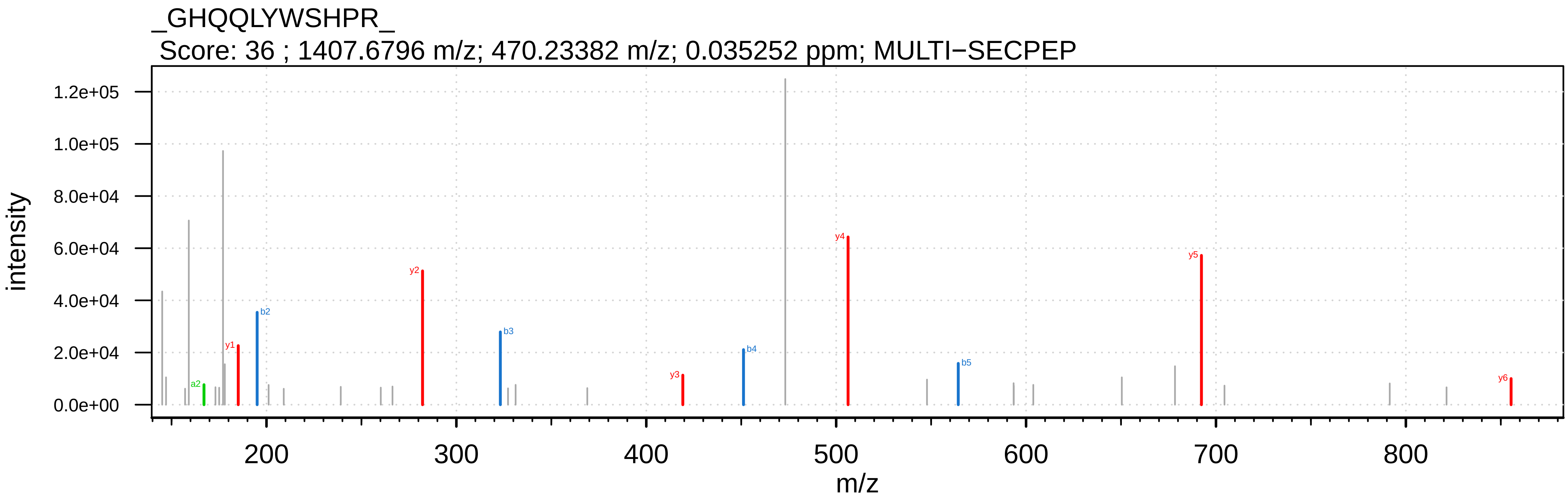

Raw File: Kermit_20111028_MSU_blast_9h_12
Scan Number: 47820
Proteins:
sp|P62274|RS29_MOUSE
ENSMUST00000037023_chr12:69157813−69159058:−
ENSMUST00000071737_chr13:73988963−73989133:−
tr|D3YYL7|D3YYL7_MOUSE

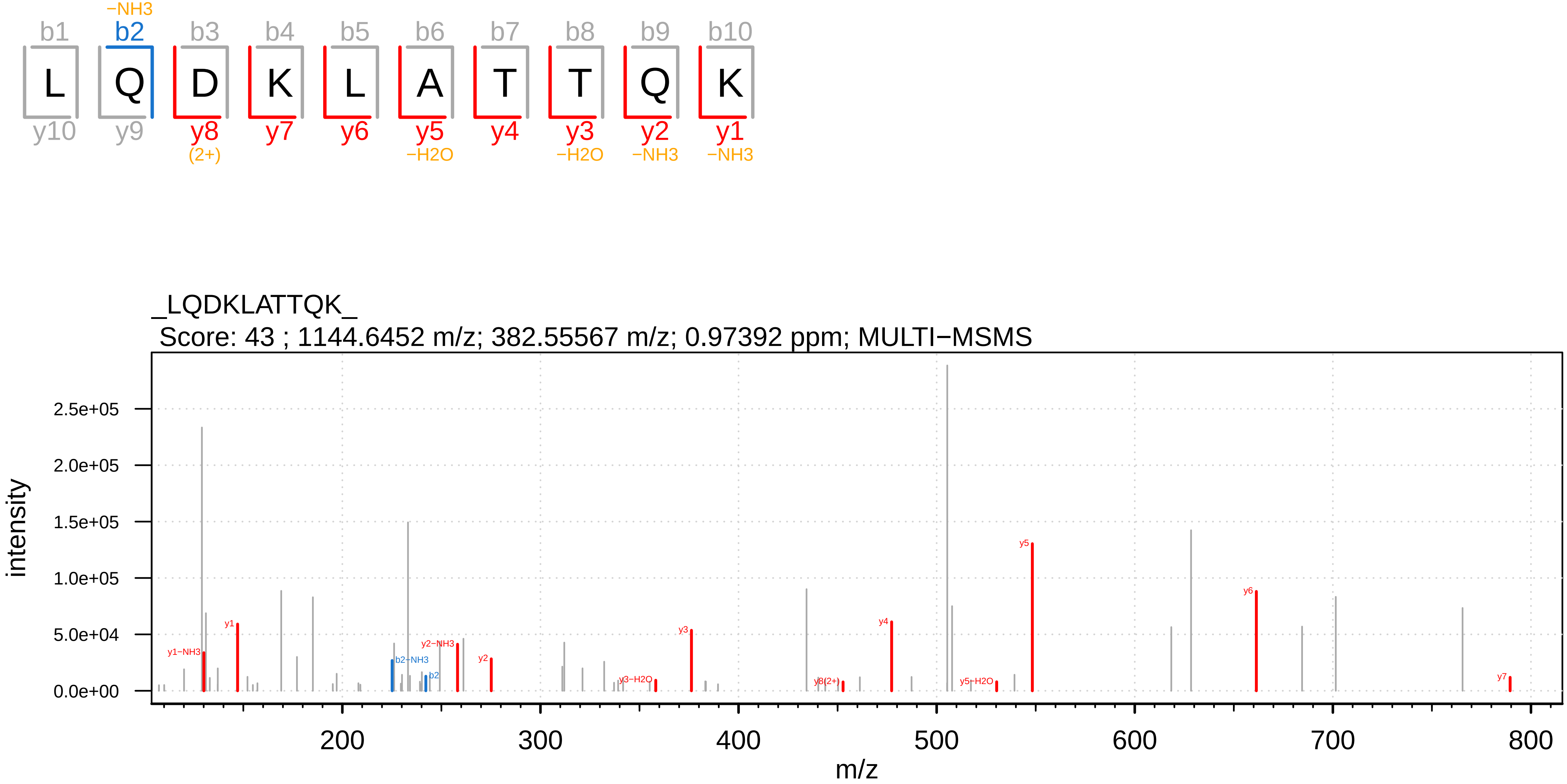

Raw File: Kermit_20111028_MSU_blast_9h_12
Scan Number: 6775
Proteins:
sp|Q8BT35|YB039_MOUSE
ENSMUST00000135091_chr2:127792297−127792467:−

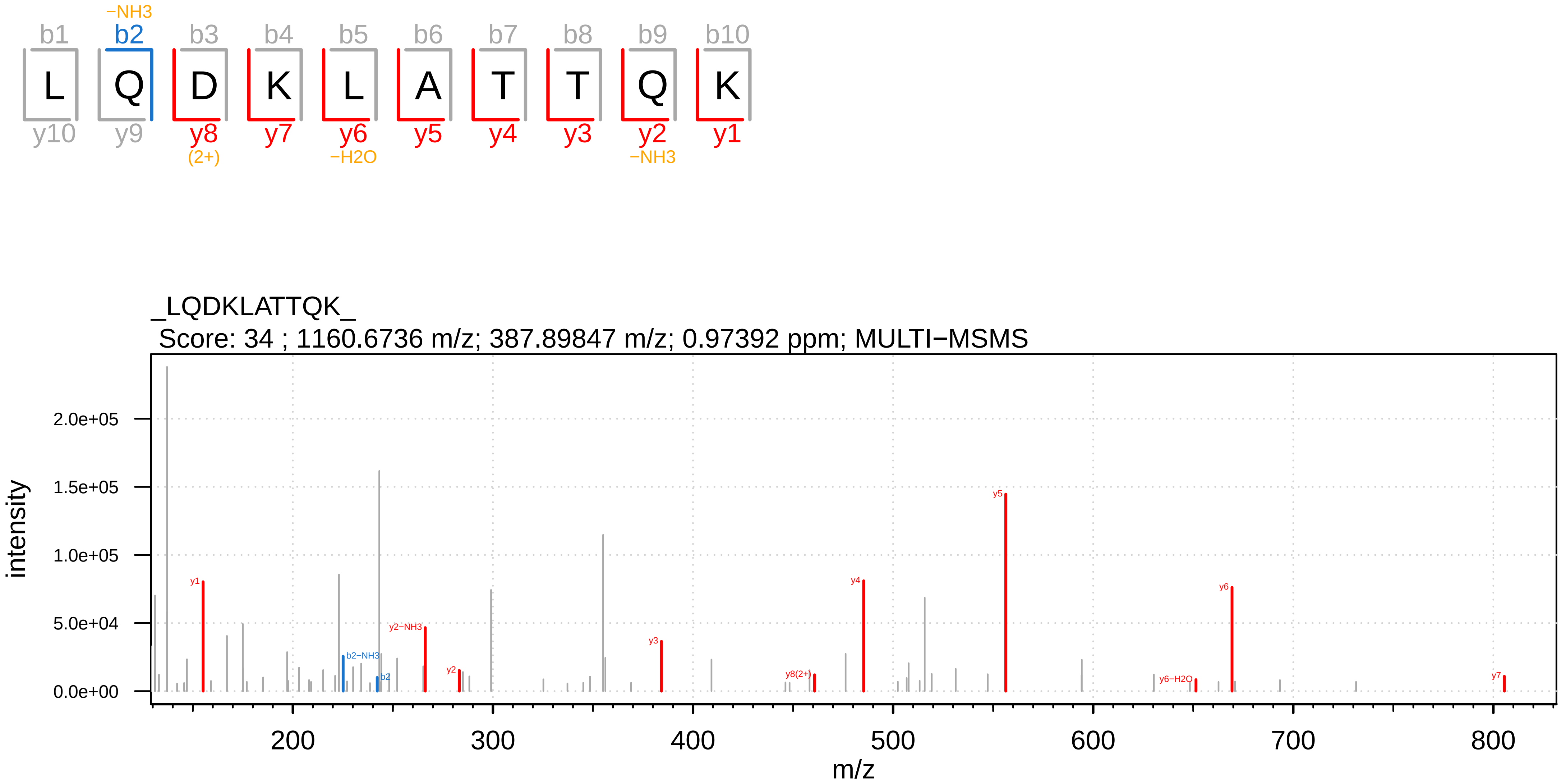

Raw File: Kermit_20111028_MSU_blast_9h_12
Scan Number: 6790
Proteins:
sp|Q8BT35|YB039_MOUSE
ENSMUST00000135091_chr2:127792297−127792467:−

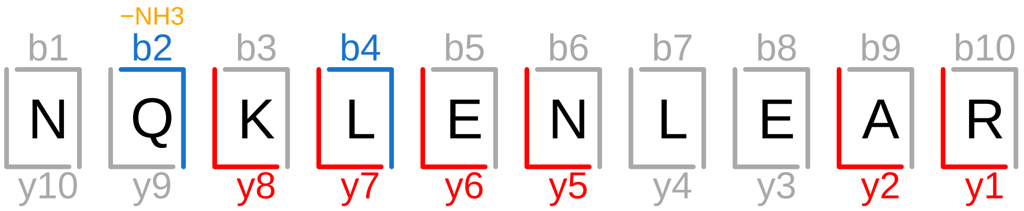

NQKLENLEAR
Score: 48 ; 1231.664 m/z; 616.83926 m/z; 0.25253 ppm; MULTI−MSMS

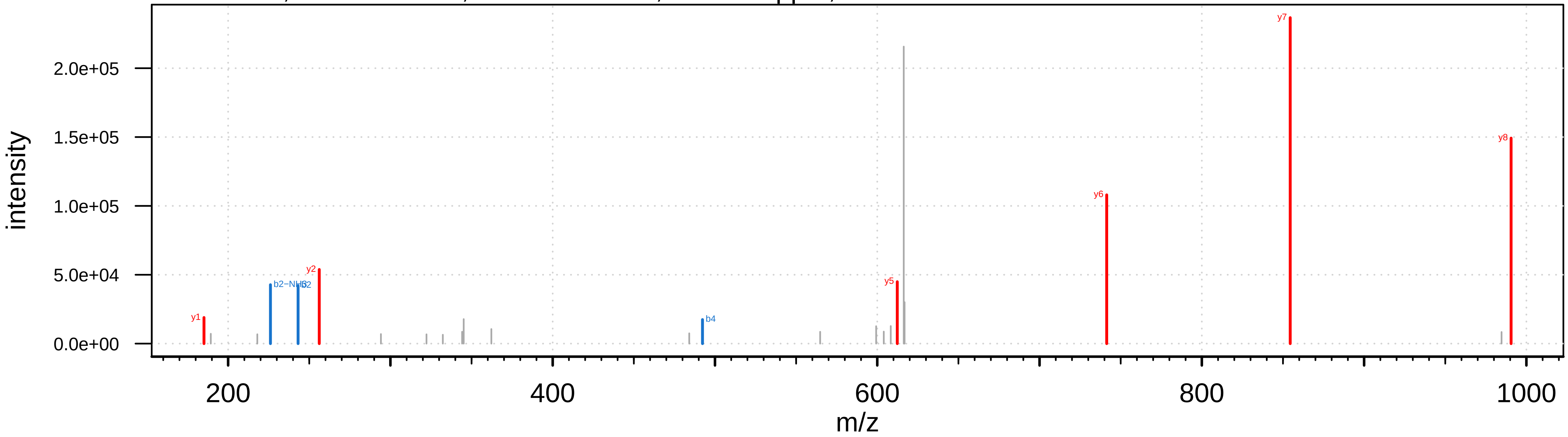

Raw File: Kermit_20111028_MSU_blast_9h_12
Scan Number: 9452
Proteins:
ENSMUST00000023048_chr15:80235835−80236047:+

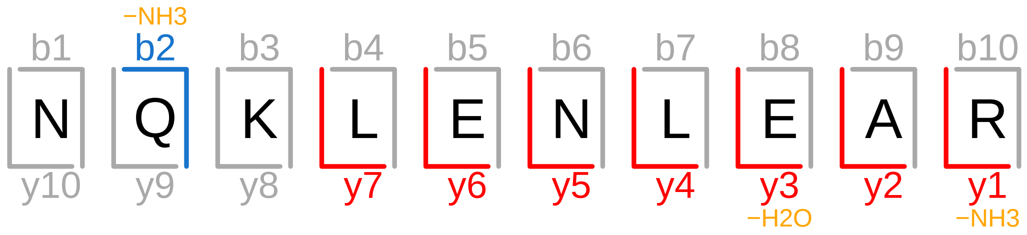

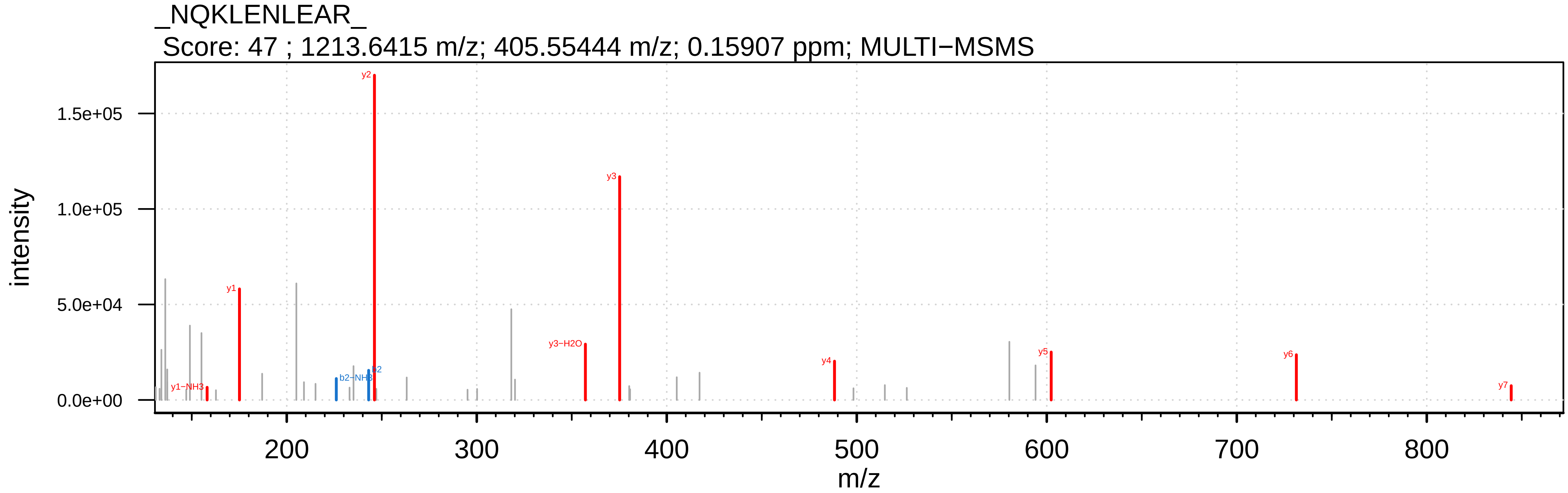

Raw File: Kermit_20111028_MSU_blast_9h_12
Scan Number: 9465
Proteins:
ENSMUST00000023048_chr15:80235835−80236047:+

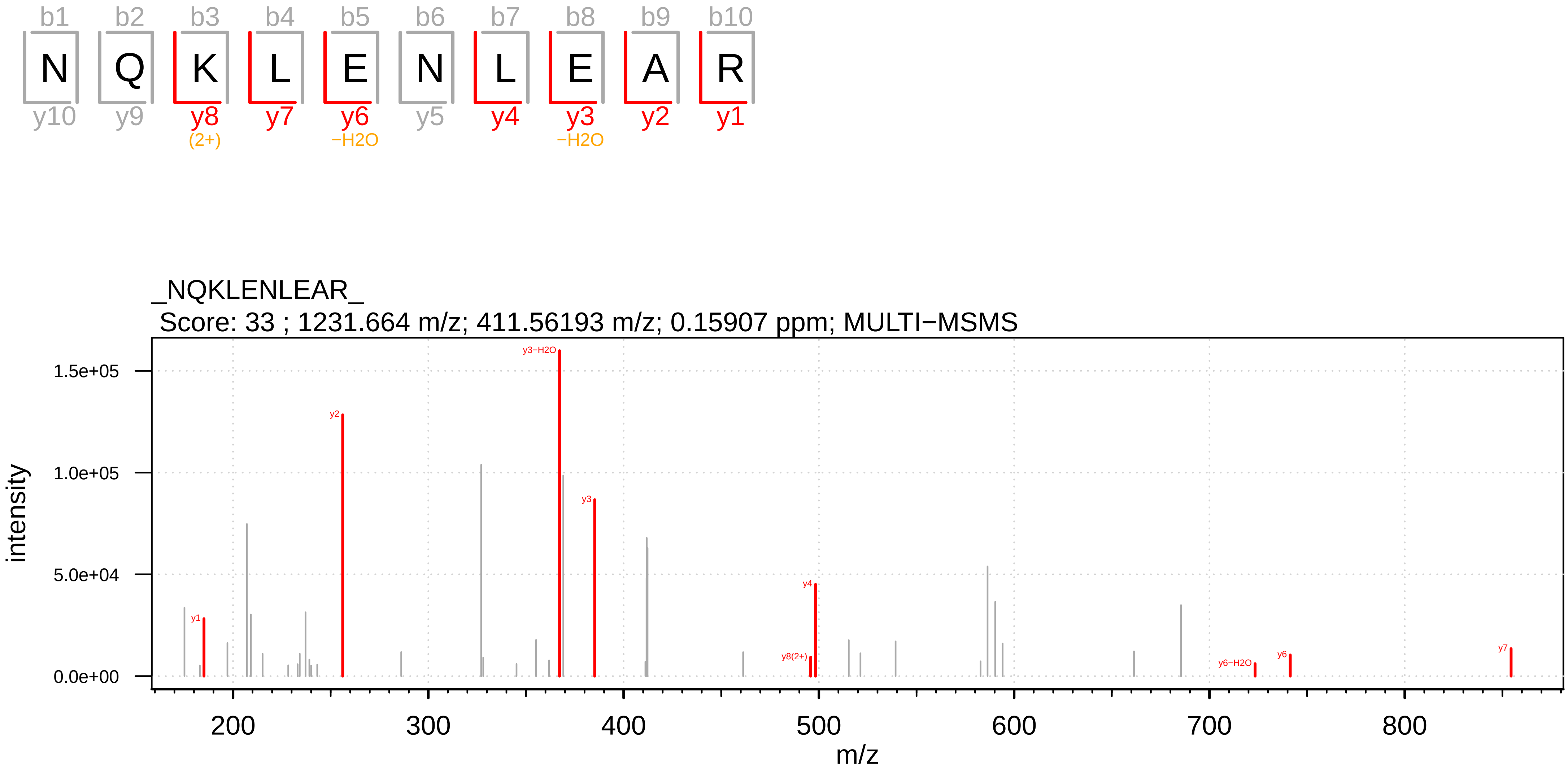

Raw File: Kermit_20111028_MSU_blast_9h_12
Scan Number: 9480
Proteins:
ENSMUST00000023048_chr15:80235835−80236047:+

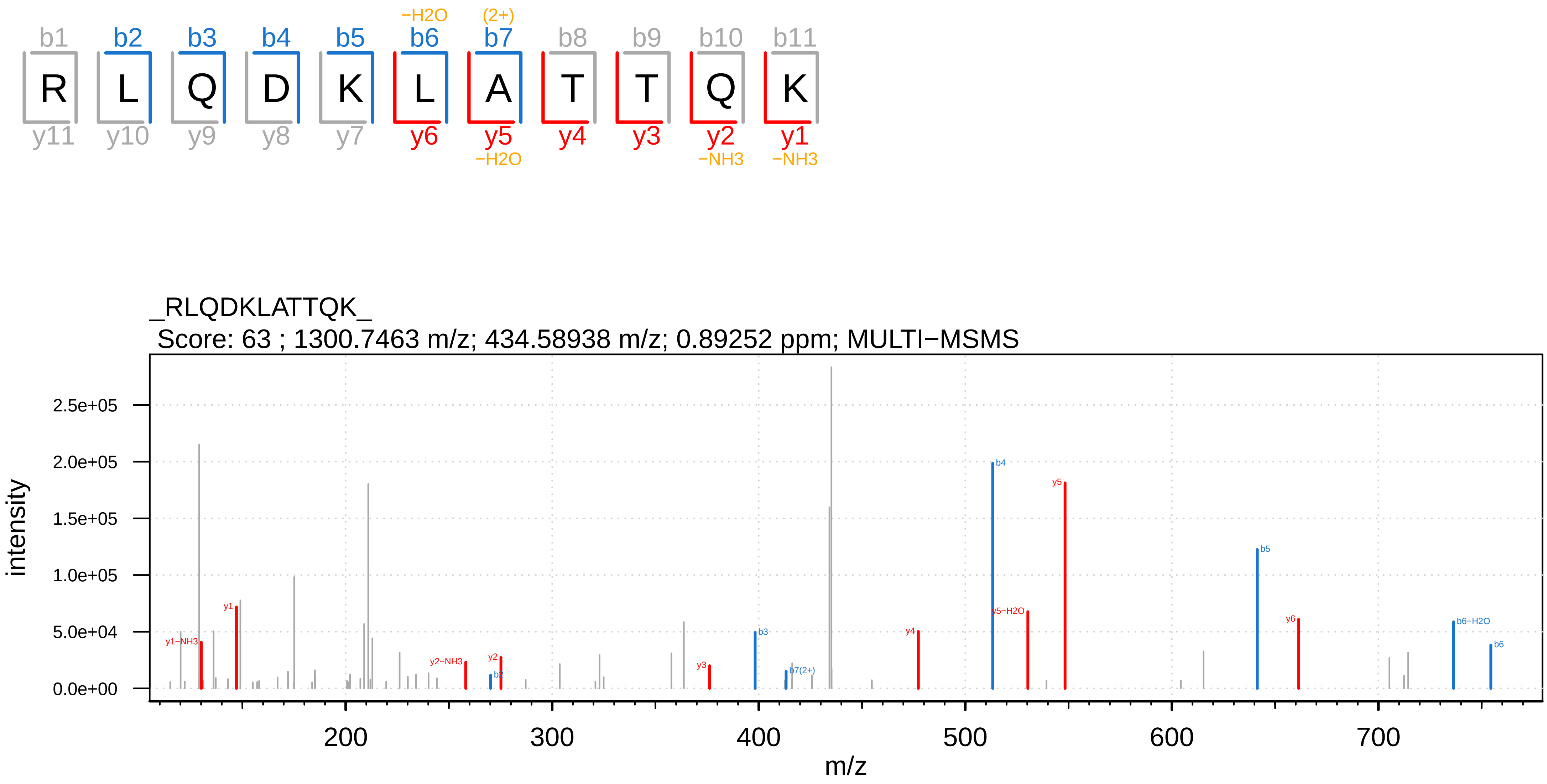

Raw File: Kermit_20111028_MSU_blast_9h_12
Scan Number: 7403
Proteins:
sp|Q8BT35|YB039_MOUSE
ENSMUST00000135091_chr2:127792297−127792467:−

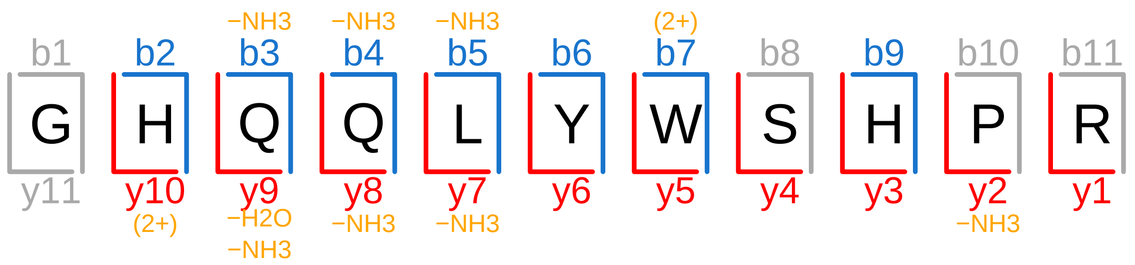

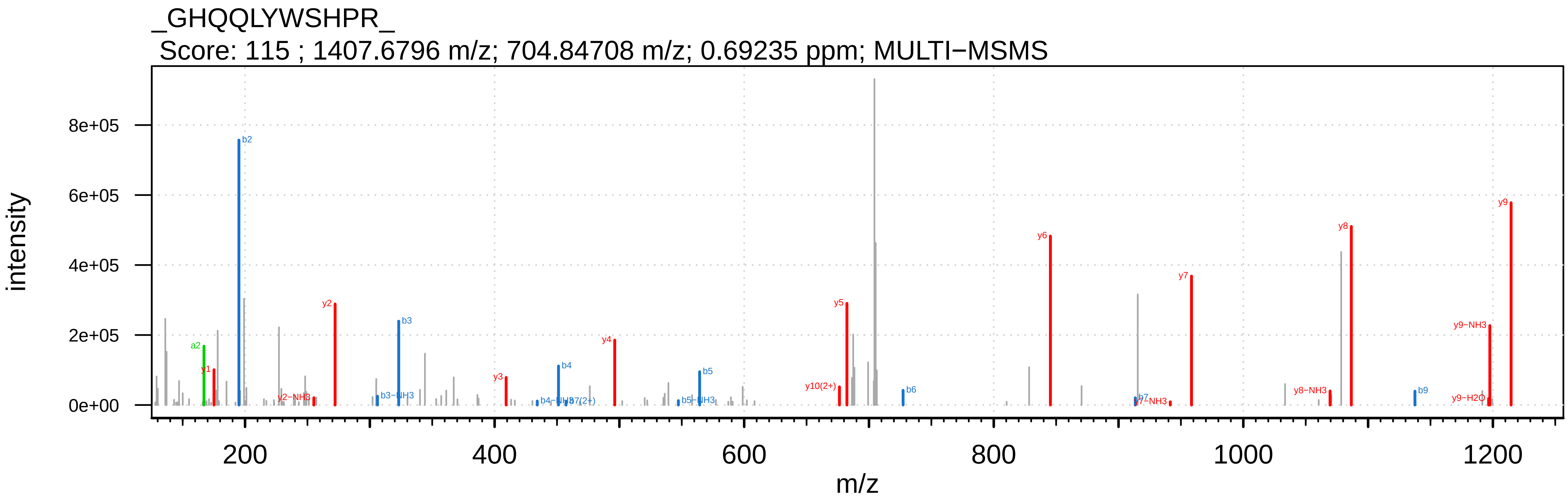

Raw File: Kermit_20111028_MSU_tube_54h_11
Scan Number: 10832
Proteins:
sp|P62274|RS29_MOUSE
ENSMUST00000037023_chr12:69157813−69159058:−
ENSMUST00000071737_chr13:73988963−73989133:−
tr|D3YYL7|D3YYL7_MOUSE

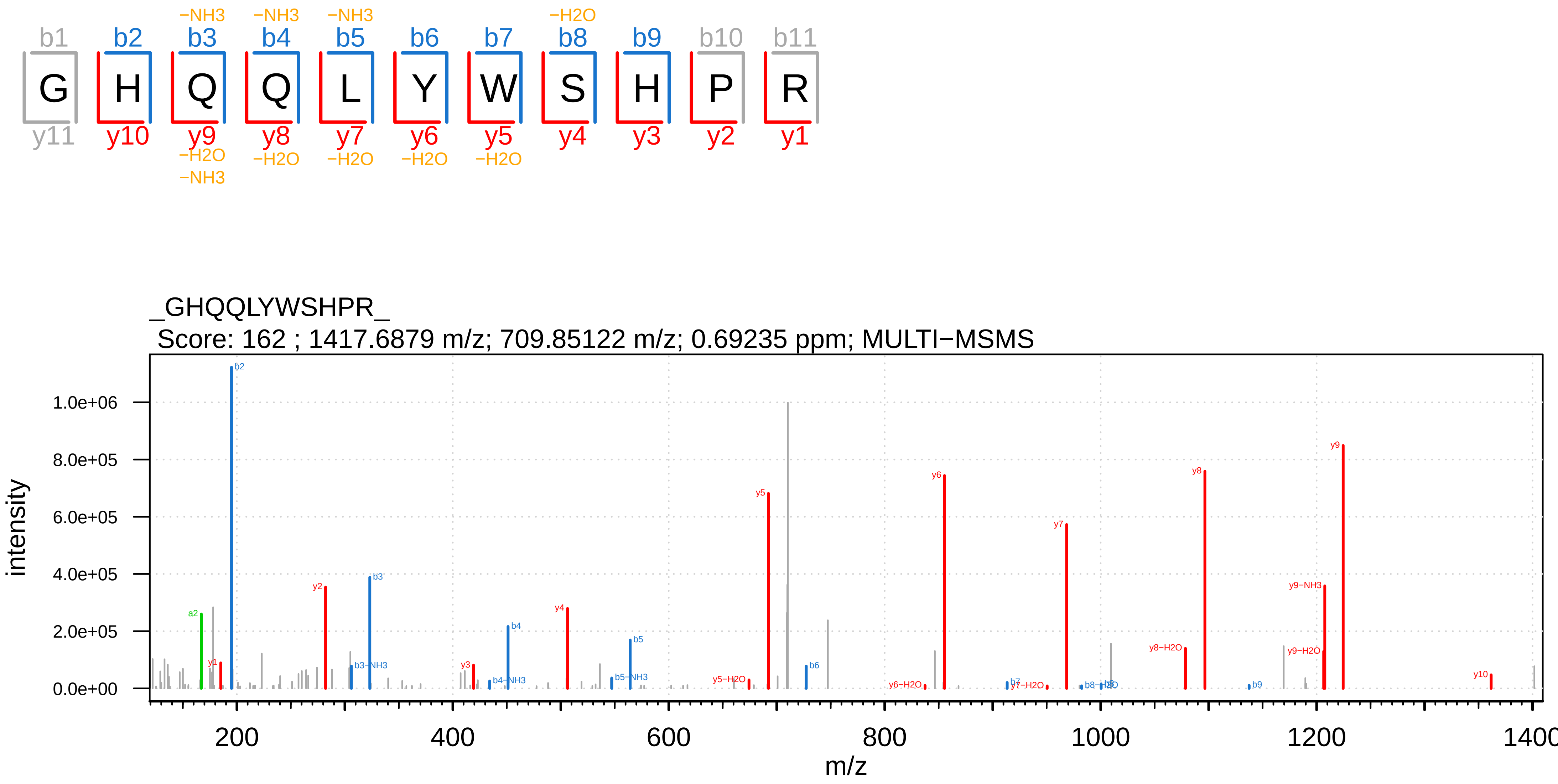

Raw File: Kermit_20111028_MSU_tube_54h_11
Scan Number: 10864
Proteins:
sp|P62274|RS29_MOUSE
ENSMUST00000037023_chr12:69157813−69159058:−
ENSMUST00000071737_chr13:73988963−73989133:−
tr|D3YYL7|D3YYL7_MOUSE

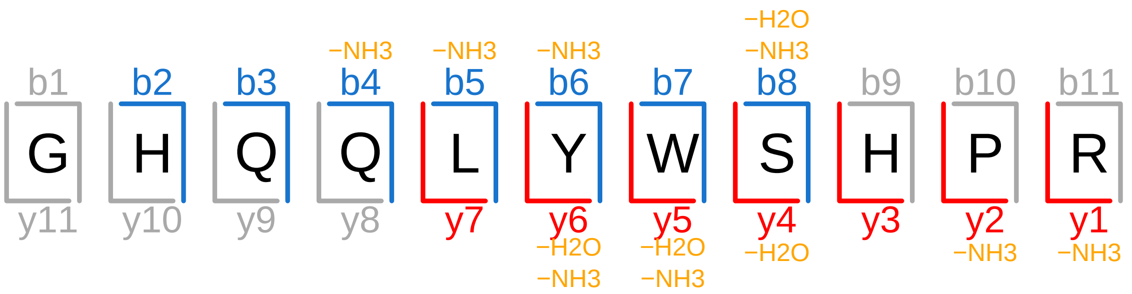

GHQQLYWSHPR
Score: 107 ; 1407.6796 m/z; 470.23382 m/z; 0.17624 ppm; MULTI−MSMS

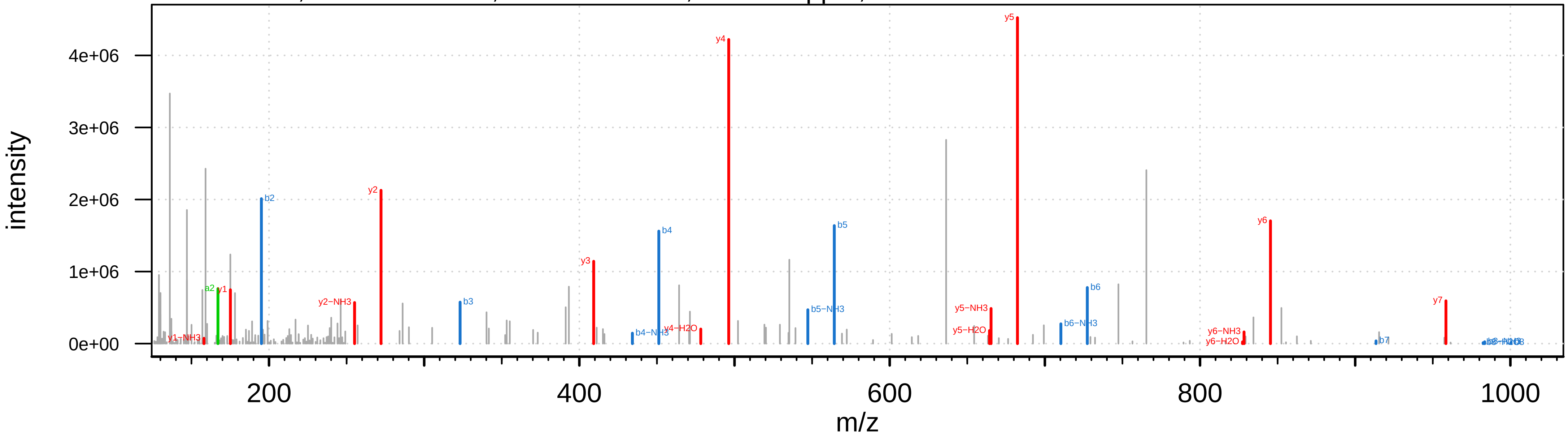

Raw File: Kermit_20111028_MSU_tube_54h_11
Scan Number: 10852
Proteins:
sp|P62274|RS29_MOUSE
ENSMUST00000037023_chr12:69157813−69159058:−
ENSMUST00000071737_chr13:73988963−73989133:−
tr|D3YYL7|D3YYL7_MOUSE

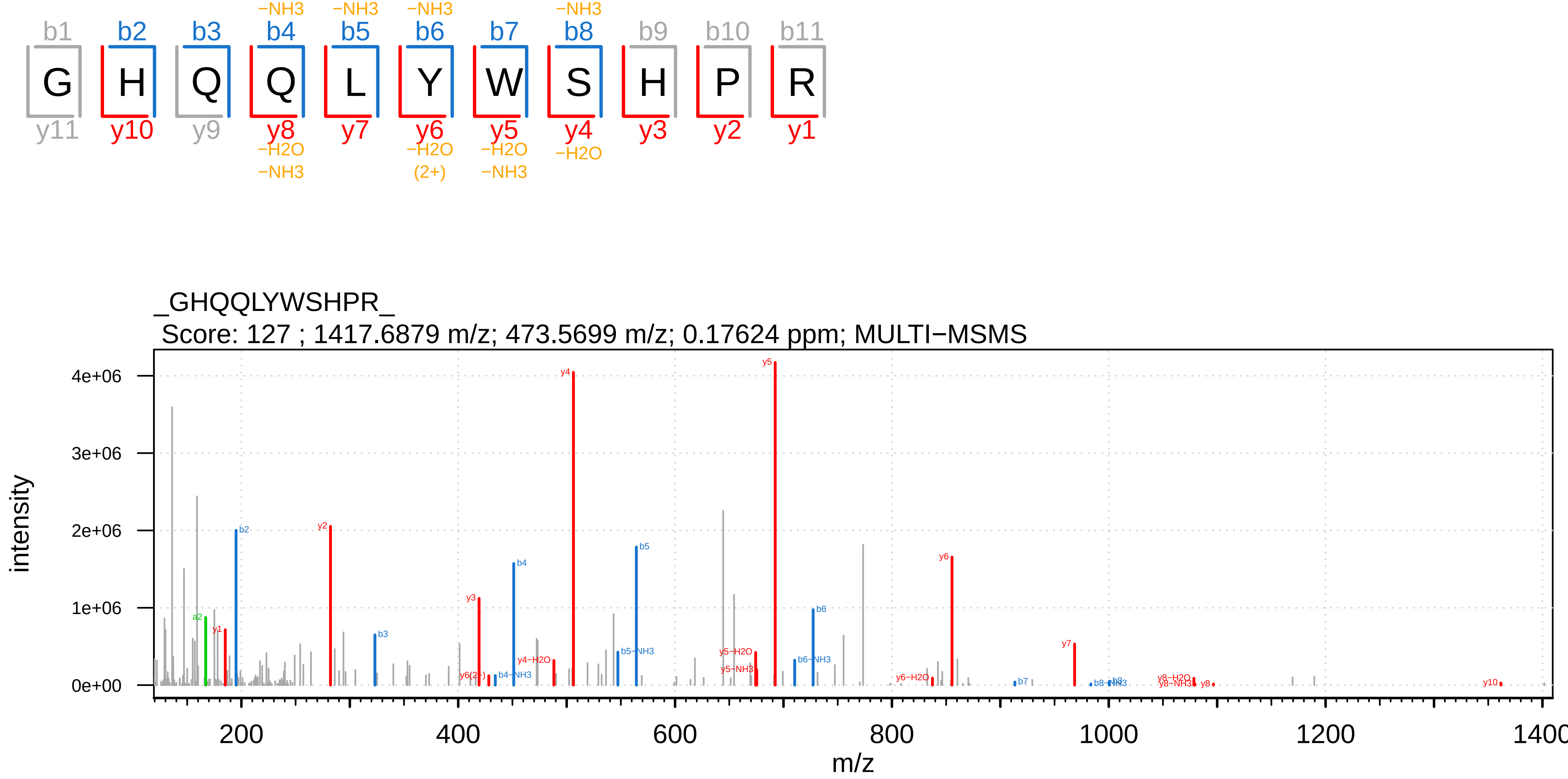

Raw File: Kermit_20111028_MSU_tube_54h_11
Scan Number: 10880
Proteins:
sp|P62274|RS29_MOUSE
ENSMUST00000037023_chr12:69157813−69159058:−
ENSMUST00000071737_chr13:73988963−73989133:−
tr|D3YYL7|D3YYL7_MOUSE

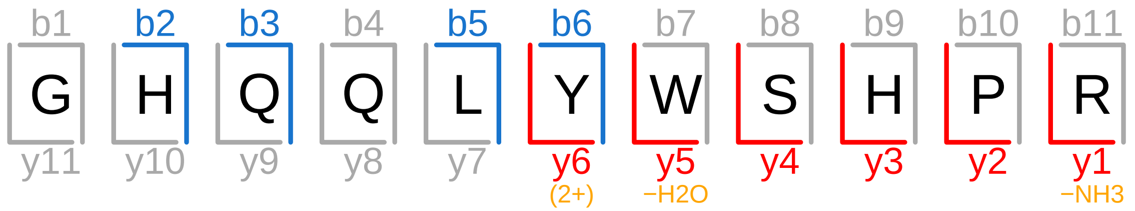

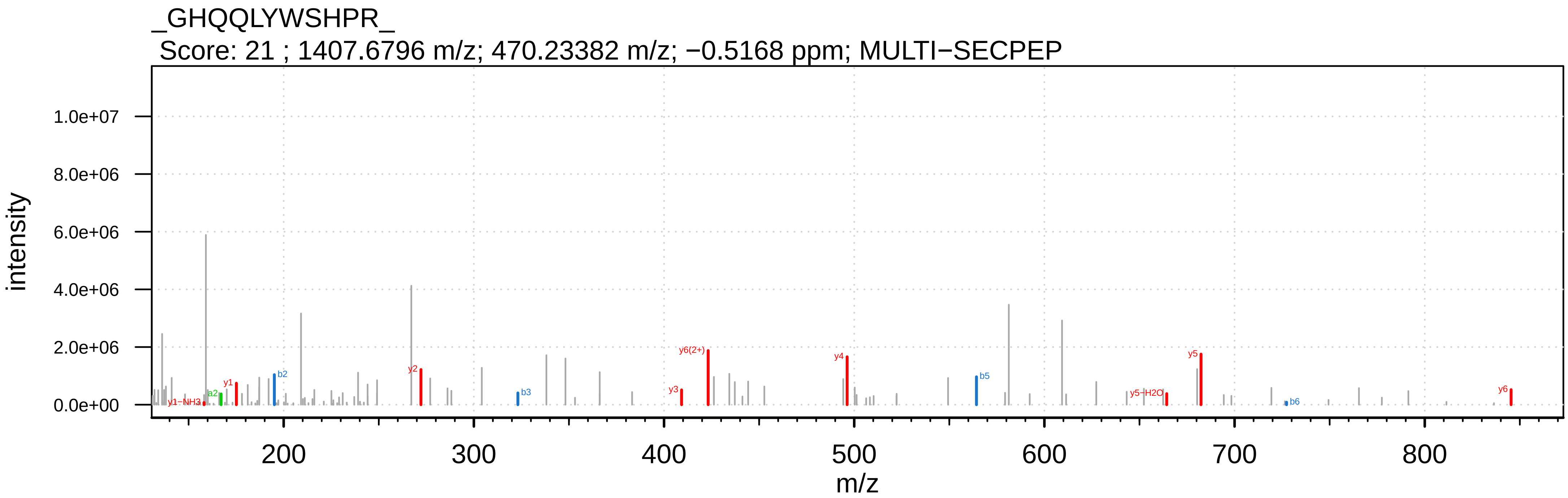

Raw File: Kermit_20111104_MSU_tube_18h_10
Scan Number: 8071
Proteins:
sp|P62274|RS29_MOUSE
ENSMUST00000037023_chr12:69157813−69159058:−
ENSMUST00000071737_chr13:73988963−73989133:−
tr|D3YYL7|D3YYL7_MOUSE

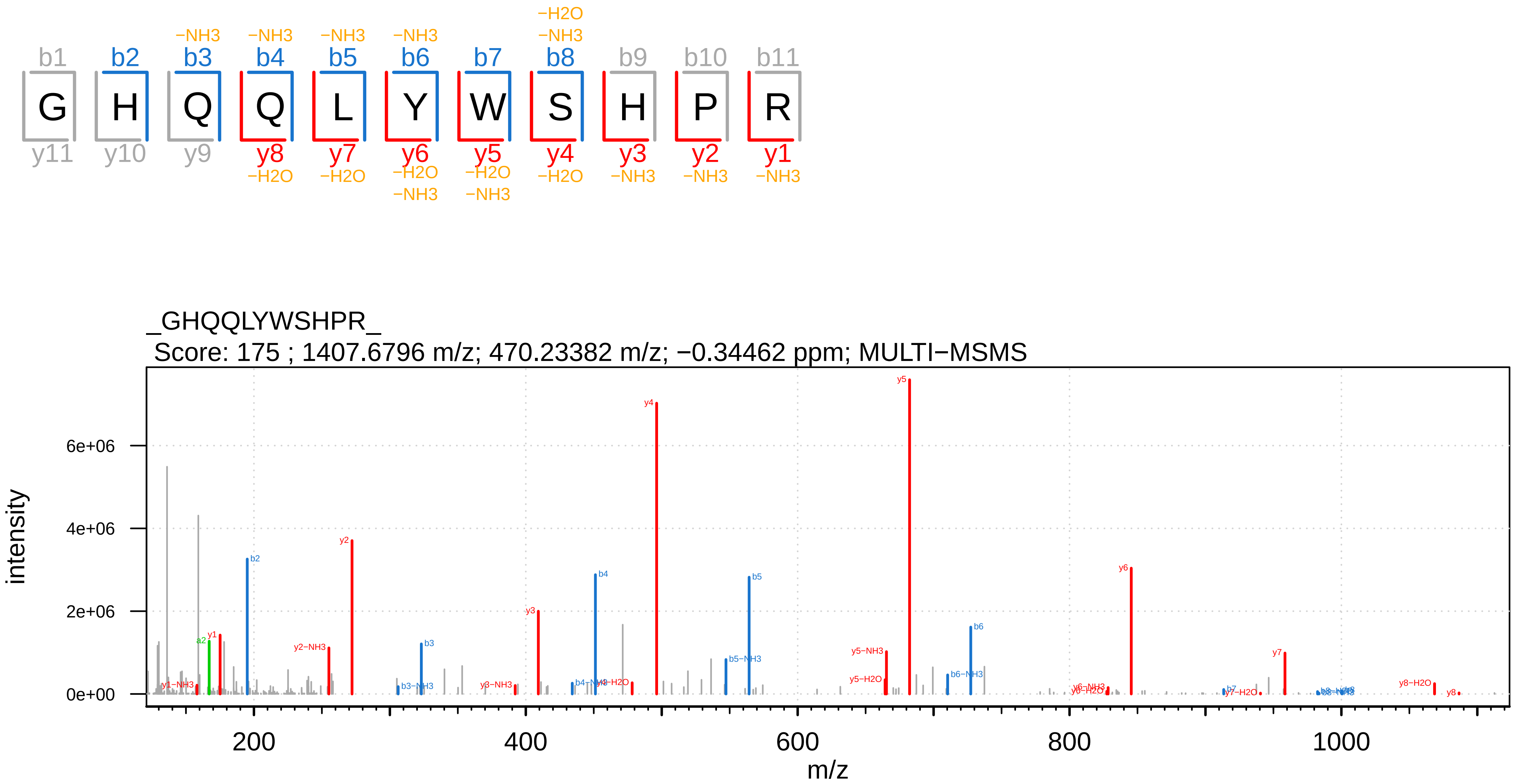

Raw File: Kermit_20111104_MSU_tube_6h_11
Scan Number: 8034
Proteins:
sp|P62274|RS29_MOUSE
ENSMUST00000037023_chr12:69157813−69159058:−
ENSMUST00000071737_chr13:73988963−73989133:−
tr|D3YYL7|D3YYL7_MOUSE

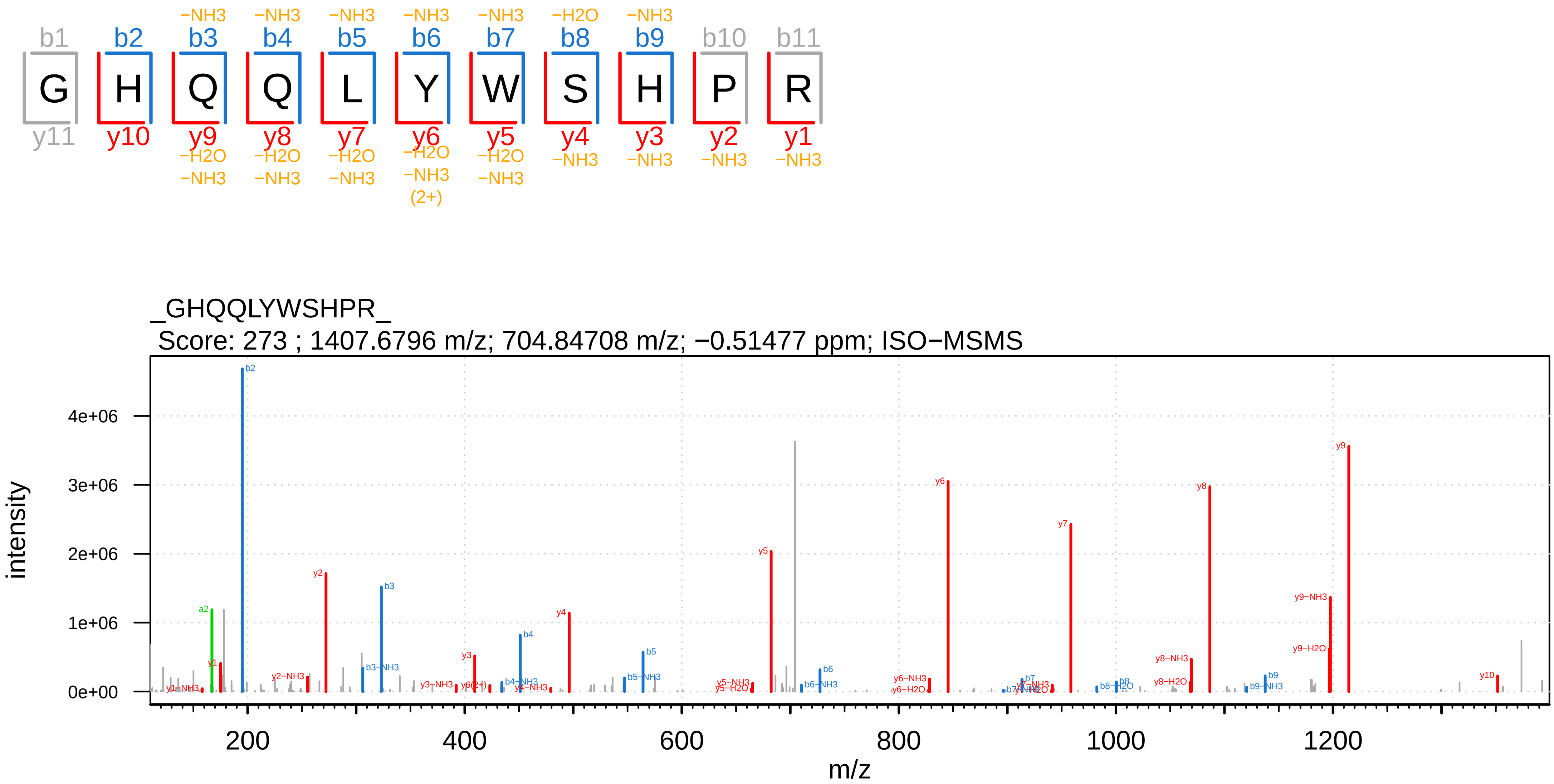

Raw File: Kermit_20111104_MSU_tube_6h_11
Scan Number: 8069
Proteins:
sp|P62274|RS29_MOUSE
ENSMUST00000037023_chr12:69157813−69159058:−
ENSMUST00000071737_chr13:73988963−73989133:−
tr|D3YYL7|D3YYL7_MOUSE

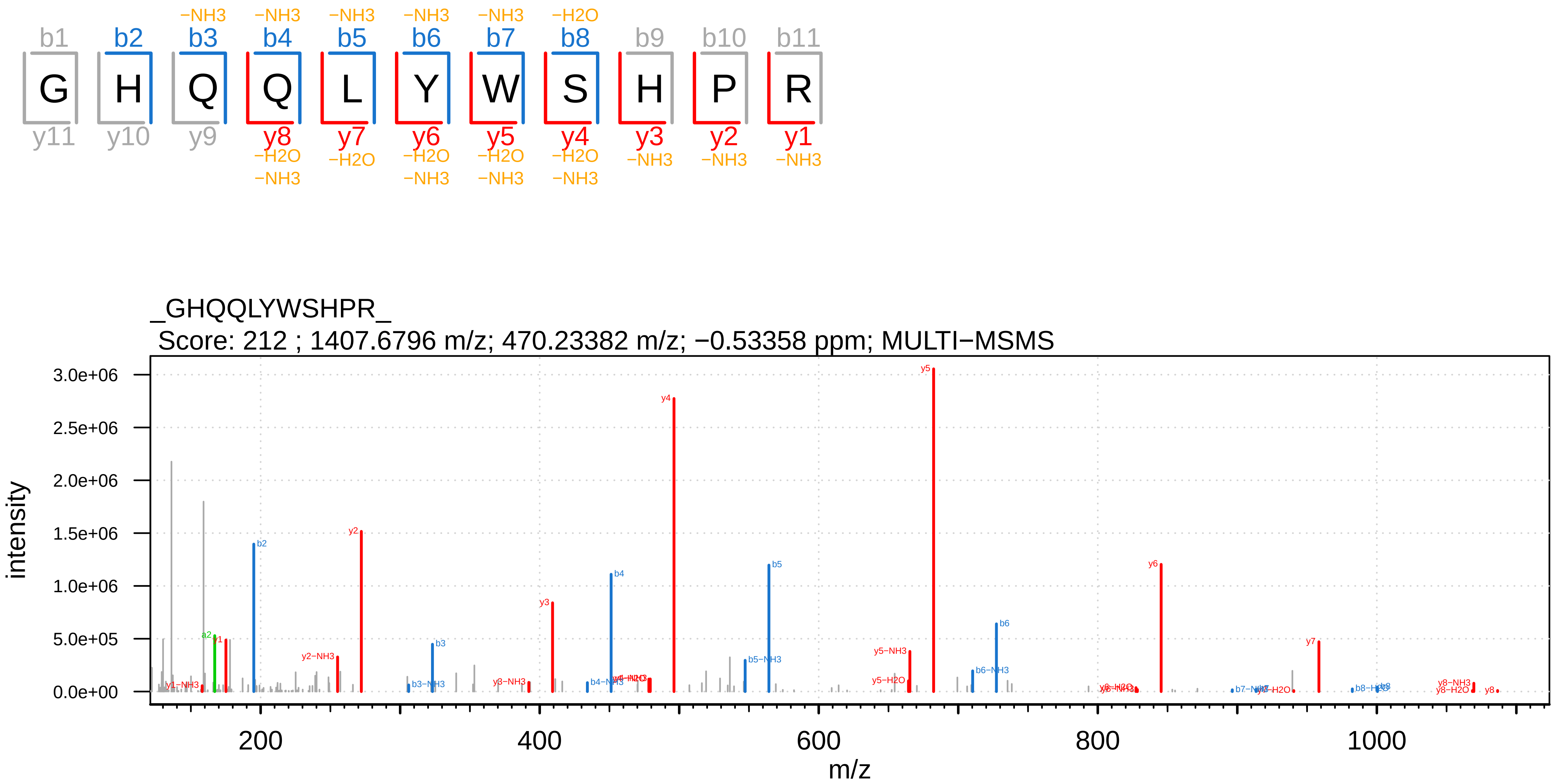

Raw File: Kermit_20111109_MSU_blast_1h_11
Scan Number: 7691
Proteins:
sp|P62274|RS29_MOUSE
ENSMUST00000037023_chr12:69157813−69159058:−
ENSMUST00000071737_chr13:73988963−73989133:−
tr|D3YYL7|D3YYL7_MOUSE

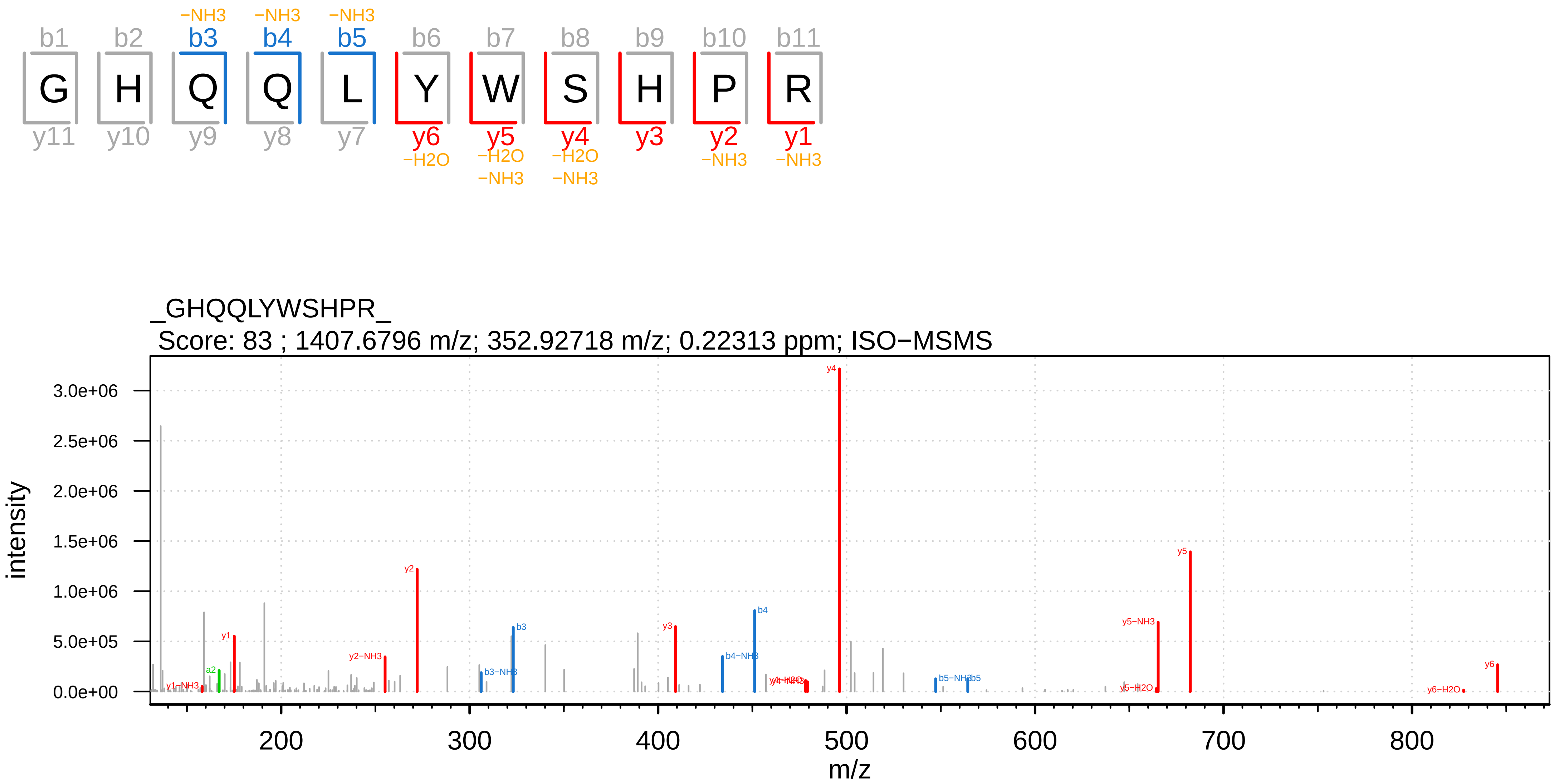

Raw File: Kermit_20111109_MSU_blast_1h_11
Scan Number: 7704
Proteins:
sp|P62274|RS29_MOUSE
ENSMUST00000037023_chr12:69157813−69159058:−
ENSMUST00000071737_chr13:73988963−73989133:−
tr|D3YYL7|D3YYL7_MOUSE

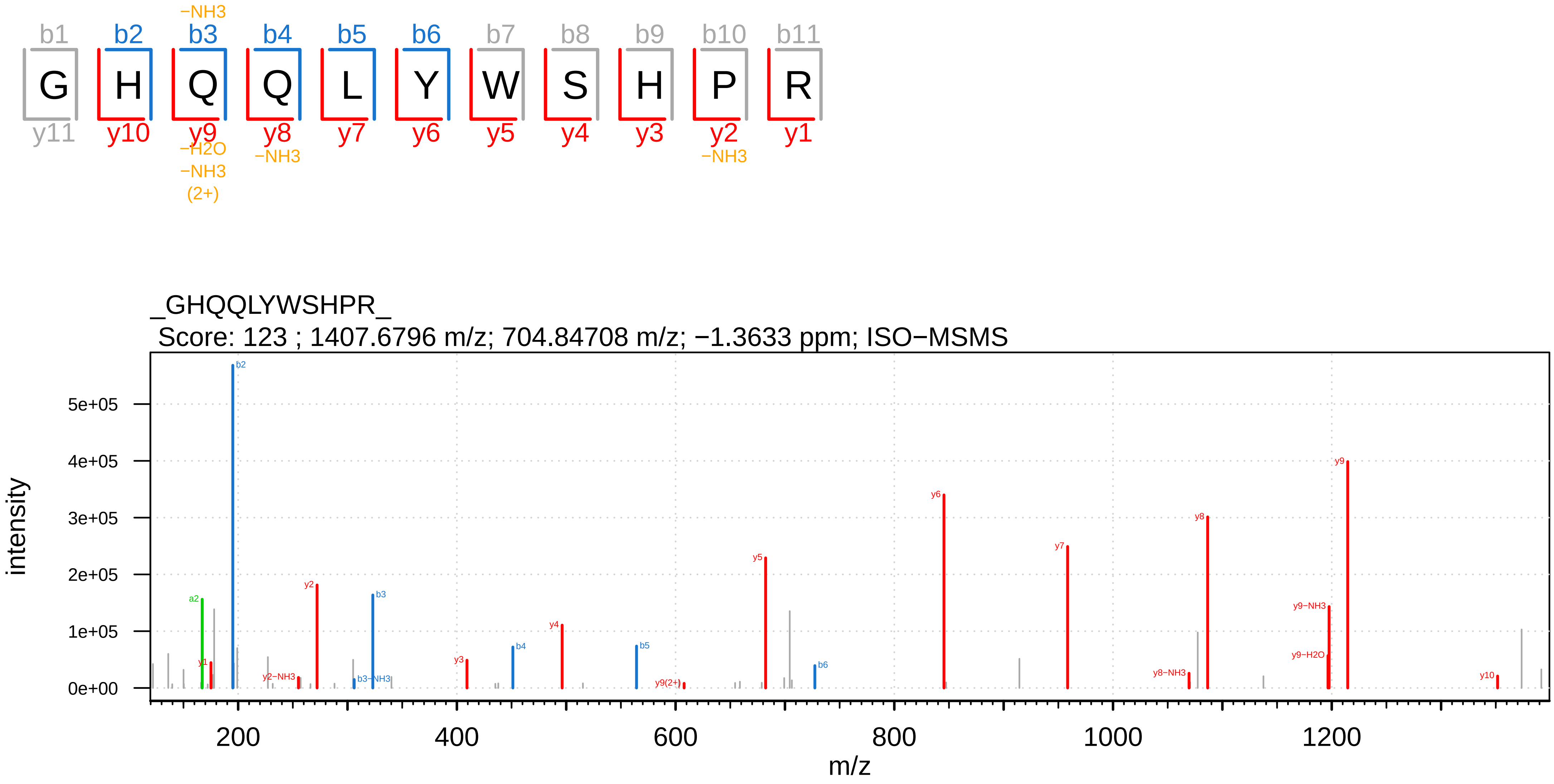

Raw File: Kermit_20111109_MSU_blast_1h_11
Scan Number: 7708
Proteins:
sp|P62274|RS29_MOUSE
ENSMUST00000037023_chr12:69157813−69159058:−
ENSMUST00000071737_chr13:73988963−73989133:−
tr|D3YYL7|D3YYL7_MOUSE

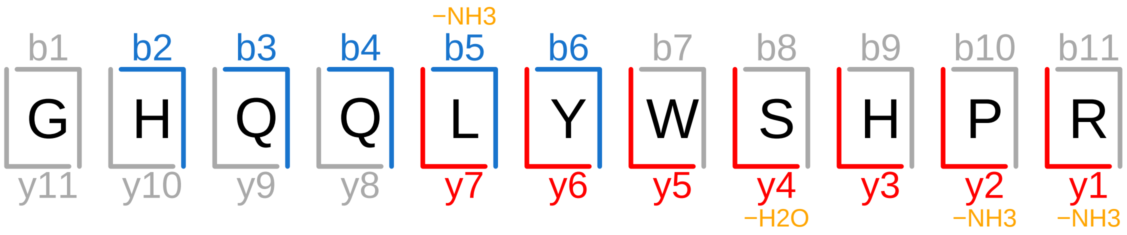

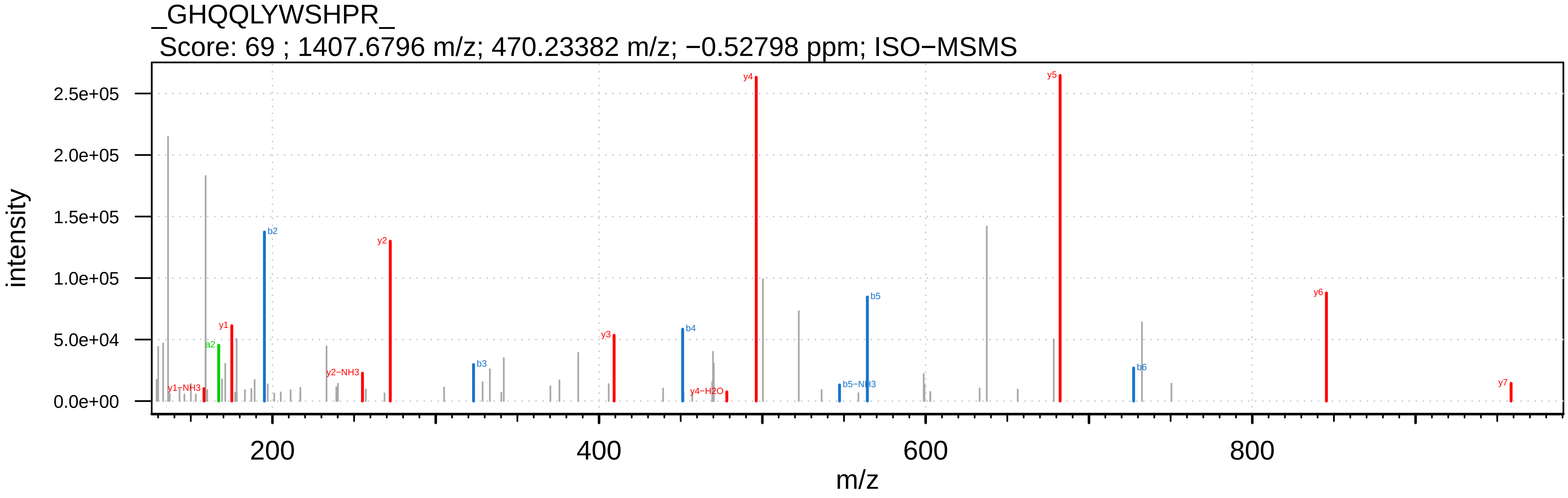

Raw File: Kermit_20111109_MSU_blast_1h_12
Scan Number: 56923
Proteins:
sp|P62274|RS29_MOUSE
ENSMUST00000037023_chr12:69157813−69159058:−
ENSMUST00000071737_chr13:73988963−73989133:−
tr|D3YYL7|D3YYL7_MOUSE

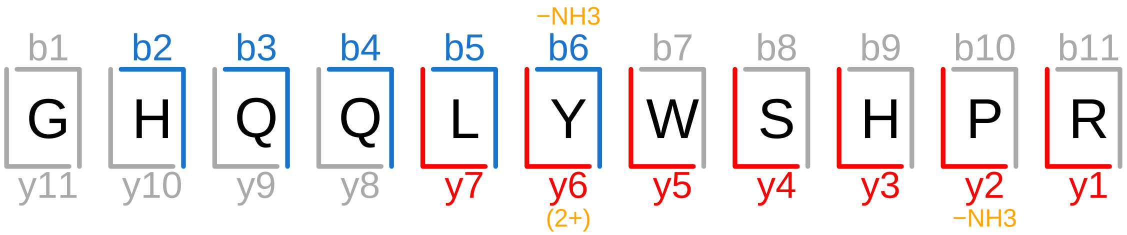

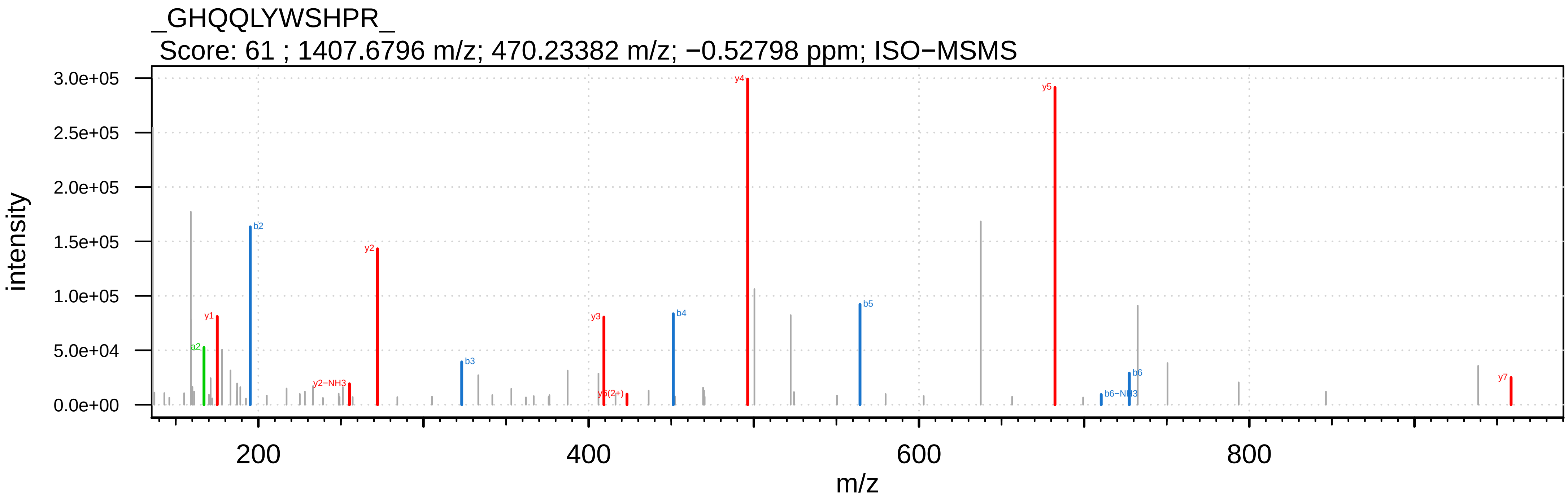

Raw File: Kermit_20111109_MSU_blast_1h_12
Scan Number: 57137
Proteins:
sp|P62274|RS29_MOUSE
ENSMUST00000037023_chr12:69157813−69159058:−
ENSMUST00000071737_chr13:73988963−73989133:−
tr|D3YYL7|D3YYL7_MOUSE

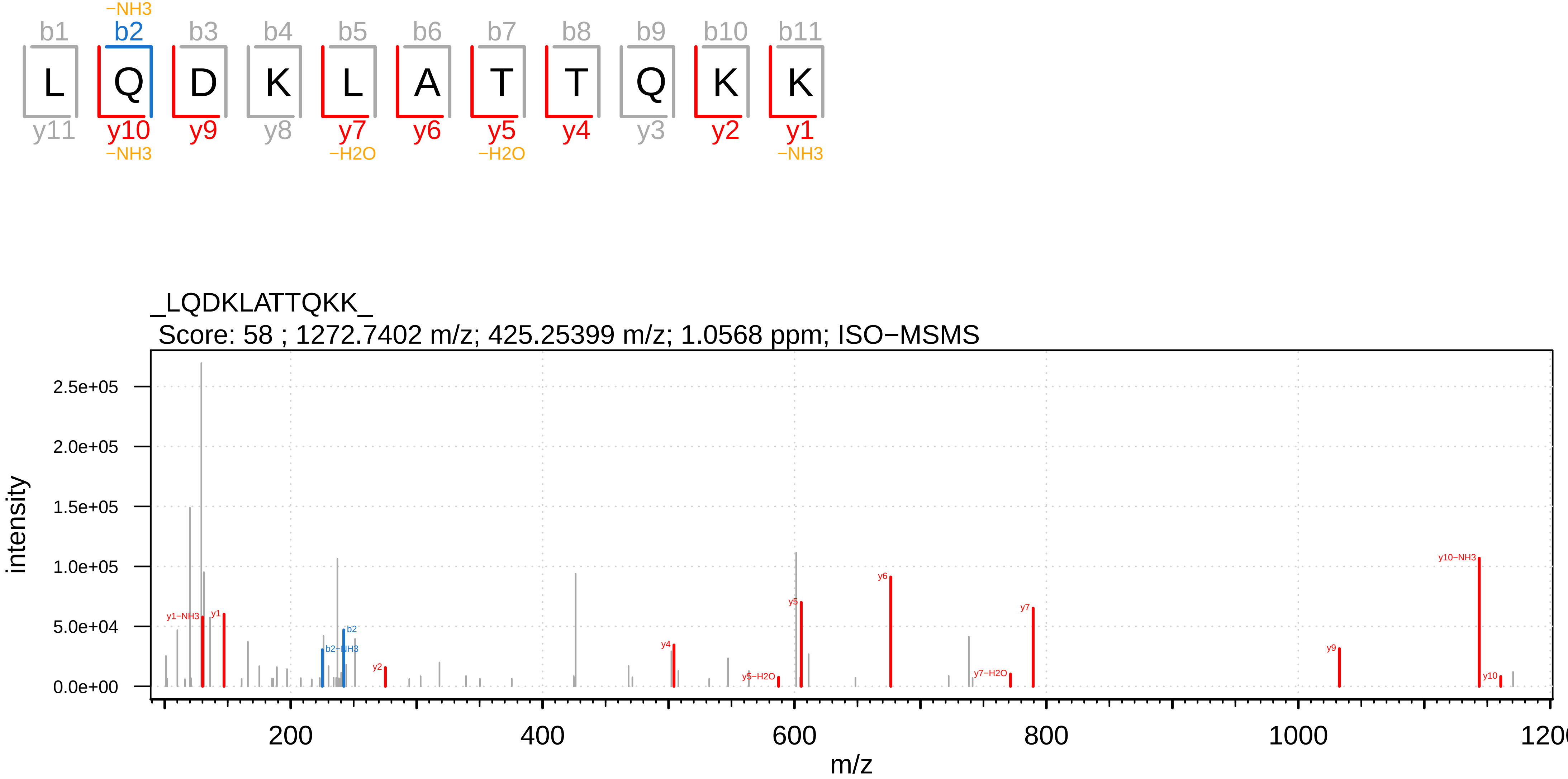

Raw File: Kermit_20111109_MSU_blast_1h_12
Scan Number: 3237
Proteins:
sp|Q8BT35|YB039_MOUSE
ENSMUST00000135091_chr2:127792297−127792467:−

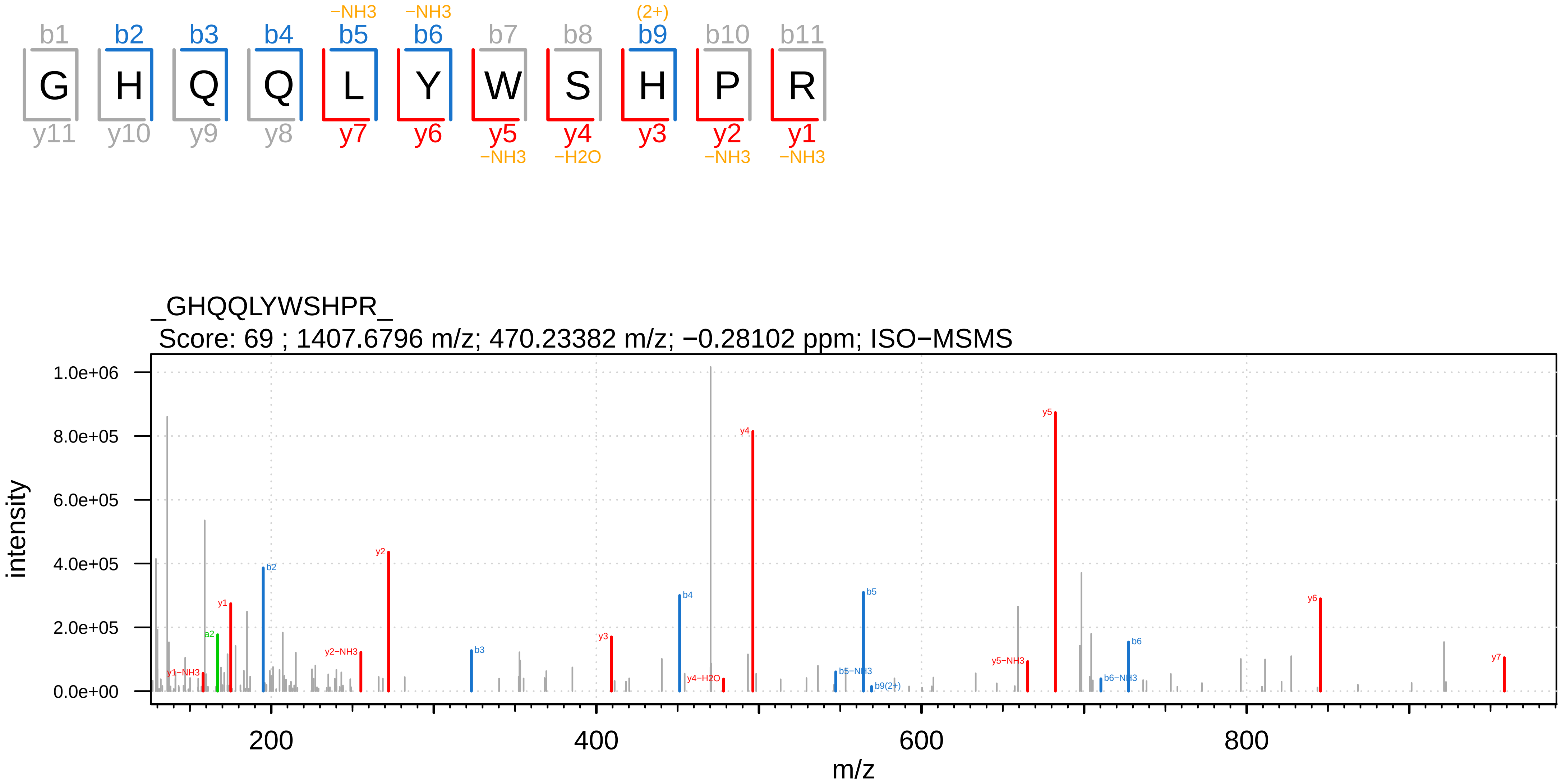

Raw File: Kermit_20111109_MSU_blast_3h_01
Scan Number: 8820
Proteins:
sp|P62274|RS29_MOUSE
ENSMUST00000037023_chr12:69157813−69159058:−
ENSMUST00000071737_chr13:73988963−73989133:−
tr|D3YYL7|D3YYL7_MOUSE

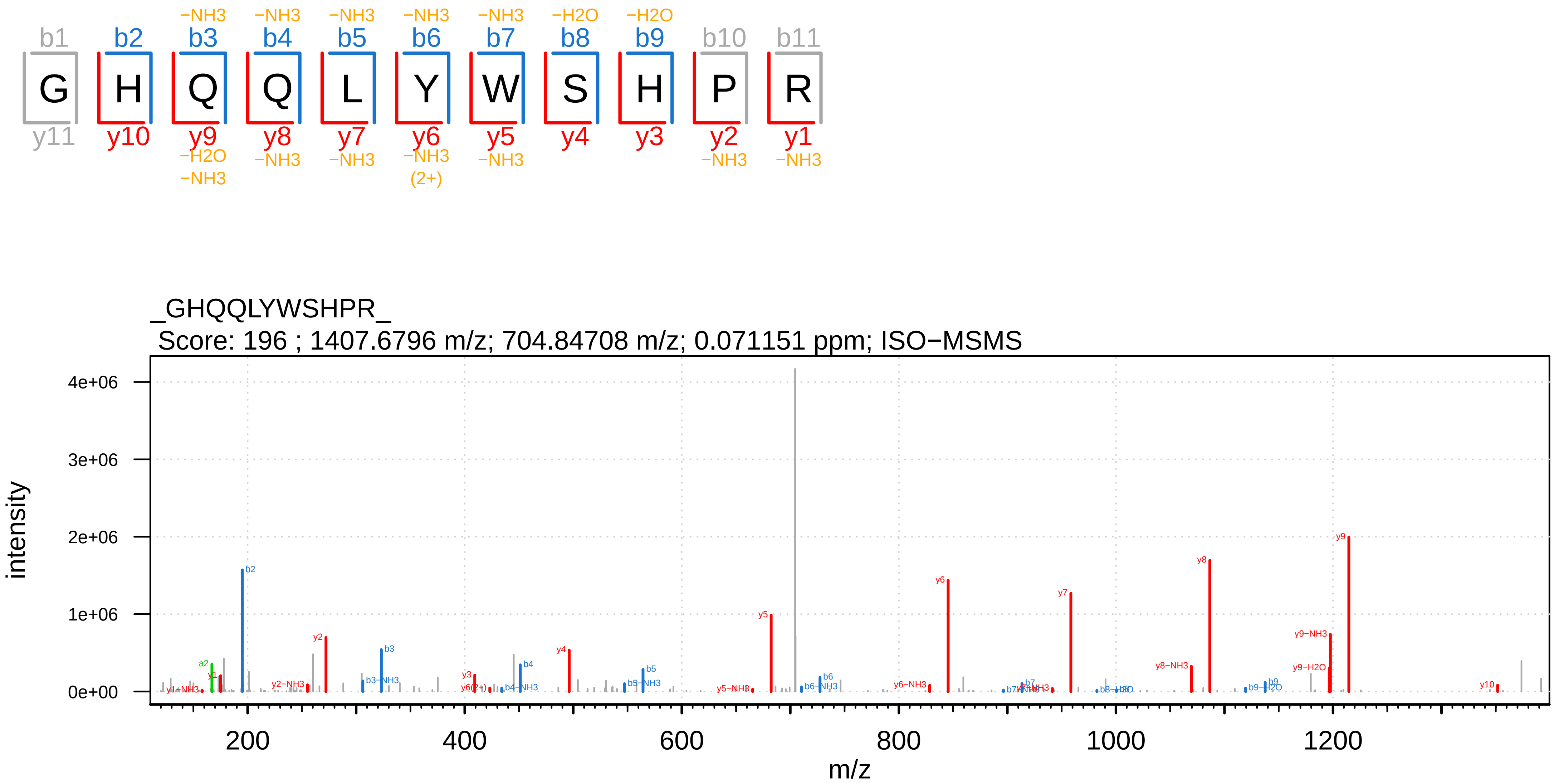

Raw File: Kermit_20111110_MSU_blast_3h_10
Scan Number: 4444
Proteins:
sp|P62274|RS29_MOUSE
ENSMUST00000037023_chr12:69157813−69159058:−
ENSMUST00000071737_chr13:73988963−73989133:−
tr|D3YYL7|D3YYL7_MOUSE

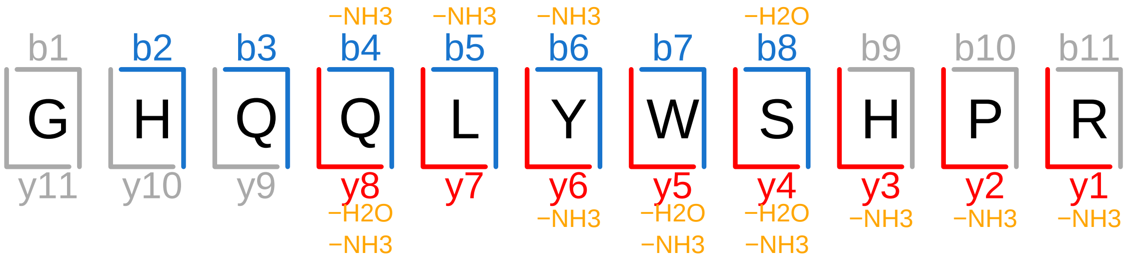

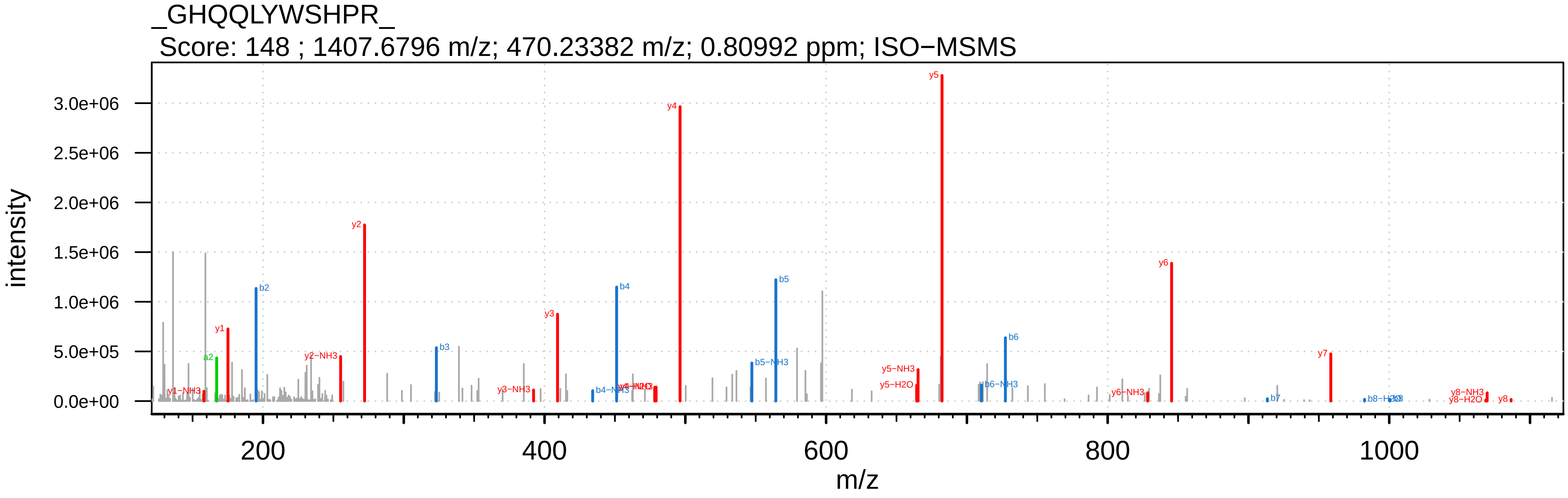

Raw File: Kermit_20111110_MSU_blast_3h_10
Scan Number: 4484
Proteins:
sp|P62274|RS29_MOUSE
ENSMUST00000037023_chr12:69157813−69159058:−
ENSMUST00000071737_chr13:73988963−73989133:−
tr|D3YYL7|D3YYL7_MOUSE

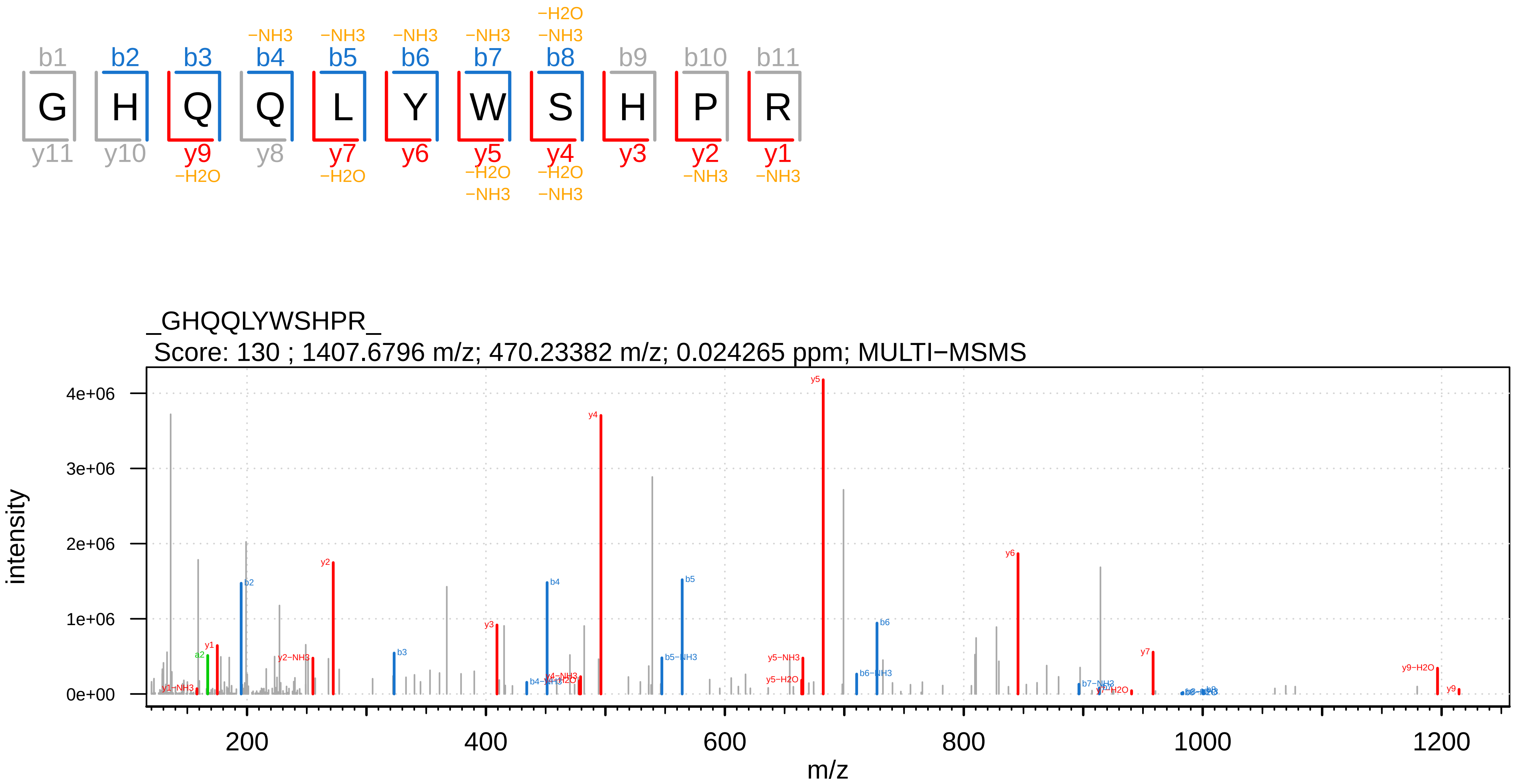

Raw File: Kermit_20111110_MSU_blast_3h_11
Scan Number: 4889
Proteins:
sp|P62274|RS29_MOUSE
ENSMUST00000037023_chr12:69157813−69159058:−
ENSMUST00000071737_chr13:73988963−73989133:−
tr|D3YYL7|D3YYL7_MOUSE

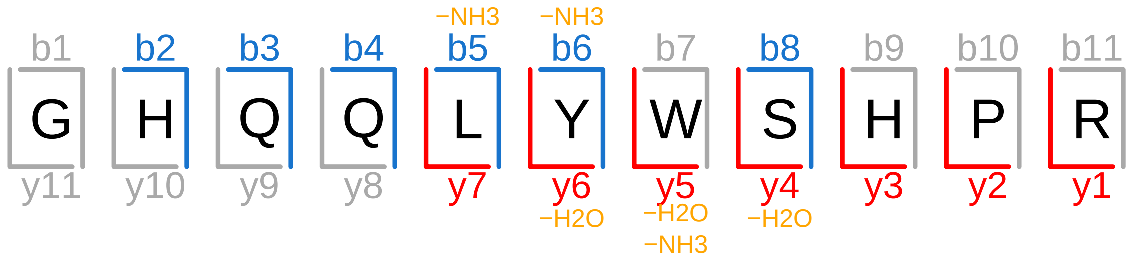

GHQQLYWSHPR
Score: 81 ; 1417.6879 m/z; 473.5699 m/z; 0.024265 ppm; MULTI−MSMS

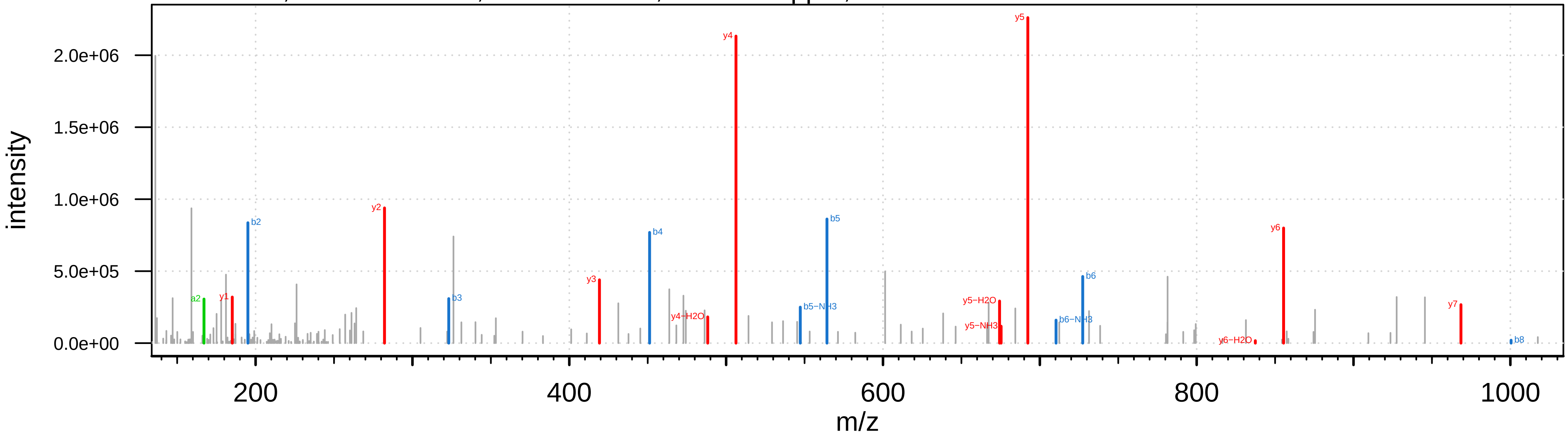

Raw File: Kermit_20111110_MSU_blast_3h_11
Scan Number: 4953
Proteins:
sp|P62274|RS29_MOUSE
ENSMUST00000037023_chr12:69157813−69159058:−
ENSMUST00000071737_chr13:73988963−73989133:−
tr|D3YYL7|D3YYL7_MOUSE

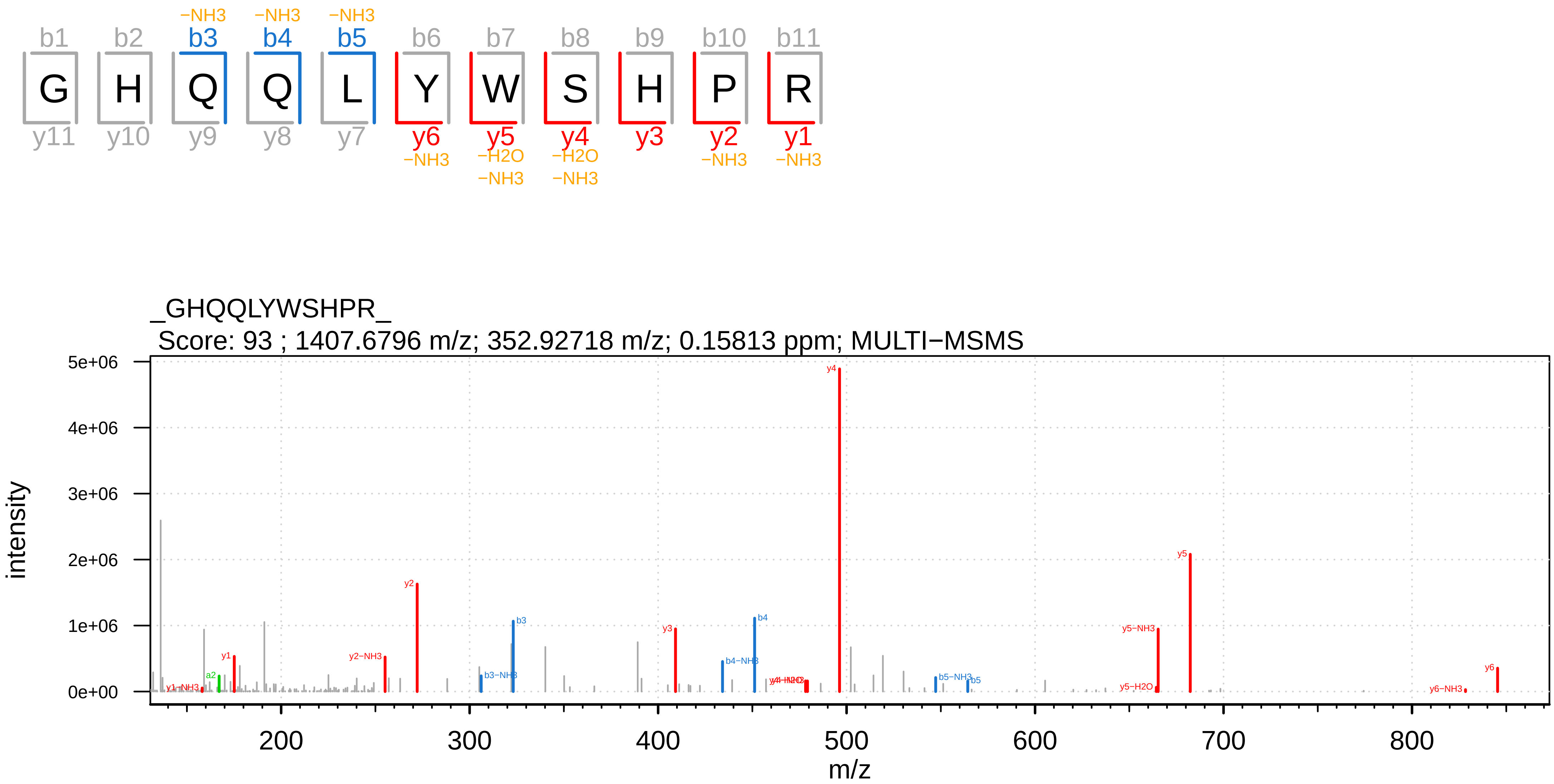

Raw File: Kermit_20111110_MSU_blast_3h_11
Scan Number: 4908
Proteins:
sp|P62274|RS29_MOUSE
ENSMUST00000037023_chr12:69157813−69159058:−
ENSMUST00000071737_chr13:73988963−73989133:−
tr|D3YYL7|D3YYL7_MOUSE

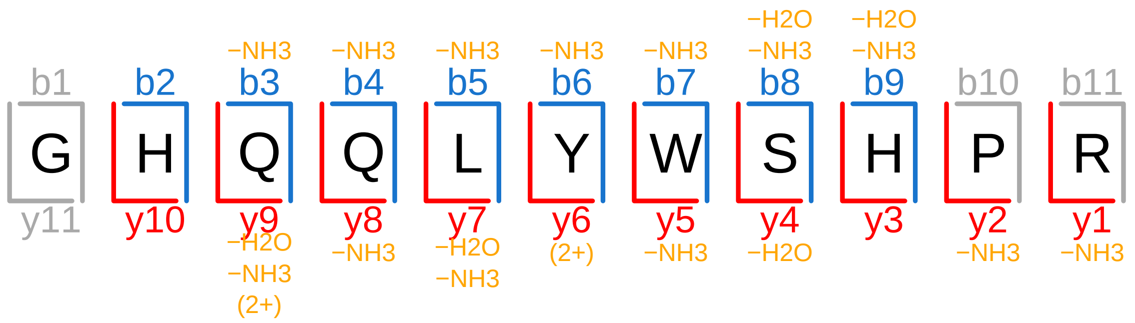

GHQQLYWSHPR
Score: 233 ; 1407.6796 m/z; 704.84708 m/z; −0.7891 ppm; ISO−MSMS

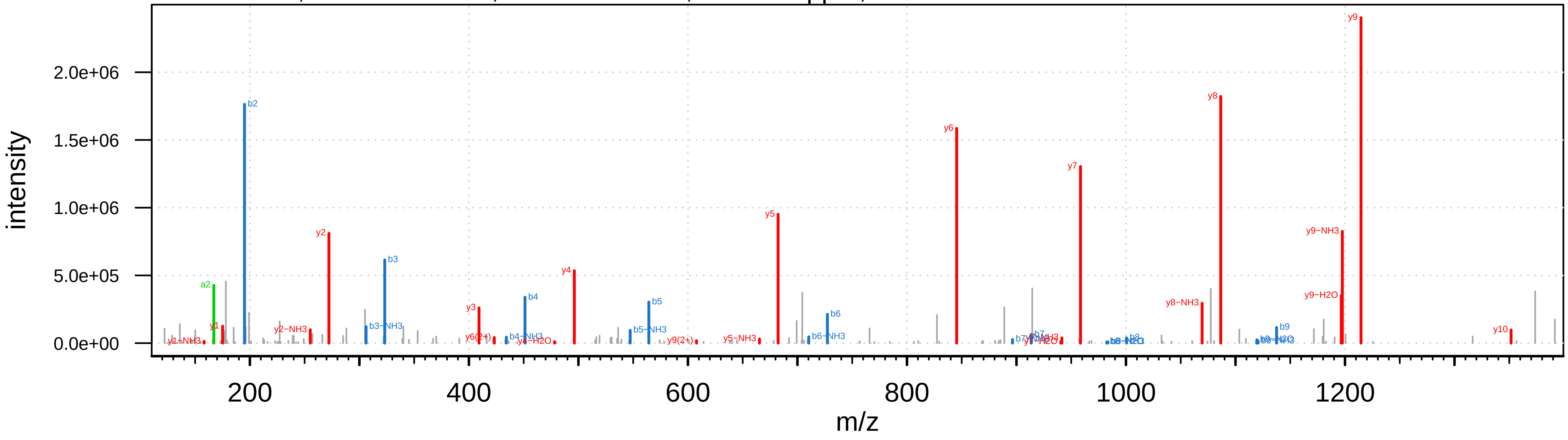

Raw File: Kermit_20111110_MSU_blast_3h_11
Scan Number: 4967
Proteins:
sp|P62274|RS29_MOUSE
ENSMUST00000037023_chr12:69157813−69159058:−
ENSMUST00000071737_chr13:73988963−73989133:−
tr|D3YYL7|D3YYL7_MOUSE

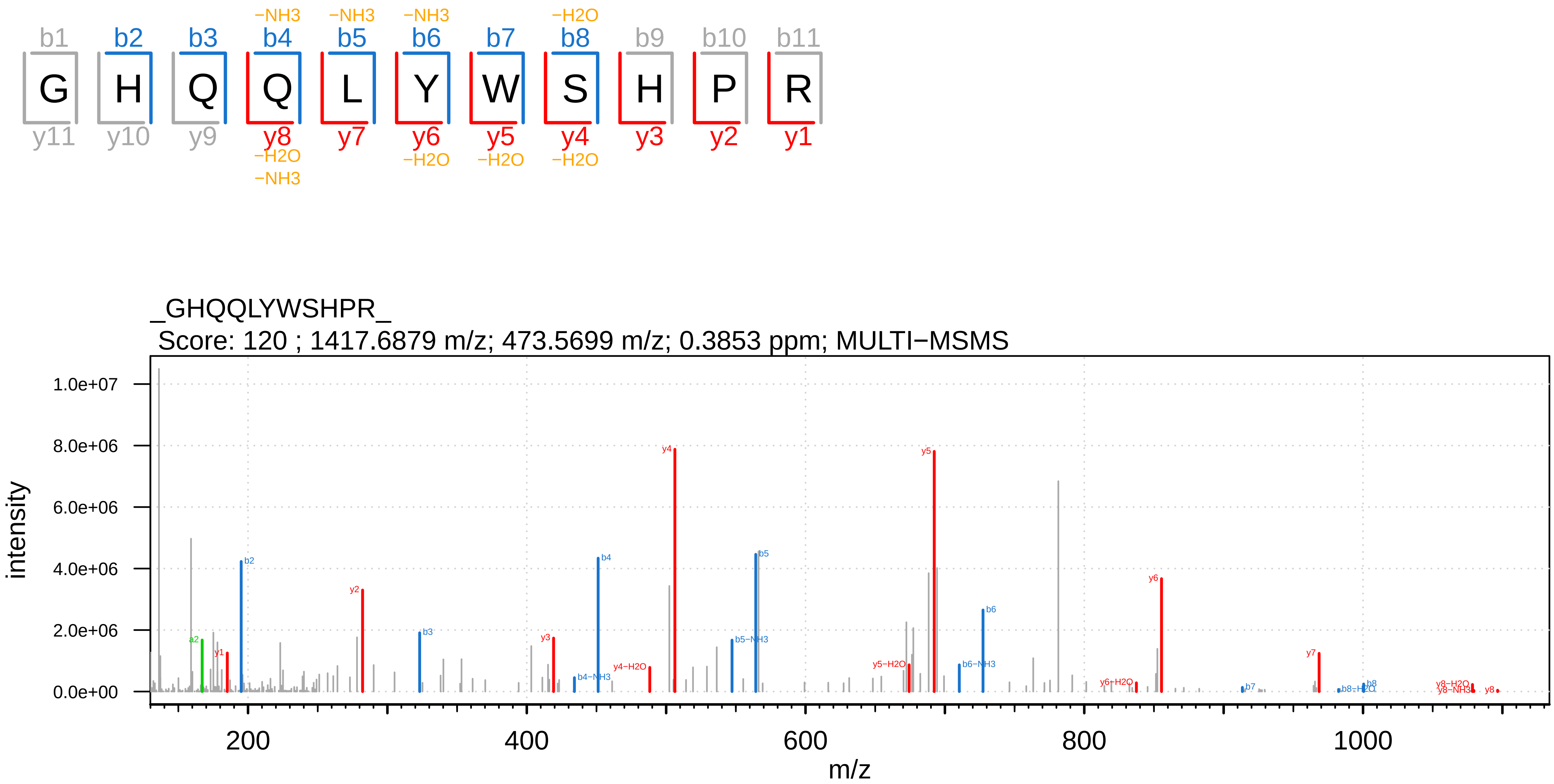

Raw File: Kermit_20111110_MSU_diff1_04
Scan Number: 6205
Proteins:
sp|P62274|RS29_MOUSE
ENSMUST00000037023_chr12:69157813−69159058:−
ENSMUST00000071737_chr13:73988963−73989133:−
tr|D3YYL7|D3YYL7_MOUSE

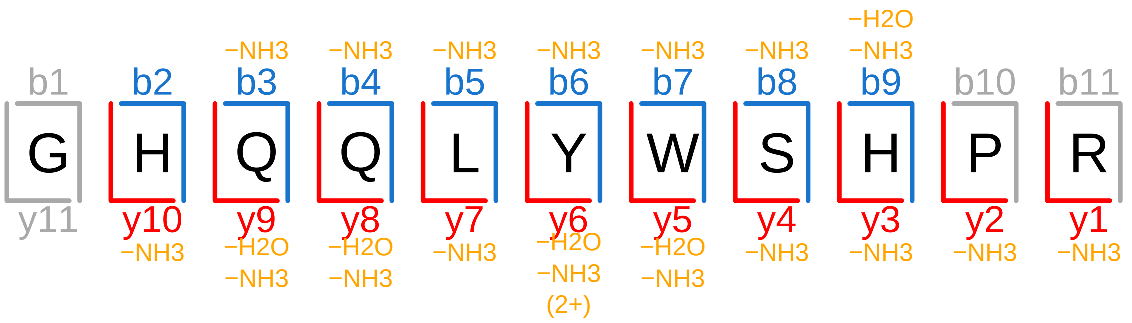

GHQQLYWSHPR
Score: 261 ; 1407.6796 m/z; 704.84708 m/z; 0.653 ppm; MULTI−MSMS

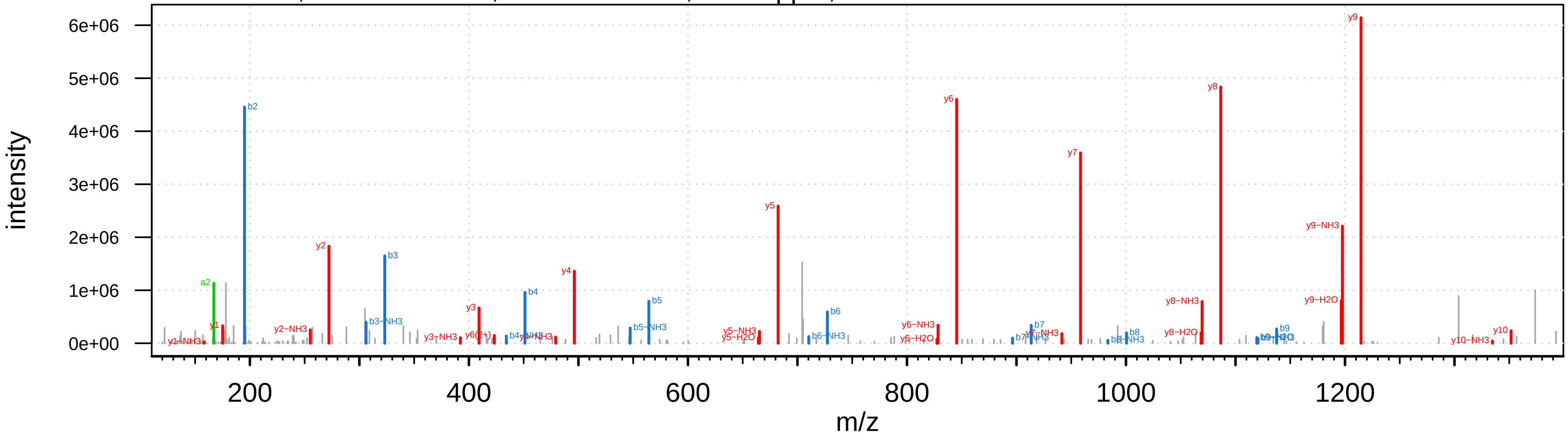

Raw File: Kermit_20111110_MSU_diff1_04
Scan Number: 6209
Proteins:
sp|P62274|RS29_MOUSE
ENSMUST00000037023_chr12:69157813−69159058:−
ENSMUST00000071737_chr13:73988963−73989133:−
tr|D3YYL7|D3YYL7_MOUSE

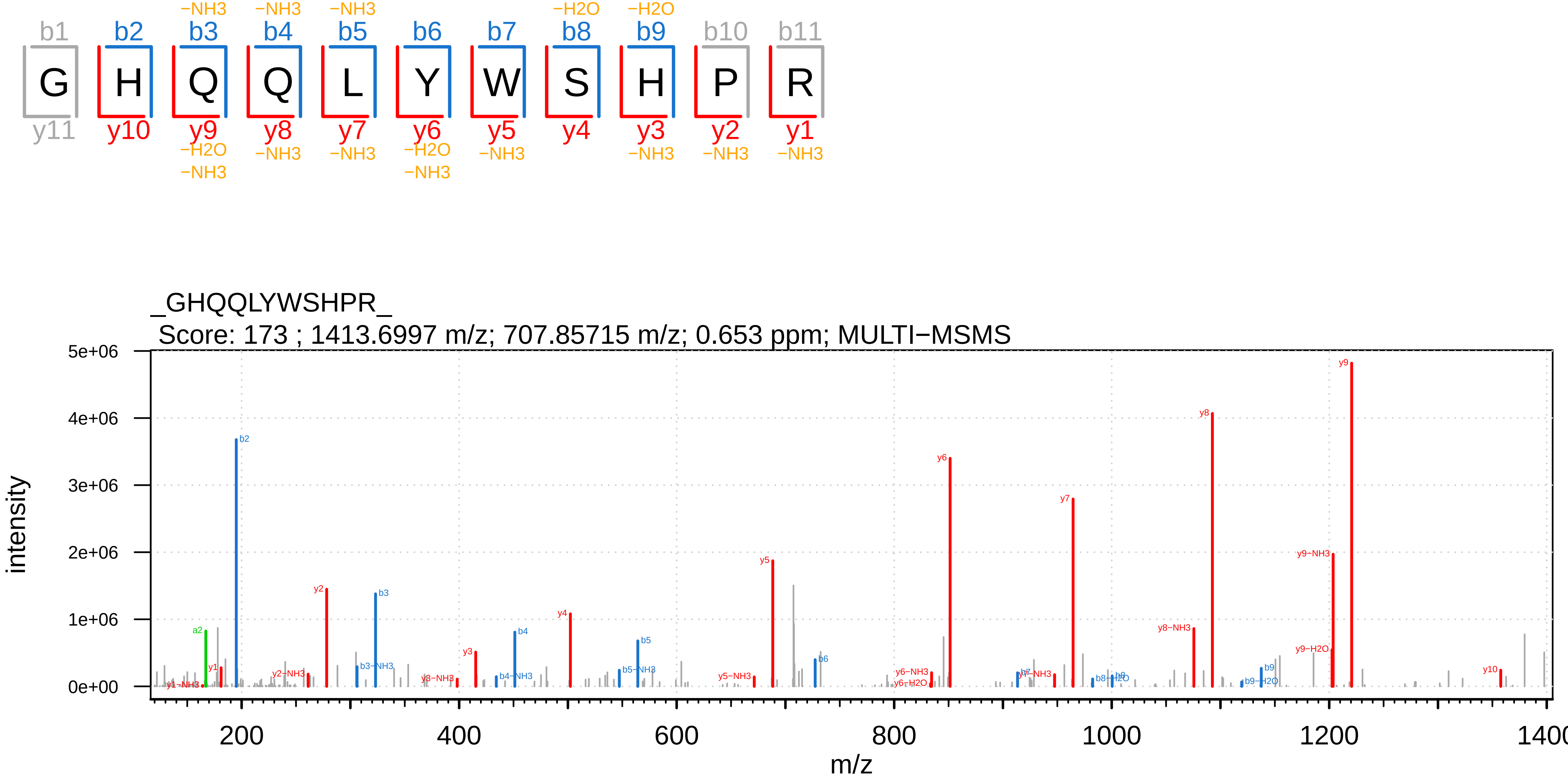

Raw File: Kermit_20111110_MSU_diff1_04
Scan Number: 6210
Proteins:
sp|P62274|RS29_MOUSE
ENSMUST00000037023_chr12:69157813−69159058:−
ENSMUST00000071737_chr13:73988963−73989133:−
tr|D3YYL7|D3YYL7_MOUSE

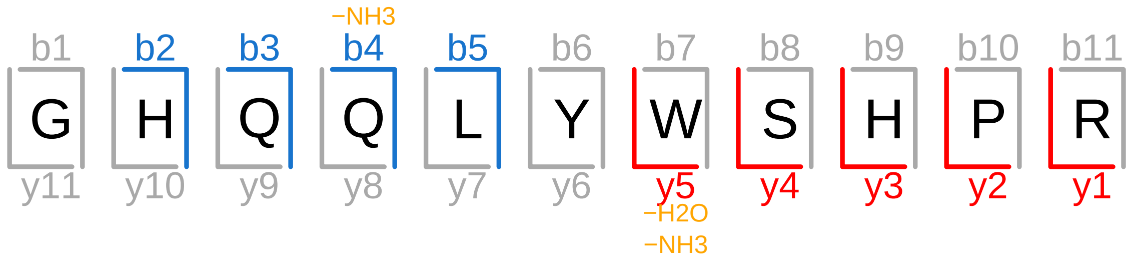

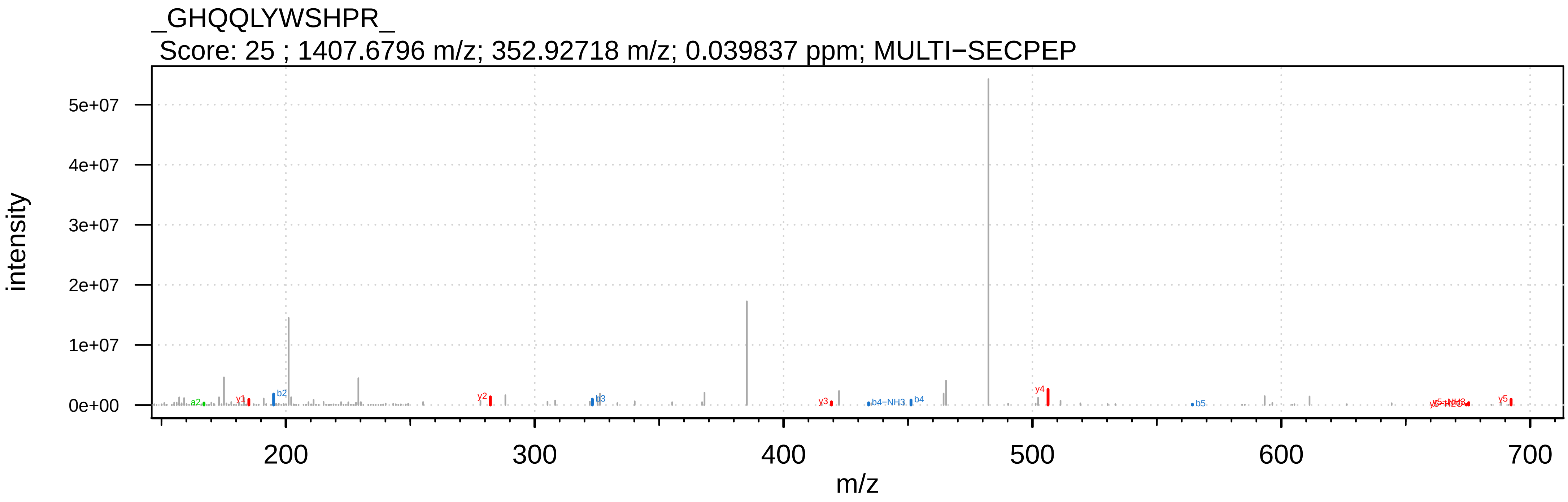

Raw File: Kermit_20111110_MSU_diff1_04
Scan Number: 6214
Proteins:
sp|P62274|RS29_MOUSE
ENSMUST00000037023_chr12:69157813−69159058:−
ENSMUST00000071737_chr13:73988963−73989133:−
tr|D3YYL7|D3YYL7_MOUSE

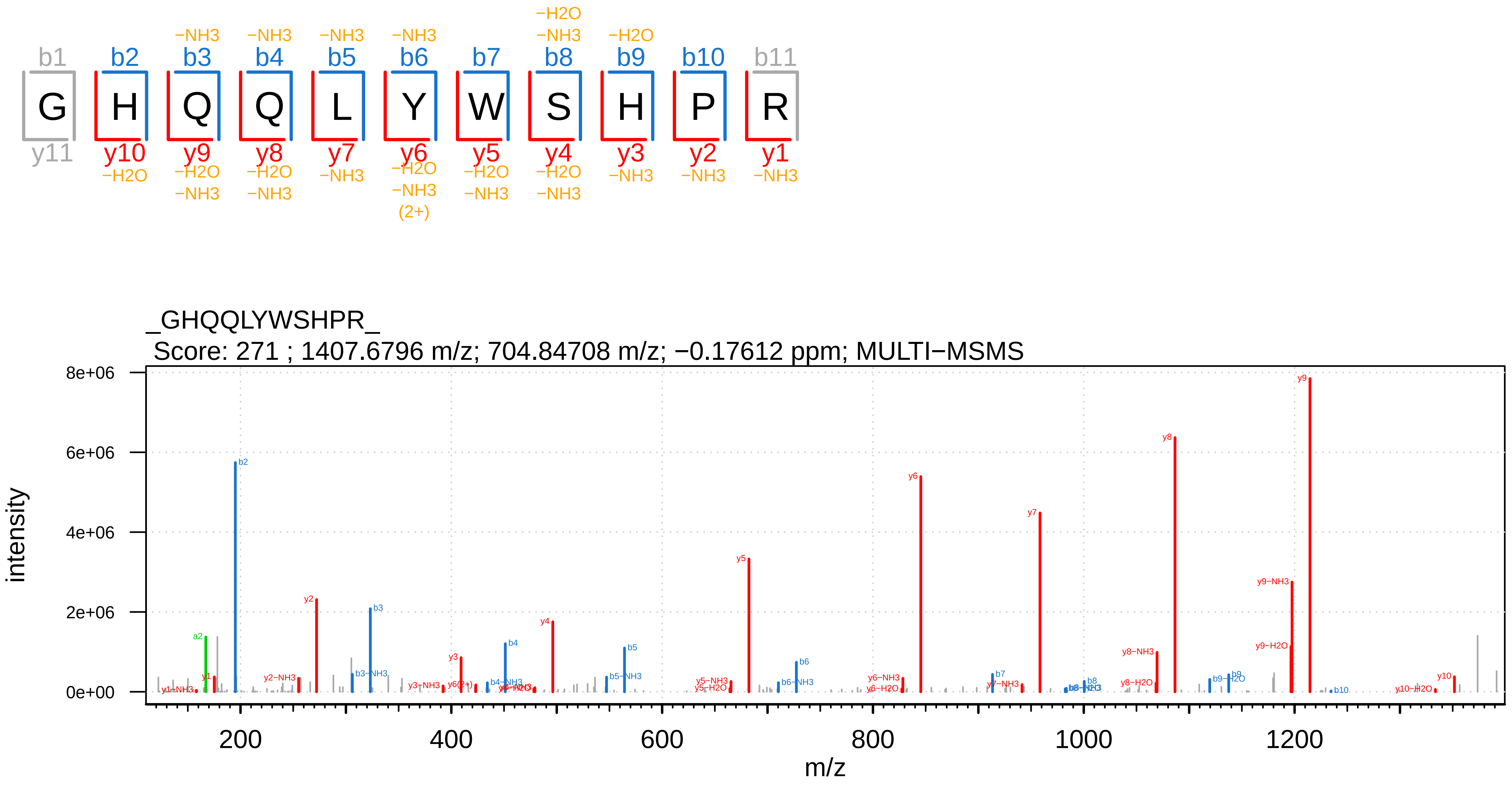

Raw File: Kermit_20111110_MSU_diff1_10
Scan Number: 6449
Proteins:
sp|P62274|RS29_MOUSE
ENSMUST00000037023_chr12:69157813−69159058:−
ENSMUST00000071737_chr13:73988963−73989133:−
tr|D3YYL7|D3YYL7_MOUSE

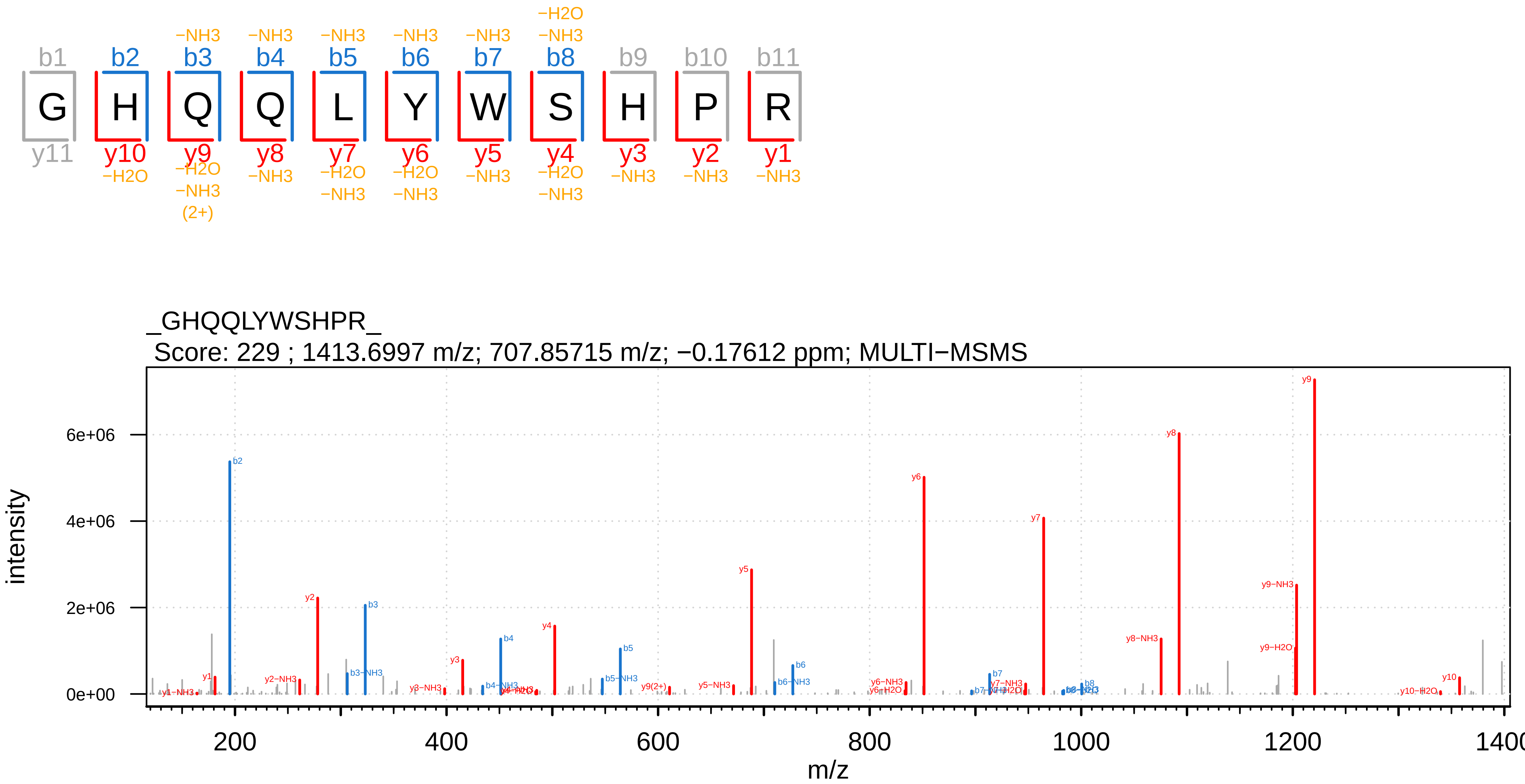

Raw File: Kermit_20111110_MSU_diff1_10
Scan Number: 6454
Proteins:
sp|P62274|RS29_MOUSE
ENSMUST00000037023_chr12:69157813−69159058:−
ENSMUST00000071737_chr13:73988963−73989133:−
tr|D3YYL7|D3YYL7_MOUSE

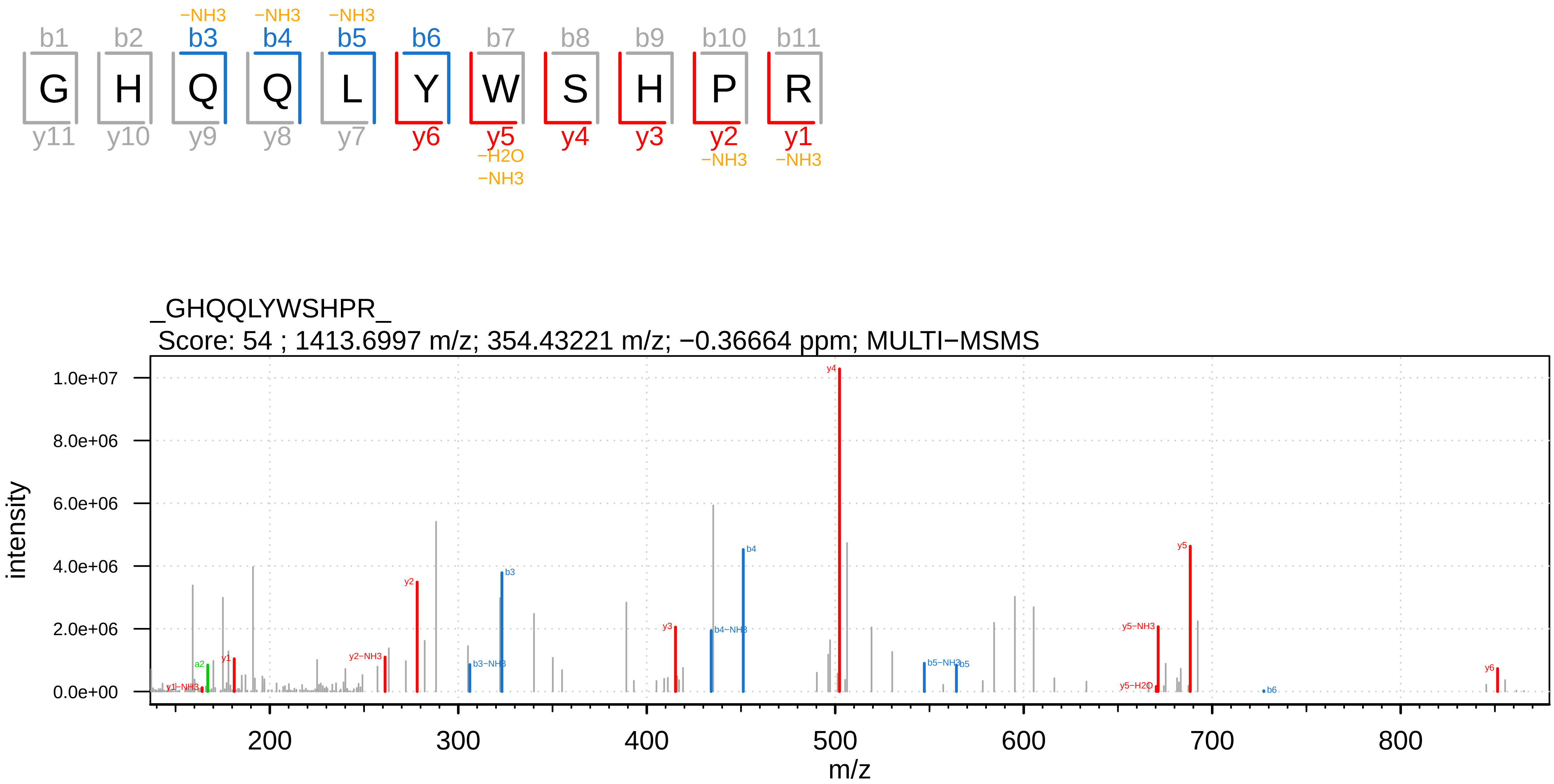

Raw File: Kermit_20111110_MSU_diff1_10
Scan Number: 6456
Proteins:
sp|P62274|RS29_MOUSE
ENSMUST00000037023_chr12:69157813−69159058:−
ENSMUST00000071737_chr13:73988963−73989133:−
tr|D3YYL7|D3YYL7_MOUSE

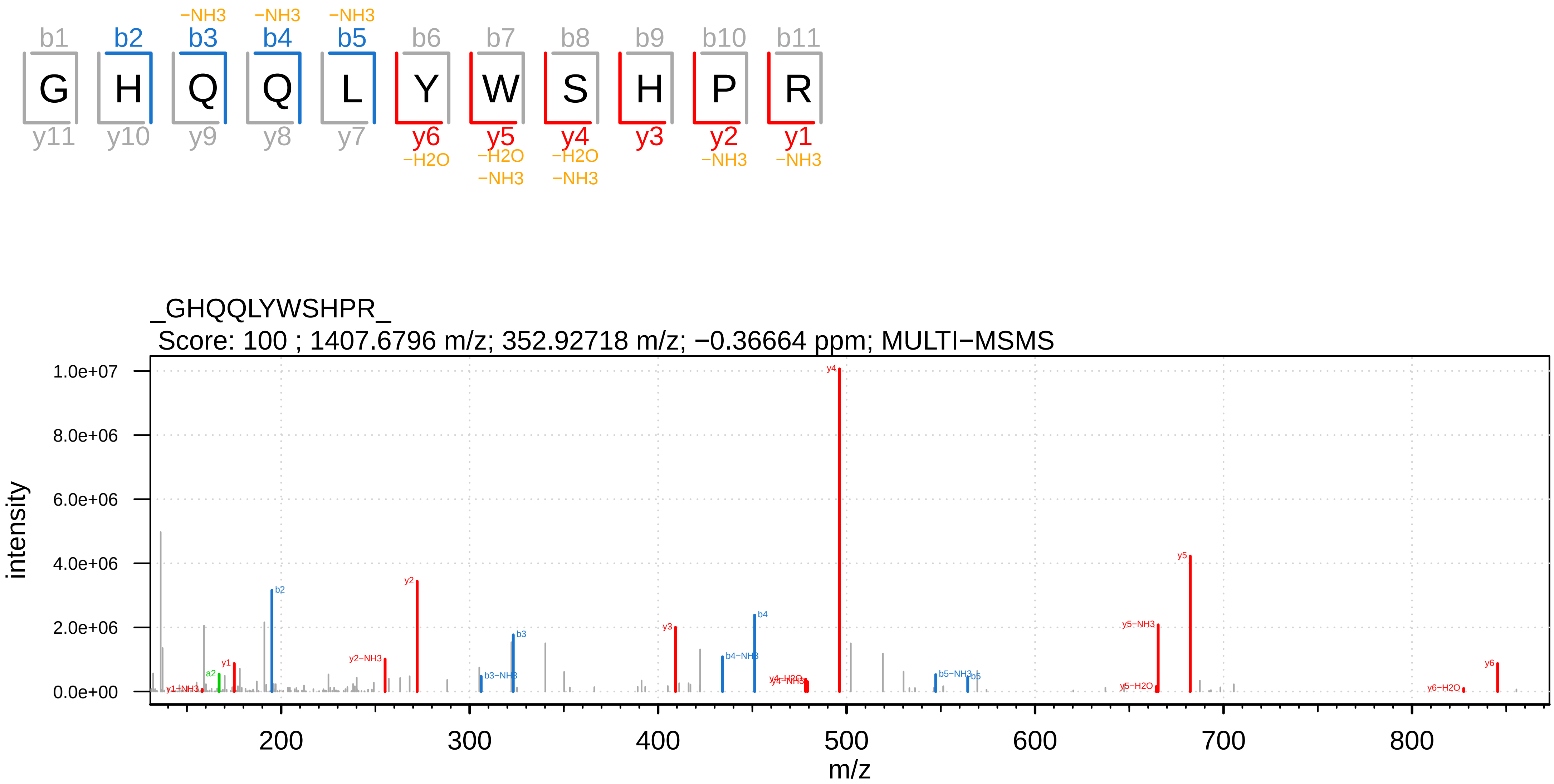

Raw File: Kermit_20111110_MSU_diff1_10
Scan Number: 6465
Proteins:
sp|P62274|RS29_MOUSE
ENSMUST00000037023_chr12:69157813−69159058:−
ENSMUST00000071737_chr13:73988963−73989133:−
tr|D3YYL7|D3YYL7_MOUSE

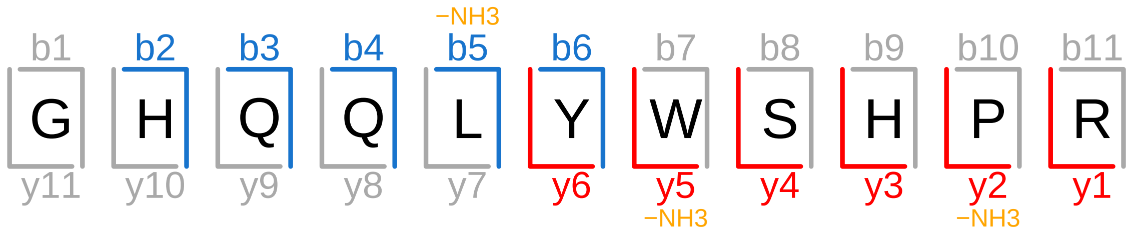

GHQQLYWSHPR
Score: 52 ; 1407.6796 m/z; 470.23382 m/z; −0.052489 ppm; MULTI−MSMS

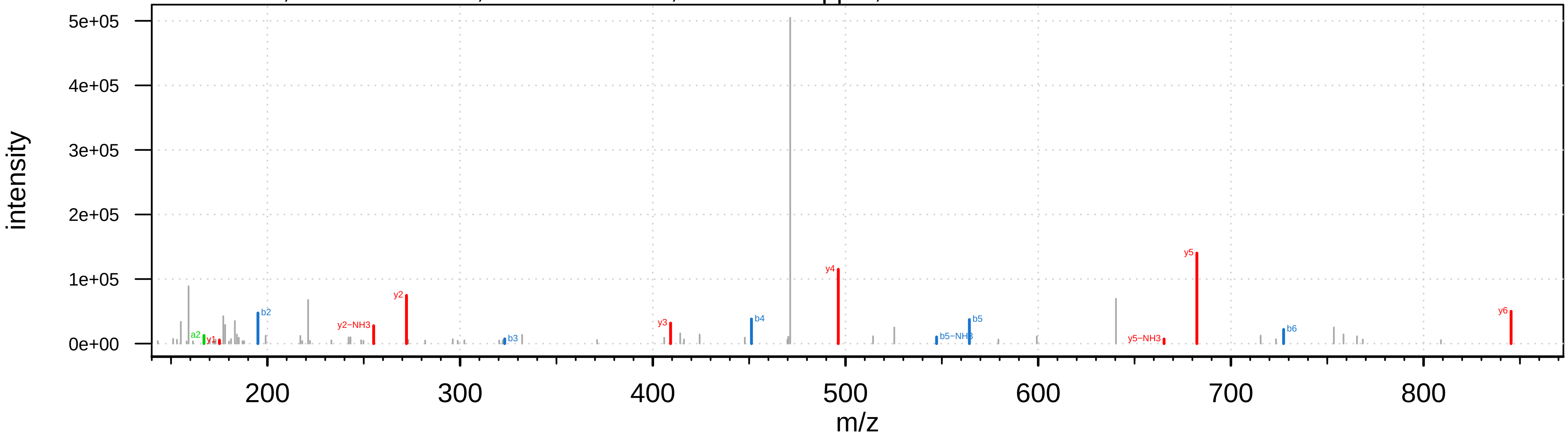

Raw File: Kermit_20111110_MSU_diff1_10
Scan Number: 65392
Proteins:
sp|P62274|RS29_MOUSE
ENSMUST00000037023_chr12:69157813−69159058:−
ENSMUST00000071737_chr13:73988963−73989133:−
tr|D3YYL7|D3YYL7_MOUSE

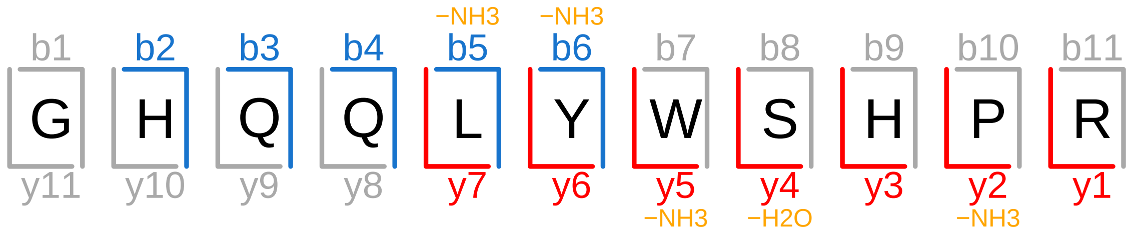

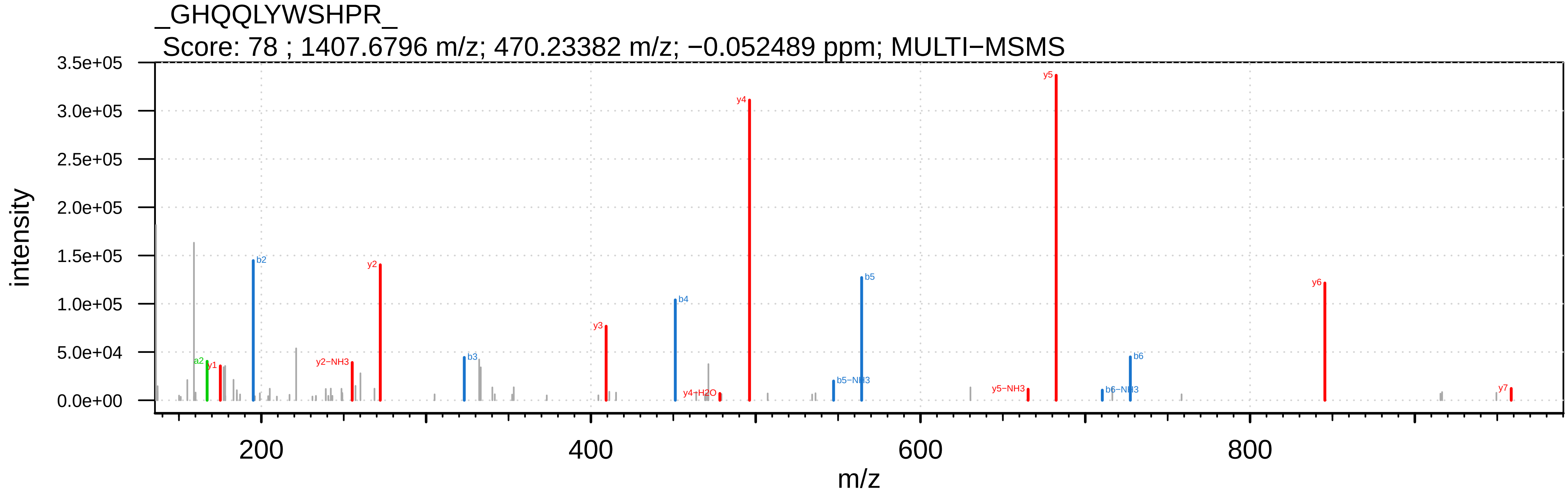

Raw File: Kermit_20111110_MSU_diff1_10
Scan Number: 65627
Proteins:
sp|P62274|RS29_MOUSE
ENSMUST00000037023_chr12:69157813−69159058:−
ENSMUST00000071737_chr13:73988963−73989133:−
tr|D3YYL7|D3YYL7_MOUSE

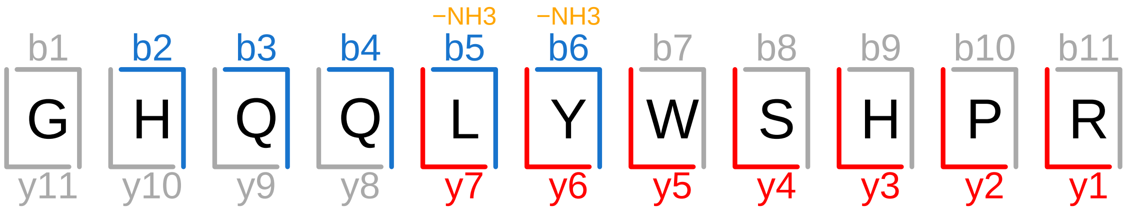

GHQQLYWSHPR
Score: 69 ; 1417.6879 m/z; 473.5699 m/z; −0.052489 ppm; MULTI−MSMS

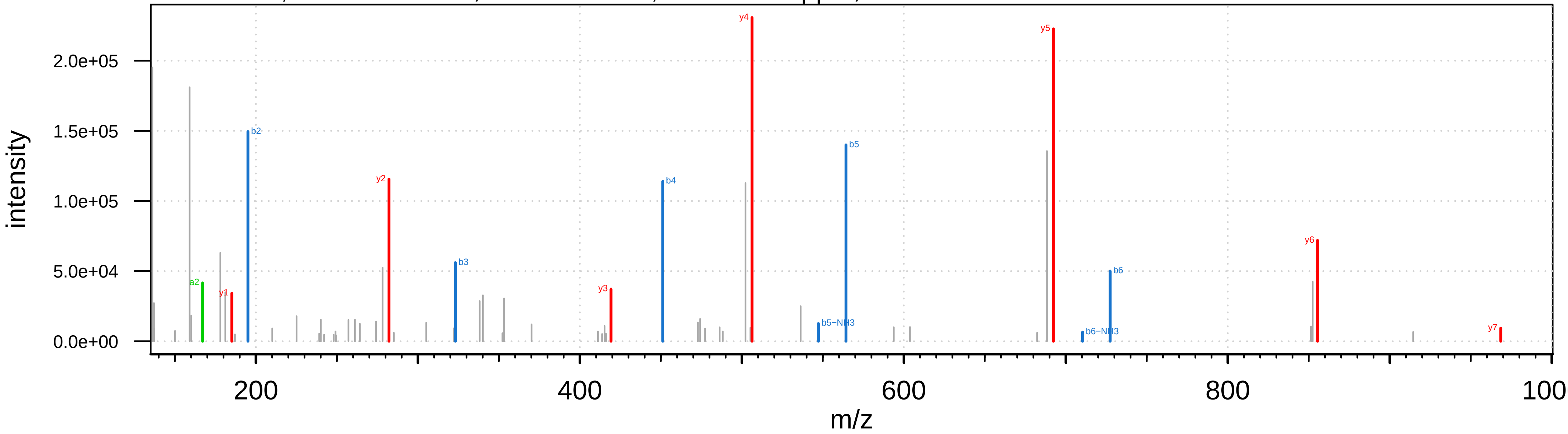

Raw File: Kermit_20111110_MSU_diff1_10
Scan Number: 65828
Proteins:
sp|P62274|RS29_MOUSE
ENSMUST00000037023_chr12:69157813−69159058:−
ENSMUST00000071737_chr13:73988963−73989133:−
tr|D3YYL7|D3YYL7_MOUSE

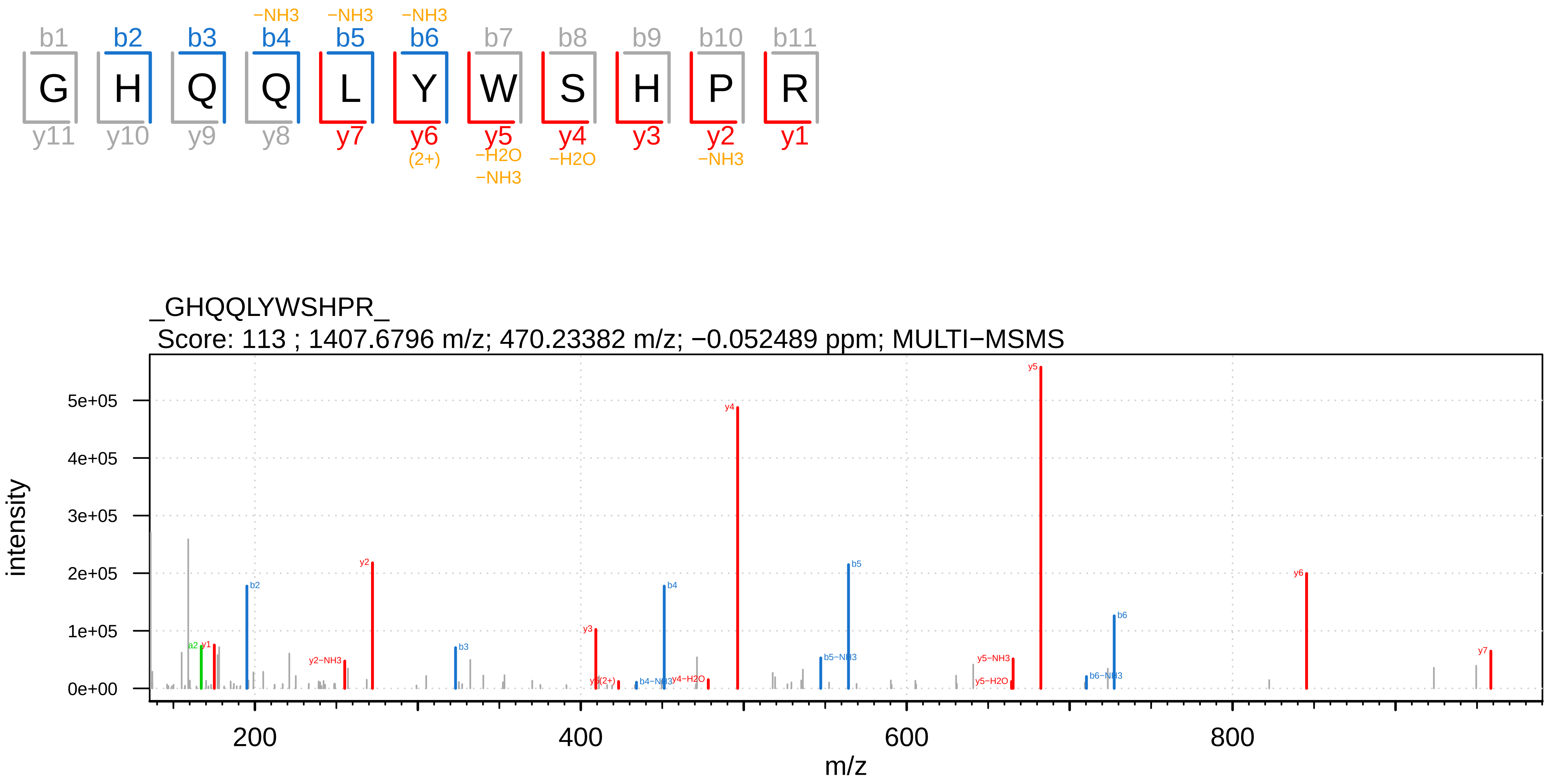

Raw File: Kermit_20111110_MSU_diff1_10
Scan Number: 65861
Proteins:
sp|P62274|RS29_MOUSE
ENSMUST00000037023_chr12:69157813−69159058:−
ENSMUST00000071737_chr13:73988963−73989133:−
tr|D3YYL7|D3YYL7_MOUSE

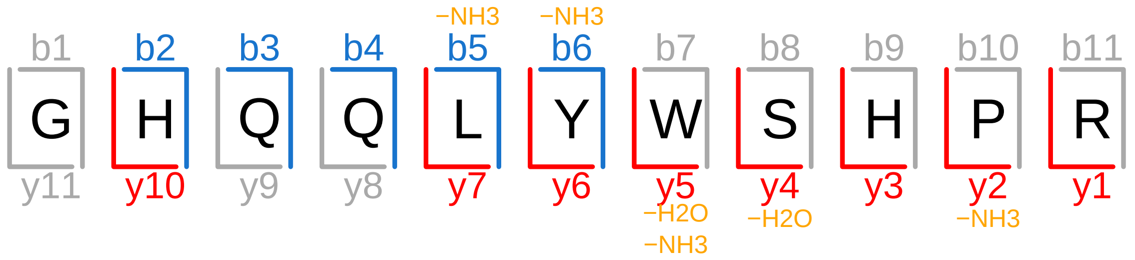

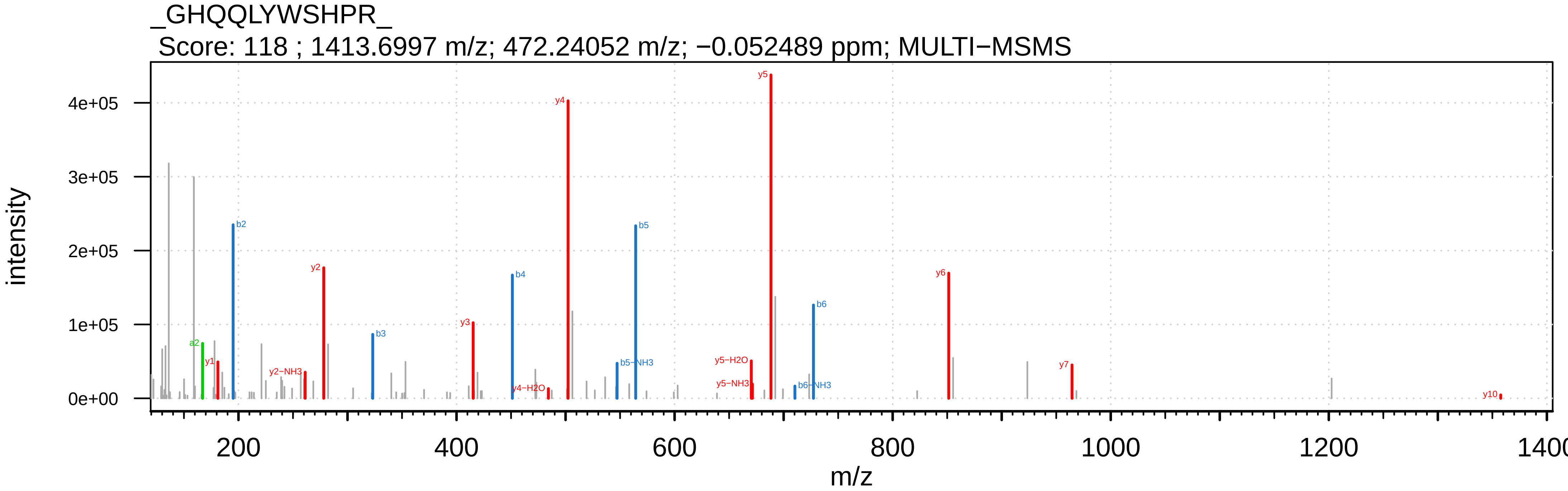

Raw File: Kermit_20111110_MSU_diff1_10
Scan Number: 65895
Proteins:
sp|P62274|RS29_MOUSE
ENSMUST00000037023_chr12:69157813−69159058:−
ENSMUST00000071737_chr13:73988963−73989133:−
tr|D3YYL7|D3YYL7_MOUSE

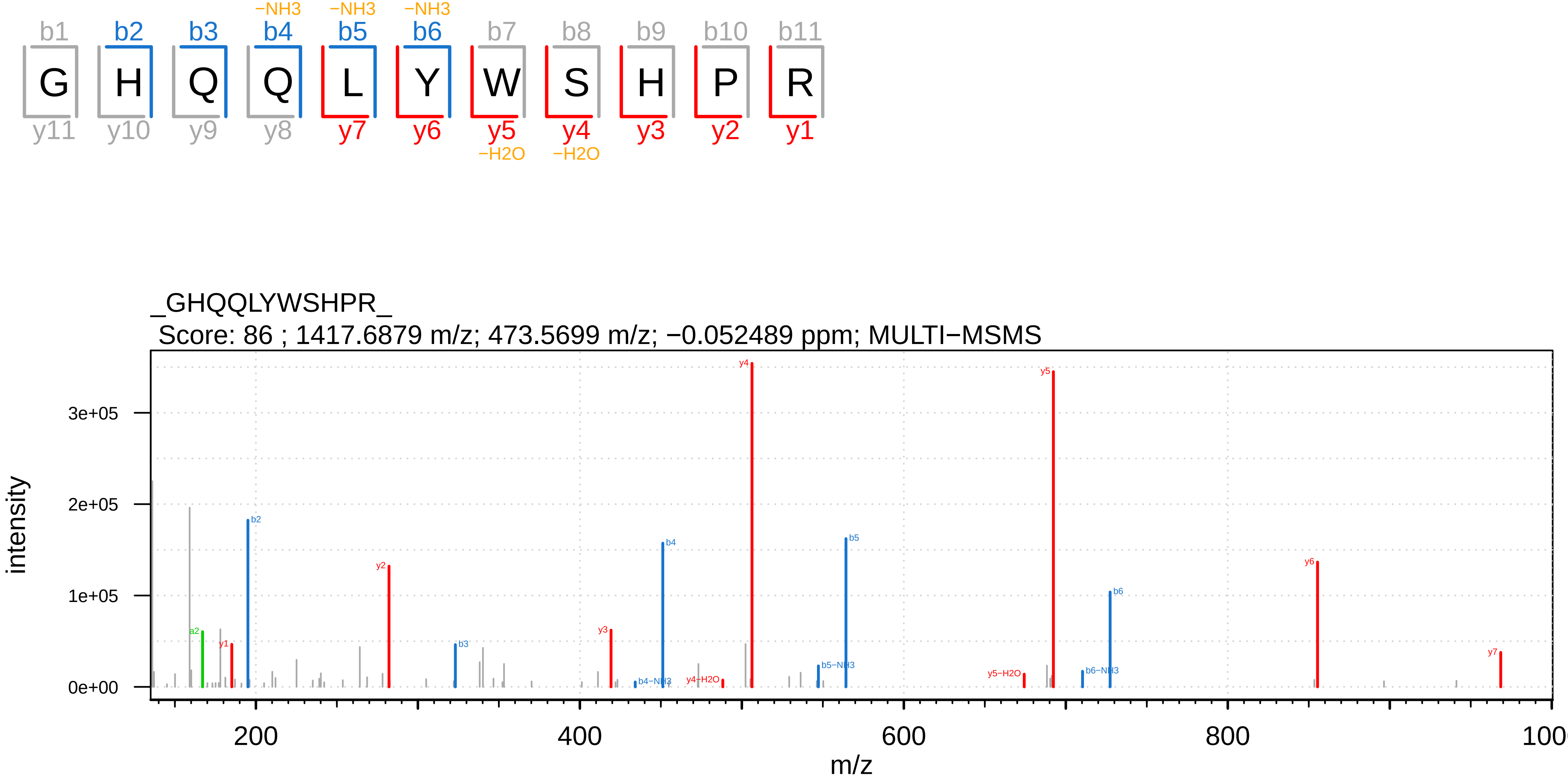

Raw File: Kermit_20111110_MSU_diff1_10
Scan Number: 66064
Proteins:
sp|P62274|RS29_MOUSE
ENSMUST00000037023_chr12:69157813−69159058:−
ENSMUST00000071737_chr13:73988963−73989133:−
tr|D3YYL7|D3YYL7_MOUSE

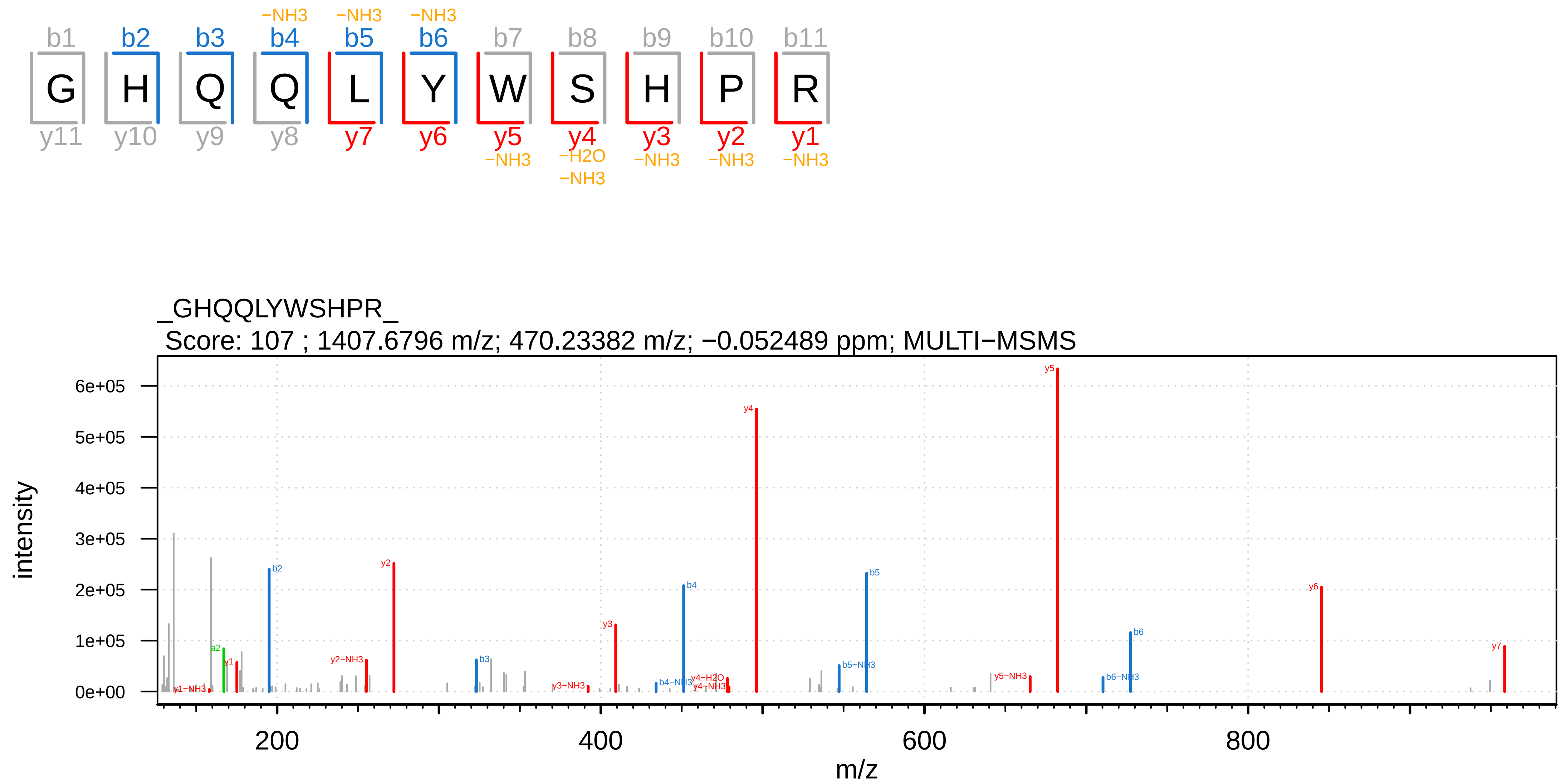

Raw File: Kermit_20111110_MSU_diff1_10
Scan Number: 66100
Proteins:
sp|P62274|RS29_MOUSE
ENSMUST00000037023_chr12:69157813−69159058:−
ENSMUST00000071737_chr13:73988963−73989133:−
tr|D3YYL7|D3YYL7_MOUSE

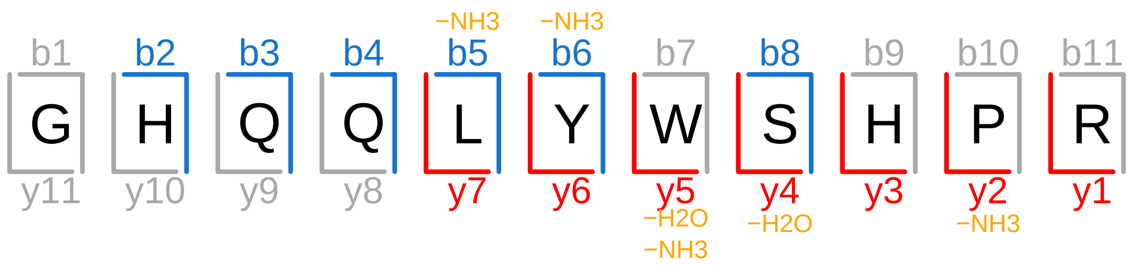

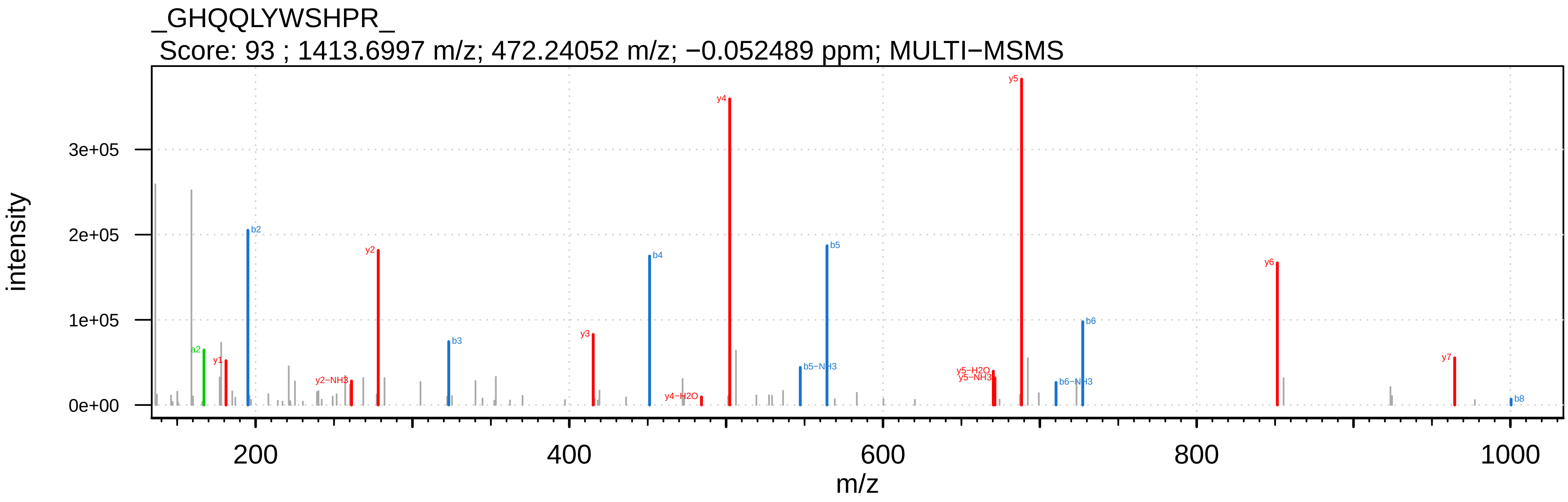

Raw File: Kermit_20111110_MSU_diff1_10
Scan Number: 66135
Proteins:
sp|P62274|RS29_MOUSE
ENSMUST00000037023_chr12:69157813−69159058:−
ENSMUST00000071737_chr13:73988963−73989133:−
tr|D3YYL7|D3YYL7_MOUSE

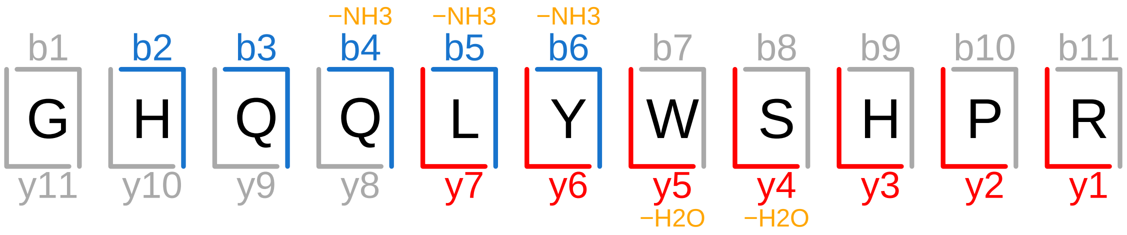

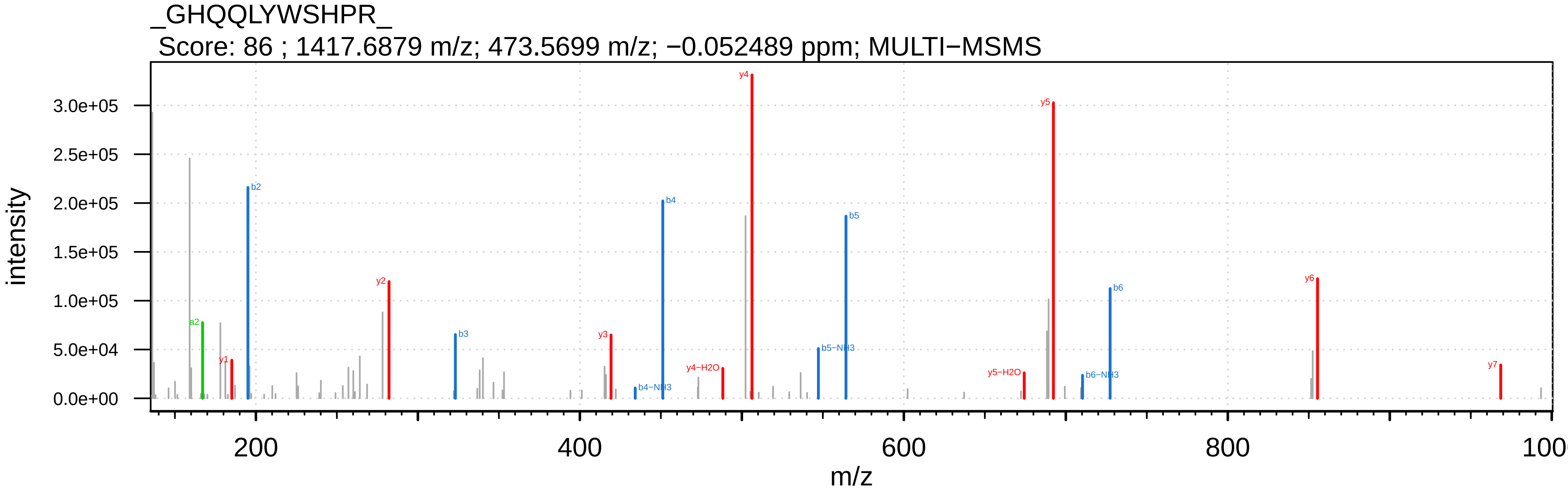

Raw File: Kermit_20111110_MSU_diff1_10
Scan Number: 66303
Proteins:
sp|P62274|RS29_MOUSE
ENSMUST00000037023_chr12:69157813−69159058:−
ENSMUST00000071737_chr13:73988963−73989133:−
tr|D3YYL7|D3YYL7_MOUSE

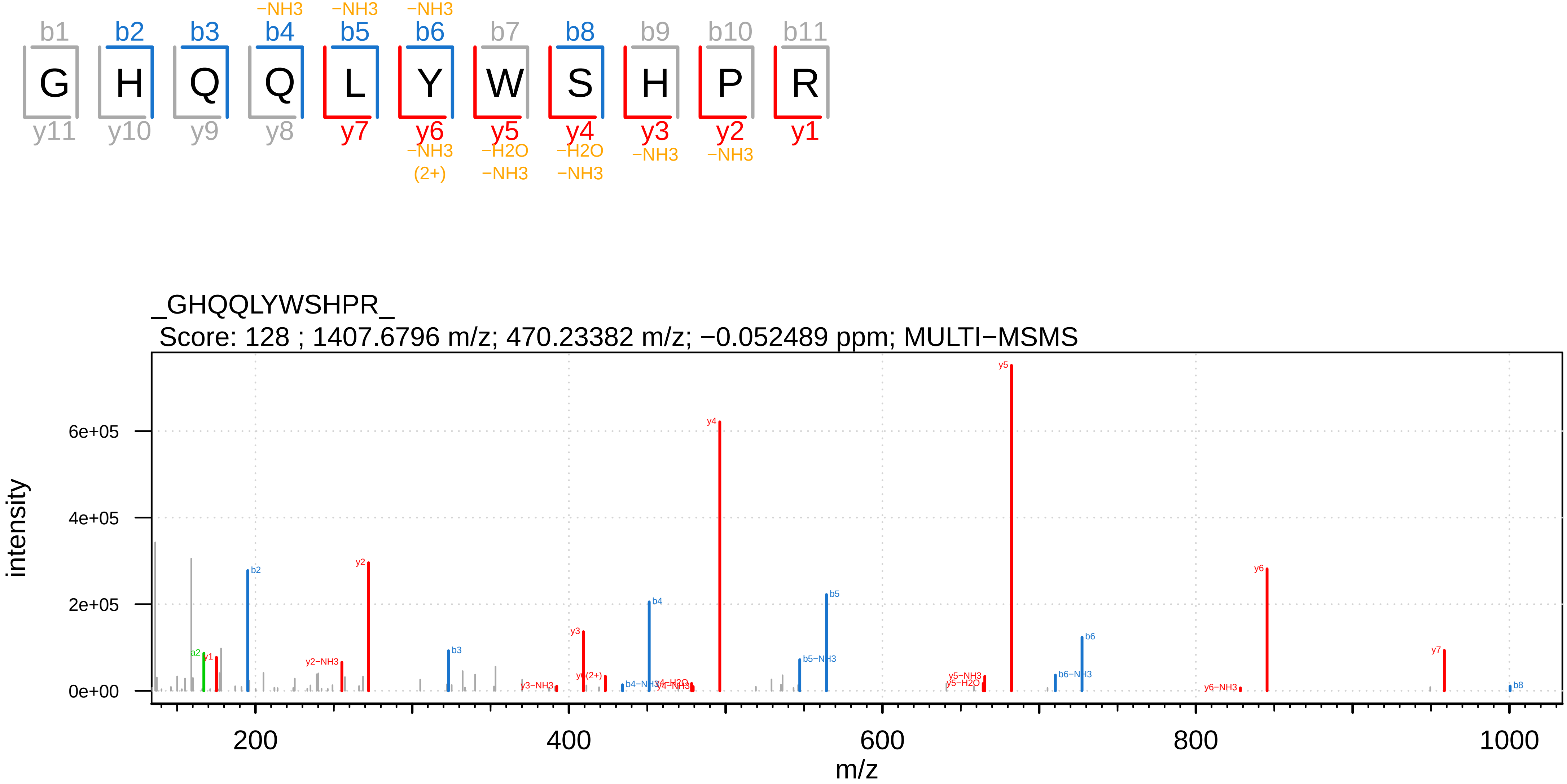

Raw File: Kermit_20111110_MSU_diff1_10
Scan Number: 66344
Proteins:
sp|P62274|RS29_MOUSE
ENSMUST00000037023_chr12:69157813−69159058:−
ENSMUST00000071737_chr13:73988963−73989133:−
tr|D3YYL7|D3YYL7_MOUSE

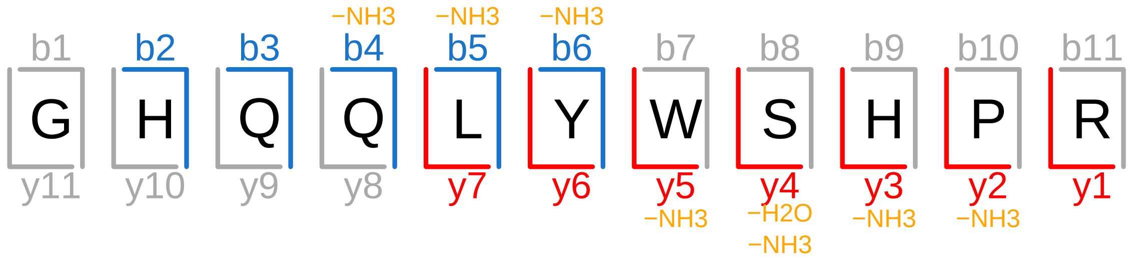

GHQQLYWSHPR

Score: 89 ; 1413.6997 m/z; 472.24052 m/z; −0.052489 ppm; MULTI−MSMS

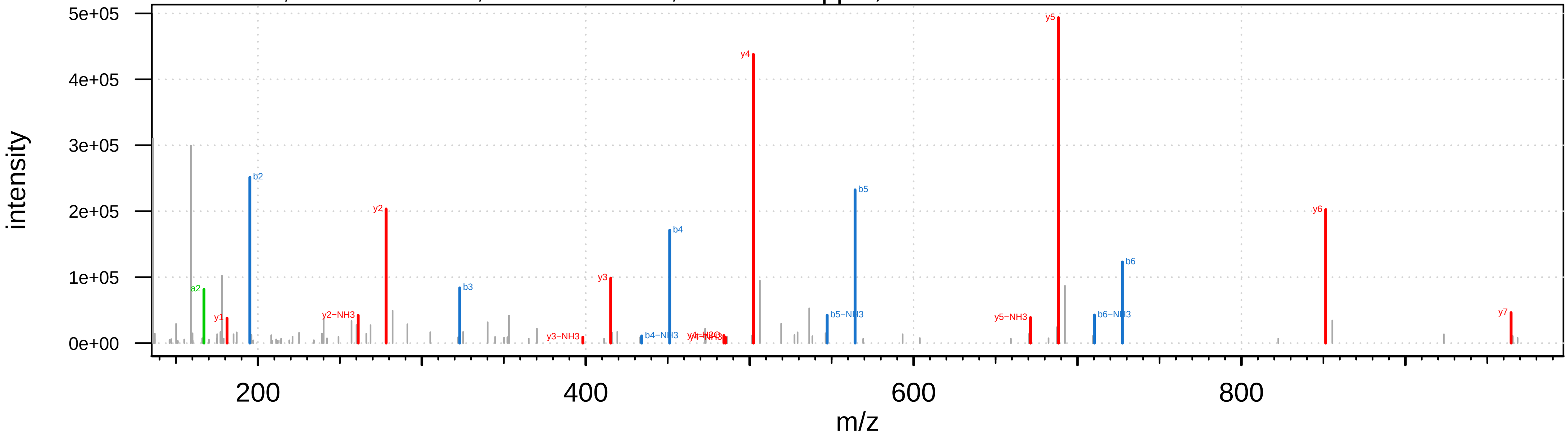

Raw File: Kermit_20111110_MSU_diff1_10
Scan Number: 66398
Proteins:
sp|P62274|RS29_MOUSE
ENSMUST00000037023_chr12:69157813−69159058:−
ENSMUST00000071737_chr13:73988963−73989133:−
tr|D3YYL7|D3YYL7_MOUSE

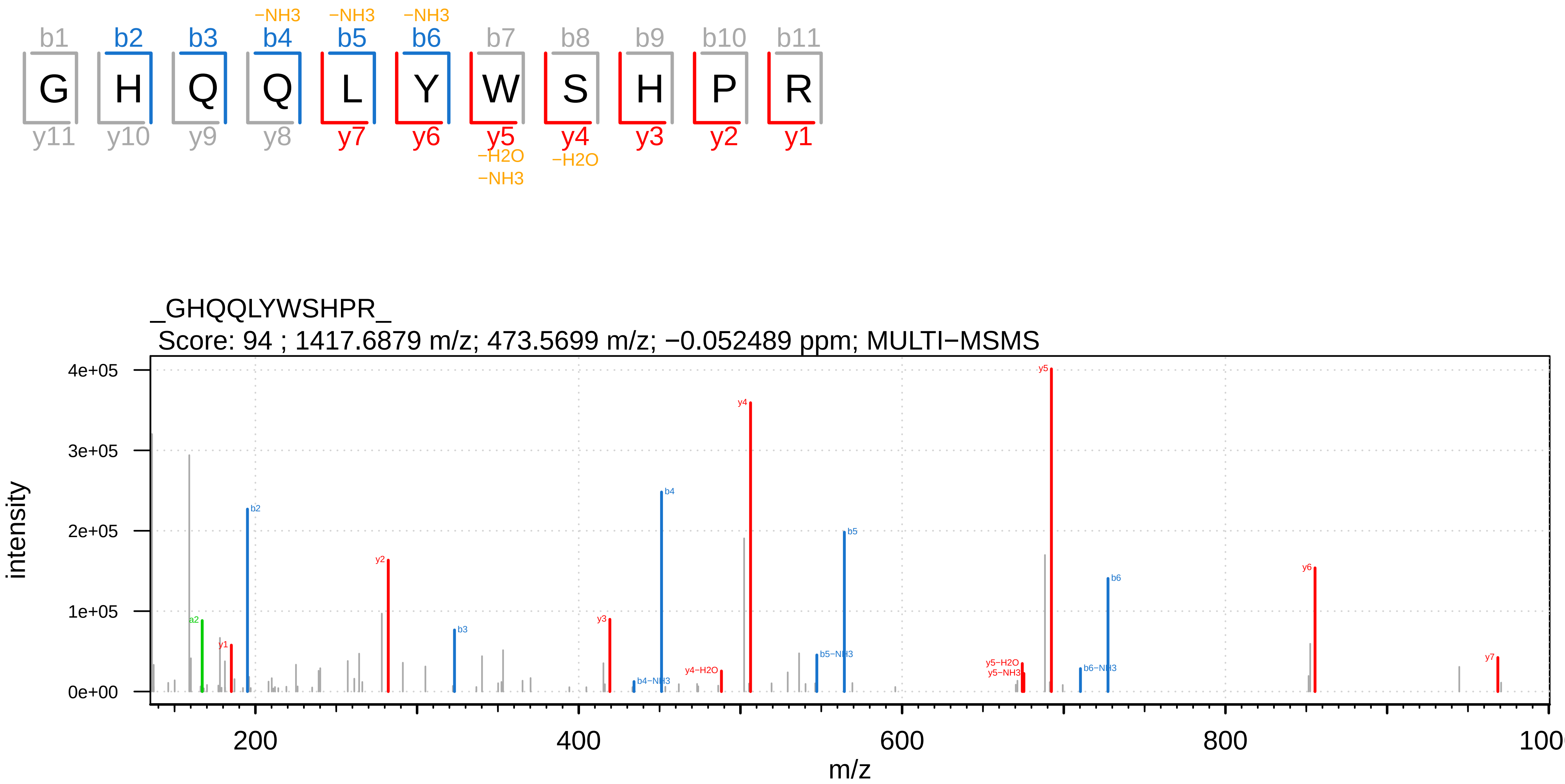

Raw File: Kermit_20111110_MSU_diff1_10
Scan Number: 66543
Proteins:
sp|P62274|RS29_MOUSE
ENSMUST00000037023_chr12:69157813−69159058:−
ENSMUST00000071737_chr13:73988963−73989133:−
tr|D3YYL7|D3YYL7_MOUSE

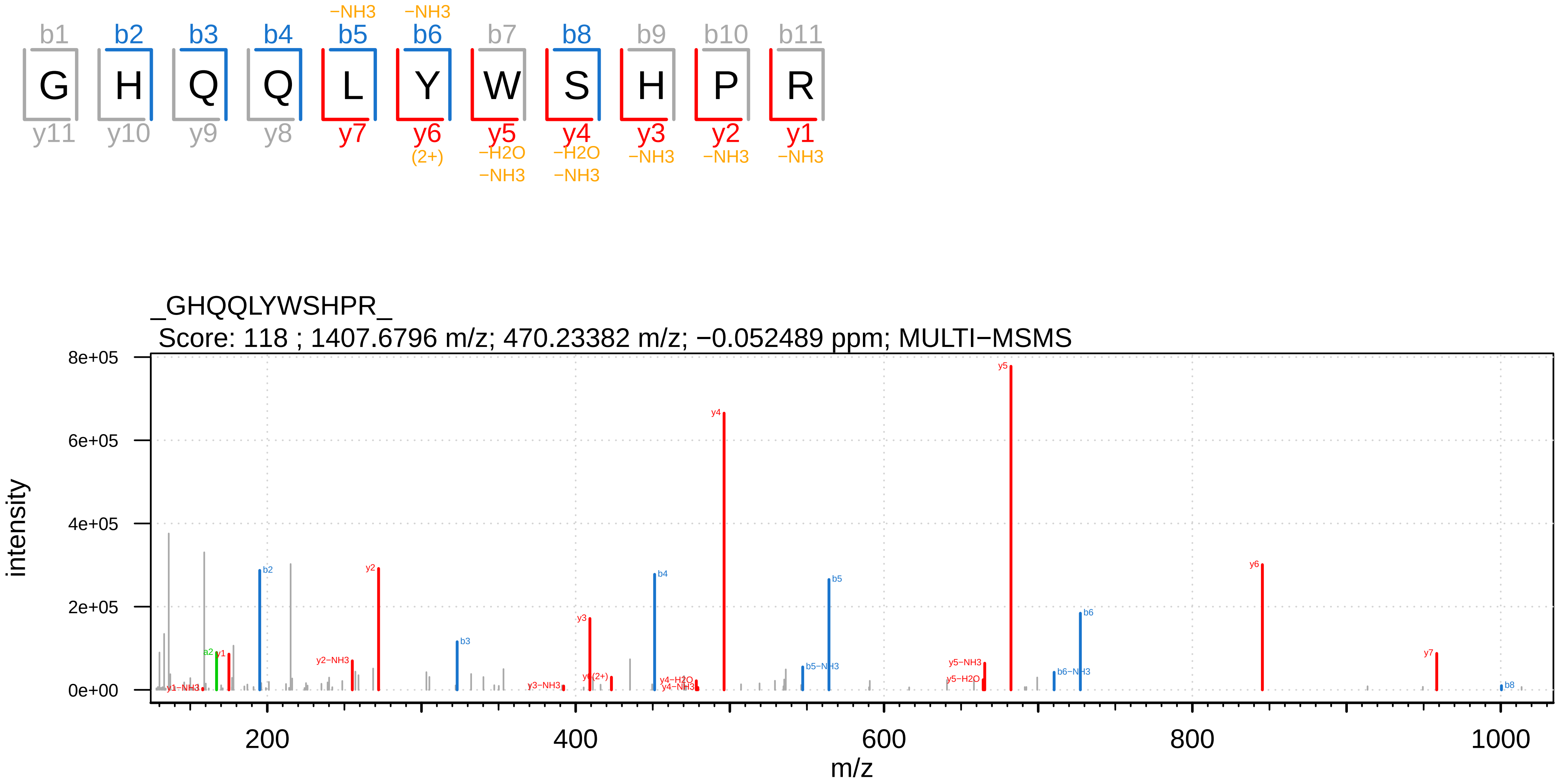

Raw File: Kermit_20111110_MSU_diff1_10
Scan Number: 66590
Proteins:
sp|P62274|RS29_MOUSE
ENSMUST00000037023_chr12:69157813−69159058:−
ENSMUST00000071737_chr13:73988963−73989133:−
tr|D3YYL7|D3YYL7_MOUSE

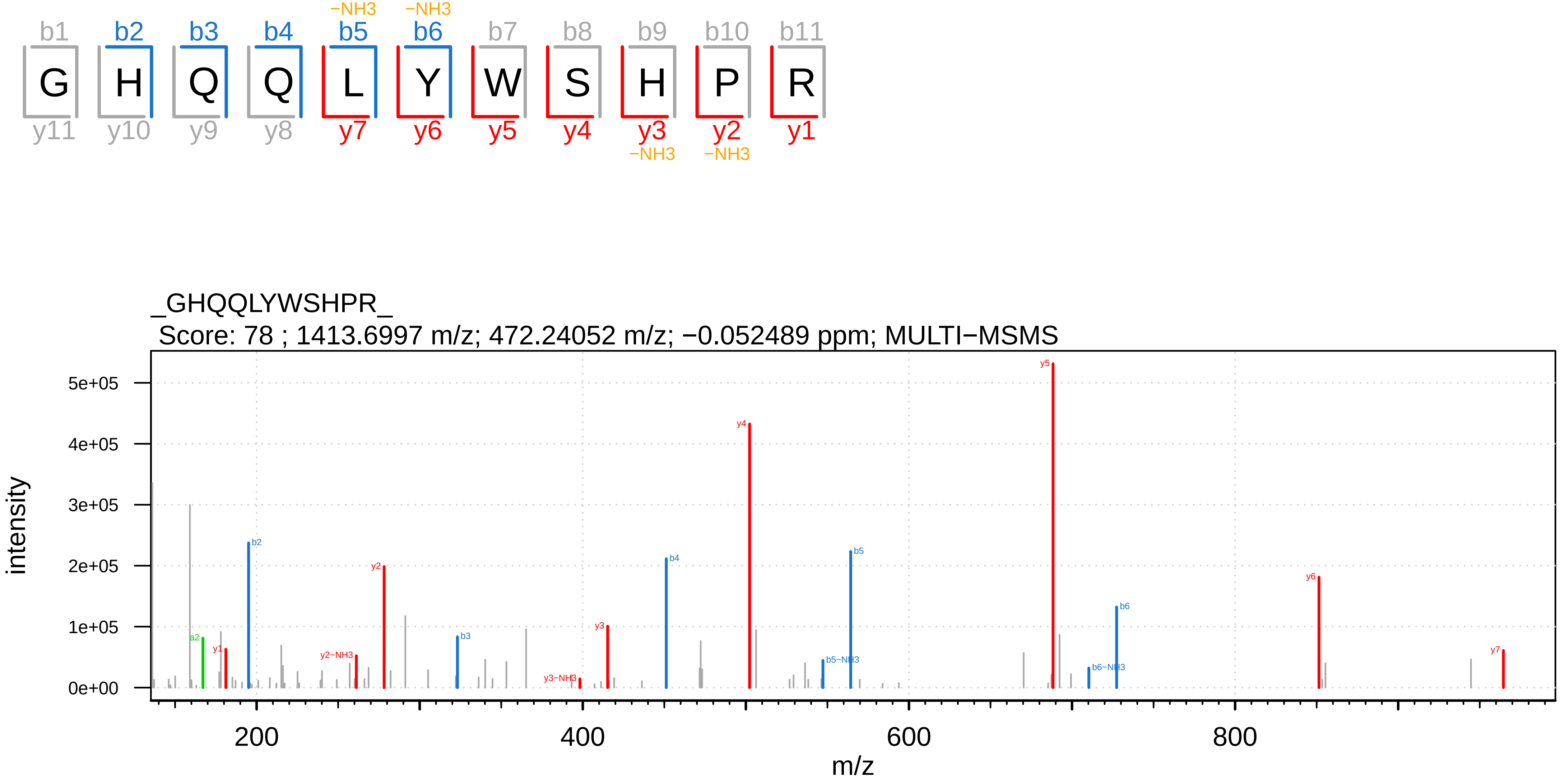

Raw File: Kermit_20111110_MSU_diff1_10
Scan Number: 66641
Proteins:
sp|P62274|RS29_MOUSE
ENSMUST00000037023_chr12:69157813−69159058:−
ENSMUST00000071737_chr13:73988963−73989133:−
tr|D3YYL7|D3YYL7_MOUSE

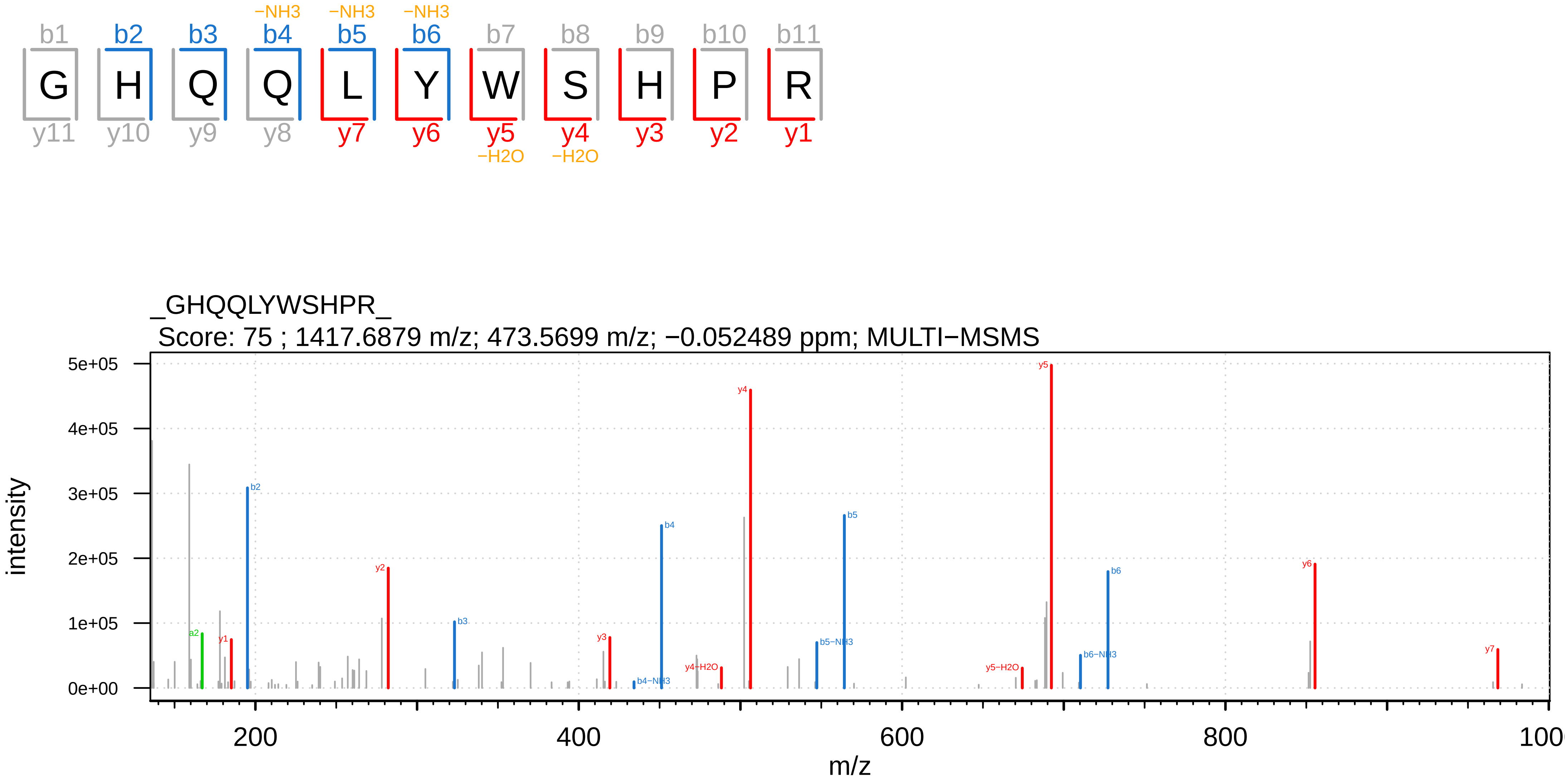

Raw File: Kermit_20111110_MSU_diff1_10
Scan Number: 66784
Proteins:
sp|P62274|RS29_MOUSE
ENSMUST00000037023_chr12:69157813−69159058:−
ENSMUST00000071737_chr13:73988963−73989133:−
tr|D3YYL7|D3YYL7_MOUSE

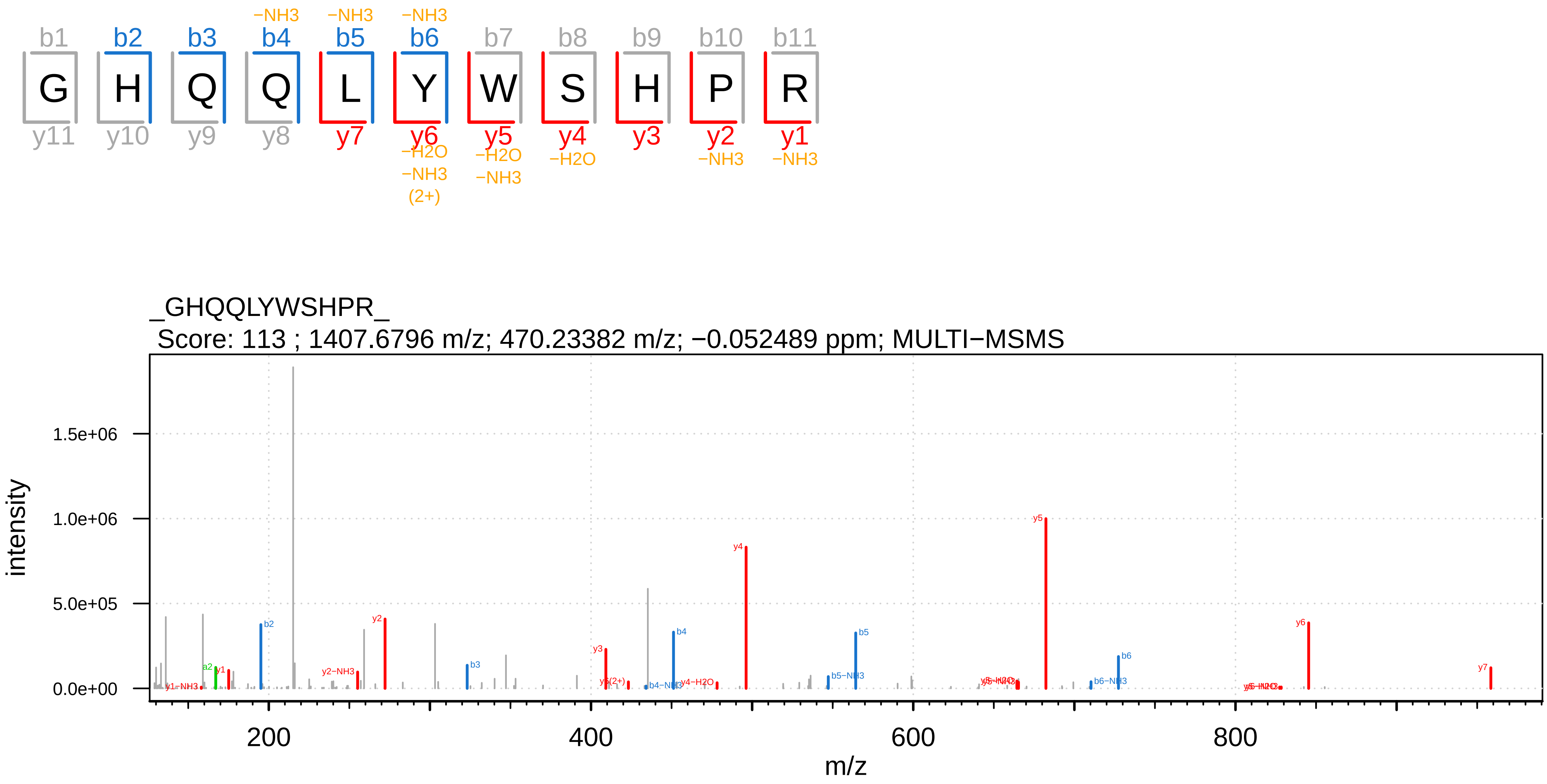

Raw File: Kermit_20111110_MSU_diff1_10
Scan Number: 66828
Proteins:
sp|P62274|RS29_MOUSE
ENSMUST00000037023_chr12:69157813−69159058:−
ENSMUST00000071737_chr13:73988963−73989133:−
tr|D3YYL7|D3YYL7_MOUSE

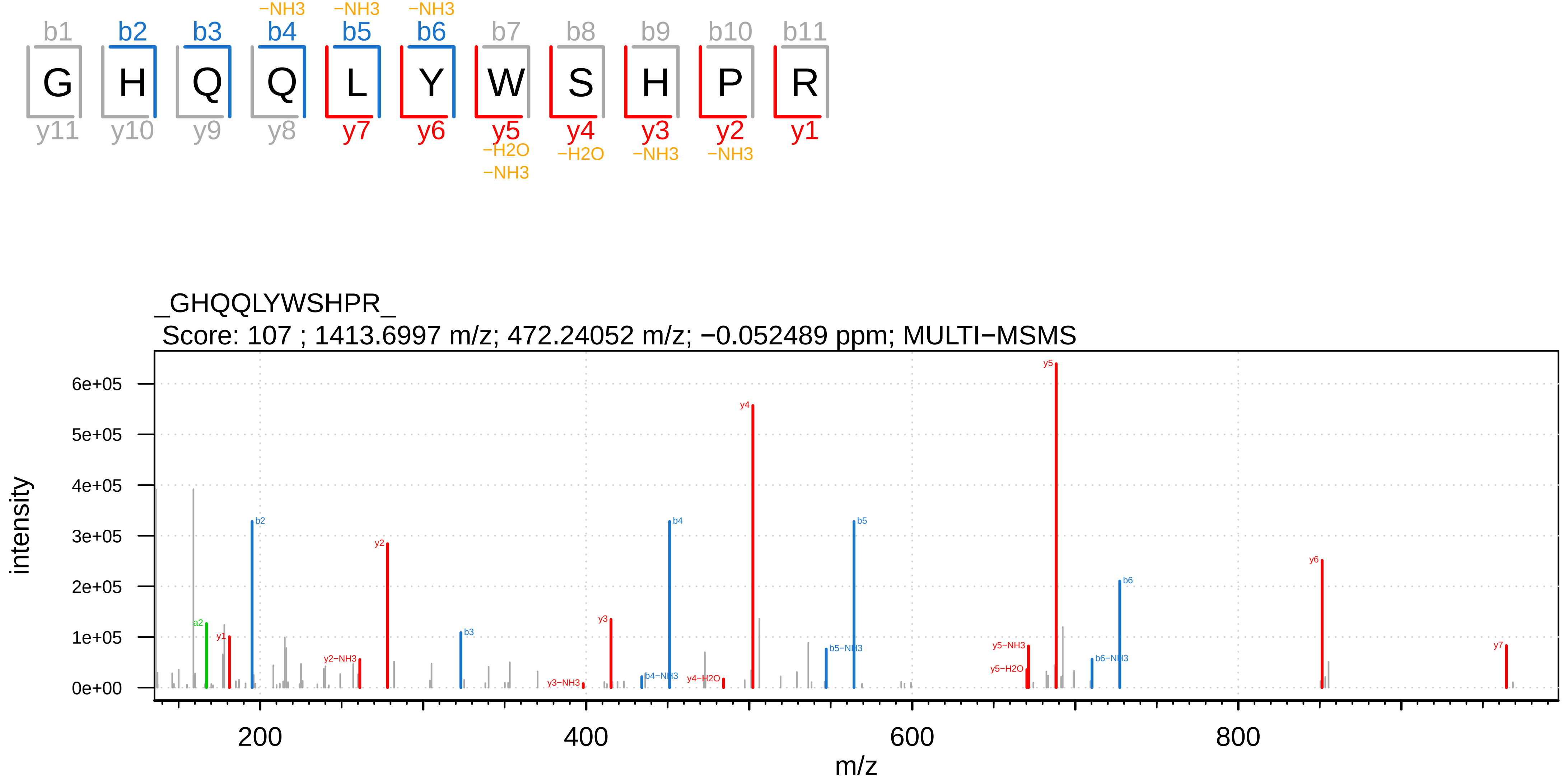

Raw File: Kermit_20111110_MSU_diff1_10
Scan Number: 66896
Proteins:
sp|P62274|RS29_MOUSE
ENSMUST00000037023_chr12:69157813−69159058:−
ENSMUST00000071737_chr13:73988963−73989133:−
tr|D3YYL7|D3YYL7_MOUSE

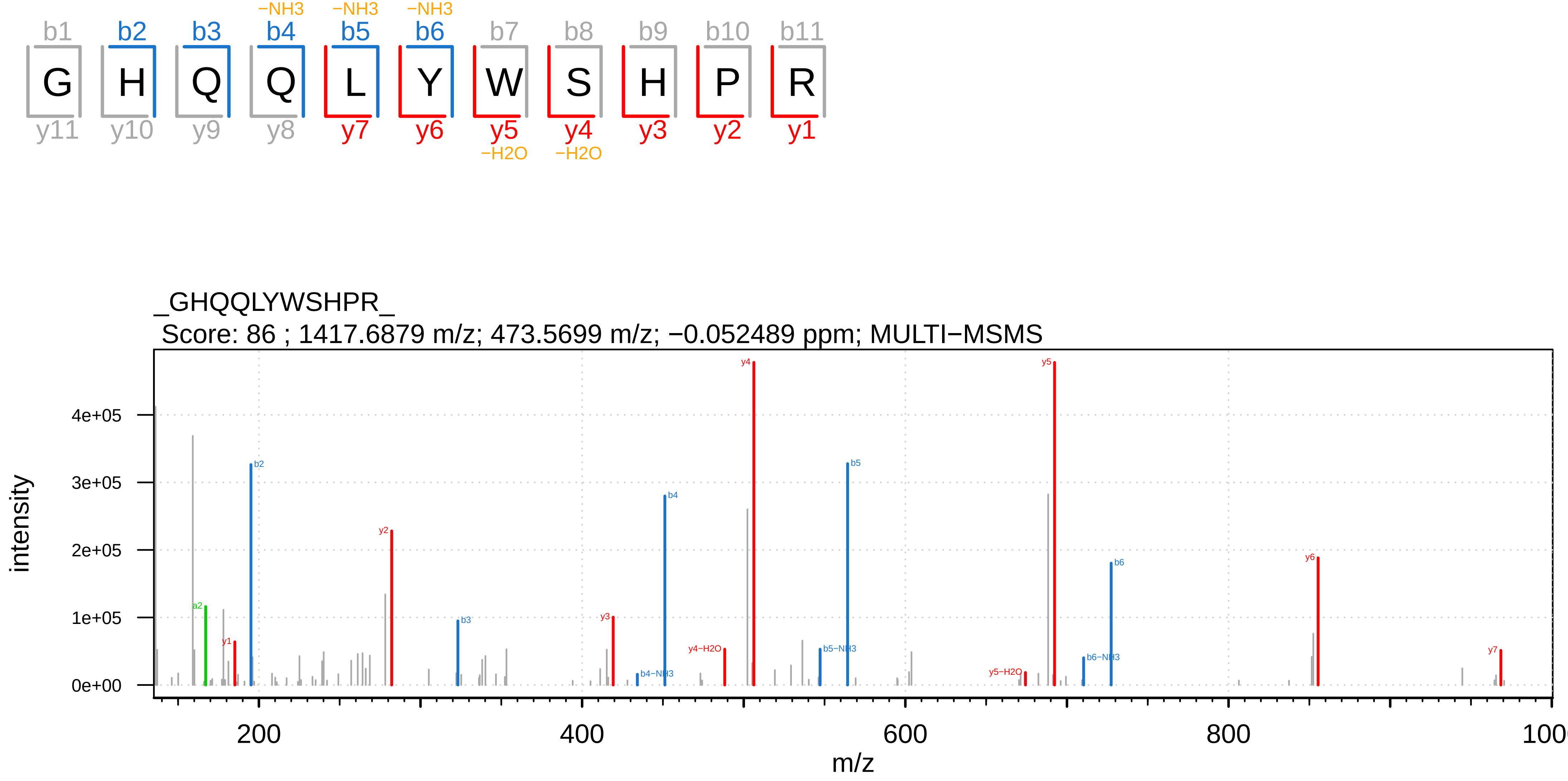

Raw File: Kermit_20111110_MSU_diff1_10
Scan Number: 67030
Proteins:
sp|P62274|RS29_MOUSE
ENSMUST00000037023_chr12:69157813−69159058:−
ENSMUST00000071737_chr13:73988963−73989133:−
tr|D3YYL7|D3YYL7_MOUSE

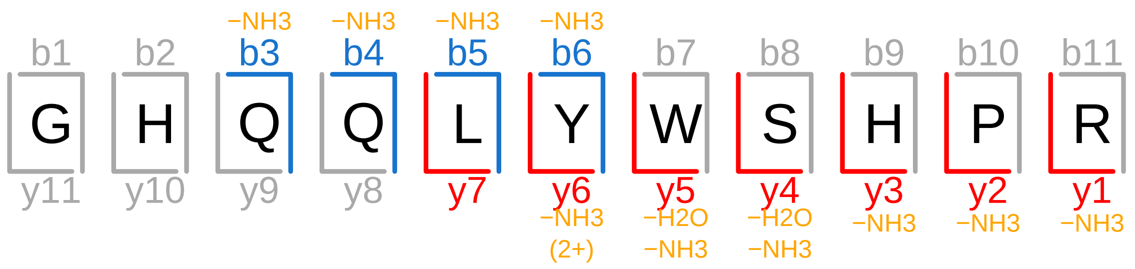

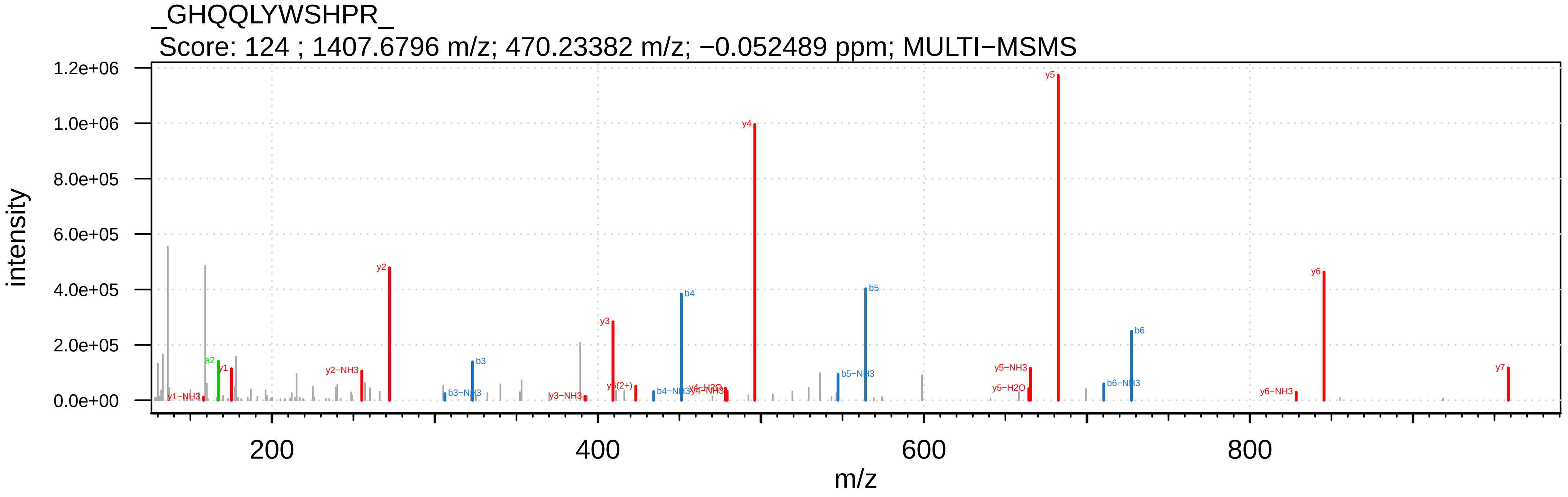

Raw File: Kermit_20111110_MSU_diff1_10
Scan Number: 67073
Proteins:
sp|P62274|RS29_MOUSE
ENSMUST00000037023_chr12:69157813−69159058:−
ENSMUST00000071737_chr13:73988963−73989133:−
tr|D3YYL7|D3YYL7_MOUSE

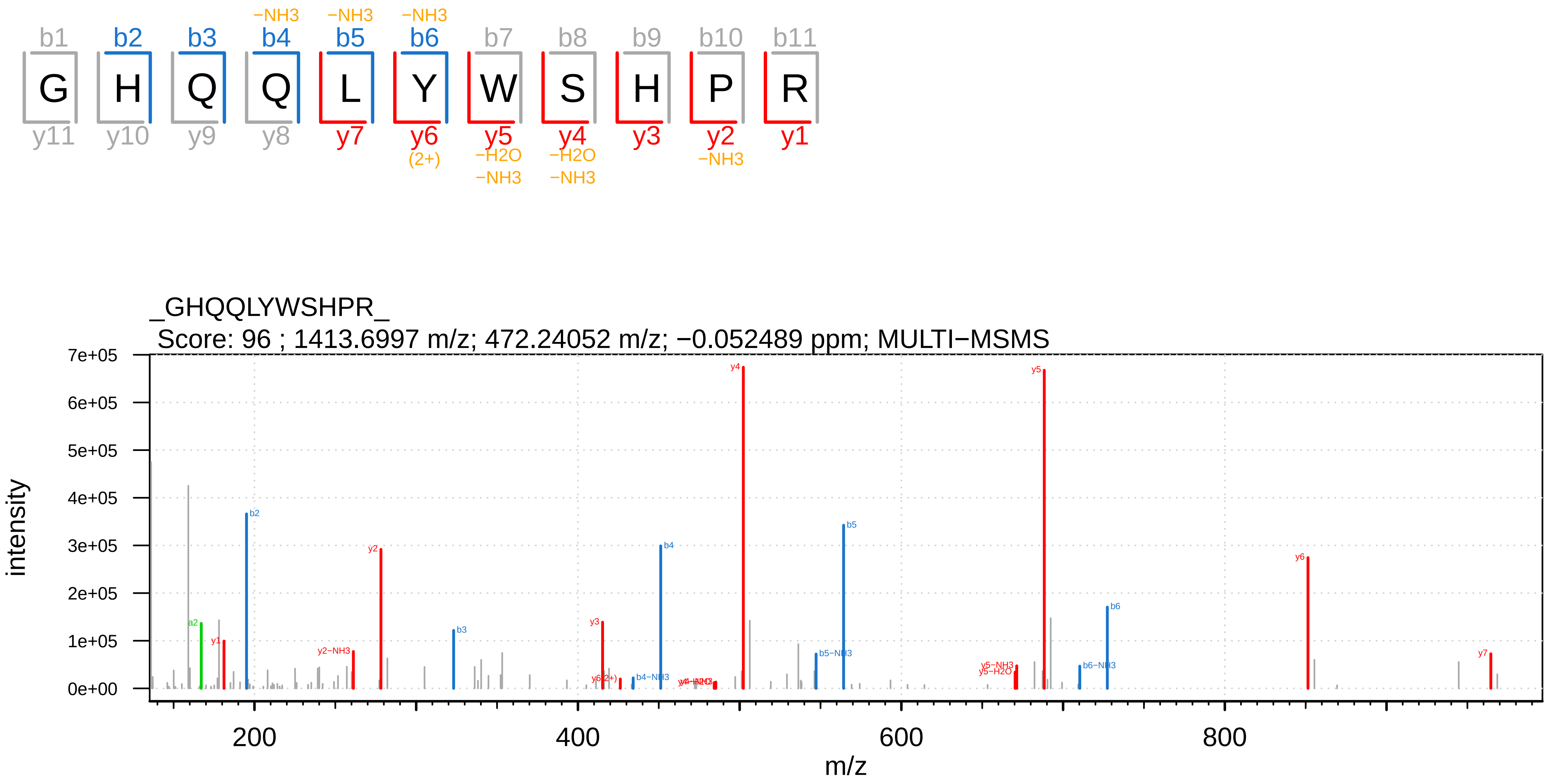

Raw File: Kermit_20111110_MSU_diff1_10
Scan Number: 67161
Proteins:
sp|P62274|RS29_MOUSE
ENSMUST00000037023_chr12:69157813−69159058:−
ENSMUST00000071737_chr13:73988963−73989133:−
tr|D3YYL7|D3YYL7_MOUSE

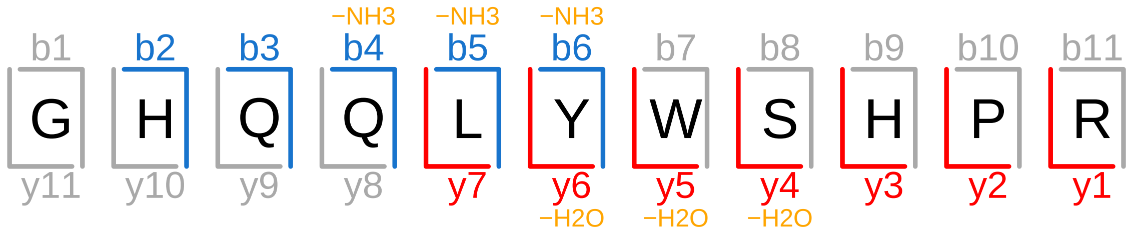

GHQQLYWSHPR
Score: 96 ; 1417.6879 m/z; 473.5699 m/z; −0.052489 ppm; MULTI−MSMS

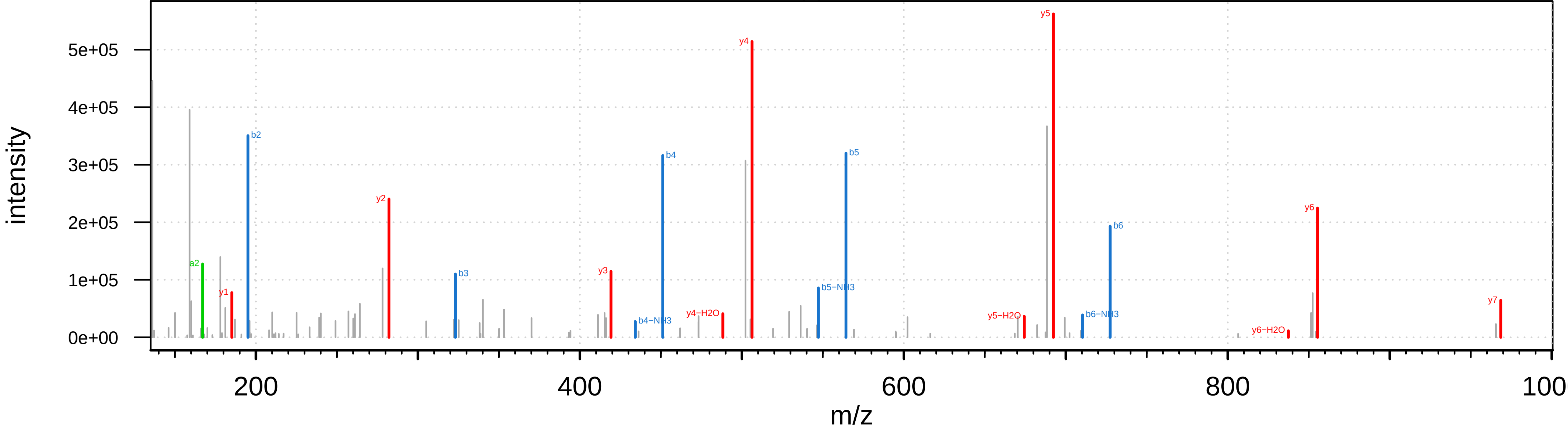

Raw File: Kermit_20111110_MSU_diff1_10
Scan Number: 67270
Proteins:
sp|P62274|RS29_MOUSE
ENSMUST00000037023_chr12:69157813−69159058:−
ENSMUST00000071737_chr13:73988963−73989133:−
tr|D3YYL7|D3YYL7_MOUSE

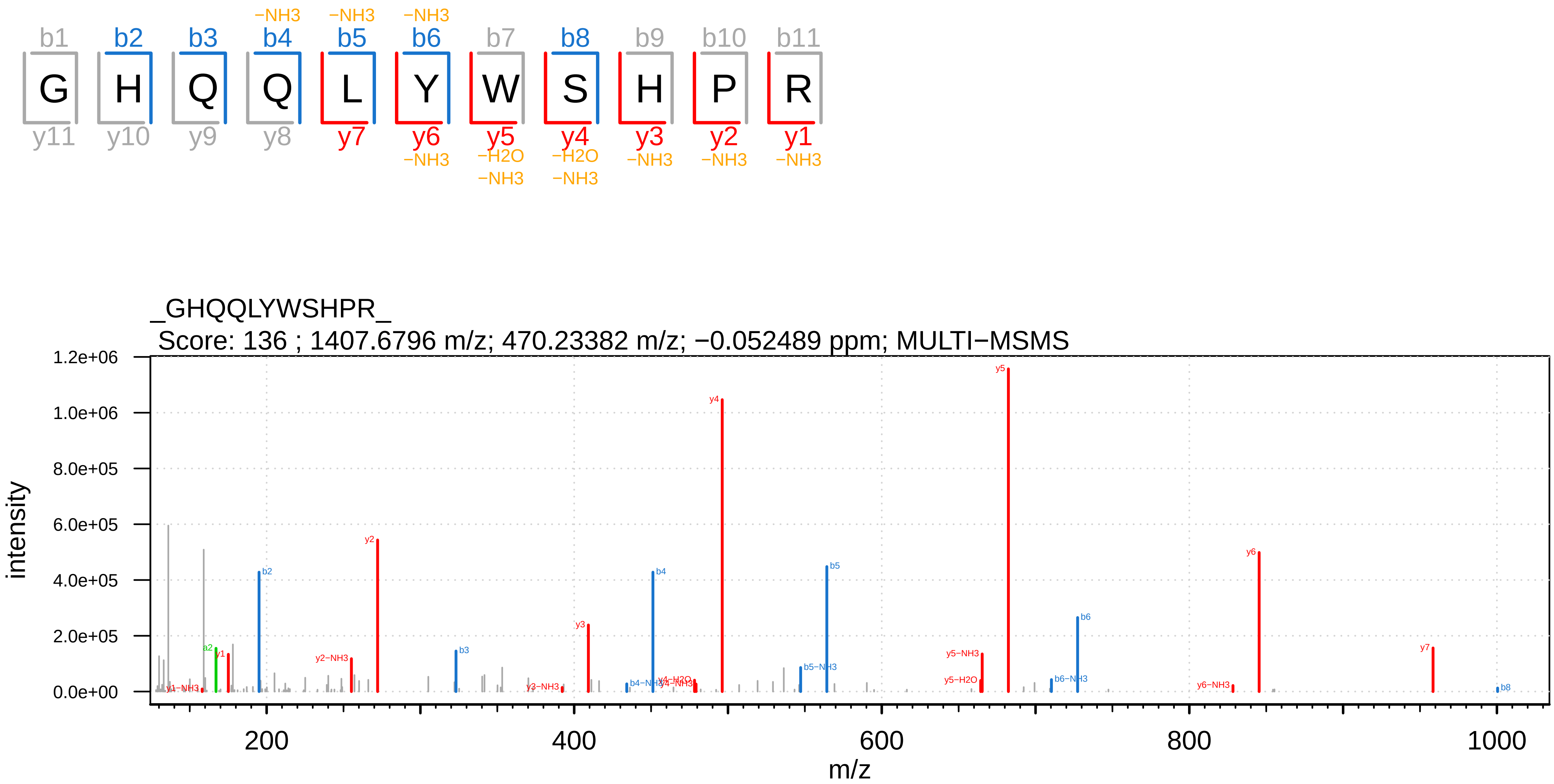

Raw File: Kermit_20111110_MSU_diff1_10
Scan Number: 67315
Proteins:
sp|P62274|RS29_MOUSE
ENSMUST00000037023_chr12:69157813−69159058:−
ENSMUST00000071737_chr13:73988963−73989133:−
tr|D3YYL7|D3YYL7_MOUSE

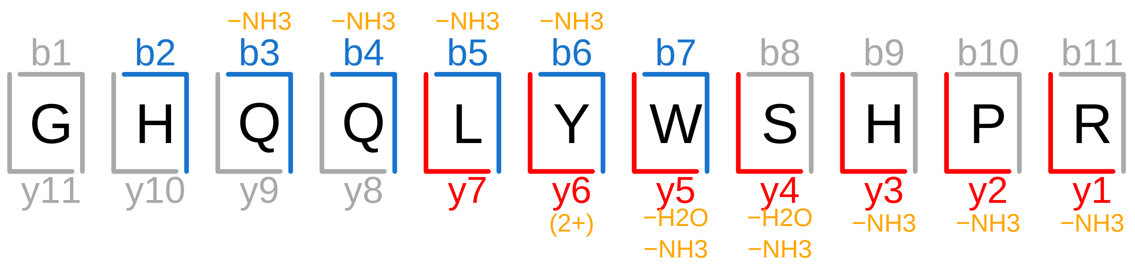

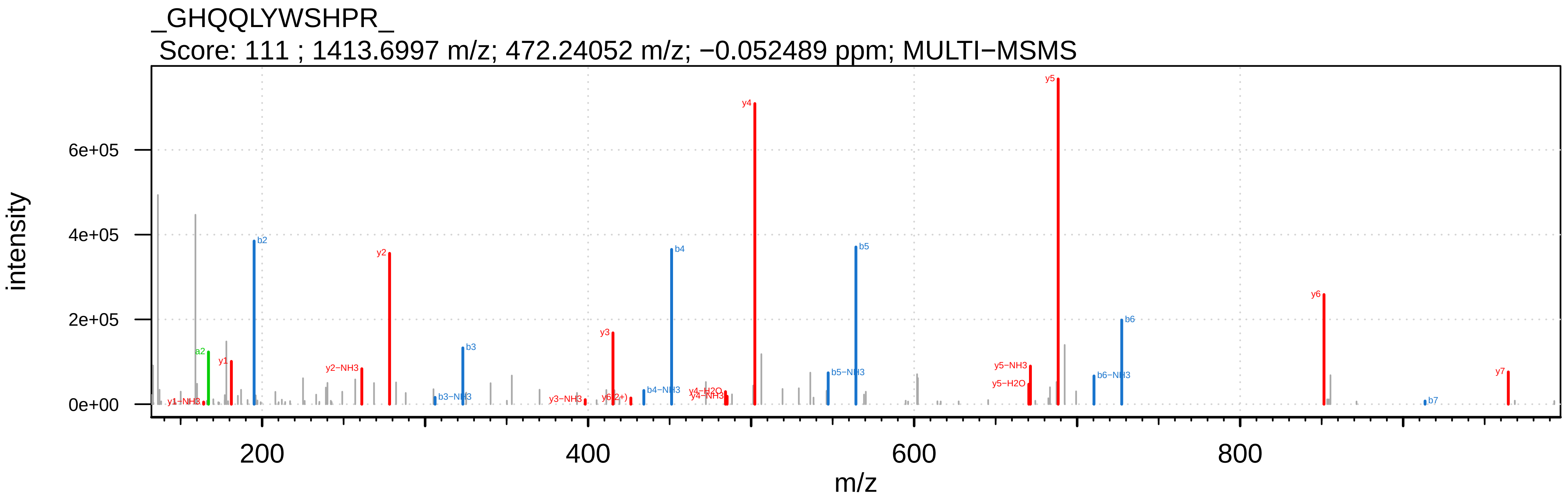

Raw File: Kermit_20111110_MSU_diff1_10
Scan Number: 67400
Proteins:
sp|P62274|RS29_MOUSE
ENSMUST00000037023_chr12:69157813−69159058:−
ENSMUST00000071737_chr13:73988963−73989133:−
tr|D3YYL7|D3YYL7_MOUSE

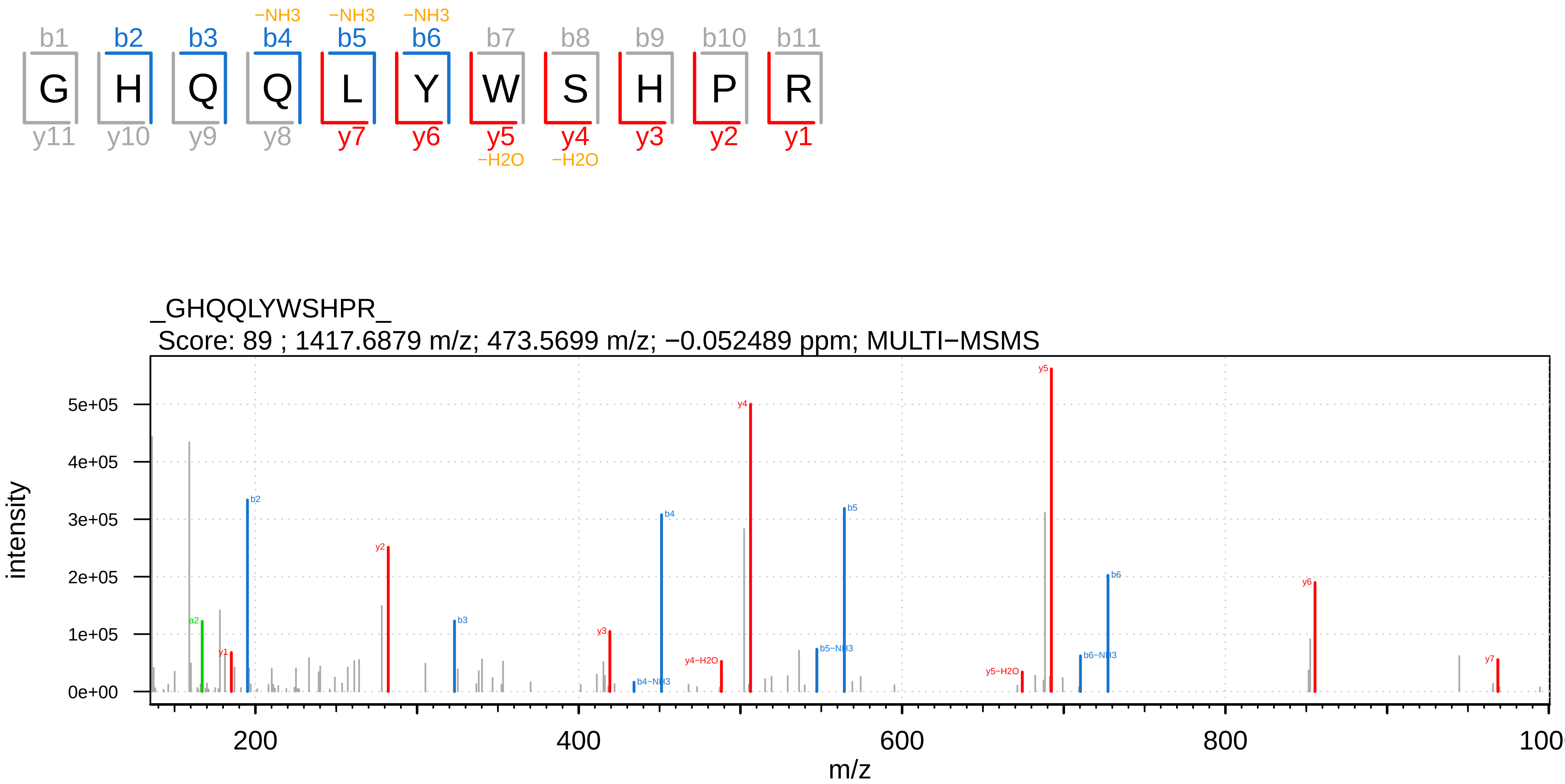

Raw File: Kermit_20111110_MSU_diff1_10
Scan Number: 67515
Proteins:
sp|P62274|RS29_MOUSE
ENSMUST00000037023_chr12:69157813−69159058:−
ENSMUST00000071737_chr13:73988963−73989133:−
tr|D3YYL7|D3YYL7_MOUSE

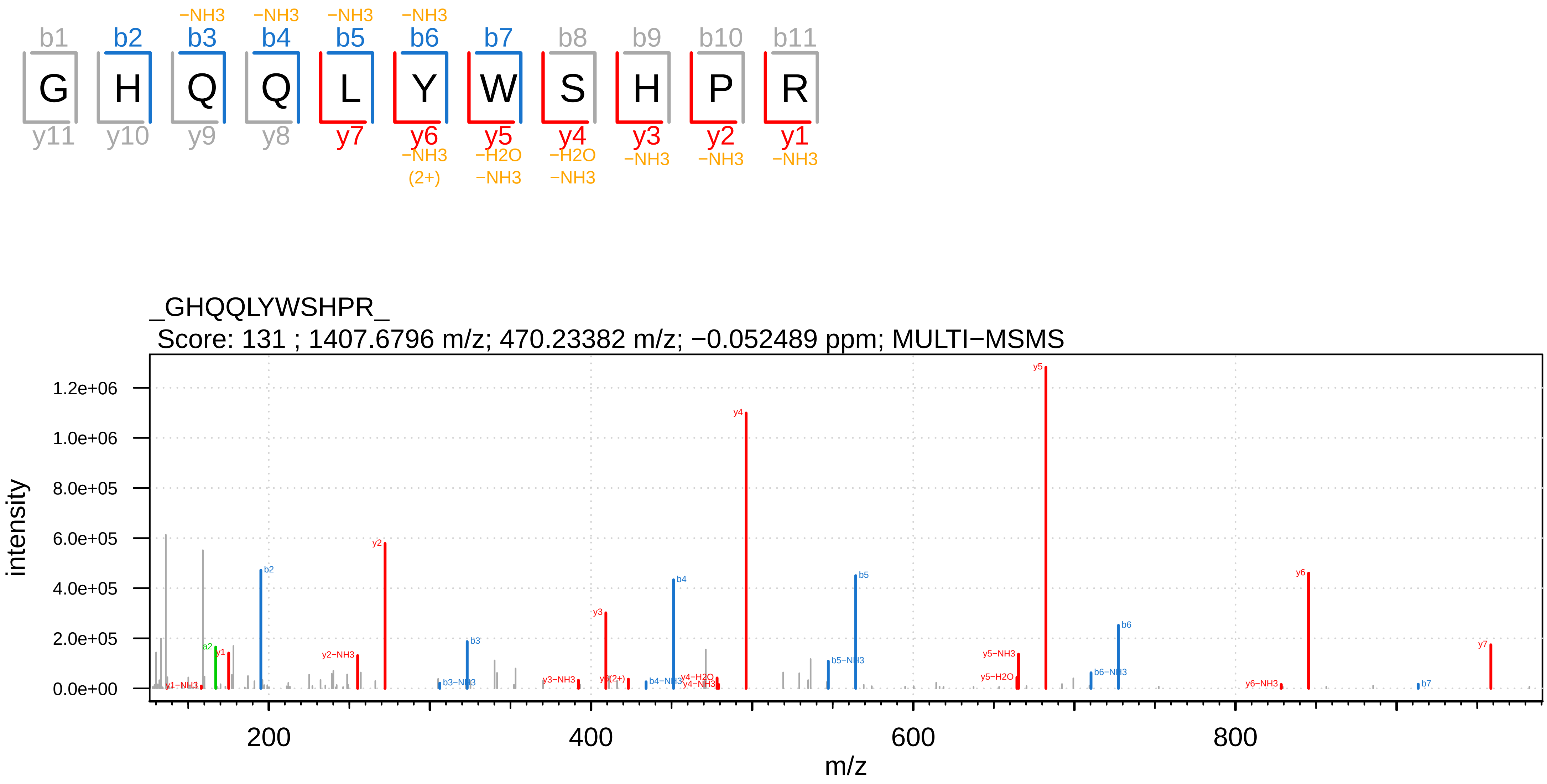

Raw File: Kermit_20111110_MSU_diff1_10
Scan Number: 67559
Proteins:
sp|P62274|RS29_MOUSE
ENSMUST00000037023_chr12:69157813−69159058:−
ENSMUST00000071737_chr13:73988963−73989133:−
tr|D3YYL7|D3YYL7_MOUSE

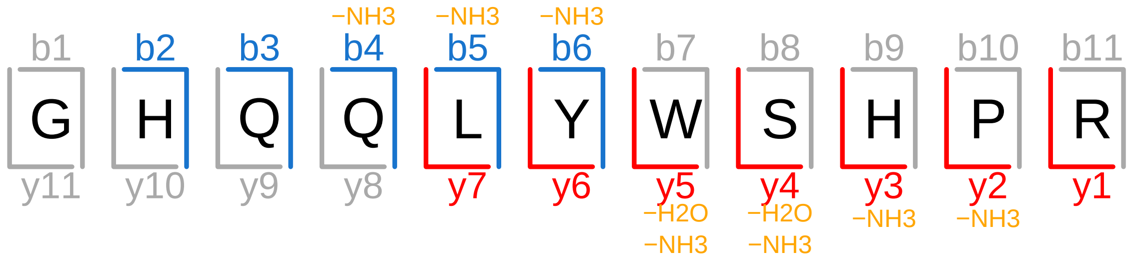

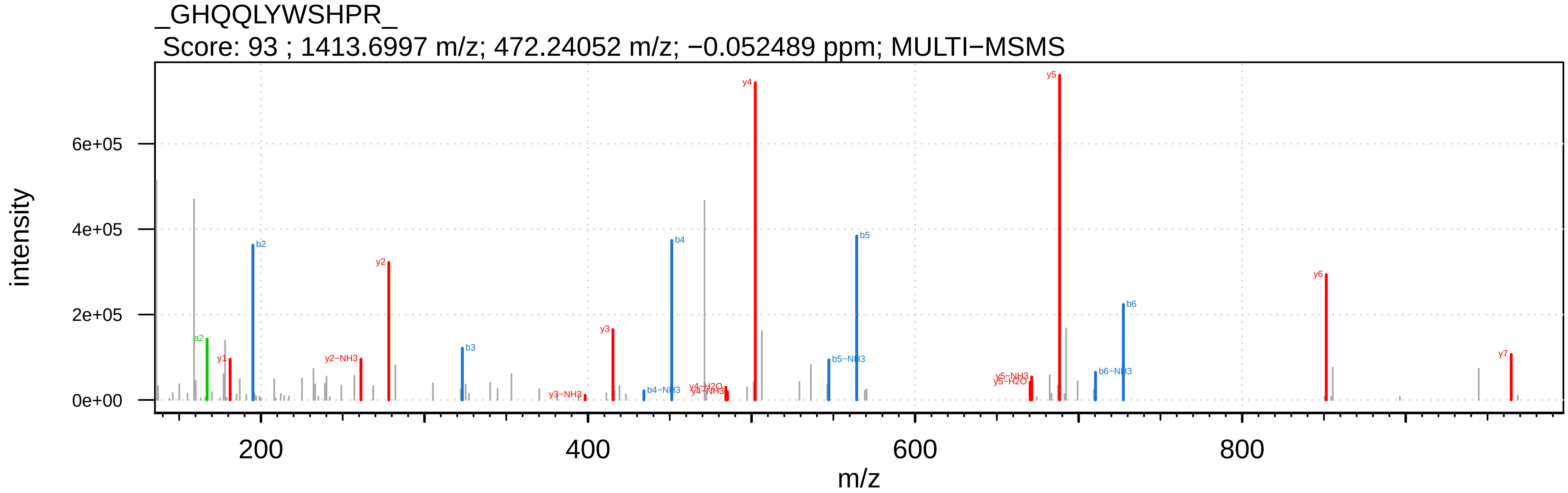

Raw File: Kermit_20111110_MSU_diff1_10
Scan Number: 67684
Proteins:
sp|P62274|RS29_MOUSE
ENSMUST00000037023_chr12:69157813−69159058:−
ENSMUST00000071737_chr13:73988963−73989133:−
tr|D3YYL7|D3YYL7_MOUSE

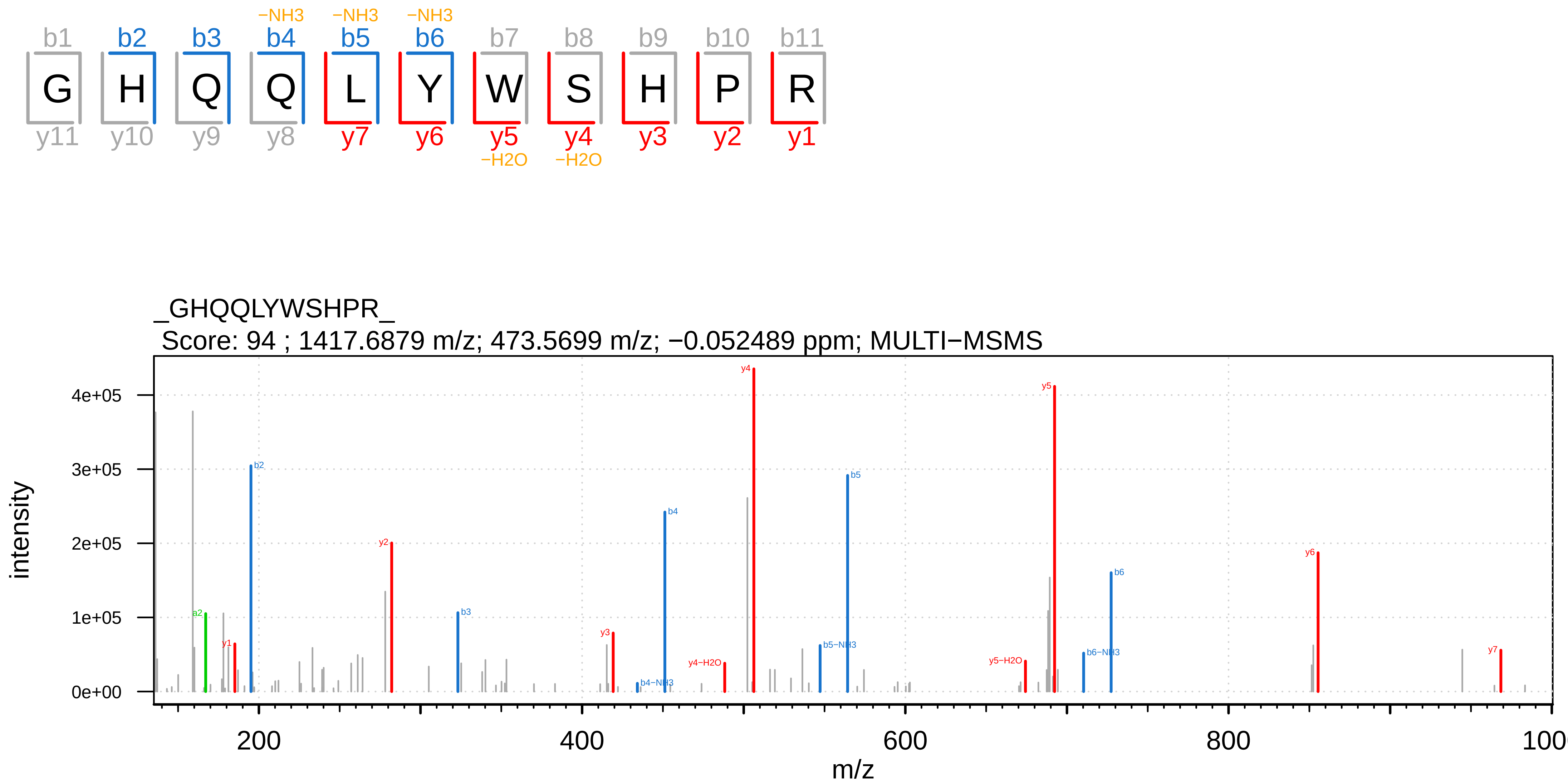

Raw File: Kermit_20111110_MSU_diff1_10
Scan Number: 67757
Proteins:
sp|P62274|RS29_MOUSE
ENSMUST00000037023_chr12:69157813−69159058:−
ENSMUST00000071737_chr13:73988963−73989133:−
tr|D3YYL7|D3YYL7_MOUSE

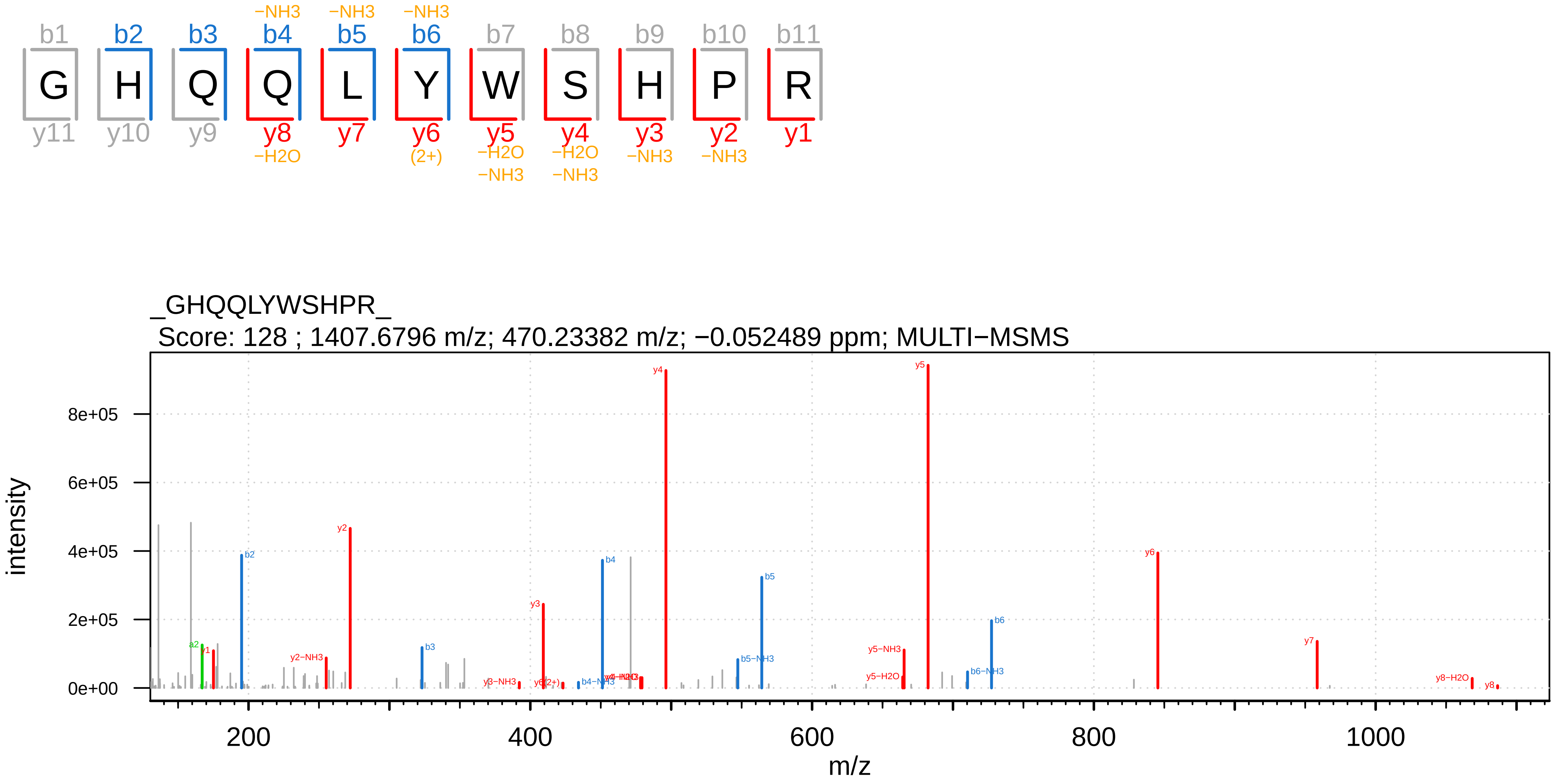

Raw File: Kermit_20111110_MSU_diff1_10
Scan Number: 67795
Proteins:
sp|P62274|RS29_MOUSE
ENSMUST00000037023_chr12:69157813−69159058:−
ENSMUST00000071737_chr13:73988963−73989133:−
tr|D3YYL7|D3YYL7_MOUSE

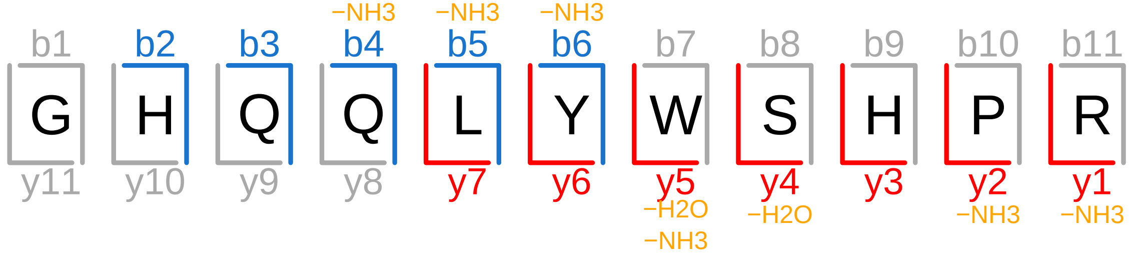

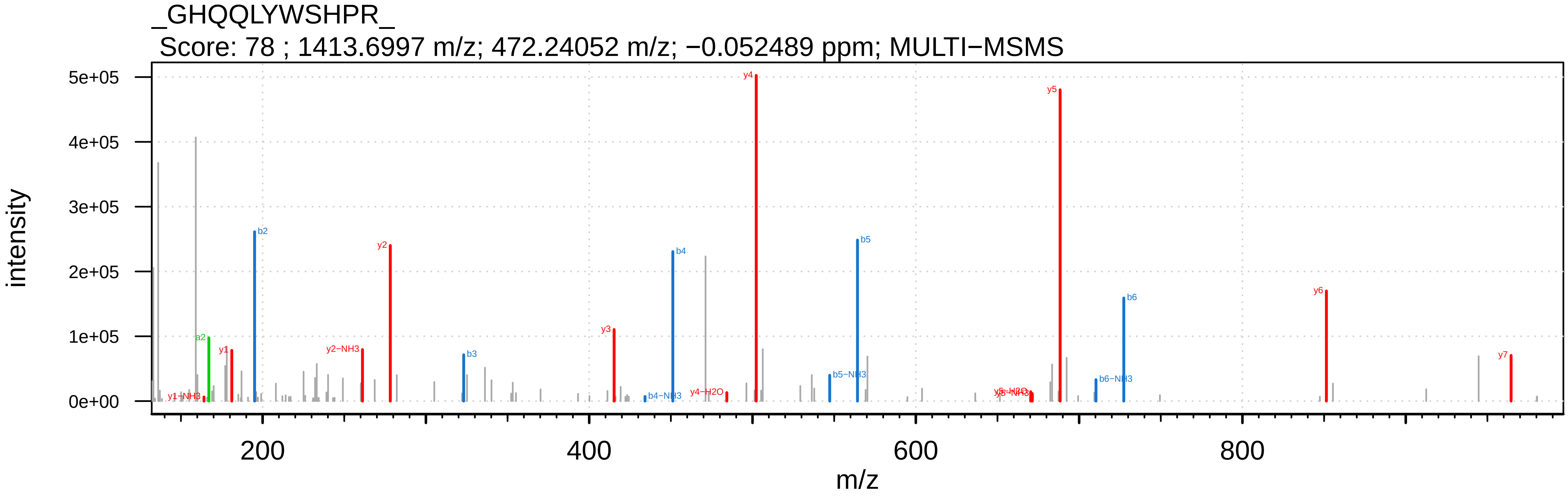

Raw File: Kermit_20111110_MSU_diff1_10
Scan Number: 67952
Proteins:
sp|P62274|RS29_MOUSE
ENSMUST00000037023_chr12:69157813−69159058:−
ENSMUST00000071737_chr13:73988963−73989133:−
tr|D3YYL7|D3YYL7_MOUSE

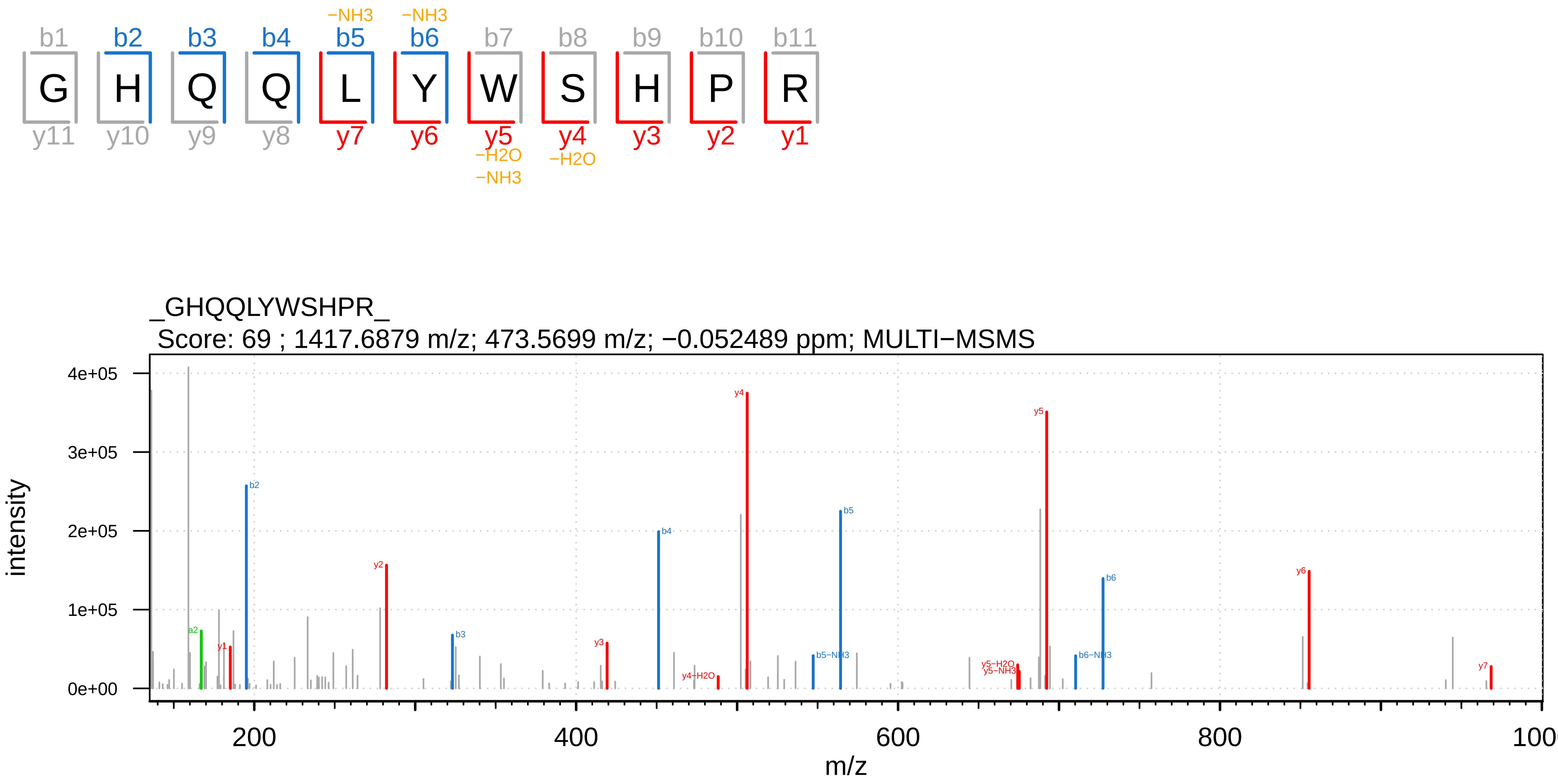

Raw File: Kermit_20111110_MSU_diff1_10
Scan Number: 68018
Proteins:
sp|P62274|RS29_MOUSE
ENSMUST00000037023_chr12:69157813−69159058:−
ENSMUST00000071737_chr13:73988963−73989133:−
tr|D3YYL7|D3YYL7_MOUSE

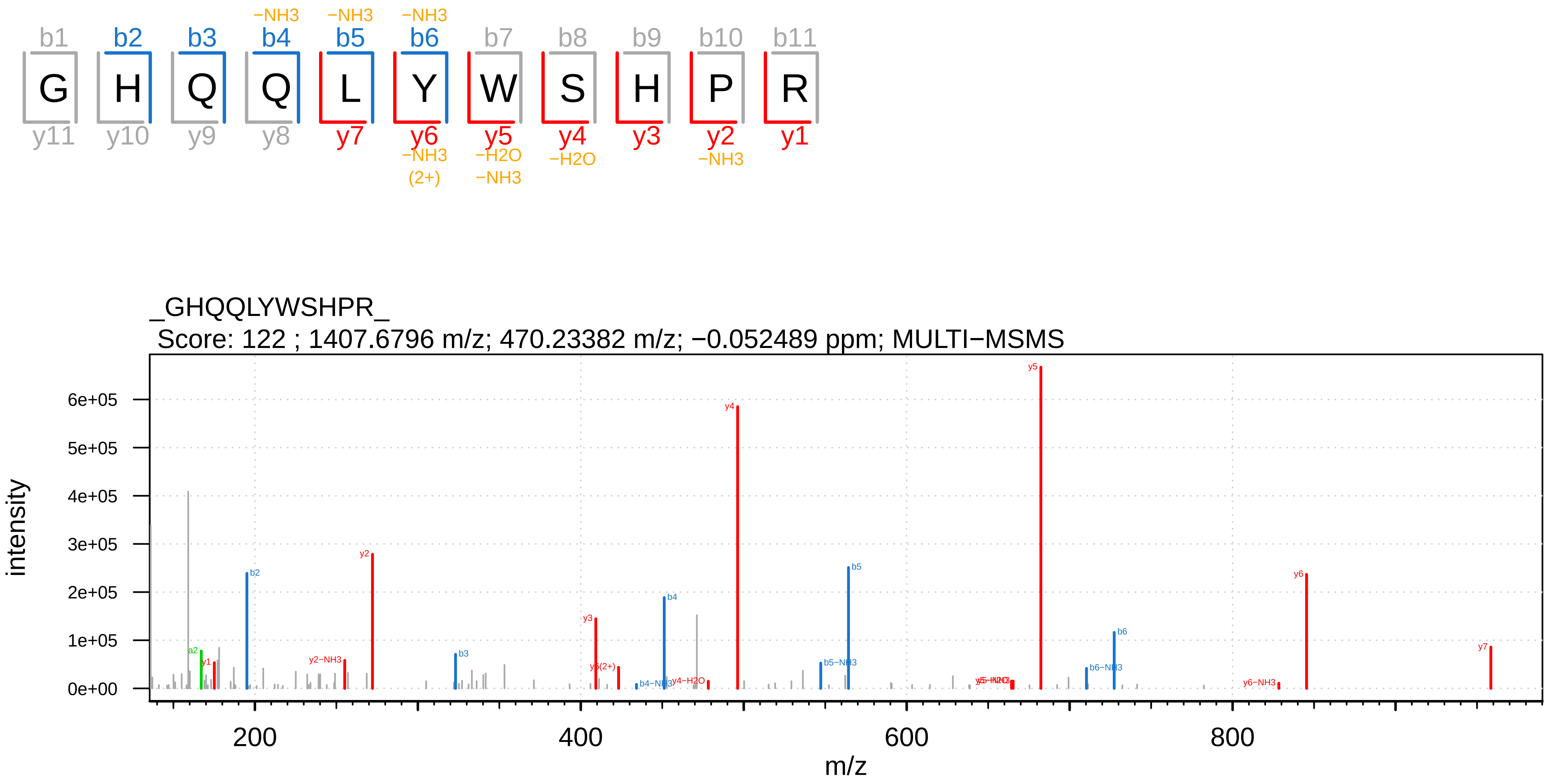

Raw File: Kermit_20111110_MSU_diff1_10
Scan Number: 68036
Proteins:
sp|P62274|RS29_MOUSE
ENSMUST00000037023_chr12:69157813−69159058:−
ENSMUST00000071737_chr13:73988963−73989133:−
tr|D3YYL7|D3YYL7_MOUSE

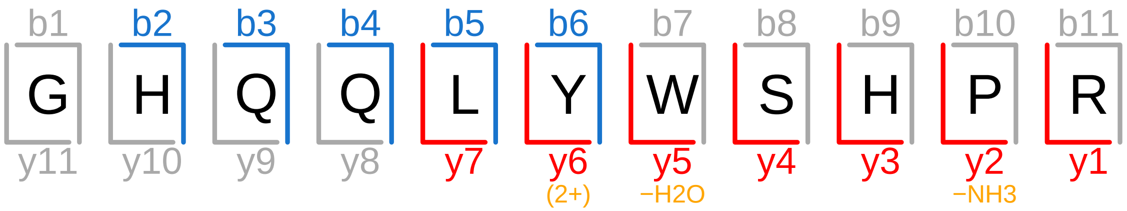

GHQQLYWSHPR
Score: 50 ; 1407.6796 m/z; 470.23382 m/z; −0.052489 ppm; MULTI−MSMS

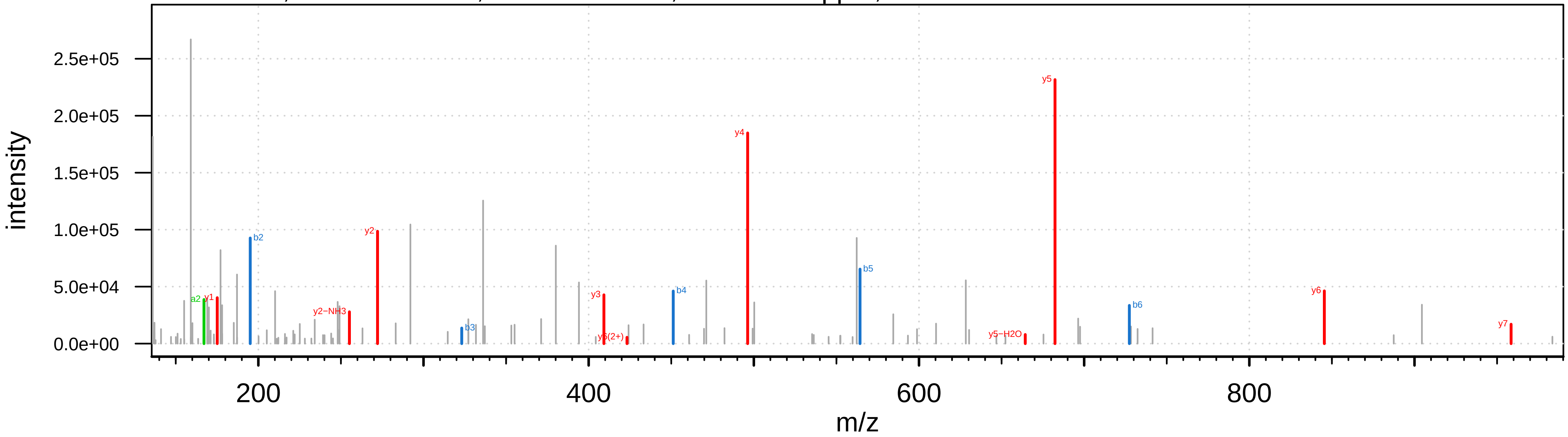

Raw File: Kermit_20111110_MSU_diff1_10
Scan Number: 68280
Proteins:
sp|P62274|RS29_MOUSE
ENSMUST00000037023_chr12:69157813−69159058:−
ENSMUST00000071737_chr13:73988963−73989133:−
tr|D3YYL7|D3YYL7_MOUSE

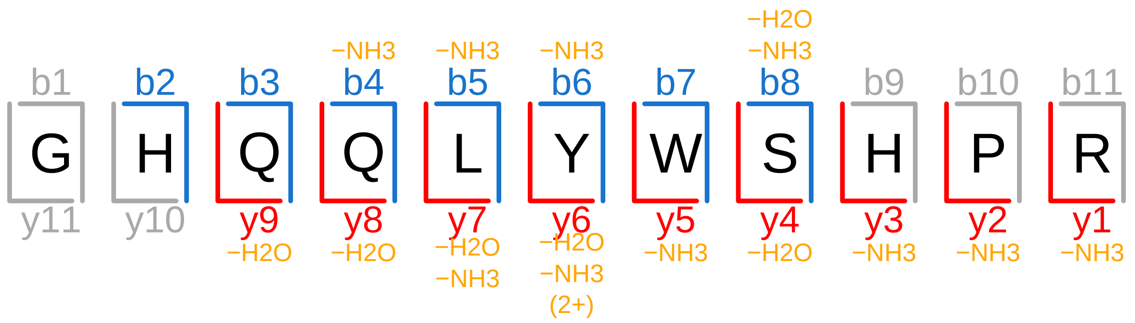

GHQQLYWSHPR
Score: 175 ; 1413.6997 m/z; 472.24052 m/z; −0.65776 ppm; ISO−MSMS

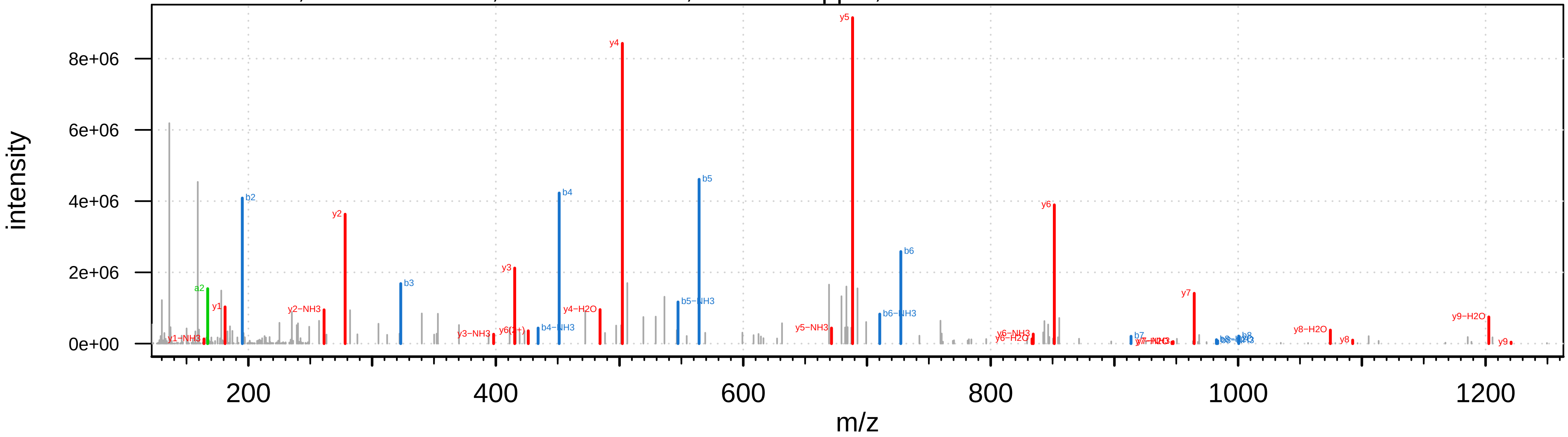

Raw File: Kermit_20111110_MSU_diff1_10
Scan Number: 6441
Proteins:
sp|P62274|RS29_MOUSE
ENSMUST00000037023_chr12:69157813−69159058:−
ENSMUST00000071737_chr13:73988963−73989133:−
tr|D3YYL7|D3YYL7_MOUSE

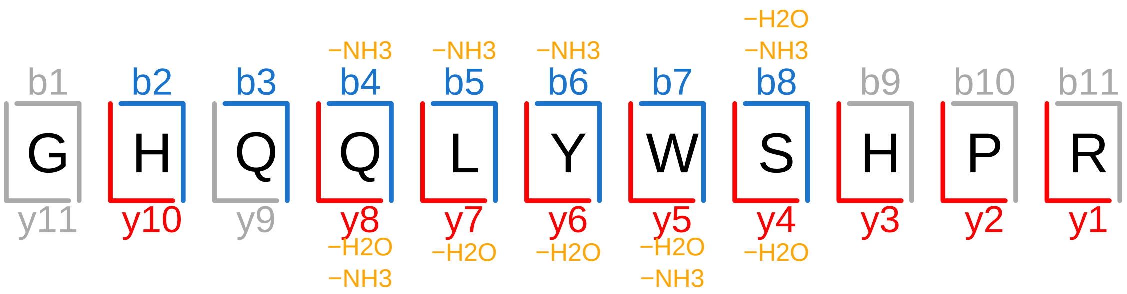

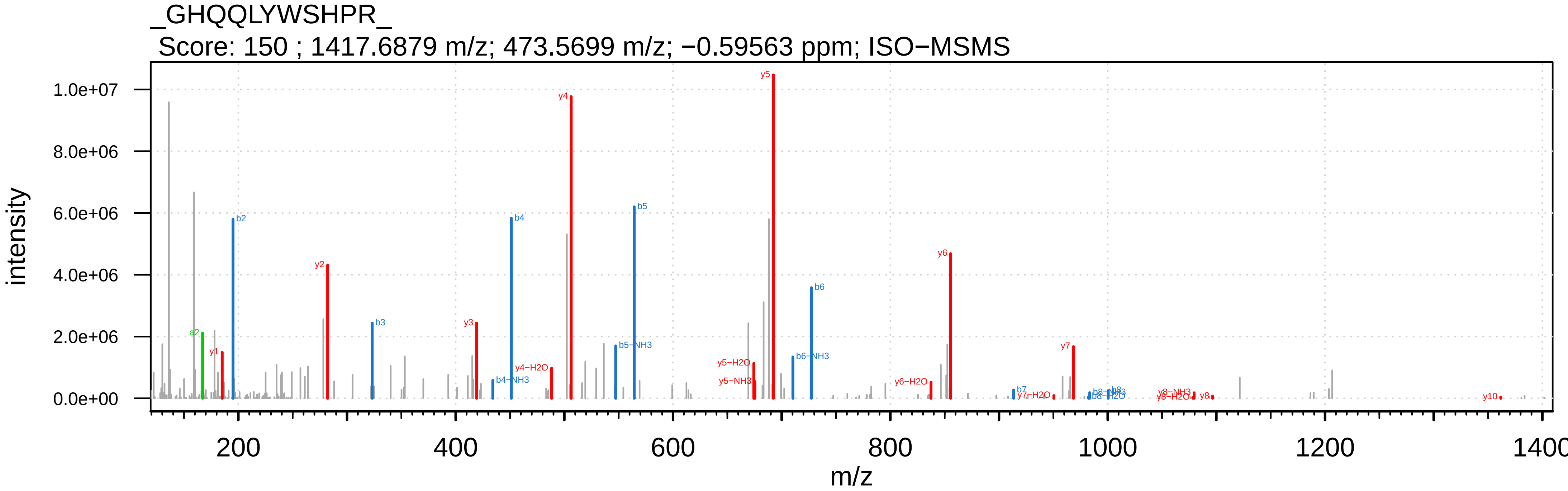

Raw File: Kermit_20111110_MSU_diff1_10
Scan Number: 6445
Proteins:
sp|P62274|RS29_MOUSE
ENSMUST00000037023_chr12:69157813−69159058:−
ENSMUST00000071737_chr13:73988963−73989133:−
tr|D3YYL7|D3YYL7_MOUSE

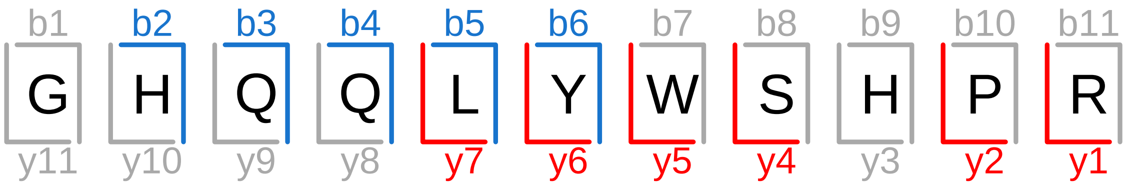

GHQQLYWSHPR
Score: 43 ; 1417.6879 m/z; 473.5699 m/z; −0.26706 ppm; ISO−MSMS

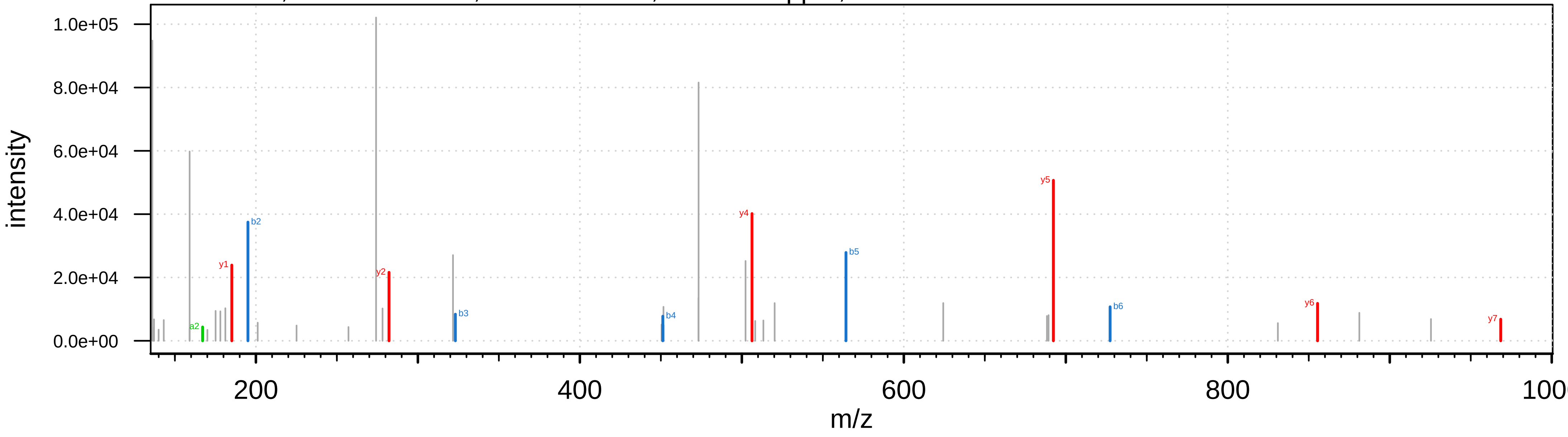

Raw File: Kermit_20111110_MSU_diff1_10
Scan Number: 65351
Proteins:
sp|P62274|RS29_MOUSE
ENSMUST00000037023_chr12:69157813−69159058:−
ENSMUST00000071737_chr13:73988963−73989133:−
tr|D3YYL7|D3YYL7_MOUSE

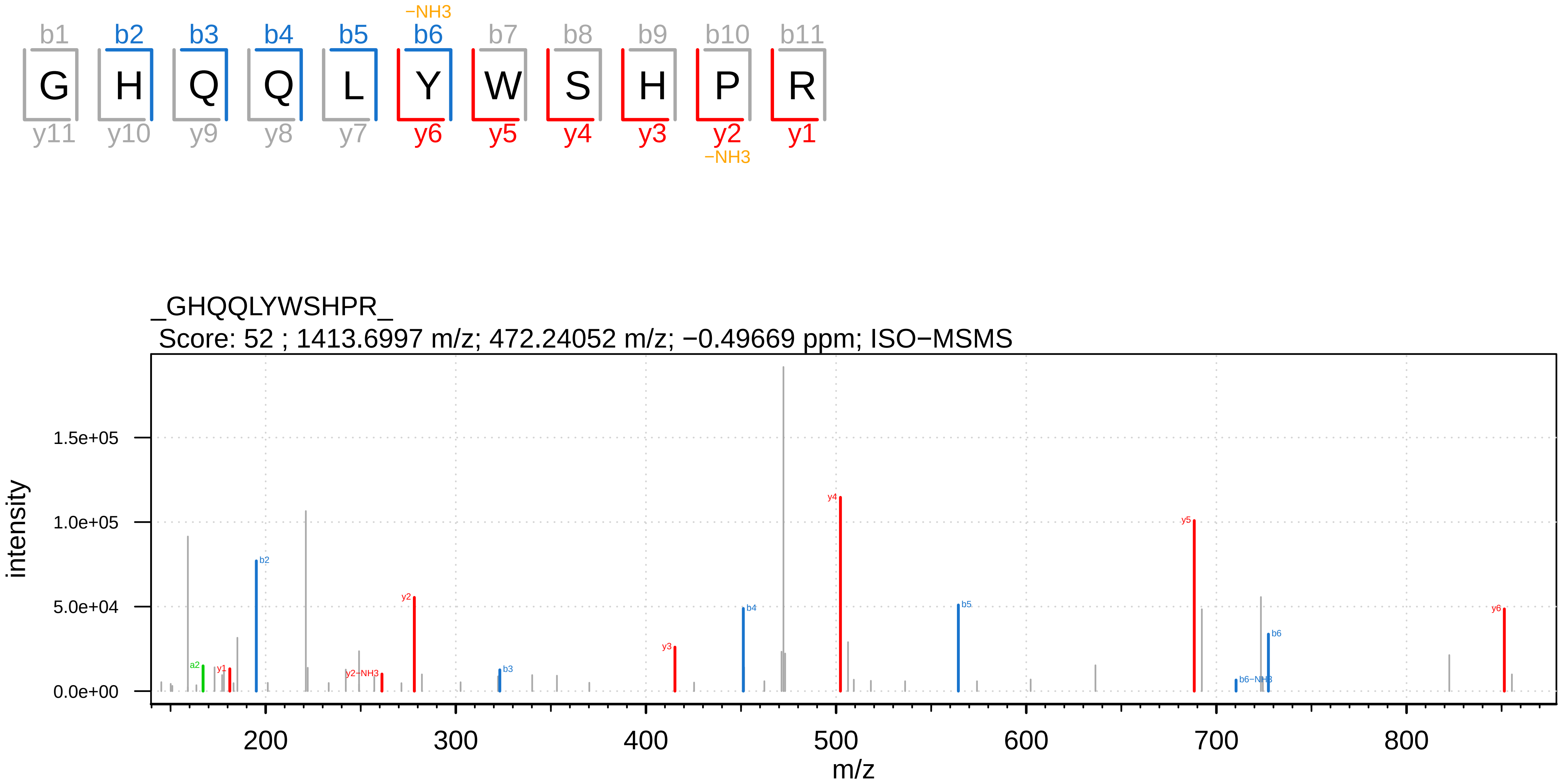

Raw File: Kermit_20111110_MSU_diff1_10
Scan Number: 65397
Proteins:
sp|P62274|RS29_MOUSE
ENSMUST00000037023_chr12:69157813−69159058:−
ENSMUST00000071737_chr13:73988963−73989133:−
tr|D3YYL7|D3YYL7_MOUSE

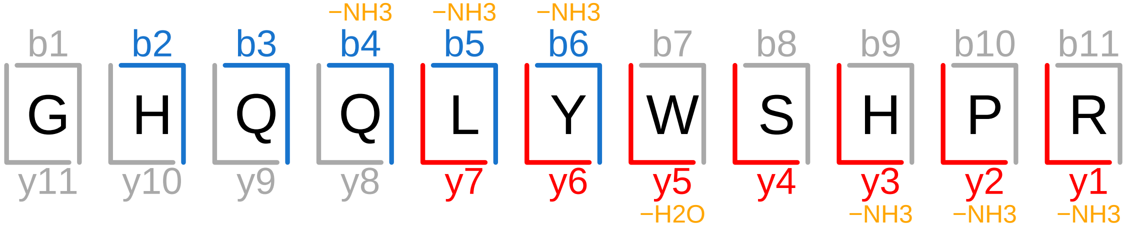

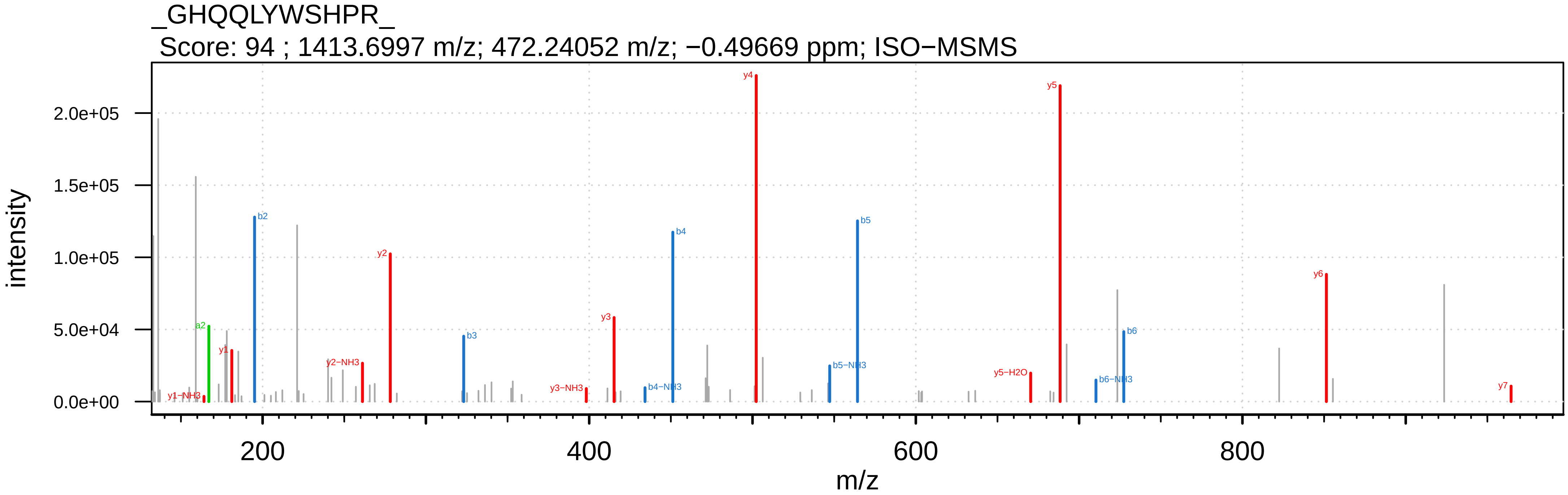

Raw File: Kermit_20111110_MSU_diff1_10
Scan Number: 65652
Proteins:
sp|P62274|RS29_MOUSE
ENSMUST00000037023_chr12:69157813−69159058:−
ENSMUST00000071737_chr13:73988963−73989133:−
tr|D3YYL7|D3YYL7_MOUSE

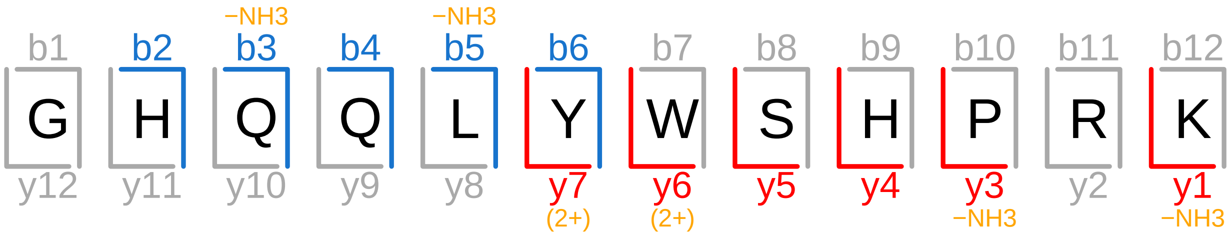

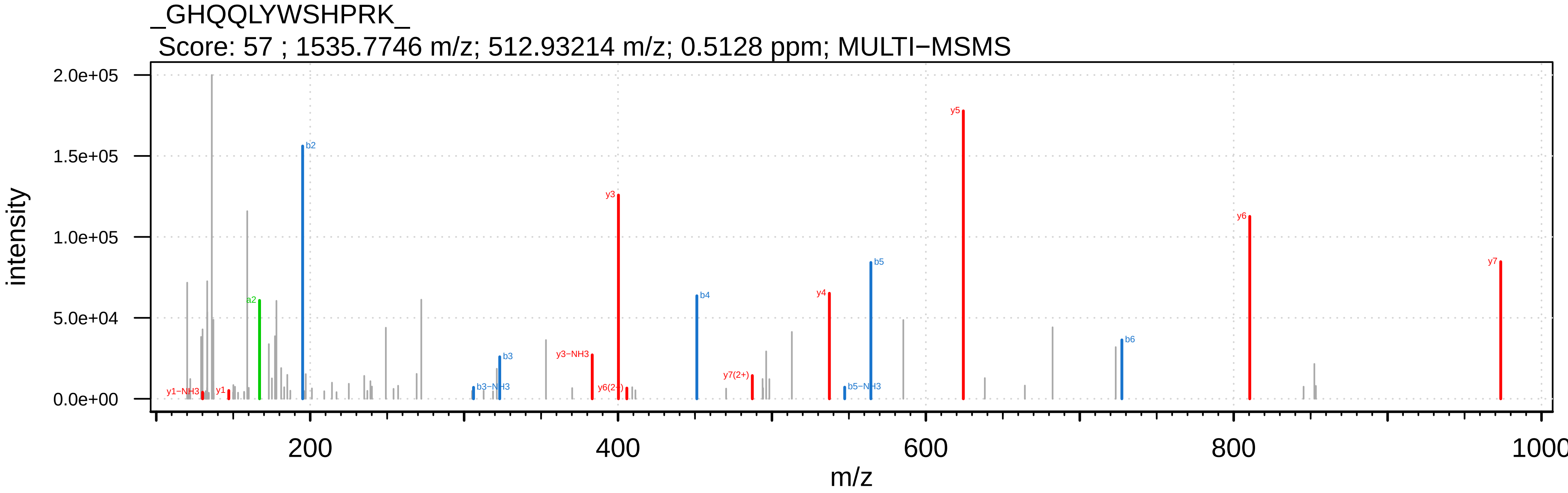

Raw File: Kermit_20111110_MSU_diff1_10
Scan Number: 78478
Proteins:
sp|P62274|RS29_MOUSE
ENSMUST00000037023_chr12:69157813−69159058:−
ENSMUST00000071737_chr13:73988963−73989133:−
tr|D3YYL7|D3YYL7_MOUSE

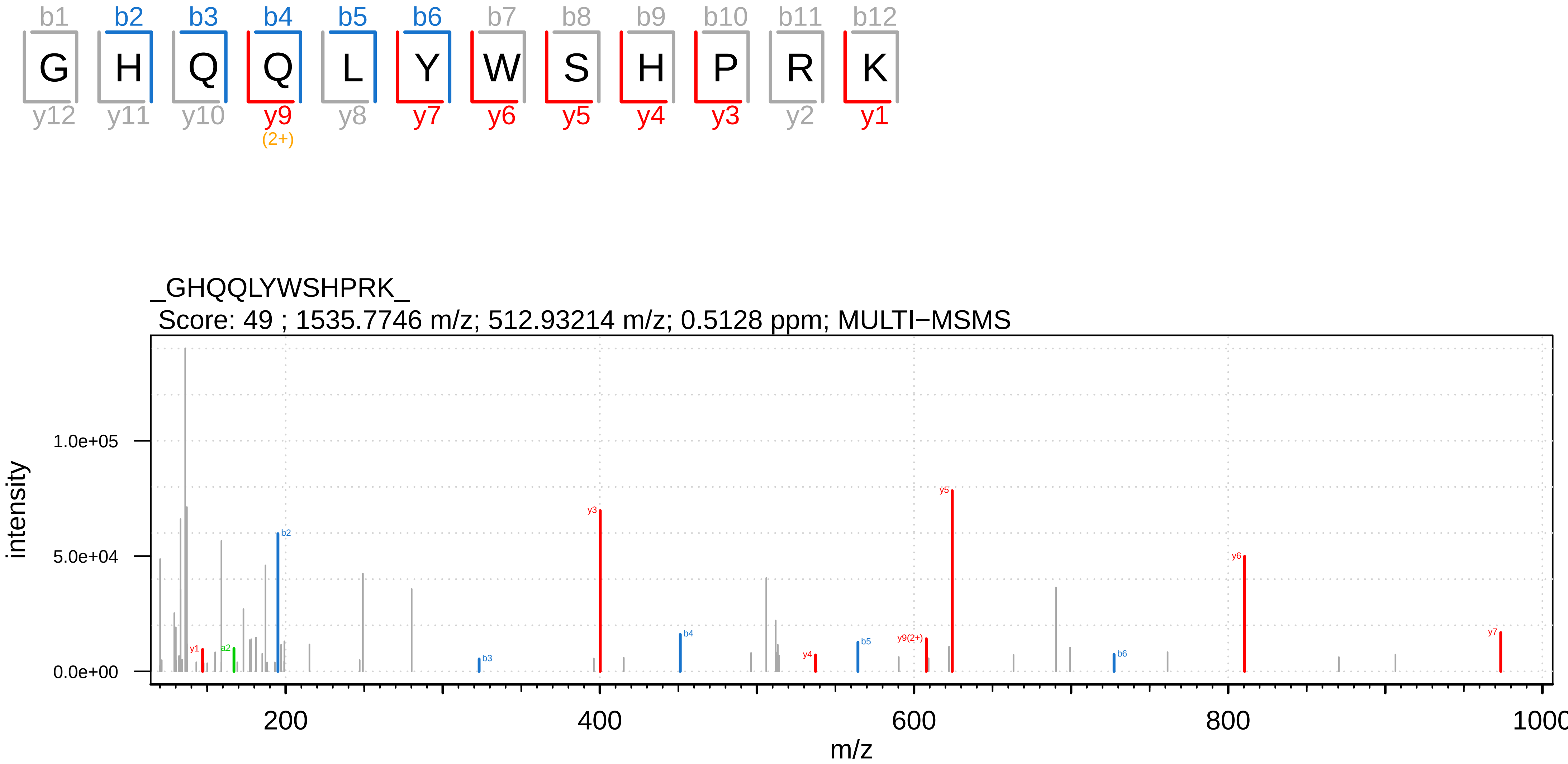

Raw File: Kermit_20111110_MSU_diff1_10
Scan Number: 78767
Proteins:
sp|P62274|RS29_MOUSE
ENSMUST00000037023_chr12:69157813−69159058:−
ENSMUST00000071737_chr13:73988963−73989133:−
tr|D3YYL7|D3YYL7_MOUSE

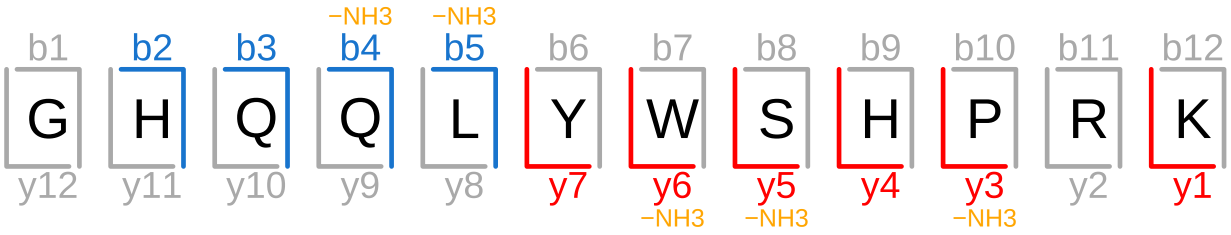

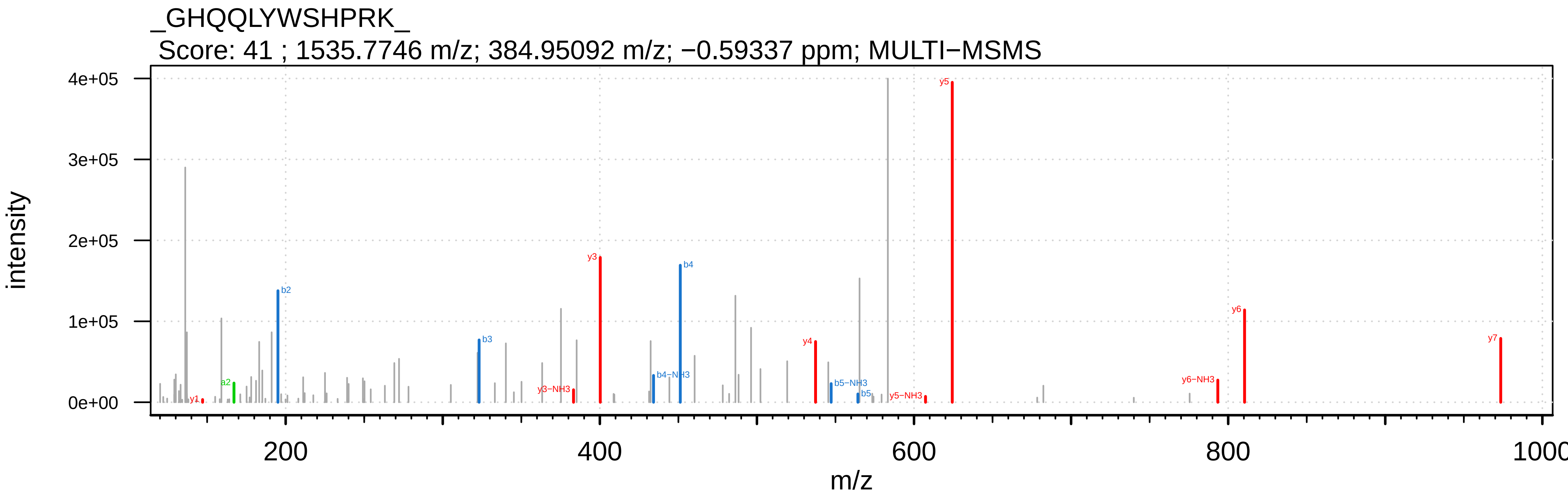

Raw File: Kermit_20111110_MSU_diff1_10
Scan Number: 78500
Proteins:
sp|P62274|RS29_MOUSE
ENSMUST00000037023_chr12:69157813−69159058:−
ENSMUST00000071737_chr13:73988963−73989133:−
tr|D3YYL7|D3YYL7_MOUSE

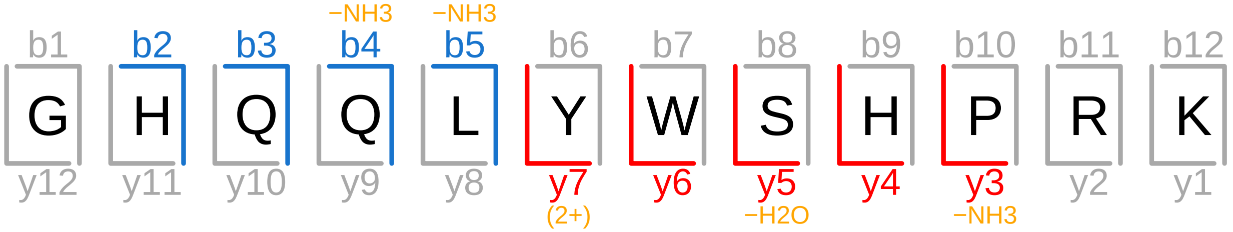

GHQQLYWSHPRK
Score: 36 ; 1545.8198 m/z; 387.46223 m/z; −0.59337 ppm; MULTI−MSMS

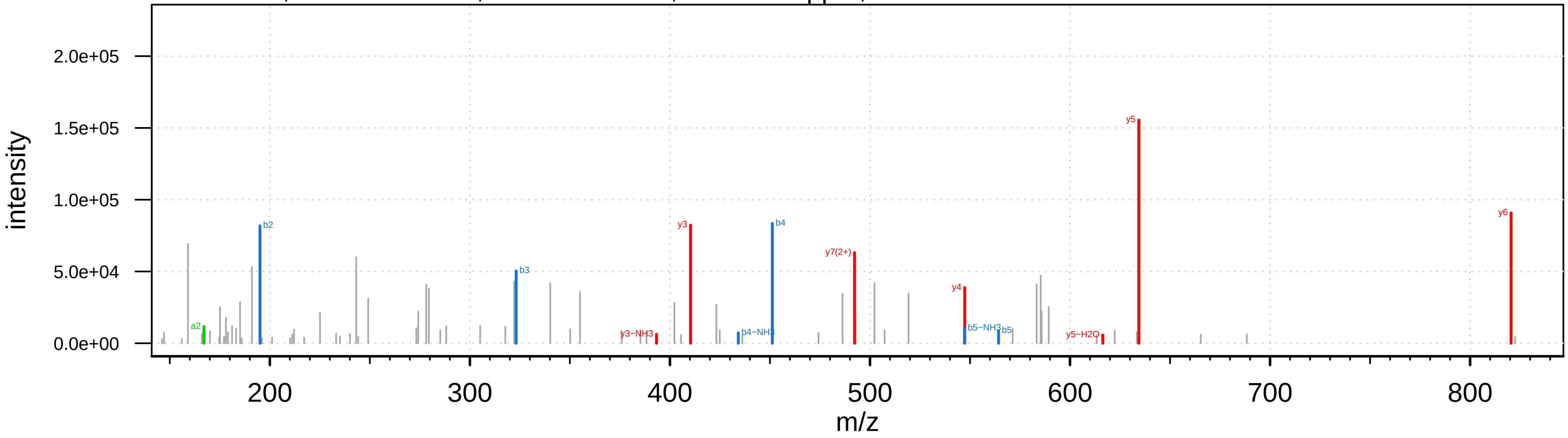

Raw File: Kermit_20111110_MSU_diff1_10
Scan Number: 78689
Proteins:
sp|P62274|RS29_MOUSE
ENSMUST00000037023_chr12:69157813−69159058:−
ENSMUST00000071737_chr13:73988963−73989133:−
tr|D3YYL7|D3YYL7_MOUSE

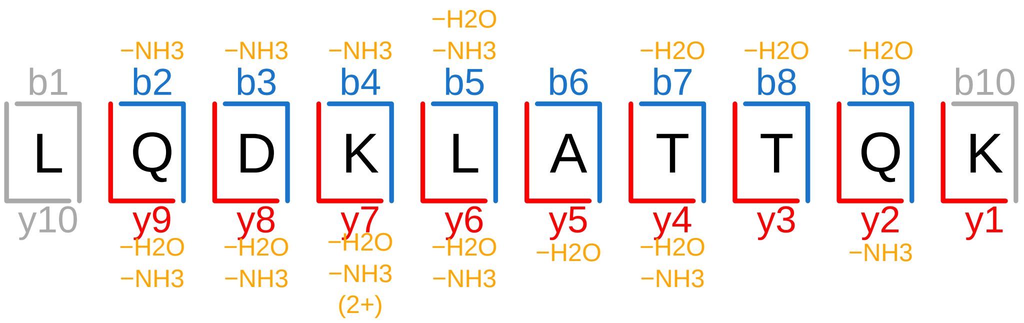

LQDKLATTQK
Score: 182 ; 1160.6736 m/z; 581.34407 m/z; −0.22103 ppm; MULTI−MSMS

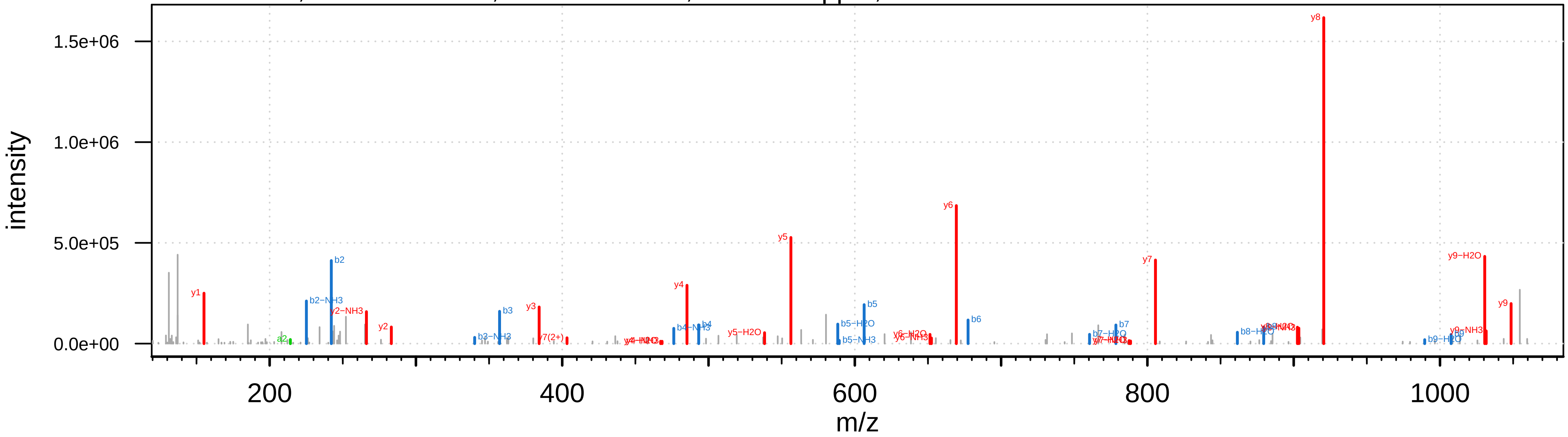

Raw File: Kermit_20111110_MSU_diff1_10
Scan Number: 4091
Proteins:
sp|Q8BT35|YB039_MOUSE
ENSMUST00000135091_chr2:127792297−127792467:−

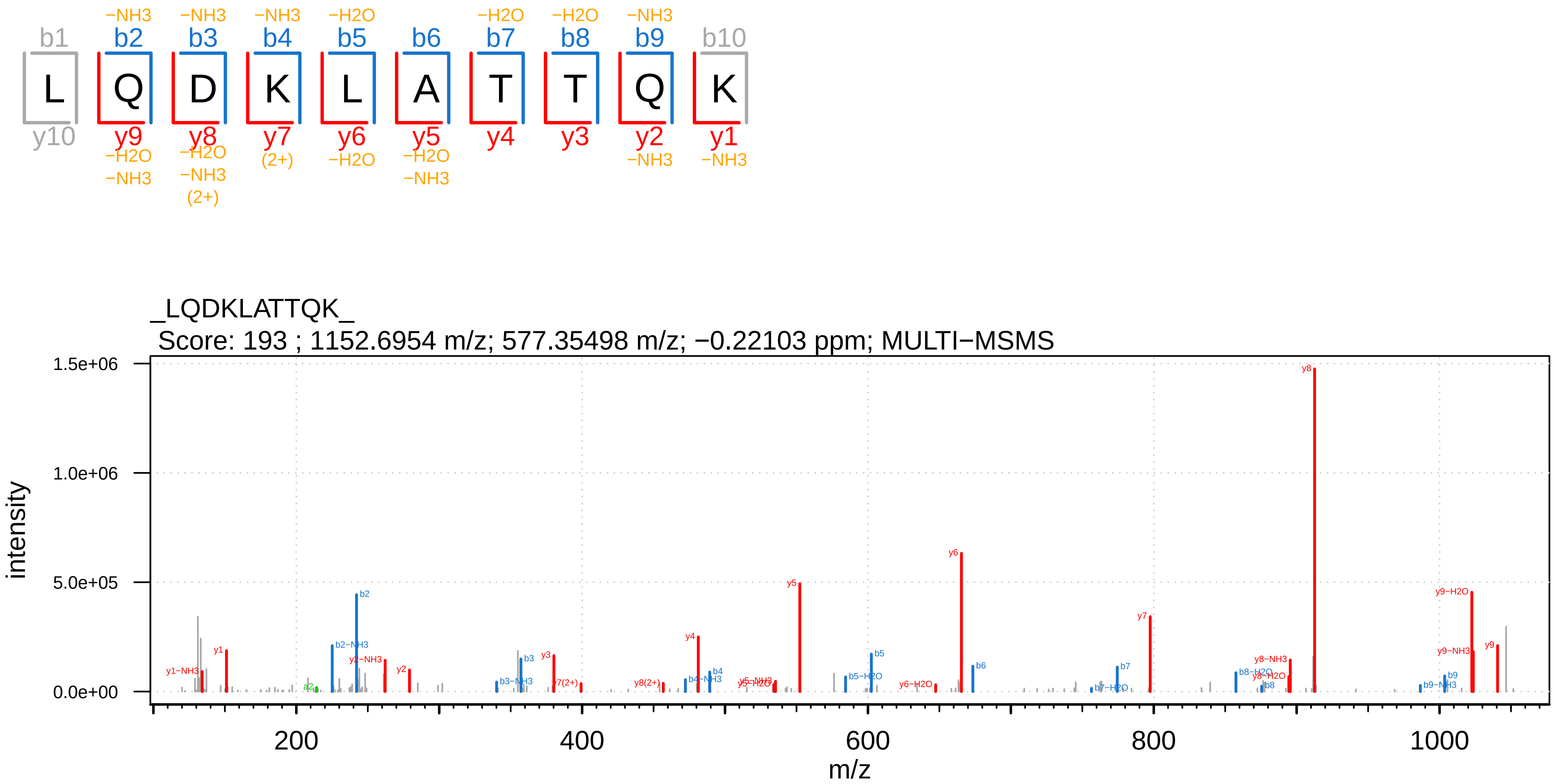

Raw File: Kermit_20111110_MSU_diff1_10
Scan Number: 4092
Proteins:
sp|Q8BT35|YB039_MOUSE
ENSMUST00000135091_chr2:127792297−127792467:−

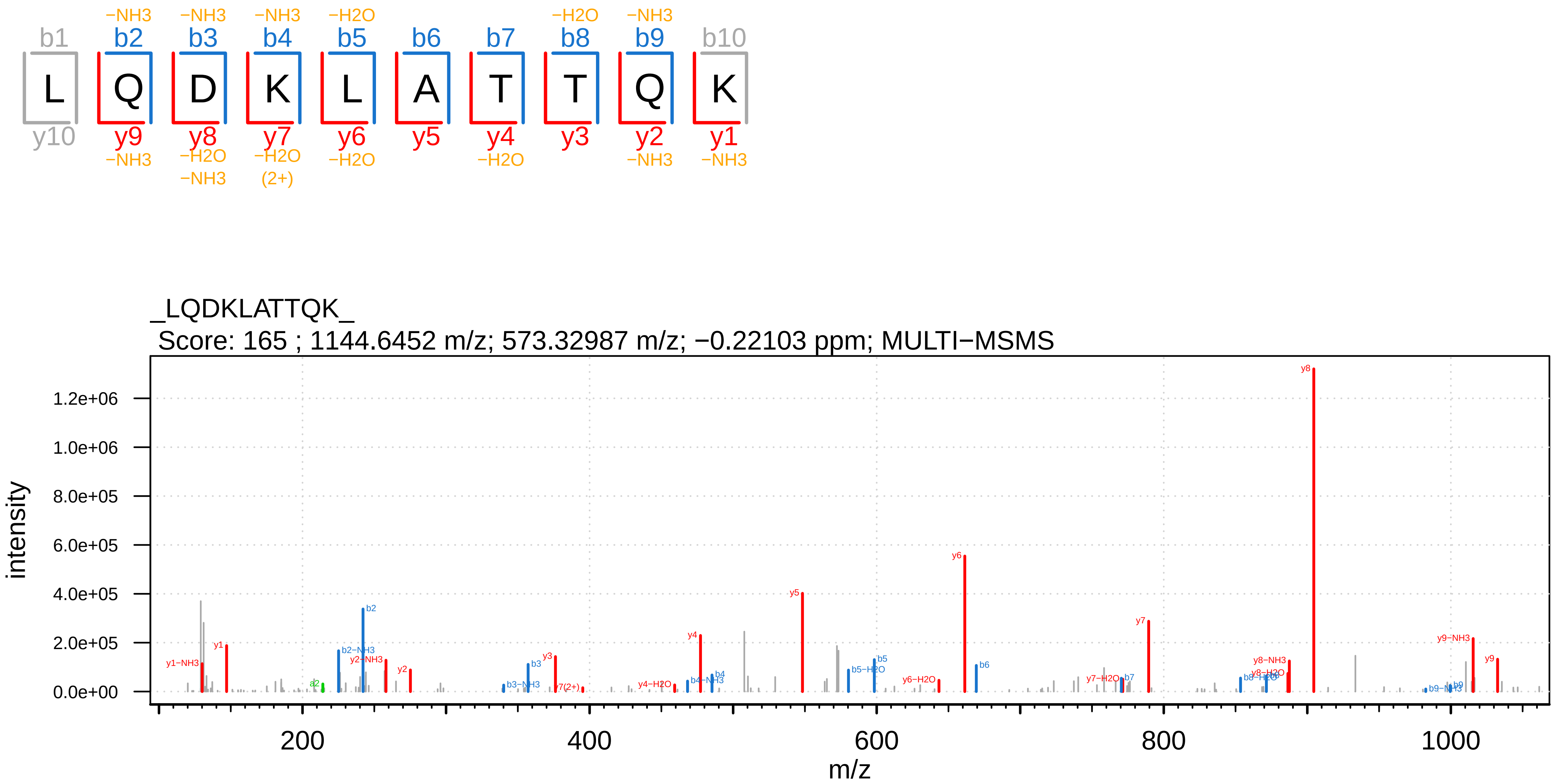

Raw File: Kermit_20111110_MSU_diff1_10
Scan Number: 4106
Proteins:
sp|Q8BT35|YB039_MOUSE
ENSMUST00000135091_chr2:127792297−127792467:−

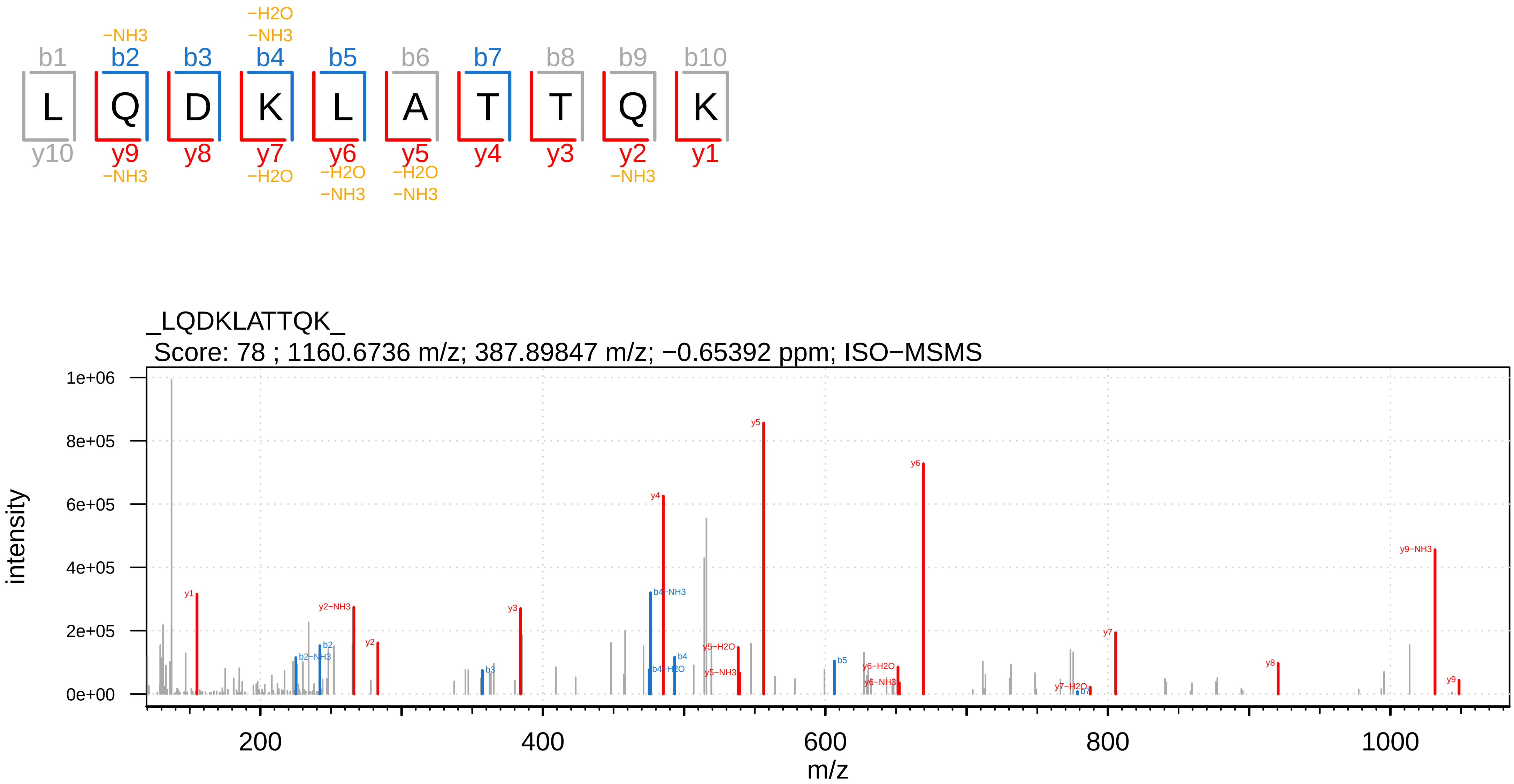

Raw File: Kermit_20111110_MSU_diff1_10
Scan Number: 4104
Proteins:
sp|Q8BT35|YB039_MOUSE
ENSMUST00000135091_chr2:127792297−127792467:−

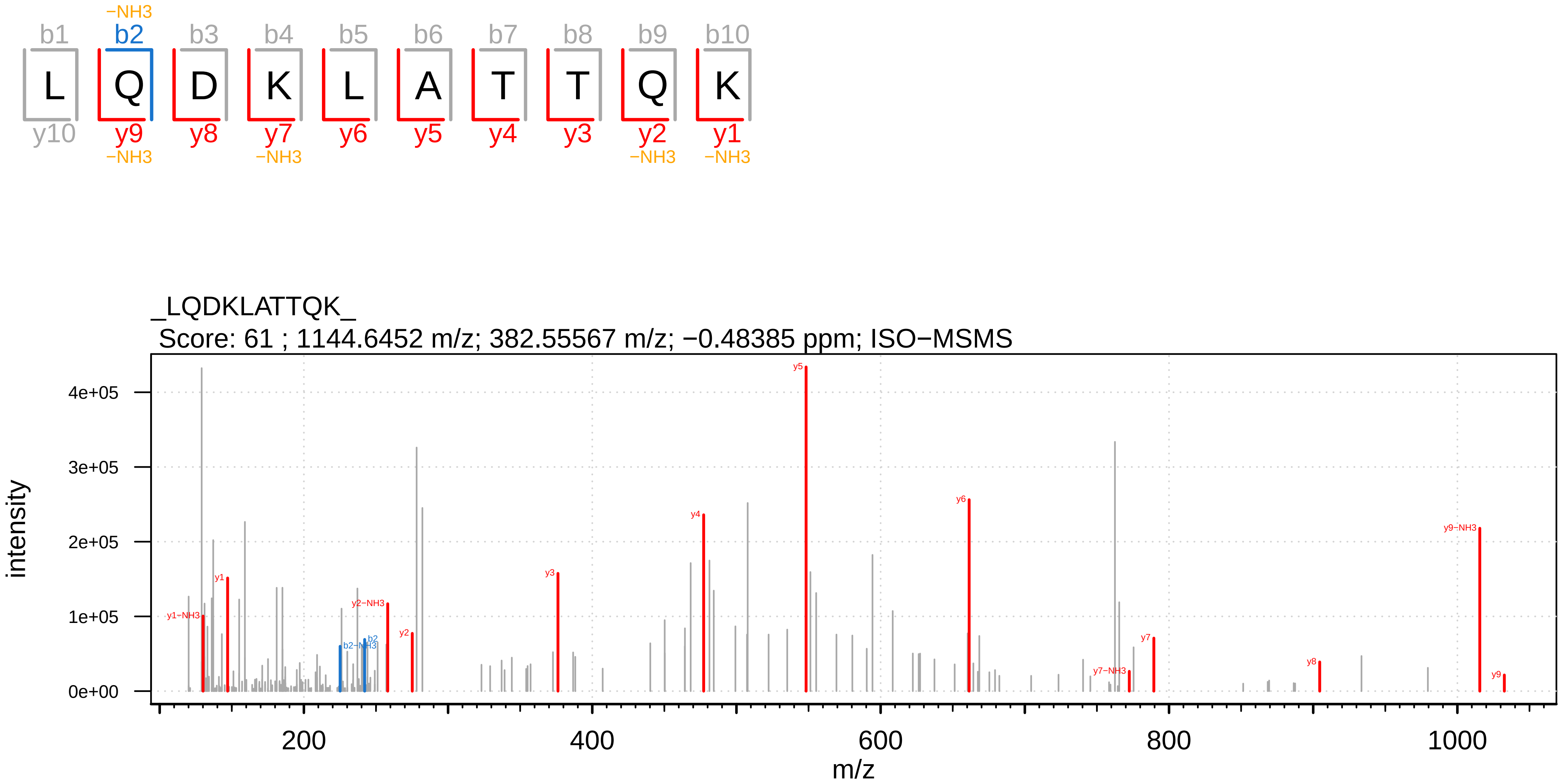

Raw File: Kermit_20111110_MSU_diff1_10
Scan Number: 4111
Proteins:
sp|Q8BT35|YB039_MOUSE
ENSMUST00000135091_chr2:127792297−127792467:−

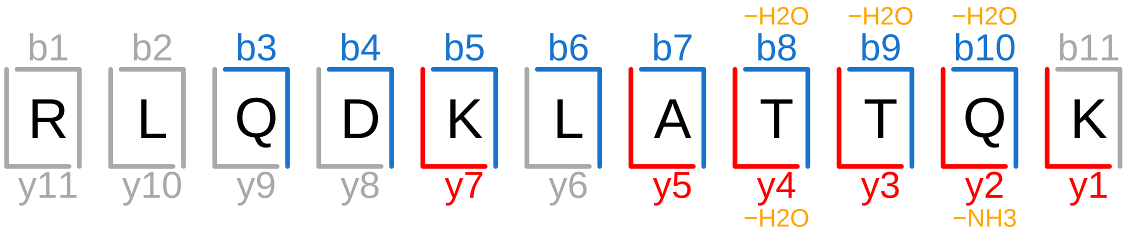

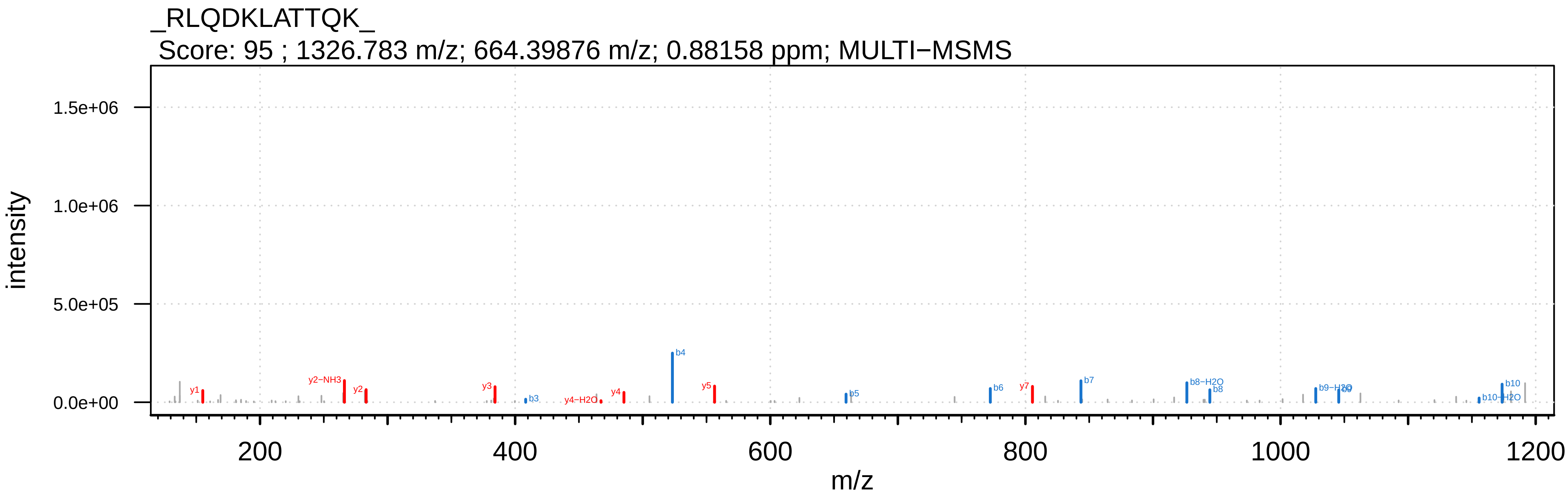

Raw File: Kermit_20111110_MSU_diff1_10
Scan Number: 4410
Proteins:
sp|Q8BT35|YB039_MOUSE
ENSMUST00000135091_chr2:127792297−127792467:−

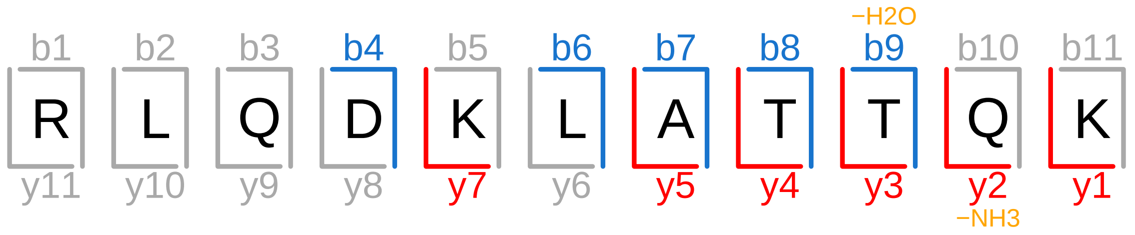

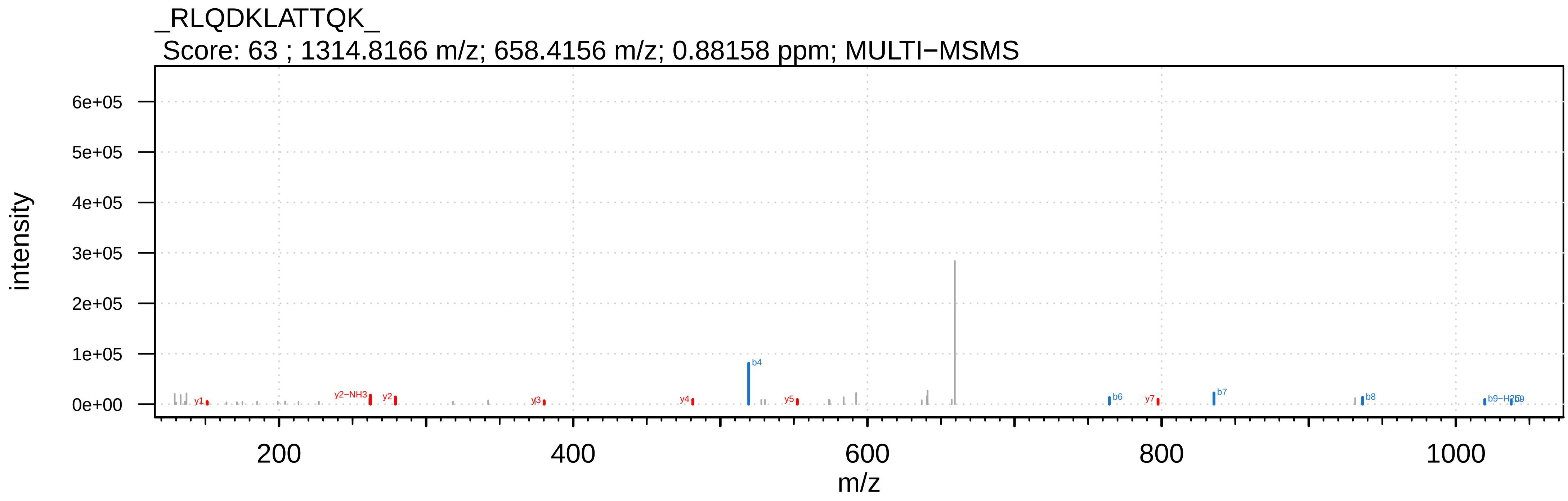

Raw File: Kermit_20111110_MSU_diff1_10
Scan Number: 4418
Proteins:
sp|Q8BT35|YB039_MOUSE
ENSMUST00000135091_chr2:127792297−127792467:−

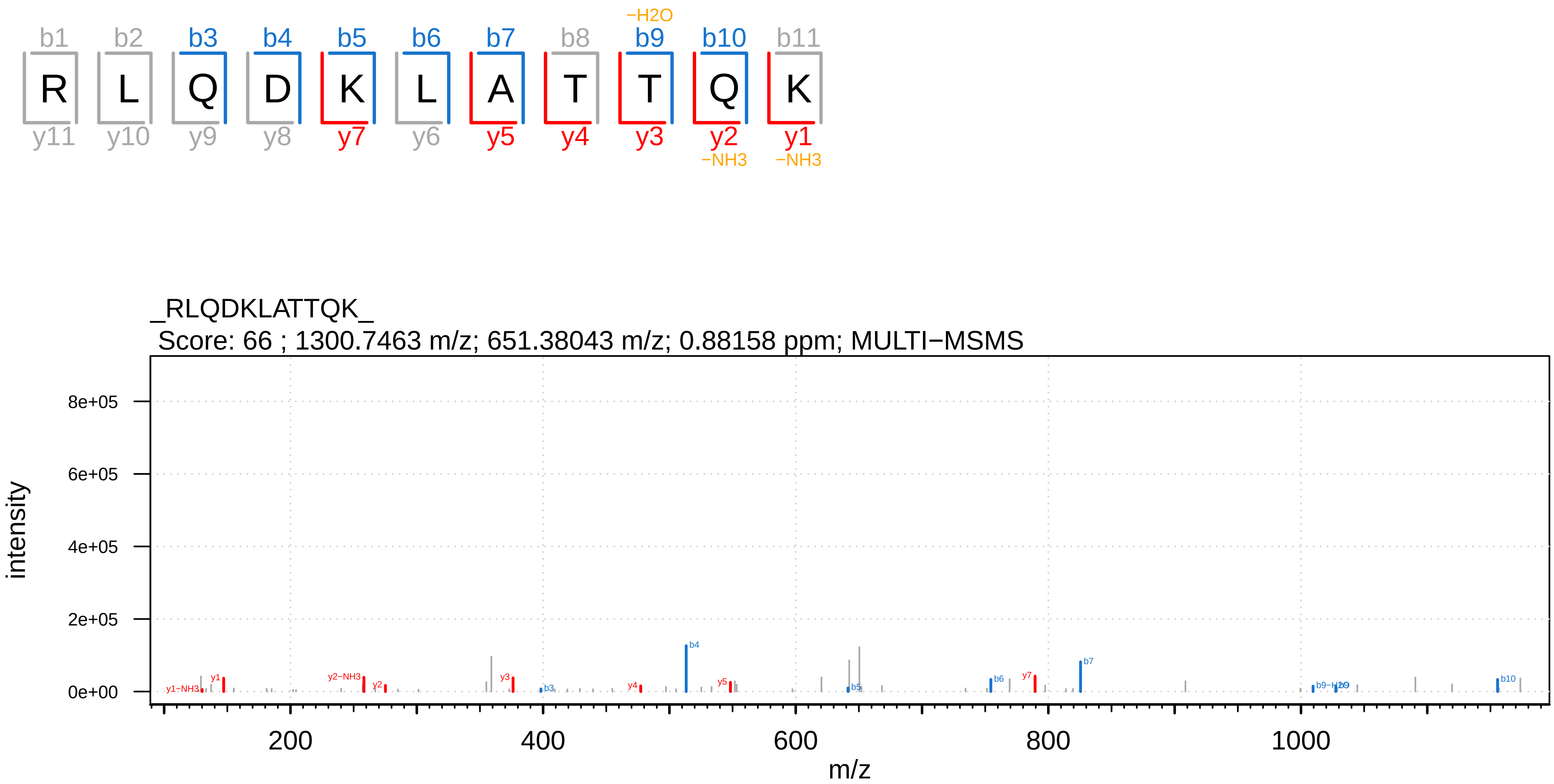

Raw File: Kermit_20111110_MSU_diff1_10
Scan Number: 4426
Proteins:
sp|Q8BT35|YB039_MOUSE
ENSMUST00000135091_chr2:127792297−127792467:−

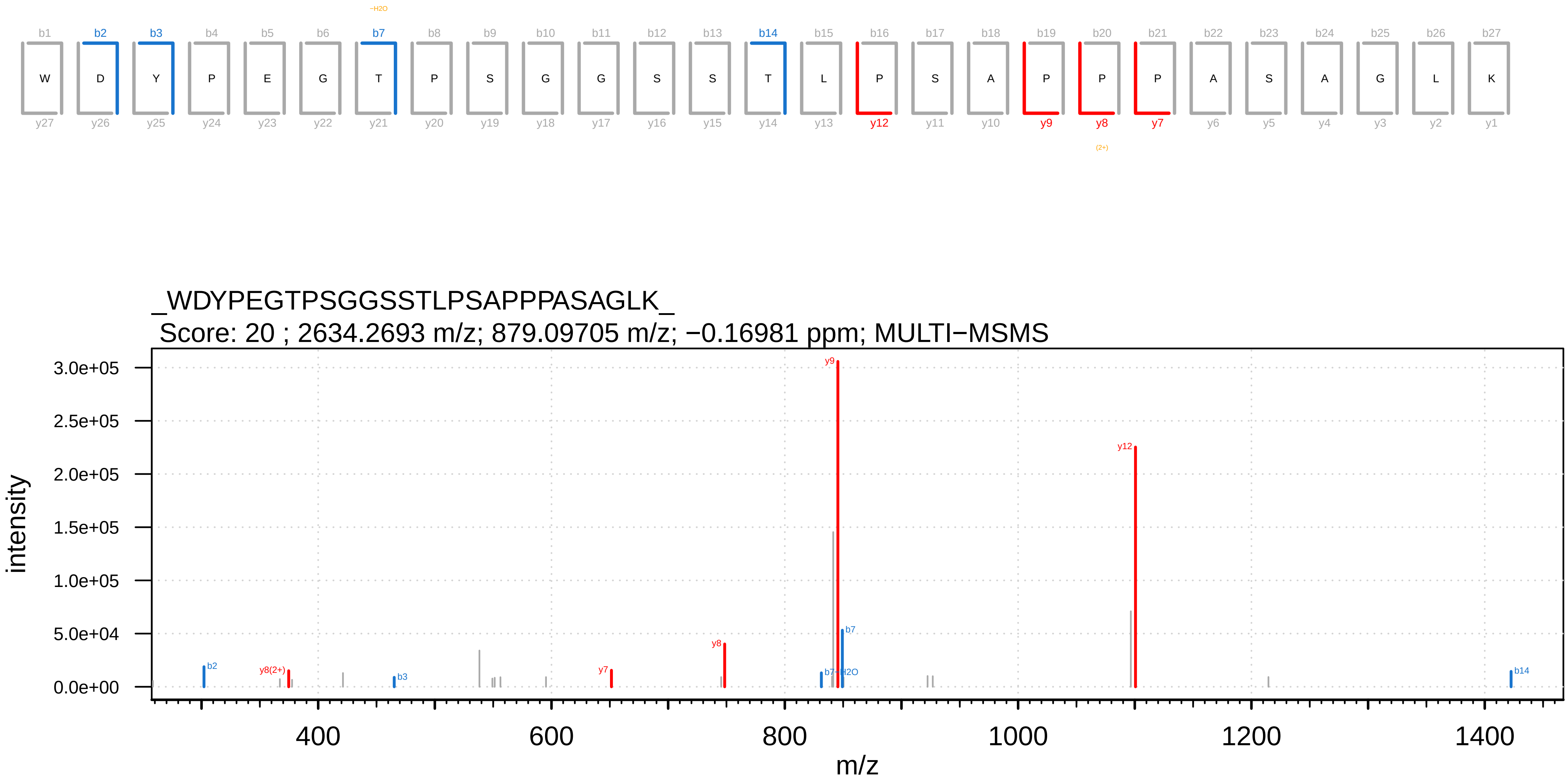

Raw File: Kermit_20111110_MSU_diff1_10
Scan Number: 38761
Proteins:
ENSMUST00000149514_chrX:153723700−153723906:+

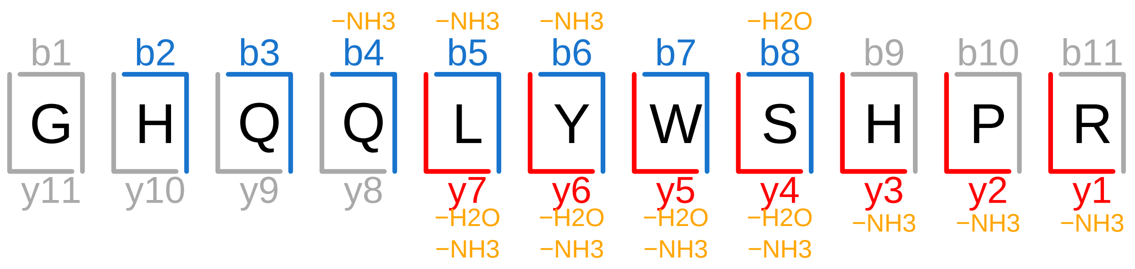

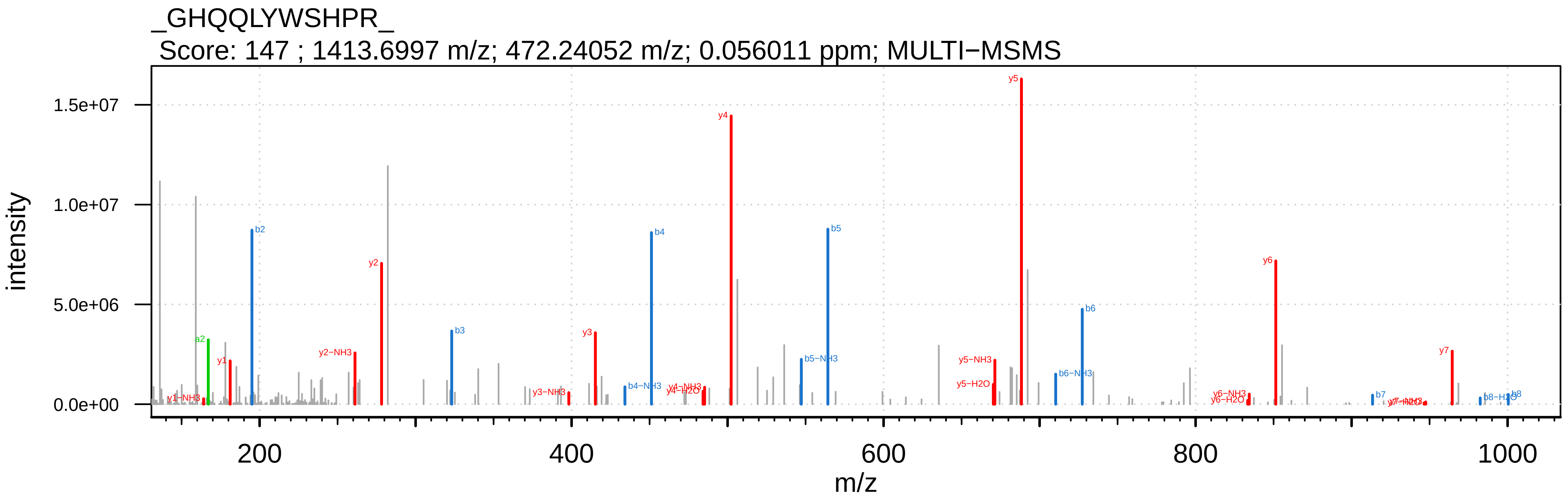

Raw File: Kermit_20111110_MSU_diff1_11
Scan Number: 6072
Proteins:
sp|P62274|RS29_MOUSE
ENSMUST00000037023_chr12:69157813−69159058:−
ENSMUST00000071737_chr13:73988963−73989133:−
tr|D3YYL7|D3YYL7_MOUSE

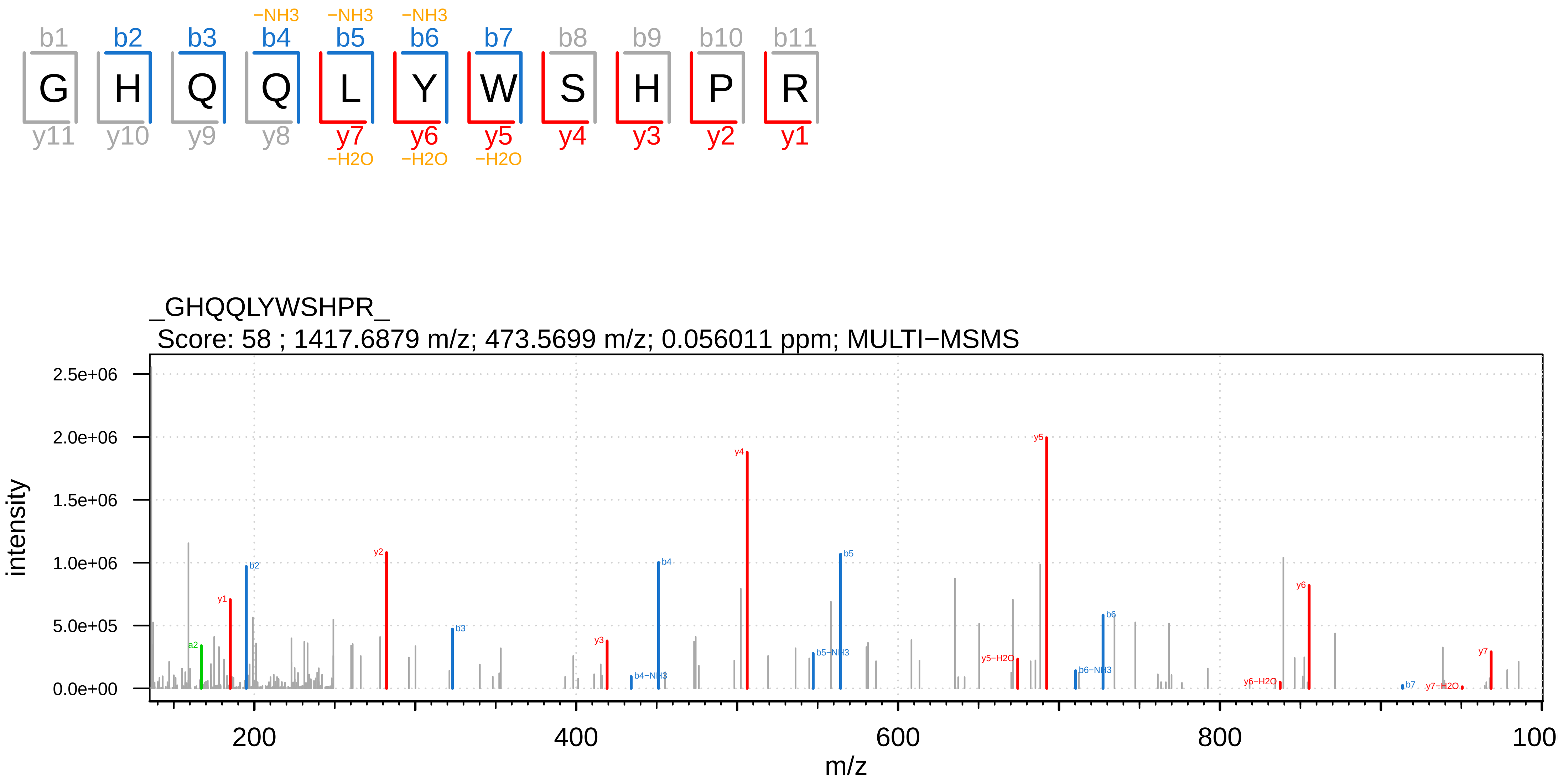

Raw File: Kermit_20111110_MSU_diff1_11
Scan Number: 6108
Proteins:
sp|P62274|RS29_MOUSE
ENSMUST00000037023_chr12:69157813−69159058:−
ENSMUST00000071737_chr13:73988963−73989133:−
tr|D3YYL7|D3YYL7_MOUSE

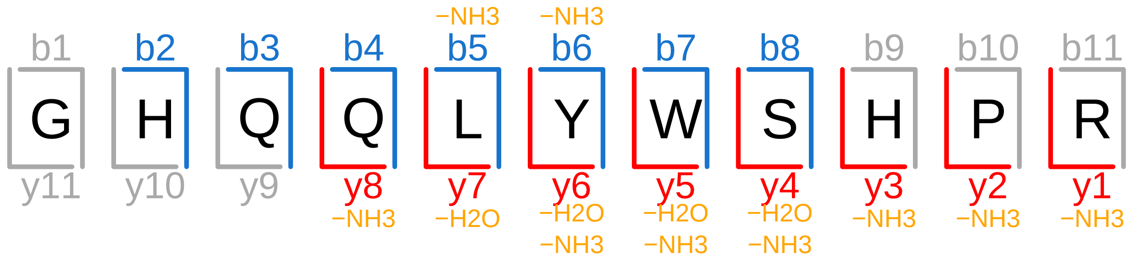

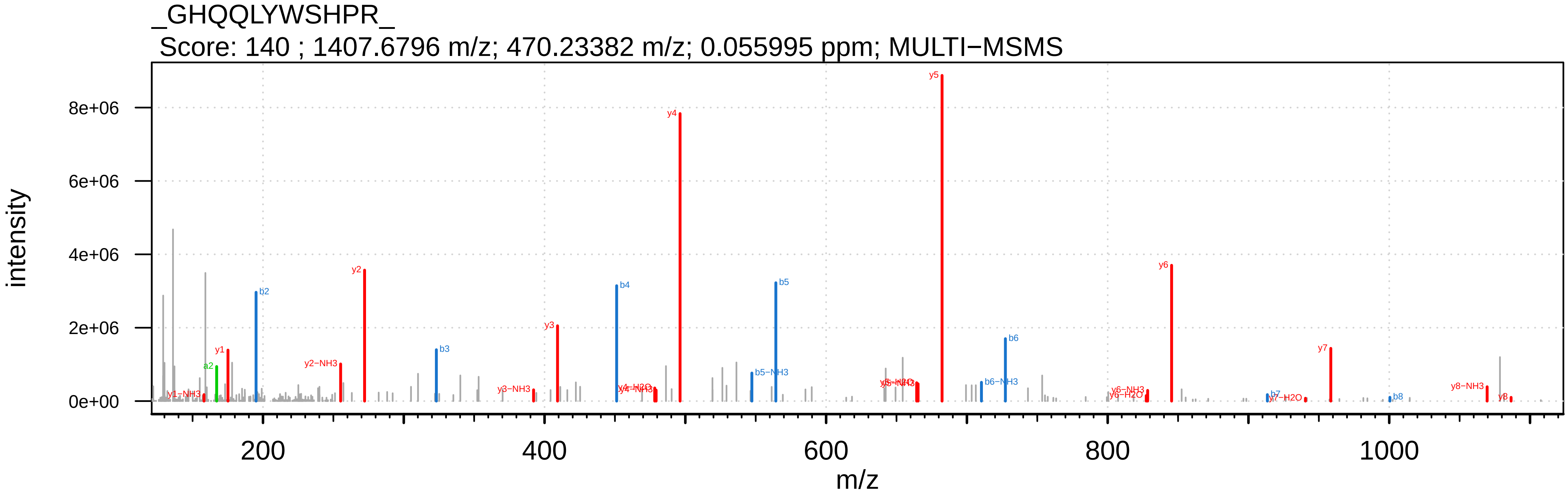

Raw File: Kermit_20111118_MSU_blast_9h_10
Scan Number: 5729
Proteins:
sp|P62274|RS29_MOUSE
ENSMUST00000037023_chr12:69157813−69159058:−
ENSMUST00000071737_chr13:73988963−73989133:−
tr|D3YYL7|D3YYL7_MOUSE

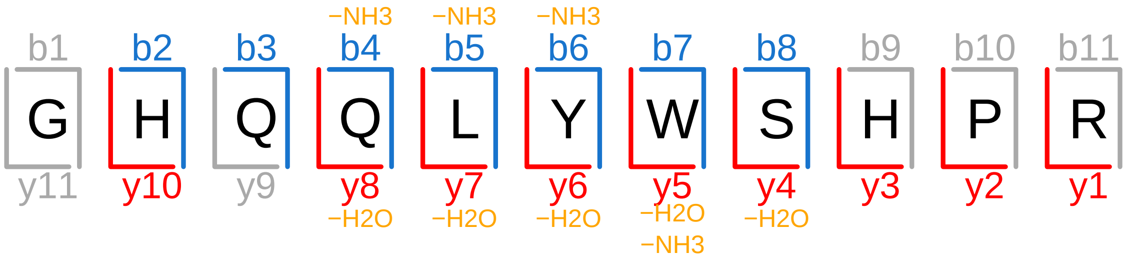

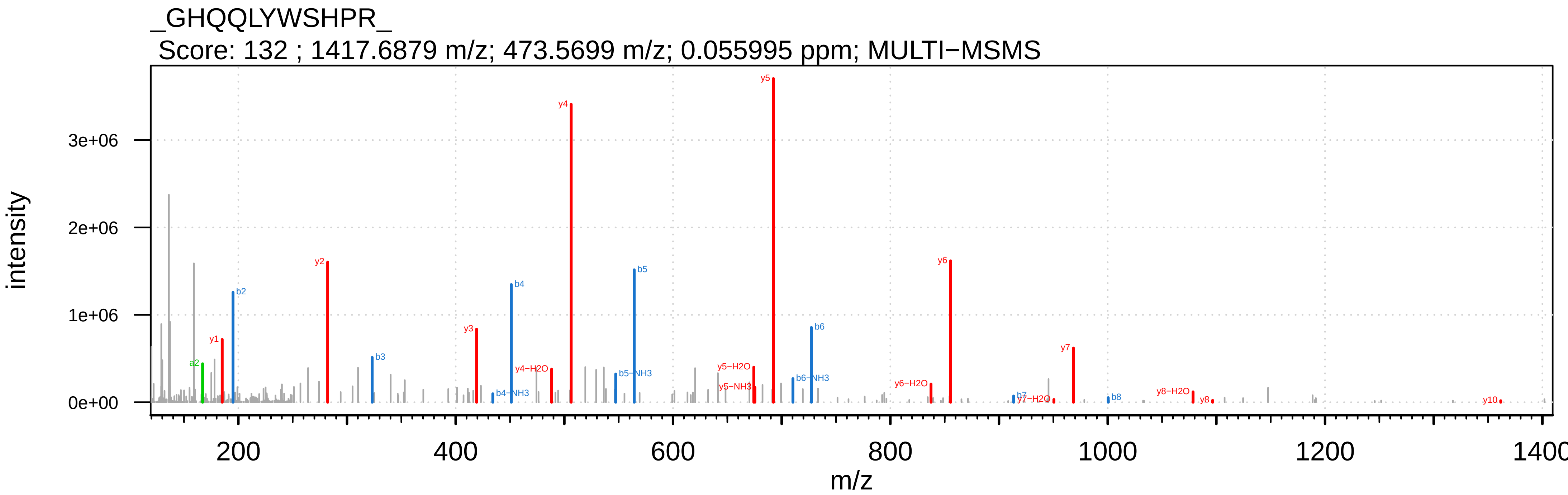

Raw File: Kermit_20111118_MSU_blast_9h_10
Scan Number: 5746
Proteins:
sp|P62274|RS29_MOUSE
ENSMUST00000037023_chr12:69157813−69159058:−
ENSMUST00000071737_chr13:73988963−73989133:−
tr|D3YYL7|D3YYL7_MOUSE

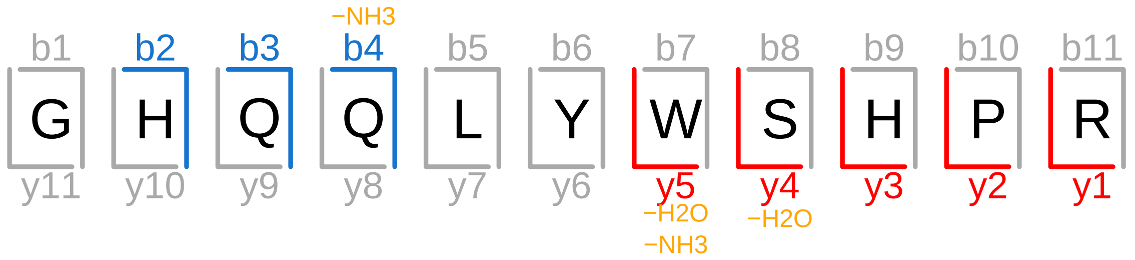

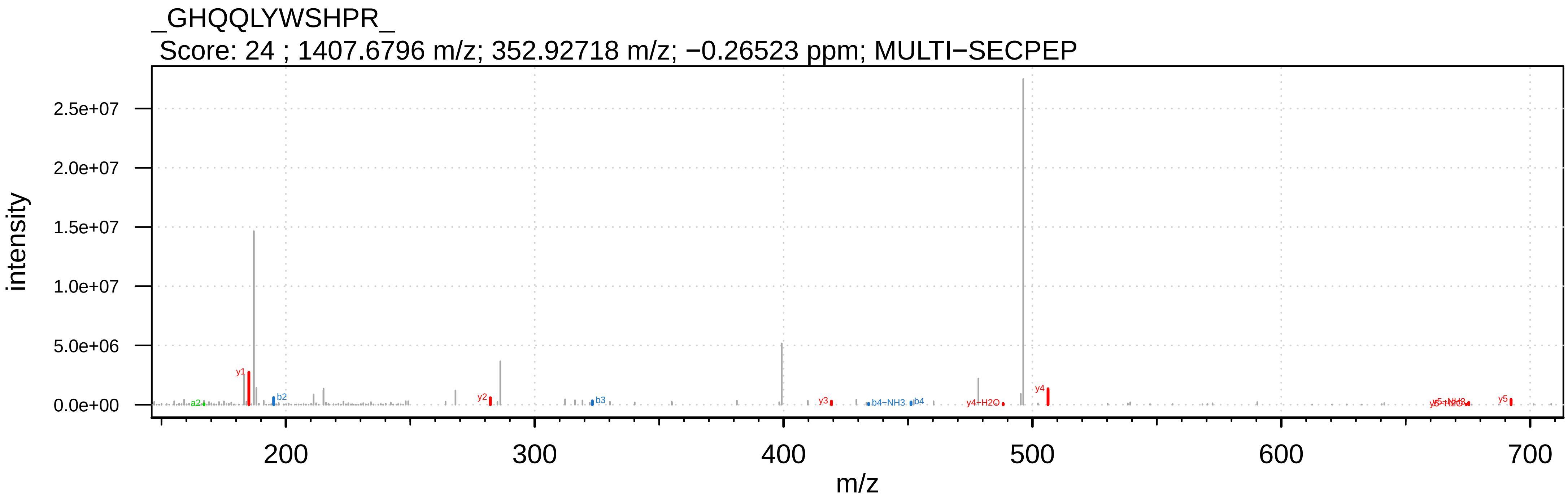

Raw File: Kermit_20111118_MSU_blast_9h_10
Scan Number: 5751
Proteins:
sp|P62274|RS29_MOUSE
ENSMUST00000037023_chr12:69157813−69159058:−
ENSMUST00000071737_chr13:73988963−73989133:−
tr|D3YYL7|D3YYL7_MOUSE

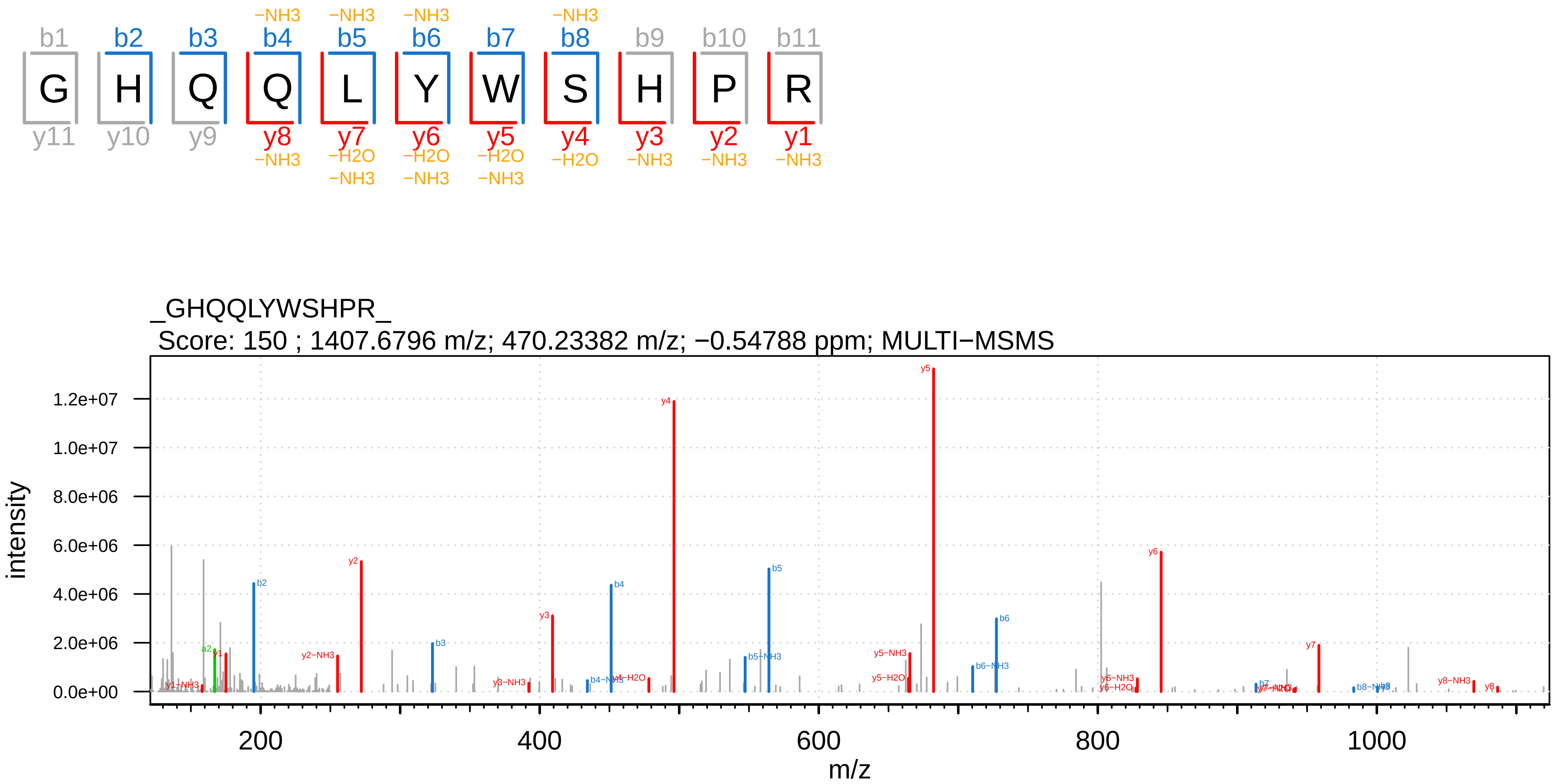

Raw File: Kermit_20111118_MSU_diff2_11
Scan Number: 4673
Proteins:
sp|P62274|RS29_MOUSE
ENSMUST00000037023_chr12:69157813−69159058:−
ENSMUST00000071737_chr13:73988963−73989133:−
tr|D3YYL7|D3YYL7_MOUSE

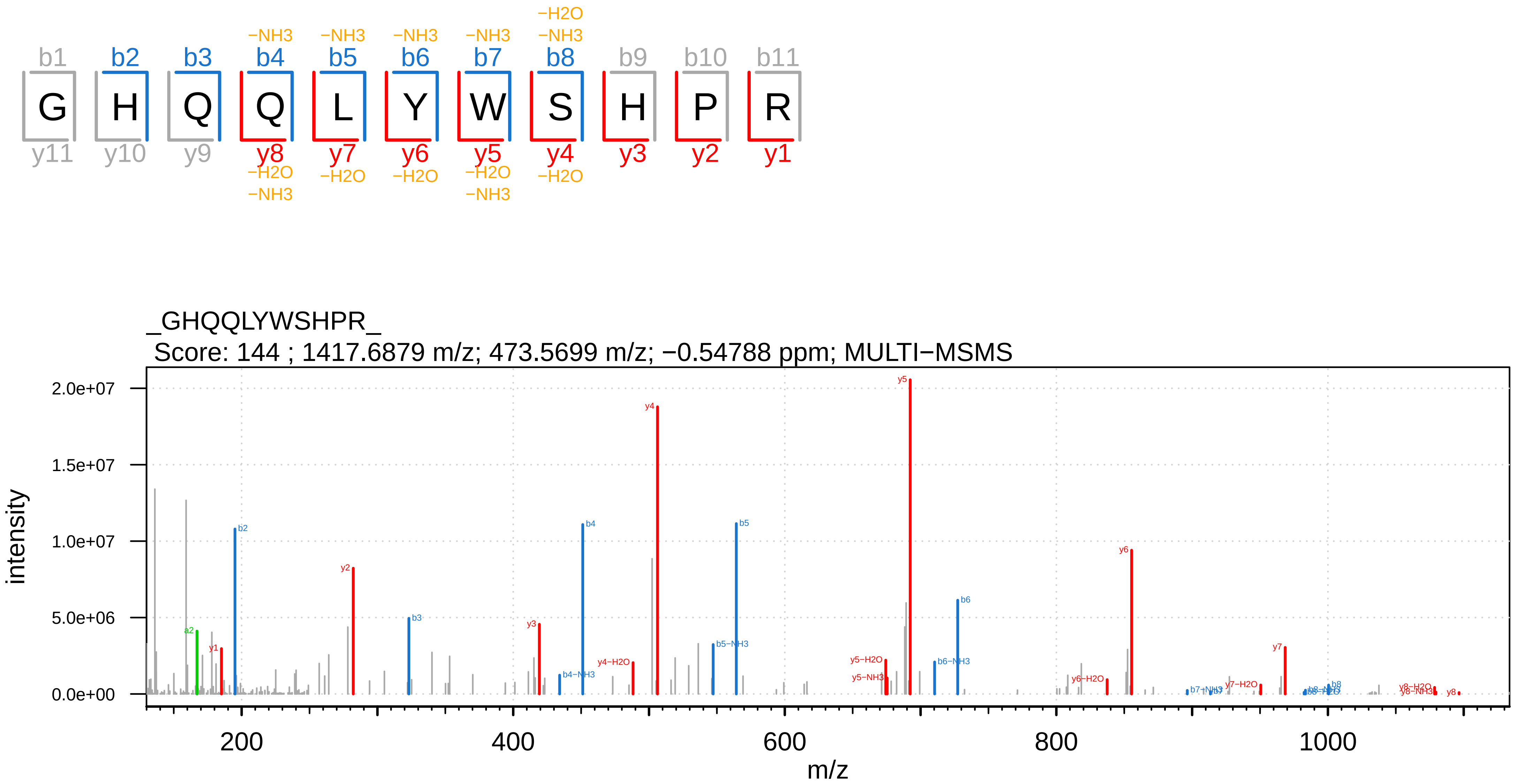

Raw File: Kermit_20111118_MSU_diff2_11
Scan Number: 4693
Proteins:
sp|P62274|RS29_MOUSE
ENSMUST00000037023_chr12:69157813−69159058:−
ENSMUST00000071737_chr13:73988963−73989133:−
tr|D3YYL7|D3YYL7_MOUSE

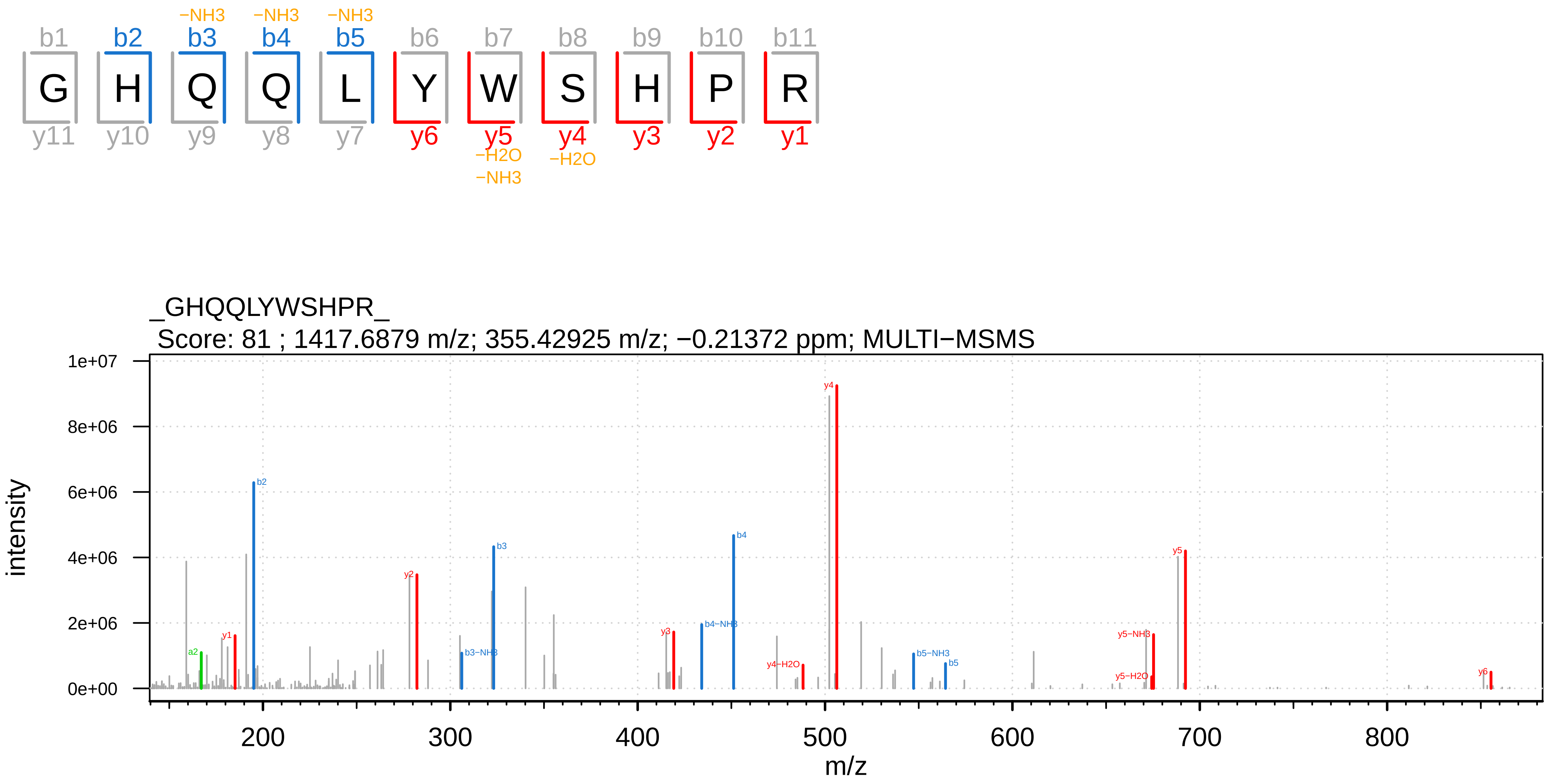

Raw File: Kermit_20111118_MSU_diff2_11
Scan Number: 4712
Proteins:
sp|P62274|RS29_MOUSE
ENSMUST00000037023_chr12:69157813−69159058:−
ENSMUST00000071737_chr13:73988963−73989133:−
tr|D3YYL7|D3YYL7_MOUSE

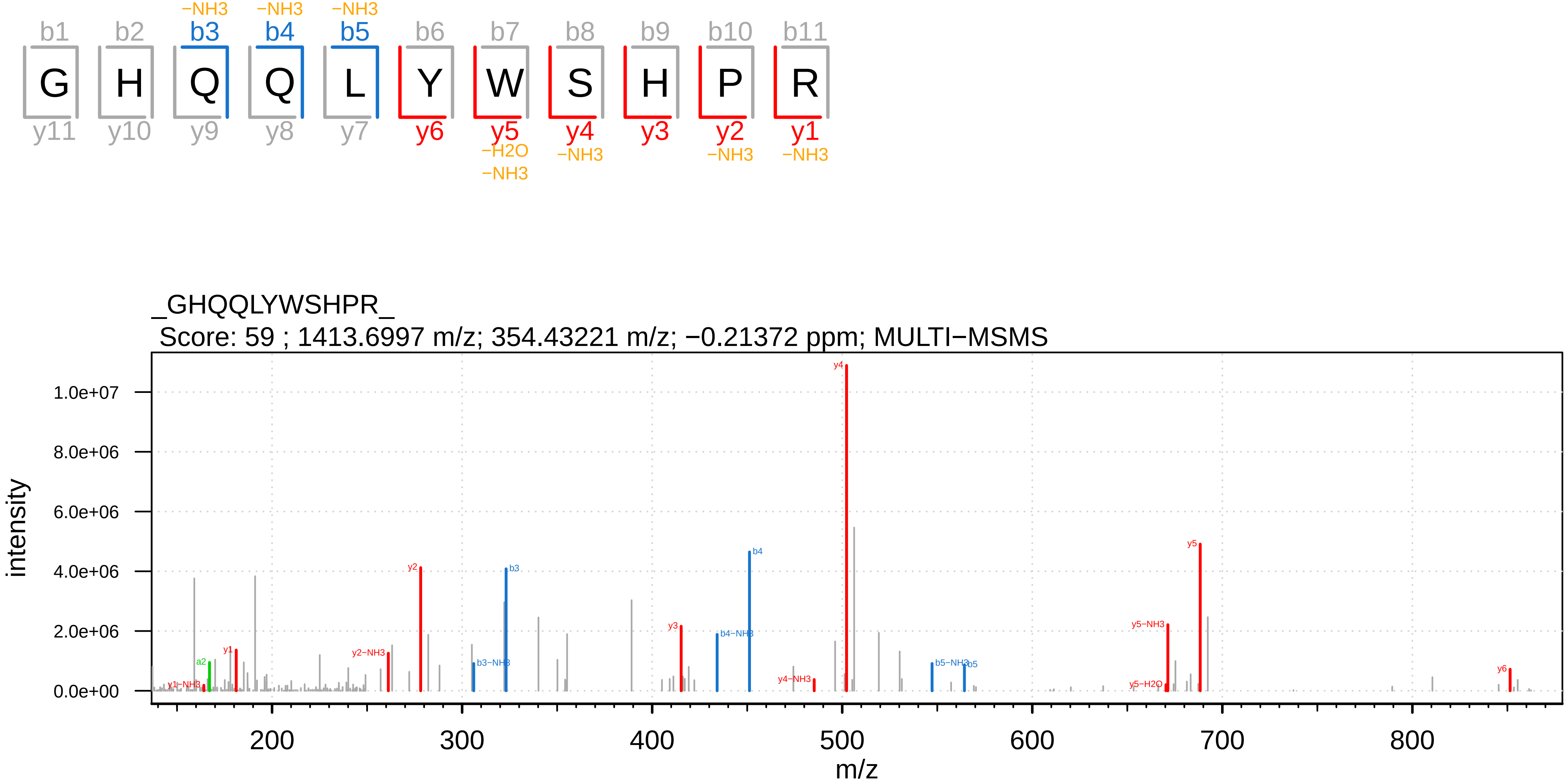

Raw File: Kermit_20111118_MSU_diff2_11
Scan Number: 4717
Proteins:
sp|P62274|RS29_MOUSE
ENSMUST00000037023_chr12:69157813−69159058:−
ENSMUST00000071737_chr13:73988963−73989133:−
tr|D3YYL7|D3YYL7_MOUSE

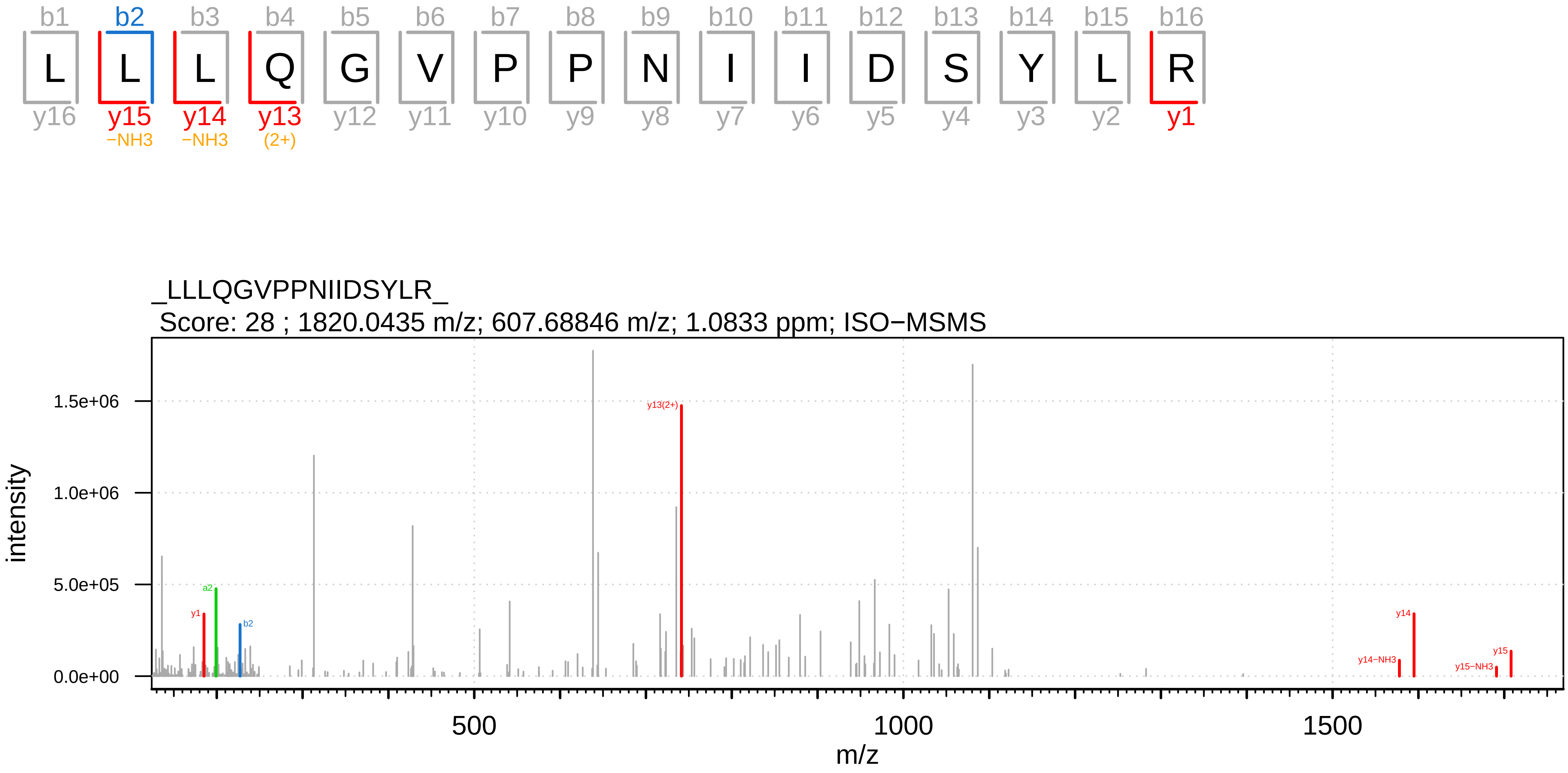

Raw File: Kermit_20111115_MSU_diff2_09
Scan Number: 27571
Proteins:
ENSMUST00000121629_chr10:88518469−88519919:−

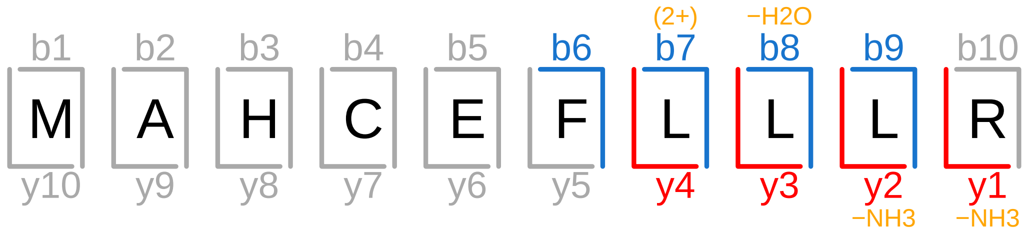

(ac)M(ox)AHCEFLLLR
Score: 44 ; 1346.6475 m/z; 674.33103 m/z; −1.2021 ppm; ISO−MSMS

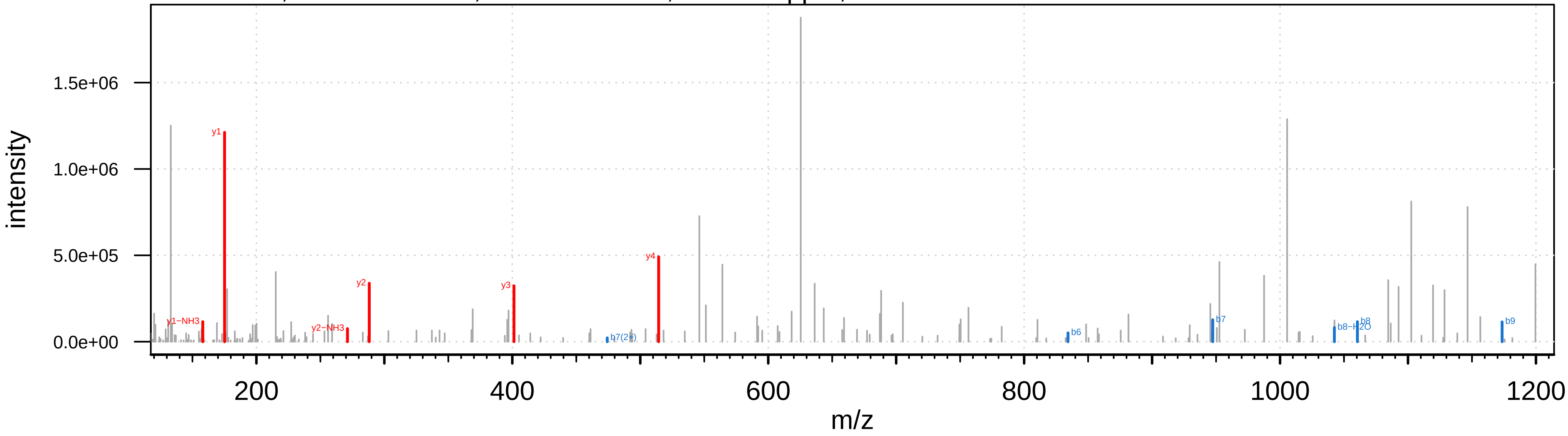

Raw File: Kermit_20111104_MSU_blast_1h_06
Scan Number: 84613
Proteins:
ENSMUST00000170323_chr16:4987743−4987790:−

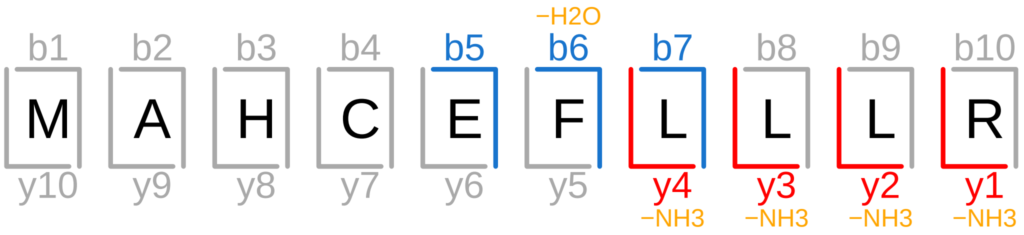

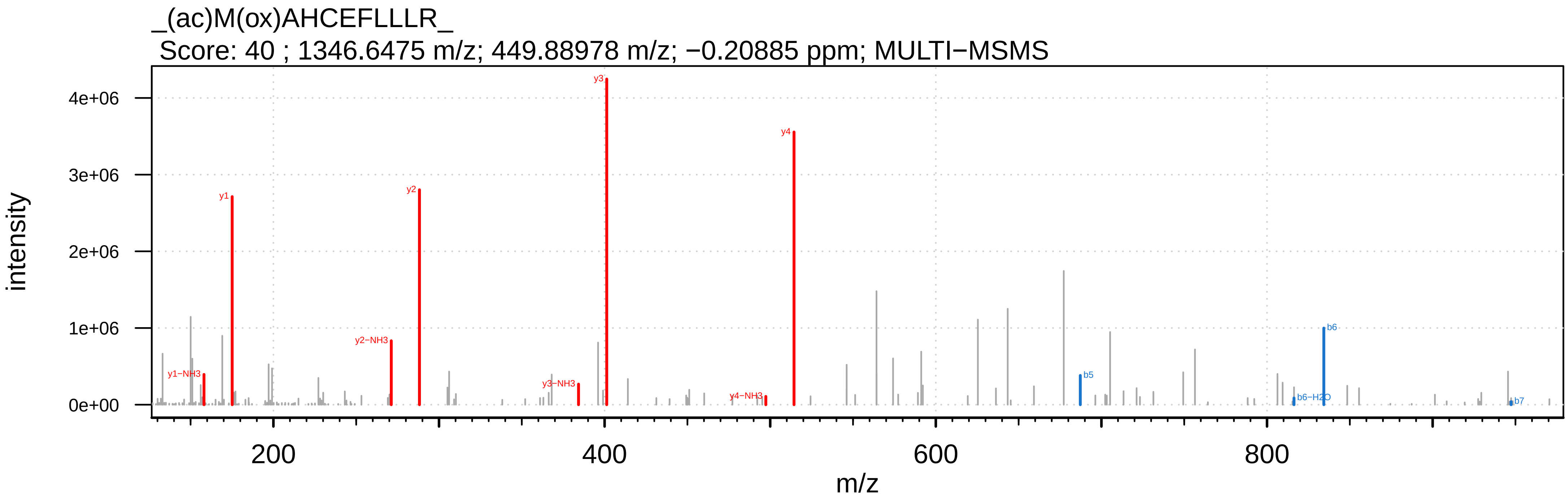

Raw File: Kermit_20111104_MSU_tube_18h_06
Scan Number: 89013
Proteins:
ENSMUST00000170323_chr16:4987743−4987790:−

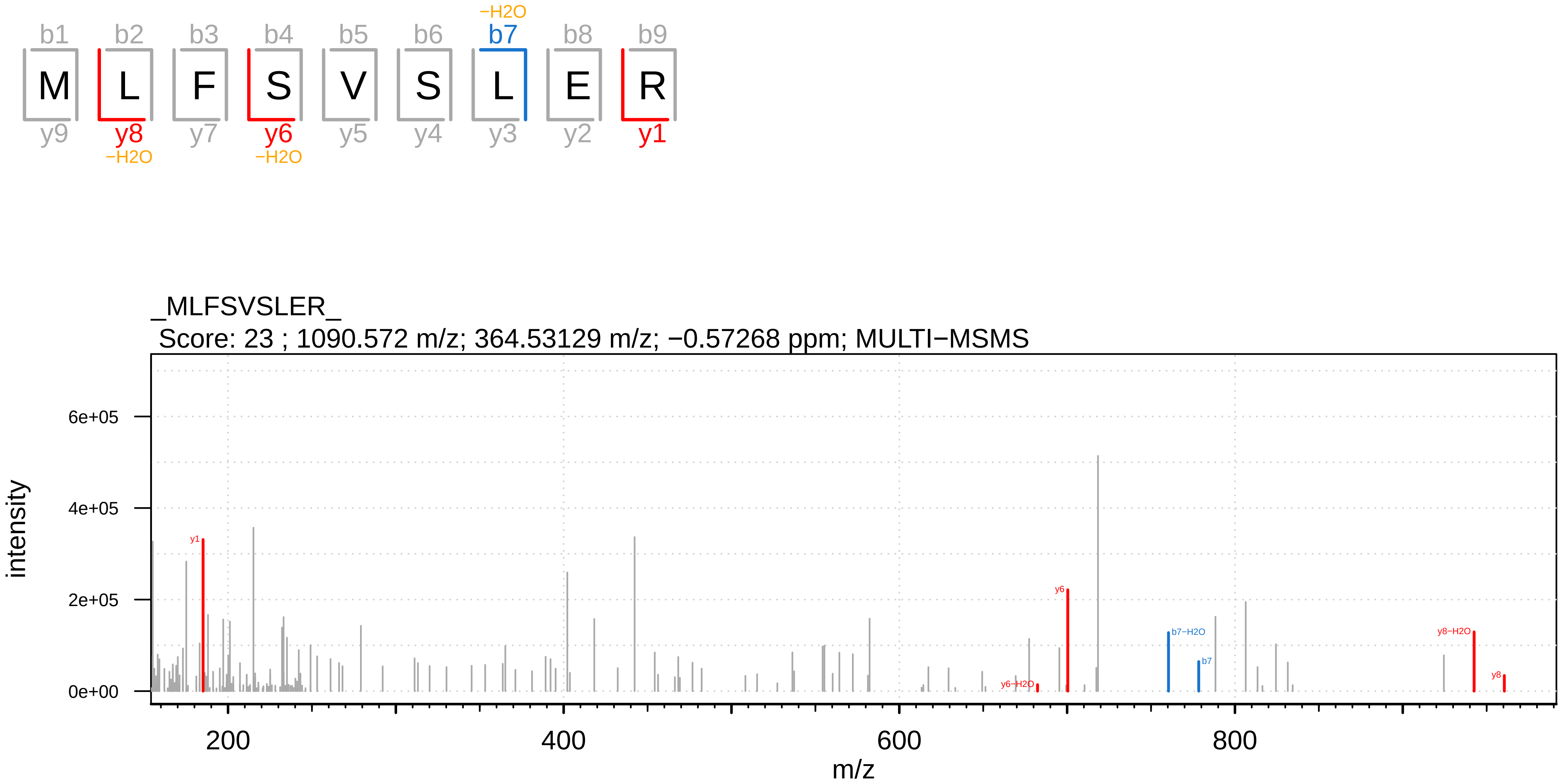

Raw File: Kermit_20111028_MSU_tube_54h_06
Scan Number: 8854
Proteins:
ENSMUST00000180503_chr12:105337548−105381530:+

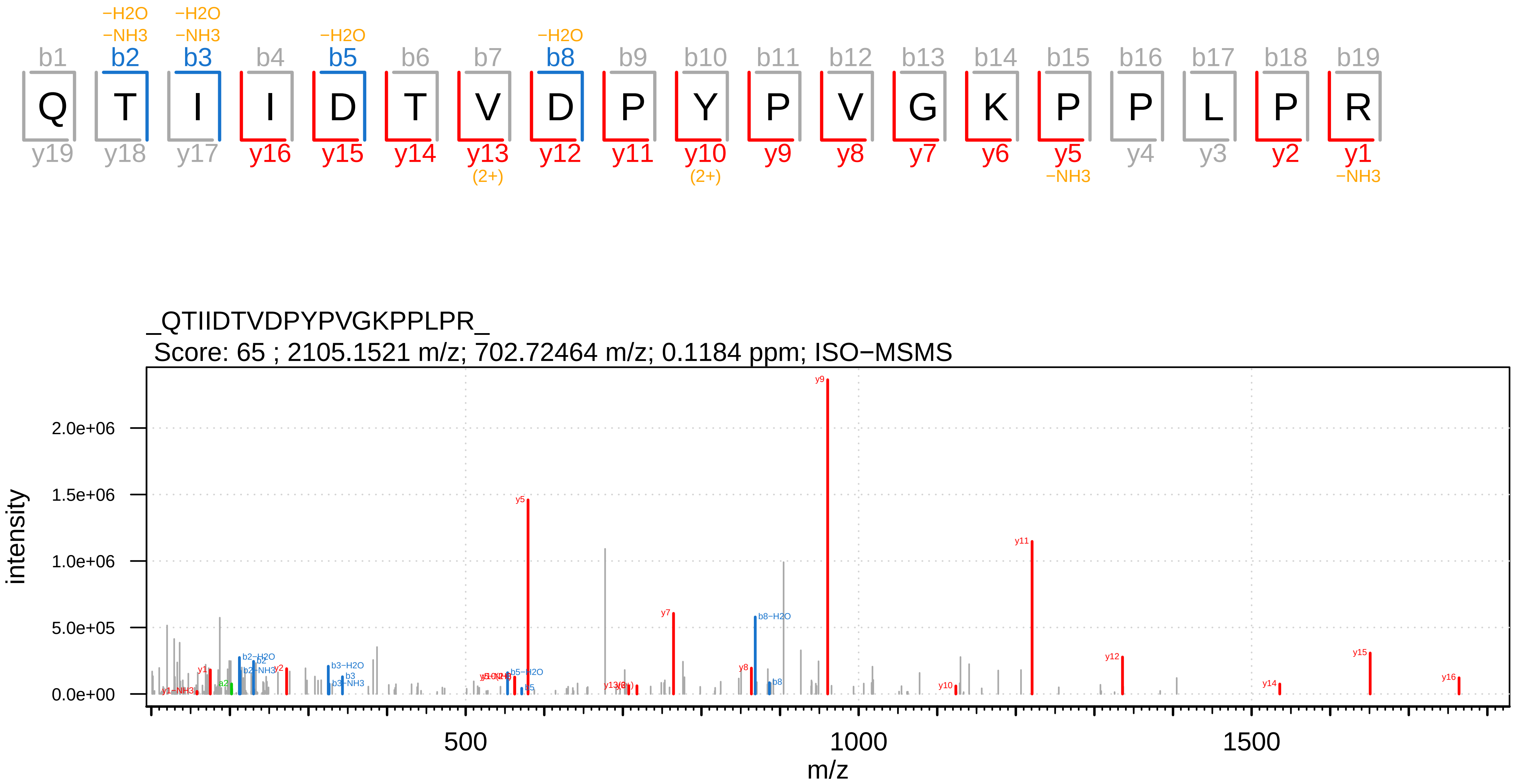

Raw File: Kermit_20111028_MSU_tube_6h_02
Scan Number: 56047
Proteins:
ENSMUST00000136126_chr3:79172848−79174982:−

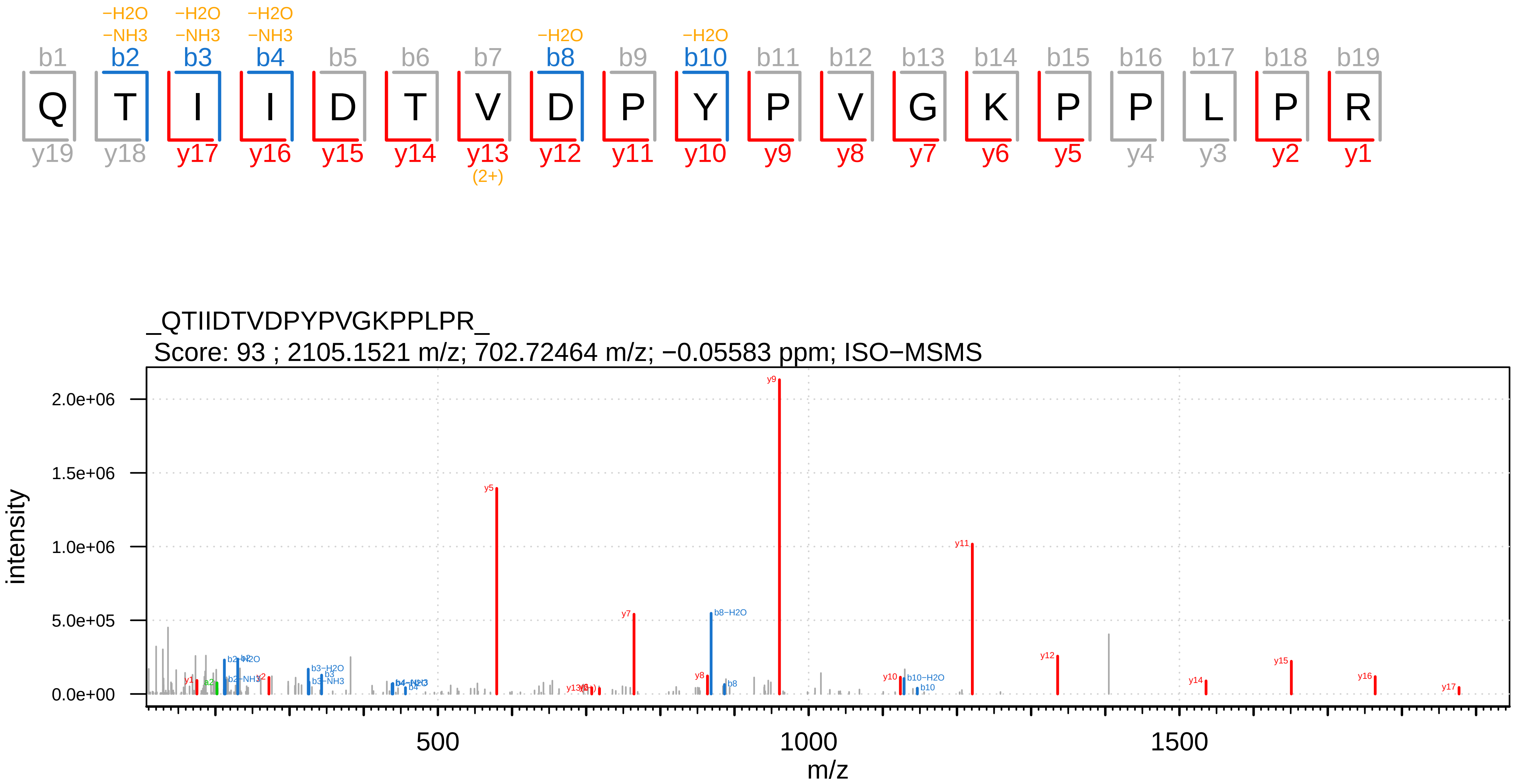

Raw File: Kermit_20111104_MSU_blast_1h_02
Scan Number: 63221
Proteins:
ENSMUST00000136126_chr3:79172848−79174982:−

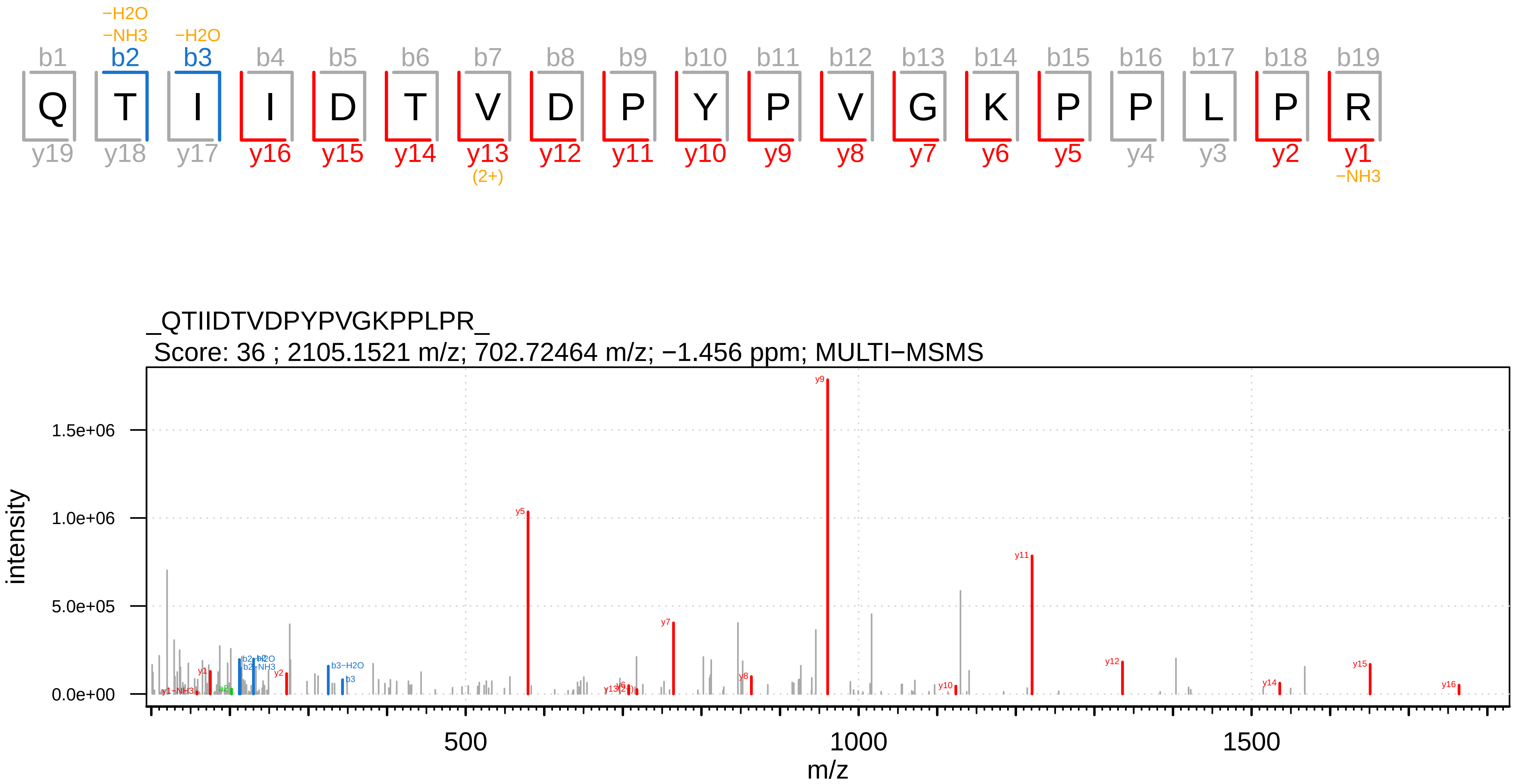

Raw File: Kermit_20111104_MSU_tube_18h_02
Scan Number: 62515
Proteins:
ENSMUST00000136126_chr3:79172848−79174982:−

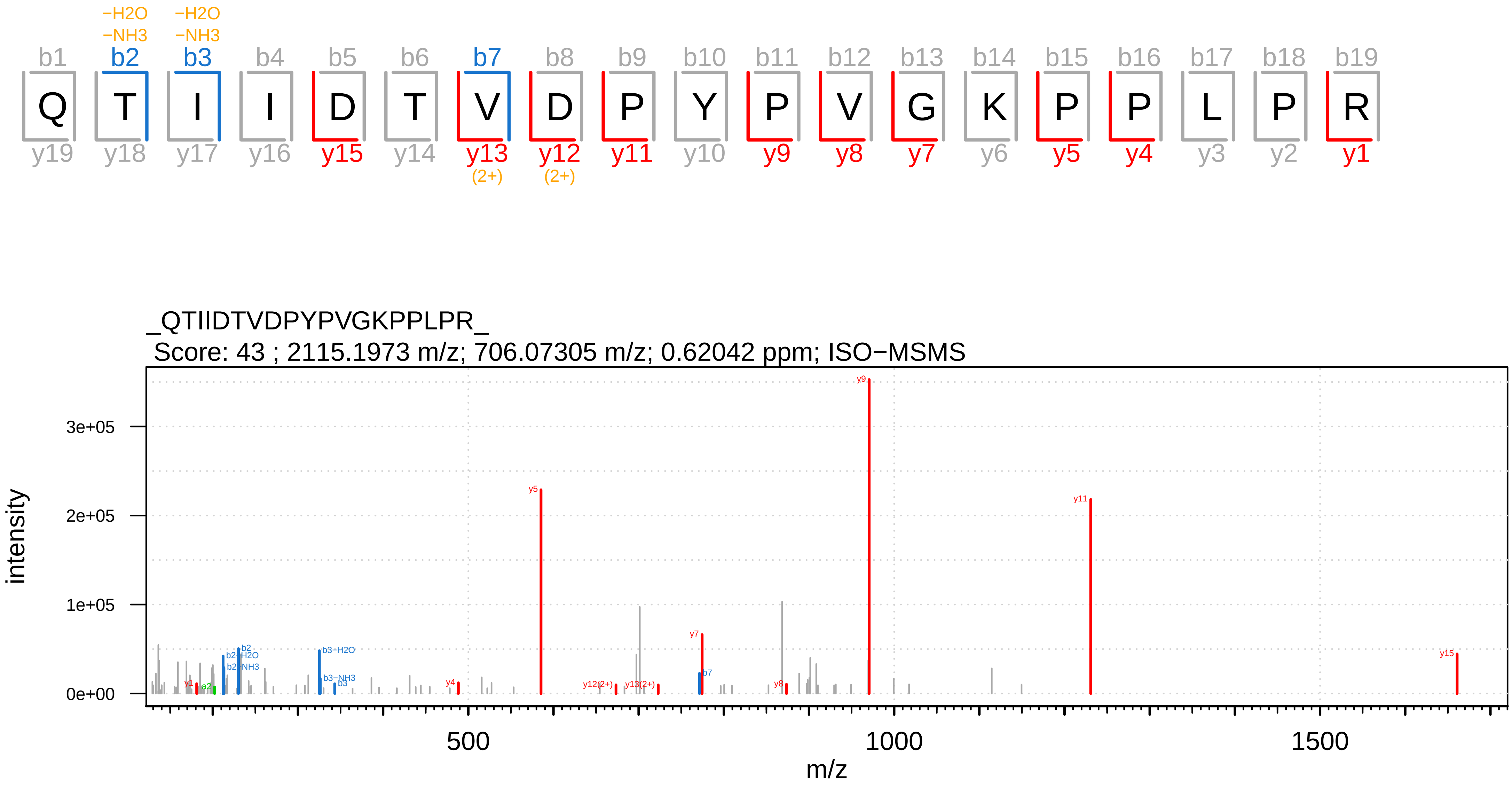

Raw File: Kermit_20111110_MSU_diff1_03
Scan Number: 49069
Proteins:
ENSMUST00000136126_chr3:79172848−79174982:−

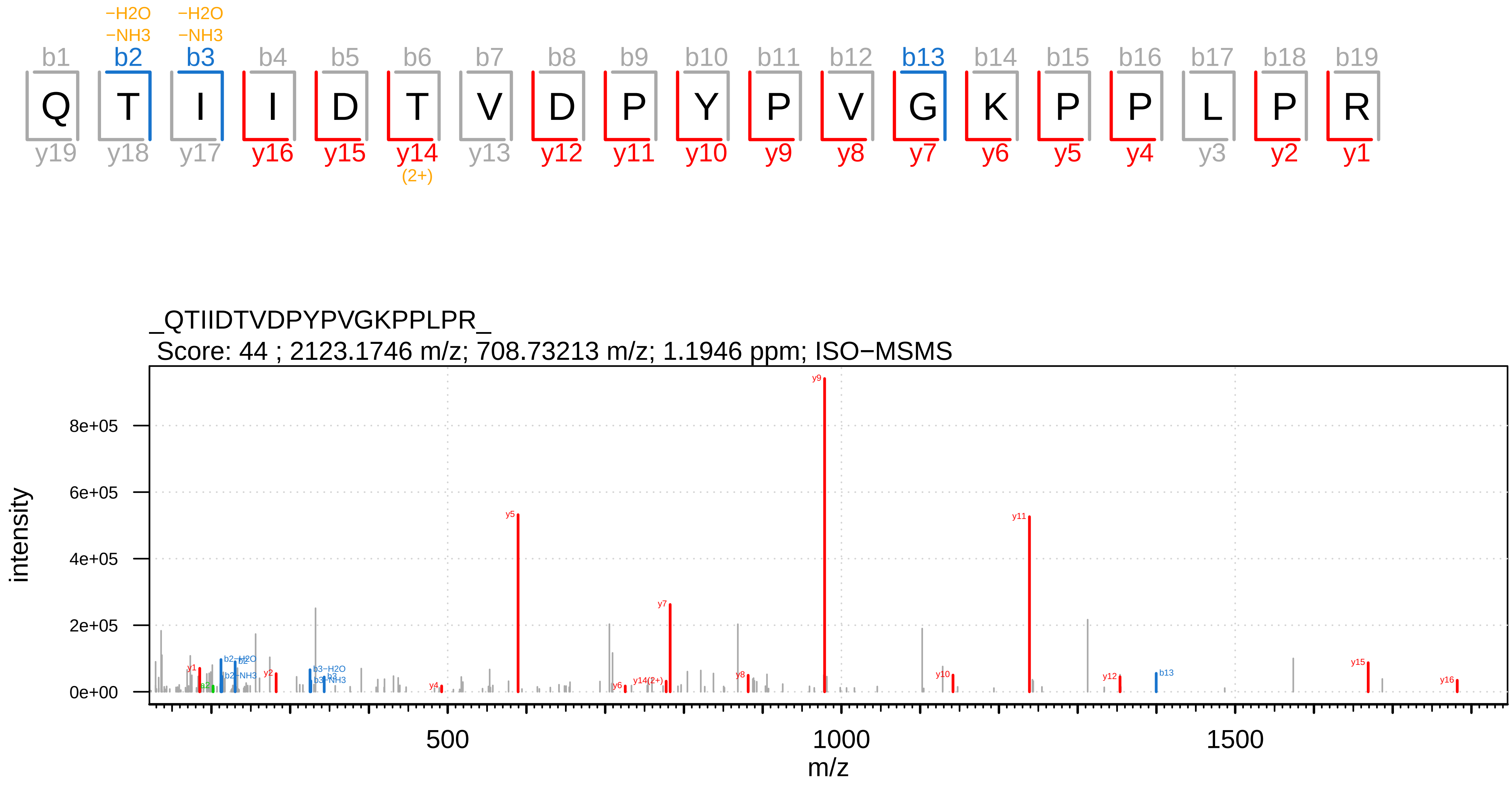

Raw File: Kermit_20111118_MSU_blast_9h_02
Scan Number: 53454
Proteins:
ENSMUST00000136126_chr3:79172848−79174982:−

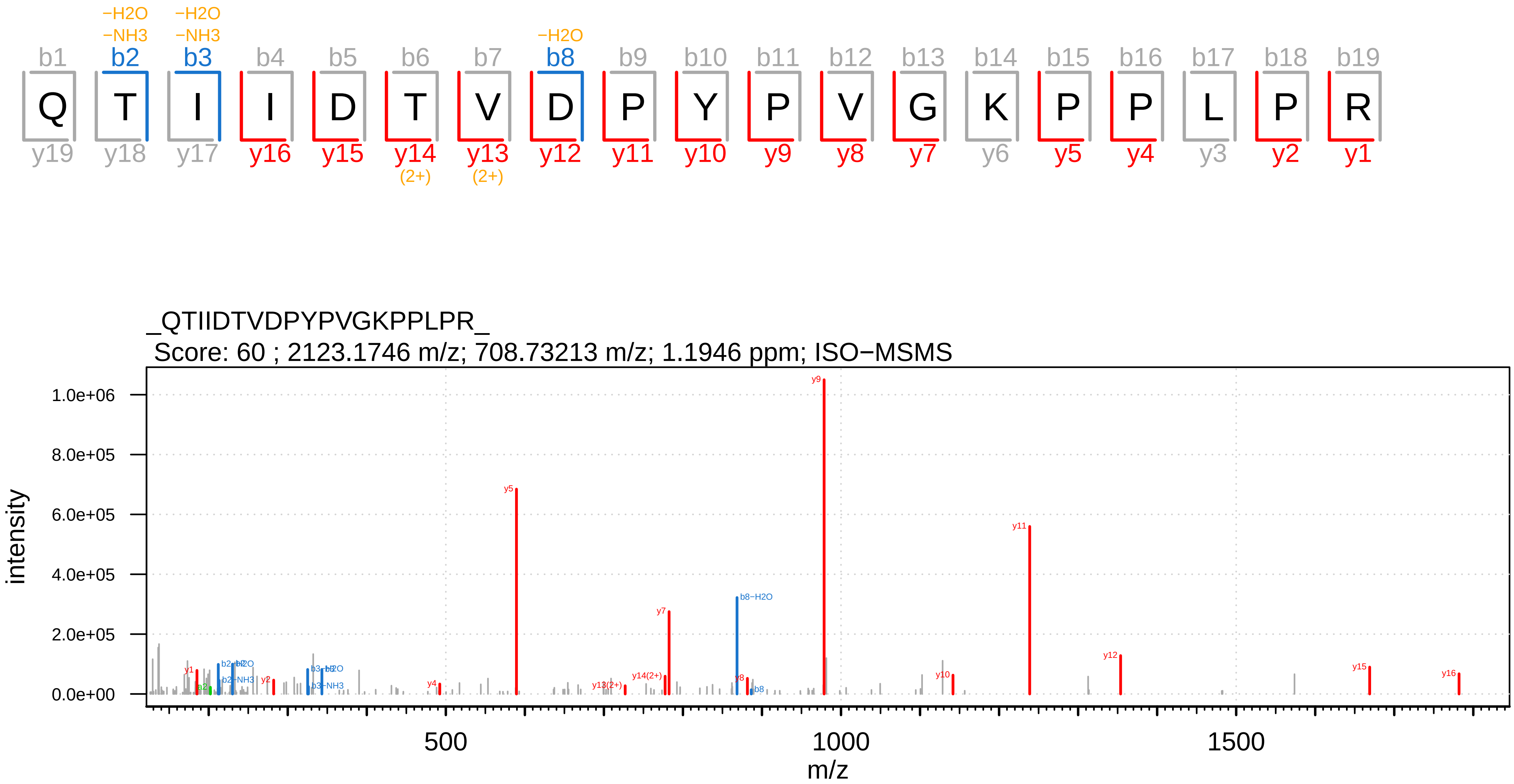

Raw File: Kermit_20111118_MSU_blast_9h_02
Scan Number: 53696
Proteins:
ENSMUST00000136126_chr3:79172848−79174982:−

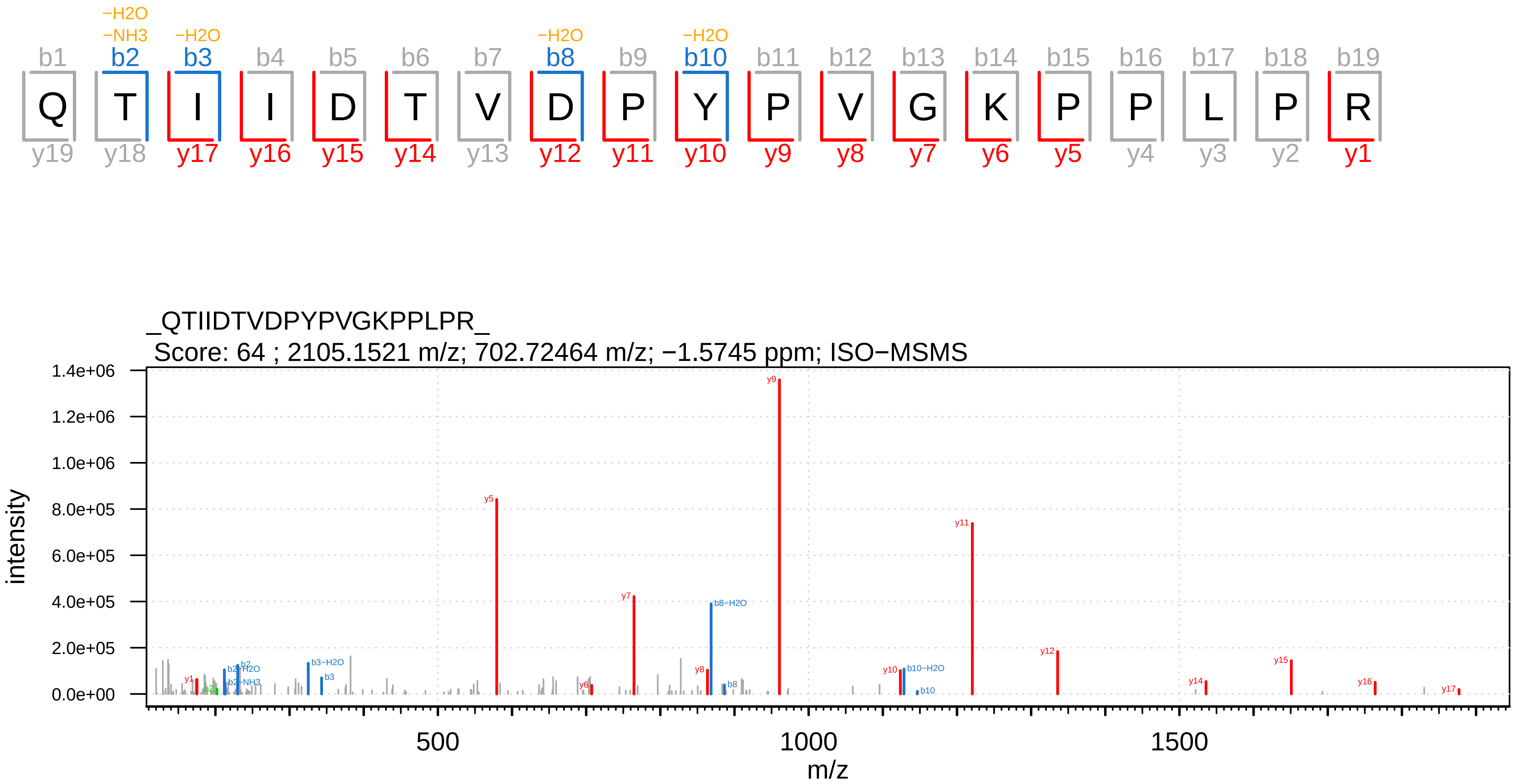

Raw File: Kermit_20111118_MSU_blast_9h_02
Scan Number: 53607
Proteins:
ENSMUST00000136126_chr3:79172848−79174982:−

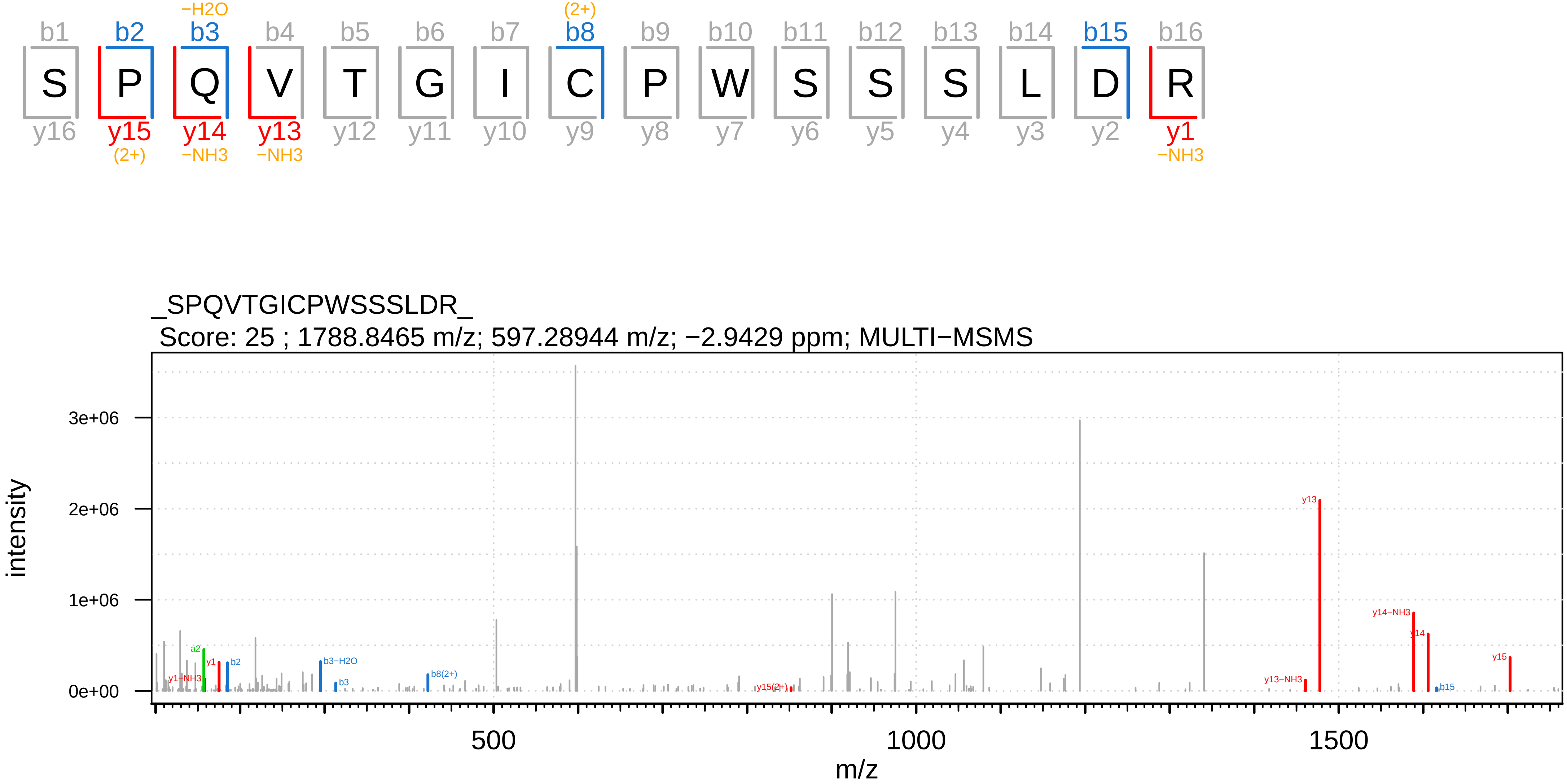

Raw File: Kermit_20111104_MSU_tube_18h_05
Scan Number: 4433
Proteins:
ENSMUST00000152360_chr5:148926733−148926927:−

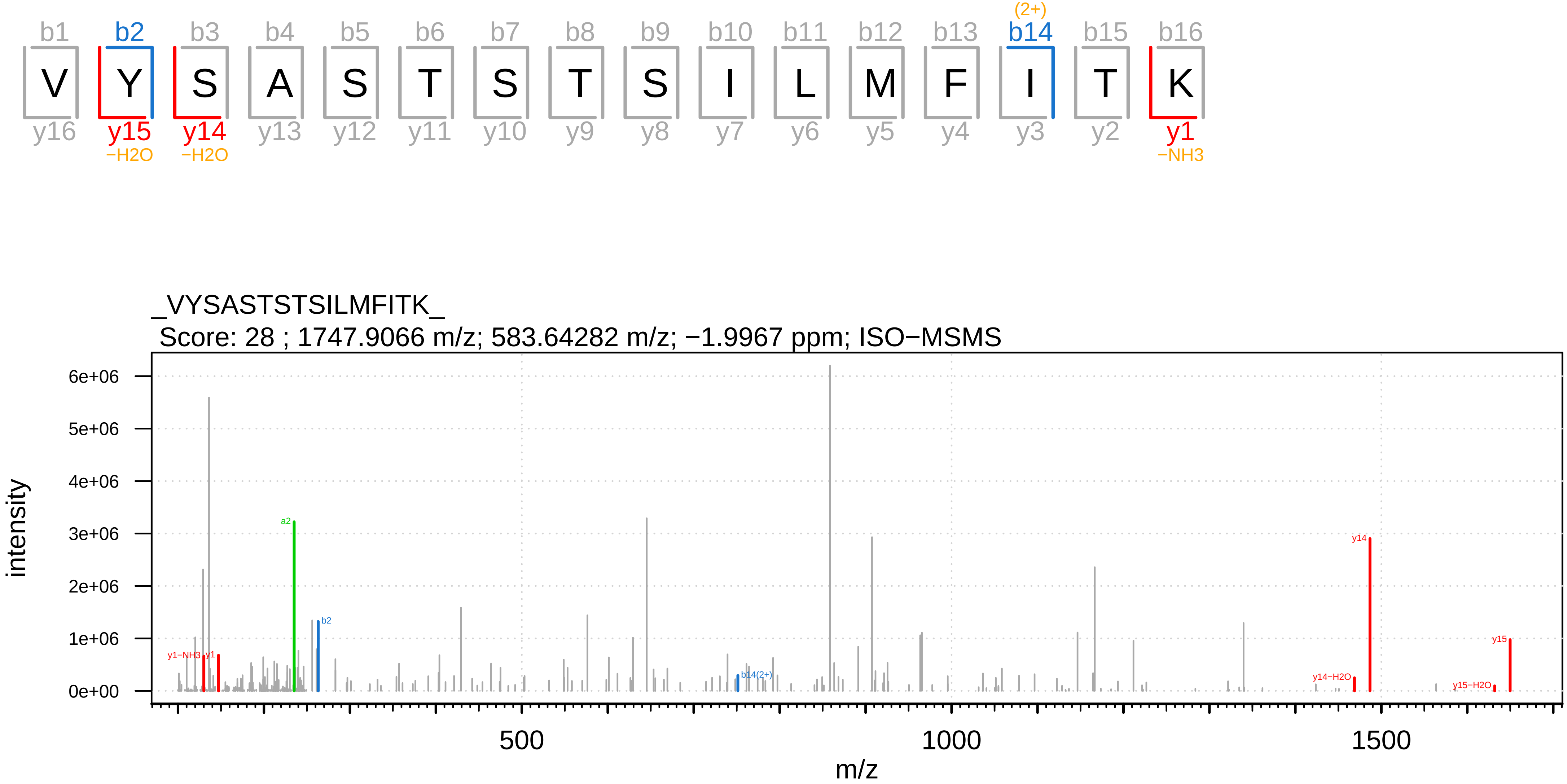

Raw File: Kermit_20111104_MSU_tube_18h_09
Scan Number: 11264
Proteins:
ENSMUST00000129513_chr1:37933668−37948683:+

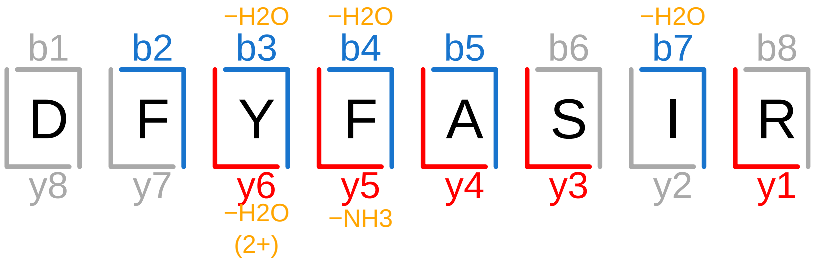

DFYFASIR
Score: 100 ; 1017.492 m/z; 509.75327 m/z; −0.067293 ppm; ISO−MSMS

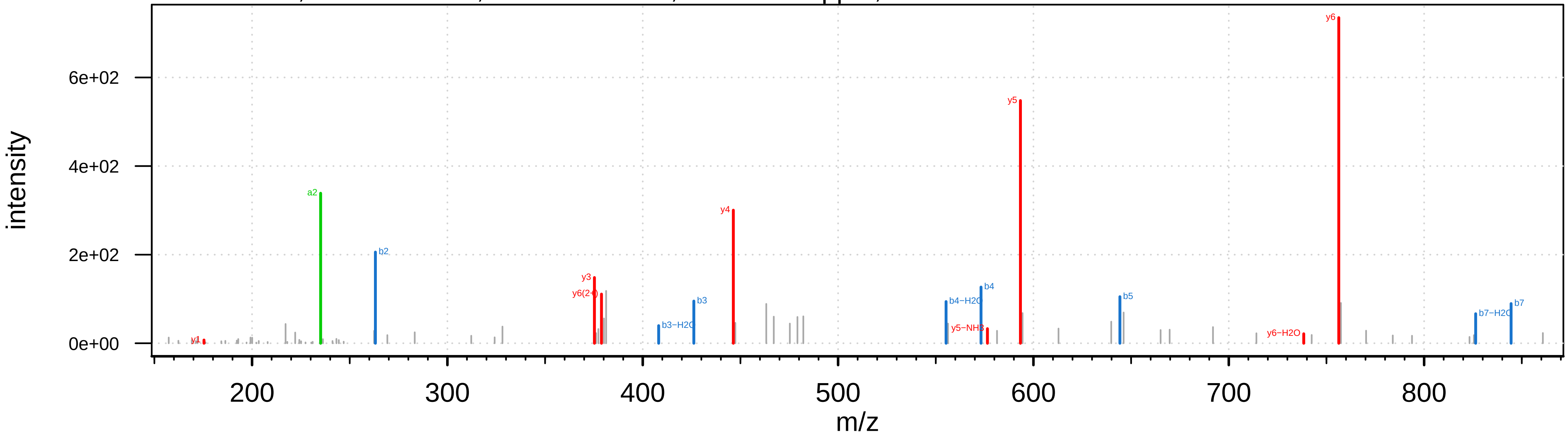

Raw File: Ernie_20090324_BJS_halflives_P1_slice01_01
Scan Number: 9632
Proteins:
ENSMUST00000023048_chr15:80235835−80236047:+

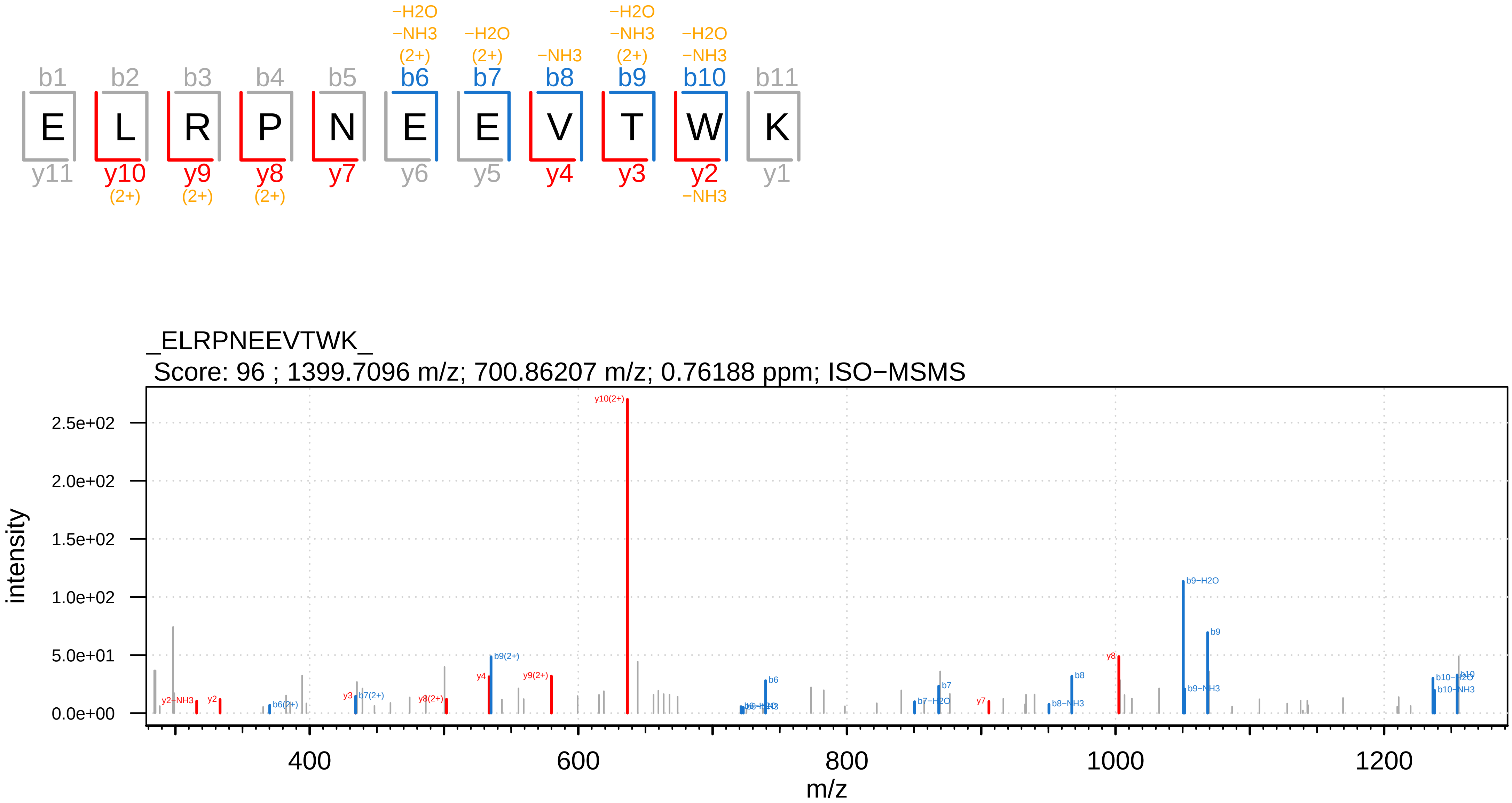

Raw File: Ernie_20090324_BJS_halflives_P1_slice01_01
Scan Number: 5372
Proteins:
chr12_103206313−103214181.T2_chr12:101968174−101969629:−

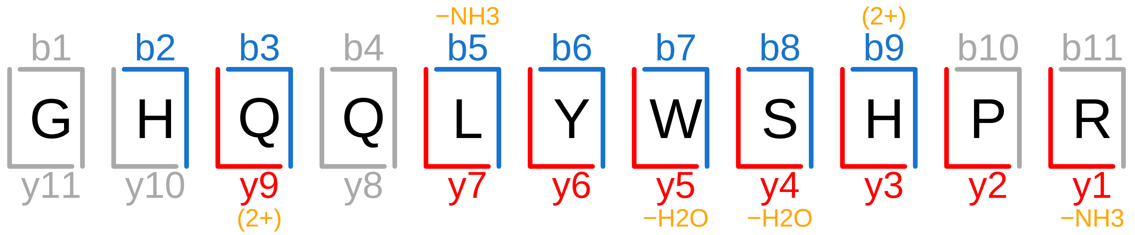

GHQQLYWSHPR
Score: 74 ; 1407.6796 m/z; 470.23382 m/z; −0.58878 ppm; MULTI−MSMS

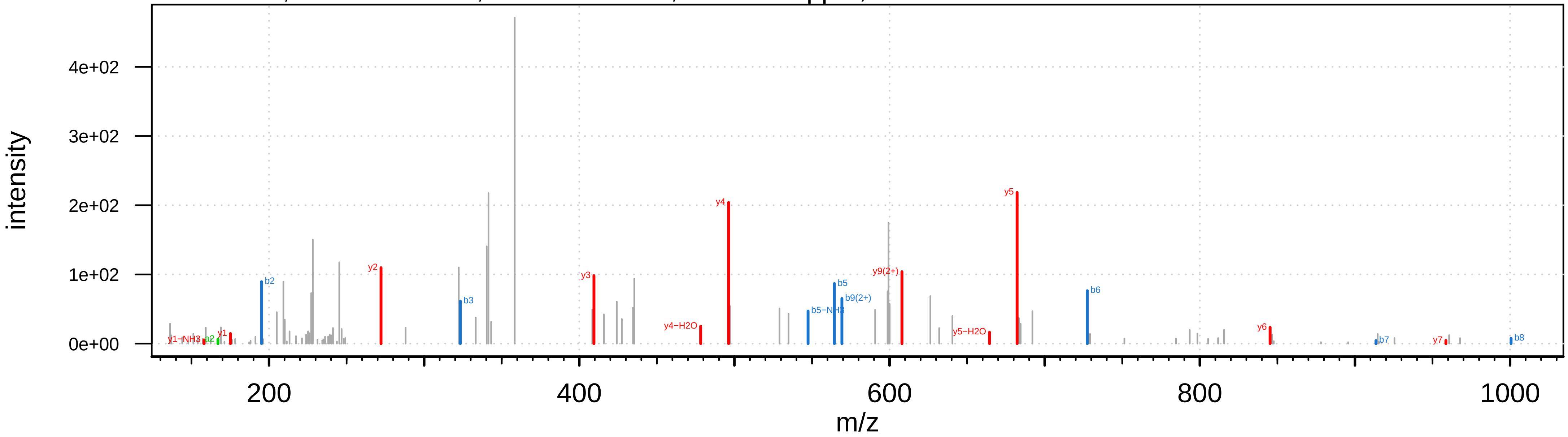

Raw File: Ernie_20090324_BJS_halflives_P1_slice01_01
Scan Number: 4300
Proteins:
sp|P62274|RS29_MOUSE
ENSMUST00000037023_chr12:69157813−69159058:−
ENSMUST00000071737_chr13:73988963−73989133:−
tr|D3YYL7|D3YYL7_MOUSE

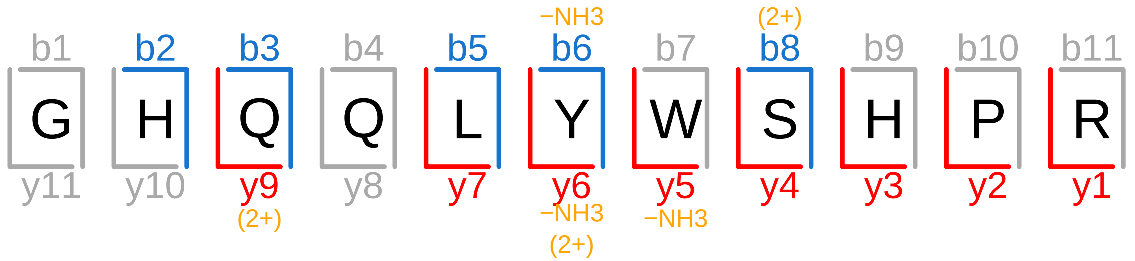

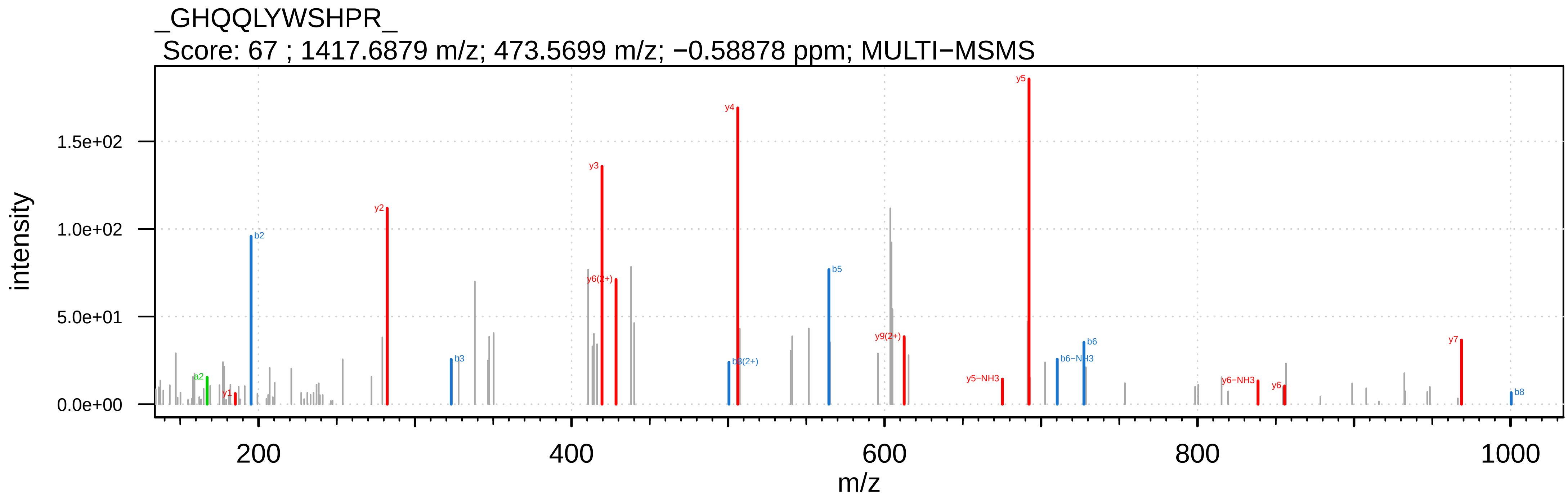

Raw File: Ernie_20090324_BJS_halflives_P1_slice01_01
Scan Number: 4323
Proteins:
sp|P62274|RS29_MOUSE
ENSMUST00000037023_chr12:69157813−69159058:−
ENSMUST00000071737_chr13:73988963−73989133:−
tr|D3YYL7|D3YYL7_MOUSE

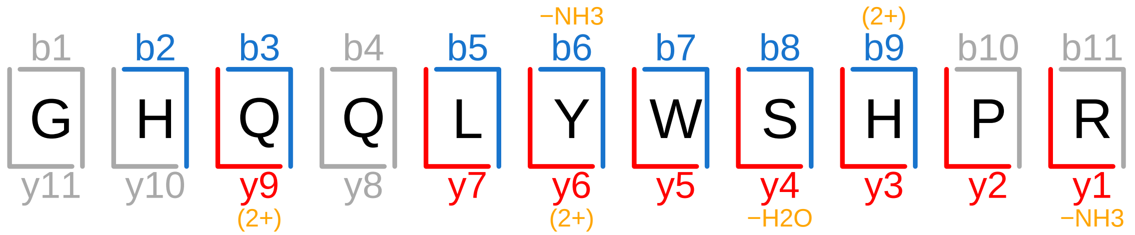

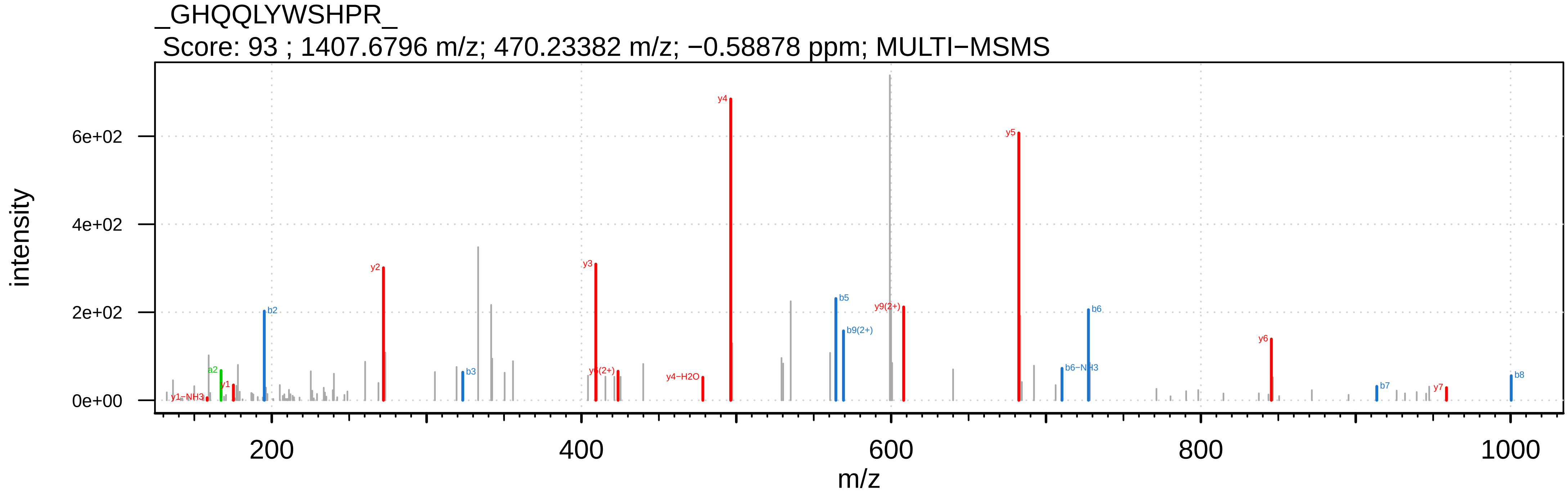

Raw File: Ernie_20090324_BJS_halflives_P1_slice01_01
Scan Number: 4424
Proteins:
sp|P62274|RS29_MOUSE
ENSMUST00000037023_chr12:69157813−69159058:−
ENSMUST00000071737_chr13:73988963−73989133:−
tr|D3YYL7|D3YYL7_MOUSE

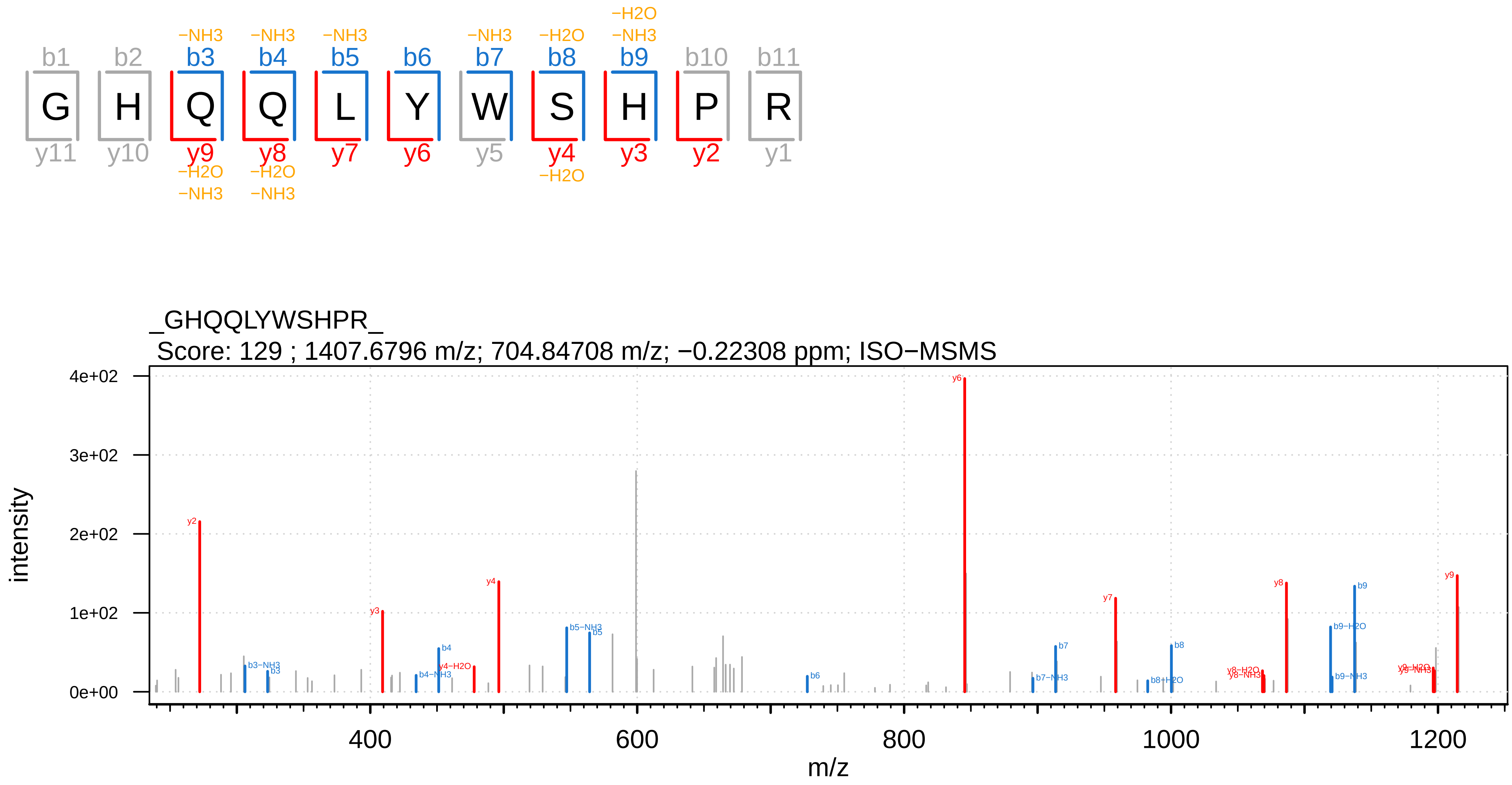

Raw File: Ernie_20090324_BJS_halflives_P1_slice01_01
Scan Number: 4317
Proteins:
sp|P62274|RS29_MOUSE
ENSMUST00000037023_chr12:69157813−69159058:−
ENSMUST00000071737_chr13:73988963−73989133:−
tr|D3YYL7|D3YYL7_MOUSE

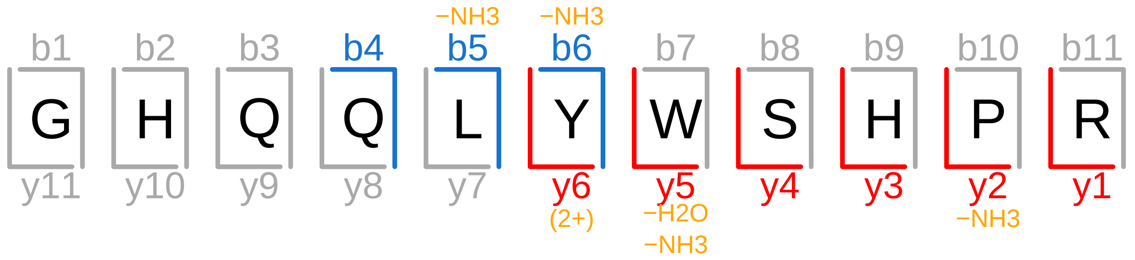

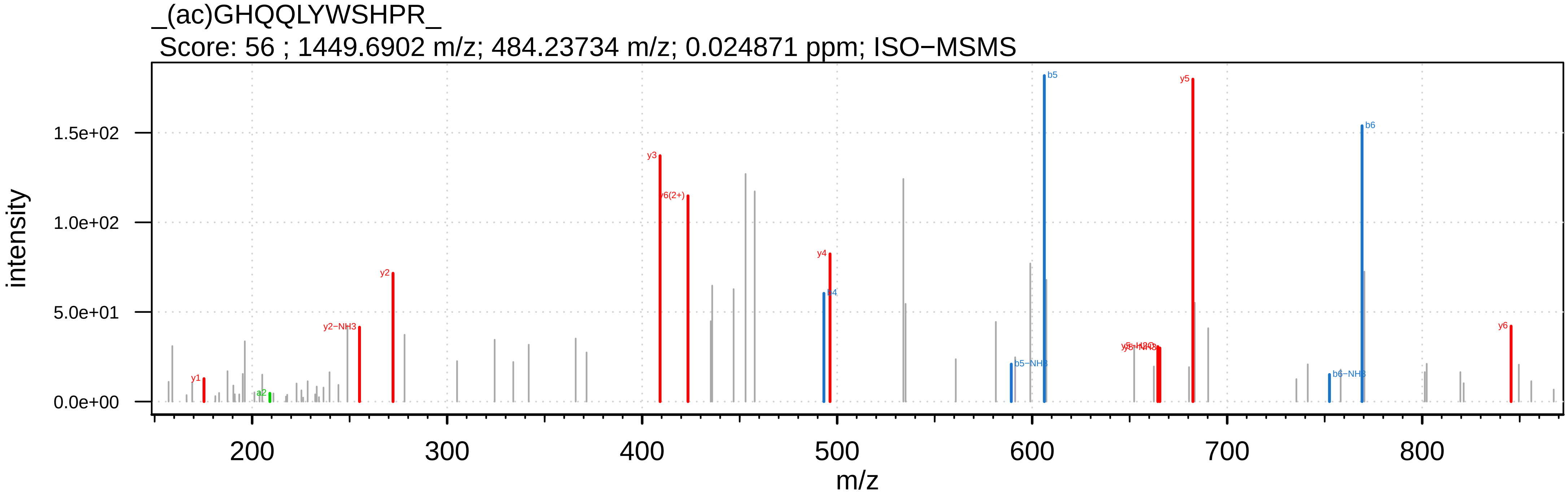

Raw File: Ernie_20090324_BJS_halflives_P1_slice01_01
Scan Number: 5286
Proteins:
sp|P62274|RS29_MOUSE
ENSMUST00000037023_chr12:69157813−69159058:−
ENSMUST00000071737_chr13:73988963−73989133:−
tr|D3YYL7|D3YYL7_MOUSE

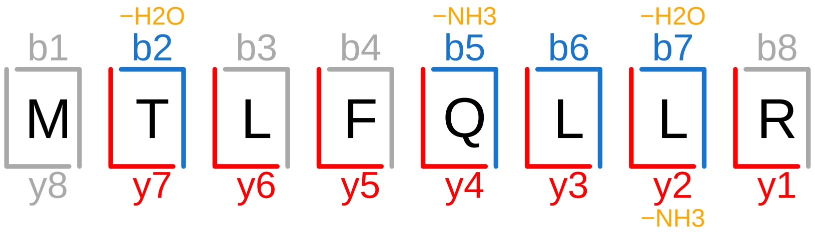

MTLFQLLR
Score: 110 ; 1020.579 m/z; 511.29679 m/z; −0.14164 ppm; MULTI−MSMS

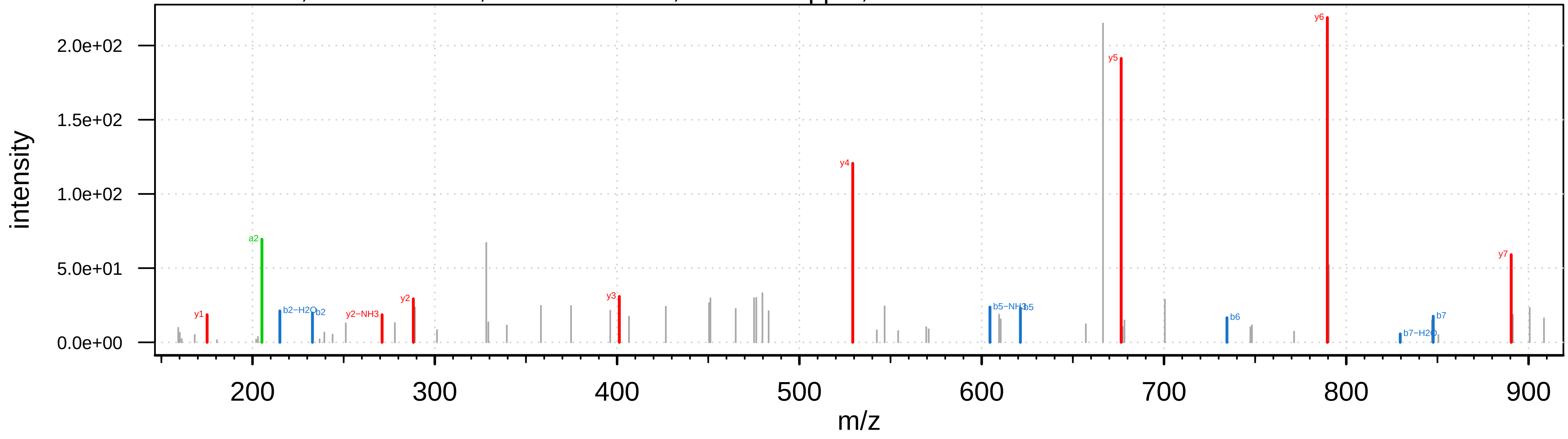

Raw File: Ernie_20090324_BJS_halflives_P1_slice01_01
Scan Number: 12601
Proteins:
chr12_103206313−103214181.T2_chr12:101968174−101969629:−

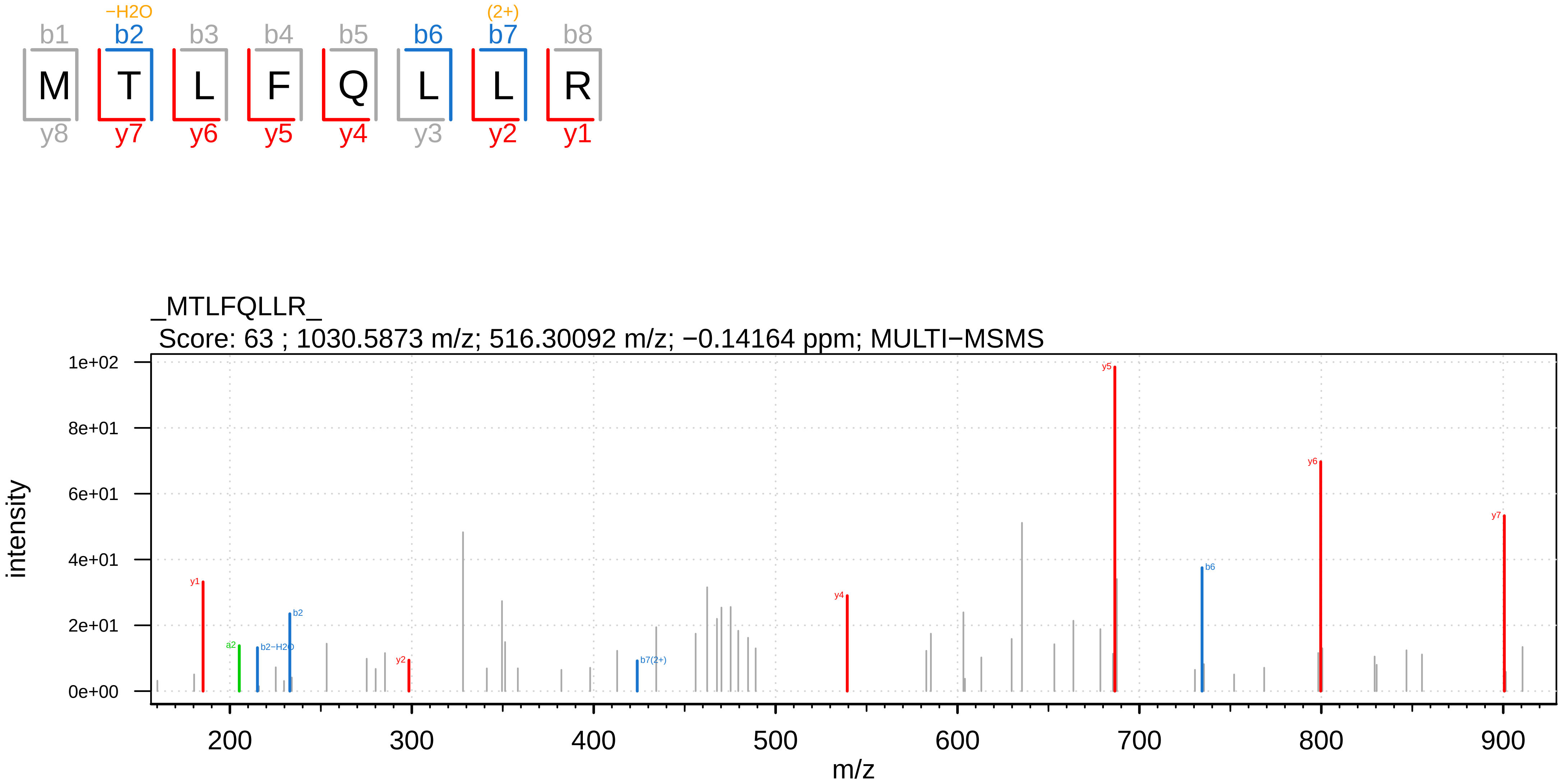

Raw File: Ernie_20090324_BJS_halflives_P1_slice01_01
Scan Number: 12617
Proteins:
chr12_103206313−103214181.T2_chr12:101968174−101969629:−

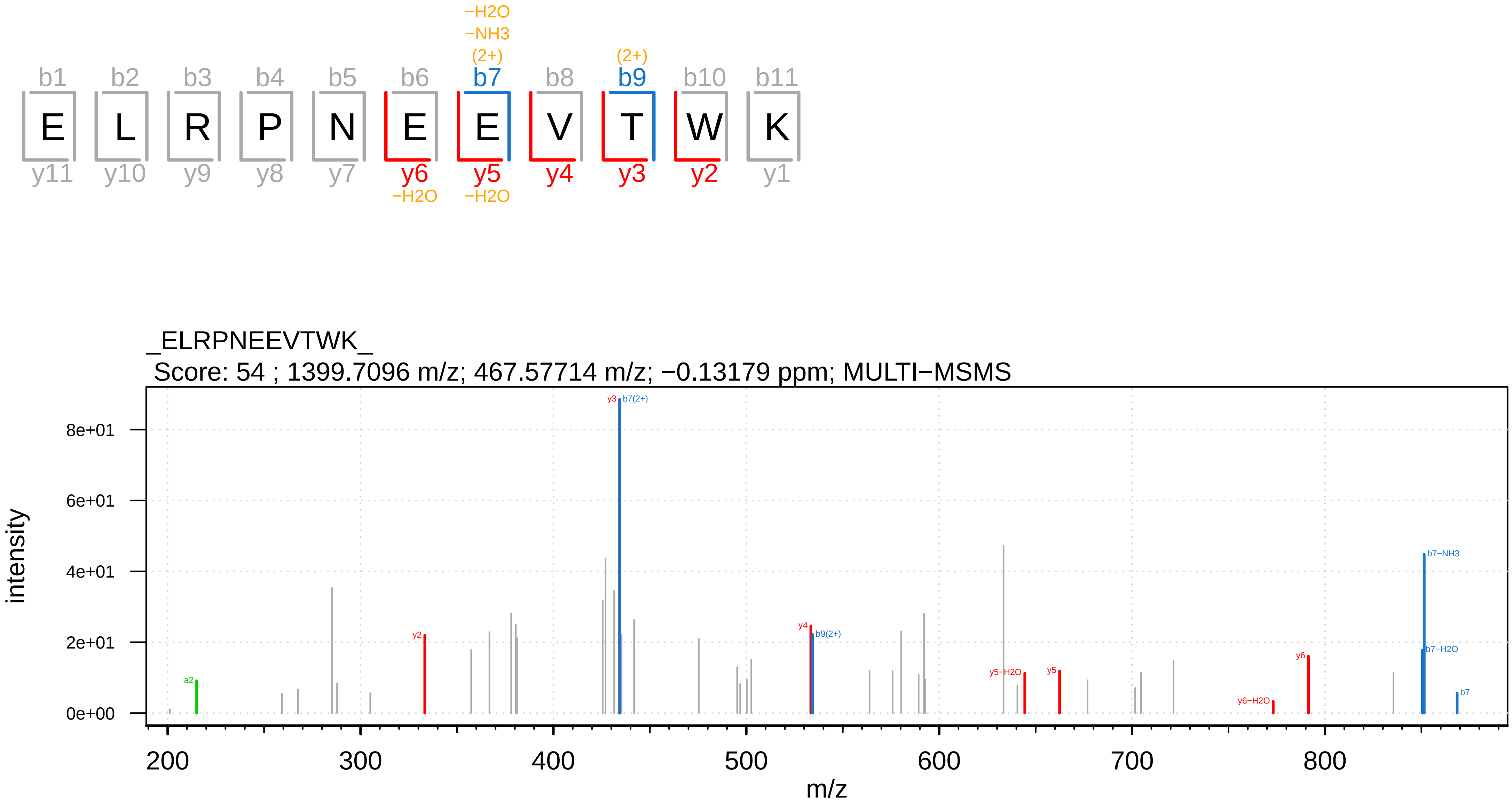

Raw File: Bert_20090403_BJS_halflives_P3_slice01_05
Scan Number: 3696
Proteins:
chr12_103206313−103214181.T2_chr12:101968174−101969629:−

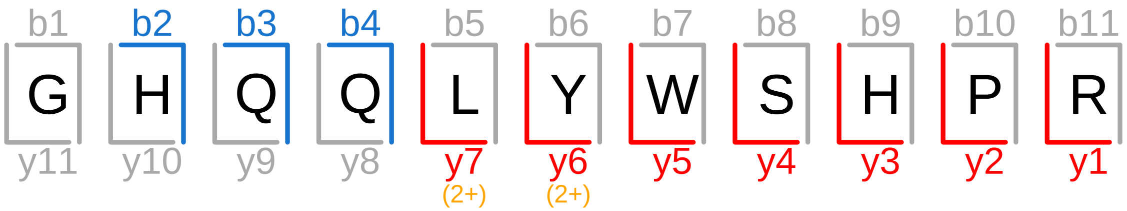

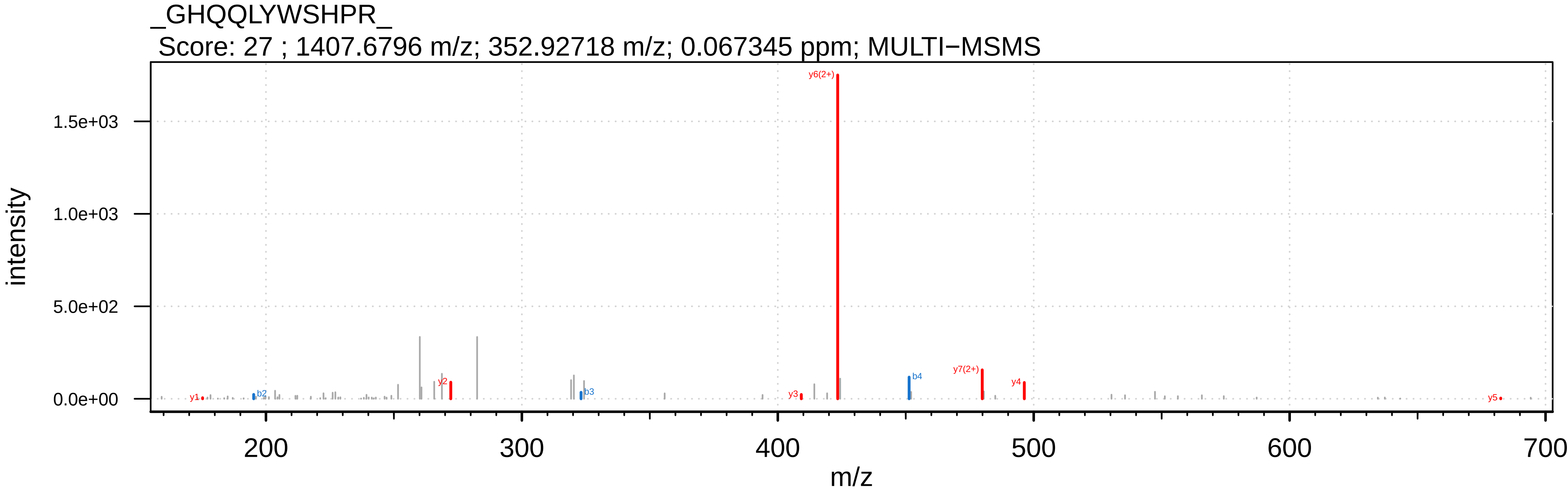

Raw File: Bert_20090403_BJS_halflives_P3_slice01_05
Scan Number: 2716
Proteins:
sp|P62274|RS29_MOUSE
ENSMUST00000037023_chr12:69157813−69159058:−
ENSMUST00000071737_chr13:73988963−73989133:−
tr|D3YYL7|D3YYL7_MOUSE

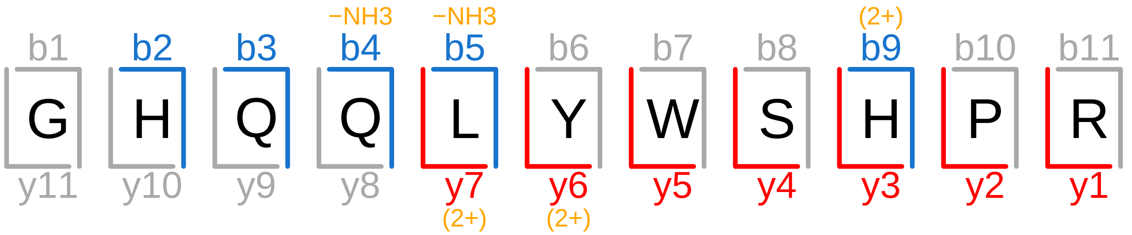

GHQQLYWSHPR
Score: 57 ; 1417.6879 m/z; 355.42925 m/z; 0.067345 ppm; MULTI−MSMS

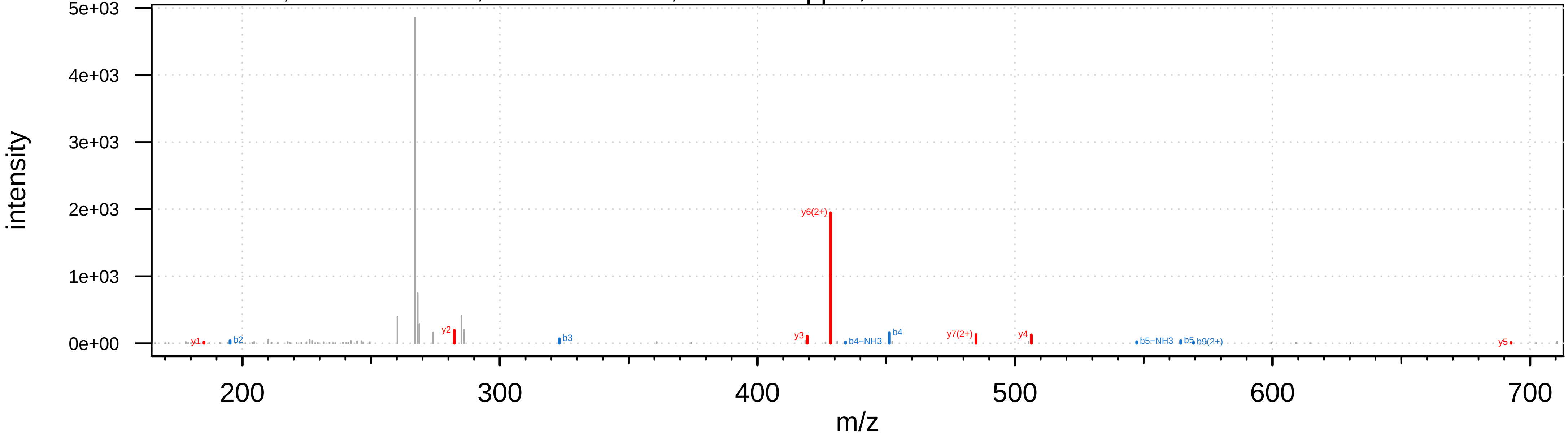

Raw File: Bert_20090403_BJS_halflives_P3_slice01_05
Scan Number: 2729
Proteins:
sp|P62274|RS29_MOUSE
ENSMUST00000037023_chr12:69157813−69159058:−
ENSMUST00000071737_chr13:73988963−73989133:−
tr|D3YYL7|D3YYL7_MOUSE

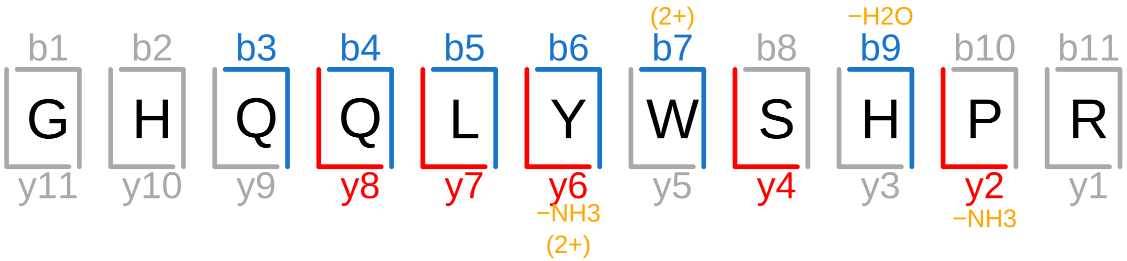

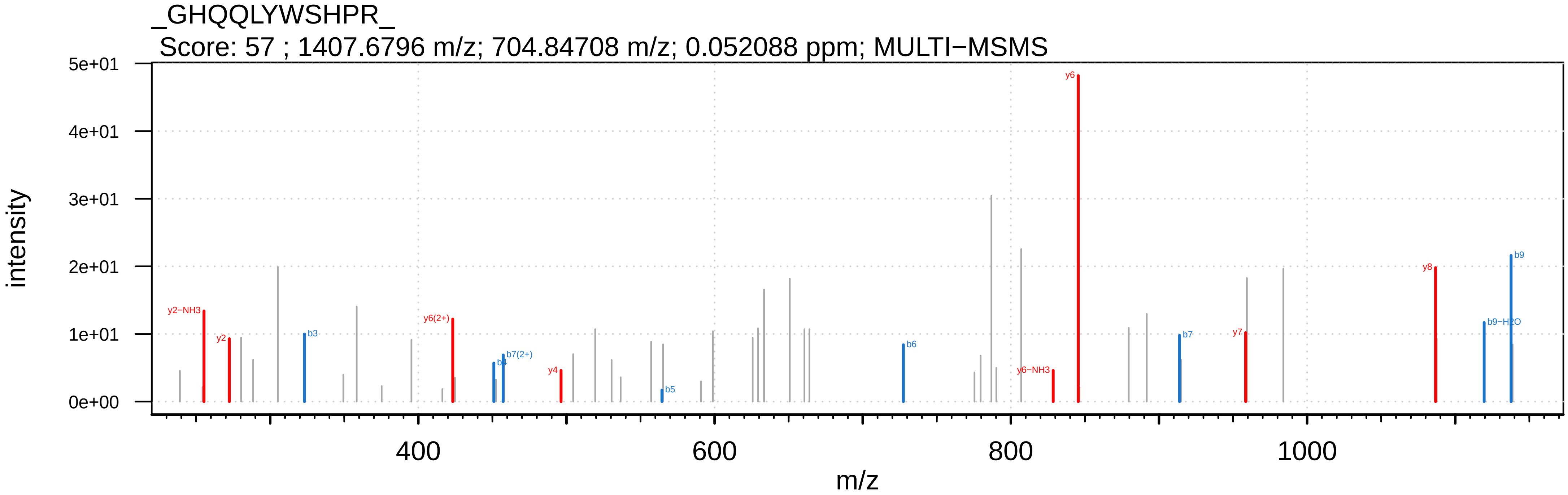

Raw File: Bert_20090403_BJS_halflives_P3_slice01_05
Scan Number: 2719
Proteins:
sp|P62274|RS29_MOUSE
ENSMUST00000037023_chr12:69157813−69159058:−
ENSMUST00000071737_chr13:73988963−73989133:−
tr|D3YYL7|D3YYL7_MOUSE

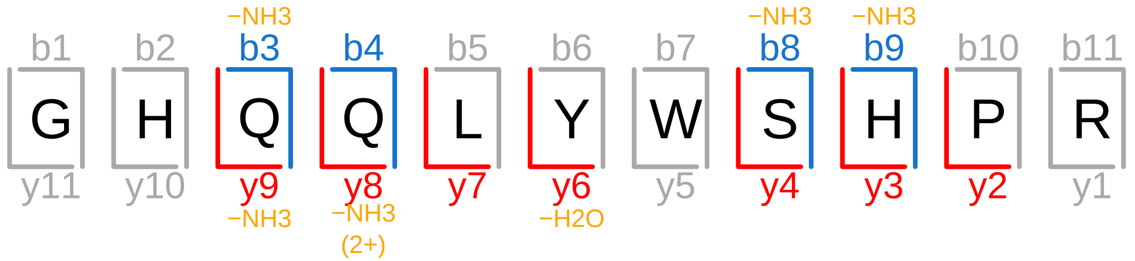

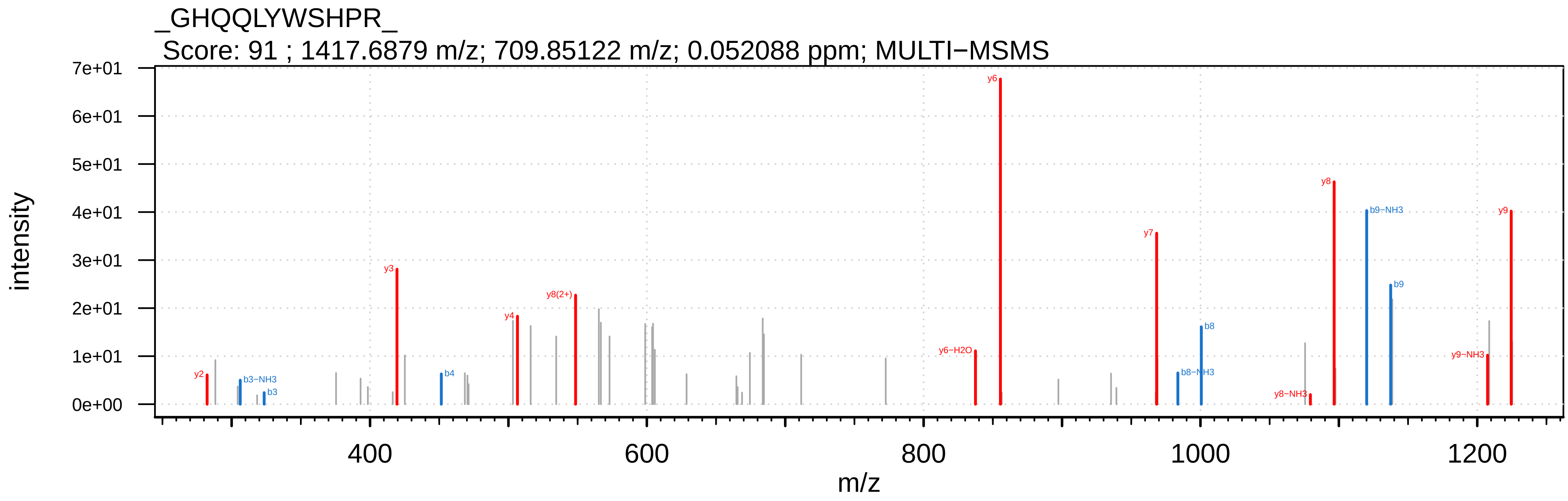

Raw File: Bert_20090403_BJS_halflives_P3_slice01_05
Scan Number: 2726
Proteins:
sp|P62274|RS29_MOUSE
ENSMUST00000037023_chr12:69157813−69159058:−
ENSMUST00000071737_chr13:73988963−73989133:−
tr|D3YYL7|D3YYL7_MOUSE

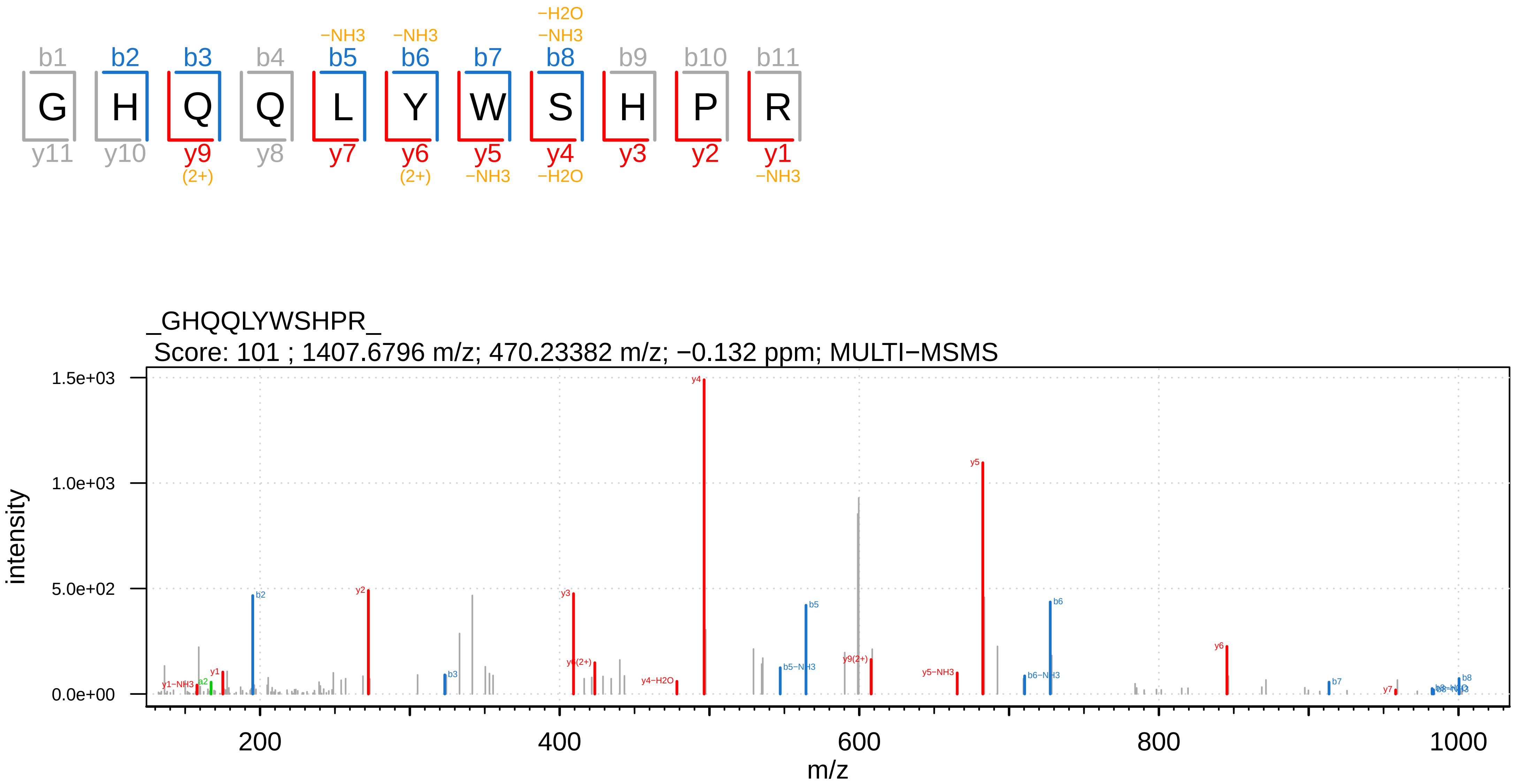

Raw File: Bert_20090403_BJS_halflives_P3_slice01_05
Scan Number: 2723
Proteins:
sp|P62274|RS29_MOUSE
ENSMUST00000037023_chr12:69157813−69159058:−
ENSMUST00000071737_chr13:73988963−73989133:−
tr|D3YYL7|D3YYL7_MOUSE

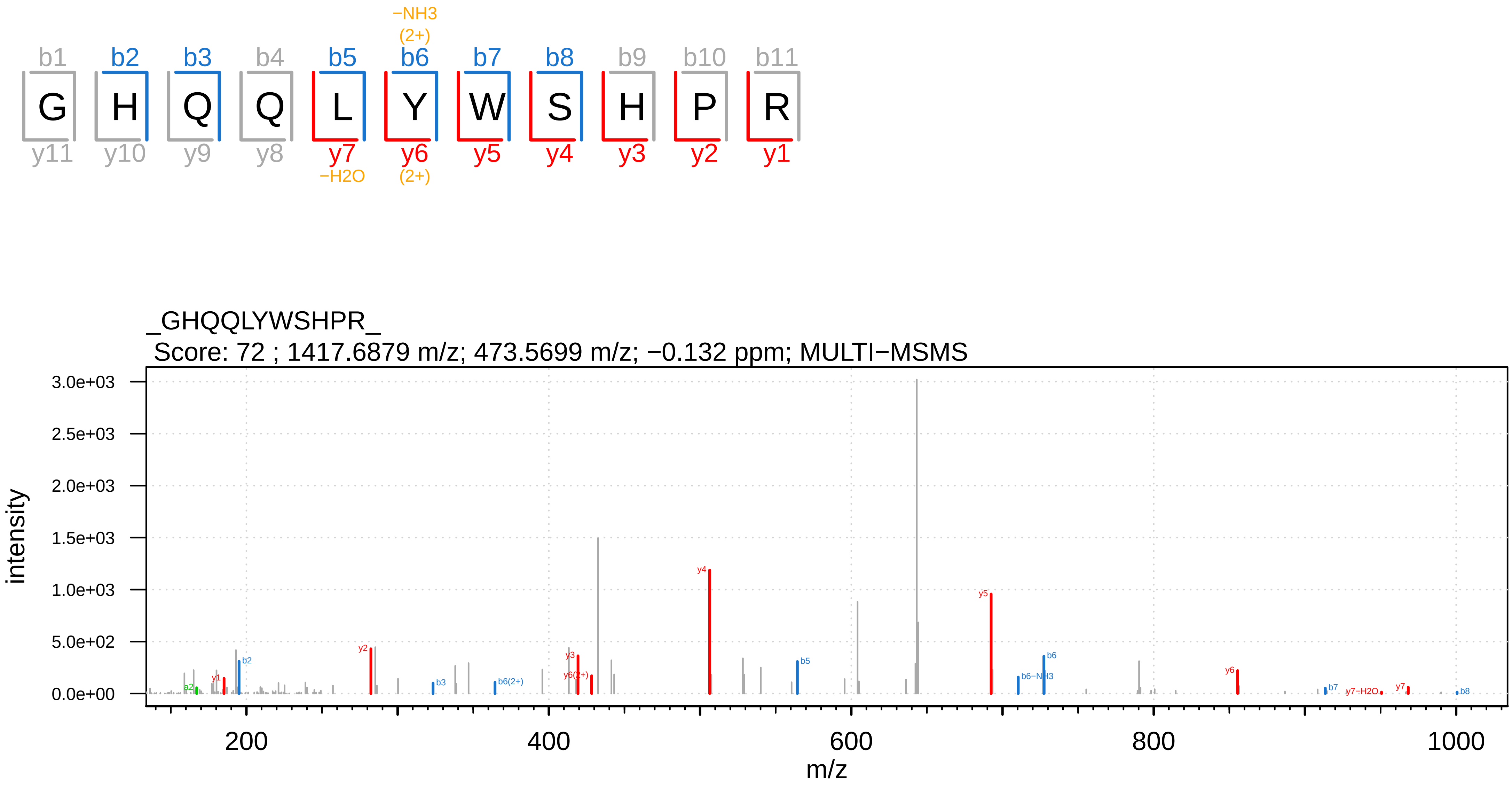

Raw File: Bert_20090403_BJS_halflives_P3_slice01_05
Scan Number: 2724
Proteins:
sp|P62274|RS29_MOUSE
ENSMUST00000037023_chr12:69157813−69159058:−
ENSMUST00000071737_chr13:73988963−73989133:−
tr|D3YYL7|D3YYL7_MOUSE

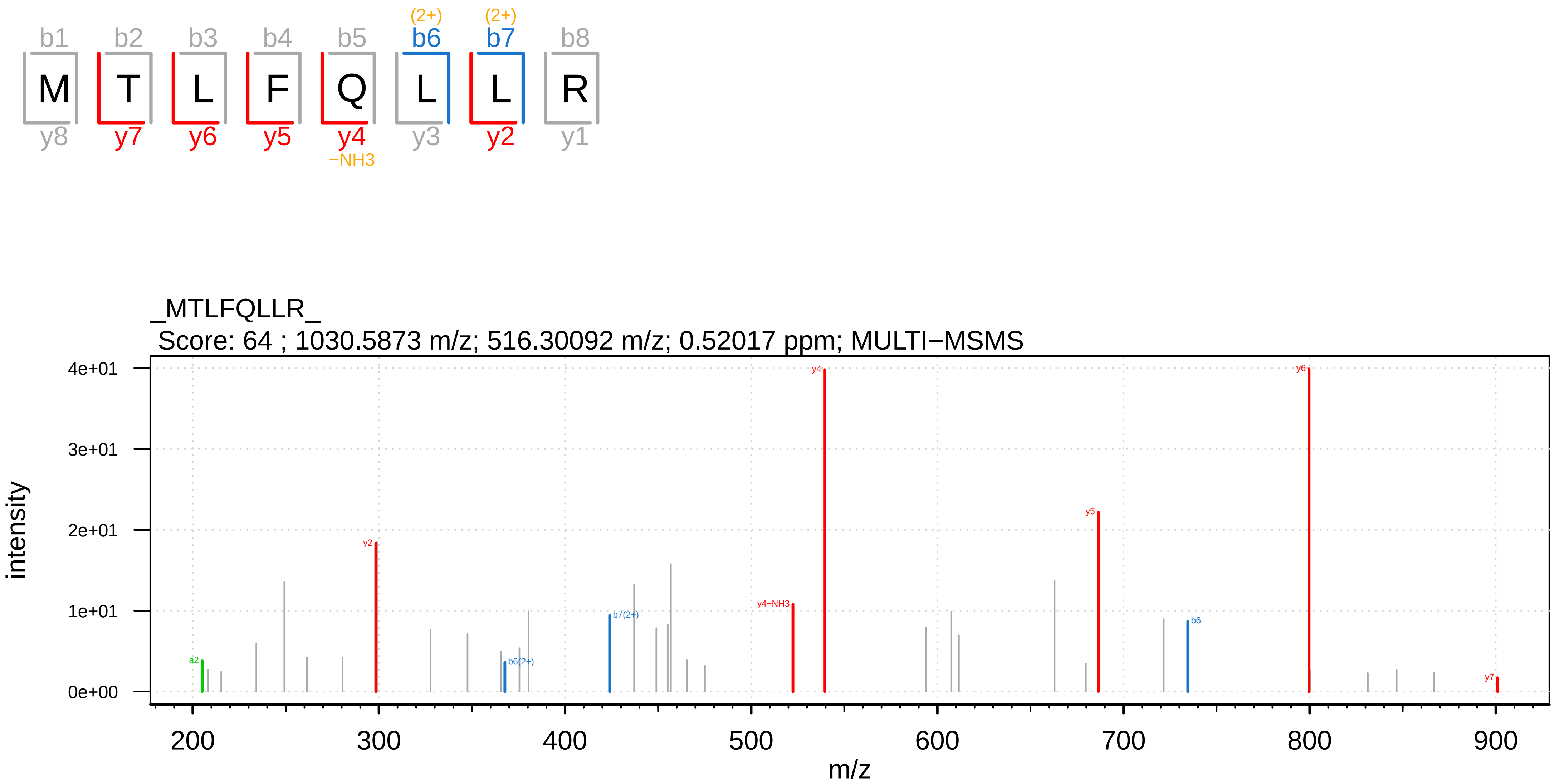

Raw File: Bert_20090403_BJS_halflives_P3_slice01_05
Scan Number: 9050
Proteins:
chr12_103206313−103214181.T2_chr12:101968174−101969629:−

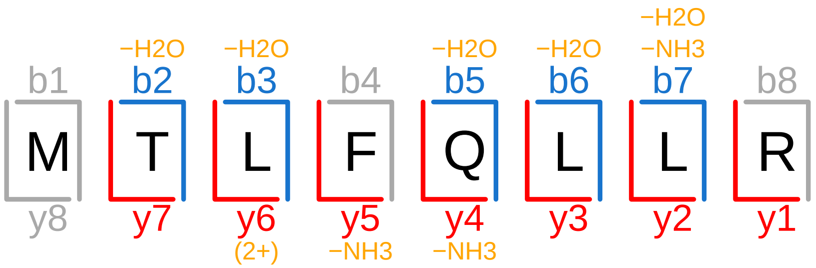

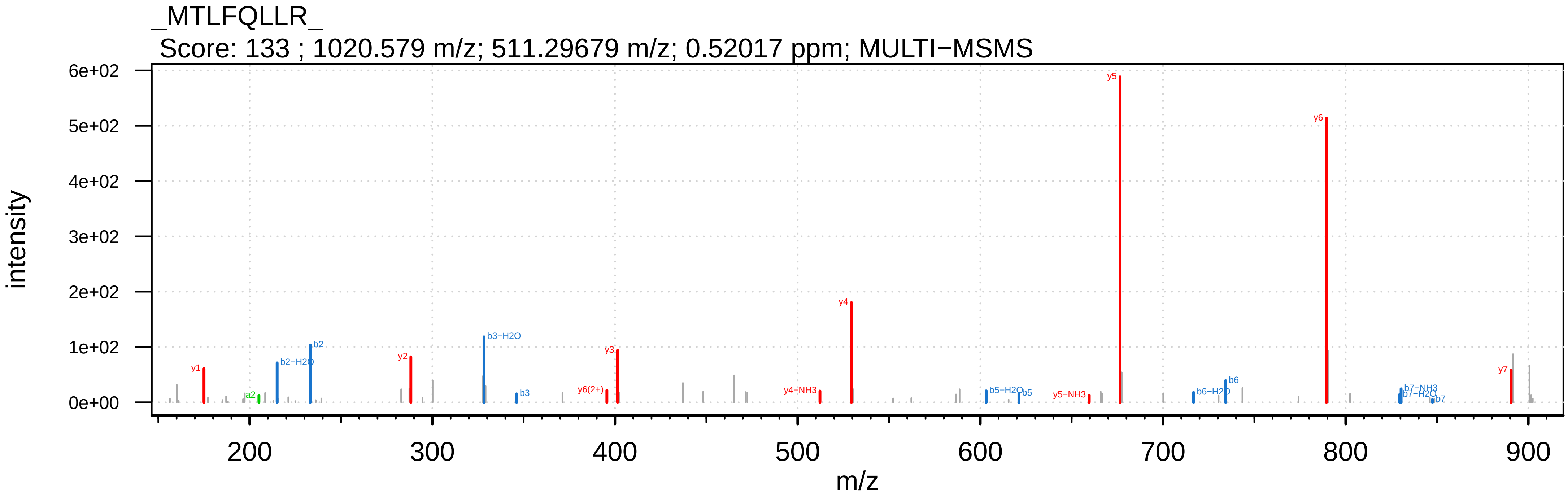

Raw File: Bert_20090403_BJS_halflives_P3_slice01_05
Scan Number: 9062
Proteins:
chr12_103206313−103214181.T2_chr12:101968174−101969629:−

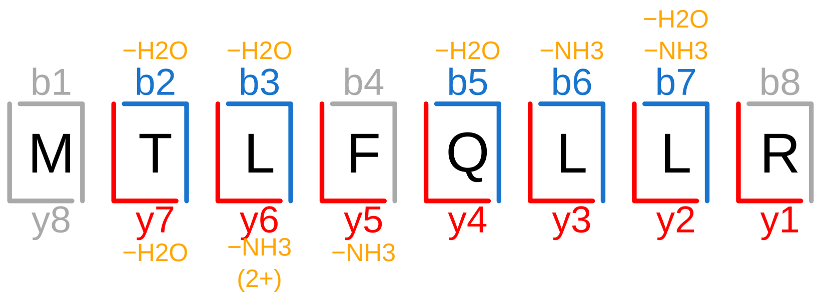

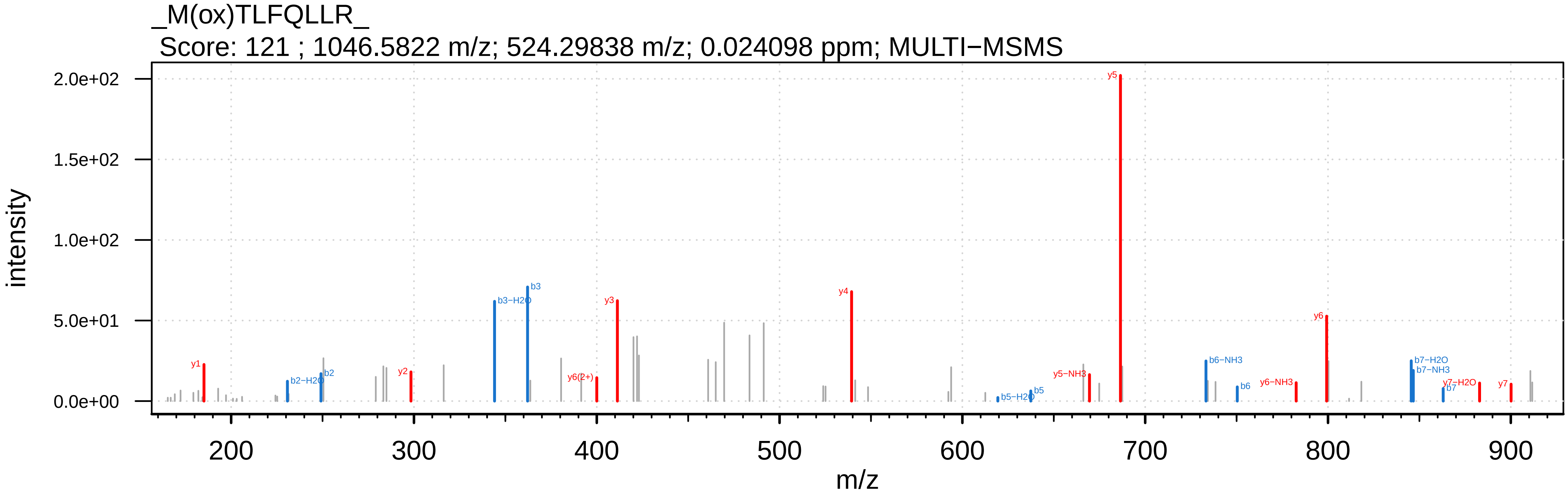

Raw File: Bert_20090403_BJS_halflives_P3_slice01_05
Scan Number: 7958
Proteins:
chr12_103206313−103214181.T2_chr12:101968174−101969629:−

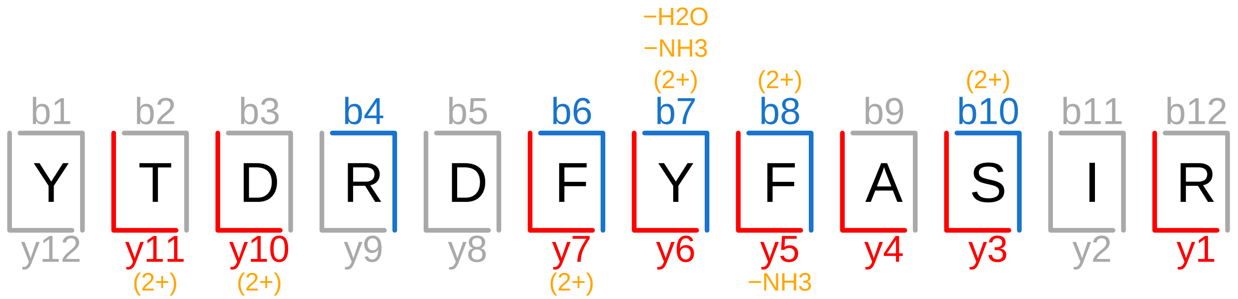

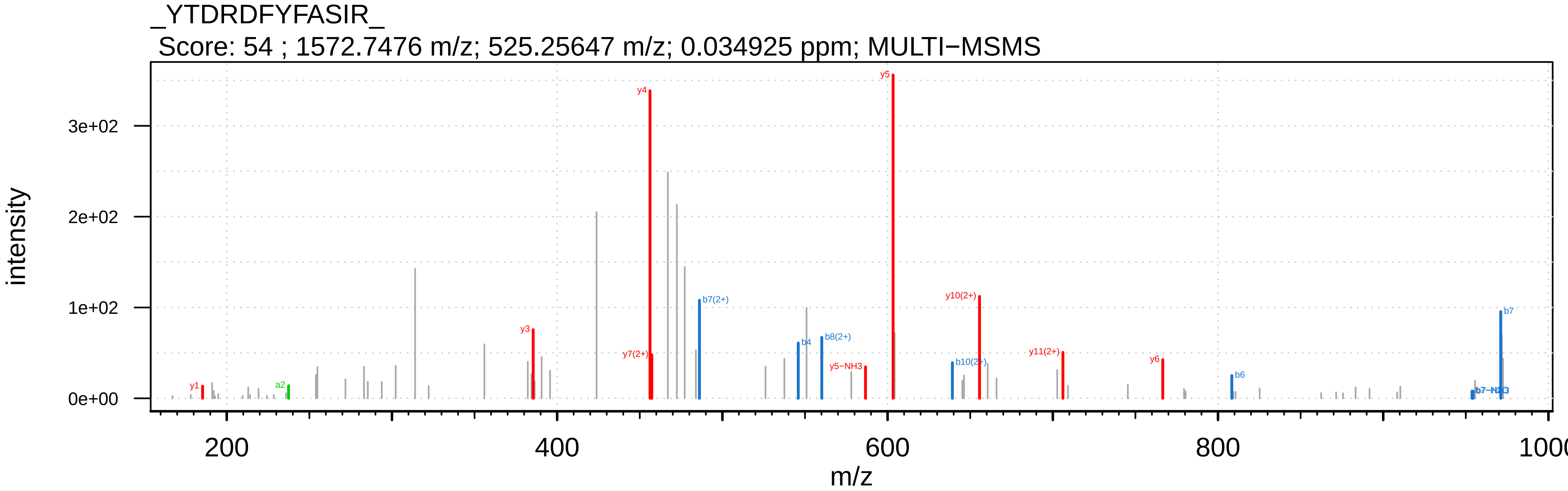

Raw File: Bert_20090403_BJS_halflives_P3_slice01_05
Scan Number: 6244
Proteins:
ENSMUST00000023048_chr15:80235835−80236047:+

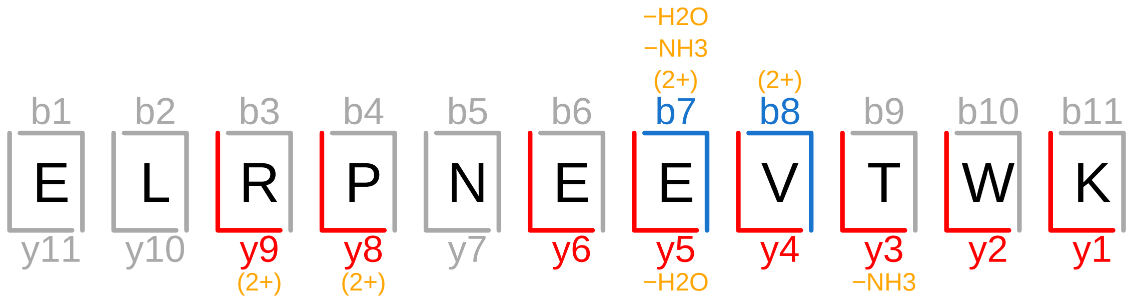

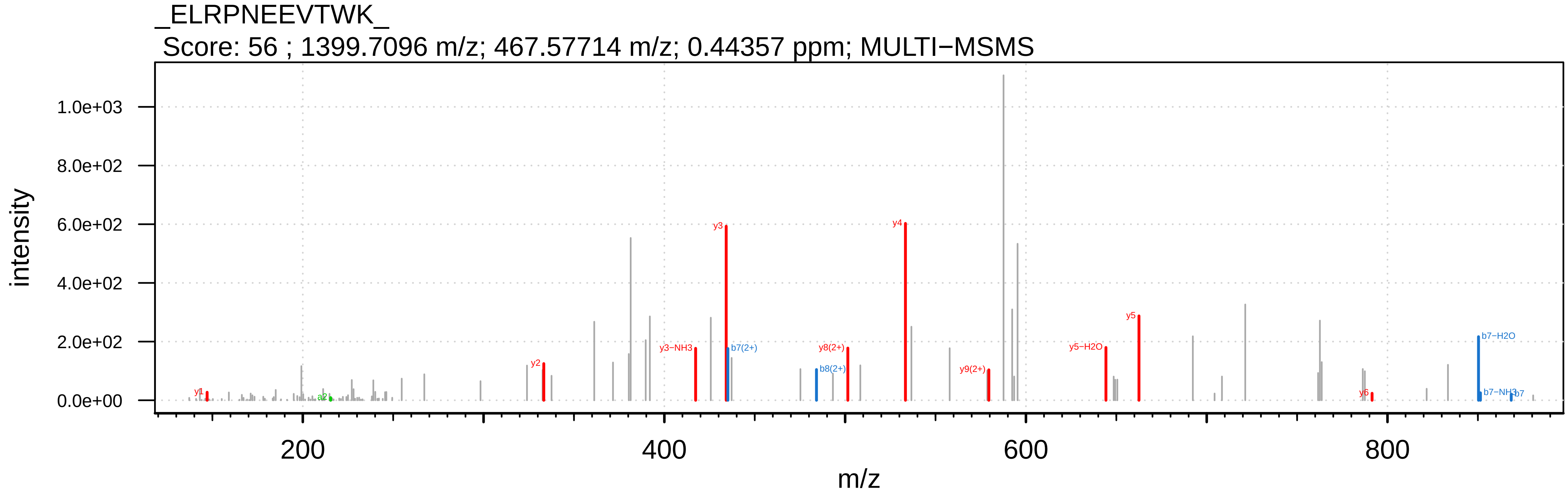

Raw File: Bert_20090403_BJS_halflives_P3_slice02_01
Scan Number: 3973
Proteins:
chr12_103206313−103214181.T2_chr12:101968174−101969629:−

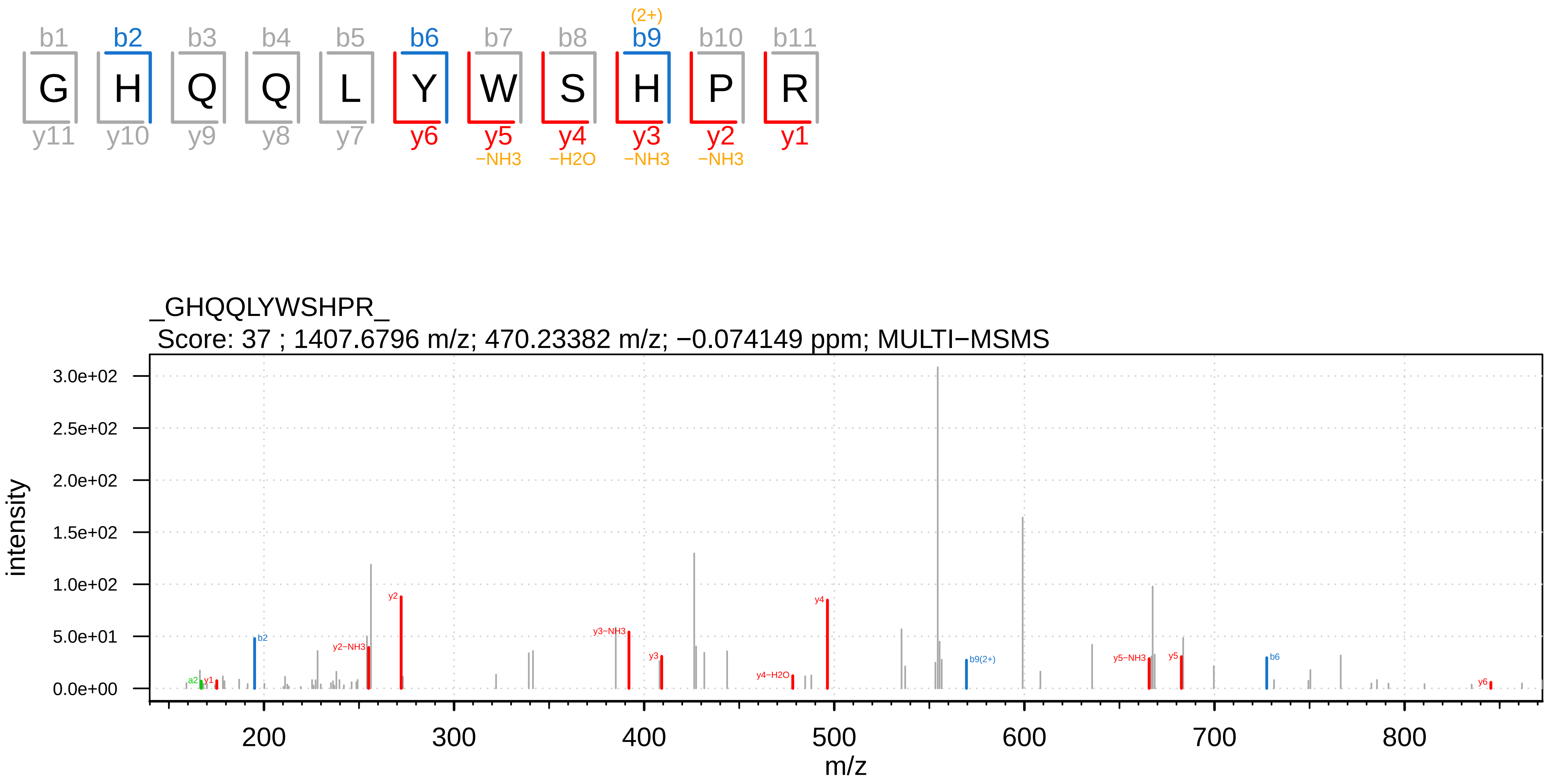

Raw File: Bert_20090403_BJS_halflives_P3_slice02_01
Scan Number: 2632
Proteins:
sp|P62274|RS29_MOUSE
ENSMUST00000037023_chr12:69157813−69159058:−
ENSMUST00000071737_chr13:73988963−73989133:−
tr|D3YYL7|D3YYL7_MOUSE

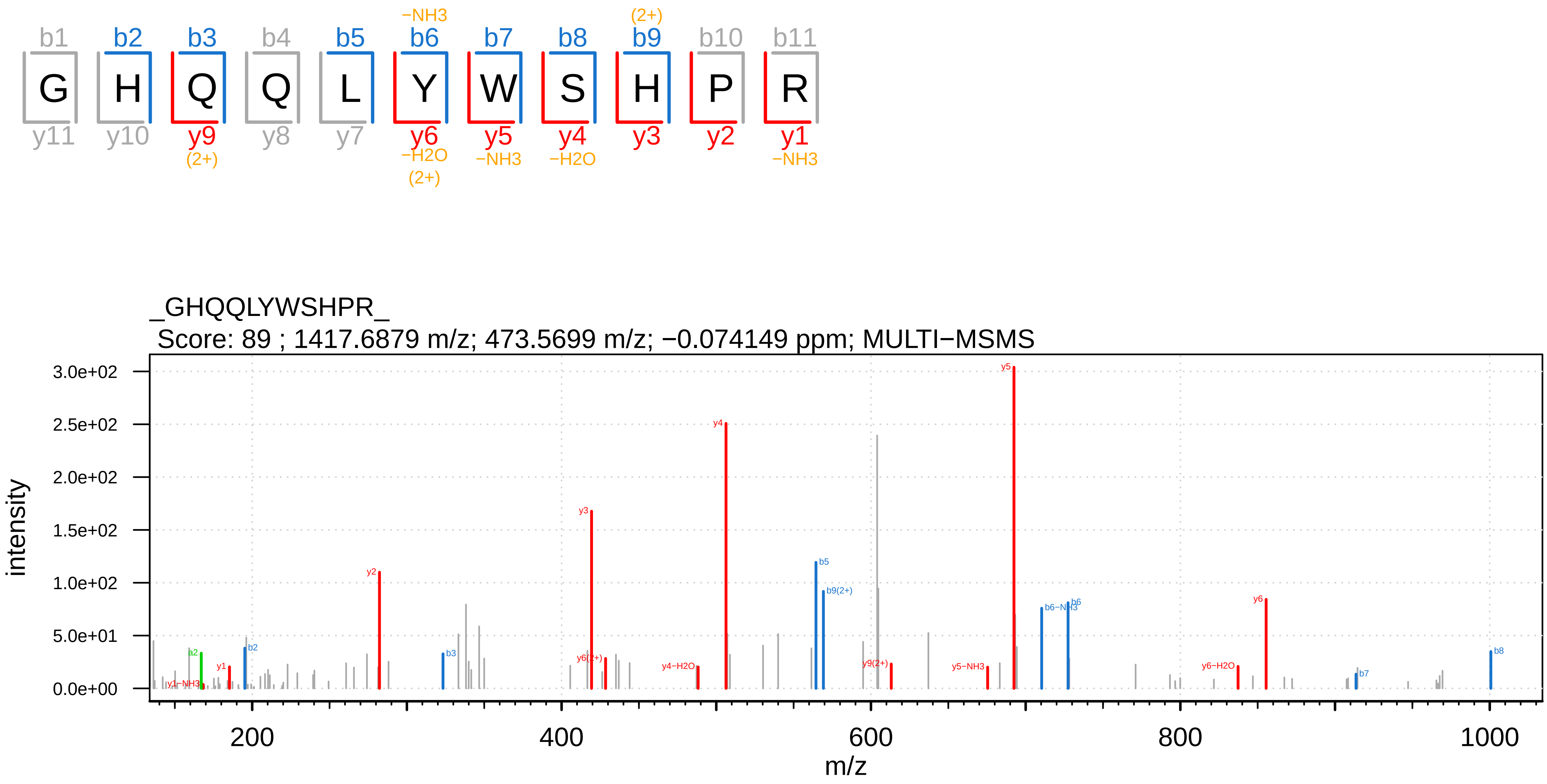

Raw File: Bert_20090403_BJS_halflives_P3_slice02_01
Scan Number: 2634
Proteins:
sp|P62274|RS29_MOUSE
ENSMUST00000037023_chr12:69157813−69159058:−
ENSMUST00000071737_chr13:73988963−73989133:−
tr|D3YYL7|D3YYL7_MOUSE

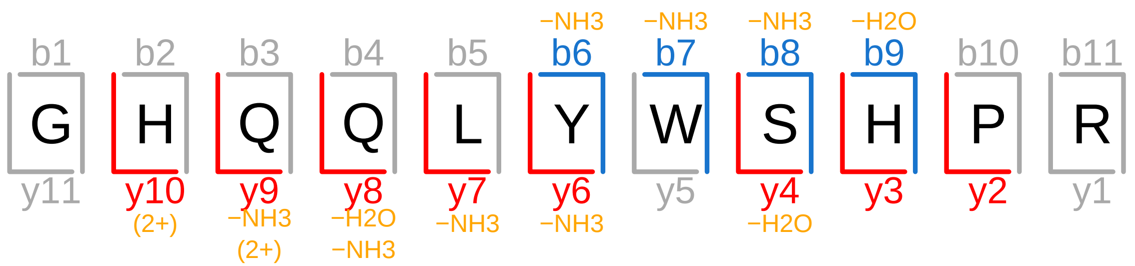

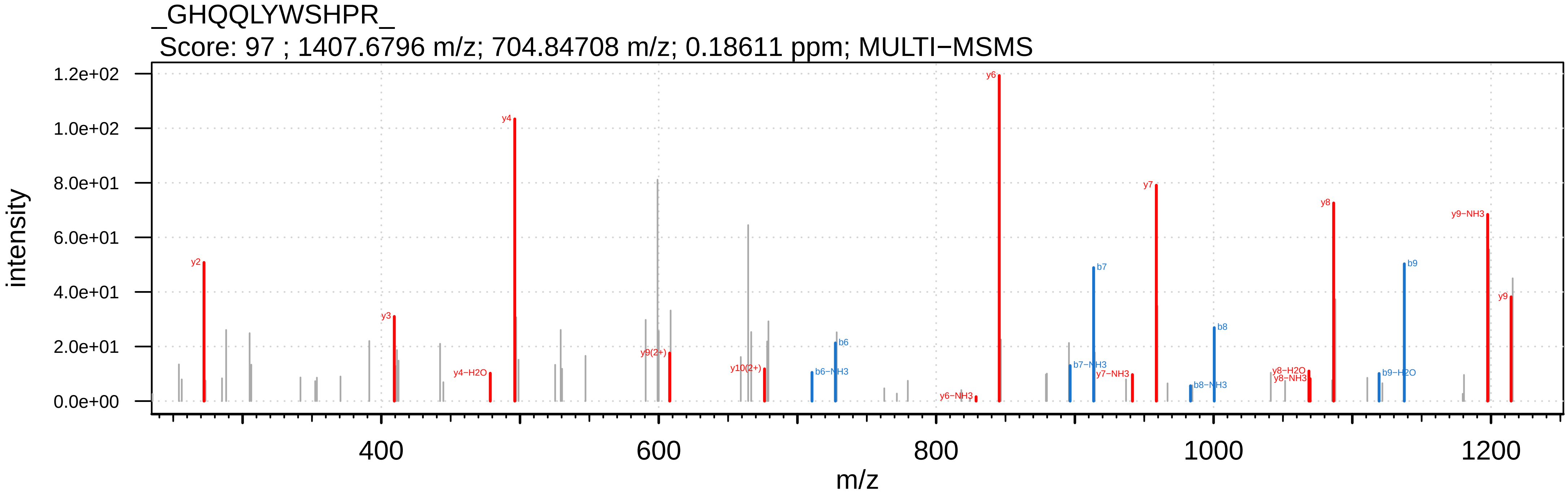

Raw File: Bert_20090403_BJS_halflives_P3_slice02_01
Scan Number: 2649
Proteins:
sp|P62274|RS29_MOUSE
ENSMUST00000037023_chr12:69157813−69159058:−
ENSMUST00000071737_chr13:73988963−73989133:−
tr|D3YYL7|D3YYL7_MOUSE

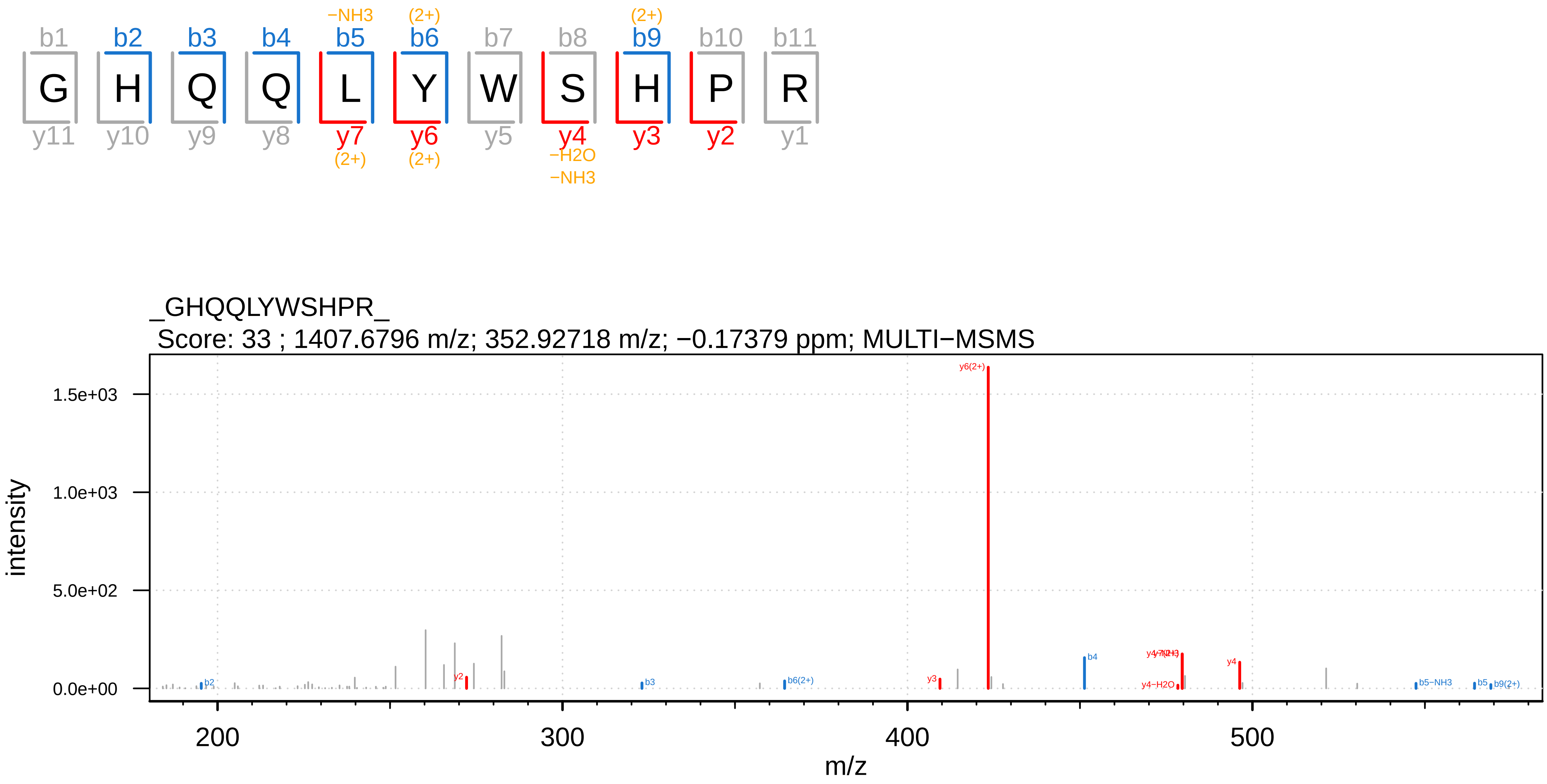

Raw File: Bert_20090403_BJS_halflives_P3_slice02_01
Scan Number: 2653
Proteins:
sp|P62274|RS29_MOUSE
ENSMUST00000037023_chr12:69157813−69159058:−
ENSMUST00000071737_chr13:73988963−73989133:−
tr|D3YYL7|D3YYL7_MOUSE

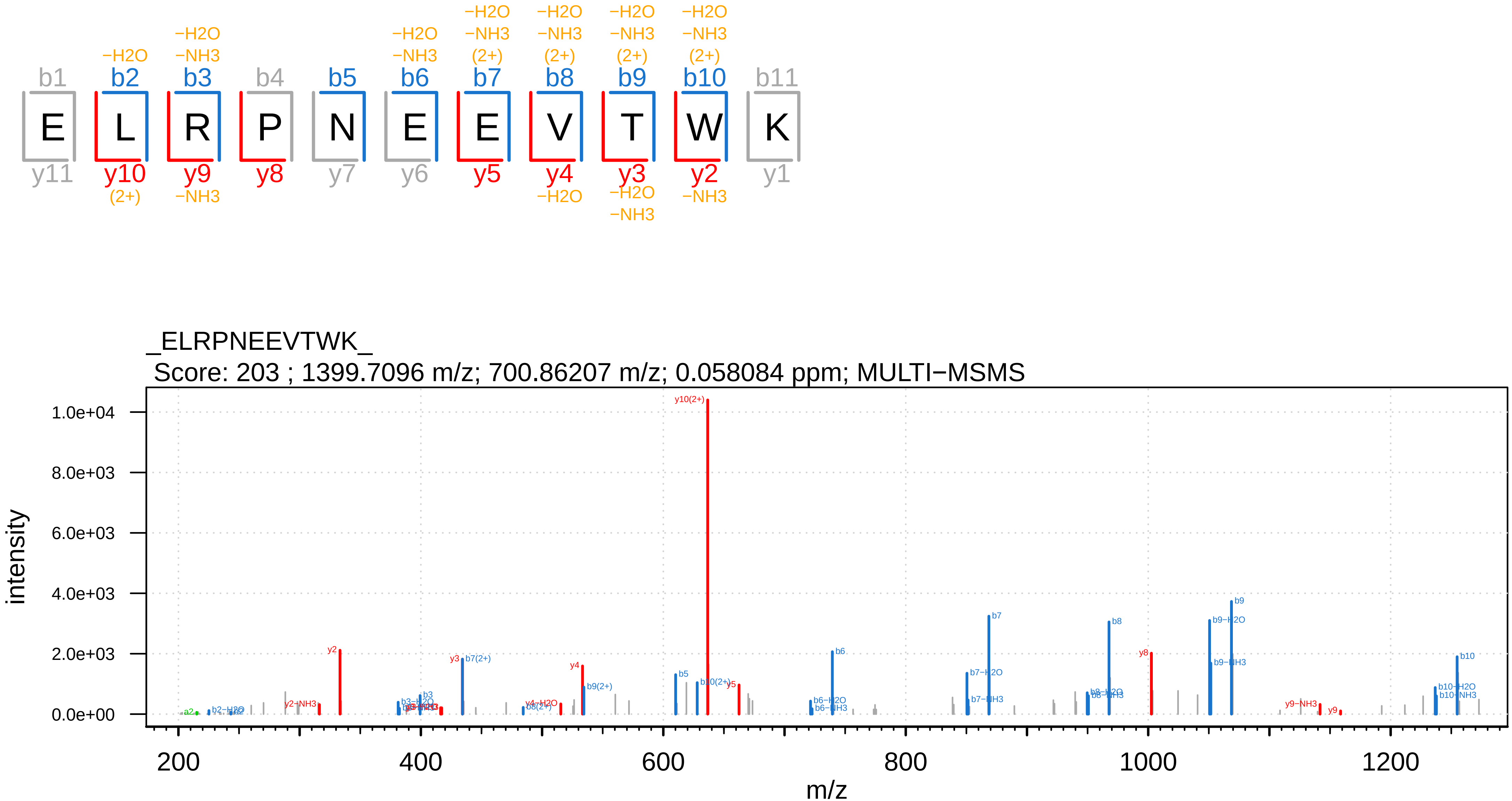

Raw File: Tiffy_20100224_BJS_Halflives_P1_01
Scan Number: 12076
Proteins:
chr12_103206313−103214181.T2_chr12:101968174−101969629:−

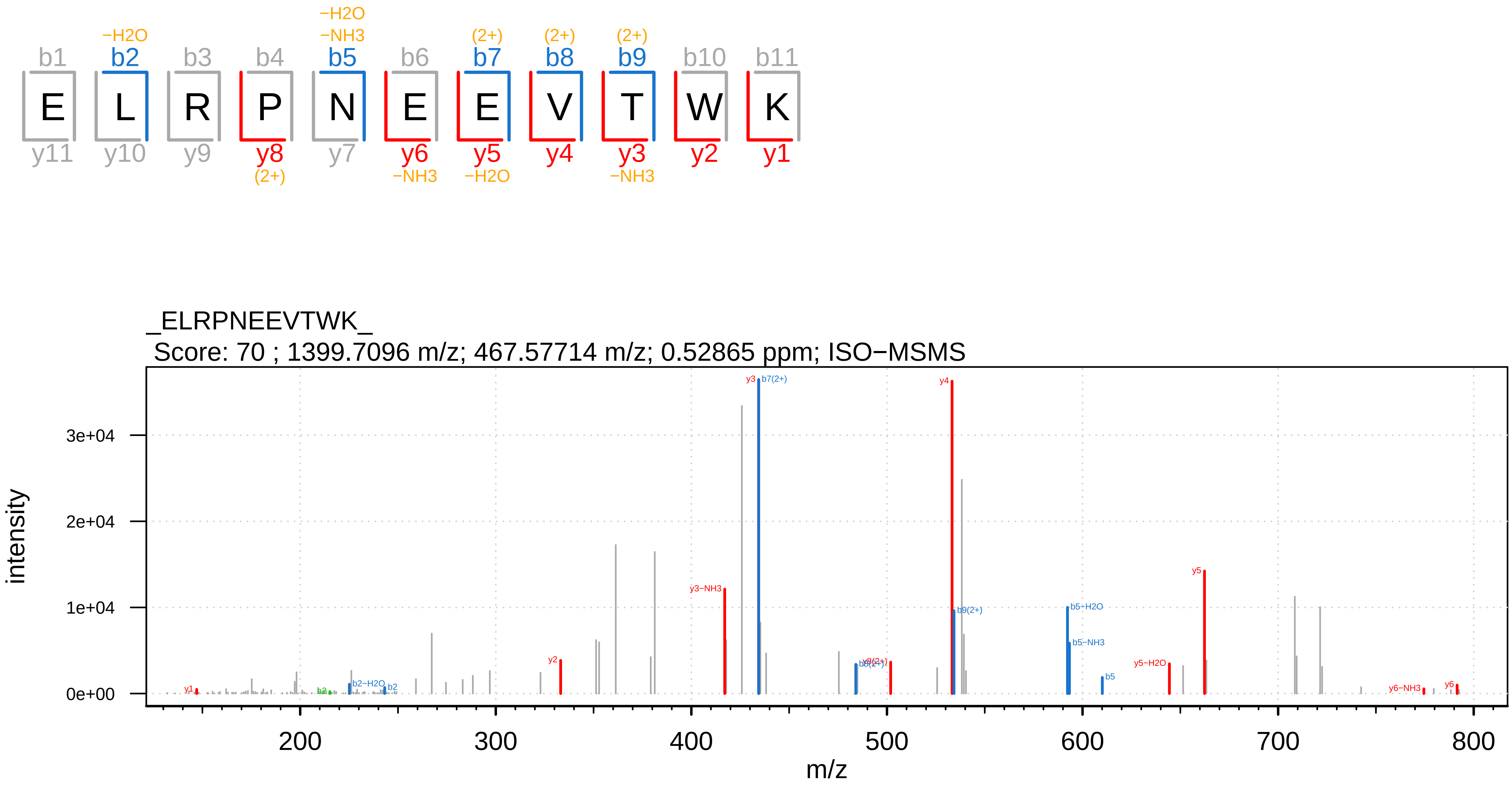

Raw File: Tiffy_20100224_BJS_Halflives_P1_01

Scan Number: 12197

Proteins:

chr12_103206313−103214181.T2_chr12:101968174−101969629:−

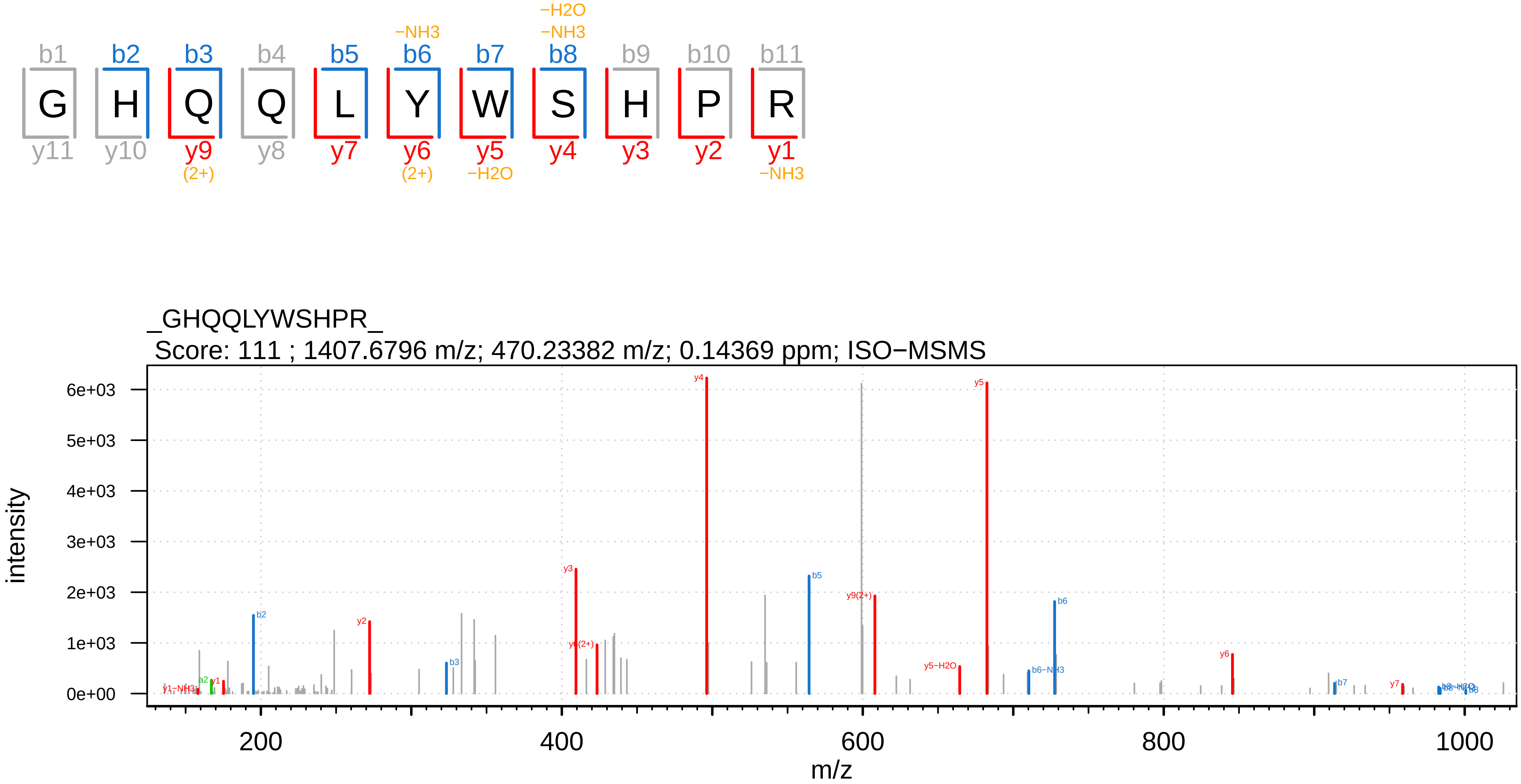

Raw File: Tiffy_20100224_BJS_Halflives_P1_01
Scan Number: 7073
Proteins:
sp|P62274|RS29_MOUSE
ENSMUST00000037023_chr12:69157813−69159058:−
ENSMUST00000071737_chr13:73988963−73989133:−
tr|D3YYL7|D3YYL7_MOUSE

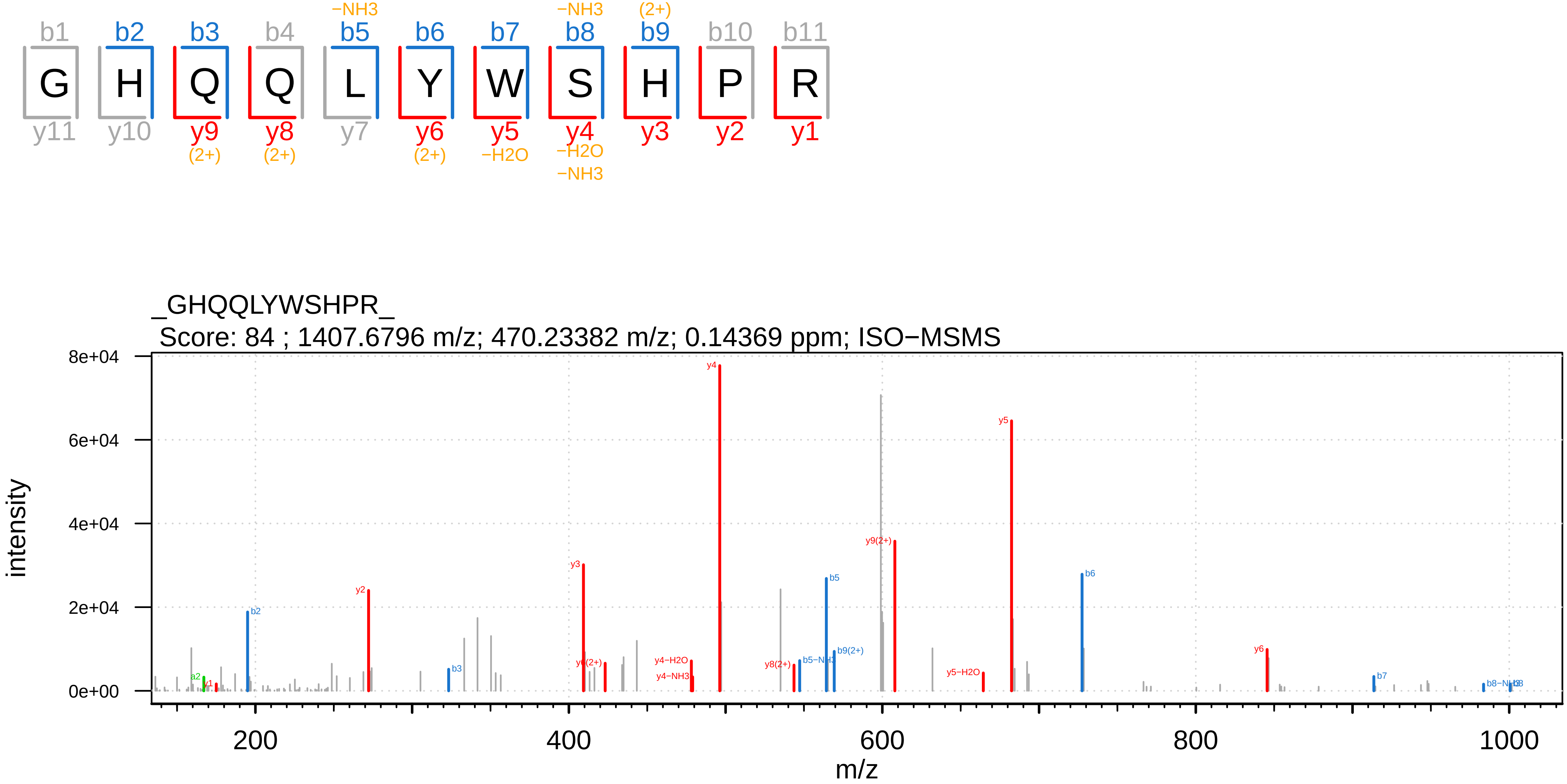

Raw File: Tiffy_20100224_BJS_Halflives_P1_01
Scan Number: 7181
Proteins:
sp|P62274|RS29_MOUSE
ENSMUST00000037023_chr12:69157813−69159058:−
ENSMUST00000071737_chr13:73988963−73989133:−
tr|D3YYL7|D3YYL7_MOUSE

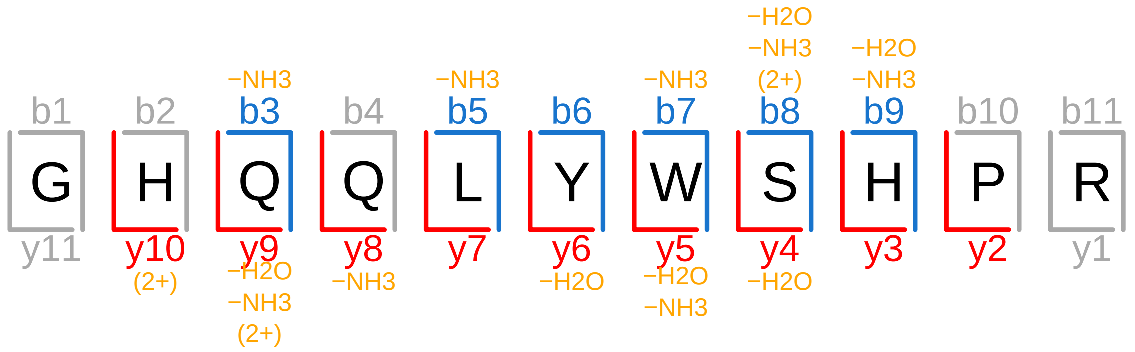

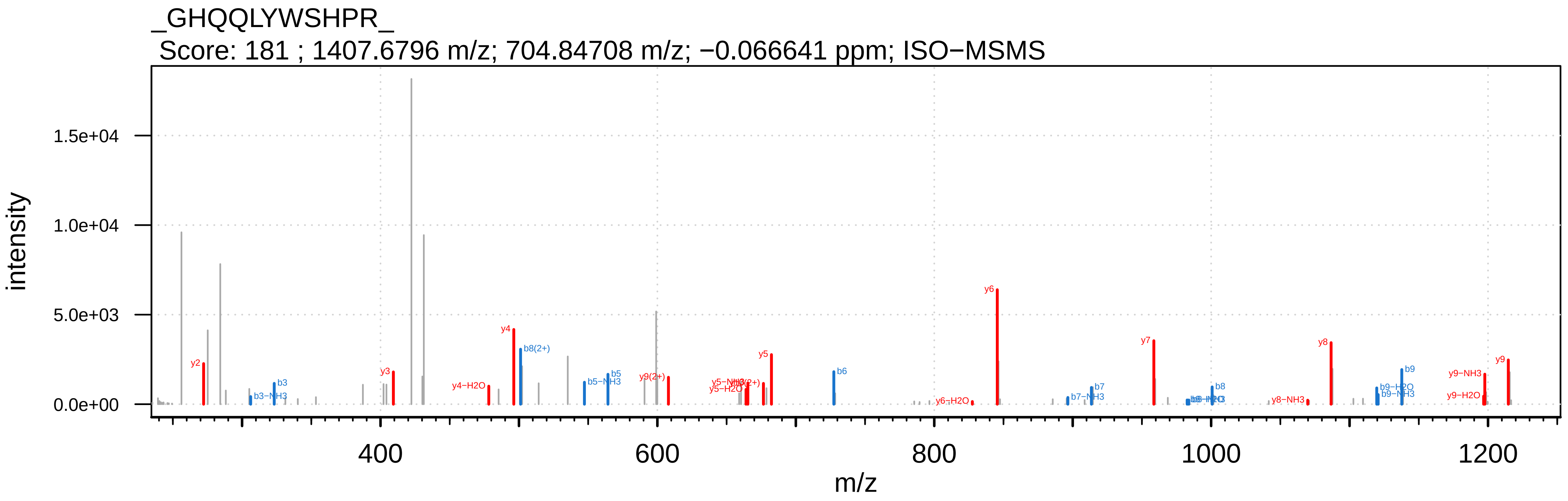

Raw File: Tiffy_20100224_BJS_Halflives_P1_01
Scan Number: 7100
Proteins:
sp|P62274|RS29_MOUSE
ENSMUST00000037023_chr12:69157813−69159058:−
ENSMUST00000071737_chr13:73988963−73989133:−
tr|D3YYL7|D3YYL7_MOUSE

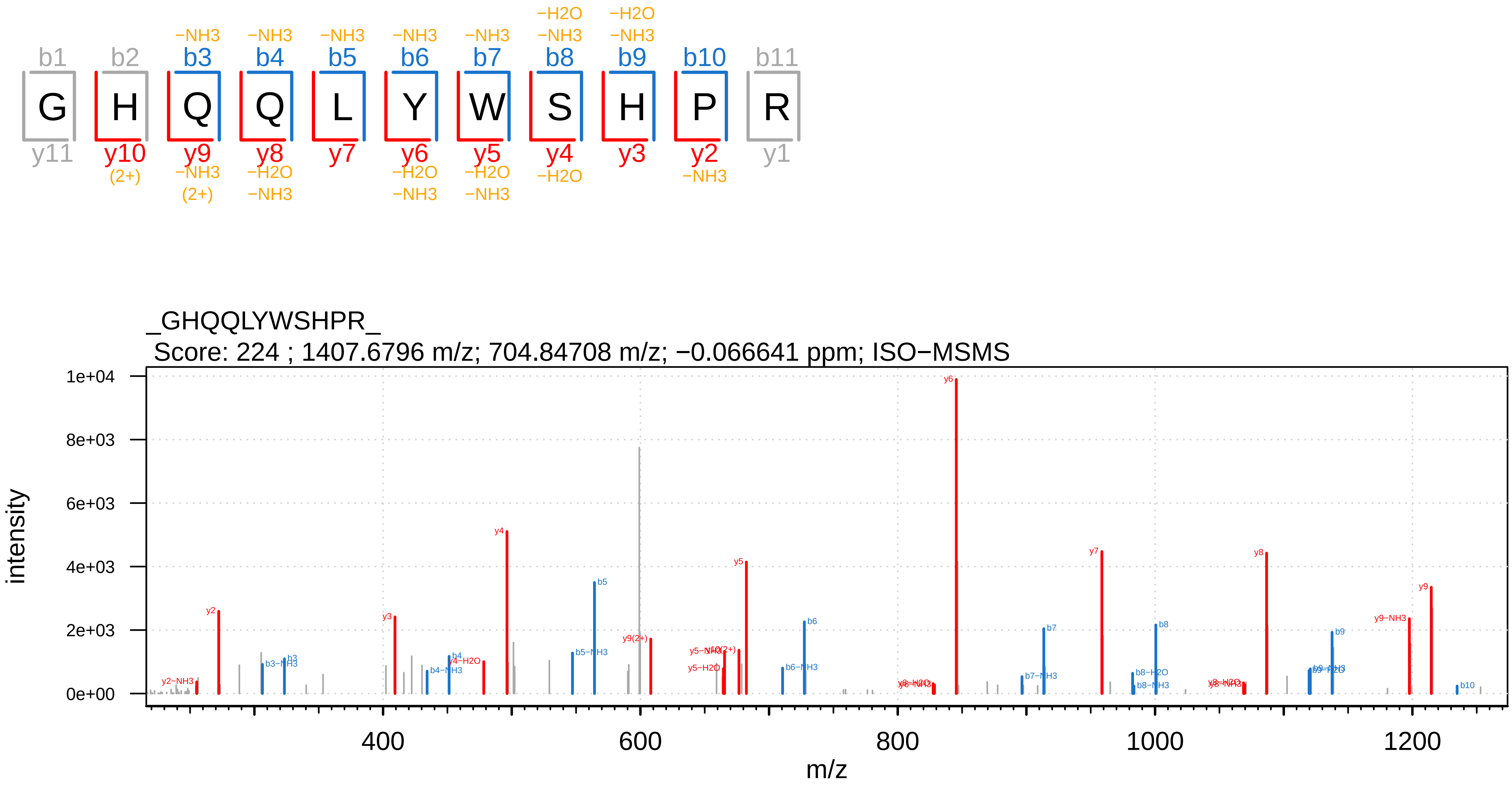

Raw File: Tiffy_20100224_BJS_Halflives_P1_01
Scan Number: 7207
Proteins:
sp|P62274|RS29_MOUSE
ENSMUST00000037023_chr12:69157813−69159058:−
ENSMUST00000071737_chr13:73988963−73989133:−
tr|D3YYL7|D3YYL7_MOUSE

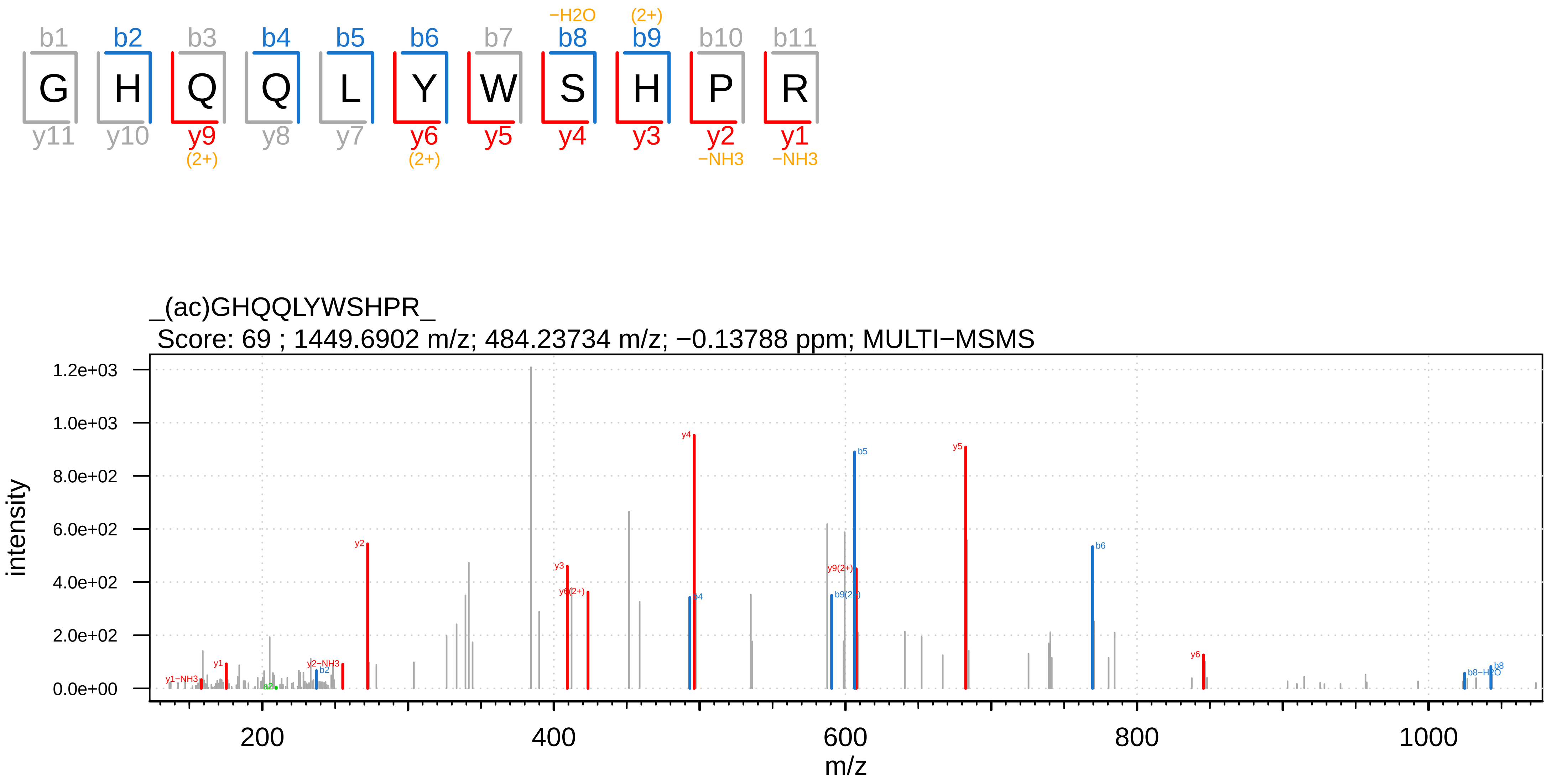

Raw File: Tiffy_20100224_BJS_Halflives_P1_01
Scan Number: 10947
Proteins:
sp|P62274|RS29_MOUSE
ENSMUST00000037023_chr12:69157813−69159058:−
ENSMUST00000071737_chr13:73988963−73989133:−
tr|D3YYL7|D3YYL7_MOUSE

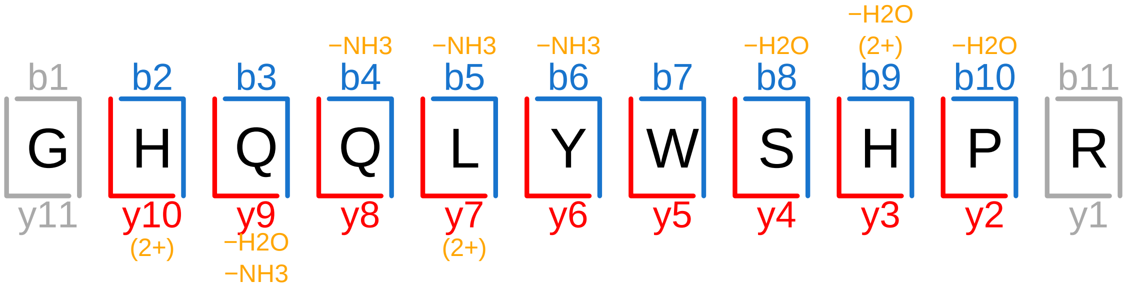

(ac)GHQQLYWSHPR
Score: 127 ; 1449.6902 m/z; 725.85237 m/z; −0.036467 ppm; MULTI−MSMS

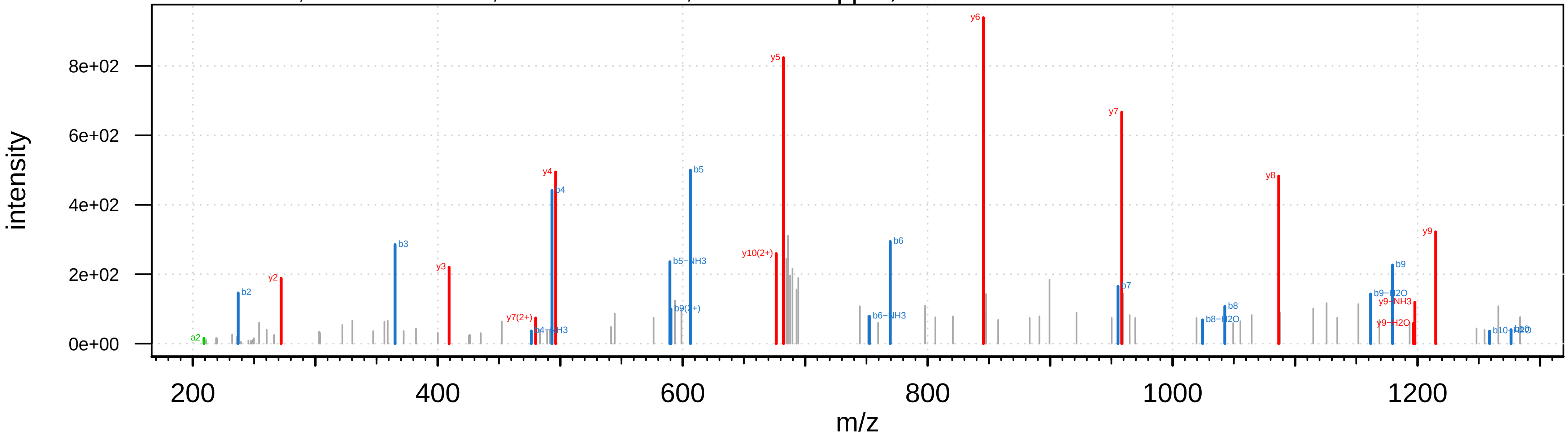

Raw File: Tiffy_20100224_BJS_Halflives_P1_01
Scan Number: 11004
Proteins:
sp|P62274|RS29_MOUSE
ENSMUST00000037023_chr12:69157813−69159058:−
ENSMUST00000071737_chr13:73988963−73989133:−
tr|D3YYL7|D3YYL7_MOUSE

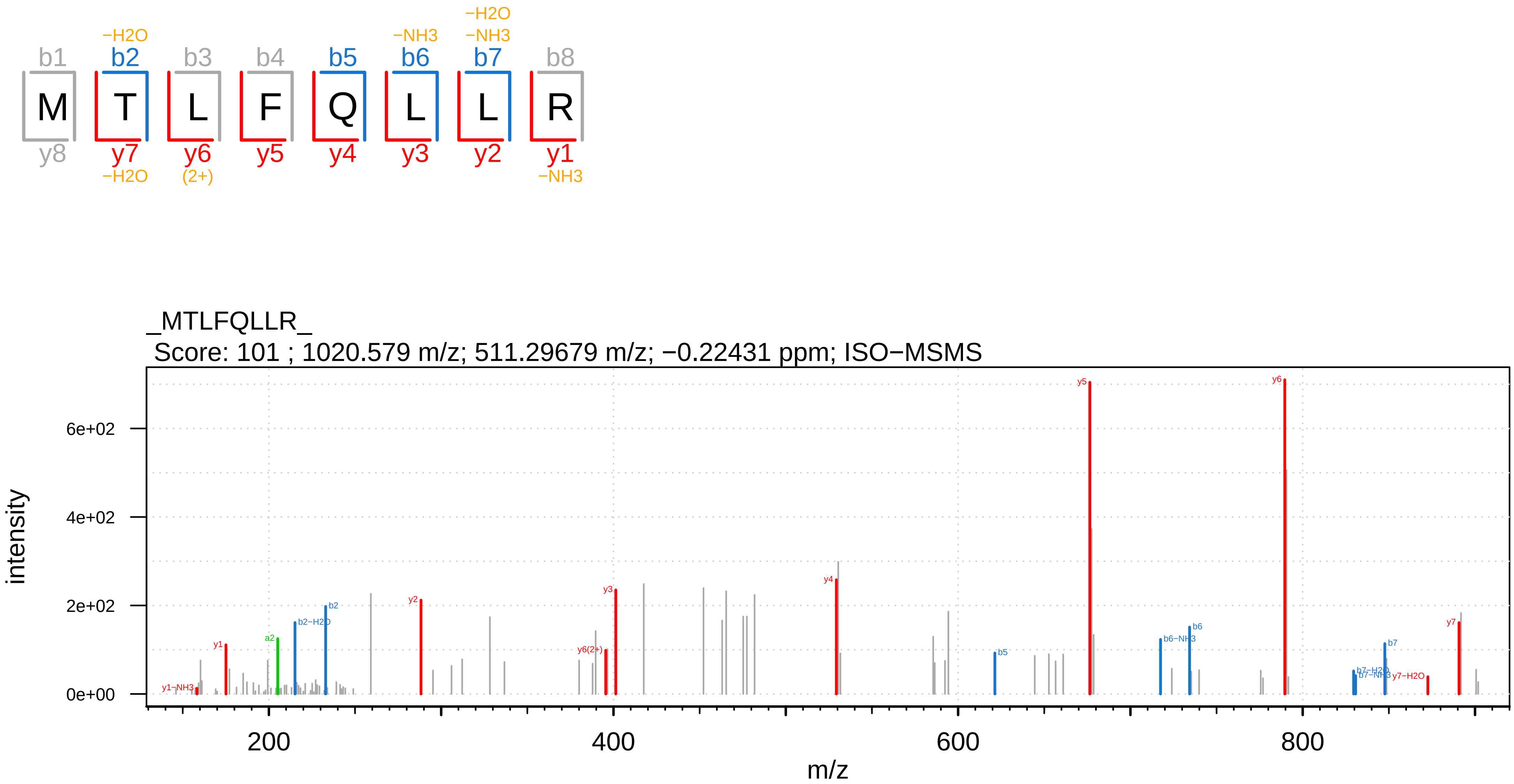

Raw File: Tiffy_20100224_BJS_Halflives_P1_01
Scan Number: 32279
Proteins:
chr12_103206313−103214181.T2_chr12:101968174−101969629:−

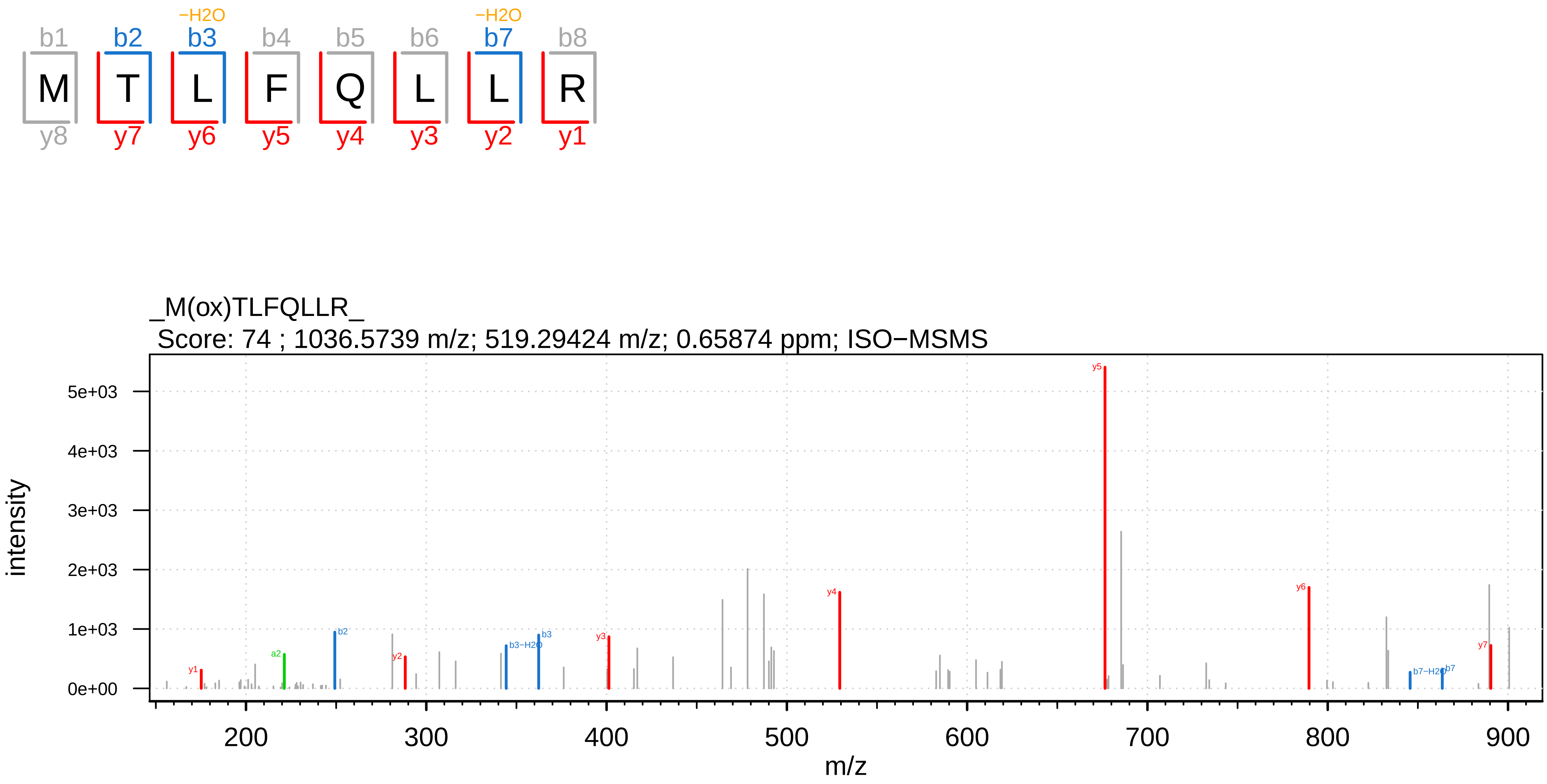

Raw File: Tiffy_20100224_BJS_Halflives_P1_01
Scan Number: 27082
Proteins:
chr12_103206313−103214181.T2_chr12:101968174−101969629:−

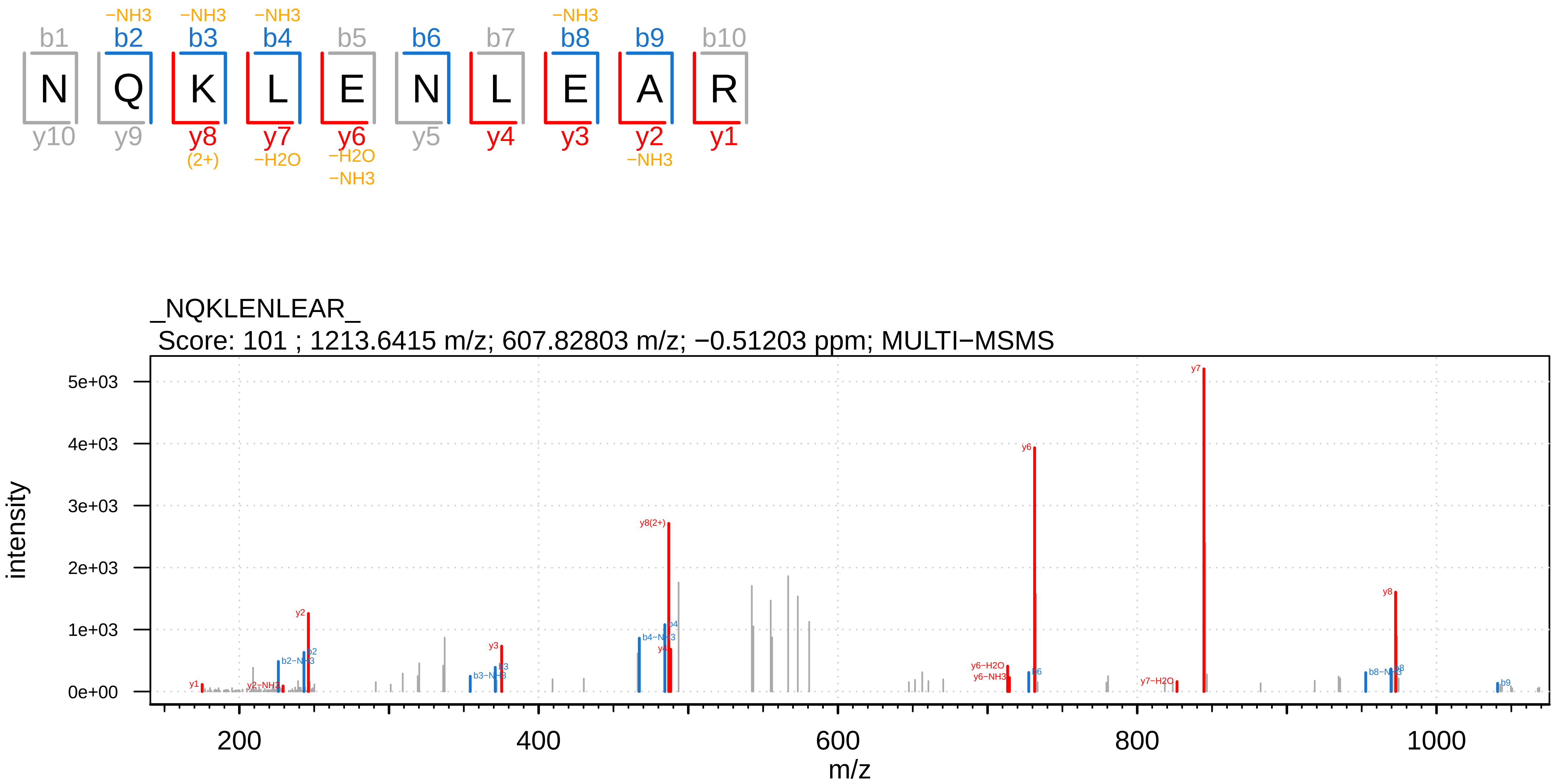

Raw File: Tiffy_20100224_BJS_Halflives_P1_01
Scan Number: 5023
Proteins:
ENSMUST00000023048_chr15:80235835−80236047:+

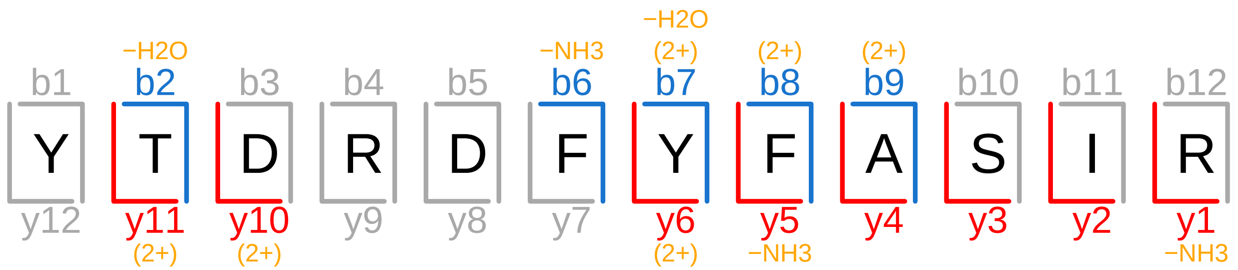

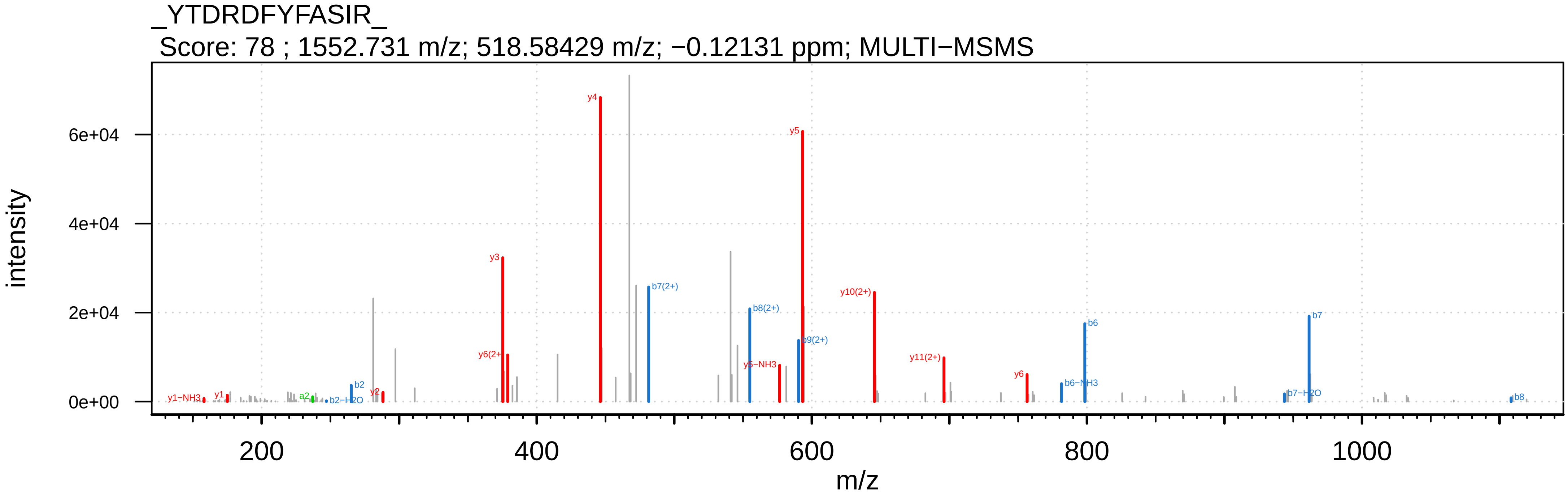

Raw File: Tiffy_20100224_BJS_Halflives_P1_01
Scan Number: 21431
Proteins:
ENSMUST00000023048_chr15:80235835−80236047:+

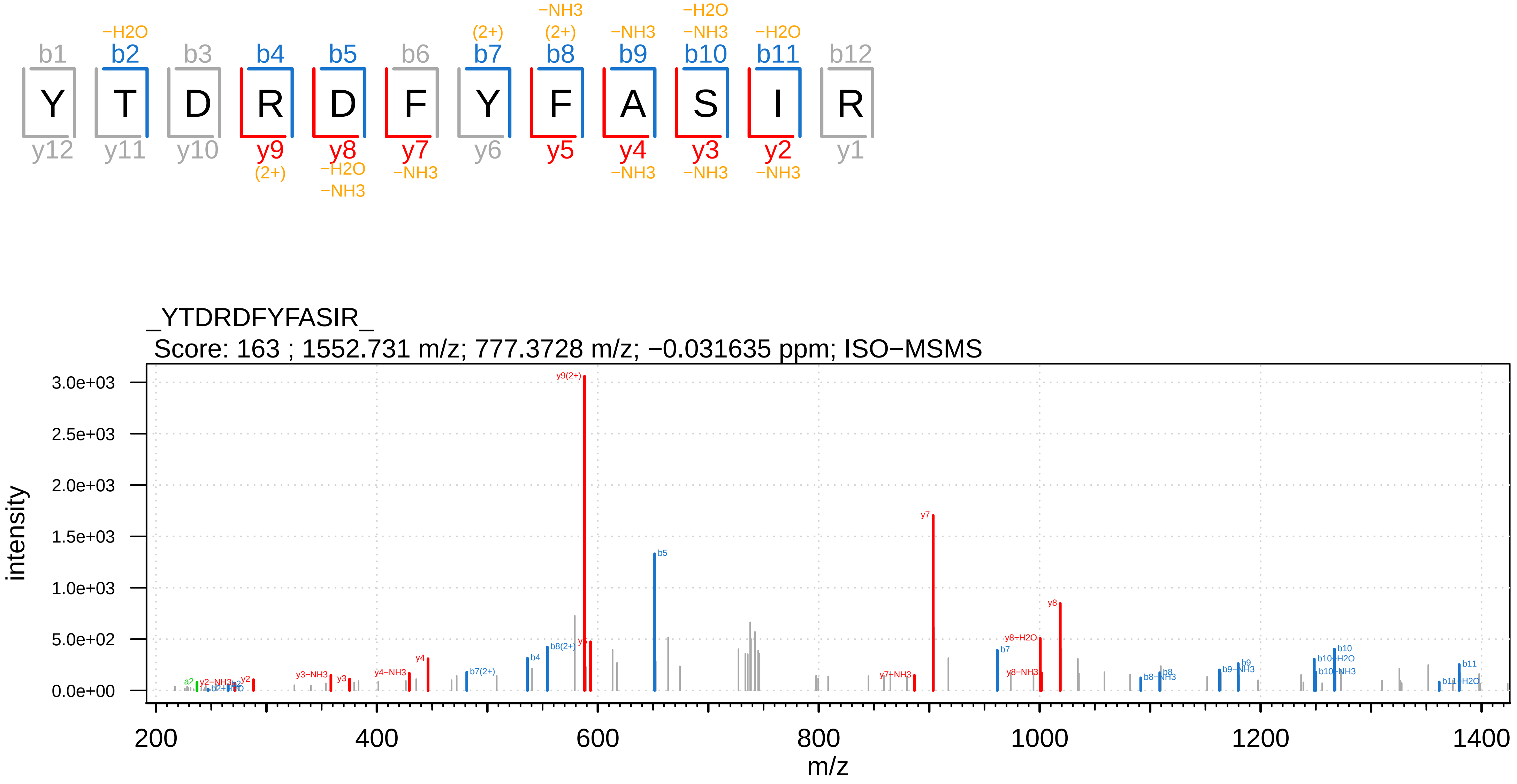

Raw File: Tiffy_20100224_BJS_Halflives_P1_01
Scan Number: 21423
Proteins:
ENSMUST00000023048_chr15:80235835−80236047:+

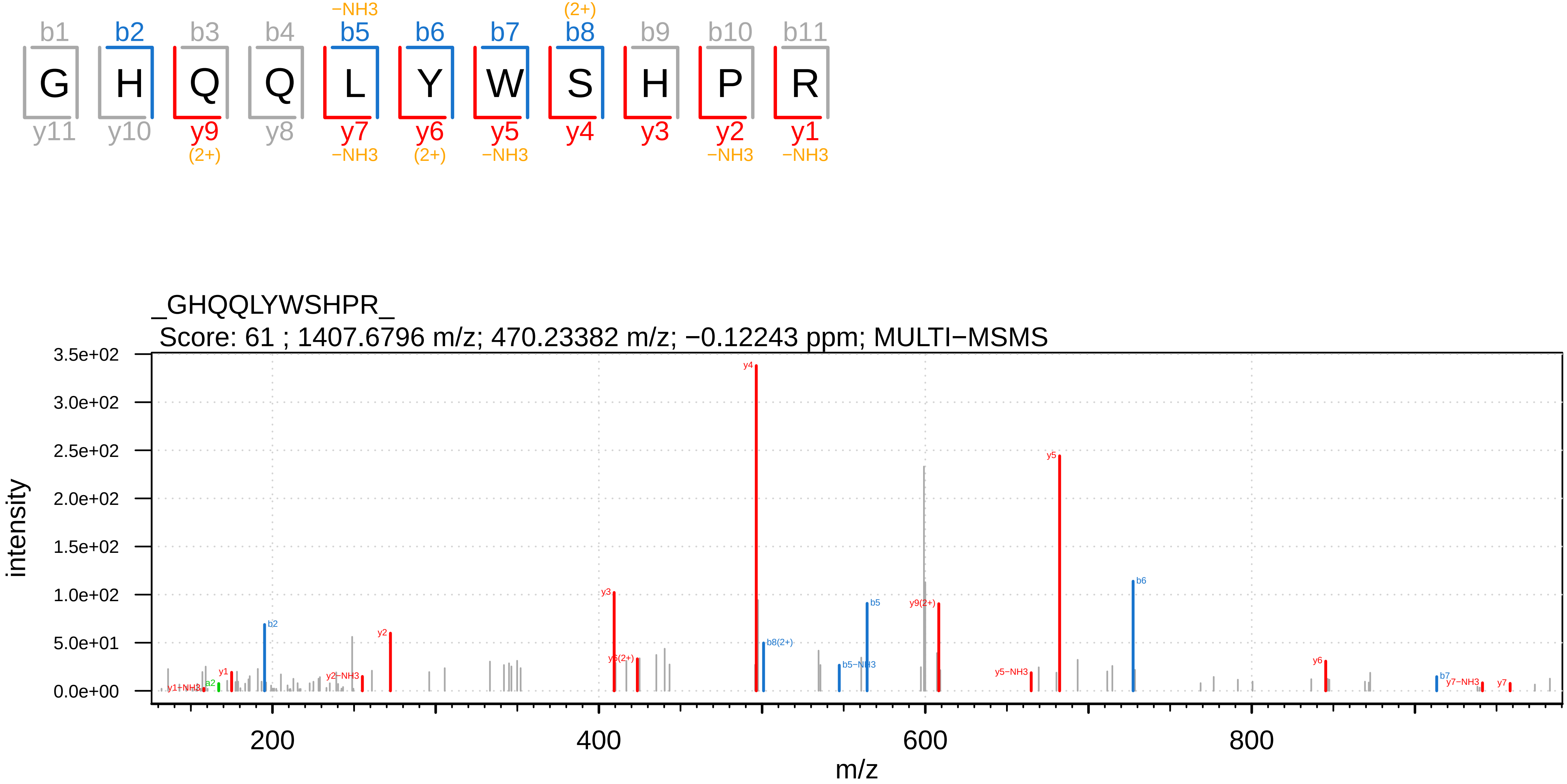

Raw File: Bert_20090331_BJS_halflives_P2_slice02_01
Scan Number: 4095
Proteins:
sp|P62274|RS29_MOUSE
ENSMUST00000037023_chr12:69157813−69159058:−
ENSMUST00000071737_chr13:73988963−73989133:−
tr|D3YYL7|D3YYL7_MOUSE

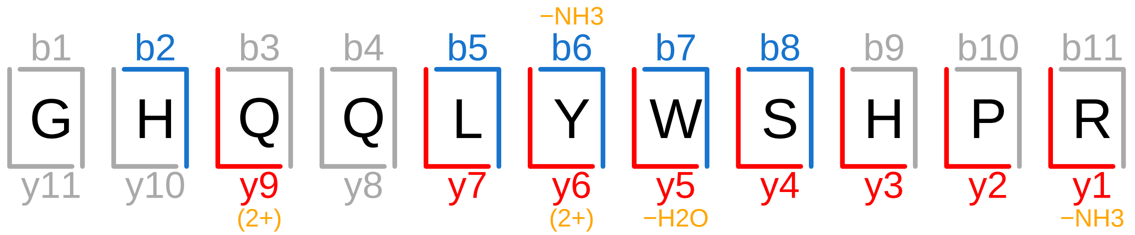

GHQQLYWSHPR
Score: 50 ; 1417.6879 m/z; 473.5699 m/z; −0.12243 ppm; MULTI−MSMS

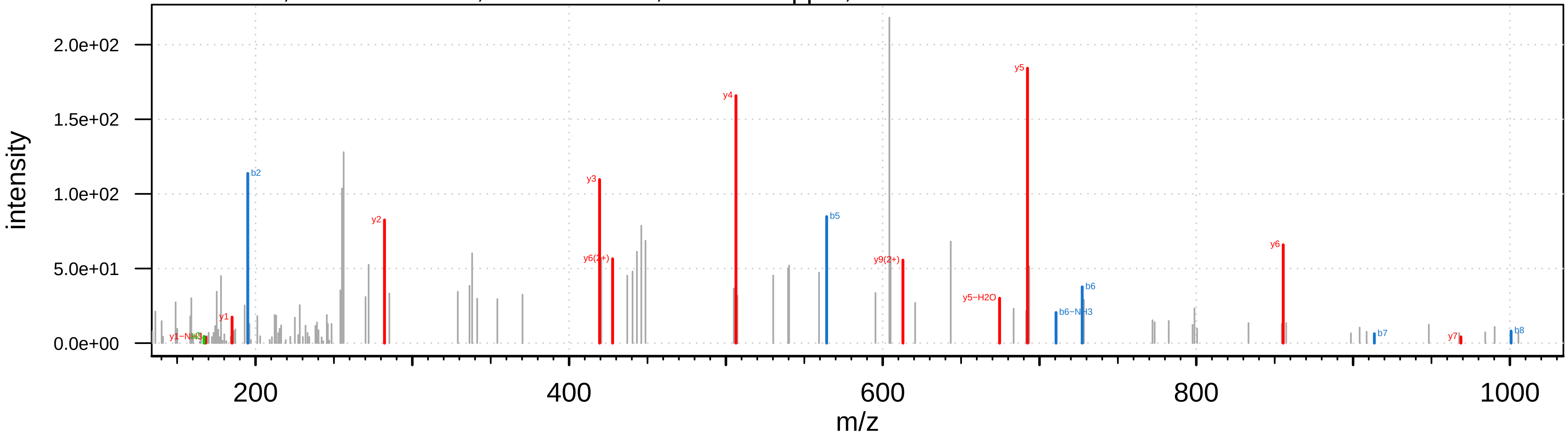

Raw File: Bert_20090331_BJS_halflives_P2_slice02_01
Scan Number: 4117
Proteins:
sp|P62274|RS29_MOUSE
ENSMUST00000037023_chr12:69157813−69159058:−
ENSMUST00000071737_chr13:73988963−73989133:−
tr|D3YYL7|D3YYL7_MOUSE

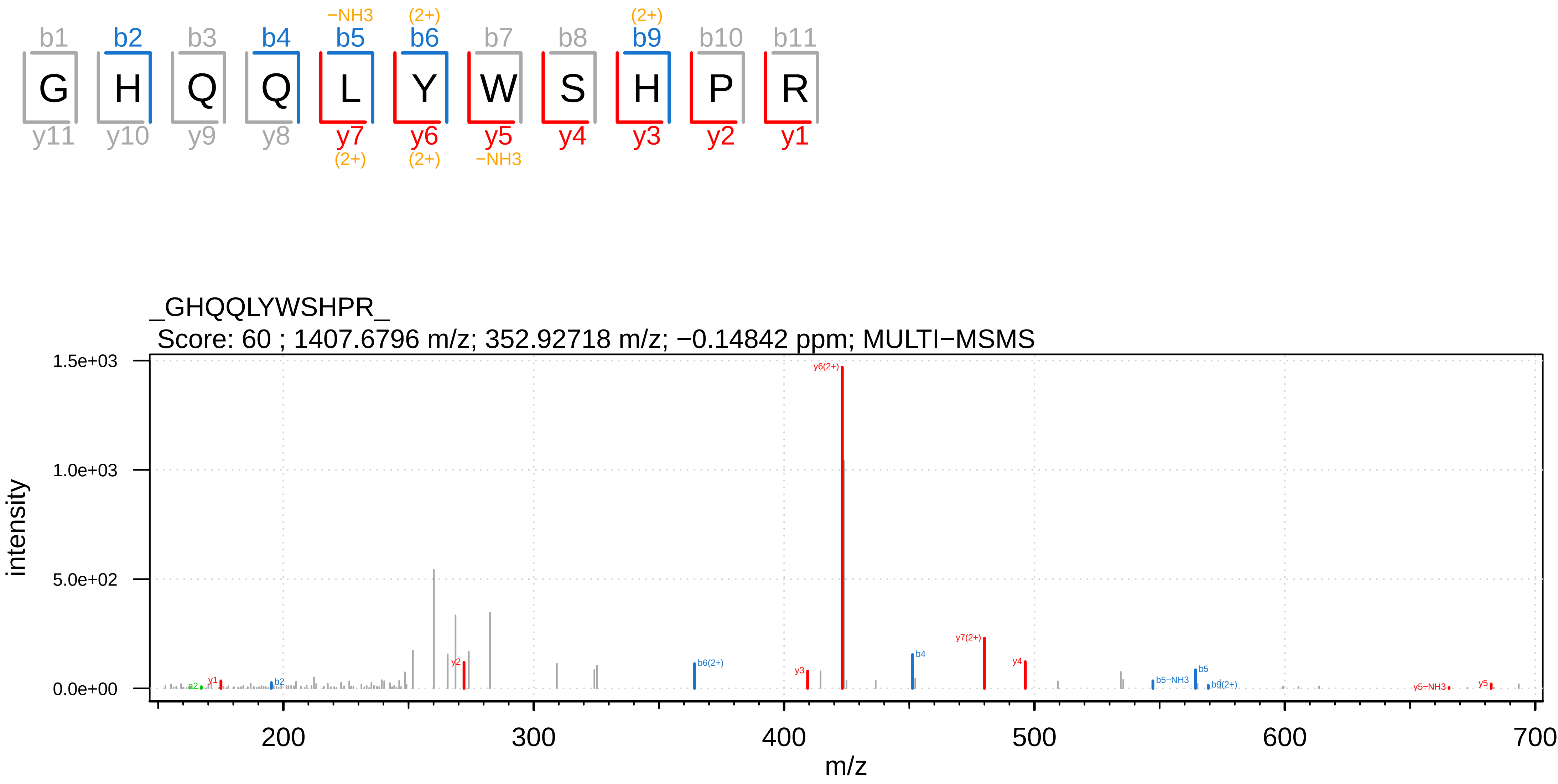

Raw File: Bert_20090331_BJS_halflives_P2_slice02_01
Scan Number: 4105
Proteins:
sp|P62274|RS29_MOUSE
ENSMUST00000037023_chr12:69157813−69159058:−
ENSMUST00000071737_chr13:73988963−73989133:−
tr|D3YYL7|D3YYL7_MOUSE

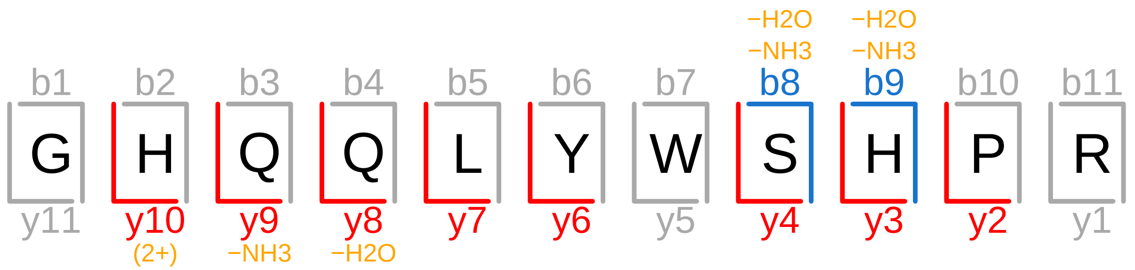

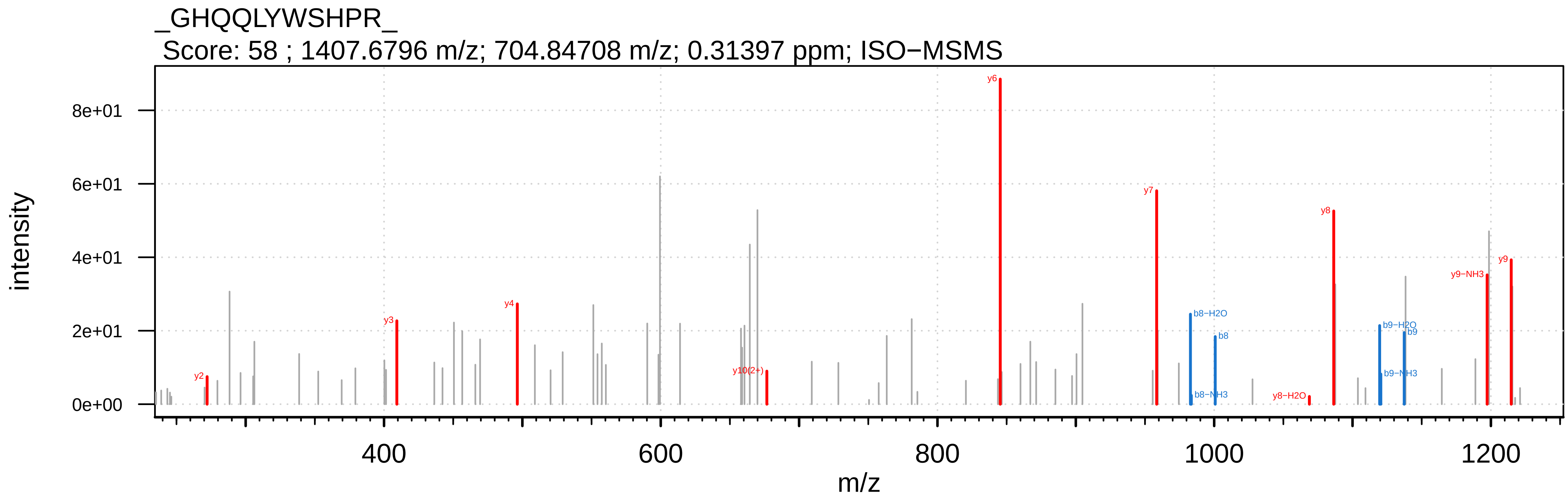

Raw File: Bert_20090331_BJS_halflives_P2_slice02_01
Scan Number: 4136
Proteins:
sp|P62274|RS29_MOUSE
ENSMUST00000037023_chr12:69157813−69159058:−
ENSMUST00000071737_chr13:73988963−73989133:−
tr|D3YYL7|D3YYL7_MOUSE

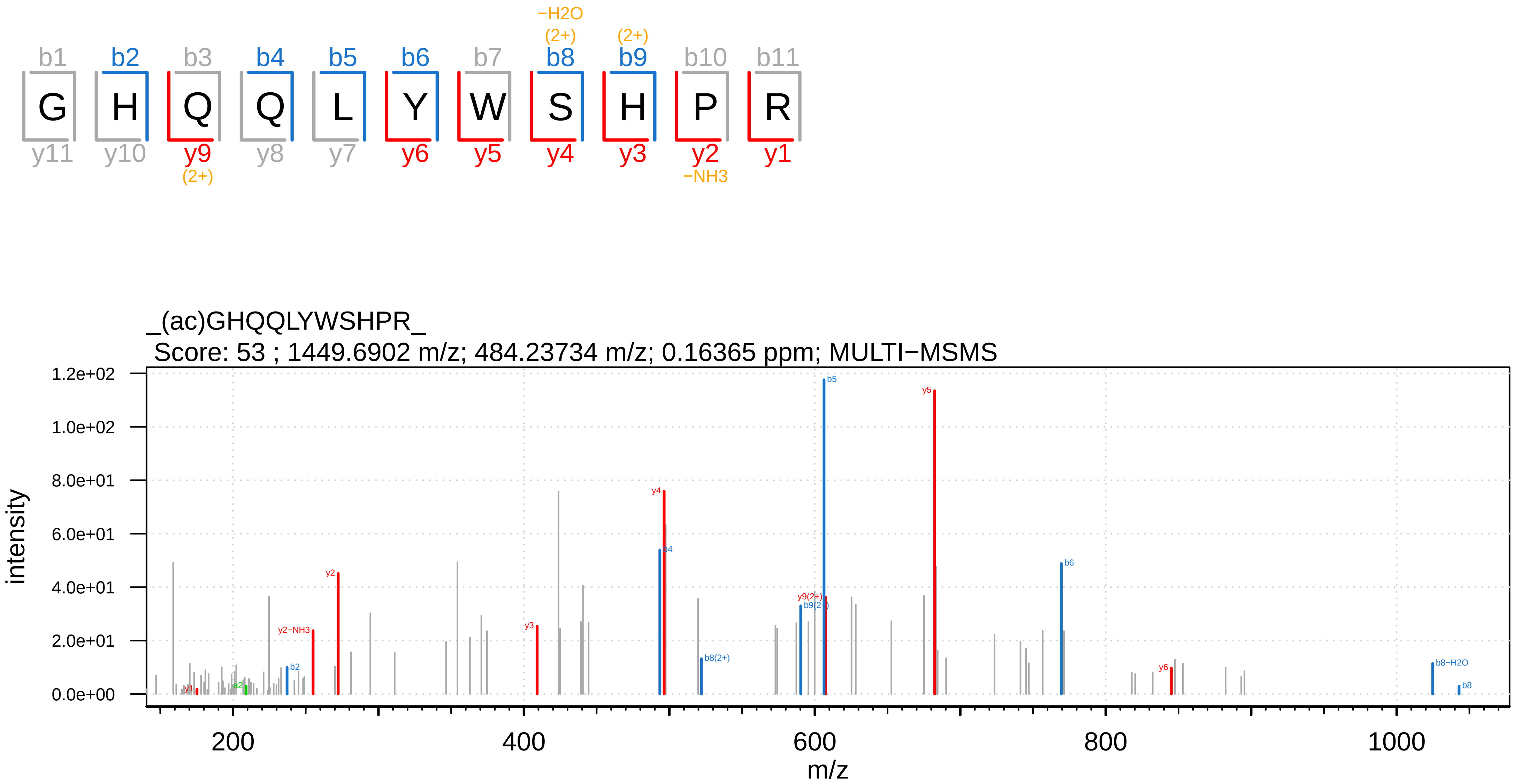

Raw File: Bert_20090331_BJS_halflives_P2_slice02_01

Scan Number: 4984

Proteins:

sp|P62274|RS29_MOUSE

ENSMUST00000037023_chr12:69157813−69159058:−

ENSMUST00000071737_chr13:73988963−73989133:−

tr|D3YYL7|D3YYL7_MOUSE

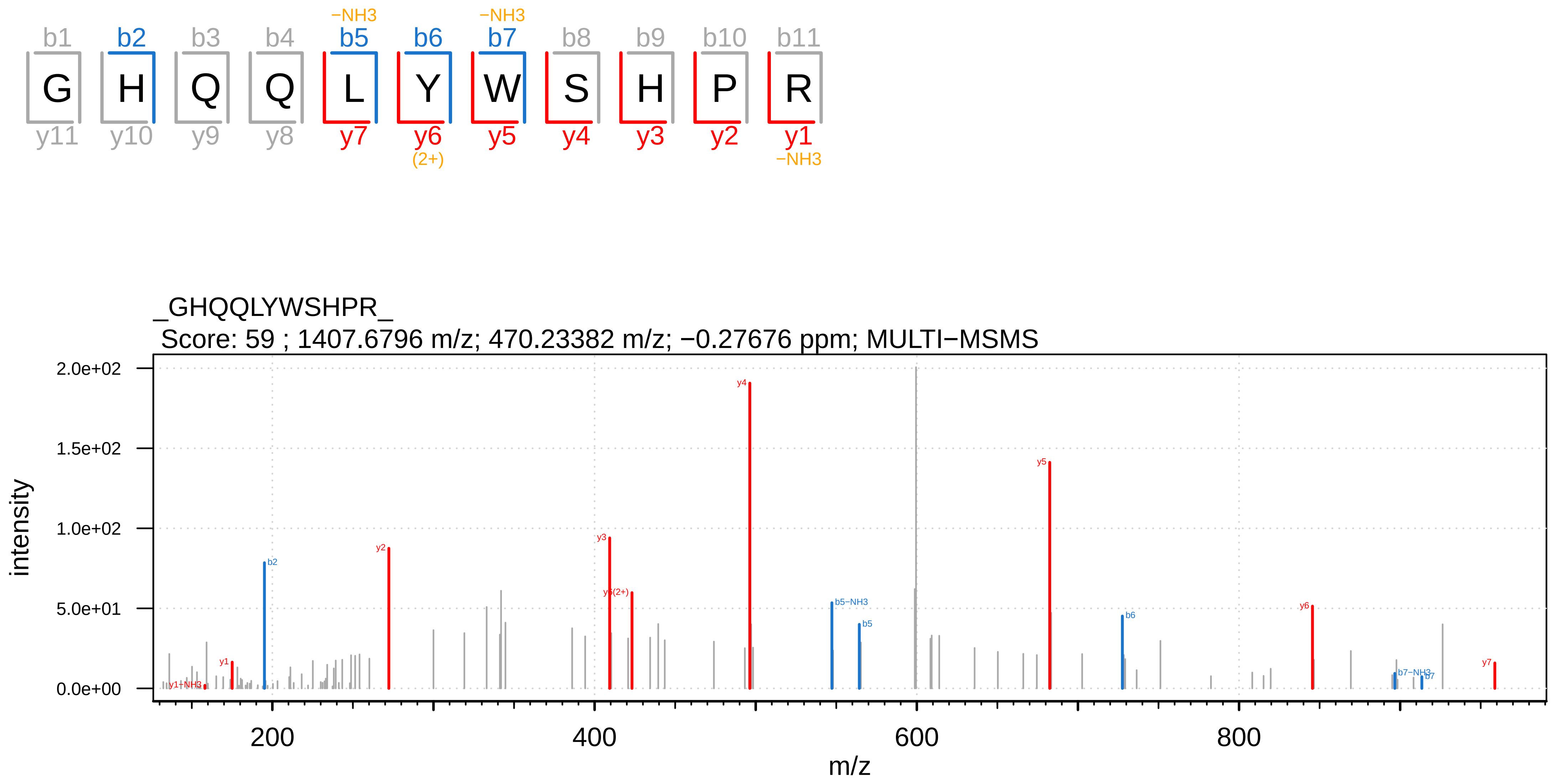

Raw File: Bert_20090331_BJS_halflives_P2_slice02_02
Scan Number: 3808
Proteins:
sp|P62274|RS29_MOUSE
ENSMUST00000037023_chr12:69157813−69159058:−
ENSMUST00000071737_chr13:73988963−73989133:−
tr|D3YYL7|D3YYL7_MOUSE

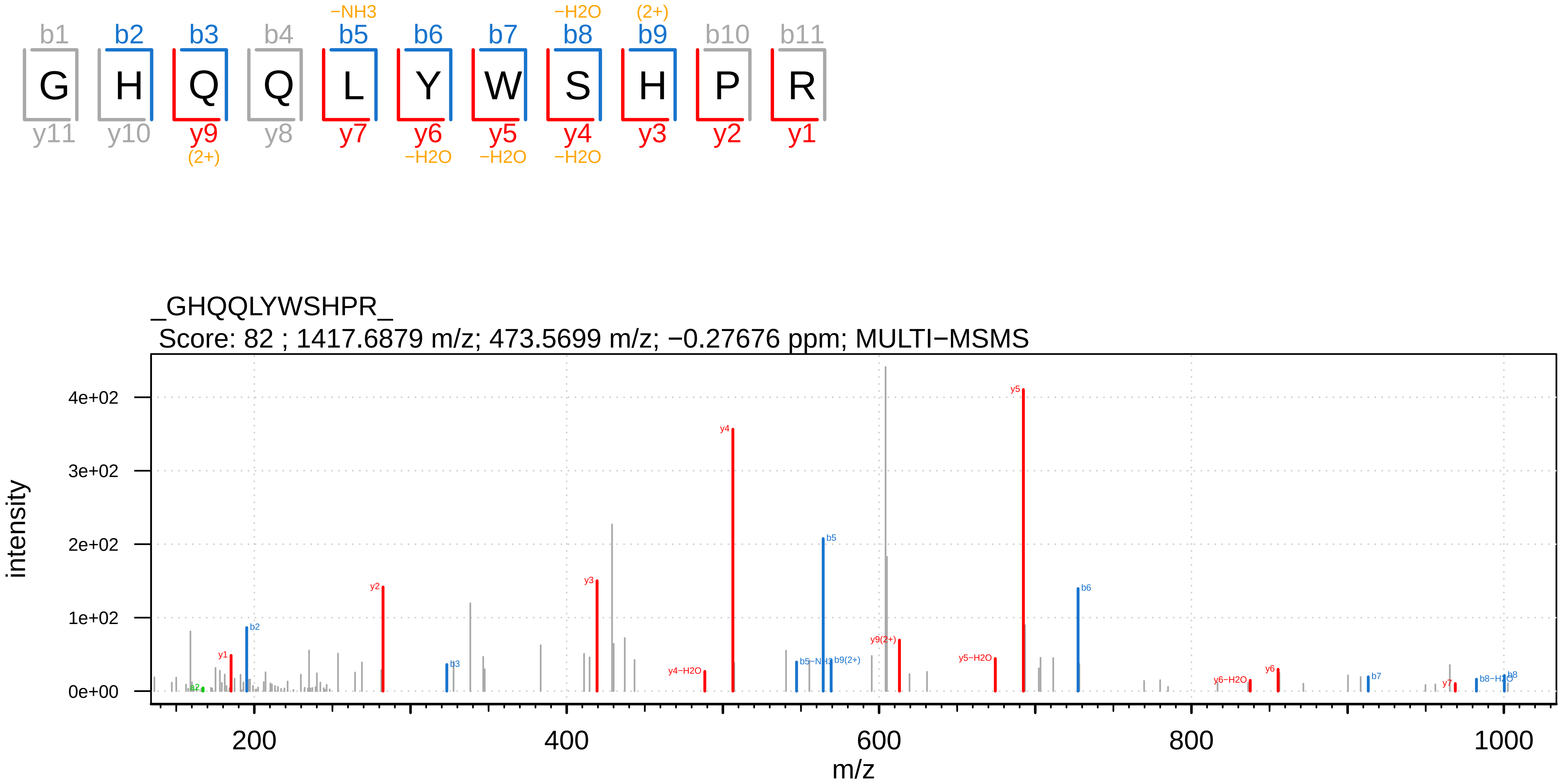

Raw File: Bert_20090331_BJS_halflives_P2_slice02_02
Scan Number: 3838
Proteins:
sp|P62274|RS29_MOUSE
ENSMUST00000037023_chr12:69157813−69159058:−
ENSMUST00000071737_chr13:73988963−73989133:−
tr|D3YYL7|D3YYL7_MOUSE

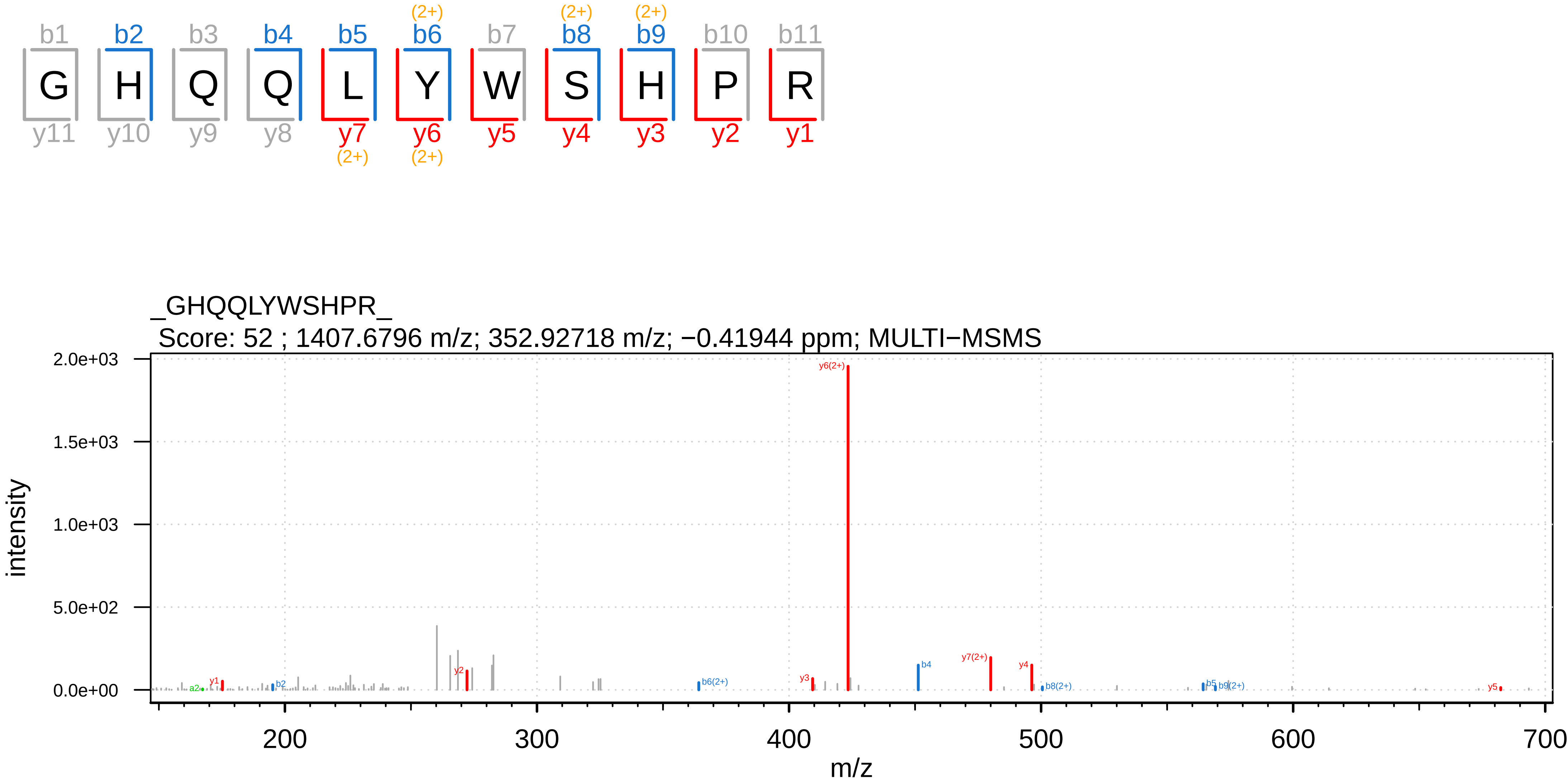

Raw File: Bert_20090331_BJS_halflives_P2_slice02_02
Scan Number: 3819
Proteins:
sp|P62274|RS29_MOUSE
ENSMUST00000037023_chr12:69157813−69159058:−
ENSMUST00000071737_chr13:73988963−73989133:−
tr|D3YYL7|D3YYL7_MOUSE

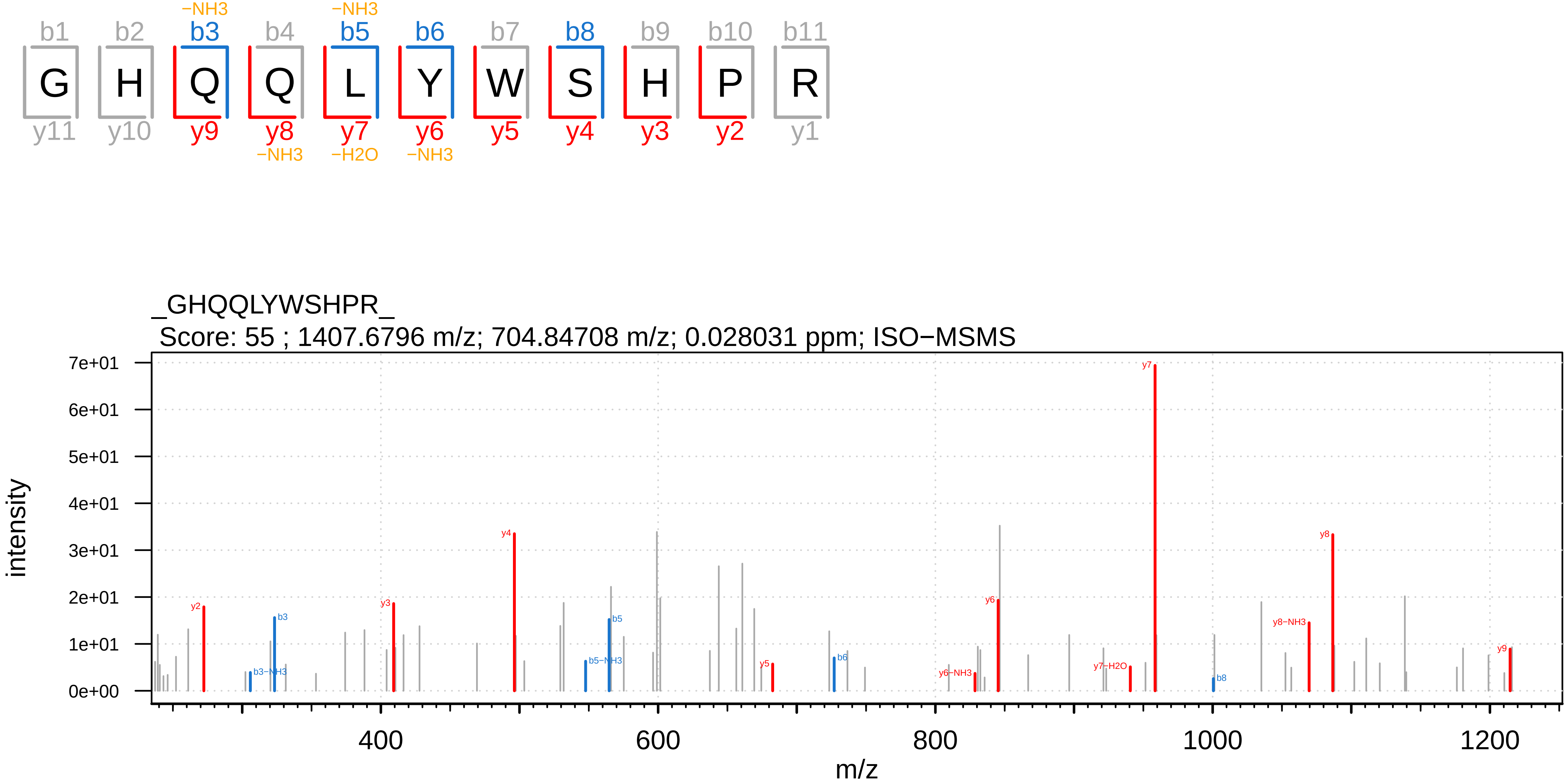

Raw File: Bert_20090331_BJS_halflives_P2_slice02_02
Scan Number: 3831
Proteins:
sp|P62274|RS29_MOUSE
ENSMUST00000037023_chr12:69157813−69159058:−
ENSMUST00000071737_chr13:73988963−73989133:−
tr|D3YYL7|D3YYL7_MOUSE

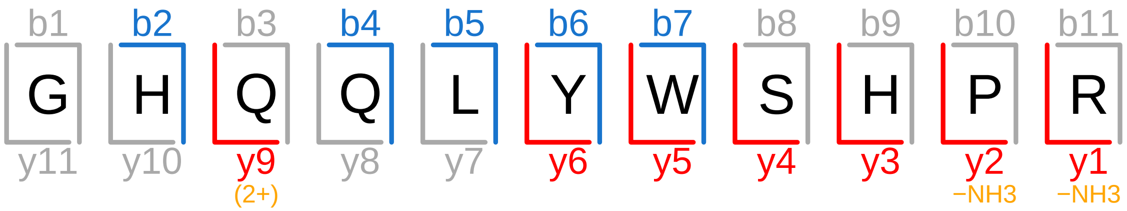

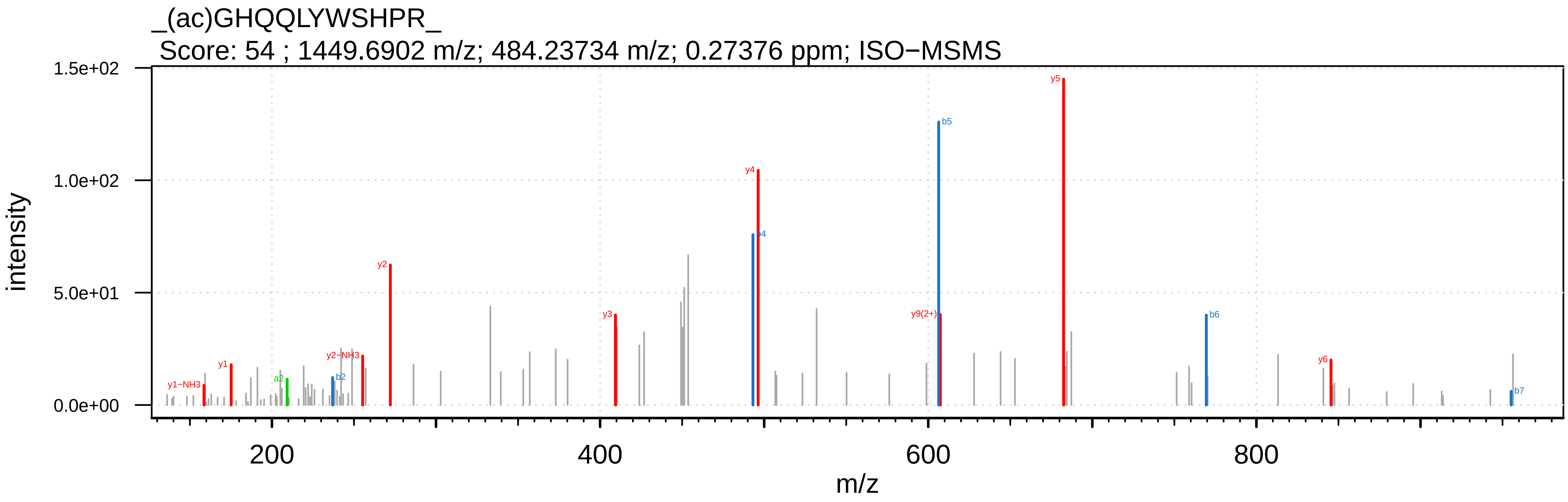

Raw File: Bert_20090331_BJS_halflives_P2_slice02_02
Scan Number: 4708
Proteins:
sp|P62274|RS29_MOUSE
ENSMUST00000037023_chr12:69157813−69159058:−
ENSMUST00000071737_chr13:73988963−73989133:−
tr|D3YYL7|D3YYL7_MOUSE

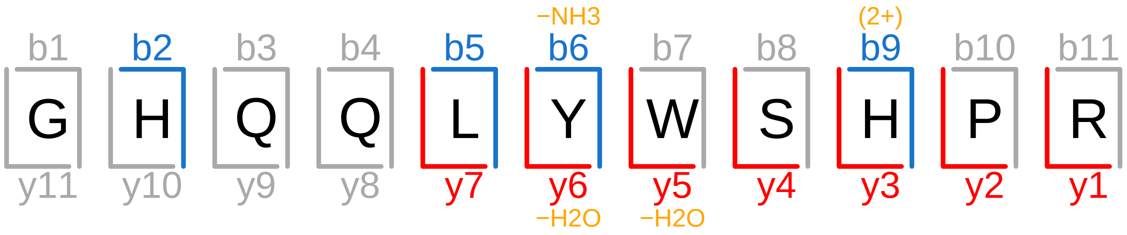

GHQQLYWSHPR
Score: 52 ; 1407.6796 m/z; 470.23382 m/z; −0.235 ppm; MULTI−MSMS

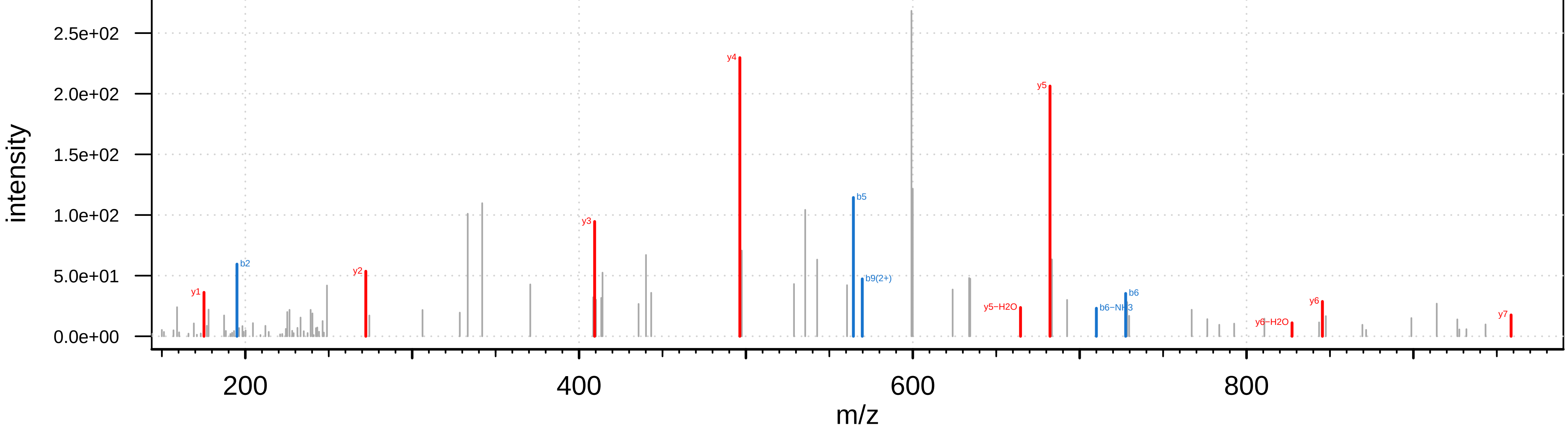

Raw File: Bert_20090331_BJS_halflives_P2_slice02_03
Scan Number: 3731
Proteins:
sp|P62274|RS29_MOUSE
ENSMUST00000037023_chr12:69157813−69159058:−
ENSMUST00000071737_chr13:73988963−73989133:−
tr|D3YYL7|D3YYL7_MOUSE

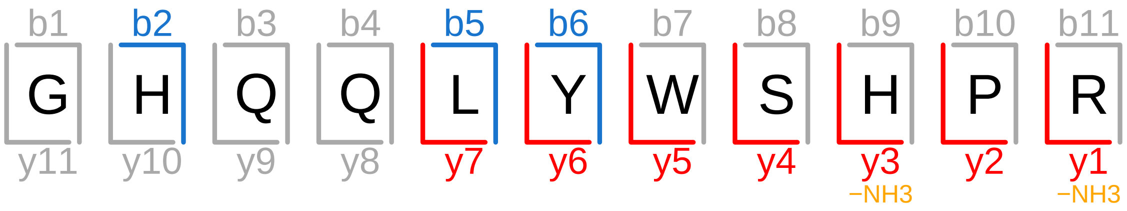

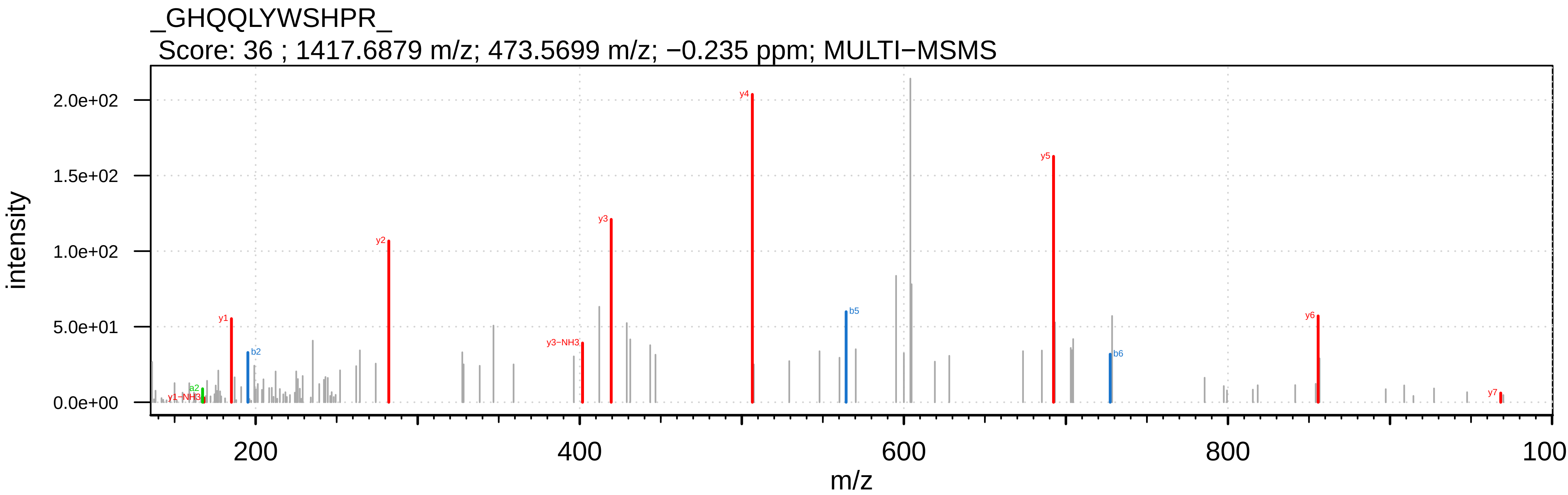

Raw File: Bert_20090331_BJS_halflives_P2_slice02_03
Scan Number: 3753
Proteins:
sp|P62274|RS29_MOUSE
ENSMUST00000037023_chr12:69157813−69159058:−
ENSMUST00000071737_chr13:73988963−73989133:−
tr|D3YYL7|D3YYL7_MOUSE

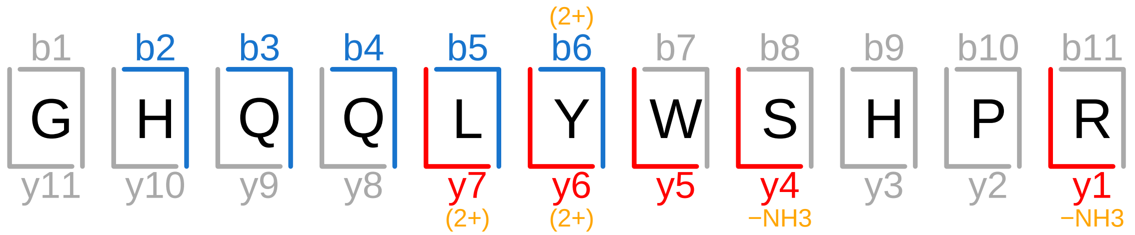

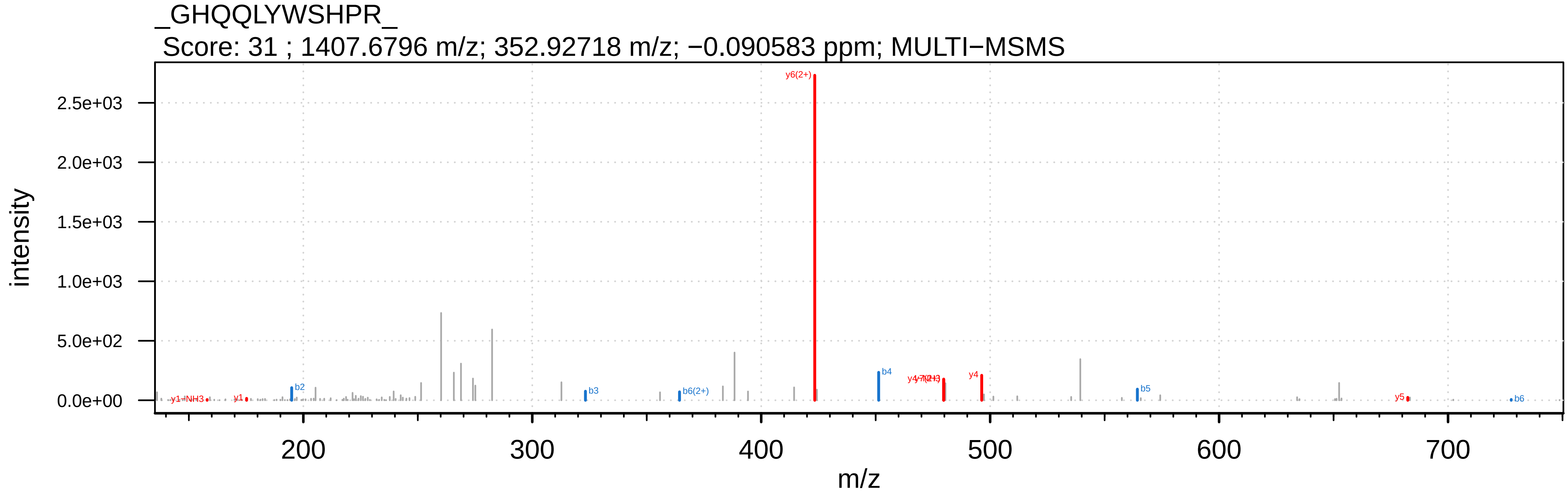

Raw File: Bert_20090331_BJS_halflives_P2_slice02_03
Scan Number: 3750
Proteins:
sp|P62274|RS29_MOUSE
ENSMUST00000037023_chr12:69157813−69159058:−
ENSMUST00000071737_chr13:73988963−73989133:−
tr|D3YYL7|D3YYL7_MOUSE

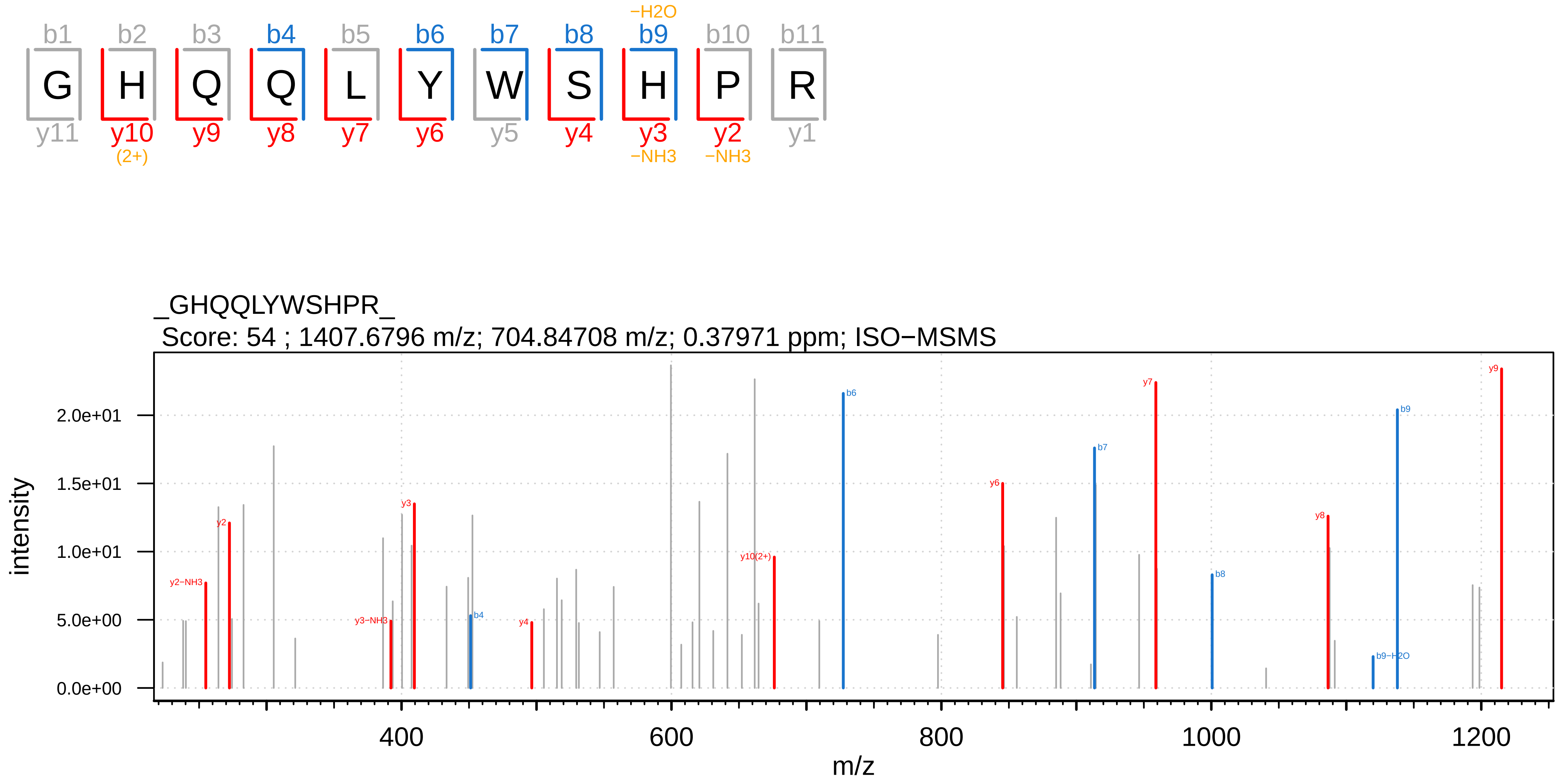

Raw File: Bert_20090331_BJS_halflives_P2_slice02_03
Scan Number: 3757
Proteins:
sp|P62274|RS29_MOUSE
ENSMUST00000037023_chr12:69157813−69159058:−
ENSMUST00000071737_chr13:73988963−73989133:−
tr|D3YYL7|D3YYL7_MOUSE

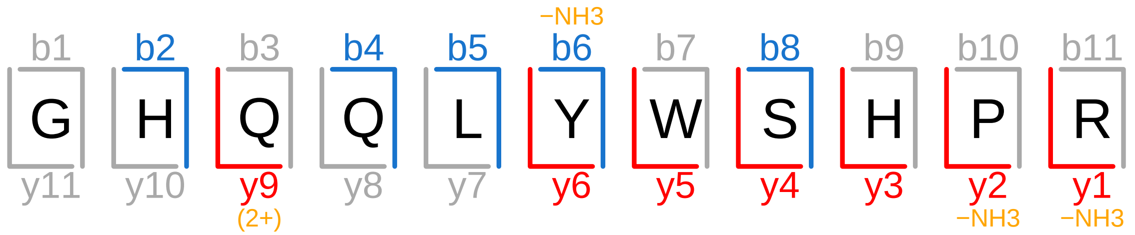

(ac)GHQQLYWSHPR

Score: 59 ; 1449.6902 m/z; 484.23734 m/z; 0.055112 ppm; MULTI−MSMS

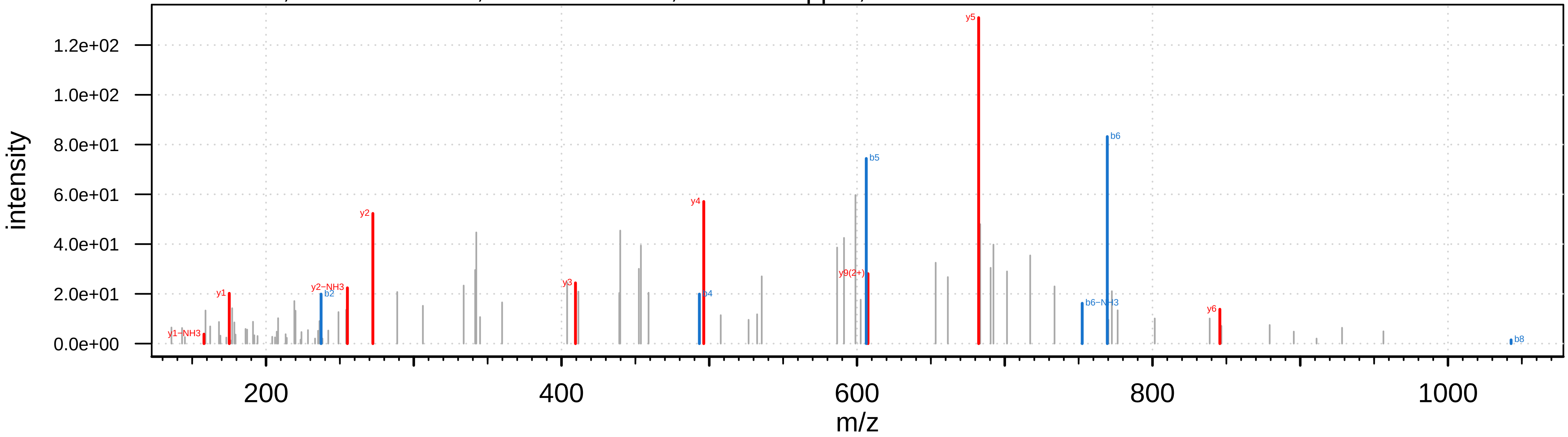

Raw File: Bert_20090331_BJS_halflives_P2_slice02_03
Scan Number: 4752
Proteins:
sp|P62274|RS29_MOUSE
ENSMUST00000037023_chr12:69157813−69159058:−
ENSMUST00000071737_chr13:73988963−73989133:−
tr|D3YYL7|D3YYL7_MOUSE

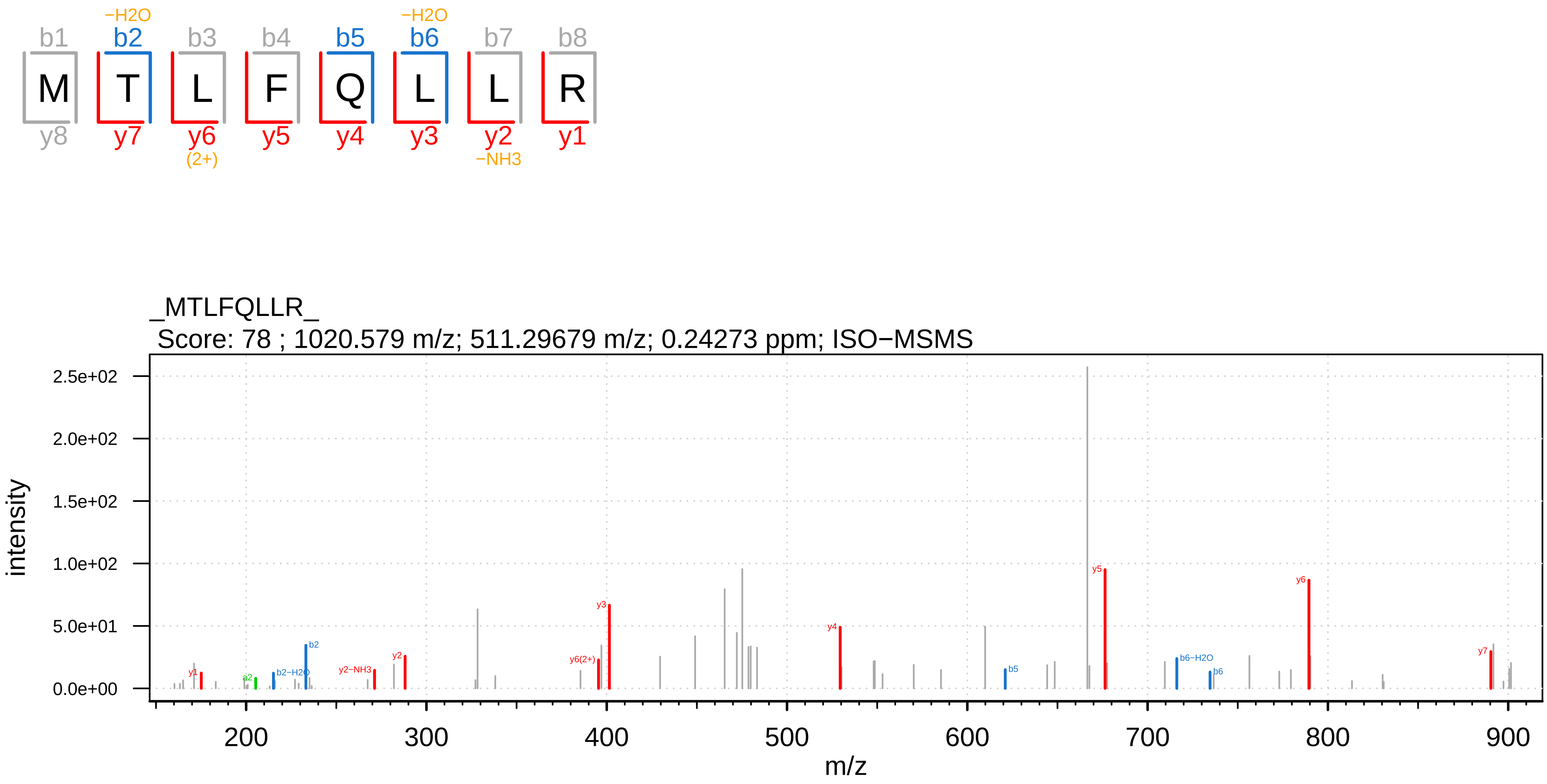

Raw File: Bert_20090331_BJS_halflives_P2_slice02_03
Scan Number: 10848
Proteins:
chr12_103206313−103214181.T2_chr12:101968174−101969629:−

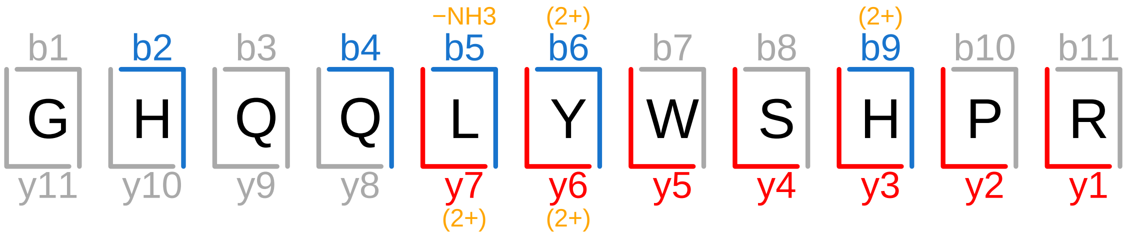

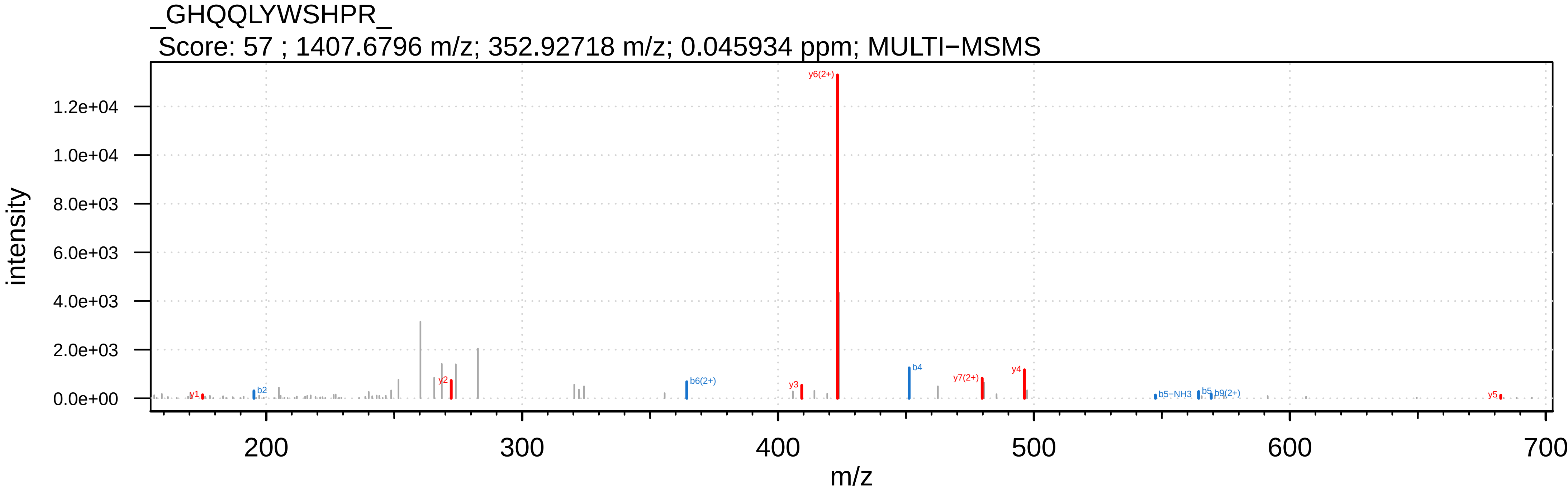

Raw File: Bert_20090331_BJS_halflives_P2_slice03_01
Scan Number: 4112
Proteins:
sp|P62274|RS29_MOUSE
ENSMUST00000037023_chr12:69157813−69159058:−
ENSMUST00000071737_chr13:73988963−73989133:−
tr|D3YYL7|D3YYL7_MOUSE

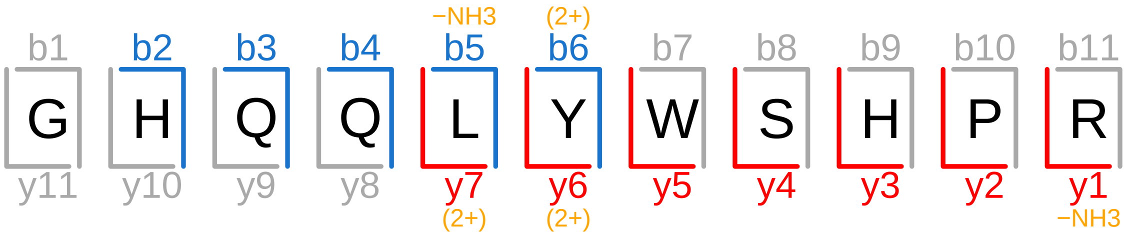

GHQQLYWSHPR

Score: 64 ; 1417.6879 m/z; 355.42925 m/z; 0.045934 ppm; MULTI−MSMS

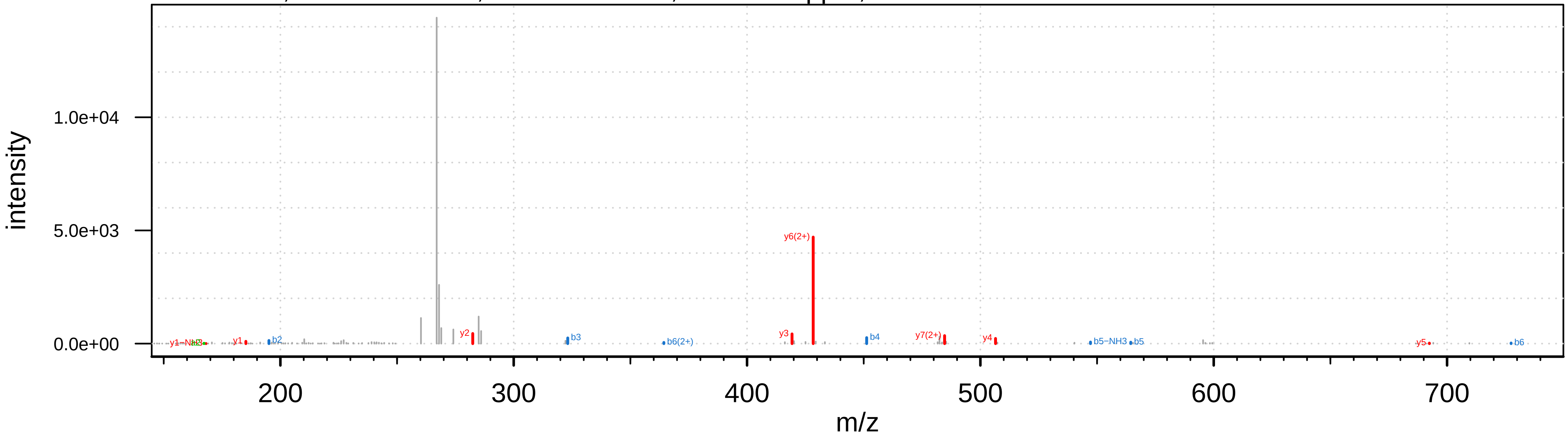

Raw File: Bert_20090331_BJS_halflives_P2_slice03_01
Scan Number: 4120
Proteins:
sp|P62274|RS29_MOUSE
ENSMUST00000037023_chr12:69157813−69159058:−
ENSMUST00000071737_chr13:73988963−73989133:−
tr|D3YYL7|D3YYL7_MOUSE

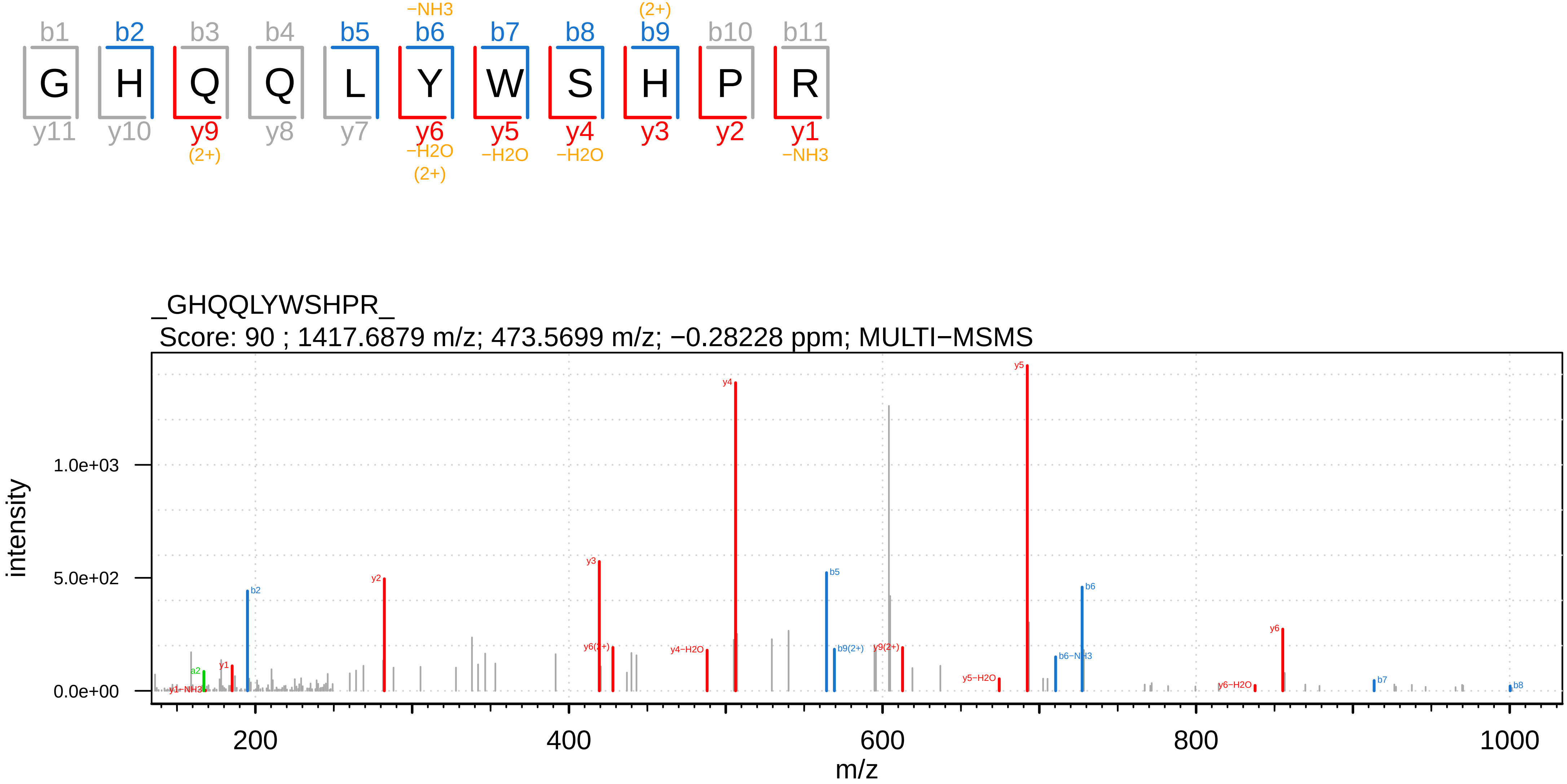

Raw File: Bert_20090331_BJS_halflives_P2_slice03_01
Scan Number: 4113
Proteins:
sp|P62274|RS29_MOUSE
ENSMUST00000037023_chr12:69157813−69159058:−
ENSMUST00000071737_chr13:73988963−73989133:−
tr|D3YYL7|D3YYL7_MOUSE

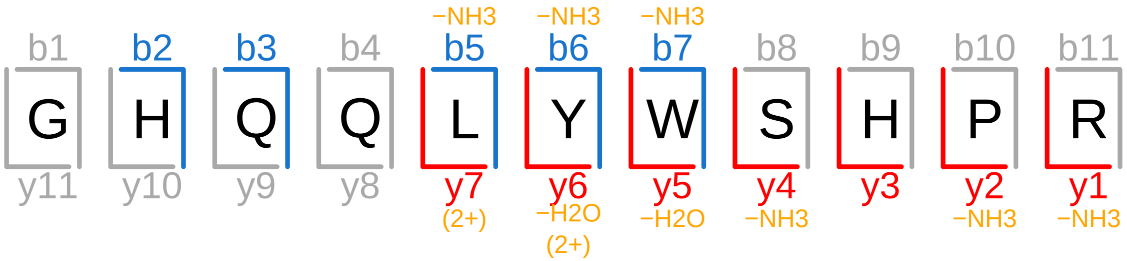

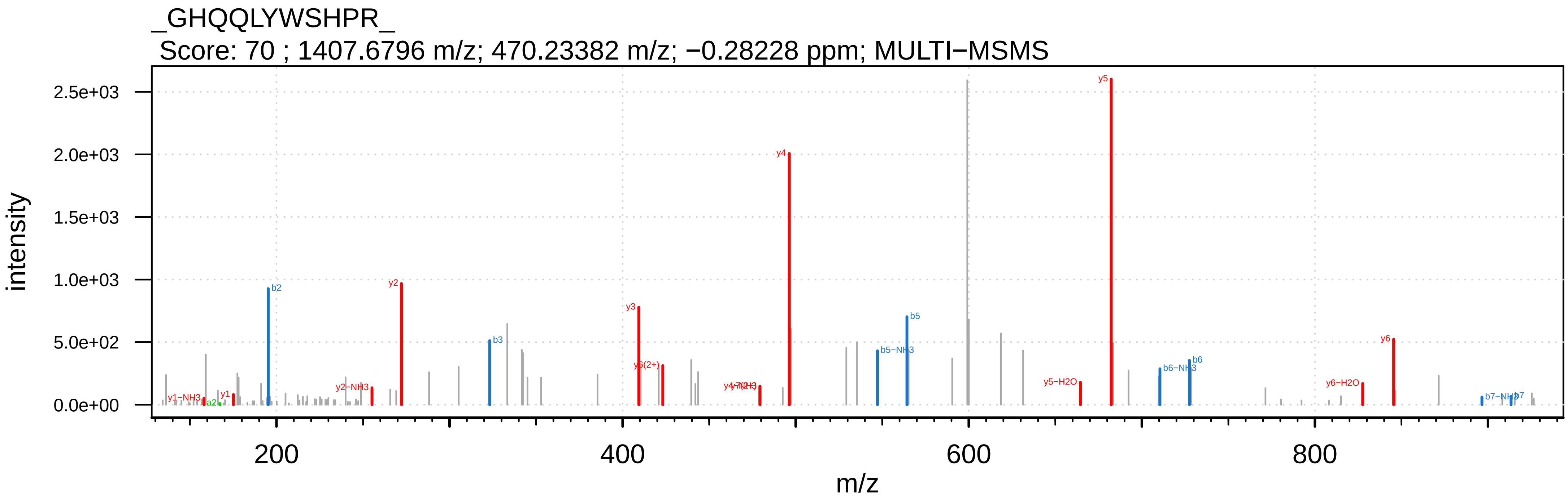

Raw File: Bert_20090331_BJS_halflives_P2_slice03_01
Scan Number: 4153
Proteins:
sp|P62274|RS29_MOUSE
ENSMUST00000037023_chr12:69157813−69159058:−
ENSMUST00000071737_chr13:73988963−73989133:−
tr|D3YYL7|D3YYL7_MOUSE

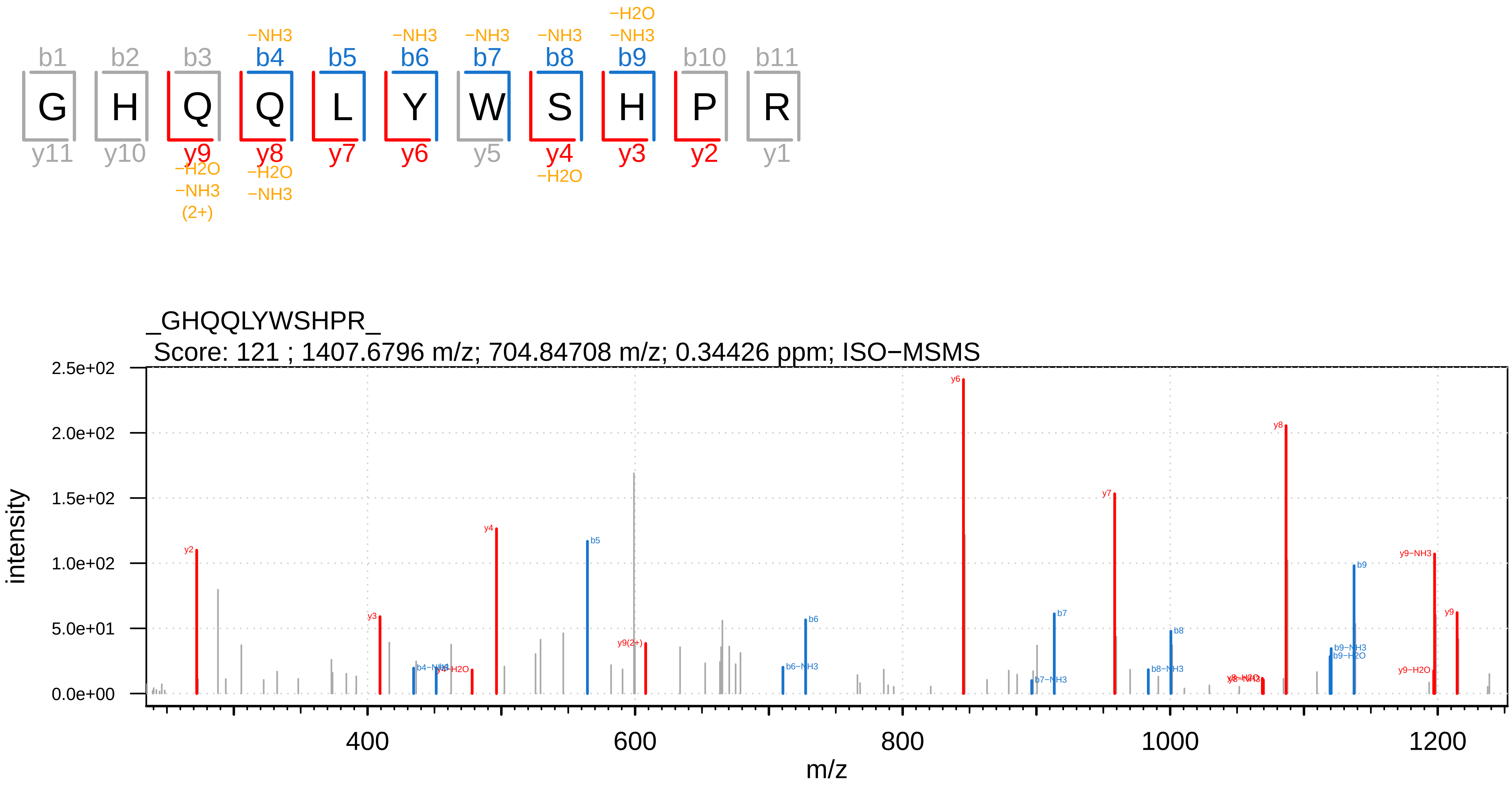

Raw File: Bert_20090331_BJS_halflives_P2_slice03_01
Scan Number: 4128
Proteins:
sp|P62274|RS29_MOUSE
ENSMUST00000037023_chr12:69157813−69159058:−
ENSMUST00000071737_chr13:73988963−73989133:−
tr|D3YYL7|D3YYL7_MOUSE

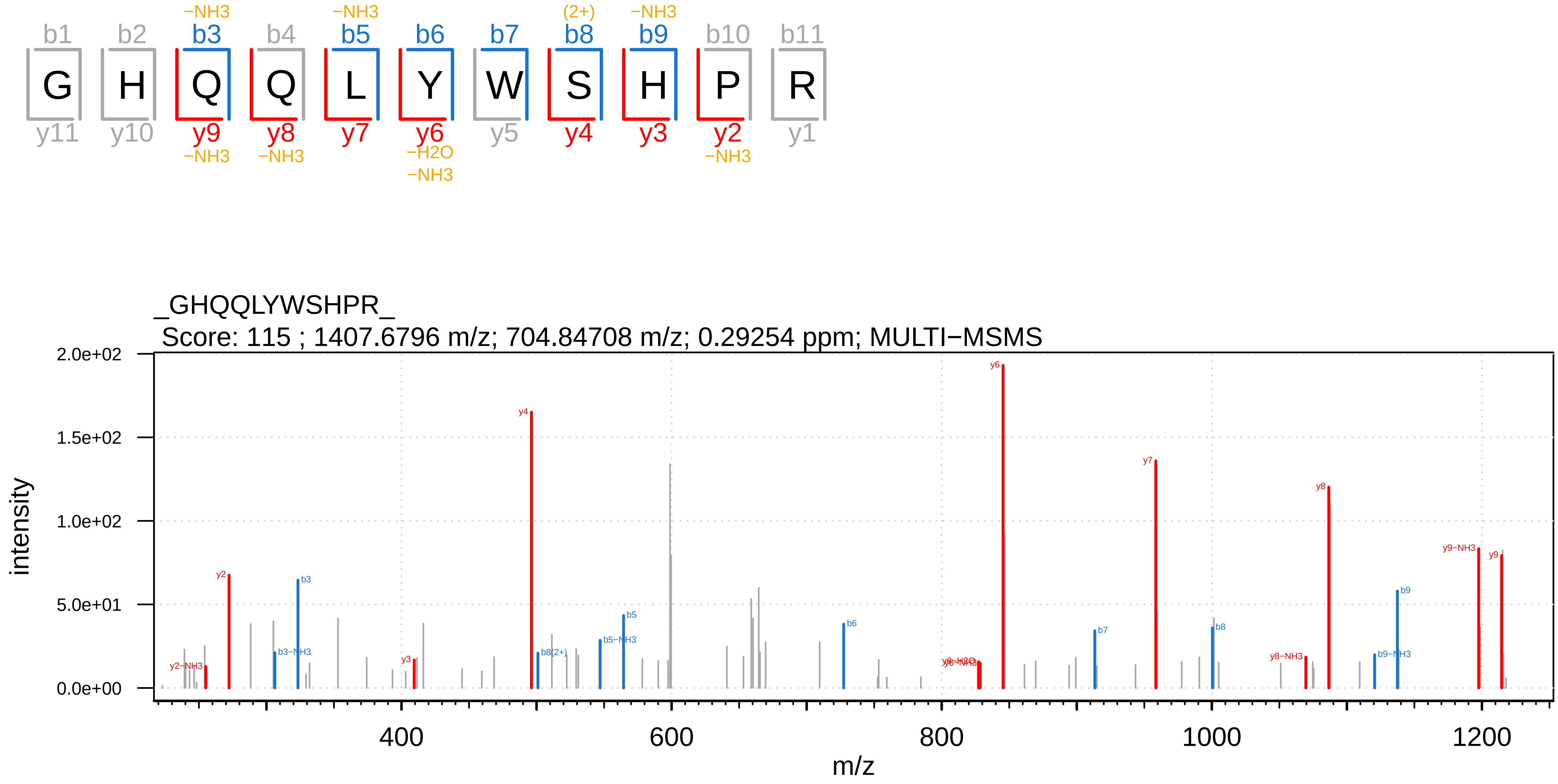

Raw File: Bert_20090331_BJS_halflives_P2_slice03_02
Scan Number: 3987
Proteins:
sp|P62274|RS29_MOUSE
ENSMUST00000037023_chr12:69157813−69159058:−
ENSMUST00000071737_chr13:73988963−73989133:−
tr|D3YYL7|D3YYL7_MOUSE

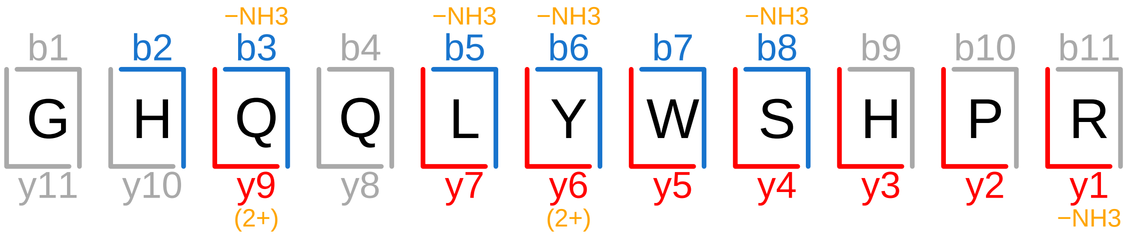

GHQQLYWSHPR
Score: 90 ; 1417.6879 m/z; 473.5699 m/z; −0.084717 ppm; MULTI−MSMS

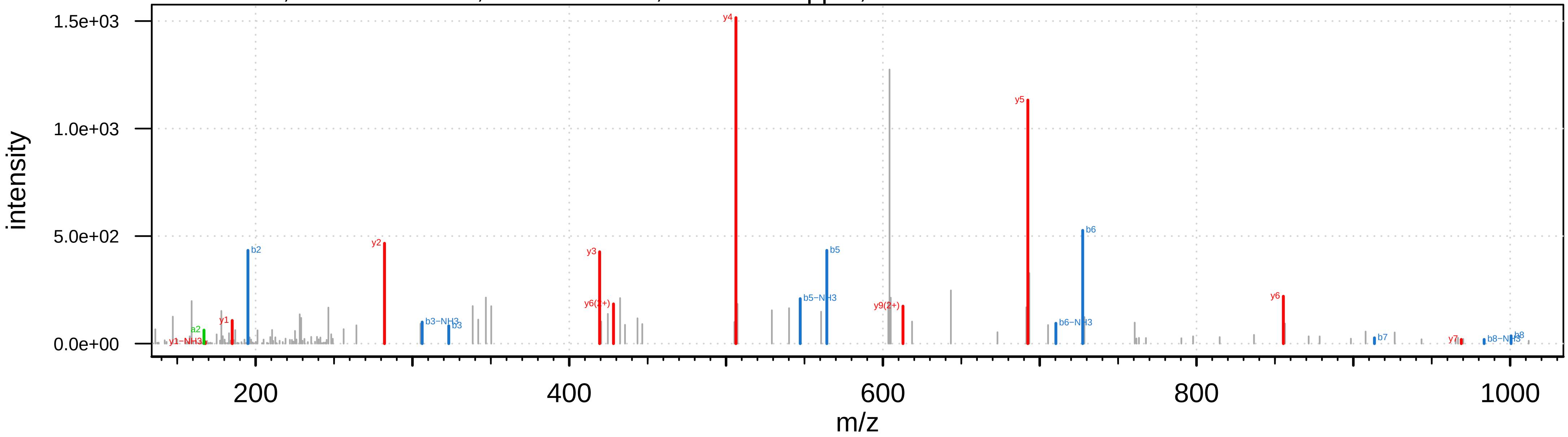

Raw File: Bert_20090331_BJS_halflives_P2_slice03_02
Scan Number: 3992
Proteins:
sp|P62274|RS29_MOUSE
ENSMUST00000037023_chr12:69157813−69159058:−
ENSMUST00000071737_chr13:73988963−73989133:−
tr|D3YYL7|D3YYL7_MOUSE

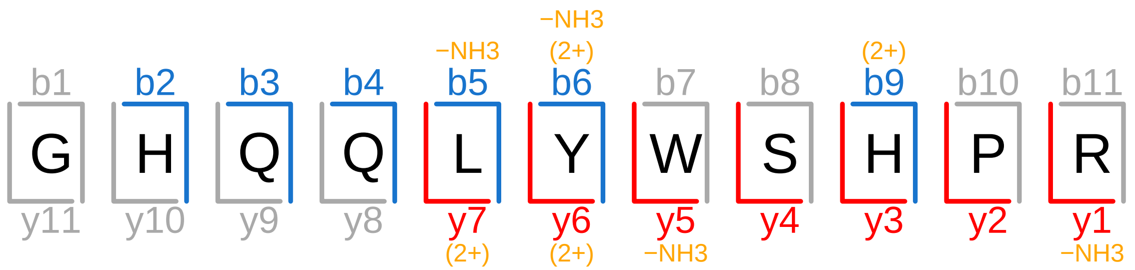

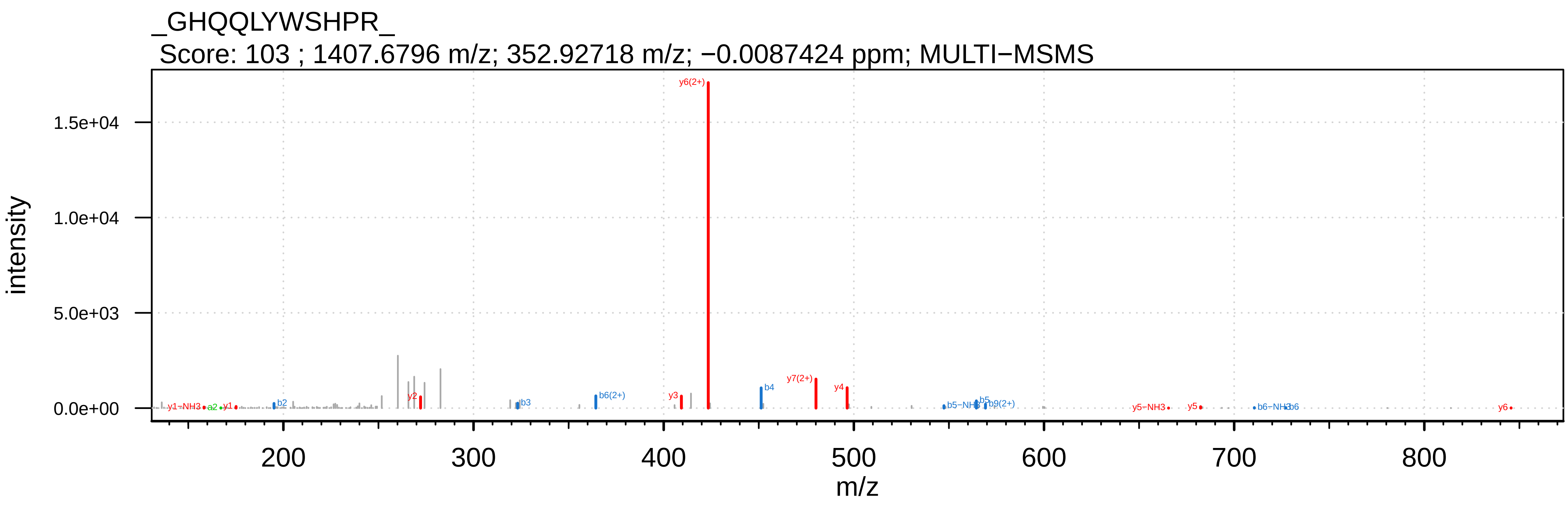

Raw File: Bert_20090331_BJS_halflives_P2_slice03_02
Scan Number: 4004
Proteins:
sp|P62274|RS29_MOUSE
ENSMUST00000037023_chr12:69157813−69159058:−
ENSMUST00000071737_chr13:73988963−73989133:−
tr|D3YYL7|D3YYL7_MOUSE

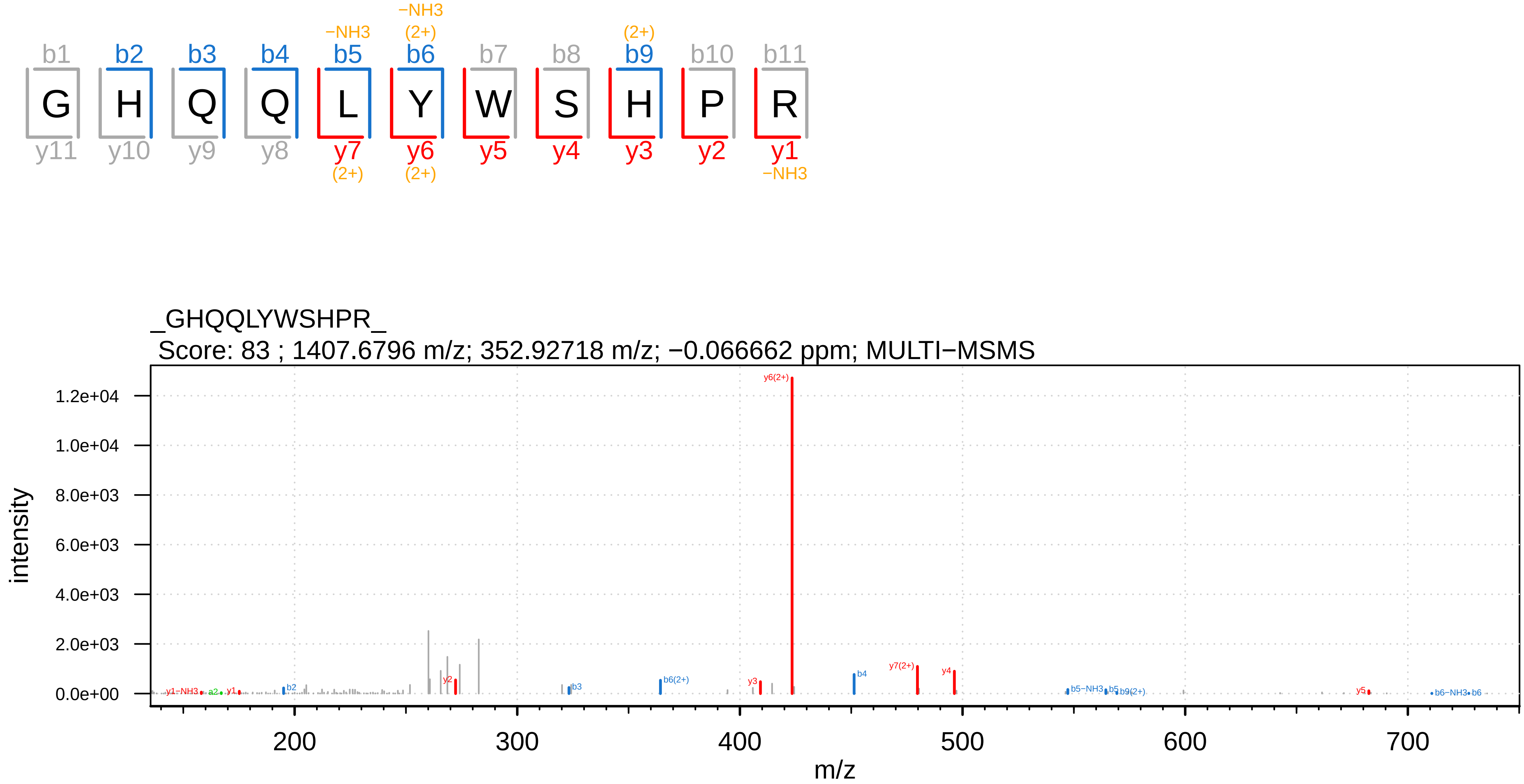

Raw File: Bert_20090331_BJS_halflives_P2_slice03_03
Scan Number: 3853
Proteins:
sp|P62274|RS29_MOUSE
ENSMUST00000037023_chr12:69157813−69159058:−
ENSMUST00000071737_chr13:73988963−73989133:−
tr|D3YYL7|D3YYL7_MOUSE

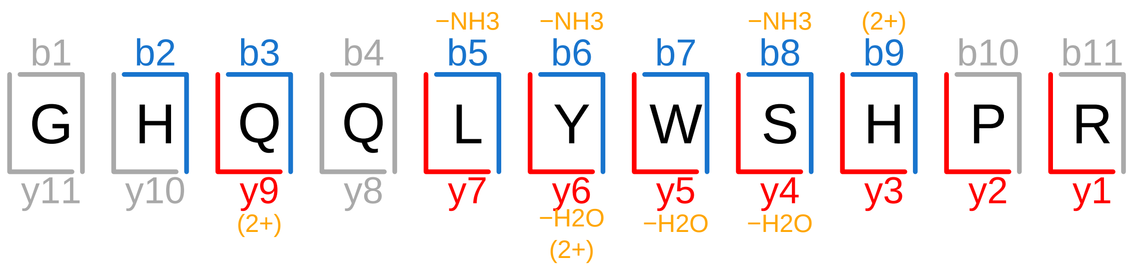

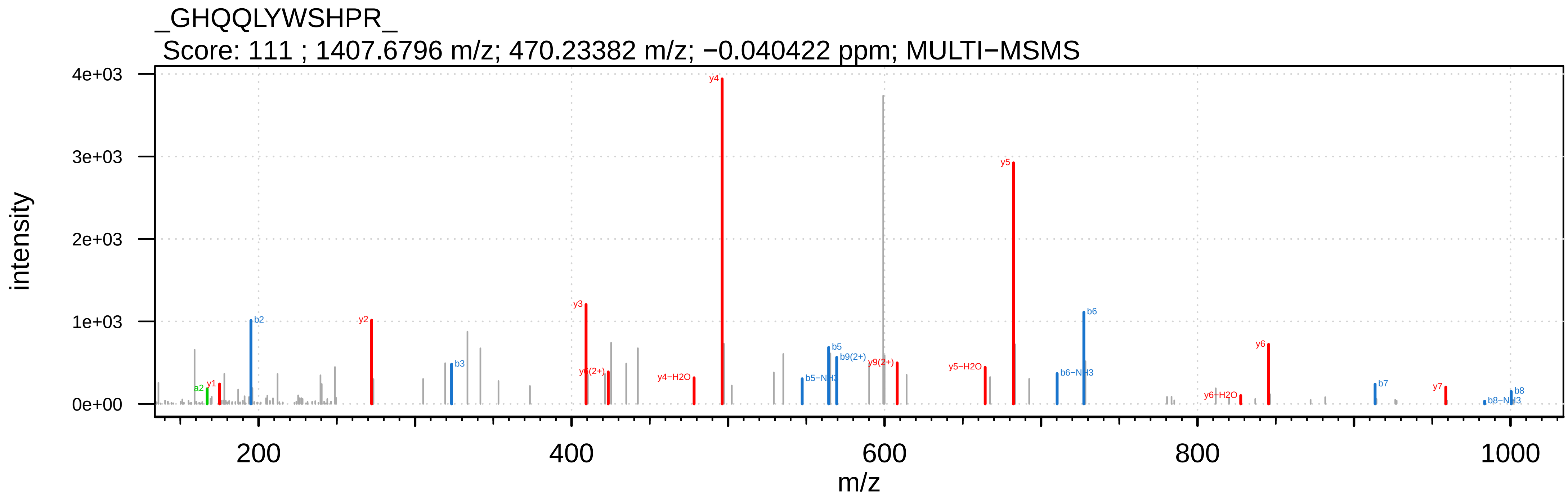

Raw File: Bert_20090331_BJS_halflives_P2_slice03_03
Scan Number: 3857
Proteins:
sp|P62274|RS29_MOUSE
ENSMUST00000037023_chr12:69157813−69159058:−
ENSMUST00000071737_chr13:73988963−73989133:−
tr|D3YYL7|D3YYL7_MOUSE

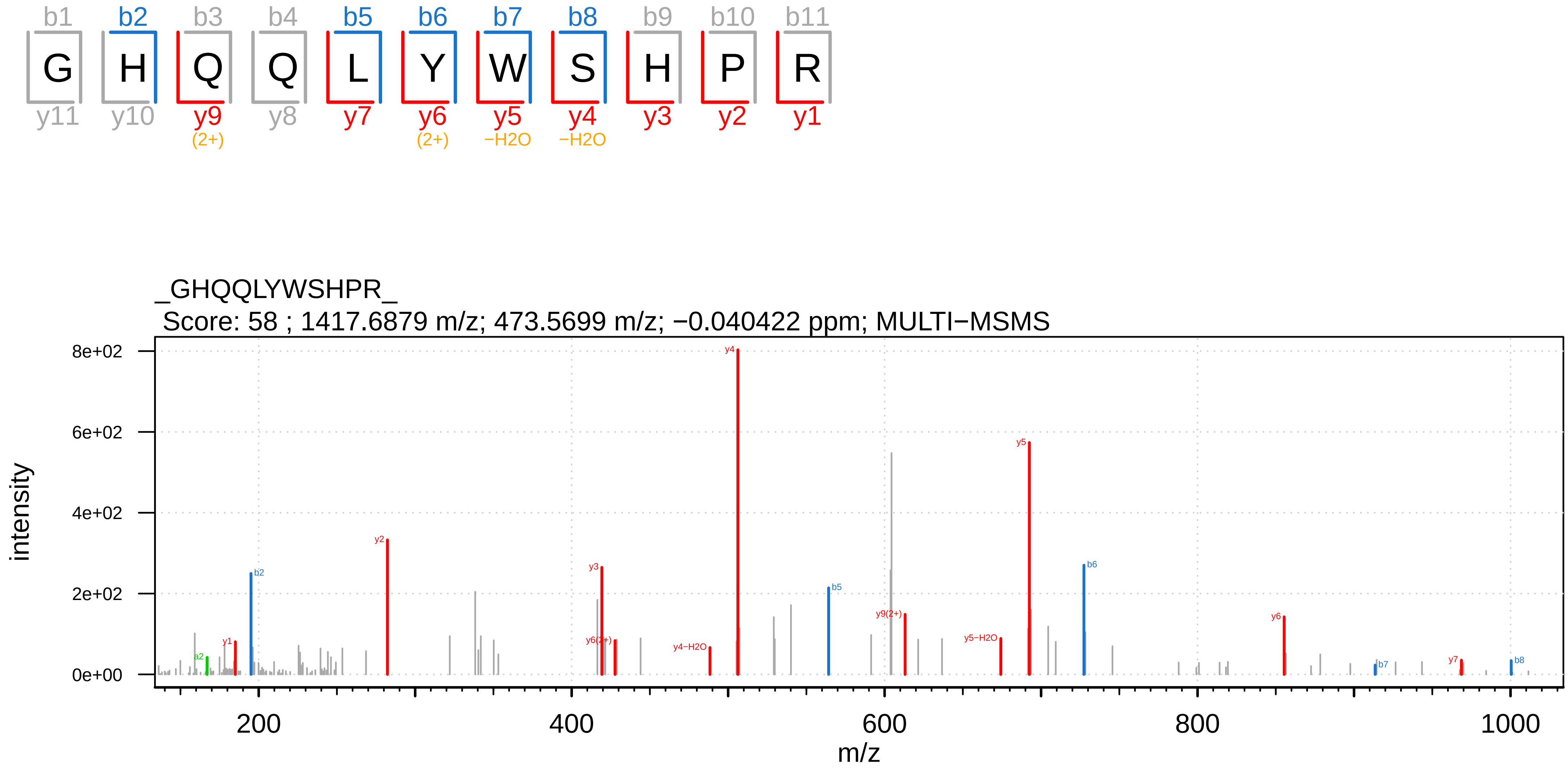

Raw File: Bert_20090331_BJS_halflives_P2_slice03_03
Scan Number: 3864
Proteins:
sp|P62274|RS29_MOUSE
ENSMUST00000037023_chr12:69157813−69159058:−
ENSMUST00000071737_chr13:73988963−73989133:−
tr|D3YYL7|D3YYL7_MOUSE

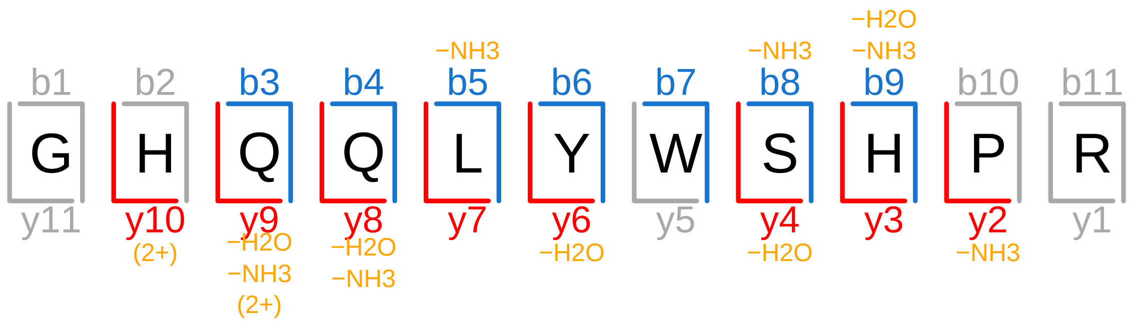

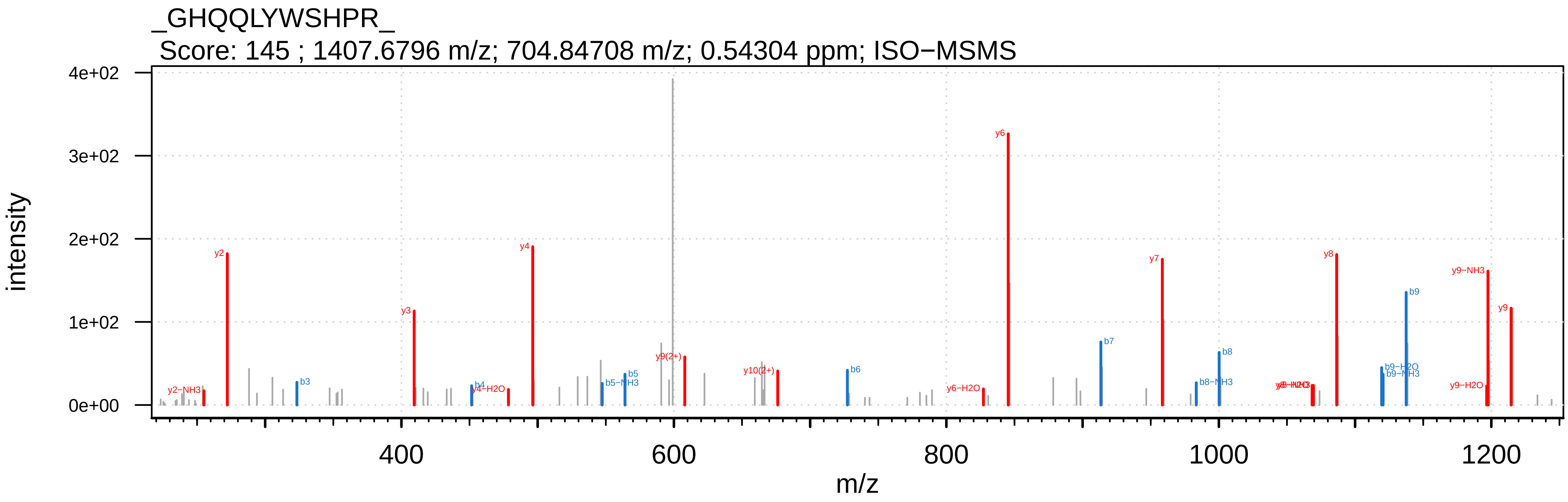

Raw File: Bert_20090331_BJS_halflives_P2_slice03_03
Scan Number: 3876
Proteins:
sp|P62274|RS29_MOUSE
ENSMUST00000037023_chr12:69157813−69159058:−
ENSMUST00000071737_chr13:73988963−73989133:−
tr|D3YYL7|D3YYL7_MOUSE

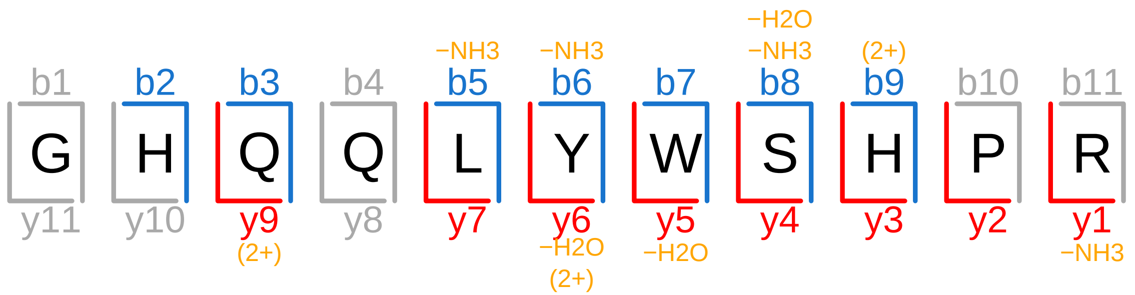

GHQQLYWSHPR
Score: 129 ; 1407.6796 m/z; 470.23382 m/z; −0.39588 ppm; ISO−MSMS

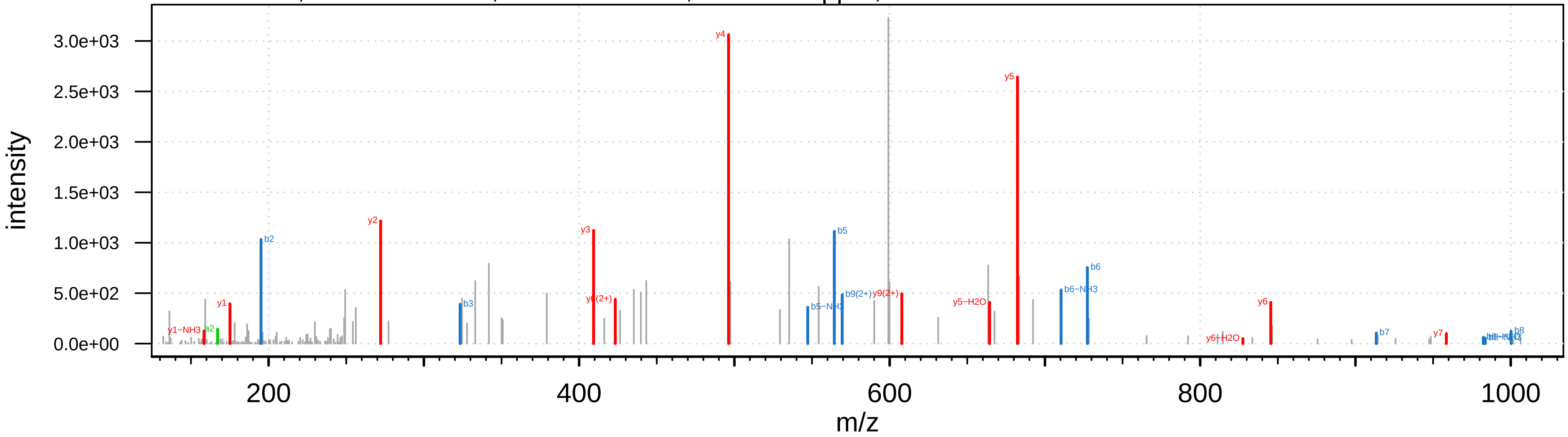

Raw File: Bert_20090331_BJS_halflives_P2_slice04_01
Scan Number: 3942
Proteins:
sp|P62274|RS29_MOUSE
ENSMUST00000037023_chr12:69157813−69159058:−
ENSMUST00000071737_chr13:73988963−73989133:−
tr|D3YYL7|D3YYL7_MOUSE

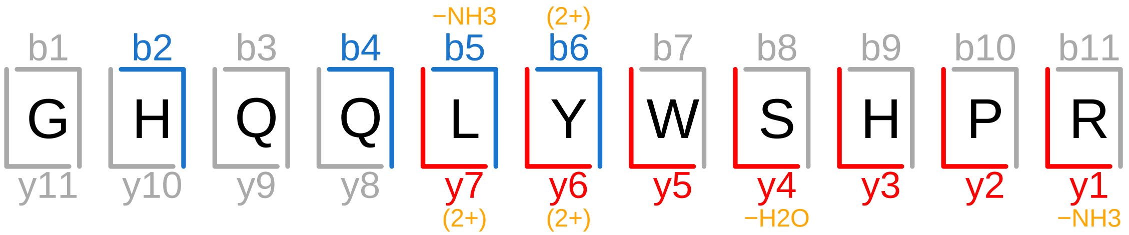

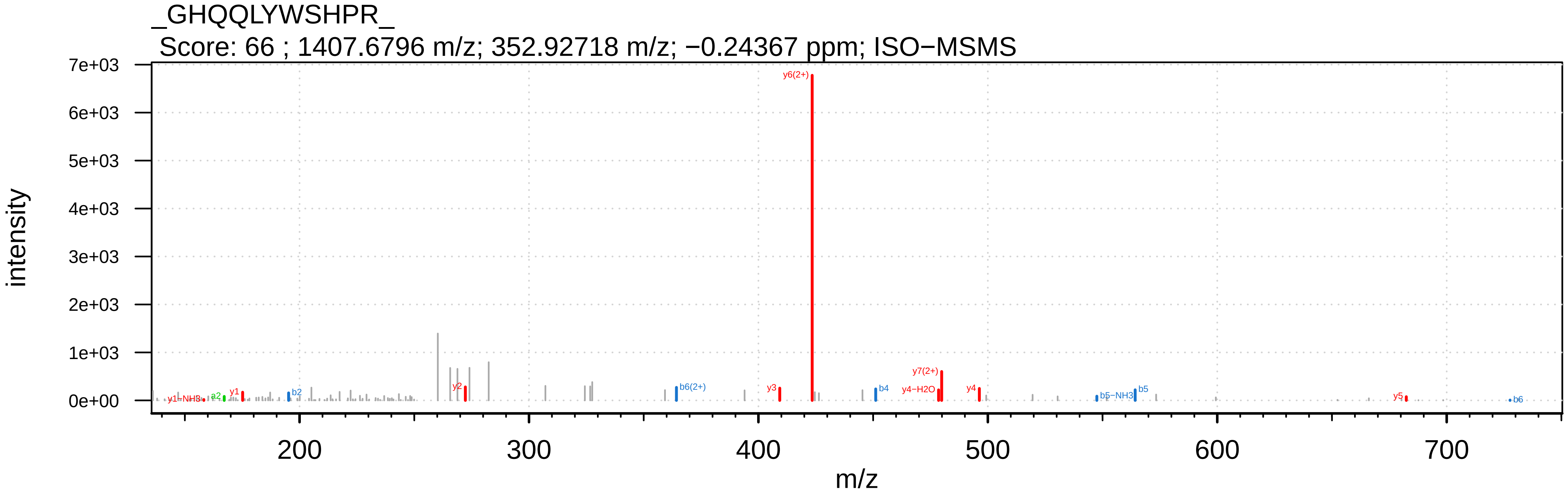

Raw File: Bert_20090331_BJS_halflives_P2_slice04_01
Scan Number: 3943
Proteins:
sp|P62274|RS29_MOUSE
ENSMUST00000037023_chr12:69157813−69159058:−
ENSMUST00000071737_chr13:73988963−73989133:−
tr|D3YYL7|D3YYL7_MOUSE

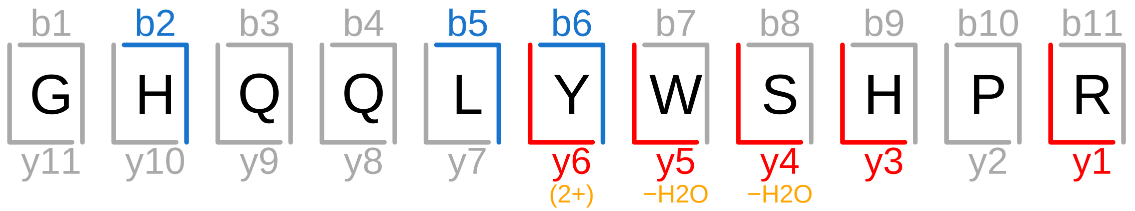

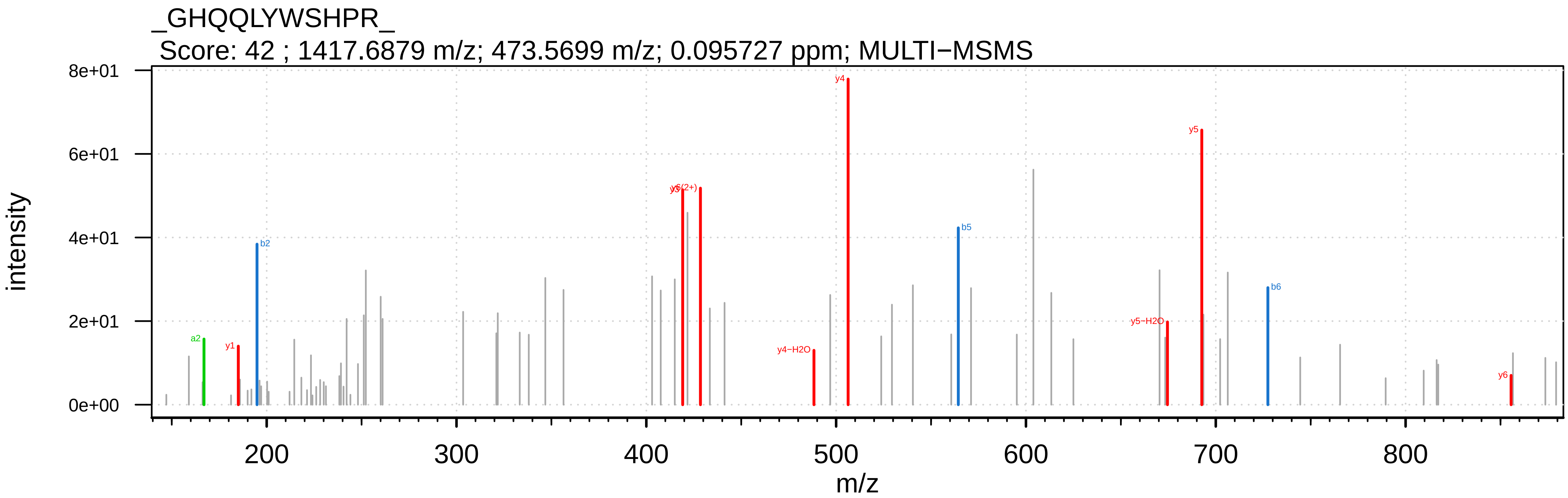

Raw File: Bert_20090331_BJS_halflives_P2_slice04_02
Scan Number: 3424
Proteins:
sp|P62274|RS29_MOUSE
ENSMUST00000037023_chr12:69157813−69159058:−
ENSMUST00000071737_chr13:73988963−73989133:−
tr|D3YYL7|D3YYL7_MOUSE

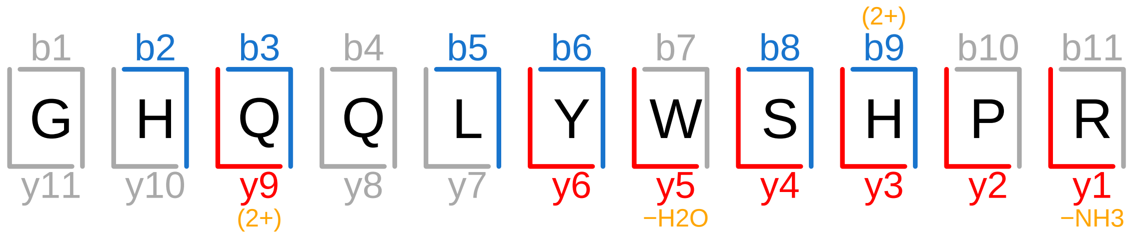

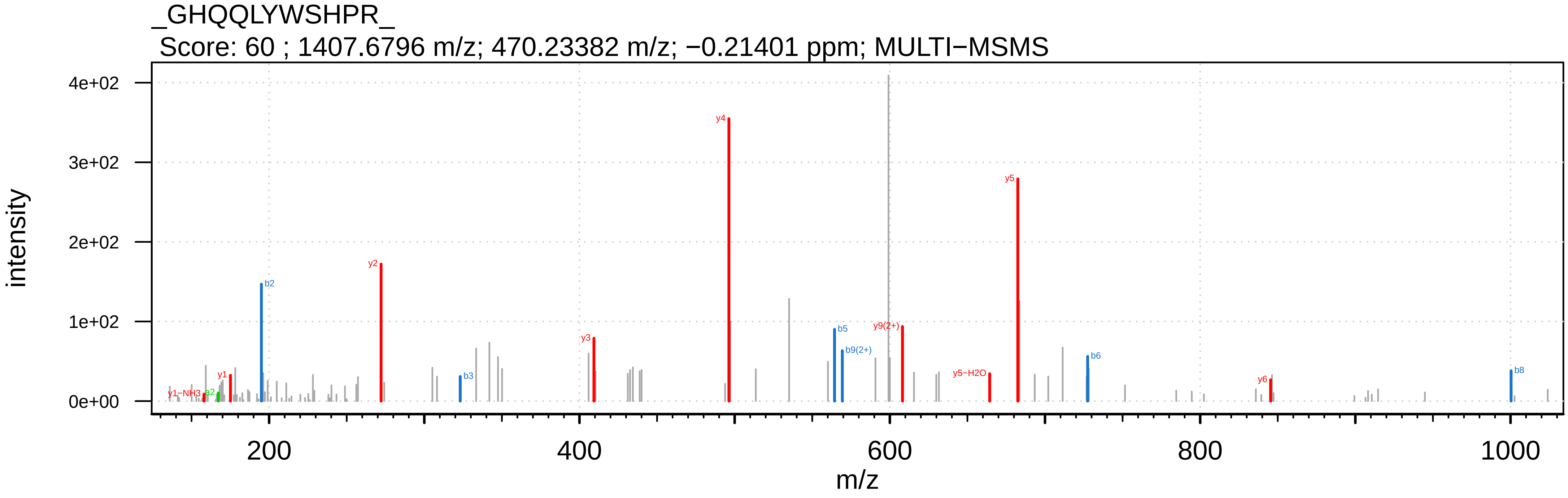

Raw File: Bert_20090331_BJS_halflives_P2_slice04_03
Scan Number: 3271
Proteins:
sp|P62274|RS29_MOUSE
ENSMUST00000037023_chr12:69157813−69159058:−
ENSMUST00000071737_chr13:73988963−73989133:−
tr|D3YYL7|D3YYL7_MOUSE

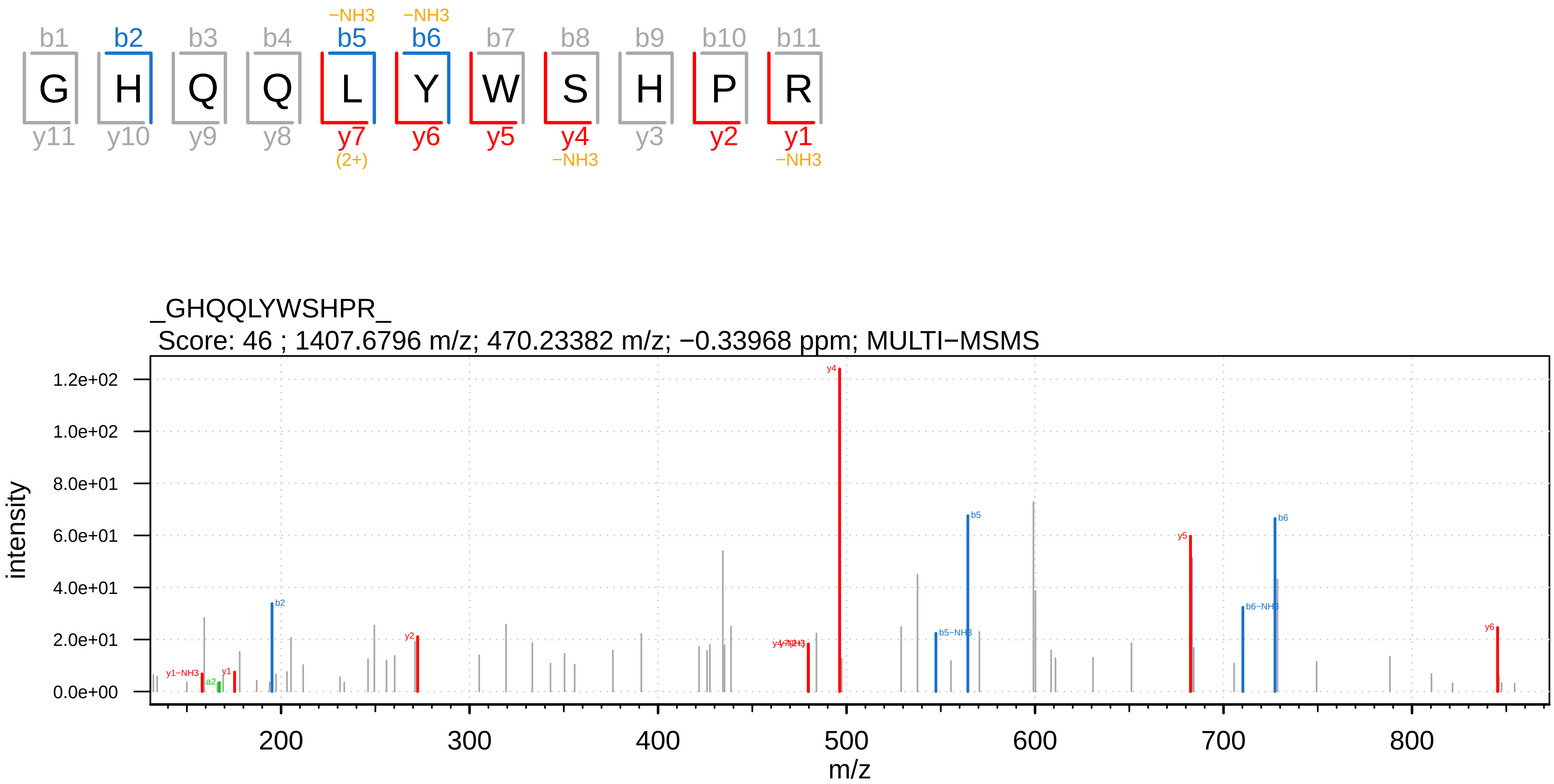

Raw File: Bert_20090403_BJS_halflives_P3_slice01_04
Scan Number: 2860
Proteins:
sp|P62274|RS29_MOUSE
ENSMUST00000037023_chr12:69157813−69159058:−
ENSMUST00000071737_chr13:73988963−73989133:−
tr|D3YYL7|D3YYL7_MOUSE

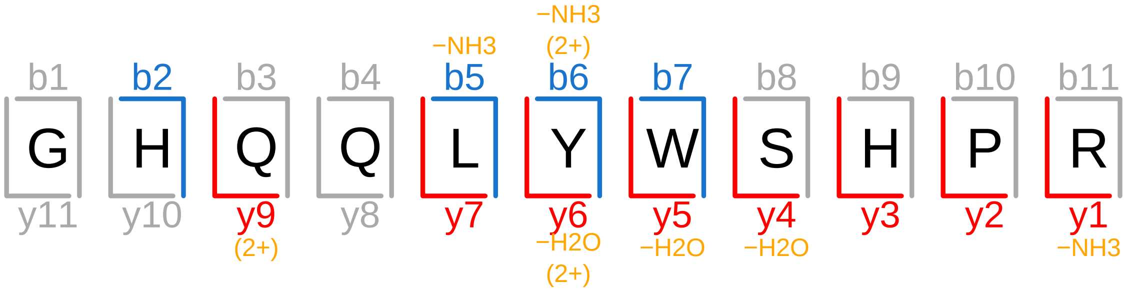

GHQQLYWSHPR
Score: 95 ; 1417.6879 m/z; 473.5699 m/z; −0.33968 ppm; MULTI−MSMS

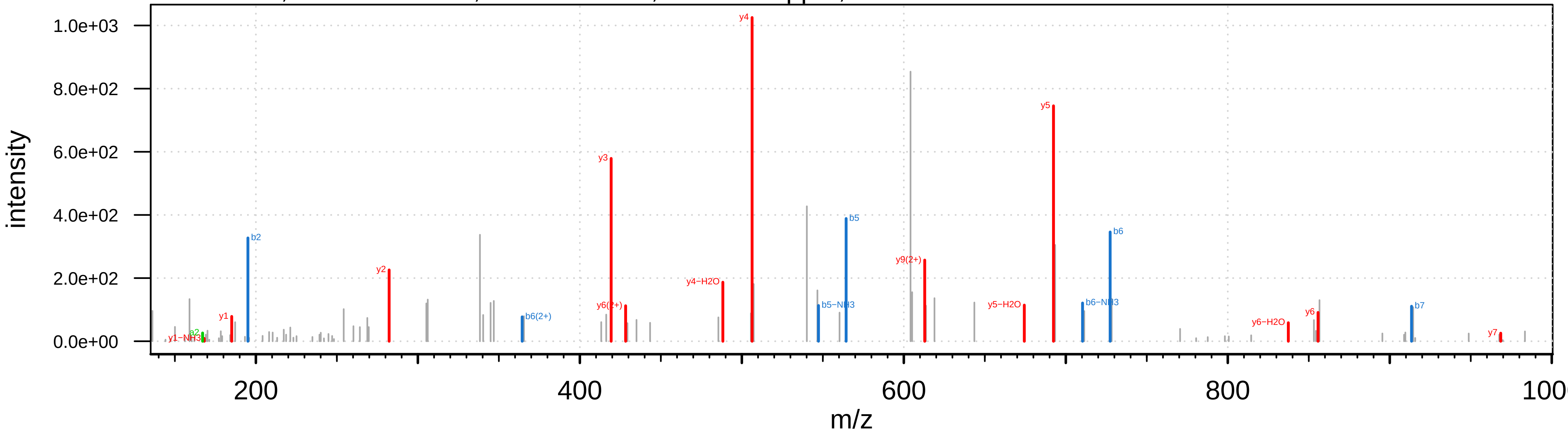

Raw File: Bert_20090403_BJS_halflives_P3_slice01_04
Scan Number: 2877
Proteins:
sp|P62274|RS29_MOUSE
ENSMUST00000037023_chr12:69157813−69159058:−
ENSMUST00000071737_chr13:73988963−73989133:−
tr|D3YYL7|D3YYL7_MOUSE

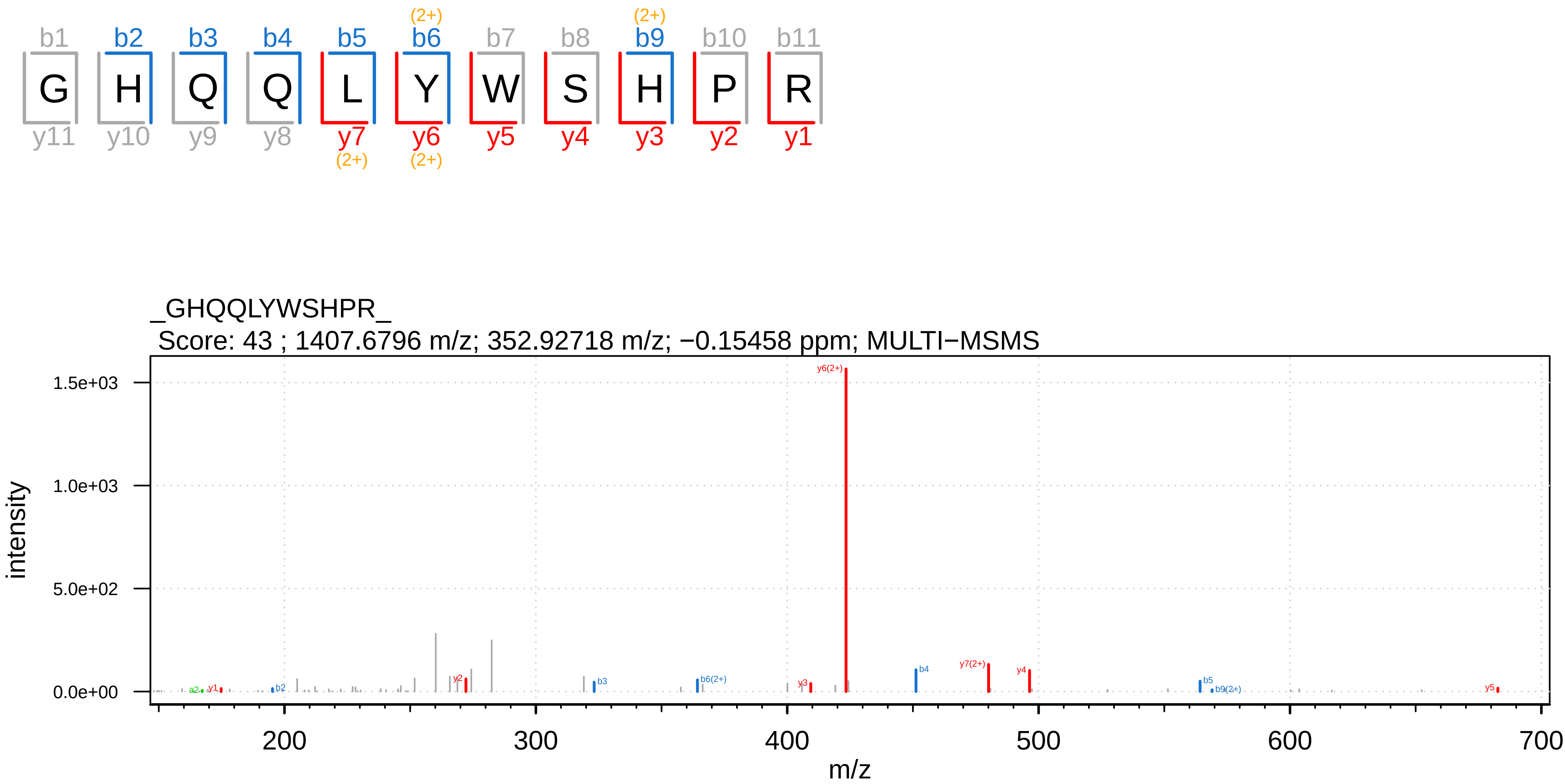

Raw File: Bert_20090403_BJS_halflives_P3_slice01_04
Scan Number: 2868
Proteins:
sp|P62274|RS29_MOUSE
ENSMUST00000037023_chr12:69157813−69159058:−
ENSMUST00000071737_chr13:73988963−73989133:−
tr|D3YYL7|D3YYL7_MOUSE

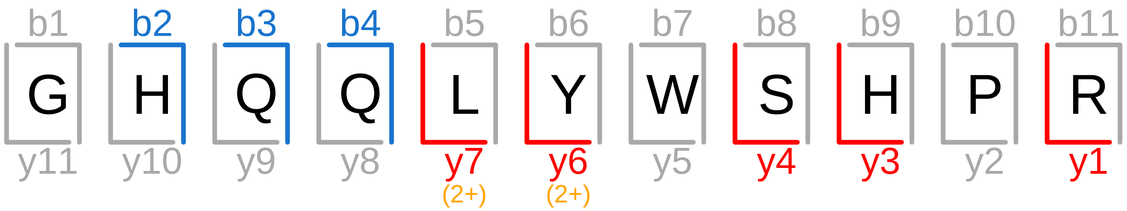

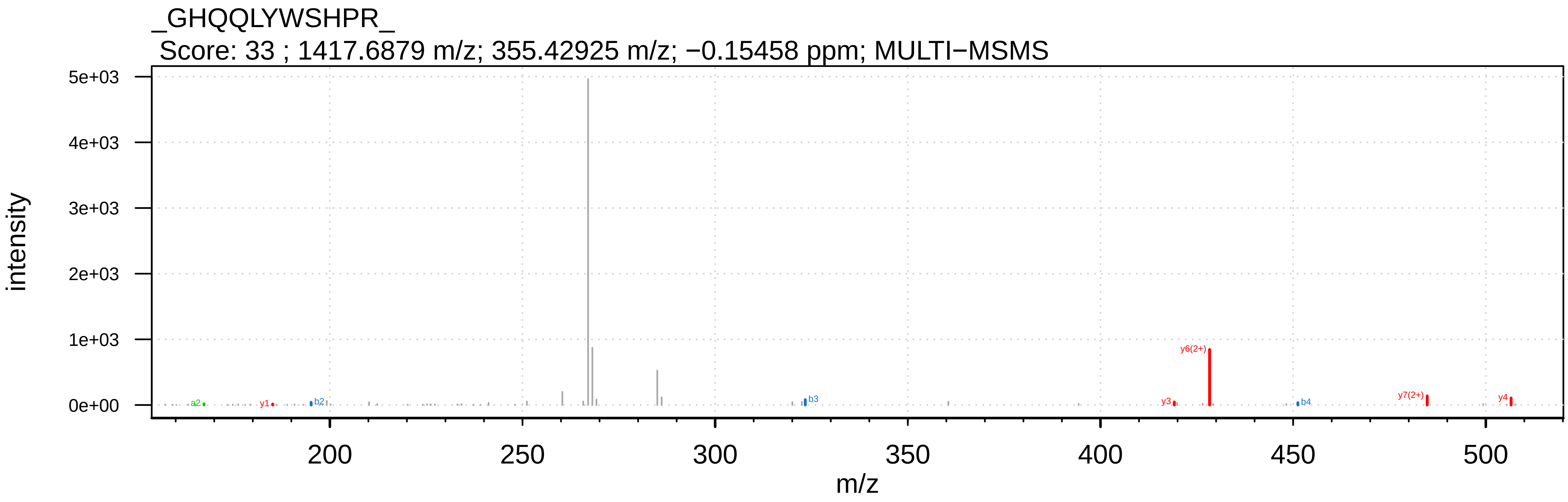

Raw File: Bert_20090403_BJS_halflives_P3_slice01_04
Scan Number: 2872
Proteins:
sp|P62274|RS29_MOUSE
ENSMUST00000037023_chr12:69157813−69159058:−
ENSMUST00000071737_chr13:73988963−73989133:−
tr|D3YYL7|D3YYL7_MOUSE

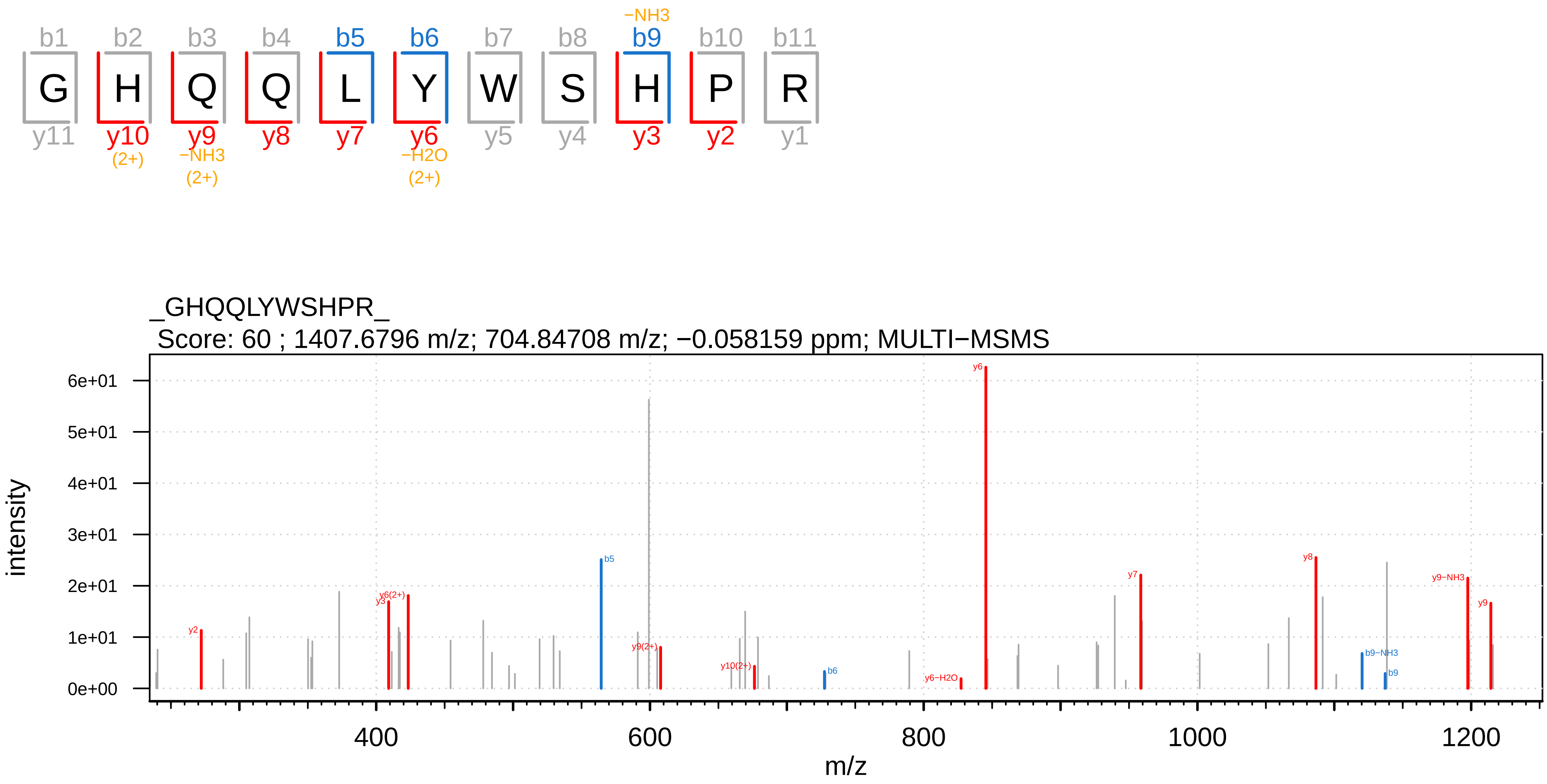

Raw File: Bert_20090403_BJS_halflives_P3_slice01_04

Scan Number: 2873

Proteins:

sp|P62274|RS29_MOUSE

ENSMUST00000037023_chr12:69157813−69159058:−

ENSMUST00000071737_chr13:73988963−73989133:−

tr|D3YYL7|D3YYL7_MOUSE

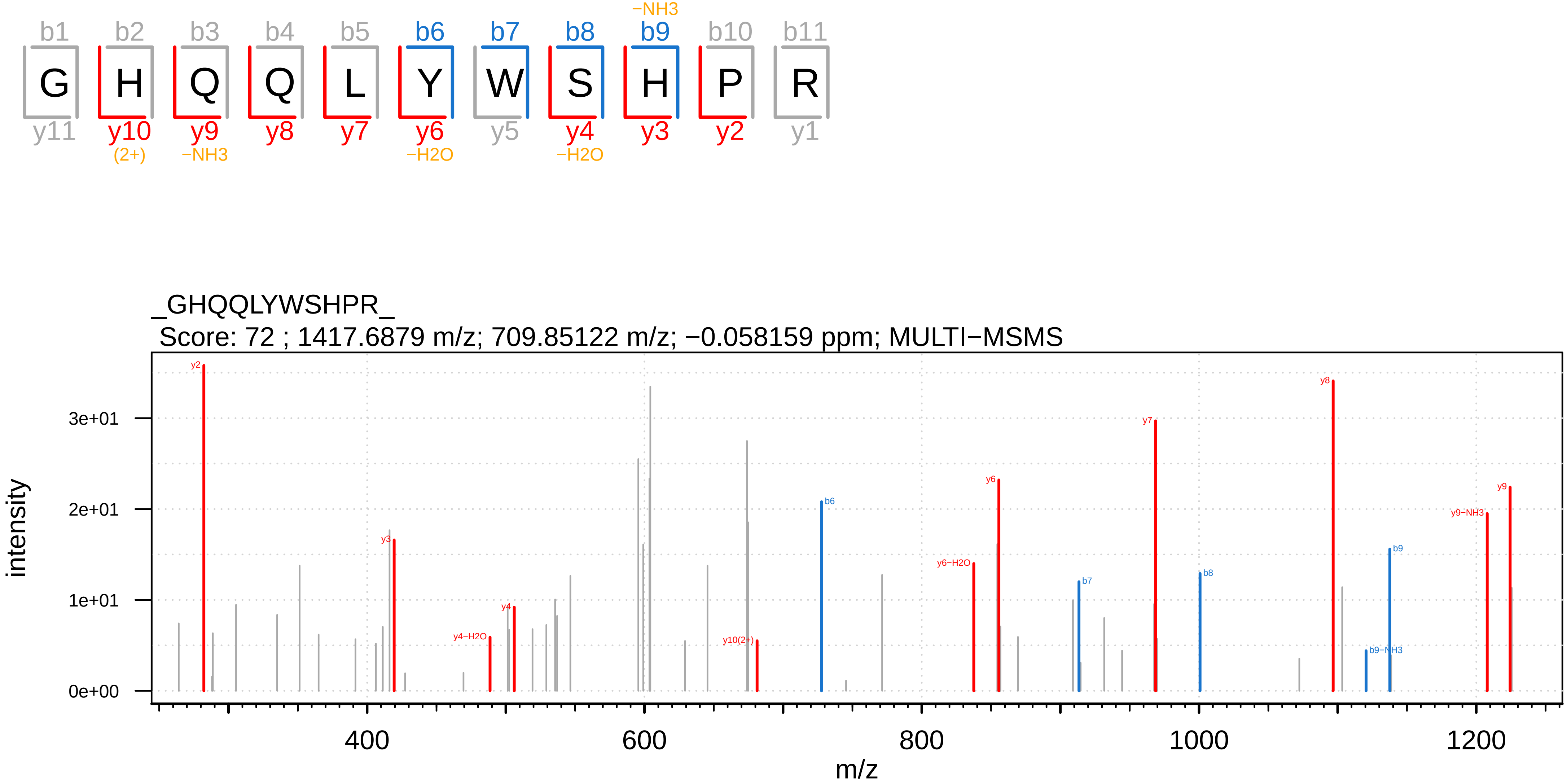

Raw File: Bert_20090403_BJS_halflives_P3_slice01_04
Scan Number: 2875
Proteins:
sp|P62274|RS29_MOUSE
ENSMUST00000037023_chr12:69157813−69159058:−
ENSMUST00000071737_chr13:73988963−73989133:−
tr|D3YYL7|D3YYL7_MOUSE

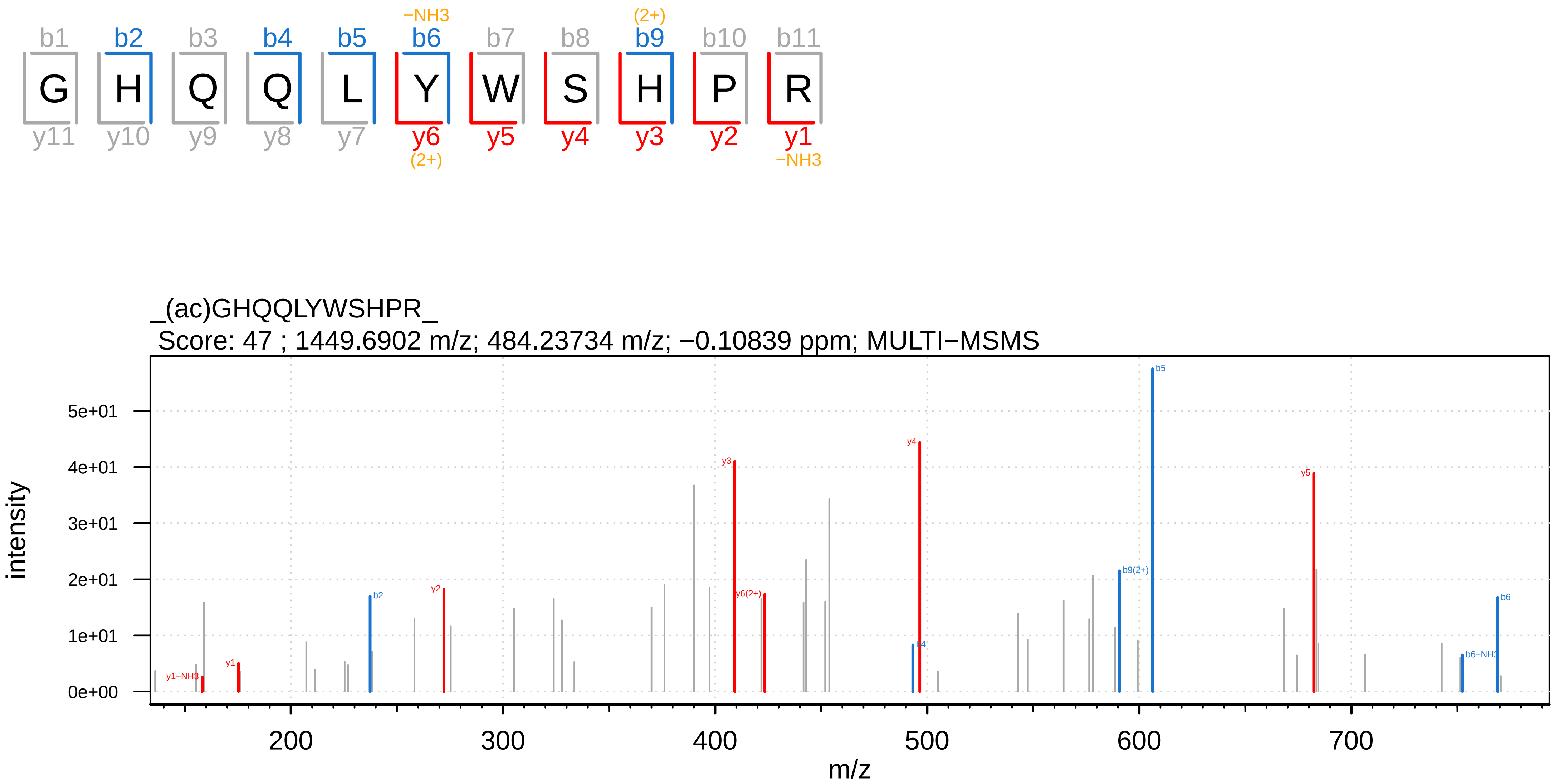

Raw File: Bert_20090403_BJS_halflives_P3_slice01_04
Scan Number: 3723
Proteins:
sp|P62274|RS29_MOUSE
ENSMUST00000037023_chr12:69157813−69159058:−
ENSMUST00000071737_chr13:73988963−73989133:−
tr|D3YYL7|D3YYL7_MOUSE

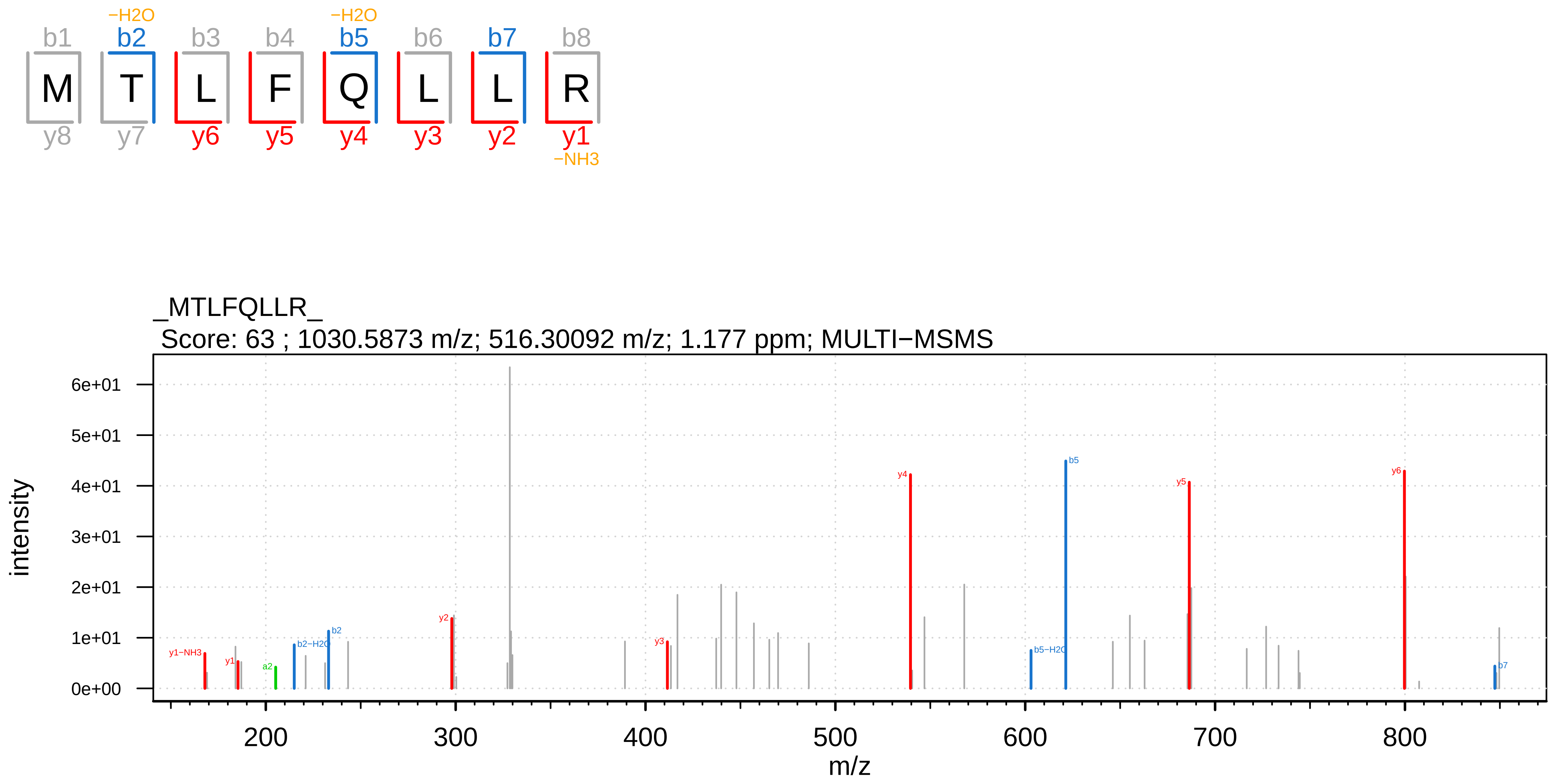

Raw File: Bert_20090403_BJS_halflives_P3_slice01_04
Scan Number: 9176
Proteins:
chr12_103206313−103214181.T2_chr12:101968174−101969629:−

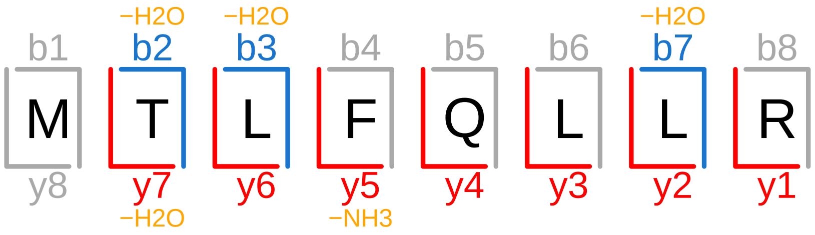

MTLFQLLR
Score: 58 ; 1020.579 m/z; 511.29679 m/z; 1.177 ppm; MULTI−MSMS

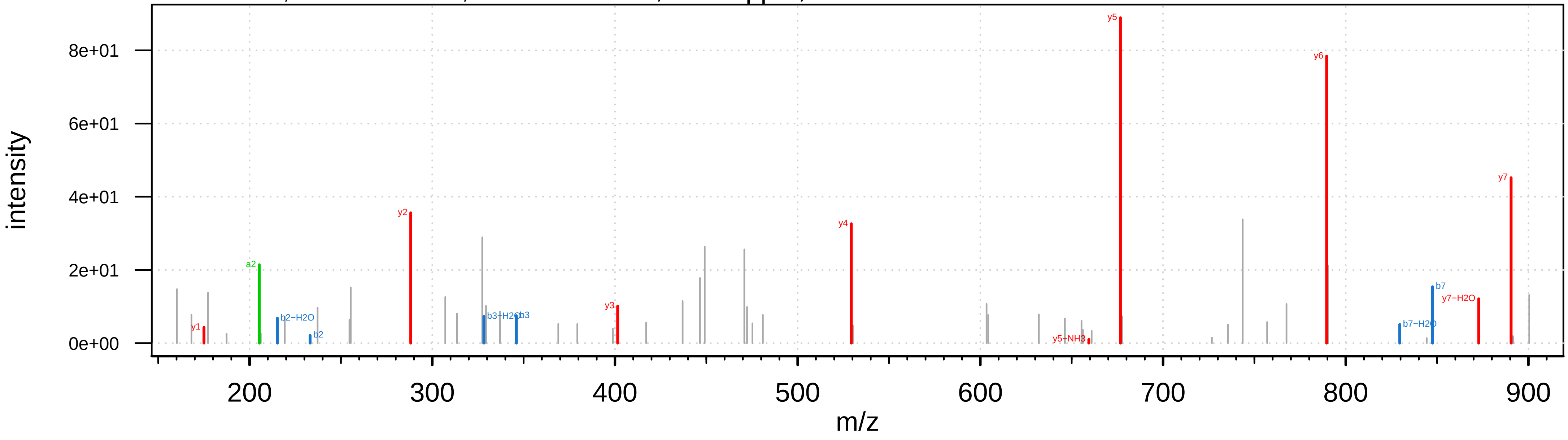

Raw File: Bert_20090403_BJS_halflives_P3_slice01_04
Scan Number: 9185
Proteins:
chr12_103206313−103214181.T2_chr12:101968174−101969629:−

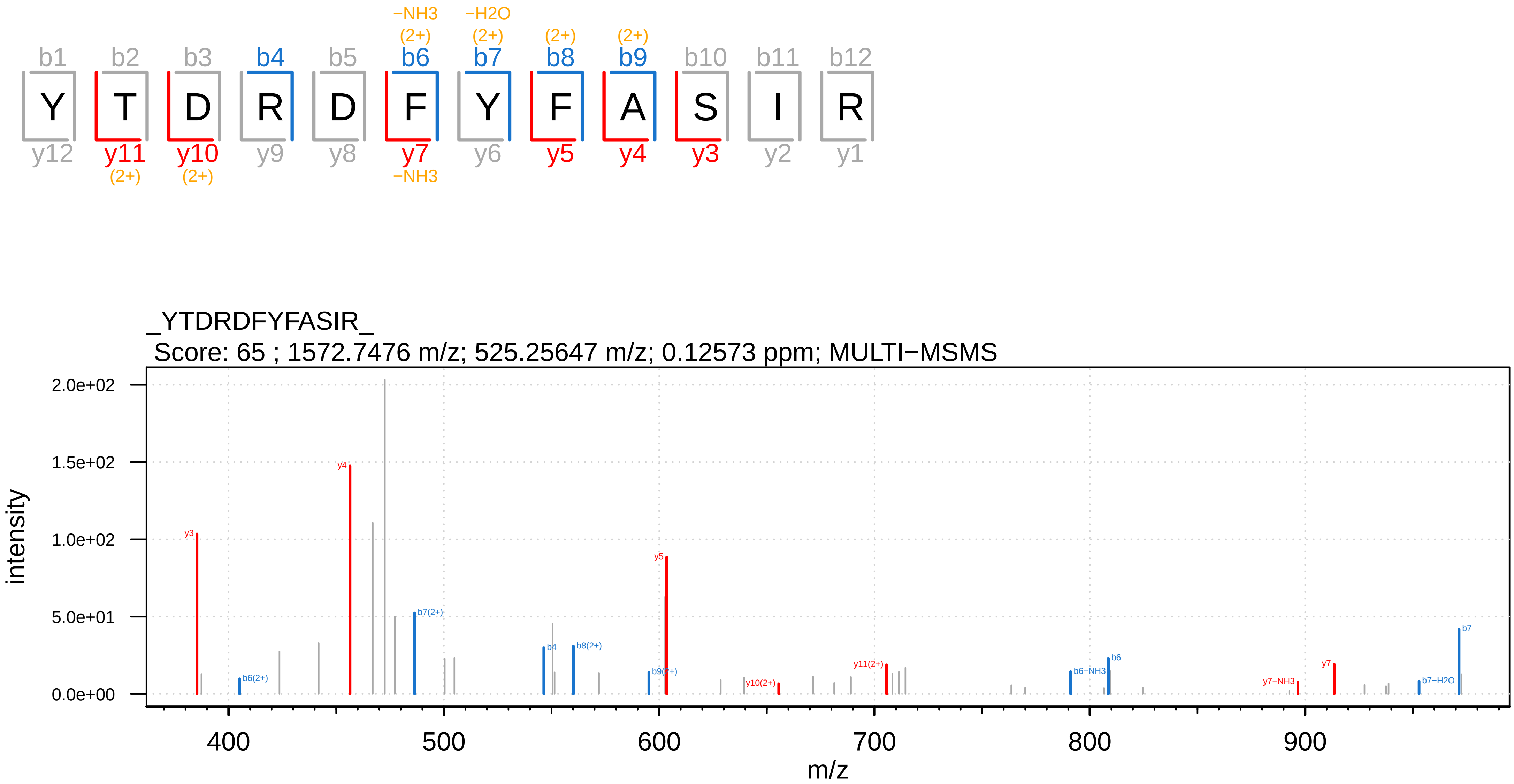

Raw File: Bert_20090403_BJS_halflives_P3_slice01_04
Scan Number: 6386
Proteins:
ENSMUST00000023048_chr15:80235835−80236047:+

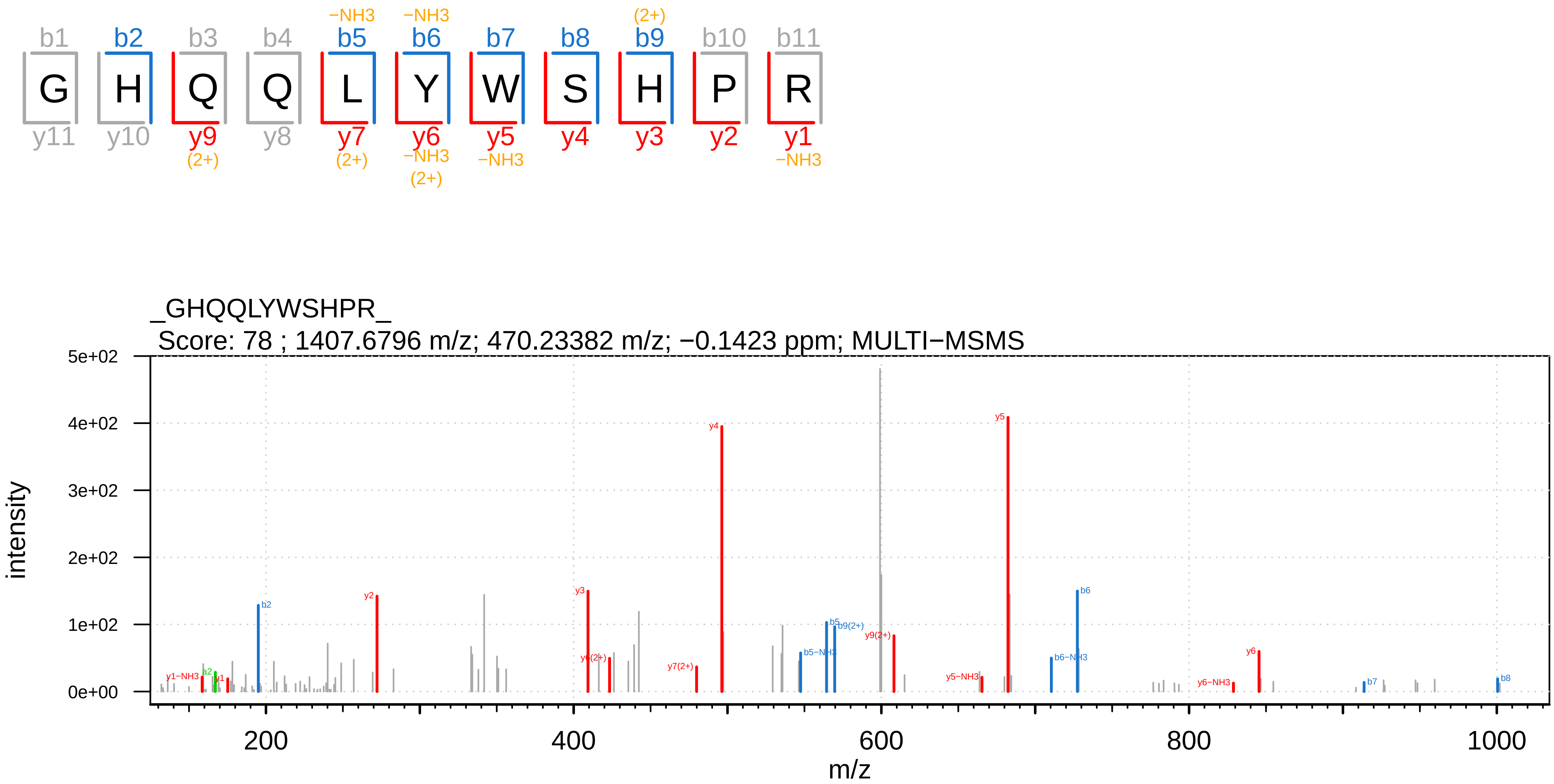

Raw File: Bert_20090403_BJS_halflives_P3_slice02_04
Scan Number: 3396
Proteins:
sp|P62274|RS29_MOUSE
ENSMUST00000037023_chr12:69157813−69159058:−
ENSMUST00000071737_chr13:73988963−73989133:−
tr|D3YYL7|D3YYL7_MOUSE

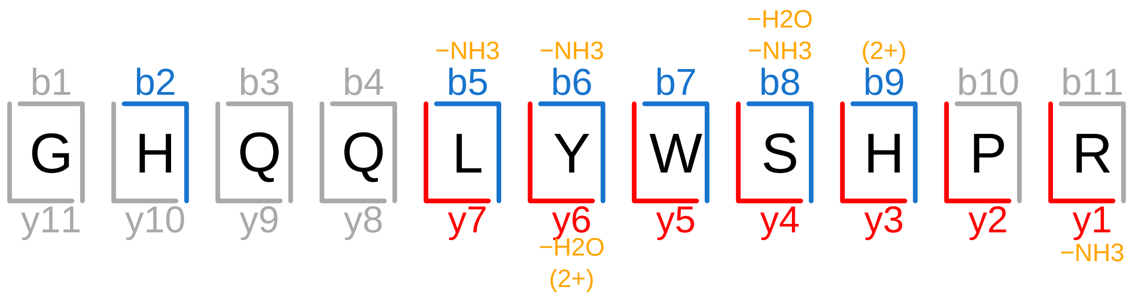

GHQQLYWSHPR
Score: 87 ; 1417.6879 m/z; 473.5699 m/z; −0.1423 ppm; MULTI−MSMS

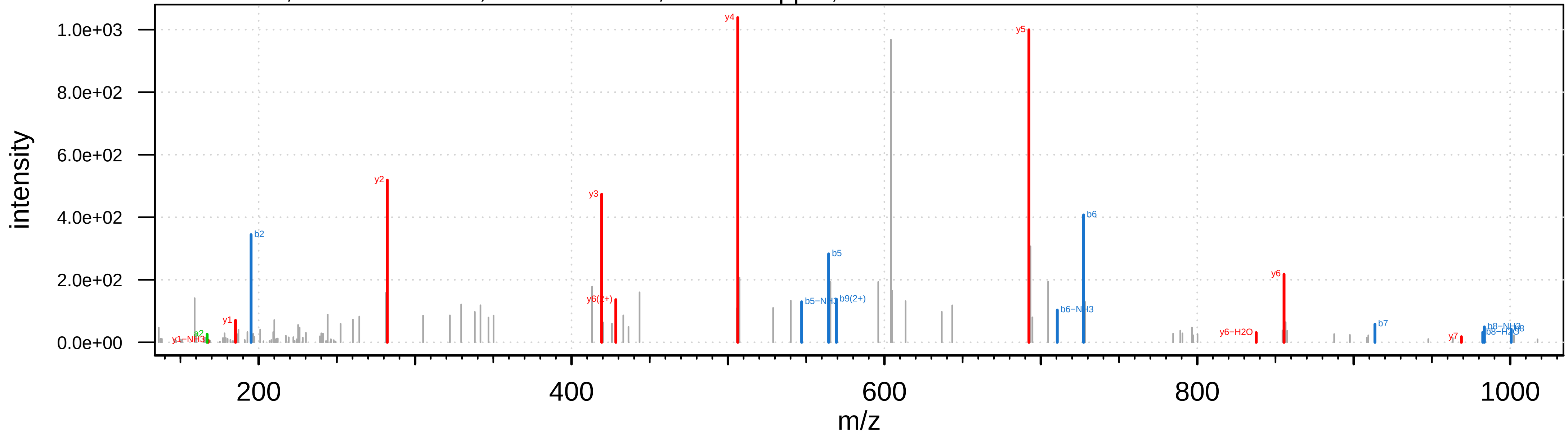

Raw File: Bert_20090403_BJS_halflives_P3_slice02_04
Scan Number: 3413
Proteins:
sp|P62274|RS29_MOUSE
ENSMUST00000037023_chr12:69157813−69159058:−
ENSMUST00000071737_chr13:73988963−73989133:−
tr|D3YYL7|D3YYL7_MOUSE

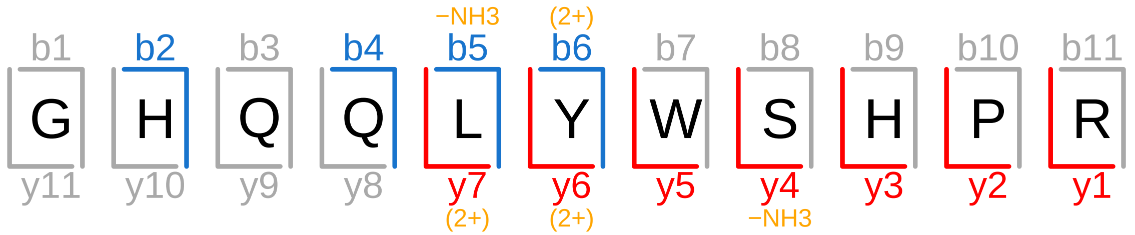

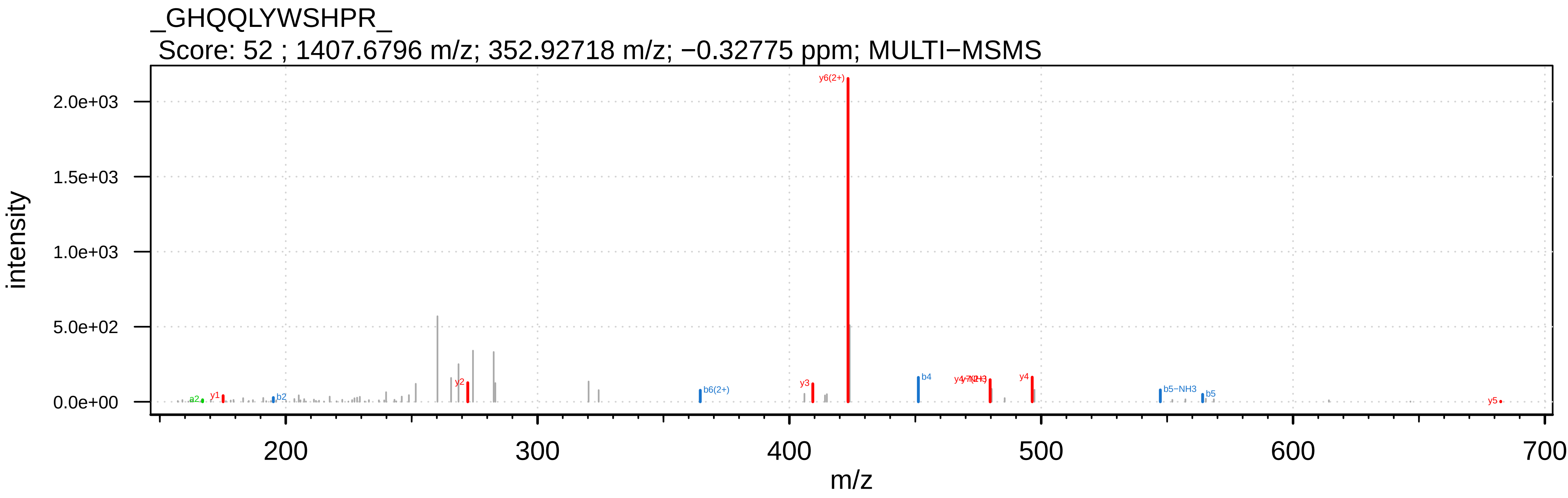

Raw File: Bert_20090403_BJS_halflives_P3_slice02_04
Scan Number: 3402
Proteins:
sp|P62274|RS29_MOUSE
ENSMUST00000037023_chr12:69157813−69159058:−
ENSMUST00000071737_chr13:73988963−73989133:−
tr|D3YYL7|D3YYL7_MOUSE

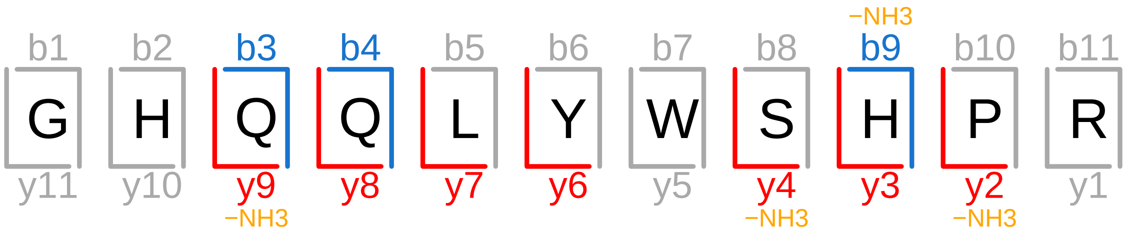

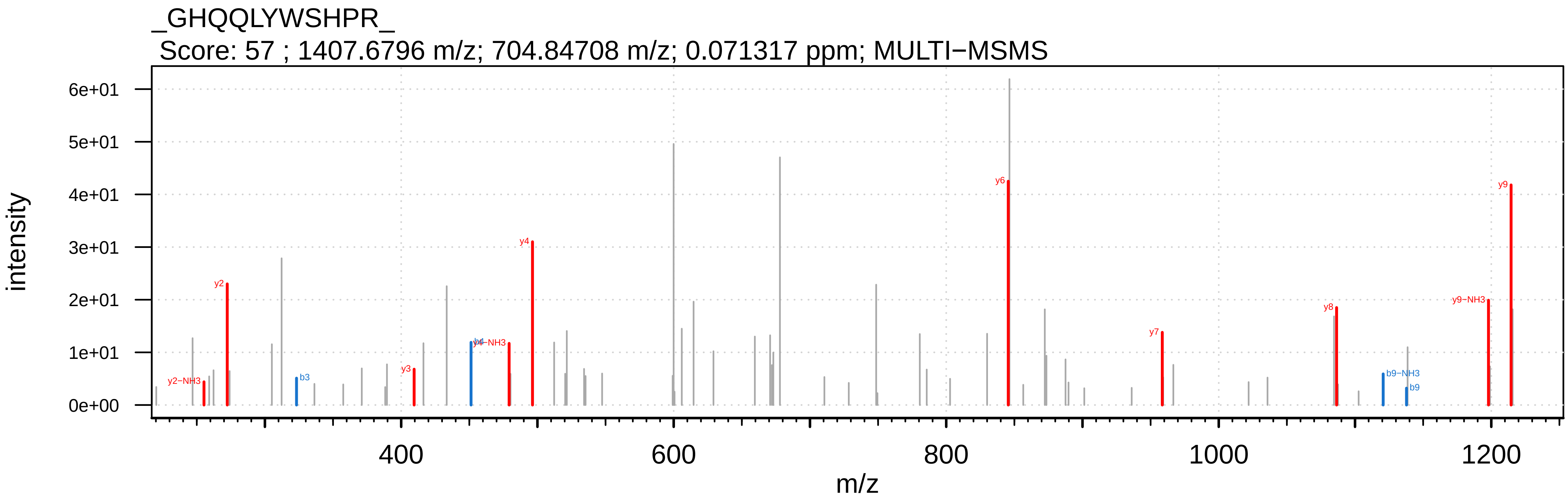

Raw File: Bert_20090403_BJS_halflives_P3_slice02_04
Scan Number: 3409
Proteins:
sp|P62274|RS29_MOUSE
ENSMUST00000037023_chr12:69157813−69159058:−
ENSMUST00000071737_chr13:73988963−73989133:−
tr|D3YYL7|D3YYL7_MOUSE

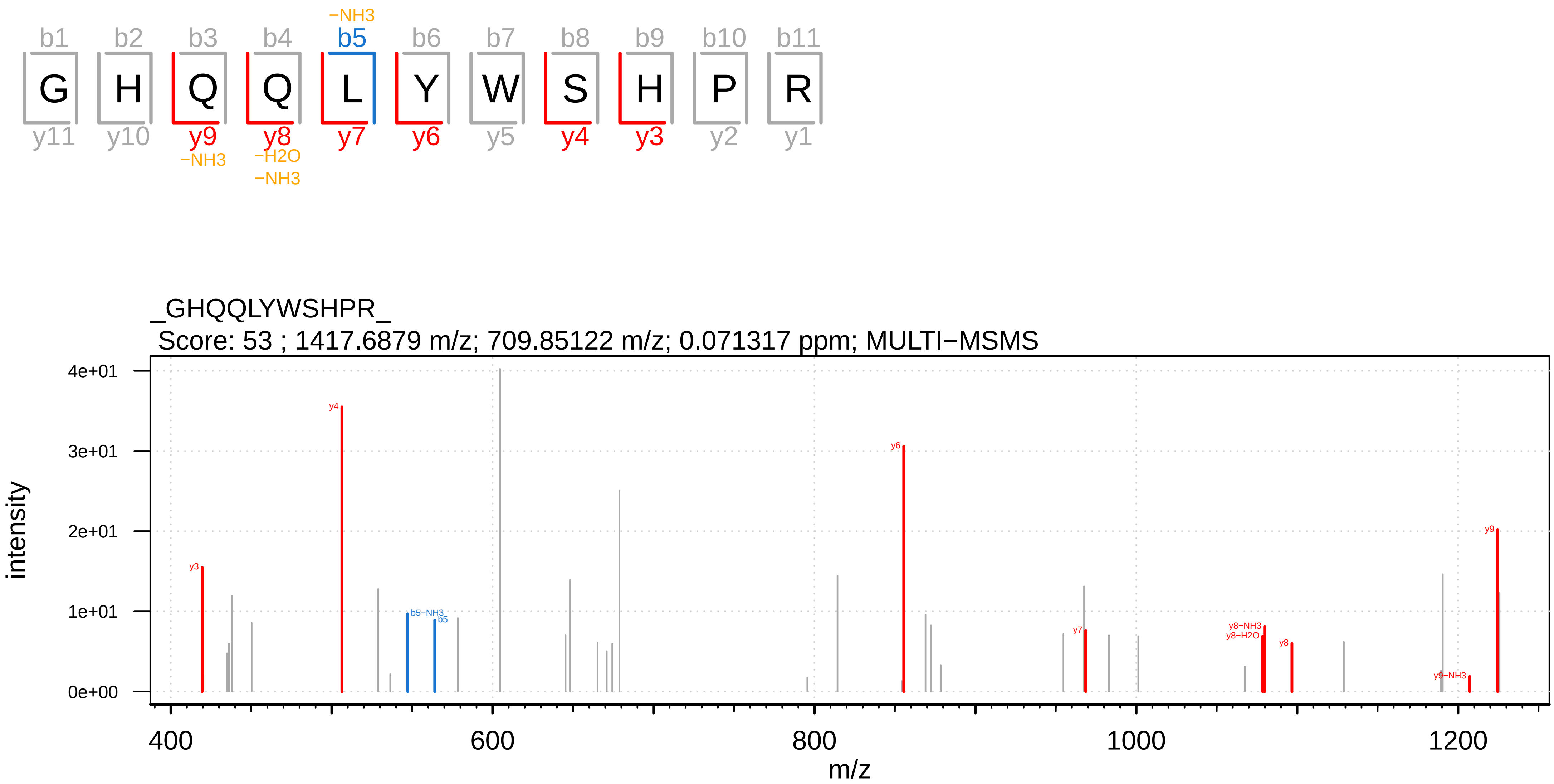

Raw File: Bert_20090403_BJS_halflives_P3_slice02_04
Scan Number: 3417
Proteins:
sp|P62274|RS29_MOUSE
ENSMUST00000037023_chr12:69157813−69159058:−
ENSMUST00000071737_chr13:73988963−73989133:−
tr|D3YYL7|D3YYL7_MOUSE

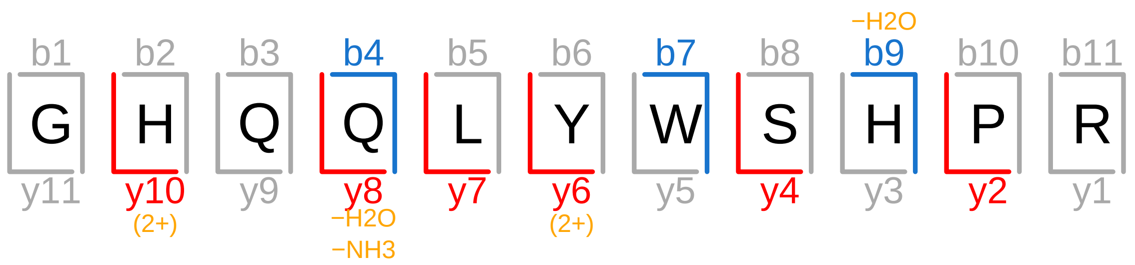

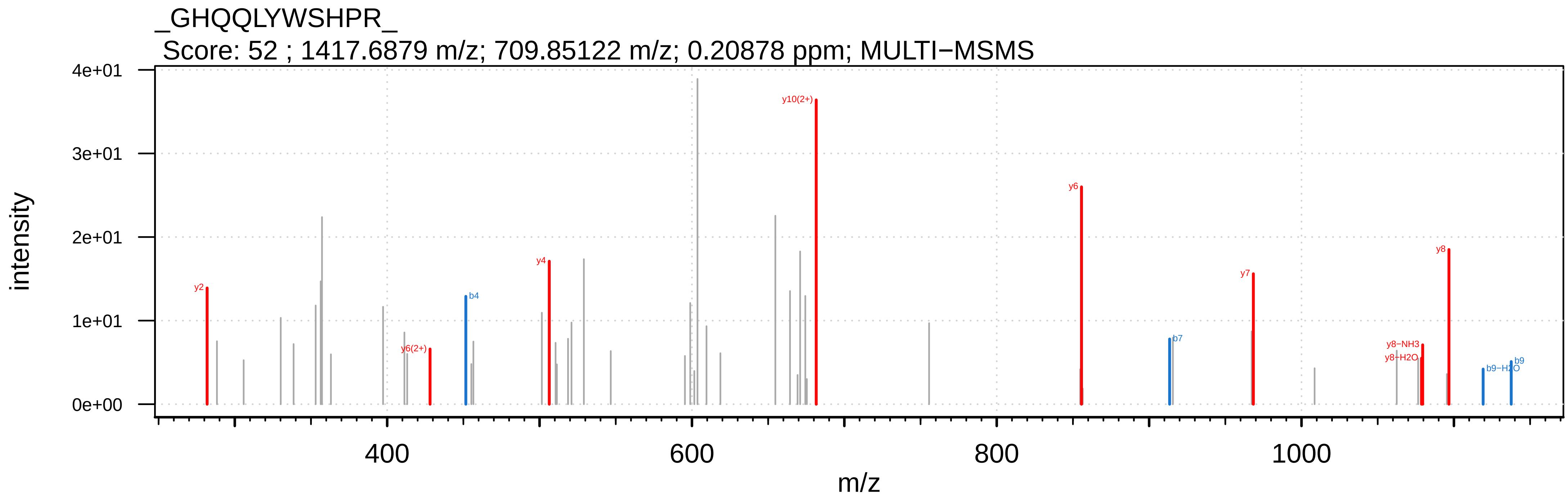

Raw File: Bert_20090403_BJS_halflives_P3_slice02_05
Scan Number: 3536
Proteins:
sp|P62274|RS29_MOUSE
ENSMUST00000037023_chr12:69157813−69159058:−
ENSMUST00000071737_chr13:73988963−73989133:−
tr|D3YYL7|D3YYL7_MOUSE

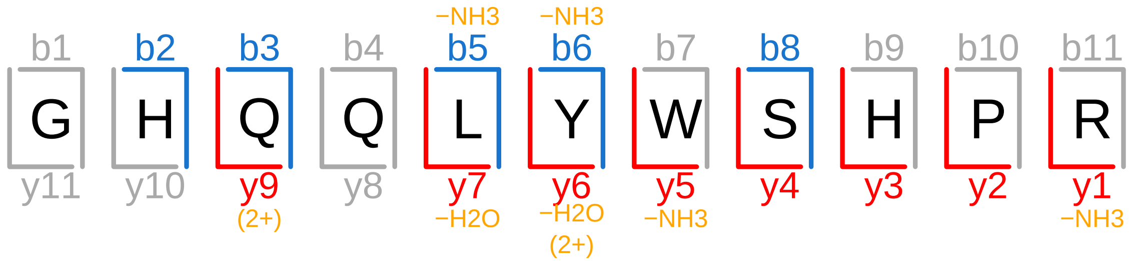

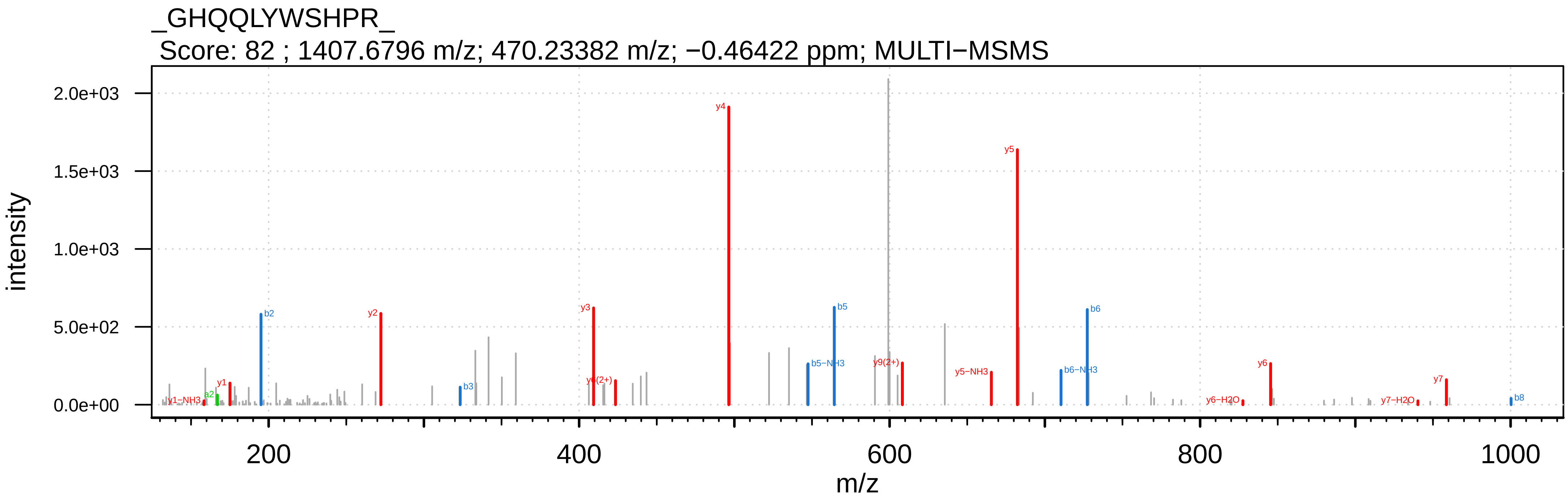

Raw File: Bert_20090403_BJS_halflives_P3_slice03_01
Scan Number: 3814
Proteins:
sp|P62274|RS29_MOUSE
ENSMUST00000037023_chr12:69157813−69159058:−
ENSMUST00000071737_chr13:73988963−73989133:−
tr|D3YYL7|D3YYL7_MOUSE

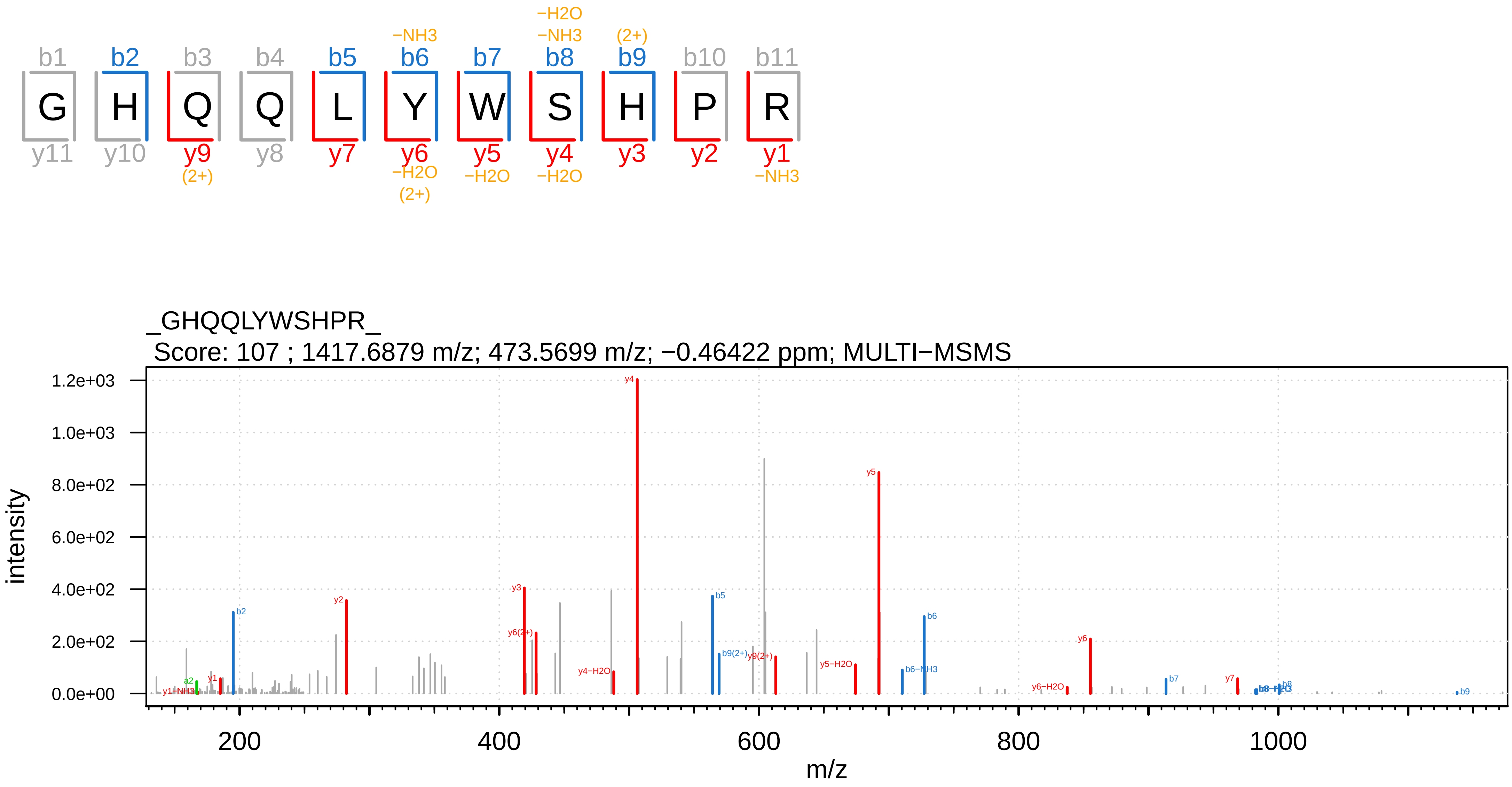

Raw File: Bert_20090403_BJS_halflives_P3_slice03_01
Scan Number: 3818
Proteins:
sp|P62274|RS29_MOUSE
ENSMUST00000037023_chr12:69157813−69159058:−
ENSMUST00000071737_chr13:73988963−73989133:−
tr|D3YYL7|D3YYL7_MOUSE

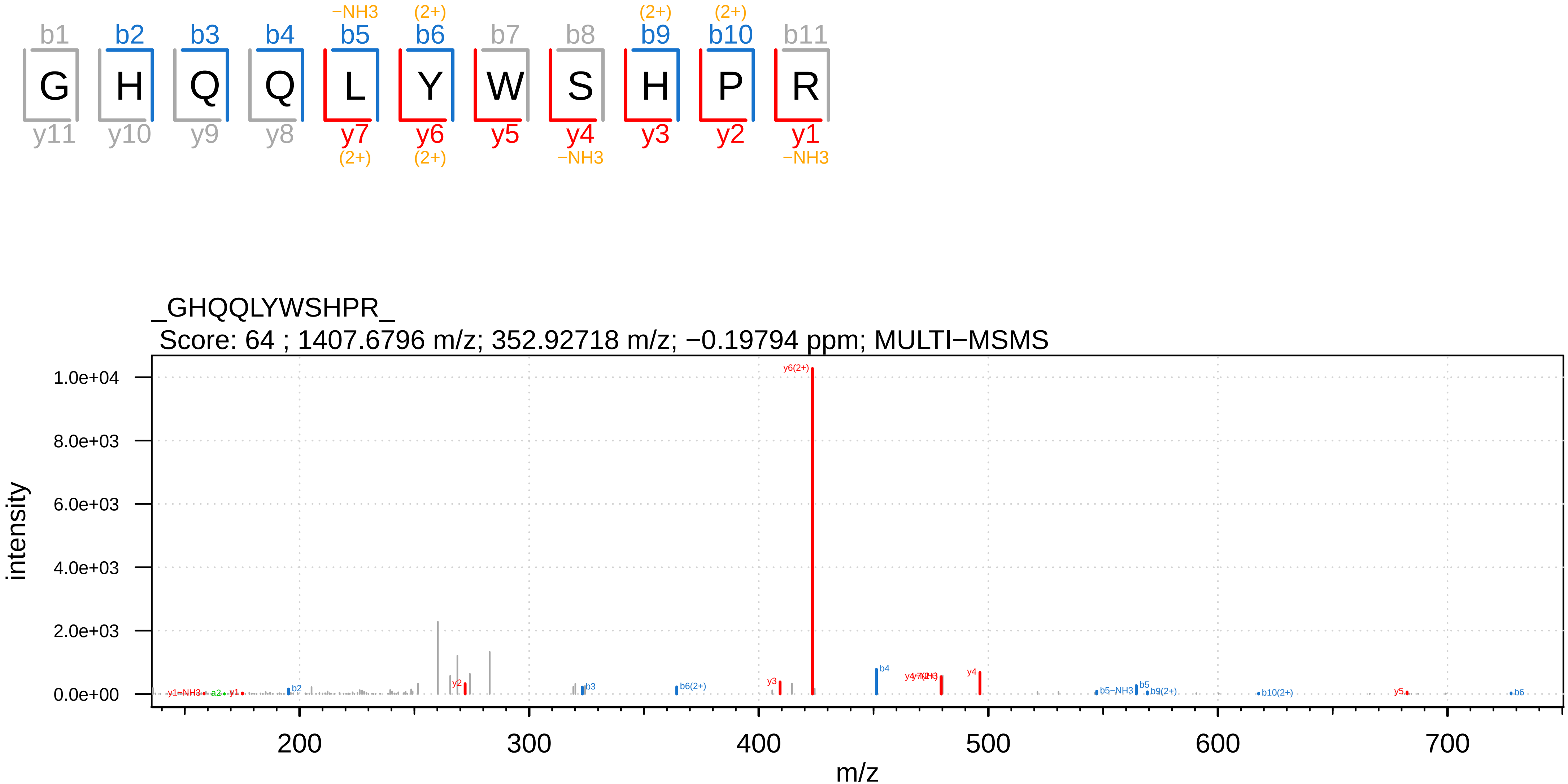

Raw File: Bert_20090403_BJS_halflives_P3_slice03_01
Scan Number: 3819
Proteins:
sp|P62274|RS29_MOUSE
ENSMUST00000037023_chr12:69157813−69159058:−
ENSMUST00000071737_chr13:73988963−73989133:−
tr|D3YYL7|D3YYL7_MOUSE

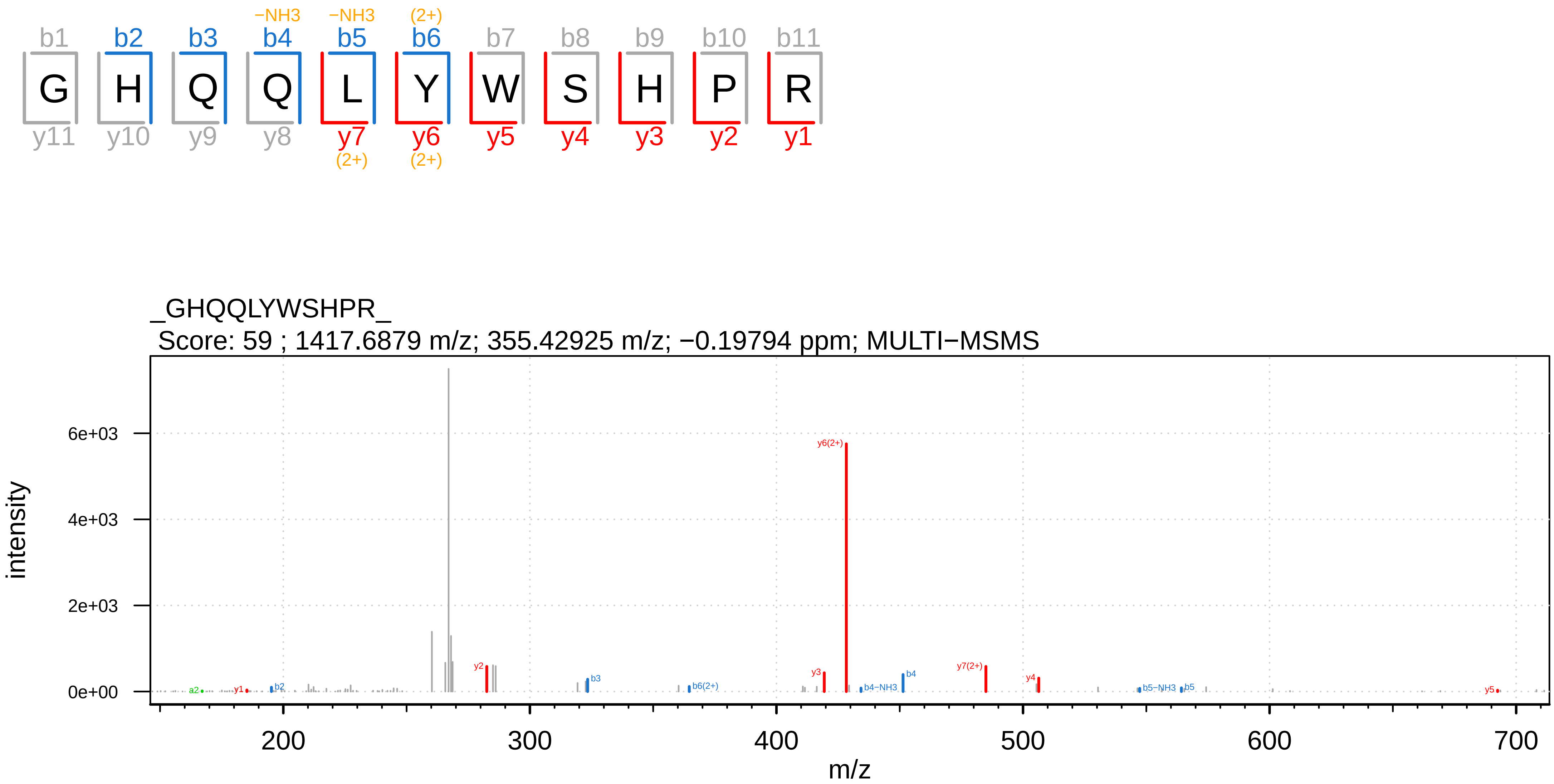

Raw File: Bert_20090403_BJS_halflives_P3_slice03_01
Scan Number: 3831
Proteins:
sp|P62274|RS29_MOUSE
ENSMUST00000037023_chr12:69157813−69159058:−
ENSMUST00000071737_chr13:73988963−73989133:−
tr|D3YYL7|D3YYL7_MOUSE

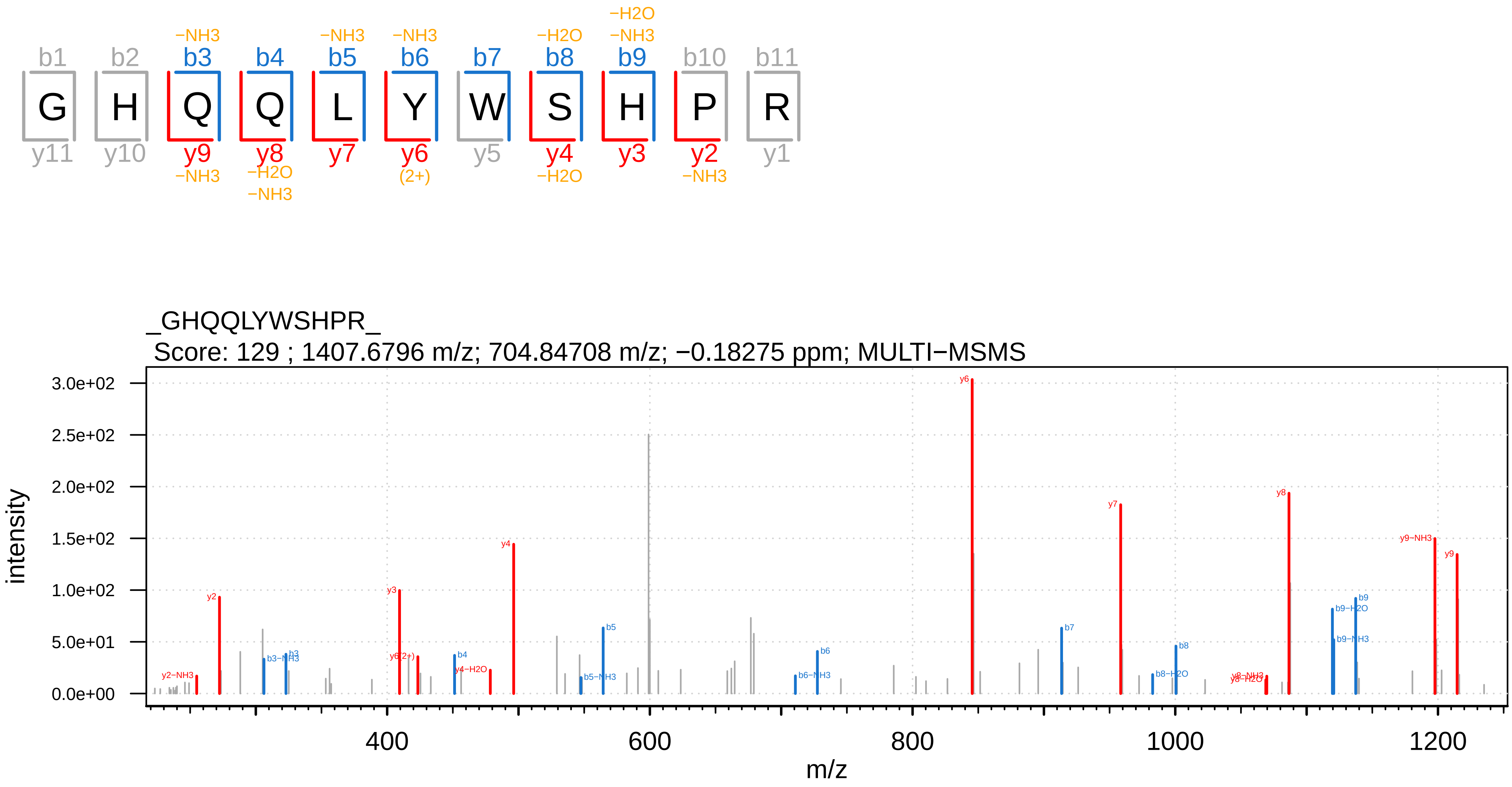

Raw File: Bert_20090403_BJS_halflives_P3_slice03_01
Scan Number: 3833
Proteins:
sp|P62274|RS29_MOUSE
ENSMUST00000037023_chr12:69157813−69159058:−
ENSMUST00000071737_chr13:73988963−73989133:−
tr|D3YYL7|D3YYL7_MOUSE

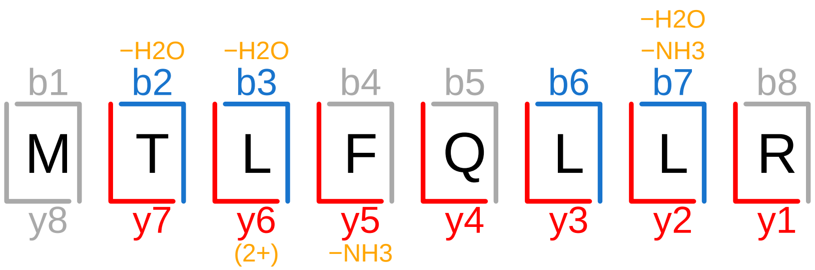

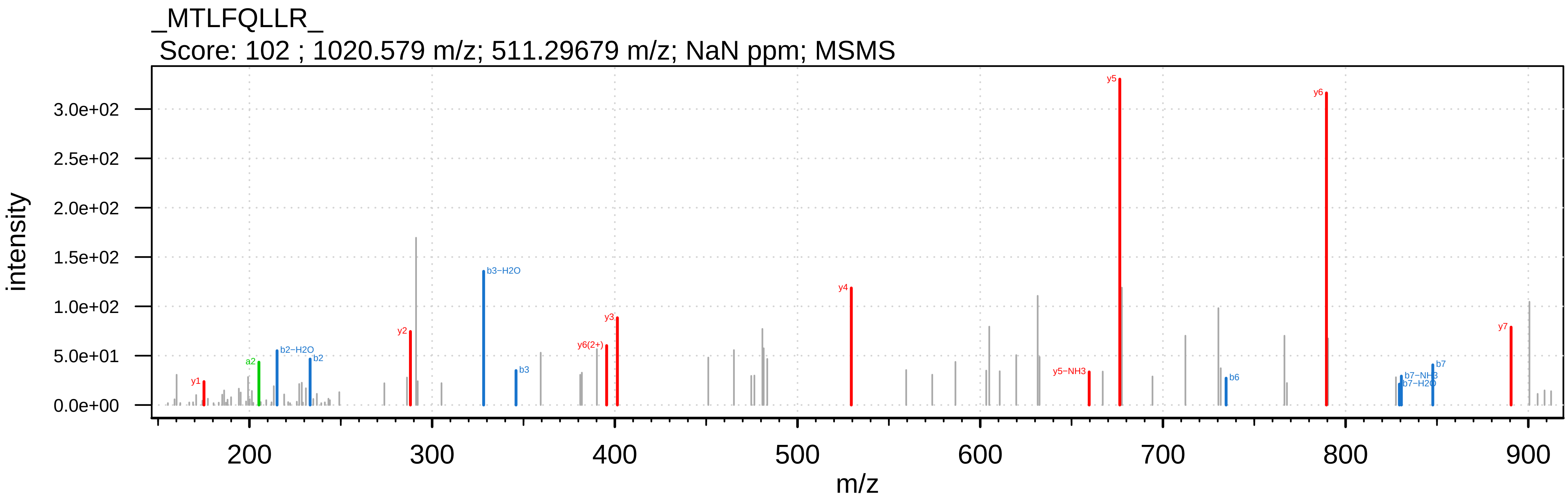

Raw File: Bert_20090403_BJS_halflives_P3_slice03_01
Scan Number: 14949
Proteins:
chr12_103206313−103214181.T2_chr12:101968174−101969629:−

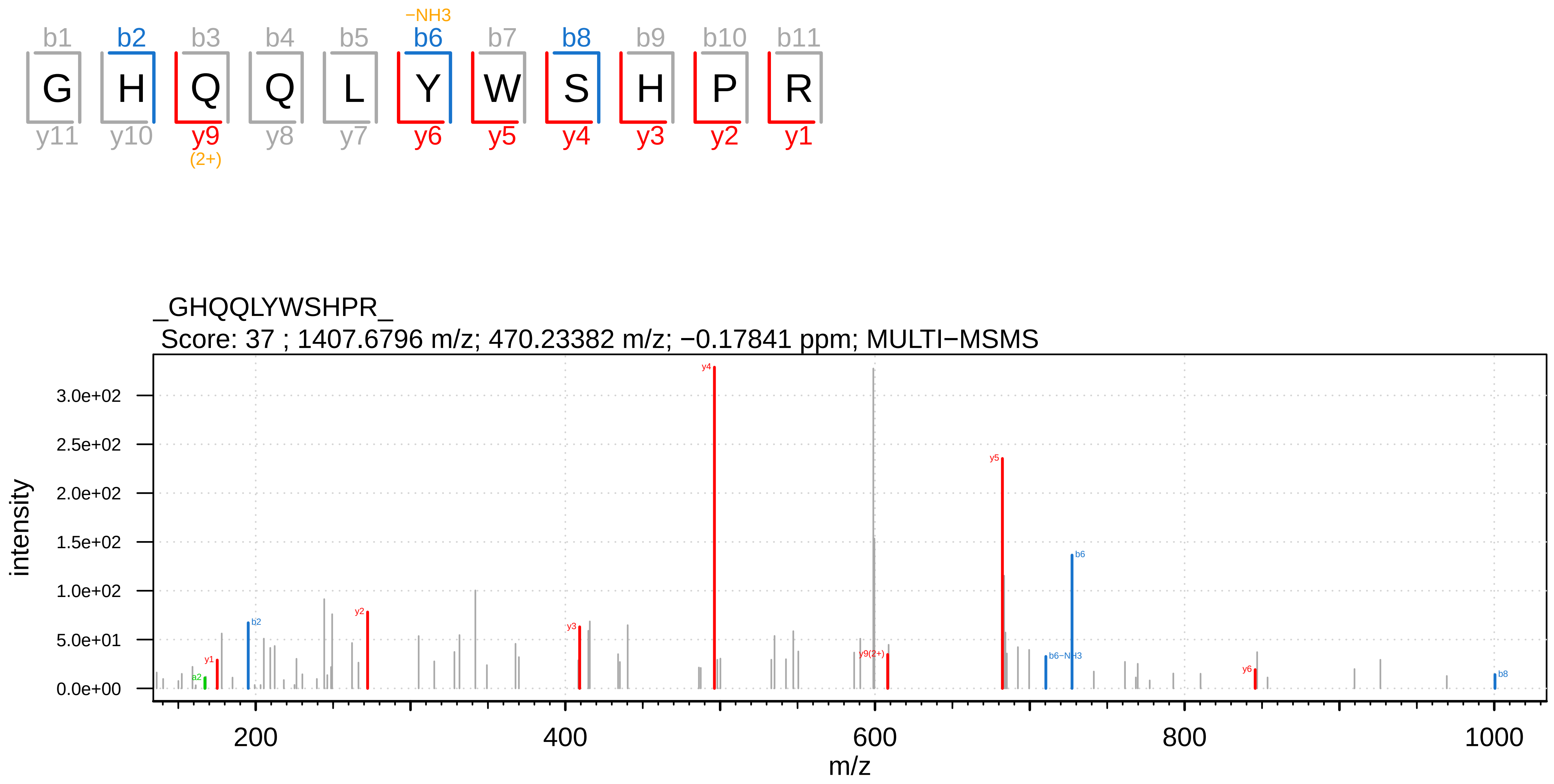

Raw File: Bert_20090403_BJS_halflives_P3_slice03_02
Scan Number: 3737
Proteins:
sp|P62274|RS29_MOUSE
ENSMUST00000037023_chr12:69157813−69159058:−
ENSMUST00000071737_chr13:73988963−73989133:−
tr|D3YYL7|D3YYL7_MOUSE

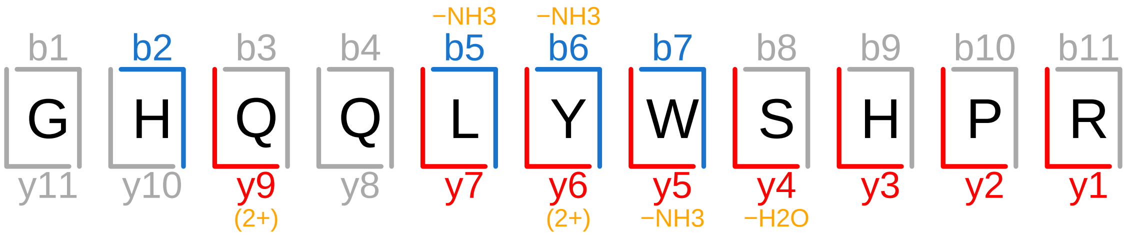

GHQQLYWSHPR
Score: 77 ; 1417.6879 m/z; 473.5699 m/z; −0.17841 ppm; MULTI−MSMS

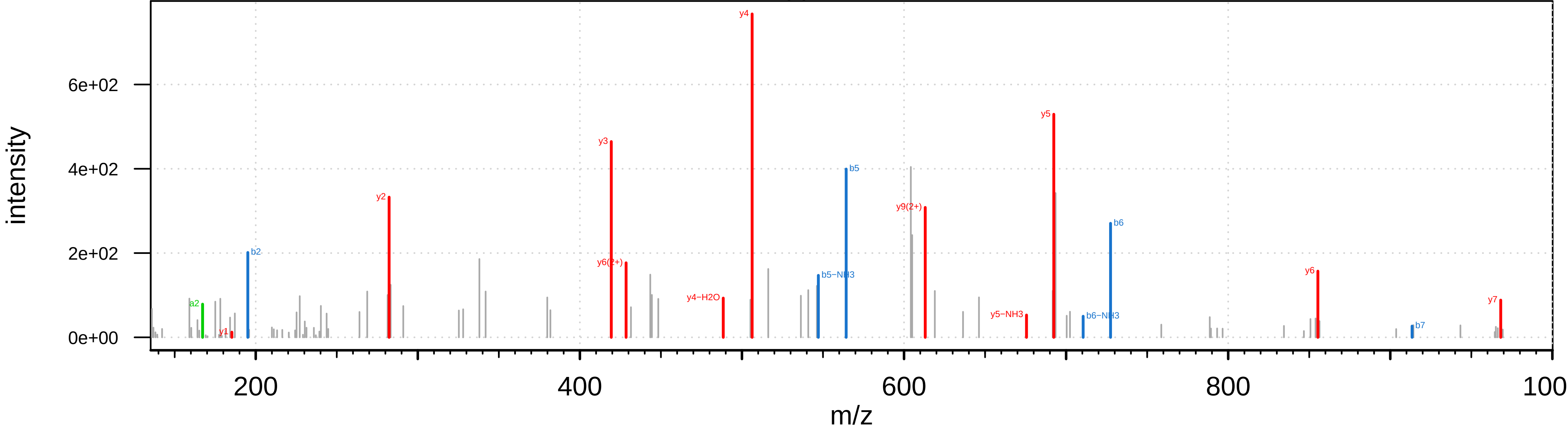

Raw File: Bert_20090403_BJS_halflives_P3_slice03_02
Scan Number: 3741
Proteins:
sp|P62274|RS29_MOUSE
ENSMUST00000037023_chr12:69157813−69159058:−
ENSMUST00000071737_chr13:73988963−73989133:−
tr|D3YYL7|D3YYL7_MOUSE

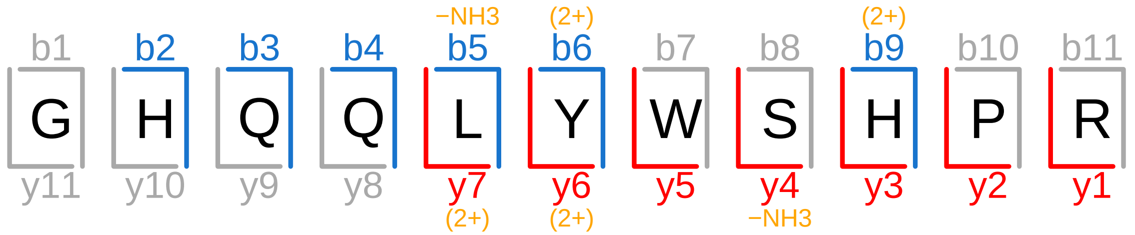

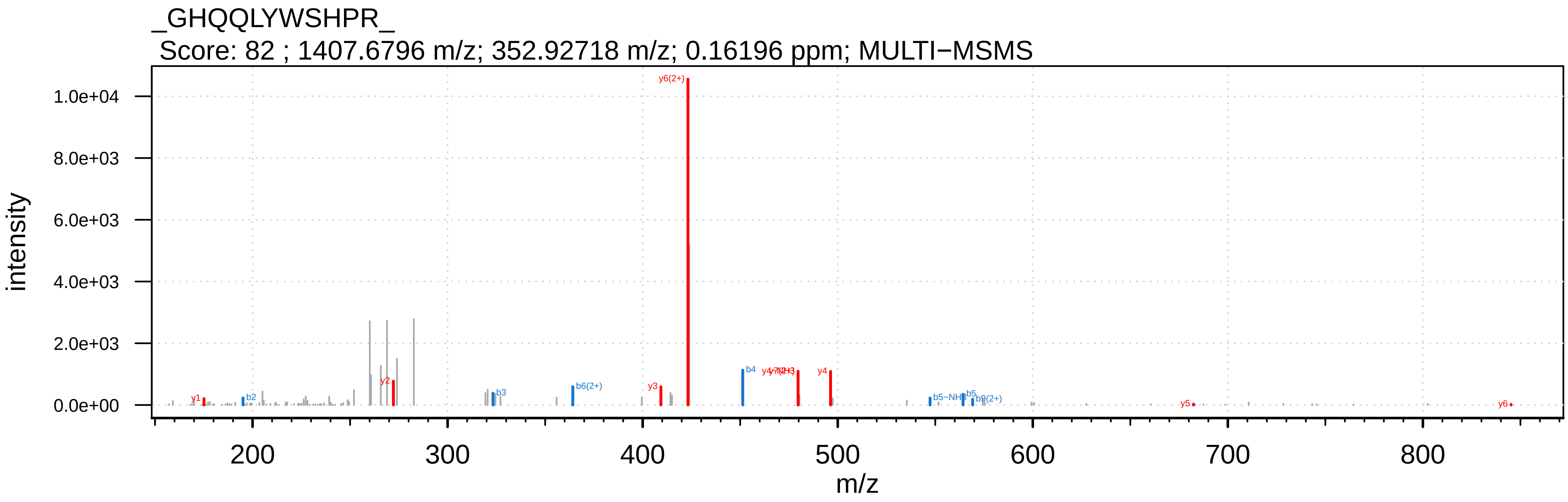

Raw File: Bert_20090403_BJS_halflives_P3_slice03_02
Scan Number: 3746
Proteins:
sp|P62274|RS29_MOUSE
ENSMUST00000037023_chr12:69157813−69159058:−
ENSMUST00000071737_chr13:73988963−73989133:−
tr|D3YYL7|D3YYL7_MOUSE

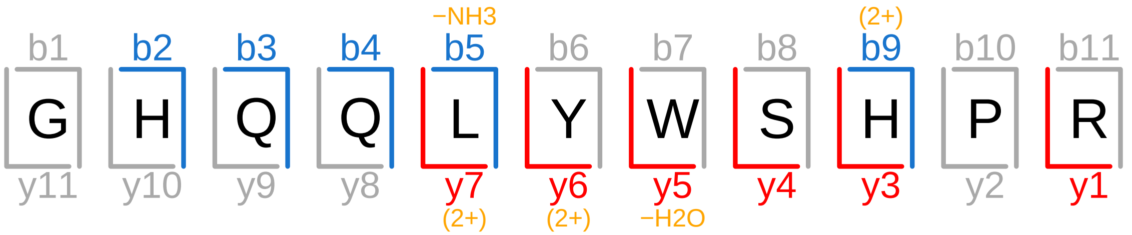

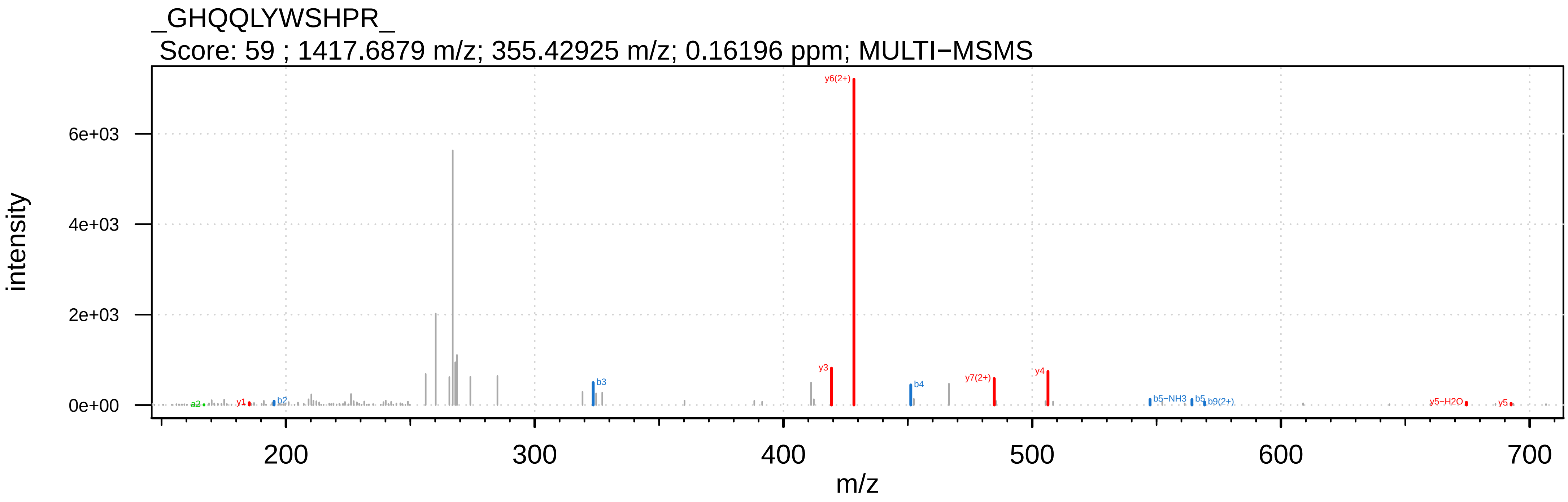

Raw File: Bert_20090403_BJS_halflives_P3_slice03_02
Scan Number: 3751
Proteins:
sp|P62274|RS29_MOUSE
ENSMUST00000037023_chr12:69157813−69159058:−
ENSMUST00000071737_chr13:73988963−73989133:−
tr|D3YYL7|D3YYL7_MOUSE

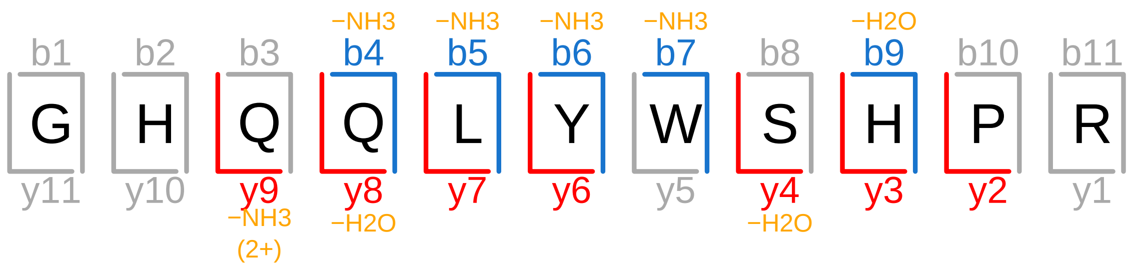

GHQQLYWSHPR
Score: 91 ; 1407.6796 m/z; 704.84708 m/z; −0.060947 ppm; MULTI−MSMS

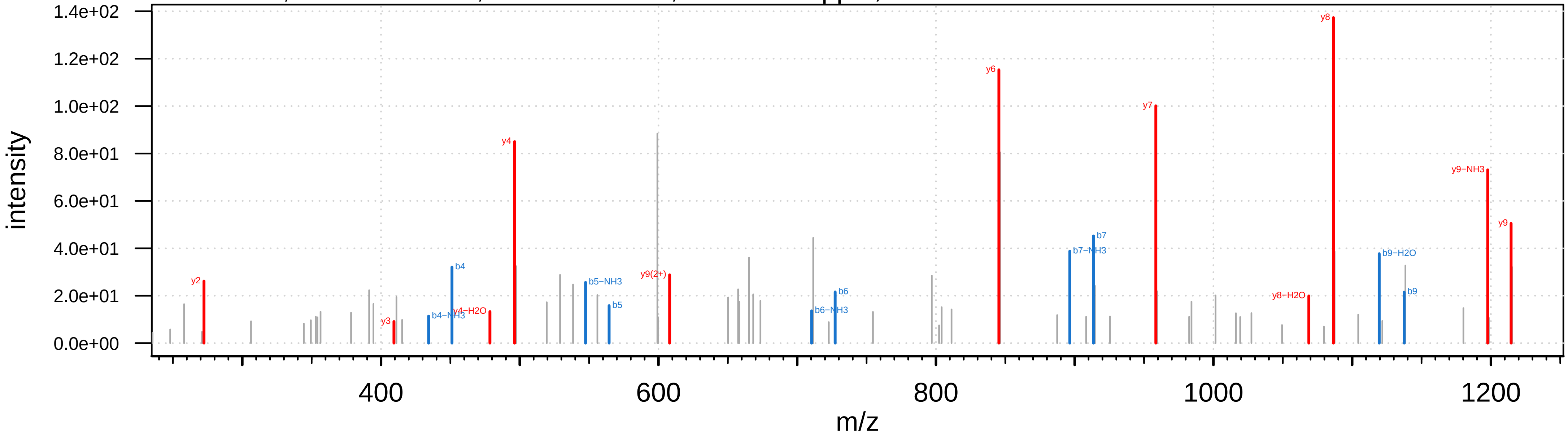

Raw File: Bert_20090403_BJS_halflives_P3_slice03_02
Scan Number: 3754
Proteins:
sp|P62274|RS29_MOUSE
ENSMUST00000037023_chr12:69157813−69159058:−
ENSMUST00000071737_chr13:73988963−73989133:−
tr|D3YYL7|D3YYL7_MOUSE

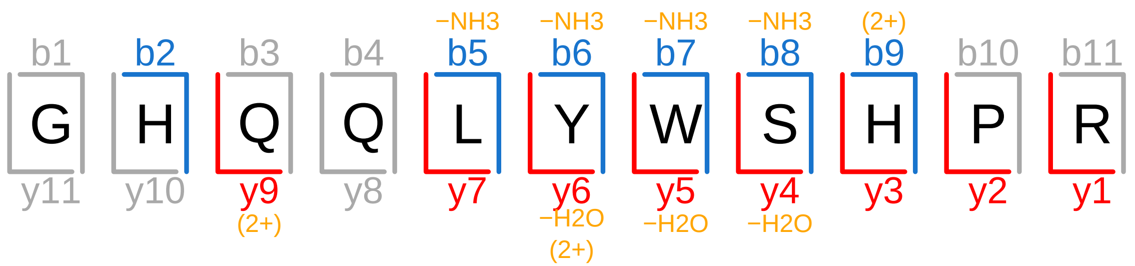

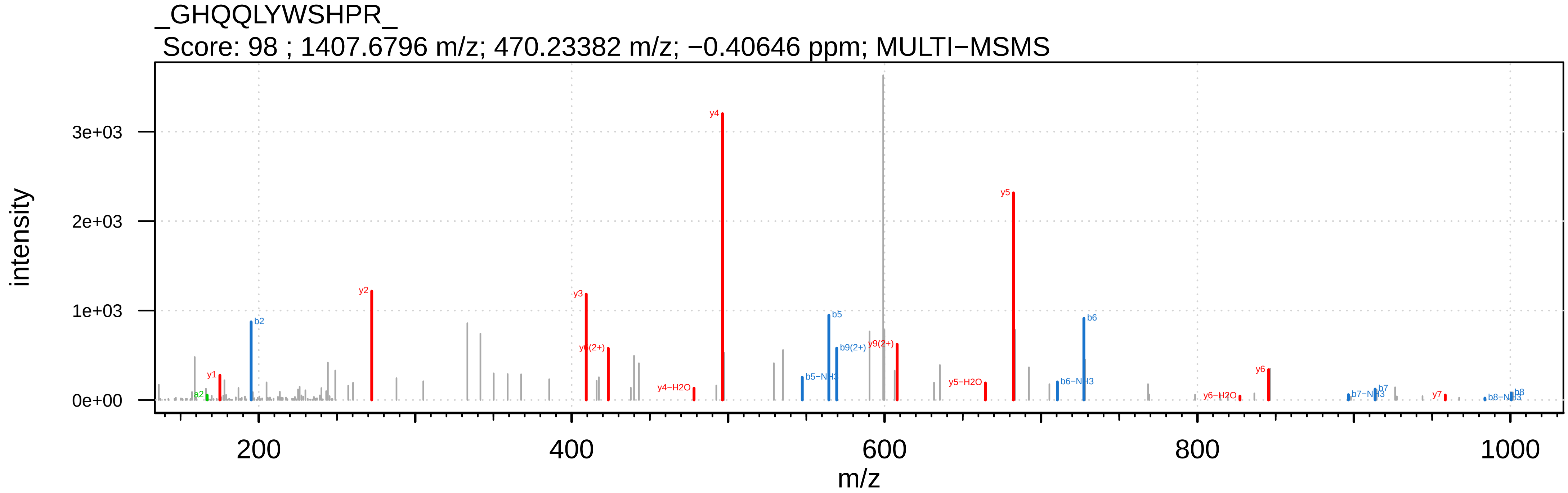

Raw File: Bert_20090403_BJS_halflives_P3_slice03_03
Scan Number: 3591
Proteins:
sp|P62274|RS29_MOUSE
ENSMUST00000037023_chr12:69157813−69159058:−
ENSMUST00000071737_chr13:73988963−73989133:−
tr|D3YYL7|D3YYL7_MOUSE

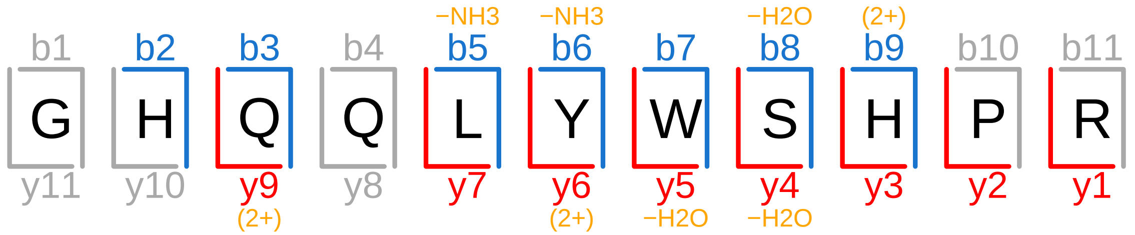

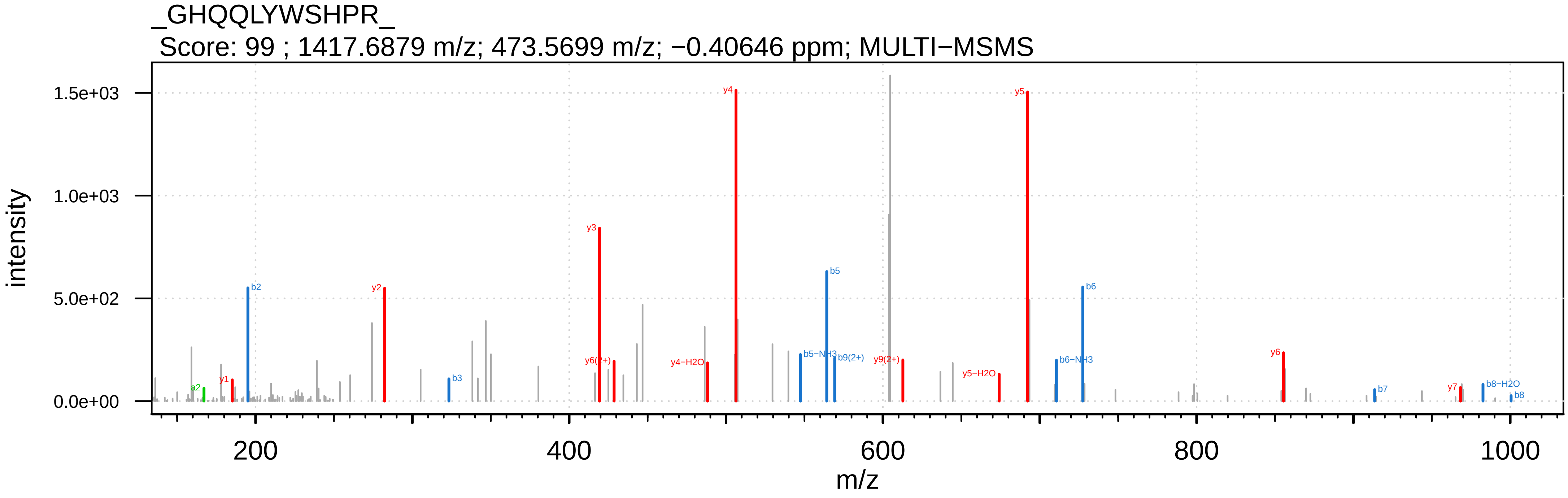

Raw File: Bert_20090403_BJS_halflives_P3_slice03_03
Scan Number: 3593
Proteins:
sp|P62274|RS29_MOUSE
ENSMUST00000037023_chr12:69157813−69159058:−
ENSMUST00000071737_chr13:73988963−73989133:−
tr|D3YYL7|D3YYL7_MOUSE

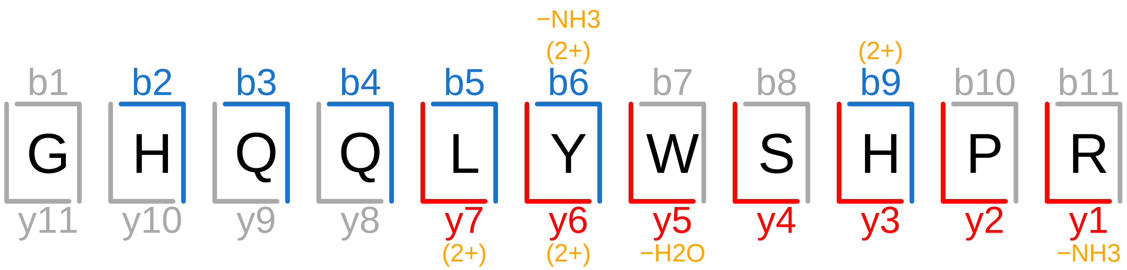

GHQQLYWSHPR
Score: 57 ; 1407.6796 m/z; 352.92718 m/z; −0.20742 ppm; MULTI−MSMS

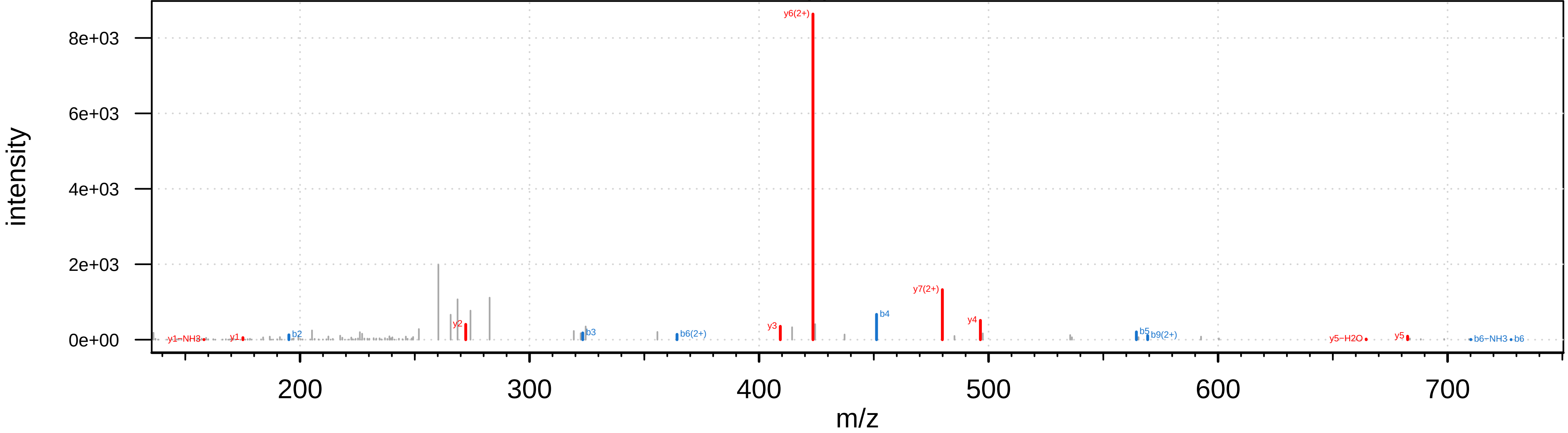

Raw File: Bert_20090403_BJS_halflives_P3_slice03_03
Scan Number: 3599
Proteins:
sp|P62274|RS29_MOUSE
ENSMUST00000037023_chr12:69157813−69159058:−
ENSMUST00000071737_chr13:73988963−73989133:−
tr|D3YYL7|D3YYL7_MOUSE

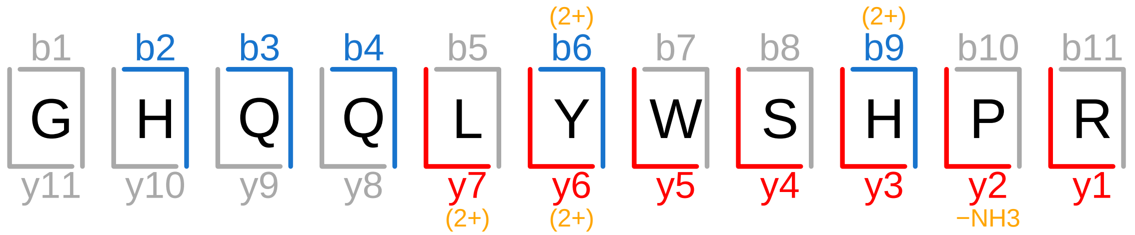

GHQQLYWSHPR
Score: 52 ; 1417.6879 m/z; 355.42925 m/z; −0.20742 ppm; MULTI−MSMS

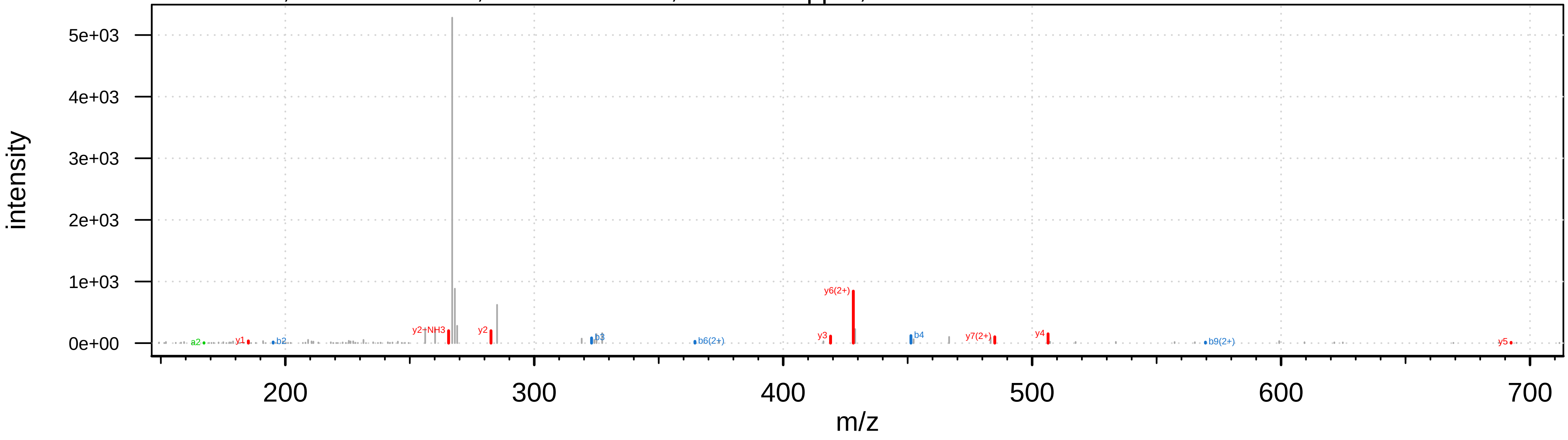

Raw File: Bert_20090403_BJS_halflives_P3_slice03_03
Scan Number: 3604
Proteins:
sp|P62274|RS29_MOUSE
ENSMUST00000037023_chr12:69157813−69159058:−
ENSMUST00000071737_chr13:73988963−73989133:−
tr|D3YYL7|D3YYL7_MOUSE

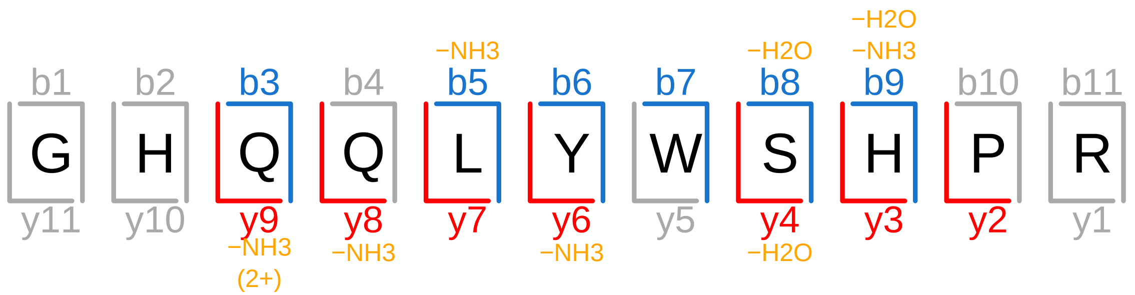

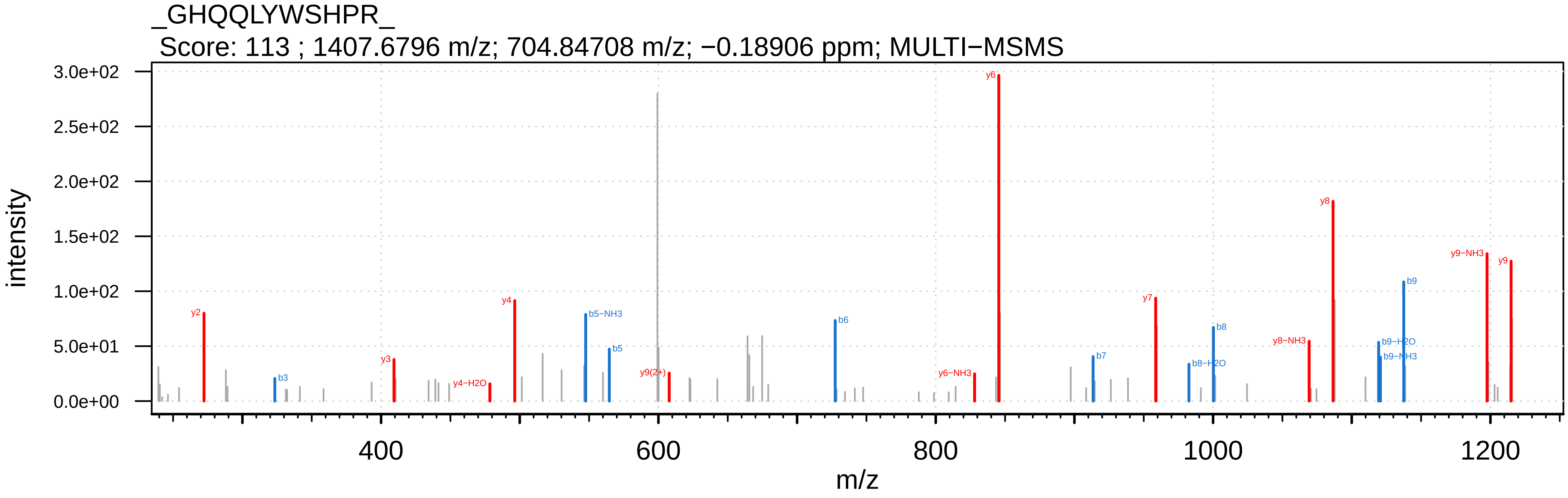

Raw File: Bert_20090403_BJS_halflives_P3_slice03_03
Scan Number: 3605
Proteins:
sp|P62274|RS29_MOUSE
ENSMUST00000037023_chr12:69157813−69159058:−
ENSMUST00000071737_chr13:73988963−73989133:−
tr|D3YYL7|D3YYL7_MOUSE

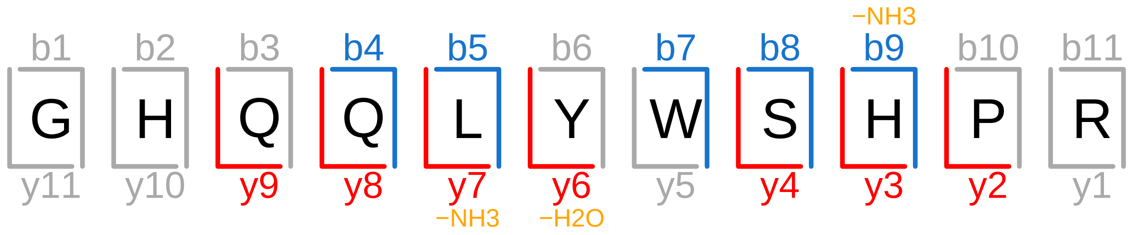

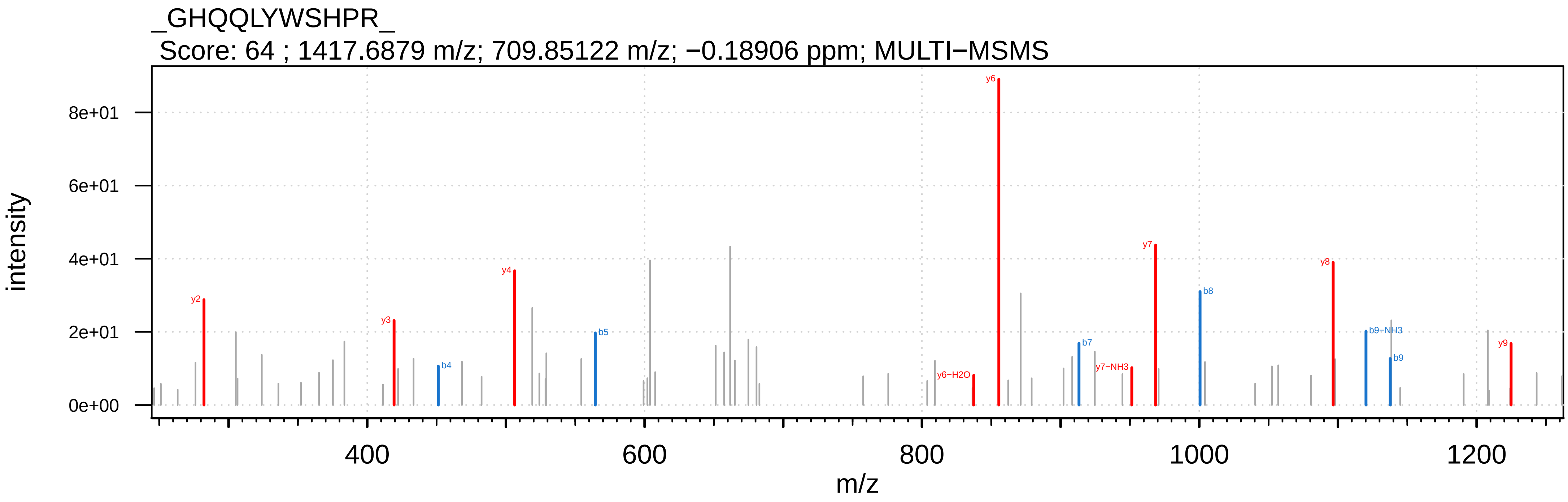

Raw File: Bert_20090403_BJS_halflives_P3_slice03_03
Scan Number: 3626
Proteins:
sp|P62274|RS29_MOUSE
ENSMUST00000037023_chr12:69157813−69159058:−
ENSMUST00000071737_chr13:73988963−73989133:−
tr|D3YYL7|D3YYL7_MOUSE

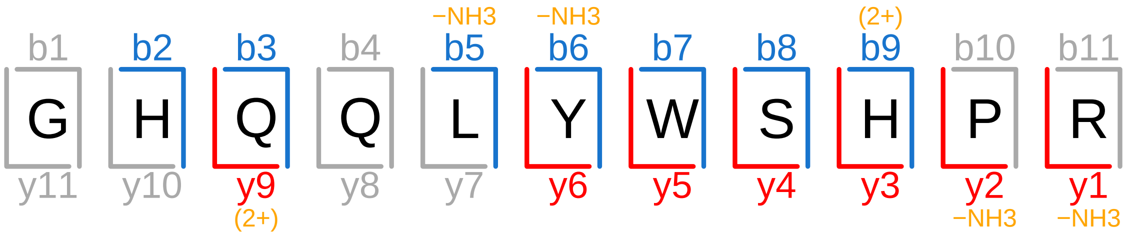

GHQQLYWSHPR
Score: 76 ; 1407.6796 m/z; 470.23382 m/z; 0.030583 ppm; MULTI−MSMS

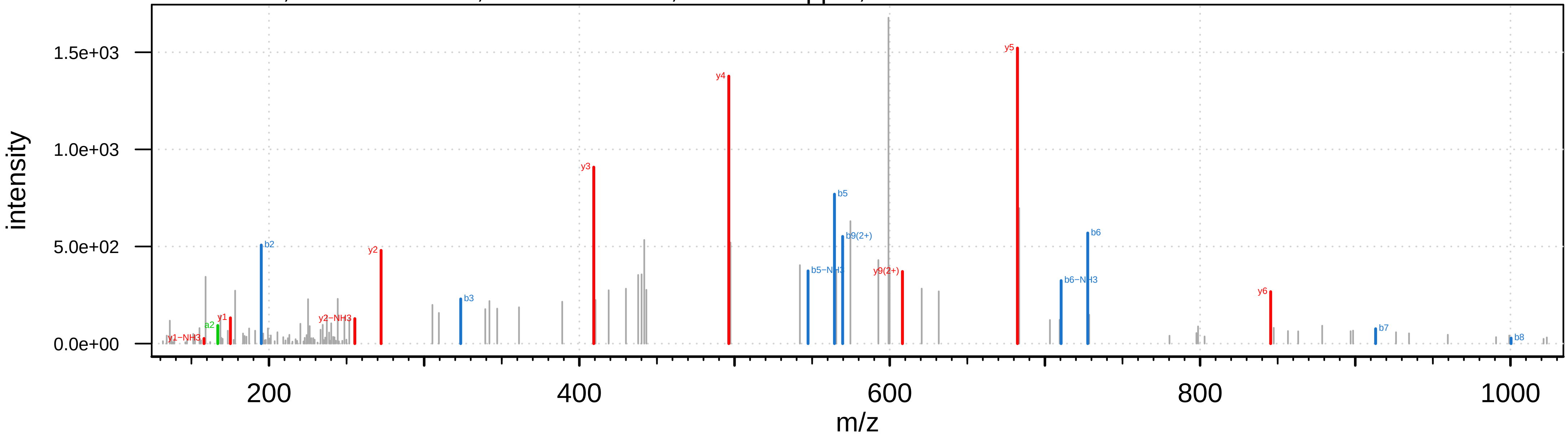

Raw File: Bert_20090407_BJS_halflives_P3_slice06_01
Scan Number: 3871
Proteins:
sp|P62274|RS29_MOUSE
ENSMUST00000037023_chr12:69157813−69159058:−
ENSMUST00000071737_chr13:73988963−73989133:−
tr|D3YYL7|D3YYL7_MOUSE

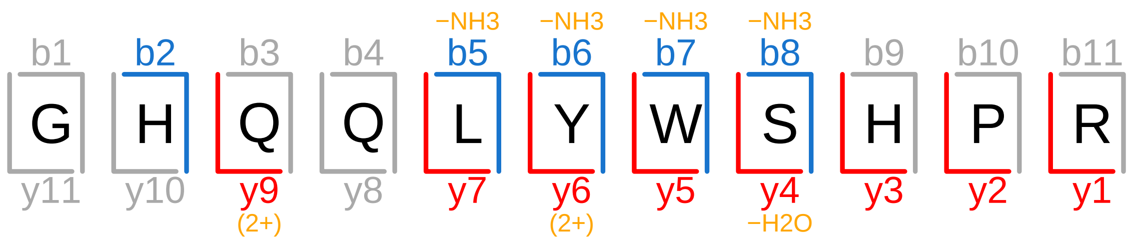

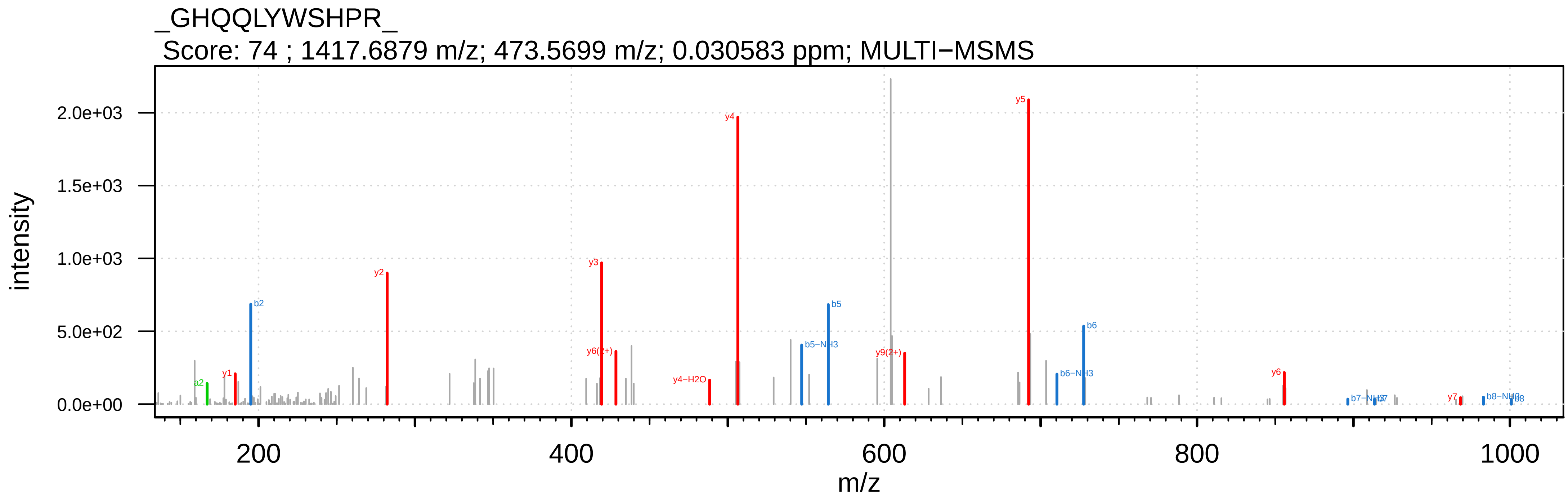

Raw File: Bert_20090407_BJS_halflives_P3_slice06_01
Scan Number: 3883
Proteins:
sp|P62274|RS29_MOUSE
ENSMUST00000037023_chr12:69157813−69159058:−
ENSMUST00000071737_chr13:73988963−73989133:−
tr|D3YYL7|D3YYL7_MOUSE

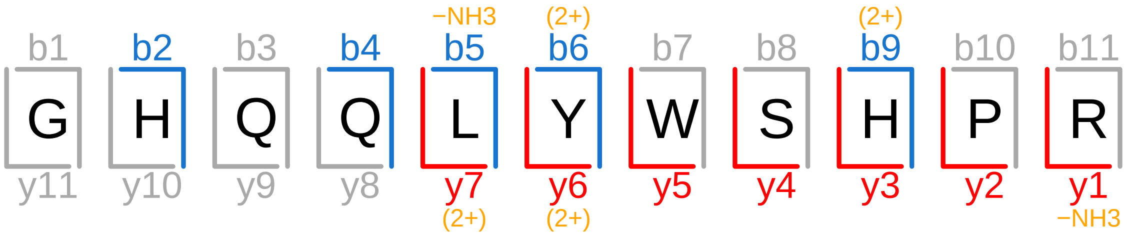

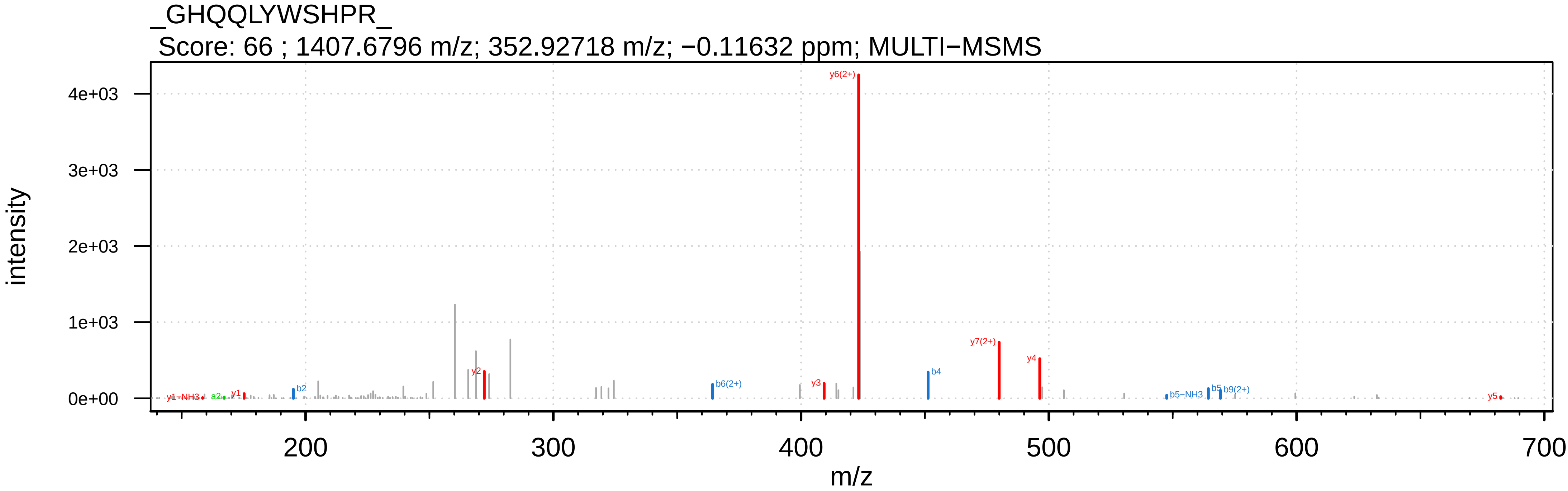

Raw File: Bert_20090407_BJS_halflives_P3_slice06_01
Scan Number: 3885
Proteins:
sp|P62274|RS29_MOUSE
ENSMUST00000037023_chr12:69157813−69159058:−
ENSMUST00000071737_chr13:73988963−73989133:−
tr|D3YYL7|D3YYL7_MOUSE

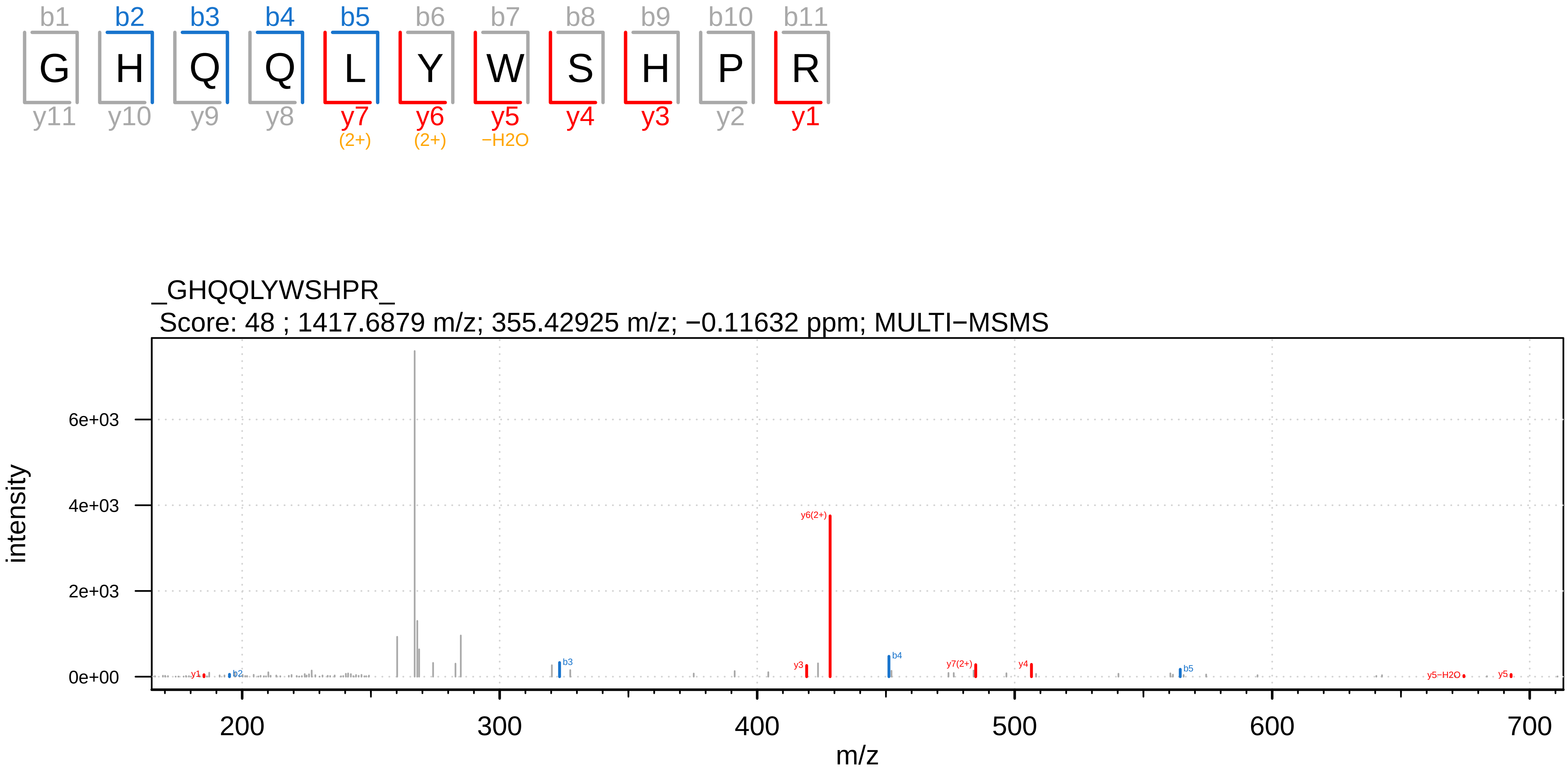

Raw File: Bert_20090407_BJS_halflives_P3_slice06_01
Scan Number: 3898
Proteins:
sp|P62274|RS29_MOUSE
ENSMUST00000037023_chr12:69157813−69159058:−
ENSMUST00000071737_chr13:73988963−73989133:−
tr|D3YYL7|D3YYL7_MOUSE

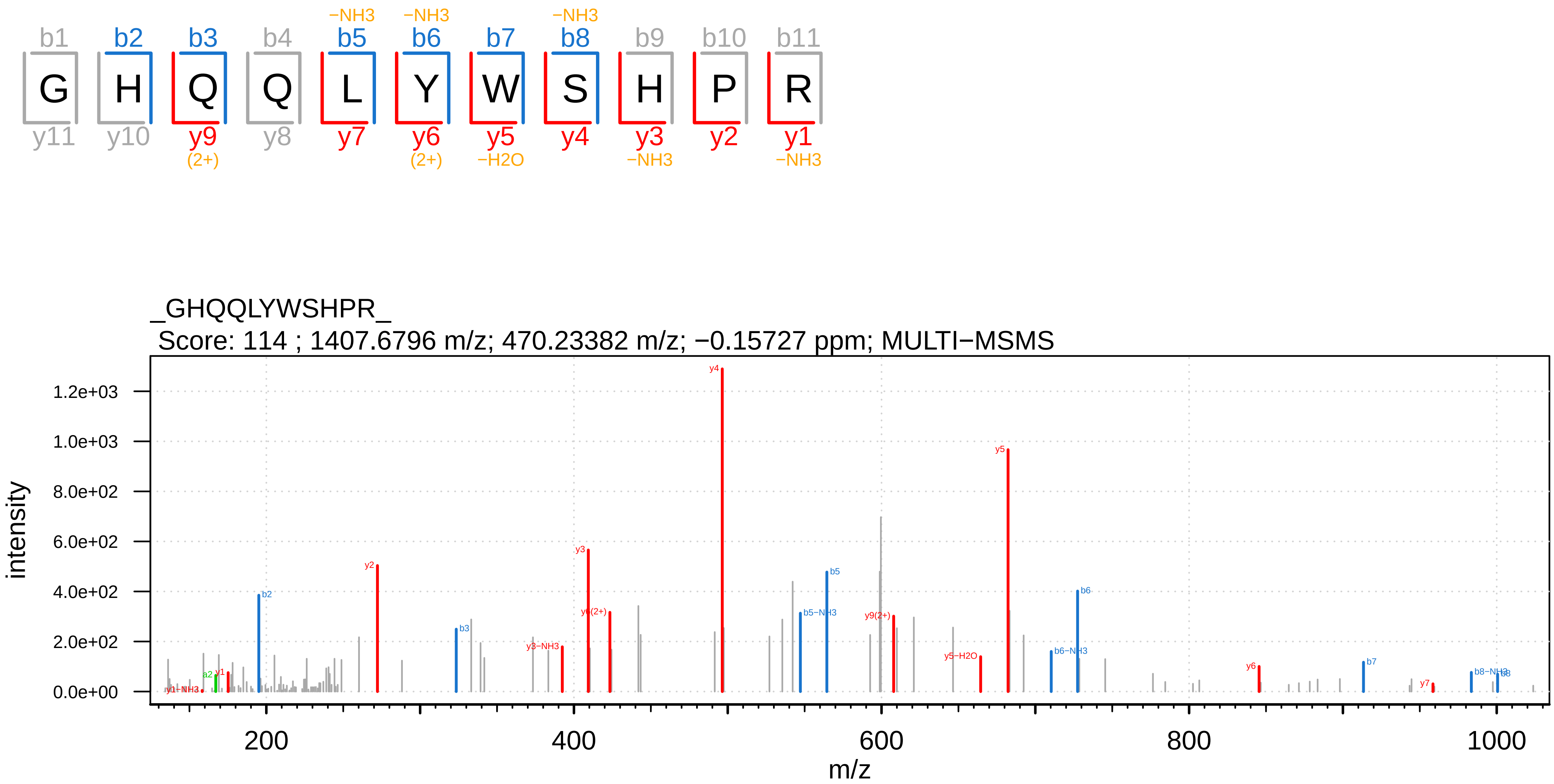

Raw File: Bert_20090407_BJS_halflives_P3_slice06_02
Scan Number: 3644
Proteins:
sp|P62274|RS29_MOUSE
ENSMUST00000037023_chr12:69157813−69159058:−
ENSMUST00000071737_chr13:73988963−73989133:−
tr|D3YYL7|D3YYL7_MOUSE

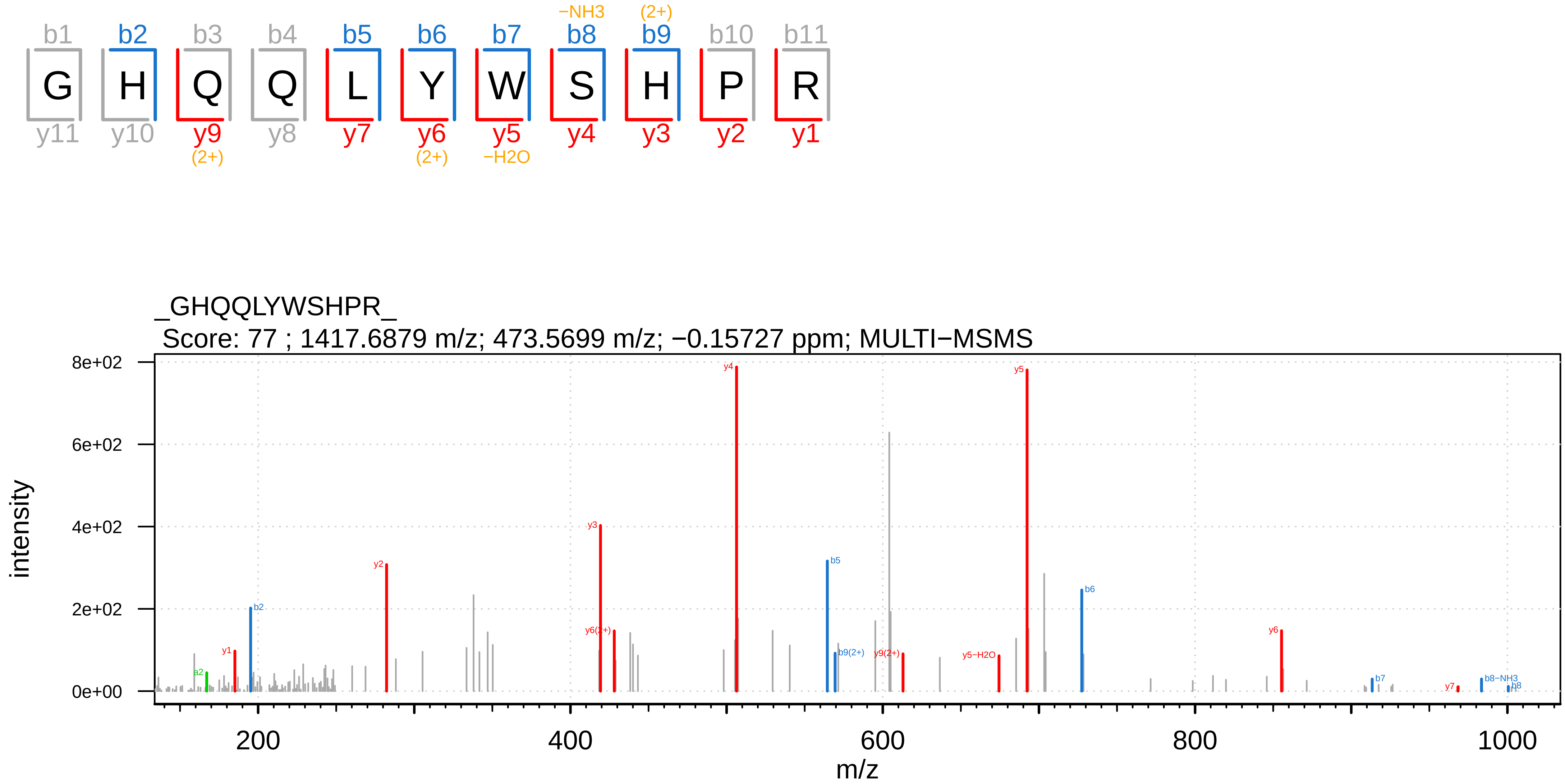

Raw File: Bert_20090407_BJS_halflives_P3_slice06_02
Scan Number: 3651
Proteins:
sp|P62274|RS29_MOUSE
ENSMUST00000037023_chr12:69157813−69159058:−
ENSMUST00000071737_chr13:73988963−73989133:−
tr|D3YYL7|D3YYL7_MOUSE

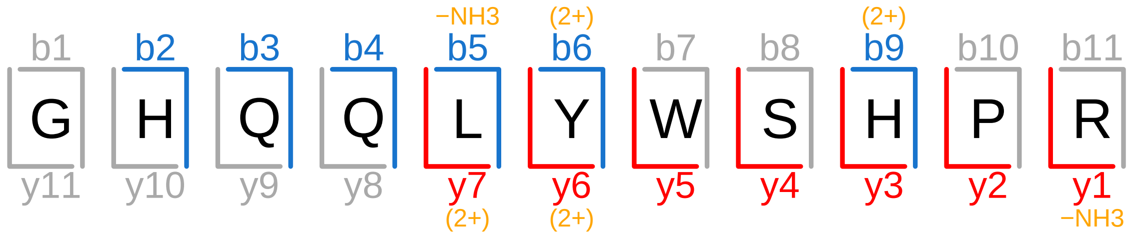

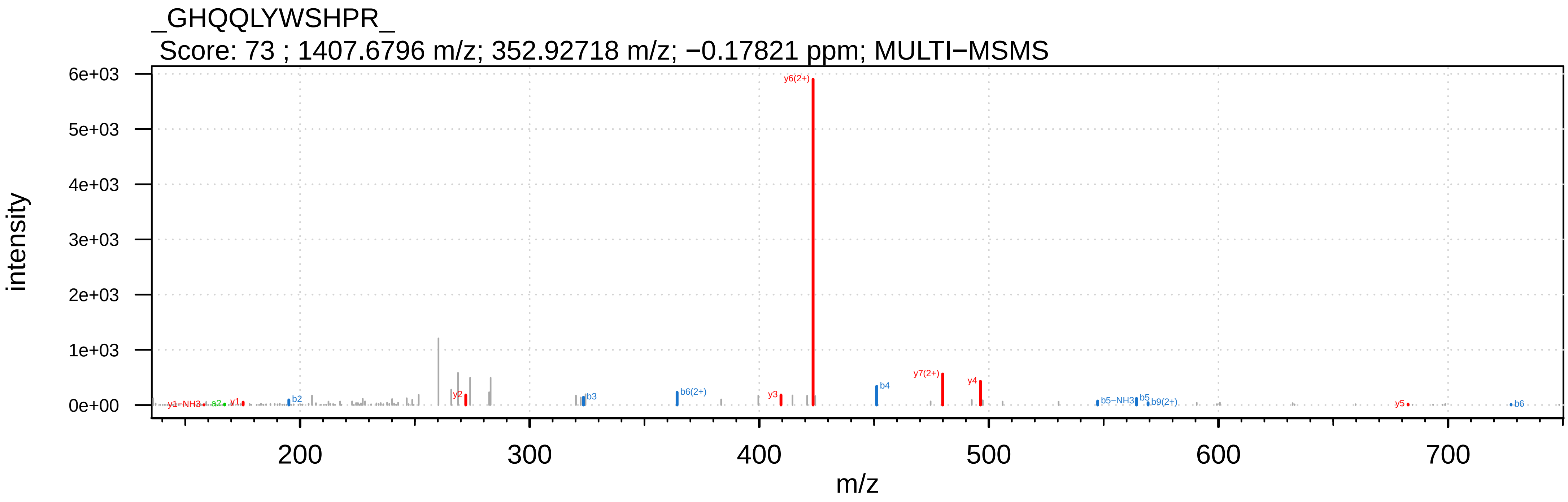

Raw File: Bert_20090407_BJS_halflives_P3_slice06_02
Scan Number: 3662
Proteins:
sp|P62274|RS29_MOUSE
ENSMUST00000037023_chr12:69157813−69159058:−
ENSMUST00000071737_chr13:73988963−73989133:−
tr|D3YYL7|D3YYL7_MOUSE

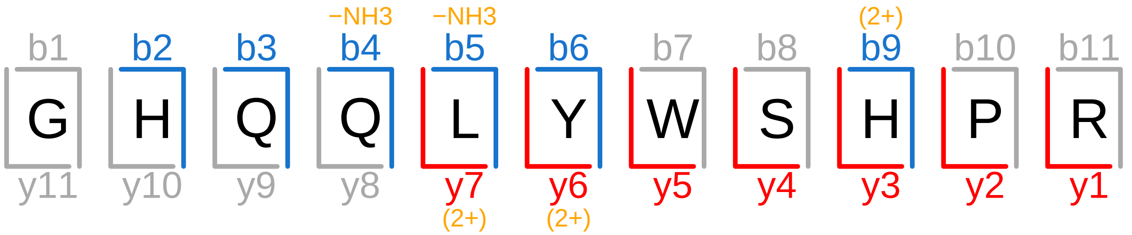

GHQQLYWSHPR
Score: 73 ; 1417.6879 m/z; 355.42925 m/z; −0.17821 ppm; MULTI−MSMS

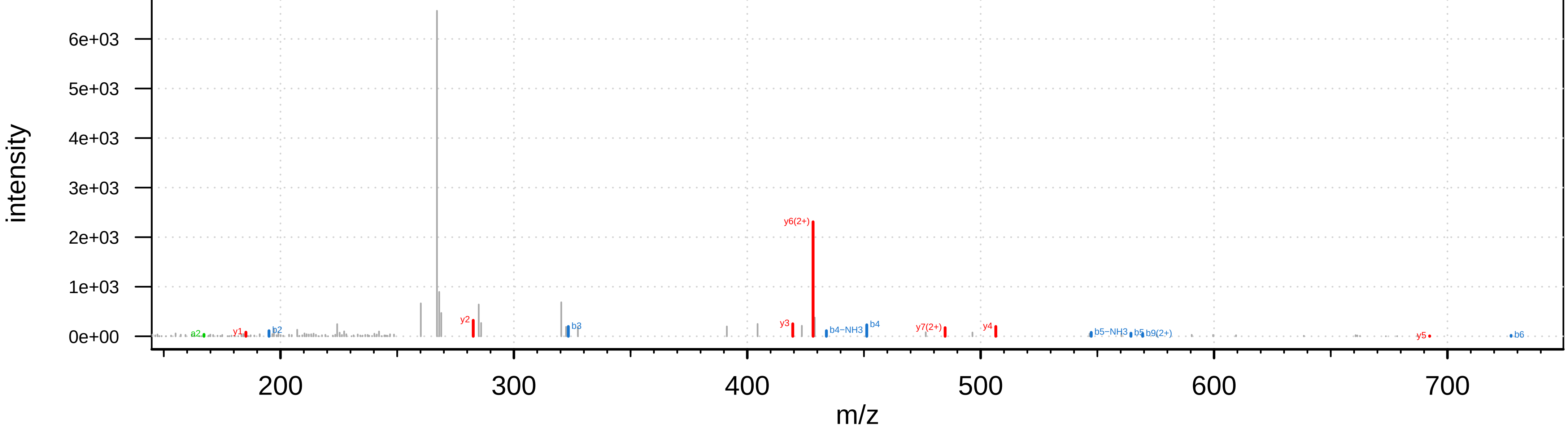

Raw File: Bert_20090407_BJS_halflives_P3_slice06_02
Scan Number: 3668
Proteins:
sp|P62274|RS29_MOUSE
ENSMUST00000037023_chr12:69157813−69159058:−
ENSMUST00000071737_chr13:73988963−73989133:−
tr|D3YYL7|D3YYL7_MOUSE

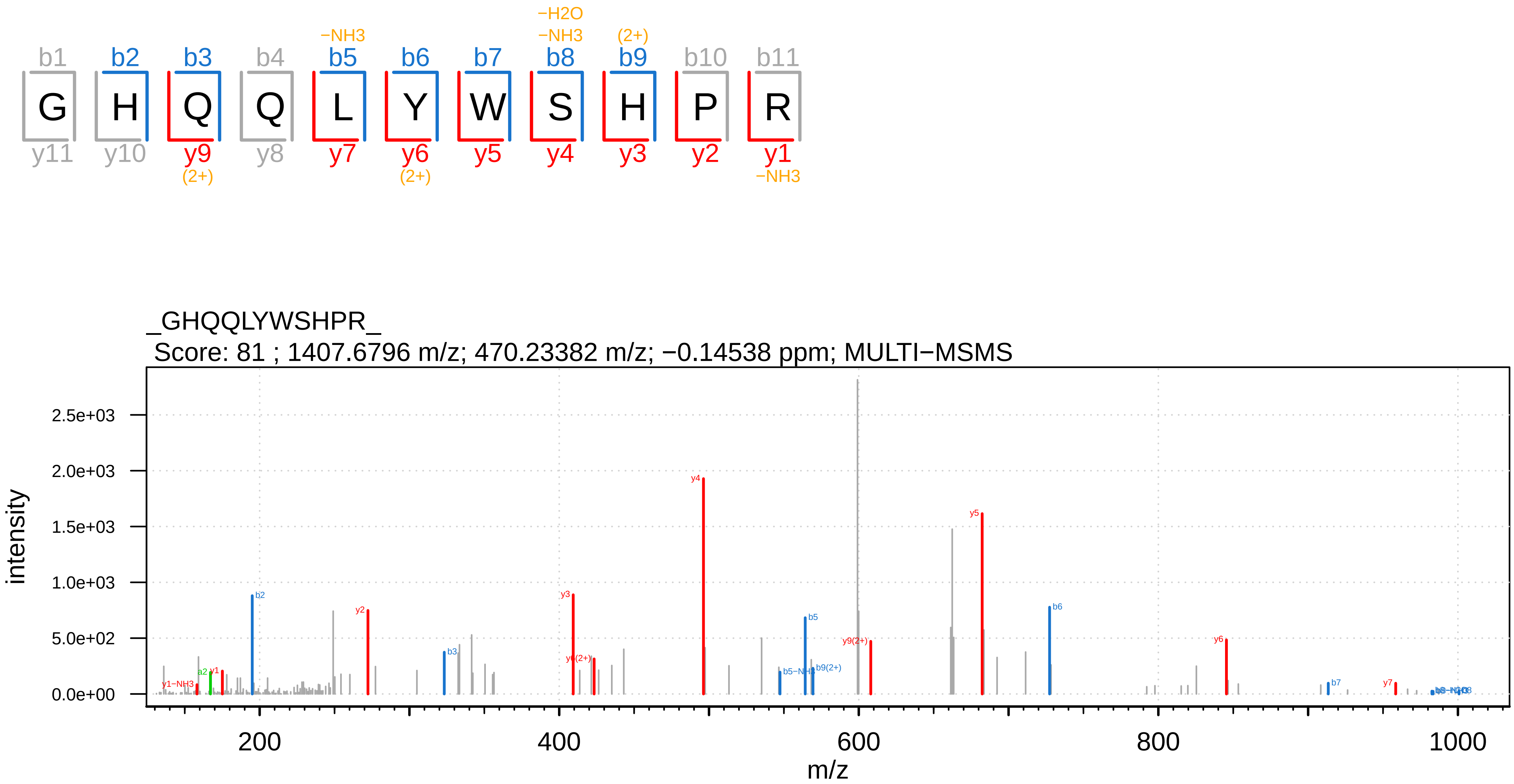

Raw File: Bert_20090407_BJS_halflives_P3_slice06_03
Scan Number: 3745
Proteins:
sp|P62274|RS29_MOUSE
ENSMUST00000037023_chr12:69157813−69159058:−
ENSMUST00000071737_chr13:73988963−73989133:−
tr|D3YYL7|D3YYL7_MOUSE

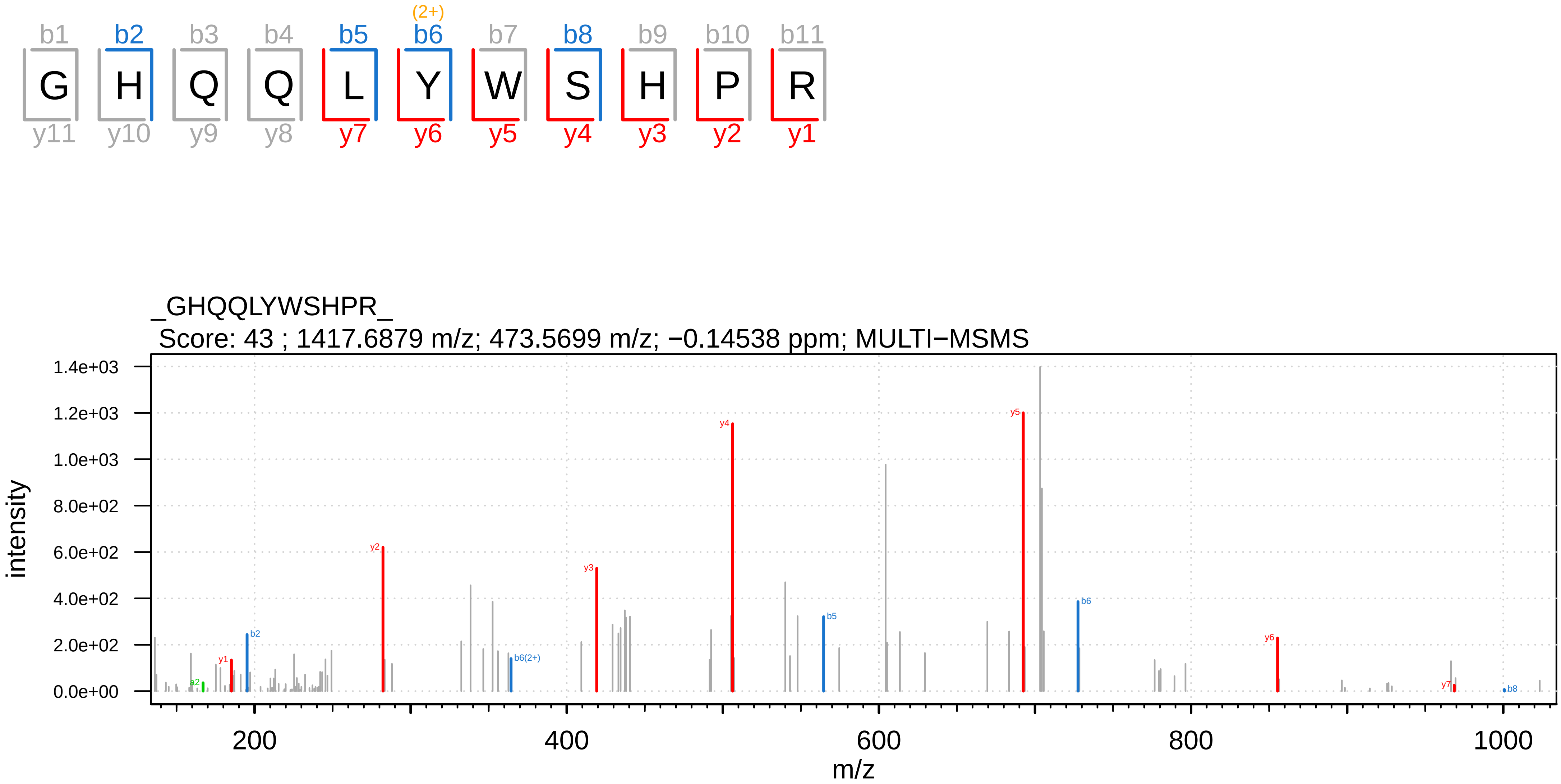

Raw File: Bert_20090407_BJS_halflives_P3_slice06_03
Scan Number: 3760
Proteins:
sp|P62274|RS29_MOUSE
ENSMUST00000037023_chr12:69157813−69159058:−
ENSMUST00000071737_chr13:73988963−73989133:−
tr|D3YYL7|D3YYL7_MOUSE

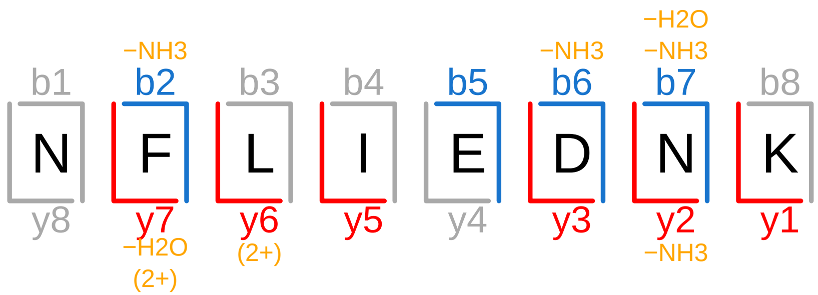

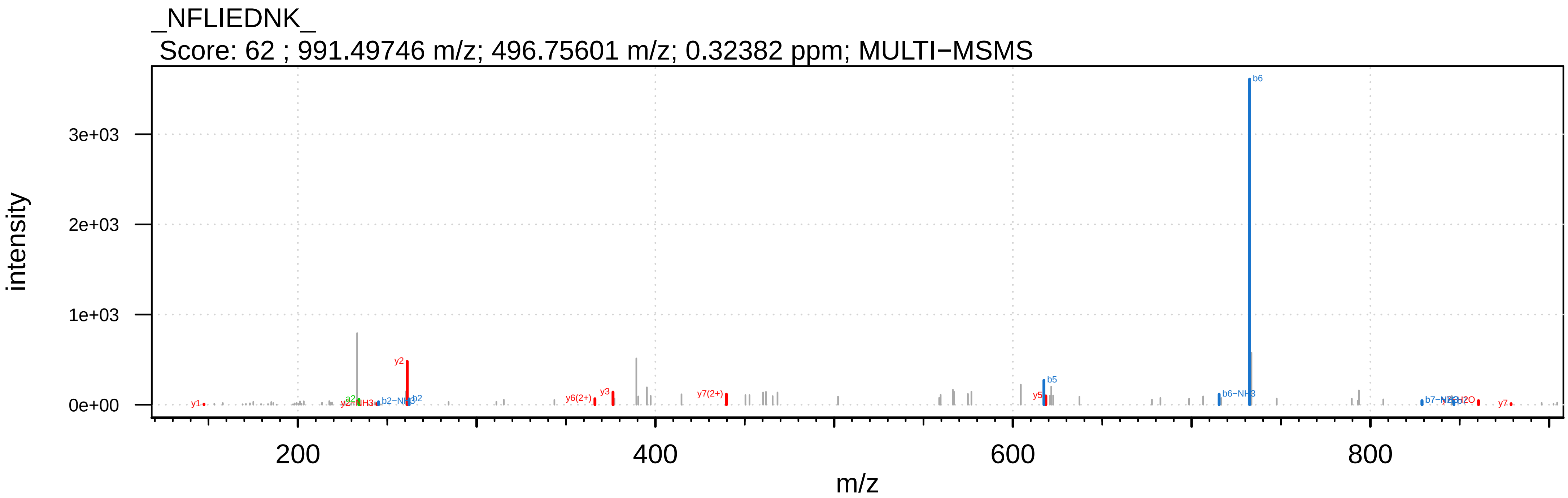

Raw File: Bert_20090407_BJS_halflives_P3_slice06_03
Scan Number: 7731
Proteins:
chr19_39127120−39129472.T1_chr19:39054658−39054798:+

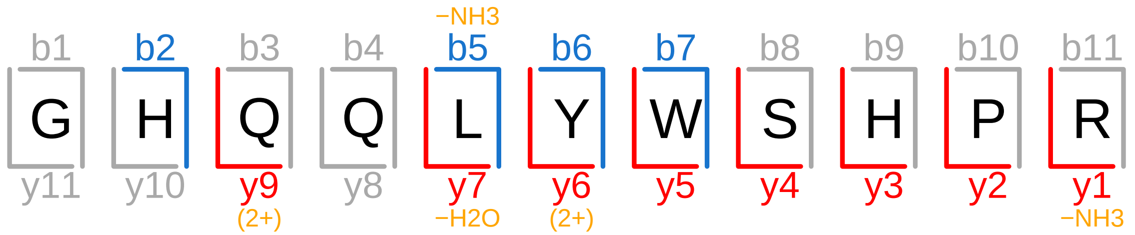

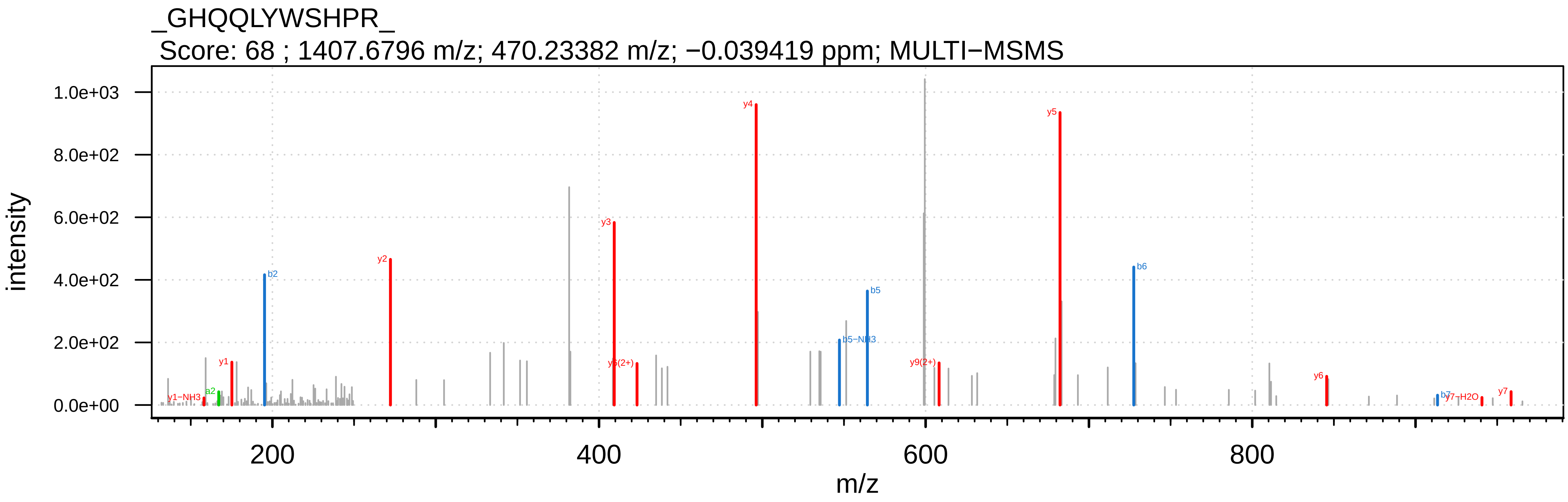

Raw File: Bert_20090407_BJS_halflives_P3_slice07_01
Scan Number: 3680
Proteins:
sp|P62274|RS29_MOUSE
ENSMUST00000037023_chr12:69157813−69159058:−
ENSMUST00000071737_chr13:73988963−73989133:−
tr|D3YYL7|D3YYL7_MOUSE

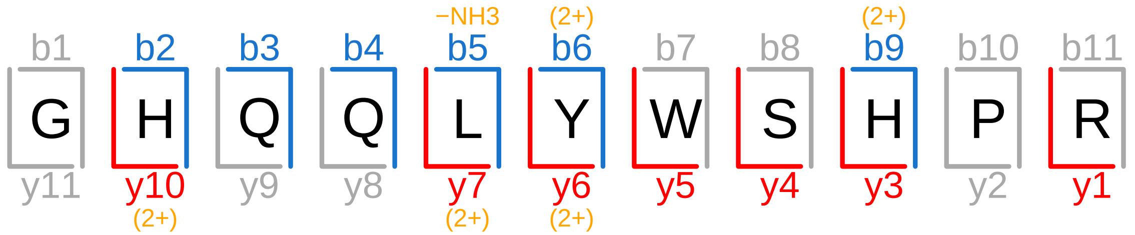

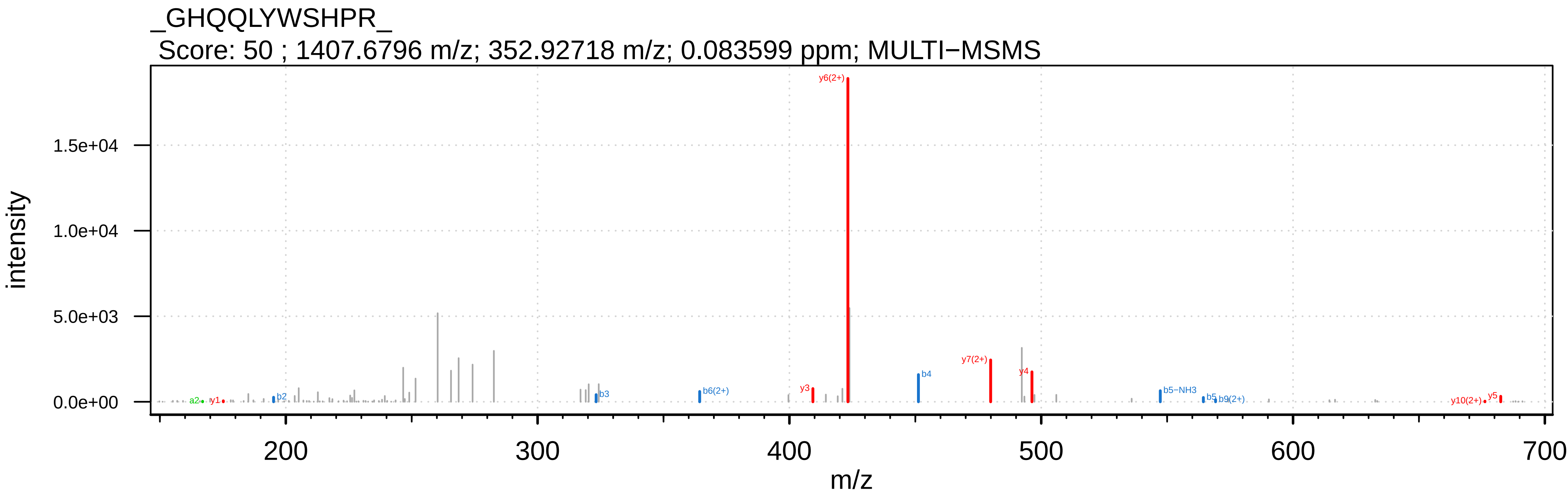

Raw File: Bert_20090407_BJS_halflives_P3_slice07_01
Scan Number: 3697
Proteins:
sp|P62274|RS29_MOUSE
ENSMUST00000037023_chr12:69157813−69159058:−
ENSMUST00000071737_chr13:73988963−73989133:−
tr|D3YYL7|D3YYL7_MOUSE

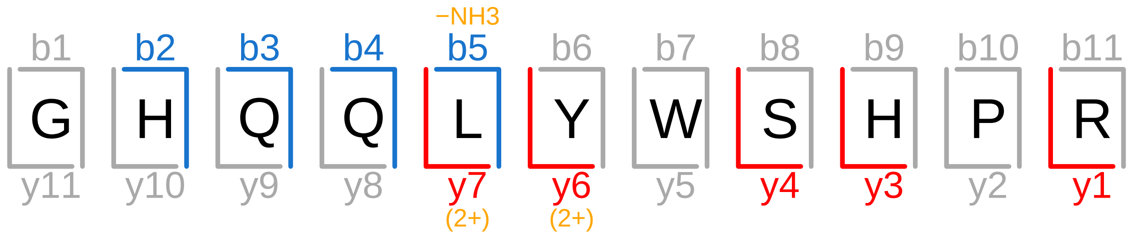

GHQQLYWSHPR
Score: 34 ; 1417.6879 m/z; 355.42925 m/z; 0.083599 ppm; MULTI−MSMS

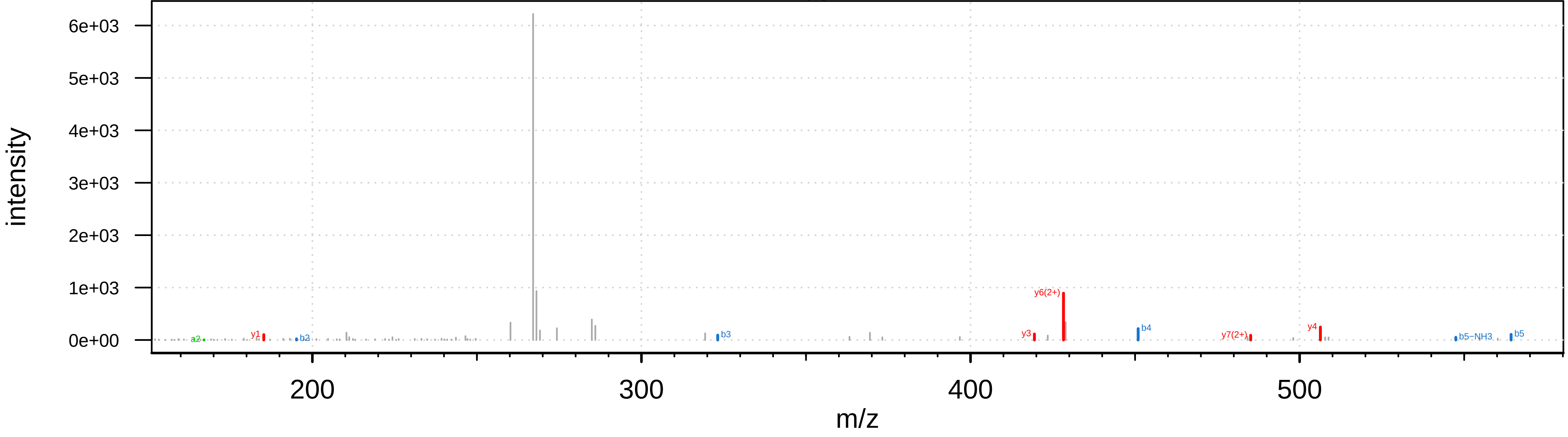

Raw File: Bert_20090407_BJS_halflives_P3_slice07_01
Scan Number: 3704
Proteins:
sp|P62274|RS29_MOUSE
ENSMUST00000037023_chr12:69157813−69159058:−
ENSMUST00000071737_chr13:73988963−73989133:−
tr|D3YYL7|D3YYL7_MOUSE

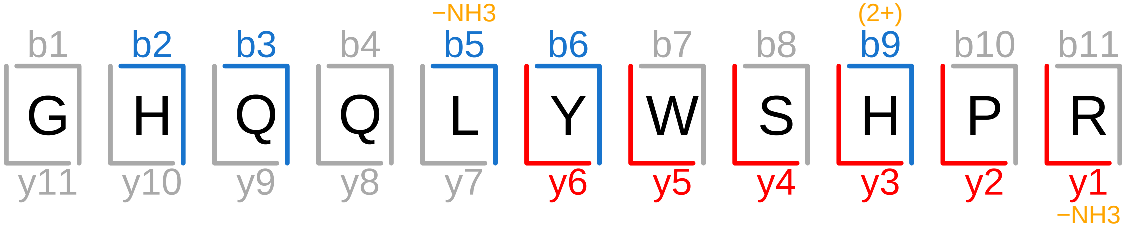

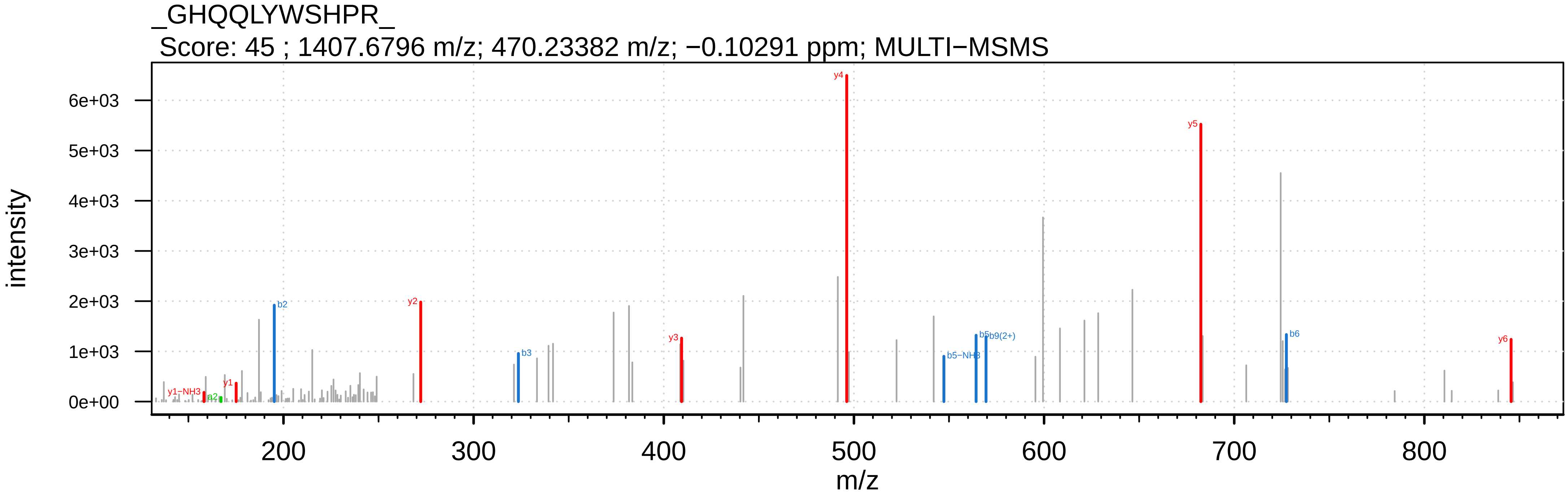

Raw File: Bert_20090407_BJS_halflives_P3_slice07_02
Scan Number: 3513
Proteins:
sp|P62274|RS29_MOUSE
ENSMUST00000037023_chr12:69157813−69159058:−
ENSMUST00000071737_chr13:73988963−73989133:−
tr|D3YYL7|D3YYL7_MOUSE

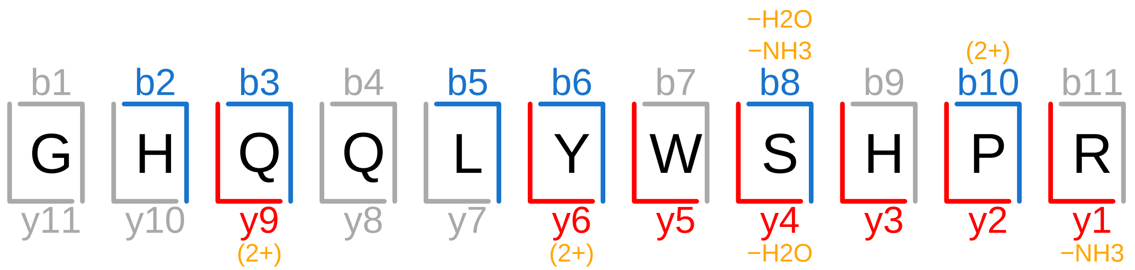

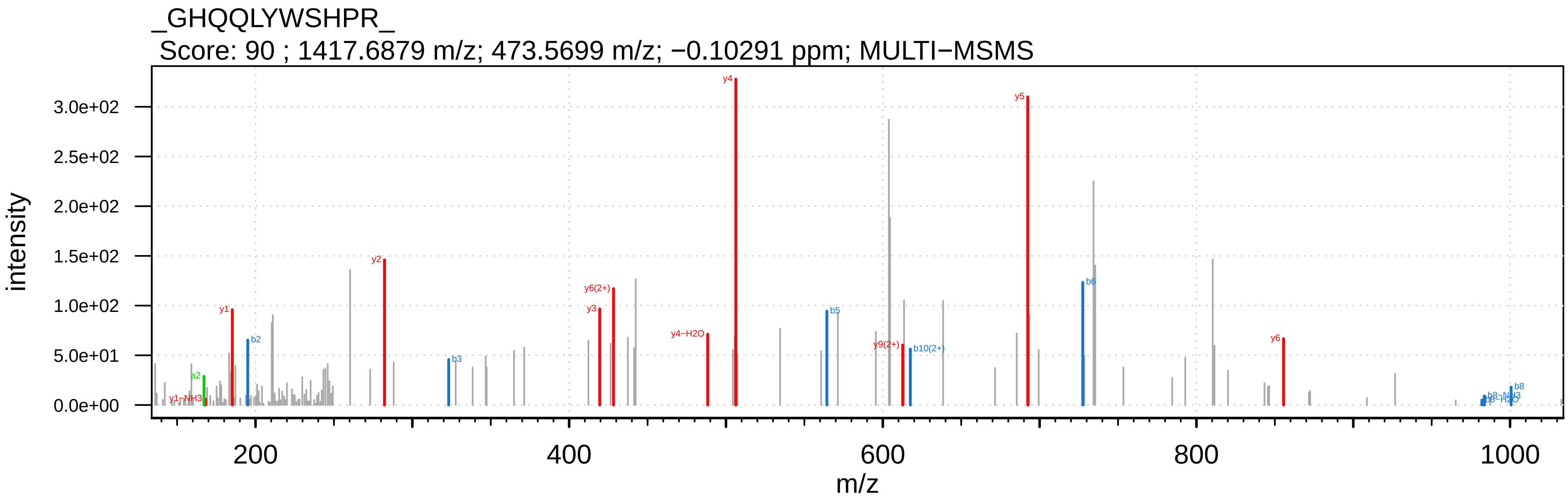

Raw File: Bert_20090407_BJS_halflives_P3_slice07_02
Scan Number: 3532
Proteins:
sp|P62274|RS29_MOUSE
ENSMUST00000037023_chr12:69157813−69159058:−
ENSMUST00000071737_chr13:73988963−73989133:−
tr|D3YYL7|D3YYL7_MOUSE

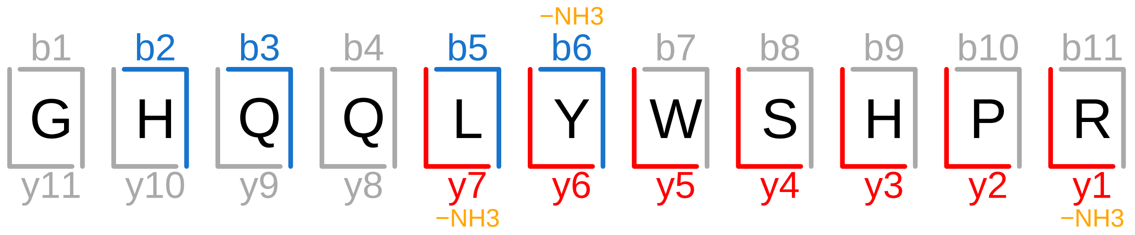

GHQQLYWSHPR
Score: 52 ; 1407.6796 m/z; 470.23382 m/z; 0.095814 ppm; MULTI−MSMS

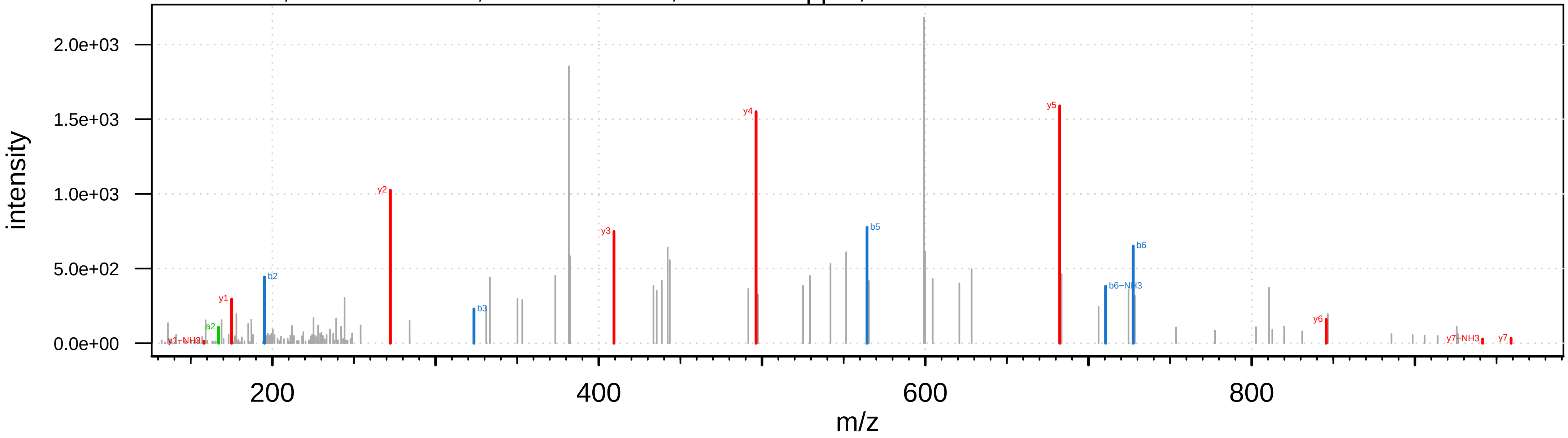

Raw File: Bert_20090407_BJS_halflives_P3_slice07_03
Scan Number: 2849
Proteins:
sp|P62274|RS29_MOUSE
ENSMUST00000037023_chr12:69157813−69159058:−
ENSMUST00000071737_chr13:73988963−73989133:−
tr|D3YYL7|D3YYL7_MOUSE

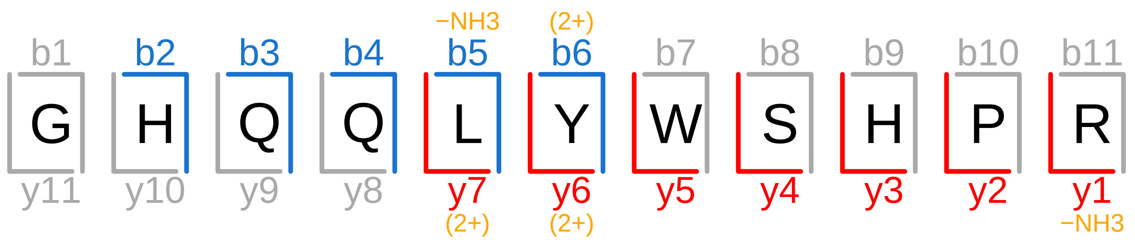

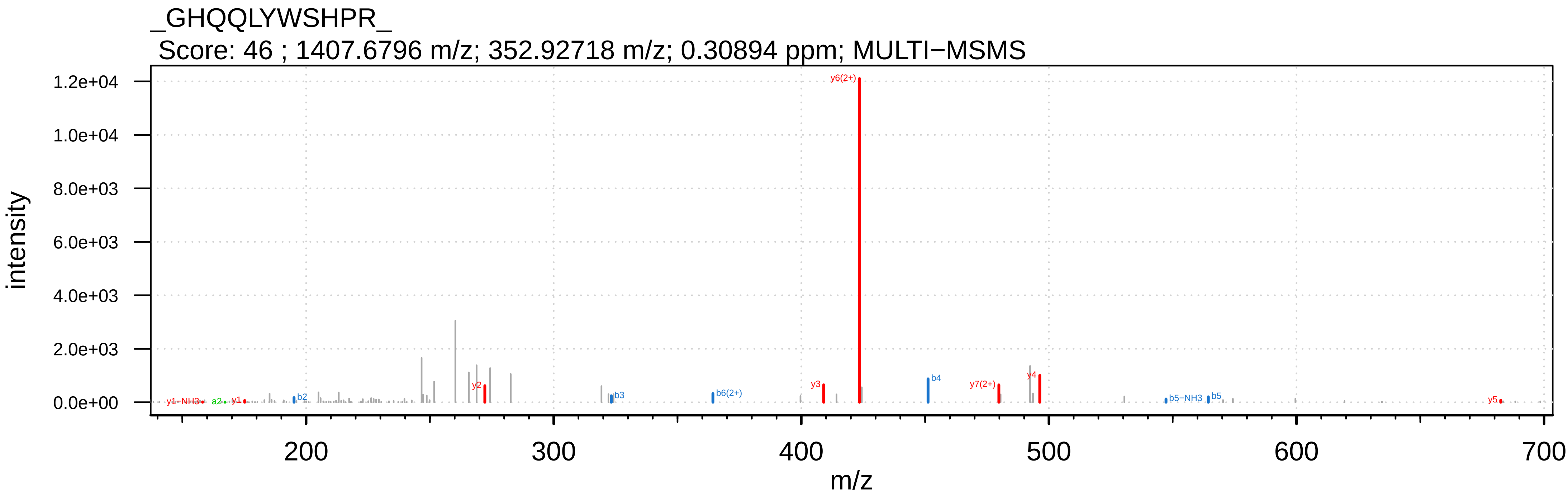

Raw File: Bert_20090407_BJS_halflives_P3_slice07_03
Scan Number: 2867
Proteins:
sp|P62274|RS29_MOUSE
ENSMUST00000037023_chr12:69157813−69159058:−
ENSMUST00000071737_chr13:73988963−73989133:−
tr|D3YYL7|D3YYL7_MOUSE

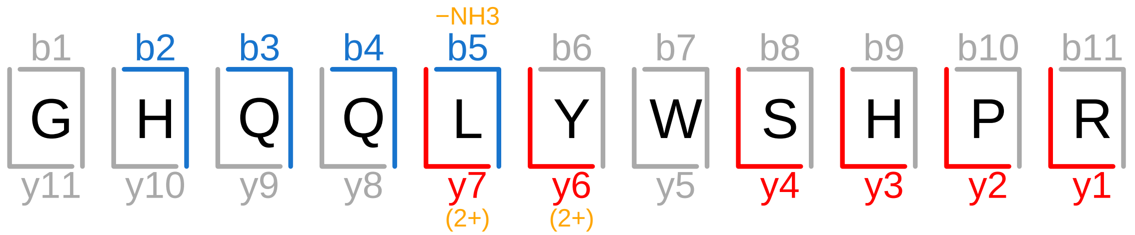

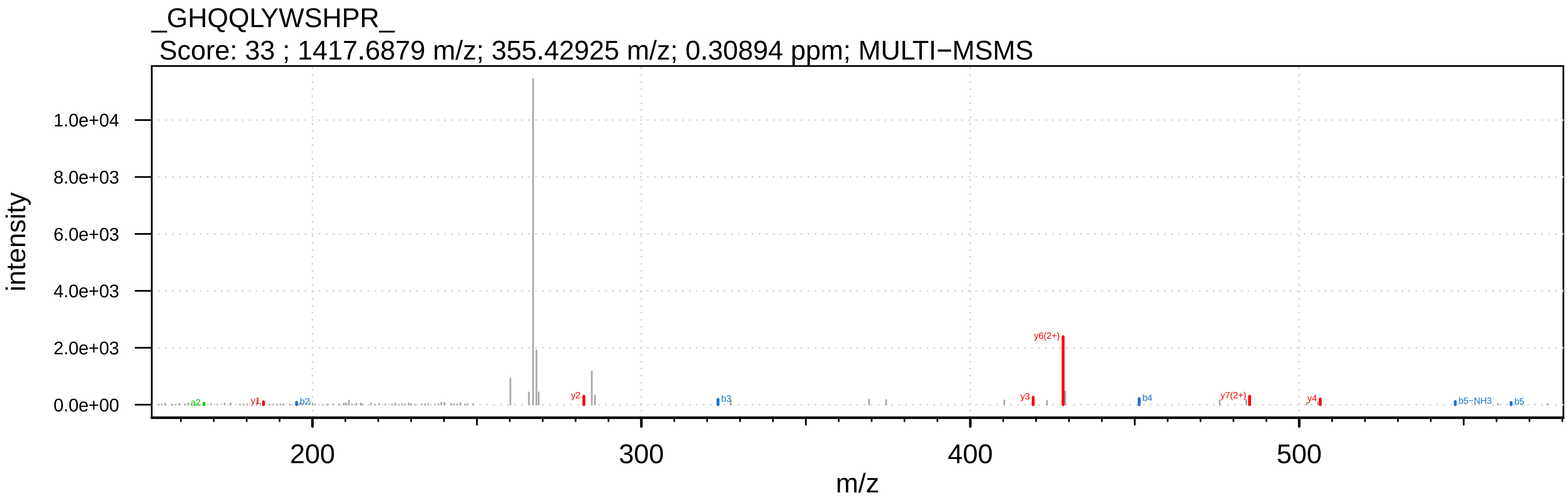

Raw File: Bert_20090407_BJS_halflives_P3_slice07_03
Scan Number: 2872
Proteins:
sp|P62274|RS29_MOUSE
ENSMUST00000037023_chr12:69157813−69159058:−
ENSMUST00000071737_chr13:73988963−73989133:−
tr|D3YYL7|D3YYL7_MOUSE

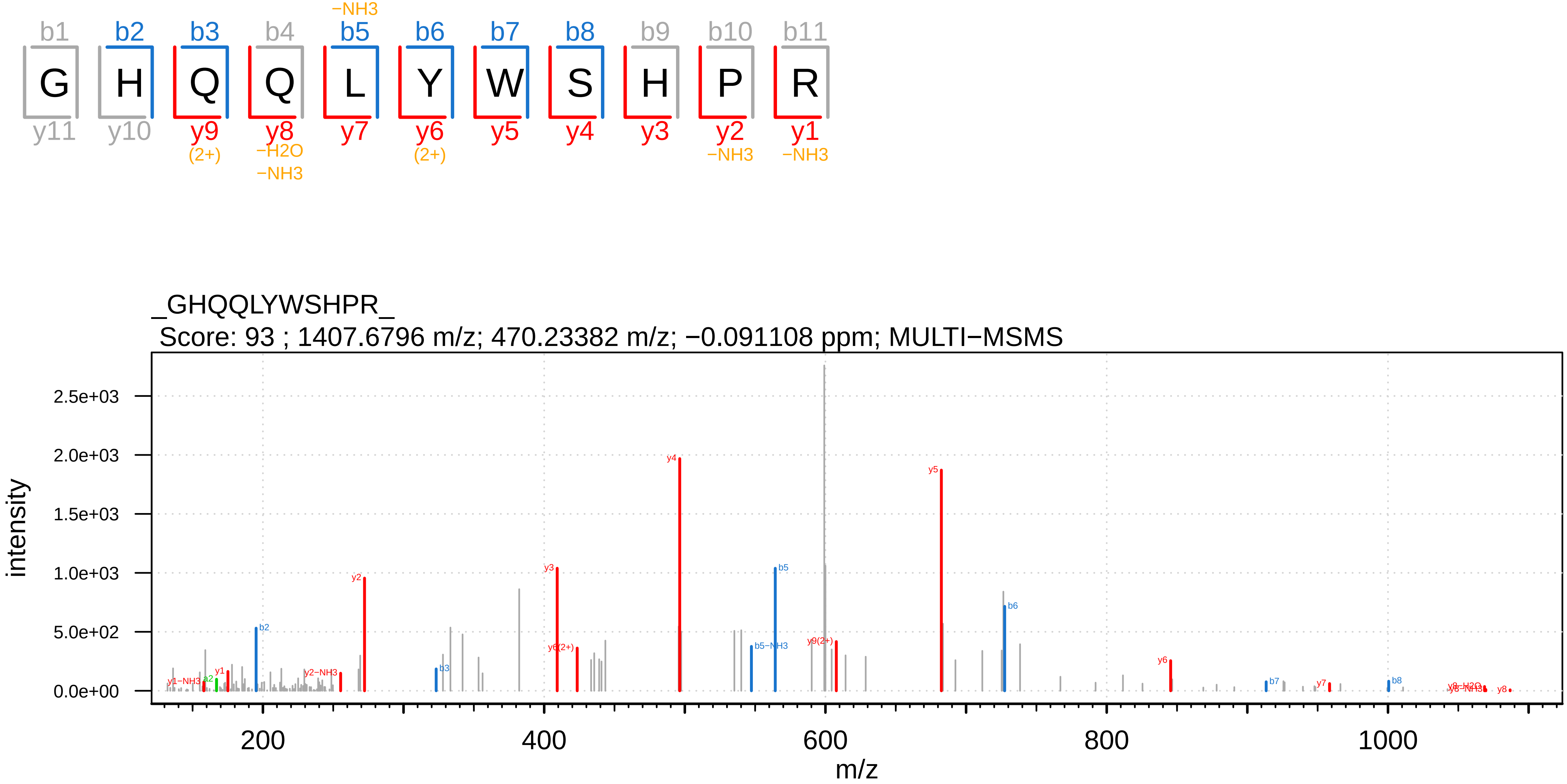

Raw File: Bert_20090407_BJS_halflives_P3_slice08_02
Scan Number: 3395
Proteins:
sp|P62274|RS29_MOUSE
ENSMUST00000037023_chr12:69157813−69159058:−
ENSMUST00000071737_chr13:73988963−73989133:−
tr|D3YYL7|D3YYL7_MOUSE

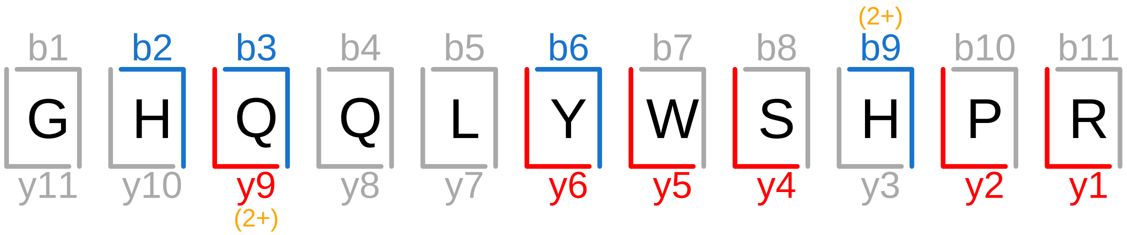

GHQQLYWSHPR
Score: 24 ; 1407.6796 m/z; 470.23382 m/z; −0.030436 ppm; MULTI−MSMS

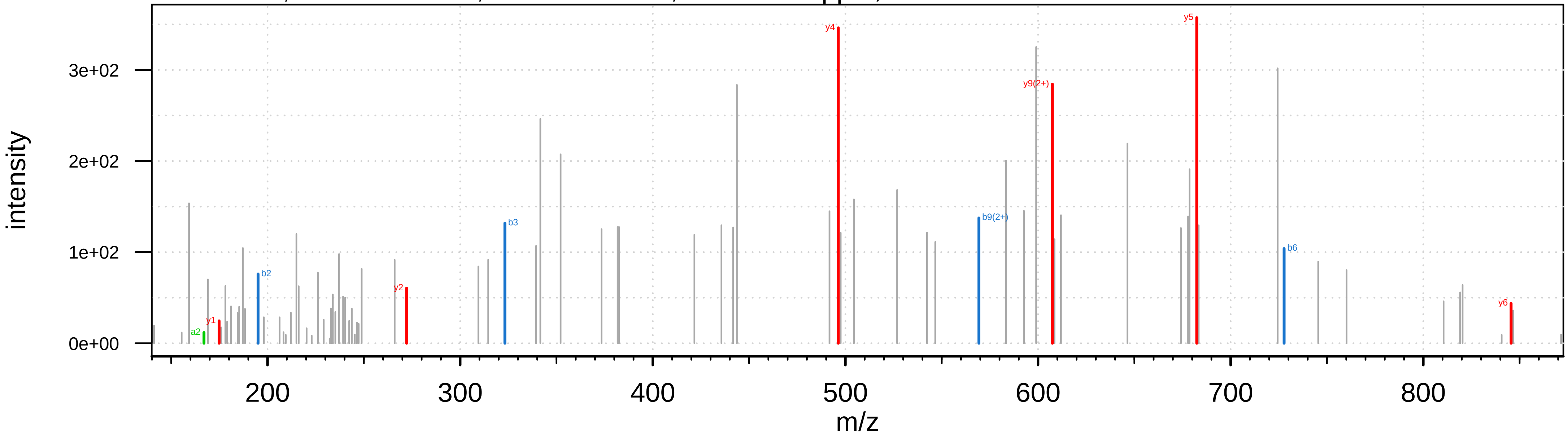

Raw File: Bert_20090407_BJS_halflives_P3_slice08_03
Scan Number: 3810
Proteins:
sp|P62274|RS29_MOUSE
ENSMUST00000037023_chr12:69157813−69159058:−
ENSMUST00000071737_chr13:73988963−73989133:−
tr|D3YYL7|D3YYL7_MOUSE

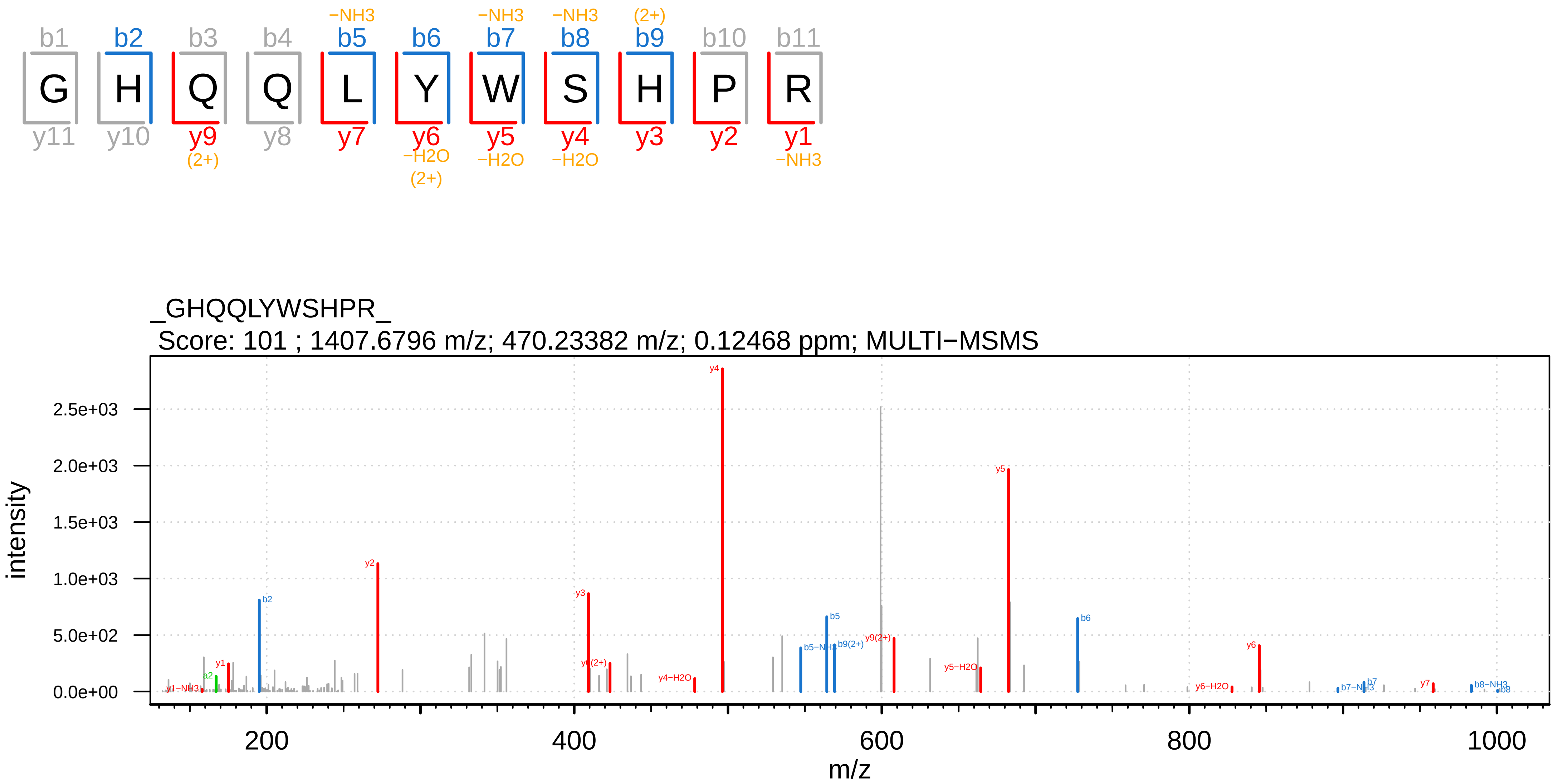

Raw File: Bert_20090408_BJS_halflives_P3_slice09_04
Scan Number: 5081
Proteins:
sp|P62274|RS29_MOUSE
ENSMUST00000037023_chr12:69157813−69159058:−
ENSMUST00000071737_chr13:73988963−73989133:−
tr|D3YYL7|D3YYL7_MOUSE

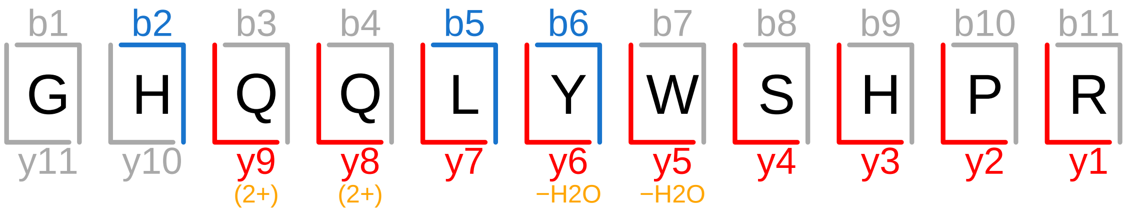

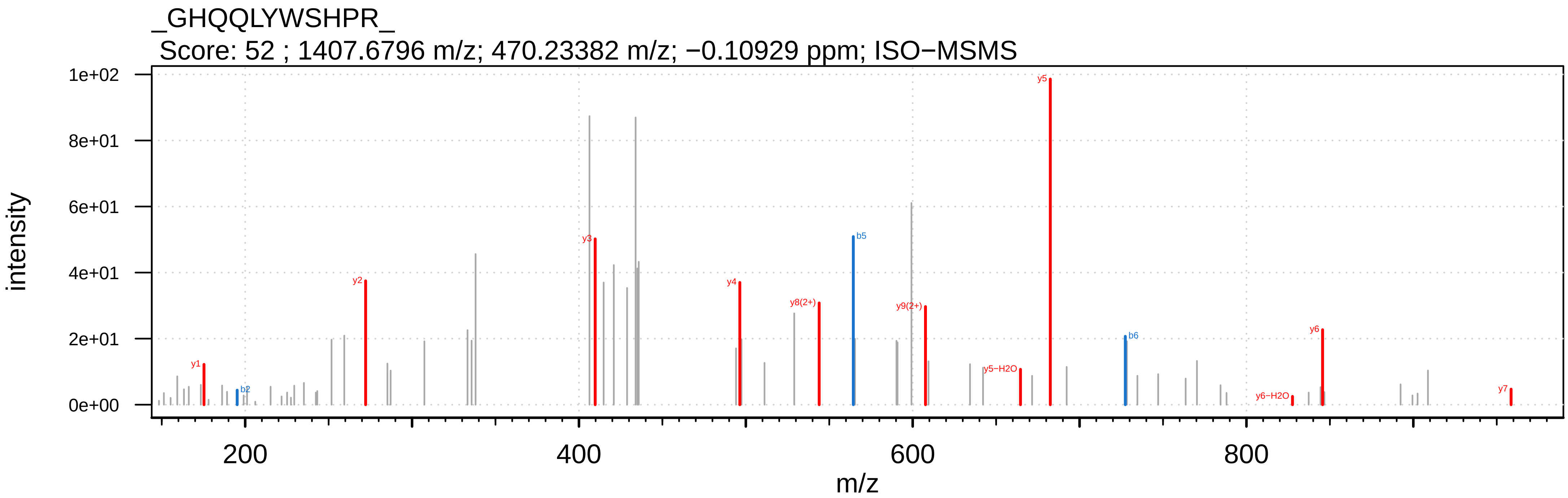

Raw File: Ernie_20090327_BJS_halflives_P2_slice01_02
Scan Number: 2950
Proteins:
sp|P62274|RS29_MOUSE
ENSMUST00000037023_chr12:69157813−69159058:−
ENSMUST00000071737_chr13:73988963−73989133:−
tr|D3YYL7|D3YYL7_MOUSE

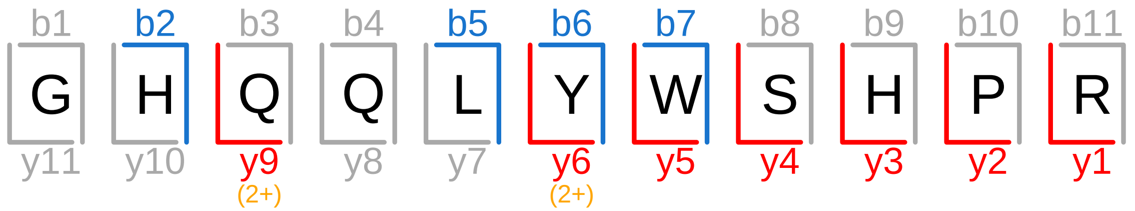

GHQQLYWSHPR
Score: 47 ; 1417.6879 m/z; 473.5699 m/z; −0.46996 ppm; MULTI−MSMS

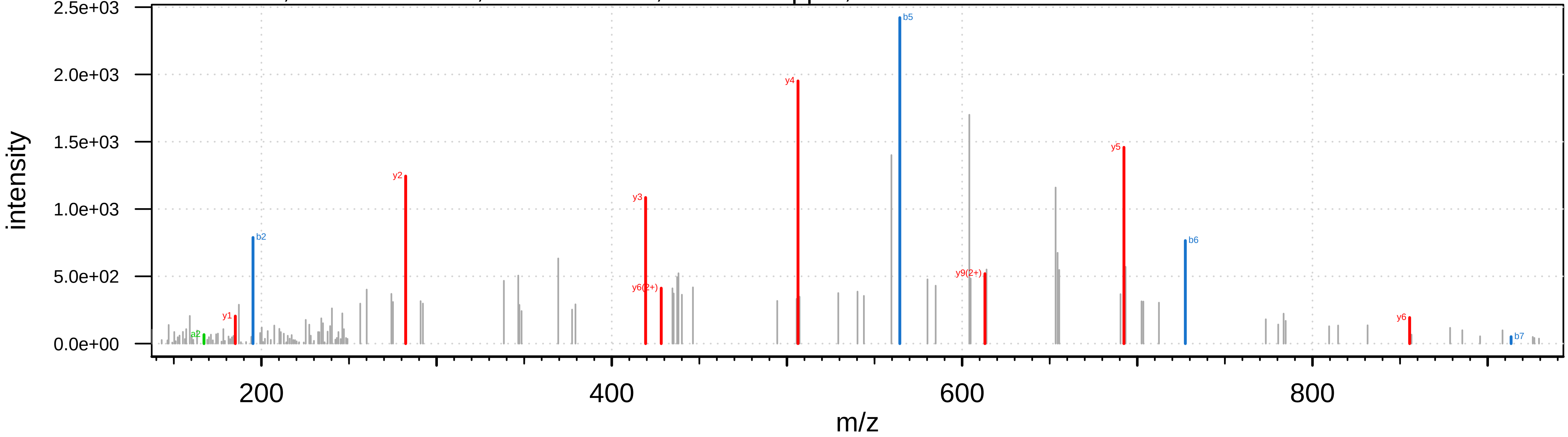

Raw File: Ernie_20090407_BJS_halflives_P3_slice04_01
Scan Number: 4614
Proteins:
sp|P62274|RS29_MOUSE
ENSMUST00000037023_chr12:69157813−69159058:−
ENSMUST00000071737_chr13:73988963−73989133:−
tr|D3YYL7|D3YYL7_MOUSE

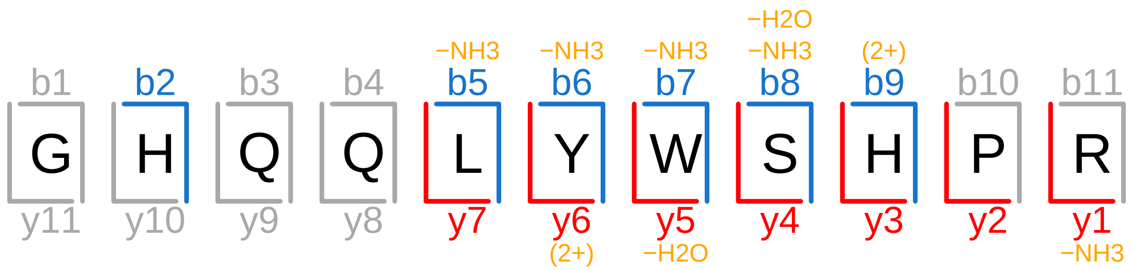

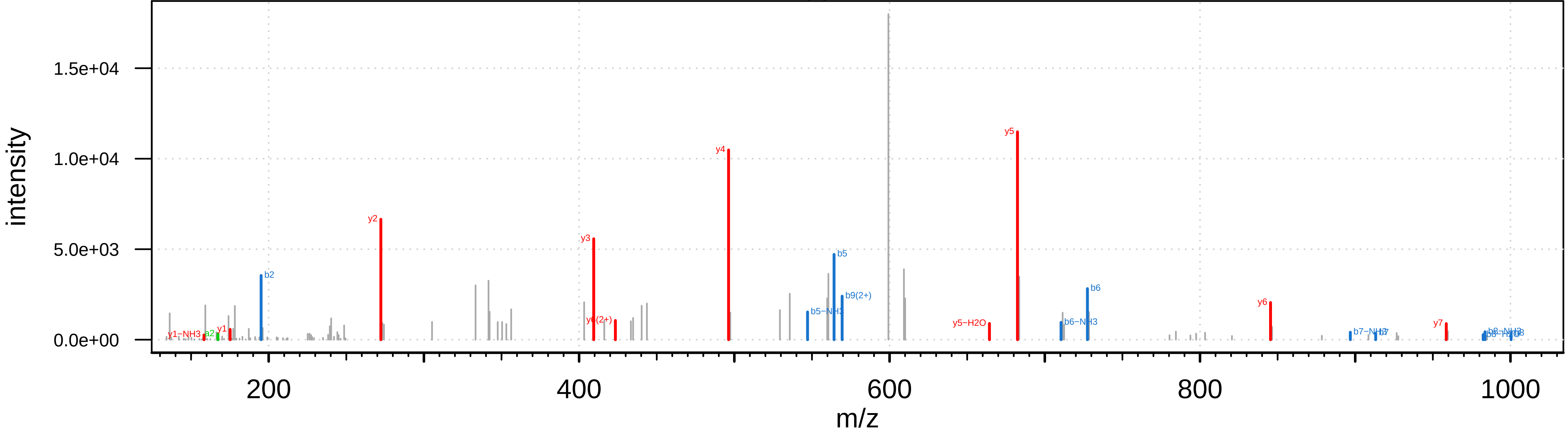

Raw File: Ernie_20090407_BJS_halflives_P3_slice04_01
Scan Number: 4635
Proteins:
sp|P62274|RS29_MOUSE
ENSMUST00000037023_chr12:69157813−69159058:−
ENSMUST00000071737_chr13:73988963−73989133:−
tr|D3YYL7|D3YYL7_MOUSE

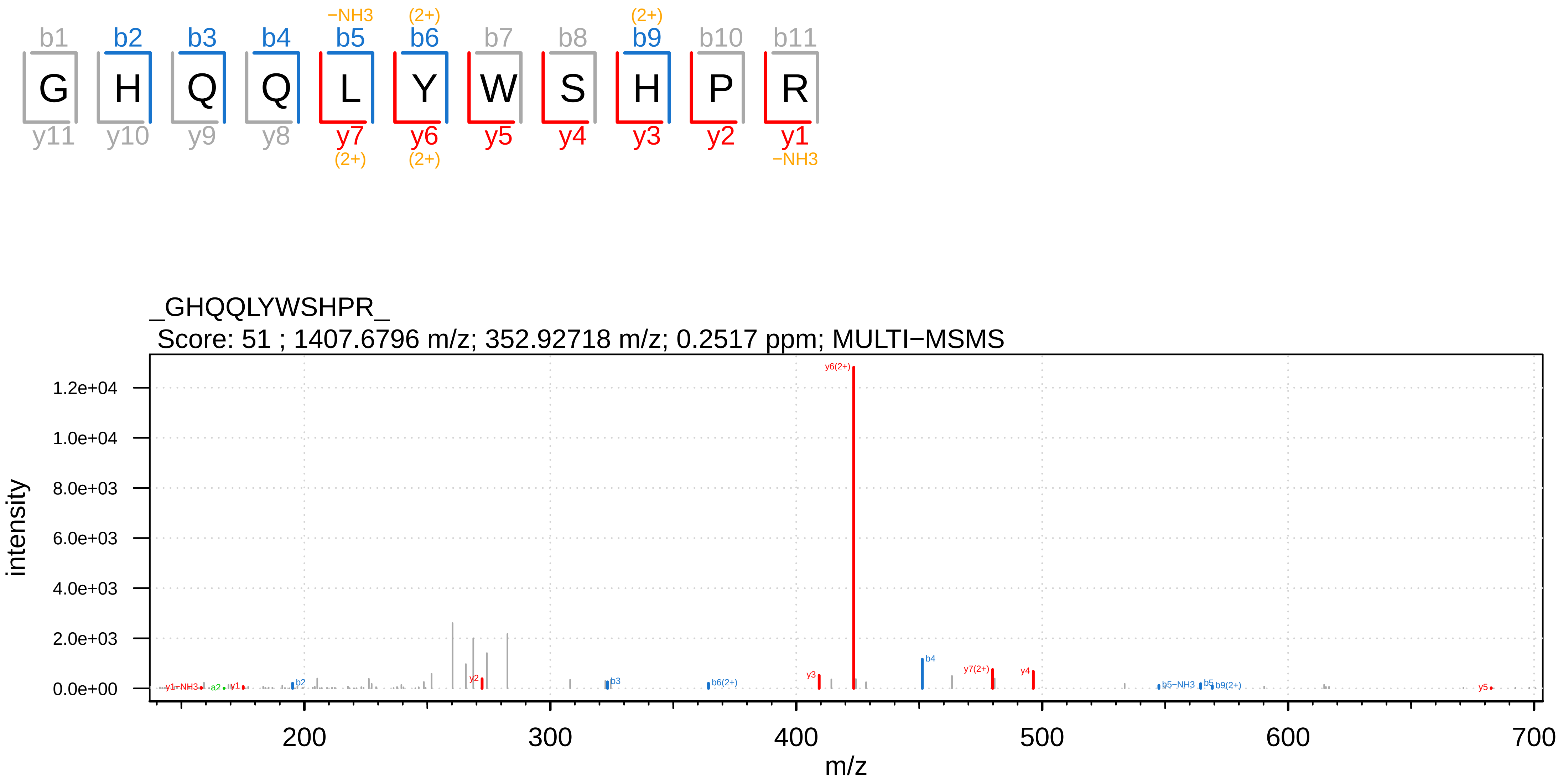

Raw File: Ernie_20090407_BJS_halflives_P3_slice04_01
Scan Number: 4628
Proteins:
sp|P62274|RS29_MOUSE
ENSMUST00000037023_chr12:69157813−69159058:−
ENSMUST00000071737_chr13:73988963−73989133:−
tr|D3YYL7|D3YYL7_MOUSE

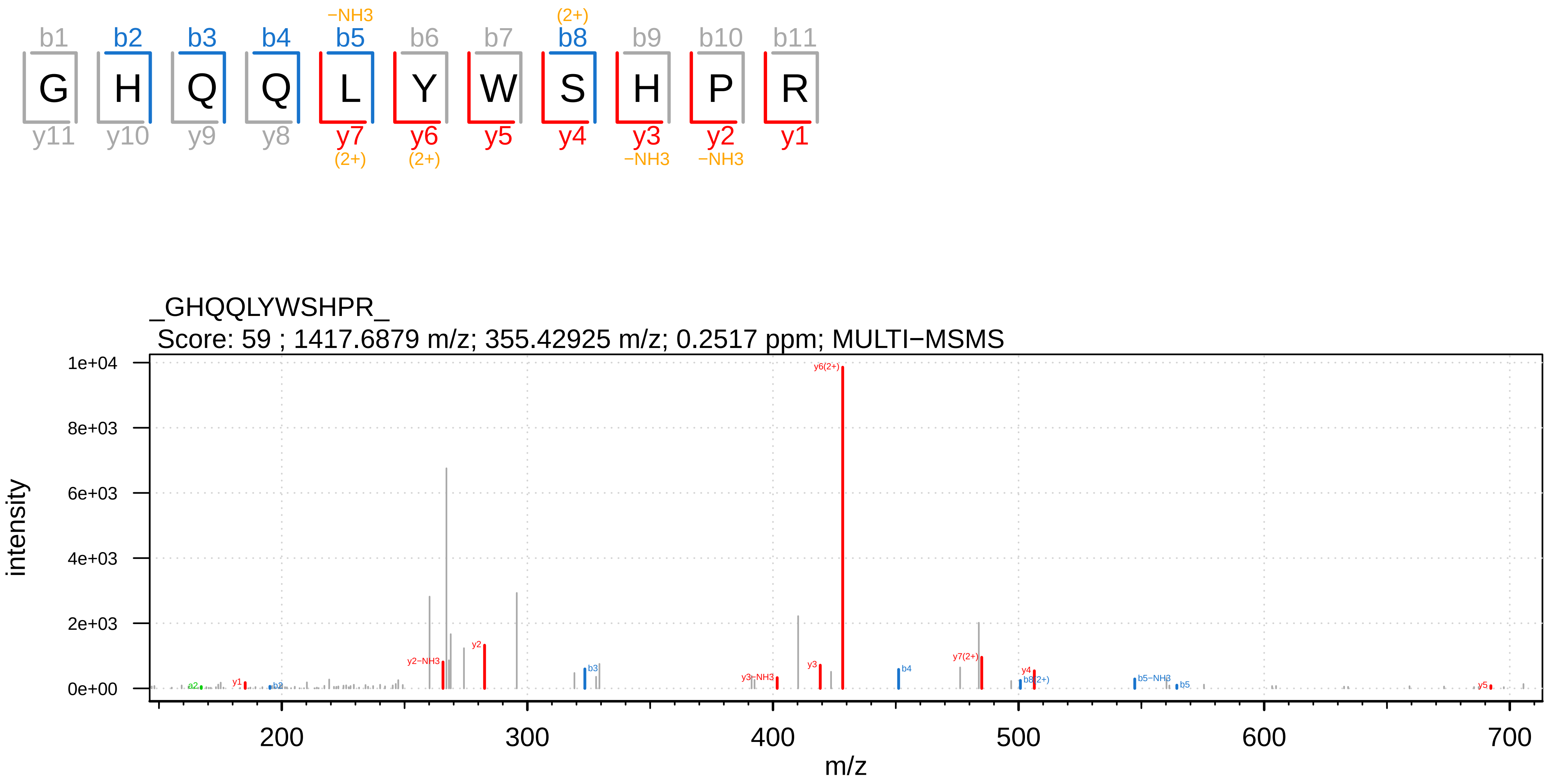

Raw File: Ernie_20090407_BJS_halflives_P3_slice04_01
Scan Number: 4641
Proteins:
sp|P62274|RS29_MOUSE
ENSMUST00000037023_chr12:69157813−69159058:−
ENSMUST00000071737_chr13:73988963−73989133:−
tr|D3YYL7|D3YYL7_MOUSE

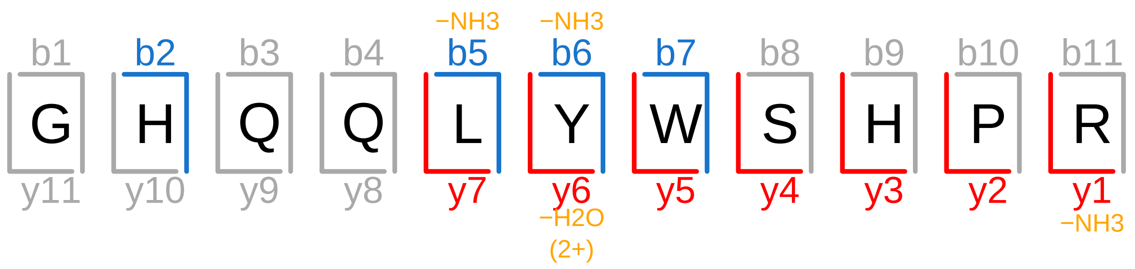

GHQQLYWSHPR
Score: 70 ; 1407.6796 m/z; 470.23382 m/z; −0.63012 ppm; MULTI−MSMS

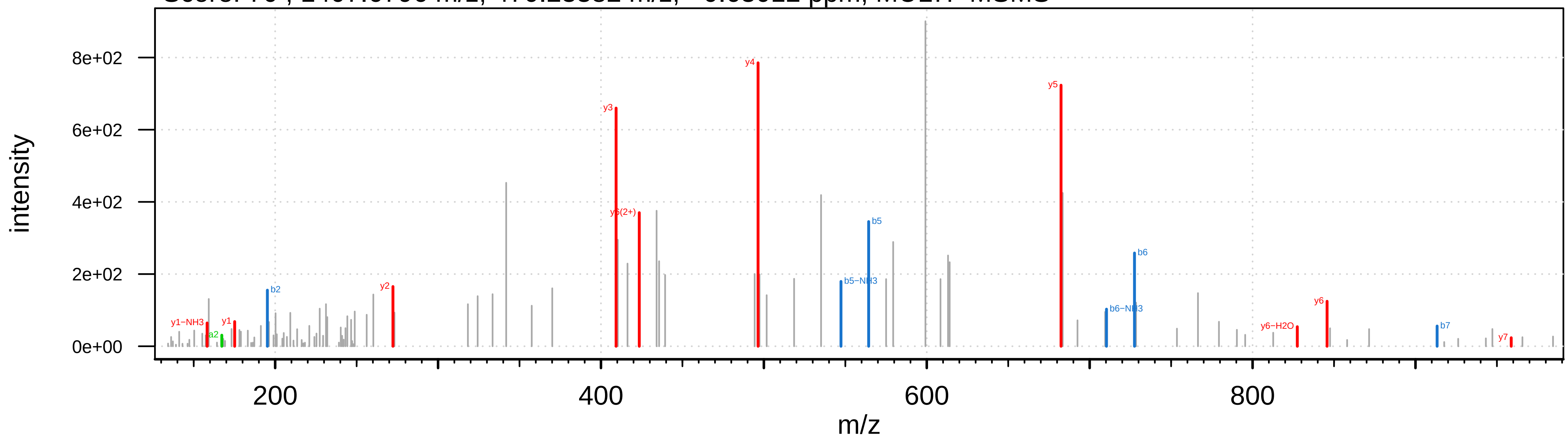

Raw File: Ernie_20090407_BJS_halflives_P3_slice04_03
Scan Number: 4279
Proteins:
sp|P62274|RS29_MOUSE
ENSMUST00000037023_chr12:69157813−69159058:−
ENSMUST00000071737_chr13:73988963−73989133:−
tr|D3YYL7|D3YYL7_MOUSE

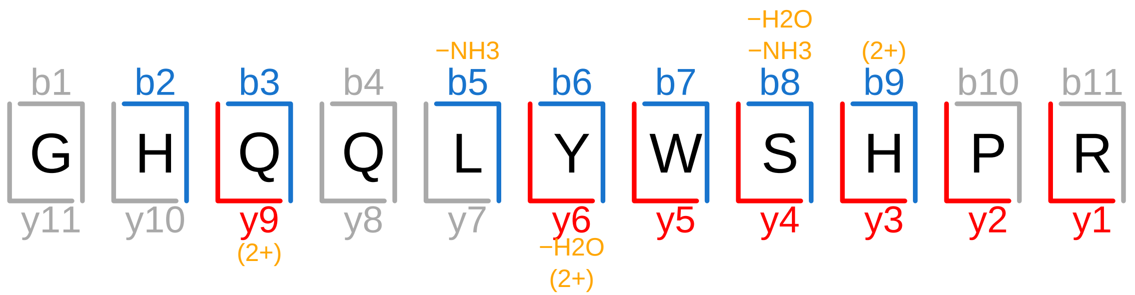

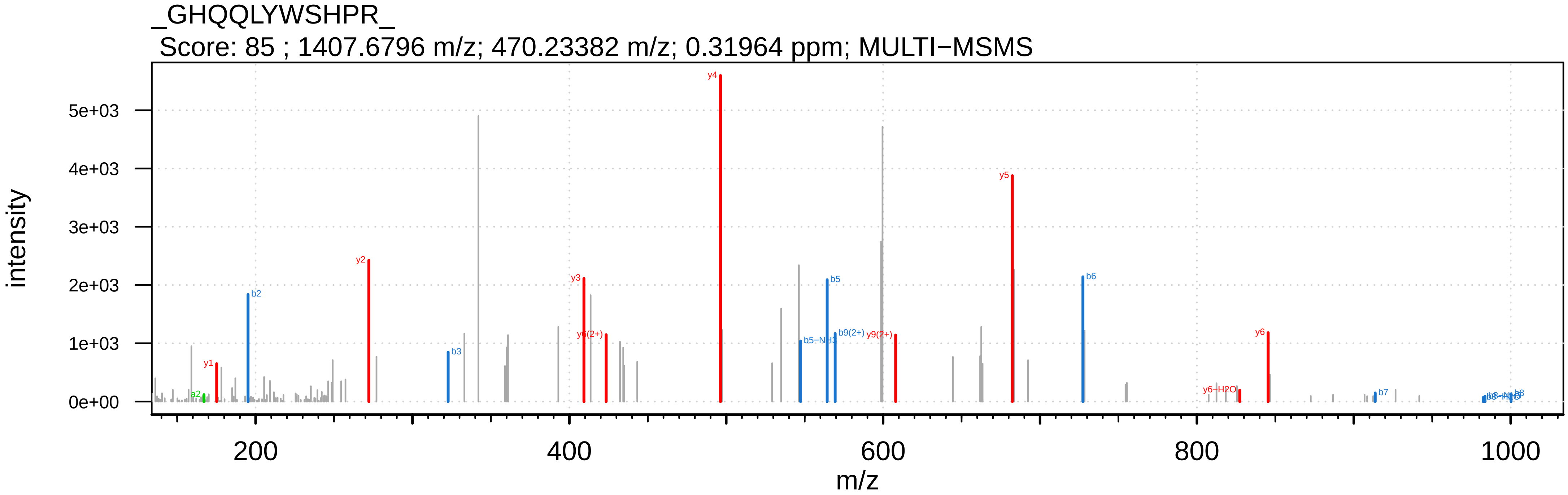

Raw File: Ernie_20090411_BJS_halflives_P3_slice10_01
Scan Number: 5317
Proteins:
sp|P62274|RS29_MOUSE
ENSMUST00000037023_chr12:69157813−69159058:−
ENSMUST00000071737_chr13:73988963−73989133:−
tr|D3YYL7|D3YYL7_MOUSE

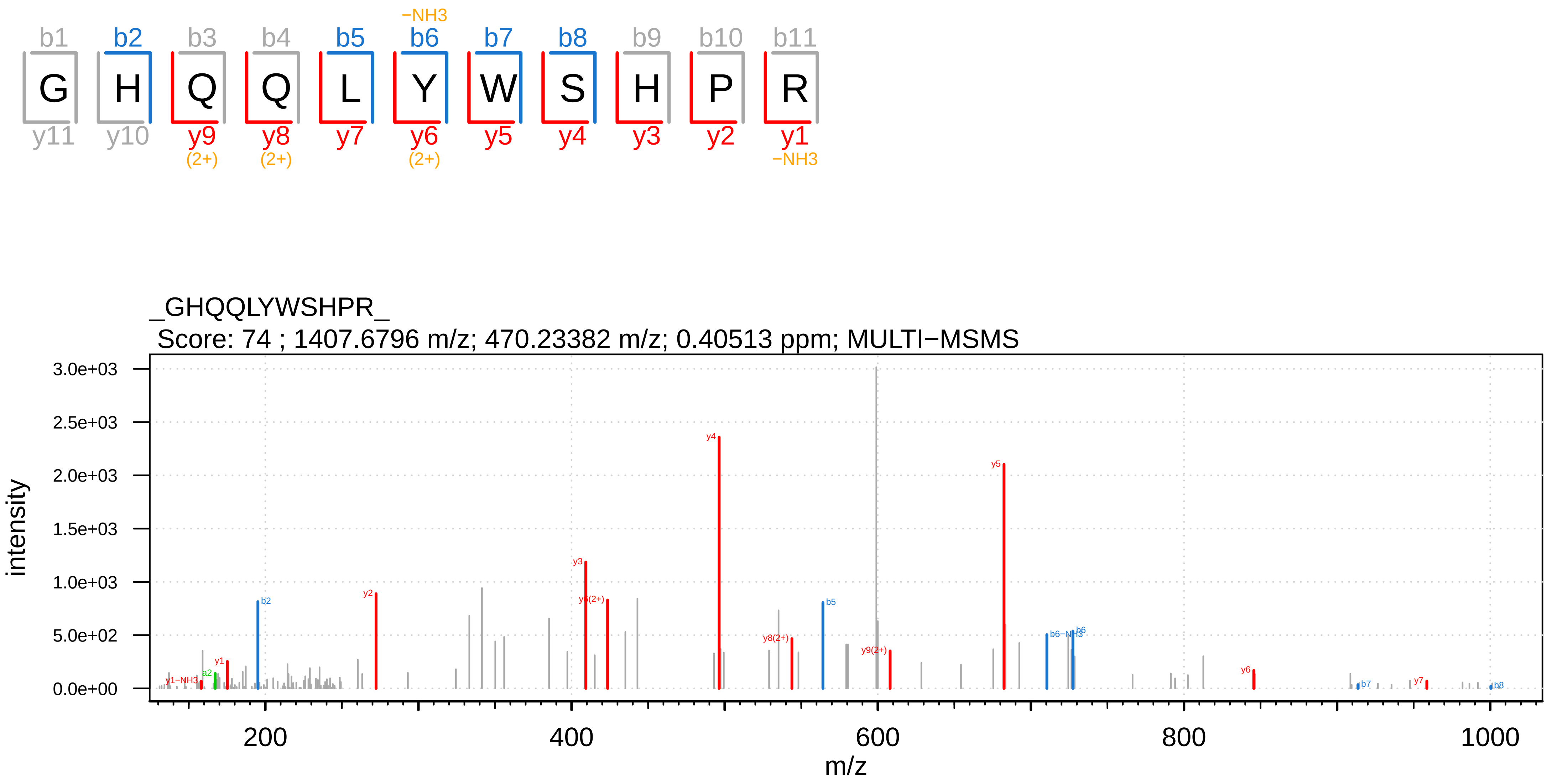

Raw File: Ernie_20090411_BJS_halflives_P3_slice10_02
Scan Number: 4782
Proteins:
sp|P62274|RS29_MOUSE
ENSMUST00000037023_chr12:69157813−69159058:−
ENSMUST00000071737_chr13:73988963−73989133:−
tr|D3YYL7|D3YYL7_MOUSE

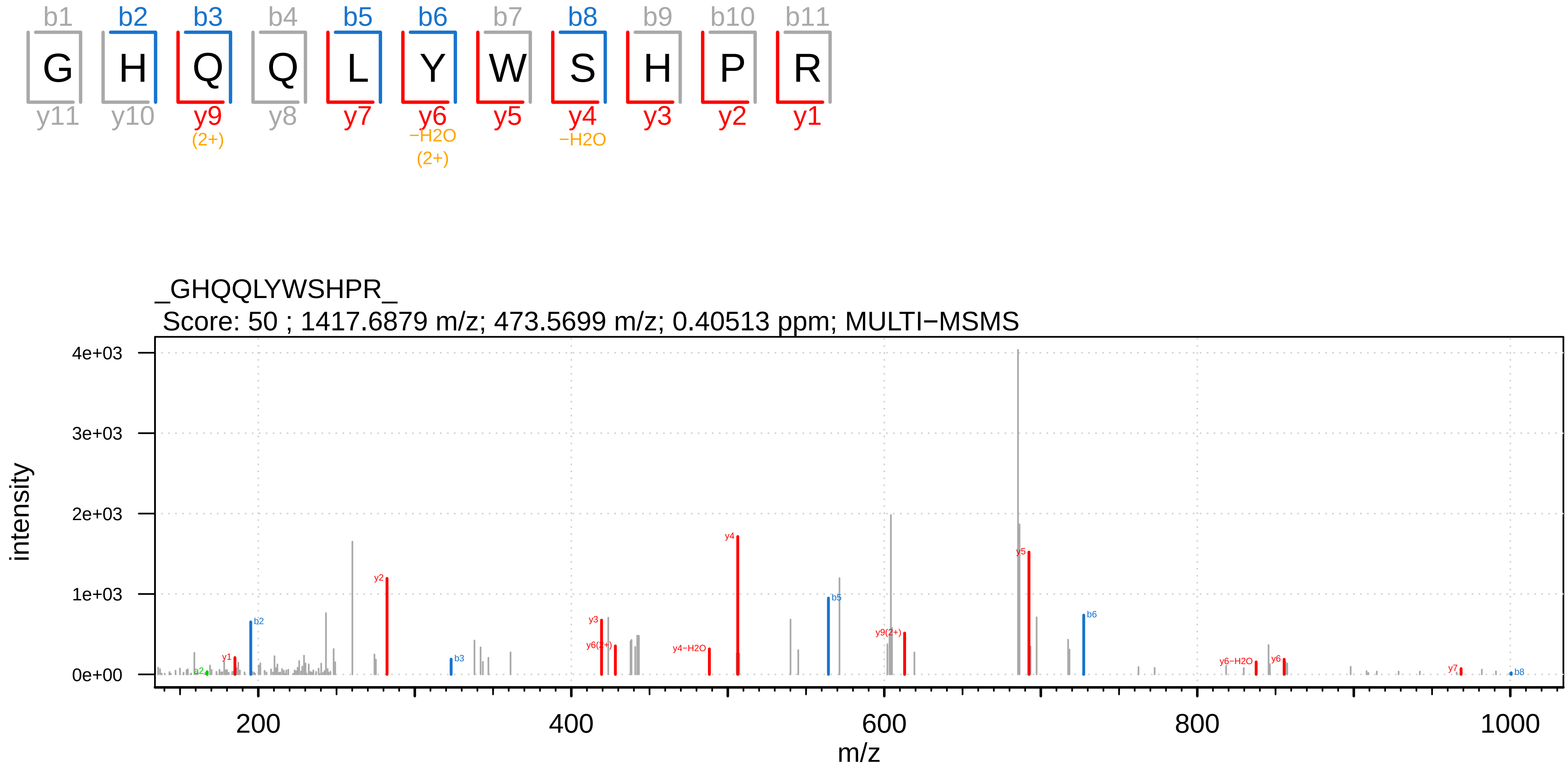

Raw File: Ernie_20090411_BJS_halflives_P3_slice10_02
Scan Number: 4792
Proteins:
sp|P62274|RS29_MOUSE
ENSMUST00000037023_chr12:69157813−69159058:−
ENSMUST00000071737_chr13:73988963−73989133:−
tr|D3YYL7|D3YYL7_MOUSE

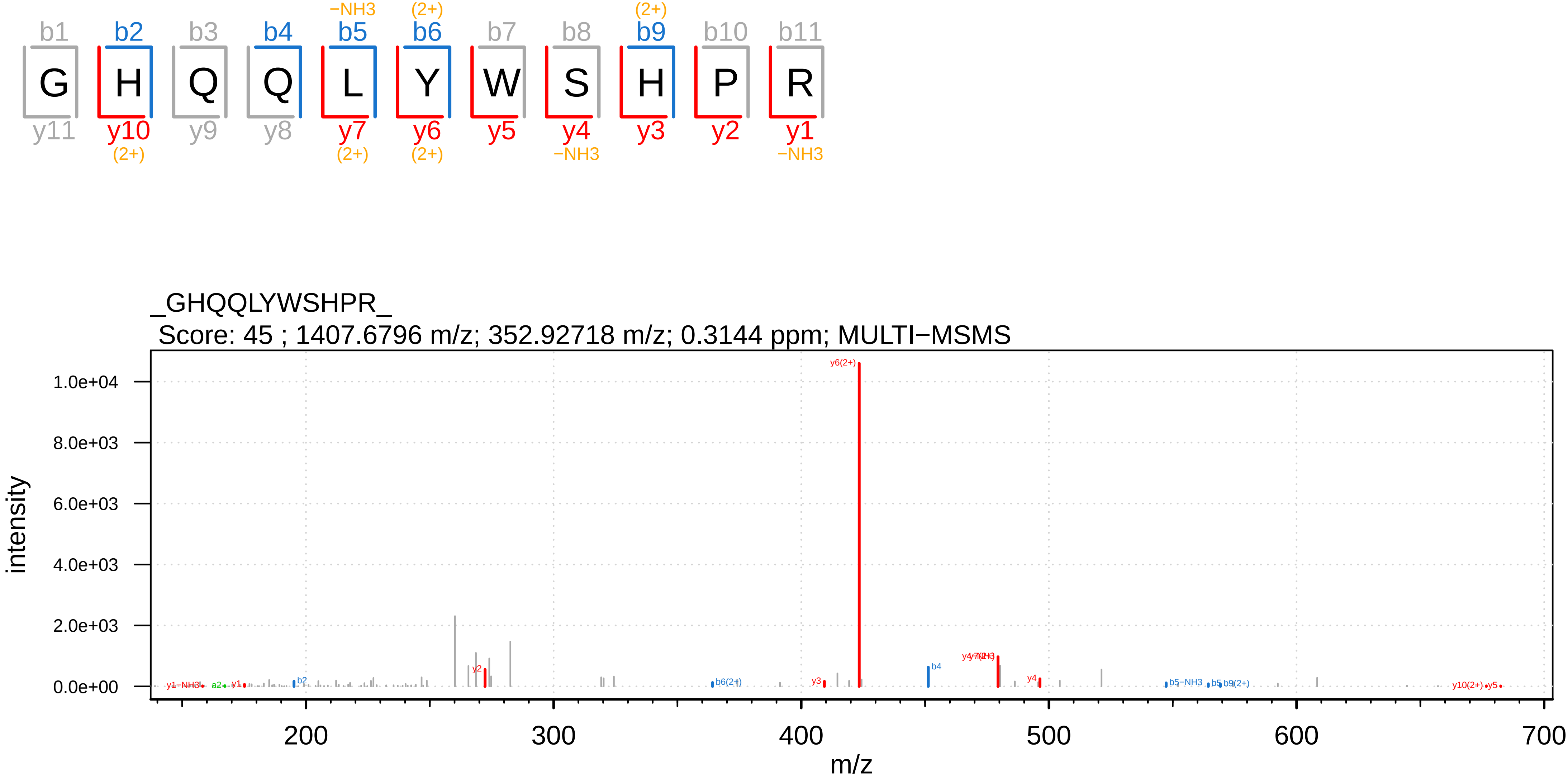

Raw File: Ernie_20090411_BJS_halflives_P3_slice10_02
Scan Number: 4799
Proteins:
sp|P62274|RS29_MOUSE
ENSMUST00000037023_chr12:69157813−69159058:−
ENSMUST00000071737_chr13:73988963−73989133:−
tr|D3YYL7|D3YYL7_MOUSE

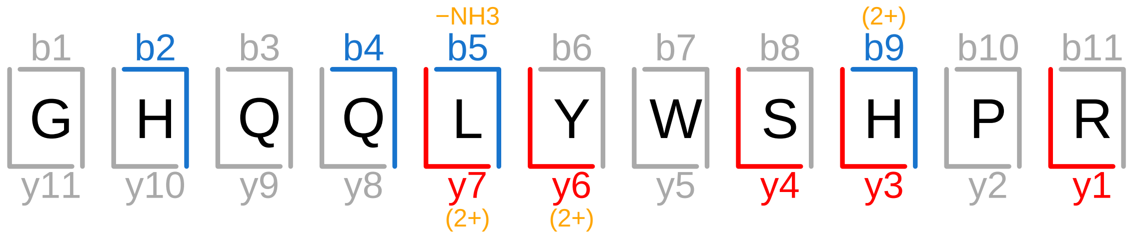

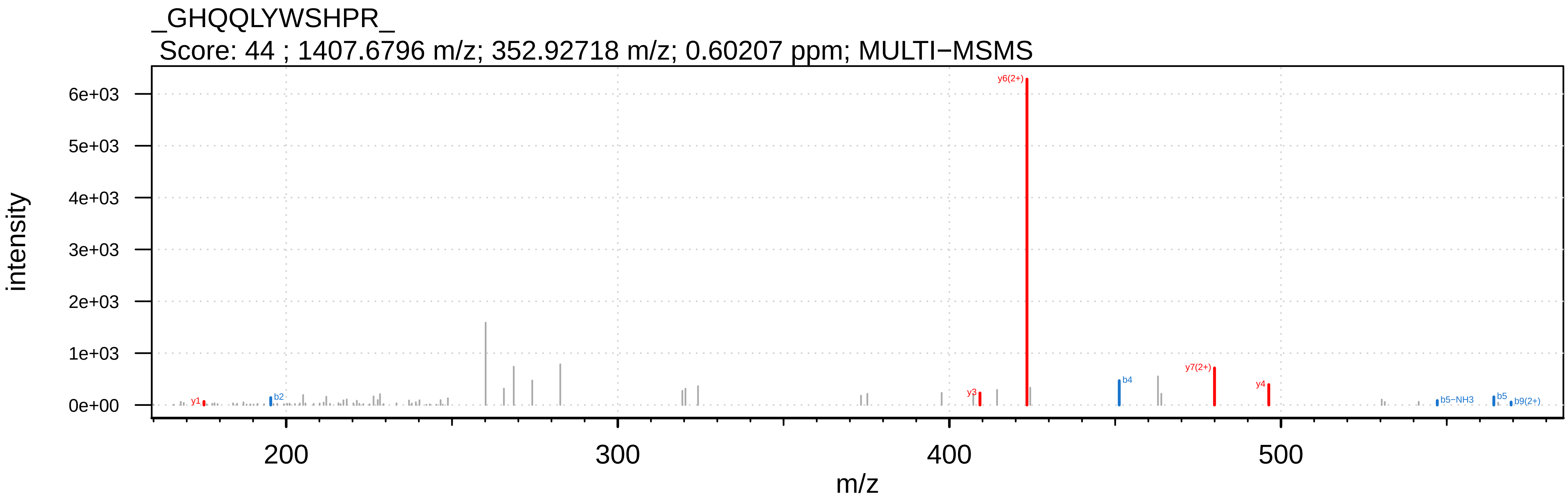

Raw File: Ernie_20090411_BJS_halflives_P3_slice11_02
Scan Number: 4467
Proteins:
sp|P62274|RS29_MOUSE
ENSMUST00000037023_chr12:69157813−69159058:−
ENSMUST00000071737_chr13:73988963−73989133:−
tr|D3YYL7|D3YYL7_MOUSE

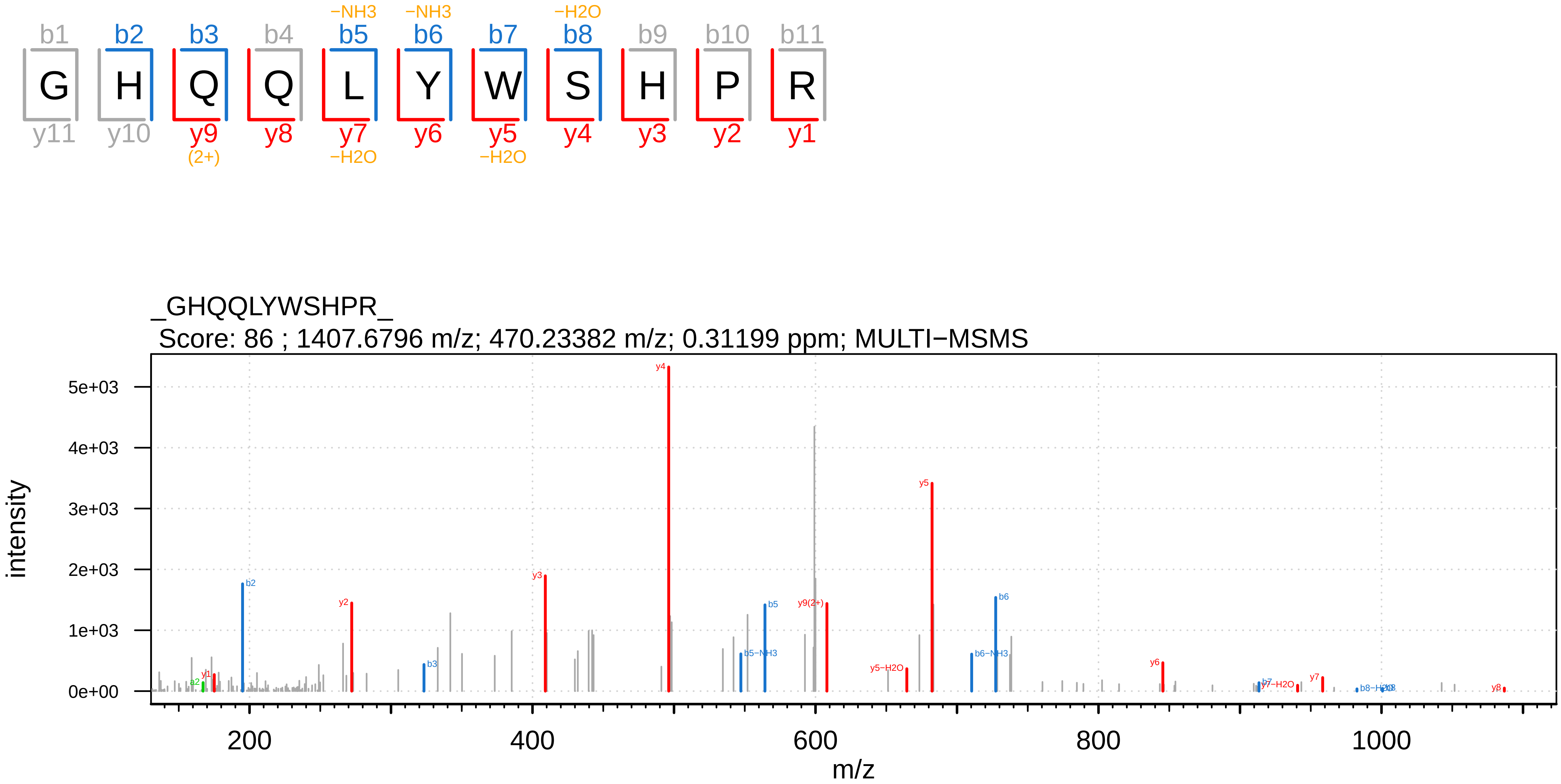

Raw File: Ernie_20090411_BJS_halflives_P3_slice11_02
Scan Number: 4473
Proteins:
sp|P62274|RS29_MOUSE
ENSMUST00000037023_chr12:69157813−69159058:−
ENSMUST00000071737_chr13:73988963−73989133:−
tr|D3YYL7|D3YYL7_MOUSE

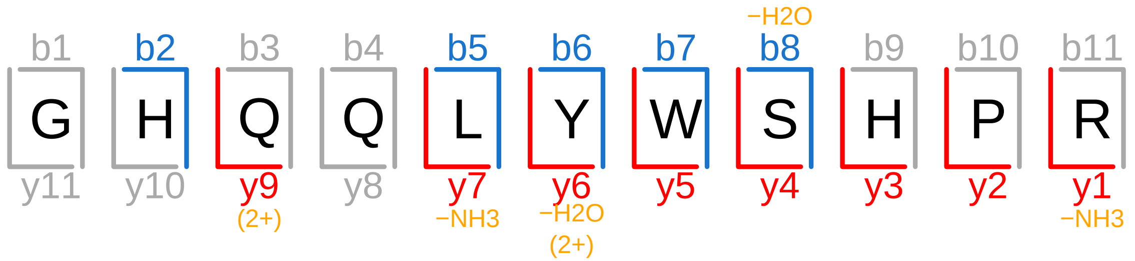

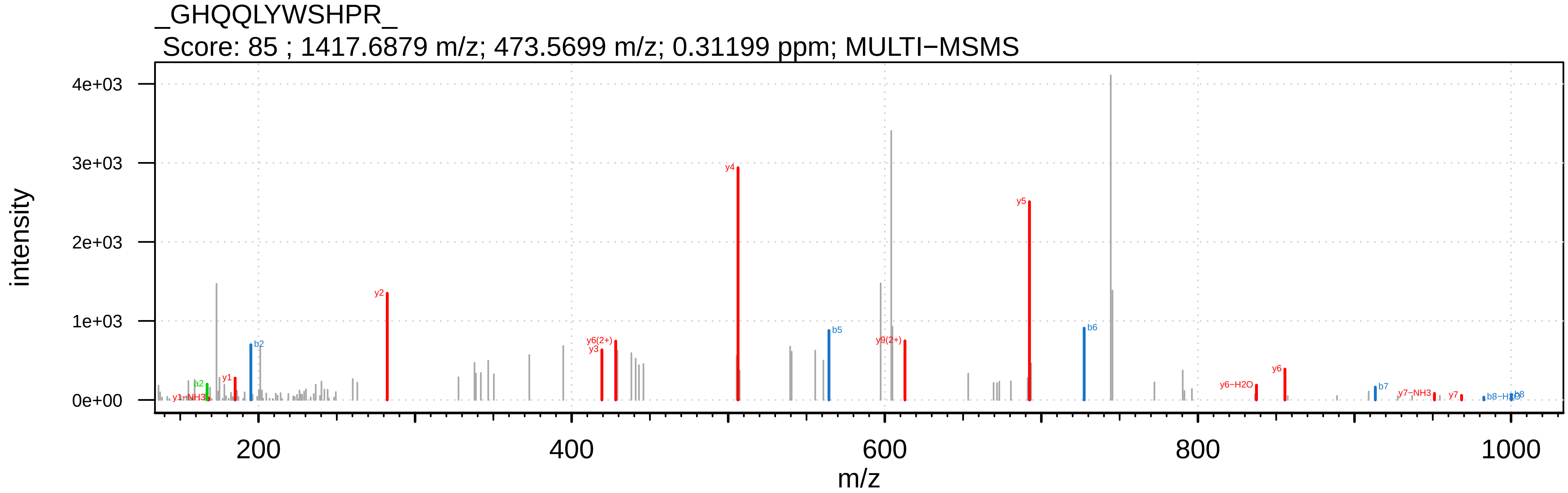

Raw File: Ernie_20090411_BJS_halflives_P3_slice11_02
Scan Number: 4474
Proteins:
sp|P62274|RS29_MOUSE
ENSMUST00000037023_chr12:69157813−69159058:−
ENSMUST00000071737_chr13:73988963−73989133:−
tr|D3YYL7|D3YYL7_MOUSE

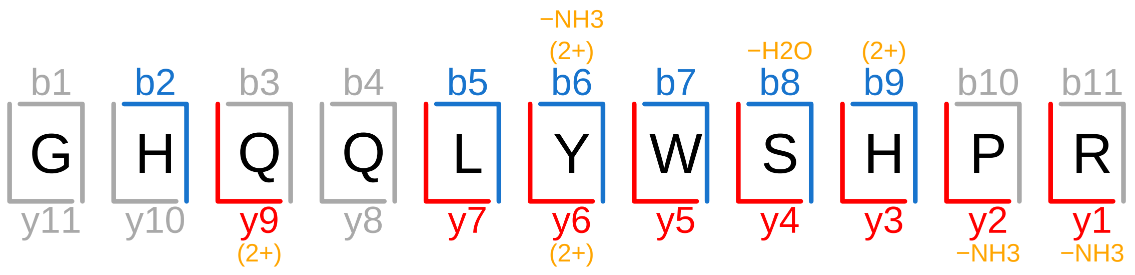

GHQQLYWSHPR
Score: 94 ; 1407.6796 m/z; 470.23382 m/z; −0.24612 ppm; MULTI−MSMS

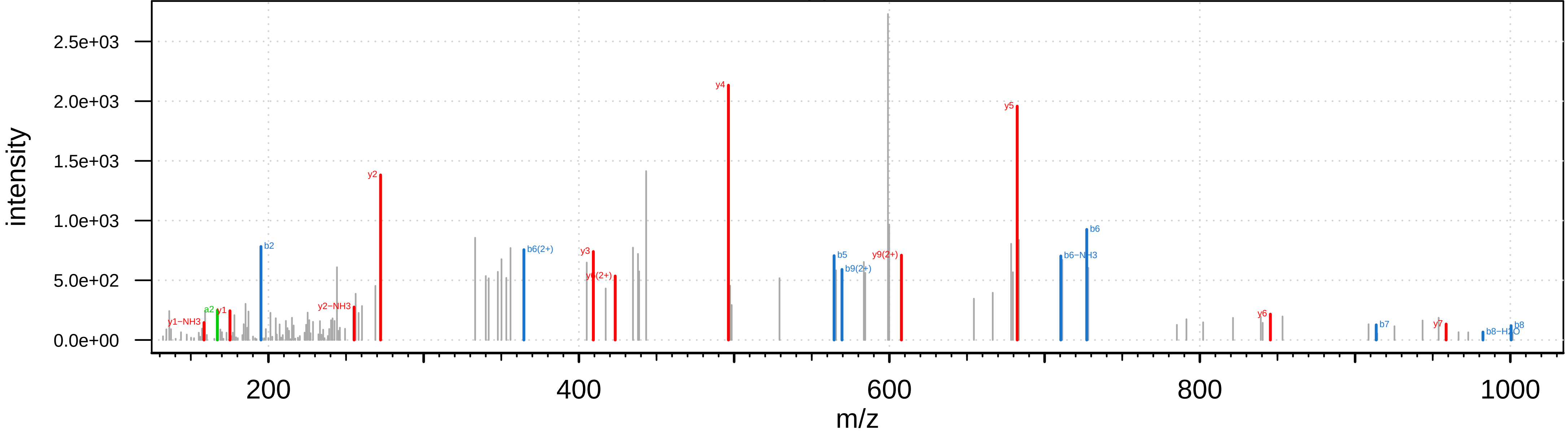

Raw File: Ernie_20090411_BJS_halflives_P3_slice11_03
Scan Number: 4145
Proteins:
sp|P62274|RS29_MOUSE
ENSMUST00000037023_chr12:69157813−69159058:−
ENSMUST00000071737_chr13:73988963−73989133:−
tr|D3YYL7|D3YYL7_MOUSE

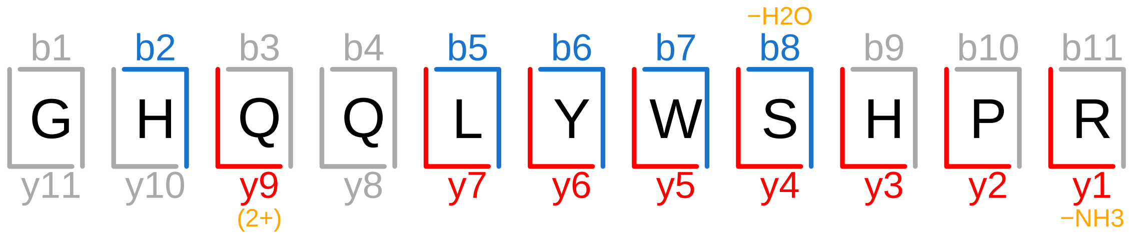

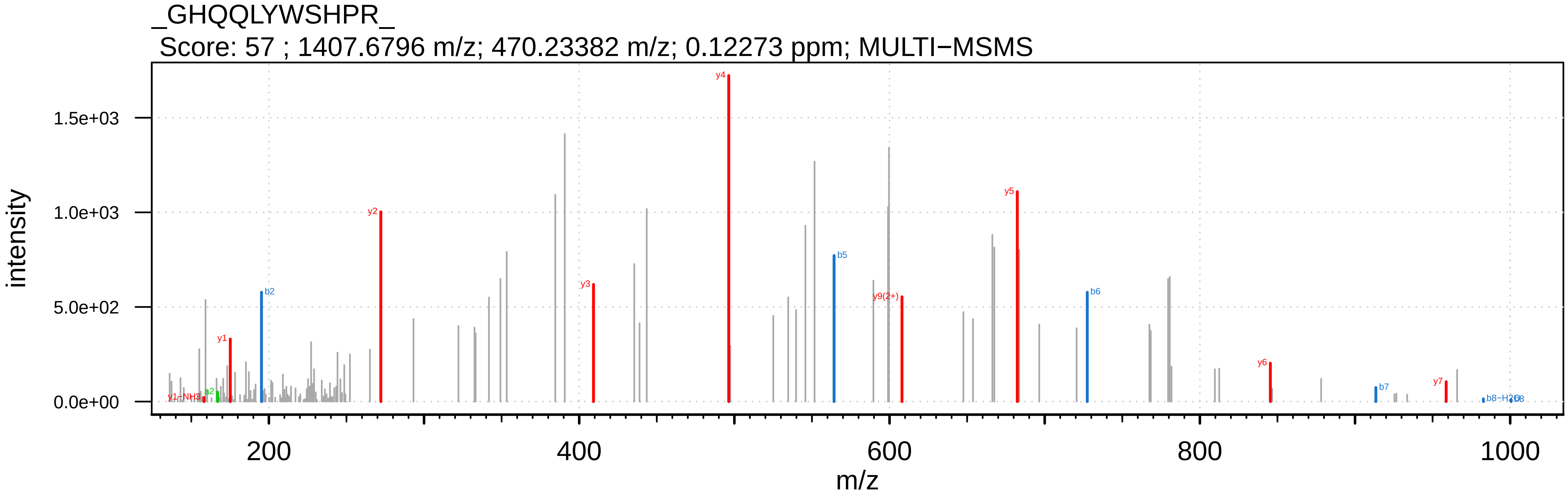

Raw File: Ernie_20090411_BJS_halflives_P3_slice12_03
Scan Number: 4207
Proteins:
sp|P62274|RS29_MOUSE
ENSMUST00000037023_chr12:69157813−69159058:−
ENSMUST00000071737_chr13:73988963−73989133:−
tr|D3YYL7|D3YYL7_MOUSE

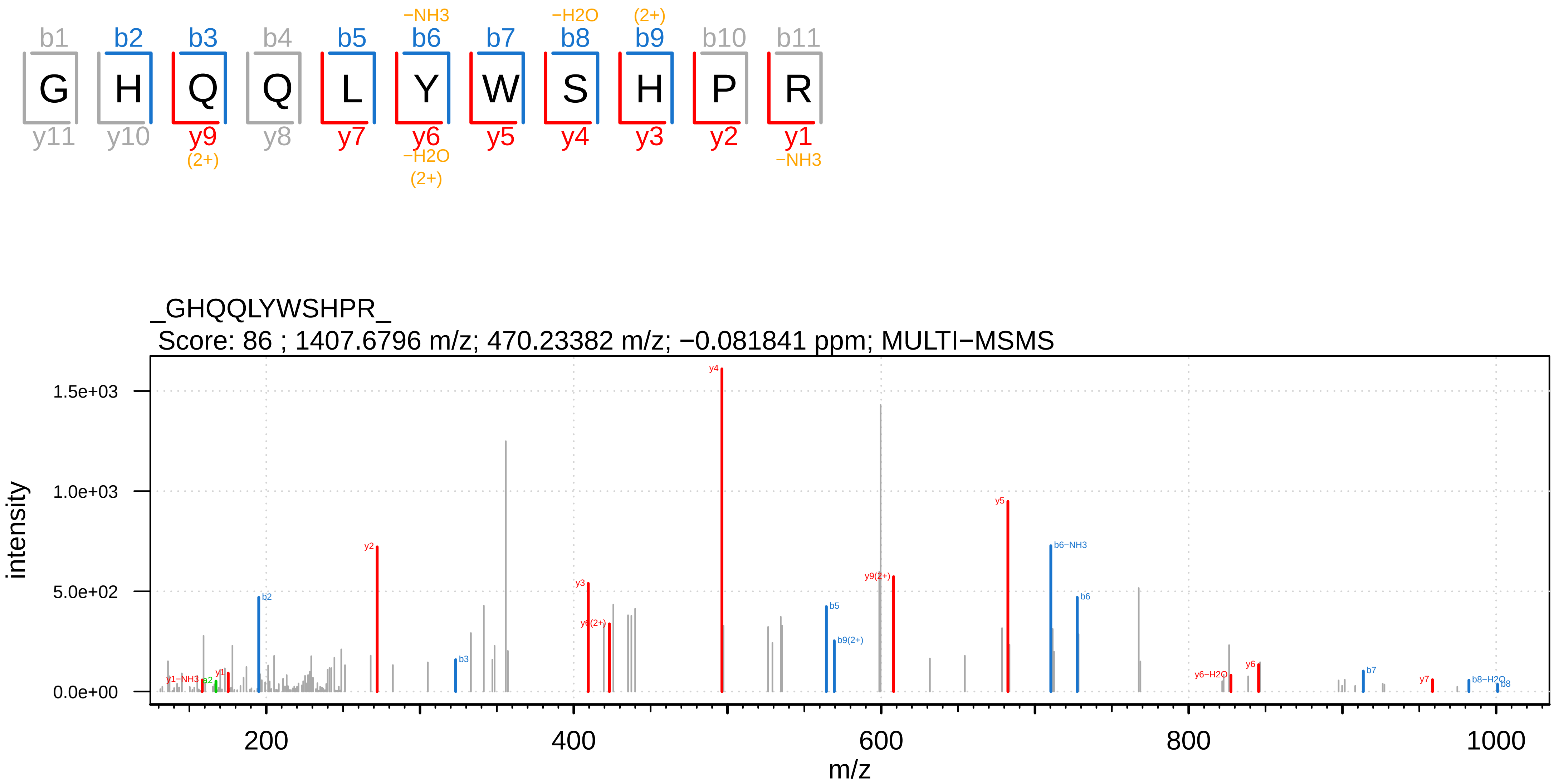

Raw File: Ernie_20090411_BJS_halflives_P3_slice13_01
Scan Number: 4351
Proteins:
sp|P62274|RS29_MOUSE
ENSMUST00000037023_chr12:69157813−69159058:−
ENSMUST00000071737_chr13:73988963−73989133:−
tr|D3YYL7|D3YYL7_MOUSE

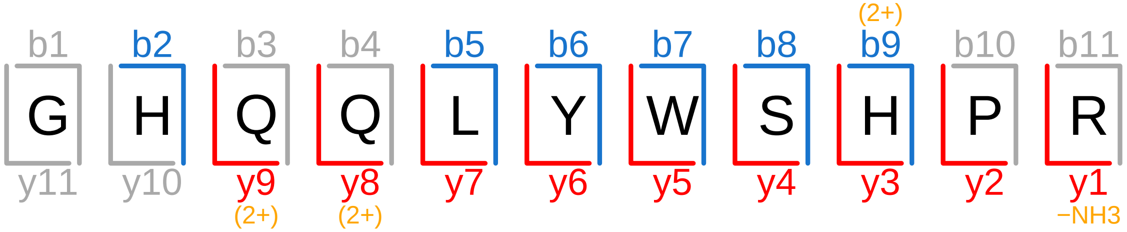

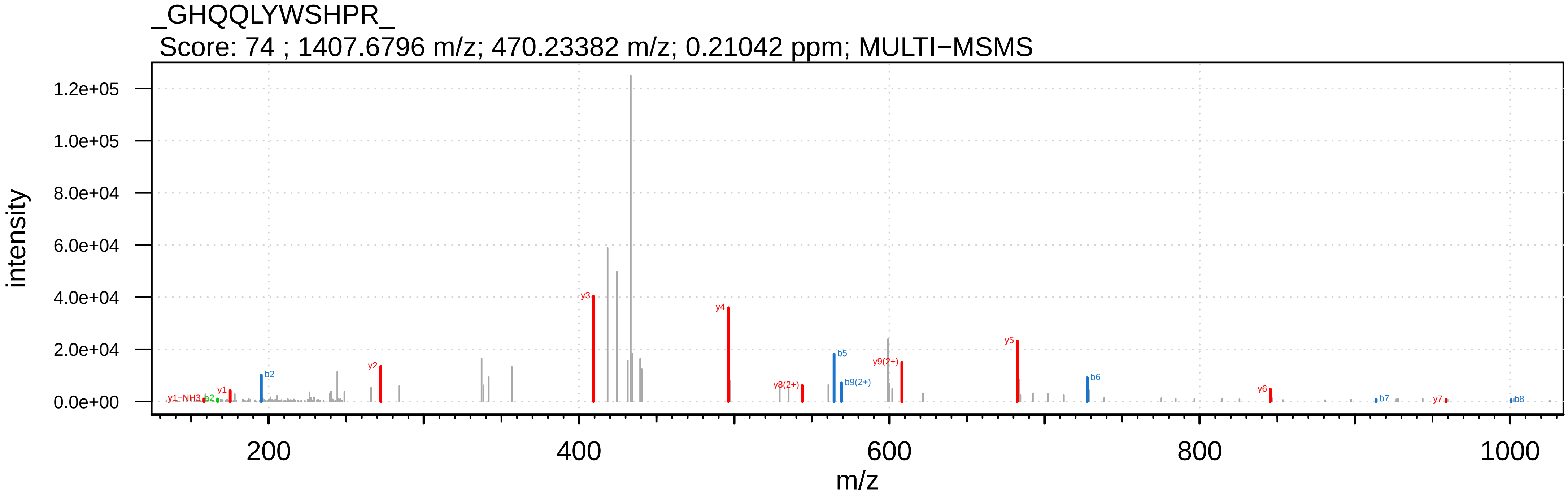

Raw File: Tiffy_20100217_BJS_Halflives_P2_01
Scan Number: 9603
Proteins:
sp|P62274|RS29_MOUSE
ENSMUST00000037023_chr12:69157813−69159058:−
ENSMUST00000071737_chr13:73988963−73989133:−
tr|D3YYL7|D3YYL7_MOUSE

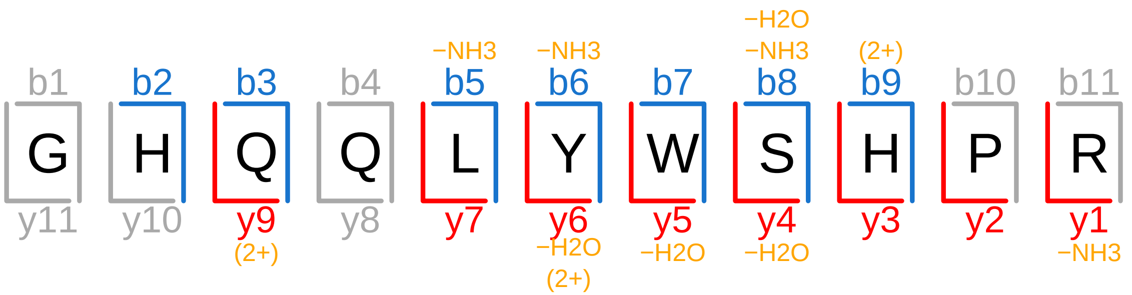

GHQQLYWSHPR

Score: 111 ; 1407.6796 m/z; 470.23382 m/z; −0.24372 ppm; MULTI−MSMS

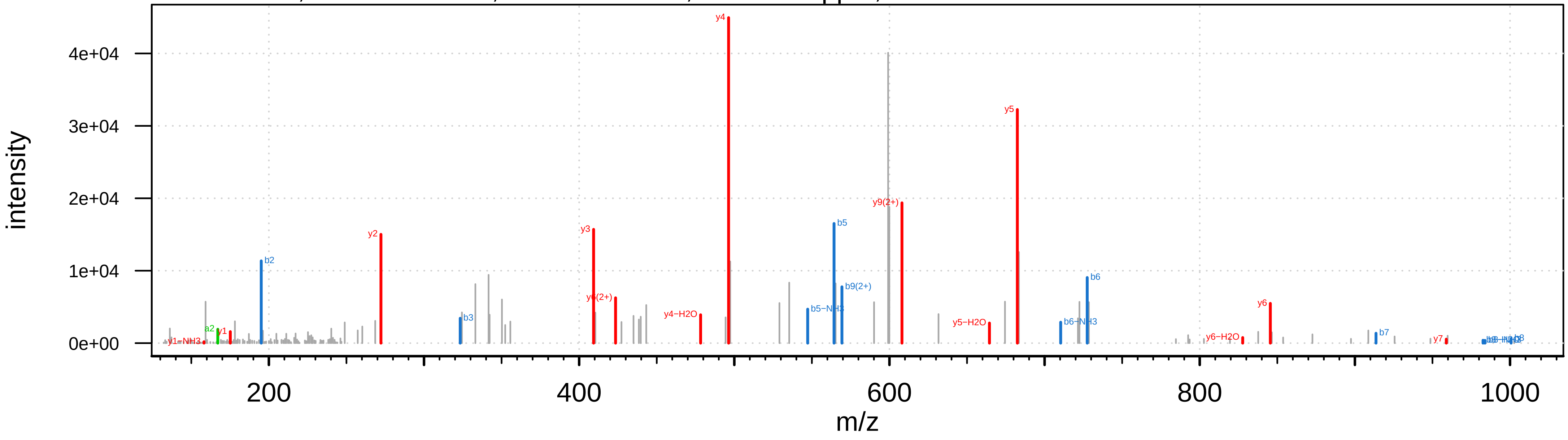

Raw File: Tiffy_20100226_BJS_Halflives_KORR_P1_02

Scan Number: 10804

Proteins:

sp|P62274|RS29_MOUSE

ENSMUST00000037023_chr12:69157813−69159058:−

ENSMUST00000071737_chr13:73988963−73989133:−

tr|D3YYL7|D3YYL7_MOUSE

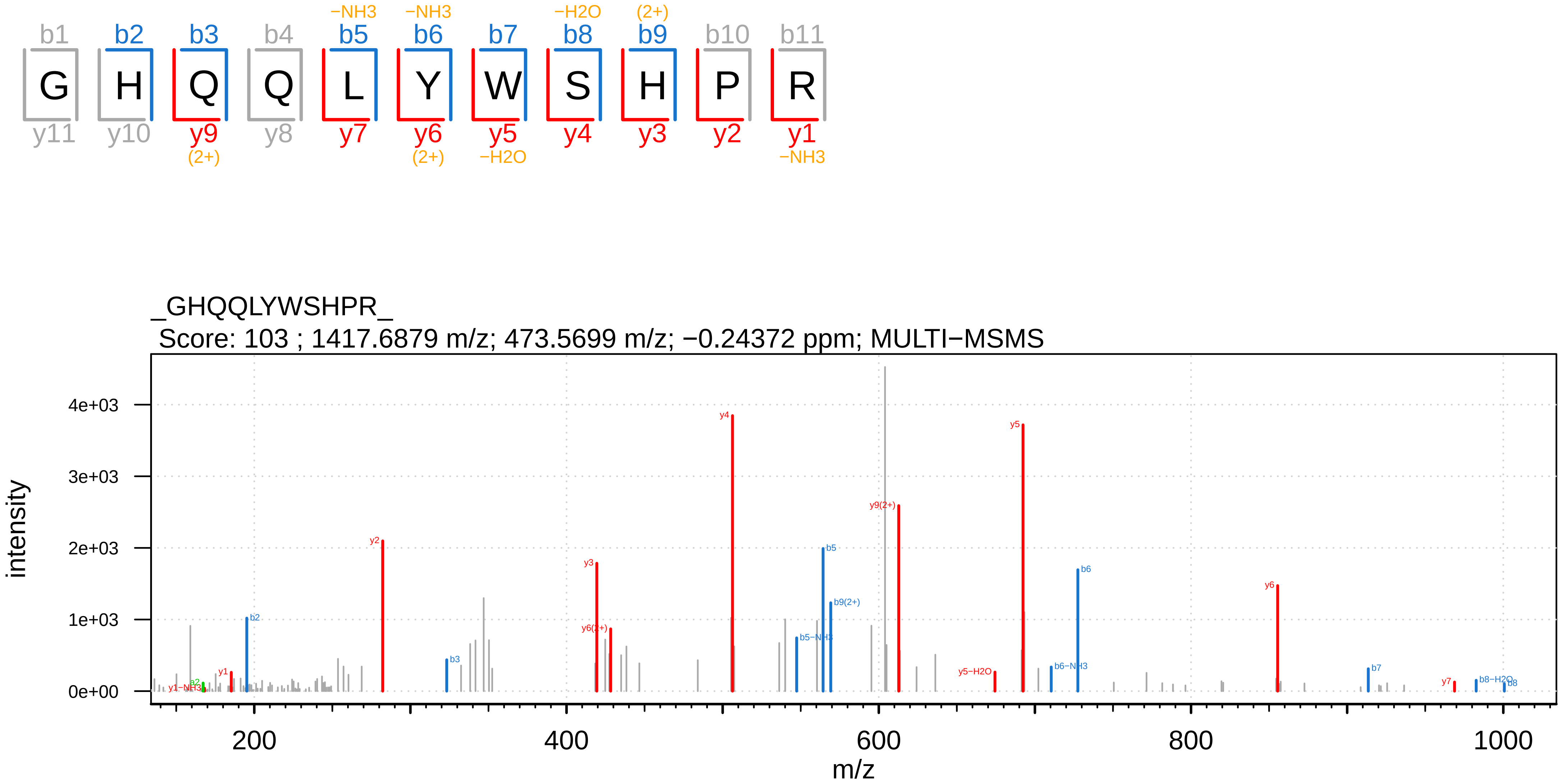

Raw File: Tiffy_20100226_BJS_Halflives_KORR_P1_02
Scan Number: 10851
Proteins:
sp|P62274|RS29_MOUSE
ENSMUST00000037023_chr12:69157813−69159058:−
ENSMUST00000071737_chr13:73988963−73989133:−
tr|D3YYL7|D3YYL7_MOUSE

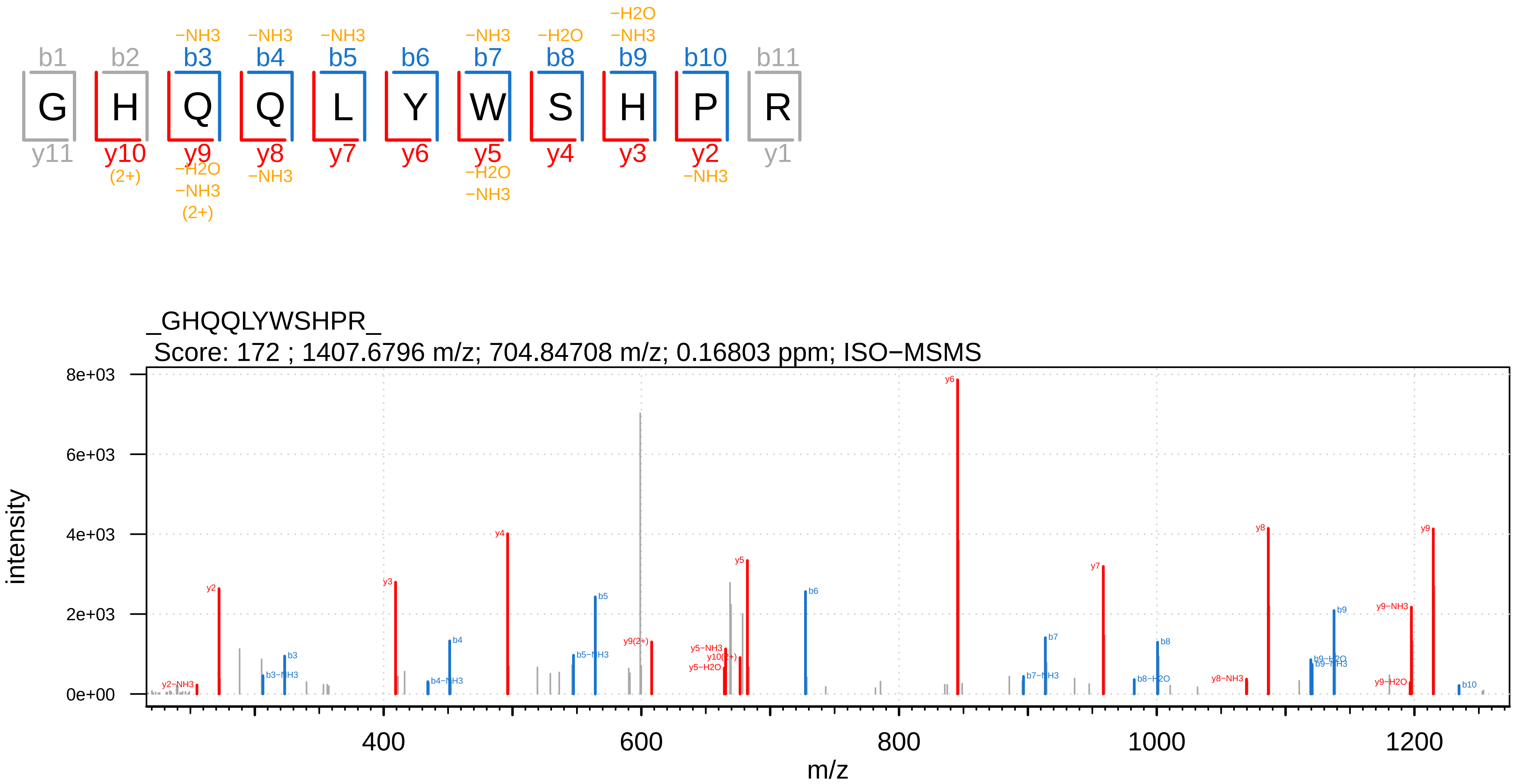

Raw File: Tiffy_20100226_BJS_Halflives_KORR_P1_02
Scan Number: 10849
Proteins:
sp|P62274|RS29_MOUSE
ENSMUST00000037023_chr12:69157813−69159058:−
ENSMUST00000071737_chr13:73988963−73989133:−
tr|D3YYL7|D3YYL7_MOUSE

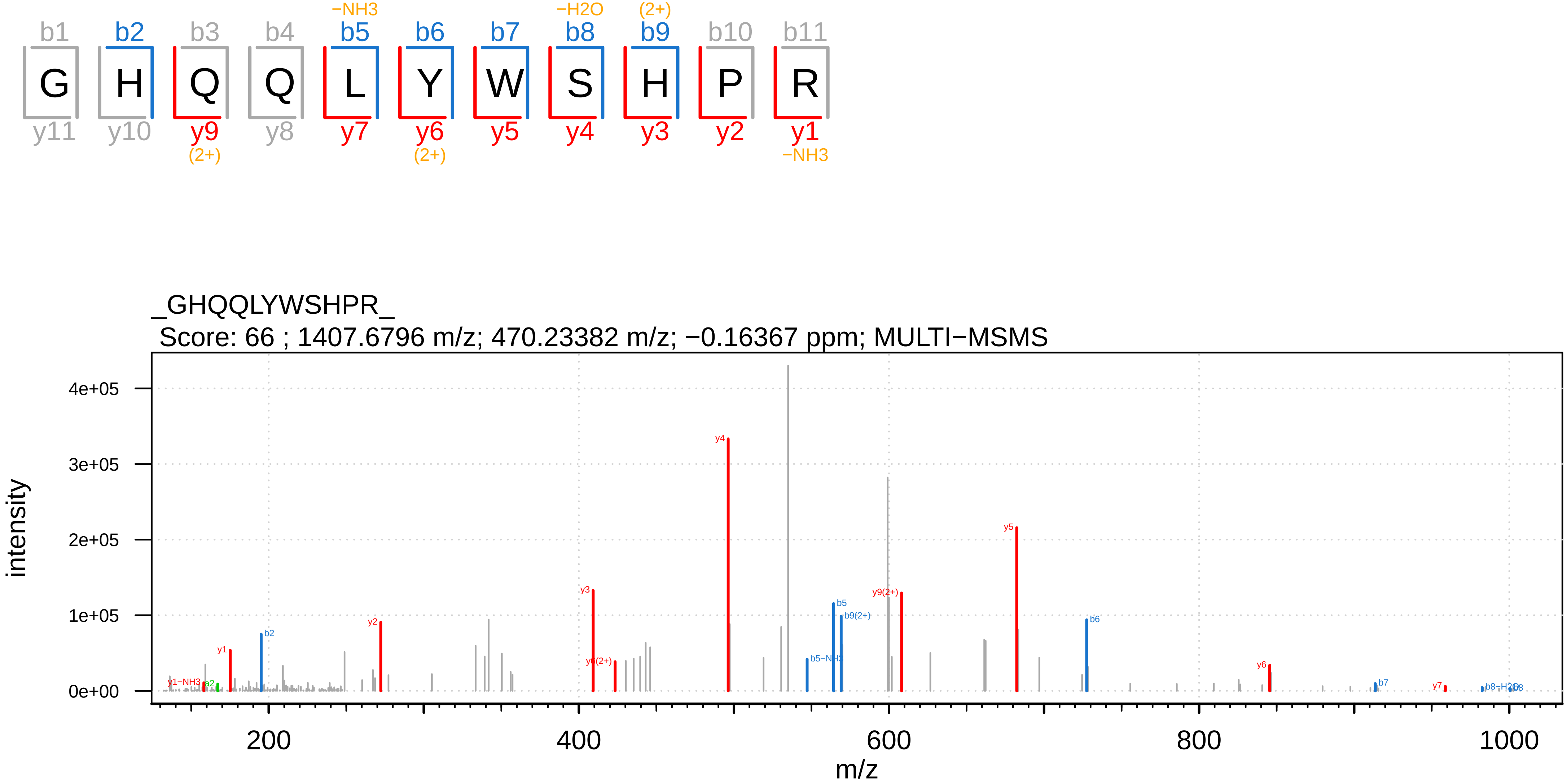

Raw File: Tiffy_20100301_BJS_Halflives_P3_Exp1_InSol_01
Scan Number: 9560
Proteins:
sp|P62274|RS29_MOUSE
ENSMUST00000037023_chr12:69157813−69159058:−
ENSMUST00000071737_chr13:73988963−73989133:−
tr|D3YYL7|D3YYL7_MOUSE

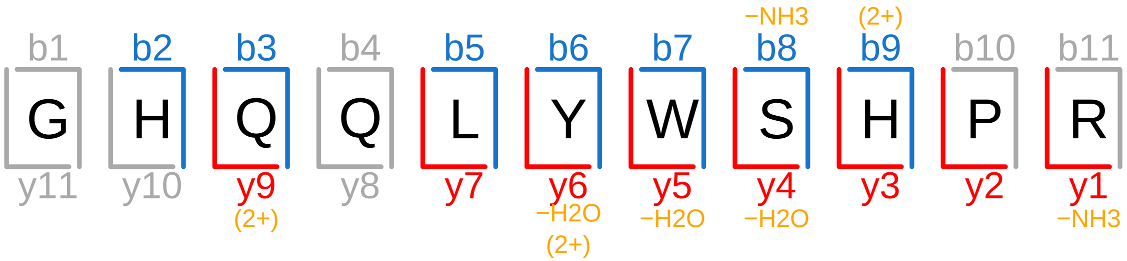

GHQQLYWSHPR

Score: 107 ; 1417.6879 m/z; 473.5699 m/z; −0.16367 ppm; MULTI−MSMS

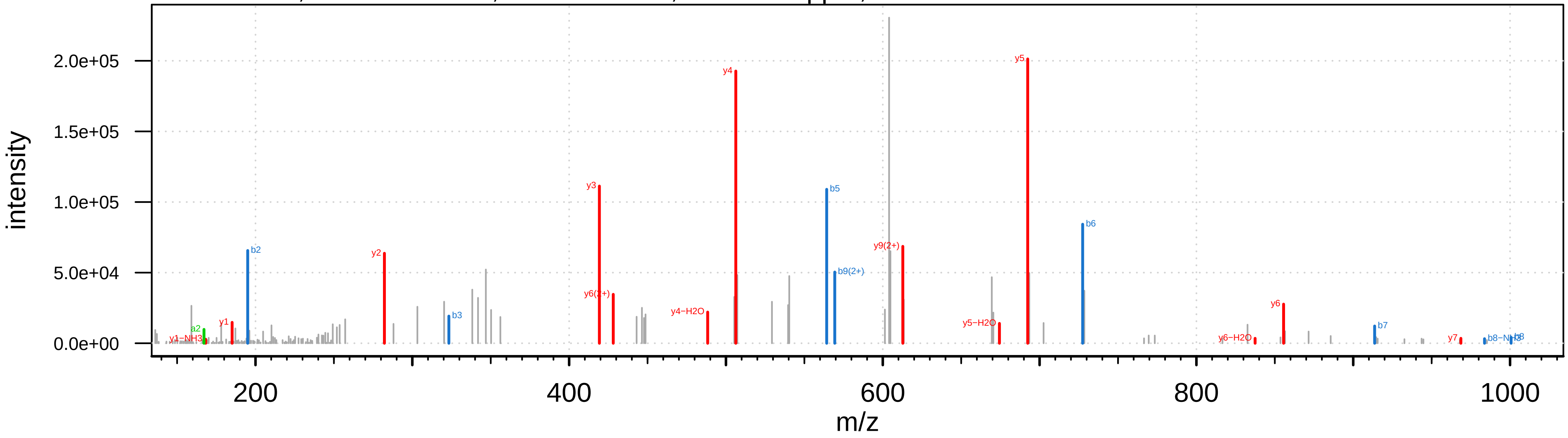

Raw File: Tiffy_20100301_BJS_Halflives_P3_Exp1_InSol_01

Scan Number: 9563

Proteins:

sp|P62274|RS29_MOUSE

ENSMUST00000037023_chr12:69157813−69159058:−

ENSMUST00000071737_chr13:73988963−73989133:−

tr|D3YYL7|D3YYL7_MOUSE

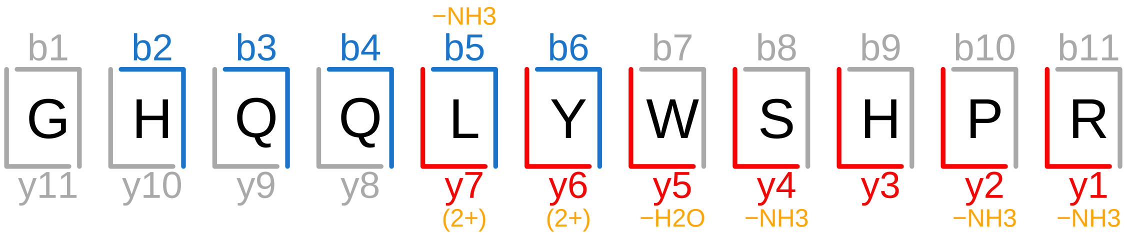

GHQQLYWSHPR
Score: 49 ; 1407.6796 m/z; 352.92718 m/z; −0.16713 ppm; MULTI−MSMS

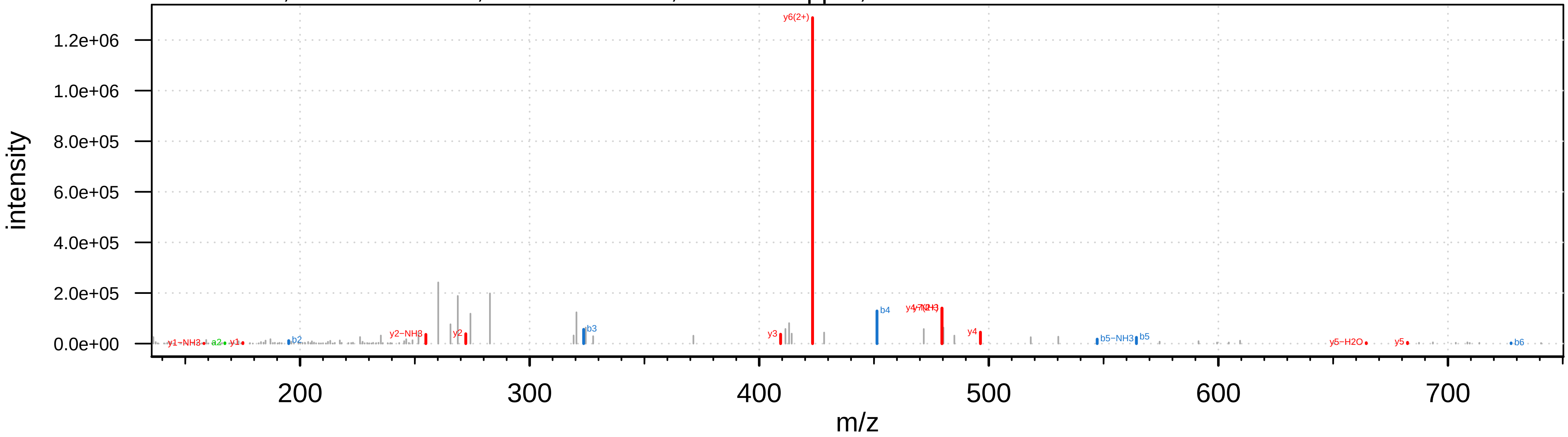

Raw File: Tiffy_20100301_BJS_Halflives_P3_Exp1_InSol_01
Scan Number: 9593
Proteins:
sp|P62274|RS29_MOUSE
ENSMUST00000037023_chr12:69157813−69159058:−
ENSMUST00000071737_chr13:73988963−73989133:−
tr|D3YYL7|D3YYL7_MOUSE

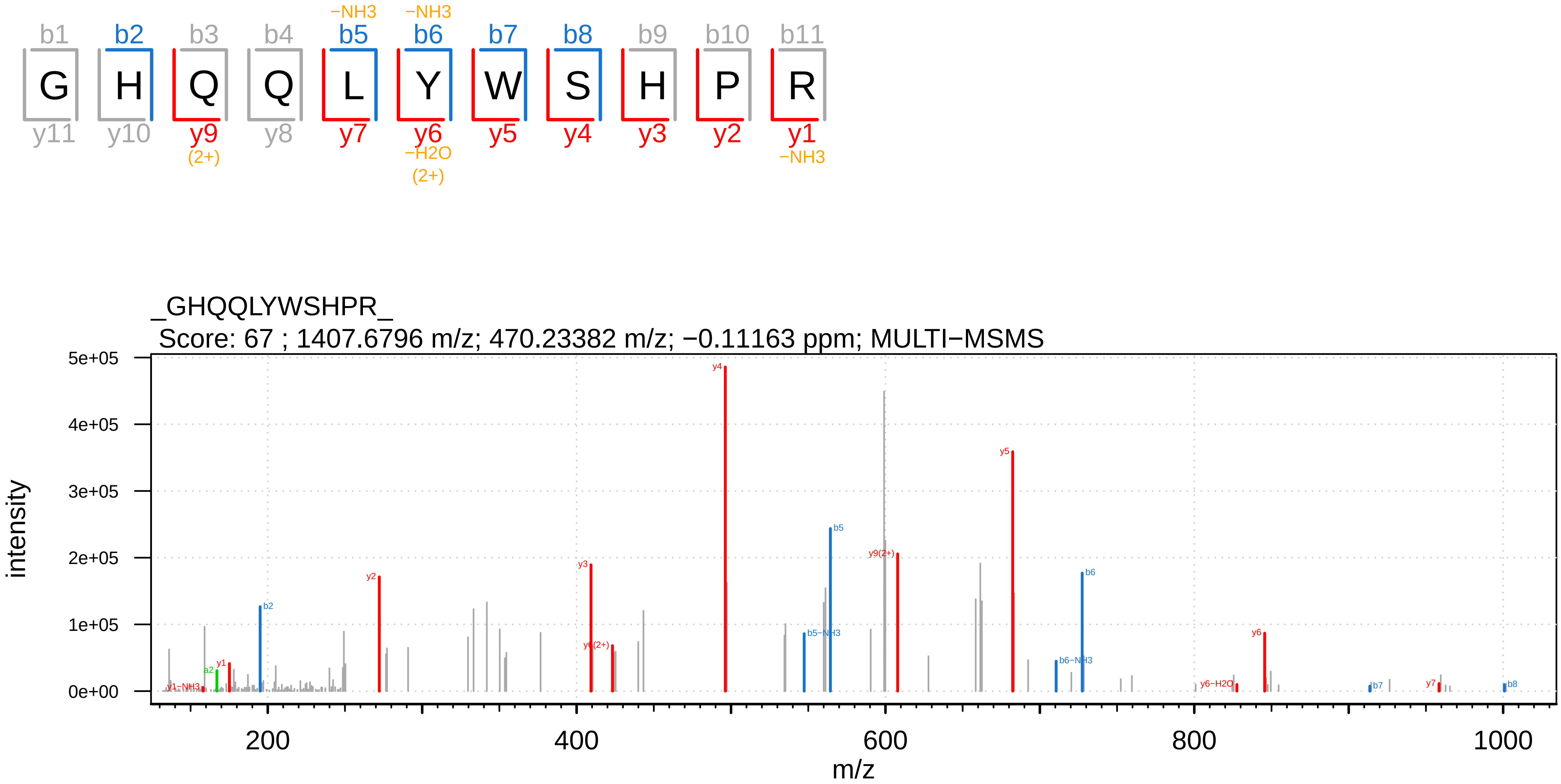

Raw File: Tiffy_20100301_BJS_Halflives_P3_Exp1_InSol_02
Scan Number: 10320
Proteins:
sp|P62274|RS29_MOUSE
ENSMUST00000037023_chr12:69157813−69159058:−
ENSMUST00000071737_chr13:73988963−73989133:−
tr|D3YYL7|D3YYL7_MOUSE

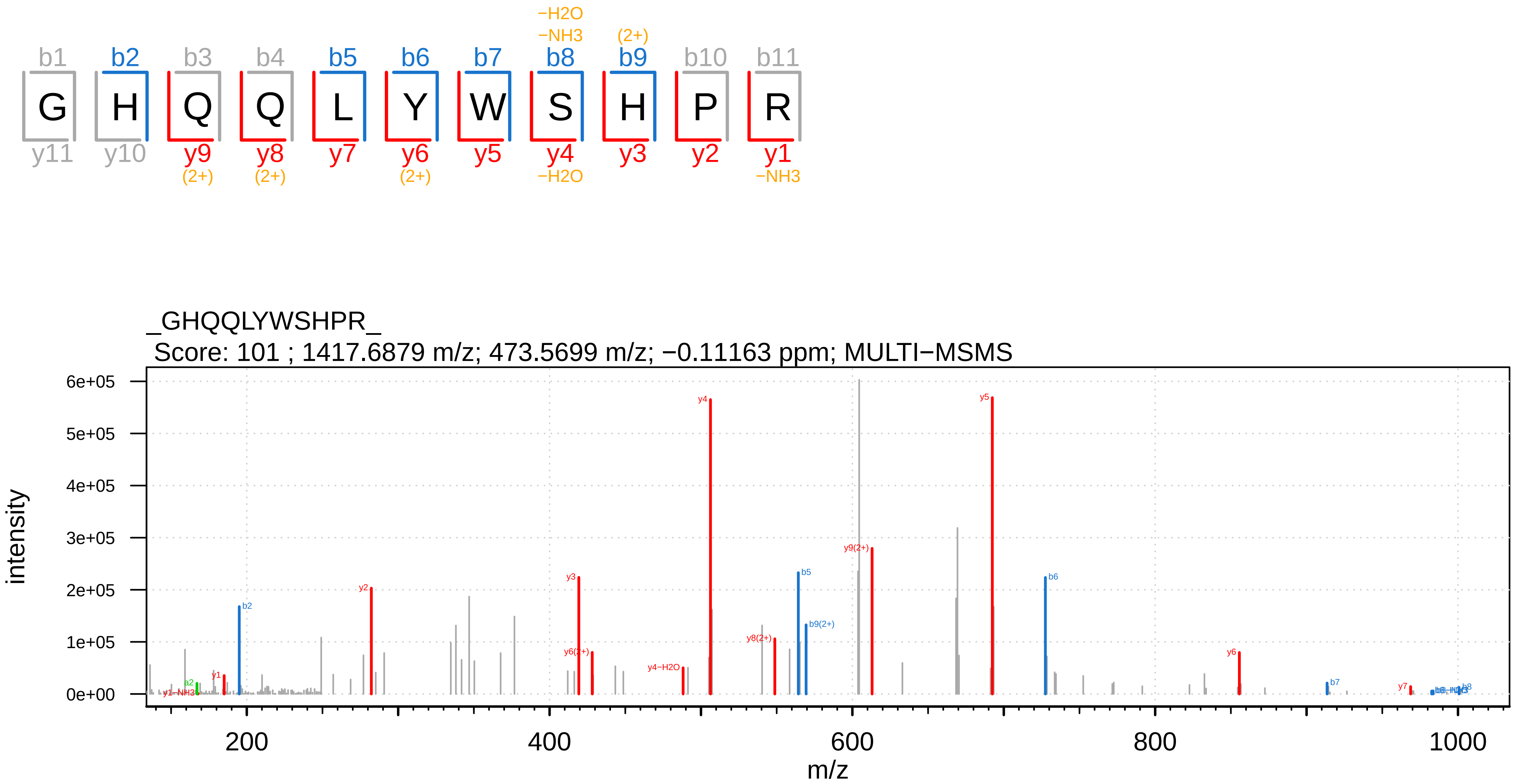

Raw File: Tiffy_20100301_BJS_Halflives_P3_Exp1_InSol_02
Scan Number: 10334
Proteins:
sp|P62274|RS29_MOUSE
ENSMUST00000037023_chr12:69157813−69159058:−
ENSMUST00000071737_chr13:73988963−73989133:−
tr|D3YYL7|D3YYL7_MOUSE

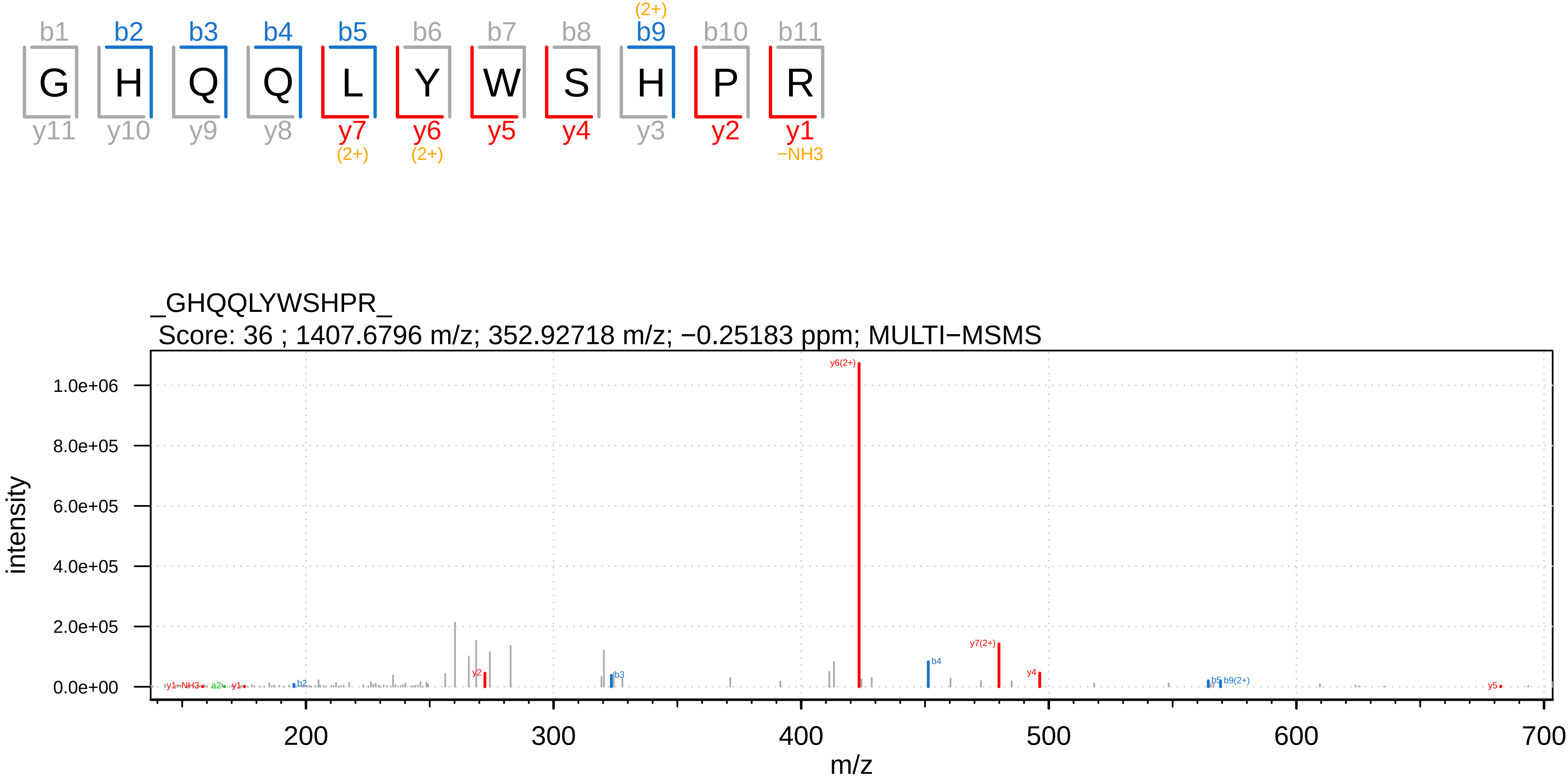

Raw File: Tiffy_20100301_BJS_Halflives_P3_Exp1_InSol_02
Scan Number: 10346
Proteins:
sp|P62274|RS29_MOUSE
ENSMUST00000037023_chr12:69157813−69159058:−
ENSMUST00000071737_chr13:73988963−73989133:−
tr|D3YYL7|D3YYL7_MOUSE

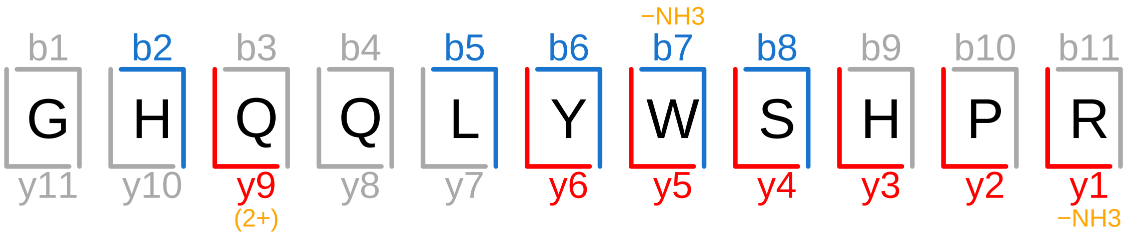

GHQQLYWSHPR

Score: 45 ; 1407.6796 m/z; 470.23382 m/z; 0.08634 ppm; MULTI−MSMS

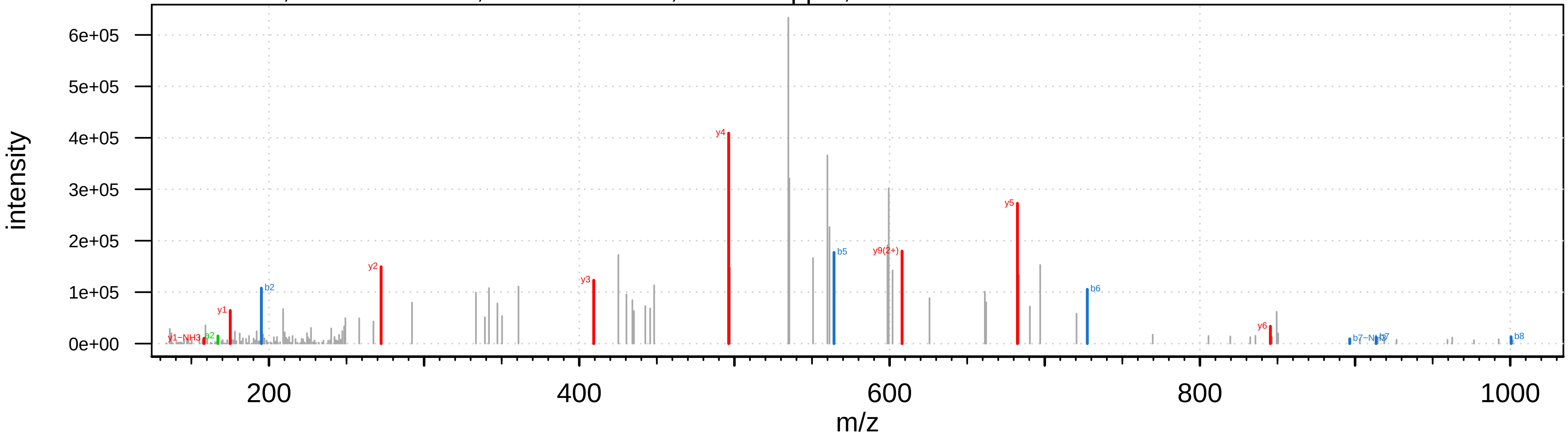

Raw File: Tiffy_20100301_BJS_Halflives_P3_Exp1_InSol_03

Scan Number: 10219

Proteins:

sp|P62274|RS29_MOUSE

ENSMUST00000037023_chr12:69157813−69159058:−

ENSMUST00000071737_chr13:73988963−73989133:−

tr|D3YYL7|D3YYL7_MOUSE

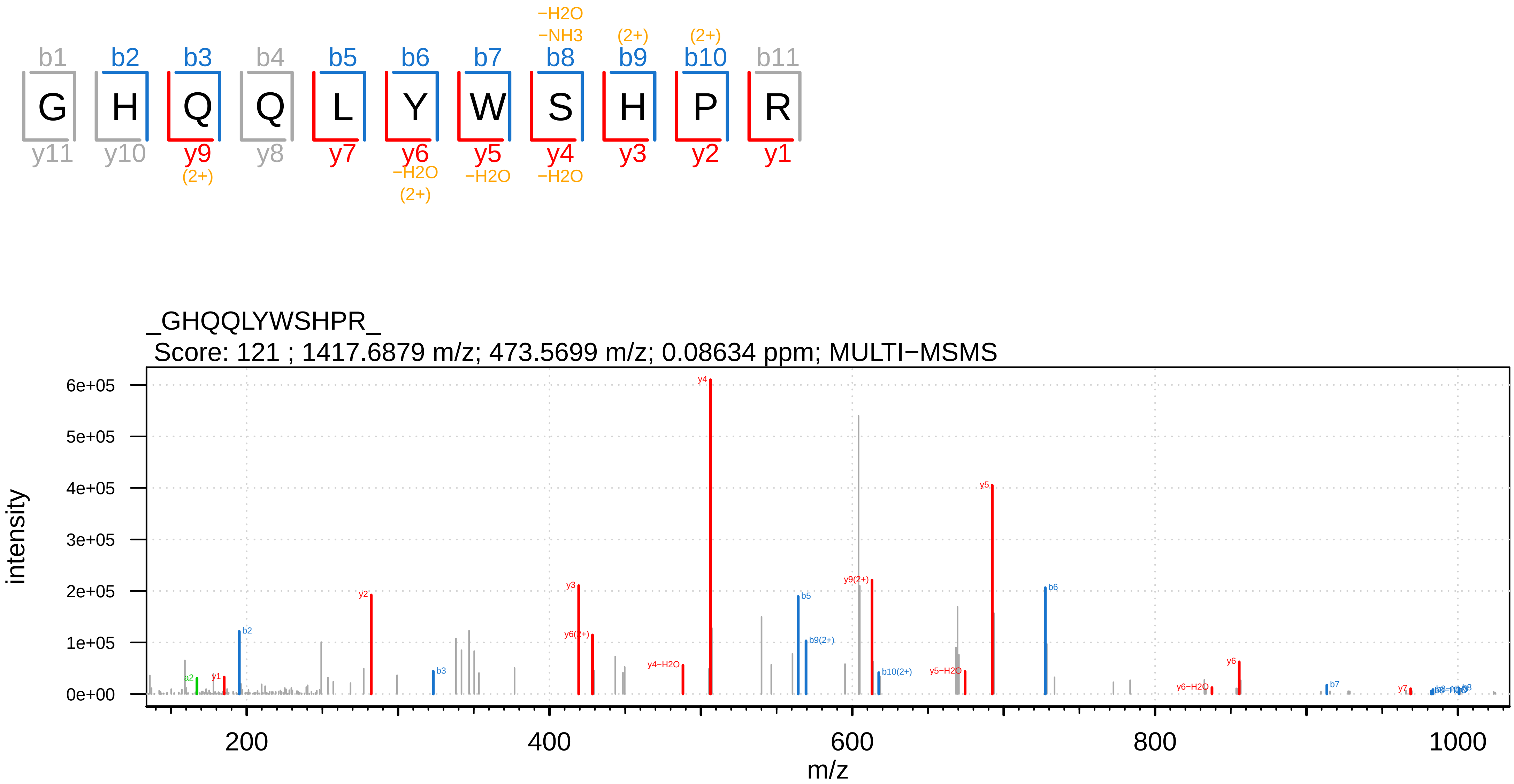

Raw File: Tiffy_20100301_BJS_Halflives_P3_Exp1_InSol_03
Scan Number: 10237
Proteins:
sp|P62274|RS29_MOUSE
ENSMUST00000037023_chr12:69157813−69159058:−
ENSMUST00000071737_chr13:73988963−73989133:−
tr|D3YYL7|D3YYL7_MOUSE

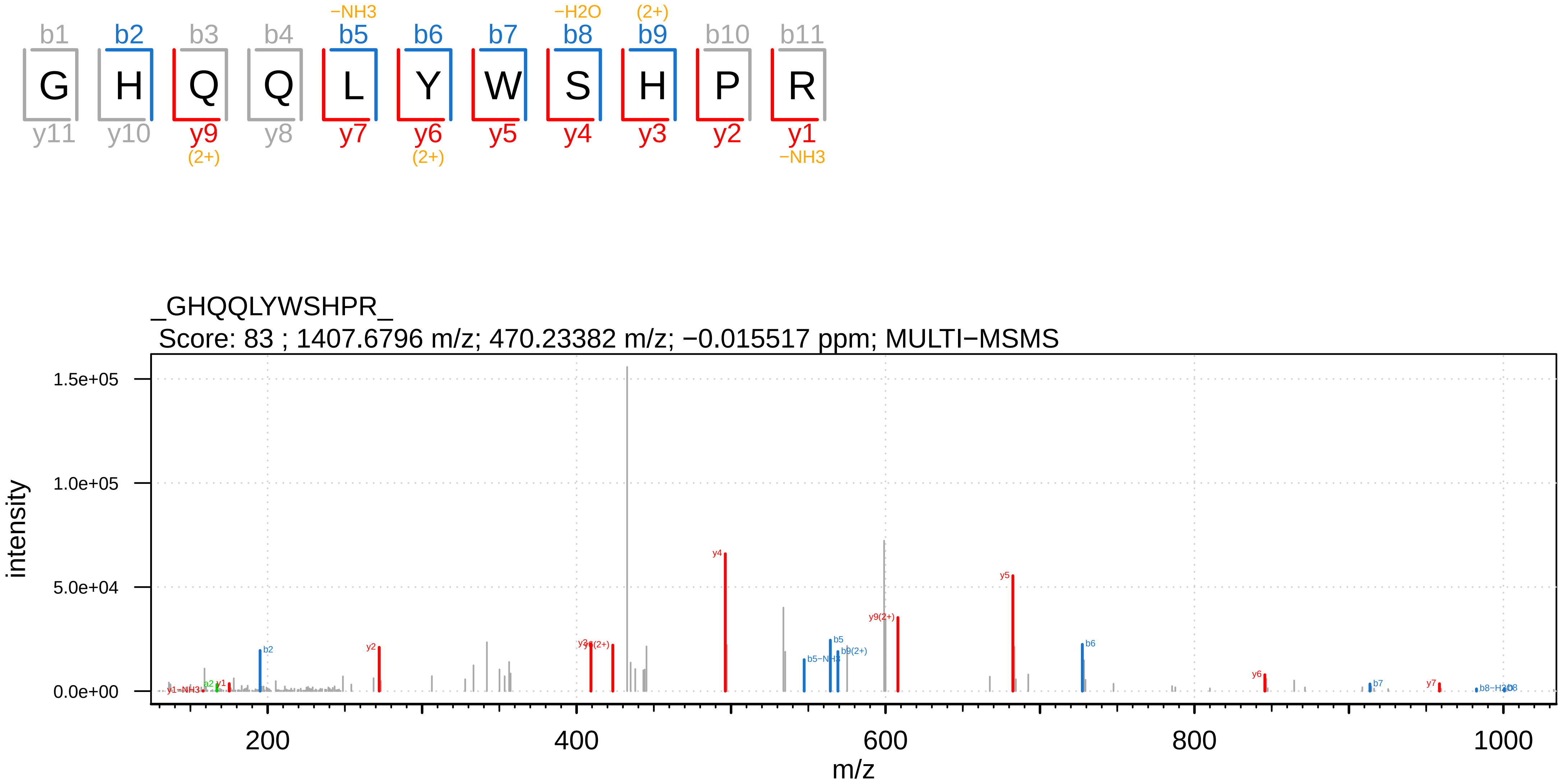

Raw File: Tiffy_20100301_BJS_Halflives_P3_Exp1_InSol_04
Scan Number: 9691
Proteins:
sp|P62274|RS29_MOUSE
ENSMUST00000037023_chr12:69157813−69159058:−
ENSMUST00000071737_chr13:73988963−73989133:−
tr|D3YYL7|D3YYL7_MOUSE

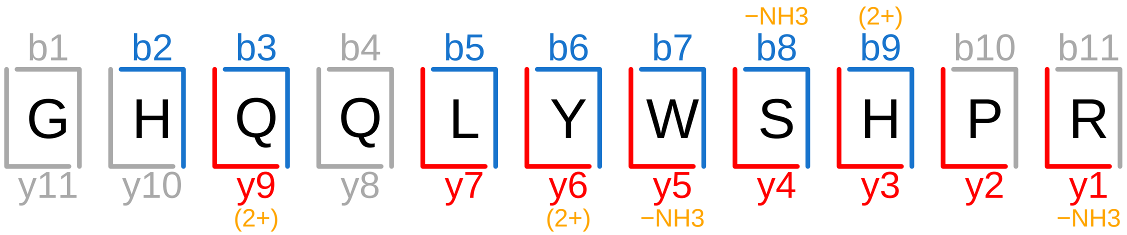

GHQQLYWSHPR
Score: 86 ; 1407.6796 m/z; 470.23382 m/z; −0.010978 ppm; ISO−MSMS

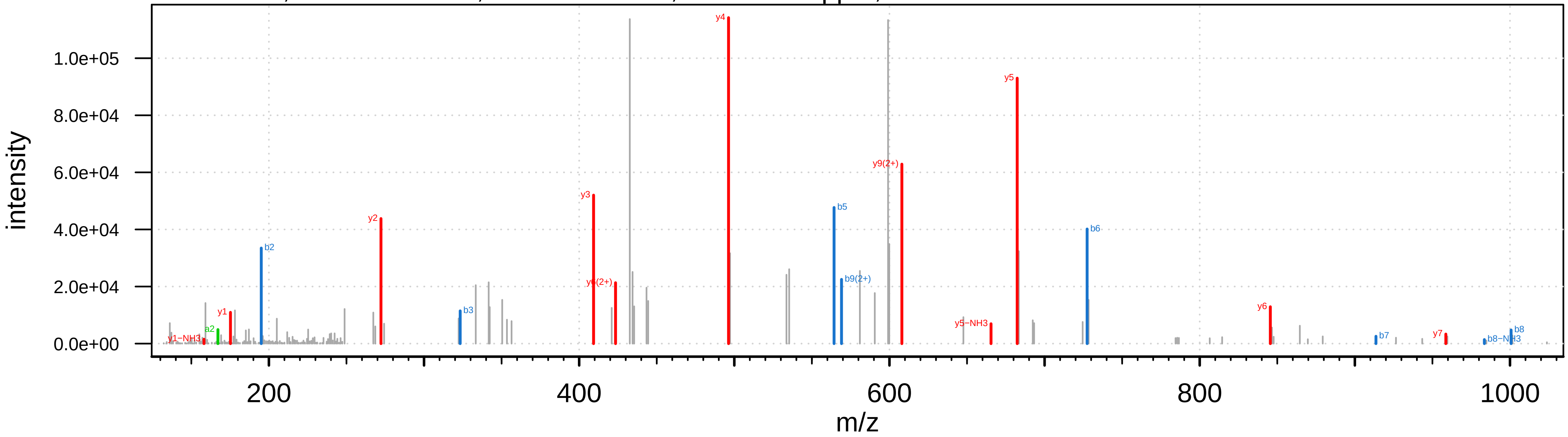

Raw File: Tiffy_20100301_BJS_Halflives_P3_Exp1_InSol_05
Scan Number: 9810
Proteins:
sp|P62274|RS29_MOUSE
ENSMUST00000037023_chr12:69157813−69159058:−
ENSMUST00000071737_chr13:73988963−73989133:−
tr|D3YYL7|D3YYL7_MOUSE

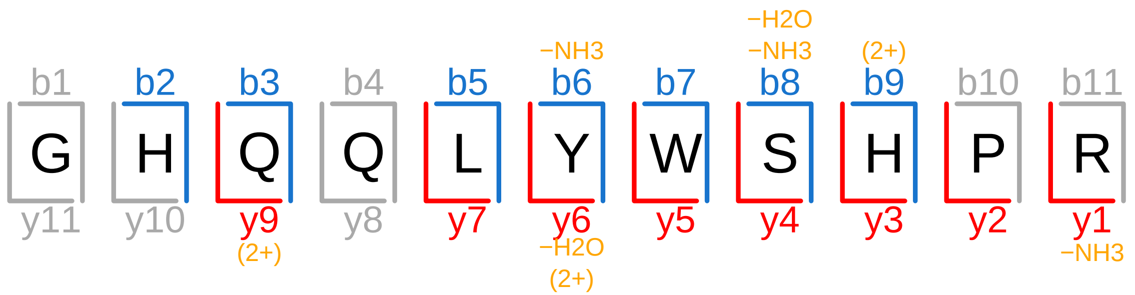

GHQQLYWSHPR
Score: 111 ; 1407.6796 m/z; 470.23382 m/z; NaN ppm; MSMS

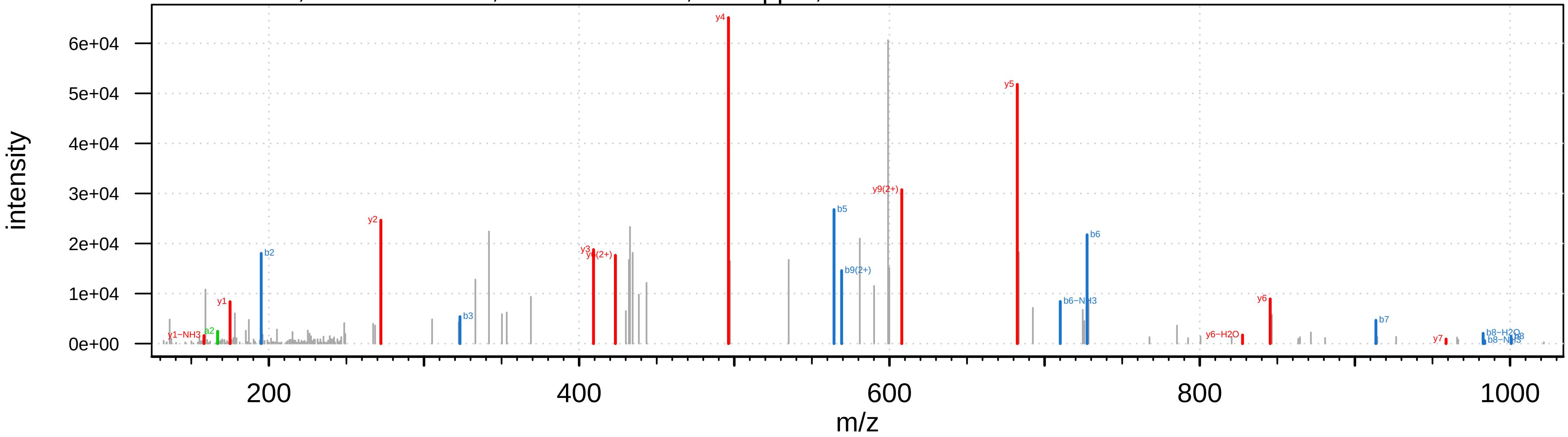

Raw File: Tiffy_20100301_BJS_Halflives_P3_Exp1_InSol_06
Scan Number: 9540
Proteins:
sp|P62274|RS29_MOUSE
ENSMUST00000037023_chr12:69157813−69159058:−
ENSMUST00000071737_chr13:73988963−73989133:−
tr|D3YYL7|D3YYL7_MOUSE

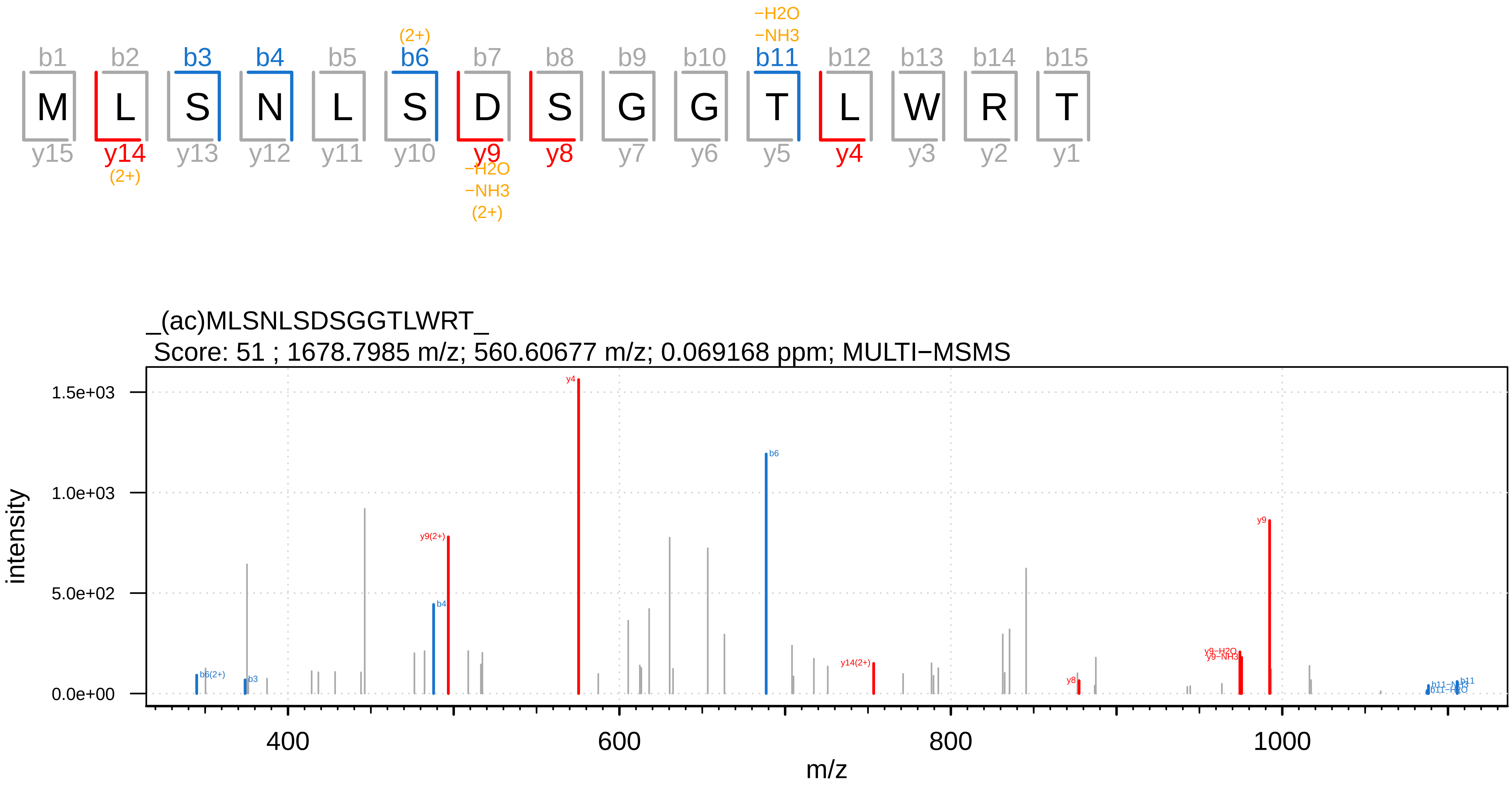

Raw File: Bert_20090331_BJS_halflives_P2_slice05_03
Scan Number: 15068
Proteins:
ENSMUST00000159701_chr1:169934095−169934142:−

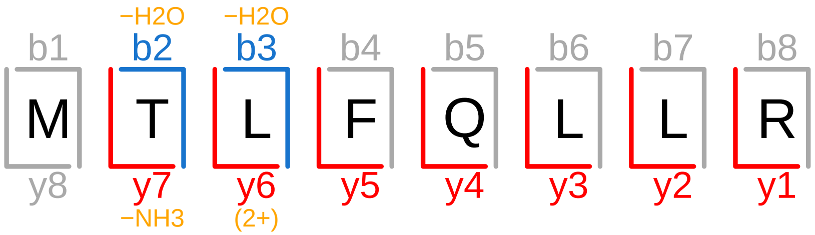

M(ox)TLFQLLR
Score: 96 ; 1046.5822 m/z; 524.29838 m/z; 0.054578 ppm; MULTI−MSMS

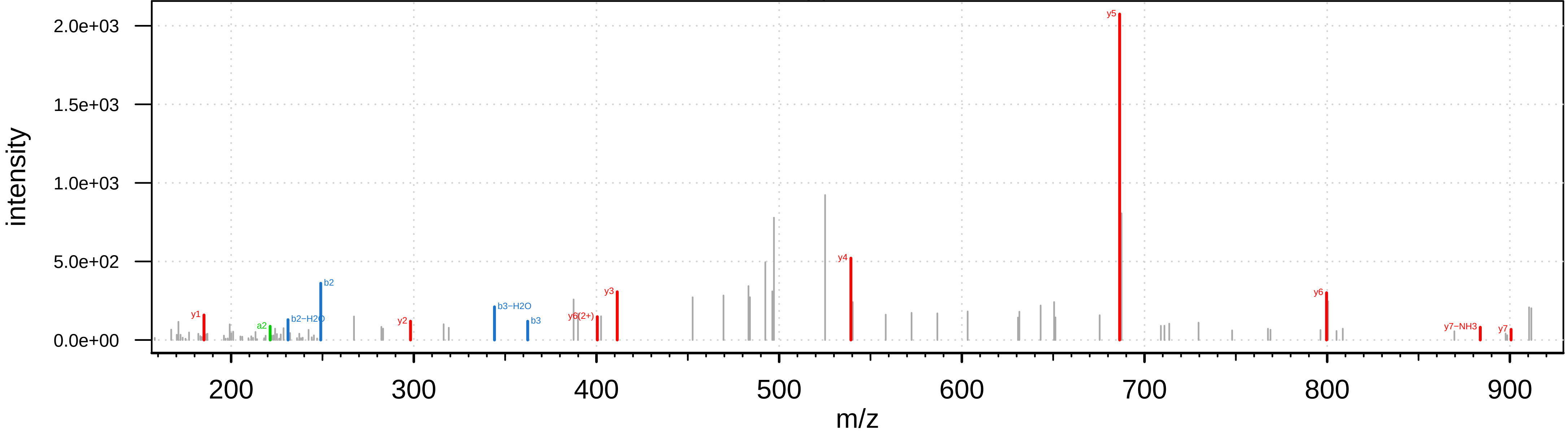

Raw File: Tiffy_20100209_BJS_Halflives_P3_01
Scan Number: 23010
Proteins:
chr12_103206313−103214181.T2_chr12:101968174−101969629:−

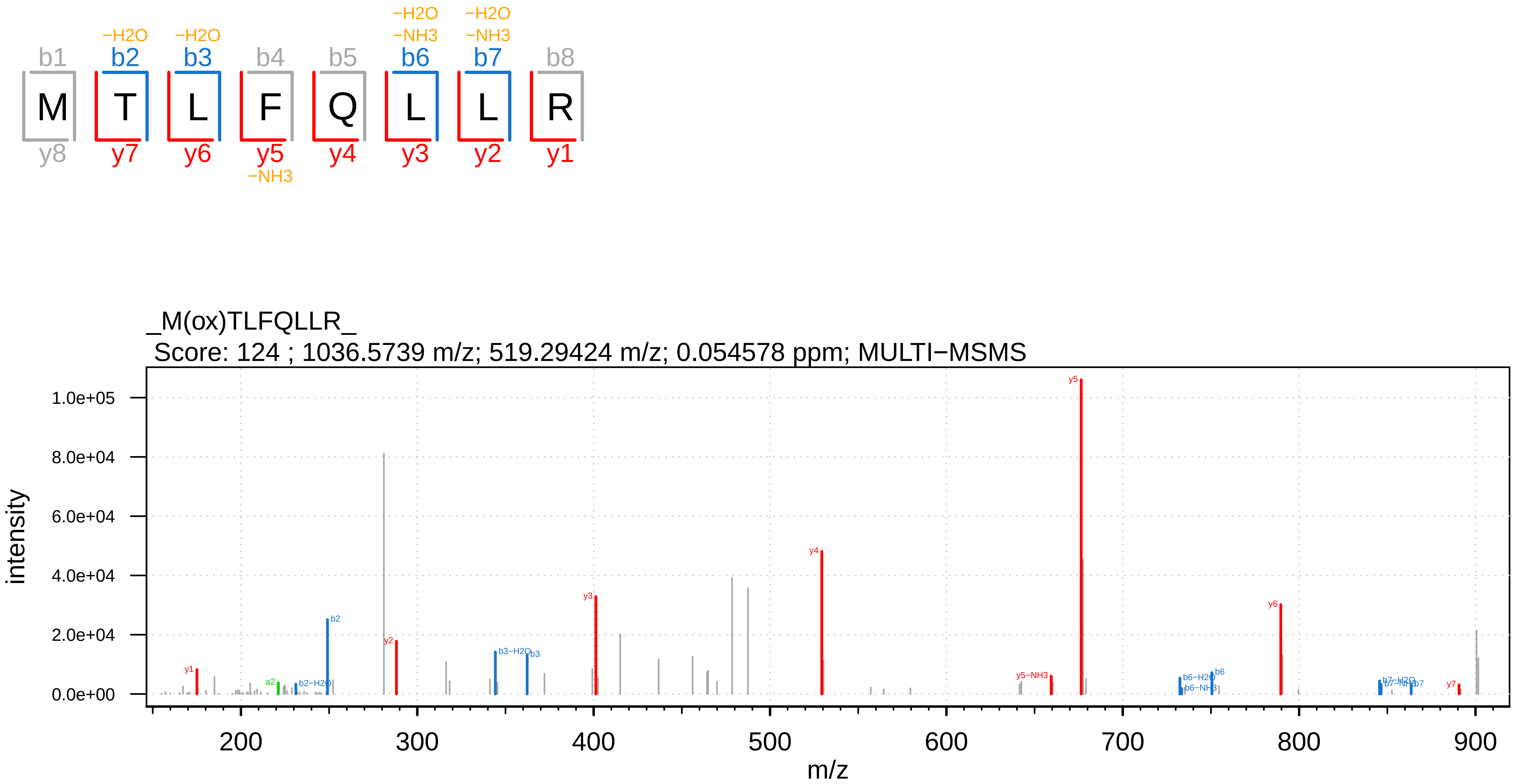

Raw File: Tiffy_20100209_BJS_Halflives_P3_01
Scan Number: 23061
Proteins:
chr12_103206313−103214181.T2_chr12:101968174−101969629:−

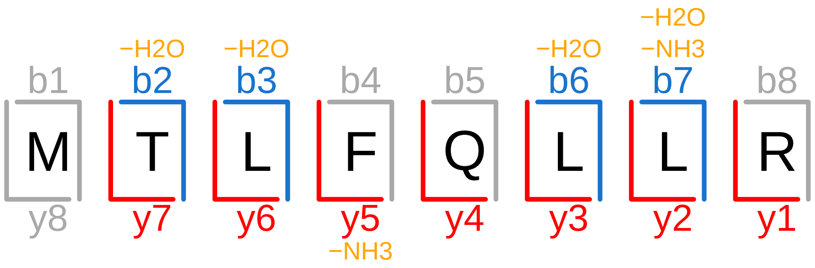

M(ox)TLFQLLR
Score: 125 ; 1046.5822 m/z; 524.29838 m/z; 0.054578 ppm; MULTI−MSMS

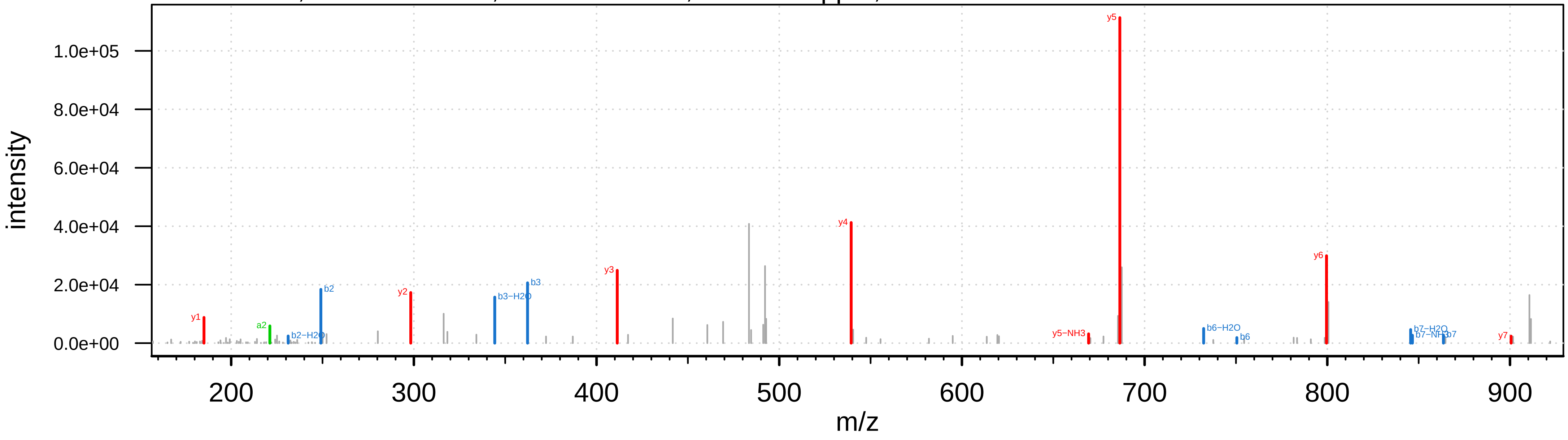

Raw File: Tiffy_20100209_BJS_Halflives_P3_01
Scan Number: 23169
Proteins:
chr12_103206313−103214181.T2_chr12:101968174−101969629:−

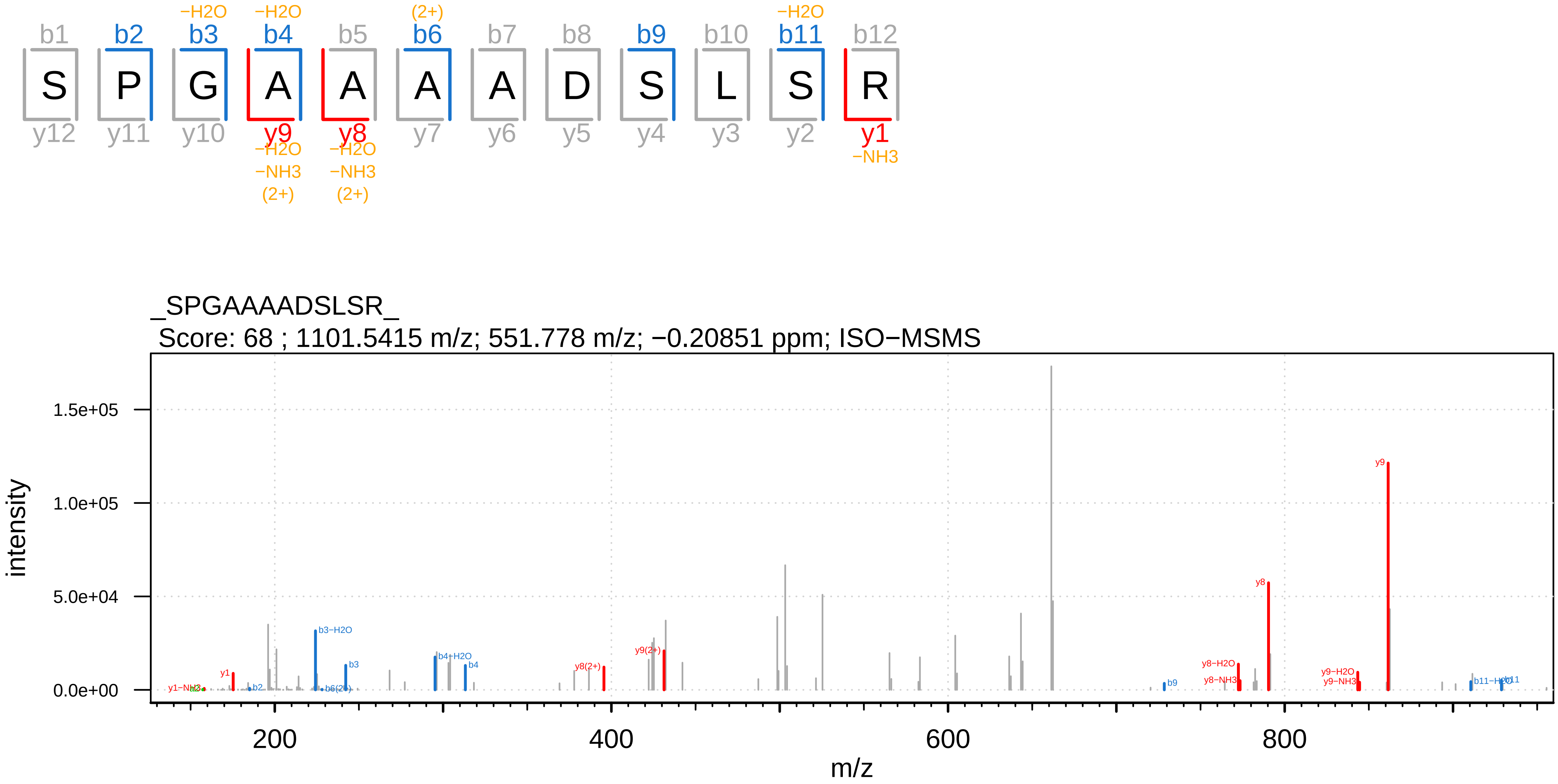

Raw File: Tiffy_20100226_BJS_Halflives_P1_19
Scan Number: 5892
Proteins:
ENSMUST00000030787_chr5:24842270−24842338:−

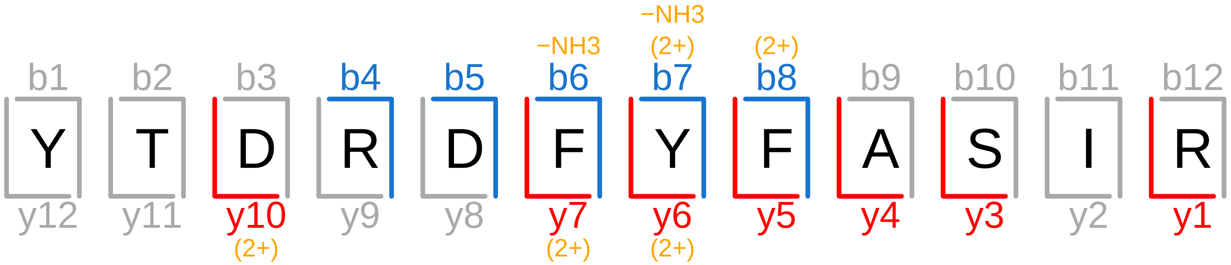

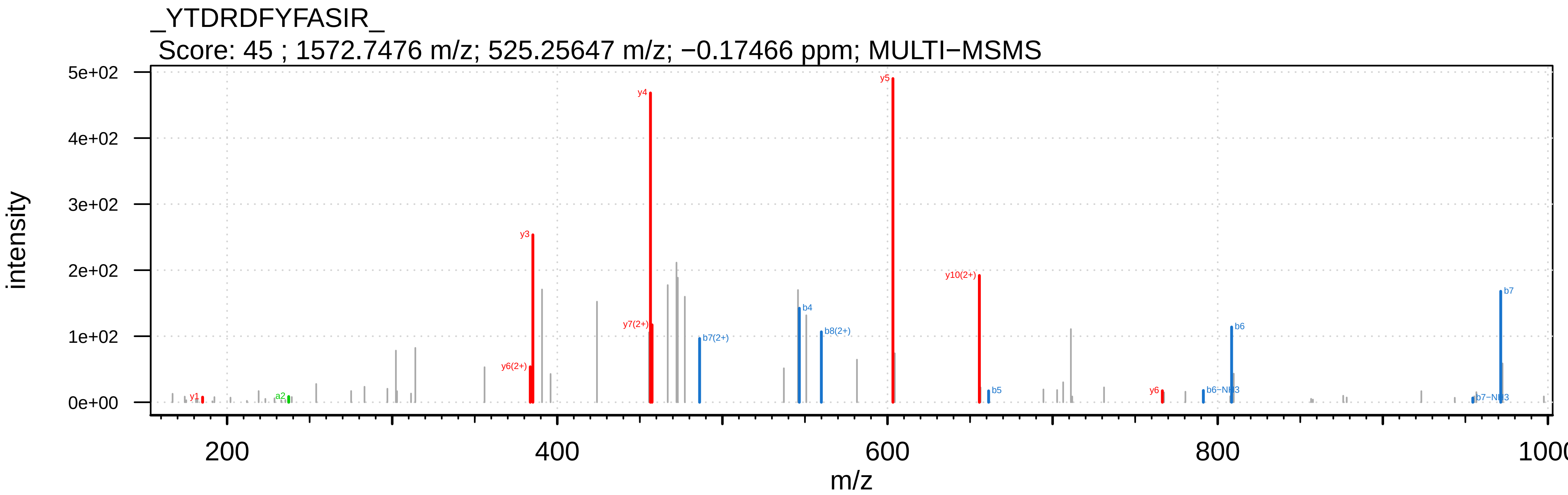

Raw File: Bert_20090403_BJS_halflives_P3_slice01_03
Scan Number: 5995
Proteins:
ENSMUST00000023048_chr15:80235835−80236047:+

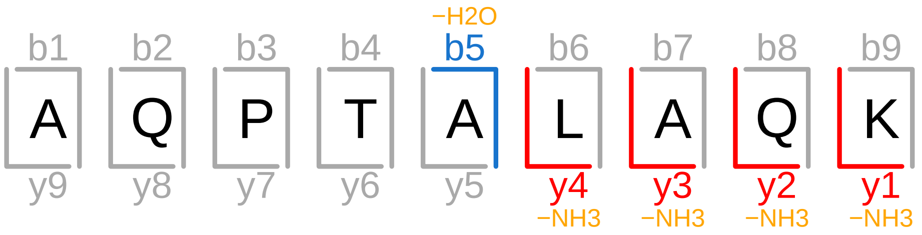

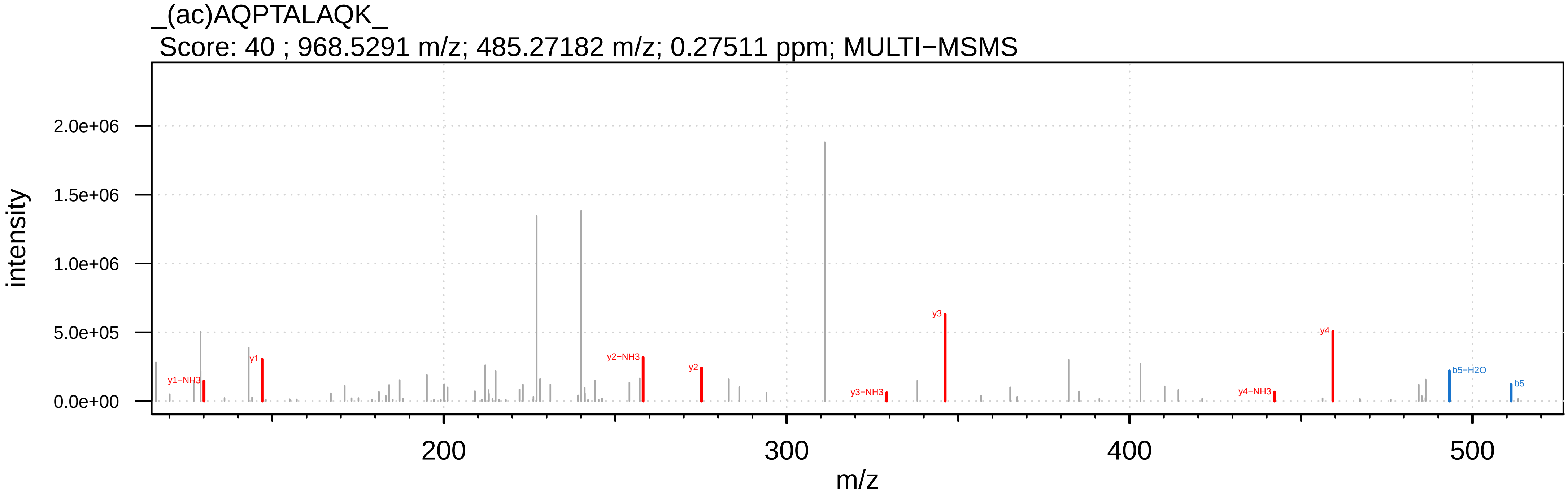

Raw File: 20130413_EXQ6_BaAz_SA_HR1_01
Scan Number: 27028
Proteins:
ENSMUST00000161069_chr14:70554856−70554960:+

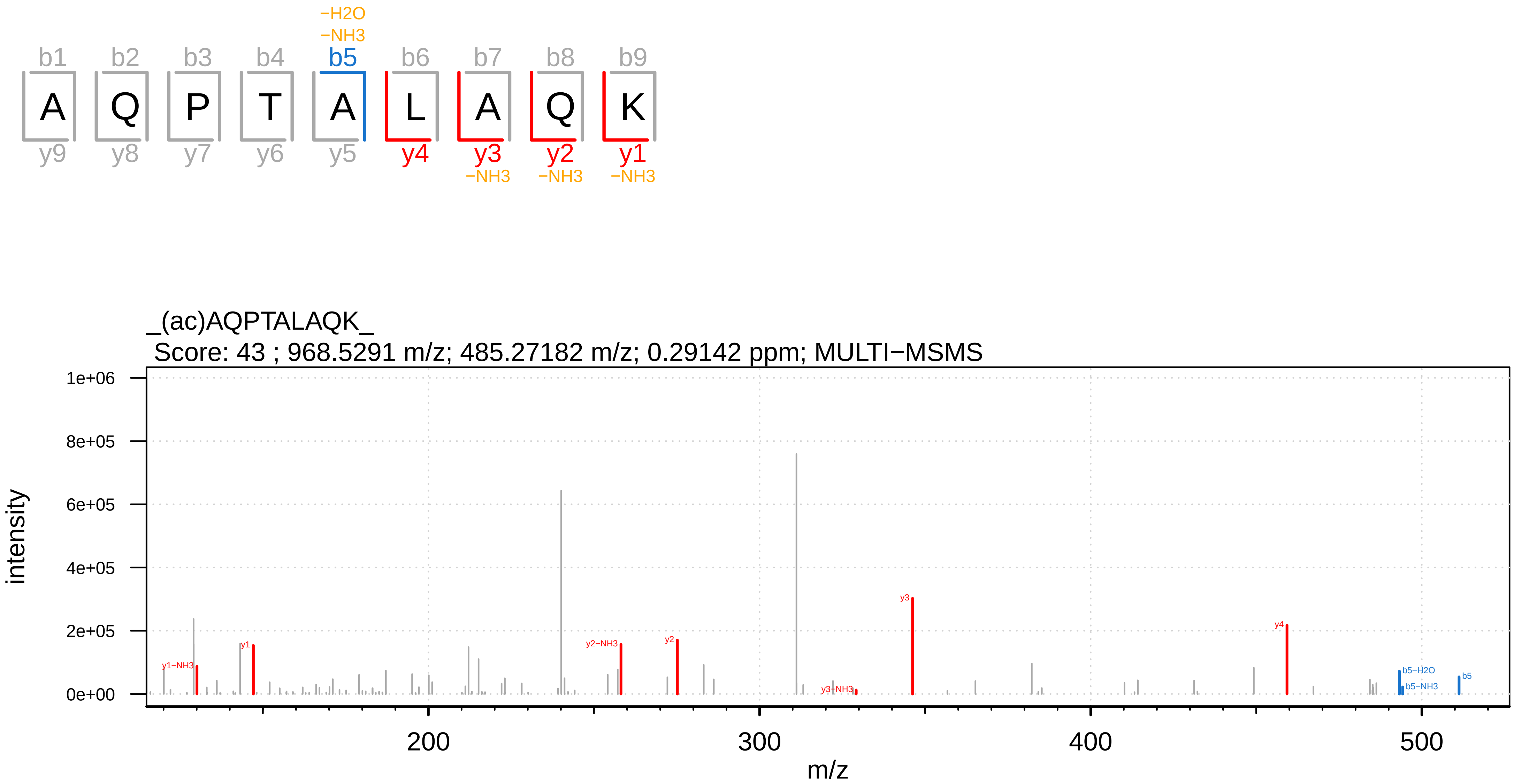

Raw File: 20130705_EXQ4_BaAz_SA_HR2_01
Scan Number: 25979
Proteins:
ENSMUST00000161069_chr14:70554856−70554960:+

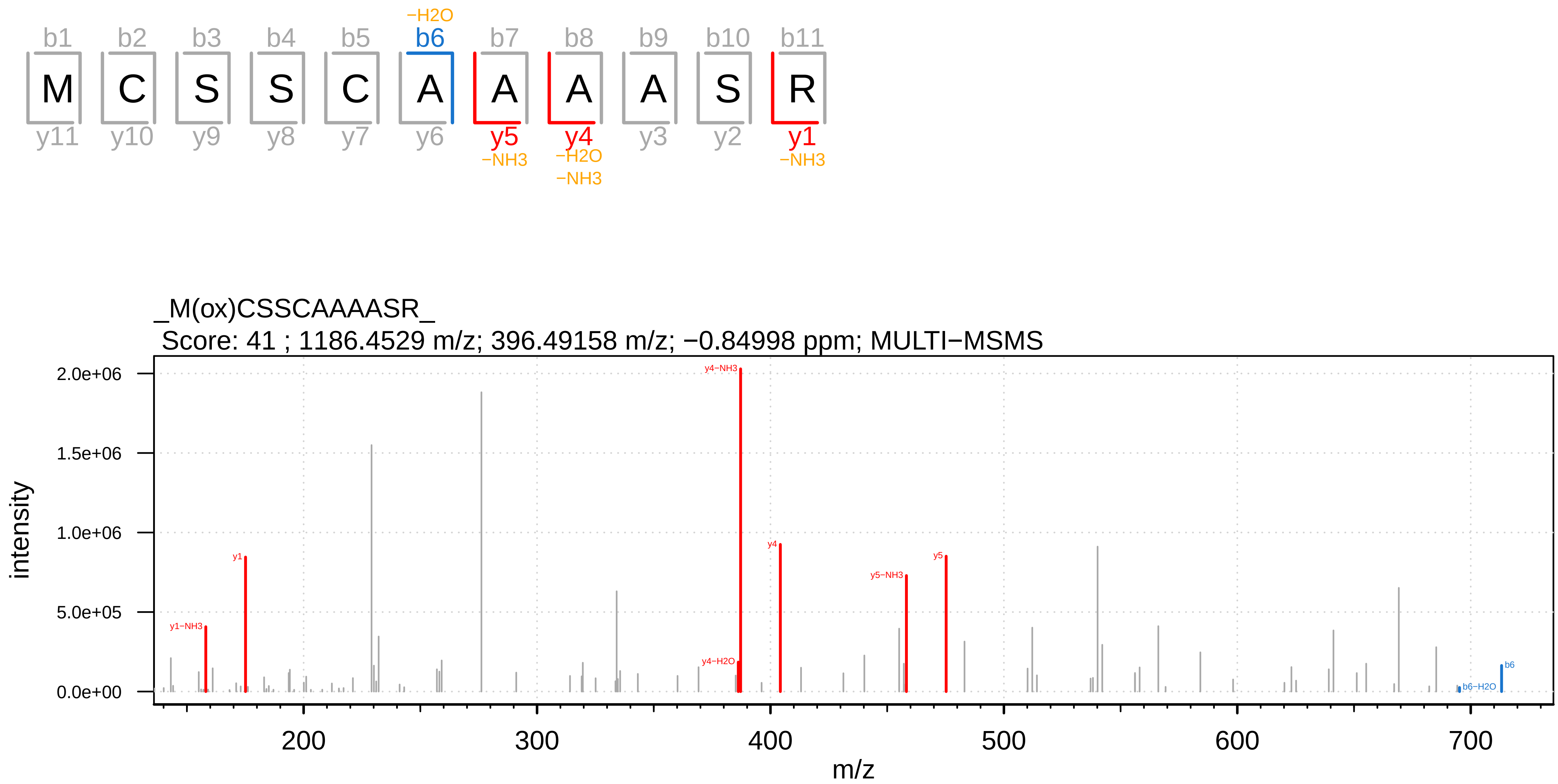

Raw File: 20130705_EXQ4_BaAz_SA_HR2_01
Scan Number: 49951
Proteins:
ENSMUST00000047876_chr3:83128571−83128696:−

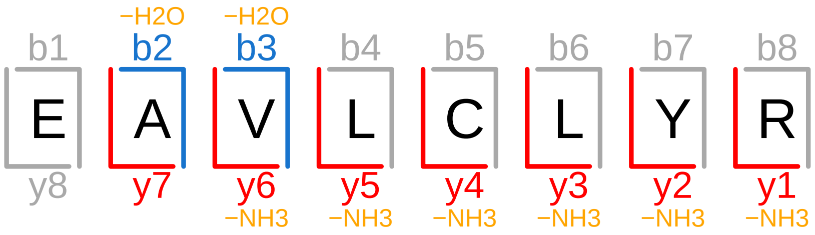

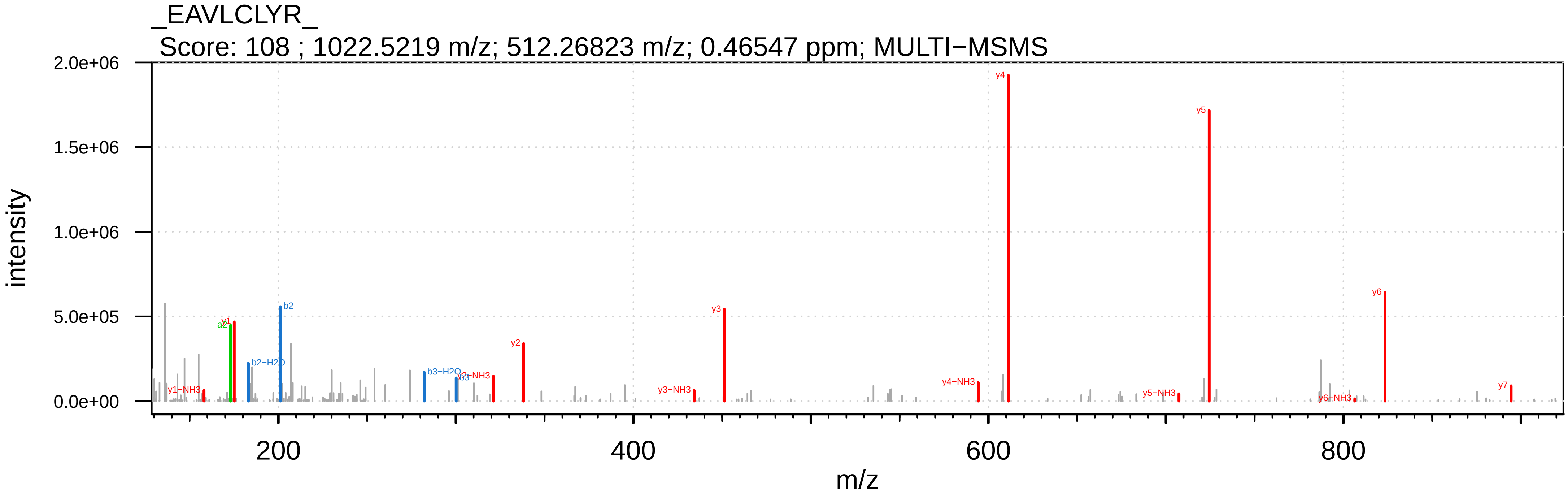

Raw File: 20130705_EXQ6_BaAz_SA_H53R1_04
Scan Number: 49225
Proteins:
ENSMUST00000023048_chr15:80235835−80236047:+

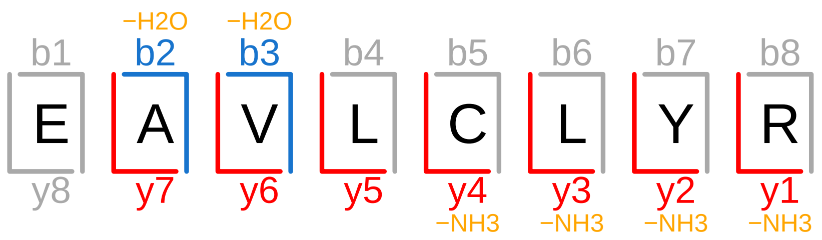

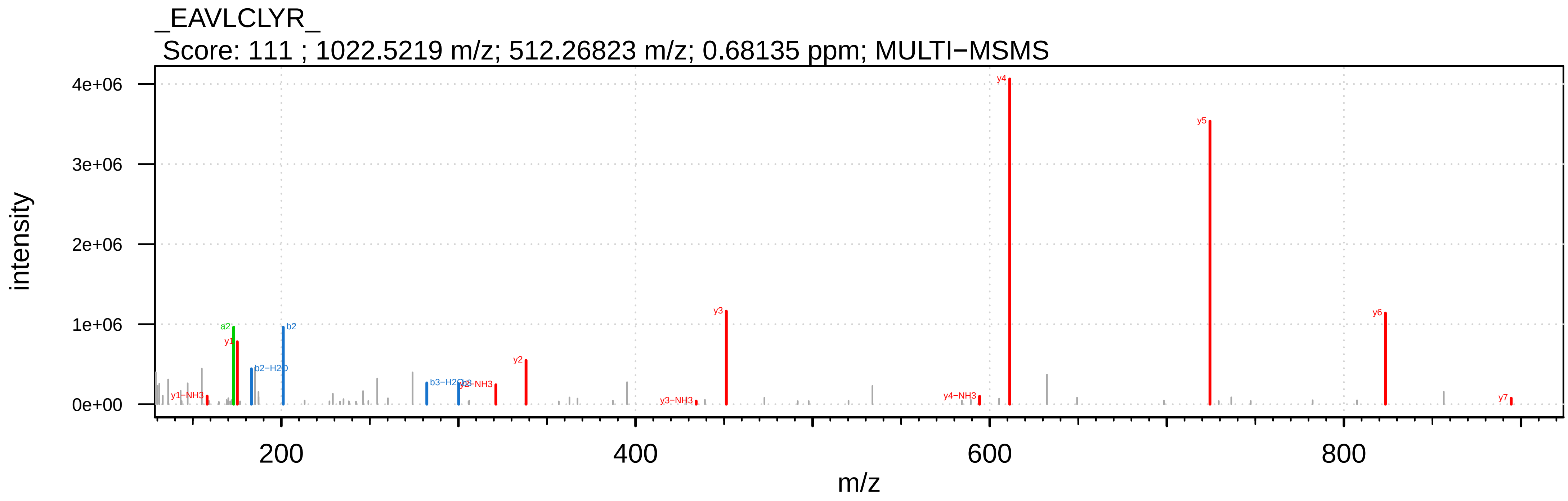

Raw File: 20130705_EXQ6_BaAz_SA_H53R1_05
Scan Number: 46993
Proteins:
ENSMUST00000023048_chr15:80235835−80236047:+

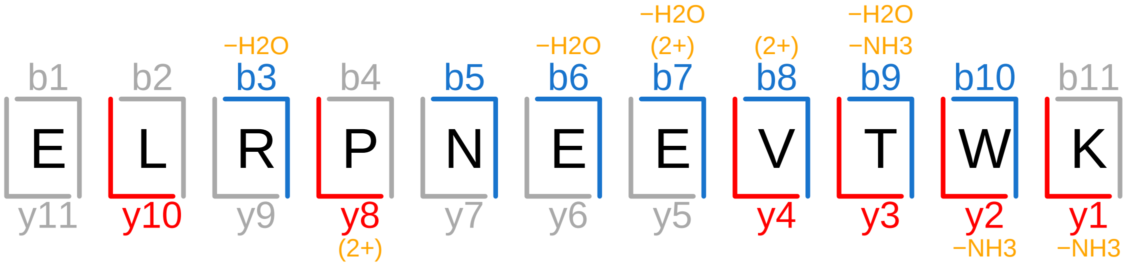

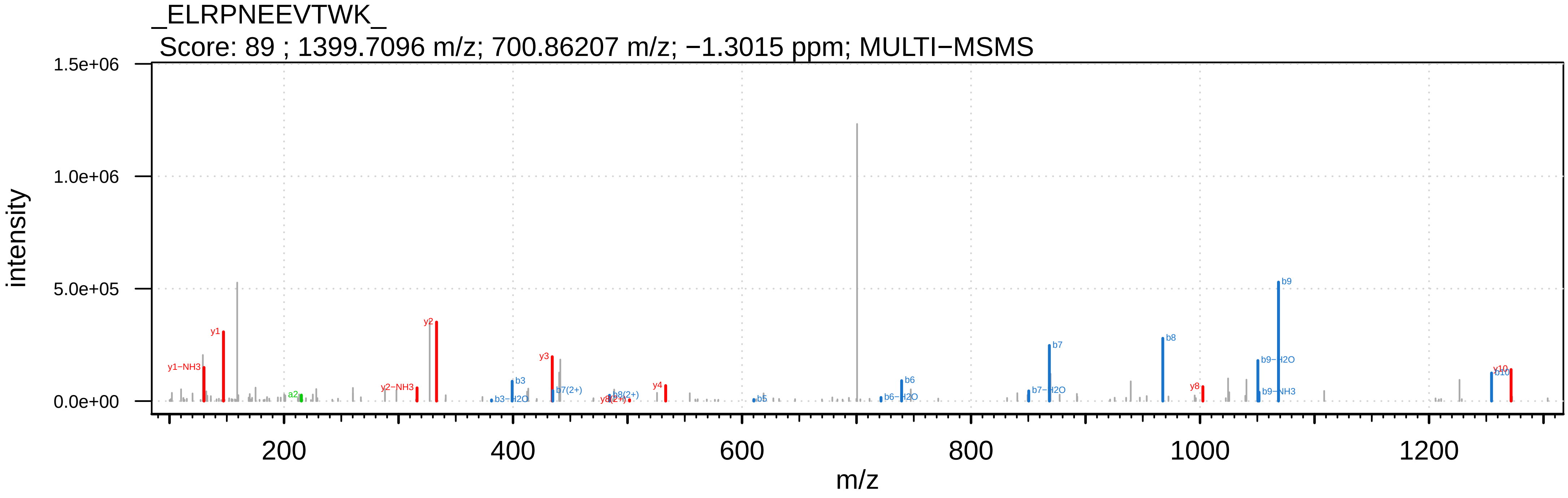

Raw File: 20130705_EXQ6_BaAz_SA_H53R1_05
Scan Number: 34180
Proteins:
chr12_103206313−103214181.T2_chr12:101968174−101969629:−

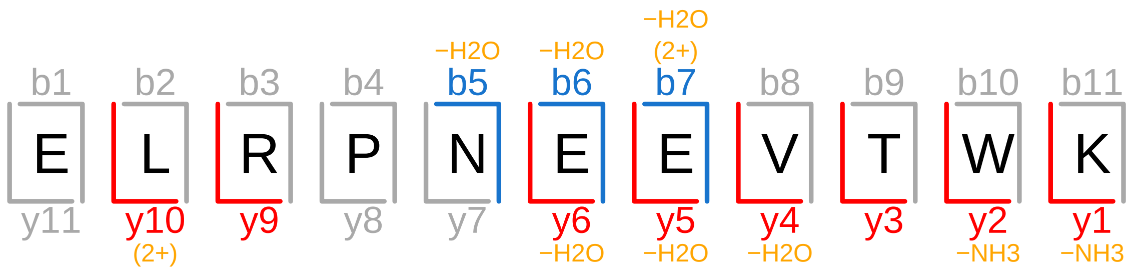

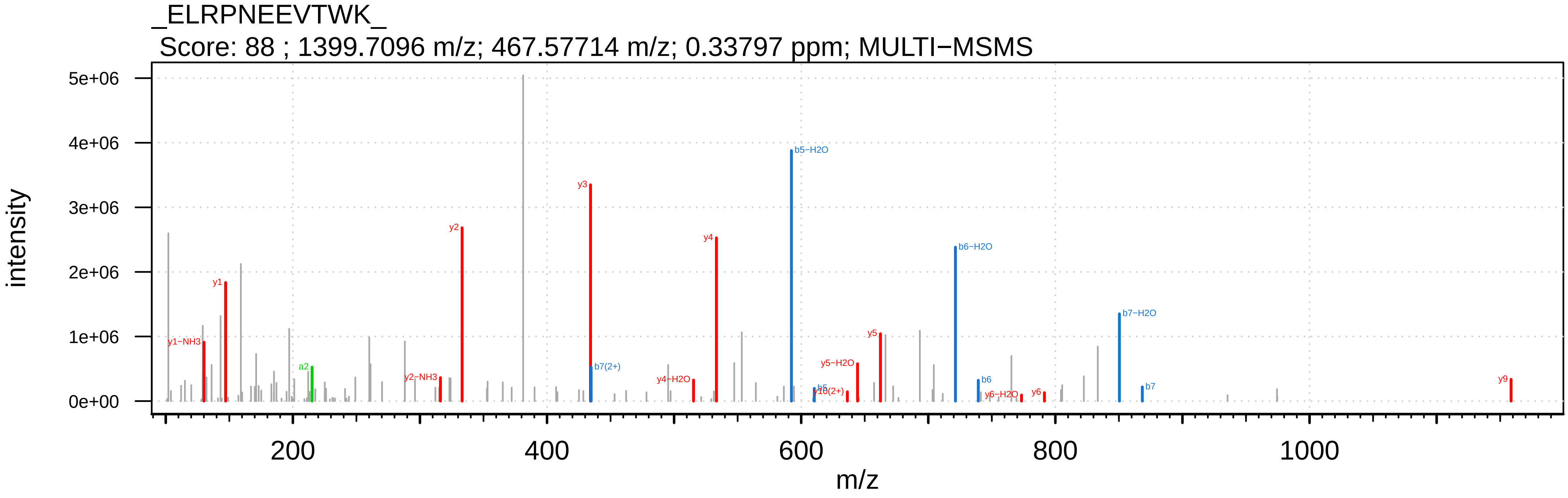

Raw File: 20130705_EXQ6_BaAz_SA_H53R1_05
Scan Number: 34192
Proteins:
chr12_103206313−103214181.T2_chr12:101968174−101969629:−

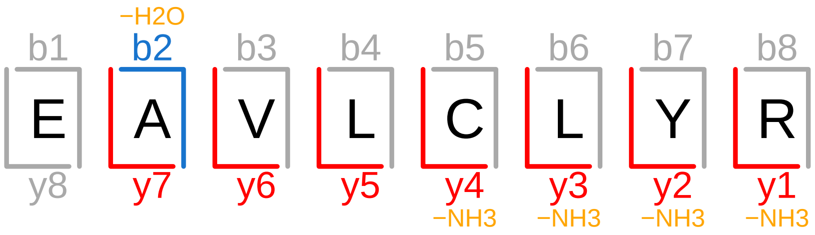

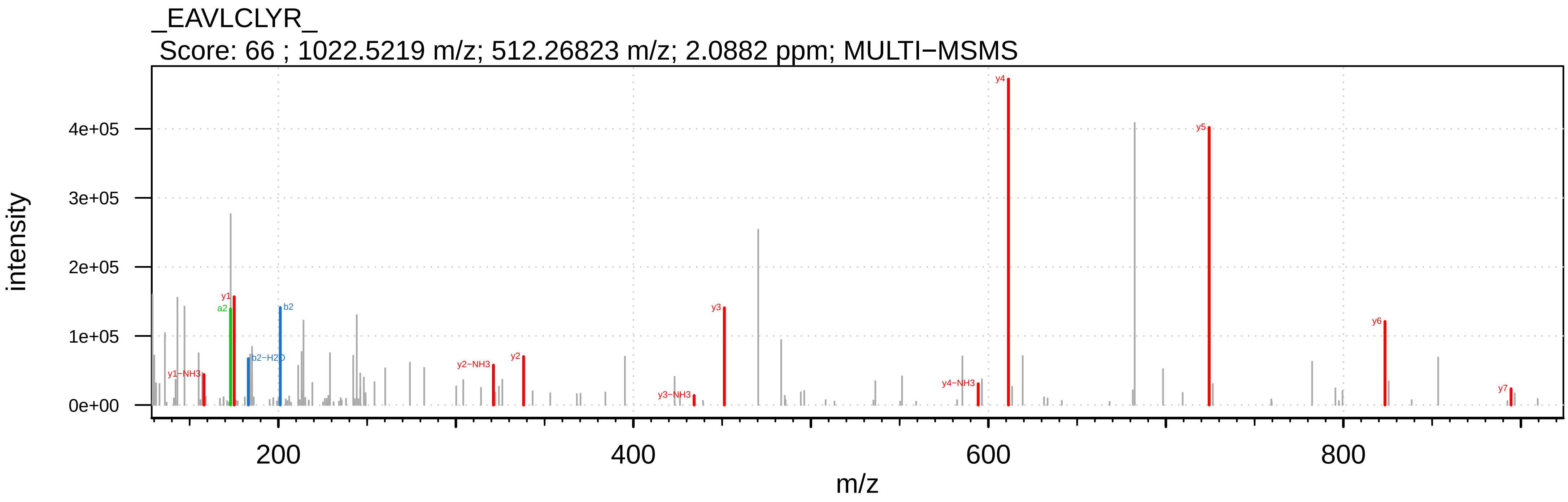

Raw File: 20130807_EXQ4_BaAz_SA_KR4_05
Scan Number: 43448
Proteins:
ENSMUST00000023048_chr15:80235835−80236047:+

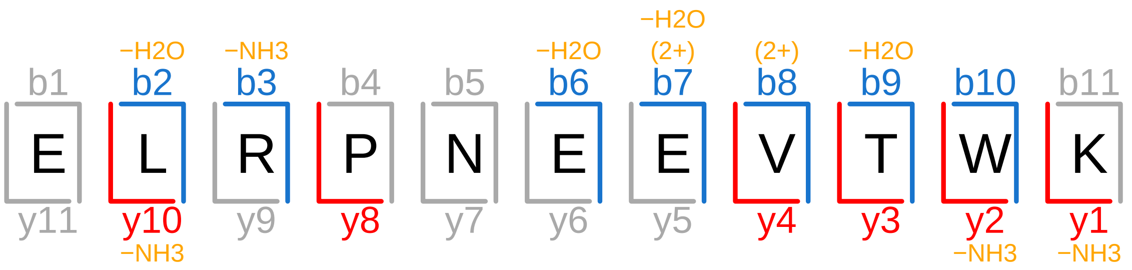

ELRPNEEVTWK
Score: 87 ; 1399.7096 m/z; 700.86207 m/z; 0.86205 ppm; MULTI−MSMS

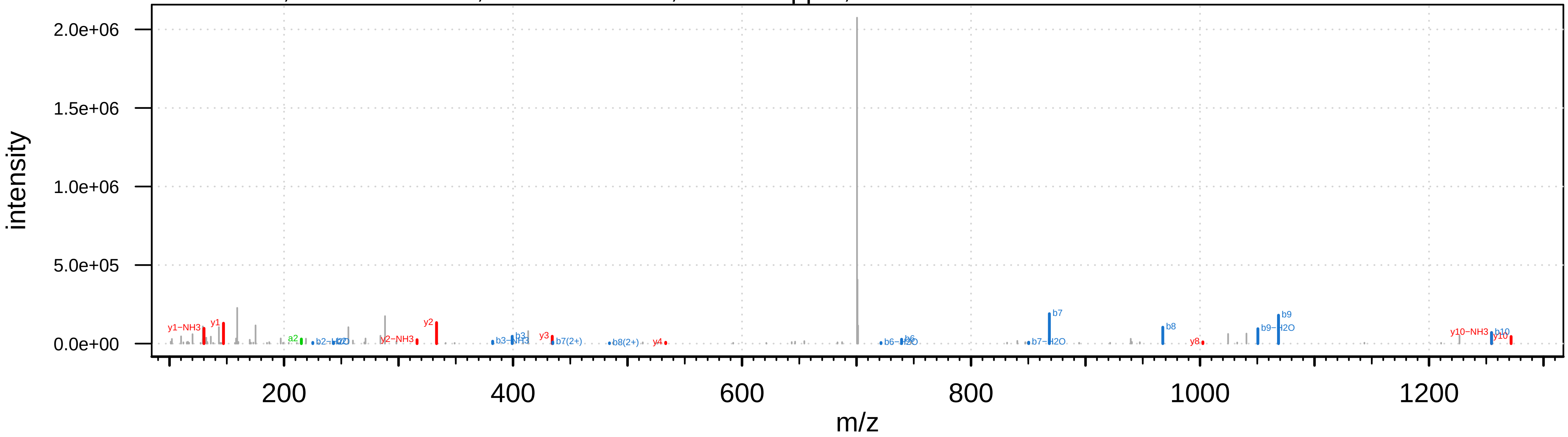

Raw File: 20130807_EXQ4_BaAz_SA_KR4_05
Scan Number: 31876
Proteins:
chr12_103206313−103214181.T2_chr12:101968174−101969629:−

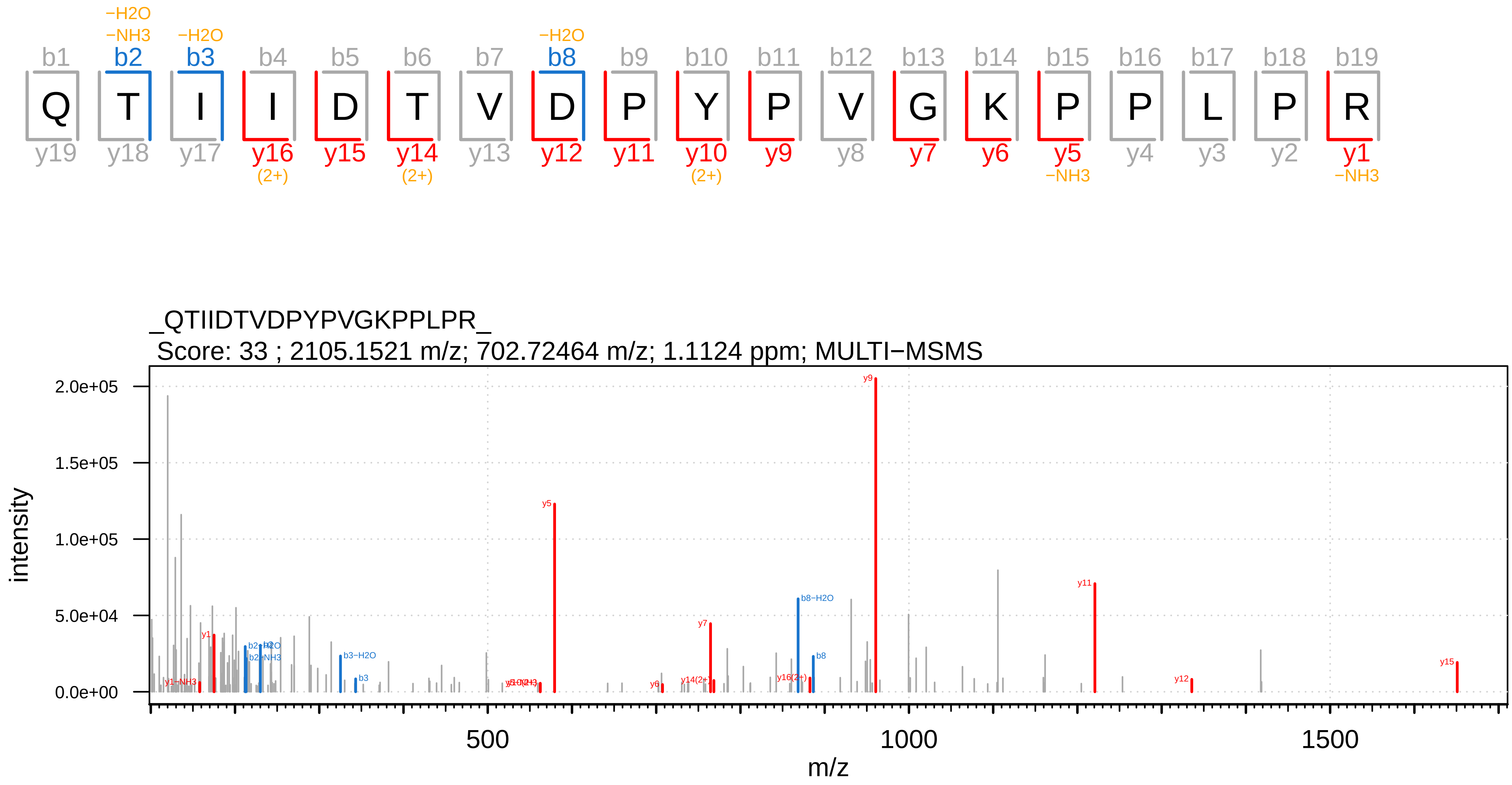

Raw File: 20130807_EXQ4_BaAz_SA_KR4_05
Scan Number: 67584
Proteins:
ENSMUST00000136126_chr3:79172848−79174982:−

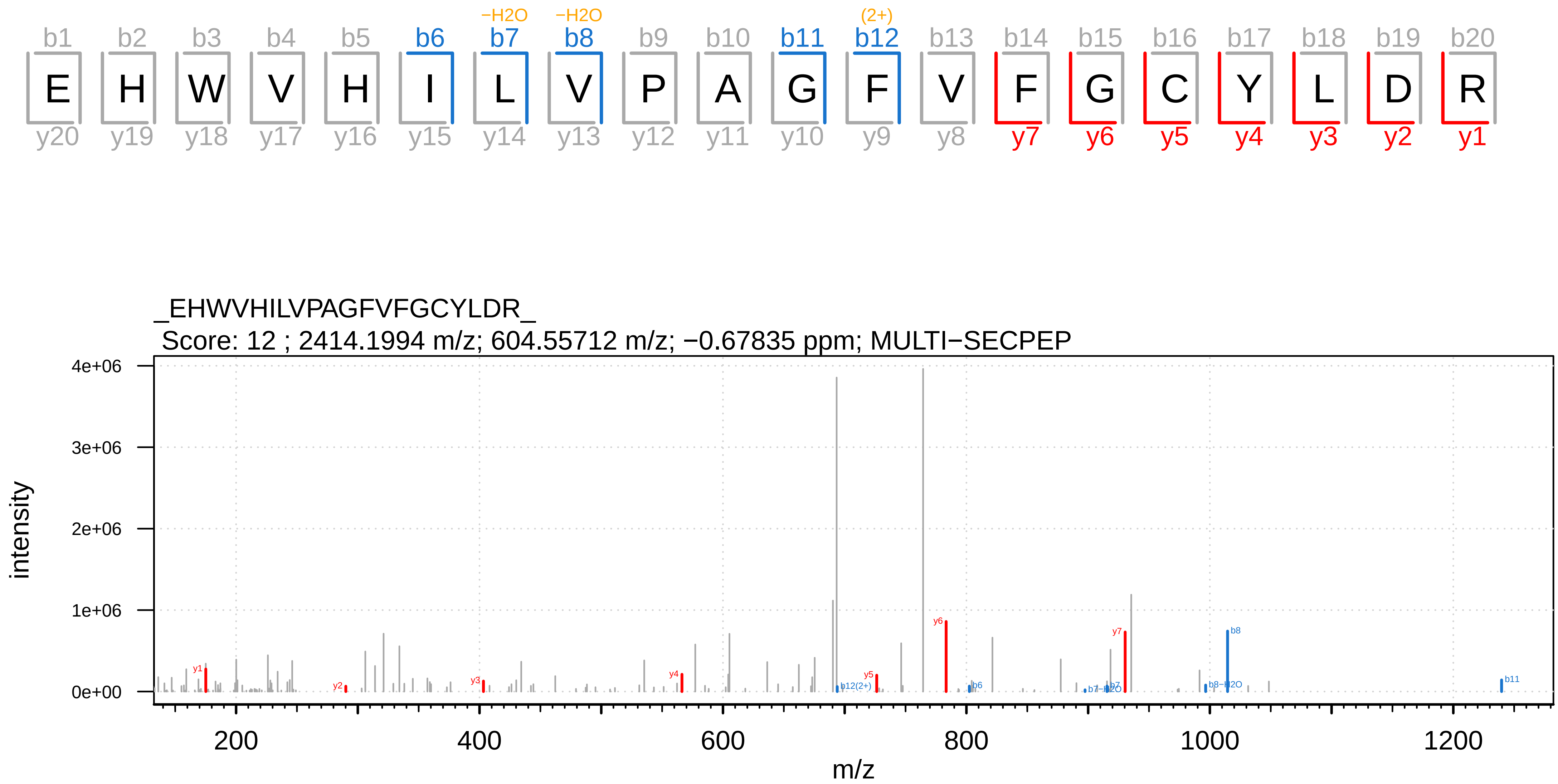

Raw File: 20140815_QEp4_BaAz_SA_HC_R1
Scan Number: 117685
Proteins:
chr12_103206313−103214181.T2_chr12:101968174−101969629:−

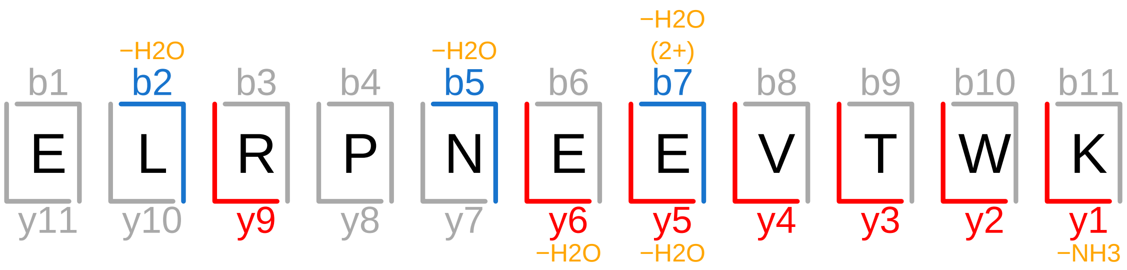

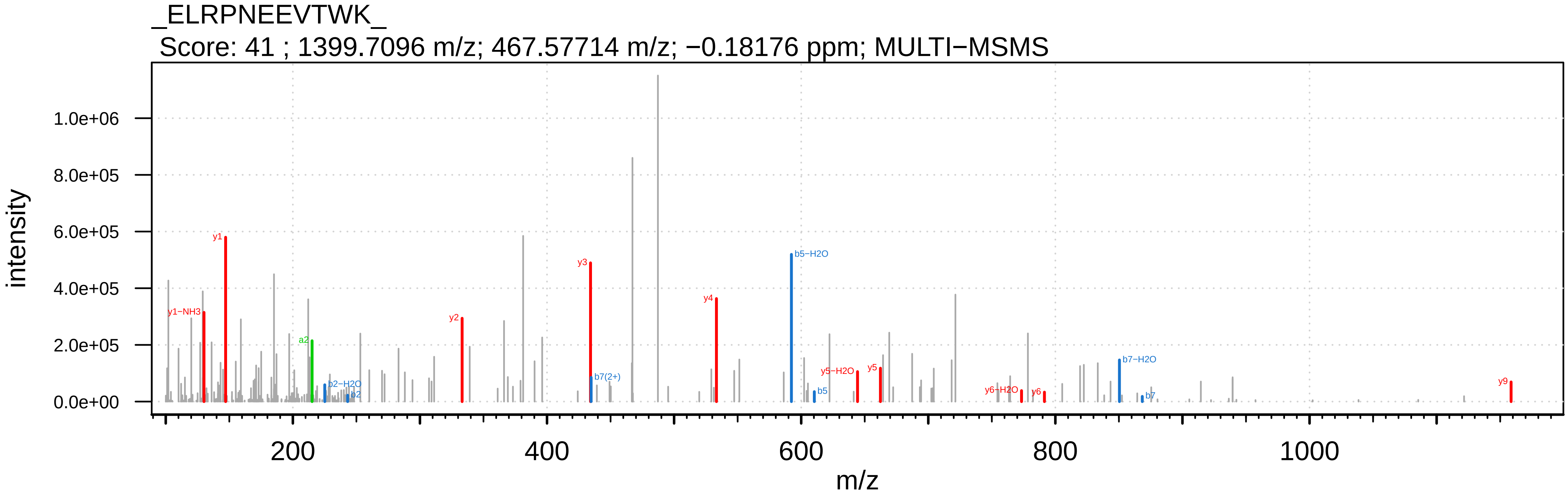

Raw File: 20140815_QEp4_BaAz_SA_HC_R1
Scan Number: 40527
Proteins:
chr12_103206313−103214181.T2_chr12:101968174−101969629:−

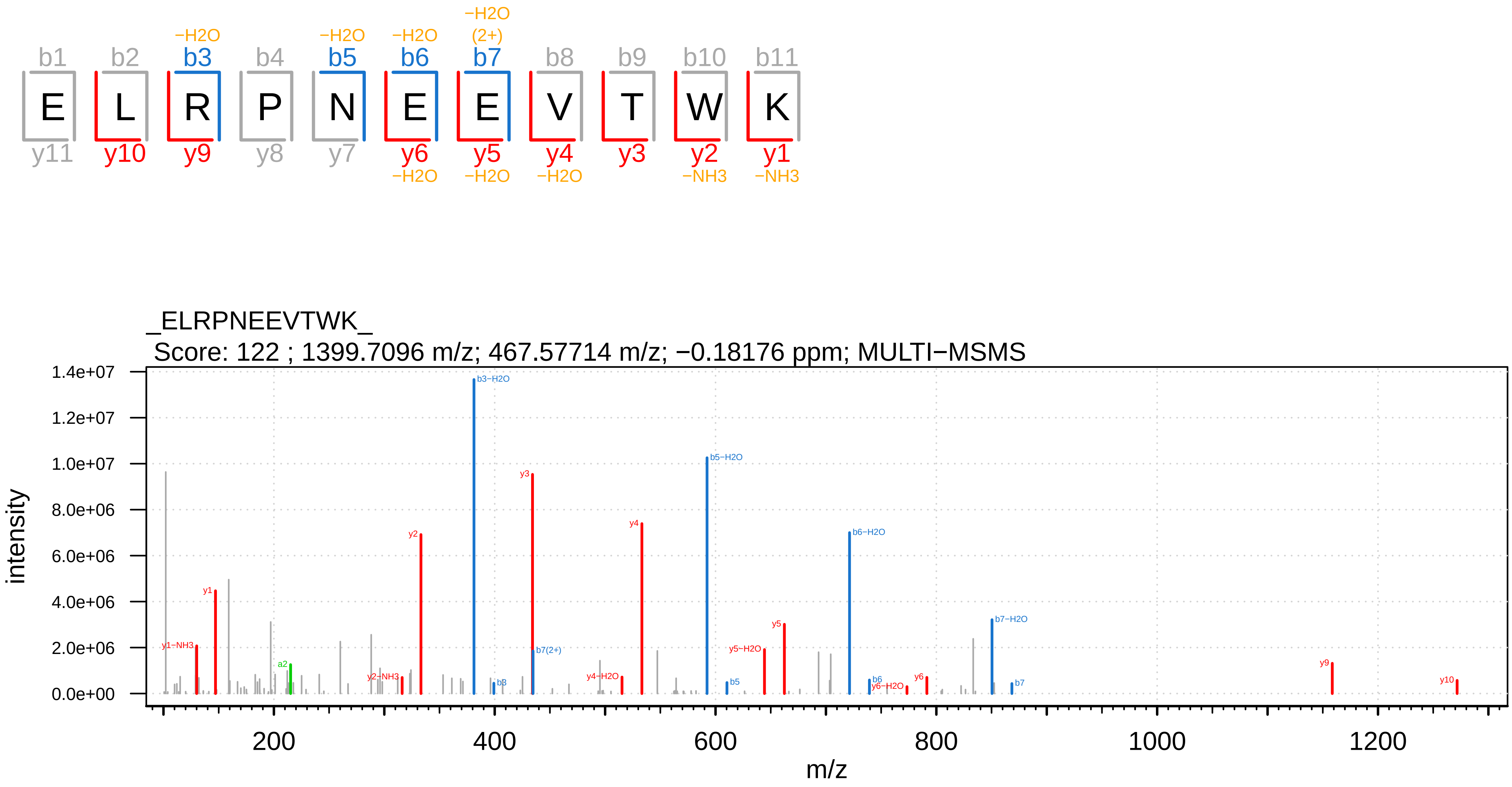

Raw File: 20140815_QEp4_BaAz_SA_HC_R1
Scan Number: 40872
Proteins:
chr12_103206313−103214181.T2_chr12:101968174−101969629:−

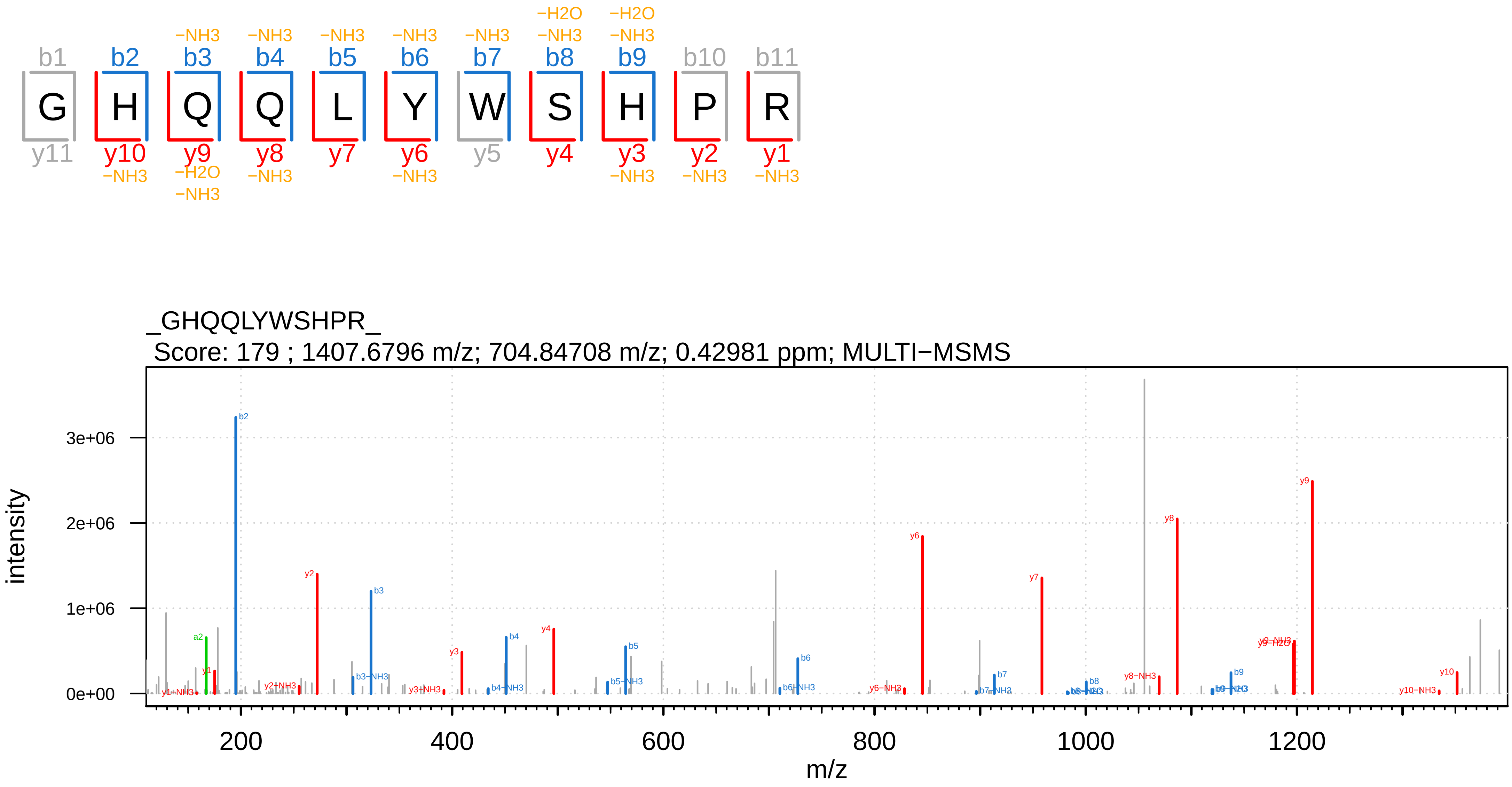

Raw File: 20140815_QEp4_BaAz_SA_HC_R1
Scan Number: 19923
Proteins:
sp|P62274|RS29_MOUSE
ENSMUST00000037023_chr12:69157813−69159058:−
ENSMUST00000071737_chr13:73988963−73989133:−

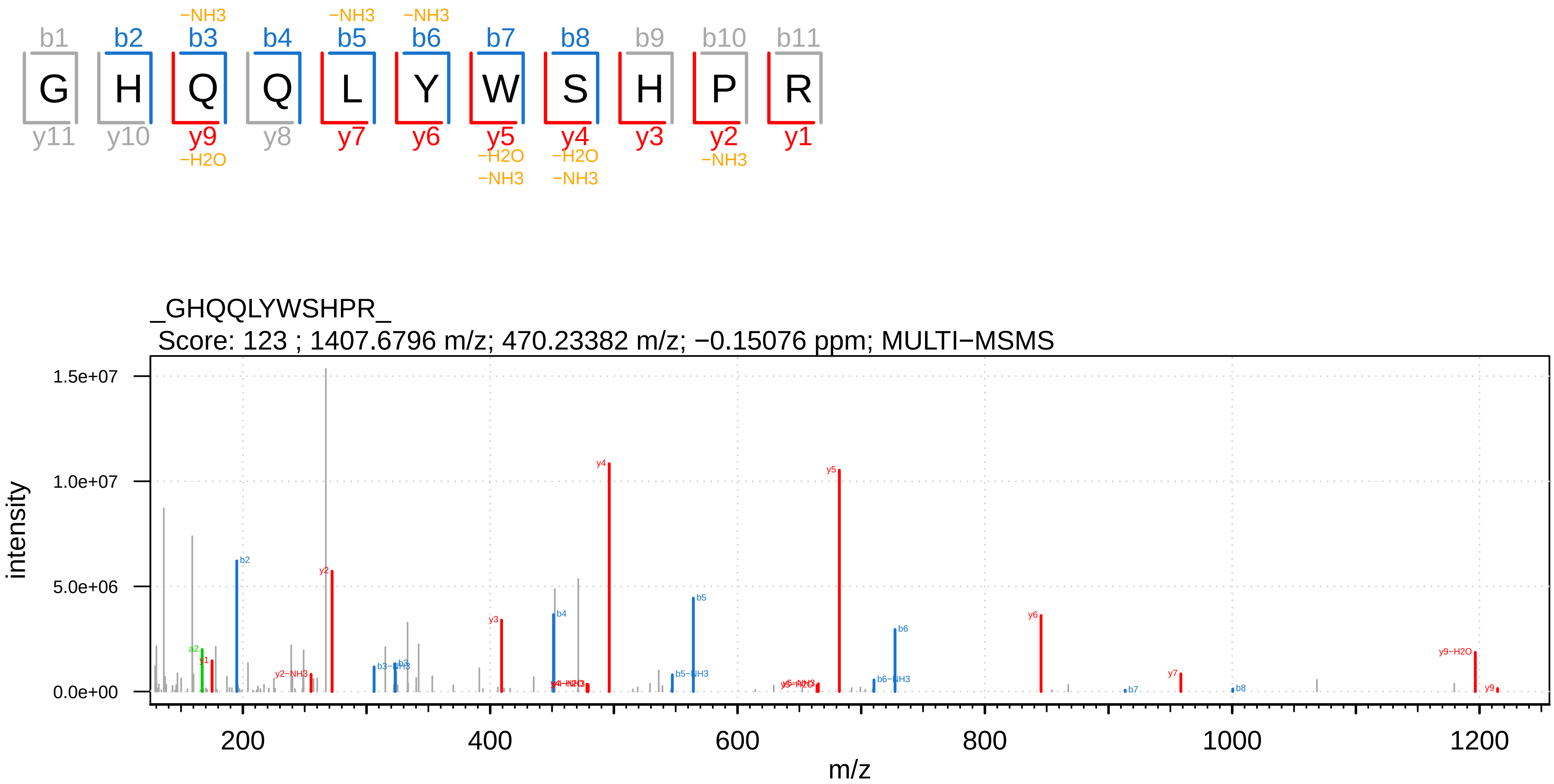

Raw File: 20140815_QEp4_BaAz_SA_HC_R1
Scan Number: 19978
Proteins:
sp|P62274|RS29_MOUSE
ENSMUST00000037023_chr12:69157813−69159058:−
ENSMUST00000071737_chr13:73988963−73989133:−

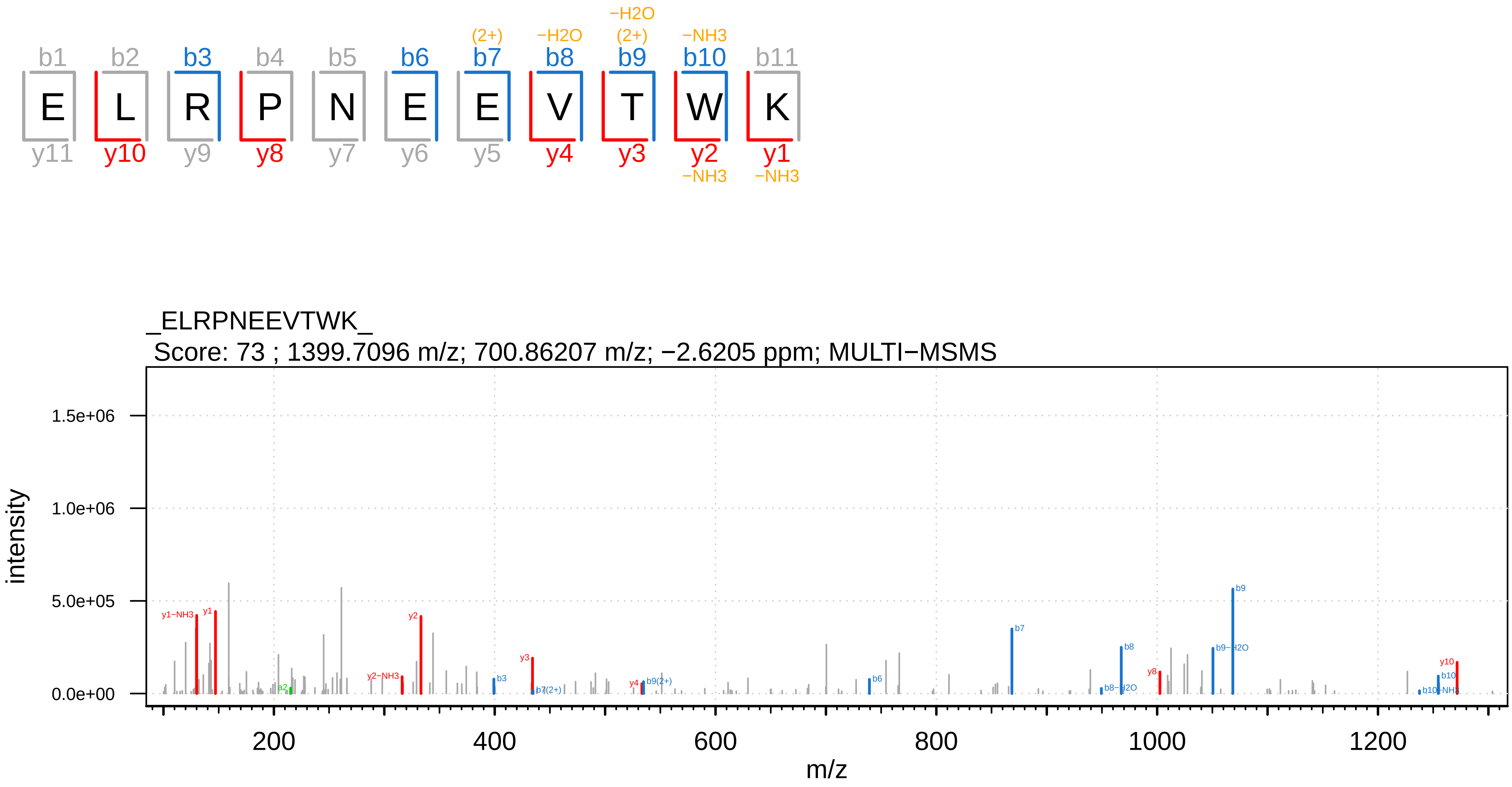

Raw File: 20130408_EXQ6_BaAz_SA_H16R1_04
Scan Number: 37444
Proteins:
chr12_103206313−103214181.T2_chr12:101968174−101969629:−

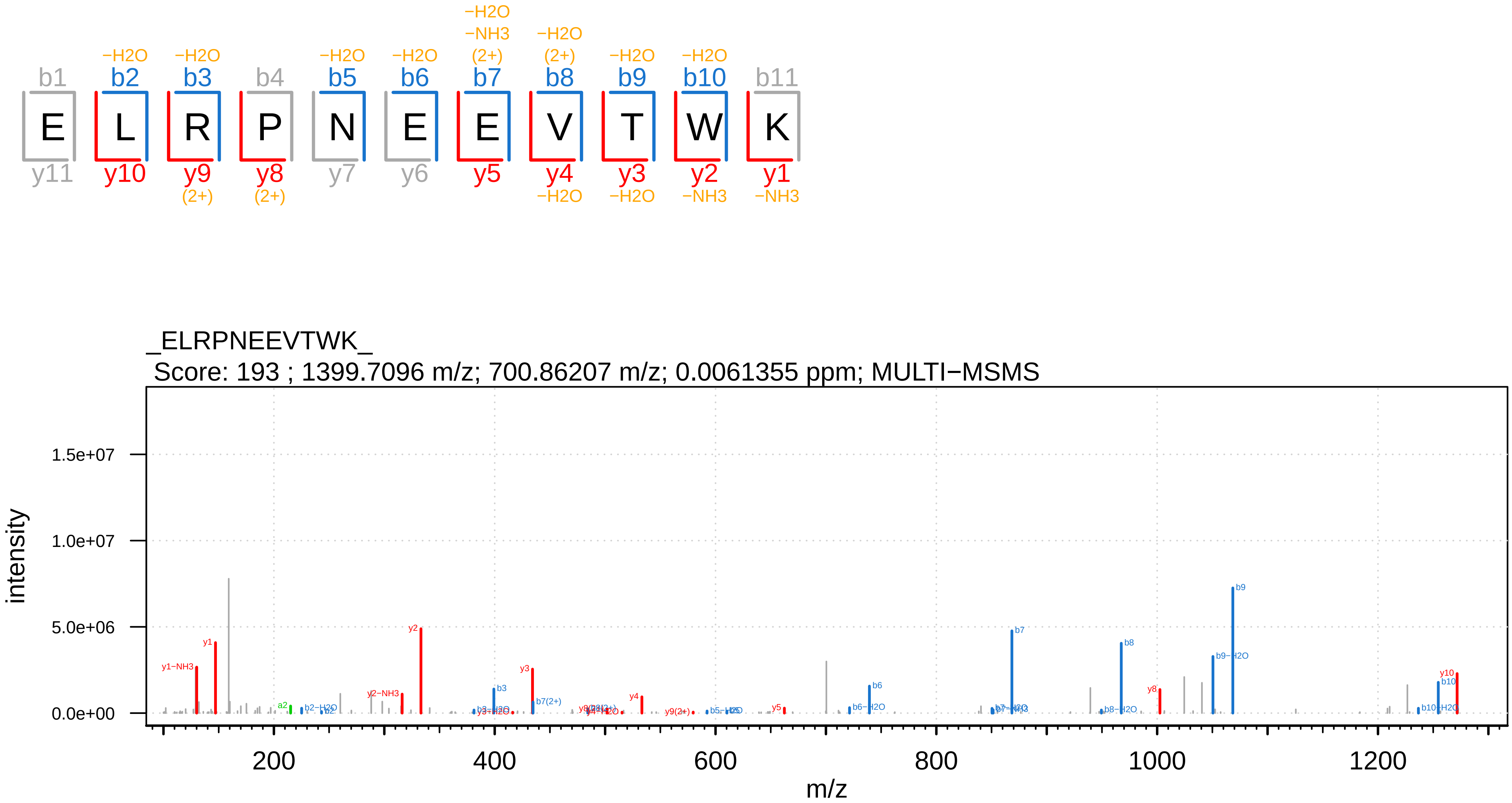

Raw File: 20130408_EXQ6_BaAz_SA_H16R1_05

Scan Number: 40396

Proteins:

chr12_103206313−103214181.T2_chr12:101968174−101969629:−

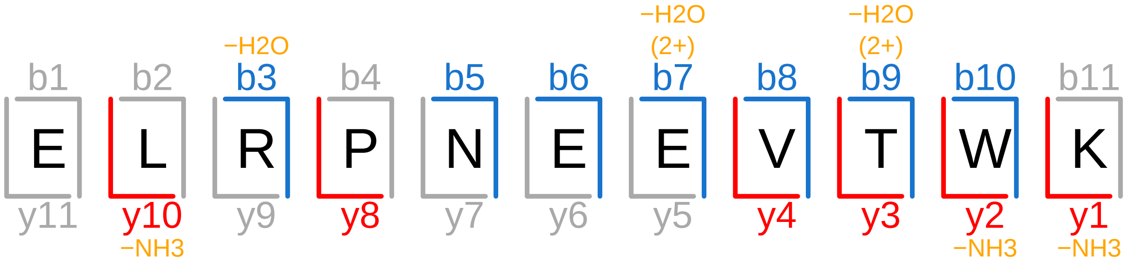

ELRPNEEVTWK

Score: 108 ; 1399.7096 m/z; 700.86207 m/z; −0.10369 ppm; MULTI−MSMS

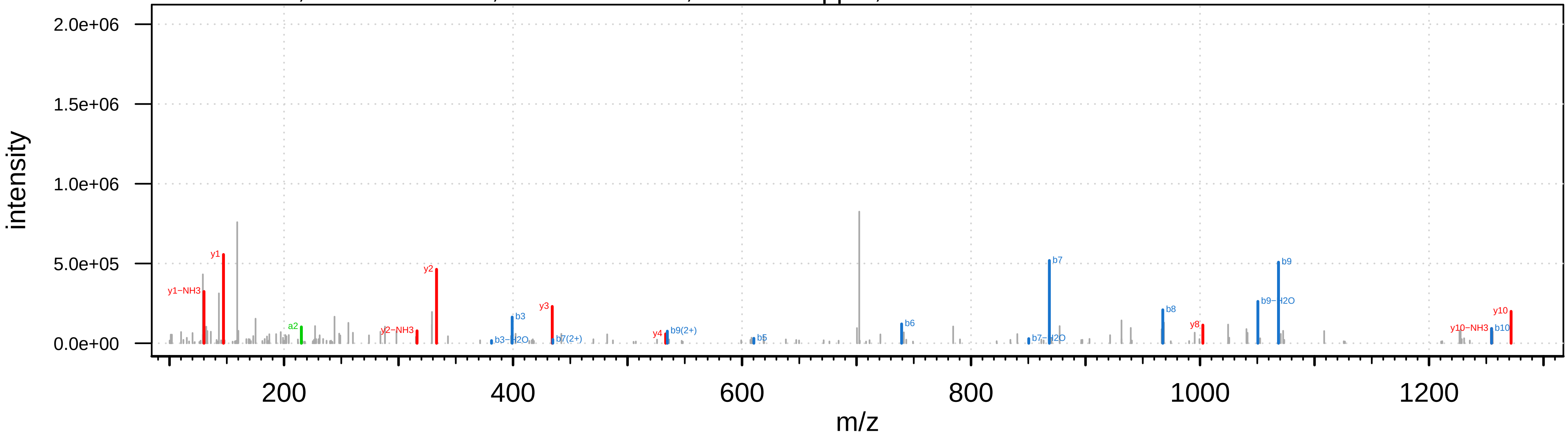

Raw File: 20130409_EXQ6_BaAz_SA_K66R1_04
Scan Number: 37368
Proteins:
chr12_103206313−103214181.T2_chr12:101968174−101969629:−

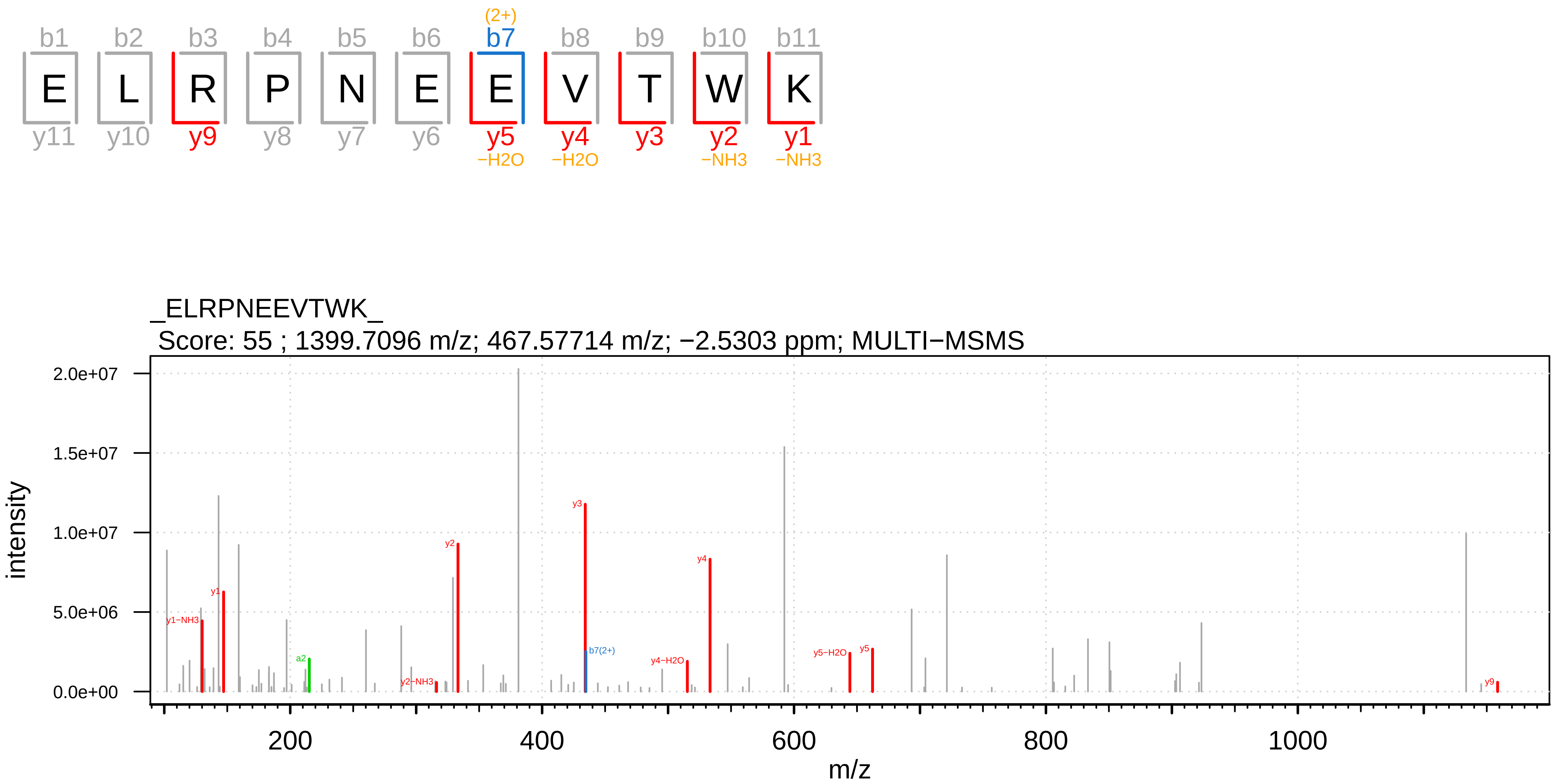

Raw File: 20130409_EXQ6_BaAz_SA_K66R1_05
Scan Number: 37782
Proteins:
chr12_103206313−103214181.T2_chr12:101968174−101969629:−

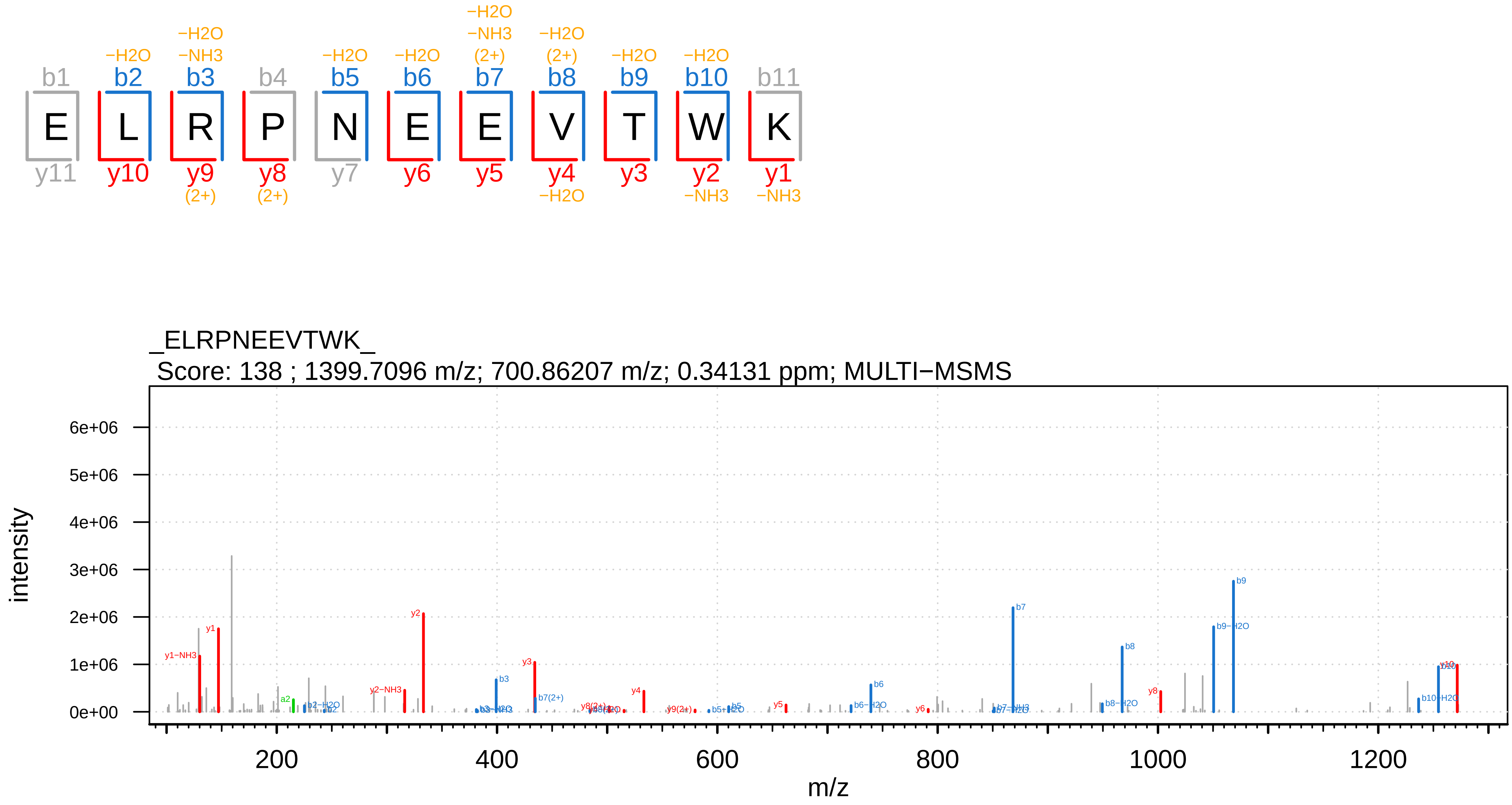

Raw File: 20130409_EXQ6_BaAz_SA_K66R1_06
Scan Number: 34656
Proteins:
chr12_103206313−103214181.T2_chr12:101968174−101969629:−

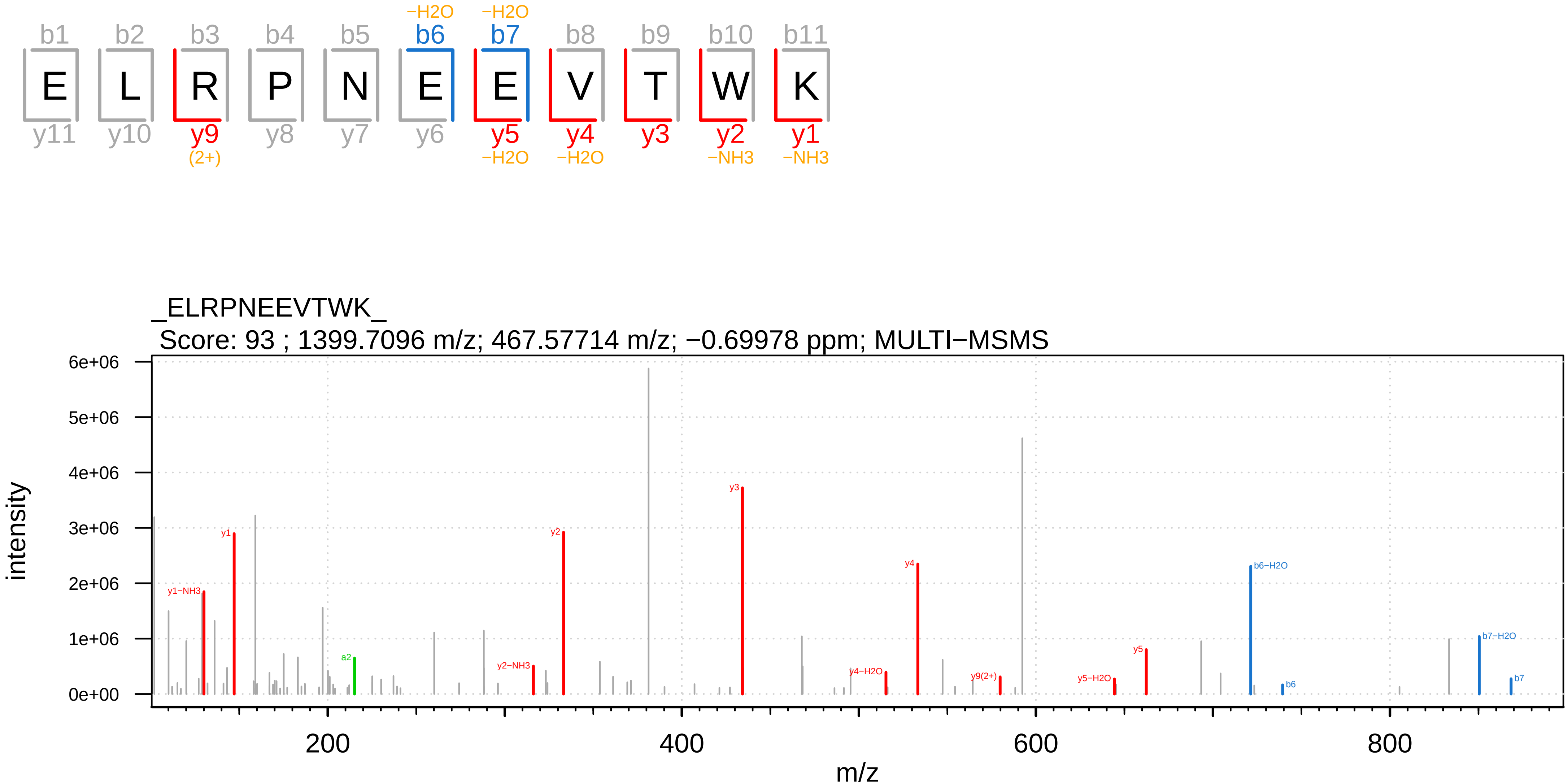

Raw File: 20130409_EXQ6_BaAz_SA_K66R1_06
Scan Number: 34671
Proteins:
chr12_103206313−103214181.T2_chr12:101968174−101969629:−

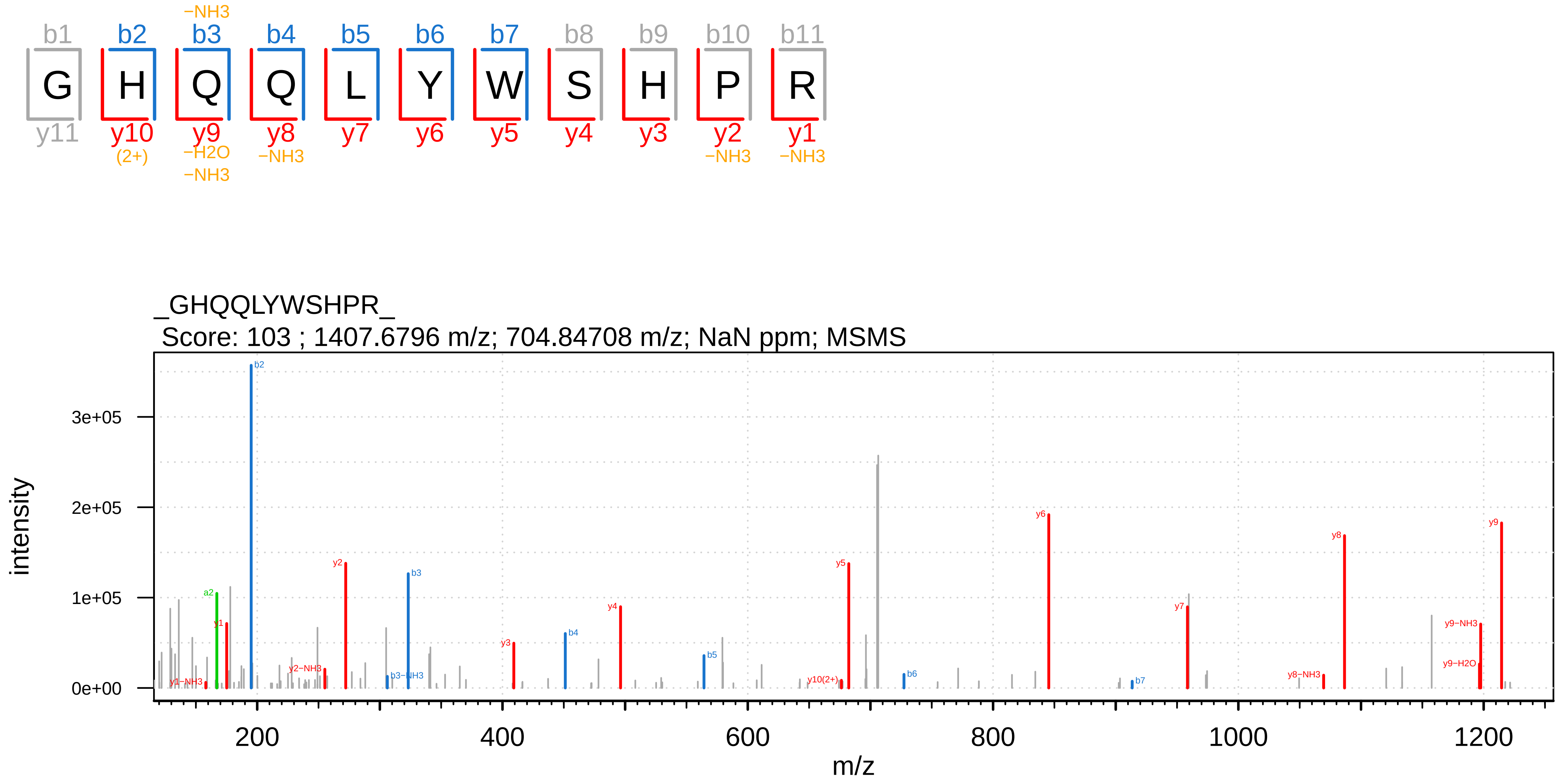

Raw File: 20130409_EXQ6_BaAz_SA_K66R1_06
Scan Number: 20808
Proteins:
sp|P62274|RS29_MOUSE
ENSMUST00000037023_chr12:69157813−69159058:−
ENSMUST00000071737_chr13:73988963−73989133:−

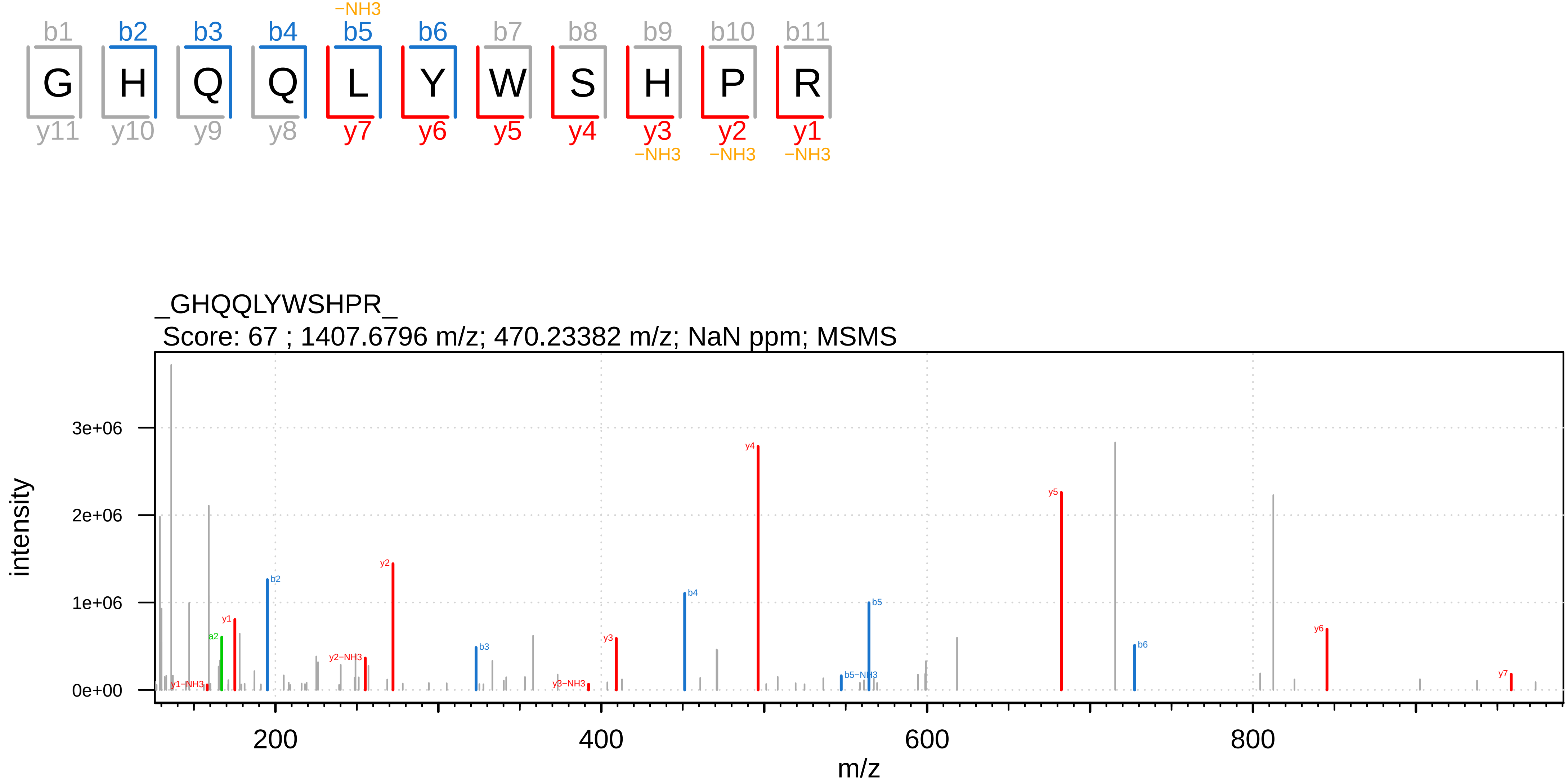

Raw File: 20130409_EXQ6_BaAz_SA_K66R1_06
Scan Number: 20825
Proteins:
sp|P62274|RS29_MOUSE
ENSMUST00000037023_chr12:69157813−69159058:−
ENSMUST00000071737_chr13:73988963−73989133:−

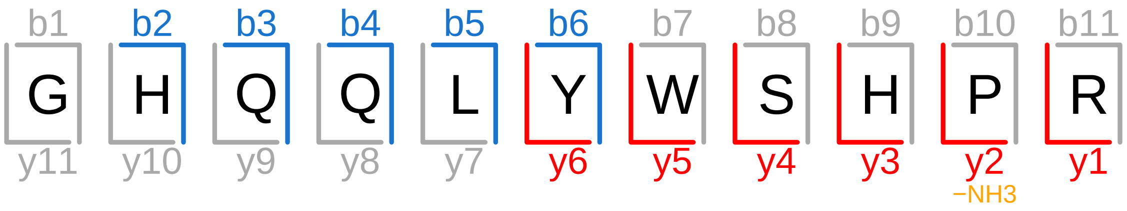

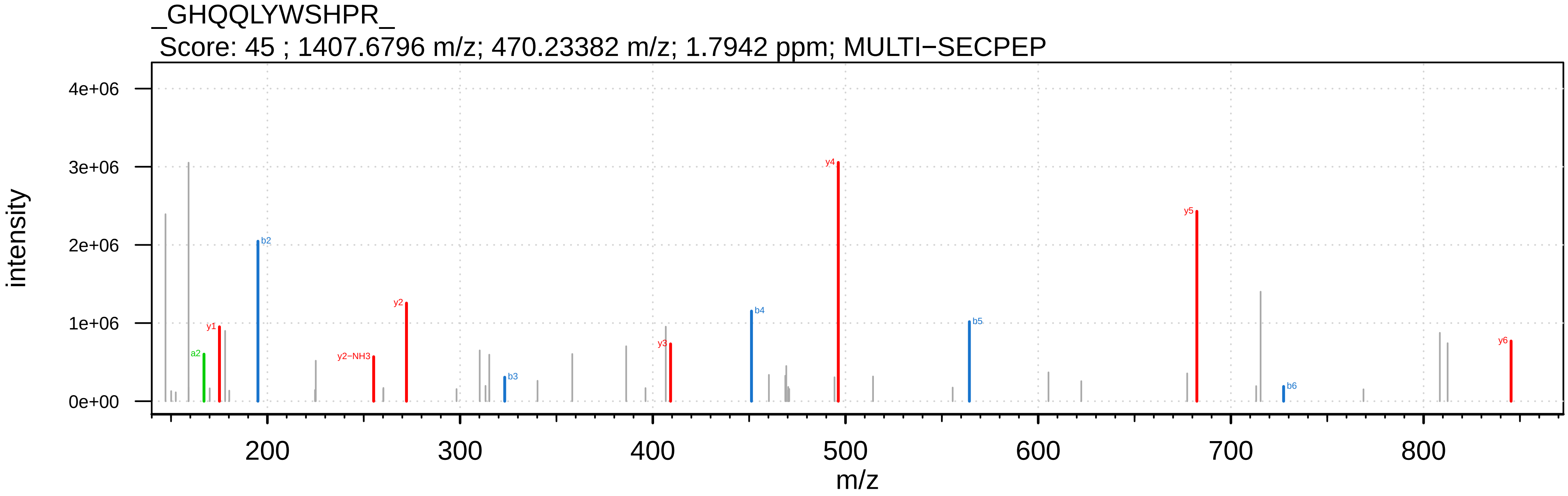

Raw File: 20130409_EXQ6_BaAz_SA_K66R1_06
Scan Number: 20979
Proteins:
sp|P62274|RS29_MOUSE
ENSMUST00000037023_chr12:69157813−69159058:−
ENSMUST00000071737_chr13:73988963−73989133:−

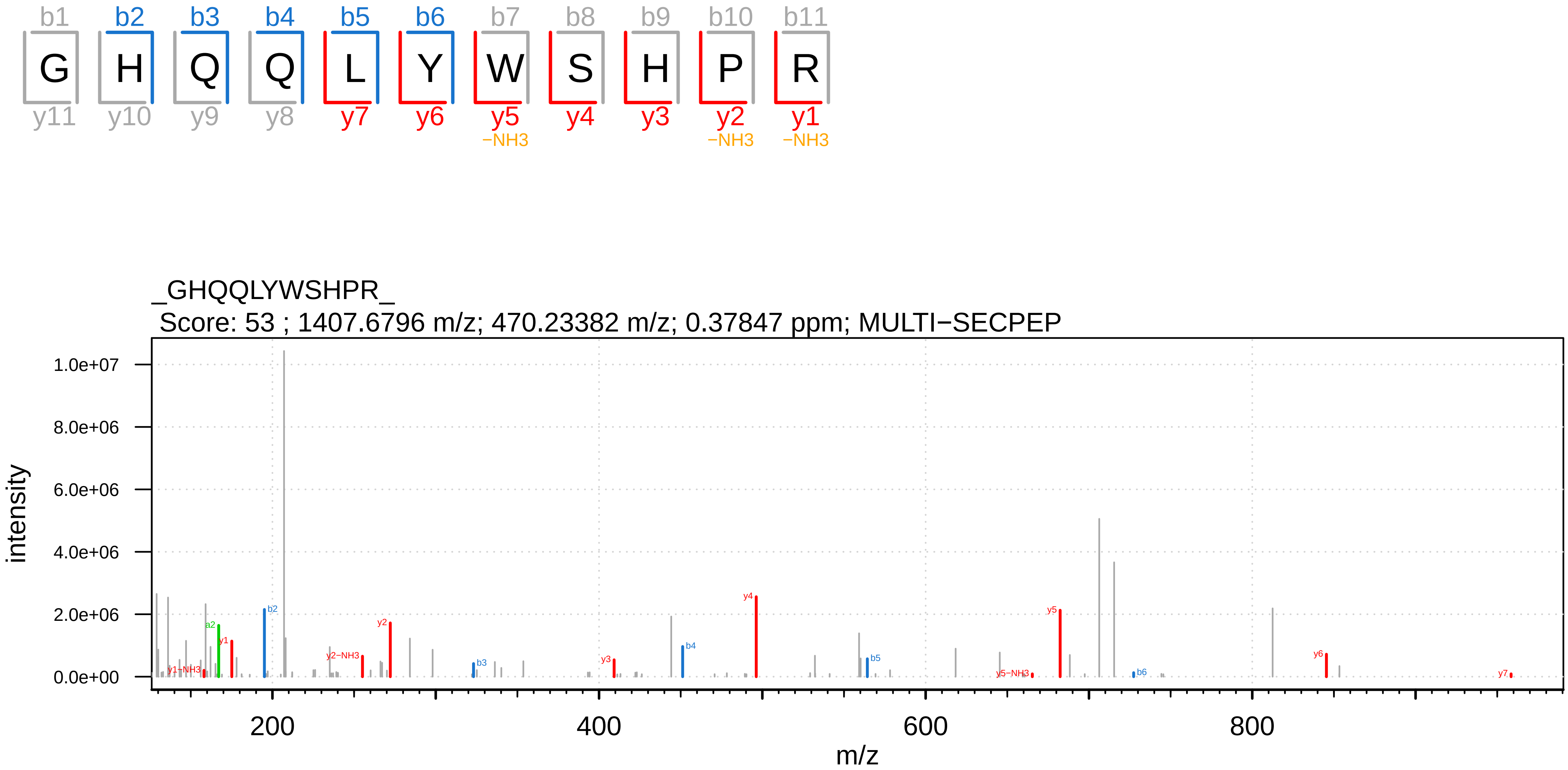

Raw File: 20130409_EXQ6_BaAz_SA_K66R1_06
Scan Number: 21340
Proteins:
sp|P62274|RS29_MOUSE
ENSMUST00000037023_chr12:69157813−69159058:−
ENSMUST00000071737_chr13:73988963−73989133:−

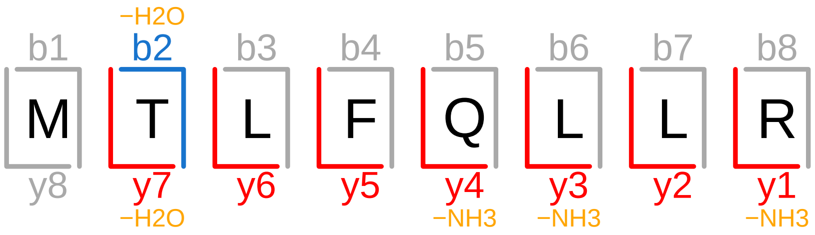

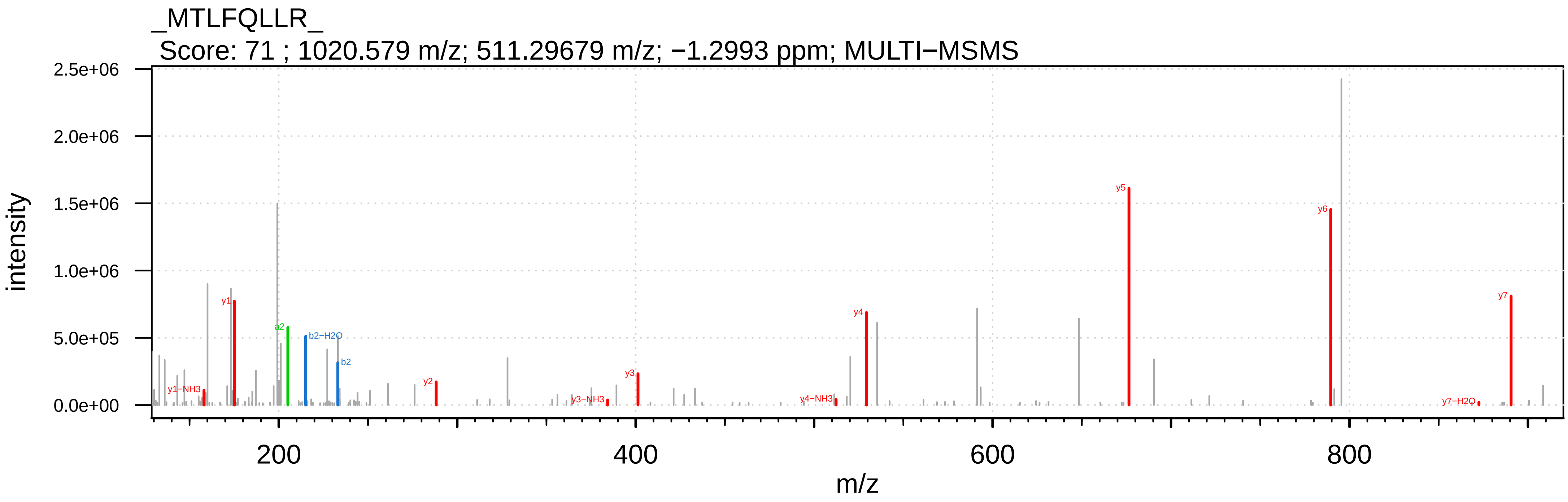

Raw File: 20130409_EXQ6_BaAz_SA_K66R1_06
Scan Number: 92980
Proteins:
chr12_103206313−103214181.T2_chr12:101968174−101969629:−

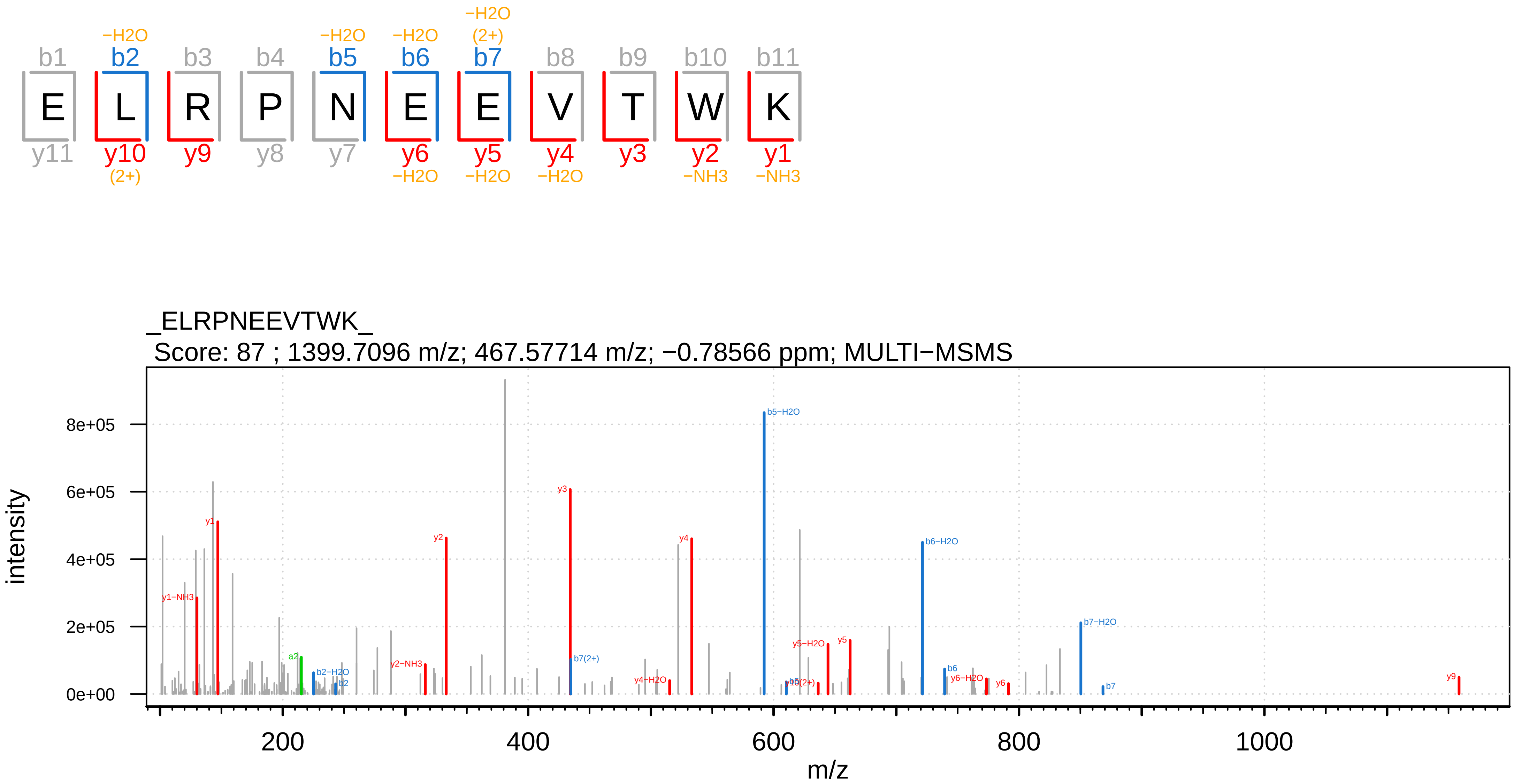

Raw File: 20130411_EXQ6_BaAz_SA_LR1_03
Scan Number: 35168
Proteins:
chr12_103206313−103214181.T2_chr12:101968174−101969629:−

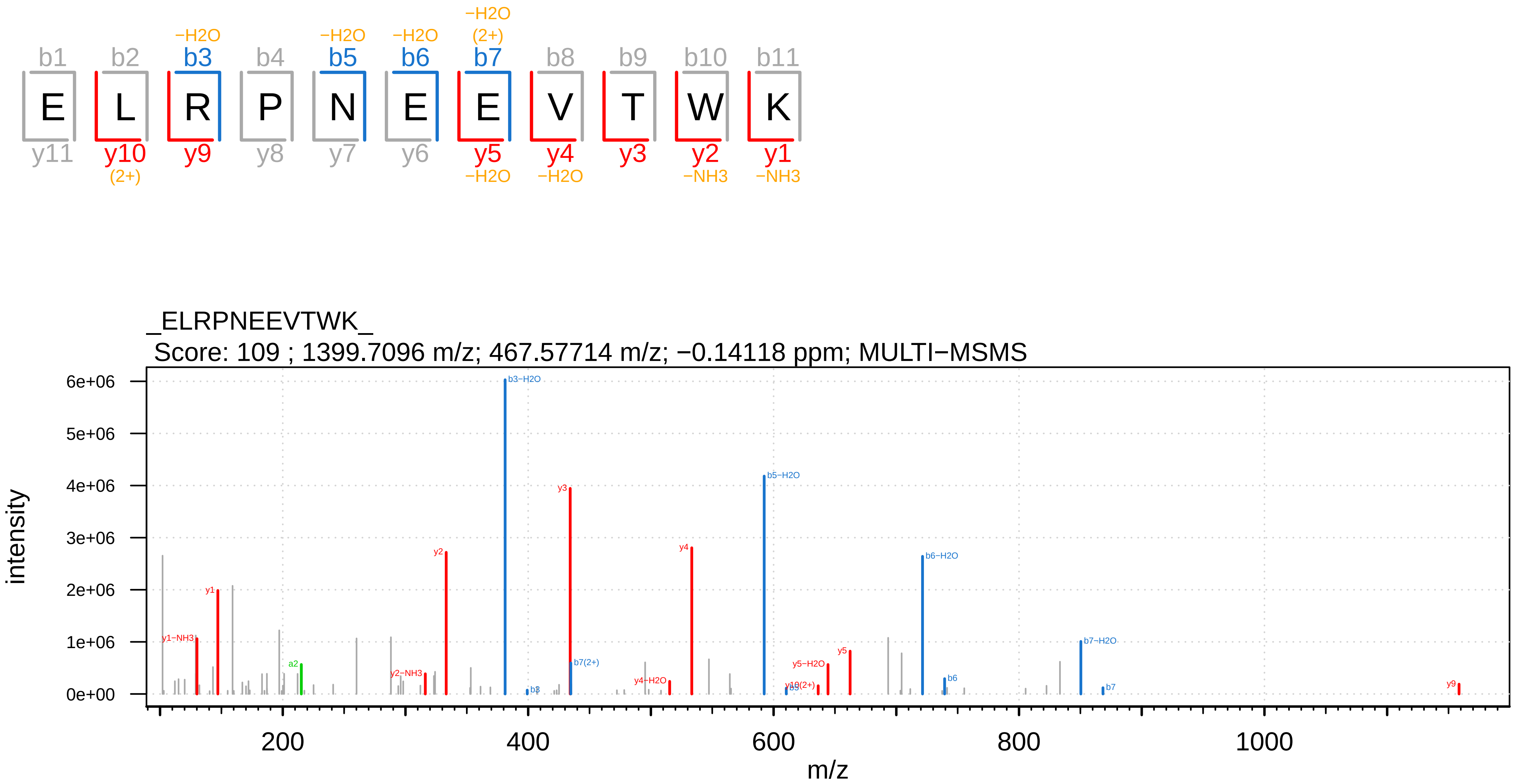

Raw File: 20130411_EXQ6_BaAz_SA_LR1_04
Scan Number: 39807
Proteins:
chr12_103206313−103214181.T2_chr12:101968174−101969629:−

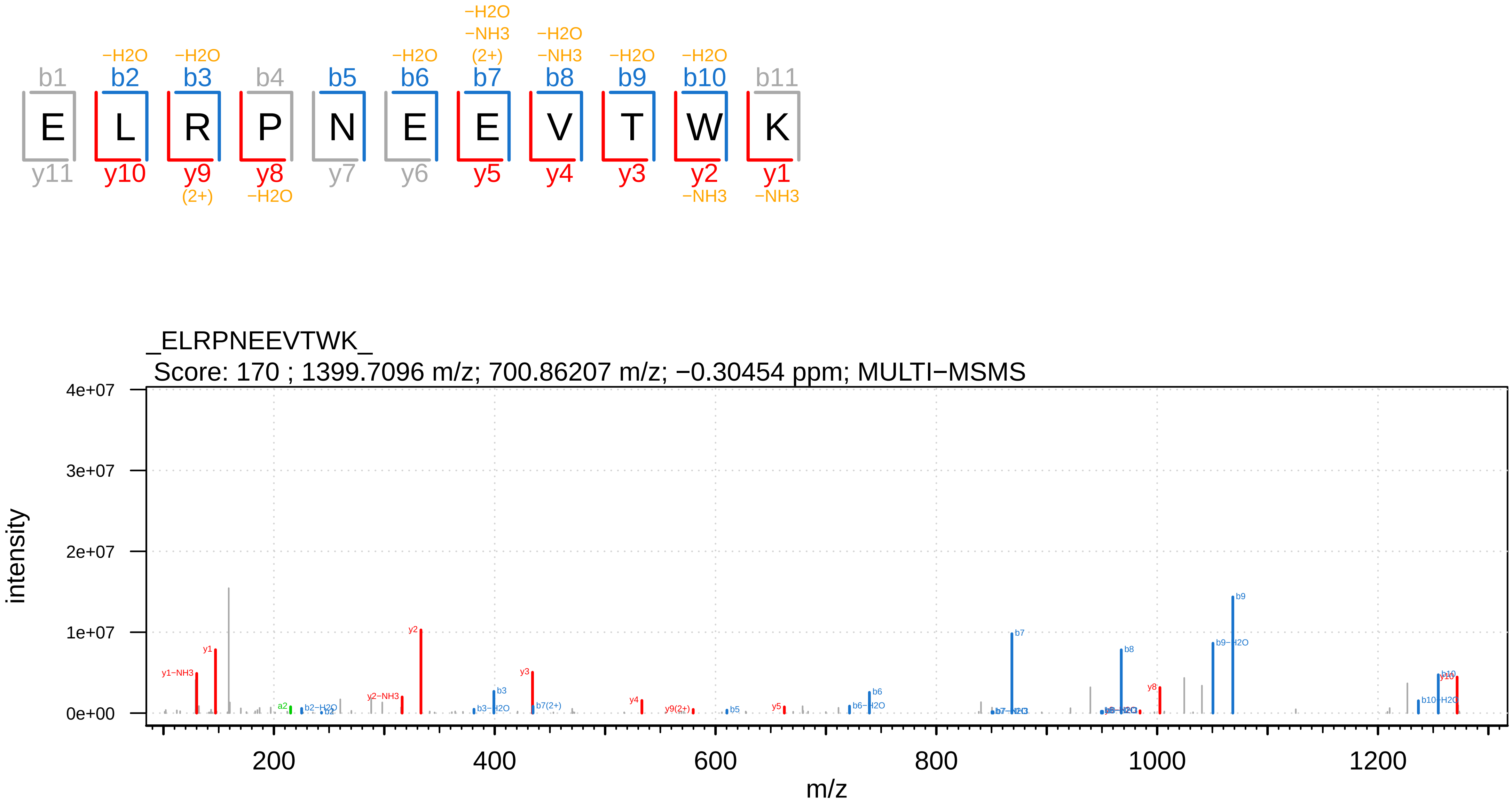

Raw File: 20130411_EXQ6_BaAz_SA_LR1_05
Scan Number: 37439
Proteins:
chr12_103206313−103214181.T2_chr12:101968174−101969629:−

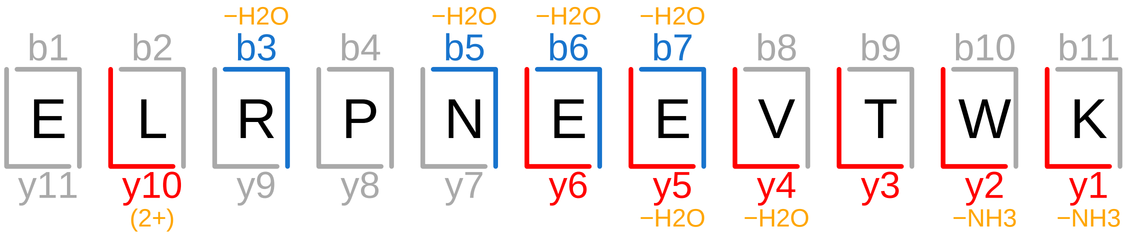

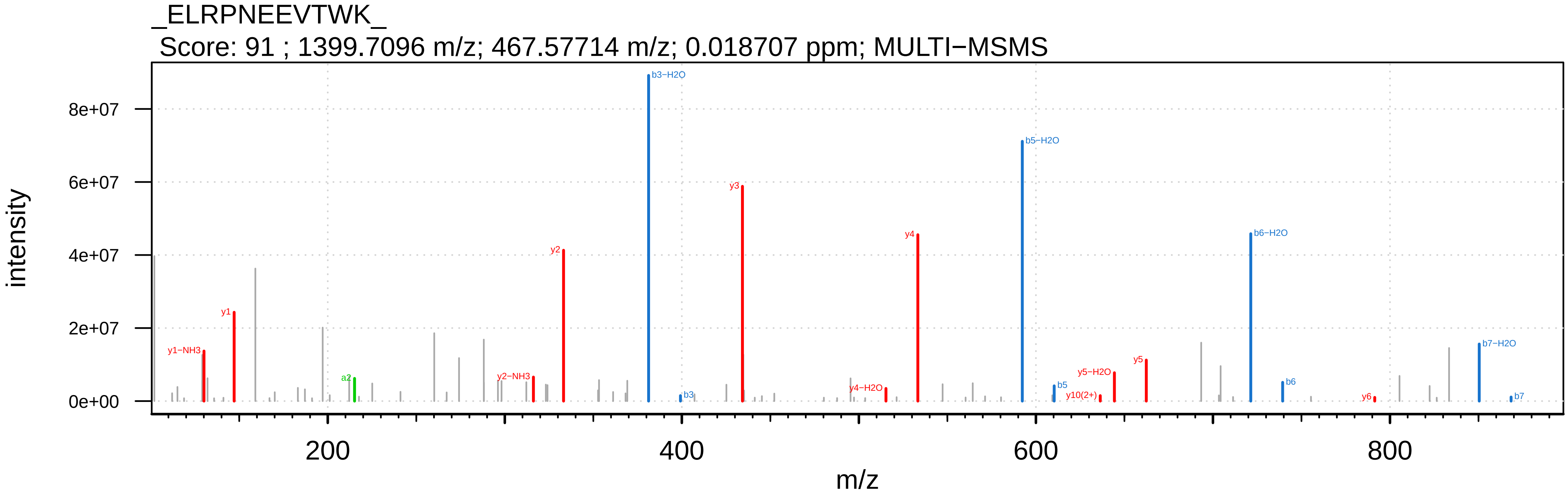

Raw File: 20130411_EXQ6_BaAz_SA_LR1_05
Scan Number: 37457
Proteins:
chr12_103206313−103214181.T2_chr12:101968174−101969629:−

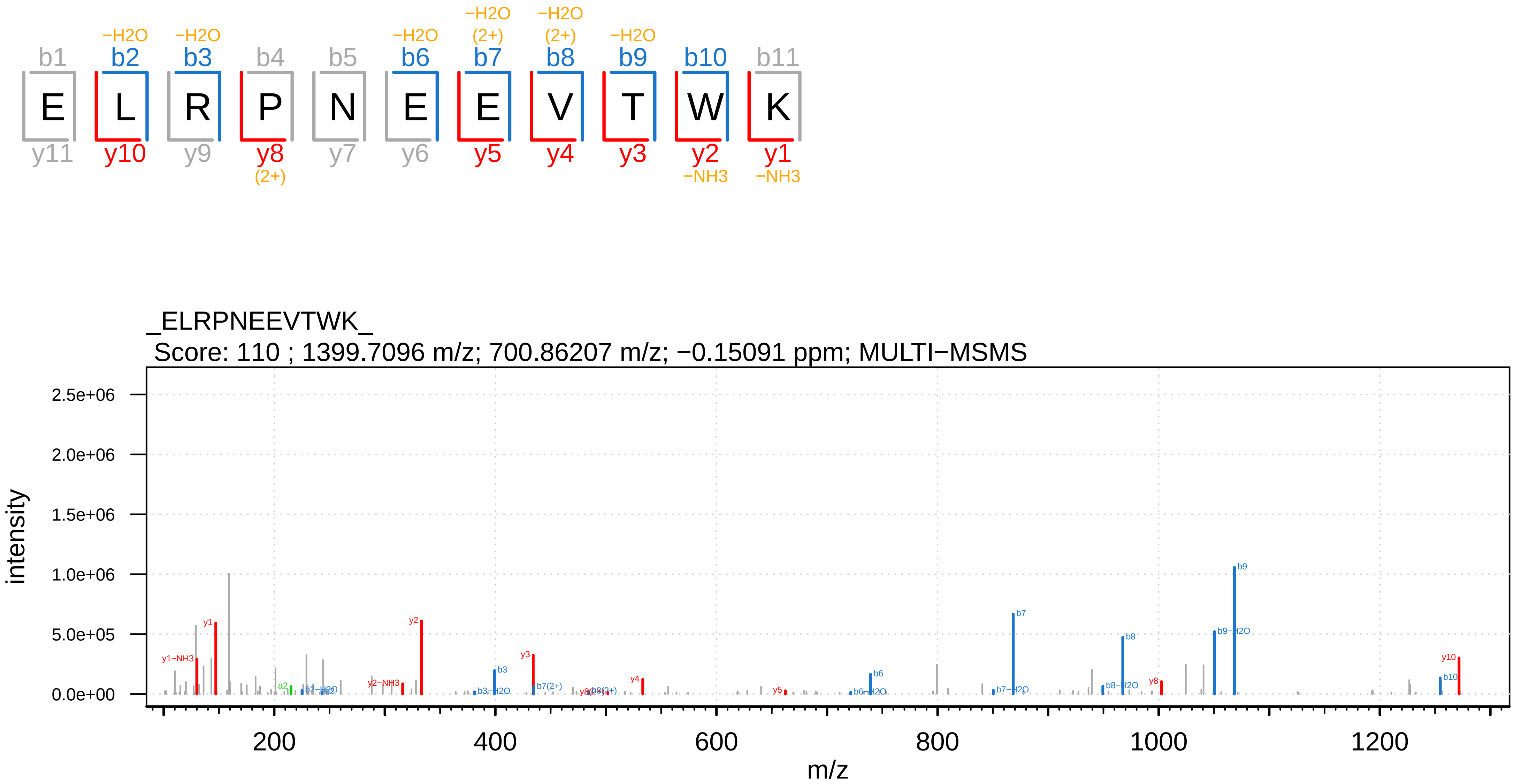

Raw File: 20130411_EXQ6_BaAz_SA_LR1_06
Scan Number: 35460
Proteins:
chr12_103206313−103214181.T2_chr12:101968174−101969629:−

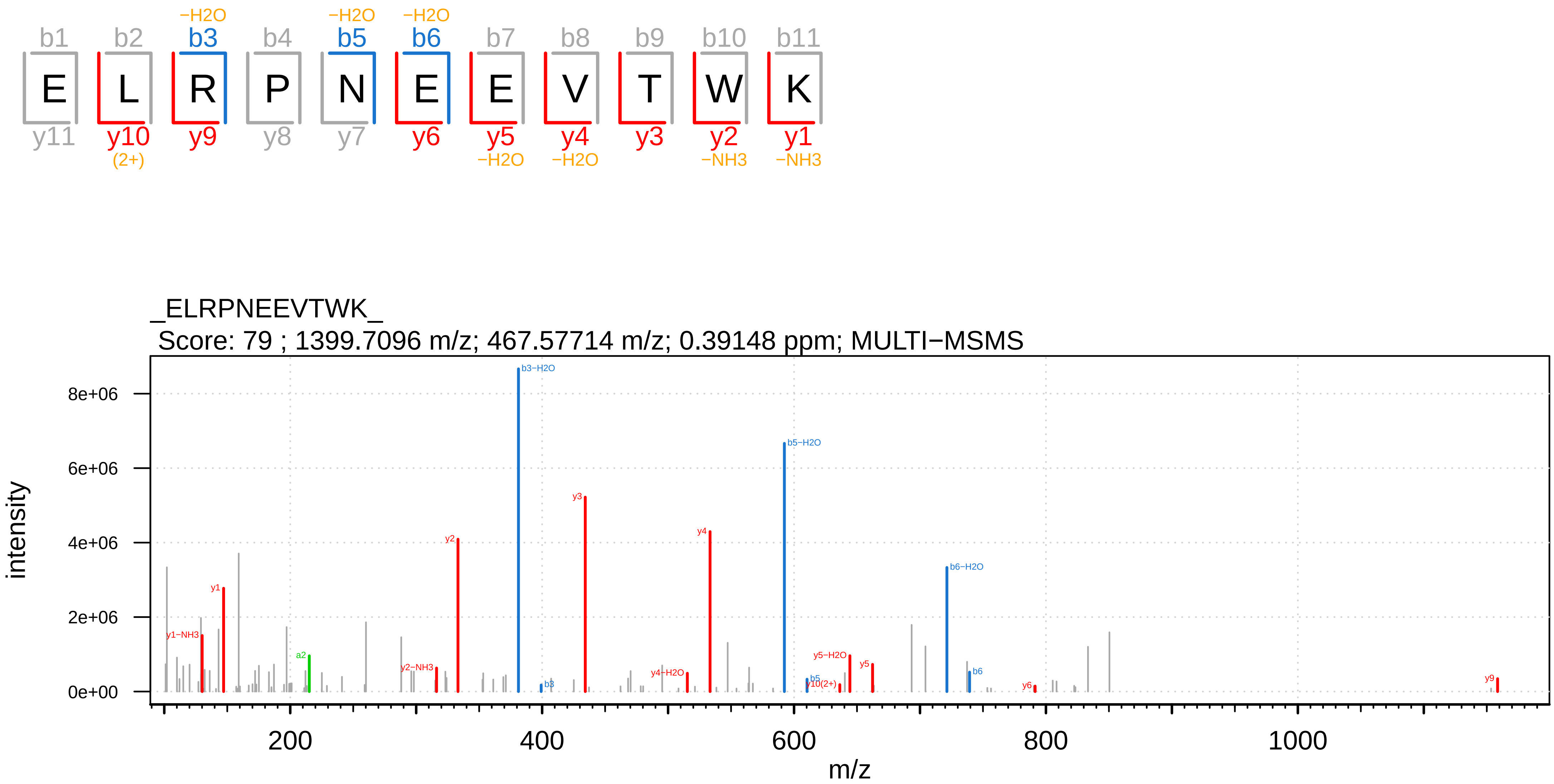

Raw File: 20130411_EXQ6_BaAz_SA_LR1_06
Scan Number: 35478
Proteins:
chr12_103206313−103214181.T2_chr12:101968174−101969629:−

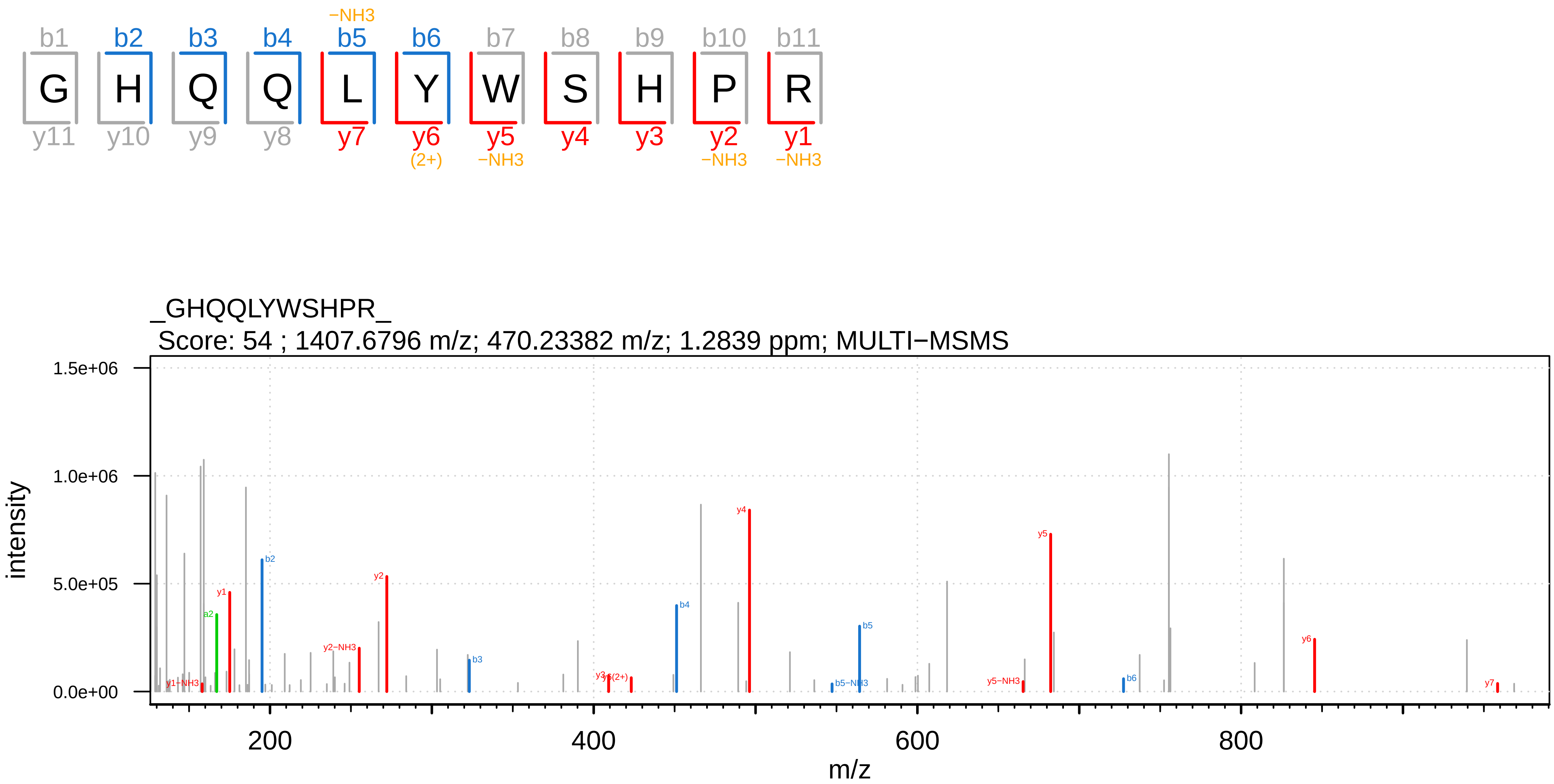

Raw File: 20130411_EXQ6_BaAz_SA_LR1_06
Scan Number: 19555
Proteins:
sp|P62274|RS29_MOUSE
ENSMUST00000037023_chr12:69157813−69159058:−
ENSMUST00000071737_chr13:73988963−73989133:−

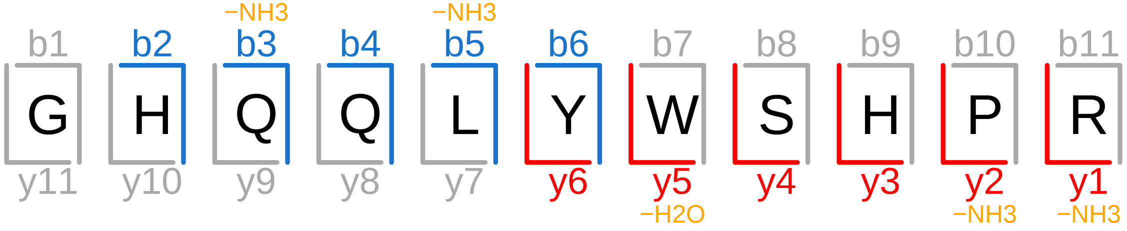

GHQQLYWSHPR

Score: 50 ; 1407.6796 m/z; 470.23382 m/z; 1.0788 ppm; MULTI−SECPEP

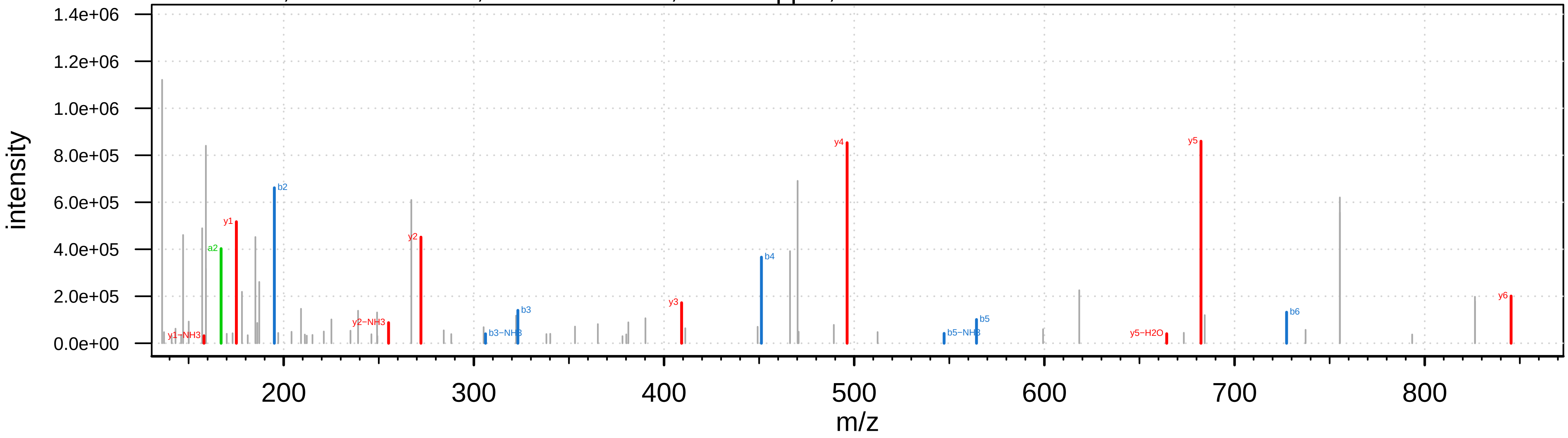

Raw File: 20130411_EXQ6_BaAz_SA_LR1_06

Scan Number: 19592

Proteins:

sp|P62274|RS29_MOUSE

ENSMUST00000037023_chr12:69157813−69159058:−

ENSMUST00000071737_chr13:73988963−73989133:−

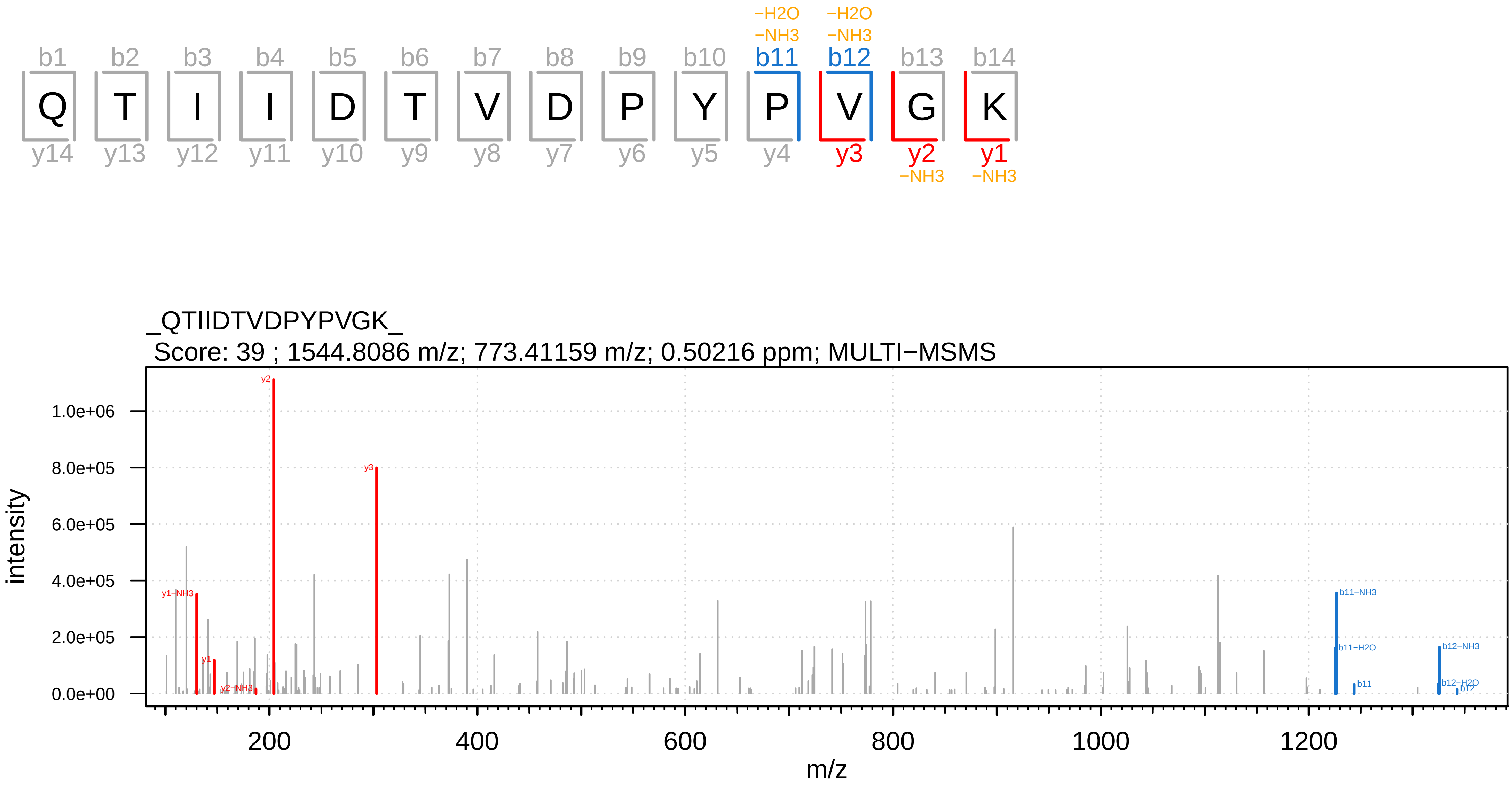

Raw File: 20130411_EXQ6_BaAz_SA_LR1_06
Scan Number: 62023
Proteins:
ENSMUST00000136126_chr3:79172848−79174982:−

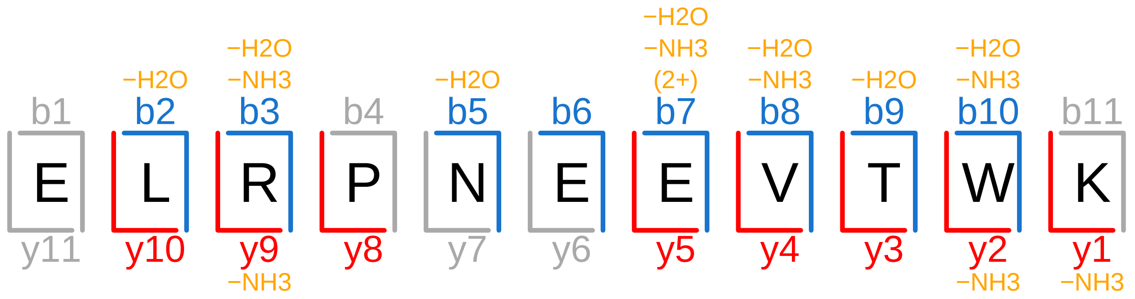

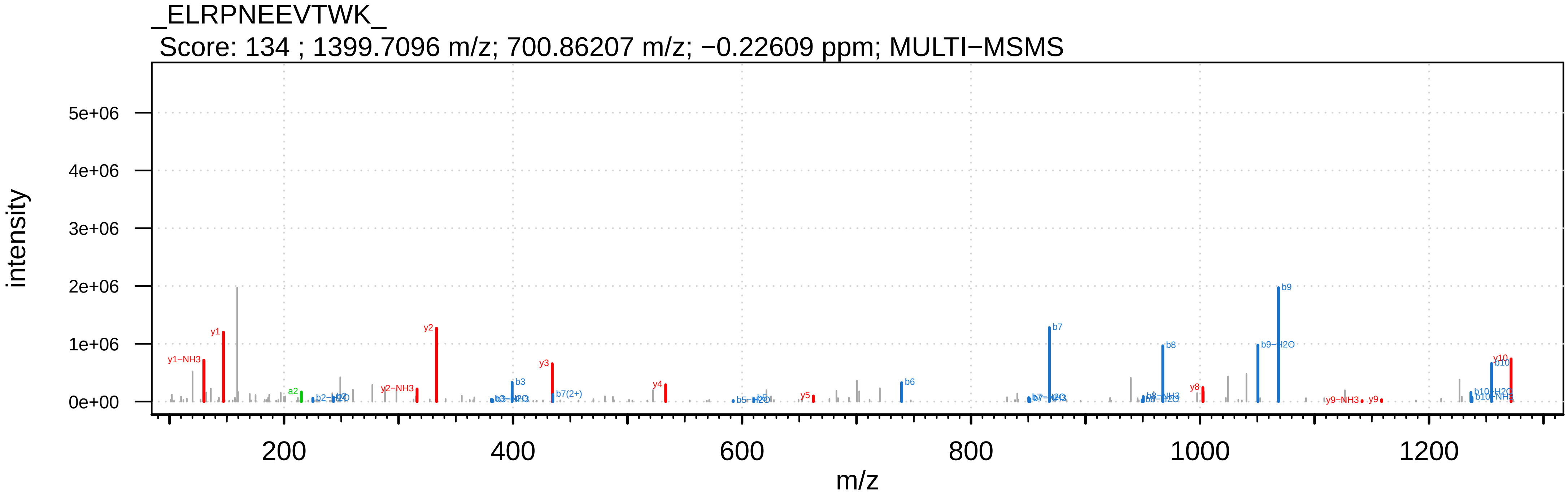

Raw File: 20130411_EXQ6_BaAz_SA_SeR1_05
Scan Number: 36950
Proteins:
chr12_103206313−103214181.T2_chr12:101968174−101969629:−

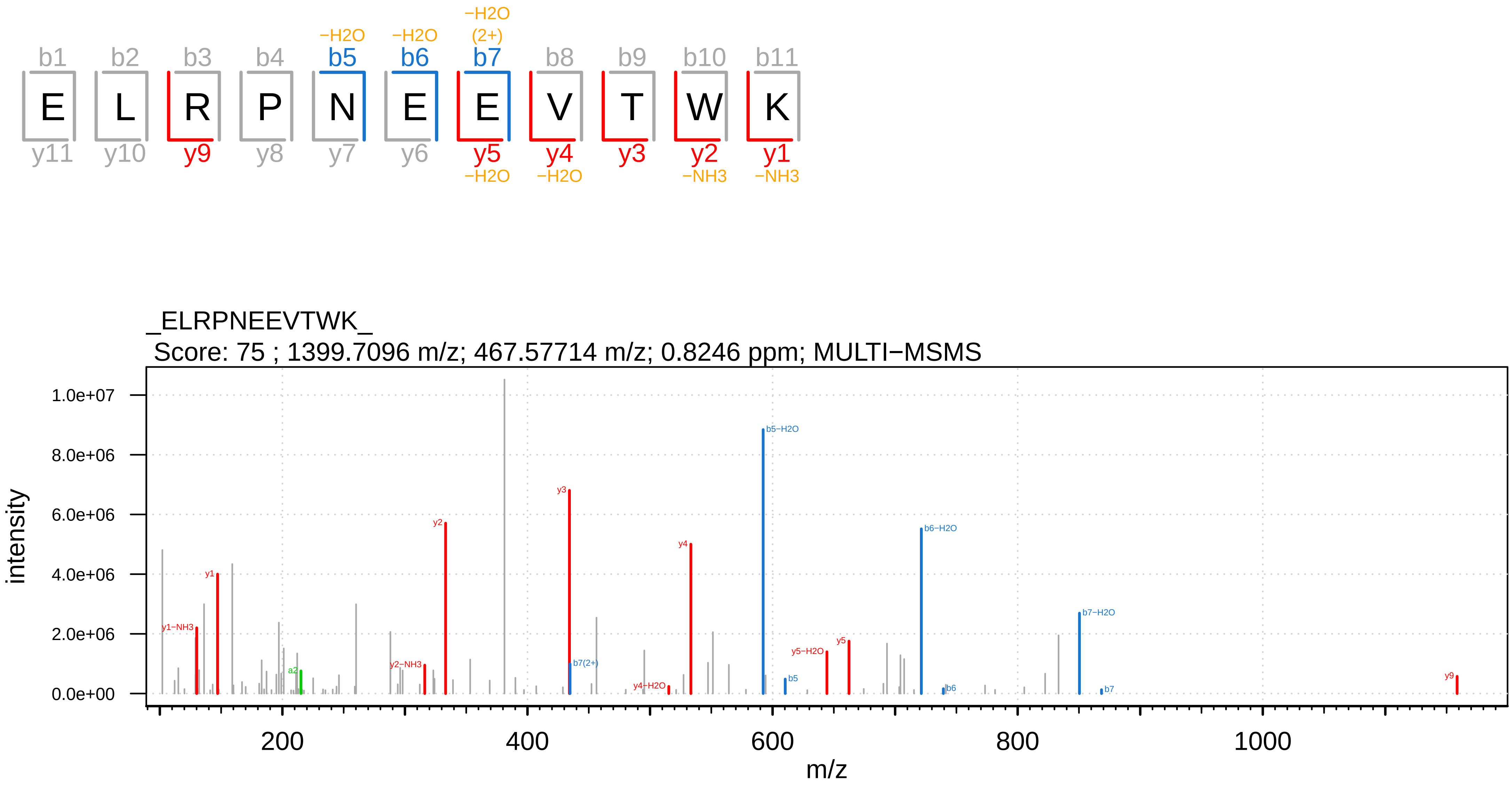

Raw File: 20130411_EXQ6_BaAz_SA_SeR1_05
Scan Number: 36969
Proteins:
chr12_103206313−103214181.T2_chr12:101968174−101969629:−

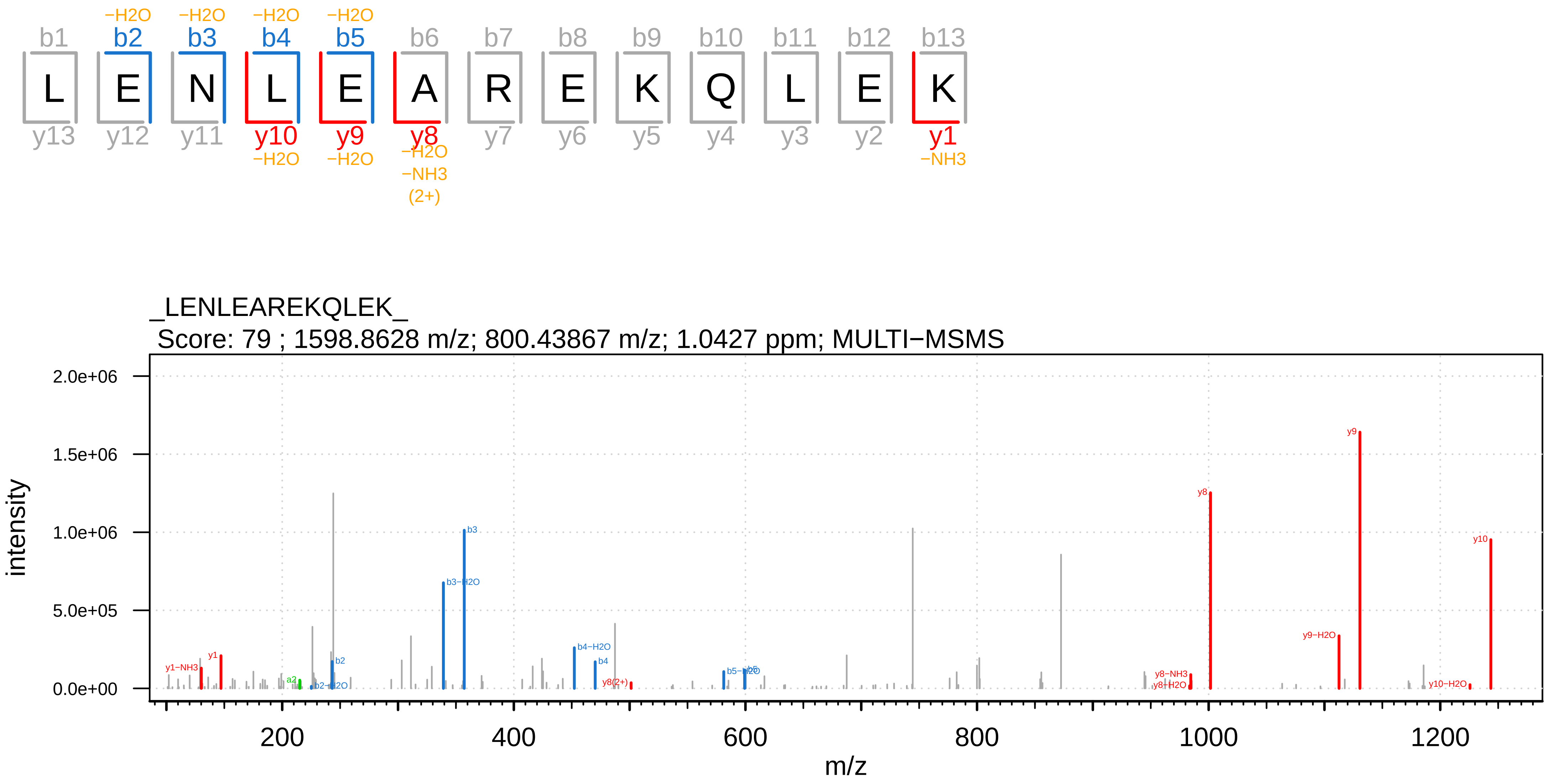

Raw File: 20130411_EXQ6_BaAz_SA_SeR1_05
Scan Number: 52154
Proteins:
ENSMUST00000023048_chr15:80235835−80236047:+

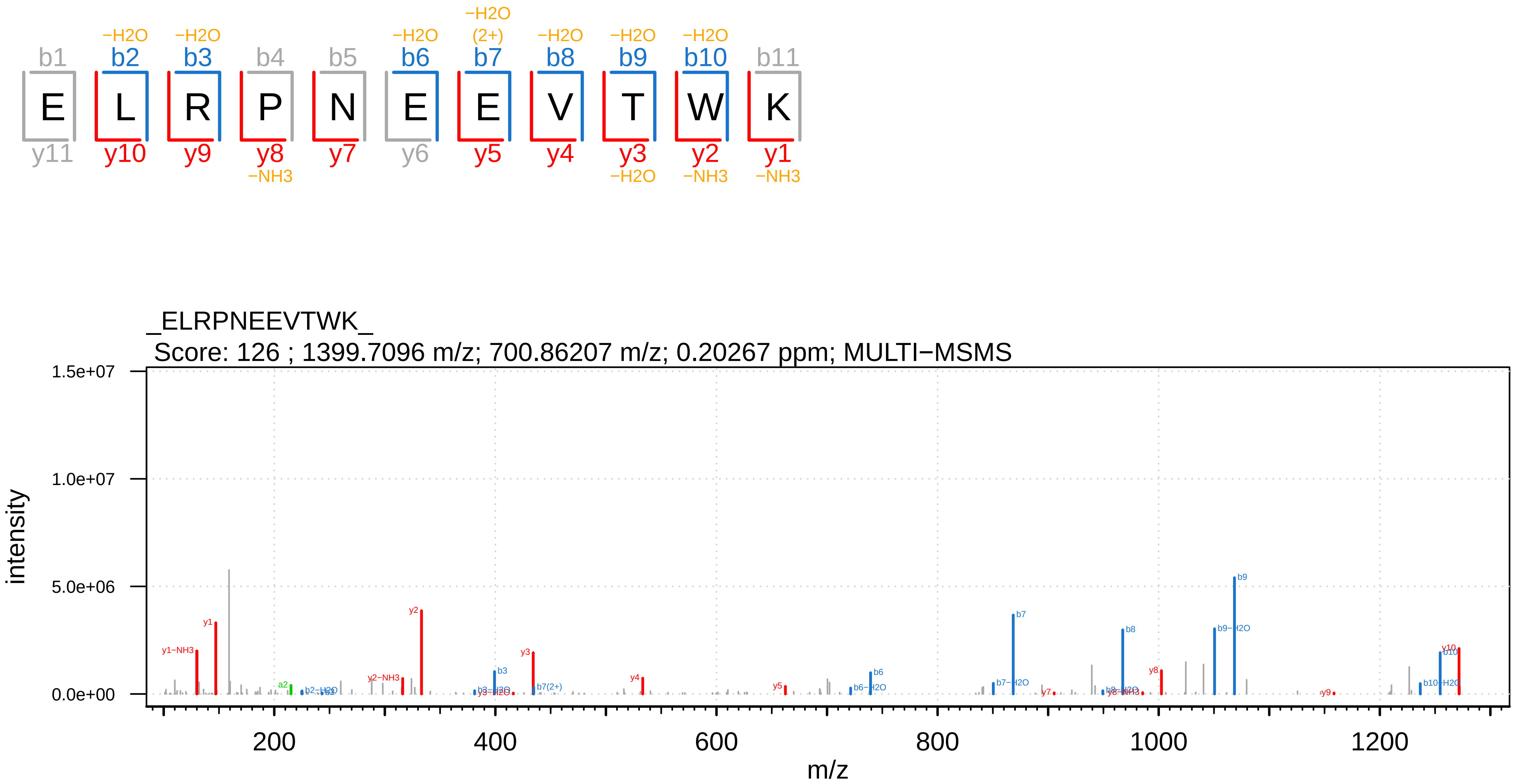

Raw File: 20130411_EXQ6_BaAz_SA_SeR1_06
Scan Number: 34504
Proteins:
chr12_103206313−103214181.T2_chr12:101968174−101969629:−

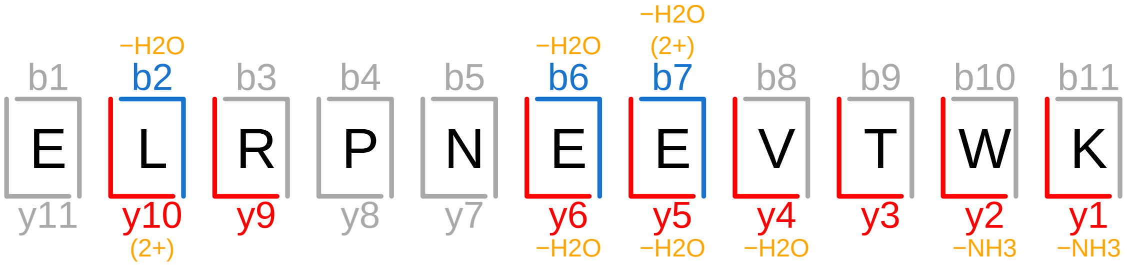

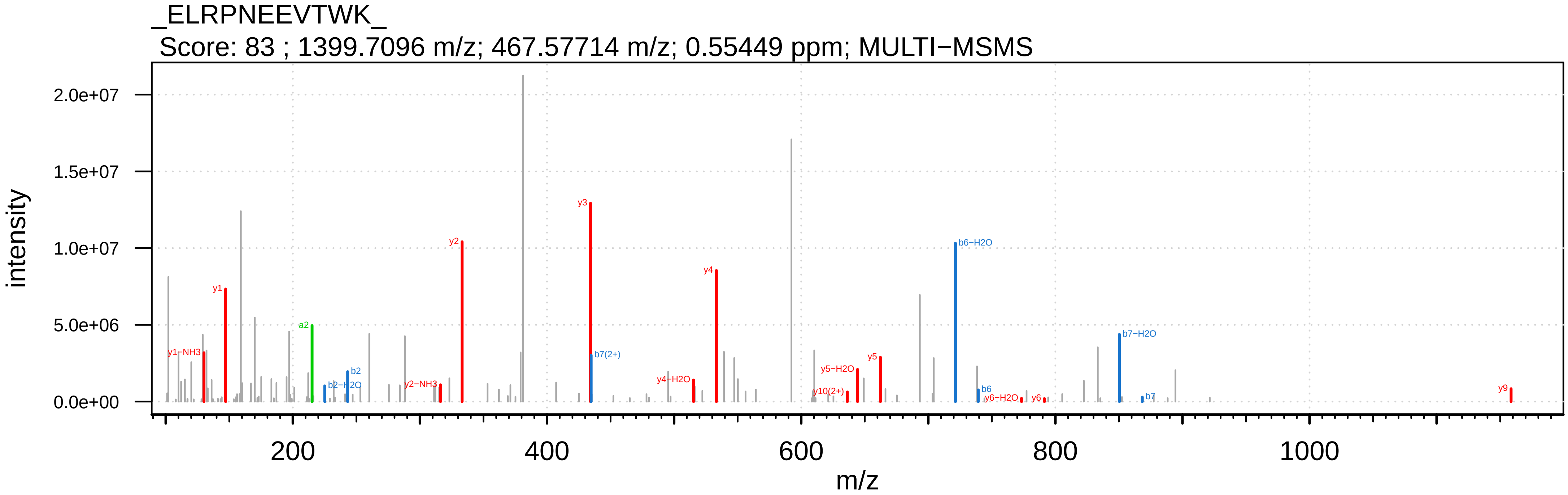

Raw File: 20130411_EXQ6_BaAz_SA_SeR1_06
Scan Number: 34510
Proteins:
chr12_103206313−103214181.T2_chr12:101968174−101969629:−

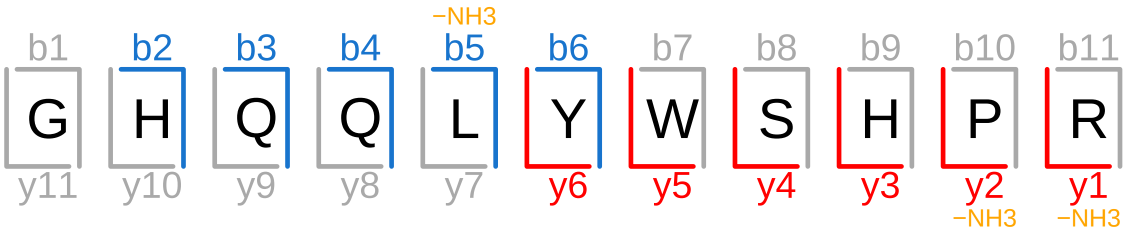

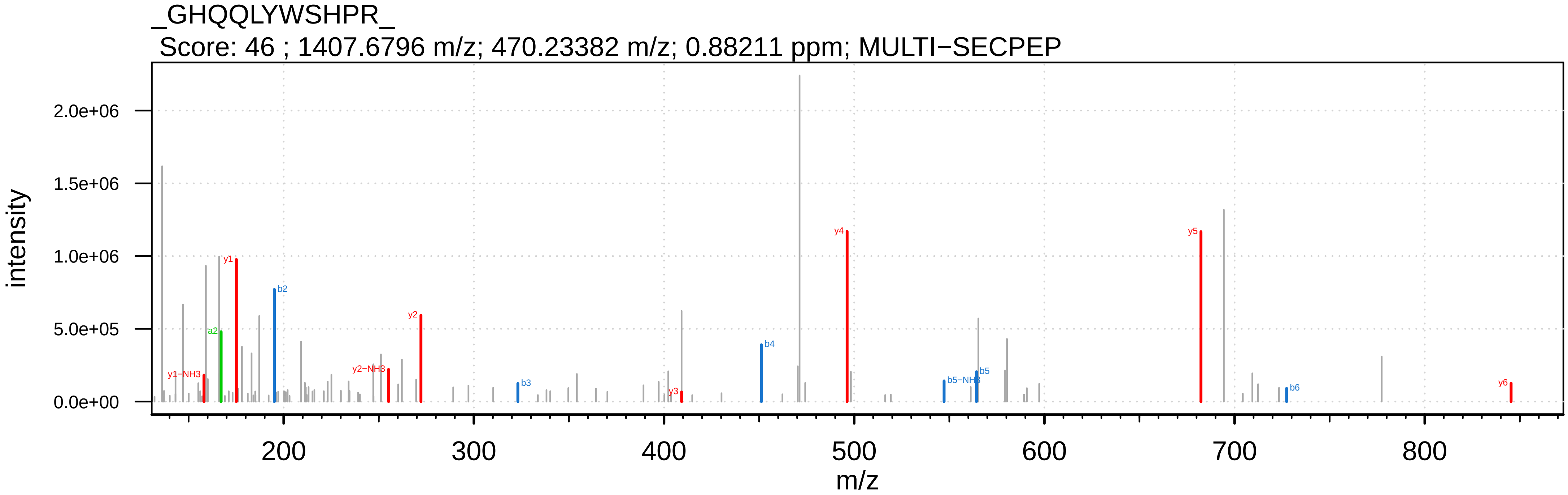

Raw File: 20130411_EXQ6_BaAz_SA_SeR1_06
Scan Number: 17562
Proteins:
sp|P62274|RS29_MOUSE
ENSMUST00000037023_chr12:69157813−69159058:−
ENSMUST00000071737_chr13:73988963−73989133:−

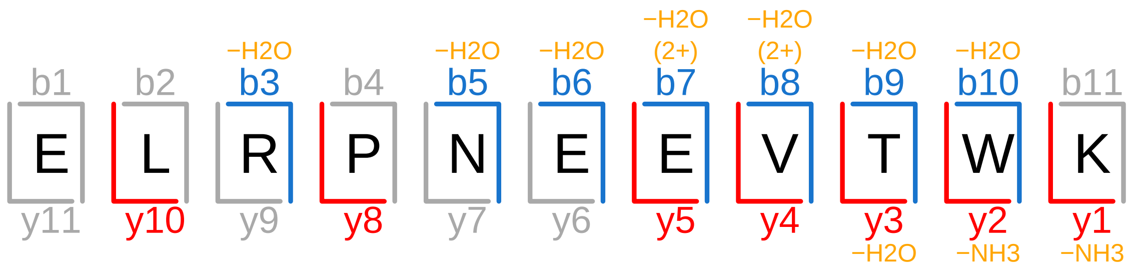

ELRPNEEVTWK

Score: 151 ; 1399.7096 m/z; 700.86207 m/z; −0.20055 ppm; MULTI−MSMS

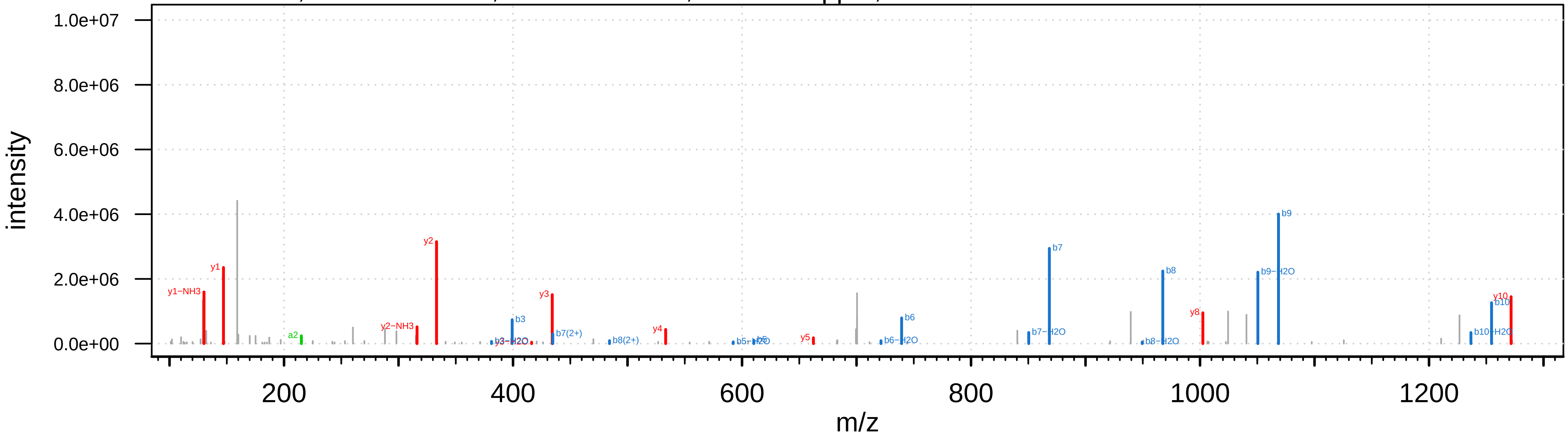

Raw File: 20130413_EXQ6_BaAz_SA_HR1_06

Scan Number: 32839

Proteins:

chr12_103206313−103214181.T2_chr12:101968174−101969629:−

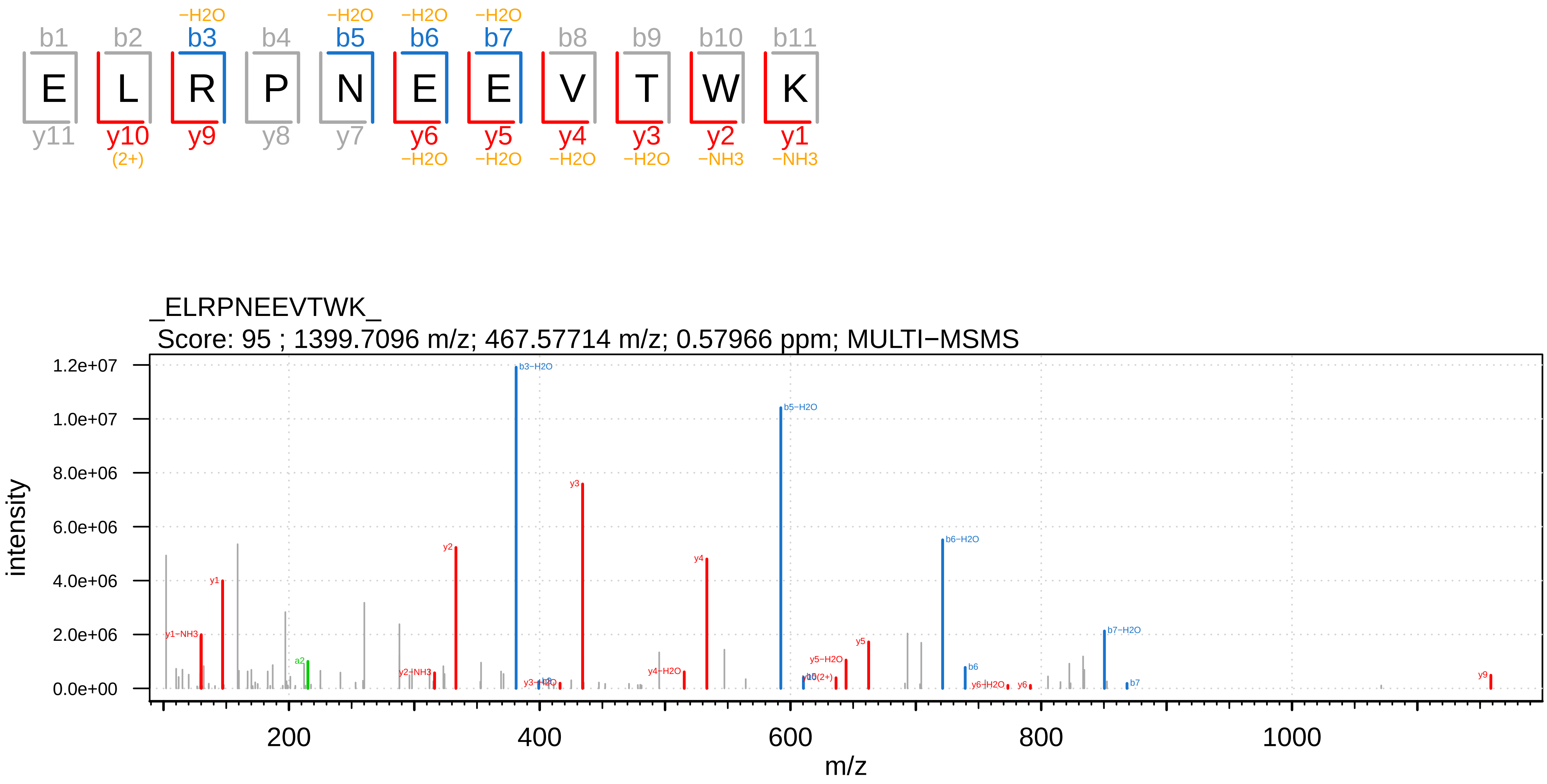

Raw File: 20130413_EXQ6_BaAz_SA_HR1_06

Scan Number: 32840

Proteins:

chr12_103206313−103214181.T2_chr12:101968174−101969629:−

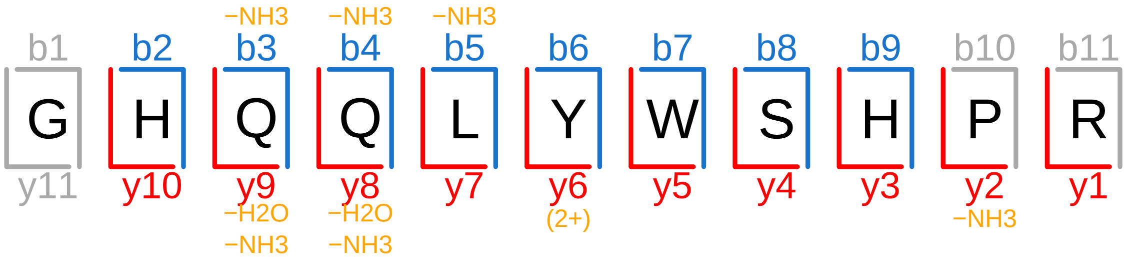

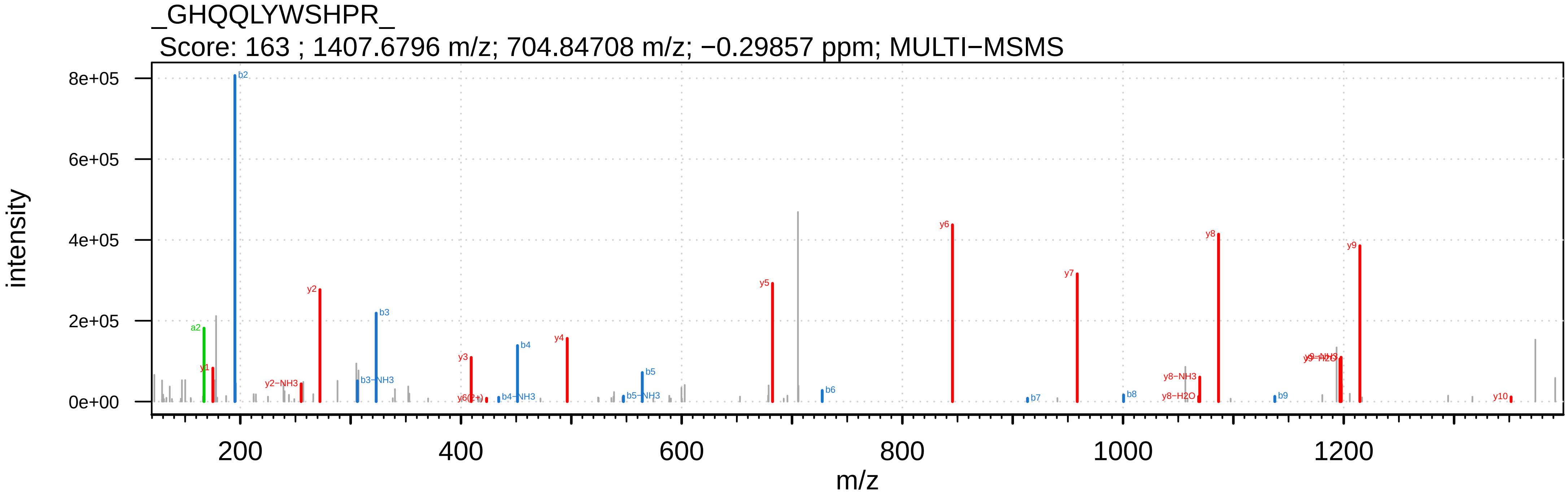

Raw File: 20130413_EXQ6_BaAz_SA_HR1_06
Scan Number: 17161
Proteins:
sp|P62274|RS29_MOUSE
ENSMUST00000037023_chr12:69157813−69159058:−
ENSMUST00000071737_chr13:73988963−73989133:−

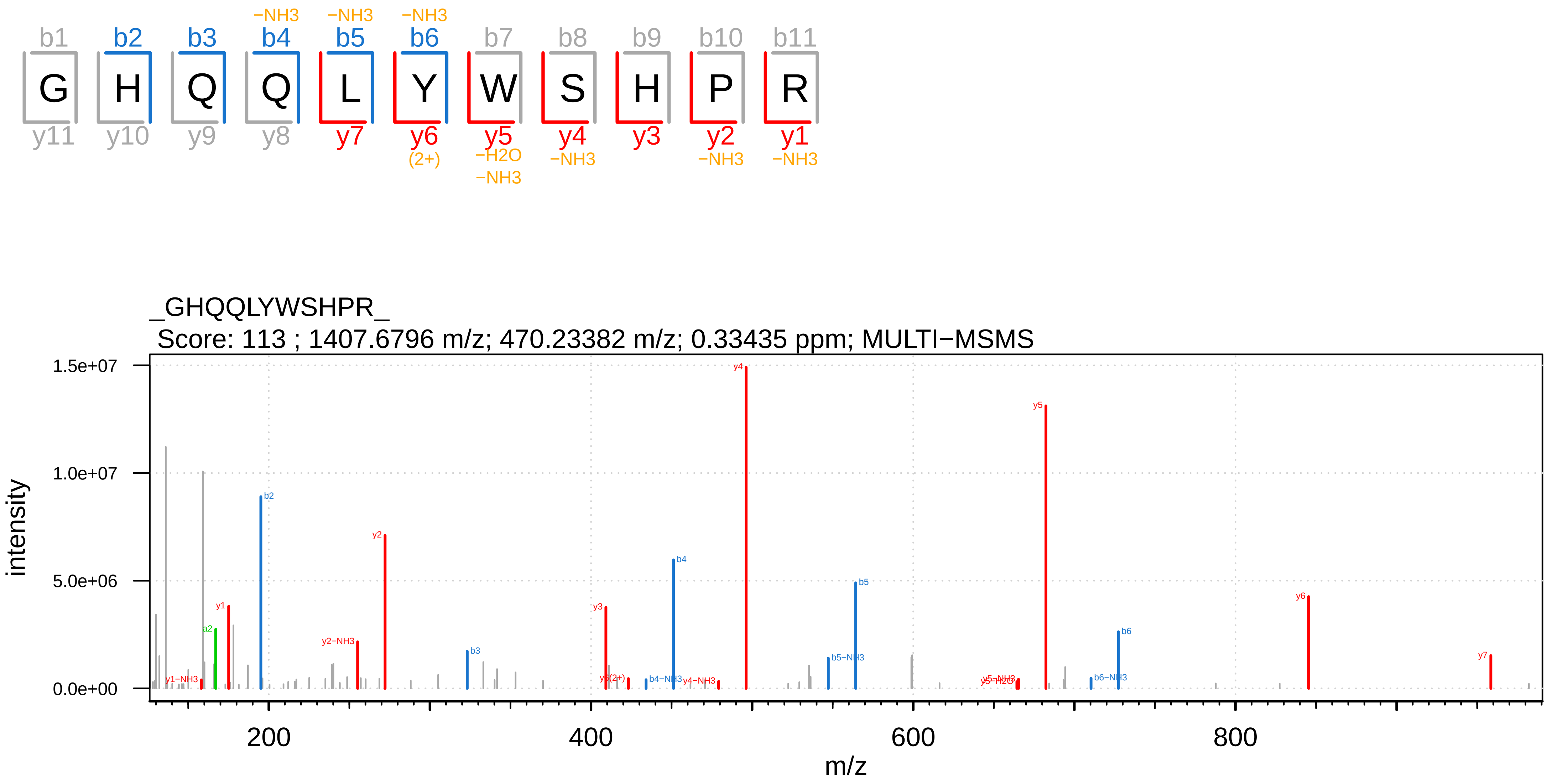

Raw File: 20130413_EXQ6_BaAz_SA_HR1_06
Scan Number: 17213
Proteins:
sp|P62274|RS29_MOUSE
ENSMUST00000037023_chr12:69157813−69159058:−
ENSMUST00000071737_chr13:73988963−73989133:−

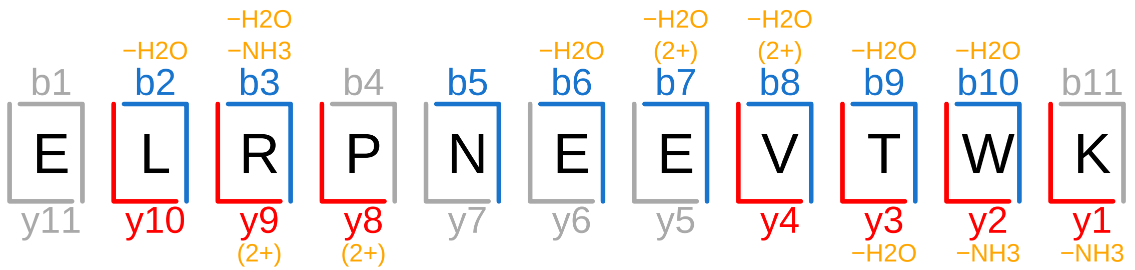

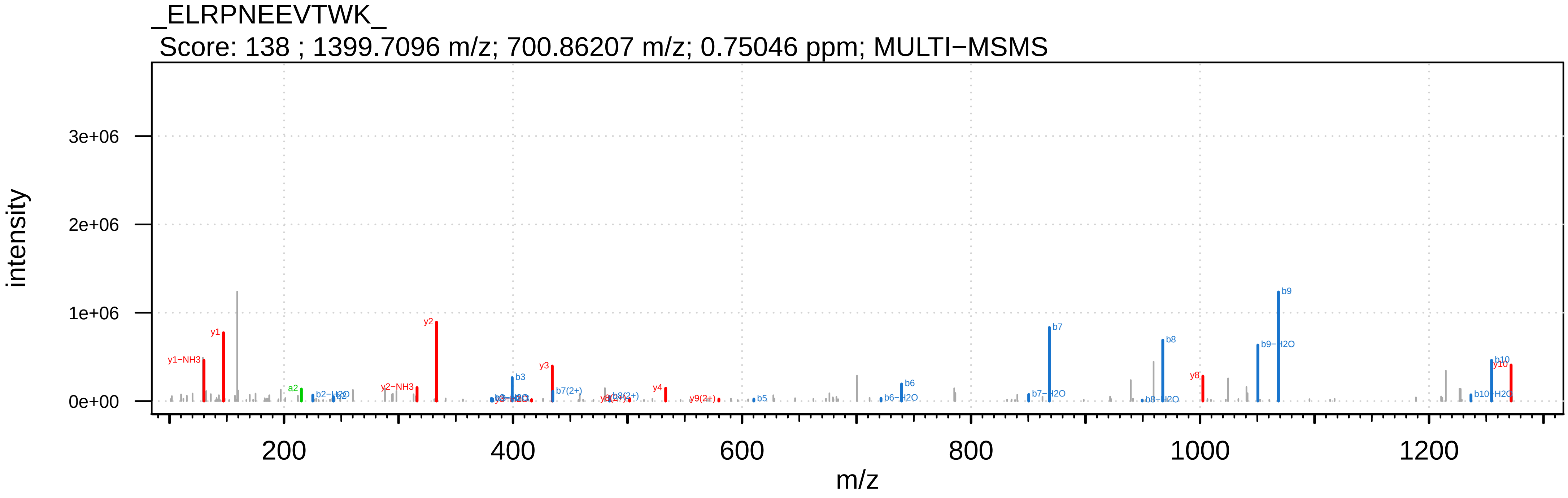

Raw File: 20130415_EXQ6_BaAz_SA_KR1_05
Scan Number: 36424
Proteins:
chr12_103206313−103214181.T2_chr12:101968174−101969629:−

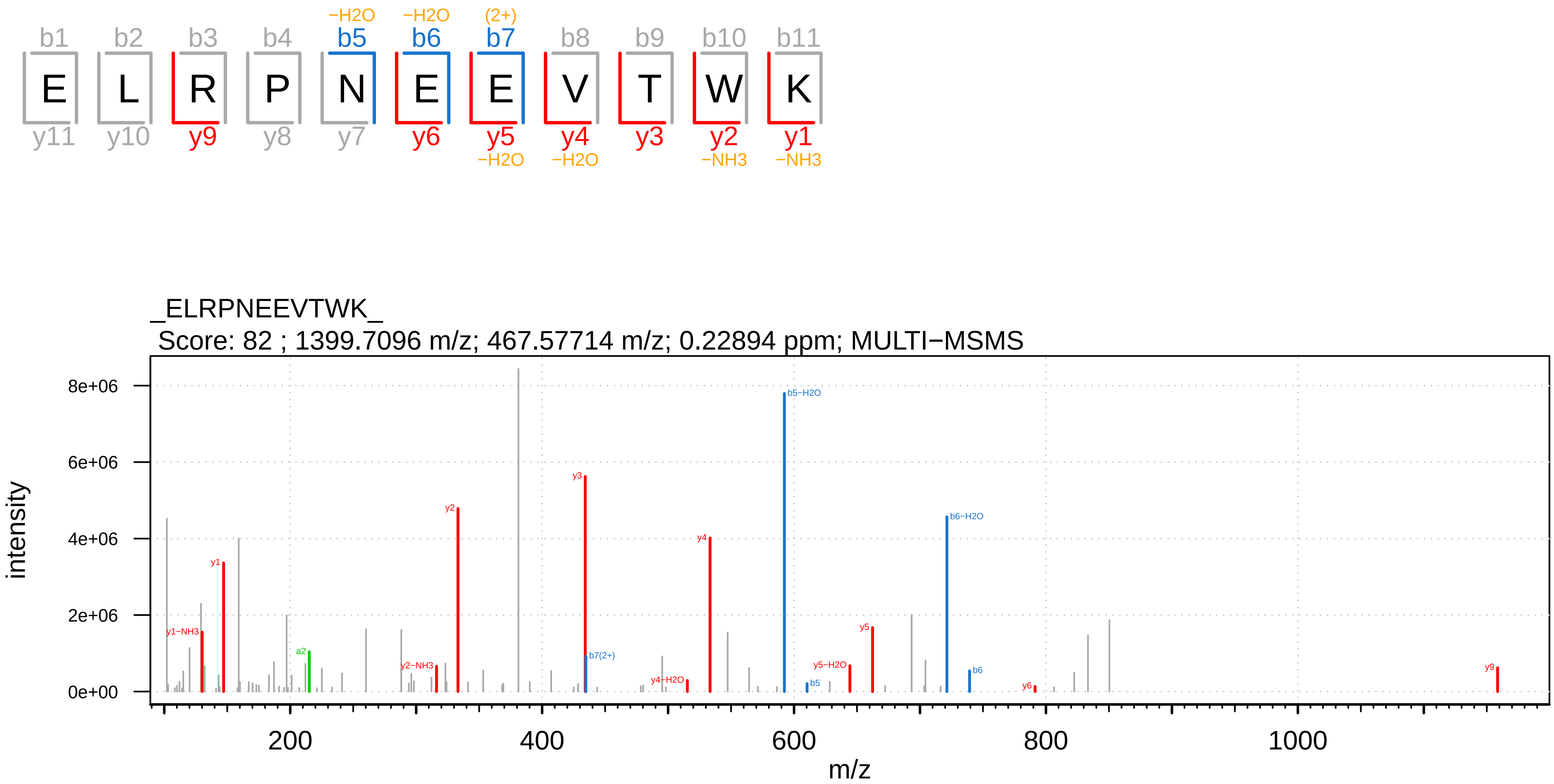

Raw File: 20130415_EXQ6_BaAz_SA_KR1_05
Scan Number: 36458
Proteins:
chr12_103206313−103214181.T2_chr12:101968174−101969629:−

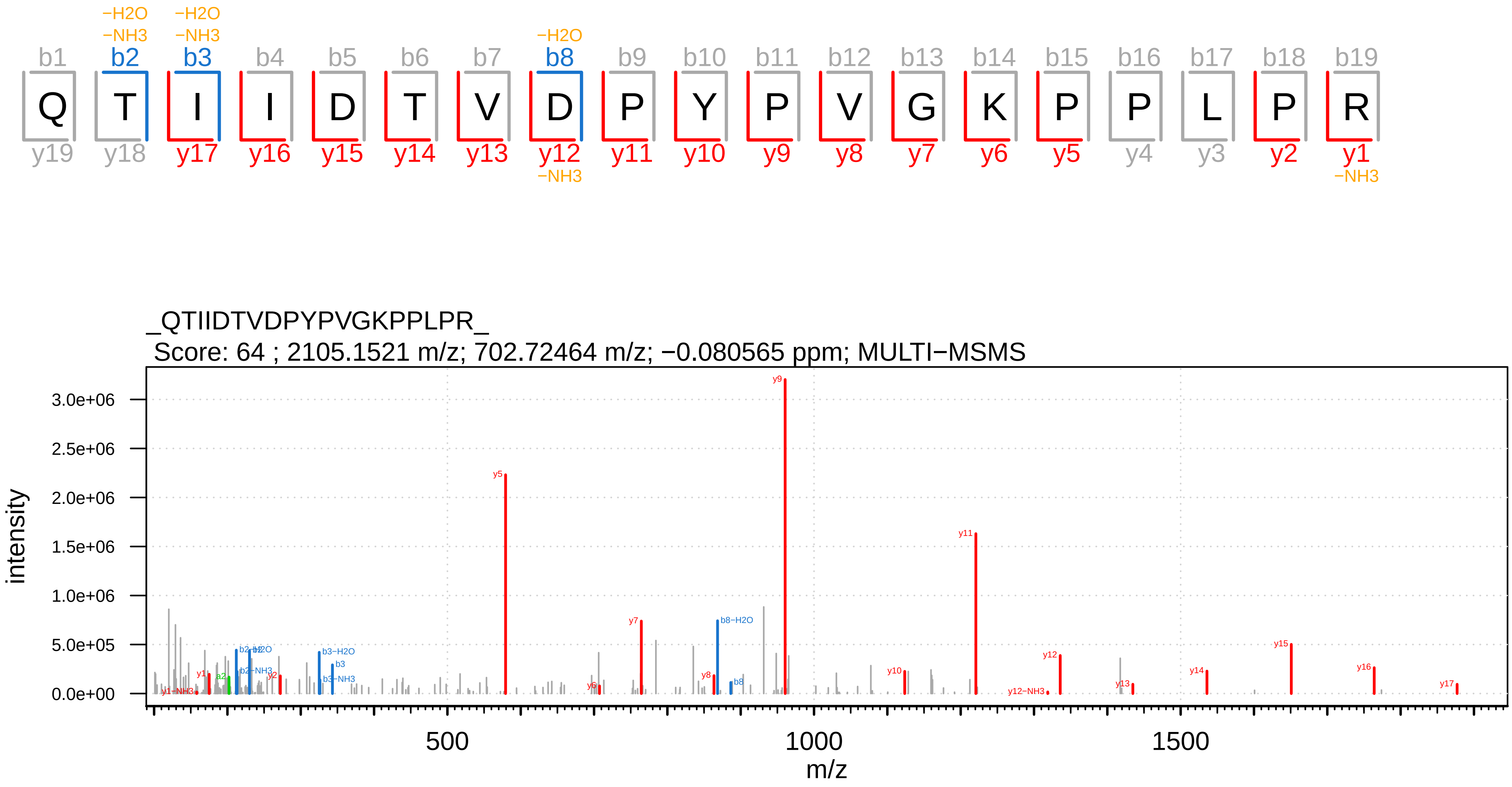

Raw File: 20130415_EXQ6_BaAz_SA_KR1_05
Scan Number: 77454
Proteins:
ENSMUST00000136126_chr3:79172848−79174982:−

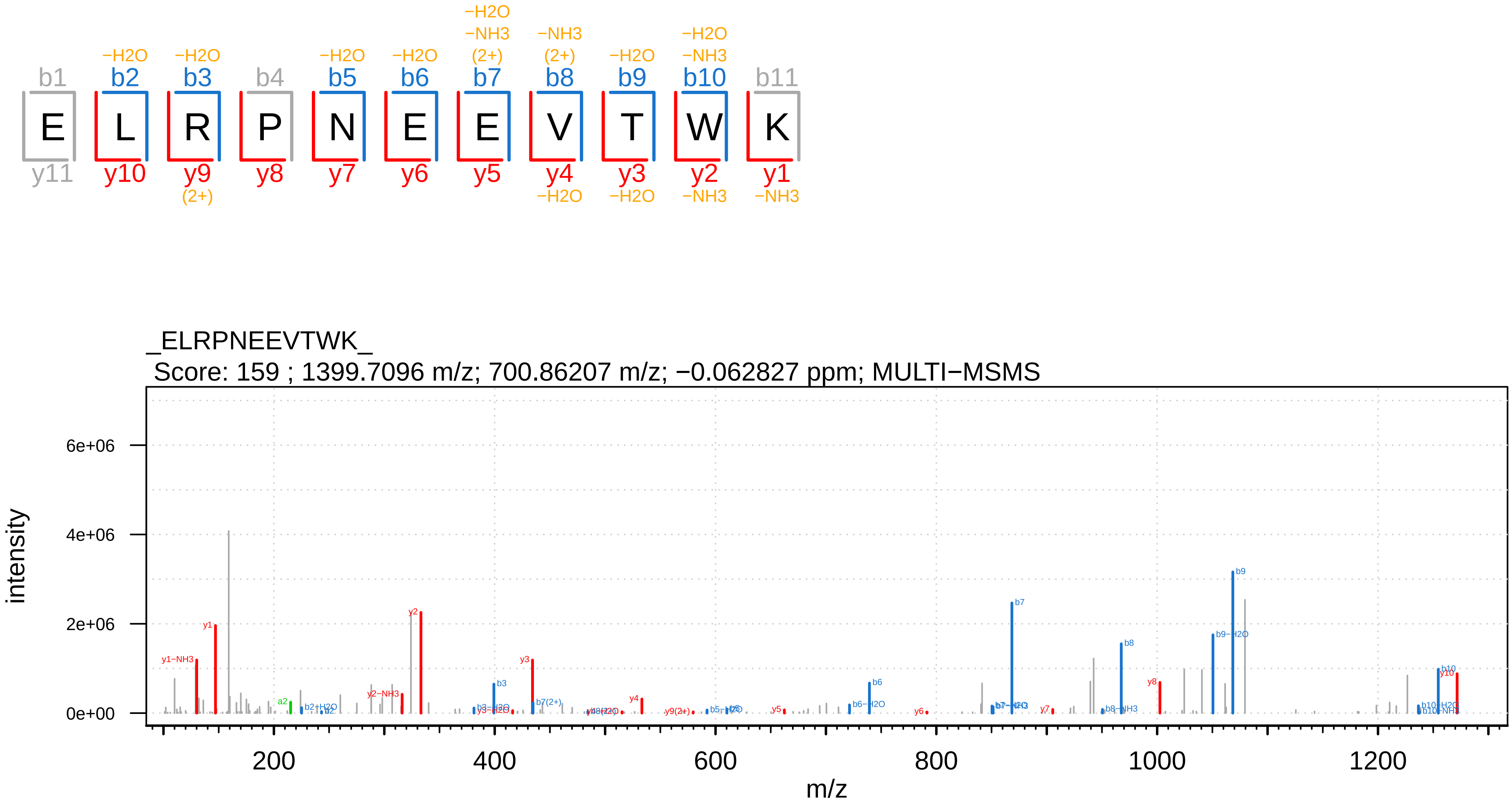

Raw File: 20130415_EXQ6_BaAz_SA_KR1_06
Scan Number: 34123
Proteins:
chr12_103206313−103214181.T2_chr12:101968174−101969629:−

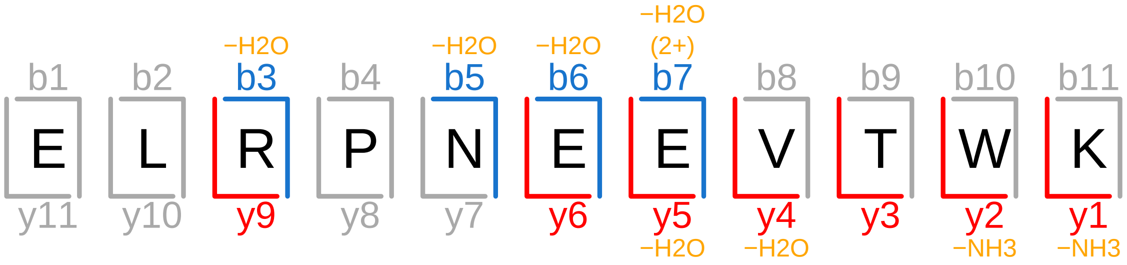

ELRPNEEVTWK
Score: 105 ; 1399.7096 m/z; 467.57714 m/z; 0.38323 ppm; MULTI−MSMS

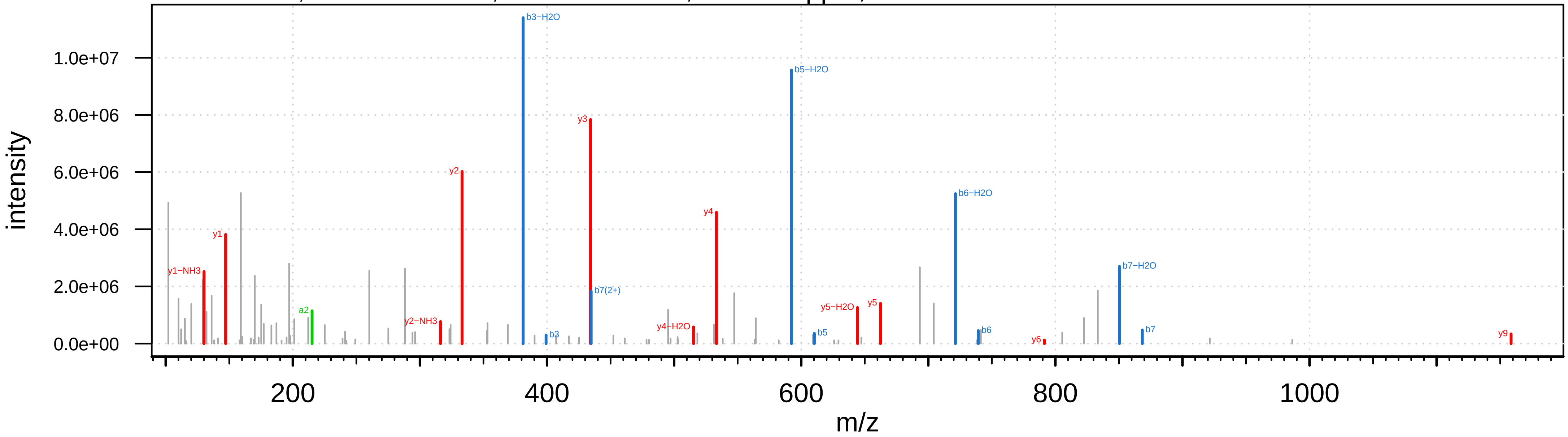

Raw File: 20130415_EXQ6_BaAz_SA_KR1_06
Scan Number: 34129
Proteins:
chr12_103206313−103214181.T2_chr12:101968174−101969629:−

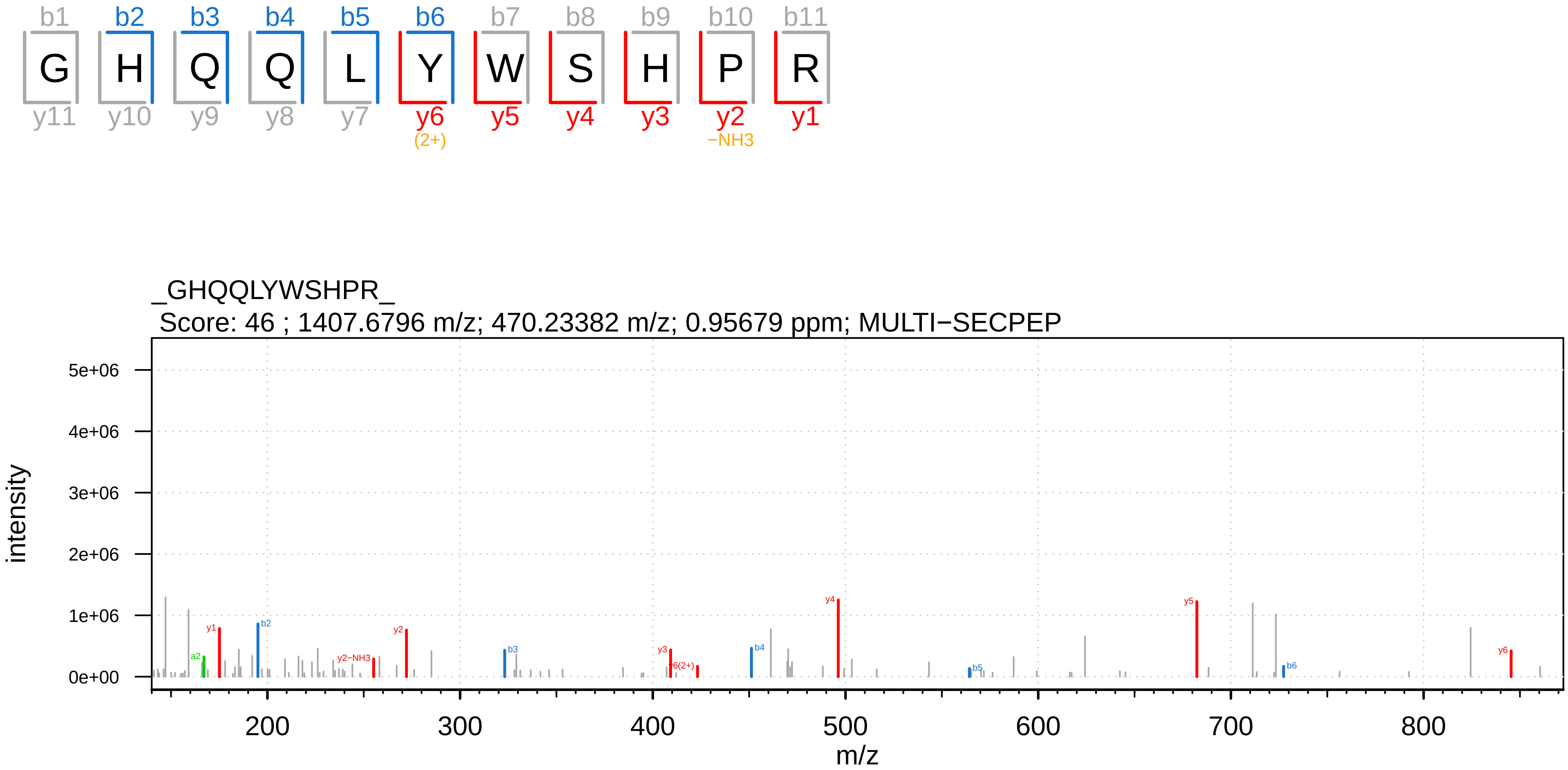

Raw File: 20130415_EXQ6_BaAz_SA_KR1_06
Scan Number: 17178
Proteins:
sp|P62274|RS29_MOUSE
ENSMUST00000037023_chr12:69157813−69159058:−
ENSMUST00000071737_chr13:73988963−73989133:−

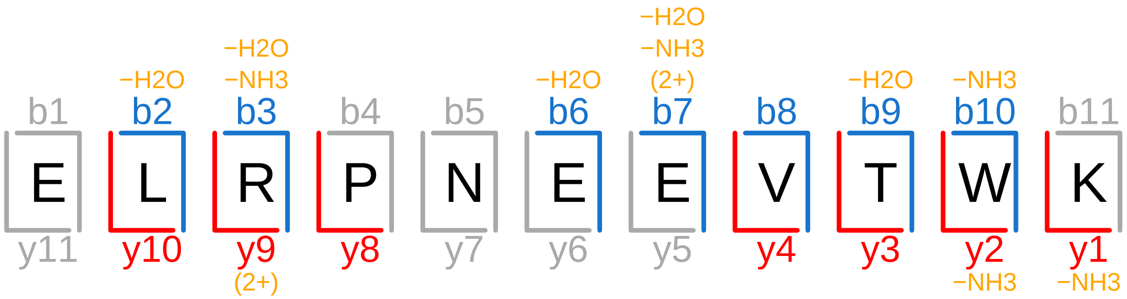

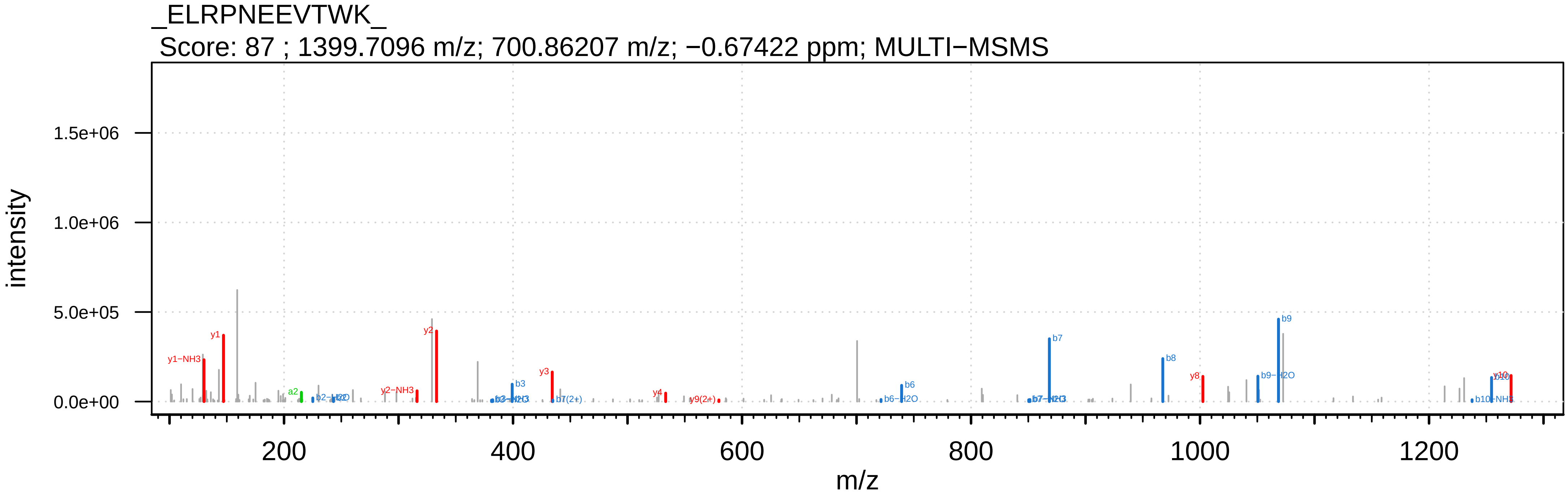

Raw File: 20130416_EXQ6_BaAz_SA_StR1_05
Scan Number: 37953
Proteins:
chr12_103206313−103214181.T2_chr12:101968174−101969629:−

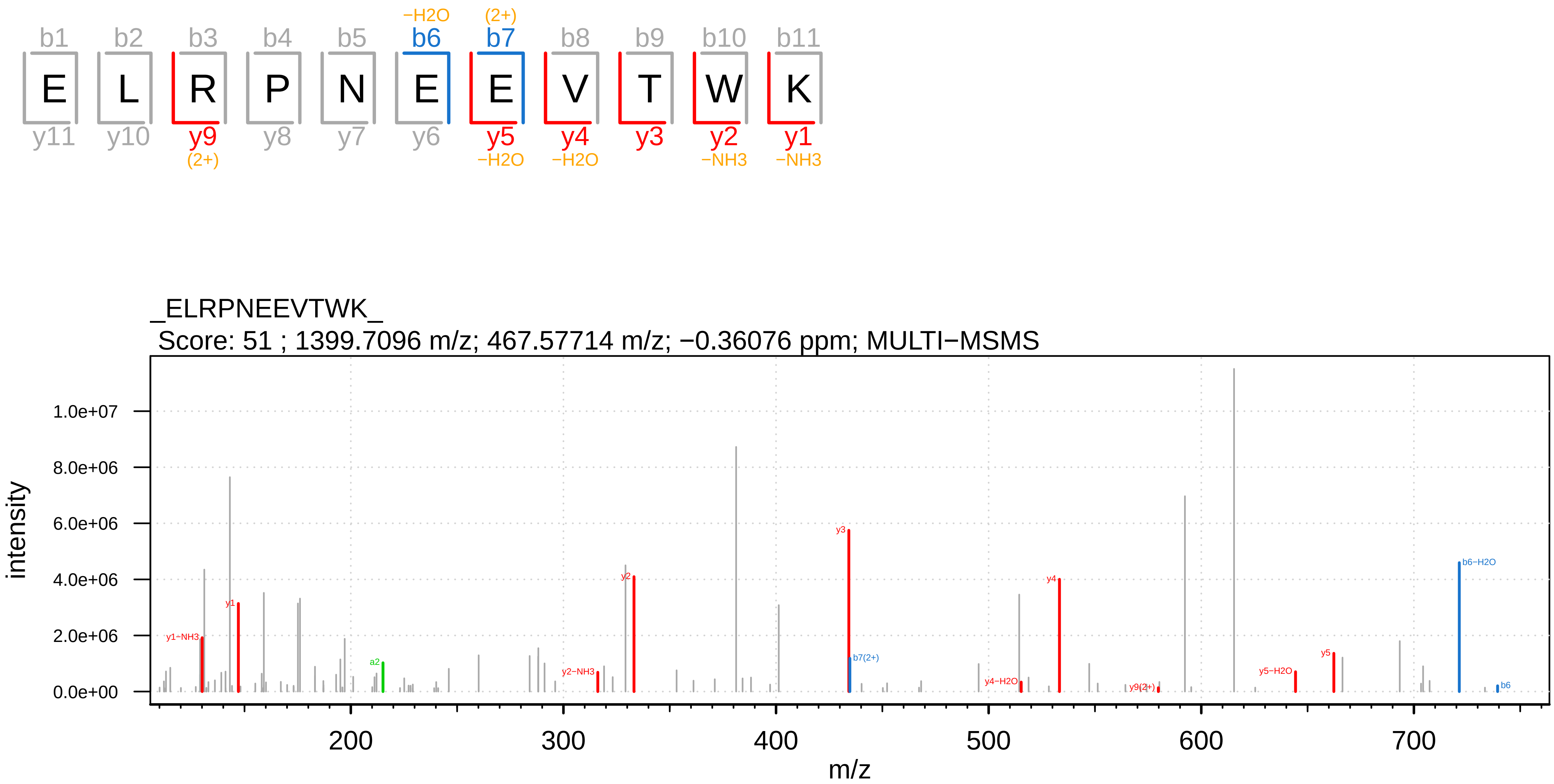

Raw File: 20130416_EXQ6_BaAz_SA_StR1_05
Scan Number: 38080
Proteins:
chr12_103206313−103214181.T2_chr12:101968174−101969629:−

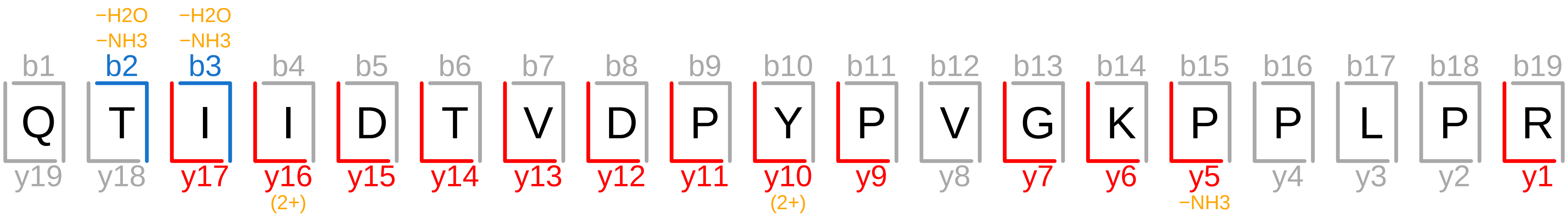

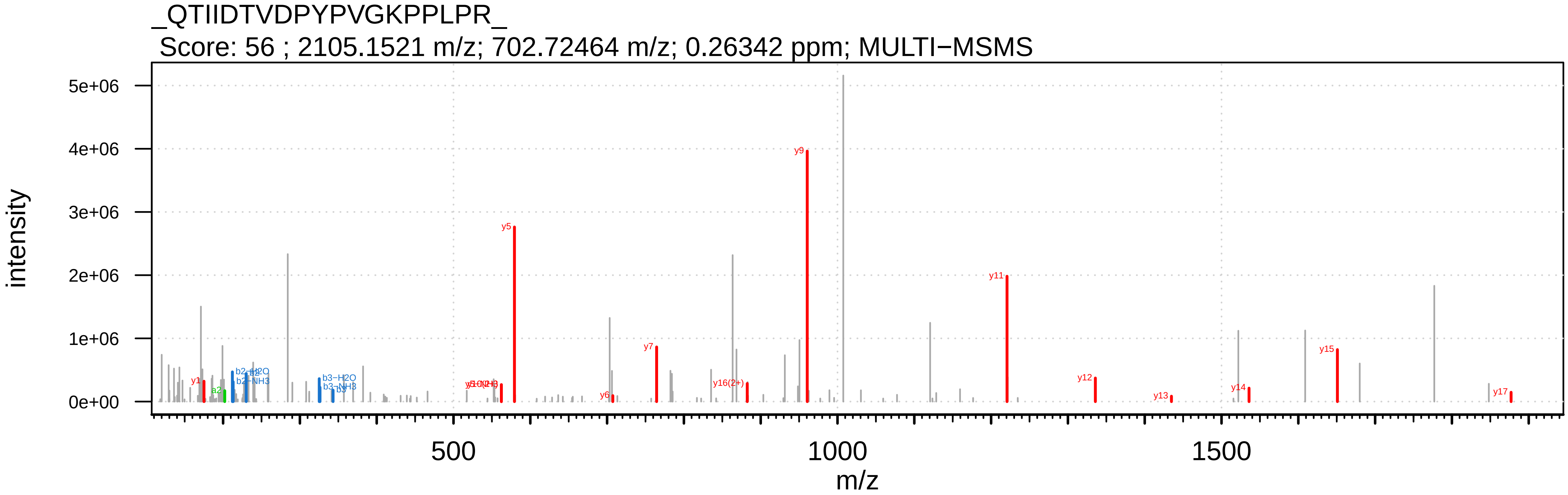

Raw File: 20130416_EXQ6_BaAz_SA_StR1_05
Scan Number: 80418
Proteins:
ENSMUST00000136126_chr3:79172848−79174982:−

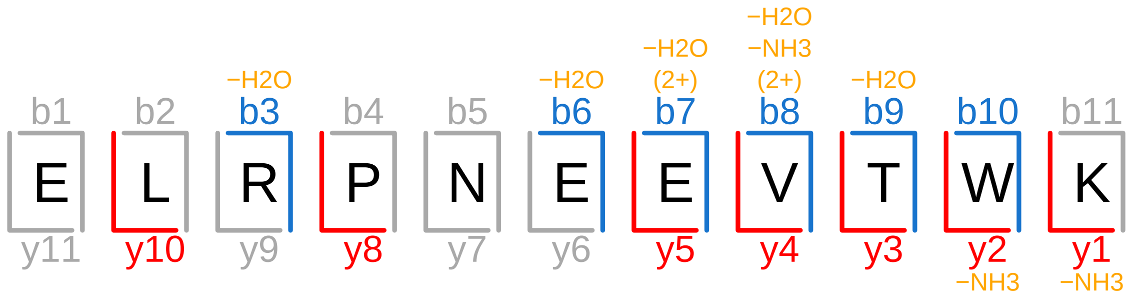

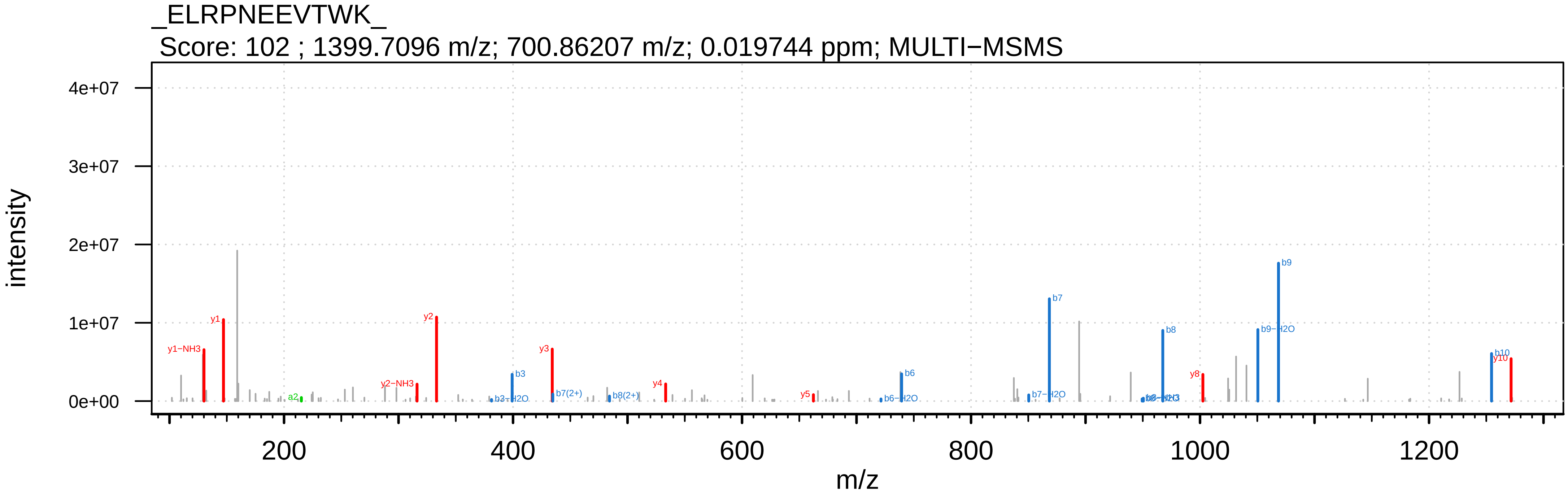

Raw File: 20130416_EXQ6_BaAz_SA_StR1_06
Scan Number: 35086
Proteins:
chr12_103206313−103214181.T2_chr12:101968174−101969629:−

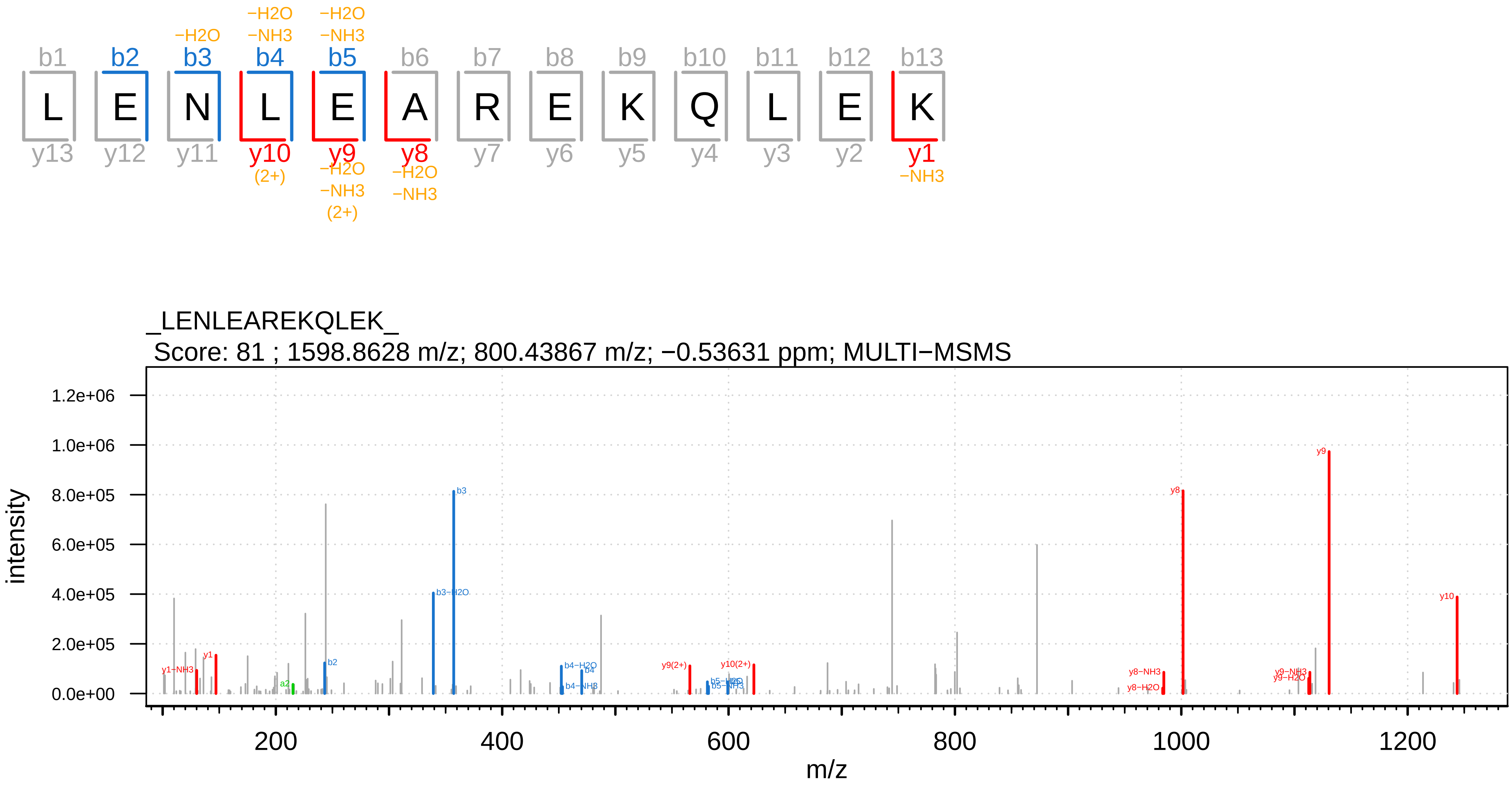

Raw File: 20130416_EXQ6_BaAz_SA_StR1_06
Scan Number: 50593
Proteins:
ENSMUST00000023048_chr15:80235835−80236047:+

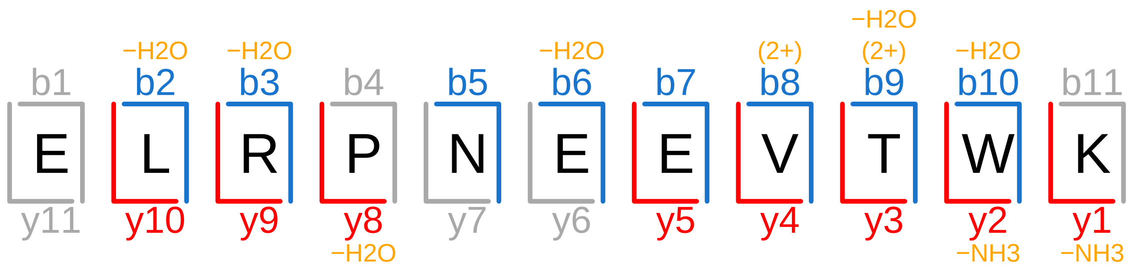

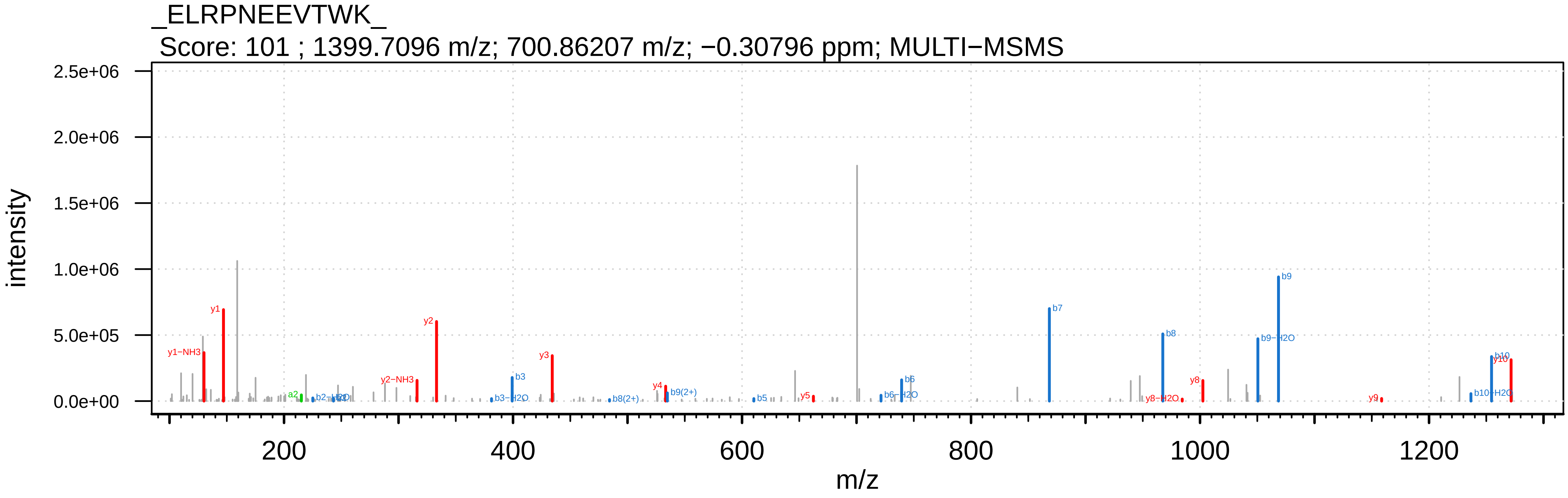

Raw File: 20130419_EXQ6_BaAz_SA_CHR1_06
Scan Number: 33914
Proteins:
chr12_103206313−103214181.T2_chr12:101968174−101969629:−

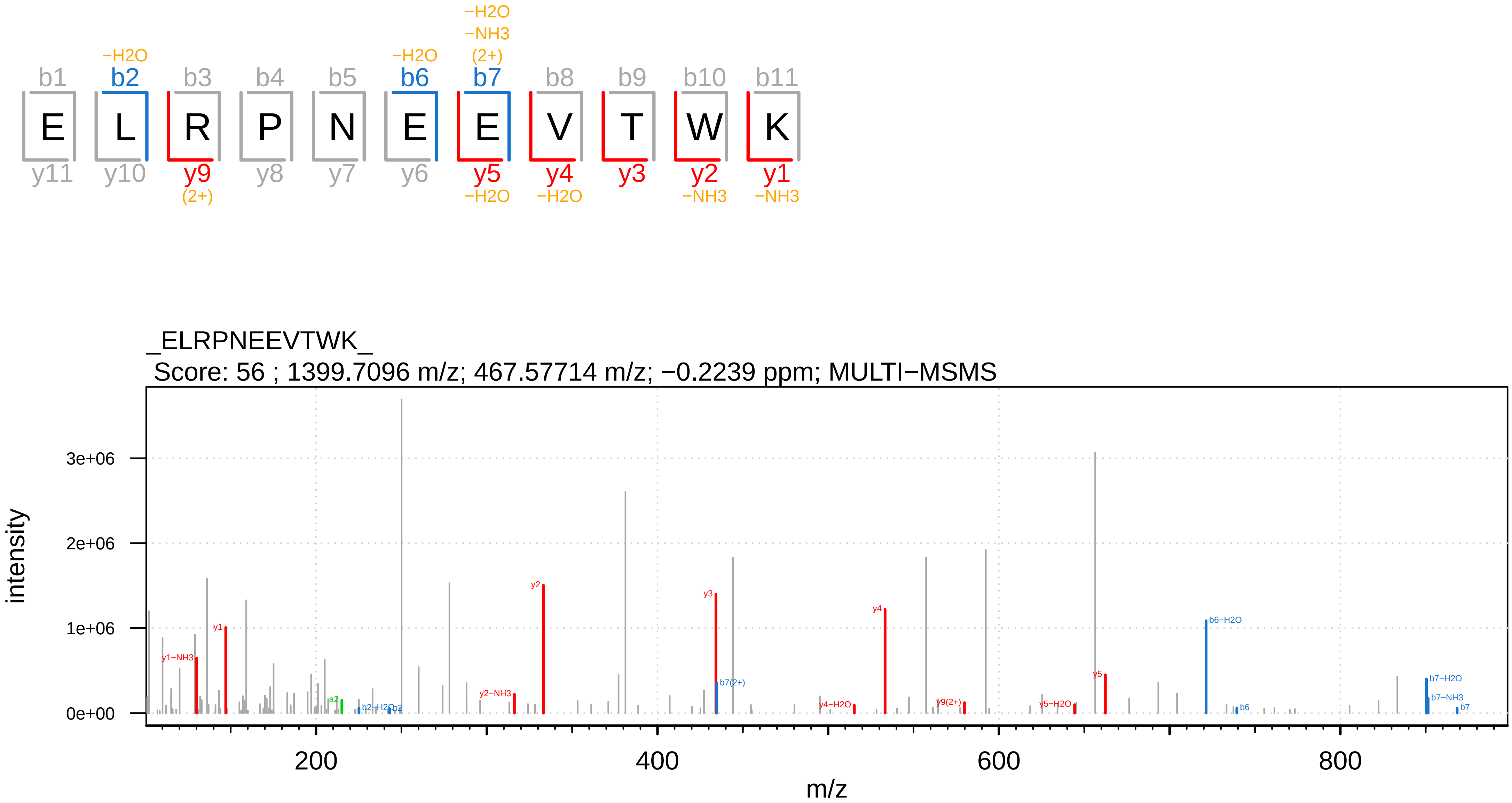

Raw File: 20130419_EXQ6_BaAz_SA_CHR1_06
Scan Number: 33921
Proteins:
chr12_103206313−103214181.T2_chr12:101968174−101969629:−

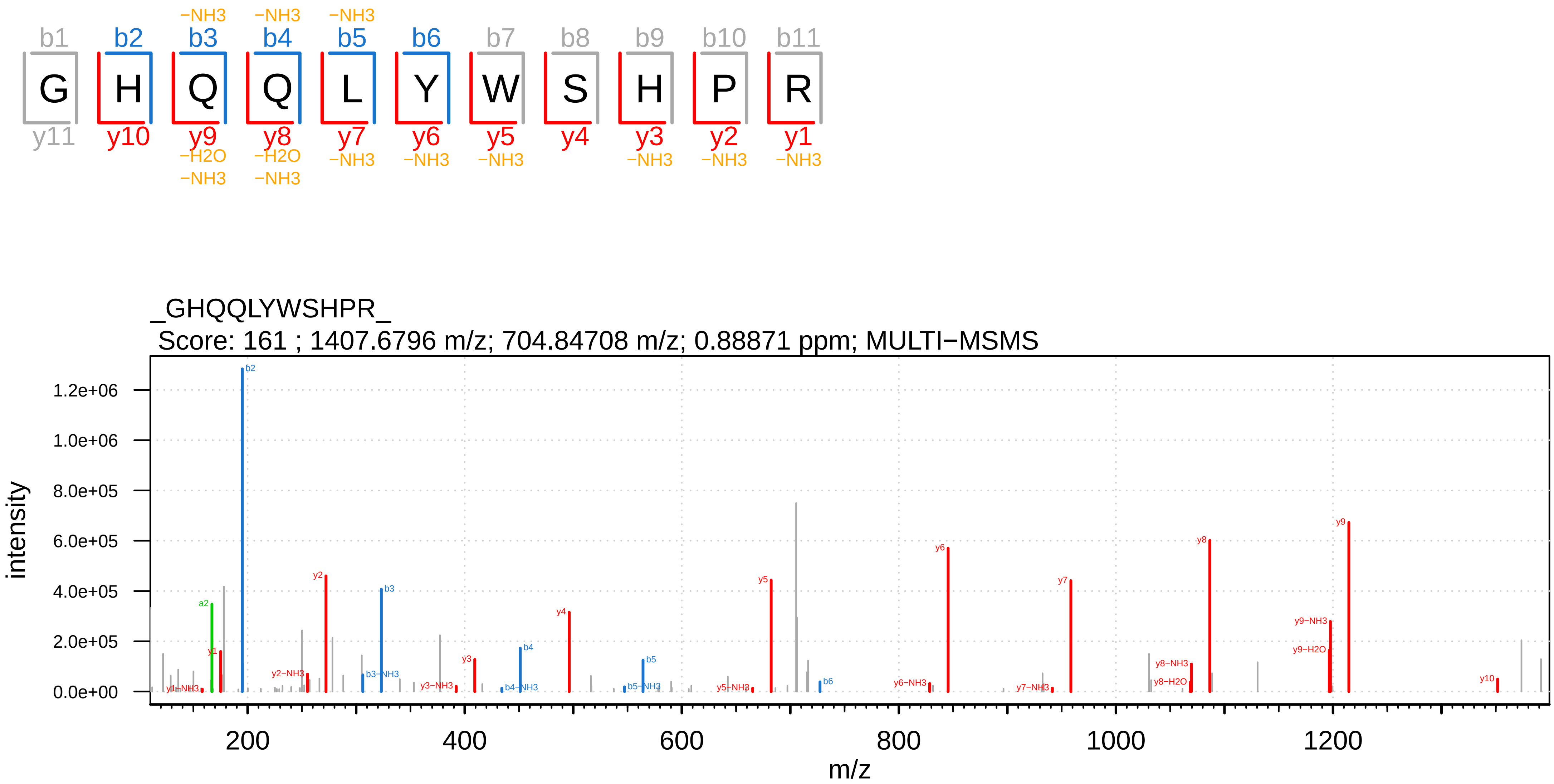

Raw File: 20130419_EXQ6_BaAz_SA_CHR1_06
Scan Number: 17941
Proteins:
sp|P62274|RS29_MOUSE
ENSMUST00000037023_chr12:69157813−69159058:−
ENSMUST00000071737_chr13:73988963−73989133:−

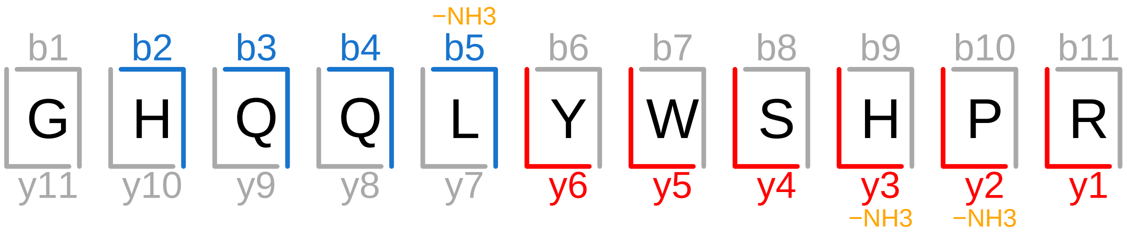

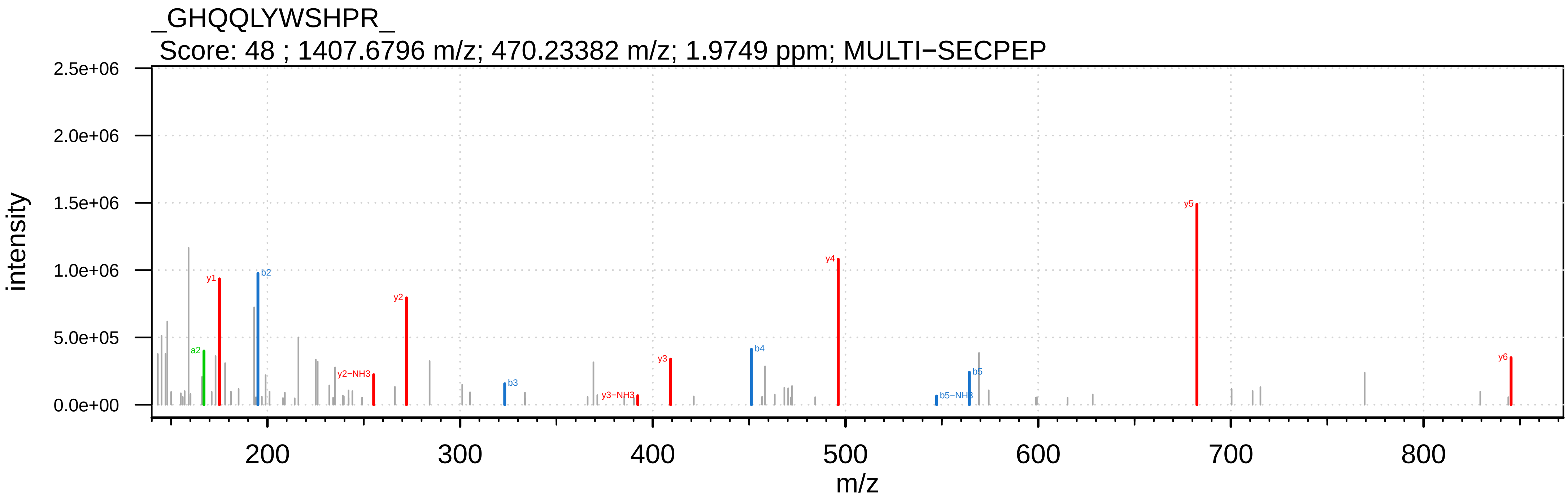

Raw File: 20130419_EXQ6_BaAz_SA_CHR1_06
Scan Number: 18010
Proteins:
sp|P62274|RS29_MOUSE
ENSMUST00000037023_chr12:69157813−69159058:−
ENSMUST00000071737_chr13:73988963−73989133:−

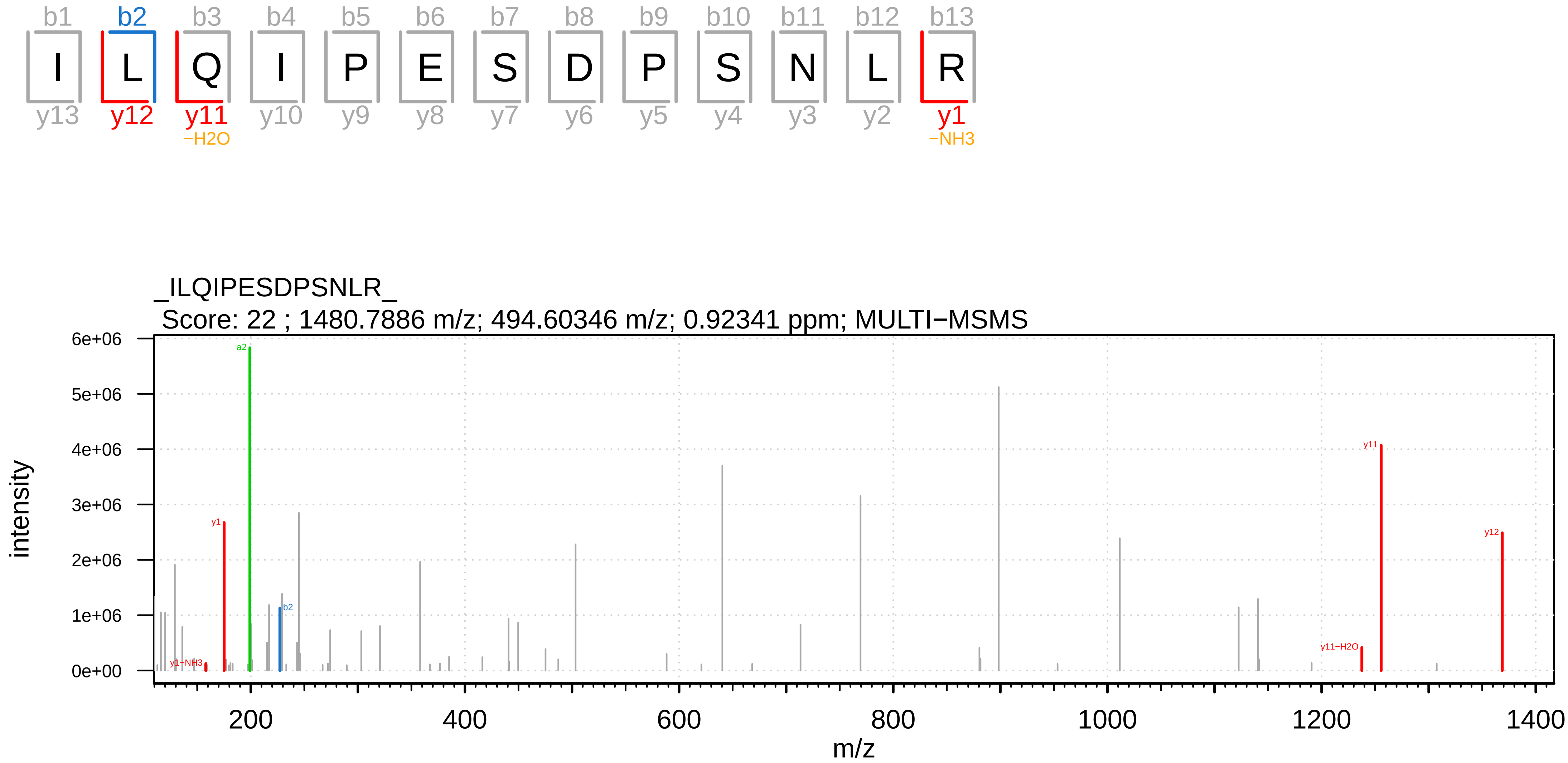

Raw File: 20130419_EXQ6_BaAz_SA_CHR1_06
Scan Number: 34575
Proteins:
ENSMUST00000161069_chr14:70554856−70554960:+

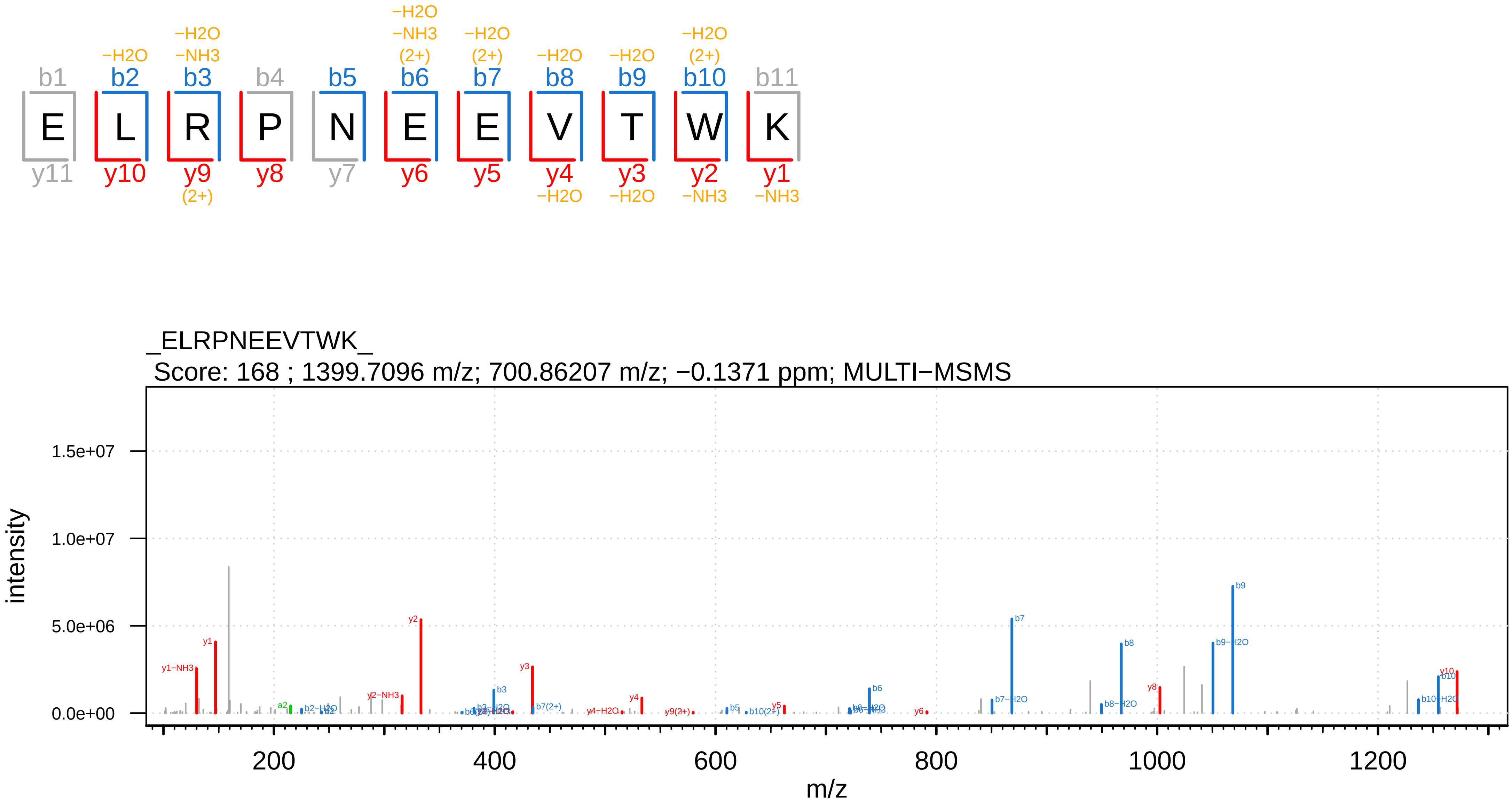

Raw File: 20130421_EXQ6_BaAz_SA_HR1_05
Scan Number: 33600
Proteins:
chr12_103206313−103214181.T2_chr12:101968174−101969629:−

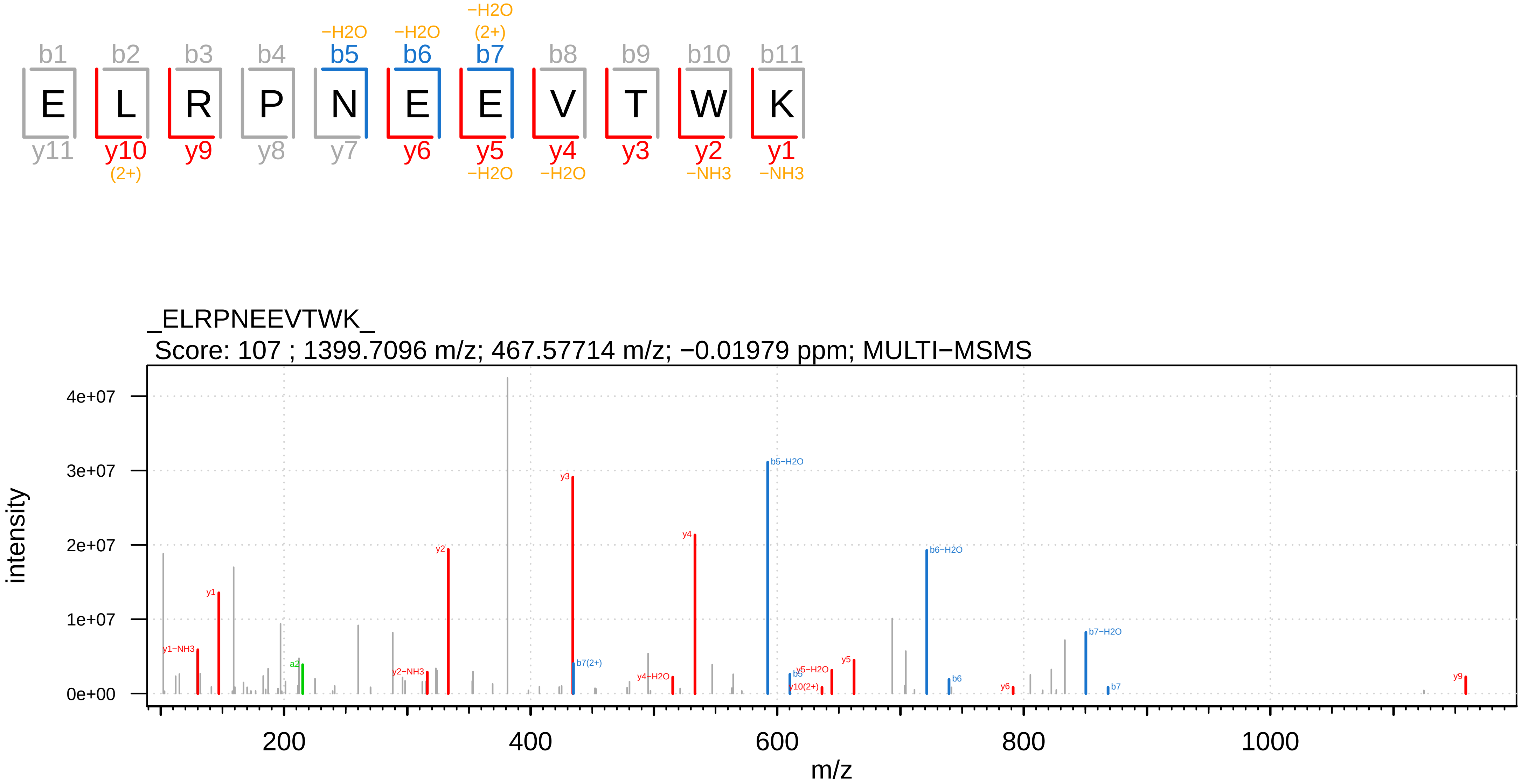

Raw File: 20130421_EXQ6_BaAz_SA_HR1_05
Scan Number: 33625
Proteins:
chr12_103206313−103214181.T2_chr12:101968174−101969629:−

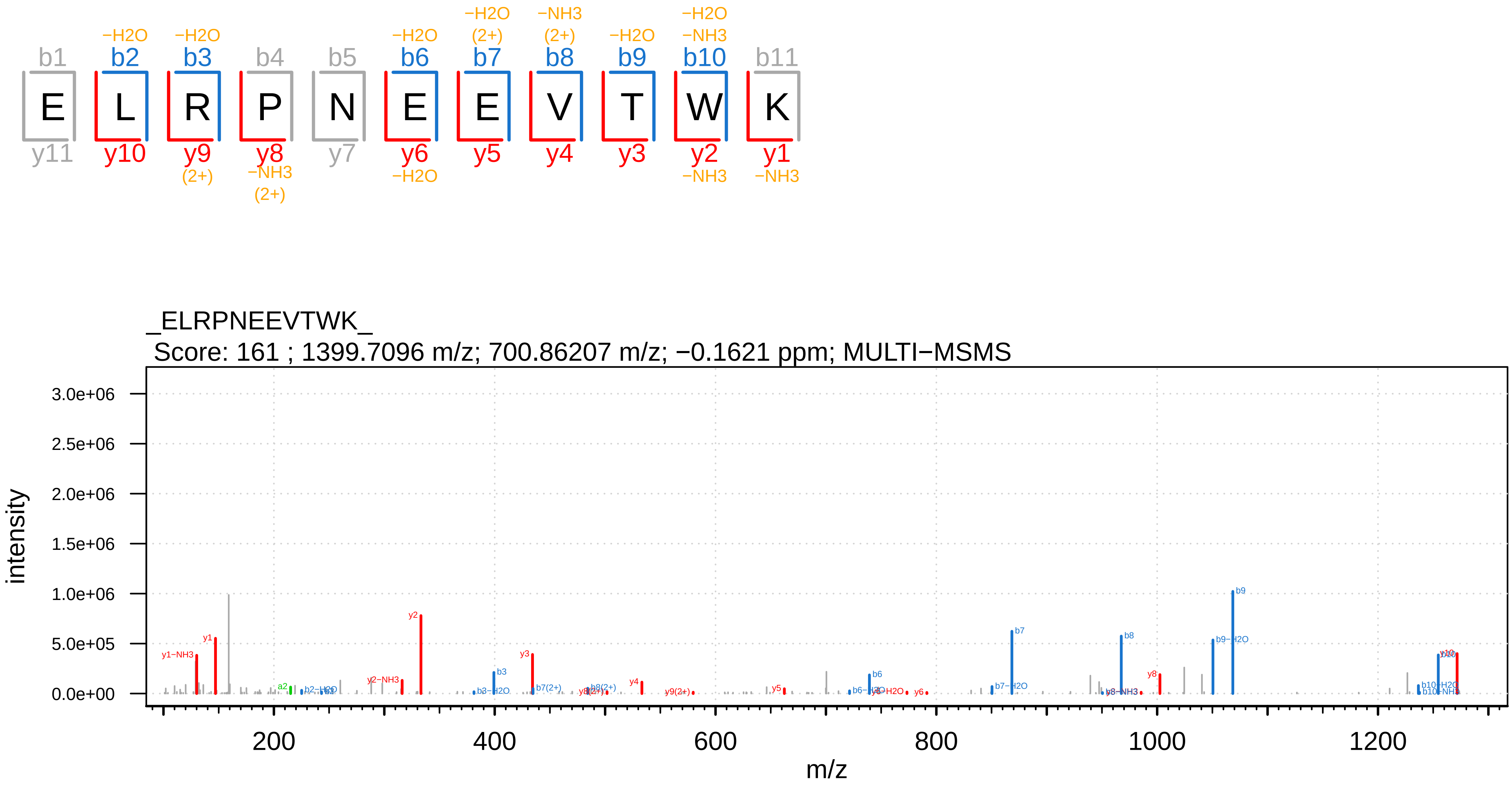

Raw File: 20130422_EXQ6_BaAz_SA_CHR1_05
Scan Number: 36002
Proteins:
chr12_103206313−103214181.T2_chr12:101968174−101969629:−

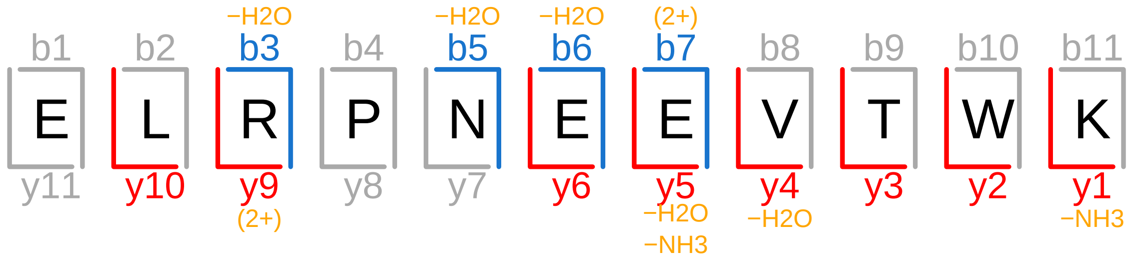

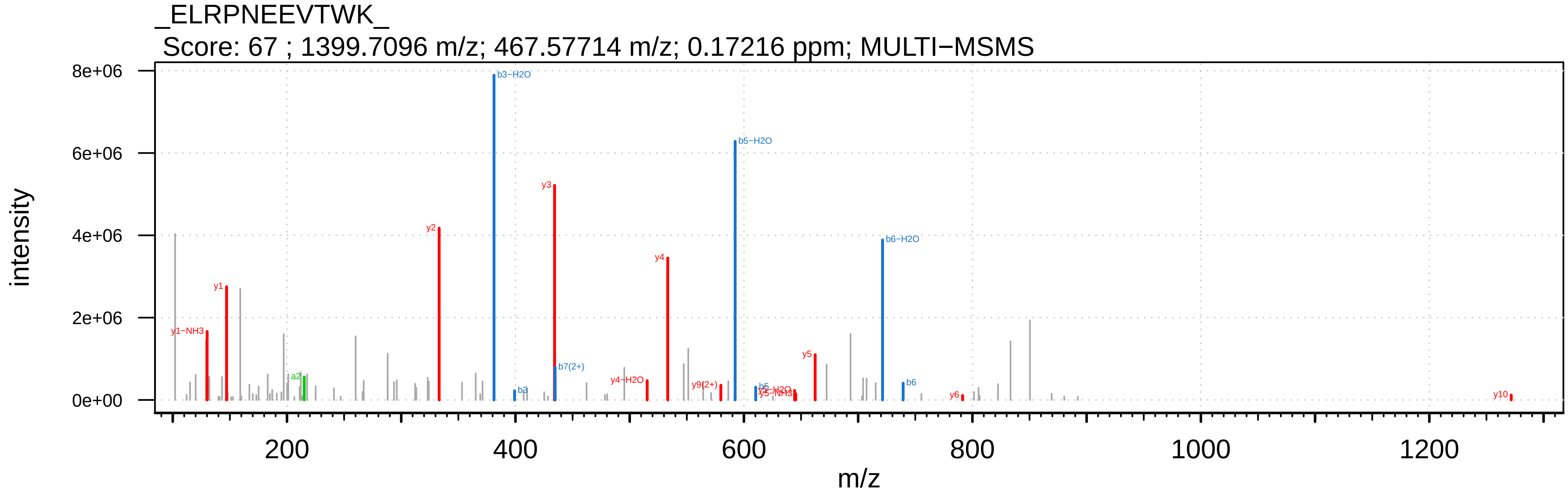

Raw File: 20130422_EXQ6_BaAz_SA_CHR1_05
Scan Number: 36010
Proteins:
chr12_103206313−103214181.T2_chr12:101968174−101969629:−

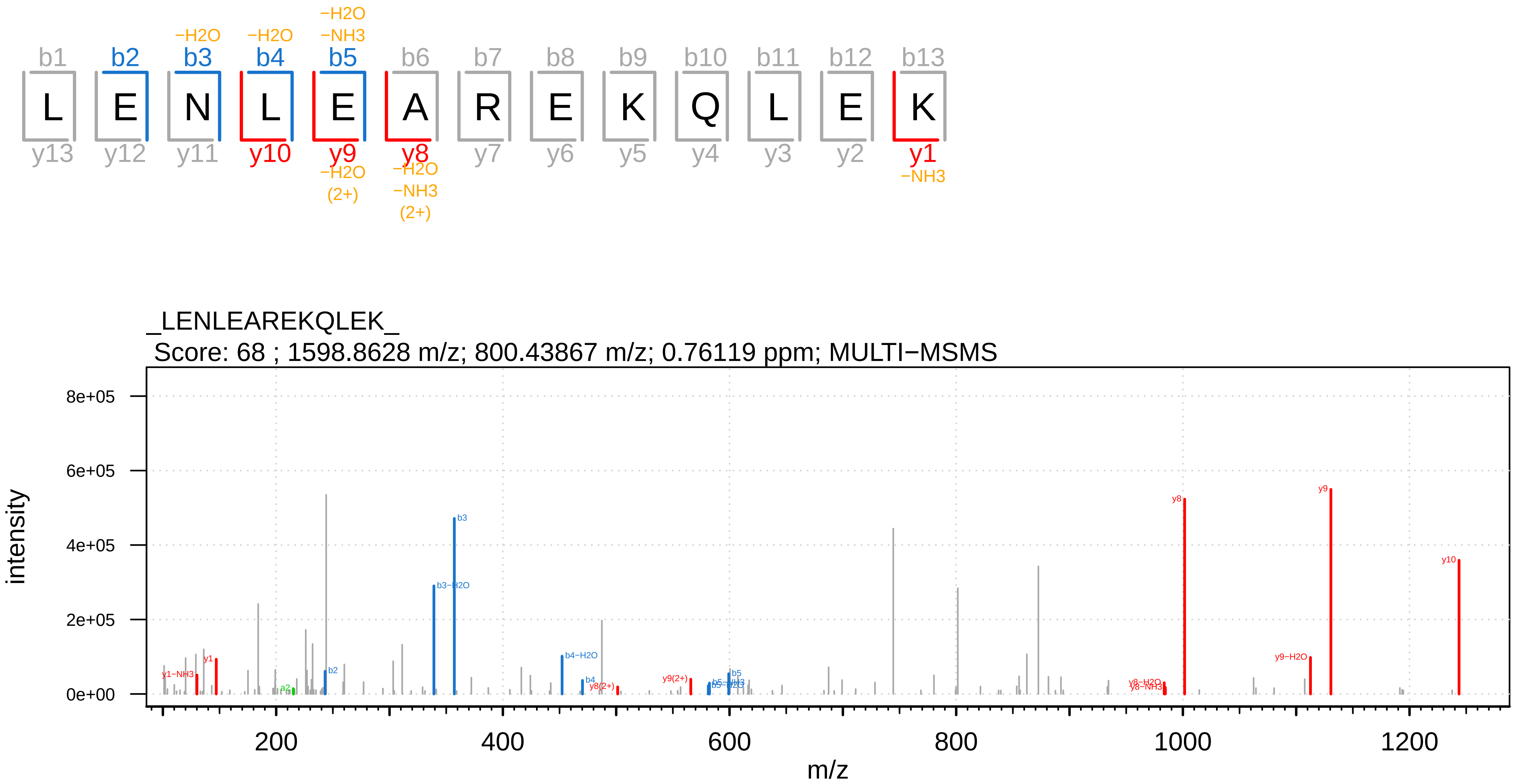

Raw File: 20130422_EXQ6_BaAz_SA_CHR1_05
Scan Number: 50669
Proteins:
ENSMUST00000023048_chr15:80235835−80236047:+

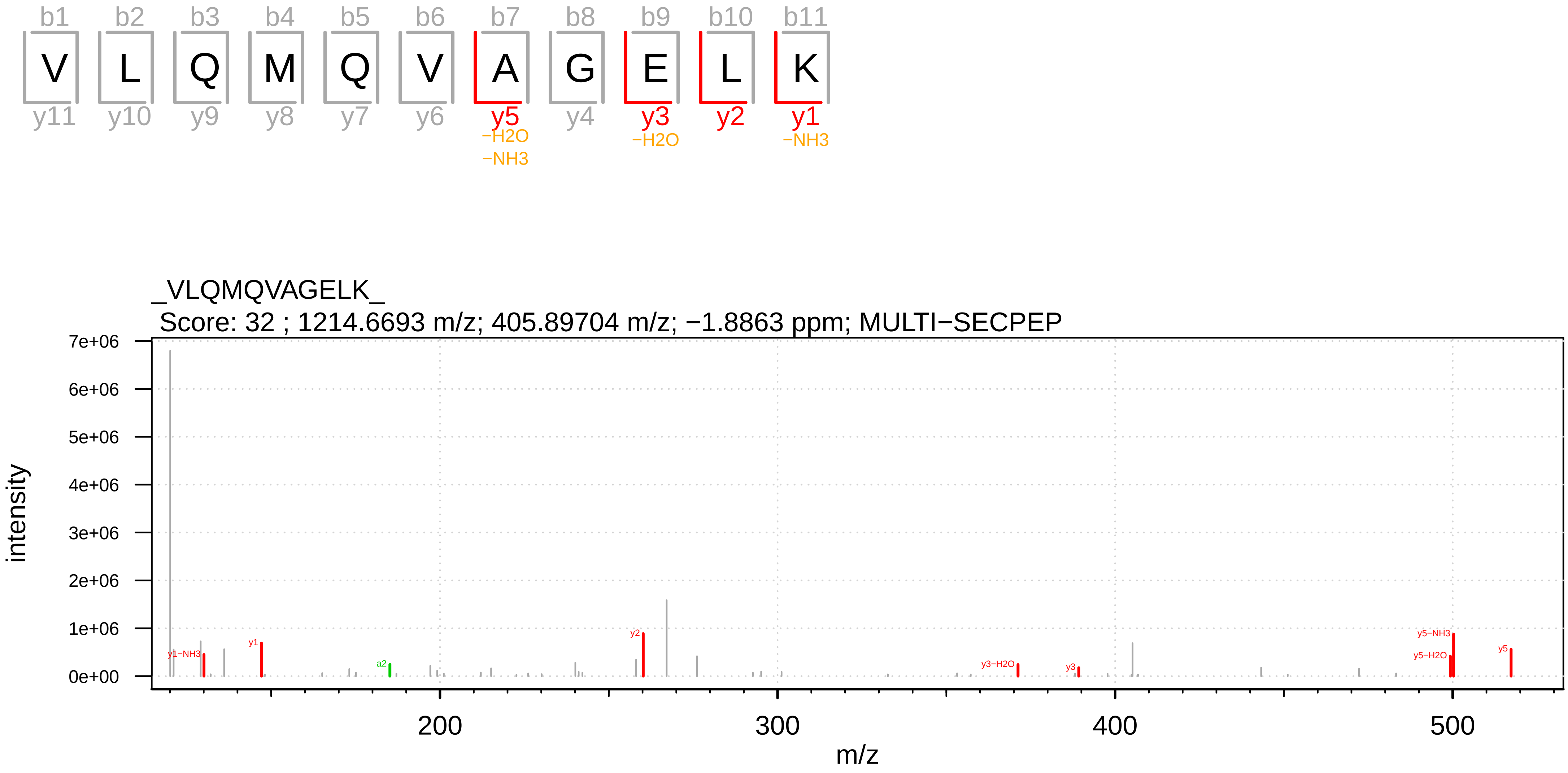

Raw File: 20130422_EXQ6_BaAz_SA_CHR1_05
Scan Number: 34639
Proteins:
ENSMUST00000131447_chr4:134243931−134244381:+

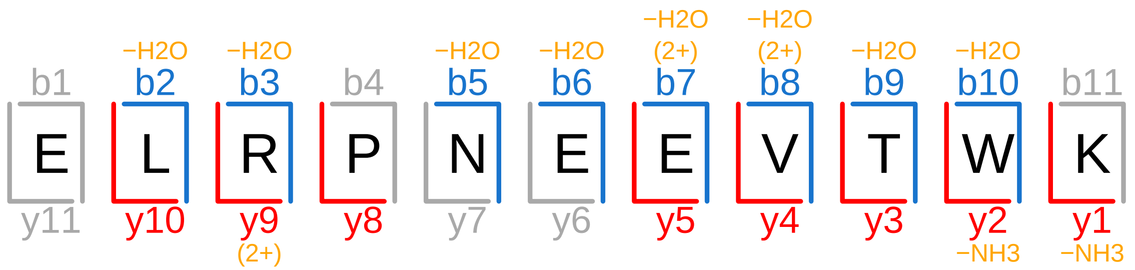

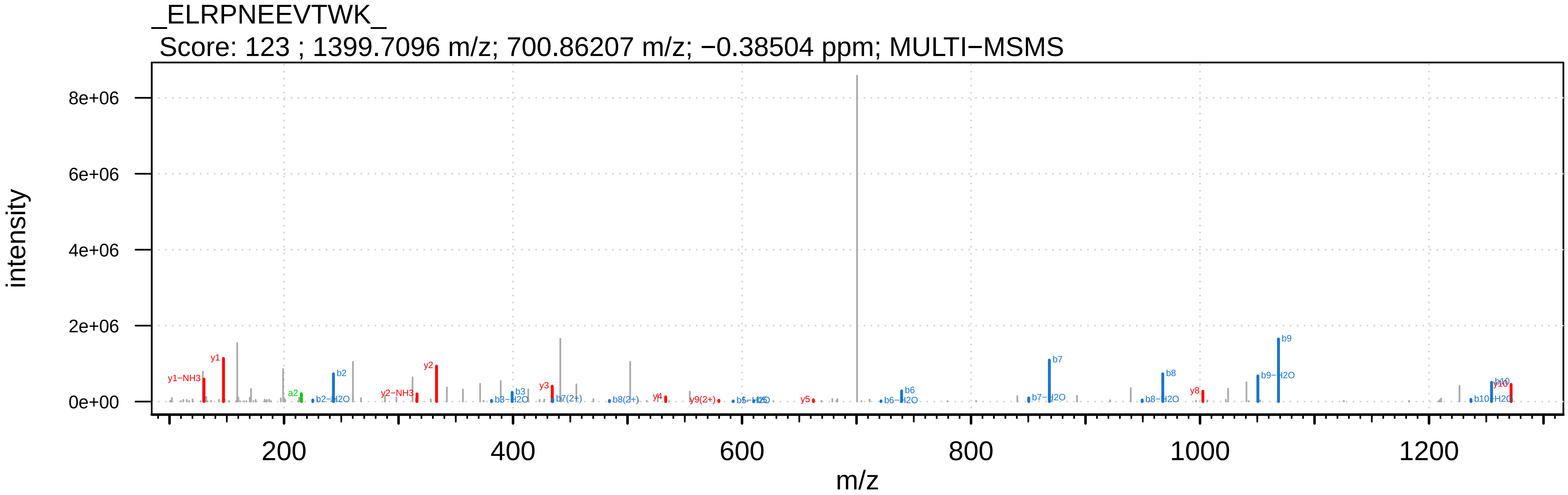

Raw File: 20130705_EXQ4_BaAz_SA_HR2_05

Scan Number: 32875

Proteins:

chr12_103206313−103214181.T2_chr12:101968174−101969629:−

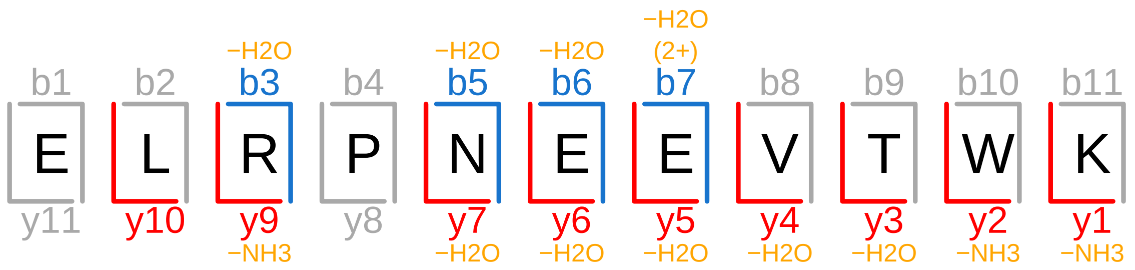

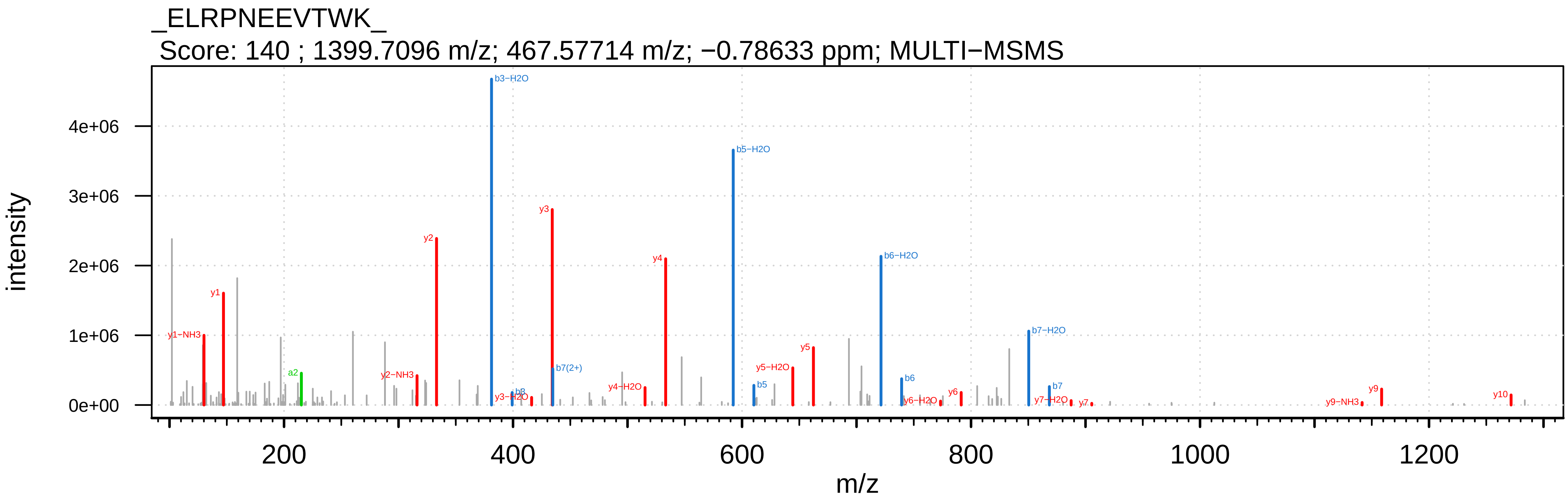

Raw File: 20130705_EXQ4_BaAz_SA_HR2_05
Scan Number: 32877
Proteins:
chr12_103206313−103214181.T2_chr12:101968174−101969629:−

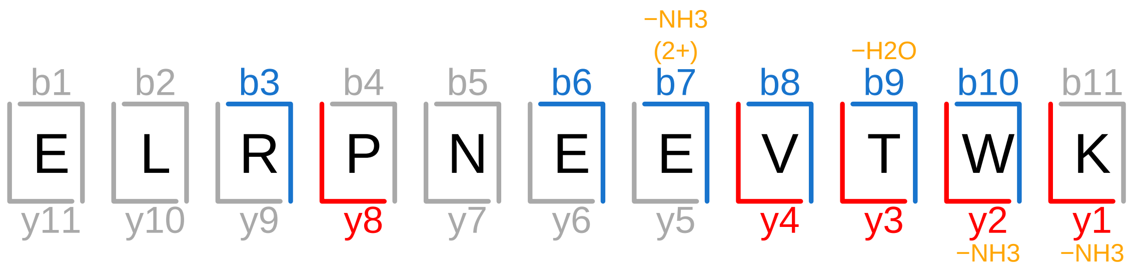

ELRPNEEVTWK
Score: 82 ; 1399.7096 m/z; 700.86207 m/z; 0.043275 ppm; MULTI−MSMS

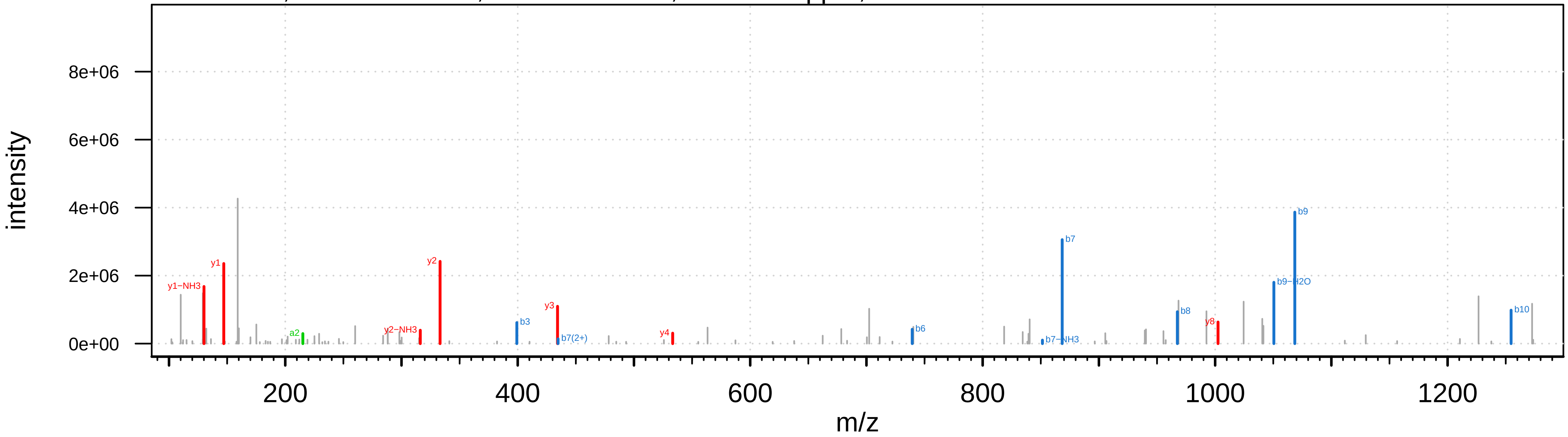

Raw File: 20130705_EXQ4_BaAz_SA_HR2_06
Scan Number: 30479
Proteins:
chr12_103206313−103214181.T2_chr12:101968174−101969629:−

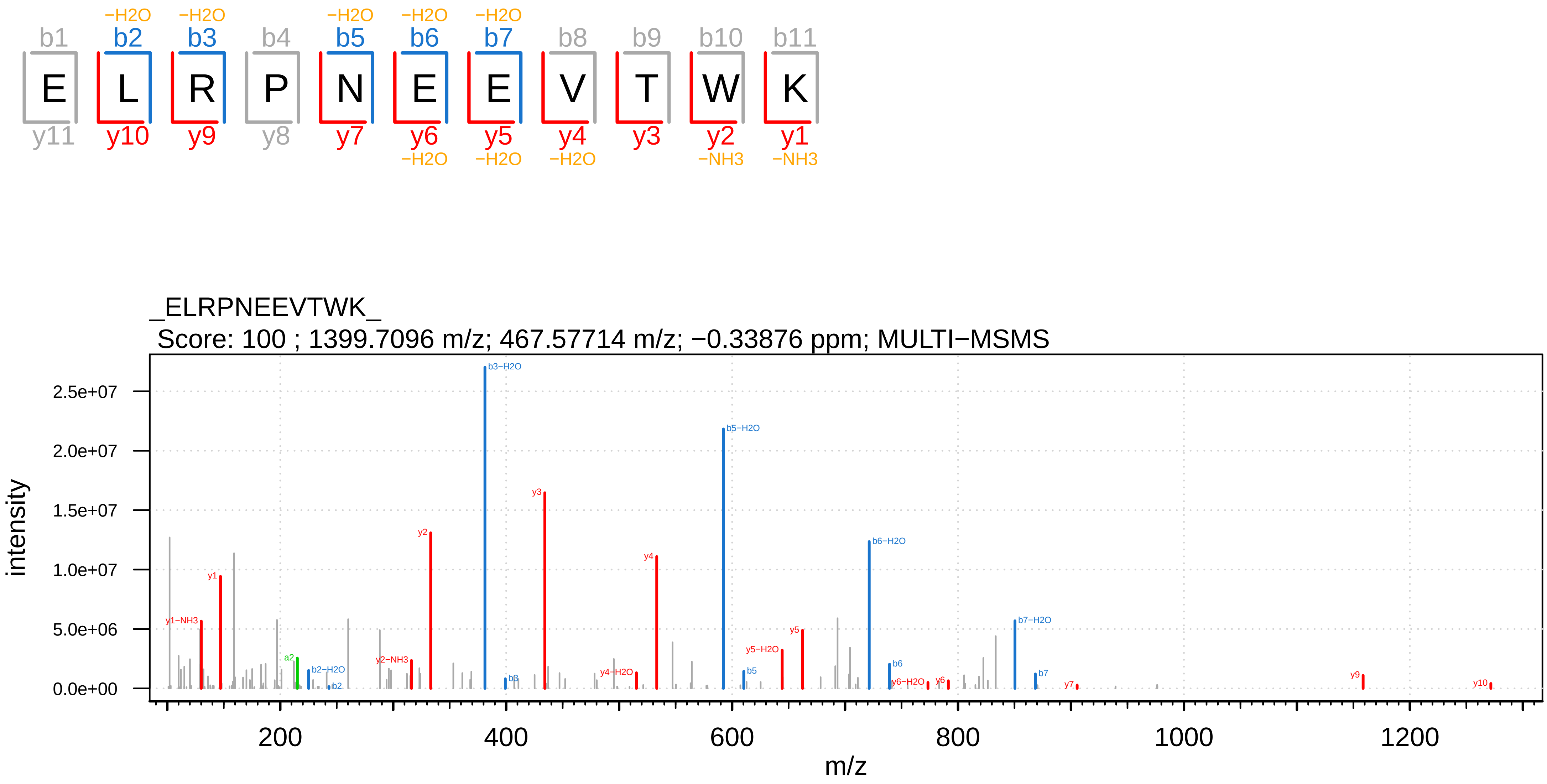

Raw File: 20130705_EXQ4_BaAz_SA_HR2_06
Scan Number: 30492
Proteins:
chr12_103206313−103214181.T2_chr12:101968174−101969629:−

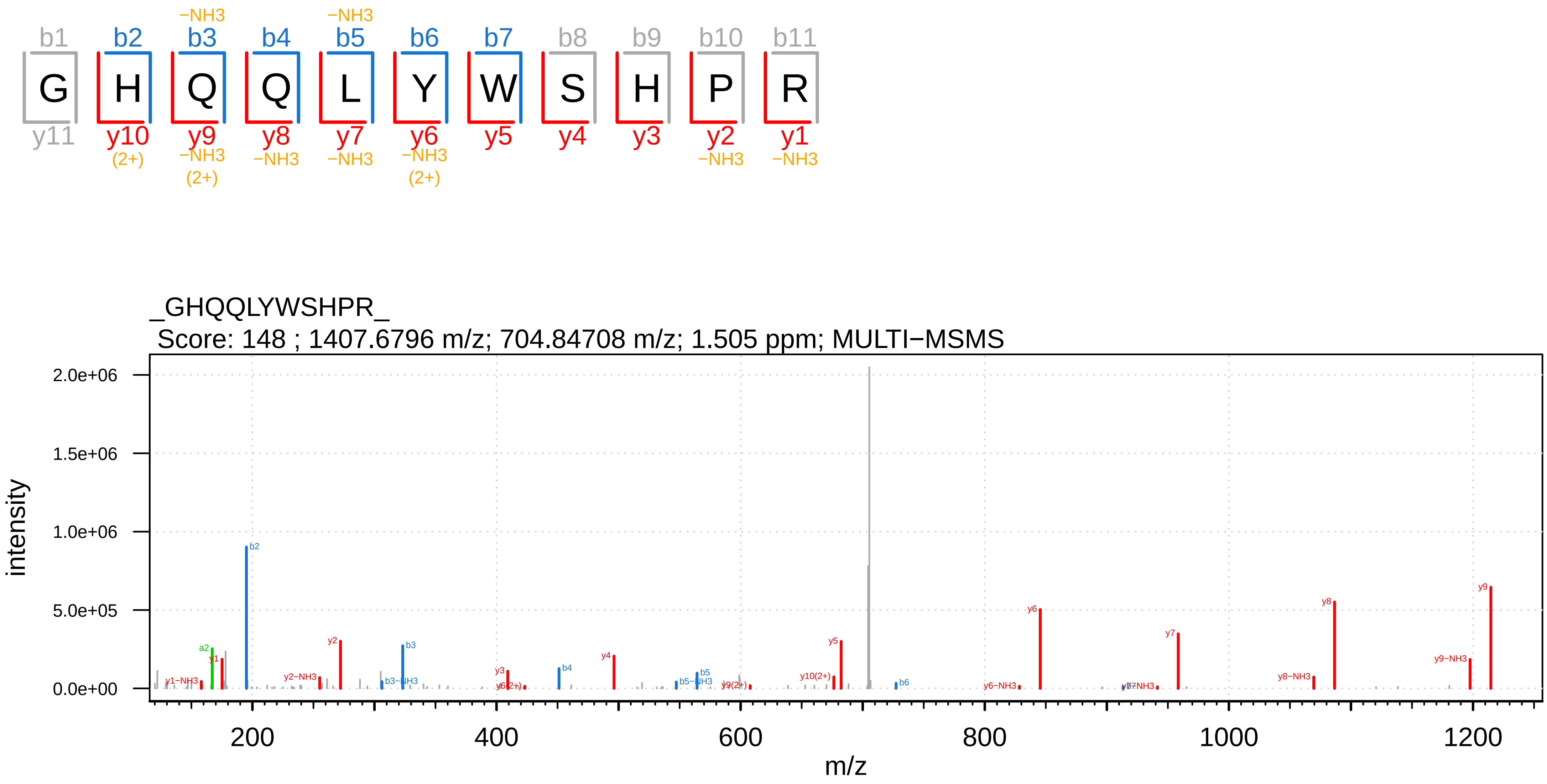

Raw File: 20130705_EXQ4_BaAz_SA_HR2_06
Scan Number: 14040
Proteins:
sp|P62274|RS29_MOUSE
ENSMUST00000037023_chr12:69157813−69159058:−
ENSMUST00000071737_chr13:73988963−73989133:−

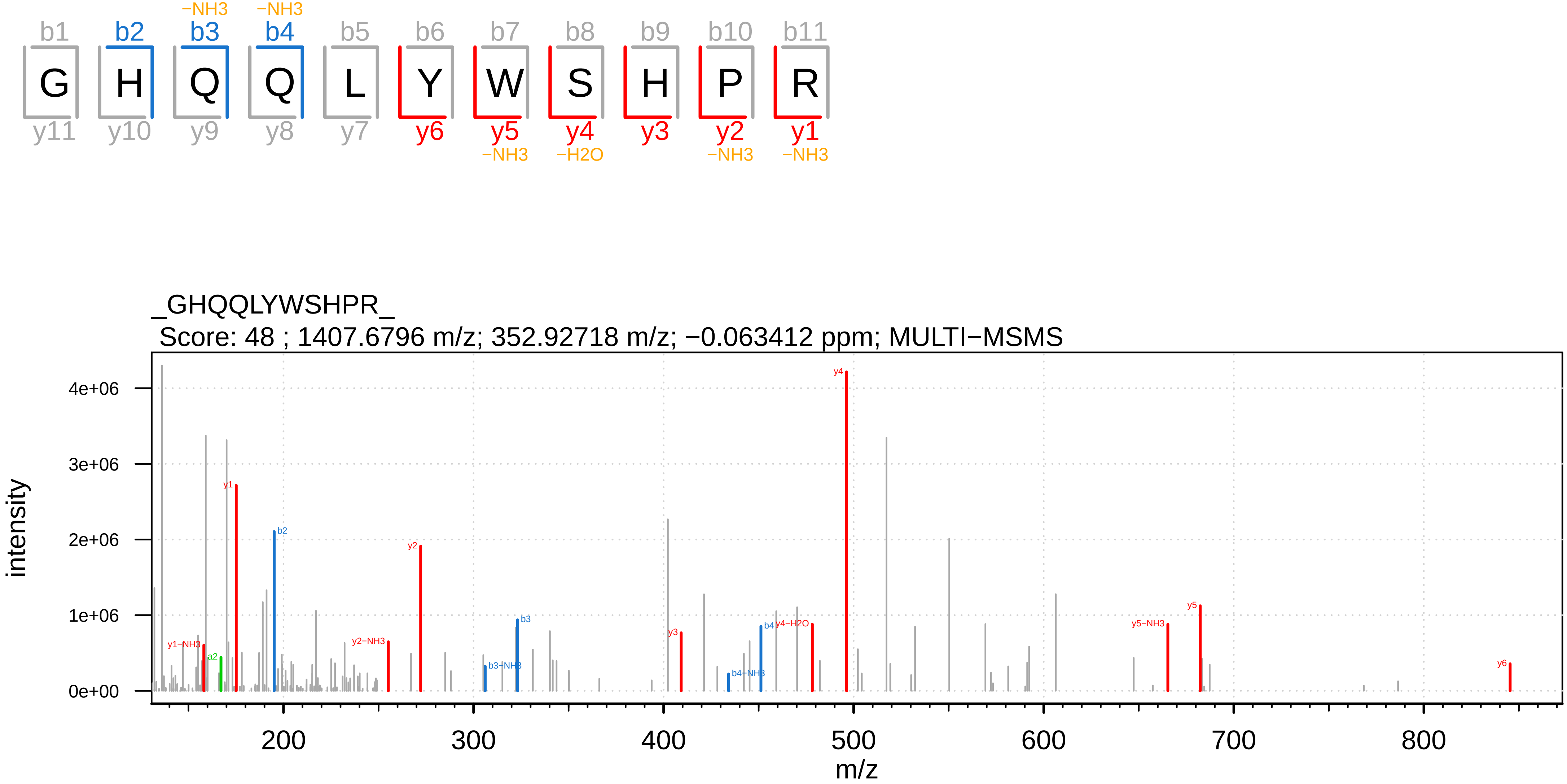

Raw File: 20130705_EXQ4_BaAz_SA_HR2_06
Scan Number: 14103
Proteins:
sp|P62274|RS29_MOUSE
ENSMUST00000037023_chr12:69157813−69159058:−
ENSMUST00000071737_chr13:73988963−73989133:−

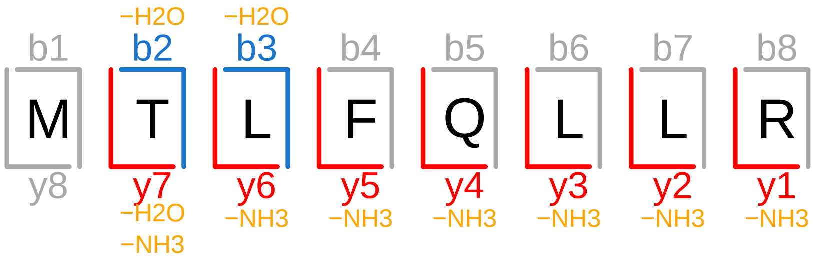

MTLFQLLR

Score: 151 ; 1020.579 m/z; 511.29679 m/z; −0.4302 ppm; MULTI−MSMS

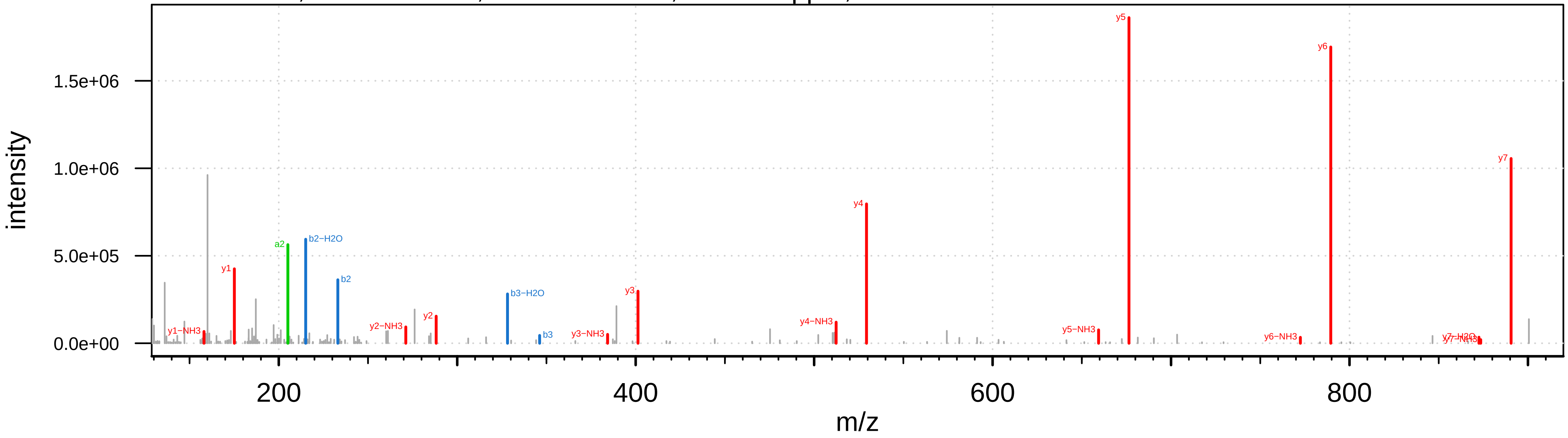

Raw File: 20130705_EXQ4_BaAz_SA_HR2_06

Scan Number: 87191

Proteins:

chr12_103206313−103214181.T2_chr12:101968174−101969629:−

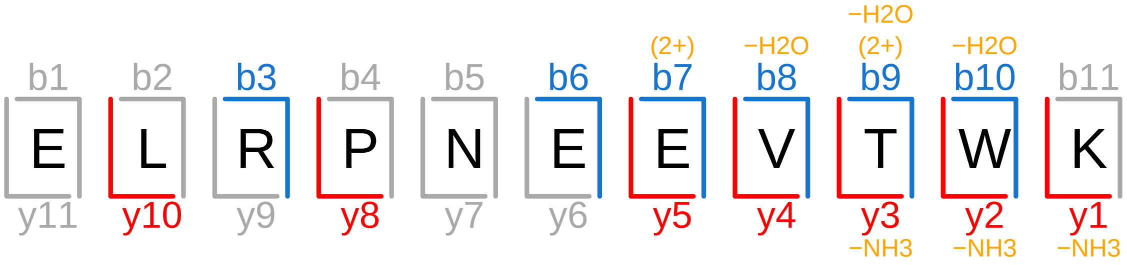

ELRPNEEVTWK
Score: 87 ; 1399.7096 m/z; 700.86207 m/z; 0.05137 ppm; MULTI−MSMS

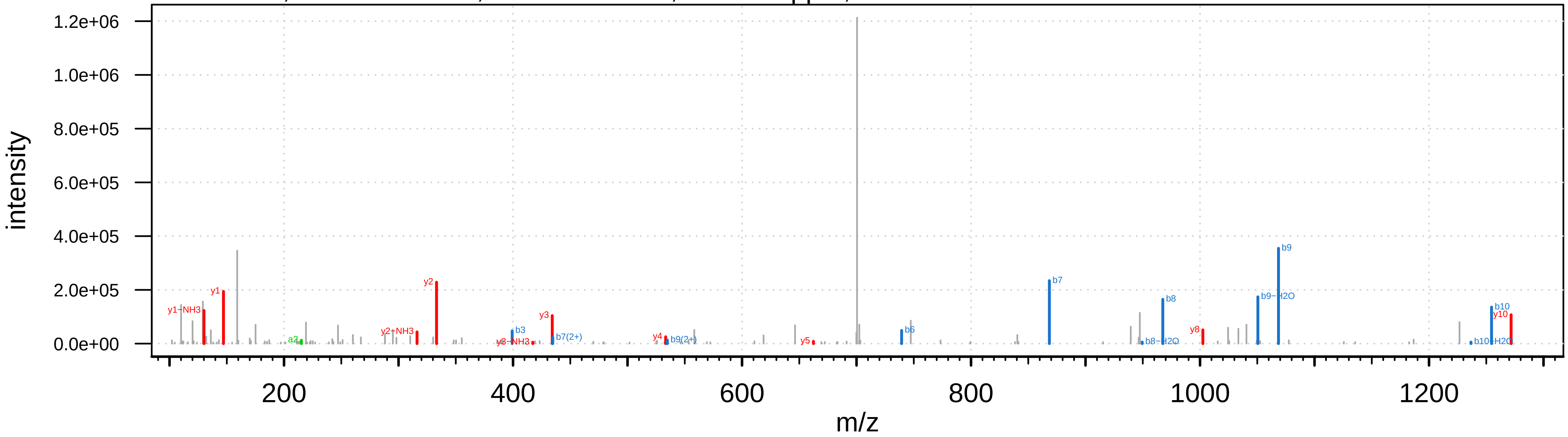

Raw File: 20130705_EXQ6_BaAz_SA_H53R1_06
Scan Number: 32481
Proteins:
chr12_103206313−103214181.T2_chr12:101968174−101969629:−

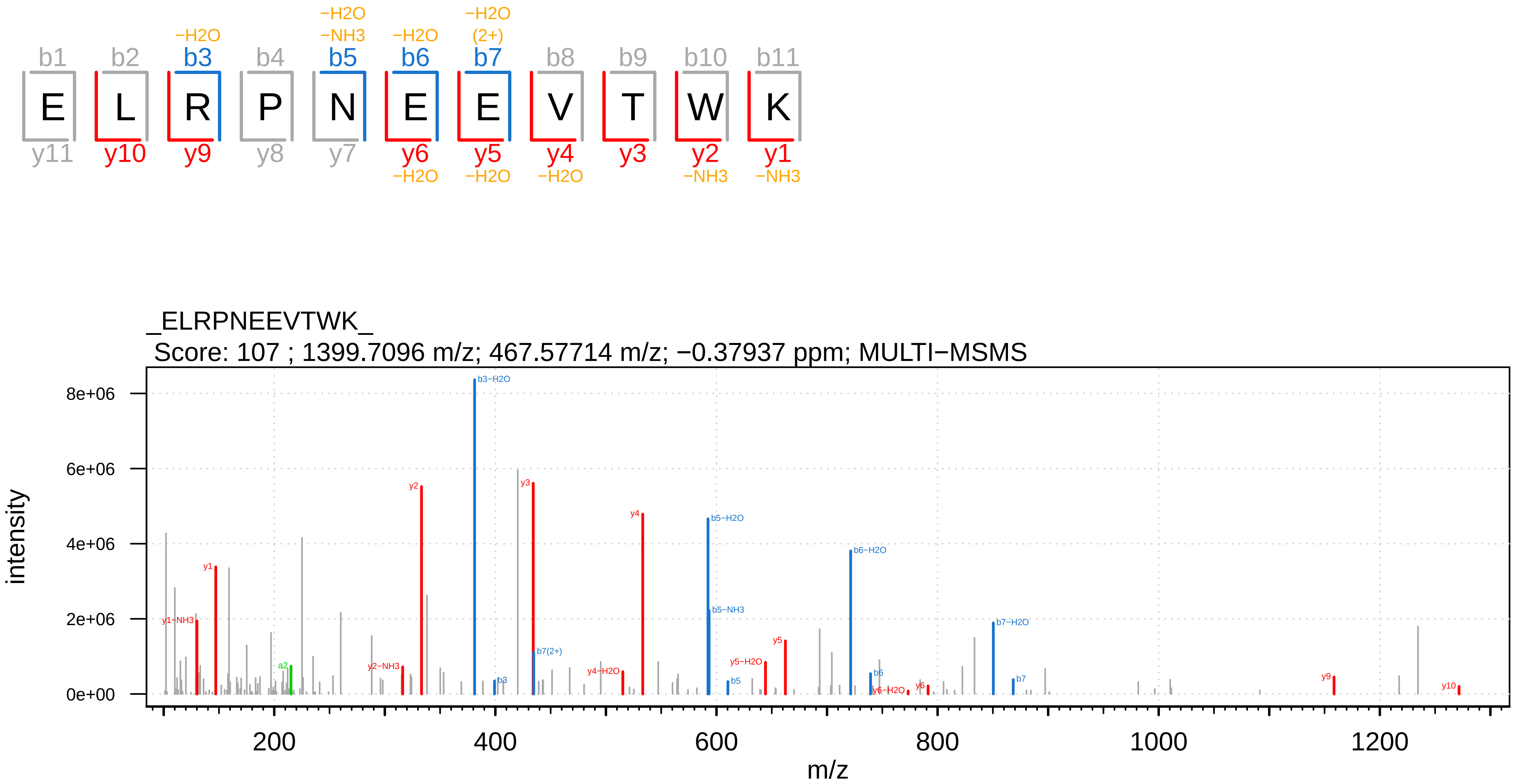

Raw File: 20130705_EXQ6_BaAz_SA_H53R1_06
Scan Number: 32509
Proteins:
chr12_103206313−103214181.T2_chr12:101968174−101969629:−

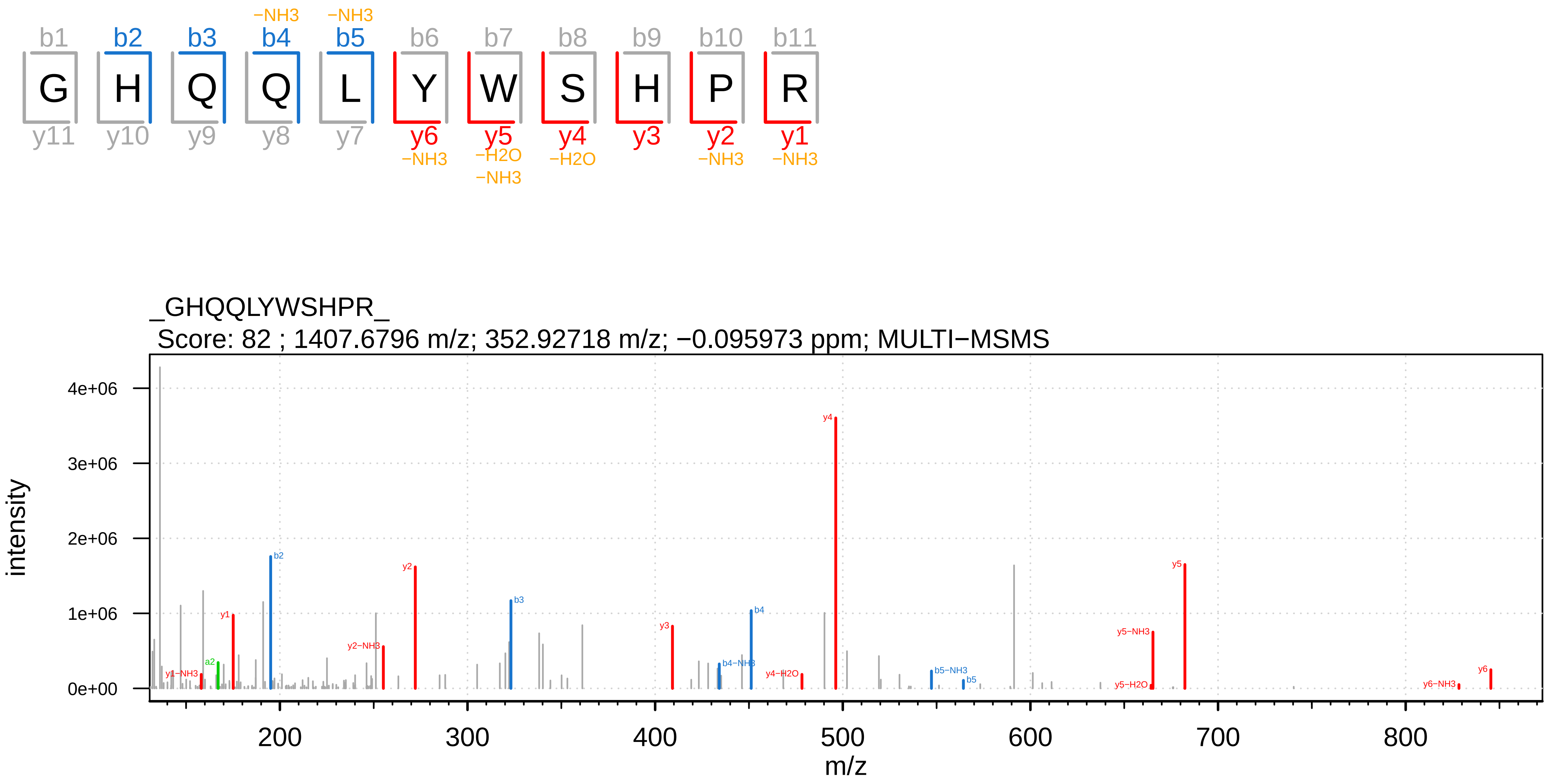

Raw File: 20130705_EXQ6_BaAz_SA_H53R1_06
Scan Number: 17535
Proteins:
sp|P62274|RS29_MOUSE
ENSMUST00000037023_chr12:69157813−69159058:−
ENSMUST00000071737_chr13:73988963−73989133:−

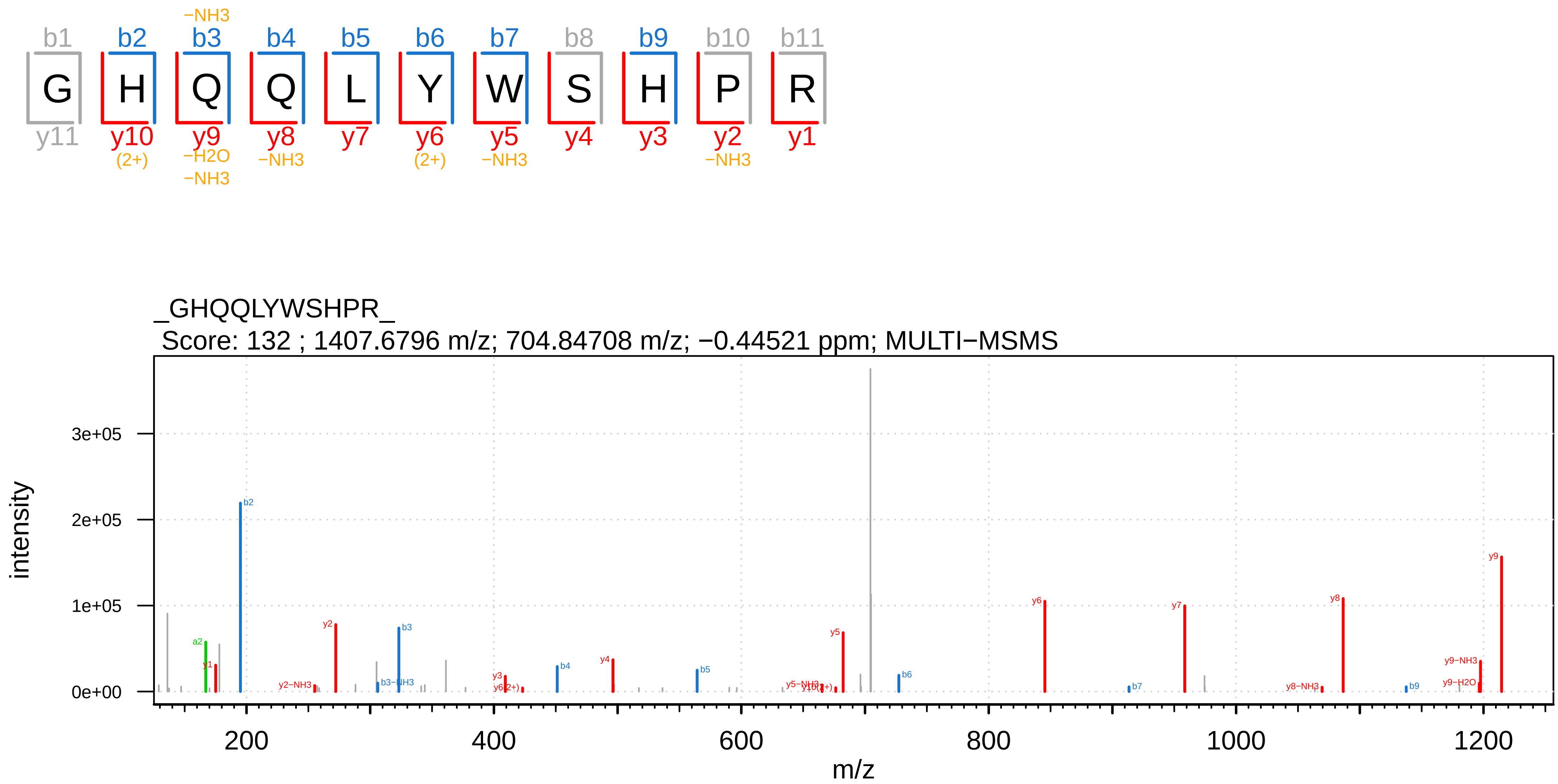

Raw File: 20130705_EXQ6_BaAz_SA_H53R1_06
Scan Number: 17541
Proteins:
sp|P62274|RS29_MOUSE
ENSMUST00000037023_chr12:69157813−69159058:−
ENSMUST00000071737_chr13:73988963−73989133:−

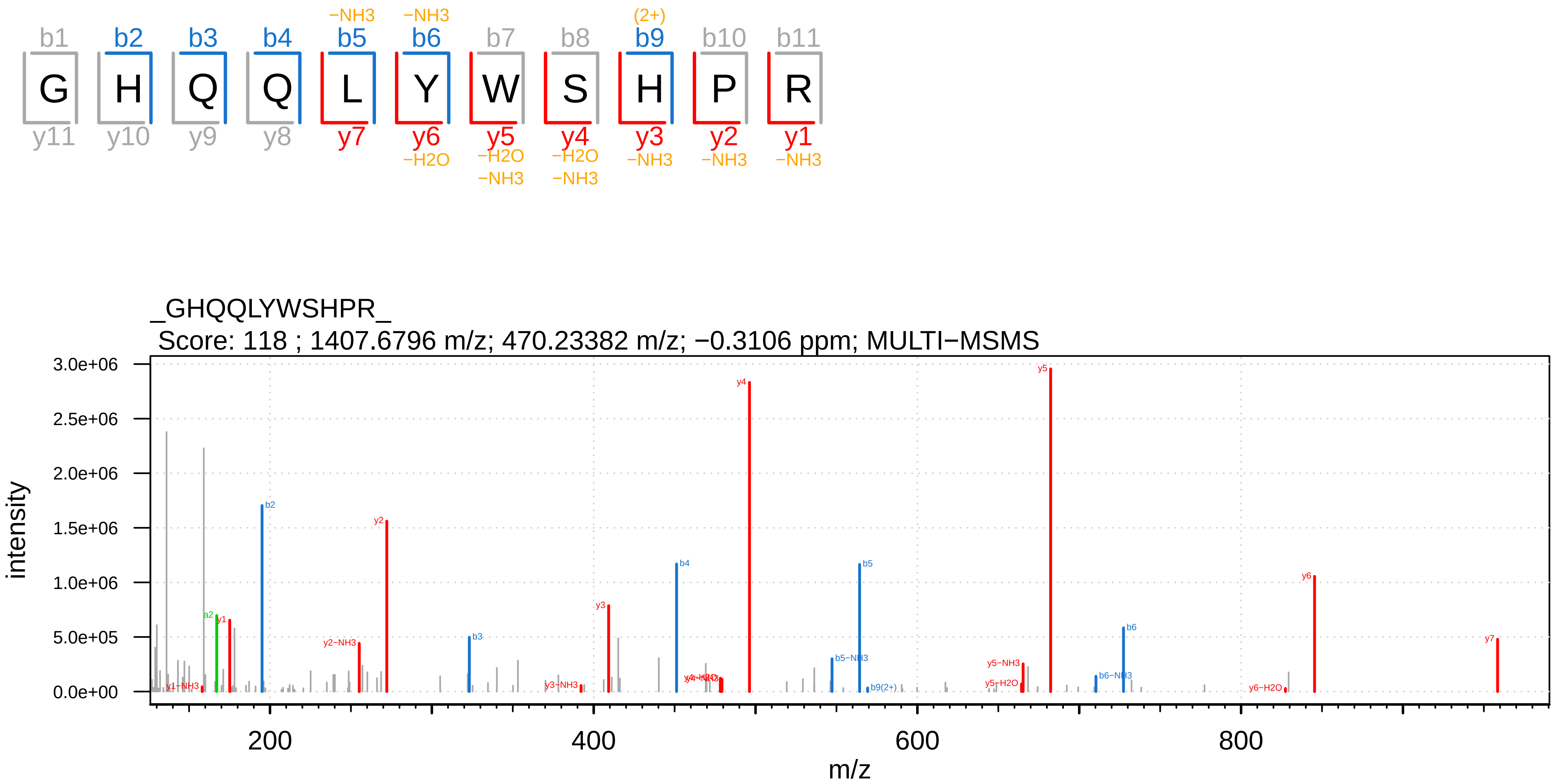

Raw File: 20130705_EXQ6_BaAz_SA_H53R1_06
Scan Number: 17783
Proteins:
sp|P62274|RS29_MOUSE
ENSMUST00000037023_chr12:69157813−69159058:−
ENSMUST00000071737_chr13:73988963−73989133:−

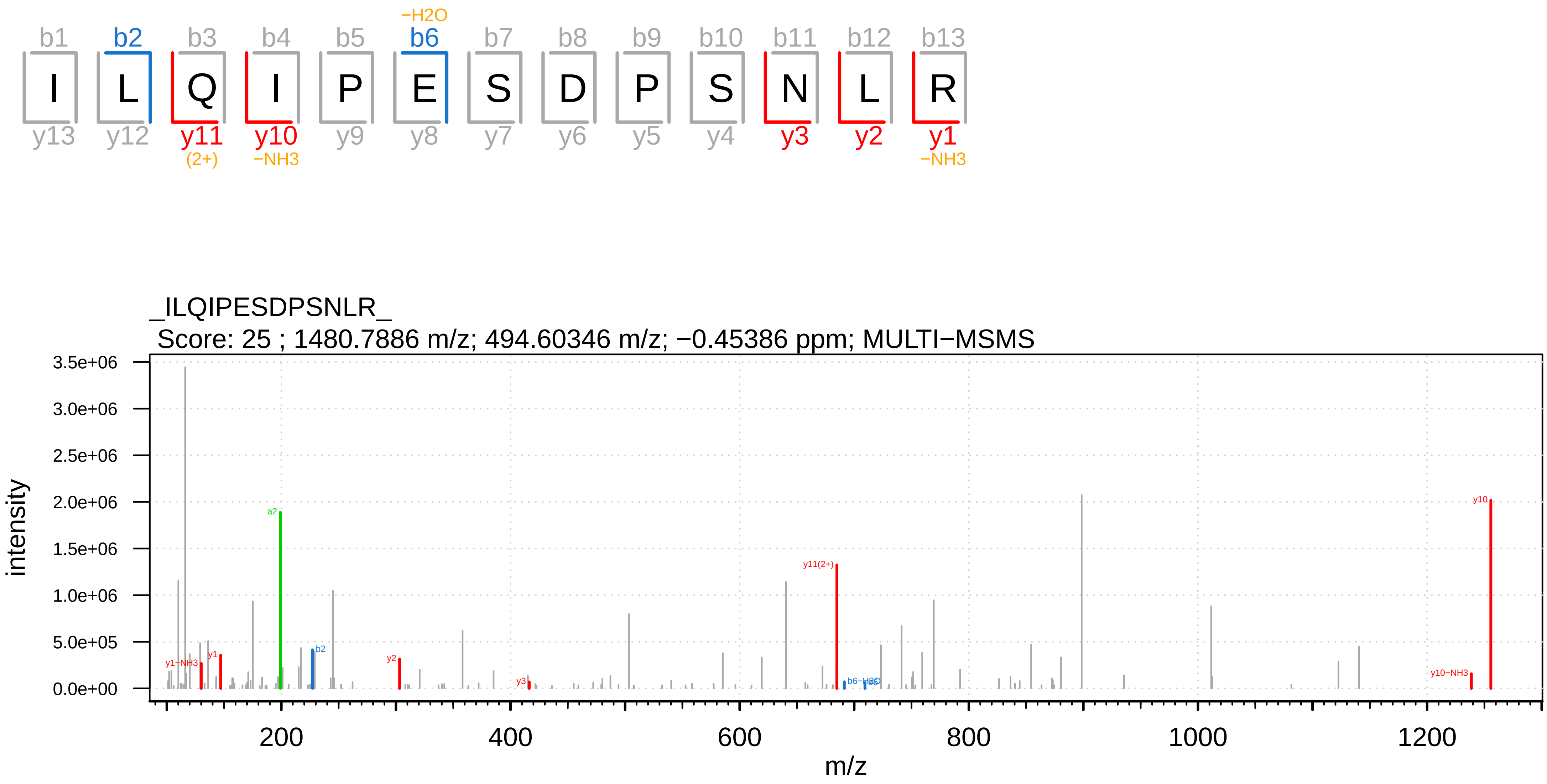

Raw File: 20130705_EXQ6_BaAz_SA_H53R1_06
Scan Number: 32915
Proteins:
ENSMUST00000161069_chr14:70554856−70554960:+

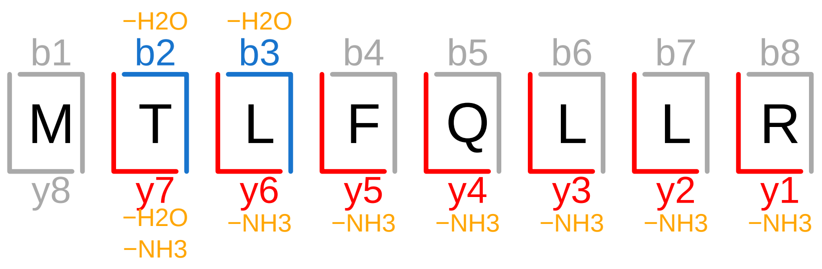

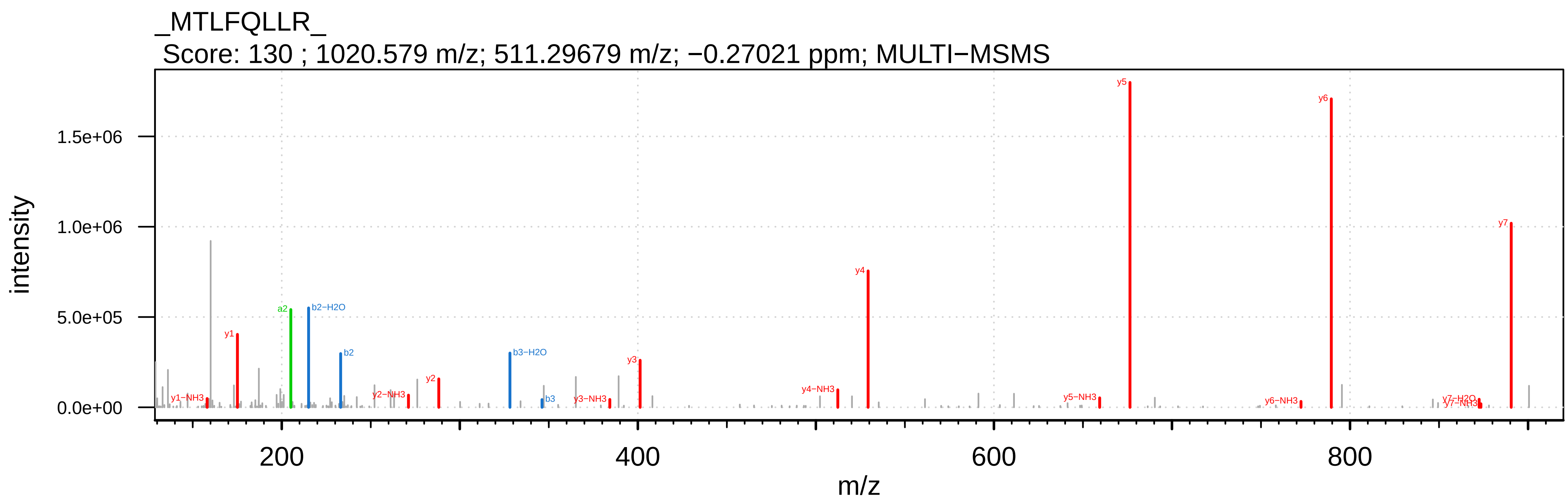

Raw File: 20130705_EXQ6_BaAz_SA_H53R1_06
Scan Number: 82821
Proteins:
chr12_103206313−103214181.T2_chr12:101968174−101969629:−

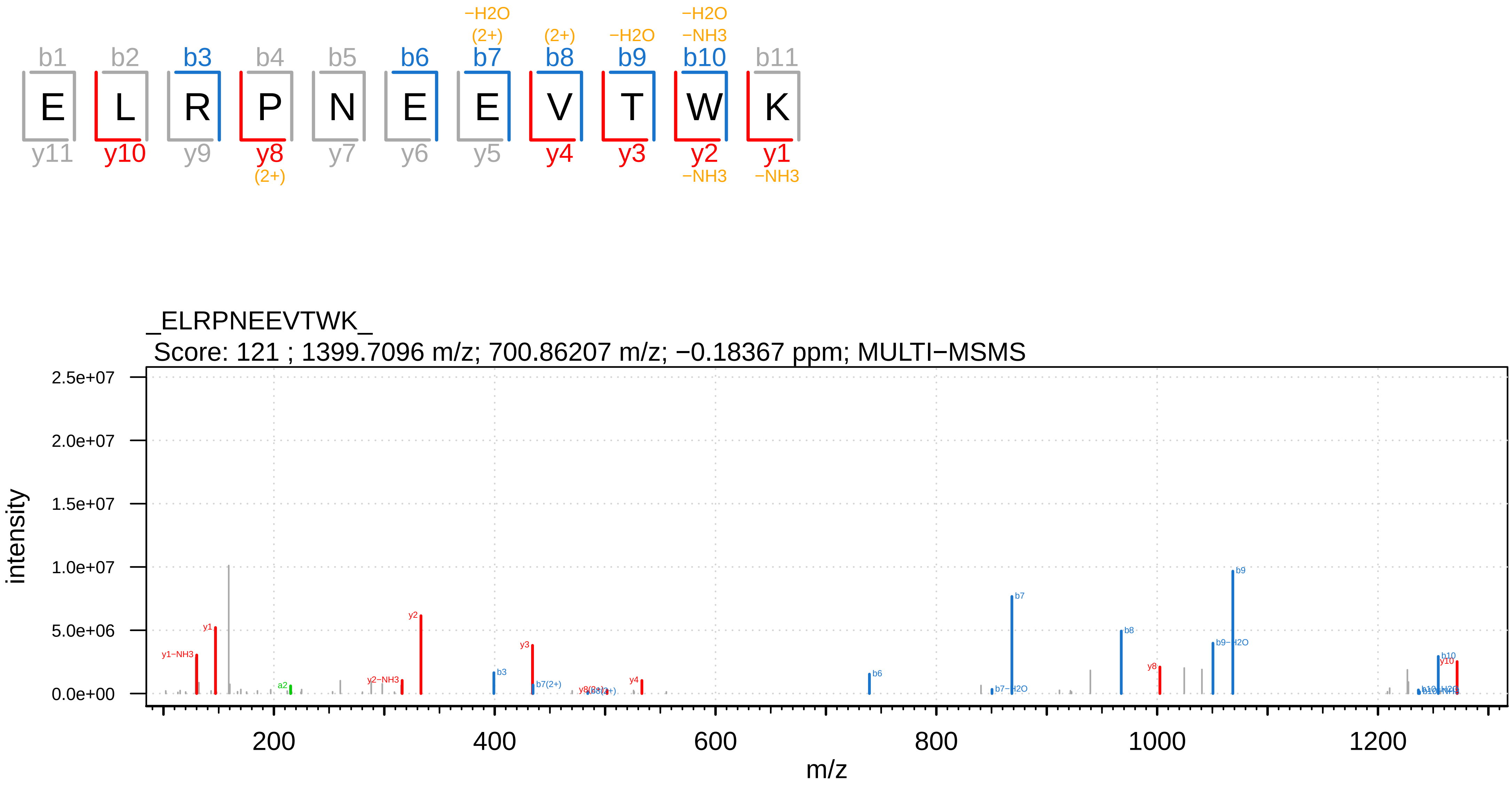

Raw File: 20130707_EXQ4_BaAz_SA_LR2_05

Scan Number: 33649

Proteins:

chr12_103206313−103214181.T2_chr12:101968174−101969629:−

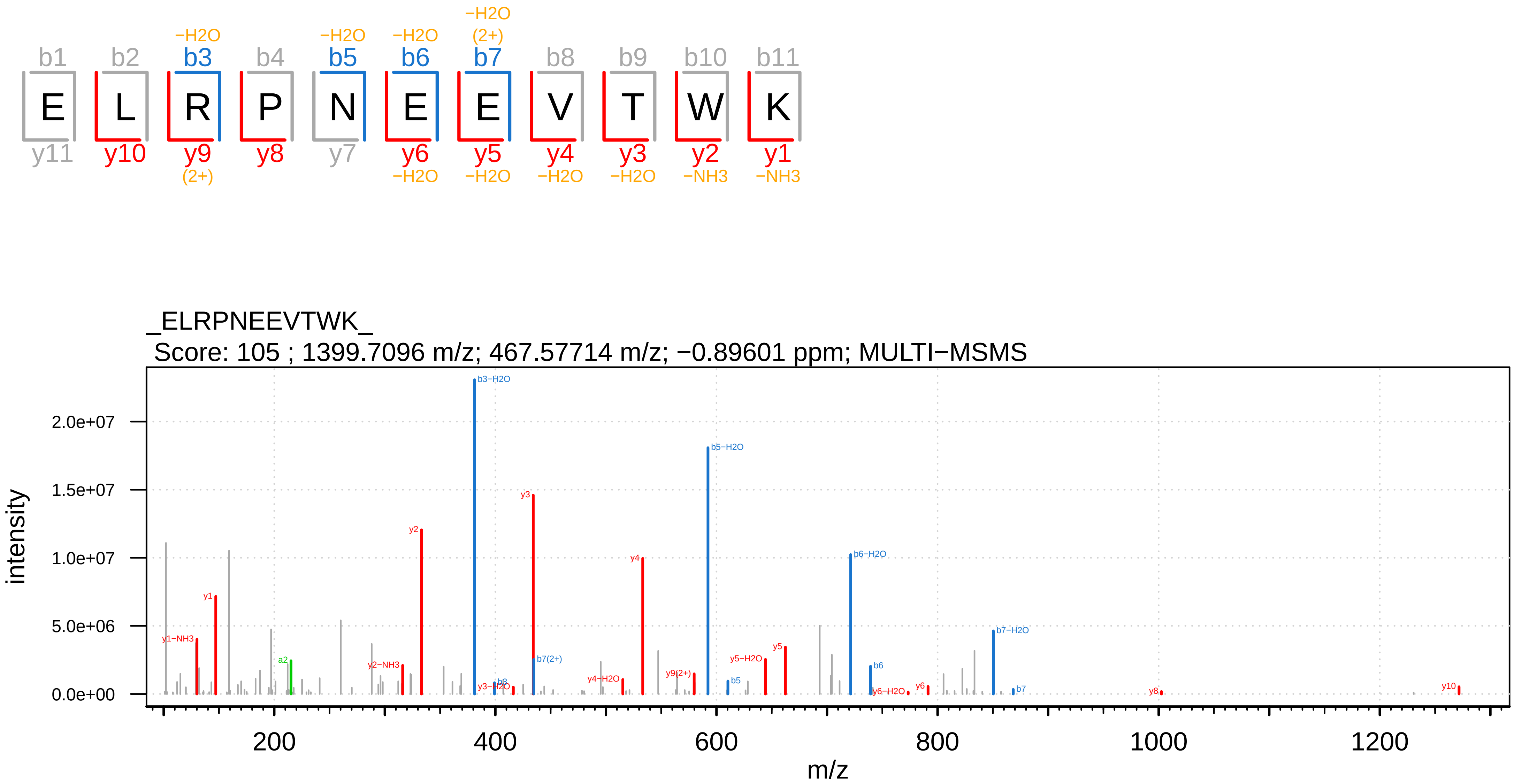

Raw File: 20130707_EXQ4_BaAz_SA_LR2_05
Scan Number: 33666
Proteins:
chr12_103206313−103214181.T2_chr12:101968174−101969629:−

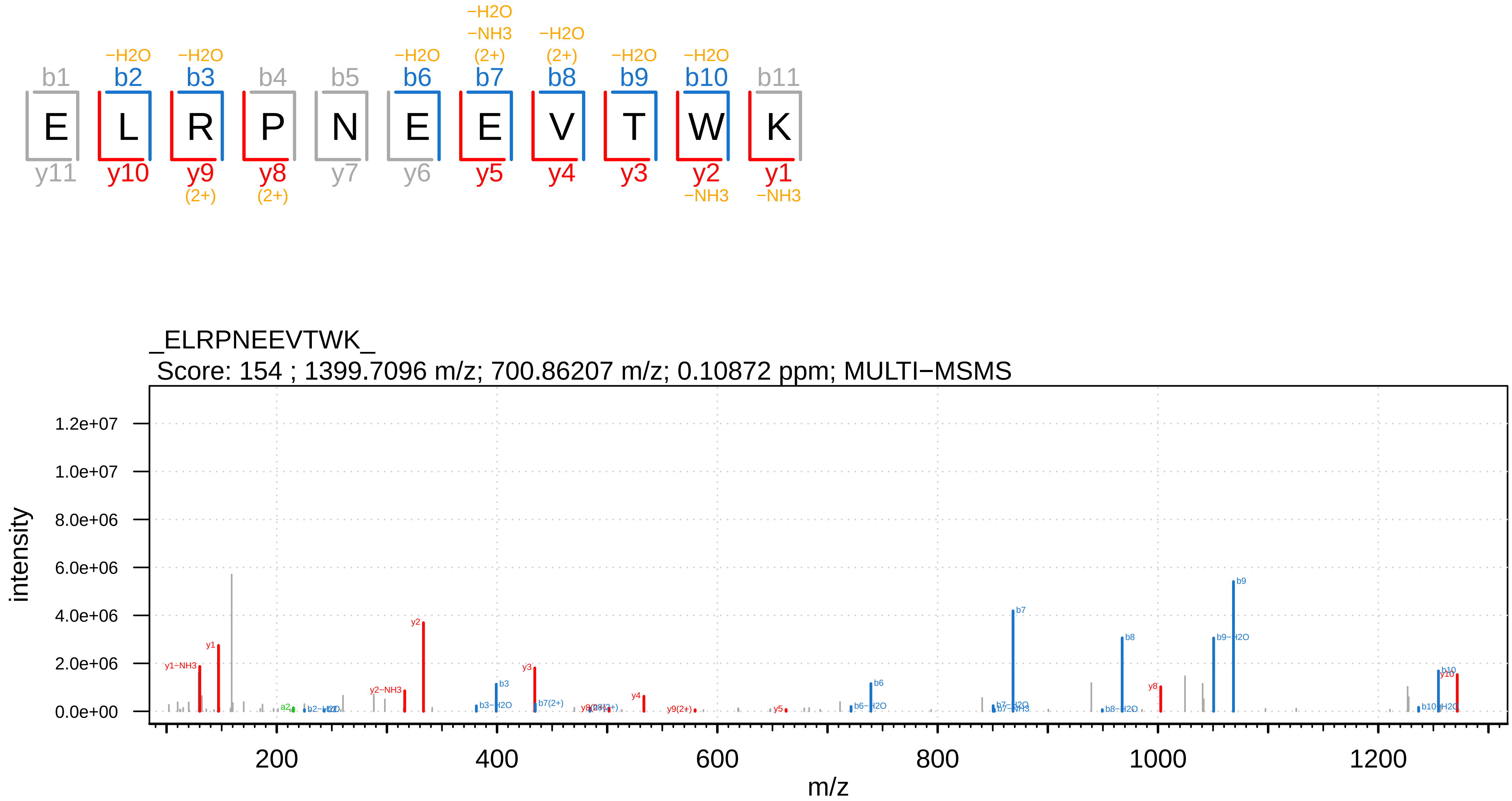

Raw File: 20130707_EXQ4_BaAz_SA_LR2_06
Scan Number: 32596
Proteins:
chr12_103206313−103214181.T2_chr12:101968174−101969629:−

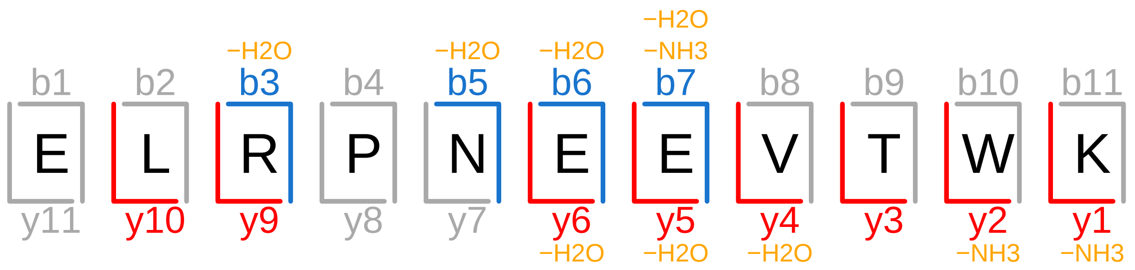

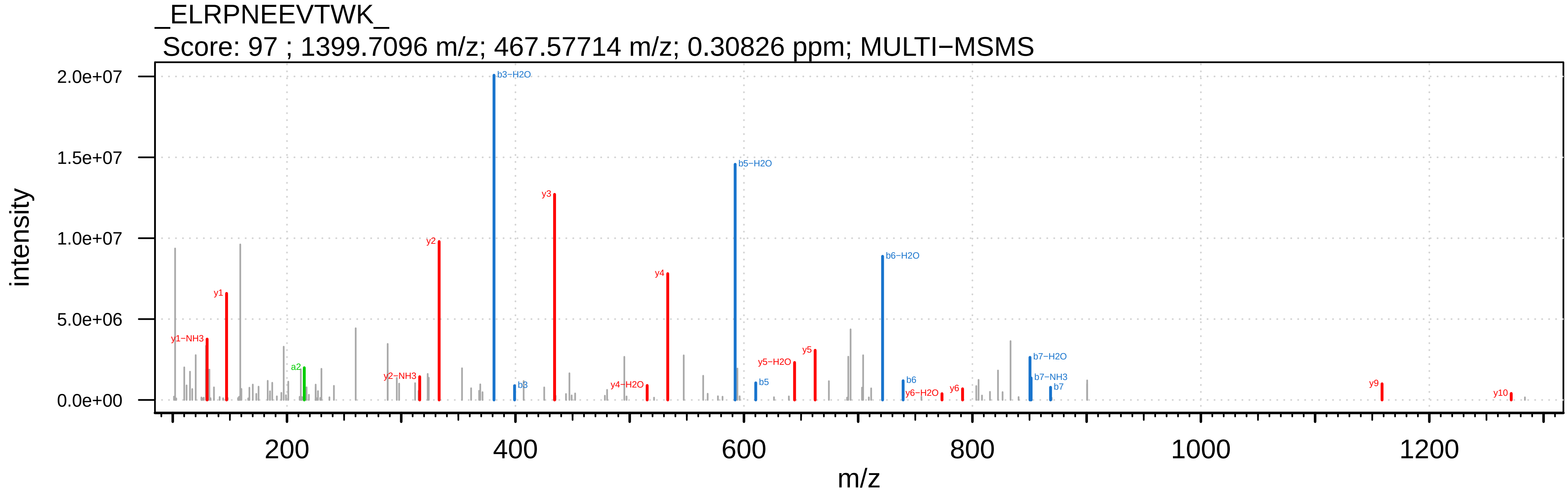

Raw File: 20130707_EXQ4_BaAz_SA_LR2_06
Scan Number: 32601
Proteins:
chr12_103206313−103214181.T2_chr12:101968174−101969629:−

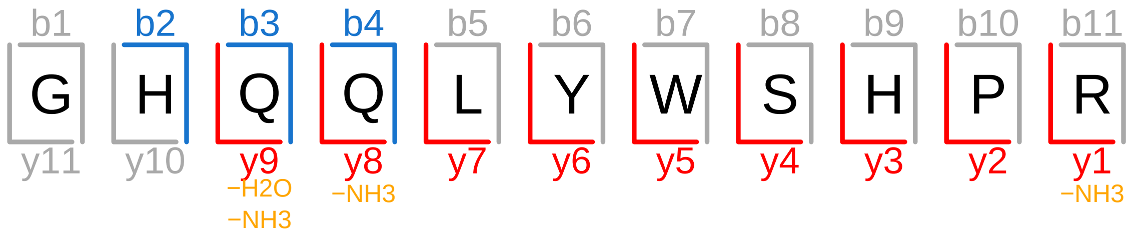

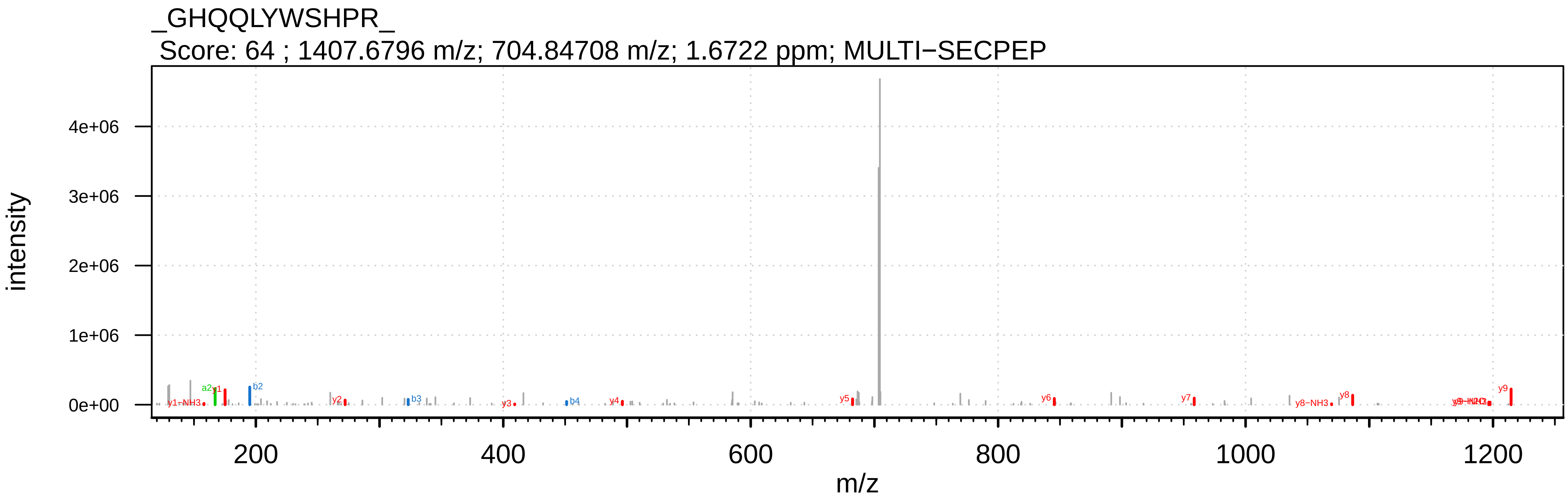

Raw File: 20130707_EXQ4_BaAz_SA_LR2_06
Scan Number: 14479
Proteins:
sp|P62274|RS29_MOUSE
ENSMUST00000037023_chr12:69157813−69159058:−
ENSMUST00000071737_chr13:73988963−73989133:−

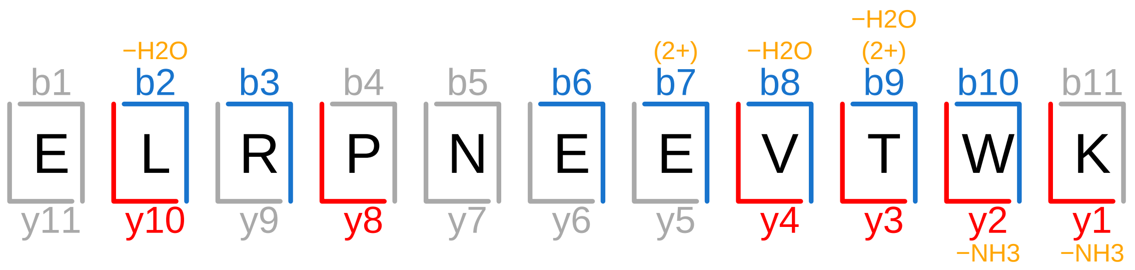

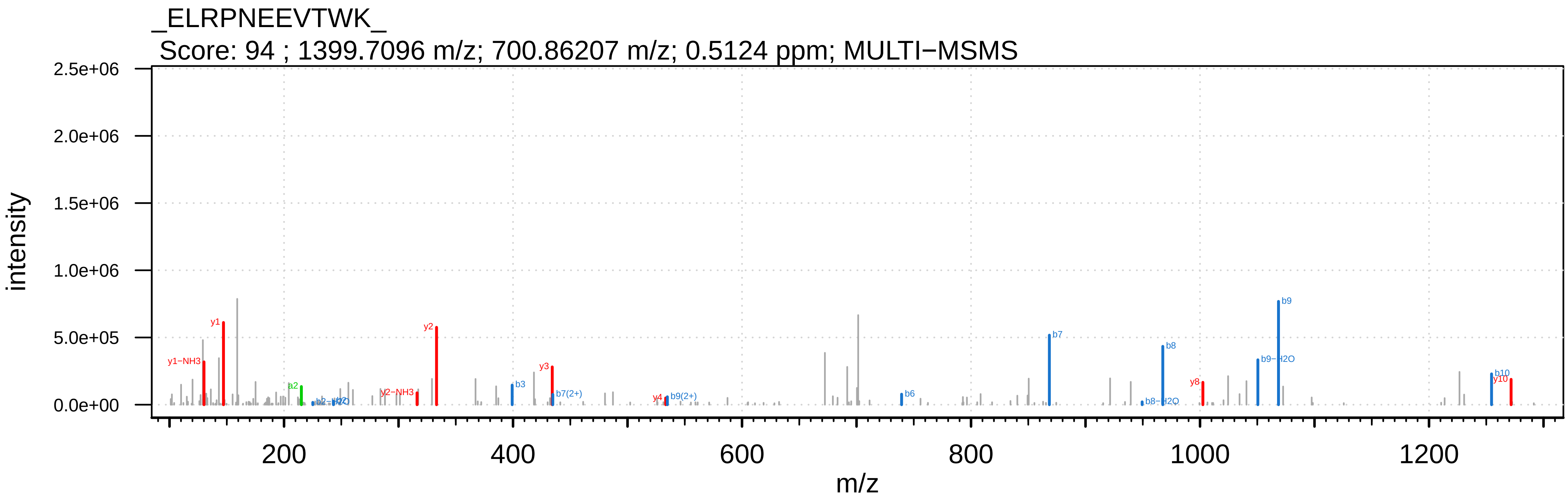

Raw File: 20130712_EXQ4_BaAz_SA_KR2_05
Scan Number: 35454
Proteins:
chr12_103206313−103214181.T2_chr12:101968174−101969629:−

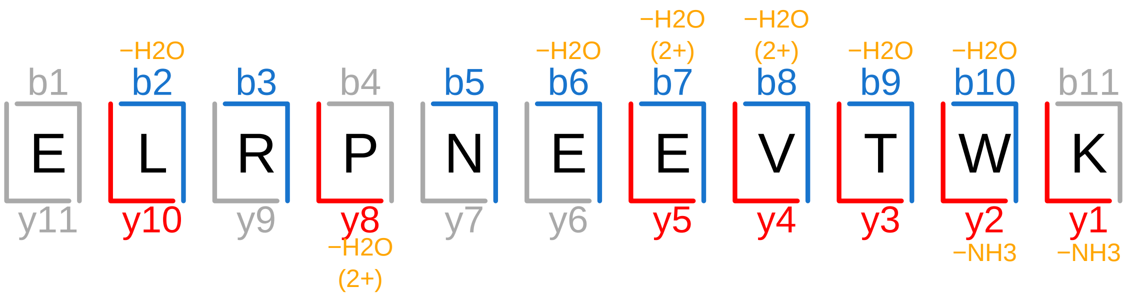

ELRPNEEVTWK
Score: 112 ; 1399.7096 m/z; 700.86207 m/z; 0.2984 ppm; MULTI−MSMS

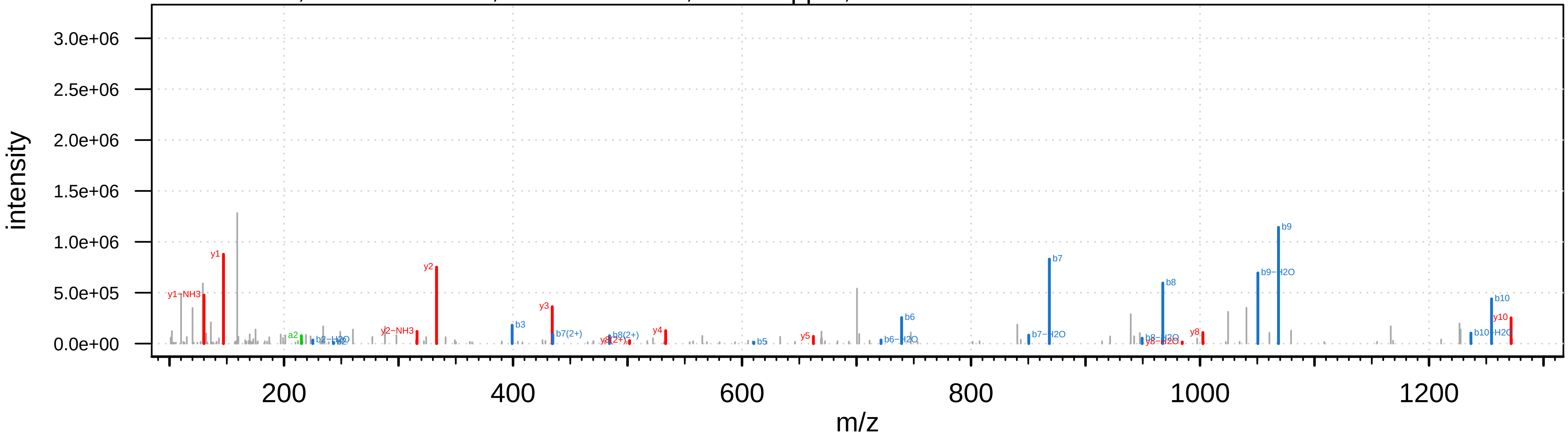

Raw File: 20130712_EXQ4_BaAz_SA_KR2_06
Scan Number: 31273
Proteins:
chr12_103206313−103214181.T2_chr12:101968174−101969629:−

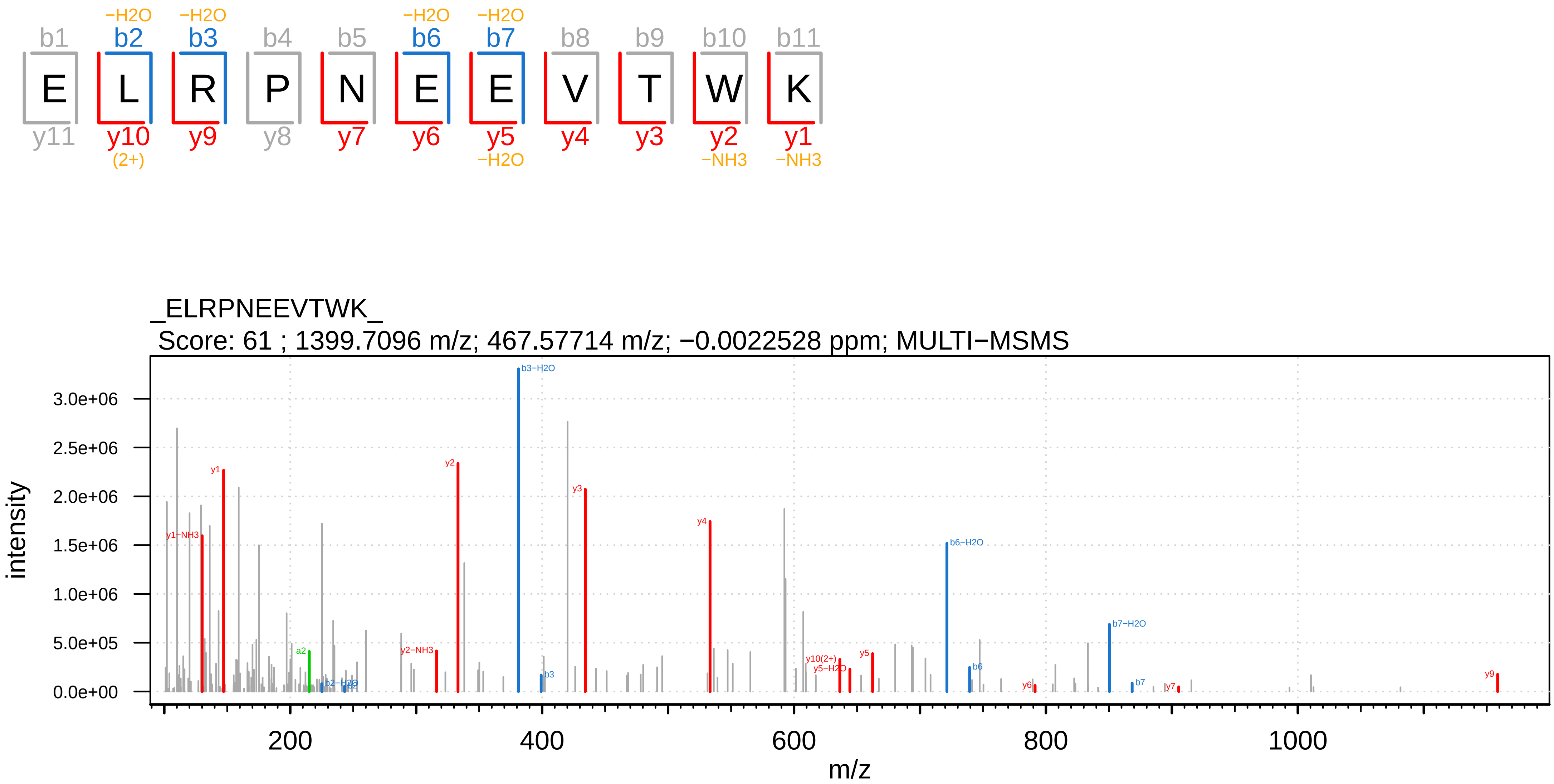

Raw File: 20130712_EXQ4_BaAz_SA_KR2_06

Scan Number: 31276

Proteins:

chr12_103206313−103214181.T2_chr12:101968174−101969629:−

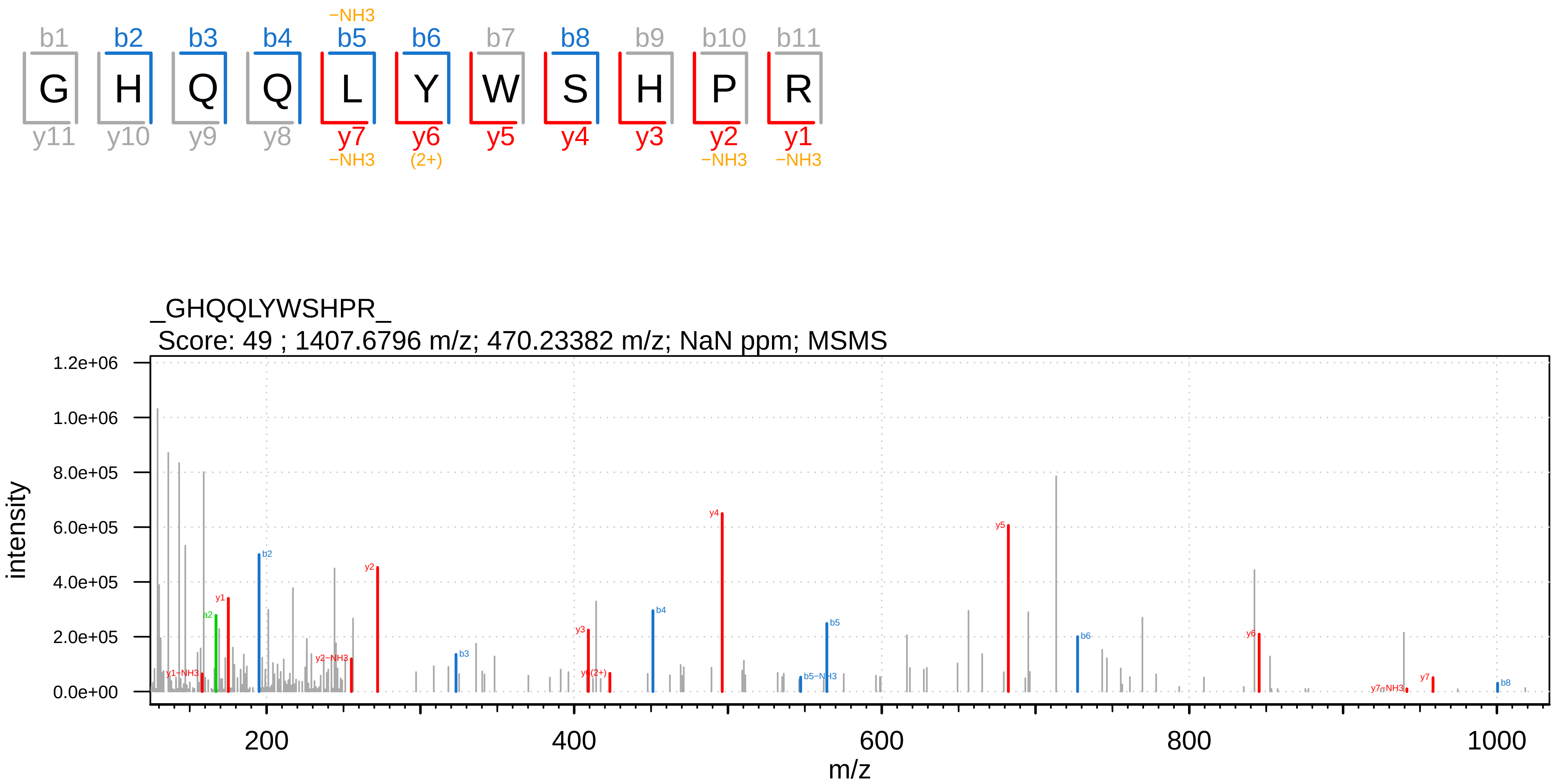

Raw File: 20130712_EXQ4_BaAz_SA_KR2_06
Scan Number: 18058
Proteins:
sp|P62274|RS29_MOUSE
ENSMUST00000037023_chr12:69157813−69159058:−
ENSMUST00000071737_chr13:73988963−73989133:−

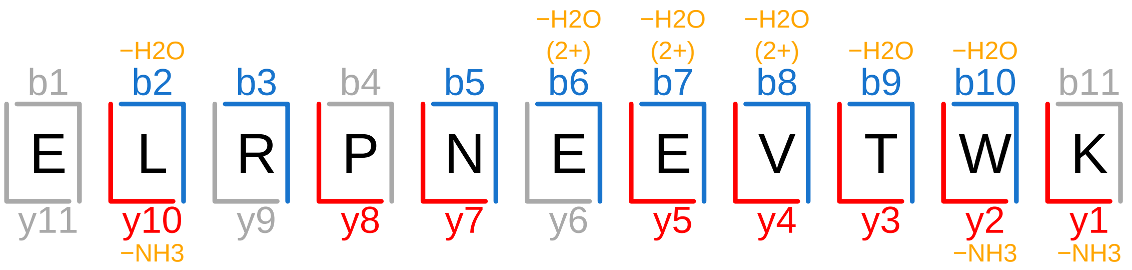

ELRPNEEVTWK

Score: 110 ; 1399.7096 m/z; 700.86207 m/z; 0.60756 ppm; MULTI−MSMS

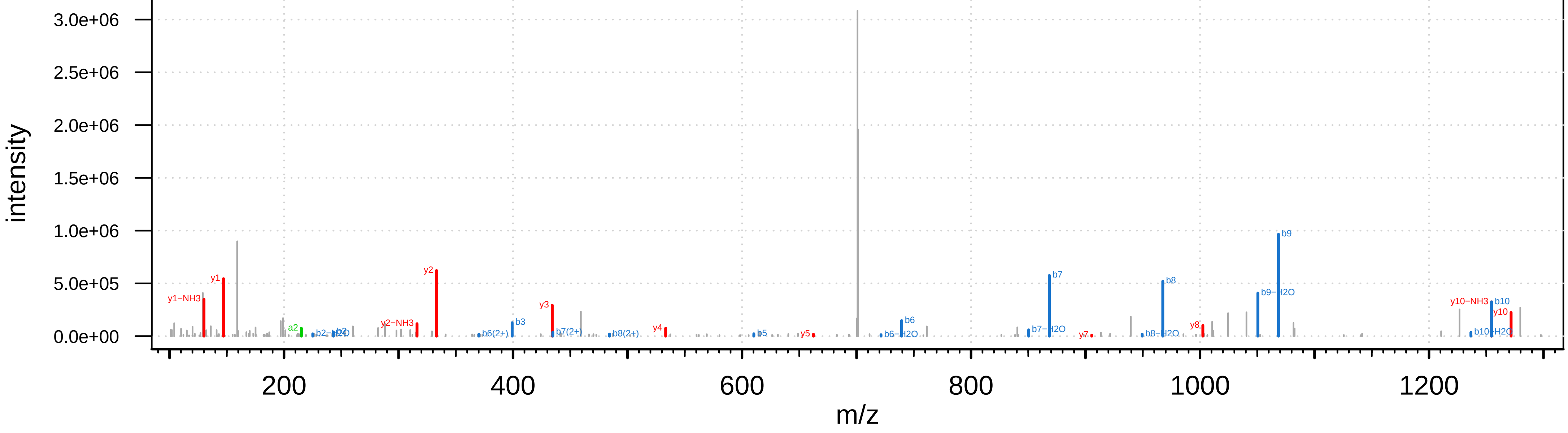

Raw File: 20130713_EXQ4_BaAz_SA_SeR2_05

Scan Number: 33550

Proteins:

chr12_103206313−103214181.T2_chr12:101968174−101969629:−

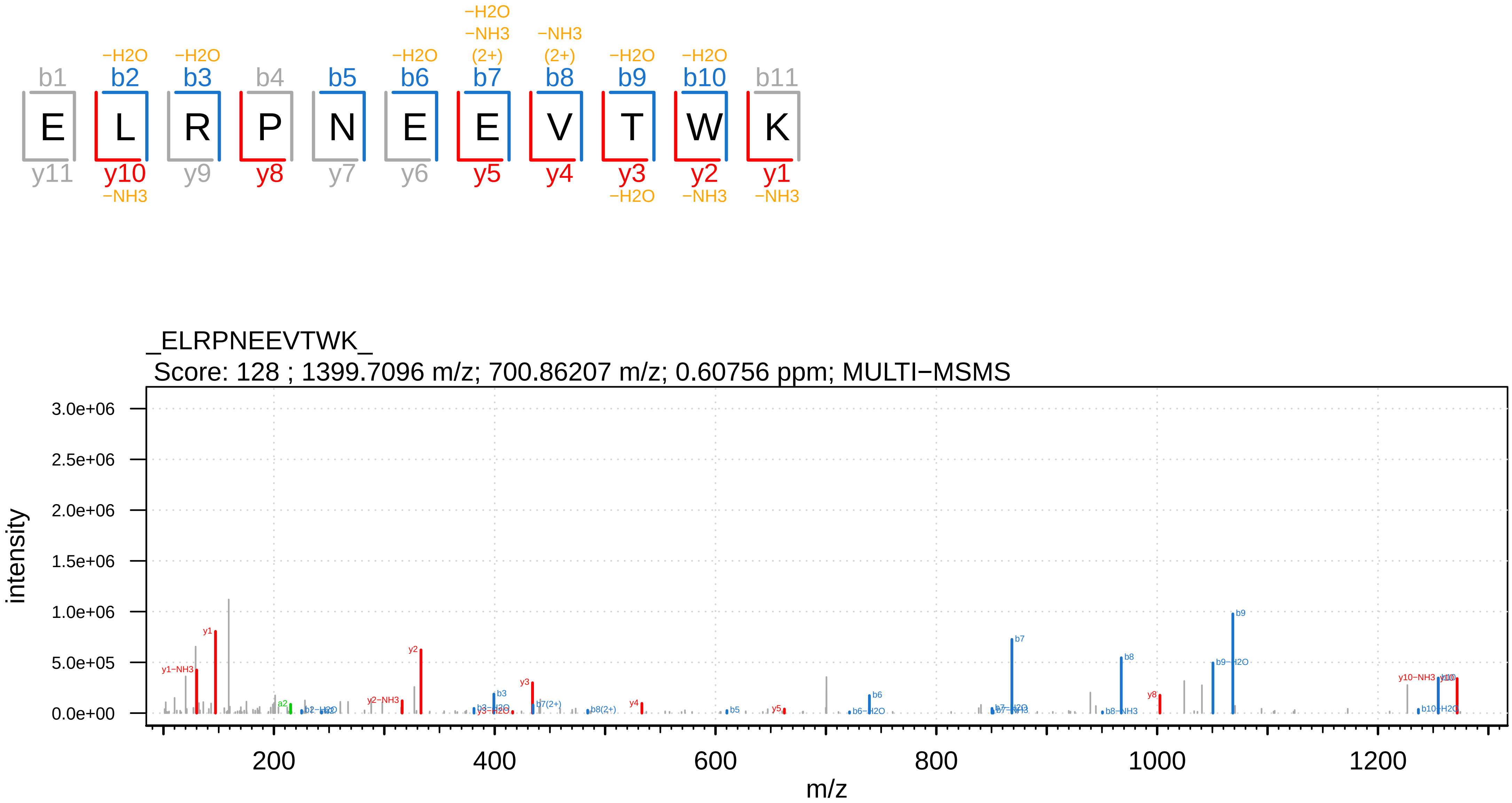

Raw File: 20130713_EXQ4_BaAz_SA_SeR2_05

Scan Number: 33896

Proteins:

chr12_103206313−103214181.T2_chr12:101968174−101969629:−

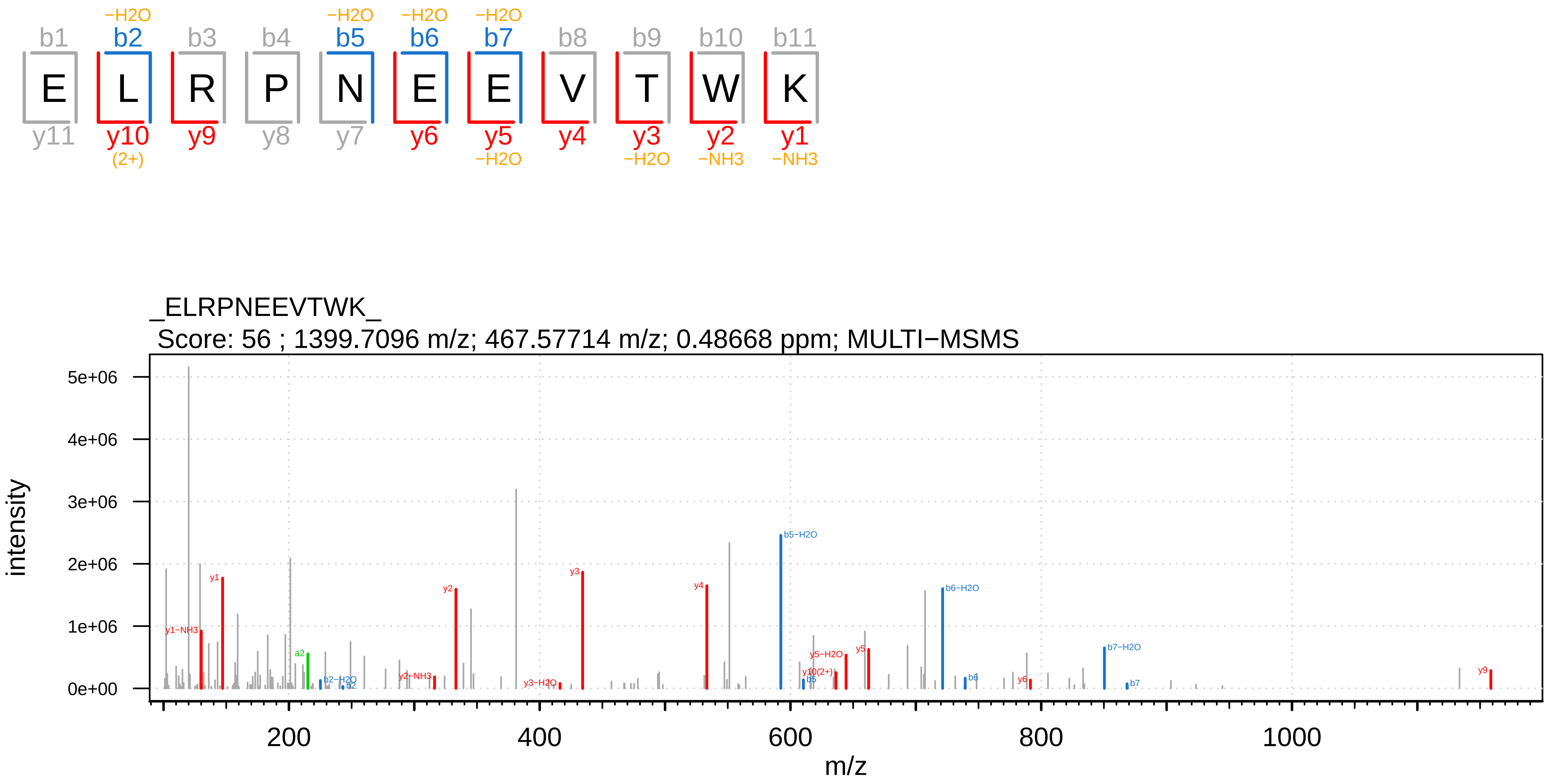

Raw File: 20130713_EXQ4_BaAz_SA_SeR2_05
Scan Number: 33846
Proteins:
chr12_103206313−103214181.T2_chr12:101968174−101969629:−

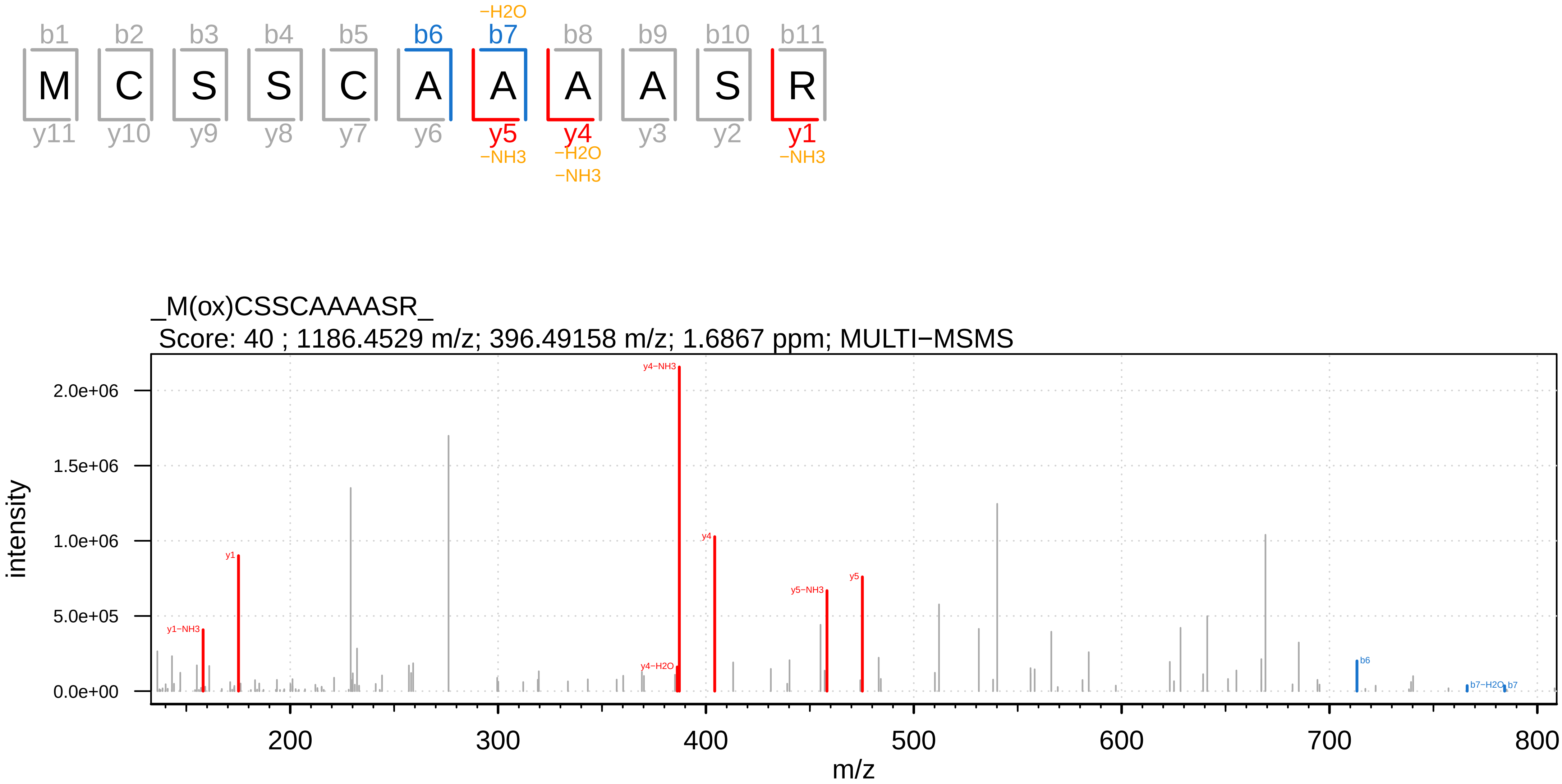

Raw File: 20130713_EXQ4_BaAz_SA_SeR2_05
Scan Number: 56033
Proteins:
ENSMUST00000047876_chr3:83128571−83128696:−

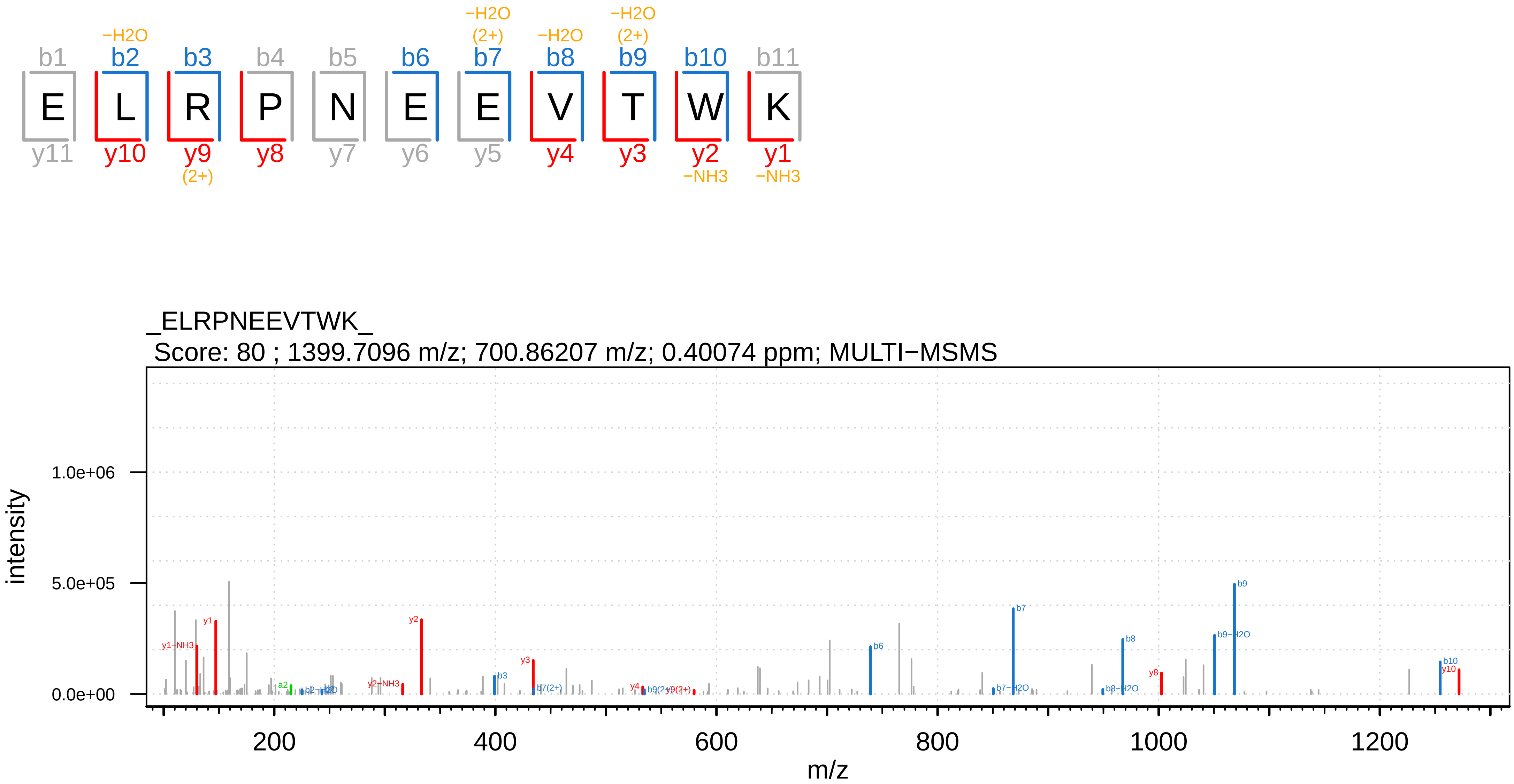

Raw File: 20130713_EXQ4_BaAz_SA_SeR2_06
Scan Number: 30395
Proteins:
chr12_103206313−103214181.T2_chr12:101968174−101969629:−

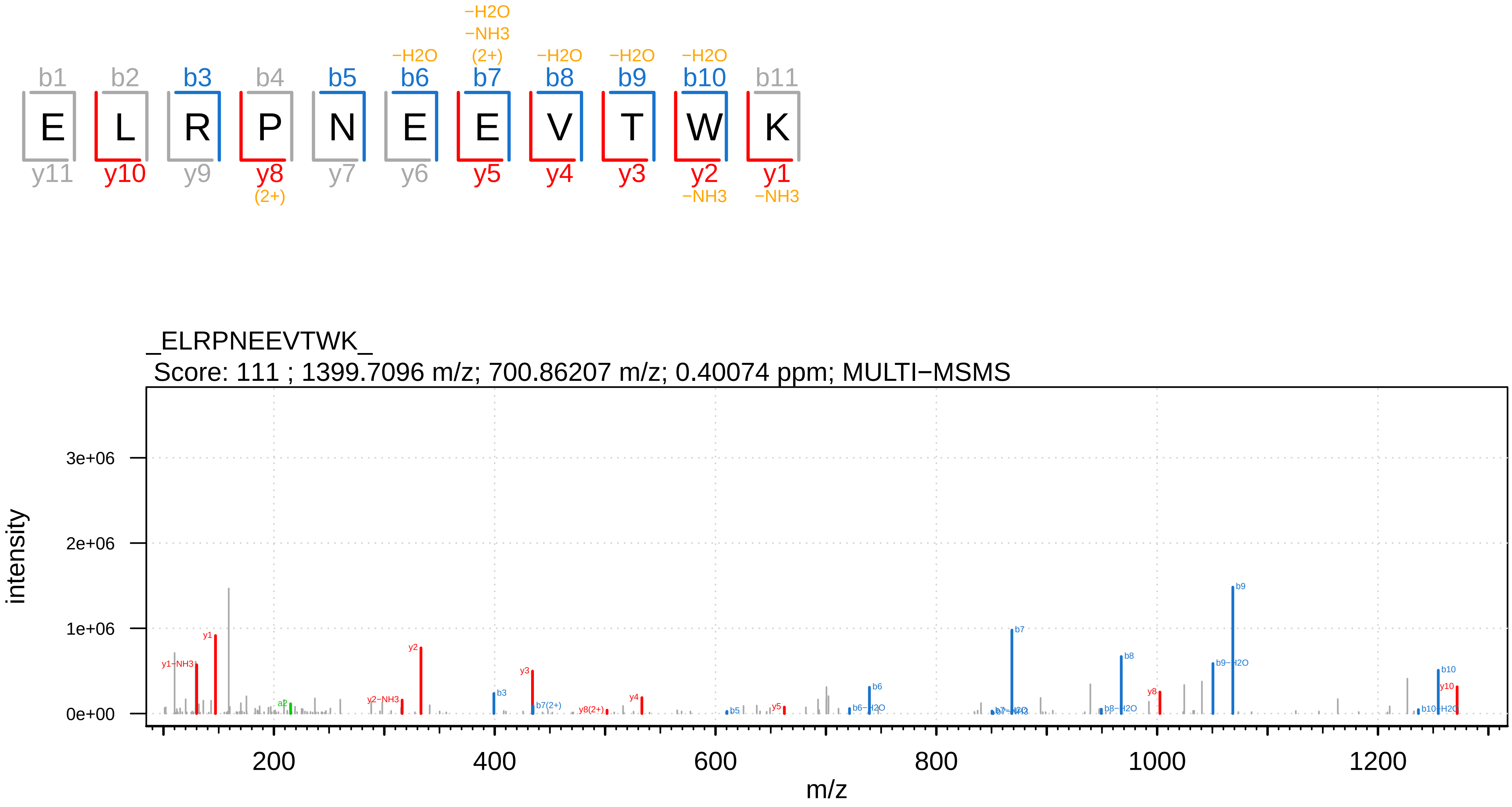

Raw File: 20130713_EXQ4_BaAz_SA_SeR2_06
Scan Number: 30754
Proteins:
chr12_103206313−103214181.T2_chr12:101968174−101969629:−

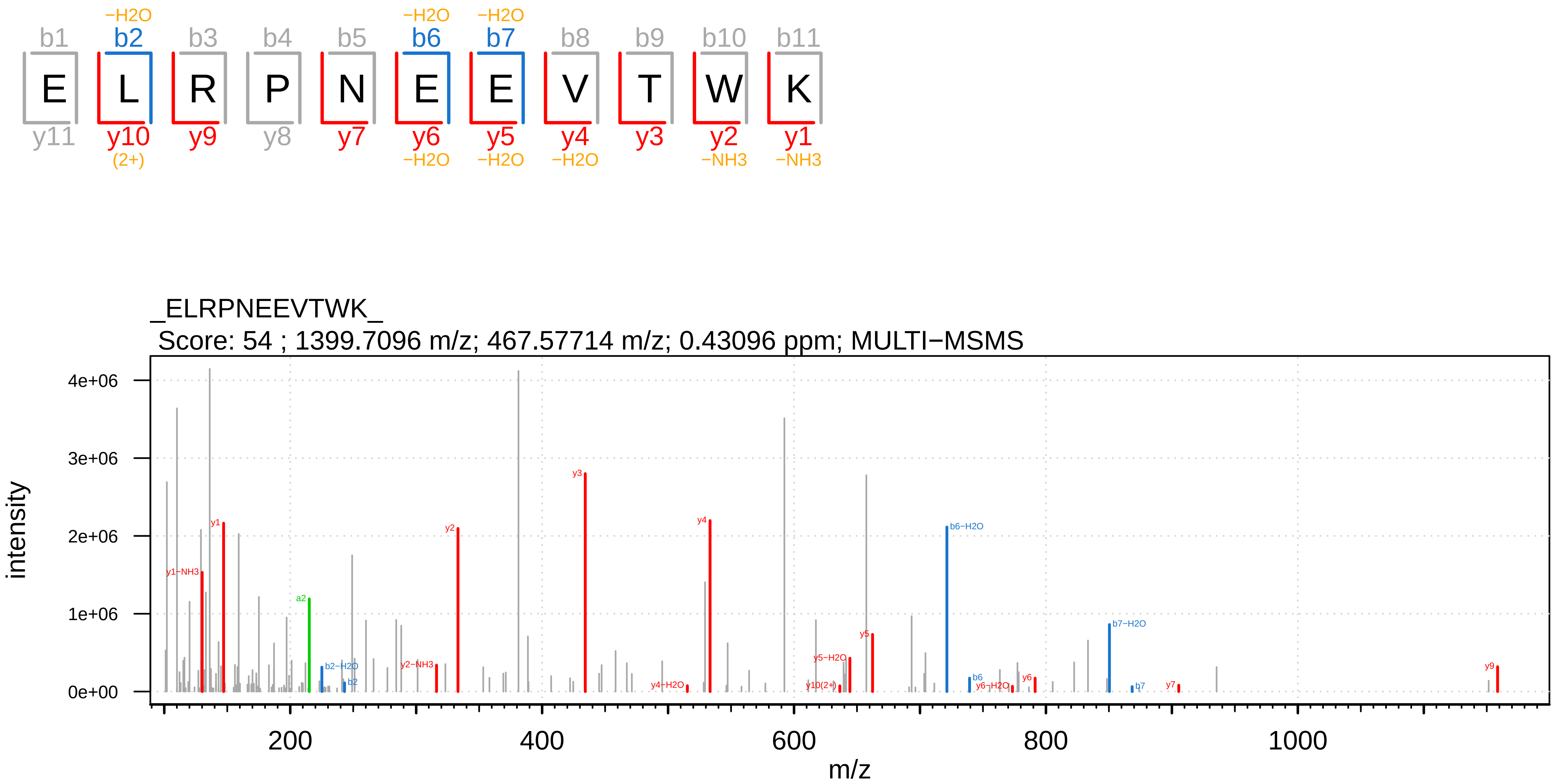

Raw File: 20130713_EXQ4_BaAz_SA_SeR2_06
Scan Number: 30414
Proteins:
chr12_103206313−103214181.T2_chr12:101968174−101969629:−

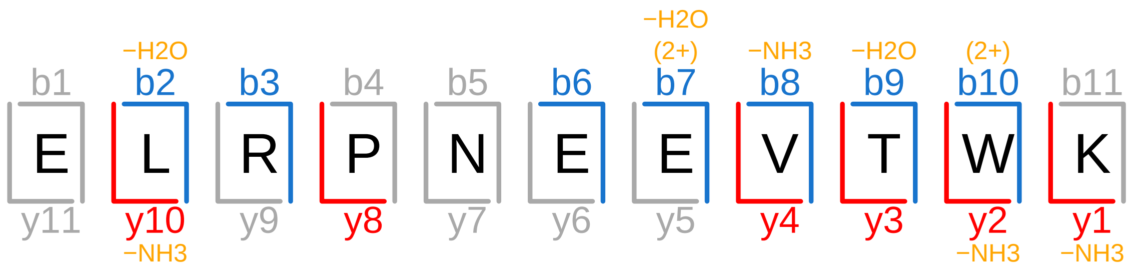

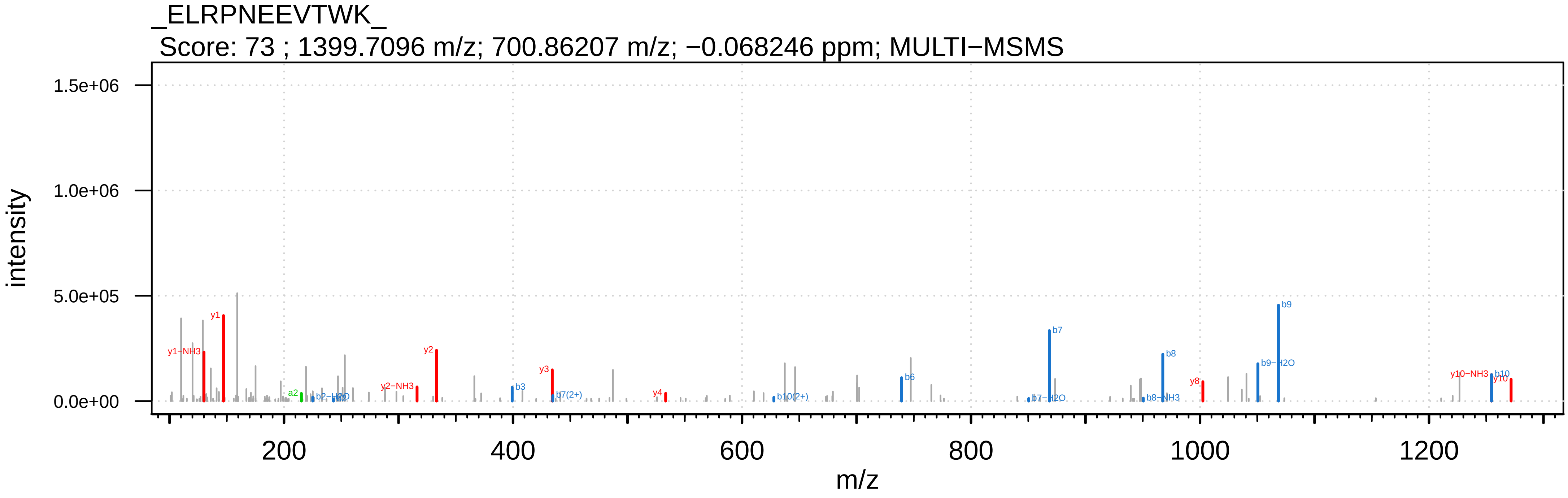

Raw File: 20130715_EXQ4_BaAz_SA_StR2_06
Scan Number: 29932
Proteins:
chr12_103206313−103214181.T2_chr12:101968174−101969629:−

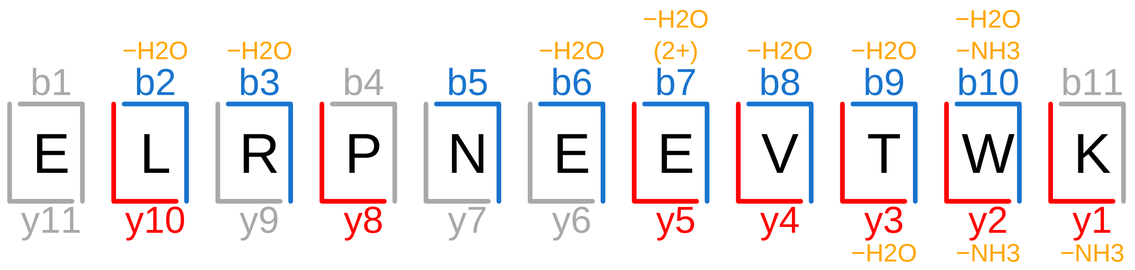

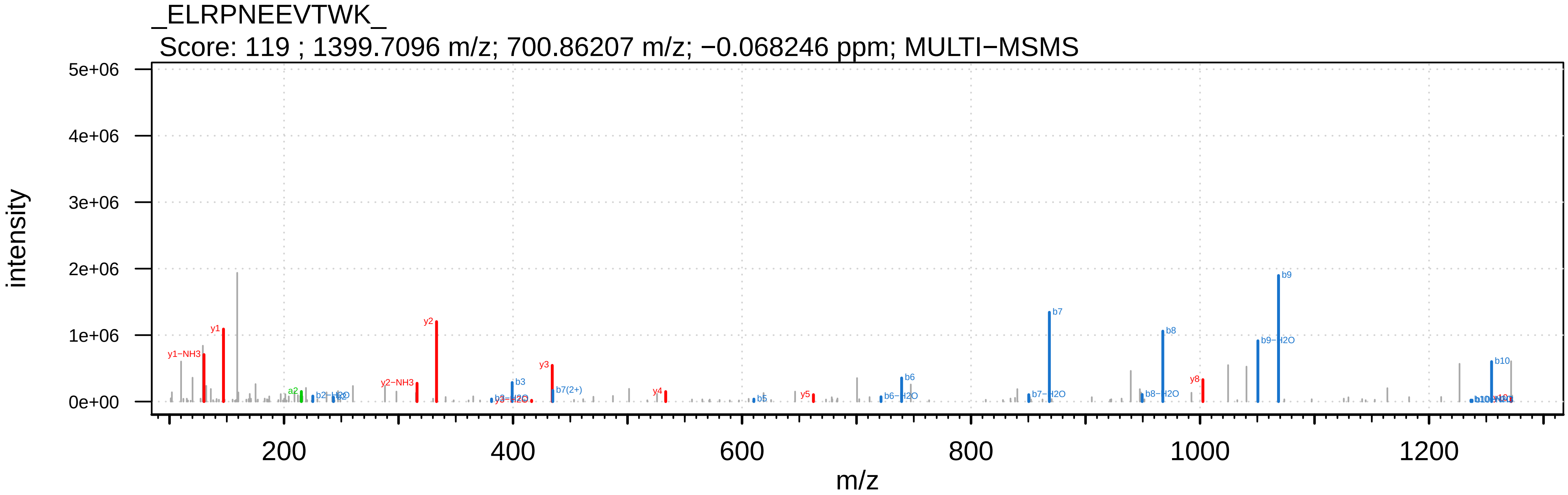

Raw File: 20130715_EXQ4_BaAz_SA_StR2_06
Scan Number: 30272
Proteins:
chr12_103206313−103214181.T2_chr12:101968174−101969629:−

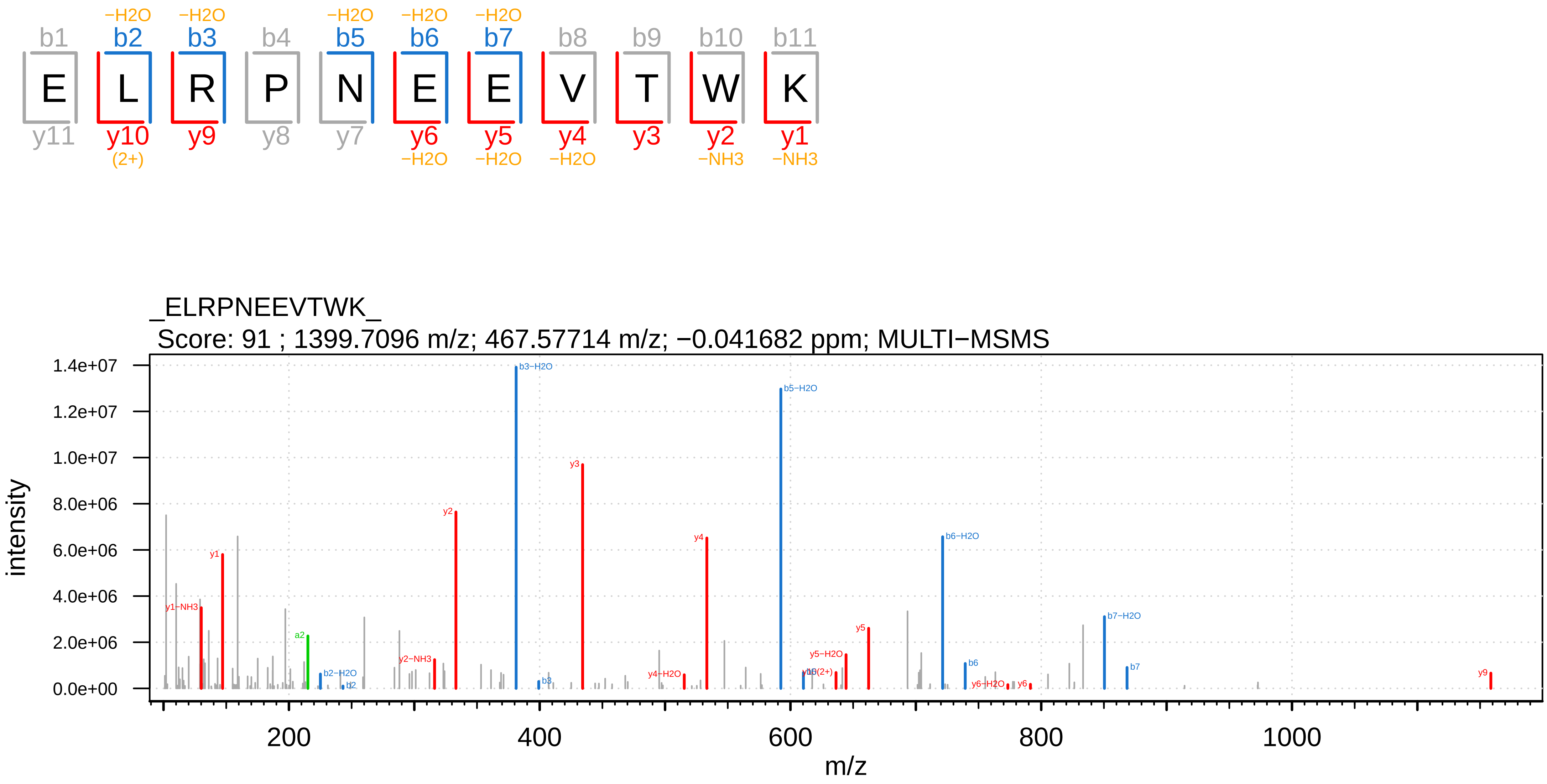

Raw File: 20130715_EXQ4_BaAz_SA_StR2_06
Scan Number: 30032
Proteins:
chr12_103206313−103214181.T2_chr12:101968174−101969629:−

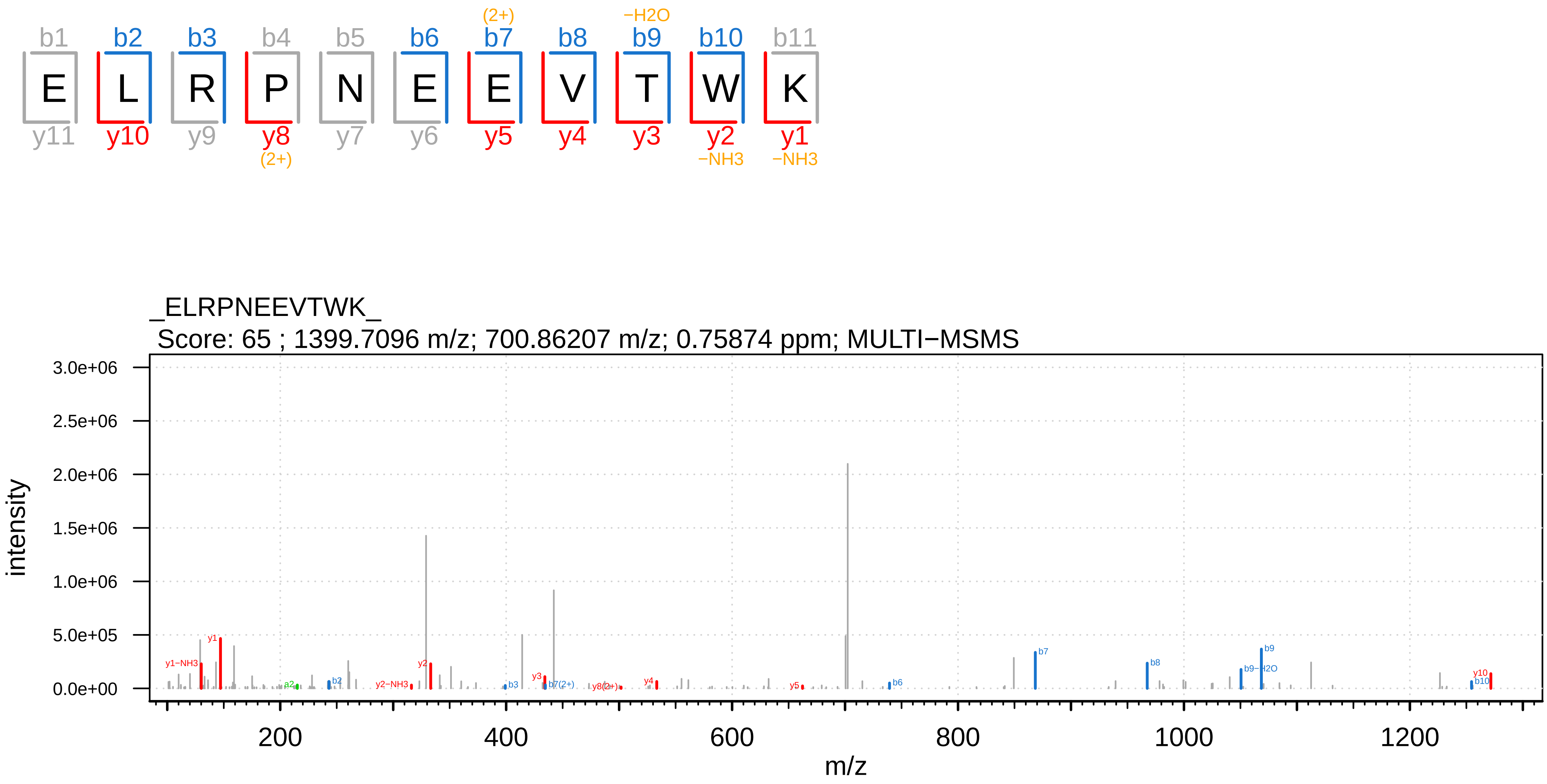

Raw File: 20130716_EXQ4_BaAz_SA_CHR2_05

Scan Number: 35684

Proteins:

chr12_103206313−103214181.T2_chr12:101968174−101969629:−

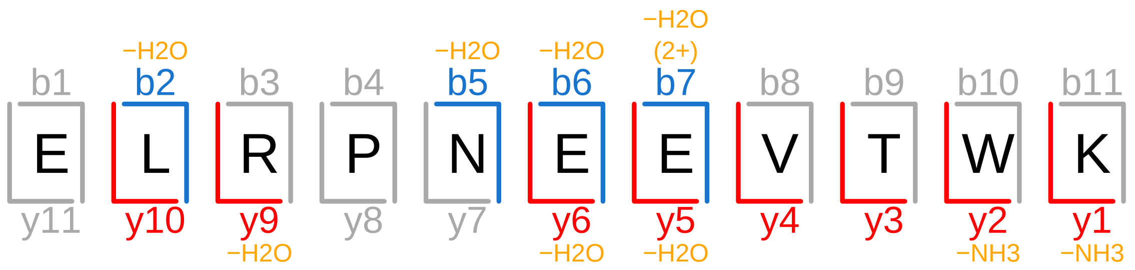

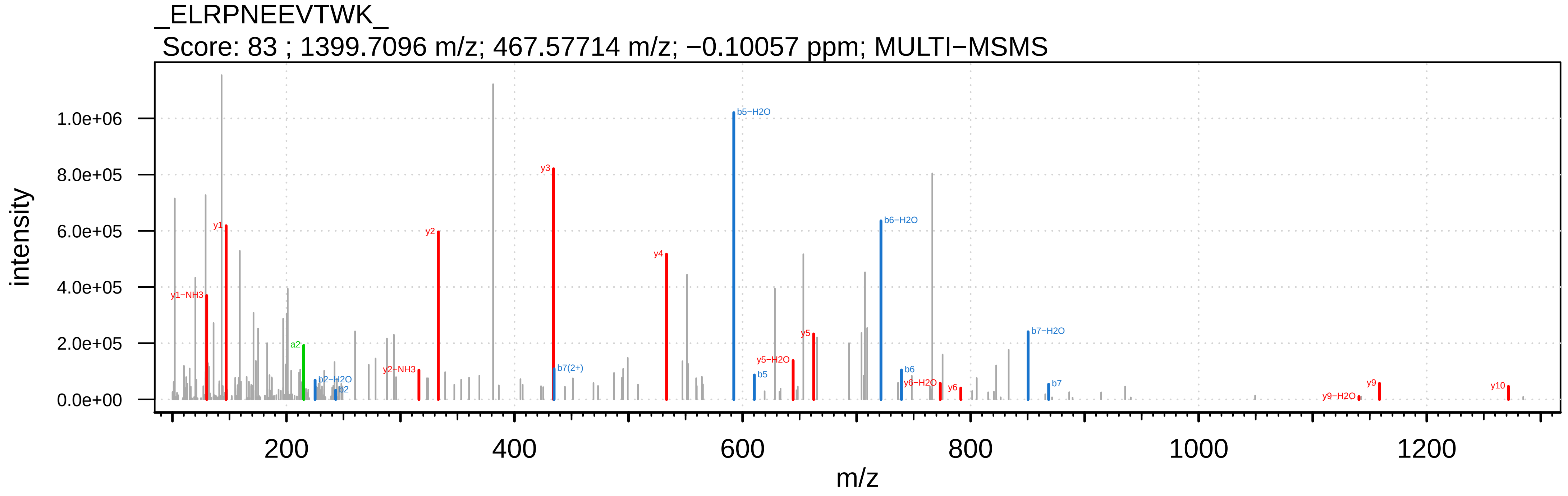

Raw File: 20130716_EXQ4_BaAz_SA_CHR2_05
Scan Number: 35956
Proteins:
chr12_103206313−103214181.T2_chr12:101968174−101969629:−

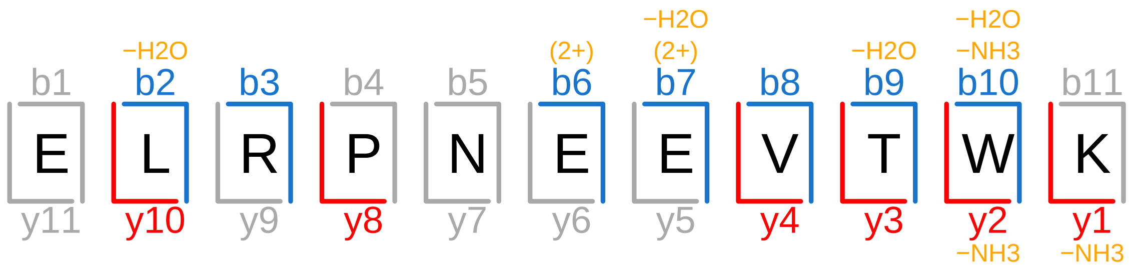

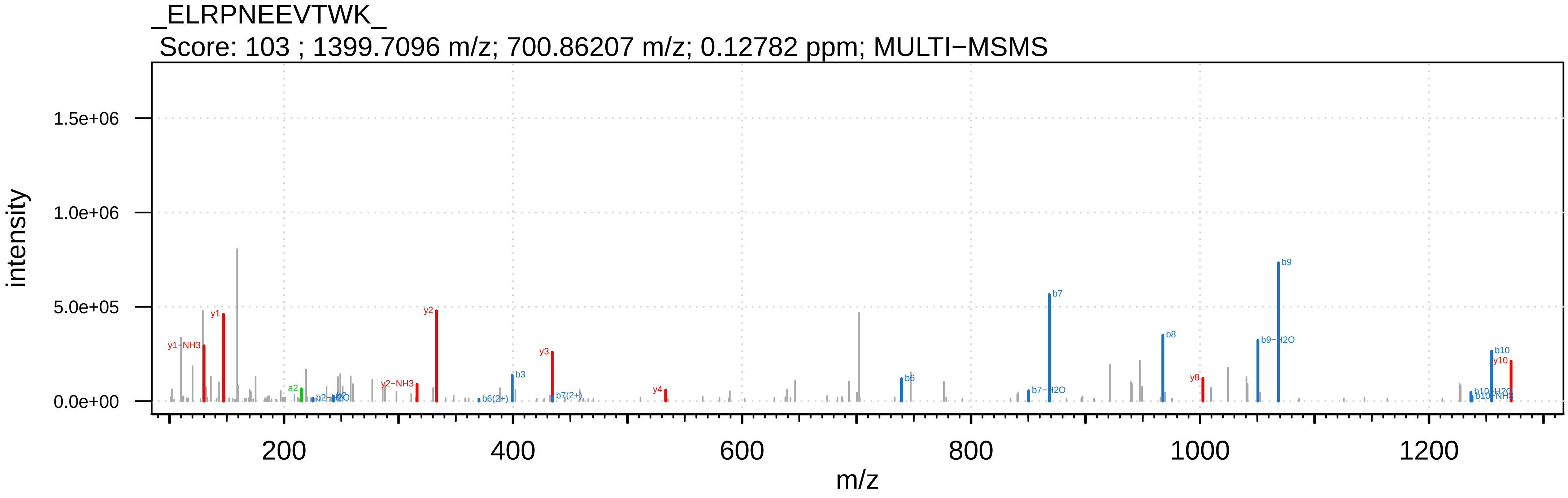

Raw File: 20130716_EXQ4_BaAz_SA_CHR2_06
Scan Number: 33231
Proteins:
chr12_103206313−103214181.T2_chr12:101968174−101969629:−

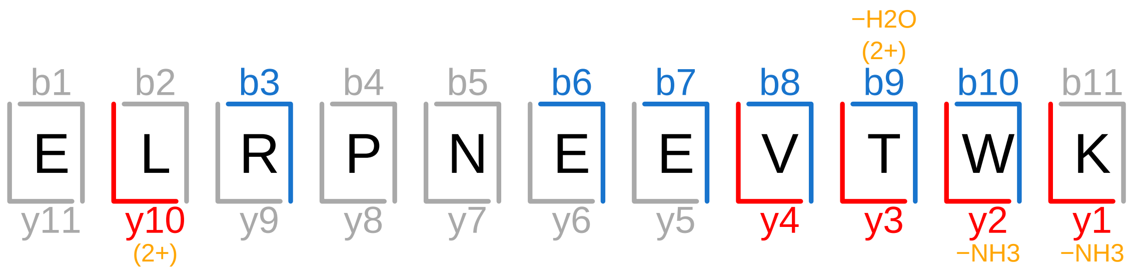

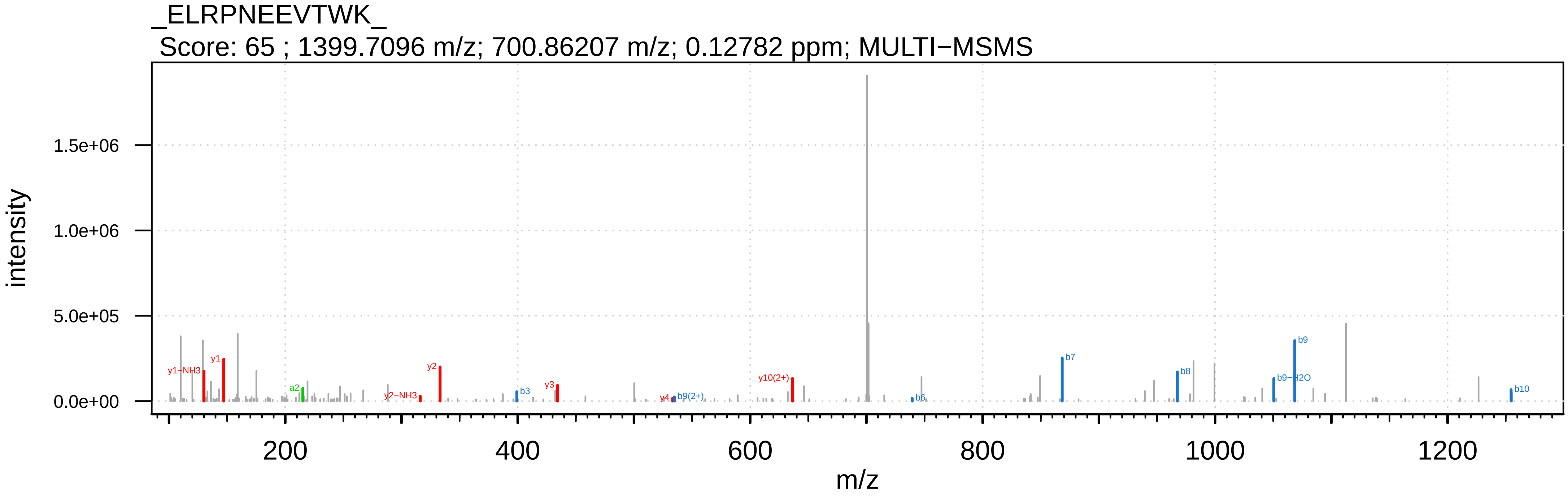

Raw File: 20130716_EXQ4_BaAz_SA_CHR2_06
Scan Number: 33605
Proteins:
chr12_103206313−103214181.T2_chr12:101968174−101969629:−

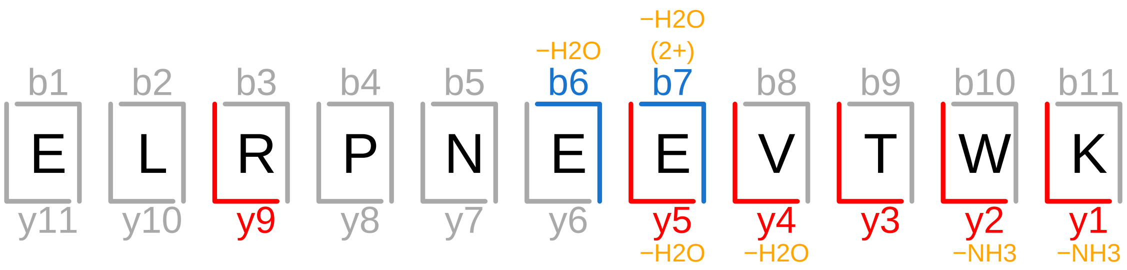

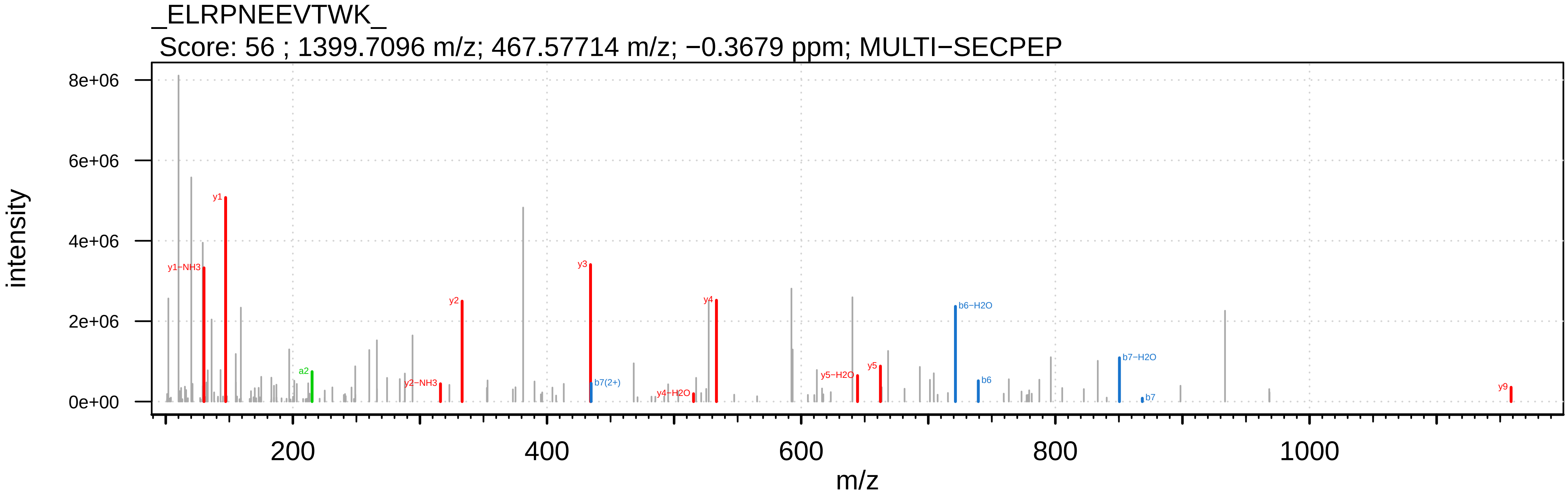

Raw File: 20130716_EXQ4_BaAz_SA_CHR2_06
Scan Number: 33239
Proteins:
chr12_103206313−103214181.T2_chr12:101968174−101969629:−

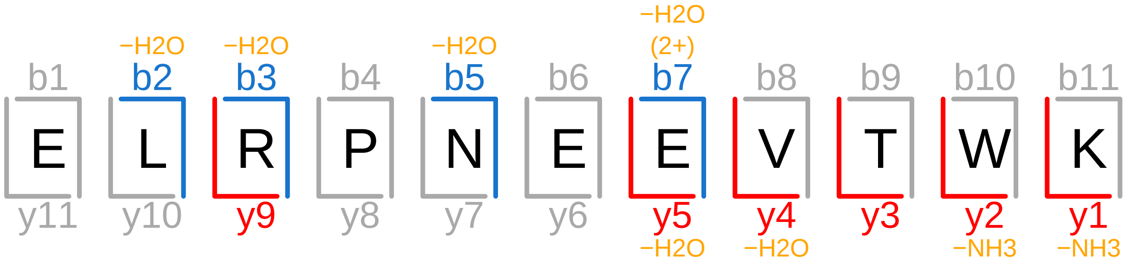

ELRPNEEVTWK
Score: 73 ; 1399.7096 m/z; 467.57714 m/z; 0.021325 ppm; MULTI−MSMS

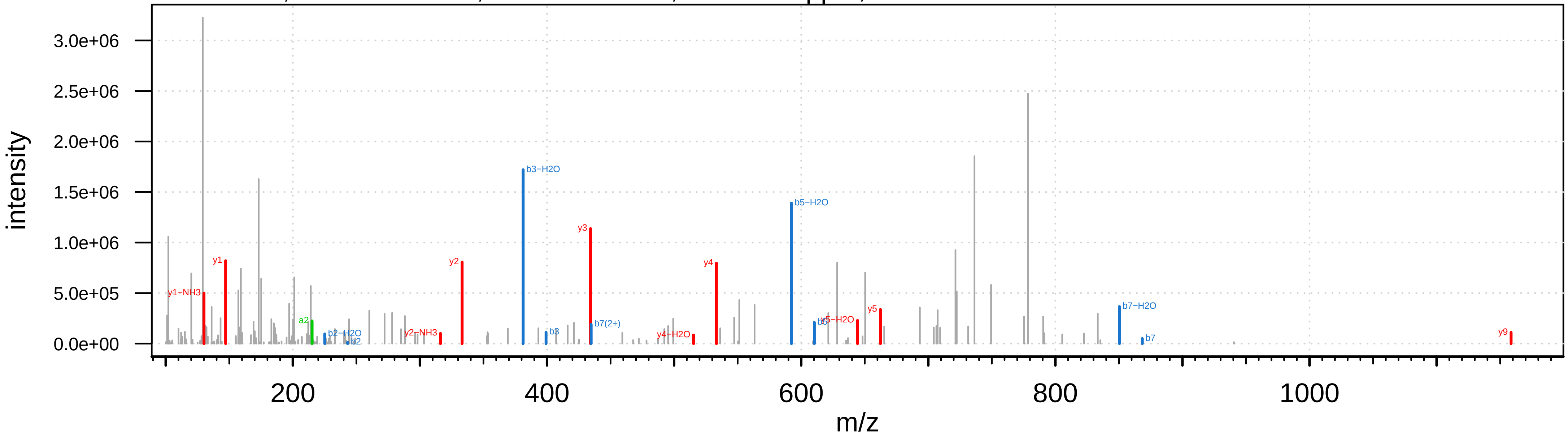

Raw File: 20130716_EXQ4_BaAz_SA_CHR3_05
Scan Number: 34358
Proteins:
chr12_103206313−103214181.T2_chr12:101968174−101969629:−

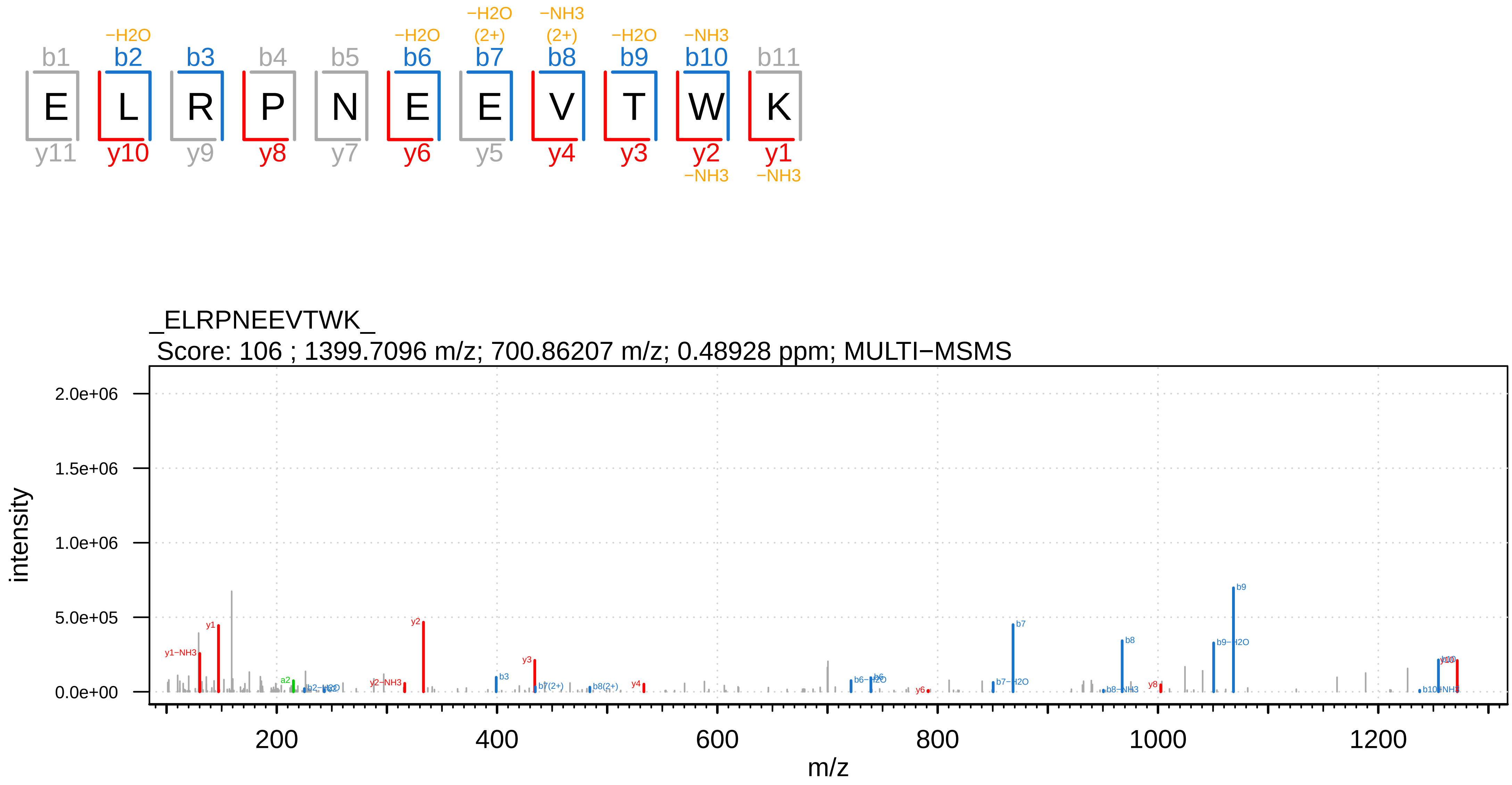

Raw File: 20130716_EXQ4_BaAz_SA_CHR3_05
Scan Number: 34479
Proteins:
chr12_103206313−103214181.T2_chr12:101968174−101969629:−

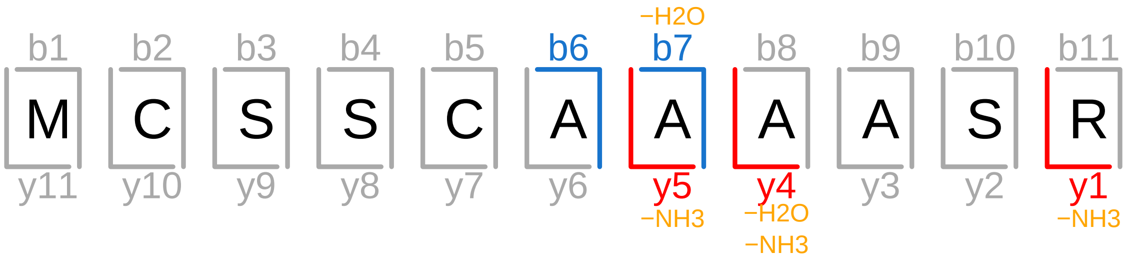

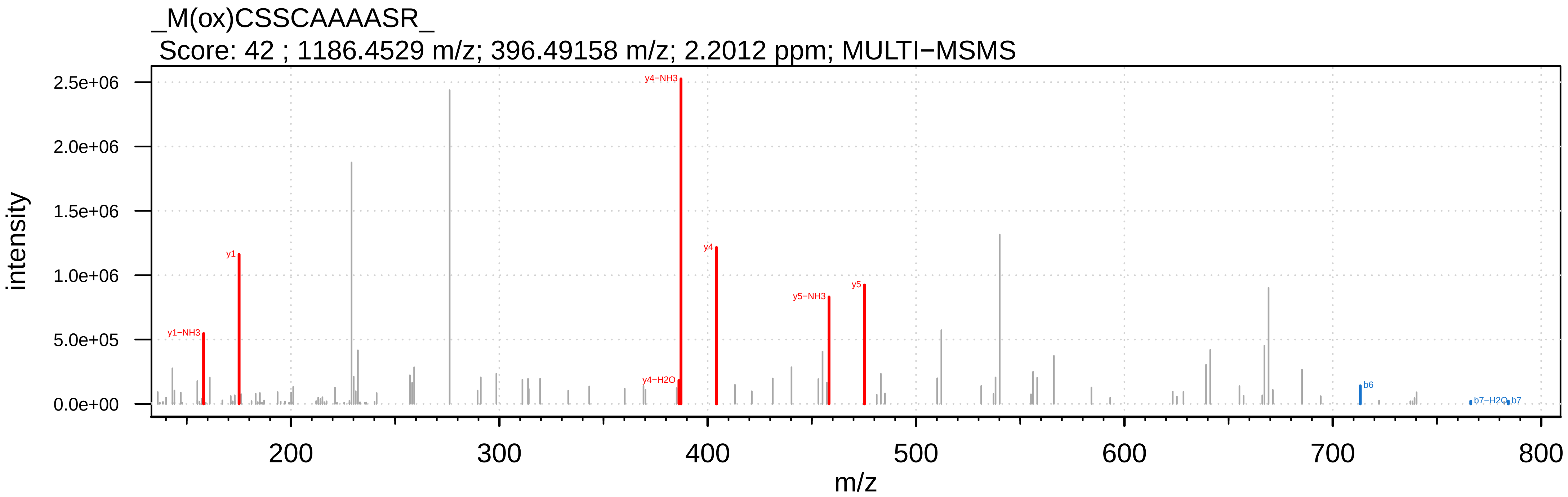

Raw File: 20130716_EXQ4_BaAz_SA_CHR3_05
Scan Number: 56628
Proteins:
ENSMUST00000047876_chr3:83128571−83128696:−

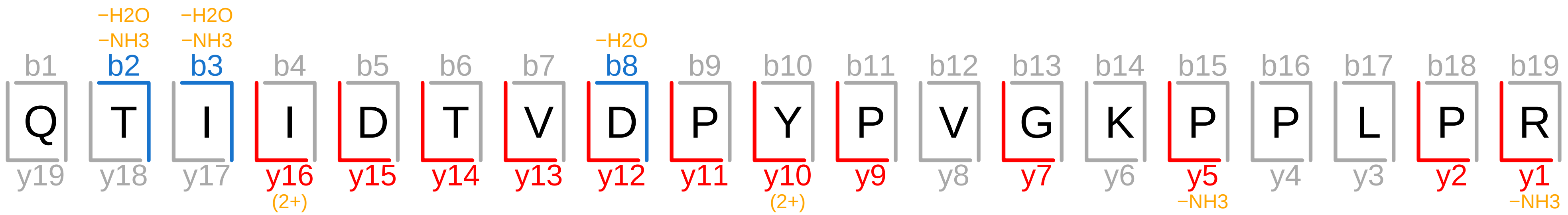

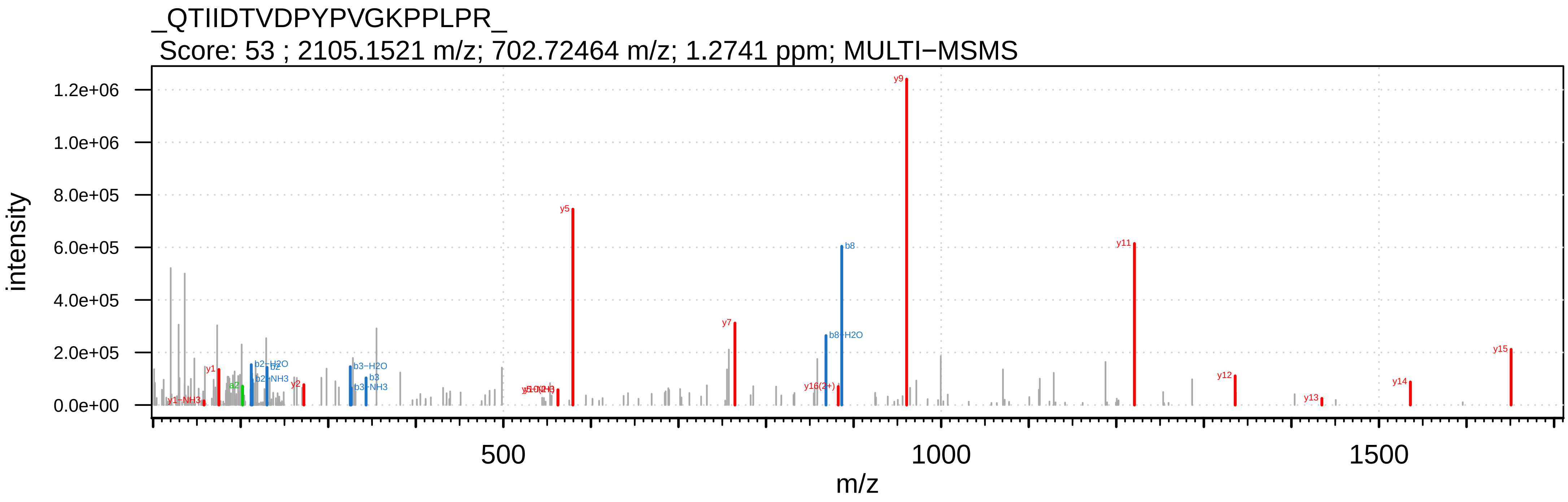

Raw File: 20130716_EXQ4_BaAz_SA_CHR3_05
Scan Number: 73115
Proteins:
ENSMUST00000136126_chr3:79172848−79174982:−

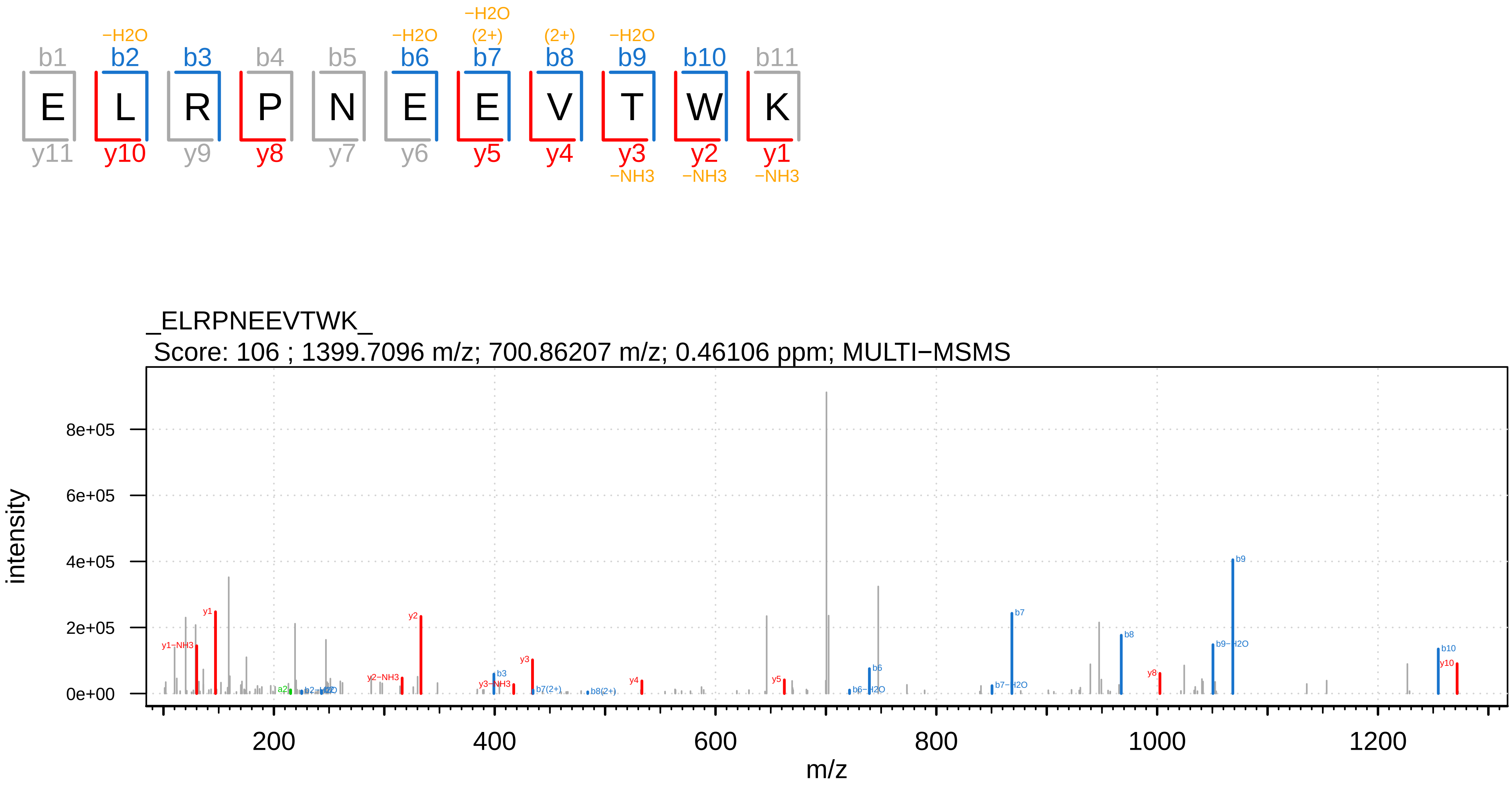

Raw File: 20130716_EXQ4_BaAz_SA_CHR32_06
Scan Number: 34831
Proteins:
chr12_103206313−103214181.T2_chr12:101968174−101969629:−

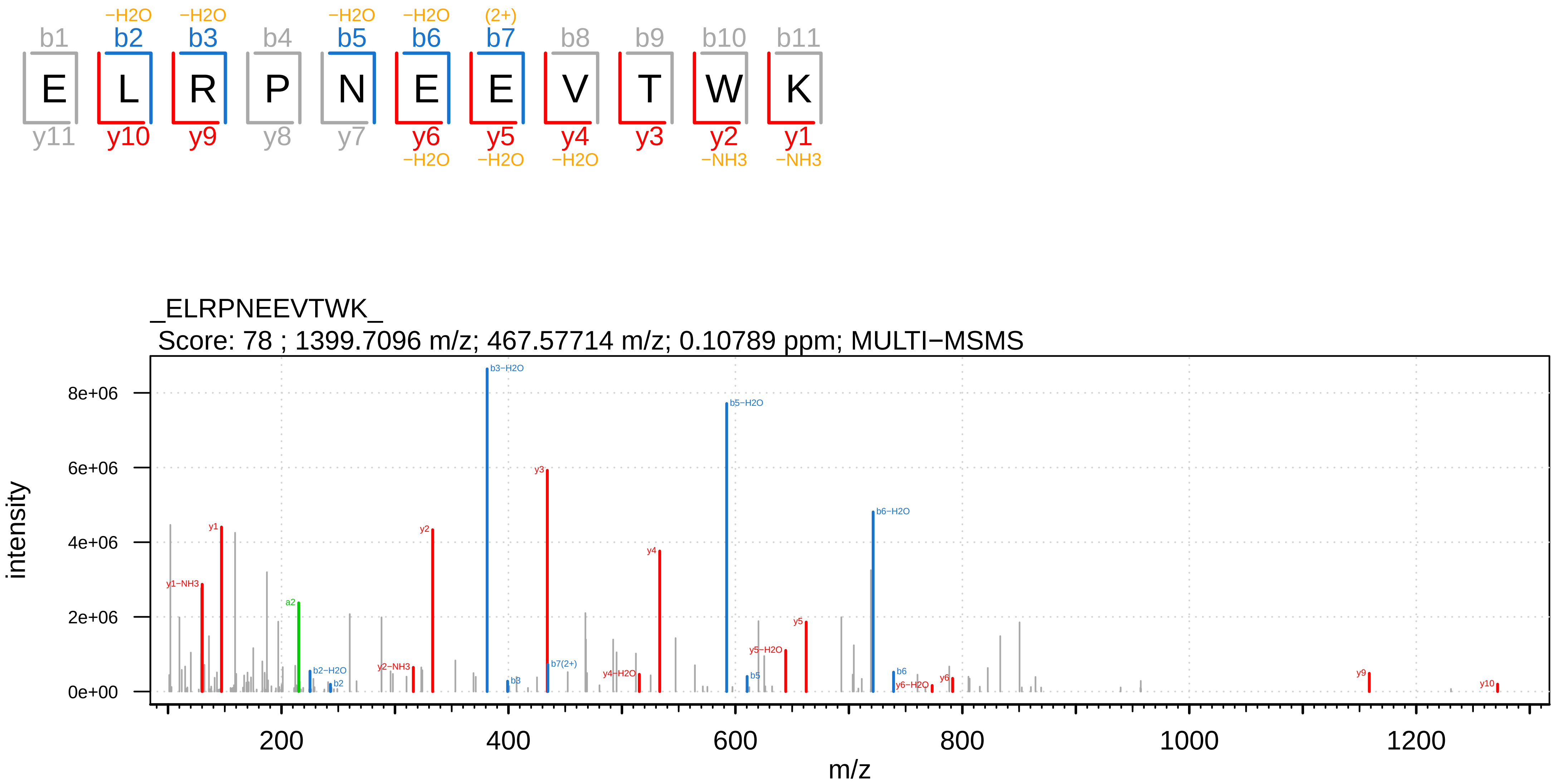

Raw File: 20130716_EXQ4_BaAz_SA_CHR32_06
Scan Number: 34887
Proteins:
chr12_103206313−103214181.T2_chr12:101968174−101969629:−

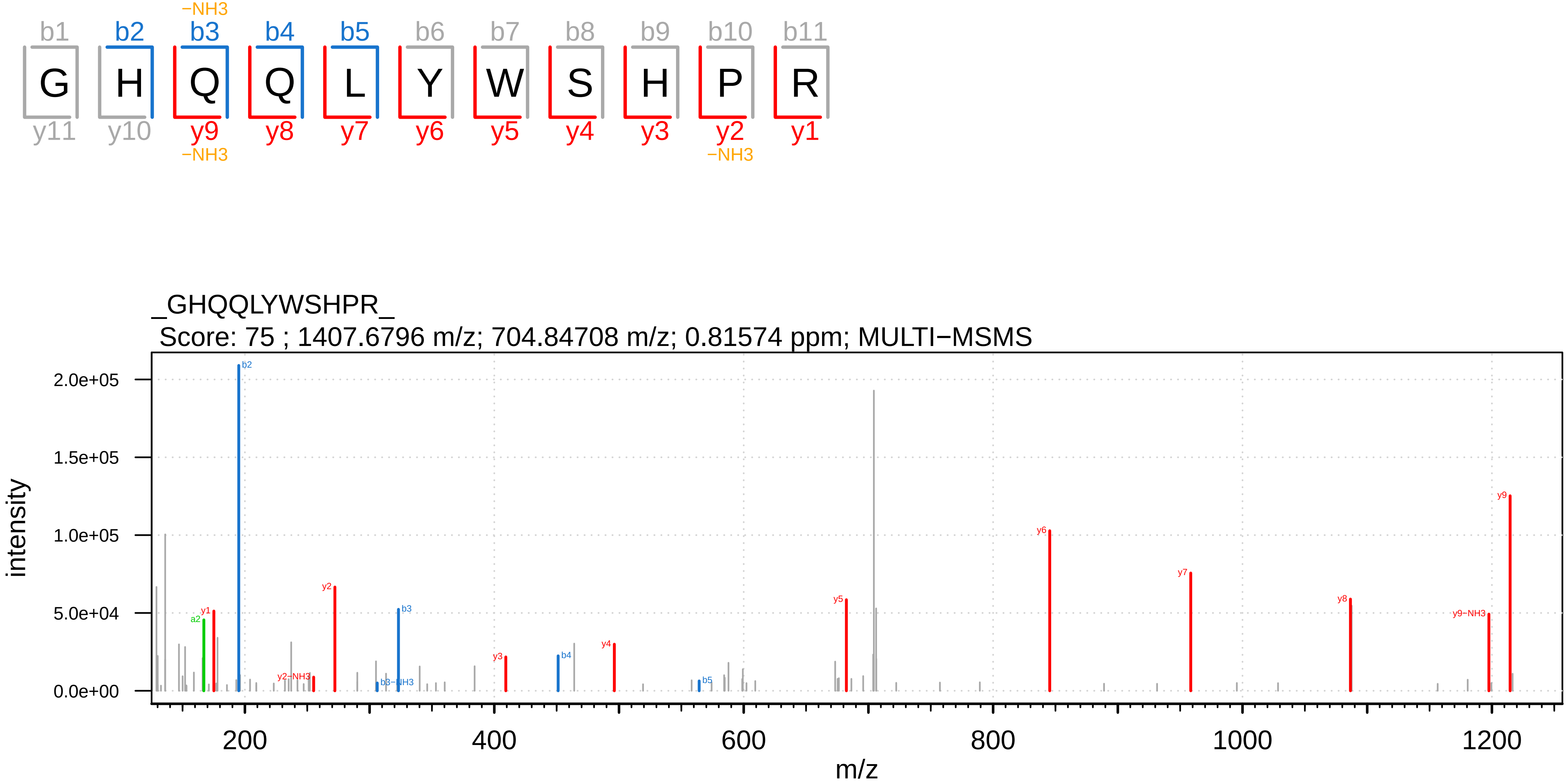

Raw File: 20130716_EXQ4_BaAz_SA_CHR32_06
Scan Number: 23434
Proteins:
sp|P62274|RS29_MOUSE
ENSMUST00000037023_chr12:69157813−69159058:−
ENSMUST00000071737_chr13:73988963−73989133:−

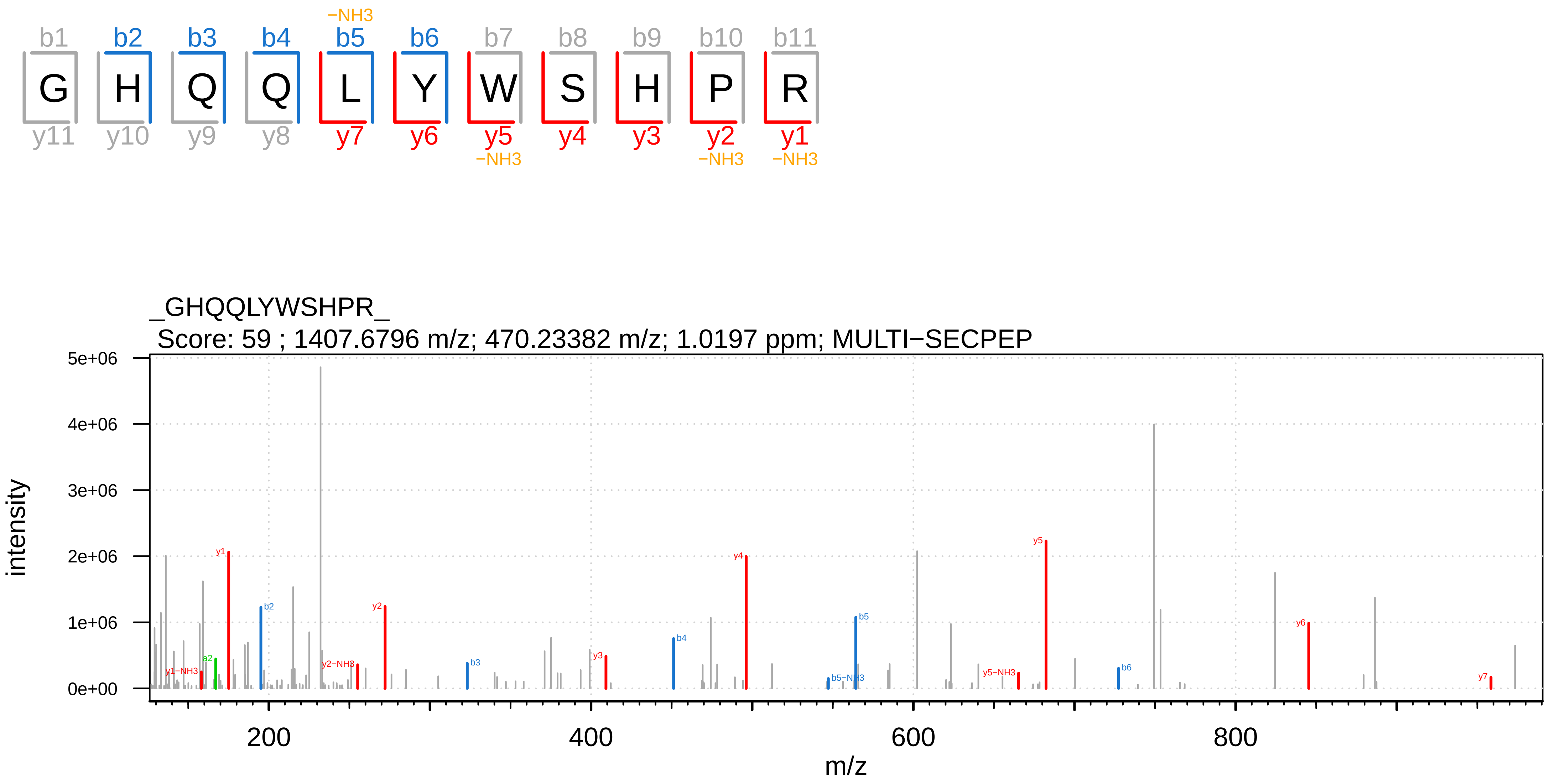

Raw File: 20130716_EXQ4_BaAz_SA_CHR32_06
Scan Number: 23566
Proteins:
sp|P62274|RS29_MOUSE
ENSMUST00000037023_chr12:69157813−69159058:−
ENSMUST00000071737_chr13:73988963−73989133:−

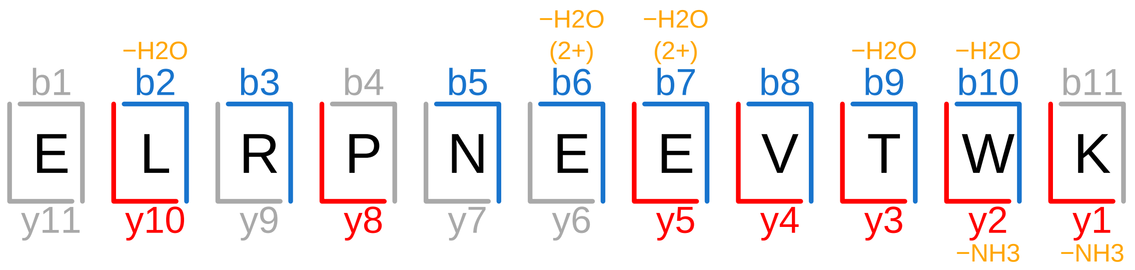

ELRPNEEVTWK
Score: 103 ; 1399.7096 m/z; 700.86207 m/z; 0.19886 ppm; MULTI−MSMS

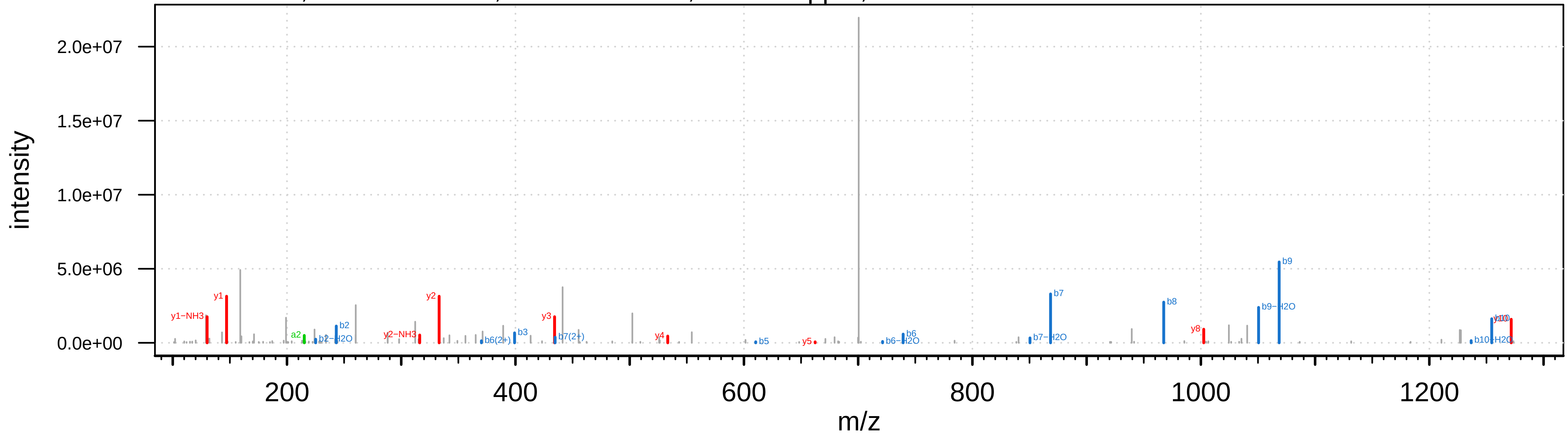

Raw File: 20130718_EXQ6_BaAz_SA_LR3_05
Scan Number: 34701
Proteins:
chr12_103206313−103214181.T2_chr12:101968174−101969629:−

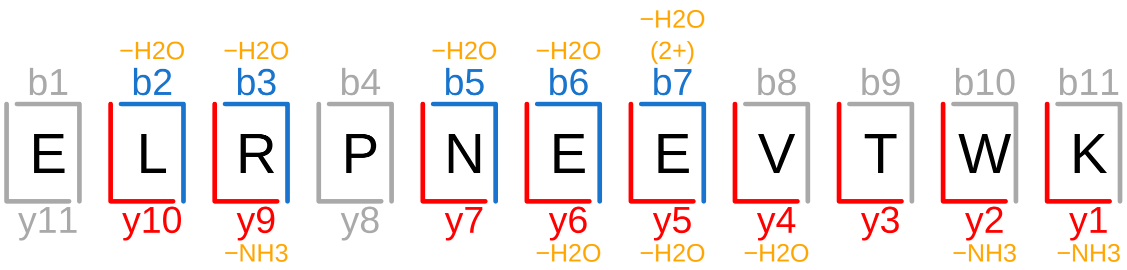

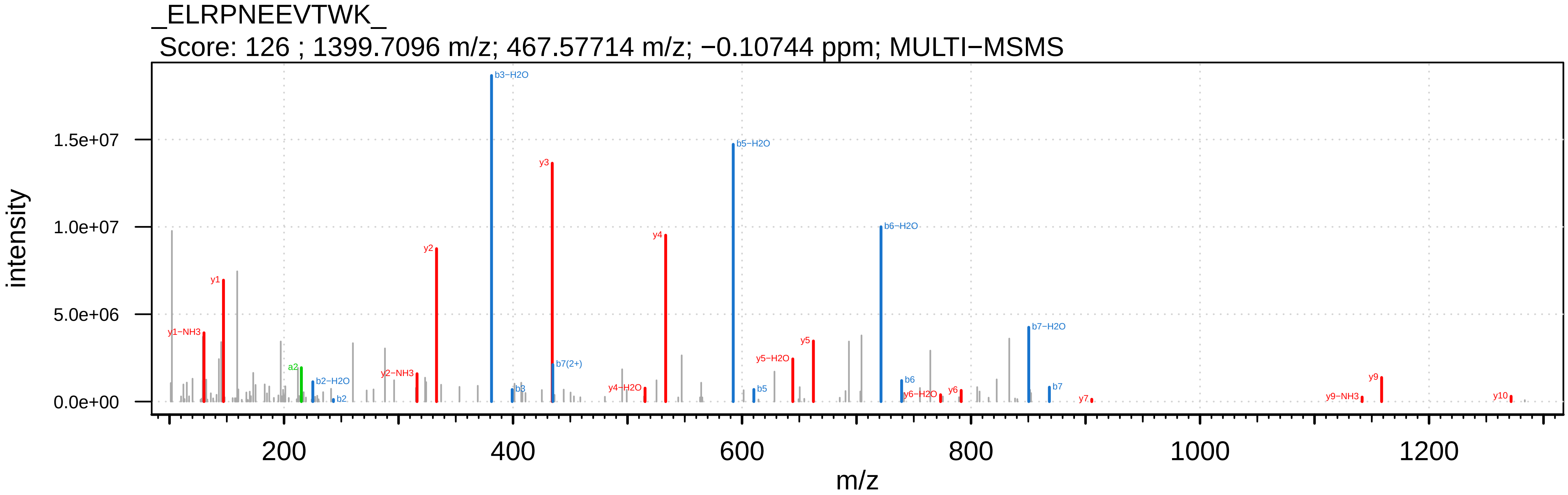

Raw File: 20130718_EXQ6_BaAz_SA_LR3_05
Scan Number: 34717
Proteins:
chr12_103206313−103214181.T2_chr12:101968174−101969629:−

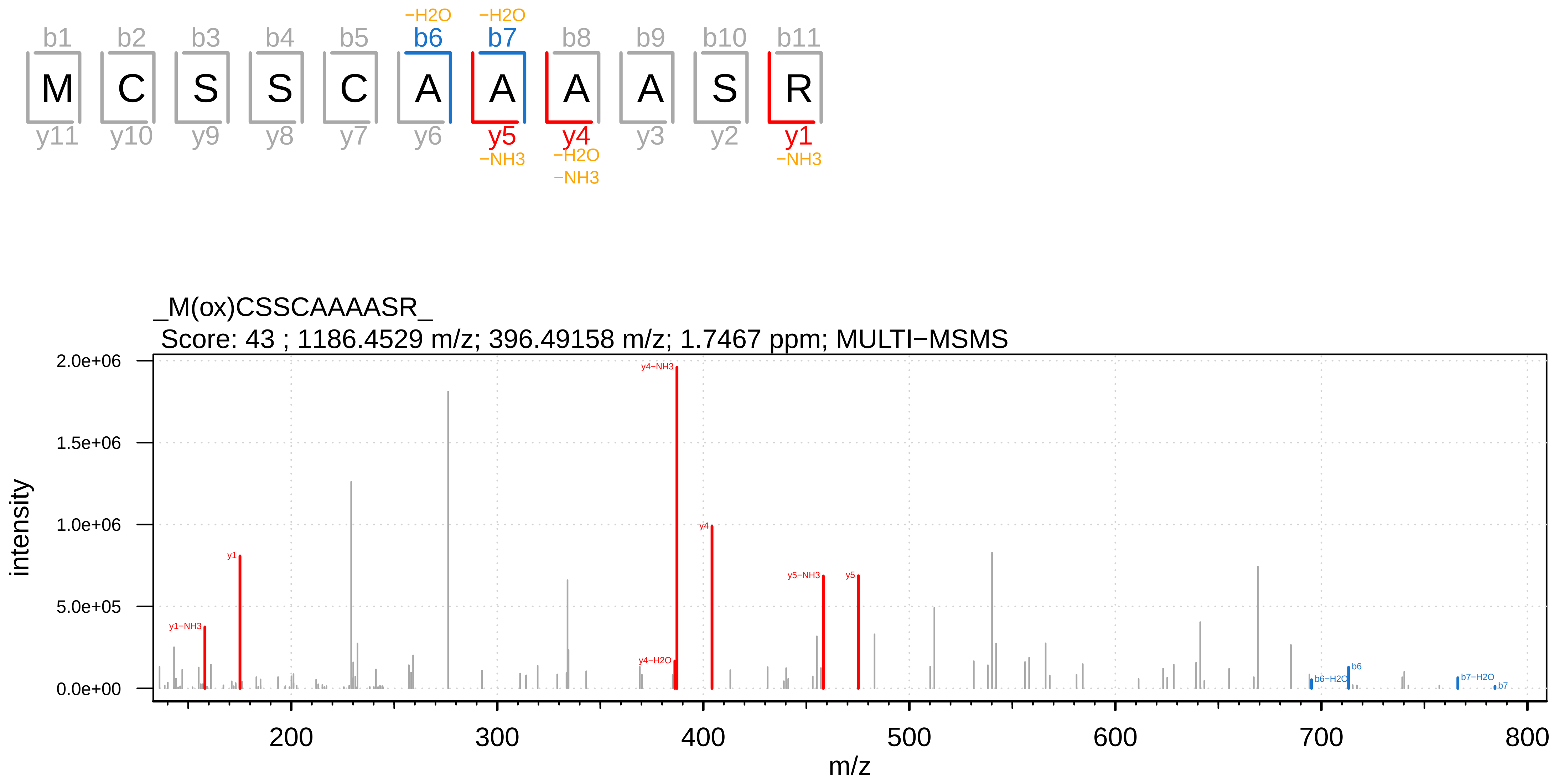

Raw File: 20130718_EXQ6_BaAz_SA_LR3_05
Scan Number: 61999
Proteins:
ENSMUST00000047876_chr3:83128571−83128696:−

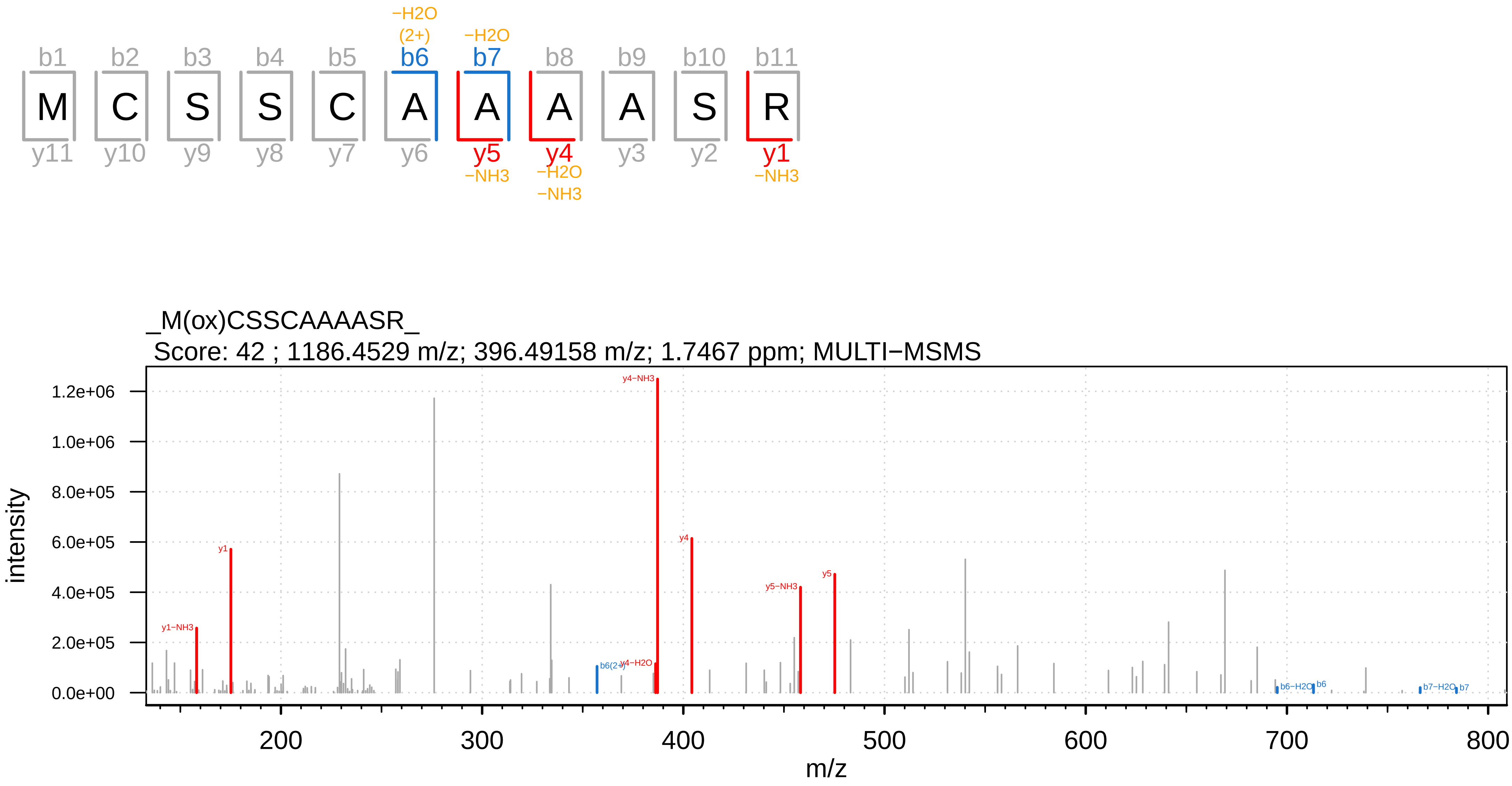

Raw File: 20130718_EXQ6_BaAz_SA_LR3_05
Scan Number: 62347
Proteins:
ENSMUST00000047876_chr3:83128571−83128696:−

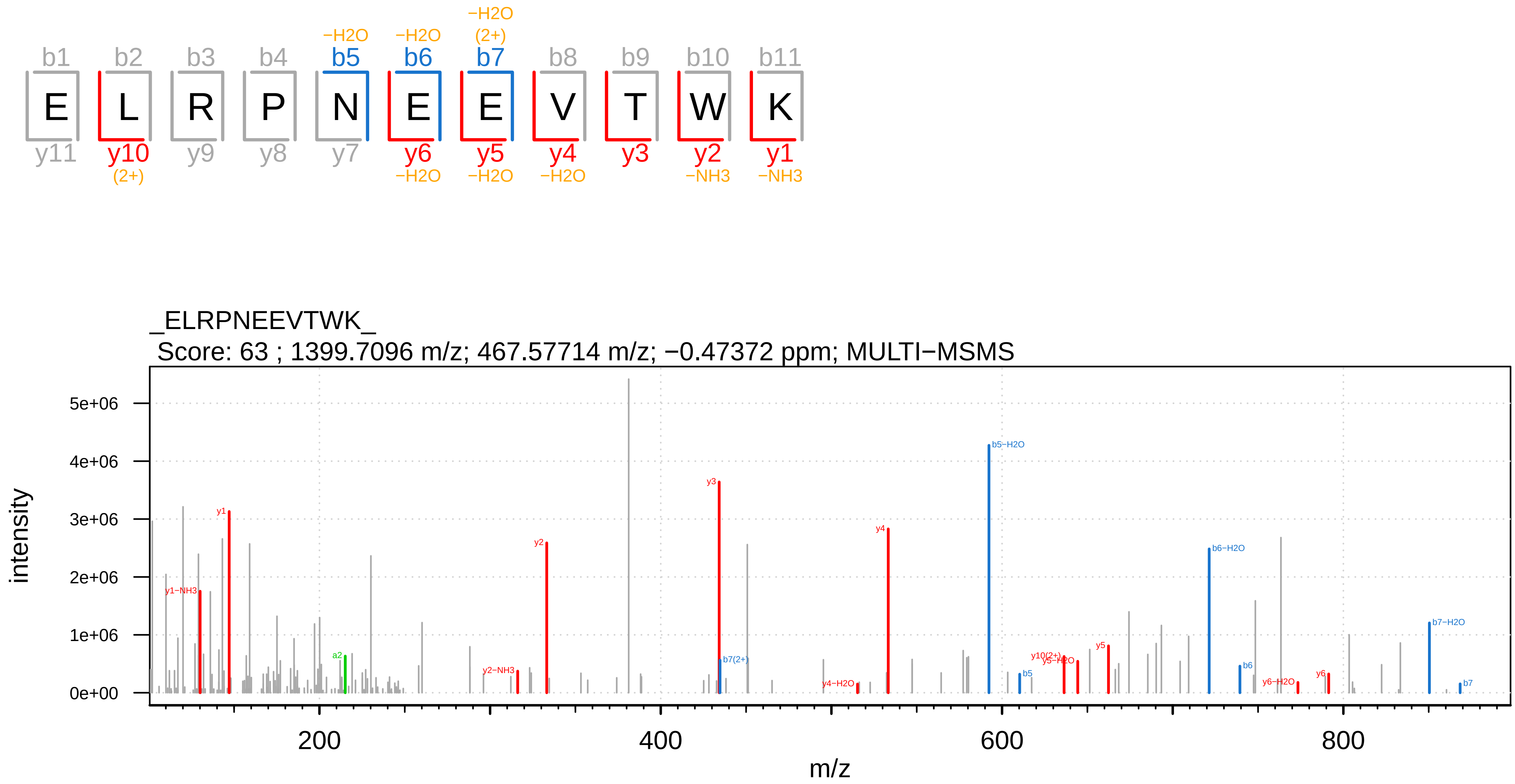

Raw File: 20130718_EXQ6_BaAz_SA_LR3_06
Scan Number: 31720
Proteins:
chr12_103206313−103214181.T2_chr12:101968174−101969629:−

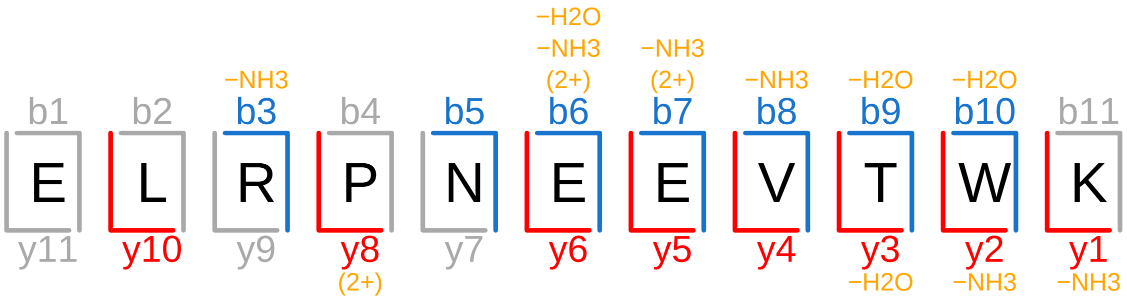

ELRPNEEVTWK
Score: 118 ; 1399.7096 m/z; 700.86207 m/z; −0.098728 ppm; MULTI−MSMS

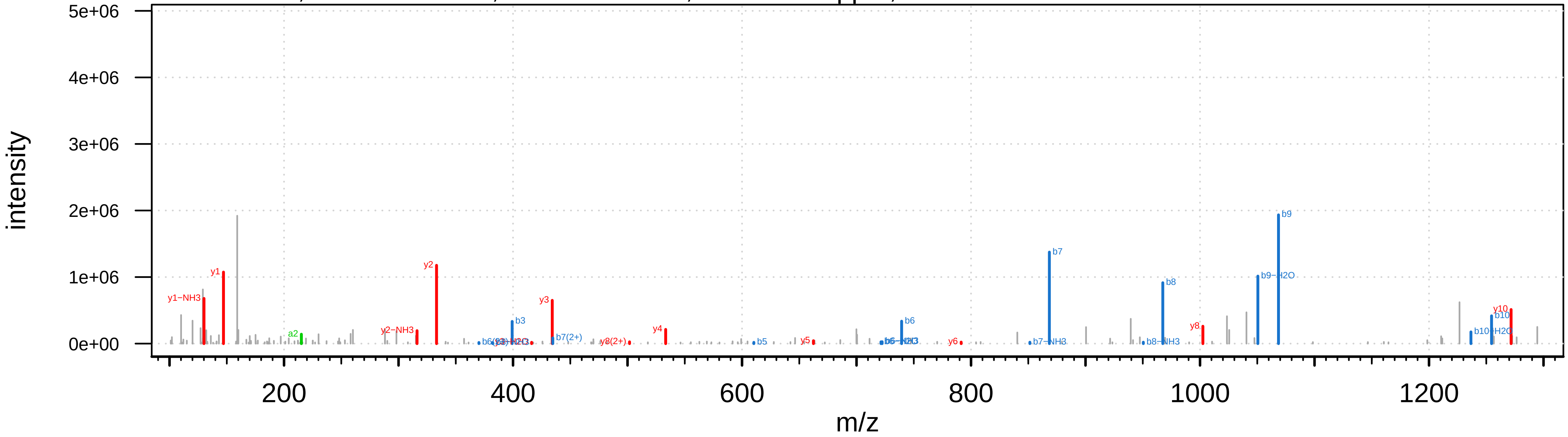

Raw File: 20130718_EXQ6_BaAz_SA_LR3_06
Scan Number: 31726
Proteins:
chr12_103206313−103214181.T2_chr12:101968174−101969629:−

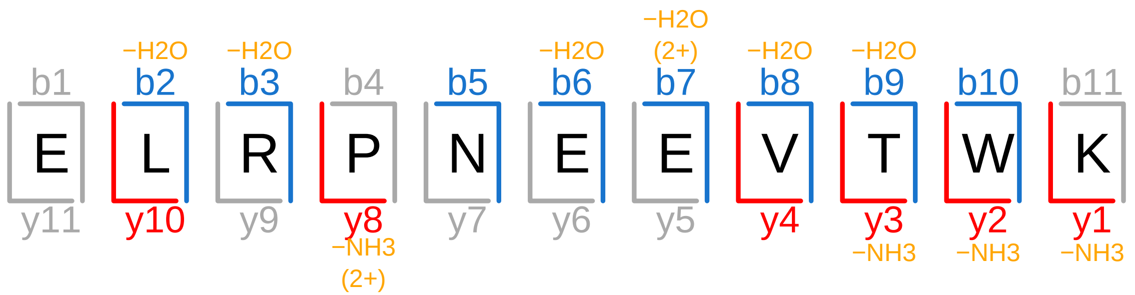

ELRPNEEVTWK

Score: 97 ; 1399.7096 m/z; 700.86207 m/z; −0.098728 ppm; MULTI−MSMS

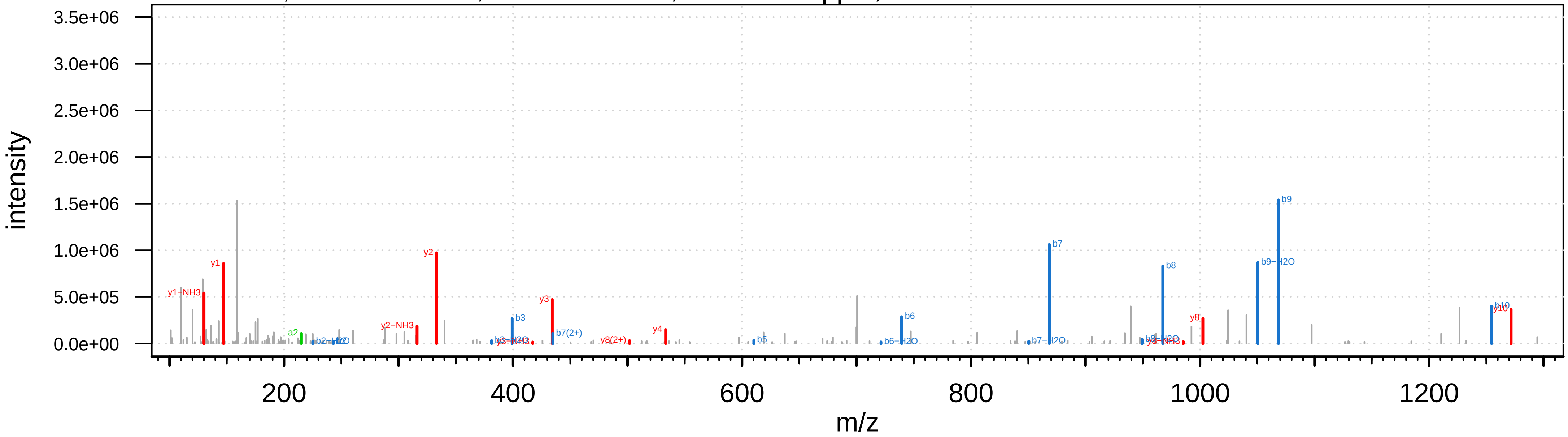

Raw File: 20130718_EXQ6_BaAz_SA_LR3_06

Scan Number: 32093

Proteins:

chr12_103206313−103214181.T2_chr12:101968174−101969629:−

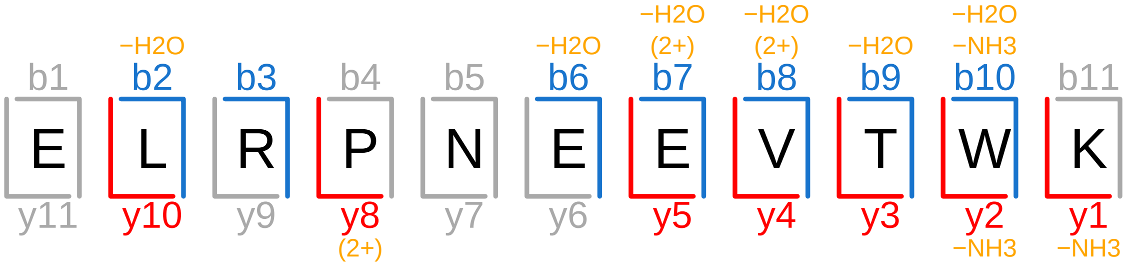

ELRPNEEVTWK
Score: 133 ; 1399.7096 m/z; 700.86207 m/z; 0.066729 ppm; MULTI−MSMS

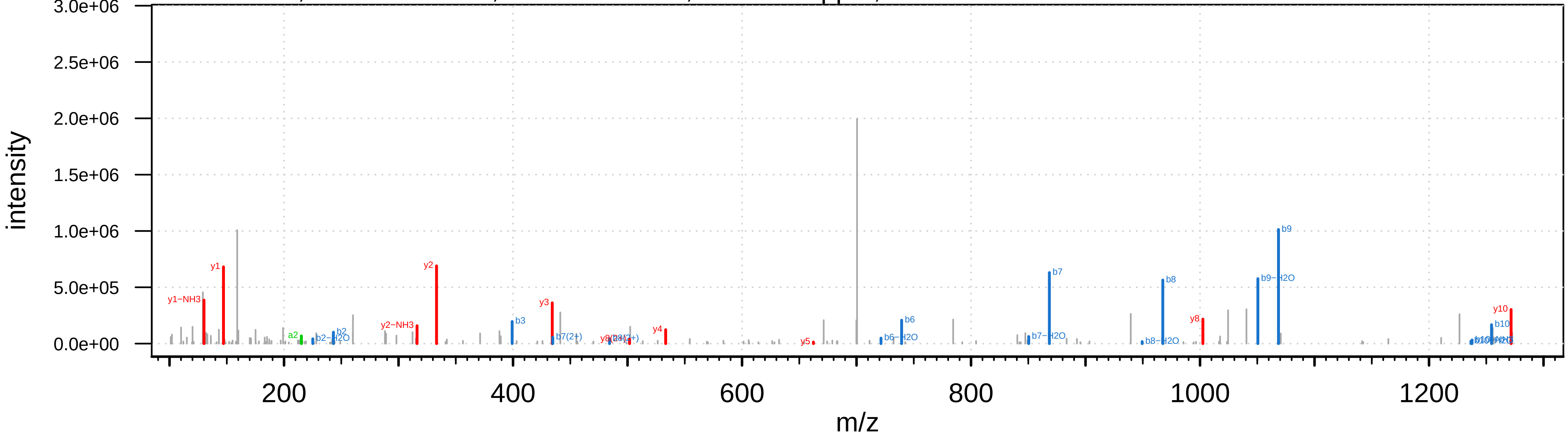

Raw File: 20130719_EXQ4_BaAz_SA_HR2_05
Scan Number: 34449
Proteins:
chr12_103206313−103214181.T2_chr12:101968174−101969629:−

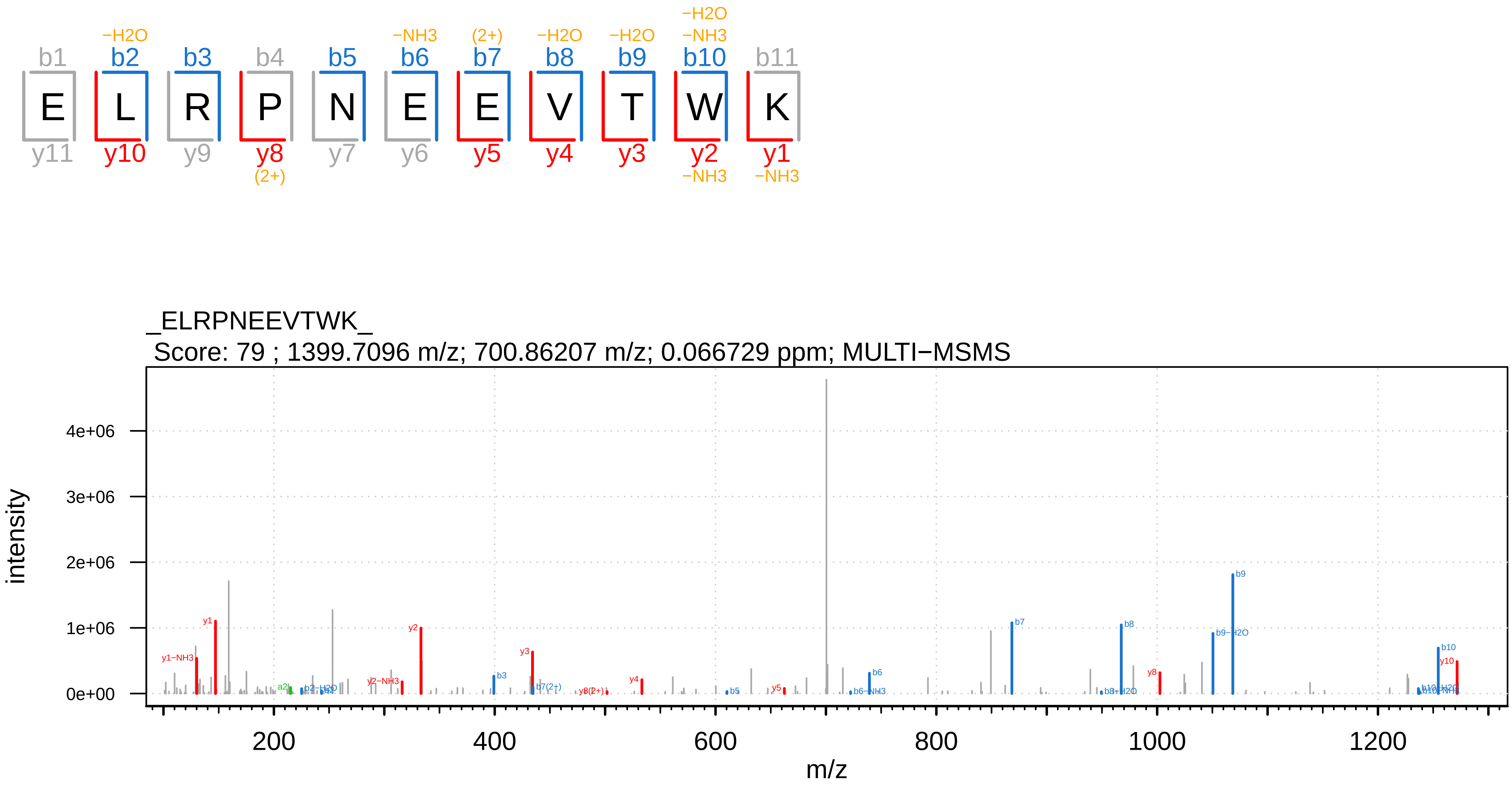

Raw File: 20130719_EXQ4_BaAz_SA_HR2_05
Scan Number: 34806
Proteins:
chr12_103206313−103214181.T2_chr12:101968174−101969629:−

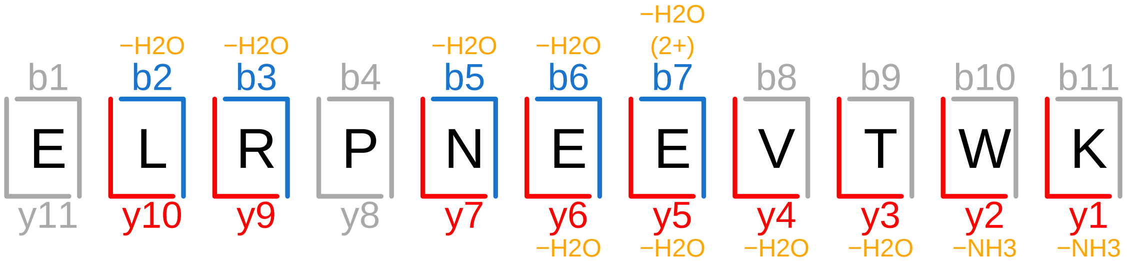

ELRPNEEVTWK
Score: 116 ; 1399.7096 m/z; 467.57714 m/z; 0.29976 ppm; MULTI−MSMS

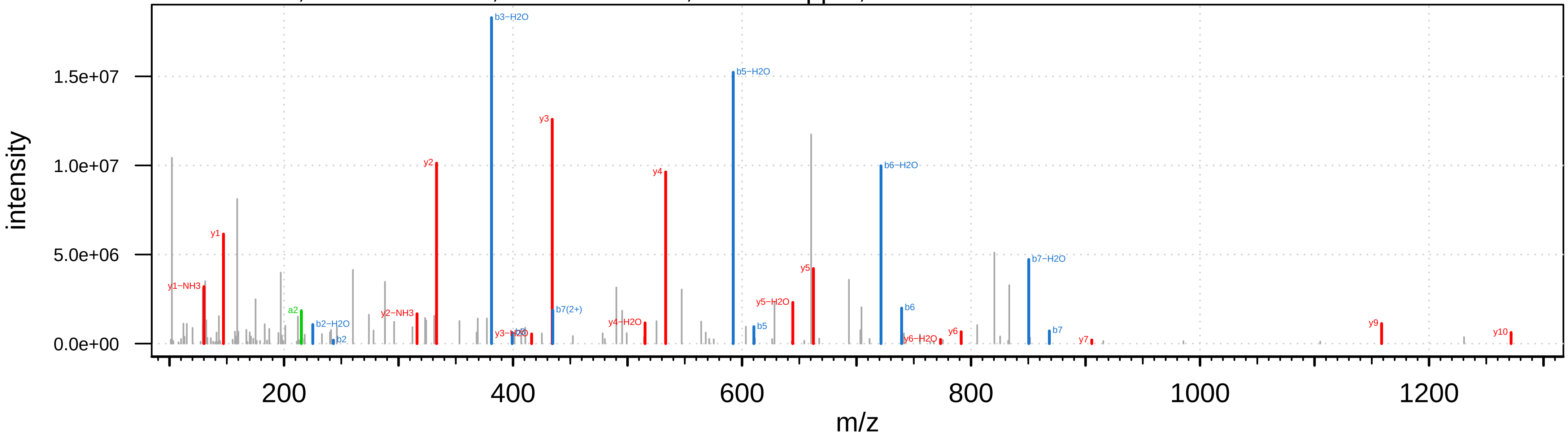

Raw File: 20130719_EXQ4_BaAz_SA_HR2_05
Scan Number: 34547
Proteins:
chr12_103206313−103214181.T2_chr12:101968174−101969629:−

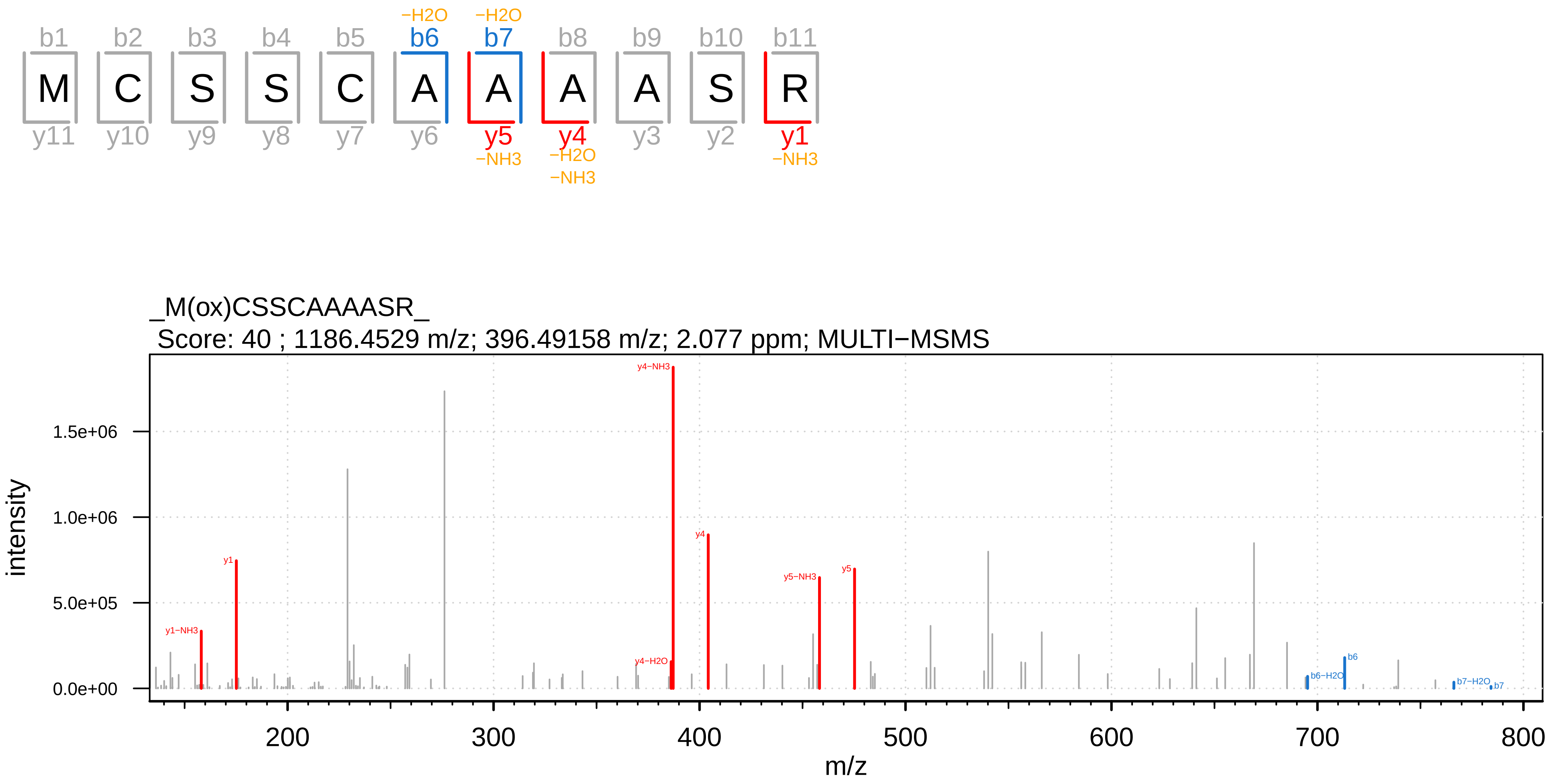

Raw File: 20130719_EXQ4_BaAz_SA_HR2_05
Scan Number: 59337
Proteins:
ENSMUST00000047876_chr3:83128571−83128696:−

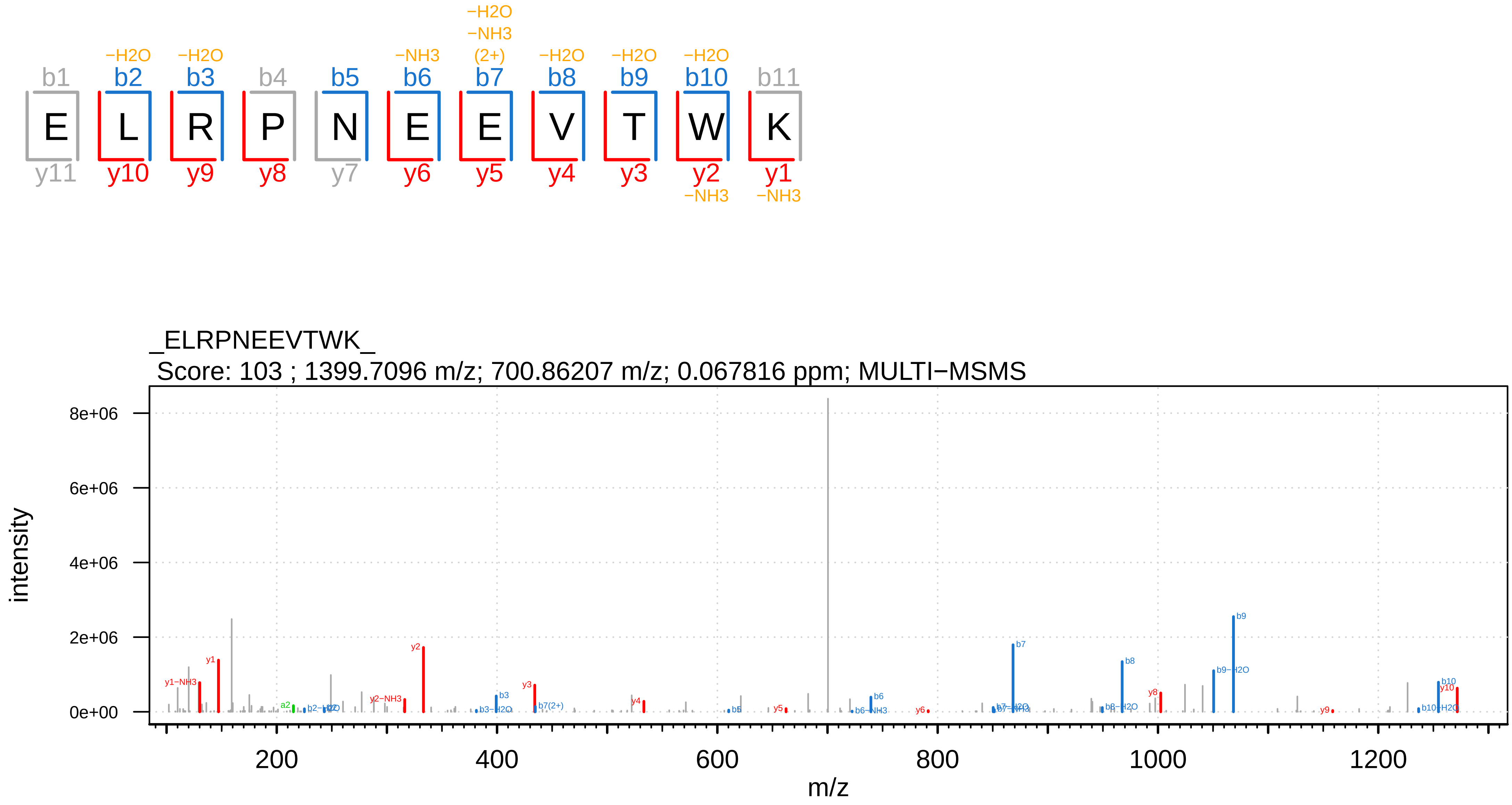

Raw File: 20130719_EXQ4_BaAz_SA_HR2_06
Scan Number: 30266
Proteins:
chr12_103206313−103214181.T2_chr12:101968174−101969629:−

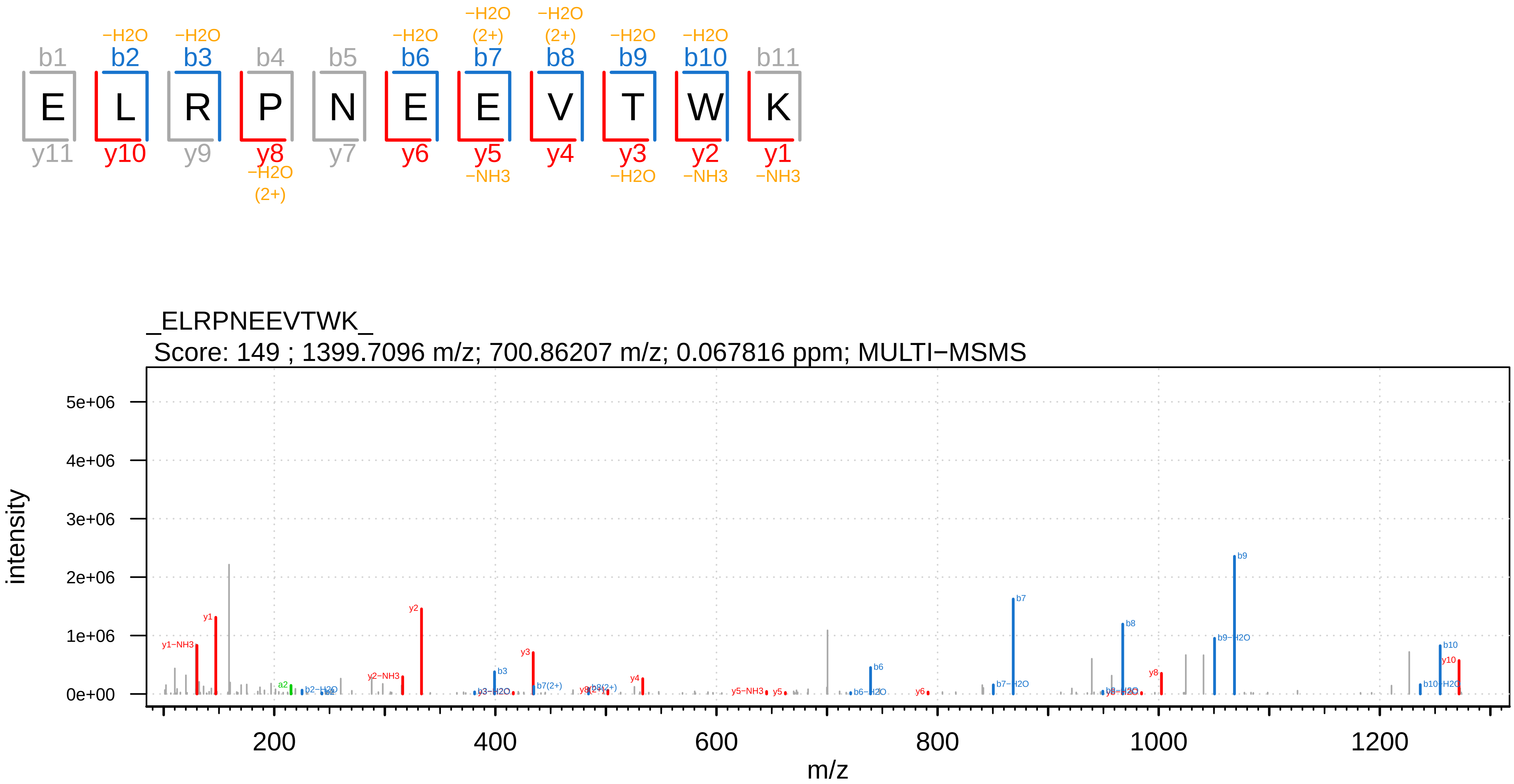

Raw File: 20130719_EXQ4_BaAz_SA_HR2_06
Scan Number: 30611
Proteins:
chr12_103206313−103214181.T2_chr12:101968174−101969629:−

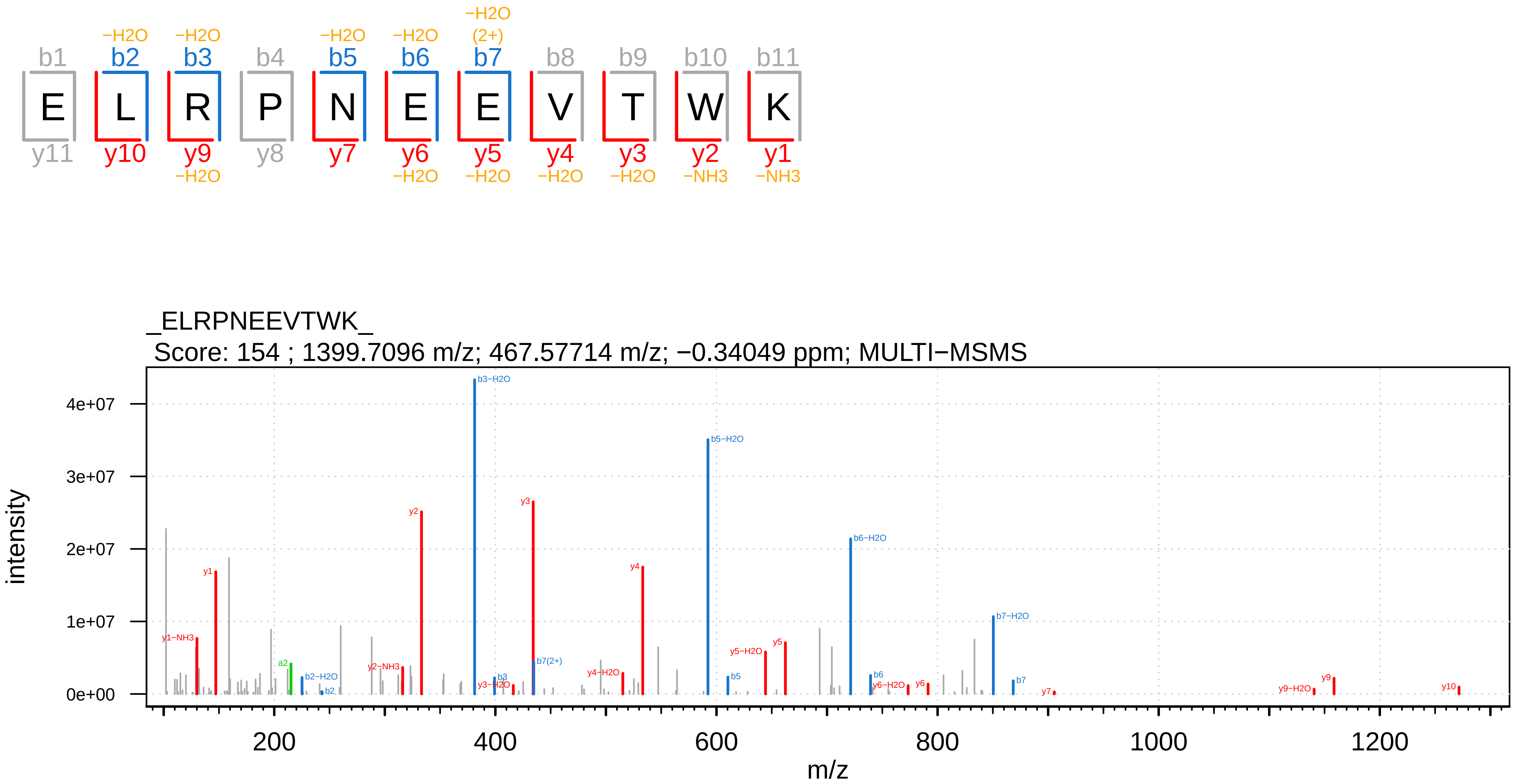

Raw File: 20130719_EXQ4_BaAz_SA_HR2_06
Scan Number: 30345
Proteins:
chr12_103206313−103214181.T2_chr12:101968174−101969629:−

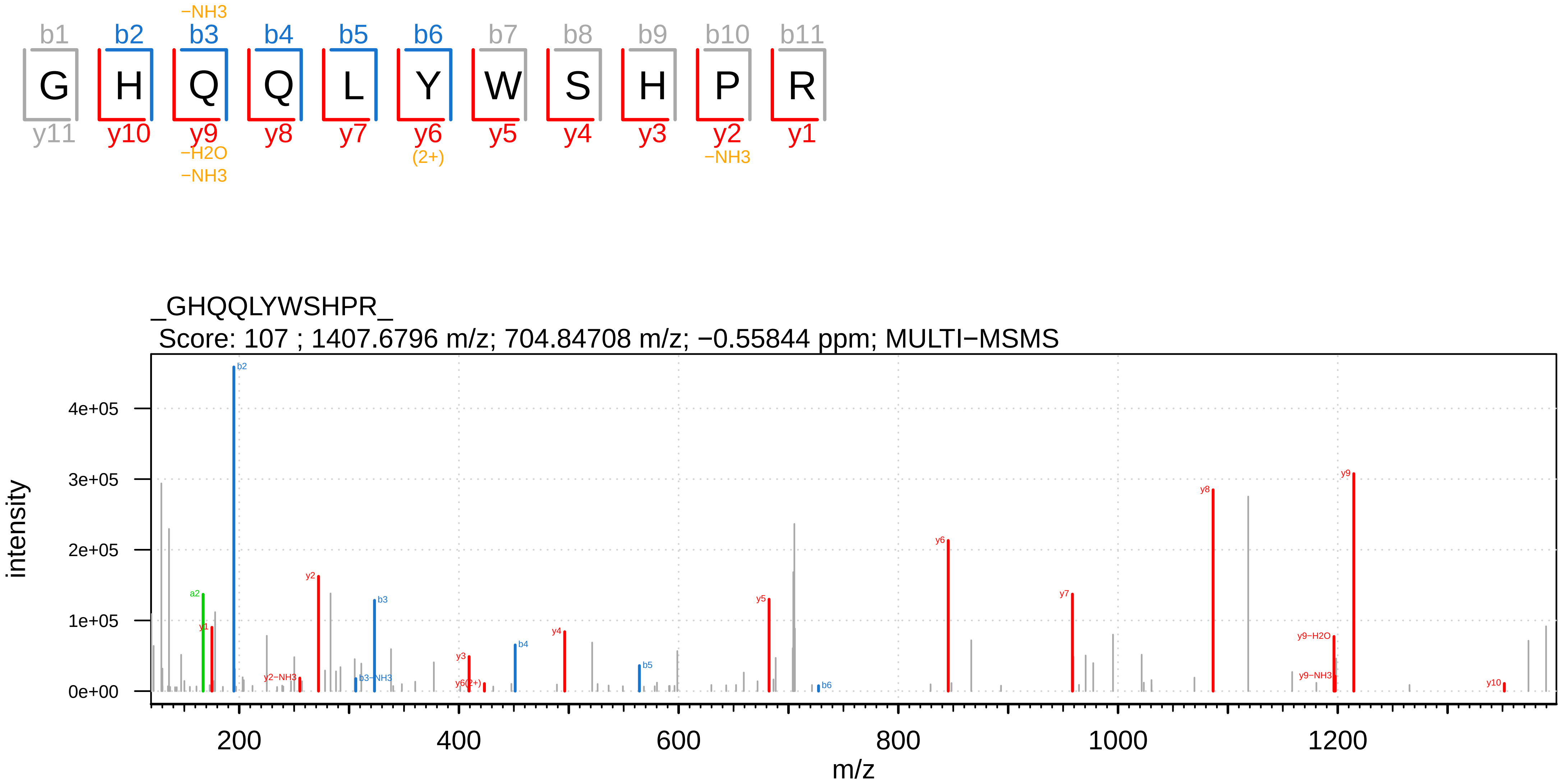

Raw File: 20130719_EXQ4_BaAz_SA_HR2_06
Scan Number: 15547
Proteins:
sp|P62274|RS29_MOUSE
ENSMUST00000037023_chr12:69157813−69159058:−
ENSMUST00000071737_chr13:73988963−73989133:−

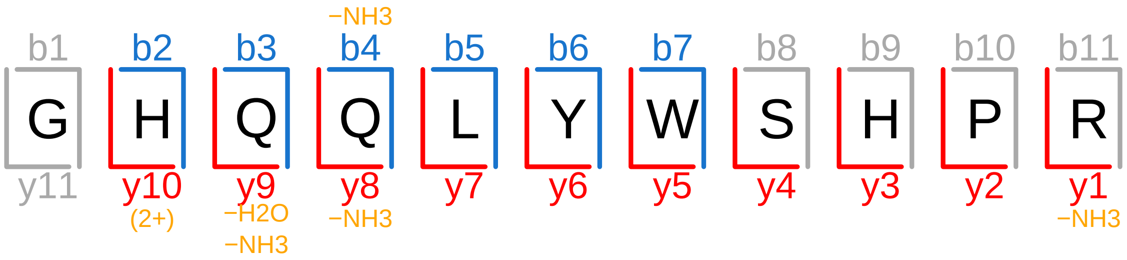

GHQQLYWSHPR
Score: 86 ; 1407.6796 m/z; 704.84708 m/z; NaN ppm; MSMS

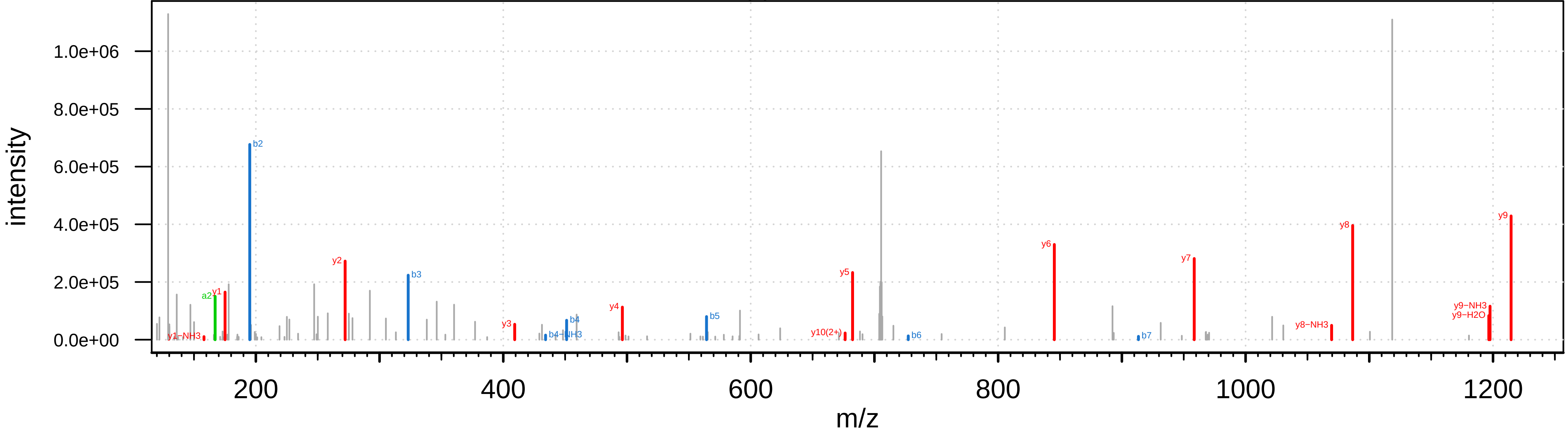

Raw File: 20130719_EXQ4_BaAz_SA_HR2_06
Scan Number: 15230
Proteins:
sp|P62274|RS29_MOUSE
ENSMUST00000037023_chr12:69157813−69159058:−
ENSMUST00000071737_chr13:73988963−73989133:−

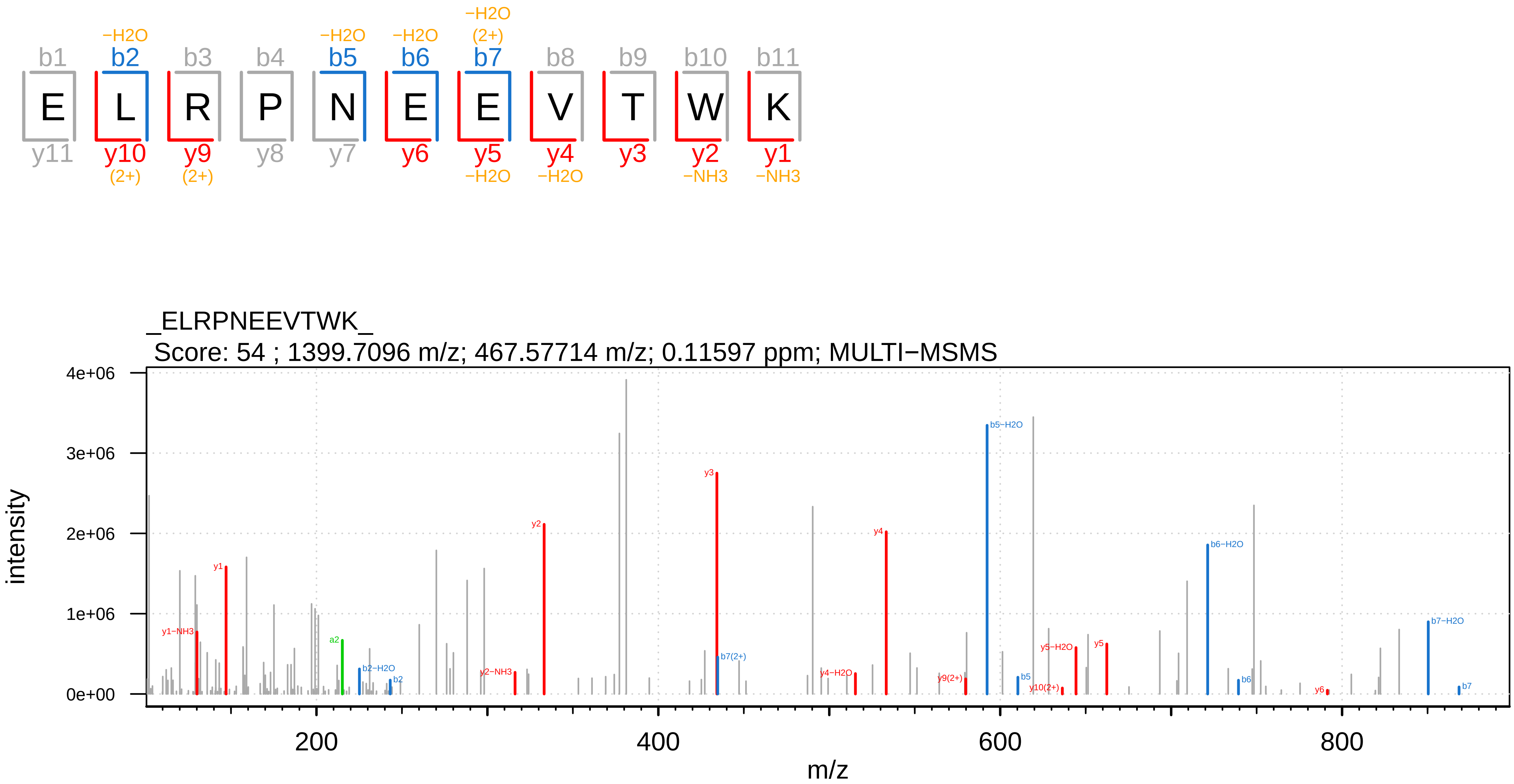

Raw File: 20130720_EXQ4_BaAz_SA_KR3_05
Scan Number: 35268
Proteins:
chr12_103206313−103214181.T2_chr12:101968174−101969629:−

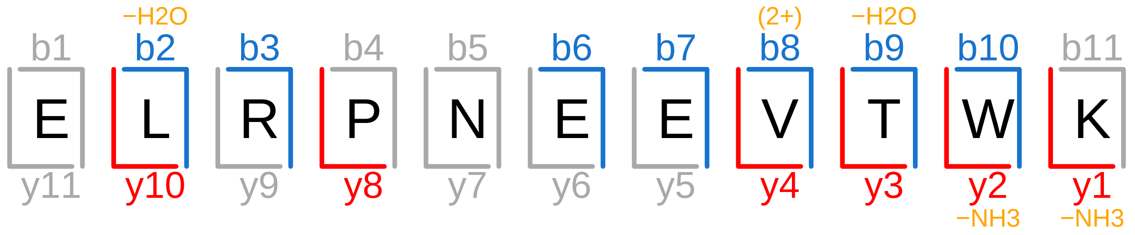

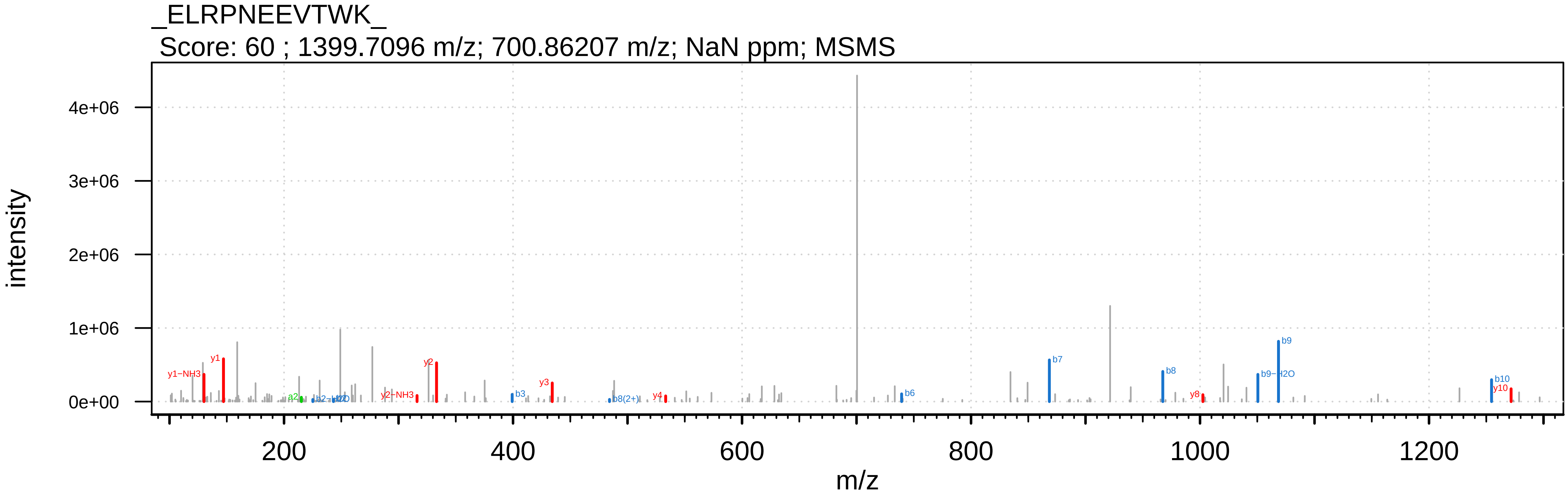

Raw File: 20130720_EXQ4_BaAz_SA_KR3_05
Scan Number: 35143
Proteins:
chr12_103206313−103214181.T2_chr12:101968174−101969629:−

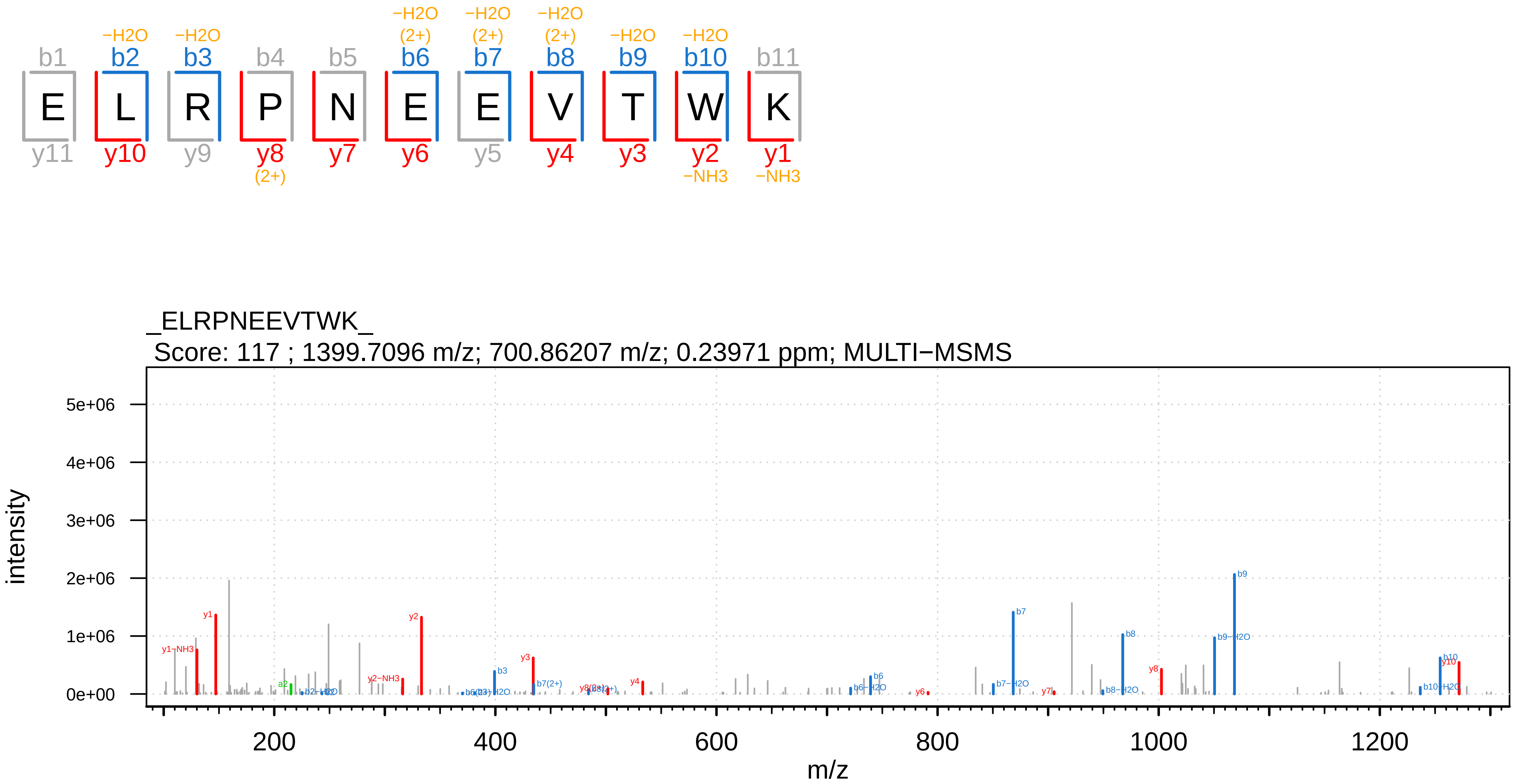

Raw File: 20130720_EXQ4_BaAz_SA_KR3_06

Scan Number: 32728

Proteins:

chr12_103206313−103214181.T2_chr12:101968174−101969629:−

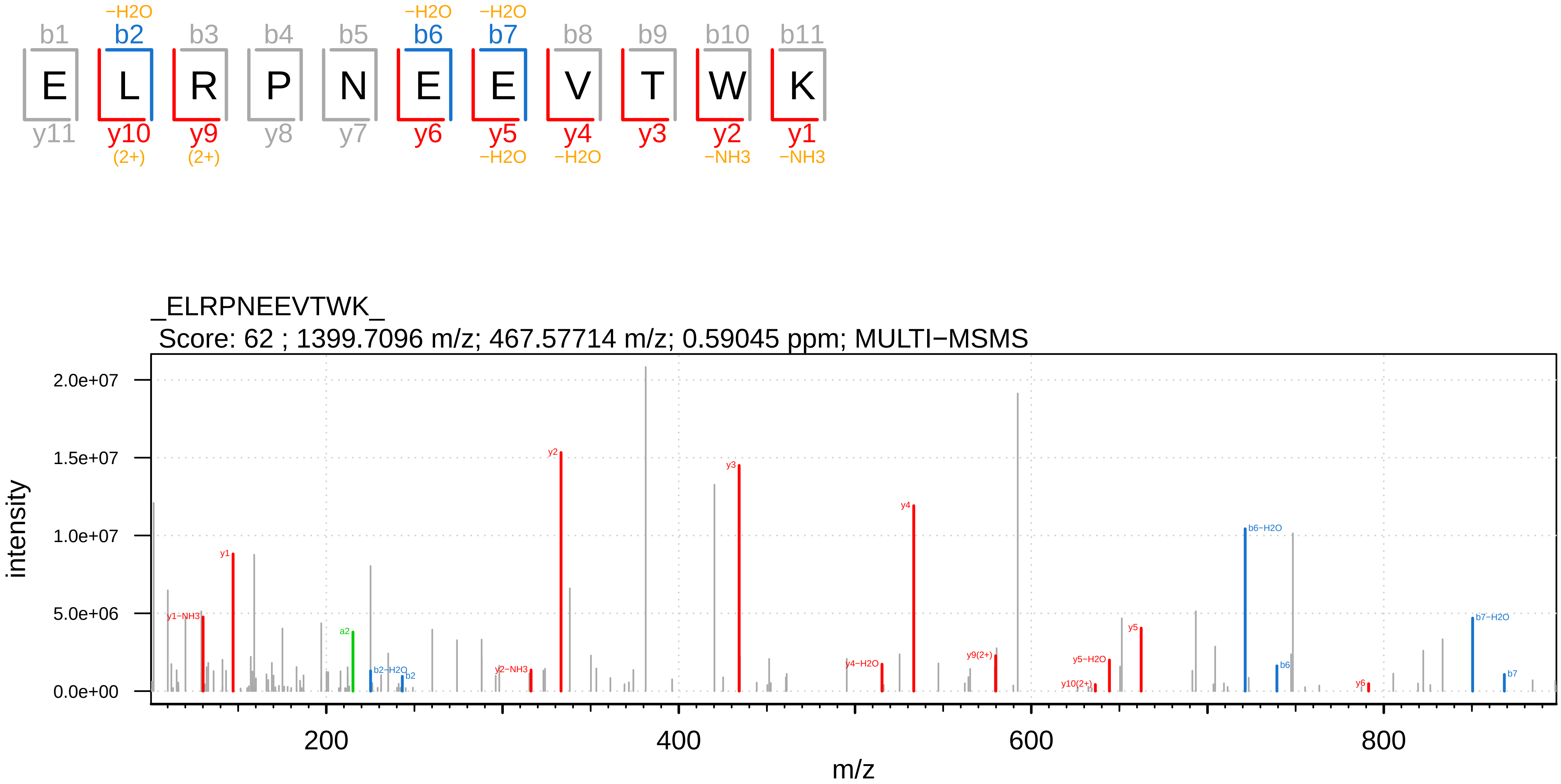

Raw File: 20130720_EXQ4_BaAz_SA_KR3_06

Scan Number: 32784

Proteins:

chr12_103206313−103214181.T2_chr12:101968174−101969629:−

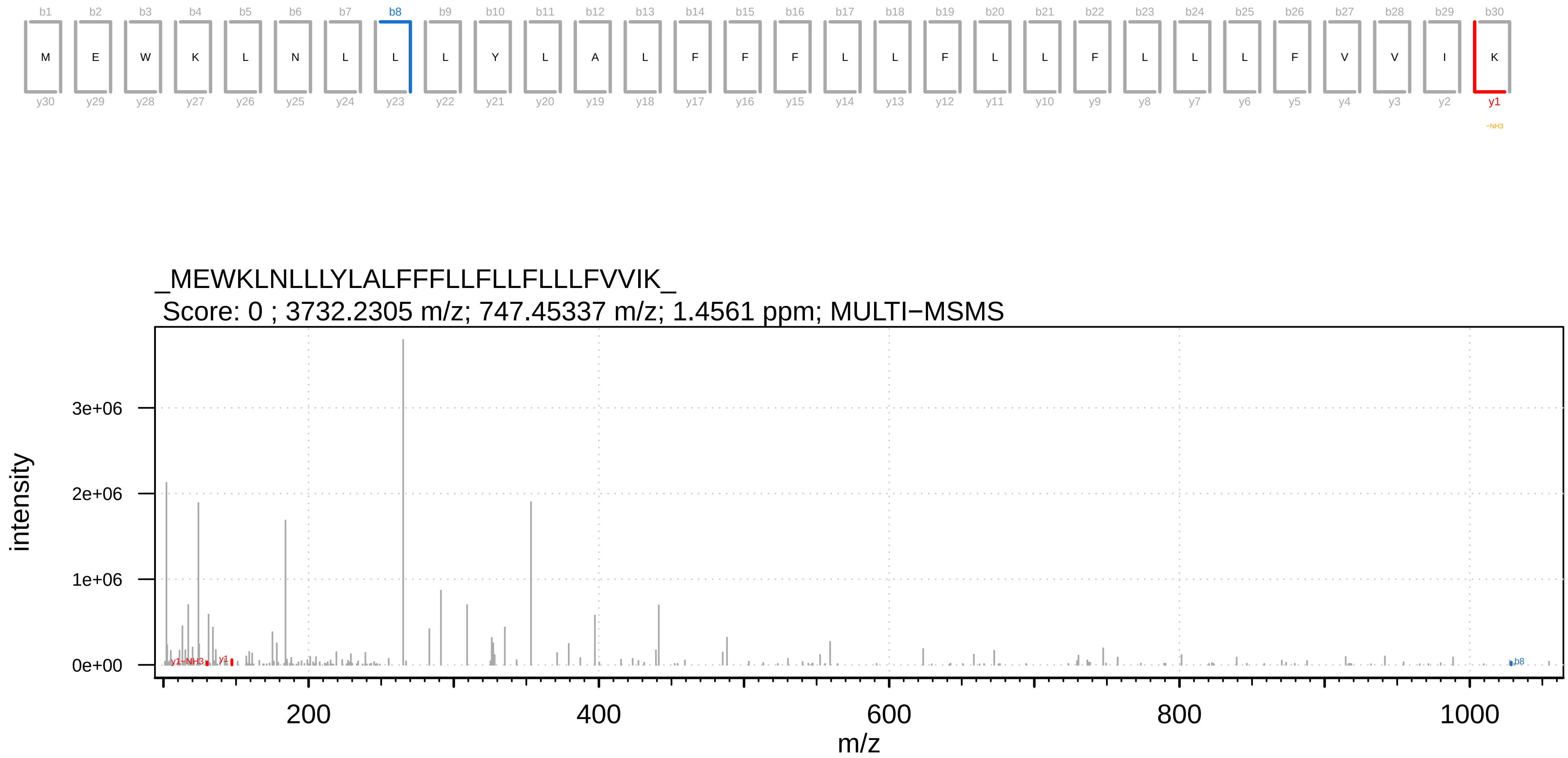

Raw File: 20130720_EXQ4_BaAz_SA_KR3_06
Scan Number: 114152
Proteins:
ENSMUST00000124606_chr3:36521200−36521391:−

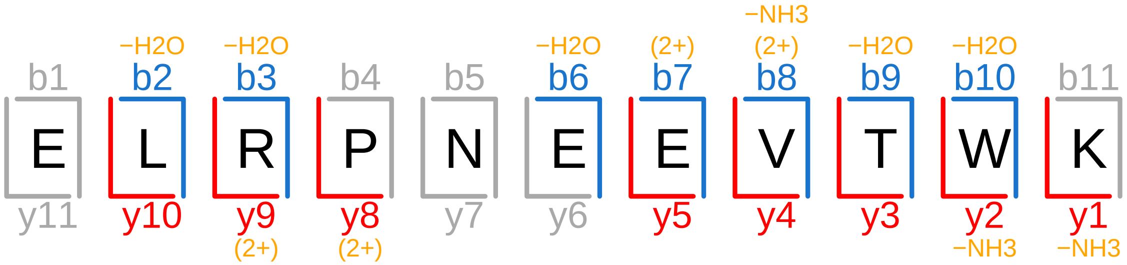

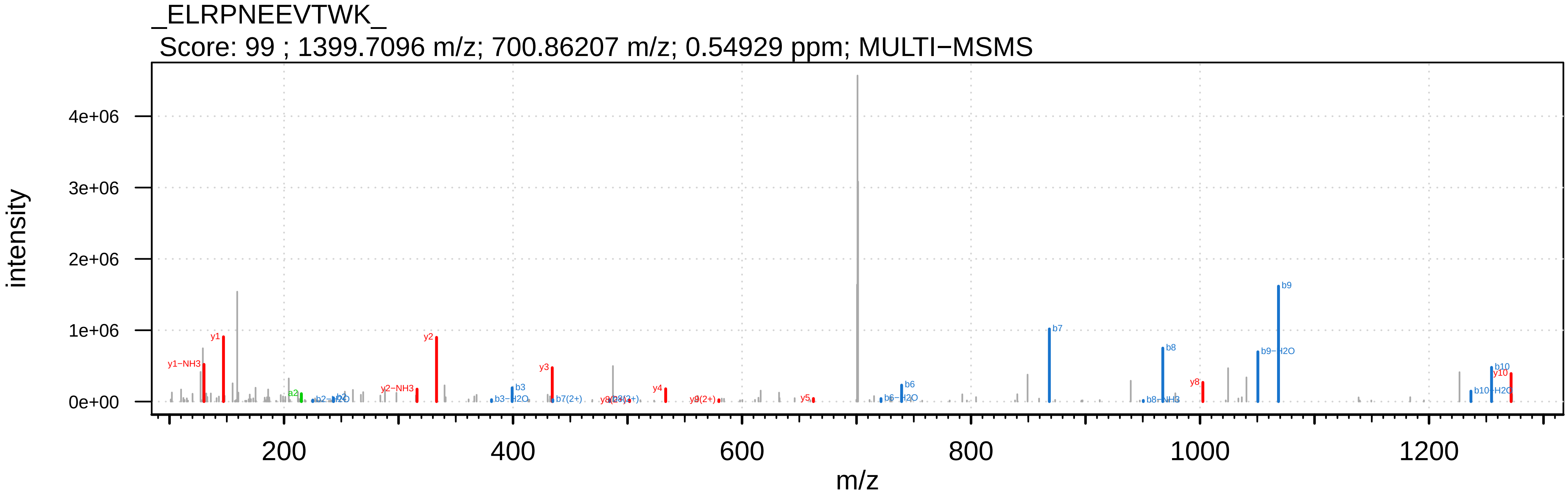

Raw File: 20130722_EXQ4_BaAz_SA_SeR3_05

Scan Number: 33275

Proteins:

chr12_103206313−103214181.T2_chr12:101968174−101969629:−

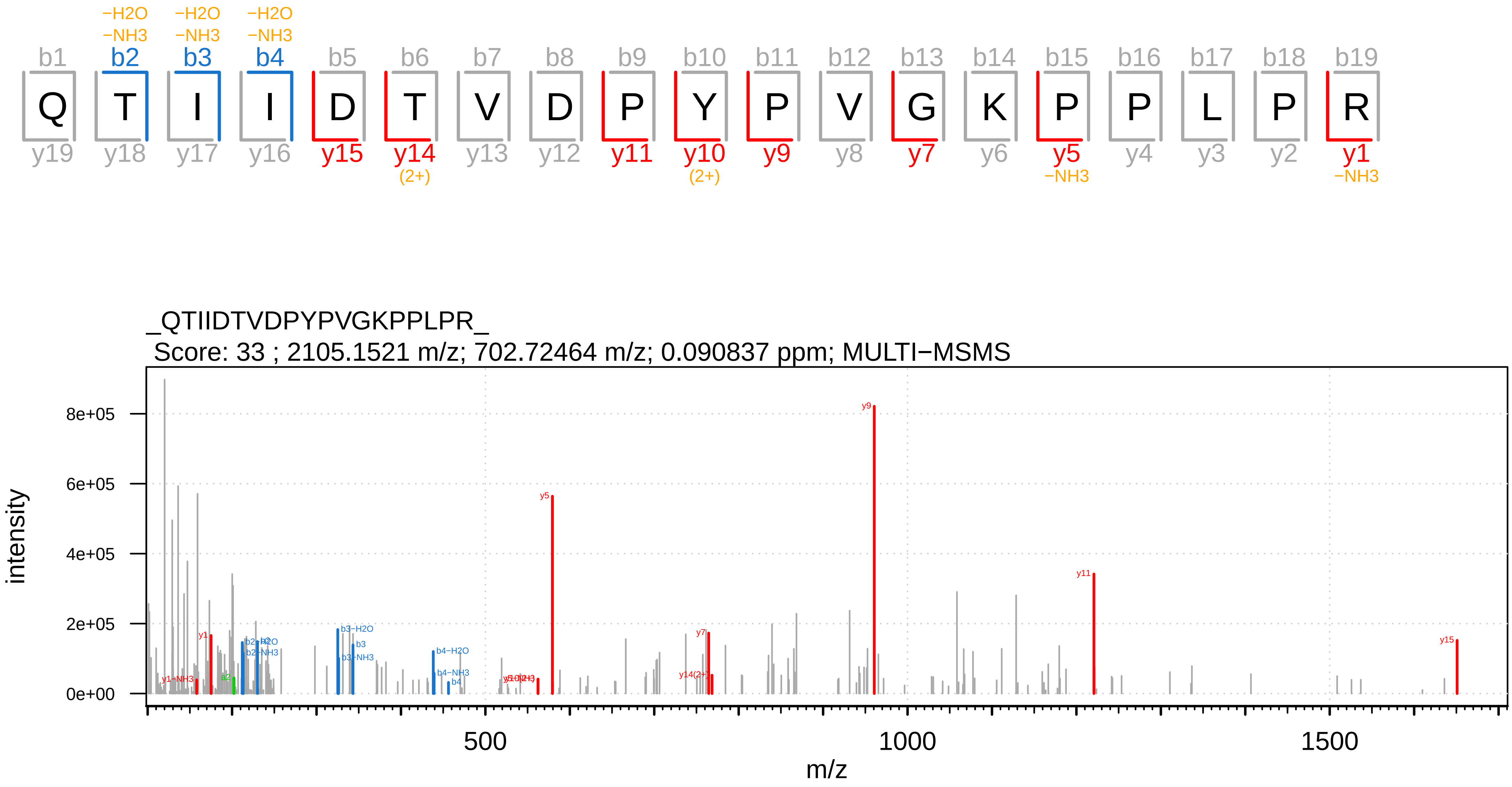

Raw File: 20130722_EXQ4_BaAz_SA_SeR3_05
Scan Number: 75237
Proteins:
ENSMUST00000136126_chr3:79172848−79174982:−

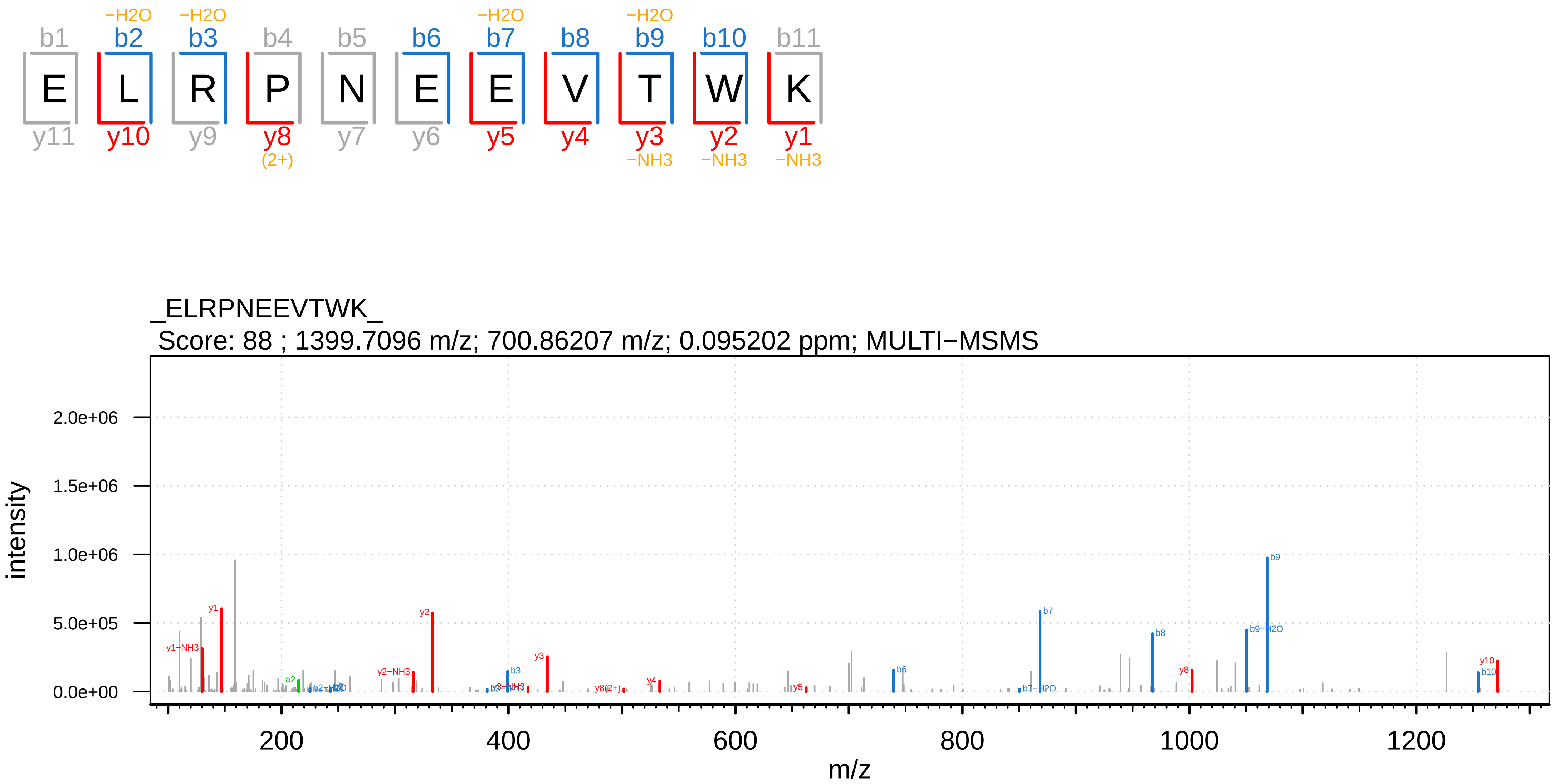

Raw File: 20130722_EXQ4_BaAz_SA_SeR3_06
Scan Number: 31473
Proteins:
chr12_103206313−103214181.T2_chr12:101968174−101969629:−

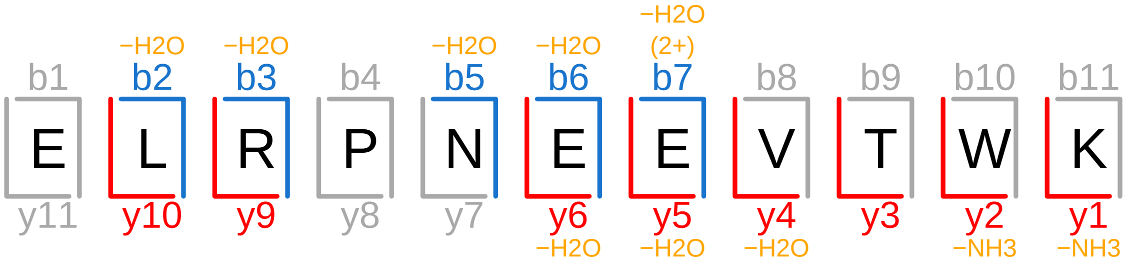

ELRPNEEVTWK

Score: 110 ; 1399.7096 m/z; 467.57714 m/z; 0.18658 ppm; MULTI−MSMS

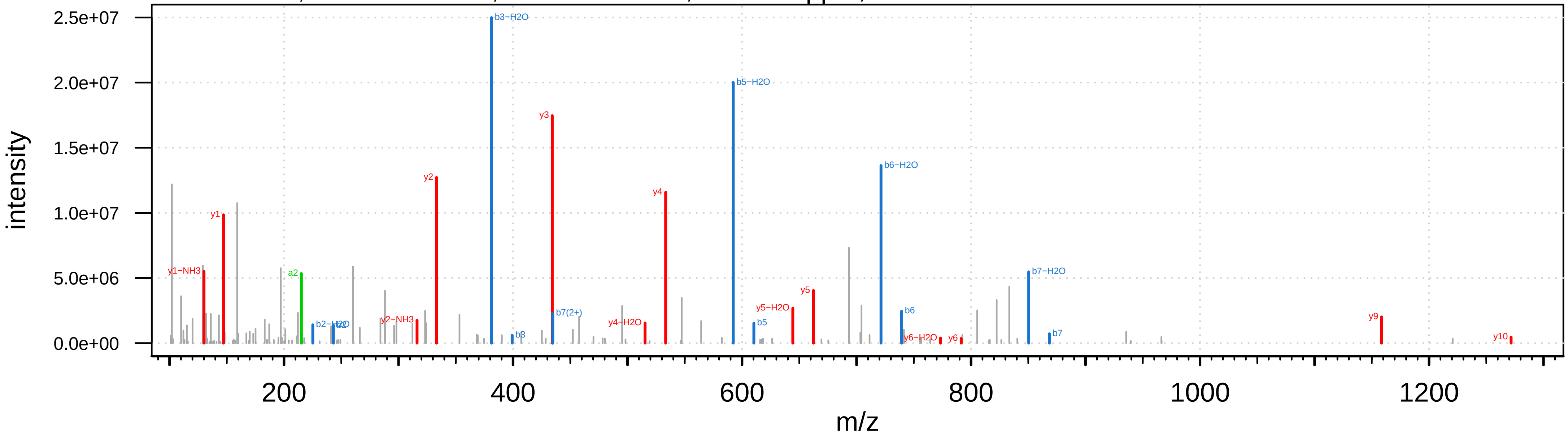

Raw File: 20130722_EXQ4_BaAz_SA_SeR3_06
Scan Number: 31513
Proteins:
chr12_103206313−103214181.T2_chr12:101968174−101969629:−

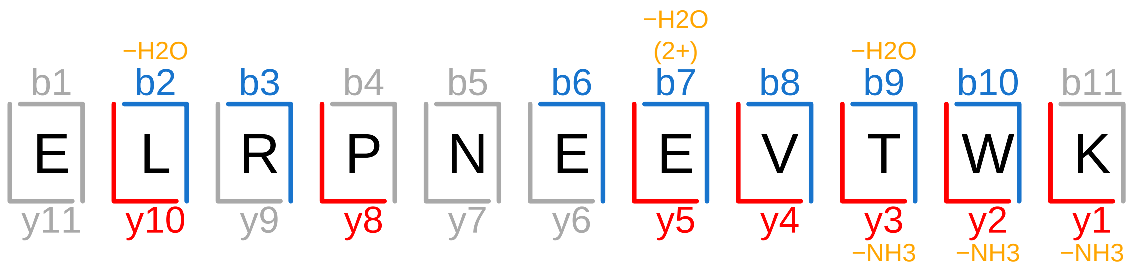

ELRPNEEVTWK
Score: 77 ; 1399.7096 m/z; 700.86207 m/z; 0.397 ppm; MULTI−MSMS

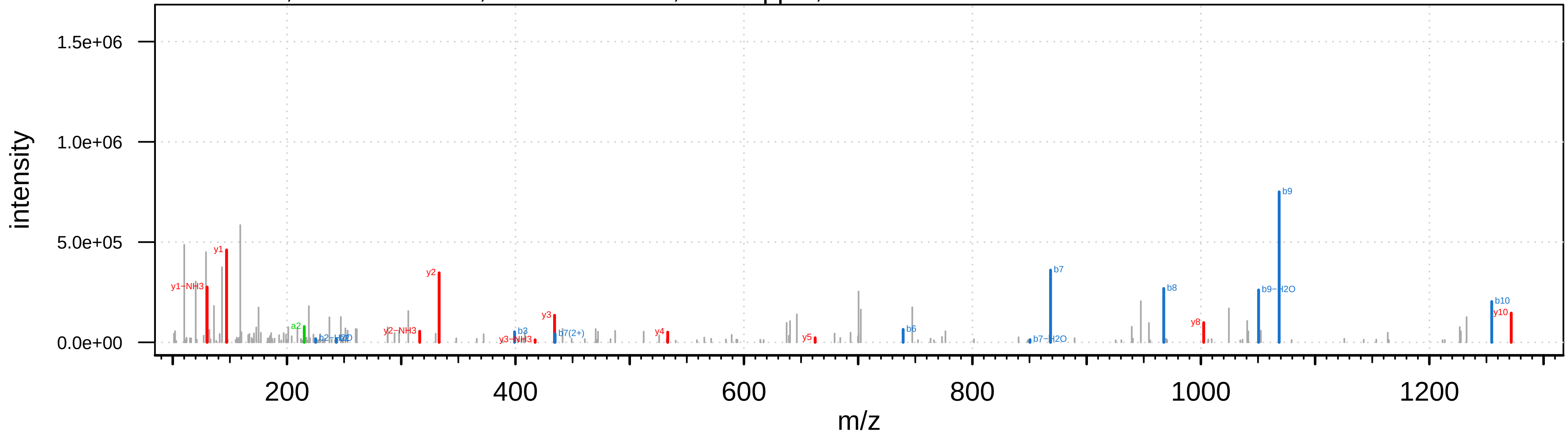

Raw File: 20130723_EXQ4_BaAz_SA_StR3_06
Scan Number: 31381
Proteins:
chr12_103206313−103214181.T2_chr12:101968174−101969629:−

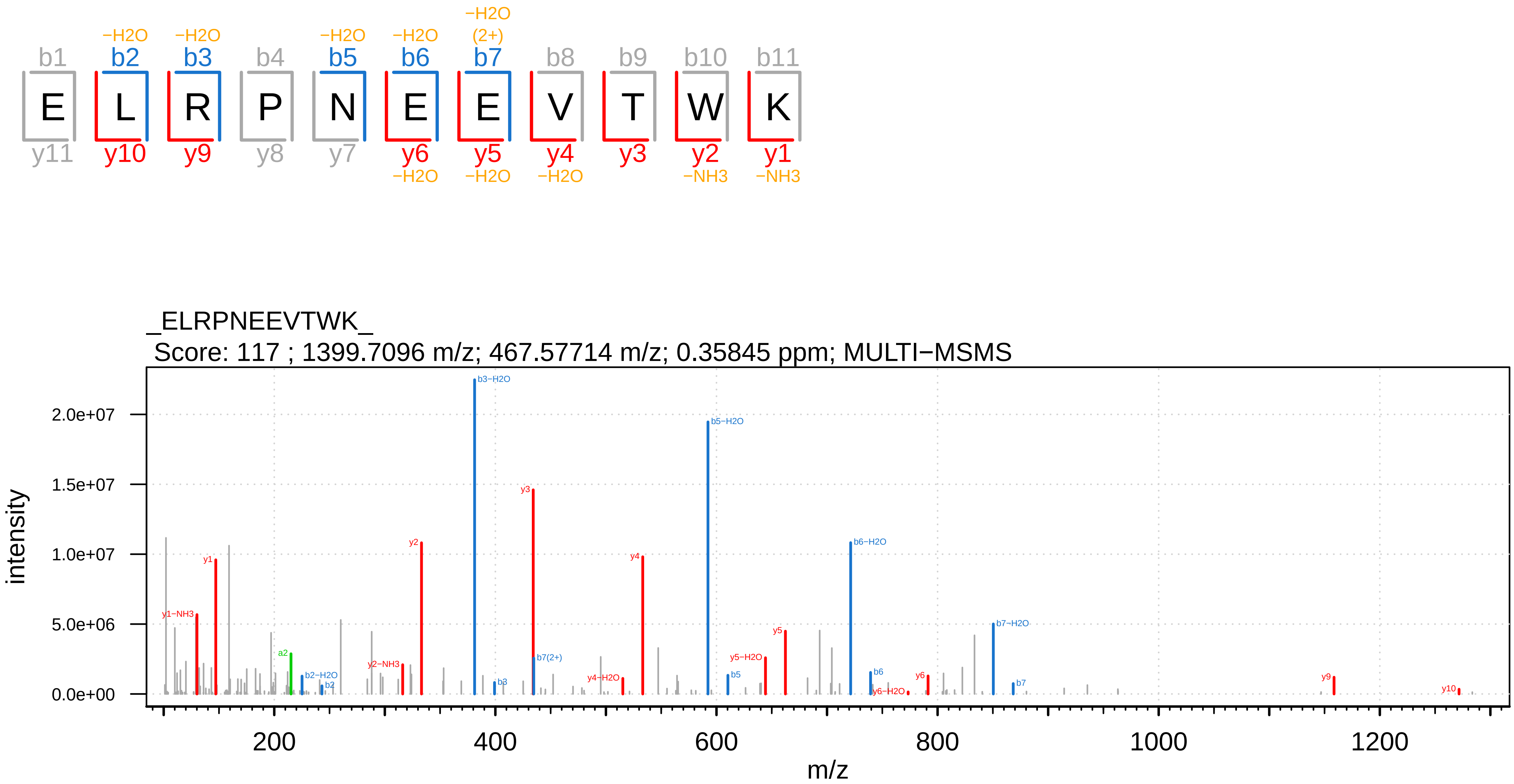

Raw File: 20130723_EXQ4_BaAz_SA_StR3_06

Scan Number: 31470

Proteins:

chr12_103206313−103214181.T2_chr12:101968174−101969629:−

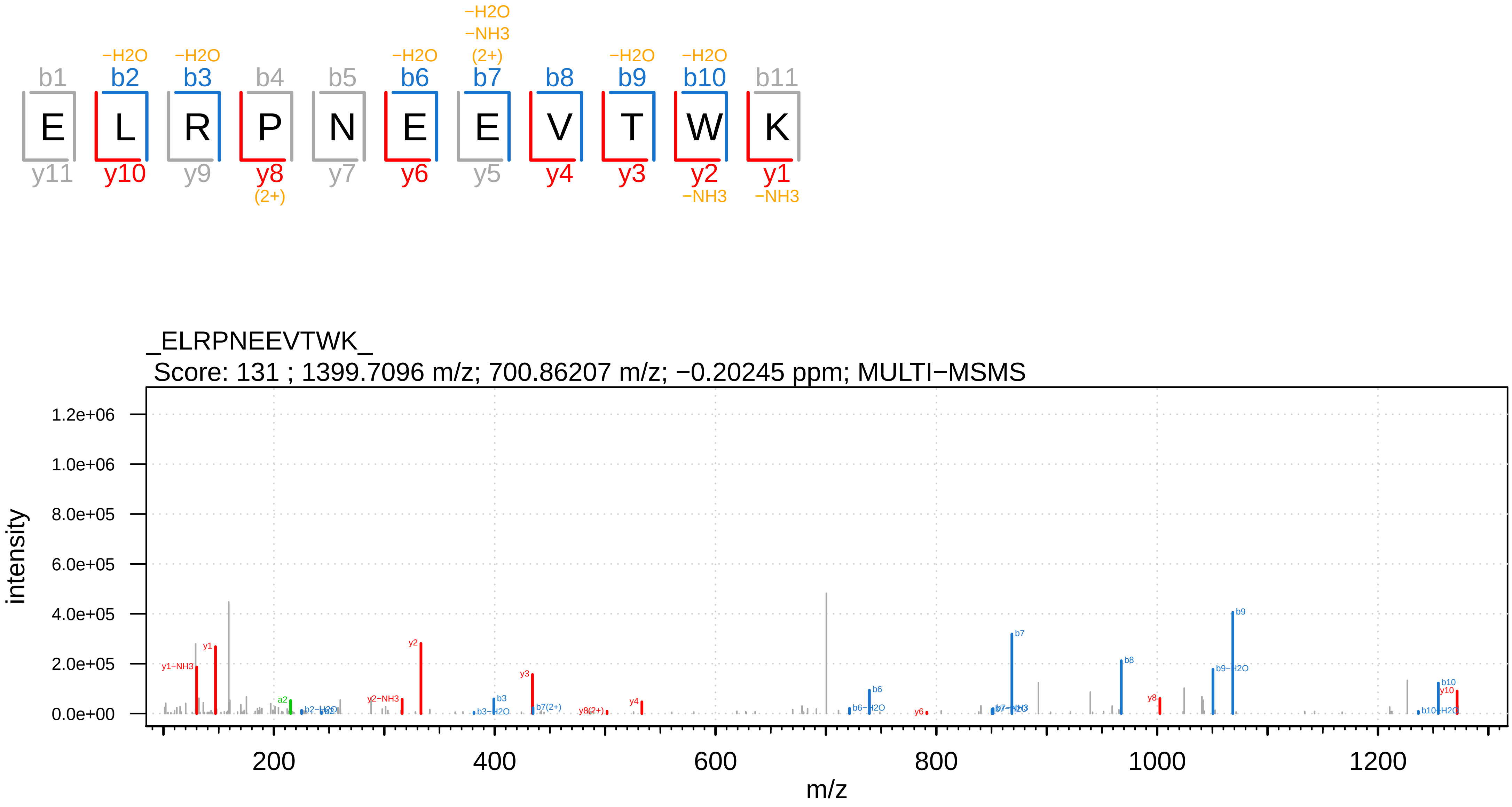

Raw File: 20130731_EXQ4_BaAz_SA_HR4_05
Scan Number: 30196
Proteins:
chr12_103206313−103214181.T2_chr12:101968174−101969629:−

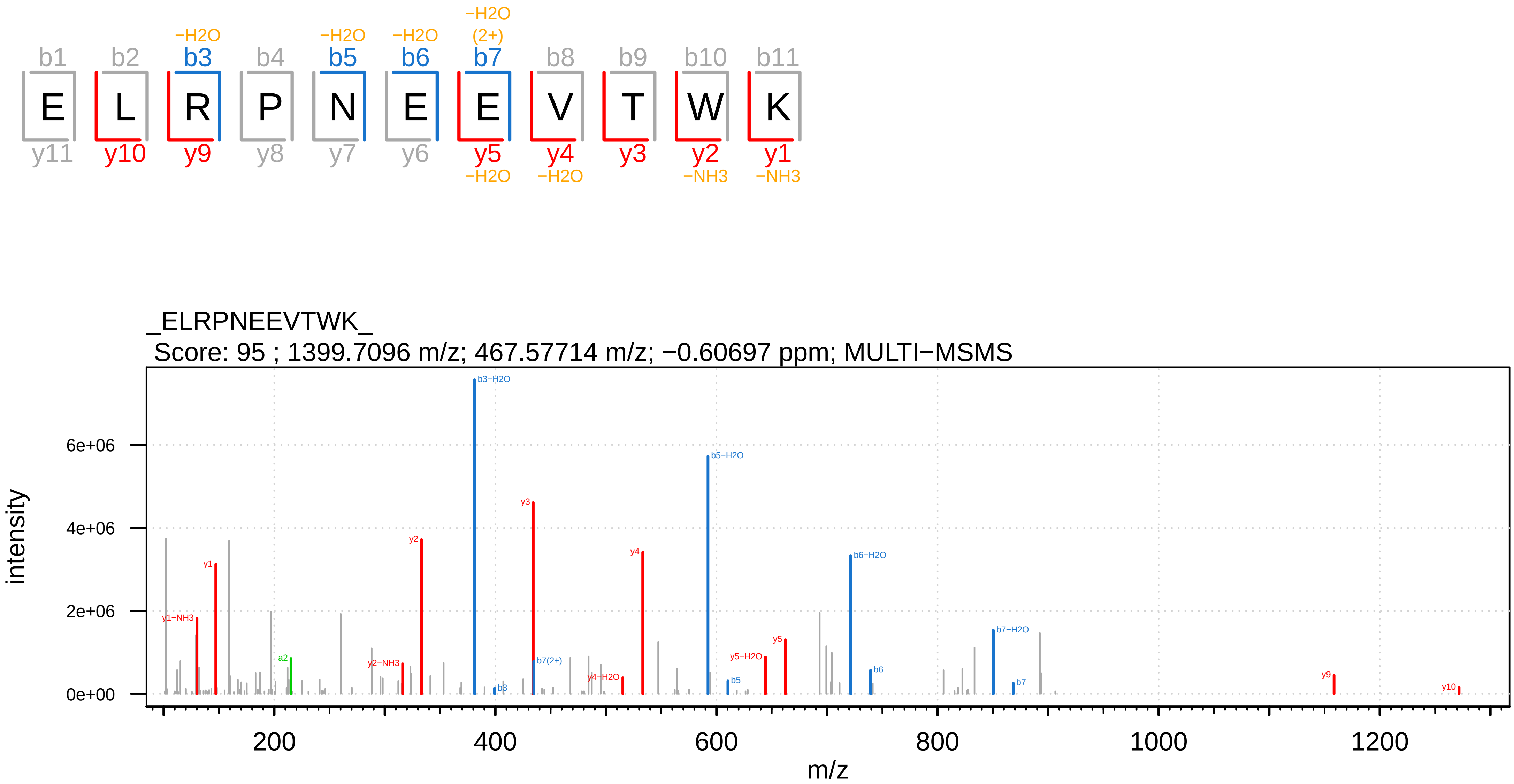

Raw File: 20130731_EXQ4_BaAz_SA_HR4_05

Scan Number: 30234

Proteins:

chr12_103206313−103214181.T2_chr12:101968174−101969629:−

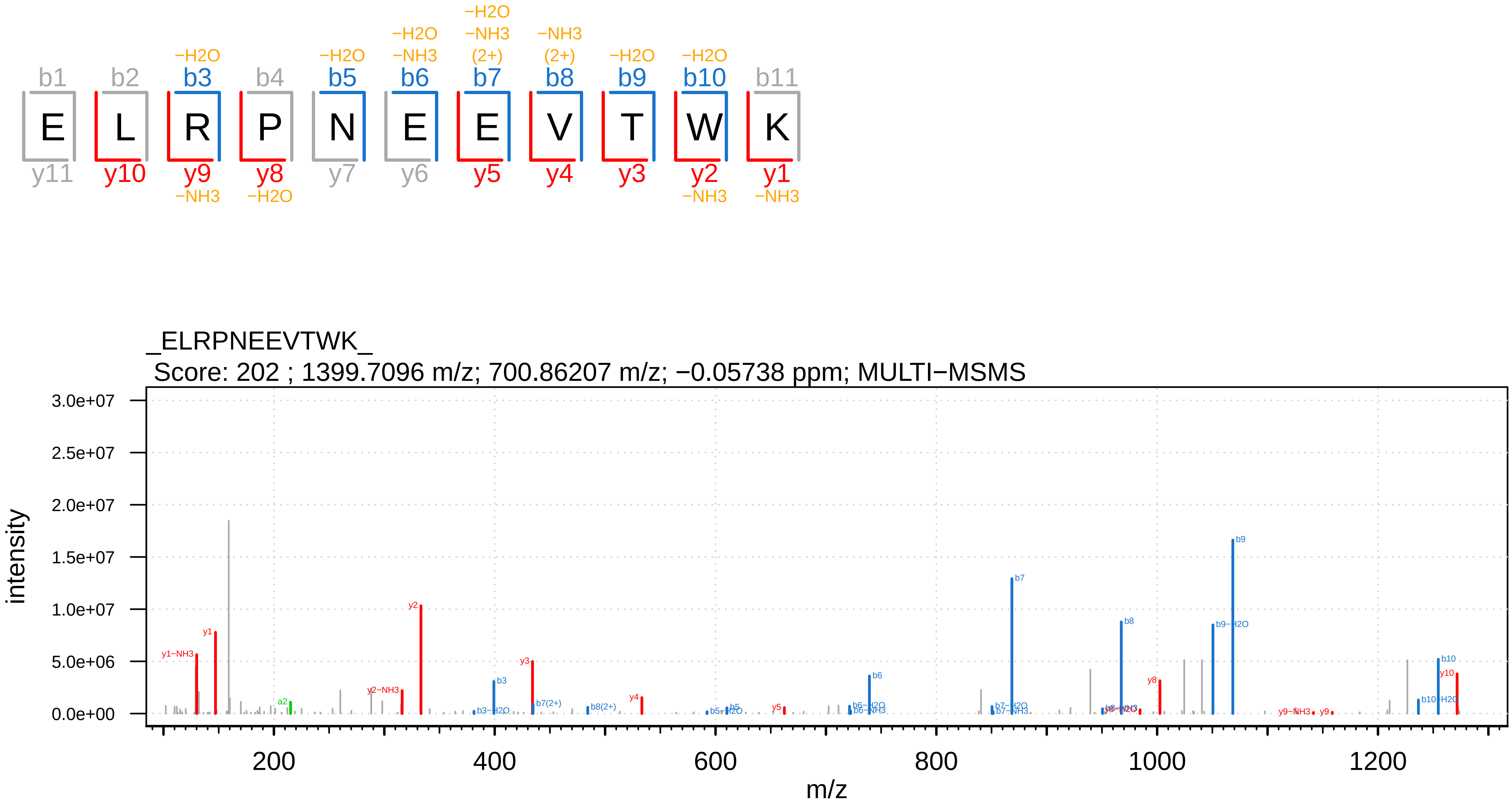

Raw File: 20130731_EXQ4_BaAz_SA_HR4_06
Scan Number: 28062
Proteins:
chr12_103206313−103214181.T2_chr12:101968174−101969629:−

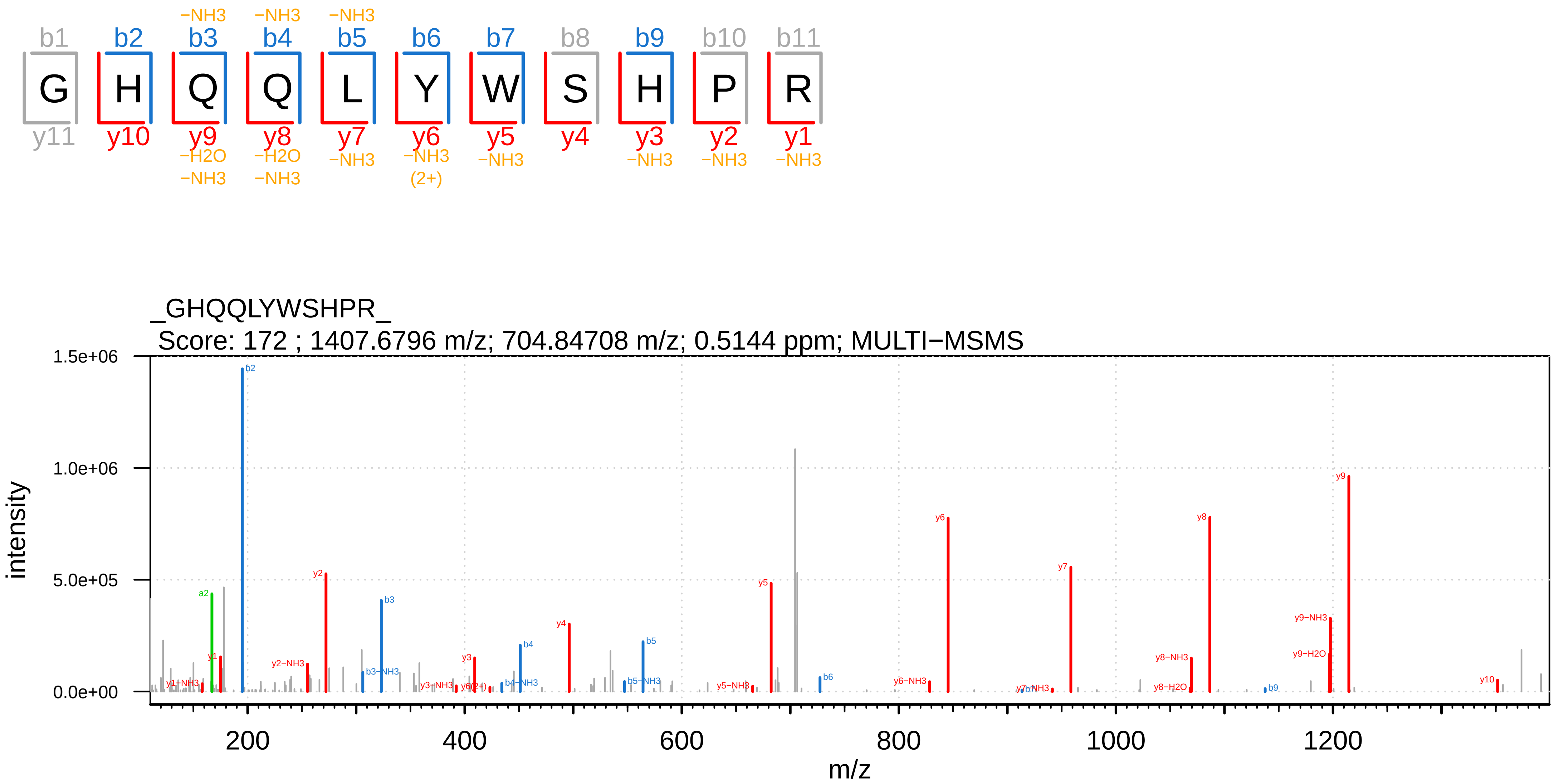

Raw File: 20130731_EXQ4_BaAz_SA_HR4_06
Scan Number: 11772
Proteins:
sp|P62274|RS29_MOUSE
ENSMUST00000037023_chr12:69157813−69159058:−
ENSMUST00000071737_chr13:73988963−73989133:−

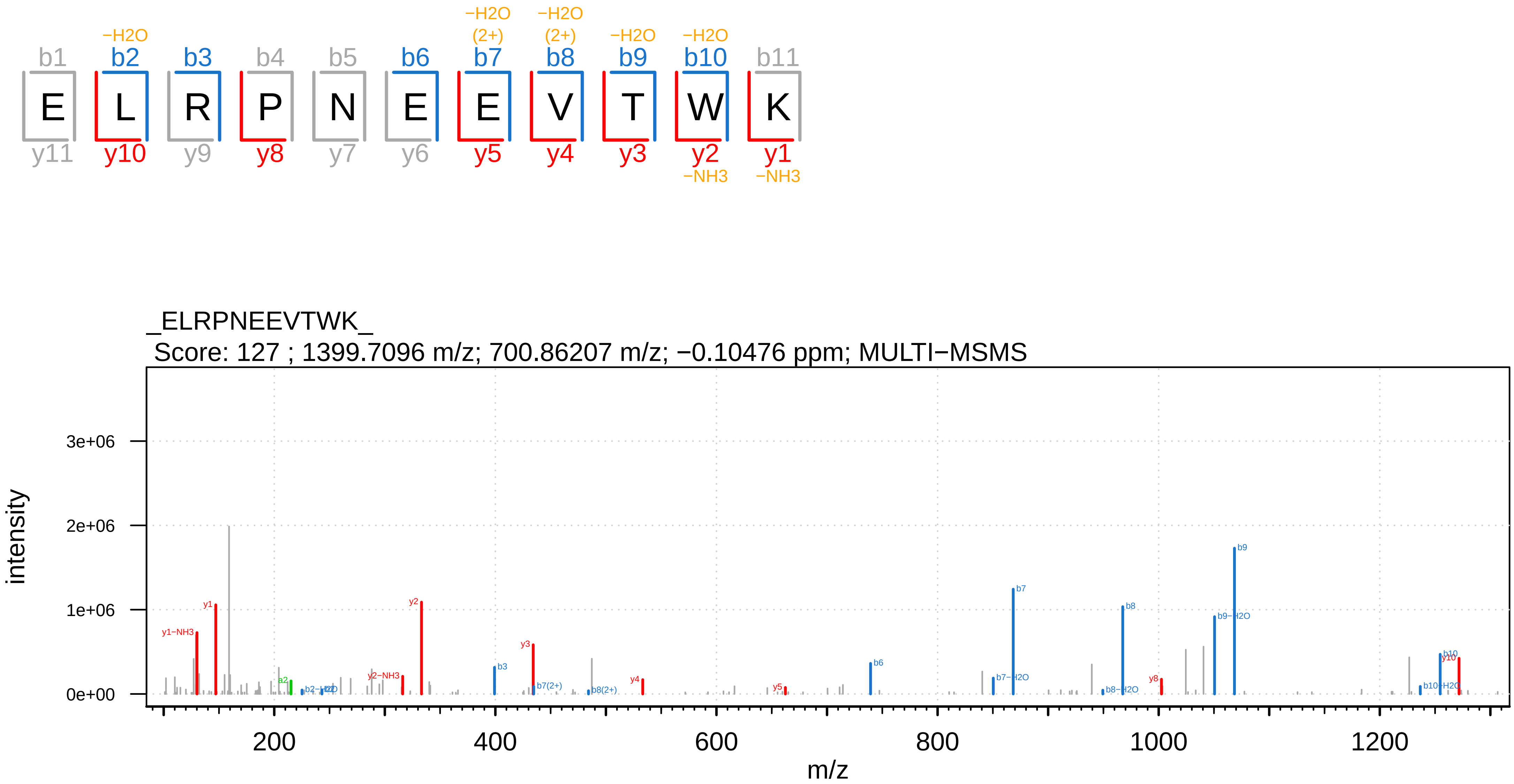

Raw File: 20130801_EXQ4_BaAz_SA_StR4_05
Scan Number: 30144
Proteins:
chr12_103206313−103214181.T2_chr12:101968174−101969629:−

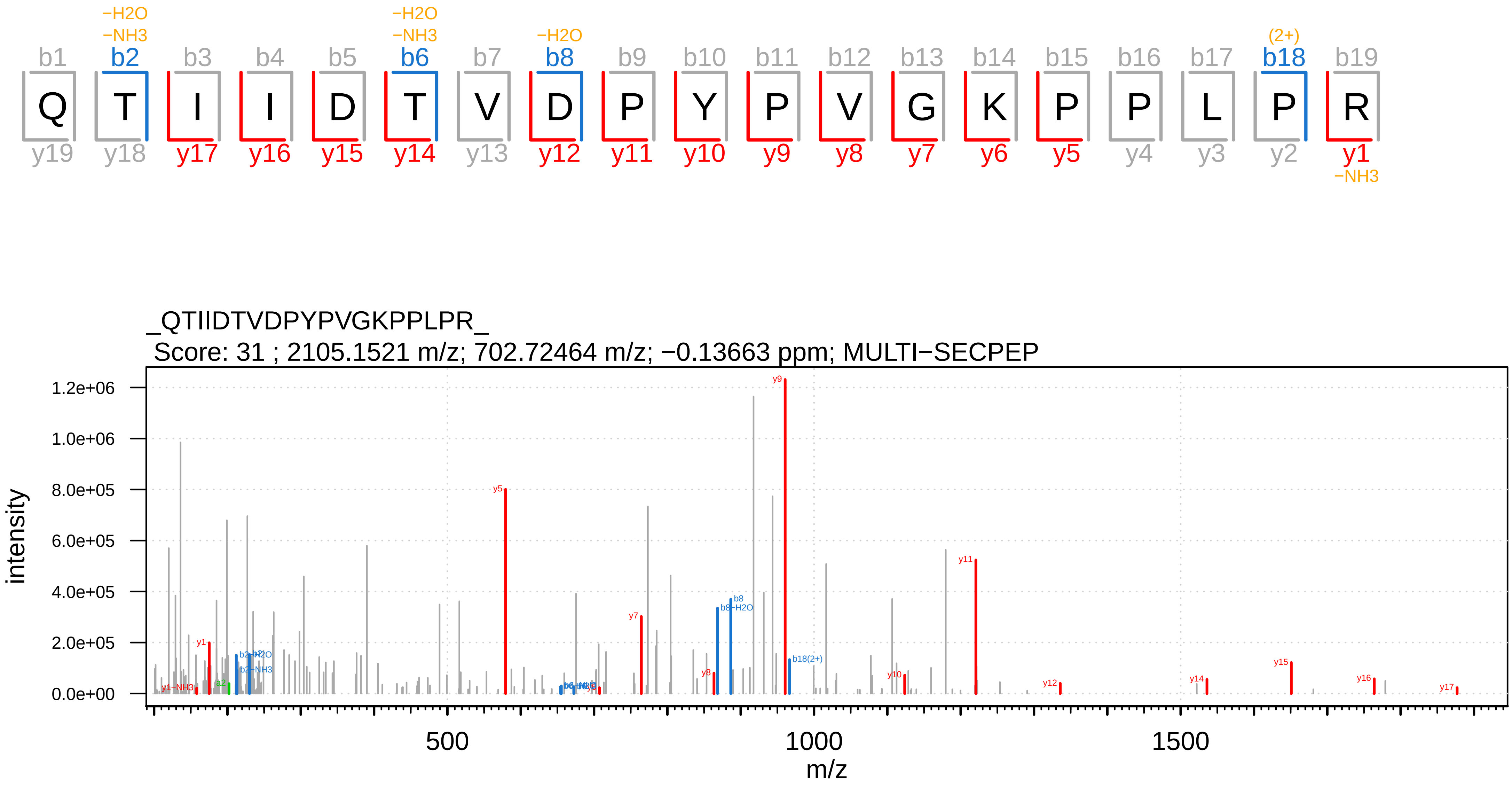

Raw File: 20130801_EXQ4_BaAz_SA_StR4_05

Scan Number: 69025

Proteins:

ENSMUST00000136126_chr3:79172848−79174982:−

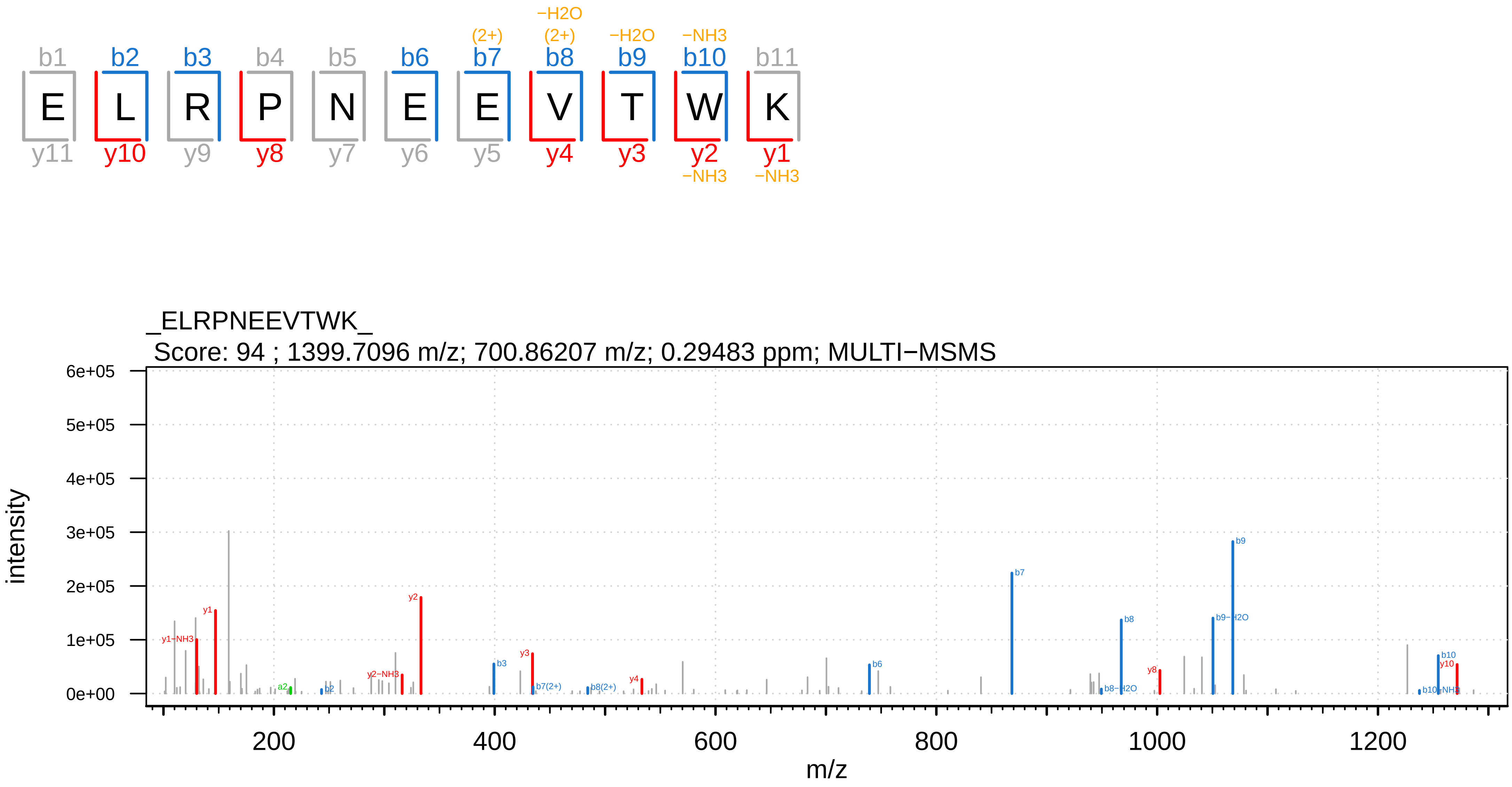

Raw File: 20130801_EXQ4_BaAz_SA_StR4_06
Scan Number: 27535
Proteins:
chr12_103206313−103214181.T2_chr12:101968174−101969629:−

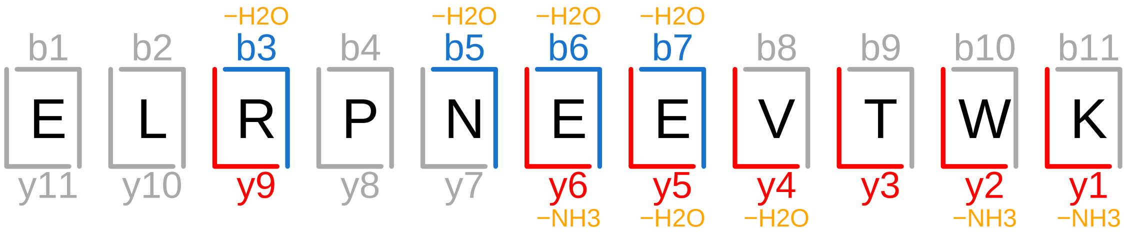

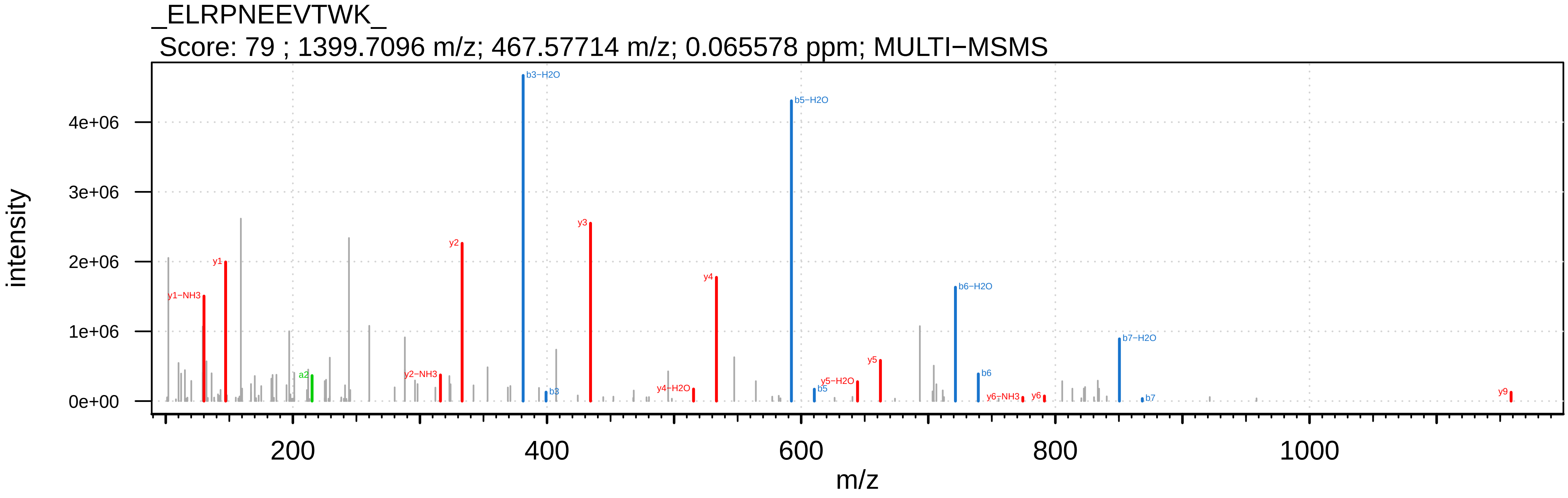

Raw File: 20130801_EXQ4_BaAz_SA_StR4_06
Scan Number: 27604
Proteins:
chr12_103206313−103214181.T2_chr12:101968174−101969629:−

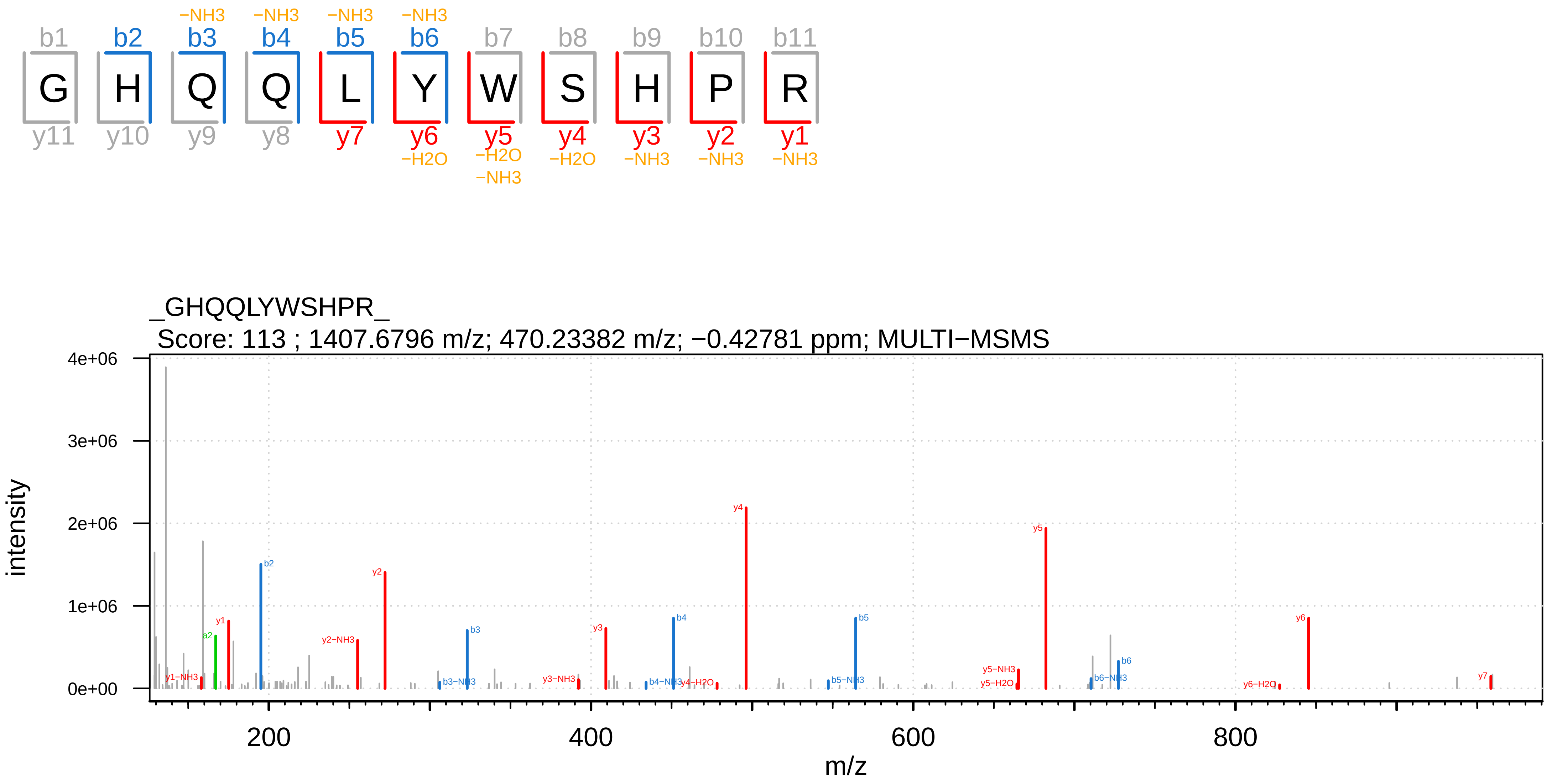

Raw File: 20130801_EXQ4_BaAz_SA_StR4_06
Scan Number: 12093
Proteins:
sp|P62274|RS29_MOUSE
ENSMUST00000037023_chr12:69157813−69159058:−
ENSMUST00000071737_chr13:73988963−73989133:−

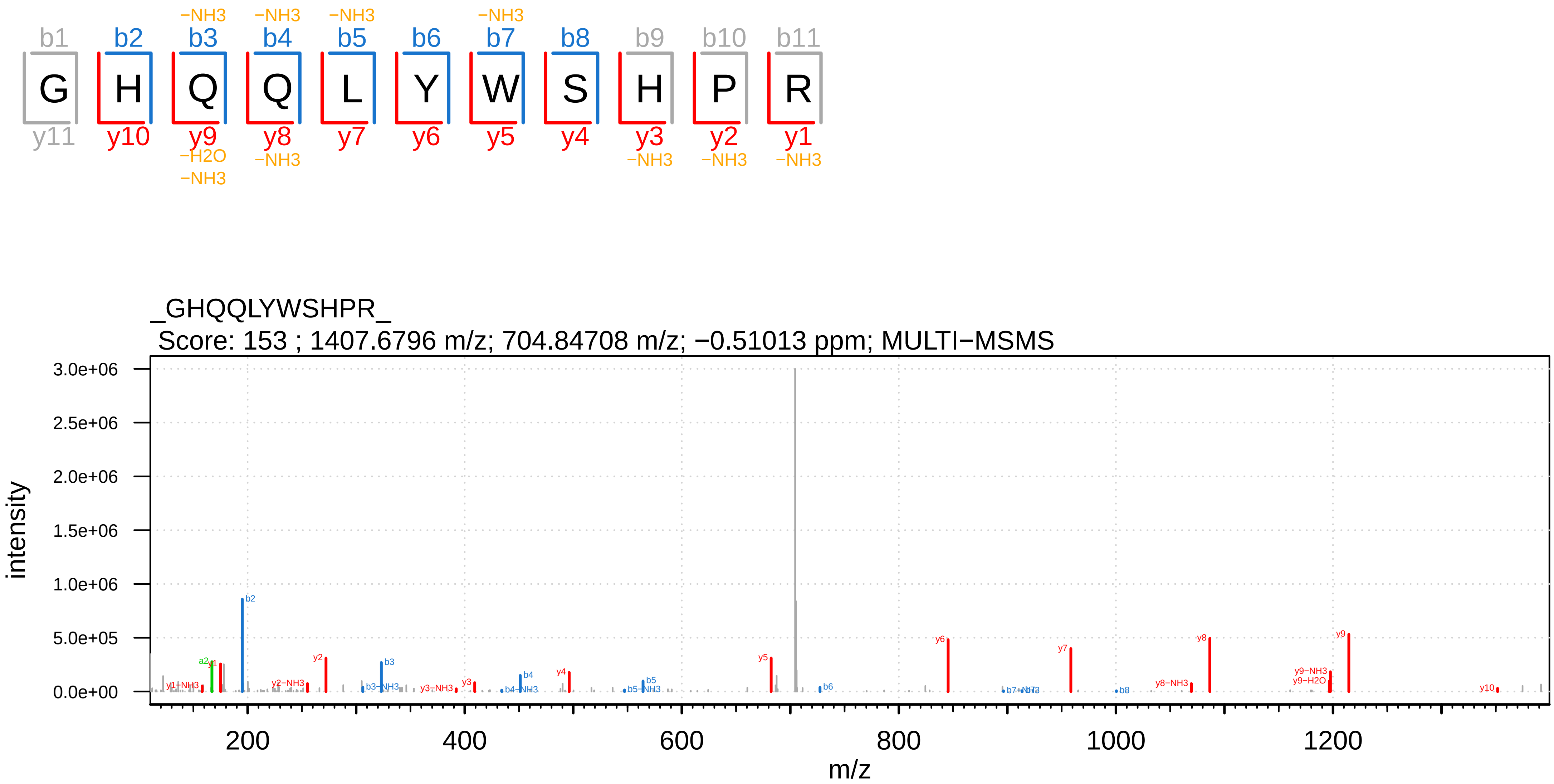

Raw File: 20130801_EXQ4_BaAz_SA_StR4_06
Scan Number: 12125
Proteins:
sp|P62274|RS29_MOUSE
ENSMUST00000037023_chr12:69157813−69159058:−
ENSMUST00000071737_chr13:73988963−73989133:−

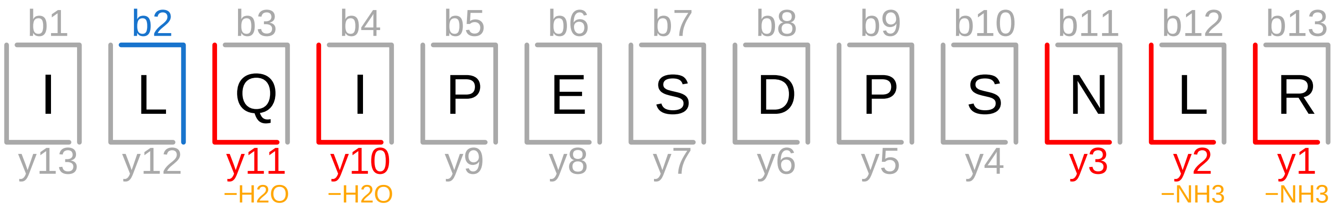

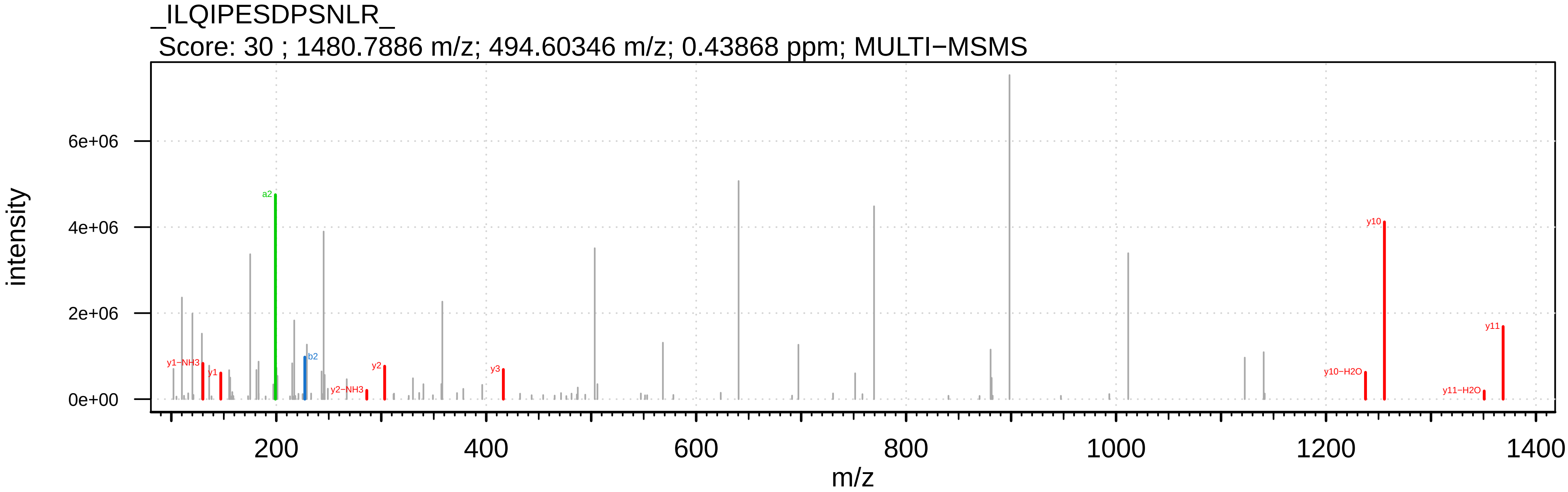

Raw File: 20130801_EXQ4_BaAz_SA_StR4_06
Scan Number: 28504
Proteins:
ENSMUST00000161069_chr14:70554856−70554960:+

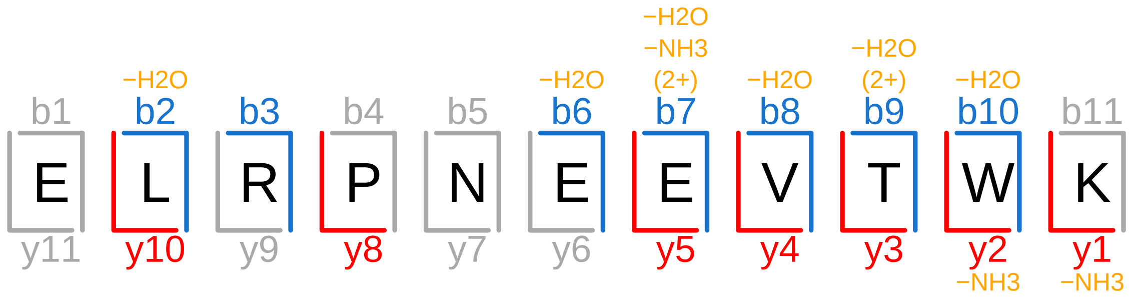

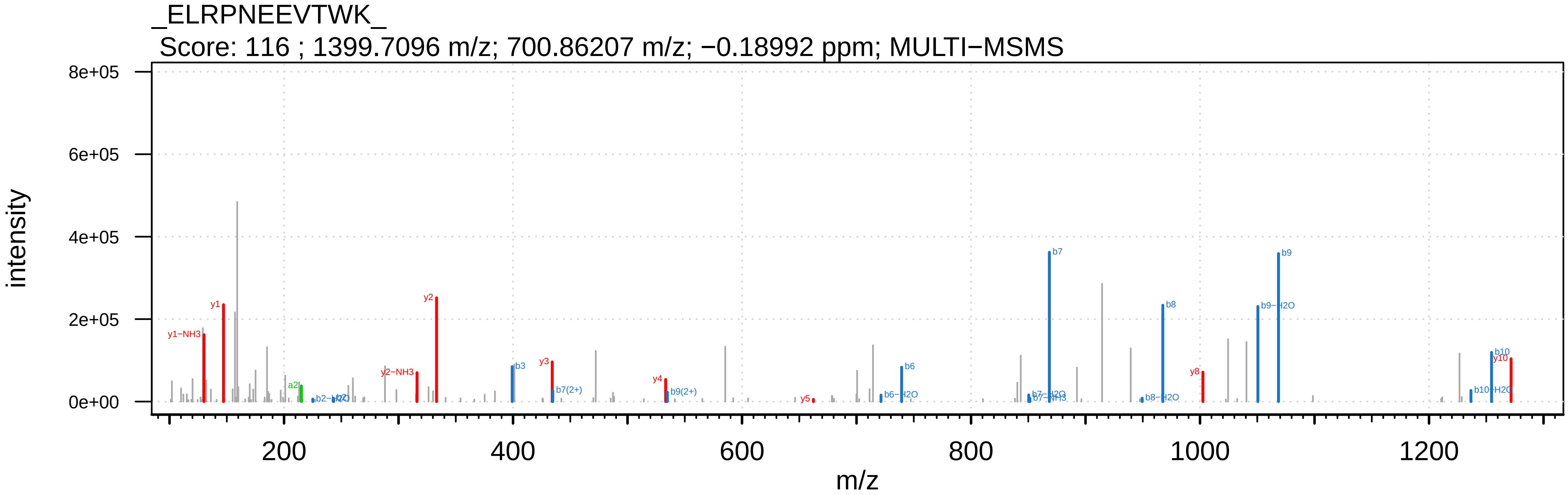

Raw File: 20130803_EXQ4_BaAz_SA_LR4_05
Scan Number: 28483
Proteins:
chr12_103206313−103214181.T2_chr12:101968174−101969629:−

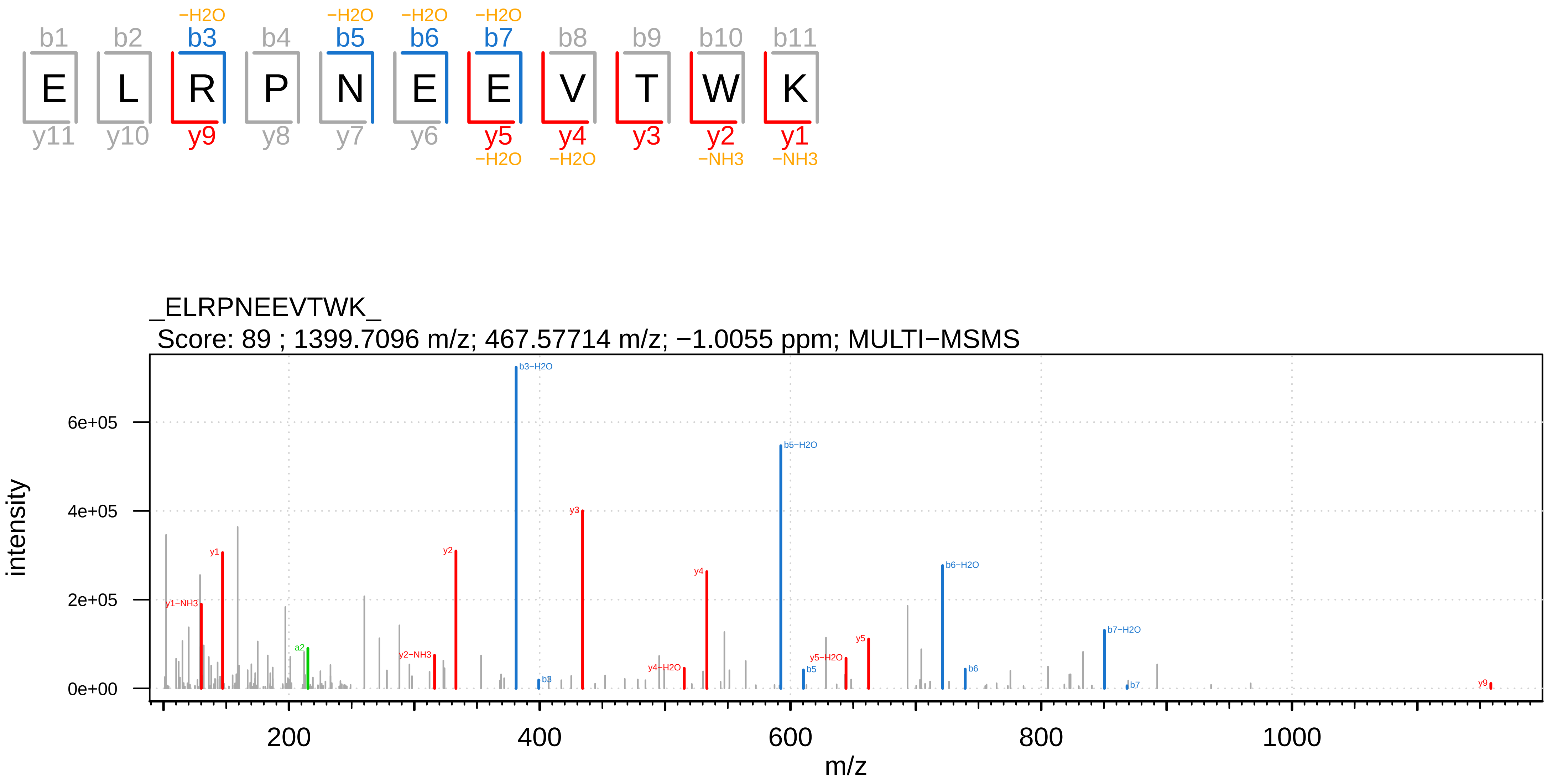

Raw File: 20130803_EXQ4_BaAz_SA_LR4_05
Scan Number: 28486
Proteins:
chr12_103206313−103214181.T2_chr12:101968174−101969629:−

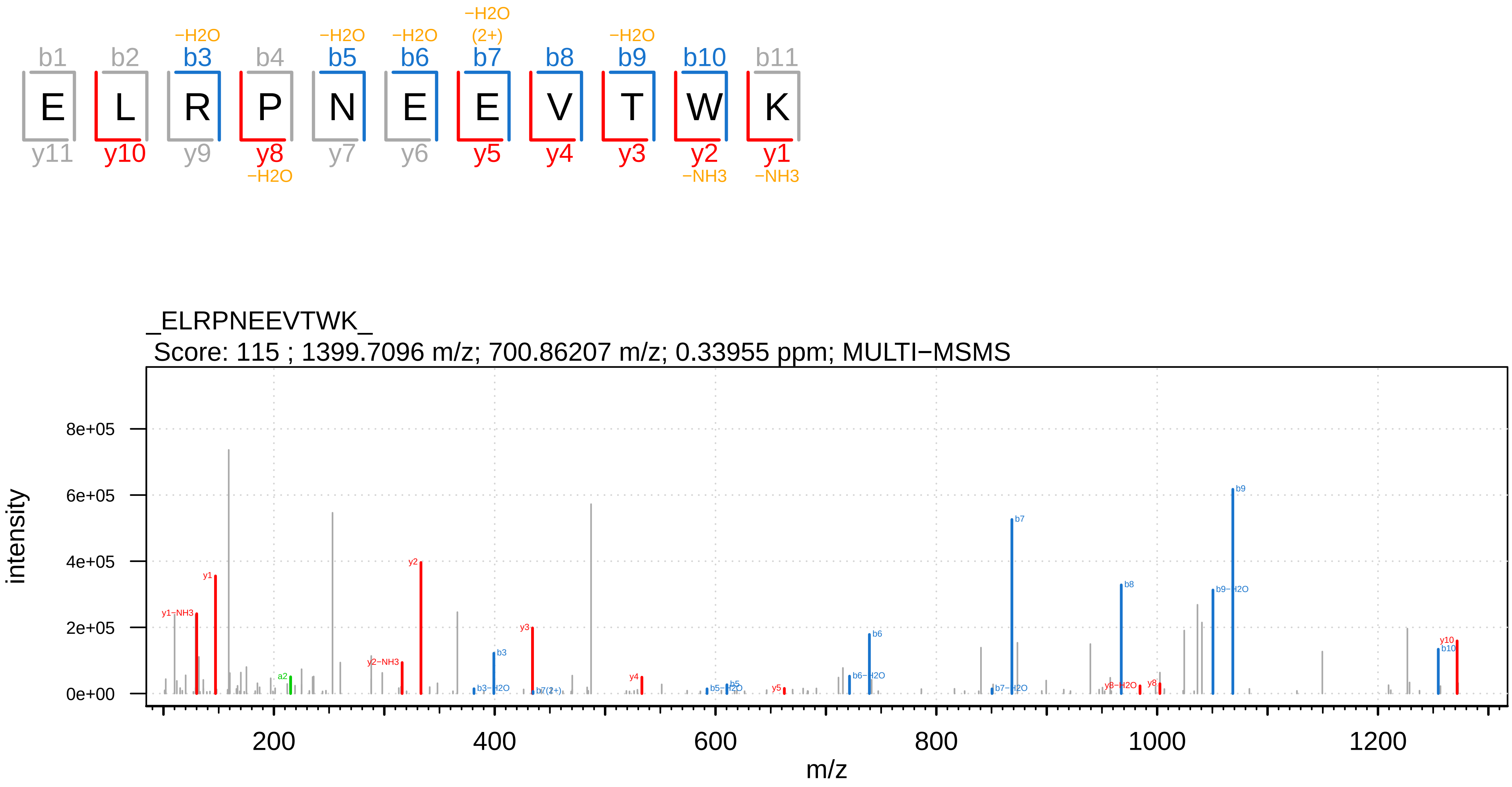

Raw File: 20130803_EXQ4_BaAz_SA_LR4_06
Scan Number: 26415
Proteins:
chr12_103206313−103214181.T2_chr12:101968174−101969629:−

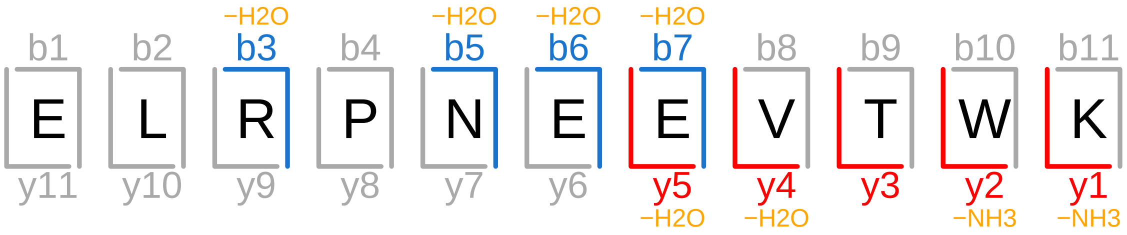

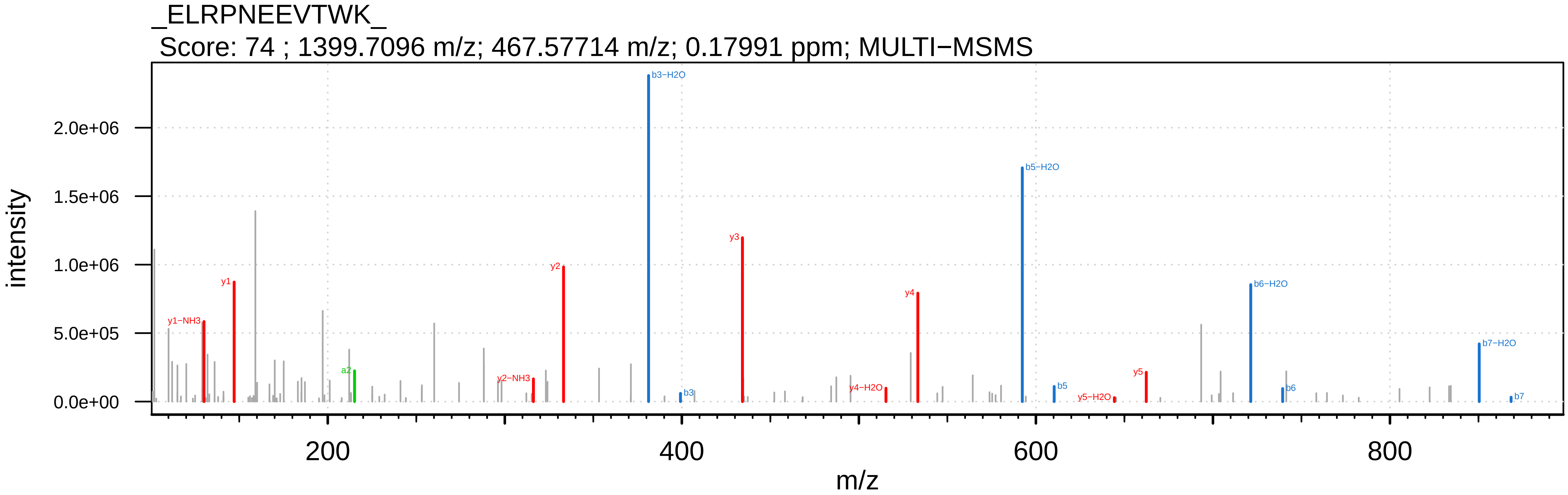

Raw File: 20130803_EXQ4_BaAz_SA_LR4_06

Scan Number: 26432

Proteins:

chr12_103206313−103214181.T2_chr12:101968174−101969629:−

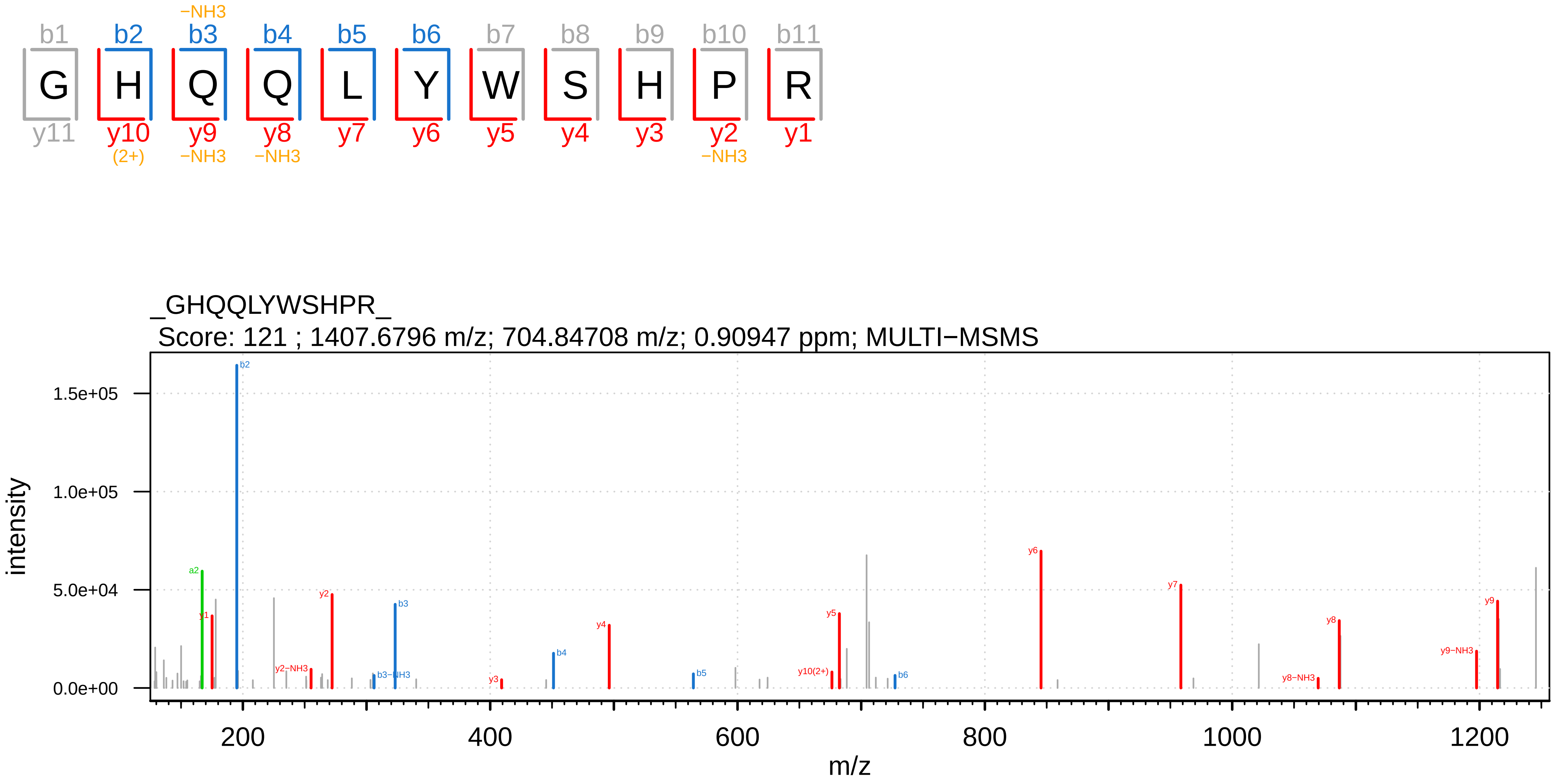

Raw File: 20130803_EXQ4_BaAz_SA_LR4_06
Scan Number: 10014
Proteins:
sp|P62274|RS29_MOUSE
ENSMUST00000037023_chr12:69157813−69159058:−
ENSMUST00000071737_chr13:73988963−73989133:−

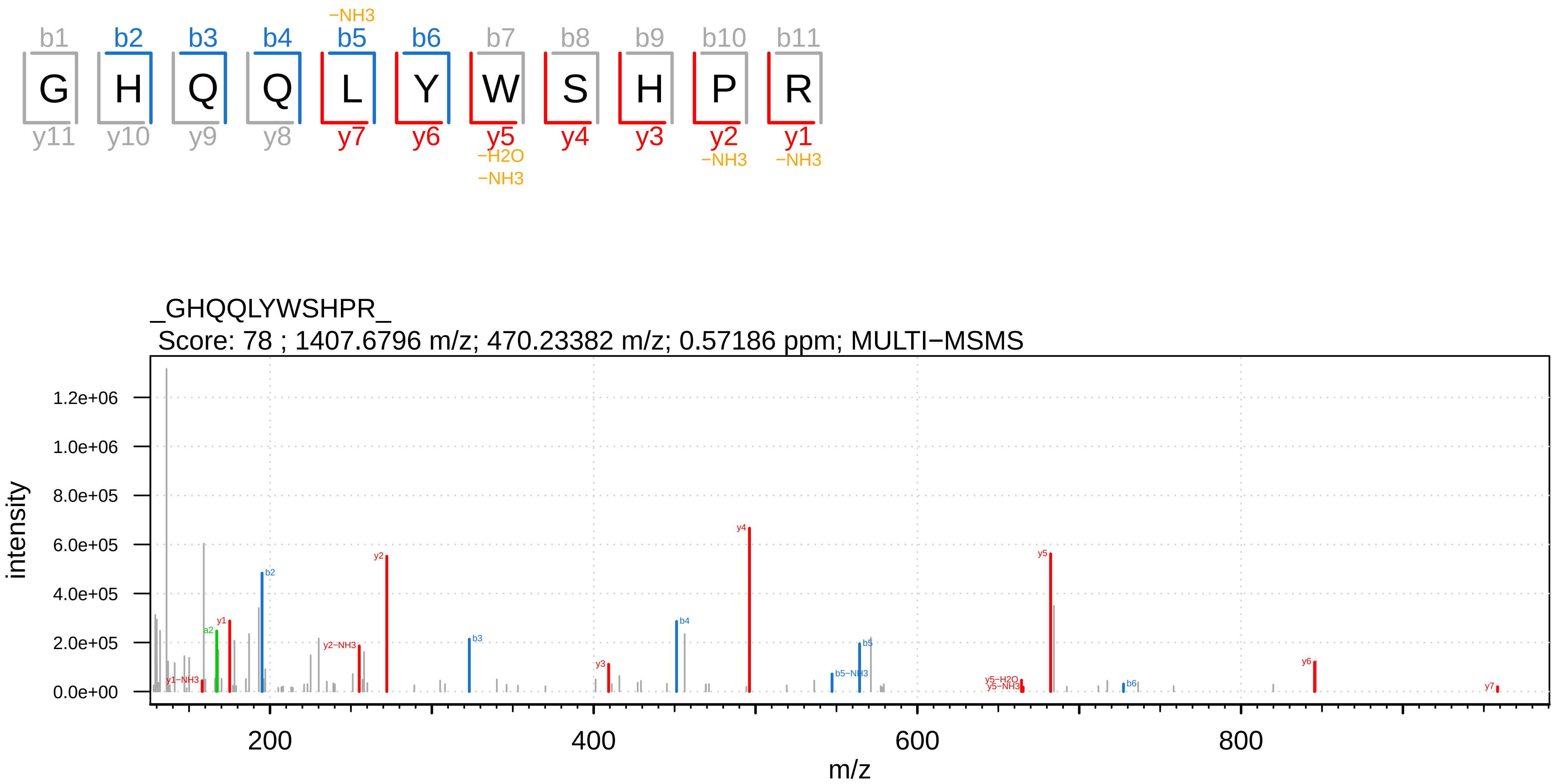

Raw File: 20130803_EXQ4_BaAz_SA_LR4_06
Scan Number: 10031
Proteins:
sp|P62274|RS29_MOUSE
ENSMUST00000037023_chr12:69157813−69159058:−
ENSMUST00000071737_chr13:73988963−73989133:−

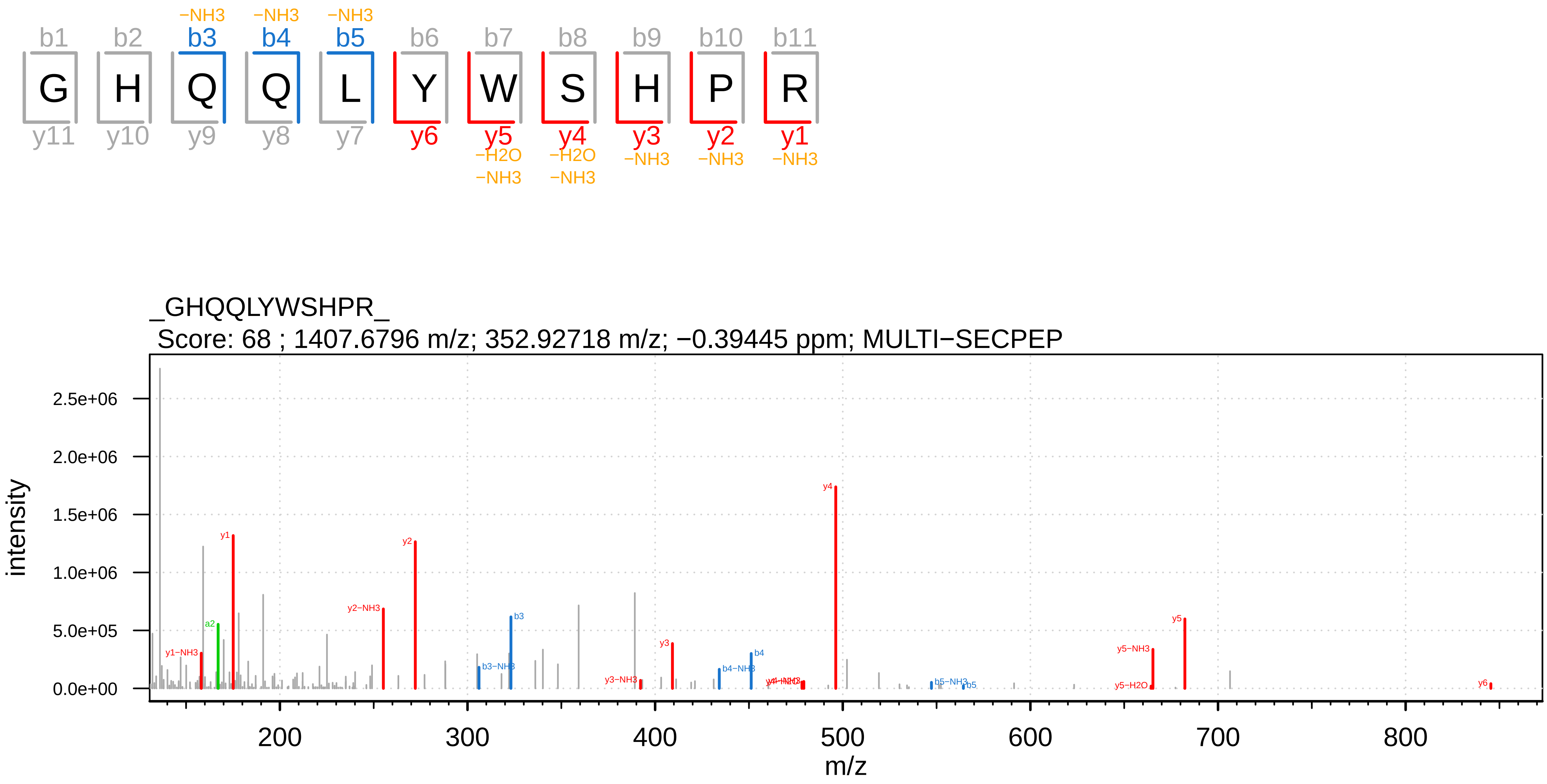

Raw File: 20130803_EXQ4_BaAz_SA_LR4_06
Scan Number: 10098
Proteins:
sp|P62274|RS29_MOUSE
ENSMUST00000037023_chr12:69157813−69159058:−
ENSMUST00000071737_chr13:73988963−73989133:−

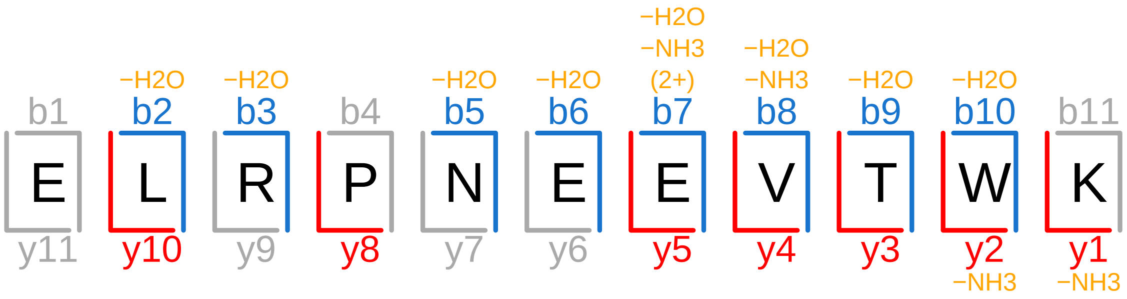

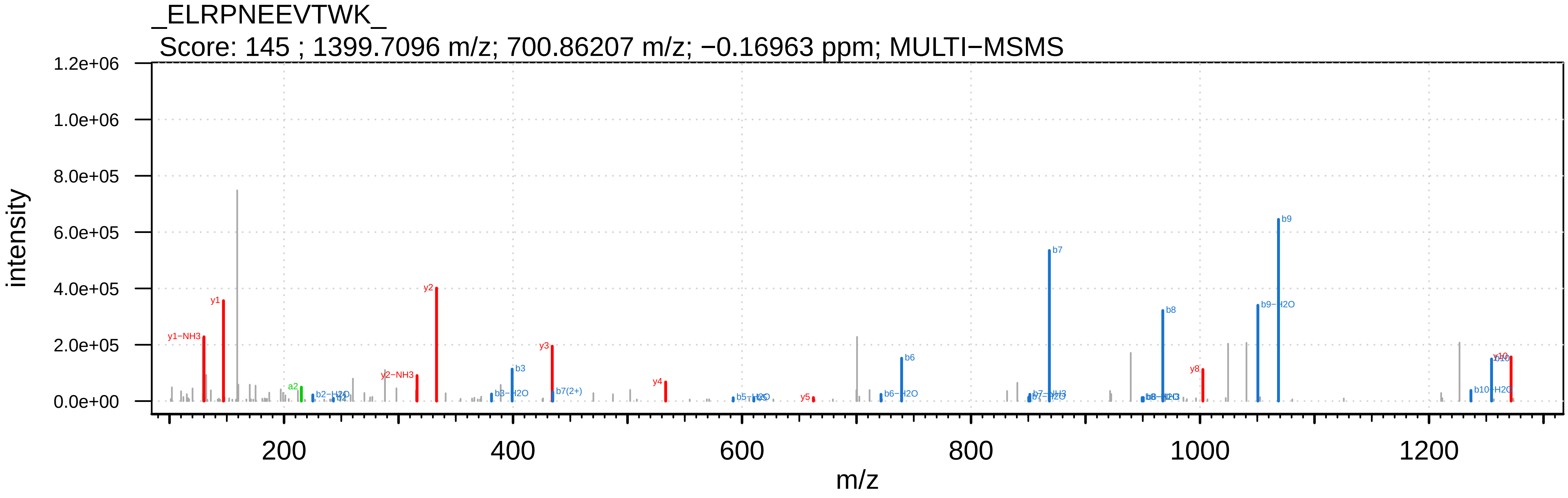

Raw File: 20130804_EXQ4_BaAz_SA_CHR4_05
Scan Number: 29455
Proteins:
chr12_103206313−103214181.T2_chr12:101968174−101969629:−

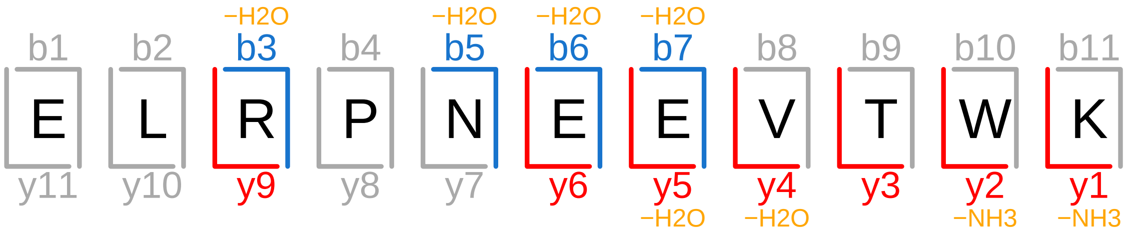

ELRPNEEVTWK
Score: 76 ; 1399.7096 m/z; 467.57714 m/z; 0.52351 ppm; MULTI−MSMS

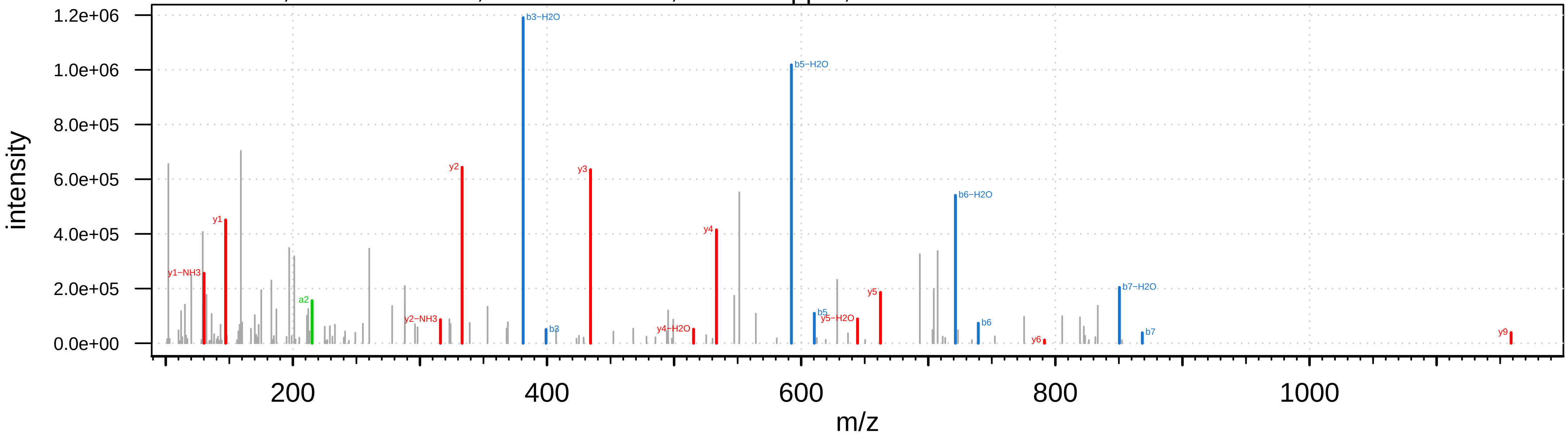

Raw File: 20130804_EXQ4_BaAz_SA_CHR4_05
Scan Number: 29460
Proteins:
chr12_103206313−103214181.T2_chr12:101968174−101969629:−

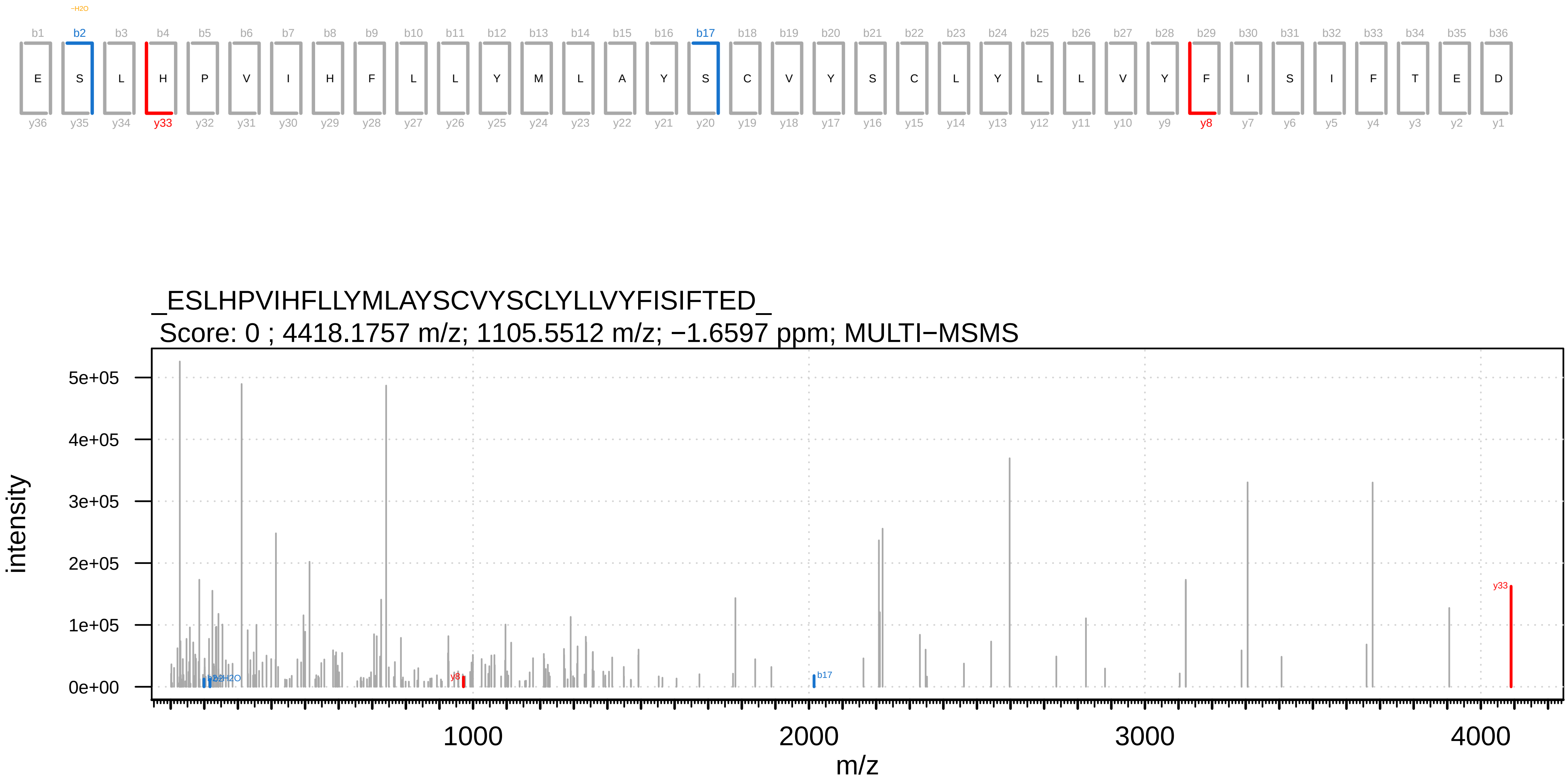

Raw File: 20130804_EXQ4_BaAz_SA_CHR4_05
Scan Number: 79023
Proteins:
ENSMUST00000140321_chr4:107707107−107707220:+

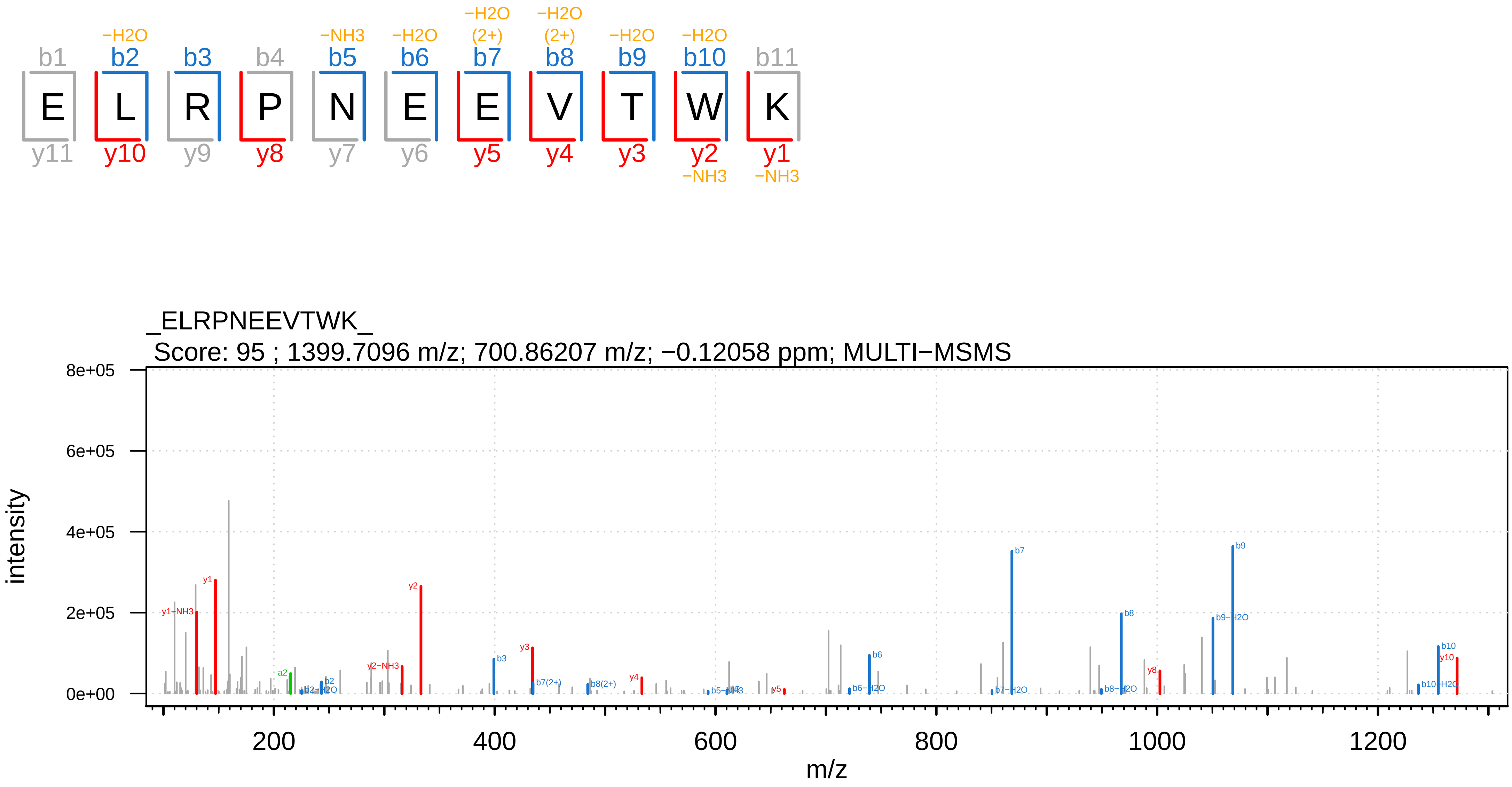

Raw File: 20130804_EXQ4_BaAz_SA_CHR4_06

Scan Number: 27402

Proteins:

chr12_103206313−103214181.T2_chr12:101968174−101969629:−

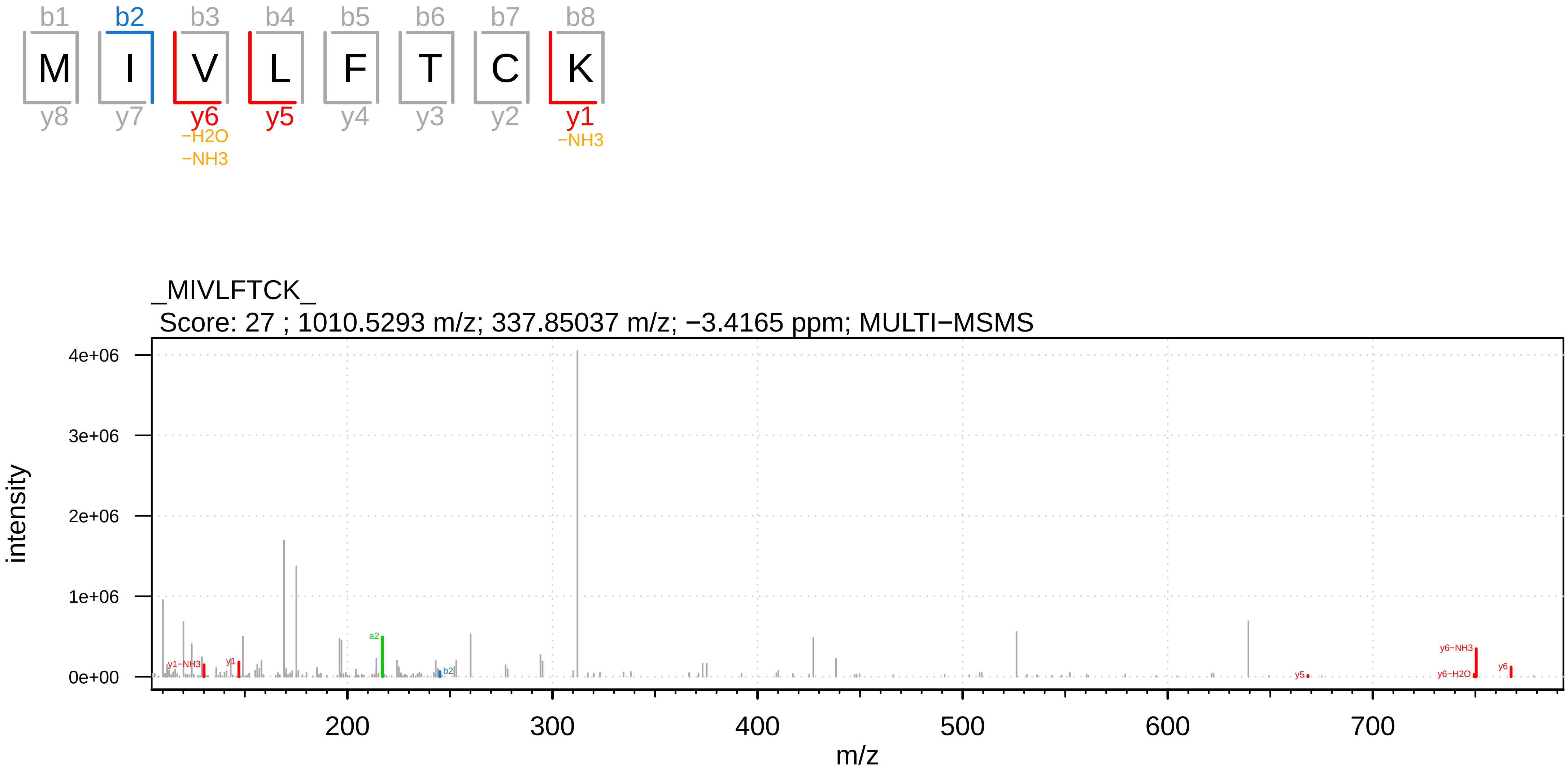

Raw File: 20130804_EXQ4_BaAz_SA_CHR4_06
Scan Number: 20325
Proteins:
ENSMUST00000123924_chr13:98273481−98273513:+

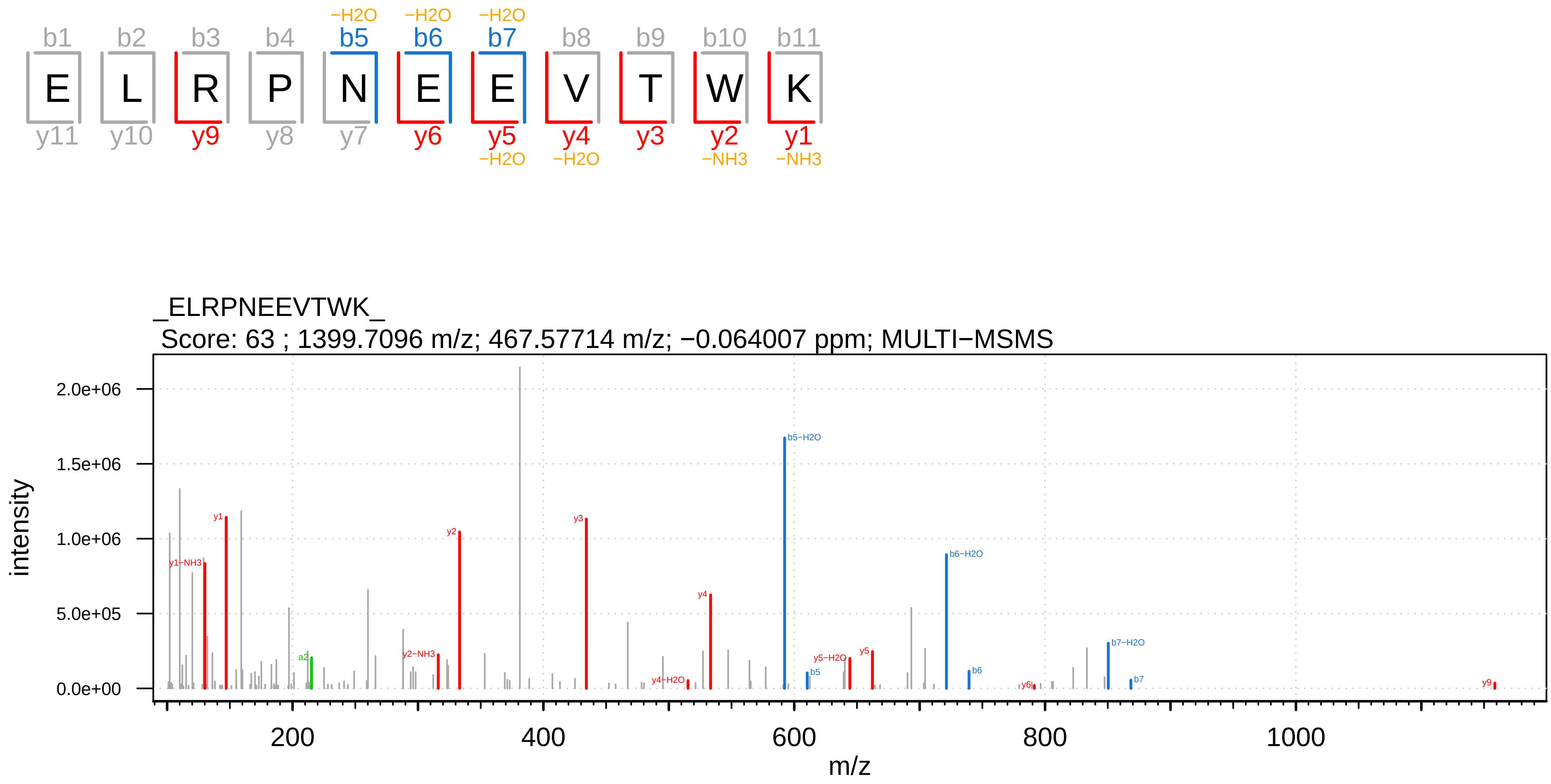

Raw File: 20130807_EXQ4_BaAz_SA_KR4_06

Scan Number: 28109

Proteins:

chr12_103206313−103214181.T2_chr12:101968174−101969629:−

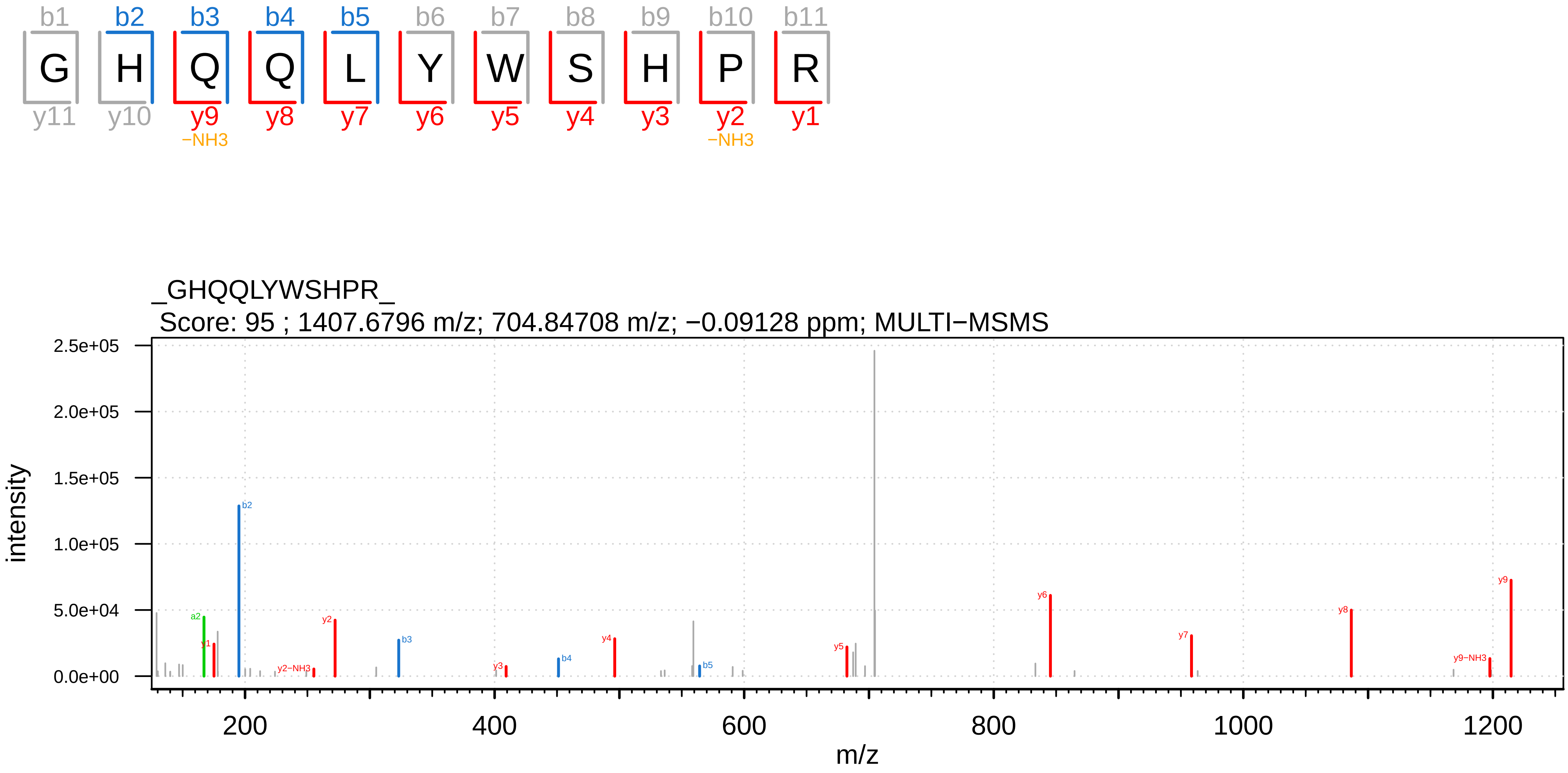

Raw File: 20130807_EXQ4_BaAz_SA_KR4_06
Scan Number: 14513
Proteins:
sp|P62274|RS29_MOUSE
ENSMUST00000037023_chr12:69157813−69159058:−
ENSMUST00000071737_chr13:73988963−73989133:−

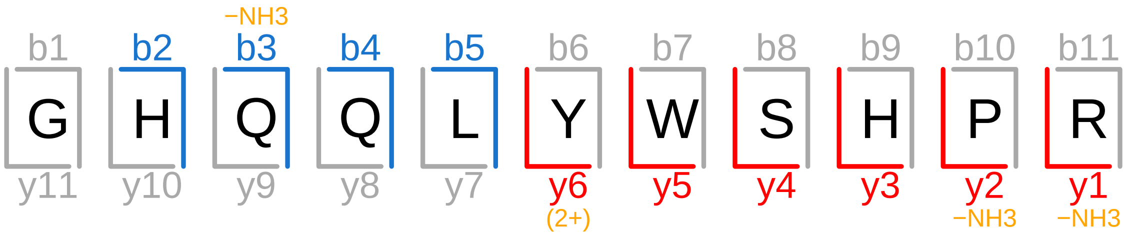

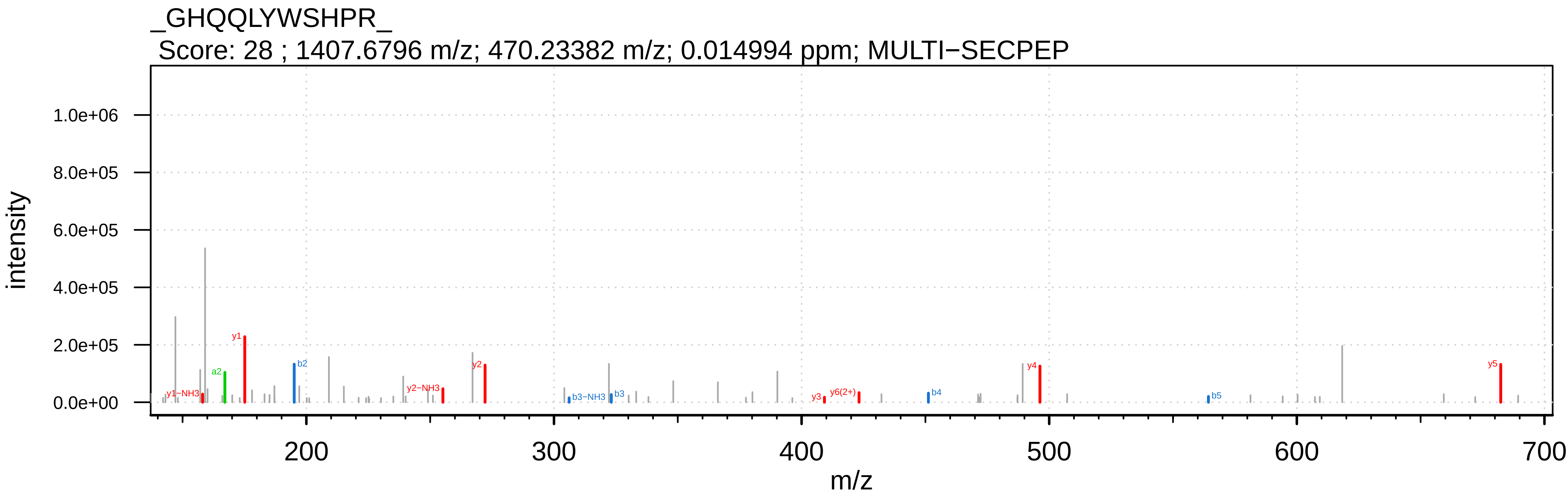

Raw File: 20130807_EXQ4_BaAz_SA_KR4_06
Scan Number: 14552
Proteins:
sp|P62274|RS29_MOUSE
ENSMUST00000037023_chr12:69157813−69159058:−
ENSMUST00000071737_chr13:73988963−73989133:−

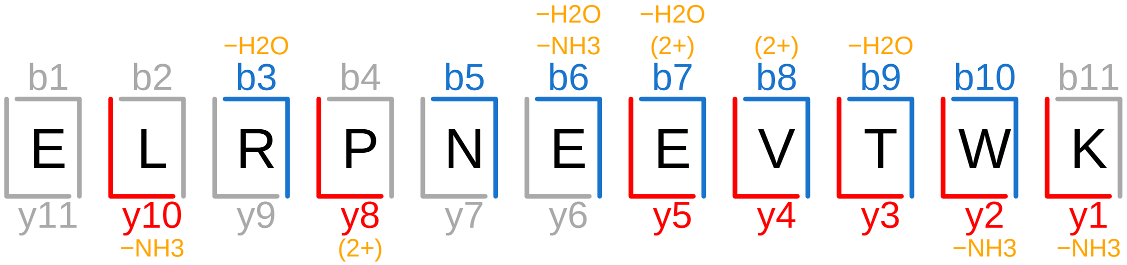

ELRPNEEVTWK
Score: 124 ; 1399.7096 m/z; 700.86207 m/z; 0.53786 ppm; MULTI−MSMS

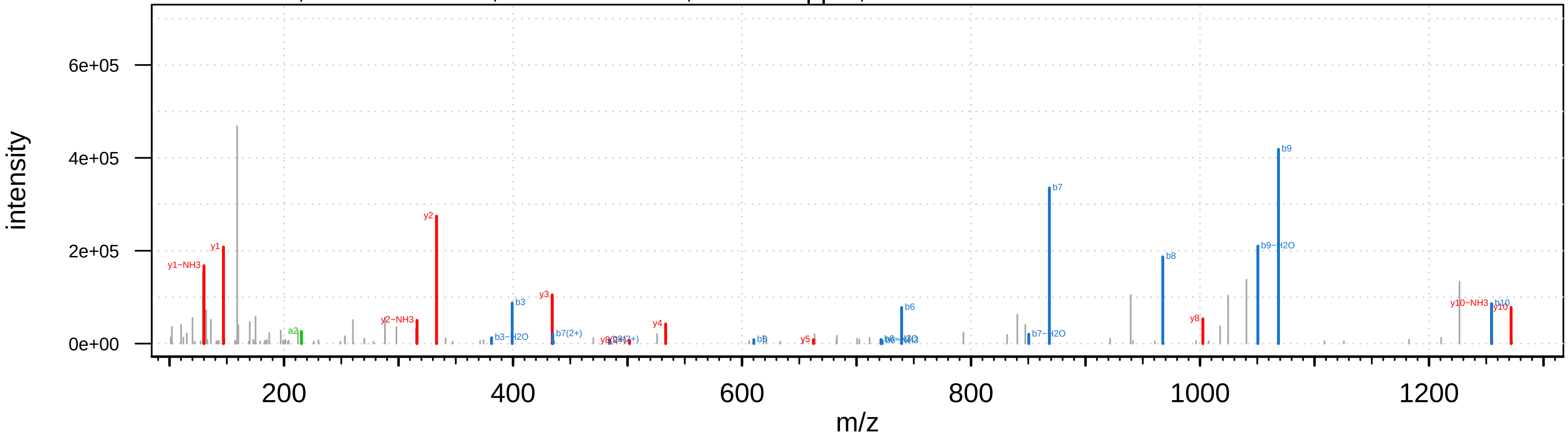

Raw File: 20130808_EXQ4_BaAz_SA_SeR4_05
Scan Number: 29603
Proteins:
chr12_103206313−103214181.T2_chr12:101968174−101969629:−

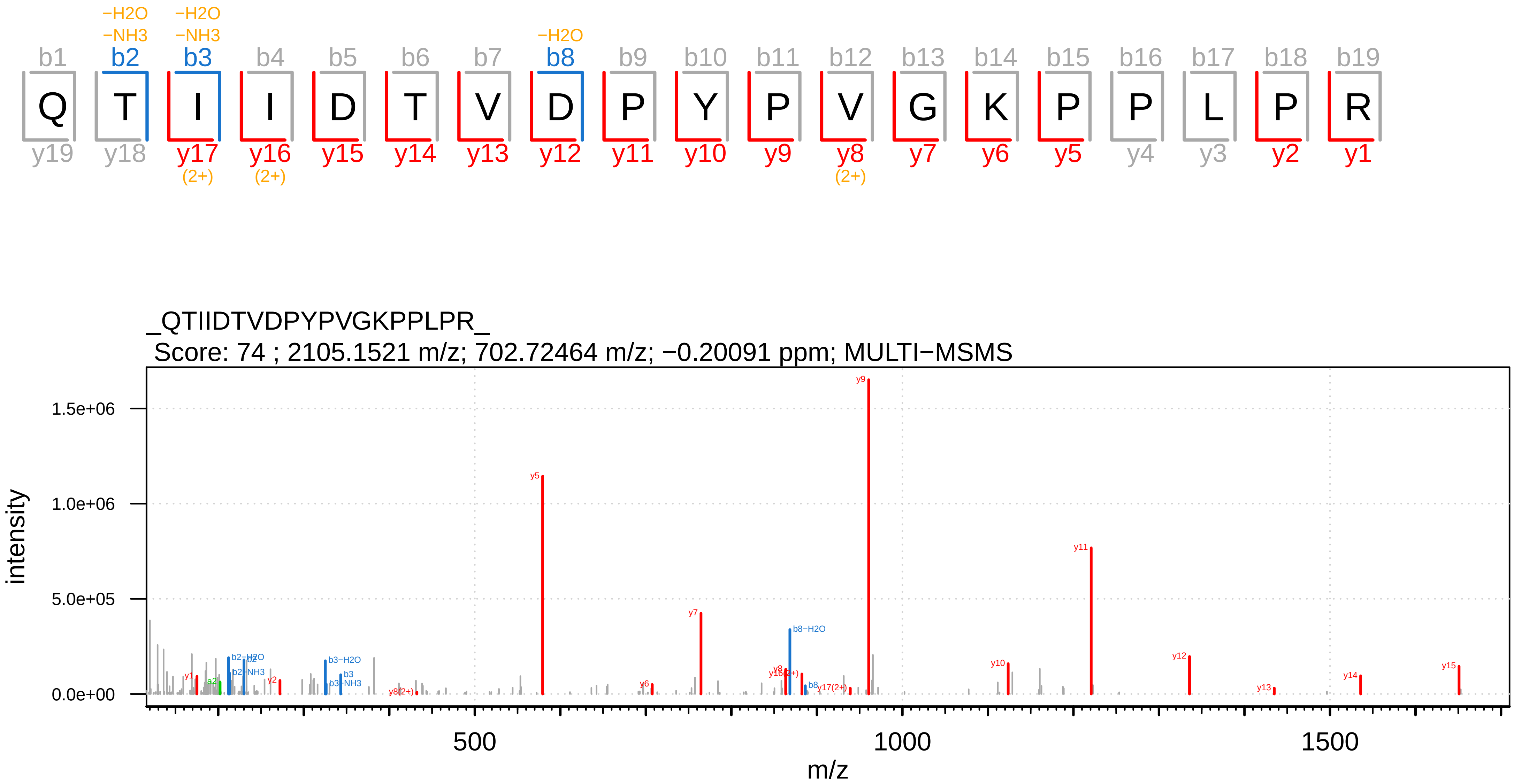

Raw File: 20130808_EXQ4_BaAz_SA_SeR4_05
Scan Number: 66527
Proteins:
ENSMUST00000136126_chr3:79172848−79174982:−

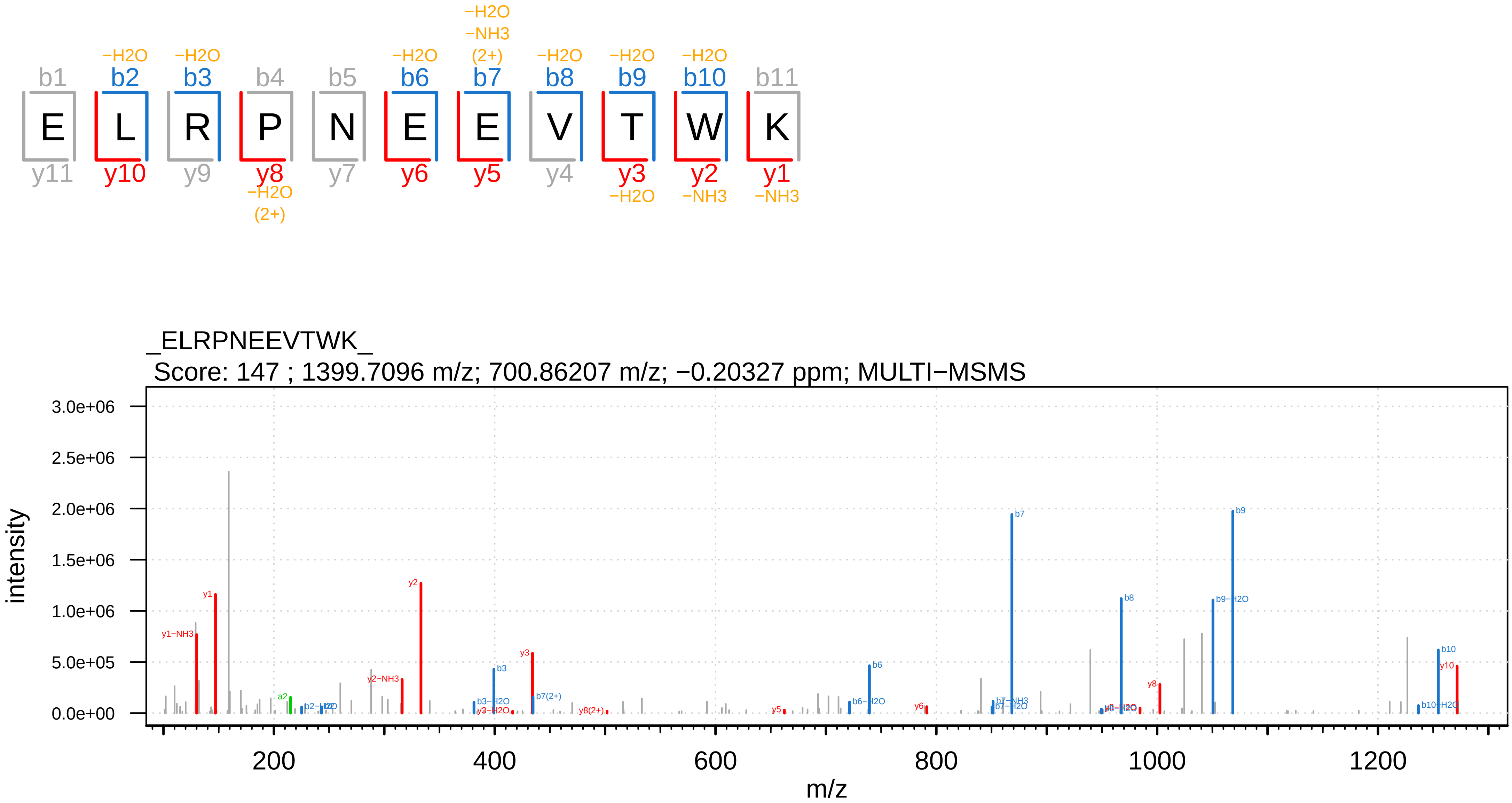

Raw File: 20130808_EXQ4_BaAz_SA_SeR4_06
Scan Number: 26866
Proteins:
chr12_103206313−103214181.T2_chr12:101968174−101969629:−

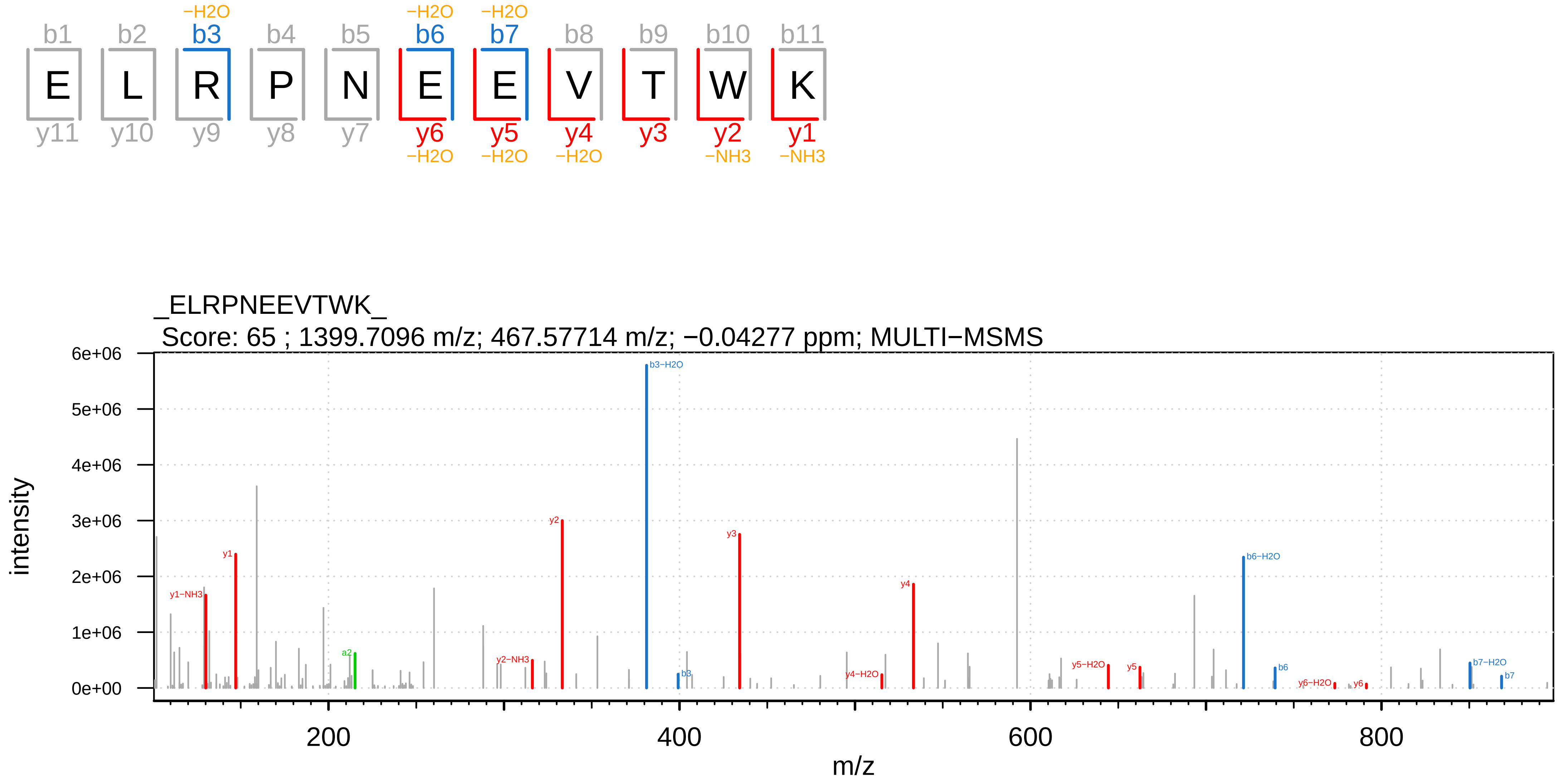

Raw File: 20130808_EXQ4_BaAz_SA_SeR4_06
Scan Number: 26883
Proteins:
chr12_103206313−103214181.T2_chr12:101968174−101969629:−

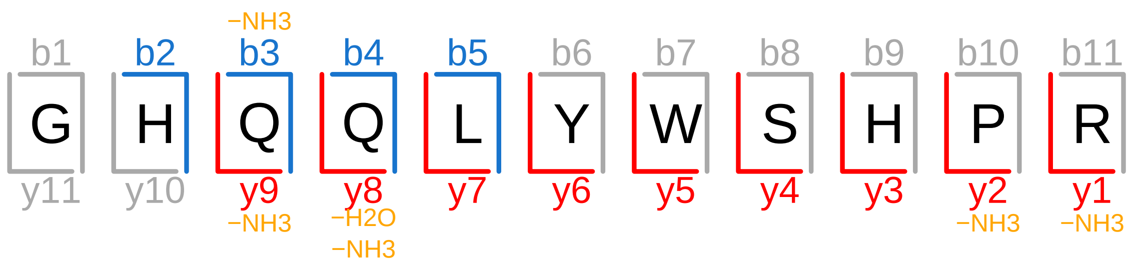

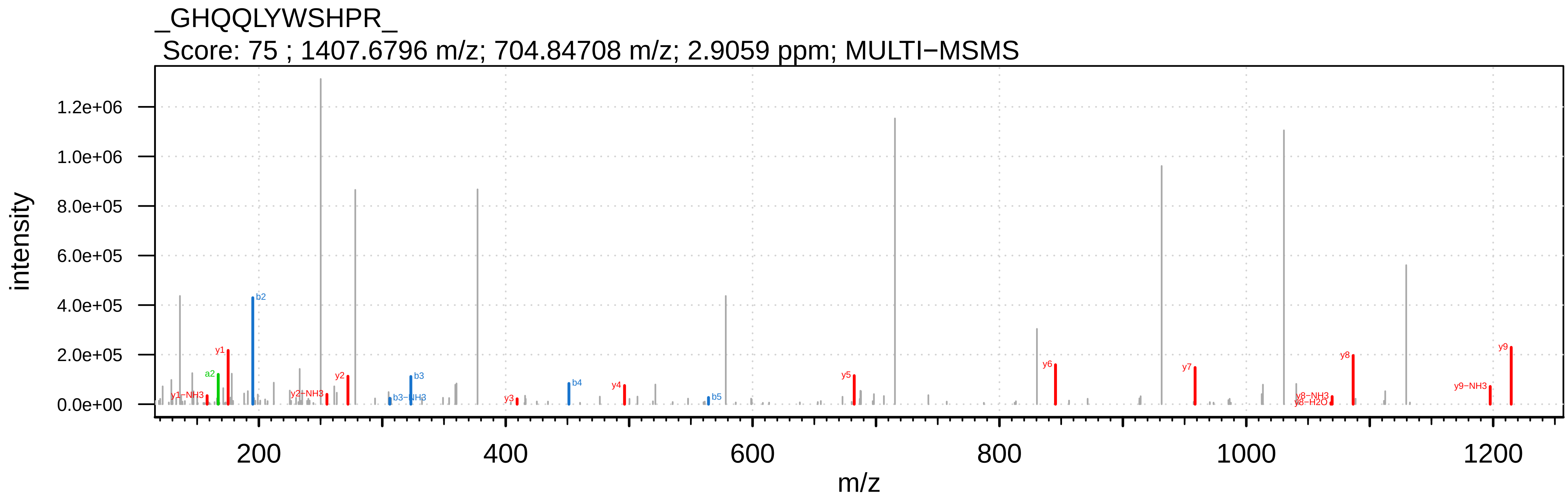

Raw File: 20130808_EXQ4_BaAz_SA_SeR4_06
Scan Number: 12776
Proteins:
sp|P62274|RS29_MOUSE
ENSMUST00000037023_chr12:69157813−69159058:−
ENSMUST00000071737_chr13:73988963−73989133:−

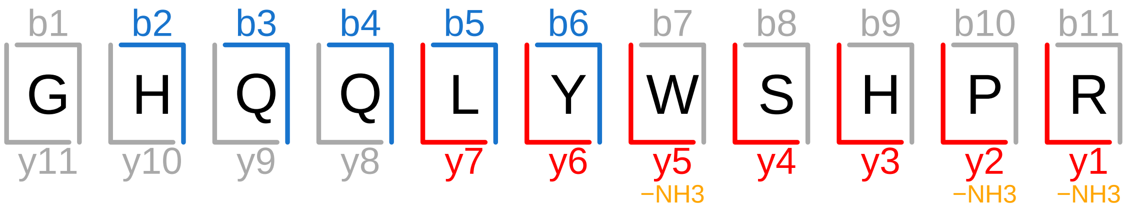

GHQQLYWSHPR
Score: 53 ; 1407.6796 m/z; 470.23382 m/z; 2.1426 ppm; MULTI−SECPEP

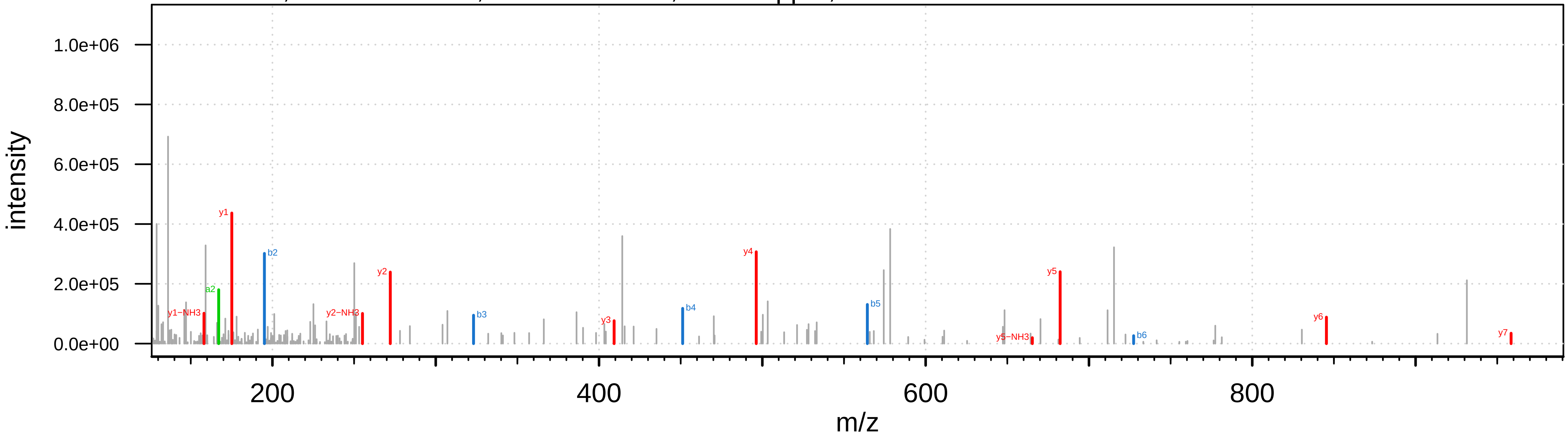

Raw File: 20130808_EXQ4_BaAz_SA_SeR4_06
Scan Number: 12757
Proteins:
sp|P62274|RS29_MOUSE
ENSMUST00000037023_chr12:69157813−69159058:−
ENSMUST00000071737_chr13:73988963−73989133:−

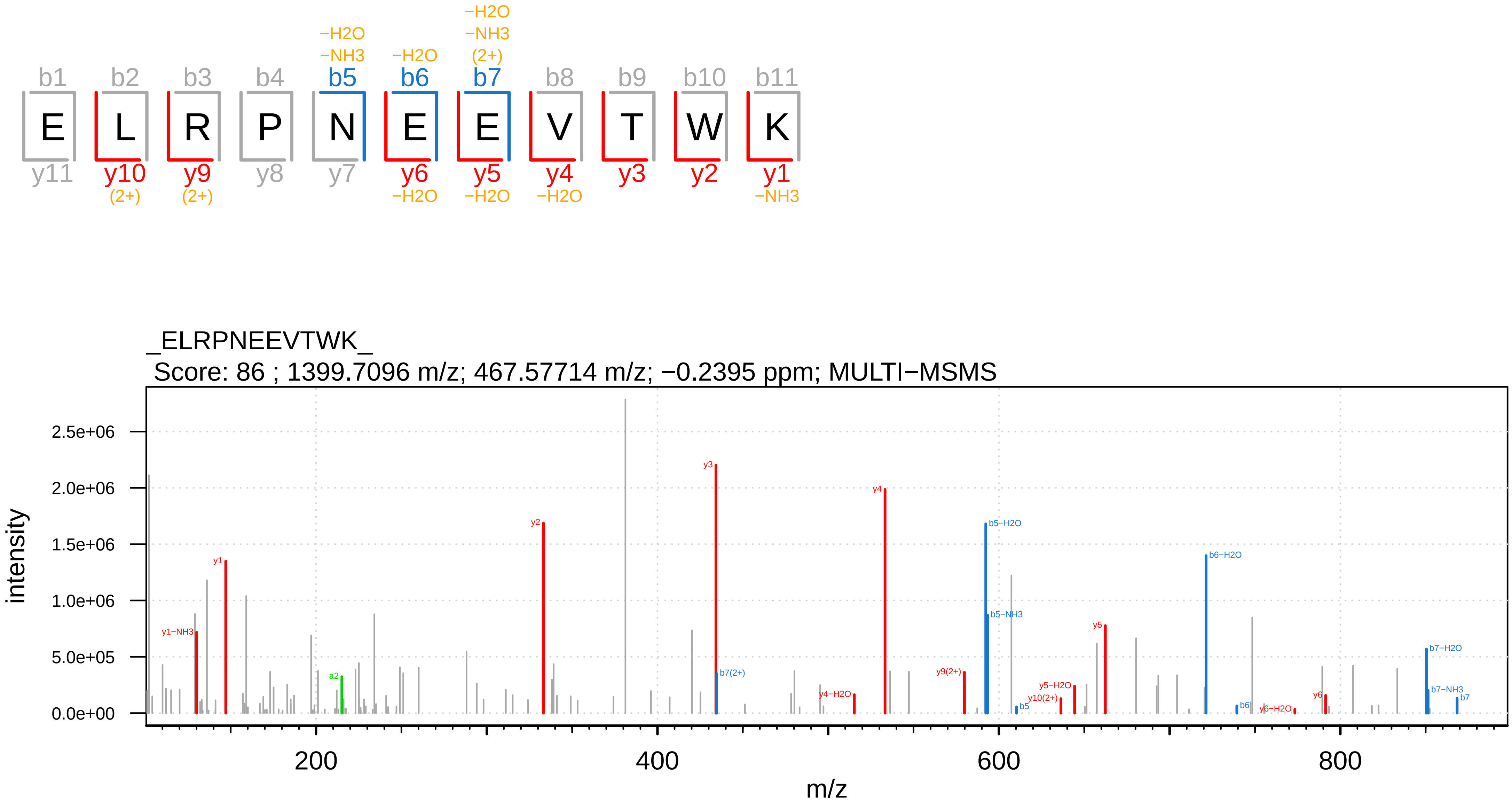

Raw File: 20140815_QEp4_BaAz_SA_DI_R1
Scan Number: 45082
Proteins:
chr12_103206313−103214181.T2_chr12:101968174−101969629:−

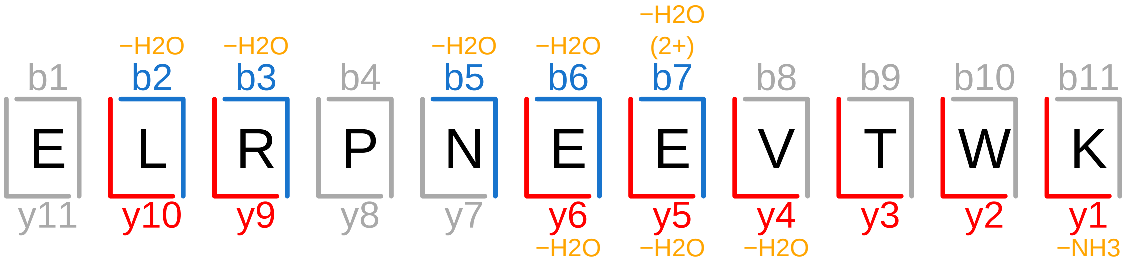

ELRPNEEVTWK

Score: 110 ; 1399.7096 m/z; 467.57714 m/z; −0.2395 ppm; MULTI−MSMS

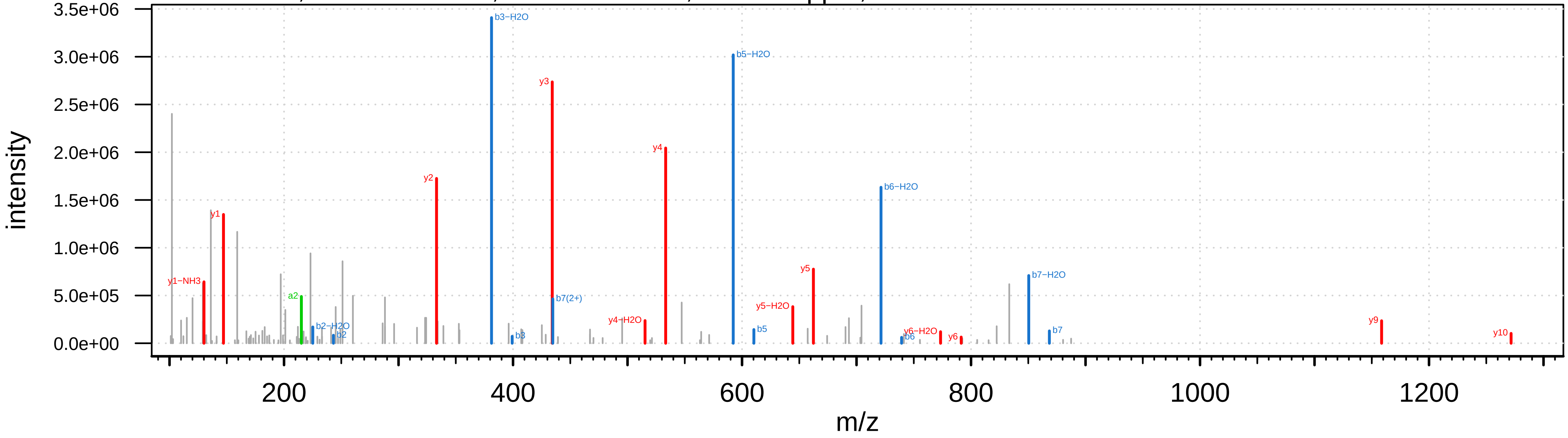

Raw File: 20140815_QEp4_BaAz_SA_DI_R1

Scan Number: 45407

Proteins:

chr12_103206313−103214181.T2_chr12:101968174−101969629:−

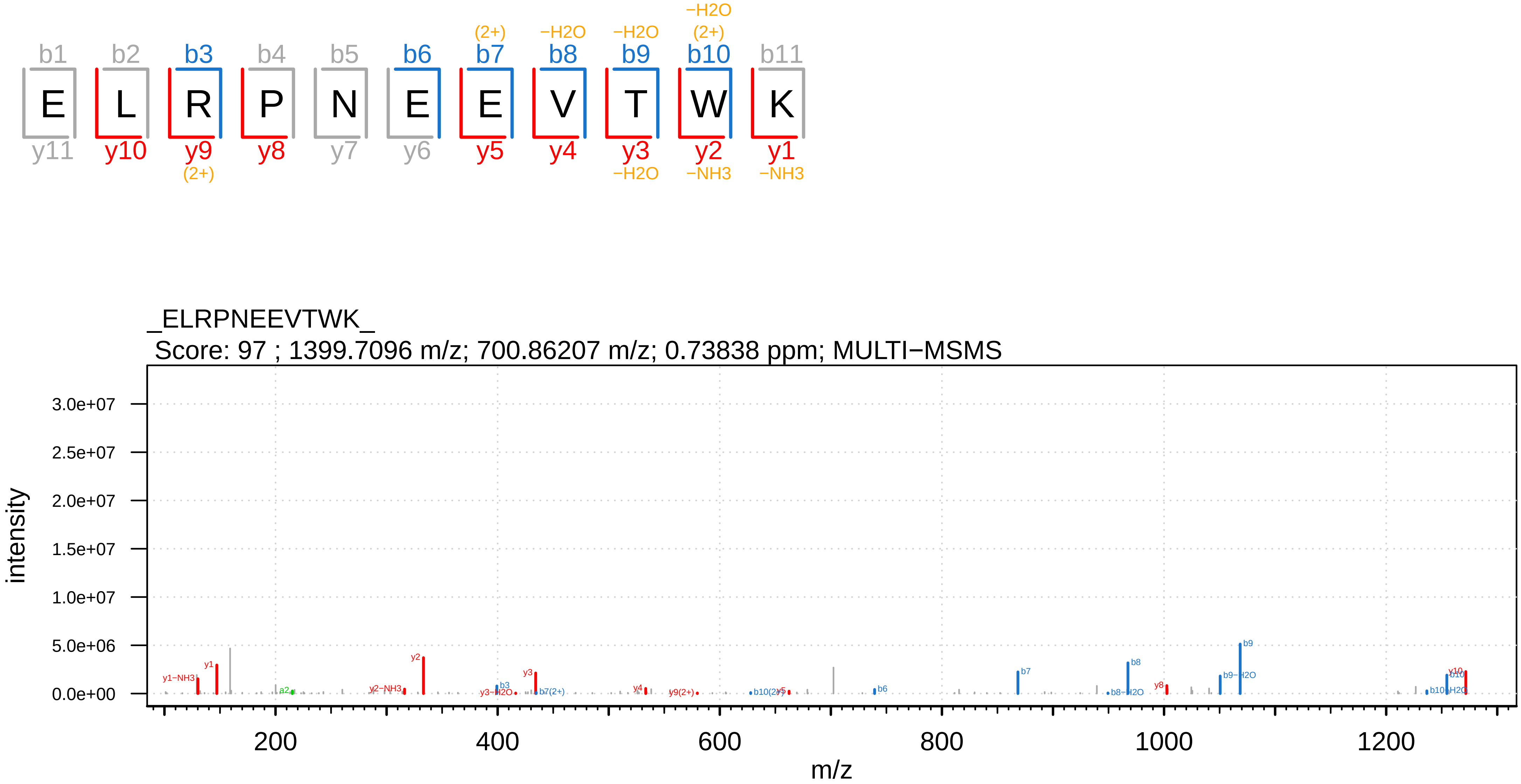

Raw File: 20140815_QEp4_BaAz_SA_DI_R1
Scan Number: 45296
Proteins:
chr12_103206313−103214181.T2_chr12:101968174−101969629:−

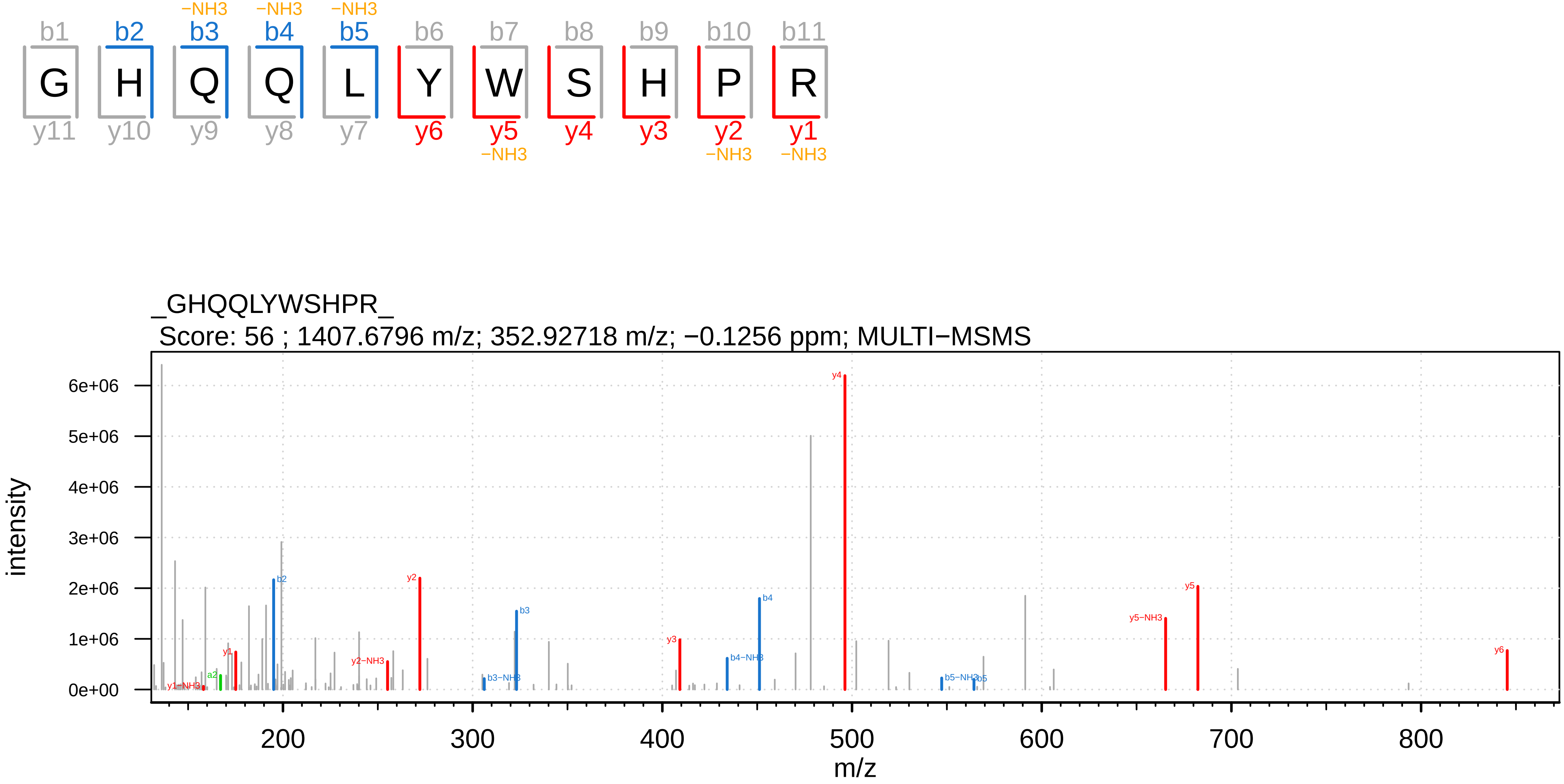

Raw File: 20140815_QEp4_BaAz_SA_DI_R1
Scan Number: 23106
Proteins:
sp|P62274|RS29_MOUSE
ENSMUST00000037023_chr12:69157813−69159058:−
ENSMUST00000071737_chr13:73988963−73989133:−

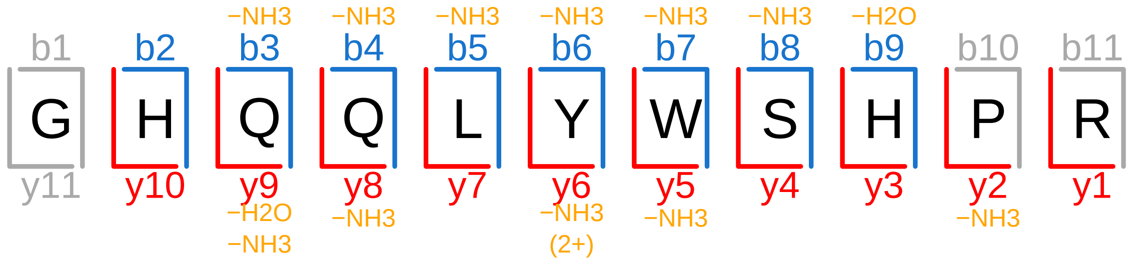

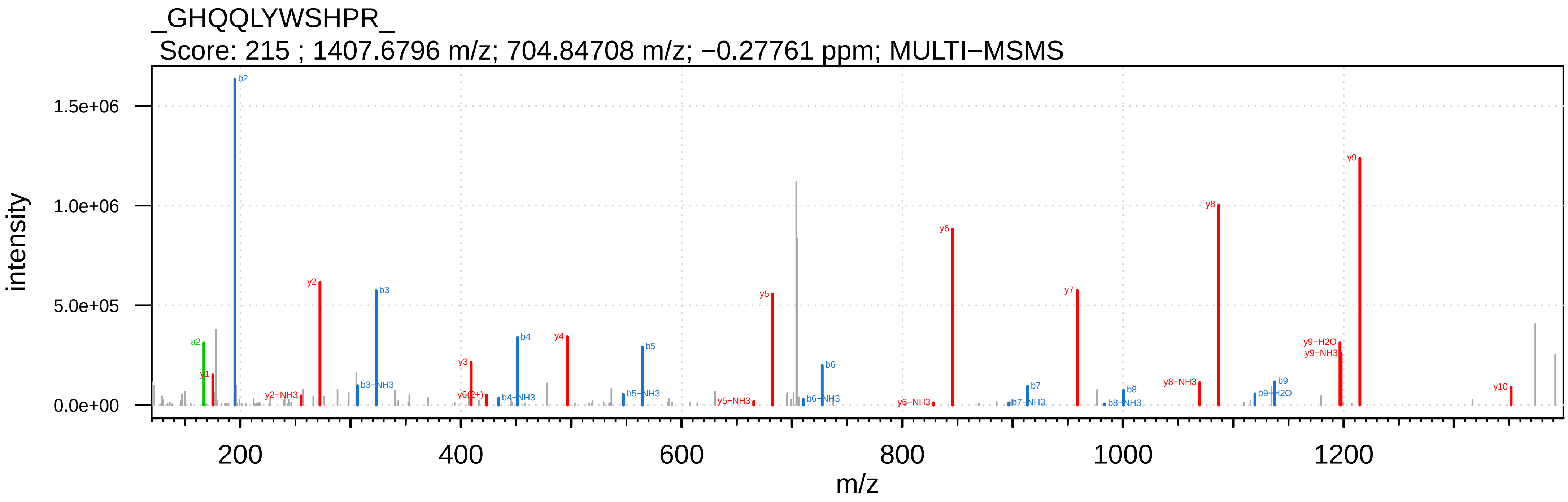

Raw File: 20140815_QEp4_BaAz_SA_DI_R1
Scan Number: 23112
Proteins:
sp|P62274|RS29_MOUSE
ENSMUST00000037023_chr12:69157813−69159058:−
ENSMUST00000071737_chr13:73988963−73989133:−

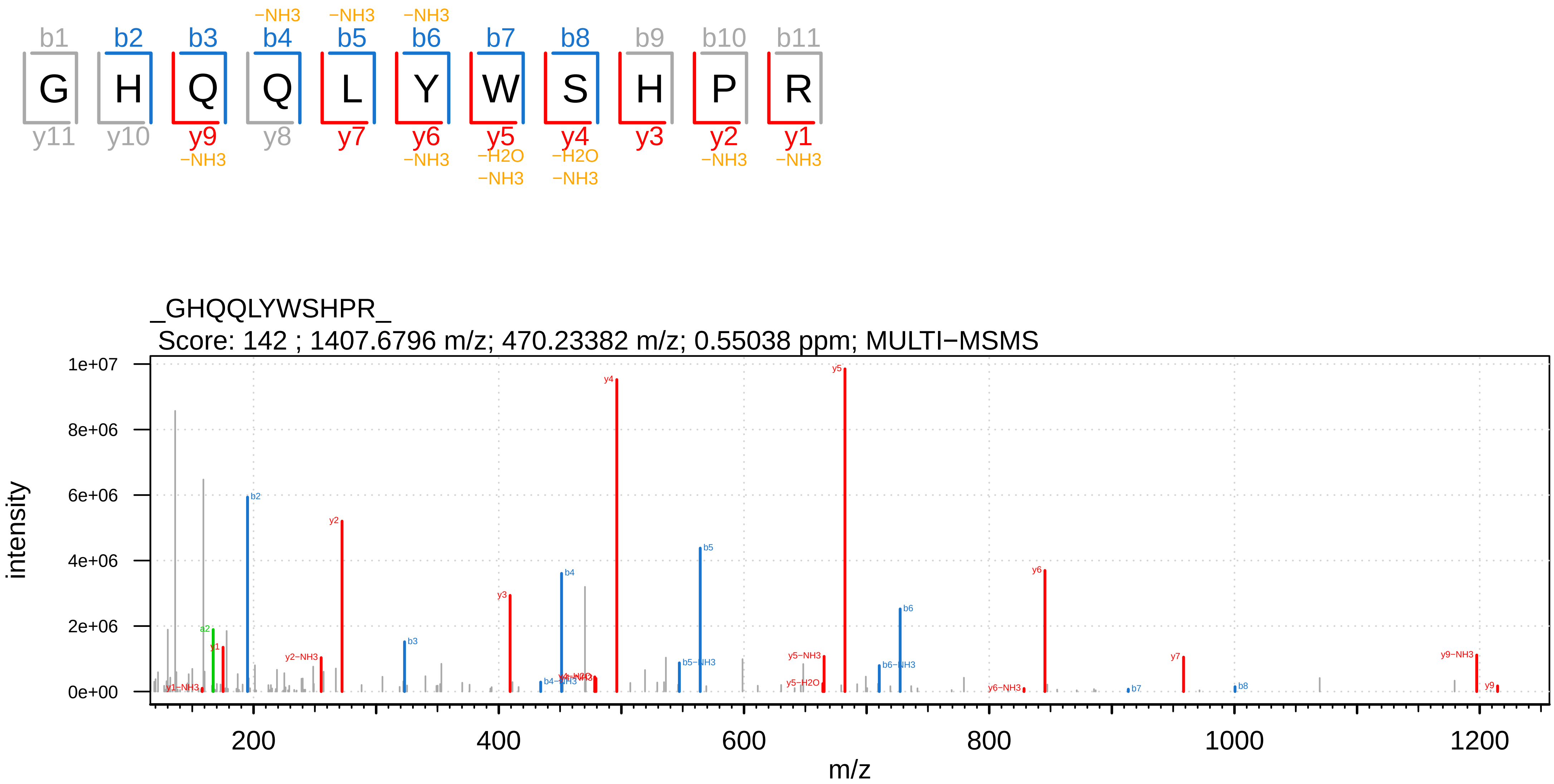

Raw File: 20140815_QEp4_BaAz_SA_DI_R1
Scan Number: 23114
Proteins:
sp|P62274|RS29_MOUSE
ENSMUST00000037023_chr12:69157813−69159058:−
ENSMUST00000071737_chr13:73988963−73989133:−

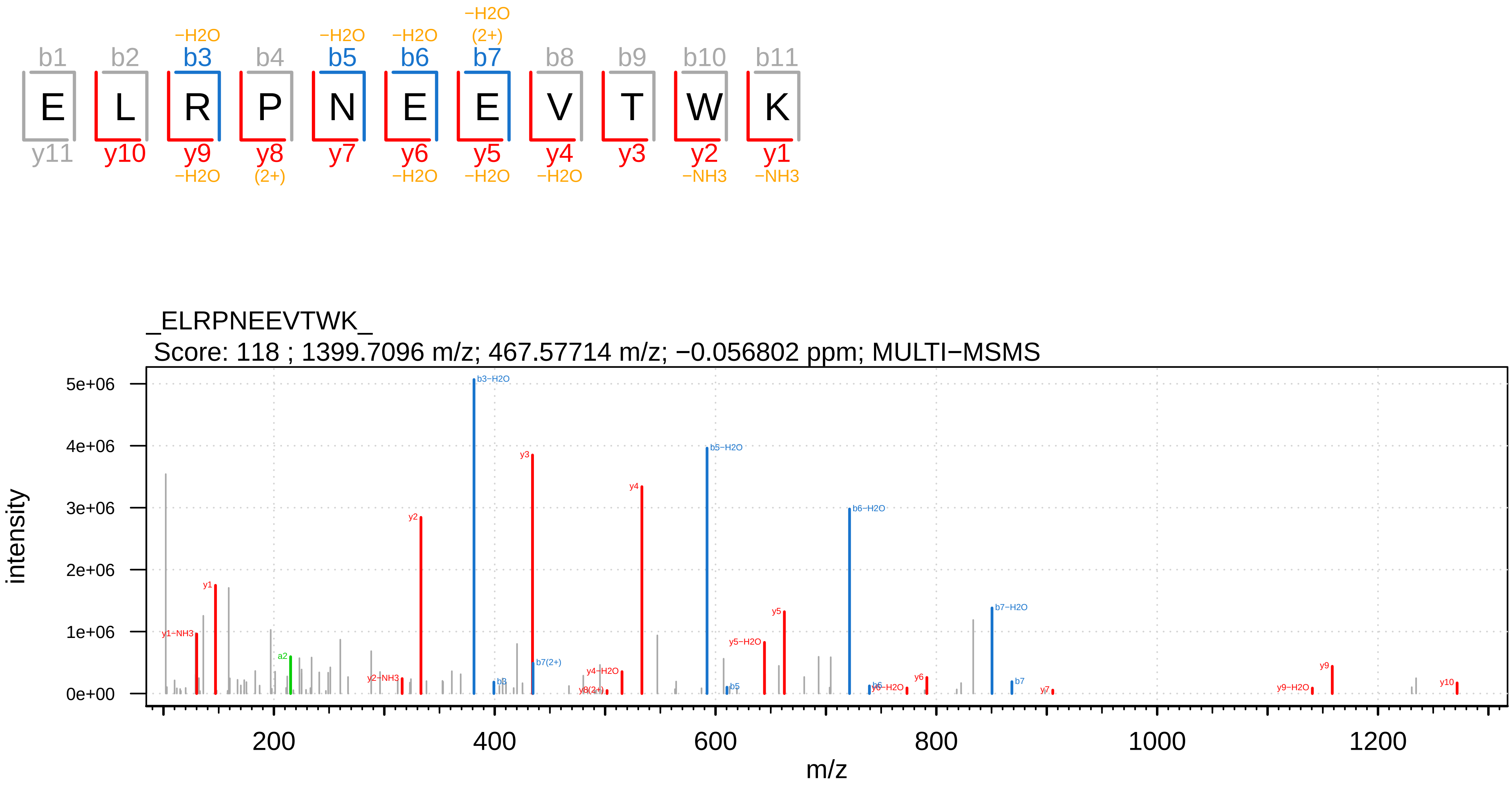

Raw File: 20140815_QEp4_BaAz_SA_DI_R2
Scan Number: 44898
Proteins:
chr12_103206313−103214181.T2_chr12:101968174−101969629:−

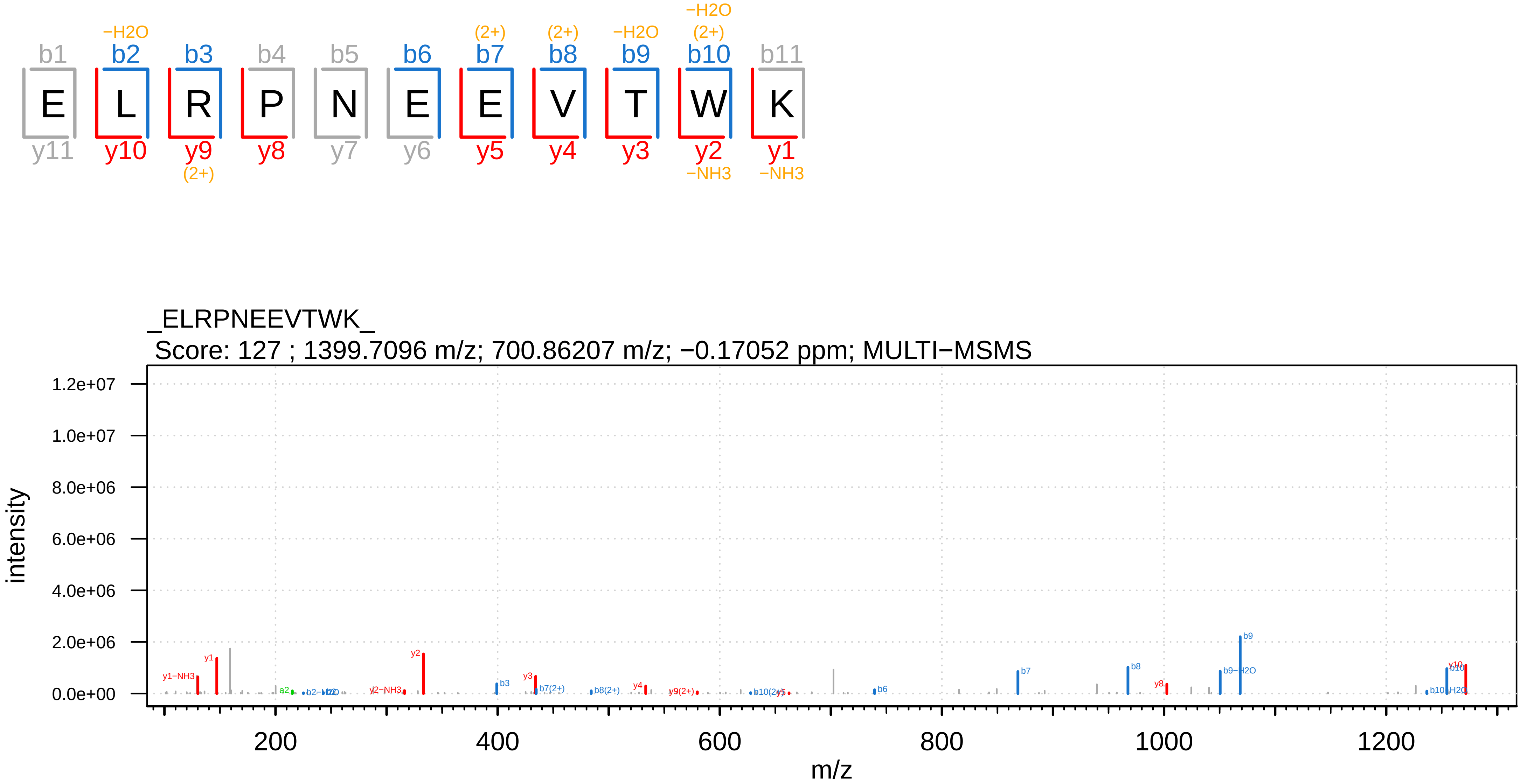

Raw File: 20140815_QEp4_BaAz_SA_DI_R2
Scan Number: 45170
Proteins:
chr12_103206313−103214181.T2_chr12:101968174−101969629:−

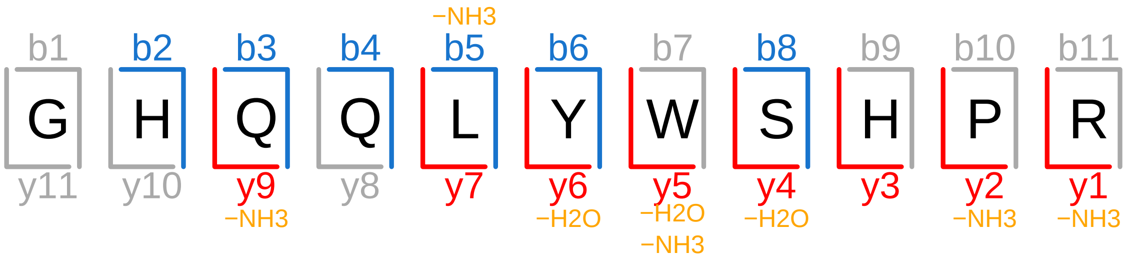

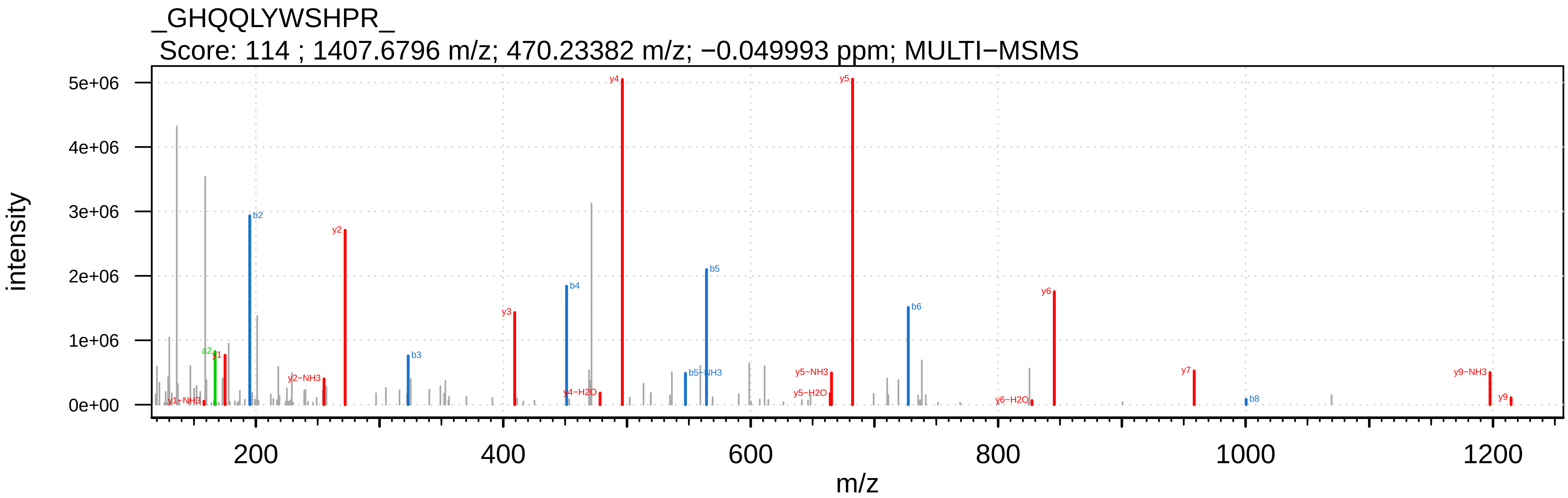

Raw File: 20140815_QEp4_BaAz_SA_DI_R2
Scan Number: 22816
Proteins:
sp|P62274|RS29_MOUSE
ENSMUST00000037023_chr12:69157813−69159058:−
ENSMUST00000071737_chr13:73988963−73989133:−

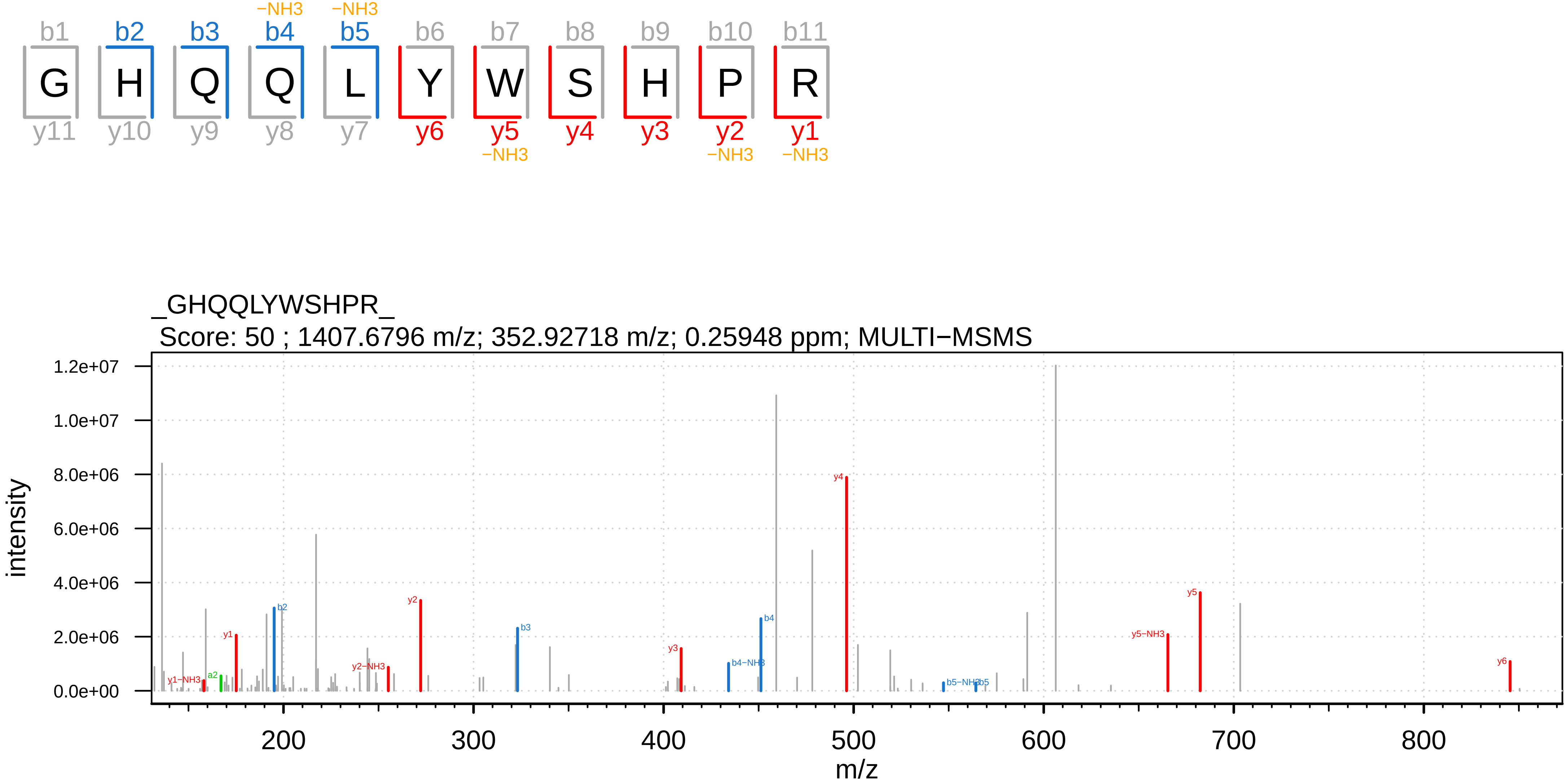

Raw File: 20140815_QEp4_BaAz_SA_DI_R2
Scan Number: 22825
Proteins:
sp|P62274|RS29_MOUSE
ENSMUST00000037023_chr12:69157813−69159058:−
ENSMUST00000071737_chr13:73988963−73989133:−

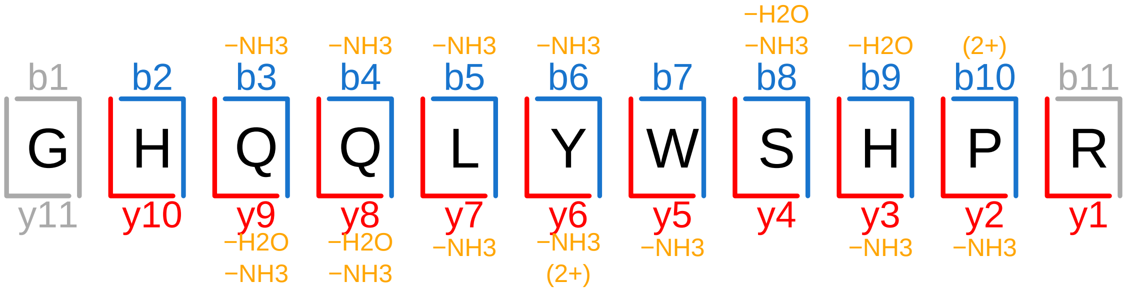

GHQQLYWSHPR
Score: 240 ; 1407.6796 m/z; 704.84708 m/z; −0.23308 ppm; MULTI−MSMS

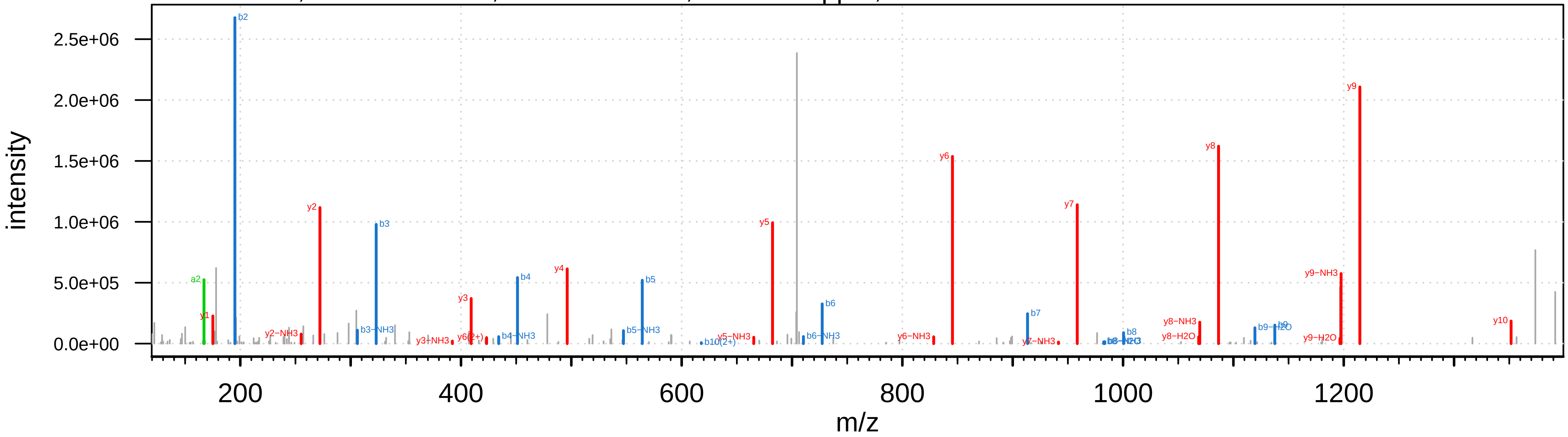

Raw File: 20140815_QEp4_BaAz_SA_DI_R2
Scan Number: 22828
Proteins:
sp|P62274|RS29_MOUSE
ENSMUST00000037023_chr12:69157813−69159058:−
ENSMUST00000071737_chr13:73988963−73989133:−

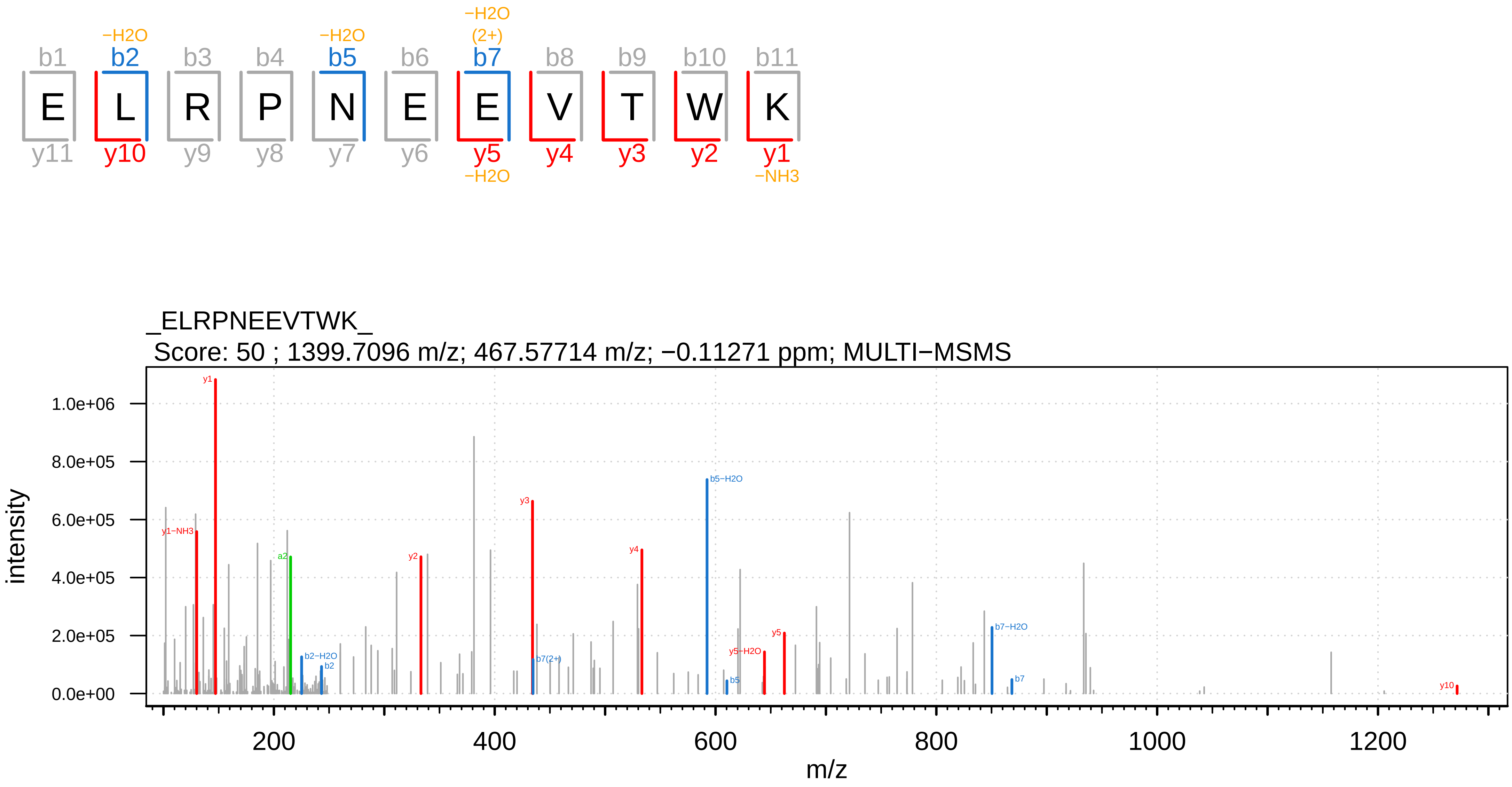

Raw File: 20140815_QEp4_BaAz_SA_HC_R2
Scan Number: 40717
Proteins:
chr12_103206313−103214181.T2_chr12:101968174−101969629:−

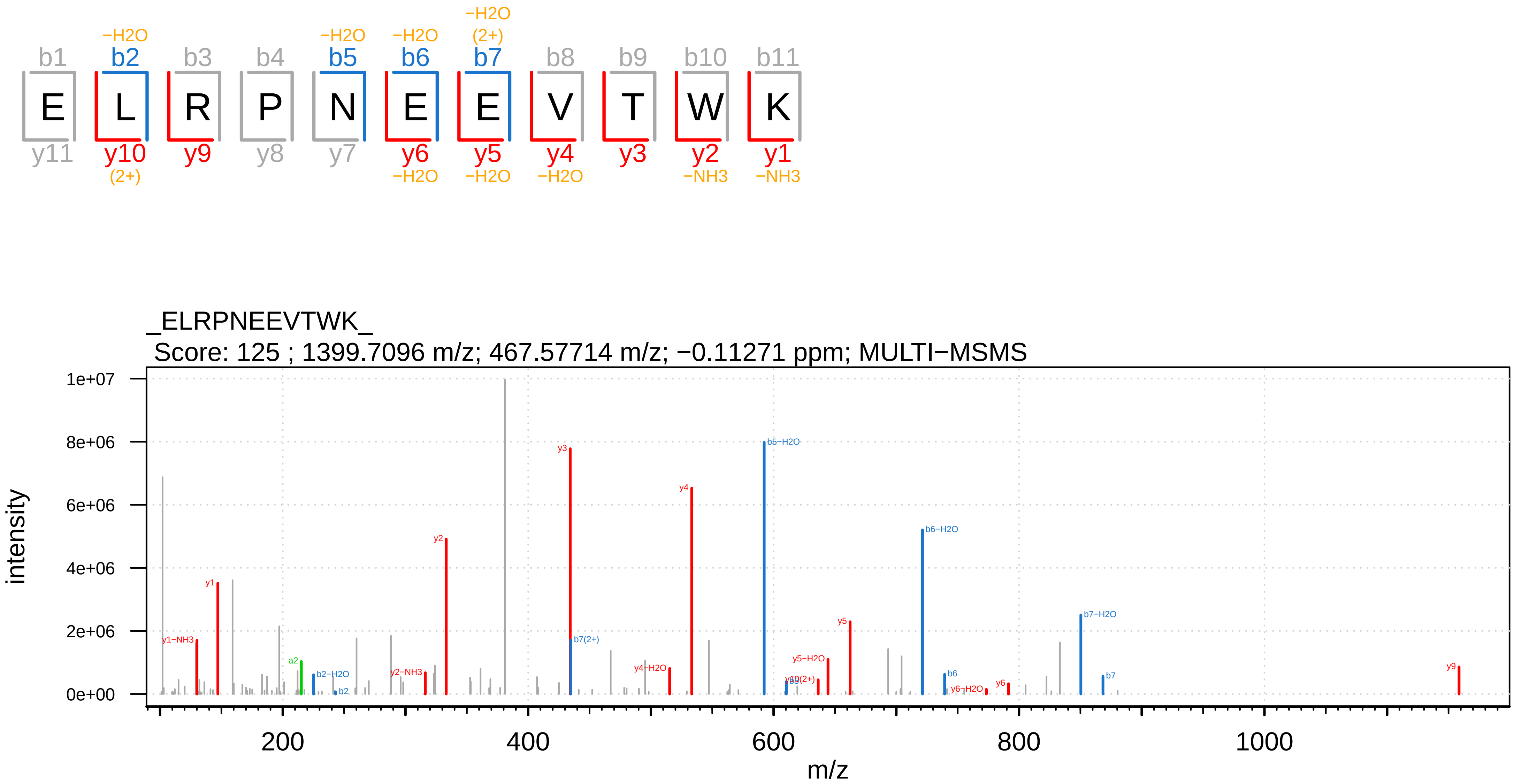

Raw File: 20140815_QEp4_BaAz_SA_HC_R2
Scan Number: 41058
Proteins:
chr12_103206313−103214181.T2_chr12:101968174−101969629:−

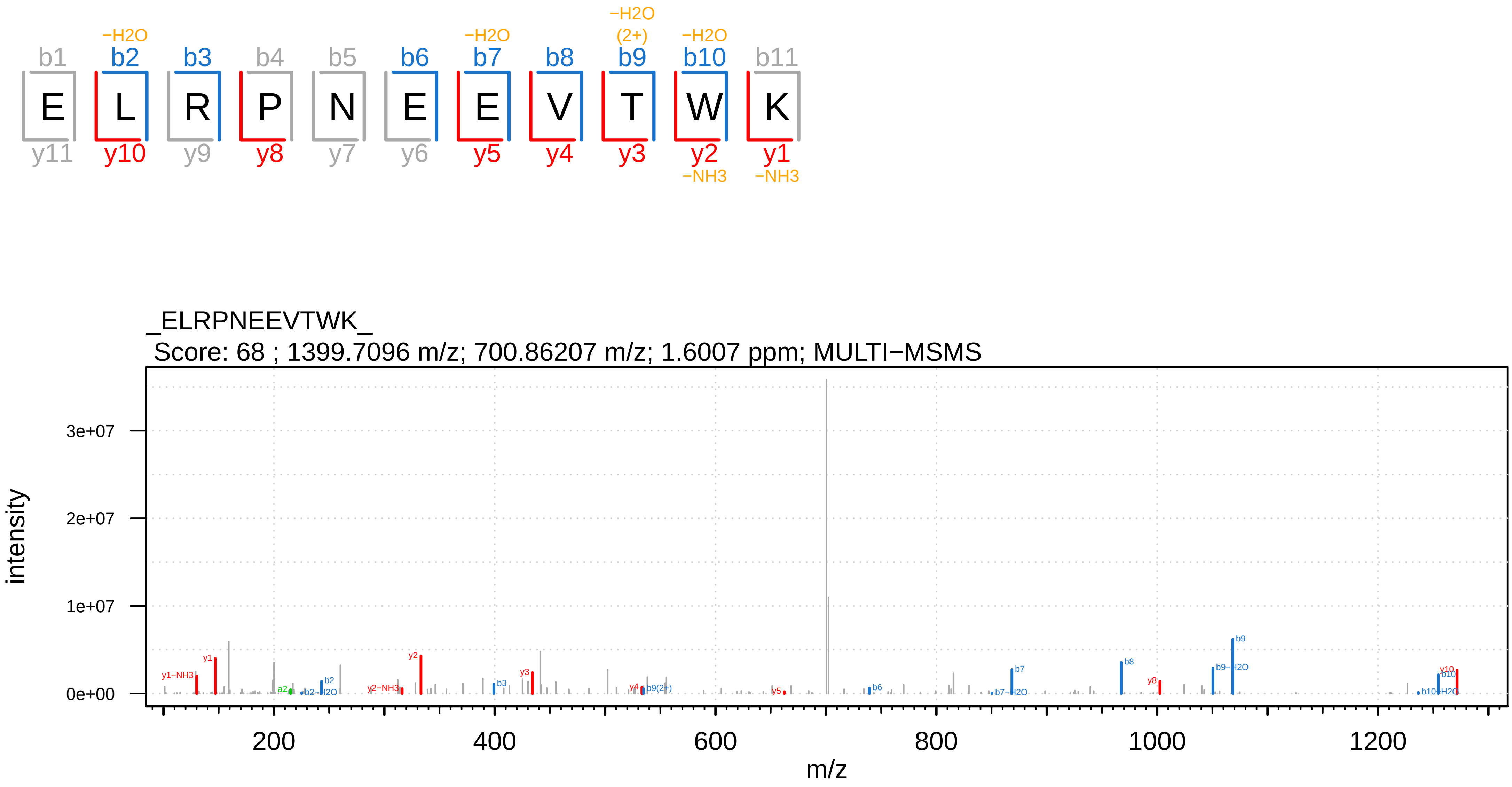

Raw File: 20140815_QEp4_BaAz_SA_HC_R2

Scan Number: 40861

Proteins:

chr12_103206313−103214181.T2_chr12:101968174−101969629:−

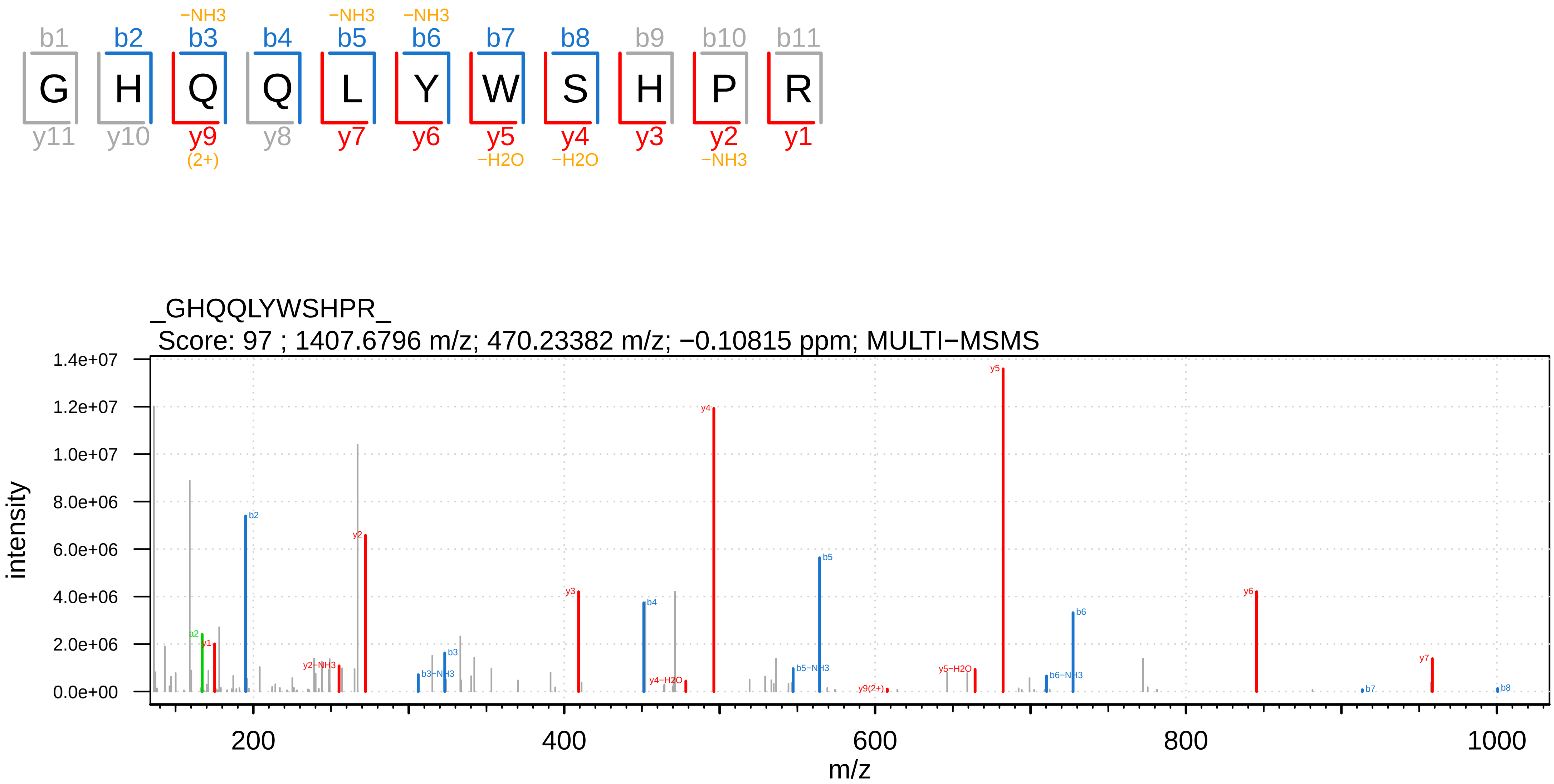

Raw File: 20140815_QEp4_BaAz_SA_HC_R2
Scan Number: 19804
Proteins:
sp|P62274|RS29_MOUSE
ENSMUST00000037023_chr12:69157813−69159058:−
ENSMUST00000071737_chr13:73988963−73989133:−

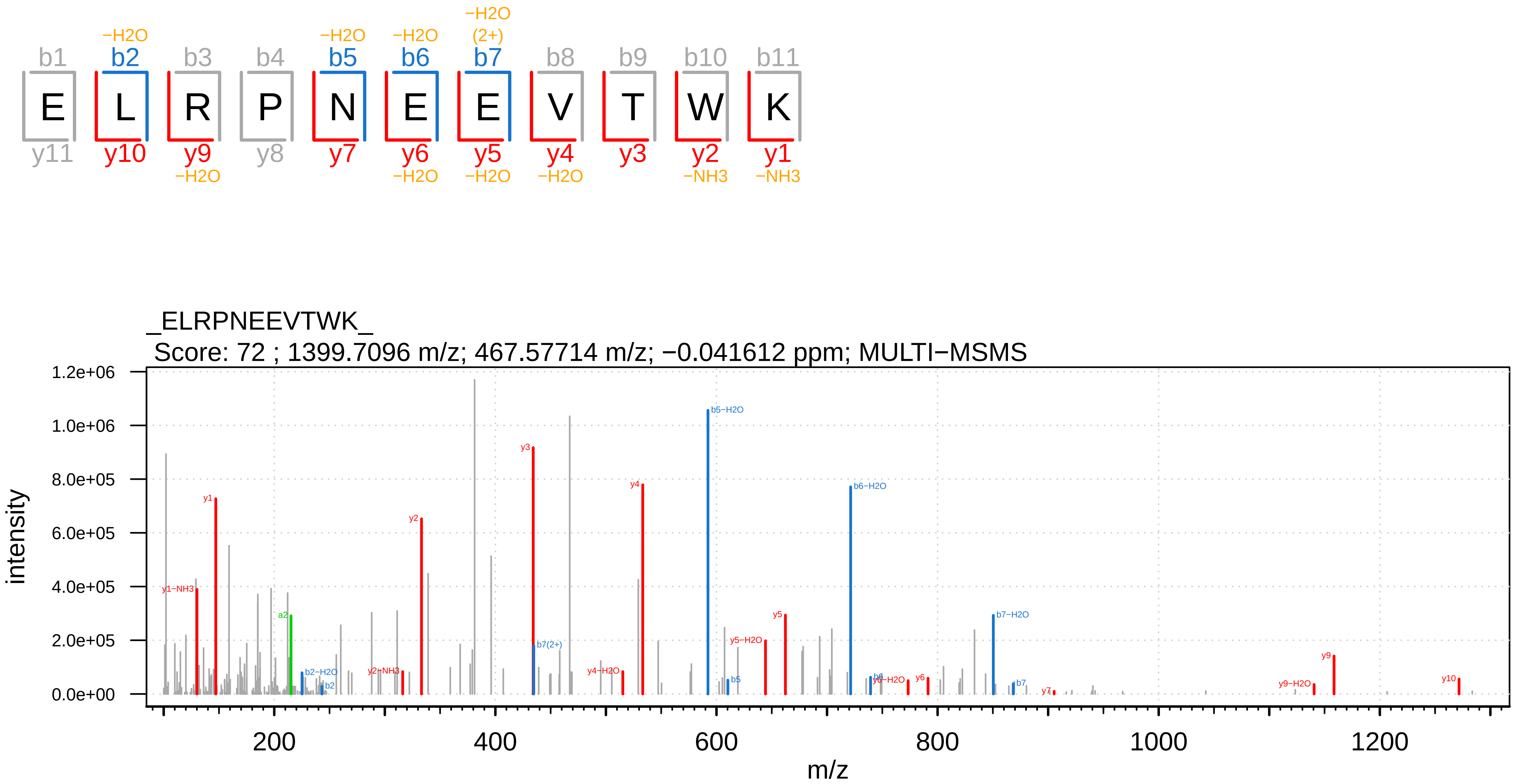

Raw File: 20140815_QEp4_BaAz_SA_HC_R3
Scan Number: 40959
Proteins:
chr12_103206313−103214181.T2_chr12:101968174−101969629:−

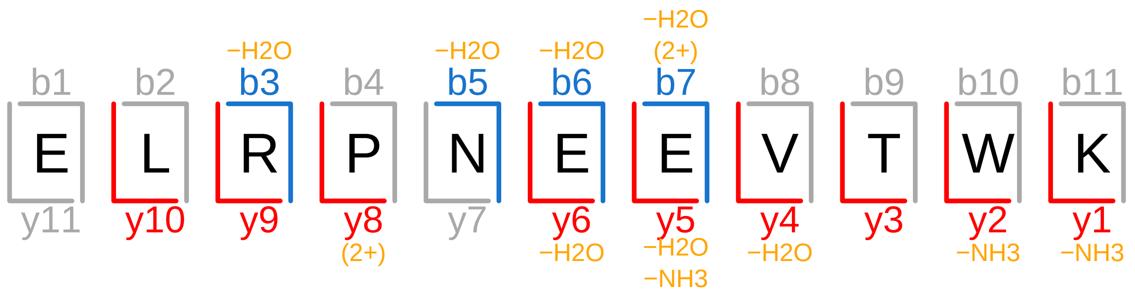

ELRPNEEVTWK
Score: 137 ; 1399.7096 m/z; 467.57714 m/z; −0.041612 ppm; MULTI−MSMS

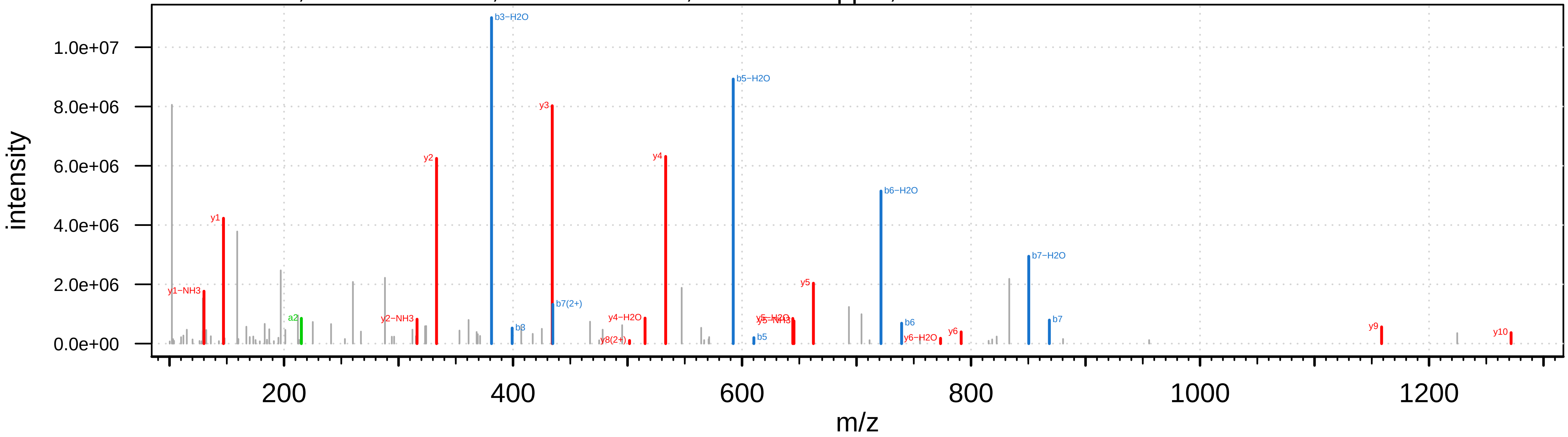

Raw File: 20140815_QEp4_BaAz_SA_HC_R3
Scan Number: 41297
Proteins:
chr12_103206313−103214181.T2_chr12:101968174−101969629:−

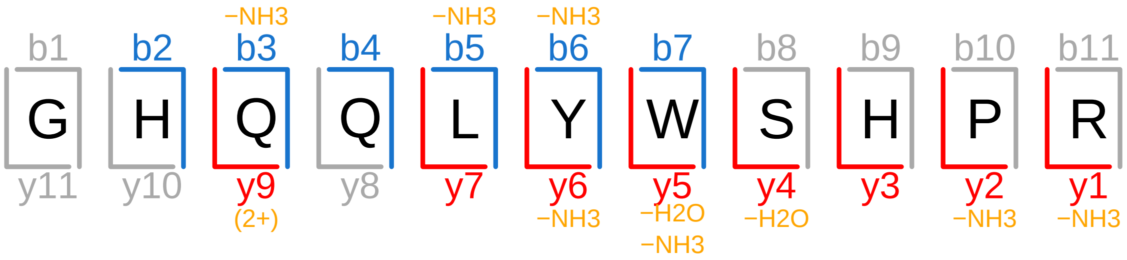

GHQQLYWSHPR
Score: 111 ; 1407.6796 m/z; 470.23382 m/z; −0.15659 ppm; MULTI−MSMS

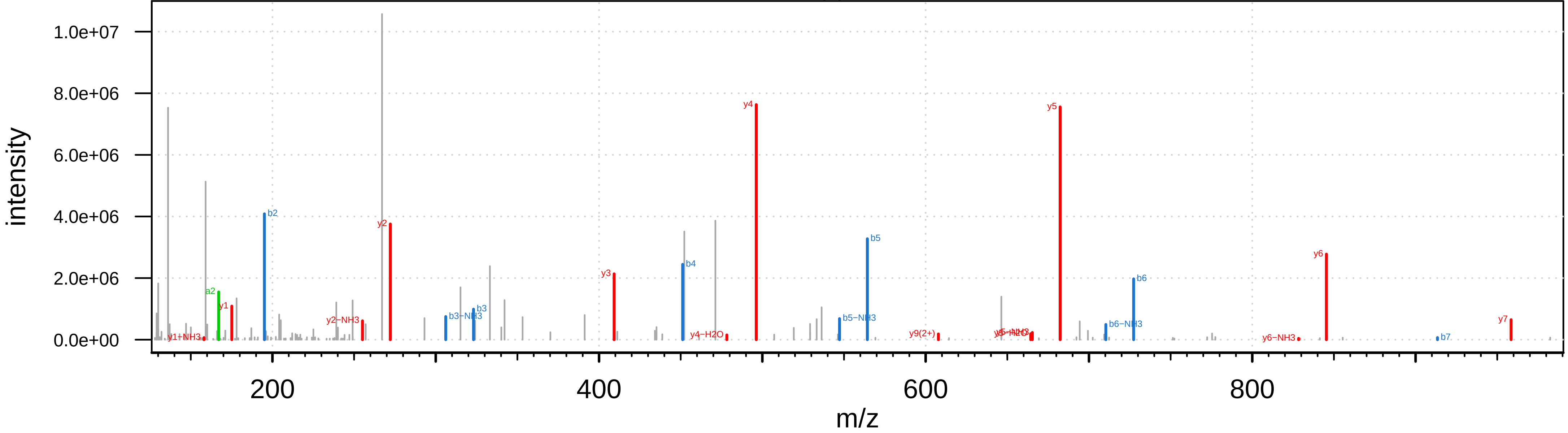

Raw File: 20140815_QEp4_BaAz_SA_HC_R3
Scan Number: 19935
Proteins:
sp|P62274|RS29_MOUSE
ENSMUST00000037023_chr12:69157813−69159058:−
ENSMUST00000071737_chr13:73988963−73989133:−

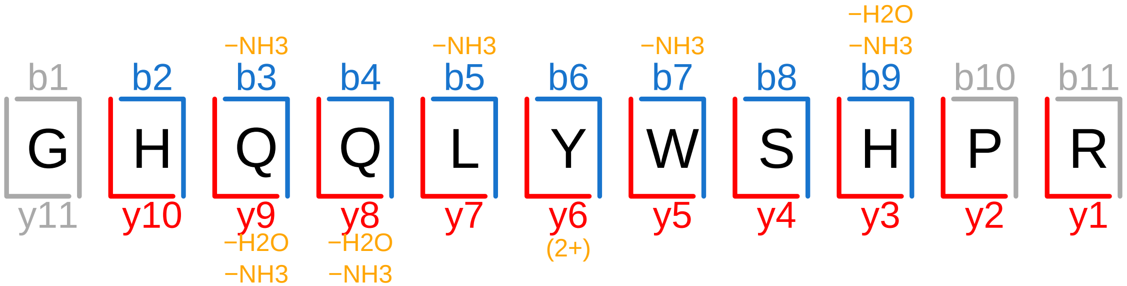

GHQQLYWSHPR
Score: 133 ; 1407.6796 m/z; 704.84708 m/z; 0.92344 ppm; MULTI−MSMS

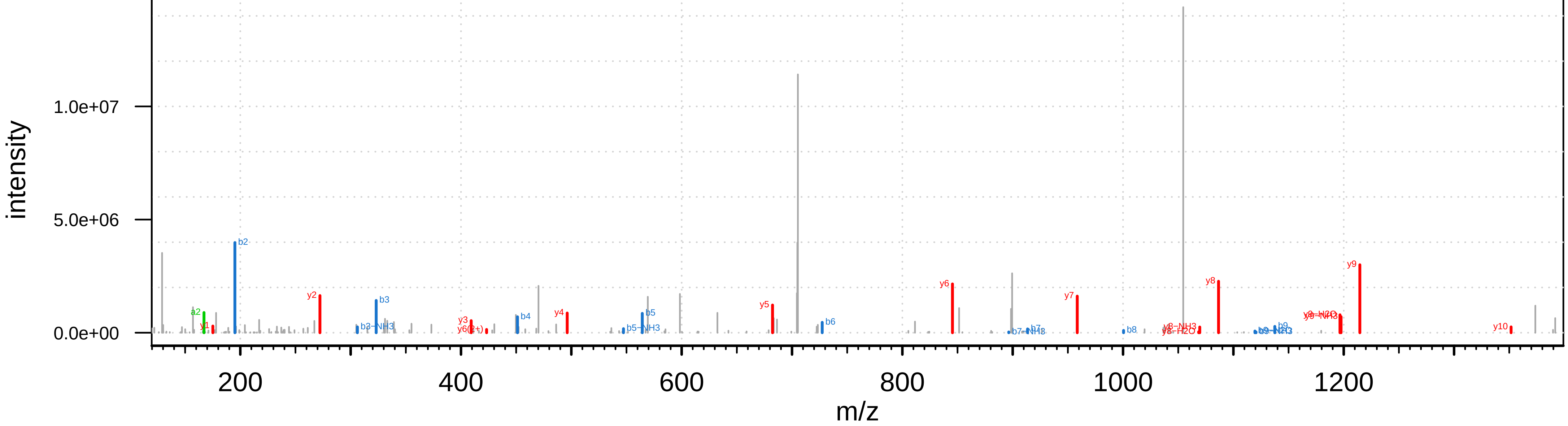

Raw File: 20140815_QEp4_BaAz_SA_HC_R3
Scan Number: 19946
Proteins:
sp|P62274|RS29_MOUSE
ENSMUST00000037023_chr12:69157813−69159058:−
ENSMUST00000071737_chr13:73988963−73989133:−

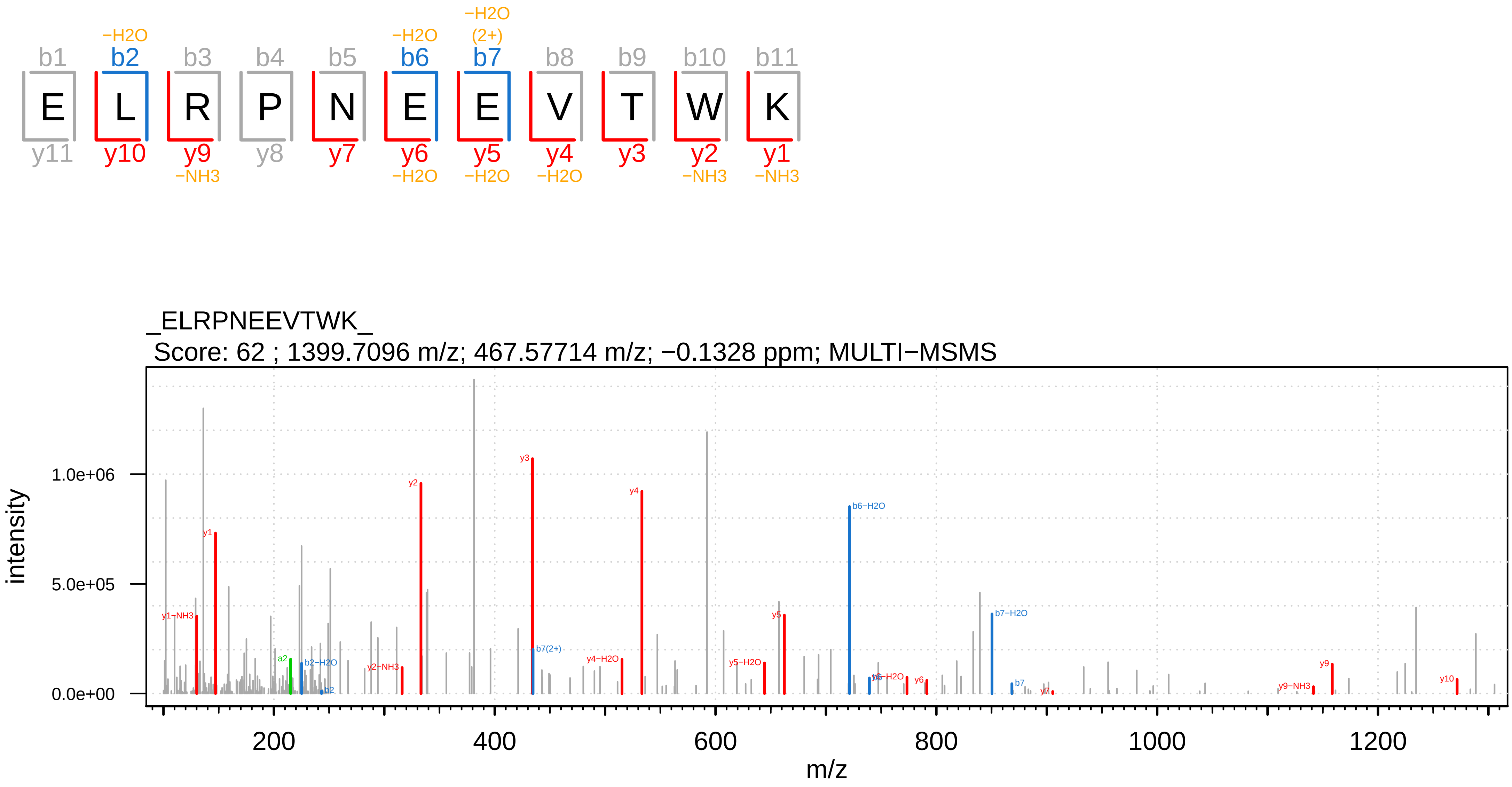

Raw File: 20140816_QEp4_BaAz_SA_DI_R3
Scan Number: 43850
Proteins:
chr12_103206313−103214181.T2_chr12:101968174−101969629:−

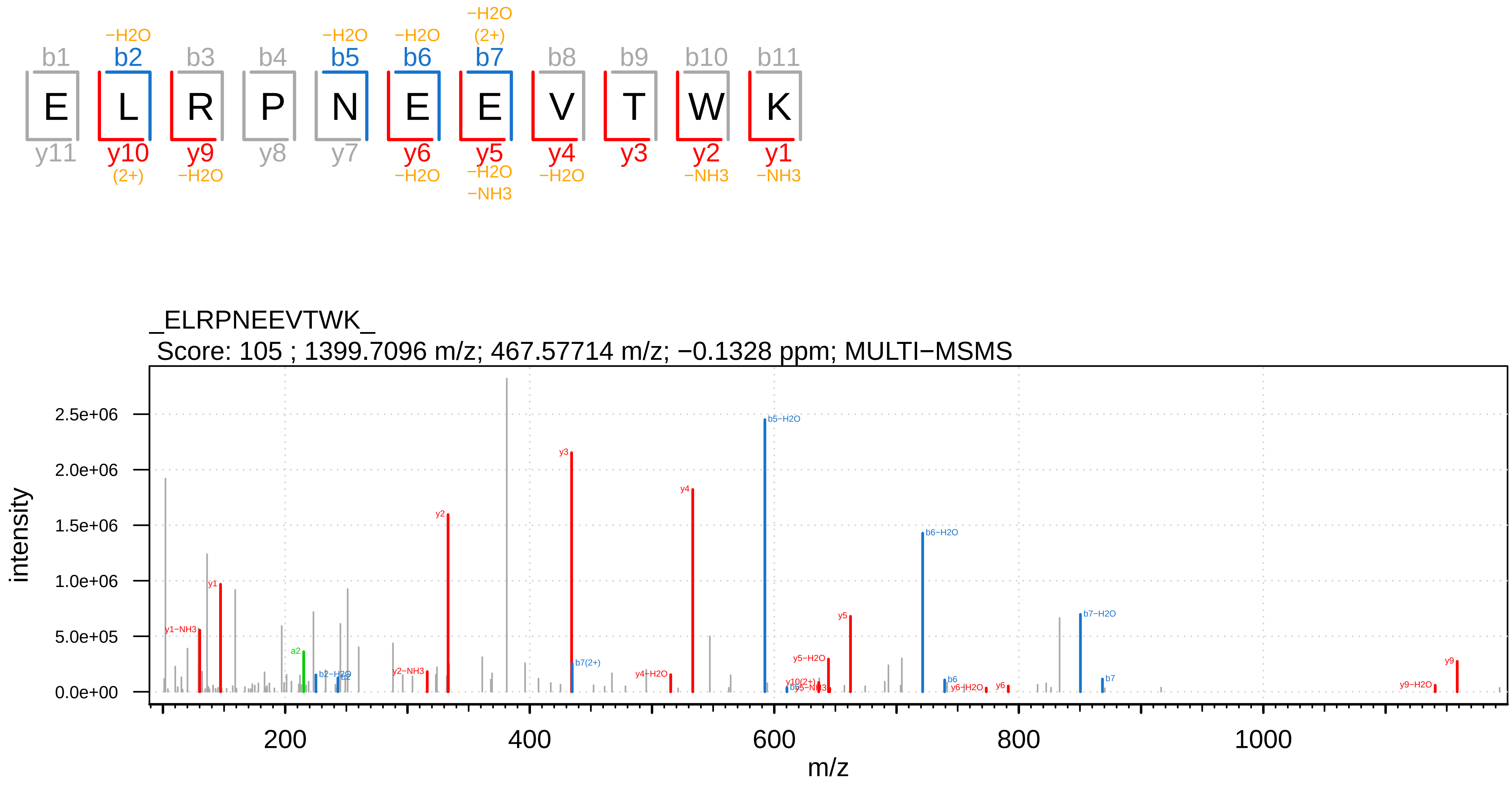

Raw File: 20140816_QEp4_BaAz_SA_DI_R3
Scan Number: 44188
Proteins:
chr12_103206313−103214181.T2_chr12:101968174−101969629:−

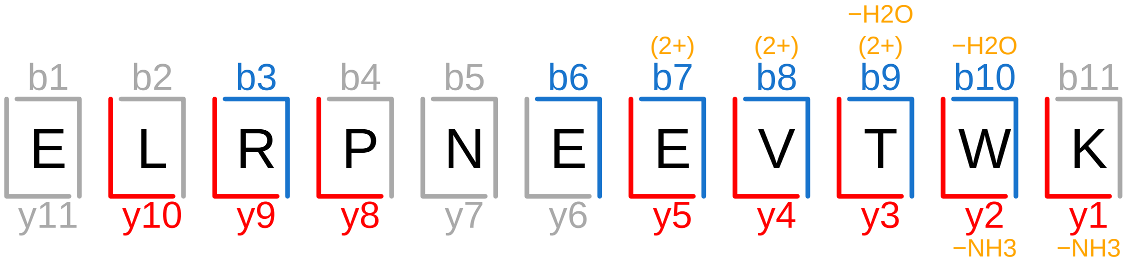

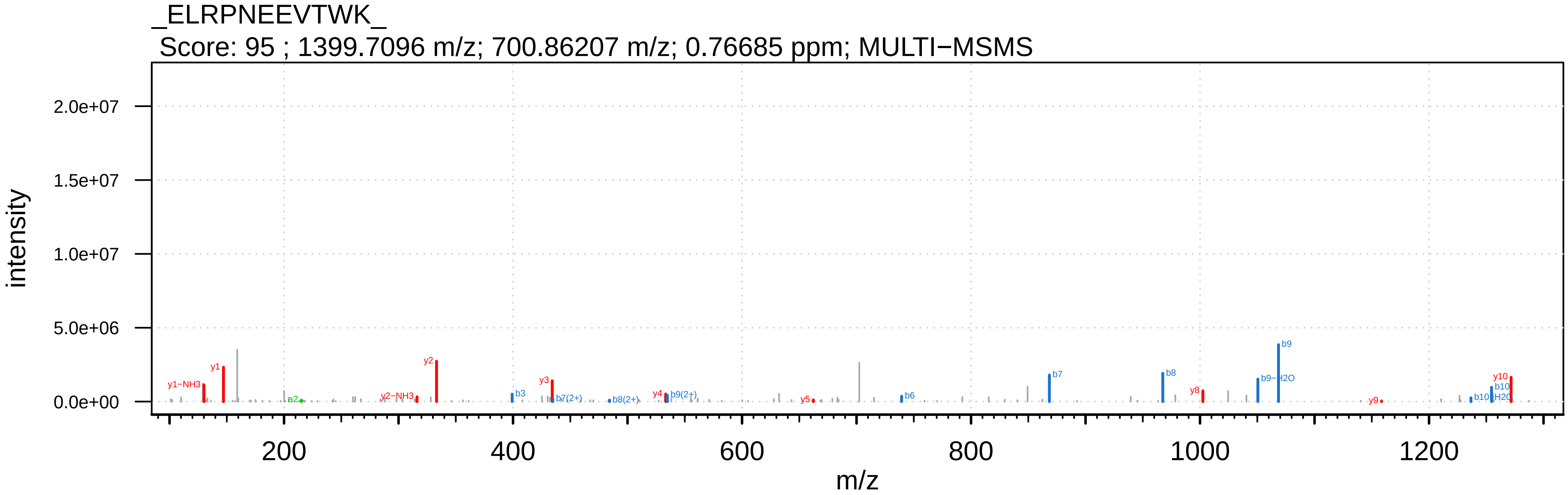

Raw File: 20140816_QEp4_BaAz_SA_DI_R3
Scan Number: 44112
Proteins:
chr12_103206313−103214181.T2_chr12:101968174−101969629:−

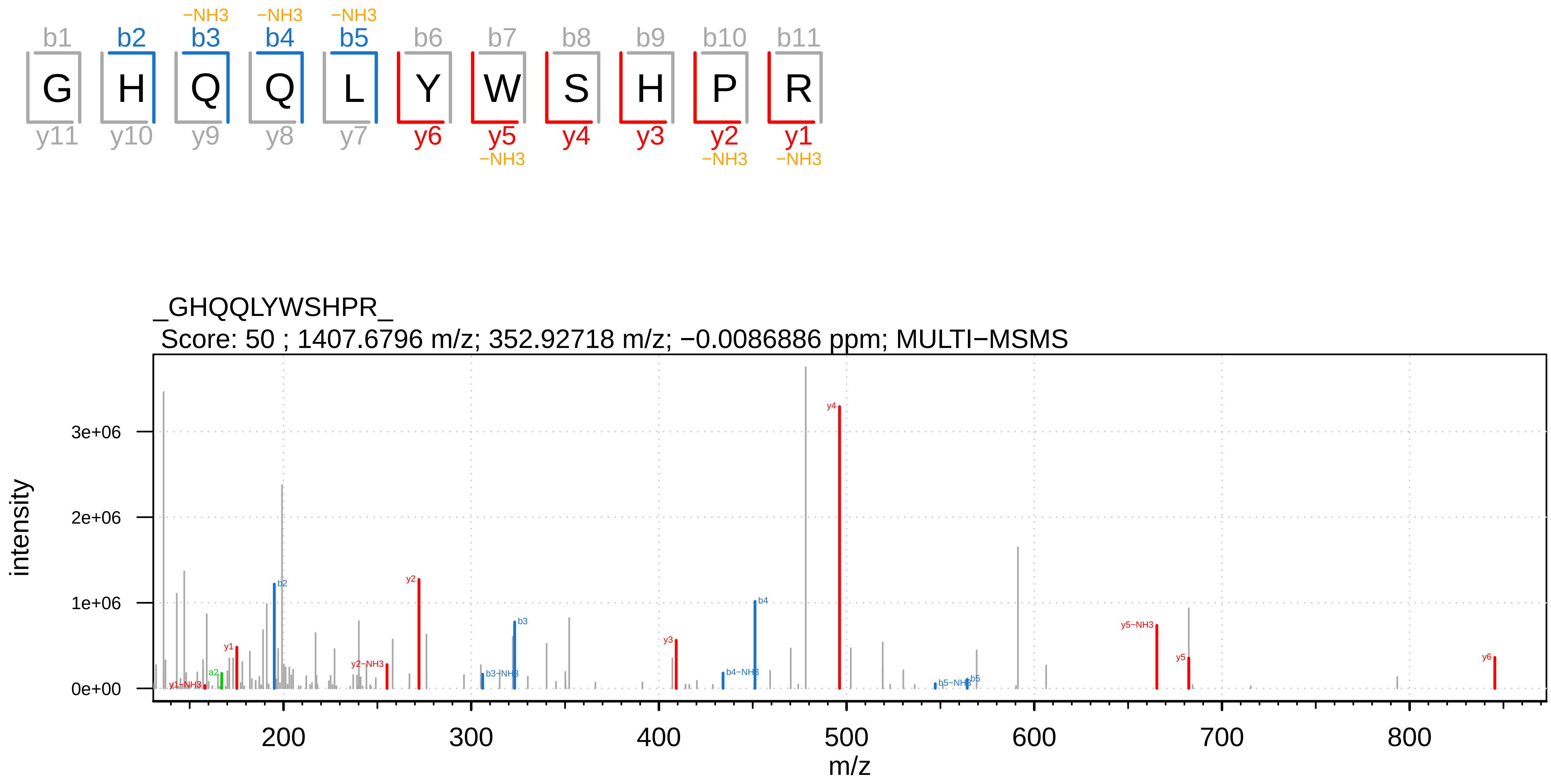

Raw File: 20140816_QEp4_BaAz_SA_DI_R3
Scan Number: 22072
Proteins:
sp|P62274|RS29_MOUSE
ENSMUST00000037023_chr12:69157813−69159058:−
ENSMUST00000071737_chr13:73988963−73989133:−

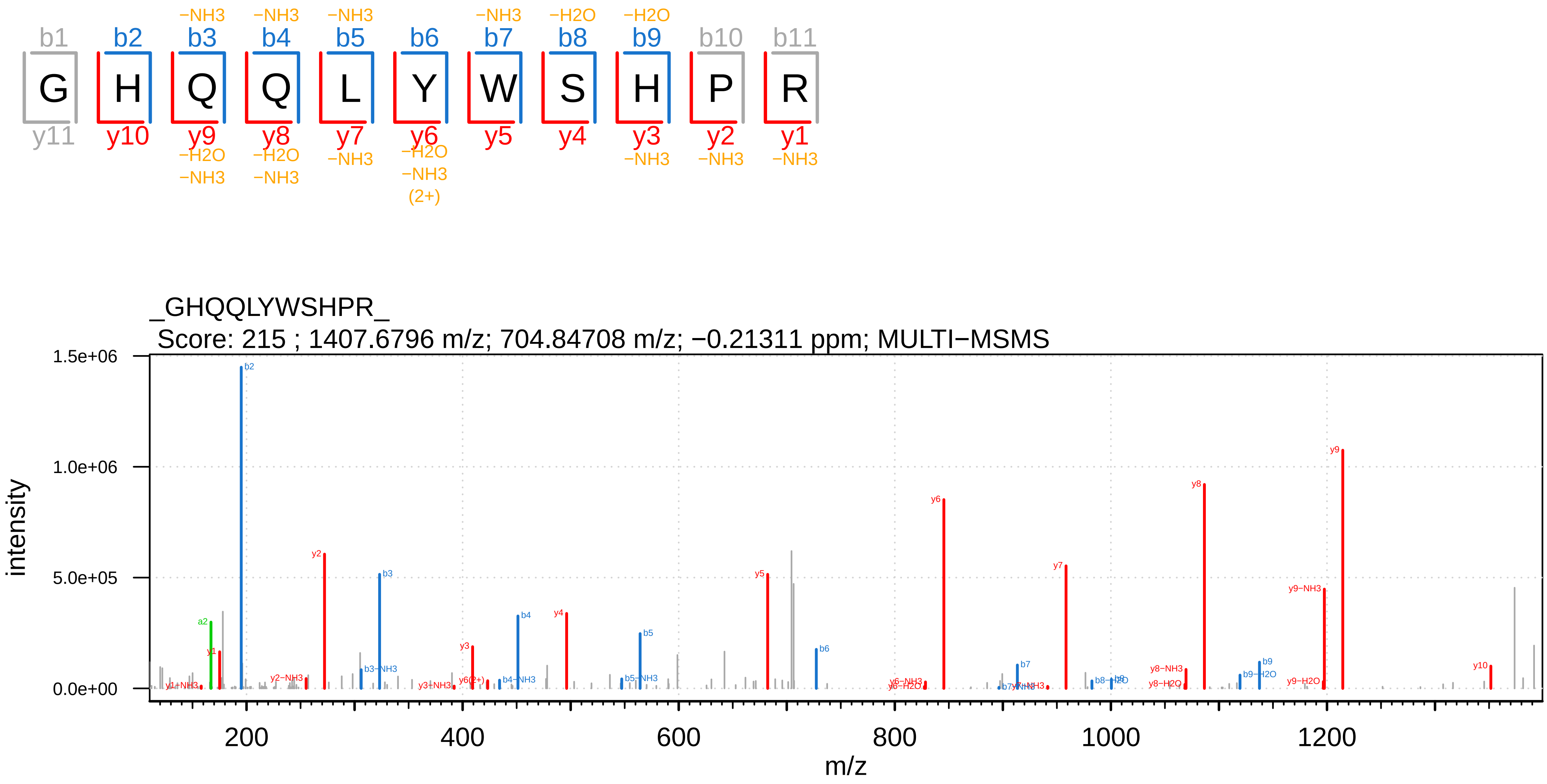

Raw File: 20140816_QEp4_BaAz_SA_DI_R3
Scan Number: 22081
Proteins:
sp|P62274|RS29_MOUSE
ENSMUST00000037023_chr12:69157813−69159058:−
ENSMUST00000071737_chr13:73988963−73989133:−

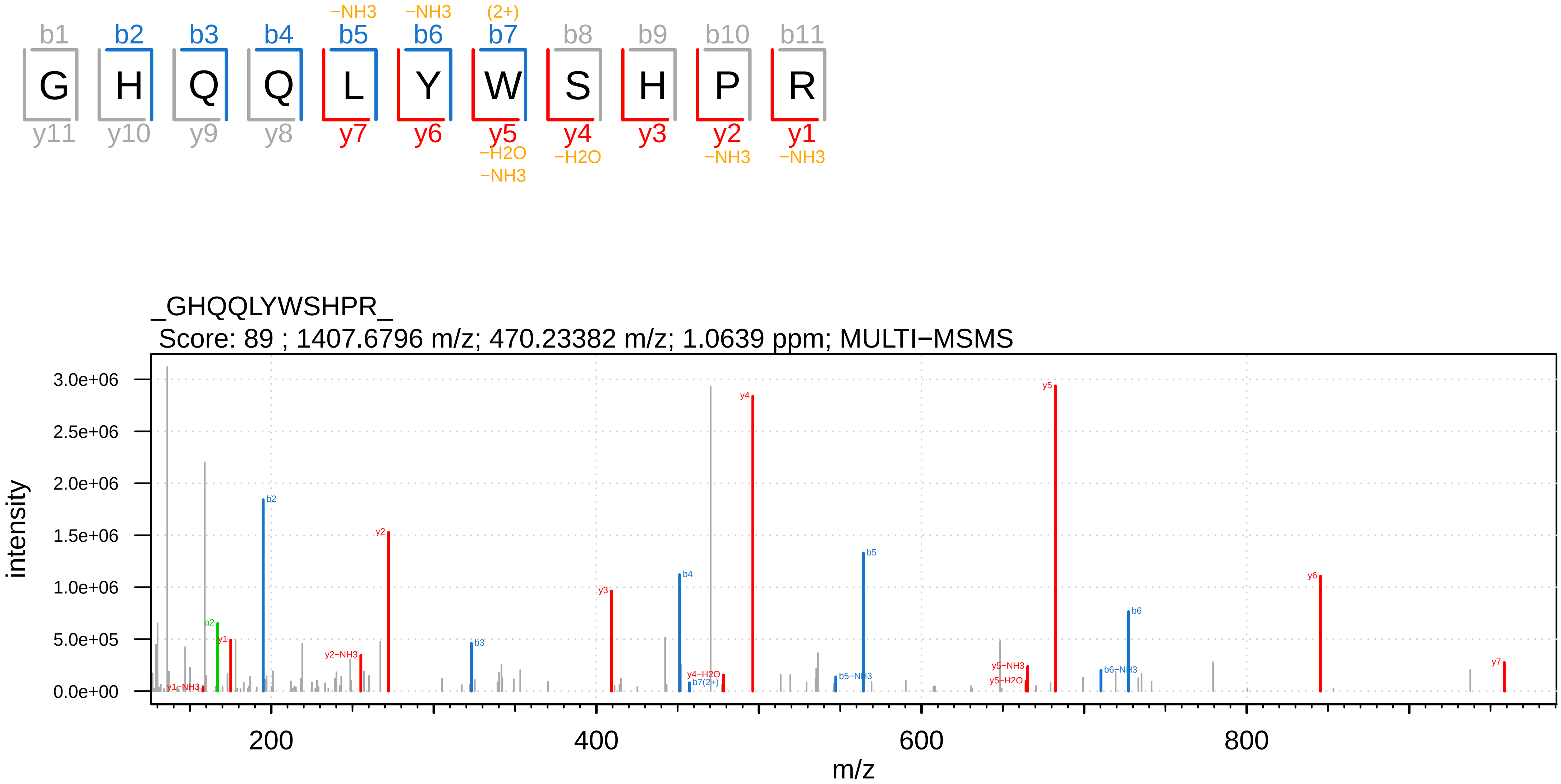

Raw File: 20140816_QEp4_BaAz_SA_DI_R3
Scan Number: 22082
Proteins:
sp|P62274|RS29_MOUSE
ENSMUST00000037023_chr12:69157813−69159058:−
ENSMUST00000071737_chr13:73988963−73989133:−

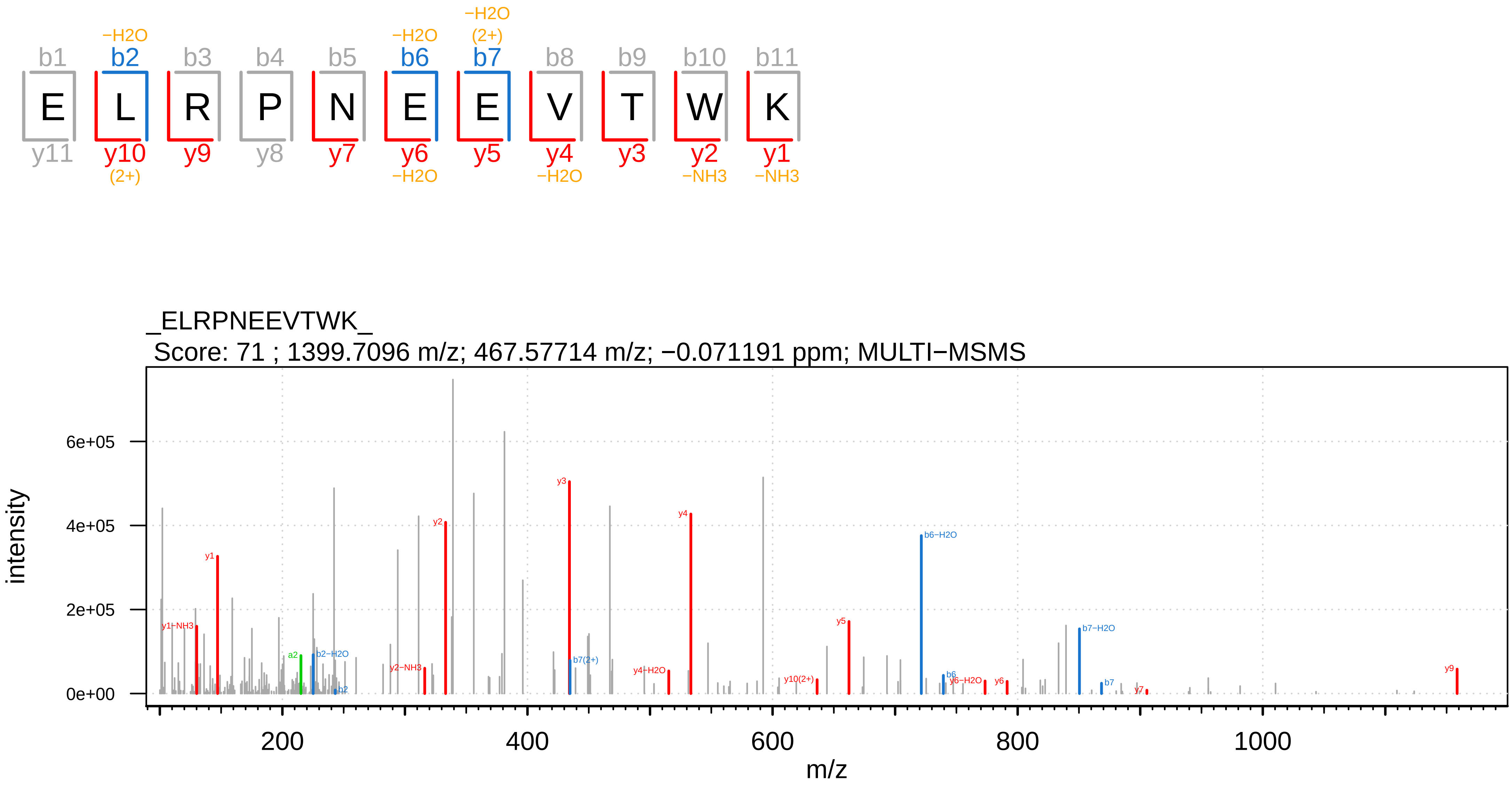

Raw File: 20140816_QEp4_BaAz_SA_DII_R1
Scan Number: 45152
Proteins:
chr12_103206313−103214181.T2_chr12:101968174−101969629:−

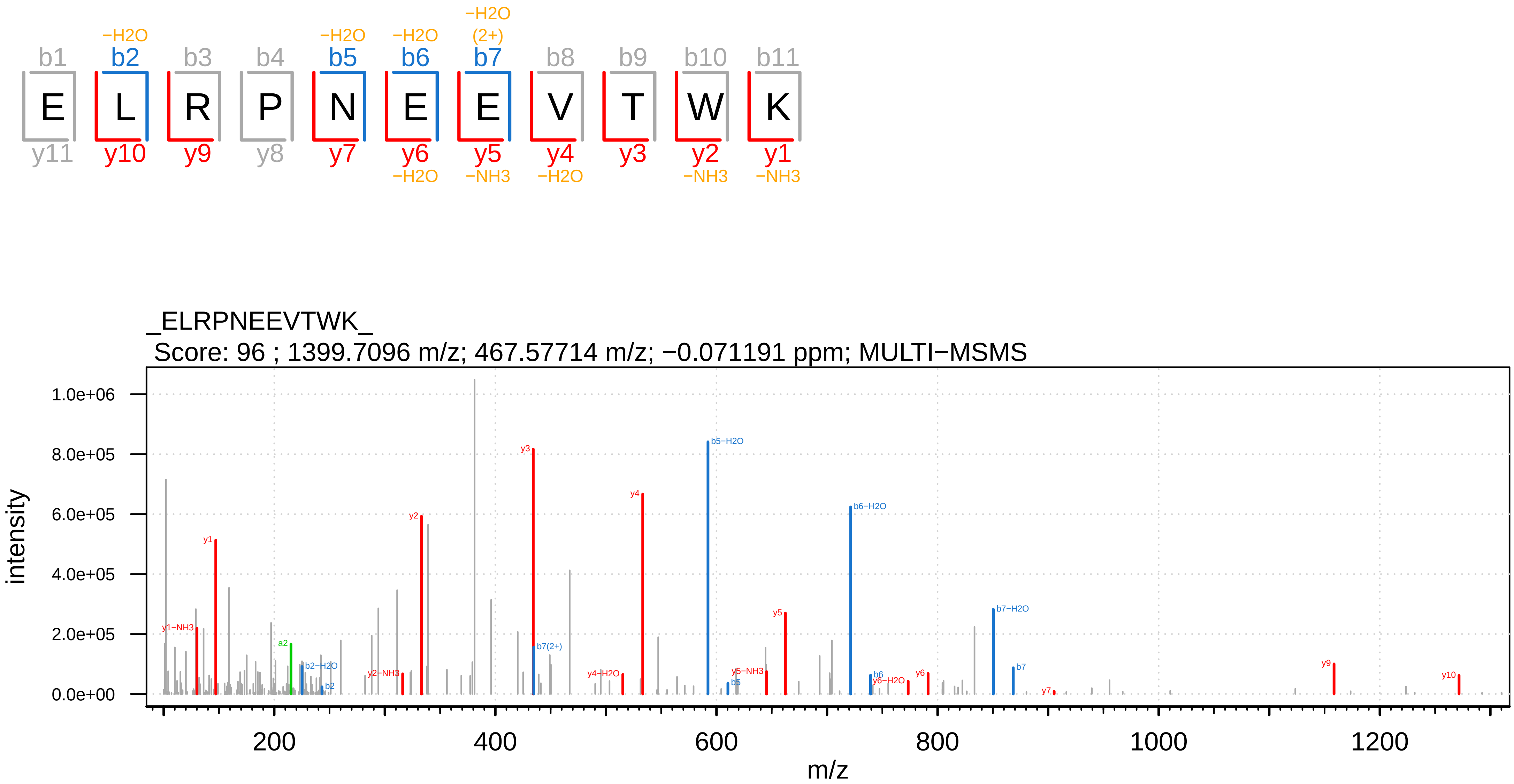

Raw File: 20140816_QEp4_BaAz_SA_DII_R1
Scan Number: 45167
Proteins:
chr12_103206313−103214181.T2_chr12:101968174−101969629:−

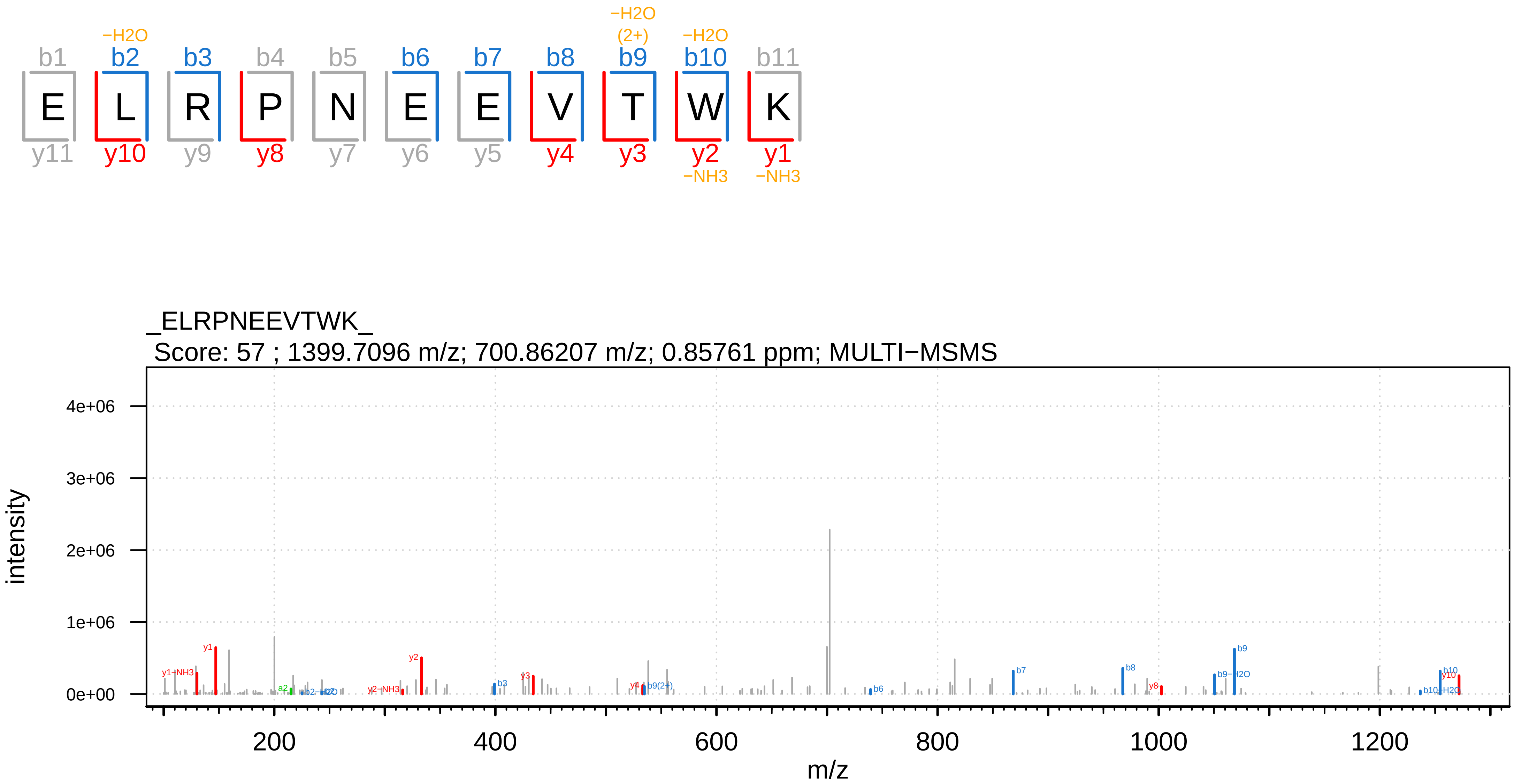

Raw File: 20140816_QEp4_BaAz_SA_DII_R1

Scan Number: 45444

Proteins:

chr12_103206313−103214181.T2_chr12:101968174−101969629:−

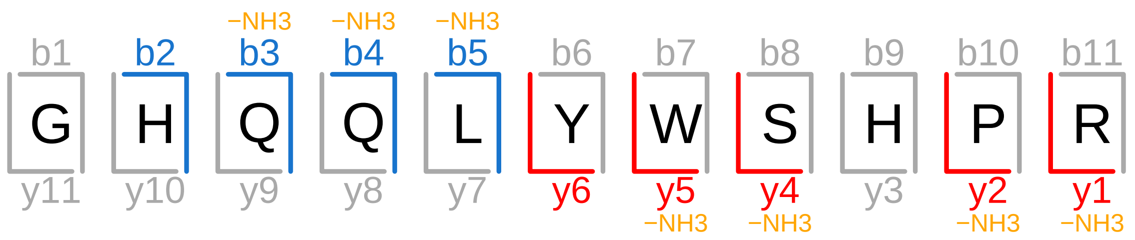

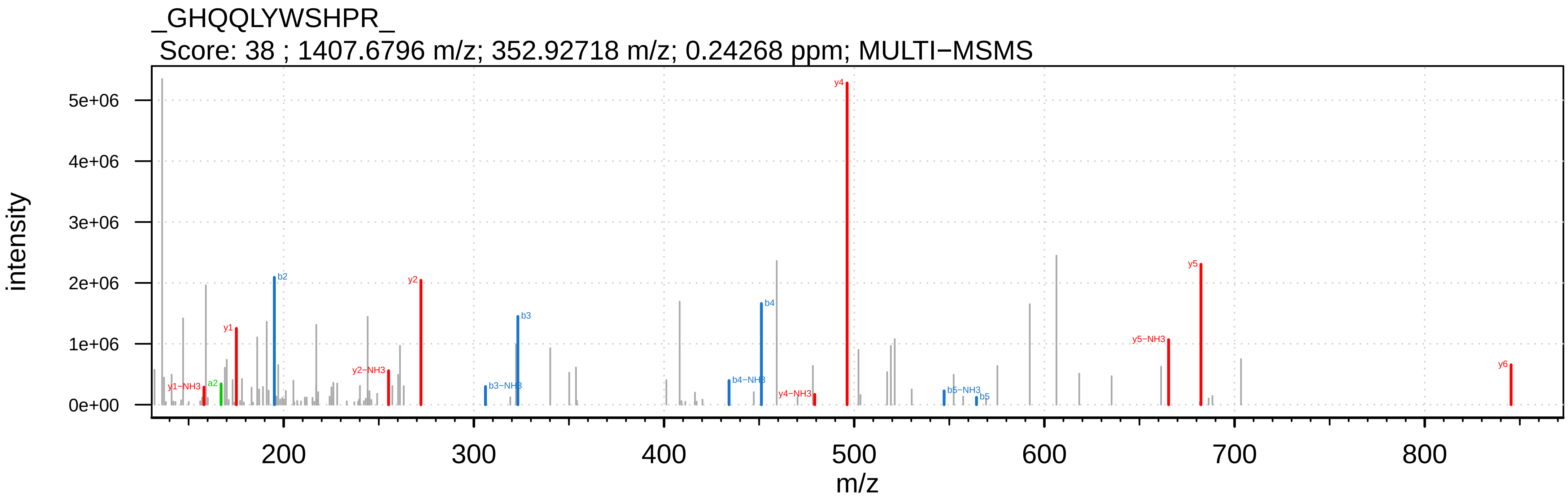

Raw File: 20140816_QEp4_BaAz_SA_DII_R1
Scan Number: 22582
Proteins:
sp|P62274|RS29_MOUSE
ENSMUST00000037023_chr12:69157813−69159058:−
ENSMUST00000071737_chr13:73988963−73989133:−

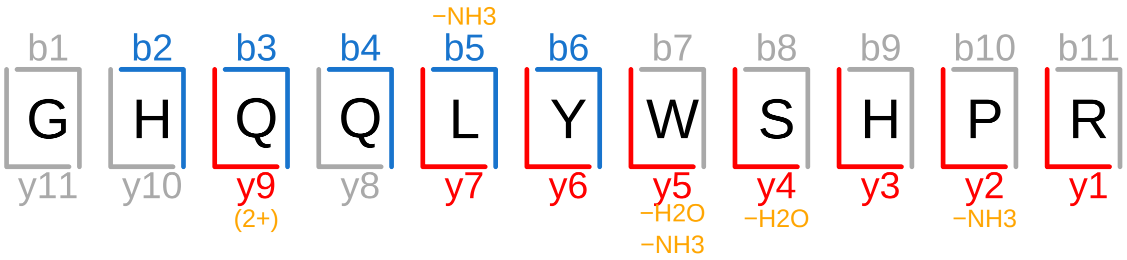

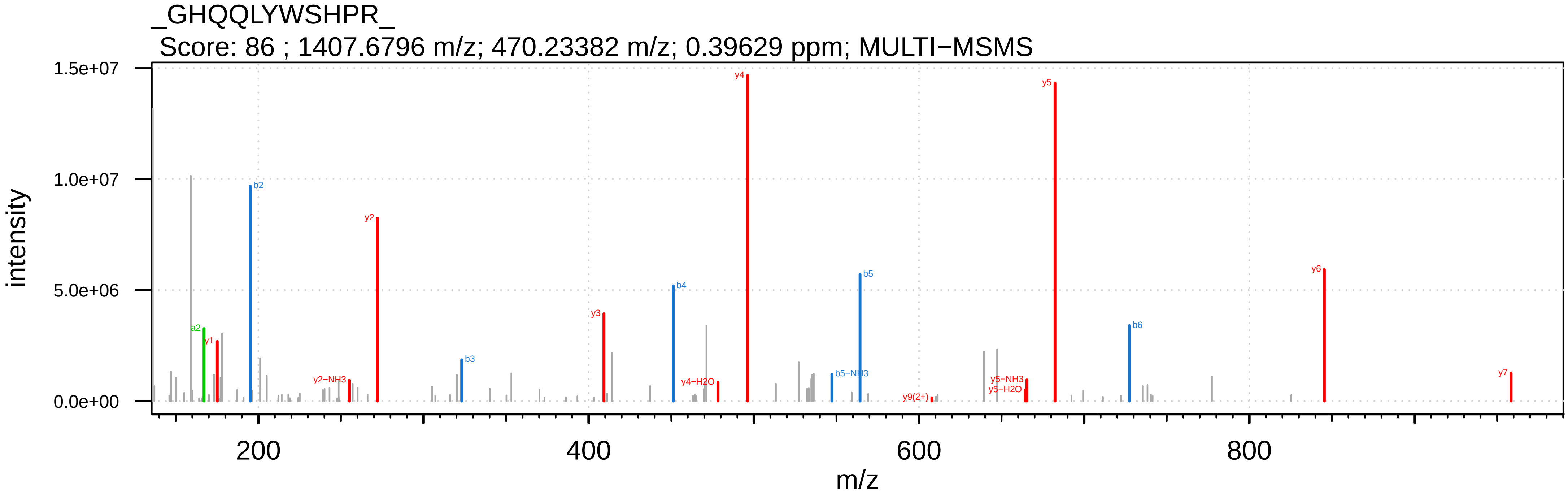

Raw File: 20140816_QEp4_BaAz_SA_DII_R1
Scan Number: 22619
Proteins:
sp|P62274|RS29_MOUSE
ENSMUST00000037023_chr12:69157813−69159058:−
ENSMUST00000071737_chr13:73988963−73989133:−

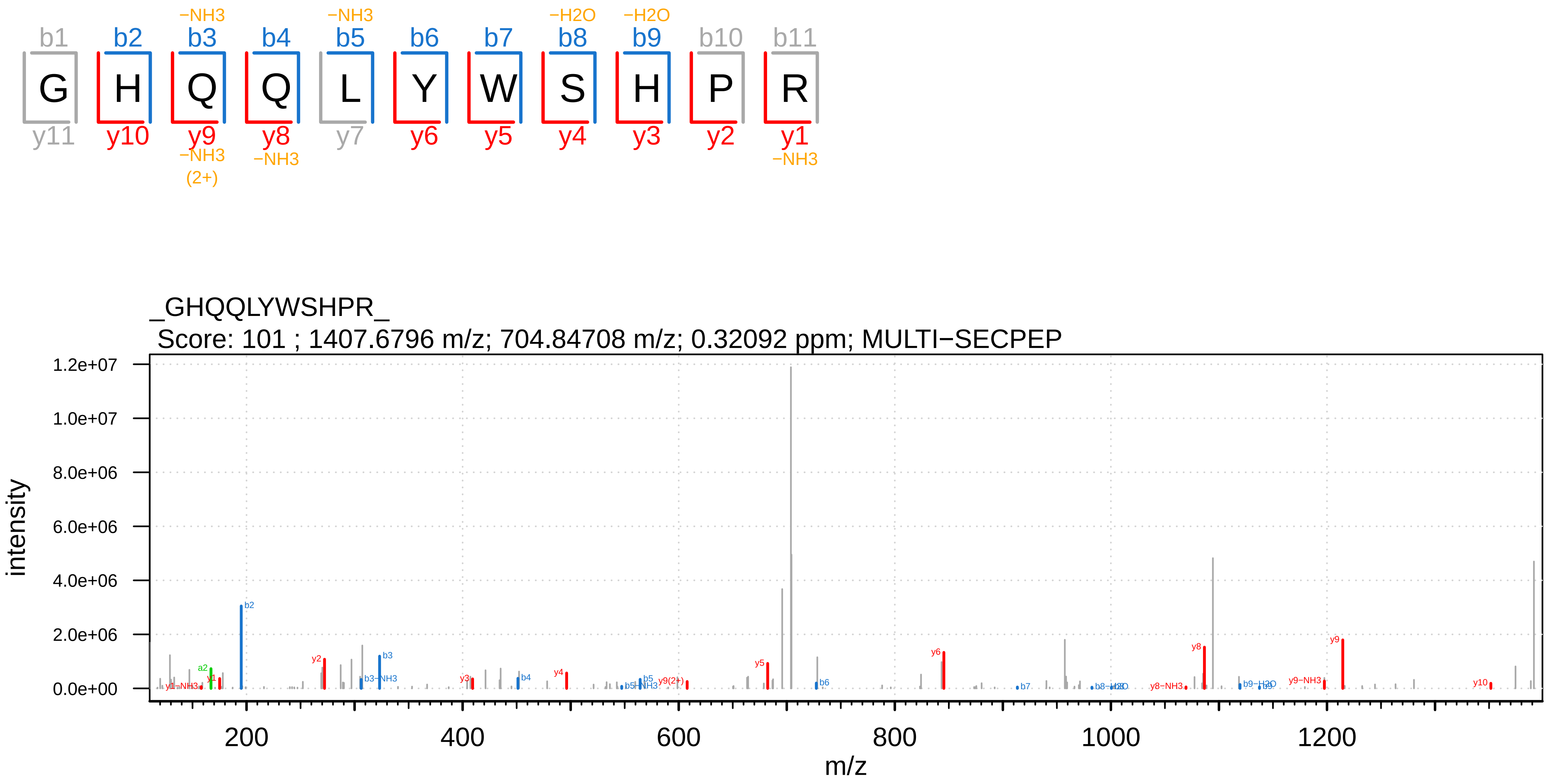

Raw File: 20140816_QEp4_BaAz_SA_DII_R1
Scan Number: 22666
Proteins:
sp|P62274|RS29_MOUSE
ENSMUST00000037023_chr12:69157813−69159058:−
ENSMUST00000071737_chr13:73988963−73989133:−

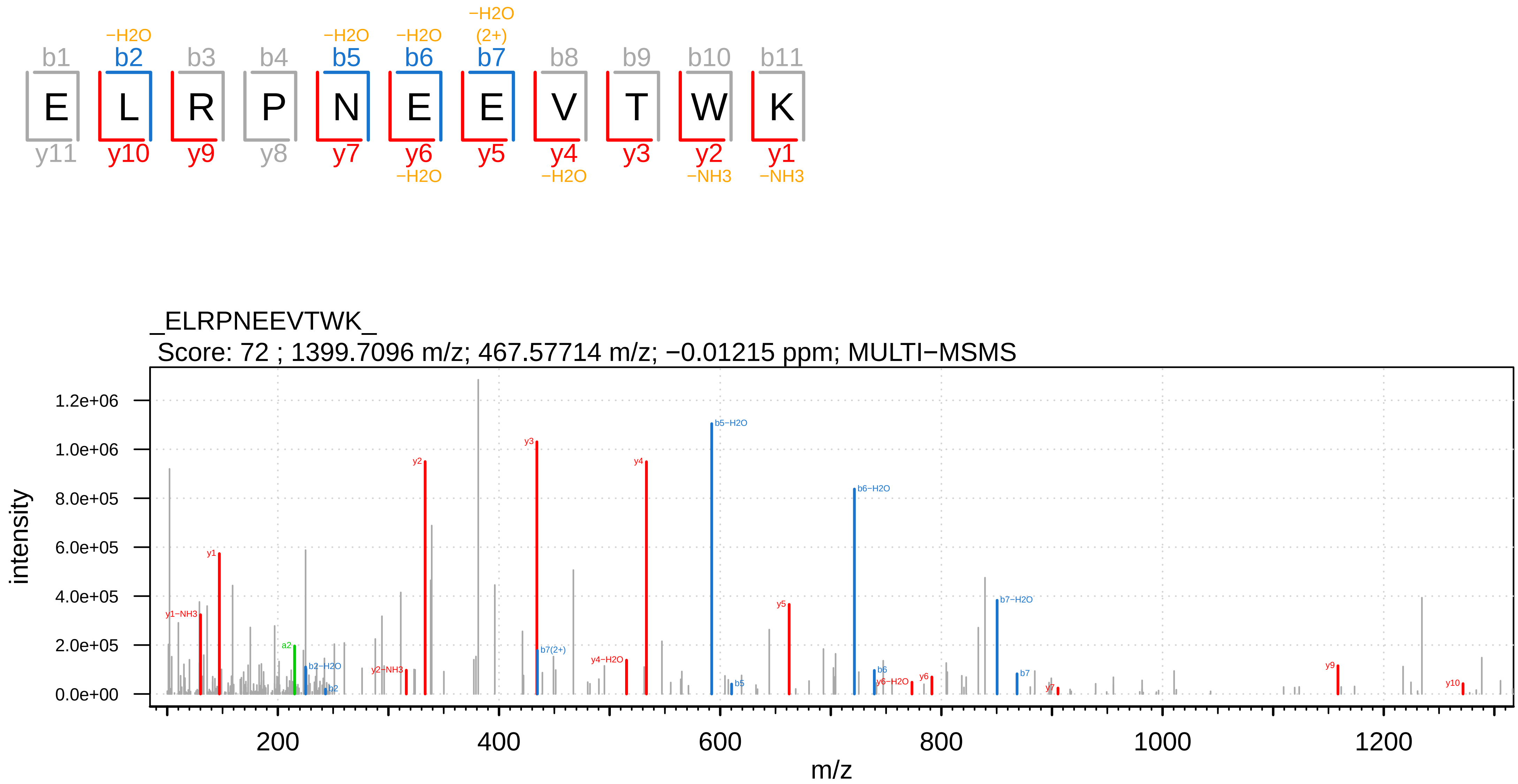

Raw File: 20140816_QEp4_BaAz_SA_DII_R2

Scan Number: 45756

Proteins:

chr12_103206313−103214181.T2_chr12:101968174−101969629:−

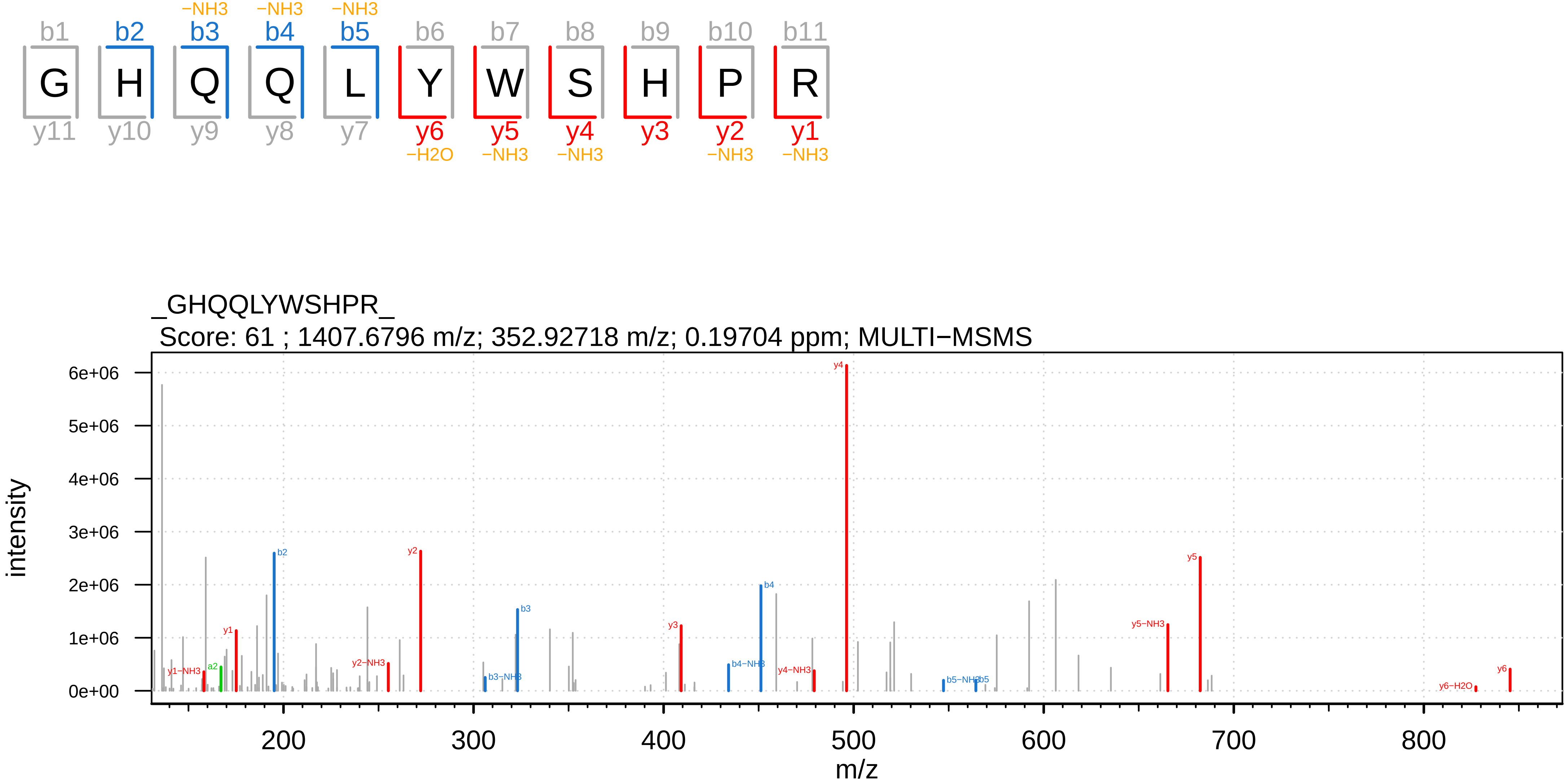

Raw File: 20140816_QEp4_BaAz_SA_DII_R2
Scan Number: 22690
Proteins:
sp|P62274|RS29_MOUSE
ENSMUST00000037023_chr12:69157813−69159058:−
ENSMUST00000071737_chr13:73988963−73989133:−

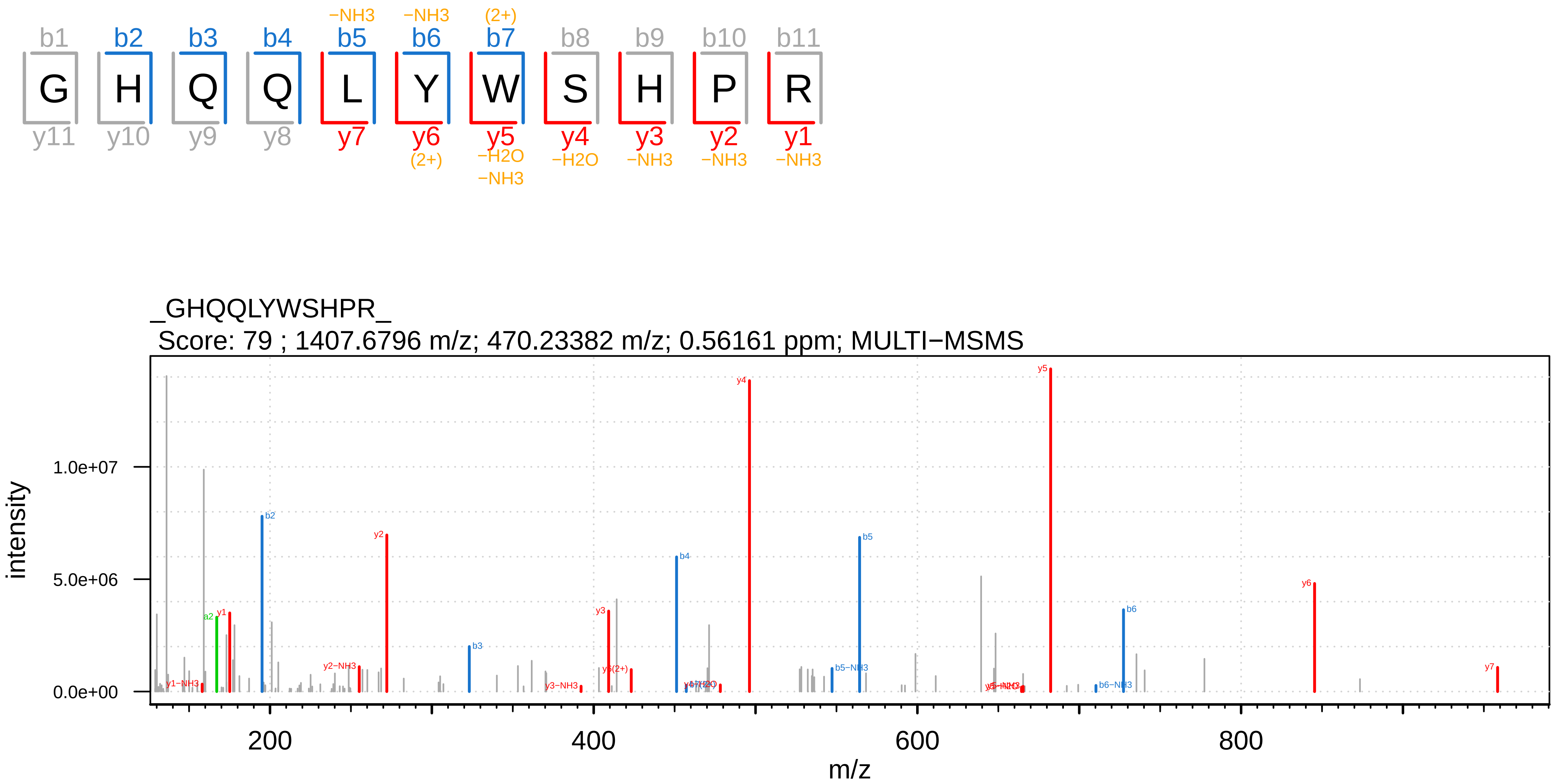

Raw File: 20140816_QEp4_BaAz_SA_DII_R2
Scan Number: 22735
Proteins:
sp|P62274|RS29_MOUSE
ENSMUST00000037023_chr12:69157813−69159058:−
ENSMUST00000071737_chr13:73988963−73989133:−

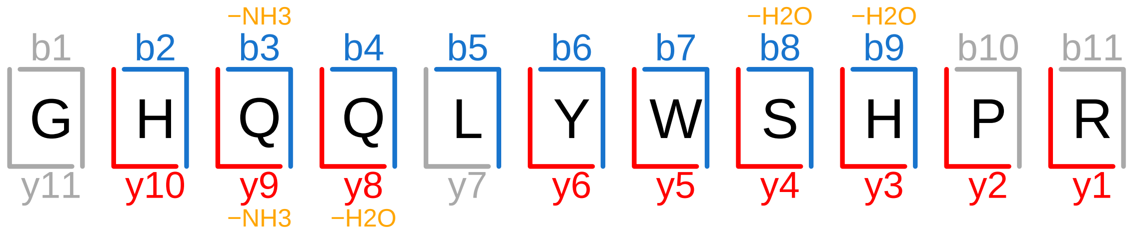

GHQQLYWSHPR

Score: 79 ; 1407.6796 m/z; 704.84708 m/z; −0.11211 ppm; MULTI−SECPEP

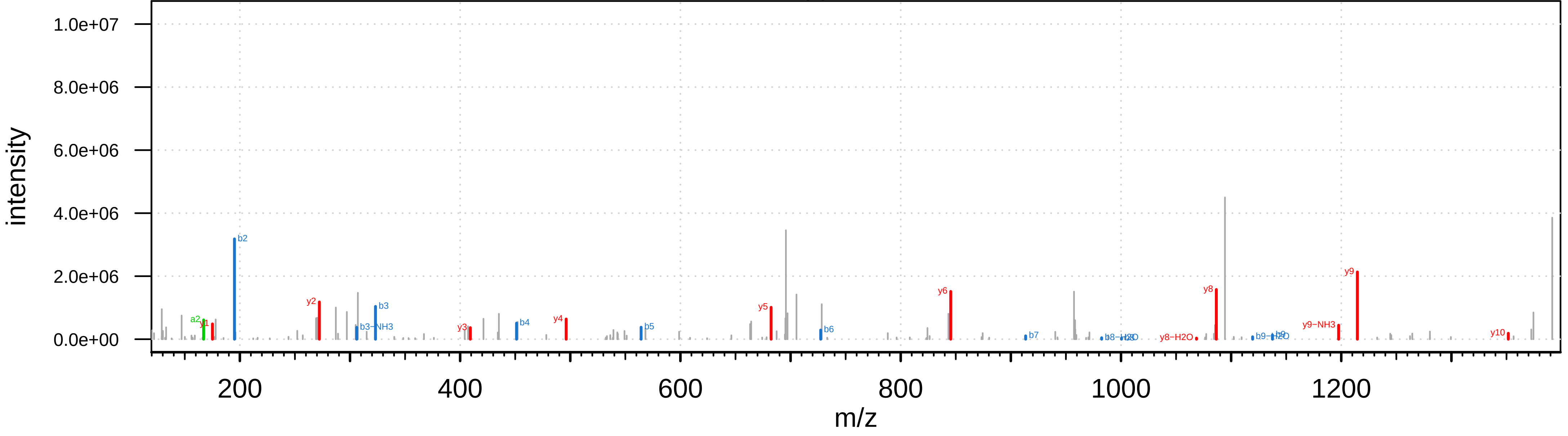

Raw File: 20140816_QEp4_BaAz_SA_DII_R2

Scan Number: 22770

Proteins:

sp|P62274|RS29_MOUSE

ENSMUST00000037023_chr12:69157813−69159058:−

ENSMUST00000071737_chr13:73988963−73989133:−

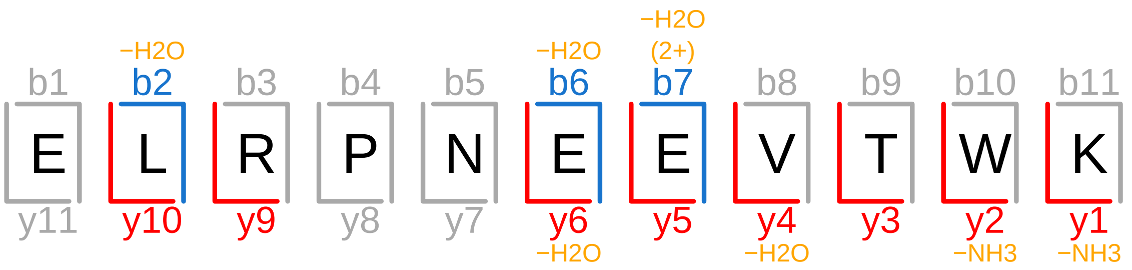

ELRPNEEVTWK
Score: 51 ; 1399.7096 m/z; 467.57714 m/z; −0.099928 ppm; MULTI−MSMS

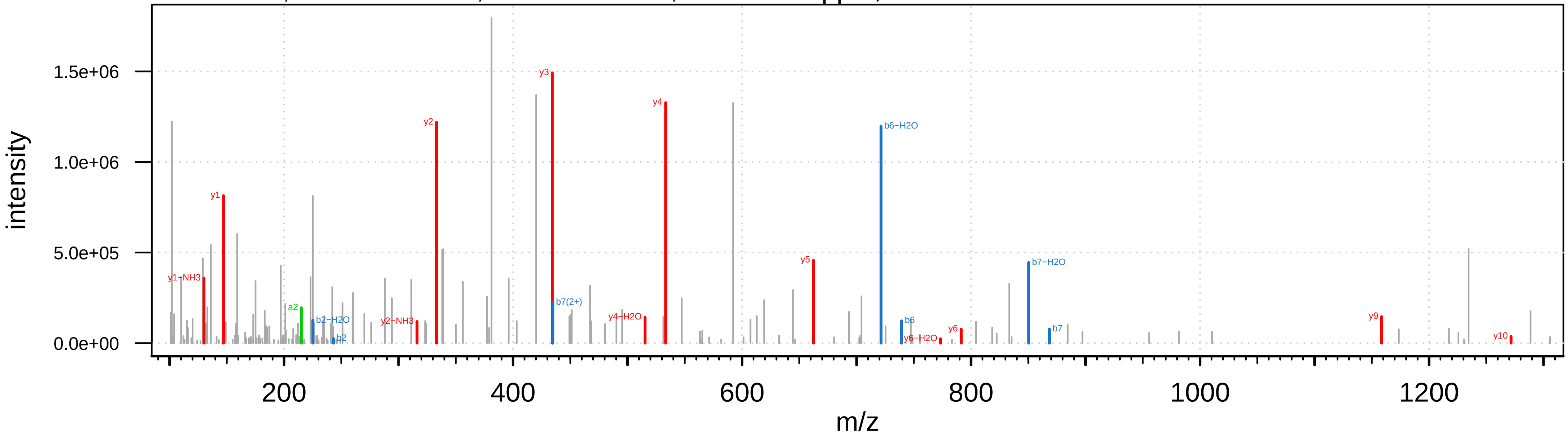

Raw File: 20140816_QEp4_BaAz_SA_DII_R3
Scan Number: 45050
Proteins:
chr12_103206313−103214181.T2_chr12:101968174−101969629:−

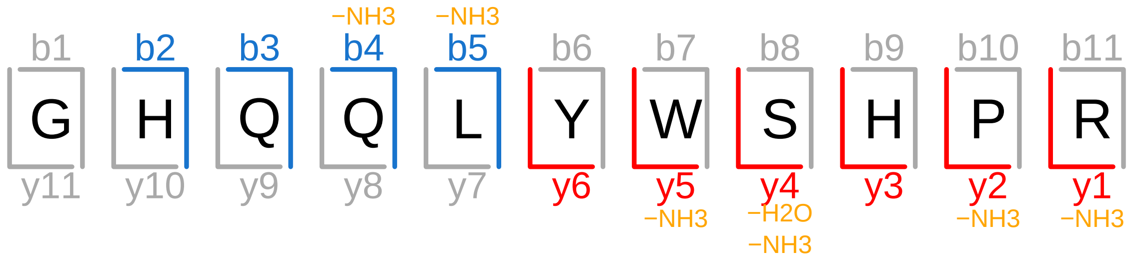

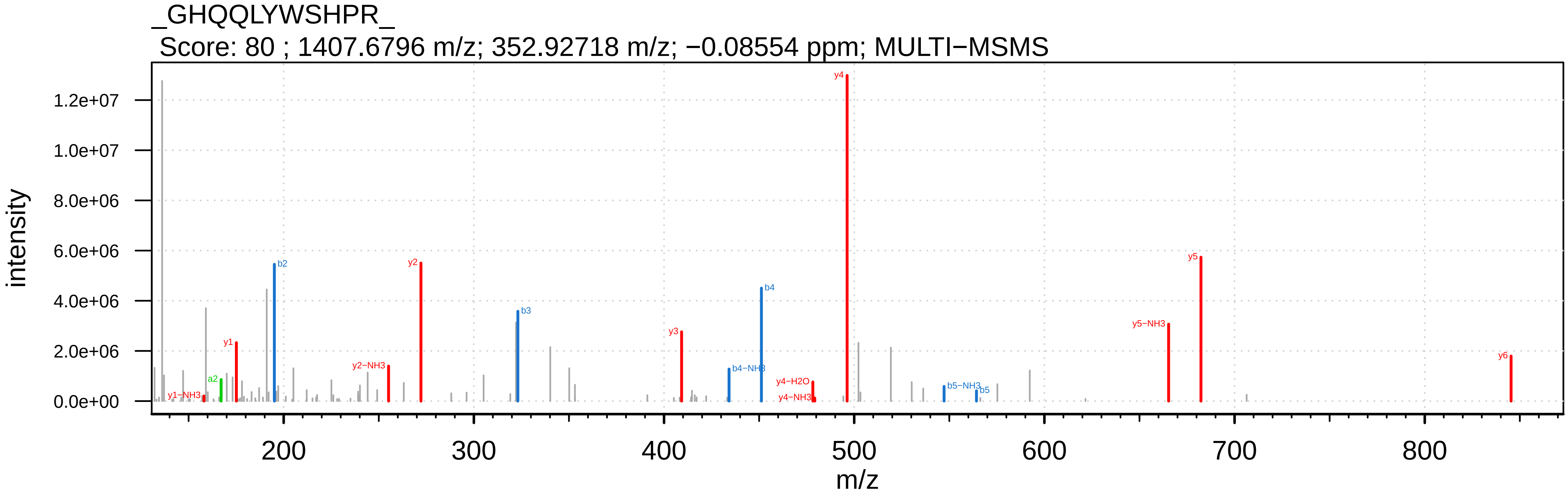

Raw File: 20140816_QEp4_BaAz_SA_DII_R3
Scan Number: 21971
Proteins:
sp|P62274|RS29_MOUSE
ENSMUST00000037023_chr12:69157813−69159058:−
ENSMUST00000071737_chr13:73988963−73989133:−

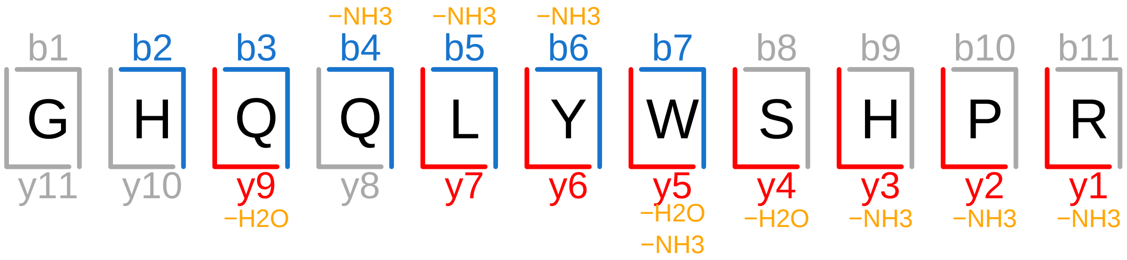

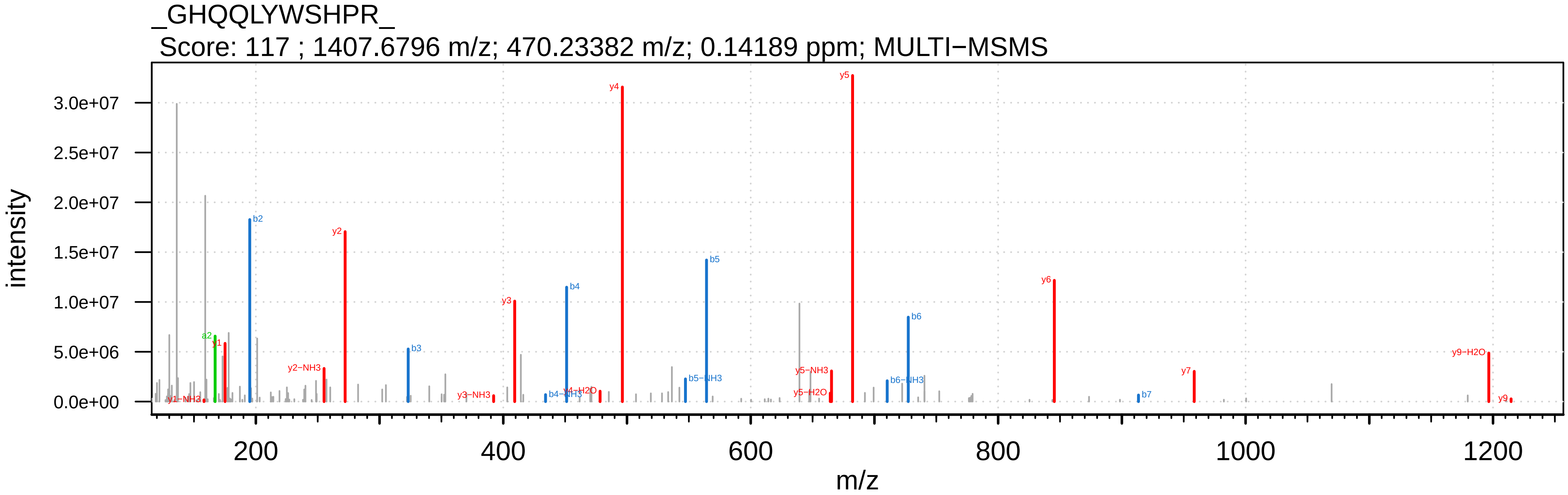

Raw File: 20140816_QEp4_BaAz_SA_DII_R3
Scan Number: 21986
Proteins:
sp|P62274|RS29_MOUSE
ENSMUST00000037023_chr12:69157813−69159058:−
ENSMUST00000071737_chr13:73988963−73989133:−

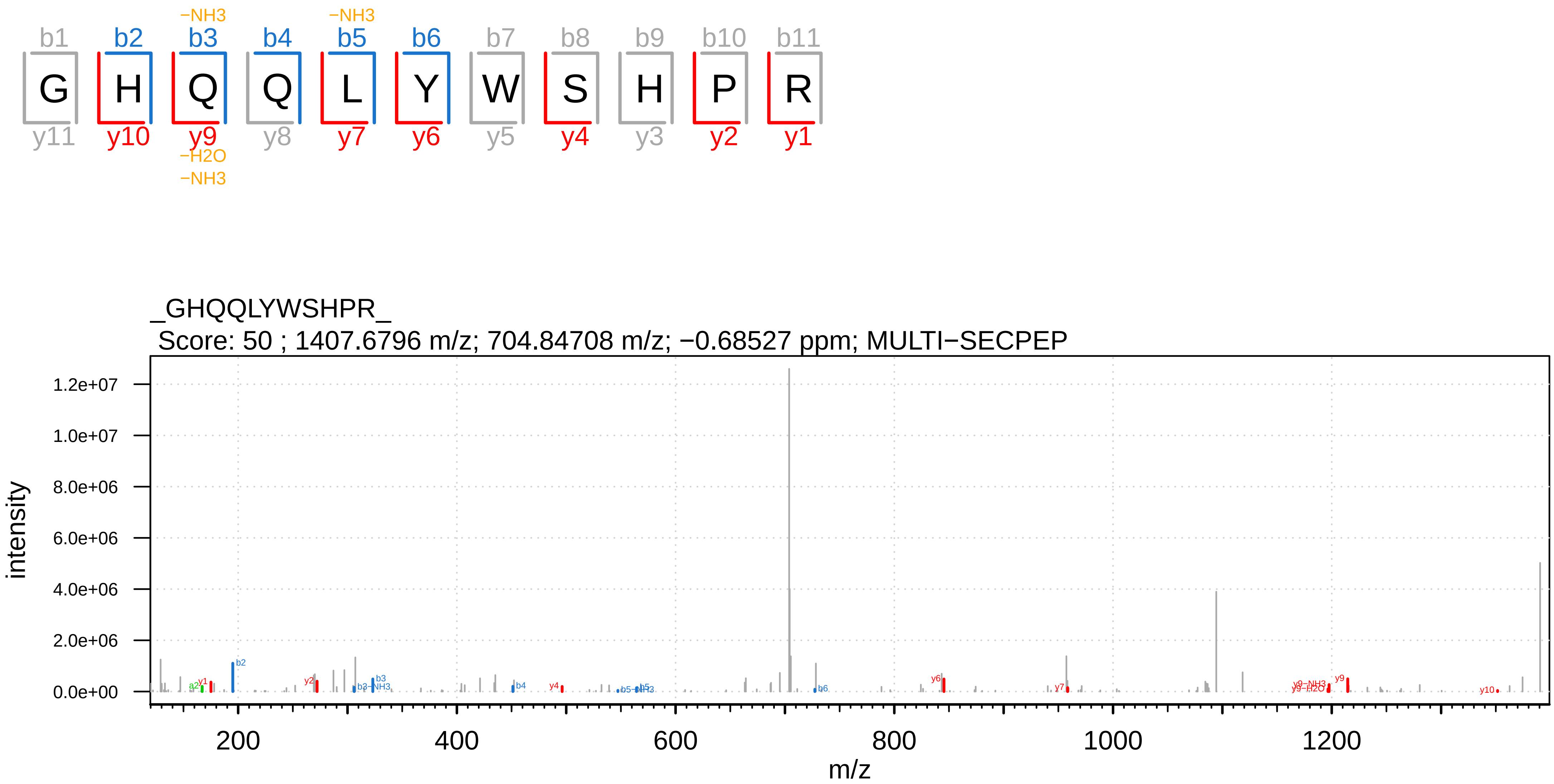

Raw File: 20140816_QEp4_BaAz_SA_DII_R3
Scan Number: 22026
Proteins:
sp|P62274|RS29_MOUSE
ENSMUST00000037023_chr12:69157813−69159058:−
ENSMUST00000071737_chr13:73988963−73989133:−

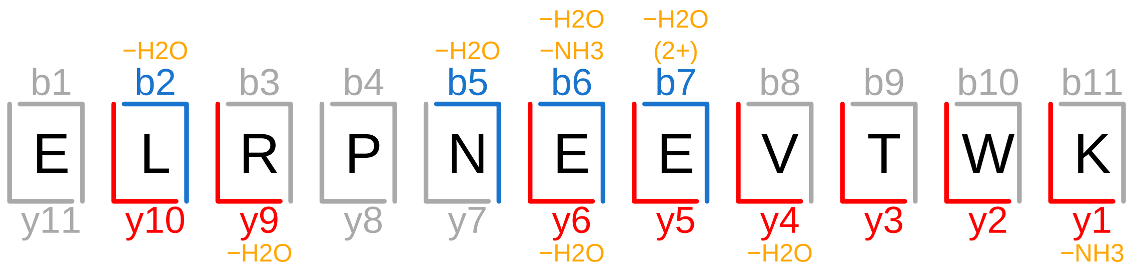

ELRPNEEVTWK
Score: 62 ; 1399.7096 m/z; 467.57714 m/z; 0.2944 ppm; MULTI−MSMS

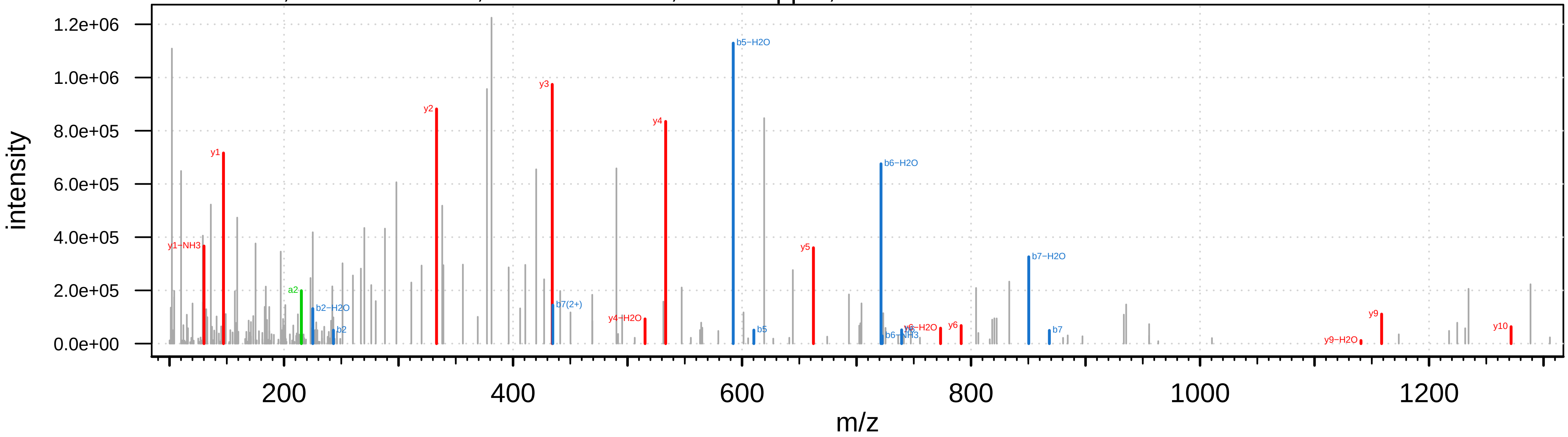

Raw File: 20140816_QEp4_BaAz_SA_DIII_R2
Scan Number: 44607
Proteins:
chr12_103206313−103214181.T2_chr12:101968174−101969629:−

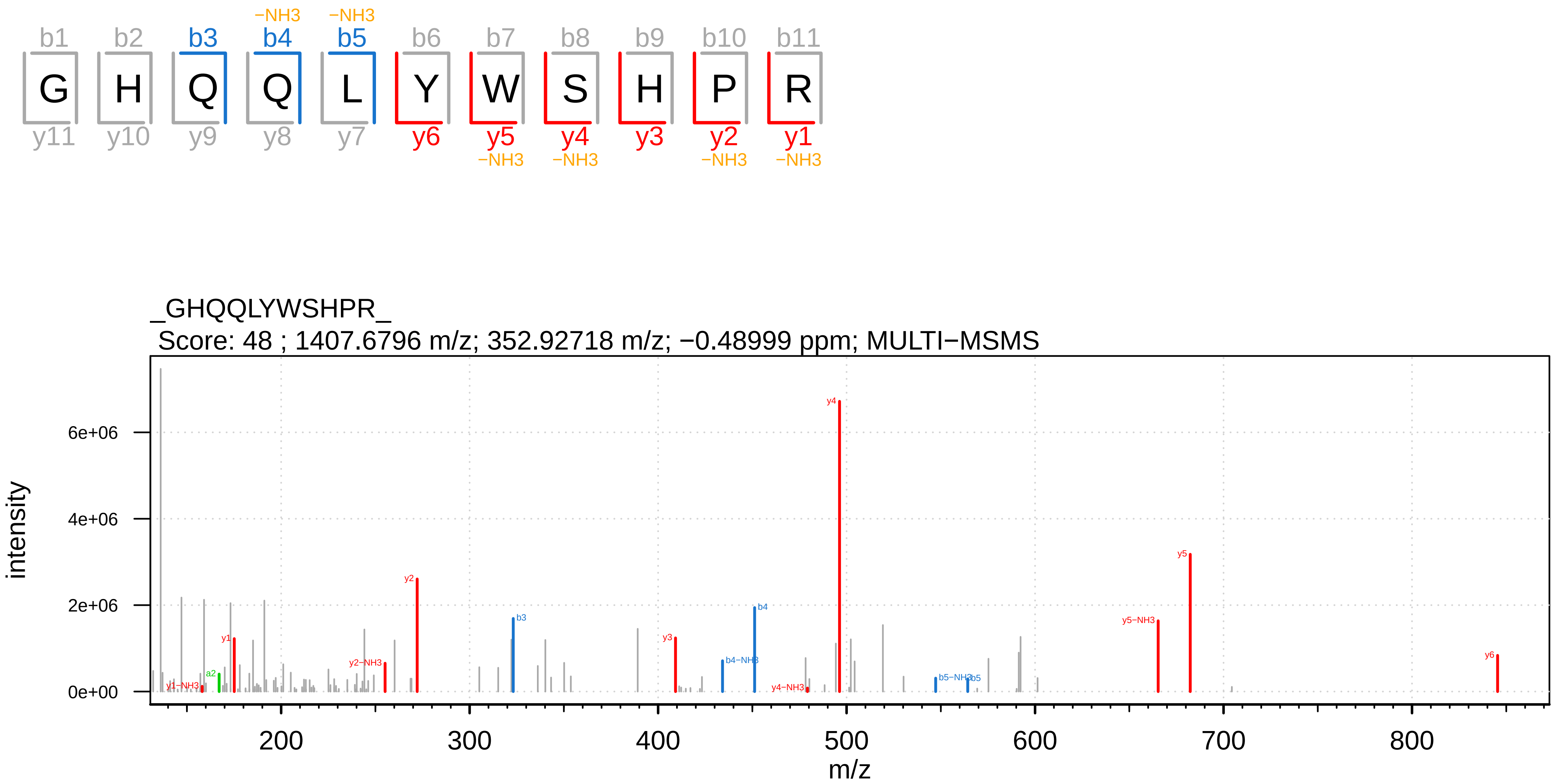

Raw File: 20140816_QEp4_BaAz_SA_DIII_R2
Scan Number: 21269
Proteins:
sp|P62274|RS29_MOUSE
ENSMUST00000037023_chr12:69157813−69159058:−
ENSMUST00000071737_chr13:73988963−73989133:−

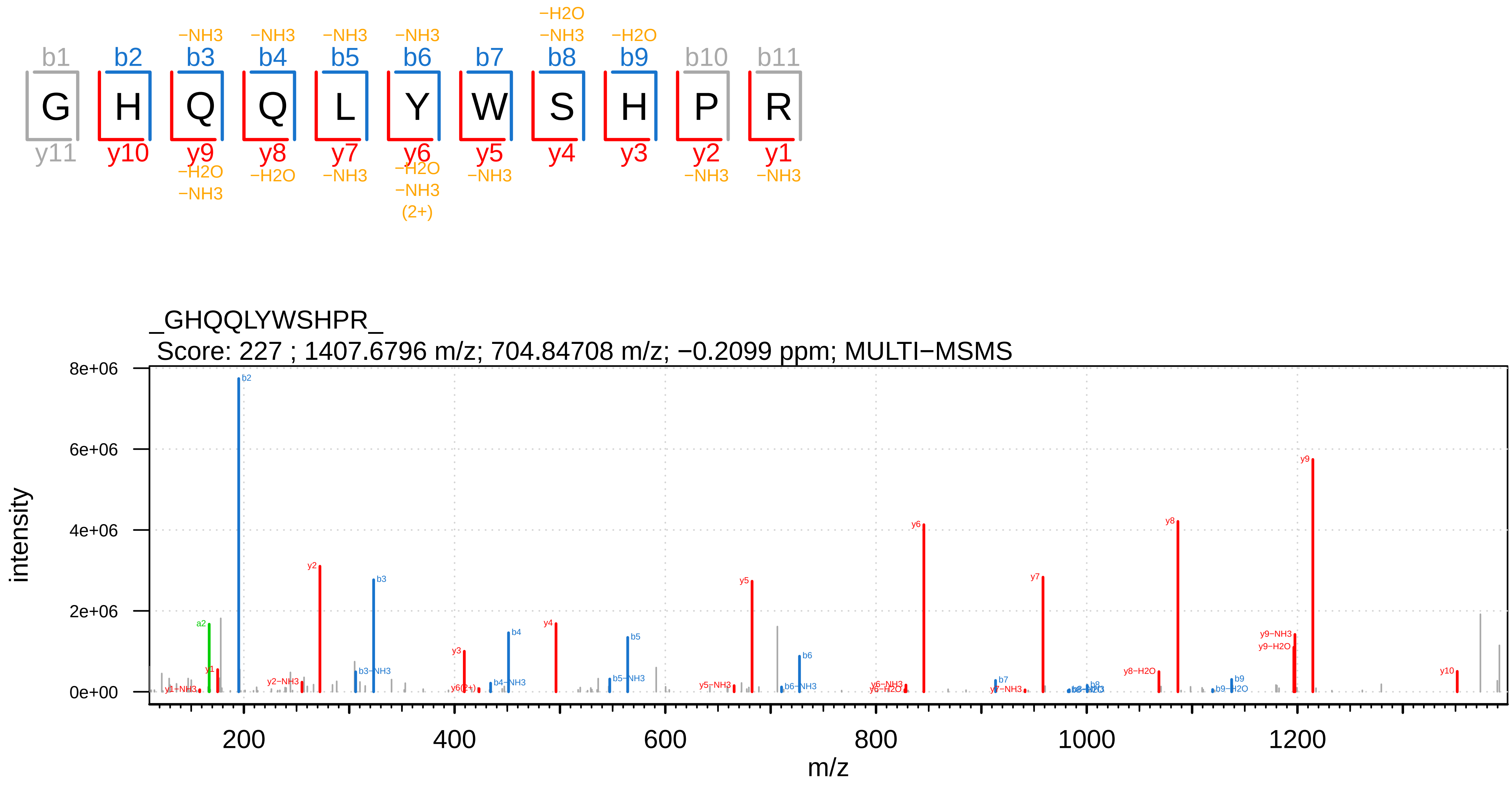

Raw File: 20140816_QEp4_BaAz_SA_DIII_R2
Scan Number: 21295
Proteins:
sp|P62274|RS29_MOUSE
ENSMUST00000037023_chr12:69157813−69159058:−
ENSMUST00000071737_chr13:73988963−73989133:−

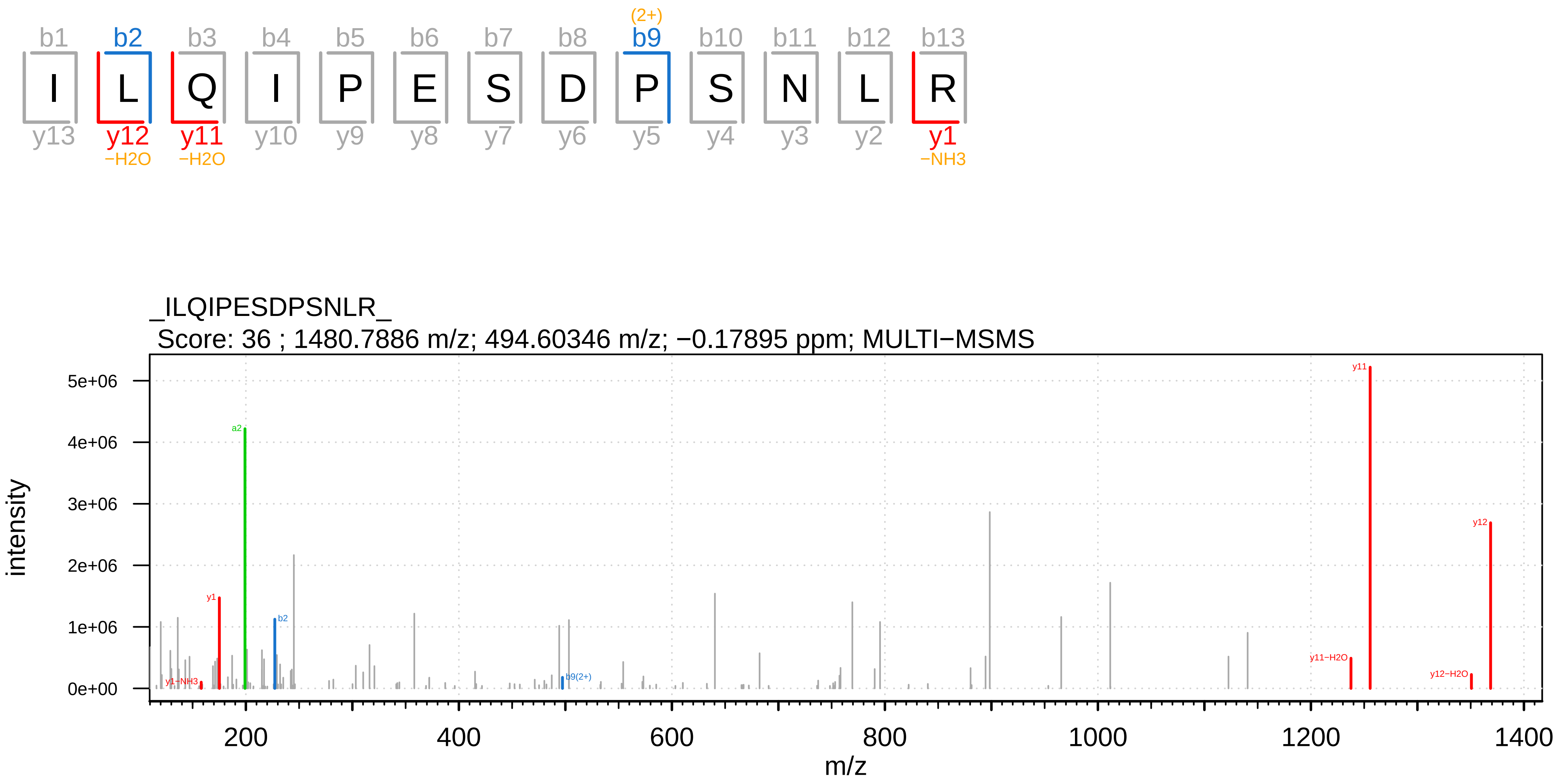

Raw File: 20140816_QEp4_BaAz_SA_DIII_R2
Scan Number: 45929
Proteins:
ENSMUST00000161069_chr14:70554856−70554960:+

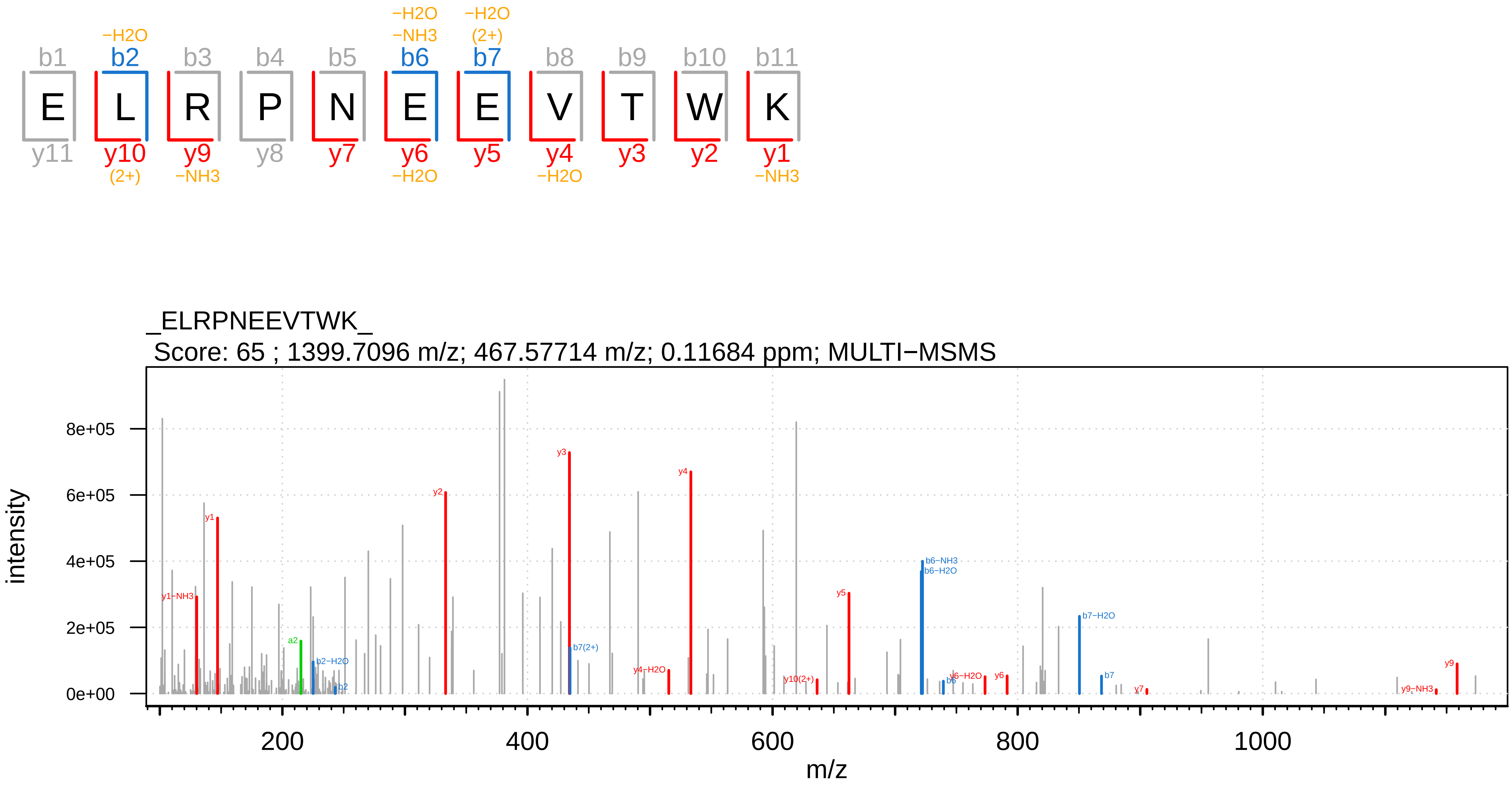

Raw File: 20140816_QEp4_BaAz_SA_DIII_R3
Scan Number: 45556
Proteins:
chr12_103206313−103214181.T2_chr12:101968174−101969629:−

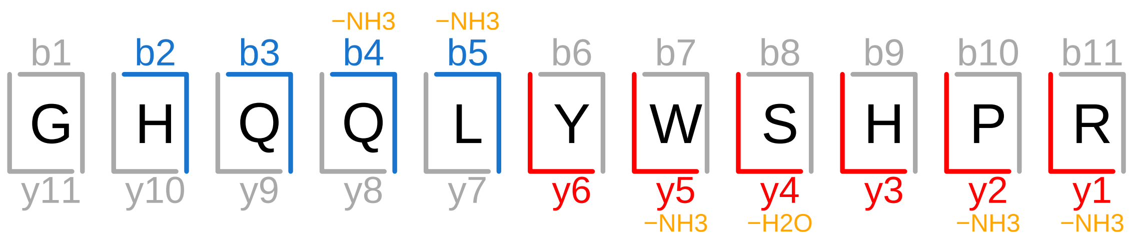

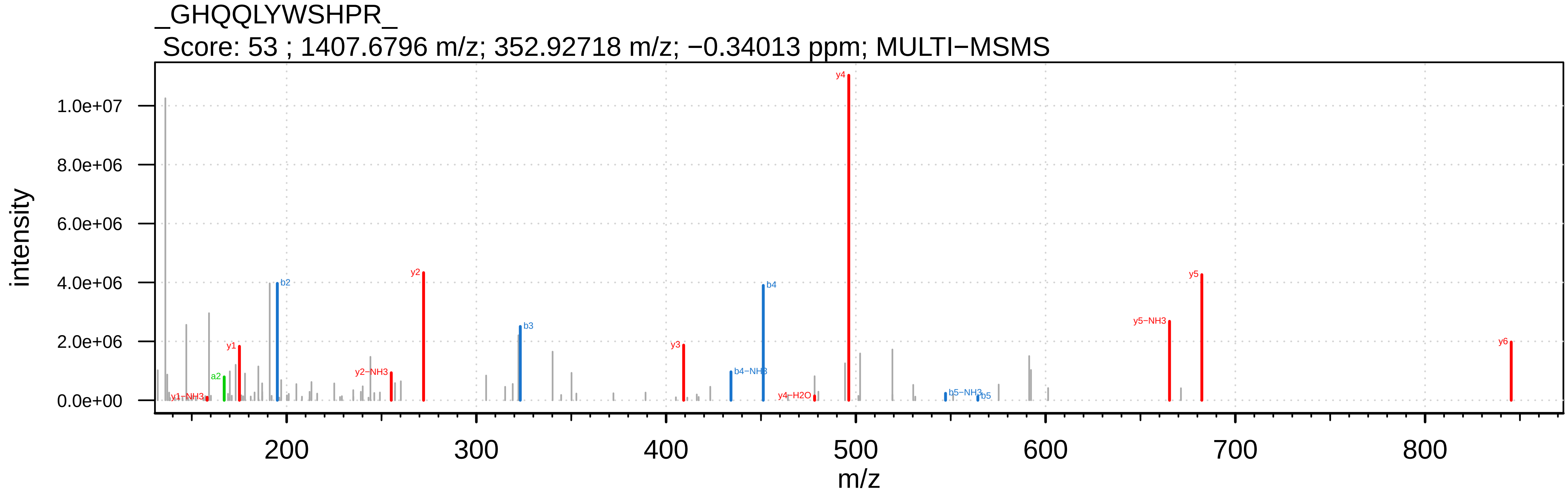

Raw File: 20140816_QEp4_BaAz_SA_DIII_R3
Scan Number: 22113
Proteins:
sp|P62274|RS29_MOUSE
ENSMUST00000037023_chr12:69157813−69159058:−
ENSMUST00000071737_chr13:73988963−73989133:−

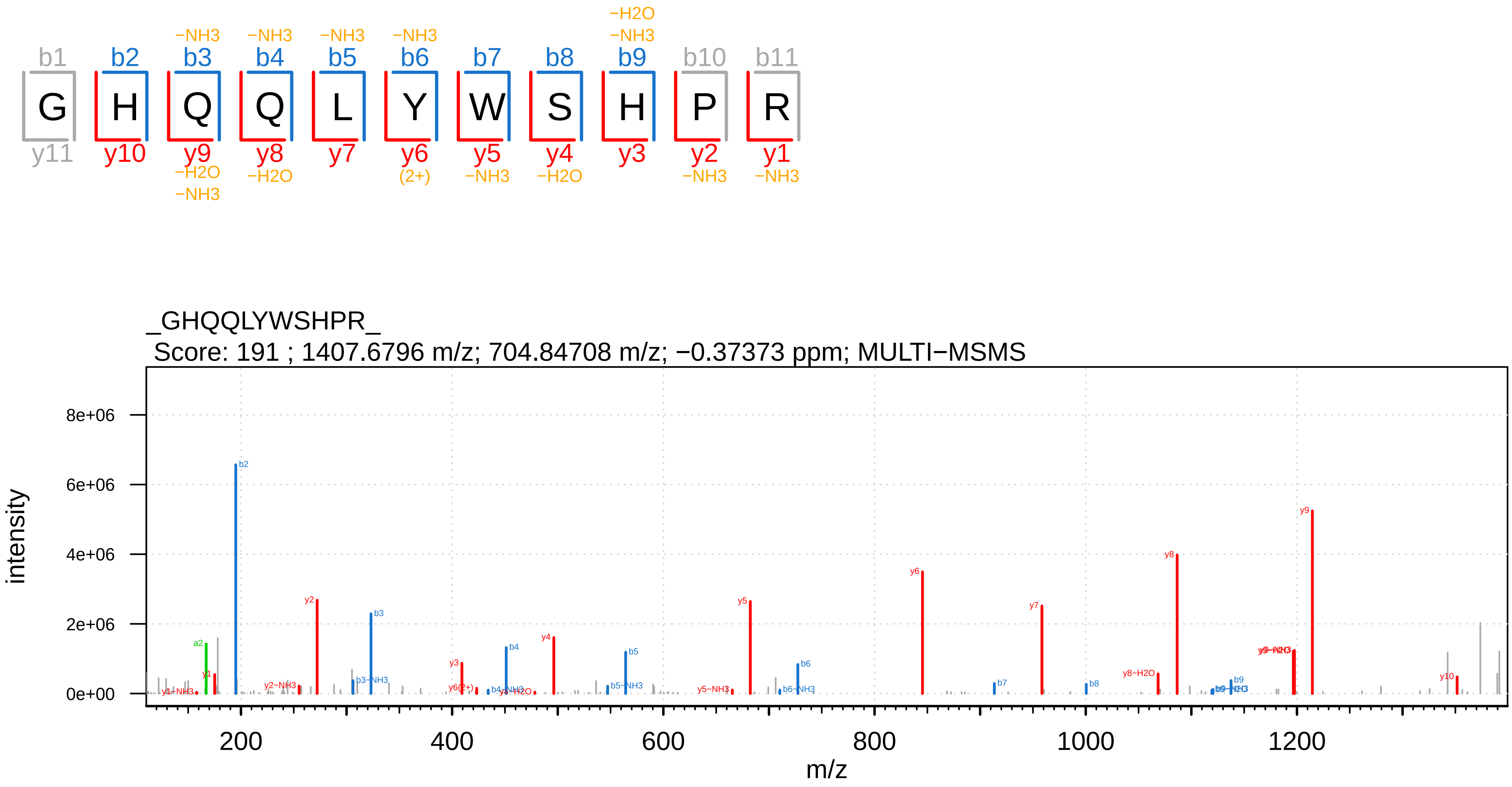

Raw File: 20140816_QEp4_BaAz_SA_DIII_R3
Scan Number: 22119
Proteins:
sp|P62274|RS29_MOUSE
ENSMUST00000037023_chr12:69157813−69159058:−
ENSMUST00000071737_chr13:73988963−73989133:−

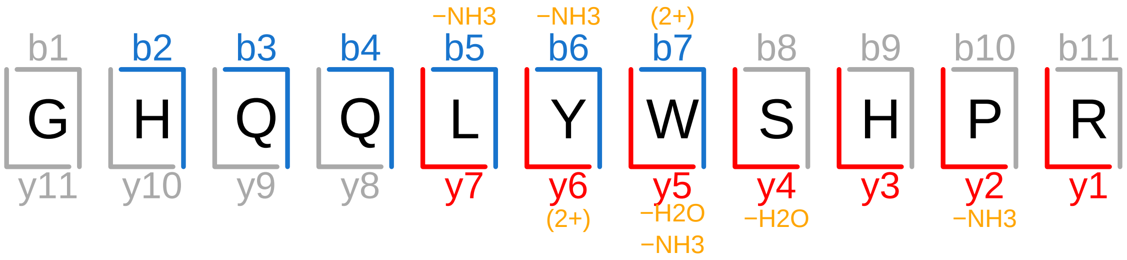

GHQQLYWSHPR
Score: 93 ; 1407.6796 m/z; 470.23382 m/z; 0.47639 ppm; MULTI−MSMS

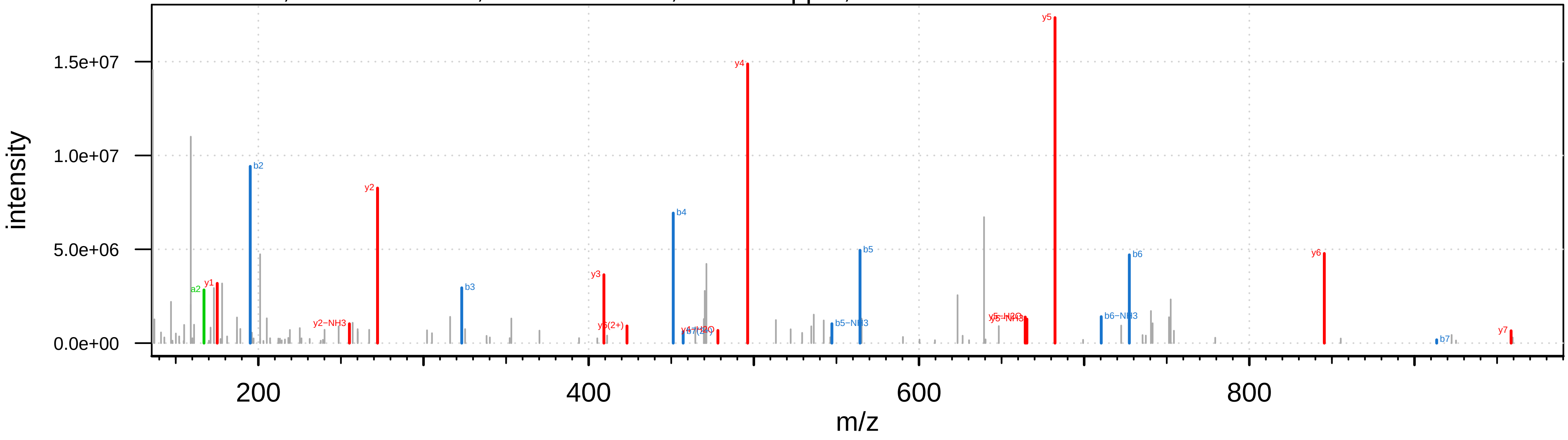

Raw File: 20140816_QEp4_BaAz_SA_DIII_R3
Scan Number: 22139
Proteins:
sp|P62274|RS29_MOUSE
ENSMUST00000037023_chr12:69157813−69159058:−
ENSMUST00000071737_chr13:73988963−73989133:−

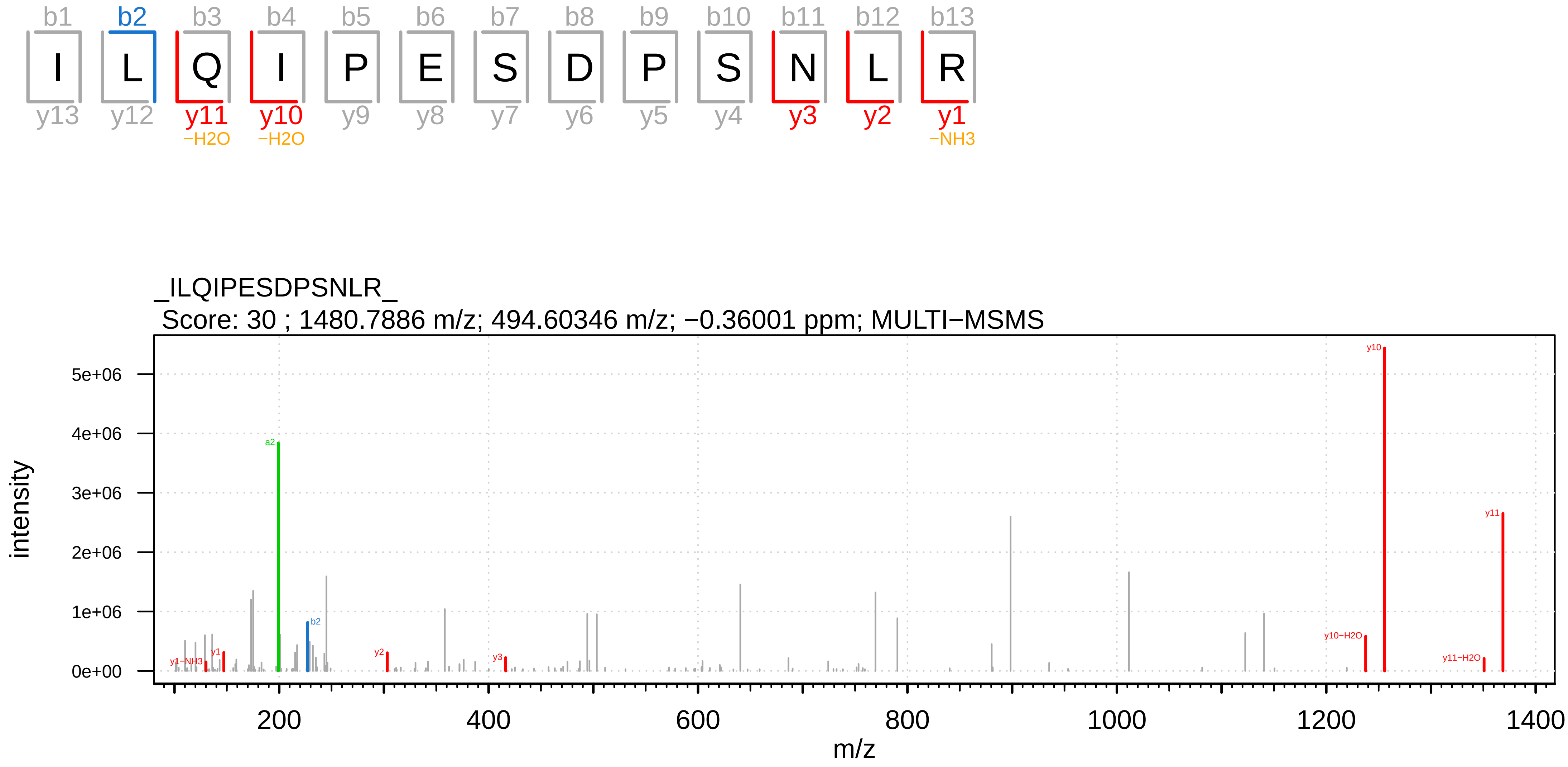

Raw File: 20140816_QEp4_BaAz_SA_DIII_R3
Scan Number: 46942
Proteins:
ENSMUST00000161069_chr14:70554856−70554960:+

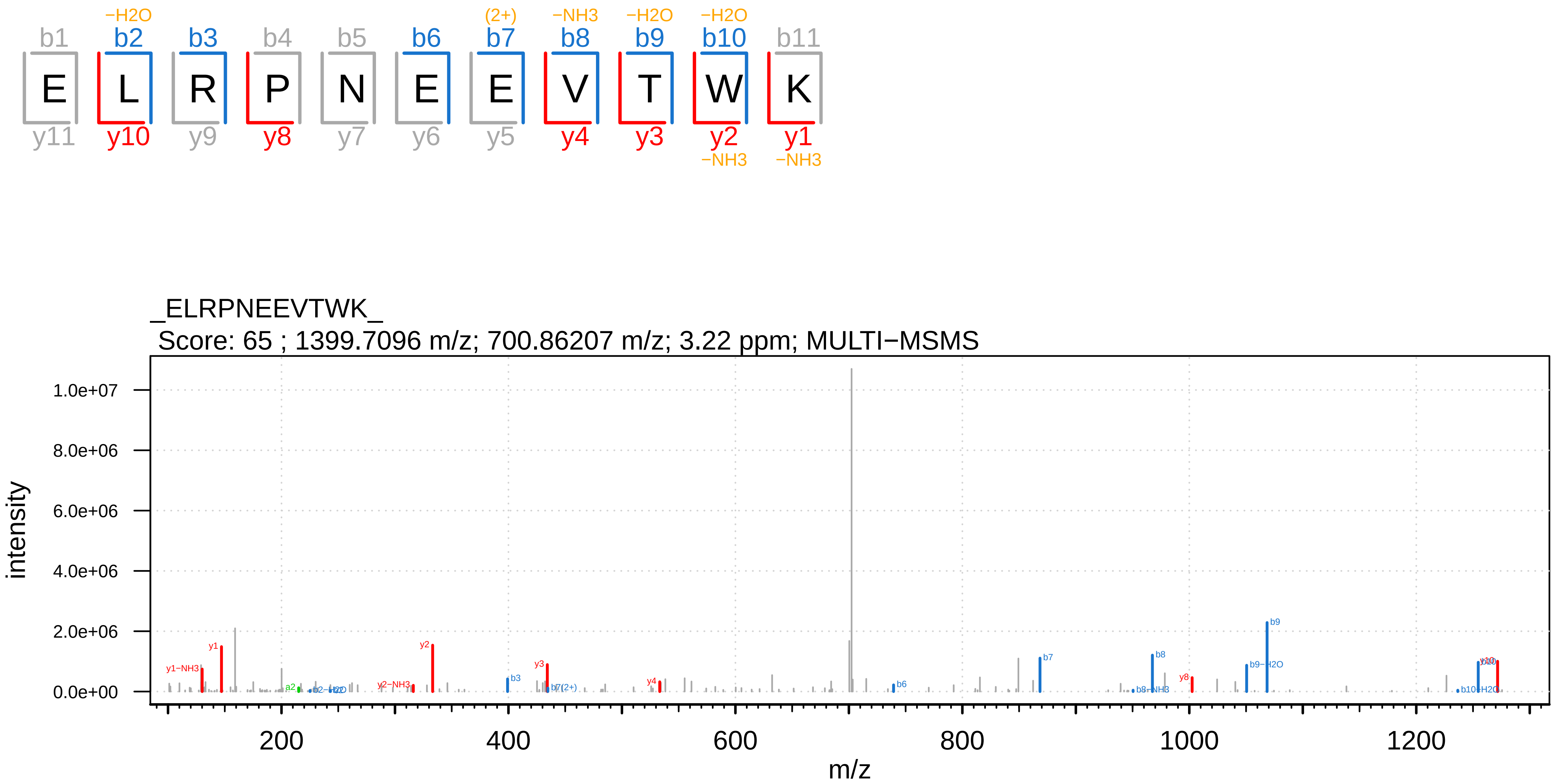

Raw File: 20140819_QEp4_BaAz_SA_H16_R1
Scan Number: 46554
Proteins:
chr12_103206313−103214181.T2_chr12:101968174−101969629:−

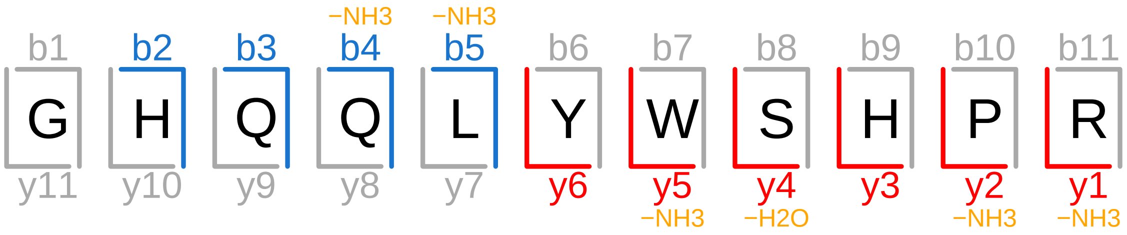

GHQQLYWSHPR

Score: 64 ; 1407.6796 m/z; 352.92718 m/z; −0.34934 ppm; MULTI−MSMS

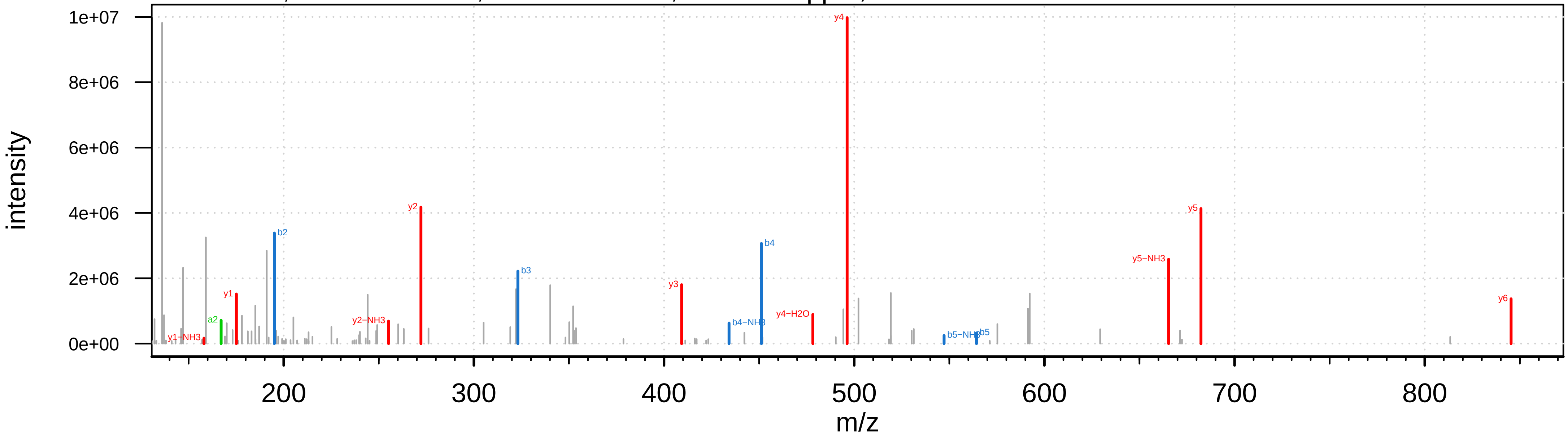

Raw File: 20140819_QEp4_BaAz_SA_H16_R1

Scan Number: 22470

Proteins:

sp|P62274|RS29_MOUSE

ENSMUST00000037023_chr12:69157813−69159058:−

ENSMUST00000071737_chr13:73988963−73989133:−

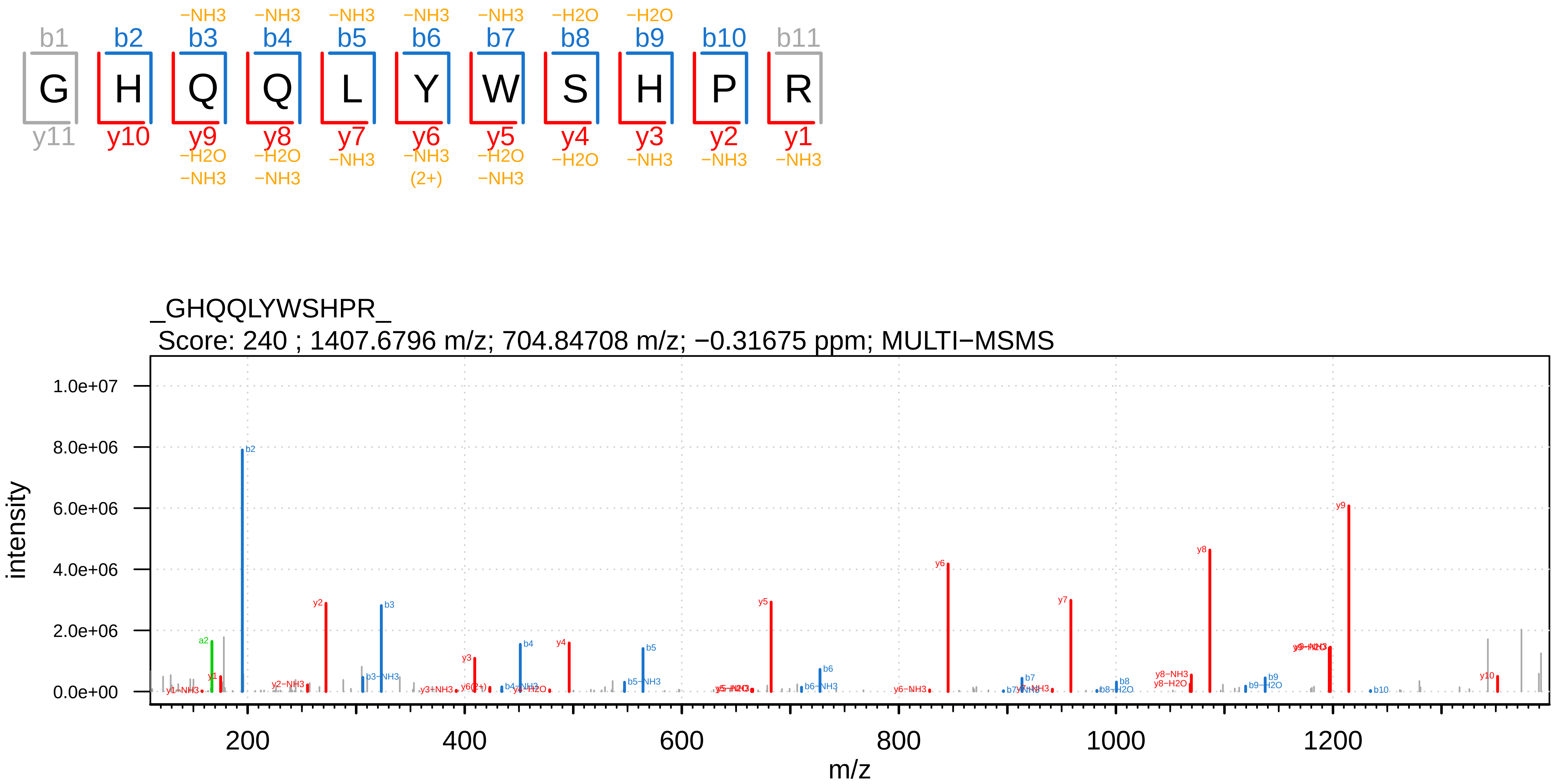

Raw File: 20140819_QEp4_BaAz_SA_H16_R1
Scan Number: 22485
Proteins:
sp|P62274|RS29_MOUSE
ENSMUST00000037023_chr12:69157813−69159058:−
ENSMUST00000071737_chr13:73988963−73989133:−

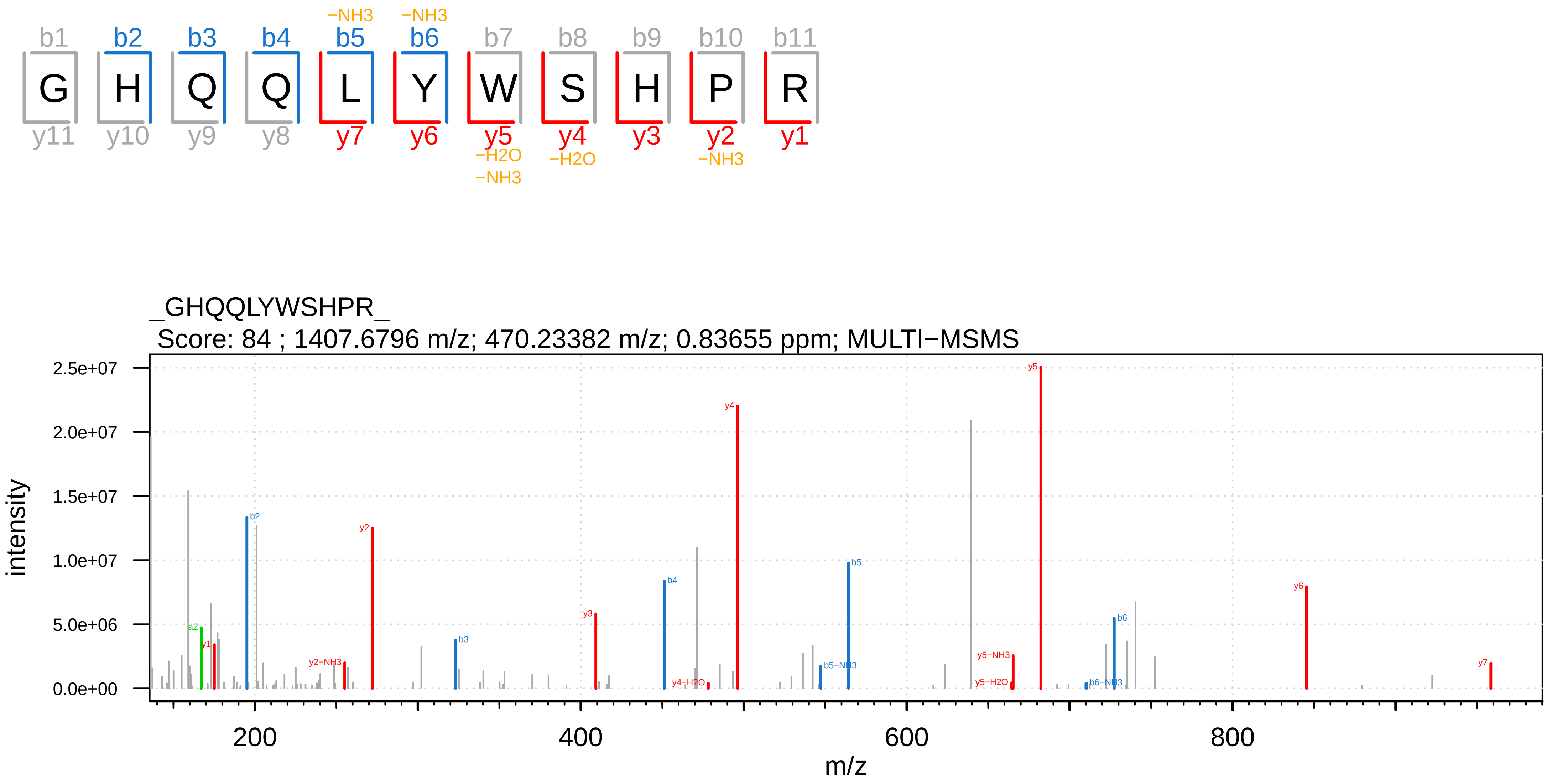

Raw File: 20140819_QEp4_BaAz_SA_H16_R1
Scan Number: 22499
Proteins:
sp|P62274|RS29_MOUSE
ENSMUST00000037023_chr12:69157813−69159058:−
ENSMUST00000071737_chr13:73988963−73989133:−

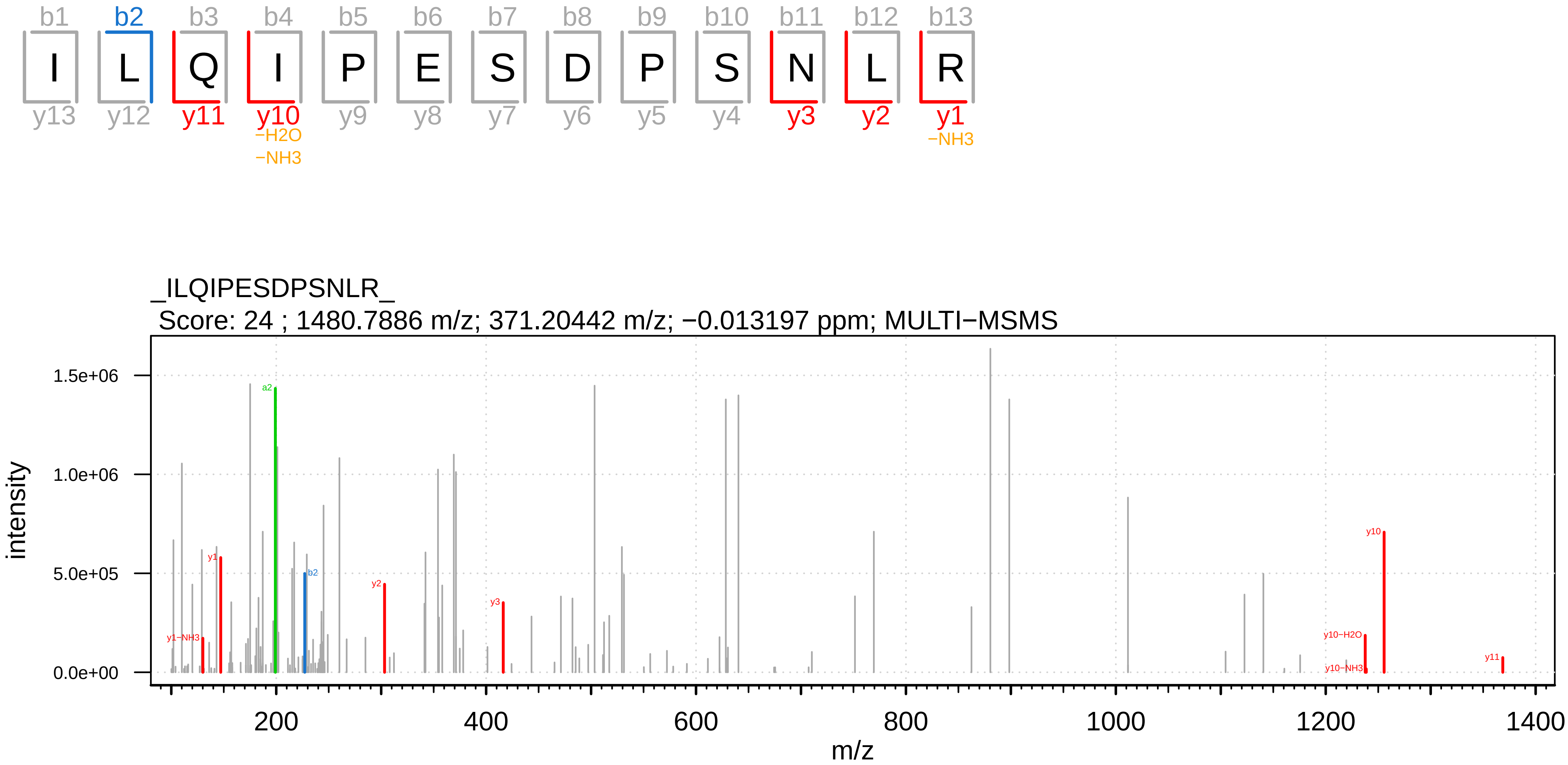

Raw File: 20140819_QEp4_BaAz_SA_H16_R1
Scan Number: 47341
Proteins:
ENSMUST00000161069_chr14:70554856−70554960:+

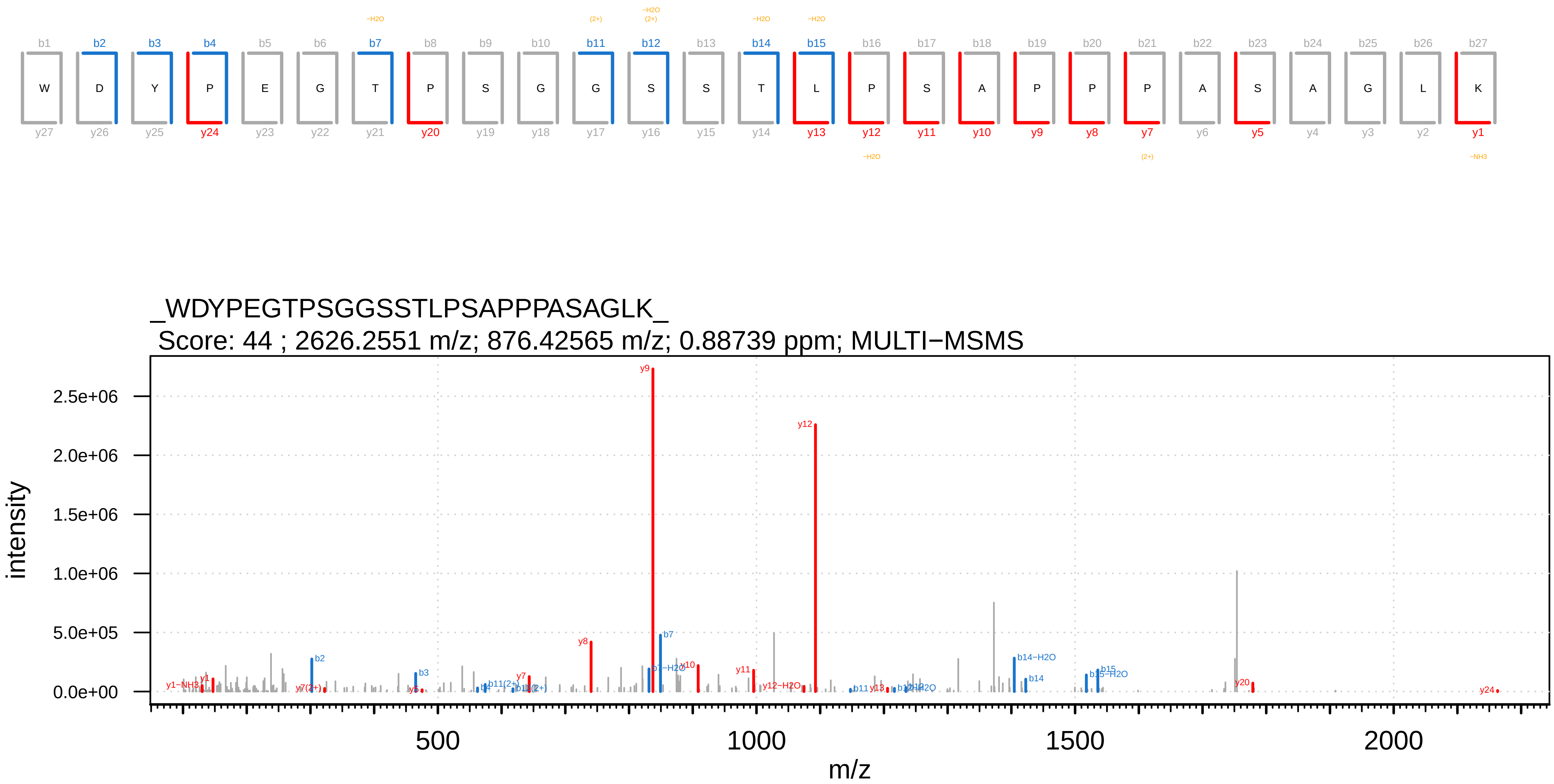

Raw File: 20140819_QEp4_BaAz_SA_H16_R1
Scan Number: 94932
Proteins:
ENSMUST00000149514_chrX:153723700−153723906:+

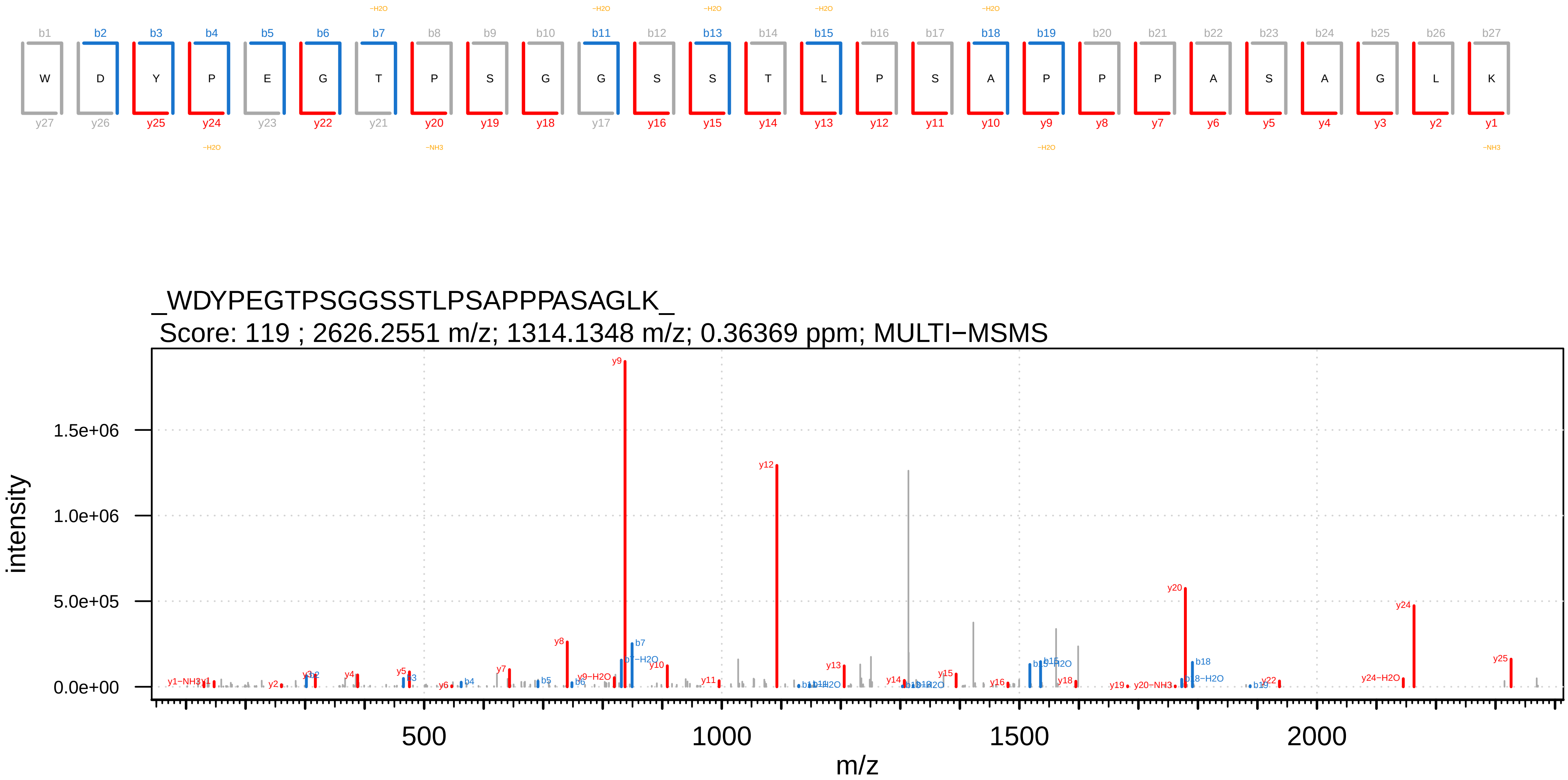

Raw File: 20140819_QEp4_BaAz_SA_H16_R1
Scan Number: 94950
Proteins:
ENSMUST00000149514_chrX:153723700−153723906:+

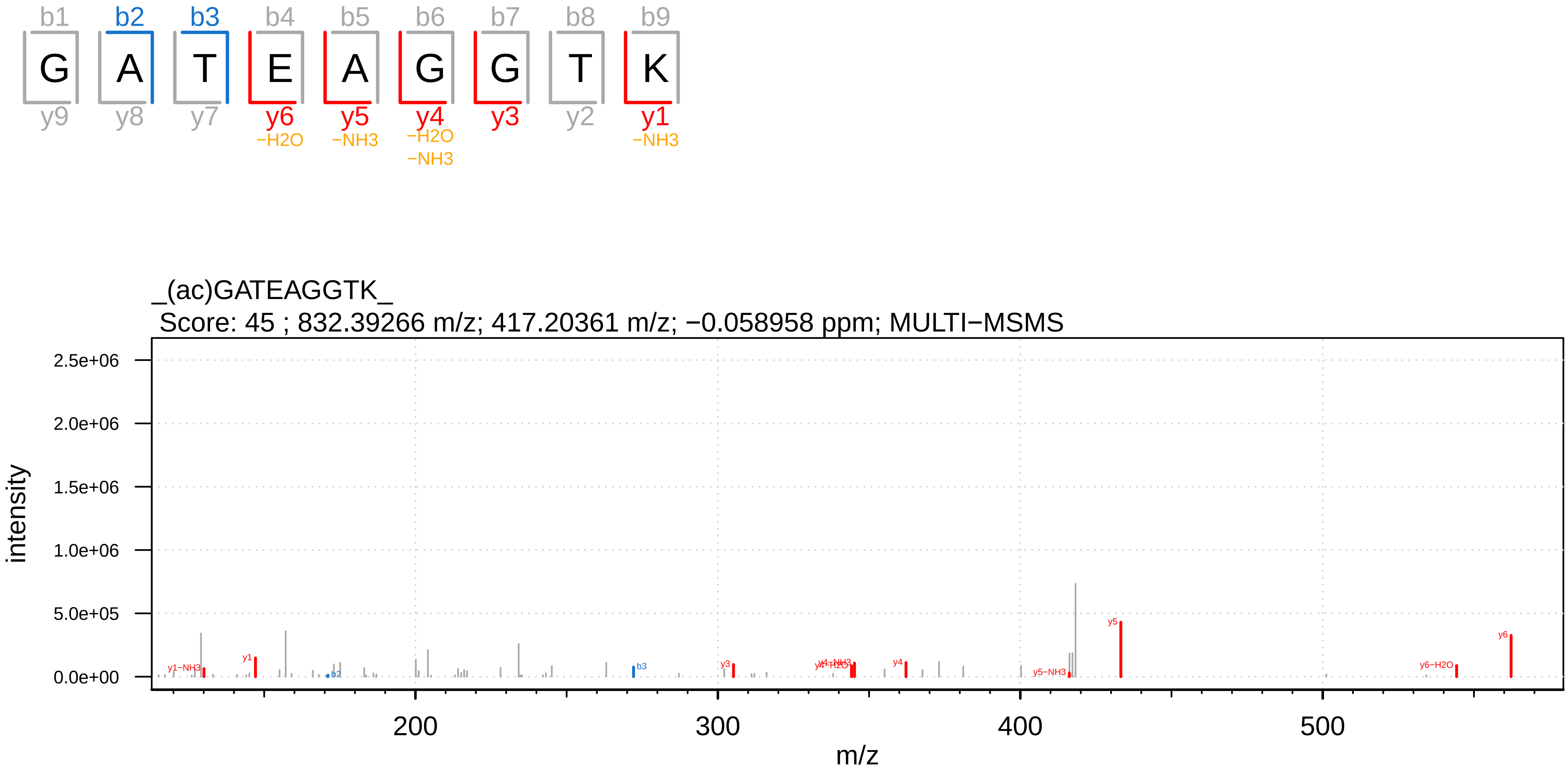

Raw File: 20130716_EXQ4_BaAz_SA_CHR2_01
Scan Number: 411
Proteins:
ENSMUST00000106930_chr4:101496658−101532002:+

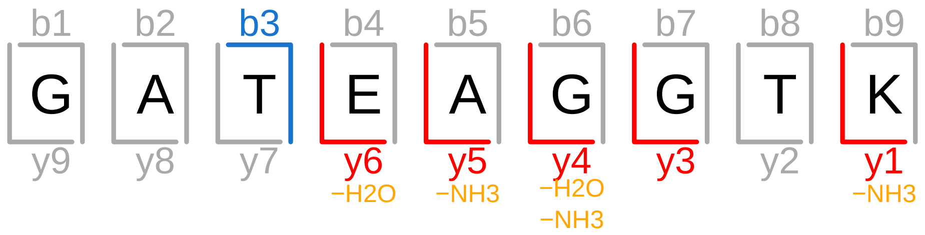

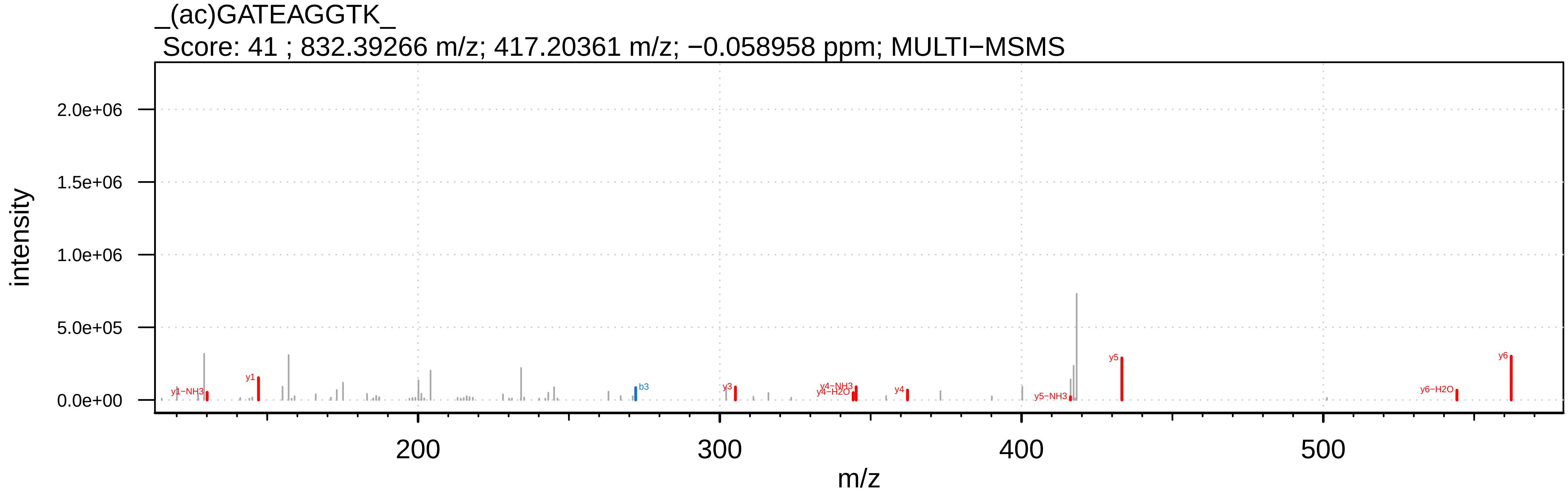

Raw File: 20130716_EXQ4_BaAz_SA_CHR2_01
Scan Number: 574
Proteins:
ENSMUST00000106930_chr4:101496658−101532002:+

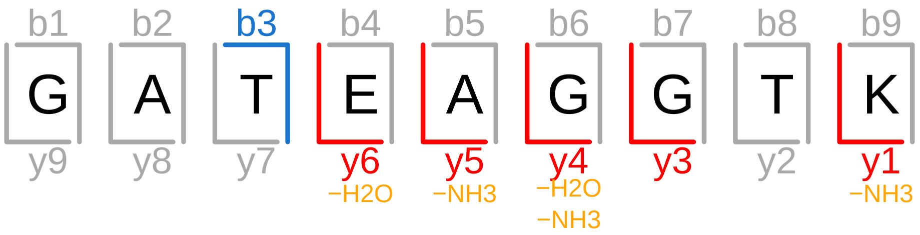

(ac)GATEAGGTK
Score: 52 ; 832.39266 m/z; 417.20361 m/z; −0.058958 ppm; MULTI−MSMS

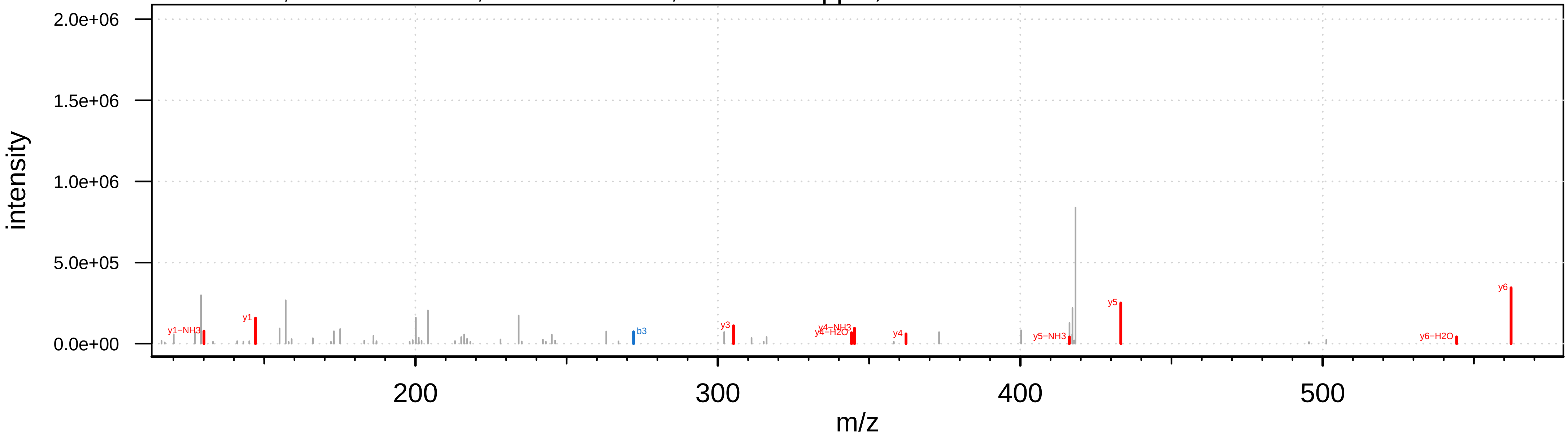

Raw File: 20130716_EXQ4_BaAz_SA_CHR2_01
Scan Number: 740
Proteins:
ENSMUST00000106930_chr4:101496658−101532002:+

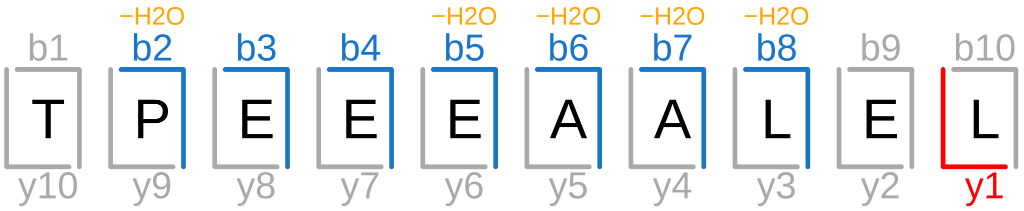

TPEEEAALEL
Score: 44 ; 1100.5237 m/z; 551.26914 m/z; −0.60512 ppm; MULTI−MSMS

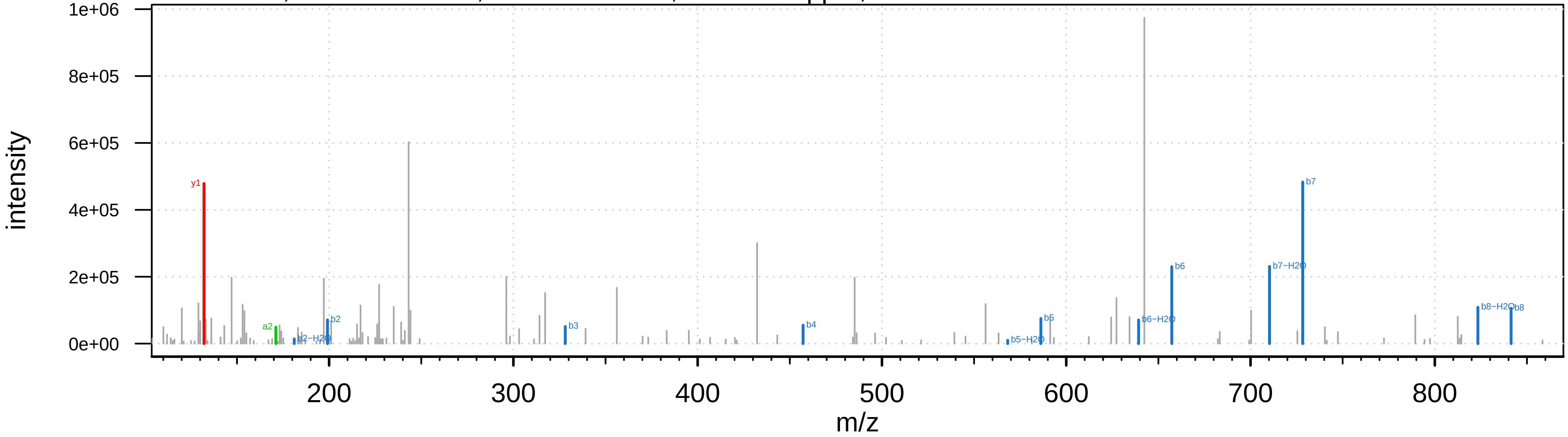

Raw File: 20130716_EXQ4_BaAz_SA_CHR2_01
Scan Number: 54394
Proteins:
ENSMUST00000172817_chr17:33905638−33905883:+

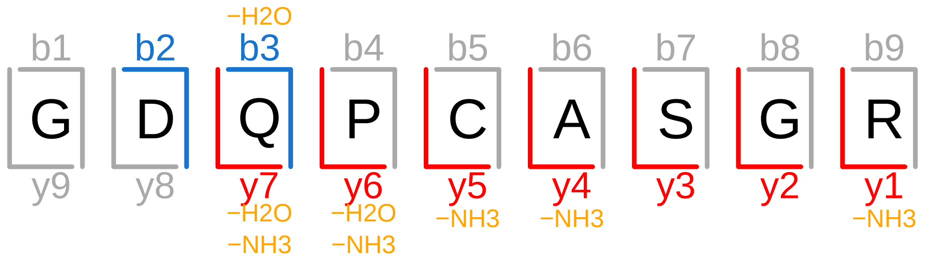

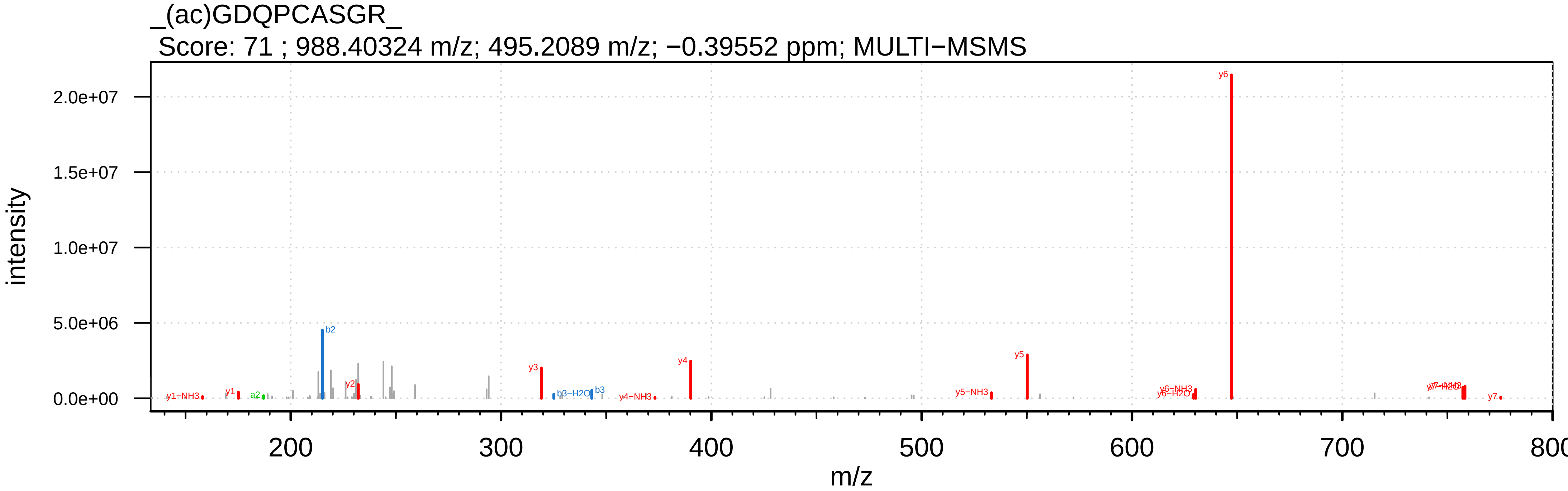

Raw File: 20130411_EXQ6_BaAz_SA_SeR1_01
Scan Number: 12788
Proteins:
ENSMUST00000149514_chrX:153723700−153723906:+

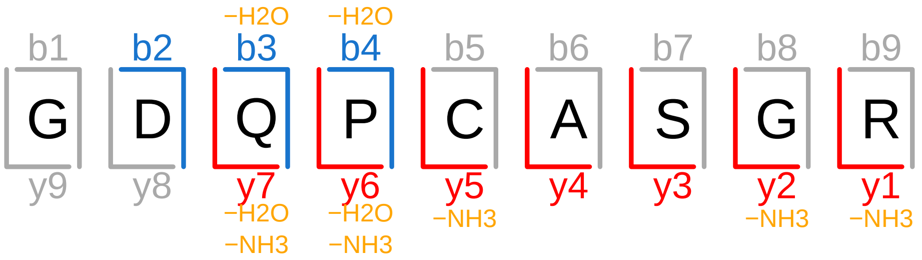

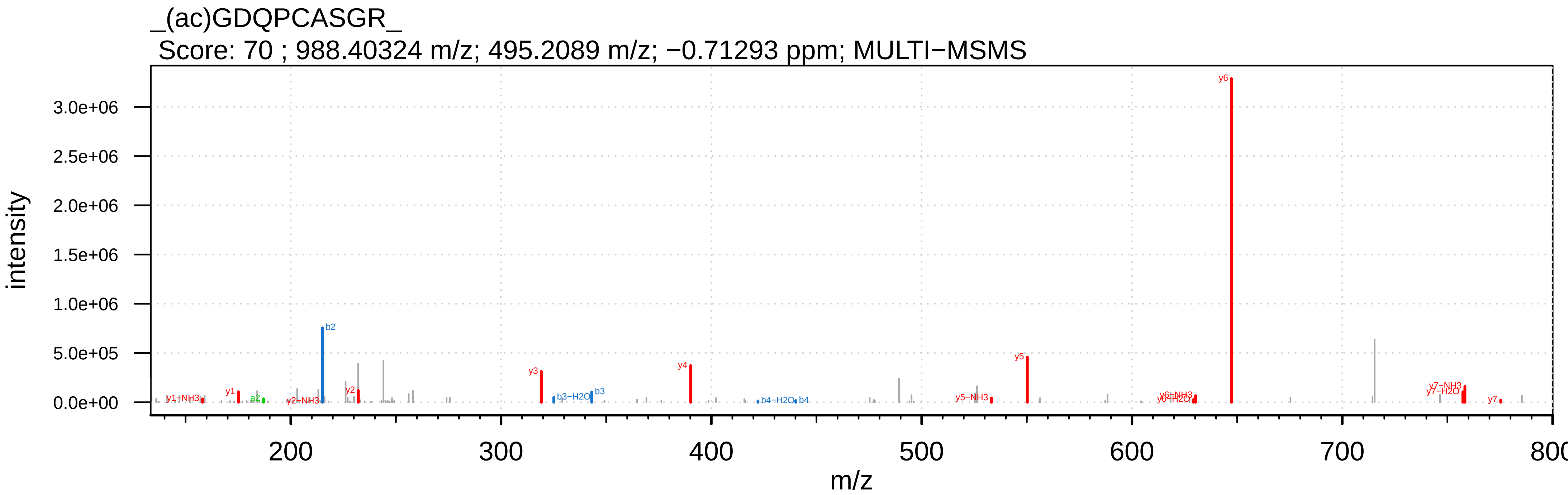

Raw File: 20130419_EXQ6_BaAz_SA_CHR1_01
Scan Number: 11131
Proteins:
ENSMUST00000149514_chrX:153723700−153723906:+

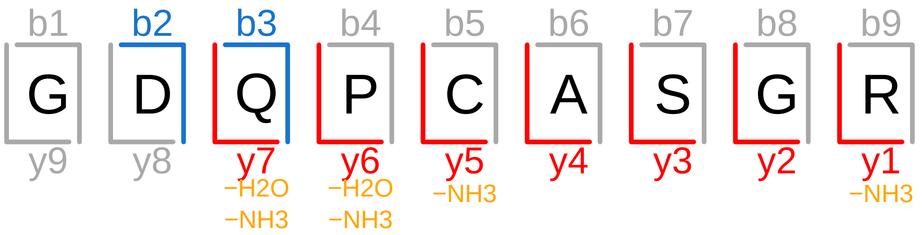

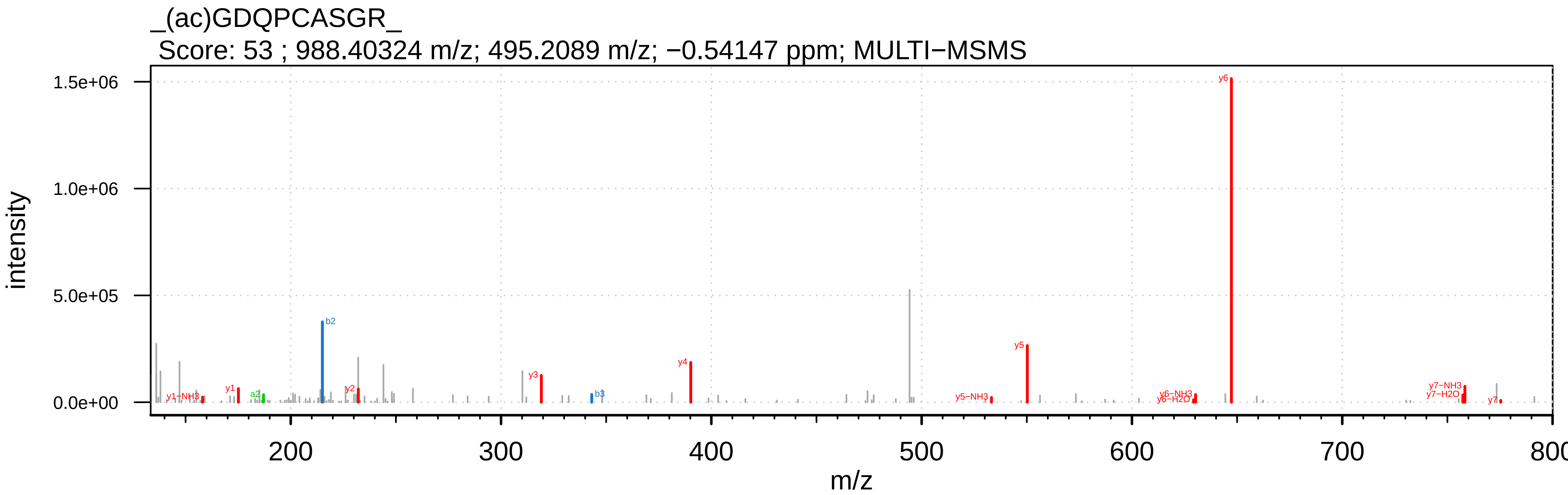

Raw File: 20130705_EXQ6_BaAz_SA_H53R1_01
Scan Number: 9980
Proteins:
ENSMUST00000149514_chrX:153723700−153723906:+

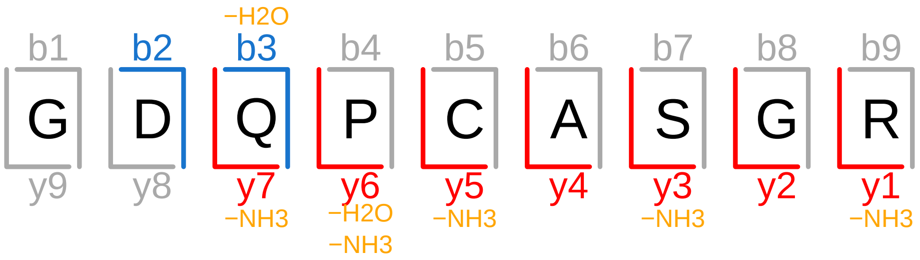

(ac)GDQPCASGR
Score: 57 ; 988.40324 m/z; 495.2089 m/z; −0.54147 ppm; MULTI−MSMS

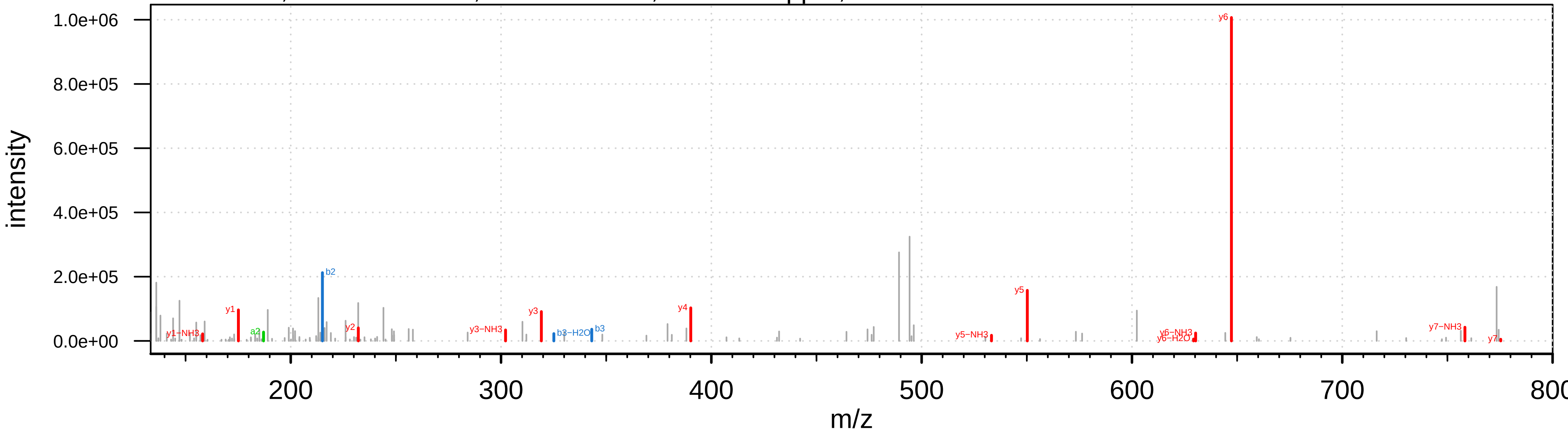

Raw File: 20130705_EXQ6_BaAz_SA_H53R1_01
Scan Number: 10229
Proteins:
ENSMUST00000149514_chrX:153723700−153723906:+

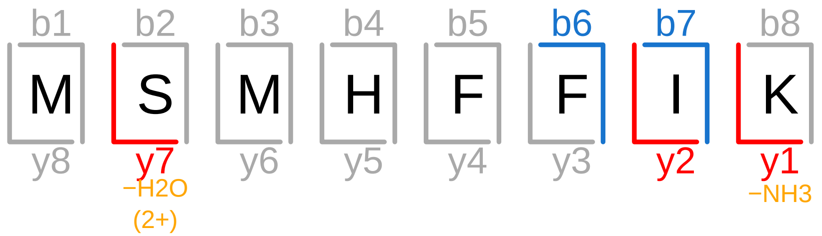

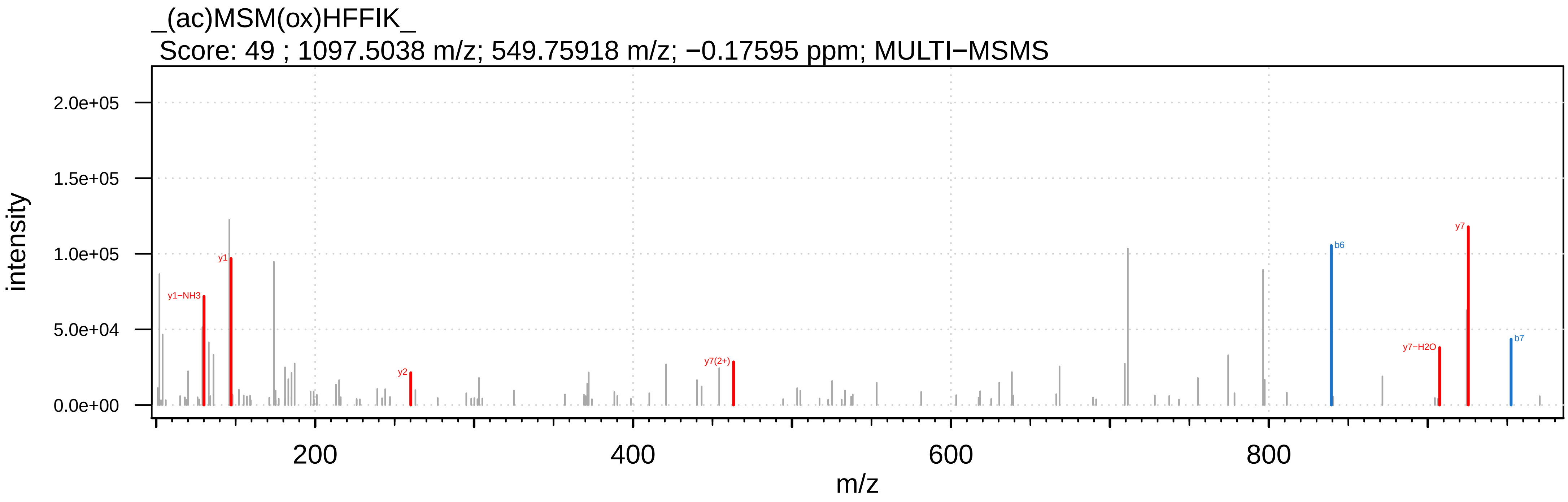

Raw File: 20130705_EXQ6_BaAz_SA_H53R1_01
Scan Number: 64322
Proteins:
ENSMUST00000092175_chr10:112463092−112463253:+

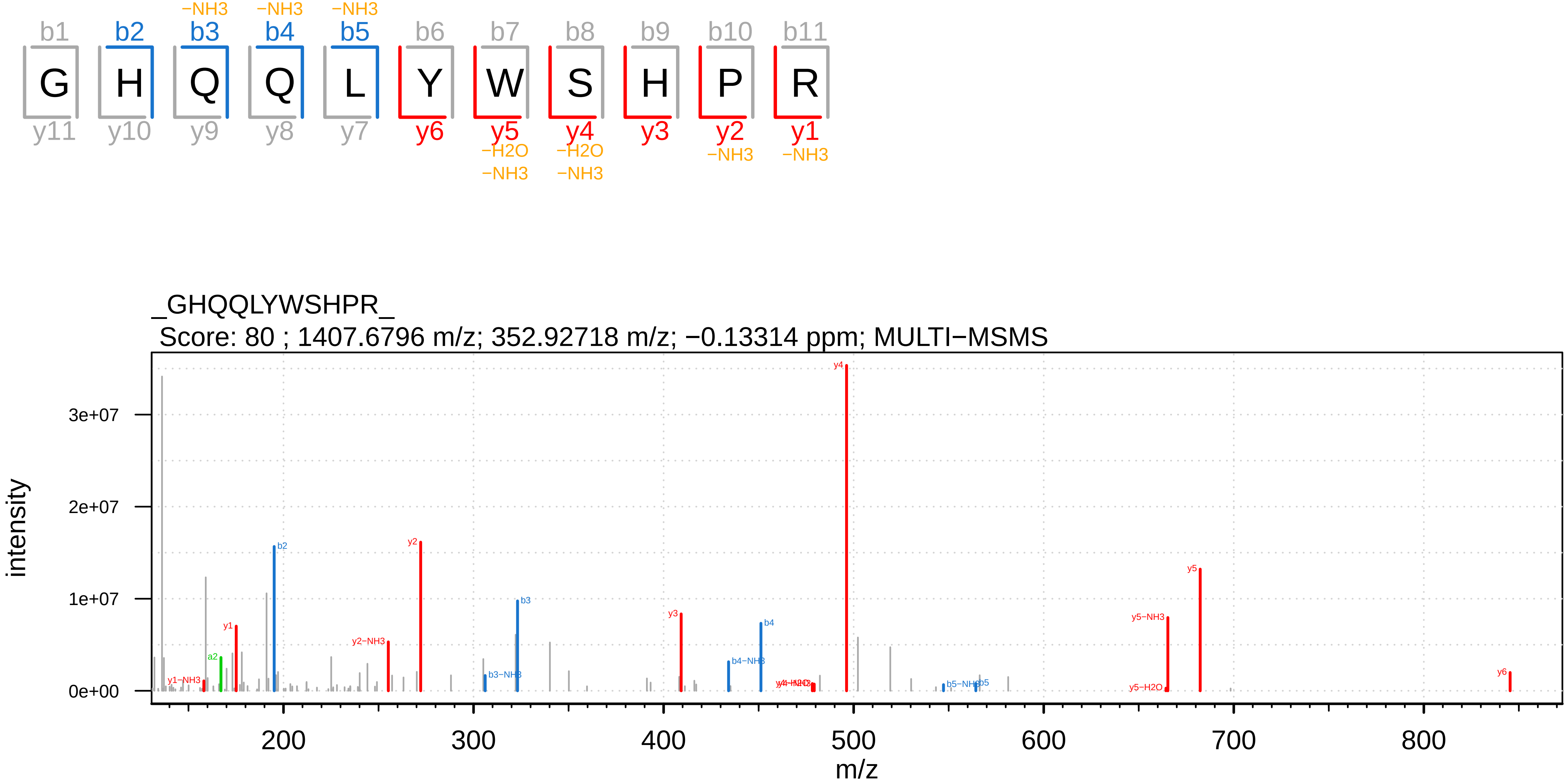

Raw File: 20130408_EXQ6_BaAz_SA_H16R1_06
Scan Number: 19124
Proteins:
sp|P62274|RS29_MOUSE
ENSMUST00000037023_chr12:69157813−69159058:−
ENSMUST00000071737_chr13:73988963−73989133:−

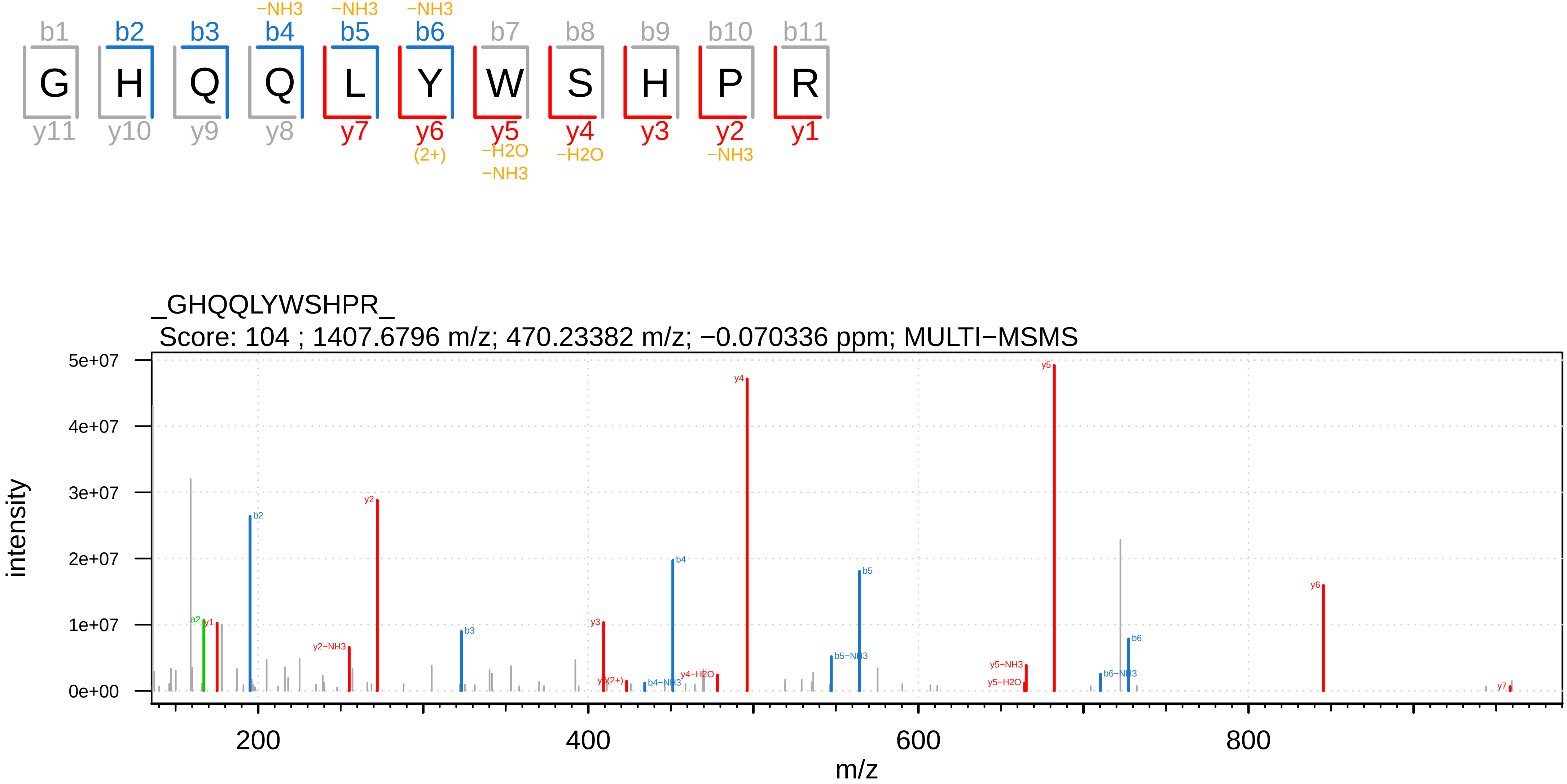

Raw File: 20130408_EXQ6_BaAz_SA_H16R1_06
Scan Number: 19130
Proteins:
sp|P62274|RS29_MOUSE
ENSMUST00000037023_chr12:69157813−69159058:−
ENSMUST00000071737_chr13:73988963−73989133:−

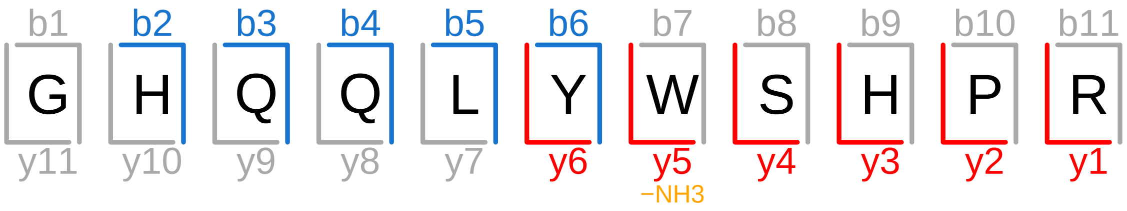

(ac)GHQQLYWSHPR
Score: 42 ; 1449.6902 m/z; 484.23734 m/z; 0.21142 ppm; MULTI−MSMS

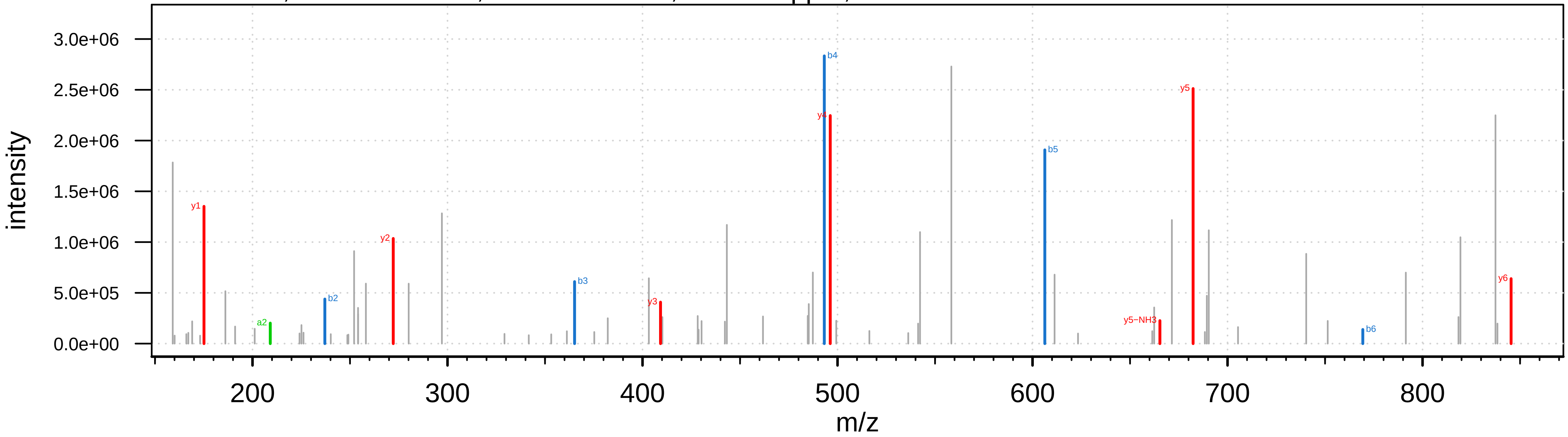

Raw File: 20130408_EXQ6_BaAz_SA_H16R1_06
Scan Number: 30327
Proteins:
sp|P62274|RS29_MOUSE
ENSMUST00000037023_chr12:69157813−69159058:−
ENSMUST00000071737_chr13:73988963−73989133:−

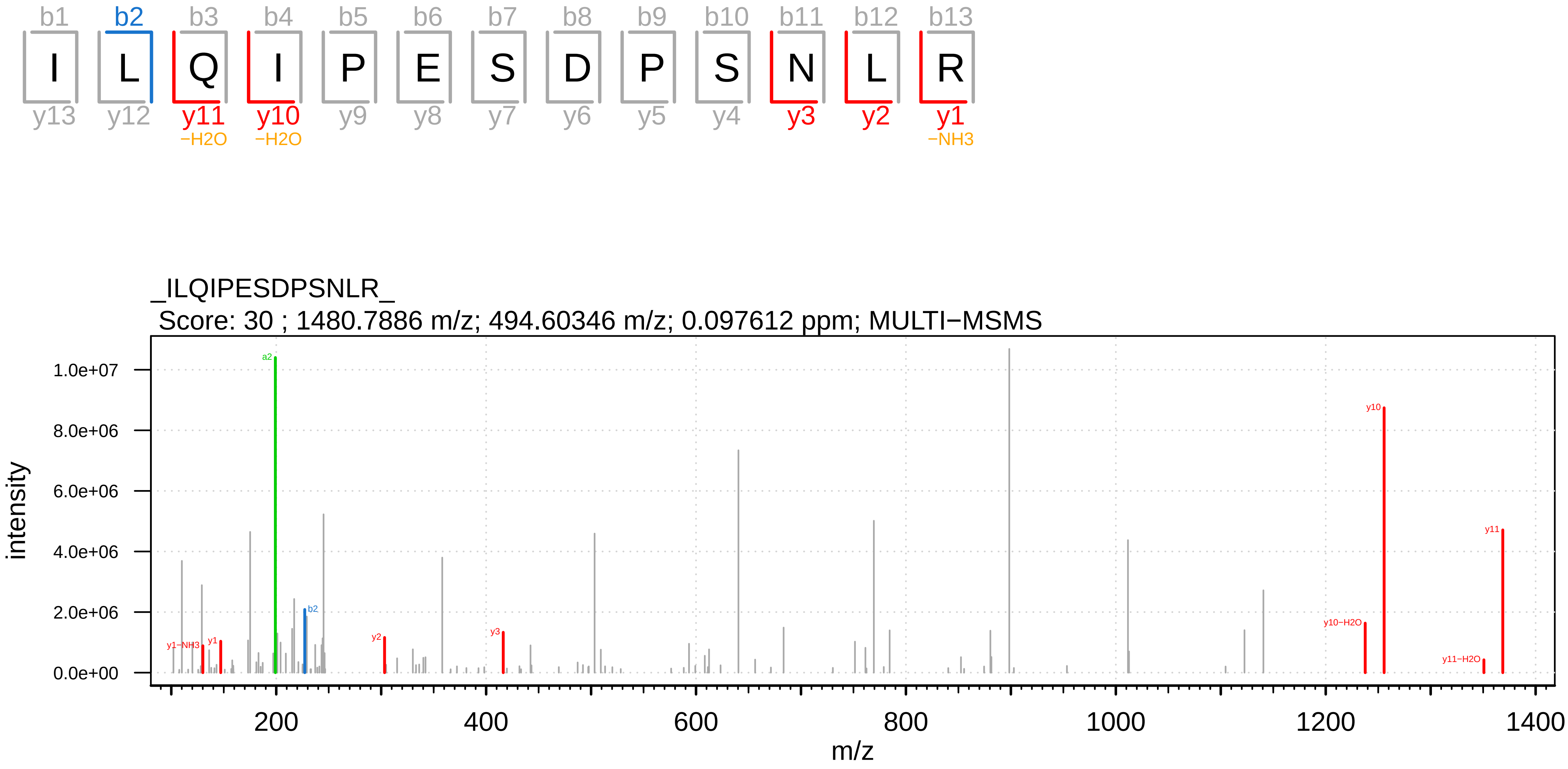

Raw File: 20130408_EXQ6_BaAz_SA_H16R1_06
Scan Number: 36491
Proteins:
ENSMUST00000161069_chr14:70554856−70554960:+

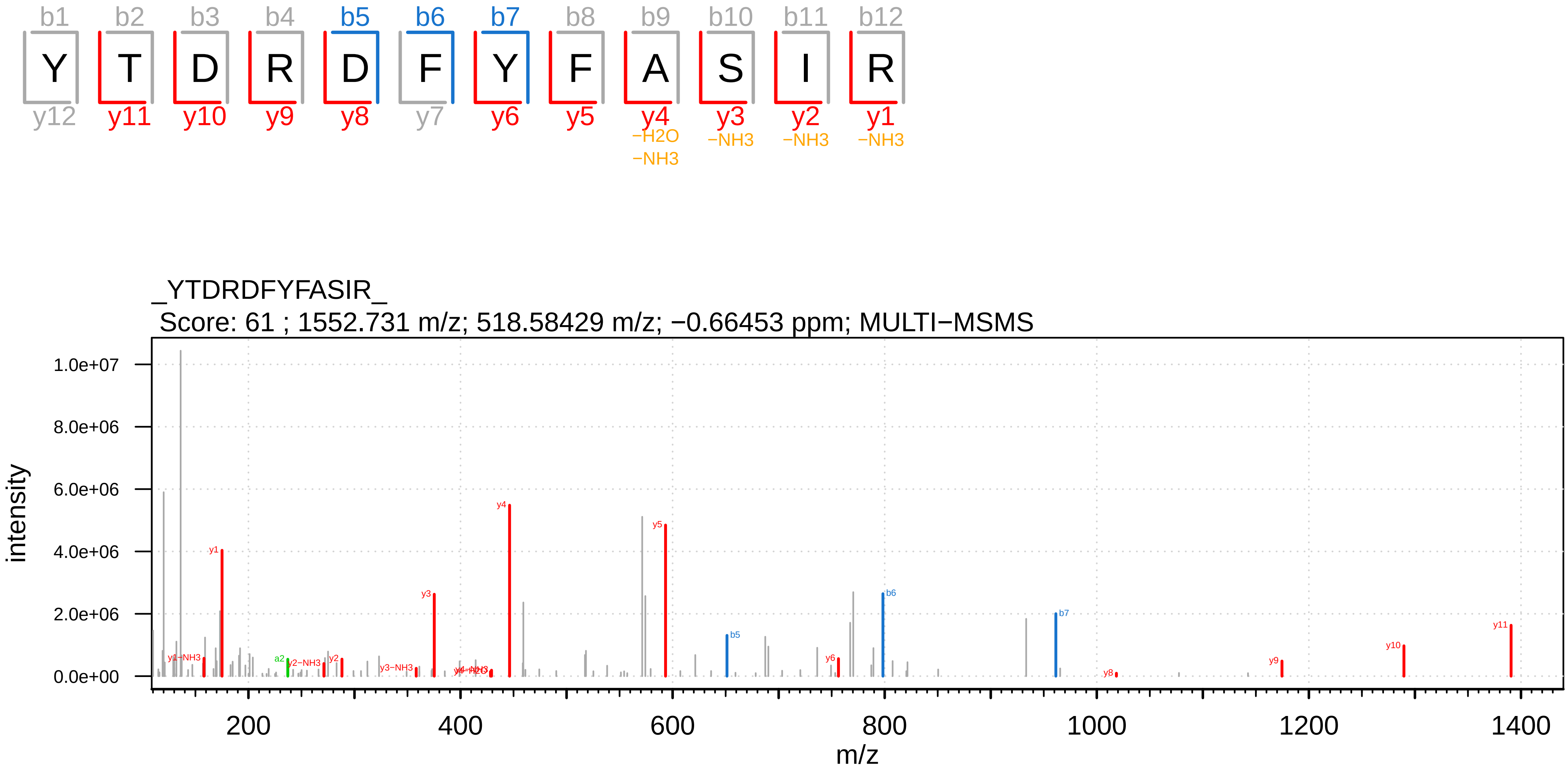

Raw File: 20130408_EXQ6_BaAz_SA_H16R1_06
Scan Number: 59947
Proteins:
ENSMUST00000023048_chr15:80235835−80236047:+

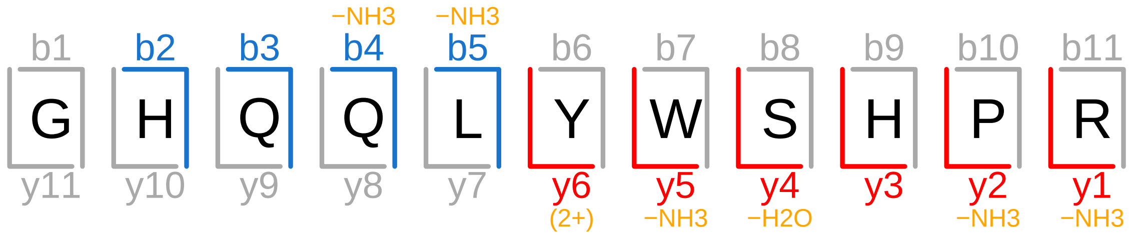

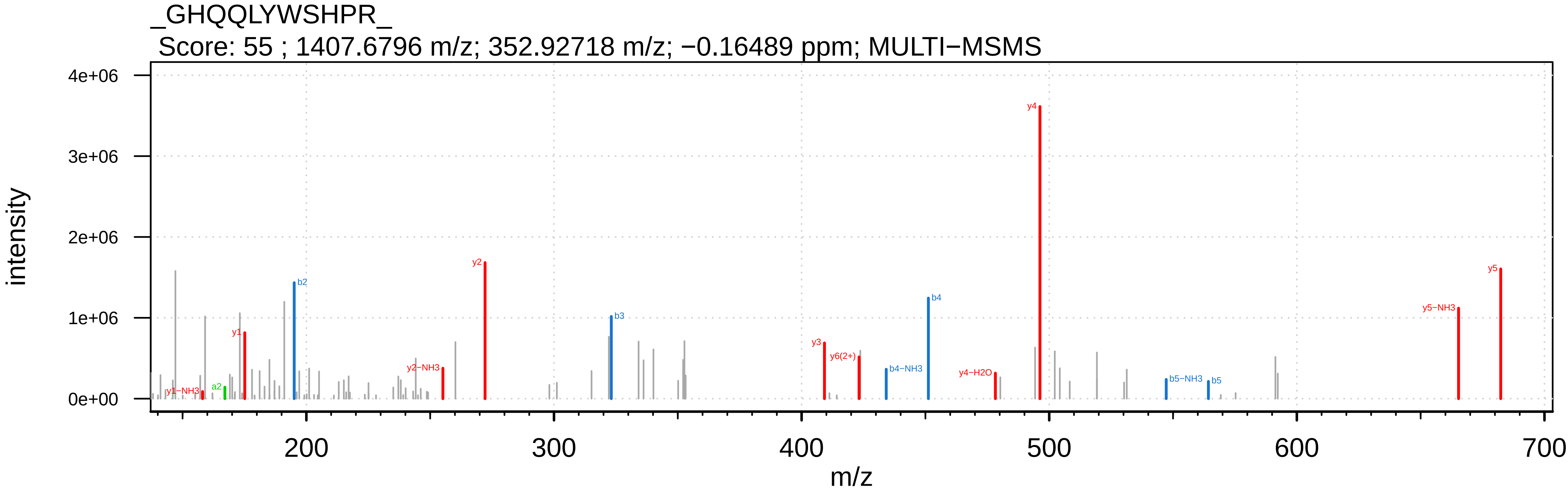

Raw File: 20140816_QEp4_BaAz_SA_DIII_R1
Scan Number: 22555
Proteins:
sp|P62274|RS29_MOUSE
ENSMUST00000037023_chr12:69157813−69159058:−
ENSMUST00000071737_chr13:73988963−73989133:−

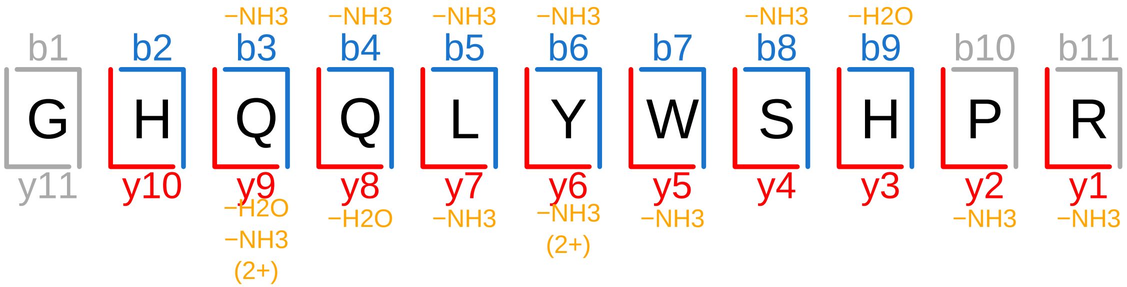

GHQQLYWSHPR
Score: 208 ; 1407.6796 m/z; 704.84708 m/z; −0.21063 ppm; MULTI−MSMS

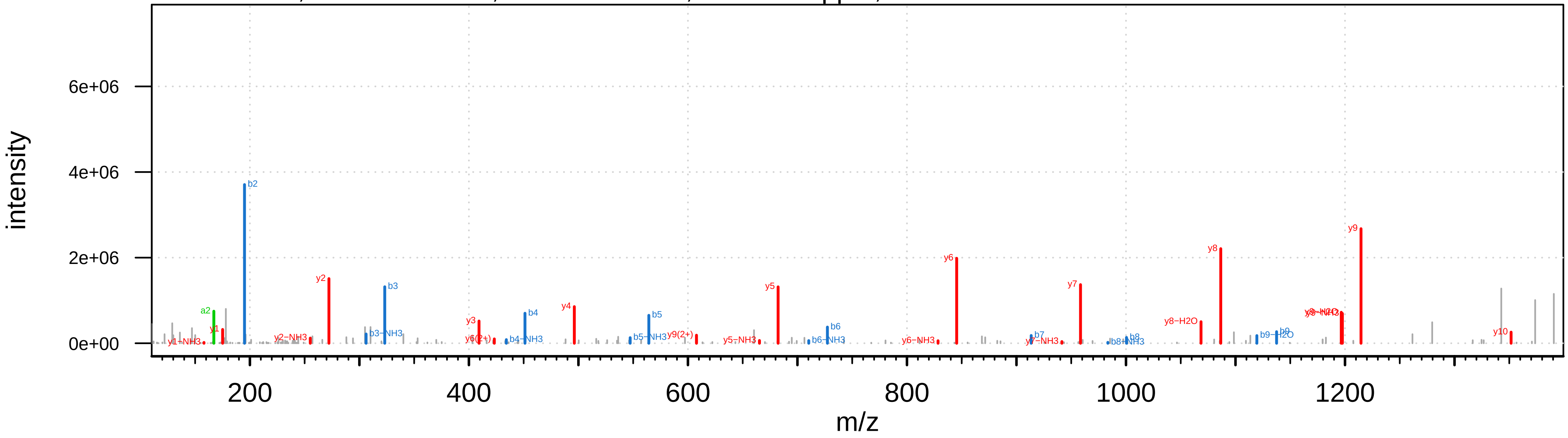

Raw File: 20140816_QEp4_BaAz_SA_DIII_R1
Scan Number: 22575
Proteins:
sp|P62274|RS29_MOUSE
ENSMUST00000037023_chr12:69157813−69159058:−
ENSMUST00000071737_chr13:73988963−73989133:−

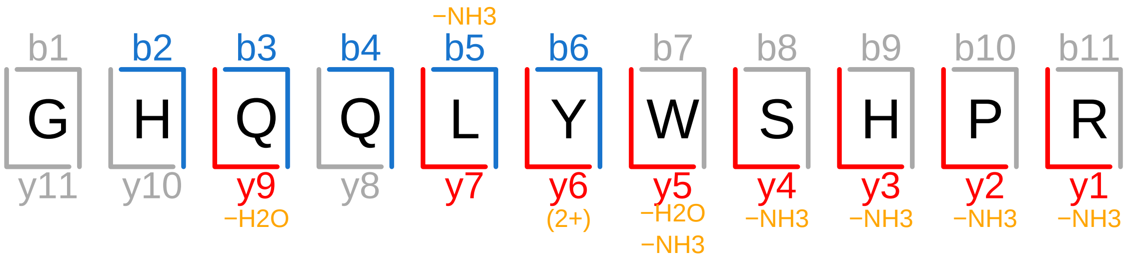

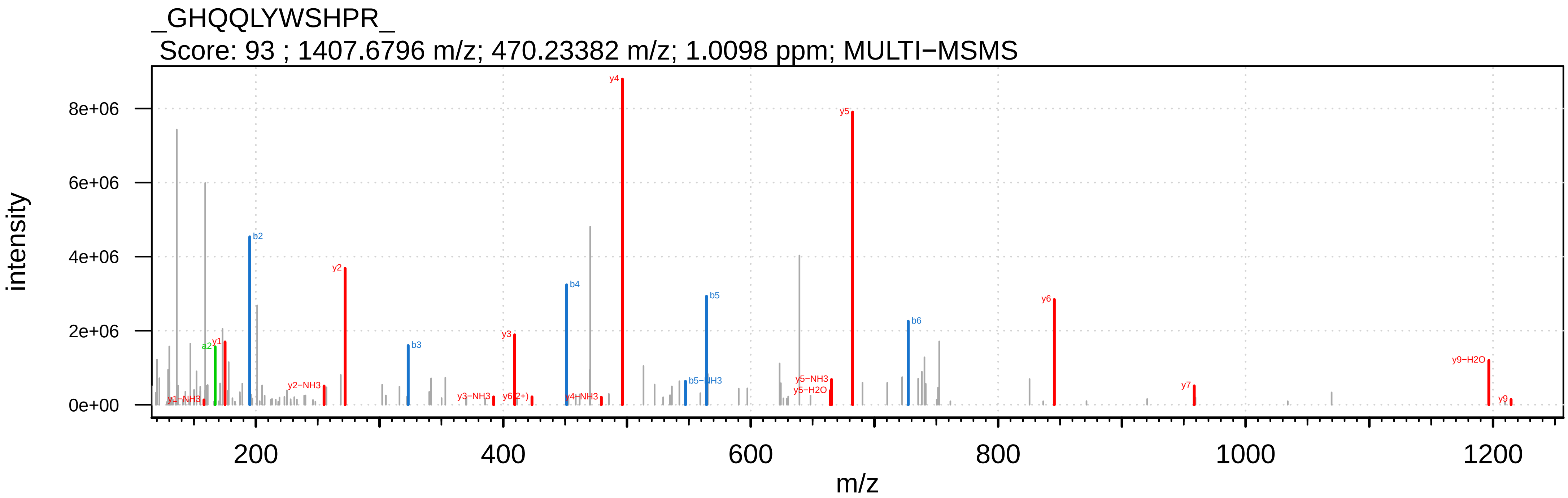

Raw File: 20140816_QEp4_BaAz_SA_DIII_R1
Scan Number: 22590
Proteins:
sp|P62274|RS29_MOUSE
ENSMUST00000037023_chr12:69157813−69159058:−
ENSMUST00000071737_chr13:73988963−73989133:−

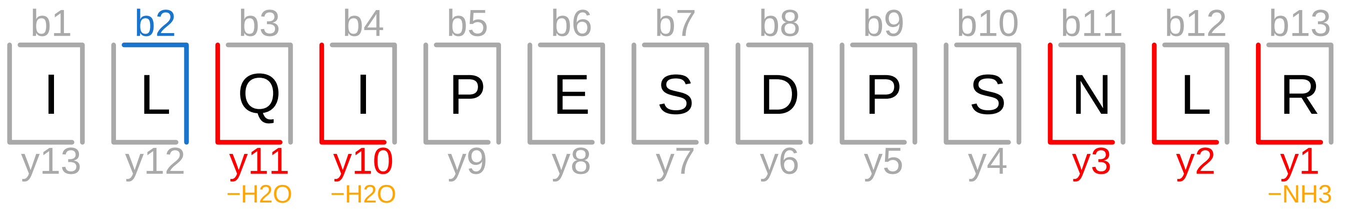

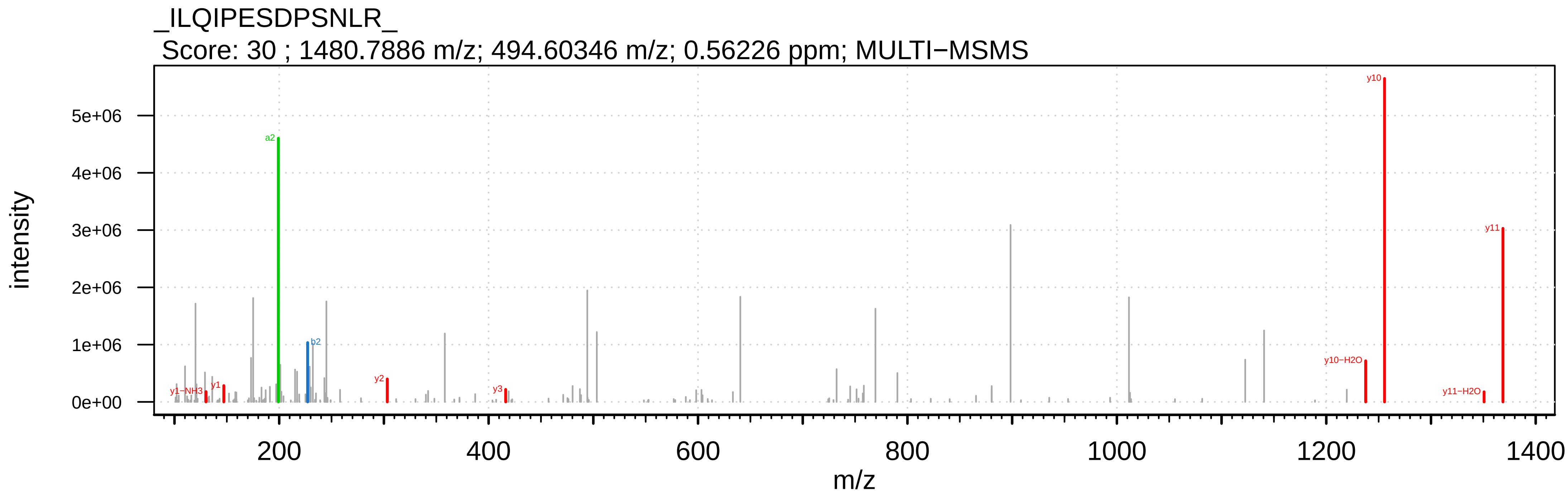

Raw File: 20140816_QEp4_BaAz_SA_DIII_R1
Scan Number: 48058
Proteins:
ENSMUST00000161069_chr14:70554856−70554960:+

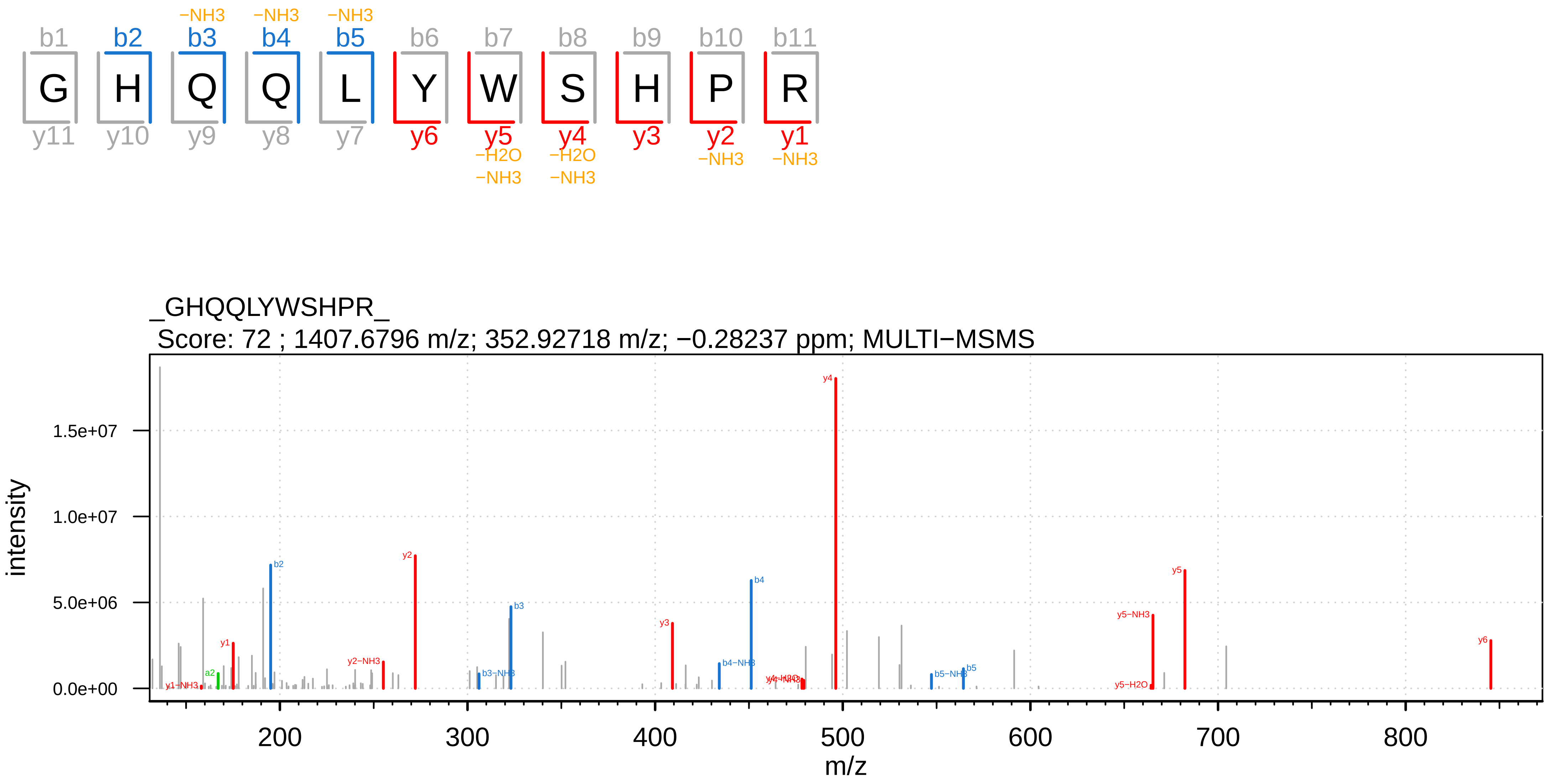

Raw File: 20140819_QEp4_BaAz_SA_H16_R2
Scan Number: 22158
Proteins:
sp|P62274|RS29_MOUSE
ENSMUST00000037023_chr12:69157813−69159058:−
ENSMUST00000071737_chr13:73988963−73989133:−

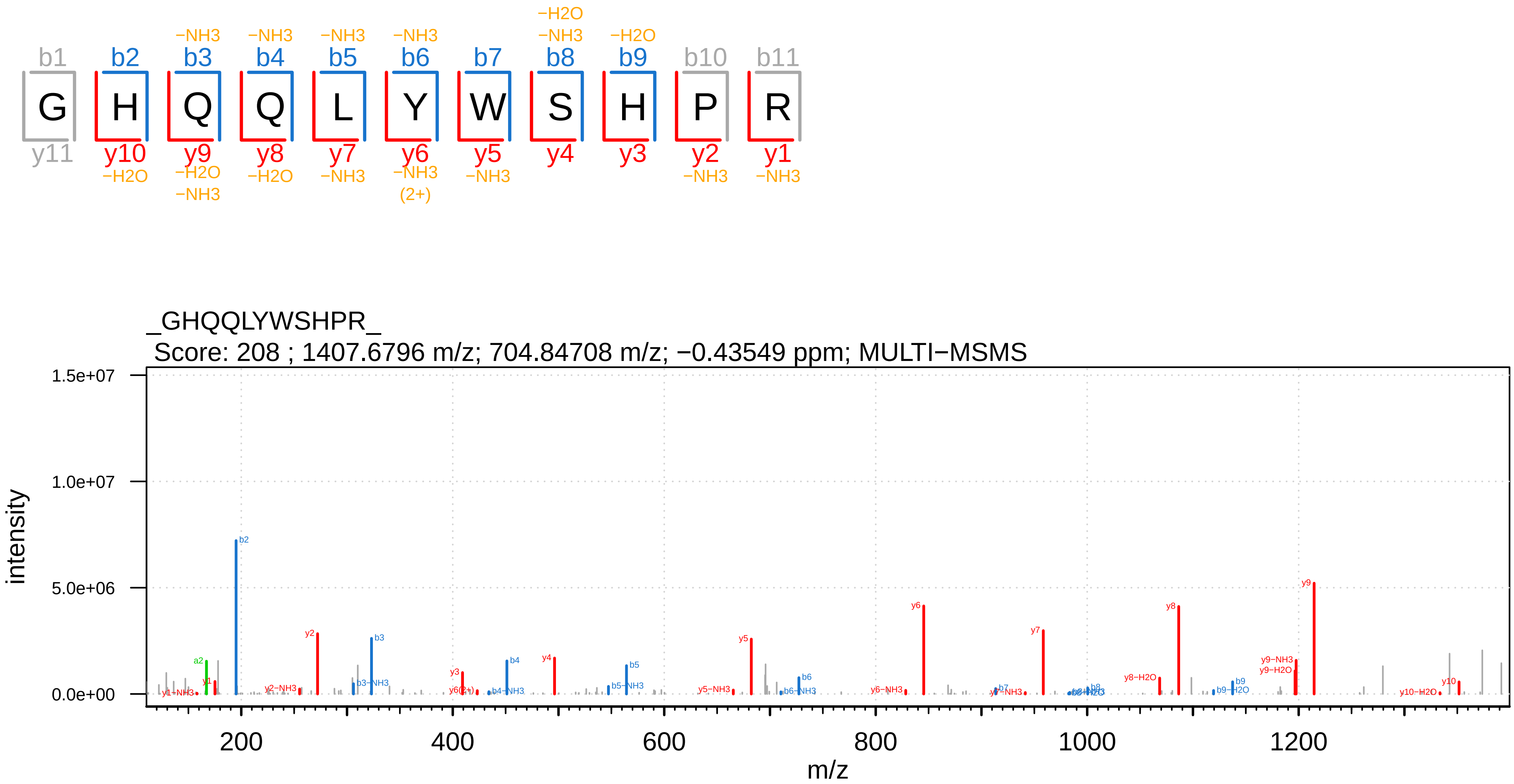

Raw File: 20140819_QEp4_BaAz_SA_H16_R2
Scan Number: 22162
Proteins:
sp|P62274|RS29_MOUSE
ENSMUST00000037023_chr12:69157813−69159058:−
ENSMUST00000071737_chr13:73988963−73989133:−

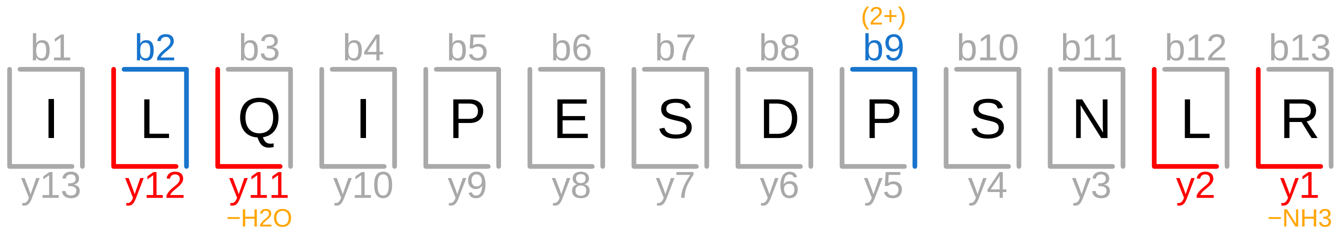

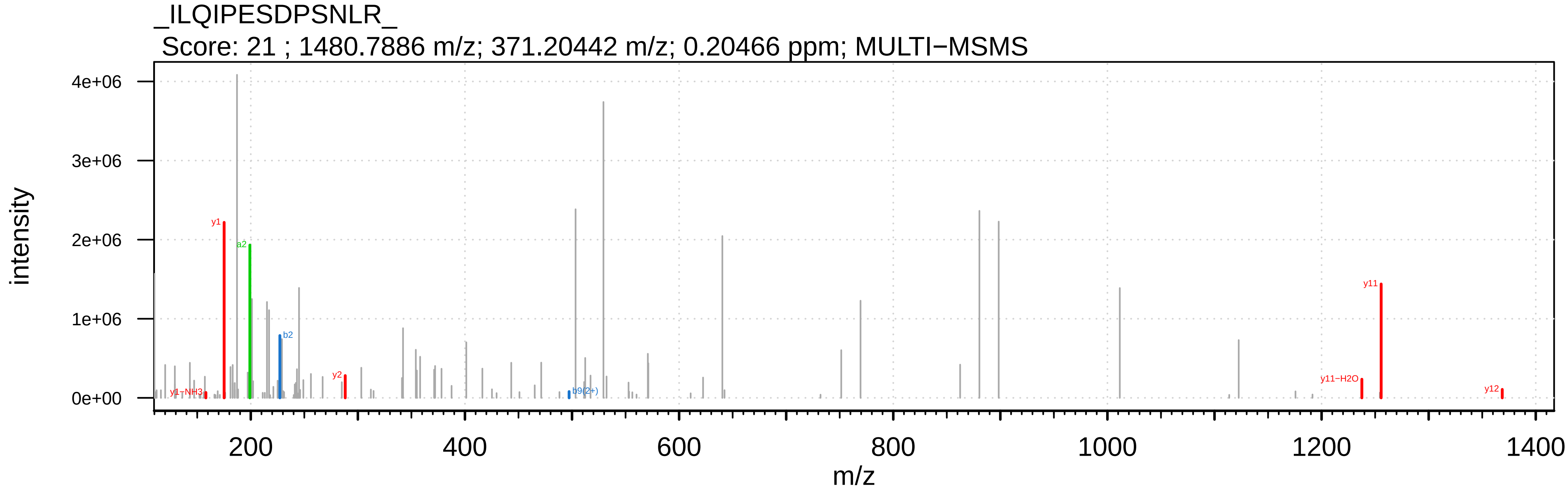

Raw File: 20140819_QEp4_BaAz_SA_H16_R2
Scan Number: 47595
Proteins:
ENSMUST00000161069_chr14:70554856−70554960:+

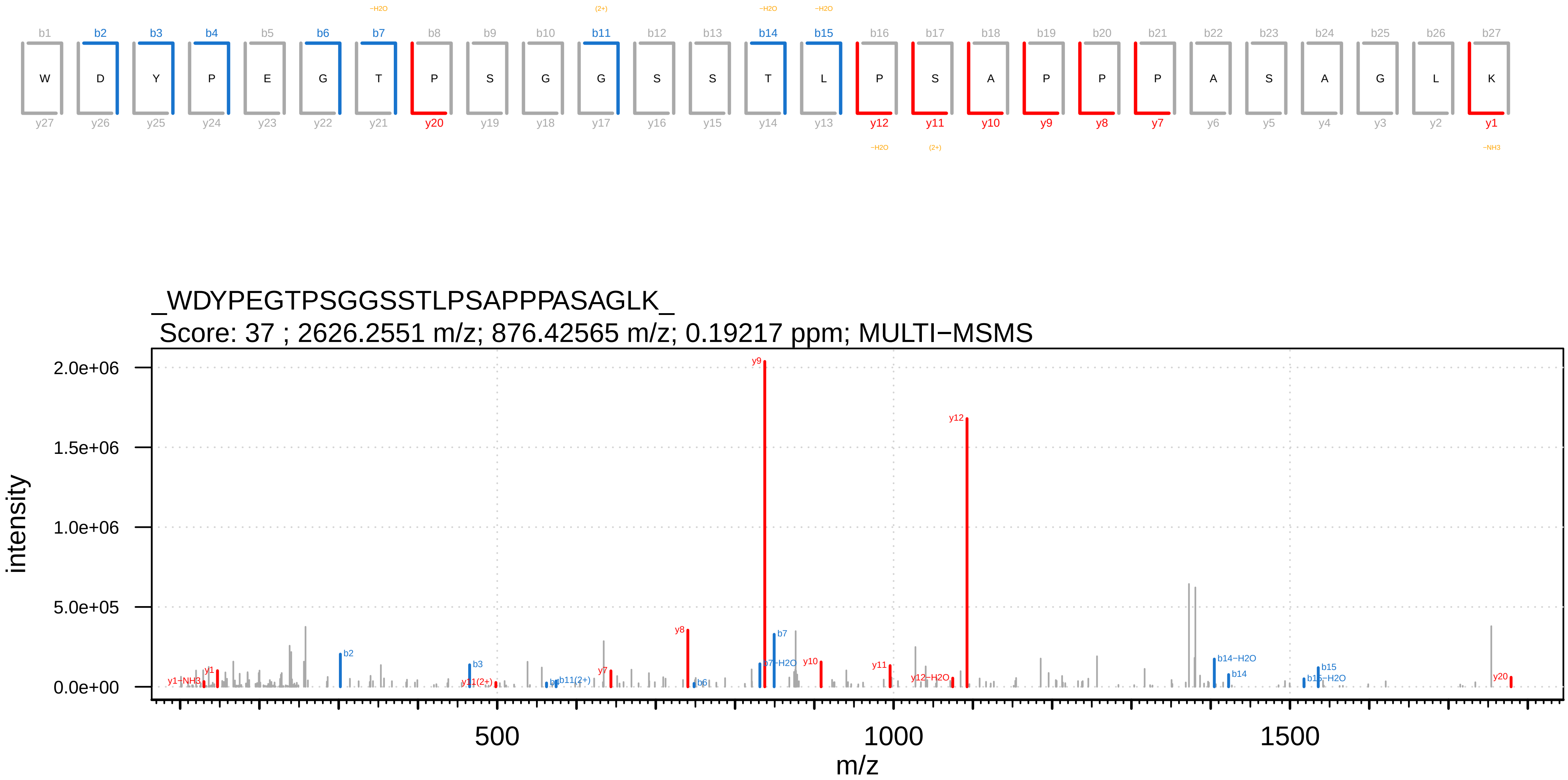

Raw File: 20140819_QEp4_BaAz_SA_H16_R2
Scan Number: 94396
Proteins:
ENSMUST00000149514_chrX:153723700−153723906:+

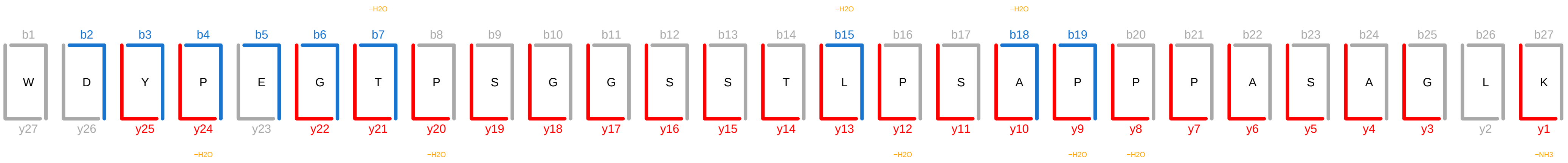

WDYPEGTPSGGSSTLPSAPPPASAGLK
Score: 94 ; 2626.2551 m/z; 1314.1348 m/z; 0.5823 ppm; MULTI−MSMS

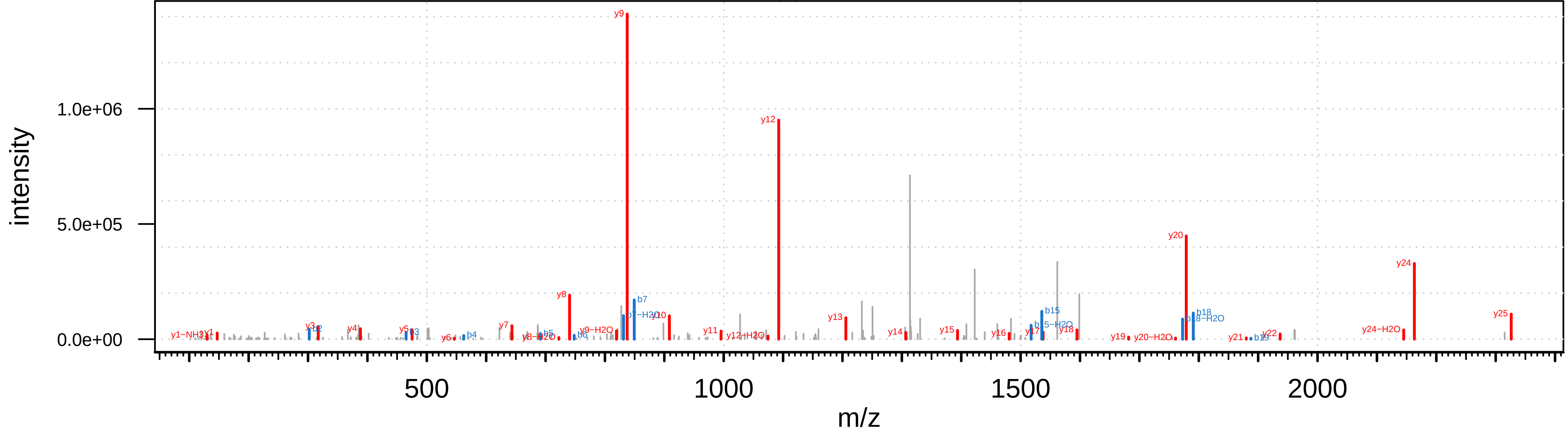

Raw File: 20140819_QEp4_BaAz_SA_H16_R2
Scan Number: 94424
Proteins:
ENSMUST00000149514_chrX:153723700−153723906:+

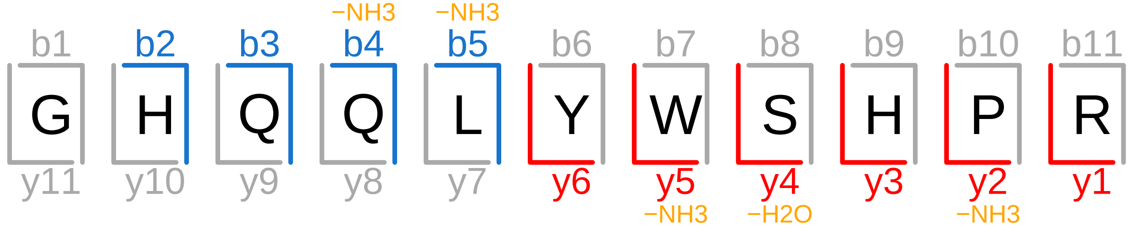

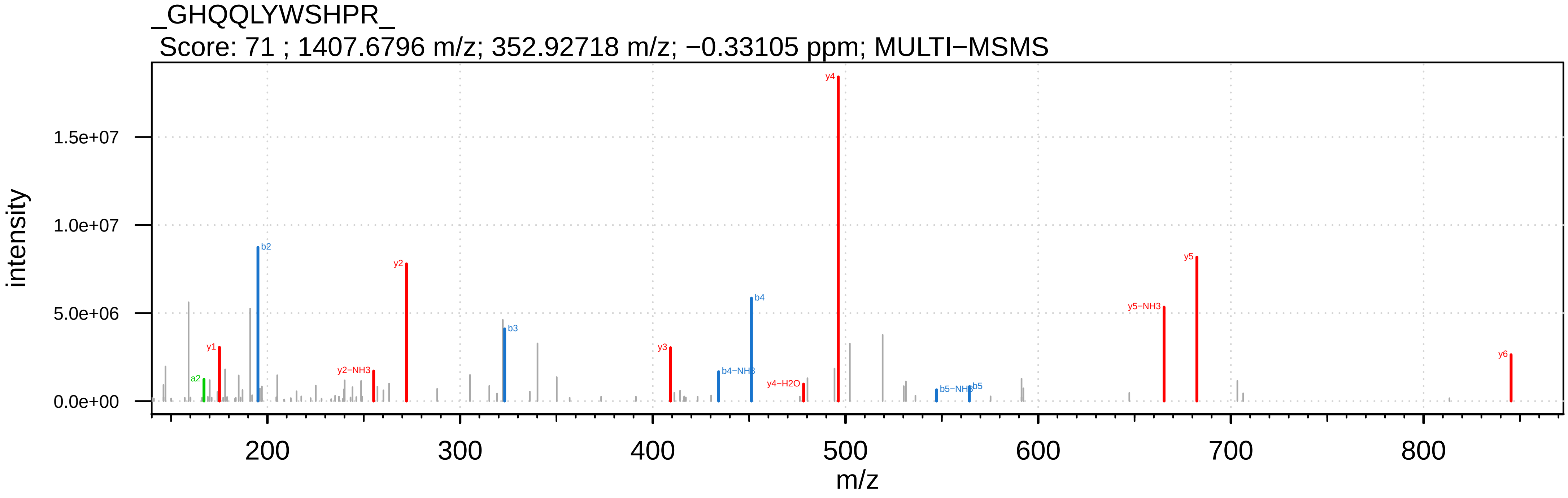

Raw File: 20140819_QEp4_BaAz_SA_H16_R3
Scan Number: 22662
Proteins:
sp|P62274|RS29_MOUSE
ENSMUST00000037023_chr12:69157813−69159058:−
ENSMUST00000071737_chr13:73988963−73989133:−

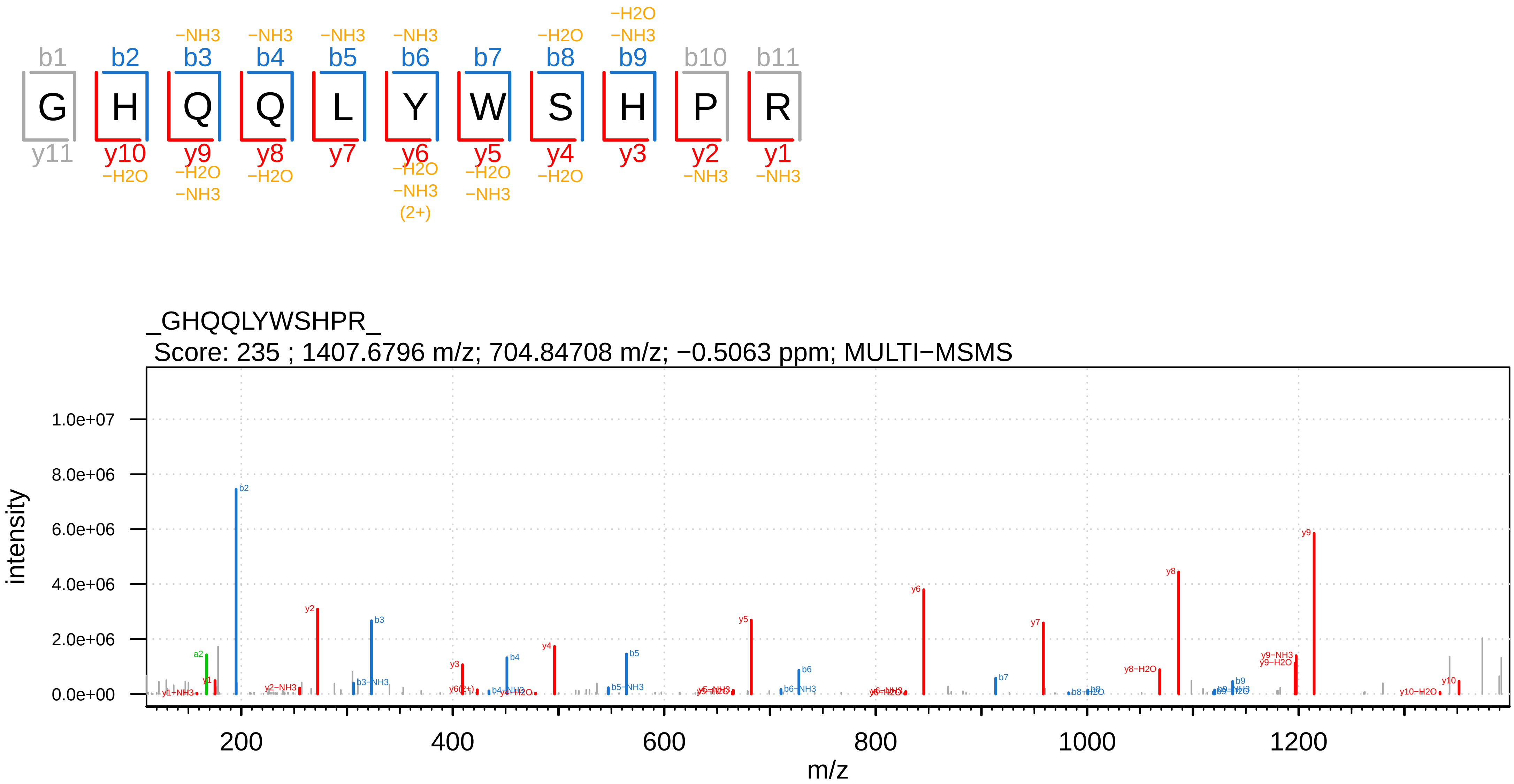

Raw File: 20140819_QEp4_BaAz_SA_H16_R3
Scan Number: 22670
Proteins:
sp|P62274|RS29_MOUSE
ENSMUST00000037023_chr12:69157813−69159058:−
ENSMUST00000071737_chr13:73988963−73989133:−

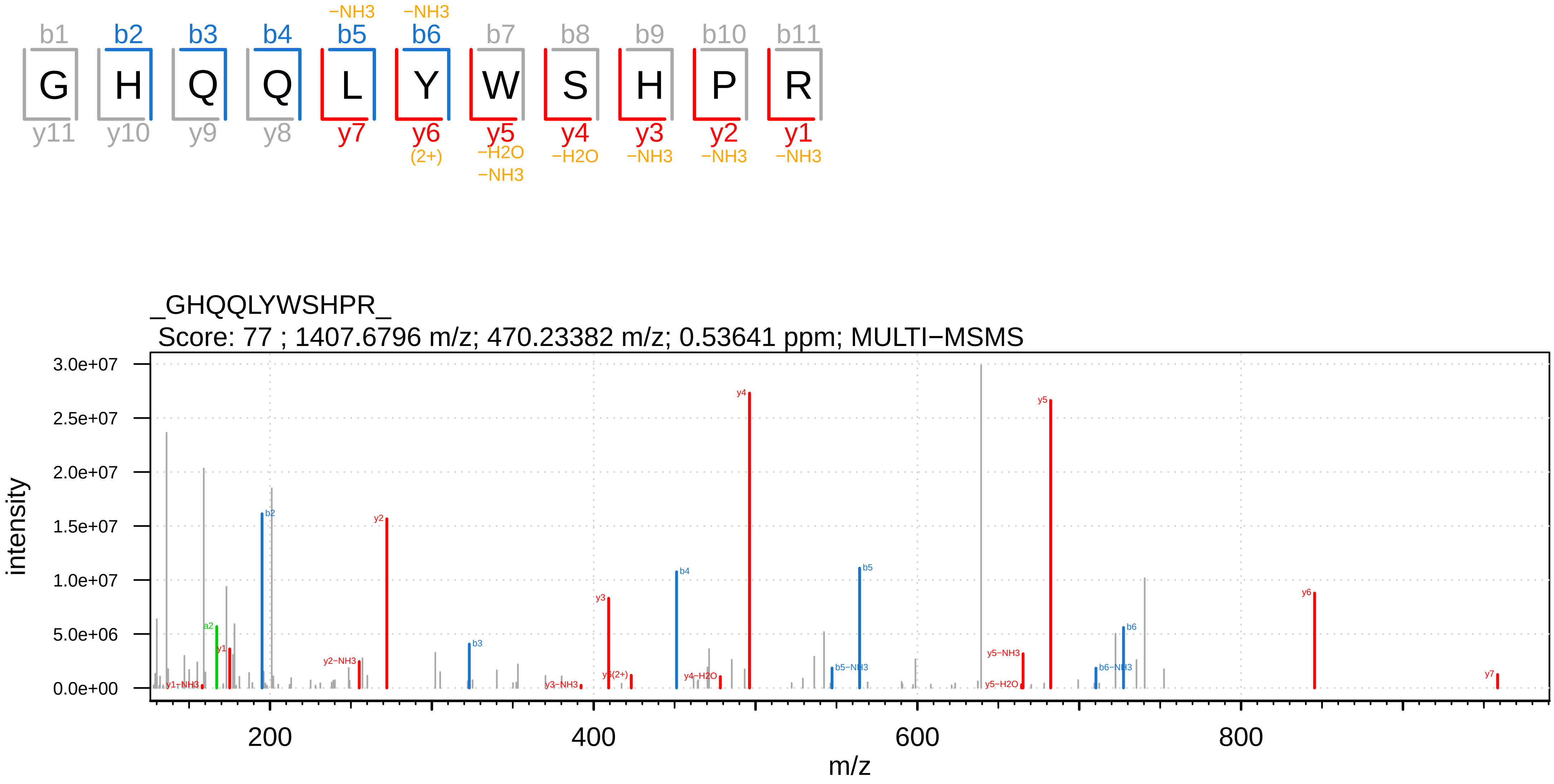

Raw File: 20140819_QEp4_BaAz_SA_H16_R3
Scan Number: 22683
Proteins:
sp|P62274|RS29_MOUSE
ENSMUST00000037023_chr12:69157813−69159058:−
ENSMUST00000071737_chr13:73988963−73989133:−

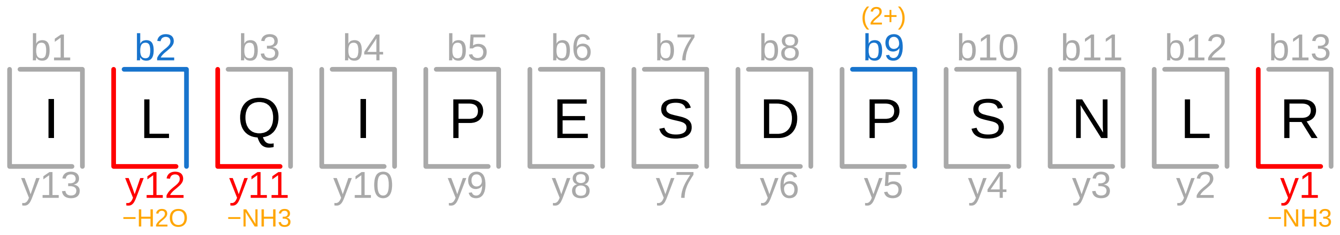

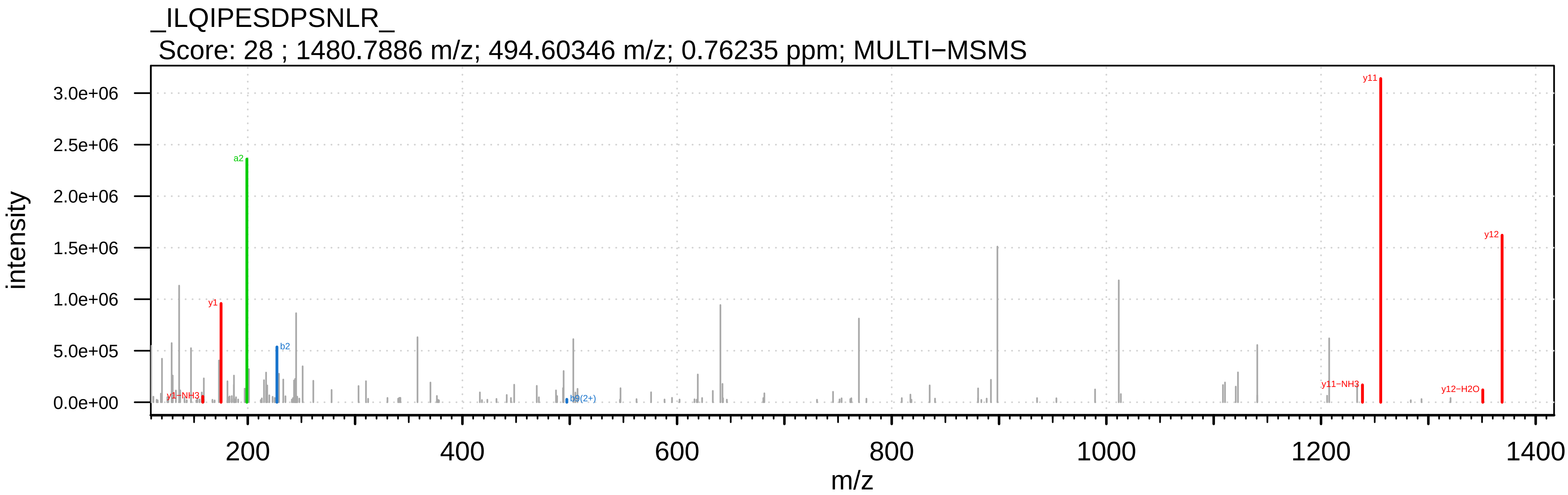

Raw File: 20140819_QEp4_BaAz_SA_H16_R3
Scan Number: 48016
Proteins:
ENSMUST00000161069_chr14:70554856−70554960:+

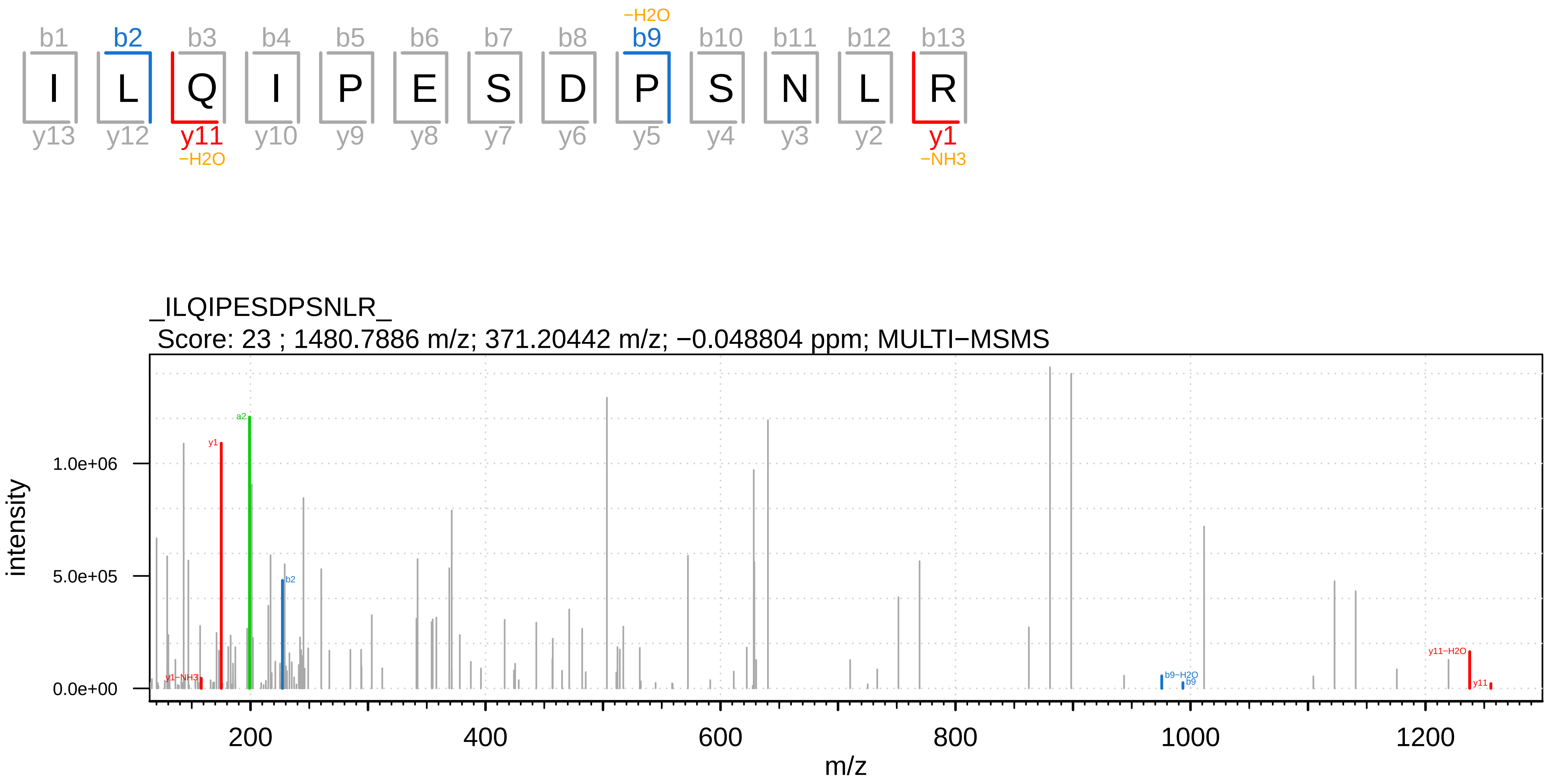

Raw File: 20140819_QEp4_BaAz_SA_H16_R3
Scan Number: 48017
Proteins:
ENSMUST00000161069_chr14:70554856−70554960:+

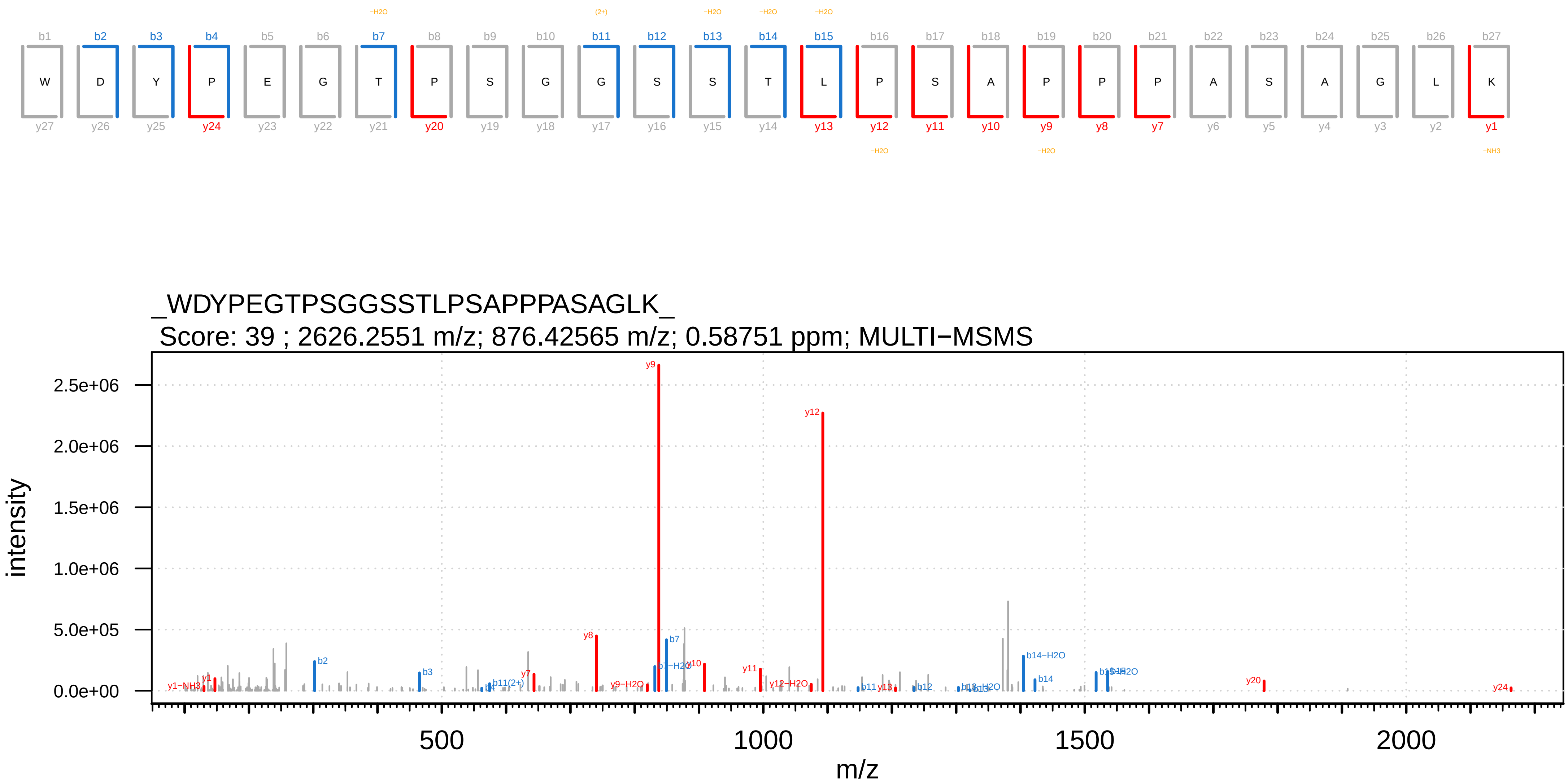

Raw File: 20140819_QEp4_BaAz_SA_H16_R3
Scan Number: 94792
Proteins:
ENSMUST00000149514_chrX:153723700−153723906:+

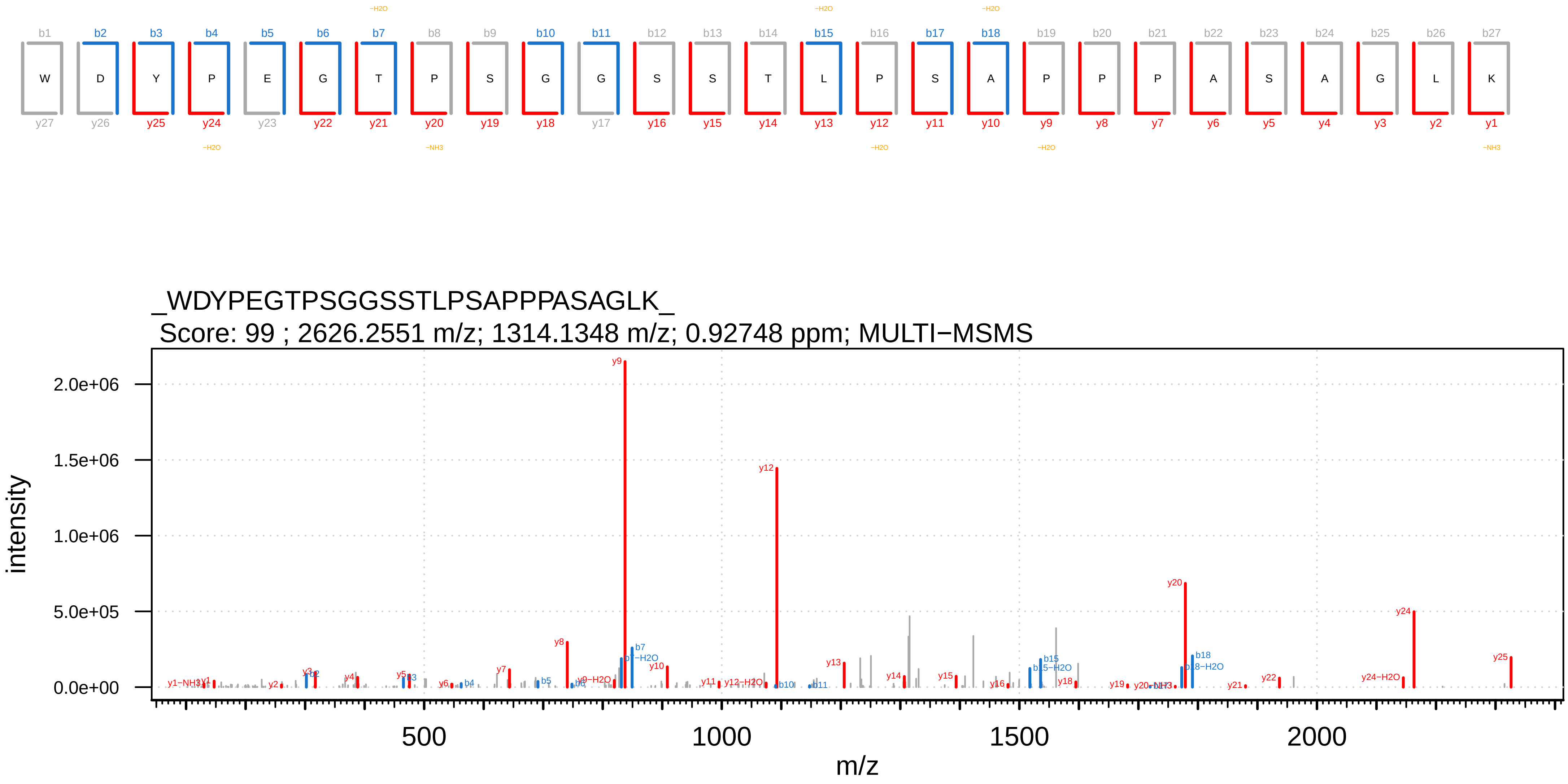

Raw File: 20140819_QEp4_BaAz_SA_H16_R3
Scan Number: 94813
Proteins:
ENSMUST00000149514_chrX:153723700−153723906:+

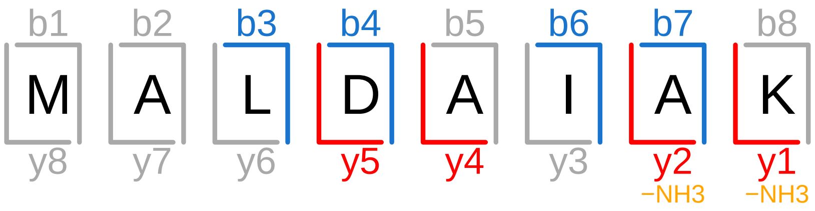

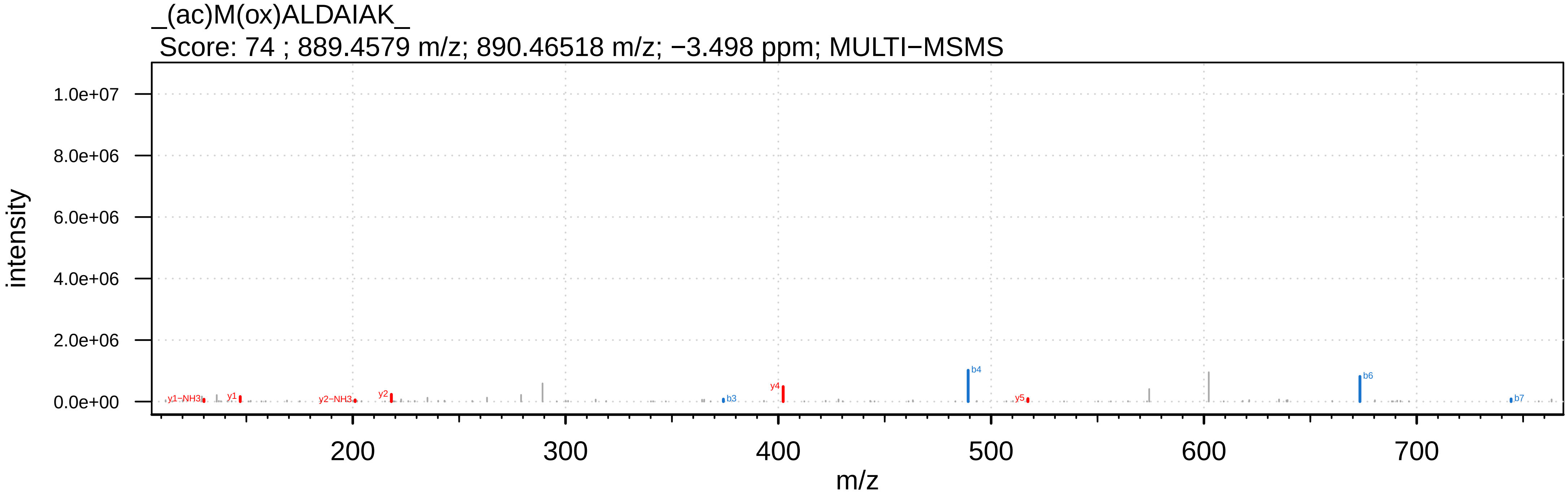

Raw File: 20130720_EXQ4_BaAz_SA_KR3_03
Scan Number: 47862
Proteins:
ENSMUST00000094227_chr3:59952394−59958606:+

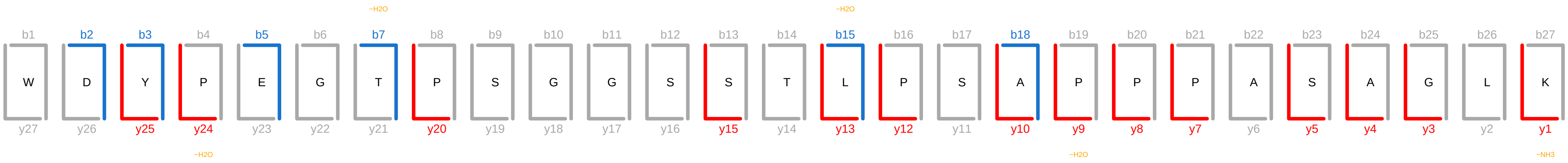

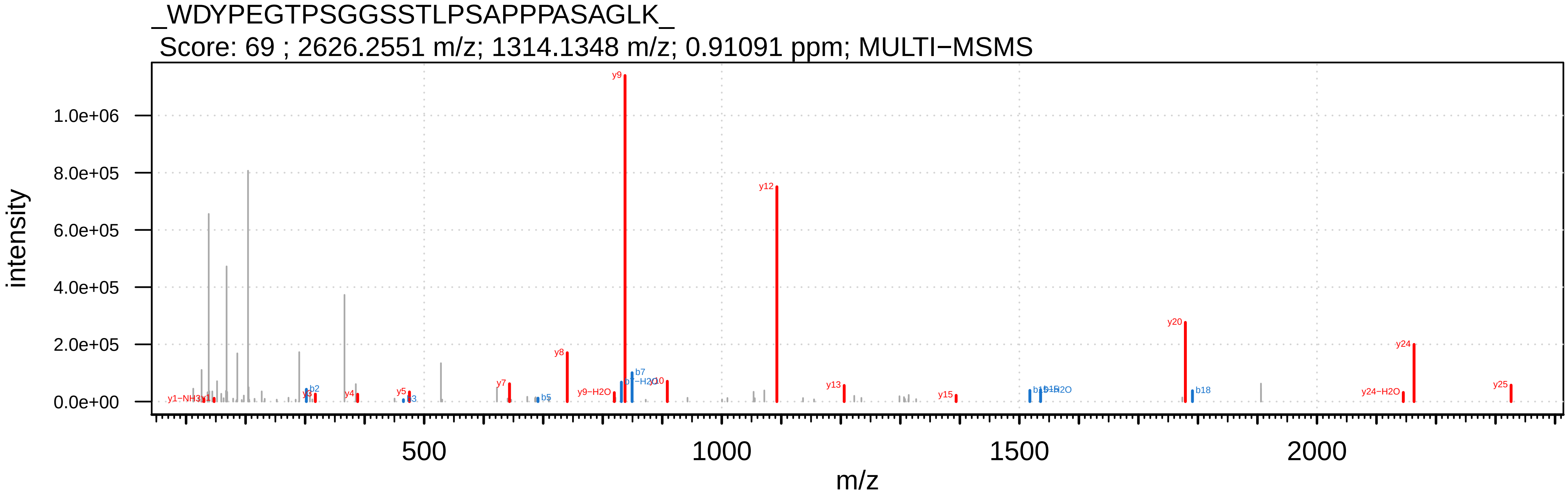

Raw File: 20130720_EXQ4_BaAz_SA_KR3_03
Scan Number: 70669
Proteins:
ENSMUST00000149514_chrX:153723700−153723906:+

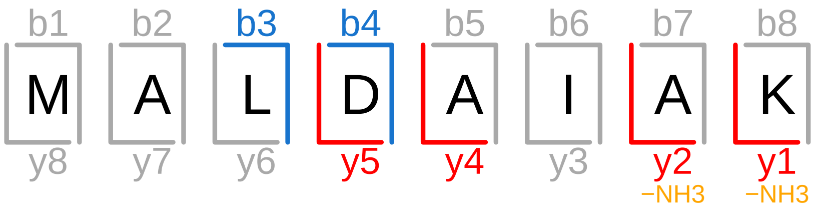

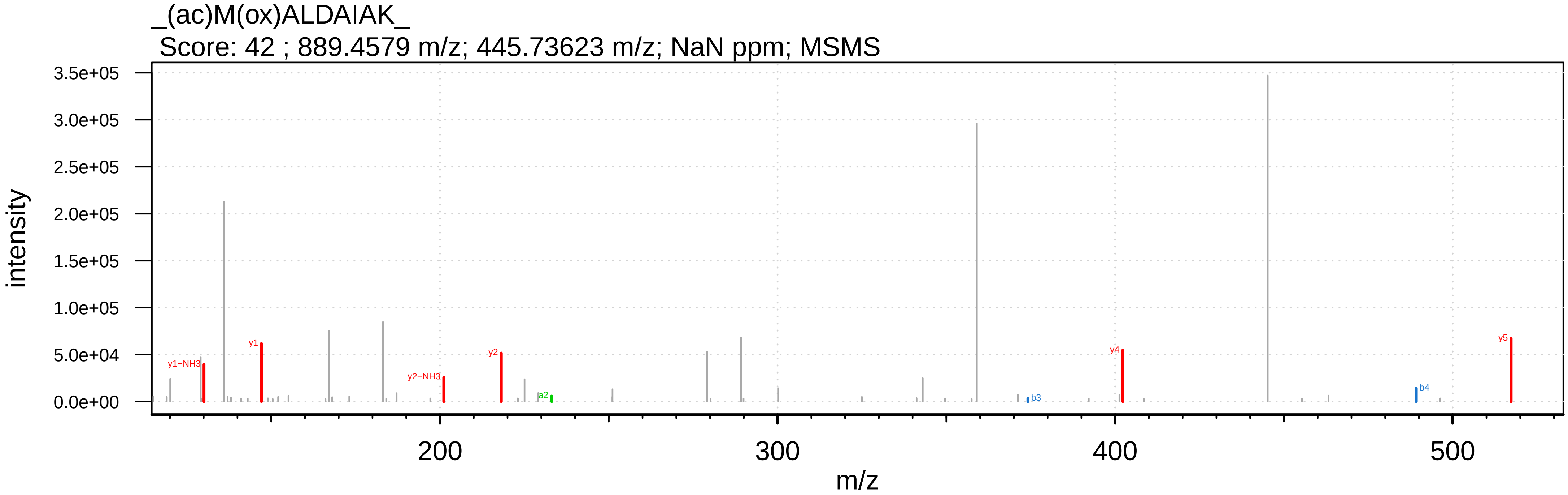

Raw File: 20130806_EXQ4_BaAz_SA_KR4_01
Scan Number: 38883
Proteins:
ENSMUST00000094227_chr3:59952394−59958606:+

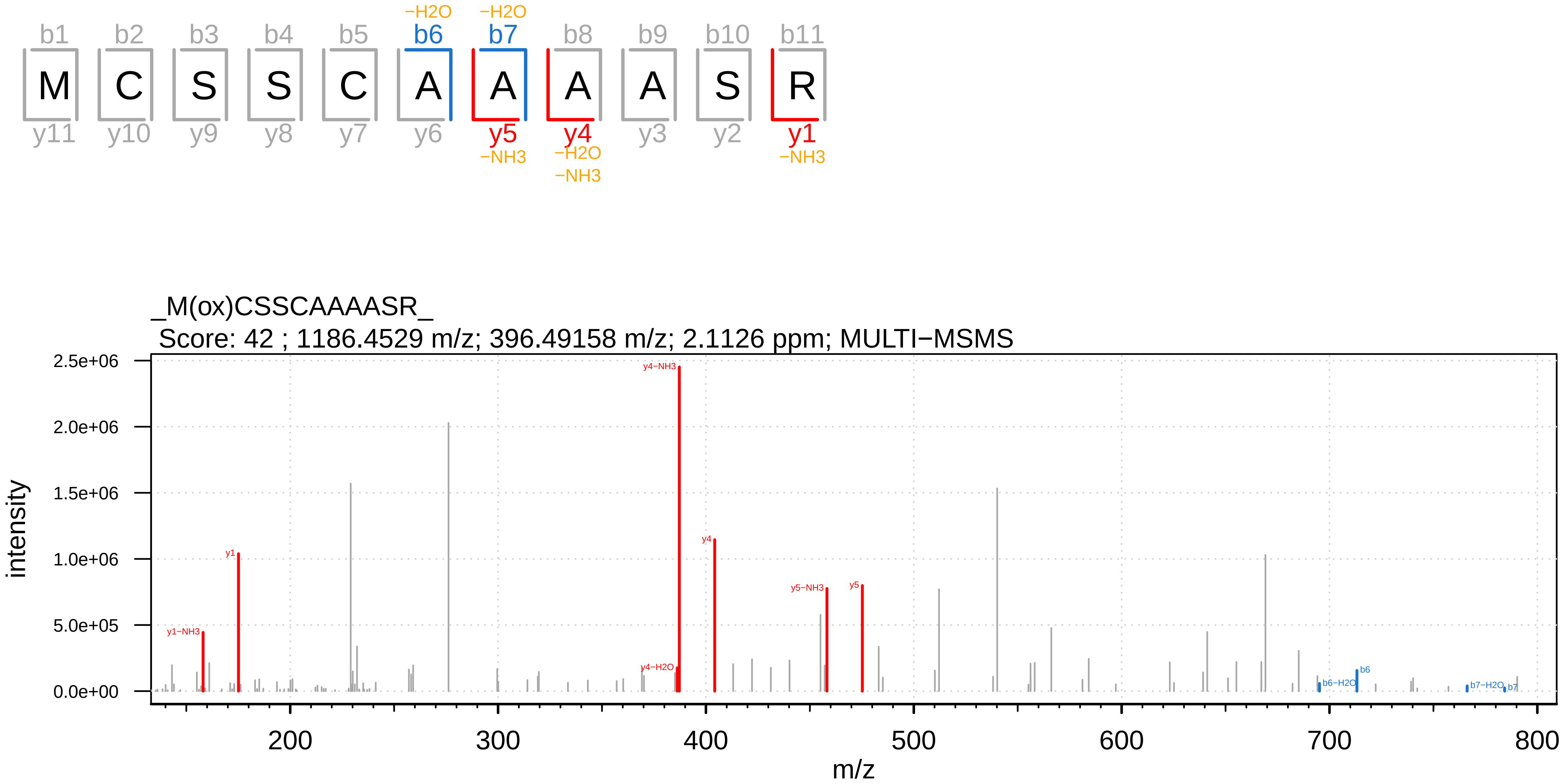

Raw File: 20130705_EXQ4_BaAz_SA_HR2_03
Scan Number: 57339
Proteins:
ENSMUST00000047876_chr3:83128571−83128696:−

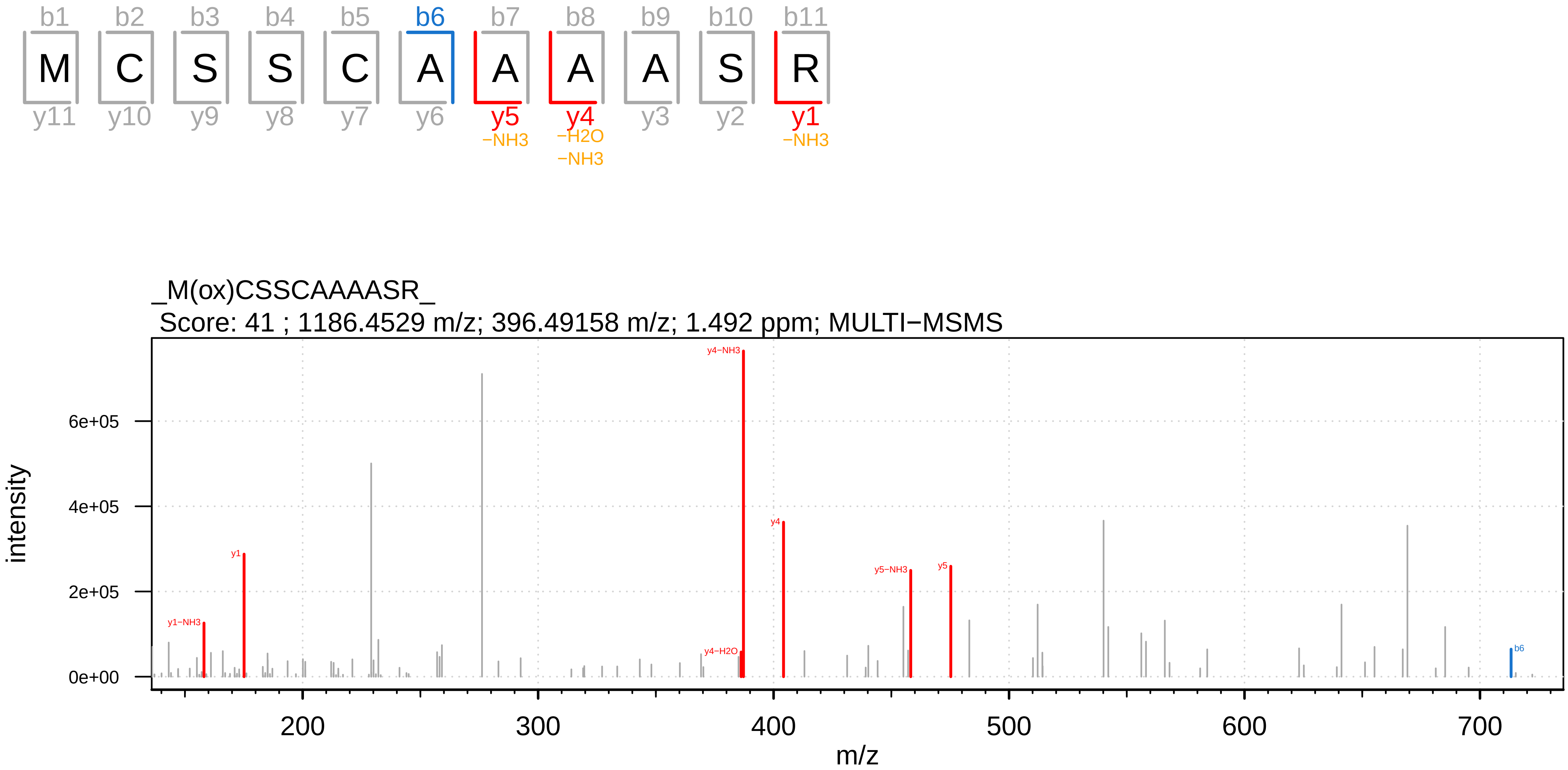

Raw File: 20130713_EXQ4_BaAz_SA_SeR2_02
Scan Number: 54511
Proteins:
ENSMUST00000047876_chr3:83128571−83128696:−

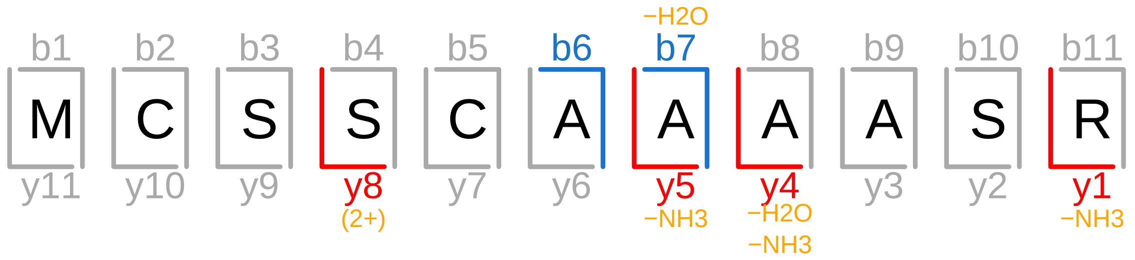

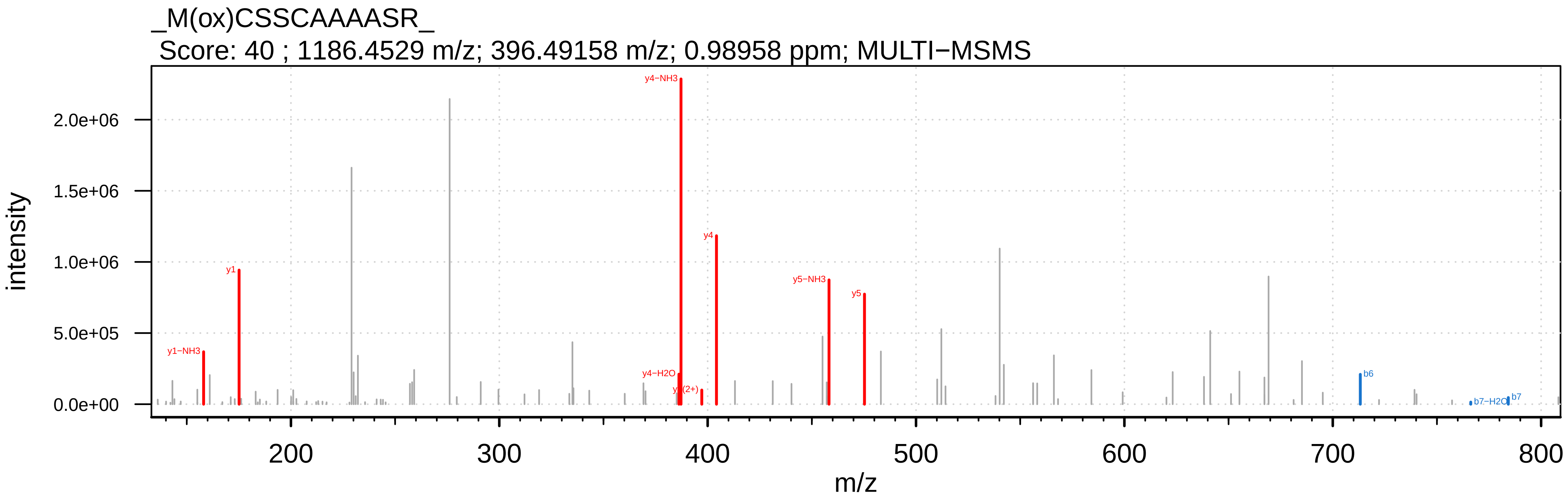

Raw File: 20130713_EXQ4_BaAz_SA_SeR2_04
Scan Number: 59856
Proteins:
ENSMUST00000047876_chr3:83128571−83128696:−

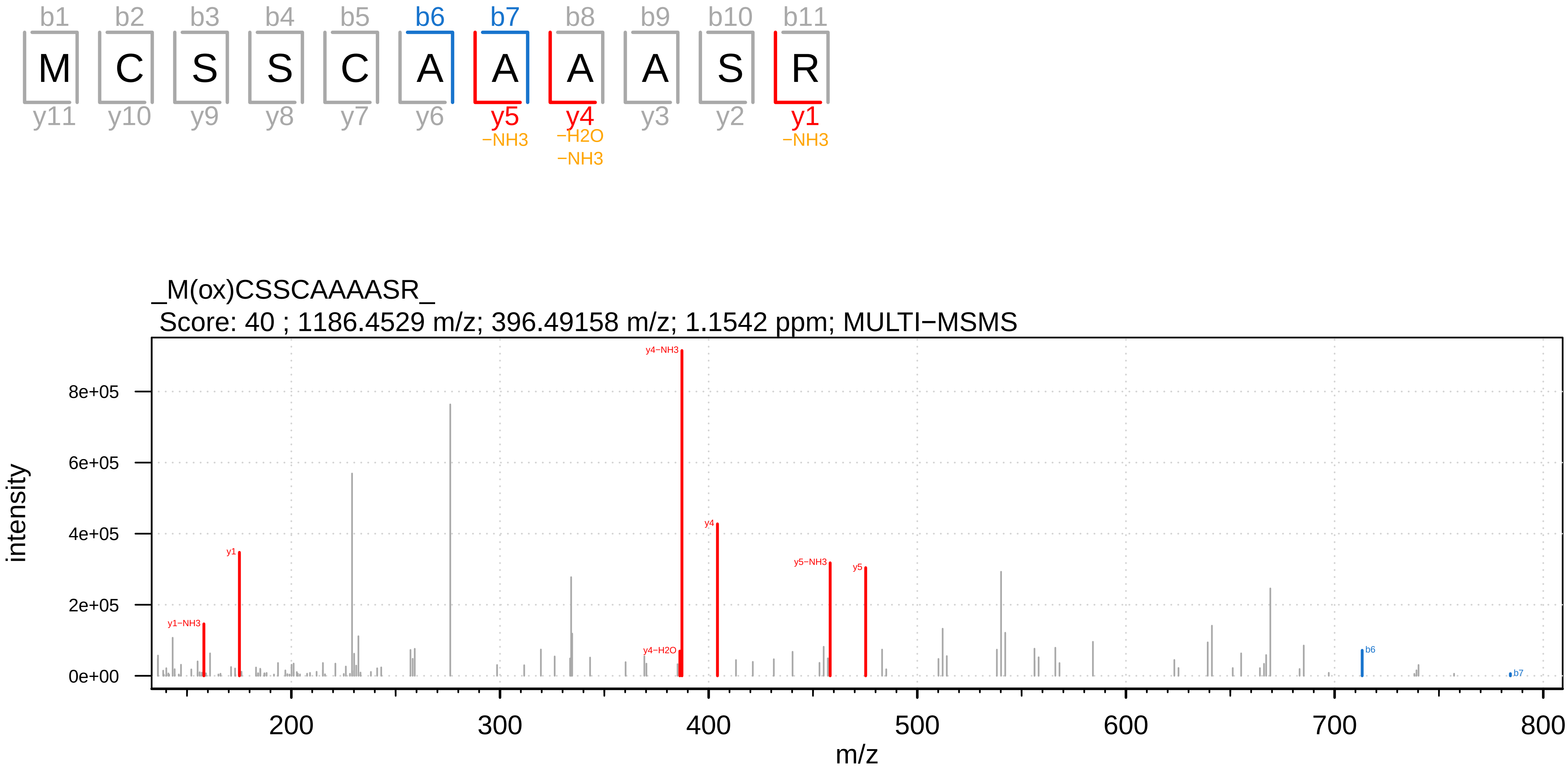

Raw File: 20130716_EXQ4_BaAz_SA_CHR3_02
Scan Number: 55680
Proteins:
ENSMUST00000047876_chr3:83128571−83128696:−

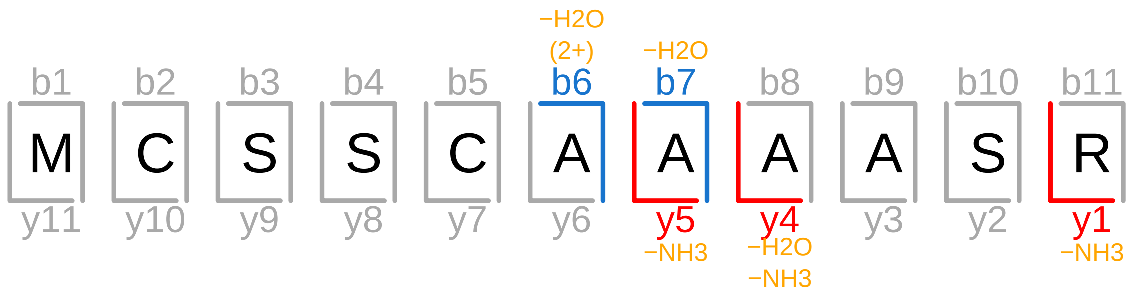

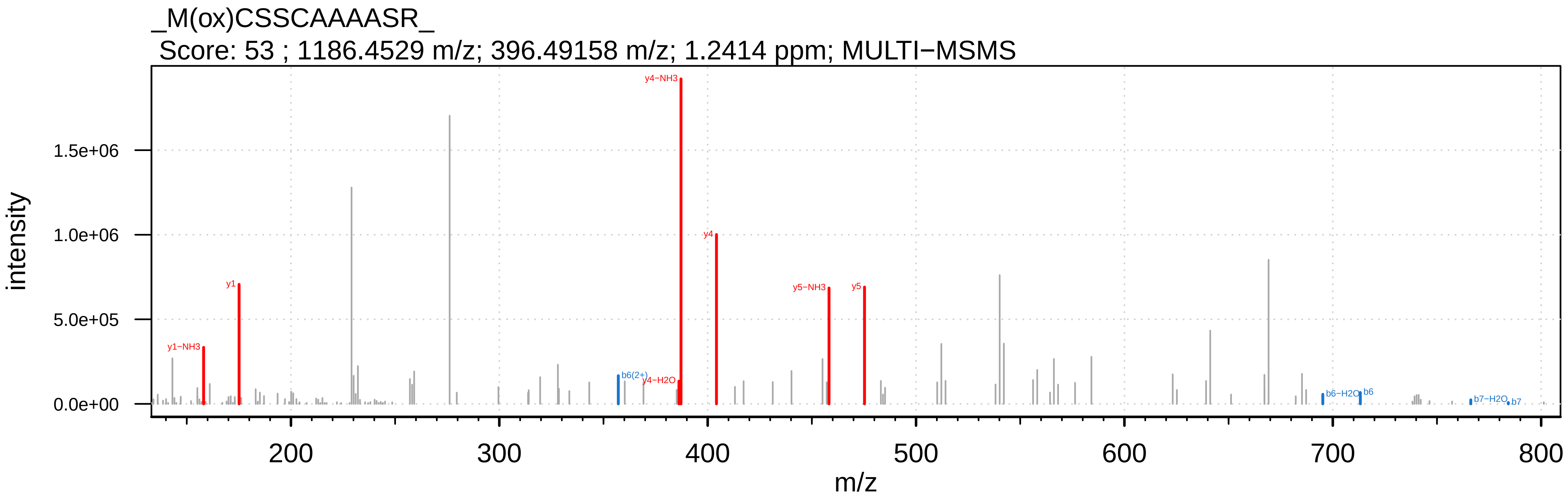

Raw File: 20130718_EXQ6_BaAz_SA_LR3_02

Scan Number: 55330

Proteins:

ENSMUST00000047876_chr3:83128571−83128696:−

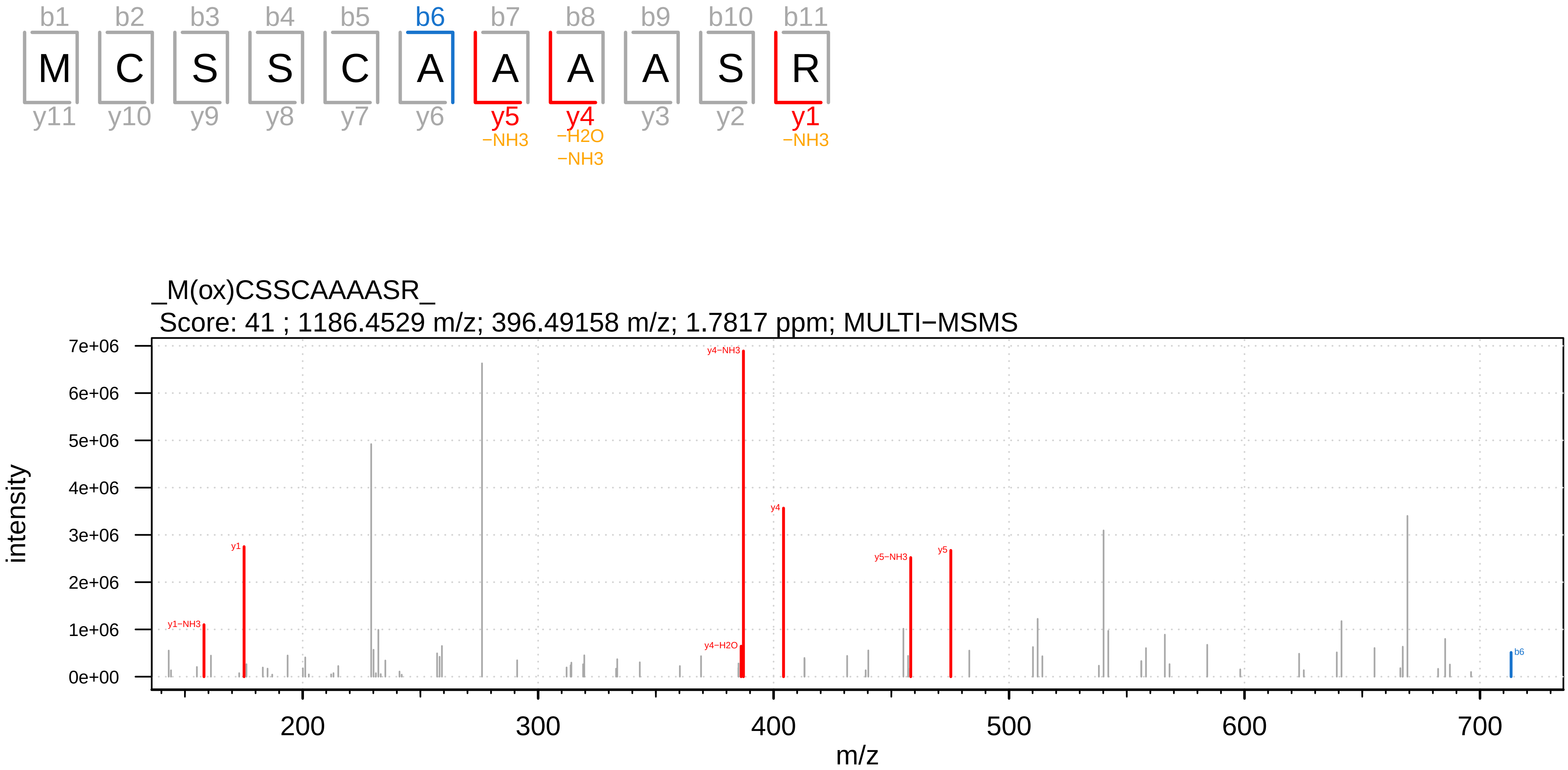

Raw File: 20130719_EXQ4_BaAz_SA_HR2_04
Scan Number: 59631
Proteins:
ENSMUST00000047876_chr3:83128571−83128696:−

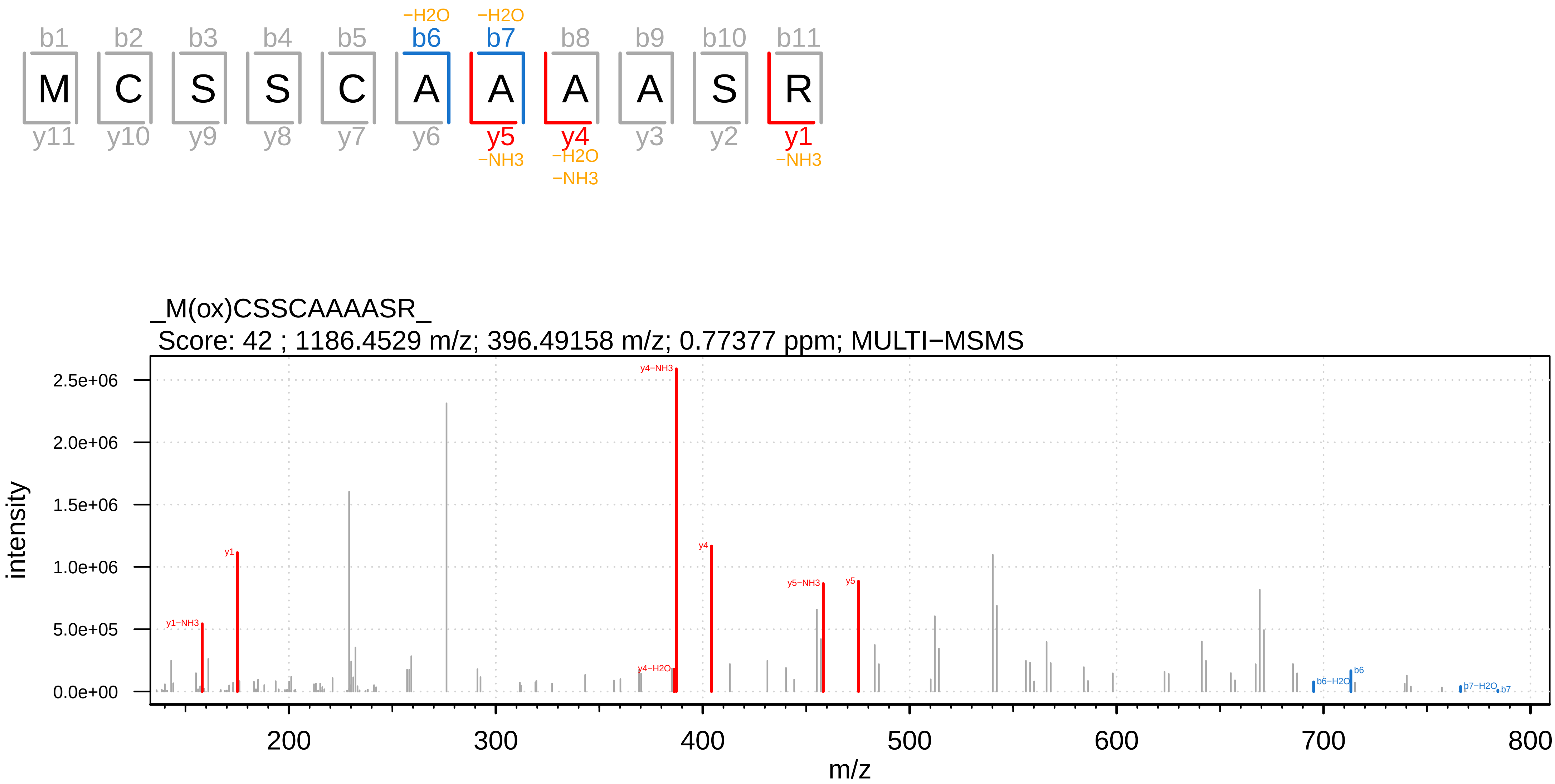

Raw File: 20130731_EXQ4_BaAz_SA_HR4_02
Scan Number: 52276
Proteins:
ENSMUST00000047876_chr3:83128571−83128696:−

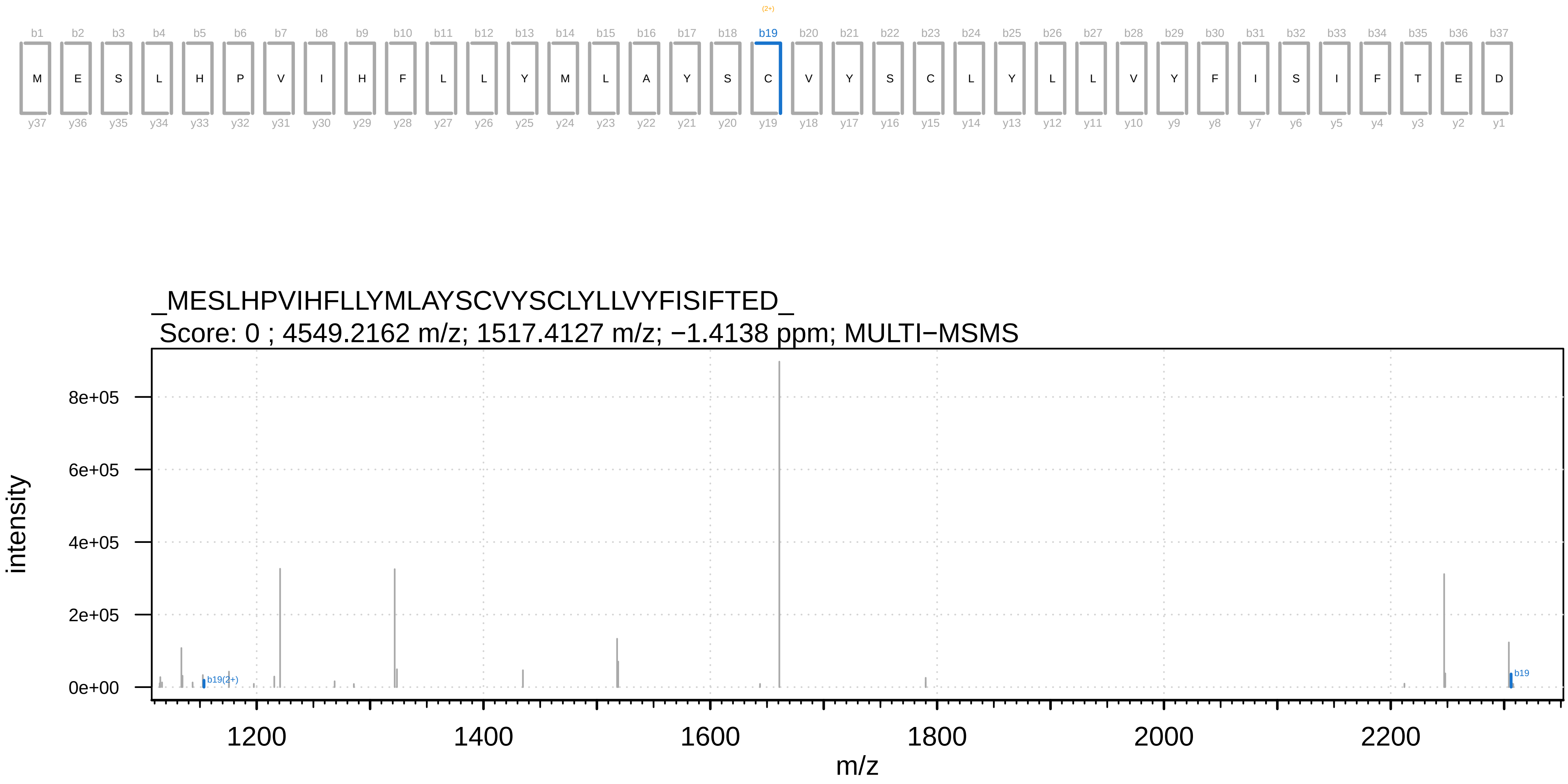

Raw File: 20130408_EXQ6_BaAz_SA_H16R1_02
Scan Number: 80477
Proteins:
ENSMUST00000140321_chr4:107707107−107707220:+

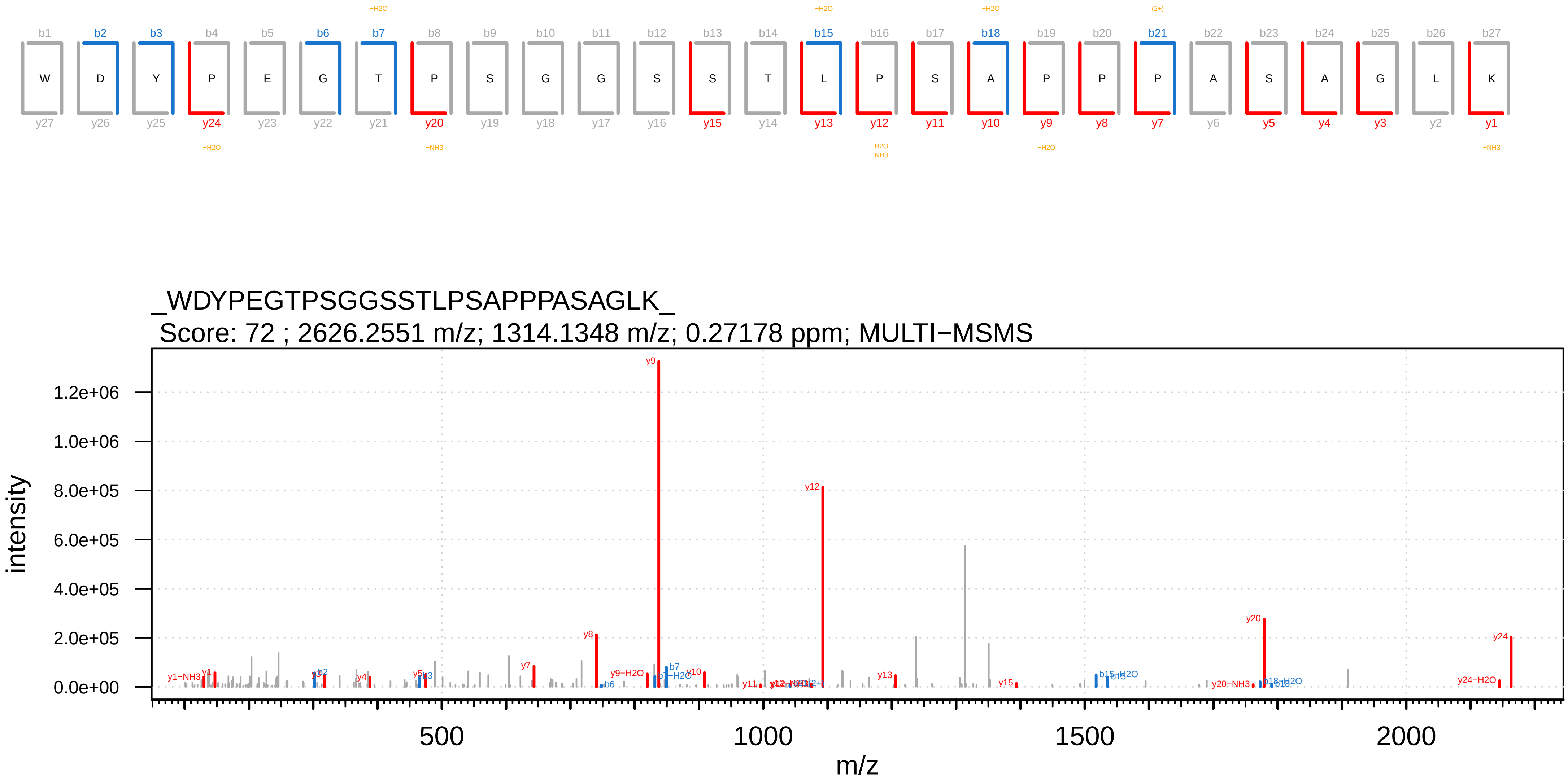

Raw File: 20130408_EXQ6_BaAz_SA_H16R1_02
Scan Number: 71695
Proteins:
ENSMUST00000149514_chrX:153723700−153723906:+

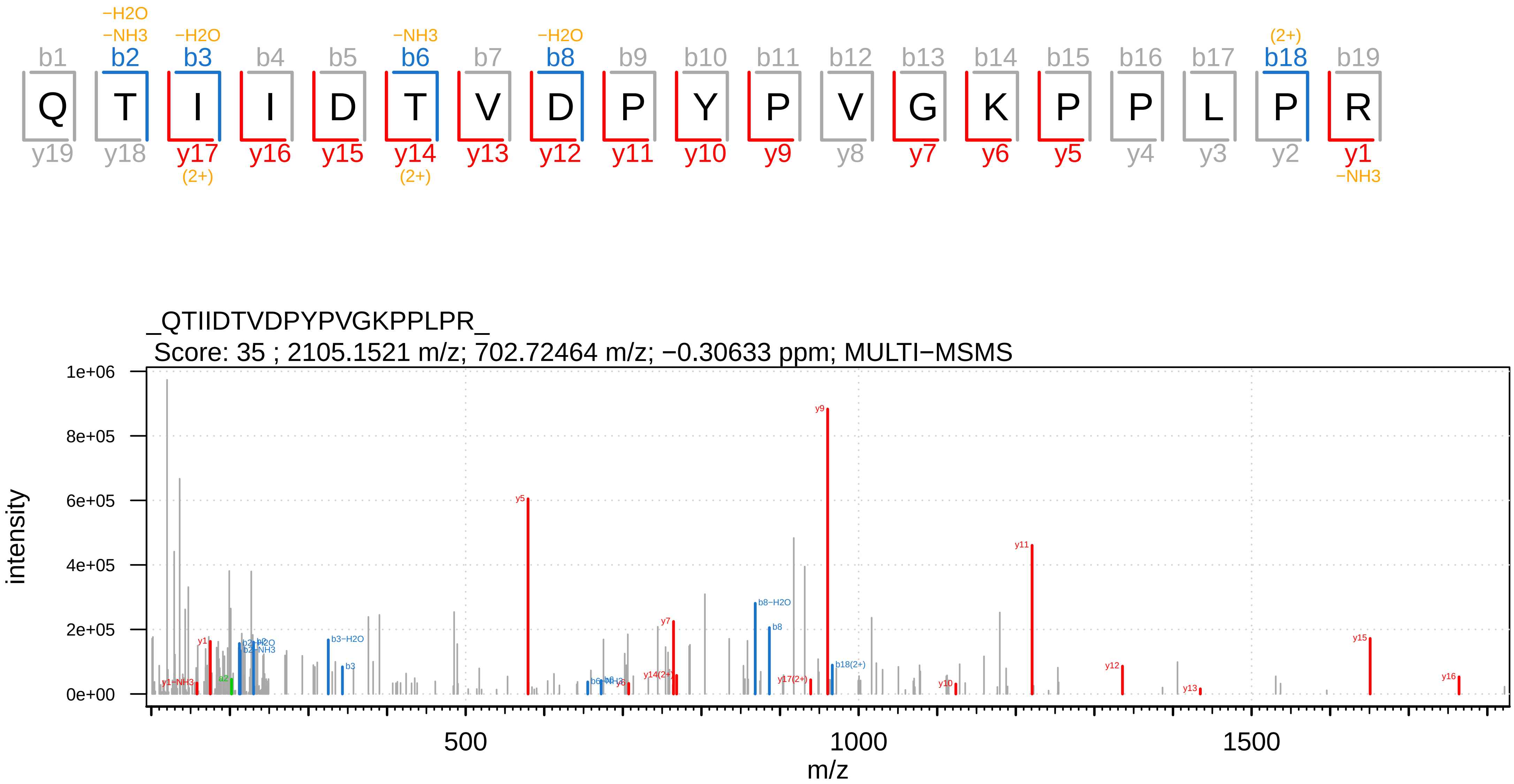

Raw File: 20130723_EXQ4_BaAz_SA_StR3_05
Scan Number: 74941
Proteins:
ENSMUST00000136126_chr3:79172848−79174982:−

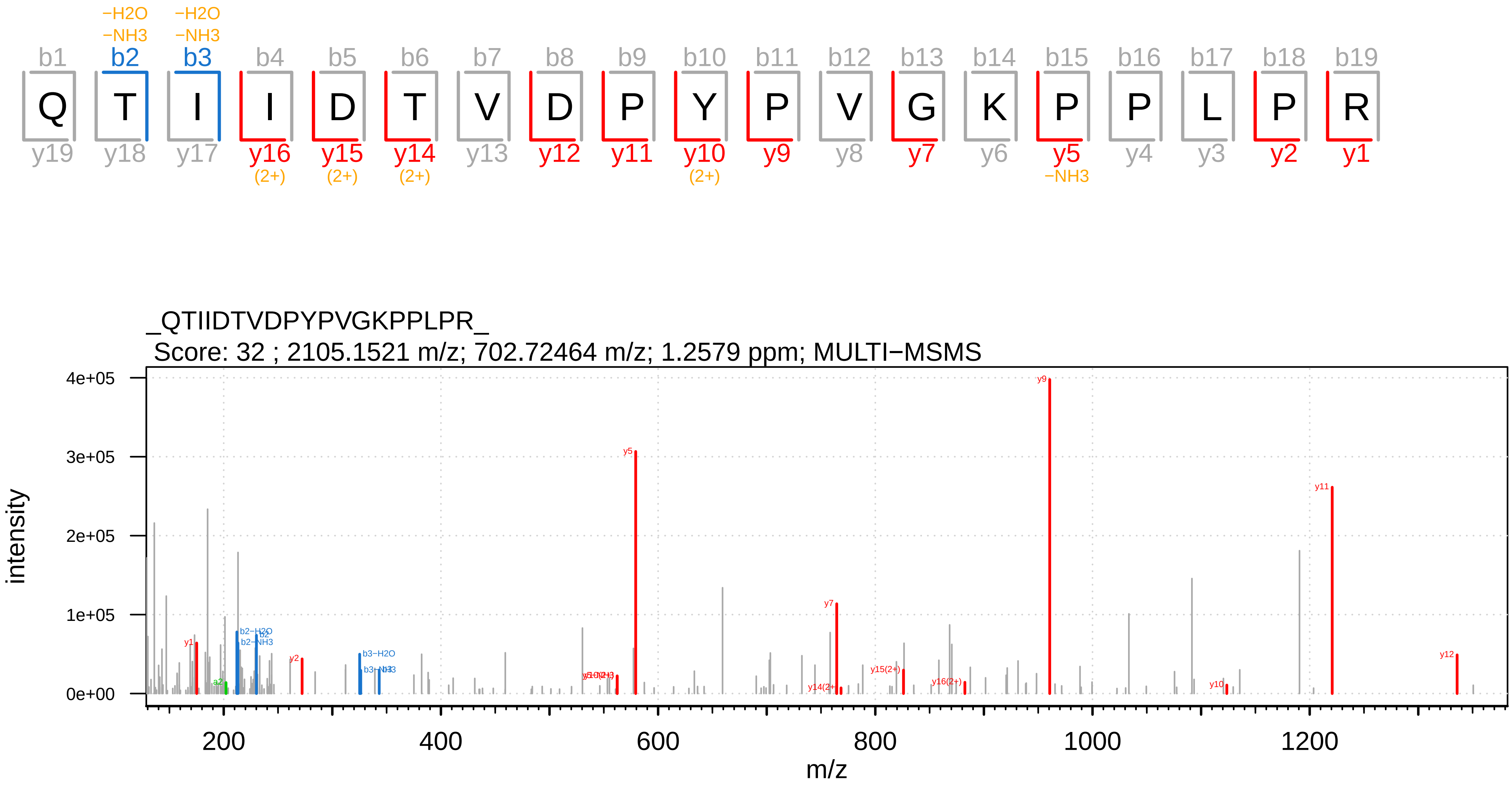

Raw File: 20130801_EXQ4_BaAz_SA_StR4_04
Scan Number: 69832
Proteins:
ENSMUST00000136126_chr3:79172848−79174982:−

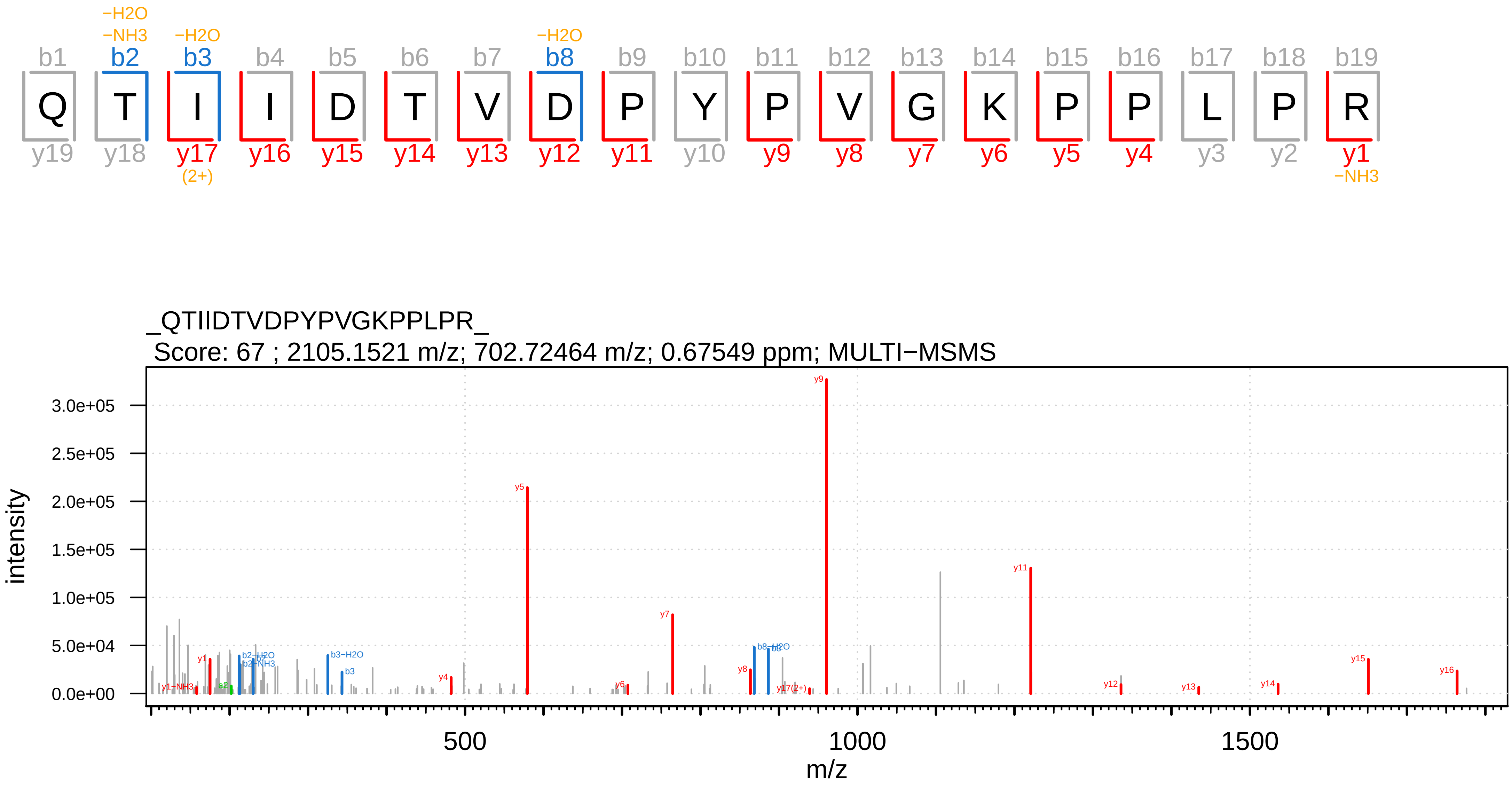

Raw File: 20130808_EXQ4_BaAz_SA_SeR4_04

Scan Number: 66909

Proteins:

ENSMUST00000136126_chr3:79172848−79174982:−

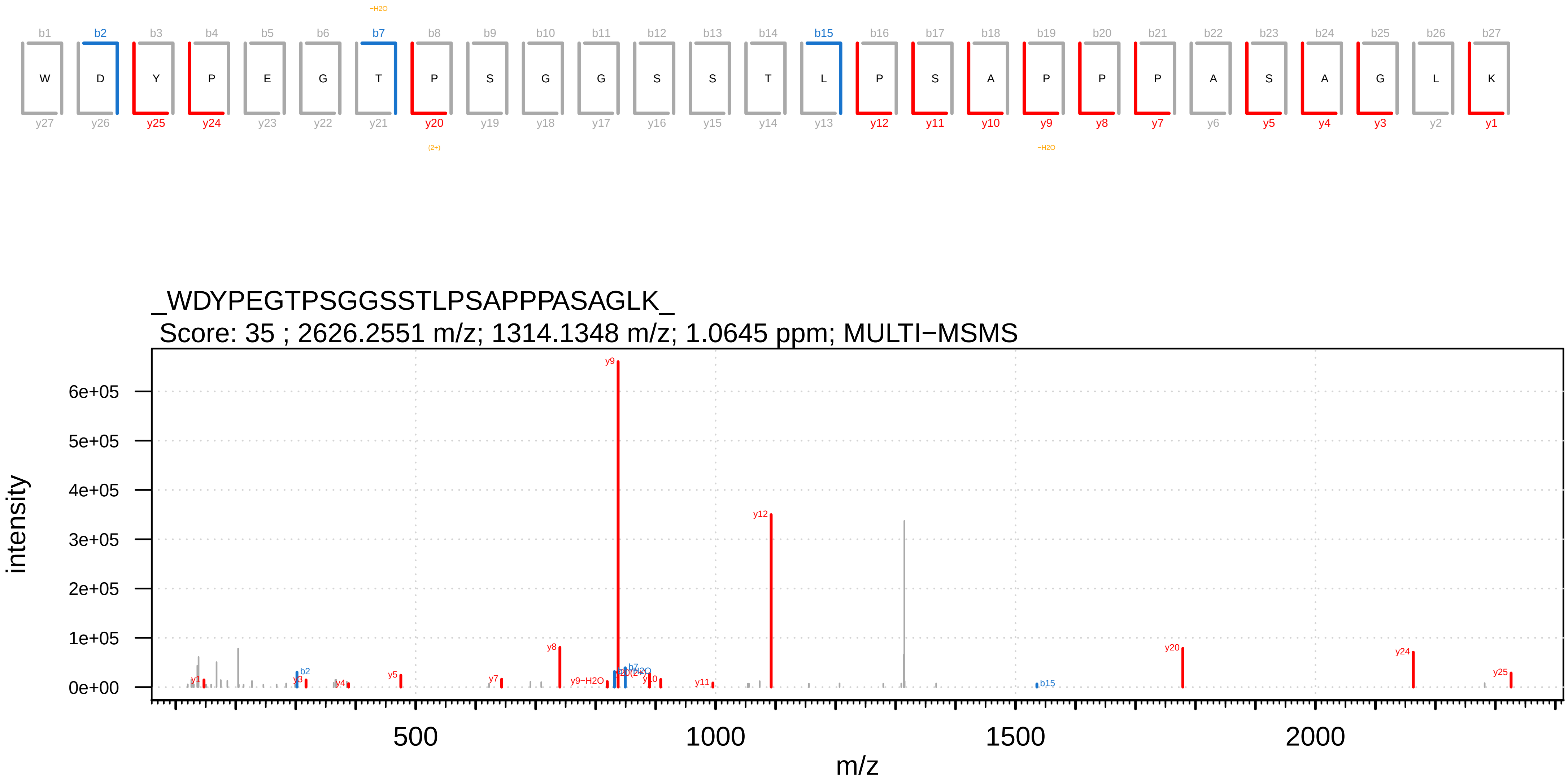

Raw File: 20130808_EXQ4_BaAz_SA_SeR4_04
Scan Number: 61266
Proteins:
ENSMUST00000149514_chrX:153723700−153723906:+

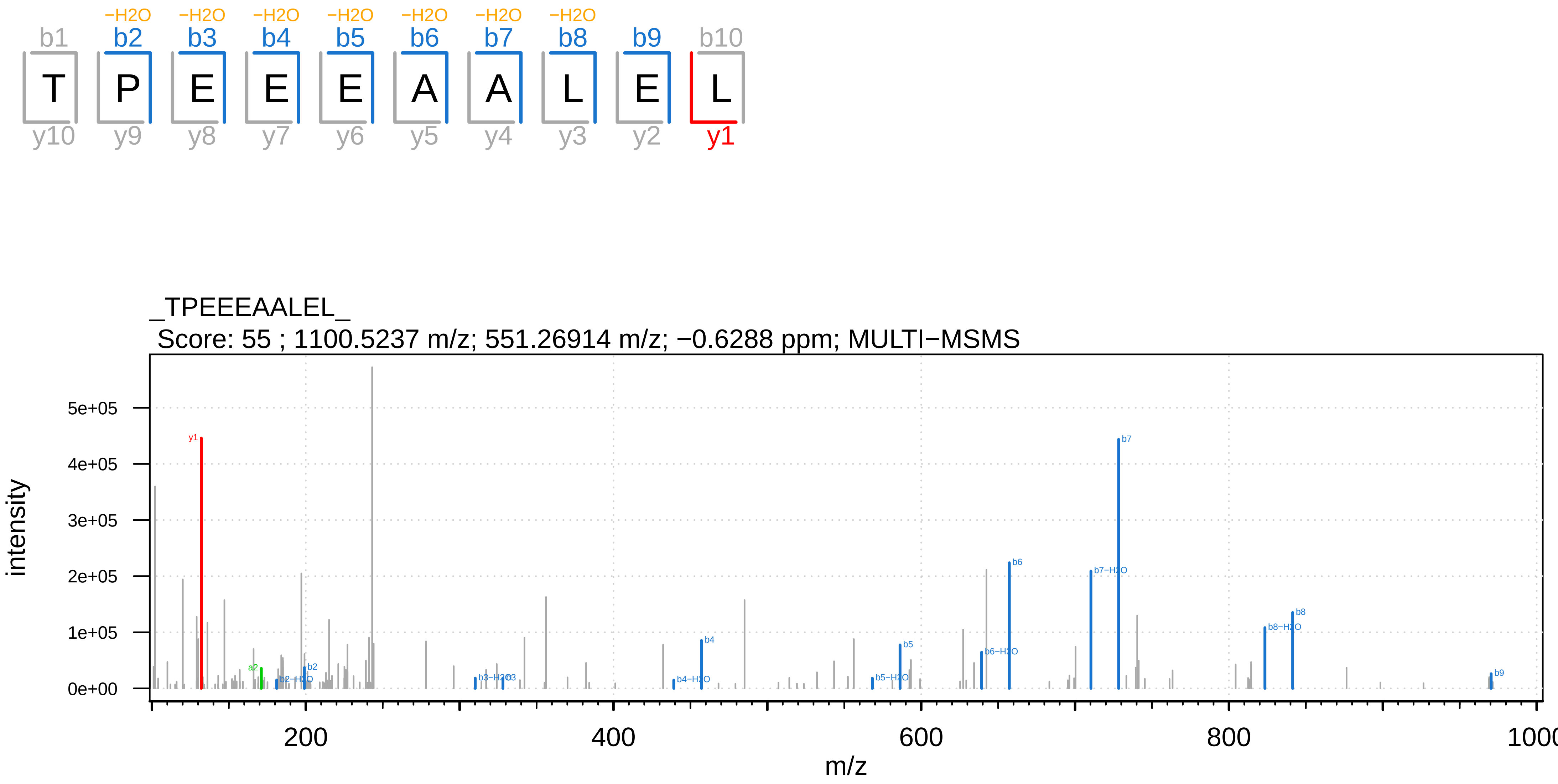

Raw File: 20130720_EXQ4_BaAz_SA_KR3_01
Scan Number: 55055
Proteins:
ENSMUST00000172817_chr17:33905638−33905883:+

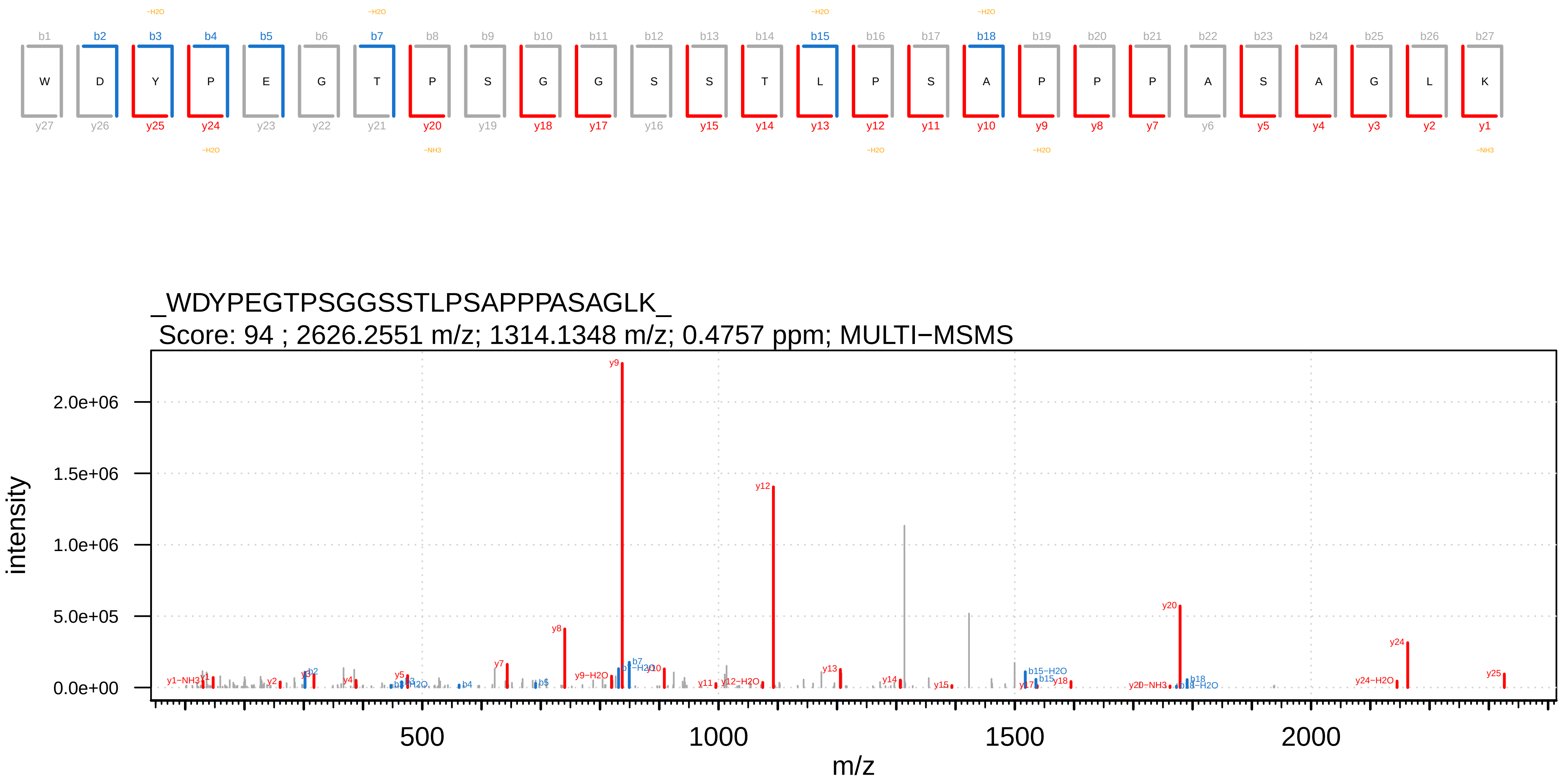

Raw File: 20130408_EXQ6_BaAz_SA_H16R1_03
Scan Number: 72861
Proteins:
ENSMUST00000149514_chrX:153723700−153723906:+

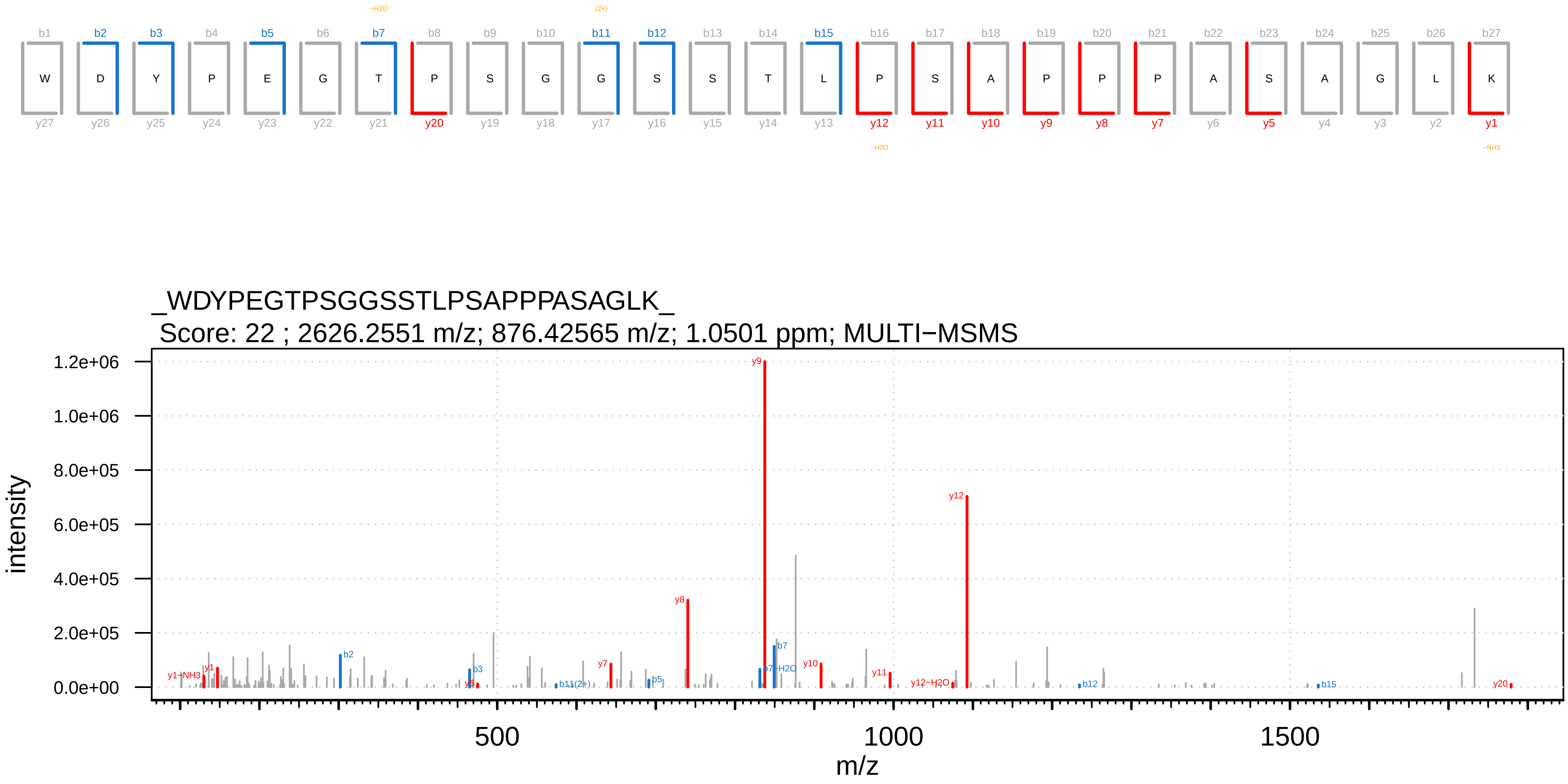

Raw File: 20130409_EXQ6_BaAz_SA_K66R1_02
Scan Number: 71309
Proteins:
ENSMUST00000149514_chrX:153723700−153723906:+

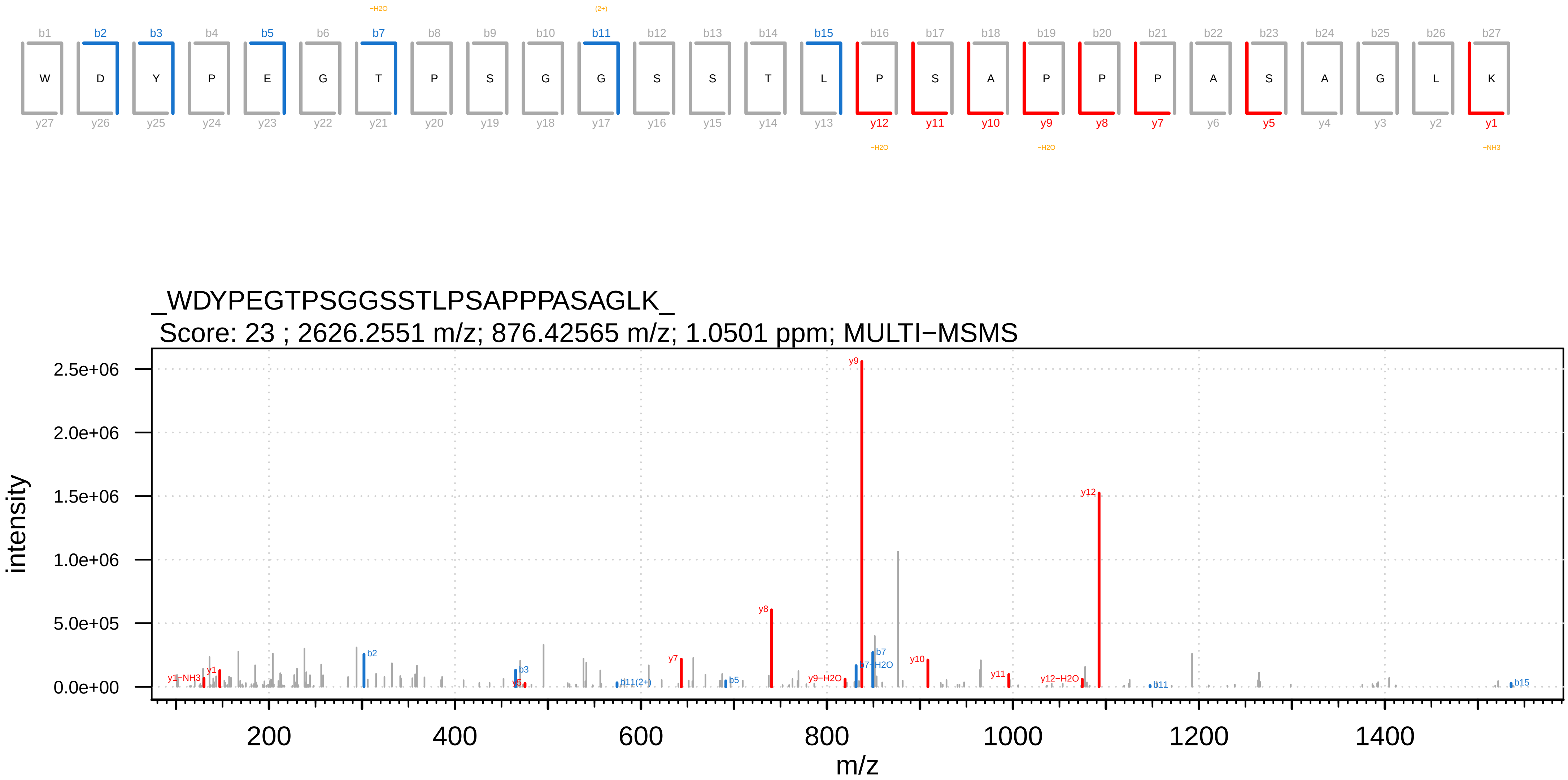

Raw File: 20130409_EXQ6_BaAz_SA_K66R1_02

Scan Number: 71382

Proteins:

ENSMUST00000149514_chrX:153723700−153723906:+

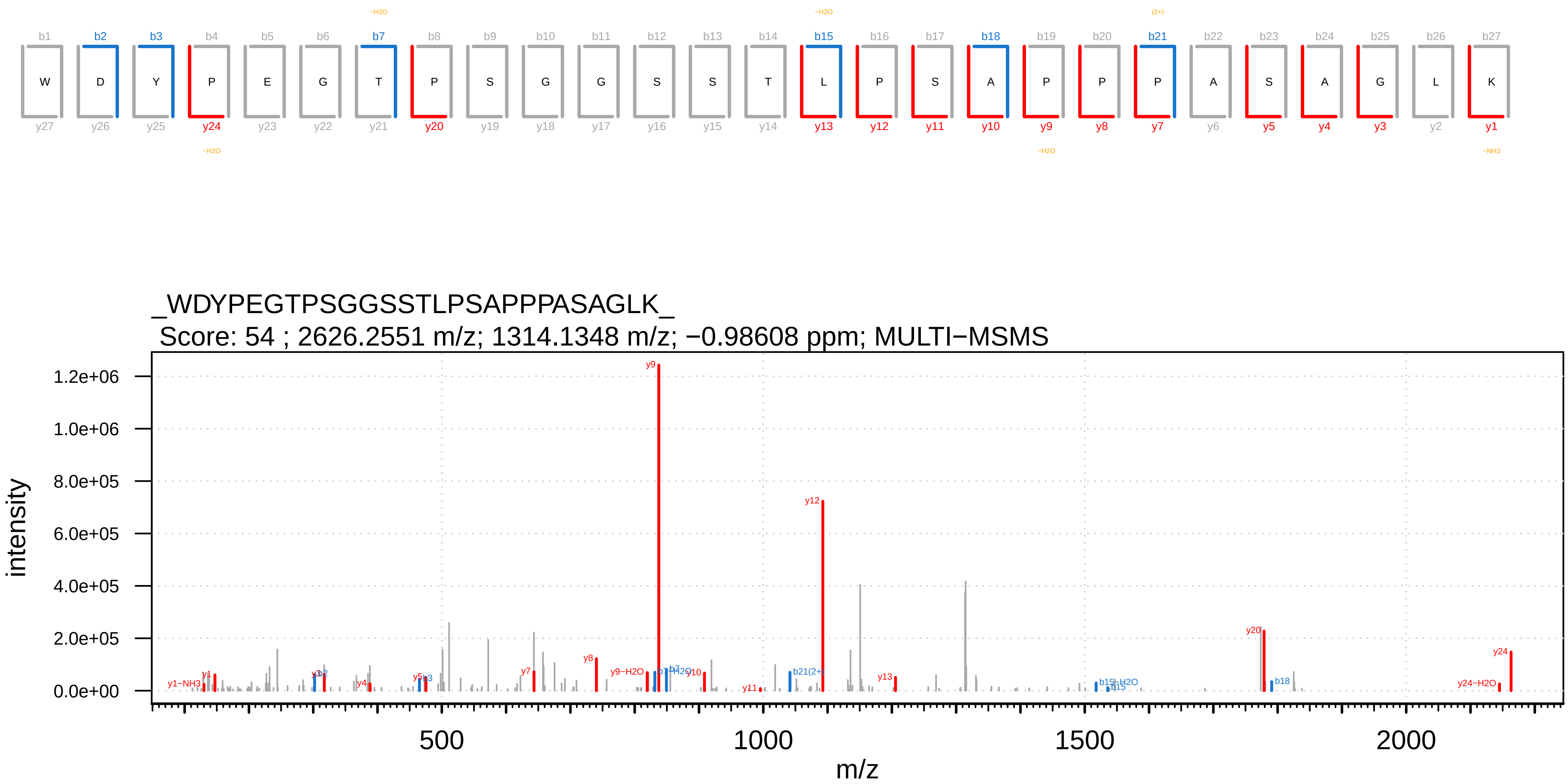

WDYPEGTPSGGSSTLPSAPPPASAGLK

Score: 54 ; 2626.2551 m/z; 1314.1348 m/z; −0.98608 ppm; MULTI−MSMS

Raw File: 20130409_EXQ6_BaAz_SA_K66R1_03

Scan Number: 72960

Proteins:

ENSMUST00000149514_chrX:153723700−153723906:+

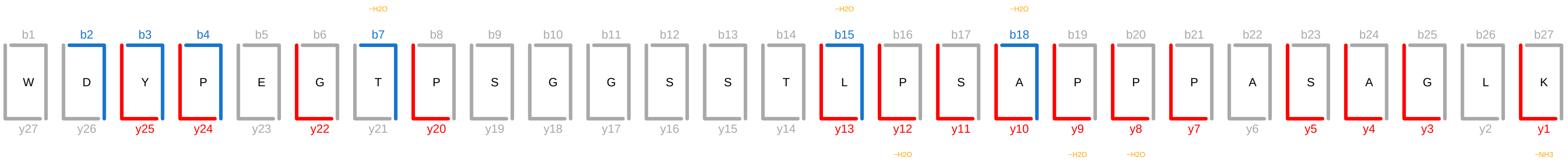

WDYPEGTPSGGSSTLPSAPPPASAGLK

Score: 67 ; 2626.2551 m/z; 1314.1348 m/z; 0.94658 ppm; MULTI−MSMS

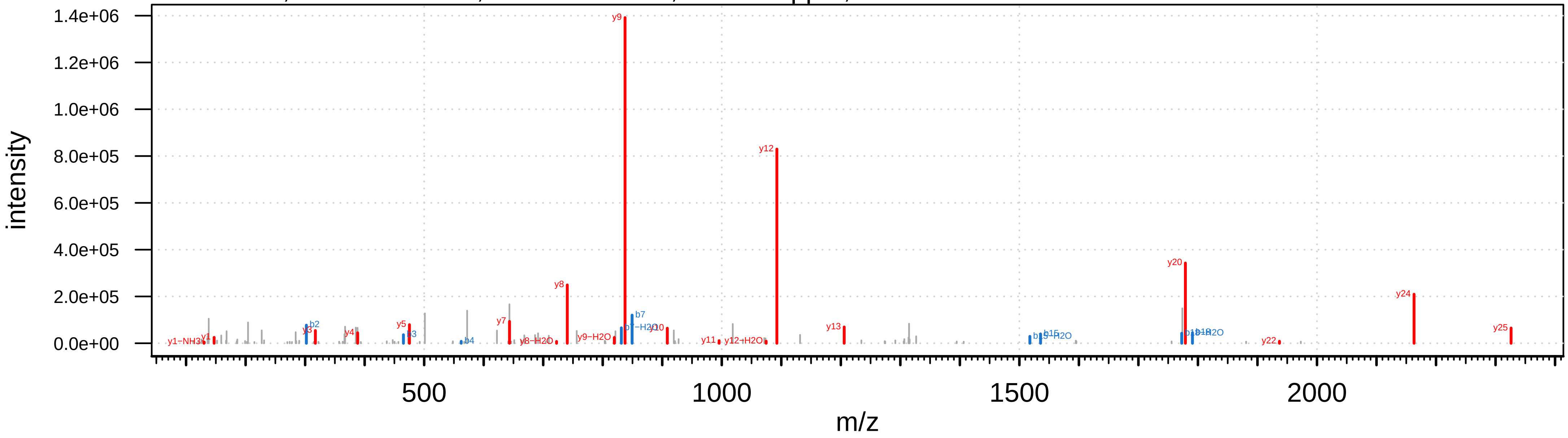

Raw File: 20130411_EXQ6_BaAz_SA_SeR1_03

Scan Number: 68306

Proteins:

ENSMUST00000149514_chrX:153723700−153723906:+

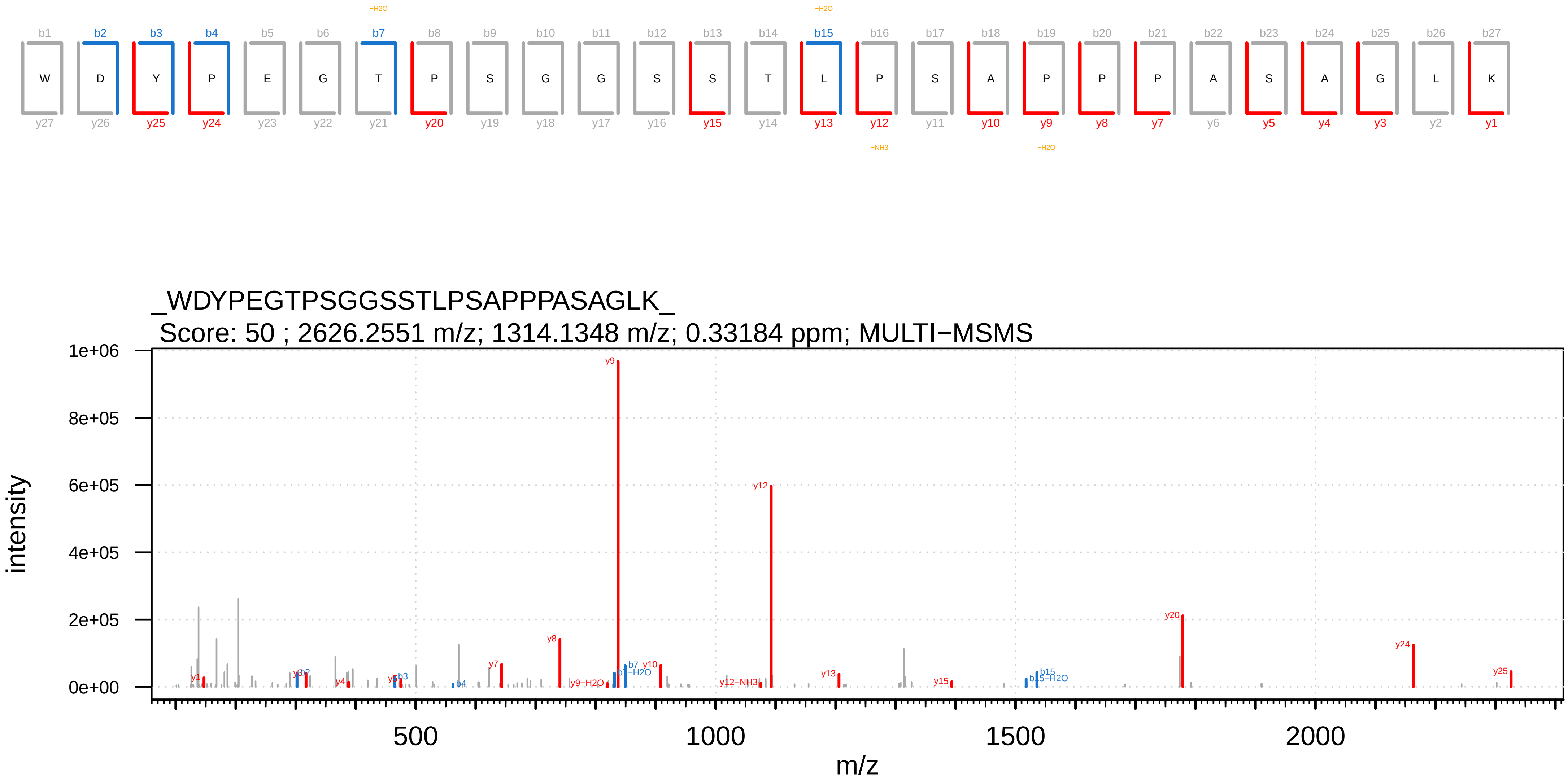

Raw File: 20130419_EXQ6_BaAz_SA_CHR1_03
Scan Number: 65722
Proteins:
ENSMUST00000149514_chrX:153723700−153723906:+

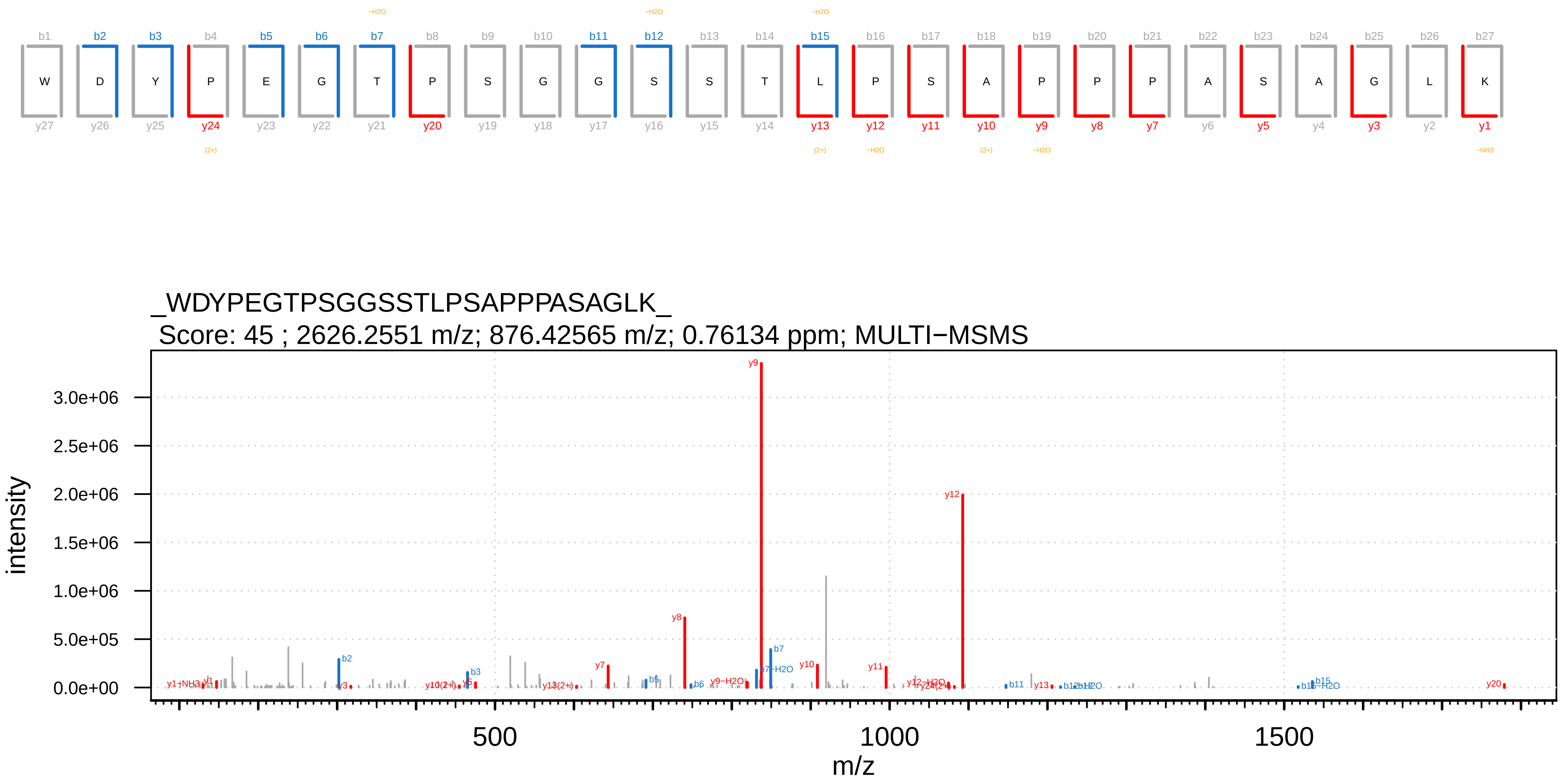

Raw File: 20130419_EXQ6_BaAz_SA_CHR1_03
Scan Number: 65733
Proteins:
ENSMUST00000149514_chrX:153723700−153723906:+

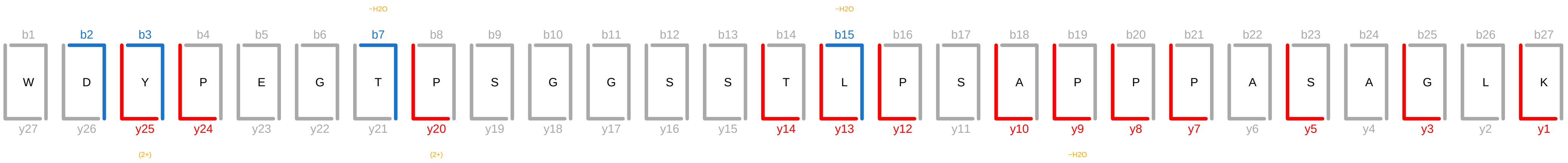

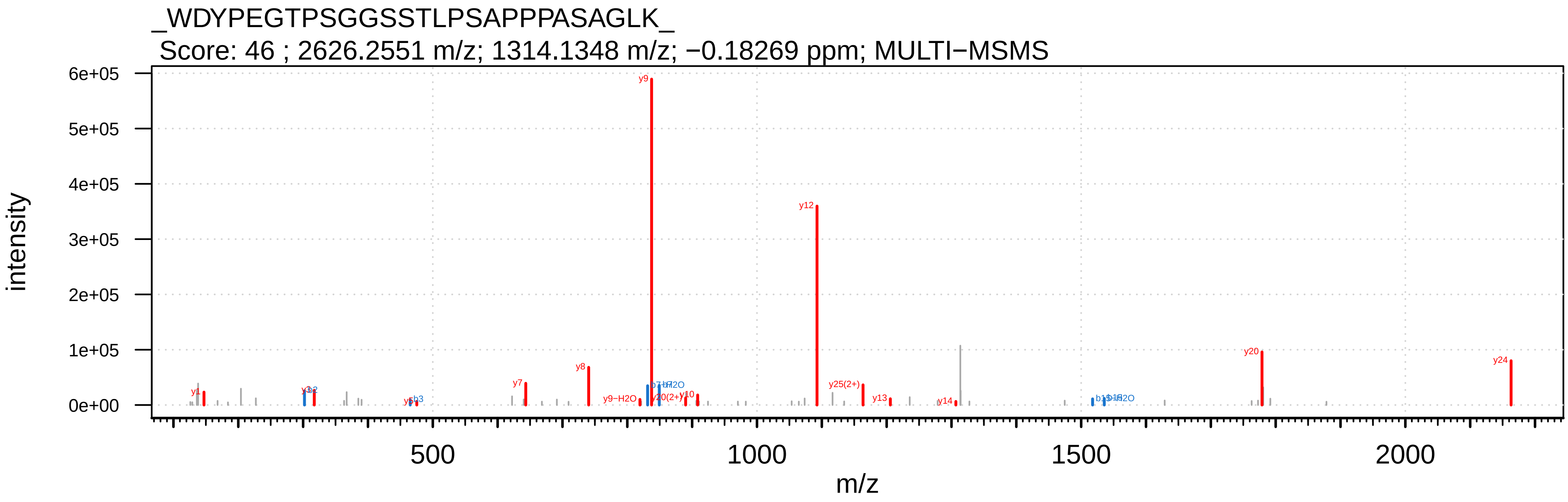

Raw File: 20130419_EXQ6_BaAz_SA_CHR1_04
Scan Number: 68713
Proteins:
ENSMUST00000149514_chrX:153723700−153723906:+

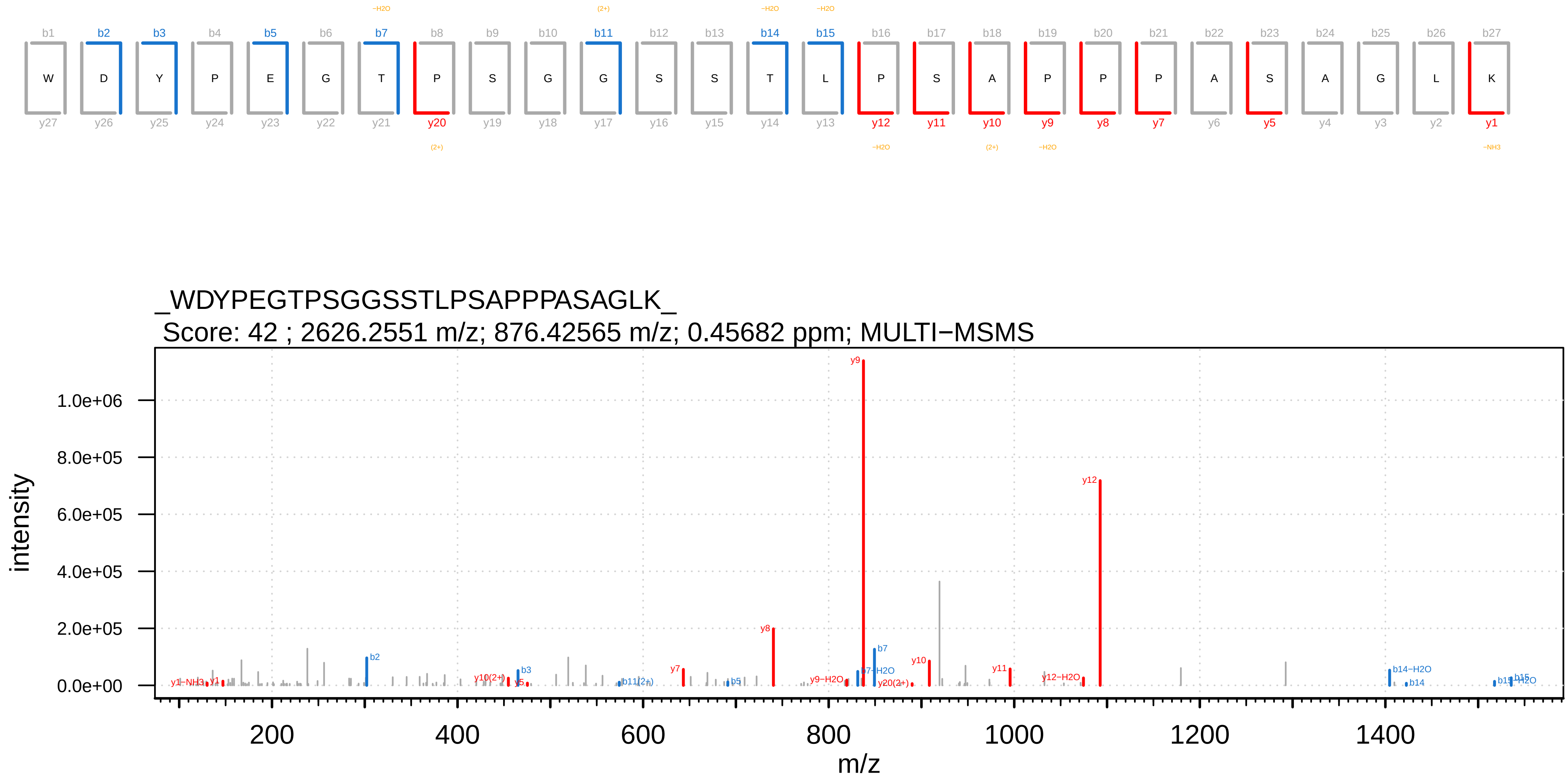

Raw File: 20130705_EXQ6_BaAz_SA_H53R1_02
Scan Number: 62173
Proteins:
ENSMUST00000149514_chrX:153723700−153723906:+

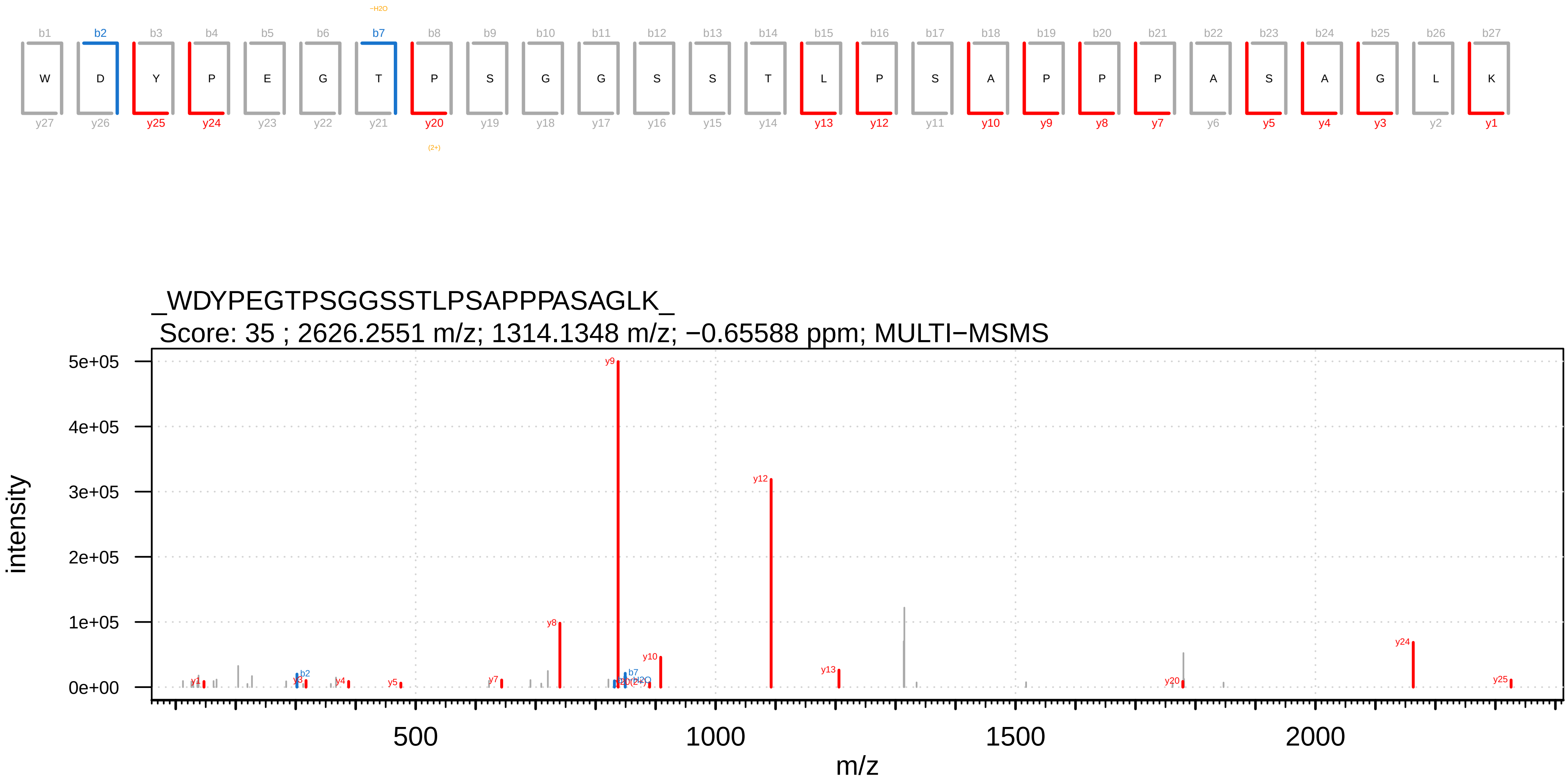

Raw File: 20130705_EXQ6_BaAz_SA_H53R1_03
Scan Number: 65896
Proteins:
ENSMUST00000149514_chrX:153723700−153723906:+

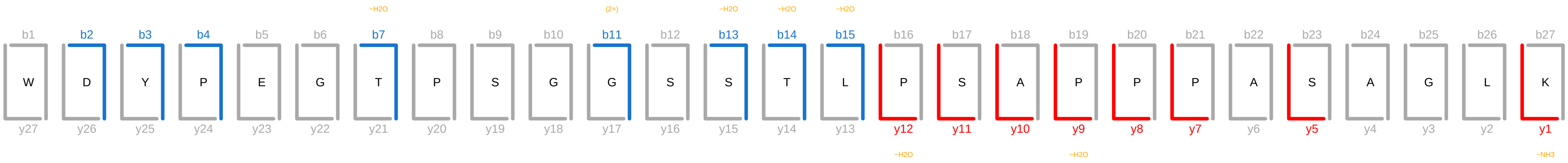

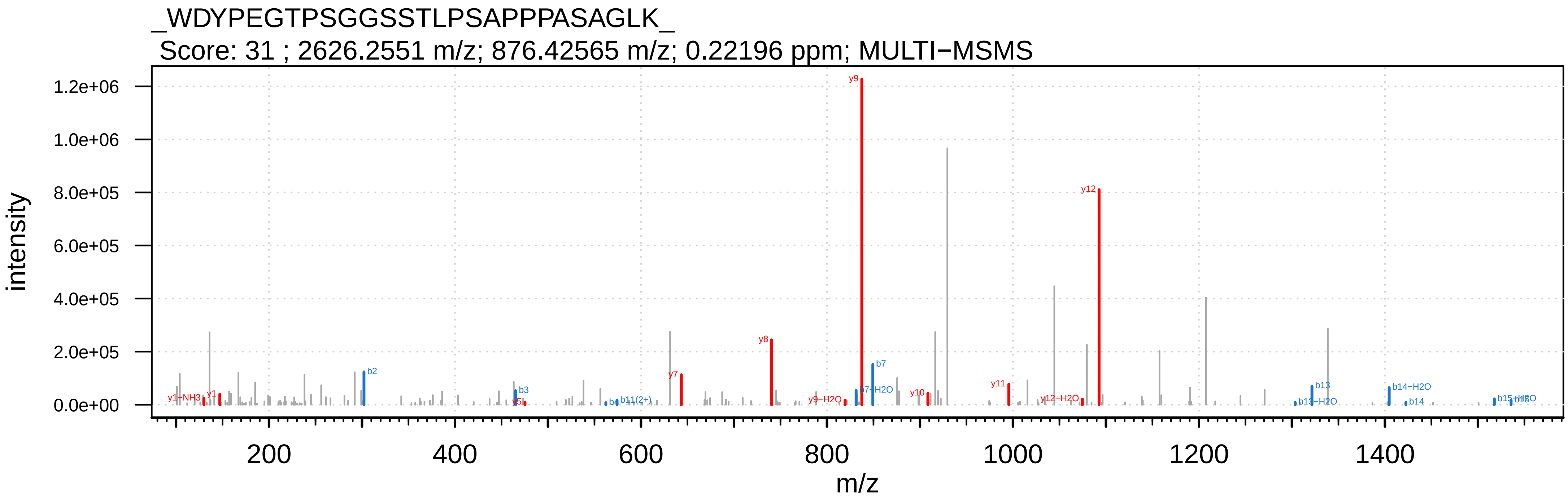

Raw File: 20130705_EXQ6_BaAz_SA_H53R1_03
Scan Number: 65900
Proteins:
ENSMUST00000149514_chrX:153723700−153723906:+

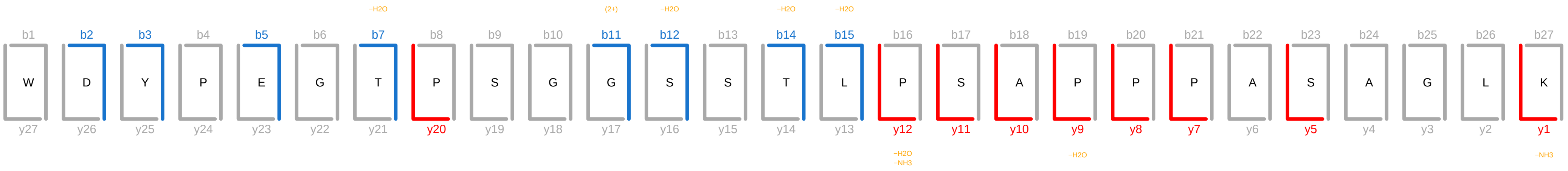

WDYPEGTPSGGSSTLPSAPPPASAGLK
Score: 66 ; 2626.2551 m/z; 876.42565 m/z; 0.1394 ppm; MULTI−MSMS

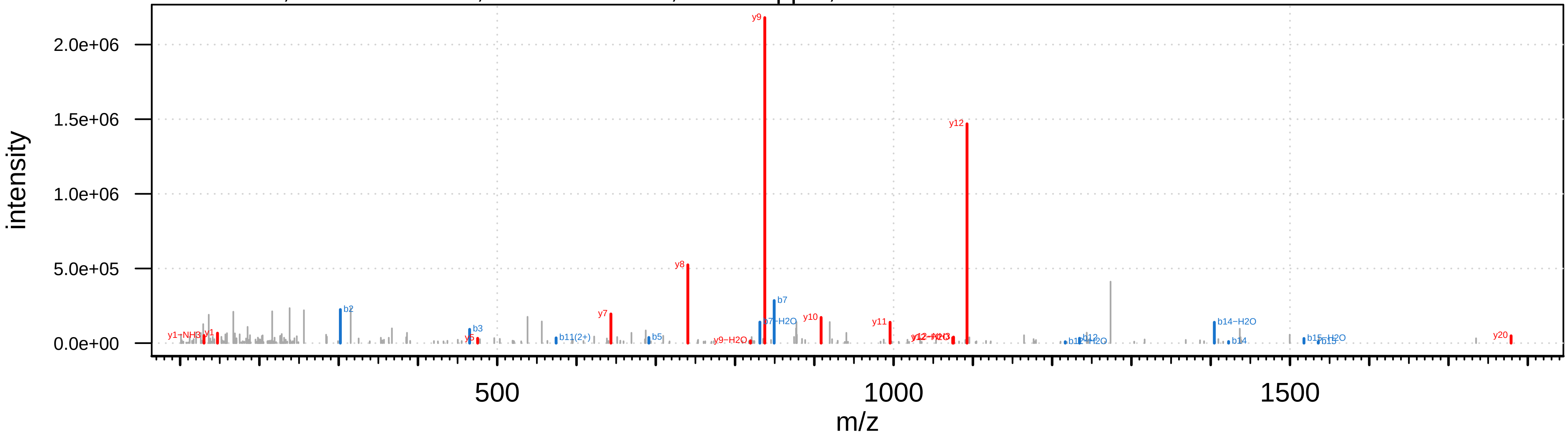

Raw File: 20130712_EXQ4_BaAz_SA_KR2_03
Scan Number: 70476
Proteins:
ENSMUST00000149514_chrX:153723700−153723906:+

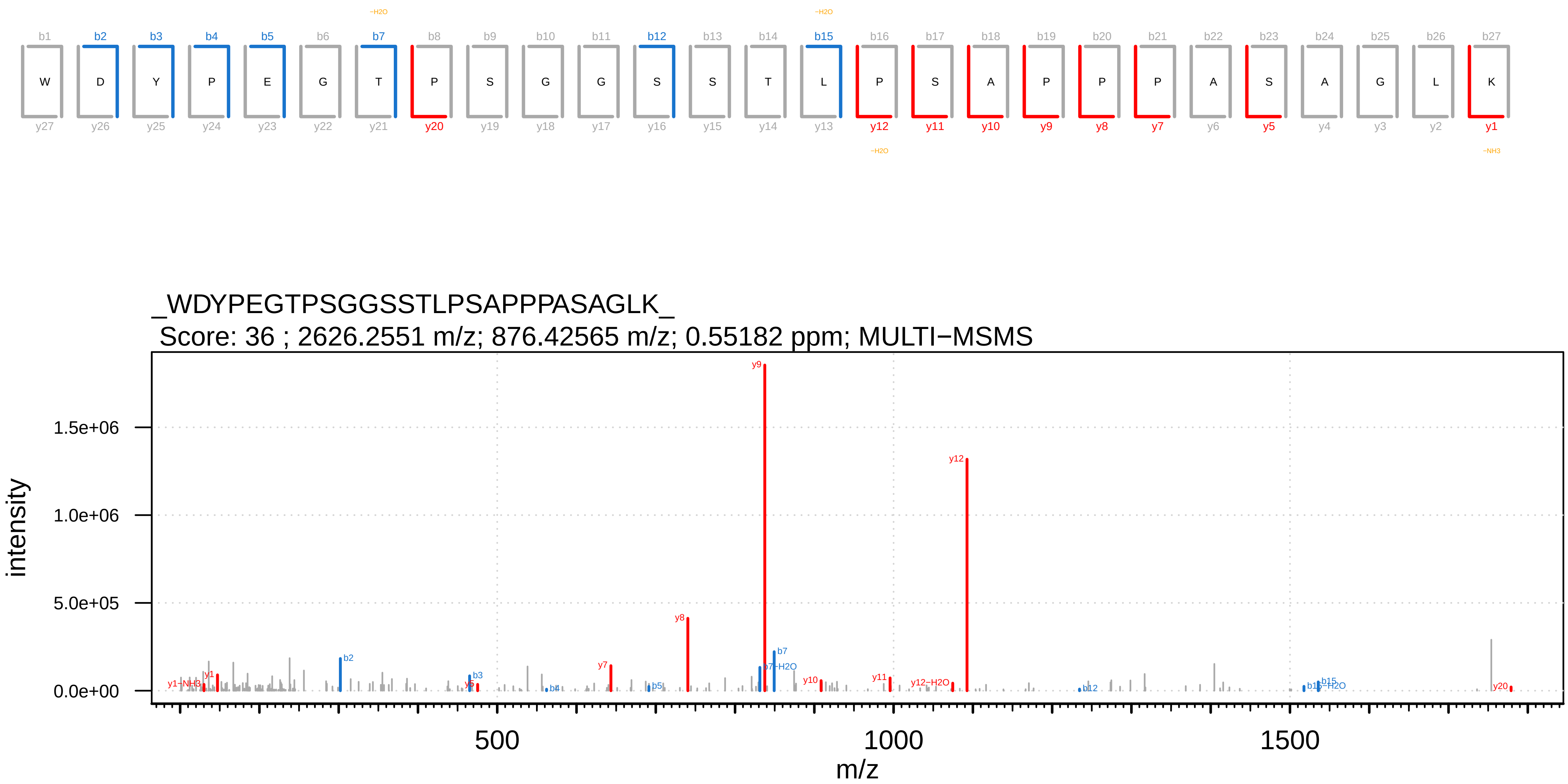

Raw File: 20130713_EXQ4_BaAz_SA_SeR2_03
Scan Number: 67066
Proteins:
ENSMUST00000149514_chrX:153723700−153723906:+

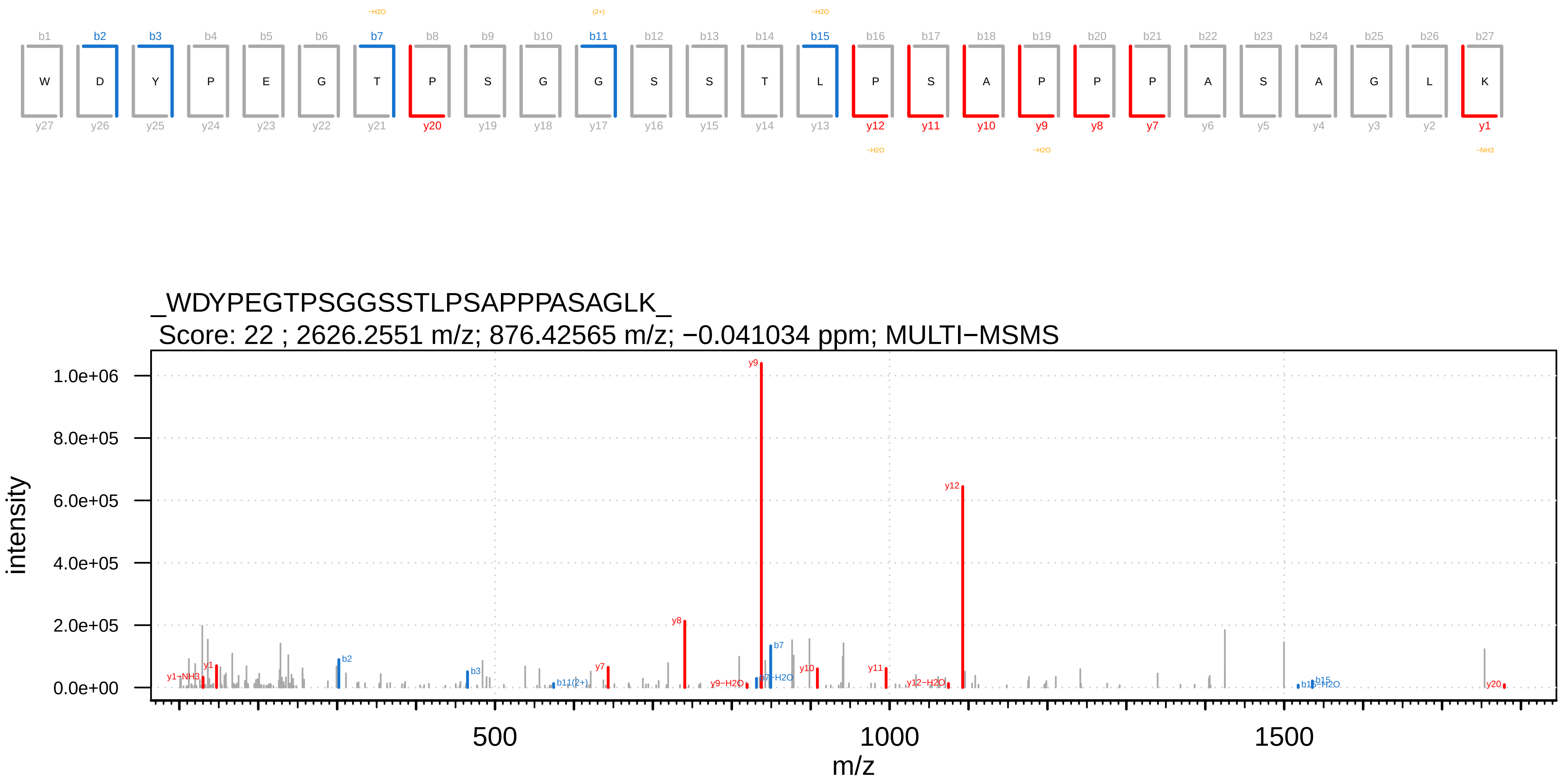

Raw File: 20130716_EXQ4_BaAz_SA_CHR3_04
Scan Number: 69509
Proteins:
ENSMUST00000149514_chrX:153723700−153723906:+

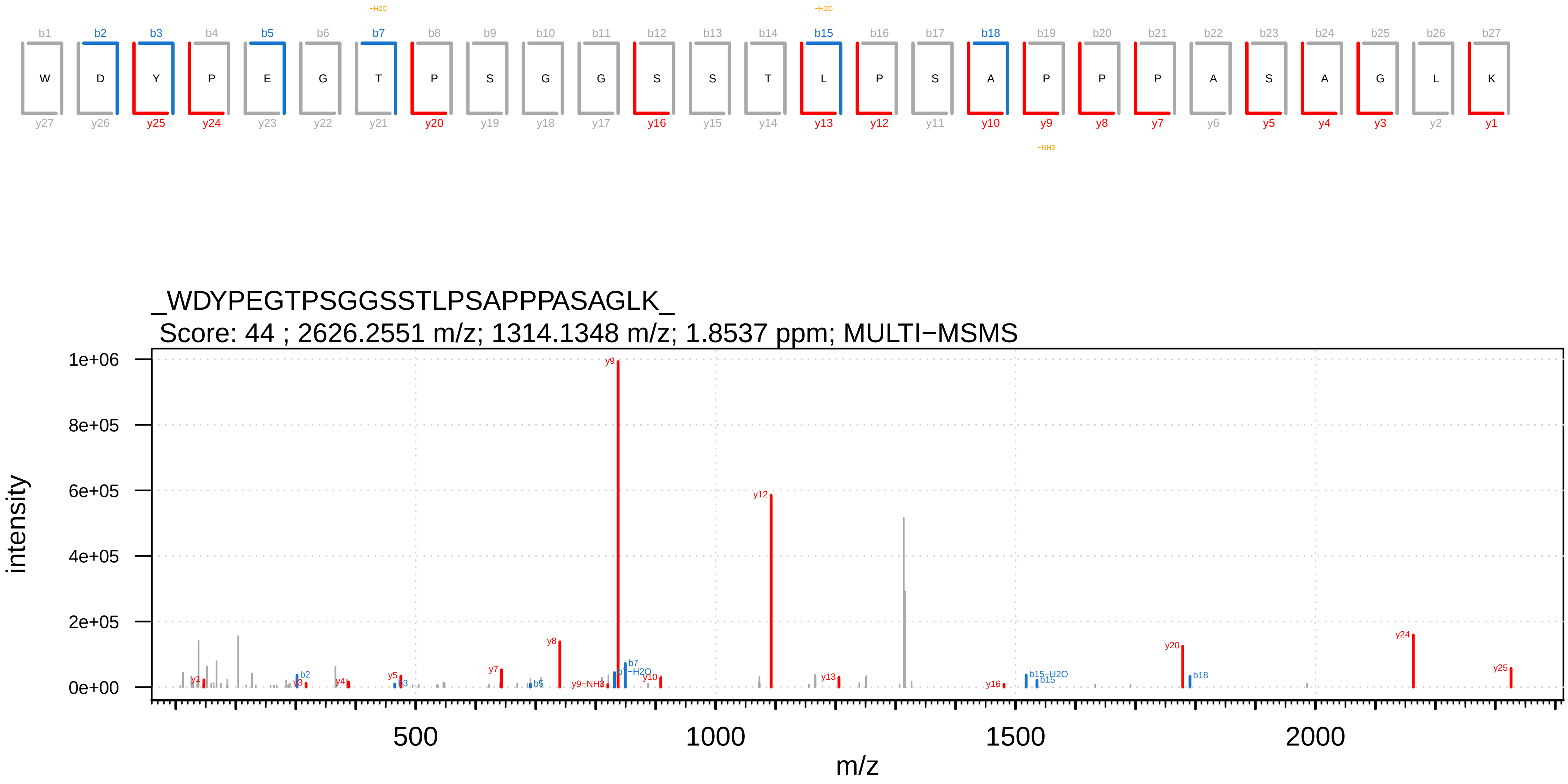

Raw File: 20130722_EXQ4_BaAz_SA_SeR3_03
Scan Number: 64688
Proteins:
ENSMUST00000149514_chrX:153723700−153723906:+

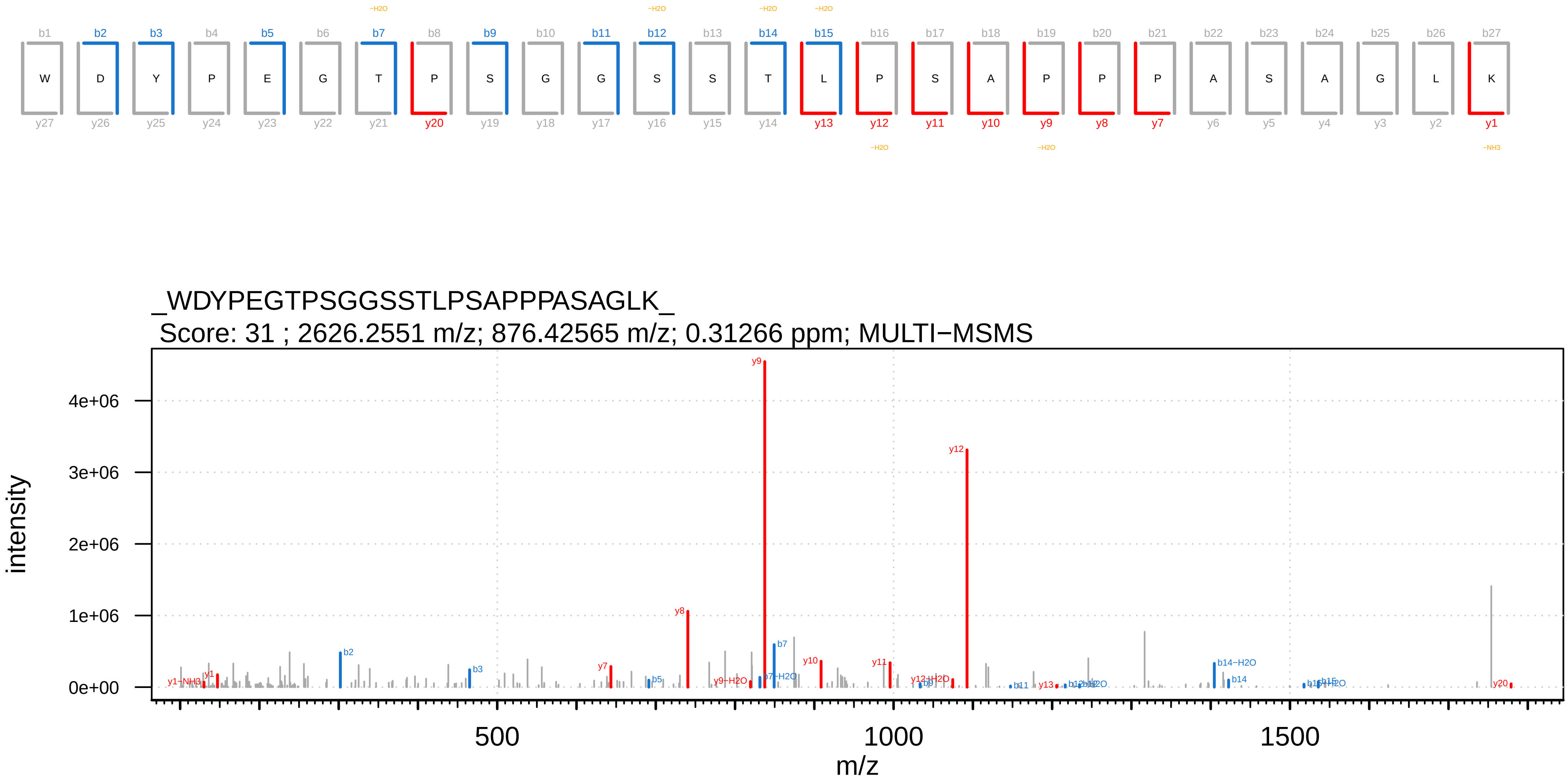

Raw File: 20130722_EXQ4_BaAz_SA_SeR3_03
Scan Number: 64706
Proteins:
ENSMUST00000149514_chrX:153723700−153723906:+

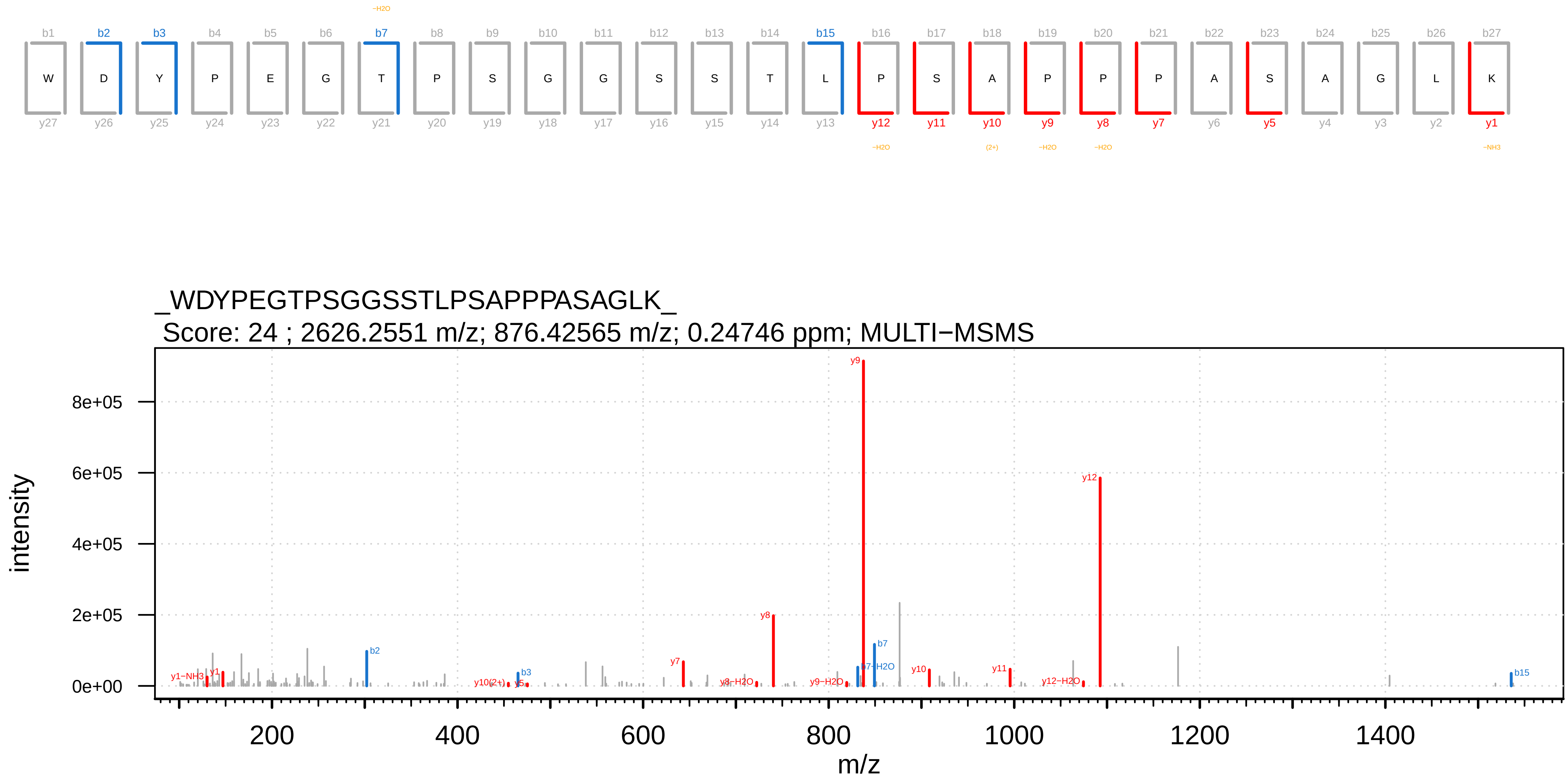

Raw File: 20130801_EXQ4_BaAz_SA_StR4_03
Scan Number: 63863
Proteins:
ENSMUST00000149514_chrX:153723700−153723906:+

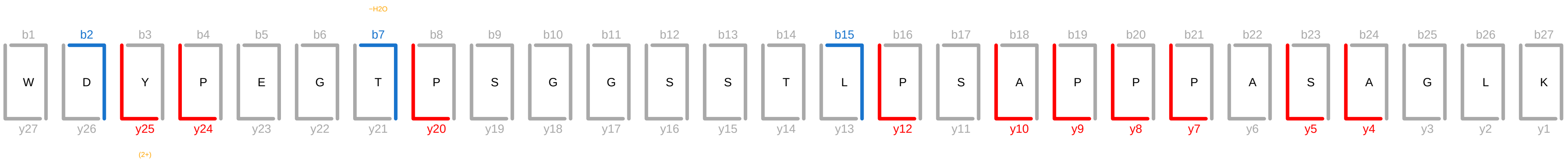

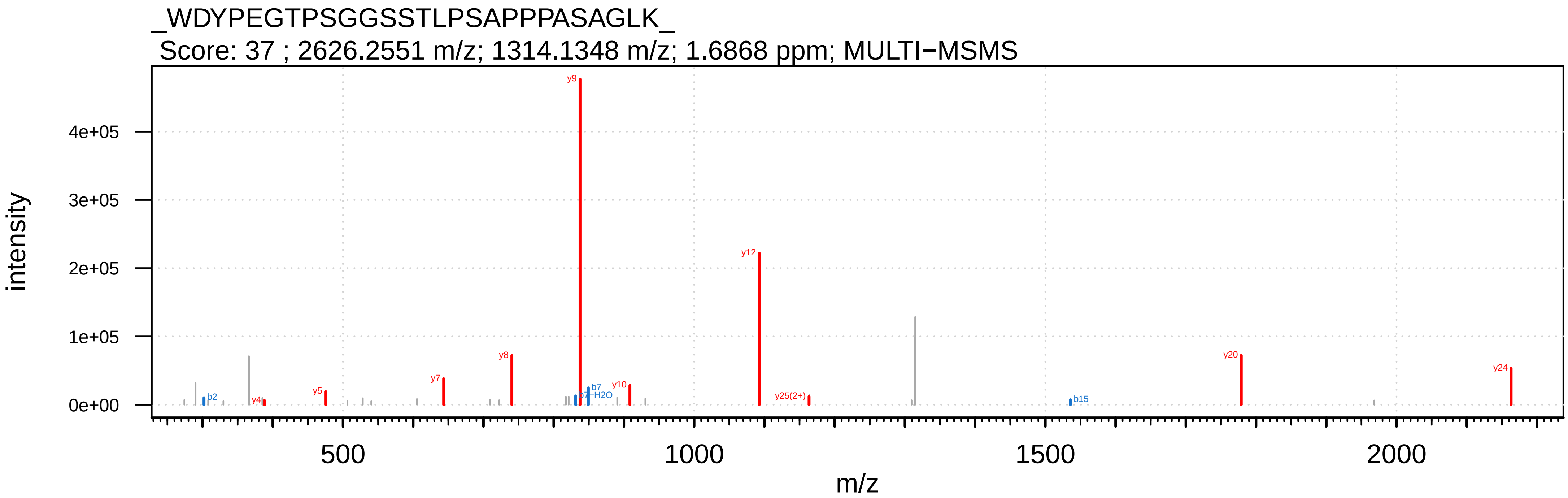

Raw File: 20130804_EXQ4_BaAz_SA_CHR4_03
Scan Number: 60787
Proteins:
ENSMUST00000149514_chrX:153723700−153723906:+

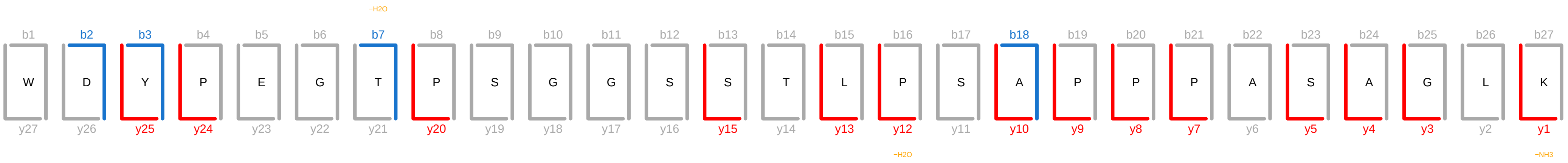

WDYPEGTPSGGSSTLPSAPPPASAGLK
Score: 45 ; 2626.2551 m/z; 1314.1348 m/z; 0.087807 ppm; MULTI−MSMS

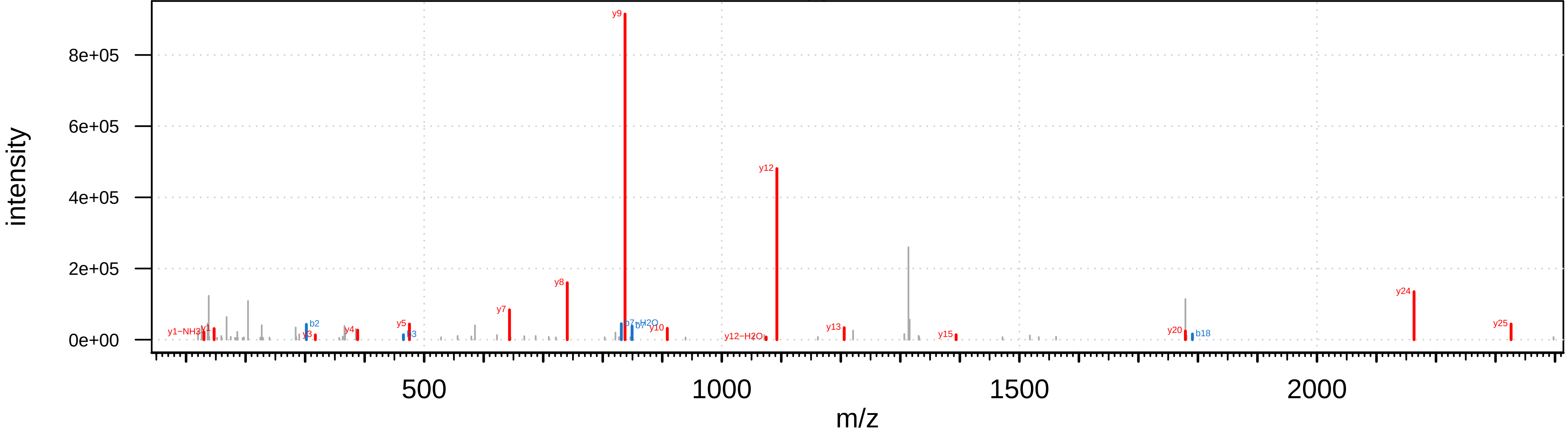

Raw File: 20130804_EXQ4_BaAz_SA_CHR4_04
Scan Number: 62305
Proteins:
ENSMUST00000149514_chrX:153723700−153723906:+
